# Foxg1 and companions: Not only transcription factors

**DOI:** 10.4103/NRR.NRR-D-25-00439

**Published:** 2025-09-03

**Authors:** Antonello Mallamaci, Osvaldo Artimagnella, Gabriele Liuzzi

**Affiliations:** 1 Laboratory of Cerebral Cortex Development, SISSA, Trieste, Italy; 2 IRCCS Centro Neurolesi “Bonino-Pulejo”, Messina, Italy

**Keywords:** BioGrid, co-transcriptional transfer, genetic pleiotropy, neuronal plasticity, polyadenylation, protein biosynthesis, splicing, transcription factors, triplosensitivity

## Abstract

Moving from the most recent results on Foxg1 biology, we first summarize the available information on some special pleiotropic effectors of neurodevelopmental interest, involved in controlling both transcription and post-transcriptional steps of gene expression. Then, after further analysis of the literature, we report evidence that, not strictly limited to neurodevelopmental effectors, such pleiotropy also applies to other transcription factors, involved in physiology and homeostasis. Furthermore, through the systematic analysis of a major public protein–protein interaction database, we gather strong evidence that the involvement of “canonical” transcription factors in post-transcriptional control of gene expression could be a pervasive phenomenon, characterizing hundreds of effectors. Finally, we discuss the biological significance of these findings and propose three evolutionary mechanisms that may have contributed to such an unexpected scenario.

## Functional Pleiotropy of Foxg1 and Other Transcription Factors Mastering Key Neurodevelopmental Processes

Classically known as a transcription factor mastering the development of the mammalian rostral brain and promoting the cytoarchitectonic maturation and activity of neocortical neurons (reviewed in Artimagnella et al., 2024), Foxg1 has been recently found to be engaged in an array of activities other than pure transcriptional control. Specifically, Foxg1 has been shown to regulate the translation of hundreds of genes, modulating the recruitment of their mRNAs to ribosomes and the progression of the latter on the former. This is associated with physical interaction between Foxg1 and specific translation factors (EIF4E and EEF1D) and can be replicated by an artificial, variant of Foxg1, confined to cytoplasm and unable to impact transcription (Artimagnella et al., 2024). Interestingly, among genes undergoing translational control by Foxg1, many are specifically involved in neuronal physiology (Artimagnella et al., 2024). Furthermore, two largely overlapping carboxyterminal fragments of Foxg1, the former generated by post-translational processing of the wild-type protein and the latter overexpressed in mutants harboring a premature stop codon, have been reported to move to mitochondria, interact with mito-ribosomal proteins and promote translation of specific mitochondrial transcripts (Bruce et al., 2024). Intriguingly, the non-transcriptional impact of Foxg1 on gene expression is not restricted to translation, but also extends to other processes, including retro-transcription (Liuzzi et al., 2024) and post-transcriptional RNA processing (Skalhegg and Tasken, 2000; Artimagnella et al., 2024).

From this point of view, Foxg1 is not a unique case. In fact, well before it, several homeodomain-TF genes, mainly involved in embryo patterning had already been shown to contribute to the post-transcriptional control of gene expression.

The first one was *bicoid* (*bcd*), which encodes for a homeodomain-TF expressed in the syncytial-blastoderm of the Drosophila embryo along an anterior^high^-to-caudal^low^ gradient, and which acts as a maternal determinant of anterior identity. As early as 1996, it was reported that, in addition to differentially promoting the transcription of three anterior gap genes, *hb*, *otd*, and *kr* (Driever and Nüsslein-Volhard, 1989; Struhl et al., 1989; Finkelstein and Perrimon, 1990), Bicoid also trans-represses the translation of the ubiquitous *cad*-mRNA, thus limiting the high expression of its protein product to the caudal-most part of the embryo (Dubnau and Struhl, 1996). Bicoid has been found to bind to a BRE-motif lying in *cad*-mRNA-3’UTR, using an arginine-rich module (ARM) located in the 3’-most part of its homeodomain (Niessing et al., 2000). Moreover, anchored to *cad*-mRNA, Bicoid further binds to EIF4E attached to *cad*-mRNA-5’cap, thanks to a YXXXXLF motif similar to those mediating the EIF4E/EIF4E-BP interaction. In this way, Bicoid prevents EIF4E interaction with EIF4G and thus impairs the translation of *cad*-mRNA (Niessing et al., 2002).

Subsequently, the pleiotropism of some vertebrate homeodomain transcription factors, often implicated in embryogenesis, has also been demonstrated. In 2005, it was reported that, specifically in myeloid cells, the Proline-Rich-Homeodomain (PRH) transcription factor binds to EIF4E through the YXXXXLF motif, which is also known to mediate the EIF4E/EIF4E-BP1 interaction. In this way it antagonizes nuclear-EIF4E-depedent, nucleocytoplasmic transport of *CyclinD1*-mRNA, reducing its translation and preventing myeloid cell transformation (Topisirovic et al., 2003). Within the same cells (and via the same YXXXXLF motif), the Hoxa9 homeobox TF can also bind to nuclear-EIF4E, outcompeting PRH and thus promoting nucleocytoplasmic transport of both *CyclinD1*- and *ODC*-mRNA. Limited to *OCD*, Hoxa9 also increases the recruitment of its mRNA to polysomes, leading to further upregulation of its post-transcriptional expression gain. All of this could contribute to the transformation of myeloid cells (Topisirovic et al., 2005).

In 2005, it was also reported that an experimental, extracellular source of midbrain-patterning, En2 homeobox TF specifically repels and attracts axonal growth cones originating from the temporal and nasal retina of amphibians, respectively, within a timeframe too short for a transcriptionally mediated mechanism. As expected, this phenomenon occurred even after axon severing, was suppressed by anysomycin and rapamycin (but not by a-amanitin), and was associated with a local increase in translation rates. Intriguingly, it was preceded by a rapid increase in EIF4E and EIF4E-BP1 phosphorylation levels, probably determinant (Brunet et al., 2005). Consistently, 4 years later, it was shown that exposure of adult mouse midbrain synapto-neurosomes (not whole cells) to the En2 paralog, En1, was followed by an *acute* selective increase of the nuclear genome-encoded, Nduf1 and Nduf3 mitochondrial complex I subunits, which resulted in increased complex I activity (Alvarez-Fischer et al., 2011). Of note, Nduf1 and Nduf3 levels were consistently reduced in *En1*^-/+^;*En2*^+/+^ mice, which are more susceptible to 6-hydroxydopamine and a-synuclein-A30P toxicity (Alvarez-Fischer et al., 2011).

Finally, substantial evidence has emerged, pointing to an involvement in the post-transcriptional control of gene expression for *Otx* and *Emx* genes, which master the early CNS patterning along the coordinated axes (Acampora and Simeone, 1999; Muzio et al., 2002). A localization in olfactory axon terminals, suggesting a likely implication in peripheral control of translation, has been described for Emx2 and its Emx1 paralog (Briata et al., 1996; Nédélec et al., 2004). In this context, Emx2 was found to bind EIF4E via a YXXXXlF domain, very similar to those mediating the reciprocal interactions among EIF4E and EIF4G, EIF4E-BPs, Bicoid, PRH, Hoxa9, and En2 (Nédélec et al., 2004). Note that, as with Hoxa9 and PRH, the involvement of Emx2 in post-transcriptional gene control appears not to be limited to translation, but also applies to other phases of mRNA metabolism, as suggested by its documented physical interaction with Cnot6l and QkI-7 (Groves et al., 2019). More recently, mass spectrometry analysis of OTX2 protein interactors in the adult retina, as well as in choroid plexus and other CNS regions, non-cell-autonomously sensitive to Otx2 manipulations (e.g., visual cortex and subventricular zone), revealed a large set of proteins implicated in splicing, nucleocytoplasmic mRNA export and translation (including U2af1, U2af2, Hnrnpk, Nxf1, Pabpc1, and EEF1a1), pointing to a likely post-transcriptional control exerted by Otx2 on these processes (Fant et al., 2015; Planques et al., 2021).

## Is the Direct Involvement of Transcription Factors in the Post-Transcriptional Modulation of Gene Expression Limited to Some Neurodevelopmental Effectors or Is It a Pervasive Phenomenon?

To assess whether, in addition to these TFs implicated in neurodevelopmental processes, other TFs might directly modulate gene expression downstream of transcription, we scanned the literature for reports documenting a functional TF impact on translation, not mediated by transcriptional control. We found almost twenty such reports, referring to as many as twelve distinctive TFs (Bmal1, Ctcfl, Eef1b2, Esr1, Hmgb3, Ilf3, Ilf3, Myf5, Phb, Stat3, Tp53, and Ybx1) (**Table 1**). This prompted us to assess if the direct contribution of transcription factors to post-transcriptional gene control might be a more general and widespread phenomenon.

**Table 1:** Selected publications reporting TF involvement in translational control

TF gene	Involvement in translation	Reference
*Ilf3*	In hypoxic in MDA-MB-435 breast cancer cells, Ilf3-encoded NF90 stimulates VEGF translation by enhancing the recruitment of its mRNAs to polysomes	Vumbaca et al., 2008
*Eef1b2*	Originally known to encode for a translation factor modulating polypeptide elongation, this gene was shown to give rise to a longer transcript, *eEF1BδL*-mRNA, in turn driving the synthesis of a transcription factor active on heat-shock-responsive elements	Kaitsuka et al., 2011
*Ilf3*	In latently *HIV1*-infected monocytic cells, Ilf3-encoded NF90 stimulates CCNT1 translation by enhancing the recruitment of its mRNAs to polysomes	Hoque et al., 2011
*Ilf3*	Specifically in actively dividing fibroblasts, Ilf3-encoded NP90 represses translation of *MCP1, GROA, IL6*, and *IL8-mRNA*	Tominaga-Yamanaka et al., 2012
*Ctcfl*	CTCFL physically associates to translating ribosomes; in hNP1 neural progenitor cells and their neuronal derivatives, anti-CTCFL precipitates 775 and 683 different mRNAs, 88 shared by the two cell types; CTCFL-OE leads to wnt-signalling upregulation	Ogunkolade et al., 2013
*Tp53*	Global repression of protein synthesis is mediated by Tp53 activation and consequent mTOR inhibition	Loayza-Puch et al., 2013
*Bmal1*	Depending on rhythmical phosphorylation by S6K1, the circadian transcription factor BMAL1 (aka Arntl) associates with the translational machinery in the cytosol and promotes protein synthesis.	Lipton et al., 2015
*Tp53*	during endoplasmic reticulum stress, Trp53 does not promote anymore anymore MDM2 and CDKN1A transcription and conversely inhibits their translation, by binding the cds's of the corresponding mRNAs.	Lopez et al., 2015
*Myf5*	In mouse C2C12 myocytes, MYF5 binds to hundreds of mRNAs implicated in myogenesis progression. in particular it binds to Ccnd1-mRNA and stimulates its translation	Panda et al., 2016
*Ilf3*	In cooperation with miR-15a, Ilf3-encoded NP90 represses translation of cytokine-encoding *BAFF*-mRNA; subtle BAFF mutation jeopardizing this regulation may ease the occurrence of autoimmune diseases like multiple sclerosis and systemic lupus erythematosus	Idda et al., 2018
*Esr1*	While repressing eIF3f transcription, estrogen-bound, Esr1-encoded ERα promotes mRNA translation, via activation of the mTORC1 pathway; this promotes binding of eIF3 to the eIF4F complex and increases rates of protein synthesis initiation.	Cuesta et al., 2019
*Ilf3*	In the human monocytic cell line THP-1 Ilf3-encoded NP90 antagonizes translation of a number of mRNAs upregulated upon exposure to *Plasmodium falciparum*, including CCL2, CCL8, CXCL10, TNF, CCL4, and IL1B mRNAs; molecular mechanisms are unknown	Idda et al., 2019
*Hmgb3*	HMGB3 and SRP14 proteins bind to TIM-TAM, a conserved RNA sequence-structure in tat mRNA that functions as a Tat IRES modulator of tat mRNA; HMGB3-KD increases Tat translation	Khouri et al., 2021
*Ybx1*	Ybx1 protein promotes polysomal engagement of selected oncogenic mRNAs, exacerbating acute myeloid leukemia aggressivenes; in a subset of cases, it was proven to bind to mRNAs differentially translated in response to its experimental manipulation	Perner et al., 2022
*Esr1*	Independently from its impact on transcription, estrogen-bound, *Esr1*-encoded ERα stimulates the PI3K-AKT-mTORC1 axis. this leads to (1) hyperphosphorylation of S6K1 and EIF4E-BPs (resulting into increased cap-dependent polypeptide initiation), and (2) hyperphosporylation of EEF2K and consequent dereppression of EEF2 (resulting into enhanced polypeptyde elongation)	Fard and Holz, 2023
*Phb*	in chronic lymphocytic leukemia (CLL) cells, PHB interacts with eIF4E, eIF4G, and eIF4A, and this interaction is needed to promote translation	Largeot et al., 2023
*Stat3*	Stat3 participates in forming a supramolecular complex (also including LRPPRC and SLIRP proteins) that is required for the stability of mature mitochondrially encoded mRNAs and their transport to the mitochondrial ribosome	Fernando et al., 2023
*Tp53*	Beyond its trascriptional impact on EIF4E and EIF4E-BP1, Tp53 inhibits EIF4E-BP1 phosphorylation, so dampening CEBPB translation	Hu et al., 2023
*Tp53*	Tp53 binds to *Mdmx*-mRNA 5'UTR and dampens its translation	Tournillon et al., 2017

**Figure 1:**
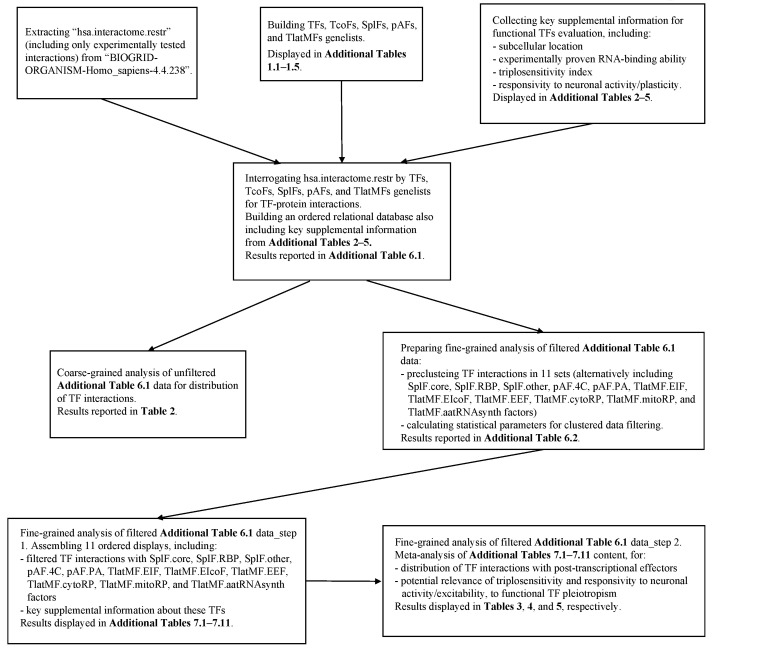
Mining the BioGrid database: operational diagram. Here shown are the structure and the concatenation of the main work packages implemented to extract key information supporting the large-scale implication of TFs in post-transcriptional control of gene expression. pAF.4C: Polyadenylation factors, “4C” set (including CFIm, CFIIm, CPSF, and CSTF elements); pAF.PA: polyadenylation factors, “PA” set (including polyadenylate-interacting elements); pAFs: polyadenylation factors; SplF.core: splicing factors, “core” set (including U1snRNP, SM proteins, U2 snRNP proteins, and related ones, A complex, LSm proteins, U5 SNP, U4/U6 SNP, PPR19 complex, B complex, Bact complex, RES complex, and C complex elements); SplF.other: splicing factors, “other” set (including protein kinase, Other SAPs, Minor, Lysine transferases, chromatin-related, Histone transferases, SWI/SNF Complex Components, and Polycomb Group Genes elements); SplF.RBP: splicing factors, “RBP” set (including (EJC/mRNP, TREX, helicases, hnRNP, SR proteins, RNA binding proteins, RNA editing, RNA modifying, RNA methylation, CBC, 3’end, RNA degradation, and RISC elements); SplFs: splicing factors; TcoFs: transcription cofactors; TFs: transcription factor; TlatMF.aatRNAsynth: translation machinery factors, “aminoacyl-tRNA-synthetases” set; TlatMF.cytoRP: translation machinery factors, “cytoplasmic ribosomal proteins” set; TlatMF.EEF: translation machinery factors, “eukaryotic elogation factors” set; TlatMF.EIcoF: translation machinery factors, “eukaryotic initiation cofactors” set; TlatMF.EIF: translation machinery factors, “eukaryotic initiation factors” set; TlatMF.mitoRP: translation machinery factors, “mitochondrial ribosomal proteins” set; TlatMFs: translation machinery factors; NB: all acronyms specifically referring to human factors. Created with Adobe Photoshop CS6 (version 13.0×64) and Microsoft PowerPoint for Mac (version 16.66.1).

To systematically address this issue, we decided to exploit the huge amount of data on protein-protein interactions stored in public databases, considering the physical interaction between a given transcription factor and the classical factors controlling the different post-transcriptional steps of gene expression (splicing, polyadenylation, translation) as a reasonable index of the possible direct control exerted by such transcription factor on these processes. Summarized in **Figure 1**, our analysis strategy is described below.

Based on pertinent literature, we compiled five specific gene lists: TFs, transcription factors; TcoFs, transcription cofactors; SplFs, splicing factors; pAFs, polyadenylation factors; TlatMFs, translational machinery factors (**Additional Tables 1.1–1.5**). By these gene lists, we mined the BIOGRID-ORGANISM-Homo_sapiens-4.4.238 database, limiting our analysis to a subset of it (“hsa.interactome.restr”), which included only experimentally verified, protein-protein interactions (*n* = 1,152,045). Specifically, we counted interaction reports involving each TF with all of the aforementioned, distinct sets of post-transcriptional factors as well as reports of “canonical” TF/TF and TF/TcoF interactions, as controls, and reported the resulting counts into a comprehensive relational table (**Additional Table 6.1**).

Starting from such table, we calculated the cumulative numbers of TF interaction reports including factors encoded by each of the five gene lists mentioned above (TFs, TcoFs, SplFs, pAFs, and TlatMFs). Furthermore, we repeated this cumulative assessment limited to TFs belonging to distinct taxa (**Table 2**). Albeit lower, the total numbers of TF/SplF and TF/TlatMF interaction reports (13,287 and 6240, respectively) were not negligible compared to those of TF/TF and TF/TcoF reports (35,728 and 31,881, respectively). Besides, upon normalization with respect to the corresponding, cumulative TF/TF and TF/TcoF values, TFs belonging to some taxa (ZF.C3H, ZF.CCHC, ZF.NFX1, IPT, HMG.WHSC2, ZF.CXXC, DBP, HSF, PUR, ZF.DM, WC.MYB, CSRNP_N, HMG, RUNT, and HMG.TOX) were found to often interact with SplFs, while TFs belonging to other taxa (ZF.CCHC, ZF.NFX1, DBP, HMG.TFAM, HMG, PUR, and GHD.CP2) were found to be preferentially connected to TlatMFs. Of note, albeit represented in the database by several hundred TF/TF and TF/TcoF interaction reports, some taxa (TEA, NF1, MADS, HB-CUT, ARID, HB-PAX, and PAX) did not display any TF/TlatMF entries. Taken together, these results suggest that: (a) TFs may be implicated in non-canonical post-transcriptional control of gene expression, sometimes to an extent comparable to their canonical involvement in transcriptional control; (b) TFs belonging to distinct families may be differentially engaged in the control of splicing and translation.

**Table 2:** Coarse-grained distribution of full BioGrid, TF-interaction reports (1)

TF taxon	*n*° of interaction reports involving TF and …								
	Absolute values						(TF+TcoF)-normalized values		
		
	TFs	TcoFs	SplFs	pAFs	TlatMFs		SplFs	pAFs	TlatMFs
All TFs	35728	31881	13287	371	6240		0.20	0.01	0.09
HB	2	3	0	0	0		0.00	0.00	0.00
HB.PHTF	1	1	0	0	0		0.00	0.00	0.00
PHDFP	12	6	0	0	0		0.00	0.00	0.00
TEA	150	161	8	3	0		0.03	0.01	0.00
HB.SINE	41	69	5	0	1		0.05	0.00	0.01
NF1	479	112	31	1	0		0.05	0.00	0.00
NRF	20	17	2	0	0		0.05	0.00	0.00
HB.LIM	345	263	37	0	17		0.06	0.00	0.03
HMG.TCF7	114	132	15	0	20		0.06	0.00	0.08
TSC22	72	88	13	0	1		0.08	0.00	0.01
HB-PAX	196	94	25	0	1		0.09	0.00	0.00
PAX	129	90	19	0	1		0.09	0.00	0.00
SNAD	7	3	1	0	0		0.10	0.00	0.00
ZF.MIZ	381	254	65	0	28		0.10	0.00	0.04
SMAD	983	781	184	4	27		0.10	0.00	0.02
HB.HOX	532	341	97	3	20		0.11	0.00	0.02
HB.TALE	293	128	47	2	5		0.11	0.00	0.01
CAAT-BF	174	45	25	1	5		0.11	0.00	0.02
HB.NK	502	336	98	2	100		0.12	0.00	0.12
HB-POU	411	220	80	1	41		0.13	0.00	0.06
HB.PD	337	254	77	0	86		0.13	0.00	0.15
FH/WH.RFX	100	60	21	3	3		0.13	0.02	0.02
WC.IRF	245	201	59	1	3		0.13	0.00	0.01
HMG.TFAM	60	41	14	1	64		0.14	0.01	0.63
GCM	40	32	10	1	0		0.14	0.01	0.00
HB-CUT	252	97	49	1	1		0.14	0.00	0.00
HMG.PBRM1	123	76	28	0	6		0.14	0.00	0.03
RHR	840	781	243	4	30		0.15	0.00	0.02
TUB	41	72	18	2	14		0.16	0.02	0.12
GHD.CP2	98	101	33	0	54		0.17	0.00	0.27
FH/WH.E2F	439	417	145	0	31		0.17	0.00	0.04
bZIP	2837	1847	805	36	318		0.17	0.01	0.07
STAT	304	358	114	0	21		0.17	0.00	0.03
FH/WH.FOX	1195	875	365	19	123		0.18	0.01	0.06
HMG.SOX	900	569	260	7	205		0.18	0.00	0.14
TB	135	106	43	0	4		0.18	0.00	0.02
ZF.CCCH	1	10	2	0	0		0.18	0.00	0.00
p53	628	1968	476	7	168		0.18	0.00	0.06
WC.ETS	708	498	222	0	22		0.18	0.00	0.02
HB-ZF.C2H2	404	307	131	3	38		0.18	0.00	0.05
HMGA	109	144	47	0	5		0.19	0.00	0.02
ARID	626	287	172	1	3		0.19	0.00	0.00
ZF.C2H2	7858	6407	2743	50	2070		0.19	0.00	0.15
HTH	29	69	19	0	4		0.19	0.00	0.04
SAND	136	142	54	1	14		0.19	0.00	0.05
ZF	12	24	7	0	0		0.19	0.00	0.00
ZF.C4_NR	3099	2966	1188	49	460		0.20	0.01	0.08
LRRFIP	10	51	12	0	0		0.20	0.00	0.00
bHLH	3751	3296	1416	48	649		0.20	0.01	0.09
RFXANK	21	28	10	2	2		0.20	0.04	0.04
ZF.C2CH	159	210	78	1	83		0.21	0.00	0.22
HB.PROS	14	9	5	0	0		0.22	0.00	0.00
MADS	173	125	65	0	0		0.22	0.00	0.00
HMG.UBF	33	63	21	0	5		0.22	0.00	0.05
GTF2I	92	126	48	1	4		0.22	0.00	0.02
ZF.C4_GATA	1002	974	469	11	37		0.24	0.01	0.02
HMG.TOX	334	228	144	4	4		0.26	0.01	0.01
RUNT	190	159	92	5	4		0.26	0.01	0.01
HMG	200	249	123	2	165		0.27	0.00	0.37
others	1028	1698	814	38	349		0.30	0.01	0.13
CSRNP_N	3	7	3	0	0		0.30	0.00	0.00
WC.MYB	1394	1468	869	15	216		0.30	0.01	0.08
ZF.DM	15	52	21	3	4		0.31	0.04	0.06
PUR	85	77	53	4	58		0.33	0.02	0.36
HSF	89	162	98	4	44		0.39	0.02	0.18
DBP	149	203	138	5	224		0.39	0.01	0.64
ZF.CXXC	410	526	368	18	138		0.39	0.02	0.15
HMG.WHSC2	68	169	115	5	10		0.49	0.02	0.04
IPT	2	4	3	0	0		0.50	0.00	0.00
ZF.NFX1	30	82	78	1	120		0.70	0.01	1.07
ZF.CCHC	47	40	90	1	110		1.03	0.01	1.26
ZF.C3H	29	22	57	0	0		1.12	0.00	0.00
HMG.WHSC1	0	0	0	0	0		na	na	na
NDT80	0	0	0	0	0		na	na	na
ZF.BED	0	0	0	0	0		na	na	na

(1) Compiled on the basis of Additional Table 6.2 data (columns E–I). pAFs: Polyadenylation factors; SplFs: splicing factors; TcoFs: transcription cofactors; TFs: transcription factors; TlatMFs: translational machinery factors.

Surprised by this scenario, we suspected that it might reflect a non-physiological TF binding to presumptive interactors, occurring upon the protein overexpression needed to implement the interaction assays indexed in BioGrid. We hypothesized that in this case, the frequencies of BioGrid interaction reports should tend to a pattern not far from that originating from the random inclusion of proteins into interactor dyads, reflecting their cumulative frequencies of representation in the database. To address this point, we decided to challenge our original inference based on a coarse-grained analysis of the entire dataset, by a new approach, based on a finer-grained investigation of it. Starting from **Additional Table 6.1** data, we counted interaction reports involving each TF and 11 smaller, distinct pools of functionally related post-transcriptional effectors (SplF.core, SplF.RBP, SplF.oth, pAF.4C, pAF.PA, TlatMF.EIF, TlatMF.EIcoF, TlatMF.EEF, TlatMF.cytoRP, TlatMF.mitoRP, and TlatMF.aatRNAsynth; see **Additional Tables 1.1–1.5**), compared the actual numbers of these reports with those expected in case of random protein/protein interaction (see **Additional Table 6.2**), and further considered only those interaction-report sets displaying a statistically significant excess of observed items over those expected by chance. In this way, we obtained 11 tables, where we synthesized “filtered” experimental evidence supporting TF implication in splicing (**Additional Tables 7.1–7.3**), polyadenylation (**Additional Tables 7.4** and **7.5**), and translation (**Additional Tables 7.6–7.11**).

Finally, we summarized the key features of the **Additional Tables 7.1–7.11** set in **Tables 3** and **4**. In the former, for each pool of post-transcriptional effector “baits,” we reported the number of filtered TF “preys” interacting with them, as well as their differential taxon allocation. In the latter, we provided the complete list of TFs engaged in “filtered” interactions with post-transcriptional effectors, as well as the distribution of their interactors among the main 11 pools considered for our analysis. Of note, where applicable, for each TF, we also annotated (a) its extranuclear localization (as documented in the Human Protein Atlas [**Additional Table 2**]), and (b) its experimentally verified capability to bind RNA (according to a full list of human RNA-binding proteins, RBPs, pre-compiled by us on the basis of primary literature [**Additional Table 3**]), i.e., two valuable pieces of information, potentially suitable to corroborate the inferred TF control over specific post-transcriptional steps of gene expression. To ease subsequent correlative analyses, this information was included in **Additional Table 6.1** as well as in its **Additional Tables 7.1–7.11** and **Table 3** derivatives.

**Table 3:** Distribution of physical interactions among distinctive pools of post-transcriptional effector and classified TFs, upon statistical filtering of primary BioGrid data (also including selected features of interacting TFs)

Bait-sets (i.e., post-transcriptional effector-sets)(1)					n° of distinctive interacting dyad reports found in BioGrid, upon statistical filtering(2)	Interacting preys (i.e., TFs)(1)	Taxon allocation of interacting preys																																																		Key features of interacting preys	
	
Reference process	Pool #	Pool acronym	Total baits number	Interacting baits number, upon statistical filtering (2)		Number of interacting preys, upon statistical filtering (2)	Taxon allocation of interacting preys																																																		Number of interacting preys with documented cytoplasmic and/or mitochondrial localization	Number of interacting preys with documented RNA-binding activity
							ARID	bHLH	bZIP	CAAT-BF	DBP	FH/WH.E2F	FH/WH.FOX	FH/WH.RFX	FH/WH.RFX	GCM	GHD.CP2	GTF2I	HB-CUT	HB-POU	HB-ZF.C2H2	HB.HOX	HB.LIM	HB.NK	HB.PD	HB.TALE	HMG	HMG.SOX	HMG.TCF7	HMG.TFAM	HMG.TOX	HSF	MADS	p53	PUR	RFXANK	RHR	RUNT	SMAD	STAT	TEA	TUB	WC.ETS	WC.IRF	WC.MYB	ZF	ZF.C2CH	ZF.C2H2	ZF.C3H	ZF.C4_GATA	ZF.C4_NR	ZF.CCHC	ZF.CXXC	ZF.DM	ZF.NFX1	others		
Splicing	1	SplF.core	116	90	192	11								+	+												+																		+			+	+					+		+	2	6
	2	SplF.RBP	117	59	180	17														+					+		+							+									+					+							+	+	8	10
	3	SplF.oth(3)	71	54	856	127	+	+	+	+		+	+			+		+	+	+	+	+		+		+	+	+			+		+	+			+	+	+	+			+	+	+	+		+		+	+		+			+	42	60
Poly-adenylation	4	pAF.4C	16	16	97	26			+		+		+	+	+												+				+					+		+							+			+		+	+		+	+		+	9	12
	5	pAF.PA	17	10	34	31		+	+	+	+		+					+			+			+		+									+	+					+	+						+		+				+		+	13	18
Translation	6	TlatMF.EIF	45	25	97	16		+														+		+	+		+																		+			+							+	+	6	7
	7	TlatMF.EIcoF	13	8	36	23					+								+			+	+	+	+		+	+							+										+			+				+			+	+	14	15
	8	TlatMF.EEF	16	7	39	25			+				+				+							+	+		+			+		+					+											+		+						+	12	9
	9	TlatMF.cytoRP	80	76	1399	63		+									+				+			+	+		+	+							+										+		+	+				+	+		+	+	27	45
	10	TlatMF.mitoRP	82	80	793	42		+	+		+		+											+			+	+	+	+																	+	+				+			+	+	22	26
	11	TlatMF.aatRNAsynth	41	15	29	17					+		+										+				+			+		+					+			+								+								+	9	8

(1) Compiled on the basis of filtered interaction data reported in Additional Tables 7.1–7.11; here, for sake of simplicity, TF are presented as “preys” captured by 11 distinctive “bait-sets,” each consisting in a specific post-transcriptional effector pool. (2) As in Additional Tables 7.1–7.11, here each TF was taken into account as a “reliable” prey, only if involved in interactions with pools' members, cumulatively satisfying the three criteria below: - chi (obs-vs-exp interactions with pool members, evaluated over all 11 pools) < 0.05; - chi (obs-vs-exp interactions with pool members, evaluated over only “reference process” pools) < 0.05; - candidate-prey interactions with members of the bait-pool in order, n(obs)/n(exp)≥2; (3) Including a large number of items known to work as epigenetic effectors. pAF.4C: (human) polyadenylation factors, “4C” set (including CFIm, CFIIm, CPSF, and CSTF elements); pAF.PA: (human) polyadenylation factors, “PA” set (including polyadenylate-interacting elements); SplF.core: (human) splicing factors, “core” set (including U1snRNP, SM proteins, U2 snRNP proteins and related ones, A complex, LSm proteins, U5 SNP, U4/U6 SNP, PPR19 complex, B complex, Bact complex, RES complex, and C complex elements); SplF.other: (human) splicing factors, “other” set (including protein kinase, Other SAPs, Minor, Lysine transferases, chromatin-related, Histone transferases, SWI/SNF Complex Components, and Polycomb Group Genes elements); SplF.RBP: (human) splicing factors, “RBP” set (including (EJC/mRNP, TREX, helicases, hnRNP, SR proteins, RNA binding proteins, RNA editing, RNA modifying, RNA methylation, CBC, 3'end, RNA degradation, and RISC elements); TlatMF.aatRNAsynth: (human) translation machinery factors, “aminoacyl-tRNA-synthetases” set; TlatMF.cytoRP: (human) translation machinery factors, “cytoplasmic ribosomal proteins” set; TlatMF.EEF: (human) translation machinery factors, “eukaryotic elogation factors” set; TlatMF.EIcoF: (human) translation machinery factors, “eukaryotic initiation cofactors” set; TlatMF.EIF: (human) translation machinery factors, “eukaryotic initiation factors” set; TlatMF.mitoRP: translation machinery factors, “mitochondrial ribosomal proteins” set.

Remarkably, as many as 312 TFs, i.e., approximately 20% of those investigated, resulted in specifically interacting with post-transcriptional controllers of gene expression (**Table 4**). As such, they are likely directly involved in the post-transcriptional control of gene expression. These TFs include 155 ones putatively involved in splicing, 57 in polyadenylation, and 186 in translation (**Table 3**, column 7), cumulatively interacting with 203, 26, and 211 post-transcriptional factors canonically implicated in these processes, respectively (**Table 3**, column 5).

**Table 4:** Allotment of filtered, transcription factor (TF)-interactors to distinctive pools of post-transcriptional effectors

TF (n = 312)	TF taxon	Interacting with factors belonging to…										
		SplF.core	SplF.RBPS	SplF.other	pAF.4C	pAF.PA	TlatMF.EIF	TlatMF.EIcoF	TlatMF.EEF	TlatMF.cytoRP	TlatMF.mitoRP	TlatMF.aatRNA synth
NFX1	ZF.NFX1		+				+	+		+	+	
FOXI2	FH/WH.FOX				+	+			+			+
HMGB2	HMG						+	+	+	+		
KLF4	ZF.C2H2			+				+		+	+	
MAEL	HMG	+	+				+	+				
ZKSCAN8	ZF.C2H2					+				+	+	+
BHLHA15	bHLH						+			+	+	
CPEB1	others		+		+	+						
DMRTB1	ZF.DM	+			+	+						
DNAJC2	WC.MYB						+	+		+		
DUX4	HB.PD						+	+		+		
FOXB1	FH/WH.FOX								+		+	+
GLI4	ZF.C2H2					+					+	+
GPBP1L1	others					+	+					+
LIN28A	ZF.CCHC							+		+	+	
MSX2	HB.NK							+	+	+		
MTF1	ZF.C2H2	+	+							+		
TFAM	HMG.TFAM								+		+	+
ZNF532	ZF.C2H2		+	+					+			
ZNF746	ZF.C2H2						+	+		+		
ATF3	bZIP			+					+			
CDC5L	WC.MYB	+			+							
CEBPD	bZIP			+					+			
CTCF	ZF.C2H2						+			+		
E4F1	ZF.C2H2									+	+	
ERG	WC.ETS	+	+									
FEZF2	ZF.C2H2			+			+					
FOXL2	FH/WH.FOX										+	+
GATA1	ZF.C4_GATA			+	+							
GATA3	ZF.C4_GATA			+	+							
GATA4	ZF.C4_GATA			+					+			
HEY1	bHLH					+				+		
KLF13	ZF.C2H2		+	+								
KLF16	ZF.C2H2						+				+	
KMT2C	HMG			+				+				
MBD4	ZF.CXXC			+						+		
MTA2	ZF.C4_GATA			+					+			
NFATC1	RHR			+								+
PAXBP1	others	+		+								
PHB	others								+			+
PURB	PUR					+				+		
PURG	PUR					+		+				
REST	ZF.C2H2		+							+		
RFX6	FH/WH.RFX	+			+							
RFXANK	RFXANK				+	+						
RUNX1	RUNT			+	+							
SON	others	+		+								
SOX15	HMG.SOX							+			+	
TADA2A	WC.MYB			+			+					
TFB1M	others									+	+	
TFCP2	GHD.CP2								+	+		
THAP3	ZF.C2CH									+	+	
TLX2	HB.NK					+					+	
YBX1	DBP							+			+	
YBX2	DBP										+	+
YBX3	DBP				+	+						
ZBTB41	ZF.C2H2		+							+		
ZEB1	HB-ZF.C2H2			+						+		
ZFPM2	ZF.C2H2			+				+				
ZNF207	ZF.C2H2	+	+									
ZNF274	ZF.C2H2									+	+	
ZNF317	ZF.C2H2									+	+	
ZNF324B	ZF.C2H2										+	+
ZNF326	ZF.C2H2		+					+				
ZNF467	ZF.C2H2					+					+	
ZNF485	ZF.C2H2									+	+	
ZNF511	ZF.C2H2	+						+				
ZNF512	ZF.C2H2					+				+		
ZNF574	ZF.C2H2									+	+	
ZNF689	ZF.C2H2									+	+	
ZNF770	ZF.C2H2									+	+	
ADNP	HB-ZF.C2H2			+								
AEBP2	ZF.C2H2			+								
ALX1	HB.PD								+			
AR	ZF.C4_NR				+							
ARID1A	ARID			+								
ARID1B	ARID			+								
ARID4A	ARID			+								
ARID5B	ARID			+								
ARX	HB.PD							+				
BACH2	bZIP					+						
BATF3	bZIP								+			
BBX	HMG.SOX									+		
BCL11A	ZF.C2H2			+								
BCL11B	ZF.C2H2			+								
BCLAF1	others							+				
CAPN15	others					+						
CC2D1A	others					+						
CDX2	HB.HOX			+								
CEBPA	bZIP				+							
CEBPB	bZIP				+							
CEBPZ	others									+		
CNBP	others									+		
CREB1	bZIP			+								
CRX	HB.PD								+			
CSDE1	others							+				
CUX1	HB-CUT							+				
E2F6	FH/WH.E2F			+								
EGR1	ZF.C2H2			+								
ELF4	WC.ETS			+								
EMX2	HB.NK						+					
ESR2	ZF.C4_NR				+							
ETV1	WC.ETS			+								
FOS	bZIP								+			
FOSL2	bZIP								+			
FOXC1	FH/WH.FOX		+									
FOXG1	FH/WH.FOX								+			
FOXL1	FH/WH.FOX				+							
FOXM1	FH/WH.FOX											+
FOXO1	FH/WH.FOX								+			
FOXO3	FH/WH.FOX			+								
FOXP3	FH/WH.FOX			+								
FOXR1	FH/WH.FOX			+								
FOXS1	FH/WH.FOX					+						
GATAD1	ZF.C4_GATA			+								
GATAD2A	ZF.C4_GATA			+								
GATAD2B	ZF.C4_GATA			+								
GCFC2	others	+										
GCM1	GCM			+								
GFI1	ZF.C2H2			+								
GFI1B	ZF.C2H2			+								
GLI1	ZF.C2H2			+								
GPBP1	others					+						
GTF2I	GTF2I			+								
GTF2IRD1	GTF2I					+						
GZF1	ZF.C2H2									+		
HHEX	HB.NK						+					
HIC1	ZF.C2H2			+								
HIF1A	bHLH			+								
HMG20A	HMG.TOX			+								
HMG20B	HMG.TOX			+								
HMGB1	HMG									+		
HNF1A	HB-POU			+								
HNF4A	ZF.C4_NR			+								
HOMEZ	HB-ZF.C2H2					+						
HOXA9	HB.HOX						+					
HSF1	HSF								+			
HSFX1	HSF											+
IKZF1	ZF.C2H2								+			
INSM1	ZF.C2H2			+								
IRF1	WC.IRF			+								
IRF2	WC.IRF			+								
JARID2	ARID			+								
KHSRP	others								+			
KLF12	ZF.C2H2										+	
KLF15	ZF.C2H2										+	
KLF7	ZF.C2H2		+									
KLF8	ZF.C2H2						+					
L3MBTL3	others			+								
LBX1	HB.NK								+			
LHX4	HB.LIM							+				
LIN28B	ZF.CCHC										+	
LMX1B	HB.LIM											+
LYAR	ZF.C2CH									+		
MAFB	bZIP										+	
MBD2	ZF.CXXC			+								
MBD3	ZF.CXXC			+								
MECOM	ZF.C2H2			+								
MECP2	ZF.CXXC				+							
MEF2A	MADS			+								
MEF2C	MADS			+								
MEF2D	MADS			+								
MEOX1	HB.HOX							+				
MESP1	bHLH			+								
MIER1	WC.MYB			+								
MIER3	WC.MYB			+								
MLLT10	others								+			
MLXIPL	bHLH			+								
MTA1	ZF.C4_GATA			+								
MTA3	ZF.C4_GATA			+								
MYB	WC.MYB			+								
MYCN	bHLH										+	
MYOD1	bHLH			+								
NCOR1	WC.MYB			+								
NCOR2	WC.MYB			+								
NFYA	CAAT-BF					+						
NFYB	CAAT-BF			+								
NKRF	others									+		
NKX2-2	HB.NK			+								
NKX2-6	HB.NK									+		
NR0B2	ZF.C4_NR			+								
NR1H2	ZF.C4_NR			+								
NR1H3	ZF.C4_NR			+								
NR1H4	ZF.C4_NR			+								
NR2C1	ZF.C4_NR			+								
OVOL1	ZF.C2H2			+								
PBX1	HB.TALE			+								
PITX3	HB.PD		+									
PLAGL1	ZF.C2H2			+								
PLSCR1	others				+							
POU3F4	HB-POU		+									
PPARA	ZF.C4_NR			+								
PPARD	ZF.C4_NR			+								
PPARG	ZF.C4_NR			+								
PRDM1	ZF.C2H2			+								
PRDM10	ZF.C2H2									+		
PRDM14	ZF.C2H2			+								
PRDM15	ZF.C2H2									+		
PURA	PUR									+		
RBPJ	RHR			+								
RCOR1	WC.MYB			+								
RCOR2	WC.MYB			+								
RCOR3	WC.MYB			+								
REL	RHR								+			
RELA	RHR			+								
RELB	RHR			+								
RERE	ZF.C4_GATA			+								
RFX5	FH/WH.RFX				+							
RREB1	ZF.C2H2			+								
RUNX2	RUNT			+								
RUNX3	RUNT			+								
SATB1	HB-CUT			+								
SFPQ	others		+									
SMAD7	SMAD			+								
SMARCC1	WC.MYB			+								
SMARCC2	WC.MYB			+								
SMARCE1	HMG.TOX			+								
SNAI1	ZF.C2H2			+								
SNAI2	ZF.C2H2			+								
SNAI3	ZF.C2H2			+								
SOX9	HMG.SOX			+								
SP1	ZF.C2H2			+								
SP3	ZF.C2H2			+								
SP7	ZF.C2H2				+							
SREBF1	bHLH			+								
SRY	HMG.SOX			+								
STAT2	STAT											+
STAT3	STAT			+								
STAT5A	STAT			+								
SUB1	others				+							
TAL1	bHLH			+								
TCF7L2	HMG.TCF7										+	
TEAD4	TEA					+						
TGIF2	HB.TALE					+						
THAP7	ZF.C2CH									+		
TLX3	HB.NK										+	
TOX4	HMG.TOX				+							
TP53	p53				+							
TP63	p53		+									
TP73	p53			+								
TRERF1	WC.MYB			+								
TRPS1	ZF.C4_GATA					+						
TTF1	WC.MYB									+		
TULP3	TUB					+						
TWIST1	bHLH			+								
USF1	bHLH			+								
WIZ	ZF.C2H2			+								
YY1	ZF.C2H2			+								
ZBED1	ZF.C2H2				+							
ZBTB1	ZF.C2H2									+		
ZBTB10	ZF.C2H2				+							
ZBTB11	ZF.C2H2									+		
ZBTB16	ZF.C2H2			+								
ZBTB2	ZF.C2H2							+				
ZBTB24	ZF.C2H2									+		
ZBTB44	ZF.C2H2					+						
ZBTB47	ZF.C2H2										+	
ZBTB48	ZF.C2H2										+	
ZBTB7A	ZF.C2H2			+								
ZEB2	HB-ZF.C2H2			+								
ZFP62	ZF.C2H2									+		
ZFP69	ZF.C2H2		+									
ZFP91	ZF.C2H2									+		
ZGPAT	ZF.C3H	+										
ZNF121	ZF.C2H2									+		
ZNF133	ZF.C2H2										+	
ZNF16	ZF.C2H2									+		
ZNF169	ZF.C2H2										+	
ZNF17	ZF.C2H2										+	
ZNF184	ZF.C2H2									+		
ZNF189	ZF.C2H2									+		
ZNF2	ZF.C2H2										+	
ZNF213	ZF.C2H2								+			
ZNF217	ZF.C2H2			+								
ZNF219	ZF.C2H2			+								
ZNF22	ZF.C2H2									+		
ZNF239	ZF.C2H2				+							
ZNF24	ZF.C2H2								+			
ZNF263	ZF.C2H2			+								
ZNF280C	ZF.C2H2			+								
ZNF280D	ZF.C2H2			+								
ZNF320	ZF.C2H2									+		
ZNF331	ZF.C2H2										+	
ZNF333	ZF.C2H2					+						
ZNF341	ZF.C2H2				+							
ZNF354A	ZF.C2H2									+		
ZNF358	ZF.C2H2									+		
ZNF436	ZF.C2H2			+								
ZNF444	ZF.C2H2											+
ZNF460	ZF.C2H2										+	
ZNF462	ZF.C2H2			+								
ZNF48	ZF.C2H2									+		
ZNF500	ZF.C2H2											+
ZNF516	ZF.C2H2			+								
ZNF528	ZF.C2H2		+									
ZNF547	ZF.C2H2					+						
ZNF556	ZF.C2H2										+	
ZNF571	ZF.C2H2				+							
ZNF592	ZF.C2H2			+								
ZNF622	ZF.C2H2							+				
ZNF629	ZF.C2H2									+		
ZNF668	ZF.C2H2									+		
ZNF677	ZF.C2H2					+						
ZNF70	ZF.C2H2									+		
ZNF707	ZF.C2H2										+	
ZNF750	ZF			+								
ZNF766	ZF.C2H2				+							
ZNF768	ZF.C2H2									+		
ZNF771	ZF.C2H2									+		
ZNF777	ZF.C2H2									+		
ZSCAN18	ZF.C2H2					+						
ZSCAN25	ZF.C2H2									+		

Compiled on the basis of filtered interaction data reported in Additional Tables 7.1–7.11. For abbreviations, see note in Table 3.

Intriguingly, in addition to the data filtering procedure per se, three emerging features of this interaction pattern rule out it could have emerged from random events involving our subjects of investigation. First, very different fractions of post-transcriptional effectors included in distinctive bait-pools were involved in filtered interactions with TFs, from as little as 36.6% of aa.tRNA.synthetases to 100% of pAF.4C members (**Table 3**, columns 4 and 5). Second, the correlation index between the number of bait-pool members engaged in filtered interactions with TFs and the number of their interactors was very low, close to 0.26 (**Table 3**, columns 5 and 7). Third, the allocation of interactors captured by distinctive bait-pools to different TF taxa was robustly diversified, being the average value of such diversification close to 33% (**Table 3**, columns 8–57). Note that such diversification is remarkably pronounced in the case of TlatMF.cytoRPs and TlatMF.mitoRPs pools (**Table 2**, **Additional Tables 7.9** and **7.10**), which, together with high TF/TlatMF.mitoRPs connectivity, rules out that high TF/TlatMF.cytoRPs connectivity could trivially reflect the geometrical contiguity of TF-proteins to ribosomes at the time of their synthesis.

Last but not least, whereas as many as 15 TFs included in **Additional Table 1.1** had been experimentally proven to be implicated in transcription-independent translation control [including 6 “neuro-developmental effectors” mentioned in the first chapter of this review (Foxg1, Hhex (aka Prh), Hoxa9, En1, En2, and Emx2) and additional 9 factors listed in **Table 1** (Ctcfl, Esr1, Hmgb3, Myf5, Phb, Tp53, Ybx1, Stat3, and Bmal1 (aka Arntl)], only 8 of them [Foxg1, Hhex, Hoxa9, Emx2, Phb, Tp53, Ybx1, Stat3] were “rediscovered” by our BioGrid-based approach as being linked to post-transcriptional gene control (**Table 4**). This suggests that our statistical filtering strategy was likely too severe, leading us to underestimate the pervasiveness of the phenomena we investigated, possibly by a factor not far from 2.

Interestingly, almost 50% of TFs interacting with the translational machinery (90/186), were detectable outside the nucleus (**Table 3**, columns 7 and 58), consistently with their direct involvement in translation. Moreover, more than 50% of TFs putatively involved in non-canonical control of post-transcriptional gene tuning (76/155, 30/57, and 110/186, in case of splicing, polyadenylation, and translation, respectively), were experimentally proven to bind to RNA (**Table 3**, columns 7 and 59). This suggests that, in principle, these TFs might modulate post-transcriptional steps of gene expression in a gene-specific way, either by connecting canonical splicing-, polyadenylation- and translation-factors to specific mRNAs or, alternatively, inhibiting this connection.

In sum, based on the protein-protein and protein-RNA interaction data available in public databases, we predict that (a) in addition to the canonical control of transcription, at least 1 TF out of 5 may be implicated in a non-canonical manner in the control of post-transcriptional steps of gene expression, with a focus on translation, and, (b) in up to half of these cases such non-canonical control might be exerted in a gene-specific way.

Albeit based on a conservative statistical evaluation of a huge body of interaction data, this prediction obviously requires caution. In fact, a large subset of TF interactions on which it is based might simply represent metabolic noise. Alternatively, they might be pertinent to processes other than post-transcriptional gene tuning. For example, massive TF “sequestration” by ribosomal proteins (> 2100 distinctive interacting dyads filtered, **Table 3**, column 6) might be instrumental in the physiological attenuation of canonical TF activity, by tightening TF confinement to extranuclear compartments. Conversely, the widespread TF ability to bind RNA might trivially reflect the emerging, pervasive employment of RNA cofactors to support specific aspects of transcriptional mechanics (reviewed in Henninger and Young, 2024). Consequently, our prediction does require functional experimental validation. In this respect, CASH (Wu et al., 2018) and ROAR (Grassi et al., 2016) (re-)analysis of RNA-Seq data from TF-OE and/or TF-LOF preparations could help to easily probe the possible involvement of TFs in splicing and polyadenylation, respectively. Similarly, a mild overexpression of cytoplasm-confined TF-chimeras followed by delayed proteome profiling might make the serial assessment of the direct TF implication in translational control a feasible task (Artimagnella et al., 2024).

## Why Are Transcription Factors Pleiotropic?

Beyond the experimental validation issues mentioned above, such a pervasive conveyance of additional control functions to molecular effectors already engaged in transcriptional dynamics is theoretically puzzling. It is commonly accepted that, during evolution, genes encoding for polypeptides with new functions have generally arisen by duplication and diversification of pre-existing genes (Holland et al., 1994; Friedman and Hughes, 2001; Blomme et al., 2006). In fact, the use of an essential gene for evolutionary experiments could pose obvious risks to the fitness of a species. Conversely, the use of duplicates of it would allow us to circumvent this problem, thanks to the release of the pressure acting on the essential ancestor gene. If so, why as many as a fifth or more of our TF genes would encode for (sophisticated) multi-task effectors?

We hypothesize that three distinctive evolutionary factors may have led to the emergence of such a scenario: (1) the triplosensitivity bottleneck; (2) the possibility of increasing the inheritability of complex, spatio-temporal gene regulation patterns; (3) the possibility of achieving rapid evolutionary transfer of pre-evolved, protein-DNA interaction specificities to protein-RNA interactions. Below, we detail these hypotheses and discuss how to assess their validity.

**Figure 2:**
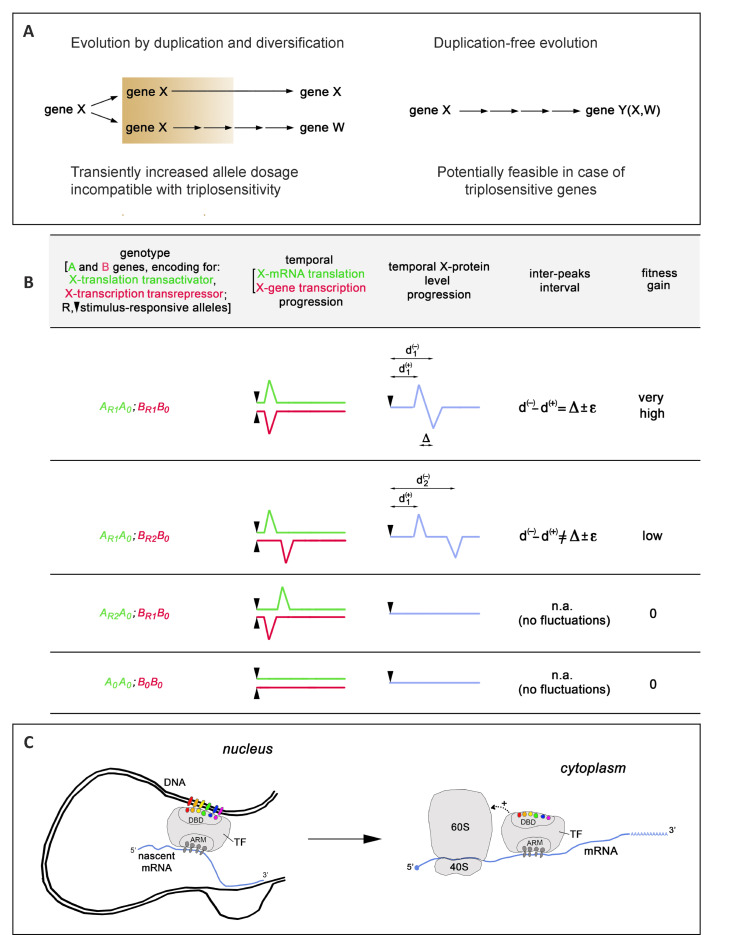
Hypothetical mechanisms conspiring to evolutionarily emergence of pleiotropic TF control over transcription and post-transcriptional steps of gene expression. (A) Implying a transient increase of allele dosage, the generation of a novel W gene by duplication and diversification of an X ancestor would lead to a corresponding decline in fitness, if the organism is X-triplosensitive. This may have pushed organisms to follow alternative, duplication-free pathways, whereby a triplosensitive X-ancestor progressively evolves into a Y derivative, encoding for a protein that co-performs both X and W functions. (B) Timed fluctuations in the translation and transcription rates of an X gene, driven by the transactive products of two stimulus-responsive genes, A and B, respectively, can lead to peculiar temporal fluctuation profiles of X protein. For example (row 1), a transient, simultaneous transactivation and transrepression of translation and transcription, respectively, generates two sequential X peaks, one positive and one negative, separated by a D interval. In a given context, this may confer on the organism harboring *A*_*R1*_ and *B*_*R1*_, stimulus-responsive alleles required for such a response, a remarkable fitness gain, paving the way for selection of such alleles. However, *partial* replacement of each of these alleles by other stimulus-responsive ones, characterized by different response-latencies (e.g., *A*_*R2*_ and *B*_*R2*_; rows 2 and 3), reduces or also abolishes this fitness gain. For this reason, the average fitness gain associated with freely recombining *A*_*R1*_ and *B*_*R1*_ can be quite lower than when they are tightly linked, so considerably delaying their selection. In such a scenario, every device that helps keep sequences encoding for *A*_*R1*_ and *B*_*R1*_ functions associated can tremendously increase the average fitness gain conferred by these alleles, speeding up their selection. Conveying transcriptional and translational control abilities on the product of the same gene can be considered just the limit strategy to achieve this goal. (C) TF binds to DNA motifs localized in 3D surroundings of specific transcription units via dedicated DBD, in a highly specific manner (interacting aa-residues and nucleotides in color). The same TFs may co-transcriptionally interact with nascent mRNA molecules, via RNA-binding domains (ARMs), supporting a less specific interaction (interacting aa-residues and nucleotides in grey), however sufficient to sustain a relatively stable association between the two molecules, which survives mRNA detachment from DNA and its nucleo-cytoplasmic translocation. In this way, specifically recruited at a given gene, TFs may selectively modulate post-transcriptional metabolism of mRNAs originating from that gene. In other words, conveying transcriptional and translational control abilities on the product of the same gene may allow to rapidly “recycle” the laborious, evolutionarily complex selection of specific DNA/protein interfaces for an advantageous, indirect control of specific RNA/protein interactions. 0: Index of not-stimulus-responsive alleles; A: translation transactivator gene; ARM: arginine-rich module; B: transcription transrepressor gene; D: optimal interpeak delay; d^(–)^: delay between stimulus delivery time and negative, X protein level peak time; d^(+)^: delay between stimulus delivery time and positive, X protein level peak time; DBD: DNA-binding domain; e: random interpeak delay fluctuation; Rx: indexes of stimulus-responsive alleles; TF: transcription factor; W: gene encoding for a protein performing W function; X: gene encoding for a protein performing X function; Y(X,W): gene encoding for a protein co-performing X and W functions. Created with Adobe Photoshop CS6 (version 13.0×64) and Microsoft PowerPoint for Mac (version 16.66.1).

Concerning the first hypothesis, it has been shown that the correct dosage of alleles is often crucial to individual health. In particular, approximately 3000 of our autosomal, polypeptide-encoding genes have been inferred to be haploinsufficient, and approximately 1000 of them are also triplosensitive (Collins et al., 2022), with a clear link between anomalous copy number variations and impaired mental health (Martin et al., 2020). Conversely, it has been estimated that, albeit rapid, the structural gene diversification that follows gene duplication requires a non-negligible evolutionary time (Assis and Bachtrog, 2015). Because of that, the evolution of triplosensitive genes can hardly exploit the “duplication and diversification strategy.” Such a strategy would expose individuals who adopt it to a lasting decline in fitness. That is why these individuals may have been “pushed” towards an alternative path, consisting of conveying novel functions to the protein product of their original gene (**Figure 2A**).

To probe the validity of this inference, we took advantage of “pTriplo,” a numerical index derived from a machine learning-based meta-analysis of clinical data from almost 1,000,000 human individuals, which provides a reliable prediction of triplosensitivity for each autosomal gene (Collins et al., 2022; **Table 5**). We found that, while equaling 0.19 across all autosomal genes, the fraction of genes with pTriplo 0.75 rose to 0.33 across all TFs and further to 0.47 across TFs involved in interactions with post-transcriptional effectors (“pt-TFs,” with observed-interaction-freq > 3*expected-interaction-freq, and *P* < 0.001). Furthermore, more and more pronounced, relative increases characterized the corresponding fractions of genes with progressively higher pTriplo (for example, fractions of genes with pTriplo 0.99 equaled 0.03, 0.06, 0.09, and 0.11, among total genes, autosomal TFs, pt-TFs with observed-interaction-freq > 2*expected-interaction-freq and *P* < 0.05, and pt-TFs with observed-interaction-freq > 3*expected-interaction-freq and *P* < 0.001, respectively). All that corroborates the hypothesis that triplosensitivity may be a major driver of gene duplication-free evolutionary pathways, such as those proposed for TFs involved in post-transcriptional control of gene expression.

**Table 5:** Incidence of triplosensitivity and responsivity to neuronal activity/plasticity among transcription factors (TFs) putatively implicated in non-canonical, post-transcriptional gene control

TF fractions satisfying specific criteria		Genes samples	#1.1	#1.2	#2	#3	#4

			All genes (1)	All autosomal genes (2)	All TFs (3)	TFs with “chi(11) < 0.05” and “at least one bait-pool with (observed interactions) / (expected interactions) > 2” (4)	TFs with “chi(11) < 0.001” and “at least one bait-pool with (observed interactions) / (expected interactions) > 3” (4)

			(*n* = 21,000)	(*n* = 18,641)	(*n* = 1620)	(*n* = 323)	(*n* = 169)
Fraction responsive to neuronal activity/plasticity(2)			0.20	na	0.16	0.20	0.23
Fractions with pTriplo index >= (3)	0.99		na	0.03	0.06	0.09	0.11
	0.95		na	0.08	0.14	0.20	0.23
	0.90		na	0.11	0.20	0.30	0.32
	0.75		na	0.19	0.33	0.47	0.47
Fractions responsive to neuronal activity/plasticity and with pTriplo index >= (2,3)	0.99		na	na	0.020	0.028	0.036
	0.95		na	na	0.044	0.059	0.059
	0.9		na	na	0.057	0.084	0.089
	0.75		na	na	0.090	0.127	0.130

(1) See https://www.gencodegenes.org/human/stats.html [Gencode v 47] for details; (2) see Joy and Carmichael (2024) doi:10.1038/s42003-024-06723-3 for details; (3) see Additional Table 2 for details; (4) see Table 3, note (1) for details.

Regarding the second hypothesis, the optimal unfolding of information-rich metabolic processes may often require a temporally structured and non-monotonic regulation of ultimate gene products involved in their implementation. For example, this is the case of homeostatic responses which follow (1) bursts of neuronal activity or (2) acute immune responses. A general strategy suitable to fulfill this requirement may consist of the orthogonal tuning of two distinct steps of gene expression (e.g., transcription and translation), exerted by two dedicated effectors, A and B. In this context, the maximal advantage can obviously emerge, only provided that the temporal gene product profiles dictated by the alleles encoding for these effectors are properly coupled. In other words, the type-X allele encoding for effector A (A_X_) elicits a robust fitness gain only if it specifically operates in concert with the type-Y allele encoding for effector B (B_Y_) (**Figure 2B**). It turns out that each device increasing the linkage between A_X_ and B_Y_ alleles can increase the average fitness-gain associated with each of them, so accelerating their positive selection. In this context, the duplication-free evolution of an original A gene toward a new hybrid A/B identity may have been implemented just as the limit-path suitable to achieve this goal.

To probe the validity of this inference, we focused on a selection of genes activated according to temporally structured patterns, *in vitro* and/or *in vivo*, in response to neuronal activity (Joy and Carmichael, 2024), and compared their prevalence among all TFs and TFs specifically involved in interactions with post-transcriptional effectors (**Table 3**). We found that equaling 0.16 in the former, such prevalence rose up to 0.23 in the latter. This suggests that the advantages offered by some temporally structured polypeptide expression profiles may have contributed to “push” some TF genes to evolve in a duplication-free manner.

Concerning the third hypothesis, three preliminary considerations led us to its formulation. First, DNA-binding specificities displayed by TFs are often very high. A preferentially-bound DNA-sequence motif has been experimentally determined (or inferred from a closely-related homolog) for approximately 1200 out of approximately 1600 human TFs (Weirauch et al., 2014), and the binding preferences displayed by TFs towards such motifs may exceed those exhibited towards other motifs by 1000-folds (Geertz et al., 2012). Second, RNA-binding proteins can also distinguish different RNA molecules based on their sequence, largely thanks to a variety of dedicated RNA-binding domains (Dominguez et al., 2018; Jolma et al., 2020; Ray et al., 2023). However, TFs rarely harbor canonical RBDs. Conversely, as many as 80% of them are provided of short, basic aminoacid-rich domains similar to the RNA-binding domain of the HIV Tat transactivator and generally proximal to the DBDs (termed ARM-like domains), which have been shown to be necessary and sufficient to mediate RNA-binding properties displayed by these TFs (Oksuz et al., 2023), but are hardly sufficient, because of their relatively poor complexity, to confer to a given TF the capability to discriminate among different mRNAs. Third, a number of yeast effectors involved in fine tuning of transcription (including Rbp4, Rbpp7, CCR4-Not, and Xnr) have been shown to be co-transcriptionally loaded onto nascent pre-mRNAs, remain specifically bound to their mRNA derivatives, even upon their nucleo-cytoplasmic translocation, and thereby impact late post-transcriptional steps of gene expression, including mRNA translation and decay (Ujvári and Luse, 2006; Goler-Baron et al., 2008; Harel-Sharvit et al., 2010; Haimovich et al., 2013; Gupta et al., 2016; Blasco-Moreno et al., 2019; Begley et al., 2019; Wilczynska et al., 2019). In other words, polypeptide effectors previously recruited to specific DNA regions can be selectively transferred to RNA emerging from transcription of these regions, likely thanks to their transient vicinity, and remain attached to these RNAs for a time sufficient to significantly affect their metabolism. Based on these premises, we propose that the conveyance of post-transcriptional functions on gene products already involved in transcriptional control was just a smart evolutionary “trick,” that made sequence-binding specificities, already laboriously distilled from distinctive DNA sequences, easily and specifically portable to the mRNA products of their transcription, where they were fruitfully “recycled,” to drive specific post-transcriptional steps of more sophisticated gene regulation programs (**Figure 2C**).

It is important to note that this third hypothesis still requires experimental validation. In this respect, a systematic inspection of TF phylogenetic trees for the possible late appearance of ARM-like domains, as well as an assessment of the necessity of nuclear co-transcriptional loading of TFs onto pre-mRNAs to achieve their further extranuclear control, might be of help.

## Conclusions

Moving from some “unorthodox” findings reported in the specialized neurodevelopmental literature, we first show that a pleiotropic involvement of TFs in translation control had been also previously documented in several other cases, not limited to the neurodevelopmental field.

Next, upon a structured query of a major public protein-protein database, we report that at least one-fifth of TFs, preferentially belonging to defined taxa, specifically interact with effectors implicated in splicing, polyadenylation, and translation, pointing to a likely large scale, functional involvement of them in post-transcriptional control of gene expression. Consistently, we show that about one-half of TFs interacting with translation machinery factors are detectable outside of the nucleus. Besides, we also show that one-half of TFs putatively implicated in post-transcriptional gene control can bind RNA, meaning that their post-transcriptional control over gene expression might be implemented in an mRNA-specific manner. Finally, we propose some experimental approaches for the systematic validation of these inferences.

If confirmed, such widespread pleiotropy is theoretically puzzling, as clashing with the standard model of gene evolution by duplication and diversification. In this regard, we propose three evolutionary factors that might have led to the high prevalence of pleiotropy among TFs (triplosensitivity bottleneck, better inheritability of temporally structured expression patterns, DNA-dependent specificity of protein-RNA interactions) and provide experimental evidence as well as methodological suggestions for the validation of these proposals.

## Methods

### Bibliographic search strategy

**Table 1** was compiled by merging information extracted from four distinct paper sets:

(1) The first set (referring to Ilf3, Ctcfl, Tp53, Mif5, Esr1, Hmgb3, and Phb) was taken from a private collection of articles, summarizing published experimental evidence on the direct and transcription-independent control exerted by canonical transcription factors on translation. This collection had been compiled by the Corresponding Author over a broad time span (2021–2024), prior to conceiving this review, through a curiosity-driven and unstructured interrogation of the PubMed database.

(2) The second set (referring to Eef1b2, Bmal1, and Esr1) originated from a structured interrogation of the PubMed database, run on May 1 to 2, 2025. Such interrogation was performed: (a) from scratch, using *ad hoc* keys (including “translation factor” AND “transcription factor”), as well as (2) starting from the PubMed webpages of the genes reported in (1) and manually scrolling the “Cited by” and “Reference” lists associated with each article. It was followed by manual filtering of the resulting candidate articles, which were retained only if reporting a direct, transcription-independent impact of a canonical transcription factor on translation.

(3) The third set (referring to Ybx1 and Stat3) originated from a structured interrogation of the free online ChatGPT tool. This was run on June 1 to 3, 2025, employing a variety of keys (“straight translation control by transcription factors, not dependent on transcription,” “transcription factors implication in translation,” “a list of specific transcription factors and their translational roles,” “direct transcription factor control of translation”). It was followed by manual filtering of the resulting 21 articles, keeping only the two ones reporting a direct, transcription-independent impact of a canonical transcription factor on translation.

(4) The fourth set (referring to Ybx1 and Ilf3) was based on a structured interrogation of the free online DeepSeek tool. This was run on June 4 and 5, 2025, by means of the same keys mentioned for (3). It was followed by manual filtering of the resulting 28 articles, retaining only the two ones reporting a direct, transcription-independent impact of a canonical transcription factor on translation.

### Preparation of hsa.interactome.restr

The “BIOGRID-ORGANISM-4.4.238.mitab.zip” file was downloaded from the www.biogrid.org website on October 6, 2024 and the “BIOGRID-ORGANISM-Homo_sapiens-4.4.238.mitab” file was extracted from it by Unarchive software. The latter was converted into a .txt file and further processed by TextEdit and Excel software.

Originally containing 1,251,627 rows (1 row = 1 protein/protein interaction report), BIOGRID-ORGANISM-Homo_sapiens-4.4.238.mitab” was trimmed to its “hsa.interactome.restr” derivative (including 1,152,045 rows), by retaining only the interactions labeled - within the “Interaction Type” column - by the “MI:0407 (direct interaction)” or “MI:0915 (physical association)” tags. These represent the large majority of the experimentally verified physical interactions available in the original database.

### Interrogation of hsa.interactome.restr and full results presentation in Additional Table 6.1

hsa.interactome.restr was interrogated by the five gene lists reported in **Additional Tables 1–5** (TFs, TcoFs, SplFs, pAFs, and TlatMFs), For each gene, (1) the total number of interaction reports involving its protein product, as well as (2) the number of interaction reports involving that product, specifically, each TF, were counted. The results of such interrogations were reported in the grids at columns G-DSJ of **Additional Table 6.1**.

To ease later formulation of correlative inferences, four additional key pieces of information pertinent to each TF, namely subcellular location, possible RNA-binding protein (RBP) function, pTriplo index and responsiveness to neuronal activity/plasticity were added to **Additional Table 6.1**, at columns DSL-DSO. They were taken from **Additional Tables 2–4**, previously compiled as detailed in the corresponding notes.

### Coarse-grained analysis of unfiltered Additional Table 6.1 data

**Additional Table 6.1** data were analyzed as follows. The distribution of all interaction reports, involving (1) each individual TF and (2) the proteins encoded by each of the TFs, TcoFs, SplFs, pAFs, and TlatMFs gene sets, was summarized (in the absence of prior filtering for statistical significance) in a grid, located in columns E–I of **Additional Table 6.2**.

Then, the information contained in this grid was employed to compile **Table 2**. Such table lists the cumulative numbers of interactions indexed by BioGrid involving (a) all TFs belonging to each taxon and, on the other side, (b) all effectors belonging to any of the five groups, TFs, TcoFs, SplFs, pAFs, and TlatMFs. Moreover, it provides an evaluation of the cumulative TF interactions putatively linked to the regulation of splicing, polyadenylation, and translation, normalized to those associated with transcriptional control.

### Fine-grained analysis of filtered Additional Table 6.1 data

Next, all **Additional Table 6.1** interaction reports involving (1) each individual TF and (2) post-transcriptional effectors from the SplFs, pAFs, and TlatMFs lists, were filtered, based on their fine-grained topological distribution, as shown in columns K–AX of **Additional Table 6.2**. Specifically, the reports including each TF and factors implicated in post-transcriptional gene regulation were partitioned into 11 groups, corresponding to the post-transcriptional factor ensembles defined in **Additional Tables 1.3–1.5** (SplF.core, SplF.RBP, SplF.other, pAF.4C, pAF.PA, TlatMF.EIF, TlatMF.EIcoF, TlatMF.EEF, TlatMF.cytoRP, TlatMF.mitoRP, and TlatMF.aatRNAsynth), and their actual numbers [real values] were reported in the grid at columns K–U. Next, a sister grid was generated, including the theoretical numbers of these reports expected as a consequence of random interaction between the corresponding dyad members [expected values]. These were calculated based on frequencies of all “hsa.interactome.restr” reports that included each of the two dyad members, taking into account the absolute size of “hsa.interactome.restr.” The resulting grid is reported in columns W–AG. Then, from these two sister grids, three sets of indices were calculated for each TF: (1) the set of “real value/expected value” ratios, referring to each of the 11 groups mentioned above (reported in columns AI–AS) and the sets of “chi-square *P* values,” including (2) values calculated across all 11 groups or (3) limited to the 3, 2 or 6 groups forming the SplF, pAF, and TlatMF ensembles, respectively (reported in columns AU–AX). Subsequently, the statistical parameters mentioned above were employed to filter **Additional Table 6.1** interaction reports to be subsequently included in **Additional Tables 7.1–7.11**. To this aim, reports had to meet three criteria: [a] p-chi_TF_ (obs-vs-exp interactions with interactor group members, evaluated over all 11 groups) < 0.05; [b] p-chi_TF_ (obs-vs-exp interactions with interactor group members, evaluated only over the groups associated with the “reference process,” i.e., splicing, polyadenylation or translation) < 0.05; [c] referring to TF interactions with members of the reference process group, n(obs)/n(exp) 2. Finally, information reported in **Additional Tables 7.1–7.11** was used as a substrate of the synopses reported in **Tables 3–5**.

In **Table 3**, to effectively condense the distribution of physical interactions among post-transcriptional effectors and TFs that emerged from statistical filtering of primary BioGrid data, selected features of “TF preys” captured by 11 distinct sets of “post-transcriptional effectors baits” were displayed. Specifically, for each “bait-set,” the number of interaction reports, the number of interacting preys and the taxon allocation of such preys were annotated. Moreover, information about the prevalence of those with non-nuclear localization and documented RNA-binding activity was also provided.

In **Table 4**, all 312 TFs resulting in significant interaction after statistical filtering of BioGrid data with post-transcriptional effectors were listed. For each of them, annotated was the taxon it belongs to, as well as the distribution of its interactors among the main 11 sets of post-transcriptional effectors considered for our analysis.

In **Table 5**, to illustrate the enrichment in triplosensitive and neuronal activity/plasticity-responsive TFs among those putatively implicated in non-canonical, post-transcriptional gene control, shown was the prevalence of TFs fulfilling specific criteria of triplosensitivity and activity/plasticity-responsivity among: (#1.1) all genes, (#1.2) all autosomal genes, (#2) all transcription factor genes, and (#3 and 4) genes encoding for transcription factors which interact with post-transcriptional effectors at progressively higher frequency than expected.

## Additional files:

**Additional Table 1.1:** *hsa-TF genes list (n = 1620).*

Additional Table 1.1 hsa-TF gene list (*n* = 1620)

**Additional Table 1.2:** *hsa-TcoF genes list (n = 958).*

Additional Table 1.2 hsa-TcoF gene list (*n* = 958)

**Additional Table 1.3:** *hsa-SplF genes list (n = 304).*

Additional Table 1.3 hsa-SplF genes list (*n* = 304)

**Additional Table 1.4:** *hsa-pAF genes list (n = 33).*

Additional Table 1.4 hsa-pAF genes list (*n* = 33)

**Additional Table 1.5:** *hsa-TlatMF genes list (n = 275).*

Additional Table 1.5 hsa-TlatMF genes list (*n* = 275)

**Additional Table 2:** *Subcellular hsa-TF localization.*

Additional Table 2 Subcellular hsa-TF localization

**Additional Table 3:** *RBP genes list (n = 4775).*

Additional Table 3 RBP genes list (*n* = 4775)

**Additional Table 4:** *Distribution of triplosensitivity indices.*

Additional Table 4 Distribution of triplosensitivity indices

**Additional Table 5:** *Gene relatedness to neuronal activity/plasticity.*

Additional Table 5 Gene relatedness to neuronal activity/plasticity

**Additional Table 6.1:** *TFs: full interaction data grid and other key information.*

Additional Table 6.1 TFs: full interaction data grid and other key information

**Additional Table 6.2:** *Full TF interact grid_statistical analysis.*

Additional Table 6.2 Full TF interact grid_statistical analysis

**Additional Table 7.1:** *Filtered TF/SplF.core interaction reports & related info.*

Additional Table 7.1 Filtered TF/SplF.core interaction reports & related info

**Additional Table 7.2:** *Filtered TF/SplF.RBP interaction reports & related info.*

Additional Table 7.2 Filtered TF/SplF.RBP interaction reports & related info

**Additional Table 7.3:** *Filtered TF/SplF.other interaction reports & related info.*

Additional Table 7.3 Filtered TF/SplF.other interaction reports & related info

**Additional Table 7.4:** *Filtered TF/pAF.4C interaction reports & related info.*

Additional Table 7.4 Filtered TF/pAF.4C interaction reports & related info

**Additional Table 7.5:** *Filtered TF/pAF.PA interaction reports & related info.*

Additional Table 7.5 Filtered TF/pAF.PA interaction reports & related info

**Additional Table 7.6:** *Filtered TF/TlatMF.EIF interaction reports & related info.*

Additional Table 7.6 Filtered TF/TlatMF.EIF interaction reports & related info

**Additional Table 7.7:** *Filtered TF/TlatMF.EIcoF interaction reports & related info.*

Additioinal Table 7.7 Filtered TF/TlatMF.EIcoF interaction reports & related info

**Additional Table 7.8:** *Filtered TF/TlatMF.EEF interaction reports & related info.*

Additioinal Table 7.8 Filtered TF/TlatMF.EEF interaction reports & related info

**Additional Table 7.9:** *Filtered TF/TlatMF.cytoRP interaction reports & related info.*

Additioinal Table 7.9 Filtered TF/TlatMF.cytoRP interaction reports & related info

**Additional Table 7.10:** *Filtered TF/TlatMF.mitoRP interaction reports & related info.*

Additional Table 7.10 Filtered TF/TlatMF.mitoRP interaction reports & related info

**Additional Table 7.11:** *Filtered TF/TlatMF.aatRNAsynth interaction reports & related info.*

Additioinal Table 7.11 Filtered TF/TlatMF.aatRNAsynth interaction reports & related info

## Data Availability

*All relevant data are within the paper and its Additional files (available at: https://doi.org/10.5281/zenodo.14443212).*
