# Supplementary material for: Foxg1 and companions: Not only transcription factors
Source: Neural Regen Res. 2025 Sep 3;21(8):3426–38. doi: 10.4103/NRR.NRR-D-25-00439 (PMC13452659; doi:10.4103/NRR.NRR-D-25-00439)
Supplement: Supplementary file 1 [file NRR-21-3426_Suppl1.pdf]

Additional Table 1.1. hsa-TF gene list (*n* = 1620)

[homo sapiens Transcription Factor genes]

NOTES

(1) harvested from TFCheckpoint2
[downloaded from: https://www.tfcheckpoint.org/index.php/download -data on 2024.11.01;
filtered as follows: column Z: "animal_tfdb_homo_sapiens" ON]

(2) harvested from Genexplain
[downloaded from: https://genexplain.com/tfclass/huTF_classification_Genera.html, on 2024.11.01;
downloaded from: http://www.edgar-wingender.de/huTF_classification.html, on 2024.11.01]

(3) harvested from TFDB4
[downloaded from: https://guolab.wchscu.cn/AnimalTFDB4/#/Download, on 2024.11.01;
column D]

(4) manually cured, on the basis of Genexplain, TFDB4 and HGNC datasets (see: https://www.genenames.org)

hsa TFs (1)	*TF family code (2)*	*TF superclass (2)*	*TF class (2)*	*TF class (3)*	*our synthetic taxon annotation (4)*
TCF25	0.0.1	Yet undefined DNA-binding domains	Nuclear localized protein 1	Others	bHLH
RFXANK	0.0.3	Yet undefined DNA-binding domains	RFXANK	Others	RFXANK
PURA	0.0.5	Yet undefined DNA-binding domains	PUR	Others	PUR
PURB	0.0.5	Yet undefined DNA-binding domains	PUR	Others	PUR
PURG	0.0.5	Yet undefined DNA-binding domains	PUR	Others	PUR
NRF1	0.0.6	Yet undefined DNA-binding domains	NRF	Nrf1	NRF
CSRNP1	0.1.1	Yet undefined DNA-binding domains	AXUD/CSRNP domain factors	CSRNP_N	CSRNP_N
CSRNP2	0.1.1	Yet undefined DNA-binding domains	AXUD/CSRNP domain factors	CSRNP_N	CSRNP_N
CSRNP3	0.1.1	Yet undefined DNA-binding domains	AXUD/CSRNP domain factors	CSRNP_N	CSRNP_N
SFPQ	0.2.1	Yet undefined DNA-binding domains	NonO domain factors	Others	others
LRRFIP1	0.3.1	Yet undefined DNA-binding domains	eucine-rich repeat flightless-interacting protei	LRRFIP	LRRFIP
LRRFIP2	0.3.1	Yet undefined DNA-binding domains	eucine-rich repeat flightless-interacting protei	LRRFIP	LRRFIP
ZNFX1	0.4.1	Yet undefined DNA-binding domains	NA	Others	ZF.NFX1
NFX1	0.4.1	Yet undefined DNA-binding domains	NFX1-type putative zinc finger factors	zf-NF-X1	ZF.NFX1
NFXL1	0.4.1	Yet undefined DNA-binding domains	NFX1-type putative zinc finger factors	zf-NF-X1	ZF.NFX1
GTF2I	0.5.1	Yet undefined DNA-binding domains	GTF2I domain factors	GTF2I	GTF2I
GTF2IRD1	0.5.1	Yet undefined DNA-binding domains	GTF2I domain factors	GTF2I	GTF2I
GTF2IRD2	0.5.1	Yet undefined DNA-binding domains	GTF2I domain factors	GTF2I	GTF2I
GTF2IRD2B	0.5.1	Yet undefined DNA-binding domains	GTF2I domain factors	GTF2I	GTF2I
CAMTA1	0.6.1	Yet undefined DNA-binding domains	CAMTA factors	CG-1	IPT
CAMTA2	0.6.1	Yet undefined DNA-binding domains	CAMTA factors	CG-1	IPT
ATF2	1.1.1	Basic domains	Basic leucine zipper factors (bZIP)	TF_bZIP	bZIP

ATF7	1.1.1	Basic domains	Basic leucine zipper factors (bZIP)	TF_bZIP	bZIP
BACH1	1.1.1	Basic domains	Basic leucine zipper factors (bZIP)	TF_bZIP	bZIP
BACH2	1.1.1	Basic domains	Basic leucine zipper factors (bZIP)	TF_bZIP	bZIP
CREB5	1.1.1	Basic domains	Basic leucine zipper factors (bZIP)	TF_bZIP	bZIP
JUN	1.1.1	Basic domains	Basic leucine zipper factors (bZIP)	TF_bZIP	bZIP
JUNB	1.1.1	Basic domains	Basic leucine zipper factors (bZIP)	TF_bZIP	bZIP
JUND	1.1.1	Basic domains	Basic leucine zipper factors (bZIP)	TF_bZIP	bZIP
NFE2	1.1.1	Basic domains	Basic leucine zipper factors (bZIP)	TF_bZIP	bZIP
NFE2L1	1.1.1	Basic domains	Basic leucine zipper factors (bZIP)	TF_bZIP	bZIP
NFE2L2	1.1.1	Basic domains	Basic leucine zipper factors (bZIP)	TF_bZIP	bZIP
NFE2L3	1.1.1	Basic domains	Basic leucine zipper factors (bZIP)	TF_bZIP	bZIP
ATF3	1.1.2	Basic domains	Basic leucine zipper factors (bZIP)	TF_bZIP	bZIP
FOS	1.1.2	Basic domains	Basic leucine zipper factors (bZIP)	TF_bZIP	bZIP
FOSB	1.1.2	Basic domains	Basic leucine zipper factors (bZIP)	TF_bZIP	bZIP
FOSL1	1.1.2	Basic domains	Basic leucine zipper factors (bZIP)	TF_bZIP	bZIP
FOSL2	1.1.2	Basic domains	Basic leucine zipper factors (bZIP)	TF_bZIP	bZIP
JDP2	1.1.2	Basic domains	Basic leucine zipper factors (bZIP)	TF_bZIP	bZIP
MAF	1.1.3	Basic domains	Basic leucine zipper factors (bZIP)	TF_bZIP	bZIP
MAFA	1.1.3	Basic domains	Basic leucine zipper factors (bZIP)	TF_bZIP	bZIP
MAFB	1.1.3	Basic domains	Basic leucine zipper factors (bZIP)	TF_bZIP	bZIP
MAFF	1.1.3	Basic domains	Basic leucine zipper factors (bZIP)	TF_bZIP	bZIP
MAFG	1.1.3	Basic domains	Basic leucine zipper factors (bZIP)	TF_bZIP	bZIP
MAFK	1.1.3	Basic domains	Basic leucine zipper factors (bZIP)	TF_bZIP	bZIP
NRL	1.1.3	Basic domains	Basic leucine zipper factors (bZIP)	TF_bZIP	bZIP
BATF	1.1.4	Basic domains	Basic leucine zipper factors (bZIP)	TF_bZIP	bZIP
BATF2	1.1.4	Basic domains	Basic leucine zipper factors (bZIP)	TF_bZIP	bZIP
BATF3	1.1.4	Basic domains	Basic leucine zipper factors (bZIP)	TF_bZIP	bZIP
XBP1	1.1.5	Basic domains	Basic leucine zipper factors (bZIP)	TF_bZIP	bZIP
ATF4	1.1.6	Basic domains	Basic leucine zipper factors (bZIP)	TF_bZIP	bZIP
ATF5	1.1.6	Basic domains	Basic leucine zipper factors (bZIP)	TF_bZIP	bZIP
ATF1	1.1.7	Basic domains	Basic leucine zipper factors (bZIP)	TF_bZIP	bZIP
ATF6	1.1.7	Basic domains	Basic leucine zipper factors (bZIP)	TF_bZIP	bZIP
ATF6B	1.1.7	Basic domains	Basic leucine zipper factors (bZIP)	TF_bZIP	bZIP
CREB1	1.1.7	Basic domains	Basic leucine zipper factors (bZIP)	TF_bZIP	bZIP
CREB3	1.1.7	Basic domains	Basic leucine zipper factors (bZIP)	TF_bZIP	bZIP
CREB3L1	1.1.7	Basic domains	Basic leucine zipper factors (bZIP)	TF_bZIP	bZIP
CREB3L2	1.1.7	Basic domains	Basic leucine zipper factors (bZIP)	TF_bZIP	bZIP
CREB3L3	1.1.7	Basic domains	Basic leucine zipper factors (bZIP)	TF_bZIP	bZIP
CREB3L4	1.1.7	Basic domains	Basic leucine zipper factors (bZIP)	TF_bZIP	bZIP
CREBL2	1.1.7	Basic domains	Basic leucine zipper factors (bZIP)	Others	bZIP

CREBZF	1.1.7	Basic domains	Basic leucine zipper factors (bZIP)	TF_bZIP	bZIP
CREM	1.1.7	Basic domains	Basic leucine zipper factors (bZIP)	TF_bZIP	bZIP
CEBPA	1.1.8	Basic domains	Basic leucine zipper factors (bZIP)	TF_bZIP	bZIP
CEBPB	1.1.8	Basic domains	Basic leucine zipper factors (bZIP)	TF_bZIP	bZIP
CEBPD	1.1.8	Basic domains	Basic leucine zipper factors (bZIP)	TF_bZIP	bZIP
CEBPE	1.1.8	Basic domains	Basic leucine zipper factors (bZIP)	TF_bZIP	bZIP
CEBPG	1.1.8	Basic domains	Basic leucine zipper factors (bZIP)	TF_bZIP	bZIP
DBP	1.1.8	Basic domains	Basic leucine zipper factors (bZIP)	TF_bZIP	bZIP
DDIT3	1.1.8	Basic domains	Basic leucine zipper factors (bZIP)	TF_bZIP	bZIP
HLF	1.1.8	Basic domains	Basic leucine zipper factors (bZIP)	TF_bZIP	bZIP
NFIL3	1.1.8	Basic domains	Basic leucine zipper factors (bZIP)	TF_bZIP	bZIP
TEF	1.1.8	Basic domains	Basic leucine zipper factors (bZIP)	TF_bZIP	bZIP
TCF12	1.2.1	Basic domains	Basic helix-loop-helix factors (bHLH)	bHLH	bHLH
TCF3	1.2.1	Basic domains	Basic helix-loop-helix factors (bHLH)	bHLH	bHLH
TCF4	1.2.1	Basic domains	Basic helix-loop-helix factors (bHLH)	bHLH	bHLH
ASCL1	1.2.2	Basic domains	Basic helix-loop-helix factors (bHLH)	bHLH	bHLH
ASCL2	1.2.2	Basic domains	Basic helix-loop-helix factors (bHLH)	bHLH	bHLH
ASCL3	1.2.2	Basic domains	Basic helix-loop-helix factors (bHLH)	bHLH	bHLH
ASCL4	1.2.2	Basic domains	Basic helix-loop-helix factors (bHLH)	bHLH	bHLH
ASCL5	1.2.2	Basic domains	Basic helix-loop-helix factors (bHLH)	bHLH	bHLH
MYF5	1.2.2	Basic domains	Basic helix-loop-helix factors (bHLH)	bHLH	bHLH
MYF6	1.2.2	Basic domains	Basic helix-loop-helix factors (bHLH)	bHLH	bHLH
MYOD1	1.2.2	Basic domains	Basic helix-loop-helix factors (bHLH)	bHLH	bHLH
MYOG	1.2.2	Basic domains	Basic helix-loop-helix factors (bHLH)	bHLH	bHLH
ATOH1	1.2.3	Basic domains	Basic helix-loop-helix factors (bHLH)	bHLH	bHLH
ATOH7	1.2.3	Basic domains	Basic helix-loop-helix factors (bHLH)	bHLH	bHLH
ATOH8	1.2.3	Basic domains	Basic helix-loop-helix factors (bHLH)	bHLH	bHLH
BHLHA15	1.2.3	Basic domains	Basic helix-loop-helix factors (bHLH)	bHLH	bHLH
BHLHA9	1.2.3	Basic domains	Basic helix-loop-helix factors (bHLH)	bHLH	bHLH
BHLHE22	1.2.3	Basic domains	Basic helix-loop-helix factors (bHLH)	bHLH	bHLH
BHLHE23	1.2.3	Basic domains	Basic helix-loop-helix factors (bHLH)	bHLH	bHLH
FERD3L	1.2.3	Basic domains	Basic helix-loop-helix factors (bHLH)	bHLH	bHLH
FIGLA	1.2.3	Basic domains	Basic helix-loop-helix factors (bHLH)	bHLH	bHLH
HAND1	1.2.3	Basic domains	Basic helix-loop-helix factors (bHLH)	bHLH	bHLH
HAND2	1.2.3	Basic domains	Basic helix-loop-helix factors (bHLH)	bHLH	bHLH
LYL1	1.2.3	Basic domains	Basic helix-loop-helix factors (bHLH)	bHLH	bHLH
MESP1	1.2.3	Basic domains	Basic helix-loop-helix factors (bHLH)	bHLH	bHLH
MESP2	1.2.3	Basic domains	Basic helix-loop-helix factors (bHLH)	bHLH	bHLH
MSC	1.2.3	Basic domains	Basic helix-loop-helix factors (bHLH)	bHLH	bHLH
MSGN1	1.2.3	Basic domains	Basic helix-loop-helix factors (bHLH)	bHLH	bHLH

NEUROD1	1.2.3	Basic domains	Basic helix-loop-helix factors (bHLH)	bHLH	bHLH
NEUROD2	1.2.3	Basic domains	Basic helix-loop-helix factors (bHLH)	bHLH	bHLH
NEUROD4	1.2.3	Basic domains	Basic helix-loop-helix factors (bHLH)	bHLH	bHLH
NEUROD6	1.2.3	Basic domains	Basic helix-loop-helix factors (bHLH)	bHLH	bHLH
NEUROG1	1.2.3	Basic domains	Basic helix-loop-helix factors (bHLH)	bHLH	bHLH
NEUROG2	1.2.3	Basic domains	Basic helix-loop-helix factors (bHLH)	bHLH	bHLH
NEUROG3	1.2.3	Basic domains	Basic helix-loop-helix factors (bHLH)	bHLH	bHLH
NHLH2	1.2.3	Basic domains	Basic helix-loop-helix factors (bHLH)	bHLH	bHLH
OLIG1	1.2.3	Basic domains	Basic helix-loop-helix factors (bHLH)	bHLH	bHLH
OLIG2	1.2.3	Basic domains	Basic helix-loop-helix factors (bHLH)	bHLH	bHLH
OLIG3	1.2.3	Basic domains	Basic helix-loop-helix factors (bHLH)	bHLH	bHLH
PTF1A	1.2.3	Basic domains	Basic helix-loop-helix factors (bHLH)	bHLH	bHLH
SCX	1.2.3	Basic domains	Basic helix-loop-helix factors (bHLH)	bHLH	bHLH
TAL1	1.2.3	Basic domains	Basic helix-loop-helix factors (bHLH)	bHLH	bHLH
TAL2	1.2.3	Basic domains	Basic helix-loop-helix factors (bHLH)	bHLH	bHLH
TCF15	1.2.3	Basic domains	Basic helix-loop-helix factors (bHLH)	bHLH	bHLH
TCF21	1.2.3	Basic domains	Basic helix-loop-helix factors (bHLH)	bHLH	bHLH
TCF23	1.2.3	Basic domains	Basic helix-loop-helix factors (bHLH)	bHLH	bHLH
TWIST1	1.2.3	Basic domains	Basic helix-loop-helix factors (bHLH)	bHLH	bHLH
TWIST2	1.2.3	Basic domains	Basic helix-loop-helix factors (bHLH)	bHLH	bHLH
BHLHE40	1.2.4	Basic domains	Basic helix-loop-helix factors (bHLH)	bHLH	bHLH
BHLHE41	1.2.4	Basic domains	Basic helix-loop-helix factors (bHLH)	bHLH	bHLH
HELT	1.2.4	Basic domains	Basic helix-loop-helix factors (bHLH)	bHLH	bHLH
HES2	1.2.4	Basic domains	Basic helix-loop-helix factors (bHLH)	bHLH	bHLH
HES3	1.2.4	Basic domains	Basic helix-loop-helix factors (bHLH)	bHLH	bHLH
HES4	1.2.4	Basic domains	Basic helix-loop-helix factors (bHLH)	bHLH	bHLH
HES5	1.2.4	Basic domains	Basic helix-loop-helix factors (bHLH)	bHLH	bHLH
HES6	1.2.4	Basic domains	Basic helix-loop-helix factors (bHLH)	bHLH	bHLH
HES7	1.2.4	Basic domains	Basic helix-loop-helix factors (bHLH)	bHLH	bHLH
HEY1	1.2.4	Basic domains	Basic helix-loop-helix factors (bHLH)	bHLH	bHLH
HEY2	1.2.4	Basic domains	Basic helix-loop-helix factors (bHLH)	bHLH	bHLH
HEYL	1.2.4	Basic domains	Basic helix-loop-helix factors (bHLH)	bHLH	bHLH
AHR	1.2.5	Basic domains	Basic helix-loop-helix factors (bHLH)	bHLH	bHLH
AHRR	1.2.5	Basic domains	Basic helix-loop-helix factors (bHLH)	bHLH	bHLH
ARNT	1.2.5	Basic domains	Basic helix-loop-helix factors (bHLH)	bHLH	bHLH
ARNT2	1.2.5	Basic domains	Basic helix-loop-helix factors (bHLH)	bHLH	bHLH
ARNTL	1.2.5	Basic domains	Basic helix-loop-helix factors (bHLH)	bHLH	bHLH
ARNTL2	1.2.5	Basic domains	Basic helix-loop-helix factors (bHLH)	bHLH	bHLH
CLOCK	1.2.5	Basic domains	Basic helix-loop-helix factors (bHLH)	bHLH	bHLH
EPAS1	1.2.5	Basic domains	Basic helix-loop-helix factors (bHLH)	Others	bHLH

HIF1A	1.2.5	Basic domains	Basic helix-loop-helix factors (bHLH)	Others	bHLH
HIF3A	1.2.5	Basic domains	Basic helix-loop-helix factors (bHLH)	bHLH	bHLH
NCOA1	1.2.5	Basic domains	Basic helix-loop-helix factors (bHLH)	bHLH	bHLH
NPAS1	1.2.5	Basic domains	Basic helix-loop-helix factors (bHLH)	bHLH	bHLH
NPAS2	1.2.5	Basic domains	Basic helix-loop-helix factors (bHLH)	bHLH	bHLH
NPAS3	1.2.5	Basic domains	Basic helix-loop-helix factors (bHLH)	bHLH	bHLH
NPAS4	1.2.5	Basic domains	Basic helix-loop-helix factors (bHLH)	bHLH	bHLH
SIM1	1.2.5	Basic domains	Basic helix-loop-helix factors (bHLH)	bHLH	bHLH
SIM2	1.2.5	Basic domains	Basic helix-loop-helix factors (bHLH)	bHLH	bHLH
SOHLH1	1.2.5	Basic domains	Basic helix-loop-helix factors (bHLH)	bHLH	bHLH
SOHLH2	1.2.5	Basic domains	Basic helix-loop-helix factors (bHLH)	bHLH	bHLH
TCFL5	1.2.5	Basic domains	Basic helix-loop-helix factors (bHLH)	bHLH	bHLH
MAX	1.2.6	Basic domains	Basic helix-loop-helix factors (bHLH)	bHLH	bHLH
MGA	1.2.6	Basic domains	Basic helix-loop-helix factors (bHLH)	T-box	bHLH
MITF	1.2.6	Basic domains	Basic helix-loop-helix factors (bHLH)	bHLH	bHLH
MLX	1.2.6	Basic domains	Basic helix-loop-helix factors (bHLH)	bHLH	bHLH
MLXIP	1.2.6	Basic domains	Basic helix-loop-helix factors (bHLH)	bHLH	bHLH
MLXIPL	1.2.6	Basic domains	Basic helix-loop-helix factors (bHLH)	bHLH	bHLH
MNT	1.2.6	Basic domains	Basic helix-loop-helix factors (bHLH)	bHLH	bHLH
MXD1	1.2.6	Basic domains	Basic helix-loop-helix factors (bHLH)	bHLH	bHLH
MXD3	1.2.6	Basic domains	Basic helix-loop-helix factors (bHLH)	bHLH	bHLH
MXD4	1.2.6	Basic domains	Basic helix-loop-helix factors (bHLH)	bHLH	bHLH
MXI1	1.2.6	Basic domains	Basic helix-loop-helix factors (bHLH)	bHLH	bHLH
MYC	1.2.6	Basic domains	Basic helix-loop-helix factors (bHLH)	bHLH	bHLH
MYCL	1.2.6	Basic domains	Basic helix-loop-helix factors (bHLH)	bHLH	bHLH
MYCN	1.2.6	Basic domains	Basic helix-loop-helix factors (bHLH)	bHLH	bHLH
SREBF1	1.2.6	Basic domains	Basic helix-loop-helix factors (bHLH)	bHLH	bHLH
SREBF2	1.2.6	Basic domains	Basic helix-loop-helix factors (bHLH)	bHLH	bHLH
TFAP4	1.2.6	Basic domains	Basic helix-loop-helix factors (bHLH)	bHLH	bHLH
TFE3	1.2.6	Basic domains	Basic helix-loop-helix factors (bHLH)	bHLH	bHLH
TFEB	1.2.6	Basic domains	Basic helix-loop-helix factors (bHLH)	bHLH	bHLH
TFEC	1.2.6	Basic domains	Basic helix-loop-helix factors (bHLH)	bHLH	bHLH
USF1	1.2.6	Basic domains	Basic helix-loop-helix factors (bHLH)	bHLH	bHLH
USF2	1.2.6	Basic domains	Basic helix-loop-helix factors (bHLH)	bHLH	bHLH
ID1	1.2.8	Basic domains	Basic helix-loop-helix factors (bHLH)	bHLH	bHLH
ID2	1.2.8	Basic domains	Basic helix-loop-helix factors (bHLH)	bHLH	bHLH
ID3	1.2.8	Basic domains	Basic helix-loop-helix factors (bHLH)	bHLH	bHLH
ID4	1.2.8	Basic domains	Basic helix-loop-helix factors (bHLH)	bHLH	bHLH
TFAP2A	1.3.1	Basic domains	Basic helix-span-helix factors (bHSH)	AP-2	bHLH
TFAP2B	1.3.1	Basic domains	Basic helix-span-helix factors (bHSH)	AP-2	bHLH

TFAP2C	1.3.1	Basic domains	Basic helix-span-helix factors (bHSH)	AP-2	bHLH
TFAP2D	1.3.1	Basic domains	Basic helix-span-helix factors (bHSH)	AP-2	bHLH
TFAP2E	1.3.1	Basic domains	Basic helix-span-helix factors (bHSH)	AP-2	bHLH
AR	2.1.1	inc-coordinating DNA-binding domain	Nuclear receptors with C4 zinc fingers	ESR-like	ZF.C4_NR
ESR1	2.1.1	inc-coordinating DNA-binding domain	Nuclear receptors with C4 zinc fingers	ESR-like	ZF.C4_NR
ESR2	2.1.1	inc-coordinating DNA-binding domain	Nuclear receptors with C4 zinc fingers	ESR-like	ZF.C4_NR
ESRRA	2.1.1	inc-coordinating DNA-binding domain	Nuclear receptors with C4 zinc fingers	ESR-like	ZF.C4_NR
ESRRB	2.1.1	inc-coordinating DNA-binding domain	Nuclear receptors with C4 zinc fingers	ESR-like	ZF.C4_NR
ESRRG	2.1.1	inc-coordinating DNA-binding domain	Nuclear receptors with C4 zinc fingers	ESR-like	ZF.C4_NR
NR3C1	2.1.1	inc-coordinating DNA-binding domain	Nuclear receptors with C4 zinc fingers	ESR-like	ZF.C4_NR
NR3C2	2.1.1	inc-coordinating DNA-binding domain	Nuclear receptors with C4 zinc fingers	ESR-like	ZF.C4_NR
PGR	2.1.1	inc-coordinating DNA-binding domain	Nuclear receptors with C4 zinc fingers	ESR-like	ZF.C4_NR
NR1D1	2.1.2	inc-coordinating DNA-binding domain	Nuclear receptors with C4 zinc fingers	THR-like	ZF.C4_NR
NR1D2	2.1.2	inc-coordinating DNA-binding domain	Nuclear receptors with C4 zinc fingers	THR-like	ZF.C4_NR
NR1H2	2.1.2	inc-coordinating DNA-binding domain	Nuclear receptors with C4 zinc fingers	THR-like	ZF.C4_NR
NR1H3	2.1.2	inc-coordinating DNA-binding domain	Nuclear receptors with C4 zinc fingers	THR-like	ZF.C4_NR
NR1H4	2.1.2	inc-coordinating DNA-binding domain	Nuclear receptors with C4 zinc fingers	THR-like	ZF.C4_NR
NR1I2	2.1.2	inc-coordinating DNA-binding domain	Nuclear receptors with C4 zinc fingers	THR-like	ZF.C4_NR
NR1I3	2.1.2	inc-coordinating DNA-binding domain	Nuclear receptors with C4 zinc fingers	THR-like	ZF.C4_NR
PPARA	2.1.2	inc-coordinating DNA-binding domain	Nuclear receptors with C4 zinc fingers	THR-like	ZF.C4_NR
PPARD	2.1.2	inc-coordinating DNA-binding domain	Nuclear receptors with C4 zinc fingers	THR-like	ZF.C4_NR
PPARG	2.1.2	inc-coordinating DNA-binding domain	Nuclear receptors with C4 zinc fingers	THR-like	ZF.C4_NR
RARA	2.1.2	inc-coordinating DNA-binding domain	Nuclear receptors with C4 zinc fingers	THR-like	ZF.C4_NR
RARB	2.1.2	inc-coordinating DNA-binding domain	Nuclear receptors with C4 zinc fingers	THR-like	ZF.C4_NR
RARG	2.1.2	inc-coordinating DNA-binding domain	Nuclear receptors with C4 zinc fingers	THR-like	ZF.C4_NR
RORA	2.1.2	inc-coordinating DNA-binding domain	Nuclear receptors with C4 zinc fingers	THR-like	ZF.C4_NR
RORB	2.1.2	inc-coordinating DNA-binding domain	Nuclear receptors with C4 zinc fingers	THR-like	ZF.C4_NR
RORC	2.1.2	inc-coordinating DNA-binding domain	Nuclear receptors with C4 zinc fingers	THR-like	ZF.C4_NR
THRA	2.1.2	inc-coordinating DNA-binding domain	Nuclear receptors with C4 zinc fingers	THR-like	ZF.C4_NR
THRB	2.1.2	inc-coordinating DNA-binding domain	Nuclear receptors with C4 zinc fingers	THR-like	ZF.C4_NR
VDR	2.1.2	inc-coordinating DNA-binding domain	Nuclear receptors with C4 zinc fingers	THR-like	ZF.C4_NR
HNF4A	2.1.3	inc-coordinating DNA-binding domain	Nuclear receptors with C4 zinc fingers	RXR-like	ZF.C4_NR
HNF4G	2.1.3	inc-coordinating DNA-binding domain	Nuclear receptors with C4 zinc fingers	RXR-like	ZF.C4_NR
NR2C1	2.1.3	inc-coordinating DNA-binding domain	Nuclear receptors with C4 zinc fingers	RXR-like	ZF.C4_NR
NR2C2	2.1.3	inc-coordinating DNA-binding domain	Nuclear receptors with C4 zinc fingers	RXR-like	ZF.C4_NR
NR2E1	2.1.3	inc-coordinating DNA-binding domain	Nuclear receptors with C4 zinc fingers	RXR-like	ZF.C4_NR
NR2E3	2.1.3	inc-coordinating DNA-binding domain	Nuclear receptors with C4 zinc fingers	RXR-like	ZF.C4_NR
NR2F1	2.1.3	inc-coordinating DNA-binding domain	Nuclear receptors with C4 zinc fingers	RXR-like	ZF.C4_NR
NR2F2	2.1.3	inc-coordinating DNA-binding domain	Nuclear receptors with C4 zinc fingers	RXR-like	ZF.C4_NR
NR2F6	2.1.3	inc-coordinating DNA-binding domain	Nuclear receptors with C4 zinc fingers	RXR-like	ZF.C4_NR

RXRA	2.1.3	inc-coordinating DNA-binding domain	Nuclear receptors with C4 zinc fingers	RXR-like	ZF.C4_NR
RXRB	2.1.3	inc-coordinating DNA-binding domain	Nuclear receptors with C4 zinc fingers	RXR-like	ZF.C4_NR
RXRG	2.1.3	inc-coordinating DNA-binding domain	Nuclear receptors with C4 zinc fingers	RXR-like	ZF.C4_NR
NR4A1	2.1.4	inc-coordinating DNA-binding domain	Nuclear receptors with C4 zinc fingers	NGFIB-like	ZF.C4_NR
NR4A2	2.1.4	inc-coordinating DNA-binding domain	Nuclear receptors with C4 zinc fingers	NGFIB-like	ZF.C4_NR
NR4A3	2.1.4	inc-coordinating DNA-binding domain	Nuclear receptors with C4 zinc fingers	NGFIB-like	ZF.C4_NR
NR5A1	2.1.5	inc-coordinating DNA-binding domain	Nuclear receptors with C4 zinc fingers	SF-like	ZF.C4_NR
NR5A2	2.1.5	inc-coordinating DNA-binding domain	Nuclear receptors with C4 zinc fingers	SF-like	ZF.C4_NR
NR6A1	2.1.6	inc-coordinating DNA-binding domain	Nuclear receptors with C4 zinc fingers	GCNF-like	ZF.C4_NR
NR0B1	2.1.7	inc-coordinating DNA-binding domain	Nuclear receptors with C4 zinc fingers	Miscellaneous	ZF.C4_NR
NR0B2	2.1.7	inc-coordinating DNA-binding domain	Nuclear receptors with C4 zinc fingers	Miscellaneous	ZF.C4_NR
GATA1	2.2.1	inc-coordinating DNA-binding domain	Other C4 zinc finger-type factors	zf-GATA	ZF.C4_GATA
GATA2	2.2.1	inc-coordinating DNA-binding domain	Other C4 zinc finger-type factors	zf-GATA	ZF.C4_GATA
GATA3	2.2.1	inc-coordinating DNA-binding domain	Other C4 zinc finger-type factors	zf-GATA	ZF.C4_GATA
GATA4	2.2.1	inc-coordinating DNA-binding domain	Other C4 zinc finger-type factors	zf-GATA	ZF.C4_GATA
GATA5	2.2.1	inc-coordinating DNA-binding domain	Other C4 zinc finger-type factors	zf-GATA	ZF.C4_GATA
GATA6	2.2.1	inc-coordinating DNA-binding domain	Other C4 zinc finger-type factors	zf-GATA	ZF.C4_GATA
GATAD1	2.2.1	inc-coordinating DNA-binding domain	Other C4 zinc finger-type factors	Others	ZF.C4_GATA
GATAD2A	2.2.1	inc-coordinating DNA-binding domain	Other C4 zinc finger-type factors	zf-GATA	ZF.C4_GATA
GATAD2B	2.2.1	inc-coordinating DNA-binding domain	Other C4 zinc finger-type factors	zf-GATA	ZF.C4_GATA
MTA1	2.2.1	inc-coordinating DNA-binding domain	Other C4 zinc finger-type factors	zf-GATA	ZF.C4_GATA
MTA2	2.2.1	inc-coordinating DNA-binding domain	Other C4 zinc finger-type factors	zf-GATA	ZF.C4_GATA
MTA3	2.2.1	inc-coordinating DNA-binding domain	Other C4 zinc finger-type factors	zf-GATA	ZF.C4_GATA
RERE	2.2.1	inc-coordinating DNA-binding domain	Other C4 zinc finger-type factors	zf-GATA	ZF.C4_GATA
TRPS1	2.2.1	inc-coordinating DNA-binding domain	Other C4 zinc finger-type factors	zf-GATA	ZF.C4_GATA
ZGLP1	2.2.1	inc-coordinating DNA-binding domain	Other C4 zinc finger-type factors	zf-GATA	ZF.C4_GATA
EGR1	2.3.1	inc-coordinating DNA-binding domain	C2H2 zinc finger factors	zf-C2H2	ZF.C2H2
EGR2	2.3.1	inc-coordinating DNA-binding domain	C2H2 zinc finger factors	zf-C2H2	ZF.C2H2
EGR3	2.3.1	inc-coordinating DNA-binding domain	C2H2 zinc finger factors	zf-C2H2	ZF.C2H2
EGR4	2.3.1	inc-coordinating DNA-binding domain	C2H2 zinc finger factors	zf-C2H2	ZF.C2H2
KLF1	2.3.1	inc-coordinating DNA-binding domain	C2H2 zinc finger factors	zf-C2H2	ZF.C2H2
KLF10	2.3.1	inc-coordinating DNA-binding domain	C2H2 zinc finger factors	zf-C2H2	ZF.C2H2
KLF11	2.3.1	inc-coordinating DNA-binding domain	C2H2 zinc finger factors	zf-C2H2	ZF.C2H2
KLF12	2.3.1	inc-coordinating DNA-binding domain	C2H2 zinc finger factors	zf-C2H2	ZF.C2H2
KLF13	2.3.1	inc-coordinating DNA-binding domain	C2H2 zinc finger factors	zf-C2H2	ZF.C2H2
KLF14	2.3.1	inc-coordinating DNA-binding domain	C2H2 zinc finger factors	zf-C2H2	ZF.C2H2
KLF15	2.3.1	inc-coordinating DNA-binding domain	C2H2 zinc finger factors	zf-C2H2	ZF.C2H2
KLF16	2.3.1	inc-coordinating DNA-binding domain	C2H2 zinc finger factors	zf-C2H2	ZF.C2H2
KLF17	2.3.1	inc-coordinating DNA-binding domain	C2H2 zinc finger factors	zf-C2H2	ZF.C2H2
KLF2	2.3.1	inc-coordinating DNA-binding domain	C2H2 zinc finger factors	zf-C2H2	ZF.C2H2

KLF3	2.3.1	inc-coordinating DNA-binding domain	C2H2 zinc finger factors	zf-C2H2	ZF.C2H2
KLF4	2.3.1	inc-coordinating DNA-binding domain	C2H2 zinc finger factors	zf-C2H2	ZF.C2H2
KLF5	2.3.1	inc-coordinating DNA-binding domain	C2H2 zinc finger factors	zf-C2H2	ZF.C2H2
KLF6	2.3.1	inc-coordinating DNA-binding domain	C2H2 zinc finger factors	zf-C2H2	ZF.C2H2
KLF7	2.3.1	inc-coordinating DNA-binding domain	C2H2 zinc finger factors	zf-C2H2	ZF.C2H2
KLF8	2.3.1	inc-coordinating DNA-binding domain	C2H2 zinc finger factors	zf-C2H2	ZF.C2H2
KLF9	2.3.1	inc-coordinating DNA-binding domain	C2H2 zinc finger factors	zf-C2H2	ZF.C2H2
SP1	2.3.1	inc-coordinating DNA-binding domain	C2H2 zinc finger factors	zf-C2H2	ZF.C2H2
SP2	2.3.1	inc-coordinating DNA-binding domain	C2H2 zinc finger factors	zf-C2H2	ZF.C2H2
SP3	2.3.1	inc-coordinating DNA-binding domain	C2H2 zinc finger factors	zf-C2H2	ZF.C2H2
SP4	2.3.1	inc-coordinating DNA-binding domain	C2H2 zinc finger factors	zf-C2H2	ZF.C2H2
SP5	2.3.1	inc-coordinating DNA-binding domain	C2H2 zinc finger factors	zf-C2H2	ZF.C2H2
SP6	2.3.1	inc-coordinating DNA-binding domain	C2H2 zinc finger factors	zf-C2H2	ZF.C2H2
SP7	2.3.1	inc-coordinating DNA-binding domain	C2H2 zinc finger factors	zf-C2H2	ZF.C2H2
SP8	2.3.1	inc-coordinating DNA-binding domain	C2H2 zinc finger factors	zf-C2H2	ZF.C2H2
SP9	2.3.1	inc-coordinating DNA-binding domain	C2H2 zinc finger factors	zf-C2H2	ZF.C2H2
AEBP2	2.3.2	inc-coordinating DNA-binding domain	C2H2 zinc finger factors	zf-C2H2	ZF.C2H2
OSR1	2.3.2	inc-coordinating DNA-binding domain	C2H2 zinc finger factors	zf-C2H2	ZF.C2H2
OVOL3	2.3.2	inc-coordinating DNA-binding domain	C2H2 zinc finger factors	zf-C2H2	ZF.C2H2
ZBTB22	2.3.2	inc-coordinating DNA-binding domain	C2H2 zinc finger factors	ZBTB	ZF.C2H2
ZBTB32	2.3.2	inc-coordinating DNA-binding domain	C2H2 zinc finger factors	ZBTB	ZF.C2H2
ZBTB33	2.3.2	inc-coordinating DNA-binding domain	C2H2 zinc finger factors	ZBTB	ZF.C2H2
ZBTB34	2.3.2	inc-coordinating DNA-binding domain	C2H2 zinc finger factors	ZBTB	ZF.C2H2
ZBTB37	2.3.2	inc-coordinating DNA-binding domain	C2H2 zinc finger factors	ZBTB	ZF.C2H2
ZBTB43	2.3.2	inc-coordinating DNA-binding domain	C2H2 zinc finger factors	ZBTB	ZF.C2H2
ZBTB46	2.3.2	inc-coordinating DNA-binding domain	C2H2 zinc finger factors	ZBTB	ZF.C2H2
ZBTB5	2.3.2	inc-coordinating DNA-binding domain	C2H2 zinc finger factors	ZBTB	ZF.C2H2
ZBTB8B	2.3.2	inc-coordinating DNA-binding domain	C2H2 zinc finger factors	ZBTB	ZF.C2H2
ZFPM2	2.3.2	inc-coordinating DNA-binding domain	C2H2 zinc finger factors	zf-C2H2	ZF.C2H2
ZNF174	2.3.2	inc-coordinating DNA-binding domain	C2H2 zinc finger factors	zf-C2H2	ZF.C2H2
ZNF396	2.3.2	inc-coordinating DNA-binding domain	C2H2 zinc finger factors	zf-C2H2	ZF.C2H2
ZNF414	2.3.2	inc-coordinating DNA-binding domain	C2H2 zinc finger factors	Others	ZF.C2H2
ZNF446	2.3.2	inc-coordinating DNA-binding domain	C2H2 zinc finger factors	zf-C2H2	ZF.C2H2
ZNF487	2.3.2	inc-coordinating DNA-binding domain	C2H2 zinc finger factors	Others	ZF.C2H2
ZNF511	2.3.2	inc-coordinating DNA-binding domain	C2H2 zinc finger factors	Others	ZF.C2H2
ZNF580	2.3.2	inc-coordinating DNA-binding domain	C2H2 zinc finger factors	zf-C2H2	ZF.C2H2
ZNF705A	2.3.2	inc-coordinating DNA-binding domain	C2H2 zinc finger factors	zf-C2H2	ZF.C2H2
ZNF740	2.3.2	inc-coordinating DNA-binding domain	C2H2 zinc finger factors	zf-C2H2	ZF.C2H2
ZSCAN1	2.3.2	inc-coordinating DNA-binding domain	C2H2 zinc finger factors	zf-C2H2	ZF.C2H2
BCL6	2.3.3	inc-coordinating DNA-binding domain	C2H2 zinc finger factors	ZBTB	ZF.C2H2

BCL6B	2.3.3	inc-coordinating DNA-binding domain	C2H2 zinc finger factors	ZBTB	ZF.C2H2
CTCF	2.3.3	inc-coordinating DNA-binding domain	C2H2 zinc finger factors	zf-C2H2	ZF.C2H2
CTCFL	2.3.3	inc-coordinating DNA-binding domain	C2H2 zinc finger factors	zf-C2H2	ZF.C2H2
FEZF1	2.3.3	inc-coordinating DNA-binding domain	C2H2 zinc finger factors	zf-C2H2	ZF.C2H2
FEZF2	2.3.3	inc-coordinating DNA-binding domain	C2H2 zinc finger factors	zf-C2H2	ZF.C2H2
GFI1	2.3.3	inc-coordinating DNA-binding domain	C2H2 zinc finger factors	zf-C2H2	ZF.C2H2
GFI1B	2.3.3	inc-coordinating DNA-binding domain	C2H2 zinc finger factors	zf-C2H2	ZF.C2H2
GLI1	2.3.3	inc-coordinating DNA-binding domain	C2H2 zinc finger factors	zf-C2H2	ZF.C2H2
GLI2	2.3.3	inc-coordinating DNA-binding domain	C2H2 zinc finger factors	zf-C2H2	ZF.C2H2
GLI3	2.3.3	inc-coordinating DNA-binding domain	C2H2 zinc finger factors	zf-C2H2	ZF.C2H2
GLI4	2.3.3	inc-coordinating DNA-binding domain	C2H2 zinc finger factors	zf-C2H2	ZF.C2H2
GLIS1	2.3.3	inc-coordinating DNA-binding domain	C2H2 zinc finger factors	zf-C2H2	ZF.C2H2
GLIS2	2.3.3	inc-coordinating DNA-binding domain	C2H2 zinc finger factors	zf-C2H2	ZF.C2H2
GLIS3	2.3.3	inc-coordinating DNA-binding domain	C2H2 zinc finger factors	zf-C2H2	ZF.C2H2
GTF3A	2.3.3	inc-coordinating DNA-binding domain	C2H2 zinc finger factors	zf-C2H2	ZF.C2H2
GZF1	2.3.3	inc-coordinating DNA-binding domain	C2H2 zinc finger factors	ZBTB	ZF.C2H2
MTF1	2.3.3	inc-coordinating DNA-binding domain	C2H2 zinc finger factors	zf-C2H2	ZF.C2H2
MYNN	2.3.3	inc-coordinating DNA-binding domain	C2H2 zinc finger factors	ZBTB	ZF.C2H2
MZF1	2.3.3	inc-coordinating DNA-binding domain	C2H2 zinc finger factors	zf-C2H2	ZF.C2H2
OSR2	2.3.3	inc-coordinating DNA-binding domain	C2H2 zinc finger factors	zf-C2H2	ZF.C2H2
OVOL1	2.3.3	inc-coordinating DNA-binding domain	C2H2 zinc finger factors	zf-C2H2	ZF.C2H2
OVOL2	2.3.3	inc-coordinating DNA-binding domain	C2H2 zinc finger factors	zf-C2H2	ZF.C2H2
PLAG1	2.3.3	inc-coordinating DNA-binding domain	C2H2 zinc finger factors	zf-C2H2	ZF.C2H2
PLAGL1	2.3.3	inc-coordinating DNA-binding domain	C2H2 zinc finger factors	zf-C2H2	ZF.C2H2
PLAGL2	2.3.3	inc-coordinating DNA-binding domain	C2H2 zinc finger factors	zf-C2H2	ZF.C2H2
PRDM1	2.3.3	inc-coordinating DNA-binding domain	C2H2 zinc finger factors	zf-C2H2	ZF.C2H2
PRDM14	2.3.3	inc-coordinating DNA-binding domain	C2H2 zinc finger factors	zf-C2H2	ZF.C2H2
PRDM5	2.3.3	inc-coordinating DNA-binding domain	C2H2 zinc finger factors	zf-C2H2	ZF.C2H2
PRDM6	2.3.3	inc-coordinating DNA-binding domain	C2H2 zinc finger factors	zf-C2H2	ZF.C2H2
RBAK	2.3.3	inc-coordinating DNA-binding domain	C2H2 zinc finger factors	zf-C2H2	ZF.C2H2
SCRT1	2.3.3	inc-coordinating DNA-binding domain	C2H2 zinc finger factors	zf-C2H2	ZF.C2H2
SCRT2	2.3.3	inc-coordinating DNA-binding domain	C2H2 zinc finger factors	zf-C2H2	ZF.C2H2
SNAI1	2.3.3	inc-coordinating DNA-binding domain	C2H2 zinc finger factors	zf-C2H2	ZF.C2H2
SNAI2	2.3.3	inc-coordinating DNA-binding domain	C2H2 zinc finger factors	zf-C2H2	ZF.C2H2
SNAI3	2.3.3	inc-coordinating DNA-binding domain	C2H2 zinc finger factors	zf-C2H2	ZF.C2H2
WT1	2.3.3	inc-coordinating DNA-binding domain	C2H2 zinc finger factors	zf-C2H2	ZF.C2H2
YY1	2.3.3	inc-coordinating DNA-binding domain	C2H2 zinc finger factors	zf-C2H2	ZF.C2H2
YY2	2.3.3	inc-coordinating DNA-binding domain	C2H2 zinc finger factors	zf-C2H2	ZF.C2H2
ZBTB11	2.3.3	inc-coordinating DNA-binding domain	C2H2 zinc finger factors	ZBTB	ZF.C2H2
ZBTB12	2.3.3	inc-coordinating DNA-binding domain	C2H2 zinc finger factors	ZBTB	ZF.C2H2

ZBTB14	2.3.3	inc-coordinating DNA-binding domain	C2H2 zinc finger factors	ZBTB	ZF.C2H2
ZBTB16	2.3.3	inc-coordinating DNA-binding domain	C2H2 zinc finger factors	ZBTB	ZF.C2H2
ZBTB18	2.3.3	inc-coordinating DNA-binding domain	C2H2 zinc finger factors	ZBTB	ZF.C2H2
ZBTB20	2.3.3	inc-coordinating DNA-binding domain	C2H2 zinc finger factors	ZBTB	ZF.C2H2
ZBTB24	2.3.3	inc-coordinating DNA-binding domain	C2H2 zinc finger factors	ZBTB	ZF.C2H2
ZBTB26	2.3.3	inc-coordinating DNA-binding domain	C2H2 zinc finger factors	ZBTB	ZF.C2H2
ZBTB40	2.3.3	inc-coordinating DNA-binding domain	C2H2 zinc finger factors	ZBTB	ZF.C2H2
ZBTB42	2.3.3	inc-coordinating DNA-binding domain	C2H2 zinc finger factors	ZBTB	ZF.C2H2
ZBTB44	2.3.3	inc-coordinating DNA-binding domain	C2H2 zinc finger factors	ZBTB	ZF.C2H2
ZBTB45	2.3.3	inc-coordinating DNA-binding domain	C2H2 zinc finger factors	ZBTB	ZF.C2H2
ZBTB47	2.3.3	inc-coordinating DNA-binding domain	C2H2 zinc finger factors	ZBTB	ZF.C2H2
ZBTB48	2.3.3	inc-coordinating DNA-binding domain	C2H2 zinc finger factors	ZBTB	ZF.C2H2
ZBTB49	2.3.3	inc-coordinating DNA-binding domain	C2H2 zinc finger factors	ZBTB	ZF.C2H2
ZBTB6	2.3.3	inc-coordinating DNA-binding domain	C2H2 zinc finger factors	ZBTB	ZF.C2H2
ZBTB7A	2.3.3	inc-coordinating DNA-binding domain	C2H2 zinc finger factors	ZBTB	ZF.C2H2
ZBTB7B	2.3.3	inc-coordinating DNA-binding domain	C2H2 zinc finger factors	ZBTB	ZF.C2H2
ZBTB7C	2.3.3	inc-coordinating DNA-binding domain	C2H2 zinc finger factors	ZBTB	ZF.C2H2
ZFP1	2.3.3	inc-coordinating DNA-binding domain	C2H2 zinc finger factors	zf-C2H2	ZF.C2H2
ZFP14	2.3.3	inc-coordinating DNA-binding domain	C2H2 zinc finger factors	zf-C2H2	ZF.C2H2
ZFP2	2.3.3	inc-coordinating DNA-binding domain	C2H2 zinc finger factors	zf-C2H2	ZF.C2H2
ZFP28	2.3.3	inc-coordinating DNA-binding domain	C2H2 zinc finger factors	zf-C2H2	ZF.C2H2
ZFP3	2.3.3	inc-coordinating DNA-binding domain	C2H2 zinc finger factors	zf-C2H2	ZF.C2H2
ZFP30	2.3.3	inc-coordinating DNA-binding domain	C2H2 zinc finger factors	zf-C2H2	ZF.C2H2
ZFP37	2.3.3	inc-coordinating DNA-binding domain	C2H2 zinc finger factors	zf-C2H2	ZF.C2H2
ZFP41	2.3.3	inc-coordinating DNA-binding domain	C2H2 zinc finger factors	zf-C2H2	ZF.C2H2
ZFP42	2.3.3	inc-coordinating DNA-binding domain	C2H2 zinc finger factors	zf-C2H2	ZF.C2H2
ZFP62	2.3.3	inc-coordinating DNA-binding domain	C2H2 zinc finger factors	zf-C2H2	ZF.C2H2
ZFP64	2.3.3	inc-coordinating DNA-binding domain	C2H2 zinc finger factors	zf-C2H2	ZF.C2H2
ZFP69	2.3.3	inc-coordinating DNA-binding domain	C2H2 zinc finger factors	zf-C2H2	ZF.C2H2
ZFP69B	2.3.3	inc-coordinating DNA-binding domain	C2H2 zinc finger factors	zf-C2H2	ZF.C2H2
ZFP82	2.3.3	inc-coordinating DNA-binding domain	C2H2 zinc finger factors	zf-C2H2	ZF.C2H2
ZFP90	2.3.3	inc-coordinating DNA-binding domain	C2H2 zinc finger factors	zf-C2H2	ZF.C2H2
ZFP91	2.3.3	inc-coordinating DNA-binding domain	C2H2 zinc finger factors	zf-C2H2	ZF.C2H2
ZFP92	2.3.3	inc-coordinating DNA-binding domain	C2H2 zinc finger factors	zf-C2H2	ZF.C2H2
ZFX	2.3.3	inc-coordinating DNA-binding domain	C2H2 zinc finger factors	zf-C2H2	ZF.C2H2
ZFY	2.3.3	inc-coordinating DNA-binding domain	C2H2 zinc finger factors	zf-C2H2	ZF.C2H2
ZIC1	2.3.3	inc-coordinating DNA-binding domain	C2H2 zinc finger factors	zf-C2H2	ZF.C2H2
ZIC2	2.3.3	inc-coordinating DNA-binding domain	C2H2 zinc finger factors	zf-C2H2	ZF.C2H2
ZIC3	2.3.3	inc-coordinating DNA-binding domain	C2H2 zinc finger factors	zf-C2H2	ZF.C2H2
ZIC4	2.3.3	inc-coordinating DNA-binding domain	C2H2 zinc finger factors	zf-C2H2	ZF.C2H2

ZIC5	2.3.3	inc-coordinating DNA-binding domain	C2H2 zinc finger factors	zf-C2H2	ZF.C2H2
ZIK1	2.3.3	inc-coordinating DNA-binding domain	C2H2 zinc finger factors	zf-C2H2	ZF.C2H2
ZIM2	2.3.3	inc-coordinating DNA-binding domain	C2H2 zinc finger factors	zf-C2H2	ZF.C2H2
ZIM3	2.3.3	inc-coordinating DNA-binding domain	C2H2 zinc finger factors	zf-C2H2	ZF.C2H2
ZKSCAN1	2.3.3	inc-coordinating DNA-binding domain	C2H2 zinc finger factors	zf-C2H2	ZF.C2H2
ZKSCAN2	2.3.3	inc-coordinating DNA-binding domain	C2H2 zinc finger factors	zf-C2H2	ZF.C2H2
ZKSCAN3	2.3.3	inc-coordinating DNA-binding domain	C2H2 zinc finger factors	zf-C2H2	ZF.C2H2
ZKSCAN4	2.3.3	inc-coordinating DNA-binding domain	C2H2 zinc finger factors	zf-C2H2	ZF.C2H2
ZKSCAN5	2.3.3	inc-coordinating DNA-binding domain	C2H2 zinc finger factors	zf-C2H2	ZF.C2H2
ZKSCAN7	2.3.3	inc-coordinating DNA-binding domain	C2H2 zinc finger factors	zf-C2H2	ZF.C2H2
ZKSCAN8	2.3.3	inc-coordinating DNA-binding domain	C2H2 zinc finger factors	zf-C2H2	ZF.C2H2
ZNF10	2.3.3	inc-coordinating DNA-binding domain	C2H2 zinc finger factors	zf-C2H2	ZF.C2H2
ZNF100	2.3.3	inc-coordinating DNA-binding domain	C2H2 zinc finger factors	zf-C2H2	ZF.C2H2
ZNF107	2.3.3	inc-coordinating DNA-binding domain	C2H2 zinc finger factors	zf-C2H2	ZF.C2H2
ZNF114	2.3.3	inc-coordinating DNA-binding domain	C2H2 zinc finger factors	zf-C2H2	ZF.C2H2
ZNF117	2.3.3	inc-coordinating DNA-binding domain	C2H2 zinc finger factors	zf-C2H2	ZF.C2H2
ZNF121	2.3.3	inc-coordinating DNA-binding domain	C2H2 zinc finger factors	zf-C2H2	ZF.C2H2
ZNF124	2.3.3	inc-coordinating DNA-binding domain	C2H2 zinc finger factors	zf-C2H2	ZF.C2H2
ZNF131	2.3.3	inc-coordinating DNA-binding domain	C2H2 zinc finger factors	ZBTB	ZF.C2H2
ZNF133	2.3.3	inc-coordinating DNA-binding domain	C2H2 zinc finger factors	zf-C2H2	ZF.C2H2
ZNF135	2.3.3	inc-coordinating DNA-binding domain	C2H2 zinc finger factors	zf-C2H2	ZF.C2H2
ZNF136	2.3.3	inc-coordinating DNA-binding domain	C2H2 zinc finger factors	zf-C2H2	ZF.C2H2
ZNF138	2.3.3	inc-coordinating DNA-binding domain	C2H2 zinc finger factors	zf-C2H2	ZF.C2H2
ZNF14	2.3.3	inc-coordinating DNA-binding domain	C2H2 zinc finger factors	zf-C2H2	ZF.C2H2
ZNF140	2.3.3	inc-coordinating DNA-binding domain	C2H2 zinc finger factors	zf-C2H2	ZF.C2H2
ZNF141	2.3.3	inc-coordinating DNA-binding domain	C2H2 zinc finger factors	zf-C2H2	ZF.C2H2
ZNF143	2.3.3	inc-coordinating DNA-binding domain	C2H2 zinc finger factors	zf-C2H2	ZF.C2H2
ZNF146	2.3.3	inc-coordinating DNA-binding domain	C2H2 zinc finger factors	zf-C2H2	ZF.C2H2
ZNF148	2.3.3	inc-coordinating DNA-binding domain	C2H2 zinc finger factors	zf-C2H2	ZF.C2H2
ZNF154	2.3.3	inc-coordinating DNA-binding domain	C2H2 zinc finger factors	zf-C2H2	ZF.C2H2
ZNF155	2.3.3	inc-coordinating DNA-binding domain	C2H2 zinc finger factors	zf-C2H2	ZF.C2H2
ZNF157	2.3.3	inc-coordinating DNA-binding domain	C2H2 zinc finger factors	zf-C2H2	ZF.C2H2
ZNF16	2.3.3	inc-coordinating DNA-binding domain	C2H2 zinc finger factors	zf-C2H2	ZF.C2H2
ZNF160	2.3.3	inc-coordinating DNA-binding domain	C2H2 zinc finger factors	zf-C2H2	ZF.C2H2
ZNF165	2.3.3	inc-coordinating DNA-binding domain	C2H2 zinc finger factors	zf-C2H2	ZF.C2H2
ZNF169	2.3.3	inc-coordinating DNA-binding domain	C2H2 zinc finger factors	zf-C2H2	ZF.C2H2
ZNF17	2.3.3	inc-coordinating DNA-binding domain	C2H2 zinc finger factors	zf-C2H2	ZF.C2H2
ZNF175	2.3.3	inc-coordinating DNA-binding domain	C2H2 zinc finger factors	zf-C2H2	ZF.C2H2
ZNF177	2.3.3	inc-coordinating DNA-binding domain	C2H2 zinc finger factors	zf-C2H2	ZF.C2H2
ZNF18	2.3.3	inc-coordinating DNA-binding domain	C2H2 zinc finger factors	zf-C2H2	ZF.C2H2

ZNF180	2.3.3	inc-coordinating DNA-binding domain	C2H2 zinc finger factors	zf-C2H2	ZF.C2H2
ZNF181	2.3.3	inc-coordinating DNA-binding domain	C2H2 zinc finger factors	zf-C2H2	ZF.C2H2
ZNF182	2.3.3	inc-coordinating DNA-binding domain	C2H2 zinc finger factors	zf-C2H2	ZF.C2H2
ZNF184	2.3.3	inc-coordinating DNA-binding domain	C2H2 zinc finger factors	zf-C2H2	ZF.C2H2
ZNF189	2.3.3	inc-coordinating DNA-binding domain	C2H2 zinc finger factors	zf-C2H2	ZF.C2H2
ZNF19	2.3.3	inc-coordinating DNA-binding domain	C2H2 zinc finger factors	zf-C2H2	ZF.C2H2
ZNF197	2.3.3	inc-coordinating DNA-binding domain	C2H2 zinc finger factors	zf-C2H2	ZF.C2H2
ZNF2	2.3.3	inc-coordinating DNA-binding domain	C2H2 zinc finger factors	zf-C2H2	ZF.C2H2
ZNF20	2.3.3	inc-coordinating DNA-binding domain	C2H2 zinc finger factors	zf-C2H2	ZF.C2H2
ZNF200	2.3.3	inc-coordinating DNA-binding domain	C2H2 zinc finger factors	zf-C2H2	ZF.C2H2
ZNF202	2.3.3	inc-coordinating DNA-binding domain	C2H2 zinc finger factors	zf-C2H2	ZF.C2H2
ZNF205	2.3.3	inc-coordinating DNA-binding domain	C2H2 zinc finger factors	zf-C2H2	ZF.C2H2
ZNF208	2.3.3	inc-coordinating DNA-binding domain	C2H2 zinc finger factors	zf-C2H2	ZF.C2H2
ZNF212	2.3.3	inc-coordinating DNA-binding domain	C2H2 zinc finger factors	zf-C2H2	ZF.C2H2
ZNF213	2.3.3	inc-coordinating DNA-binding domain	C2H2 zinc finger factors	zf-C2H2	ZF.C2H2
ZNF214	2.3.3	inc-coordinating DNA-binding domain	C2H2 zinc finger factors	zf-C2H2	ZF.C2H2
ZNF215	2.3.3	inc-coordinating DNA-binding domain	C2H2 zinc finger factors	zf-C2H2	ZF.C2H2
ZNF22	2.3.3	inc-coordinating DNA-binding domain	C2H2 zinc finger factors	zf-C2H2	ZF.C2H2
ZNF221	2.3.3	inc-coordinating DNA-binding domain	C2H2 zinc finger factors	zf-C2H2	ZF.C2H2
ZNF222	2.3.3	inc-coordinating DNA-binding domain	C2H2 zinc finger factors	zf-C2H2	ZF.C2H2
ZNF223	2.3.3	inc-coordinating DNA-binding domain	C2H2 zinc finger factors	zf-C2H2	ZF.C2H2
ZNF224	2.3.3	inc-coordinating DNA-binding domain	C2H2 zinc finger factors	zf-C2H2	ZF.C2H2
ZNF225	2.3.3	inc-coordinating DNA-binding domain	C2H2 zinc finger factors	zf-C2H2	ZF.C2H2
ZNF226	2.3.3	inc-coordinating DNA-binding domain	C2H2 zinc finger factors	zf-C2H2	ZF.C2H2
ZNF227	2.3.3	inc-coordinating DNA-binding domain	C2H2 zinc finger factors	zf-C2H2	ZF.C2H2
ZNF229	2.3.3	inc-coordinating DNA-binding domain	C2H2 zinc finger factors	zf-C2H2	ZF.C2H2
ZNF23	2.3.3	inc-coordinating DNA-binding domain	C2H2 zinc finger factors	zf-C2H2	ZF.C2H2
ZNF230	2.3.3	inc-coordinating DNA-binding domain	C2H2 zinc finger factors	zf-C2H2	ZF.C2H2
ZNF232	2.3.3	inc-coordinating DNA-binding domain	C2H2 zinc finger factors	zf-C2H2	ZF.C2H2
ZNF233	2.3.3	inc-coordinating DNA-binding domain	C2H2 zinc finger factors	zf-C2H2	ZF.C2H2
ZNF234	2.3.3	inc-coordinating DNA-binding domain	C2H2 zinc finger factors	zf-C2H2	ZF.C2H2
ZNF235	2.3.3	inc-coordinating DNA-binding domain	C2H2 zinc finger factors	zf-C2H2	ZF.C2H2
ZNF239	2.3.3	inc-coordinating DNA-binding domain	C2H2 zinc finger factors	zf-C2H2	ZF.C2H2
ZNF24	2.3.3	inc-coordinating DNA-binding domain	C2H2 zinc finger factors	zf-C2H2	ZF.C2H2
ZNF25	2.3.3	inc-coordinating DNA-binding domain	C2H2 zinc finger factors	zf-C2H2	ZF.C2H2
ZNF250	2.3.3	inc-coordinating DNA-binding domain	C2H2 zinc finger factors	zf-C2H2	ZF.C2H2
ZNF253	2.3.3	inc-coordinating DNA-binding domain	C2H2 zinc finger factors	zf-C2H2	ZF.C2H2
ZNF254	2.3.3	inc-coordinating DNA-binding domain	C2H2 zinc finger factors	zf-C2H2	ZF.C2H2
ZNF257	2.3.3	inc-coordinating DNA-binding domain	C2H2 zinc finger factors	zf-C2H2	ZF.C2H2
ZNF26	2.3.3	inc-coordinating DNA-binding domain	C2H2 zinc finger factors	zf-C2H2	ZF.C2H2

ZNF260	2.3.3	inc-coordinating DNA-binding domain	C2H2 zinc finger factors	zf-C2H2	ZF.C2H2
ZNF263	2.3.3	inc-coordinating DNA-binding domain	C2H2 zinc finger factors	zf-C2H2	ZF.C2H2
ZNF264	2.3.3	inc-coordinating DNA-binding domain	C2H2 zinc finger factors	zf-C2H2	ZF.C2H2
ZNF266	2.3.3	inc-coordinating DNA-binding domain	C2H2 zinc finger factors	zf-C2H2	ZF.C2H2
ZNF267	2.3.3	inc-coordinating DNA-binding domain	C2H2 zinc finger factors	zf-C2H2	ZF.C2H2
ZNF268	2.3.3	inc-coordinating DNA-binding domain	C2H2 zinc finger factors	zf-C2H2	ZF.C2H2
ZNF273	2.3.3	inc-coordinating DNA-binding domain	C2H2 zinc finger factors	zf-C2H2	ZF.C2H2
ZNF274	2.3.3	inc-coordinating DNA-binding domain	C2H2 zinc finger factors	zf-C2H2	ZF.C2H2
ZNF275	2.3.3	inc-coordinating DNA-binding domain	C2H2 zinc finger factors	zf-C2H2	ZF.C2H2
ZNF276	2.3.3	inc-coordinating DNA-binding domain	C2H2 zinc finger factors	zf-C2H2	ZF.C2H2
ZNF28	2.3.3	inc-coordinating DNA-binding domain	C2H2 zinc finger factors	zf-C2H2	ZF.C2H2
ZNF280A	2.3.3	inc-coordinating DNA-binding domain	C2H2 zinc finger factors	Others	ZF.C2H2
ZNF280B	2.3.3	inc-coordinating DNA-binding domain	C2H2 zinc finger factors	Others	ZF.C2H2
ZNF280C	2.3.3	inc-coordinating DNA-binding domain	C2H2 zinc finger factors	Others	ZF.C2H2
ZNF280D	2.3.3	inc-coordinating DNA-binding domain	C2H2 zinc finger factors	Others	ZF.C2H2
ZNF281	2.3.3	inc-coordinating DNA-binding domain	C2H2 zinc finger factors	zf-C2H2	ZF.C2H2
ZNF282	2.3.3	inc-coordinating DNA-binding domain	C2H2 zinc finger factors	zf-C2H2	ZF.C2H2
ZNF283	2.3.3	inc-coordinating DNA-binding domain	C2H2 zinc finger factors	zf-C2H2	ZF.C2H2
ZNF284	2.3.3	inc-coordinating DNA-binding domain	C2H2 zinc finger factors	zf-C2H2	ZF.C2H2
ZNF285	2.3.3	inc-coordinating DNA-binding domain	C2H2 zinc finger factors	zf-C2H2	ZF.C2H2
ZNF286A	2.3.3	inc-coordinating DNA-binding domain	C2H2 zinc finger factors	zf-C2H2	ZF.C2H2
ZNF286B	2.3.3	inc-coordinating DNA-binding domain	C2H2 zinc finger factors		ZF.C2H2
ZNF287	2.3.3	inc-coordinating DNA-binding domain	C2H2 zinc finger factors	zf-C2H2	ZF.C2H2
ZNF3	2.3.3	inc-coordinating DNA-binding domain	C2H2 zinc finger factors	zf-C2H2	ZF.C2H2
ZNF30	2.3.3	inc-coordinating DNA-binding domain	C2H2 zinc finger factors	zf-C2H2	ZF.C2H2
ZNF300	2.3.3	inc-coordinating DNA-binding domain	C2H2 zinc finger factors	zf-C2H2	ZF.C2H2
ZNF302	2.3.3	inc-coordinating DNA-binding domain	C2H2 zinc finger factors	zf-C2H2	ZF.C2H2
ZNF311	2.3.3	inc-coordinating DNA-binding domain	C2H2 zinc finger factors	zf-C2H2	ZF.C2H2
ZNF317	2.3.3	inc-coordinating DNA-binding domain	C2H2 zinc finger factors	zf-C2H2	ZF.C2H2
ZNF32	2.3.3	inc-coordinating DNA-binding domain	C2H2 zinc finger factors	zf-C2H2	ZF.C2H2
ZNF320	2.3.3	inc-coordinating DNA-binding domain	C2H2 zinc finger factors	zf-C2H2	ZF.C2H2
ZNF322	2.3.3	inc-coordinating DNA-binding domain	C2H2 zinc finger factors	zf-C2H2	ZF.C2H2
ZNF324	2.3.3	inc-coordinating DNA-binding domain	C2H2 zinc finger factors	zf-C2H2	ZF.C2H2
ZNF324B	2.3.3	inc-coordinating DNA-binding domain	C2H2 zinc finger factors	zf-C2H2	ZF.C2H2
ZNF329	2.3.3	inc-coordinating DNA-binding domain	C2H2 zinc finger factors	zf-C2H2	ZF.C2H2
ZNF331	2.3.3	inc-coordinating DNA-binding domain	C2H2 zinc finger factors	zf-C2H2	ZF.C2H2
ZNF333	2.3.3	inc-coordinating DNA-binding domain	C2H2 zinc finger factors	zf-C2H2	ZF.C2H2
ZNF337	2.3.3	inc-coordinating DNA-binding domain	C2H2 zinc finger factors	zf-C2H2	ZF.C2H2
ZNF33A	2.3.3	inc-coordinating DNA-binding domain	C2H2 zinc finger factors	zf-C2H2	ZF.C2H2
ZNF33B	2.3.3	inc-coordinating DNA-binding domain	C2H2 zinc finger factors	zf-C2H2	ZF.C2H2

ZNF34	2.3.3	inc-coordinating DNA-binding domain	C2H2 zinc finger factors	zf-C2H2	ZF.C2H2
ZNF343	2.3.3	inc-coordinating DNA-binding domain	C2H2 zinc finger factors	zf-C2H2	ZF.C2H2
ZNF345	2.3.3	inc-coordinating DNA-binding domain	C2H2 zinc finger factors	zf-C2H2	ZF.C2H2
ZNF347	2.3.3	inc-coordinating DNA-binding domain	C2H2 zinc finger factors	zf-C2H2	ZF.C2H2
ZNF35	2.3.3	inc-coordinating DNA-binding domain	C2H2 zinc finger factors	zf-C2H2	ZF.C2H2
ZNF350	2.3.3	inc-coordinating DNA-binding domain	C2H2 zinc finger factors	zf-C2H2	ZF.C2H2
ZNF354A	2.3.3	inc-coordinating DNA-binding domain	C2H2 zinc finger factors	zf-C2H2	ZF.C2H2
ZNF354B	2.3.3	inc-coordinating DNA-binding domain	C2H2 zinc finger factors	zf-C2H2	ZF.C2H2
ZNF354C	2.3.3	inc-coordinating DNA-binding domain	C2H2 zinc finger factors	zf-C2H2	ZF.C2H2
ZNF358	2.3.3	inc-coordinating DNA-binding domain	C2H2 zinc finger factors	zf-C2H2	ZF.C2H2
ZNF362	2.3.3	inc-coordinating DNA-binding domain	C2H2 zinc finger factors	zf-C2H2	ZF.C2H2
ZNF366	2.3.3	inc-coordinating DNA-binding domain	C2H2 zinc finger factors	zf-C2H2	ZF.C2H2
ZNF383	2.3.3	inc-coordinating DNA-binding domain	C2H2 zinc finger factors	zf-C2H2	ZF.C2H2
ZNF384	2.3.3	inc-coordinating DNA-binding domain	C2H2 zinc finger factors	zf-C2H2	ZF.C2H2
ZNF391	2.3.3	inc-coordinating DNA-binding domain	C2H2 zinc finger factors	zf-C2H2	ZF.C2H2
ZNF394	2.3.3	inc-coordinating DNA-binding domain	C2H2 zinc finger factors	zf-C2H2	ZF.C2H2
ZNF397	2.3.3	inc-coordinating DNA-binding domain	C2H2 zinc finger factors	zf-C2H2	ZF.C2H2
ZNF398	2.3.3	inc-coordinating DNA-binding domain	C2H2 zinc finger factors	zf-C2H2	ZF.C2H2
ZNF404	2.3.3	inc-coordinating DNA-binding domain	C2H2 zinc finger factors	zf-C2H2	ZF.C2H2
ZNF408	2.3.3	inc-coordinating DNA-binding domain	C2H2 zinc finger factors	zf-C2H2	ZF.C2H2
ZNF41	2.3.3	inc-coordinating DNA-binding domain	C2H2 zinc finger factors	zf-C2H2	ZF.C2H2
ZNF410	2.3.3	inc-coordinating DNA-binding domain	C2H2 zinc finger factors	zf-C2H2	ZF.C2H2
ZNF415	2.3.3	inc-coordinating DNA-binding domain	C2H2 zinc finger factors	zf-C2H2	ZF.C2H2
ZNF416	2.3.3	inc-coordinating DNA-binding domain	C2H2 zinc finger factors	zf-C2H2	ZF.C2H2
ZNF419	2.3.3	inc-coordinating DNA-binding domain	C2H2 zinc finger factors	zf-C2H2	ZF.C2H2
ZNF420	2.3.3	inc-coordinating DNA-binding domain	C2H2 zinc finger factors	zf-C2H2	ZF.C2H2
ZNF425	2.3.3	inc-coordinating DNA-binding domain	C2H2 zinc finger factors	zf-C2H2	ZF.C2H2
ZNF426	2.3.3	inc-coordinating DNA-binding domain	C2H2 zinc finger factors	zf-C2H2	ZF.C2H2
ZNF429	2.3.3	inc-coordinating DNA-binding domain	C2H2 zinc finger factors	zf-C2H2	ZF.C2H2
ZNF43	2.3.3	inc-coordinating DNA-binding domain	C2H2 zinc finger factors	zf-C2H2	ZF.C2H2
ZNF430	2.3.3	inc-coordinating DNA-binding domain	C2H2 zinc finger factors	zf-C2H2	ZF.C2H2
ZNF431	2.3.3	inc-coordinating DNA-binding domain	C2H2 zinc finger factors	zf-C2H2	ZF.C2H2
ZNF432	2.3.3	inc-coordinating DNA-binding domain	C2H2 zinc finger factors	zf-C2H2	ZF.C2H2
ZNF433	2.3.3	inc-coordinating DNA-binding domain	C2H2 zinc finger factors	zf-C2H2	ZF.C2H2
ZNF436	2.3.3	inc-coordinating DNA-binding domain	C2H2 zinc finger factors	zf-C2H2	ZF.C2H2
ZNF439	2.3.3	inc-coordinating DNA-binding domain	C2H2 zinc finger factors	zf-C2H2	ZF.C2H2
ZNF44	2.3.3	inc-coordinating DNA-binding domain	C2H2 zinc finger factors	zf-C2H2	ZF.C2H2
ZNF440	2.3.3	inc-coordinating DNA-binding domain	C2H2 zinc finger factors	zf-C2H2	ZF.C2H2
ZNF441	2.3.3	inc-coordinating DNA-binding domain	C2H2 zinc finger factors	zf-C2H2	ZF.C2H2
ZNF442	2.3.3	inc-coordinating DNA-binding domain	C2H2 zinc finger factors	zf-C2H2	ZF.C2H2

ZNF443	2.3.3	inc-coordinating DNA-binding domain	C2H2 zinc finger factors	zf-C2H2	ZF.C2H2
ZNF444	2.3.3	inc-coordinating DNA-binding domain	C2H2 zinc finger factors	zf-C2H2	ZF.C2H2
ZNF449	2.3.3	inc-coordinating DNA-binding domain	C2H2 zinc finger factors	zf-C2H2	ZF.C2H2
ZNF45	2.3.3	inc-coordinating DNA-binding domain	C2H2 zinc finger factors	zf-C2H2	ZF.C2H2
ZNF454	2.3.3	inc-coordinating DNA-binding domain	C2H2 zinc finger factors	zf-C2H2	ZF.C2H2
ZNF460	2.3.3	inc-coordinating DNA-binding domain	C2H2 zinc finger factors	zf-C2H2	ZF.C2H2
ZNF461	2.3.3	inc-coordinating DNA-binding domain	C2H2 zinc finger factors	zf-C2H2	ZF.C2H2
ZNF468	2.3.3	inc-coordinating DNA-binding domain	C2H2 zinc finger factors	zf-C2H2	ZF.C2H2
ZNF471	2.3.3	inc-coordinating DNA-binding domain	C2H2 zinc finger factors	zf-C2H2	ZF.C2H2
ZNF479	2.3.3	inc-coordinating DNA-binding domain	C2H2 zinc finger factors	zf-C2H2	ZF.C2H2
ZNF480	2.3.3	inc-coordinating DNA-binding domain	C2H2 zinc finger factors	zf-C2H2	ZF.C2H2
ZNF483	2.3.3	inc-coordinating DNA-binding domain	C2H2 zinc finger factors	zf-C2H2	ZF.C2H2
ZNF484	2.3.3	inc-coordinating DNA-binding domain	C2H2 zinc finger factors	zf-C2H2	ZF.C2H2
ZNF485	2.3.3	inc-coordinating DNA-binding domain	C2H2 zinc finger factors	zf-C2H2	ZF.C2H2
ZNF490	2.3.3	inc-coordinating DNA-binding domain	C2H2 zinc finger factors	zf-C2H2	ZF.C2H2
ZNF491	2.3.3	inc-coordinating DNA-binding domain	C2H2 zinc finger factors	zf-C2H2	ZF.C2H2
ZNF492	2.3.3	inc-coordinating DNA-binding domain	C2H2 zinc finger factors	zf-C2H2	ZF.C2H2
ZNF493	2.3.3	inc-coordinating DNA-binding domain	C2H2 zinc finger factors	zf-C2H2	ZF.C2H2
ZNF496	2.3.3	inc-coordinating DNA-binding domain	C2H2 zinc finger factors	zf-C2H2	ZF.C2H2
ZNF497	2.3.3	inc-coordinating DNA-binding domain	C2H2 zinc finger factors	zf-C2H2	ZF.C2H2
ZNF500	2.3.3	inc-coordinating DNA-binding domain	C2H2 zinc finger factors	zf-C2H2	ZF.C2H2
ZNF501	2.3.3	inc-coordinating DNA-binding domain	C2H2 zinc finger factors	zf-C2H2	ZF.C2H2
ZNF502	2.3.3	inc-coordinating DNA-binding domain	C2H2 zinc finger factors	zf-C2H2	ZF.C2H2
ZNF506	2.3.3	inc-coordinating DNA-binding domain	C2H2 zinc finger factors	zf-C2H2	ZF.C2H2
ZNF514	2.3.3	inc-coordinating DNA-binding domain	C2H2 zinc finger factors	zf-C2H2	ZF.C2H2
ZNF517	2.3.3	inc-coordinating DNA-binding domain	C2H2 zinc finger factors	zf-C2H2	ZF.C2H2
ZNF524	2.3.3	inc-coordinating DNA-binding domain	C2H2 zinc finger factors	zf-C2H2	ZF.C2H2
ZNF525	2.3.3	inc-coordinating DNA-binding domain	C2H2 zinc finger factors	zf-C2H2	ZF.C2H2
ZNF528	2.3.3	inc-coordinating DNA-binding domain	C2H2 zinc finger factors	zf-C2H2	ZF.C2H2
ZNF529	2.3.3	inc-coordinating DNA-binding domain	C2H2 zinc finger factors	zf-C2H2	ZF.C2H2
ZNF530	2.3.3	inc-coordinating DNA-binding domain	C2H2 zinc finger factors	zf-C2H2	ZF.C2H2
ZNF534	2.3.3	inc-coordinating DNA-binding domain	C2H2 zinc finger factors	zf-C2H2	ZF.C2H2
ZNF540	2.3.3	inc-coordinating DNA-binding domain	C2H2 zinc finger factors	zf-C2H2	ZF.C2H2
ZNF543	2.3.3	inc-coordinating DNA-binding domain	C2H2 zinc finger factors	zf-C2H2	ZF.C2H2
ZNF544	2.3.3	inc-coordinating DNA-binding domain	C2H2 zinc finger factors	zf-C2H2	ZF.C2H2
ZNF546	2.3.3	inc-coordinating DNA-binding domain	C2H2 zinc finger factors	zf-C2H2	ZF.C2H2
ZNF547	2.3.3	inc-coordinating DNA-binding domain	C2H2 zinc finger factors	zf-C2H2	ZF.C2H2
ZNF548	2.3.3	inc-coordinating DNA-binding domain	C2H2 zinc finger factors	zf-C2H2	ZF.C2H2
ZNF549	2.3.3	inc-coordinating DNA-binding domain	C2H2 zinc finger factors	zf-C2H2	ZF.C2H2
ZNF550	2.3.3	inc-coordinating DNA-binding domain	C2H2 zinc finger factors	zf-C2H2	ZF.C2H2

ZNF551	2.3.3	inc-coordinating DNA-binding domain	C2H2 zinc finger factors	zf-C2H2	ZF.C2H2
ZNF554	2.3.3	inc-coordinating DNA-binding domain	C2H2 zinc finger factors	zf-C2H2	ZF.C2H2
ZNF555	2.3.3	inc-coordinating DNA-binding domain	C2H2 zinc finger factors	zf-C2H2	ZF.C2H2
ZNF556	2.3.3	inc-coordinating DNA-binding domain	C2H2 zinc finger factors	zf-C2H2	ZF.C2H2
ZNF557	2.3.3	inc-coordinating DNA-binding domain	C2H2 zinc finger factors	zf-C2H2	ZF.C2H2
ZNF558	2.3.3	inc-coordinating DNA-binding domain	C2H2 zinc finger factors	zf-C2H2	ZF.C2H2
ZNF559	2.3.3	inc-coordinating DNA-binding domain	C2H2 zinc finger factors	zf-C2H2	ZF.C2H2
ZNF560	2.3.3	inc-coordinating DNA-binding domain	C2H2 zinc finger factors	zf-C2H2	ZF.C2H2
ZNF561	2.3.3	inc-coordinating DNA-binding domain	C2H2 zinc finger factors	zf-C2H2	ZF.C2H2
ZNF562	2.3.3	inc-coordinating DNA-binding domain	C2H2 zinc finger factors	zf-C2H2	ZF.C2H2
ZNF563	2.3.3	inc-coordinating DNA-binding domain	C2H2 zinc finger factors	zf-C2H2	ZF.C2H2
ZNF564	2.3.3	inc-coordinating DNA-binding domain	C2H2 zinc finger factors	zf-C2H2	ZF.C2H2
ZNF565	2.3.3	inc-coordinating DNA-binding domain	C2H2 zinc finger factors	zf-C2H2	ZF.C2H2
ZNF566	2.3.3	inc-coordinating DNA-binding domain	C2H2 zinc finger factors	zf-C2H2	ZF.C2H2
ZNF567	2.3.3	inc-coordinating DNA-binding domain	C2H2 zinc finger factors	zf-C2H2	ZF.C2H2
ZNF568	2.3.3	inc-coordinating DNA-binding domain	C2H2 zinc finger factors	zf-C2H2	ZF.C2H2
ZNF569	2.3.3	inc-coordinating DNA-binding domain	C2H2 zinc finger factors	zf-C2H2	ZF.C2H2
ZNF57	2.3.3	inc-coordinating DNA-binding domain	C2H2 zinc finger factors	zf-C2H2	ZF.C2H2
ZNF570	2.3.3	inc-coordinating DNA-binding domain	C2H2 zinc finger factors	zf-C2H2	ZF.C2H2
ZNF571	2.3.3	inc-coordinating DNA-binding domain	C2H2 zinc finger factors	zf-C2H2	ZF.C2H2
ZNF572	2.3.3	inc-coordinating DNA-binding domain	C2H2 zinc finger factors	zf-C2H2	ZF.C2H2
ZNF575	2.3.3	inc-coordinating DNA-binding domain	C2H2 zinc finger factors	zf-C2H2	ZF.C2H2
ZNF576	2.3.3	inc-coordinating DNA-binding domain	C2H2 zinc finger factors	zf-C2H2	ZF.C2H2
ZNF577	2.3.3	inc-coordinating DNA-binding domain	C2H2 zinc finger factors	zf-C2H2	ZF.C2H2
ZNF581	2.3.3	inc-coordinating DNA-binding domain	C2H2 zinc finger factors	zf-C2H2	ZF.C2H2
ZNF582	2.3.3	inc-coordinating DNA-binding domain	C2H2 zinc finger factors	zf-C2H2	ZF.C2H2
ZNF583	2.3.3	inc-coordinating DNA-binding domain	C2H2 zinc finger factors	zf-C2H2	ZF.C2H2
ZNF584	2.3.3	inc-coordinating DNA-binding domain	C2H2 zinc finger factors	zf-C2H2	ZF.C2H2
ZNF585A	2.3.3	inc-coordinating DNA-binding domain	C2H2 zinc finger factors	zf-C2H2	ZF.C2H2
ZNF585B	2.3.3	inc-coordinating DNA-binding domain	C2H2 zinc finger factors	zf-C2H2	ZF.C2H2
ZNF586	2.3.3	inc-coordinating DNA-binding domain	C2H2 zinc finger factors	zf-C2H2	ZF.C2H2
ZNF589	2.3.3	inc-coordinating DNA-binding domain	C2H2 zinc finger factors	zf-C2H2	ZF.C2H2
ZNF594	2.3.3	inc-coordinating DNA-binding domain	C2H2 zinc finger factors	zf-C2H2	ZF.C2H2
ZNF595	2.3.3	inc-coordinating DNA-binding domain	C2H2 zinc finger factors	zf-C2H2	ZF.C2H2
ZNF596	2.3.3	inc-coordinating DNA-binding domain	C2H2 zinc finger factors	zf-C2H2	ZF.C2H2
ZNF599	2.3.3	inc-coordinating DNA-binding domain	C2H2 zinc finger factors	zf-C2H2	ZF.C2H2
ZNF600	2.3.3	inc-coordinating DNA-binding domain	C2H2 zinc finger factors	zf-C2H2	ZF.C2H2
ZNF605	2.3.3	inc-coordinating DNA-binding domain	C2H2 zinc finger factors	zf-C2H2	ZF.C2H2
ZNF607	2.3.3	inc-coordinating DNA-binding domain	C2H2 zinc finger factors	zf-C2H2	ZF.C2H2
ZNF610	2.3.3	inc-coordinating DNA-binding domain	C2H2 zinc finger factors	zf-C2H2	ZF.C2H2

ZNF611	2.3.3	inc-coordinating DNA-binding domain	C2H2 zinc finger factors	zf-C2H2	ZF.C2H2
ZNF613	2.3.3	inc-coordinating DNA-binding domain	C2H2 zinc finger factors	zf-C2H2	ZF.C2H2
ZNF614	2.3.3	inc-coordinating DNA-binding domain	C2H2 zinc finger factors	zf-C2H2	ZF.C2H2
ZNF615	2.3.3	inc-coordinating DNA-binding domain	C2H2 zinc finger factors	zf-C2H2	ZF.C2H2
ZNF616	2.3.3	inc-coordinating DNA-binding domain	C2H2 zinc finger factors	zf-C2H2	ZF.C2H2
ZNF619	2.3.3	inc-coordinating DNA-binding domain	C2H2 zinc finger factors	zf-C2H2	ZF.C2H2
ZNF620	2.3.3	inc-coordinating DNA-binding domain	C2H2 zinc finger factors	zf-C2H2	ZF.C2H2
ZNF621	2.3.3	inc-coordinating DNA-binding domain	C2H2 zinc finger factors	zf-C2H2	ZF.C2H2
ZNF623	2.3.3	inc-coordinating DNA-binding domain	C2H2 zinc finger factors	zf-C2H2	ZF.C2H2
ZNF624	2.3.3	inc-coordinating DNA-binding domain	C2H2 zinc finger factors	zf-C2H2	ZF.C2H2
ZNF625	2.3.3	inc-coordinating DNA-binding domain	C2H2 zinc finger factors	zf-C2H2	ZF.C2H2
ZNF626	2.3.3	inc-coordinating DNA-binding domain	C2H2 zinc finger factors	zf-C2H2	ZF.C2H2
ZNF627	2.3.3	inc-coordinating DNA-binding domain	C2H2 zinc finger factors	zf-C2H2	ZF.C2H2
ZNF641	2.3.3	inc-coordinating DNA-binding domain	C2H2 zinc finger factors	zf-C2H2	ZF.C2H2
ZNF648	2.3.3	inc-coordinating DNA-binding domain	C2H2 zinc finger factors	zf-C2H2	ZF.C2H2
ZNF649	2.3.3	inc-coordinating DNA-binding domain	C2H2 zinc finger factors	zf-C2H2	ZF.C2H2
ZNF652	2.3.3	inc-coordinating DNA-binding domain	C2H2 zinc finger factors	zf-C2H2	ZF.C2H2
ZNF653	2.3.3	inc-coordinating DNA-binding domain	C2H2 zinc finger factors	zf-C2H2	ZF.C2H2
ZNF655	2.3.3	inc-coordinating DNA-binding domain	C2H2 zinc finger factors	zf-C2H2	ZF.C2H2
ZNF66	2.3.3	inc-coordinating DNA-binding domain	C2H2 zinc finger factors	zf-C2H2	ZF.C2H2
ZNF660	2.3.3	inc-coordinating DNA-binding domain	C2H2 zinc finger factors	zf-C2H2	ZF.C2H2
ZNF662	2.3.3	inc-coordinating DNA-binding domain	C2H2 zinc finger factors	zf-C2H2	ZF.C2H2
ZNF664	2.3.3	inc-coordinating DNA-binding domain	C2H2 zinc finger factors	zf-C2H2	ZF.C2H2
ZNF665	2.3.3	inc-coordinating DNA-binding domain	C2H2 zinc finger factors	zf-C2H2	ZF.C2H2
ZNF667	2.3.3	inc-coordinating DNA-binding domain	C2H2 zinc finger factors	zf-C2H2	ZF.C2H2
ZNF669	2.3.3	inc-coordinating DNA-binding domain	C2H2 zinc finger factors	zf-C2H2	ZF.C2H2
ZNF670	2.3.3	inc-coordinating DNA-binding domain	C2H2 zinc finger factors	zf-C2H2	ZF.C2H2
ZNF672	2.3.3	inc-coordinating DNA-binding domain	C2H2 zinc finger factors	zf-C2H2	ZF.C2H2
ZNF675	2.3.3	inc-coordinating DNA-binding domain	C2H2 zinc finger factors	zf-C2H2	ZF.C2H2
ZNF676	2.3.3	inc-coordinating DNA-binding domain	C2H2 zinc finger factors	zf-C2H2	ZF.C2H2
ZNF677	2.3.3	inc-coordinating DNA-binding domain	C2H2 zinc finger factors	zf-C2H2	ZF.C2H2
ZNF678	2.3.3	inc-coordinating DNA-binding domain	C2H2 zinc finger factors	zf-C2H2	ZF.C2H2
ZNF679	2.3.3	inc-coordinating DNA-binding domain	C2H2 zinc finger factors	zf-C2H2	ZF.C2H2
ZNF680	2.3.3	inc-coordinating DNA-binding domain	C2H2 zinc finger factors	zf-C2H2	ZF.C2H2
ZNF681	2.3.3	inc-coordinating DNA-binding domain	C2H2 zinc finger factors	zf-C2H2	ZF.C2H2
ZNF682	2.3.3	inc-coordinating DNA-binding domain	C2H2 zinc finger factors	zf-C2H2	ZF.C2H2
ZNF683	2.3.3	inc-coordinating DNA-binding domain	C2H2 zinc finger factors	zf-C2H2	ZF.C2H2
ZNF684	2.3.3	inc-coordinating DNA-binding domain	C2H2 zinc finger factors	zf-C2H2	ZF.C2H2
ZNF689	2.3.3	inc-coordinating DNA-binding domain	C2H2 zinc finger factors	zf-C2H2	ZF.C2H2
ZNF691	2.3.3	inc-coordinating DNA-binding domain	C2H2 zinc finger factors	zf-C2H2	ZF.C2H2

ZNF692	2.3.3	inc-coordinating DNA-binding domain	C2H2 zinc finger factors	zf-C2H2	ZF.C2H2
ZNF695	2.3.3	inc-coordinating DNA-binding domain	C2H2 zinc finger factors	zf-C2H2	ZF.C2H2
ZNF696	2.3.3	inc-coordinating DNA-binding domain	C2H2 zinc finger factors	zf-C2H2	ZF.C2H2
ZNF7	2.3.3	inc-coordinating DNA-binding domain	C2H2 zinc finger factors	zf-C2H2	ZF.C2H2
ZNF70	2.3.3	inc-coordinating DNA-binding domain	C2H2 zinc finger factors	zf-C2H2	ZF.C2H2
ZNF701	2.3.3	inc-coordinating DNA-binding domain	C2H2 zinc finger factors	zf-C2H2	ZF.C2H2
ZNF705D	2.3.3	inc-coordinating DNA-binding domain	C2H2 zinc finger factors	zf-C2H2	ZF.C2H2
ZNF705E	2.3.3	inc-coordinating DNA-binding domain	C2H2 zinc finger factors	zf-C2H2	ZF.C2H2
ZNF705G	2.3.3	inc-coordinating DNA-binding domain	C2H2 zinc finger factors	zf-C2H2	ZF.C2H2
ZNF707	2.3.3	inc-coordinating DNA-binding domain	C2H2 zinc finger factors	zf-C2H2	ZF.C2H2
ZNF709	2.3.3	inc-coordinating DNA-binding domain	C2H2 zinc finger factors	zf-C2H2	ZF.C2H2
ZNF71	2.3.3	inc-coordinating DNA-binding domain	C2H2 zinc finger factors	zf-C2H2	ZF.C2H2
ZNF710	2.3.3	inc-coordinating DNA-binding domain	C2H2 zinc finger factors	zf-C2H2	ZF.C2H2
ZNF713	2.3.3	inc-coordinating DNA-binding domain	C2H2 zinc finger factors	zf-C2H2	ZF.C2H2
ZNF716	2.3.3	inc-coordinating DNA-binding domain	C2H2 zinc finger factors	zf-C2H2	ZF.C2H2
ZNF718	2.3.3	inc-coordinating DNA-binding domain	C2H2 zinc finger factors	zf-C2H2	ZF.C2H2
ZNF721	2.3.3	inc-coordinating DNA-binding domain	C2H2 zinc finger factors	zf-C2H2	ZF.C2H2
ZNF726	2.3.3	inc-coordinating DNA-binding domain	C2H2 zinc finger factors	zf-C2H2	ZF.C2H2
ZNF727	2.3.3	inc-coordinating DNA-binding domain	C2H2 zinc finger factors	zf-C2H2	ZF.C2H2
ZNF729	2.3.3	inc-coordinating DNA-binding domain	C2H2 zinc finger factors	zf-C2H2	ZF.C2H2
ZNF730	2.3.3	inc-coordinating DNA-binding domain	C2H2 zinc finger factors	zf-C2H2	ZF.C2H2
ZNF732	2.3.3	inc-coordinating DNA-binding domain	C2H2 zinc finger factors	zf-C2H2	ZF.C2H2
ZNF736	2.3.3	inc-coordinating DNA-binding domain	C2H2 zinc finger factors	zf-C2H2	ZF.C2H2
ZNF737	2.3.3	inc-coordinating DNA-binding domain	C2H2 zinc finger factors	zf-C2H2	ZF.C2H2
ZNF74	2.3.3	inc-coordinating DNA-binding domain	C2H2 zinc finger factors	zf-C2H2	ZF.C2H2
ZNF746	2.3.3	inc-coordinating DNA-binding domain	C2H2 zinc finger factors	zf-C2H2	ZF.C2H2
ZNF747	2.3.3	inc-coordinating DNA-binding domain	C2H2 zinc finger factors	zf-C2H2	ZF.C2H2
ZNF749	2.3.3	inc-coordinating DNA-binding domain	C2H2 zinc finger factors	zf-C2H2	ZF.C2H2
ZNF75A	2.3.3	inc-coordinating DNA-binding domain	C2H2 zinc finger factors	zf-C2H2	ZF.C2H2
ZNF75D	2.3.3	inc-coordinating DNA-binding domain	C2H2 zinc finger factors	zf-C2H2	ZF.C2H2
ZNF76	2.3.3	inc-coordinating DNA-binding domain	C2H2 zinc finger factors	zf-C2H2	ZF.C2H2
ZNF763	2.3.3	inc-coordinating DNA-binding domain	C2H2 zinc finger factors	zf-C2H2	ZF.C2H2
ZNF764	2.3.3	inc-coordinating DNA-binding domain	C2H2 zinc finger factors	zf-C2H2	ZF.C2H2
ZNF765	2.3.3	inc-coordinating DNA-binding domain	C2H2 zinc finger factors	zf-C2H2	ZF.C2H2
ZNF766	2.3.3	inc-coordinating DNA-binding domain	C2H2 zinc finger factors	zf-C2H2	ZF.C2H2
ZNF768	2.3.3	inc-coordinating DNA-binding domain	C2H2 zinc finger factors	zf-C2H2	ZF.C2H2
ZNF77	2.3.3	inc-coordinating DNA-binding domain	C2H2 zinc finger factors	zf-C2H2	ZF.C2H2
ZNF771	2.3.3	inc-coordinating DNA-binding domain	C2H2 zinc finger factors	zf-C2H2	ZF.C2H2
ZNF774	2.3.3	inc-coordinating DNA-binding domain	C2H2 zinc finger factors	zf-C2H2	ZF.C2H2
ZNF776	2.3.3	inc-coordinating DNA-binding domain	C2H2 zinc finger factors	zf-C2H2	ZF.C2H2

ZNF777	2.3.3	inc-coordinating DNA-binding domain	C2H2 zinc finger factors	zf-C2H2	ZF.C2H2
ZNF780A	2.3.3	inc-coordinating DNA-binding domain	C2H2 zinc finger factors	zf-C2H2	ZF.C2H2
ZNF780B	2.3.3	inc-coordinating DNA-binding domain	C2H2 zinc finger factors	zf-C2H2	ZF.C2H2
ZNF781	2.3.3	inc-coordinating DNA-binding domain	C2H2 zinc finger factors	zf-C2H2	ZF.C2H2
ZNF782	2.3.3	inc-coordinating DNA-binding domain	C2H2 zinc finger factors	zf-C2H2	ZF.C2H2
ZNF785	2.3.3	inc-coordinating DNA-binding domain	C2H2 zinc finger factors	zf-C2H2	ZF.C2H2
ZNF789	2.3.3	inc-coordinating DNA-binding domain	C2H2 zinc finger factors	zf-C2H2	ZF.C2H2
ZNF79	2.3.3	inc-coordinating DNA-binding domain	C2H2 zinc finger factors	zf-C2H2	ZF.C2H2
ZNF790	2.3.3	inc-coordinating DNA-binding domain	C2H2 zinc finger factors	zf-C2H2	ZF.C2H2
ZNF791	2.3.3	inc-coordinating DNA-binding domain	C2H2 zinc finger factors	zf-C2H2	ZF.C2H2
ZNF793	2.3.3	inc-coordinating DNA-binding domain	C2H2 zinc finger factors	zf-C2H2	ZF.C2H2
ZNF799	2.3.3	inc-coordinating DNA-binding domain	C2H2 zinc finger factors	zf-C2H2	ZF.C2H2
ZNF80	2.3.3	inc-coordinating DNA-binding domain	C2H2 zinc finger factors	zf-C2H2	ZF.C2H2
ZNF805	2.3.3	inc-coordinating DNA-binding domain	C2H2 zinc finger factors	zf-C2H2	ZF.C2H2
ZNF808	2.3.3	inc-coordinating DNA-binding domain	C2H2 zinc finger factors	zf-C2H2	ZF.C2H2
ZNF81	2.3.3	inc-coordinating DNA-binding domain	C2H2 zinc finger factors	zf-C2H2	ZF.C2H2
ZNF813	2.3.3	inc-coordinating DNA-binding domain	C2H2 zinc finger factors	zf-C2H2	ZF.C2H2
ZNF816	2.3.3	inc-coordinating DNA-binding domain	C2H2 zinc finger factors	zf-C2H2	ZF.C2H2
ZNF823	2.3.3	inc-coordinating DNA-binding domain	C2H2 zinc finger factors	zf-C2H2	ZF.C2H2
ZNF829	2.3.3	inc-coordinating DNA-binding domain	C2H2 zinc finger factors	zf-C2H2	ZF.C2H2
ZNF83	2.3.3	inc-coordinating DNA-binding domain	C2H2 zinc finger factors	zf-C2H2	ZF.C2H2
ZNF835	2.3.3	inc-coordinating DNA-binding domain	C2H2 zinc finger factors	zf-C2H2	ZF.C2H2
ZNF836	2.3.3	inc-coordinating DNA-binding domain	C2H2 zinc finger factors	zf-C2H2	ZF.C2H2
ZNF837	2.3.3	inc-coordinating DNA-binding domain	C2H2 zinc finger factors	zf-C2H2	ZF.C2H2
ZNF84	2.3.3	inc-coordinating DNA-binding domain	C2H2 zinc finger factors	zf-C2H2	ZF.C2H2
ZNF841	2.3.3	inc-coordinating DNA-binding domain	C2H2 zinc finger factors	zf-C2H2	ZF.C2H2
ZNF844	2.3.3	inc-coordinating DNA-binding domain	C2H2 zinc finger factors	zf-C2H2	ZF.C2H2
ZNF845	2.3.3	inc-coordinating DNA-binding domain	C2H2 zinc finger factors	zf-C2H2	ZF.C2H2
ZNF846	2.3.3	inc-coordinating DNA-binding domain	C2H2 zinc finger factors	zf-C2H2	ZF.C2H2
ZNF85	2.3.3	inc-coordinating DNA-binding domain	C2H2 zinc finger factors	zf-C2H2	ZF.C2H2
ZNF852	2.3.3	inc-coordinating DNA-binding domain	C2H2 zinc finger factors	zf-C2H2	ZF.C2H2
ZNF853	2.3.3	inc-coordinating DNA-binding domain	C2H2 zinc finger factors	zf-C2H2	ZF.C2H2
ZNF860	2.3.3	inc-coordinating DNA-binding domain	C2H2 zinc finger factors	zf-C2H2	ZF.C2H2
ZNF875	2.3.3	inc-coordinating DNA-binding domain	C2H2 zinc finger factors	zf-C2H2	ZF.C2H2
ZNF878	2.3.3	inc-coordinating DNA-binding domain	C2H2 zinc finger factors	zf-C2H2	ZF.C2H2
ZNF879	2.3.3	inc-coordinating DNA-binding domain	C2H2 zinc finger factors	zf-C2H2	ZF.C2H2
ZNF880	2.3.3	inc-coordinating DNA-binding domain	C2H2 zinc finger factors	zf-C2H2	ZF.C2H2
ZNF891	2.3.3	inc-coordinating DNA-binding domain	C2H2 zinc finger factors	zf-C2H2	ZF.C2H2
ZNF90	2.3.3	inc-coordinating DNA-binding domain	C2H2 zinc finger factors	zf-C2H2	ZF.C2H2
ZNF91	2.3.3	inc-coordinating DNA-binding domain	C2H2 zinc finger factors	zf-C2H2	ZF.C2H2

ZNF92	2.3.3	inc-coordinating DNA-binding domain	C2H2 zinc finger factors	zf-C2H2	ZF.C2H2
ZNF93	2.3.3	inc-coordinating DNA-binding domain	C2H2 zinc finger factors	zf-C2H2	ZF.C2H2
ZNF98	2.3.3	inc-coordinating DNA-binding domain	C2H2 zinc finger factors	zf-C2H2	ZF.C2H2
ZNF99	2.3.3	inc-coordinating DNA-binding domain	C2H2 zinc finger factors	zf-C2H2	ZF.C2H2
ZSCAN10	2.3.3	inc-coordinating DNA-binding domain	C2H2 zinc finger factors	zf-C2H2	ZF.C2H2
ZSCAN12	2.3.3	inc-coordinating DNA-binding domain	C2H2 zinc finger factors	zf-C2H2	ZF.C2H2
ZSCAN16	2.3.3	inc-coordinating DNA-binding domain	C2H2 zinc finger factors	zf-C2H2	ZF.C2H2
ZSCAN2	2.3.3	inc-coordinating DNA-binding domain	C2H2 zinc finger factors	zf-C2H2	ZF.C2H2
ZSCAN21	2.3.3	inc-coordinating DNA-binding domain	C2H2 zinc finger factors	zf-C2H2	ZF.C2H2
ZSCAN22	2.3.3	inc-coordinating DNA-binding domain	C2H2 zinc finger factors	zf-C2H2	ZF.C2H2
ZSCAN23	2.3.3	inc-coordinating DNA-binding domain	C2H2 zinc finger factors	zf-C2H2	ZF.C2H2
ZSCAN25	2.3.3	inc-coordinating DNA-binding domain	C2H2 zinc finger factors	zf-C2H2	ZF.C2H2
ZSCAN26	2.3.3	inc-coordinating DNA-binding domain	C2H2 zinc finger factors	zf-C2H2	ZF.C2H2
ZSCAN29	2.3.3	inc-coordinating DNA-binding domain	C2H2 zinc finger factors	zf-C2H2	ZF.C2H2
ZSCAN30	2.3.3	inc-coordinating DNA-binding domain	C2H2 zinc finger factors	zf-C2H2	ZF.C2H2
ZSCAN31	2.3.3	inc-coordinating DNA-binding domain	C2H2 zinc finger factors	zf-C2H2	ZF.C2H2
ZSCAN32	2.3.3	inc-coordinating DNA-binding domain	C2H2 zinc finger factors	zf-C2H2	ZF.C2H2
ZSCAN4	2.3.3	inc-coordinating DNA-binding domain	C2H2 zinc finger factors	zf-C2H2	ZF.C2H2
ZSCAN5A	2.3.3	inc-coordinating DNA-binding domain	C2H2 zinc finger factors	zf-C2H2	ZF.C2H2
ZSCAN5B	2.3.3	inc-coordinating DNA-binding domain	C2H2 zinc finger factors	zf-C2H2	ZF.C2H2
ZSCAN9	2.3.3	inc-coordinating DNA-binding domain	C2H2 zinc finger factors	zf-C2H2	ZF.C2H2
ZXDA	2.3.3	inc-coordinating DNA-binding domain	C2H2 zinc finger factors	zf-C2H2	ZF.C2H2
ZXDB	2.3.3	inc-coordinating DNA-binding domain	C2H2 zinc finger factors	zf-C2H2	ZF.C2H2
ZXDC	2.3.3	inc-coordinating DNA-binding domain	C2H2 zinc finger factors	zf-C2H2	ZF.C2H2
BCL11A	2.3.4	inc-coordinating DNA-binding domain	C2H2 zinc finger factors	zf-C2H2	ZF.C2H2
BCL11B	2.3.4	inc-coordinating DNA-binding domain	C2H2 zinc finger factors	zf-C2H2	ZF.C2H2
BNC1	2.3.4	inc-coordinating DNA-binding domain	C2H2 zinc finger factors	zf-C2H2	ZF.C2H2
BNC2	2.3.4	inc-coordinating DNA-binding domain	C2H2 zinc finger factors	zf-C2H2	ZF.C2H2
CASZ1	2.3.4	inc-coordinating DNA-binding domain	C2H2 zinc finger factors	Others	ZF.C2H2
E4F1	2.3.4	inc-coordinating DNA-binding domain	C2H2 zinc finger factors	zf-C2H2	ZF.C2H2
FIZ1	2.3.4	inc-coordinating DNA-binding domain	C2H2 zinc finger factors	zf-C2H2	ZF.C2H2
HIC1	2.3.4	inc-coordinating DNA-binding domain	C2H2 zinc finger factors	ZBTB	ZF.C2H2
HIC2	2.3.4	inc-coordinating DNA-binding domain	C2H2 zinc finger factors	ZBTB	ZF.C2H2
HINFP	2.3.4	inc-coordinating DNA-binding domain	C2H2 zinc finger factors	zf-C2H2	ZF.C2H2
HIVEP1	2.3.4	inc-coordinating DNA-binding domain	C2H2 zinc finger factors	zf-C2H2	ZF.C2H2
HIVEP2	2.3.4	inc-coordinating DNA-binding domain	C2H2 zinc finger factors	zf-C2H2	ZF.C2H2
HIVEP3	2.3.4	inc-coordinating DNA-binding domain	C2H2 zinc finger factors	zf-C2H2	ZF.C2H2
IKZF1	2.3.4	inc-coordinating DNA-binding domain	C2H2 zinc finger factors	zf-C2H2	ZF.C2H2
IKZF2	2.3.4	inc-coordinating DNA-binding domain	C2H2 zinc finger factors	zf-C2H2	ZF.C2H2
IKZF3	2.3.4	inc-coordinating DNA-binding domain	C2H2 zinc finger factors	zf-C2H2	ZF.C2H2

IKZF4	2.3.4	inc-coordinating DNA-binding domain	C2H2 zinc finger factors	zf-C2H2	ZF.C2H2
IKZF5	2.3.4	inc-coordinating DNA-binding domain	C2H2 zinc finger factors	zf-C2H2	ZF.C2H2
INSM1	2.3.4	inc-coordinating DNA-binding domain	C2H2 zinc finger factors	zf-C2H2	ZF.C2H2
INSM2	2.3.4	inc-coordinating DNA-binding domain	C2H2 zinc finger factors	zf-C2H2	ZF.C2H2
MAZ	2.3.4	inc-coordinating DNA-binding domain	C2H2 zinc finger factors	zf-C2H2	ZF.C2H2
MECOM	2.3.4	inc-coordinating DNA-binding domain	C2H2 zinc finger factors	zf-C2H2	ZF.C2H2
PATZ1	2.3.4	inc-coordinating DNA-binding domain	C2H2 zinc finger factors	ZBTB	ZF.C2H2
PEG3	2.3.4	inc-coordinating DNA-binding domain	C2H2 zinc finger factors	zf-C2H2	ZF.C2H2
PRDM10	2.3.4	inc-coordinating DNA-binding domain	C2H2 zinc finger factors	zf-C2H2	ZF.C2H2
PRDM13	2.3.4	inc-coordinating DNA-binding domain	C2H2 zinc finger factors	zf-C2H2	ZF.C2H2
PRDM15	2.3.4	inc-coordinating DNA-binding domain	C2H2 zinc finger factors	zf-C2H2	ZF.C2H2
PRDM16	2.3.4	inc-coordinating DNA-binding domain	C2H2 zinc finger factors	zf-C2H2	ZF.C2H2
PRDM2	2.3.4	inc-coordinating DNA-binding domain	C2H2 zinc finger factors	zf-C2H2	ZF.C2H2
PRDM4	2.3.4	inc-coordinating DNA-binding domain	C2H2 zinc finger factors	zf-C2H2	ZF.C2H2
PRDM8	2.3.4	inc-coordinating DNA-binding domain	C2H2 zinc finger factors	zf-C2H2	ZF.C2H2
PRDM9	2.3.4	inc-coordinating DNA-binding domain	C2H2 zinc finger factors	zf-C2H2	ZF.C2H2
REST	2.3.4	inc-coordinating DNA-binding domain	C2H2 zinc finger factors	zf-C2H2	ZF.C2H2
RLF	2.3.4	inc-coordinating DNA-binding domain	C2H2 zinc finger factors	zf-C2H2	ZF.C2H2
RREB1	2.3.4	inc-coordinating DNA-binding domain	C2H2 zinc finger factors	zf-C2H2	ZF.C2H2
SALL1	2.3.4	inc-coordinating DNA-binding domain	C2H2 zinc finger factors	zf-C2H2	ZF.C2H2
SALL2	2.3.4	inc-coordinating DNA-binding domain	C2H2 zinc finger factors	zf-C2H2	ZF.C2H2
SALL3	2.3.4	inc-coordinating DNA-binding domain	C2H2 zinc finger factors	zf-C2H2	ZF.C2H2
SALL4	2.3.4	inc-coordinating DNA-binding domain	C2H2 zinc finger factors	zf-C2H2	ZF.C2H2
VEZF1	2.3.4	inc-coordinating DNA-binding domain	C2H2 zinc finger factors	zf-C2H2	ZF.C2H2
WIZ	2.3.4	inc-coordinating DNA-binding domain	C2H2 zinc finger factors	zf-C2H2	ZF.C2H2
ZBTB1	2.3.4	inc-coordinating DNA-binding domain	C2H2 zinc finger factors	ZBTB	ZF.C2H2
ZBTB17	2.3.4	inc-coordinating DNA-binding domain	C2H2 zinc finger factors	ZBTB	ZF.C2H2
ZBTB2	2.3.4	inc-coordinating DNA-binding domain	C2H2 zinc finger factors	ZBTB	ZF.C2H2
ZBTB21	2.3.4	inc-coordinating DNA-binding domain	C2H2 zinc finger factors	ZBTB	ZF.C2H2
ZBTB25	2.3.4	inc-coordinating DNA-binding domain	C2H2 zinc finger factors	ZBTB	ZF.C2H2
ZBTB38	2.3.4	inc-coordinating DNA-binding domain	C2H2 zinc finger factors	ZBTB	ZF.C2H2
ZBTB39	2.3.4	inc-coordinating DNA-binding domain	C2H2 zinc finger factors	ZBTB	ZF.C2H2
ZBTB4	2.3.4	inc-coordinating DNA-binding domain	C2H2 zinc finger factors	ZBTB	ZF.C2H2
ZBTB41	2.3.4	inc-coordinating DNA-binding domain	C2H2 zinc finger factors	ZBTB	ZF.C2H2
ZFAT	2.3.4	inc-coordinating DNA-binding domain	C2H2 zinc finger factors	zf-C2H2	ZF.C2H2
ZFP57	2.3.4	inc-coordinating DNA-binding domain	C2H2 zinc finger factors	zf-C2H2	ZF.C2H2
ZFPM1	2.3.4	inc-coordinating DNA-binding domain	C2H2 zinc finger factors	zf-C2H2	ZF.C2H2
ZNF101	2.3.4	inc-coordinating DNA-binding domain	C2H2 zinc finger factors	zf-C2H2	ZF.C2H2
ZNF132	2.3.4	inc-coordinating DNA-binding domain	C2H2 zinc finger factors	zf-C2H2	ZF.C2H2
ZNF134	2.3.4	inc-coordinating DNA-binding domain	C2H2 zinc finger factors	zf-C2H2	ZF.C2H2

ZNF142	2.3.4	inc-coordinating DNA-binding domain	C2H2 zinc finger factors	zf-C2H2	ZF.C2H2
ZNF195	2.3.4	inc-coordinating DNA-binding domain	C2H2 zinc finger factors	zf-C2H2	ZF.C2H2
ZNF211	2.3.4	inc-coordinating DNA-binding domain	C2H2 zinc finger factors	zf-C2H2	ZF.C2H2
ZNF217	2.3.4	inc-coordinating DNA-binding domain	C2H2 zinc finger factors	zf-C2H2	ZF.C2H2
ZNF219	2.3.4	inc-coordinating DNA-binding domain	C2H2 zinc finger factors	zf-C2H2	ZF.C2H2
ZNF236	2.3.4	inc-coordinating DNA-binding domain	C2H2 zinc finger factors	zf-C2H2	ZF.C2H2
ZNF248	2.3.4	inc-coordinating DNA-binding domain	C2H2 zinc finger factors	zf-C2H2	ZF.C2H2
ZNF251	2.3.4	inc-coordinating DNA-binding domain	C2H2 zinc finger factors	zf-C2H2	ZF.C2H2
ZNF256	2.3.4	inc-coordinating DNA-binding domain	C2H2 zinc finger factors	zf-C2H2	ZF.C2H2
ZNF277	2.3.4	inc-coordinating DNA-binding domain	C2H2 zinc finger factors	zf-C2H2	ZF.C2H2
ZNF292	2.3.4	inc-coordinating DNA-binding domain	C2H2 zinc finger factors	zf-C2H2	ZF.C2H2
ZNF296	2.3.4	inc-coordinating DNA-binding domain	C2H2 zinc finger factors	zf-C2H2	ZF.C2H2
ZNF304	2.3.4	inc-coordinating DNA-binding domain	C2H2 zinc finger factors	zf-C2H2	ZF.C2H2
ZNF316	2.3.4	inc-coordinating DNA-binding domain	C2H2 zinc finger factors	zf-C2H2	ZF.C2H2
ZNF319	2.3.4	inc-coordinating DNA-binding domain	C2H2 zinc finger factors	zf-C2H2	ZF.C2H2
ZNF334	2.3.4	inc-coordinating DNA-binding domain	C2H2 zinc finger factors	zf-C2H2	ZF.C2H2
ZNF335	2.3.4	inc-coordinating DNA-binding domain	C2H2 zinc finger factors	zf-C2H2	ZF.C2H2
ZNF341	2.3.4	inc-coordinating DNA-binding domain	C2H2 zinc finger factors	zf-C2H2	ZF.C2H2
ZNF37A	2.3.4	inc-coordinating DNA-binding domain	C2H2 zinc finger factors	zf-C2H2	ZF.C2H2
ZNF382	2.3.4	inc-coordinating DNA-binding domain	C2H2 zinc finger factors	zf-C2H2	ZF.C2H2
ZNF407	2.3.4	inc-coordinating DNA-binding domain	C2H2 zinc finger factors	zf-C2H2	ZF.C2H2
ZNF417	2.3.4	inc-coordinating DNA-binding domain	C2H2 zinc finger factors	zf-C2H2	ZF.C2H2
ZNF418	2.3.4	inc-coordinating DNA-binding domain	C2H2 zinc finger factors	zf-C2H2	ZF.C2H2
ZNF423	2.3.4	inc-coordinating DNA-binding domain	C2H2 zinc finger factors	zf-C2H2	ZF.C2H2
ZNF438	2.3.4	inc-coordinating DNA-binding domain	C2H2 zinc finger factors	zf-C2H2	ZF.C2H2
ZNF445	2.3.4	inc-coordinating DNA-binding domain	C2H2 zinc finger factors	zf-C2H2	ZF.C2H2
ZNF462	2.3.4	inc-coordinating DNA-binding domain	C2H2 zinc finger factors	zf-C2H2	ZF.C2H2
ZNF467	2.3.4	inc-coordinating DNA-binding domain	C2H2 zinc finger factors	zf-C2H2	ZF.C2H2
ZNF469	2.3.4	inc-coordinating DNA-binding domain	C2H2 zinc finger factors	zf-C2H2	ZF.C2H2
ZNF48	2.3.4	inc-coordinating DNA-binding domain	C2H2 zinc finger factors	zf-C2H2	ZF.C2H2
ZNF507	2.3.4	inc-coordinating DNA-binding domain	C2H2 zinc finger factors	zf-C2H2	ZF.C2H2
ZNF510	2.3.4	inc-coordinating DNA-binding domain	C2H2 zinc finger factors	zf-C2H2	ZF.C2H2
ZNF512	2.3.4	inc-coordinating DNA-binding domain	C2H2 zinc finger factors	zf-C2H2	ZF.C2H2
ZNF512B	2.3.4	inc-coordinating DNA-binding domain	C2H2 zinc finger factors	zf-C2H2	ZF.C2H2
ZNF513	2.3.4	inc-coordinating DNA-binding domain	C2H2 zinc finger factors	zf-C2H2	ZF.C2H2
ZNF516	2.3.4	inc-coordinating DNA-binding domain	C2H2 zinc finger factors	zf-C2H2	ZF.C2H2
ZNF519	2.3.4	inc-coordinating DNA-binding domain	C2H2 zinc finger factors	zf-C2H2	ZF.C2H2
ZNF521	2.3.4	inc-coordinating DNA-binding domain	C2H2 zinc finger factors	zf-C2H2	ZF.C2H2
ZNF526	2.3.4	inc-coordinating DNA-binding domain	C2H2 zinc finger factors	zf-C2H2	ZF.C2H2
ZNF532	2.3.4	inc-coordinating DNA-binding domain	C2H2 zinc finger factors	zf-C2H2	ZF.C2H2

ZNF536	2.3.4	inc-coordinating DNA-binding domain	C2H2 zinc finger factors	zf-C2H2	ZF.C2H2
ZNF552	2.3.4	inc-coordinating DNA-binding domain	C2H2 zinc finger factors	zf-C2H2	ZF.C2H2
ZNF574	2.3.4	inc-coordinating DNA-binding domain	C2H2 zinc finger factors	zf-C2H2	ZF.C2H2
ZNF579	2.3.4	inc-coordinating DNA-binding domain	C2H2 zinc finger factors	zf-C2H2	ZF.C2H2
ZNF587	2.3.4	inc-coordinating DNA-binding domain	C2H2 zinc finger factors	zf-C2H2	ZF.C2H2
ZNF592	2.3.4	inc-coordinating DNA-binding domain	C2H2 zinc finger factors	zf-C2H2	ZF.C2H2
ZNF597	2.3.4	inc-coordinating DNA-binding domain	C2H2 zinc finger factors	zf-C2H2	ZF.C2H2
ZNF606	2.3.4	inc-coordinating DNA-binding domain	C2H2 zinc finger factors	zf-C2H2	ZF.C2H2
ZNF618	2.3.4	inc-coordinating DNA-binding domain	C2H2 zinc finger factors	zf-C2H2	ZF.C2H2
ZNF628	2.3.4	inc-coordinating DNA-binding domain	C2H2 zinc finger factors	zf-C2H2	ZF.C2H2
ZNF629	2.3.4	inc-coordinating DNA-binding domain	C2H2 zinc finger factors	zf-C2H2	ZF.C2H2
ZNF639	2.3.4	inc-coordinating DNA-binding domain	C2H2 zinc finger factors	zf-C2H2	ZF.C2H2
ZNF646	2.3.4	inc-coordinating DNA-binding domain	C2H2 zinc finger factors	zf-C2H2	ZF.C2H2
ZNF654	2.3.4	inc-coordinating DNA-binding domain	C2H2 zinc finger factors	zf-C2H2	ZF.C2H2
ZNF658	2.3.4	inc-coordinating DNA-binding domain	C2H2 zinc finger factors	zf-C2H2	ZF.C2H2
ZNF668	2.3.4	inc-coordinating DNA-binding domain	C2H2 zinc finger factors	zf-C2H2	ZF.C2H2
ZNF671	2.3.4	inc-coordinating DNA-binding domain	C2H2 zinc finger factors	zf-C2H2	ZF.C2H2
ZNF674	2.3.4	inc-coordinating DNA-binding domain	C2H2 zinc finger factors	zf-C2H2	ZF.C2H2
ZNF687	2.3.4	inc-coordinating DNA-binding domain	C2H2 zinc finger factors	zf-C2H2	ZF.C2H2
ZNF697	2.3.4	inc-coordinating DNA-binding domain	C2H2 zinc finger factors	zf-C2H2	ZF.C2H2
ZNF711	2.3.4	inc-coordinating DNA-binding domain	C2H2 zinc finger factors	zf-C2H2	ZF.C2H2
ZNF717	2.3.4	inc-coordinating DNA-binding domain	C2H2 zinc finger factors	zf-C2H2	ZF.C2H2
ZNF770	2.3.4	inc-coordinating DNA-binding domain	C2H2 zinc finger factors	zf-C2H2	ZF.C2H2
ZNF772	2.3.4	inc-coordinating DNA-binding domain	C2H2 zinc finger factors	zf-C2H2	ZF.C2H2
ZNF775	2.3.4	inc-coordinating DNA-binding domain	C2H2 zinc finger factors	zf-C2H2	ZF.C2H2
ZNF778	2.3.4	inc-coordinating DNA-binding domain	C2H2 zinc finger factors	zf-C2H2	ZF.C2H2
ZNF784	2.3.4	inc-coordinating DNA-binding domain	C2H2 zinc finger factors	zf-C2H2	ZF.C2H2
ZNF786	2.3.4	inc-coordinating DNA-binding domain	C2H2 zinc finger factors	zf-C2H2	ZF.C2H2
ZNF787	2.3.4	inc-coordinating DNA-binding domain	C2H2 zinc finger factors	zf-C2H2	ZF.C2H2
ZNF788P	2.3.4	inc-coordinating DNA-binding domain	C2H2 zinc finger factors		ZF.C2H2
ZNF792	2.3.4	inc-coordinating DNA-binding domain	C2H2 zinc finger factors	zf-C2H2	ZF.C2H2
ZNF800	2.3.4	inc-coordinating DNA-binding domain	C2H2 zinc finger factors	zf-C2H2	ZF.C2H2
ZNF814	2.3.4	inc-coordinating DNA-binding domain	C2H2 zinc finger factors	zf-C2H2	ZF.C2H2
ZSCAN20	2.3.4	inc-coordinating DNA-binding domain	C2H2 zinc finger factors	zf-C2H2	ZF.C2H2
ZBED1	2.3.5	inc-coordinating DNA-binding domain	C2H2 zinc finger factors	zf-BED	ZF.C2H2
ZBED2	2.3.5	inc-coordinating DNA-binding domain	C2H2 zinc finger factors	zf-BED	ZF.C2H2
ZBED3	2.3.5	inc-coordinating DNA-binding domain	C2H2 zinc finger factors	zf-BED	ZF.C2H2
ZBED4	2.3.5	inc-coordinating DNA-binding domain	C2H2 zinc finger factors	zf-BED	ZF.C2H2
ZBED6	2.3.5	inc-coordinating DNA-binding domain	C2H2 zinc finger factors	zf-BED	ZF.C2H2
DMRT1	2.5.1	inc-coordinating DNA-binding domain	DM-type intertwined zinc finger factors	DM	ZF.DM

DMRT2	2.5.1	inc-coordinating DNA-binding domain	DM-type intertwined zinc finger factors	DM	ZF.DM
DMRT3	2.5.1	inc-coordinating DNA-binding domain	DM-type intertwined zinc finger factors	DM	ZF.DM
DMRTA1	2.5.1	inc-coordinating DNA-binding domain	DM-type intertwined zinc finger factors	DM	ZF.DM
DMRTA2	2.5.1	inc-coordinating DNA-binding domain	DM-type intertwined zinc finger factors	DM	ZF.DM
DMRTB1	2.5.1	inc-coordinating DNA-binding domain	DM-type intertwined zinc finger factors	DM	ZF.DM
DMRTC1B	2.5.1	inc-coordinating DNA-binding domain	DM-type intertwined zinc finger factors	Others	ZF.DM
DMRTC2	2.5.1	inc-coordinating DNA-binding domain	DM-type intertwined zinc finger factors	DM	ZF.DM
MBD1	2.6.1	inc-coordinating DNA-binding domain	CXXC zinc finger factors	MBD	ZF.CXXC
MYT1	2.7.1	inc-coordinating DNA-binding domain	C2HC zinc finger factors	zf-C2HC	ZF.C2CH
MYT1L	2.7.1	inc-coordinating DNA-binding domain	C2HC zinc finger factors	zf-C2HC	ZF.C2CH
ST18	2.7.1	inc-coordinating DNA-binding domain	C2HC zinc finger factors	zf-C2HC	ZF.C2CH
L3MBTL1	2.7.4	inc-coordinating DNA-binding domain	C2HC zinc finger factors	zf-C2HC	ZF.C2CH
LYAR	2.7.5	inc-coordinating DNA-binding domain	C2HC zinc finger factors	Others	ZF.C2CH
ZGPAT	2.8.2	inc-coordinating DNA-binding domain	C3H zinc finger factors	Others	ZF.C3H
THAP1	2.9.1	inc-coordinating DNA-binding domain	C2CH THAP-type zinc finger factors	THAP	ZF.C2CH
THAP10	2.9.1	inc-coordinating DNA-binding domain	C2CH THAP-type zinc finger factors	THAP	ZF.C2CH
THAP11	2.9.1	inc-coordinating DNA-binding domain	C2CH THAP-type zinc finger factors	THAP	ZF.C2CH
THAP12	2.9.1	inc-coordinating DNA-binding domain	C2CH THAP-type zinc finger factors	THAP	ZF.C2CH
THAP2	2.9.1	inc-coordinating DNA-binding domain	C2CH THAP-type zinc finger factors	THAP	ZF.C2CH
THAP3	2.9.1	inc-coordinating DNA-binding domain	C2CH THAP-type zinc finger factors	THAP	ZF.C2CH
THAP4	2.9.1	inc-coordinating DNA-binding domain	C2CH THAP-type zinc finger factors	THAP	ZF.C2CH
THAP5	2.9.1	inc-coordinating DNA-binding domain	C2CH THAP-type zinc finger factors	THAP	ZF.C2CH
THAP6	2.9.1	inc-coordinating DNA-binding domain	C2CH THAP-type zinc finger factors	THAP	ZF.C2CH
THAP7	2.9.1	inc-coordinating DNA-binding domain	C2CH THAP-type zinc finger factors	THAP	ZF.C2CH
THAP8	2.9.1	inc-coordinating DNA-binding domain	C2CH THAP-type zinc finger factors	THAP	ZF.C2CH
THAP9	2.9.1	inc-coordinating DNA-binding domain	C2CH THAP-type zinc finger factors	THAP	ZF.C2CH
CDX1	3.1.1	Helix-turn-helix domains	Homeo domain factors	Homeobox	HB.HOX
CDX2	3.1.1	Helix-turn-helix domains	Homeo domain factors	Homeobox	HB.HOX
CDX4	3.1.1	Helix-turn-helix domains	Homeo domain factors	Homeobox	HB.HOX
EVX1	3.1.1	Helix-turn-helix domains	Homeo domain factors	Homeobox	HB.HOX
EVX2	3.1.1	Helix-turn-helix domains	Homeo domain factors	Homeobox	HB.HOX
GBX1	3.1.1	Helix-turn-helix domains	Homeo domain factors	Homeobox	HB.HOX
GBX2	3.1.1	Helix-turn-helix domains	Homeo domain factors	Homeobox	HB.HOX
GSX1	3.1.1	Helix-turn-helix domains	Homeo domain factors	Homeobox	HB.HOX
GSX2	3.1.1	Helix-turn-helix domains	Homeo domain factors	Homeobox	HB.HOX
HOXA1	3.1.1	Helix-turn-helix domains	Homeo domain factors	Homeobox	HB.HOX
HOXA10	3.1.1	Helix-turn-helix domains	Homeo domain factors	Homeobox	HB.HOX
HOXA11	3.1.1	Helix-turn-helix domains	Homeo domain factors	Homeobox	HB.HOX
HOXA13	3.1.1	Helix-turn-helix domains	Homeo domain factors	Homeobox	HB.HOX
HOXA2	3.1.1	Helix-turn-helix domains	Homeo domain factors	Homeobox	HB.HOX

HOXA3	3.1.1	Helix-turn-helix domains	Homeo domain factors	Homeobox	HB.HOX
HOXA4	3.1.1	Helix-turn-helix domains	Homeo domain factors	Homeobox	HB.HOX
HOXA5	3.1.1	Helix-turn-helix domains	Homeo domain factors	Homeobox	HB.HOX
HOXA6	3.1.1	Helix-turn-helix domains	Homeo domain factors	Homeobox	HB.HOX
HOXA7	3.1.1	Helix-turn-helix domains	Homeo domain factors	Homeobox	HB.HOX
HOXA9	3.1.1	Helix-turn-helix domains	Homeo domain factors	Homeobox	HB.HOX
HOXB1	3.1.1	Helix-turn-helix domains	Homeo domain factors	Homeobox	HB.HOX
HOXB13	3.1.1	Helix-turn-helix domains	Homeo domain factors	Homeobox	HB.HOX
HOXB2	3.1.1	Helix-turn-helix domains	Homeo domain factors	Homeobox	HB.HOX
HOXB3	3.1.1	Helix-turn-helix domains	Homeo domain factors	Homeobox	HB.HOX
HOXB4	3.1.1	Helix-turn-helix domains	Homeo domain factors	Homeobox	HB.HOX
HOXB5	3.1.1	Helix-turn-helix domains	Homeo domain factors	Homeobox	HB.HOX
HOXB6	3.1.1	Helix-turn-helix domains	Homeo domain factors	Homeobox	HB.HOX
HOXB7	3.1.1	Helix-turn-helix domains	Homeo domain factors	Homeobox	HB.HOX
HOXB8	3.1.1	Helix-turn-helix domains	Homeo domain factors	Homeobox	HB.HOX
HOXB9	3.1.1	Helix-turn-helix domains	Homeo domain factors	Homeobox	HB.HOX
HOXC10	3.1.1	Helix-turn-helix domains	Homeo domain factors	Homeobox	HB.HOX
HOXC11	3.1.1	Helix-turn-helix domains	Homeo domain factors	Homeobox	HB.HOX
HOXC12	3.1.1	Helix-turn-helix domains	Homeo domain factors	Homeobox	HB.HOX
HOXC13	3.1.1	Helix-turn-helix domains	Homeo domain factors	Homeobox	HB.HOX
HOXC4	3.1.1	Helix-turn-helix domains	Homeo domain factors	Homeobox	HB.HOX
HOXC5	3.1.1	Helix-turn-helix domains	Homeo domain factors	Homeobox	HB.HOX
HOXC6	3.1.1	Helix-turn-helix domains	Homeo domain factors	Homeobox	HB.HOX
HOXC8	3.1.1	Helix-turn-helix domains	Homeo domain factors	Homeobox	HB.HOX
HOXC9	3.1.1	Helix-turn-helix domains	Homeo domain factors	Homeobox	HB.HOX
HOXD1	3.1.1	Helix-turn-helix domains	Homeo domain factors	Homeobox	HB.HOX
HOXD10	3.1.1	Helix-turn-helix domains	Homeo domain factors	Homeobox	HB.HOX
HOXD11	3.1.1	Helix-turn-helix domains	Homeo domain factors	Homeobox	HB.HOX
HOXD12	3.1.1	Helix-turn-helix domains	Homeo domain factors	Homeobox	HB.HOX
HOXD13	3.1.1	Helix-turn-helix domains	Homeo domain factors	Homeobox	HB.HOX
HOXD3	3.1.1	Helix-turn-helix domains	Homeo domain factors	Homeobox	HB.HOX
HOXD4	3.1.1	Helix-turn-helix domains	Homeo domain factors	Homeobox	HB.HOX
HOXD8	3.1.1	Helix-turn-helix domains	Homeo domain factors	Homeobox	HB.HOX
HOXD9	3.1.1	Helix-turn-helix domains	Homeo domain factors	Homeobox	HB.HOX
MEOX1	3.1.1	Helix-turn-helix domains	Homeo domain factors	Homeobox	HB.HOX
MEOX2	3.1.1	Helix-turn-helix domains	Homeo domain factors	Homeobox	HB.HOX
MNX1	3.1.1	Helix-turn-helix domains	Homeo domain factors	Homeobox	HB.HOX
PDX1	3.1.1	Helix-turn-helix domains	Homeo domain factors	Homeobox	HB.HOX
HDX	3.1.10	Helix-turn-helix domains	Homeo domain factors	Homeobox	HB-POU
HMBOX1	3.1.10	Helix-turn-helix domains	Homeo domain factors	Homeobox	HB-POU

HNF1A	3.1.10	Helix-turn-helix domains	Homeo domain factors	Homeobox	HB-POU
HNF1B	3.1.10	Helix-turn-helix domains	Homeo domain factors	Homeobox	HB-POU
POU1F1	3.1.10	Helix-turn-helix domains	Homeo domain factors	Pou	HB-POU
POU2F1	3.1.10	Helix-turn-helix domains	Homeo domain factors	Pou	HB-POU
POU2F2	3.1.10	Helix-turn-helix domains	Homeo domain factors	Pou	HB-POU
POU2F3	3.1.10	Helix-turn-helix domains	Homeo domain factors	Pou	HB-POU
POU3F1	3.1.10	Helix-turn-helix domains	Homeo domain factors	Pou	HB-POU
POU3F2	3.1.10	Helix-turn-helix domains	Homeo domain factors	Pou	HB-POU
POU3F3	3.1.10	Helix-turn-helix domains	Homeo domain factors	Pou	HB-POU
POU3F4	3.1.10	Helix-turn-helix domains	Homeo domain factors	Pou	HB-POU
POU4F1	3.1.10	Helix-turn-helix domains	Homeo domain factors	Pou	HB-POU
POU4F2	3.1.10	Helix-turn-helix domains	Homeo domain factors	Pou	HB-POU
POU4F3	3.1.10	Helix-turn-helix domains	Homeo domain factors	Pou	HB-POU
POU5F1	3.1.10	Helix-turn-helix domains	Homeo domain factors	Pou	HB-POU
POU5F1B	3.1.10	Helix-turn-helix domains	Homeo domain factors	Pou	HB-POU
POU5F2	3.1.10	Helix-turn-helix domains	Homeo domain factors	Pou	HB-POU
POU6F1	3.1.10	Helix-turn-helix domains	Homeo domain factors	Pou	HB-POU
POU6F2	3.1.10	Helix-turn-helix domains	Homeo domain factors	Pou	HB-POU
PHTF1	3.1.11	Helix-turn-helix domains	Homeo domain factors	Others	HB.PHTF
BARHL1	3.1.2	Helix-turn-helix domains	Homeo domain factors	Homeobox	HB.NK
BARHL2	3.1.2	Helix-turn-helix domains	Homeo domain factors	Homeobox	HB.NK
BARX1	3.1.2	Helix-turn-helix domains	Homeo domain factors	Homeobox	HB.NK
BARX2	3.1.2	Helix-turn-helix domains	Homeo domain factors	Homeobox	HB.NK
BSX	3.1.2	Helix-turn-helix domains	Homeo domain factors	Homeobox	HB.NK
DBX1	3.1.2	Helix-turn-helix domains	Homeo domain factors	Homeobox	HB.NK
DBX2	3.1.2	Helix-turn-helix domains	Homeo domain factors	Homeobox	HB.NK
DLX1	3.1.2	Helix-turn-helix domains	Homeo domain factors	Homeobox	HB.NK
DLX2	3.1.2	Helix-turn-helix domains	Homeo domain factors	Homeobox	HB.NK
DLX3	3.1.2	Helix-turn-helix domains	Homeo domain factors	Homeobox	HB.NK
DLX4	3.1.2	Helix-turn-helix domains	Homeo domain factors	Homeobox	HB.NK
DLX5	3.1.2	Helix-turn-helix domains	Homeo domain factors	Homeobox	HB.NK
DLX6	3.1.2	Helix-turn-helix domains	Homeo domain factors	Homeobox	HB.NK
EMX1	3.1.2	Helix-turn-helix domains	Homeo domain factors	Homeobox	HB.NK
EMX2	3.1.2	Helix-turn-helix domains	Homeo domain factors	Homeobox	HB.NK
EN1	3.1.2	Helix-turn-helix domains	Homeo domain factors	Homeobox	HB.NK
EN2	3.1.2	Helix-turn-helix domains	Homeo domain factors	Homeobox	HB.NK
HHEX	3.1.2	Helix-turn-helix domains	Homeo domain factors	Homeobox	HB.NK
HLX	3.1.2	Helix-turn-helix domains	Homeo domain factors	Homeobox	HB.NK
HMX1	3.1.2	Helix-turn-helix domains	Homeo domain factors	Homeobox	HB.NK
HMX2	3.1.2	Helix-turn-helix domains	Homeo domain factors	Homeobox	HB.NK

HMX3	3.1.2	Helix-turn-helix domains	Homeo domain factors	Homeobox	HB.NK
LBX1	3.1.2	Helix-turn-helix domains	Homeo domain factors	Homeobox	HB.NK
LBX2	3.1.2	Helix-turn-helix domains	Homeo domain factors	Homeobox	HB.NK
MSX1	3.1.2	Helix-turn-helix domains	Homeo domain factors	Homeobox	HB.NK
MSX2	3.1.2	Helix-turn-helix domains	Homeo domain factors	Homeobox	HB.NK
NANOG	3.1.2	Helix-turn-helix domains	Homeo domain factors	Homeobox	HB.NK
NKX1-1	3.1.2	Helix-turn-helix domains	Homeo domain factors	Homeobox	HB.NK
NKX1-2	3.1.2	Helix-turn-helix domains	Homeo domain factors	Homeobox	HB.NK
NKX2-1	3.1.2	Helix-turn-helix domains	Homeo domain factors	Homeobox	HB.NK
NKX2-2	3.1.2	Helix-turn-helix domains	Homeo domain factors	Homeobox	HB.NK
NKX2-3	3.1.2	Helix-turn-helix domains	Homeo domain factors	Homeobox	HB.NK
NKX2-4	3.1.2	Helix-turn-helix domains	Homeo domain factors	Homeobox	HB.NK
NKX2-5	3.1.2	Helix-turn-helix domains	Homeo domain factors	Homeobox	HB.NK
NKX2-6	3.1.2	Helix-turn-helix domains	Homeo domain factors	Homeobox	HB.NK
NKX2-8	3.1.2	Helix-turn-helix domains	Homeo domain factors	Homeobox	HB.NK
NKX3-1	3.1.2	Helix-turn-helix domains	Homeo domain factors	Homeobox	HB.NK
NKX3-2	3.1.2	Helix-turn-helix domains	Homeo domain factors	Homeobox	HB.NK
NKX6-1	3.1.2	Helix-turn-helix domains	Homeo domain factors	Homeobox	HB.NK
NKX6-2	3.1.2	Helix-turn-helix domains	Homeo domain factors	Homeobox	HB.NK
NKX6-3	3.1.2	Helix-turn-helix domains	Homeo domain factors	Homeobox	HB.NK
NOTO	3.1.2	Helix-turn-helix domains	Homeo domain factors	Homeobox	HB.NK
TLX1	3.1.2	Helix-turn-helix domains	Homeo domain factors	Homeobox	HB.NK
TLX2	3.1.2	Helix-turn-helix domains	Homeo domain factors	Homeobox	HB.NK
TLX3	3.1.2	Helix-turn-helix domains	Homeo domain factors	Homeobox	HB.NK
VAX1	3.1.2	Helix-turn-helix domains	Homeo domain factors	Homeobox	HB.NK
VAX2	3.1.2	Helix-turn-helix domains	Homeo domain factors	Homeobox	HB.NK
VENTX	3.1.2	Helix-turn-helix domains	Homeo domain factors	Homeobox	HB.NK
ALX1	3.1.3	Helix-turn-helix domains	Homeo domain factors	Homeobox	HB.PD
ALX3	3.1.3	Helix-turn-helix domains	Homeo domain factors	Homeobox	HB.PD
ALX4	3.1.3	Helix-turn-helix domains	Homeo domain factors	Homeobox	HB.PD
ARGFX	3.1.3	Helix-turn-helix domains	Homeo domain factors	Homeobox	HB.PD
ARX	3.1.3	Helix-turn-helix domains	Homeo domain factors	Homeobox	HB.PD
CRX	3.1.3	Helix-turn-helix domains	Homeo domain factors	Homeobox	HB.PD
DMBX1	3.1.3	Helix-turn-helix domains	Homeo domain factors	Homeobox	HB.PD
DPRX	3.1.3	Helix-turn-helix domains	Homeo domain factors	Homeobox	HB.PD
DRGX	3.1.3	Helix-turn-helix domains	Homeo domain factors	Homeobox	HB.PD
DUX4	3.1.3	Helix-turn-helix domains	Homeo domain factors	Homeobox	HB.PD
DUXA	3.1.3	Helix-turn-helix domains	Homeo domain factors	Homeobox	HB.PD
ESX1	3.1.3	Helix-turn-helix domains	Homeo domain factors	Homeobox	HB.PD
GSC	3.1.3	Helix-turn-helix domains	Homeo domain factors	Homeobox	HB.PD

GSC2	3.1.3	Helix-turn-helix domains	Homeo domain factors	Homeobox	HB.PD
HESX1	3.1.3	Helix-turn-helix domains	Homeo domain factors	Homeobox	HB.PD
HOPX	3.1.3	Helix-turn-helix domains	Homeo domain factors	Homeobox	HB.PD
ISX	3.1.3	Helix-turn-helix domains	Homeo domain factors	Homeobox	HB.PD
LEUTX	3.1.3	Helix-turn-helix domains	Homeo domain factors	Homeobox	HB.PD
MIXL1	3.1.3	Helix-turn-helix domains	Homeo domain factors	Homeobox	HB.PD
NOBOX	3.1.3	Helix-turn-helix domains	Homeo domain factors	Homeobox	HB.PD
OTP	3.1.3	Helix-turn-helix domains	Homeo domain factors	Homeobox	HB.PD
OTX1	3.1.3	Helix-turn-helix domains	Homeo domain factors	TF_Otx	HB.PD
OTX2	3.1.3	Helix-turn-helix domains	Homeo domain factors	TF_Otx	HB.PD
PHOX2A	3.1.3	Helix-turn-helix domains	Homeo domain factors	Homeobox	HB.PD
PHOX2B	3.1.3	Helix-turn-helix domains	Homeo domain factors	Homeobox	HB.PD
PITX1	3.1.3	Helix-turn-helix domains	Homeo domain factors	Homeobox	HB.PD
PITX2	3.1.3	Helix-turn-helix domains	Homeo domain factors	Homeobox	HB.PD
PITX3	3.1.3	Helix-turn-helix domains	Homeo domain factors	Homeobox	HB.PD
PROP1	3.1.3	Helix-turn-helix domains	Homeo domain factors	Homeobox	HB.PD
PRRX1	3.1.3	Helix-turn-helix domains	Homeo domain factors	Homeobox	HB.PD
PRRX2	3.1.3	Helix-turn-helix domains	Homeo domain factors	Homeobox	HB.PD
RAX	3.1.3	Helix-turn-helix domains	Homeo domain factors	Homeobox	HB.PD
RAX2	3.1.3	Helix-turn-helix domains	Homeo domain factors	Homeobox	HB.PD
RHOXF1	3.1.3	Helix-turn-helix domains	Homeo domain factors	Homeobox	HB.PD
RHOXF2	3.1.3	Helix-turn-helix domains	Homeo domain factors	Homeobox	HB.PD
RHOXF2B	3.1.3	Helix-turn-helix domains	Homeo domain factors	Homeobox	HB.PD
SEBOX	3.1.3	Helix-turn-helix domains	Homeo domain factors	Homeobox	HB.PD
SHOX	3.1.3	Helix-turn-helix domains	Homeo domain factors	Homeobox	HB.PD
SHOX2	3.1.3	Helix-turn-helix domains	Homeo domain factors	Homeobox	HB.PD
TPRX1	3.1.3	Helix-turn-helix domains	Homeo domain factors	Homeobox	HB.PD
UNCX	3.1.3	Helix-turn-helix domains	Homeo domain factors	Homeobox	HB.PD
VSX1	3.1.3	Helix-turn-helix domains	Homeo domain factors	Homeobox	HB.PD
VSX2	3.1.3	Helix-turn-helix domains	Homeo domain factors	Homeobox	HB.PD
IRX1	3.1.4	Helix-turn-helix domains	Homeo domain factors	Homeobox	HB.TALE
IRX2	3.1.4	Helix-turn-helix domains	Homeo domain factors	Homeobox	HB.TALE
IRX3	3.1.4	Helix-turn-helix domains	Homeo domain factors	Homeobox	HB.TALE
IRX4	3.1.4	Helix-turn-helix domains	Homeo domain factors	Homeobox	HB.TALE
IRX5	3.1.4	Helix-turn-helix domains	Homeo domain factors	Homeobox	HB.TALE
IRX6	3.1.4	Helix-turn-helix domains	Homeo domain factors	Homeobox	HB.TALE
MEIS1	3.1.4	Helix-turn-helix domains	Homeo domain factors	Homeobox	HB.TALE
MEIS2	3.1.4	Helix-turn-helix domains	Homeo domain factors	Homeobox	HB.TALE
MEIS3	3.1.4	Helix-turn-helix domains	Homeo domain factors	Homeobox	HB.TALE
MKX	3.1.4	Helix-turn-helix domains	Homeo domain factors	Homeobox	HB.TALE

PBX1	3.1.4	Helix-turn-helix domains	Homeo domain factors	Homeobox	HB.TALE
PBX2	3.1.4	Helix-turn-helix domains	Homeo domain factors	Homeobox	HB.TALE
PBX3	3.1.4	Helix-turn-helix domains	Homeo domain factors	Homeobox	HB.TALE
PBX4	3.1.4	Helix-turn-helix domains	Homeo domain factors	Homeobox	HB.TALE
PKNOX1	3.1.4	Helix-turn-helix domains	Homeo domain factors	Homeobox	HB.TALE
PKNOX2	3.1.4	Helix-turn-helix domains	Homeo domain factors	Homeobox	HB.TALE
TGIF1	3.1.4	Helix-turn-helix domains	Homeo domain factors	Homeobox	HB.TALE
TGIF2	3.1.4	Helix-turn-helix domains	Homeo domain factors	Homeobox	HB.TALE
TGIF2LX	3.1.4	Helix-turn-helix domains	Homeo domain factors	Homeobox	HB.TALE
TGIF2LY	3.1.4	Helix-turn-helix domains	Homeo domain factors	Homeobox	HB.TALE
ISL1	3.1.5	Helix-turn-helix domains	Homeo domain factors	Homeobox	HB.LIM
ISL2	3.1.5	Helix-turn-helix domains	Homeo domain factors	Homeobox	HB.LIM
LHX1	3.1.5	Helix-turn-helix domains	Homeo domain factors	Homeobox	HB.LIM
LHX2	3.1.5	Helix-turn-helix domains	Homeo domain factors	Homeobox	HB.LIM
LHX3	3.1.5	Helix-turn-helix domains	Homeo domain factors	Homeobox	HB.LIM
LHX4	3.1.5	Helix-turn-helix domains	Homeo domain factors	Homeobox	HB.LIM
LHX5	3.1.5	Helix-turn-helix domains	Homeo domain factors	Homeobox	HB.LIM
LHX6	3.1.5	Helix-turn-helix domains	Homeo domain factors	Homeobox	HB.LIM
LHX8	3.1.5	Helix-turn-helix domains	Homeo domain factors	Homeobox	HB.LIM
LHX9	3.1.5	Helix-turn-helix domains	Homeo domain factors	Homeobox	HB.LIM
LMX1A	3.1.5	Helix-turn-helix domains	Homeo domain factors	Homeobox	HB.LIM
LMX1B	3.1.5	Helix-turn-helix domains	Homeo domain factors	Homeobox	HB.LIM
SIX1	3.1.6	Helix-turn-helix domains	Homeo domain factors	Homeobox	HB.SINE
SIX2	3.1.6	Helix-turn-helix domains	Homeo domain factors	Homeobox	HB.SINE
SIX3	3.1.6	Helix-turn-helix domains	Homeo domain factors	Homeobox	HB.SINE
SIX4	3.1.6	Helix-turn-helix domains	Homeo domain factors	Homeobox	HB.SINE
SIX5	3.1.6	Helix-turn-helix domains	Homeo domain factors	Homeobox	HB.SINE
SIX6	3.1.6	Helix-turn-helix domains	Homeo domain factors	Homeobox	HB.SINE
PROX1	3.1.7	Helix-turn-helix domains	Homeo domain factors	HPD	HB.PROS
PROX2	3.1.7	Helix-turn-helix domains	Homeo domain factors	HPD	HB.PROS
ADNP	3.1.8	Helix-turn-helix domains	Homeo domain factors	Homeobox	HB-ZF.C2H2
ADNP2	3.1.8	Helix-turn-helix domains	Homeo domain factors	Others	HB-ZF.C2H2
HOMEZ	3.1.8	Helix-turn-helix domains	Homeo domain factors	Homeobox	HB-ZF.C2H2
NANOGNB	3.1.8	Helix-turn-helix domains	Homeo domain factors	Homeobox	HB-ZF.C2H2
TSHZ1	3.1.8	Helix-turn-helix domains	Homeo domain factors	zf-C2H2	HB-ZF.C2H2
TSHZ3	3.1.8	Helix-turn-helix domains	Homeo domain factors	zf-C2H2	HB-ZF.C2H2
ZEB1	3.1.8	Helix-turn-helix domains	Homeo domain factors	zf-C2H2	HB-ZF.C2H2
ZEB2	3.1.8	Helix-turn-helix domains	Homeo domain factors	zf-C2H2	HB-ZF.C2H2
ZFHX2	3.1.8	Helix-turn-helix domains	Homeo domain factors	Homeobox	HB-ZF.C2H2
ZFHX3	3.1.8	Helix-turn-helix domains	Homeo domain factors	Homeobox	HB-ZF.C2H2

ZFHX4	3.1.8	Helix-turn-helix domains	Homeo domain factors	Homeobox	HB-ZF.C2H2
ZHX1	3.1.8	Helix-turn-helix domains	Homeo domain factors	Homeobox	HB-ZF.C2H2
ZHX2	3.1.8	Helix-turn-helix domains	Homeo domain factors	Homeobox	HB-ZF.C2H2
ZHX3	3.1.8	Helix-turn-helix domains	Homeo domain factors	Homeobox	HB-ZF.C2H2
CUX1	3.1.9	Helix-turn-helix domains	Homeo domain factors	CUT	HB-CUT
CUX2	3.1.9	Helix-turn-helix domains	Homeo domain factors	CUT	HB-CUT
ONECUT1	3.1.9	Helix-turn-helix domains	Homeo domain factors	CUT	HB-CUT
ONECUT2	3.1.9	Helix-turn-helix domains	Homeo domain factors	CUT	HB-CUT
ONECUT3	3.1.9	Helix-turn-helix domains	Homeo domain factors	CUT	HB-CUT
SATB1	3.1.9	Helix-turn-helix domains	Homeo domain factors	CUT	HB-CUT
SATB2	3.1.9	Helix-turn-helix domains	Homeo domain factors	CUT	HB-CUT
PAX3	3.2.1	Helix-turn-helix domains	Paired box factors	PAX	HB-PAX
PAX4	3.2.1	Helix-turn-helix domains	Paired box factors	PAX	HB-PAX
PAX6	3.2.1	Helix-turn-helix domains	Paired box factors	PAX	HB-PAX
PAX7	3.2.1	Helix-turn-helix domains	Paired box factors	PAX	HB-PAX
PAX1	3.2.2	Helix-turn-helix domains	Paired box factors	PAX	PAX
PAX2	3.2.2	Helix-turn-helix domains	Paired box factors	PAX	PAX
PAX5	3.2.2	Helix-turn-helix domains	Paired box factors	PAX	PAX
PAX8	3.2.2	Helix-turn-helix domains	Paired box factors	PAX	PAX
PAX9	3.2.2	Helix-turn-helix domains	Paired box factors	PAX	PAX
FOXA1	3.3.1	Helix-turn-helix domains	Fork head / winged helix factors	Fork_head	FH/WH.FOX
FOXA2	3.3.1	Helix-turn-helix domains	Fork head / winged helix factors	Fork_head	FH/WH.FOX
FOXA3	3.3.1	Helix-turn-helix domains	Fork head / winged helix factors	Fork_head	FH/WH.FOX
FOXB1	3.3.1	Helix-turn-helix domains	Fork head / winged helix factors	Fork_head	FH/WH.FOX
FOXB2	3.3.1	Helix-turn-helix domains	Fork head / winged helix factors	Fork_head	FH/WH.FOX
FOXC1	3.3.1	Helix-turn-helix domains	Fork head / winged helix factors	Fork_head	FH/WH.FOX
FOXC2	3.3.1	Helix-turn-helix domains	Fork head / winged helix factors	Fork_head	FH/WH.FOX
FOXD1	3.3.1	Helix-turn-helix domains	Fork head / winged helix factors	Fork_head	FH/WH.FOX
FOXD2	3.3.1	Helix-turn-helix domains	Fork head / winged helix factors	Fork_head	FH/WH.FOX
FOXD3	3.3.1	Helix-turn-helix domains	Fork head / winged helix factors	Fork_head	FH/WH.FOX
FOXD4	3.3.1	Helix-turn-helix domains	Fork head / winged helix factors	Fork_head	FH/WH.FOX
FOXD4L1	3.3.1	Helix-turn-helix domains	Fork head / winged helix factors	Fork_head	FH/WH.FOX
FOXD4L3	3.3.1	Helix-turn-helix domains	Fork head / winged helix factors	Fork_head	FH/WH.FOX
FOXD4L4	3.3.1	Helix-turn-helix domains	Fork head / winged helix factors	Fork_head	FH/WH.FOX
FOXD4L5	3.3.1	Helix-turn-helix domains	Fork head / winged helix factors	Fork_head	FH/WH.FOX
FOXD4L6	3.3.1	Helix-turn-helix domains	Fork head / winged helix factors	Fork_head	FH/WH.FOX
FOXE1	3.3.1	Helix-turn-helix domains	Fork head / winged helix factors	Fork_head	FH/WH.FOX
FOXE3	3.3.1	Helix-turn-helix domains	Fork head / winged helix factors	Fork_head	FH/WH.FOX
FOXF1	3.3.1	Helix-turn-helix domains	Fork head / winged helix factors	Fork_head	FH/WH.FOX
FOXF2	3.3.1	Helix-turn-helix domains	Fork head / winged helix factors	Fork_head	FH/WH.FOX

FOXG1	3.3.1	Helix-turn-helix domains	Fork head / winged helix factors	Fork_head	FH/WH.FOX
FOXH1	3.3.1	Helix-turn-helix domains	Fork head / winged helix factors	Fork_head	FH/WH.FOX
FOXI1	3.3.1	Helix-turn-helix domains	Fork head / winged helix factors	Fork_head	FH/WH.FOX
FOXI2	3.3.1	Helix-turn-helix domains	Fork head / winged helix factors	Fork_head	FH/WH.FOX
FOXI3	3.3.1	Helix-turn-helix domains	Fork head / winged helix factors	Fork_head	FH/WH.FOX
FOXJ1	3.3.1	Helix-turn-helix domains	Fork head / winged helix factors	Fork_head	FH/WH.FOX
FOXJ2	3.3.1	Helix-turn-helix domains	Fork head / winged helix factors	Fork_head	FH/WH.FOX
FOXJ3	3.3.1	Helix-turn-helix domains	Fork head / winged helix factors	Fork_head	FH/WH.FOX
FOXK1	3.3.1	Helix-turn-helix domains	Fork head / winged helix factors	Fork_head	FH/WH.FOX
FOXK2	3.3.1	Helix-turn-helix domains	Fork head / winged helix factors	Fork_head	FH/WH.FOX
FOXL1	3.3.1	Helix-turn-helix domains	Fork head / winged helix factors	Fork_head	FH/WH.FOX
FOXL2	3.3.1	Helix-turn-helix domains	Fork head / winged helix factors	Fork_head	FH/WH.FOX
FOXM1	3.3.1	Helix-turn-helix domains	Fork head / winged helix factors	Fork_head	FH/WH.FOX
FOXN1	3.3.1	Helix-turn-helix domains	Fork head / winged helix factors	Fork_head	FH/WH.FOX
FOXN2	3.3.1	Helix-turn-helix domains	Fork head / winged helix factors	Fork_head	FH/WH.FOX
FOXN3	3.3.1	Helix-turn-helix domains	Fork head / winged helix factors	Fork_head	FH/WH.FOX
FOXN4	3.3.1	Helix-turn-helix domains	Fork head / winged helix factors	Fork_head	FH/WH.FOX
FOXO1	3.3.1	Helix-turn-helix domains	Fork head / winged helix factors	Fork_head	FH/WH.FOX
FOXO3	3.3.1	Helix-turn-helix domains	Fork head / winged helix factors	Fork_head	FH/WH.FOX
FOXO4	3.3.1	Helix-turn-helix domains	Fork head / winged helix factors	Fork_head	FH/WH.FOX
FOXO6	3.3.1	Helix-turn-helix domains	Fork head / winged helix factors	Fork_head	FH/WH.FOX
FOXP1	3.3.1	Helix-turn-helix domains	Fork head / winged helix factors	Fork_head	FH/WH.FOX
FOXP2	3.3.1	Helix-turn-helix domains	Fork head / winged helix factors	Fork_head	FH/WH.FOX
FOXP3	3.3.1	Helix-turn-helix domains	Fork head / winged helix factors	Fork_head	FH/WH.FOX
FOXP4	3.3.1	Helix-turn-helix domains	Fork head / winged helix factors	Fork_head	FH/WH.FOX
FOXQ1	3.3.1	Helix-turn-helix domains	Fork head / winged helix factors	Fork_head	FH/WH.FOX
FOXR1	3.3.1	Helix-turn-helix domains	Fork head / winged helix factors	Fork_head	FH/WH.FOX
FOXR2	3.3.1	Helix-turn-helix domains	Fork head / winged helix factors	Fork_head	FH/WH.FOX
FOXS1	3.3.1	Helix-turn-helix domains	Fork head / winged helix factors	Fork_head	FH/WH.FOX
E2F1	3.3.2	Helix-turn-helix domains	Fork head / winged helix factors	E2F	FH/WH.E2F
E2F2	3.3.2	Helix-turn-helix domains	Fork head / winged helix factors	E2F	FH/WH.E2F
E2F3	3.3.2	Helix-turn-helix domains	Fork head / winged helix factors	E2F	FH/WH.E2F
E2F4	3.3.2	Helix-turn-helix domains	Fork head / winged helix factors	E2F	FH/WH.E2F
E2F5	3.3.2	Helix-turn-helix domains	Fork head / winged helix factors	E2F	FH/WH.E2F
E2F6	3.3.2	Helix-turn-helix domains	Fork head / winged helix factors	E2F	FH/WH.E2F
E2F7	3.3.2	Helix-turn-helix domains	Fork head / winged helix factors	E2F	FH/WH.E2F
E2F8	3.3.2	Helix-turn-helix domains	Fork head / winged helix factors	E2F	FH/WH.E2F
TFDP1	3.3.2	Helix-turn-helix domains	Fork head / winged helix factors	E2F	FH/WH.E2F
TFDP2	3.3.2	Helix-turn-helix domains	Fork head / winged helix factors	E2F	FH/WH.E2F
TFDP3	3.3.2	Helix-turn-helix domains	Fork head / winged helix factors	E2F	FH/WH.E2F

RFX1	3.3.3	Helix-turn-helix domains	Fork head / winged helix factors	RFX	FH/WH.RFX
RFX2	3.3.3	Helix-turn-helix domains	Fork head / winged helix factors	RFX	FH/WH.RFX
RFX3	3.3.3	Helix-turn-helix domains	Fork head / winged helix factors	RFX	FH/WH.RFX
RFX4	3.3.3	Helix-turn-helix domains	Fork head / winged helix factors	RFX	FH/WH.RFX
RFX5	3.3.3	Helix-turn-helix domains	Fork head / winged helix factors	RFX	FH/WH.RFX
RFX6	3.3.3	Helix-turn-helix domains	Fork head / winged helix factors	RFX	FH/WH.RFX
RFX7	3.3.3	Helix-turn-helix domains	Fork head / winged helix factors	RFX	FH/WH.RFX
RFX8	3.3.3	Helix-turn-helix domains	Fork head / winged helix factors	RFX	FH/WH.RFX
HSF1	3.4.1	Helix-turn-helix domains	Heat shock factors	HSF	HSF
HSF2	3.4.1	Helix-turn-helix domains	Heat shock factors	HSF	HSF
HSF4	3.4.1	Helix-turn-helix domains	Heat shock factors	HSF	HSF
HSF5	3.4.1	Helix-turn-helix domains	Heat shock factors	HSF	HSF
HSFX1	3.4.1	Helix-turn-helix domains	Heat shock factors	HSF	HSF
HSFX3	3.4.1	Helix-turn-helix domains	Heat shock factors	HSF	HSF
HSFY1	3.4.1	Helix-turn-helix domains	Heat shock factors	HSF	HSF
CDC5L	3.5.1	Helix-turn-helix domains	Tryptophan cluster factors	MYB	WC.MYB
DMTF1	3.5.1	Helix-turn-helix domains	Tryptophan cluster factors	MYB	WC.MYB
DNAJC1	3.5.1	Helix-turn-helix domains	Tryptophan cluster factors	MYB	WC.MYB
DNAJC2	3.5.1	Helix-turn-helix domains	Tryptophan cluster factors	MYB	WC.MYB
MIDEAS	3.5.1	Helix-turn-helix domains	Tryptophan cluster factors	Others	WC.MYB
MIER1	3.5.1	Helix-turn-helix domains	Tryptophan cluster factors	MYB	WC.MYB
MIER3	3.5.1	Helix-turn-helix domains	Tryptophan cluster factors	MYB	WC.MYB
MYB	3.5.1	Helix-turn-helix domains	Tryptophan cluster factors	MYB	WC.MYB
MYBL1	3.5.1	Helix-turn-helix domains	Tryptophan cluster factors	MYB	WC.MYB
MYBL2	3.5.1	Helix-turn-helix domains	Tryptophan cluster factors	MYB	WC.MYB
NCOR1	3.5.1	Helix-turn-helix domains	Tryptophan cluster factors	MYB	WC.MYB
NCOR2	3.5.1	Helix-turn-helix domains	Tryptophan cluster factors	MYB	WC.MYB
RCOR1	3.5.1	Helix-turn-helix domains	Tryptophan cluster factors	MYB	WC.MYB
RCOR2	3.5.1	Helix-turn-helix domains	Tryptophan cluster factors	MYB	WC.MYB
RCOR3	3.5.1	Helix-turn-helix domains	Tryptophan cluster factors	MYB	WC.MYB
SMARCC1	3.5.1	Helix-turn-helix domains	Tryptophan cluster factors	MYB	WC.MYB
SMARCC2	3.5.1	Helix-turn-helix domains	Tryptophan cluster factors	MYB	WC.MYB
SNAPC4	3.5.1	Helix-turn-helix domains	Tryptophan cluster factors	MYB	WC.MYB
TADA2A	3.5.1	Helix-turn-helix domains	Tryptophan cluster factors	MYB	WC.MYB
TADA2B	3.5.1	Helix-turn-helix domains	Tryptophan cluster factors	MYB	WC.MYB
TRERF1	3.5.1	Helix-turn-helix domains	Tryptophan cluster factors	zf-C2H2	WC.MYB
ZNF541	3.5.1	Helix-turn-helix domains	Tryptophan cluster factors	zf-C2H2	WC.MYB
EHF	3.5.2	Helix-turn-helix domains	Tryptophan cluster factors	ETS	WC.ETS
ELF1	3.5.2	Helix-turn-helix domains	Tryptophan cluster factors	ETS	WC.ETS
ELF2	3.5.2	Helix-turn-helix domains	Tryptophan cluster factors	ETS	WC.ETS

ELF3	3.5.2	Helix-turn-helix domains	Tryptophan cluster factors	ETS	WC.ETS
ELF4	3.5.2	Helix-turn-helix domains	Tryptophan cluster factors	ETS	WC.ETS
ELF5	3.5.2	Helix-turn-helix domains	Tryptophan cluster factors	ETS	WC.ETS
ELK1	3.5.2	Helix-turn-helix domains	Tryptophan cluster factors	ETS	WC.ETS
ELK3	3.5.2	Helix-turn-helix domains	Tryptophan cluster factors	ETS	WC.ETS
ELK4	3.5.2	Helix-turn-helix domains	Tryptophan cluster factors	ETS	WC.ETS
ERF	3.5.2	Helix-turn-helix domains	Tryptophan cluster factors	ETS	WC.ETS
ERG	3.5.2	Helix-turn-helix domains	Tryptophan cluster factors	ETS	WC.ETS
ETS1	3.5.2	Helix-turn-helix domains	Tryptophan cluster factors	ETS	WC.ETS
ETS2	3.5.2	Helix-turn-helix domains	Tryptophan cluster factors	ETS	WC.ETS
ETV1	3.5.2	Helix-turn-helix domains	Tryptophan cluster factors	ETS	WC.ETS
ETV2	3.5.2	Helix-turn-helix domains	Tryptophan cluster factors	ETS	WC.ETS
ETV3	3.5.2	Helix-turn-helix domains	Tryptophan cluster factors	ETS	WC.ETS
ETV3L	3.5.2	Helix-turn-helix domains	Tryptophan cluster factors	ETS	WC.ETS
ETV4	3.5.2	Helix-turn-helix domains	Tryptophan cluster factors	ETS	WC.ETS
ETV5	3.5.2	Helix-turn-helix domains	Tryptophan cluster factors	ETS	WC.ETS
ETV6	3.5.2	Helix-turn-helix domains	Tryptophan cluster factors	ETS	WC.ETS
ETV7	3.5.2	Helix-turn-helix domains	Tryptophan cluster factors	ETS	WC.ETS
FEV	3.5.2	Helix-turn-helix domains	Tryptophan cluster factors	ETS	WC.ETS
FLI1	3.5.2	Helix-turn-helix domains	Tryptophan cluster factors	ETS	WC.ETS
GABPA	3.5.2	Helix-turn-helix domains	Tryptophan cluster factors	ETS	WC.ETS
SPDEF	3.5.2	Helix-turn-helix domains	Tryptophan cluster factors	ETS	WC.ETS
SPI1	3.5.2	Helix-turn-helix domains	Tryptophan cluster factors	ETS	WC.ETS
SPIB	3.5.2	Helix-turn-helix domains	Tryptophan cluster factors	ETS	WC.ETS
SPIC	3.5.2	Helix-turn-helix domains	Tryptophan cluster factors	ETS	WC.ETS
IRF1	3.5.3	Helix-turn-helix domains	Tryptophan cluster factors	IRF	WC.IRF
IRF2	3.5.3	Helix-turn-helix domains	Tryptophan cluster factors	IRF	WC.IRF
IRF3	3.5.3	Helix-turn-helix domains	Tryptophan cluster factors	IRF	WC.IRF
IRF4	3.5.3	Helix-turn-helix domains	Tryptophan cluster factors	IRF	WC.IRF
IRF5	3.5.3	Helix-turn-helix domains	Tryptophan cluster factors	IRF	WC.IRF
IRF6	3.5.3	Helix-turn-helix domains	Tryptophan cluster factors	IRF	WC.IRF
IRF7	3.5.3	Helix-turn-helix domains	Tryptophan cluster factors	IRF	WC.IRF
IRF8	3.5.3	Helix-turn-helix domains	Tryptophan cluster factors	IRF	WC.IRF
IRF9	3.5.3	Helix-turn-helix domains	Tryptophan cluster factors	IRF	WC.IRF
TEAD1	3.6.1	Helix-turn-helix domains	TEA domain factors	TEA	TEA
TEAD2	3.6.1	Helix-turn-helix domains	TEA domain factors	TEA	TEA
TEAD3	3.6.1	Helix-turn-helix domains	TEA domain factors	TEA	TEA
TEAD4	3.6.1	Helix-turn-helix domains	TEA domain factors	TEA	TEA
ARID1A	3.7.1	Helix-turn-helix domains	ARID domain factors	ARID	ARID
ARID1B	3.7.1	Helix-turn-helix domains	ARID domain factors	ARID	ARID

ARID2	3.7.1	Helix-turn-helix domains	ARID domain factors	ARID	ARID
ARID3A	3.7.1	Helix-turn-helix domains	ARID domain factors	ARID	ARID
ARID3B	3.7.1	Helix-turn-helix domains	ARID domain factors	ARID	ARID
ARID4A	3.7.1	Helix-turn-helix domains	ARID domain factors	ARID	ARID
ARID4B	3.7.1	Helix-turn-helix domains	ARID domain factors	ARID	ARID
ARID5A	3.7.1	Helix-turn-helix domains	ARID domain factors	ARID	ARID
ARID5B	3.7.1	Helix-turn-helix domains	ARID domain factors	ARID	ARID
JARID2	3.7.1	Helix-turn-helix domains	ARID domain factors	ARID	ARID
BBX	4.1.1	her all-α-helical DNA-binding domai	High-mobility group (HMG) domain factors	HMG	HMG.SOX
CIC	4.1.1	her all-α-helical DNA-binding domai	High-mobility group (HMG) domain factors	HMG	HMG.SOX
HBP1	4.1.1	her all-α-helical DNA-binding domai	High-mobility group (HMG) domain factors	HMG	HMG.SOX
SOX1	4.1.1	her all-α-helical DNA-binding domai	High-mobility group (HMG) domain factors	HMG	HMG.SOX
SOX10	4.1.1	her all-α-helical DNA-binding domai	High-mobility group (HMG) domain factors	HMG	HMG.SOX
SOX11	4.1.1	her all-α-helical DNA-binding domai	High-mobility group (HMG) domain factors	HMG	HMG.SOX
SOX12	4.1.1	her all-α-helical DNA-binding domai	High-mobility group (HMG) domain factors	HMG	HMG.SOX
SOX13	4.1.1	her all-α-helical DNA-binding domai	High-mobility group (HMG) domain factors	HMG	HMG.SOX
SOX14	4.1.1	her all-α-helical DNA-binding domai	High-mobility group (HMG) domain factors	HMG	HMG.SOX
SOX15	4.1.1	her all-α-helical DNA-binding domai	High-mobility group (HMG) domain factors	HMG	HMG.SOX
SOX17	4.1.1	her all-α-helical DNA-binding domai	High-mobility group (HMG) domain factors	HMG	HMG.SOX
SOX18	4.1.1	her all-α-helical DNA-binding domai	High-mobility group (HMG) domain factors	HMG	HMG.SOX
SOX2	4.1.1	her all-α-helical DNA-binding domai	High-mobility group (HMG) domain factors	HMG	HMG.SOX
SOX21	4.1.1	her all-α-helical DNA-binding domai	High-mobility group (HMG) domain factors	HMG	HMG.SOX
SOX3	4.1.1	her all-α-helical DNA-binding domai	High-mobility group (HMG) domain factors	HMG	HMG.SOX
SOX30	4.1.1	her all-α-helical DNA-binding domai	High-mobility group (HMG) domain factors	HMG	HMG.SOX
SOX4	4.1.1	her all-α-helical DNA-binding domai	High-mobility group (HMG) domain factors	HMG	HMG.SOX
SOX5	4.1.1	her all-α-helical DNA-binding domai	High-mobility group (HMG) domain factors	HMG	HMG.SOX
SOX6	4.1.1	her all-α-helical DNA-binding domai	High-mobility group (HMG) domain factors	HMG	HMG.SOX
SOX7	4.1.1	her all-α-helical DNA-binding domai	High-mobility group (HMG) domain factors	HMG	HMG.SOX
SOX8	4.1.1	her all-α-helical DNA-binding domai	High-mobility group (HMG) domain factors	HMG	HMG.SOX
SOX9	4.1.1	her all-α-helical DNA-binding domai	High-mobility group (HMG) domain factors	HMG	HMG.SOX
SRY	4.1.1	her all-α-helical DNA-binding domai	High-mobility group (HMG) domain factors	HMG	HMG.SOX
HMG20A	4.1.2	her all-α-helical DNA-binding domai	High-mobility group (HMG) domain factors	HMG	HMG.TOX
HMG20B	4.1.2	her all-α-helical DNA-binding domai	High-mobility group (HMG) domain factors	HMG	HMG.TOX
HMGXB3	4.1.2	her all-α-helical DNA-binding domai	High-mobility group (HMG) domain factors	HMG	HMG.TOX
SMARCE1	4.1.2	her all-α-helical DNA-binding domai	High-mobility group (HMG) domain factors	HMG	HMG.TOX
TOX	4.1.2	her all-α-helical DNA-binding domai	High-mobility group (HMG) domain factors	HMG	HMG.TOX
TOX2	4.1.2	her all-α-helical DNA-binding domai	High-mobility group (HMG) domain factors	HMG	HMG.TOX
TOX3	4.1.2	her all-α-helical DNA-binding domai	High-mobility group (HMG) domain factors	HMG	HMG.TOX
TOX4	4.1.2	her all-α-helical DNA-binding domai	High-mobility group (HMG) domain factors	HMG	HMG.TOX
LEF1	4.1.3	her all-α-helical DNA-binding domai	High-mobility group (HMG) domain factors	HMG	HMG.TCF7

TCF7	4.1.3	her all-α-helical DNA-binding domai	High-mobility group (HMG) domain factors	HMG	HMG.TCF7
TCF7L1	4.1.3	her all-α-helical DNA-binding domai	High-mobility group (HMG) domain factors	HMG	HMG.TCF7
TCF7L2	4.1.3	her all-α-helical DNA-binding domai	High-mobility group (HMG) domain factors	HMG	HMG.TCF7
HMGXB4	4.1.4	her all-α-helical DNA-binding domai	High-mobility group (HMG) domain factors	HMG	HMG.PBRM1
PBRM1	4.1.4	her all-α-helical DNA-binding domai	High-mobility group (HMG) domain factors	HMG	HMG.PBRM2
NSD2	4.1.5	her all-α-helical DNA-binding domai	High-mobility group (HMG) domain factors	HMG	HMG.WHSC1
SSRP1	4.1.5	her all-α-helical DNA-binding domai	High-mobility group (HMG) domain factors	HMG	HMG.WHSC2
UBTF	4.1.6	her all-α-helical DNA-binding domai	High-mobility group (HMG) domain factors	HMG	HMG.UBF
UBTFL1	4.1.6	her all-α-helical DNA-binding domai	High-mobility group (HMG) domain factors	HMG	HMG.UBF
TFAM	4.1.7	her all-α-helical DNA-binding domai	High-mobility group (HMG) domain factors	HMG	HMG.TFAM
NFYA	4.2.1	her all-α-helical DNA-binding domai	Heteromeric CCAAT-binding factors	NF-YA	CAAT-BF
NFYB	4.2.1	her all-α-helical DNA-binding domai	Heteromeric CCAAT-binding factors	NF-YB	CAAT-BF
NFYC	4.2.1	her all-α-helical DNA-binding domai	Heteromeric CCAAT-binding factors	NF-YC	CAAT-BF
MEF2A	5.1.1	α-Helices exposed by β-structures	MADS box factors	SRF	MADS
MEF2B	5.1.1	α-Helices exposed by β-structures	MADS box factors	SRF	MADS
MEF2C	5.1.1	α-Helices exposed by β-structures	MADS box factors	SRF	MADS
MEF2D	5.1.1	α-Helices exposed by β-structures	MADS box factors	SRF	MADS
SRF	5.1.2	α-Helices exposed by β-structures	MADS box factors	SRF	MADS
AIRE	5.3.1	α-Helices exposed by β-structures	SAND domain factors	SAND	SAND
DEAF1	5.3.2	α-Helices exposed by β-structures	SAND domain factors	SAND	SAND
GMEB1	5.3.3	α-Helices exposed by β-structures	SAND domain factors	SAND	SAND
GMEB2	5.3.3	α-Helices exposed by β-structures	SAND domain factors	SAND	SAND
SP110	5.3.4	α-Helices exposed by β-structures	SAND domain factors	SAND	SAND
SP100	5.3.5	α-Helices exposed by β-structures	SAND domain factors	SAND	SAND
SP140	5.3.5	α-Helices exposed by β-structures	SAND domain factors	SAND	SAND
SP140L	5.3.5	α-Helices exposed by β-structures	SAND domain factors	SAND	SAND
NFKB1	6.1.1	Immunoglobulin fold	Rel homology region (RHR) factors	RHD	RHR
NFKB2	6.1.1	Immunoglobulin fold	Rel homology region (RHR) factors	RHD	RHR
REL	6.1.1	Immunoglobulin fold	Rel homology region (RHR) factors	RHD	RHR
RELA	6.1.1	Immunoglobulin fold	Rel homology region (RHR) factors	RHD	RHR
RELB	6.1.1	Immunoglobulin fold	Rel homology region (RHR) factors	RHD	RHR
NFAT5	6.1.3	Immunoglobulin fold	Rel homology region (RHR) factors	RHD	RHR
NFATC1	6.1.3	Immunoglobulin fold	Rel homology region (RHR) factors	RHD	RHR
NFATC2	6.1.3	Immunoglobulin fold	Rel homology region (RHR) factors	RHD	RHR
NFATC3	6.1.3	Immunoglobulin fold	Rel homology region (RHR) factors	RHD	RHR
NFATC4	6.1.3	Immunoglobulin fold	Rel homology region (RHR) factors	RHD	RHR
RBPJ	6.1.4	Immunoglobulin fold	Rel homology region (RHR) factors	CSL	RHR
RBPJL	6.1.4	Immunoglobulin fold	Rel homology region (RHR) factors	CSL	RHR
EBF1	6.1.5	Immunoglobulin fold	Rel homology region (RHR) factors	COE	RHR
EBF2	6.1.5	Immunoglobulin fold	Rel homology region (RHR) factors	COE	RHR

EBF3	6.1.5	Immunoglobulin fold	Rel homology region (RHR) factors	COE	RHR
EBF4	6.1.5	Immunoglobulin fold	Rel homology region (RHR) factors	COE	RHR
STAT1	6.2.1	Immunoglobulin fold	STAT domain factors	STAT	STAT
STAT2	6.2.1	Immunoglobulin fold	STAT domain factors	STAT	STAT
STAT3	6.2.1	Immunoglobulin fold	STAT domain factors	STAT	STAT
STAT4	6.2.1	Immunoglobulin fold	STAT domain factors	STAT	STAT
STAT5A	6.2.1	Immunoglobulin fold	STAT domain factors	STAT	STAT
STAT5B	6.2.1	Immunoglobulin fold	STAT domain factors	STAT	STAT
STAT6	6.2.1	Immunoglobulin fold	STAT domain factors	STAT	STAT
TP53	6.3.1	Immunoglobulin fold	p53 domain factors	P53	p53
TP63	6.3.1	Immunoglobulin fold	p53 domain factors	P53	p53
TP73	6.3.1	Immunoglobulin fold	p53 domain factors	P53	p53
RUNX1	6.4.1	Immunoglobulin fold	Runt domain factors	Runt	RUNT
RUNX2	6.4.1	Immunoglobulin fold	Runt domain factors	Runt	RUNT
RUNX3	6.4.1	Immunoglobulin fold	Runt domain factors	Runt	RUNT
TBX19	6.5.1	Immunoglobulin fold	T-Box factors	T-box	TB
TBXT	6.5.1	Immunoglobulin fold	T-Box factors	T-box	TB
TBX1	6.5.3	Immunoglobulin fold	T-Box factors	T-box	TB
TBX10	6.5.3	Immunoglobulin fold	T-Box factors	T-box	TB
TBX15	6.5.3	Immunoglobulin fold	T-Box factors	T-box	TB
TBX18	6.5.3	Immunoglobulin fold	T-Box factors	T-box	TB
TBX20	6.5.3	Immunoglobulin fold	T-Box factors	T-box	TB
TBX21	6.5.3	Immunoglobulin fold	T-Box factors	T-box	TB
TBX22	6.5.3	Immunoglobulin fold	T-Box factors	T-box	TB
TBX2	6.5.4	Immunoglobulin fold	T-Box factors	T-box	TB
TBX3	6.5.4	Immunoglobulin fold	T-Box factors	T-box	TB
TBX4	6.5.4	Immunoglobulin fold	T-Box factors	T-box	TB
TBX5	6.5.4	Immunoglobulin fold	T-Box factors	T-box	TB
TBX6	6.5.5	Immunoglobulin fold	T-Box factors	T-box	TB
MYRF	6.6.1	Immunoglobulin fold	NDT80 domain factors	NDT80_PhoG	NDT80
GRHL1	6.7.1	Immunoglobulin fold	Grainyhead domain factors	CP2	GHD.CP2
GRHL2	6.7.1	Immunoglobulin fold	Grainyhead domain factors	CP2	GHD.CP2
GRHL3	6.7.1	Immunoglobulin fold	Grainyhead domain factors	CP2	GHD.CP2
TFCP2	6.7.2	Immunoglobulin fold	Grainyhead domain factors	CP2	GHD.CP2
TFCP2L1	6.7.2	Immunoglobulin fold	Grainyhead domain factors	CP2	GHD.CP2
UBP1	6.7.2	Immunoglobulin fold	Grainyhead domain factors	CP2	GHD.CP2
SMAD1	7.1.1	-Hairpin exposed by an α/β-scaffo	SMAD/NF-1 DNA-binding domain factors	MH1	SMAD
SMAD2	7.1.1	-Hairpin exposed by an α/β-scaffo	SMAD/NF-1 DNA-binding domain factors	MH1	SMAD
SMAD3	7.1.1	-Hairpin exposed by an α/β-scaffo	SMAD/NF-1 DNA-binding domain factors	MH1	SMAD
SMAD4	7.1.1	-Hairpin exposed by an α/β-scaffo	SMAD/NF-1 DNA-binding domain factors	MH1	SMAD

SMAD5	7.1.1	-Hairpin exposed by an α/β-scaffo	SMAD/NF-1 DNA-binding domain factors	MH1	SMAD
SMAD6	7.1.1	-Hairpin exposed by an α/β-scaffo	SMAD/NF-1 DNA-binding domain factors	MH1	SMAD
SMAD7	7.1.1	-Hairpin exposed by an α/β-scaffo	SMAD/NF-1 DNA-binding domain factors	MH1	SMAD
SMAD9	7.1.1	-Hairpin exposed by an α/β-scaffo	SMAD/NF-1 DNA-binding domain factors	MH1	SMAD
NFIA	7.1.2	-Hairpin exposed by an α/β-scaffo	SMAD/NF-1 DNA-binding domain factors	CTF_NFI	NF1
NFIB	7.1.2	-Hairpin exposed by an α/β-scaffo	SMAD/NF-1 DNA-binding domain factors	CTF_NFI	NF1
NFIC	7.1.2	-Hairpin exposed by an α/β-scaffo	SMAD/NF-1 DNA-binding domain factors	CTF_NFI	NF1
NFIX	7.1.2	-Hairpin exposed by an α/β-scaffo	SMAD/NF-1 DNA-binding domain factors	CTF_NFI	NF1
GCM1	7.2.1	-Hairpin exposed by an α/β-scaffo	GCM domain factors	GCM	GCM
GCM2	7.2.1	-Hairpin exposed by an α/β-scaffo	GCM domain factors	GCM	GCM
HMGA1	8.2.1	β-Sheet binding to DNA	A.T hook factors	HMGA	HMGA
HMGA2	8.2.1	β-Sheet binding to DNA	A.T hook factors	HMGA	HMGA
YBX1	9.1.1	β-Sheet binding to DNA	Cold-shock domain factors	CSD	DBP
YBX2	9.1.1	β-Barrel DNA-binding domains	Cold-shock domain factors	CSD	DBP
YBX3	9.1.1	β-Barrel DNA-binding domains	Cold-shock domain factors	CSD	DBP
AFF1				AF-4	others
AFF2				AF-4	others
AFF3				AF-4	others
AFF4				AF-4	others
AHCTF1				Others	others
AKNA				Others	others
ANHX				Homeobox	HB
ANKRD30A				Others	others
ARID3C				ARID	ARID
ATMIN				zf-C2H2	ZF.C2H2
BAZ2A				MBD	ZF.CXXC
BAZ2B				MBD	ZF.CXXC
BCLAF1				Others	others
BLZF1				Others	others
CAPN15				Others	others
CARF				Others	others
CARHSP1				CSD	others
CBFB				CBF	others
CC2D1A				Others	others
CCDC88A					others
CDIP1				zf-LITAF-like	others
CEBPZ				Others	others
CENPX					others
CIZ1				Others	others
CNBP				Others	others

CPEB1				Others	others
CPHXL				Homeobox	HB
CREBRF				Others	others
CSDC2				CSD	others
CSDE1				CSD	others
DACH1				DACH	others
DACH2				DACH	others
DUXB				Homeobox	HB
EOMES				T-box	TB
FAM170A				Others	others
GABPB1				Others	others
GABPB2				Others	others
GCFC2				GCFC	others
GLMP				NCU-G1	others
GON4L				Others	others
GPBP1				Others	others
GPBP1L1				Others	others
HES1				bHLH	bHLH
HMGB1				HMG	HMG
HMGB2				HMG	HMG
HMGB3				HMG	HMG
HMGB4				HMG	HMG
HSFX4				HSF	HMG
JAZF1				zf-C2H2	ZF.C2H2
KHSRP				Others	others
KIAA1549				Others	others
KLF18				zf-C2H2	ZF.C2H2
KMT2C				HMG	HMG
L3MBTL3				Others	others
L3MBTL4				zf-C2HC	ZF.C2H2
LCOR				HTH	HTH
LCORL				HTH	HTH
LIN28A				CSD	ZF.CCHC
LIN28B				CSD	ZF.CCHC
LITAF				zf-LITAF-like	others
LTF				Others	others
MAEL				HMG	HMG
MAF1				Others	others
MBD2				MBD	ZF.CXXC
MBD3				MBD	ZF.CXXC

MBD4				MBD	ZF.CXXC
MECP2				MBD	ZF.CXXC
MEIOSIN				HMG	HMG
MIS18BP1				MYB	WC.MYB
MLLT10				Others	others
MYEF2				Others	others
MYRFL				NDT80_PhoG	others
MYSM1				MYB	WC.MYB
NANOGP8				Homeobox	HB
NFRKB				Others	others
NHLH1				bHLH	bHLH
NKRF				Others	others
NME2				Others	others
PARP12				Others	others
PAXBP1				GCFC	others
PHB				Others	others
PIAS1				zf-MIZ	ZF.MIZ
PIAS2				zf-MIZ	ZF.MIZ
PIAS3				zf-MIZ	ZF.MIZ
PIAS4				zf-MIZ	ZF.MIZ
PLEK				Others	others
PLSCR1				Others	others
PMS1				HMG	HMG
PRDM11				Others	others
PRDM12				zf-C2H2	ZF.C2H2
PREB				Others	others
RAG1				Others	others
RBCK1				Others	others
REPIN1				zf-C2H2	ZF.C2H2
SAMD11				SAND	SNAD
SETBP1				Others	others
SETDB1				MBD	ZF.CXXC
SETDB2				MBD	ZF.CXXC
SLC2A4RG				Others	others
SMARCAL1				Others	others
SON				Others	others
SPZ1				Others	others
SUB1				PC4	others
TAX1BP1				Others	others
TBR1				T-box	TB

TCF19				Others	PHDFP
TCF24				bHLH	bHLH
TERB1				MYB	WC.MYB
TERF1				MYB	WC.MYB
TERF2				MYB	WC.MYB
TET2				Others	others
TFB1M				Others	others
THYN1				Others	others
TMF1				Others	others
TSC22D1				TSC22	TSC22
TSC22D2				TSC22	TSC22
TSC22D3				TSC22	TSC22
TSC22D4				TSC22	TSC22
TTF1				MYB	WC.MYB
TUB				Tub	TUB
TULP1				Tub	TUB
TULP3				Tub	TUB
TULP4				Tub	TUB
USF3				bHLH	bHLH
WDHD1				HMG	HMG
XPA				Others	others
YEATS4				Others	others
ZBTB10				ZBTB	ZF.C2H2
ZBTB3				ZBTB	ZF.C2H2
ZBTB8A				ZBTB	ZF.C2H2
ZFP36L1				zf-CCCH	ZF.CCCH
ZFTA				zf-BED	ZF.BED
ZMIZ1				zf-MIZ	ZF.MIZ
ZMIZ2				zf-MIZ	ZF.MIZ
ZNF112				zf-C2H2	ZF.C2H2
ZNF12				zf-C2H2	ZF.C2H2
ZNF207				Others	ZF.C2H2
ZNF318				Others	ZF.C2H2
ZNF326				Others	ZF.C2H2
ZNF367				zf-C2H2	ZF.C2H2
ZNF385A				zf-C2H2	ZF.C2H2
ZNF385B				zf-C2H2	ZF.C2H2
ZNF395				Others	ZF.C2H2
ZNF470				zf-C2H2	ZF.C2H2
ZNF473				zf-C2H2	ZF.C2H2

ZNF486				zf-C2H2	ZF.C2H2
ZNF488				Others	ZF.C2H2
ZNF503				Others	ZF.C2H2
ZNF527				zf-C2H2	ZF.C2H2
ZNF573				zf-C2H2	ZF.C2H2
ZNF578				zf-C2H2	ZF.C2H2
ZNF587B				zf-C2H2	ZF.C2H2
ZNF608				Others	ZF.C2H2
ZNF609				Others	ZF.C2H2
ZNF622				zf-C2H2	ZF.C2H2
ZNF630				zf-C2H2	ZF.C2H2
ZNF688				zf-C2H2	ZF.C2H2
ZNF69				zf-C2H2	ZF.C2H2
ZNF699				zf-C2H2	ZF.C2H2
ZNF700				zf-C2H2	ZF.C2H2
ZNF704				Others	ZF.C2H2
ZNF705B				zf-C2H2	ZF.C2H2
ZNF706				Others	ZF.C2H2
ZNF708				zf-C2H2	ZF.C2H2
ZNF714				zf-C2H2	ZF.C2H2
ZNF720				zf-C2H2	ZF.C2H2
ZNF723				zf-C2H2	ZF.C2H2
ZNF728				zf-C2H2	ZF.C2H2
ZNF735				zf-C2H2	ZF.C2H2
ZNF750				Others	ZF
ZNF761				zf-C2H2	ZF.C2H2
ZNF773				zf-C2H2	ZF.C2H2
ZNF783				zf-C2H2	ZF.C2H2
ZNF8				zf-C2H2	ZF.C2H2
ZNF821				zf-C2H2	ZF.C2H2
ZNF827				zf-C2H2	ZF.C2H2
ZNF831				zf-C2H2	ZF.C2H2
ZNF850				zf-C2H2	ZF.C2H2
ZNF865				zf-C2H2	ZF.C2H2
ZNF883					pseudogene
ZNF888				zf-C2H2	ZF.C2H2
ZSCAN18				zf-C2H2	ZF.C2H2
ZZZ3				MYB	WC.MYB
