# Supplementary material for: Foxg1 and companions: Not only transcription factors
Source: Neural Regen Res. 2025 Sep 3;21(8):3426–38. doi: 10.4103/NRR.NRR-D-25-00439 (PMC13452659; doi:10.4103/NRR.NRR-D-25-00439)
Supplement: Supplementary file 2 [file NRR-21-3426_Suppl10.pdf]

Additional Table 6.1. TFs: full interaction data grid and other key information

NOTES

(1) To left (columns G-DSJ), shown is the full interaction pattern among TFs and all factors belonging to the following categories: TF, TcoF, SplF.core, SlpF.RBP, SplF.others, pA.4C, pA.PA, TlatF.EIF, TlatF.EIcoF, TlatF.EEF, TlatF.cytoRB, TlatF.mitoRB, TlatF.aatRNAsynth; numerbs refer to the numer of indepedent BioGrid enties documenting the interaction in order;

(2) To right (columns DSL-DSO), shown are four key pieces of information referring to each TF: subcellular localization, RNA-binding ability, pTriplo index, sensitiveness of mRNA levels to neuronal activity/plasticity

Total interaction reports within hsa.interactome.restr
1152045

Total TF-interaction reports within hsa.inter-actome.restr	TF-taxon	TFs	ARID1A	ARID1B	ARID2	ARID3A	ARID3B	ARID4A	ARID4B	ARID5A	ARID5B	JARID2	ARID3C	TCF25	TCF12	TCF3	TCF4	ASCL1	ASCL2	ASCL3	ASCL4	ASCL5	MYF5	MYF6	MYOD1	MYOG	ATOH1	ATOH7	ATOH8	BHLHA15	BHLHA9	BHLHE22	BHLHE23	FERD3L	FIGLA	HAND1	HAND2	LYL1	MESP1	MESP2	MSC	MSGN1	NEUROD1	NEUROD2	NEUROD4	NEUROD6	NEUROG1	NEUROG2	NEUROG3	NHLH2	OLIG1	OLIG2	OLIG3	PTF1A	SCX	TAL1
		Total factor-interaction reports within hsa.interactome.restr	350	248	209	135	130	102	214	145	82	104	10	117	214	313	478	41	10	19	20	2	34	28	244	91	82	1	2	98	12	2	20	27	19	19	39	15	11	13	16	36	44	12	10	4	20	4	195	28	34	22	36	18	7	170
		Total factor/TF-interaction reports within hsa.interactome.restr	127	101	84	67	63	20	82	33	21	24	4	4	110	165	143	13	1	14	11	0	16	10	94	25	4	1	1	16	0	0	0	11	14	9	16	8	4	9	8	11	21	1	3	0	8	2	31	13	6	9	8	4	5	45
			ARID																																																					
350	ARID	ARID1A		4	1																				1																															
248		ARID1B	4		1																				1																															
209		ARID2	1	1																																																				
135		ARID3A					3						2												1																															
130		ARID3B				3							1												1																								2							
102		ARID4A							1																																															
214		ARID4B						1																	1																															
145		ARID5A								2																																											1			
82		ARID5B																																																						
104		JARID2																																																						
10		ARID3C				2	1																																																	
117		TCF25																																																						
214		TCF12													2	2	3	1		3	4			3	2	3	1							3	2	1	2			3	2	2	4		1		2		5	2			1			6
313		TCF3													2	8	3	4		3	1		2	3	7	6	1		1	1				2	1	2	2	3	2	3	1	1	6		1		3	2	2	2	1	1		1	2	11
478		TCF4													3	3		3		4	2			2	3	3		1		1				4	2	1	2	1		3	2	2	4		1		2		3	2				1	1	6
41		ASCL1													1	4	3																																							
10		ASCL2																																																						
19		ASCL3													3	3	4			2																																				
20		ASCL4													4	1	2																																							
2		ASCL5																																																						
34		MYF5														2																			1							1								1					1	
28		MYF6													3	3	2																																							
244		MYOD1	1	1		1	1		1						2	7	3								2										1																					
91		MYOG													3	6	3													1					1																					
82		ATOH1													1	1																																								
1		ATOH7															1																																							

2		ATOH8														1																																							
98		BHLHA15														1	1									1																								1	1						
12		BHLHA9																																																					
2		BHLHE22																																																					
20		BHLHE23																																																					
27		FERD3L													3	2	4																								1														
19		FIGLA													2	1	2						1		1	1																													
19		HAND1													1	2	1																																						
39		HAND2													2	2	2																				2																			
15		LYL1														3	1																																						1
11		MESP1														2																																							
13		MESP2													3	3	3																																						
16		MSC													2	1	2																																						
36		MSGN1													2	1	2						1											1																						
44		NEUROD1													4	6	4																																						
12		NEUROD2																																																					
10		NEUROD4													1	1	1																																						
4		NEUROD6																																																					
20		NEUROG1													2	3	2																																						
4		NEUROG2														2																																							
195		NEUROG3					2								5	2	3													1																				2							
28		NHLH2													2	2	2						1							1																											
34		OLIG1														1																																							
22		OLIG2														1																																			2				
36		OLIG3								1					1																																					2			
18		PTF1A														1	1																																						
7		SCX														2	1						1																																		
170		TAL1													6	11	6																					1																		2
13		TAL2													2	1	3																																						1
13		TCF15													1	2	1																																						
19		TCF21													4	3	3								1																																
14		TCF23													3	1	2																		1																					
126		TWIST1													1	5	4								1																																
26		TWIST2													4	3	3																	1																						
145		BHLHE40														3									1																																
45		BHLHE41																							2																																
12		HELT																																																					
3		HES2																																																					
4		HES3																																																					
23		HES4																																																					
3		HES5																																																					
53		HES6																																																2							
9		HES7																																																					
162	bHLH	HEY1																							1																											1				
67		HEY2																																		1																				
36		HEYL																																		1																				
226		AHR																																																					
11		AHRR																																																					
423		ARNT																																																					
103		ARNT2																																																					
151		ARNTL																																																					
40		ARNTL2																																																					
128		CLOCK																																																					
243		EPAS1		1																																																		
1186		HIF1A																																																					
12		HIF3A																																																					
382		NCOA1	1																																																				
257		NPAS1																																																					
57		NPAS2																																																					
9		NPAS3																																																					
8		NPAS4																																																					
12		SIM1																																																					
48		SIM2																																																					

114	SOHLH1							1																																															
1	SOHLH2																																																						
4	TCFL5																																																						
508	MAX						2																																																
304	MGA																						1		1																														
80	MITF																																																						
98	MLX																																																						
30	MLXIP																																																						
63	MLXIPL																						1																																
49	MNT																																																						
84	MXD1					3	3																																																
72	MXD3						2																																																
23	MXD4																																																						
83	MXI1																																																						
5563	MYC	3	3	1		2	1							2		1																																							
87	MYCL																																																						
1430	MYCN	1	1											1	1	1																			1	1																			
190	SREBF1																																								2													
170	SREBF2																																																						
197	TFAP4	1		1											1																																								
70	TFE3																																																						
64	TFEB																																																						
17	TFEC																																																						
140	USF1														3																																								
61	USF2																																																						
105	ID1													4	8	7			1	1		2		8	1																1								1						
151	ID2													7	10	7				2		1		3																1	1									2	1				
71	ID3													4	8	8						1		1																	1														
32	ID4													1	2	3			1				2		2									1							1							2		2	2				2
160	TFAP2A																																																						
23	TFAP2B																																																						
33	TFAP2C																																																						
77	TFAP2D																																																						
1	TFAP2E																																																						
97	HES1																																															3						
93	NHLH1				1			1						2	3	2																																							
7	TCF24														1	4																																							
0	USF3																																																						
390	ATF2																																																						
84	ATF7																																																						
298	BACH1			1												3																																							
108	BACH2																																																						
227	CREB5																																																						
880	JUN					1																		2																															
291	JUNB																																																						
104	JUND																																																						
32	NFE2																																																						
67	NFE2L1																																																						
451	NFE2L2																																																						
31	NFE2L3																																																						
185	ATF3																																																						
413	FOS	1	1	1	1	1																																																	
57	FOSB																																																						
97	FOSL1																																																						
169	FOSL2																																																						
37	JDP2																																																						
80	MAF																																																						
19	MAFA																																								2													
263	MAFB																																																						
99	MAFF																																																						
77	MAFG																																																						
51	MAFK																																																						
14	NRL																																																						

46		BATF																																																				
38	bZIP	BATF2																																																				
117		BATF3		1																																																		
66		XBP1																																																				
180		ATF4																																																				
29		ATF5																																																				
70		ATF1																																																				
160		ATF6																																																				
90		ATF6B																																																				
313		CREB1																							1																													
300		CREB3																																																				
279		CREB3L1																																																				
27		CREB3L2																																																				
64		CREB3L3																																																				
13		CREB3L4																																																				
12		CREBL2																																																				
43		CREBZF																																																				
85		CREM																																																				
1366		CEBPA	1	1	1	1	1								1	1																																						
2823		CEBPB				1										1																																						
68		CEBPD																																																				
69		CEBPE																																																				
68		CEBPG																																																				
18		DBP																																																				
194		DDIT3	1	1	1																																																	
11		HLF																																																				
44		NFIL3																																																				
7		TEF																																																				
163		NFYA																																																				
64	CAAT-BF	NFYB																																																				
203		NFYC			2				1																																													
30		CSRNP1																																																				
16	CSRNP_N	CSRNP2																																																				
3		CSRNP3																																																				
856		YBX1																											2																										
305	DBP	YBX2																																																				
380		YBX3																											2																										
442		E2F1				2																																																
54		E2F2				1																																																
88		E2F3																																																				
560		E2F4				1																																																
33		E2F5																																																				
321	FH/WH.E2F	E2F6																																																				
49		E2F7																																																				
26		E2F8																																																				
236		TFDP1																																																				
97		TFDP2																																																				
65		TFDP3																																																				
551		FOXA1											1																																									
30		FOXA2																																																				
53		FOXA3																																																				
133		FOXB1				1	1																																															
0		FOXB2																																																				
256		FOXC1	1			1																																																
44		FOXC2																																																				
6		FOXD1																																																				
29		FOXD2								1																																													
28		FOXD3																																																				
84		FOXD4																																																				
31		FOXD4L1																																																				
16		FOXD4L3																																																				
1		FOXD4L4																																																				
0		FOXD4L5																																																				

76		FOXD4L6																																																						
79		FOXE1					1																																																	
7		FOXE3																																																						
47		FOXF1																																																						
67		FOXF2																																																						
96		FOXG1																																																						
121		FOXH1				1	1			1					1	1																																								
91		FOXI1	1	1					1																																													
52		FOXI2																																																						
2	FH/WH.FOX	FOXI3																																																						
42		FOXJ1																																																						
85		FOXJ2																																																						
33		FOXJ3																																																						
288		FOXK1						2	1																																														
327		FOXK2						2	1																																														
203		FOXL1				2	2		1		1																																												
72		FOXL2																																																						
198		FOXM1															2																																						
37		FOXN1																																																						
29		FOXN2																																																						
62		FOXN3																																																						
7		FOXN4																																																						
433		FOXO1																																																						
169		FOXO3																																																						
52		FOXO4																																																						
16		FOXO6																																																						
274		FOXP1																																																						
116		FOXP2																																																						
118		FOXP3																																																						
123		FOXP4																																																						
139		FOXQ1																																																						
91		FOXR1																																																						
97		FOXR2																																																						
96		FOXS1																																																						
86		RFX1																																																						
31		RFX2																																																						
55		RFX3																																																						
4	FH/WH.RFX	RFX4																																																						
67		RFX5																																																						
51		RFX6																																																						
27		RFX7																																																						
1		RFX8																																																						
87	GCM	GCM1	1	1					1																																													
46		GCM2																																																						
9		GRHL1																																																						
17		GRHL2																																																						
18		GRHL3																																																						
513	GHD.CP2	TFCP2					1																																		1															
8		TFCP2L1																																																						
76		UBP1																																																						
366		GTF2I																																																	1						
129	GTF2I	GTF2IRD1																																																						
13		GTF2IRD2																																																						
19		GTF2IRD2B																																																						
0		ANHX																																																						
0	HB	CPHXL																																																						
17		DUXB																																																						
0		NANOGP8																																																						
227		CUX1																							1																															
9		CUX2																																																						
13		ONECUT1																																																						
6	HB-CUT	ONECUT2																																																						
4		ONECUT3																																																						

175		SATB1																																																				2
179		SATB2																																																				
68	HB-PAX	PAX3																																																				
19		PAX4																																																					
382		PAX6	1	1	1	1	1		1																																													
131		PAX7	1	1		1			1															1																														
29	HB-POU	HDX																																																				
154		HMBOX1																																																					
99		HNF1A				1																			1																													
204		HNF1B	1	1	1	1	1		1		1																																											
38		POU1F1								1																																												
218		POU2F1																																																					
36		POU2F2																																																					
8		POU2F3																																																					
14		POU3F1																																																					
21		POU3F2																																																					
14		POU3F3																																																					
1		POU3F4																																																					
10		POU4F1																																																					
78		POU4F2																																																					
12		POU4F3																																																					
378		POU5F1		1								1					1																																					
0		POU5F1B																																																					
1		POU5F2																																																					
3		POU6F1																																																					
147		POU6F2																																														1							
231	HB-ZF.C2H2	ADNP	1																																																			
71		ADNP2																																																					
177		HOMEZ																																																					
2		NANOGNB																																																					
13		TSHZ1																																																					
87		TSHZ3																																																					
257		ZEB1																						1																															
124		ZEB2																						1																															
7		ZFHX2																																																					
101		ZFHX3																																																					
76		ZFHX4																																																					
116		ZHX1																																																					1
76		ZHX2																																																					
54		ZHX3																																																					
120		CDX1																																																					
41		CDX2																																																					
10		CDX4																																																					
1		EVX1																																																					
9		EVX2																																																					
7		GBX1																																																					
4		GBX2																																																					
46		GSX1																																																					
3		GSX2																																																					
549		HOXA1								1																																												
48		HOXA10																																																					
14		HOXA11																																																					
14		HOXA13																																																					
24		HOXA2																																																					
16		HOXA3																																																					
10		HOXA4																																																					
91		HOXA5																																																					
1		HOXA6																																																					
21		HOXA7																																																					
38		HOXA9																																																					
34		HOXB1																																																					
20		HOXB13																																																					
35		HOXB2		1			1																																															

7	HB.HOX	HOXB3																																																				
6		HOXB4																																																				
159		HOXB5																																																				
64		HOXB6																																																				
36		HOXB7																																																				
16		HOXB8																																																				
148		HOXB9																																																				1
21		HOXC10																																																				
22		HOXC11																																																				
5		HOXC12																																																				
22		HOXC13																																																				
58		HOXC4																																																				
45		HOXC5																																																				
16		HOXC6																																																				
127		HOXC8																																																				
62		HOXC9																																																				
2		HOXD1																																																				
13		HOXD10																																																				
10		HOXD11																																																				
18		HOXD12																																																				
82		HOXD13																																			2																			
14		HOXD3																																																				
28		HOXD4																																																				
11		HOXD8																																																				
10		HOXD9																																																				
118		MEOX1																																																				
771		MEOX2																																																				
27		MNX1																																																				
24		PDX1																																																				
58	HB.LIM	ISL1																																																			
65		ISL2																																																				
110		LHX1	1	1	1	1																																														
190		LHX2	1	1	1	1	1		1																																										
196		LHX3	1	1	1	1																													1																				
220		LHX4	1	1	3	1											1																																					
0		LHX5																																																				
100		LHX6			1		3																												1																				
59		LHX8			1																														2																				
24		LHX9																																																				
13		LMX1A														2																																						
69		LMX1B																																																				
3		BARHL1																																																				
2		BARHL2																																																				
9		BARX1																																																				
2		BARX2																																																				
1		BSX																																																				
1		DBX1																																																				
7		DBX2																																																				
7		DLX1																																																				
21		DLX2																																																				
5		DLX3																																																				
6		DLX4																																																				
21		DLX5																																																				
32		DLX6																																																				
10		EMX1																																																				
36		EMX2																																																				
119		EN1				1	1		1																																													
7		EN2																																																				
54		HHEX																																																				
48		HLX																																																				
0		HMX1																																																				
6		HMX2																																																				
10		HMX3																																																				

27	HB.NK	LBX1																																																				
7		LBX2																																																				
29		MSX1																																																				
201		MSX2																																																				
273		NANOG		1		1	1					1					1																																						
1		NKX1-1																																																				
2		NKX1-2																																																				
185		NKX2-1																																																				
5		NKX2-2																																																				
4		NKX2-3																																																				
12		NKX2-4																																																				
91		NKX2-5										4																																											
1		NKX2-6																																																				
0		NKX2-8																																																				
65		NKX3-1																																																				
13		NKX3-2																																																				
4		NKX6-1																																																				
1		NKX6-2																																																				
0		NKX6-3																																																				
34		NOTO																																																				
180		TLX1	1	1		1	1		1																																													
147		TLX2	1	1		1																																																
265		TLX3	1	1	1	1	1		1																																													
15		VAX1																																																				
11		VAX2																																																				
113		VENTX								1																																												
19	HB.PD	ALX1																																																			
33		ALX3																																																				
19		ALX4																																																				
8		ARGFX																																																				
29		ARX																																																				
285		CRX	1	1	1	1			1																																													
2		DMBX1																																																				
17		DPRX				1																																																	
0		DRGX																																																				
295		DUX4																																		1																			
11		DUXA				1																																																
3		ESX1																																																				
81		GSC		1					1		1																																											
60		GSC2																																																				
17		HESX1																																																				
18		HOPX												1																																								
32		ISX																																																				
46		LEUTX																																																				
0		MIXL1																																																				
5		NOBOX																																																				
9		OTP																																																				
192		OTX1								1																																											
57		OTX2																																																				
9		PHOX2A																																		2																			
4		PHOX2B																																																				
138		PITX1								1																																											
75		PITX2																																																				
5		PITX3																																																				
64		PROP1																																																			1			
9		PRRX1																																																				
0		PRRX2																																																				
3		RAX																																																				
0		RAX2																																																				
3		RHOXF1																																																				
141		RHOXF2								1																																											
0		RHOXF2B																																																				
0		SEBOX																																																				

9		SHOX																																																					
8		SHOX2																																																					
20		TPRX1																																																					
3		UNCX																																																					
77		VSX1				1	1																																																
32		VSX2																																																					
19	HB.PHTF	PHTF1																																																					
37	HB.PROS	PROX1																																																					
2		PROX2																																																					
64	HB.SINE	SIX1																																																					
46		SIX2																																																					
23		SIX3																																																					
46		SIX4																																																					
28		SIX5																																																					
12		SIX6																																																					
2	HB.TALE	IRX1																																																					
6		IRX2																																																					
1		IRX3																																																					
7		IRX4																																																					
4		IRX5																																																					
11		IRX6								1																																													
55		MEIS1																																																					
115		MEIS2																																																					
26		MEIS3																																																					
16		MKX																																																					
105		PBX1																							1																														
99		PBX2																																								1															
115		PBX3																																																					
92		PBX4																																																					
94		PKNOX1																																																					
51		PKNOX2																																																					
47		TGIF1																																																					
21		TGIF2						1	1																																													
37		TGIF2LX						1	1																																													
53		TGIF2LY																																																					
512	HMG	HMGB1																																																					
287		HMGB2																																																					
75		HMGB3																																																					
7		HMGB4																																																					
0		HSFX4																																																					
188		KMT2C																	1						1																														
29		MAEL																																																					
0		MEIOSIN																																																					
122		PMS1																																																					
126		WDHD1																																																					
127	HMG.PBRM1	HMGXB4							1																																														
283		PBRM1	2	1	4																				1																														
132	HMG.SOX	BBX						1																																														
717		CIC						1	1																																													
142		HBP1						1	1																																													
9		SOX1																																																					
79		SOX10	1	1	1				1								1																																						
6		SOX11																																																					
8		SOX12																																																					
47		SOX13																																																					
13		SOX14																																																					
148		SOX15	1	1	1																																																		
80		SOX17	1	1	1	1	1																																																
6		SOX18																																																					
1645		SOX2	4	2	1	3	3		1		1				1	3																																						
14		SOX21																																																					
19		SOX3																																																					
20		SOX30																																																					

82		SOX4																																																				
187		SOX5							1	2																																												
164		SOX6							1																																													
80		SOX7	1	1	1		1				1																																											
11		SOX8																																																				
129		SOX9	1	1	1	1	1		1																																												1	
33		SRY													1																																				1				
119	HMG.TCF7	LEF1																																																				
26		TCF7																																																				
12		TCF7L1																																																				
313		TCF7L2																					1																															
447	HMG.TFAM	TFAM			1																																																	
325	HMG.TOX	HMG20A															2																																		1			
153		HMG20B																																																				
9		HMGXB3																																																					
502		SMARCE1	12	7	9												1								1																													
13		TOX																																																				
34		TOX2																																																				
27		TOX3																																																					
200		TOX4																																																					
277	HMG.UBF	UBTF																																																				
30		UBTFL1																																																				
0	HMG.WHSC1	NSD2																																																				
826	HMG.WHSC2	SSRP1	1																																																			
483	HMGA	HMGA1																																																				
151		HMGA2																																																				
717	HSF	HSF1																																																				
114		HSF2																																																				
68		HSF4																																																				
6		HSF5																																																				
25		HSFX1																																																				
0		HSFX3																																																				
51		HSFY1																																																				
273	HTH	LCOR										1																																										
24		LCORL																																																				
10	IPT	CAMTA1																																																				
25		CAMTA2																																																				
116	LRRFIP	LRRFIP1																																																	1				
137		LRRFIP2																																																				
104	MADS	MEF2A																3								1										2																				
9		MEF2B																																																				
61		MEF2C																1							2	1																													
114		MEF2D																1																																				
163		SRF			1		1																			2																													
21	NDT80	MYRF																																																				
188	NF1	NFIA	1	1		1	1		1																1																													
176		NFIB	1	1		1	1																																															
227		NFIC	1	1		1	1		1																																													
222		NFIX	1	1	1	1	1		1																																													
84	NRF	NRF1																																																				
605		SFPQ																																																				
147		AFF1																																																				
5		AFF2																																																				
21		AFF3																																																				
197		AFF4																																																				
181		AHCTF1																																																				
7		AKNA																																																				
8		ANKRD30A																																																				
363		BCLAF1																																																				
193		BLZF1																																																				
42		CAPN15																																																				
4		CARF																																																				
50		CARHSP1																																																				

161	others	CBFB													1										2																																
159		CC2D1A																																																					
276		CCDC88A																																																					
18		CDIP1																																																					
290		CEBPZ																																																					
0		CENPX																																																					
80		CIZ1																																																					
348		CNBP																																																					
159		CPEB1																																																					
12		CREBRF																																																					
5		CSDC2																																																					
261		CSDE1																																																					
124		DACH1														1																																							
16		DACH2																																																					
43		FAM170A																																																					
62		GABPB1																																																					
17		GABPB2																																																					
86		GCFC2																																																					
0		GLMP																																																					
51		GON4L																																																					
94		GPBP1																																																					
133		GPBP1L1																																																					
274		KHSRP																																																					
97		KIAA1549																																																					
175		L3MBTL3																																																					
117		LITAF								1																																													
165		LTF																																																					
53		MAF1																																																					
72		MLLT10																																																					
141		MYEF2																																																					
1		MYRFL																																																					
188		NFRKB																																																					
292		NKRF																																																					
193		NME2																																																					
53		PARP12																												2																										
123		PAXBP1																																																					
1230		PHB																							1																															
11		PLEK																																																					
203		PLSCR1																																																					
20		PRDM11																																																					
191		PREB																																																					
67		RAG1																																																					
364		RBCK1																																																					
37		SETBP1																																																					
5		SLC2A4RG																																																					
58		SMARCAL1																																																					
177		SON														1											1																													
34		SPZ1																																																					
200		SUB1																																																					
463		TAX1BP1																																																					
177		TET2														1																																							
91		TFB1M																																																					
25		THYN1																																																					
108		TMF1																																																					
137		XPA				1																																																	
331		YEATS4																																																					
5340	p53	TP53	3			2	2				1						2																																						
249		TP63															1																																						
350		TP73																																																					
5	PAX	PAX1																																																					
83		PAX2		1					1																																														
73		PAX5																																																					
117		PAX8	1	1																																																			

133		PAX9	1	1					1																																													
43	PHDFP	TCF19																																																				
1	pseudogene	ZNF883																																																				
253	PUR	PURA																																																				
151		PURB																																																				
222		PURG																																																				
170	RFXANK	RFXANK																																																				
533	RHR	NFKB1																																			3																		
185		NFKB2																																																				
502		REL																																														1							
986		RELA															1																																					
131		RELB																																																				
46		NFAT5																																																				
204		NFATC1	1																																																			
229		NFATC2	1												1																																							
44		NFATC3																																																				
45		NFATC4																																																				
383		RBPJ					2																																													1		
9		RBPJL																																																			1		
16		EBF1																																																				
58		EBF2																																																				
21		EBF3																																																				
4		EBF4																																																				
403	RUNT	RUNX1	1			1					1				1	2									2																												1			1
122		RUNX2																																																				
83		RUNX3																																																				
139	SAND	AIRE																																																				
54		DEAF1																																																				
58		GMEB1																																																				
63		GMEB2																																																				
132		SP110																																																				
256		SP100				3									1																																							
10		SP140																																																				
10		SP140L																																																				
305	SMAD	SMAD1																																											1									
616		SMAD2	1	1											1																																							
909		SMAD3	1												1																																							
799		SMAD4																							1																															
111		SMAD5																																																				
91		SMAD6																																																				
270		SMAD7																							1																															
150		SMAD9		1																																																		
50	SNAD	SAMD11																																																				
447	STAT	STAT1																																																				
156		STAT2																																																				
671		STAT3																							3																															
32		STAT4																																																				
155		STAT5A													1																																		1					
106		STAT5B																																																				
89		STAT6																																																				
17	TB	TBX19																																																				
0		TBXT																																																				
11		TBX1																																																				
1		TBX10																																																				
15		TBX15																																																				
7		TBX18																																																				
37		TBX20																																																				
14		TBX21																																																				
11		TBX22																																																				
25		TBX2																																																				
72		TBX3																																																				
4		TBX4																																																				
157		TBX5				1																																																	

46		TBX6																																																					
2		EOMES																																																					
117		TBR1	1	1					1																																													
203	TEA	TEAD1	1	1	1	1	1		1		1																																											
146		TEAD2					1																																															
46		TEAD3																																																					
138		TEAD4																																																					
115	TSC22	TSC22D1																																																				
74		TSC22D2			1																																																	
98		TSC22D3																						2																														
201		TSC22D4																																																					
26	TUB	TUB																																																				
3		TULP1																																																				
437		TULP3											1																																									
22		TULP4																																																					
15	WC.ETS	EHF																																																				
121		ELF1			1				1																																													
164		ELF2						1	1																																													
64		ELF3																																																				
118		ELF4			1				1																																													
54		ELF5	1	1	1																																																	
166		ELK1																																																				
119		ELK3							1							2																																						
87		ELK4											1																																									
129		ERF																																																				
315		ERG	1	1		1			1																																	1												
126		ETS1	1	1	1				1																																													
81		ETS2																																																				
41		ETV1																																																				
4		ETV2																																																				
133		ETV3			1																																																	
1		ETV3L																																																				
80		ETV4	1	1																																																		
49		ETV5																																																				
140		ETV6																																																				
34		ETV7																																																				
202		FEV	1	1	1	1			1																																													
71		FLI1	1	1	1																																																	
142		GABPA																																																				
55		SPDEF																																																				
94		SPI1																																																				
19		SPIB																																																				
5		SPIC																																																				
126	WC.IRF	IRF1	1	1	1																																																	
137		IRF2																																																				
345		IRF3																																																				
94		IRF4	1	1	1																																																	
70		IRF5																																																				
14		IRF6																																																				
151		IRF7																																																				
72		IRF8							1																																													
56		IRF9					1																																															
1041		CDC5L	1																																																			
8		DMTF1																																																				
418		DNAJC1																																																				
253		DNAJC2																																																				
0		MIDEAS																																																				
90		MIER1									1																																											
32		MIER3																																																				
176		MYB				1	1																																															
25		MYBL1																																																				
217		MYBL2							1																																													
579		NCOR1																						2																														

466		NCOR2																							1																															
603		RCOR1									1					1	1																																						1
44		RCOR2																																																					
235	WC.MYB	RCOR3																																																					
487		SMARCC1	11	7	3																				1																															
461		SMARCC2	10	8	5												1								1																															
82		SNAPC4																																																					
287		TADA2A														1																																								
183		TADA2B																							1																															
43		TRERF1																																																					
3		ZNF541																																																						
87		MIS18BP1																																																					
62		MYSM1																																																					
0		TERB1																																																					
488		TERF1																																																					
487		TERF2																																																					
128		TTF1																																																					
198		ZZZ3																							1																															
48	ZF	ZNF750																																																					
0	ZF.BED	ZFTA																																																					
11		MYT1																																																					
7		MYT1L																																																					
5		ST18																																																					
143		L3MBTL1																																																					
444		LYAR																																																					
183		THAP1																																																					
30		THAP10																																																					
165		THAP11																																																					
0	ZF.C2CH	THAP12																																																					
22		THAP2																																																					
74		THAP3																																																					
79		THAP4																																																					
7		THAP5																																																					
21		THAP6																																																					
139		THAP7																																																					
33		THAP8																																																					
11		THAP9																																																					
82		EGR1			1																																																		
168		EGR2	1	1	1	1			1																																														
1		EGR3																																																					
4		EGR4																																																					
43		KLF1	1																																																				
54		KLF10							1																																														
26		KLF11																																																					
220		KLF12				1	1		1																																														
26		KLF13																																																					
0		KLF14																																																					
308		KLF15			1	1			1																																														
451		KLF16			1				1																																														
3		KLF17																																																					
22		KLF2																																																					
287		KLF3	1	1	2	1	1	1	2		2																																												
221		KLF4	1	1																																																			
272		KLF5	1	1	1	1	1		1																																														
99		KLF6			1																																																		
8		KLF7																																																					
340		KLF8			1				1																																														
90		KLF9			1			1	1																																														
693		SP1							1																1	2																													3
44		SP2																																																					
103		SP3																																																					
54		SP4																																																					
3		SP5																																																					

33	SP6																																																						
305	SP7	1	1	1	1	1		1		1																																											
3	SP8																																																						
1	SP9																																																						
105	AEBP2										7																																												
6	OSR1																																																						
0	OVOL3																																																						
22	ZBTB22																																																						
84	ZBTB32																																																						
174	ZBTB33																																																						
32	ZBTB34																																																						
6	ZBTB37																																																						
61	ZBTB43																																																						
55	ZBTB46																																																						
71	ZBTB5																																																						
11	ZBTB8B																																																						
41	ZFPM2																																																						
97	ZNF174																																																						
51	ZNF396																																																						
152	ZNF414																																																						
150	ZNF446																																																						
2	ZNF487																																																						
59	ZNF511																																																						
32	ZNF580																																																						
0	ZNF705A																																																						
24	ZNF740																																																						
75	ZSCAN1																																																						
330	BCL6																																																						
38	BCL6B																																																						
541	CTCF	1																																				1																	1
46	CTCFL																																																						
28	FEZF1																																																						
5	FEZF2																																																						
40	GFI1																																																						
136	GFI1B																																																						
192	GLI1																																																						
82	GLI2																																																						
97	GLI3																																																						
127	GLI4																																																						
17	GLIS1														1																																								
111	GLIS2								1																																														
40	GLIS3																																																						
12	GTF3A																																																						
86	GZF1																																																						
116	MTF1																																																						
13	MYNN																																																						
35	MZF1																																																						
8	OSR2																																																						
16	OVOL1					1																																																	
141	OVOL2																																																						
9	PLAG1																																																						
28	PLAGL1																																																						
60	PLAGL2								1																																											1			
106	PRDM1																																																						
132	PRDM14																																																						
81	PRDM5																																																						
94	PRDM6								1							1																																							
67	RBAK																																																						
9	SCRT1																																																						
9	SCRT2																																																						
728	SNAI1																																																						
80	SNAI2																																																						
14	SNAI3																																																						

92	WT1																																																						
693	YY1			1				1							1																																								
15	YY2																																																						
121	ZBTB11																																																						
8	ZBTB12																																																						
103	ZBTB14																																																						
305	ZBTB16																																																						
77	ZBTB18																																																						
40	ZBTB20																																																						
198	ZBTB24																																																						
47	ZBTB26																																																						
24	ZBTB40																																																						
77	ZBTB42																																																						
81	ZBTB44																																																						
6	ZBTB45																																																						
146	ZBTB47																																																						
234	ZBTB48																																																						
39	ZBTB49																																																						
17	ZBTB6																																																						
193	ZBTB7A	1																																																					
81	ZBTB7B																																																						
23	ZBTB7C																																																						
61	ZFP1																																																						
8	ZFP14																																																						
18	ZFP2																																																						
5	ZFP28																																																						
3	ZFP3																																																						
12	ZFP30																							1																															
6	ZFP37																																																						
124	ZFP41																																																						
16	ZFP42																																																						
84	ZFP62																																																						
51	ZFP64																																																						
1	ZFP69																																																						
13	ZFP69B																																																						
12	ZFP82																																																						
30	ZFP90																																																						
152	ZFP91																												2																			1							
2	ZFP92																																																						
17	ZFX																																																						
14	ZFY																																																						
152	ZIC1								1																																														
43	ZIC2																																																						
25	ZIC3																																					1																	
2	ZIC4																																																						
5	ZIC5																																																						
3	ZIK1																																																						
57	ZIM2																																																						
3	ZIM3																																																						
91	ZKSCAN1																																																						
8	ZKSCAN2																																																						
43	ZKSCAN3																																																						
131	ZKSCAN4																																																						
16	ZKSCAN5																																																						
27	ZKSCAN7																																																						
166	ZKSCAN8																																																						
25	ZNF10																																																						
25	ZNF100																																															2							
33	ZNF107																																																						
37	ZNF114																																																						
1	ZNF117																																																						
54	ZNF121																							1																															
70	ZNF124															2																																							

149		ZNF131																																																				
37		ZNF133																																																				
10		ZNF135																																																				
45		ZNF136																																																				
25		ZNF138																																																				
7		ZNF14																																																				
11		ZNF140																																																				
2		ZNF141																																																				
55		ZNF143																																																				
101		ZNF146																																																				
141		ZNF148																																																				
27		ZNF154																																																				
10		ZNF155																																																				
8		ZNF157																																																				
37		ZNF16																																																				
8		ZNF160																																																				
64		ZNF165																																																				
85		ZNF169																																																				
36		ZNF17																																																				
13		ZNF175																																																				
24		ZNF177																																																				
21		ZNF18																																																				
9		ZNF180																																																				
8		ZNF181																																																				
4		ZNF182																																																				
75		ZNF184																																																				
60		ZNF189																																																				
30		ZNF19																																																				
32		ZNF197																																																				
120		ZNF2																																																				
48		ZNF20																																																				
14		ZNF200																																																				
35		ZNF202																																																				
22		ZNF205																																																				
1		ZNF208																																																				
70		ZNF212																																																				
42		ZNF213																																																				
22		ZNF214																																																				
22		ZNF215																																																				
146		ZNF22																																																				
11		ZNF221																																																				
0		ZNF222																																																				
48		ZNF223																																																				
24		ZNF224																																																				
6		ZNF225																																																				
11		ZNF226																																																				
19		ZNF227																																																				
4		ZNF229																																																				
28		ZNF23			1																																																	
11		ZNF230																																																				
102		ZNF232																																																				
2		ZNF233																																																				
7		ZNF234																																																				
14		ZNF235																																																				
11		ZNF239																																																				
222		ZNF24																																																				
5		ZNF25																																																				
161		ZNF250																					1																																	
6		ZNF253																																																				
14		ZNF254																																																				
4		ZNF257																																																				
13		ZNF26																																																				
15		ZNF260																																																				

213		ZNF263																																																				
58		ZNF264																																																				
17		ZNF266																																																				
16		ZNF267																																																				
17		ZNF268																																																				
27		ZNF273																																																				
30		ZNF274																																																				
24		ZNF275																																																				
34		ZNF276																																																				
10		ZNF28																																																				
16		ZNF280A																																																				
5		ZNF280B																																																				
70		ZNF280C																																																				
30		ZNF280D																																																				
197		ZNF281																						1																								1							
28		ZNF282																																																				
1		ZNF283																																																				
16		ZNF284																																																				
1		ZNF285																																																				
37		ZNF286A																																																				
1		ZNF286B																																																				
12		ZNF287																																																				
41		ZNF3																																																				
7		ZNF30																																																				
5		ZNF300																																																				
29		ZNF302																																																				
11		ZNF311																																																				
80		ZNF317																																																				
16		ZNF32																																																				
28		ZNF320																																																				
4		ZNF322																																																				
68		ZNF324																																																				
149		ZNF324B																																																				
33		ZNF329																																																				
102		ZNF331																																																				
22		ZNF333																																																				
15		ZNF337																																																				
13		ZNF33A																																																				
8		ZNF33B																																																				
53		ZNF34																																																				
25		ZNF343																																																				
6		ZNF345																																																				
0		ZNF347																																																				
48		ZNF35																																																				
41		ZNF350																																																				
50		ZNF354A																																																				
4		ZNF354B																																																				
41		ZNF354C																																																				
22		ZNF358																																																				
42		ZNF362																																																				
9		ZNF366																																																				
19		ZNF383																																																				
73		ZNF384																																																				
7		ZNF391																																																				
21		ZNF394																																																				
126		ZNF397																																																				
60		ZNF398																																																				
2		ZNF404																																																				
222		ZNF408																																																				
11		ZNF41																																																				
62		ZNF410																																																				
6		ZNF415																																																				
13		ZNF416																																																				

10	ZNF419																																																						
46	ZNF420																																																						
1	ZNF425																																																						
56	ZNF426																																																						
0	ZNF429																																																						
1	ZNF43																																																						
37	ZNF430																																														2							
27	ZNF431																																																						
8	ZNF432																																																						
13	ZNF433																																																						
20	ZNF436																																																						
36	ZNF439																																																						
19	ZNF44																																																						
51	ZNF440																																																						
9	ZNF441																																																						
2	ZNF442																																																						
3	ZNF443																																																						
155	ZNF444																																																						
13	ZNF449																																																						
9	ZNF45																																																						
0	ZNF454																																																						
156	ZNF460																																																						
8	ZNF461																																																						
7	ZNF468																																																						
19	ZNF471																																																						
8	ZNF479																																																						
9	ZNF480																																																						
80	ZNF483																																																						
13	ZNF484																																																						
31	ZNF485		1																																																				
65	ZNF490																																																						
29	ZNF491																																																						
11	ZNF492																																																						
4	ZNF493																																																						
85	ZNF496																																														2							
46	ZNF497																																																						
10	ZNF500																																																						
14	ZNF501																																																						
4	ZNF502																																																						
2	ZNF506																																																						
21	ZNF514																																																						
45	ZNF517																																																						
69	ZNF524																																																						
3	ZNF525																																																						
4	ZNF528																																																						
6	ZNF529																																																						
2	ZNF530																																																						
3	ZNF534																																																						
2	ZNF540																																																						
21	ZNF543																																																						
19	ZNF544																																																						
2	ZNF546																																																						
16	ZNF547																																																						
4	ZNF548																																																						
5	ZNF549																																																						
95	ZNF550																																																						
1	ZNF551																																																						
13	ZNF554																																																						
15	ZNF555																																																						
21	ZNF556																																																						
18	ZNF557																																																						
33	ZNF558																																																						
18	ZNF559																																																						

0	ZF.C2H2	ZNF560																																																				
14		ZNF561																																																				
36		ZNF562																																																				
17		ZNF563																																																				
55		ZNF564																																																				
3		ZNF565																																																				
17		ZNF566																																																				
14		ZNF567																																																				
7		ZNF568																																																				
12		ZNF569																																																				
17		ZNF57																																																				
2		ZNF570																																																				
7		ZNF571																																																				
71		ZNF572																																																				
26		ZNF575																					1																																	
38		ZNF576																																																				
5		ZNF577																																																				
143		ZNF581																																																				
6		ZNF582																																																				
3		ZNF583																																																				
11		ZNF584																																																				
4		ZNF585A																																																				
6		ZNF585B																																																				
6		ZNF586																																																				
17		ZNF589																																																				
6		ZNF594																																																				
7		ZNF595																																																				
2		ZNF596																																																				
10		ZNF599																																																				
3		ZNF600																																																				
2		ZNF605																																																				
12		ZNF607																							1																														
9		ZNF610																																																				
13		ZNF611																																																				
6		ZNF613																																																				
8		ZNF614																																																				
5		ZNF615																																																				
19		ZNF616																																																				
2		ZNF619																																																				
85		ZNF620																																																				
17		ZNF621																																																				
10		ZNF623																																																				
50		ZNF624																																																				
14		ZNF625																																																				
3		ZNF626																																																				
28		ZNF627																																																				
21		ZNF641																																																				
64		ZNF648																																																				
13		ZNF649																																																				
6		ZNF652																																																				
53		ZNF653																																																				
190		ZNF655																																																				
8		ZNF66																																																				
7		ZNF660																																																				
10		ZNF662																																																				
30		ZNF664																																																				
2		ZNF665																																																				
8		ZNF667																																																				
77		ZNF669																																																				
31		ZNF670																																																				
11		ZNF672																																																				
13		ZNF675																																																				
1		ZNF676																																																				

26		ZNF677																																																				
3		ZNF678																																																				
7		ZNF679																																																				
4		ZNF680																																																				
1		ZNF681																																																				
3		ZNF682																																																				
9		ZNF683																																																				
20		ZNF684																																																				
99		ZNF689																																																				
36		ZNF691																																																				
64		ZNF692																																																				
33		ZNF695																																																				
81		ZNF696																																																				
29		ZNF7																																																				
46		ZNF70																																																				
14		ZNF701																																																				
1		ZNF705D																																																				
0		ZNF705E																																																				
1		ZNF705G																																																				
147		ZNF707																																																				
11		ZNF709																																																				
108		ZNF71																																																				
4		ZNF710																																																				
5		ZNF713																																																				
3		ZNF716																																																				
14		ZNF718																																																				
10		ZNF721																																																				
11		ZNF726																																																				
3		ZNF727																																																				
1		ZNF729																																																				
1		ZNF730																																																				
4		ZNF732																																																				
2		ZNF736																																																				
2		ZNF737																																																				
38		ZNF74																																																				
346		ZNF746																						1																															
54		ZNF747																																																				
5		ZNF749																																																				
9		ZNF75A																																																				
1		ZNF75D																																																				
112		ZNF76																																																				
5		ZNF763																																																				
124		ZNF764																																																				
23		ZNF765								1																																												
11		ZNF766																																																				
135		ZNF768																																																				
25		ZNF77																																																				
72		ZNF771																																																				
83		ZNF774																																																				
16		ZNF776																																																				
114		ZNF777																																																				
26		ZNF780A																																																				
1		ZNF780B																																																				
1		ZNF781																																																				
1		ZNF782																																																				
152		ZNF785																																																				
4		ZNF789																																																				
12		ZNF79																																																				
4		ZNF790																																																				
22		ZNF791																																																				
6		ZNF793																																																				
2		ZNF799																																																				
10		ZNF80																																																				

9	ZNF805																																																						
9	ZNF808																																																						
7	ZNF81																																																						
17	ZNF813																																																						
26	ZNF816																																																						
5	ZNF823																																																						
27	ZNF829																																																						
12	ZNF83																																																						
68	ZNF835																					1																																	
5	ZNF836																																																						
80	ZNF837																																																						
14	ZNF84																																																						
4	ZNF841																																																						
15	ZNF844																																																						
35	ZNF845																																																						
4	ZNF846																																																						
8	ZNF85																																																						
13	ZNF852																																																						
0	ZNF853																																																						
4	ZNF860																																																						
0	ZNF875																																																						
3	ZNF878																																																						
4	ZNF879																																																						
1	ZNF880																																																						
4	ZNF891																																																						
2	ZNF90																																																						
15	ZNF91																																																						
70	ZNF92																																																						
9	ZNF93																																																						
1	ZNF98																																																						
3	ZNF99																																																						
3	ZSCAN10																																																						
86	ZSCAN12																																																						
18	ZSCAN16																																																						
8	ZSCAN2																																																						
132	ZSCAN21																																																						
45	ZSCAN22																																																						
49	ZSCAN23																																																						
90	ZSCAN25																																																						
65	ZSCAN26																																																						
37	ZSCAN29																																																						
24	ZSCAN30																																																						
23	ZSCAN31																																																						
131	ZSCAN32																																																						
17	ZSCAN4																																																						
57	ZSCAN5A																																																						
5	ZSCAN5B																																																						
25	ZSCAN9																																																						
18	ZXDA																																																						
20	ZXDB																																																						
37	ZXDC																																																						
164	BCL11A							1																																															
122	BCL11B							1		1																																													
6	BNC1																																																						
12	BNC2																																																						
42	CASZ1																																																						
165	E4F1																																																						
21	FIZ1																																																						
70	HIC1	2																																																					
70	HIC2																																																						
55	HINFP																																																						
212	HIVEP1																							1																															1
38	HIVEP2																																																						

21		HIVEP3																																																					
467		IKZF1																																																					
36		IKZF2																																																					
421		IKZF3																																																					
52		IKZF4																																																					
124		IKZF5																																																					
18		INSM1																																																					
4		INSM2																																																					
112		MAZ																																																					
470		MECOM		1																																																		
187		PATZ1																																																					
15		PEG3																																																					
75		PRDM10																											2																										
10		PRDM13																																																					
85		PRDM15																																																					
205		PRDM16																																																					
51		PRDM2																																																					
24		PRDM4																																																					
5		PRDM8																																																					
8		PRDM9																																																					
312		REST										1																																										
65		RLF																																																					
99		RREB1																																																					
117		SALL1					1																																															
118		SALL2																						1																														
19		SALL3																																																					
40		SALL4																																																					
99		VEZF1																																																					
282		WIZ																						1																														
149		ZBTB1																																																					
104		ZBTB17																																																					
500		ZBTB2																																																					
189		ZBTB21																																																					
63		ZBTB25																																																					
177		ZBTB38									1																																											
30		ZBTB39																																																					
38		ZBTB4																																																					
14		ZBTB41																																																					
11		ZFAT																																																					
14		ZFP57																																																					
21		ZFPM1																																																					
41		ZNF101																																																					
0		ZNF132																																																					
2		ZNF134																																																					
19		ZNF142																																																					
66		ZNF195																																																					
24		ZNF211																																																					
121		ZNF217																																																					
143		ZNF219							1																																													
9		ZNF236																																																					
11		ZNF248																																																					
20		ZNF251																																																					
3		ZNF256																																																					
38		ZNF277																																																					
67		ZNF292																																																					
33		ZNF296																																																					
6		ZNF304																																																					
58		ZNF316																																																					
26		ZNF319																																																					
3		ZNF334																																																					
31		ZNF335																																																					
19		ZNF341																																																					
3		ZNF37A																																																					

9		ZNF382																																																				
11		ZNF407																																																				
218		ZNF417															1						1																																	
22		ZNF418																																																				
49		ZNF423																																																				
72		ZNF438																																																				
78		ZNF445																																																				
80		ZNF462																																																				
174		ZNF467																																																				
7		ZNF469																																																				
158		ZNF48																																																				
65		ZNF507																																																				
22		ZNF510																																																				
335		ZNF512																																																				
230		ZNF512B							1																																															
8		ZNF513																																																				
70		ZNF516																																																				
1		ZNF519																																																				
46		ZNF521																																																				
80		ZNF526																																																				
22		ZNF532																																																				
64		ZNF536																																																				
44		ZNF552																																																				
250		ZNF574																																																				
90		ZNF579																												1																			2							
160		ZNF587															2						1																																	
91		ZNF592																																																				
45		ZNF597																																																				
5		ZNF606																																																				
48		ZNF618																																																				
15		ZNF628																																																				
131		ZNF629																																																				
36		ZNF639																																																				
46		ZNF646																																																				
2		ZNF654																																																				
11		ZNF658																																																				
278		ZNF668																																																				
3		ZNF671																																																				
8		ZNF674																																																				
146		ZNF687																																																				
14		ZNF697																																																				
39		ZNF711																																																				
2		ZNF717																																																				
106		ZNF770																																																				
8		ZNF772																																																				
27		ZNF775																																																				
10		ZNF778																																																				
31		ZNF784																																																				
25		ZNF786																																																				
45		ZNF787																																																				
0		ZNF788P																																																				
68		ZNF792																																																				
51		ZNF800																																																				
1		ZNF814																																																				
160		ZSCAN20																																																				
144		ZBED1																																																				
5		ZBED2																																																				
10		ZBED3																																																				
31		ZBED4																																																				
9		ZBED6																																																				
21		ATMIN																																																				
97		JAZF1																																																				
0		KLF18																																																				

23		L3MBTL4																																																				
3		PRDM12																																																				
132		REPIN1																																																				
170		ZBTB10																																																				
62		ZBTB3																																																				
137		ZBTB8A																																																				
11		ZNF112																																																				
19		ZNF12																																																				
116		ZNF207																																																				
154		ZNF318																																																				
230		ZNF326																																																				
0		ZNF367																																																				
20		ZNF385A																																																				
4		ZNF385B																																																				
29		ZNF395																																																				
2		ZNF470																																																				
30		ZNF473																																																				
2		ZNF486																																																				
25		ZNF488																																																				
64		ZNF503		1						1																																										
2		ZNF527																																																				
7		ZNF573																																																				
20		ZNF578																																																				
8		ZNF587B																																																				
93		ZNF608																						1																															
158		ZNF609										1												1																															
152		ZNF622																																																				
0		ZNF630																																																				
120		ZNF688												1		1																																						
20		ZNF69																																																				
13		ZNF699																																																				
0		ZNF700																																																				
40		ZNF704						2	2																																													
0		ZNF705B																																																				
5		ZNF706																																																				
19		ZNF708																																																				
17		ZNF714																																																				
6		ZNF720																																																				
0		ZNF723																																																				
1		ZNF728																																																				
1		ZNF735																																																				
10		ZNF761																																																				
13		ZNF773																																																				
14		ZNF783																																																				
48		ZNF8																																																				
13		ZNF821																																																				
30		ZNF827																																																				
5		ZNF831																																																				
6		ZNF850																																																				
57		ZNF865																																																				
10		ZNF888																																																				
56		ZSCAN18																																																				
246	ZF.C3H	ZGPAT																																																				
201		GATA1	1	1																																																		2
223		GATA2	1	1	1	1	1		1		1																																											1
206		GATA3	1	1		1	1																																															1
457		GATA4	1				1					5			1																						2																			
11		GATA5								1																																												
25		GATA6																																																					
190		GATAD1						1	2																																													
315	ZF.C4_GATA	GATAD2A							1																																													
358		GATAD2B							1																																													
520		MTA1							1																																													

586		MTA2							1																																														
254		MTA3							1																																														
106		RERE																																																						
166		TRPS1																							1																														
0		ZGLP1																																																						
1689	ZF.C4_NR	AR	4	4	1		2			1	3																																													
3355		ESR1	1		1					5		1													2																														
2403		ESR2			1	1				1	1			1											1																														
92		ESRRA																																																						
121		ESRRB																																																						
108		ESRRG																																																						
1749		NR3C1	2	2	1		2		1		1																																												1
48		NR3C2																																																						
134		PGR	1																																																					
30		NR1D1																																																						
72		NR1D2																																																						
101		NR1H2																																																						
89		NR1H3																																																						
47		NR1H4																																																						
99		NR1I2																																																						
80		NR1I3																																																						
123		PPARA																																																						
159		PPARD																																																						
689		PPARG		1	1												1																																						
367		RARA	1							1																																													
36		RARB	1																																																					
59		RARG	1																				1																																	
48		RORA																							1																														
44		RORB																																																						
47		RORC																																																						
165		THRA								1																																													
135		THRB																																																						
254		VDR																																																						
344		HNF4A	1	1	1	1	1		1																																														
17		HNF4G																																																						
119		NR2C1																							1																														
1448		NR2C2																																																						
72		NR2E1																																																						
26		NR2E3																																																						
75		NR2F1																																																						
189		NR2F2																							1																														
54		NR2F6																																																						
344		RXRA								1															2																														
96		RXRB																																																						
67		RXRG																																																						
277		NR4A1															1																																						
33		NR4A2																																																						
51		NR4A3																																																						
55		NR5A1																																																						
51		NR5A2																																																						
8		NR6A1																																																						
47		NR0B1																																																						
155		NR0B2																																									3													
33	ZF.CCCH	ZFP36L1																																																						
475	ZF.CCHC	LIN28A																																																						
248		LIN28B																																																						
93	ZF.CXXC	MBD1																																																						
88		BAZ2A																																																						
65		BAZ2B																																																						
247		MBD2							1																																														
416		MBD3							1												1					1																													
112		MBD4																																																						
1462		MECP2	1	1	1																																																		

275		SETDB1										3																																											
29		SETDB2																																																						
5	ZF.DM	DMRT1																																																						
3		DMRT2																																																						
101		DMRT3														1																																							
3		DMRTA1																																																						
0		DMRTA2																																																						
142		DMRTB1																																																						
1		DMRTC1B																																																						
2		DMRTC2																																																						
505	ZF.MIZ	PIAS1																																																						
255		PIAS2																																																						
126		PIAS3																																																						
252		PIAS4														4																																							
52		ZMIZ1																																																						
69		ZMIZ2																																																						
61	ZF.NFX1	ZNFX1																																																						
498		NFX1																																																						
119		NFXL1																																																						

											3	1	2													TAL2	bHLH	7	13
											1	2	1													TCF15		5	13
			1								3	3	4													TCF21		13	19
											2	1	3													TCF23		7	14
			1								4	5	1													TWIST1		43	126
											3	3	4													TWIST2		19	26
			1									3														BHLHE40		22	145
			2																							BHLHE41		16	45
																										HELT		1	12
																										HES2		0	3
																										HES3		0	4
																										HES4		0	23
																										HES5		2	3
																										HES6		11	53
																										HES7		0	9
			1																							HEY1		26	162
																										HEY2		15	67
																										HEYL		6	36
																										AHR		66	226
																										AHRR		4	11
																										ARNT		208	423
																										ARNT2		31	103
																										ARNTL		47	151
																										ARNTL2		14	40
																										CLOCK		42	128
																								1		EPAS1		61	243
																										HIF1A		190	1186
																										HIF3A		9	12
																									1	NCOA1		271	382
																										NPAS1		20	257
																										NPAS2		28	57
																										NPAS3		5	9
																										NPAS4		5	8
																										SIM1		8	12
																										SIM2		25	48
																		1								SOHLH1		3	114
																										SOHLH2		0	1
																										TCFL5		3	4
																			2							MAX		232	508
	1		1																							MGA		77	304
																										MITF		32	80
																										MLX		44	98
																										MLXIP		7	30
			1																							MLXIPL		16	63
																										MNT		31	49
																			3	3						MXD1		30	84
																			2							MXD3		20	72
																										MXD4		12	23
																										MXI1		18	83
											1		2							1	2		1	3	3	MYC		456	5563
																										MYCL		9	87
											1	1	1											1	1	MYCN		120	1430
																										SREBF1		30	190
																										SREBF2		27	170
												1											1		1	TFAP4		69	197
																										TFE3		28	70
																										TFEB		15	64
																										TFEC		8	17
												3														USF1		56	140
																										USF2		33	61
		1	8		2		1	1			7	8	4													ID1		45	105
			3		1		2				7	10	7													ID2		50	151
			1		1						8	8	4													ID3		40	71
		2		2				1			3	2	1													ID4		23	32
																										TFAP2A		36	160
																										TFAP2B		10	23
																										TFAP2C		8	33
																										TFAP2D		10	77
																										TFAP2E		0	1
																										HES1		21	97
											2	3	2						1			1				NHLH1		26	93
											4	1														TCF24		5	7
																										USF3		0	0
																										ATF2		93	390
																										ATF7		41	84
											3												1			BACH1		92	298
																										BACH2		58	108
																										CREB5		58	227
			2																		1					JUN		298	880
																										JUNB		88	291
																										JUND		47	104

					1																																																																										
			1																																																							1																	
															1	1																																		1																													
																																																			1																													
																																																											1																			
																																																										1	1	1	1																	
																																																				2																												
													2																																																		2																	
																																																													1									3											
																																																														2		2																	
															1																																															1		2																	
1																																																																2																	
					1								1																									1																																																	
				1																																															2																												
						2	1																																																																										
						1	6																	1		1																																																									
				1									2																																																											2											
															2	7				1																																																				4											
															7					1																																																				1											
																		2		32	1	2				1		4																																																											
																				3																																																																
															1	1		32	3	4				2	15	70	5	3	2	3	1	2	5	13															1																													2		
																		1							2	3			2	1	2	2	2	6																																														
																		2				2	2	14	1	2				8		1						1																																												1		
																						2		4	1					4																																																							
							1													2		14	4							2								1																1																											
																				15	2	1	1		4	1	2											1		2																		2					2													1		
							1											1		70	3	2			1	2	2	1										1										3																													3		
																				5					2	2																																																						
																		4		3						1		2																																																					4		
																				2	2																																																												
																				3	1	8	4	2																																																									
																				1	2																																																												
																				2	2	1																																																										
																				5	2																																																												
																				13	6																																																												

																																																										3																						
				1																		1		1	1	1												8	7					9	14	9	8	14	79	4	13							1						1														1		
																																						7			2				1				4																					1										
																									2																														3	4	1																							
																																							2		8	7	8	6	2		2															1													2	1				
																																									7																																							
																																									8																																							
																																						9			6			4	1	2			2																															
																																						14	1		2			1		1	1		1																															
																																						9						2	1			2																																
																																						8			2				1				1																															
																																						14								2			1																															
																				1						3												79	4					2	1		1	1	18		2									2				2		2												1	2	
																																						4																																										
																																						13											2		2														1		1												1	
					2																																															2	1					1			1	1																		
																																																				1	4																											
																								1																																			2																					
																																								3															2	4	1																							
																																								4															4	4	2																							
																																								1															1	2	2																							
																																					3	1														1						14	12																					
																									2																													2				12	6																					
																																																	2																															
																																																				1									2		2																	
																																									1											1										2															1			
																																																													2																			
																									2													1											2																2	1	2											1		
																																																			1													2			1													
																																																	2															1			1													
																																																			1													2	1	1														
													2		4	1																																																					2											
																																							1																																									
																																																																									6	2	2	1	3	13	2	2
																																																																									2		3	1	3	3	2	
																																									2																																2	3	6	1	3			
																																									1																																1	1	1		1	1		
																																																														1											3	3	3	1	4	8	3	3
																				2		1			1	3		4										1											1															1									13	3		1	8	8	2	1
																																																	2		1																						2	2			3	2		2
																																																																									2				3	1	2	
																																																	1																											1		2		
																																																	3																													5		
																																																																												1				
																																																	1													1											3	1				9	5	3
																												5												1																		1	1														10	4			5	45	9	9
																																																																									2	1			2	4	5	1
																																																	1									7															2	1			3	7	6	1
																																																											2														4	4		4	6	8	8	4
																																																																									3	1			1	3		1
																		1																																									1																					
																																																																											2	1				
																																																	2																										9	5				
																										2																																																	11	4				
																										1																							1																										11	3				

																									1																																																2					1	11	
																									1																																																		1			2	3	
																									1																																	2															3	2	2	4	3	7	6	3
																																																																									2					4	1	
																																																						2																			1							
																																																					3																											
																				1						1																									1		1																									3		1
																																																																														1		
																									1	3																																																						
															1																																																																																													2					
						1																																																																									
																				3						4		1										6						2					1					1				1	1					1									5	1	1			2	1	1
							1																														1										2					2				1	1														3	2	1		1	2	2	1
																																																																														1		
																																																																									2	1			1	2		1
																								1	2																																																						
																									2																		1																														1	1	1	1		3	1	3
																				1																																																											
																																																					1																			1					2		
																																																	2																														
																																							1										4				2																										
															1											1																							6		3													1																
																																																	2		1																												
																		1																				1										1																															
																																							1										1						3			2																						
																																							1															1							1	1																		
																																						7	5						1				2																														
																										1																																																									
																																						7	2						1				2																														
																																						1	1																																								
																																																	1																														
																																																						1																									
																												1																																																							
																																																																1															
																																						1																																									

																																																						1										1																
															1																																																															
																																																										1																					
																													1									2												1	1			2																1					1					
																													1									2						1					2		1			3																1										
																										1																							1																															
																				1														1																																														
																				1								1																												1				2																				
																																																		1																															
																																																		1																															
																																																		1																															
																																							1											4		1																													
																				4														3				3											4																															
																					1																																																													
																													1																								1																										
																																																							1																										
																																																							1																										
																										1																							8		1			1				4																1					
																																																											1																						
																																							1																																												

																																																																												1		
																																																																												1		
																																			1					1	1														1																																		
																				1																																																																	
																													2																																																																	
																																									1																																																									
																																																																																					1		
																																																																												1							
																																								1																																					1					
						1																																																																												
																																								1										5		1																									1					
																																																		1																																		
																																																											1																						
																																																																							1										
						1																																																																												
						1																																																																												
																																																			1					1																																		
																1																																																			1															1			
				4																																																																															
																																																																																									1	
																													1																																																															

		1																																																																													
																																																1																										2					
																																																																													1		
																																																		1																													
																																																		1																													
													1																																						1																													
																																																																1													
																															1																																														1		
																																									1																																													
																					1		1																		1																																													
																																											1																																										
																																																		1																													
																																								1																																																
																																								1																																																
																																								1																																																
																																								1																																																
																																																																								1							
																																																																													1			
																																																		1																													
																																								1																																																
																																																	1		1																													

																				2						2																																																	1					
																												3																																													1	1					1	
																																																												1	1	1																1		
																																																						1																										
																																																			1																													
																																																							1																									
						1																																																																										
																				1								1		1									1															1																										
																				1																																																												
																														1																																																		
																																																																														1		
																																																																			1													
															1																																				1																													
																																																	1																															
																														1																																																		

																																																				1																									
																																																																											1		
																					1																																																											
																																															1		1																														
																																																				1																										
																																																																												2		
																				1																													1		1			2																										
																																																		1		1			1																										
																																																				1			1																										
																																																																												1		
																															1																																																										
																														1																																																											
																																																		1		1																														
																																																		1																																
																																																		1		1																														
							1													1														1					1				1											1				1	1															1			1	1		
																																																	3																																
																										2																																																															
						1																																																1																										
				1																1				1																1														2	1									1											1			1		1
																																																		1																																

																																												1					1																															
																				1																																																										
																												1																																															1				
																																																																										1		
																																								4																																							
																																																		1																												
													1																1						1																																																		
																																																	3		1																														
																						1		1														1											1		1																									1				
																												5																					3		1			1	1																							1		
																																																	1																															
																																																	3		1																														
																							1																										4		1													1											1			1		
																						1				1																							2		1																						1					2		
																																																	2																																
																													1										1																																																		
																																																																								1					
																																																	1																															
																																																																									1				2		1
					1																				1																								1																															
																																																																										1		
																												2																																																									
																																																	2					1																2										
																																																																					1										
																																							1										2					2																										
																																							1															1																								1		
																																																	3		1																														
																																																	1																															
																																																	2																															
																																																	2		1																														
																																																	1																															

																																																1																															
																																																																										1					
														1																																																												1				
																																																2		1																													
																			1																												2		1																													
																																																2		1																													
																																																1																												3		
																																																3		1																										1		
																									3																						1																				1										
																																																																													1			
																																																1																															
																																																1		1																													
																																																2																															
																																																3		1																		1								1		
																																																1																															
																																																1		1																													
																									1																								3																								1							
																1					1																																																										
																					1																																																						3		1			
																																																3		1																													
																																																1		1																													
																																																1								2																							
																																																1																															
																																																1					1																									
																						1		1														3											7	2	2														4															
				5		1									2					2						11		2			1							1	1										3					1										5	1	1									7	1		1		
																									1																																																									
																									1																						5		1																										2		
																																						1																																									
																																																													1																	

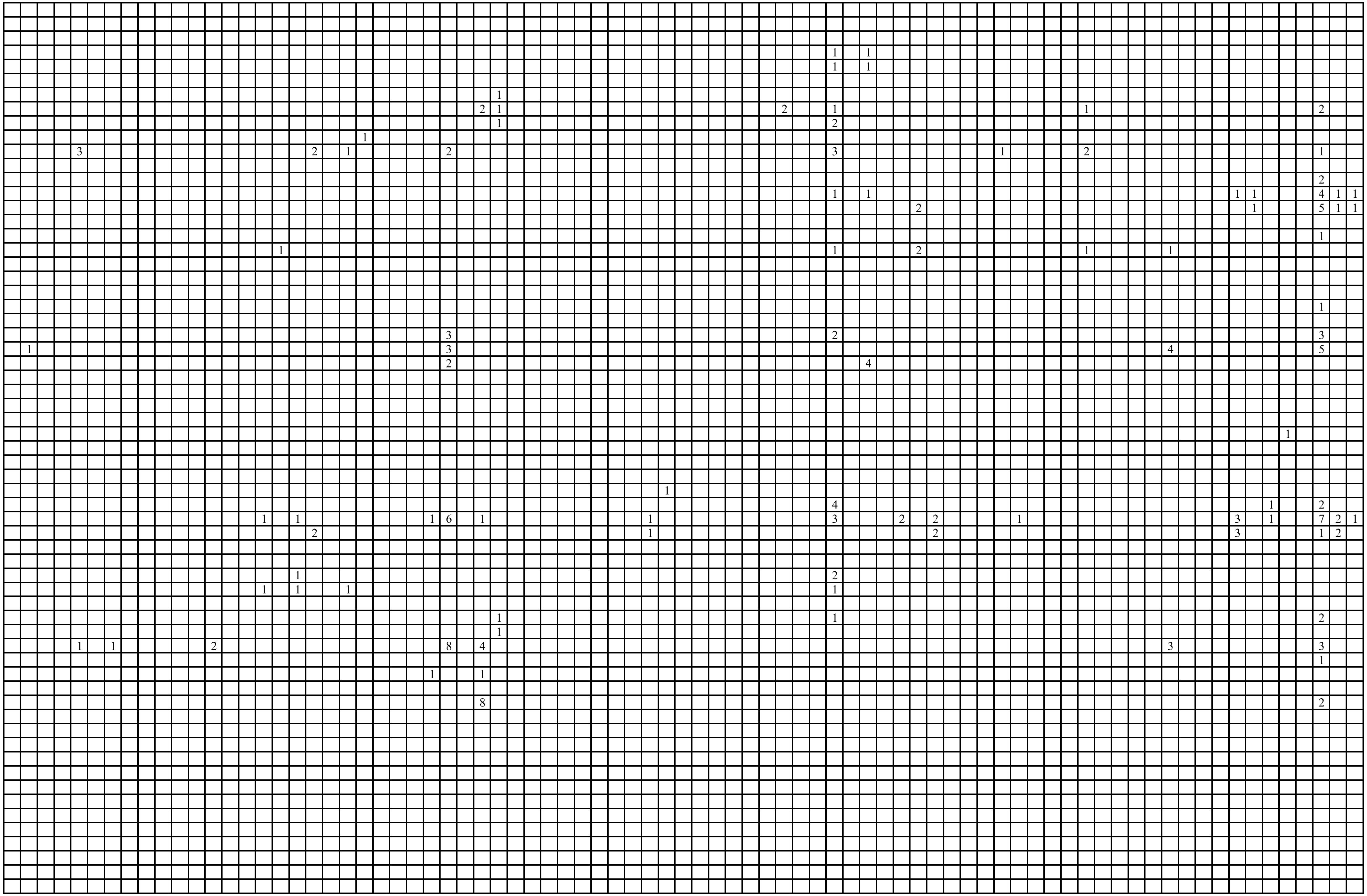

																											1										2	1														1																							1		
																			1						1		2																					1										1																					
																																																																														1		
																																							1	1																																					4				
																																								1										1																																
																																								1										2		1																													
																																																		1																															1		
																																								1																																										
																									1	1																																																								
																																								1																																										
													1																1																																																							
																																									1																																						2		
																									1			1											1																																					1					2		
					2	2																						1																																																					2		
																																																																																	1		
																													1						1				1										1																																
																									1	2																																																						1		
																																																			1																																		
																				1																			1	1																																														
																																																		1																																		
																																									1																																						1		
																																																																																	1		
																																								1																																										
																																																								1																													
																																																		5		1																																	
																																							1																																											
																																								1																																										
																									2																								2																																			
																2												1																																																1	1					

																		4		1									1																				2																											1		4		
																						1		1														1	1										1																															
																																														2			1																															
																									1			2																					5		1	2																										1		
																									1																								3		1			1																								1		
								1													1																												2																															
																																																	3			1																												
															1																																		2																															
																																																						1																										
																																																	1		1			1																										
																																																																1																
																																																	1																															
																																																	3		1																													
																						1		1														1											1																															
																																																					1																										1	1
																												1											1	1																																								
																																																	1																															
																																							1																1																									
																																																				1	1																											
		1																										1											2																																									
																																						1	1										1					1	1																									
																																																	1																															
																																							1																1																									
																																							1													1			1																							2		
																																							1																																									
																																						1	1					1											1																									
																																						1	1					1																																				
																		1		1					2	5																							7		2	1	2	2										4														6		
																																																						1																										

																				1								1										1	1	1									1		1																															
																		1																																																																
																																									1																											1														
																																																		1																																
																																																																												2				
																						1																																																															
																															1																																																								
					1																	1																																																								1		2		3	1	1
																																																		1		1			2																1											
																																																		1																																
																				1																													1																																
						1														1						1																								1																																
																																																																	1															1			

						1																																											1																															
				7																																		2	1										2		1	1									2			1									2	1					1	1
				1																																																																												
																																																	1																															
					1																																																																													
																																																																										1	2				
																																																																												1				
																																							1													2		1																										
																																																						1																										
																																																				2																								1		3		
				1																																																																												
																																																							1																													
																																																			1																															
																																																																													1				

																													1																																															3	1				
																																																	1																														
																										1																																																					
																																																	1		1																													
																																																																						1										

1

5

1

1

1 1

2 1

1

1

																																																																														1	
																																								1																																								
																																																												1																			
																																																	1																																
																																																		1																															
																																																				1																													
																																																				1																													

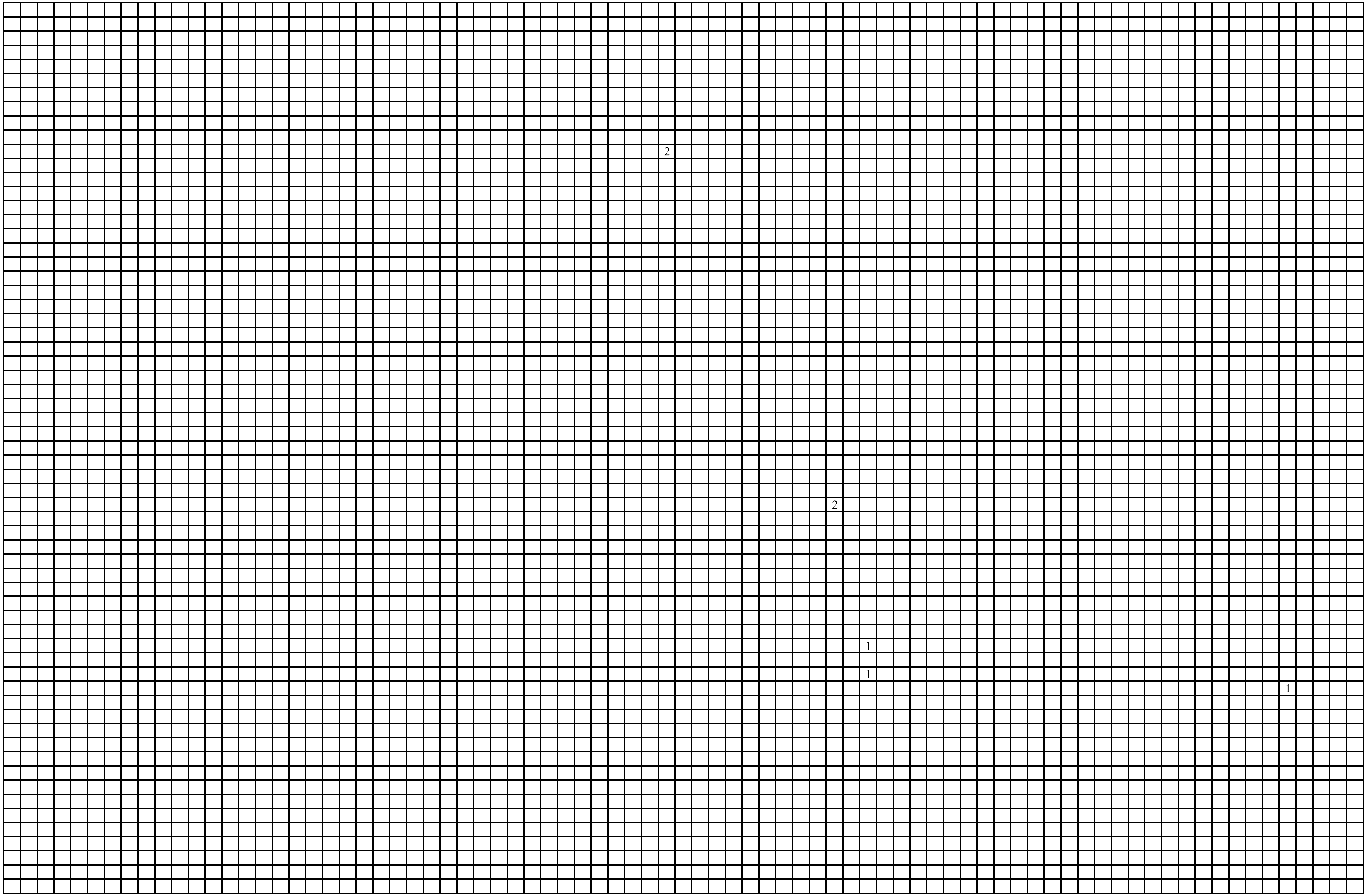
2
2
1
1
1

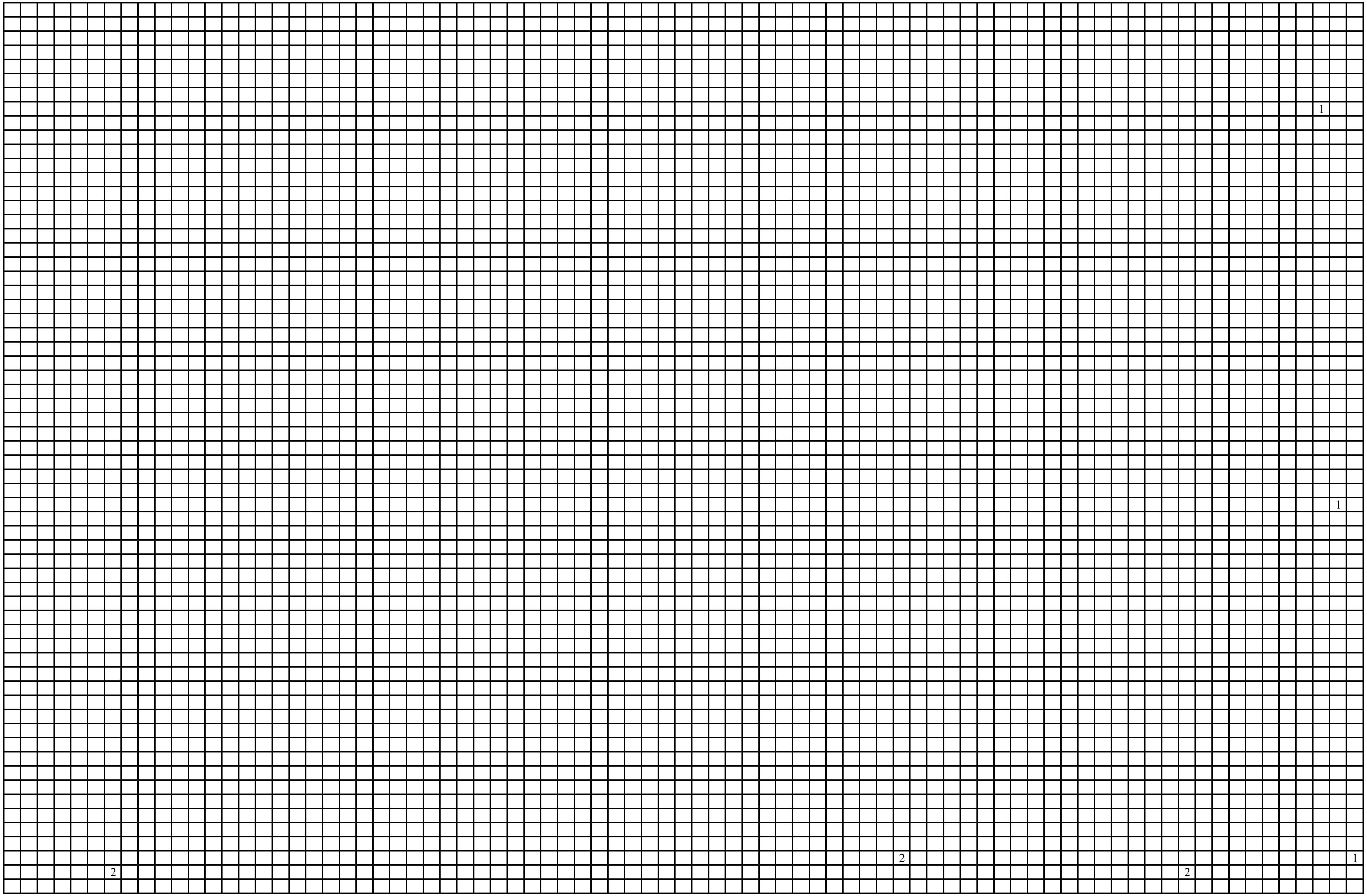
1
1
2
1
2
2

																					1																																											1													
																																																	2		1																												2		
																																																			1																											1	1	
																																								3																																			1	2				
																																																						1																													
																																																	1																																		
																																																	1																																		
																																																			1																																
																																							1											1					1																1										
																																							1											19		4																									1				
																																																																							1										
																																								1																																												
													1																																																																							
																																																	2																																			
																																																							1																													
																					1																																																																

																																																																											1				
																																																			1																										
																																														1		1																													
																														1																				1																															
																																														2		1																			1										
																		1																																			1																													
																																														1																															
																																														1																															
																																																1																													
																																																1																													
																																														1																															
																																														1		1																													
																																																																													1				
																																															2																														

																																																	1																													
																																																	1																															
																						1																												2		1																													
																																																	1																															
																																																	3		1																													
																																																	1		1																													
																																																		1																													
																		1																																																															
																				1																																																															
																																																	1												1																			
																																																		1																													1		
																										2																									1																												
						2																																					1																	2	3	3																		
																																																	1																															
																																																	2																															1	
																																																	1																																
																										5																							1																													1		

				3																																													2																											1				
																				1																																		1																								1		
																																																																						1										
																		1		1						6		10											1													2												1									1					3		
																		9	1	2					3	4		38																					1																	2	1											4	1	
																				1								10											1				1															1	1						1												1			
																										2		2																										1																										
																										2																																																						
																										2		2																																																				
																						1						9																											1									2														2		
																												2																																																				
																												6																																																				
																																																																											1					
																								2																																																							
																												1																																																				
																												1																																																				
																												2															1																																					
																												11																																						1														
																												4																																																				
																												5																																																				
																												4																																																				
																												26																																				1														1		1
																						1		3				11		2																																																		
																												3																																																				
																												2																																																				
							1																			1		2																																																				
																								2				2																																																				
							1															2	1	2						1																																																		
																												6																																																				
																												9																																													1							
																												10																	1				3																													1		
																				3						4		1											1													2	1																											
																												2																																																				
																																																	1																															
																		2																																				1																										
																																																						1																										
																												1																										1																								1		
						1	1															1		2				13		2																																																		
																												1																																																				
																										2		2																																2									1											
																												1																																																				
																												1																																																		1		
																												2																								1	3																											
																												1																																																				
																																																	1			2																												
																				1																																									2																			
																																																	1		1																													
																																																	1																															
																																																	1																															
																																						1					1						1					1																										
																																																	2																									1				3		
																																																	1																															
																														1			1					1											1		1																													

					1																																																																												
																									1														1												1							1																		2		
																			1																				2	1			1					2				1																			1					2		
																																							5													1																											
																																						1										1																															
																																																	1																														
																																																1																															

32	67	451	31	185	413	57	97	169	37	80	19	263	99	77	51	14	46	38	117	66	180	29	70	160	90	313	300	279	27	64	13	12	43	85	1366	2823	68	69	68	18	194	11	44	7	163	64	203	30	16	3	856	305	380	442	54	88	560	33	321	49	26	236	97	65	551	30	53	133	0	256	44	6	29	28	84	31	16	1	0	76
10	20	117	20	107	175	24	43	57	21	25	7	39	56	51	35	8	39	24	66	23	51	3	18	28	10	87	17	12	8	8	6	6	18	18	287	285	22	39	43	15	92	6	12	4	59	33	82	3	0	0	81	34	34	100	18	28	47	11	89	12	3	85	37	9	60	17	13	17	0	88	26	2	9	9	7	7	0	0	0	6
bZIP																																													CAAT-BF			CSRNP_N			DBP			FH/WH.E2F																											
NFE2	NFE2L1	NFE2L2	NFE2L3	ATF3	FOS	FOSB	FOSL1	FOSL2	JDP2	MAF	MAFA	MAFB	MAFF	MAFG	MAFK	NRL	BATF	BATF2	BATF3	XBP1	ATF4	ATF5	ATF1	ATF6	ATF6B	CREB1	CREB3	CREB3L1	CREB3L2	CREB3L3	CREB3L4	CREBL2	CREBZF	CREM	CEBPA	CEBPB	CEBPD	CEBPE	CEBPG	DBP	DDIT3	HLF	NFIL3	TEF	NFYA	NFYB	NFYC	CSRNP1	CSRNP2	CSRNP3	YBX1	YBX2	YBX3	E2F1	E2F2	E2F3	E2F4	E2F5	E2F6	E2F7	E2F8	TFDP1	TFDP2	TFDP3	FOXA1	FOXA2	FOXA3	FOXB1	FOXB2	FOXC1	FOXC2	FOXD1	FOXD2	FOXD3	FOXD4	FOXD4L1	FOXD4L3	FOXD4L4	FOXD4L5	FOXD4L6
					1																														1						1																													1										
					1														1																1						1																																								
					1																														1						1						2																																					
					1																														1	1																		2	1		1										1		1										
					1																														1																																		1													
																																															1																																					
																																																																													1							
																																																																			1															
																																			1																																																
																																			1	1																																												
																									1																																																									

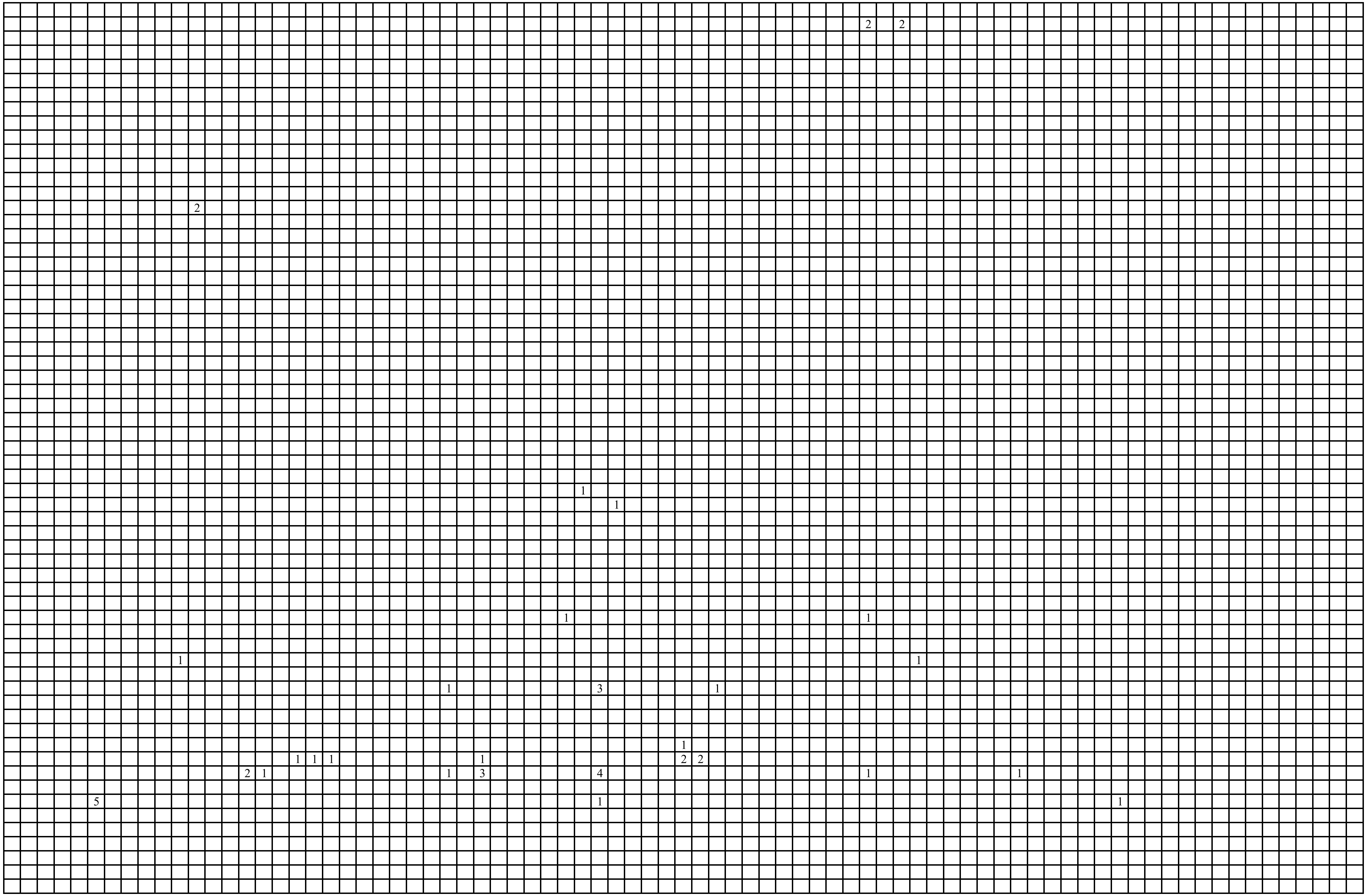

2 2
2
1
1
1 1
1 1
1 3 1
1
1 1 1 1 2 2
2 1 1 3 4 1 1
5 1 1

																																			6	1																							7			7	1							1										
																																														1						1		1	1		5			2	1																	
					1																																																																											
																																									1																																														
																																			2																																																	
																																																											1			1																			
	1	3		1			1						2		1																				1	2										2	4				6		2	1		1			2			2		1																
																										1																									3		1																														
																								3		1																			1		2																																		
																							2												1	2																					1								1															
																																																								3																									
					1		7												2																1	1																				2																								
					1			2		1																									1	1																																													
																																																									1																							
				1																																																					1																							
																																			1																1																	1												
				3	10	2	2	4	3								2		3		2		1												5	3			2		1				1																																			
				1	4	1	1	4	1										2																1	2			1		1																																									
												2	9	11	11			1	2														2		1	1					1																																									
	1		1					4				1	5	4	3				4																						1																																									
					5	2	3	6	1										3																	1			1																																											
	2	5		9	45	4	7	8	3								1	2	7		4					3	1								2	2		1	2		3				2																																			
				5	9	5	6	8									11	3	6		1														1	2					1																																									
				3	9	1	1	4	1										3							1									1	1			1		3																																									
4		1	2											1							1																																																													
	2		1										6	2	1																		1																																																	
1			1	3		3		3		1			7	9	6						4						1					3	3						3		3			3																																				
2	1	1	2										5	3	3												1																																																						
		3		6	2		1						2	1			2		2		3						1								3	2		2	2	1	11																																									
				2	2	1				2		2	1							1	2														2			1	1		6																																									
		3			1																																																																														
				1						1		1							1		2					1									2	1		1	1		1																																									
		3																																	1	1					3																																									
									2												1														1						1																																									
		1			2		1			4		2		1							2														1																																																
					2		1			2		12									1																																																														
	6	7	5	2	1								4	2				2	2																						1		2																																						
1	2	9	3	1						1			2	4	1				2																1						1		1																																						
	1	6	3											1																					1	1																																														

				2													2		2		2														2	1		2	2	2	5		1																																					
													2								1														2			1	2	1	2																																							
				2			1						2	2			2		2		2														2	1		2	2	2	3																																							
					1															2				2	2								2				2								1	1	1																																	
1		4		3	2		2		1	2		1					2	1	2		2						1						1		2	1	2	2	3		3																																							
																																			1			1	1																																									
																										1								1								1																																					
																				2				4	1	1										2								1		2																																	
																				2				1	2								1											1		1																																	
							1																1	1		2								2	1	4							2		1	1																			1					1	1									
		1	1	1																1						4	1	3	1	1								1		1																																							
																											1	2	2	2										1																																							
																											3	2		1																																																		
																											1	2	1	2	1		1																																															
																											1			1				1																																													
		3																																						1																																							
	1	3																		2	1				1				1			2																																															
																										2				1			2																																													
				3	2		2	1	1	1				1	1		2	2	2		2	1	1			1									10	5	1	4	3	1	4		1								1						2				1								2									
				2			1	1							1		1		1		1					4									5	8	2		2		8		1	1		1	2				2		2																											
																				2	2			2											1	2																			1																									
				2	1		1										2	1	2		2	1													4			2	3		2													2																1									
		3		2	1		1										2	2	2		3	1					1								3	2		3	2	1	4																																							
				1													2	1	2																1				1		3	1																																						
		3		11	6		1	3	1				1	1			5	2	3		3						1	1				1			4	8		2	4	3	2		1																																					
																																								1																																								
													2	1			1						1			2									1	1					1		2																																					
		3																																		1																																											
																				1				1	1	1																				8	11												1																					
																				1						1										1									8		9																																	
																				1				2	1											2									11	9																																		
																																			1	2																3	4																											
																																																			3		3																											
																																				2															4	3																												
																																					1	2																	1	2	1	1	1			20	7																	
																																																						1		1	1	1	1			3	3																	
																																																						2	1		1	1	1			3	3	3																
																																			2																		1	1	1		1	1			9	5	3																
																																																						1	1	1	1		1			3	3																	
																																													1								1	1	1	1	1				16	13																	
																																																												6																				
																																			1																																																	
																																																						20	3	3	9	3	16				1																	
																																																						7	3	3	5	3	13			1																		
																																																								3	3																							
																										1																																							2		1													
																																																																	1															
																										1									2			1																																		2				1					
																										1																																													2	2									
																																																																										1	1					
																																																																											2				3
																																																																								1								
																																																																						1		1	2			3				
																																																																											3	2				

																																																																								3							
																									1																																																						
																		1																																																							1							
																		1																																																	1										
																																													1																							1										
																																			1	1																																												
																																			2	1																																												
																																			1												1																																	
																																																																								1										
																																		1																																														
																																																																		1														
																																				1																																												
																																			1	2	2																								1								1										
																																																												1								1										
						1																																																										1													
																																																													1								1										
																																																																							1										
																																			1	2																																												
																									1																																																										
																									1									1												1																																										
																																															1																																										
																																																																			1															
																																				3											1				1										1				1					2	1									
																																				1																														1					1	1			1						
																								1											2	1																														1					1										
																																			1	1																																							1						
																																																															1	2														

																																																																	1			1		1	1			1						
																																		1																															1	1	1	1		1	1			1						
																																														1																																					
											1	1																																			1											1			1								1										
																																																																								1										
					1																																																																											
																							1			1																																													1										
																																					1																																															
																										3								1	1	2		1							1																											1	1									
																																																																									1									
																																													1	1	1																																					
																			2	2			2																										1																							2						
																																														1																																					
						1																																									1																																					
																																			1	1																																							1										
																																															1																																					
														1																					1																																																	
																																				1																																																	
																																																		1		1																														
																																			2	1																																																	
																																																																							1												
																										1																		4																																						
																																			1																																																		
																																			3																																																		
																		1																																																																				
																																																																							1		1										
																																			1																																																		
																																								1																									2														
																																																																			1					1									

																																																													1					1	1			1						
																																																																					1	1									
										1		1	1	1	1																																																															
					1																					1									1																															1	1	1	1		1	1			1						
								1																																						1															1																			
				2							2																																																																						
											1	1																																																																					
																																			1																																																	
																																																																		1														
																																																											1																					
																																																											1																					
																																																											1																					
								1																	1																																																									
																																																																				1													
																																																																		1															
																																																																		1					1	1									
																																																																							1										
																																																																			1														
																																					1																																																	

																																			1																																															
							1		1				1																							1																															6	1														
																																				1																																															
																																																														1																
																																																																								1							
																																																																				1										
						1																																																														1										
																		1																1																																																		
																																						1																											1		1													
																4																															1												1																					
																																																	1		1																													
																																																																1														
																																																																2														
																																			1																																															
																																																																							1									
																		1																																																																		
																			1															1																																																		

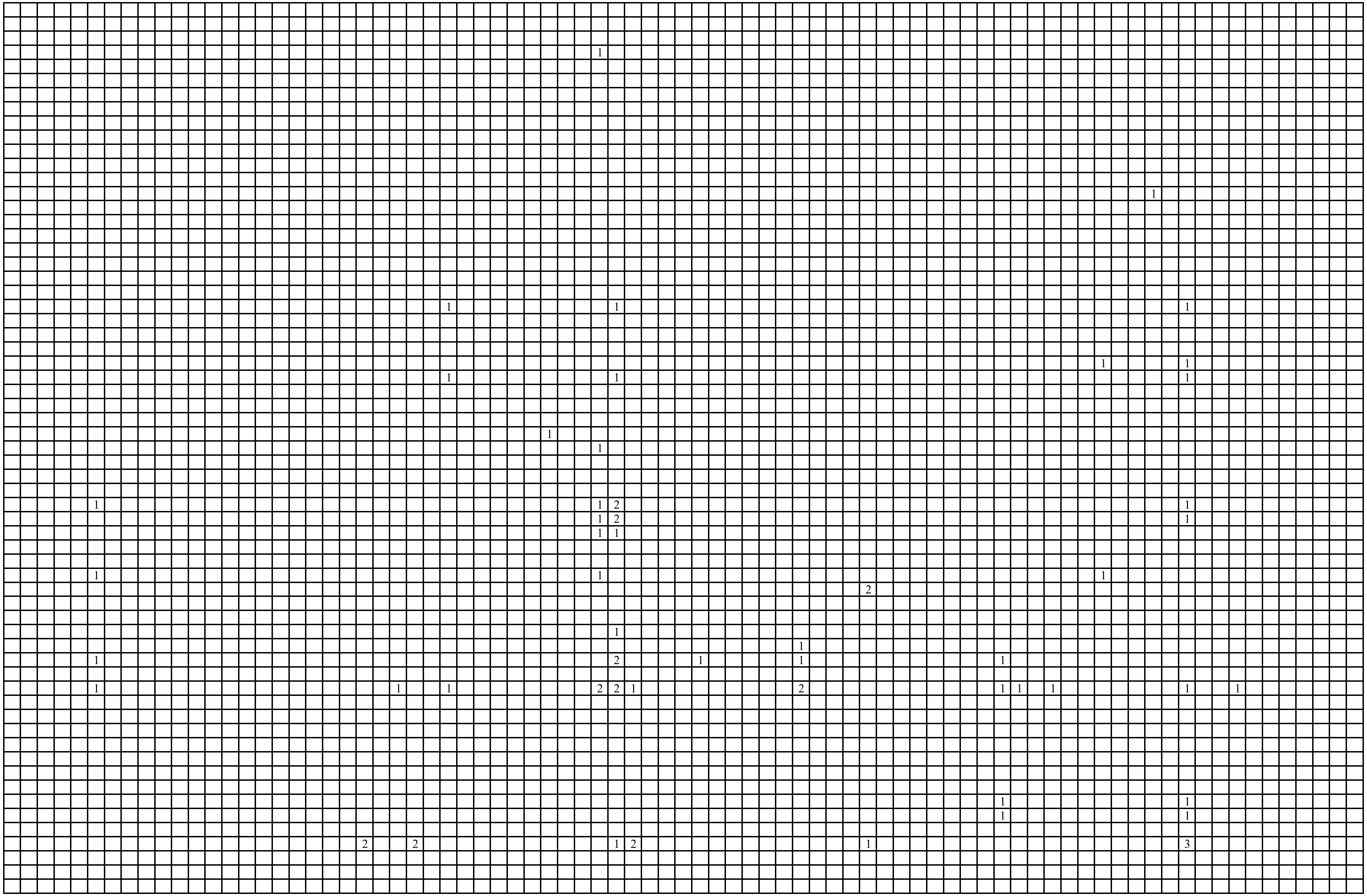

1
1
1 1 1
1 1
1 1 1
1
1
1 1 2 1
1 2 1
1 1
1 1 1
2
1
1
1 2 1 1 1
1 1 1 2 2 1 2 1 1 1 1 1
1 1
1 1
2 2 1 2 1 3

																																																																						1										
																																																																						1										
										5																					1																																																						
																																			1																																																							
		2																																																				1																																										
																																				1																																																						
																																			1																																																							
												1																								1																				1									1																									
																																				1																																						1						1					
																																			1	1																							1																																																
					2																														2	2					1																1														1										
																										1										1																																																							
																																			1	2																														1																																																	
																																			1	3																				1								1						1										
												1														1									1	4								1												1								2															
												1														1																																																										
																																				2														1																																																						
																																									1																																																											
																																																1													2																					
																																		1		1	1																																																								
																																			1																																																								
																																				1																																																						
				1																														1																																																								
																																			1																																																								
																								2												3									2		1																																																				
					2																					1																																	1						1					1	1									
					1																																																													1					1										
					1																					1									1	2																							1			1			1					2										
					1																					1																																	1			1			1					2										
	1												1																							3																																																							
																																			2	2																																						1										
																																				1																																																						
																																				1																																																						
																																				2																																																						
																																			1	2																																																																														

																																			1	1																																		2										
																																			1	1																																												
																																																																2																
																																														2											1																							
																																				1																	1																											
																										1																									1		1																											
																							2									1																																																
												1																							1		2															1																													
																										1									1																															1				1		1									
																																			2																			4																										
																													1																																																			
																																															1																																	
					1																														2		2																																		1										
																																			1																			1																										
																																				1																																												
																																			1	1											1																																	
												1																								1											1					1					1								1															
																																			1	1																																												
					1																															1															1		1	9																										
																																				1																																												
																																				2																																												
																																			1	2																																		1										
																																																																							1									
																																			1							2																																						
																																																																	1															
																																			1																																													
																																				1																																												
																																				1																																												
					3		1	3																												2																																												
		2		10	2				1						1											5							2		2										7	2					7		1	5	1	1				1		5					1	1		1										
				4																																1									1																																			
				3																																										2	2																																	
																																															1																																	

																																																																						1										
																																																											2																					
												1																																								1		3																										
																																			1																1	1																												
																																																			1	1	1																											
																																				1											1																																	
																																				2											2							2																										
		3																																																																														
		3		2	1										1			1																		8	1																																											
																																				1																																												
		2			2																															1																																												
				1	2																		1			3																																																						
					2									1	1											3												1								1	1																																	
																																			1																																													
																							1											1																																														
																																			1																																				1									
					3																														1	2																																		2										
					5																														1																																													
																																				1																																												
																										2									1																																													
																																			1																																													
																																			1																																													
																																			1																																													
												1																						1																																				1										
		3																																																																														
		1																																																																														
				2																																											1																																	
				4	3										1																				1	1	2										1										2																							
																																			1	1	2																																											
				2																															1	1																		1													1													
		3		1							1								1							1										4																																		1										
																																				1																																												
																																						1																																										
																																						1																																										

																																																														1								1										
				1																																																																													
																																																					1		1																											
																																					1																																															
					1																																																																												
	1																																																										1																					
		1																					1												1										1		1												1											1										
																																			1																								1																					
		2																																																																															
		1																																																									1																					
1		1																																																									1																					
		3																																																																																
																																																											1																					
																																																				1																													
										1																1									1																																																
		2			1																															1																																														
		2																																																																																
		3																																																																																
																																			1																																																
		3																																	2																																			1										
																							1			1																																					1											1										
																																			1																																																
																							1												2	1																																													
					1																		1											1	2	1									1																										1										
																																																						1	1	1	1																							
		1																																																																																
		2																																																		2								1																					
		1																																		1																																														
												2																																																																								
																	1																			1																								1																					
												1																																																																							
																																				1																																																
												1																								2															1																															
																								1	1																																																										
										2																									1			1				1																	1																					
																																			1																			1		1																								
		1																								1									1	2																					1													1										

		3			3																														1	3																		1			2																							
																																		2	1																						1																					
																																																													1															
					2																														1	5					1															1		1																					
		1			2																														2	2					1															1								1					1										
		2																																																			1																										
																																			1																																																	
																																				2																																																	
																																																							1																										
																																				1																																																	
												1																								2																																						1									
																																			1																																																		
																																																				1		1																											
																																			1																																																		
								1																											1	4																																																		
				1																																																																														
																																																1																																					
																																																												1			1																		
																																																1												1																					
																																																													1			1					1			1										
																																																													2			1																		
																																			3	3			3																																																	
																																																							1																										
																																																													1			1																		
																																																													1																					
																																			1		6									9	1	1							8	1	1																												
																																																2							2																										
																																					2																		2																										
																																				1												1							2																										

																																																																				1										
																																																								1																						
																																					1																															1	1													
														1																					1																																							1									
																																																									1																						
																																				1																																																	
		1																																																																															
																																				1																																																	
																				1																																																																
																																															1																																					
																																																							1																										
																																					1															4	1	1																														
																																				1																																																	
																				1																																																																	
																										1																																																															
																																																				1																																
						1																												1																																																		
																																																				1	1																																
													1																																																																						
																																				1																																																	
																																																					2																																
																																																																										1				
																																				1																																																	
																																																				1		1							1																					

																																			1																																													
		2																						4		2									2	1																										1						1												
																																																				1																												
																																			2																																													
		1												1	1																																					1																												
																																			1																																	1												
																																																			1																													
																																				2																		1																										
																																				1																																												
																																						1																																										
																																																																	1															
												1																																																																				
																																			1																	1			3		1								2															
																																				1																																												
																																			1																																													
																																																				1																												
																																			1																																													

																										1									1											1																				1												
				1																																																																									
																																			1																																															
																																			1	1										1																																	
																																			1	1																																													
																																			1																1	1																													
				1																	1								1																																																							
																																					1															1																													
																				1																																																															
																																			1	1																																													

																																			1	1																																											
																																			1																																													
																																			1										1																																			
																																																			1																													
																																															1																																	
																										1																																																							
																																																				1																												
																																			1	1																																											
																																			1	1		1																																								

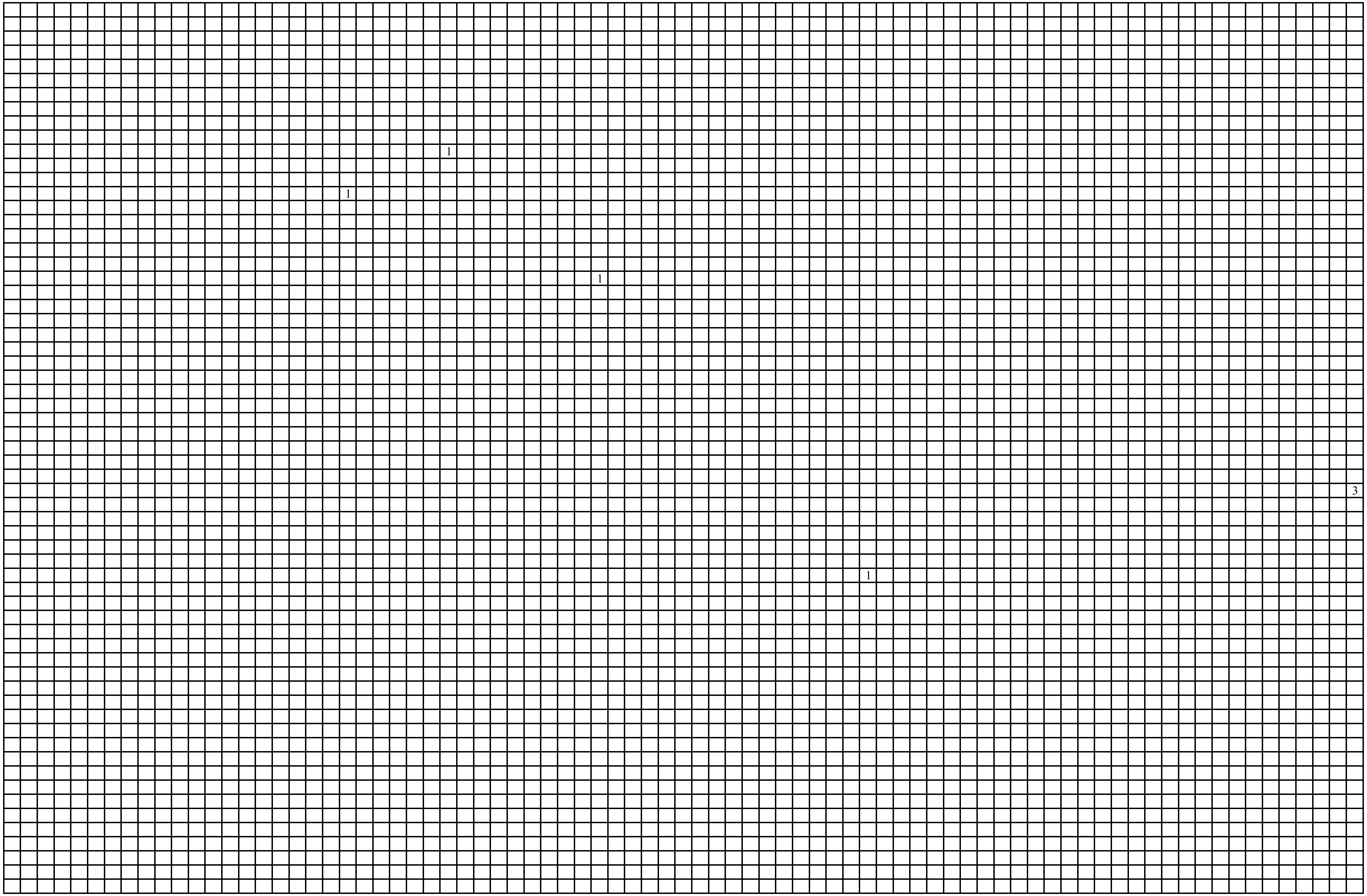
1
1
1
3
1

1

1

1

																																		1																																													
																																																					1																											
																																																				1																												
																																																		1	1																												

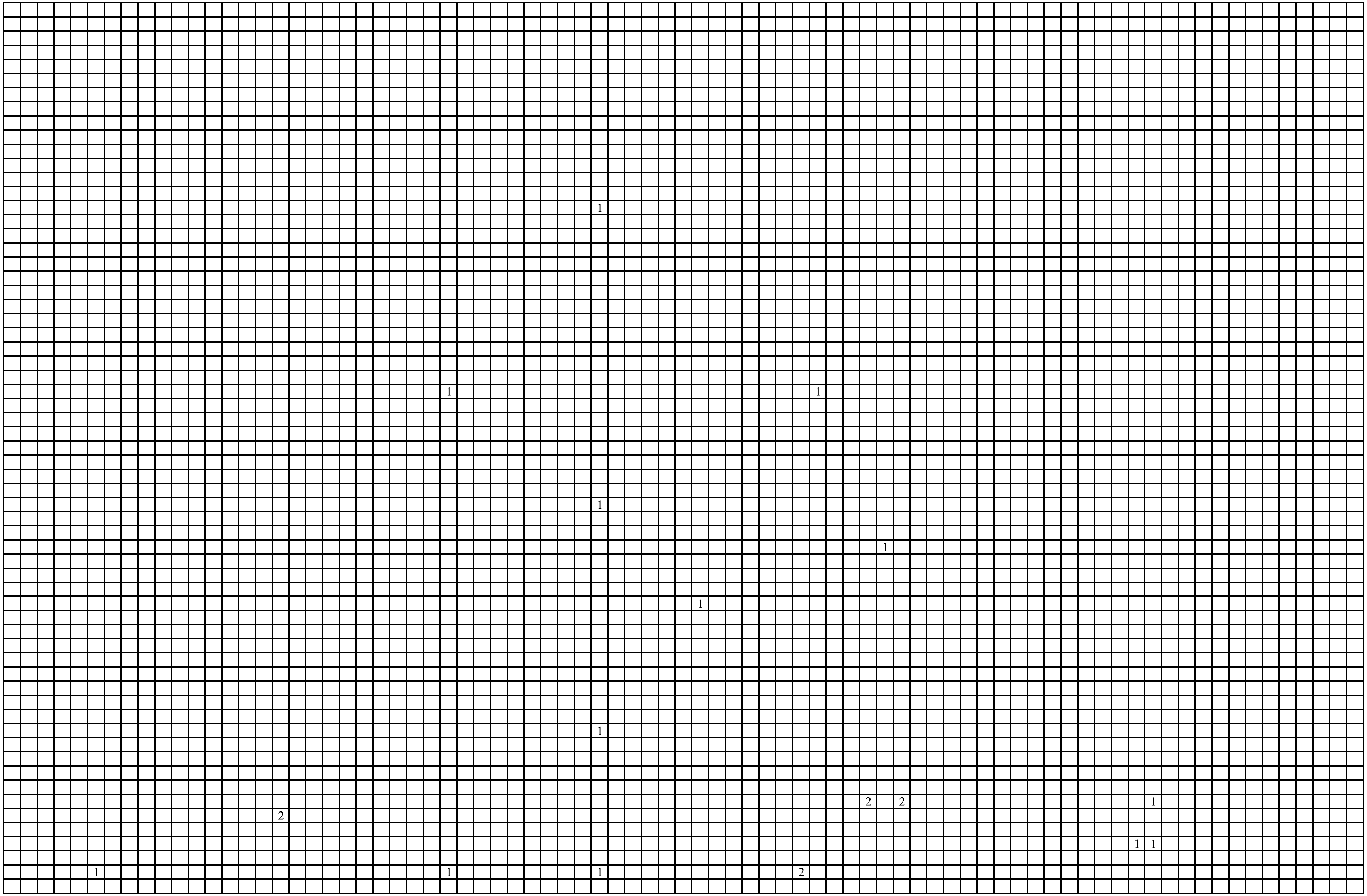
1
1
1
1
1
1
1
2
2
1
2
1
1
1
1
1
2

																																			1	1																																				1										
																																			1																																																		
					2																															1																																																		
								1																										2																				1																												
																																																							1																														
																																																							1																														
																																			1																			1																												
																																																				1																															
																																																				1																															
																																			1																																																		
																																			1																																																		
																																																																			1		1		1	1									
						1																													1	1									1																																										
					1																														1	1																																																				
																																				1																						1																					
																										1																											1																											
																										1									1																																																	
																																									1																																												
																																			1																																																		
																																			1																																																		
																																			1	1																																																	
																																			1																																																		
																																				1																																																	

																																																1																															
																																				1																																												
																																		1																																														
																																				1																																												
																																																																												1									
																																					1															1	1																													
																																																	1																															
																																				1																	1																												
																																																					1	1																												
																																				1																																												
																																																						1																												
																																				1																																												
																																					1																																											
																																					1																																											

																																				1																																												
																			2																																																												
																																			1																																													
																																			1	2																																												
																																			1																																													
																																				1														1																												
																																																																	1														
																																			1																																													
																																			1	1																																												
																																				1																																											
		2																																1			1																																									
																																																																						1										
																																				1																												1					1										
																																																			1													1														
	1																																													1							1																									
																																			2	2																																												
																																			1	2																																	2										
																																			1	2																		3																									

																																			2	2																					1													1										
																																			1																																													
																																																																	1															
								1																		1									1	1																		2			2								9															
					1			1												2			1								1				1	5									1						2		2	2					1						2															
																										1					1																1					1	1																										
																																				2																																											
				1									1													2									1	2										1																																	
									5										3																																																													
				4																														2																																													
																																																																			1													
		1	1										1																						1	2																																											
																																			3																			2																									
																																			1	1																																											
																																				1																																											
																										2																																																						
					1																													2																																													
																																																											1											1										
																																										1																																					
																																			1												1																																	
																							1												1	1															1																													
																2																																																																
					1																																																										1				1									
																																				1																												1															
					1																																																																										
																																				1																																											
																																																			1	1																												
																										3																																																						
																				2																																																												
																																																			2		1																											
												1																																						1	1																												
																																				1																																											
																																			1																																												
																																			1	1																																											
																																			1	1																																											
																																																			1																												
																										1								1																3																		1										

																									1									2			3																										1															
																									1																																																										
																																		1																																																
																																					1																																													
																																			1																																																	
											1																																									2																													

																					1					FOXE1	FH/WH.FOX	33	79
																										FOXE3		0	7
																										FOXF1		12	47
																										FOXF2		7	67
																										FOXG1		19	96
													1	1				1			1	1				FOXH1		45	121
																			1					1	1	FOXI1		23	91
																										FOXI2		8	52
																										FOXI3		0	2
																										FOXJ1		13	42
																										FOXJ2		34	85
																										FOXJ3		11	33
																			1	2						FOXK1		68	288
																			1	2						FOXK2		86	327
																	1		1		2	2				FOXL1		49	203
																										FOXL2		9	72
												2														FOXM1		19	198
																										FOXN1		18	37
																										FOXN2		6	29
																										FOXN3		10	62
																										FOXN4		1	7
																										FOXO1		47	433
																										FOXO3		21	169
																										FOXO4		10	52
																										FOXO6		6	16
																										FOXP1		118	274
																										FOXP2		60	116
																										FOXP3		38	118
																										FOXP4		90	123
																										FOXQ1		25	139
																										FOXR1		13	91
																										FOXR2		19	97
																										FOXS1		16	96
																										RFX1	FH/WH.RFX	31	86
																										RFX2		15	31
																										RFX3		19	55
																										RFX4		4	4
																										RFX5		16	67
																										RFX6		9	51
																										RFX7		6	27
																										RFX8		0	1
																			1					1	1	GCM1	GCM	22	87
																										GCM2		18	46
																										GRHL1	GHD.CP2	7	9
																										GRHL2		12	17
																										GRHL3		8	18
																					1					TFCP2		43	513
																										TFCP2L1		5	8
																										UBP1		23	76
																										GTF2I	GTF2I	62	366
																										GTF2IRD1		17	129
																										GTF2IRD2		5	13
																										GTF2IRD2B		8	19
																										ANHX	HB	0	0
																										CPHXL		0	0
																										DUXB		2	17
																										NANOGP8		0	0
				1																						CUX1	HB-CUT	59	227
																										CUX2		1	9
																										ONECUT1		6	13
																										ONECUT2		1	6
																										ONECUT3		0	4
																										SATB1		80	175
																										SATB2		105	179
																										PAX3	HB-PAX	13	68
																										PAX4		1	19
																			1		1	1	1	1	1	PAX6		131	382
				1															1			1		1	1	PAX7		51	131
																										HDX	HB-POU	2	29
																										HMBOX1		31	154
			1																			1				HNF1A		25	99
																	1		1		1	1	1	1	1	HNF1B		84	204
																		1								POU1F1		23	38
																										POU2F1		89	218
																										POU2F2		20	36
																										POU2F3		2	8
																										POU3F1		1	14
																										POU3F2		8	21
																										POU3F3		3	14
																										POU3F4		0	1
																										POU4F1		2	10

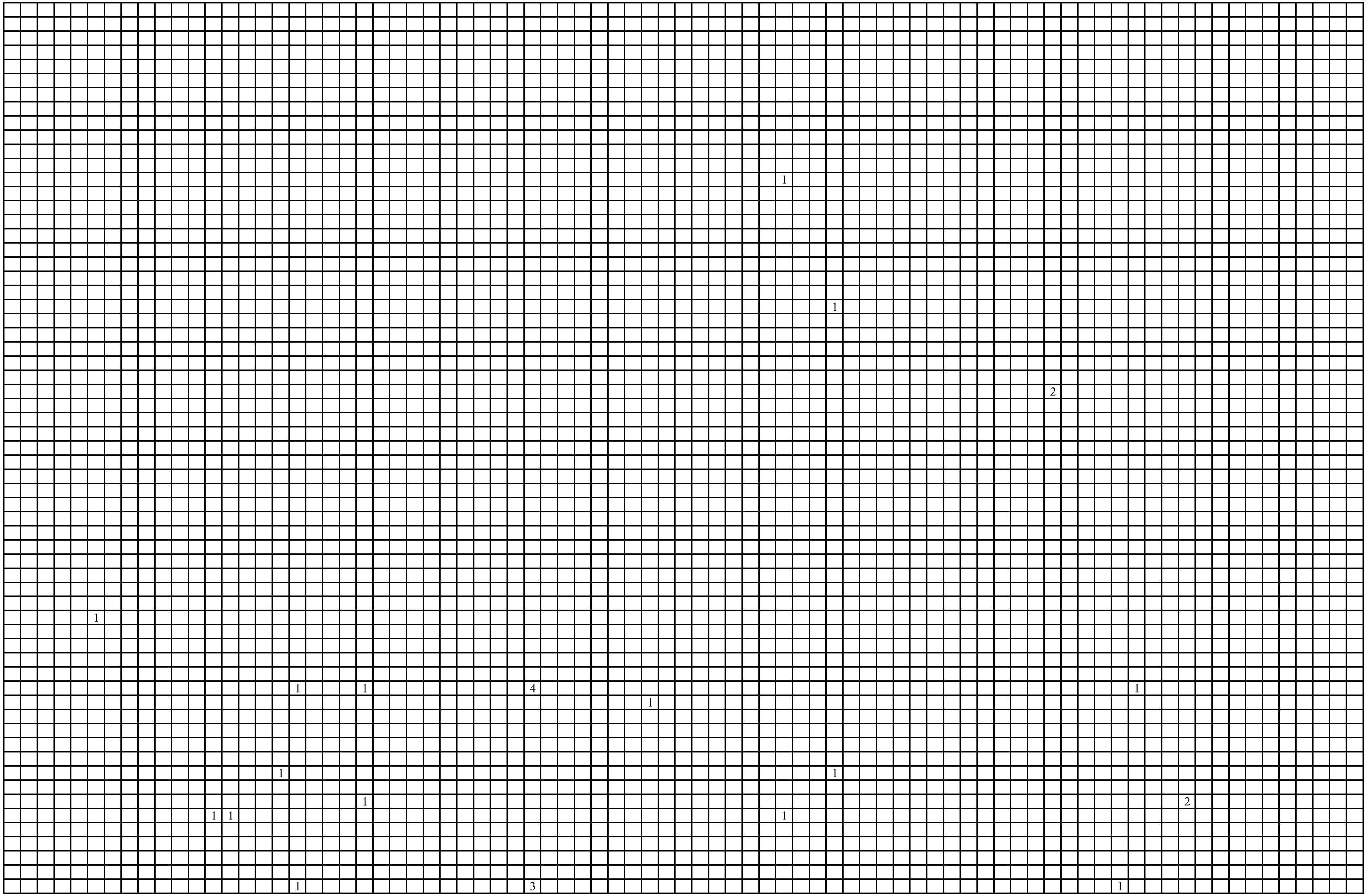

											2	2																	1	3																									1																							
																																																														1					1									
																																																														1														
												1																																																																			
												2			1						1		1	1					4	4																			8																																			
											1																																																																					
											1	1																	1																			1																																			
1												2	3																																	1	1	1	1																	1														
																					1																																																												
																																																			4	1																																
										1																																																																							
																					2																																																												
1																																																																																
											1	1																																																																				
																																																																													1							
											1																																					1																																			
																																																																1	1									1								
																																																																										1										
																												1																																																										
																																																																					1														
																																																																					1														

					1	1																																																																							
																																																															1									
																																																	1																																
1																																		1		1																																			1		3							
															1																																																											1							
											1	2	1												1								1				1												2								1						1										1							
											1	1									1				2								2													3		1	1								1																2							
																									2																																															1								
																																																																									1							
																																																																										1				1			
																																																																													1			
										1			1																								1		1							1																			1	1											1			
																																														1																																			
																																																																	1															
																																														1																																			
																									1	1		1																																																1															
																																												1		1		1	1										1			1	1																		
																		1																																														2				1																		
																											1																																												1																		
																																																																	1	1																		
						1				1				1											1	1		1				1														2		1	1													1	1			1	1				1		1							
																																														1		1														1	1										1	1						
					1																																																																													
																																																	1									1					1	1																		

													1																																	1		1									1					1	1	1																
		2	1																						1																																					1	2																	
		1								1																																																																						
					2																																																				1					1	1																	
				2																																																																												
							1																		1			1																																		1	1																	
						1																																																									1																	
											1																						3	4	4																																													
			1																														1	1	1																						1						1											1						
									1																																																																							
													1									1																																												1														
	1												1																																													1						1			1					1									
																									1			1																													1					2	2										1							
																																														1		1									1					1	1																	
																																	3	2																																														
																															1		2	2	2																																													
																																		1																																														
																					4		1	1																																	1					1	1	1																
												1												1																																																								
																					1			1																																																								
																					1	1	1								1																																																	
		1				1								1											4	8	3	5					1								1					1		1									1					3	3			1	1				1									
																									8	4	3	7					1																													2	2																	
																									3	3		3		1																1		1														1	1																	
						1								1											5	7	3	4					1																													2	2				1													
																																														1											1					1	1																	
																											1																																																									
																			1					1																																																								
																																																														1	1										1	1						
									3	1								3	2						1	1		1							2																																													
									4	1								2	2	1															1	1		2																																										
									4	1									2														2	1		1		1																																										
																																		1	1	2																																												
																																							2																											1														
																																		2	1																																													
																																					2																																											
																									1																																																							
																																																																		1														
																																												2	2																																			
																																											2		2																					1														
																																											2	2	4																																			
	1																	1								1		1		1																	2	2	4	1																												1			
																																														2		1																																
	1																	1								1		1																			4	1																																	
																																														1					1	3																												
																																																			1	2																												
																																																	1	1		3																												
																																																	3	2	3																													
																																																															1																	
	1				1						1			1	1			1				1				1				1																												2	1				2				1					1									
																																																									1																							

1		1		1		1								2			1				1				3	2	1	2	1			1																										2						4			1	1				1									
1		2		1		1	1				1			1	2			1				1				3	2	1	2	1			1																									1								4				1	1				1									
1																					1																																																									1			
												1	1													1													1													1		1																	1									1	1			6			1		1		1						
																											1			1																																											1	1									1		1						
																																																																						1			4		1									
																																																																																		6	5										
													1												1																															1													1	1			1	1		1	5	4										
														1																			1																																									1	1							4						
										1																					1																																																	2					
																																														1																			1																2			
																																																																								1						1		
																																										1																													1		1						
1				1						1			1																1			1																																																	
																																																		2																1													
										1																																																																						
																																																																		1	1													
														1																																																				1	1				1								
														1																																																				1						1								
														1																																																				1	1				1		1						
						1				1				2										1	1		1	1																										1										1	1				1								
																																																																		1													
																																																																		1													
1																																																																		4				1									
					1												1																									1																							1													
1																																																																																	
																					1																																																												
																					1																																																												
																																																																		1													
1		1		1																										1																																									1								
																												1																																		1																

1		1											1																			1																																																	
1				1			1			1							1										1					1																																																	
																																																																										3							
																																																																1																
																																																																1							1							
1		1		1			1			1			1				1				1				1		1		1	1		1																																																	
																																																															2					1											
																													1					1							1																			1		2					1											
																																																																	1	1													
																											1																																					1	1																	
											1												1			1																													1					1	1			1														
											1												1			1														1															1						1																	
											1															1																																		1	1								1	1							
																																																																	3																	
																1																																																														
																														1																																																				
1							1			1											1																																																														
																																																											1								1			1											
					1																																																																												

																																																															1																
					1																																																																1		1						
																																																													1					1												
																																														1																	1																	
1																																																																																			
					1																																																																													
												1													1			1																													1					1	1																	
																												1																													1					1	1								1		1							
					1							1													1			1																													1					1	1								1		1							
																																																																							1											
1																																																																																				
					1							1	1												1	1		1														4																					1								1		1							
																																																																1																	
																												1	1		1																																					1									1							
																																											1																											1											
																																																																											1									
																						1																	1																																													2								
																																																																													1								
																																																																				2														

																									1			1																																		1	1																	
																																																																		1														
																																																																		1														
1															1																																																																	
																																																																		1														
														1																																																				1	1				1									
														1																																																																		
1																													1																																												2							
1							1																									1																																																
1																																																																																
																																			1																															1							1							
					1																																																																											
												1		1																																																																	
																																																																						3										
																																																																										2						
																																																																		1														
						1								1																											1																									1	1			1	1									
										1																						1																																																
													1																																																																			
																																																																		1					1									
										1																																																																						
												1	2	1															1				1								1					1		1	1								1					1	1	1		2	1		1	1	1									
																																																																3							1						1			
																									1	1		1																																											1									
					1																																																																											
												1													1			1																													1					1	1								1		1							
																												1																																			1								1		1							
												2	2												1			2					1		1														1								5					2	1			2					1		2							

					1																																																				1																							
												1													3	2		3																													1					1	1			1					1		1							
																									1			1																													1					1	1								1		1							
																									1			1																																			1																	
																																																									1									1					1									
																																																																1														1		
			1			1								1																											1																1									1	1			1	1									
																																																									1																							
														1															1												1																									1	1				1									
																													1												1																									1	1				1									
																	1																																																	1														
																									1	1		1																																																				
																																										1																																						
																																																															1																	
1																																																	1																								2	2		1				
																																																	1																															
																																																	1																															
					1	1																																																																										
																																										3																					2																	
																					1																																																											
																					1																																																											
																																																																			1				1									
				1								2																1																		1		1	1								1												1											
						1								2											1			1													1																						1			1	1				1									
														2											1			1					1																														1			1	1				1									
1														1											1			1					1																								1						1			1	2						1							
													1				1								1								1				1																				1						1			1	1						1							
										1			1														1																																																					
																																																																							1									

			1																																																																															
																									1																																																												
1														1							1																																																1	1				1									
		1																			1																									1																																						
																																																																		1														
																			1																																																															
						1																																																																1		1							
					1		1																																																																								
																																																					1																	1					1									
															1														1																																																							
														1																																1																																						
																																											1																																	1									
													1																																																																							
																								1						4	3																																																								
				1							1	2	1			1	1					5	2		2	1		1	1			1														1			2													1	1																						
												2	2																																																		2																					
																																																																			1														
																									1			1																																							1								1		1							
																																												1																										1											
																									1			1														1																						1								1									

												1													1			1																																		1	1																	
																									1																																																							
												1	1															1									5		2										1																															
																																																	1																															
																							1				1																																																					
																2											1																																																2							
																									1	1		1																																																				
												1	1														2																														1					2																		
												1																																																		2																		
																									2																																1					2	1																	
			1																								3																					1																																
															1																																																																	
															1										1			1																																																				
				1																		1											1																																	1														
					12																																												1	1																														
					6										1	2					1	2	1																																											1														
					2																2	4	2										1														1																			1														
																																																																		1														
																																	1																																															
																																																	1																															
																																																	1																					1			1							
																																																																									1							
																																										1																					1																	

												1														1		1																																			1								1									
													1												1																																1					1	1																	
													1																																		1																1	1										1	1						
												2	3																																				1																														
												1	1																																																																			
												1																																						1																1							1							
												1																																																																				
																																																														1	1																	
												1													1			1																																			1										1							
																									1			1																																			1																	
																																																														1																		
																																																															1																	
												1													1	1		1					1				1																				1					1	1										1							
																																																																			1													
										1																																																																							
																																																															1																	
												4	5																																											1																							
																					3																																																												
																												1																																		1	1																	
																																			1																																													
																1																																																																	
														1																																																										1							
																									1	1		1																																																					
			1																																																																													
																																																									1						1																	
																3																																																			1														
												1	2	1																										1																										1	1				1	3								

						1							3	1											2				1												1																									1	1				1		5							
													3																																											1					1	1				1				1									
						1								1															1												1																									1	1		1											
																																									1																									1					1									
																		1																								1																									1															
																																																																												1							
											1					1																																																																
											1																																																																						
															1																																																																	
																																																													2																	
																					1																																																														
																										1	1		1																																																							
												1																							1																																																					
												1																1																						1																																						
										1			1												1																								1																									1							
												2	1												1			1											1																									1																		
																																																									1																							
												1													1			1																						1																																
												1	1												1			1																						1																									1							
												1																																																																					
																2																																																											5							
																											2																																																							2							

												1													1			1					1																								1					1	1								1		1							
																																																																						2											
				1	1											1											1		1																																																				
1										1									2								1																																																								
																											1																																																							
																1																																																										1					1					
																																																																		1			1														
																																																																		1																	
																									1																							1																					1									

																																																																						2											
										1				1			1								2	2		2																				2			1																	1					2									
																																																																				1												
			1																																																																													
																																																															1						1											
																																																																						1											
																					1																																																											
																																																		1																															
					1																																																																											
																																																																		1	1													
																																																																		1															
																																																																		1															

										1																																																																							
																													1																																																								
										1															1	1		1																																											1														
																																												1																																
																																														1																														
																																																														1													
																																																																1											
																																								1																														1									

													1																																																		1	1				1									
																																																																											1											
					1																																				1																																							
																																																																	1														

1

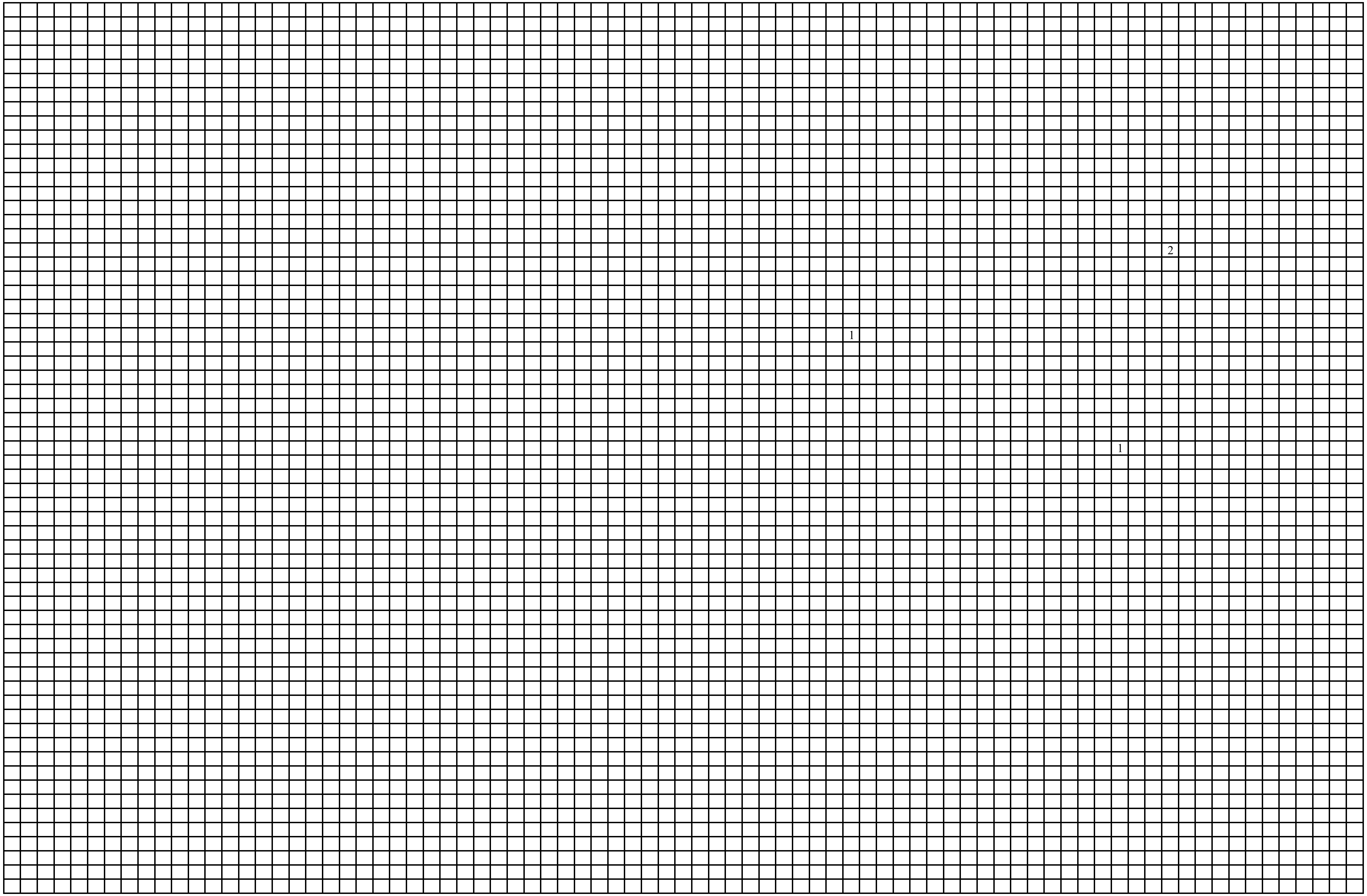
2
1
1

1 1

2

																																								1																										1													
																									1	1		1																																																								
										1															1	1		1	1																																																								
														1																											1																									1	1				1		1							

												2	2												3																																																							
																											2																																							1	1													
													1														5																																																					
										1																																							2																															
										1																												1																																										
													1																																																																			
													2																																																																			
																																																																		1														
																																	1																																	1	1													
1				1						1															1																																									1														
1				1																																																										1					1									
											1														1	1		1																																																				
		1																																																																														
												1	2												1	1		1																																																				
																																																																			1				1									

																																																		1																														
																																																																								2											
																																																															1														
																																																															1														
																																																1									1									1														
																																																															1														
																																																																1														
						1				1				1																												1																									1					1									
																																																																								1											
																											1	1		1																																																							
																																																																1														

											1		1												1				1																																		1														
													1			1																																																															
																												1		1																																																									
					1	1								1																											1																									1	1													
						1								1															1																																							1	1				1									
						1								1															1												1																									1	1				1									
																																							1																																		1											
																																																																			1																	
																									1																																	1					1	1									5								
																											1																																				1	1																	
1																																																	1																																				
												1																																																					1					1									
				1						1															2	3		1	1			1																																																			
													2												1																																																									

													2																						1																											1	1																	
													3																																																																			
						1				1				1															1																																															1	1				1									
				2								1									3				2			1									1						1	2					1								2									7							2	1						
											1	1	5		1						5		1																					1				1	1																								2	2	1					2
										1	1	1										1			1			1					1					1						1				1	1								1																							
																																																																		1														
										1	1			1																			1															1									1		1				1									1	5	3				1		
																																																																											1	1						
																																															1																																		
																																																																2																	
																						1																																																														
																						1																																																														
																																																																										1								
																																																																										1								
																																																																										1								
													1																																																																						
																					3																2												1																							1								
																					1																												1																								1							
									1																																																																					1							
																						3	1																																																									1	1							
													1								2				1																																1						1																	
															1																																																								1	1				1									
														1																																			1																	1	1													
				1										1											2	2		2				1																																																2	1						
																									2	2		2																																																								
																	1																																																																			
																																																																											2	1						
																																															1																																		
																																																											2																					
																									1																																																							1										
																																			1																																																						
1							1																																												1										1																				

																	1																						1																																																		
																																													3				2												7									1									
																																			1										1					3	3																			1											
																										1																																					2																		
																																																														2																		
																																																														1																		
																																											1																																														
														1																																																																		

																										POU4F2		5	78
																										POU4F3		0	12
											1					1								1		POU5F1		70	378
																										POU5F1B		0	0
																										POU5F2		0	1
																										POU6F1		1	3
																										POU6F2		45	147
																									1	ADNP		50	231
																										ADNP2		9	71
																										HOMEZ		34	177
																										NANOGNB		1	2
																										TSHZ1		4	13
																										TSHZ3		18	87
			1																							ZEB1	HB-ZF.C2H2	35	257
			1																							ZEB2		55	124
																										ZFHX2		1	7
																										ZFHX3		41	101
																										ZFHX4		48	76
																										ZHX1		51	116
																										ZHX2		35	76
																										ZHX3		22	54
																										CDX1		12	120
																										CDX2		10	41
																										CDX4		1	10
																										EVX1		0	1
																										EVX2		0	9
																										GBX1		2	7
																										GBX2		1	4
																										GSX1		0	46
																										GSX2		0	3
																		1								HOXA1		76	549
																										HOXA10		14	48
																										HOXA11		6	14
																										HOXA13		4	14
																										HOXA2		0	24
																										HOXA3		2	16
																										HOXA4		1	10
																										HOXA5		21	91
																										HOXA6		0	1
																										HOXA7		2	21
																										HOXA9		10	38
																										HOXB1		8	34
																										HOXB13		14	20
																					1			1		HOXB2		12	35
																										HOXB3		1	7
																										HOXB4		0	6
																										HOXB5	HB.HOX	13	159
																										HOXB6		3	64
																										HOXB7		5	36
																										HOXB8		5	16
																										HOXB9		31	148
																										HOXC10		1	21
																										HOXC11		8	22
																										HOXC12		0	5
																										HOXC13		16	22
																										HOXC4		7	58
																										HOXC5		7	45
																										HOXC6		2	16
																										HOXC8		37	127
																										HOXC9		13	62
																										HOXD1		2	2
																										HOXD10		1	13
																										HOXD11		3	10
																										HOXD12		7	18
																										HOXD13		34	82
																										HOXD3		6	14
																										HOXD4		9	28
																										HOXD8		1	11
																										HOXD9		7	10
																										MEOX1		15	118
																										MEOX2		77	771
																										MNX1		19	27
																										PDX1		6	24
																										ISL1		24	58
																										ISL2		22	65
																					1	1	1	1		LHX1		30	110
																		1		1	1	1	1	1		LHX2		61	190
																					1	1	1	1		LHX3		64	196
											1										1	3	1	1		LHX4		64	220
																										LHX5	HB.LIM	0	0
																				3		1				LHX6		26	100

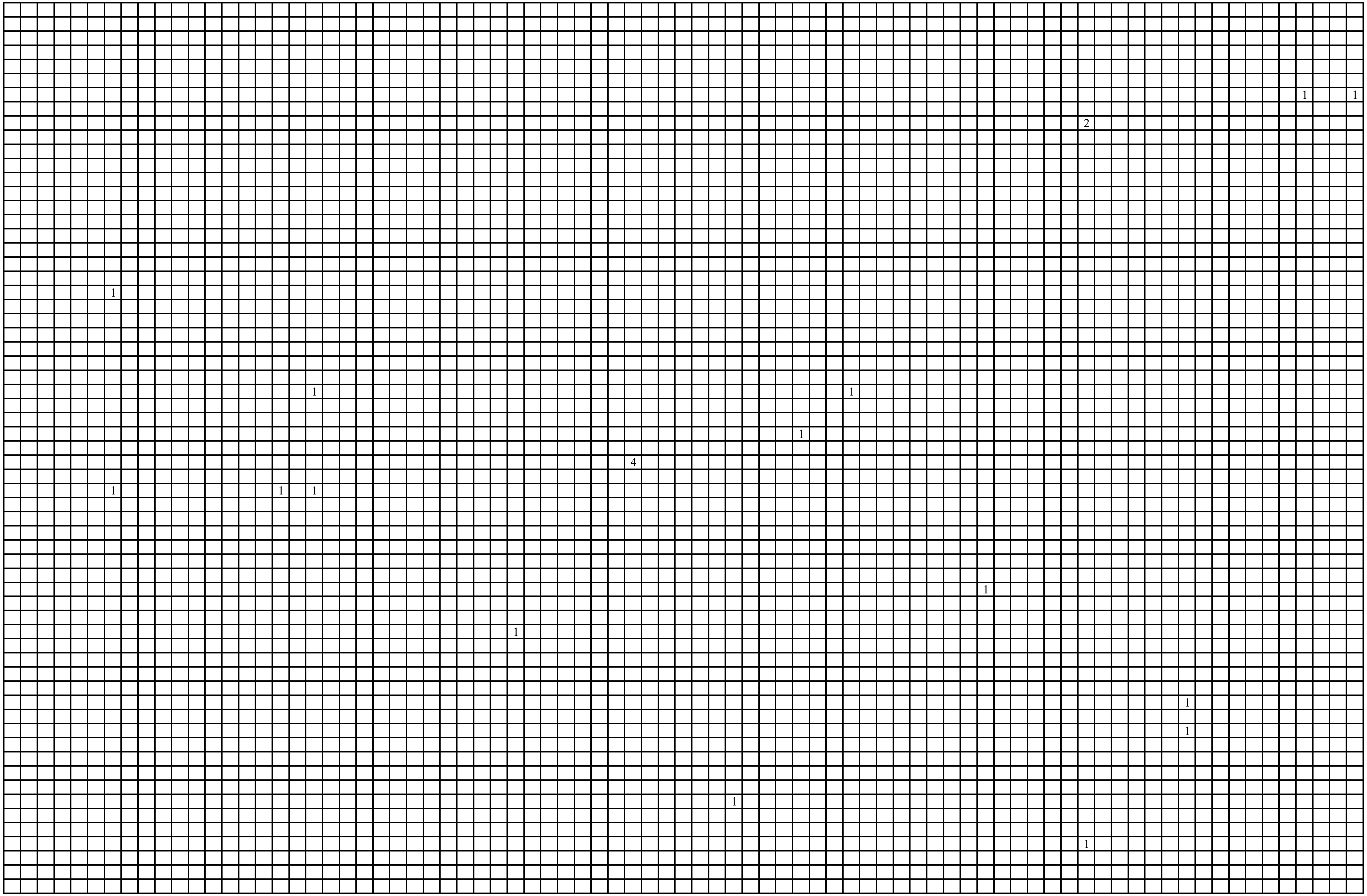
1
1
2
1
1
1
1
4
1
1
1
1
1
1
1
1
1

		1																																																																																	
							1																																																															1	1	1	1		
																																																																				1	1										
																																																																								1								
							5	1												1																														1																																		
							1																																																	1		1	1															1							
																				1																																																																	
									1																																																																											
																															1																																																													
																																																																	1																		
													1																																																																									
		1					1																																										2																																		
																															1																																																												
																																																				1														1																	
																																									1																																														
																																																																											2								
																																																																		1																	
						1																																																																																
																																																																										1										
																																																																1																		
																																																																														2	1							
																																																																1										1							
																																																																1																		
									1																																																						1																		
																																																																1																		

																														1																																																		
		2																																																																															
		2																																																																															
		2																																																																															
																		1																																																1																
							1		1					2						1	3												1																															1									1						
							1					1		1																																																																			
																																					1																																															
																		4																																																																	
																																																																										1										
					1	1		1																																																																									
		1											1																																																																				
													1																																																																							
																																																																														1	1	1		
																																																																										1										
																																																			1														1																
																																					2					1																					1										1					
																																																																1																
																	1														1																																	1																
							1																								1																			1				1										1																
																																										1								1				1										1																
		2																																																1														1																

							1															1									1											1							1				1									1																
																																										1							1													1																
							1																																			1											1									1																
																													1																																																		
																	1																																																															
																																																						1									1																
							1				1						1																																							1									1																
																																																																												1	1	1		
							1																																											1													1																
													1	1		1	2																																																													
																													1																							1									1																
																															1					1																											1																
																	1																																														1											1	1				
																	1																																																															
																																																						1									1																
																	1																									1																																1	1	1	1	
							1									1																																																1																
																																																																1					1											
							1																																		1							1				1									1																
																																																																						1											
						1																						1																																								1						1				
									2																																																																							
																	1																																																															
																																																																												1	1				

																																																																						1					1	1		1		
																																											1																																1	1	1	1		3
																																																																					2	2										
									1			1	1	1		1	1	1	1			4								1											1																	1	1												1					1				
												1	1			1	1																																																						1									
																																																																					1	1										
																						1																																																										
						1							1	1		1	1																																																													1		
		1																																																																												1		
						1										1																									1													3				1																					
		1																																																																													
																																																																												1				
		4																																																																													
															1	1																																								1		1						1																
								1																																																																								
							1																																																																									
																																																																												1				
												1																																																																				
											1																																																																	1				
																																																																																1
																																											1																																		1	1			
						1																																																																									
						1										1																																																										1				
																1																																																										1	1	1			
																		10	1	3																																																								1	1		
																		1		3																																																							1	1	1		
																		3	3	4																																																												
																											1																																																						
																										1																																																							
																														12											1									1															3															
																																																1																		1														
																																																1																																
																														1																														1																				
																																																																		1														
														1																													2																																					

																																																							3															1										
																																					1		1																																									
																														1																																																		
																																																											1																					
																																														3																																		
						1																																																																							1	1		
						1																																																											1				1	1						1	1			
																																																						1						1								1												
																																									1																		1																					
						1																																																																										
																														3																												1																						
																																					1					1																																						
																																																											1																					
																																																										1												3										
																																														1												1											3											
																																																																													1			
																																																																									2	1			1	4		
																																																																									1		2	1	4	6		
																	1																																																									2			1			
1									1			1		1		1	1		1																																							1																1						
														1			1	1	1																																					1		1													1		1	4	1					
																		1	1																																					1																	4	6						
													1																																																																			
																																																																										1						1
																		3																																																														
																																																																									1				2			
																														1																																												1						1
																																																																				1												
																																																										1																						
1																																																																																
																																																										1																						
																															1																																																	
													1		1			1																																																															

																																																									1																		1				
						1										1																																										1																					
																1																														1																																			
						1																																																																											
1																																																																																	
									1					1		1	1																																			1																					1									
						1										1																																																										1									
						1			1							1	1																																			1		1							1												1									
						1																																																																											
						1																																																		1																						
																																			1							1					1																		1															
									1					1		1	1	1	1																																			1																					1			1						
																1	1																																																															
														1		1	1																																																														
																																																									1																						
																																																		1																														
						1											1																																																															
																														1																																																								
		1																																																																									1					
																							1							1																																																								
																														4																																																							
						1																								1																																																							
																																																1																											

												1		1		1	1																																	1																														
																																																		1																														
						1																																																																										
																																																																													1			
						1																																																																										
																														1																																								1										
																																								4												1																1												
						1					1																																																																					
																														1	1									1	1					1		2	1							1		2								1		1				1								
																														1							1			2						1										1		1	1																					
											1																													1									1																															
																																											1			1										1		1								1														
																														1																																																		
		2																													1										1			1													1				1	1			1		1	1								1		1		
		1																																																																												1		
																																																																					1	1						1				
																																																																											1	1	1	1		
		1																																																																										1	1	1	1		1
							1									1	1														1	1	1				1													1				1					1			1		1												1	1	1		1
						1																																																																										
																			1																															2																					1									
																																					1																																											
		4					1		1			1	1	1		1	2				1																2													1																					1			1						
		1																																																																														

										1																																																																					
												1				1	1	1	1	1																																																									1			
												1				1	1		1	1																																																												
								1						1			1																																															1															
																																																				1																											
																					2																																																											
		1																																																																													
																																																																											1					
																																																																												1		1		
																																																																						2						1				
																																																																												1	1			
							1																																																																									
		1																																																																													
		1																																																																													
																														2																																																			
																																																																					1											
																																																																											1	1	1	1		
								1					1						1	1																																																												
																																																		1																				1					2	2	1		
																																																																											1	2	1	1		
									1					1				1																																																					1				1	1	1		
													1	1		1	1		1																																																											1		
																																																																													1	1		1
																														2																												1																							

																																																																								1										
																																																1																																	
																																																																											1				
						1																																																																								
								1																																																																							
																																																																												1	1		
																														3										1							1																																					
																														4																											1																						
																																																		1																														
												1																																																																			
																																																		2																														
																																																																									1										
		1																																																																													
							2						2					1																																														1																	
																																																																								2	2										
																	1	1	1																																																													
																																																							1																						
														1			1																																				1								1	1												1									

									1							1	1		1																																											1									1									
																														1																																																		
		1																																																																														
		1																																																																														
																			1			1																																																										
																			1																																																													
						1													1			3																																																2			1							
																			1																																																													
							1																								1																																	1									1							
							1																																																																									
							1																								1	1	1																	1				1										1																
																																																																						1										
		1				1			2																																																																							
						1																																																																										
																	1																																																															
													1	3																							1																					3																						
													1	2																																																																		
													1	4																																						1																		2			1							
																																								1																		1																				3		
						1																																																																										
													2																																													3																						
													3																																																																			
														1																																																																		
																1																																				1																												
																																																										1																						
																														1																																																		
																																																				1																												
																																																										1																						
							1																														1																																											

						1																																																																										1										
																1	1													1																																																										1									
												1	1	1		1	1		1												1																			1																																												
							1							1			1														1											1								1														1																									
									2																																																																																		
																																																																																2		
																			1																																			2																																																																																																																																																																																																																

		1																																																																								1	1	1	1		
												1		1		1	1																																																													
																																																																														1			
																																																																												1				
		1					1																																																																			1				
																																			1																																													
																		1																	1																																																	
																														3																																																						
																																																																				1																
																		1																																																																				
						1																																																																															
																														1																																																							
									1					1		1	1																																																													1						
													1	1				1	1	1																																																																	
												1						1																																																																	
														1				1	1																																																																
												1	2	2		1		1	1	1																																																																				1
														1																																																																								
																			1																																																																			
							1						1	1				1	1																		1																																																		
																																																				1																															

									1			1		1			1														1																																								1			1						
																													2																																						1														
																																																																			2			1				
									1																																																																	
																																																																					1	1				
								1																																																																				
						1																																																																						
																																																																				1							
																	1																																																														
																																														1												1																						
																																																											1																						
																																																																		1														

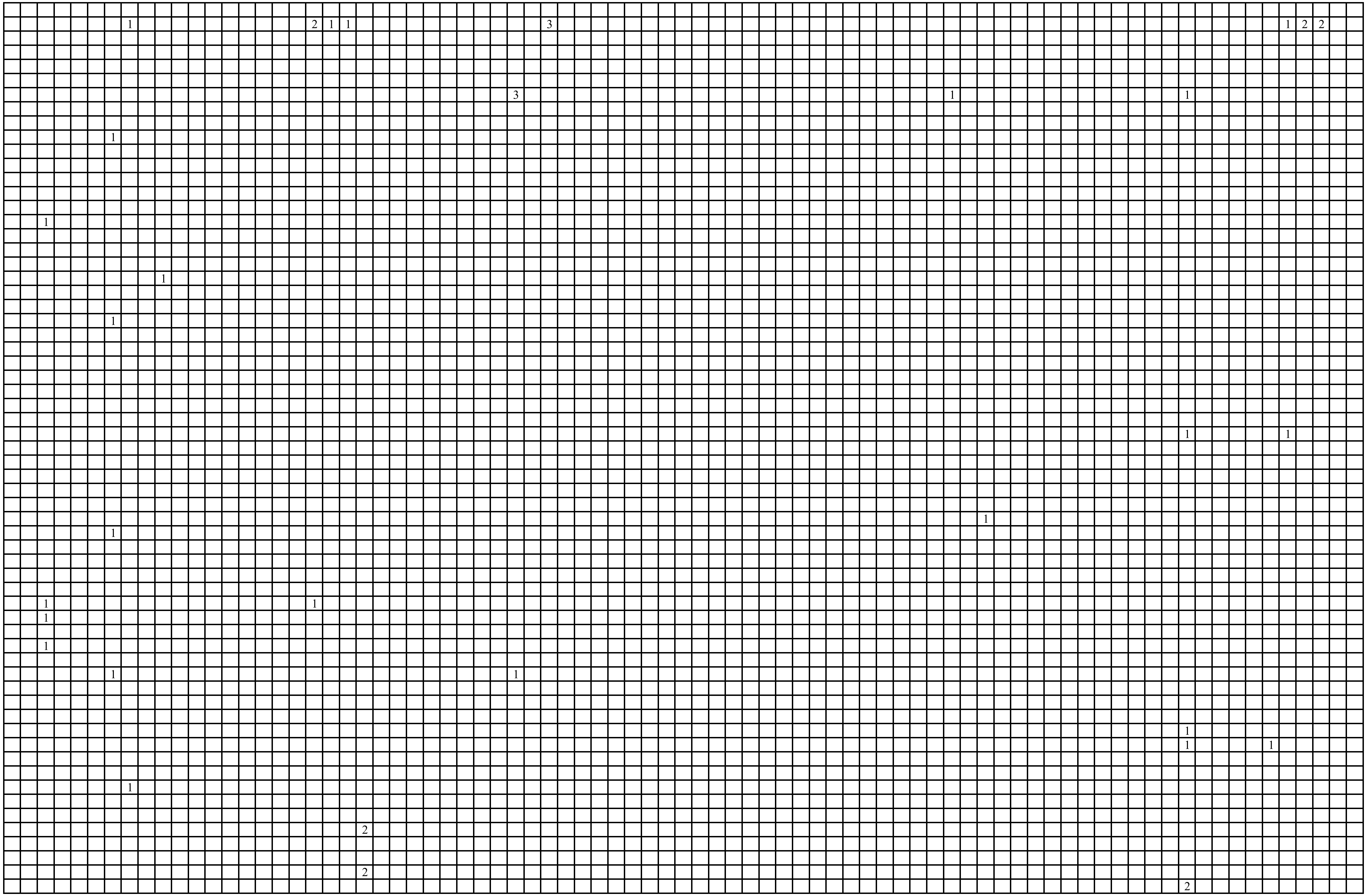

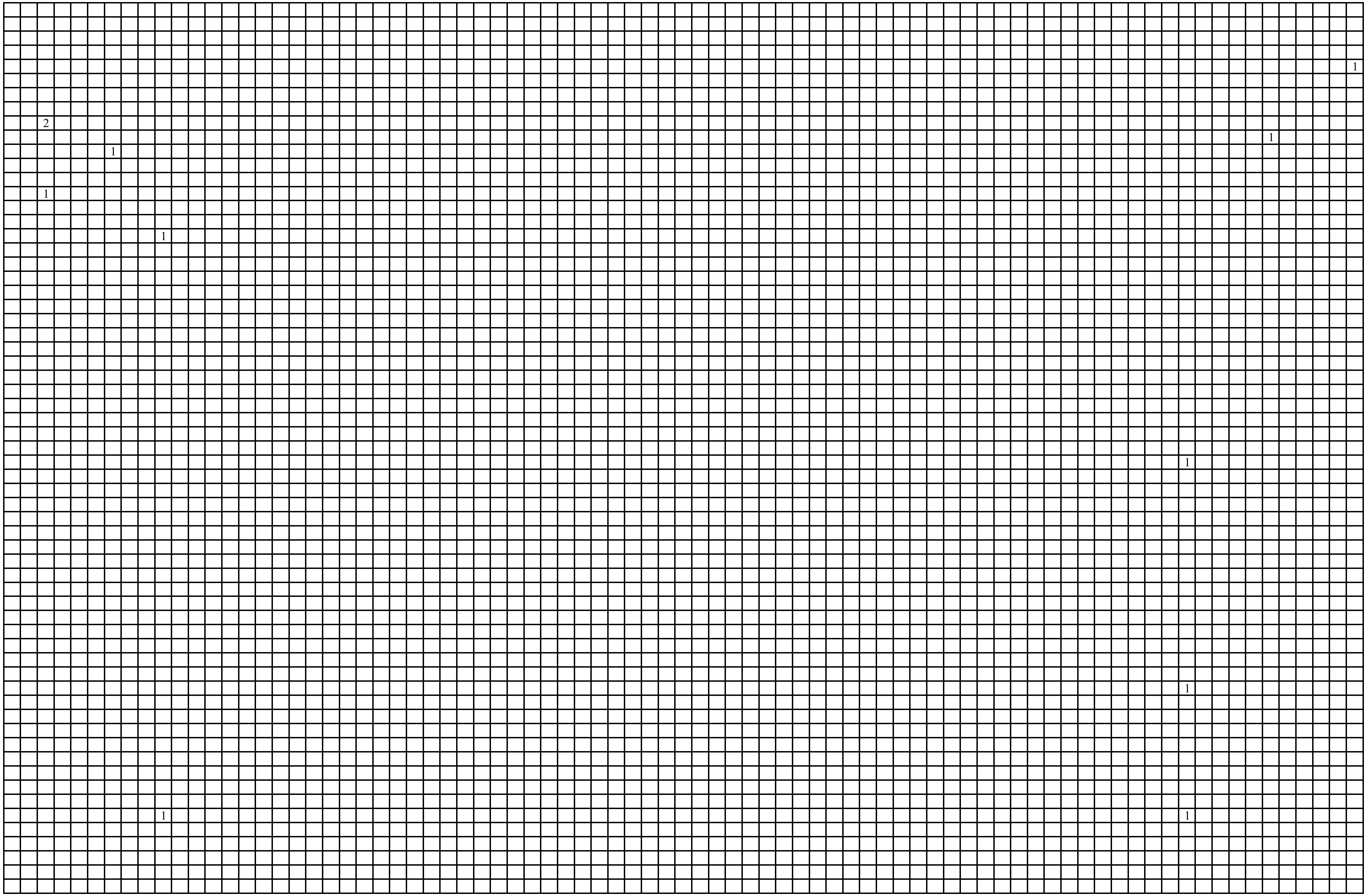
1
2
1
1
1
1
1
1
1

																																																																			1										
									1																																																																					
		1																																																																													
																																													1																																								
																																																																			1										
																		1																																																														
						1																																															1																						
									1																																																																					
																																																																								1	1		
						1																																																1																					
																																																																			1										
																																					1													1																														
						1																																					1																										1	1										

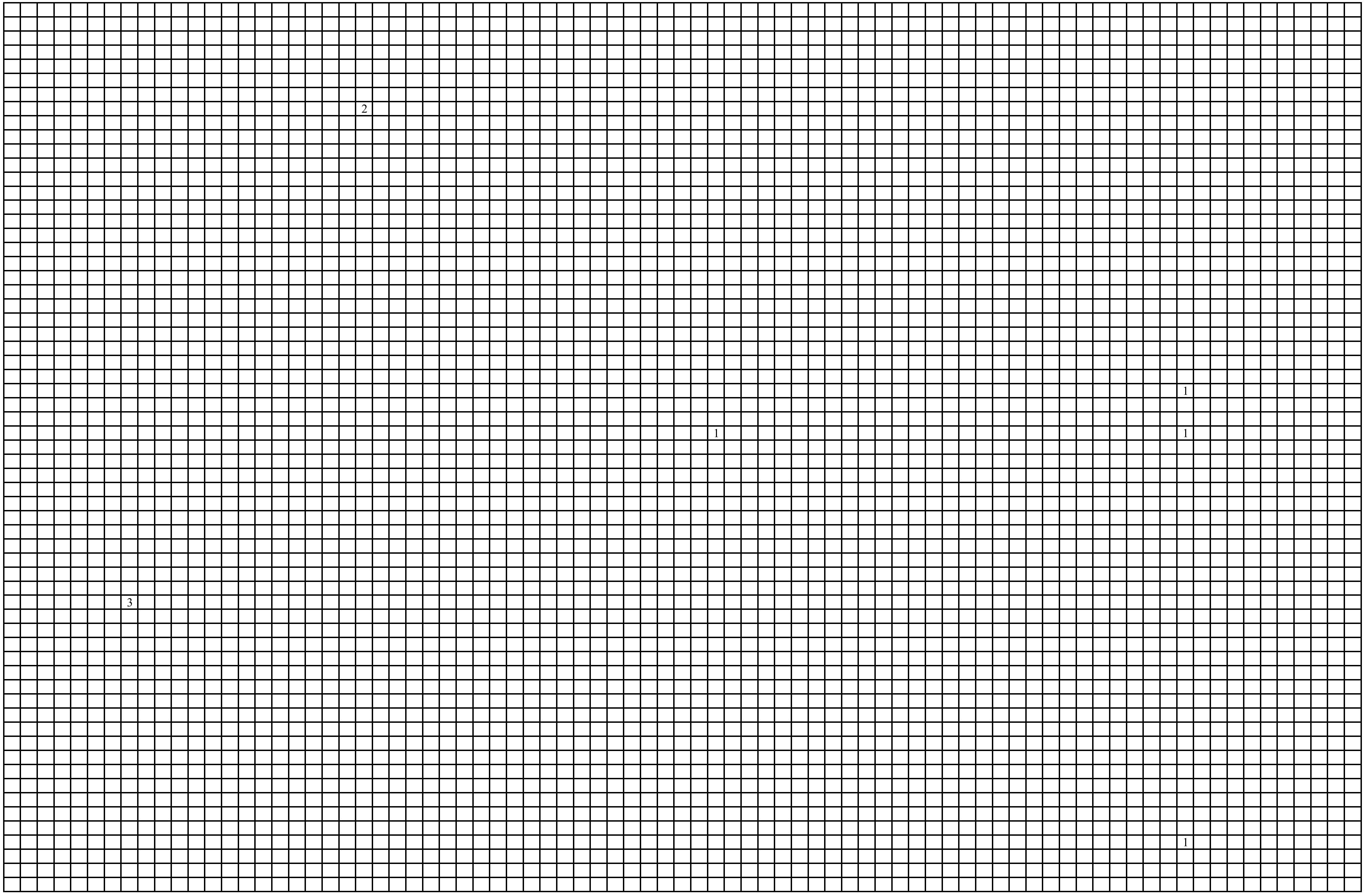
2
1
1
1
3
1

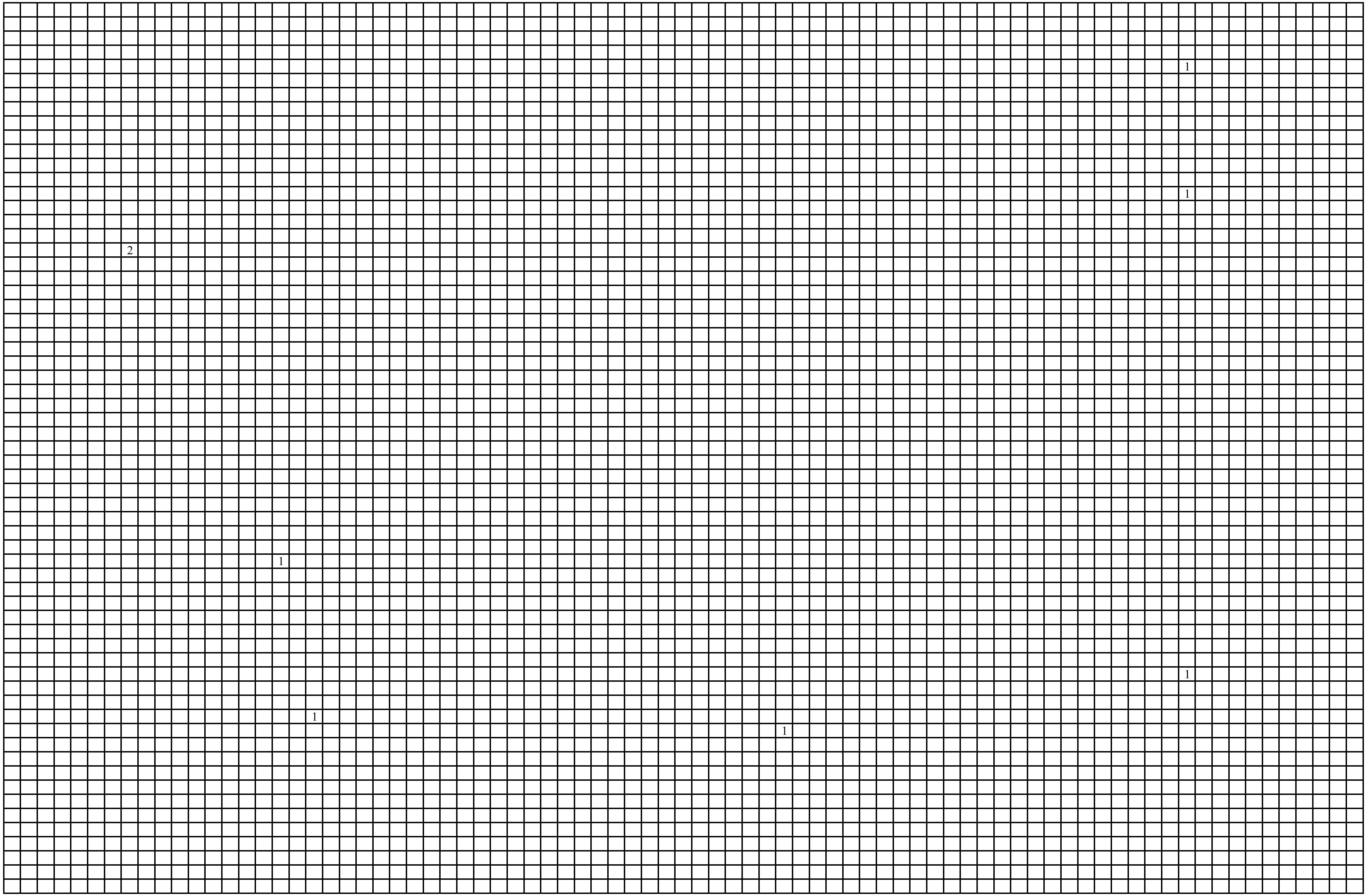
1
1
2
1
1
1
1

																																																																						1										
																																							1																																												
										1																																																																			1	1		1
																															1																1																																						

																																								1																																								
																															1																																																									
																																																															1											
																																																																					1					1
									1																																																																								
													1																																																																			
						1										1																																																						1	1	1	1		1

																																																																	1								1		
																													3																																																								
																																																							1																						
																																																																									1			
							1																																																																									
		1																																																																													
																																											1																																												
																																																																														1				
		1																																																																											1			
		1																																																																														2
		1																																																																																
		2																																																																																
							1			1																																																							1							1			
																																																																											1	1				
		1							1																																																								1								1		
									1																																																																							
						1																																																																											
																																																																								1										
							1	1																																																																										
						1																																																																											

									1																				1																																														2							1			
		1																																																																																
																																																																																			3
																																																																										1	1	1			
																																																																				1					1	1	1			
													1																																																																			1
																																																																												1				1
																																														1																																			
						1																																																													1							1	2		
																														1																																																2							1			
									1																																																																										
																																																																																1		1		
																																																																						1										
							3																																																																													
																		1																																																																	
																																																																						1										

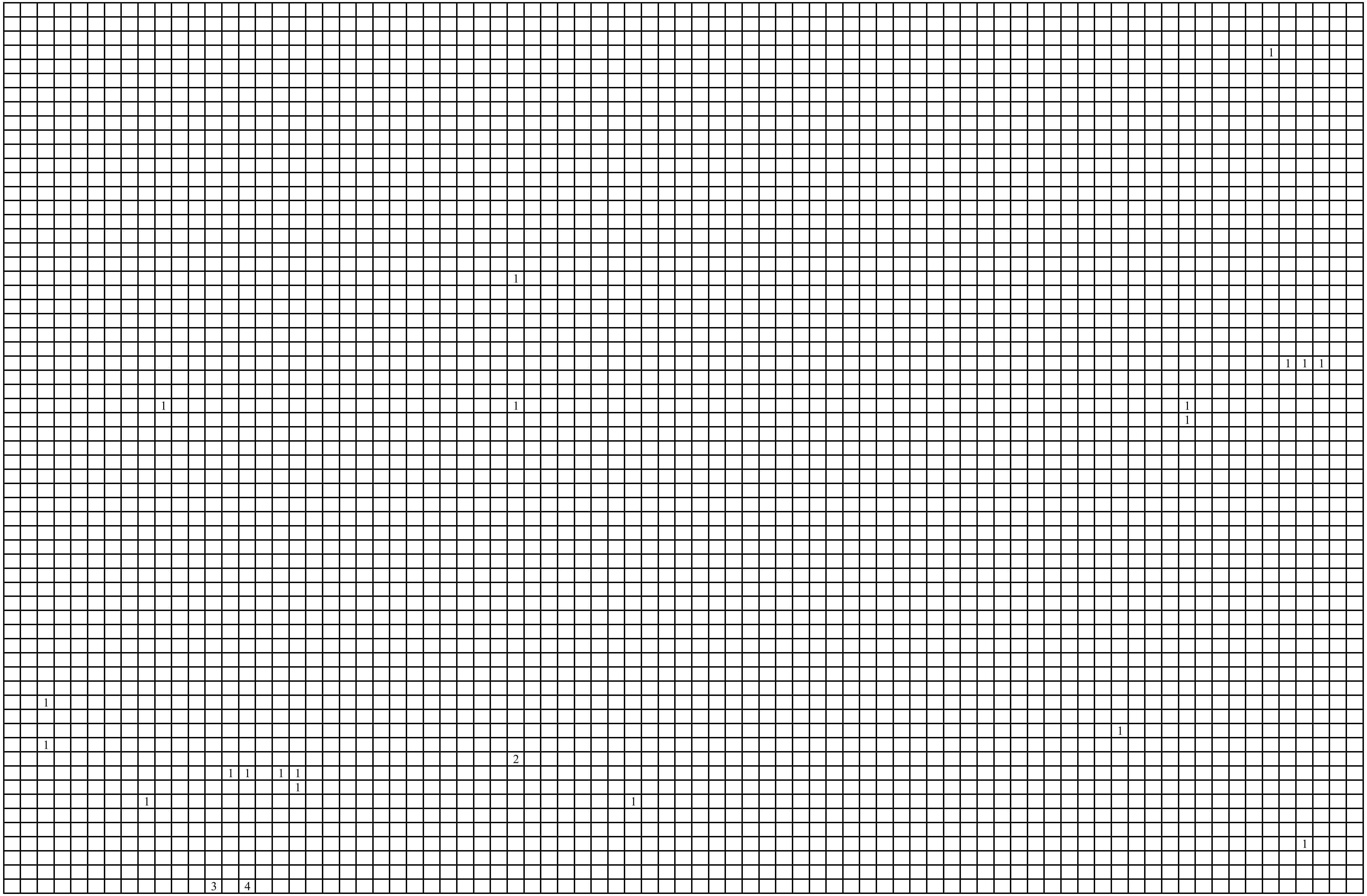
1
1
1 1 1
1 1 1
1
1
1
1
2
1 1 1 1
1
1 1
1
3 4

														3																																																																
																1																																																										1	1	1	1	
							1									1				1													1									1																																												
2		2					1									1		1	1																																1																	1					2							
							1									1			1	1										1																																										1										
																																																																					1										
																				1																					2																																														
																													1																																										1										
																																																							1																							
																																																																					1										
																																																																					1										
																																																																					1										
							1							1				1	1																																																								3							
																																																																											1	1	1	
							1																																																																				1	1	1	
																																																				1																										
																																																																									1							
		1																																																																												
																																																														1														
																													1																																																					
		4																																																																												
							1																																																																									
		1											2																																																						1	1										
							1																																																																						1				

		2																																																																												
																																																																			1										
																3																																																													
																		1																																																										
													1																																																																		
																																												1																																													

																							1			LHX8			15	59
																										LHX9			6	24
												2														LMX1A			9	13
																										LMX1B			24	69
																										BARHL1	HB.NK		0	3
																										BARHL2			1	2
																										BARX1			3	9
																										BARX2			0	2
																										BSX			0	1
																										DBX1			0	1
																										DBX2			0	7
																										DLX1			2	7
																										DLX2			10	21
																										DLX3			1	5
																										DLX4			0	6
																										DLX5			19	21
																										DLX6			3	32
																										EMX1			5	10
																										EMX2			2	36
																			1		1	1				EN1			32	119
																										EN2			1	7
																										HHEX			1	54
																										HLX			2	48
																										HMX1			0	0
																										HMX2			1	6
																										HMX3			2	10
																										LBX1			2	27
																										LBX2			0	7
																										MSX1			16	29
																										MSX2			36	201
											1					1					1	1		1		NANOG			50	273
																										NKX1-1			0	1
																										NKX1-2			1	2
																										NKX2-1			44	185
																										NKX2-2			1	5
																										NKX2-3			2	4
																										NKX2-4			10	12
																4										NKX2-5			35	91
																										NKX2-6			0	1
																										NKX2-8			0	0
																										NKX3-1			18	65
																										NKX3-2			6	13
																										NKX6-1			2	4
																										NKX6-2			0	1
																										NKX6-3			0	0
																										NOTO			7	34
																			1		1	1		1	1	TLX1			59	180
																						1		1	1	TLX2			34	147
																			1		1	1	1	1	1	TLX3			71	265
																										VAX1			3	15
																										VAX2			1	11
																		1								VENTX			19	113
																										ALX1	HB.PD		4	19
																										ALX3			1	33
																										ALX4			14	19
																										ARGFX			0	8
																										ARX			0	29
																			1			1	1	1	1	CRX			97	285
																										DMBX1			0	2
																						1				DPRX			4	17
																										DRGX			0	0
																										DUX4			7	295
																						1				DUXA			2	11
																										ESX1			0	3
																	1		1					1		GSC			41	81
																										GSC2			5	60
																										HESX1			4	17
													1													HOPX			4	18
																										ISX			4	32
																										LEUTX			1	46
																										MIXL1			0	0
																										NOBOX			0	5
																										OTP			1	9
																		1								OTX1			7	192
																										OTX2			10	57
																										PHOX2A			7	9
																										PHOX2B			0	4
																		1								PITX1			31	138
																										PITX2			21	75
																										PITX3			2	5
																										PROP1			9	64

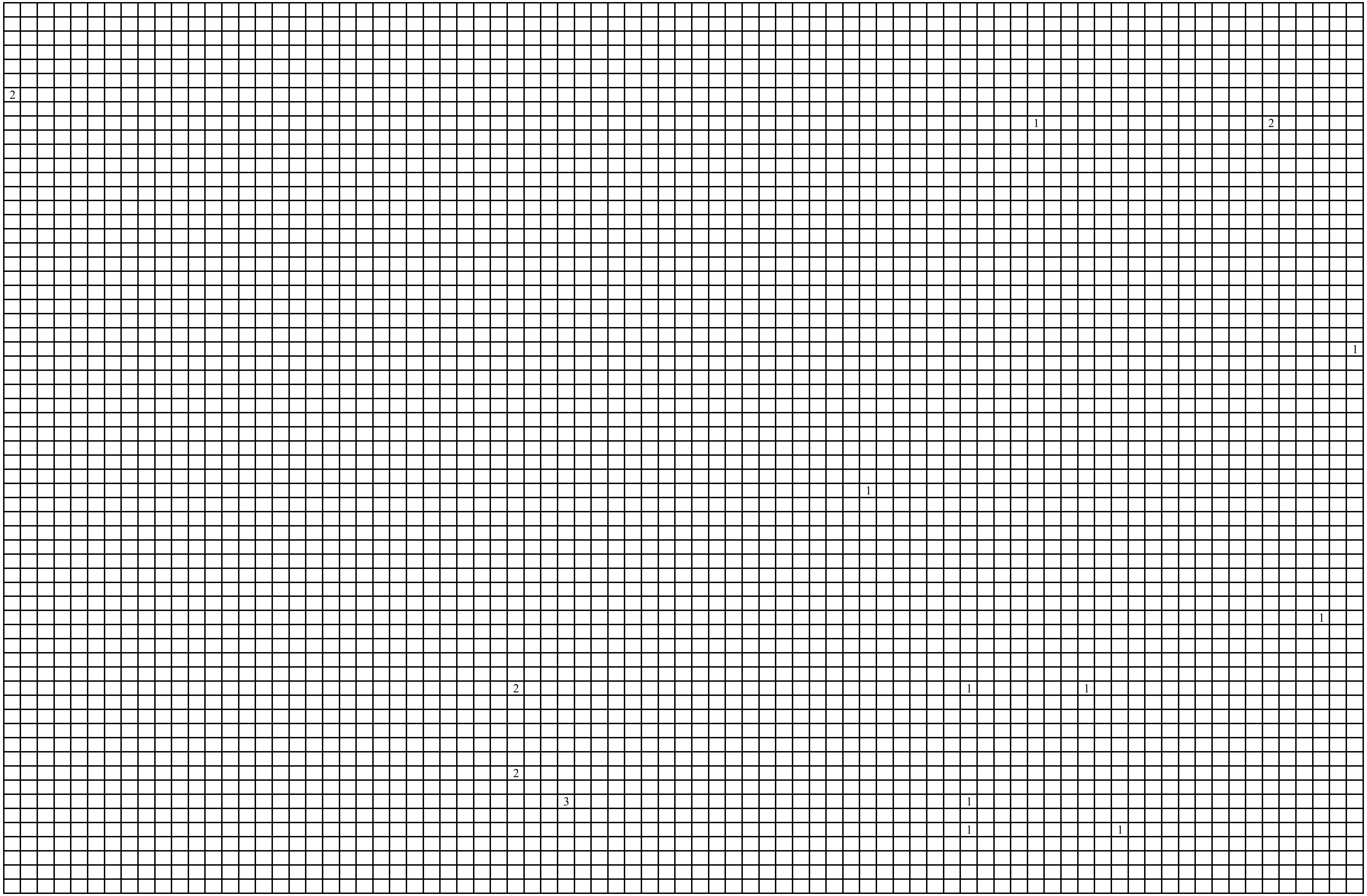
2
1
2
1
1
1
2
1
1
2
3
1
1
1

																			1																																							1																							
																									1																																																												
																1									1																						1																															1		
																																															1										1																							
																																																	1																																
																																						1																																															
																																						1																																															
																																						1																																															
																																																																												1			
1																																	1																																															
																																	1																																															
																														1																																																			
			1																																																																																
																																						1																																									1					
																																	1																																															
																																																	1																																
																																	1																																															
1																																																																																	
																																	1																																															
																																	1																																															
																																																								4																							

																																																			1																													1
1																																																																																	
																																																			1																													
																													1																																																				1			
																						1											1	1																																														
																																																						1																										
																																																									1																							
																																																													1																			
																																																													1																			
																																																									1																							
											1				1																		6				1																1																										
																				1													1																															1										2						
						1																																															1																										
															1				1																											1		1																																
															1																																																																1		
																																													1																																			

														1																						1																	1																										
																					1							1																1			1									1																							
														1																																																																			
														1																																																																			
																																														1		1									1																							
																																																										1																							
1																																																																																		
														1																																																														1			
																																														1		1									1							1																
																																																										1							1																
																																														1	1	1									1							1																
						1																																																																												
																																																																															1			
																																																										4								1															
																																	1																																																	
																		1																															1	1	1																																

																																														1	1	1																																
																													1			1													1	1	1									1		1					1																
																												1																																																			
																			1																																																													
																														1																																																				
																			1																															1																1															
																																															1	1									1																	1						
																													1																																																2	1		
																																															1	1									1							1																
																													1																																																					
													1																															1																																								
																																																																						1						
																													1												1						1	1		1	1																	1												1
																																														1		1									1																							
																			1																																																																
																			1																											1											1							1																
																													1				1													1	1	1									1		1					1																
																			1																											1		1									1		1					1				1												
	3																																																								1																							
																																																										1																							
																																																																												1			
			1																																																																				1				1	4		1
																	1																																																															
																																																								1																										
																																																									1																										

																																	1																																																						
																																																			1																										
																																														1		1										1										1													
																																																1																																
												1			1													1																							1														1														
																													1																			1																																
																																																				1																										
						1																																																																											
																																														1	1	1									1																							
		1																																																																														
1			1																																																					1																							
																																																																														1						
																												1																																																							
		2																																																																															
1			1																																																																													
		4																																																																															
												2			2													3	2																																																								
												2			2													2	2																																																							
																																																				1																										

												3			2													4	3																																																	
												2			2													3	2																			1																														1			
																														2																																													1						
																																	2																																																
																																					1								1																																							
																																				1												1																																		
																																				1																																														
																																														2																																			
																													1								1											2								1									1									1			
																																																			2																													
																		1																																				2																										
																																																	1																																
																																																																												1												
																																																	1																																
																																																																							1															
																														1																																																	2						
																																																																														2					
																													1																			1																															1		
																																																																													1	6		
																																																																																			2
																																																						1																											
																													1	1		1								1											1							1																			1			

																																					1																																										
																																														1																																		
																																																									1																						
			3															1	1																											1	1																																
			3																																									1	1																																		
			3																																																					1																						
			3																1																											1											1							1																
			3																1																																																												
			3																																																																												
																															1																																1																			
																			1																											1	1	1									1							1																
1																														1																1	1	1									1				1																			
			1																1																	1	1									1	1	1																									1				1	1		
																																					1																				1																						
																																																																1																
																																																			1						1																						
																																				1																																												
		1																													1																								1																								2			
																														1																																																			
																																																									1																						

																													1																												3							1																		
														1						1																																																										
																																																																							2		
																														1																																																		
																													1				1												1		1									2							1																		
1																			1														1				1									1		2																																		
																																												1	1	1																																		
																																								1							1																	1																		
																																															1																																		
	1																									1																			1																																							
																		1															1																																																
																													1	1			1																																																
																																																												1																			
																																																																										1			
																																					1																																																	
																																													1																																				
																																					2			1																																													
																			1																											1	1	1									1							1																		
																			1																		1											1	1	1																																		
																																	1													1	1	2									1							1																		
																																														1	1																																		
																																	1																													1																	2	
																														1																																																		

																																	1																																															
																													1																																																								
																			1																											1																																		
																														1																																																							
																																																						1																													
																																																												1																							
1																																	1																																																				
																																																																												1			
																													1																																																									
																																																	1									1																							
																	1																																																																	1			
																																				1																																																			
																																		2			1																																																								

																																					1																																																
					1																																											1																																				
																																	1																																																			
																																	1																																																			
														1	1																					1					2												1																									1		
																																	1																																																				
																													1																																																								
																														1																																																					
																																									3																																															
	1																																																																																				
																																																								1							1												1	1		
											1																			1							2				2																																													
																																																																		1																
																																																		1																											1			
																																																		1																																
																																																1			2																											1			1
																																																																1																		
																																					3																																																

																																																									1																							
																																																																								1								
																																				1																																									1			
																																	1																								1																							
															1															1																																																		
																																					1																																											
																	1																																																															
																																																									1																							
																																													1																																			
																																	1																																															
																																				1																																												
																																																									1							1																
																																								3																																								
																																					1																																											
																																															1																																	
																																															1	1			1																													
																													1																																																			
																			1																											1	1	1																		1														

																			1											1																1	1	1									1							1																
																																	1													1		1																																
																																														1		1	1																															
																													1																	1											1																1							
																														1																											1																							
																																																																													1			
																																																																													1			
1																																																																										1						
																												1												1								1									1																		2					
																																								2																																								
																																								1																	1																							
																																								1																	2																							
																																								1																																								

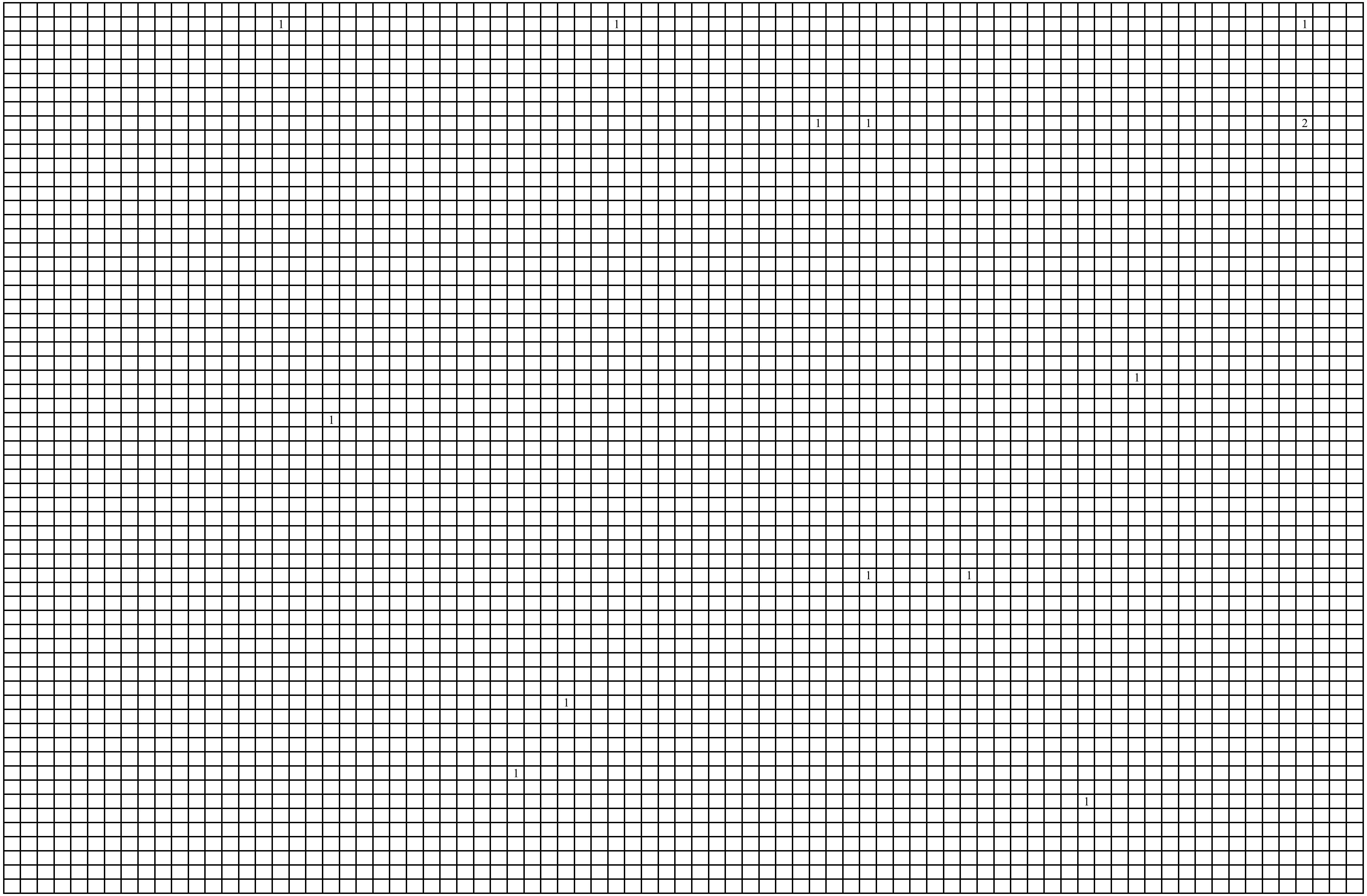
1
1
1
1
1
2
1
1
1
1
1
1
1

																														1																																																					
1																																																																																
																																																																								1							
																																																				1																							
																														1																																																						
																														1																																																							
																																												1																																		

																			1																																																										1																							
																															1																																																										
																																					1																																																														
																			1																											1																																						

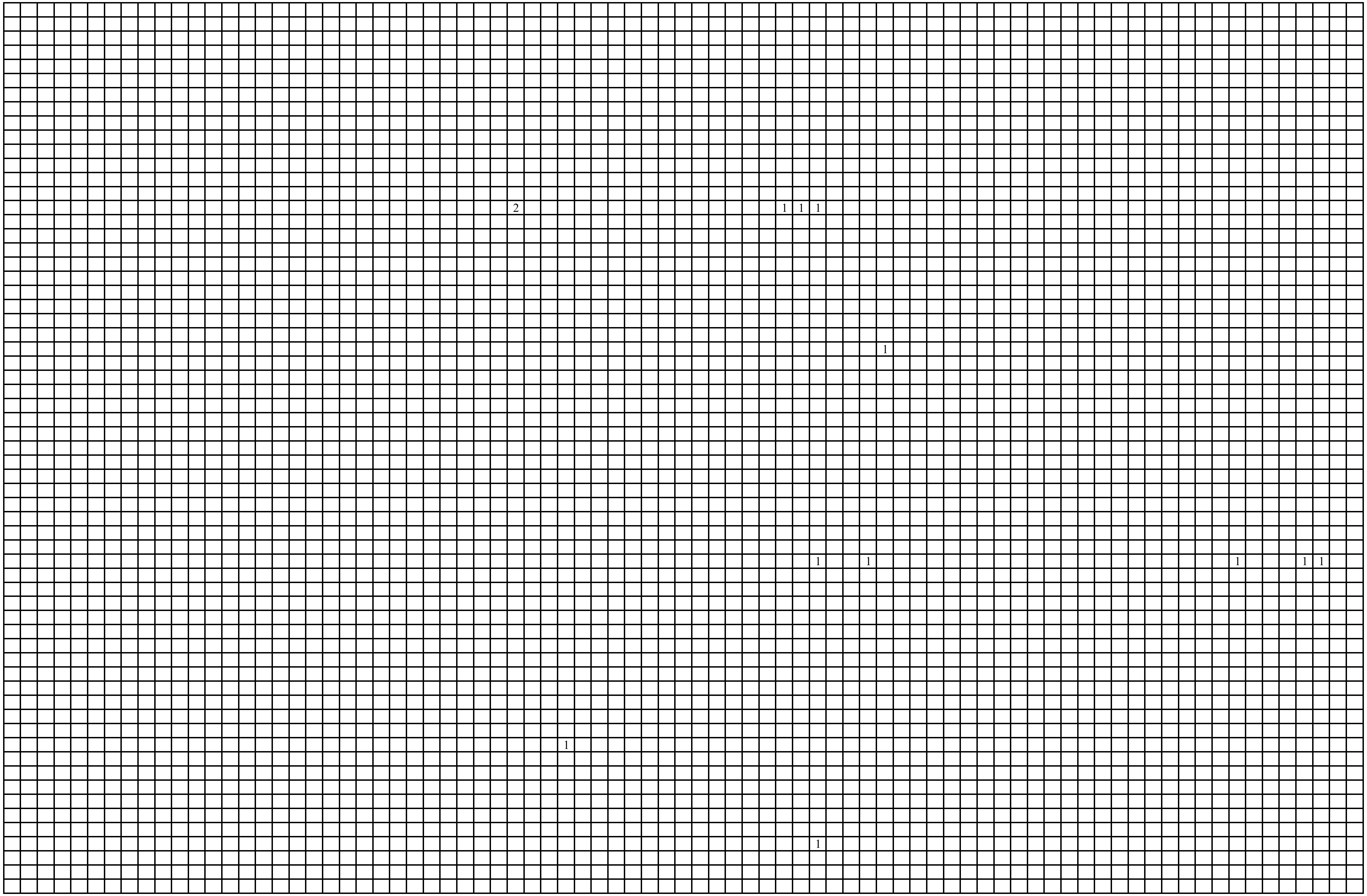

2
1 1 1
1
1 1 1 1 1
1
1

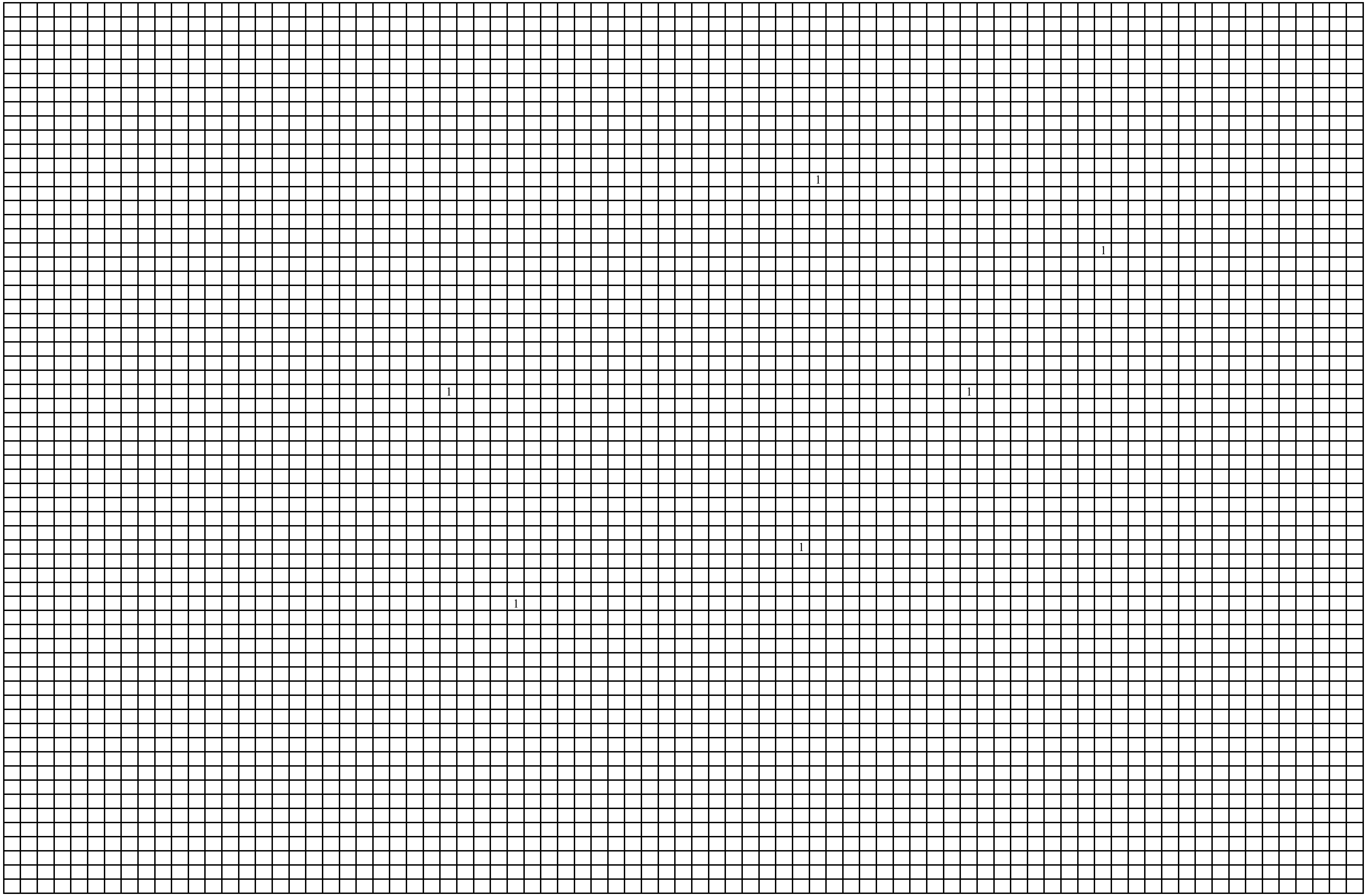
1
1
1
1
1
1

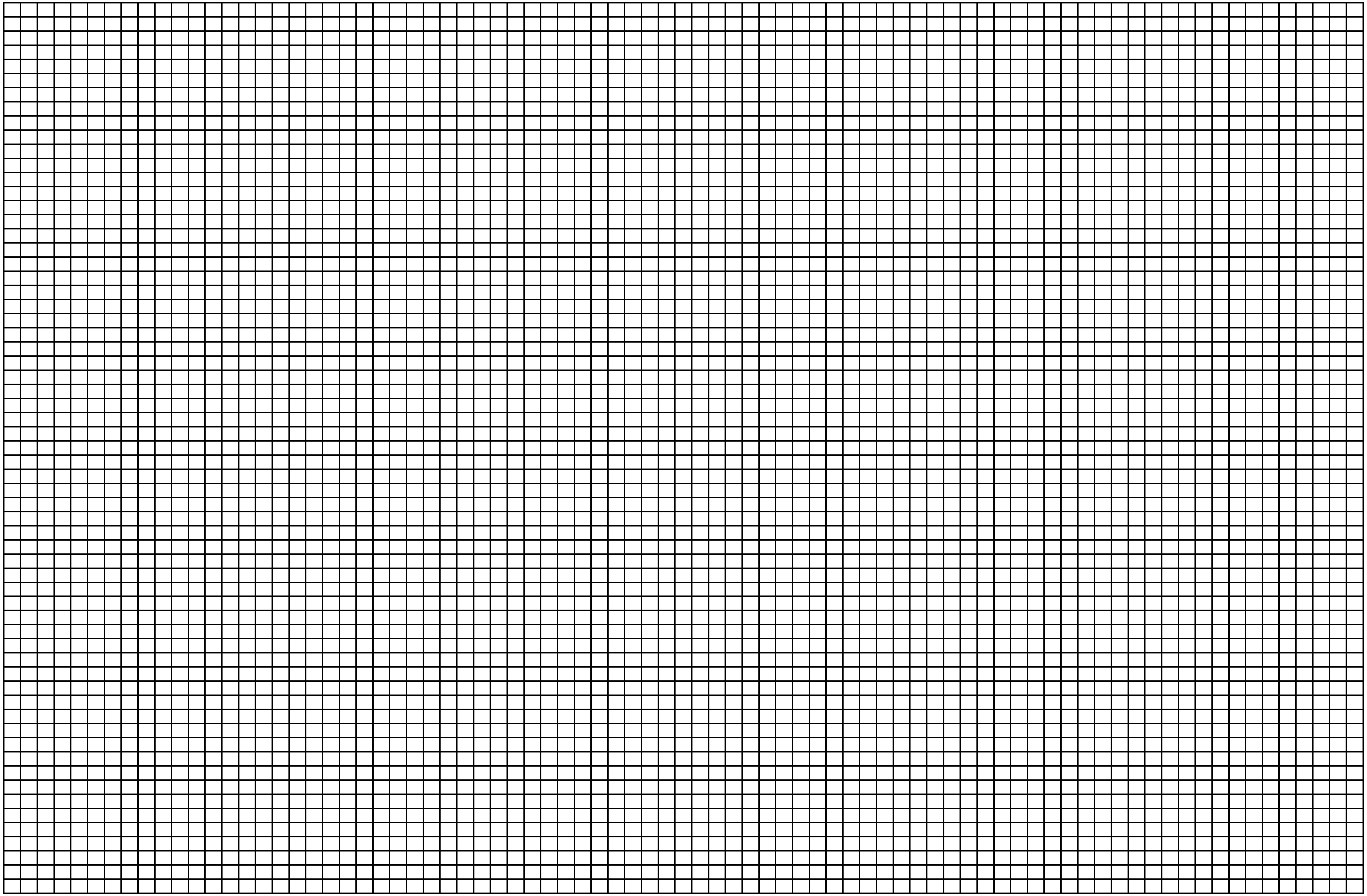

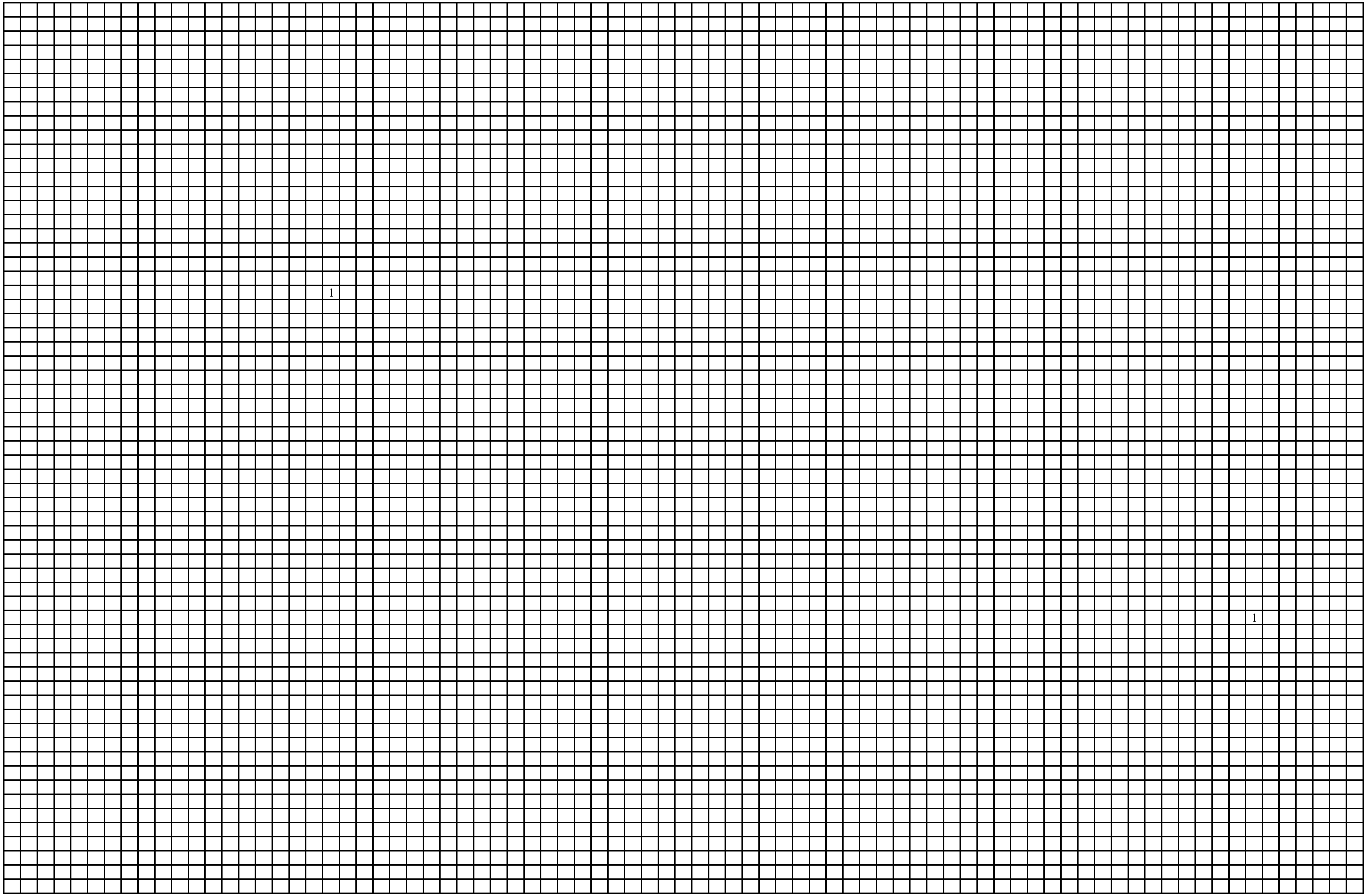
1
1

																																									1																																		
																																																													1																
																			1																											1		1															1								1							

																																																									1							1																
																													1																																																			
																																																									1							1																
																	1																																																															
																																																									1																							
																																														1											1							1																
																														1																1		1									1							1																
																														1																																																	
																														1																																																	
																														2																																																	
																													1																																																			
																			1																											1	1	1									1																						
																																																				1																											
																																														1											1																							

																																							1																																								
																																													1											1							1																
																														1																																																									
																																														1	1																																	
																																																						1					1																		
																																													1																		1																
																																																																	1																
																		1																														1											1							1																
																																																																		1															
																																																																								1											

																																															1		1																1																1
																			1																												1		1									1							1																
1																			1											1																1	1	1									1							1				1												
																																																								1																							
																													1																																																									
																													1																																																									
																																																																											1			
																												1																																																										
																																					7																	1			1																							
																													1																																																									

																														1			1																																																
																														1																																																	
																																																1									1							1																	
																			1																										1		1									1							1																	
																																								3																																														
																													1											1																																														
			1																																1																																																		
																																																	1																																				
																													1																						1																																				
																																																																																			1
																													1																																																										
																																	1																			1																																				
																																								1																																															
																																														1	1	1									1																											
																																																									1																											
																																																									2																											
																																																																															1			
																														1																																																									
																																	1																																																					

																								1																						1	1										1																							
																													2																																																								
																																																						1																							
																														1																											1																							
																																																1									1																							

	PRRX1	2	9																										
	PRRX2	0	0																										
	RAX	2	3																										
	RAX2	0	0																										
	RHOXF1	1	3																										
	RHOXF2	21	141								1																		
	RHOXF2B	0	0																										
	SEBOX	0	0																										
	SHOX	5	9																										
	SHOX2	0	8																										
	TPRX1	1	20																										
	UNCX	1	3																										
	VSX1	27	77				1	1																					
	VSX2	1	32																										
HB.PHTF	PHTF1	1	19																										
HB.PROS	PROX1	14	37																										
	PROX2	0	2																										
HB.SINE	SIX1	5	64																										
	SIX2	0	46																										
	SIX3	9	23																										
	SIX4	22	46																										
	SIX5	4	28																										
	SIX6	1	12																										
HB.TALE	IRX1	0	2																										
	IRX2	1	6																										
	IRX3	0	1																										
	IRX4	4	7																										
	IRX5	0	4																										
	IRX6	5	11								1																		
	MEIS1	30	55																										
	MEIS2	35	115																										
	MEIS3	6	26																										
	MKX	1	16																										
	PBX1	53	105																							1			
	PBX2	41	99																										
	PBX3	22	115																										
	PBX4	25	92																										
	PKNOX1	33	94																										
	PKNOX2	10	51																										
	TGIF1	13	47																										
	TGIF2	4	21						1	1																			
	TGIF2LX	7	37						1	1																			
	TGIF2LY	3	53																										
HMG	HMGB1	79	512																										
	HMGB2	34	287																										
	HMGB3	18	75																										
	HMGB4	0	7																										
	HSFX4	0	0																										
	KMT2C	51	188																1						1				
	MAEL	4	29																										
	MEIOSIN	0	0																										
	PMS1	7	122																										
	WDHD1	7	126																										
HMG.PBRM1	HMGXB4	27	127							1																			
	PBRM1	96	283	2	1	4																			1				
HMG.SOX	BBX	17	132						1																				
	CIC	206	717						1	1																			
	HBP1	14	142						1	1																			
	SOX1	4	9																										
	SOX10	31	79	1	1	1				1								1											
	SOX11	3	6																										
	SOX12	1	8																										
	SOX13	23	47																										
	SOX14	7	13																										
	SOX15	41	148	1	1	1																							
	SOX17	30	80	1	1	1	1	1																					
	SOX18	3	6																										
	SOX2	247	1645	4	2	1	3	3		1		1				1	3												
	SOX21	8	14																										
	SOX3	10	19																										
	SOX30	1	20																										
	SOX4	14	82																										
	SOX5	83	187							1	2																		
	SOX6	55	164							1																			
	SOX7	39	80	1	1	1		1				1																	
	SOX8	3	11																										
	SOX9	41	129	1	1	1	1	1		1																			
	SRY	19	33													1													
HMG.TCF7	LEF1	33	119																										
	TCF7	6	26																										
	TCF7L1	3	12																										

																																		1																																																
																																																																									1			
																																																																								1				
																																																														1													
																																																												1																
																																																				1																								
																																													1												1											1						1							
																															1																																																										
																																																														1													
																																																								2																					
																																																	1																						1						
																																															1																														
				1																																																																												
																																																						1																								

																																																				1																								
																																																															1											4		
																																																				1																								
																																																																							1									
1																																			1								1	1									1	1	1		3												1		1									
																																			1								1	1	1								1		1																											
																																																																																1
																																					1						2	1	1											1								1			2														
												1																																																						1													
																																																				1																								
																																																				1																								
																																																																				1													
																																																				1																								
																																																																					1													
																																																																												1						
																																																				1																								
																				1																			2									1								1											1										1			
																																																																				1													
																																																																														2		
																																											1					1						1		1																										
																																																																													5				

					1																																																																									
																																																												2														
																																																					1																								
																																																																2														
																														1					1																						1																								
																																																																								1								
																																								1																																															
					1																																																																									
											1																												1				1	1	1			1								2																					1			
																														1					1									2	2	1							1		2		2											1												1
																																																									1											2														
																																																								1																										
																																																									1	1		2																								
																																																			2																		1														
																																																																														1		
																																																									1		1								1	1																
																																																											1																								
																																																											1																								
																																		1															1																																
																					1																																																																
																														1				1	1									1	1												1								1	1		3					1	1								
																																																											1																								

																	1																1	1	1																																													
																																							1																								1								1									
																																																1																																
																																		1																																																	
																																																				1			1																									
																																									1															1								1			2					1								
																																									1												1			2											2													
																				1	1																											1								1																								
																	1																																																															
												1																																																		1		1			1					3	1	1						
																																																														1										2								
												1																																																		1		1	1		2					3	1	1						
																																	1																						1																									
																																		1																	1																												
																																																								1											1													
																																					1																														1													
																																																1								1																									
																																																								1																									
																																																								1																									
																																																								1											1													
																																																								1								1			5				1	1	1			1				

												1																																												1								1			2						1	1							
												1																																												1								1	1		1					1	1	1						
																																																								1			3																		1			
		2											1		1				1	1																	1								1			1						1		2														2						1				1				
																				1																												1								1																									
																																																								1																									
																																											3					1								1																									
																				1																												1						1		1			1			1		1	1		1					1	1			1				
																																	2				1																											1	1		2					1	1							
																																											2																																								
																																																											1																							
																																																																												1			
																																											2	1										1													4	1												1
																	1									1				1																													1																						
																																																								1											1													
																																																																								1						
																																																																			1													
																																																																							1									
																														1					1																																														
												1																																																							1					1	1							
																																																																				1													
												1																																																							1							1						
												1																																											1											1					1	1							
												1																																										1											2					1	1	1						
																																																																								1								
																																																															1								1	1							
																																																																									1	1							
																																																																				1											2		
																										1					1	1			1																																																		
																																	1										1													1																								
																																																								1																								
																																																								1																								
																																		1																						1									1		2													
																													4				1	2	1																																																	
																																	1										1																																															
																																				1																																																			

																																											1																																					
																																	1	1		1																																												
																																	2																																															
																																	1		1																																													
												1		1																																										1								2			1													
																													1																																														1					
					1																																																			1																								
																																	1	1		1																																												
																																											1																																					
																																	2	1		1																																												
																																		1																						1																								
																																											1																																					
																																											1													1																								
																																																								1																		1						
																																											1																																					
																																	1			1																																												
																																											1																																					
																													1				1										1																																					
																																													1																																			
																												1																	1																																			
																																																																1			1													
																																	1																																															
																																																																			1													
																																																1						1																										
																																											1		1			1						1		1																								
																				1																												1						1		1																1								
																																											1	1					1						1		1																								
																																																						1		1																								
																																																						1																										
																																																																			1													
																													3	3			3	3	3			3																		1																								
																																																																													1			
																													1																																																			
																													1					1	1													1								1																								
																																																																													1			

					1																																							1																														1								
					1																																																		1													1	1												1
					1																																																																														
																																																										1									1															
								1																																																1			1																					
					1																																																																															
																														1																																																						
																				1									1	1				1														1						1		1																								
																													1																			1						1		1																								
																																																1						1		1																								
					1																																																							1																	
1																																																																																				
																																																																								1													
					1																						1					1	1														1						1					1				1						1			3								
																																												1										1																											
																																		1														1														1										1								
																																																										1																								
					1																																																		1										2													
																																																									1																					2		
					4																																																																															

																																	1	1			1																																												
																																																								1																								1	
																																																								1																								
																						1																																																										
																																																								1								1			2														
																			1																																																													
																														3			5		2	1																																													
																													3	2	2		6	3	3	4																																1														
																														2						1																																														
								1																					5	6							8	1																		1																									
								1																						3						1	4	1																		1											1														
																													2	3					2		1	1																		1																								
																													1	4	1			1		2	4	2																														1														
								1																									8	4	1	4								2																																					
																																	1	1	1	2																																													
																																																								1																								
																																										3																																							
																																									3																																								
																																											2	6	4																																					
																																					2					6		2																																							
																																											4	2																																						
																																																																	1		1							1		1				
																																																			2																														
																																																					2																												
																																																											1					1	1		1				1			1		1				
												1					1			1													1	1	1					1																			1					1	1		2					1	1			1				
																																																									2																						
																																																																			2					2								
																																																						1		1																							
																																																														1											2								
																																																													1		2										3	2								
																																																																			4												
																				1																																	1		1																							
																																																1						1		1																							
																				2										1				1		1												1						1		2		2					4				6	5	1			1	1		1					
																																																																			5							1						
																																																																			1		2												

																																																						1																									
																																																								1		2		2		3					1					2	2	1						
																																																								1						2					1					2								
																																																1						1														1				1								
																																																																			1													
																																																1						1		1																				2				
												1																																																																		
																																										1														1										1							1				1		
										1																																1													1			1					1	1		2				1	2	1							
																																																												1					1			1					1	1							
																																																																										1	1							
																																																								7											1		2													
																																																																				1										1		
																																				1																																																		
																																										1									1	1	1		1											1													
																																										1	1	1									1																			1							
																																										1	1																																									
																																																													1																					
																																																																			1																
																																																																						2													
																																																																					2	1													
																																																																						1						1							
																				1	1																																																														
												1								1																																				2			1					1	1		2					1	1			1				
												1																																				1								2			1					1	1		3					1	1	1						
																				1																																				2			1					1	1		3		1			2	2			1				
																				1																												1						1		2			1					1	1		2					1	1			2				
																																																										1																								
																																			2																																																		
																																											1													1										1													
																																																																										1									

																																																																													1	
															1																																																																
																																																															1		1		1	1								
																																																					1																								
																																																2																																	
																																																																			2					1								
																																																		1																													
																																																																			3													
																																																							1										1													
																																																																											1							
																																												2	2																																									
																																																							1	1																							
																																													1																																											
																																					2																																	1							1						
																																													1																																											
																																													5								1					2						1		1							3									
																																																																				1													
																																													1													1											1													
																																																			1								1																								
																																															1																																							
																																																		1						1		1																								

																																																1						1		1																								
																																																			1																													
																																											1																																					
																	1																																																															
																																											1																																					
																																																								1																								
																																																								1																						1		
																																																								1																						1		
																																																								1																								
																																																								1																								
																																	1	1																						1																		1						
																																																								1																								
																																																								1											1													
																																																								1											1									1				
																																																								1																								
																																											5	3																																				
																																							1																		1															1						1		
																																							4									1																												2		3		
															1																								2	1																1																				3	1	5		
																																																								1					1																	1		
																																																																								1		1						
																																																														1										1		1				2		
																																					1																																																																																					
																																																																														1		
																																					1																																												
																												1																																																							
																														1																									1										3													
																																																																			1													
																													1	1			1	1	1		1																																										

																																																1								1																1								
																																																1						1		2											2													
																																																																							1											
																																																1					1																												
																																																							1																										
																																																																			1													
																																																							1																						1				
																																																							1		1																								
																																																1						1																										
																																																							1		1																								
																																																1								1			1														1							
																														2																									1																										
																																													1											1													1													
																																																																					1													
																				1	1													1											1			1						1		1						1										1								
																																																							1										1			1			1										
																																																							1																										
															1																																	1						1		1																								
																																																									1																								
																																																				1					1																								
																																																																				1		2													
																																																									1																					1		
																																																							1														1													
																																																														1						1		4					1	1							

																																																											1					1	1		3					1	1	1						
																																																							2											1					1	1		1					
																																																																			1					1	1							
																																																				4		1											3							1		1				
																																																				8													2							1		1				
																																																																							1									
																																																																			1													
																																											1																																					
																																																																				1													
																				1																													1																								1					1			
																																																				1		1																								
																																																1						1		1																								
																																																					1																	1								
																																																				1		1																2								
																																																1																																
																																																1						1		1																								
																																																					1																										
																																											1											1		1																1								
																																																				1	1																									
																														1																																																	1			
																																																																			1													

																				1													1	1														1						1		1																				1								
					5																																																																											
																																																				1																											
																																																				1																											
																																																														1																
																																																																									1							
																																																				1																											
																																																				1																											
																																				1																																																
																																																				1											1																
																																																														1																
																																																																1														
								1																																																							1														
																																																														1																
																								1																																																															
																																																				1																											

																																																																							1									
																																											1										1	1		1											1													
																																																					2																								
																																																					1																								
																																																																								1								
																																																					1																								
																																																		1																										
																																																					1											1									1				
																																																					1																								
																																																					1																								
																																																					1																								
												1																																																			1																
																																																					1																								
																																																																											2			

																																																								1											1													
																																																																			1													
																																																																									1							
																																																								1																								
																																																									1																							
																																											1													1											1													

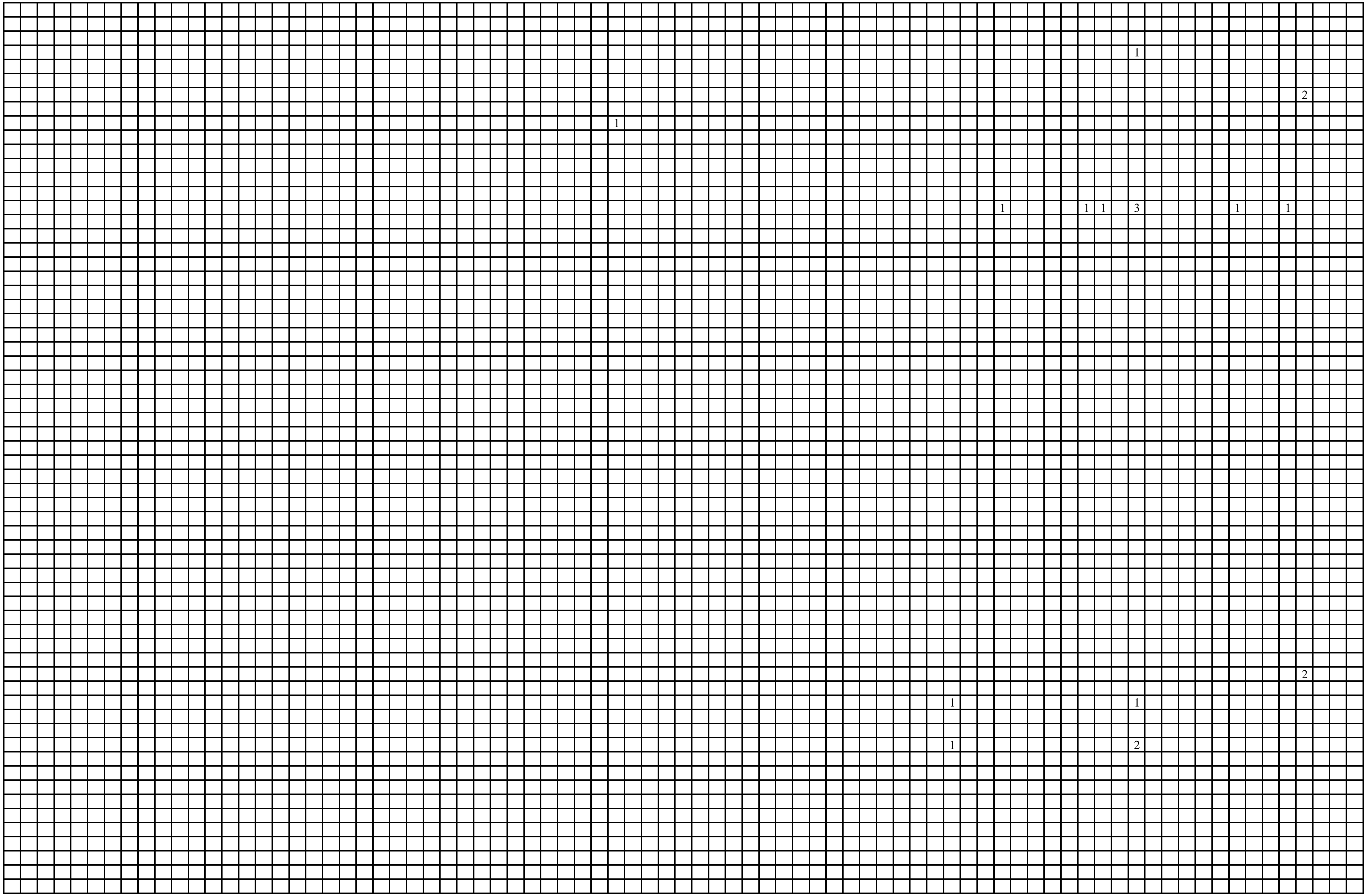
1
2
1
1 1 1 3 1 1
2
1 1
1 2

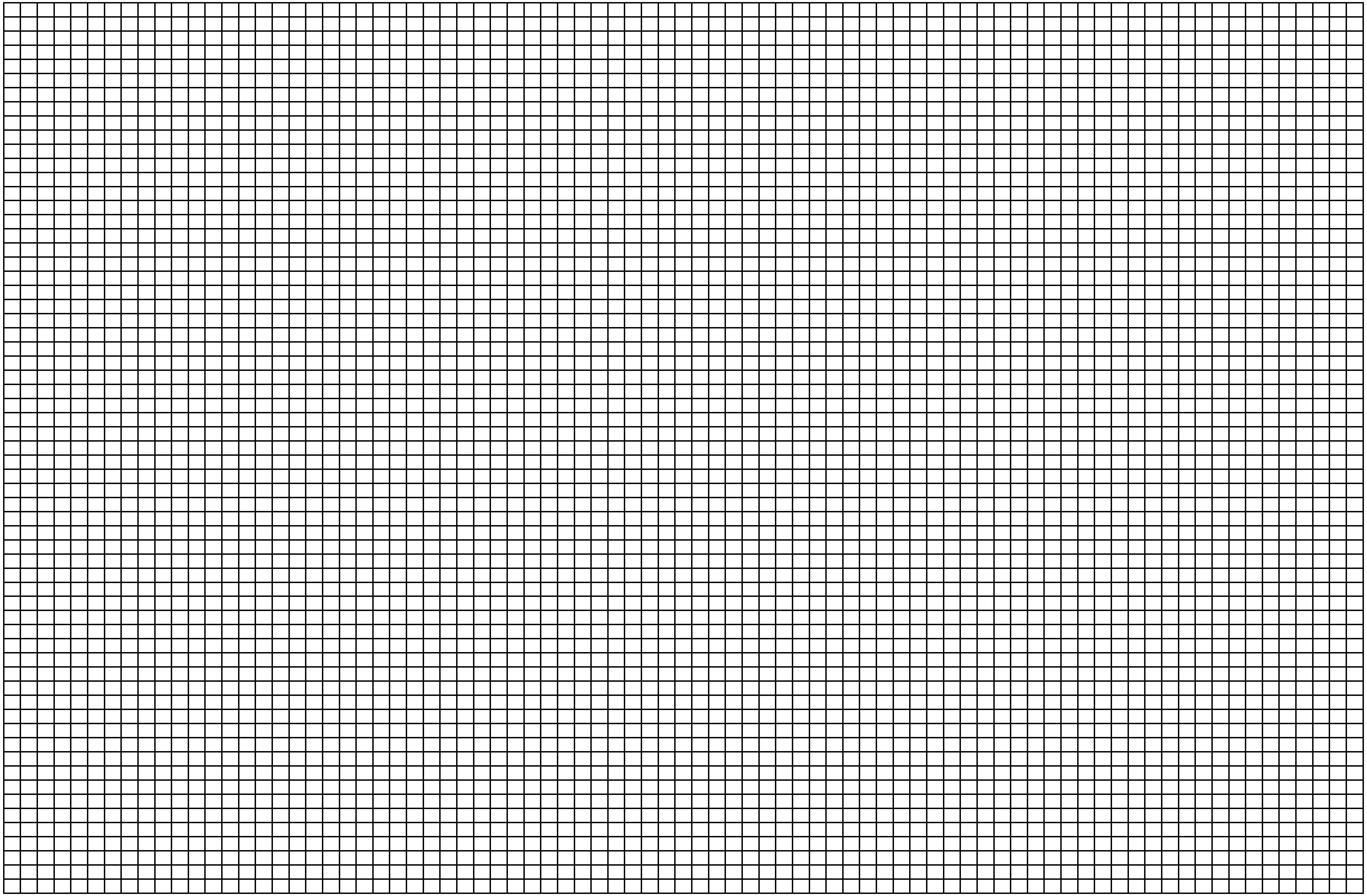

1

1

1

1

1 1

1

																																																			1																								
																																																		2																									
																																																	1																											
																																																			1																		1					1	
																																																																									1						
																																																			1																								
																																																																1													

																																											1																																					
																																											1																																					
																																																								1																								
																																																								1																								
																																																														1					1						1							
																																																																			1													
																																																																										1	1						
												1																																																							1													
												1																																											1																			1						
																																																								1									1		2							1						
																																																																			1													
																																																																			1													
																															1																																1																		
												1																									1																			1								1	1		2							1						
																																																																										1							
																																																								1																								
																																																							1								1																		
																																																								1																								
																																																								1																								
																																																																												1							
																																																							1																											
																																																																					1							1						
																																																							1																											

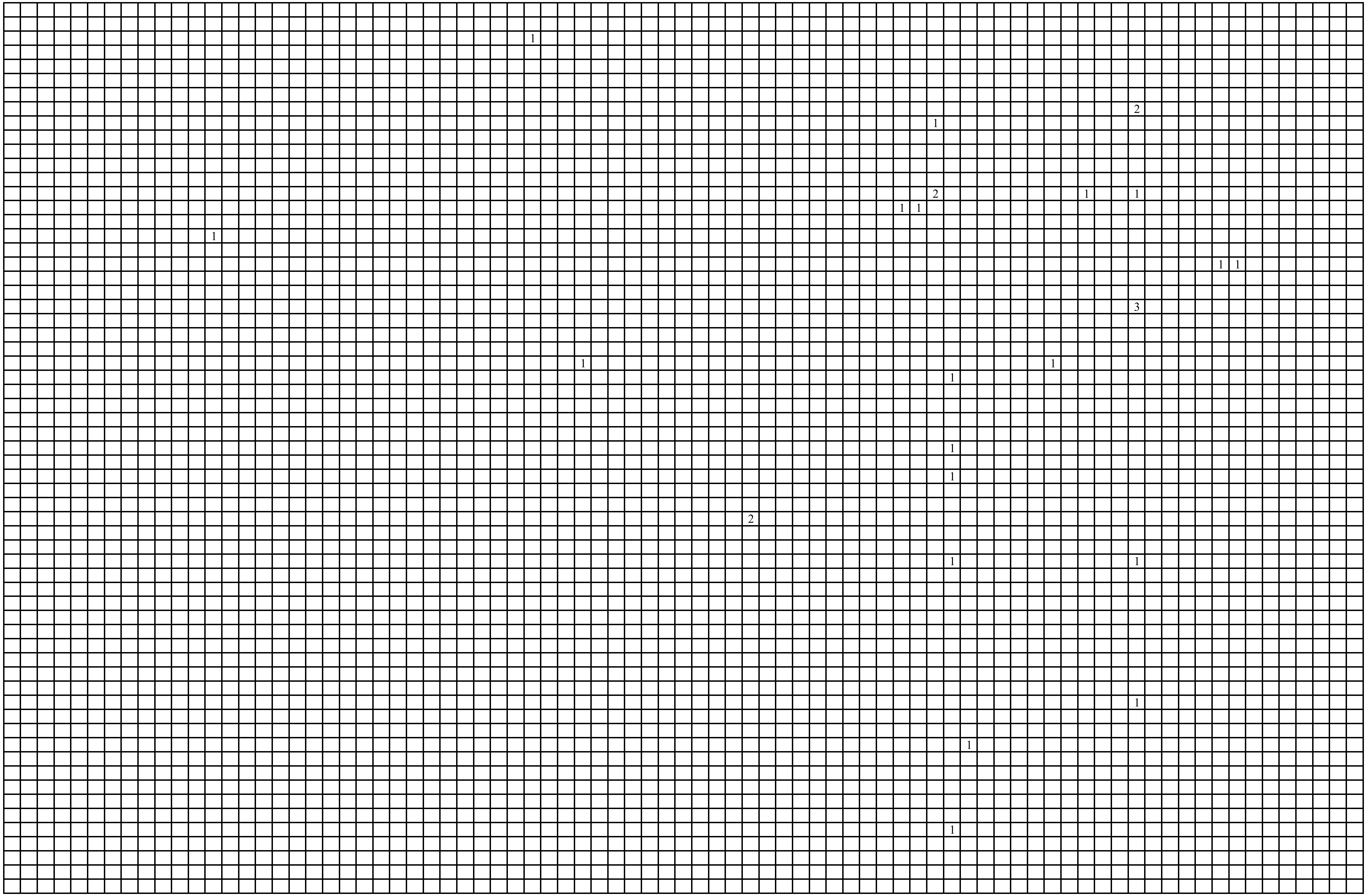
1
2
1
2 1 1
1 1
1
1 1
3
1 1
1
1
1
2
1 1
1
1
1

																																																						1											1													
														1																						1																																										1		
																																										1																																						
																																										1													1																								
																																																																								1	1							
												1																																												1					1			2					1		1						
												1																																												1					1	1		2					1	1	1		1				
																																											1																																					
																																																													1																		
																																																							1																								
																			1																													1						1		1																								
																			1																																				1		2																								
																																	1										1																							2													
																																																							1																								
																																																						1																											
																																																						1			1											2					1	1							
																																																						1			1											1						1							
																		1																																						1			1											2						1							

																																																					1			1											2													
																																																					1			1											1					1	1							
												1																																														1			1					1	1		2					1	1	1		1				
																																							1				1	1				1						2		1																					2			
																				1																												1					1	1	1	1	1																							
																				1													1				1		1									1				1		1	1	1						1																	
															1																																								1																							
															2																																																																	
																																	1										1	1								1		1		1																							1	
															1																																																																	
																																											1	1																																				
																																																					1																											
															1																																																																	
															1																																	1						2																										
																										3																																																						
															1																																																																	
															1																																1						1		1																								
												1																																																				1			2						1							
												1																															1											1		1											2													
																																																									1																							
																																																									1																							
																																																									1																							
																			6																																																												
																																																																												3				
																																																									1																							
																																																									1																							
																																																																				1													
																																																					1														1					1	1							
																															1					1		1															1			1											1													
															1																		1											2		1								1		3											1	2													

												1																																																																						
																																																																		2						1							
																																																			1			1																1								
																																																																													3		
																																																																														1	
																																																														1			1						1	1						

			1																							TCF7L2	HMG.TFAM	72	313
																							1			TFAM		60	447
											2															HMG20A	HMG.TOX	90	325
																										HMG20B		55	153
																										HMGXB3		0	9
			1								1												9	7	12	SMARCE1		154	502
																										TOX		6	13
																										TOX2		6	34
																										TOX3		1	27
																										TOX4		22	200
																										UBTF	HMG.UBF	24	277
																										UBTFL1		9	30
																										NSD2	HMG.WHSC1	0	0
																									1	SSRP1	HMG.WHSC2	68	826
																										HMGA1	HMGA	75	483
																										HMGA2		34	151
																										HSF1	HSF	47	717
																										HSF2		16	114
																										HSF4		11	68
																										HSF5		0	6
																										HSFX1		0	25
																										HSFX3		0	0
																										HSFY1		15	51
																1										LCOR	HTH	27	273
																										LCORL		2	24
																										CAMTA1	IPT	0	10
																										CAMTA2		2	25
																										LRRFIP1	LRRFIP	5	116
																										LRRFIP2		5	137
		1								3																MEF2A	MADS	32	104
																										MEF2B		4	9
		1	2							1																MEF2C		22	61
										1																MEF2D		34	114
		2																			1		1			SRF		81	163
																										MYRF	NDT80	0	21
			1																1		1	1		1	1	NFIA	NF1	124	188
																					1	1		1	1	NFIB		106	176
																			1		1	1		1	1	NFIC		128	227
																			1		1	1	1	1	1	NFIX		121	222
																										NRF1	NRF	20	84
																										SFPQ	others	63	605
																										AFF1		20	147
																										AFF2		0	5
																										AFF3		0	21
																										AFF4		16	197
																										AHCTF1		12	181
																										AKNA		0	7
																										ANKRD30A		0	8
																										BCLAF1		25	363
																										BLZF1		21	193
																										CAPN15		2	42
																										CARF		0	4
																										CARHSP1		3	50
			2										1													CBFB		53	161
																										CC2D1A		7	159
																										CCDC88A		9	276
																										CDIP1		0	18
																										CEBPZ		43	290
																										CENPX		0	0
																										CIZ1		5	80
																										CNBP		41	348
																										CPEB1		9	159
																										CREBRF		3	12
																										CSDC2		1	5
																										CSDE1		18	261
												1														DACH1		48	124
																										DACH2		4	16
																										FAM170A		6	43
																										GABPB1		15	62
																										GABPB2		4	17
																										GCFC2		2	86
																										GLMP		0	0
																										GON4L		7	51
																										GPBP1		2	94
																										GPBP1L1		3	133
																										KHSRP		30	274
																										KIAA1549		0	97
																										L3MBTL3		38	175
																		1								LITAF		10	117
																										LTF		12	165
																										MAF1		2	53

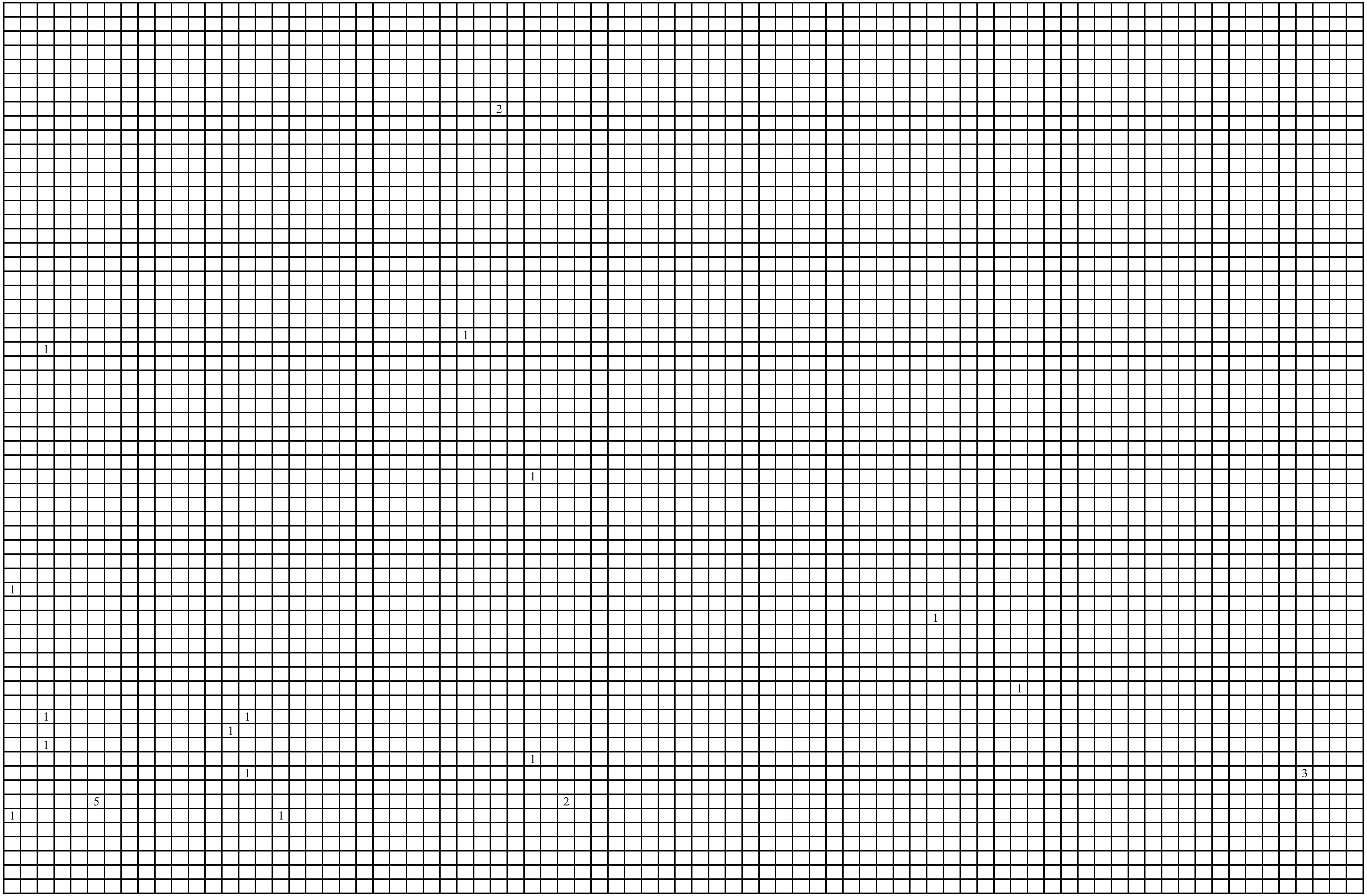
2
1
1
1
1
1
1
1
1
1
1
1
1
3
5
2
1
1

1																																																																																	
		1																																																																															
																1																					1	1																																											
	3	1			3				1	3			4	2	2													1				1				2		2			3				1	2			2				1	1				2			2				2	1								3		1		1	
	1	1			1					1			1	1																														1								1									1			1				1											1					
					1																														2		2	1																																										
					1																																																																											
													1																																																																					
																																			2	1																																										1			
														1																																																																				
																													1																																																				
													1														1																											1																											
		1																																																				1																										
																																																																														1		
					1							1	2															2		1						1																												3										1					
																													1																																																				
																																							1																																										
																													1																																																				
					2																													2	1	1	1																																						1					
	1													1	1																																																1																	
																																							1																																										

																																																														2																		
																																	2																																															
									1					1	1																				1		1	1																							1				1															
																																																																						1										
																																																														1																		
																							1																																																									
1			1		2					1			1	1									1	1					1			1					1			2								1					1	1										1	1			2							2		1			
	1	1	1		2				1	2			3	4		2							1					1					3				2		3	2	1			1	2			2					1	1						1				2											2				1	
					1												1																																																															
														1																			2																																															
																																																									2																							
																						1											1																																							1								
																1																																													1			1																
																																																												1	1																			
																																																																				4									1			
	1				1								1	1																																											1																							
			1																																1		1	1																																										
																						2																																																										
																																					1	1																																										
																																																							2																									
	1									1			1	2																					1	1	1	1																																										
																																																																	1															
		1			1								1																						1	1	2	2		1													2												1										1					
																																			1																														1															
		1																																																																														

														1																							1																													1															
																																																																						1									
1																																																			1																											
																																	1																																															
																		1																																																															
1																		1																	1																																													
																																								1																																														
																																	2																																															
																																						1	1																																														
1		1																																2	2	1																												1															
					1																																	1																																															
																																																																														1	
																							1				1																																										1										1						
						1																													1	1	1	1																											1																
						1																																																																											
																																							1																																													
						1																											1		1	1	1																																														
		1	1																																																																														
																																				1	1	1																																													
																																						1																																														
1		1	1																															1																																																
								1														3																																																														
																																	1																																											1					
																																	1																																															
														1	1	1																	1																																														
1	1																																1				1	1																																																

													1									2													1	1	1	1																																											
1		1	1		1																														1	1	1	1																											1												1			
1		1	1																													1			1	1	2	1																											1															
																																	1																																															
1																																																																																
1		1	1																													1			1	1												1																1															
														2																						1	1																																											
														2																																																																		
														1																																																																		
														1	1																																																																		
													1																																																																			
																																	1																																															
																																					1																																											
																																	1					1																																											
																																					1	1																																											
																																						1																																											
																																						1																																											
																																					1																																											
																																	1					1																																											
																																	1																																															
																						2																											2																															

																																																																						1										
																																			1																																													
																																																	1																															
																													1																																																			
					2																																																									1																	
																																			1		1																																										
1																																1				1																																											
	1				1						1																				1			2	2	1																																								1			
											1																				1			2	1	1		1																																									
	1																														1			1	1	1	1	1																																									
																																							1																																									
	1																																																																															
											1																																																																					
													1																																																																			
	1																																		1	1																													1															

											1																																																																					
1														1																																																		1																				
														1																																		1																											1					
1	1											1	1																							1			1													1																											
																																					1																																												
	1																									1							2																																																		
			1																														1																																																	
											1																																																																					
1	1	1																														1			1	1	1																												1															
		1	1																																	1	1	1	1																																											
1	2	1					1																													1	1	2	1																																											
																																																																											1		
2																																			1		1																																															
															1																									1																																												
1			1																																1		1																																																
																		1																																																																			
																																								2																																												

	1																																																																															
																																			1	1																																																	
																																																					1																								
																																	1		1		1	1																																															
																																	1																																																	
																																											2																																								
										1																																																																								
1	1												1	1	1																																	1																																
														1	1																																																															
														1																																																																
																																				1		1																																															
																																																																									2					
																																																																															1	
													1																																																																		
													1																																																																		
					7								1	1																								1																																																
	1												1																						2	2	2	2		1							1																					1												
1																																																																																	
	1	1																1																	1	1	1	1																																														
	1	1																				1													1	1	1	1																																													
	1				1																														1	1	1	1																																															
																															2																																																									
1	2	1			2					1			1																2		1	1			2	3	3	2										1																	1											2			
																																					1																												1															

	1																																																	1																1																		
	2	1	1																																	1	1	2	1																											1												1				
	1	1	1											1																		1			1	1	2	1																																											
1																																			1																																																
																																			1		1	2																																														
1										1																																																																						
																																																					1																													
										1																							1		1		1	1																			1																								
																																		1																1									1								1																
		10	8											1																					1	1	1																																				1									
		8																																		1																																													
														1																								1																																												
							2																																																																											
						2			2													1																																																												
							2		4				1																																																																					
1																																																																																
									1						1	1																	2																1																																		
		1			1										2								1										1												1																																							
													1	2																																																																			
													1			8	9	3																						2								1							1																									1	
																9		3																																																															
																3	3																																																																
							1																																																																												
														1																																															1																		
																														2	2	5																																																				
																													2		1	1																																																			
																													2	1	2																																																				
																													5	1																																																					
1	1												2	1																			6					1																																															
1		1																																	7	4	4																																															
		1	1																															7		7	8																																														
1		1																															4	7	2	4																											1																
1					1																												1		4	8	4	2																											1												1				
																																																																											1				
																2																								4																																												
																																									6			6																																								
																																									6																																											
														1																																																																					
	1												1			1																																																																				

																																																					2																											
																1																																												1																			
																																																							2																									
1	1																																																																														
																																																											2																				
																																																						1							2																			
																						1																																																										
	1																																			1	1																														4	2														
																																																																		2															
		1																																																																													
																																							1	1																																											6			
																																																																														2		
																1																																																																	
																																																																																2
												1																												1																																															
																																								1																																																		
																																																													1	1																			
																2																																																																							
	1																																								1																									1											1					
															1																																									1																															
												1																																																																								
												1																																																																								
										1			1																																																1													1		
																																				1											1																																1	
	3																																																																																		
																																																											1																							
		1																																			1																																																
								1				1	2	3	2	1																						1					1											2							1	2										1					
																																																											2																							
			1																															1	1	1	1																																																			
		1	1																													1			1	1	1	2																																																			

		1	1																													1			1	1	1	1																																												
																																																	1																															
																																																												1	1																			
																																																													1																			
																																																									1																							
1																																						1																																													
														1	2																		1							1																																												
																																	1																																																		
															1																		1				1				2																1																							
					1																																																																												
					1									1	1																																					1																											
	1				1								1																			2																																																
	1																																																																																
			3		1																		1									1			1		1	1																																									3			
																																																																																1	
																																								1													15																							1					
									2																																													5																											
																																																				7																											
													2																																																																				
													1																																																																				
																																	1																																																		
																																												1																																								
																																								1																																					1			
					1										1																																																	1															
																												2		1				1																																																
																																			1		1												2																1															
		2																																	2			1																											3															
															1																									1																																								
1																																	2																																																		
															1																										1																																							
																1																																																																	
					1																																																																												
																												1																																																				
	1																																																																																	
																																								3																	1																					2		

									3													3																																																										
1		1	1																															1	1	1	1																																														
1		1																													2	1	3		1	1	1																												1												1			
																																					1																													1															
																																																				1																												
																																																																											1							
																																																	1																															
																												1				1																																																		
														1																					1			1																																															
																															1				1		1	1																																												1			
	1																																3																																																	
1	1																																		2	2	1																												1															
																																	1															1																																
1		1			2																											1			2	2	1			2																																												
					1																																																1																											
																																			1	1																														1															
	1																																																																																		
																																			2	2	1																																																
1		1	1																																1	1	1																												1												1			
					1																												1																																																		
																																						1																														6	3											
													1																																																																					
	1																																																																																		
																																																								1																									
																																1			1																																																	
																																			1																																																		
																																																																			1														
									1																														2																																														
																																													1									1																										
		1																																																															3					1								
																																			1																														1															
																																						1																																																	
													1																						1	1	1	1															1												2															

														1																					4		1	1	1	1																																										
		5	7		3				1					1																										1																			1																				1		1	
		2	3																																																																												
		3	3																																	1	1																																									1		1	
					19								1	1																									1		2																																							
					17																																		1		1																																							
																																								1																																							
										1																																																																							
									1																																																																							
									1				1																																																																			
																																																												2																							
																																						1										1																															
1										1			1																																1												1																								
		1																																																																															
																							1																																																									
																																																									3																										
1																																			1		1																																											
					1																																																																												
																																																												1																							
	1																																		1	1																																								1			
2																																						1																																										
1	1									1																						1			2	1	1																																			1					1			
1	2																															1			3	3	2																								1																					
																																			1																																												
1					1																										1			1																																												
																							4																																					1																							
1										1																						1			2	1	1																																								1			
																																																												1											1													
														4																2	2	1							1																																							
															1																		1						1																																								
																																								1																																							
																																							2																																								

1		1	1		1																								1			1			1	1	2	1																																											
																							1																																																								1	
3																																																																																
																	1																																	1																													1		
																																						1																																																
																																						1																																																
			1										1																																			1																																
1		1	1																																1		1	1																																													
															1																																																																			
		4	3																																																																													

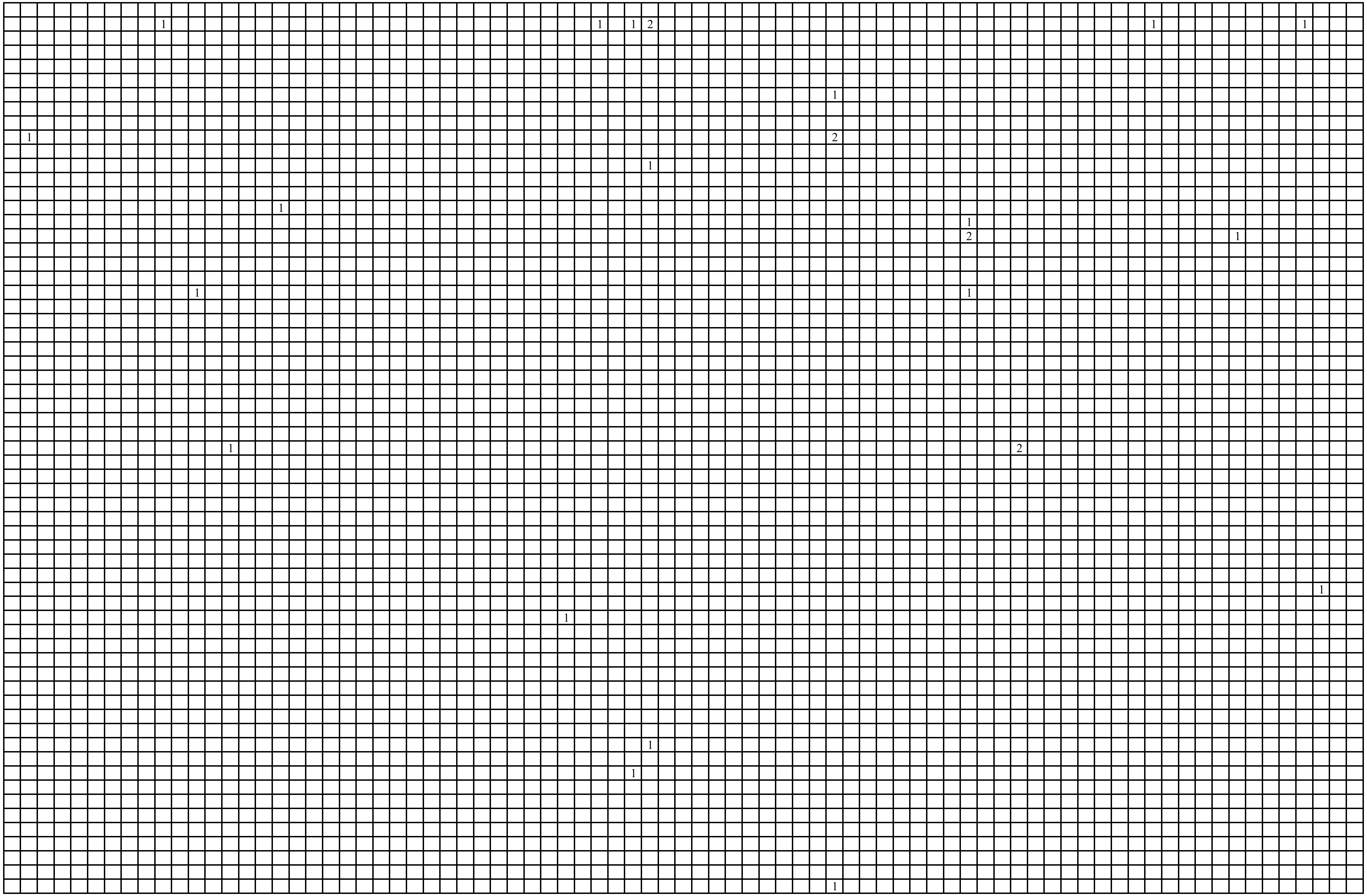
1
1
1
2
1
1
1
1
2
1
1
1
2
1
1
1
1
2
1
1
1
1
1

																																						1																																												
																																						1																																												
																																		1	1	1	1																																												
																																																													2																				
																																																						1																							
												1																																																			1																				
														1																																																																		
																																																																																2	
												1																																																																				

														1	1																					1				1																	1																							
																																																									2																				
														1																																																																		
																																		1	1	1	1																																																
1																																																																																						
																																																																					3													
													1																																																																					
																																																3																															

																													1																																																									
1																																																																																	
										1																																																																					
																										1																																																										
										1																																																																					
											1																																																																					

1

1

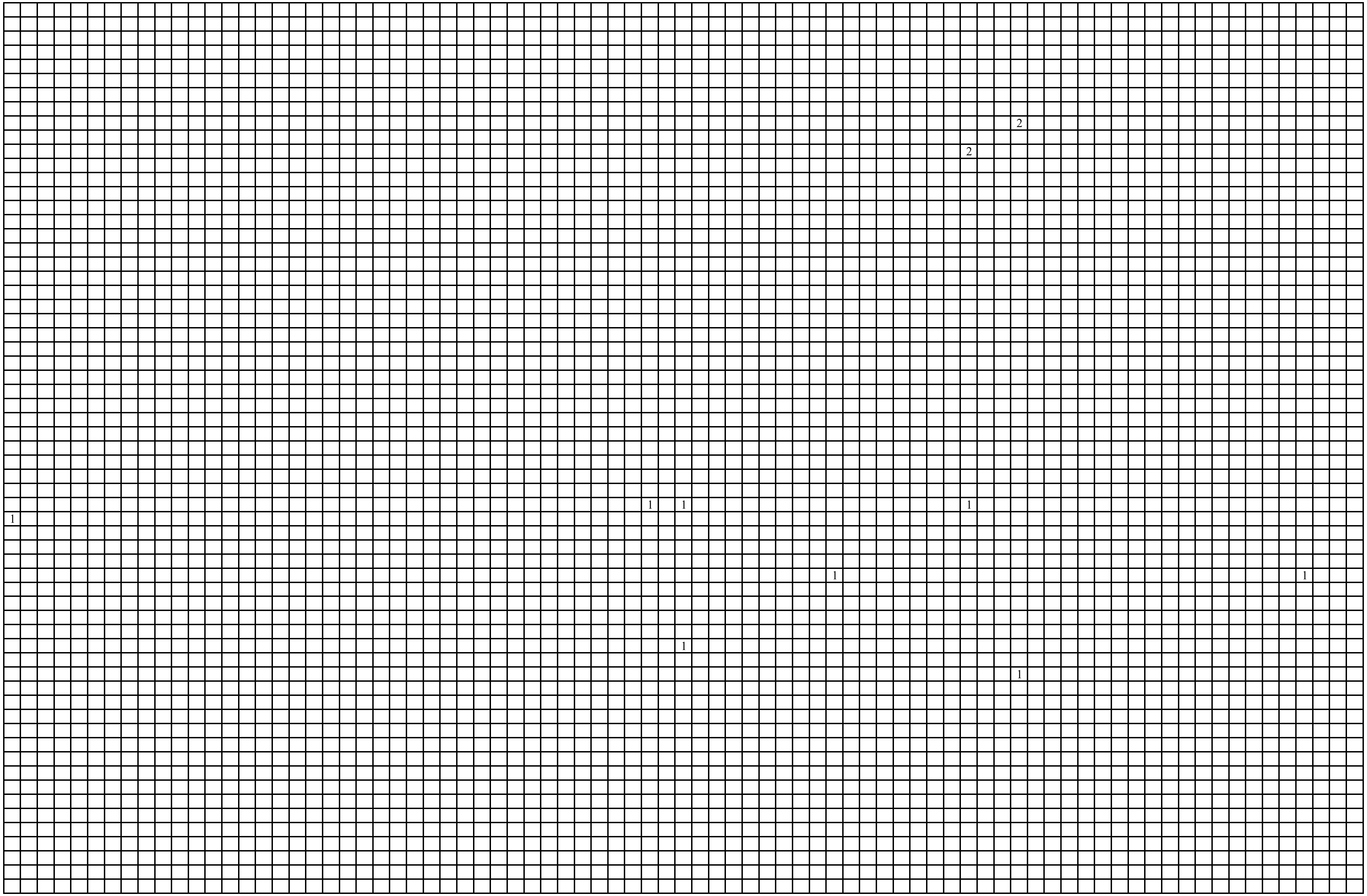

2
2
1 1 1
1
1 1
1
1

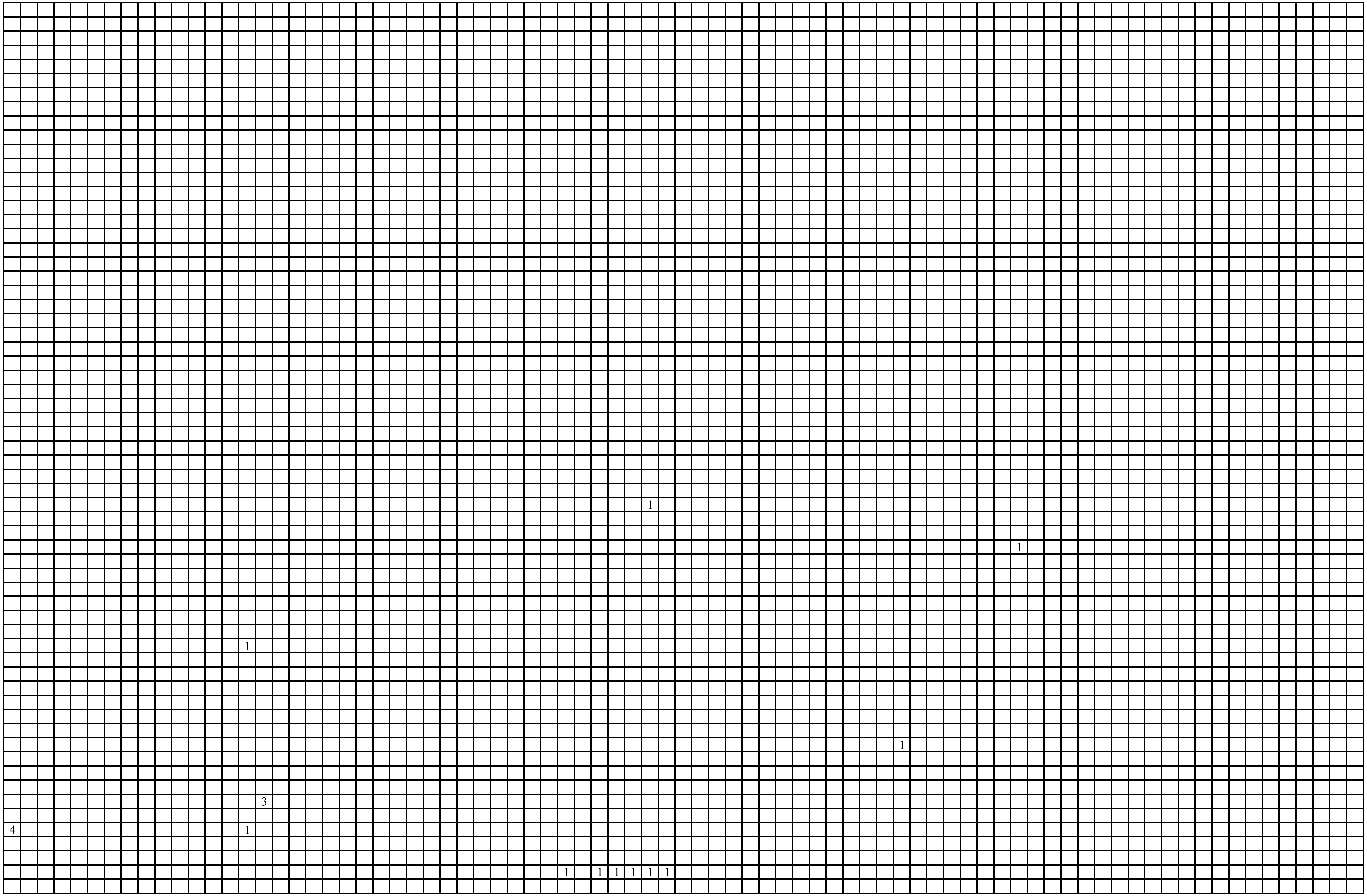
1
1
1
1
3
4
1
1 1 1 1 1 1

					1									1																										1													3																						1					
																																																					1																													
														1																										1									1				3																						1					
2																																																																																	
1																																																																																	
					2								1				1																														1								1																											
													1	1																																																																		
					1									1																										1																																								
													1																							1	1	1																																											
																																						1	1																																											
																																							1																											1	1															
																																								1																																																			
	1															1	1																			1		1	1																																											
																																																1					1																					2						
	1	1								1			1																										1										1									1																								
																																						1																																																					
																																							1																																																		
																																	1																																																								

																																																			1																							
																																																			1			1																				
	1												1	1																								1																			2			2																				
	1								1						1																																																													
																						2																																																								
																																							1																																																
																																										1																																															
																																							1																																																
												1																																																																						
																																																												1																							
												1			1																																																																		
					1																																																																											
																																																												2																				

																																																																													2			
	1																																																											2																				
									1																													1																																										
													1			1																																																																
																																			1	1	1	1																																										
																																			1	1	1	1																																										
																																																	1																															
																																																	2																															
		1			1																																																																											
1		1	1		1																														2	2	2																																											
3		1							1																										2	1	2																																											
	1				1																			1									5							1					1			1																																
																																	2																																															
																																						1																																										
		1											1			1																																																																
		1																															1																																															
		1											1	1		3																																																																

1		1											1	1		1																																																																
		1	1										1																																																																			
																																	1		1	1	1	1																																									
2			1		5					1			1										2						1			1			1	2	1	1		4				1																1															1					
	1	1	1		8					1			1										5													1				1				1	1			2									1		1	2				1								1		1	1					
		1			2																						1									1	1	1			1				1									1			1		1	1				1				1	1			1			1					
																							1																																																									
					2																		1												1	1			1	1																				2																				
					1																																																																											
1																																																																																
														3																										3																																			1					
														2																			2																																													
					1																																																																											
																							1																																																									
																													3											1																																					1			
																							1																		1																																								
1			1		1																														1	1																													1												1			
																																			1	1	1	1		1																																							
									1				1																								1	1		1					1												1							1											1					
																																																											3																					
																												1					1							1																																							
																																												1																																			
																																	2																							1																							
					2																																																																					1					
																																								1																																							
					1																																																																											
																																																									1																							
	1																																																																															
		1																																																																														
													1																																																																			
	1	1			2								1	2	1																										1				1				1									2																		1					

						1																																		1																																								
														1														1												1																																								
		1	1																																																																														
															2																																					1																													
																																																1																																
					1																																																																												
																	1																																															2																
										1																																					1							1																				1					

																										MLLT10		18	72
																										MYEF2		17	141
																										MYRFL		0	1
																										NFRKB		37	188
																										NKRF		40	292
																										NME2		13	193
																										PARP12		5	53
																										PAXBP1		8	123
			1																							PHB		55	1230
																										PLEK		0	11
																										PLSCR1		23	203
																										PRDM11		1	20
																										PREB		6	191
																										RAG1		11	67
																										RBCK1		42	364
																										SETBP1		2	37
																										SLC2A4RG		2	5
																										SMARCAL1		6	58
	1											1														SON		22	177
																										SPZ1		1	34
																										SUB1		31	200
																										TAX1BP1		15	463
												1														TET2		38	177
																										TFB1M		11	91
																										THYN1		4	25
																										TMF1		13	108
																						1				XPA		9	137
																										YEATS4		64	331
											2						1				2	2			3	TP53	p53	542	5340
											1															TP63		35	249
																										TP73		51	350
																										PAX1	PAX	4	5
																			1					1		PAX2		28	83
																										PAX5		11	73
																								1	1	PAX8		38	117
																			1					1	1	PAX9		48	133
																										TCF19	PHDFP	12	43
																										ZNF883	pseudogene	0	1
																										PURA	PUR	27	253
																										PURB		17	151
																										PURG		41	222
																										RFXANK	RFXANK	21	170
																										NFKB1	RHR	165	533
																										NFKB2		56	185
																										REL		72	502
											1															RELA		236	986
																										RELB		45	131
																										NFAT5		15	46
																									1	NFATC1		61	204
													1												1	NFATC2		64	229
																										NFATC3		5	44
																										NFATC4		5	45
																					2					RBPJ		97	383
																										RBPJL		2	9
																										EBF1		7	16
																										EBF2		3	58
																										EBF3		7	21
																										EBF4		0	4
			2									2	1				1					1			1	RUNX1	RUNT	102	403
																										RUNX2		50	122
																										RUNX3		38	83
																										AIRE	SAND	12	139
																										DEAF1		14	54
																										GMEB1		13	58
																										GMEB2		22	63
																										SP110		11	132
													1									3				SP100		61	256
																										SP140		3	10
																										SP140L		0	10
																										SMAD1	SMAD	89	305
													1											1	1	SMAD2		186	616
													1												1	SMAD3		306	909
			1																							SMAD4		284	799
																										SMAD5		17	111
																										SMAD6		23	91
			1																							SMAD7		46	270
																								1		SMAD9		32	150
																										SAMD11	SNAD	7	50
																										STAT1		78	447
																										STAT2		28	156
			3																							STAT3		120	671

						2																																																																											
																																										3																																										
																																																																						1											
																																												1																																						
																																																							1																							
																																																		1	1																											
																																																							1																							
																																																								1																						
																												5																	3																																			1
																												1																																																								1
																																																																																			2
																												2																																														1					1					
										1																																									1																													
																																																																								1				1	1					
																																													2																												2									
																												2																	1																															1				
										1				1																														1																																				
																											1																																																								
																											1																																																								
								1																																																														1									
																												11	1	1															2													3	3	2											6									8
																												2														2																															1									4
																																									1	1	1																																					1	1	
																												1																																																							

																											3	1																																											1	1								
																												1				1																																					1											
																																										2																																						
	1		2	3		1	1	3										3		1		1	1	1			7	3		5								1	1			1	2		3			1				1						2												4	3				2	1		1		
																											2																																																					
	1			1			1											1		1							2			1								1	1									1												4																				
																																																																							2									
																								1				1																					2			2																												
																																																																							2	2								
																						2																																																										
																																													1																																			
																																																																							1									
																																	1																																															
																												5														1			2							1																												
																											4	1																																																				
																												1																																																				
																																																				1							4																					3
				1																																																																												
								1																																								1																							3	3								
																																																1	1																															
														3														7																																										1	1									
																												1																																					1															
														1																																																																		
				1																								1		2												2			1		2	4	5		1					1		3	5											2	7	1						2		3
																																																1	1																						2	2								
																																																1	1																						1									
																												2																3	3		2																				3		1											3
																												10	4	3															2			1																						2	4							2		1
								1																			3	2																	1		2	2	2									3	5												3									
																											1																																																					
																											3																																																					
																												1																																																				
																																																																																1
				1																																		1																												1														
																																																	1																															
																												1																	1				1																						1									

																																												1																																				
																																																																																1
																																																1				1																												
																												5																			3	3													2																		1
																												2																																																					
																																																				1														1														
			1		1															1		1		1				2											1													1					1		1	1			1	1	1	1						1	1						1		
			1	1	1			1										1	2		2					1	1	2		1													1	2			8	1	1											2		1											1	1						1		4
																																													1																									2	2								
																																																	1																																
																						2																																																										
																												7	1																																																		
																												2		2																		1																																
			1	1																										2			1								1	2							1																					1	1										
								1																					7											1	1																																									
				1																																		1	1	1																																							
								1																					1													1																																							
								9																					5											3				2																																				1		
																												1																																																					
																												1																																																					
				1																																																																		2										
																																				2																																													
																												1																																																					
																												5																																																					
				1																		1																																																											
																												1																																																	1		
																												1																																																					
																				1								1						1																						2							1														1
																					1																																			1																									

																																																					1																						
																											1																																																			1											
	1																																																																						12	6	2								
1																																																																																	
	1																																																																															
																											1																																																														
																											2	2						1						1							1	1																															
																								1			1	2												1							1																																
								1																																																																									
				1																																																											1			1					1									
																											1																	2																													2									
																											1																																																														
																																																																								1	2								
																											5																																										1		2	4								
																											2															1																												1	2								
																										1																																																															
																											2				1		1	1			1									1					2														1														
																											1																		1																																									
																																												1	1			2										3																						
																											1				1		1	1						1						1																		1														
				1																							1																																																													
																										4																																																															
																										3																																																														
																											1																																																															
																																																																						1			1				1				
																																									5																																														
																																									2																																														
													1																																																																						
																																		1																																																	
																																	1																																																		
								1																			1																																																												
																																																																										1								
																																																													1																						
			1																								2													1	1																													1								1		1
																																																																										1										
																																																	1				1																													

																												1							1													2	2			2																												
																												1	2			1		1	1																	1																												
			1																										1																																												1		1	1	1							
																																1																																																			
																																																																																		1
1			1																		1									1		1																																																				
1																															1												2																																											1
																											1											1	1															1																											
1																																													1									1										1									1							
																												2																				1	1			1																												
	1																																																																																				
																																			1																		2																										
																					1																																																														
																												2																																												1	1	1			2	3					
																																		1																																												3	2	4					1				
																																			1																																																				1
																																1		1	1																																								1														
																												1				1																																																										
																																1			1							1	1		1	1																																										
																																										1			3																																														
										3				4																						1																																																			
																																																1				1																												
																																																					1																												
																																																					1																												
																																																																					1											
										1																																																																1								

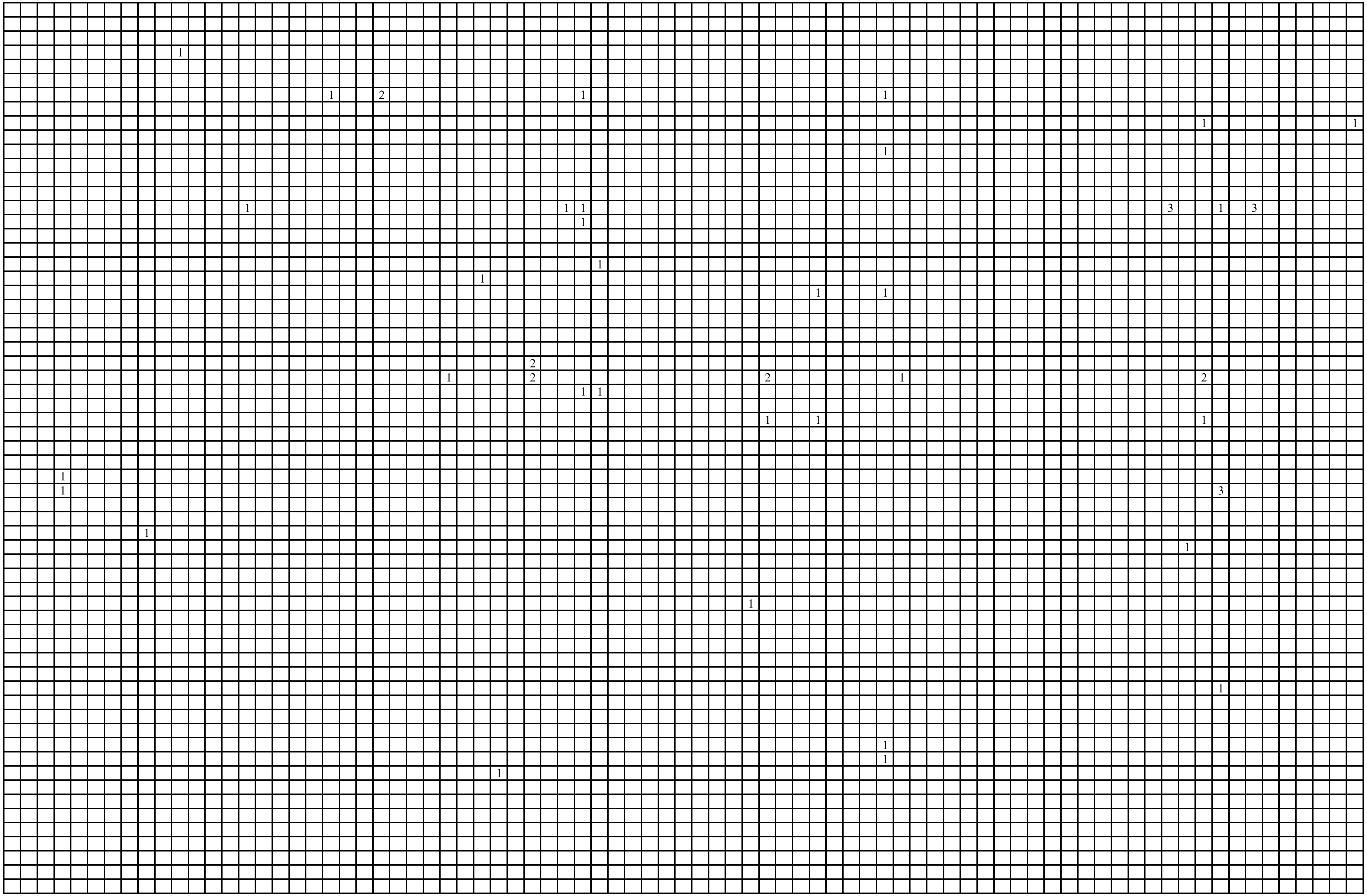
1
1 2 1 1
1 1
1
1 1 1 3 1 3
1
1
1
1 1
2
1 2 2 1 2
1 1
1 1 1
1
1 3
1
1
1
1
1
1
1

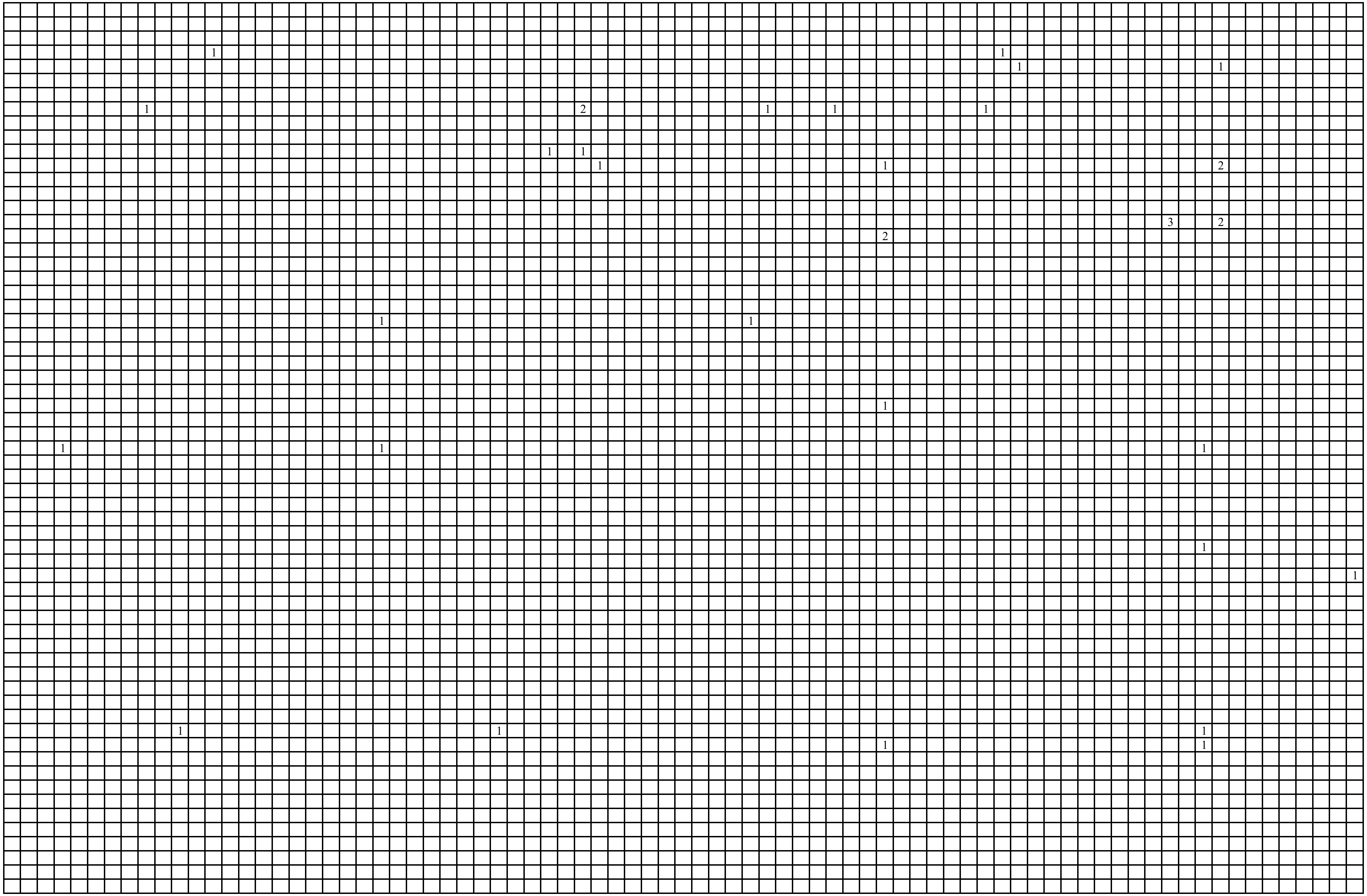

																																																																							1									
																																												1																																				
																																																				1																												
																																																				1																												
																						2																																																								1		
																																																																					1	4	2									
																																																																							1									
													2								1					1		5		1												1			1																					5														
													2																																																																					3														
																																	1																																															
																																1		1	1																																			1										
																												1													1																																							
																																		1	1																																													
																		1										2		1		1		1	1												1	1	1	1	1	1				1		1	1	1											1	1								
				1														1																																																			1											
																																																																								1								
																												1																																															1					
																												1																																																				
	3			1																		1							1		1																												1		1																					

																												3																																																				
																																																																				1				1		1					
								1																																																																							
																						1																														1																					1		1					
																																																								1											2	3									
																																																																					1									
																																															1	1																				1	3	5	1			2					
																																									1																																			1					
								1															3																										1		1																														
																											1							1	1																																									2								
																																1		1	1																	3																													
																																													1		1	1			1																	1										1	
																												1																																																						
																																																											2																					
																					1																																																												
1																		1		1								1																					1												2		1																		
																					1							2														1						1																																	
														1														3														2			1			1																					1				1			1				
					2																							2																																																			1		
																												1																																																					
																																																				1																															
																																																																																		1
																																																																							2										
																																																																								1										
																																		1	1														2			1																														
																																										1		1	1																			1											2					
																																1		1	1																	1																					1	1	2								
																																1		1	1																																															
																						1										1		1	1										1							1																			1									
			1																								1					1		2	1						1											1																				1								
								1																				1														1																1								1								1							
1																																												2																															1					
																																																															1																
																												1																																																						
																						1																																																												
																																				1																																				2									
														1																																																																				

																																																1										15	5	7																					
																									1			2		2									1					1																																				
				1																																	1																																										
				1																																	1	1																																									
								1													1							1																																																					
																												2																																									1		1	3									
								1																			1																													1																							
																																																				3														1														
																					1																																																										
																						1																																1																									
2																											4																																																				
																																																																		1														
																												1																																																				
																												1											1		1	1	1	1																																				
								2										1					1					2																																													1						
																												1																																																				
												2																																																																				
													4															1																													2																							
														10														3																3																																			
															2																																																																
																2																																																															
																																																				2																												
								1																																																																								
																				6		1						3																																		1																		
																					4																								1																																	
																				1								1						1																				1														2										
								1																																																																								
																									2																																																						
																										2																																											1									
4																											1																																																				
			1	1				2			1		1	3						3		1					1	54	5	8													1	1	1	3			2		1		3							2	3					1	1			2	3	2				1			2		1
																												5	2	5											1			2																																				
																												8	5	4																																																	
																																		1																																													
																																	2	1																								1																							
																						1										1	1																																														

																																						2		1																																								
																																								1																																								
				1																																		1	1																																									
																																									2		1																																				
				1																								1	1													24	11	10	46	10																										1								2
				1																								1													1	11	4	8	10	9																																		1
				1																								1														10	8		14	4																															1			
				1										3														3	2													46	10	14	12	10																									1	1								6
																					1																					10	9	4	10																									1	1	1								
																																															4																																	
																												2																					1			2						2																						
																																																1	2									2																						
																												1																								1																												
																	2											3																				2		1										2						1														
																						1																																																										
																																																								2																							
																																																							2																								
													2																				1															2	2									4	1										1	1	2		1							
																												2																														1		1									4	1	4		1							1
																												3																								2							1						1				1	1	3		1							
																																																													10																			
																				1																																										2																	
																																																															2	4					1		1									
																																																															4	4																
																												1																																1																			
	1																											1																								1														10														
																						2						2																														1	4	1			1						4	4	3	15	1	5	1					1
																										1		3																		1												1	1	1									4	8	18	61	1	1	2	2				
																												2																	1	1												2	4	3			1						3	18	10	73	1		2	3				
																																										1			1	1																							15	61	73	12	3	2	5	5		1	1	
								1																																																			1	1	1									1	1	1	3		1						
																																																																					5	1		2	1		4					
																												1																																									1	2	2	5		4		1				
																																																																						2	3	5			1	2				
																																												1																																				
																												2																																												1						8	11	10
																																																																								1						11		
																												1														2	1		6														1										1									10		12
																																																																														3		
																						2																				1																	1																					
																												1														2																																					1	1
																																																																		1													
																																																																		1				1								
																												1																							1																											

																						1																																																					1					
																	1											1																				1										1																						
																																										2			3													1																						
																												2																																					1									1	2					
																												3														2	2	1																																				
																																																																							2									
	1																																																																															
																							1																																																									
			1																								1															2		1														1																						
			1																																																							1						1																
																																										3			1																																			
			1																																																							2																						
																																																																1																
1																						1																																																										
			1																									4														1																	1							2														
																												3		1																																																		
																													1																																									2		1								
																																																	1																															
1			1																									1																																				1																
																												1							1																																				1					1				
																											3																																																									
																																																										1																						
																																																																														3		
																																										1			1																																			
																																																														1								1		1								
																																																	2																															
																												2																					1													1								2	3	2								
																																										1			1																																			
																																										1			1																																	2	8	
								1														1																		1																																								
																												3																																																				
												1																																																																				
																																																																								1								
																																																																								1								
																																	1																																	1														
																												1																																																				
								1																				3						1	1							1														1														1								

																												1				1		1	1										7							3																												
																				2					1		1																						1																	1														
																																																																	1															
																																																				1														1														
										1																		1																		2		1	1			2						2												2	1								1	
																																														2		2	1			2						1												2	2								1	
																																																																													1			
	1																											1																																																				
																												1																																																				
																																																																		1										1				
			1				1																																																											1														
																																								1																													1						1					
																												1																																																				
																												2		2																																															2			
	1			1																														1				1																																										
																											1																																																					
				1																																																																												
																												1																																																				
																												4																	1			1	1																															
			1																			1																										1																																
																												2																																				1																
			2					1																																																							1	2													1			1
			1		1			1																			1																																			1		1																
			1					1																																																								1																
																												1							1										1																																			
1				1				1														1						2														2																												2	1	1								
																											1																															1																						
			1					1																																																						1		1													1			
																				2								26		1								1				4	1	1	8																	2								5	9	6								4
																				1								3		1															1																	2																		

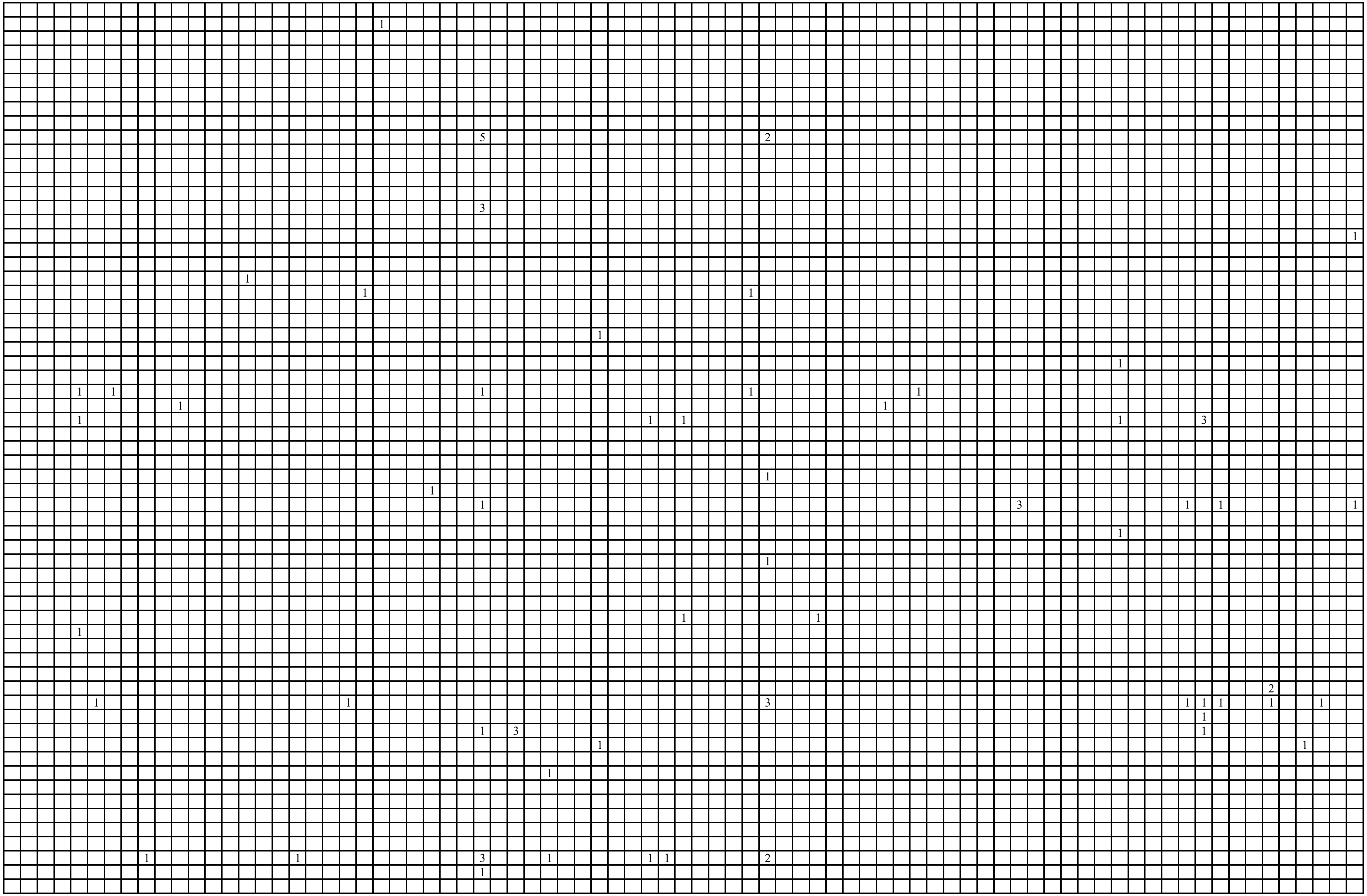

1
5 2
3
1
1
1 1
1
1
1 1 1 1 1
1 1
1 1 1 1 1 3
1
1
1 1 3 1 1 1
1
1
1 1
1
2
1 1 3 1 1 1 1 1
1
1 3 1
1 1
1
1 1 3 1 1 1 2
1

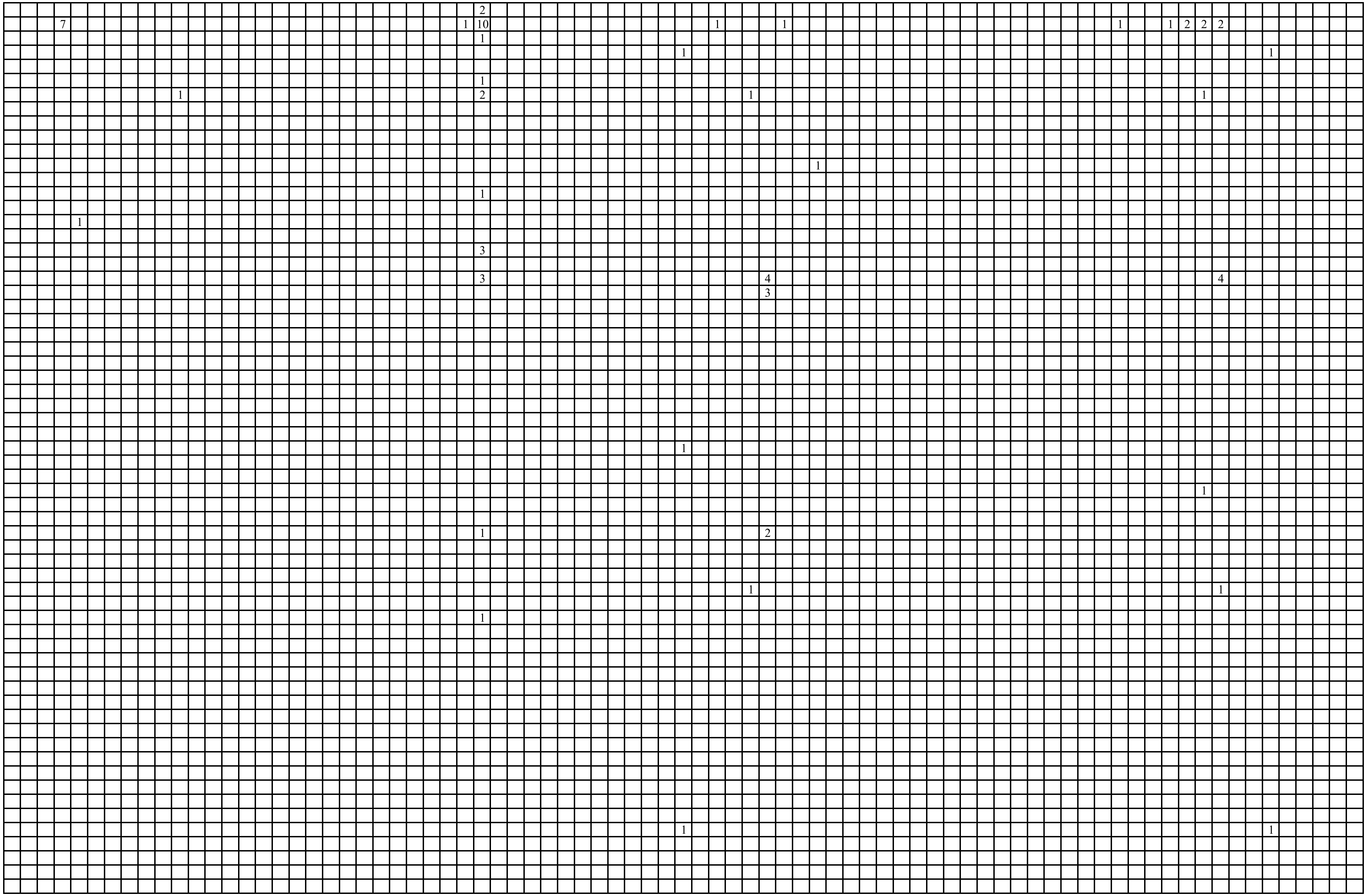
2
7 1 10 1 1 1 1 2 2 2
1
1 1
1
1 2 1 1
1
1
1
3
3 4 4
3
1
1
1 2
1 1
1
1 1

																																																	1									1																						
																																																	1																									1				
																												1																																																										
																												2																					1																															
																												1																																																									
																												1																																																									
																																								1																																															
																																																																																			1								
																												1																					1																															

																																						1																																																
																												1																																																												
																																																			4																												
																																								1																																														
																																								1																																														
																												1																																																												
																																																																		1														1
																												1																																																												
																																								1																																														
																												1																																															1									
																																				1																																																				
																												1																					1			1																												
																												3																																																												
																																																																						1										

																											6												1																																								
																																		1																																																	
																																								1																																								
																																								1																																								
																																								1																																								
																																							1																																									
																													1																																																											
																													1																																																											
																																												1																																				
																																																																											1				1

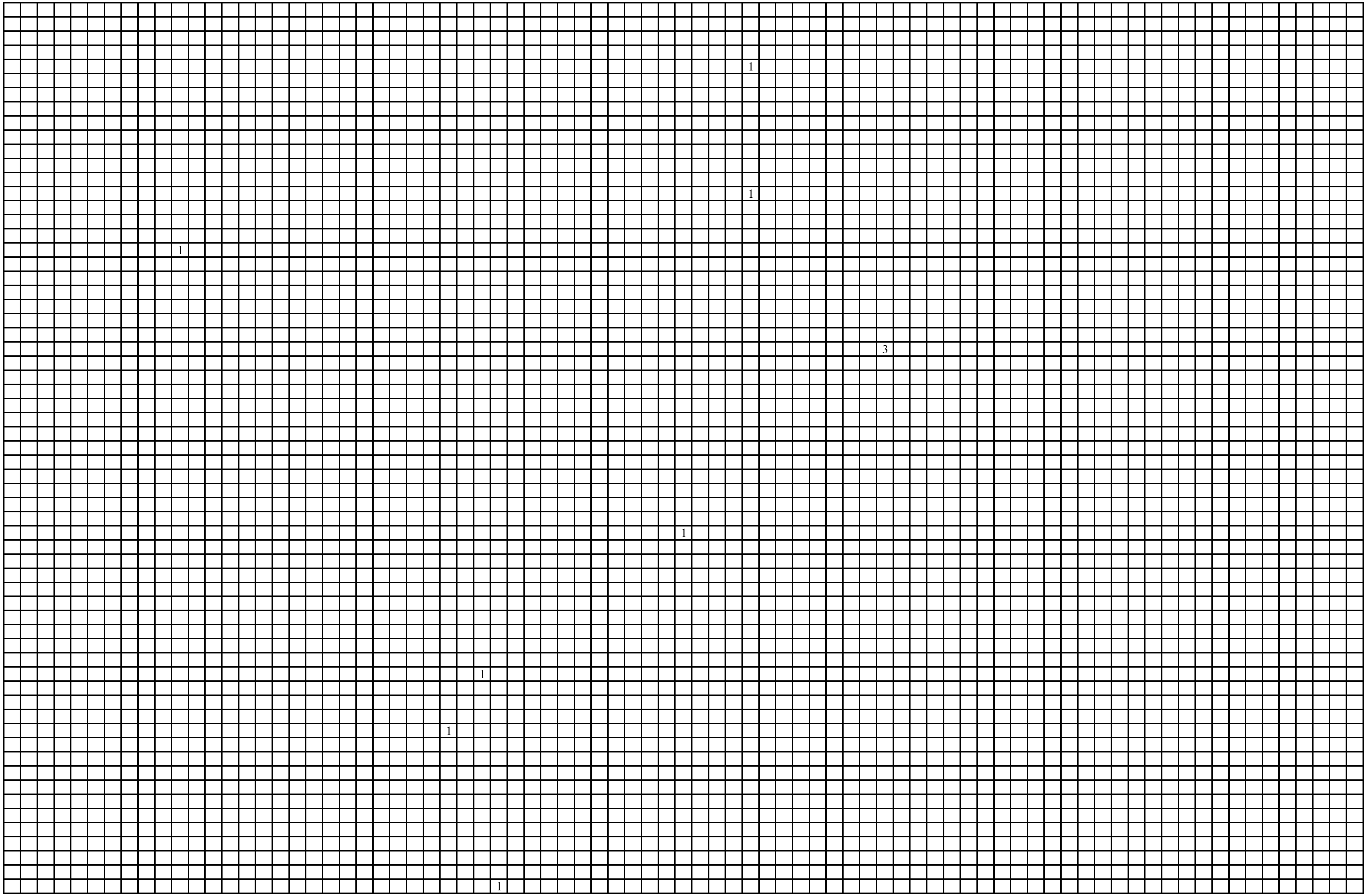
1
1
1
3
1
1
1
1

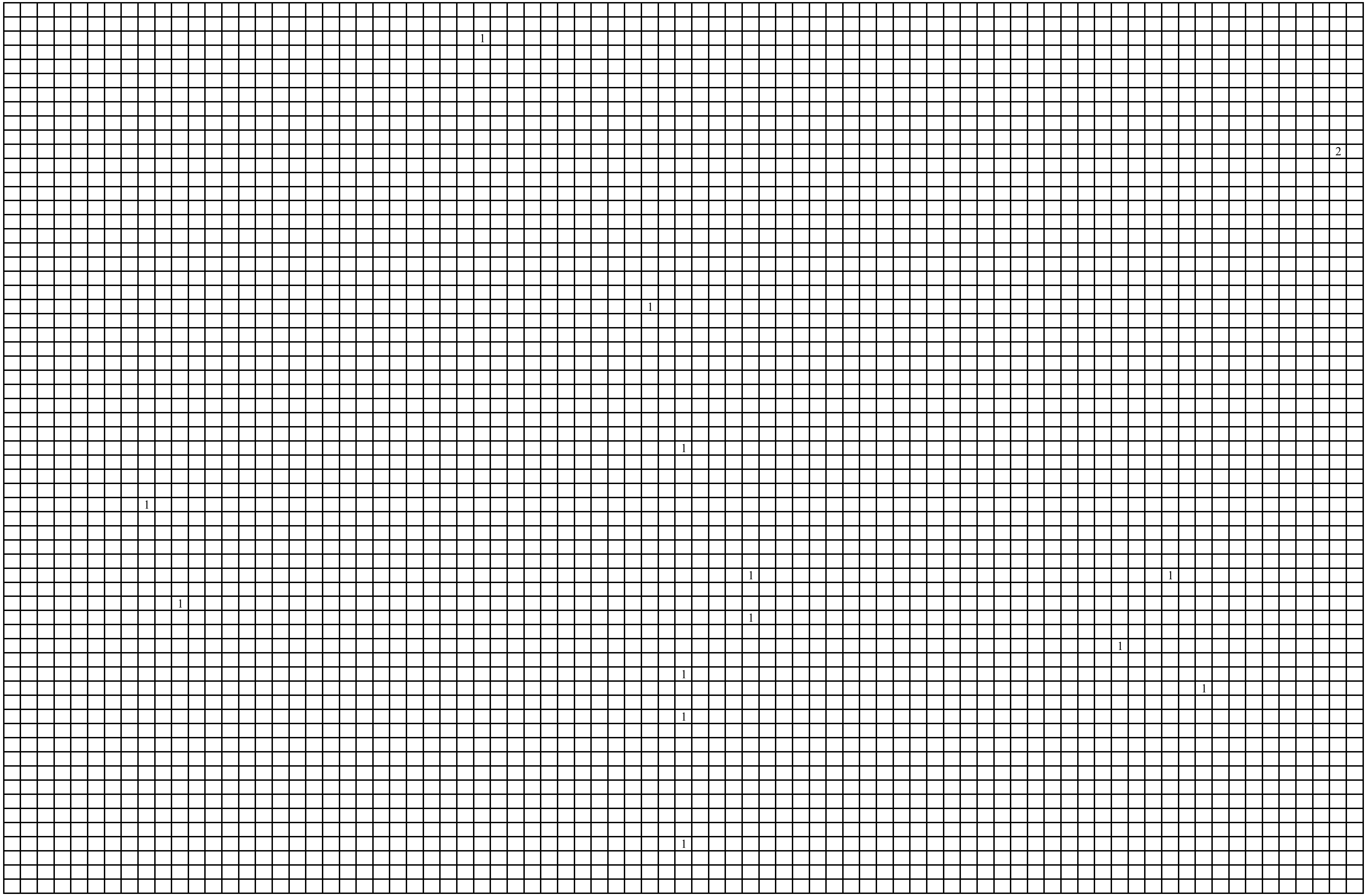

																																								1																																								
																																																																																1
																																																																							1					1				
																																								1																																								
																																								1																																								
																												1												1																																								
																																								1																																								
																																																																	1															
																																																																					1							1				
																																																																	1															
																																			1																															1														
																																																1		1																	1														
																												1																																																				
																												5																																																1				
																																																																		1														
																																																																																6
																																																										1																						
																												1																																																				
																												1				1		1																																					2									1

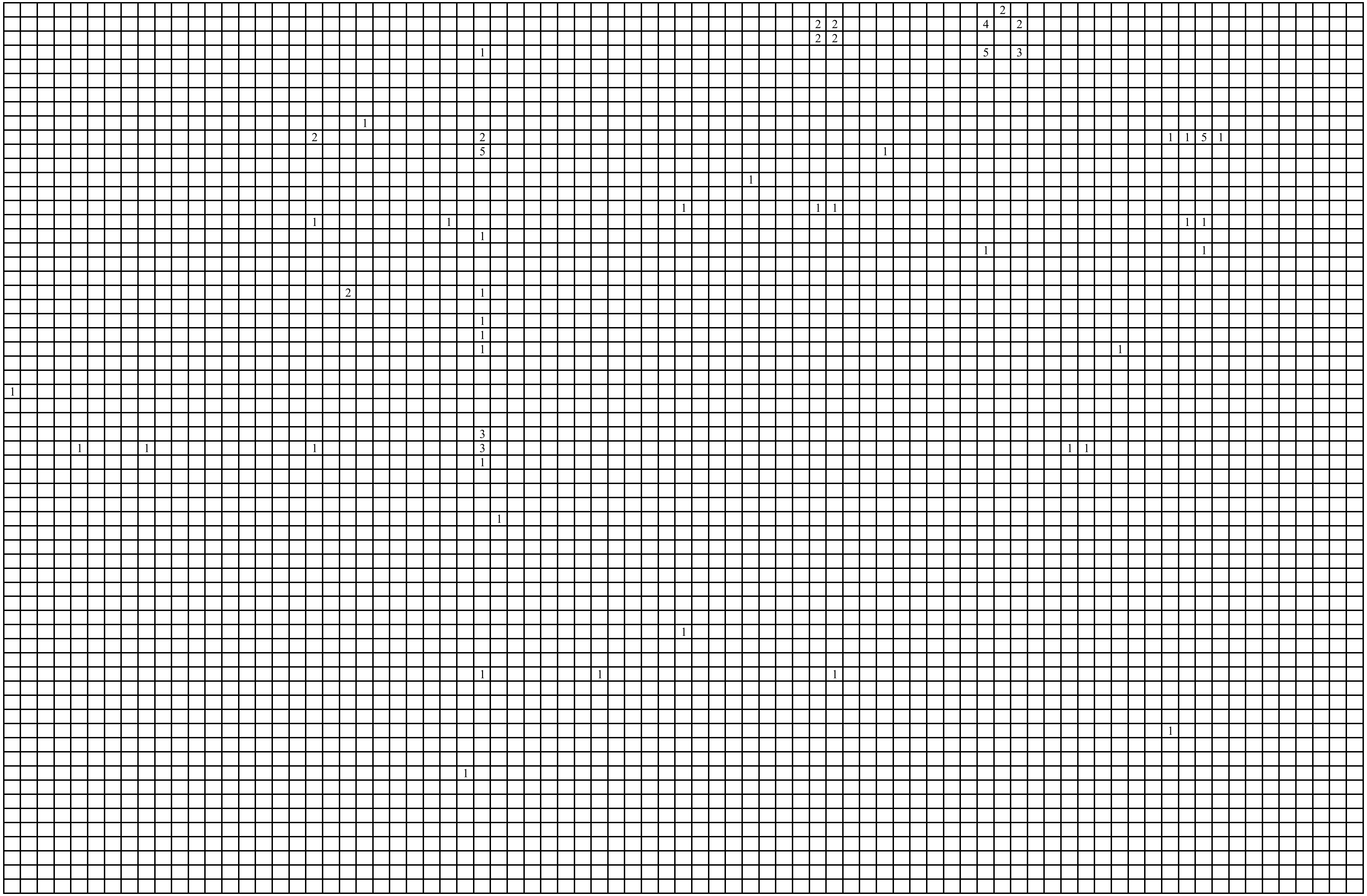

										1																																		1																																				
																																																								1															2			2								
																																									1																																								
				1																			1																1																																													
																													1																																																								
																																																																						1	1		1								
																																																																						1											
																													1																																																								
																																																																						1			1								
1																																																																																				
																																				1																																1														
																													1												1																																														
										1																																																																										
																																																																												1				
																													1																																																									
				1																								2										1		1																																																			
																												1																																																											
																													1																																																											
																													1																																																											
																												6																																																												

								1																																																																								
																																																				1																												
																																																																							1									
																												3																																																				
																						2																																																										
																												1																																																				
																												1											1																	1																						
																																			1																																												
																																																																												1				
																												1			1		1	1																																												
																																1		1	1																															1													
										1																																	1																																			
																																																																		1															
																																								1																																									
																																																																		1															
				1																																																																											
																																																																								1								
																												1																									1																											
								1									1									1						1																1														1													
																																																				1																												
																												1																				1			1						1																					
																												1							1											1	1									1											1									
1																												6							1												1																														

																												4								2													1									1																						
																																				2																1																												
										1																																																																						
																																1		1	1													1																		1														
	1		1			1	1	1										1			1	1			3																			2								1																	4		12	4								5
	1		1	1	2		1	1				1		8				1					1		1			5										2	2						6				1																						4	1							1		2
	1		1		1		1	2		3		1						1		1					1		1												1				1							1		1													1	1												1	1	1
																												1																1																																					
								1																																																																									
																												1																1																																					
	1																1								1			4														2	1		3			2																							4									3
																																													1																																		1
																																																																														1		
																																																																														2		
														4														2																							1																												
																																													1																																1		
																																													1																														1		1		1
																												3														2			10																										3							2		
																																																										1													1									
																																																																						1										
					2																																																																											
					2																																																																											
																												1																																																				
																												2																																																				
																												2														1	1		1													3		1											4							2		
			1																	1							1	5													1											1																			2	1								
																																			1																																													
			1	1			1	1						1				1		1												1		1	1																													1														1		1
																																																				1																													
																																										1			2																								1								2		
										1																																																																						
																												4														1																												3				2					2
																																																										2																						
										1																		5							1																								1													1								
				1																																		2	1																																									
																																						2	1																																									
																																																				1																										1		
																																							1													2																												
																						1														2								1					1			1																												
	1			1			1	1										1		1			2	1				1																								2				1										1														

																												1																																																				
																																																																1															
										1																																	2																																								
																												9		4					1												1																				1			2			1			8		
																												7							1																																			1	1						1		
																												1																	5																									2	2	2		2						8
																												3																								1																	3	3	6	5		2	3					
																												3																						1		2																		4	4								
																												1							1																																																		
																																								1																																														
				1				1												1																				1																																													

STAT4	STAT	7	32																										
STAT5A		25	155														1												
STAT5B		14	106																										
STAT6		32	89																										
TBX19	TB	8	17																										
TBXT		0	0																										
TBX1		2	11																										
TBX10		0	1																										
TBX15		5	15																										
TBX18		2	7																										
TBX20		3	37																										
TBX21		7	14																										
TBX22		6	11																										
TBX2		11	25																										
TBX3		7	72																										
TBX4		1	4																										
TBX5		33	157																						1				
TBX6		12	46																										
EOMES		1	2																										
TBR1		37	117																			1						1	1
TEAD1	TEA	79	203																		1		1		1	1	1	1	1
TEAD2		51	146																						1				
TEAD3		3	46																										
TEAD4		17	138																										
TSC22D1	TSC22	21	115																										
TSC22D2		9	74																								1		
TSC22D3		23	98				2																						
TSC22D4		19	201																										
TUB	TUB	4	26																										
TULP1		1	3																										
TULP3		36	437															1											
TULP4		0	22																										
EHF	WC.ETS	1	15																										
ELF1		43	121																			1					1		
ELF2		53	164																			1	1						
ELF3		15	64																										
ELF4		23	118																			1					1		
ELF5		14	54																								1	1	1
ELK1		18	166																										
ELK3		50	119												2							1							
ELK4		6	87															1											
ERF		8	129																										
ERG		58	315																			1				1		1	1
ETS1		41	126																			1					1	1	1
ETS2		24	81																										
ETV1		4	41																										
ETV2		0	4																										
ETV3		19	133																								1		
ETV3L		1	1																										
ETV4		22	80																									1	1
ETV5		3	49																										
ETV6		35	140																										
ETV7		18	34																										
FEV		98	202																			1				1	1	1	1
FLI1		43	71																								1	1	1
GABPA		43	142																										
SPDEF		11	55																										
SPI1		46	94																										
SPIB		9	19																										
SPIC		2	5																										
IRF1	WC.IRF	25	126																								1	1	1
IRF2		18	137																										
IRF3		72	345																										
IRF4		37	94																								1	1	1
IRF5		8	70																										
IRF6		9	14																										
IRF7		23	151																										
IRF8		29	72																			1							
IRF9		24	56																					1					
CDC5L		54	1041																										1
DMTF1		3	8																										
DNAJC1		9	418																										
DNAJC2		4	253																										
MIDEAS		0	0																										
MIER1		15	90																	1									
MIER3		1	32																										
MYB		43	176																					1	1				
MYBL1		0	25																										
MYBL2		49	217																			1							
NCOR1		251	579				2																						
NCOR2		243	466				1																						

																																										1																																								
	1																																																																																	
																																													2																																						
																																													2																																						
																																										1																																									
																																																																																		2	
																																																																																			4
																					1																																1																											1
	1																																				1					1						1																														2		
																				1																	1										2																																
	1		8																1	2																						1	1																																						1	
																																								1						1																																				1

																																															1																																	
																				2										1																							1																					1						
																				1										1			1	1		1			1				1				1						1							1															1				
																																										1															1																							
																					1											1	2	1											1				1				1													5								2		2
																																		1																																						1											
																				1																																																												
																																																																				1													
																					1																																																										
																																													1																																								
																																																																														1	
																														4																																																				1	1
1			2																	1						1																																																									
																																						1																																																	
																														1																																																							
																																		1		2	1	1	3						2	2				3		3								1	2	1																		1	3
																					1																																																											
																										1																						1												1																						3
																																													1																																	2				
																																																														2				1			1											

																																																															1																	
																																	1																				1		1		1																							
																																																																							1									
																																																																							1									
																																											1										1																										1	
																																																									1																							
																																	1	1									1							1	2			1	2		2																			1		1	1	1
		1																					1																					1											1		1				1		1				1		2										2	3
			1										1																																																															1				
																																																																												1				
																																	1																								1																							
																																	1																																															
																																																												2									1											
																																								1																																								
																						1																																				1																				1		1
																																																										1																						
																						1																																				1																				1		
																																																										1																					1	2
																														1			1	1		1	1		1														1							1			1													1				
																				1																																																												
																				1													1																		1		1				1																						1	

																																																																						1						
																																																																														1
																																																			1																													
																			1											2			1	1		1			1														1								4																		1	
																				1	1									3			1																												5																		2	3
																																																																				1											1	1
																																																																		1											3		
																																																													3																		
																				1																			1			1											1																1											2
																			1																																	1																1											
																			1																			1			1											1									1						1											
																																																																																1
																																																					1																											
																																																																1															
																																																					1																											
																																																																																1	1
				1																																																																												
																					1																																																													
																															1																																																					
																																			1																																														
																				1																																	1											1														1					

																				1	1																1										1						1										1																	
				1																1	1	1																1		1			1										1	1							1			1													1				
																																		1																																											1	1	1
																																																						1																								1	1
																			1																																																											1	1
																																																																															3	
		1																			1													1					1														1															1												5
																					1																																																											
																																												2																									1											1
																	1																																																															
																1					1																																																											
																							2																														1																											
																				1																																																												
																				1														1																			1																							1				
																				1	1																		1														1																							1				
																			1	1																																	1																											
																			1	1	1																		1			1											1																											
																																				1			1														1																											
																																	1	1		1			1														1																											
																																																							1																									
								1											1																																																													
																				1	1																																				2																							
																1																																																																	
																					1																1																								1																	

																				1	1																																1																												
											1																																																				1																	
																																	2																																																		
				1								1																																																																				
																																																					1															1												
																					1																																																														
																																																		1																																			
																																																			1																																		
																																																											1																					
																	1																								1								1																																			
																			1																				1												1	1										1																	
																																										1																																													
																																																																																		1
																																		1																																																		1	1
																																																							1																								1	1	1
																											2							1																					1																									1	1
																					1																																																															
																																								1																																																
																																																																																		1	1

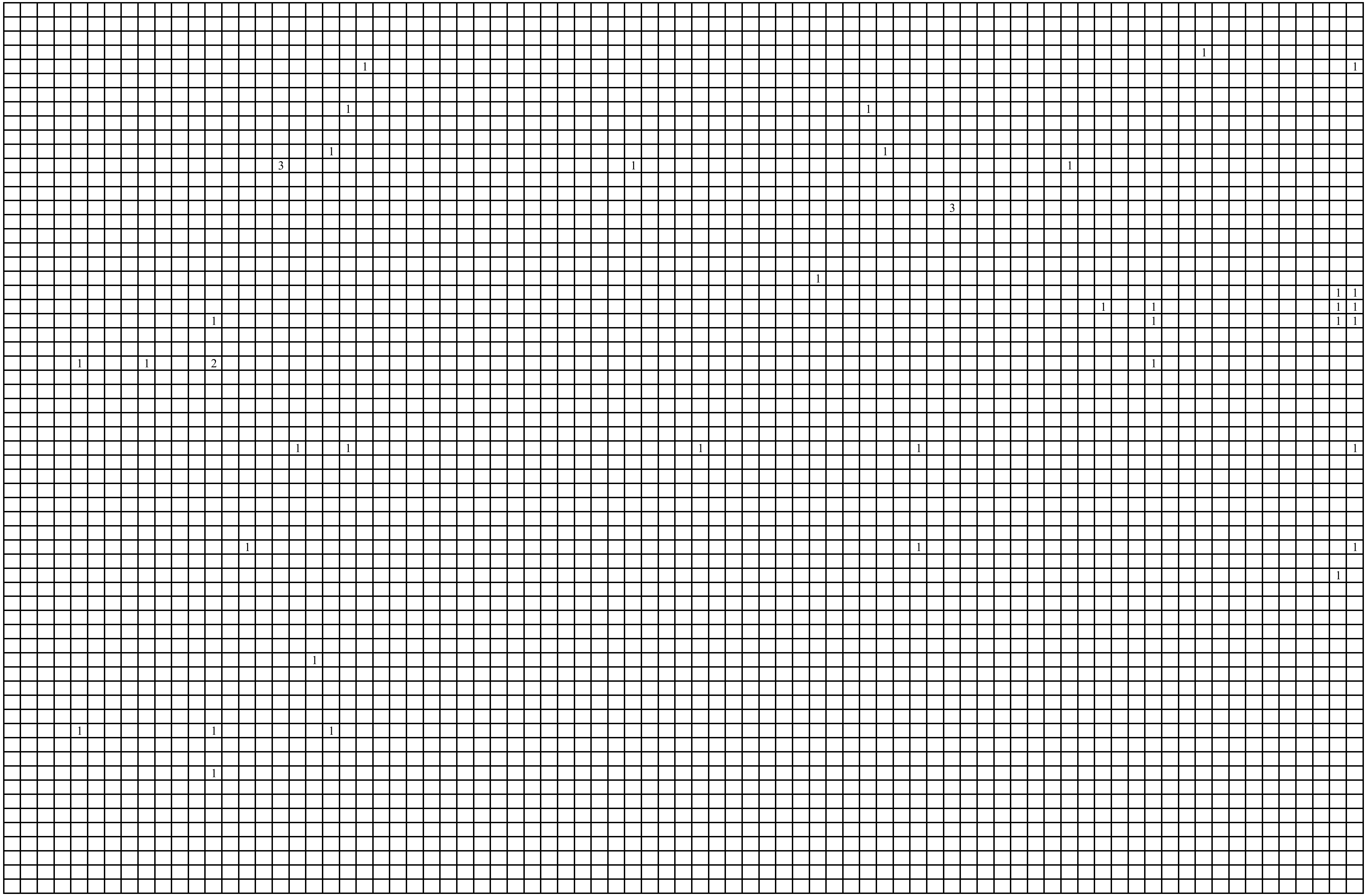

																																																															1																	
																																																					1																											
																																																					1																											
				1																																																																												
																1																											2																																					
													1			1																																																																
																1																																																																
																1																																					1																											
																1																																																																
			1													1																																																																
																																															1																																	
																																																					1																											
																			1	1				1													1					1											1										1																	
																																																																					1											
																								1																																																						1		
																				1													1		1	1	1		1				1				1						1	1						1			1																	
													1						1	2																1			1			1											1										1				1		1							1				
																																										1																																					1	1
																																																					1																											
																																																						1																										1
																																																																										1					1	1
													3	1						2														1													1				1			1																				2				1	4	3
																					1																																																											
																																																						1																										

																			1																																		1																									1	1
																																										1																																						1	1
																																																																																1
																																			1																																																	
	1																																																																											1				
																			1	1																			1			1											1																											
1																																						1	1										1												1																			
																			1	1																						1											1																					1						
																			1																																		1																											
																																										2	1											1																										
																	3																																																														
																																																																				1											
																																																																																1	
																																	1																											1																				1
																	3																																																														
																														1																																																			
																				2										1				1																																																		
																				1																						1																					1																	
																				3																		3		1														1																										4
																			1	1												1	1					2			2					1					2	1									1				1								1			1	1
																			1	1																		2			2					1					2	1																									1	1
																			1	1	1												1					1			1										1	1																									1	1
																			1													1	1																					1																							1	1	1
			3																																						2																											2											
																																																																								1									
																																								1																																												
																												1																																																										
																								1																																																																

																																											1																																				1	
																																																																							1									
																																																														1																		
			1																																																																													
																				1	1																		1								1						1																						1			2	
																																																																				1												
																																																																												3						
																																																							6																									
																																																							3																									
																																																																												1						
																									1																																																										
																				1														1																			1																											
			2																																																																															
																																										1											1																											
																												1																																																								
																																			1	1		1							1									1																											
																																																																							1										1	
																																																																										1									
																					1																																																														
	2																		1																							1																											1											
																														1																																																						
																																		1																					3																									
			1												1					1			2		3																		4	3								1	1												2				3								1	3	1
																																																1																															
																																												1																																				
																																																																																			1
																																																																										1				
																																																																																1	1

																																																						1																											1	1
																																																																	1											
	1		2																				2			2							2		3								1																		1						1	1											1	
																										2																																																					
																										1							1																																																	
																							3												1																										1						1	1												7
																					1																																																													
																																																			1												2			1															
																1																																																																3
																					1		1										1	1		2																				1																						1	
	1																																										1																																							
																																																													1				1														
																																		1					1														1																											
																								1																																																								
													1	1																													2																																1				
																																																	2												1				2															
														1													2																											1											3														
																																																	1												1				2					1		1					1	
																								1																																																								
																			1					2																																																									
																																																						1																													
3																																																												3								2												
			1																																																																			8												
			1																																																																															
	2	2																																																																									1				
	2	4																																																																														
																																																														3																	
																	1																																																															
								2									1																																																																
										2																																																																					
									2																																																																						
																1																																																															
																					1																																																												
											1					2																																																				1											

				1				1																																																																								
																																																																															1	1
																					2		3											1																																														1
													1							2	2		3																																																						1		
																				3	3																																																										1	
																								4		2	3																																																					
																										2	3																																																					
																								2	2	2	4																																																					
																								3	3	4																																																						
																														2																																																	
																														1																																																	
																												2	1																																																		1	
																																			1																																													
																																		1																		1		1																									
																				1													1			1																		1																						1		
																																1			2																																													
																																		1																				1																						1		
																																						4																1																									
																																																							1																								1
																																								2																																							
																																																	1																														
																																												2										1															2										1	1
																																												1													1																						1	
																																										2	1														1																						1	
																																																									1																						
																																																							1																								
																																									1													1																										1
																																																			6	5		3													1												4	
																																																			5												1																	
																																	1																						1																						1	1	1
																																						1				1							1		3																1													
																																	1	1		1			1								1						1																										
																																											1	1	1													1		2	1		1				3													
																																																									1	2																						
																																																									2				1						3	2												
																																																									1			1							1													
																																																														56		1		4														
			3																																																1					1																						1
																																																														1		2	1														1	2
																																																																1			4													
																																																														4				4														
																																																			1			1			3			3	1				4		2											1		
																																																												2																				
																1																										2																										8											
	1																																																																														3	
																					1													1		1																	1														1										4		
																			1				1							1												1	1	1							4		1												1													3				1

																			1	1																			1			1							1				1										1	2															1	
																1																																																														
																				1										1																																																					
																																										1	1											1																										
																1																										2	1											1															1											
																																		1			1																																		1											
						1																																																																									
						1																																																																										
																																		1			1																																																
																																																																										1											
																																																				2	1																												
																																																																										1											
																																																																																			1	
													1																																																																			
																																																					1																									1	1
																																																					2																												
																														1																																																						
																																			1																					1																									
																																										2																																													1	1	1
																																	1	1																					1																						1		
													1	1																			1	1																					1																							1	1
																																						2																																																
																																																																																		3	3
																																	1	1																					1																						1		1
																																																							1																									
										2																					1									1											2																							3	3
																																			1			1													1							2																				
																																																								2																									
																																							1																																																

																			1															1																				1	1																							1	1	1
																																		1																																																				
															1																																																												1			4	
																																																																															3	3
																												1																																																									
										1																																																												1											
																																																									4						4															5	13
																				1																																	1																							1				
																																																										3																							
																					1																																																													
																					1																																																													
																																																																											1								
																																																																																		3	
																																																																	3																1	1
																							1																																																															
																																																																										1											

																																																				1																												
																				1													1	1		1			1														1		1																					1				
																																							1														1																											
																																																																														5	9
																					1																																																											
																											2																																																					
																																		1																																																		
																																										1		1																																								
																																											2										1																										3	3
																																																																					1											
																																		1																																																		
				1																																																																											
																					1																																																											
																																																					1																											
																				1																																		1																											

																																	1	1					1														1																							1				
1																																																																														
																									1									1																			1																											
																																																			1																													
																				1													1	1		1			1			1					1						1																							1				
																														1																																																							
																																																			1																									
																1														1																							1													1														

																																																																					1											
																																																																														1	
																				1														1			1		1			1											1																							1				
														1			1																																																														
																																																																									1											

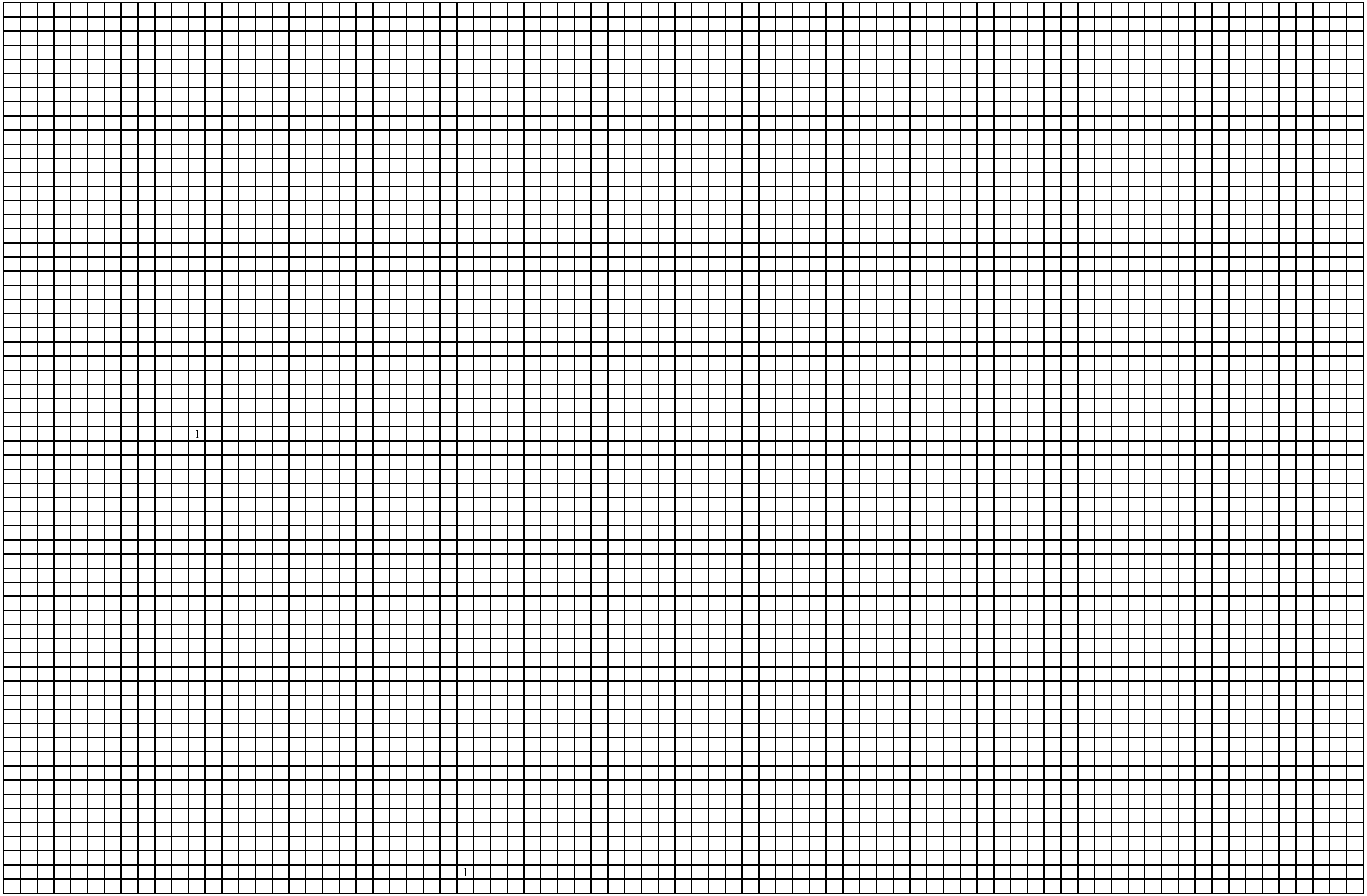
1
1

	1																																																																																	
																														1																																																						
																				1																																																										1				

																																																		1																											
																																																																										1				
																																								1			1											1																1											
																														1																																																												

																														1																																																						
			1																																																																															
																				1																																				1																												
																														2																																																						
																																																																											1		
																																																																												2	2
																																																	1																									1		
																																																																											1		
																																																												1															
																					1																																																																				
																																																	1																												
			1																	1	1													1	1					1			1	1									1	1											1			1									1				

																																																																																1
																																		2																																														
																1				1																																																												
																																							1														1			3													1											
																																							1																	3													1											
																																																																															1	
																				1																																	1																							1				
																				1																			1														1																											
																				1	1																																1																											
																1																																														1																		
																																							1														1																											
																				1				1													1		1														1																					2	1	1				
																																																																			1													
			1																																																																		1											
																																																																										1						
																														1																																																			
																			1	1																			1														1																											
																																																																														1		
																									1																																																						
																														1																																																			
																																																																														1		

			1																																																																												
																				1										1																							1																											
																																			1																																														
																																																1						1																											
																																						1																1														1											1		
																				1																																																															
																																											1											1																											
																			1	1	1																																																						1				
																																		1			1																																													
																															1																																																									

																1																																																																
																																							1																1																	1													1				
																																																																	1																
																			1																																	1																														
																			1	1																						1										1										1																		
																			1	1																						1							1			1										1			1														
																																																																							1	1						3		
																																																						7			2	1																						1
																																																									6																						1	1
											2																																													1																		1			1	1
																3																										1										1																										1	1
																1																																				1																												
																																	1	1		1			1				1																																1		1		
																1																																																														1		
																1																																																														1		
																																																																														1		

																1										1																																																						1		
																																																																													1		
																																									1																																										
																			1	1	1																		1			1	1										1																							1				
														1								1												1	2							3					1			1						1													1										21	15
	2													1																																						1				1														1		1	1							5	7
	1																																	1																	1				1	1															1								1	2
																															1																																																						
		2													1						1												1											1												1																						1	9	4
	2	2																																																																													1	4
																																																																													1	1
																																																																													1	
																																																																													2	3
																																																																													3	
																																																																													1	1
																																																																													2	5
																																																																														1
																																																																													7	1
																																									1						1																																			5	4
	4		3																																																																												18	14
																																																									2																					18	9
																																																																														1
																																																																													2	1
																												1																																																							
																																																																														9	3
																																																																													9	6
																																																																													7	2
																																		1																																															1	1	2
																				1													1	1					1								1					1	1																							1				
	1																																	1					1								1						1		1												1		1										2	
																																																																				1												
																					1																																																													1
																					1																																																												1
																																																																															3	4
																																																																					1											
																																																																																1
																																																																																4
																																																																																1
																																																																						2											
																1																																																																				
																																																																															1		
																																																																															1		
																					1																																			3												1										4	

																								2																																																							
		1																																																																												
																																																												1												
																																							1			2											1	1																						3			3	
1																				1																			1												1		1	5																										
																																										1																																						
																																											2																			1				1														
																			1																								1						1																															
																																																	1														1																	

603	151	TFs	WC.MYB	RCOR1									1				1	1												
44	13			RCOR2																										
235	62			RCOR3																										
487	154			SMARCC1	11	7	3																				1			
461	136			SMARCC2	10	8	5											1									1			
82	3			SNAPC4																										
287	37			TADA2A													1													
183	18			TADA2B																							1			
43	11			TRERF1																										
3	0			ZNF541																										
87	8			MIS18BP1																										
62	3			MYSM1																										
0	0			TERB1																										
488	35			TERF1																										
487	40			TERF2																										
128	16			TTF1																										
198	35			ZZZ3																							1			
48	12		ZF	ZNF750																										
0	0		ZF.BED	ZFTA																										
11	1		ZF.C2CH	MYT1																										
7	1			MYT1L																										
5	1			ST18																										
143	30			L3MBTL1																										
444	53			LYAR																										
183	20			THAP1																										
30	0			THAP10																										
165	21			THAP11																										
0	0			THAP12																										
22	1			THAP2																										
74	8			THAP3																										
79	5			THAP4																										
7	0			THAP5																										
21	4			THAP6																										
139	12			THAP7																										
33	2			THAP8																										
11	0			THAP9																										
82	22			EGR1			1																							
168	58			EGR2	1	1	1	1			1																			
1	0			EGR3																										
4	0			EGR4																										
43	7			KLF1	1																									
54	8			KLF10							1																			
26	3			KLF11																										
220	54			KLF12				1	1		1																			
26	2			KLF13																										
0	0			KLF14																										
308	63			KLF15			1	1			1																			
451	76			KLF16			1				1																			
3	0			KLF17																										
22	3			KLF2																										
287	107			KLF3	1	1	2	1	1	1	2		2																	
221	34			KLF4	1	1																								
272	79			KLF5	1	1	1	1	1		1																			
99	20			KLF6			1																							
8	0			KLF7																										
340	77			KLF8			1				1																			
90	29			KLF9			1			1	1																			
693	293			SP1							1																1	2		
44	21			SP2																										
103	39			SP3																										
54	18			SP4																										
3	1			SP5																										
33	0			SP6																										
305	87			SP7	1	1	1	1	1		1		1																	
3	3			SP8																										
1	1			SP9																										
105	13			AEBP2										7																
6	0			OSR1																										
0	0			OVOL3																										
22	1			ZBTB22																										
84	26			ZBTB32																										
174	35			ZBTB33																										
32	17			ZBTB34																										
6	2			ZBTB37																										
61	9			ZBTB43																										
55	19			ZBTB46																										
71	19			ZBTB5																										
11	0			ZBTB8B																										
41	19			ZFPM2																										
97	57			ZNF174																										
51	36			ZNF396																										

1																																																									3																							
																																										1																																		
						1																																																																										
										1																																																																					
																																																												1																
																																																										1																							
																																																										1						1																	
						1																																																																										
1																										1																																																							1	
1																										1																																																								
			1	1																																																			2																							
																																																									5																							
			2																																		1									1																	1																	

1																										1																						1								1	1							1																	
1																																					1						1			2	1			1		1	1		1	1							1																	
																																					1																										1																	
																																																																						1										
																																																						1	1																										
		2																																																																														
1		1	5	3		2	3			2						1						1	3			1															1						1		1								7							1									1									
			1	1												1							1																																		2							1																	
			2				1																																				1								1						1																									
																																			1								1													2																									
				1										1		1																													1										2		1																					
																																										1				1			1		1			1																												
																	1																																									4																								
																																																																								1										
																																																																										2								
			1	1																																															2					6																								
																																				1																																																
																																				1																																																
				1		2																																																																												1
																																					1																																															
			2	2																																																																															
																																				1																																																
																							1																																																													
																																																																										1								

																																																							1																								
2			1	2				1																		1				1						1																3								1												1							1	
1			5	2										2							1		2													4																3					6		2												1									
																																																			3																													
			1	1																																																																												
																																																							9																								
																																																								1																								
																																											1				1											1	2		1																				
																													1																																																				
																													1																																																				
						1										1																																					1				8	2	2	2																				
																																																										1																								
																																																										1																								
			1	1																																																																												
1			1																																								1			1	1			2					1	1														1								1	
																																											1				1			1					1																									
		1		1																																																																												
																																															1																								1									
																																																																							1									
				1																																											1																1																	
																							1																																																				1								

																																																																						1								
																																																																					1									
																																																																				1									
		1																																																																														
																																																1																									1								
													1	1																																																																		
																																												1			1				2		1			1	1							1																	
3																																															1			1					1																									
		1																																																																														
															1																																																																
													1																																											2														1									
				1																																																																											
																																																																								2								
																							1																																																								
																																						1										1			1		1			1								1																	
																																						1																							2																				
																																																																						1	1	1							
																																						1									1				1		1			1								1																	
		1																																																																				1									
																																												1																				1																	
																																																				1																														
		1	1	1																																																																												
																																																1	1					1			1																										
1																																																			1												1																	

1																																																														1																	
1																					2																												1												1																	
		1	1	1																																																																									
1		1																																																																													
		1																																																															2														
1			1																																																											1																	
					1																																										1							1		5			2			1																	
				1																																																																													
																								1																																																									
				1																																																		1																									
																																					1																										1																	
1																																														1				1												1																	
																																											1						2					1																									
1																																				1					1				1			2	1				1								1																	
1																																				1												1																													
1																																				1																									1																	
													1										1																			1			1	1			1					1																									
																																											1				1			1		1			1																									
																																											1						1																													
																3																	1																																				2									
																																																																1																	
													1																																																																					
					1																																																																													
																																																							1																									

																																																						1																							
																						1																																																														
																																																																									1										
																																																												1																	
																																						1																										1																	
			1	1																																																																										
		1																																																																															
																																																				1																															
																																																				1																															
																																																													1																	

																																																				1																							
			1																																																																													
								1																																																																							
1																																																																																
																																																												1																	
																																																				1	2	1	1	1																			
1		1	1																																																																												
1		1																																																					1													1										
		1																																																																														
																																																																					1										
			1					1																																																	1		1	2																				
			1																																																																													
																																																		1																														
																																																										2																							
																																				1											1																1							2										
																																																																						5										

																																						1																									1																	
																																																							1																							
																																																														1																	
																																																															1																	
														1																																								1																									
																																						1									1					1	1											1																	
			4	8																																							1			1				1		1	1		1	1							1																	
																																																1									1																								
2			1																																								1			1				1		1			1								1								1			1						
1		1	3	2				1															1																																			1																	1				

							1																																																																										
1		1																																1										1			2					1									1																			
1		1																																																																												
			1	1																																																																										1		
1																																																																																
			1	1																																																																													
																																						1																				1																							
																							1														1								1	1			1		1			1									1								3										
																																										1				1			2																															
5	2	3																							1																																					1																		
7	3	3																																																													1																		
3			19	17																																			1												1											1																		
1														1	1																																																																	
											1												1																								1								1																									
			1										1										1																																																									
1			1																																																					4																							
																																																										1																							
																														1																						4													1														
																																																															1																		
																																																										2																							
																																															1			1		1			1		2						1																		
																																																										1	1																							
																																						1						1				2			3	1	1			2								1																		
1		1																																									1				1			3					1								1																		
		1																																			1										1			2					1								2																		
			1	1											1																																														1																		
																																														1													1		2																				
			2	1		1																																																			1		1																				
																							1																																																										
																1																																																																	

1																																																																																
																							1										3																																															
															2																										1									1			1			1																								
																																															1									1																								
1		1																																									1				1								1																									
1		1																																																																1														
																																																				1																												
						1																	1																																																									
														1																							1									2	1			1					1																									
																							1						1																							1																												
																																															1																																	
														1																																																																		
																																														1	1			1		1			1																									
			1																																																																													
2																																																								2		1																					
																																					1															1											1																	
1																																																																																
1																										1																					1						1																											
			1			1				1						1						2												1		4							2								1	2					26		3												5					3				
																						2																																			1		1																					
																							1																																																									

																																																		1																														
																						1																																	1																								
														1																																																																		
																																																			2					4																								
																																																								1																								
																																																								1																								
																																			1															1						8		1											2										
			2	2																																																																											
			1	2																														1	1																																												
1			1	1																														1																																											
		1	2	2																																																																											
			2	1																																															1																												
																																															1							1		2		2																					
																																															1																																	
																																											1			2	1		1					1																								
	1																																																																															
1		1										1	1																																																																	
															1																																																																		
			2	2																																													2					5																										
			1	2																																													1					9																										
																																																			1					6																								
															1																																																																		
													1																																																																				
					1																2																								1								1																										
			1	1																																																																											
																																														1										4																				1		
													1	1																																																																		
																																																									2																								
																																				1														1																													
																																																		1																													
1				1																																																																1										

																																																															1																	
		1																																																																														
		1																																							1																																							
						1										1																															1			1					1		1																							
																																											1				1			1					1								1							1										
						1										1																																										1																						
																																																			2																													
																																																										1		1																				
																																														2																																		
			1	2																																																																												
			1	1																																																					1																							
																						2																																																										
																						1																																																										
																																																										1																						
			1	1																																	1			2																							1																	
																																											1				1			1					1	1	2		2				1																	
																																																										2																						
				1		1														1				1																																																								
																																																																							1									
																																														1	1								1								1																	
																																	1				1									1				1		3					3						1								4					3				

																																					1									1				1		3			1		3						1													3				
2		3		1		1										1	5		1							1																	1							1	2												1								1					1			1	
																																																																															1	
3																																											1							1													1																1	
			2	14																																	1			1																	1						1																	
1			14										1																											1																	1						1																	
1						2	1									4																											1							2																														
						1								3		1																																					1		1																									
																																					1																										1																	
										2																																																																						
				1									14	3									1																																																									
							3						3	6									1																																																									
																							1																																																									
1						4	1																																				1							1					1																									
5																																																			5																													
1																																																																																
																						2																																																		1								
													1	1	1								2																																																									
																								10																			1																							1														
1																										2			1																																																			
																										1																																																						
																														2																																																		
																																2																																																
																																	4																																															
																																																									1																							
			1					1																																																																								
			1	1																																																																												
																																																									2																							
																																										2																																						
1		1				1										1								1																																																								
																																																										1																						
																																																																							1									
1		1				2										1																																																																
2																	5																																				1				3																							
							1																																												1						4																							
							1									1																																									1																							
																																																									2																							
			1	1																																1					2										3		4		1	2	2	1	12																	3				
																																														1											1																							
																																																									12																							
																																																												2																				

1		1	1	1				1																																																		1															
																																																															1																	
																								1																																																							
																																																																						2										
1																																														1																																
																						1																																																		1							
																																																																								1								
																																																																										2						
1																																																								3																						
1	1	1																																																																											4	3
																																																																															3	
																																																																															1	5
																																																																															3	1
																																																									1																						
																							1						1														1			1	2								1	1															1									
																																																									2																						
1																																																																																
5	2	2																																																																														
				1																																																																											
																																																																															1	
1		1																																																																														
1		1																																																																				1										
		1																																																																														
																										2																																																							
5		3																																	1	3																																										

																								1												1																																																
						1							1	1		1							1			1																	1				1								1	1	7		1																					
1																																																																																
																																										1			1	1			2					1	1									1																	
												1											1																																1													2										
																																													3																											1		1						
																																																																								1								
																1																																																															
																							1	1																																																									
				1																																																				2																	1						
																																																									1		1																	2				
																																																																														1				
																							1																																																											
																							1																																																										
																																															1																																		
																						1																																				1																									1	
																																																1																																	1	3
														1																													1				1								1	1							1																2	3
																										1																																																							2	3
																																																1																																		

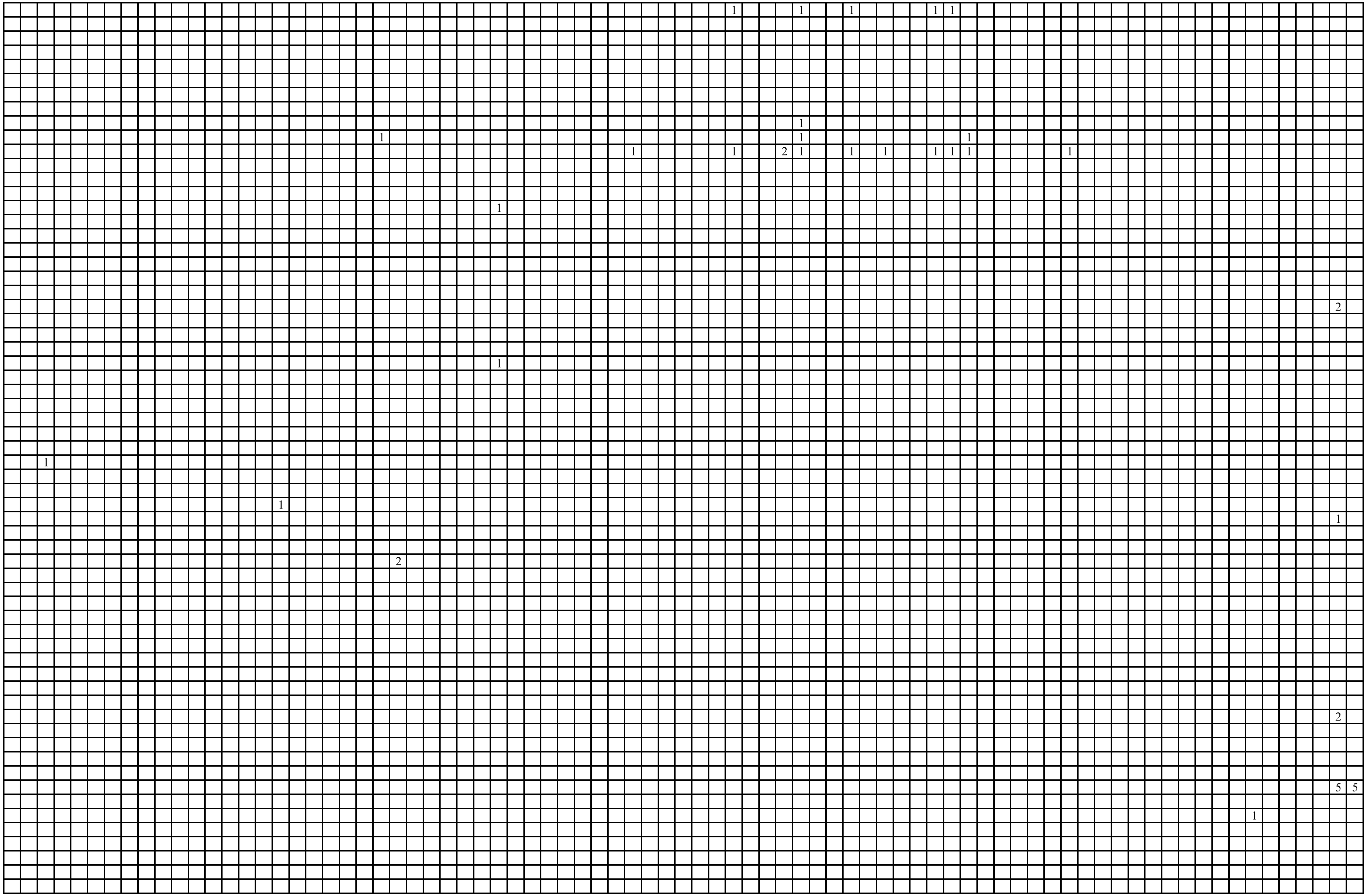

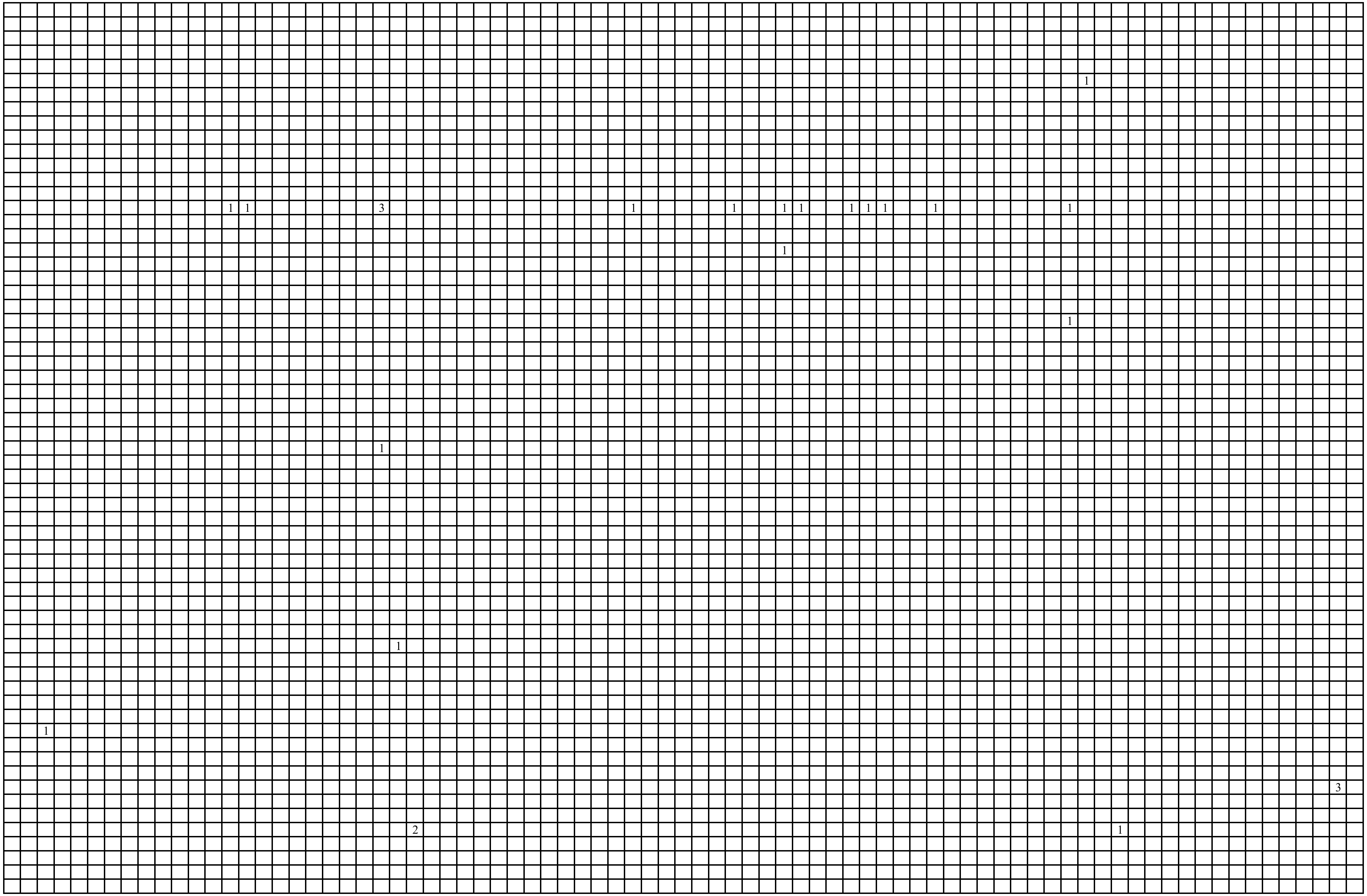
1
1 1 3 1 1 1 1 1 1 1 1 1
1
1
1
1
1
3
2 1

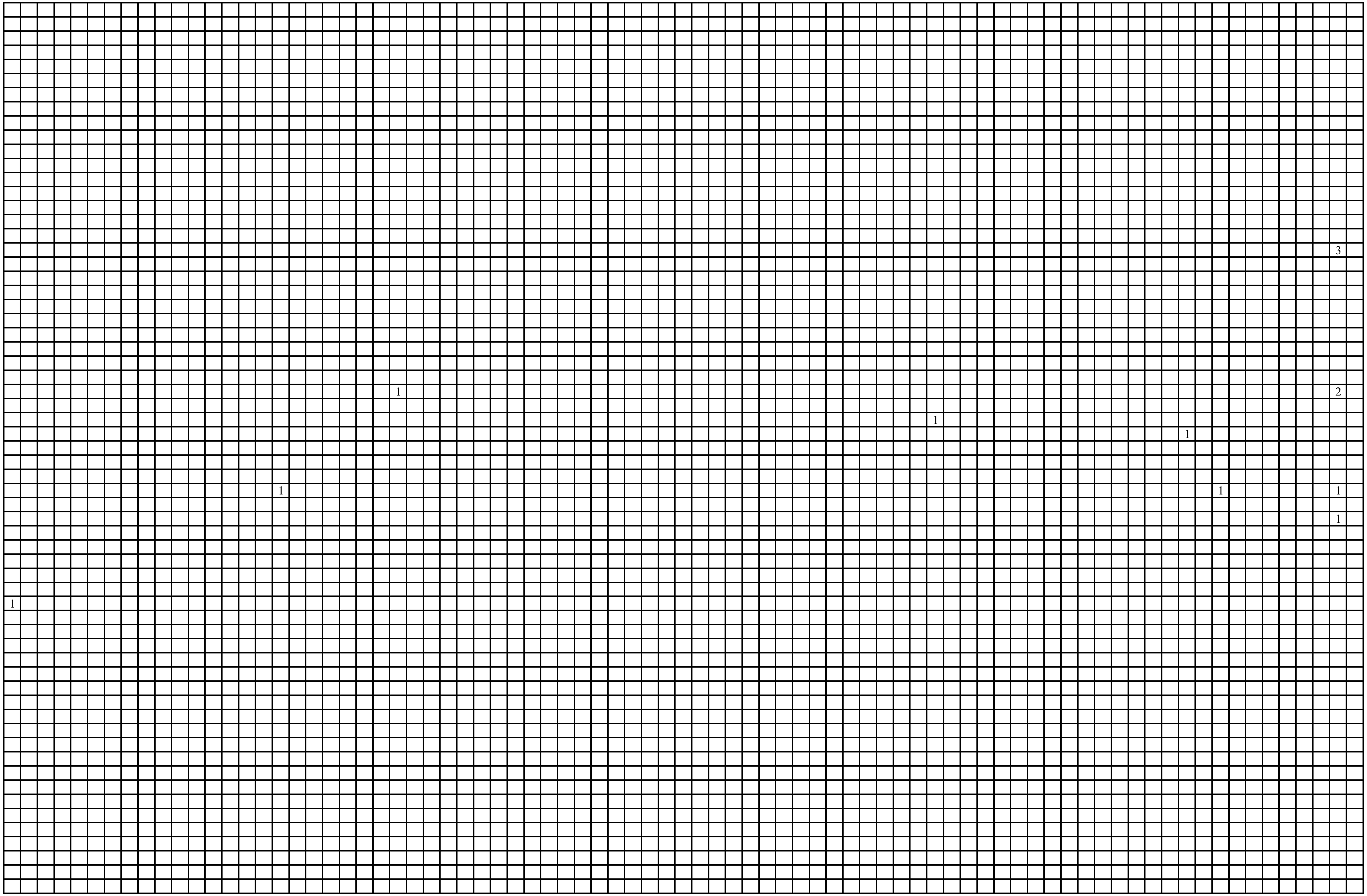
3
1
2
1
1
1
1
1
1
1

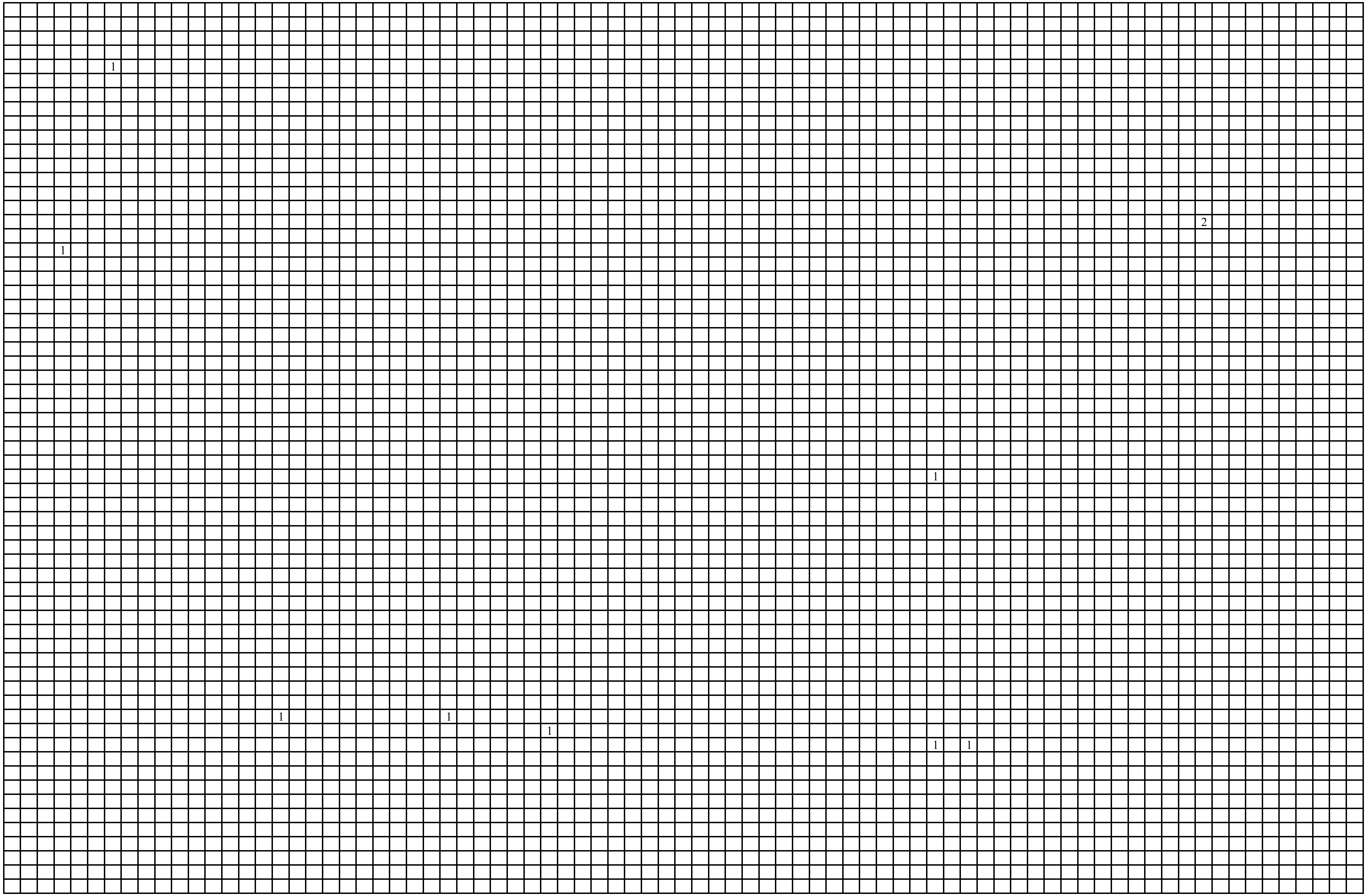
1
2
1
1
1
1
1
1
1
1

																																																										1																	
																												1																																																									
																																															1																									
																							1														1																																																				
																																										1																																														
																																										1	1								1																									
																																										1									1																									
																							1																																																																		
												1																																																																																				

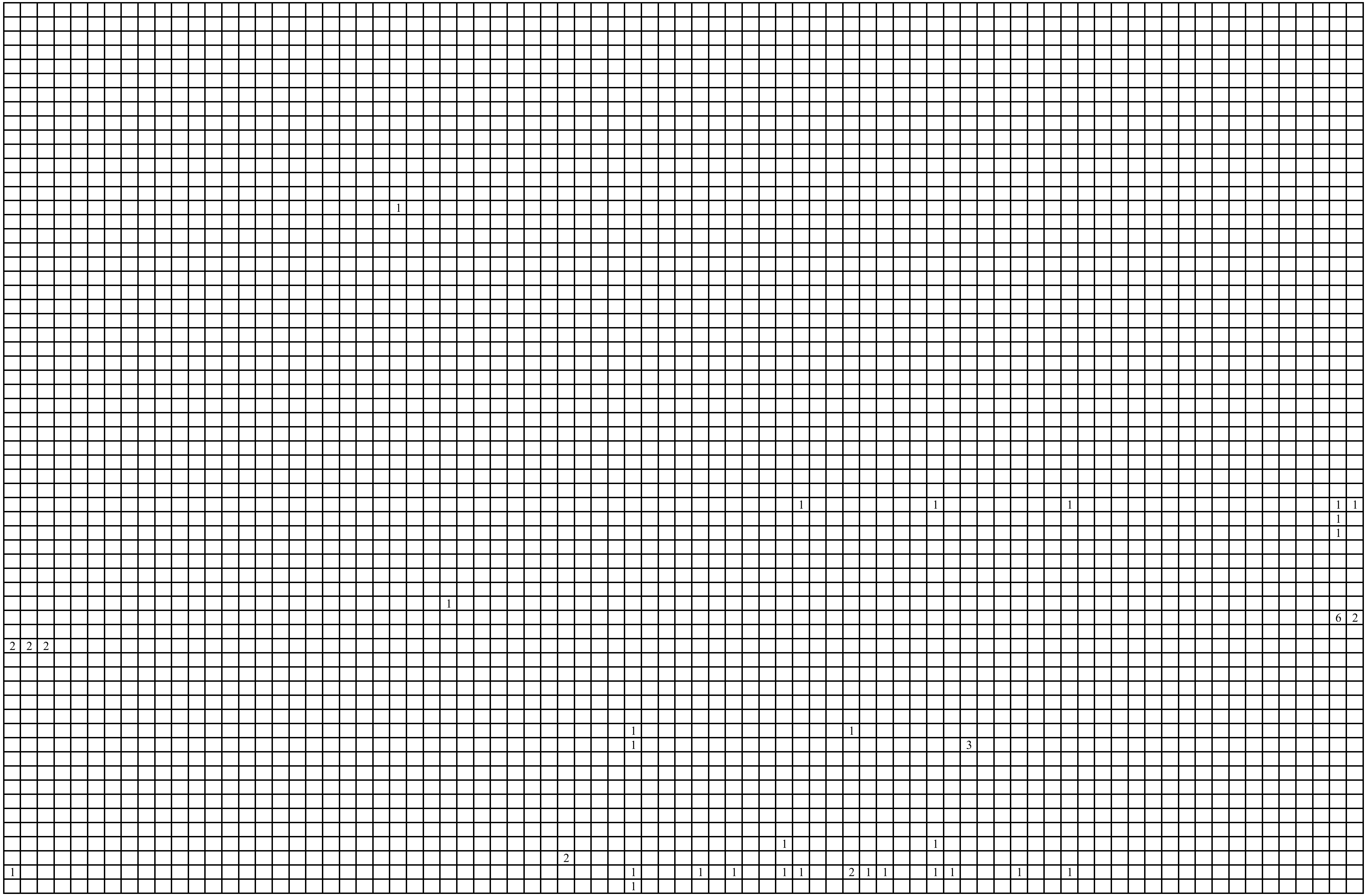
1
1 1 1 1 1
1
1
1
6 2
2 2 2
1 1
1 3
1 1
2
1 1 1 1 1 1 2 1 1 1 1 1 1
1

			1																																																																											
		2	1																																												1																																	
						2				2						2										2																																																						
																																														2						1			1																									
1				2																		2															1						1				1			2					1																									
																																								1																														1										
																							1																																																												
1	1																																				1						1							2																														
																																											1							2					1																									
5				1										2									1																																		2																							
1																																															1			1					1	1																								
2																																					1						1				1			1					1																									
1													2																																																																				
1																						1																																																													
																																														1													1																				
																						2															1						1				1			2					1								1												3					
1																																																																																				
																																																				1																													
																							1																																		3																							
1																																																																																					
																																															1																																				
																						2																																																													
9		1																																			1						1							2					1								1																	
																																															1																																				
																																															1																																				
				1																																																																																	

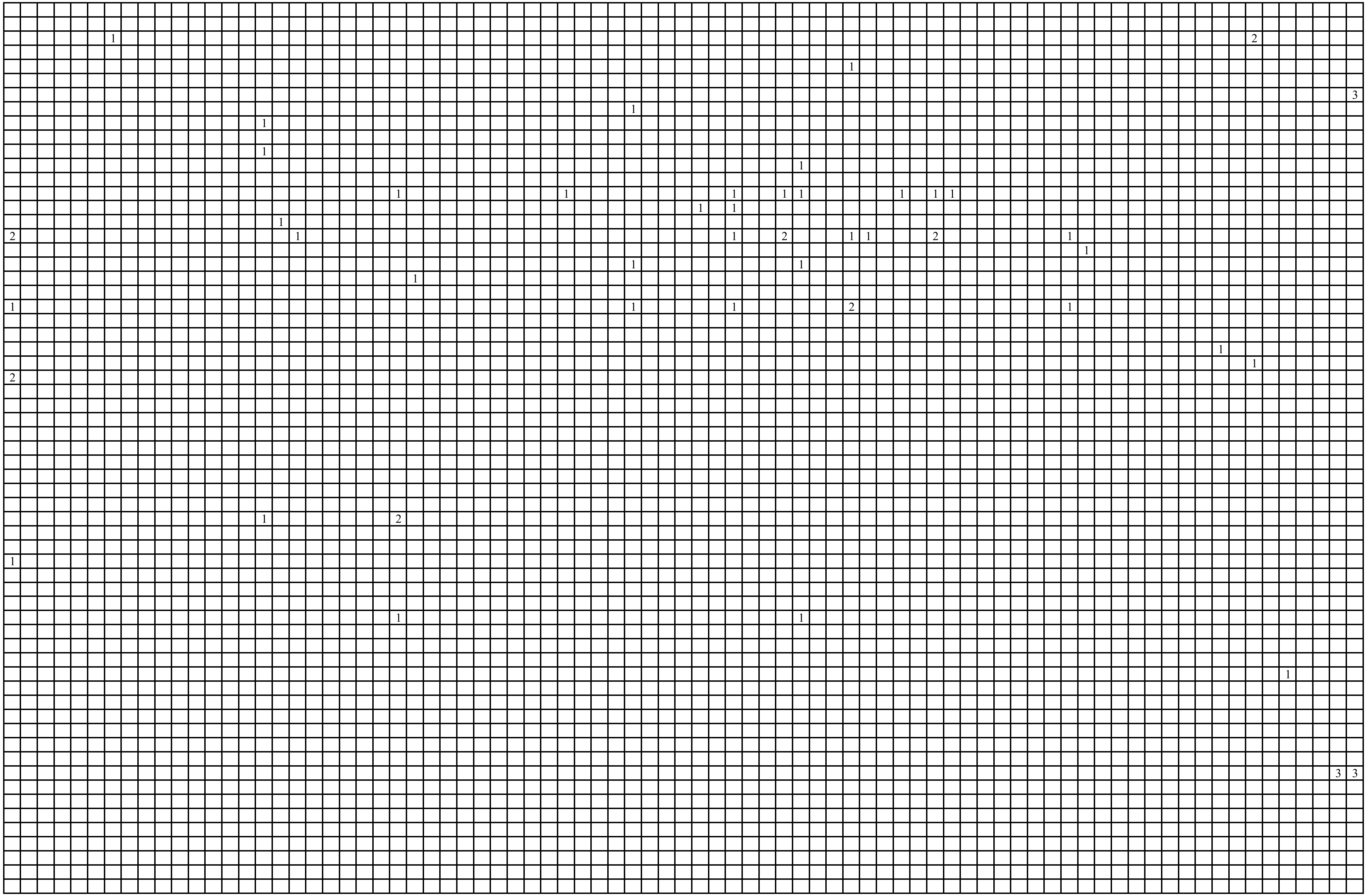
1 2
1
3
1
1
1
1
1 1 1 1 1 1 1 1
1 1
1
2 1 1 2 1 1 2 1
1
1 1
1
1 1 1 2 1
1
1
2
1 2
1
1 1
1
3 3

																						1																																																									
														1																																																																		
														1																																	1					1																							3						
1																																																													1												1						
																																																		1																														
																																															1																1																	
																1																					1									1				1		1											1																	
																																					1									1				2		1			1								1																	
		1				1																																																																											
1																																																																																		
1																																																																																		
																																															1	1																																		
																																																																																	2	3
							1							2										1								1																																																			
		1	1	1												1																																								1												1									3		
1		1	1	1																																																																	1								1		
2		1	1																																																				2																					1		
			2	1		1																																																	3														1							6		
																																																1																																
																																																1								4																							
																																					1						1			1	1			1					1	1																								
																																											1							1																									3					
																																																			1																					1				3					
																																																																													3			2		

																																																																														2		
1		1																																									1							1																												1		
																1																					1																										1																	
1																																					1						1			1	1			1		1			1								1																	
3		1	10	3			1	1			2																																								2	1					2													2	1									
							1							1	1								3																												3	3					13	1	2																					
1							1							1	1								1							1																						3					2		1																					
																																																									1																							
																																																									1																							
								1																																																	1																							
1		1	3	2		1																						1																													1																							
																																																								2	2																							
			1			1										1																																			2	2					7																			1				
																																																				6					3																							
																																																									1																							
		1	1	1		3	1									1																																									4																							
1																																					1						1				1			2		1			1	1																								
			2												1								1			1											1						1			2	1			2					1	1		1																						
2																																																									2																							
																																																									2																							
																																																				4					2																							
			3	2																																																																												
								3																																																	2																							
			1																																																		2					2																						
													1																																																																			
																																																									1																							
															2																																																																	
																																																			1																													
																																																																											3					
1	1		1	1									1	1									2			1																															2		2																					

																																		1																																								1									
														4																																						2									2																					
2																																																		2													1																	
																1																																																											1								
																																																												1																	
																																						1																											1																	
																							1																																																														

																										ZNF414	2	152
																										ZNF446	85	150
																										ZNF487	0	2
																										ZNF511	5	59
																										ZNF580	6	32
																										ZNF705A	0	0
																										ZNF740	3	24
																										ZSCAN1	36	75
																										BCL6	84	330
																										BCL6B	6	38
																									1	CTCF	58	541
																										CTCFL	3	46
																										FEZF1	0	28
																										FEZF2	0	5
																										GFI1	13	40
																										GFI1B	11	136
																										GLI1	15	192
																										GLI2	9	82
																										GLI3	14	97
																										GLI4	29	127
												1														GLIS1	10	17
																		1								GLIS2	18	111
																										GLIS3	2	40
																										GTF3A	0	12
																										GZF1	16	86
																										MTF1	3	116
																										MYNN	0	13
																										MZF1	10	35
																										OSR2	0	8
																					1					OVOL1	4	16
																										OVOL2	20	141
																										PLAG1	4	9
																										PLAGL1	6	28
																		1								PLAGL2	9	60
																										PRDM1	32	106
																										PRDM14	15	132
																										PRDM5	17	81
											1							1								PRDM6	12	94
																										RBAK	21	67
																										SCRT1	1	9
																										SCRT2	0	9
																										SNAI1	43	728
																										SNAI2	7	80
																										SNAI3	0	14
																										WT1	18	92
												1							1				1			YY1	167	693
																										YY2	6	15
																										ZBTB11	18	121
																										ZBTB12	1	8
																										ZBTB14	15	103
																										ZBTB16	69	305
																										ZBTB18	12	77
																										ZBTB20	6	40
																										ZBTB24	48	198
																										ZBTB26	12	47
																										ZBTB40	9	24
																										ZBTB42	7	77
																										ZBTB44	7	81
																										ZBTB45	3	6
																										ZBTB47	32	146
																										ZBTB48	39	234
																										ZBTB49	11	39
																										ZBTB6	8	17
																									1	ZBTB7A	64	193
																										ZBTB7B	23	81
																										ZBTB7C	20	23
																										ZFP1	2	61
																										ZFP14	0	8
																										ZFP2	3	18
																										ZFP28	0	5
																										ZFP3	0	3
			1																							ZFP30	3	12
																										ZFP37	0	6
																										ZFP41	4	124
																										ZFP42	5	16
																										ZFP62	15	84
																										ZFP64	7	51
																										ZFP69	0	1
																										ZFP69B	1	13
																										ZFP82	1	12
																										ZFP90	1	30

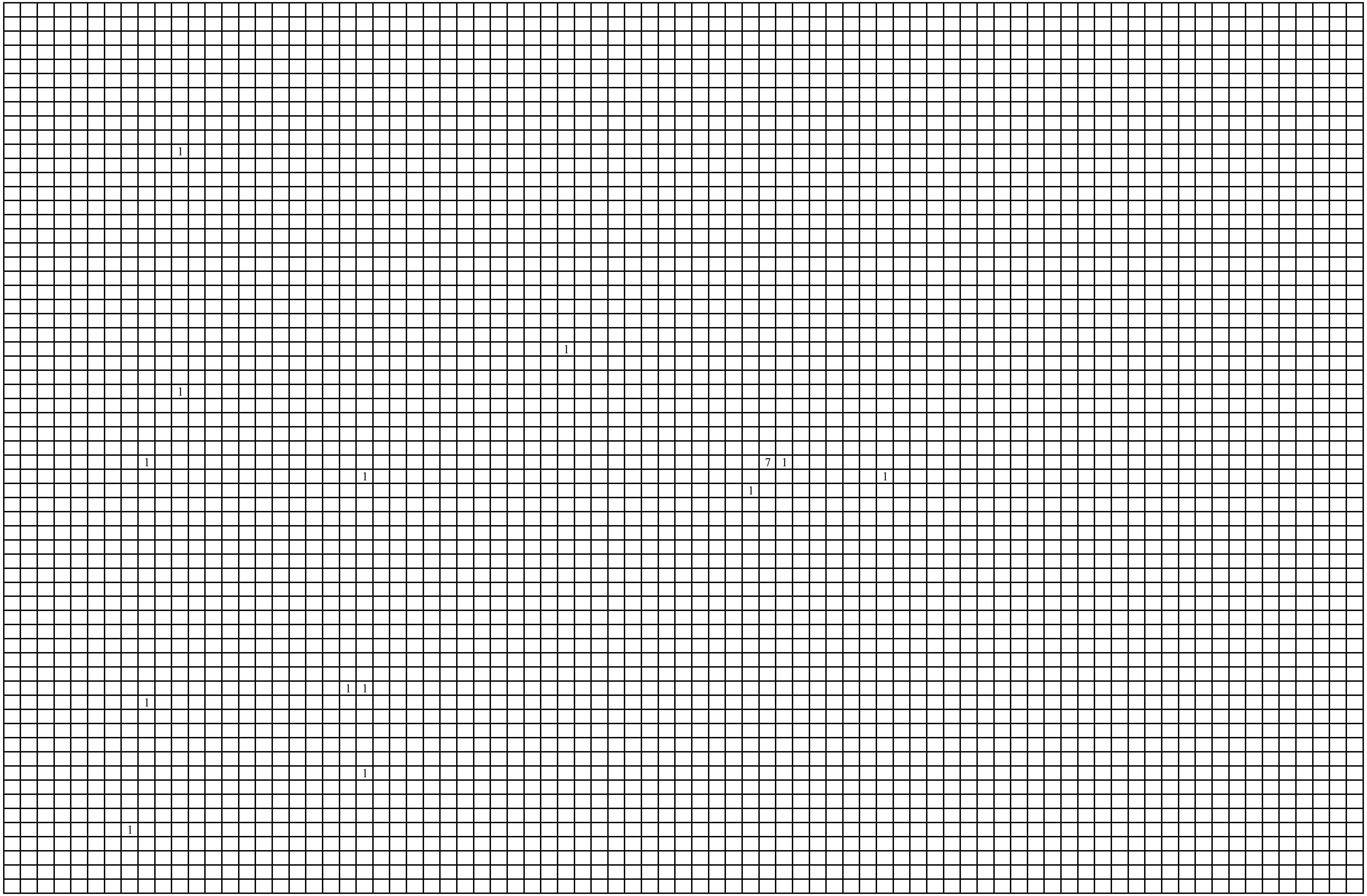
1
1
1
1 7 1
1 1
1
1 1
1
1
1

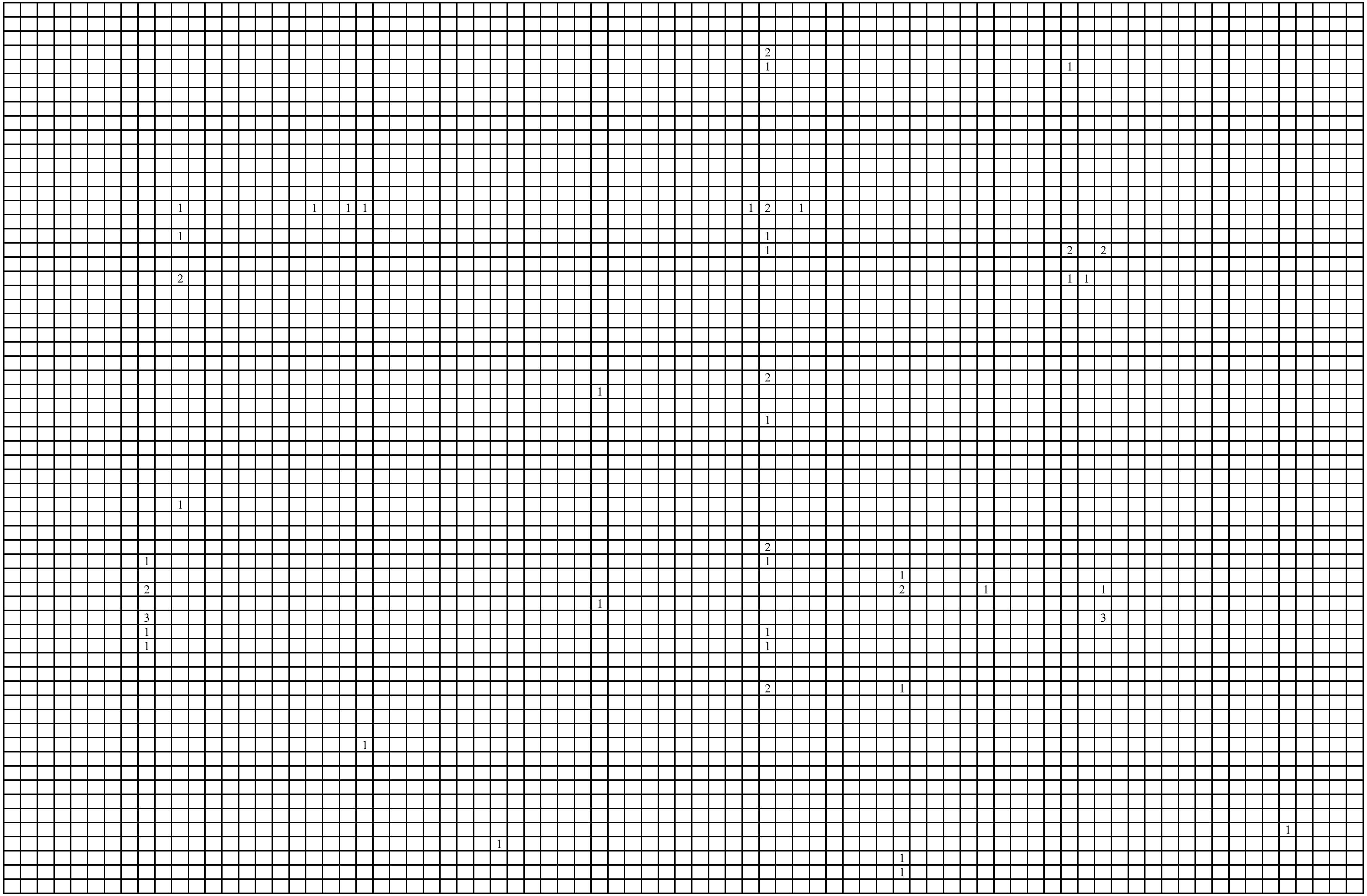

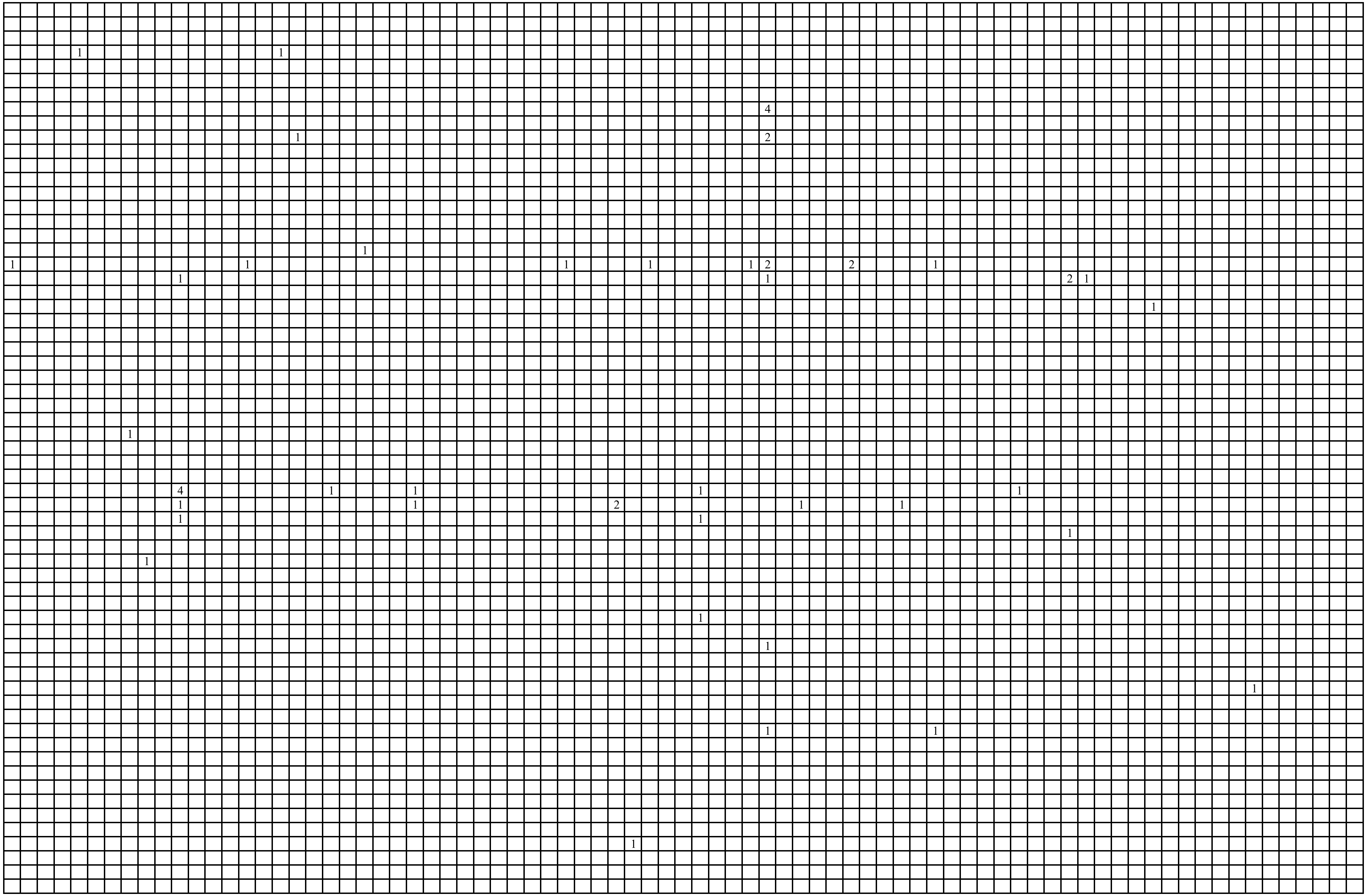

1
1
4
1
2
1
1
1
1
1
1
2
2
1
1
1
2
1
1
1
4
1
1
1
1
1
1
2
1
1
1
1
1
1
1
1
1
1
1
1

																																																			1																												
																																												1																																			
																																												1																																			
								1																																																																							
																																												1																																			
																																																							1																									
																																				1											2																																			
																																														2																																			
																																															2																																			
																																															2																																			
																																				1											1																		1																	

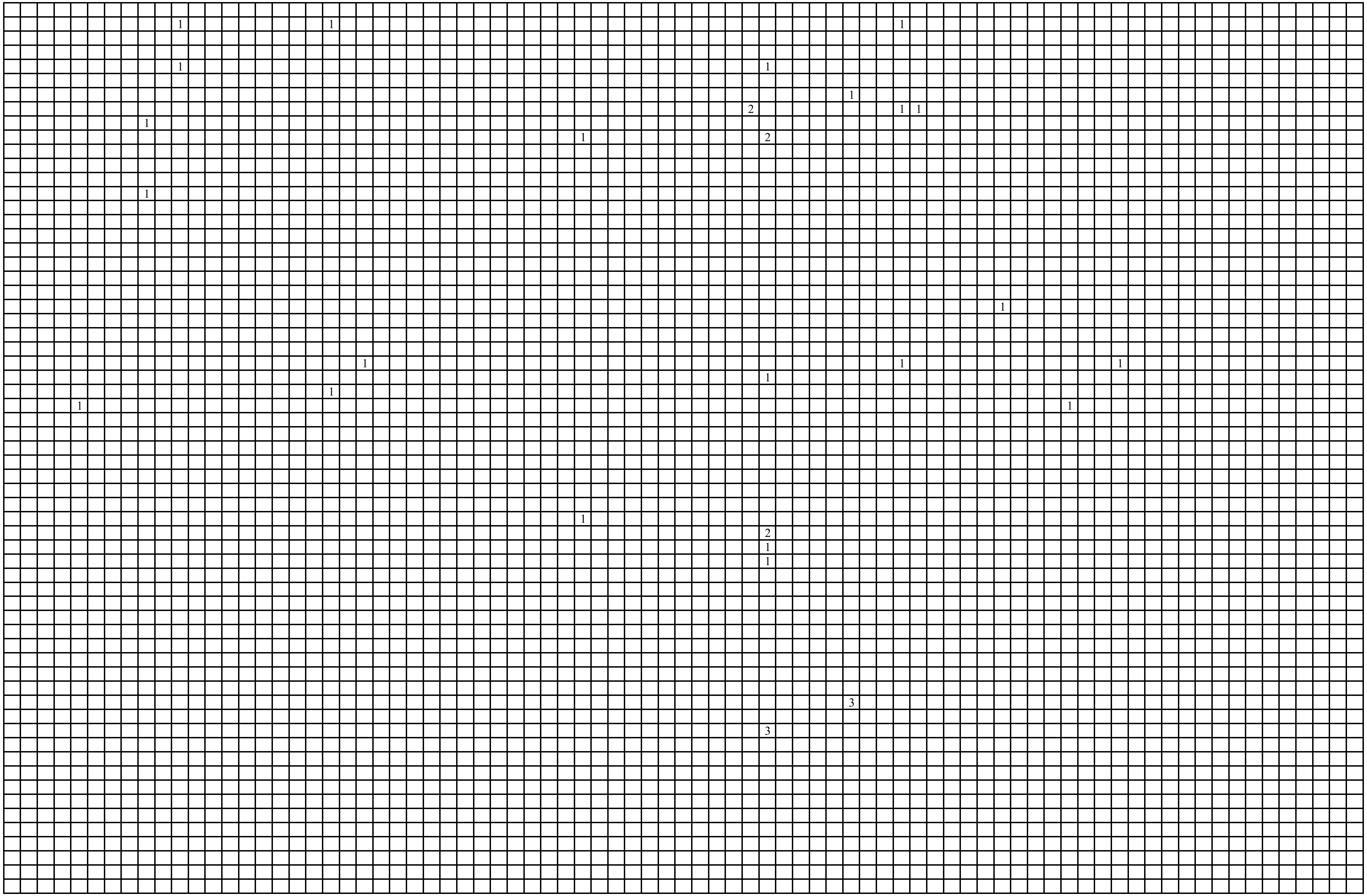

1 1 1
1 1
1
2 1 1
1
1 2
1
1
1 1 1
1
1 1
1
1
2
1
1
3
3

																																		1																																																		
																																																			1																														
																																		1		1																																																	1
																																										1																																														
																																																			1																								1						
			2																			1																																																														
										1																																																																					
			1							1																																			1																													1						
																																														2																																			
																																														2																																			
																																																							1																											
										1																																																																					

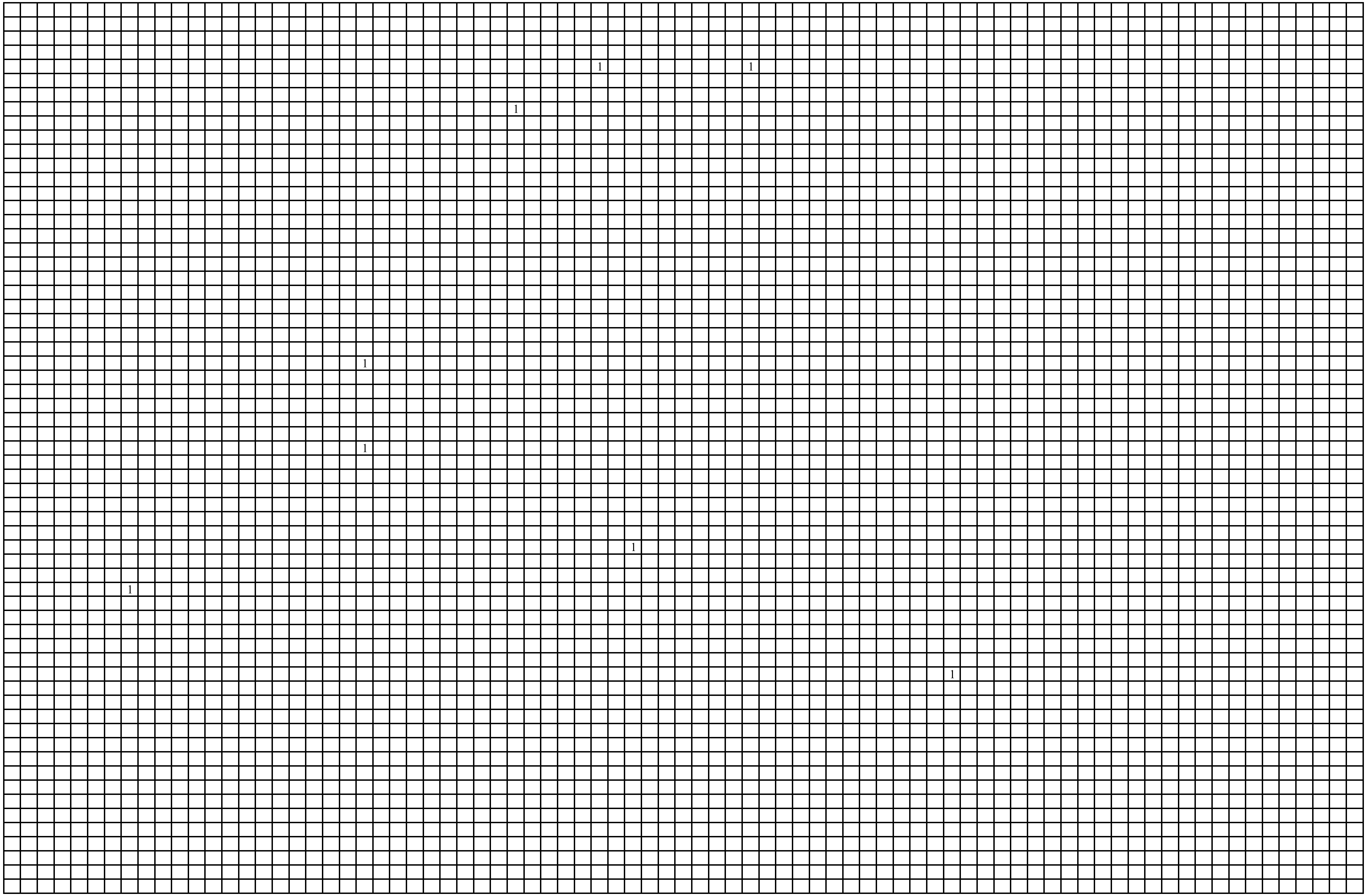
1
1
1
1
1
1
1
1

																					1																																																												
																																	1																																															
								1																																																																						
																																												1																																			
																																												1																																			
																																												1															1																				
	1					1				1																								1											1						2		1				1						1													1				
										1								1				1																							1																	1																	
																				1	1																																																													

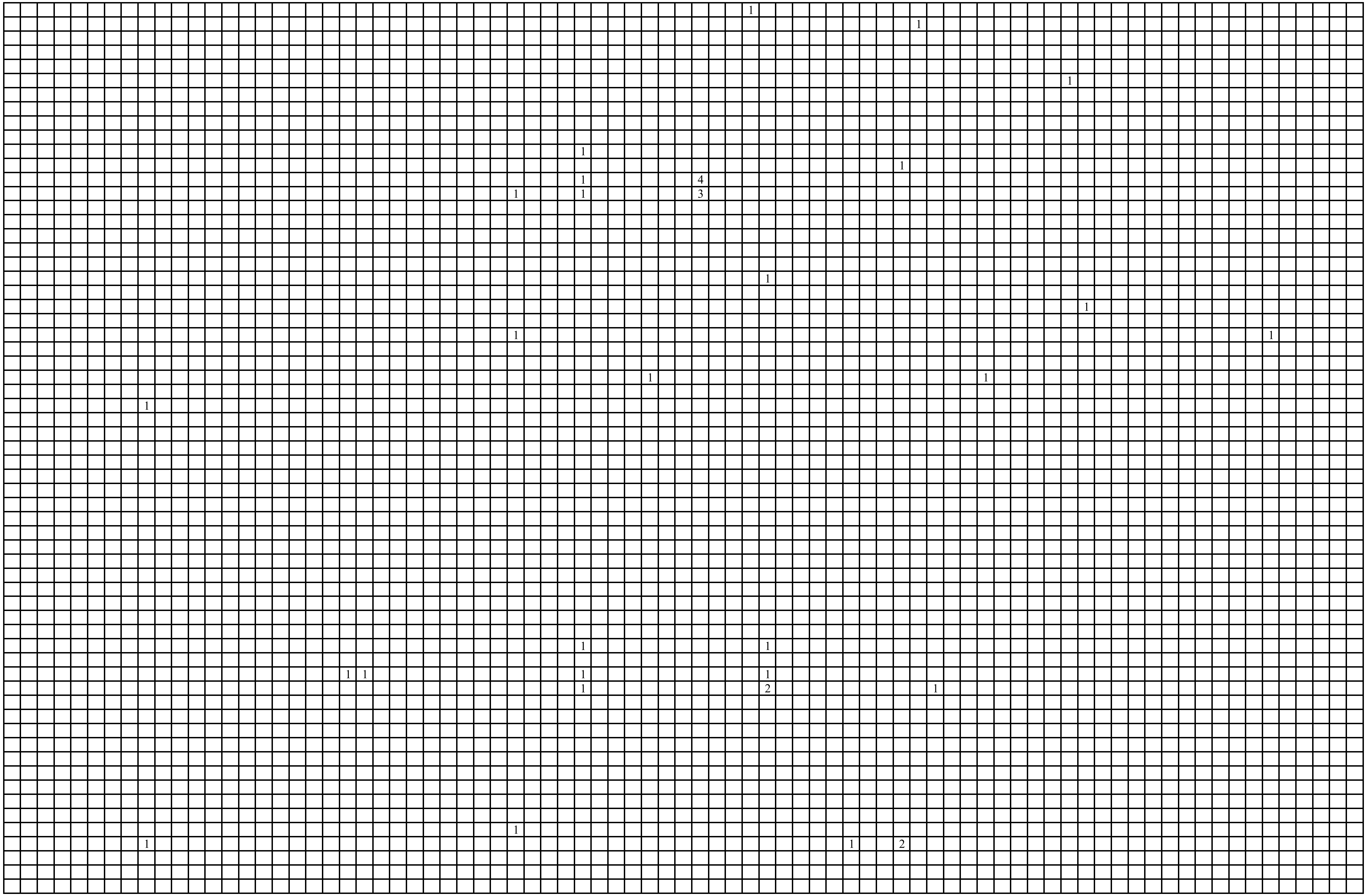
1
1
1
1
1
1
4
1
1
3
1
1
1
1
1
1
1
1
1
1
1
1
1
1
1
1
2
1
1
1
1
2

																																																									1	2				1																
																																																																											2					
																																															1																																			
																																																															1																				
																																															1																																			
								1																																																																								
																																															7																																			
								1		1														1																																						1																					
																														1																																																							
								1																																																																							
																																										1																																													
									1																																								1																														
1																																																																																		
																																										1																																													
																														1																																																							
	1																																																																																	
														1																																																																		
																																															1																																			
								1							1															1									3	1		2	10	1			1	2							1				3		3																	
																																3																																																							
																																				1						1																																														

				1																													1																																															
										1																															1																																							
																																									1																																							
										1														1																							1																												1					
																																													1																																			
	1							1																																										1																														
														1						1										3											2																						4	3																
																																													1																																			
																								1																															1																									
									1																																																																							
								1																																																																								
																3																																																																
						1				1								1																											1																																			
																																													1																																			
																1														1															2																																			
										3																				1	1	1													2					1																												1		
																1														1															2																		4																	
																													2	1																	1																																	
																																	1																																															
																														1																																																		
																1																																																																
							1																																																																									

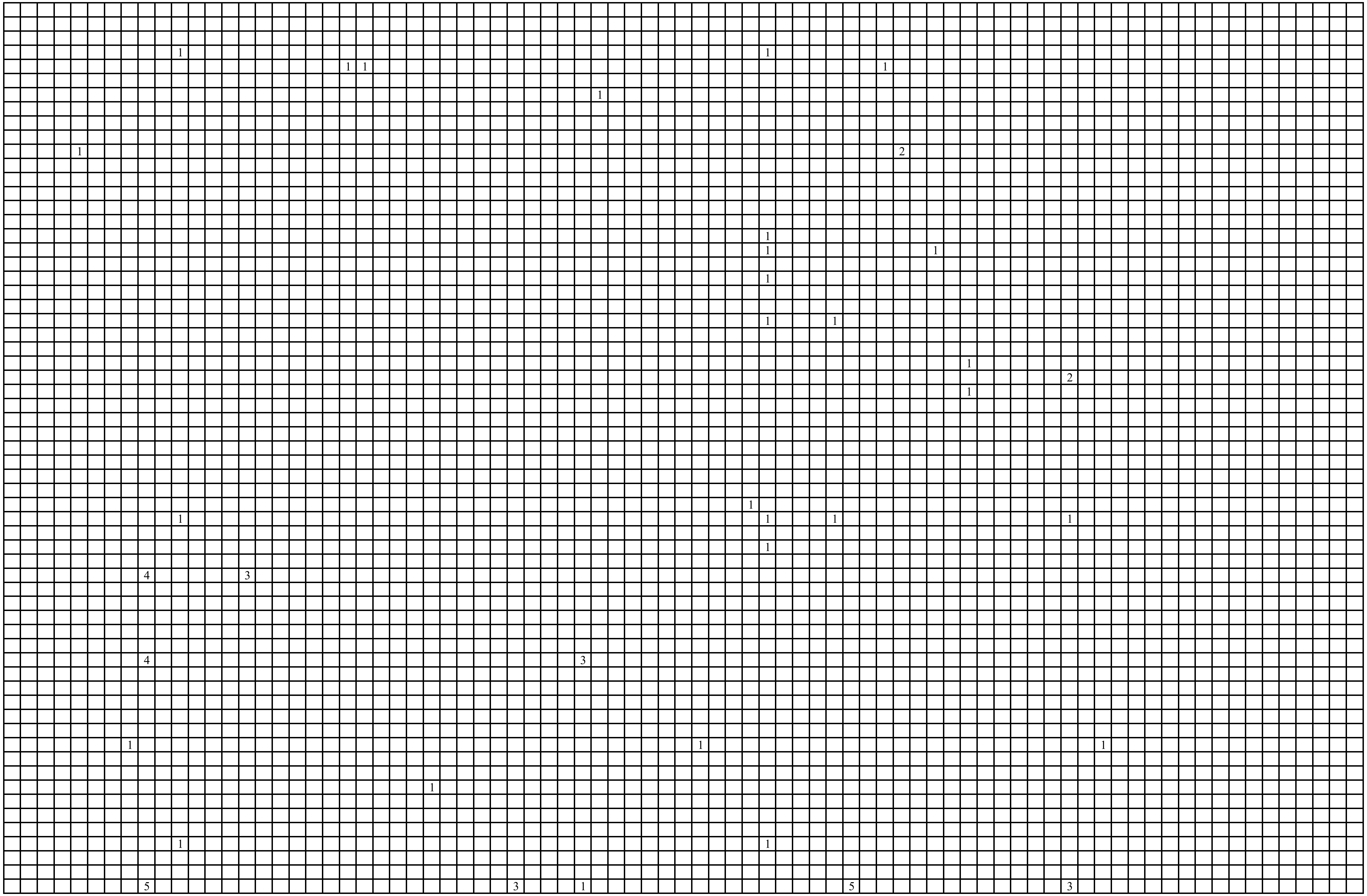
1 1
1 1 1
1
1 2
1
1 1
1
1 1
1
2
1
1
1 1 1 1
1
4 3
4 3
1 1 1
1
1 1
5 3 1 5 3

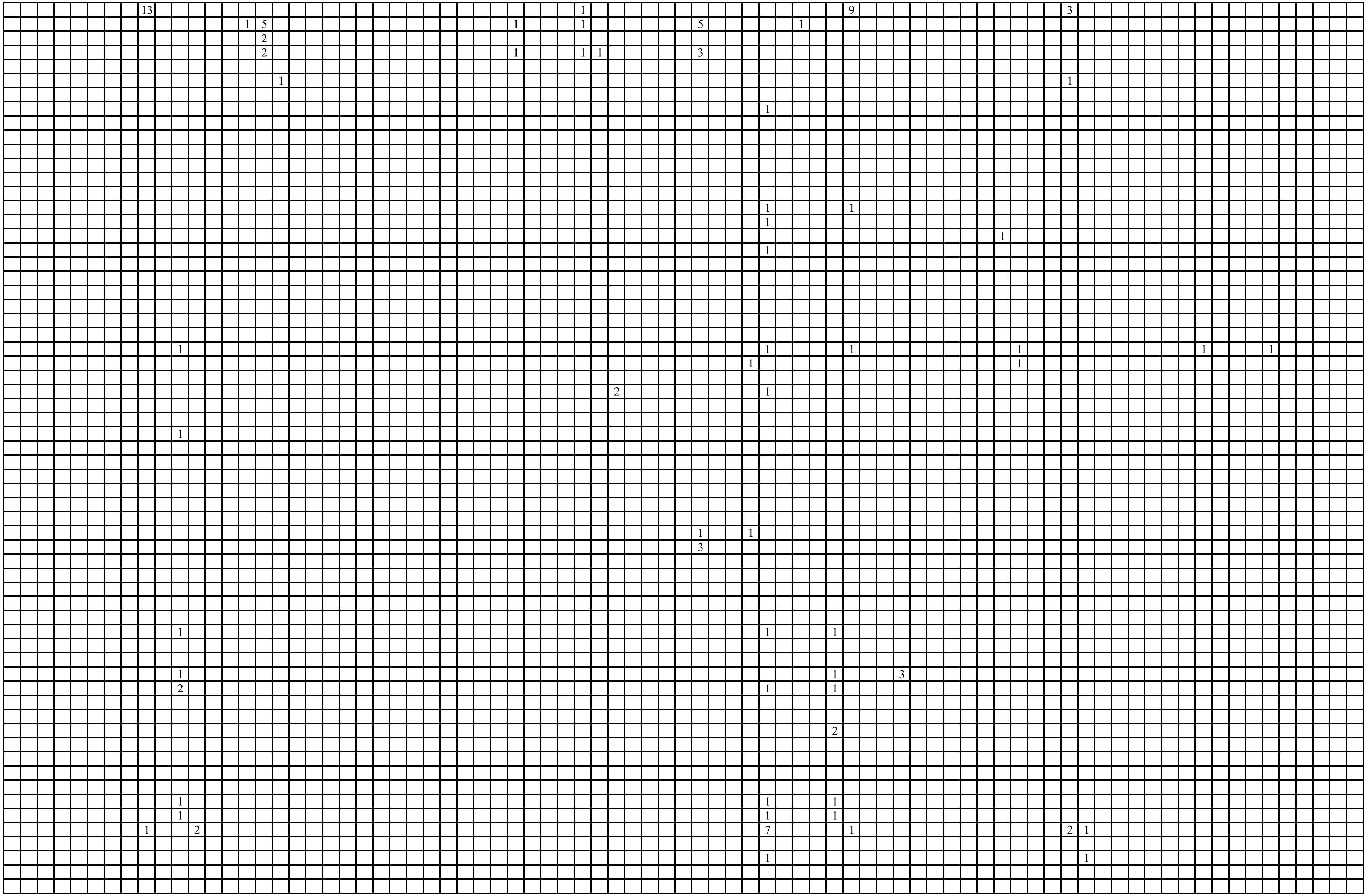
13 1 9 3
1 5 1 1 5 1
2
2 1 1 1 3
1 1
1
1 1
1
1
1
1 1 1 1 1 1
1 1
2 1
1
1 1
3
1 1 1
1 1 3
2 1 1
2
1 1 1
1 1 1
1 2 7 1 2 1
1 1

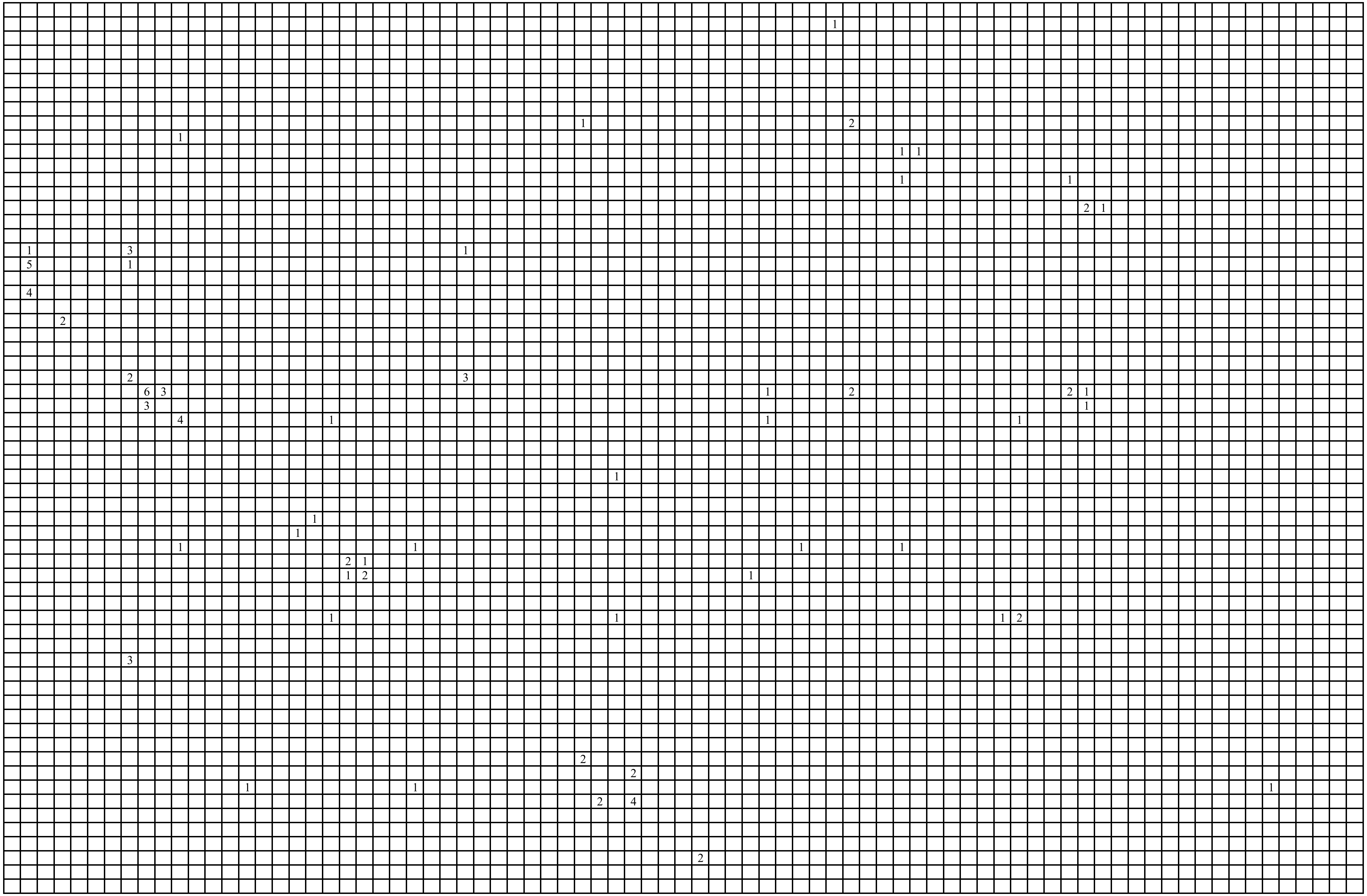
1
1 2
1
1 1
1 1
2 1
1 3 1
5 1
4
2
2 3
6 3 1 2 2 1
3 1
4 1 1 1
1
1
1
1 1 1 1
2 1
1 2 1
1 1 1 2
3
2
2
1 1 1
2 4
2

																					1																							2																																							
								1		1																																			6	3																																						
																																													3																																							
																			1																																										1																					
																																																						1																										
																																																	2																															
								2																																										12						1																								
																																																			2					2																								
																			1																																										1	2																				
																																																1						4						1		2																		
																																																		1	2													1																
																																																									2																							
																								1																							1						1							1															1					
										1														2																													2	1					1	6		1	3																	
																																																							2						1		4																		
								2																																																			3			6	2	1															
								1	1																																															1							2	2	1															
																																																															1	1																
																																				1																						1																					
																																				2																																												2							
																2	2	3																																																																	
																1	1	2																																																																	
																		2																																																																		
	1																									1																																																									
																		1																																																																
	4																																																																																				
						1																																																																														
	1																																																																																				
	1																		1																																																																	
																			1																																																																		
																			1																				1																					1	2																				

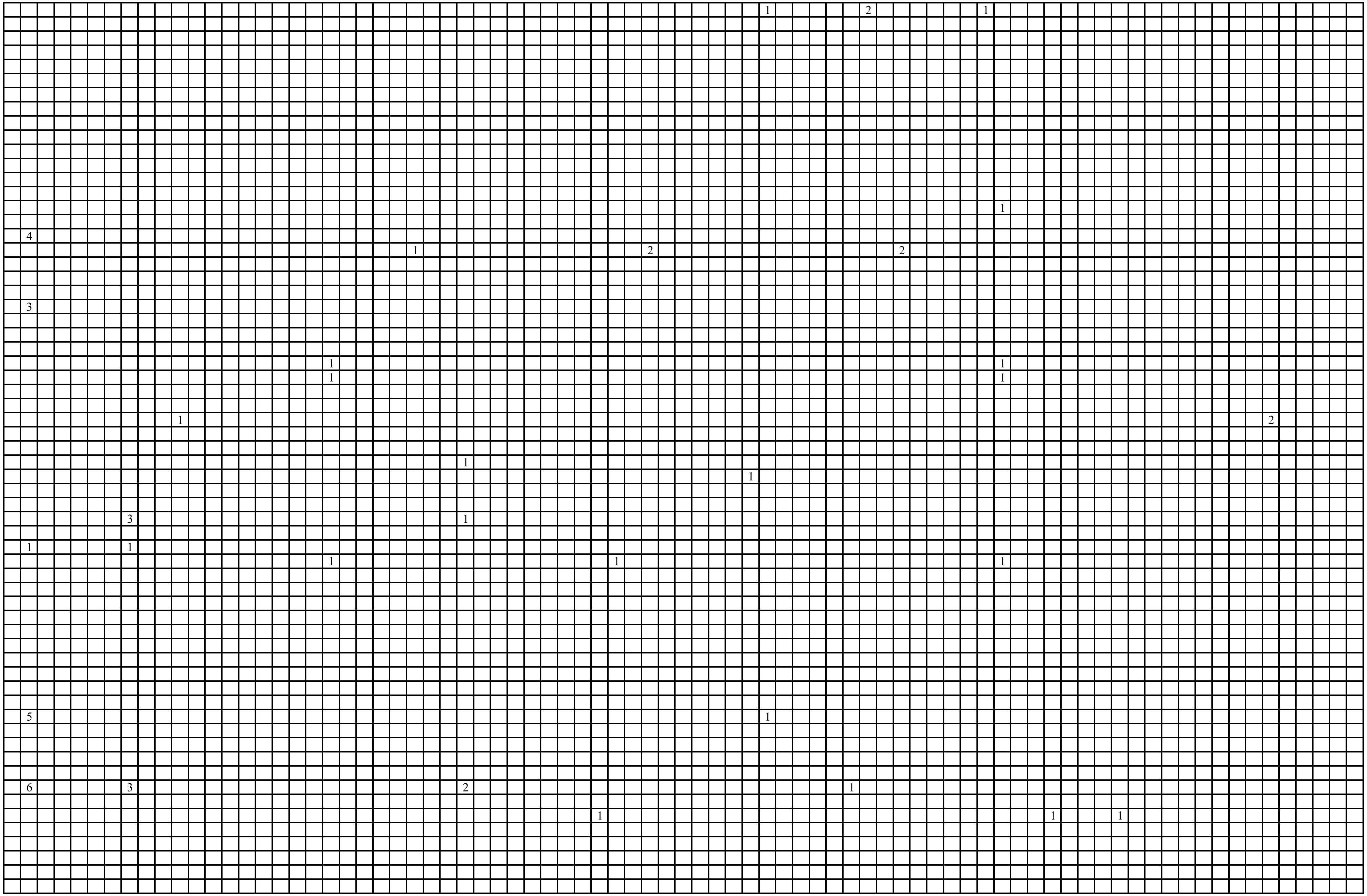
1 2 1
1
4
1 2 2
3
1 1
1 1
1 2
1
1
3 1
1 1
1 1 1
5 1
6 3 2 1
1 1 1

	3						1																																																																										
																																																		1																														
																			1																																								1	1																				
																																						2									2						1																											
																																																											1																					
	7																		1																																																													
																																																						1						1																				

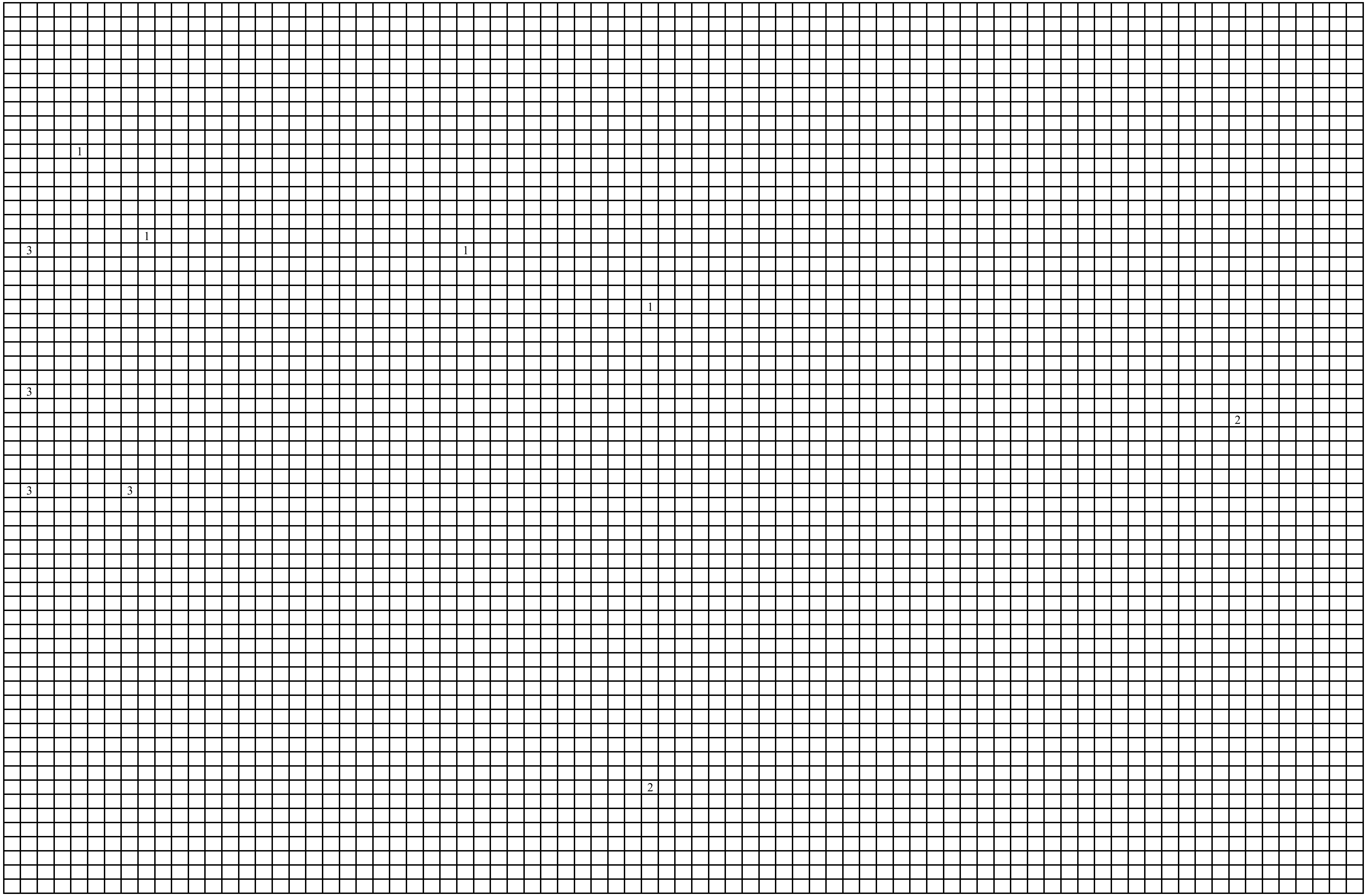

1
1
3
1
1
3
2
3
3
2

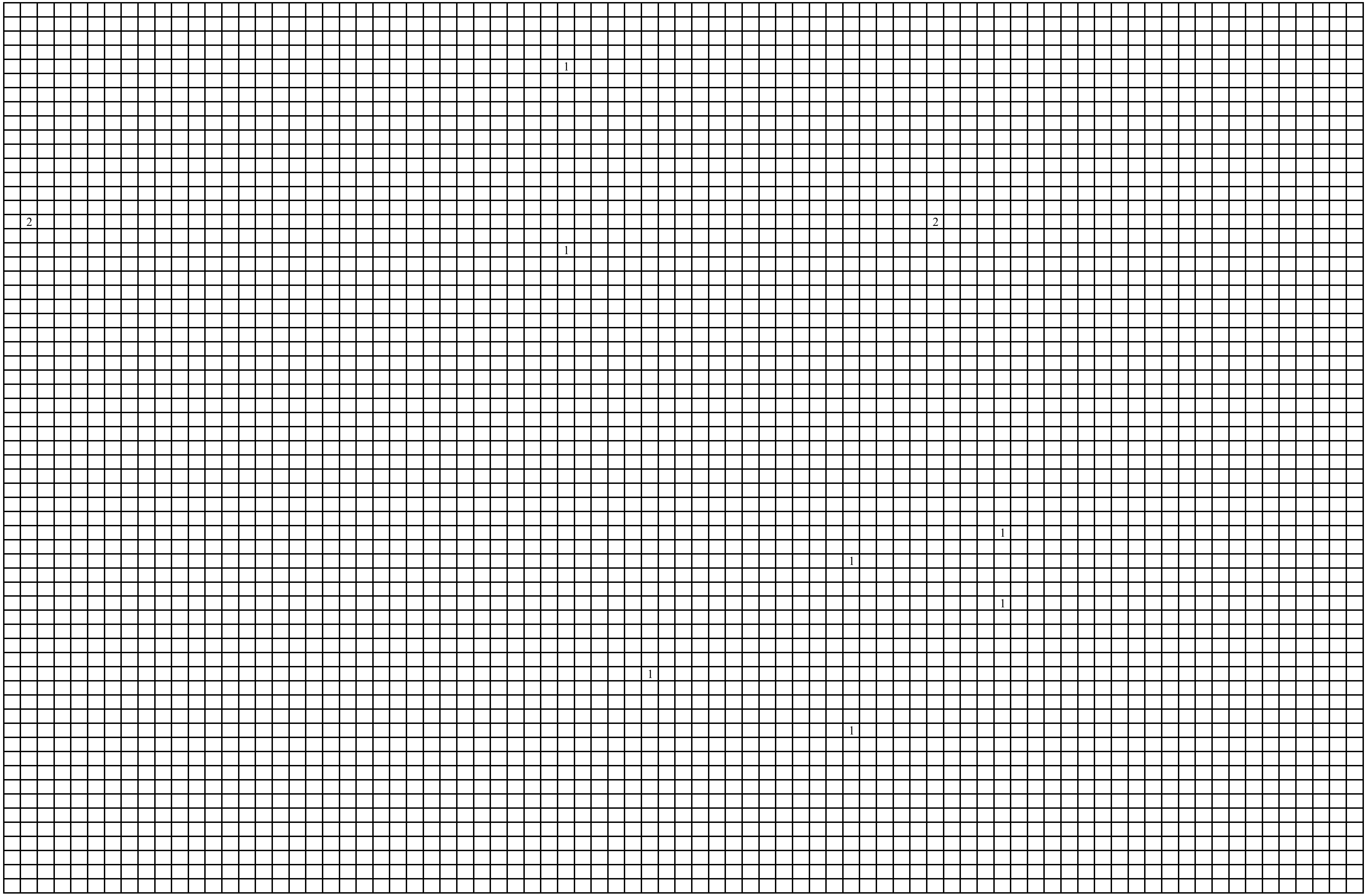
1
2
2
1
1
1
1
1
1

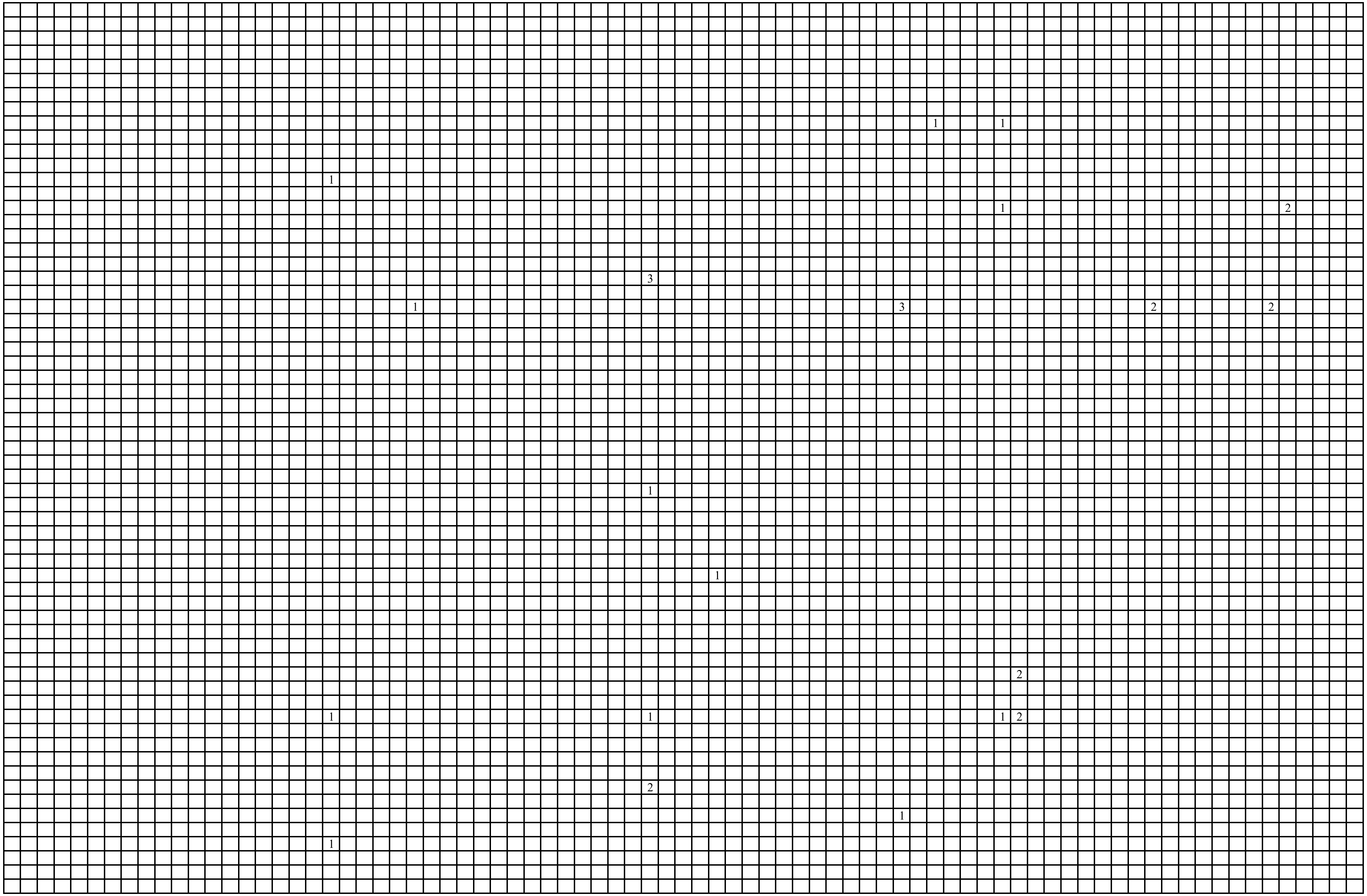

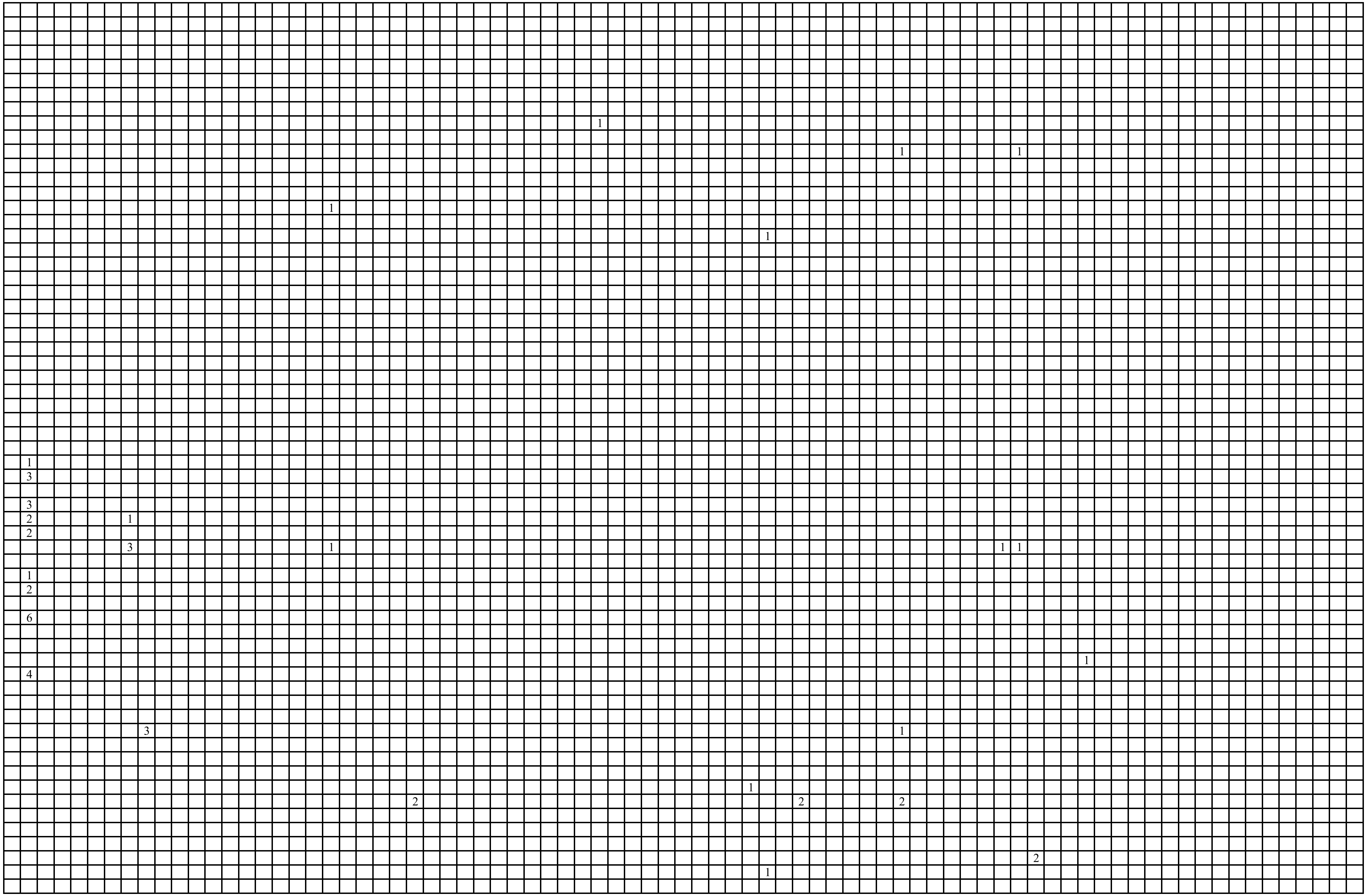
1
1 1
1
1
1
3
3
2 1
2
3 1 1 1
1
2
6
1
4
3 1
1
2 2 2
2
1

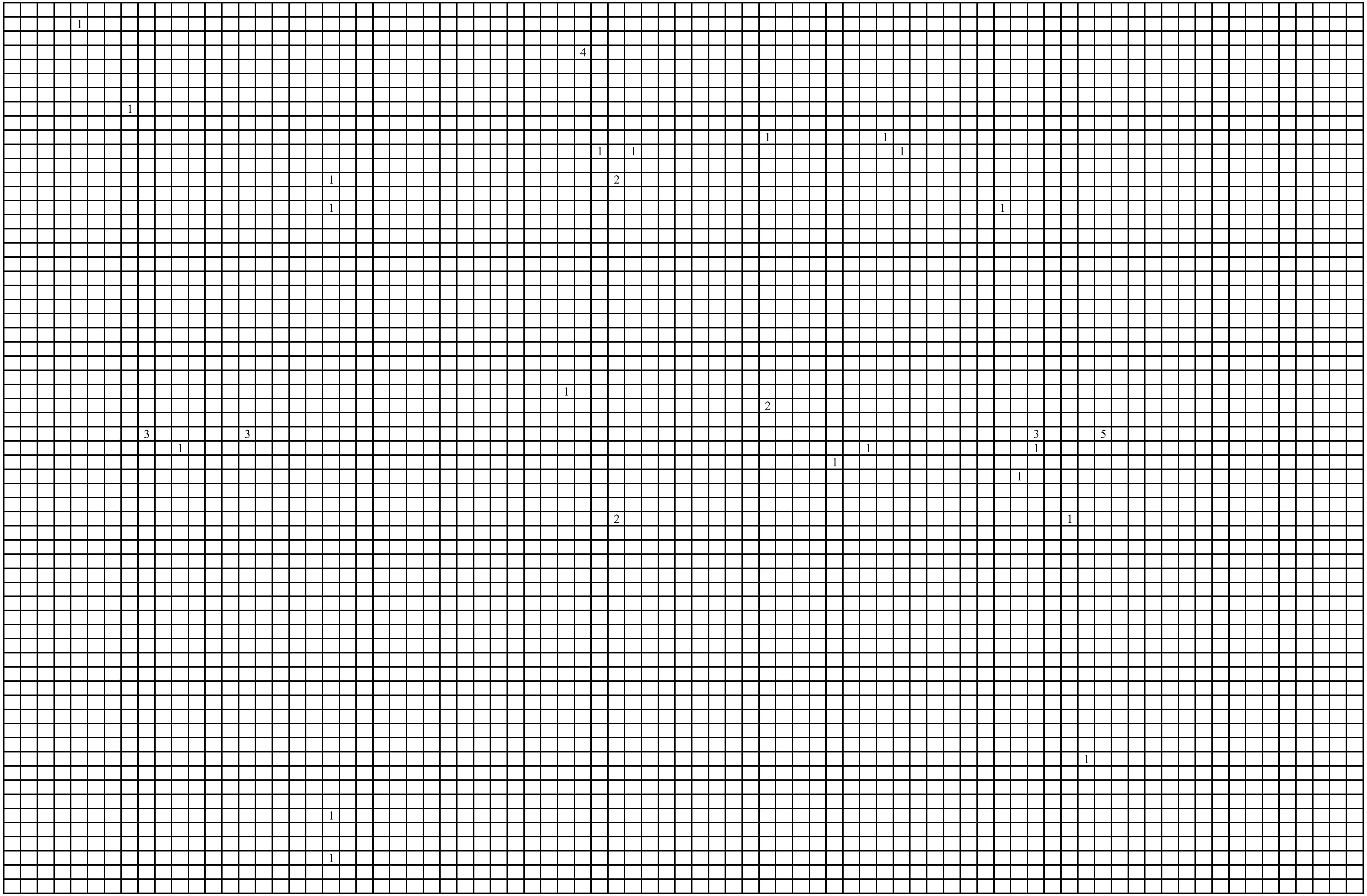
1
4
1
1
1
1
1
1
1
2
1
1
1
2
3
3
3
5
1
1
1
1
1
2
1
1
1
1

							1																																																																								
																			1																																							1																					
																								1													1														1																	1			1					
											1																																															1															1					
																			1																									1							1					1																				
																																													1																																		
																																																																												1				
																																																			1				2																							
																2	1																																																														
																														1																						1																										
																																																		1																													
																			1																											1											1																				
																																							1																																								
																																			1																																												
																																													1																																		
																																																											1																				
																																																					1																										
																																																											1																				
										1																																				1					1					1															1					
																																				2								1																																		
																			1																																							1																					
																																																					1																										

																																																									1	1																				
																																																		2		1												1															
																																																			2							3	2																			
										1																																																																				
																																			1																																																
																																			1																																																
																																			1																																																
	1						3										1																																																														
																																																								1																							
								1																																					2																															
																																																	2																															
										1																																																																											
																																												1																																			
																																																																1																	
																																																																			1															
																																										3																																															

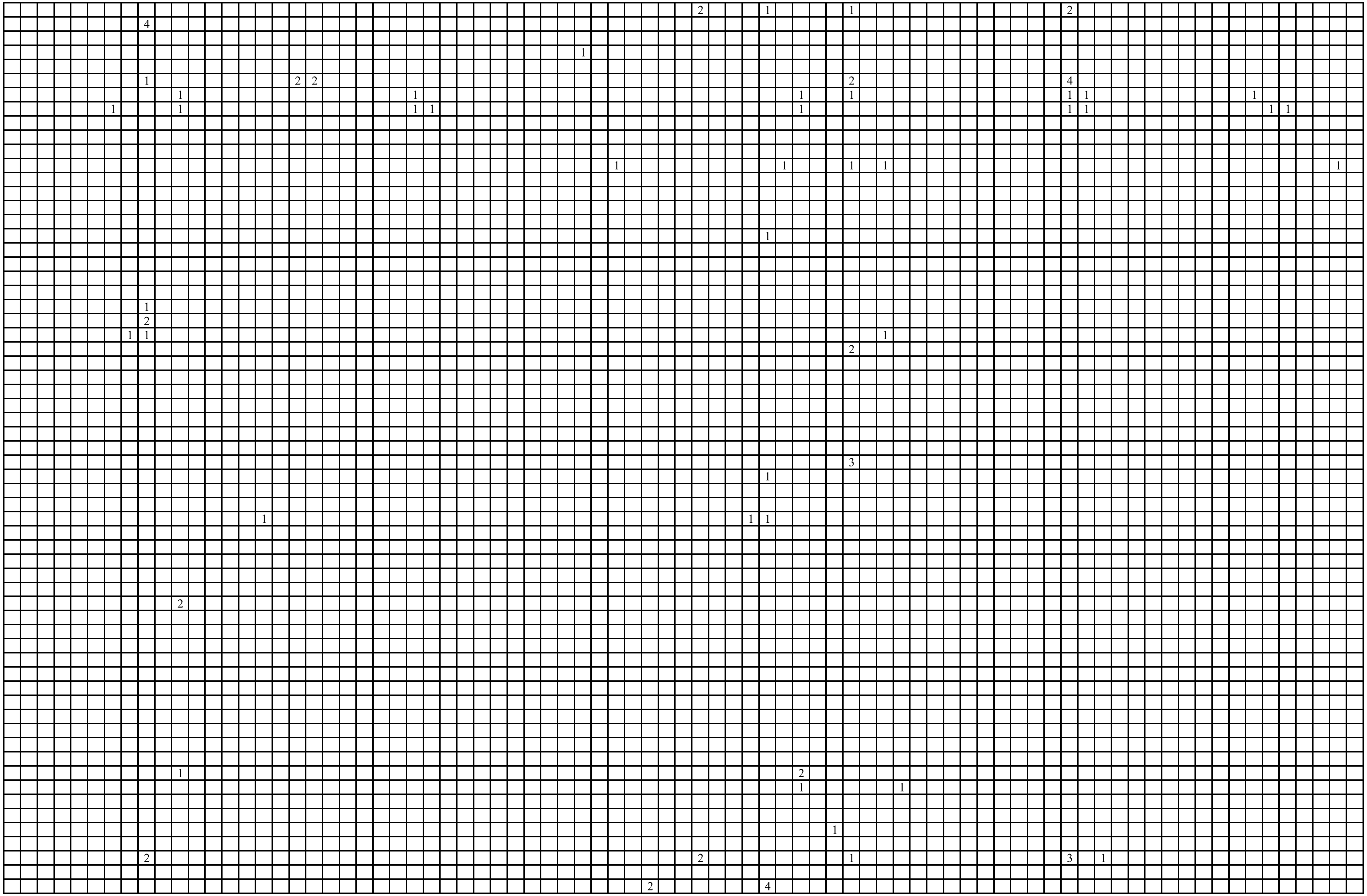
2 1 1 2
4
1
1 2 2 2 4
1 1 1 1 1 1 1
1 1 1 1 1 1 1 1 1
1 1 1 1 1
1
1
2
1 1 1
2
3
1
1 1 1
2
1 2
1 1
1
2 2 1 3 1
2 4

																																																																													1				
																																			1																																																	
																1		1													1			3								1																							2															
																															1	1										1								1																								1						
														3																												1																																										
																															1											2			4																																											

																										ZFP91	26	152
																										ZFP92	0	2
																										ZFX	4	17
																										ZFY	3	14
																		1								ZIC1	17	152
																										ZIC2	9	43
																										ZIC3	8	25
																										ZIC4	0	2
																										ZIC5	2	5
																										ZIK1	0	3
																										ZIM2	5	57
																										ZIM3	0	3
																										ZKSCAN1	30	91
																										ZKSCAN2	2	8
																										ZKSCAN3	30	43
																										ZKSCAN4	77	131
																										ZKSCAN5	7	16
																										ZKSCAN7	13	27
																										ZKSCAN8	66	166
																										ZNF10	2	25
																										ZNF100	4	25
																										ZNF107	9	33
																										ZNF114	5	37
																										ZNF117	0	1
			1																							ZNF121	25	54
											2															ZNF124	12	70
																										ZNF131	40	149
																										ZNF133	1	37
																										ZNF135	0	10
																										ZNF136	1	45
																										ZNF138	5	25
																										ZNF14	0	7
																										ZNF140	0	11
																										ZNF141	1	2
																										ZNF143	17	55
																										ZNF146	10	101
																										ZNF148	45	141
																										ZNF154	0	27
																										ZNF155	0	10
																										ZNF157	2	8
																										ZNF16	6	37
																										ZNF160	1	8
																										ZNF165	19	64
																										ZNF169	34	85
																										ZNF17	4	36
																										ZNF175	3	13
																										ZNF177	2	24
																										ZNF18	8	21
																										ZNF180	1	9
																										ZNF181	0	8
																										ZNF182	1	4
																										ZNF184	22	75
																										ZNF189	6	60
																										ZNF19	1	30
																										ZNF197	15	32
																										ZNF2	22	120
																										ZNF20	1	48
																										ZNF200	1	14
																										ZNF202	9	35
																										ZNF205	1	22
																										ZNF208	0	1
																										ZNF212	27	70
																										ZNF213	24	42
																										ZNF214	0	22
																										ZNF215	9	22
																										ZNF22	26	146
																										ZNF221	4	11
																										ZNF222	0	0
																										ZNF223	8	48
																										ZNF224	1	24
																										ZNF225	3	6
																										ZNF226	1	11
																										ZNF227	2	19
																										ZNF229	0	4
																							1			ZNF23	3	28
																										ZNF230	0	11
																										ZNF232	54	102
																										ZNF233	0	2
																										ZNF234	0	7
																										ZNF235	1	14
																										ZNF239	0	11

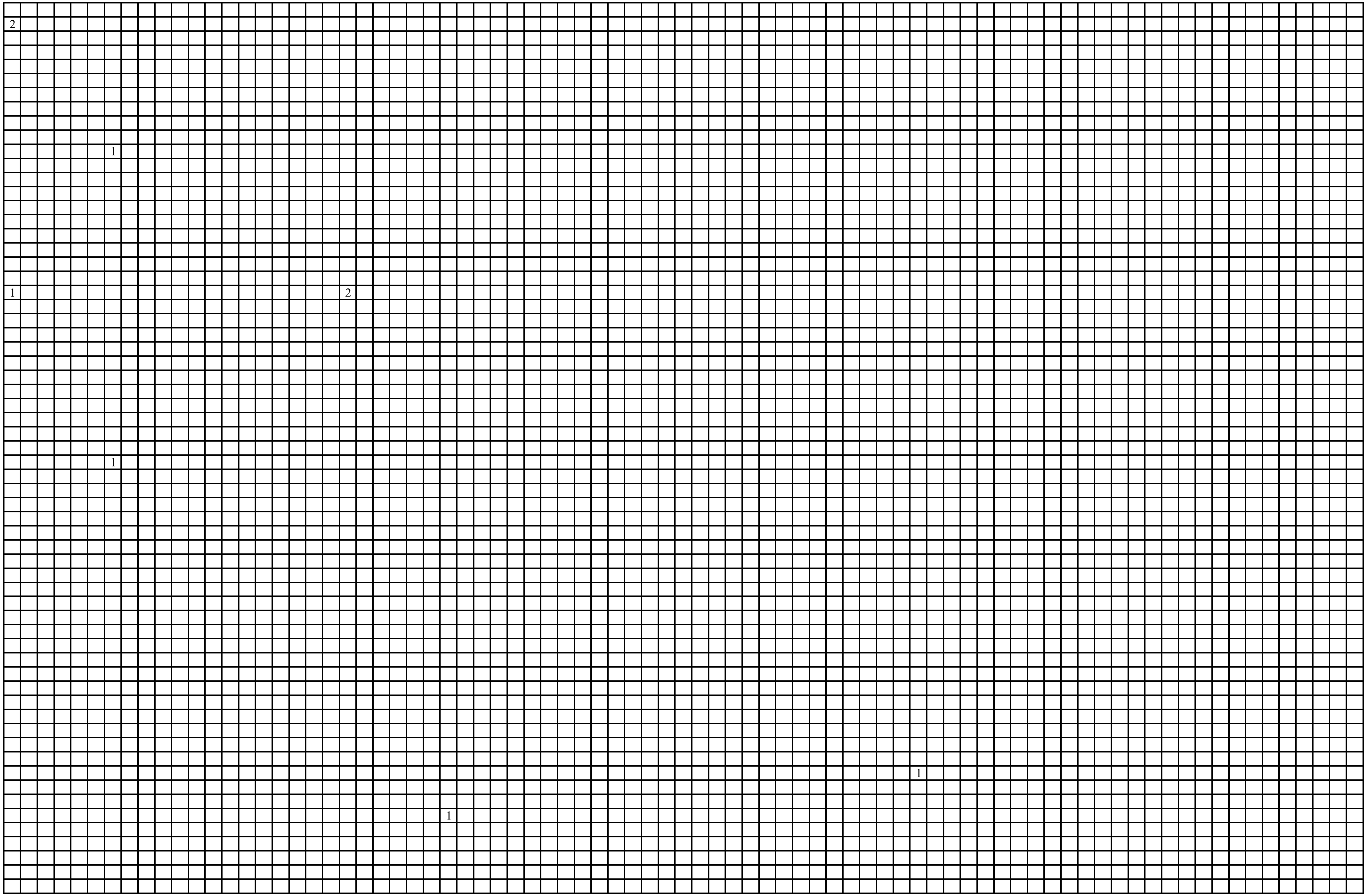

2
1
1
2
1
1
1

1 1 1

1 1

3

1 1

1

1

																																																													1										1									
																										1																																																						
																																																													1																			
1															1									1		1							1	1		1															1																													
		1																																1		1																													1																	
																										1								1																																														
																																																			1																													
1																		1																																	1														1															
3																																																																																
1																																																																																
2																																																																																
																										1																																																						

				1																																																																											
																									1										1																																																
																																					1																																																
																																					1																																																
																																					1																																																
																																			1																																																		
																																																		1																													
																																																																											1						

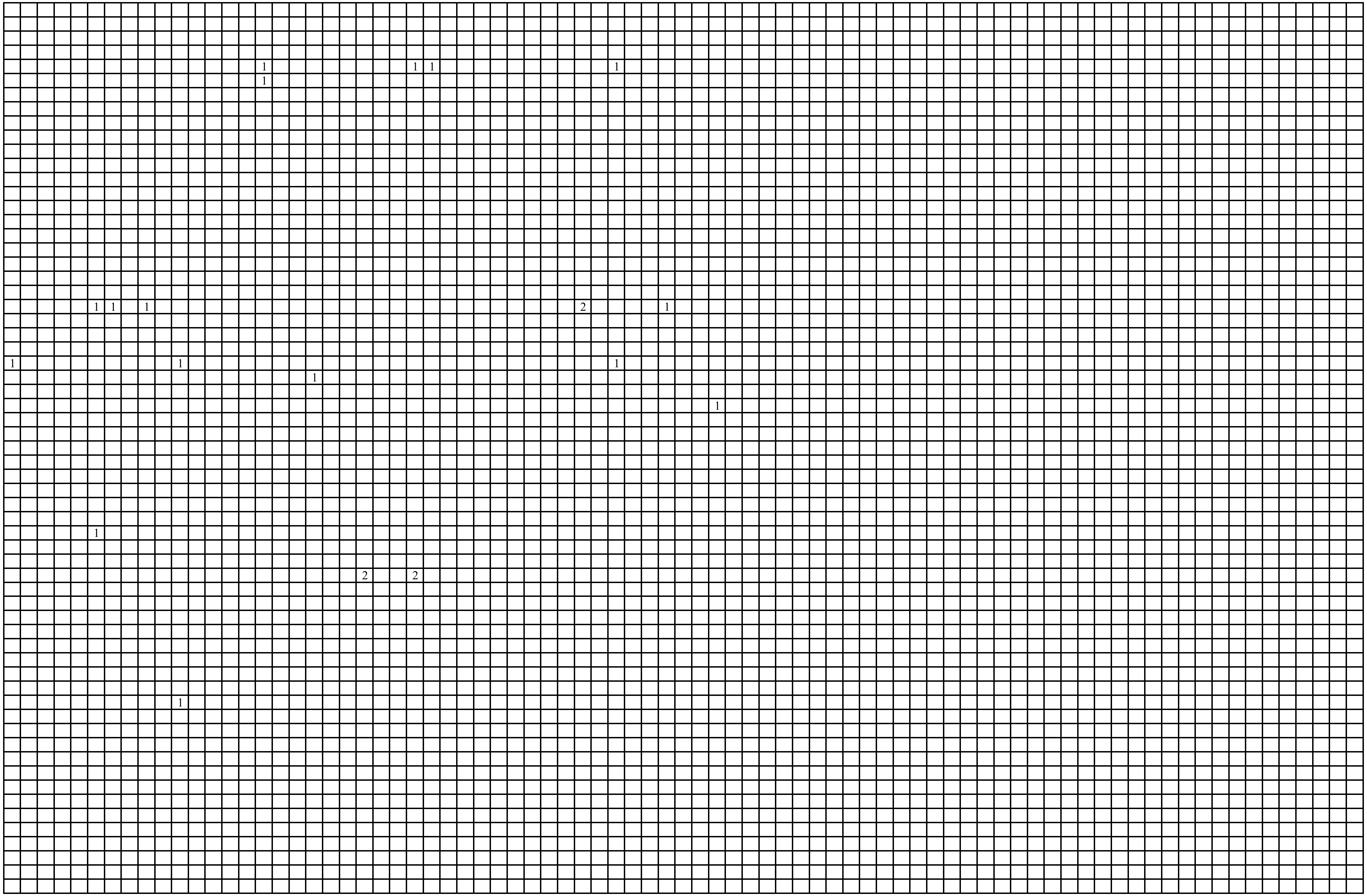
1
1
1
1
1
1
1
1
2
1
1
1
1
1
1
1
2
2
1

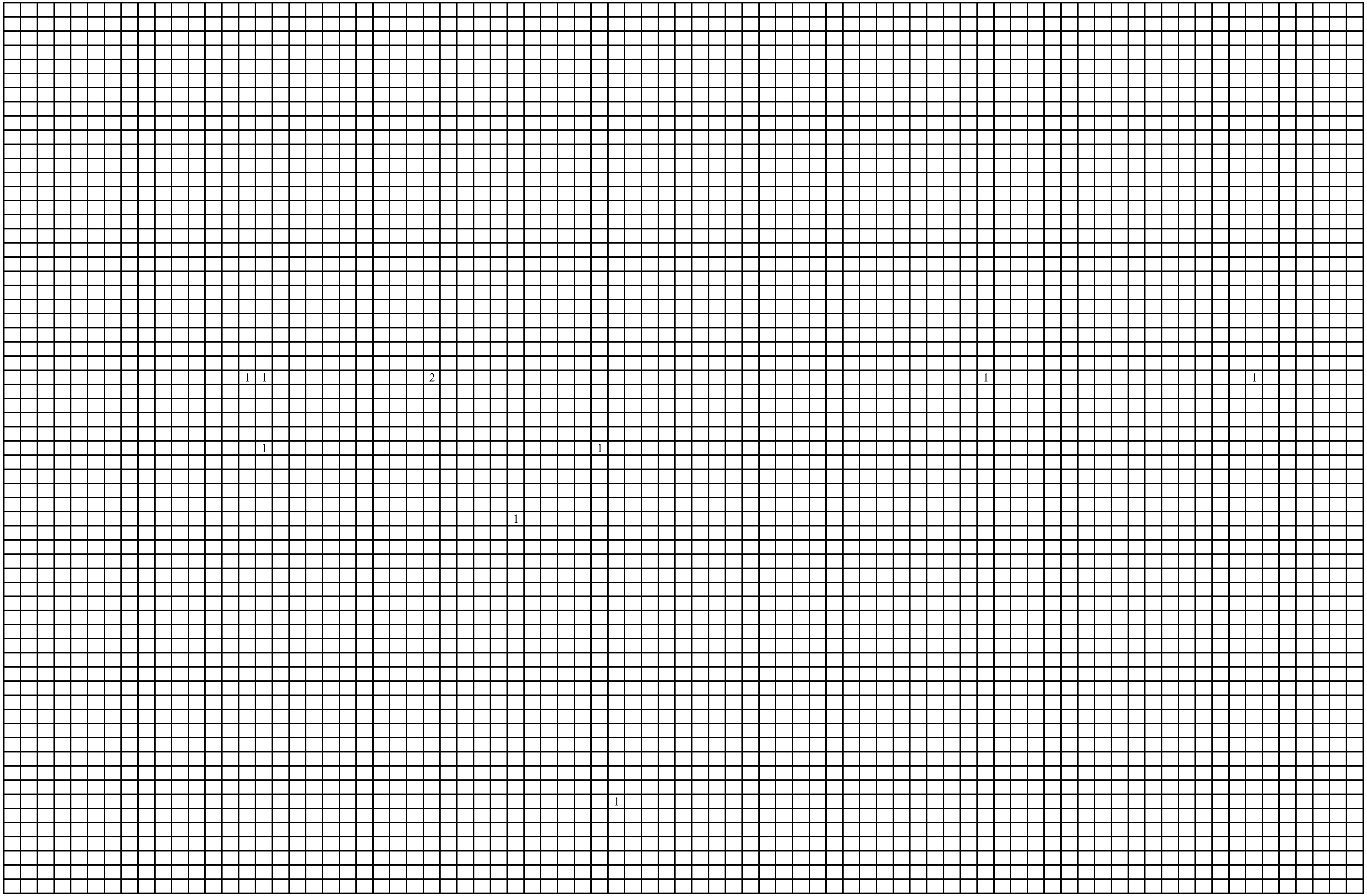
1 1 2 1 1
1 1
1
1

					1	1																																	1																																													
																																																										1																			
															1																																																																							
				1																																1																																																		

														1																																																																	
				1			1										1																		1																1																												
																																																														1																			
														1																																																																	
																																				1							1																																						

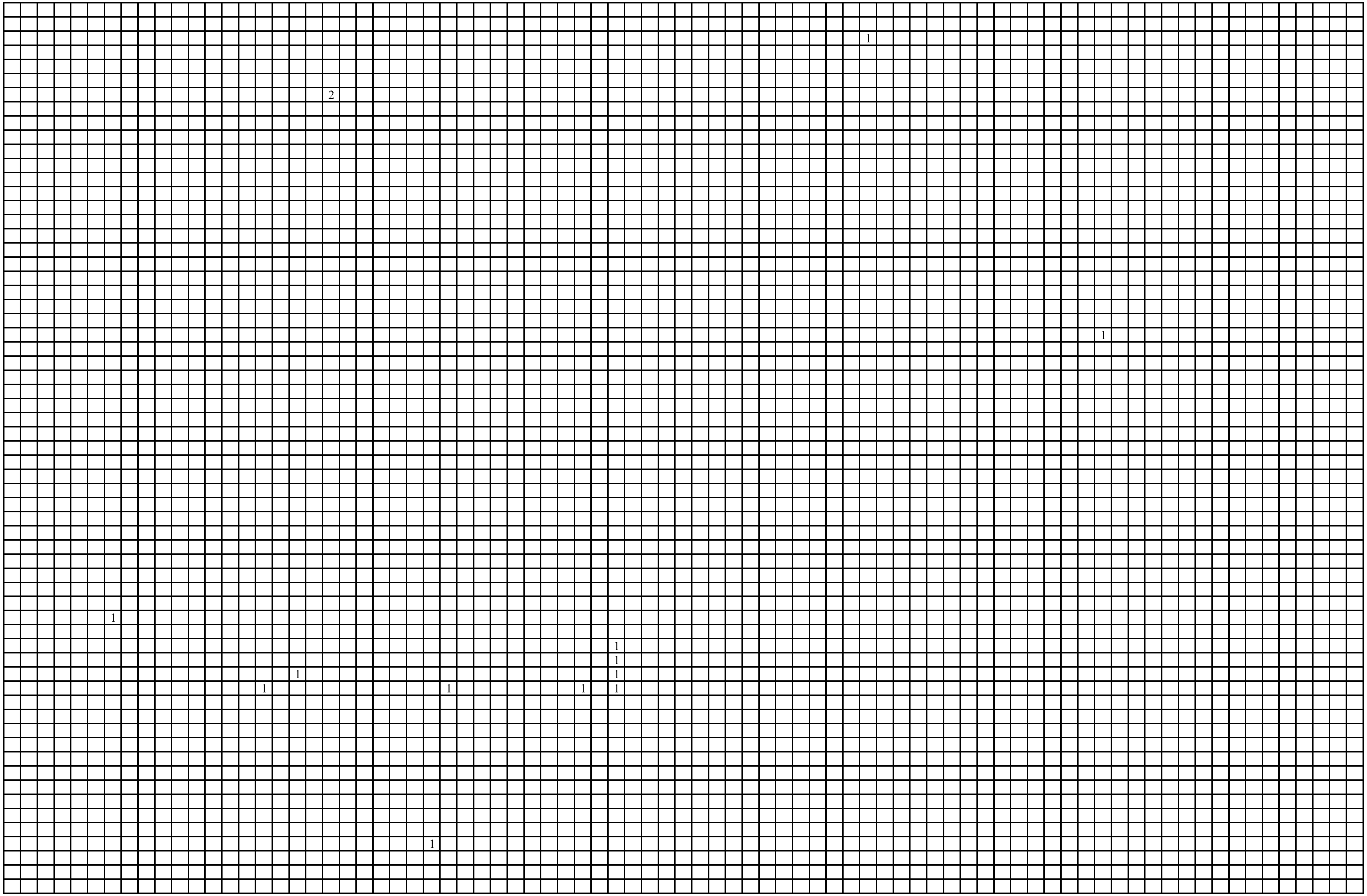
1
2
1
1
1
1
1
1
1
1
1
1
1
1

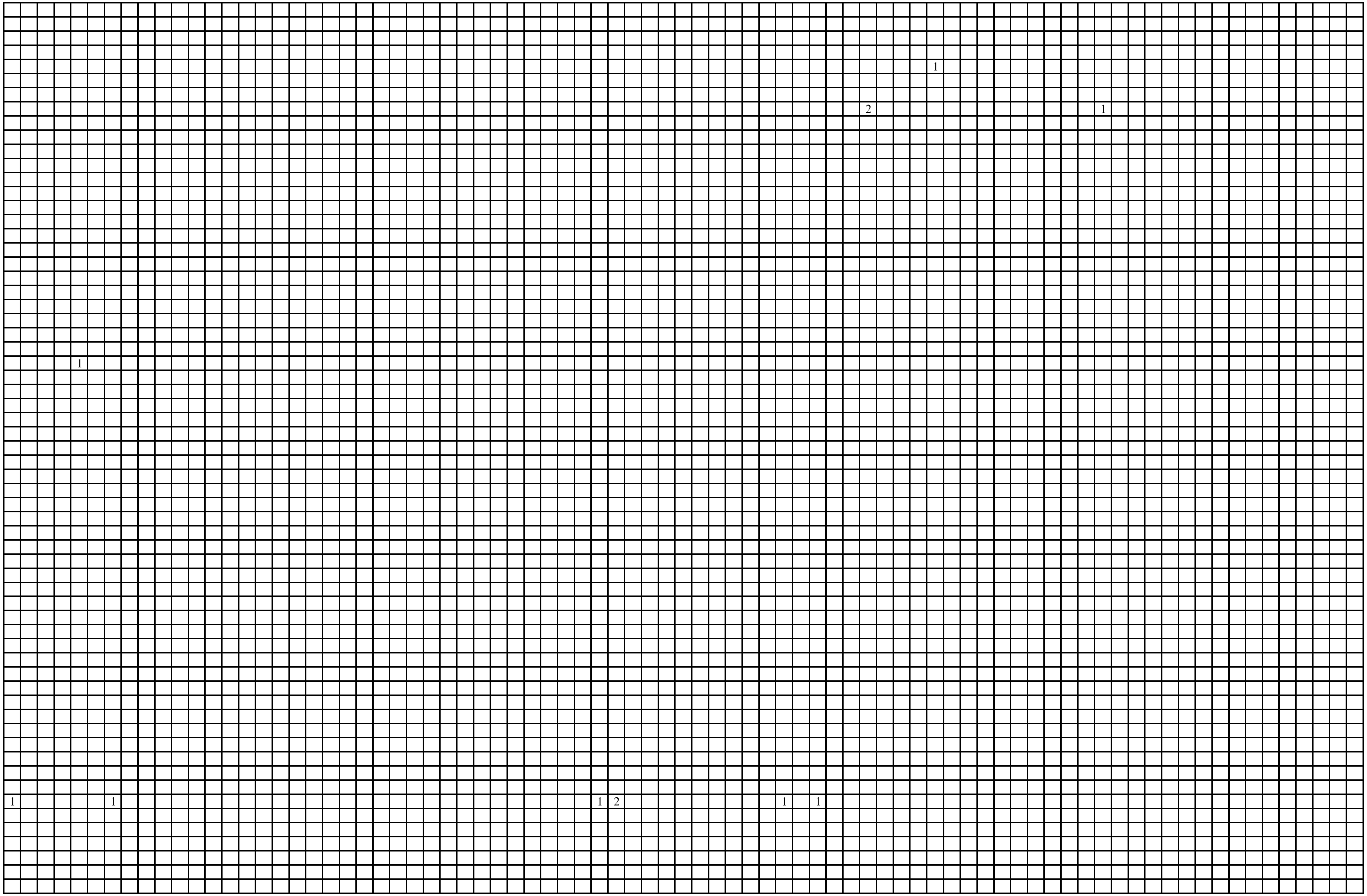
1
2
1
1
1
1
1
2
1
1

																					1																														1																													
				1																																																																												
2																																																																																	
																											1										1																																										
																																			1																																														
																											1																																																										
				1																																											1																												
																					1																																																													
																																			1																																													
																														1																																																							
				1																																																																													

															1																					1																																														
					1																																																																												
																																		1																																																		
																																									1																																									
																										1										1																																																	
1																										1								1		1																																																	
																																				1																																																	
																										1										1																																																	
																																				1																																																	
																																				1																																																	
																																			1																																																	
														1	1											1								1		1																																																	
																																																						1																							
																										1										1																																																	

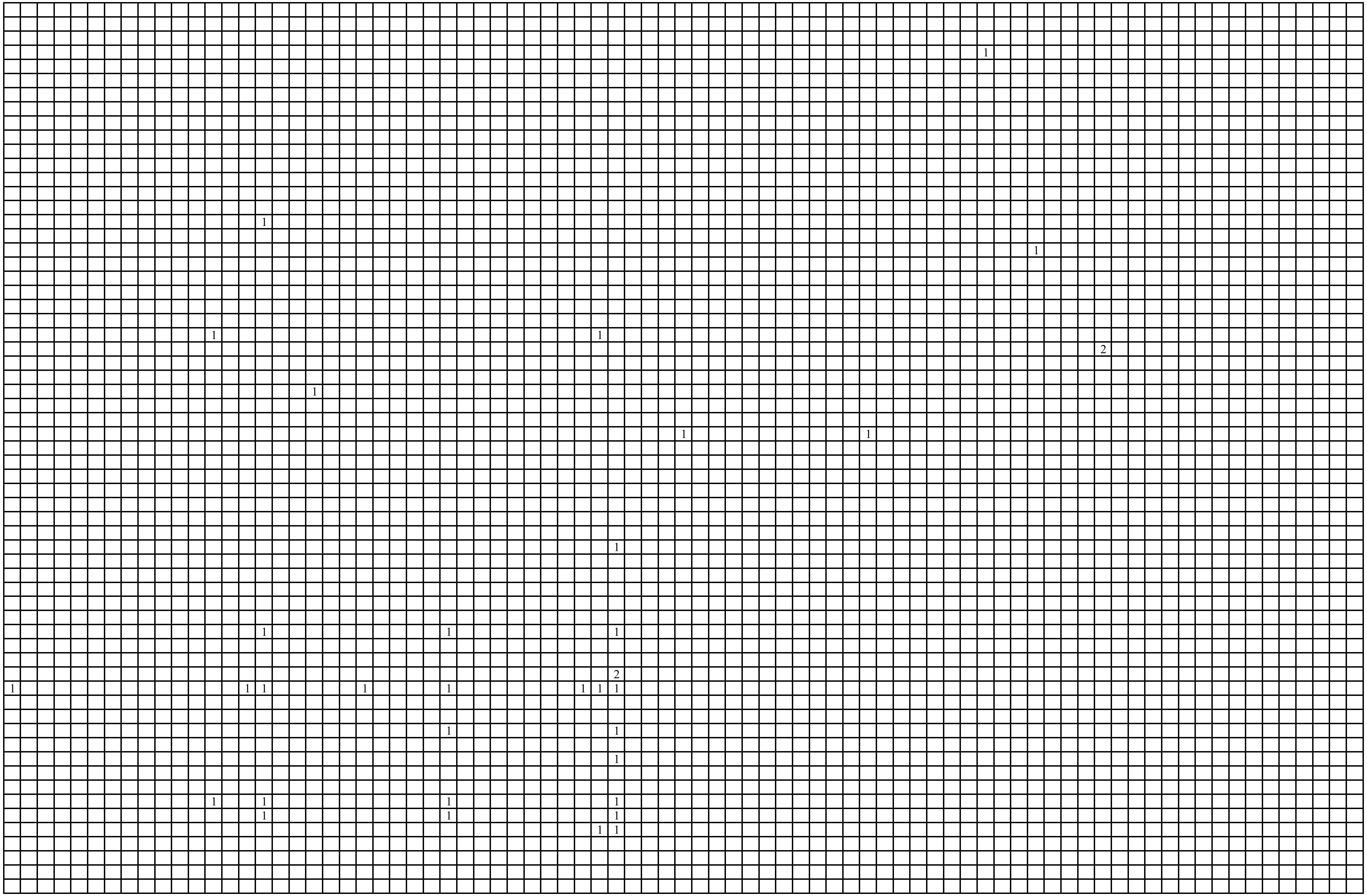
1
1
1
1 1
2
1
1 1
1
1 1 1
2
1 1 1 1 1 1 1 1
1 1
1
1 1 1 1
1 1 1
1 1

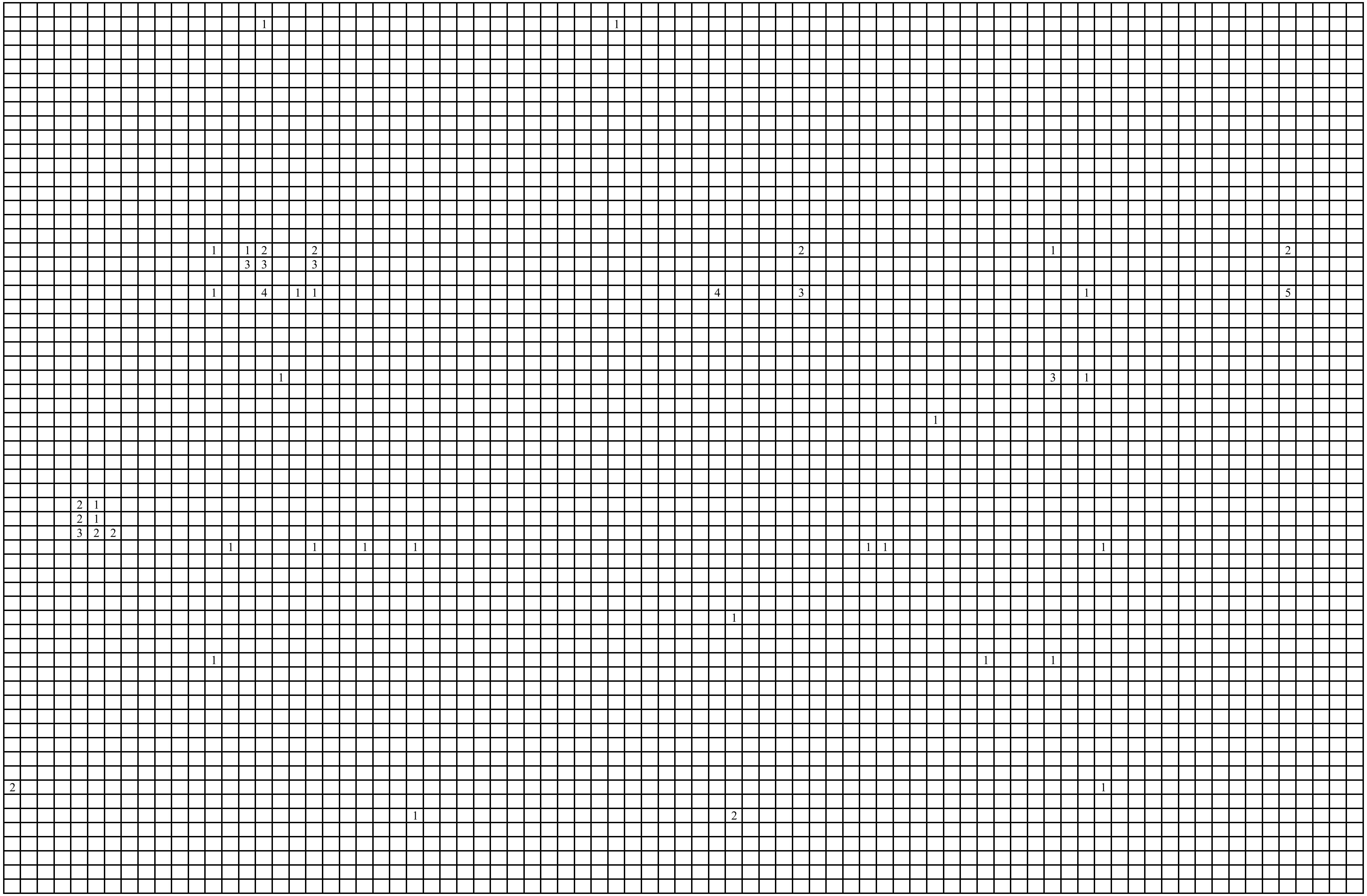
1 1
1 1 2 2 2 1 2
3 3 3
1 4 1 1 4 3 1 5
1 3 1
1
2 1
2 1
3 2 2
1 1 1 1 1 1 1 1
1
1 1 1
2 1
1 2

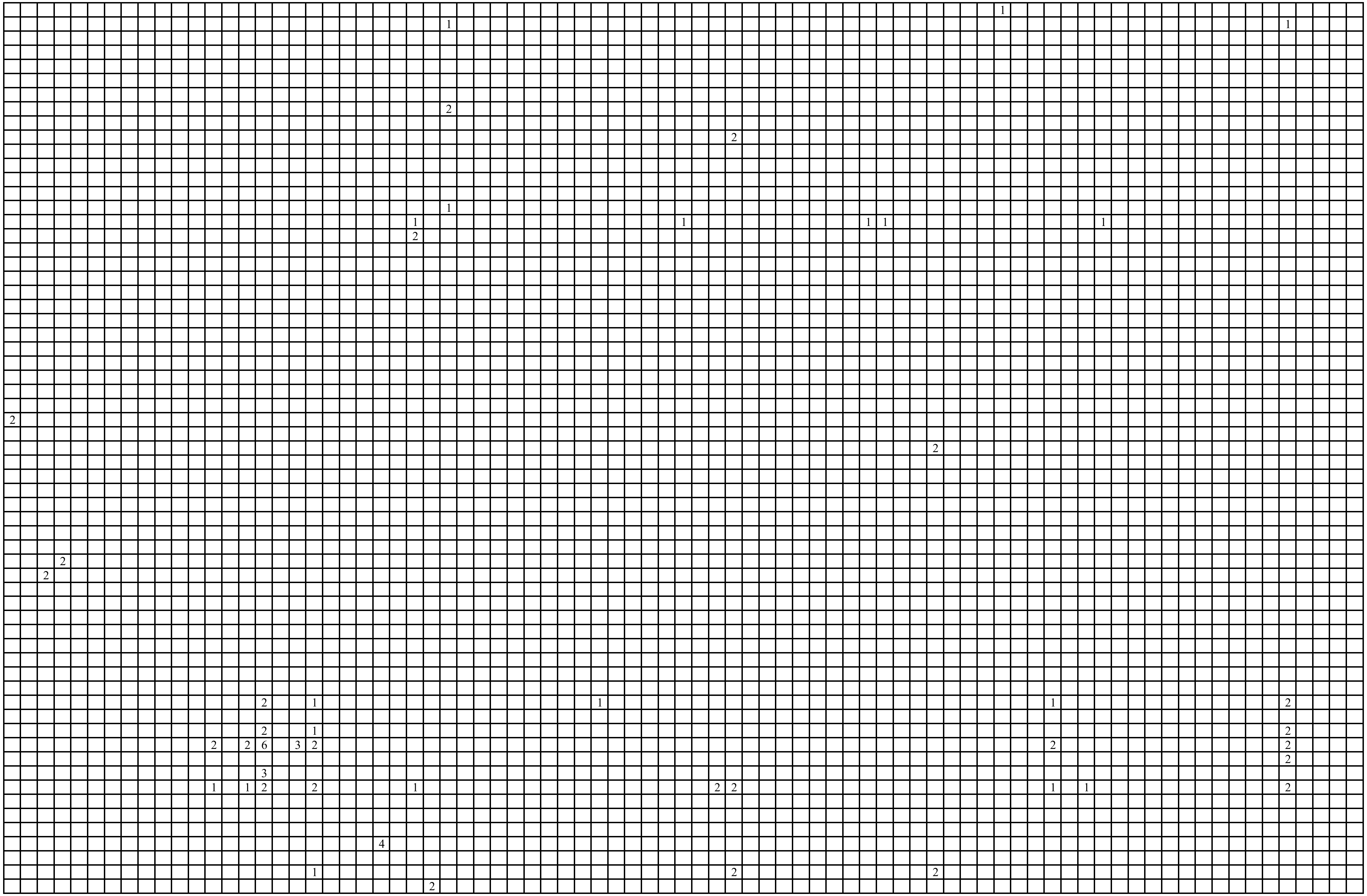
1
1 1
2
2
1
1 1 1 1 1
2
2
2
2
2
2 1 1 1 2
2 1 2
2 2 6 3 2 2 2
2
3
1 1 2 2 1 2 2 1 1 2
4
1 2 2
2

																										2																																																						
																														2																																																		
												1																																																																				
																		2																								2																																						
																		2						2																											1																													
																																											1												2																									
																																																							2																									
																																																																												2				
																								2																											2		2													2															
																																																													4																			
												1			2			1																																																										2				
																		1																																																														
																																																							2																									
																																																																						2										
																																																																		2														
												2		2		2	2			2																																				2								2																		
												2		2	3			2																								2					2															1		1												3				
																																										2			1													1																						

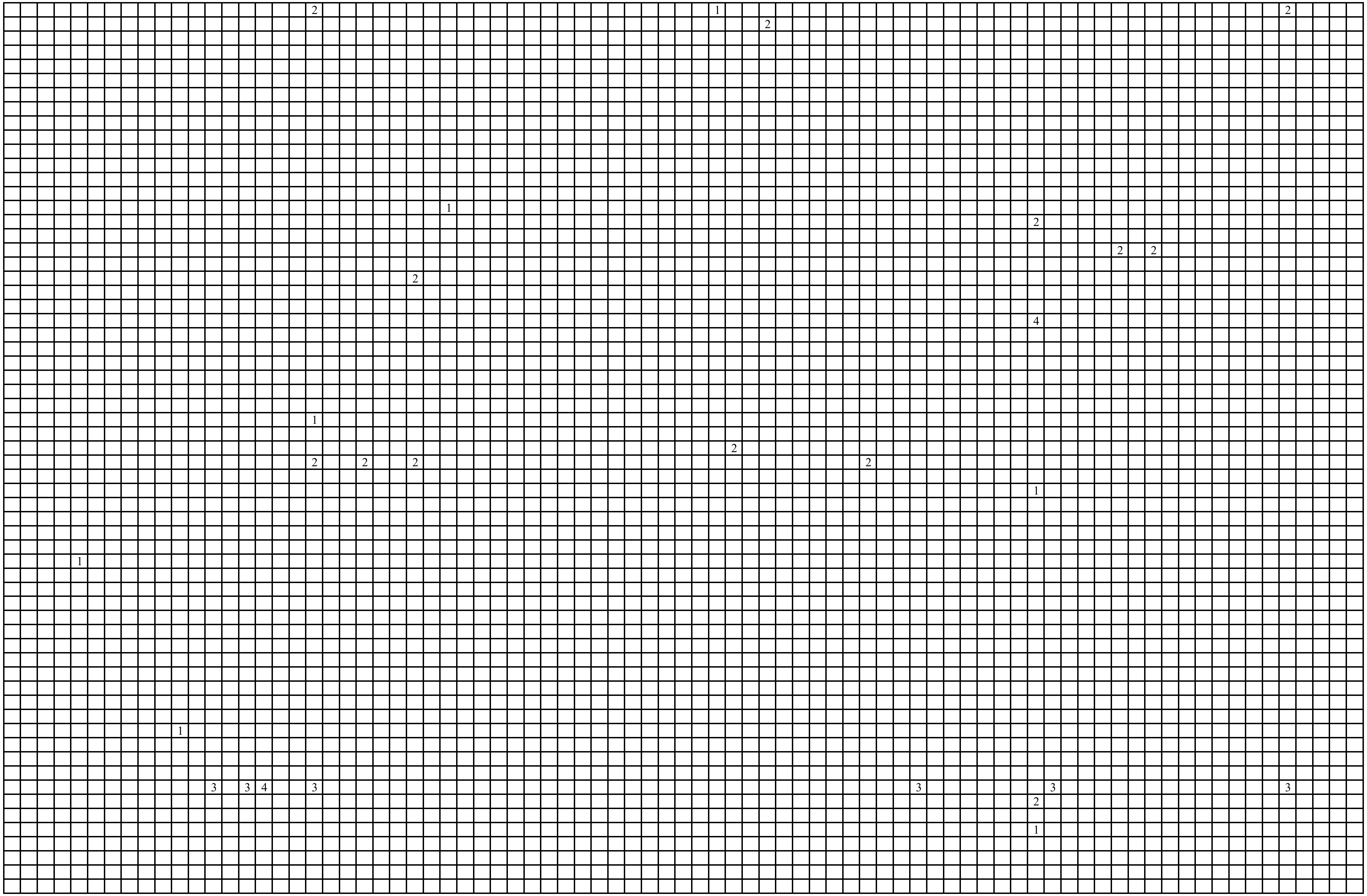

2

1 3 3 4 2

2

2 2 3 2 1 2 1 1

3 1 2

1

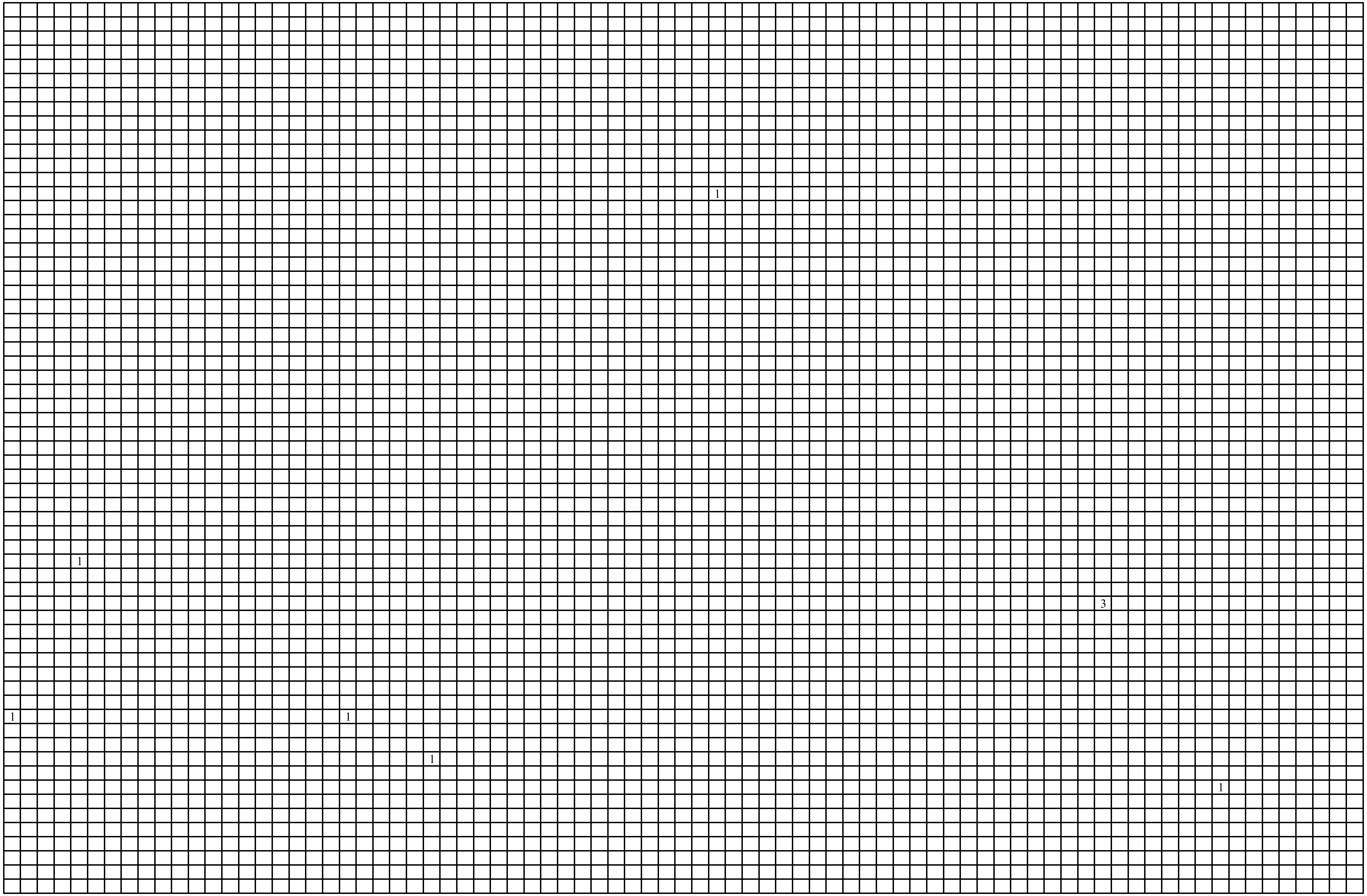
1
1
3
1
1
1
1

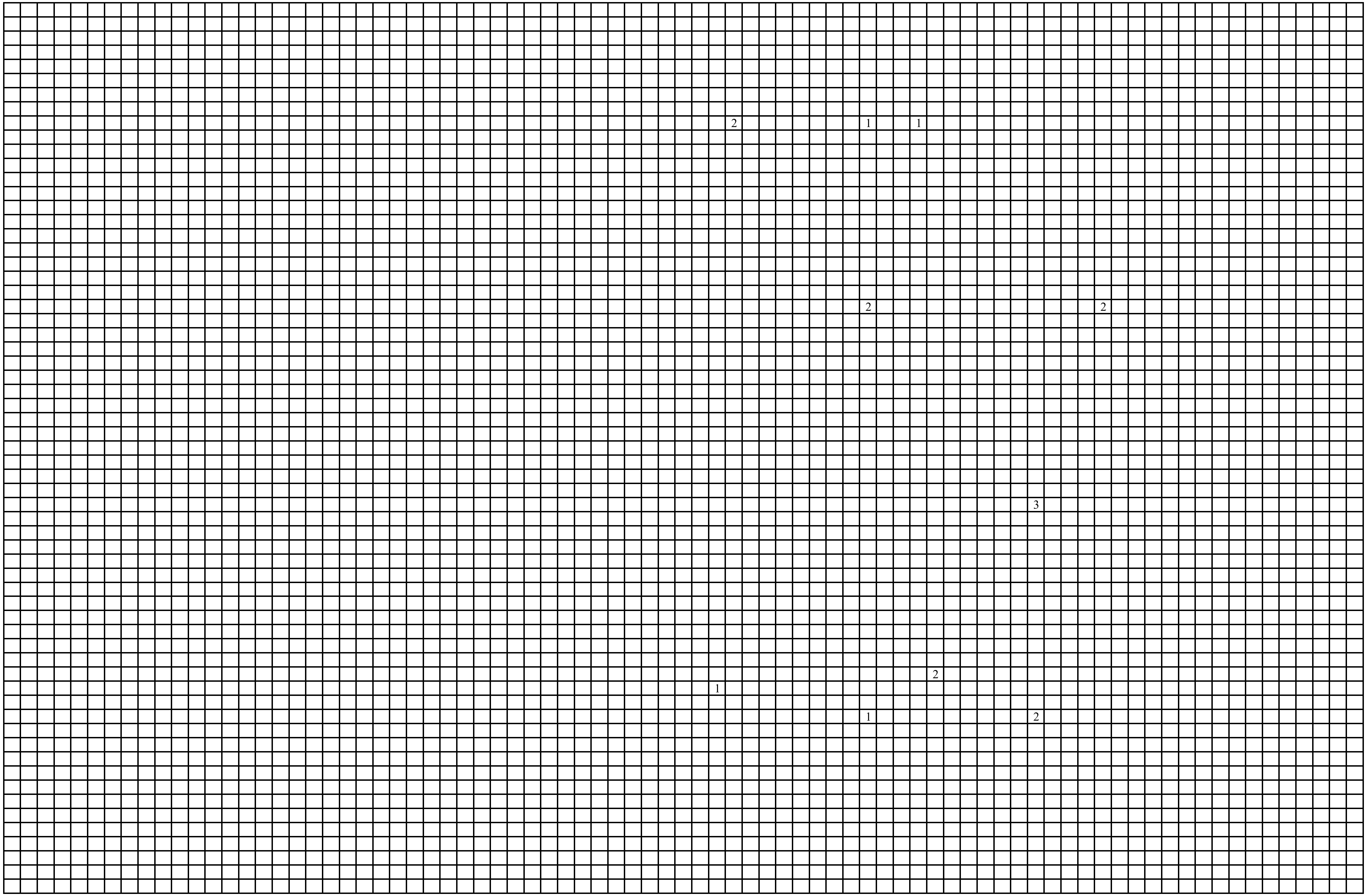
2
1
1
2
2
3
2
1
1
2

																																																													3															
																																																																			1											
																								1																																		1																						
																								1	1																	1		1														1																						
																		1																																																												
																		1																									2																									2												
																	2																																																														
												1			3	1	2	1																																														2														2				
															1		1																																																														
												1		1	1		1	1																																																											1				
																		1																									2												2																										
																		1																																																													
																																																																												2				
															1																																																														2				
												4	1	3	6		2	3																																							3								3														4				
																		1																																																													
																																											2																																										

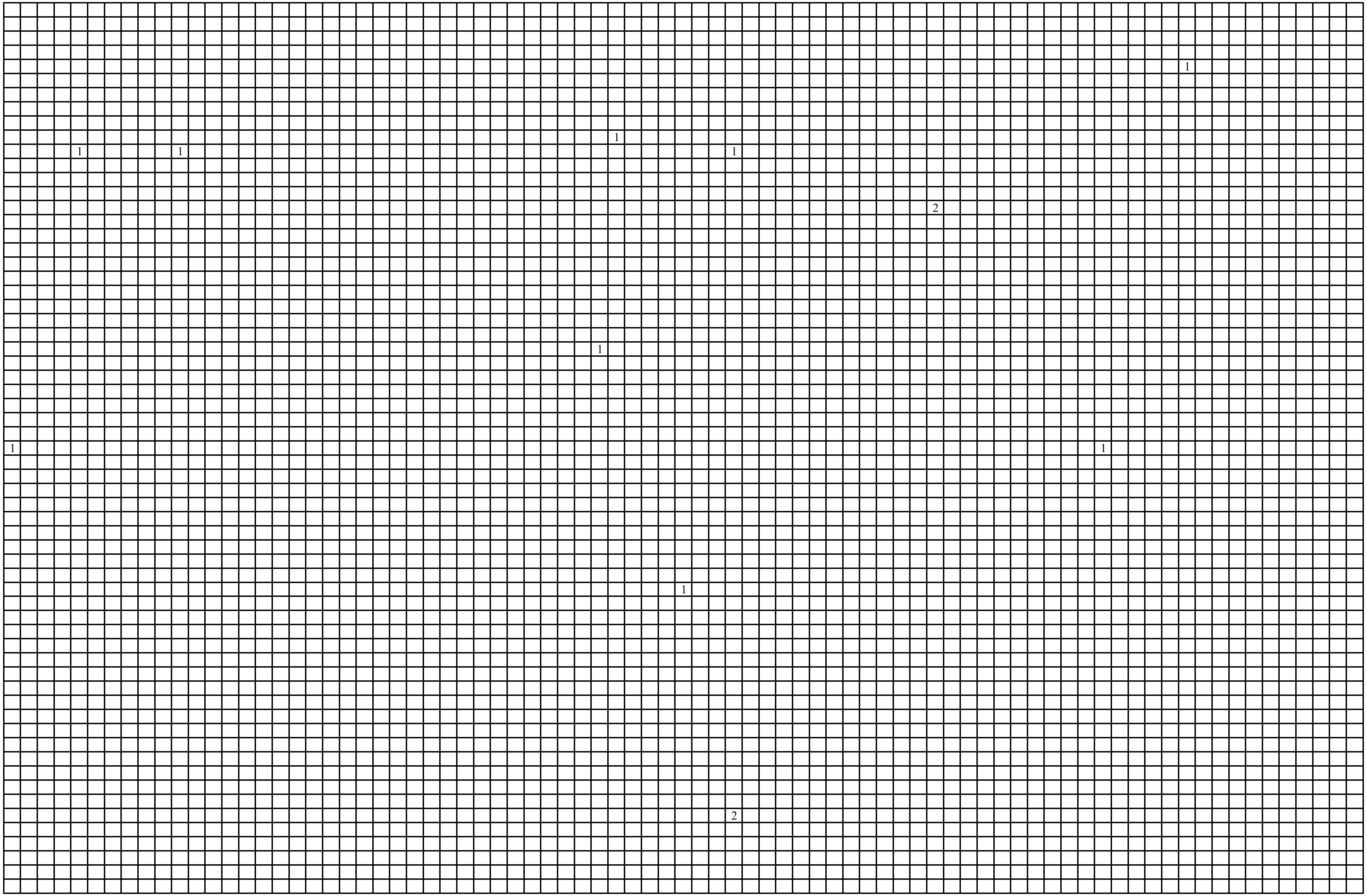
1
1
1
1
1
2
1
1
1
1
2

																																										1											1			1																								
																																												1																																				
																																		1										1														1																						
																		1																									2																																	2				
																		1		1	1			1																1											1														1															
																								1																																									1															
																								1																																																								
																																		1																																														
																																												1																																				
1																									1																																																							
										1								1																																																														
																		1																									2																																					
																		1																								1									1																													
																																																							2																									
																																																							2																									
												3		3	4	3		3																																				3				1				3		3												3				

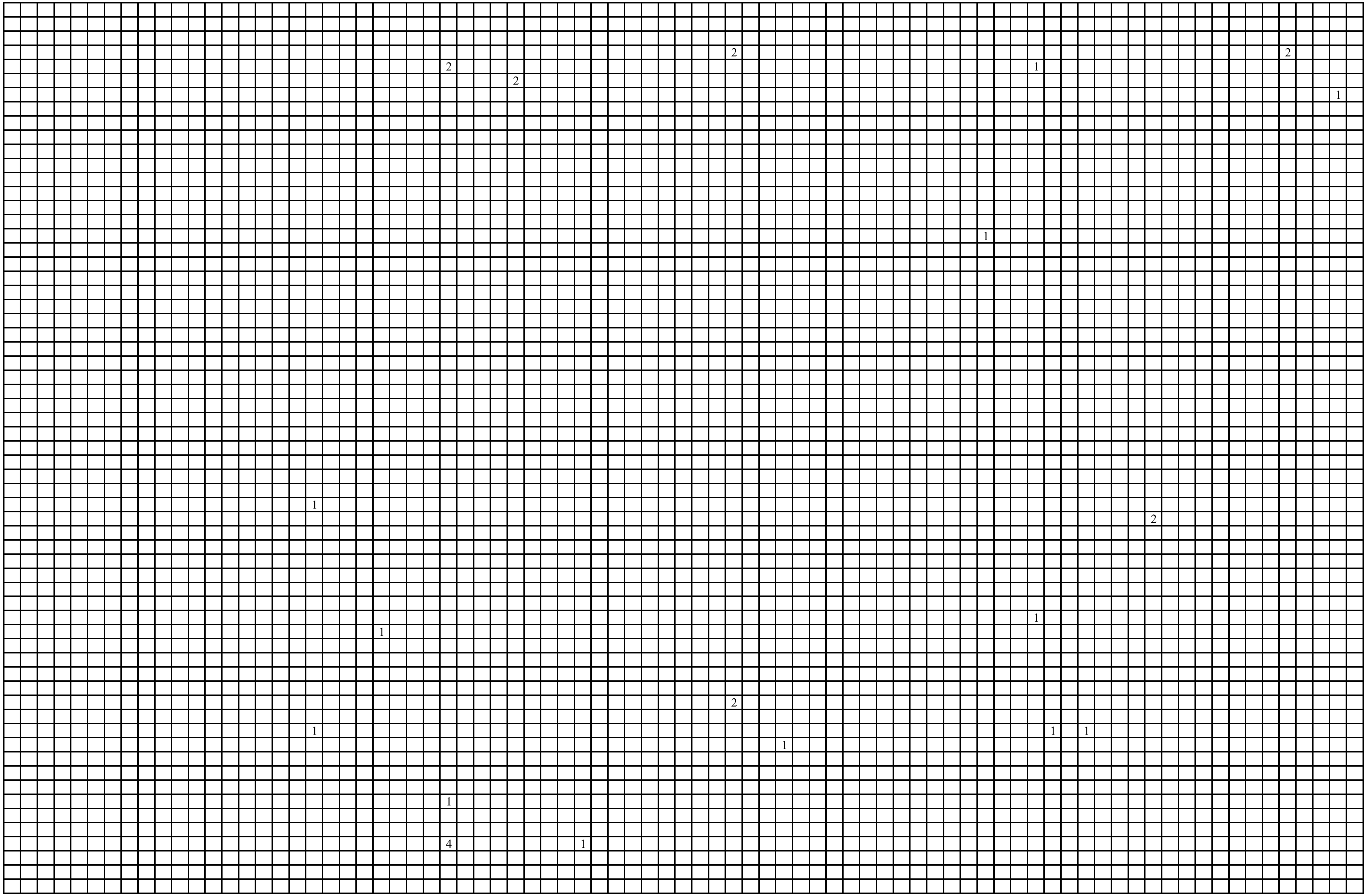
2
2
2
1
2
1
1
1
2
1
1
2
1
1
1
1
1
4
1

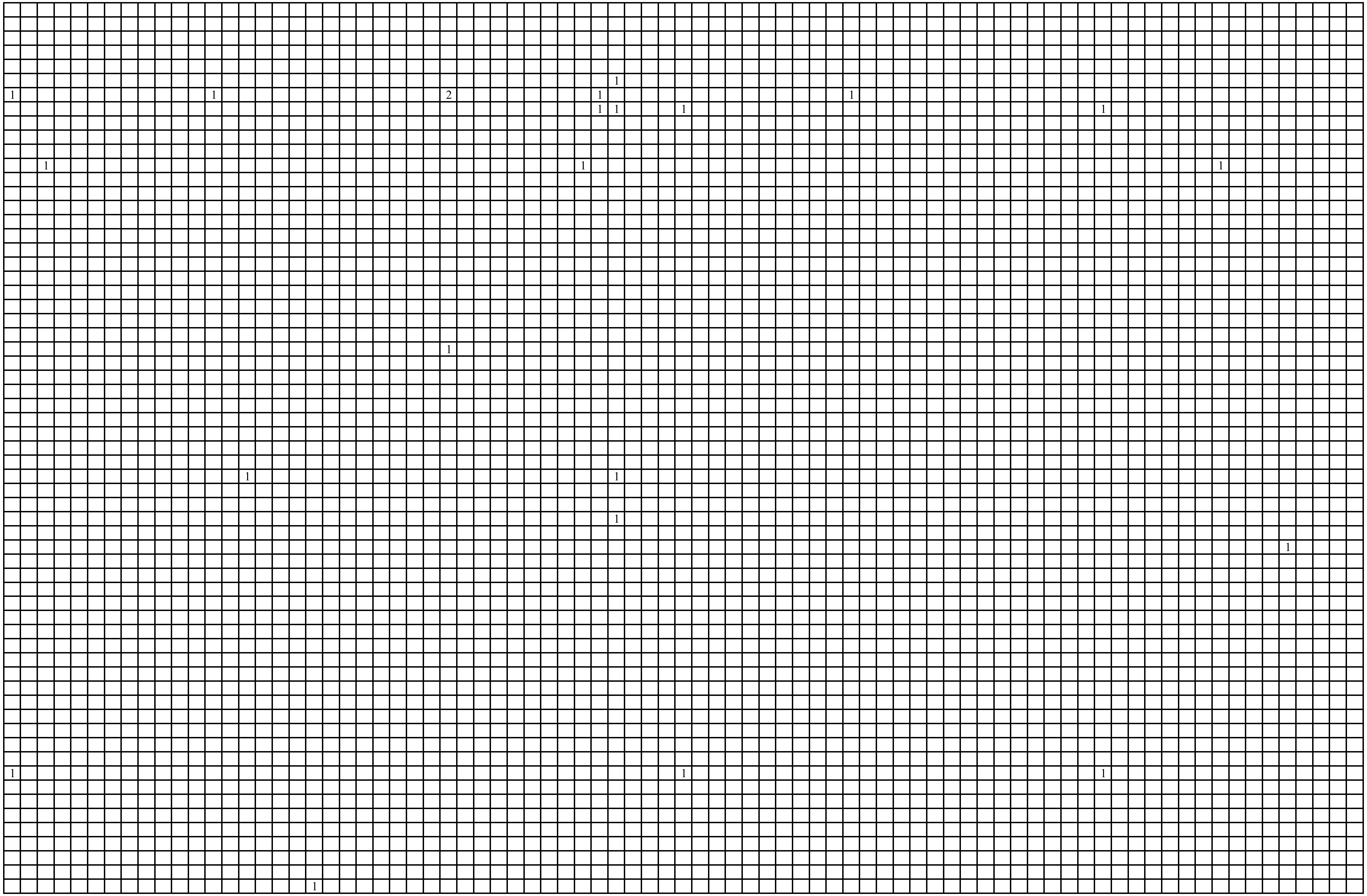
1
1 1 2 1 1
1 1 1 1
1 1 1
1
1 1
1
1
1 1 1
1

1

																										ZNF24	96	222
																										ZNF25	0	5
					1																					ZNF250	32	161
																										ZNF253	0	6
																										ZNF254	3	14
																										ZNF257	1	4
																										ZNF26	1	13
																										ZNF260	6	15
																										ZNF263	38	213
																										ZNF264	7	58
																										ZNF266	0	17
																										ZNF267	4	16
																										ZNF268	4	17
																										ZNF273	7	27
																										ZNF274	4	30
																										ZNF275	7	24
																										ZNF276	8	34
																										ZNF28	2	10
																										ZNF280A	1	16
																										ZNF280B	1	5
																										ZNF280C	5	70
																										ZNF280D	2	30
			1																							ZNF281	68	197
																										ZNF282	5	28
																										ZNF283	0	1
																										ZNF284	6	16
																										ZNF285	0	1
																										ZNF286A	8	37
																										ZNF286B	0	1
																										ZNF287	2	12
																										ZNF3	12	41
																										ZNF30	0	7
																										ZNF300	1	5
																										ZNF302	1	29
																										ZNF311	0	11
																										ZNF317	9	80
																										ZNF32	0	16
																										ZNF320	5	28
																										ZNF322	0	4
																										ZNF324	15	68
																										ZNF324B	39	149
																										ZNF329	6	33
																										ZNF331	4	102
																										ZNF333	1	22
																										ZNF337	2	15
																										ZNF33A	0	13
																										ZNF33B	1	8
																										ZNF34	14	53
																										ZNF343	4	25
																										ZNF345	1	6
																										ZNF347	0	0
																										ZNF35	6	48
																										ZNF350	6	41
																										ZNF354A	9	50
																										ZNF354B	0	4
																										ZNF354C	7	41
																										ZNF358	4	22
																										ZNF362	7	42
																										ZNF366	0	9
																										ZNF383	4	19
																										ZNF384	18	73
																										ZNF391	0	7
																										ZNF394	10	21
																										ZNF397	71	126
																										ZNF398	15	60
																										ZNF404	2	2
																										ZNF408	34	222
																										ZNF41	2	11
																										ZNF410	10	62
																										ZNF415	0	6
																										ZNF416	2	13
																										ZNF419	0	10
																										ZNF420	11	46
																										ZNF425	0	1
																										ZNF426	2	56
																										ZNF429	0	0
																										ZNF43	0	1
																										ZNF430	8	37
																										ZNF431	4	27
																										ZNF432	1	8
																										ZNF433	0	13

1

2

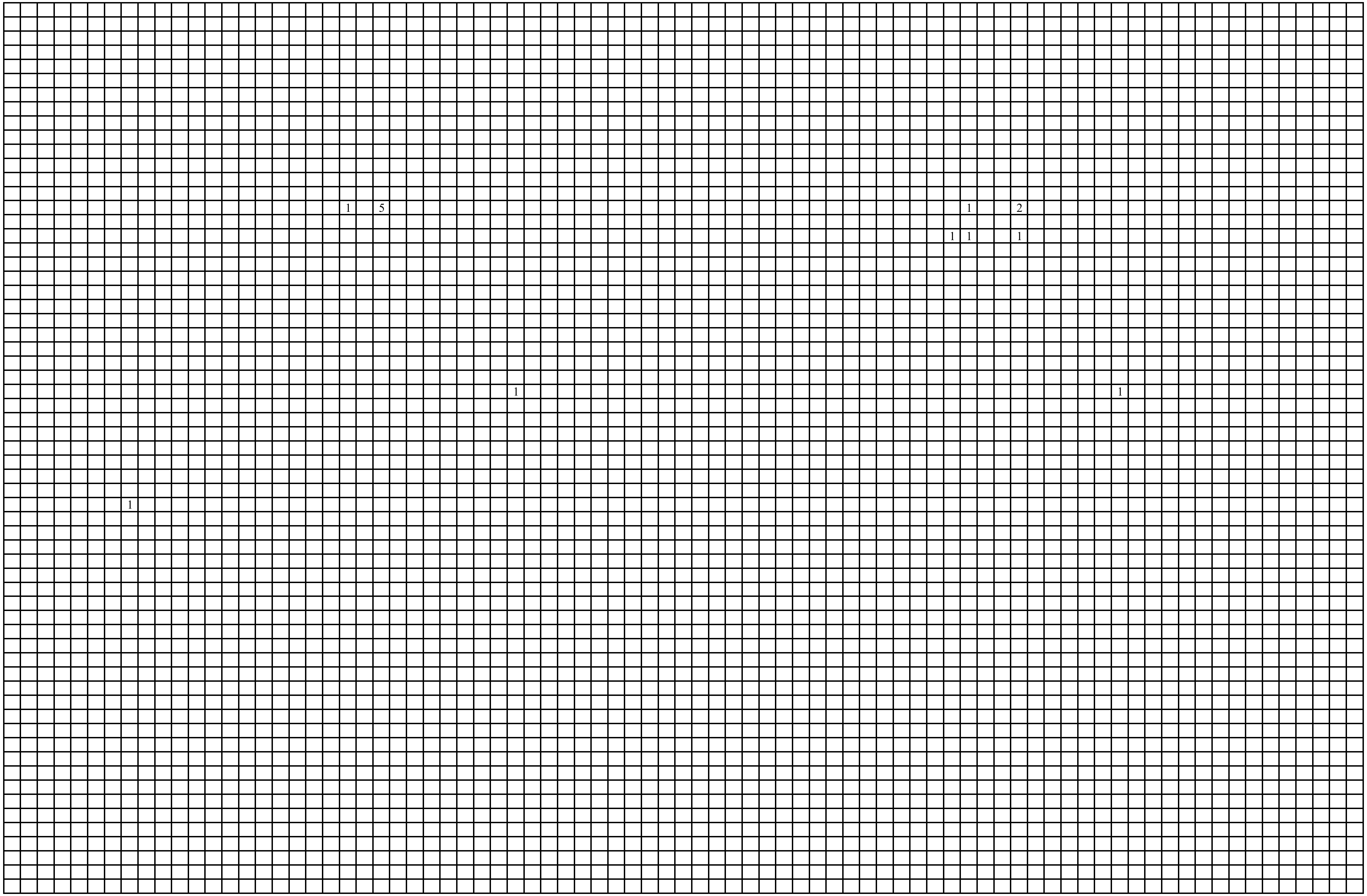
1 5
1 2
1 1 1
1 1
1

																																														1																													
1																						1					1			1																											1			1																				
1																						1																																			1			1																				
																																																									1																				
																													1																																																						
																																															1																																					
																																									1																																														
																																																					1																											

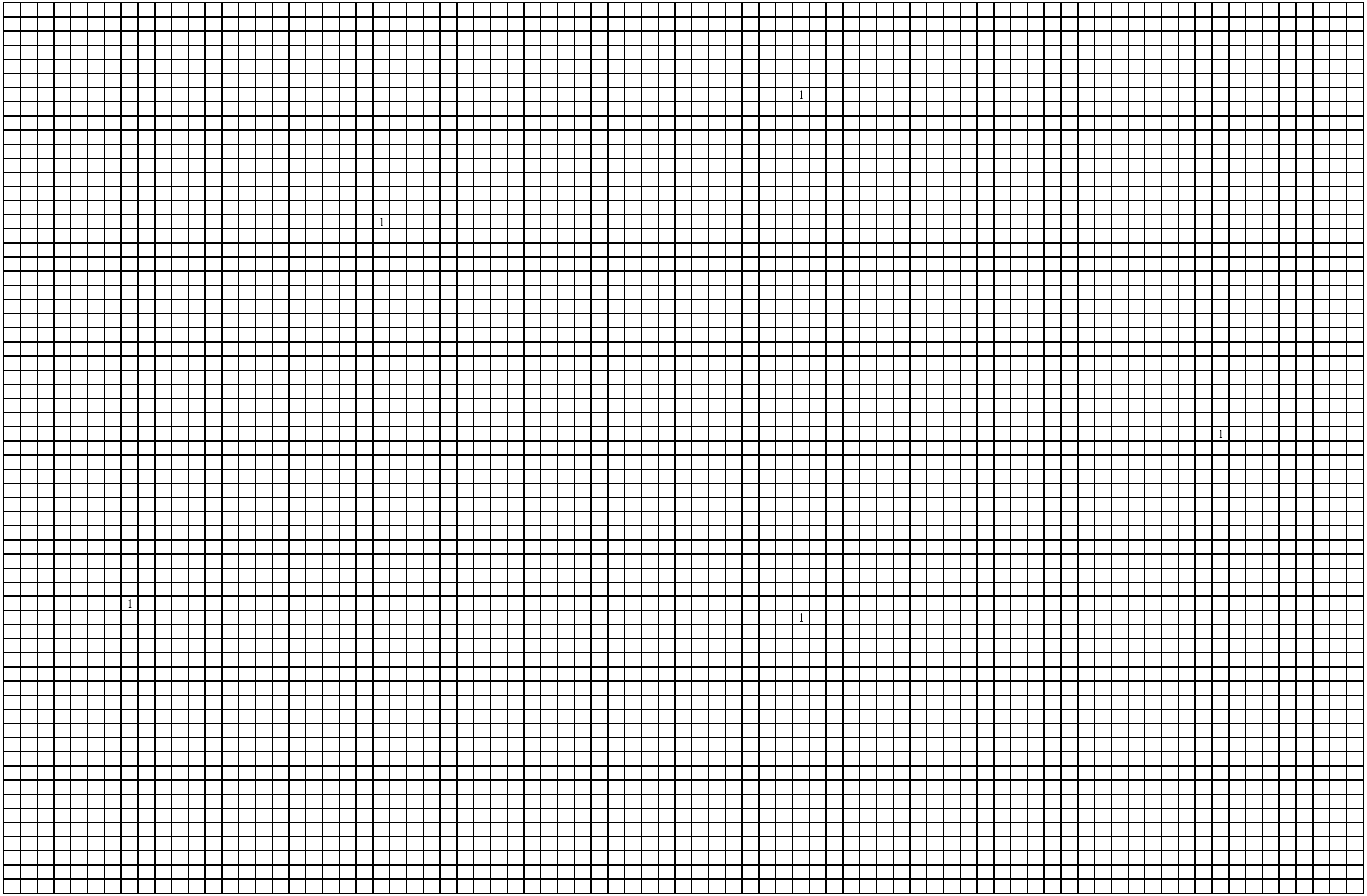
1
1
1
1
1

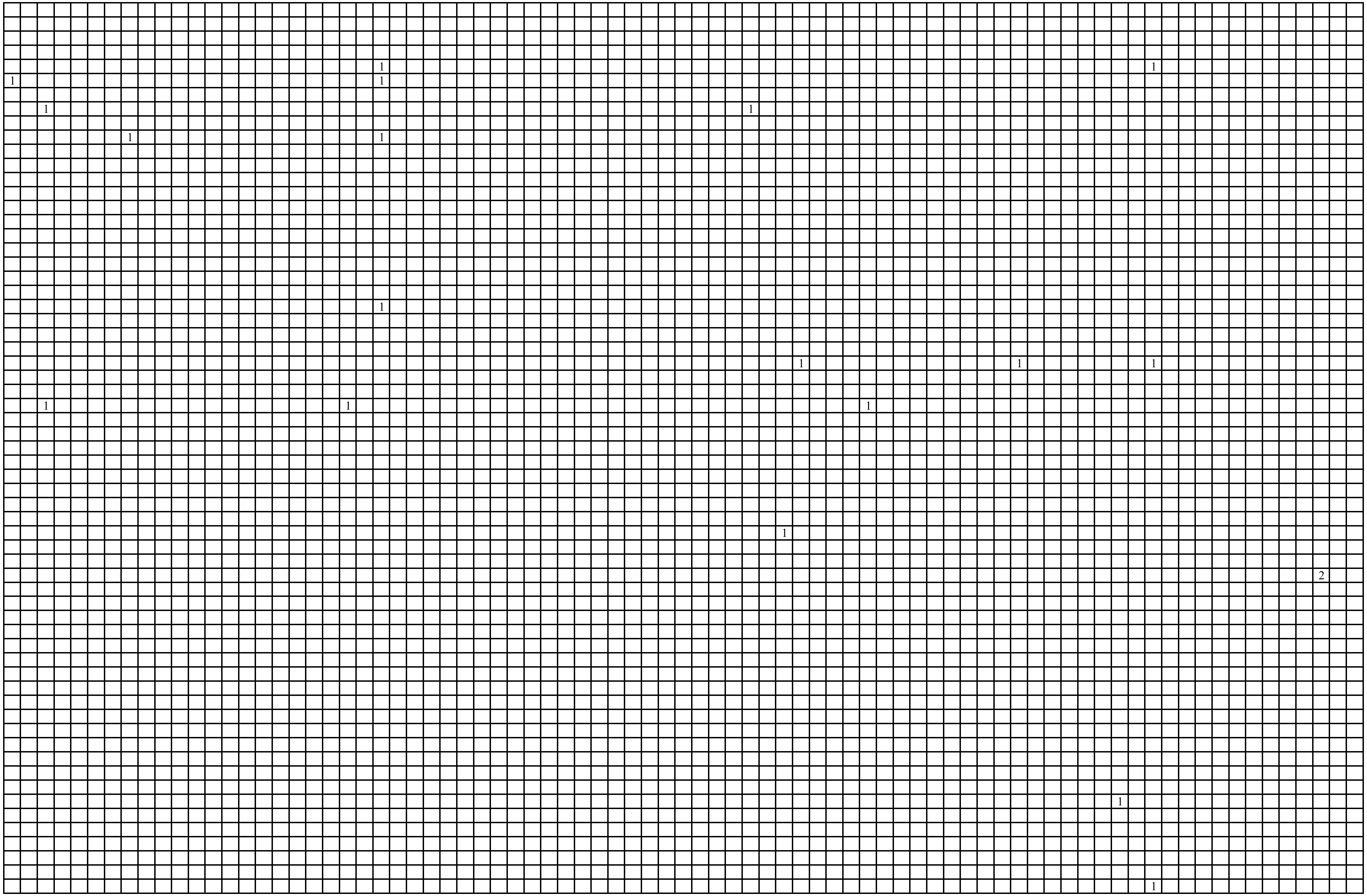
1
1
1
1
1
1
1
1
1
1
1
1
1
1
1
1
1
2
1
1

																											1																																																							
																																																																1														
																																														1																																	
																																																													1																				
																																																																						1												
		1														1																									1																								1			1												
																																																					1																									
																																																					1																									
								1																																																																								

																						2																																																										
																																																									1																				
							1															1																																																										
																						1																																																										
																						1																									1																				1													
																																											1																																				
																																1																																																				
																																											1																																				
																																											1																																				
																																											1																																				

																1																																																																
1																																																																															
1																																																									1			1																				
																						1																																																													
																						1																																																													
																						1																																																													
1											1											3																																		1			2																				

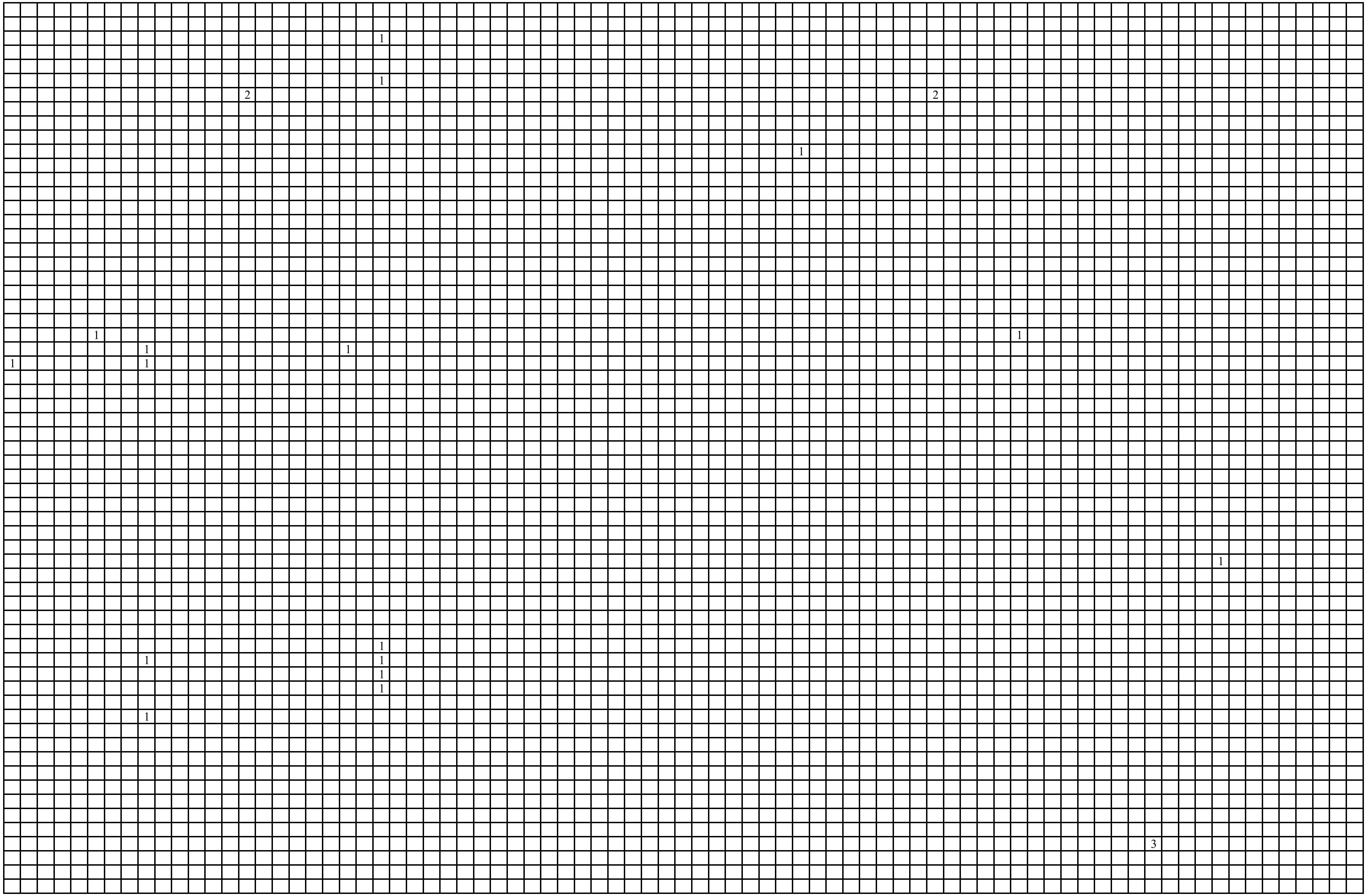
1
1
2
2
1
1
1
1
1
1
1
1
1
1
1
1
1
1
3

1

2

3

2

1 1 1 1 1 1 3 6

																																																1																																
								1																																																																						
													1				1																				1																																							1								
1																																																												1																				
												4																																																1																				
																						1																																																											
																																																																			1													
																																										1																																														
																						1																																																											
																																																	1																																	
1																																																																																

																																															1																																	
																					1																																																											
1																																																																																
																					1																																																													
																					1																																																													
																					1																																																														
																					1																																																													
1																					1																																																													
1																																																																																
								1																																							1																													
																					1																																																													
															1																																																																		

																																																											1																					
																						1																																																										
																						1																																																										
																						3																	1																																									
																																																					1																											
																																																																		2														
																						1																																																										
																						1																																																										
																						1			1																																																							
																						1																																																										
																						1																																																										
																						1																																																										
																						1																																																										
																						1																																																										

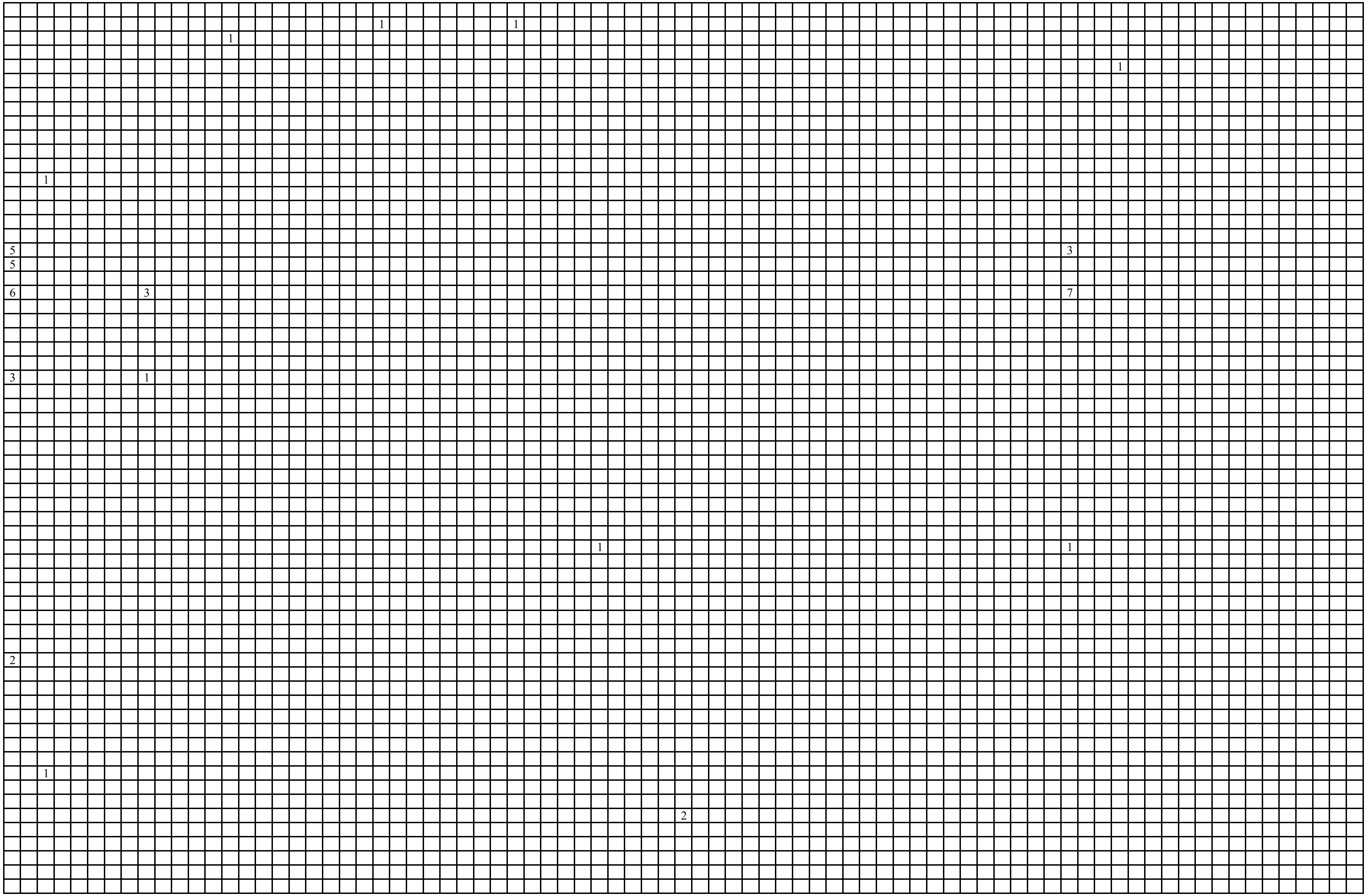
1
1
1
1
1
5
3
5
6
3
7
3
1
1
1
2
1
2

2
1 1
1
1
1 1
1 1
1
1
1
1
2 3
2 3
3 4
2 2 1 2 3
2
2 2

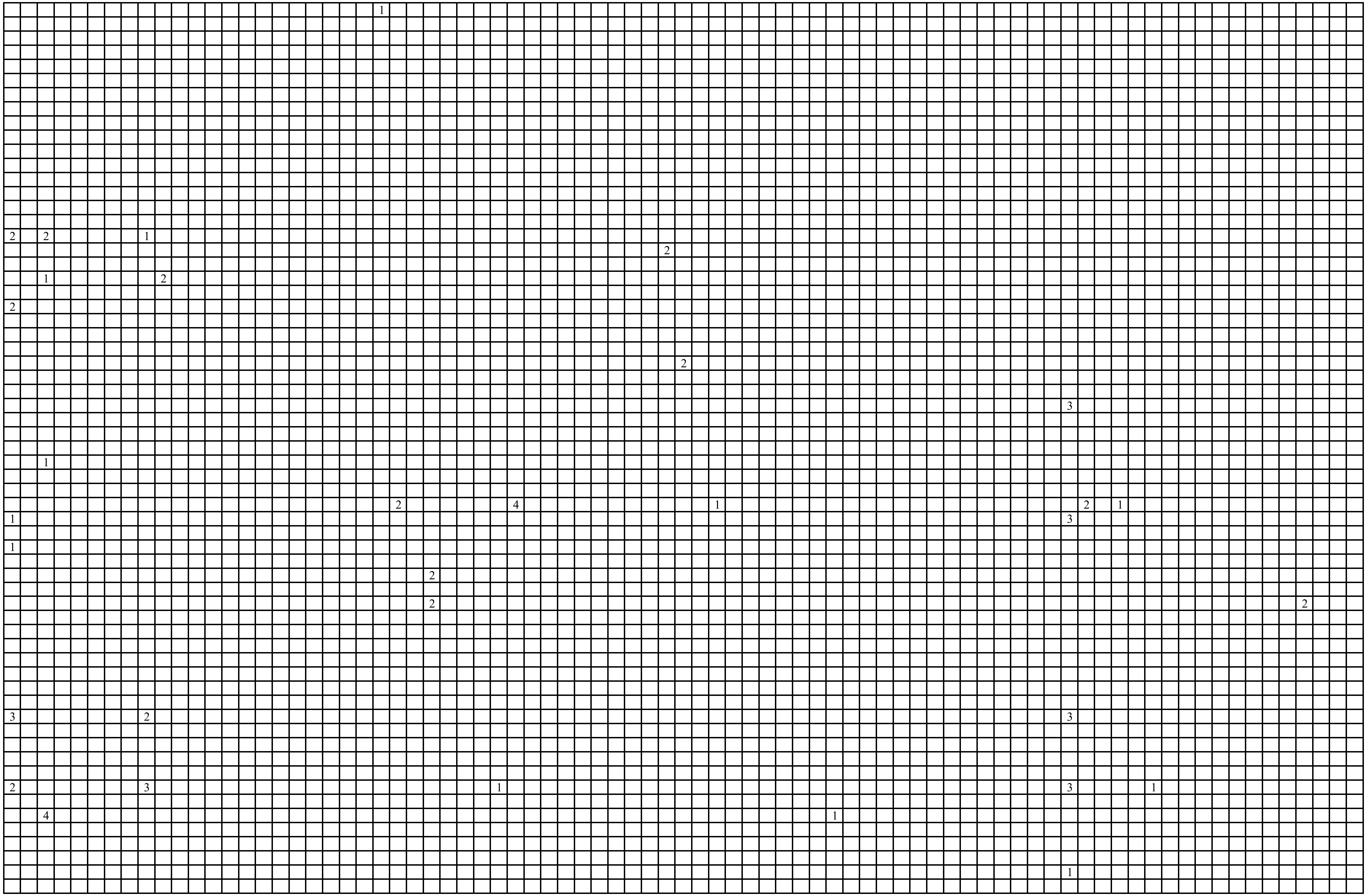

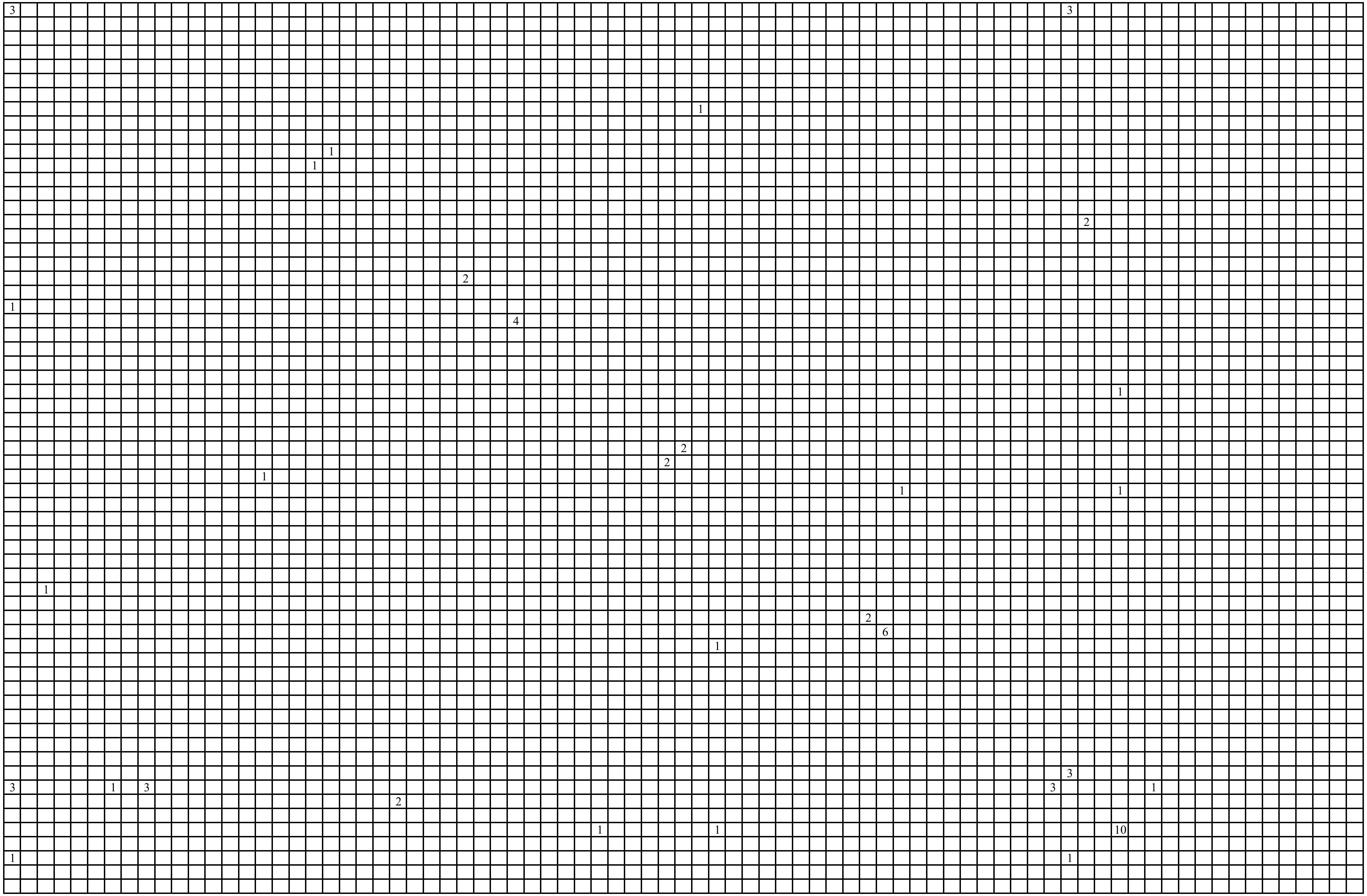
3
3
1
1
1
2
2
1
4
1
2
2
1
1
1
1
2
6
1
3
3
1
3
3
1
2
1
1
10
1
1

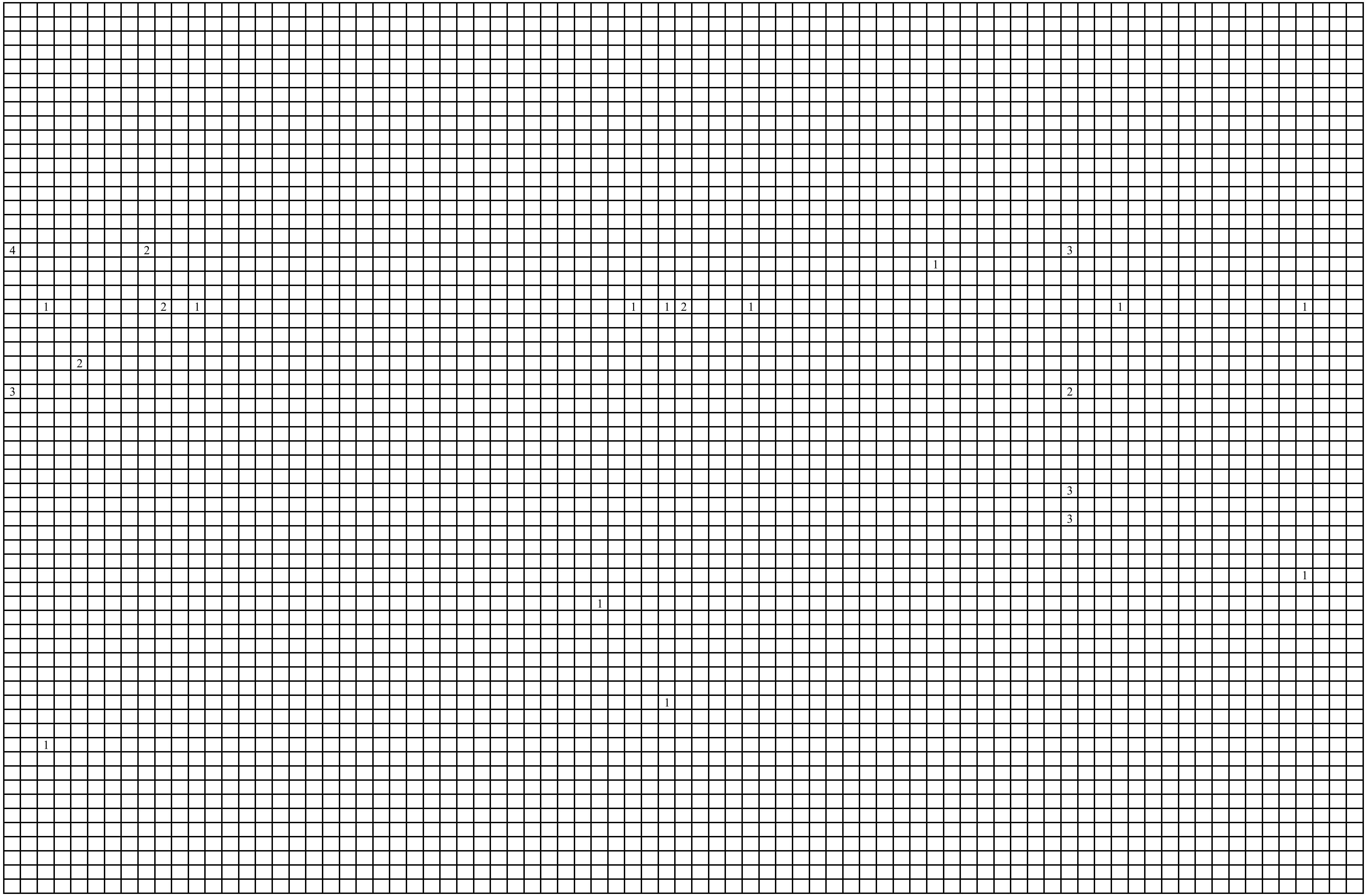
4 2 3
1
1 2 1 1 1 2 1 1 1
2
3 2
3
3
1
1
1
1

		1																																																																														
		1																																																							1																					
1																																																																																
																																							2																																										
		1					1								1																				1													1																																

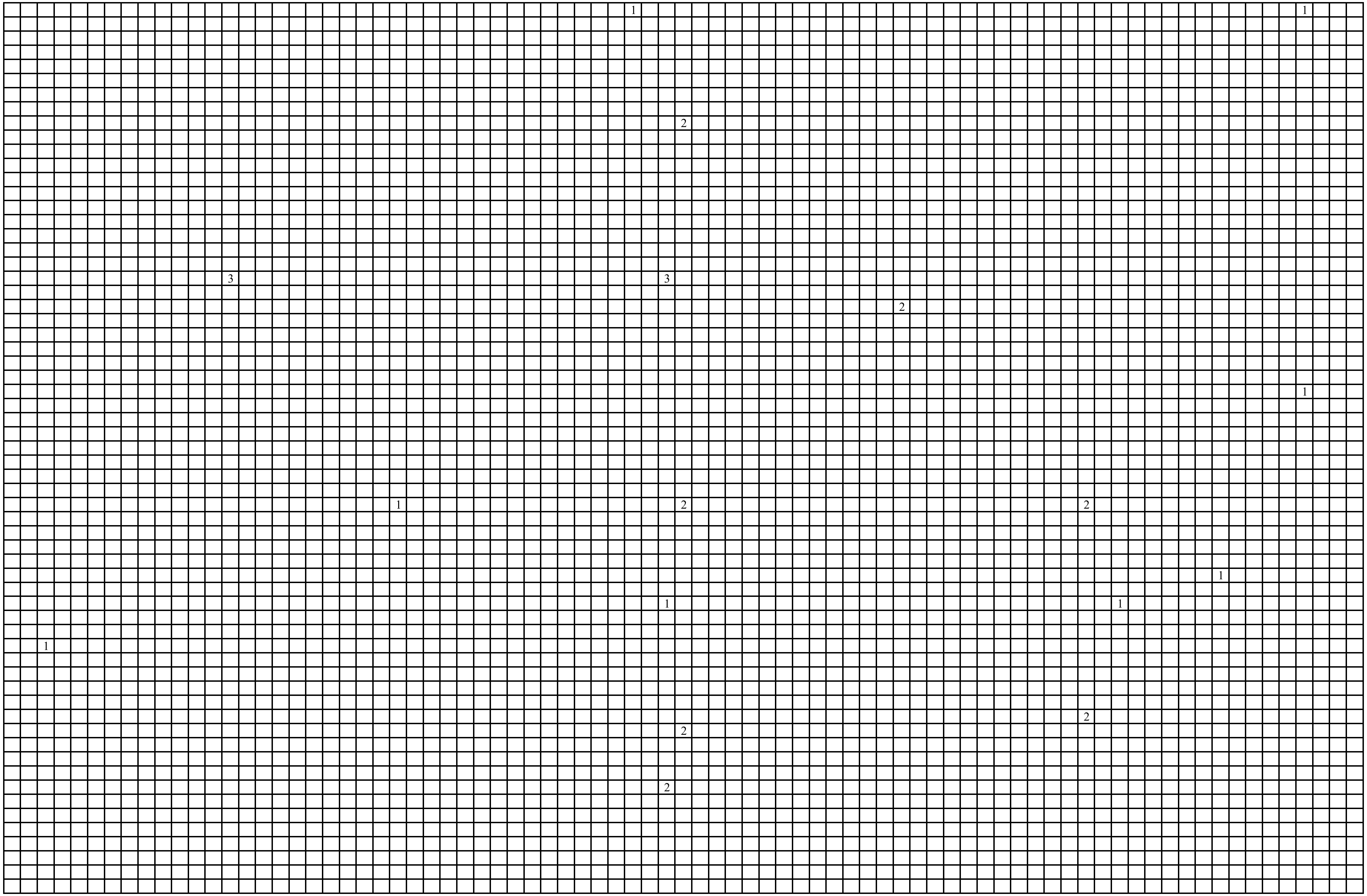
1
1
2
3
3
2
1
1
2
2
1
1
1
1
2
2
2

									2																																																																							
																																							1																																								
																																														1																																
		1													1																																																						1														
																																							2																																								
1																																																															1																	
1																																																																																
3																																																															3																	
1								1																																																						1																	
2								1																																																																1						
																																							2																																								
																																																														1																	
																																																														3																	
7																																																														3	3																	
2																																																																																	

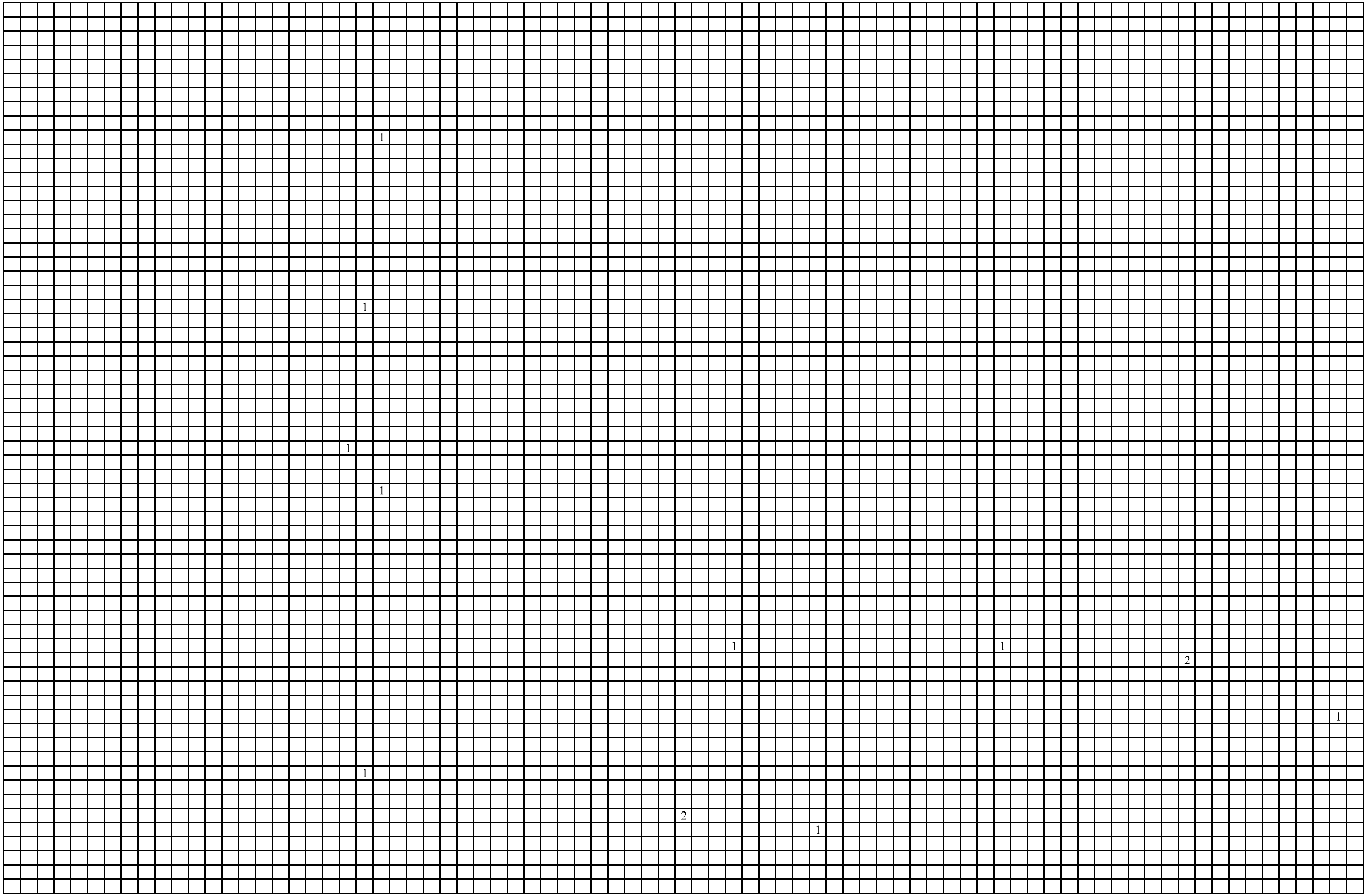
1
1
1
1
1
1
2
1
1
2
1

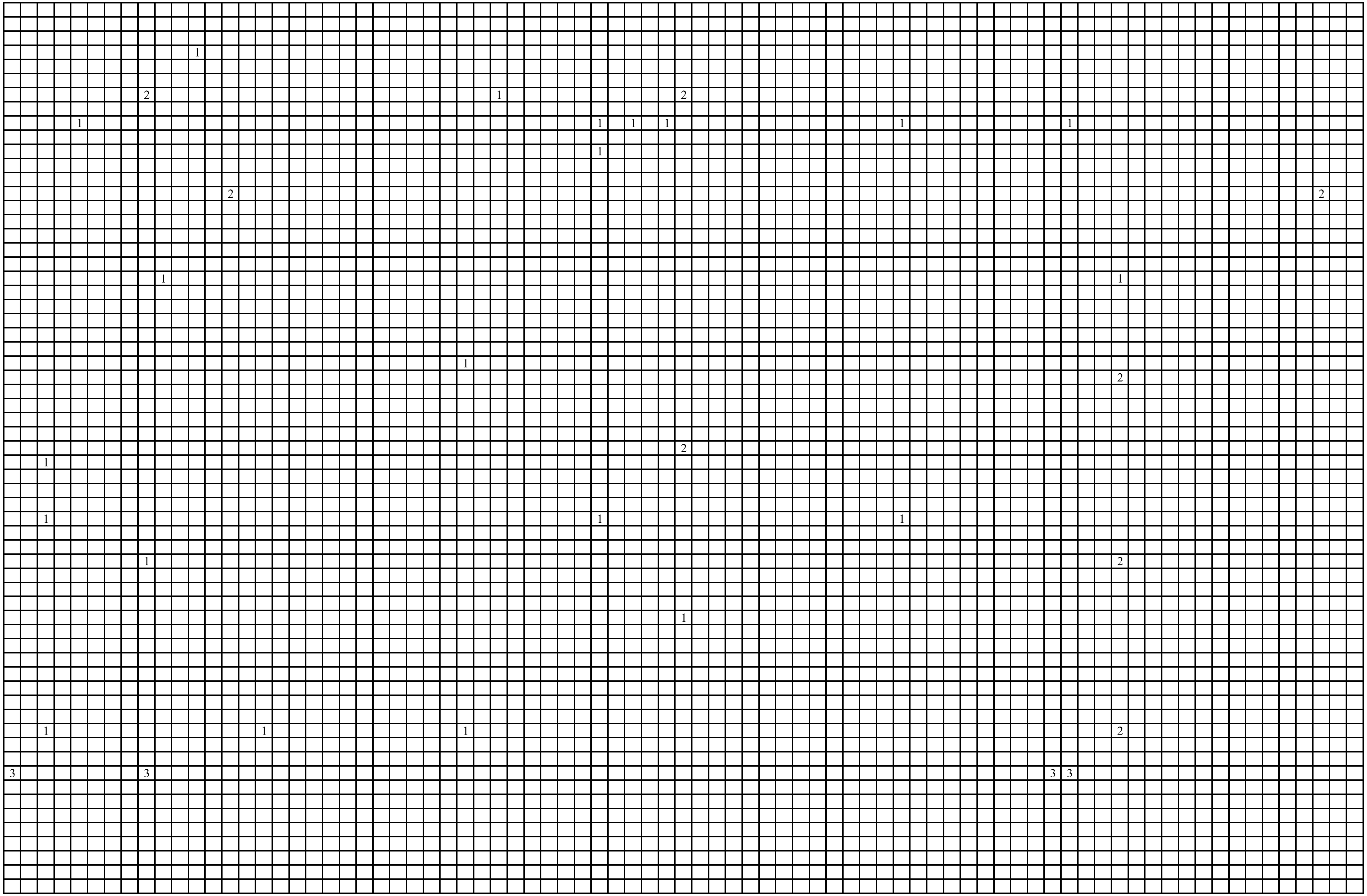
1
2 1 2
1 1 1 1 1 1
1
2 2
1 1
1
2
2
1
1 1 1
1 2
1
1 1 1 2
3 3 3 3

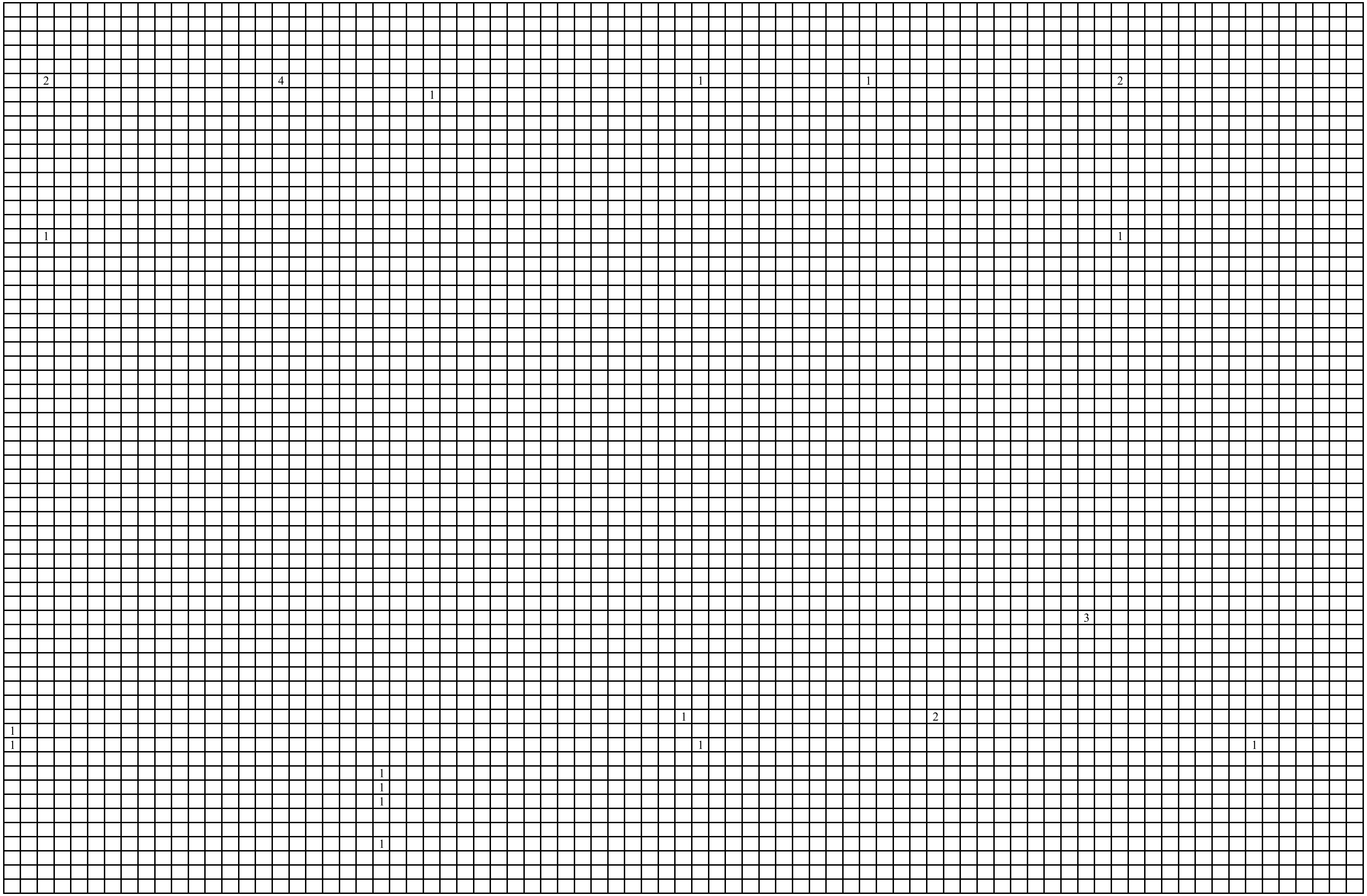
2 4 1 1 2
1
1 1
3
1 2
1
1 1 1
1
1
1
1

								2																																																																								
																						1																																		1																							
1																						1																																											1																
1																1	1					1																																																												
																						1																																																					1								
																																																																					1															
																						1																																																																
																																									1																																						
															1																																												1																				
																																																			1																											
																																																										1																				
								1														1																1														1																											

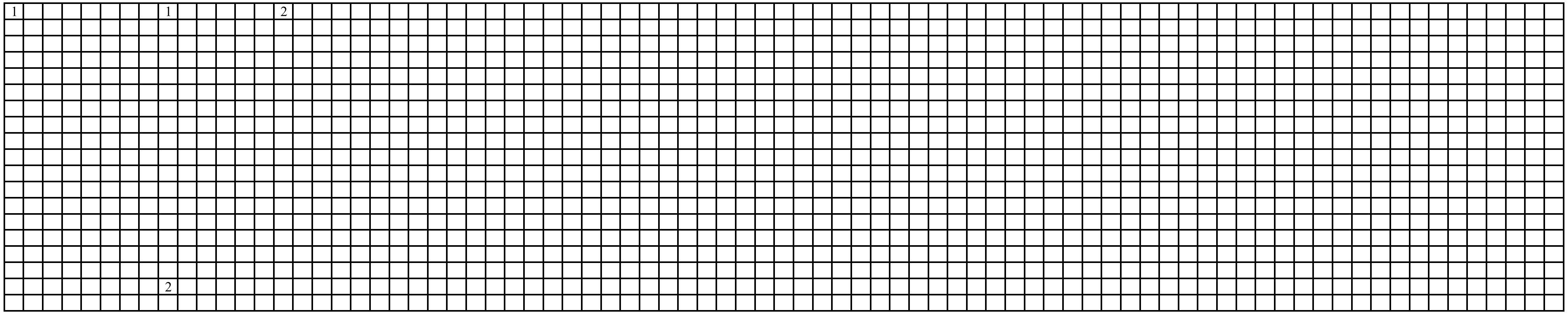
1
1
2
2

																										ZNF436		2	20
																										ZNF439		0	36
																										ZNF44		4	19
																										ZNF440		2	51
																										ZNF441		0	9
																										ZNF442		1	2
																										ZNF443		1	3
																										ZNF444		39	155
																										ZNF449		5	13
																										ZNF45		3	9
																										ZNF454		0	0
																										ZNF460		31	156
																										ZNF461		0	8
																										ZNF468		0	7
																										ZNF471		5	19
																										ZNF479		2	8
																										ZNF480		1	9
																										ZNF483		41	80
																										ZNF484		1	13
																								1		ZNF485		6	31
																										ZNF490		7	65
																										ZNF491		0	29
																										ZNF492		3	11
																										ZNF493		0	4
																										ZNF496		33	85
																										ZNF497		12	46
																										ZNF500		5	10
																										ZNF501		0	14
																										ZNF502		1	4
																										ZNF506		0	2
																										ZNF514		4	21
																										ZNF517		0	45
																										ZNF524		11	69
																										ZNF525		0	3
																										ZNF528		0	4
																										ZNF529		0	6
																										ZNF530		0	2
																										ZNF534		0	3
																										ZNF540		0	2
																										ZNF543		4	21
																										ZNF544		0	19
																										ZNF546		2	2
																										ZNF547		3	16
																										ZNF548		0	4
																										ZNF549		0	5
																										ZNF550		9	95
																										ZNF551		0	1
																										ZNF554		1	13
																										ZNF555		0	15
																										ZNF556		2	21
																										ZNF557		2	18
																										ZNF558		2	33
																										ZNF559		0	18
																										ZNF560		0	0
																										ZNF561		3	14
																										ZNF562		4	36
																										ZNF563		0	17
																										ZNF564		4	55
																										ZNF565		0	3
																										ZNF566		0	17
																										ZNF567		0	14
																										ZNF568		1	7
																										ZNF569		0	12
																										ZNF57		2	17
																										ZNF570		0	2
																										ZNF571		0	7
																										ZNF572	ZF.C2H2	13	71
					1																					ZNF575		2	26
																										ZNF576		13	38
																										ZNF577		0	5
																										ZNF581		13	143
																										ZNF582		0	6
																										ZNF583		0	3
																										ZNF584		0	11
																										ZNF585A		0	4
																										ZNF585B		0	6
																										ZNF586		1	6
																										ZNF589		3	17
																										ZNF594		0	6
																										ZNF595		1	7
																										ZNF596		1	2

2

1

1

			1																																																																													
1																																																																																
									1																																																																									
																														1																																																								

3

1

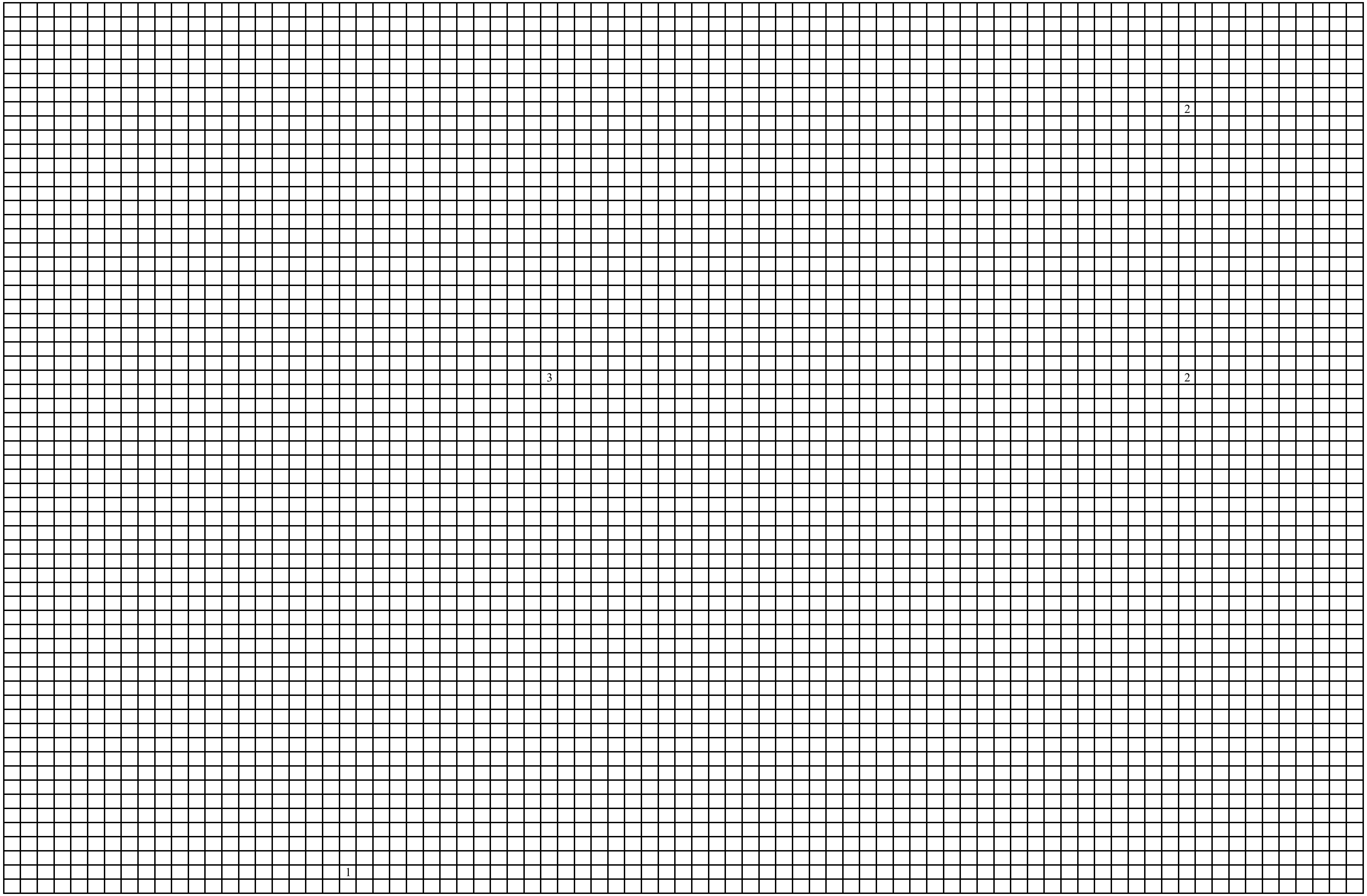
2
3
2
1

1 1 1 1 1

																	1																																																																	
																																	1																																																
																													1																																																					
		1																																																																														
																	1																																																																		
							1																																																																									

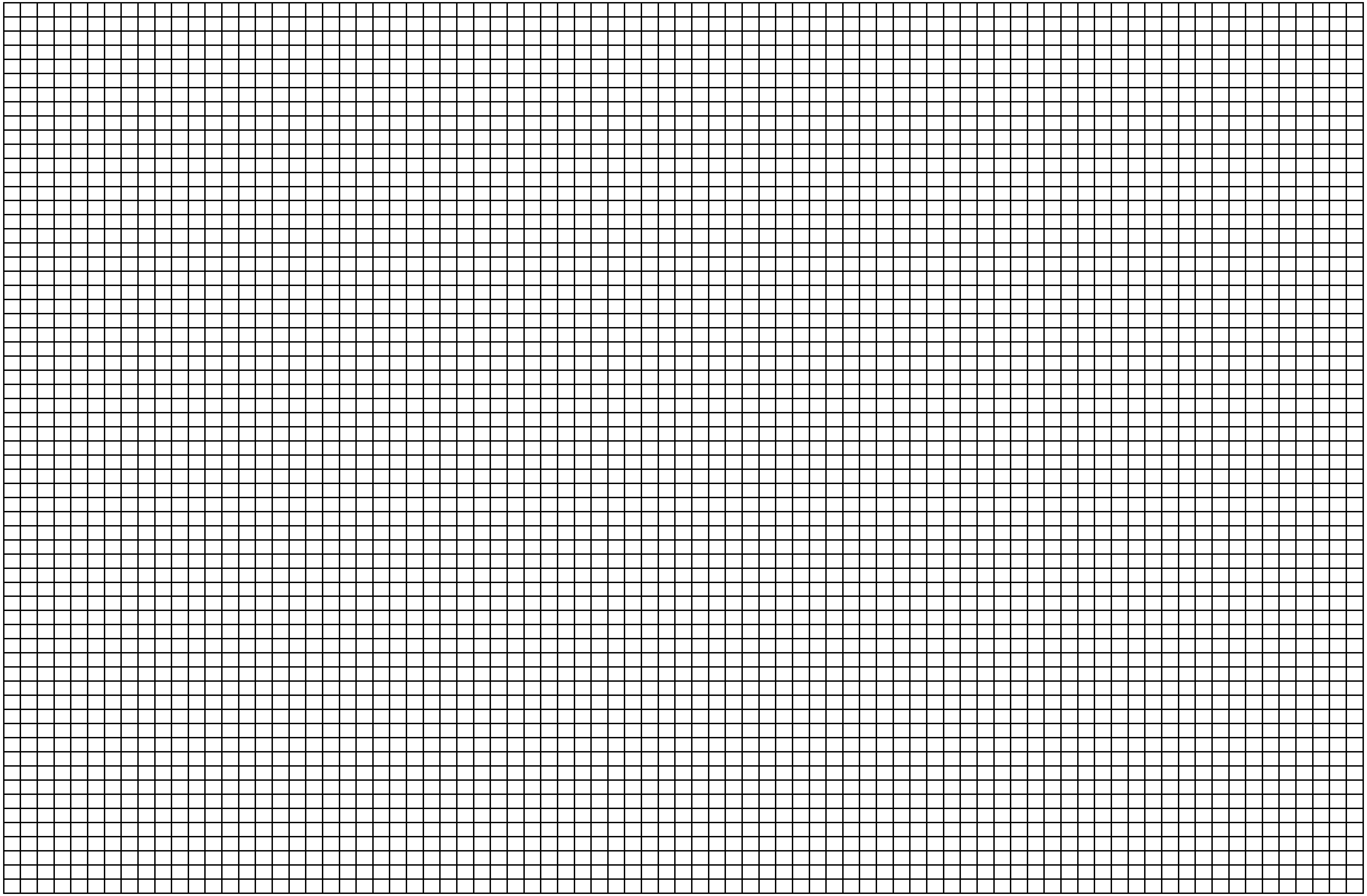

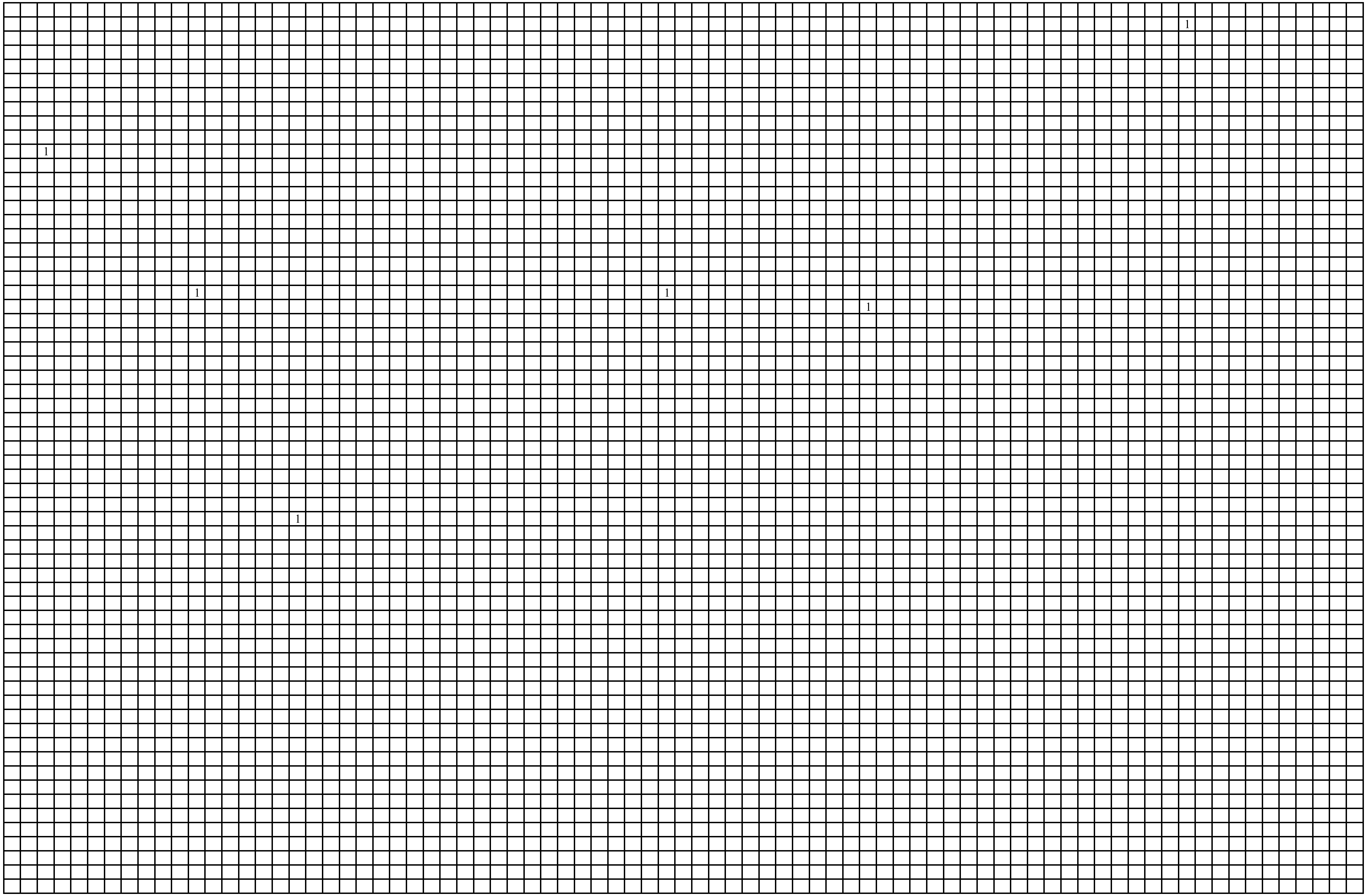
1
1
1
1
1
1

1

1 1

			1																																																																													
																									1																																																						
																	1		1			1																																																										
																																												1												1									1														
																																																																											3			
																																																1																														
																																																1																														
																																																																																1
																				1																																																													

1

																															1																																														
																																																																				1										
																																																				1																								
																										1																																																													
																		1																																																																										
																				1																																																																					

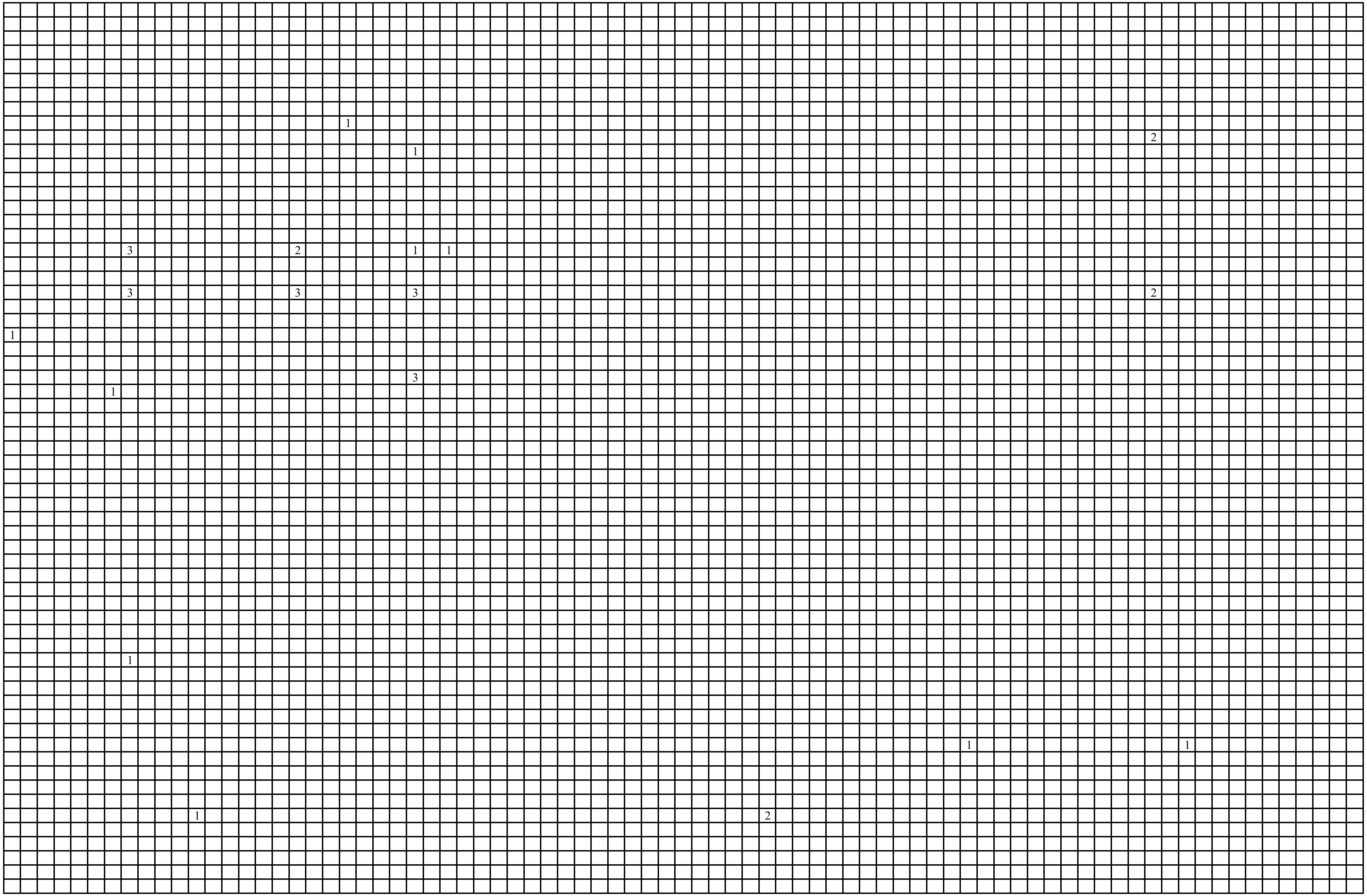
1
2
1
3
2
1
1
3
3
3
2
1
3
1
1
1
1
1
2

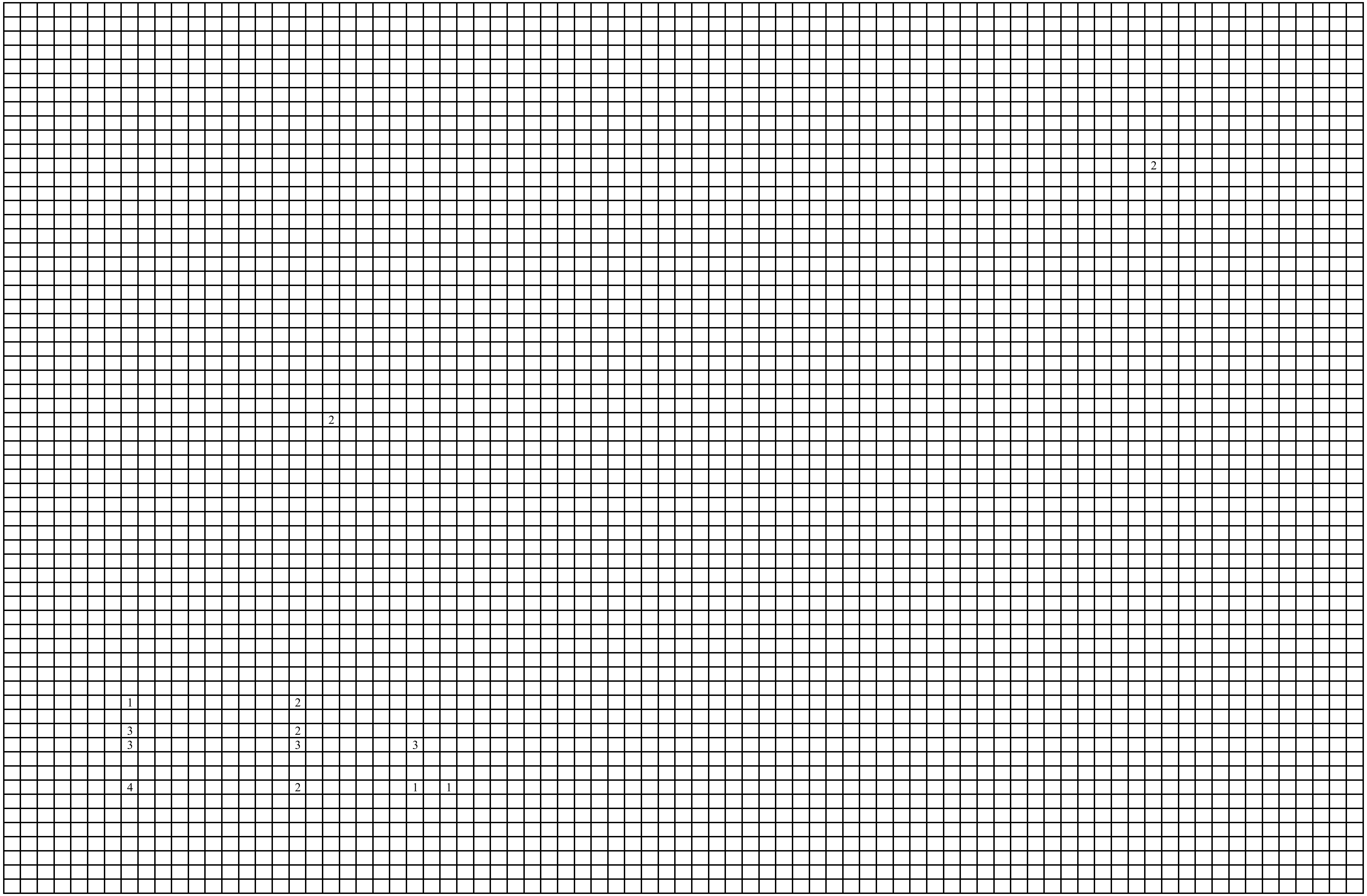
2
2
1 2
3 2
3 3 3
4 2 1 1

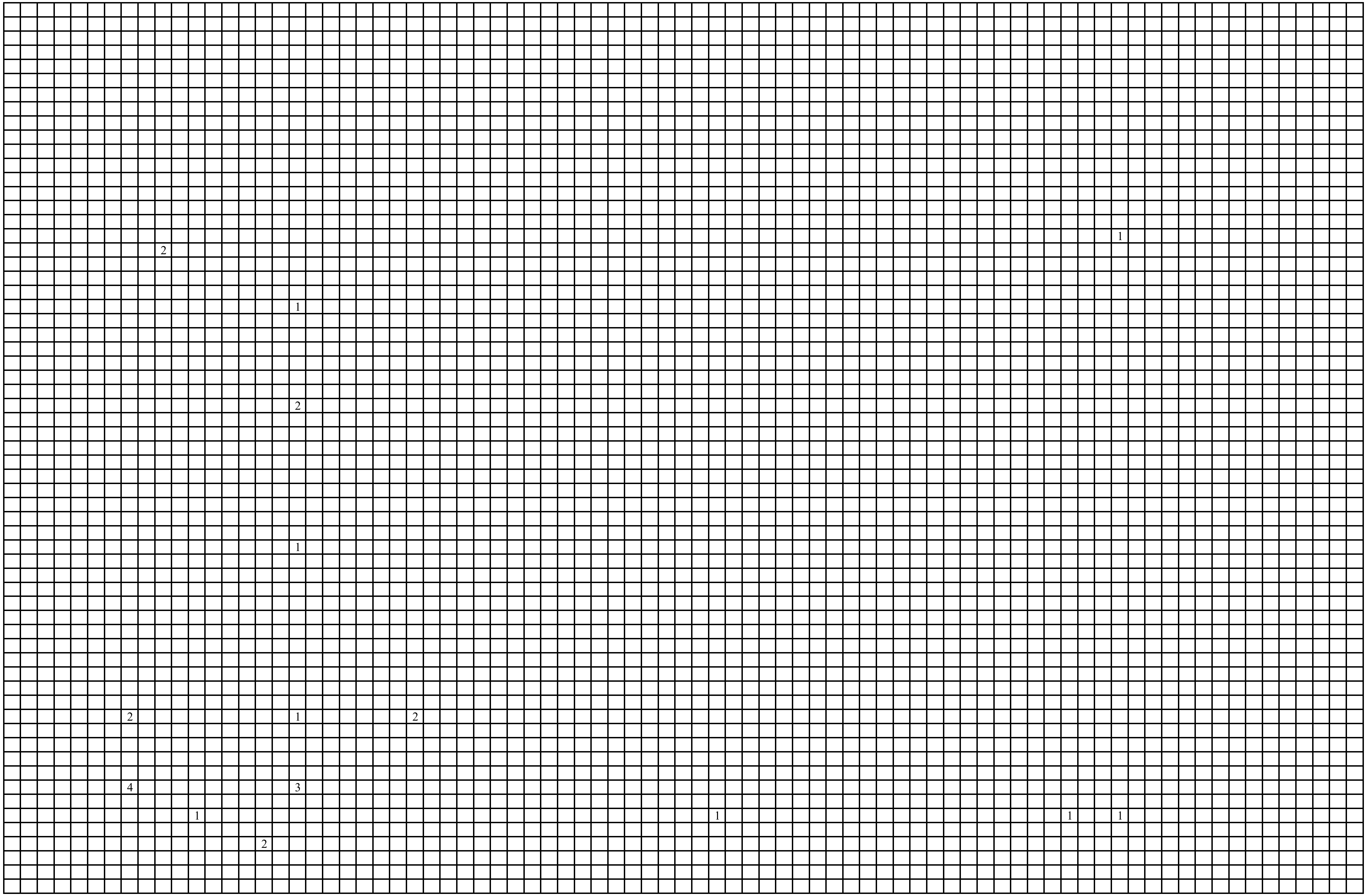
1
2
1
2
1
2 1 2
4 3
1 1 1 1
2

							2																																																																								
											2																																																																		
											1																																																																		
																																	1																																																
											1																																																																						
											1																											1																																											
											2																																																																						
											1																																																																					
								1																																																																								
																																																															1														
							3									2							3		3																																																							
											1																																																																							

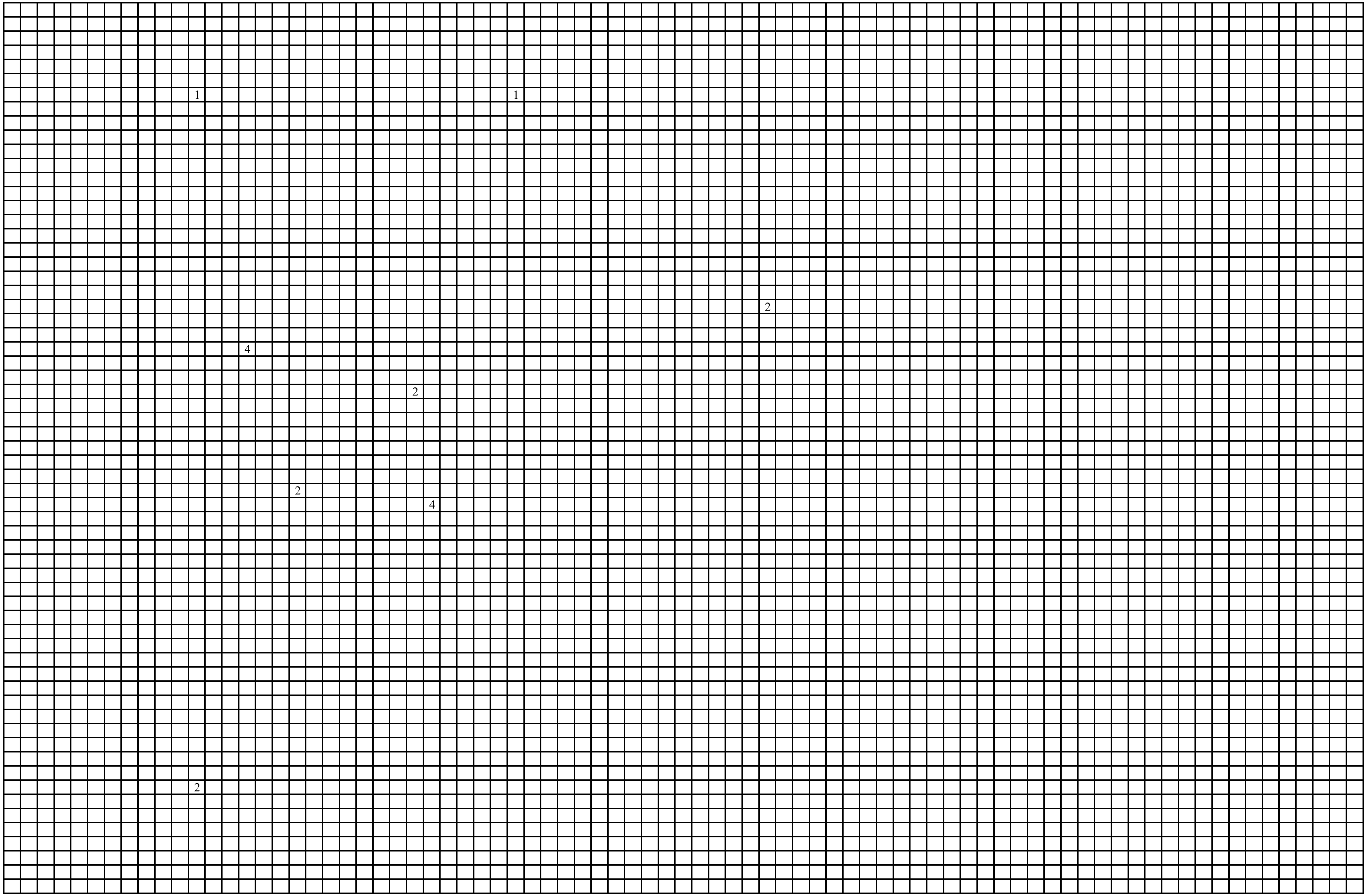
1
1
2
4
2
2
4
2

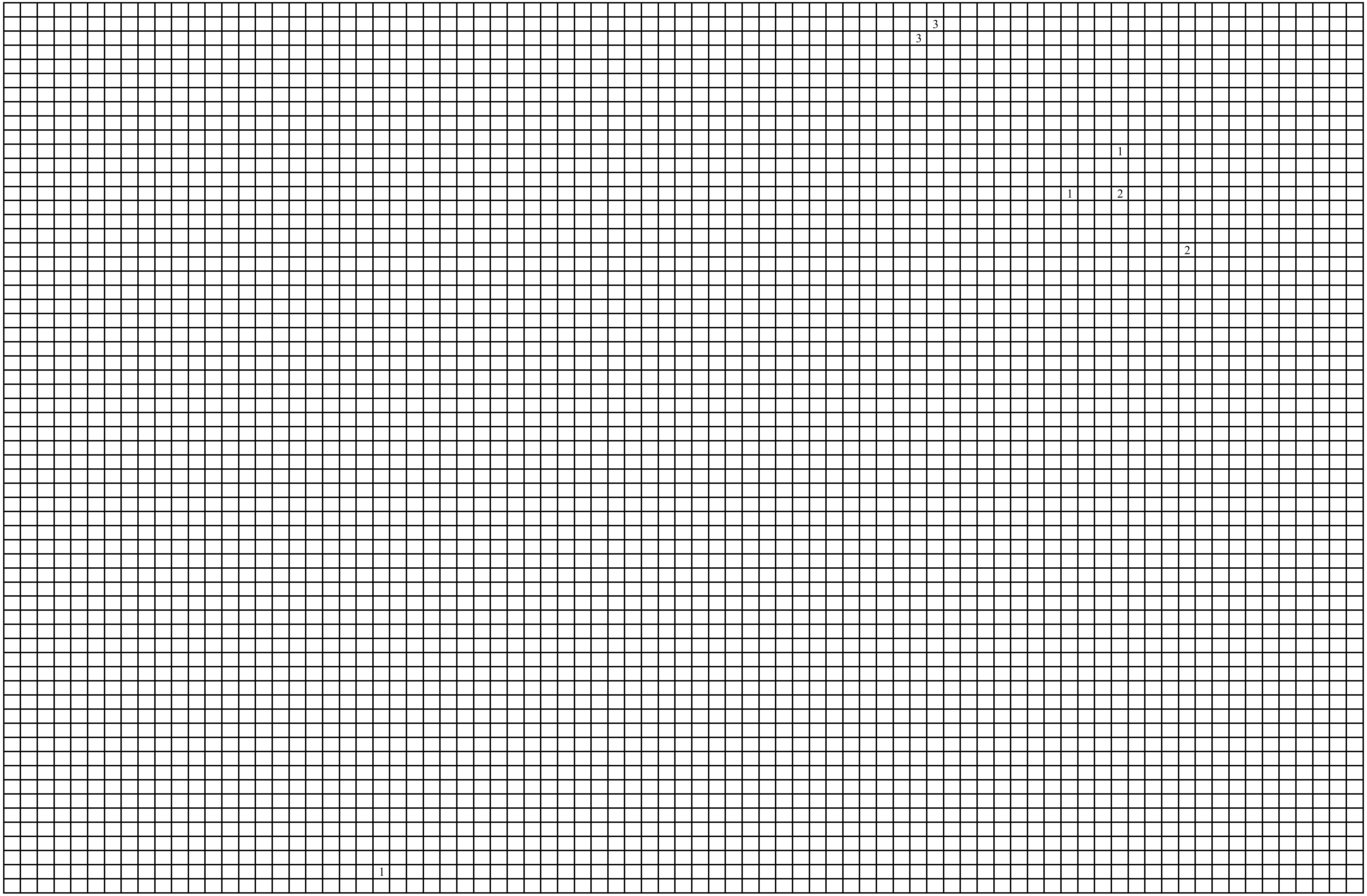
3
3
1
1
2
2
1

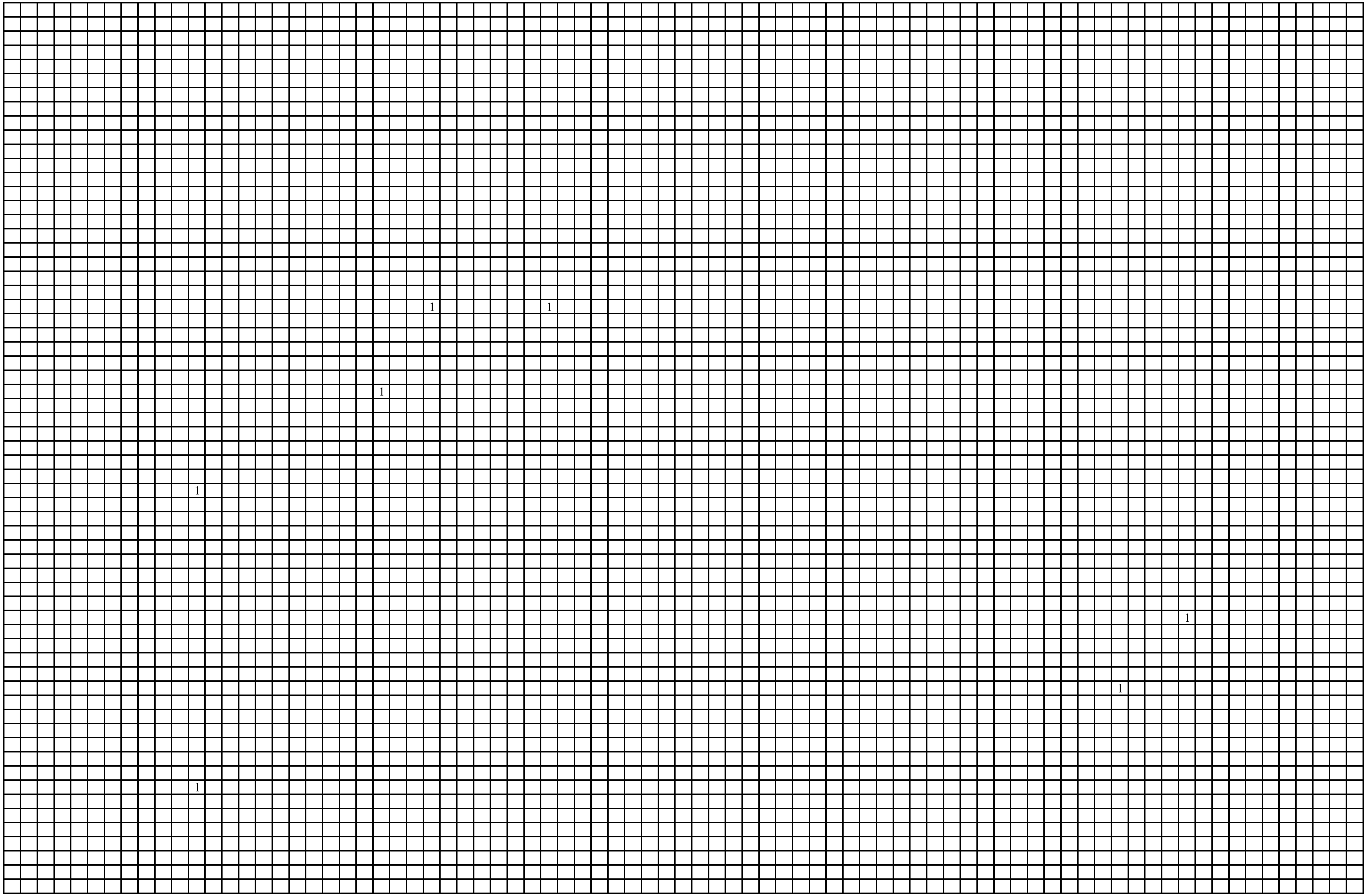
1
1
1
1
1
1
1

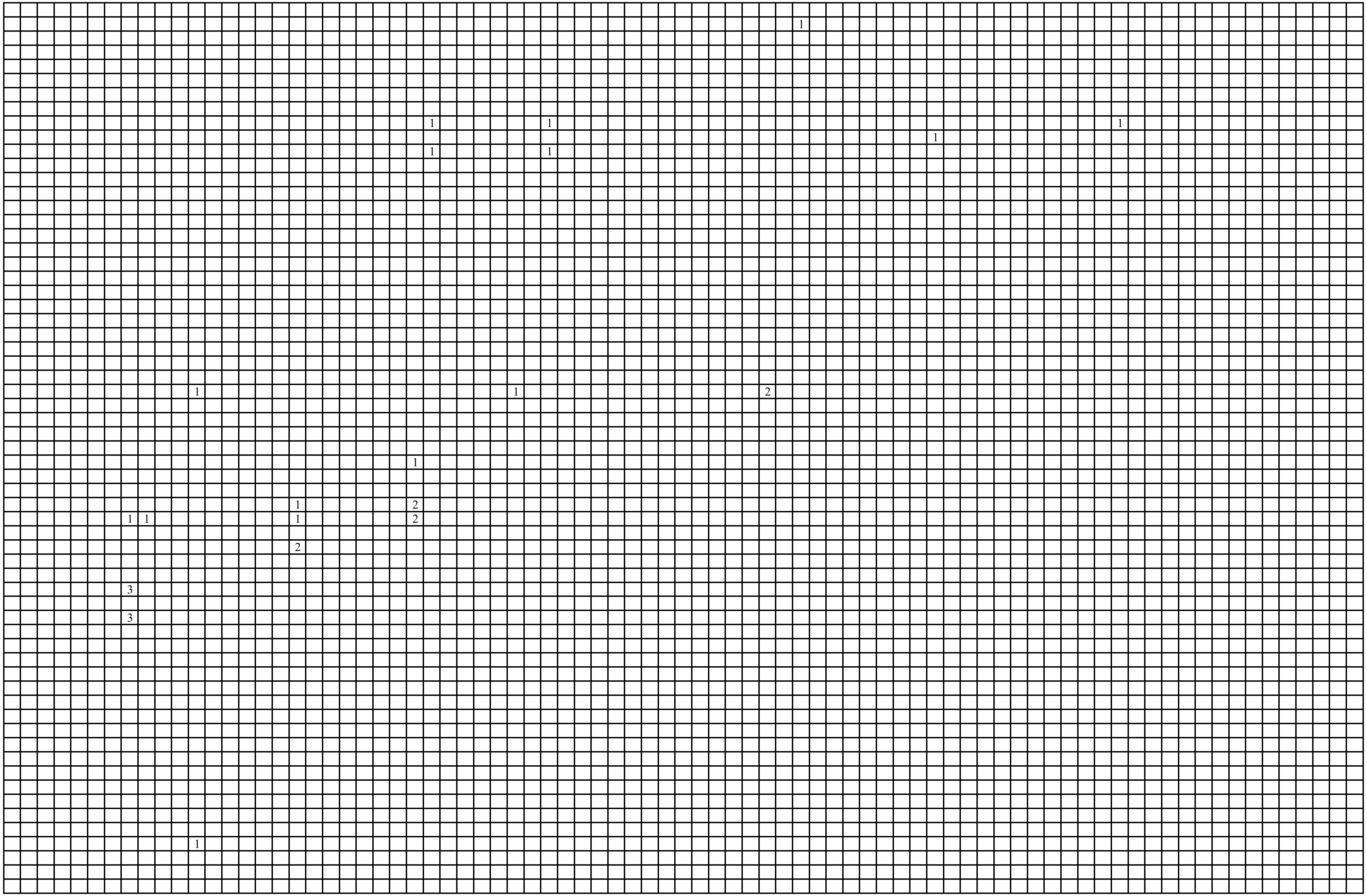
1
1 1 1
1
1 1
1 1 2
1
1 2
1 1 1 2
2
3
3
1

																																																																			1										
										1																																																																			
																			2																																																												
										1																																													1														
										1																																																																			
		1								1						1																																																													
										1																																																																			
										1																																																																			
										1																																																																				

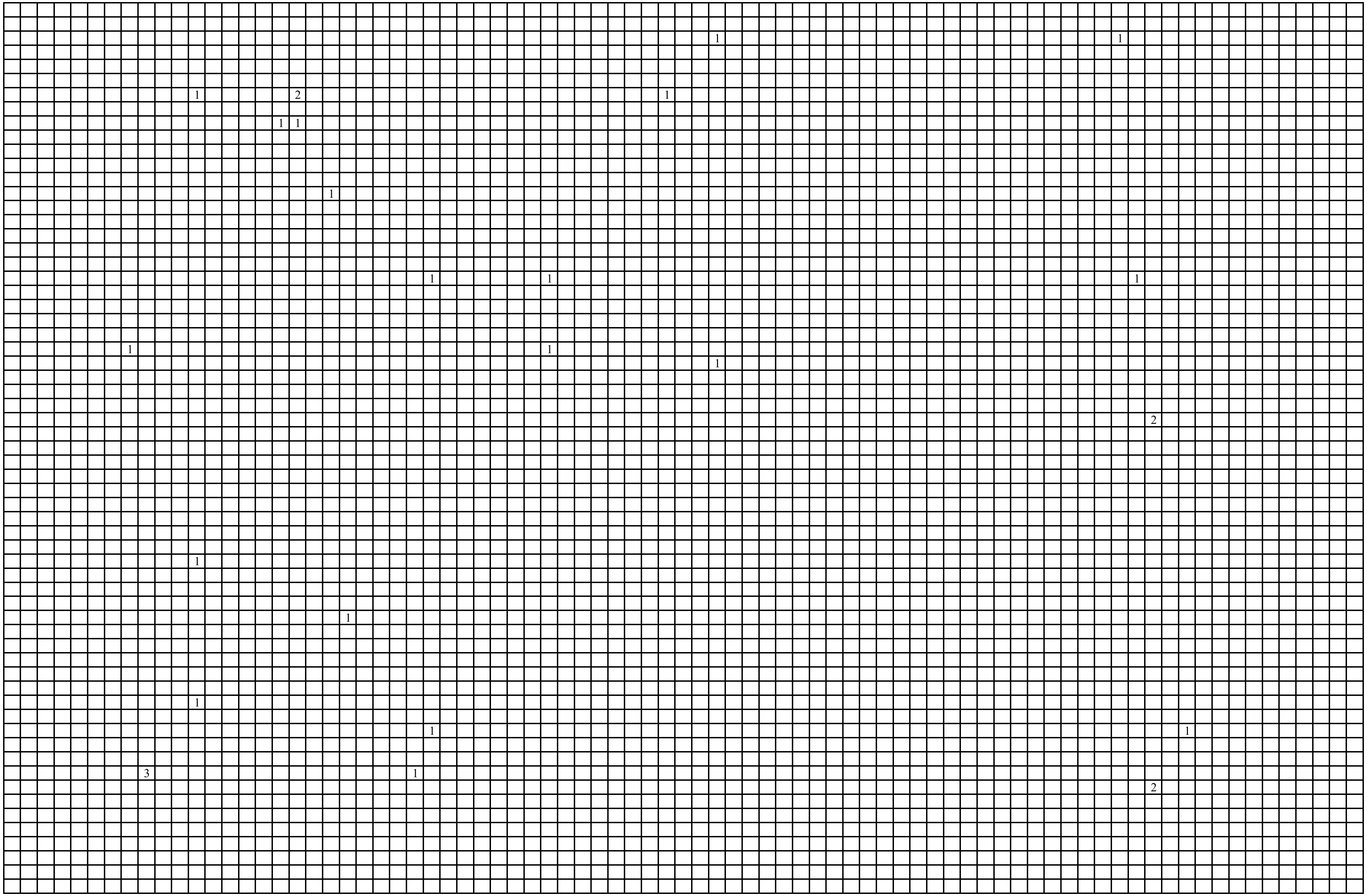
1
1
1
2
1
1
1
1
1
1
1
1
1
2
1
1
1
1
1
3
1
2

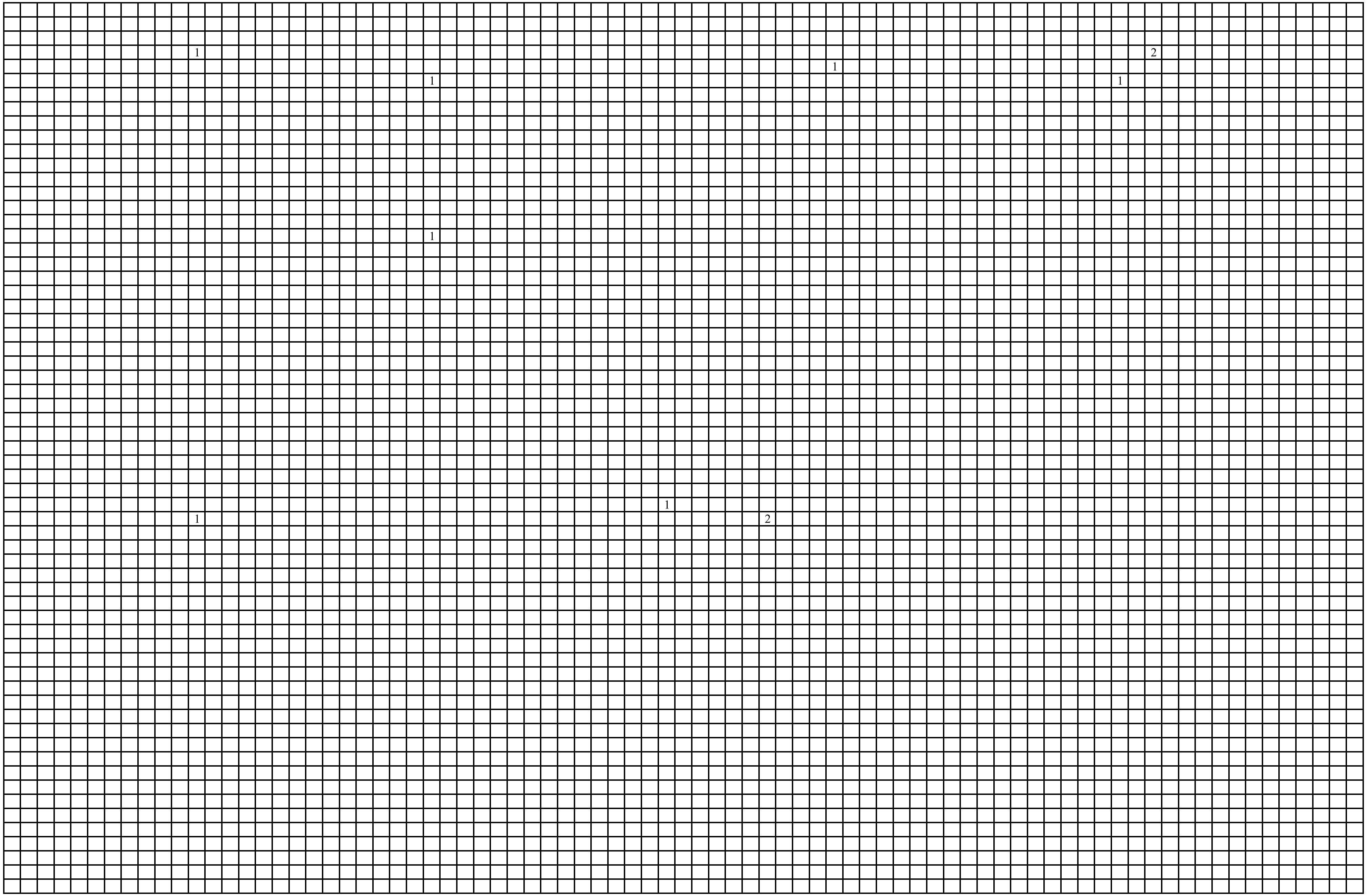
1
2
1
1
1
1
1
1
2

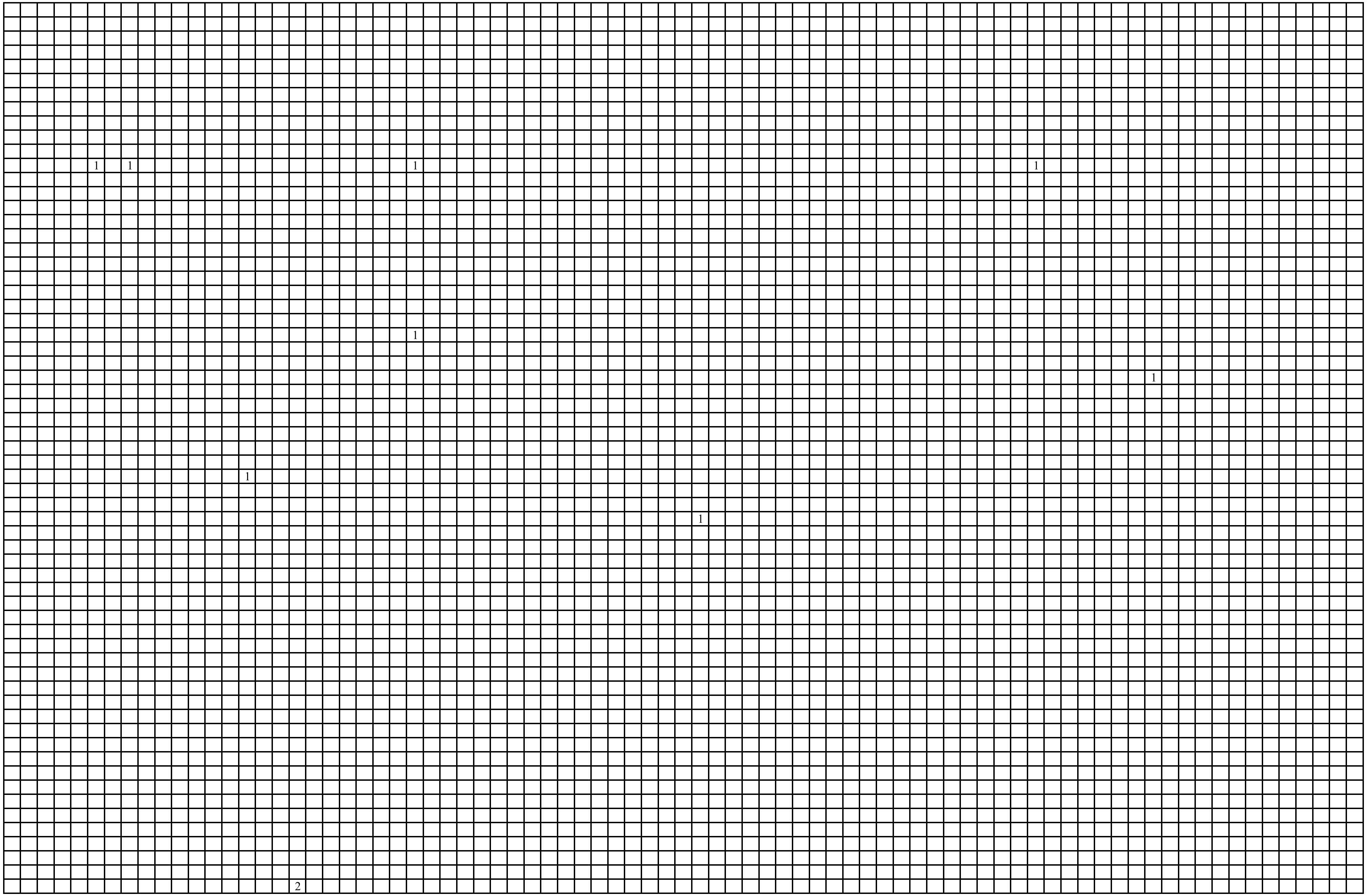
1
1
1
1
1
1
1
1
1
2

																										ZNF599	0	10
																										ZNF600	0	3
																										ZNF605	0	2
			1																							ZNF607	2	12
																										ZNF610	0	9
																										ZNF611	1	13
																										ZNF613	0	6
																										ZNF614	1	8
																										ZNF615	0	5
																										ZNF616	3	19
																										ZNF619	1	2
																										ZNF620	7	85
																										ZNF621	0	17
																										ZNF623	1	10
																										ZNF624	8	50
																										ZNF625	0	14
																										ZNF626	1	3
																										ZNF627	0	28
																										ZNF641	0	21
																										ZNF648	17	64
																										ZNF649	0	13
																										ZNF652	3	6
																										ZNF653	11	53
																										ZNF655	13	190
																										ZNF66	6	8
																										ZNF660	2	7
																										ZNF662	0	10
																										ZNF664	2	30
																										ZNF665	1	2
																										ZNF667	0	8
																										ZNF669	0	77
																										ZNF670	0	31
																										ZNF672	3	11
																										ZNF675	1	13
																										ZNF676	1	1
																										ZNF677	3	26
																										ZNF678	0	3
																										ZNF679	1	7
																										ZNF680	1	4
																										ZNF681	0	1
																										ZNF682	0	3
																										ZNF683	1	9
																										ZNF684	0	20
																										ZNF689	20	99
																										ZNF691	4	36
																										ZNF692	6	64
																										ZNF695	2	33
																										ZNF696	3	81
																										ZNF7	3	29
																										ZNF70	9	46
																										ZNF701	0	14
																										ZNF705D	0	1
																										ZNF705E	0	0
																										ZNF705G	0	1
																										ZNF707	29	147
																										ZNF709	3	11
																										ZNF71	32	108
																										ZNF710	0	4
																										ZNF713	0	5
																										ZNF716	1	3
																										ZNF718	1	14
																										ZNF721	2	10
																										ZNF726	3	11
																										ZNF727	0	3
																										ZNF729	0	1
																										ZNF730	0	1
																										ZNF732	1	4
																										ZNF736	0	2
																										ZNF737	0	2
																										ZNF74	3	38
			1																							ZNF746	37	346
																										ZNF747	6	54
																										ZNF749	0	5
																										ZNF75A	2	9
																										ZNF75D	0	1
																										ZNF76	21	112
																										ZNF763	1	5
																										ZNF764	9	124
																		1								ZNF765	5	23
																										ZNF766	0	11
																										ZNF768	20	135

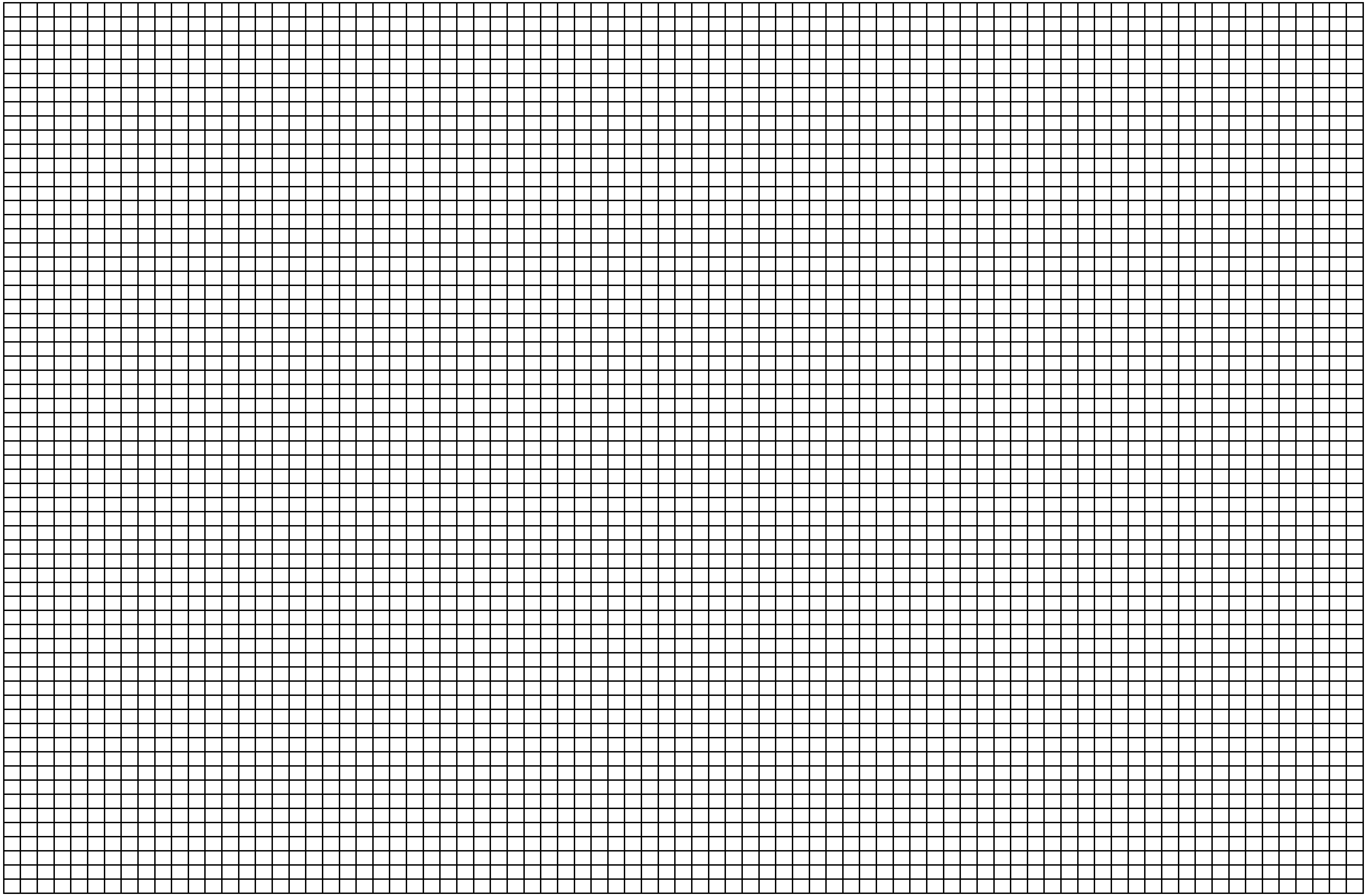

																																									2																																			
											1																																																																	
																						1																																																							
																							1																																																2										
																								1		1																																																							1
																1																																																												

																				1		1														1																																														
													1																																																														1
																																																																	1										

2

			1																																																																													
																																																																						1											
																																																																											1					
											1																																																																					
																						1																																																										
																																																						1																										

																							1																																																							
																			1																											1																																			
																																																																												1					
																																																																												1					
																																																																												1					
																																																						1																									

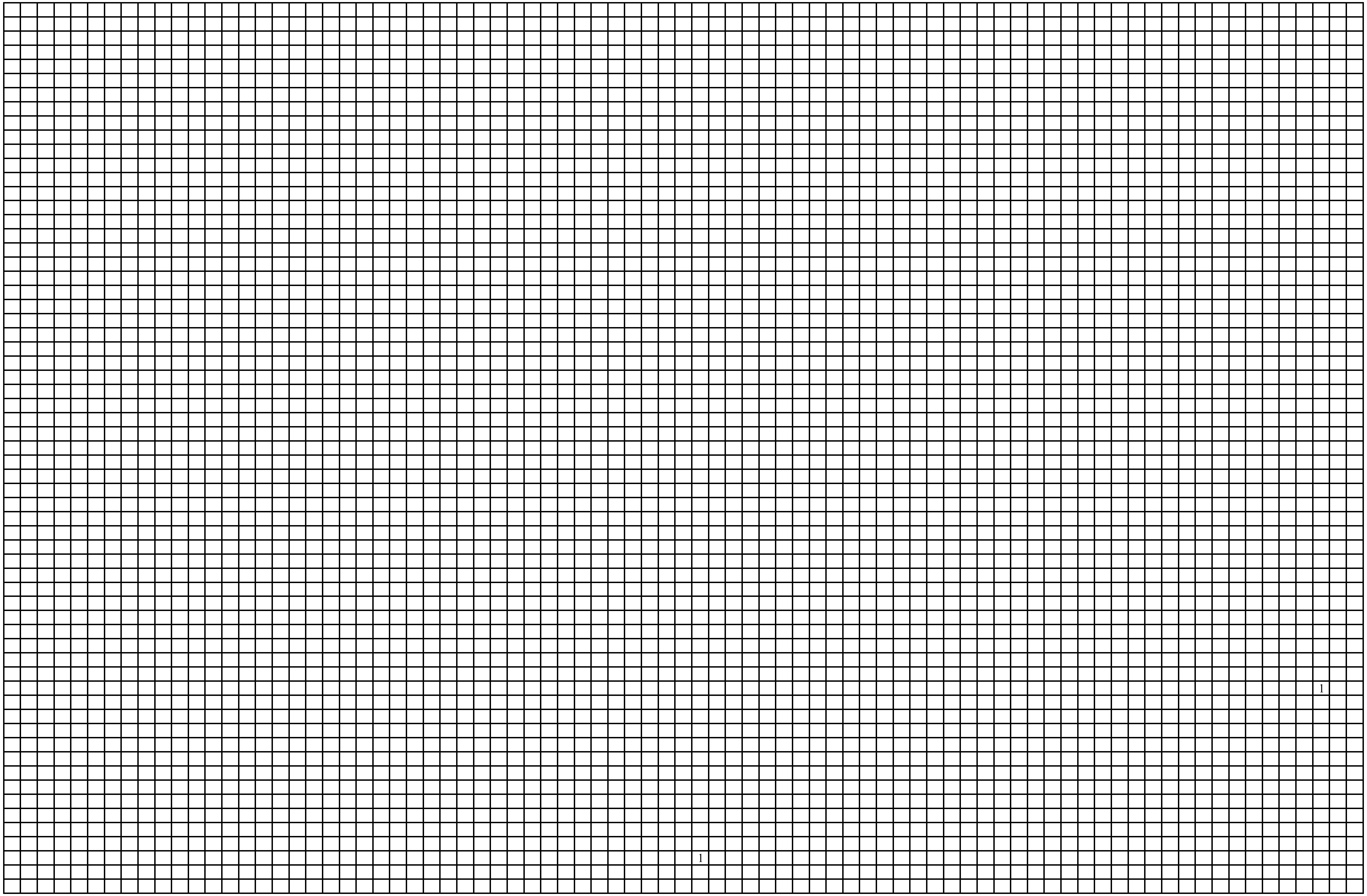
1
1

																							1																																																								
																						1																																																										
																																											1																																	
																																																			1																								

																																															1																																
																																																																						1									
																							1																																																												
							1																																																											1										
																																																																						1										1
																																																																											1					

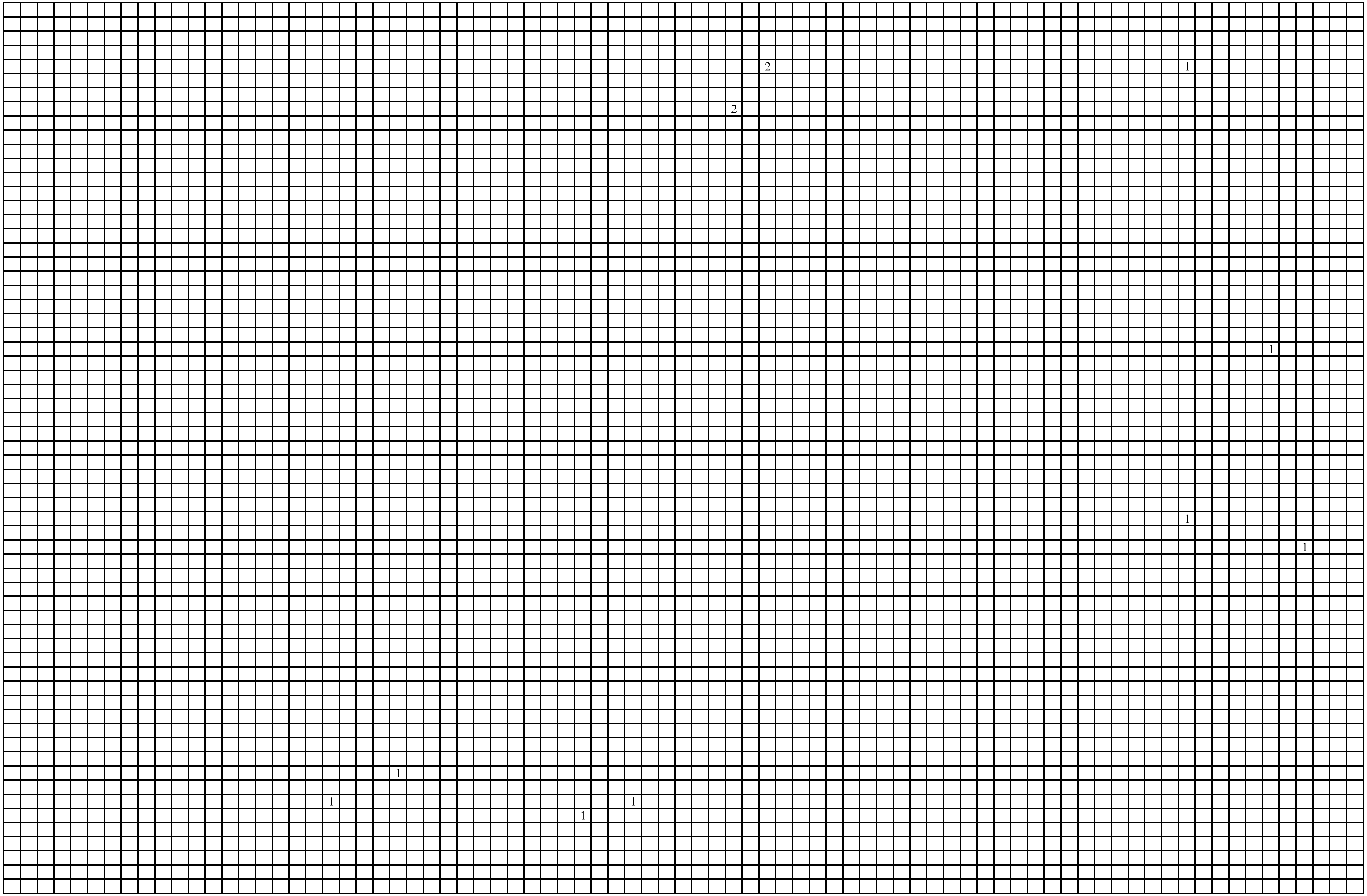
2
1
2
1
1
1
1
1
1
1

																																																								1																								
									1																																																									1														
																																																																											1			1		
																																																																																1
																																																																											1					
																																													2																																			

																								1																																																												
										1																																																																										
																																																																						1										
																																																																						1										
																																																1																											1										
																																																																						1										
																								1																															1																									

																						1																																																										
																																																																	1										
																						1																																																										
																																										1																																	
																							1																																																									
																																																																	1										
																																																																							1					1
																																																																															1
					1																			1																																													1																			1
																								1																																																									

																																											1																																				
																																														1																																		
																																																						1																								
																			1																																			3															1											
																																																																											1					

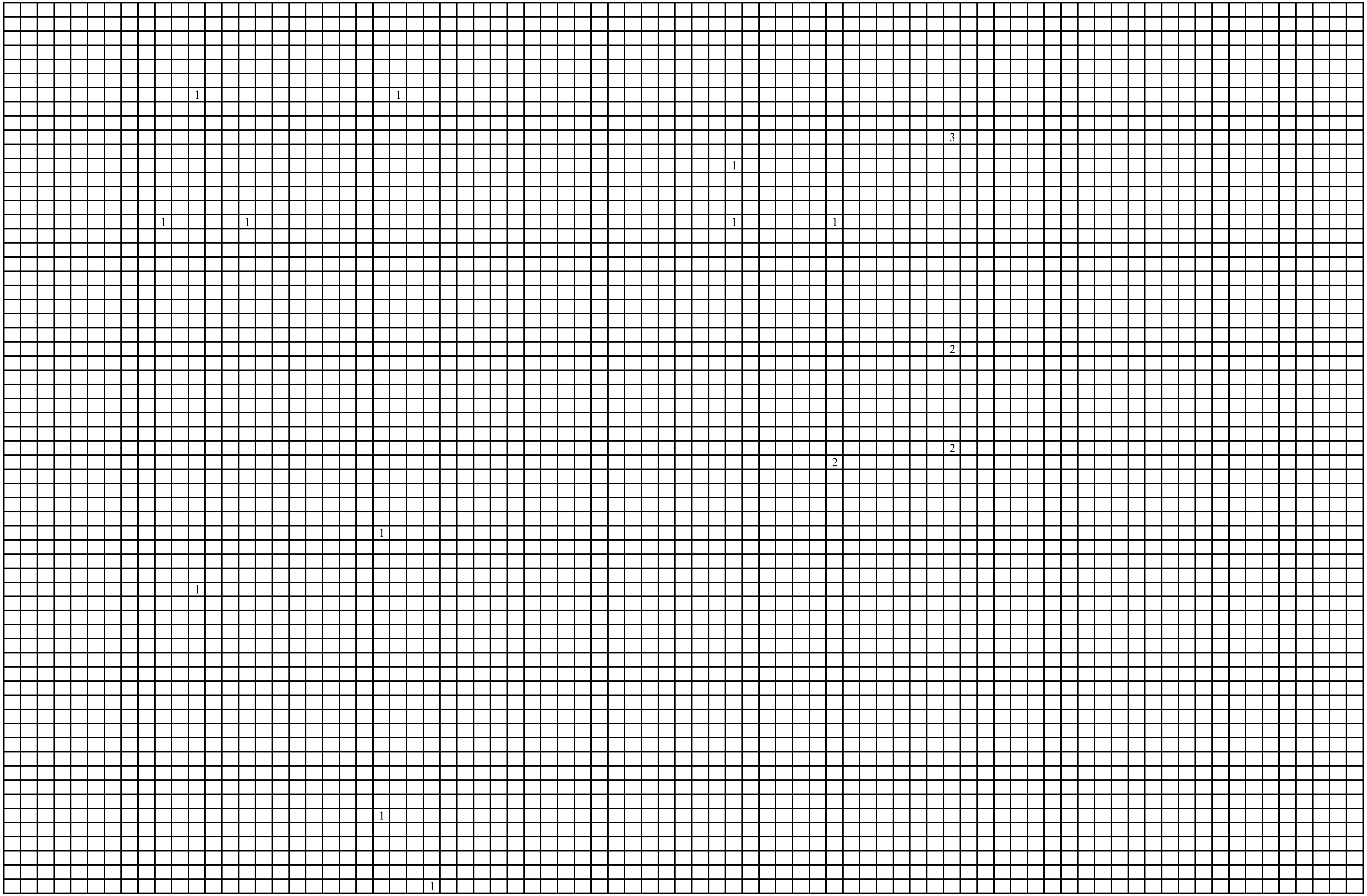
1
1
3
1
1
1
1
1
2
2
2
1
1
1
1

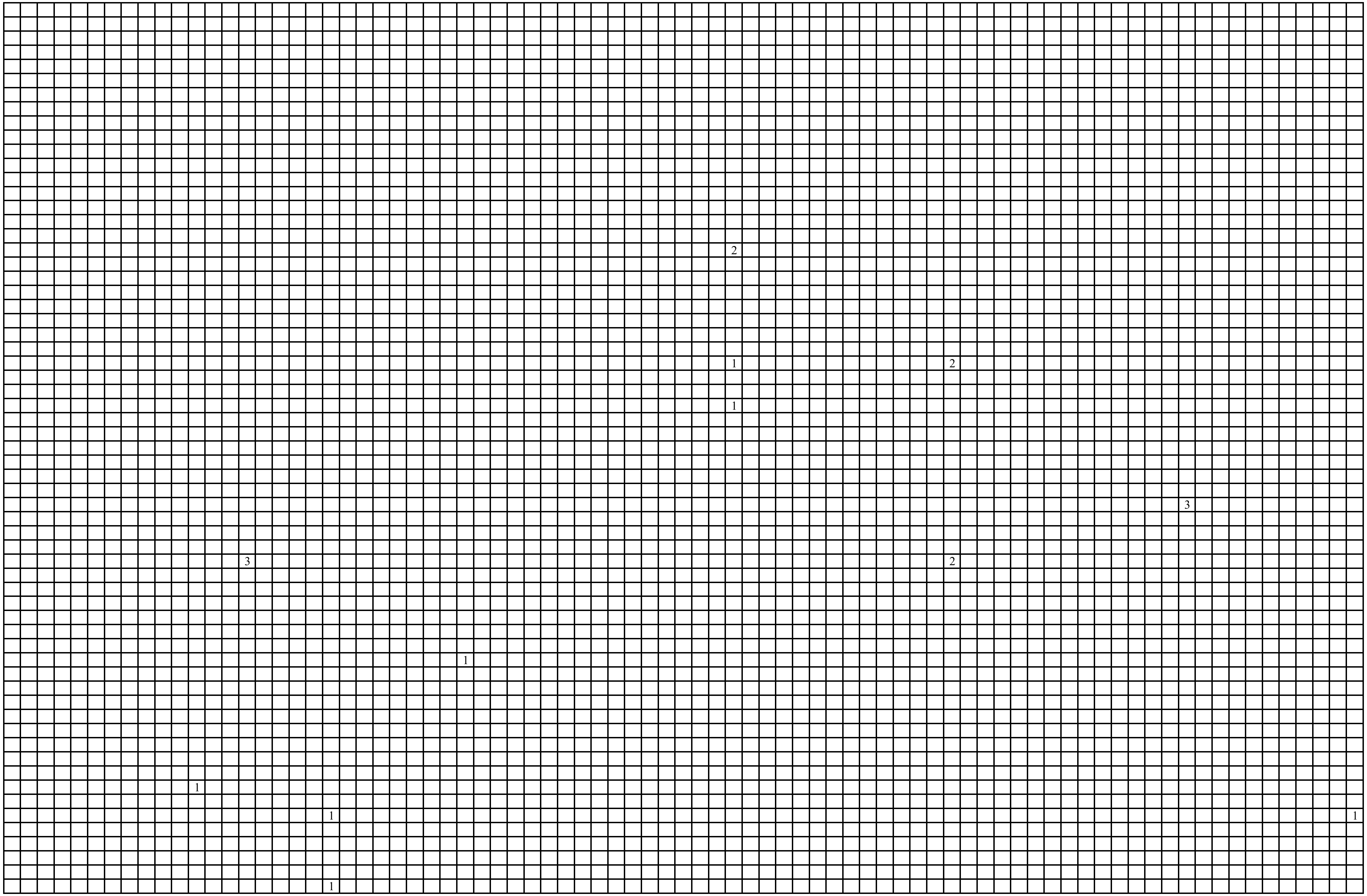
2
1
2
1
3
3
2
1
1
1
1
1

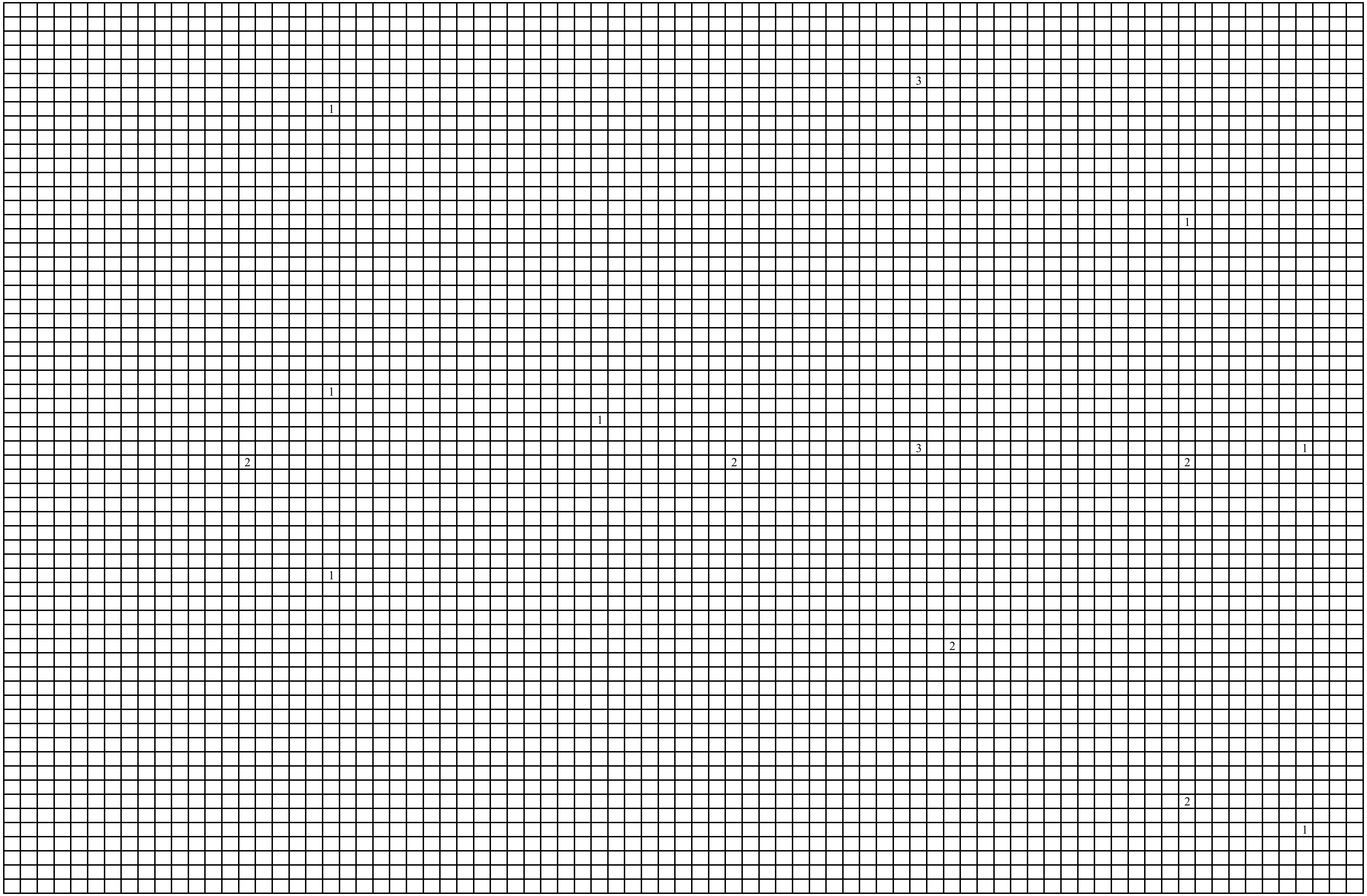
3
1
1
1
1
3
1
2
2
2
1
2
2
1

																																																																							1					
																																				1																											1																		
																																																																						1											
																																		1																													1																		
																																																						1																								
																																																						1																								

																																																																										1		
																		1																																																																
																																																																										1					
										1												1																																					1																							1
																		1																																																															

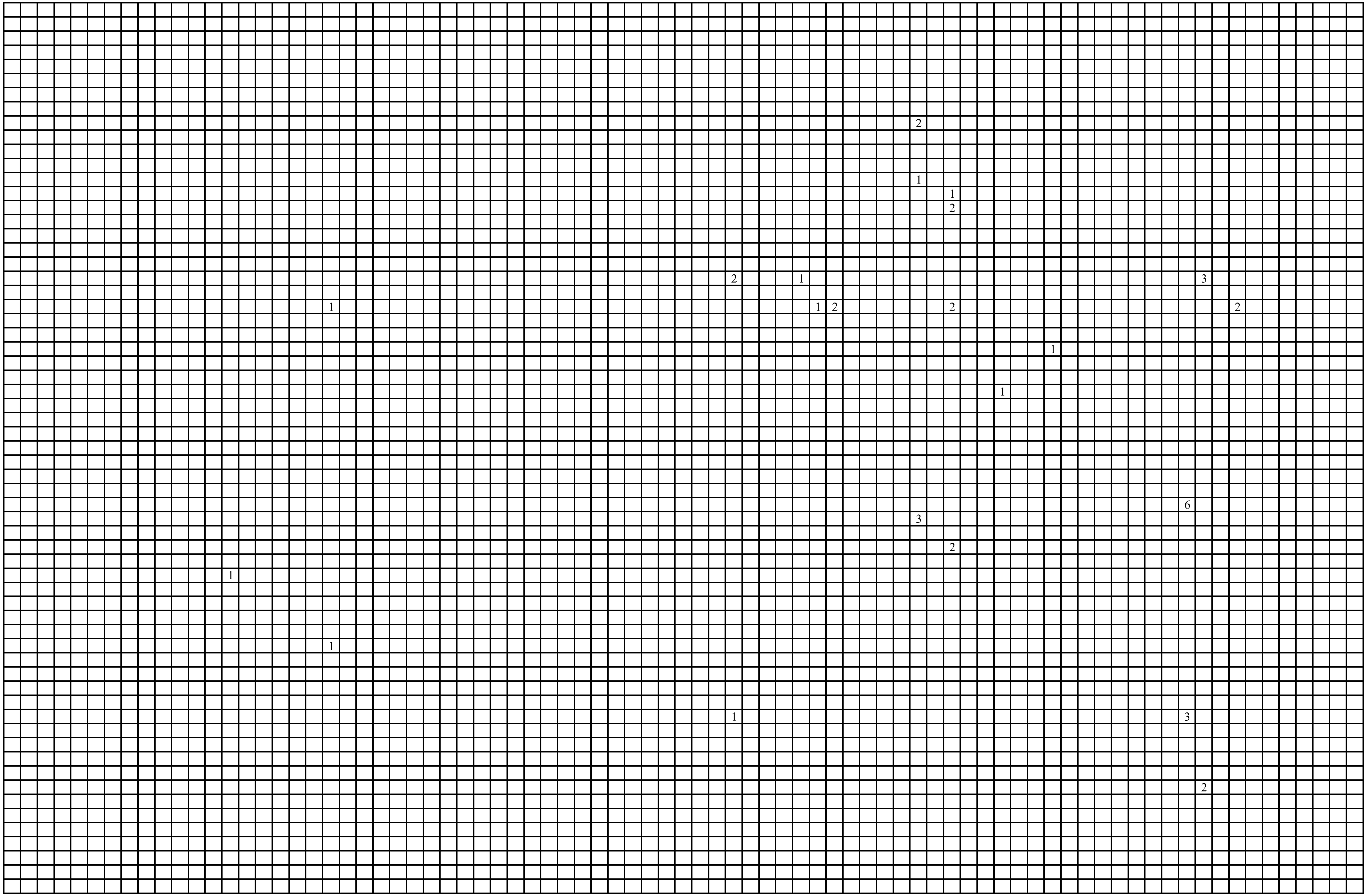
2
1
1
2
2
1
3
1
1
2
2
2
1
1
6
3
2
1
1
1
3
2

																						1																																																										1			
																																																						1																							1
																			1				1																																																									1			
																																																					3																							1			
									1																																																																								
																																																	2																															

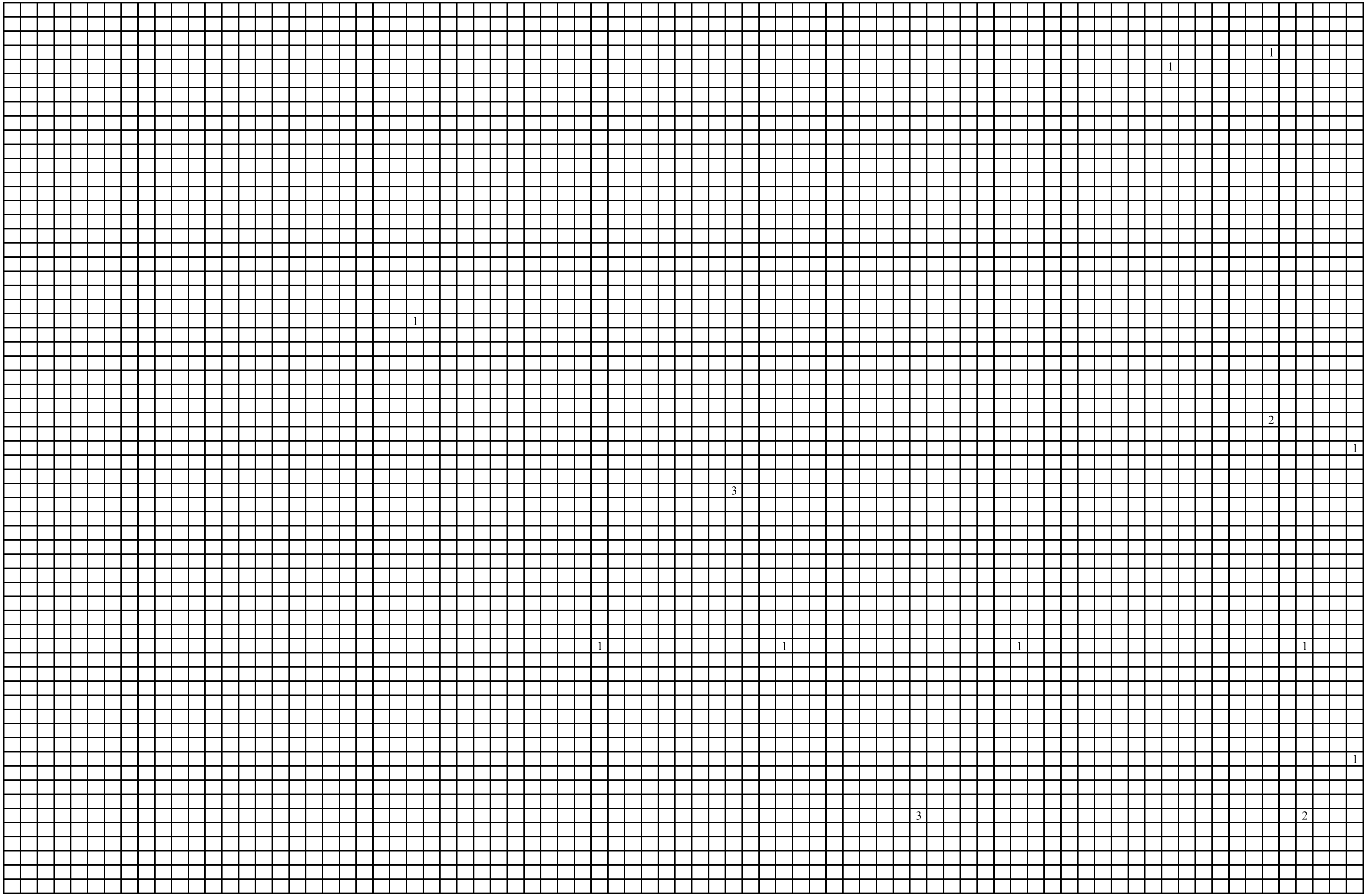
1
1
1
2
1
3
1
1
1
1
1
3
2

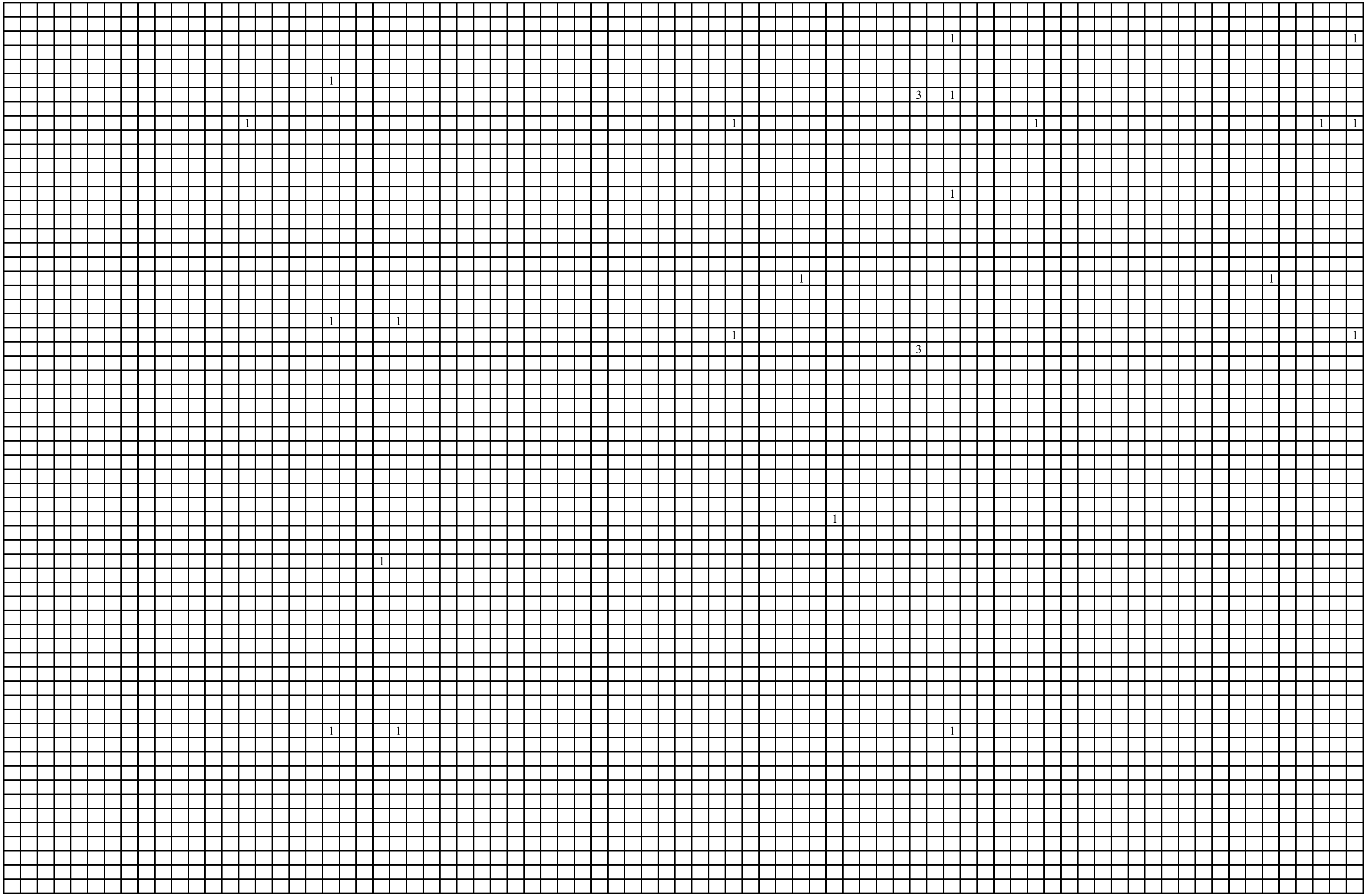
1
1
1
3
1
1
1
1
1
1
1
1
1
1
1
1
1
1
3
1
1
1
1
1

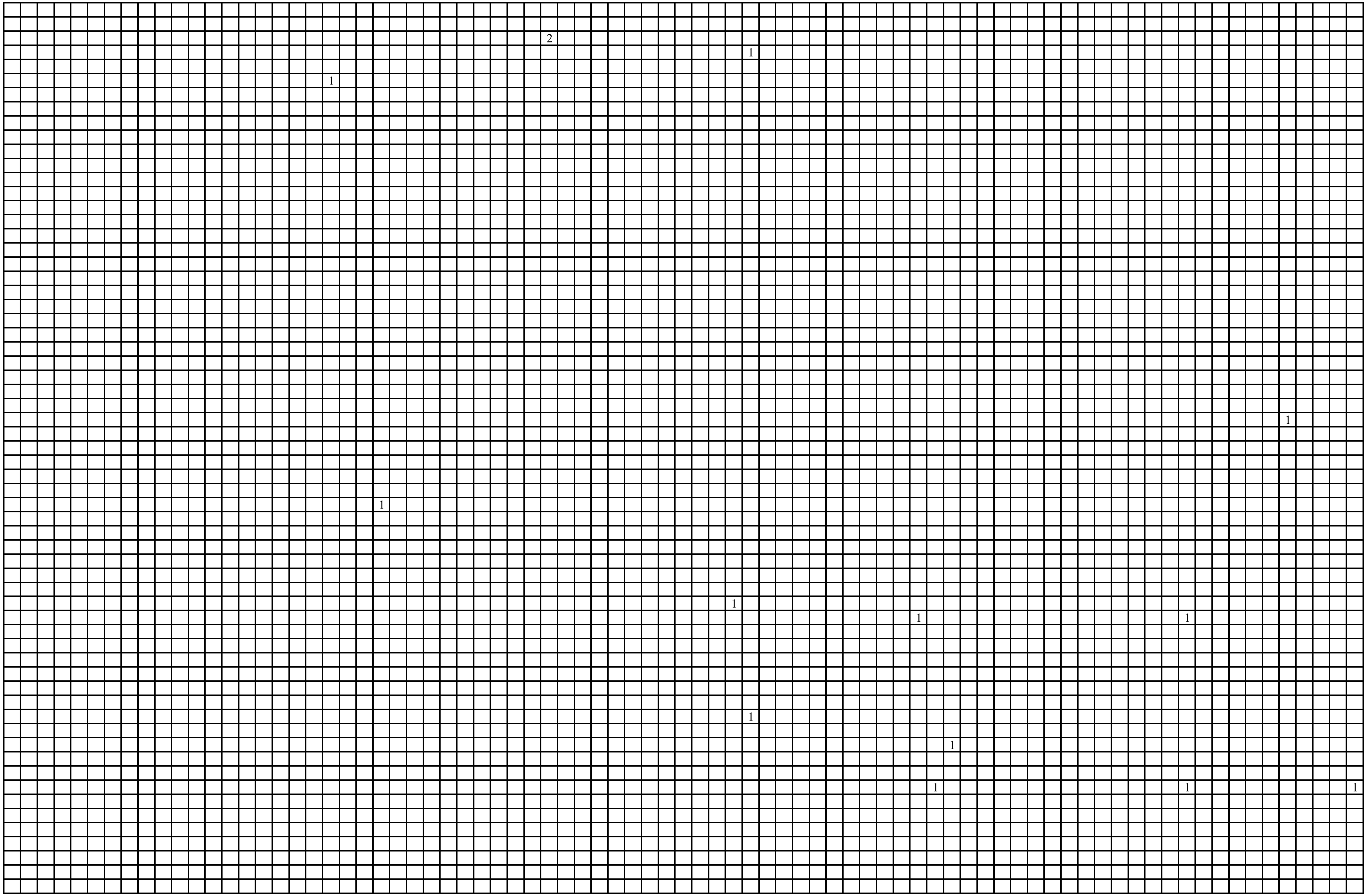

2
1
1
1
1
1
1
1
1
1
1
1
1
1

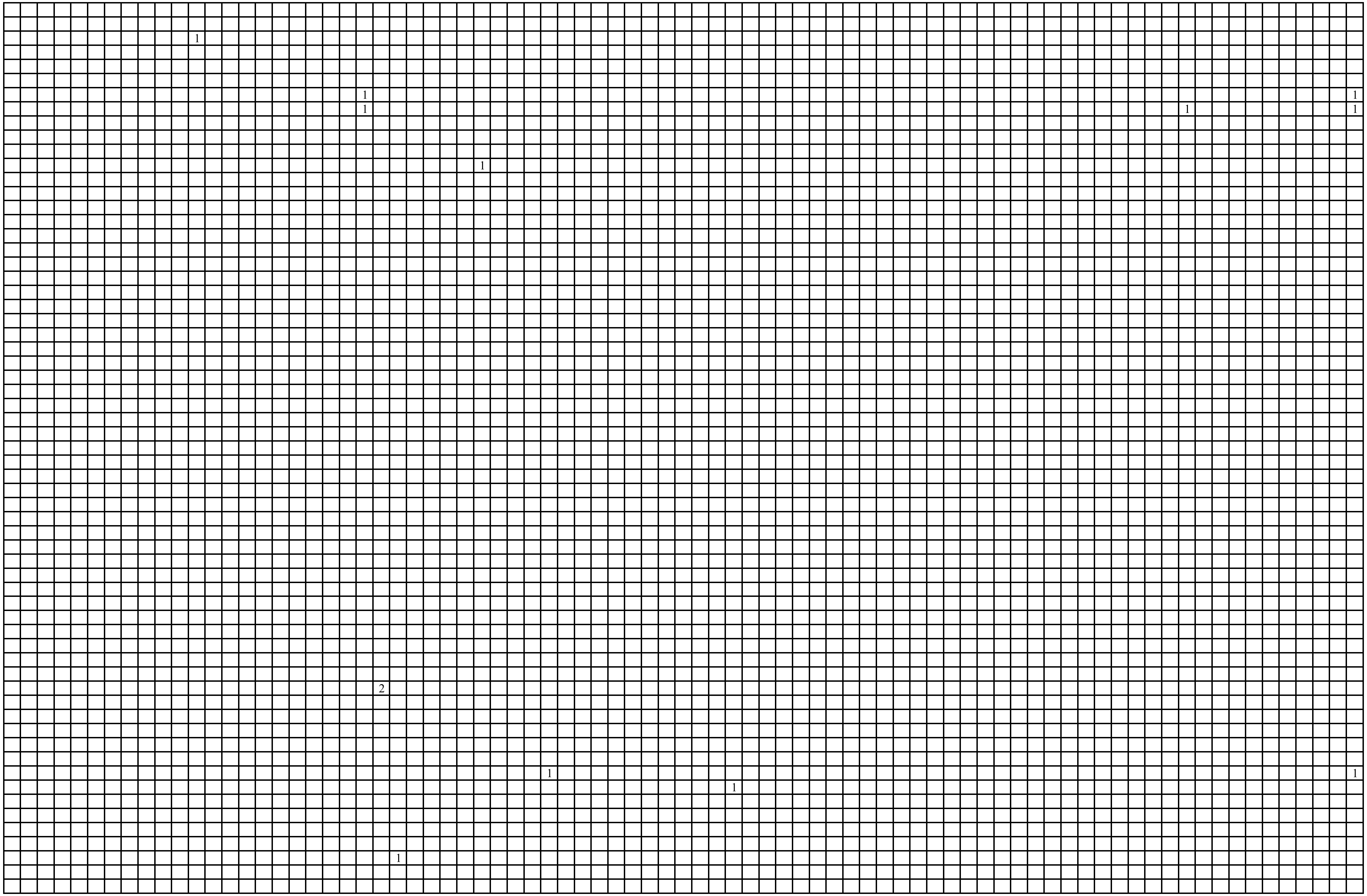
1
1
1
1
1
1
1
2
1
1
1
1

																																																																								3					
																																																																	1										
																																																																																	1

																										ZNF77	0	25
																										ZNF771	13	72
																										ZNF774	12	83
																										ZNF776	0	16
																										ZNF777	21	114
																										ZNF780A	2	26
																										ZNF780B	0	1
																										ZNF781	0	1
																										ZNF782	0	1
																										ZNF785	11	152
																										ZNF789	0	4
																										ZNF79	1	12
																										ZNF790	1	4
																										ZNF791	3	22
																										ZNF793	1	6
																										ZNF799	0	2
																										ZNF80	0	10
																										ZNF805	2	9
																										ZNF808	4	9
																										ZNF81	0	7
																										ZNF813	8	17
																										ZNF816	2	26
																										ZNF823	1	5
																										ZNF829	2	27
																										ZNF83	3	12
					1																					ZNF835	17	68
																										ZNF836	1	5
																										ZNF837	29	80
																										ZNF84	0	14
																										ZNF841	0	4
																										ZNF844	0	15
																										ZNF845	7	35
																										ZNF846	0	4
																										ZNF85	2	8
																										ZNF852	0	13
																										ZNF853	0	0
																										ZNF860	0	4
																										ZNF875	0	0
																										ZNF878	0	3
																										ZNF879	0	4
																										ZNF880	0	1
																										ZNF891	0	4
																										ZNF90	0	2
																										ZNF91	2	15
																										ZNF92	23	70
																										ZNF93	0	9
																										ZNF98	0	1
																										ZNF99	0	3
																										ZSCAN10	0	3
																										ZSCAN12	12	86
																										ZSCAN16	9	18
																										ZSCAN2	0	8
																										ZSCAN21	56	132
																										ZSCAN22	31	45
																										ZSCAN23	31	49
																										ZSCAN25	26	90
																										ZSCAN26	3	65
																										ZSCAN29	13	37
																										ZSCAN30	19	24
																										ZSCAN31	4	23
																										ZSCAN32	96	131
																										ZSCAN4	5	17
																										ZSCAN5A	12	57
																										ZSCAN5B	2	5
																										ZSCAN9	10	25
																										ZXDA	0	18
																										ZXDB	1	20
																										ZXDC	4	37
																			1							BCL11A	62	164
																	1		1							BCL11B	46	122
																										BNC1	0	6
																										BNC2	5	12
																										CASZ1	9	42
																										E4F1	37	165
																										FIZ1	3	21
																									2	HIC1	28	70
																										HIC2	22	70
																										HINFP	11	55
			1																							HIVEP1	79	212
																										HIVEP2	1	38
																										HIVEP3	2	21

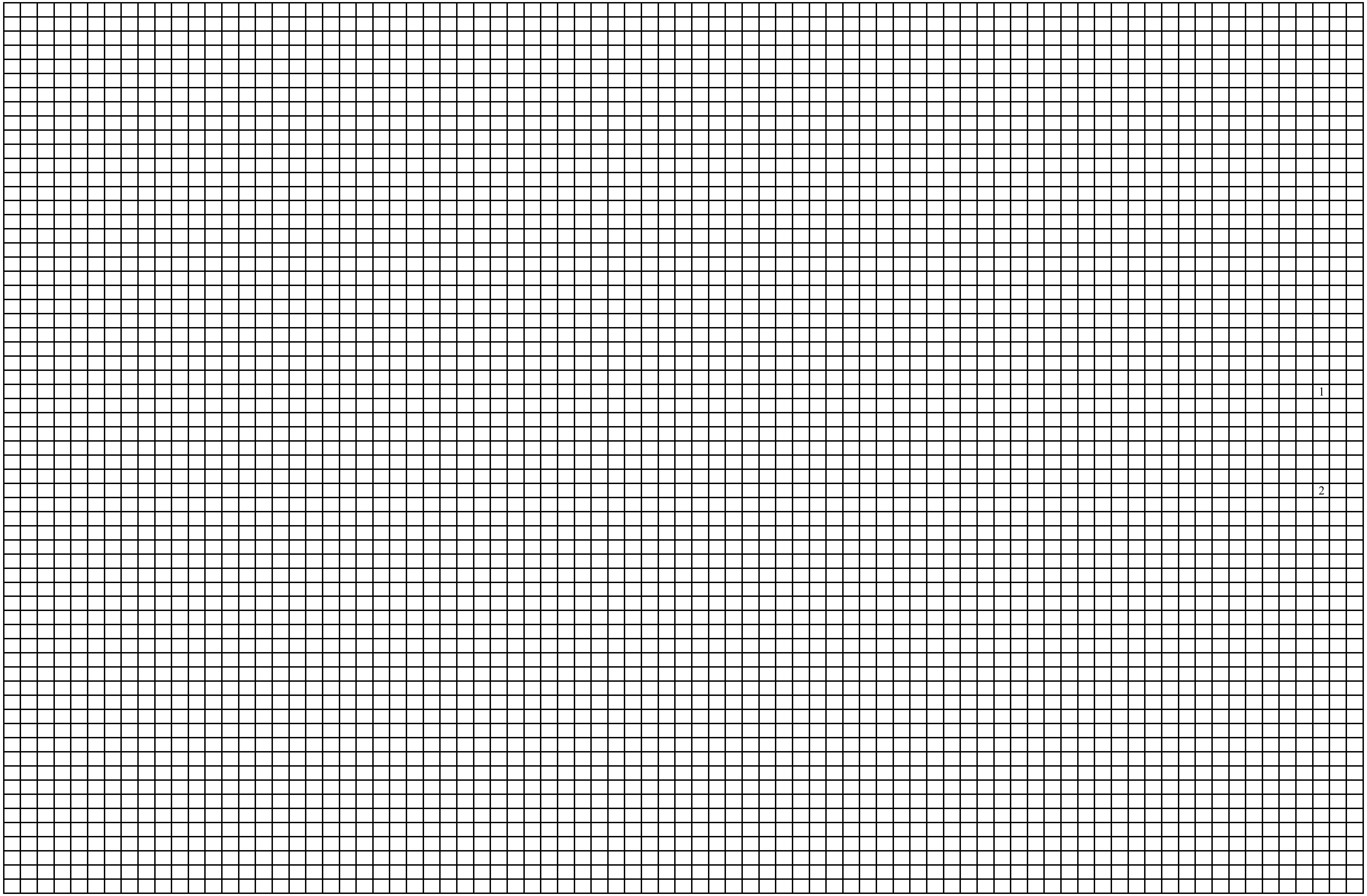
1
2

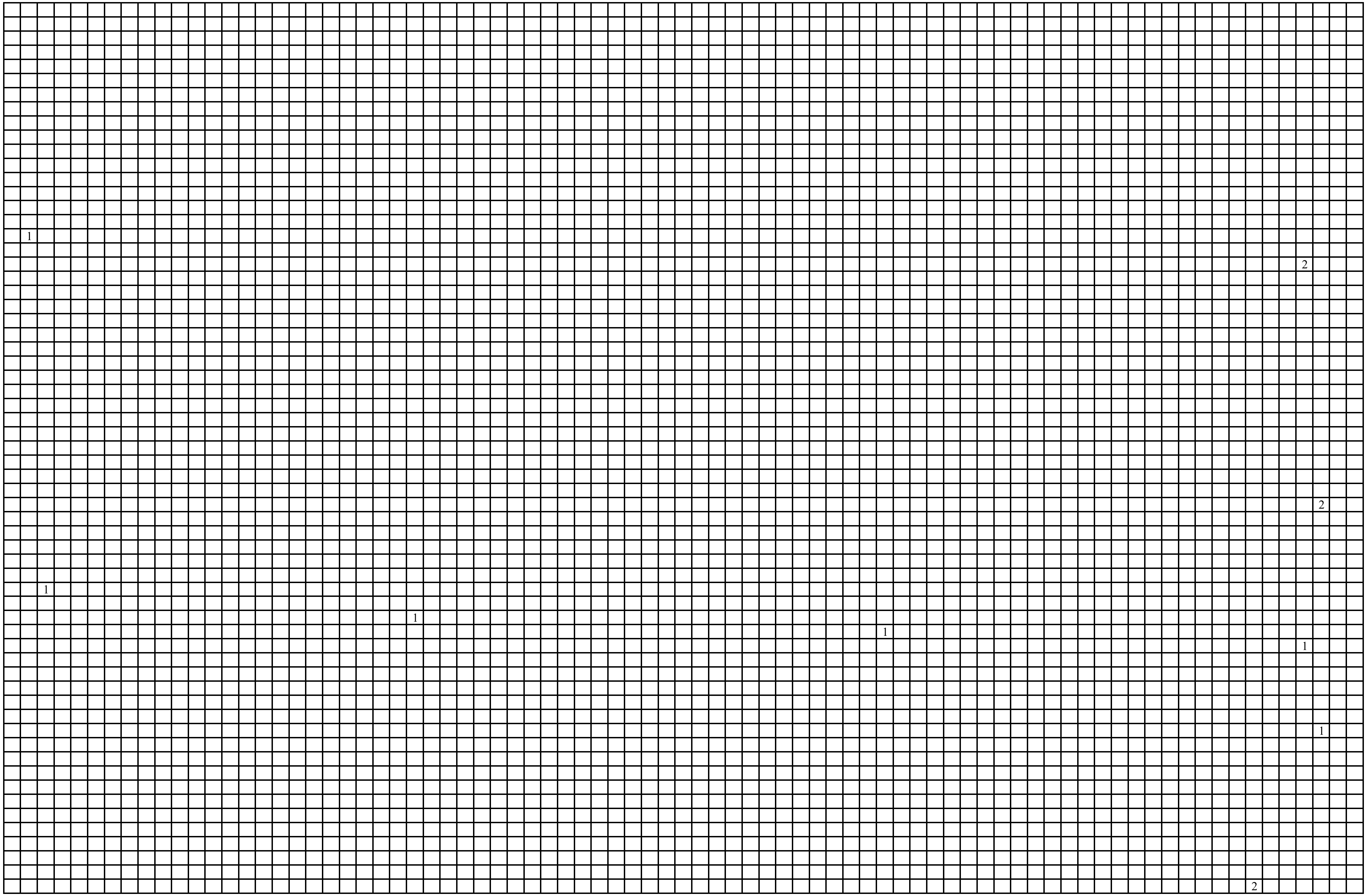
1
2
2
1
1
1
1
1
2

																																											1																																			1		
																															1																					1																1										1		
																																																								1																						
																																																																													2		
																																												1																																						
				1																																																																			2						
				1																																															1																											
																																																																											2						
																																																																											1				
																																																																										1			1				

																																																																											1				
																																																																													1		
																																																																					1					1				
																																																																					1					1				
																																																																					1					1				
																																																																												1				
																																																				1																										1		

																																																																												1		
																																																			1																										1		
																																																																												1		
																																																																												1		
																																																																														1		
																																																										1																	
																																																																							1							
																																																																														1		
		1																								1																																																							
																									1																																																											

		1																																																																															
																																											1																																				
																																																		1																										1		
																																																																															1		
																																																																																1		
																																																																																1		
																																																		1																										1		
																																																																																1		

																																															1																									1		
																																																																														1		
																																																																						1										1		
																																																																																	1		

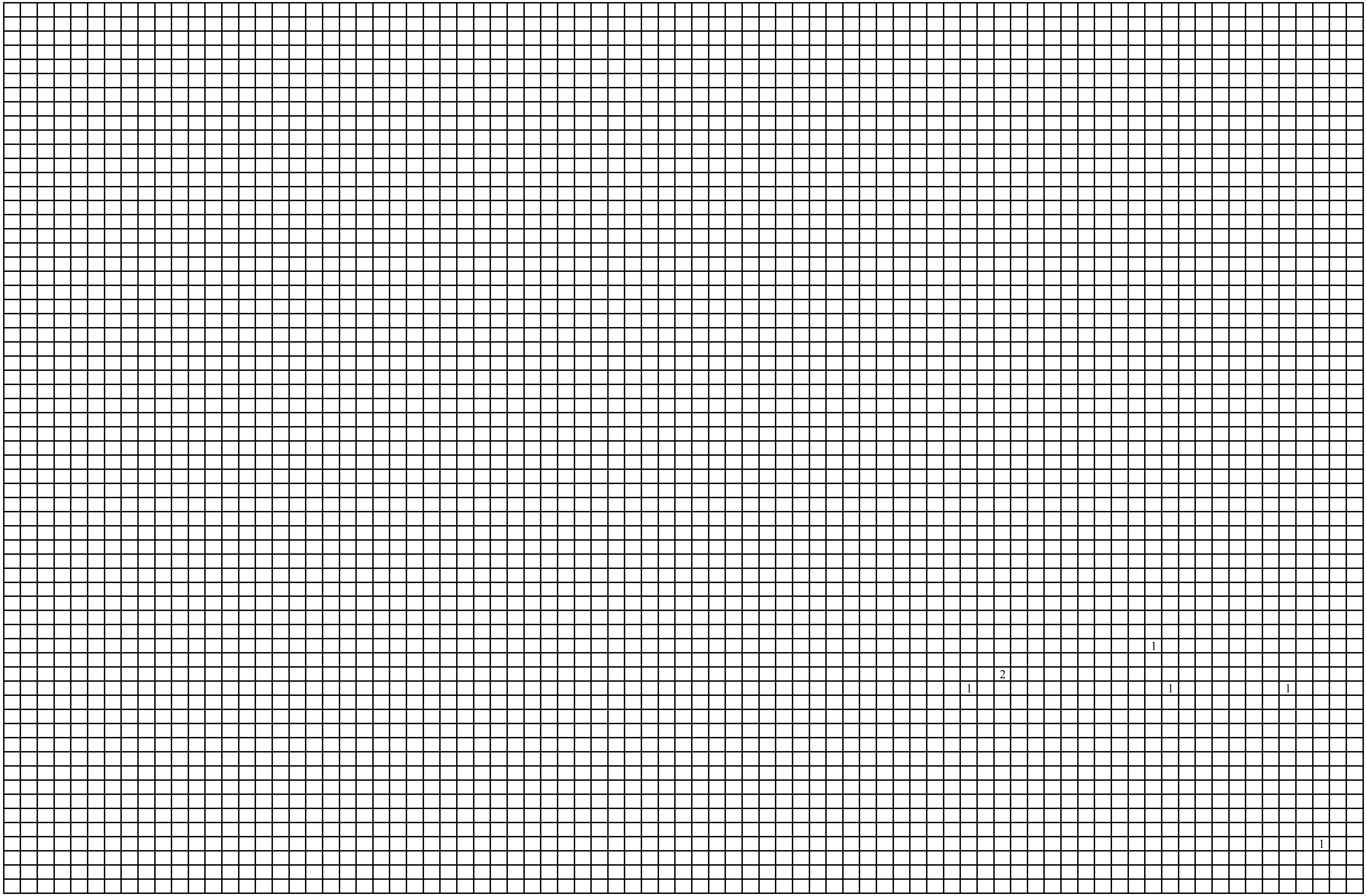
1
2
1
1
1
1

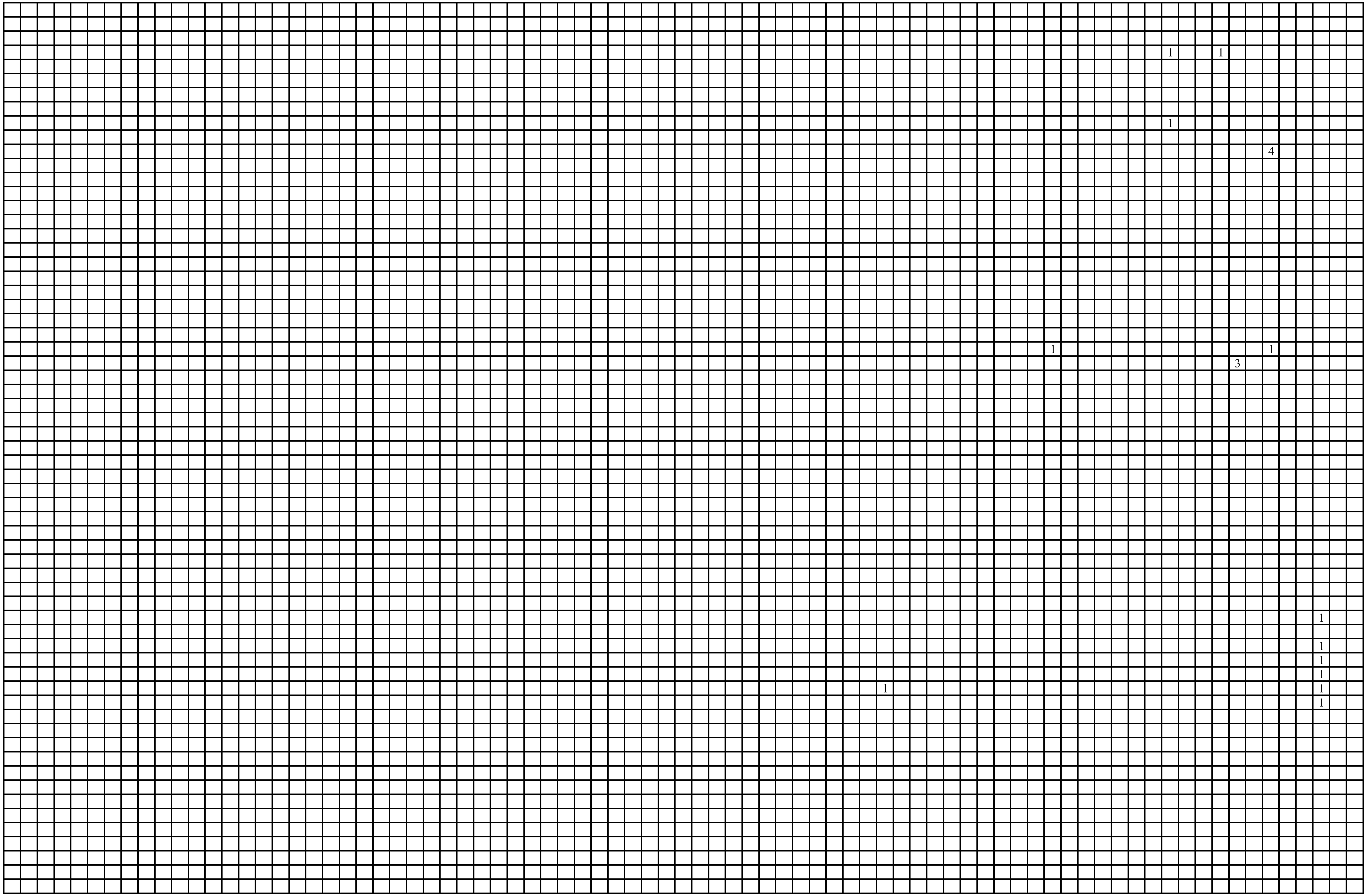
1
1
1
4
1
1
3
1
1
1
1
1
1
1

																																																																						1											
	1																																																					1																									
																																												1																											1		5				1	1		
																																																																																1		
																																																																																1		

																																																																				1												
	1			1									1							1											1												1	1											1																													
																																																																						1											
																																																																							1											
																																																																														1					
																																																																																			2
																																																							1								1																									
																																																																					1	1					1							
																																																												1																				
		1																							1																																																								2		
																									1																																										1												1							
																								1																																																							6			1		
																																																																																	1		
																																												1																																														

																																																																														1		
																																																				1																										1		
																																																																								1								
														1											1																													2																										
																																																																														1		
																																																																														1		
																																																																														1		
																																																																														1		
																																																																														1		
																																																																														1		
																																																				1																1								1		1		
																																																																														1		
																																																																							1									
																																																																														1		
																																																																														1		
																																																								1												1	1											
																																																														2																		

																																																													2																		
																																																													2																1		
																																																													2																		
																																																													2																		
											1																																																																				
			1																										1																																																	
																																																									1																					
																																																																										2				
																																																															1	1								1	1	
																																																																												1		
																																																																												1		
	1																																																																								1		1		
																																																			1																										1		
																																																															1										2		
																																																																												1		
																																																																												1		
	1																																																		1																								1		1		
																																																																												1		
																																																																3											
																																																																												1		

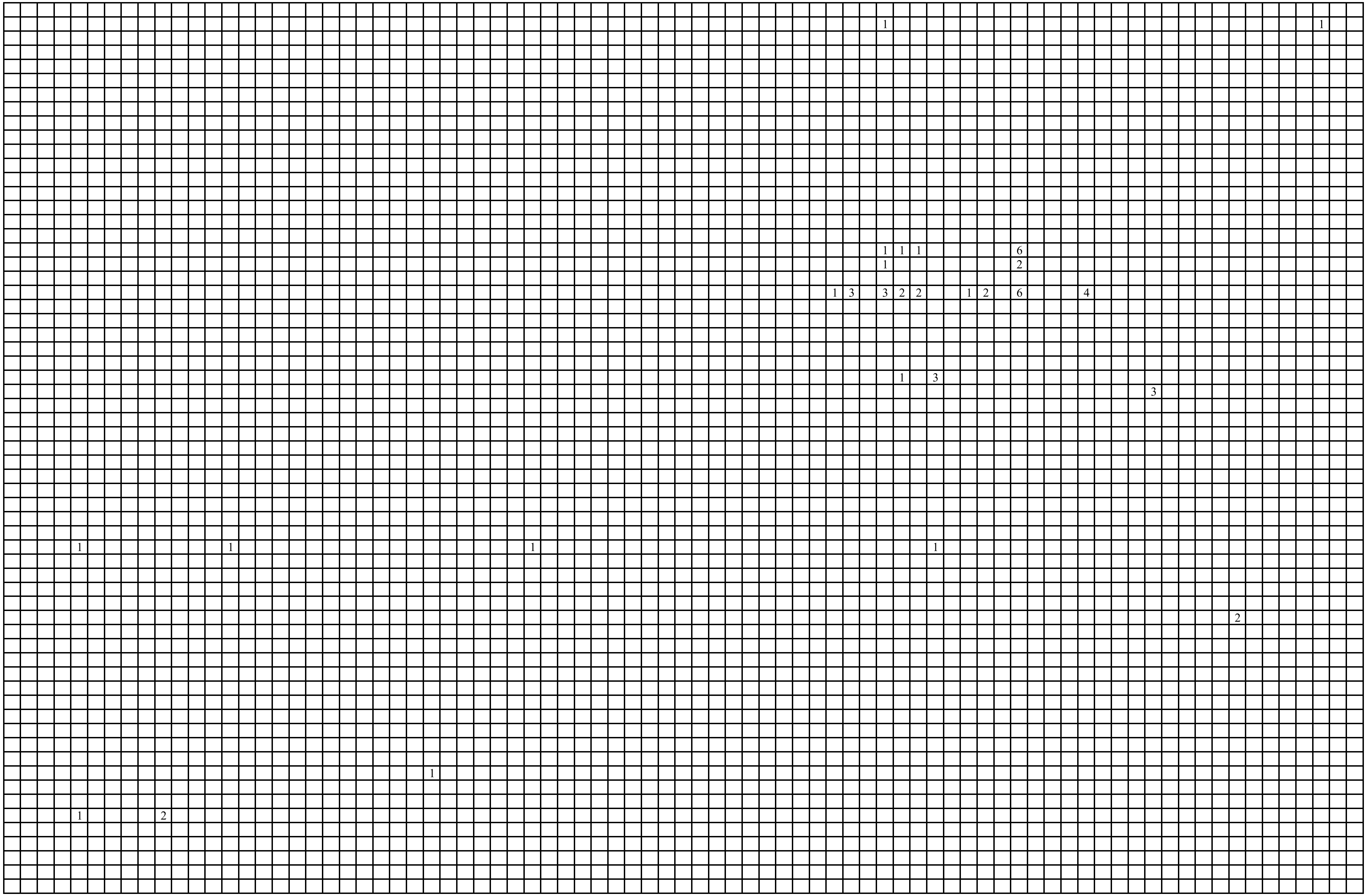
1 1
1 1 1 6
1 2
1 3 3 2 2 1 2 6 4
1 3
3
1 1 1 1
2
1
1 2

																																																																								1								
																																	1																																													1		
																																																																									2							
											1																1																																								1					2							
				1																																																			1																									
	2			2																							1																												1																									
																																																																													2			
																																																															1																	
																																																				1		1						4																				
																																																												1																				
																																																						1						3																				
																																																				3	1	1				1		6																				
																																																				1																												
																																																	2			2	1	1						2																				
																																											1	1								1		1	1	1				3				1																
																									1		1																																																				
																											1																																																				

		1																									1																																																					
																																												2											2																					2				
																											1																																																					
				1																																																																												
																																																												3																				
	2																																																						2																									
																									1		1																																																					
				2																																																																												
																																																				2								3																				
																		3																																																														
																																												2																																				
																				1																																																												
																																																				2		1			2	2		4																				
																																																	1	1		3	1	2						7				2																
																											1																																																					

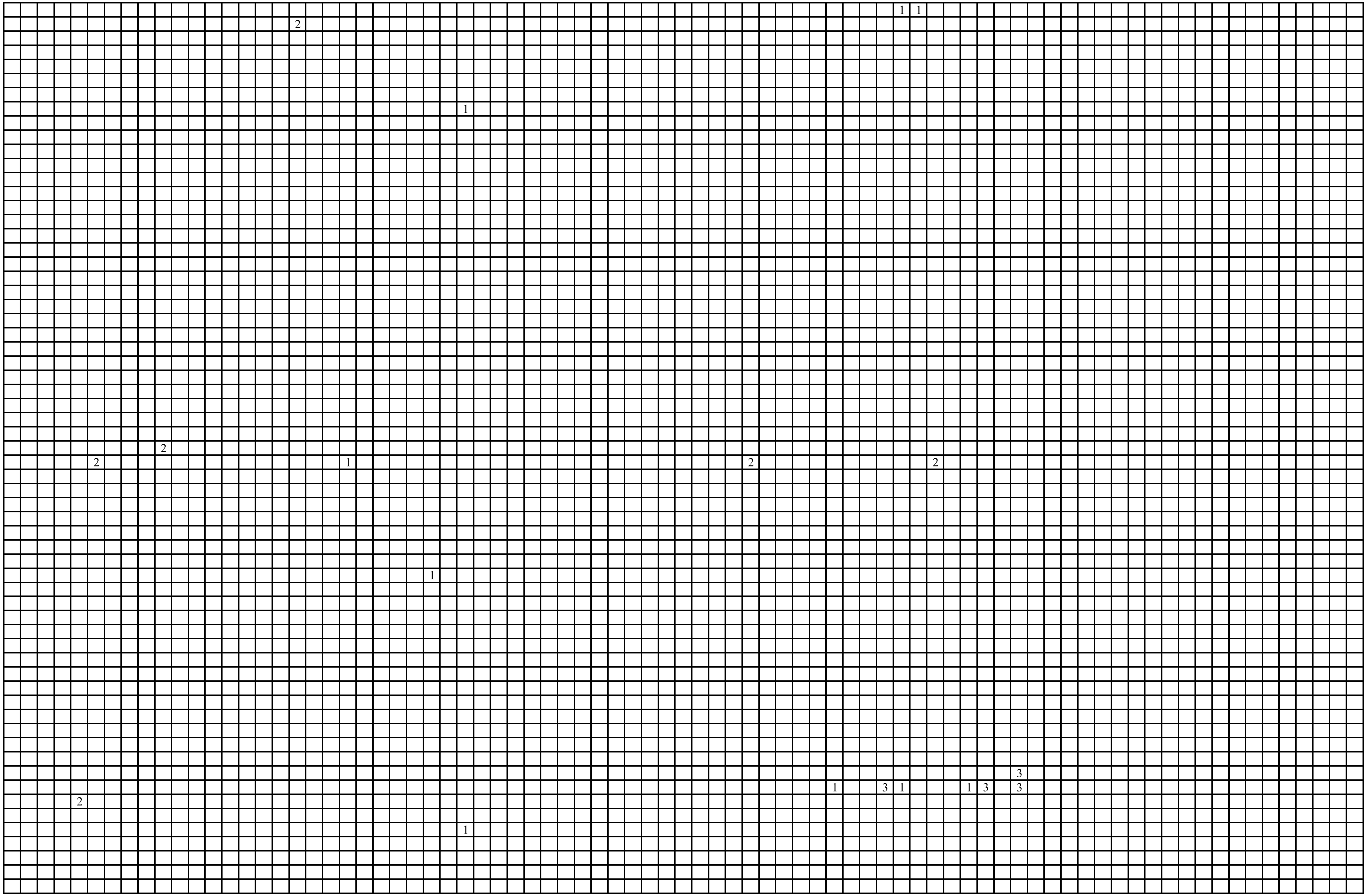

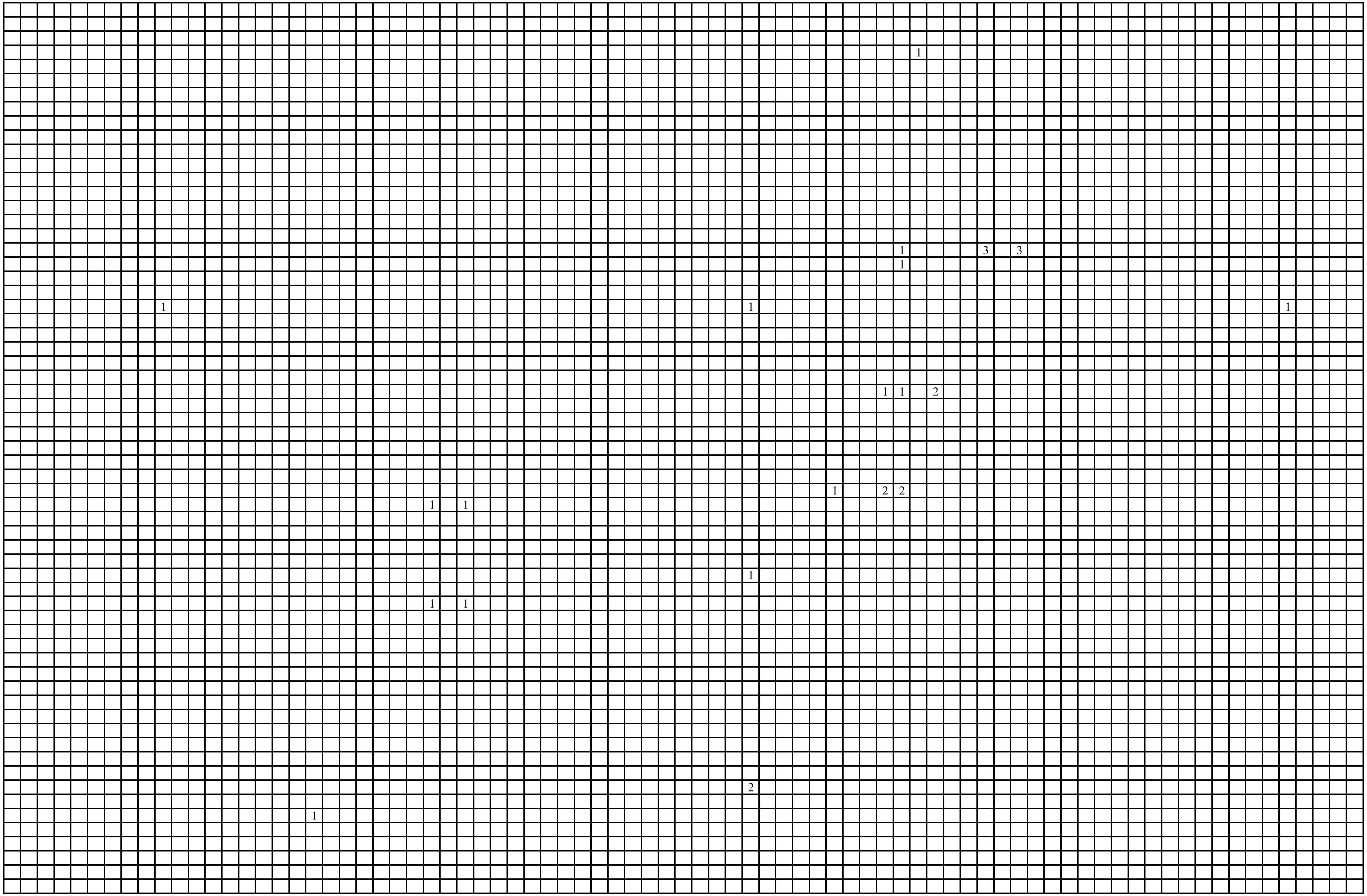
1
1 3 3
1
1 1 1
1 1 2
1 2 2
1 1
1
1 1
2
1

																										1																																																				
		1																							1																																																								
																																																																1																		
																											1																																																											
																				1																																																																
																											1																																																											

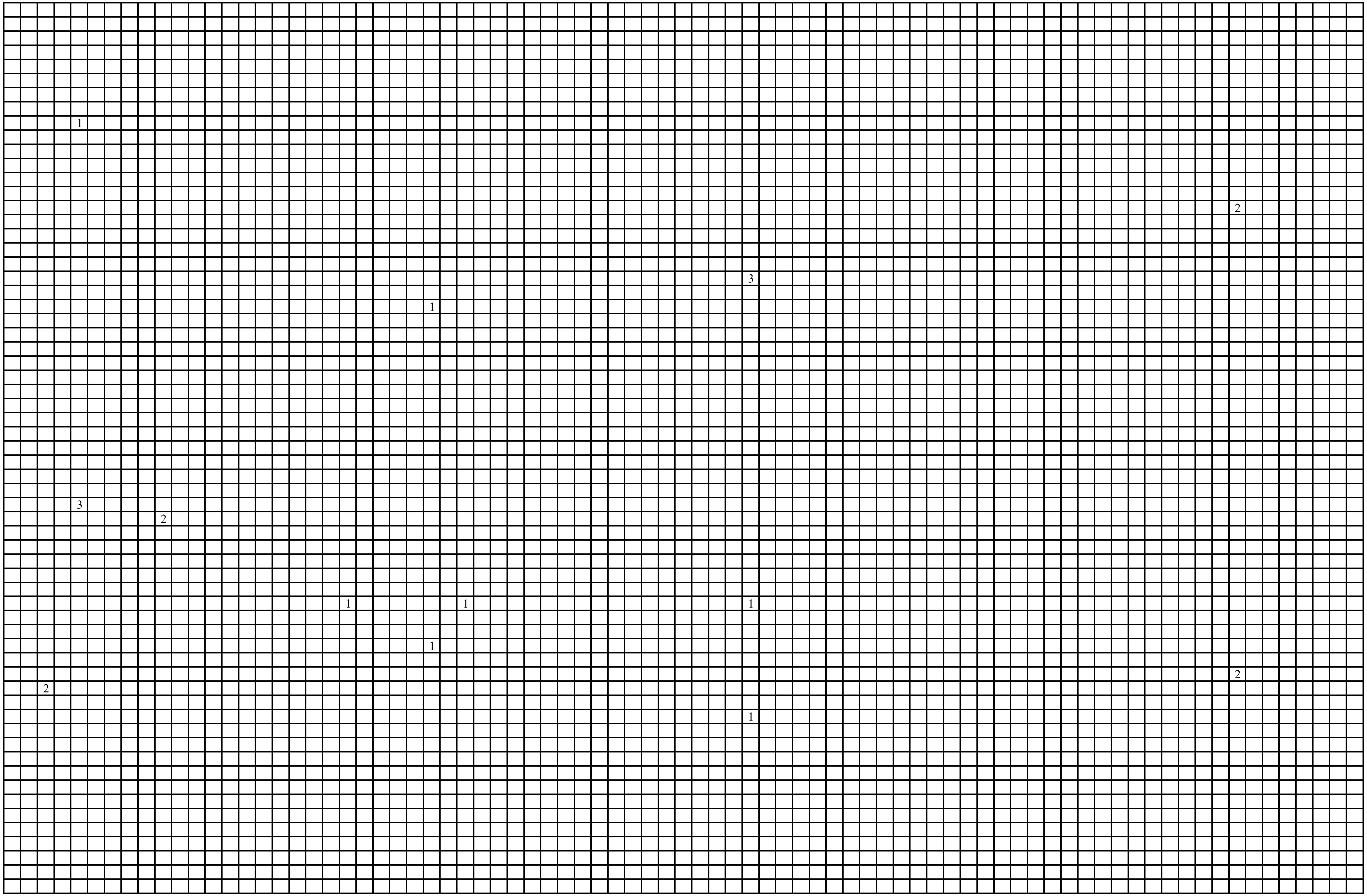
1
2
3
1
3
2
1
1
1
1
2
2
1

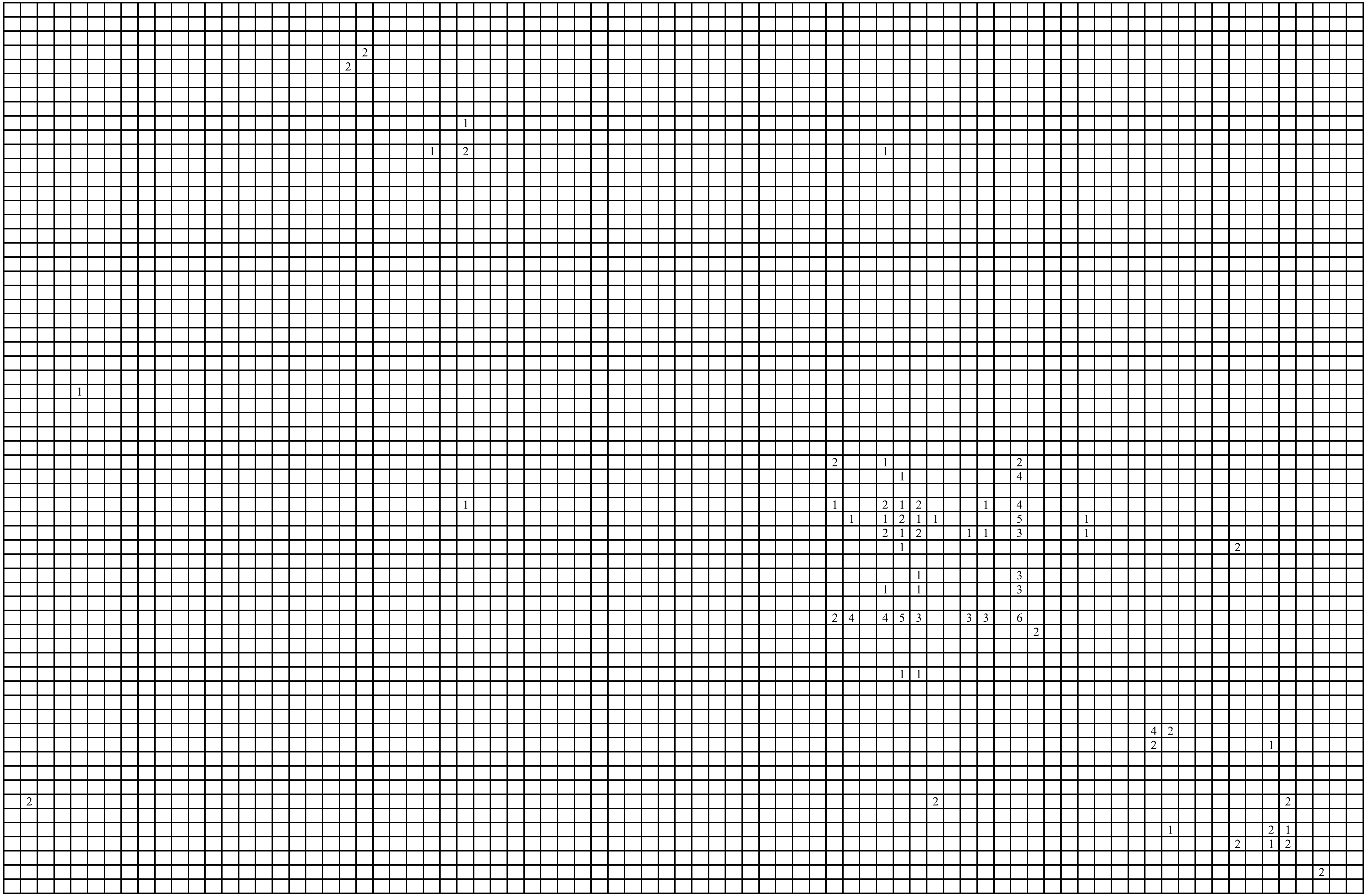
2
2
1
1 2 1
2 1 2
1 4
1 1 2 1 2 1 4
1 1 2 1 1 5 1
2 1 2 1 1 3 1
1 2
1 3
1 1 3
2 4 4 5 3 3 3 6
2
1 1
4 2
2 1
2 2 2
1 2 1
2 1 2
2

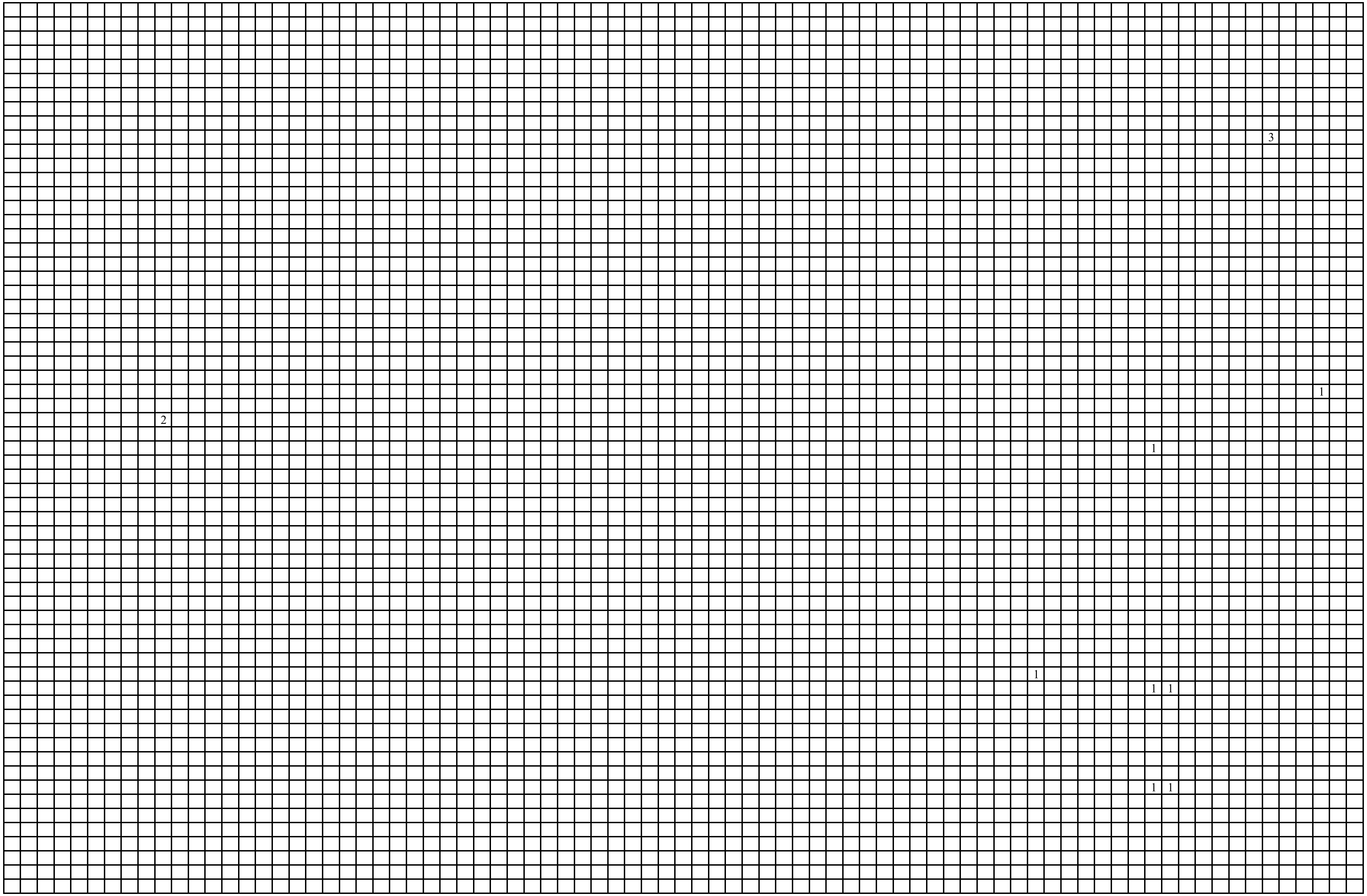
3
1
2
1
1
1 1
1 1

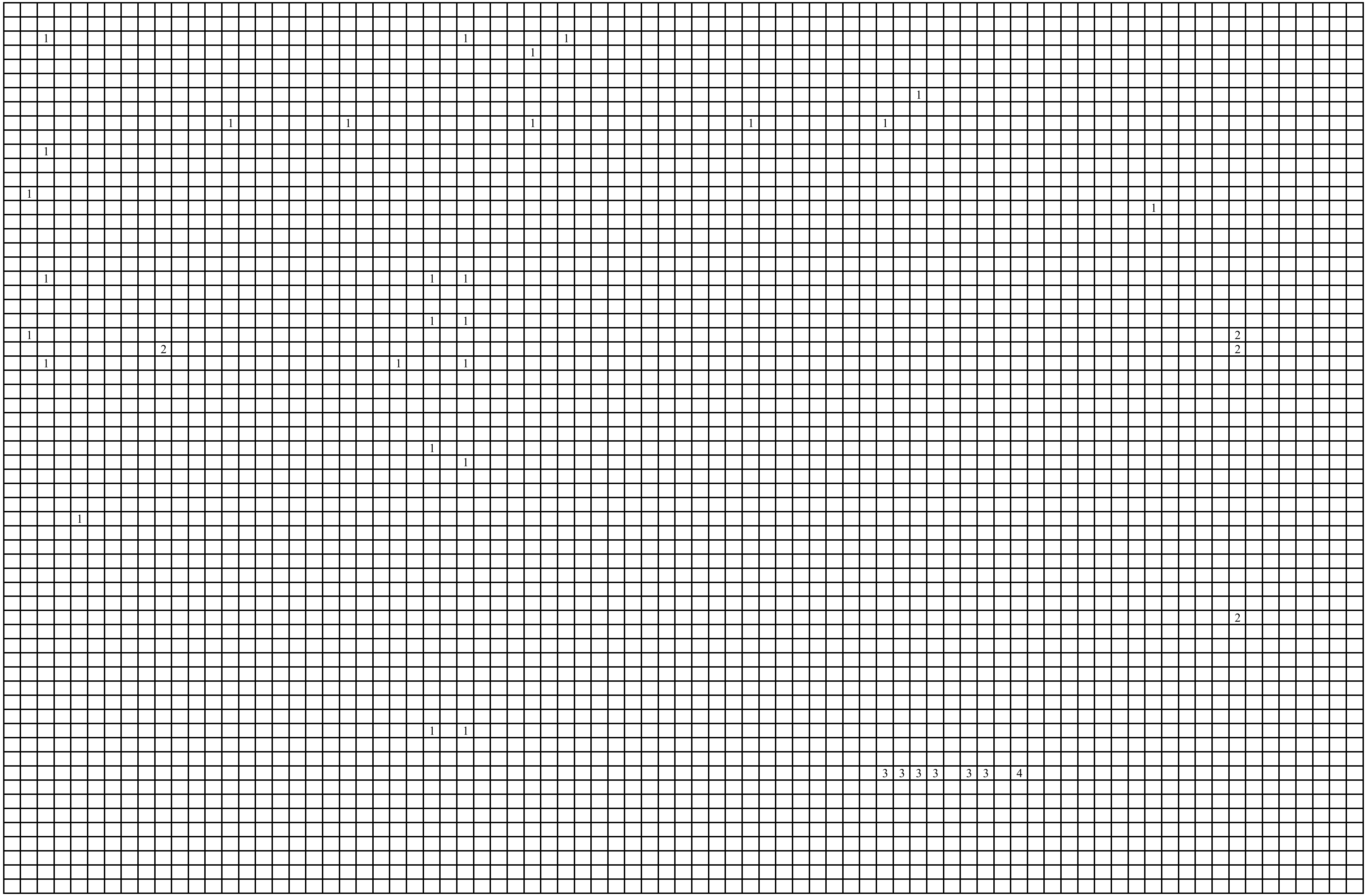

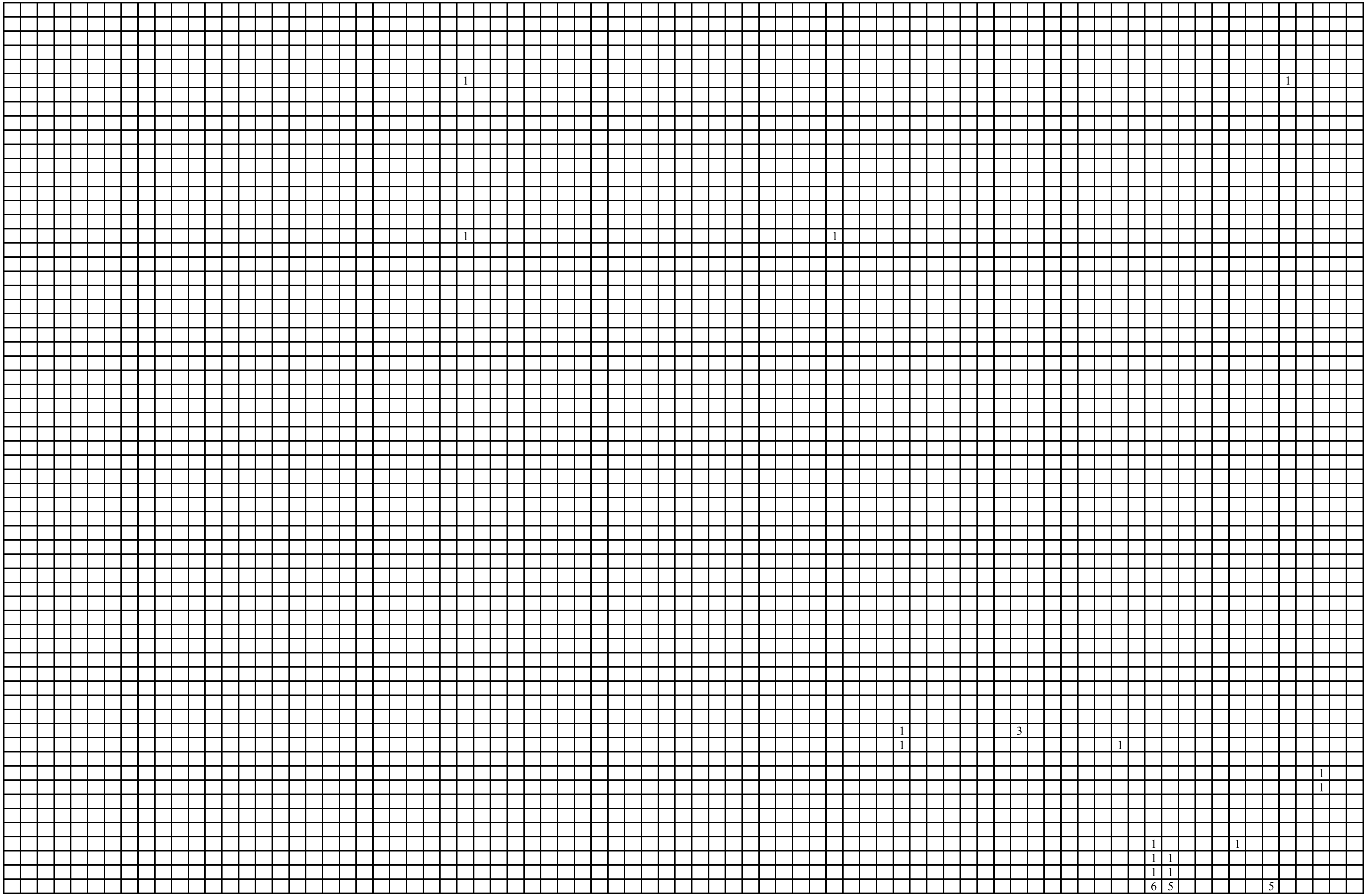

																																																																					2	5											
																																																																					1	2											
																																																				1																				2						1		
																																																																										1	1							
				1																											1																																																						1			2								
																							1																																																							1						1		
																																																																						1													
																																																																						2													
																																																						1																											
																												1																																																												
																																																																																1		
																																																																						1													
																																																																																1		
																																																																							7												
																																																																							4												
																																																																							5	5											
																																																																							4												
																																																																							2												
																																																								1																									
																																																																							1	2								3			
																																																																							2	5						1					
																																																								1		1																			1				

																																																																										1					
																																																																										1					
																																																																				1												

																										IKZF1	84	467
																										IKZF2	18	36
																										IKZF3	69	421
																										IKZF4	23	52
																										IKZF5	41	124
																										INSM1	0	18
																										INSM2	1	4
																										MAZ	20	112
																								1		MECOM	83	470
																										PATZ1	38	187
																										PEG3	0	15
																										PRDM10	10	75
																										PRDM13	0	10
																										PRDM15	14	85
																										PRDM16	33	205
																										PRDM2	14	51
																										PRDM4	5	24
																										PRDM8	0	5
																										PRDM9	3	8
																1										REST	25	312
																										RLF	19	65
																										RREB1	29	99
																					1					SALL1	51	117
			1																							SALL2	21	118
																										SALL3	6	19
																										SALL4	15	40
																										VEZF1	23	99
			1																							WIZ	72	282
																										ZBTB1	22	149
																										ZBTB17	53	104
																										ZBTB2	77	500
																										ZBTB21	12	189
																										ZBTB25	11	63
																	1									ZBTB38	12	177
																										ZBTB39	8	30
																										ZBTB4	7	38
																										ZBTB41	0	14
																										ZFAT	1	11
																										ZFP57	0	14
																										ZFPM1	6	21
																										ZNF101	2	41
																										ZNF132	0	0
																										ZNF134	0	2
																										ZNF142	3	19
																										ZNF195	13	66
																										ZNF211	5	24
																										ZNF217	37	121
																			1							ZNF219	40	143
																										ZNF236	0	9
																										ZNF248	2	11
																										ZNF251	4	20
																										ZNF256	0	3
																										ZNF277	4	38
																										ZNF292	14	67
																										ZNF296	6	33
																										ZNF304	0	6
																										ZNF316	15	58
																										ZNF319	5	26
																										ZNF334	0	3
																										ZNF335	3	31
																										ZNF341	2	19
																										ZNF37A	0	3
																										ZNF382	1	9
																										ZNF407	2	11
						1						1														ZNF417	39	218
																										ZNF418	3	22
																										ZNF423	16	49
																										ZNF438	12	72
																										ZNF445	32	78
																										ZNF462	18	80
																										ZNF467	50	174
																										ZNF469	0	7
																										ZNF48	25	158
																										ZNF507	9	65
																										ZNF510	3	22
																										ZNF512	57	335
																			1							ZNF512B	50	230
																										ZNF513	4	8
																										ZNF516	29	70
																										ZNF519	1	1
																										ZNF521	24	46

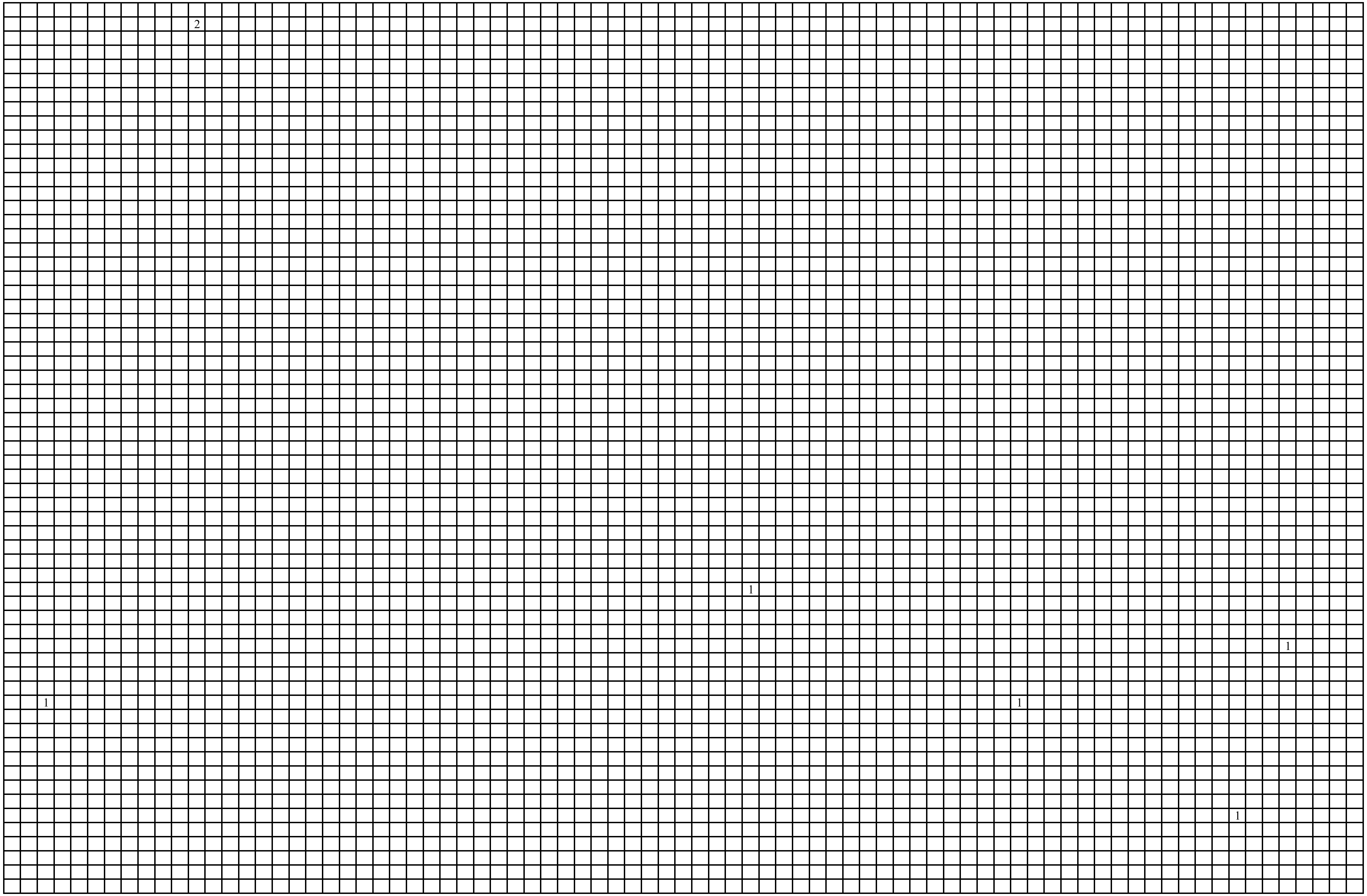
2
1
1
1
1
1

																											1		1																																																			
																																	1																																															
									3																																																																							
							2													1			1				1		19																	2																										1	1		2					
							1																			1			4																																											1			1	1				
								1																																																																								
														1													1																																1										1											
		1																																																																														
																											1				1																																												1					
									1																																																																							
									2																				1																																						1													
							2	1																																																																								
								1																																																																								
							2																				1																																																					
																										1																																																						
								1																																																																								

																														1	1																																																
																																																																										1				
								2																																																																						
1				1											1					1	1					1	1				1			1												1							1			1																1					1		
1							1																			1	1		1																								1				1																							
																																1																																																
																										1																																																								
																																																																1																
																		1	1																																																														
											1		1																																																																			
								1																						1																																																					
														1																																																																		
																													1																																																							
																						1																																																														
																						1																																																														
1																						1																																																													
																						1																																																														

																						1					1																																																					
																																1																																																				
																						1					1																																																					
								1	1													1																																																											
																														1																																																						
2																																		1																																																		
2			1								1								2															2																																																		
3																						1								1				1																																																		
																														1				1																																																		
		2	5																																																																																
																														1				1																																																		
																					1																																																														
									1																																																																									
																																																																									1				
								2																																																																										
																																																														1																		
																																																																												1				

		1																		1	1	1					1																																										1						1	1		1		1
		1																			1																									1																																						
																																																																2																	
																												1																			1																																						
									1													1	1	1	2					1																																												1										
																											1									1																						1																							
								1																																														1																											
																																																								1																											
																											1				1		1																																		1																	
																																																																													1		
	3																																																															1																	
														1																																																																							

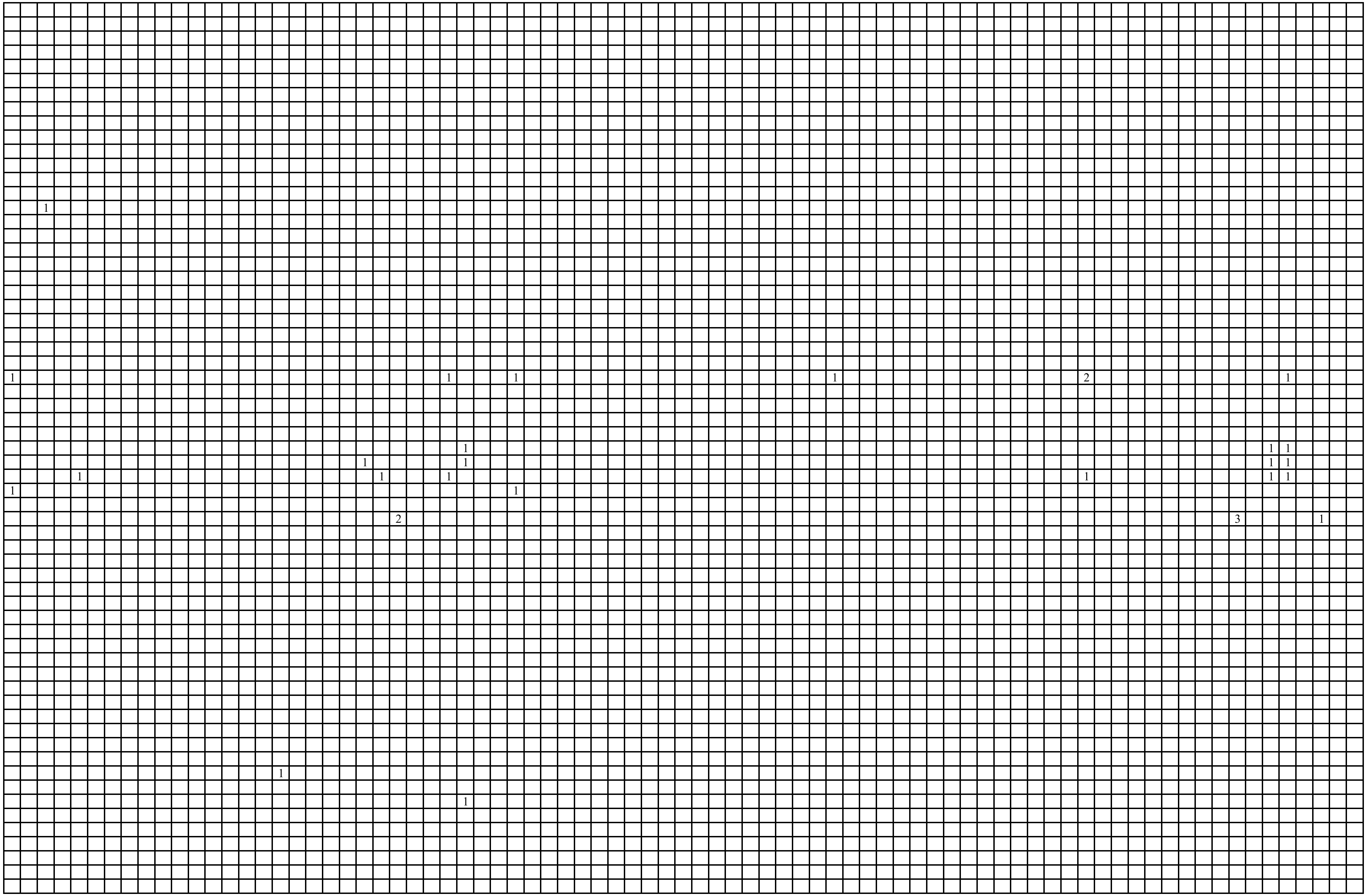
1
1 1 1 1 2 1
1 1 1
1 1 1 1
1 1 1 1 1 1
1 1
2 3 1
1
1

									1																	1																																																								
																						1	1	1	2																																															1										
																																																														1																
																					1	1					1																		1																						1								1		
																											1																																														1					
																						1					1																																														1					
																																		1																																																		
								1						1						1	1	1					1																		1																						1							1				
																																																																											1				
								1						1							1	1																																														1								1		1

																				1	1						1																																																								1		
																										1																																														1																
																											1																																																											
1		1																																																																															
																																														1							1																					1				
																														1																																																	1				
																																																																						1					2					
				1			1														1	1					1		1		1			1																																																
								1																		1				1																																																								
																												1																																																			1					
																						1					1																																																											
								1	1											1		2	1	1			2																													1																2						1					

																																																																																1
								1						1														1																		1																																		1
														1							1	1					1																										1																											
				2			1																																																																									
																											1			1																																													1	1				
																														1																																																		
1								2											1																																																													
																																																																												1				
																														1																																																		
								1						1						1										1																																													1					
1		1												1					1																																																								1					
																											1																																																	1				
																											1																																																					
								1																																																																								
																																																																														2		
																																																					1																											
																											1																																																					
																				1																																																												
																				1	1						1						1																																															
																				1	1	1					1																			1																													1					1
																										1				1																																																		
1		1																	1																																																													
								1																																																																								
																												1		1																																																		
		1																																																																														

3	1	3						1																				1																																																				
																														1																																																1		1			2					
																																																																											1			2					
																					1																																																												
																					1																																																												
																													2																																																						
1		1																																																																															
																										1																																																									
																														1																																															1										
																														1																																																										
																																																																1																
								2						1																1																																																								
																		2																																																															
							1																																																																										
																																																																									1										
														1																																																																					
																																																						1																											
		1						2	5						1				1		1	1	1						3	3	1															1																															1					1		
																																			1																																																		

																																														1																																		
																																																																						1										
													1																														1																									1												
											1																																																					1																
2	2												1																																																																			
2	2												1																																	1																																		
									1																																																																							
																																																																		1														
4		5														1																																																																
2		3																																																																														
																														1																																																		
																														1																																																		
																							1																																																									
								1																																										1																2								1		1				1
								1						1																																																												1						
								5						1		1																																																																
								1																																																				2								1						1
																														1																																	1																	
							1																1																																																									

																																												1																																			
							1													1	1	1					1																			1																							1									1		
																						1																																																										
																											1																																																					
																																																		1																															
																																					1																1															1											
				2																																																																					1							
																											1																																																	1				
								1						1							1					1	1																			1																																			
																																																																															1
																																																																										1					
								1											1	1	1				1	1																			1																							1						1	1				1
								3						3																																																																		
																							1																																																										
																													1																																																		1				
								1						1																1																																																					
																											2						1																																																			
																											1																																																								
																				1							1																																																								
																																																1							1																						1				
																			1																																																															

1																																																																																
								1						1					5	1	2	1	1					1			1																	9																																			2		
														1																																																																		
		2																																											1																																		
1		1																																																																													
								2											1																																									1																			
				2																																																												1															
				2																																																																											
																						2																																																										
																			2																																																												
																																																																						1		1									
				2																																																																								1				
																																																																														1		
								2															1				2																	2																																				
													1						1												1																																												1				
				2																																																																											
																																																																											1				
								1						1						1						1																			1																						1											1
									1																																																																						
																																																																												1				
								1						1	1						1						1																			1																													1	1		1		
							2																			1																											1				1																				1			2		
		1						1												1	1						1						1																																									1		1					1
								2						2	2						1	1						2																			2																				1												1		
																																																																														1		
							1																						1																																																			
																																																																											1				
							1	1							1					1	1						1																			1																													1			2		
																				1																																																						1				
																			2										3																																																	
																										1																																																					

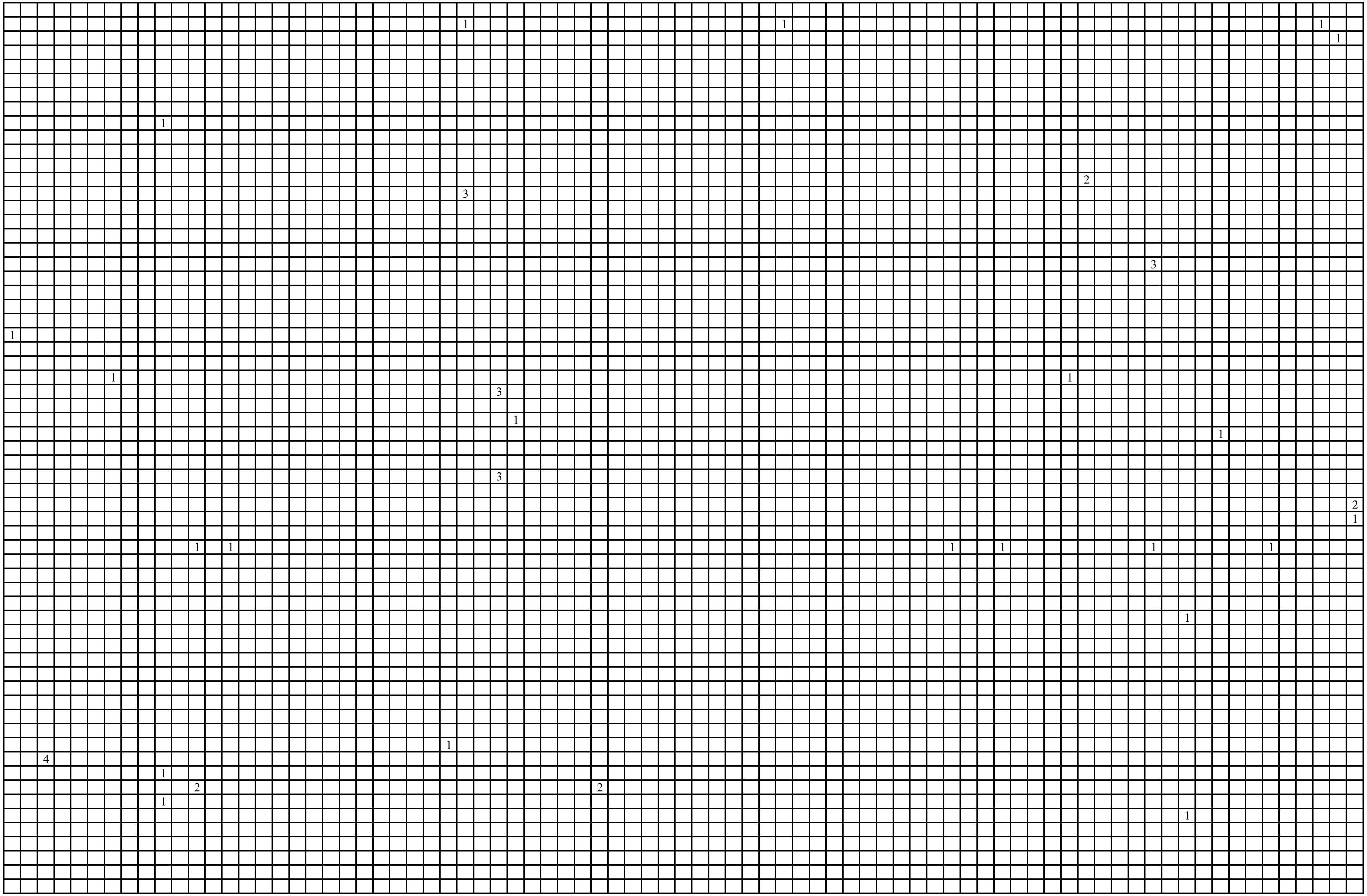
1 1 1
1
1
2
3
3
1
1 1
3
1
1
3
2
1
1 1 1 1 1 1
1
1
4
1
2 2
1
1

								1																			2																																															1	1				
																															1																																																		
																														1																																																	1		
								1																																																																								
									1																																																													1					1					
																																																																												2		
													1																																																								1				1			1					
																																1																																																	
																													3	1																																																			
																																				1																																																
																																																				1																																	
																													5																																																						
																																																																						1										
																																																																						1		1								
																																																																												1			
																														1																																																						
									1																																																																								
									1																																																																									
																																																																				1		1										
																																																																						1										
																																																																						1										
																																																																						1		1			1					

																																																						1									1				
																							1																																																												
								1																																																																							
																																								1																														1										
																																																												1																
									1																																									2												2												
																																																														1	1													1
																																																																					1										
																																																												1																
													2																																																																			
																																																												1																
																																																																1													
																														1																																															1		1								
			1																																																																															
																																																																	2												
																																																																					1										

																																																																				2												
																																																																			1															
																																																																												2					
																														1																																																									
																				1																																			1																											
								1																									1																																																
																																																																										1												
																																																																								1		1								
																																																																									1										
																																																																									1										
																																																							2												2												
																																												1																																					
																																																									1																							
																																																																									1										
																																												1																																					
																																																																									1										
																																													2																																				

																																																																										2					
																																																	1																															
																																												1																																				
									1																			1															1	1	1					1						1												1												
																																																																								1										
																																																																					2		1										
																																												1																																				
																																																																											1					
													2																																																																		
																																																																								1												
																																																																			1																

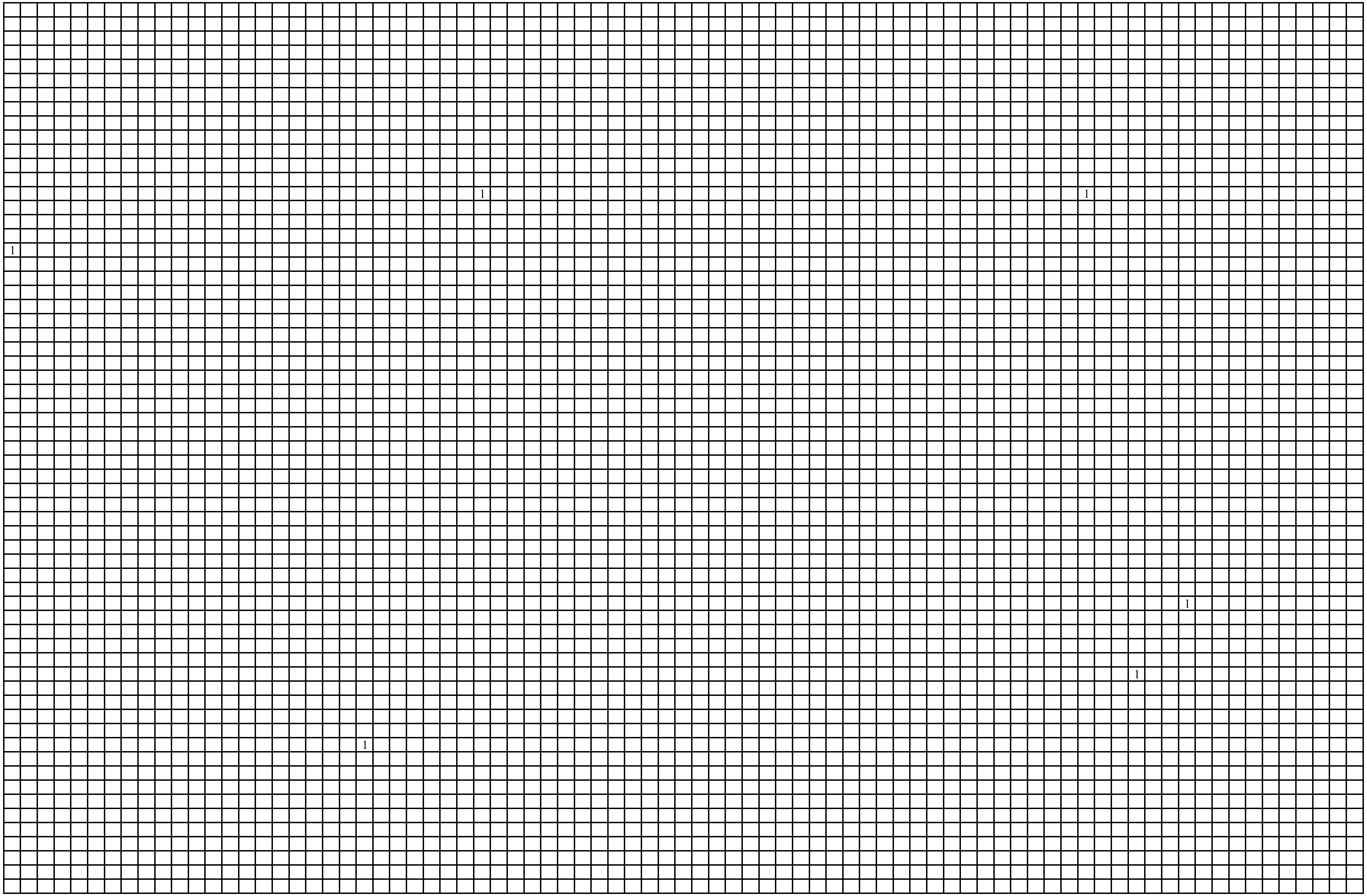
1
1
1
1
1
1

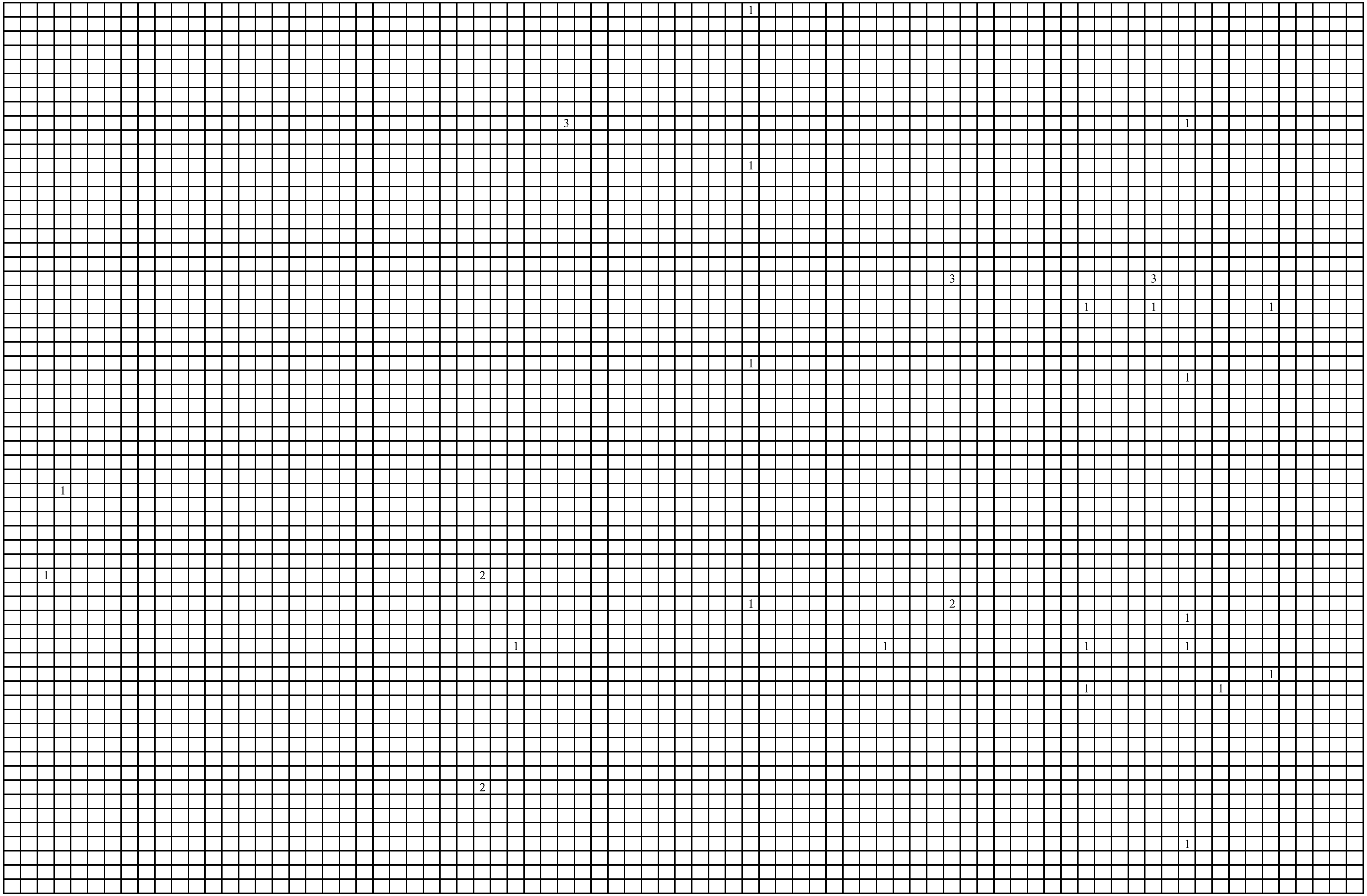
1
3 1
1
3 3
1 1 1
1
1
1
1 2
1 2
1
1 1 1 1
1
1 1
2
1

																																																																					1										
																																																														1																
																																																														1					1										
																																																													1																
																																																																						1										
																																																																					1										
																																																																				1												
																																															1																																			
																															1																		1							1																						1				
																																																1							1																										
								3																																																																							
																										1																																																										

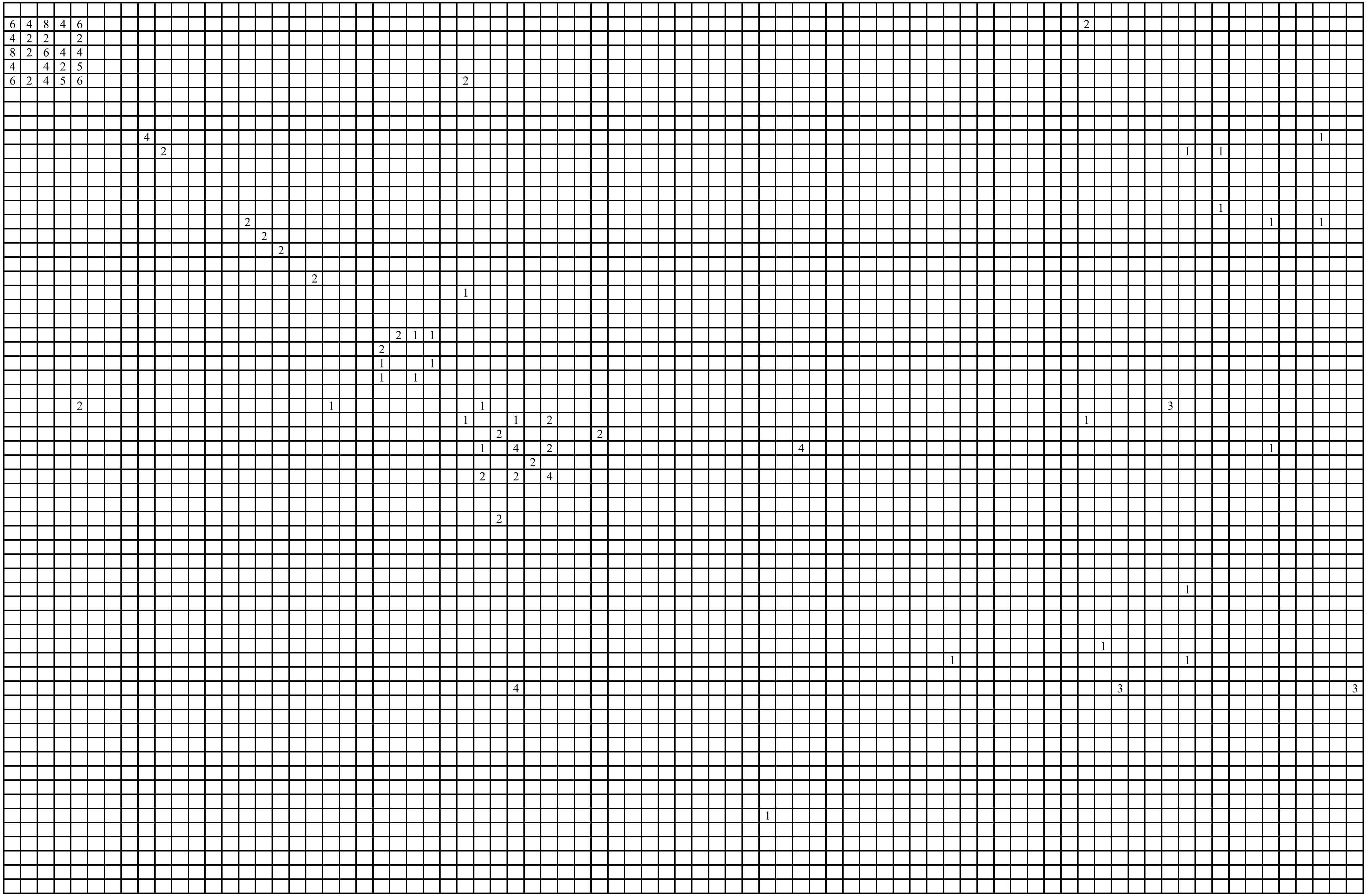

2																												1																																				2																	
																																												1																																								
																																																3																																	1
																																																																					2													
																																																																											1										
																											3																																																								
									1																															1					1																							1				1			1					
									1				1																																																									1		2			1					
														1																1																																														1		1								
																																																																														2			
								1						1																																																																		
																																																3																			1														
																																																																						1													
																																																																										1										
1		1																																																															1																
																														1																																																							
																																																																						1			1		1								
																														6																																																						
																																																																										1					3					
																														1																																																							
																																																																										1		1								
																																																																													1								
																																																																												1								
																																																																								3												

																																																																								1					
																													1																																		2																
																													1																																			2			1													
																														1																																																				
																																																																	1										
																																																												1																
																																																																						1								
																																																																	1										
		2																																																																														
1								1																														2																																														
		1																				1					1												3																																										1		
																											1																																																										
									1													1	1	1	1		1				1								1								1																			1			1											
																																																													1																	1				
2		1						2						1								2			1					3																	3																													2				
1		1						3						1								2			1			1		1																	3																													2				
5		2						3						2								2			2					2																	3																			3										4				3

3		1						2						1								2			1			2		1																	3						1																							2				
2																						1			1					1																	4							1																						2				
																			1																																																														
								1																																																																									
									2												1						2	1			1														1																						1							1				
							1		1						4											1	1	1	1	1															2						1																				1				1				
									1		1		1													1	1																		1							1			1																	1			1					
																																																																			1													
																					1																				1				1			1																									2			1		
															1																																																																	
																			1																																																													
																																																																		1																
																					1						1				1																																										1										1
1									1												1																																																						1					
																						2																																																												
									1																																																																									
											1																																																															1					
									1				1																	1																										1																								
																																																																											1				
																														1																																																		
2		2																				1			1					1																	4							1																						2				
1		1						5						1								1			1					1																	3					1																									2				
							1																			1	1			1			1																				1												1			1							1				1

																																																																			2												
																																																																		1													
							1																																																																								
				1																		1																																																										
1																																																										1																							
								2													1																																																			1								1				
																							2																																																																

80	22	64	44	250	90	160	91	45	5	48	15	131	36	46	2	11	278	3	8	146	14	39	2	106	8	27	10	31	25	45	0	68	51	1	160	144	5	10	31	9	21	97	0	23	3	132	170	62	137	11	19	116	154	230	0	20	4	29	2	30	2	25	64	2	7	20	8	93	158	152	0	120	20	13	0	40	0	5	19	17
21	1	34	9	25	16	31	15	2	0	6	0	22	11	4	0	0	38	0	0	32	0	1	0	15	1	2	0	2	0	10	0	24	16	1	82	20	0	1	6	1	1	16	0	3	0	15	37	20	41	2	0	13	12	24	0	4	0	0	0	7	0	0	24	0	0	1	2	44	66	13	0	14	5	0	0	4	0	1	4	6
ZNF526	ZNF532	ZNF536	ZNF552	ZNF574	ZNF579	ZNF587	ZNF592	ZNF597	ZNF606	ZNF618	ZNF628	ZNF629	ZNF639	ZNF646	ZNF654	ZNF658	ZNF668	ZNF671	ZNF674	ZNF687	ZNF697	ZNF711	ZNF717	ZNF770	ZNF772	ZNF775	ZNF778	ZNF784	ZNF786	ZNF787	ZNF788P	ZNF792	ZNF800	ZNF814	ZSCAN20	ZBED1	ZBED2	ZBED3	ZBED4	ZBED6	ATMIN	JAZF1	KLF18	L3MBTL4	PRDM12	REPIN1	ZBTB10	ZBTB3	ZBTB8A	ZNF112	ZNF12	ZNF207	ZNF318	ZNF326	ZNF367	ZNF385A	ZNF385B	ZNF395	ZNF470	ZNF473	ZNF486	ZNF488	ZNF503	ZNF527	ZNF573	ZNF578	ZNF587B	ZNF608	ZNF609	ZNF622	ZNF630	ZNF688	ZNF69	ZNF699	ZNF700	ZNF704	ZNF705B	ZNF706	ZNF708	ZNF714
																																																															1																	
																																																																												2				
																																																																												2				
																																																															1																	
																																																																					1											
																																																																								1								
						2																																																																		1								
						1																																																																											
																																																																						1	1											

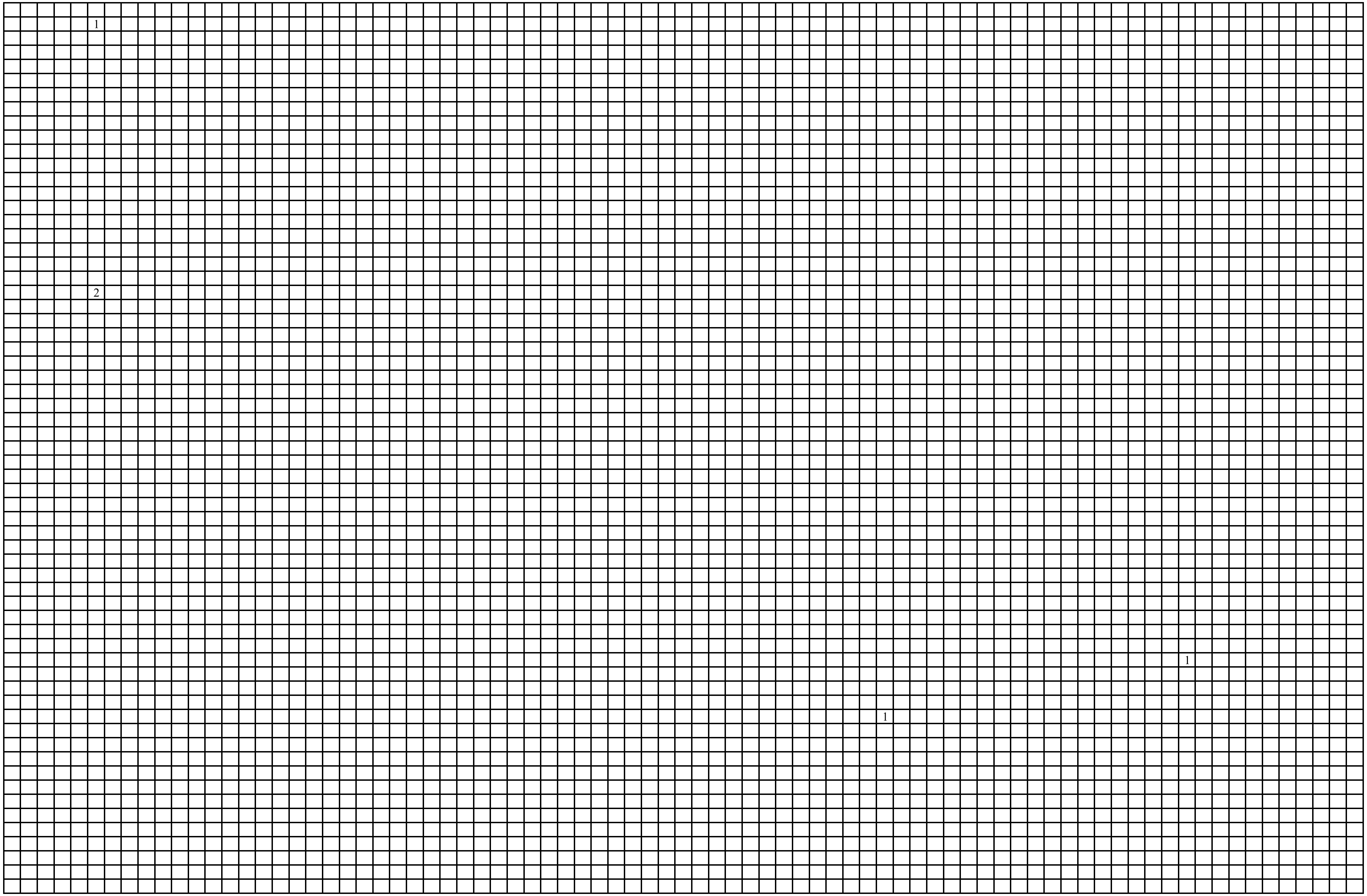
1
2
1
1

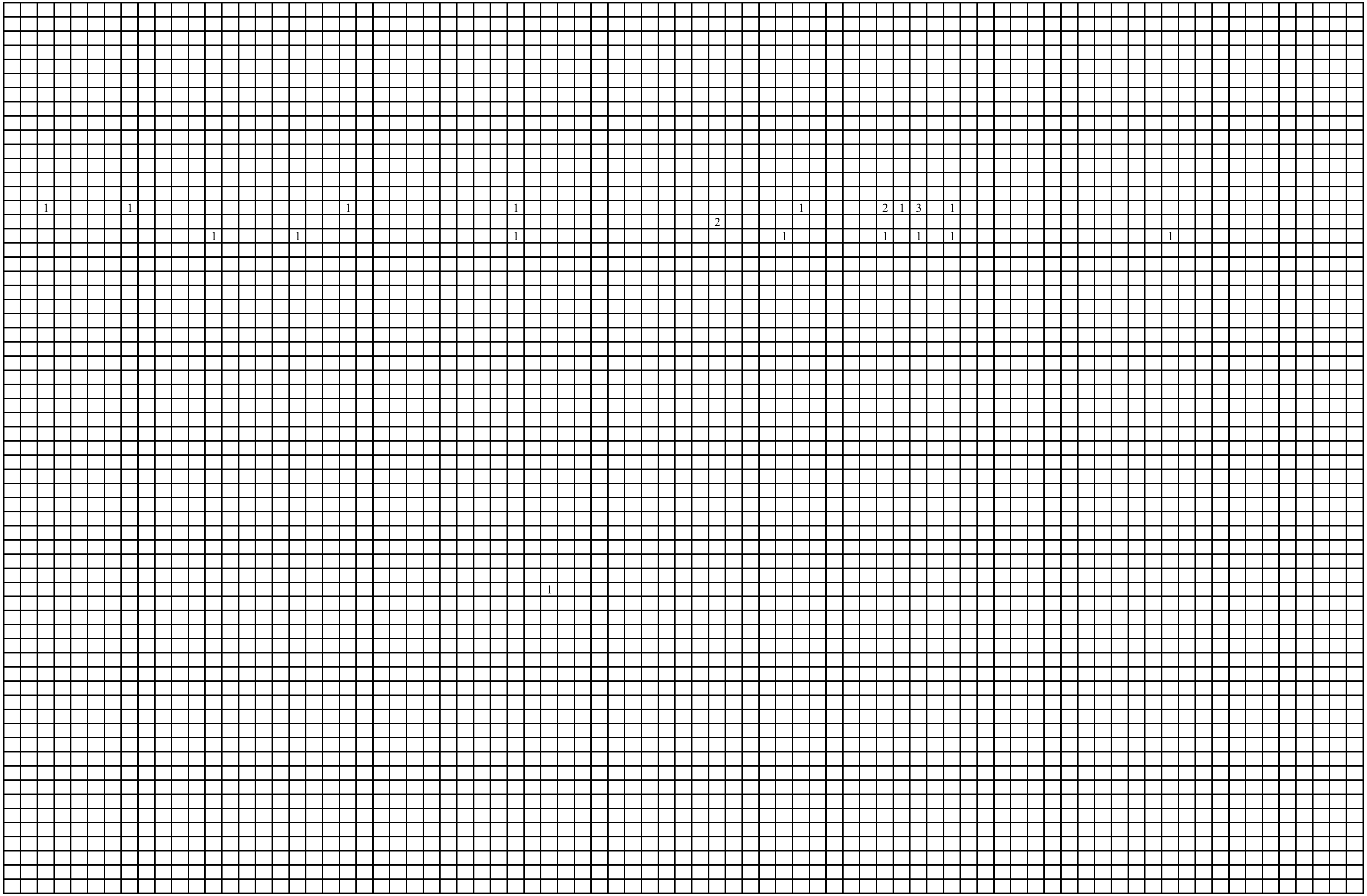
1 1 1 1 1 2 1 3 1
2
1 1 1 1 1 1 1 1
1

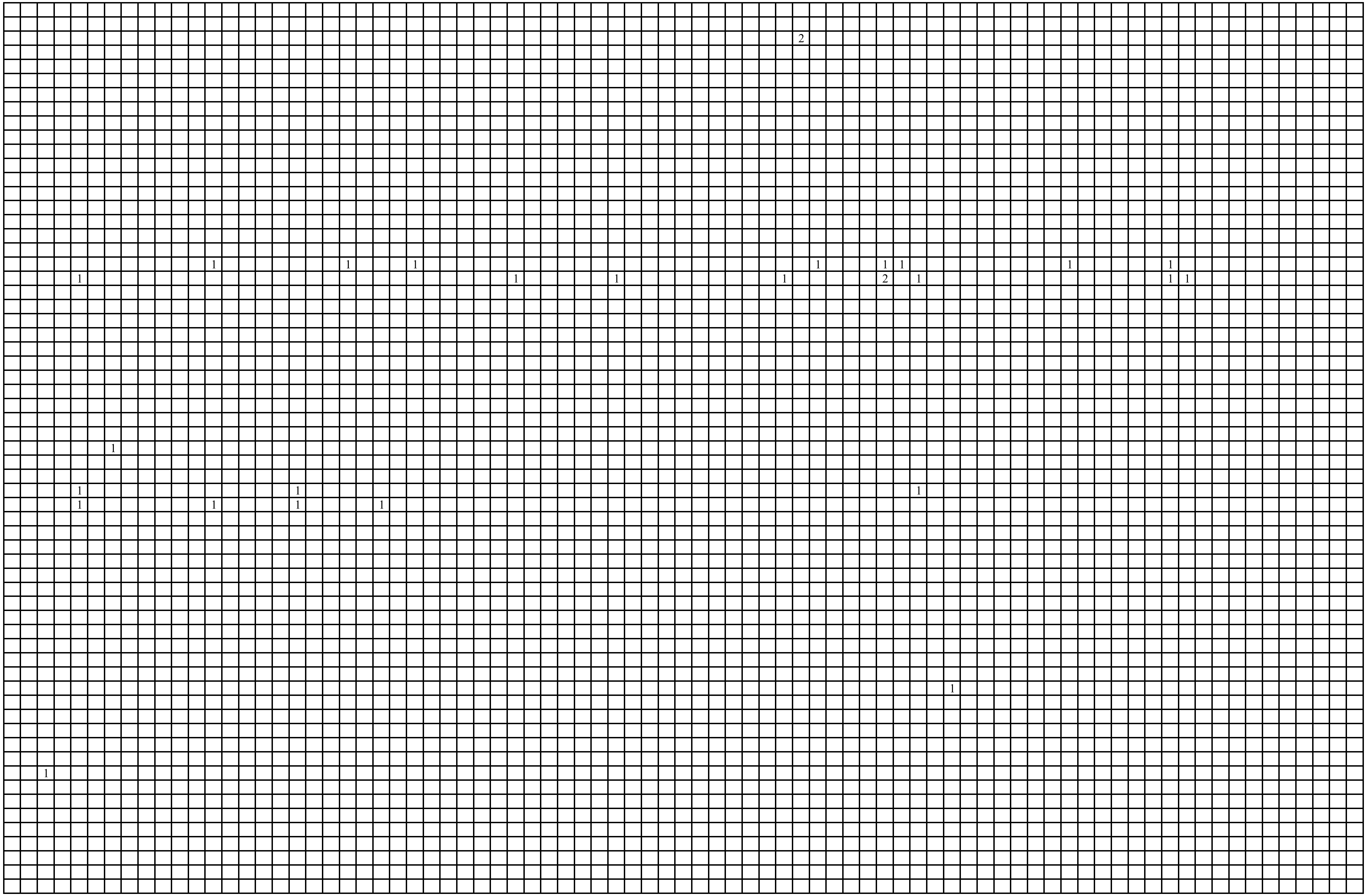
2
1 1 1 1 1 1 1 1 1
1 1 1 1 1 2 1 1 1
1
1 1 1
1 1 1 1
1
1

																																																												1																		
		1																																																										1					1	1											
		1																																																																															
																																															1																																			
																																															1	1																																	
		1																																																										1					1	1											
																																																1																																	
																				1																											1																																			
																				1																																																													
																																																						1																											
																				1																																																													
																																															1							1														1	1											
																																																																									1									
		1																																																										1						1											

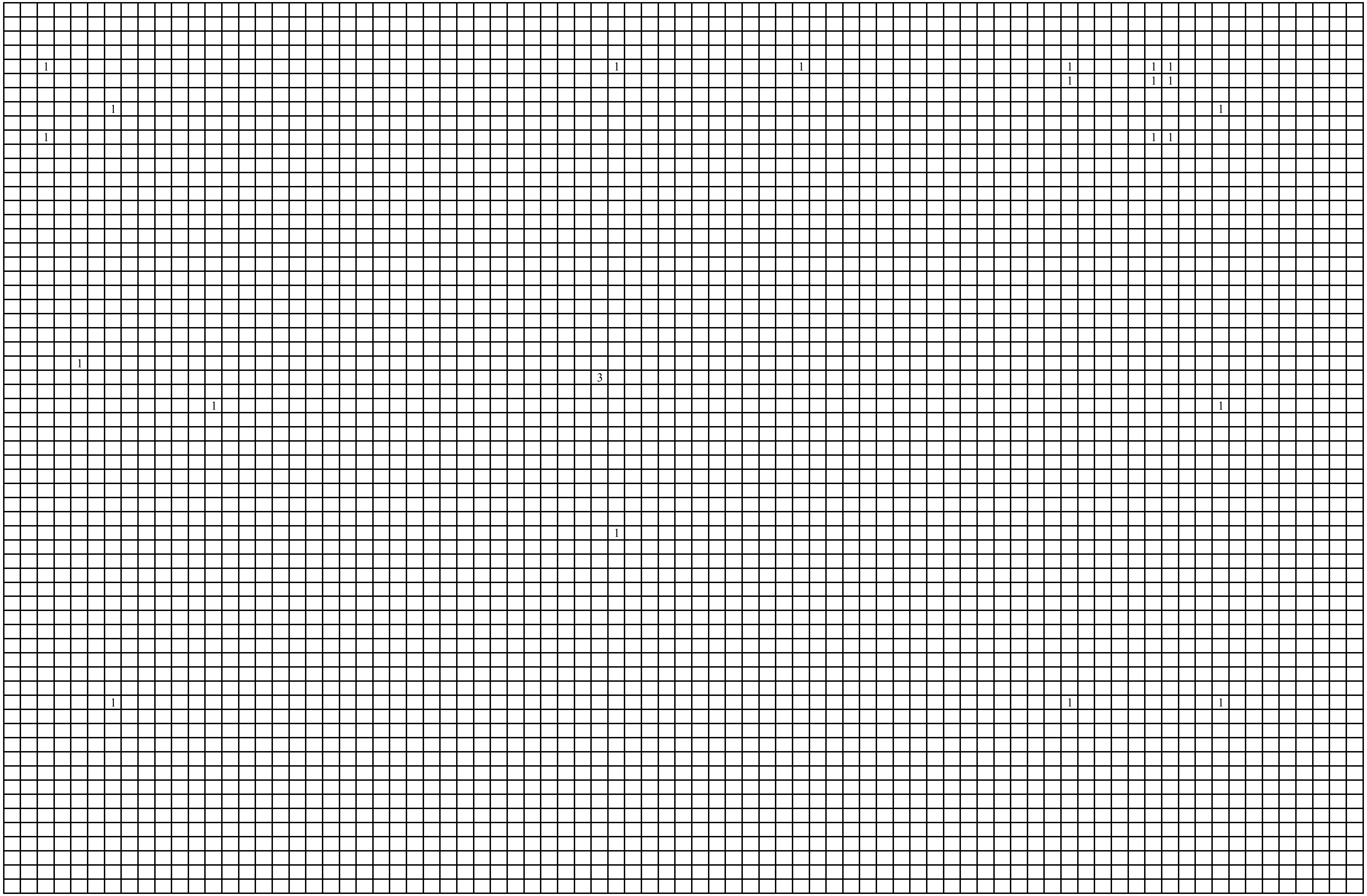
1 1 1 1 1 1
1 1 1
1 1
1 1 1
1
3
1 1
1
1 1 1

			1																																																																							
				1		2																			1														1																																			1	1							
																																														1																																		
		1																		1																																																			1											
				1		1																																																															1											
				2																1																																																				1											
		1																																																																									
																																																																					1											
		1																																																																					1	1											

																																																																					1											
		1																																																											1					1	1											
																																																																					1											
																																																															1					1	1											
		1																																																																		1	1			1								
		1																																																											1					1	1											
						1																																																																													
																																																																					1											
														1																																																																			
																																																																1																	

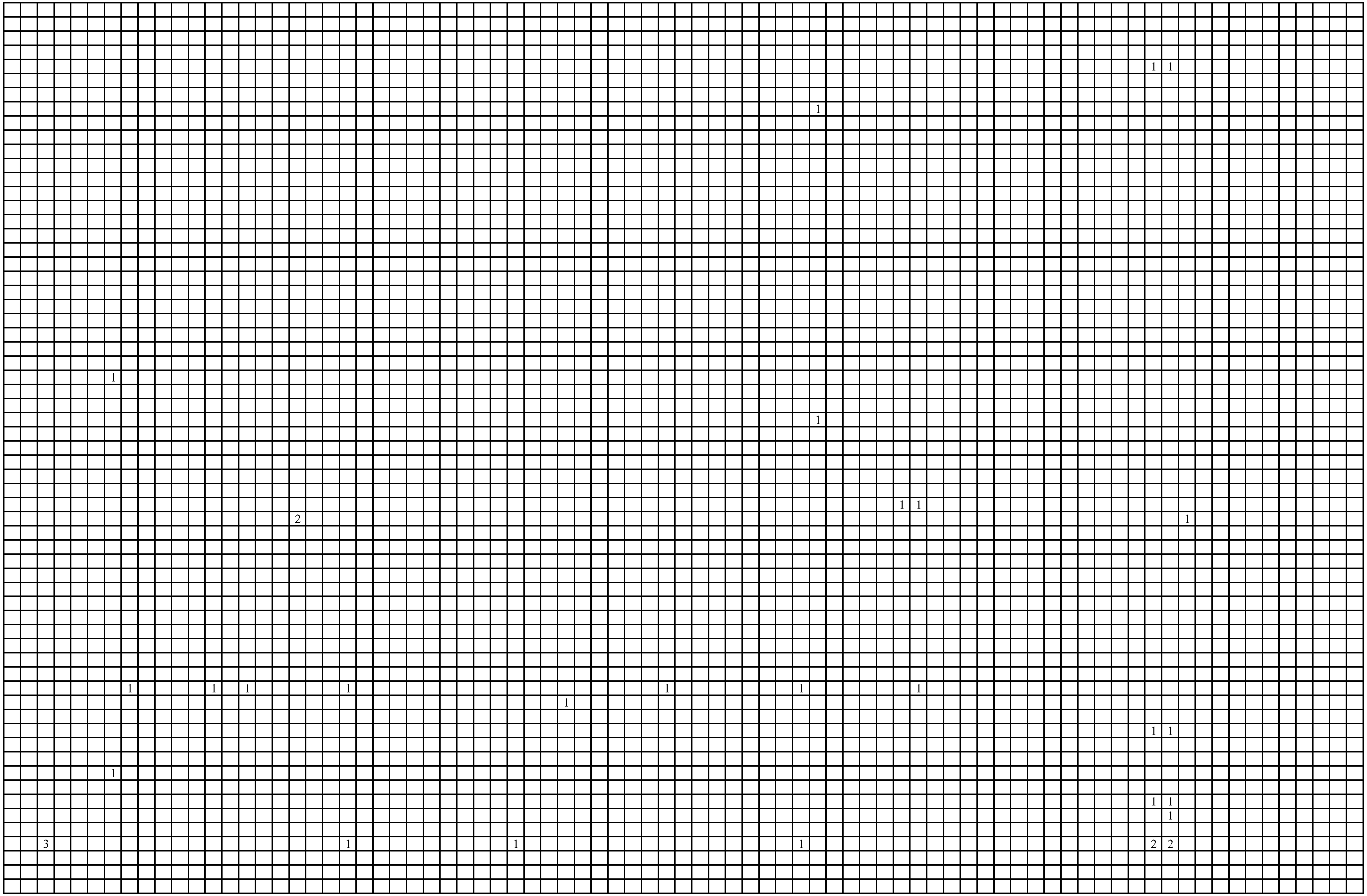

1 1
1
1
1
1 1
2 1
1 1 1 1 1 1 1 1
1
1 1
1
1 1
1
3 1 1 1 2 2

																																																													1					1	1											
																																																													1						1											
																																																																			1	1											
																																																																					1											
																																																	1																																
																																														1																																		
																														1																																																					
																																																1																																	
							1													1																																					1																										
																				1																																					1																										
																																																																						1	1											
																																																																						1	1											
																																																																						1	1											
		1																																												1																					1	1											
1																																																																																				
																																																																													1								

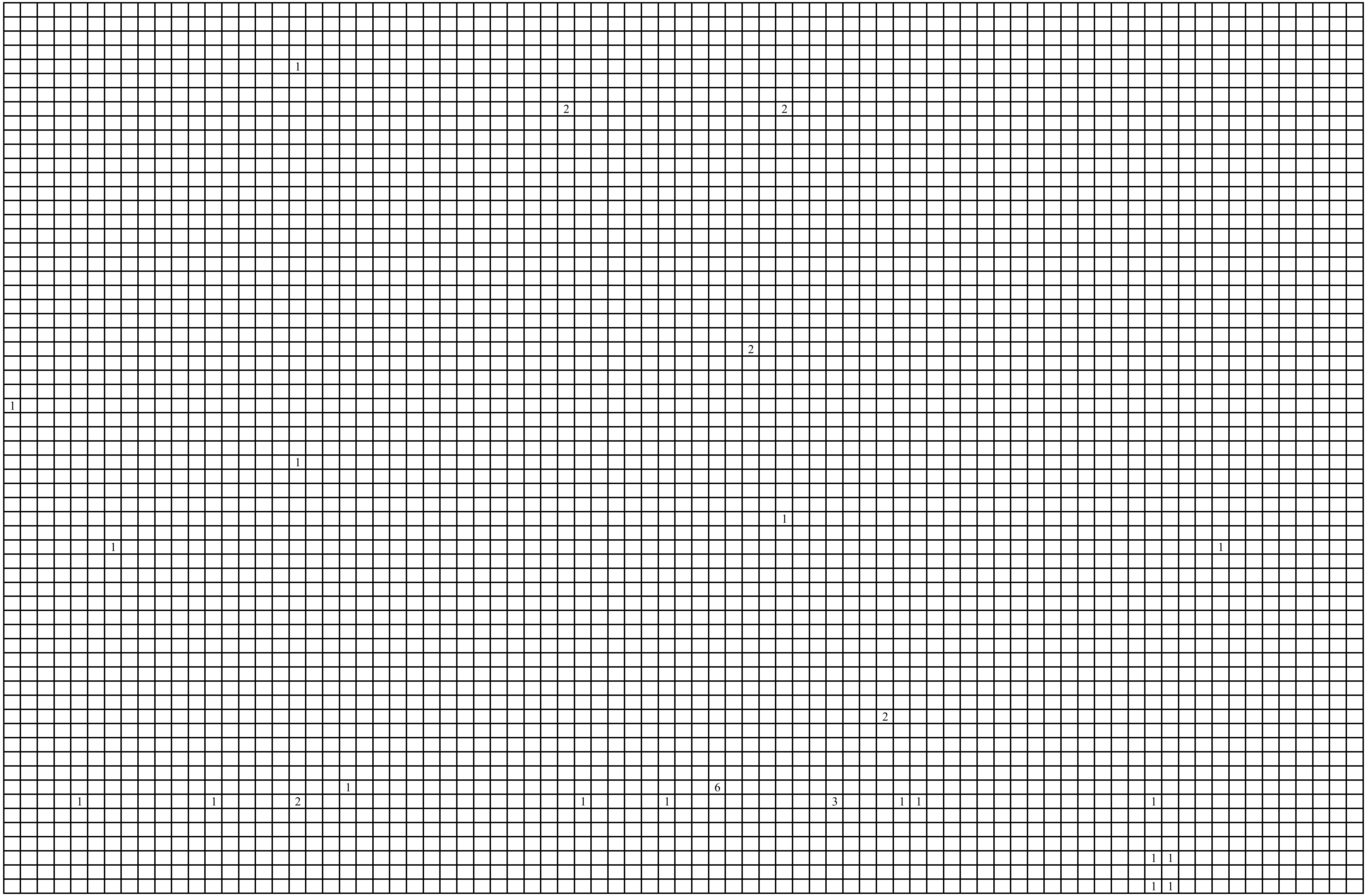
1
2 2
2
1
1
1 1
2
1 6
1 1 2 1 1 3 1 1 1
1 1
1 1

		1																																																													1					1	1											
																		1																																																														
				1														1																																			1																													1	
																																																																						1									
																																												1																																							
																																																				1																													
		1																																																																				1										1		
																																													1																																						
							1																																																											1														
																																											1																																								

		1																																																											1					1	1											
		1																																																																	1	1											
		1																																																																													
																																					1																																																				
																				1																																																																	
																				1																																																																
																																																	1																																									
																																																																									1	1											
																																																																										1											
																																														1																1					1	1											
																																																																										1	1											
																																																1																																
																																															1																							1											
																																																																											1										
																																																																											1										
		1																																												1																																									
																																																																											3										

		1					2													1																																	1																											
																																																																								1								
																																																																								1								
																																												1	1																																					
																	1																																																																	
																																																																					1												
																																											1																																								
																	2							1																																																												
1																																																																																
		1																																																																	1	1											
		1																																																																														
																																																																						1	1											
																							1																							1																1																	
		2																																																																	1	2											
																																																				1																										
																																														1																				1	1											
																																																																							1											

		1																																																			1										1					1	1											
					1																																																																											
						1																																																																										
																																										3						1																											
																												1																																																						
																																				3																																																	
																																				3																																																	
																1																																									1																										
				1																				1																																																											
1																																																																																			
																																																												1					1	1											
						1																																																																											
																				2																																																																
					1																																																																											

							1													1																																																										
				1													1																																																													
			1																																																																													
																																																2																																
1													1				1															1																1	2																															
				1								1		1			1							1																						1																																		
																																													1			3																															
																																																	2																															
																																																1																																
																	1																																																																	
1																																																																																	
						1																																																																											
																																				3																																																	
																																				3																																																	
																																				4																																																	
																																				3																																																	
						1						1					1																			3																																																		1	
1																																																																																	

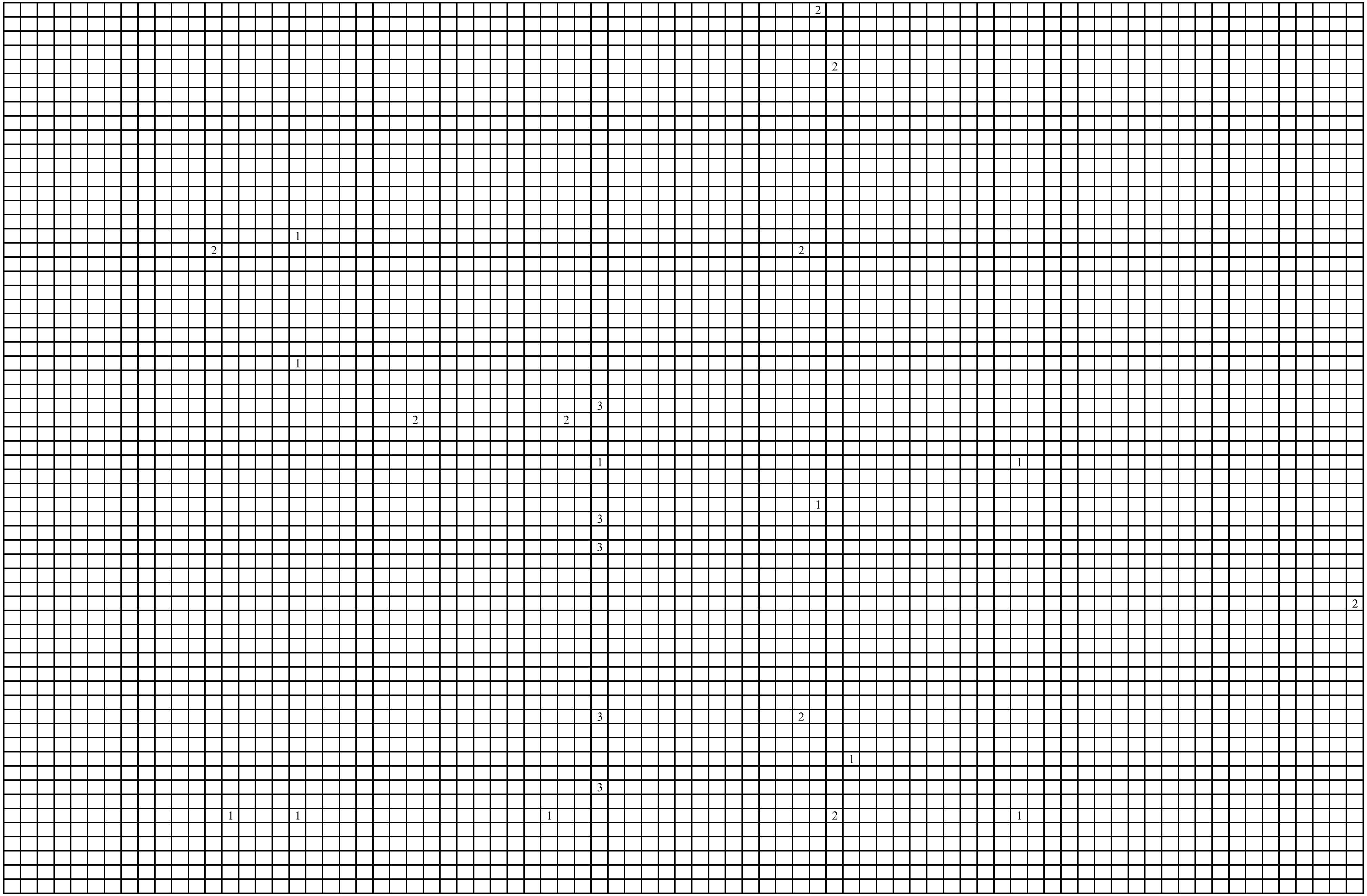
2
2
1
2 2
1
3
2 2
1 1
1
3
3
2
3 2
1
3
1 1 1 2 1

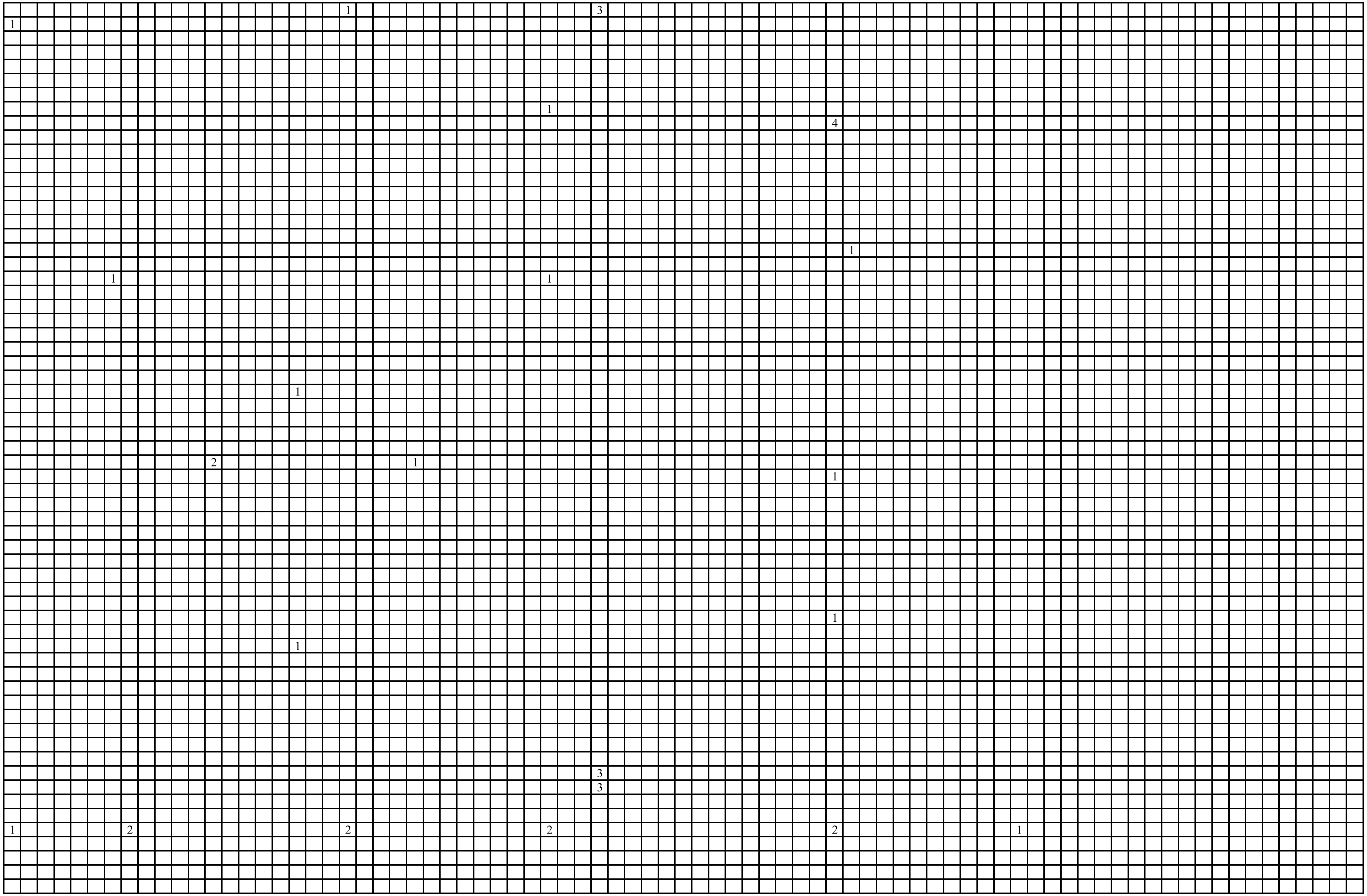
1
3
1
1
4
1
1
1
1
2
1
1
1
1
3
3
1
2
2
2
2
1

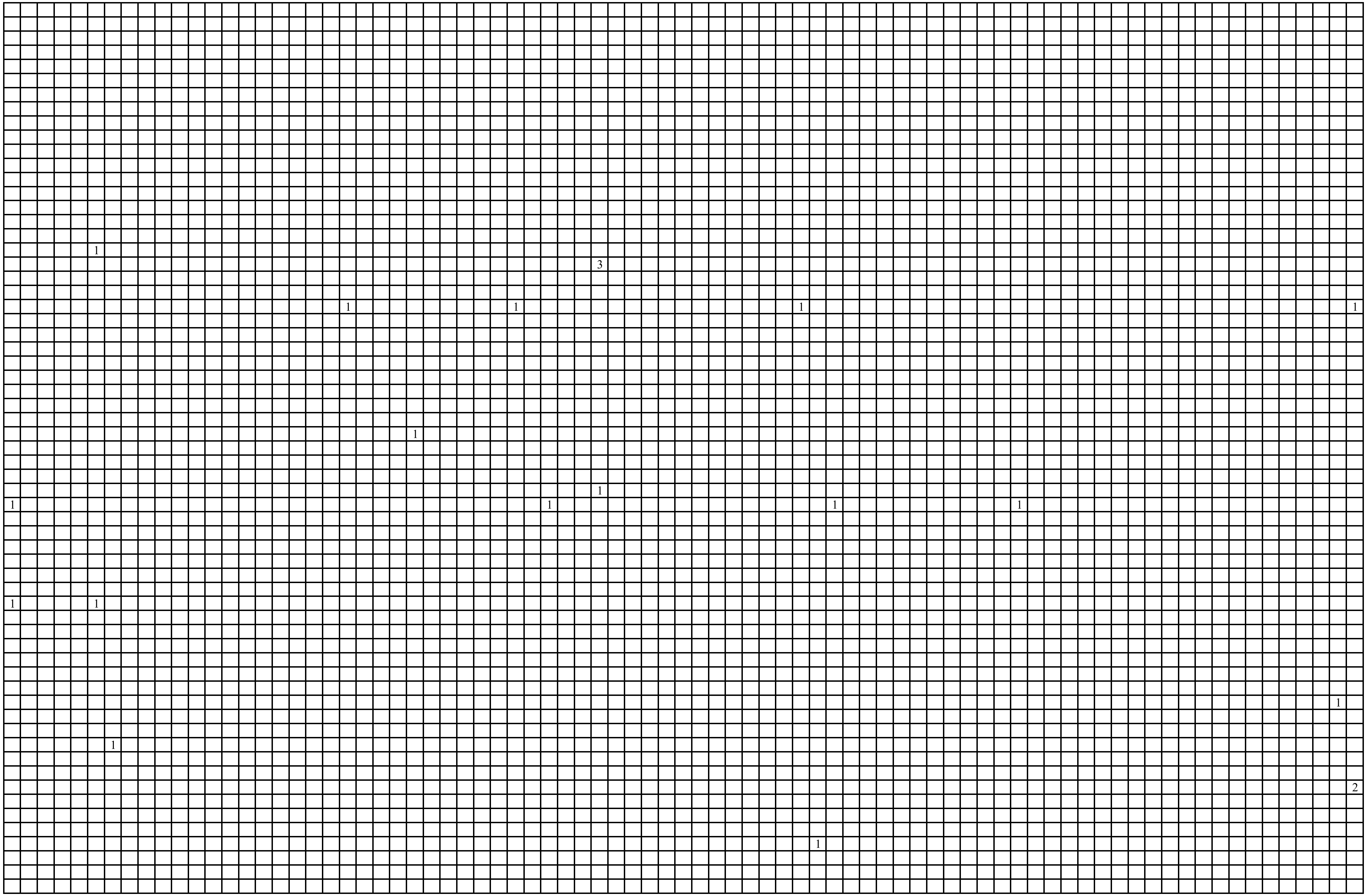
1
3
1
1
1
1
1
1
1
1
1
1
1
1
1
1
2
1

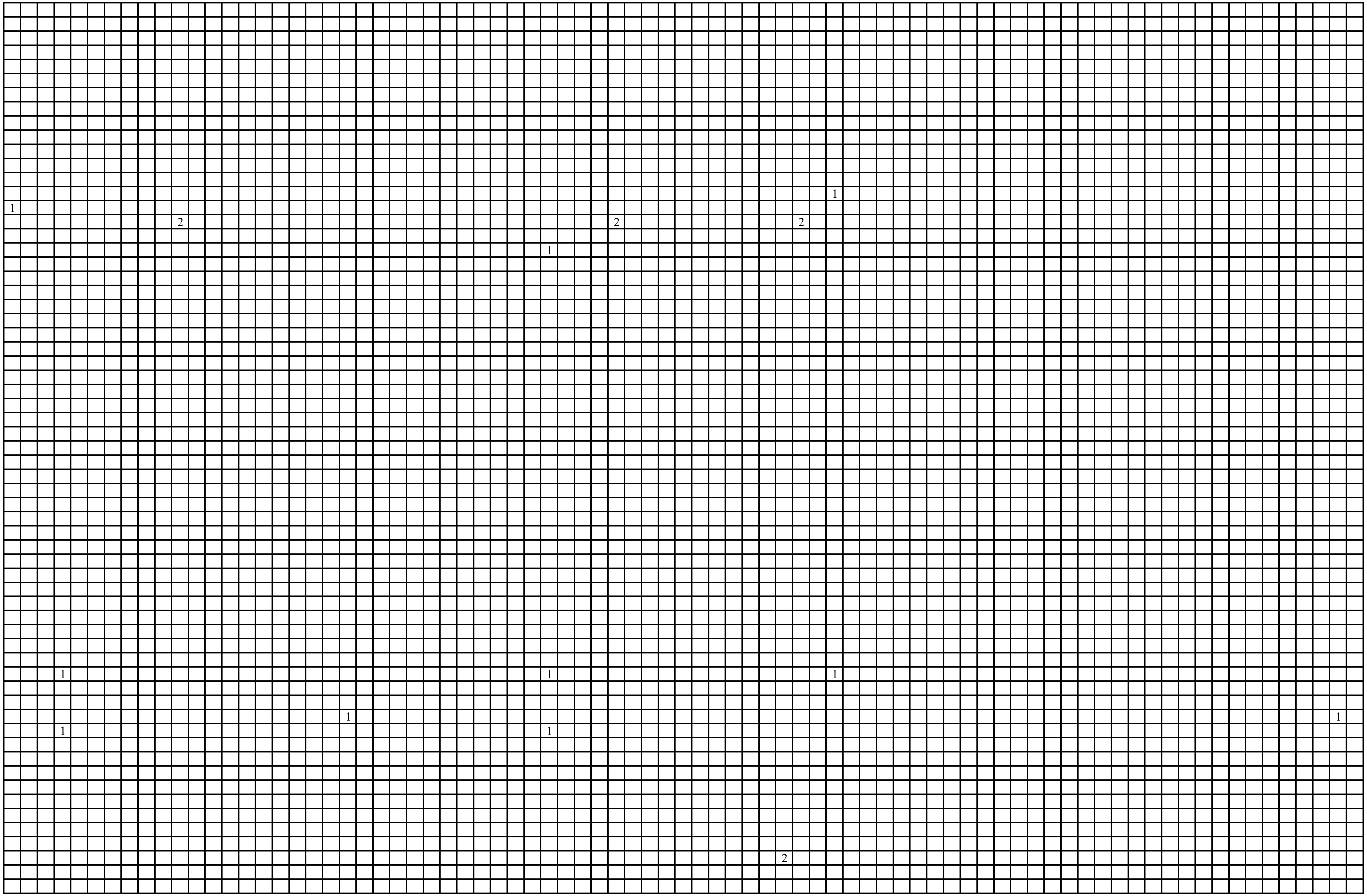
1
1
2 2 2
1
1 1 1
1 1
1 1
2

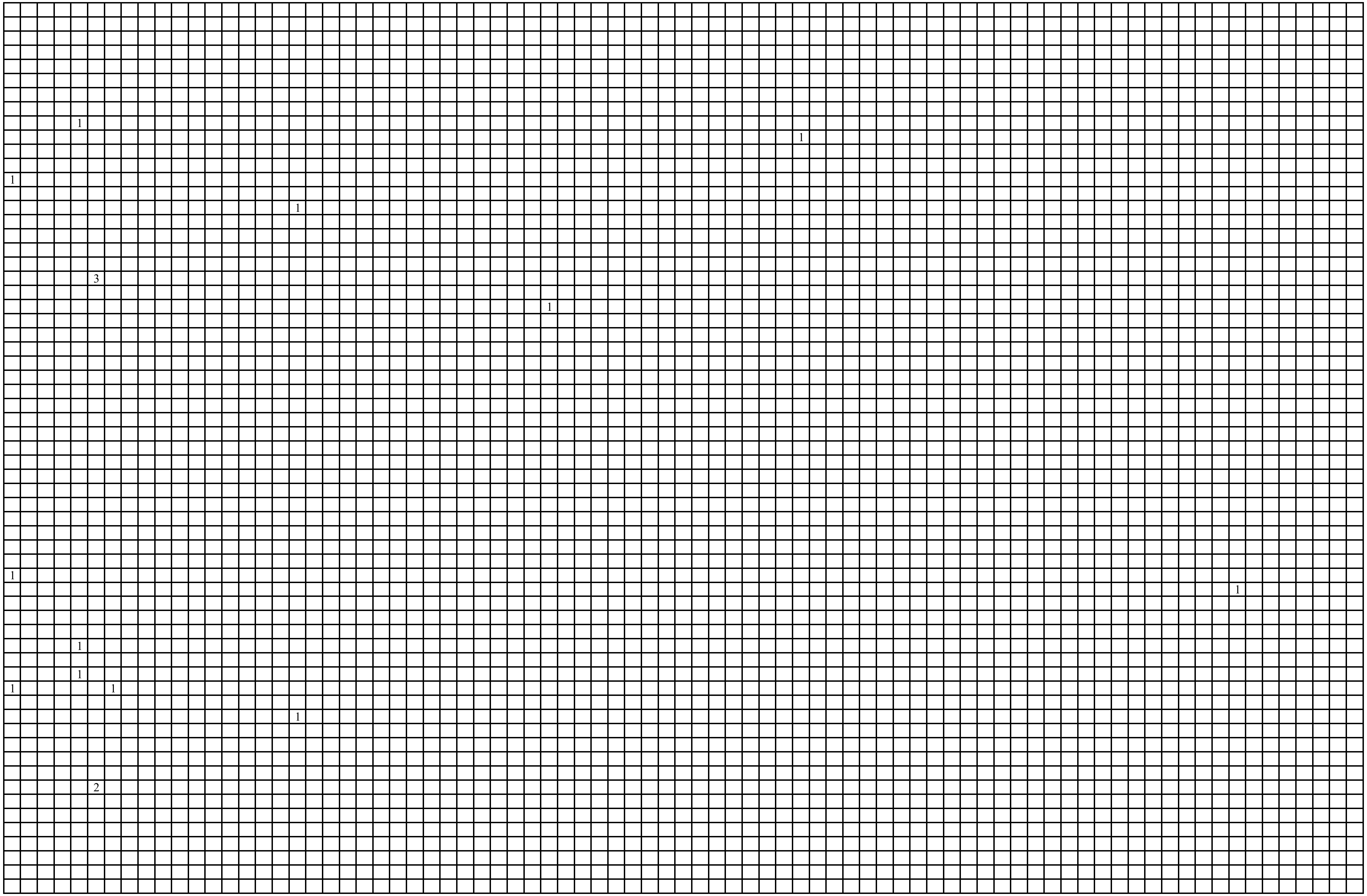
1
1
1
1
3
1
1
1
1
1
1
1
1
2

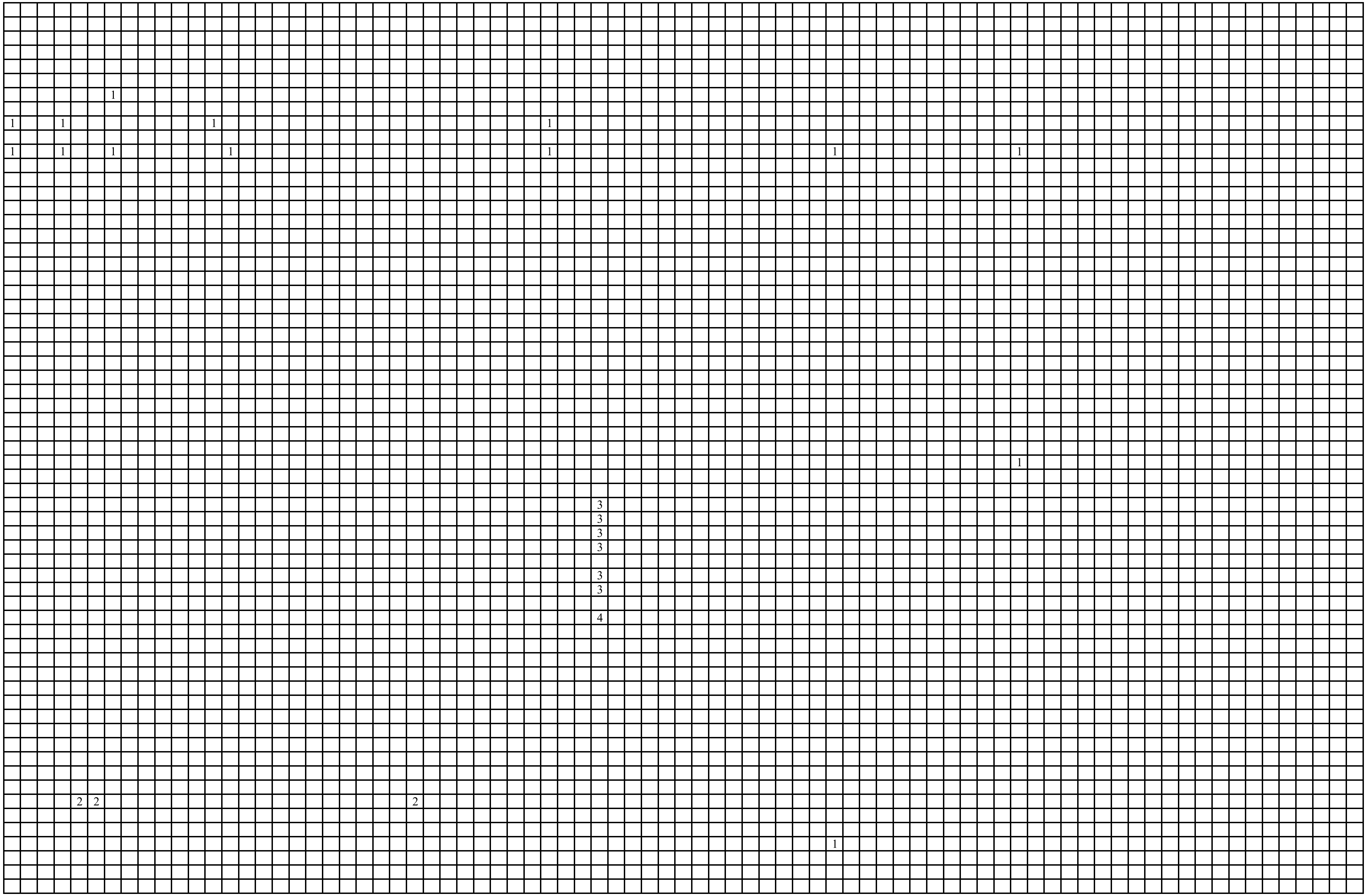

1
1 1 1 1
1 1 1 1 1 1 1
1
3
3
3
3
3
3
4
2 2 2
1

1

1

1

1 6 1 1 1

						1																																			2		2																								1							
1												1																																					1																															
																																			3																																																			
				1								1					1							1																																											1													
												1												1								1	1																																																		1
																	3																															1																																						
				1																												1																																					1														
			2																														1																																																			
1																	1																																																																
																																																		2																																
																				1																																																													
								2																																																																								
										2																																																																							
																	1																																																																
																																	1																																																			
																	1																																																																
				1								1		1										1								1																																																				
							1																																																																												
																	1																																																																
																										2																																																								
1			1										1				1															4																												1																				
																																					6																																																	
																																								2																																													

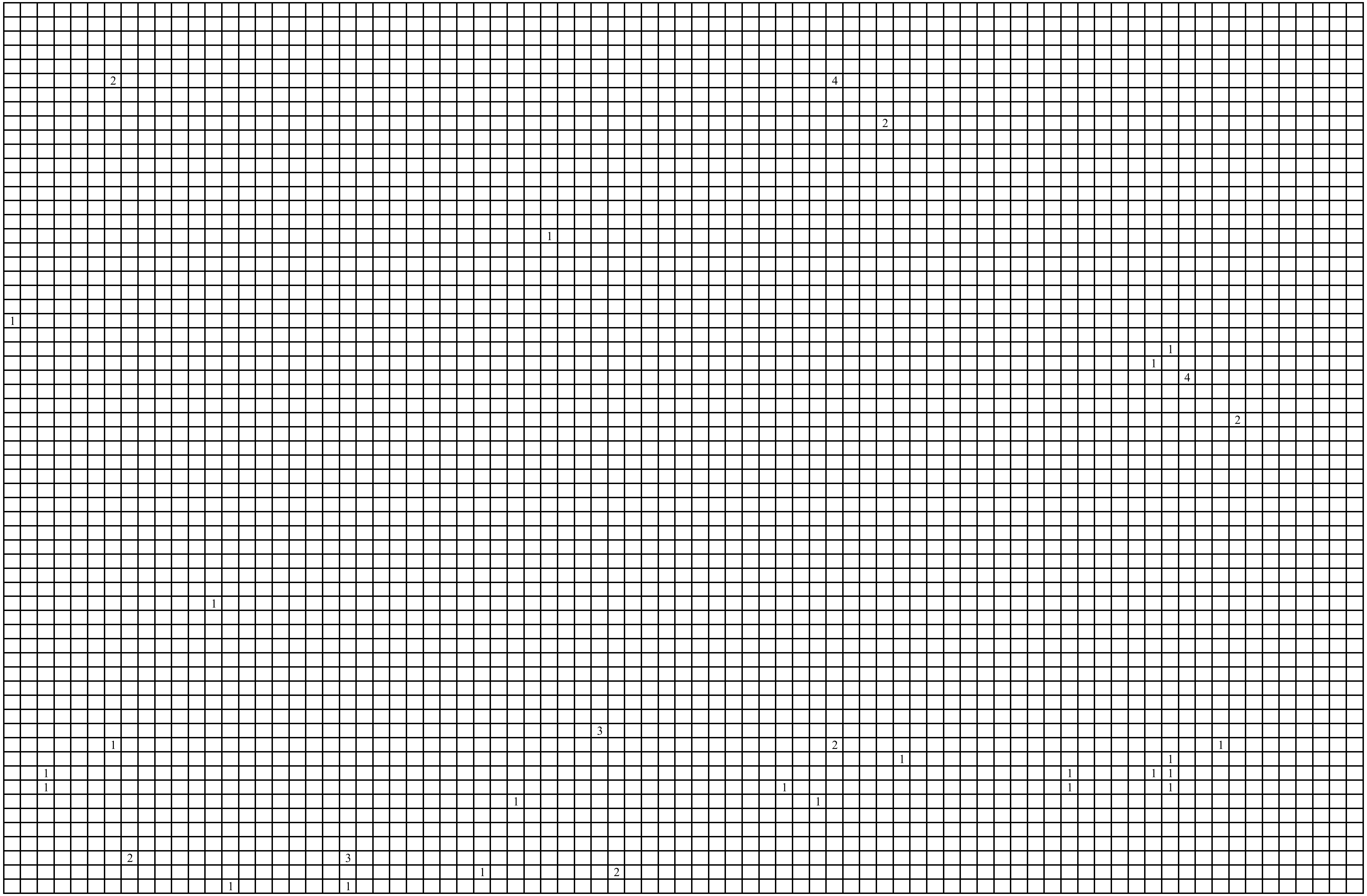

																																	2			2																																																			
																																	2																																																				
																																																																					1	1											
																																														1						2	1														1	2											
				1	1		1													1										1			1							1						1						1	1	1		1														1										
				1			1			1							1													1			1														1						1	1															1											
		1																		1																		1										1																																				
						1																																																																											
						1																																																																											
		1																																						1	1						1																																				
						1																																																																											
																																																	1																																				
												1																																																							1	1											
												1																																																																					
																																										1																																										
																				1																						5											1																											
																																										1																																										
					1																																																	1																											
				1								1					1							1																																																										
				1																																																																														
																																																		1																																			
																																	2			2																																																			
	1									1		1																		1			2						1													1		1																											

																																				1																																												
																																				1													1																															

																										ZNF720		1	6
																										ZNF723		0	0
																										ZNF728		0	1
																										ZNF735		0	1
																										ZNF761		1	10
																										ZNF773		3	13
																										ZNF783		8	14
																										ZNF8		3	48
																										ZNF821		0	13
																										ZNF827		9	30
																										ZNF831		0	5
																										ZNF850		2	6
																										ZNF865		5	57
																										ZNF888		4	10
																										ZSCAN18		23	56
																										ZGPAT	ZF.C3H	29	246
																								1	1	GATA1	ZF.C4_GATA	46	201
																	1		1		1	1	1	1	1	GATA2		75	223
																					1	1		1	1	GATA3		60	206
													1			5					1				1	GATA4		105	457
																		1								GATA5		3	11
																										GATA6		11	25
																			2	1						GATAD1		53	190
																			1							GATAD2A		90	315
																			1							GATAD2B		104	358
																			1							MTA1		148	520
																			1							MTA2		143	586
																			1							MTA3		73	254
																										RERE		15	106
			1																							TRPS1		76	166
																										ZGLP1		0	0
																	3	1			2		1	4	4	AR	ZF.C4_NR	339	1689
			2													1		5					1		1	ESR1		402	3355
			1											1			1	1				1	1			ESR2		195	2403
																										ESRRA		29	92
																										ESRRB		10	121
																										ESRRG		43	108
																	1		1		2		1	2	2	NR3C1		187	1749
																										NR3C2		7	48
																									1	PGR		42	134
																										NR1D1		8	30
																										NR1D2		13	72
																										NR1H2		40	101
																										NR1H3		41	89
																										NR1H4		16	47
																										NR1I2		49	99
																										NR1I3		17	80
																										PPARA		36	123
																										PPARD		41	159
											1												1	1		PPARG		216	689
																		1							1	RARA		137	367
																									1	RARB		21	36
					1																				1	RARG		20	59
			1																							RORA		13	48
																										RORB		13	44
																										RORC		10	47
																		1								THRA		38	165
																										THRB		46	135
																										VDR		98	254
																			1		1	1	1	1	1	HNF4A		119	344
																										HNF4G		5	17
			1																							NR2C1		59	119
																										NR2C2		105	1448
																										NR2E1		16	72
																										NR2E3		19	26
																										NR2F1		38	75
			1																							NR2F2		37	189
																										NR2F6		22	54
			2															1								RXRA		201	344
																										RXRB		52	96
																										RXRG		50	67
											1															NR4A1		61	277
																										NR4A2		10	33
																										NR4A3		7	51
																										NR5A1		22	55
																										NR5A2		19	51
																										NR6A1		3	8
																										NR0B1		35	47
																										NR0B2		92	155
																										ZFP36L1	ZF.CCCH	1	33
																										LIN28A	ZF.CCHC	28	475

																		2																																																														
																																																																													3		
																2	1	1																				1																																															
																										3																																																										
																			2																																																		1												
																																																					1		1													1												
																															1	9																																		2															
																																1																																																						
														1													1				1	2	1																									3																			1		
																																					1													1					2													1												
																																																								1																									
																																										2									3				2	2													2												
																																3																																																						
																		2							5						6	4		2	2	2																					1						4												2									
																															10	38	10	2		2	9	2	6			1	1	2	11	4	5	4	26	11	3	2	2	2		6	9	10	1	2							1	13	1		2	1		1	2	1				
																																																			2					1													2												

																														1		1																											1																						
																		1														1										1																																								
																																																							1																							
														1							1	2	1	1	2						1																									3			1																1			
																1	1																																																														
																														2																											2															1		2			
																																																										1															3					
																										1						1																															1	1	1													
																																				1																																														
																																1																																																
																																1																																																
																		2																																																				2									
														1				3																																																											2		
																		3																																																													
																														1						2												1																																	
																																1																																																
																															2												1																																						
																															1																																																		
																																																																						1									
																													1																																																					
																														1																									1																								
																																								1																																												
																									1																																																										
																																1																																																
																1									1		1				3	4					2											1									1									1							1						
																						1									1																																																			
																																																1																															
																					1																																																															
															2																																	1																															
																																																	1																															
																																						1								4																																			
																																1																									1							1		1													
																														1	1																																																		
																																							5																																														
																																						1												1																																	
																																																																			2																

																																							3																																																	
																																2																																																						2		
																																1																														1																				
																															1		1				2																				2																			3						
																																1	1																																																		
																1							2	1	1	2	1				1	1					1								2				1	3	1							2			1	1																			
																		1					2	2	2	2					1	5		2			2												2		1		1									1				1			1											
																1																																																																
																																																													1																				
																																1																																																						
																																					1																																															
																					1												1																													1																				
																			1													2																														1								1									2
																																	1																																						1									
																																2	1																																															1
																						1			3						2	2																		2																															
																										1					2																																																			
																																	1																										1																					
																		1	1										1		9	2																																			1														
																																																1																		1															
																	1	1						2		1																																	1																					
																																																																	1															

																			1																																																												
																								1							2																																		1															
																													1																																																			
																																																								1																								
																								1					1				1				1																																											
																																1	1				1																																											
																						1									1	1	1																																															
																									2	2	3					5																1											1																					
																													1								1																									1			1															
																																1																													1																			
																																																																			1													
																															3	5																	3	1									2																					
																																	1									1	1															3																						
																																1																										1																						
																	1							2	1						2		1																										1						2	2														
																								3																																									2	2														
																		1																																																														
																								1							1		1																																2	2														
																								1					1																																																			
																								1																																									1															
																																	1				1																																											
																										1																																																						
																															1																		2																															
																																	1																																															
																															1																																																	
																															2	1	1																																															
																																								1																													1											
																																1	1				1																																											
																			1												1	1	1																1	1												1																		
																	1														2		1				1																						1																					
																																					1																																									2		

																		1	1								1																																																										
																	1	1	1								1											1				1																			1																					
																							1							1		1					1																									1	1																		
																															1																																1	1																		
																							1							1																																1																			
																	5																				1								1	1	1		1									1																						
																															2	2					5		1											1						1		1							2			2												
																															1	2					3		1																										1			1												
																																1																																																
																																					1																																											
																																2																																																
																																2																																																
												1			1																2																																		1										4			
																															1	1	1																										1			1																		1
																			1																																																													
																									3																																																							
																	1																																																															
																	1								4	3																																	1																				
																	1												1		1	1	1																																															
																	1	1																																																												
																																1																										1																				
																																1	1																									1																				
																															1		1				1																																											
																2																	1								1																														1									
																															1																																																	
																			1																																																													
																																					2																																											
																															1																																																	

																																1																																																
																																																														1																
																																														1																																	
															1																																																				1										
																																1																																																
																																	1			1					1										1					1		1																							
																																2																											3						1															
																													1																																																					
																													1																																1	1																		
																						1							1																																1	1																		
																													1																																1	1																		
																																	1																																																	
																													1																																																					

																	1															1					1												1																															
												1			1										1	1	1																																																				
																										1																							1																													
																																	1																																														
																			7																																																												
																															3	1																				1																													
																													1																																1																			
																																																													1																			
																												1	1																																1																			
																																				1																																													
																																					1													1																													
																			1																																																												
																			1									1	1																																1	1		2																
																												1	1																																																		
																1																																																										1						
																																									1																																						

																													1																																1	1																		
																																		1		2		1										1	1				1						1																					
																									1																																																6							
																	1	1														1	1																																																	
																																																		3																														
																			1														1				1																																														
																																		1																																																
																														1		1																																																
																			1												1						1		1																							1																		
																														1						1		1																																														
																	1														1	1	1																1									1																					
																																		1				1																																														
																					1	1	1	1	1	1					1									1																																												
																	1	1													2	1	1				1												2									1			1																		
																															1	1																																															
												1					1	2			1		1	1	1	1	1		1		1	1	1	1			1																							1			1	1		1	1														
																																1																																																
																													1																																																							
						1																												1																																																
																													1																																1																			
																													1																																																							
																			2				2	1	2	2	1		2																																	2	2																		

																							1				1		1																																																				
																							1	1	1		1		1																															1																				
																													1																																																				
																													1																																										3						
																															2																																																
																																					1																																												
																	1	3								1					2																	1											1																						
																			1													1																																																	
																1	1	1					1	1	1	1	1					1	1																																															
																	1										1				1	1																											1																						
																1	1		1												5	8	2				2		1												1								1												2							1		
																		1																																											1																			
																															1	1																																																
																							1		1	1	1				1	1																														1																			
																									1	1																						3	2																																
																							1		3	1																																																									
																															2	5			1		1																		1				1																							
																			1																																																															
																																	1																																																
																																																																							1												
																															1																									3																								
																															1																																																			
																			5		2			1					1																					2																			1		2										
																	2	2											1		1						1																						1		1																				
																	2	1											1		2	1	1				1																						1		1																				
																	2	2											1		1		1																												1	1																			
																						1							1		1		1																												1	1																			
																																					1																																															
																			1												4	1					1												3							1					1	1						1				1									
																																	1																								1																						
																															1	1																																						1											
																			1													1	1																													1																			
																			1													2																																																		
															2																																																																		

																																	1																																															
																																1	1																													1								1										1
																																1	1																														3																	
																															1	2	1				2																																											
																																1	1																													1																		
																																																											1																					
																																	1																																															
																																	1																																															
																																1	1																																															
																																1																																																
																															1	1	1																1													1									1									
																																																								1			1																					
																									1																																																							
																															1	1	1				1																																											
																															1	1	1																										1			1																		
																1																1																														1																		1
																																2	1																				2	2																										
																															1																																																	
																															1	1	1																													1																		
																			1												1	1	2		1																										1																		
																												1					3																																				1									1		
																																1	1																																															
																																8													4																	1																		
																																					1																																											
																			1												1	1	1																													1																		
																																	1																										1			1																		
																															1																																																	
																															1																																																	
																																1																																																
																															3	1	1				1																																											
																																	1																										1																					
																		1	1				1	1	6	4						5		1		1	4								2				3						1		2	2	5												4							5		
																													1																																	1																		
																													1																																	1																		

																			1										1																																1	1																1		
																								1	1	2	2																																																					
																																2																																																				2
																																2	1																																																	1
																																																										1																					
																																					2												2									1										1			1									
																																	1				1																					1																						
																															2			1		1																																																								
																																6					3		1								1	1	10									1										2												
																								1					1								2																																																								
																							1	1	1	1						1																																																											
																																	1																																																							
																																												1																																							
																		1	1		1		1				1				1		1																										1							1														
																							1	1		1																								1								3														2								
																																																																												1		
																																																											1																						
																																																														1																		
																																	1																																																				
									1										1										1				1																																																			
																															4																																																					
																								1								4																			1																	1												
																															12	1					4												3	1								4	2												3									
																	3														4																												1																			1		
																																																																								2									
																																																	1																																		
																																1	1									1	2				1	1	2									2				1						2												
																																	1																																																				
																															5	2	1				3		1									1														1									2									
																																2	1						2										4													1																		
																																					2		2																																																						
																																																		3																																	
																		2																																																																								
																																1																																																				
																															1																																																					
																																					1																																																		
																			3		1		1	1		1																																																														

																													1																																																		
																													1																															1																		
																													1							1																											1	1														
																															1																																																
																										1																																																					
																																																						1																									
																																				1																																											
																						1														1																							1																		
																						1								1		1																									1		1	1																	
																															2																																																
																						1																																																									
																						1						1																															1	1																	
																																																1																															
																			1								1	1		3																																																
																						1						1																																																		
																																					1																																										
																															1																1													1	1																	
																															1																																																
																																1	1																																														
																																																													1																		
																			1		1								1																															1	1																	
																7																																																															
																																1	1																												1																	
																															1		1																																														
																2	6	1																		1													2																													
																1																																																															
																																																														1																	
																																																																1															
																															1	1																													1							1											2
																																1	1																																														
																																1																																															
																		1				1						1																															1																		
																						1	1	1	1	1	1										1																						1																				
																	1	1	1												21	5	1				9		1	1	1	2	3	1	2		7	5	18	18		2				9	9	7	1			2						3												

																1	1	1	1												15	7	2				4		4	1		3		1	5	1	1	4	14	9	1	1				3	6	2	2						1	1		4		1	4	1							
							1		1								1	2								1		1		3		1				1																								1		2																	
																1	1	1									1				1						1																								1																					
																1	1	1	2											10						3												1										1			2										3							1		
																1	1		1											3						2																								1												2									
																			1																		1												1										3																					
															1																	1	1	1																										1																					
																															1				1																																								3					
																															2																																																	
																																																																														1		
															2																		1	1																																															
																																1	1																														1																	
																1											1																				1										1																					
																																3	1																														1																	
															1																																																																
																																																																1																	
																																					1																																											
																																	1																																															
															1																																																																
																						1						1	1																																	1	1																	
																						1	1				1		1																																	1	1																	
											1											1						1																																			2																	
											1											1						1																																		1	1																	
																				1	1																																																										
																						1	1	1			1		1																																	2	2																	
																															2	3																	2																															
																													1		1	3	3																	2	6										1							4													
																																																																														2		
																						1						1																																		1	1																	
																						1																	2																							1	1																	
															1		2	3		4									2	13	2	1	1	1	1		2										7	3			1						4				2		2			2						2					
																																1																															1																	2		
																																2	1																																															

																												1	1																																																			
																1																																																																
																	1														2																																																
																			1					1							1																																																
																							3	3	3																																																							
																																																	1																															
																3	1	1	6						2	2	1																																																					
														2																																																																	
														3																																																																	
														1																																																																	
																																	1																																															
														3																																			1																															
																1											4				1																1	2	1																															
																		1														1	1																																			2												1
																																																														1																		
														1																2																																																
																															2																																																
																																1	1																																															
																																	1																																															
																													1																																																			
																																					1																																										
																									3	2																																																					

																																																												1																		
																					1				1															1																1			1																		
																																				1																																															
																																1	1																																																			2
															2	2												1					2	1					1													2								3																						
																																				1												1																																
														1																																																																		
																						1			2					4	1	1																																																		
																																1	1																																																		
																							1																																																										
																																1																																																		
																																	1																																															
																																	1																																															
																																				1																																																
																																1																																																			1
																																				1																																																
																																1																																																			
																																																										1																					
													1																																																																					
							1																																																																										

																			1			4										2																		1																															
																						1															1																																																		
																																1	1																																																			
																															1		1																									1			1																			
																																	1																																																1
												2																																																																				
															1																																																																	
																																1																																																	
							1																																																																										
														1																																																																		
														1																																																																			
																																	1																																																1
																																						1																																																	
																																																													1																				
														1	1																	1	1																																																			

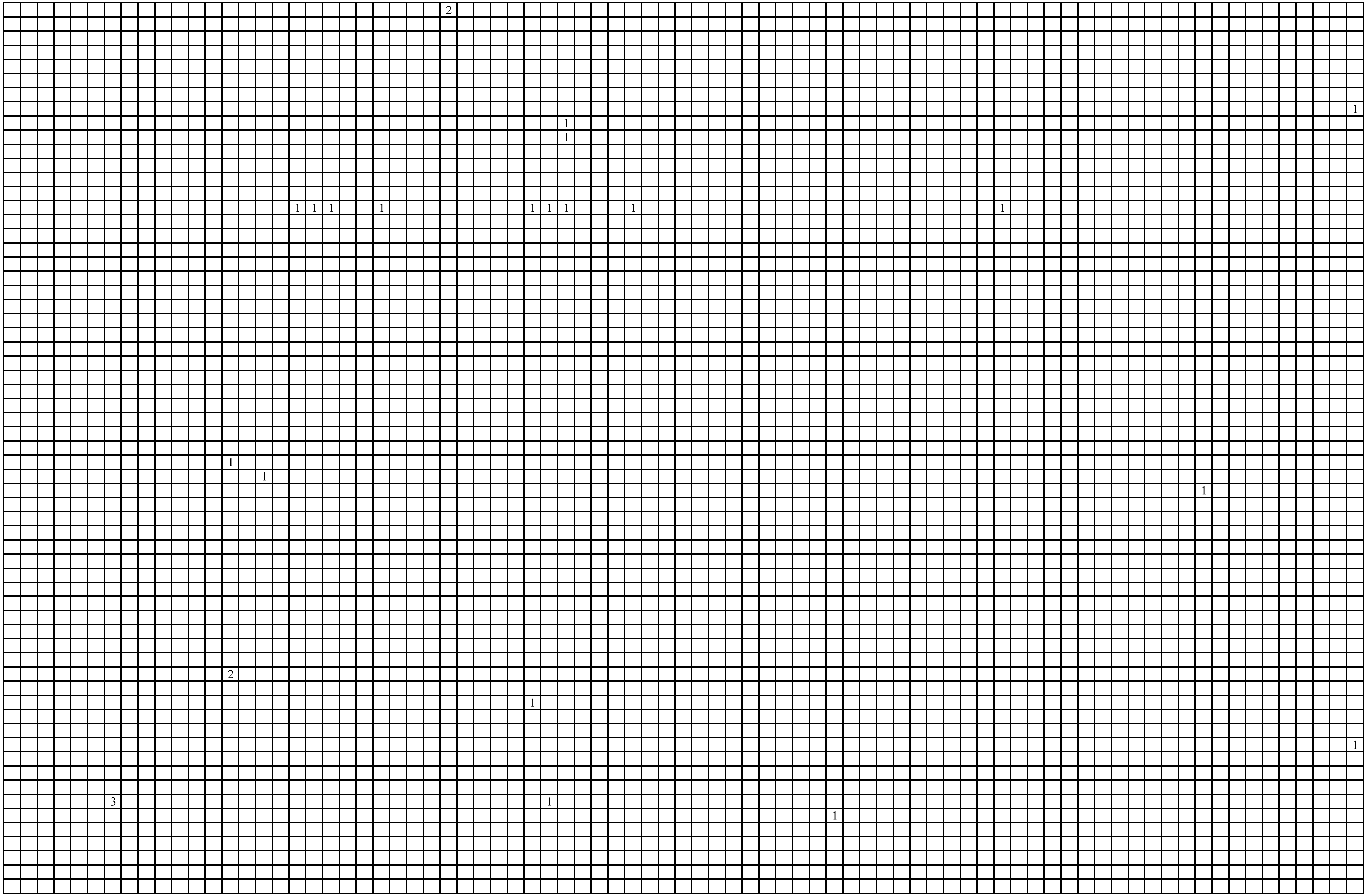
2
1
1
1
1 1 1 1 1 1 1 1 1
1
1
1
2
1
1
3 1
1

																																					1																																											
															1																																																																	
																																					1																																											
																																					1																																											
																																																											1																					
																																					1												1																															
																																																															1																		

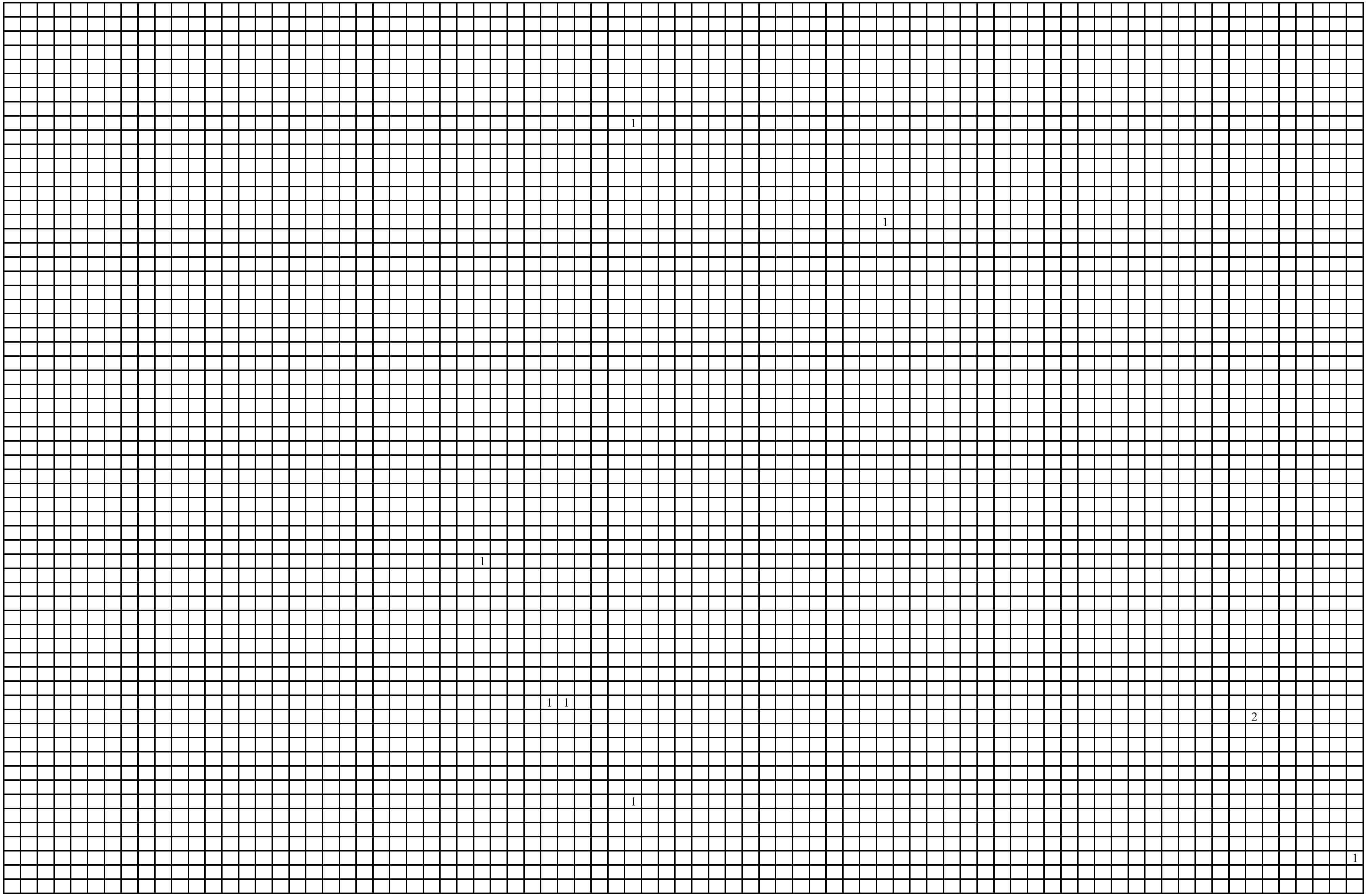
1
1
1
1 1
2
1
1

					1																																																																									
													1																																																														
						1																																																																							
																		1																																																												
															1																																																															
						1											1															1																																																
																		1														1	1																																															1
																																		1																																															

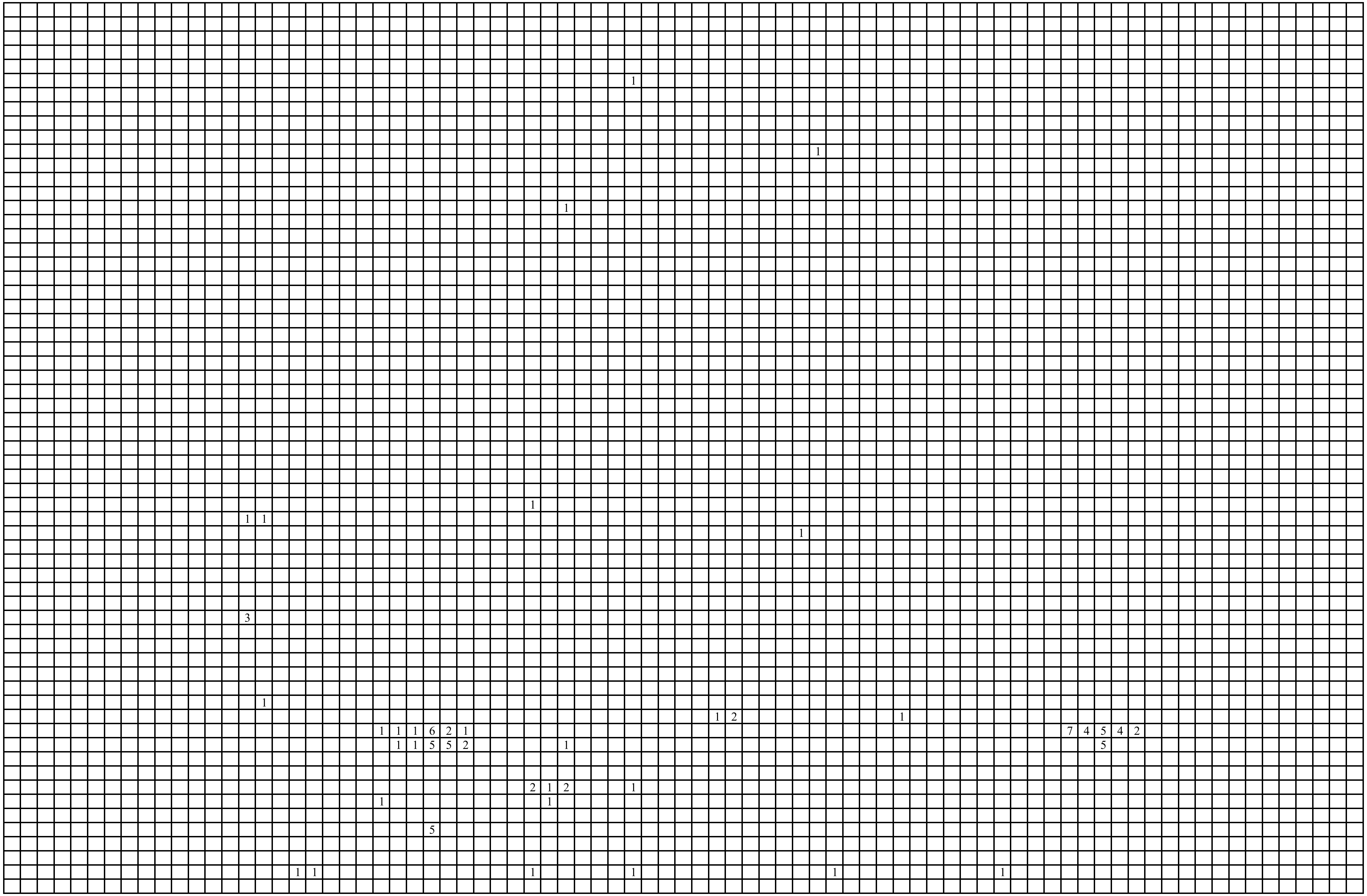

1
1
1
1
1 1
1
3
1
1 2 1
1 1 1 6 2 1 7 4 5 4 2
1 1 5 5 2 1 5
2 1 2 1
1 1
5
1 1 1 1 1 1

																1							2	1	5	3	2																																								1																		
															2		1						1	1	2	1																																																										
																																1																																																						
																1							2	3	3	2			1																																																											
																			1												2	1	1																														1									1									
																																		1																																																				1
																																		1																																																					
																							1	1	2	1																																																										
																																4							1																																																	
																												1													1																																															
																															1																												1			1																		
																	1		1				2	2	2	2	1										1																																																	
																			1																																																2														
																			1																																																													
																			1				1	1	2	1	1																																																									
																																1	1																																																					
																	1	1	1												2	1	1																									1																					
																								1		2					1	1																																																				
																																1																																																					
																							3	1	2	1	1					1																																																					
																			1												1																												1																					
																2	3		1																																																																
																																					1																																																	
																															1	2	1																																																				
																			1				3	3	3	3	4										1																																																
																																					1																																																	
																																1																																																						
																										1							1																																																							
																											1																																																										
																																		1																																																				
																						1																																																															

																																																						1																									
																			1						3																																																							
																																				1																																													
																			1												1																												1																					
				1																																																																											
																																1	1																																															
																																	1				2																										1																		1
																						1	2	2	4	2	2				1	1																																															
																	1																				1																																											
																									3																																		1																					
																	1	1																			1												1																															
																																1	1																																														1
																																1																																						1									
															1																										1						1							1																										
																							2									1	1																																															
																																	1																																															
					1																																																						1	1																				1
																									1																																																							
																																	1																																															1
																							3		1							1					1																									1																		
																																																																																1
																								1																																																							
																			1													1	1																																															
																										2	2					1	1																																															
														3																																																																	
																								2		2																																																							
																																					1																																											
																																1																																															
																																																	1																														
																																																	1												1	5						1												

																		1														1																																																
																															1		1																																															
																			1																		1												1							1																							
															2																																																																	
																																1																																																	
																1															2	1	1																																															
																															1	1	1																													1									1									
																																1																																																	
																	1	1																																																													
																	1											1			1																												1																					
																1	1	1										1			2		1																										1																					
																																1																																																	
															1																																																																	
						2																																																																									
																							1	2	1	2	1																																																					
																								1		1						1	1																																															
																		1											1																																1																			
																	1												1			1																		1																													
																			2			1	1		1	1	1									1																																						1						
																																																	1																															
																			1			2	1	1	1	2	1				1	1	1																										1																					
									1										1			1		1	4	5	4		1								1																										1																			
									2						1							1	1		5	6	5		1		1	1	1																																															
									1										1			1	4	5		8	2		2		1	7	1																												3	1																			

									2						1				1			2	5	6	8	2	3		1			3	1																	1											2	1									2									
									1										1			1	4	5	2	3			1		1	1	1																																															
																															2																																1																	
																	1	1					1	1	2	1	1				1	2					1												1										1																					
																						1		1	1		1	2	1		28																														2	3															2	3		
															1			1				1		1	7	3	1		2			24	8	3					2					1	1	1										1					2	2			4	2	1	2						1				5		
															1							1		1	1	1	1					8	8		1																															1	1													
																																3			2	7													1																			1									1	1		
																																	1	2		2																																												
																			1															7	2	10																																									3	5		
																							1						1								8	3					1						1															1		5	1		1		1		1				2	2		
																																					3																															1												
																																2																																													3			
																																									2																							2																
																																								2															1																									
																																															2	2	2	1								1										5	4	7								2		
																																					1										4	2	2																			4	6	5								6		
																																1																																				4	4	2										
																																1																											5									4	2	1					1			2		
																																1																											1									4									2	3		
																																										2	4																									6	1	2										
																																										2	2					2																				5	2	2								1		
																				1									1					1			1					2	2						2	3								2						1				37	4	1	5	1					6	3		
																										1																1							3	8	2	2									1			1				16	9	8	1							4		
																		1																																2																			4	4										
																																																		2																	1	1	4	2								1		
																																																																													2			
																																																						2	1																									
																																									1													1																										
																																1																								2												3										2		
																																																									6										3	8	1	1										
																																										1							2									4	2									29	2	3								1		
																						1							1																5	1												2	4		1	4																9		
																																																																														1		
																									3	2					2	2																		1									1			3																		
																	1						1		1	1					3	2																											4		3	4				1			1											
																												1																																																				
																																					1			2									1	1														2				1												
																																4																																																
																																2	1				5																									1				2	5													
																																1	1				1															1					3									5		1		2										
																																2		1				1				5	4	4	4	4	6	5	37	16		1				3	8	29						1			1	4		2	6	3						3		
																																					1					4	6	4	2		1	2	4	9	4	4					1	2				1										1								
																																										7	5	2	1		2	2	1	8	4	2					1	3									2	2			1							2		
																										2											1												5	1																		6		1	2									
																																																	1																			3	1											
																																					1																																											
																			1													1																																													7			
																																													1																																	9		
																																																																												2				
																															2			1		3	2		3							2			6				2																					7						
																															3	5		1		5	2					2	6		2	3		1	3	4		1				2		1	9	1								3		2					9					
																																																																																1
																																1																																																
																			1													1																														1																		
																						1	10	8	3	8	3		1		1	1																																																
																			1			1	7	12	4	9	2		1			1																		1											1																			
																		1														1	1																																															
							1																			1						1																	1																															

																																																											1																					
1																	1		1												4	2					1					3	2						1										1												1							2		
																															4	1	1				2		1																																	1									
																															1		1																																							2									
																2															2			1																									1																					
																															2																																																		
																															2																																																		
																																					1																																													
																								1		1						1	1																																																			
																																	1																																																

LIN28B	MBD1	BAZ2A	BAZ2B	MBD2	MBD3	MBD4	MECP2	SETDB1	SETDB2	DMRT1	DMRT2	DMRT3	DMRTA1	DMRTA2	DMRTB1	DMRTC1B	DMRTC2	PIAS1	PIAS2	PIAS3	PIAS4	ZMIZ1	ZMIZ2	ZNFX1	NFX1	NFXL1
248	93	88	65	247	416	112	1462	275	29	5	3	101	3	0	142	1	2	505	255	126	252	52	69	61	498	119
19	17	10	7	89	131	5	125	23	3	0	0	10	1	0	3	0	1	124	81	53	70	27	26	5	22	3
ZF.CCHC	ZF.CXXC									ZF.DM								ZF.MIZ						ZF.NFX1		
							1																			
							1																			
							1																			
				1	1																					
								3																		
												1									4					
					1																					
					1																					

AGO1	AGO2	AIM2	ASCC1	AUTS2	BBS7	BEX1	BRD2	BRD3	BTRC	CAMK1	CCAR1	CLU	CNOT1	CNOT2	CNOT6	COMMD6	CRYM	CSRP3	DUSP22	DVL2	DYRK1B	EGLN1	EIF2AK2	FBXW11	FGF2	GPS2	HAX1	HCK	HRAS	HSPA1A	HSPA1B	IKBKB	INSR	IRAK2	LMO1	LMO2	LOXL3	MAP2K3	MED23	MED25	MED7	MKL2	MTOR	NAB2	NCK1	NIF3L1	NLRP3	NOTCH1	NRG1	NSD1	PCBD2
240	641	112	127	84	136	55	521	600	1653	113	184	367	411	305	97	134	12	18	161	742	156	184	565	959	91	236	573	199	954	1931	388	629	460	78	171	375	31	311	342	169	233	62	854	151	349	169	217	541	174	138	53
19	31	36	9	5	10	4	60	74	198	1	23	12	23	14	3	13	1	7	1	49	12	38	33	117	1	65	15	9	19	89	16	39	10	3	54	68	2	27	48	22	12	7	27	11	4	9	6	80	4	50	13
TcoF.1																																																			
								1	2																																										
							1																																												
																																				1															
																				1																															
							1																							1																					
1																																																			
																																																1			
																																			1																
																	1																																	
																		3																																	
																		1																																	
1	1																																																		

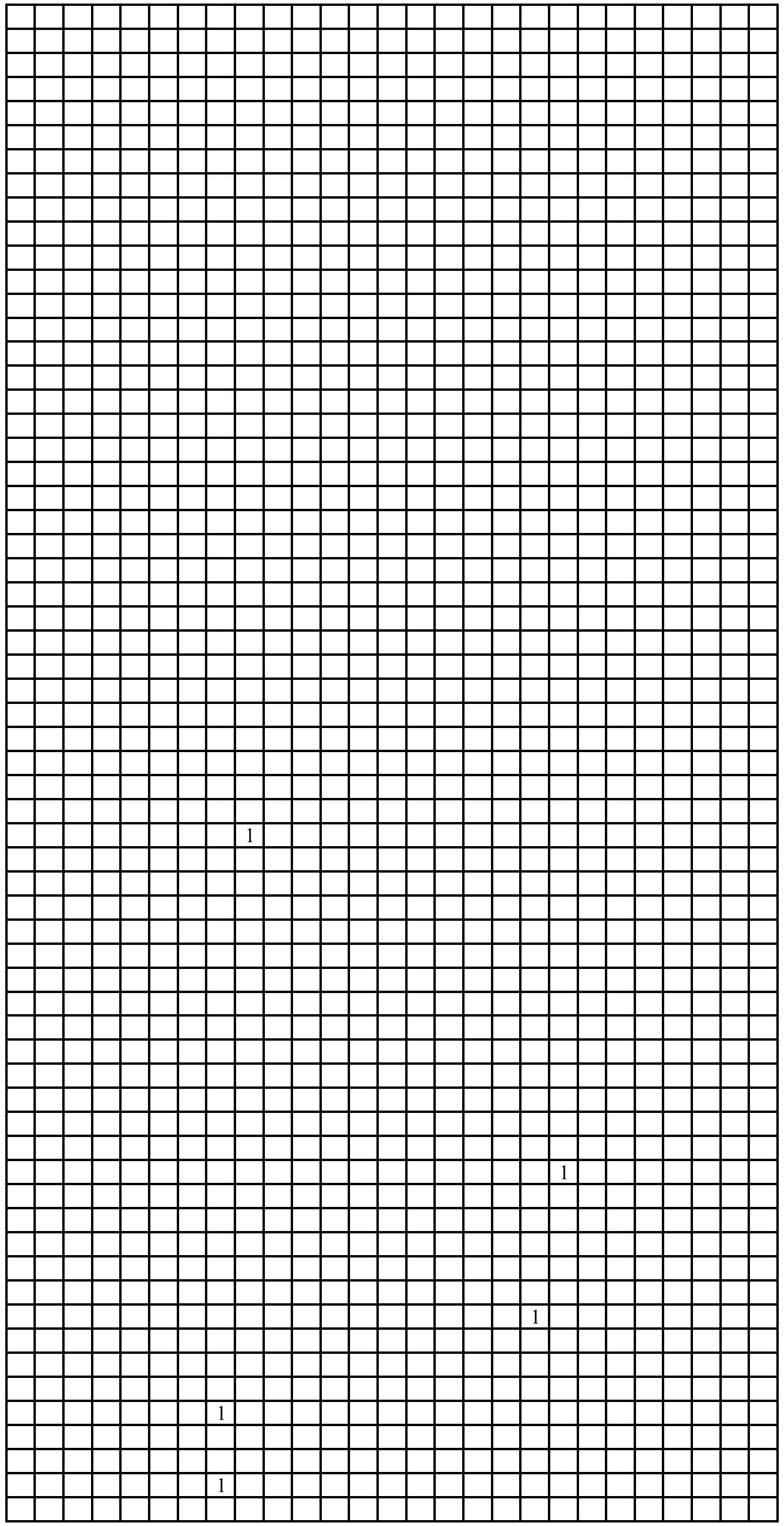
1
1
1
1
1
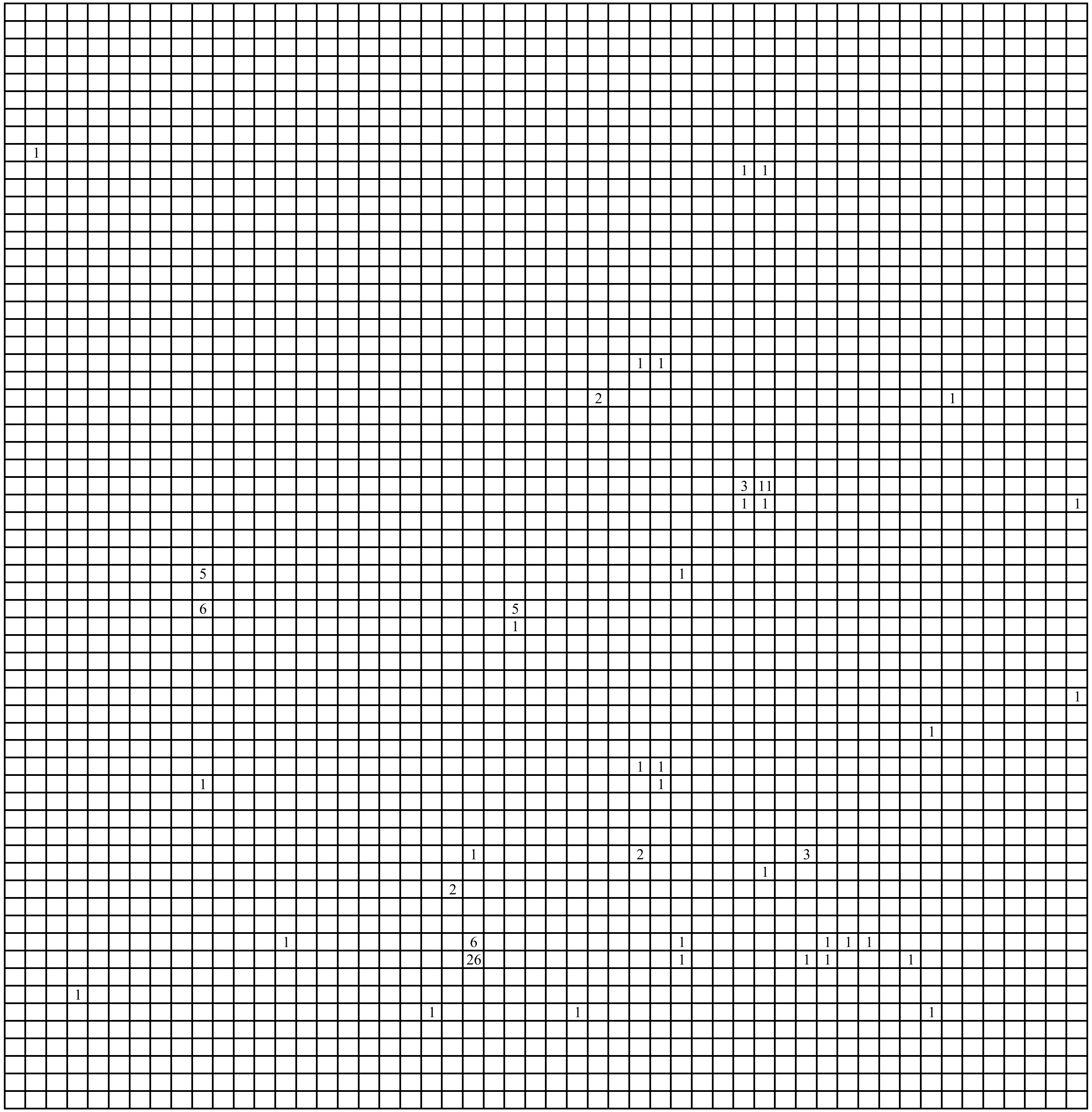
1
1 1
1 1
2 1
3 11
1 1 1
5 1
6 5
1
1
1
1 1
1 1
1 2 3
1
2
1 6 1 1 1 1
26 1 1 1 1
1
1 1 1

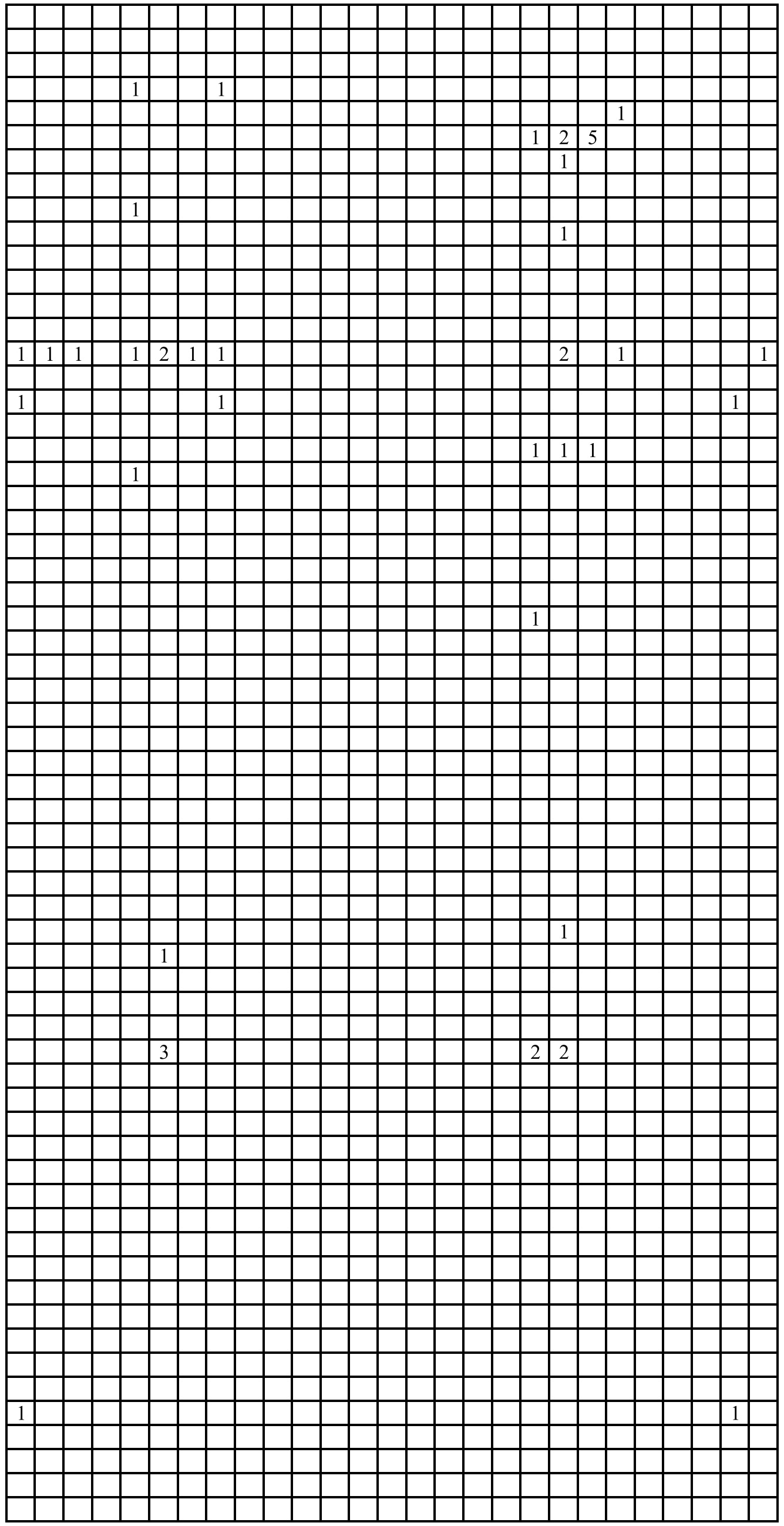
1 1
1
1 2 5
1
1
1
1 1 1 1 2 1 1 2 1 1
1 1 1
1 1 1
1
1
1
1
3 2 2
1 1

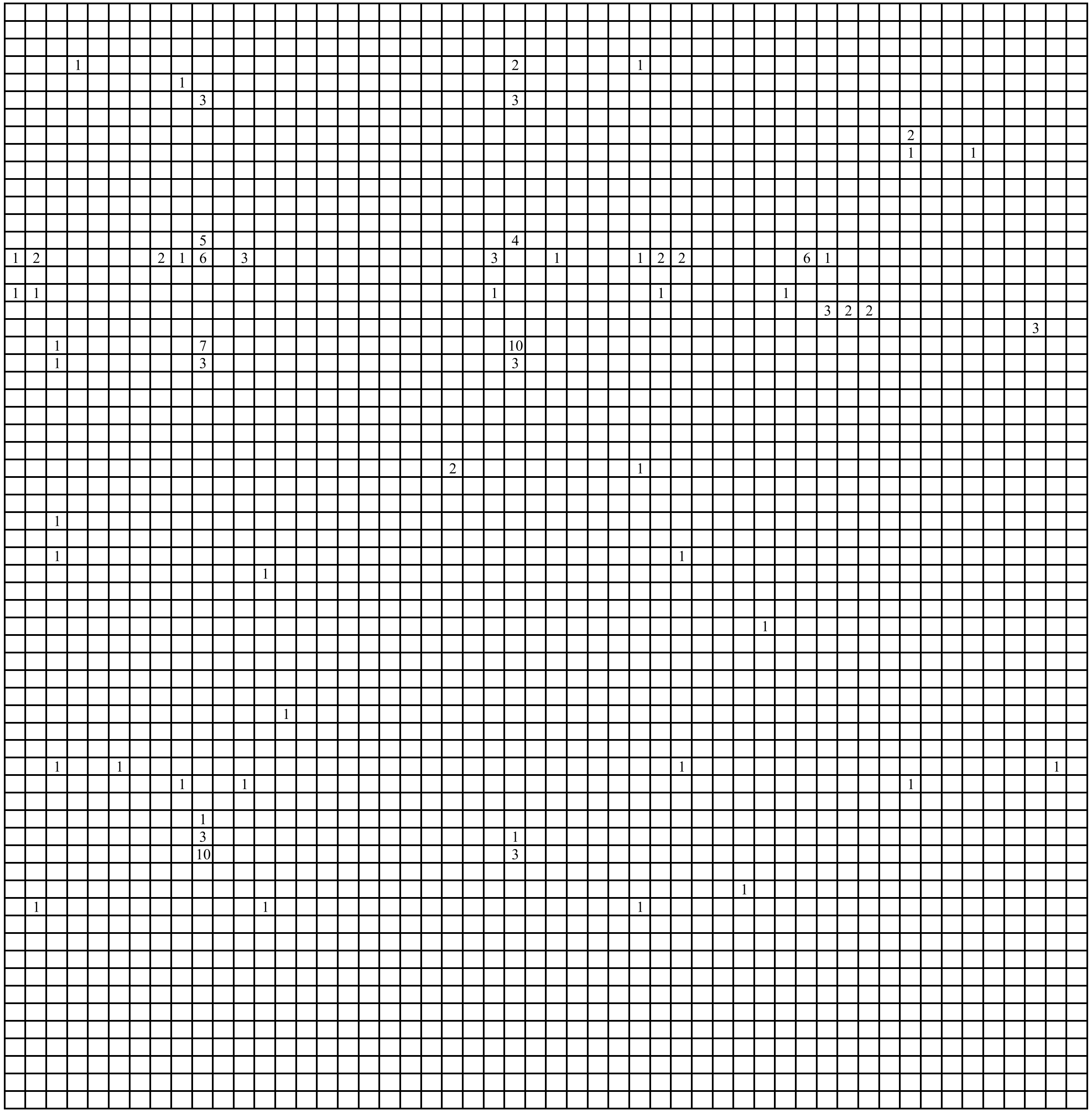
1 2 1
1
3 3
2
1 1
5 4
1 2 2 1 6 3 3 1 1 2 2 6 1
1 1 1 1 1
3 2 2
3
1 7 10
1 3 3
2 1
1
1 1
1
1
1
1 1 1 1
1 1 1
1
3 1
10 3
1
1 1 1

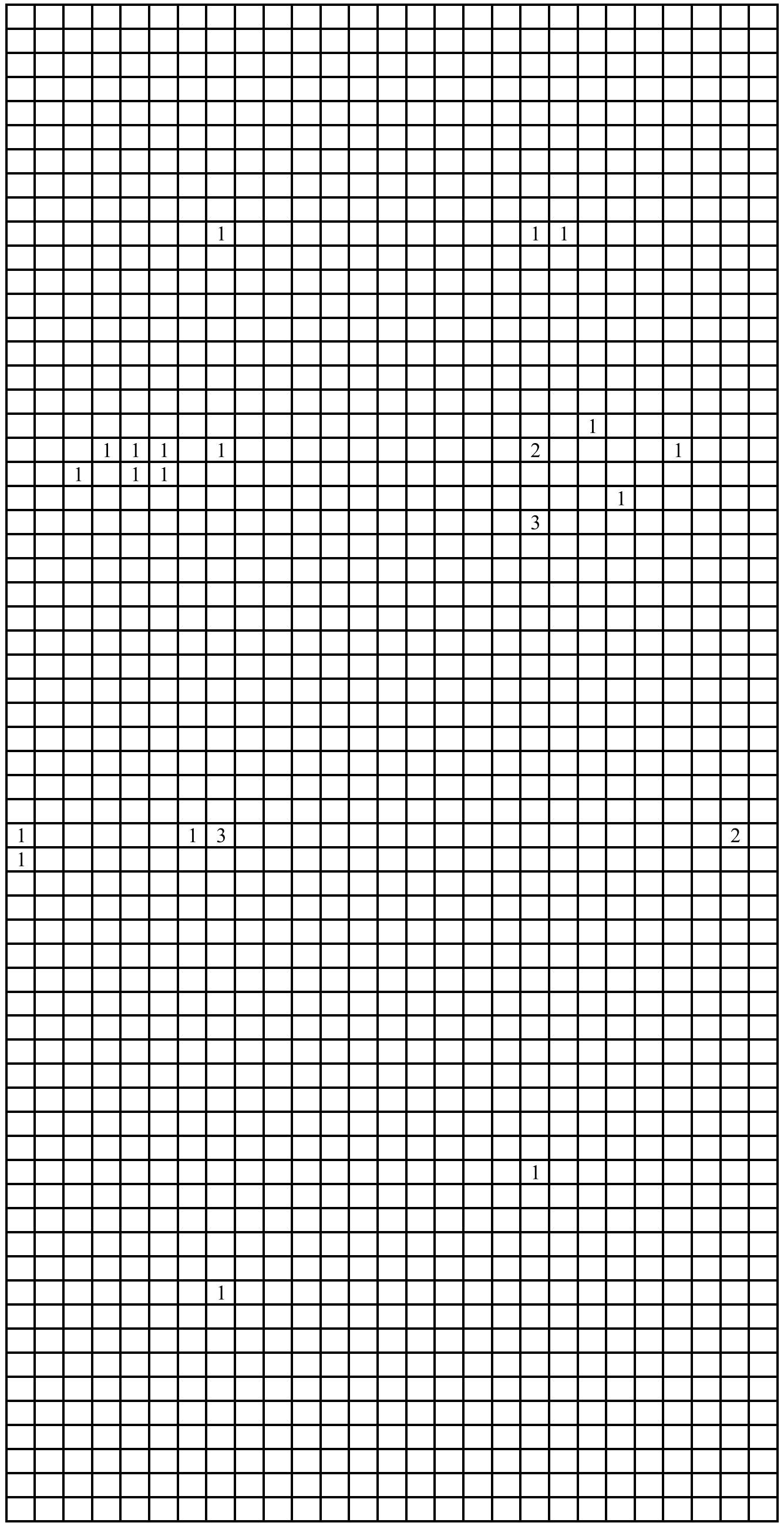
1 1 1
1
1 1 1 1 2 1
1 1 1
1
3
1 1 3 2
1
1
1

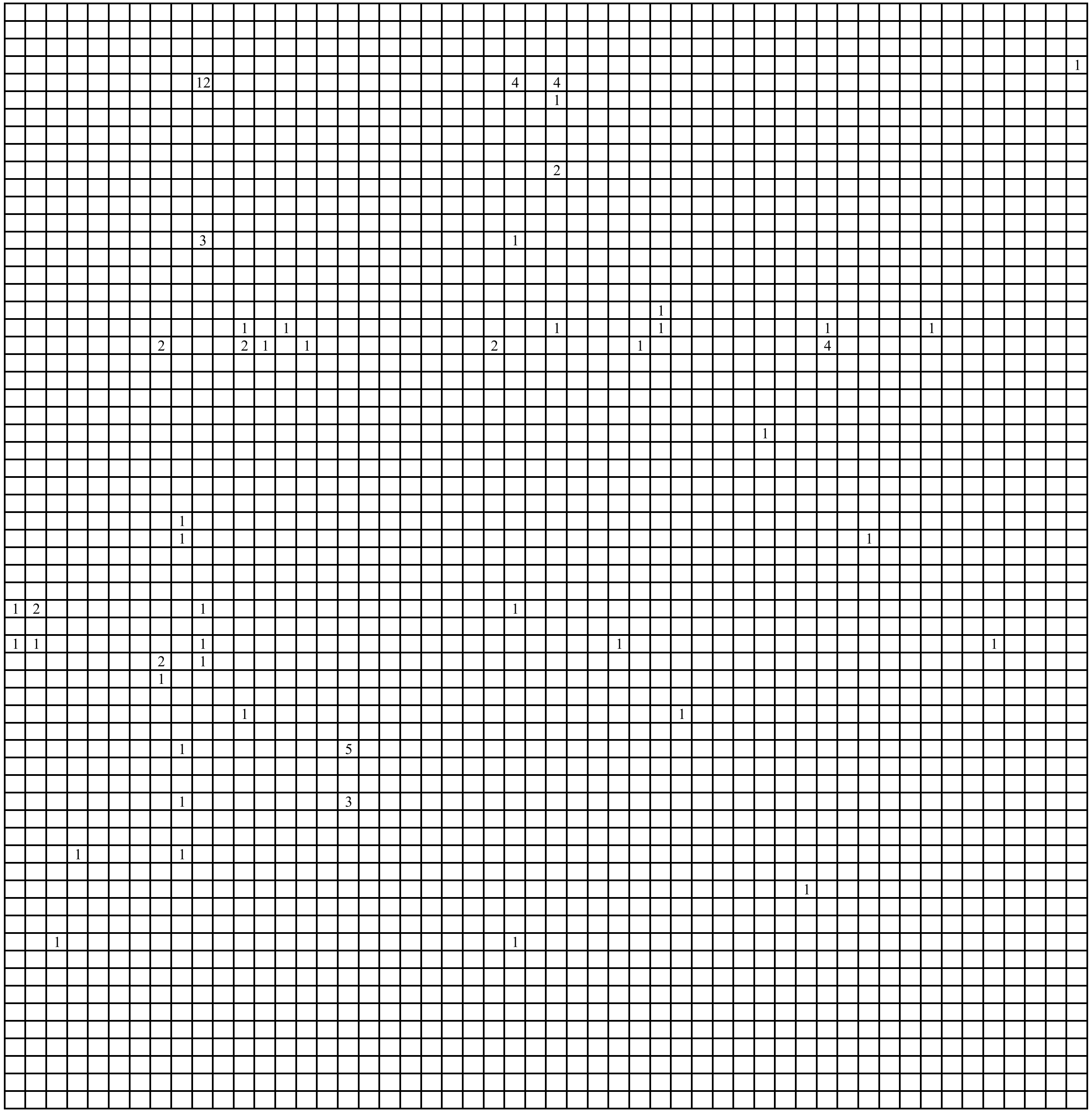
1
12 4 4
1
2
3 1
1
1 1 1 1 1 1
2 2 1 1 2 1 4
1
1
1 1
1 2 1 1
1 1 1 1 1
2 1
1
1 1
1 5
1 3
1 1
1
1 1

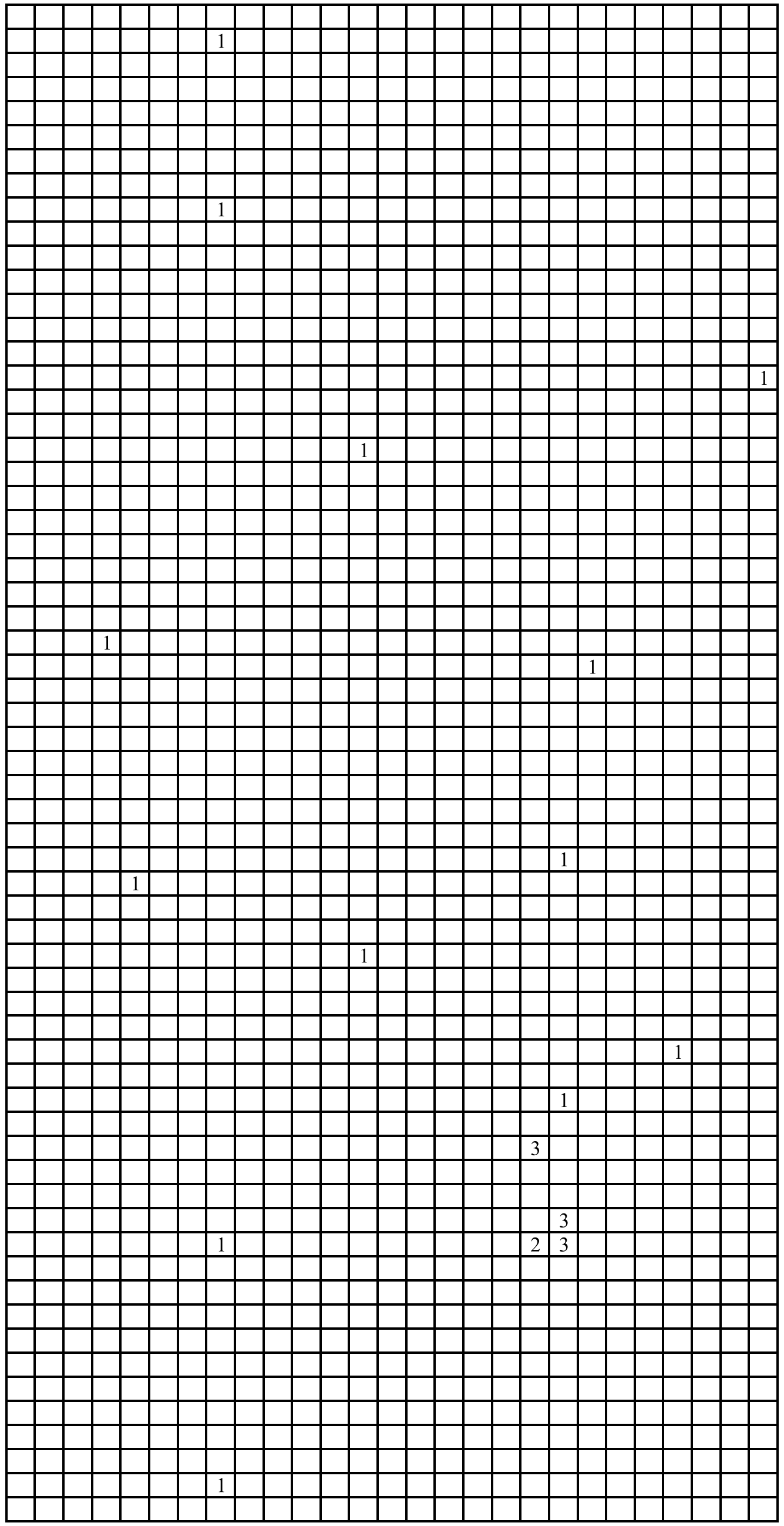

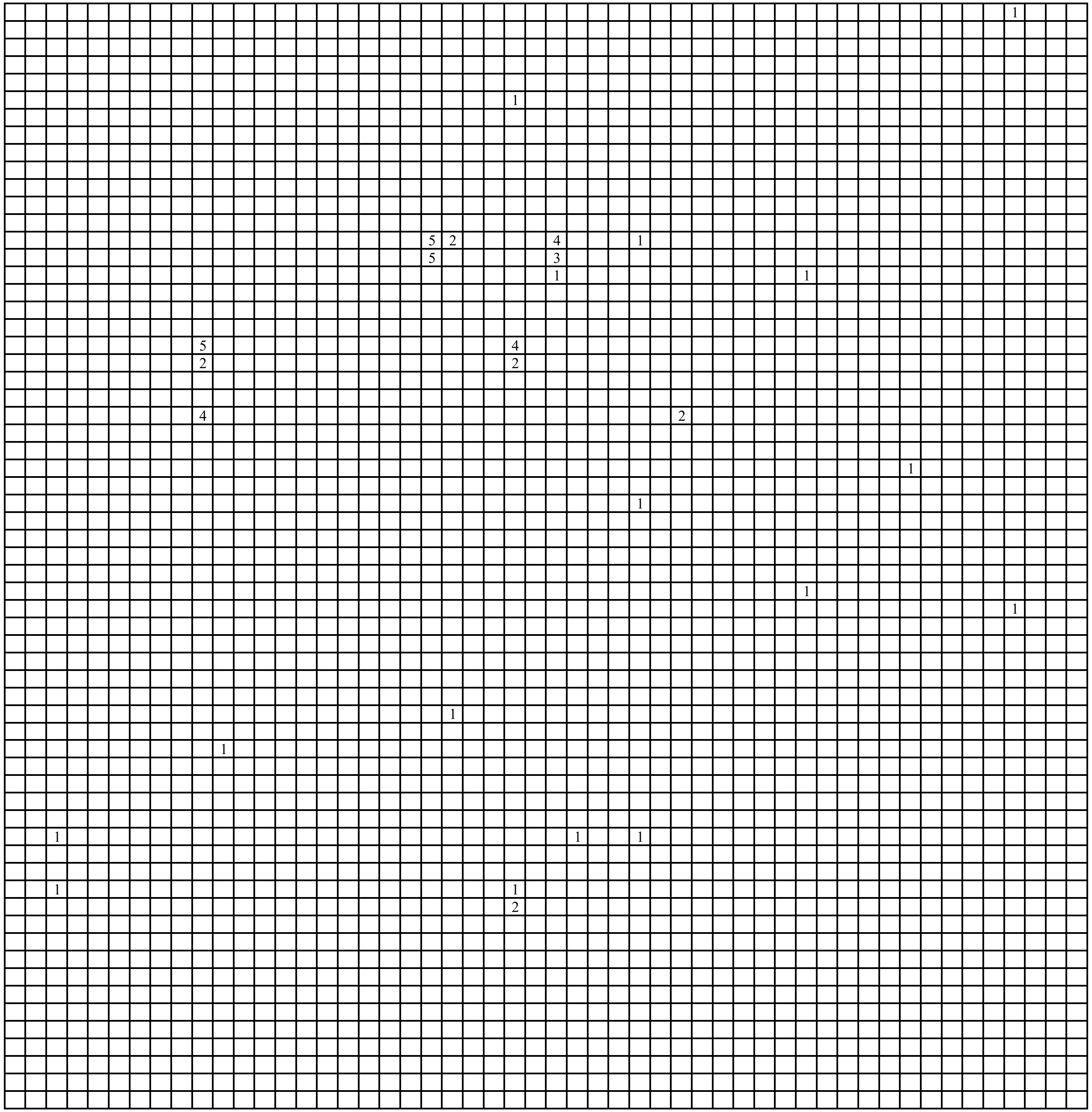

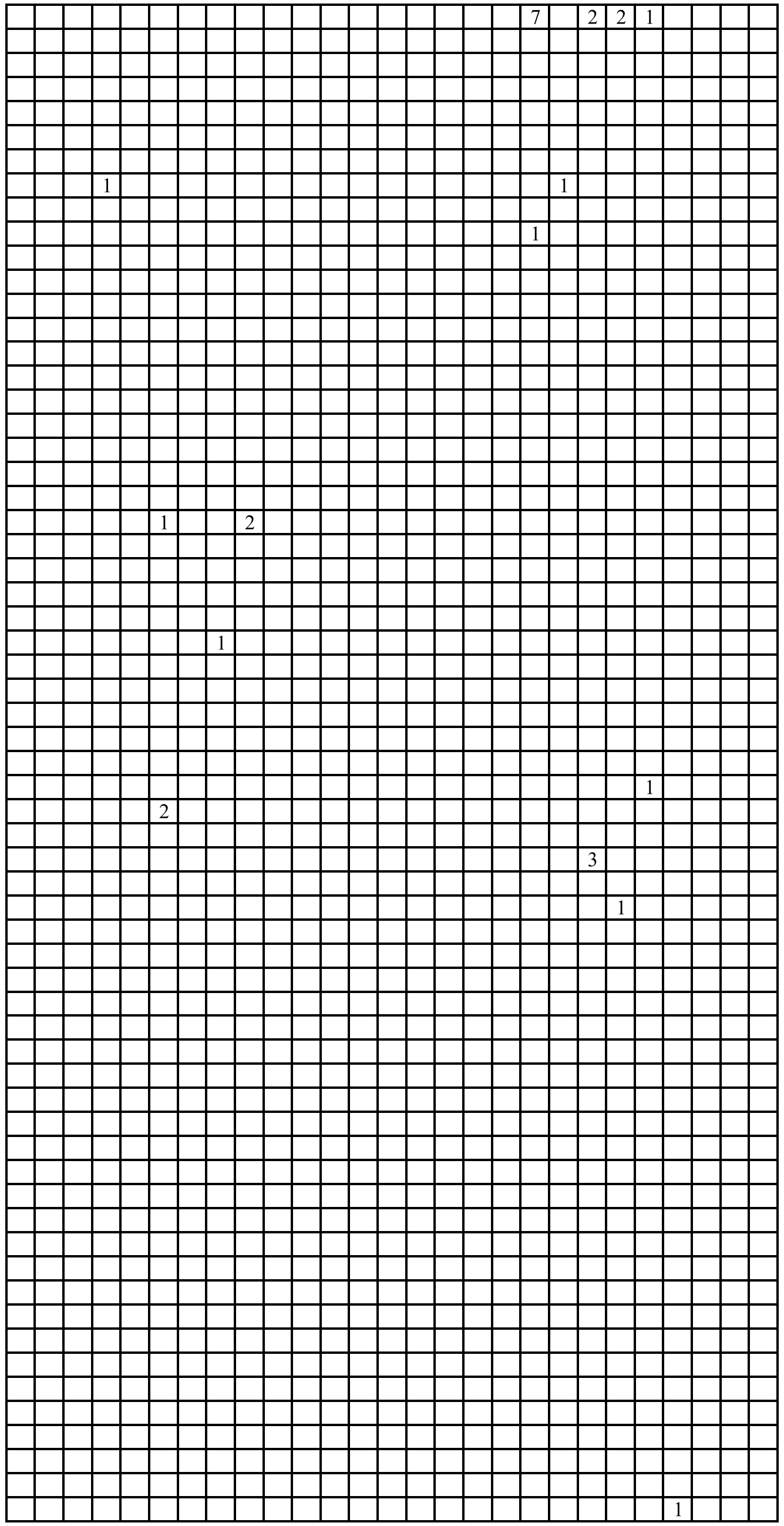

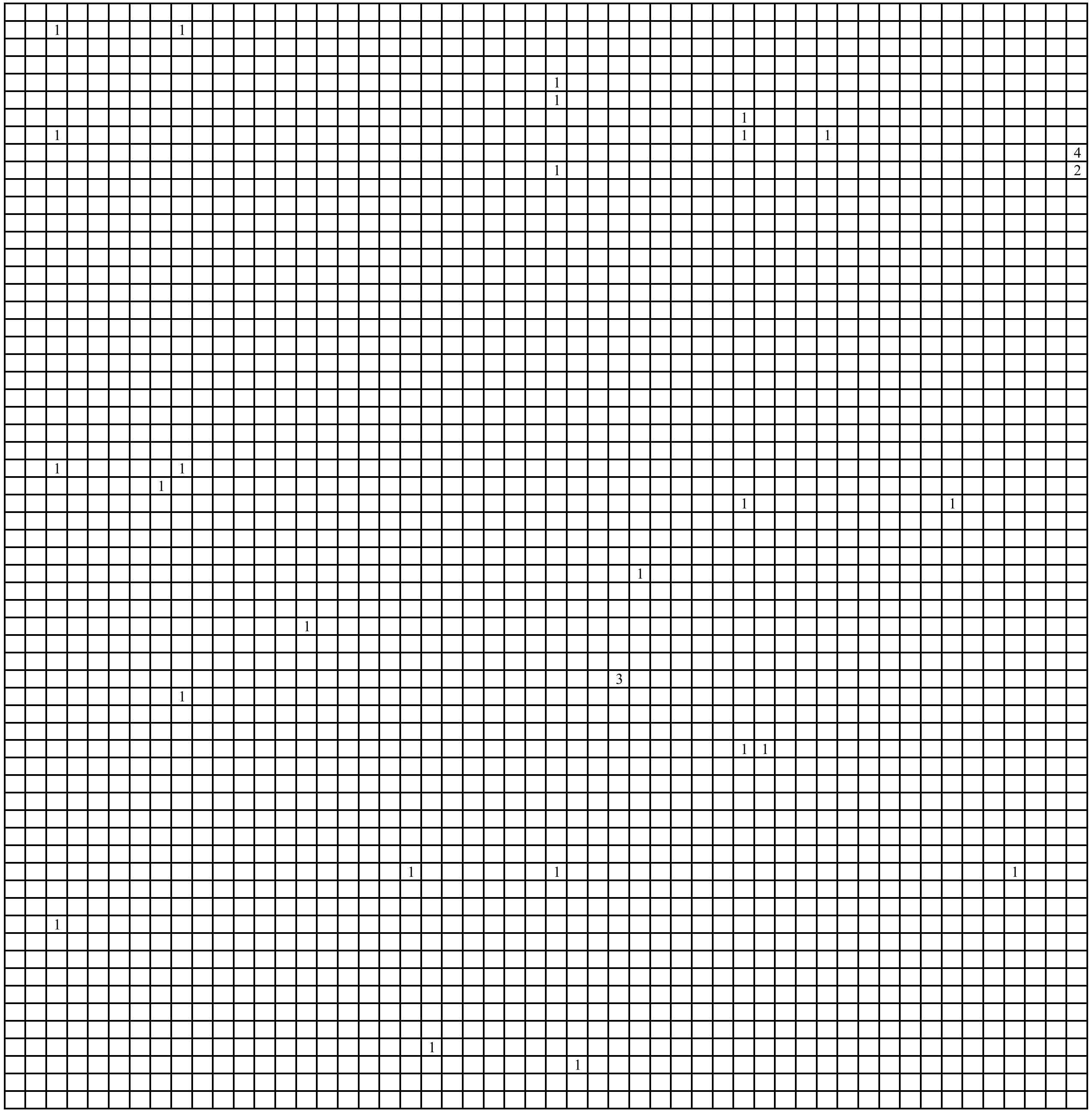

					1																					
					1							1														
							1																			
																		1								

																													2																						
																																							1													
																																												2					
						1																									2			1																
																																					1															
																																				1	2															
																										1																									
																																				1	1															
																																									1										

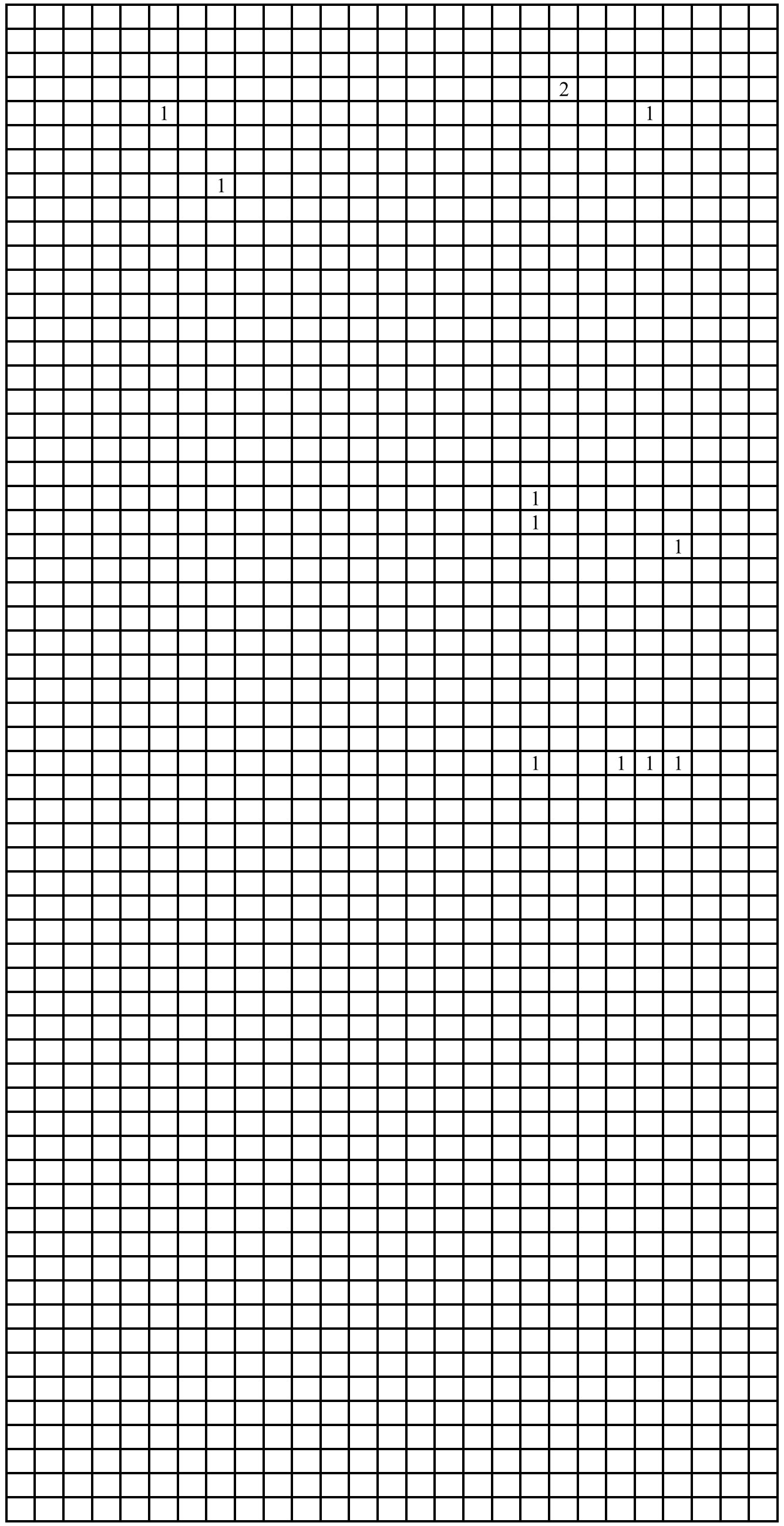
2
1
1
1
1
1
1
1
1
1
1

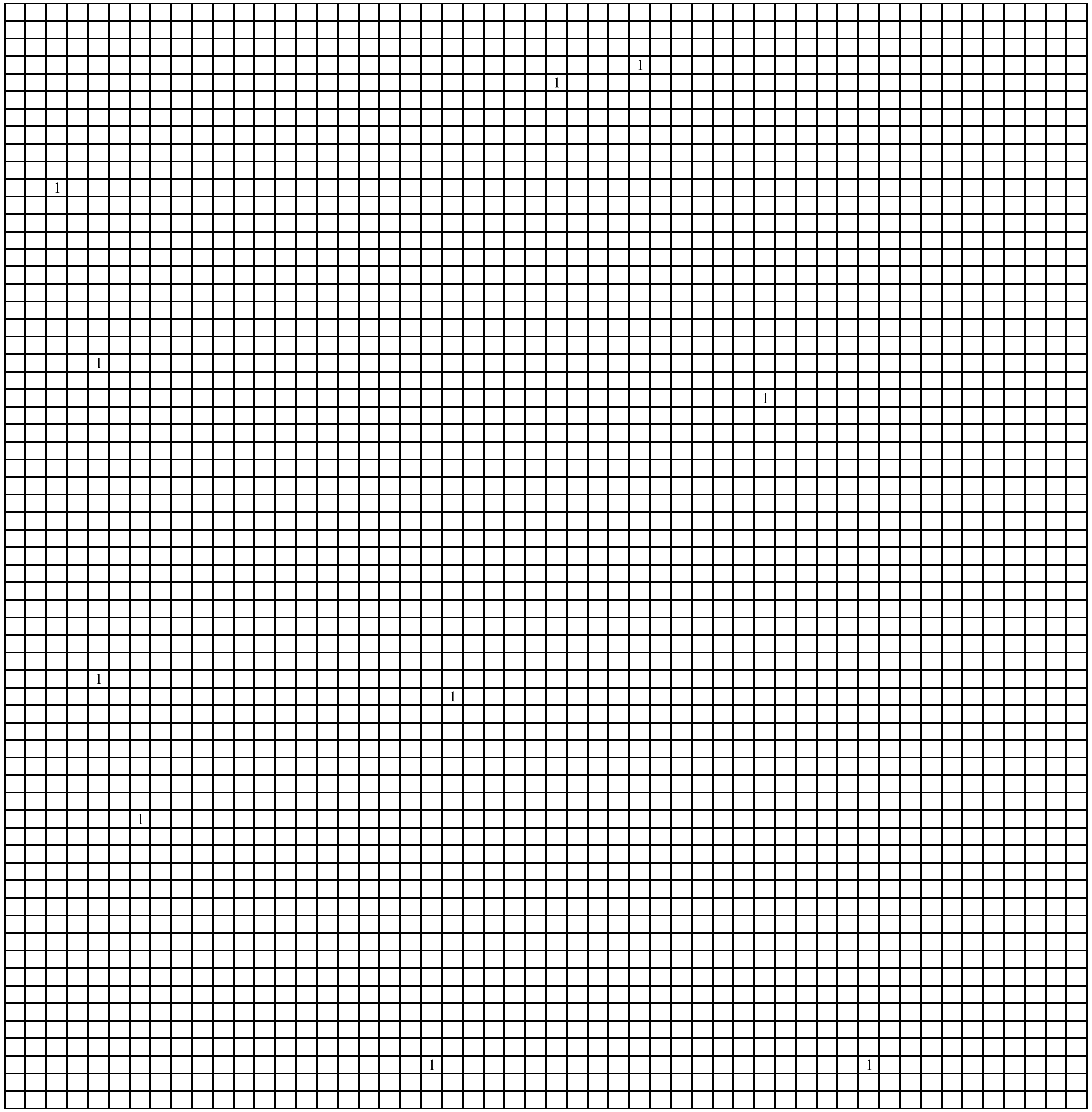
1
1
1
1
1
1
1
1
1
1

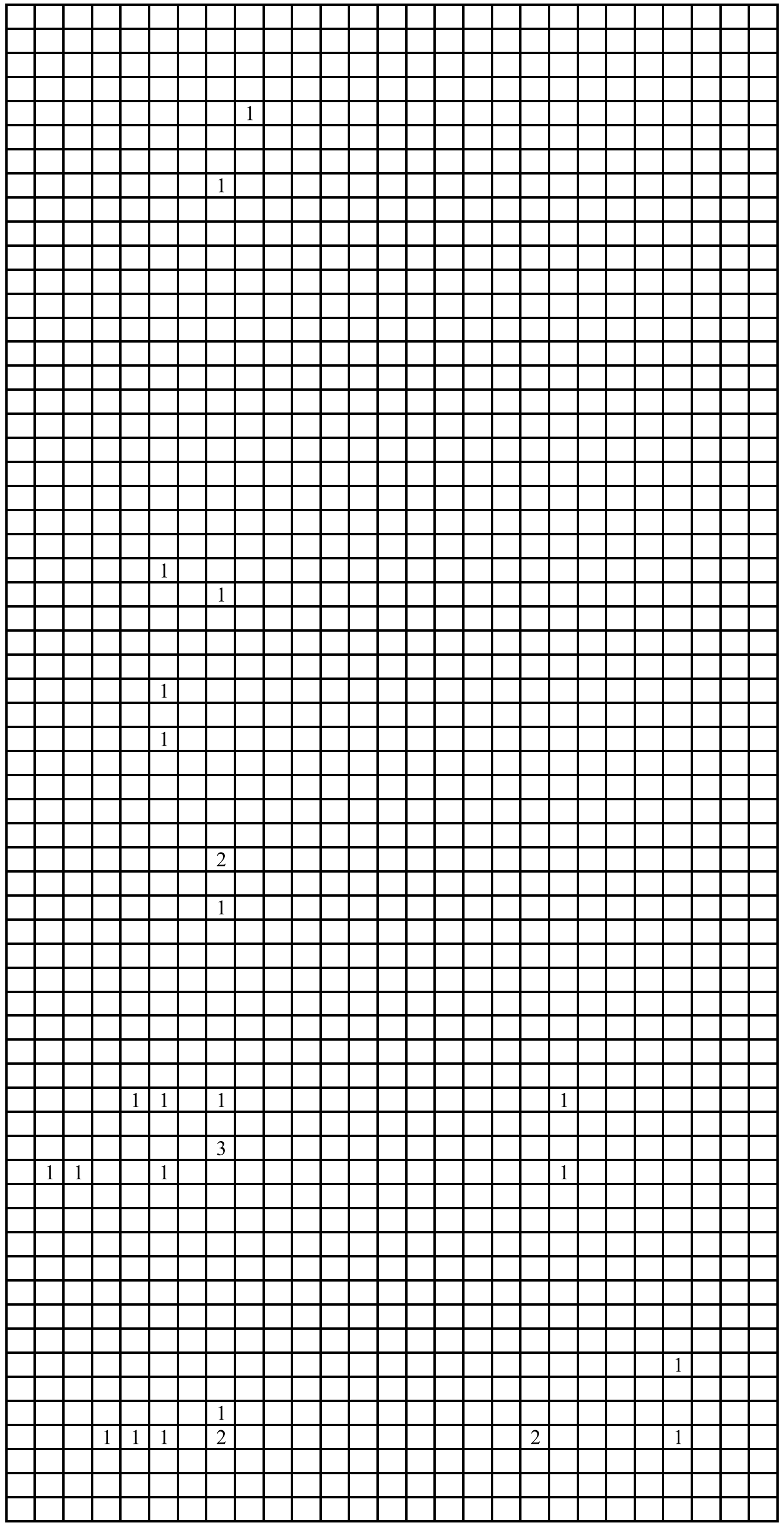
1
1
1
1
1
1
2
1
1 1 1 1
3
1 1 1 1
1
1
1 1 1 2 2 1

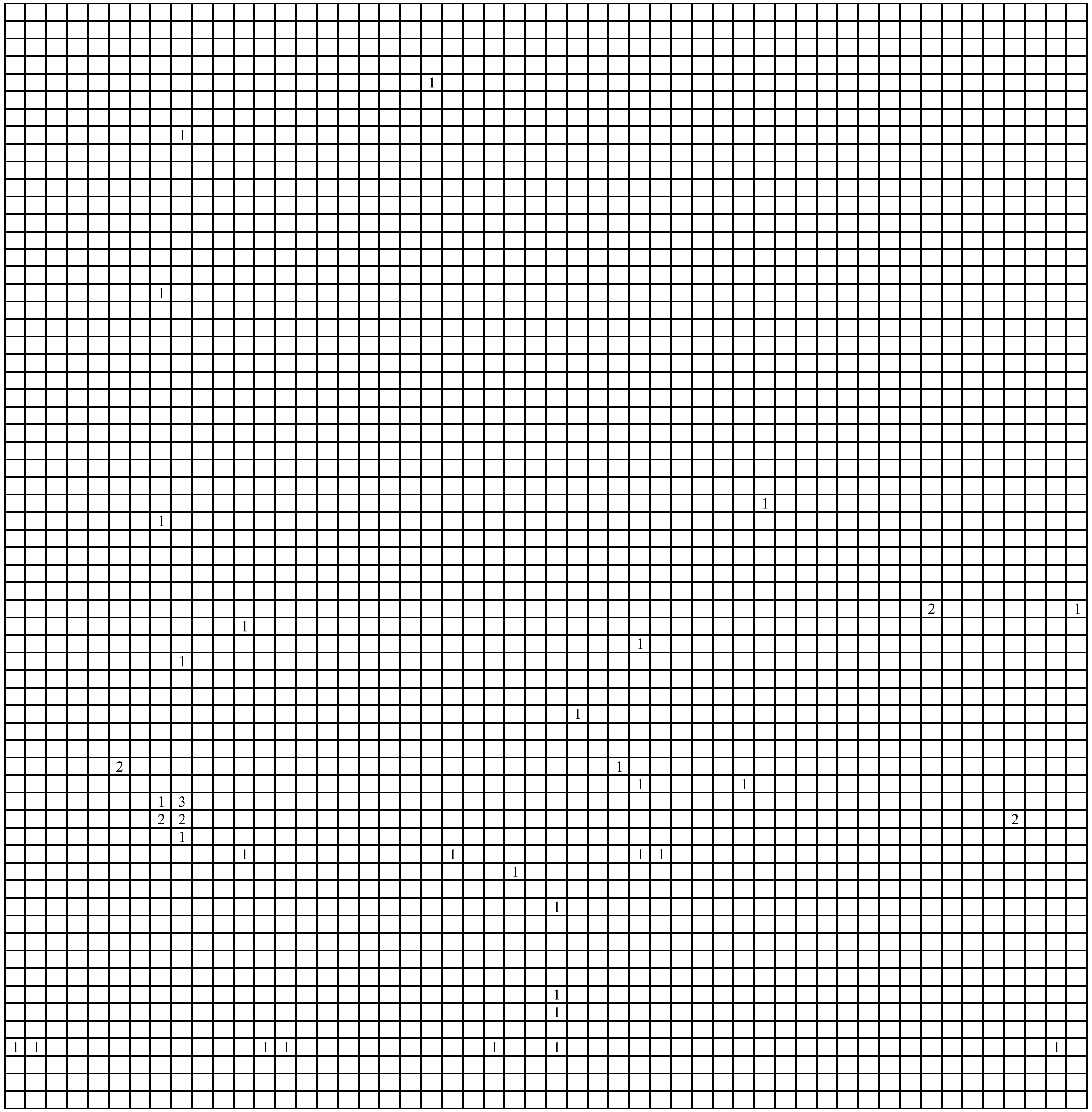
1
1
1
1
1
2 1
1
1
1
1
2 1
1 1
1 3
2 2 2
1
1 1 1 1 1
1
1
1
1
1 1 1 1 1 1 1

				1															1							
				1														1					1			
																							1			
																					3					
																						1				
		1					1																			
				1			1												1							
																			1							
							2																1			
															1											
																									1	
					1		1																			
							2											1								
							1													2						
																								1		
																		1								
							1								1			1								
							1																			
							1														1			1	
																				1						

																																			2	1															
									1																					1																					
																																															1			
								1															1					1																						
								1																										1																
							2	1																																										
																													1																							
																												1																								
							1	1	1				1																																						
							1	1			1					1																																			
																															1																			1	
							1																																												
									2																					7									1												
																														1																					
							1					1		1																																				
																																											2									
								1																		1																									
																										1												1													
							1																																												
									1																																										
																																																	1	
													1																																						
																																			1																
									1																																										
																																			2	1															

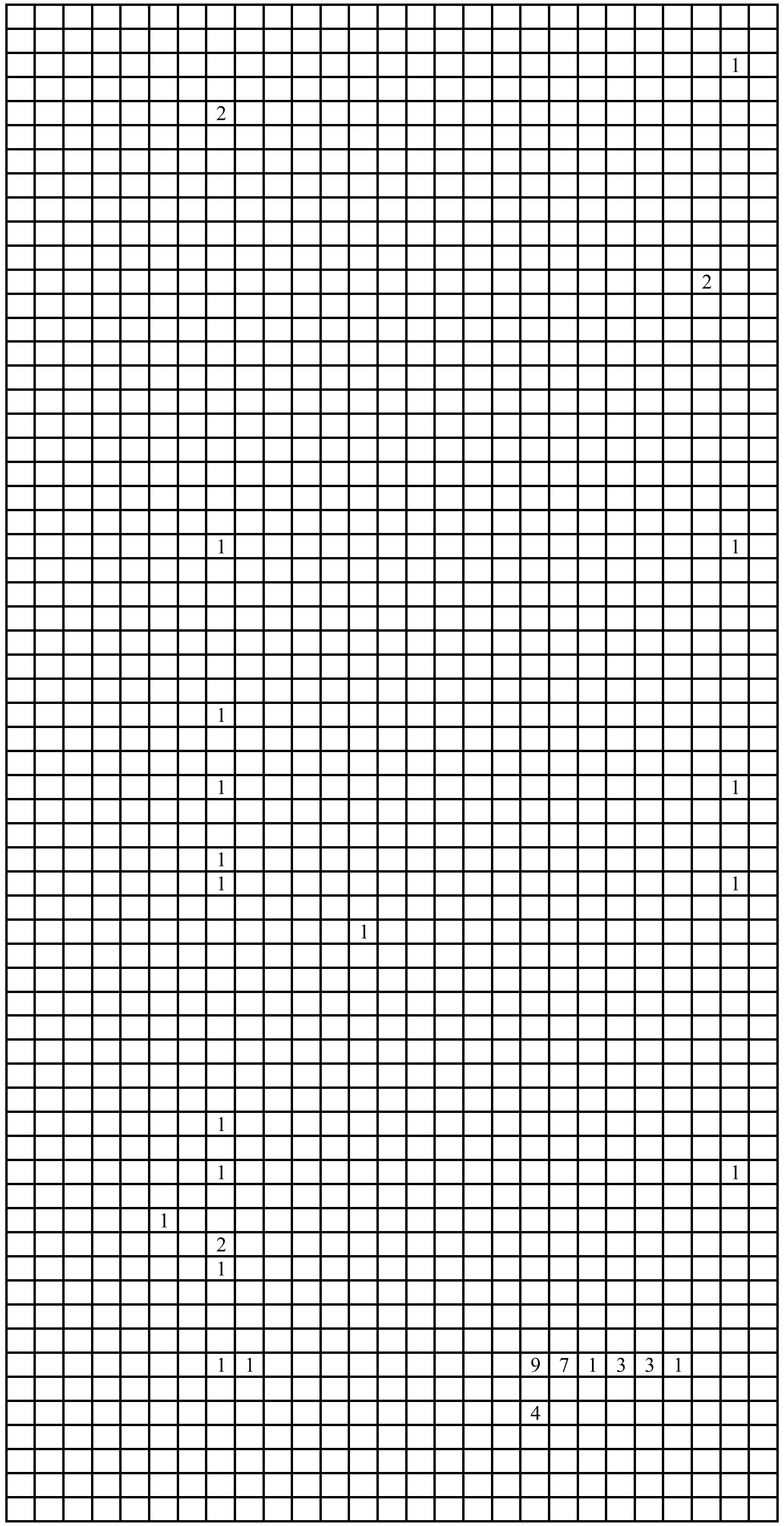
1
2
2
1 1
1
1 1
1
1 1
1
1
1 1
1
2
1
1 1 9 7 1 3 3 1
4

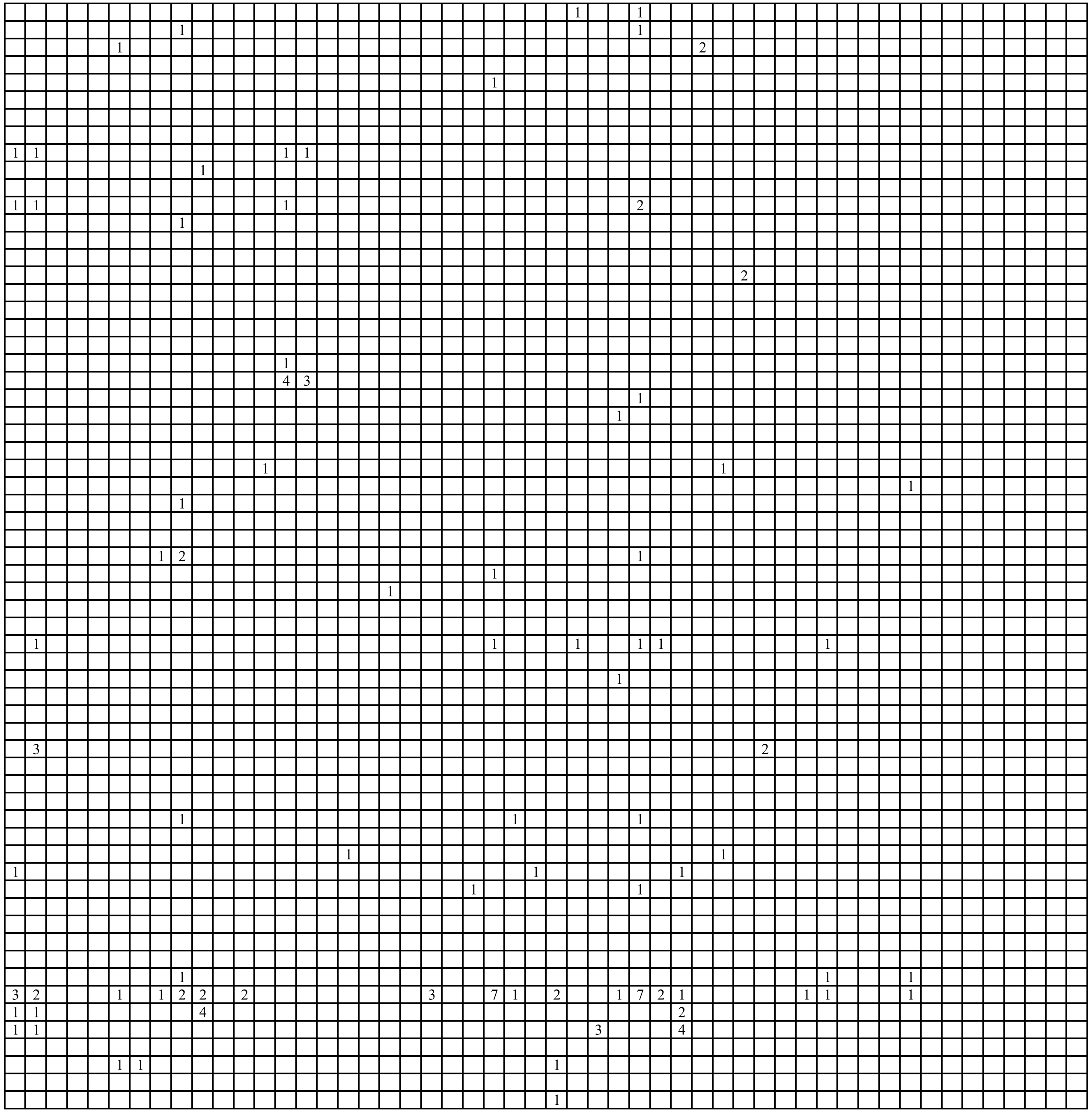
1 1
1 1
1 2
1
1 1 1 1
1
1 1 1 2
1
2
1
4 3
1
1
1 1
1
1
1 2 1
1
1
1 1 1 1 1 1
1
3 2
1 1 1
1 1
1 1 1
1 1
1 1 1
3 2 1 1 2 2 2 3 7 1 2 1 7 2 1 1 1 1
1 1 4 2
1 1 3 4
1 1 1
1

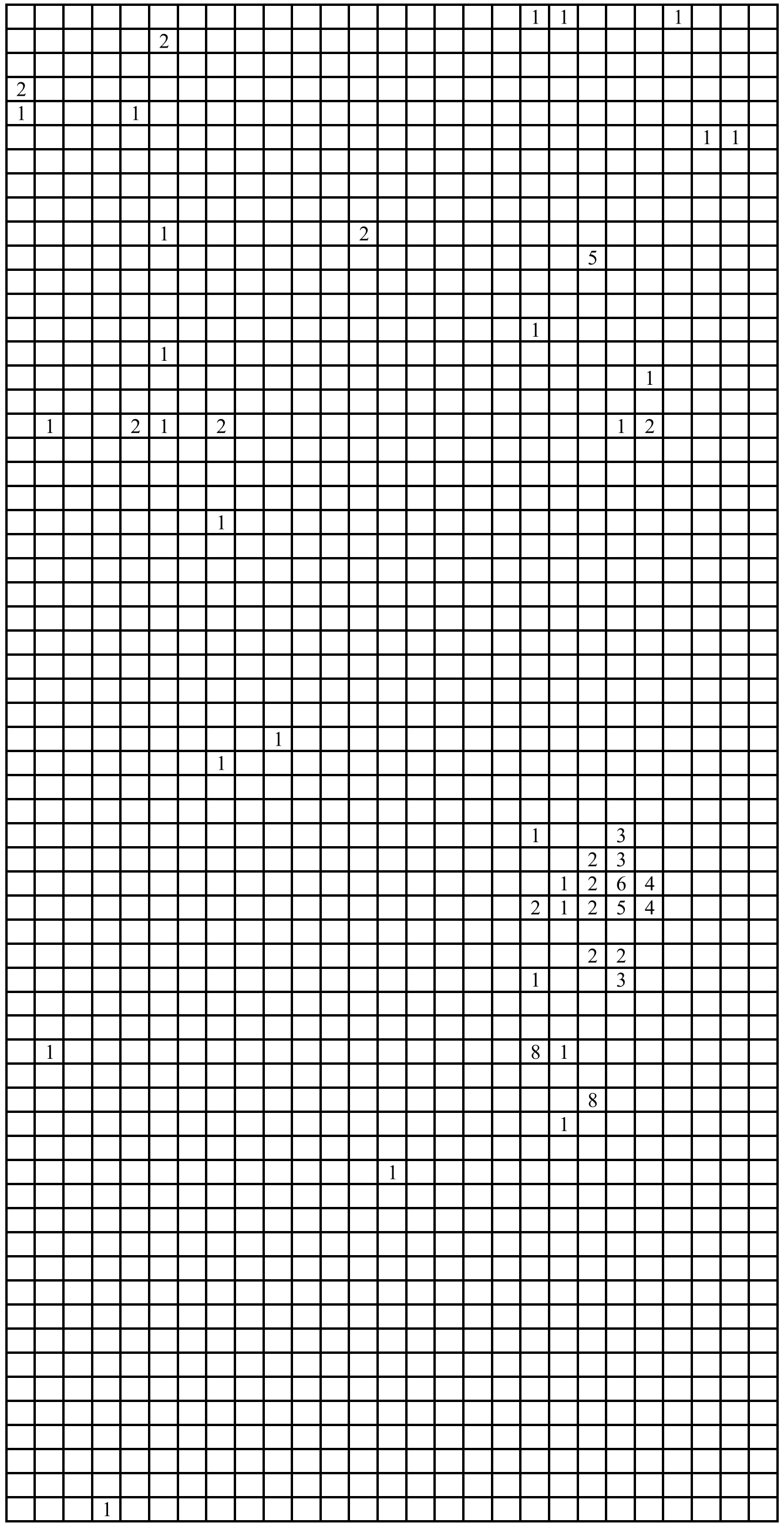

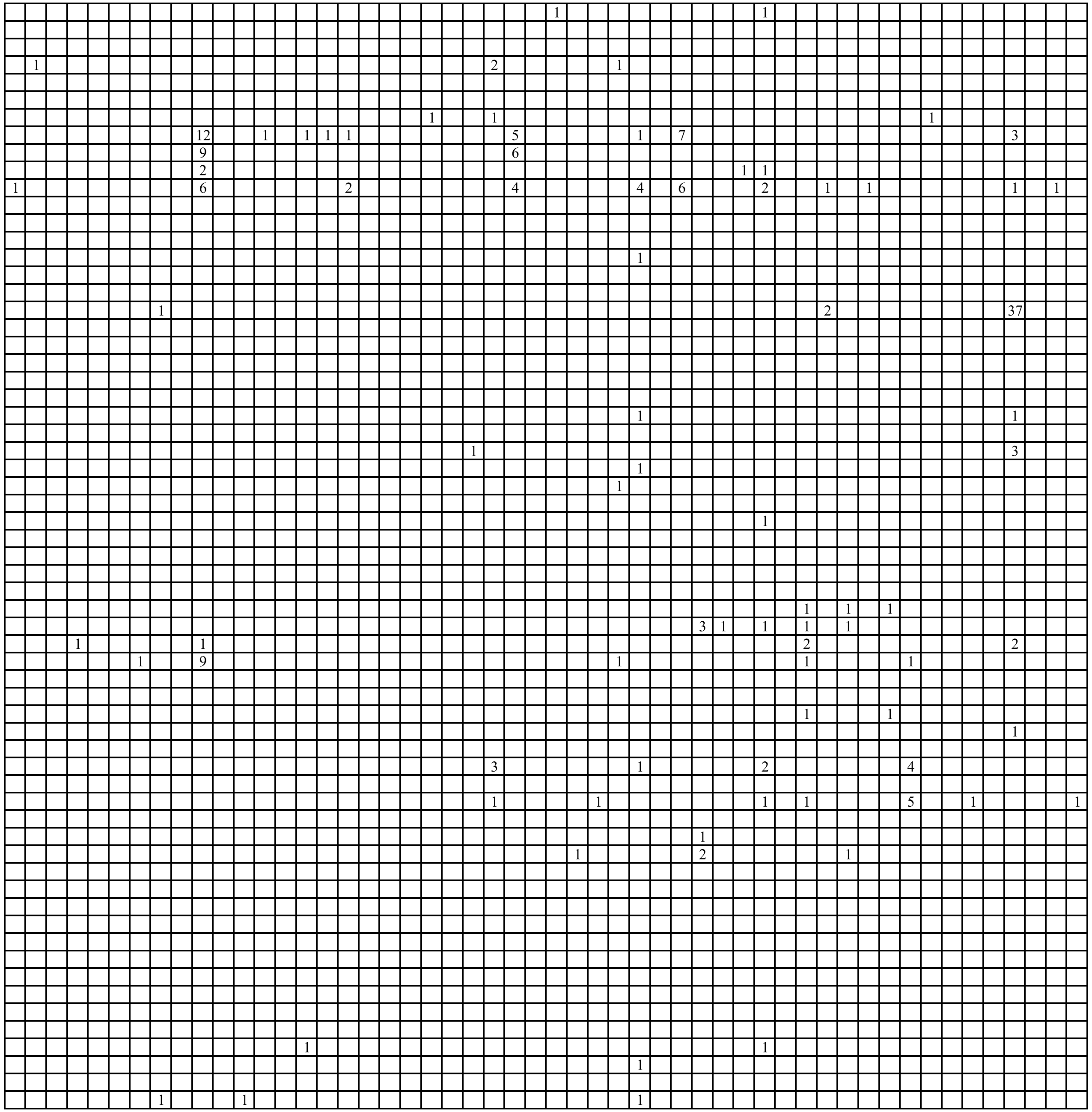

																						1				
																			1							
							1																			
								2																		
																		1	1							
																		2		1						
																					2	1				
																						1	1			
																			1							
																		1	1							
																		1	5							
							3																			
																					1					
																							1			
																					1					
																	1									
							1																			
																		3								
				1	1																					
							4											3								

							1	1																																											
																																	1																		
																																						1													
													1																																						
									3			1												3											1	1										1					
																													1																						
																														1																					
									1																														5												
																														1																					
							1																																												
					1																																														
							1	1																																											
																																							1												
												1								1																															
																				1																															
																														1																					
																																	1																		
							1						1										1													1													1		1
														1												9				1																					

							1												2							
							1																			
							1																			
							1																			
							1																			
							1											4								
		2																								
																					1					
							2																		1	
							1																			
								1																		
																							1			
																		2	2							
				1																						
	1						2																			
							2											2								

									2					1												5				1																					
																				1										1	1								1			1									
							2	1																						1																			1			
							2	1																																									1			
																																				1				1	1											
																				1																															
																														1																					
									2								1																										1								
							1	1																																											
																																				2															1
													1																																						
							1	1														2																													
																				1																															
																				1																															
																																				1															
																																												1							
																																												2							
												1																																							
							1																																												
																										1																									
									1																																									
												1					1																																		
							1																																												
									2																										1															

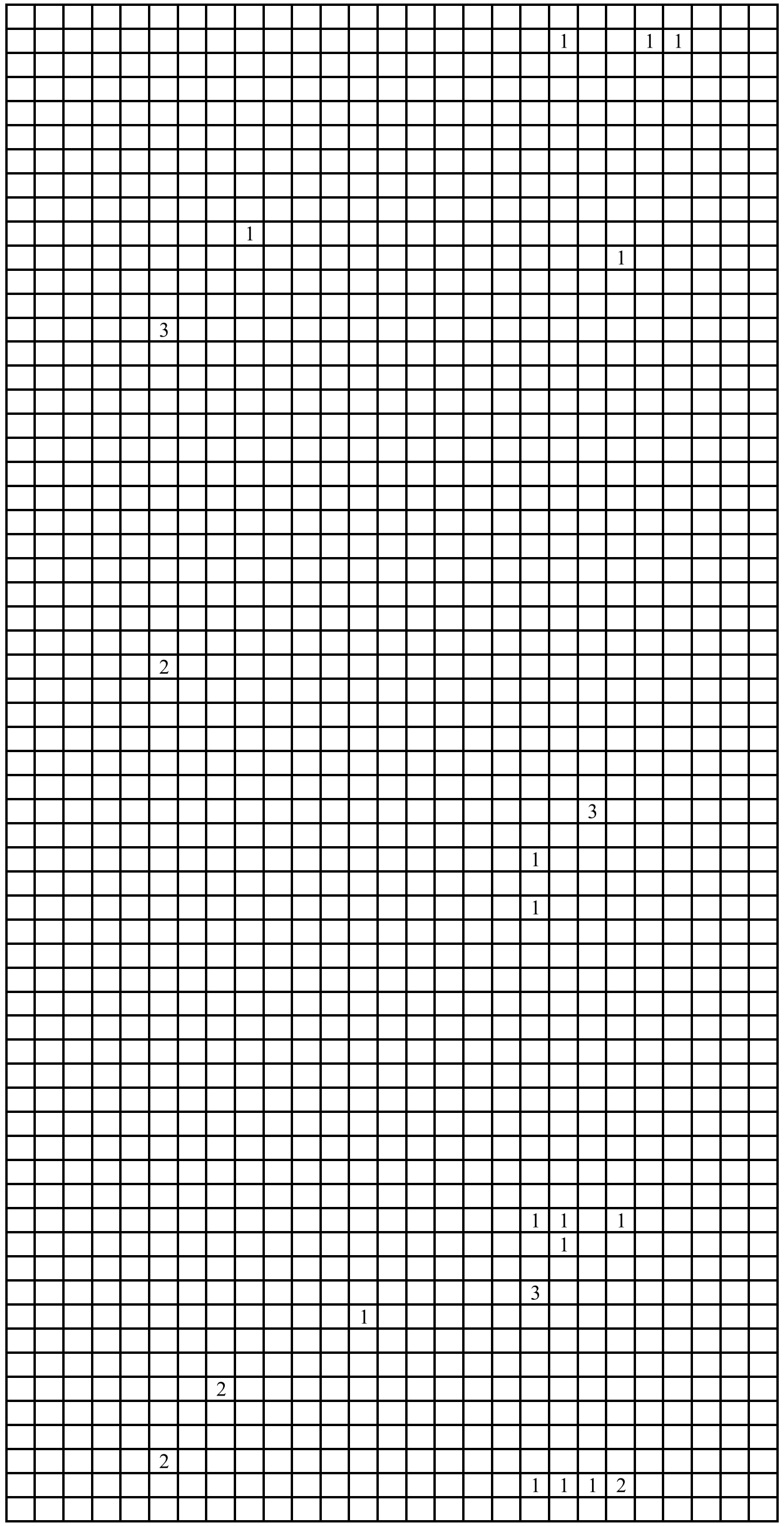
1 1 1
1
1
3
2
3
1
1
1 1 1
1
3
1
2
2
1 1 1 2
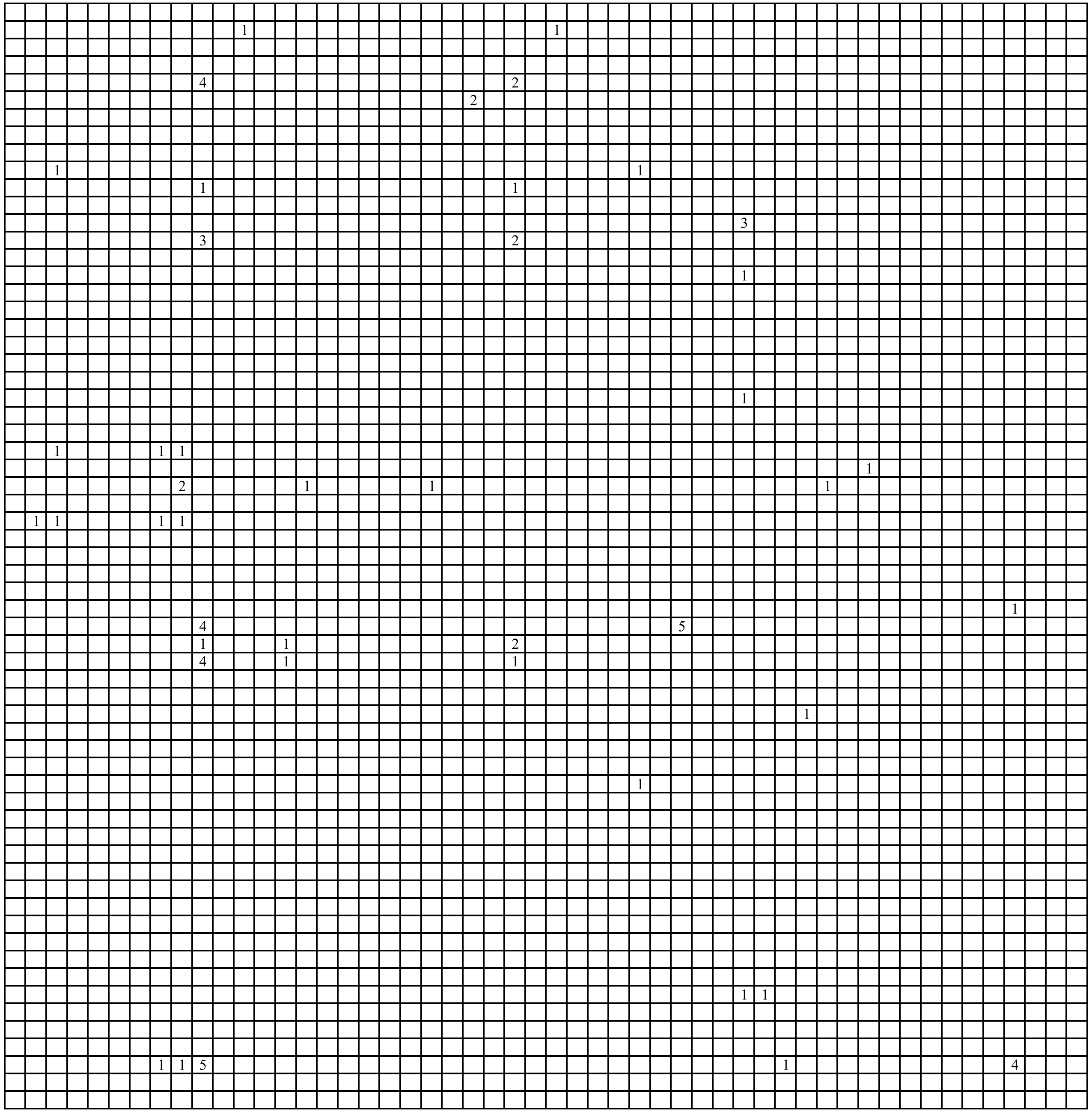
1 1
4 2
2
1 1
1 1
3
3 2
1
1
1 1 1
1
2 1 1 1
1 1 1 1
1
4 5
1 1 2
4 1 1
1
1
1 1
1 1 5 1 4

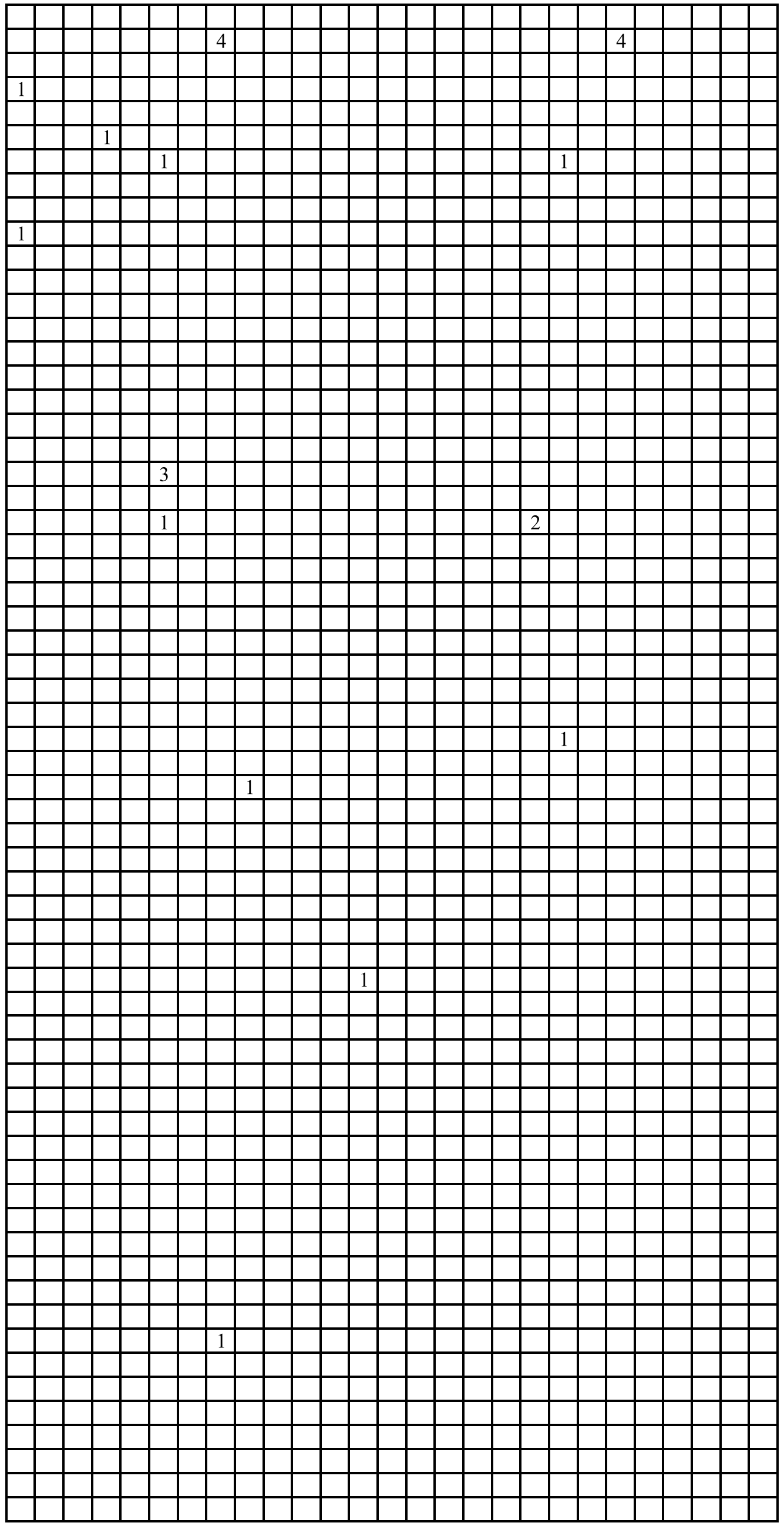
4 4
1
1
1 1
1
3
1 2
1
1
1
1

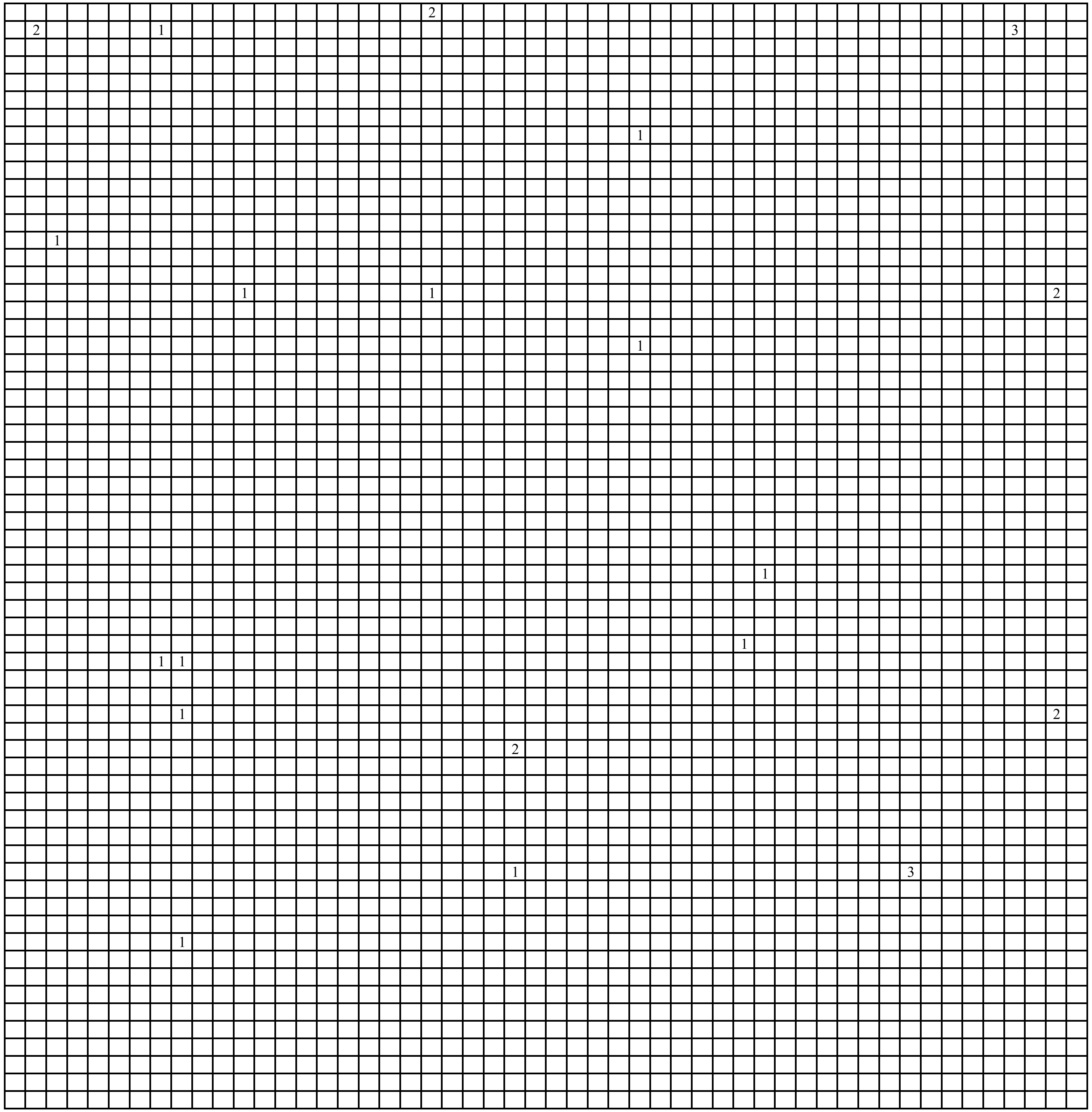
2
2 1 3
1
1
1 1 2
1
1
1
1 1
1 2
2
1 3
1

								1																		

																																			1																
		1						1																																										
																				2																															
																																			1																
																																			1	1															
																						1												1	2															
																				2																1															

							1	1																	2	
								2																		
							1																			
							1																			
1							1																			
	1																									

	1						1	1			1									1																															
								1														1		4																											
																				1																															
																				1																															
																																				1															
																																				1																
								1																																											
		1																											1																					
																				1																1															
	1																																																		

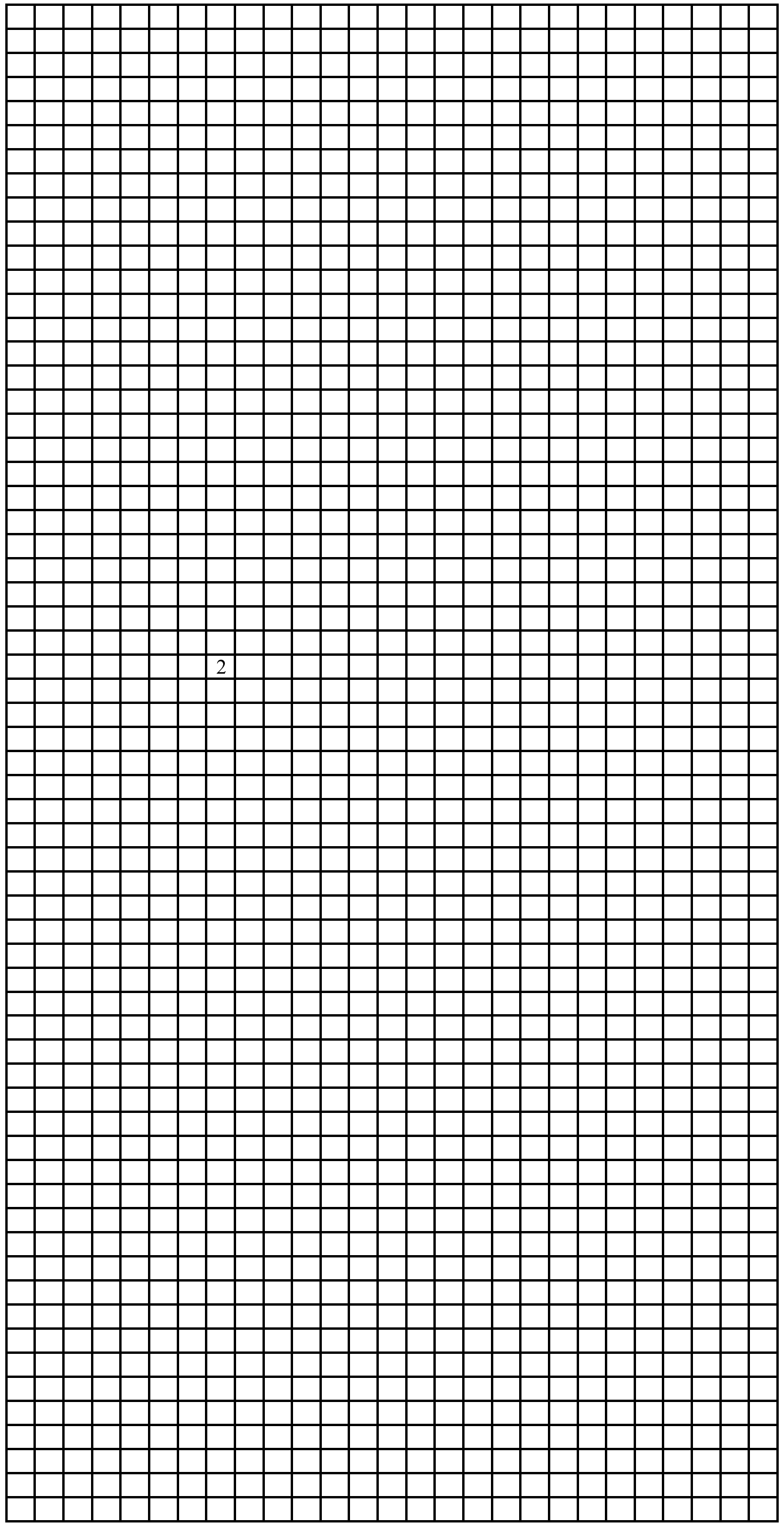
2

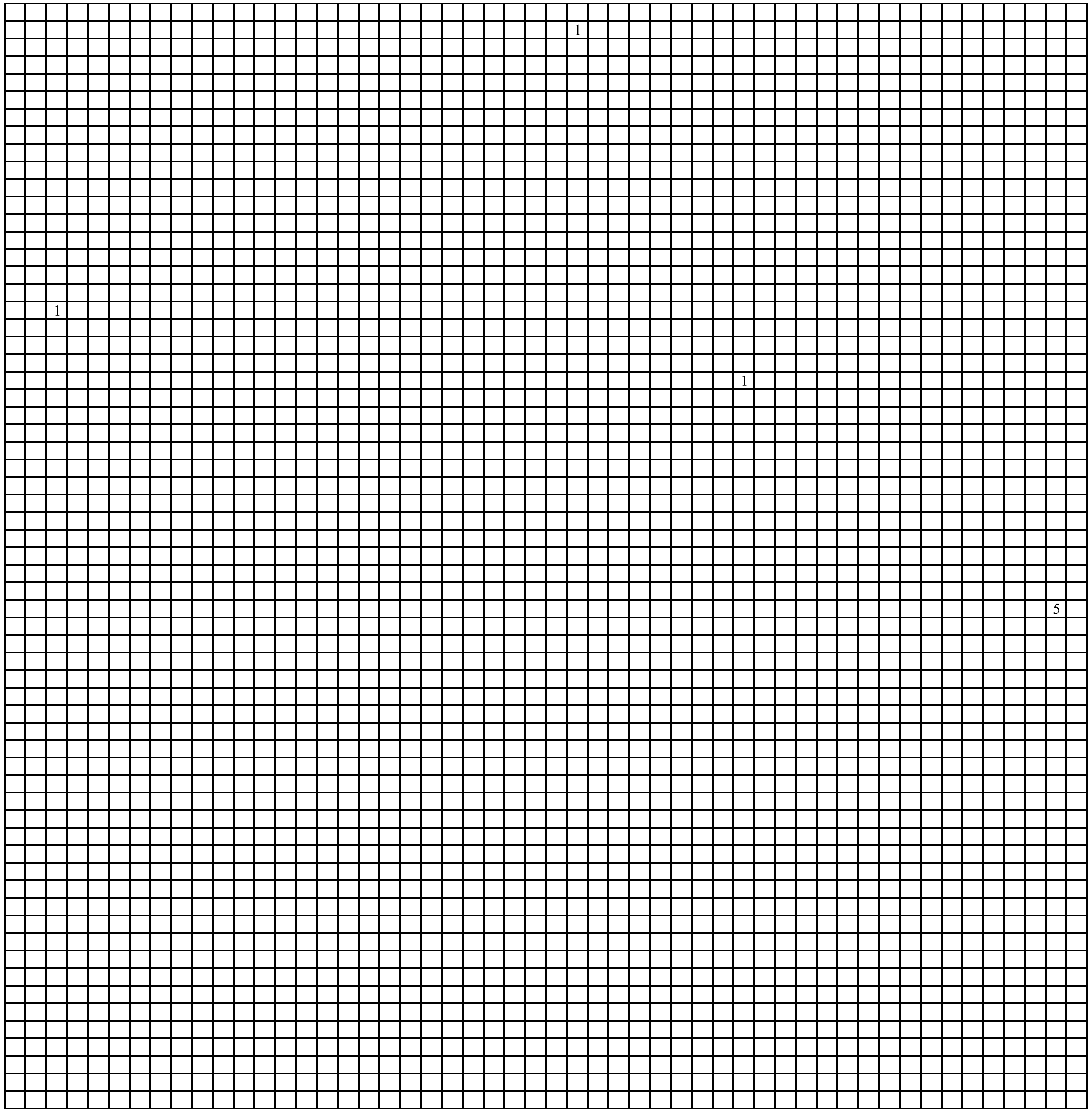
1
1
1
5

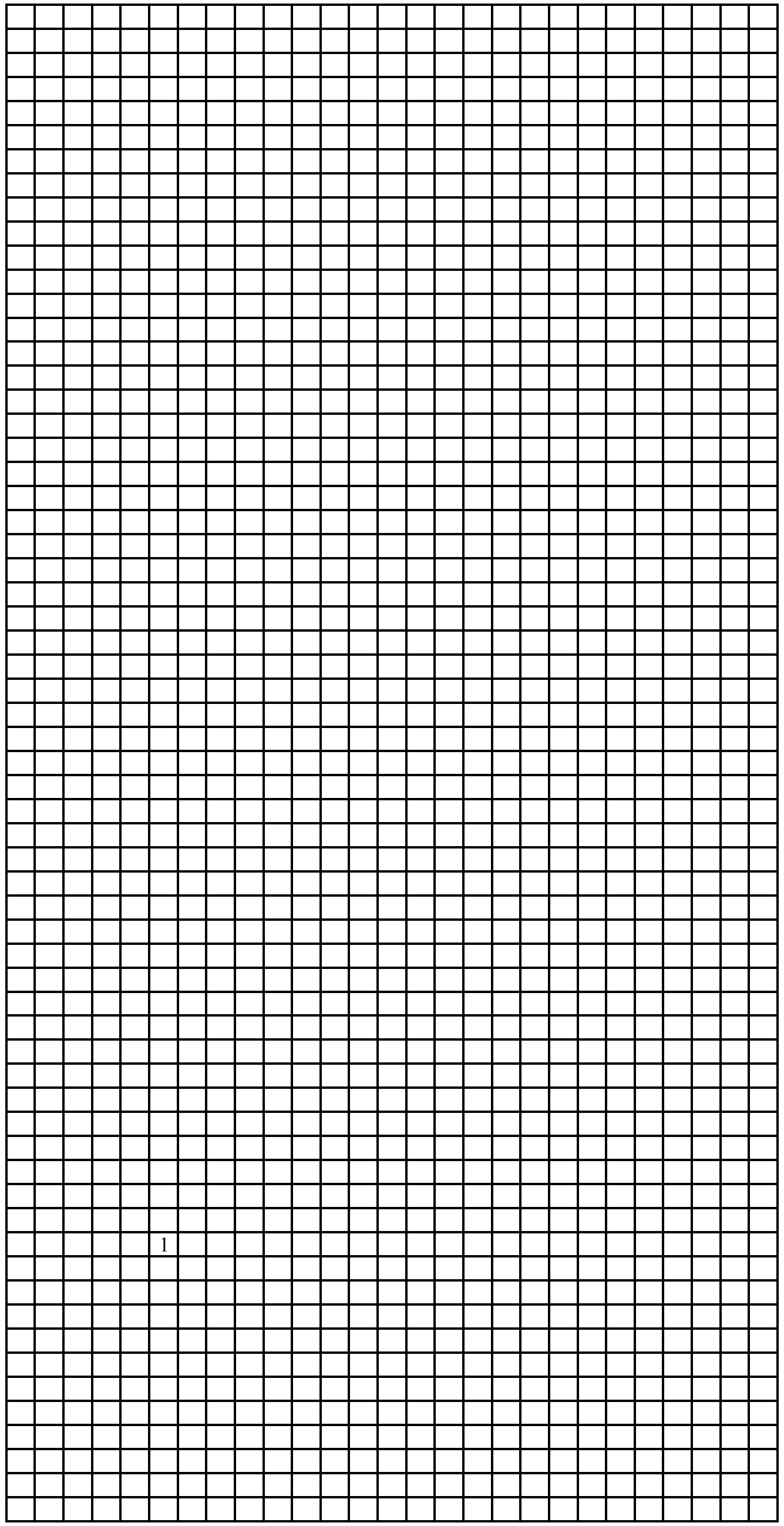
1

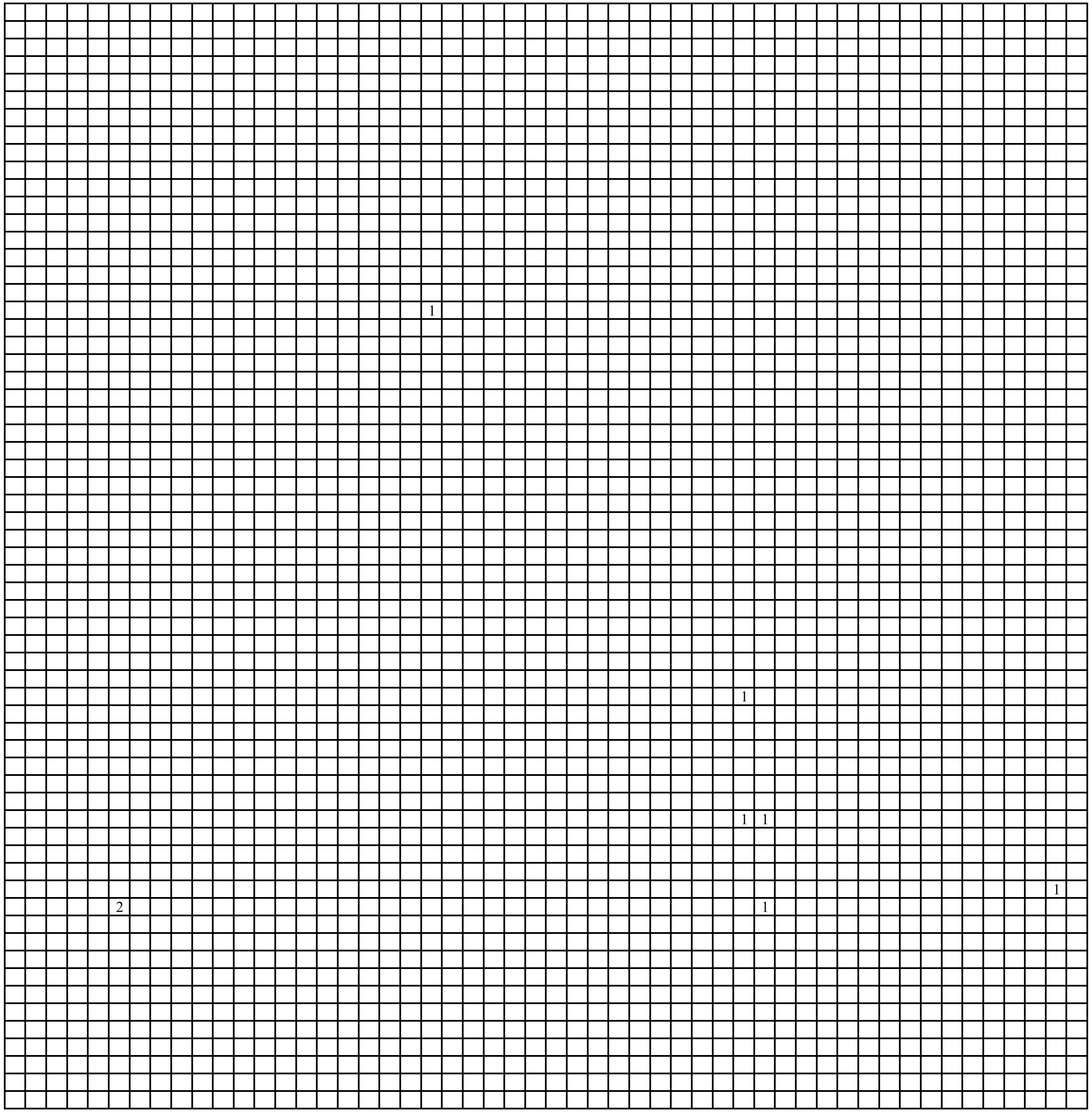
1
1
1 1
1
2 1

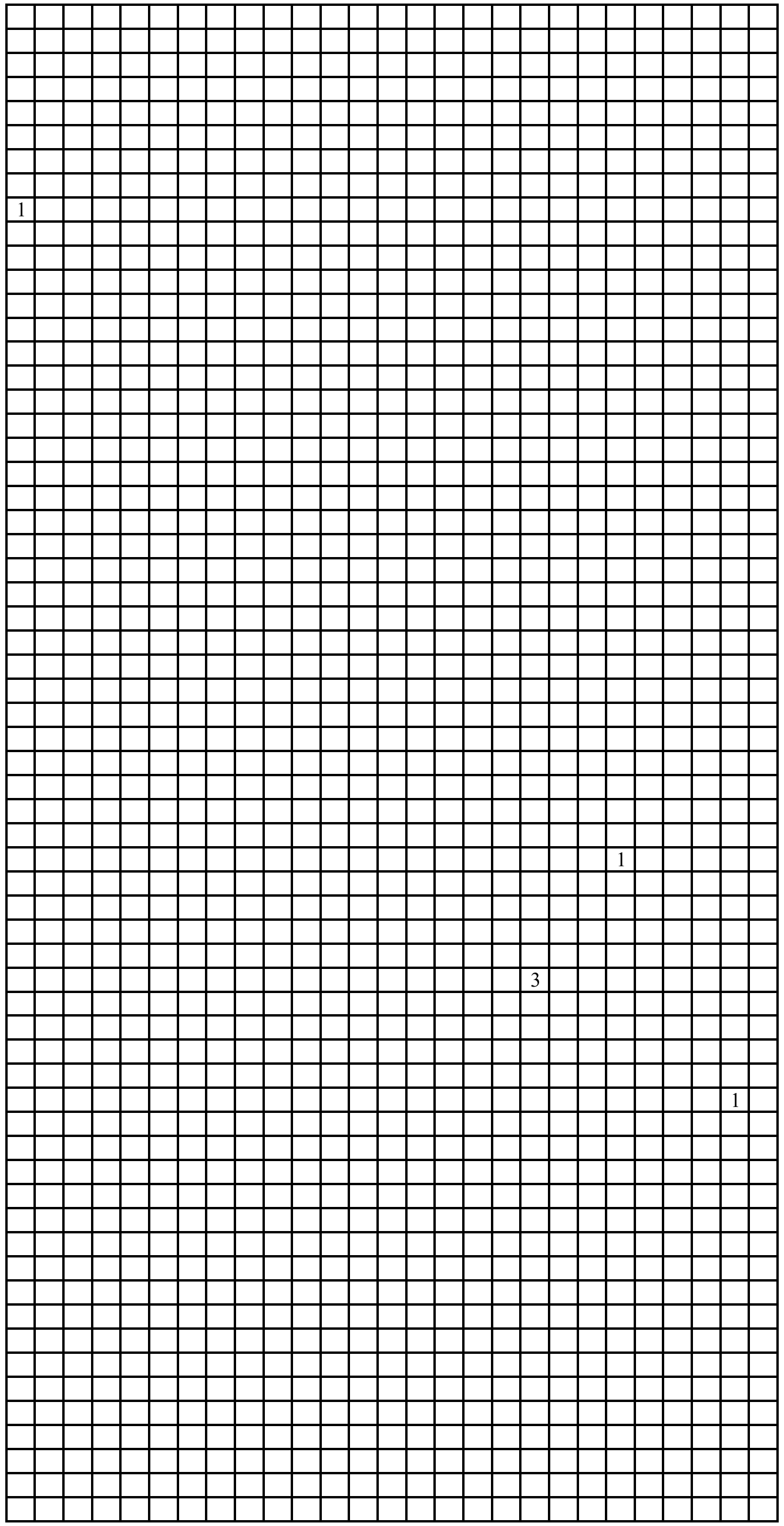
1
1
3
1

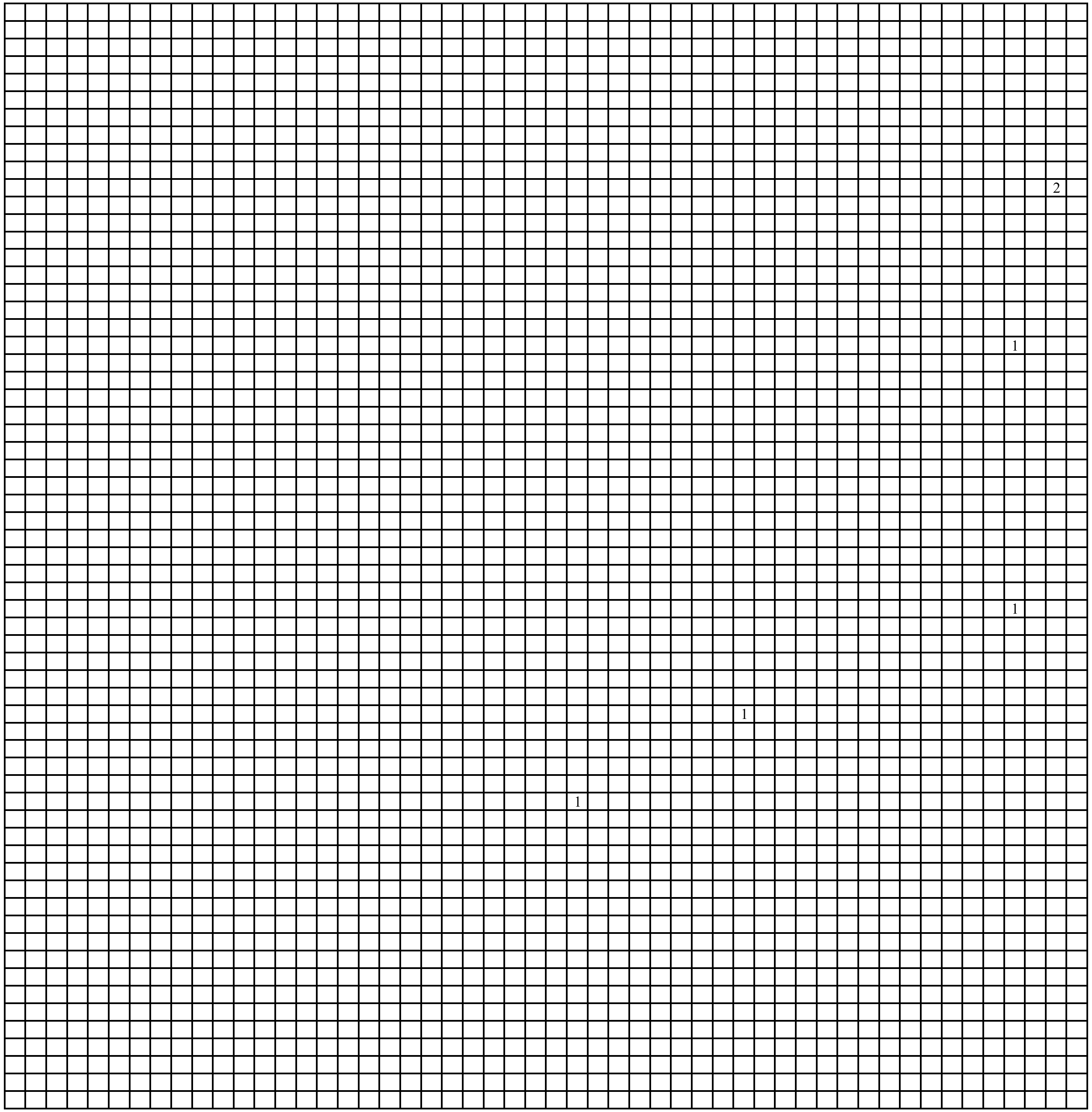
2
1
1
1
1

1							1																			
							1																			
				1	2															1						
				2	5																					
					1													1	1							
							1																			
				3																						

																																				1																
		1																																																
																																											1								
																												1																							
		1																																1																
		1																																													2			
																																				1																
									6															4						2																					
									5															5																											

				2	1														1							
				2	1																					
																		1								
							1								1											
					5																2					
1																										
1																										
					1																					
																					1					
				1	1													1								
																							2			
				1	1																					
							1																			
							1																			
1		1		1	1		1																			
							1																			
				4	3																					
					1																					
							1																			
				1																						
1																										
																		1								

																																			1	1												1			
																																																1			
																													3						1	2												1			
		1																																																
		1																																																
							1																			1											1												
																																				1															
									9											1				7						1																					
		1																																																
																																				1															
							1	1															1																							1			
																				2																															
																																															1			
		1																																																
																					1																														
																																			1																
																				1																															

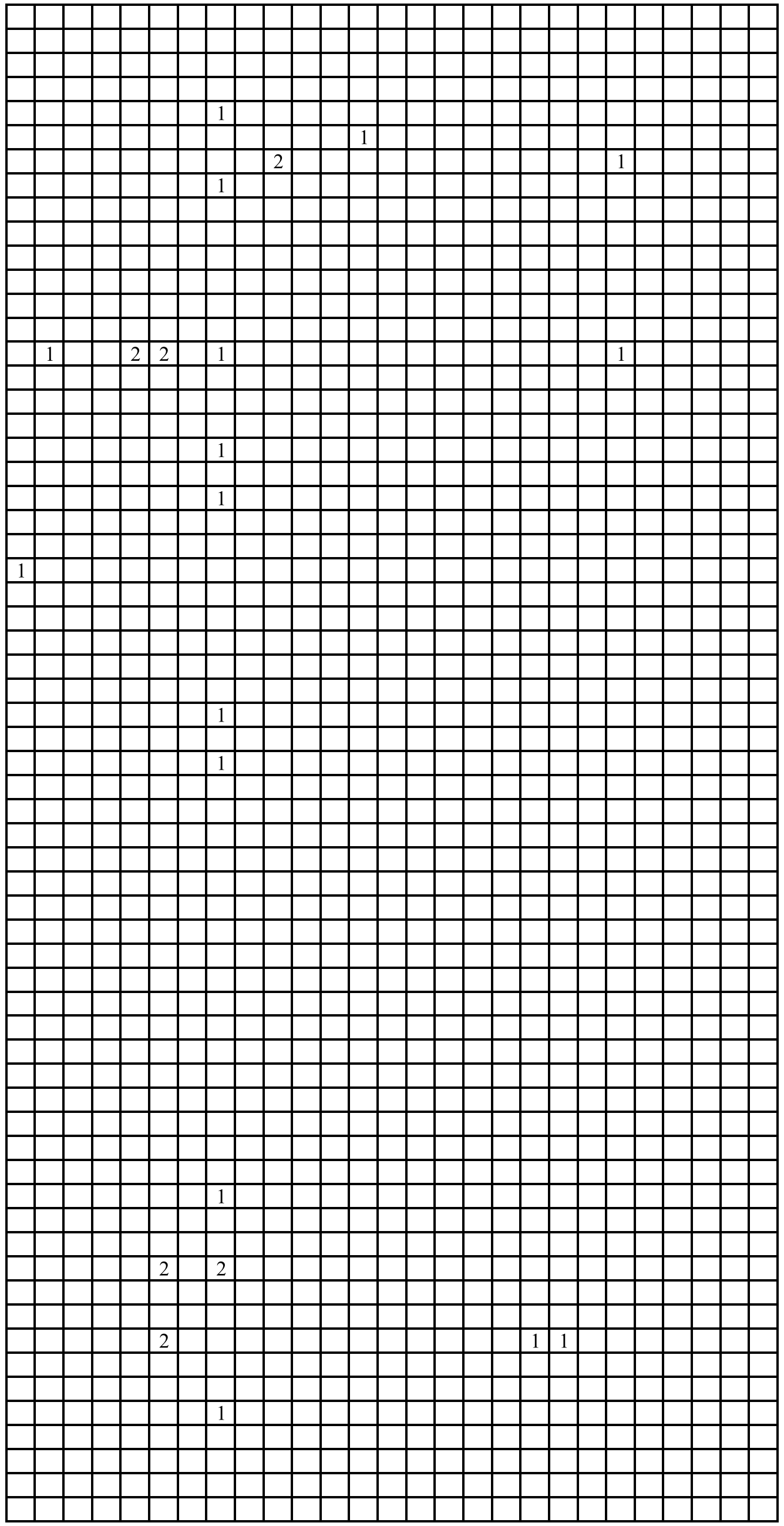

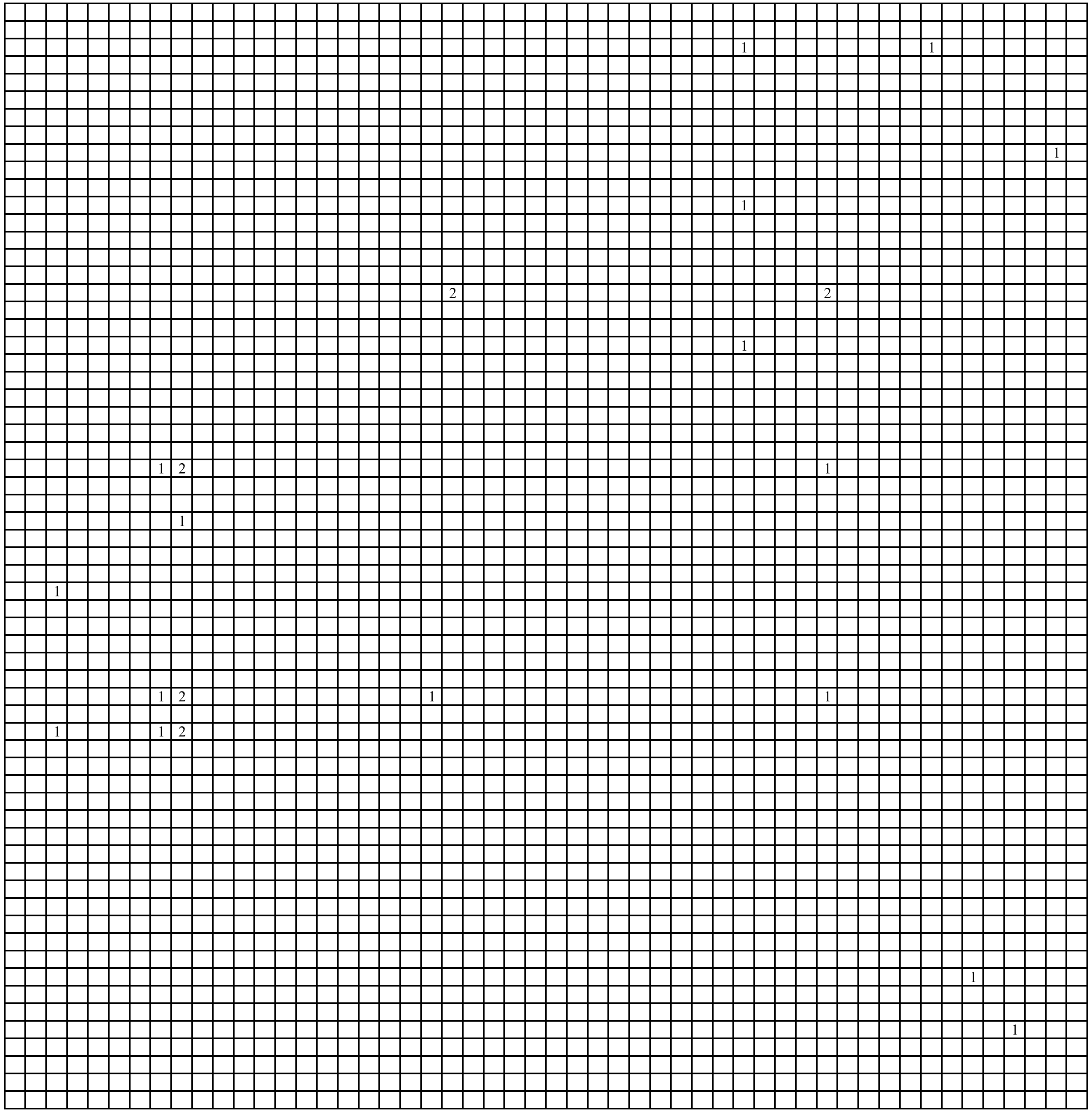

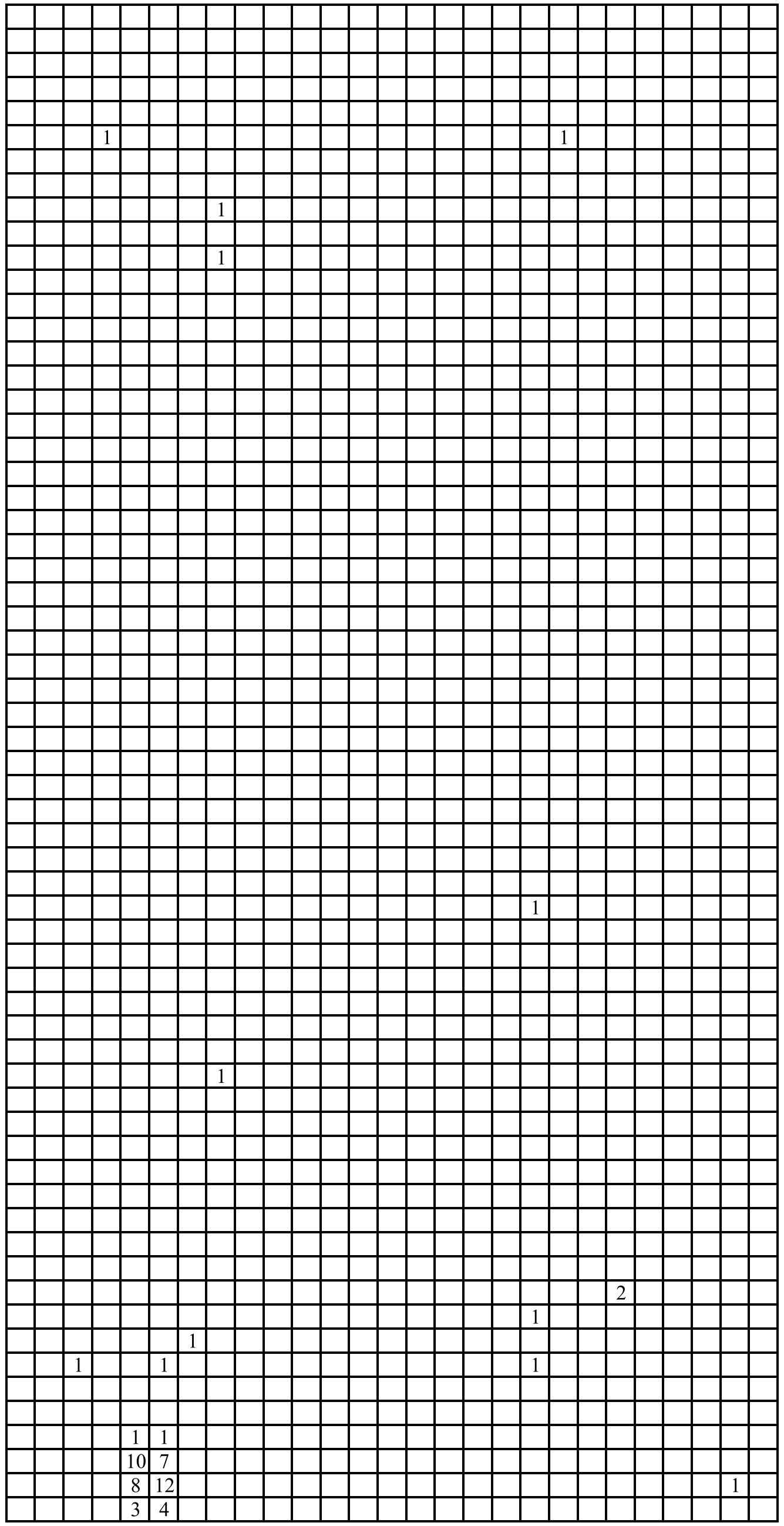

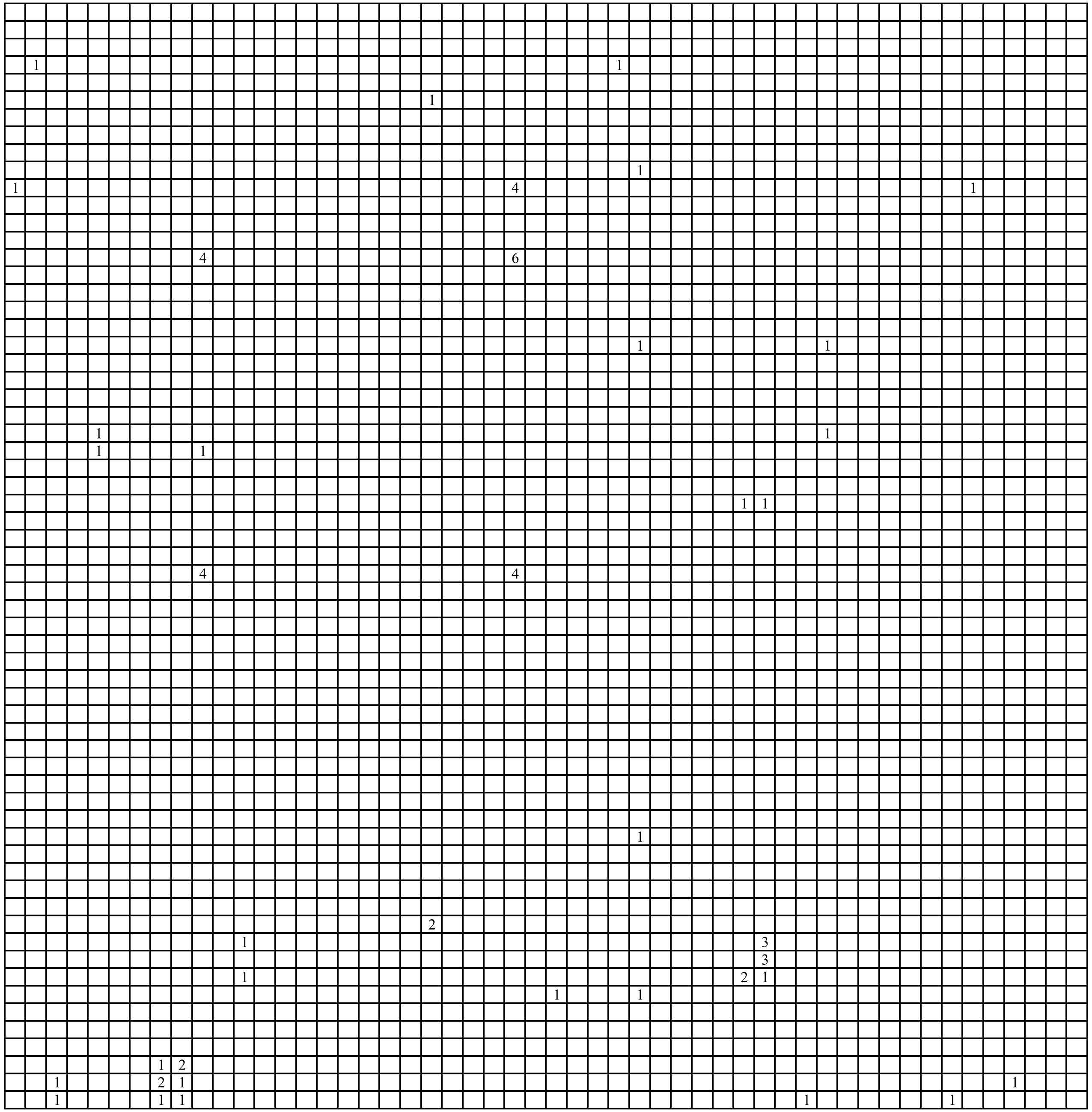

				8	9		1																		1	
				3	2																					
				1	1																					
				1														4	4	1	2	2	2			
	1	1		1	1	1	1											2	1						1	
						1													1	1					1	1
																					1					
																		1	2					1		
																			1							
																		3								
																		2								
							1											1								
					1																					
								1										1			1					
					1																					
		1																								
																		1	1	2						
																		2								
1																										
								4										3	2							
				4	11																					
				11	6		1																			
					1		2																			

		1																												1																					
							1	1																																					1						
				1																																															
							1	1			1															1	1	1		2									1											3	
			3				1	1			1		1	1	1								1							2									3	4	1	1	3							5	
1	2		1									1	1	1									1				1												2	1	1	1								1	
																																																		1	
																														1																					
					1								1																	2										1											
																														1																					
																										1																									
																																							3												
																				1																								1							
	1																									1					1	1																		1	
																																								4										4	
																																																		1	
																																								1										1	
																														1																					
																										1														1										3	
																										1																								1	
																																							2		1						5			1	
							1																			2													1		1										
																											1																							1	
		1									1									1			1							1								1													
																																																		1	
																																																		1	
		1																																																1	
																																																		1	
													1																											3										1	
	1																														1																				
																										1																								1	
																																																		1	
																										5																									
															1																																				
		1																																														1			
							1																																												
							1	1	3																																										
1		1					1	3			1		1										2				1												1											2	

	4																				1					
	3																	2					1			
	2																		4		1					
																						1	4			
								1											1		2		1			
																				1						
																		1		4	1					
																									2	

																										1				1																					
																																				1															
																																													2					
																														1																					
								2																																											
																																														3			
																				1																															
			1																											1	1																				

																										PFDN5	42	348
													1													PHIP	33	254
																										PIK3R1	21	832
																										PIM1	12	118
																										PPP1R12A	18	308
																										PPP3CB	7	97
																										PPP3R1	5	104
																										PRAME	14	148
																										PRKCQ	5	86
																										PRKD1	12	223
													1													RNASEL	2	42
																								1		RNF10	7	103
																										RPS6KA3	14	254
																										RQCD1	16	327
																										RWDD3	3	14
																										SMURF1	116	856
																										SMYD2	10	84
																										SNX6	7	248
			1																				4	4	7	SS18	57	199
																										SSU72	4	48
																										STK3	13	367
																										SUV39H2	13	107
																										TGFB1I1	15	85
																										TRAPPC2	10	192
																										TRIM32	24	337
																										TRIM5	6	445
																										TTC21B	4	27
																										UBE2V1	4	130
																							3			USP2	34	349
	1																									USP47	15	239
																										ZMYM5	5	43
											1												1	1	3	ACTL6A	80	385
																										ACTL6B	7	100
																										ACTR5	20	125
																										ACTR8	17	107
																										AIP	23	228
																										AKAP17A	9	119
																										AKT2	11	145
																										ANP32A	12	122
																										ARGLU1	13	180
																	1						1	1	1	ASF1A	42	400
																										ASF1B	1	212
																										ATF7IP2	7	21
																										ATXN3	26	643
																										AXIN2	10	92
																										BAZ1A	24	141
																										BAZ1B	26	248
																										BAZ2A	10	88
																										BRD8	50	236
																										BRWD1	5	54
																										BTG1	2	38
																	1							1		CAND1	42	1025
		1	1																						1	CARM1	46	298
																										CASK	21	275
			1																							CBX1	84	591
										1										1						CCNA2	24	474
																				1						CCNE1	18	342
																								1		CDC45	7	157
																										CDC6	9	196
																										CDCA7L	17	103
																1				1						CDK1	73	1091
																										CDK11A	20	219
																										CDK11B	22	219
										1										1						CDK2	88	1535
																			1							CDK8	40	598
																										CDKN2B	7	106
																										CENPU	13	175
																										CHAF1B	29	236
																							1		1	CHD7	88	165
																										CHEK1	17	373
																										CHTOP	25	300
																										CNOT6L	7	171
																										CNOT7	14	274
																										COPRS	6	99
																										COPS2	25	439
																										CRABP2	6	54
																										CRTC2	4	91
																			1							CSNK2A1	178	1477
																			1							CSNK2A2	104	1079
																										CTDP1	3	222
																										CTDSPL2	3	97

																																																																					1										
																																																																												1	1		
																																																																												1			
1																																																																																
																																																																				1												
								1																																																																						
1																																																																																
																																																																												1	1		
																																																																												1			
																																																			1																		1			
																																																	2																												
				1																														10																																																	
																																			1					1										1					1									1															
																																																1																										2			
																																																																							1								
													2																	1																			1						2		3			1	1	1															
																																																	2																												
																	1																1	1																												1																		

1																							1																																	1																						
1			2									1																			2	1													1			4						2																								2			
													1																										1													3										1													1	1		
																																																					1																							2	2		
																																																				1																												
9	1		4	1	1								1					1						1				1			11					1		2	1						1	2	1	3			1	4	1	2		1	1	1	3	2	1	1		1	1		1	2		2							4	1		
																															1																2					2																										
																		1						3							1								1							1						1								1	1					1		1		1							1			
															3																																												1				2																
																																																					2																										
																					1																																						1			2																
																																																															1																
									1																																																		1																		1			
			1																																																																												
																		3																																																										2			
																													1		1																																																
					2				2																																																									1			3			
				2											2																																																															
								1																																																																							
															2																																																																
				1														1	1																																	2																									2			
																																																				2																												
																																																																						1				1			1			

		1																									1																																																						
																																																																												4			
																									1																	2					1				1								2																1	1			
																																																																												1			
																																																			1																													
1																															1				1															1									1			5	2				1	1	1							1	1		
1												1					1										1				2				1			2	1						3	1	1	1				3		1				1				1		2				2		2							1			
																																																																	1																	
				1														1													1																																																	2			
																																																								1								4														3			
																																																							1	1						2																	
																																																																	1																	
		1										1			1														3																						1			1																							1			
																																																			1												1														1		
																																																							6	1			1			6																	
																												6									1															1	1	1							3														1			
																																																																	2																	
																																																							3												1														
														1																																																																			
																																																			1			2	1				1			4																	
																																																							1	1						1																	
																																																													2																				
																																			1																																																	
																																																																										1								
							2																																																																										

							1																																																																									
																																											1																																						
							1																																																																									
																													1																																																											
																													1											1																					1																	1		
																																												1																							1												
																																																					2				5			3																		
																															1																																																						
												2																																																																					
1																																							1																					4																				
																																											2																																							
																															1																																																						
	1																																																																																	
	1																																																																																	
																2																																																																	
	1																																																																																	
																															1																	4																																				
																																																3																																				
																													1																																																											
																		1																																																															
																																																														1																
																																			1									1																				1																	
																														2																																																											
																																																																			1												
																																									1																				1							1													

																													2																2																																								
	1																																																																															
																																		1																																										1									1			
																		1																																																										1									1			
																														1																																																									
																																								1																												1												
																																																																	2																		
																			1																																																																
																																																																													1					
																																																																								1												
																																																																															1			
																																																						7													1										1	1		
																																																							7													1													
																																																																										1								1			
																																																																															1	1		
	1																																																																																
	1																																																																																	
																																																																																	1			
																																																																		1																	
																																																																															1					
		1																			1																																																															

																																												1																																					
1																																																		2																																
																																																																										1				
					1	1																																																																											
1																																																																																		
									1																																																																								
																																																																				1									
																																																																										1				
											3																												1																						1																		1				
																																																														1															
																																																																			1												
																		1																													1																						1												
																												1										2					1																										1									1				
																												1																																											1												
																																																																											1				
															1																																																														1				
												1																																																																							
		1																																																																																
																			1																																																								1									1				
1																																																																																		
																																																																											2	2			

																																						1																																						1			
																																																																		1												
																																																																		1									1	2		
																																																																		1										1		
																																																																		1										1		
1																																																																															
																																																																			1												
																																1																				1																														
																																														1	1																						1												
																																																																												1			
																																																																1																	
1																							2																																																											
1																																																																																

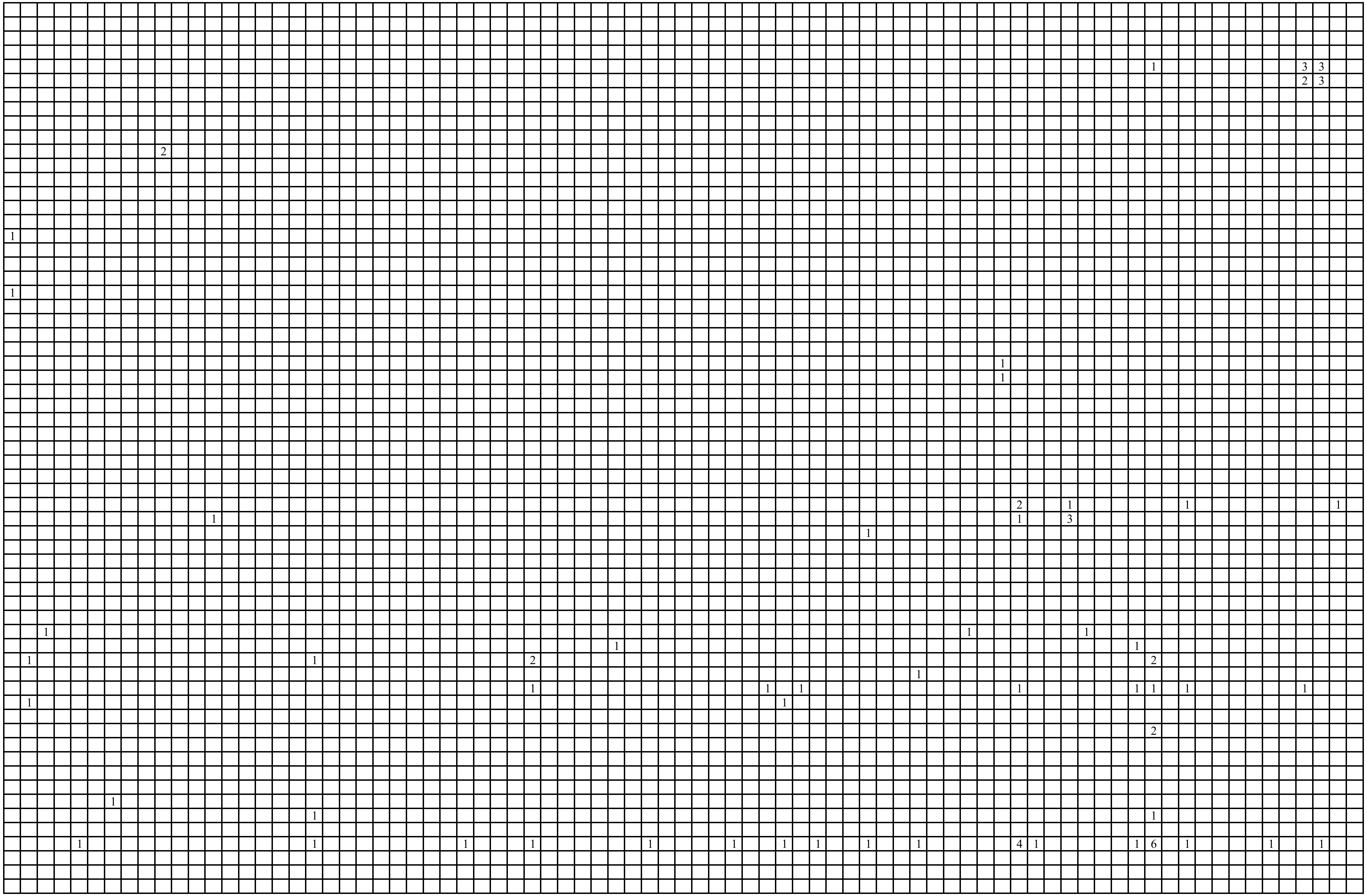
1 3 3
2 3
2
1
1
1
1
2 1 1 1
1 1 3
1
1 1 1
1 1
1 1 2 2
1
1 1 1 1 1 1 1 1
1 1
2
1
1 1
1 1 1 1 1 1 1 1 1 1 4 1 1 6 1 1 1

																																																																			1												
																																								1																												1												
																																																																					1												
																																																																				1												
																																																																													1		
																																														1	1	1			1									1																				
																																								1																																				1			
																			5													6																				1	1																2												
															1																																							1		1							1													2			
	2	1													1																1																		1		1																		1							4	2		
	1											1																			1													1	1								1						2			1	1													2			
																																																											1																2	1		
																			1																				1												1															1			1										
																																																																						1										
																																				1					1																																															
																																																																		1																	
																																								1												1																												
																																																																														1	1		
															2																																																																		
																																																			1																												2			
		1							1																																		1																			1													1			
																																												1									1															1													
																																																															1																				
																																																								1																										
															1																								1												1							1					1							1							4	3		
																					1																																																															

																																																																			1							
															1																																																																		
	1																																																																	1										
																																																						1			2																
													1																																																					1								
									1																																																																								
																																																												1													3	3		
																																																																									1								
													2																																																								3	2								
		1																		1																															1												1																	
																																																																															1		
																																																																															3	3		
																		2																																																														
	1																																																																																
																															1		3	3						1																																														
	1														1																																							1																										1			
												1																																																																					
																																																						1																											
																									1															1			1								1						1			1				1				1														
																																						1																																													
															1													1																																																										
															1																																																2														1			
																												1							1																																																	1		
																																																						1																													
																															1	3																5																															1			
				1		1	1		1	1		1			2	3								2							2				1				1	1			5					2				2			1			1		4	1	1	2	1	1			1	5					1		1	7	3		
																																																1				1																									1	1		
3																								2																1								1							4	2				3			1														3			
																																																																				1												
																														1																																																		1			
																																																																					1												

1																																																																				1										1		
1																																																																																
																																																			1																													
																																																																								3					1			
																																																														1																		
																																																																					1		1	1								
																												1																																																				
																																					1																										1														1			
																																											1									6																	1								3	2		
																															1																													1																				
			2			2						1																			1																																						2											
																															1																							1													1		2											
																																																												1																				
																													2																									2													1	1												
					1																				1						1								1																					2			1										1							
															1																																																																	
				2																																												1						1																								2		
																																																						2																1										
															29																1													1																				3																
															3													1																	1																		1	1																
															7							4						1																																	1		1	1																
				1											2							1																																													1		1											
															12						1																																											1																
															8																																																																	
															15							2						4																2																																				
																																													1																																			
															3													2																																																				
		1										1												1																									1																											1				
																																																				2																												
																												1													1																																							
1																																																																1																
																																																																						1										1

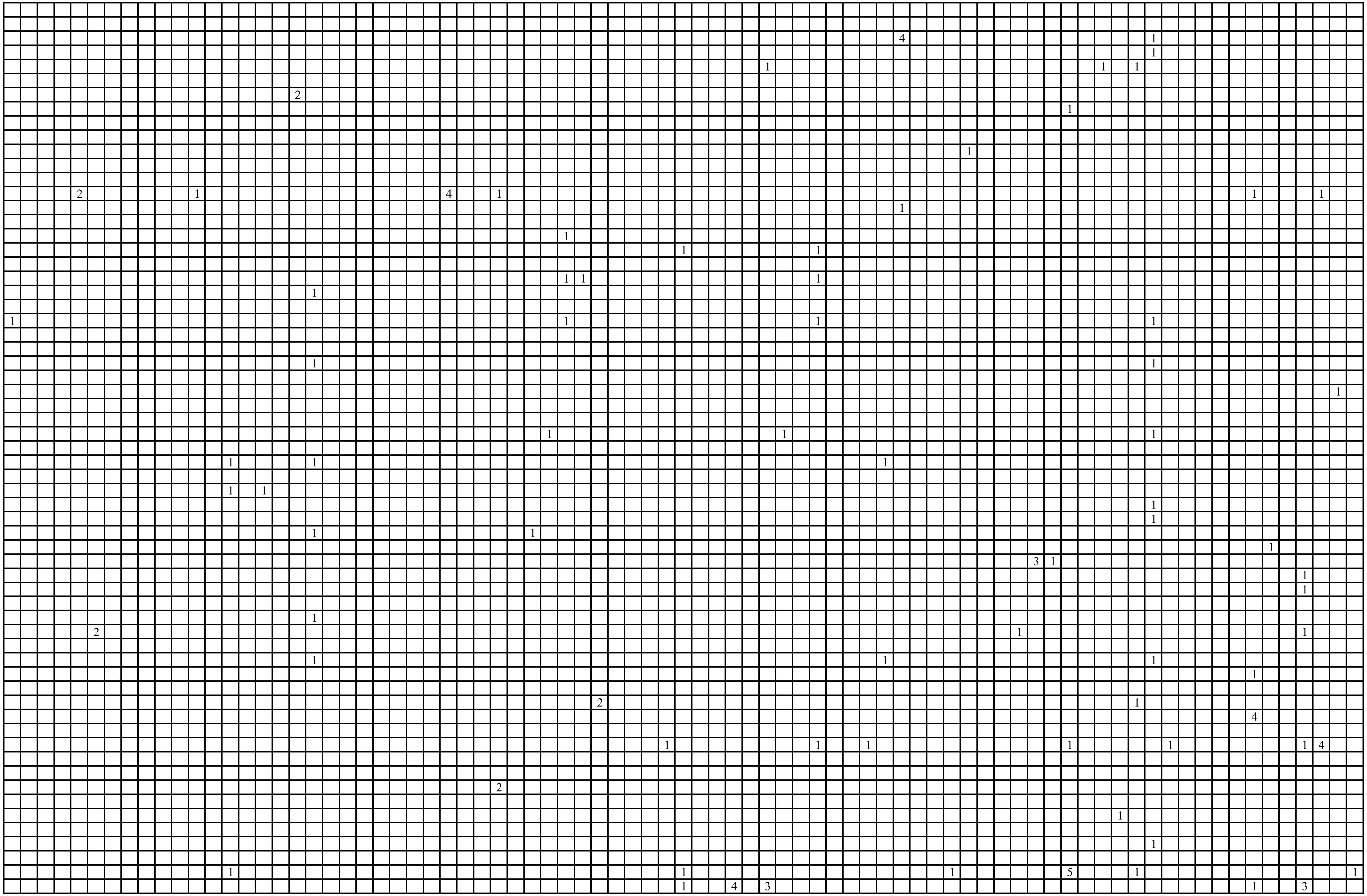
4 1
1
1 1 1
2
1
1
2 1 4 1 1 1
1
1
1 1
1 1 1
1
1 1 1 1
1 1
1
1 1 1
1 1 1
1 1
1
1
1 1
1
3 1
1
1
1
2 1 1
1 1 1
1
2 1
4
1 1 1 1 1 1 4
2
1
1
1 1 1 5 1 1
1 4 3 1 3

	1																																							1																							2				1													
	1																																																					2														1									2	1		
	1																																																					1																										
																		4													4																				1	1				1												1												
		1																4													6	2																				1																3									1			
																																																												1																	1	1		
																																																											1					2																
																																																												1																				
																																																																1																
											1																																															1		1																		1		
																																																				1																1										1		
																																															2																																	
																																																																1																
																																																									1																							
																																				1										1					1																											1		
																																				2																																									3			
																																																															1																	
1	1																																																																															
																																																																				1												
																																																																													2	2		
																																																								2							4																	
																																																						1																										
																																	1																																			1												
																																																1																																
																																														1																						1												1
															3																																																																	
																		1			1																																																											
																																											2																																					
																		1															1																																															
																																																1																																
																																	1	1												1		1																				1												
																																														1																																		
1																																										2													4					5			1																	

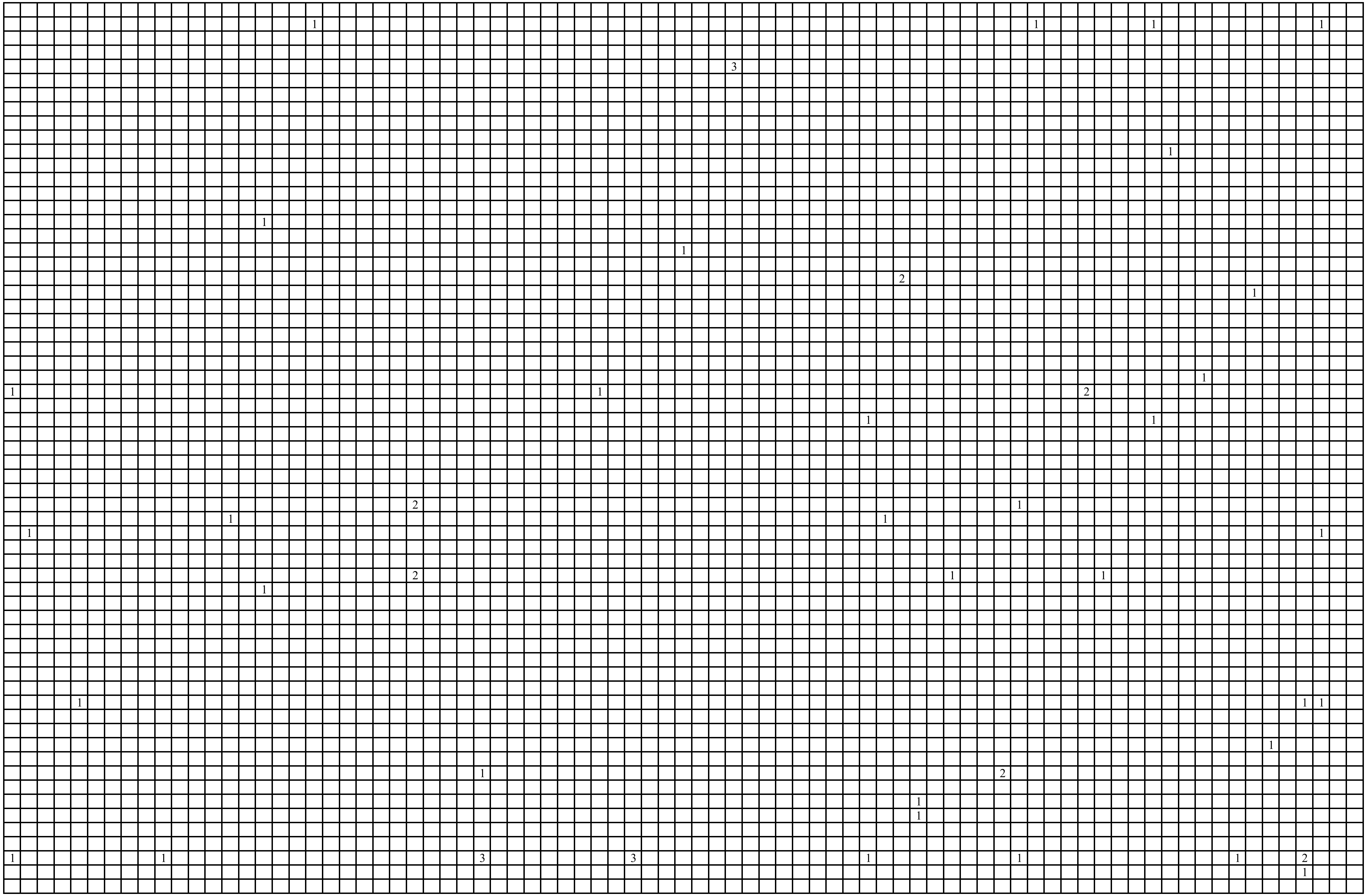

1 1 1 1
3
1
1
1
2
1
1
1 1 2
1 1
2 1
1 1
1 1
2 1 1
1
1 1 1
1
1 2
1
1
1 1 3 3 1 1 1 2
1

																															6		7	8														1			1			1														1												
																																																										1																				
																																																														1					1												
																														1																																																							
																																																																				1														
																																																																									1										
																															1																																																								
																																																				1																											
																																																														1																			
																																																													1																				
	1																																																																																	
																				2								1																																																												
																																											1																																							

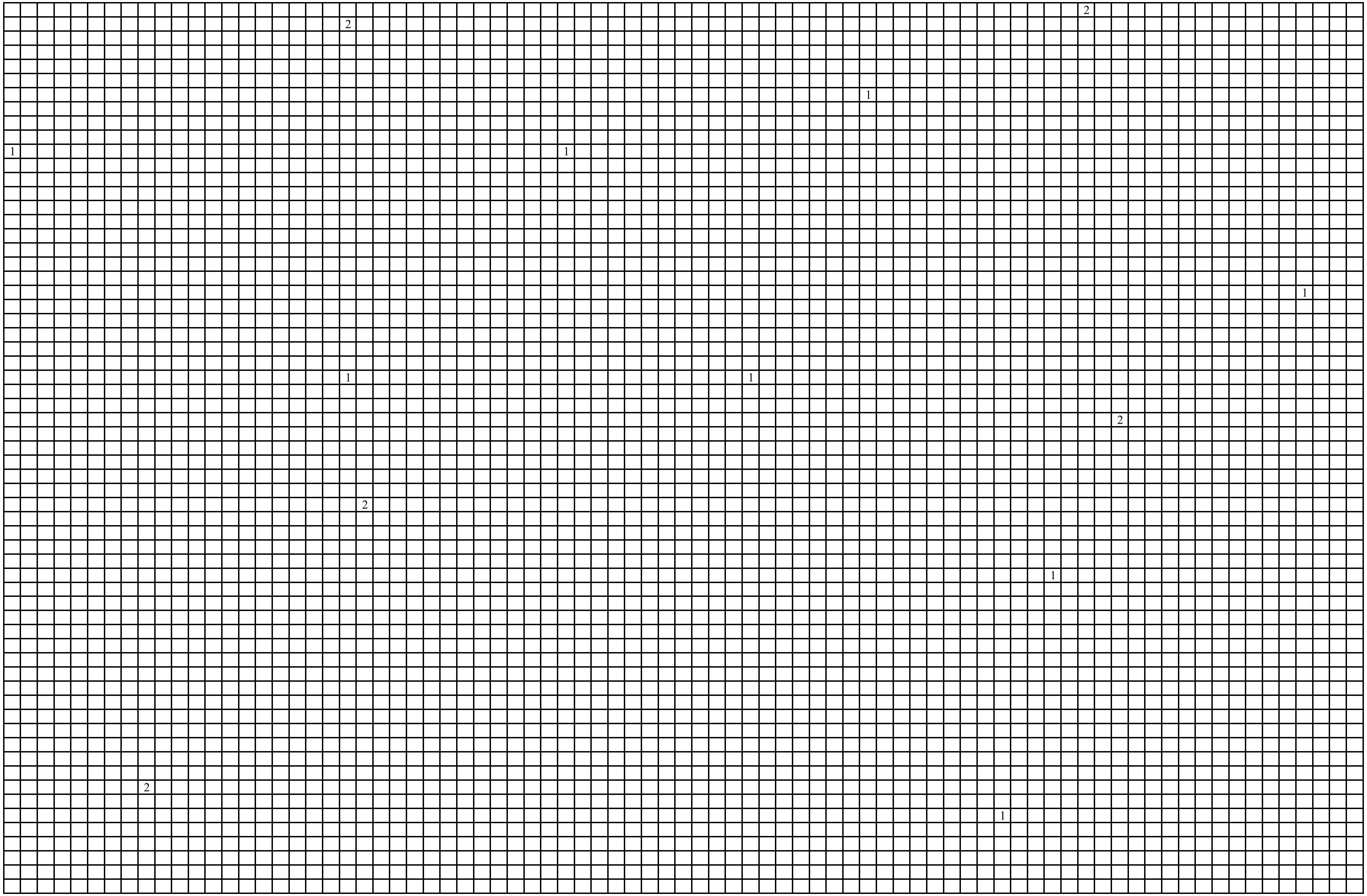
2
2
1
1
1
1
1
1
2
2
1
2
1

				2																																									1	1																								1										
																																																											1																				
																																																			4																										
																																																			4																										
	1																																							1																								1														1		
																																																1																													
																																																											1																				
																																																														1																
																																								1																																															
																																								1																																															
							1									2																																																																			

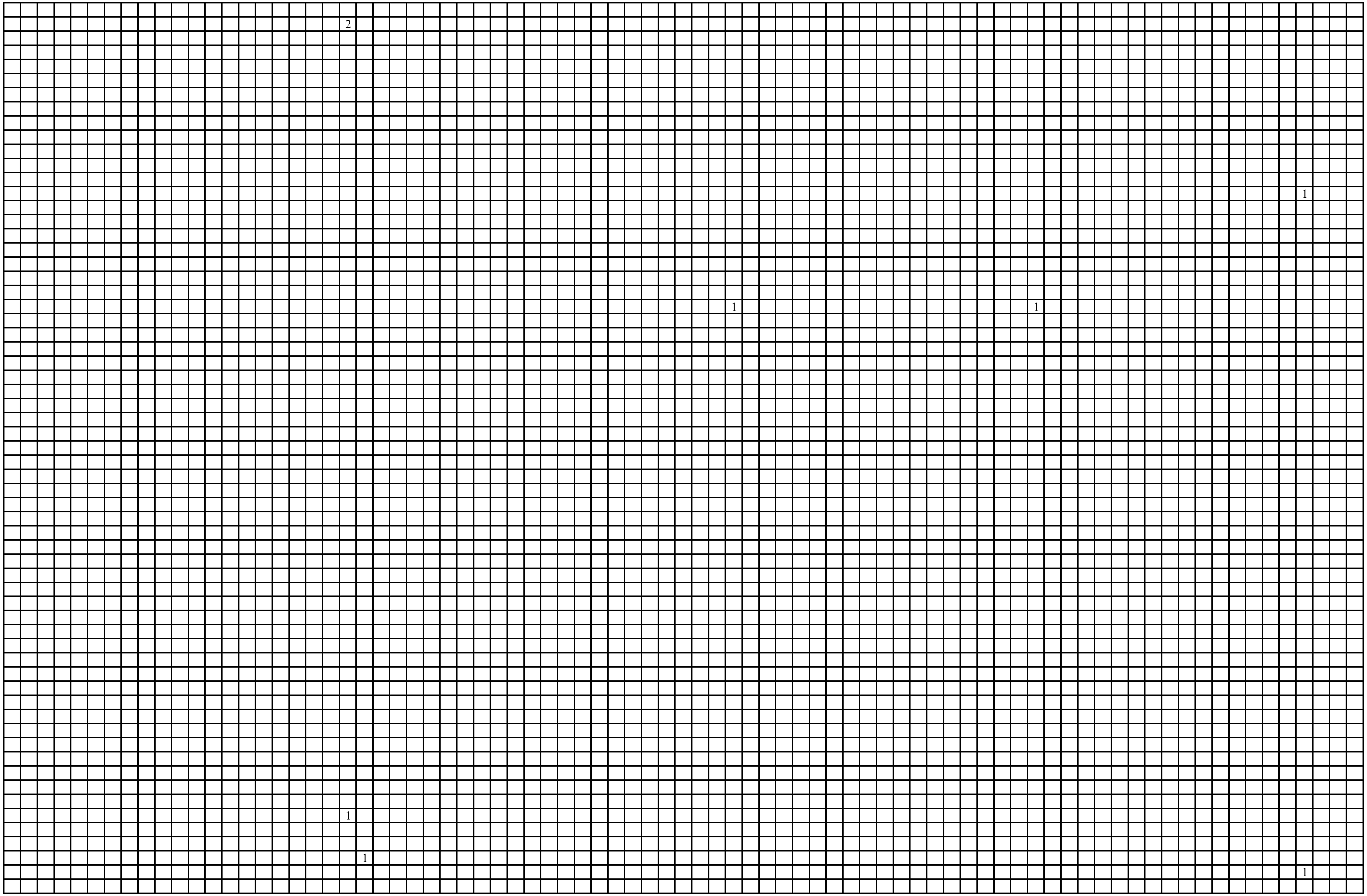
2
1
1
1
1
1
1

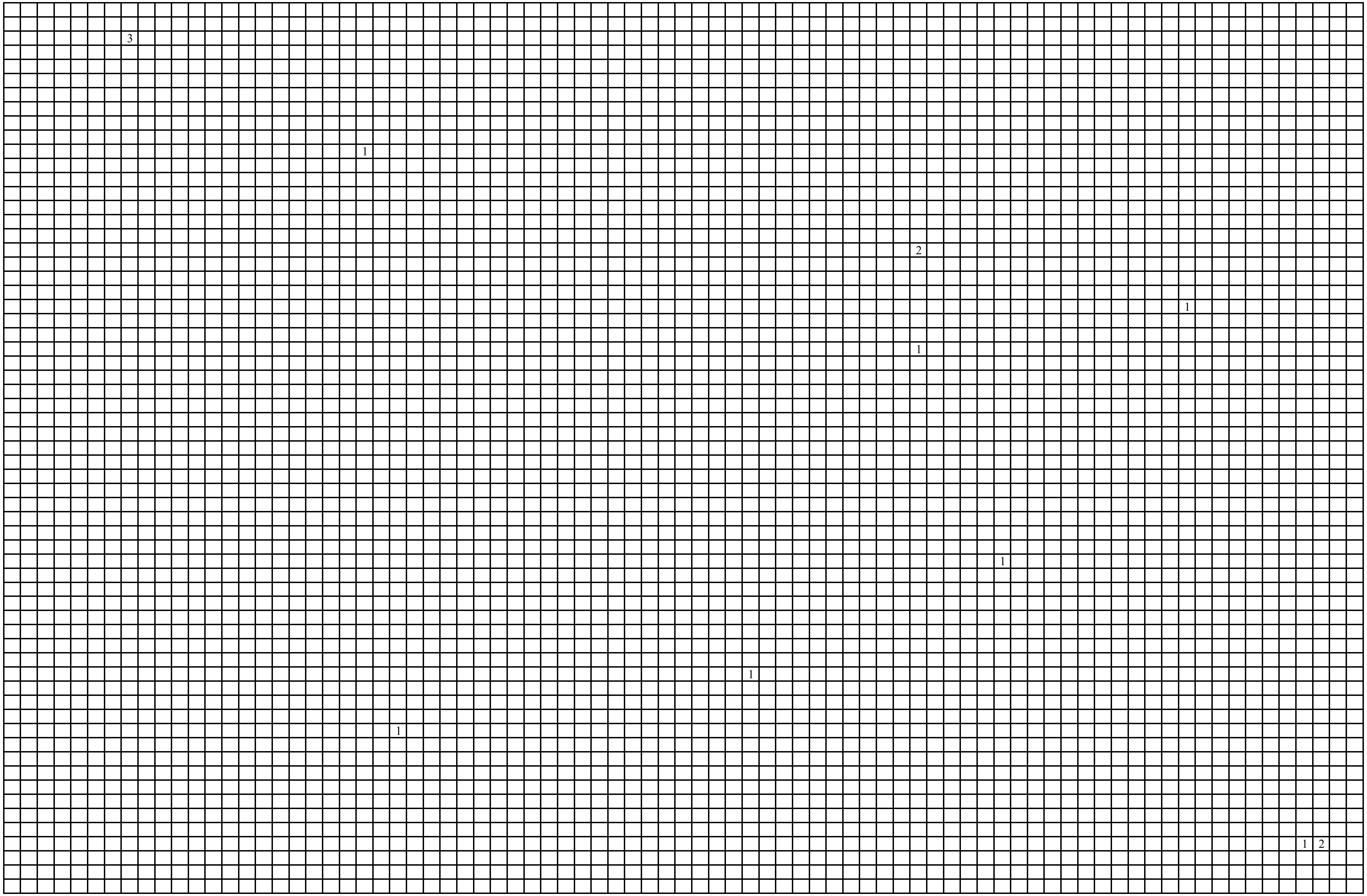
3
1
2
1
1
1
1
1
1 2

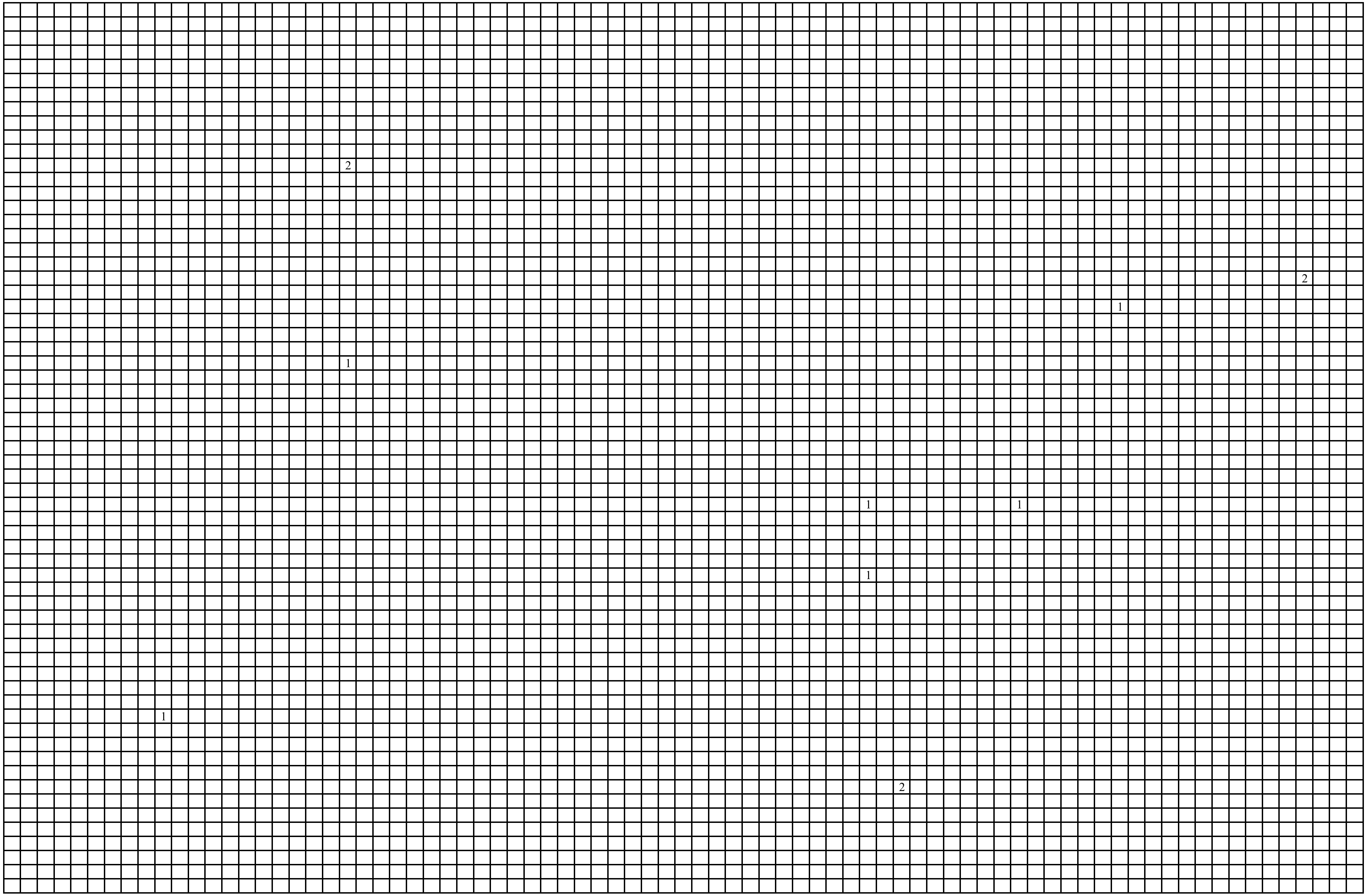
2
2
1
1
1
1
1
1
2

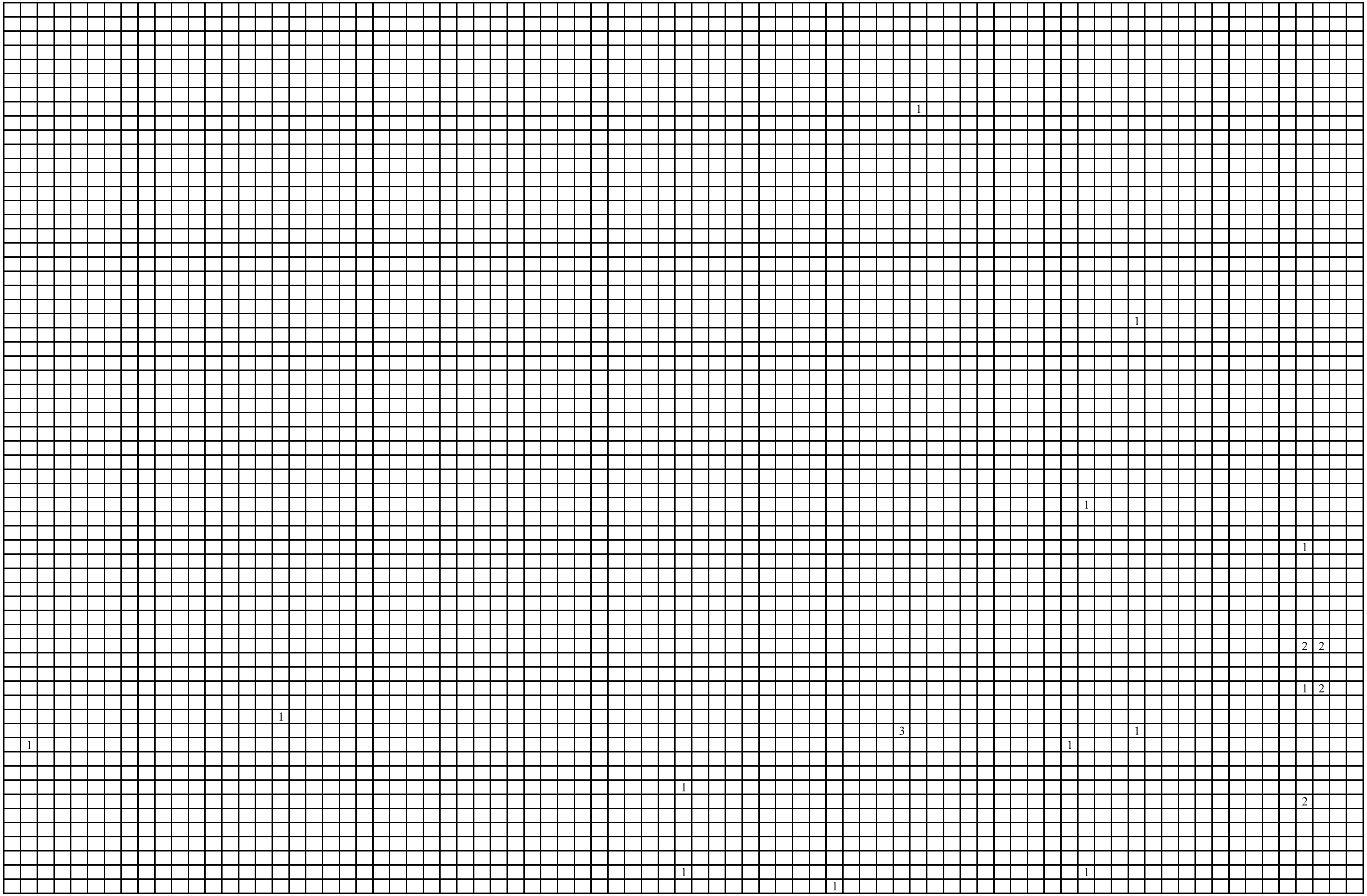
1
1
1
1
2 2
1 2
1
3 1
1 1
1
2
1 1
1

																														1																																																			
2		1																										1			1																																																			
																																																												1																				
																																																																										1			
	1																																													1																																		4	1		
1																																																																																
	1																																													1																																		1	1		
																																							1																																													
																																																				2																										
																																							1																																													
													1																																1																																						
																																							1																																													
																																																																		1													
1																																																																																
																				1																			1														4										1			1						2							
			1																																																																													
	1			1																																										1	1							1								1																			
																																																																				1							1				
																						1																																																													
																																																																												1			
																																																																													1		
																																																				2																										
													3																																																																							

																																																						1																										
																																																			1																													
																																												1																																				
																							1																																				1																					
																																																						1																										
																																																						2																										
																																													1																					1														
																																																																		1														
																																																													1																			
																																																						1												2														
																																								1																																								
																																								1																																								
	1																																							1																																								
																																																																						1										
																																												1																																				
																																								1																																					1	4		
																	1																							1																																					4	7		
																							3					1																																																				
																																																4																																

																																													1																																		
																																																																														1	1		
																1	1																																																												
													1																																																																		
															1																																				1																													
	1																																																																																
	1																																																																																
																																																										1																					
																																																			1							3																					
																		1													1																								2					1			1					1												
																																																														1					1												
																		1													1															1	1													1								1		1							1	1		
																																																	1																																
																																								1																					1	1					1	1									3	2		
																																								1																											1										3	3		
																																								1														1														1									1	2		

																																								1														1							1	1					2	1									2	2		
																																																												1	1		1			1													
	1																																																																														
	1																																							1																																								
		1					1		1												4	5		1				1			3																	1			1			1		1				1				1				2							3			2			
		4		1								1	1		5	1	1							1									1			1	2	1	1	1					1	1	1	1			1	1	1	1						1	1	3	2	1				1		1	1	1					1	1		
													1															1				1		1	1		1											1					1												1		1			1	1		1	1	1					1			
																		1					1						1	1																																								1										1		
																																			1																1																																	
																																						1																										3																		
																																																																													1		
																																																			1																																
																																				2																																														
																								1																																																										
															1								1																1																																					1							
		2					3																		2																																	1																					
																																																				1																																
		1																																																												1										4						
																																						1										2																											2						
																																																											1			2												2						
																		1																															1																				1												
	1							1				1																			1						1			1							1	1				1	1		1						1		1	1					1									1	1	
															1																																																																	
																																																																										1						
																																					2																																													
																									1																														1																										
		2																						2																								2																																
																				1																								1																																	2			
																																														1																																			
																							2																																																											
																																																																										2						
																																																		1																																
																																										2																																										
1																																																																																	
																																																																	1														
																																																													1	1					1										2	3		
														1																																																												1					
															1									1							1					1			1						3						1		1	2									1						1									1			

											1																									1					3												1													1												1	
1																																																																																
			1																						2																																															1					1			
			1																					1																																																							
							1																	1																																																							

																										CTH	7	67
																										CTNND1	26	385
																								3	1	CUL2	35	906
																										DHX36	36	302
																										DNTTIP2	17	156
																										DPPA2	1	45
																										DPY30	43	242
																										DYNLL1	66	829
																			1							EAF1	19	228
																										ECD	12	196
																6			1						1	EED	90	829
	1																									EEF1A1	43	726
																										EGLN2	10	51
		2									1	1	1	1			1									EGLN3	93	1360
			1													1										EHMT1	64	218
																										ELL2	12	65
																										ELP2	8	87
																										EMSY	0	0
																									2	EWSR1	100	941
																										EYA1	15	62
																										EYA3	9	76
																										FBXO5	1	132
														1					1							FBXW7	232	1547
																										FGFR2	20	296
																										FLII	32	273
																										GSK3B	118	1350
																										GTF2B	26	179
																										GTF2E1	1	100
																										GTF2H2	8	99
																										GTF2H3	7	90
																										GTF2H4	8	100
																			1		1	1	1			H3F3A	153	852
																										H3F3B	4	125
																										HABP4	12	167
											1								1							HAT1	31	242
																										HCFC2	26	151
																										HDAC11	17	188
																										HDAC6	56	1301
																		1								HDAC7	48	241
																										HDAC8	5	45
																										HEATR1	17	243
																										HIPK1	7	43
																										HIPK3	9	37
																										HIRA	41	199
						1																				HIST1H1C	61	795
																										HIST1H1D	36	341
																										HIST1H1E	61	777
																										HMGN3	9	63
																			1							HNRNPA2B1	46	777
																										HNRNPAB	32	378
																										HNRNPD	58	732
																										HNRNPUL1	49	376
																										HSFX2	2	10
																										HSPA5	76	1789
																										HSPH1	40	372
																										HTATIP2	0	58
														1												HTATSF1	9	181
		1																								IL16	24	126
																										ING3	33	175
																										INO80B	23	214
																										INO80C	12	74
												1														INO80E	21	140
																										INPP5K	5	85
																										IRF2BP1	40	99
											1	1	1													IRF2BP2	48	140
																									1	ITCH	82	790
																										ITGB3BP	24	110
																										JAK2	33	271
																										JMJD6	17	306
																										KANK1	0	50
																										KDM4B	12	94
																										KEAP1	184	783
																										KHDRBS3	11	135
																										KMT5C	0	0
																										LDB2	5	37
																		1								LITAF	10	117
																					1	1				LMNA	105	1632
																										LRIF1	36	288
																										LRPPRC	31	769
																										MAP3K7	17	798
																										MAPK12	2	71

																																												2																																			
																		1																																																															
																																																																		1														
																																																																												1		
																																																																1																
																																																					1																											
																								1																																																											
																																																																				1													
																		1																															1		1			1																											
																																										1																																										
													1																																																																									
		2											1																																	1																																										
		1				1						2	3					1																																																															
		7										4	13									2		1	1												1	4		1																																					1							1		
																											1																											1												1															
																																																																1																	

																																																									1																							
			1																			1									1																											2																						
									1			1																	1																																													1			
																		1																																																											
																										1																																																				
	4		3	3		2	1			2	1							2	3			53		2	6					1	2		1	1			1			3			2	3		1		3	4	7	3		3	7				3						2	1							1				5	1	4		
																																																										2																						
			1	1		1	1			2	1							1				5									1		1											1		1		1	1	1	1		1	1					1													1				1		2		
																						12			2																																																				4				
																																																						1																											
														1																													2																			1																		
		1																1																																																													
																		1													1																																																	
																																																						2																							1			
									1								2							1						2																																																		
																																					1																																					1													
														1																																																		1																		
	1																																																					1									1																	
							5			1			1								1																																														1										1		
													1					1																																																												1					
													1					2				14			1	2																	1																						3															
									1																																							1								1									2															
																																																																				4															
																						2			2																																																	8									
																									3																												1																					##					2				
																						1			2										1																																																	
																		1							1												3																																															1			
																																																				2																															
							1																																																									3	3													1			
																																					3																										3																	
				1																																																1																											1		

																						2																																																										
												2	3													1																																																						
																																																																							1									
																									1														2			1												1																										
																																			3																																									1				
																						5																																																										
							1															4																																																										
						1																			1										1																																													
											1			1				2		1		2			2						1			1										1				1	1	1	2			1									2	2	2												1			
	1		2	1		3	1				2			1				2						2	2					1	1	1								1				2	1	2	1	2	2	2	2		2	1		2							1													2		1		
																						3			1																																																							
																		1																			1																2																											
		1																1				1			2								2				1							4	1	1			1	5			1																							1				
			1																			1														1								1	1	2																																		
		1																							1											1										1													1																					
		2																							1																																																							
																															1																																																	
			1																															1																														1														1		
										2																																																	1																		1			
																		1																																																														
																															1																																																	
				1		1				1																												3													1																							1						
	1																																							1																																								
													1					1													1																				1													1																

																																							1																																								
																										1																																																									
																																							1																																								
																																							1																																								
																																							1																																								
1																																																																																
												2																																																																				
																														1																																																				
												1																		1																																																				
						1																								1												1																																									
																																				1			1																																								
																					1			1																																																													
												1																																																																				
																																															1	1	1			1																						1		1		
												1																																																																				
																																											1																																								
																																													1				1	1																													
																					1																																																																	
																																					1			1																																											
																																							1																																								
																																				1																					4																						
																																																									3																						
																														1																																2																		
																	1																																																1																		
																	1													1																																																								
								1																																																																									
								1			1																																							1			1	1																										
																																			1																																	2													
						2				1			1					1												1												1																			1						1												1
																																																	1																														
																														1																																																								

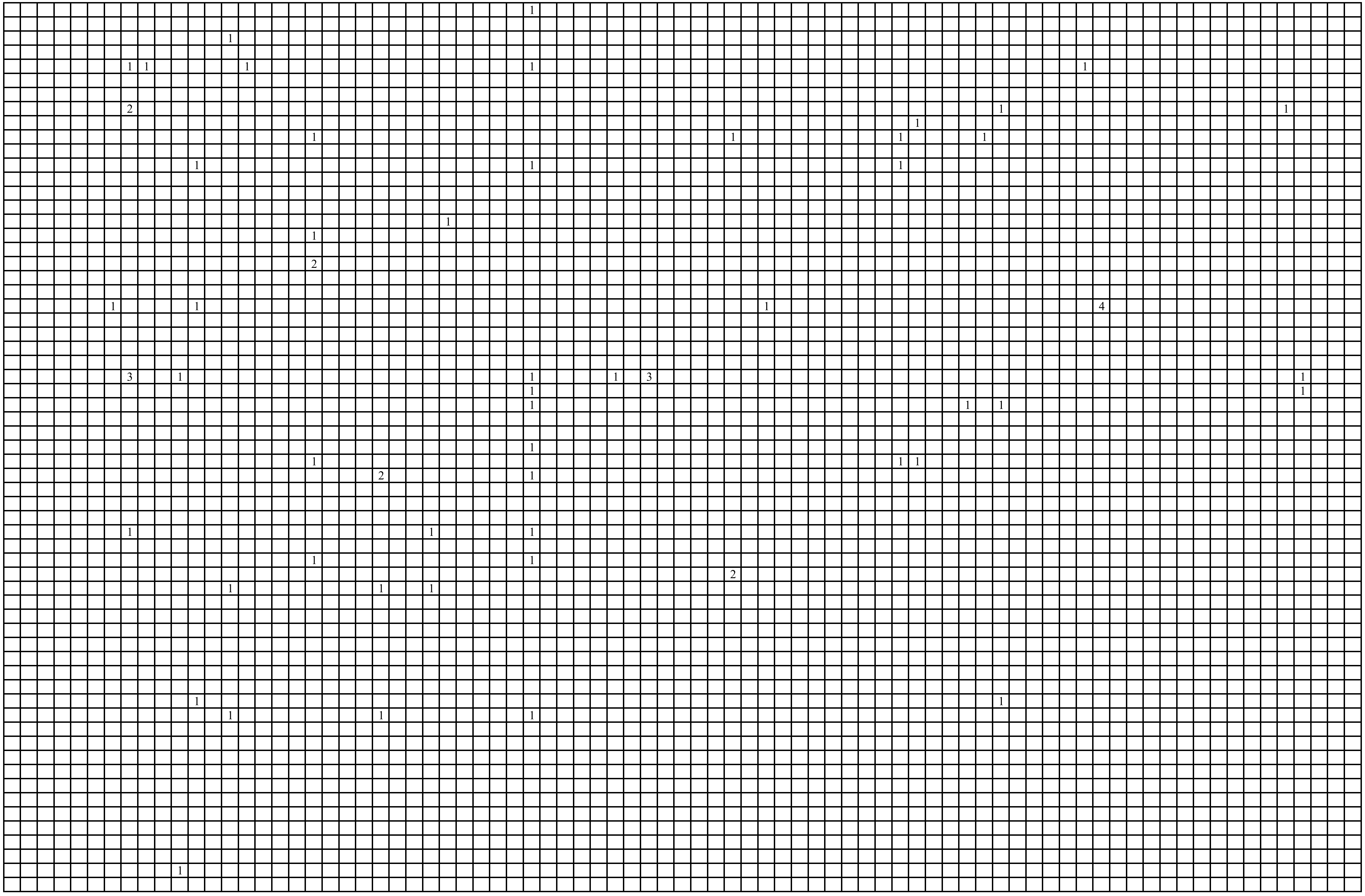

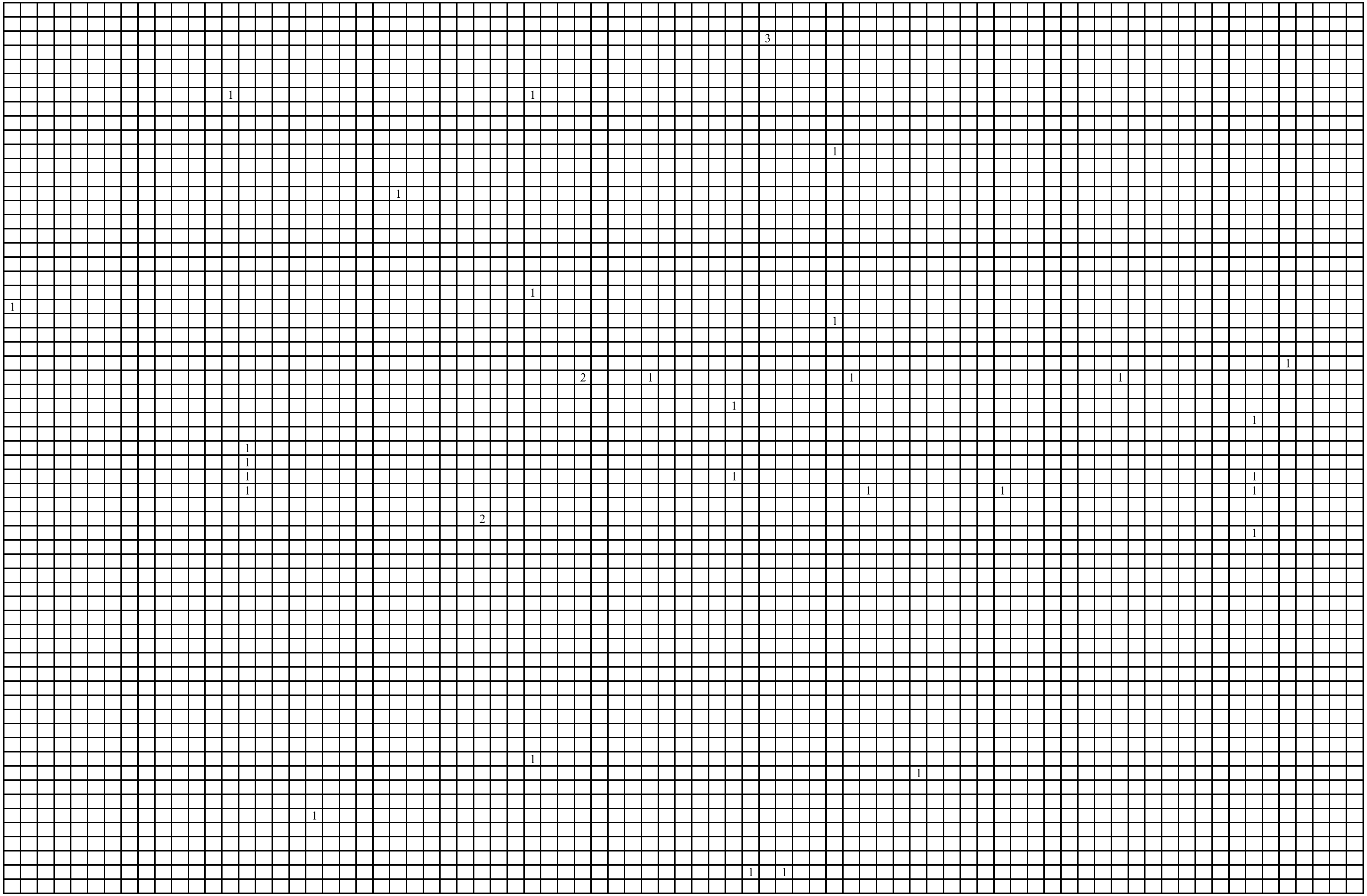
3
1
1
1
1
1
1
1
2
1
1
1
1
1
1
1
1
1
1
1
1
1
1
1
1
1
1
2
1
1
1
1
1
1

																																														1	1																																				
						1																																																																											
																														1																					1													1	1											1				
																		1																																																															
																																											1																																						
														1						1																																											1	1															
														1																																																																			
														1																																				1																															
								1																																																																									
																																																																											1					
																																																																												1				
														1																												1																																		1			
			1																															1																																										1				
													1																																																																				
						1											1																																																															
													1					1												1																																																	
																		1																																																															

																																																																									1				
														1																																																															
																									1																																																								
																			6																																			1																											
																			3	2																																																												
																			1																																																													
																			1	2																																																												
																																																																1														
																										1																																																								
																																																													1				1														
																																																																	1														
											1																																																													1			
																						1																																																										
											1							1													2															1							1																								1		1
										1													1								1													1																																			1
																		1													1																																													1				
						2	2																								1				1																																							1										
																																																1																																
																															1														1																																			
													1																																																																			
										1																					1		1	1																									1									1												
										1																					1																																																				
																															1															1																														1				
											1			1				1						1							1																						1	1									1													1				
																		1																																																														
																						2			2																																																							
																																																												1																					
														1																																						1																														
	1													1				1						1																1								1															1	1	1															
																		1																																																															

																		1																																																													
																																											1								1												1	1																
																		1																																																													
		1																				4			2						1																																								1									
													1																																																																			
																		1				1									1												1																																					
										1													1																						2																															1		1		
																						1									1																																	1																
																		1													1																			1															1															
																																										1																																		1				
																		1				1									1															2																														1				
						1				2												2	1		1						3	1					1											1		1																										1				
						1				1						1		1											1		1												1	2	2	2			1	1																		1												
				1																														1										1		1																																		
											2							1				4		1	3	2																		1				2		2	1		1	1									1															1		
																																																				2																												
																																																																			1													
										2														1													4																																											
																															1																																																	
																								10																																																								
																								4													1																																											
																																						4					1																																					
																																					1																																											
																																						3																																										
																																											1																																					
						1								1																	1																																	1												1				
						1				1				1																																																	1	1																
						1								1																	1																																													1				
														1																																												1					1	1				1												
							4																																																																									
										1								3				1			1												1											1																												1				
								4							4																																																																	
								5							8																1																																																	
										1																					1																1																													5				
																						1														1												2																																
																		1																																							1																							

		1																																																				1																									
													1										1		1																																																								
										1												1			2											1								1		2																																										
							5																																																																							
																																																1		1															2														
			1																																															1			1																										
																									1																							1																																	
																		1																																																														
																																																																										1				
																																																								2																							
									1																											3														1																																
													1																																								1																														
									2				1					1																																																															
		1								1															1																																																										
																																																								1																							
																							2																																															3										2					
							1																		1																																																													
										1																																									1																															
		1																1															1																																3	3	5																				
																																												1																																			
																																					1																																														
																																				3																																		2												
			1							1	2							1																1			1									1		1	1	1	1		2	1											1											2		1		
																		2																																																																		
																													1	1																																																							
													1																																																																							
																																																								1																						1	
										1																										1																																1												
																						1									1																																						1														1	
		1																																														1															3						1				1					
		1							2		1		2																																																									2									
																																	1													1																																		
																															1																																																					
																						1																						1																																				
																																																									6																						
		1	1				1		1		2		7	2		1		1		1		8			4					1			3								5	2		1	1			2		1	1		4	2		1										4											2	1	2		
											1											1																							1				5	1			1												6															
																																										1																							10													1		
																																																				1												1																	

																		1																																													1																	
																					1											1											1	1	2																																										
																																											1		1																																										
			1																																																																														
																																																															1												1				
																					1			1																																											3												1		
																					9			1																																																										
													2													1								2				2																																																	
		2											1	3							1			1	2										1															2													1					6									
																					1			3											1																																																						
													1											2																												1																													
																								2			1																1										1	1									1	4																
													1																																																																				
																							1	1						1																																						1												
			1				1				1							1																		1																	1																							1				
																																					2	2																																														
												1																																																																										
																																																					2																													
																								3						1																																																													
																		1																																																																			
								1																																																																														
																																												1		1				1																																						
																																												1																																														
																					1			1																																										1												1				
											1																																																								2															
																								3																																												2															
											1					1		1							2												1																	2												1															
		1																																																														1														2	
																																					2																												6														1	
						1																																																																												
																1						2																																																5													
																					4			1																																																														
																3						3	2														2																1																12												4	
																					1	2																																																5													
																1							2																									1																			3													
											1																																																																	2					
																																												1	1	1																																											
																		1																																																										1				
																		1																																														1																					

													1																	1													1																	1	1											2				
																																										2																																	1				
																																																																												1				
																																													1																															1				
										1																																																																			1			
																																														1							1																	1									
																						1																										1																							5									
																																																																				1													
				1																				1										1																																						3												
																															1				1																1																									1			
													1																	1				1																																												1				
																																																		1																													
																																											1								1									1																	1			
													1																																																																			
																	1																																				1	1																											
													1																													1																			1	1																
																															1																			1																													
																																									1							3	2	2	2																																	
												1						1																																																																
													1				1																																																																	
																																																			1																													
																																				1																																																
													1																							1														1																									1				
																																										1								1																													
														1																																																																			
																		1													1																																																		
																															1																																								3									
																						3			2																																																											
																						2			4																																																	2									
						1																													3							1																			7	7																
																		1																																																					3												
																																		1																																																	
													1																																																																				
																																			1																											1																	
	1									1																																						1					1																							2				
									1																																												2																							2				
									1																																														1																								
													1							7																																																																		
						1				1			1					1													1			1											1																																								
													1					1								2											3																									1						1										

												1																													5																			1									1		1																		
							3																	1																																																	1									
							2																																			1																																												
							3																																																																										
										1								2													1						1																1															1																	
										1								2				1								1																								1															1																	
																															1																								1																															
																																																									1																												
																															1																																																						
																																																																	1						1										
											1																			1																																													1				
																																															1						1																	1										1
																																															1						1																													
																																														1																																		
																																			1																																														
							1						2																																																																	
																						1			1						1																																																	
																																																											1																						
							1																																																																									
																						1																																																									
																																			3																																														
																														1					1																																														
							1																																																																									
																						2																																																									
																																											1																		1																			
																						3																																								1	1	2																	
														1																																				1																														
														1																																				1							1																							
													1	1																								1											1	1			1				1																				1			
																						2			1																																																							
							1						1																												1																	1		1	1														1			
			1																			1															2												1																										1		
													1								10			3	1																																																					
																																																										1																					
														1																																				1			1				1																							
																									1																													1																2										
																																											2																																						

														1						1		3			1																																							1	1																
										8																																		1																								1												
	16												1																																																														1				
														3																																																																	
													1																																																																		
													1																																																																		
																															1				1																																																				
																																																																								3										
																																																																										1								
																											1																																																									
														1																							2																																																		1					
										1												1																					1		1	2							1																							1				
																																					1																																																							
																						4			1																																																															
														1																																																							1										
																																					1																											7															
													1																							2																	1																																								
													1																							2																																																													
																																											1																							5															
											1																																				1																																								
	1																							1																																																																	
																																																											1																					
																																																				1																											
										2																																											4																											
														1																																											1																							
																															1																																																									
											1											2			8																			1				1		1			1	1																								1	
																									4																																																												

										4			1	1								1									1				1																								5	7	8				1												1			
																														1															1																																							
								1																																																																									
											3																									2	1																																														
																																																				1																												
																														1																																																				
																																										2	2																	1																		1	
											3																																				1																												1				
																																																					1																										
																														1																																																				
										1																																																																						
																																												1	1																																							
																																																		1																																
																														1																																																					
									2																1						1																		1																																							
																																																																													1						
													1																																																																					
																																																										1																								
																						1																																																								2					
																																																																														1					
																														1																																																2					
																																						1																																															
																																										1																																													
																																														1																									1									

						1																									1				1																																																
																								1																																																							
																														1																																																	
																																																																												1				
								1																																																1																							
																														1																																																	
																														1																										2																			1				
																																														2																																			
																		2																																										1																							
																		1													1													1		2																																			
				1																																																																														
								1																																																																								
																																					1																																																		
																																																																											1									
																																												3	1																																						
																														1																																																			
																								1																																																									
																											2																																																											
																																																																													2				
										1		1																			1																																								2									1			
																																																																															1				
					1																																																																														

			1																																																																											
																									1																																																			
										1																					1																																													2				
																															1																																														1			
													1												3						1																																													2				
													1																																																															
																																																			1																													
																																																																									1				
																																											1																																			
																																																	1																																
																																					1									2																																		
																																					1																																											
																															1																																																		
																		1																																																																
																															1																																																1				
																																																																				2								
																																																																									1			

																																																																								1				
																																																																								1				
																																																																									1				
																															1																																																				
																																																																												1				

																																																																									1				
																															1																																																		
																																																																									1				
																															1																																																			
																																																									1																								
																																																																									1								
																																																									1																								
								1																																										1																								
																															1																																																				
													1																																																																		
																																														3																																			

																																											1																																1				
																																											1																																					
							2																																																																								
																								1																			1																																		1	1	
																																					1																																											
																	1																												1												1																				
																																													1																																		
																																																								1																						
																														1																																																					
			1																																																						1																						
																														1																																																					

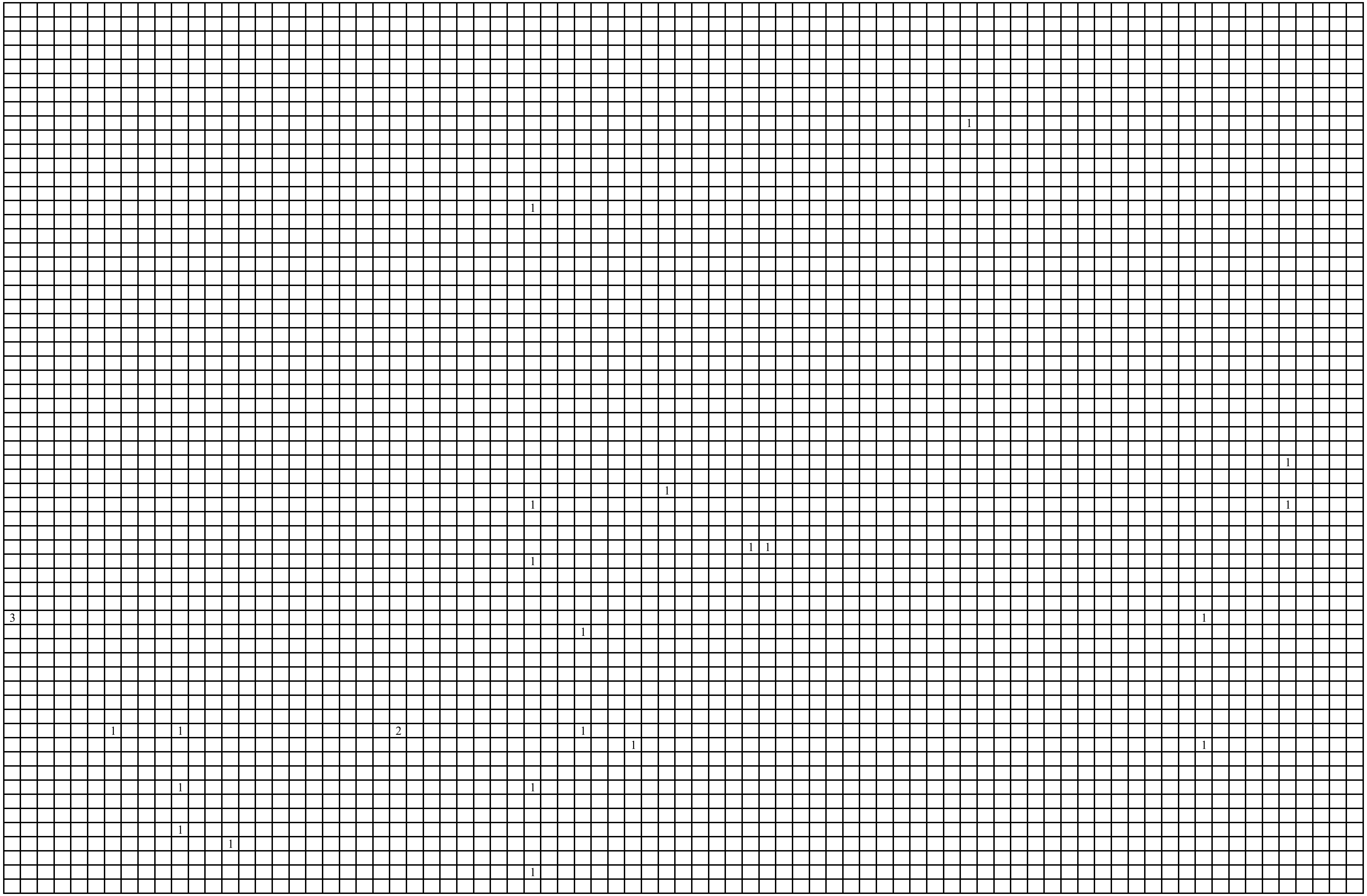
1
1
1
1
1
1
1
1
1
3
1
1
1
1
2
1
1
1
1
1
1
1
1

		2									1															1										1		2						1	1	1		1					1																											
											1		1																									1						1	1	1		1					1																											
																																						1																																																		
																																																																															1			
																		1							1						1																																													1									
																						1																						1					1																															2				
																																												1		1																																						
																																												1	1																																							
																																												1																																								
																															1													1	1	1																																						
																																																					1																											
										1																					1																																																							
																		1				1				2																		1					1	1			1			1																									
																															1																																																								
																															1																																																								
																		1																																																																		
																															1																																																								
										1				3				1													1	1																																															1	1			
													1					1																																																															
							1			1				1																														1		1																																						
																															1							1						1																																								
																																																					1																											
			3																																																																														
																																																										1																							
																									2																																																															
																																																																													1				
																		1																																																																				
																															1																																																		1			
																																												1																																								
																																														1																																						
																																												1																																								

														3																																																												
																																												2																																		
													1																																																														
																						1																																																							
																															1														1	1																																		
						1				1																				1			2												1									1																			1				
																																																					1		1																					
																																																							1																					
			1																																											1																																		
																															1																																																	
																																			1																																														
			1																																										1	2																																		
										1																																																																			
																															1																																																	
																															1															1																																		
																													1																																																			
																									1																																																										
							2																																																																										
							1																	1																																										2																						

																						1																																																										
													1																1	1												1																													1								
																															1																																																		
						1												1																																1	2			1																										
													1																																																																			
										1								1				1												1												1																							1								
2																																																				1																											
							2																												1																																												
							4																																																																									
																																																																								1								
																																																																												1				
																															1																																																		
																							1								1																																																		
													1																				1																																															
			4																																																																												
														1							3																																																											
																						2																																																											
		1								1								1													1																		1	1	1			1																										
													1				1																																																																
										1																					1			1																																													
						1			1																					1			1																																													
						1			2																					1			1																																													
						1	1			2																					1			1																													3															

						1				1																					1			1																2																												1		
						1				1													1								1			1																																														
						1	1																													1																																													
							7																																																																									
		1				2				3														1	2			1	2		1							1					1						1	1	1		4											2																
			1	3		1								1				2						3		1			1	1	1	1	1	1	1			2		1			1	1				2	1	1	1		1	2				1	1		1			1						5						1	1	1		
			1	2						1	2									1				1				1							1		1			1			1	1	1			1						1		1				1			1							1	1							1	1	
																																							2																																									
											1																																										1																									1		
						1			1					1								3							1								3													1			1										1	2													1	1		
													1																																																																			
																							2																																																									
																										1																																																						
																																																										1																						
																																																										1																						
																																					1																																											
																																						1																																										
				1																																				1													1																								1	1	1	
																																																																													3			
																																																																	4												1			
																										1																																								5														
																																															2																														1			
																																															1																																	
							1			2				1												1																	1															1					1	2																
																															1																																																	
			1	1		1	1			1	1							1										1		1	2			1						1				1	1	1		1	1	1	1		1	1														1			1					1	1	1		
																																						1																																										
																										1																																																						
																																										1																																						
				1																					1																																									2														
																																																																		2														
											1								1																		1							1		1		1			1		1																		1									
																										1																																																						
																																					1																																											
													1																																																																			
			2																																									1		1			1																															
			1																																									1	1																																			
																						1																																																										
										2																					1																																													1				
										1																																																																						
			1			1				1																								1																																														
		1				1				1																					1			1																																														
																															1																	1																																
			1								1			1				1								1					1						1						3					1	1	1	1		1													1						1				2		1		

														1																																																															1			
																														1																																																	
																																																																									1								
																		1																																	1						2																								
							1																		2						2																	1	1							2																							1				
																														1																				1																										3				
																																1																																														
																														1																				1													1												1	1			
																																																	1																														
																																																					1																										
			1																			1																											1	1	1	1		1	1																									1	1	
													1									1																																																											

				2																						MAPK14		105	663
																										MAPK8		164	482
																										MCRS1		64	347
																										MEAF6		25	196
																										MED10		10	339
																										MED11		1	164
																										MED15		30	203
																										MED18		7	259
																										MED19	TcoF.2	8	400
																										MED22		9	233
																										MED29		9	314
			1																							MED8		12	225
																						1				MORF4L1		58	447
																						1				MORF4L2		41	267
			1																							MRGBP		22	201
																										MYBBP1A		73	543
																										N4BP2L2		10	99
																										NELFA		8	135
																										NELFB		13	128
																										NELFCD		9	136
																										NELFE		16	251
																										NMI		20	155
																										NOTCH2		131	460
																										NOTCH3		18	150
																										NUP107		21	374
																										NUP133		18	226
																								1	1	NUP35		42	521
																										NUP85		11	185
																										ORC2		20	223
																										PADI4		8	45
																										PCBD1		30	115
																								1		PCGF1		65	374
																						1				PCGF3		11	174
																										PCNA		39	1121
																										PER2		15	193
																										PFN1		20	541
																1		1	2							PHF1		59	283
																							1	1	1	PHF10		56	187
																1										PHF19		14	127
																										PIDD1		3	66
														1												PINK1		17	851
																										PKN2		11	206
																										PLCB1		2	65
																										PNRC2		12	32
																										POLR3F		7	97
																										POLR3G		2	60
																										POLR3GL		4	77
																										PPHLN1		15	175
																										PRKAA1		25	543
																										PRMT1		85	1142
		1	2									1												1		PRMT5		73	600
																										PRMT7		12	62
																										PRMT8		8	174
																										PSMC1		29	500
	1																									PSMC2		22	501
																										PSMC3		55	670
																										PSMC4		21	561
	1																									PSMC5		73	754
																										PSMC6		19	395
																										PTOV1		9	89
																										PTRF		31	322
																										RAN		35	578
																										RB1CC1		15	481
																3	1		3	2						RBBP4		222	950
			1						1																1	RBBP5		76	508
																2			4	4						RBBP7		182	812
																				1						RBL1		48	181
												1														RBM39		75	1342
																										REXO4		51	321
																										RITA1		4	54
																										RLIM		29	444
																										RPS27A		53	506
																										RRM2		6	118
																										RRN3		1	52
																										RUVBL1		73	919
																										S100A1		2	70
																										S100A8		18	200
																										S100A9		23	238
												1														SAFB		30	246
																										SAP18		25	266
																										SAP30BP		15	131

																																				2																													1				2									
		1																																																																																
																																								1																																								
															1																					1																					1																					
															1																																																		1															
																																																																					3															
		1																																		1											2																																
																																		1																																														
																														1																																																							
																1																																																								1										
																																													1									1	1	1																								
1										1																1																																																						1		1	1			
																																		1																					1		1	1		1																				
																																		1																															1																				
				1	1	1	1		1	1	1																																																								1																		1		1	1			
						1																																																	1														1				3						
																																																1																																
																1																																																									1										

			1									1	1	1																																														1					1									1						
																															1	2																	1						3																									
																																																																			1													1
																																																													1																			
																																																		4																														
1	3	2	2	1							1	3	2	2	6		2			2	3		1	1	2						1		3		3						1						1		2				3	4	4	3	4	3	1	4	3		2	1	1	1	4	3		2	2	1		7		1	3	3	2	2
			2									2		2																																																											2							
			1										1		1						1												1						1								1		3				1				1	1			1						1	1			1						1	1	2	1
						5																																											4																														
		1																														2																											1														1				
																																																	2														2	3	1															
																																																																												1	1			
	1																																																	1																1														
																										1																																																						
																																					1																															1												
28	15																																1		1																																													
	1																																																								2																							
															1																															1														1						1				1									
		1																																																						1																							
1	87																	2																1																	1									1							2													
	1					1				1																														1																																							
	2																									1																																																					
	1																																																																															
																																																																						1										
	3																																																																															
											1							2																																							2							1																
																																				1																																												
																																																							3																									
																																																					1	1	1	1	1	1																					
1																																																																1																
													1		1																																																1			1													
																																							1																																										
1																																																																																

1																																																																															
1																																																															1									1								
		1																																																																														
	3			1		2			1	1	2	1					2	1		1																					1								1				1		1	1		1					1				1											1		
				1		1			1		1	1	1		1					1				1	1	1	1						1		2									1			1		1	1			2	1	1	1	1				1		2		2		2				1	1		2		1	1	1	2	1
																		1																																																														
1																																																																															
		2																																																																														
															1																		2		1					1									1																	1	1												
															1																																																				1												
																																																	1																		2							1						
																																																															1			9														
																																																																		1														
																																	1																																				2														
												1			1									1	1																									1														1			10	1												1	
																																																																		1														
																															1	1																								3																									
																																																	1																									1						
																																																															1			1														
																																																															1			2														
																																																															1					1										1		
																										1																																																									
																								1																																																											
1																					1																													1																1		1													

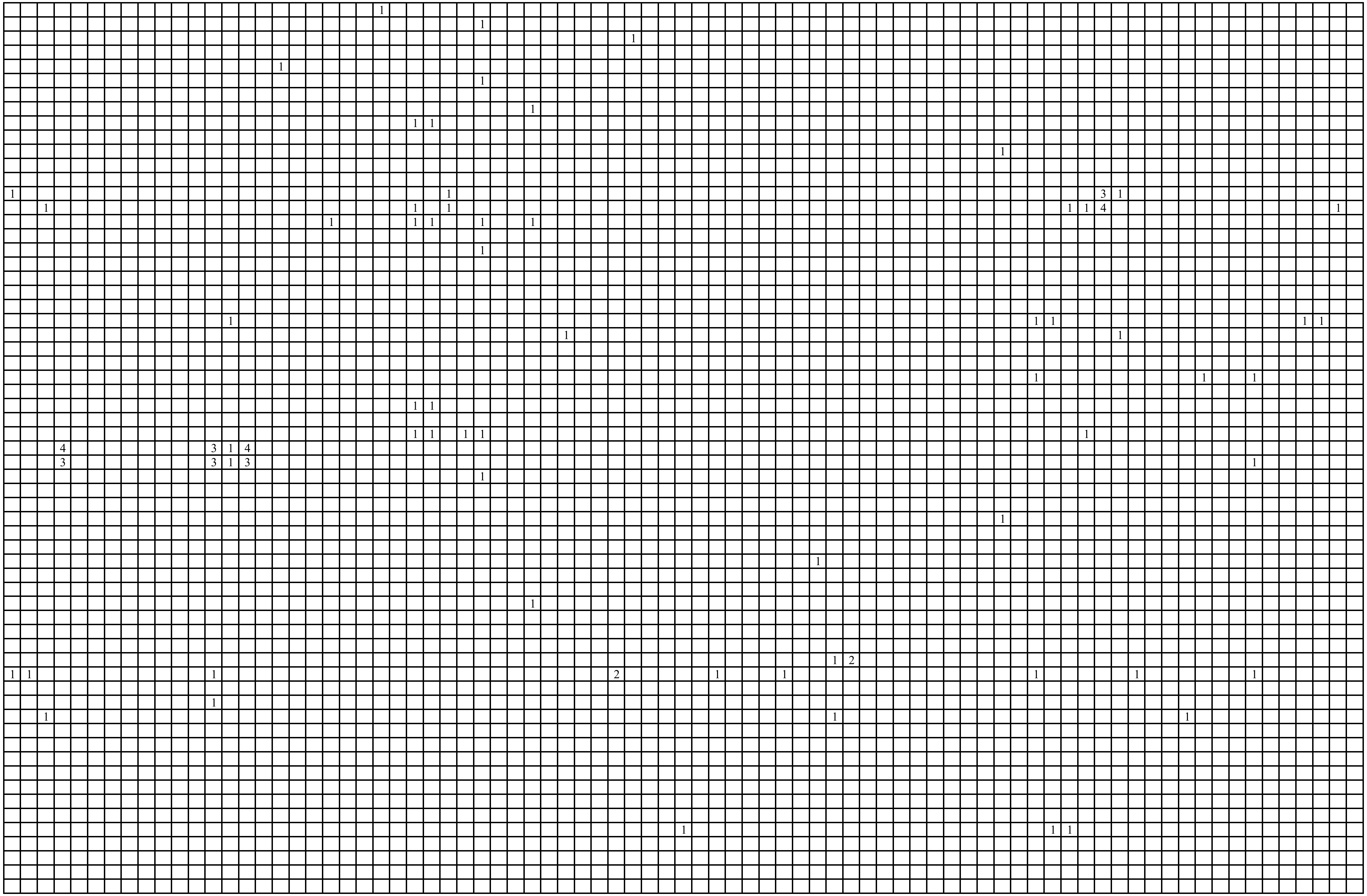

1
1
1
1
1
1
1 1
1
1 1 3 1
1 1 1 1 1 4 1
1 1 1 1 1
1
1 1 1 1 1
1 1
1 1 1
1 1
1 1 1 1 1
4 3 1 4
3 3 1 3 1
1
1
1
1
1 2
1 1 1 2 1 1 1 1 1
1
1 1 1
1 1 1

													2																		1																																																	
																																																				1																											
																															1						1									1															1			1				1												
																															1																																																		
																																																	1																															
		1									2	1																																				1																																
																														8																																																					
																										1				6	1																																					1										1						
																																																			1																														
2																															1																																																			
													1																1																																														1													
							2						1																							1		1										1																			1													
																																																														1																	
																																																														2																	
																																																														1																	
																																																														3		1															
																																																														2		1															
																																																														2		1															
															1																																																																				
																																																	1																																
																										1																																																										
																3							1																																																													
3																																																																																			
1																																																																																		
																																																				3																														

																										2												1																																										
3																																																																																
3															1																																																																
2																																																																																
																																																		1																														
3																					1																																																										
		1										1																																		1																																	
												1	1																						1										1							1																											
1																																																																																
																																					1																															1												
												1																									1																											1				1												
																																					1																											1						2										
																															1						1																																										
																																					1																																										
																															1						1																																										
1																																																																																
																															1																																																

																																				1																																																	
								1																																																																									
																															1																																1		1																	
																								1	1	1	1						2																						1					1	1		1											1						
1																																																																																			
																																																																1		1															
																															1																																																						
																															1																																																				
																										1																																																														
						1																										1					1																												1																	
															2																																																1	2				1											1			
												1																																																																								

																		1																																																															
																																																															1																			
		1																																																																															
																																1																														1	1	1																	
																				1																																														1											1		1
																																																																			1															
1																										1																																							10																
																																																					1		2																												
																																				1																																															
																																				1																										2																			
																																					2																																														
																																																															1																			
1														1									1		1	1	1																						1											1	1		1		1							1							
															1																																														1	1			1																
																					3										1						1																																												
																																																	1																																
																															1						1																																												
									1																						2						1																1	1		1	1			1		2	3	1			1			1			1							

																														1																																																			
																														1																																																			
																																							1																																													
																			2																	1																																															
																																																																											1						
																										1																																											1																	
																																																																							1	1												
																																																													1			1		1															
																										1											7													1								1			1																				
																																				1																																																				
		1													3																																																													1	1						
																	1	1		1												1				1									1															1	2			1										1	1	
						1																						1			1			1			1																													1	1	1						1									
																																1																																																					
	1																							1			1						1																					1		1						1	1									1			1		1	1		1	
																																																	1																																				
2																												1								3		2																										2		1															
																																								1																																													
																																																																									1										
2																																																																																			
1																																																																																				
4																										1																								1														1																
						1																																																															1										
																										1					1																																													1										
1																										1					1																																													1										
		1																													1																																					1																
															1											1								1	1					1									1	1																	1													
																																				1																																																				
								1																											1																																																			
																								3	4	2																																																													
																				1																				1							1	1	2			3															2			1										

																																																	1											1				1																
														1															1										1																													1												
3																																																	1																																
1			1																																														1											1																			
																																																	1																																
		2											1																																																																				
		2																																																																																
																				1																				1									1																		1														
												2	2																																			1																																	
																																																																						1									
																																																																													1	1			
																																												4	1																																							
																																				1																																																	
1															1											1																								1																	1													
		3																																																					3																			6						
																																																	1																		1													
																																			1						1																																														
																																																	1																		1													
													1																				1															1	2	1					1	1				1		1			2	2							1						
						1																																																												1														
																										1																																																													
																																									1																												2											1	
																				1																												1																																	
																						1																																								1		3									1									
																																																	1																																
																																				1																										1																		
																																		2																																																		
			3									4	3	5																																														1														6						
9	10														1		1							1	1				5				6	6		6												1	2	3	1				1	2	4			1	1	1	2	3	1		2				1	1			1	3		2		
		1																																																																												1		
						3																																																																										
	1																														1																																																				
																															1																																		1																

																															1																																																				
															1																																		1																		1	1												
																																																																			1			1									
	1															2																																																					1										
																														1							1																																												
	2											1	1																																																					1								1					
1						1									1						2																1												3	4				1					1																				1	
																												1																																																					
	1	1																																																													1																	
	1																																																																															
	3																																																																																
																						6	5																					1															2										4											
																															1																																																		
																		1									1	1																						4	1														1			1													
															2																		1																																										2			
																																																																				1													
						1		1	1													1																																																										
						2		1	1																																																							1							2									
						1																																																										1		2														
																			1																													1																		1					1									
										1																																																																							
2																																																			1																			2										
										1																																																																					
																					1																												2						1																										
																																			1																																													
	3																				1																			2																						1																		
																															1																																																		
																					3																												2																																
																					1																																		1								1																	
																																																																							1										
																																																																									1									
																										1																																	1																							

																																																	1																															
																														1																																		1			1													
																									1											1																											1																
																											1																																																										
																																						1																																															
																																																																		1												1	
																																															1																																
		3										1																														1							1																																
		1										1																										1																											1												1				
		1										1																																														3									1															
		1										1																										1																											1															
	4																												2																																																								
		1	1									1																			1																																		1															
	1																																																	1																															
3		1																																																																														
																															1																																																				
																																						1																																															
1	1																																																																															
																																																																						1												
																															1																																																			
																															1	1					1																																																				
																																						1																																															
																																																																												1	1			
																																																					2																												
	1																																																																																					
	1																																																																																					
																																						1																																															
																																			1																			1								1															
	2																																							1																									2													
																															1																																																				
																				3																																																																	
											1			1								1				2												1							1				1	1	1	1	1	1					1	1	1		3						1						
									1				1							1		1	1																																																										
														1																																																																	
		1																																																																															
																																																															1				1															
																																																																	1																
																																				1																										8			4													
																			1						1																																					1																		5		

																										1																																							1															
																			1					1																																				1		1																		
																																						1																																										
																										1											3													2																														
																															1						6																								1																			
																1																																																																
						1		1			1		1		1																			1																									3												1													
		1						1			1																																																																						
																														1																																																		
																																	2																	1																														
																																																	1		1																				4						1				1	
																												3			1																		3		1										2																1				1	
															1																																													1								1												
		1																												1																									3									1																
																																																																	1															
																																	1				1										2						1		1		1		1		1		1									1													
															3																																																					1												
												1		1																							2		1										1																			2													1	
																															1																										3			1																				
																																																																							1									
																																						1										1																							3									
																					1																														3																													
		1		1																												1		1					1																											1																
																															1																																					1												
																														1																																																		
						1									1																1																								1																									
															1																1		1																1				1		1		1		1		1		1																						
																															1		1																																1																
																															1						1													1														1																
			1																																																																													
			1																												1		1																1				1		1		1		1		1		1																						
1	2																					1																																			4						1		1	2														
																						1																																																										
																																																															1																	
		1																																																																														

1																															1	1					1																																											
																																				1		1																									6		5																						
		2																																																																1														
																						1																																																									
												1	1																																																																		
												1																																																																				
																																																														2		2																
																						1																																																									
																											1																																																				
																																																				2																											
																															1																			4																													
															2																1																													1																		1	
																																																			3																												
																																																	1																														
																																																																										2					
																										1																						4	3	1																													1	
																																																						1	1																								
																								1																																																							
																						1																																																									
																										1																							1																													
															1																																		1																													
																						1																																																									
																																																		1																				1			1					
																															1																		4	1																													
																						1																																																									
															1																	1																		1			1	1	1	1	1						1										1					
																				2																																1																										

		6																																															2															1							1			10						
													1																																				1																														
																																				1																																												
																																																															1																
																						1																													1																		2											
												1	1																																																																		
																						1																																																										
																						1																																																										
																																																																				1											
																						1																					1																																				
															1																																																		1		1							1						
													1		1																																																																	
																																																												1																			
																																																1																															
																						3	3																																																							1		
															1																																																					1											
																						1																																																										
																						1																																																										
																																																																													2			
																																				1																																												
																						1																																																										
																						1																																																										
																						1																																																	1									
																						1																																																										
																						1																																																										

												1	1																							1																																															
																																																																												2									
																						1																																																														
																						1																																																														
																															1																													2																																												
																															1																																																																									
																						1																																																					1												
																																																			1																		1												
																														1																																																																												
																																																																																		1
														1																																																																																1												
																							2																											3																																																				
		1																											1																																																																																					
																						1																																																														
																						1																																																														
																						1																																																														

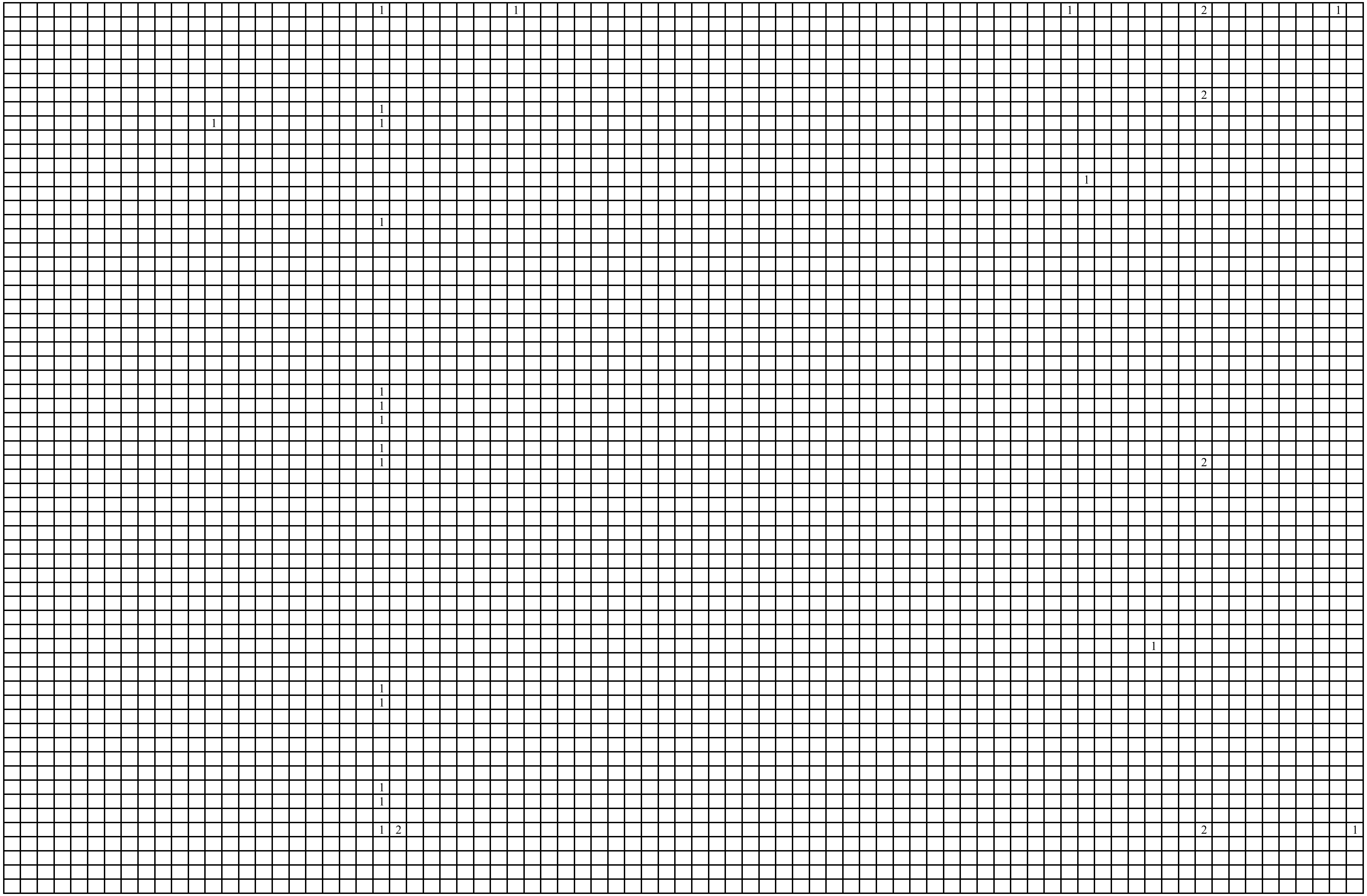
1 1 1 2 1
2
1 1
1
1
1
1
1
1
1
1 2
1
1
1
1
1
1 2 2 1

																						1																																																												
																						1																																																											
																						1																																																											
																																																			1																														
																						1																																																											
												1																																																																				
																						1																																																											
																																																																				1													
																						1																																																											
																						1																																													1												
																																																																								1									
																						1																																																											
																						1																																																											
																																				1																																																
																																																		1																															
																																											1																																												
																																																																				1													
																																																																													1			
																						1																																																											

																																																					1																											
																														1																																																		
																						1																																																										
	1																																																																															
																						1																																																										
																						1																																																										
																						1																																																										
																						1																																																										
																						1																																																										
																						1																																																										
																						1																																																										

																																															1																																	
																						1																																																													
																																																																							2												
																						1																																																													
																						1																																																	1												
																						2	1																																																										
																						1																																																													
																																					1																																										2									
																							1																																																											
															2							1		1																														1														1													
																						1																			1																																														
								1																																																																									
																																																																												2									
																						1																																																													
																						1																																																														
																						1																																																	1												
																						1																																																														
															1							1																																																													
																						1																																																						1									
																											1														2																																																		
																						1																																																														

																						1																																																											
																						1																																																											
																																							1																																																
																											1																																																												
																						1																																																											
																						1																																																											
																						1																																																			1																				
																						1																																																											
																						1																																																											
																																					1																																																		
																						1																																																											
													1																							1																											5		5															
																																																															5		4															
																						1																																																					2									
																						1																																																											
																																					3		3																										1																	
												1																																																																					
1	1																																																															1															

																										1																																																							
		4										1	1																																																4		2									1					1	
		1																																														1														2		2									1					1	
		1																																																																														
1																						1																																																									
															1										2																								1				1	1		1	1	1		3	1	1	6		1		1				1			1						
															1							1									1					1																																														
																																																																						1												
																																																						1									3		1		1				1									
													1																																																			1															
																																																																	2		4															
																																																																			1					1										
																																																																	1																	
																																																																	1																	
																					1																																																												
																																																		1	1																														
																																	1																											2																				
																					1																																																		2										
															1																										1																						3		4														1	1	1
																					1																																																												
																														1																																																		
															1																		1																						1								1		1		1													
																					1																																																												
																																																																	2																	
1																						1																																																										
																														1																																																			
																																				1																																												
	1																					1																																										3		3															
																																																																	2		1															
																					1																																																												
																					1																																												1																
																					1																																																													

																						1																																																										
																						1																																																										
																																																													1		3															
												1																																						1																													
																						1																																																										
																																																													1		1															
																																																																			1												
													1		1							1																																														1												
															1							1																																												1				3												
																																				1																											5		3						1									
																																																													1		3															
																						1														1																																																	
																																																													1	1																
																						1																																														1												
																						1																																																												
																						1																																																												
																																															3																																			
																																		1																																																			
																						1																																																												
																						1																																																												
		1																																																											1																	
																						1																																														1												
															1							1																																														2												
																						1																																																													
															1																																																																						
																						1																																																													
																						1																																																													
															1																																																																							
																																																	1																																		
			4										1	1																																																													3						

																																																					1																											
															1							1																																														1												
												1	1																							2																																												
																																																					1																											
																										1																																																			1			
																										1					1									1									1	2																	2													
																										1																																																						
																						1																																																										
		1																																																																														
		1																								1					1																																																	
																																																												2																				
																														1						1																																												
																						1																																																										
																														1																																																		
																						1																																																										
																						1																																																										
		1																																							1																																							
																																																															4		2															
																						1																																																										
																						1																																																										
																															1																																		1															
																															1																	1																																
																															1																																	1																
1																															1																																																	
																																					1										1														1				1									1				1	1	
												8	3	1																						1																											3		8															
																																				1				1																							7	1	7															
																																				1																											10		10															
																			1																	4																											21		12															

																																				2				1																			1				15		16															
																						1														3																										6		7															
		1																																																														2																	
																																																															2																	
		1		1		1	1		1		1				1		1		4	1								1			1				1														2				1	1	3	1	2	1			2			1		1						1		1					1	
		1		2		1		1			1	1	1		3			3								1								3								1				1		3	2			1				1	1			1		3	5	4		5	2		3	1			1		1		4		1
		1		1		1	1							1			1	1	1			1	1		1	1	1	1				1			1								1				1		1	2				1	1		2	1			2				1		2	2									1	1	1
																																																									2																							
																																																																							1									
																																											3													2																							
	1	1				1			1		1																														1																		1														1	1			
																																																									1																							
																																																																			1													
																																																									1																							
																																																	1																						1								
																																																	1								1								2															
						1																																																	1	2																							
																																																	1			1																											
																1																																								1																							
																																																1					1											1										1			1			
	1	1																																																						4														1			1						
																																																									2																							
																																											1													1																							
																																											1							1							4																	1						
																																																								1	2																							
																																		1																						2																							
																																																	1								3																							
																																																									2																							
		1		1							1																				1	1						1						1						3															1															
																																											1													1																							
																																																															3		1		1													
								1							1							1					1	1					1		1														1	1			1	1	1	1	1	1			1		2		1		1				1			1			1	1	1
																																																															1																	
																																																									2																							
																																												1																																			
	2														2																																						1													1				1										
																																											1																																				
																																											1																																				
																																																		1																													
3																																																																																
							1																																																											1	1			1									
															1																																																				1													
																							1									1																																														
																																																																1	1															
																																				1													2	2													6		7															
																																				1																										7		7															
				1								1	1		2																																			1											1		1	1			1	1			1			1			1	1	1	

																																	1				1																																																				
																																			1																																																			1	
																																	1																																																			1		
2	4																																1																																																				
																																																						1																											
																									1																																																												
												1		1																																					1					1	1	1		1				1	1											1		1	1			

																										Gene		
																										SAV1	12	237
																										SCAND1	84	117
																										SENP2	4	224
																										SETD1A	37	309
																										SETD1B	9	128
			2																							SETD7	34	121
																										SF1	26	332
																										SGF29	0	0
																										SGK1	6	116
																										SIAH2	26	216
																							1	1	1	SIRT6	56	744
																										SLTM	20	160
																										SNAPC2	6	16
					1																					SNAPC5	8	59
																										SNCA	14	1215
																								1	1	SNIP1	46	563
																										SQSTM1	89	2260
																										SRSF10	18	303
											1															SRSF2	21	222
																										SSX2	7	56
																										SSX2B	1	11
																										STAG1	6	163
																										SUPT16H	58	517
																										SUPT20H	27	184
																14										SUZ12	129	923
																										TAB1	10	370
																										TAB3	8	132
																										TAF1C	12	140
																										TAF5	23	117
																										TARDBP	34	589
																										TCEAL1	15	136
											1															TDG	29	101
																										TDP2	12	94
																						1			1	TERF2IP	127	644
																										TGS1	4	126
																										THRSP	9	69
																										TLE4	46	122
														1												TNIP1	59	1423
																										TNRC18	14	99
																										TRIM11	25	275
																										TRIM29	18	278
																										TRIP11	12	219
																										TSPYL2	27	123
														1												TULP3	36	437
																										TXLNG	7	127
																										TXN	22	306
																										UBA3	4	81
																										UBA52	14	421
																										UBB	25	562
																						1				UBC	125	4148
																										UBE2B	2	136
																										UBR2	8	168
																										UCHL5	50	621
																										USP21	42	221
																										VGLL1	9	19
																										VPS25	5	110
																										WDR5	155	1637
																										WWOX	60	759
														2												XIAP	13	683
												1	1	1												XPO1	105	1610
																										XRN2	44	377
																										YLPM1	17	194
																										YY1AP1	22	77
																										ZMIZ1	27	52
																						1				ZMYM2	121	345
																										ZMYND11	19	141
																										ZNF638	27	284
																										ZRANB2	12	179
																										ADRB2	11	418
																										ANKRA2	11	50
																										ASCC2	7	146
																										ASCC3	11	213
																										ATXN3L	6	14
																										BAHCC1	9	43
			1																							BCL9	50	99
																										BRF1	4	109
																										BRF2	0	35
			1																1	1						BRMS1L	65	170
																										BRWD3	6	108
																										BTF3	59	821
																						2				BTK	18	154

																																																														2														
																					1																																																												
																																												1																																		
																																							1																						2				2														
																																																						1																							
																						2																											1																															
																																																					1																								
																																																				1																											
																																																					1																								
																																						1																																																	
																																																													1																			
										1																							1																									2																					
																																																																					2											
																																		1							3																																														
																																																							1				2																							1	
																1																	1																1										1																					
					2					1						1																																			12								1		1																					
						1																																					1																																														
1																																																																																	

															1																												1	1												4			1																				1			
																																		1										1													6								1																	
																2																																										1									1												
																																																											1																					
																																																											2																					
																																																																									3			
																																										2																																			2			
		1	3	1		3					3				4	4	3	2				2	3	1			1		2		1	1	1												4		1		1		1					9			6	5	1	1		3		2	2				1						1		1	
																							2																																																								
						1					1				1		1	1			1			2					1																1		1		1							3			1	1						1	1													1	
																																																	1																		2														
																																																																1																
																																		1																																														
																														1																															1																					1	
																																																											1																					
			3																																					2																	2																								
																1																																	1																															
											1					1																																																																
																																						1																															1												
																																																									3																							
																																																		1																															
																			3																																																														
																																																		1																															
																						1		1																		1																		4																					
																																												4																																					
														1																																											1								1															
																2																														1			2																															
						1																									1																																																	
	1																																												1																																			
																																												1																																					
									2																																										1																															
																																																				1																													
																																																																									1						
																																																																									2							
																		1																																												1																				
																						1																																																													

													1																																																																				
																																																	1																															
																								1																1																										1															
						1																										1						1						1	1														1	1		2		1			2							1					1	
					1	2					1					1	2	2				3		1					2				3											1	2			1				1				4			1	2	2					2	1				1								2	
									2																																							1										1																						
																																																																2																
													1																																																																				
			1																									1																																		1																	
																2								1											2	1																			1			1																				1	
																								1					1								1			1																																							
					3																																											1			4						2																					
																																																				1																											
																																																				1																											
											1											1											1																						1			2																					
																																								1			1														7								1	1															
																																																	1							3								1			1													
						1											1												1																										2			1	2	1																		1	
																																				1																																															
																						1							1				1																																															
																																																									3																											
																																								1																																								
																																																									1																										

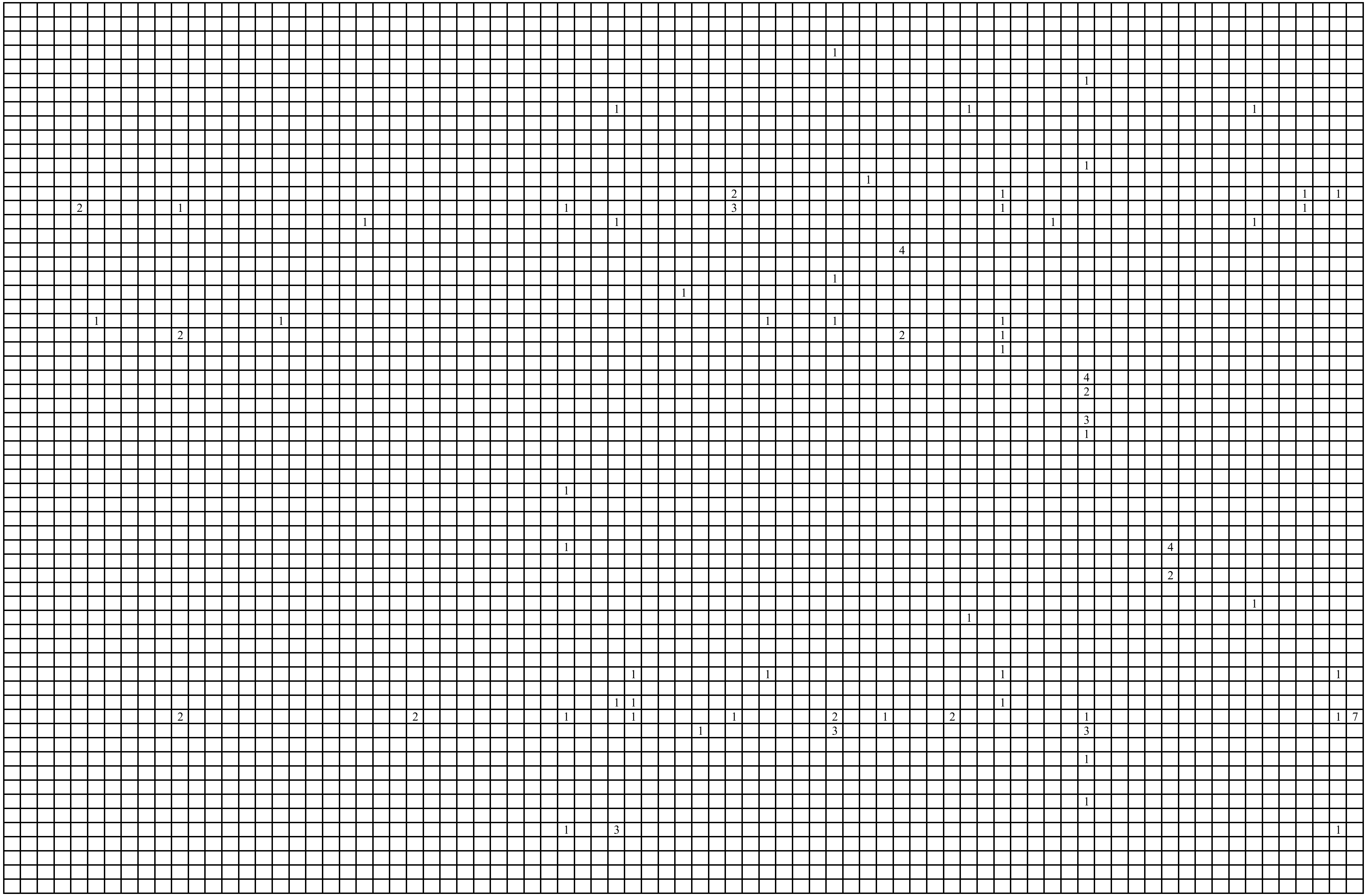
1
1
1 1 1
1
1
2 1 1 1
2 1 1 3 1 1
1 1 1 1
4
1
1
1 1 1 1 1
2 2 1
1
4
2
3
1
1
1 4
2
1
1
1 1 1 1
1 1 1
2 2 1 1 1 2 1 2 1 1 7
1 3 3
1
1
1 3 1

																																																														1																	
																																																															1																
																																				1			1																									1										1			1			
																																				1																				3																		1			1			
																																																																								1			1			
														1																																																																	
																																				2																																																		
																							1																					1										3																								
										1																						1		1																				1							2																
																																																															1																
																																																															1																
																																																																				1											
									1																							1																														1																	
																													1																				1															2															
																																		1																																																		
																																																																														1	
																																																																														1	
																																																																														1	
																																								1																																															
																																																							1																							
														1																								1								1																																					
																																																1																													

																																							2																																														
																														1																																																								
																														3																																																								
																																																				1																											
																						1																																																														
																																																							1																									
																																																									1																							
																																																															1															
																																																								1																							1
																																																																								1						
																																																																								2						
																							1																																																									1						
																																																																								1						
																																																																								1			1			
	1																																																																									2						
																																																																								1						
																																																																								1						
																																																				1																															
																																	1																																																				
																						1																																																														
																						1																									1																																					
																																				3																																														1			
																																				2																																																				

														1																																																																	
																1						1		1																					1								13										1																	
																	1																																																														
																														1																																																	
																																																																															1	
																																				1																																						1						
																																				1																								1														1						
																																				1																				1																		1			1			
																																				2																					1																							
																						2																																																										
																																						1																									1										1	1						
																																																	1																														
																																																	1																														
																																				1		1															1																				1	1						
																																				2													1																														
																																																									1																						
																																																											1																				
																																																								2	1																						
										1																																																																					
																																																							1																								

																																							1																																										
																																				1																																															
																																																																														1	
																						1																																																											
																																																															1																
																																				1																																															
																1																																						1																										
																																																			1																													1			
																																																																															1			
																																																											1																							
																																																																															1			
																																							1																																				1				1			
											1					1	1					1											1						1										2							1			1								1													
																1																						1																			1																								
																																	1																																															
			1	1																																																			9																								
																																		1																1																																	
										1														1									1																									1																					
																								1									1									1																																								1			
										1				1										1																																									1															
																																	1																																																
																						1						1	1				1															1							1				1				1													1			
																																																																															1			
																																																																														1			1			
																																																													1																					
																																																																														1						
																																																																														1						
						2						1					3						2							1									1		1																4			1		1			2		1					1			2			1			

																								1					1																																																								
																																																												1																	1			
																																1																											1																	1			
																																						1																																		1							
																																																																										1						
																																																			1					1																								
																																																	1																														1	
																																																				4	2										1																1	
																																																																															1	
																						1																																			2																					1	
														1										1																																1																							1	
																						1		1																																																							
													1																																														1																					
															1																																										1		1																					
															1																																																																
										1														1			2						1				1																											1														1	1	
										1	1				1							19		1					1				1				1																			1			2		1					1												1	1	
																								1													1	1		1							1	1								2																1							1	
																						1		1																																																							
																		1																					1	1	1			1			1		1			1								1																				
																																																								3																								
																							1	4																																																								
																																					1						1																																1					
																																					1																																										1	
																																																											1																				
																																											1																																				
														1																			1																										1																				
																																	1																1										1															1						
																																																																										1						
																																																	1																												1			
			1													1												1																																																				1			
									4																																																																							
						2								1															1								1												4							1	2				2			2															1	1
									3																																																																						1	
																																	1																																														
														1	1									1									1				1																			1																							
																																																	1																														
										1					4									1					1								1																			1			1				1																1	
																			1																																								1																				
																																																1	2																															
								1																																																																							

																																																																														1	
																2																																										1																					
																																																											1																					
																																					2											1	1																																
										1																											1																							1		2																	1	
																																	1																																																	
						1																		1																																1																						1	
																																																												1																				
															1																						1																				1		1																				1	
																																	1																																																	
																																																		3																														
										1	1					1																																																																
																1																																										1																					
															1									1					1																				1							1	1				1																		1	
																								1																																			1					1																
																																					1										1	2									1																							
								1																													1												1							1																								
										1				1																																																																			
																																	1																			13																												
										1					1									1					1								1																						3																				
																																																									2																							
																																																											1																					
																								2									1																																																	
														1		1	1	1						1					1																				1	1							1		5	3									1												
						1																																																													1														
																																																									1																							
																																																	3																																
																										1										1											17			1	1																											
																																																						1																								
																																															1																															1	
															1									1													1																																													
														1																																	1								1			1																					
											1					5			1																		8									1	1	9																															1	
															5																																									3																								
															1																						1						1																																								
																																																	1																															
																																																									1																							
																																																											1																					
																1																											1																1																					
			6	1	4											8						1	1	1	1		1	1		1	4	2								2					2		1	2	3	2			1			2	6		3	2		1	3	2	1										2				1	1
																															3																										2																							
																					1										4																										5		1																					
																																																																											1			1			
																																				4																																																
																																				1																																									1						

																																				1																					1																		1						
																													1																			1																																
																													1																																																			
																	1										1																																												1	1									
						2																		1													7																						1																					
																																																												1																					
																1																																							1				1									1												
					6					5					1	2								2																									1										1																					
																								1						1																													1																					
																																																																															1	
																																																																1																
																																																															1																	
																								1																																			1				2																	
						1																																																						1																			1	
																																																																															1	
																		2											1																			1											1																					
																																																																						1										
																																																																1																
																																	1																																																
															1													1					1							1																																								
															1													1																					1										1																					
															1	1												1																			1					1													1															
																3								1								1																				2					3						4		1															
															2	1															1				1													1									2		1				4	1						1										
																																																												1																					
																									1																																																						
					1																				2	2																										2																												
																																								1																																							
										1																																																																					
														1		1																							1																				2																					
																																																												1																					
					2				1														1			2				4	1								1																													1												
																																																																				2												
																1																																																																4
																																				3																																												
																																																	1																														
																																																																1															

																																				1						1																				1											1						
																																																			2																										
																					2																																		2																										
																																																		2																										
																																																		2																										
																																																													1																
								4		1																																								1																															
																																											2																																					
																																											1																																					
																																																																						1							1			
			1																								1																																																					1			
			2																								1					1																																																1			
			1																			1					1																								1																																					
										4																																																																							
														2																																																																			
			1																			1					1																								3																											1			
																																																											1																					
																																																												2				1										1						
																																4																																		1																	
																																	1																1										1					5															
																																																												1																					
																																																																1											1						
																										1																																															1																
			1																			1					1								1																													1												1			1			
																					1																																																													
															5																																										1	1																					
															2																																																												1						
														1			1						1					2								1																1					1			1	1					1														
		1																																																																																	
																																													1																																				
			1																																																				1									2															
																								1									1																																				1													1			
																1																				1										1																						1															

						1																									1																	1																			1											1		
																1								1								1				1												1								1						5								1						1				
																																																																2																	
																								1																			1																					2																	
										1														1								1																							2																									
										1					1									1								2																																														
												5	2																																																																		
																								1																										1							1	7																									
																							7										3				1																																										1	
																																	1																1																														
																					1												7																										1	2																	1		
											1																						31																							1				2																					
			1	1																				1										1		1																					7								1																	
																													1																																																		
					3																	2																																																									
																																					1	1																			1																						1	1	
																					1																																																										
																																										1																																						
																																						1																																		1	1							
																																											1																																	1			
			1																																1																				1								1														1			
																																					1																																										
																1											1																																1						1								1			1			
																												1	1								1																			1				1																	1			
			1																																																				1								1														2			
					1																															1																																				1							
																																																											1	1												4		1							
																							1																																																								
			1																			1					1																														1	1				1		1												1			
																																																																													1			

																																				1																							1	1		1			1								1			1			
																								13																		1																																													
																																																						1																							
																																																						2																							
																																																													1																
	2																																																														2																
	4																																																																																
																																	1																								1																							
	1																																																																																
			1																					5									1																																									1													
																																				1																																																	
																																										1																																																					
																																																					2																											
																																																					1																											
																																										1																																																				
																																																						1																							
																																																		1														1																
																					1																																																																				
	3																																																																																
																																									1																																																					
																																				1																																																	
																								1																																																																	
																																							2							1																																														
																		1						1	1	1																							1				1						1																					
		1																																																																																

																								2											1																																													
			1												1									1				1																			1	1				10	2			1								1													1			
										1																																																																						
																																																																1																
																																																1																																
																																										1																																						
																																																																1																
																																							2							2																																		
																																										1														1		1	1																				1	
																																										1																																						
					1	1																																																																										
																																					1					3																																						
																																																									1																							
2																																																																																
	1																																																															1																
	1																																																																															
	4																													3																																																		
	3																																																																															
	1																																																															2																

																																	1																															1																
2																																																																																
																																																									1																							
																																																																1																
																								1																									1																															
																								1									1																1																															
																								1																																																								
																																											1																																					
	2																																																																															
													1																																																																			
	2																																																																															
	2																																																															2																
	2																																																1																															
	1																																															1																																
	2																																																																															
	5																																										1																		1			1																
																																																																				1												
																																																																				1												

	5										1																																													1								2							2									
																																																													1																	
																																	1									1																							1																	
																																																																	1																	
																							2									1																																																	
																																											1																																																			
	1																			1																																																																				
																																											2																																																		
																																											1																																																			
																																																					1																																	
																																	1																																1																	
																1																																																													1																								
	3																																																																																				
	5																																																															1																	

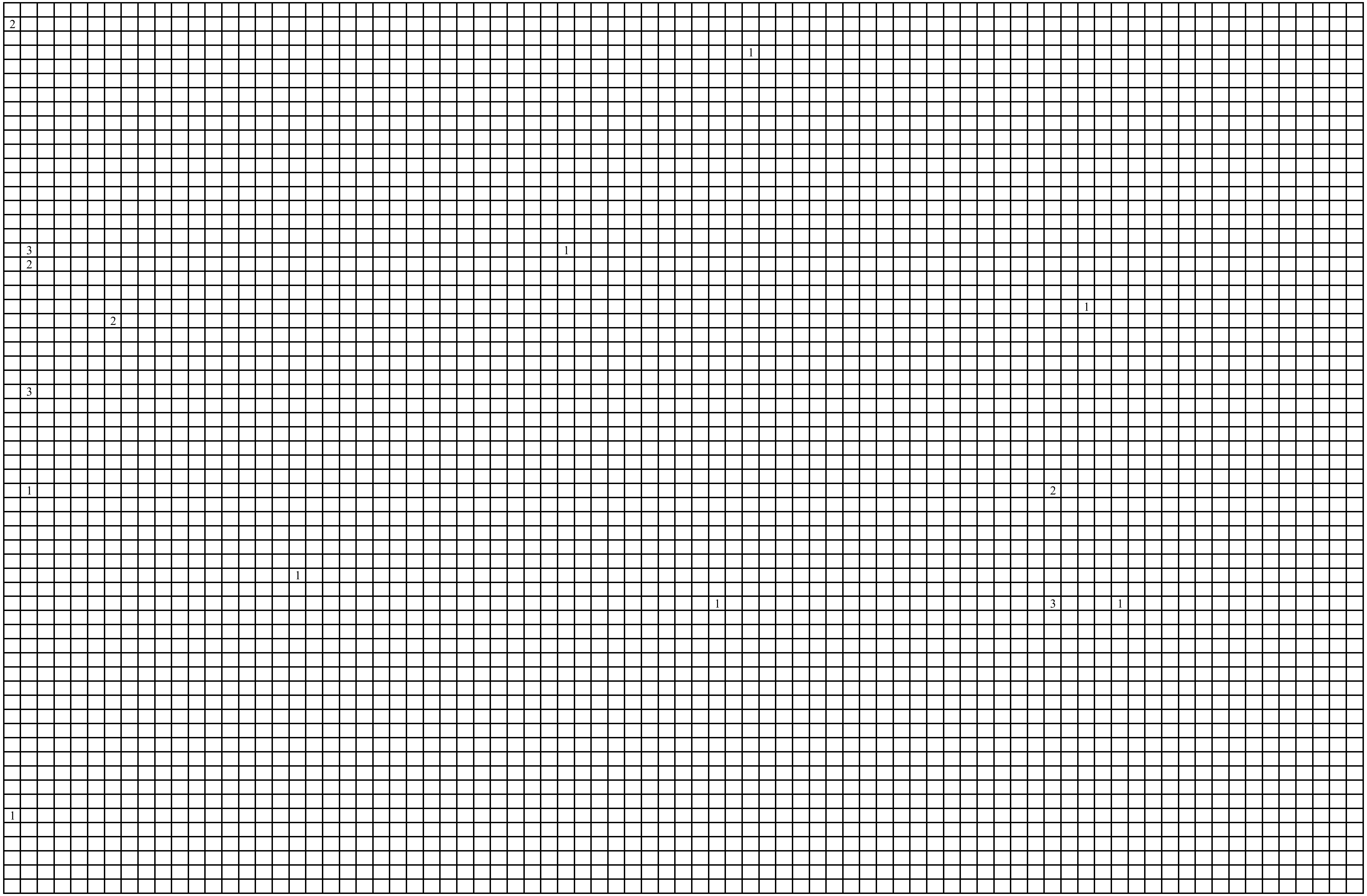
2
1
3
1
2
1
2
3
1
2
1
1
3
1
1

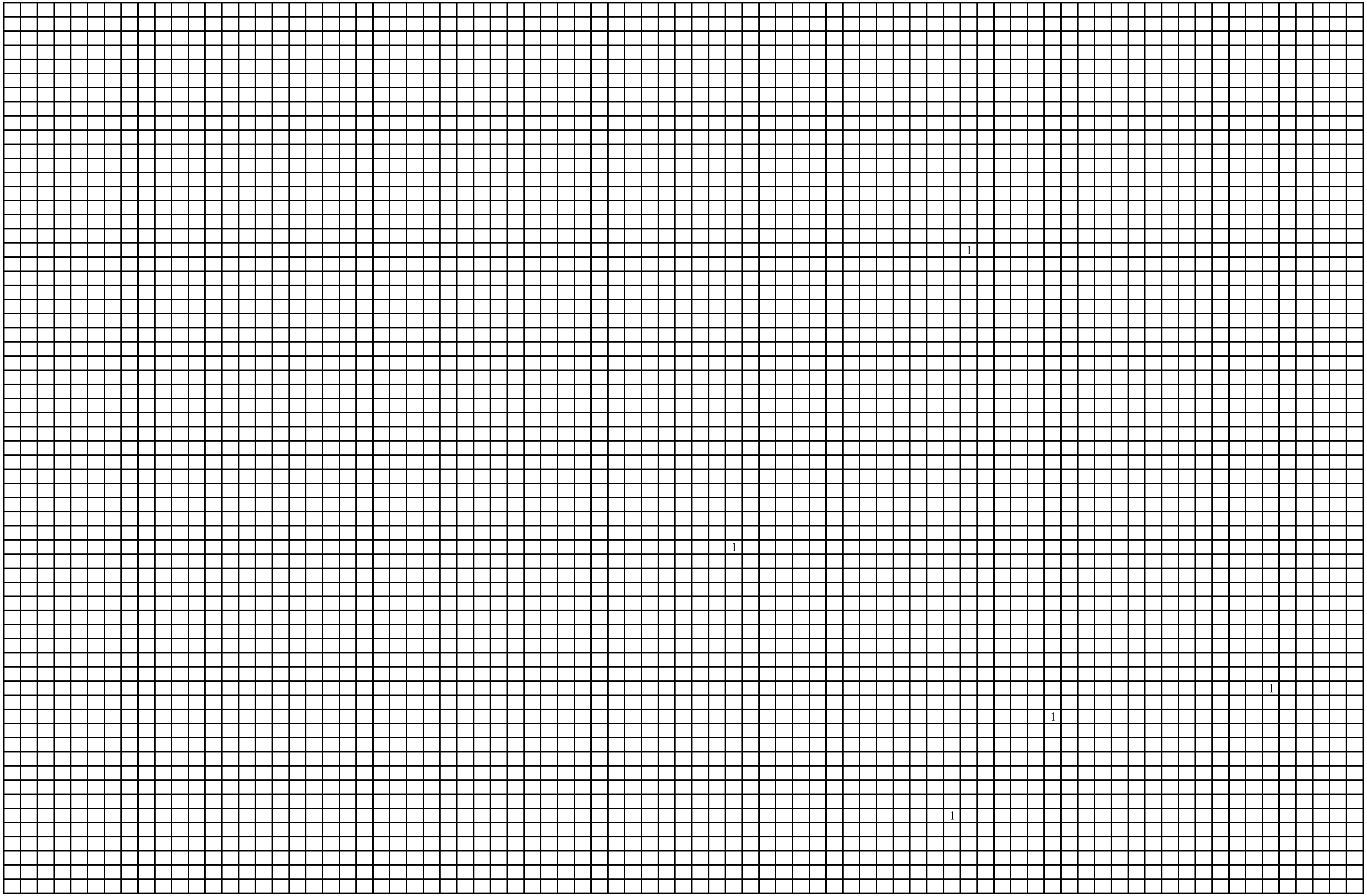
1
1
1
1
1

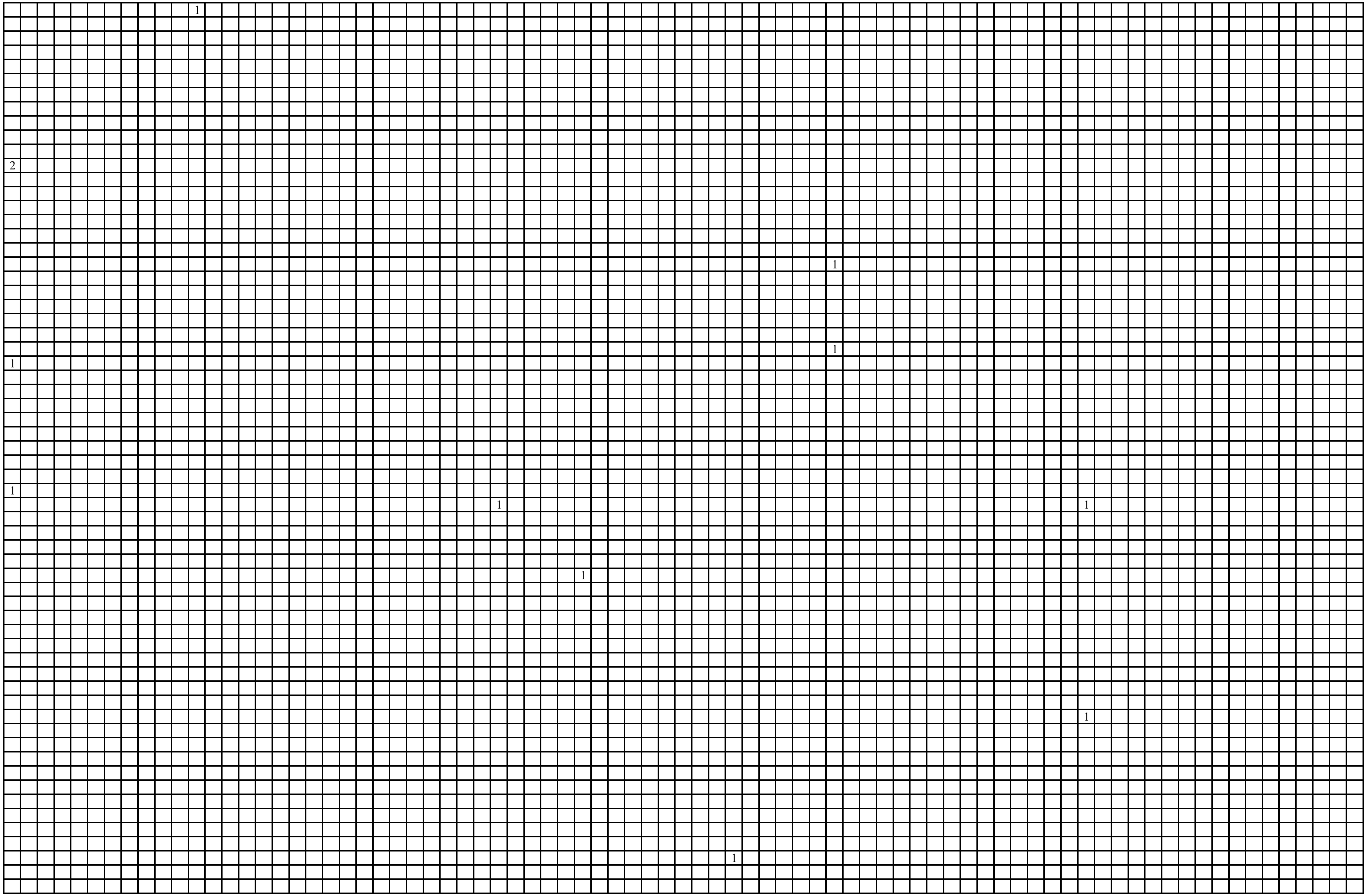
1
2
1
1
1
1
1
1
1
1
1

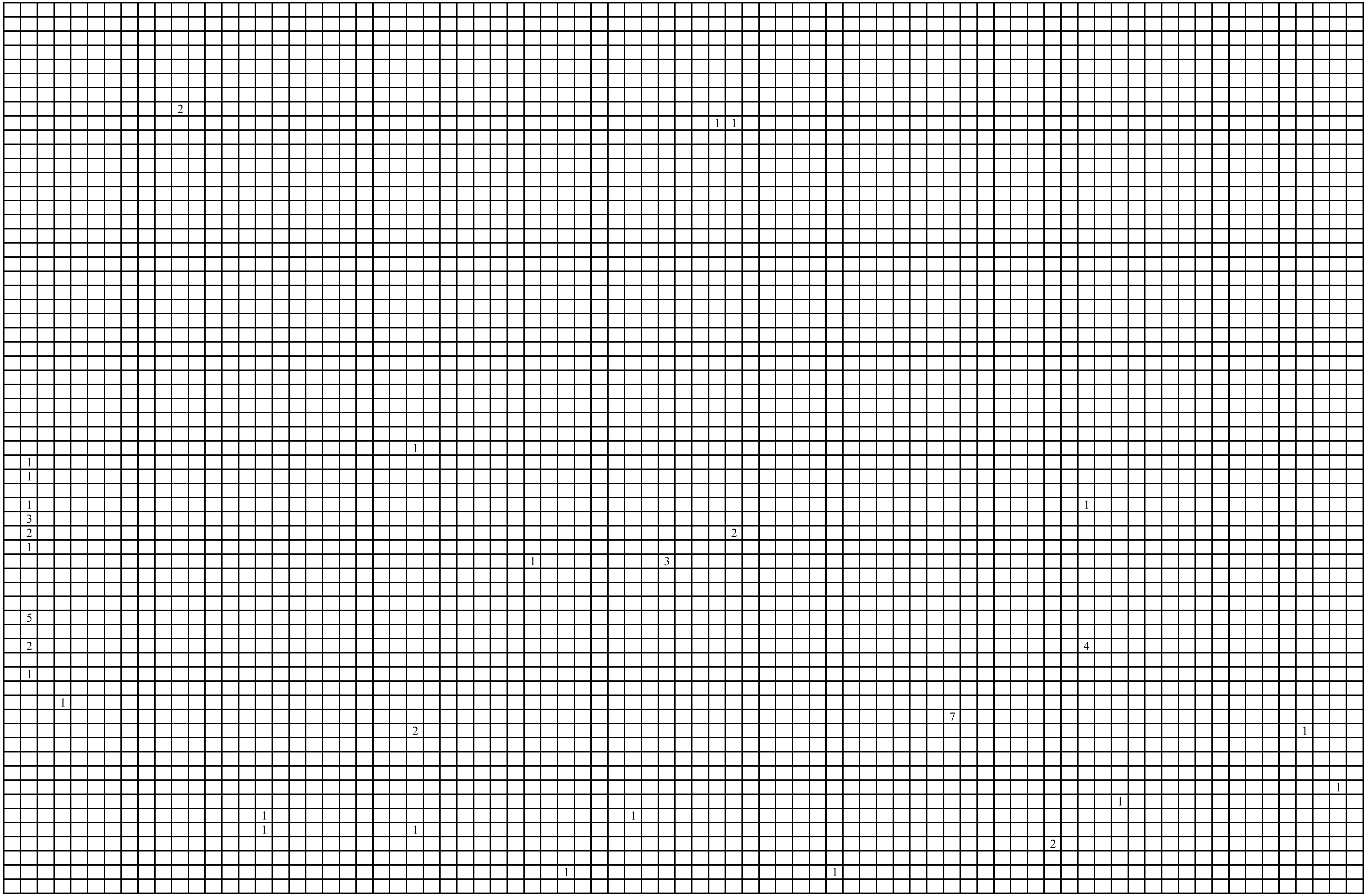
2
1
1
1
1
1
1
1
3
2
2
1
1
3
5
2
4
1
1
7
2
1
1
1
1
1
1
1
2
1
1

																																																	1																														
																												1			1																																																
																												1																					1															1																
											2																																																																				
																																					1																																										
																1						1																	1																																								
										1																																														1																						
									2																																																																						
																						1																																																									
																1																																																															
																								1																								1																														
																																							2																																								
														1																			1																															1																
																																	1																																														
																								2																																																							
																																																									1																						
										1																						1																																														
										1																																						2										1					1																
																	1	1				1		1									1																								1			1																				
																																	1																				1											1		1														
																																					2																																										
																																								1																																							
																																											1																			1																		
										1																																																																					
																																																																1																
																																	1										1																																				
																																																																													1			
																																																								1																							

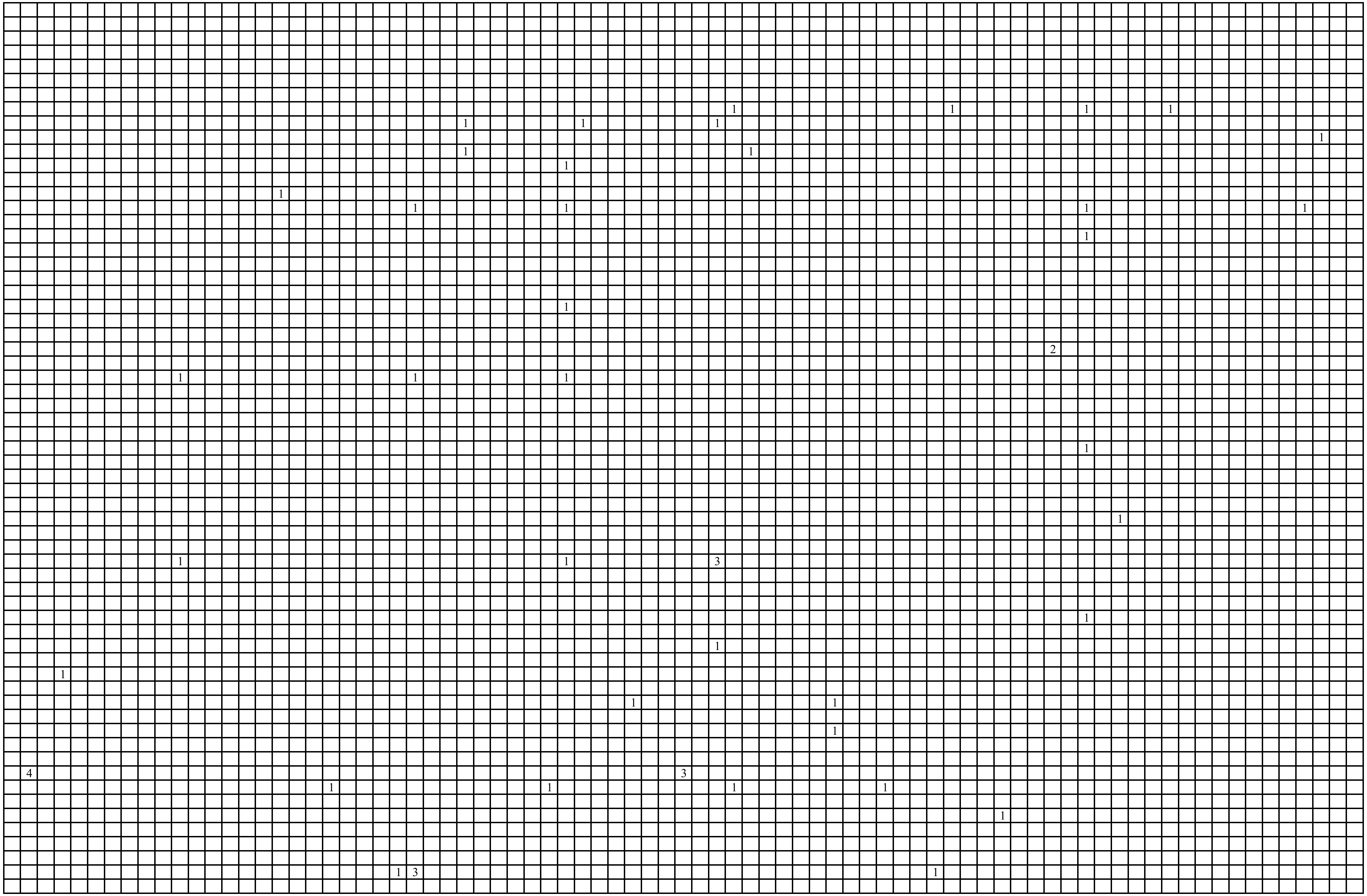

1 1 1 1
1 1 1
1
1 1
1
1
1 1 1 1
1
1
2
1 1 1
1
1
1 1 3
1
1
1
1 1
1
4 3
1 1 1 1
1
1 3 1

																								1																									1																															
										1																							2																																															
																																																														1																
			1																					1													1																																													1	
										2																							1																																															
																								1													1																																															
																														1																																																						
			1	1																													1																							1																								
				1																													1																																															
																																																						1																								
																																																																												2			
																								1																																																													
										1																																																																								
	2																																1																																																
																																	1																							1																								
																																								1																										1								1						
																																					1																																								1			1			
																													1																								4																					1						
											1							1						1					1																																	1			1		1														
																																							1																																						1			2			
																								2									1		1																																															1			
										1														2									1		1																					1																					1			
														1										3											1																																															1		1	

			1							1														1													1																																			1					1		1	
																								2									1		1																																										1		1	
			1	1																																																				7																								
																																																																1																
		1			1				3							1						1		1					2		1					1		1										1				1				2			1	2	2		2	1										1						1
					3	1									1	3	1	2				1	1	2	1	1	1	1	1		3		1						2						1				1							5			1	1				1			1			1	1								4	
			1								1				1	2		1						1	1		1	1					1					1							1											1			1					1						1	1								1	
																																																																		1														
															1																												1																																					
																															1										1				3		1																	1										1					1	
																															1																																																	
																																																3																																
																																																3																																
									1	2																																							1										1																					
																4								1							2						1																				1																							
																								1													1																																											
																															1										2																																						1	
																																									1																																						1	
																5															1																																																	
			1																					2				1						1																						1			1					1													1			
																																	1																																															
			1			1									1		1	1				1			1			1	1																			2								1				1											1								1	1
																																																																1																
																																																								1																								
																																																	1																															
					1											2																																																																
																1															1																																																	
																3									1																																		1																					
										1																																																																						
																																																											1																					
																	1																	1																										1						1														
																																																	1																															
												1																																																																				
																																	1																				1															1												
																								1																																																					1			
																								2											1																																										1			
						1					1						1	1				1	1				2		1																														1	1	1			1		1	1												1	

										1																			1																														1					1																
																																								1																																											
																																																										1																							
						1												1																					2																																																
									5	3					1		1																1																1															1	2				1				1							
									1						1																																																	1																
									1						1																																																1																	
																												1																																									1																
																				1																																																															
																																																										1			1																						
						1										1									1																					1				1										1	1							1			1	1									

																										C14orf169	8	68
																										CAMK2D	12	278
																										CBL	29	1423
																										CC2D1A	7	159
																										CCDC59	12	138
																										CCNA1	10	139
																										CCNC	22	356
																										CIDEA	1	22
																										CIPC	10	27
																										CITED4	3	8
																										CNBP	41	348
																										CNOT10	6	185
																										CNOT11	8	173
																										CNOT3	10	224
																										CNOT8	5	124
																										COMMD10	5	94
																										COMMD3	6	144
																										COMMD4	16	160
																										COMMD5	5	79
																										COMMD8	12	125
																										COMMD9	2	70
							1																			CRK	19	574
																										CTNND2	3	32
																									1	CUL3	277	4168
																										DAPK3	5	119
																										DHFR	8	131
																										DNAJB5	9	151
							1																			DNM2	14	304
																										DPPA4	13	183
																										DTX1	1	45
																										DYDC1	5	50
													1													EDRF1	7	134
																										EFCAB6	3	11
																										EID3	2	66
																										EPC2	32	112
																										ERN1	2	97
																										ESF1	11	137
																								1	1	EYA2	19	135
											1	1														EYA4	40	270
																										FABP4	3	24
																										FAM208A	5	107
																										FAM58A	1	25
																										FGFR1	16	841
																										FHIT	2	34
																										FHL5	43	127
																										GMCL1	11	270
																										GTF2H2C	6	40
																										GTF2H5	1	64
																										HELLS	24	249
																										HIP1	5	140
																										HMGB3	18	75
																										HMOX1	4	151
																										HSBP1	6	89
																										IFT74	8	177
																										INO80D	11	55
																										IRAK1BP1	3	15
																										JMJD1C	22	98
																										JMY	2	50
																										KDM3B	12	111
																										KDM4D	7	46
																										KDM6B	6	99
																							1			KHDRBS2	12	198
																										KMT5B	0	0
																										KPNA6	20	210
																										LBH	1	26
																										LMCD1	3	20
																										LPIN1	4	41
																										MAFIP	1	6
																		1								MAGED1	46	357
																										MAP2K5	14	171
																										MAP2K6	5	114
																										MAPK10	16	94
																										MAPK11	2	58
																										MAPK8IP1	4	102
																										MAPK9	69	312
																										MBTD1	35	144
		2	3		3													2								MDFI	177	653
																										MED12L	2	87
																										MED13L	6	139
																										MED20	22	352
																										MED28	3	325

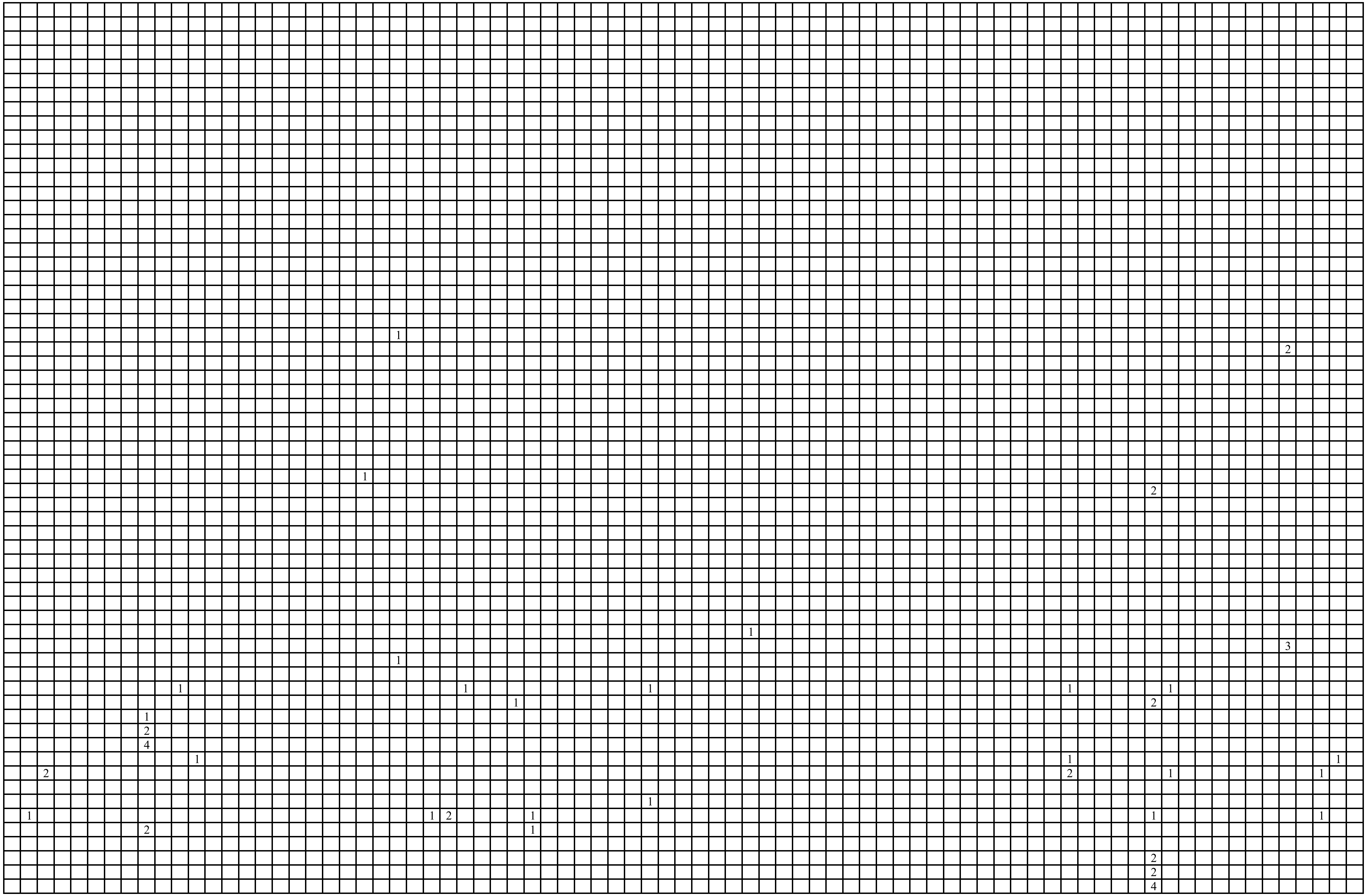

																																		2																																									2					
																																																																												1				
3	2			1						2						1	1																	4		3				1						1		2								3		2										1	1						3					
																																		2																																									2					
				1						1														1													1														1																														
																																																		1																														
													1																																																																			
																							1																																																									
									1																												1																																											
									1																																																																							
									1																																																																							
																							1																																																3	2		8						
																																																																															1	
			1																				1																																																									
																							1																																																									
																																																																												3				
																1																						1																																8		1	20						
																																																																										2						
																																																																										2						
																							3																																														3				1						
																					1																																																											
																																																																										1						
																																													1																																		
																																																																										1						
																																																																			1													
																							1																																																									
																							1																																																									

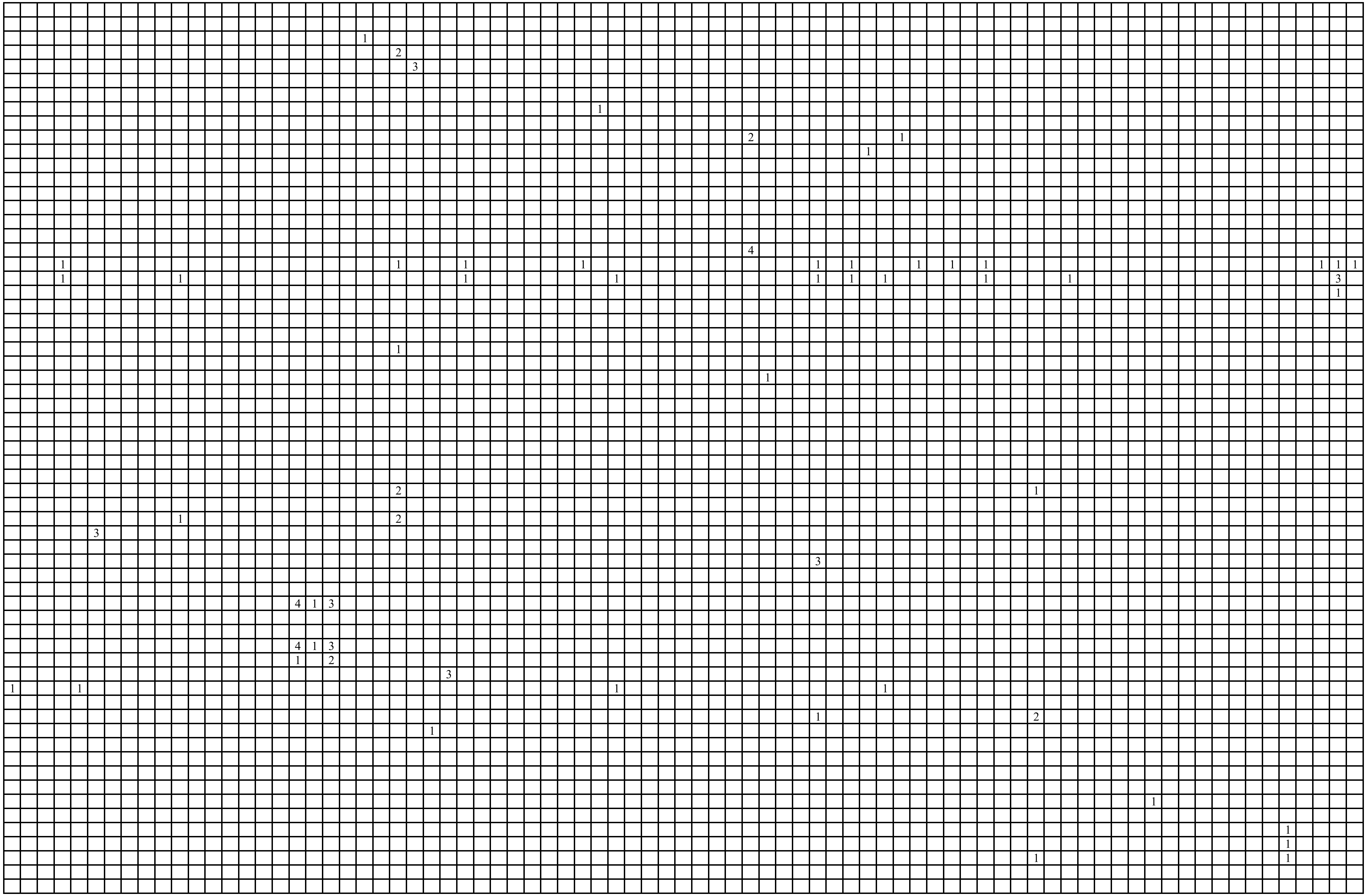

																																																																											2				
																																															1															1																	
																																															1																																	
																																																																	1												
																																																																	1												
																																						1																																														
																																						1																																															
																									1																							1																																	
				1																																																																												
																																							1																																													
						1																																																																									
																																															1																																	
																												1						4											2																													4					
						1																											3																																															3					
																																																																				1												
																																					1																																												1				
																																						1																																															
																																																							1																									
		1																				2																																																										
					1																																																																										

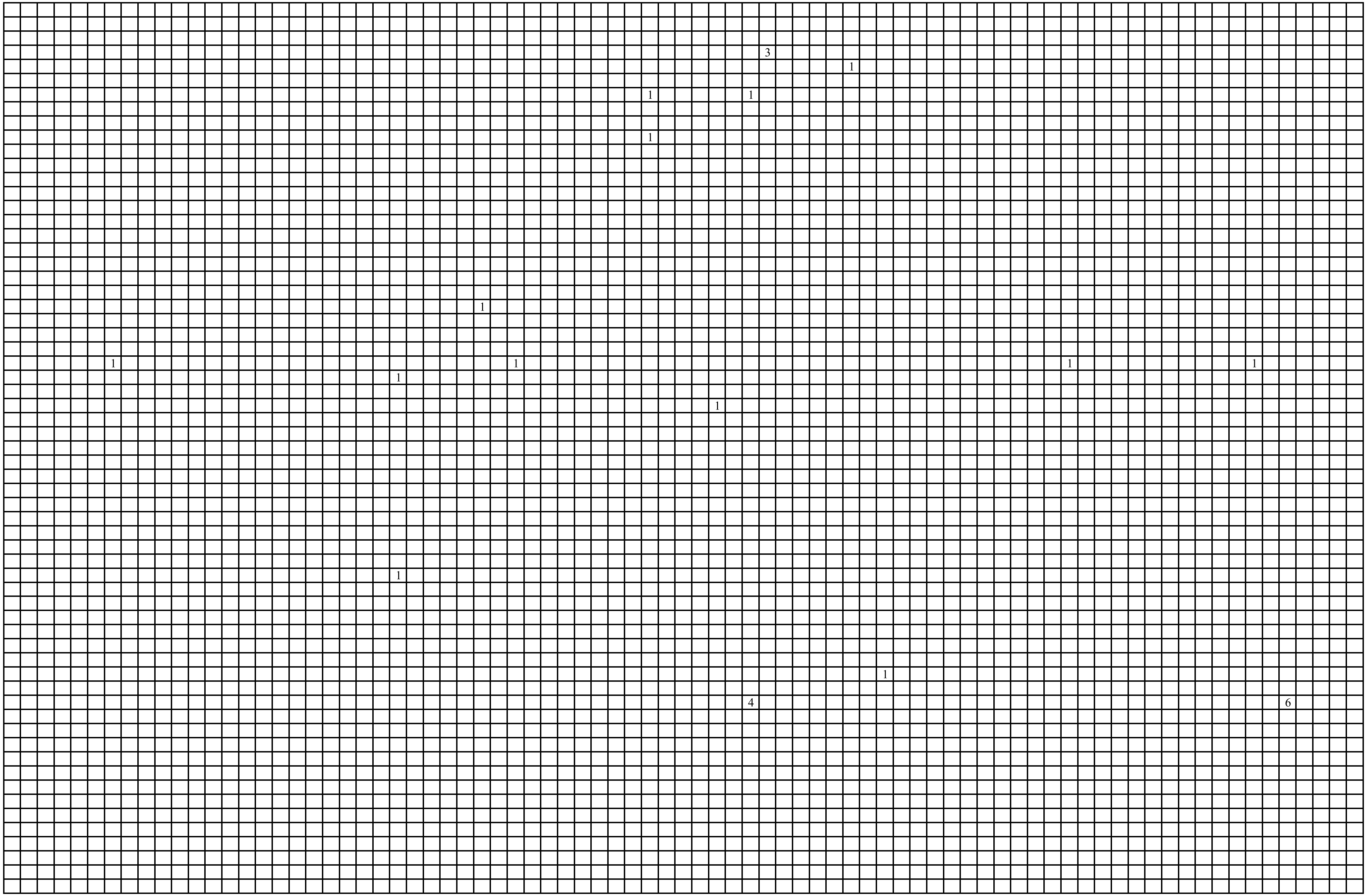
3
1
1
1
1
1
1
1
1
1
1
1
1
1
4
6

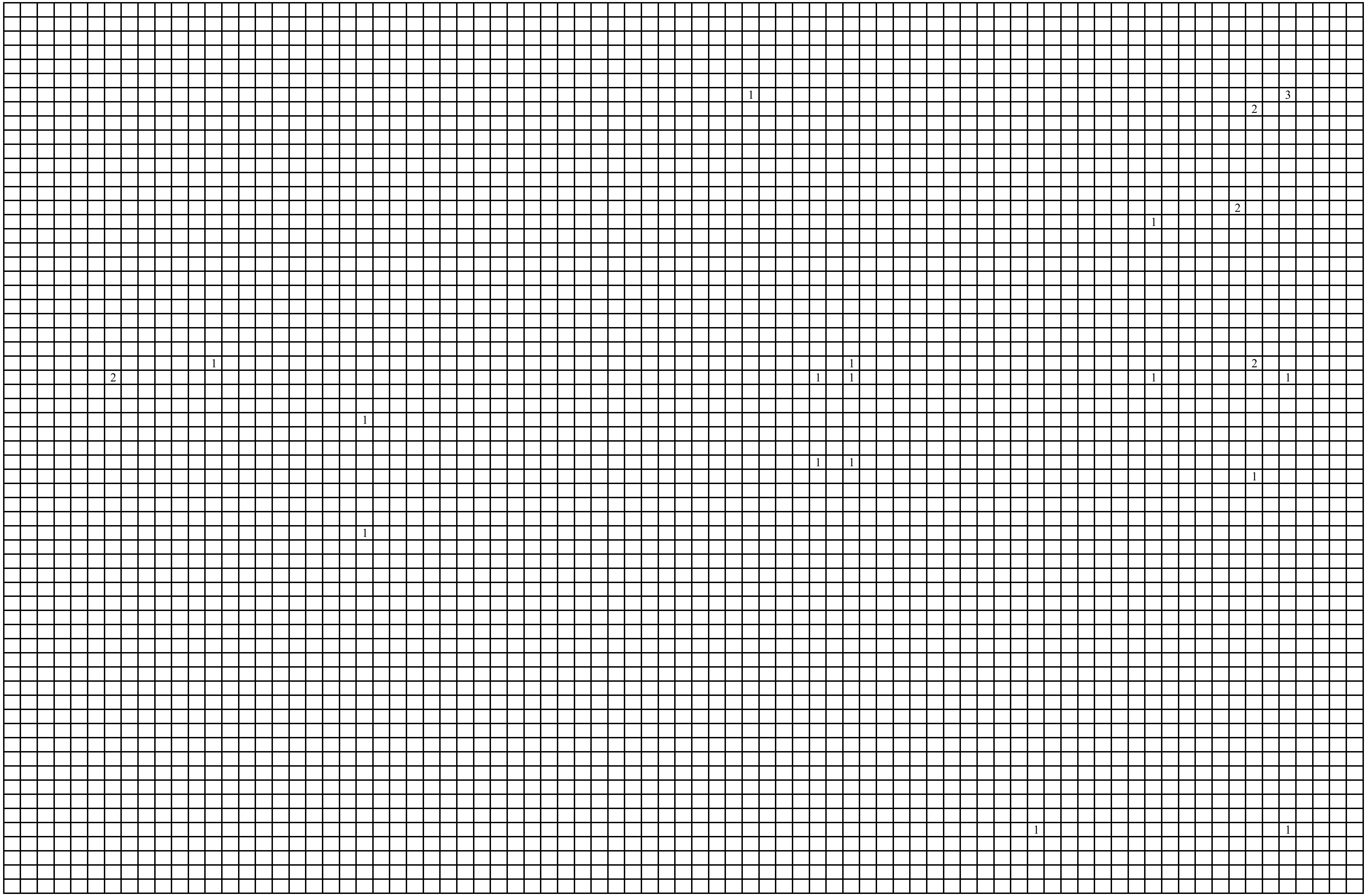

1 3
2
2
1
1 1 2
2 1 1 1 1
1
1 1
1
1
1 1

										1																																																																						
																							1																																															1												
																																																					1																								
																																																													1															
																																																																		1												
																																							1																																																
																																																																		1												
																																																																		1												
																											1																																																													
						1																												1																																																				1					
																																																															2															
																																							1																																																
																																															1																											1							
																																												1																																	5					
																																																																		2												
																																							1																																																	
																																																																		1												
																																																																		2							1					

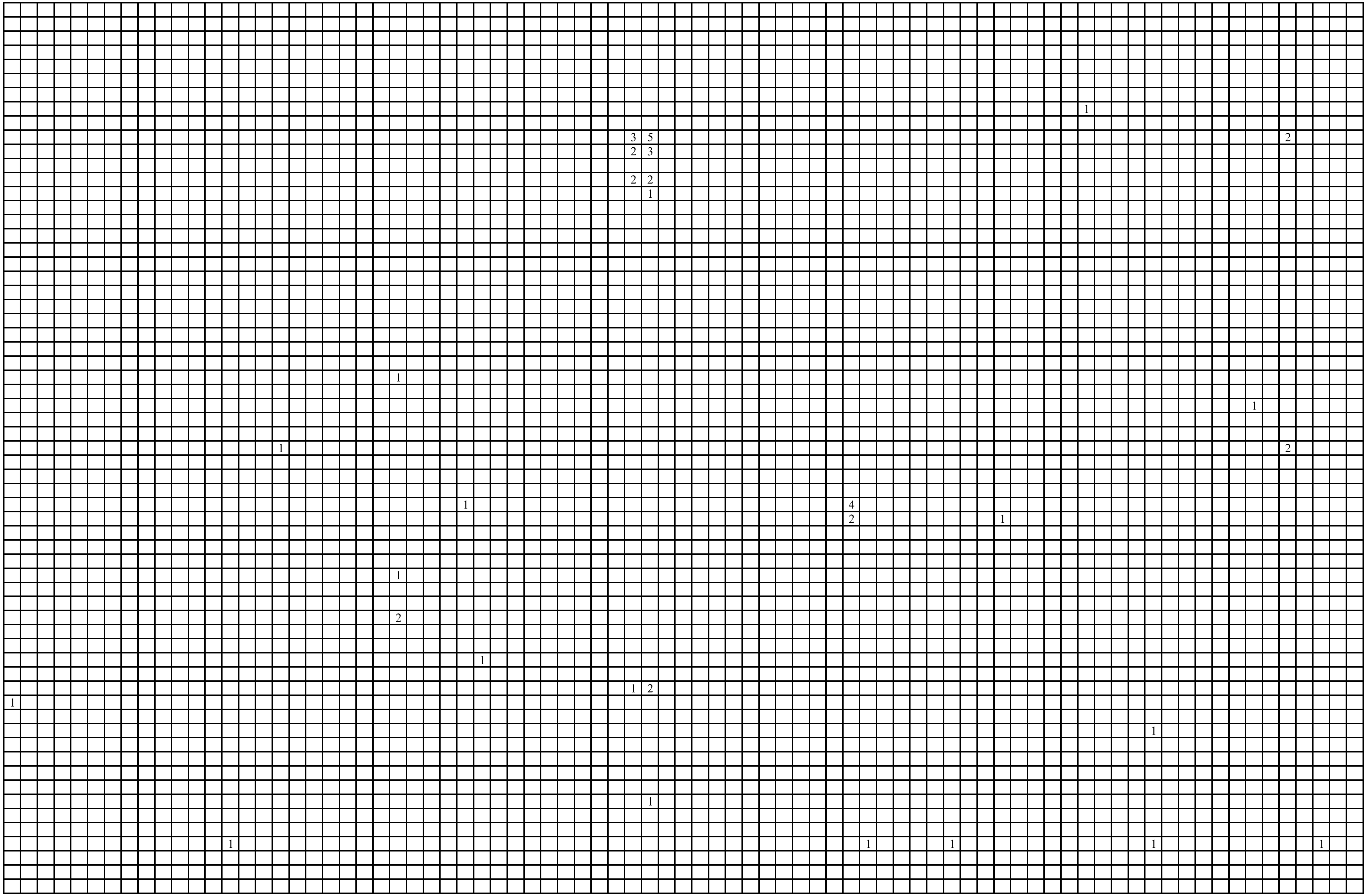

																																																																					1												
																																																				1																								
																							1																																																										
																																											1																																											
																																																																											1				
																							1																																																										
																							1																														1																											
																	1																								1																			1																				
																							1					1																						1																			1											
																																																																											1											
				1																																																2																											1	
																							4																																																									
																																																						2																											
	1																																																																																
																							1														1																																																
																																																										1																							
																							1																																																											
																							1																																														1											
																							2																																																											
																							1																																																											

		1																																																																													
																																										1																																										
																																							1				1																																										
				2																																																																													
																																							1																																																		
										2													1																																														1																					
			1								1	1	1	1																																																											1												
																							2																																																													
																																																																														1	
																					1																																																															
											2	3	1	2																																																																			
																							3																																																													
																							1					3																																																		2						
																																										2							1																																							
																																																					3																														
										1													1																																																															
1		1		1																			3				1																1																										1											1	
																					1																																																																
																																														1																																						
																							1		1																																																											
																							1																																																																
																	1						1																																																															
													1										1																		1			3				1																																							
																																							1																																																		
																																		4																																																				6					
		2																			1		1	1	1		1								1													3											3				1						1		1			3	1					
						1																																																																												
																																																																										1							
						1																																														1																							1						

																																					1																																																	
																							1																																																										
										1													1																																																									
																							1																																																									
																									1																																																							
												1			1	1	1	1	1	1			1																																																										
																							1																																																								
						1																																						1						1																															
															3		1	1	1																																													1								2									
		1													1		1	1	1	1																																												2																	
																																														1																													2						
																							1																																																							1						
																																																																			2								3						
	2																						1																																	2																									
																										1																																																						
													1																																																																				
																							1																																																										
																																																																1																	
																																																										1		1																			1	
																										1																																		1																			1	
		2																								1																																		1																				
																																																														1																				
		3																																																																					1										
		1																					1																			3																																																		
																					1																																																																		
		3																																																																																			
																																																																1																				

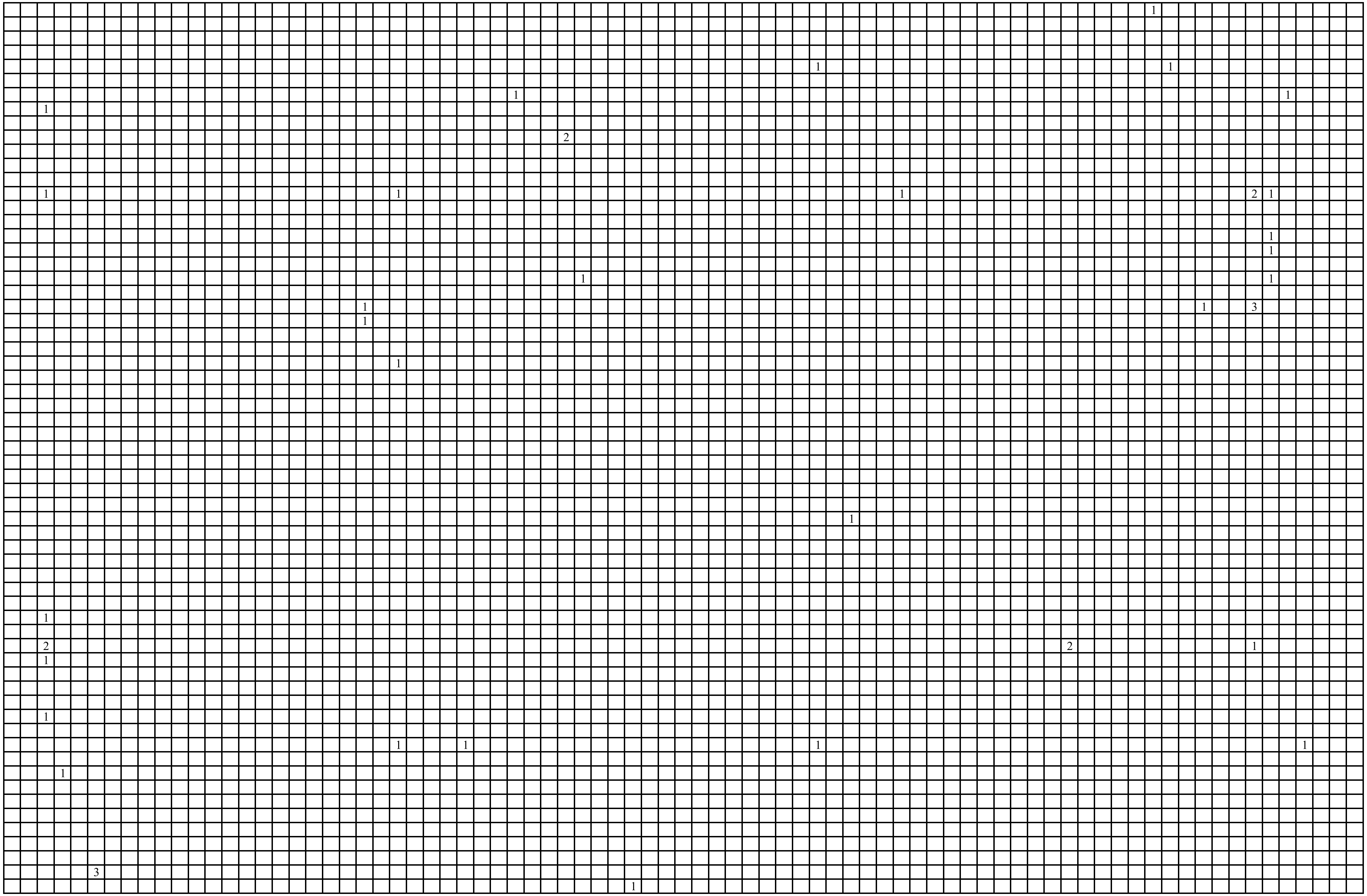
1
1 1
1 1
1
2
1 1 1 2 1
1
1
1 1
1 1 3
1
1
1
1
2 2 1
1
1
1 1 1 1
1
3
1

																																					1	1																																															
																					1		1																	1												1			1																								1
																							2																																																									
																							2					1																																																					
																																																																														1	1
																																																																1																	
																							1																																																									
																																																3																																
																																																				1																													
										2																																																																					
	1																																																																															
																							2																																								1																	
																																																																										1				
																													1																																																							
										1																																																																					
																					1													1																																																		
																																																																										1					
										1																								1																					1																		2					
																																																																														1	
										1																								1																																												1					
				1																														1																																															1					
										1																																																																						
					1																		1																																																										
																							1																																																									

		3																															1				1																																										
																																																																												1				
																						1	1																					1																																				
																															3																																											2		1				
																																												1																																1				
																																																																												1				
																1																																																																
																							1																																																				1				
																																												1																																1				
																							1																																																									
																																												1									1																								1				
																																																										1																						
																																																					1																											
													1										3																																																									
													1																																																							1												
																																																																					1	1										
																							1																																																									
		1																																																																														
	1																																																																															
																							1																																																									
																							1																																																									
																																																																					1											
																																																																					1											

																							1																						1									5																						1			1	
																							1																																																									
							1																				1																	1																																				
																																													1																															4				
																																																																												1				
																																				1																																																
																							1				1																																																									
																							1																										1																																
																							1																																																											
																							1																																																											
										2																																																																							
																							1																																																											
																							1																																																											
						1																	1																																																											
																							1																																																											
																																																																					1												
																																																																													1				
																							1																																																											
																							1																																																											
																							1																																																											
																					1																																																													
																					1																																																														
																							1																																																											
																							1																																																											
																							1																					1																															2				

																							1																					4																				1																	
																							1																																																									5				
																																																																								1		2				
																							1																																																													
																							1																																																													
																							1																																																													
																							1																																																													
																							1																																																													
																							1																																																													
																																																																												2				
																												1																																																					
						2																																																																											
																							1																																																													
										2													1																																																													
																																																																												1				
																							1																																																														
										1																																																																							
																																																																												1				
																																																																												3				
																							1																																																													
																							1																																																														
																							1																																																														
																							1																																																														
																							1																					1	1																																	1				
																							1																																																									1				
																							1																																																														
																							1																																																														
																							1																																																														
																							1																					1																																								
																							1																																																														
																							1																					1	1																																							

																							1																																																							2				
																																																																												2				
																							1																																																												
																			1				1																																																											
																							1																																																													
										2																																																																											
																							1																																																															
																							1																																																														
																							1																																																													
																							1																																																													
																							1																																																														
																							1																																																													
																							1																																																													
																							1																																																														
																1							1																																																												
																							1																																																														
																							1																																																														
						1																	1																																																													
																							1																																																														
																							1																																																													
																							1																																																													
																							1																																																													
																							1																																					1																									
																																													1																																3				
																																																						1																										

																							1																																																									
						1																	1																																														1							1				
																							1																																																									
																																																																												1				
																																																																												4				
																																												1																																5				
																																																																												1				
																							3																																																									
																							1																																																									
																							1																																																									
																																																																												1				
																																																																												1				
																							1																																																									
				1																			1																																																									
																							1																																																									
																																																																												3				
																																																																												1				
																																																													1															1				
																																																																												1				
																							1																																																									
																							1																																																									
																							1																																																									
																							1																																																									
																							1																																																									
																					1		1																																																									
																							1																																																									
																																																																										1		5				

																							1																																																									
																							1																																																									
																							1																					1																																2				
																							1																																																									
																							1																																																									
																							1																																																									
																							1																																																									
																							1																																																									
																																																																											1				
																																																																											1				
																							1																					1																	1															3				
																							1																																																									
																							1																																																									
																							1																																																									
																							1																																																									
																							1																																																									
																																																																											2				
																							1																																																									
																							1																					1																																1				
																							1																																																									
																							1																																																									
						1																	1																																																1											
																							1																																																									
																							1																																																									
																																																																												1				
																							1																																																									
																							1																																																									
																																																																												1				
																							1																																																									
																																																																												1				
																																																																												2				

																																																																											2				
																							1																																																										
										2													1																																																									
																																																																											2				
																							1																																																										
																																																																												3				
1																																																																																	
																							1																																																										
																							1																					1																																		2				
																																														1																																							
																							1																																																											
																							1																																																											
										1													1																																																											
																							1																																																											
																							1																																																											
																																																																												1				
																																																																												1				
																							1																																																											

																							1																																																								
																							1																																																								
																							1																																																								
																							1																																																								
																																																																1															1				
																							1																																																								
																																																																											2				
																							1																																																								
																							1																																																								
																							1																																																								
										1																																																																					
																							1																																																								
																							1																																																								
																							1																																																								
																																																																				1												

																					1																																																											
																							1																																				1																					
																							1														1																																						1				
																							1																																																									
																							1																																																									
																							1																																																									
																							1																																																									
																							1																																																									
	2																											1																																																				
																							1																																																									
																																																				1																						1		2				
																																																																												3				
																							1																																																									
																							1																																																									
																																												1																																				
																							1																																																									
																							1																																																									

																							1																					1																																3				
																																																																												1				
																							1																																																									
																												1																																																				
										1																																																																						
																							1																																																									
										2																																																																						
																																																										1																		1				
																							1																																																									
																							1																																																									
																							1																																																									
																																												1																																2				
																							1																																																									
																							1																																																									
																							1																																																					2				
																							1			1																																																					
																							1																																																									
																							1																																																									
																							2																																																									
																							1																																																									
																																																																												1				
																																																																												1				
																							1																																																									
						1																																						1																																				
										2																																																																						
																																																			1																													
																																							1																																									
																							1																																																									
																																		3																																										3				

										2																																																																					
																							2																																																								
																							1																																																			1				
																							1																																																								
																																																																			1												
																																							1																																															
																							1																																																								
																							1																																																									
																							1																																																			2				
																																							1																																															
																							1														1	1																																															
						1																	1																					1																	1							1								1				
																							1																																																								
																							1																																																									
																							1																																																									
																							1																																																									
																							1																																																									
																							1																																																									
																							1																																																									
																												1		1																																																										
																																																																												2				
																																							1																																												1				
																												1																																						1																	
																																																																												1												
																																																														1																			

																							2																																																							
																							1																																																							
																							1														1																																											
										1											1				1							3														1										5		2	1									1									1	1	1	
				1		3				2	1	1	1	1								1	1								1					1				1						1				1					1														1												3	
1	1		1	1		1				1	1	1	1										1	1			1							1		1				1										1					1		1		1										1							1			1	2	
																							1																																																								1												
	1	1		1						2				1								1	1				1			1						1												1								2		1																						1	
					2																																																																										
																							1																																																							
																							1																																																				4				
																																																																												3				
																																																																		2														
																																							1																2																		1						
																																										3																												1			1						
																																																																										1						
								1																																																																							
																																																								1																								
																							1																																																							
																																		1																																								1					
																	1								1		1																												1			1					1																	
																							1																																																							
																																										2																																					
																							1																																																							
																																						1																								1																	
																																										2																																					
																																														1																																	
																																																																												1				
						1																1																																																							
																																				1														1											1																			

																							1																																																									
																																																																				1												
																																											1																																2				
																																					1																																															
																																						1																																														
																																						1																																														
																							1																																																														
																							1																																																														
																							1																																																														

241	265	61	8	117	319	32	35	456	640	499	72	21	110	20	126	9	99	45	269	272	127	133	70	102	126	352	14	15	25	908	1040	22	332	568	1182	182	153	27	2413	94	47	178	1330	31	36	174	64	161	92	172	32	100	217	95	478	91	424	204	93	993	76	126	151	148	12	44	10	2	10	306	45	98	62	30	4	45	11	553	574	22
13	6	3	2	10	21	1	8	34	37	16	7	6	34	5	6	3	7	1	11	4	14	6	53	14	53	7	1	1	9	52	54	2	32	18	89	11	6	7	93	3	4	18	37	2	1	12	11	6	7	18	2	11	71	13	115	11	29	9	10	69	6	42	36	66	1	6	5	0	2	39	0	6	13	2	0	1	3	28	32	1
TcoFs																																																																																
TcoF.3																																																																																	
MED31	MED9	MEGF8	MSL3P1	MTPN	MYCBP2	NAA16	NAB1	NDN	NEDD4L	NEDD8	NFATC2IP	NFE4	NLK	NOTCH4	NRBF2	NRIP2	PAK6	PARP14	PBXIP1	PDPK1	PELI1	PER1	PGBD1	PHF14	PIAS3	PIK3R2	PKIA	PKIG	PNRC1	PPP2CA	PPP2R1A	PPRC1	PRKAA2	PRKCZ	PRNP	PSMD9	PTTG1	PYGO1	RAF1	RALGAPA1	RASD1	RBMXL1	RBX1	RCBTB1	RGS14	RGS20	RIPPLY1	SAMD4B	SERTAD1	SETD2	SETD3	SFSWAP	SIN3B	SIRT5	SMURF2	SMYD3	SND1	SORBS3	SPAG8	SRC	SRFBP1	SS18L1	SSBP2	SSBP3	SSX1	SSX3	SSX4	SSX4B	SSX5	STRAP	TAF7L	TARBP1	TBL1Y	TCEAL2	TCEAL3	TCEAL4	TCEAL5	TCEB1	TCEB2	TENM1
																																																														2																			
																																											1																				1																	2		
																																																															1																			
																																																					3																												
																																																					2																												
																				1																																																																	
																																	1																																																		
																																																				2											1																			
					1																																																																													

																																							1																																									
																																															1																																	
																																																						1									3	3																
																														1																																																		
																																									1																																							
																																																												1																				
					2																																																																											
										2																													1				1																	1																				
																															1								1																																						1	1	2	
																																																										1						1																
																																				1																																												
																						1																																																										
																																				1																																												
								3																																																								1														1	2	
								2		3												1									1												1												2	2				2							3											6	4	
																						1																	1																																									

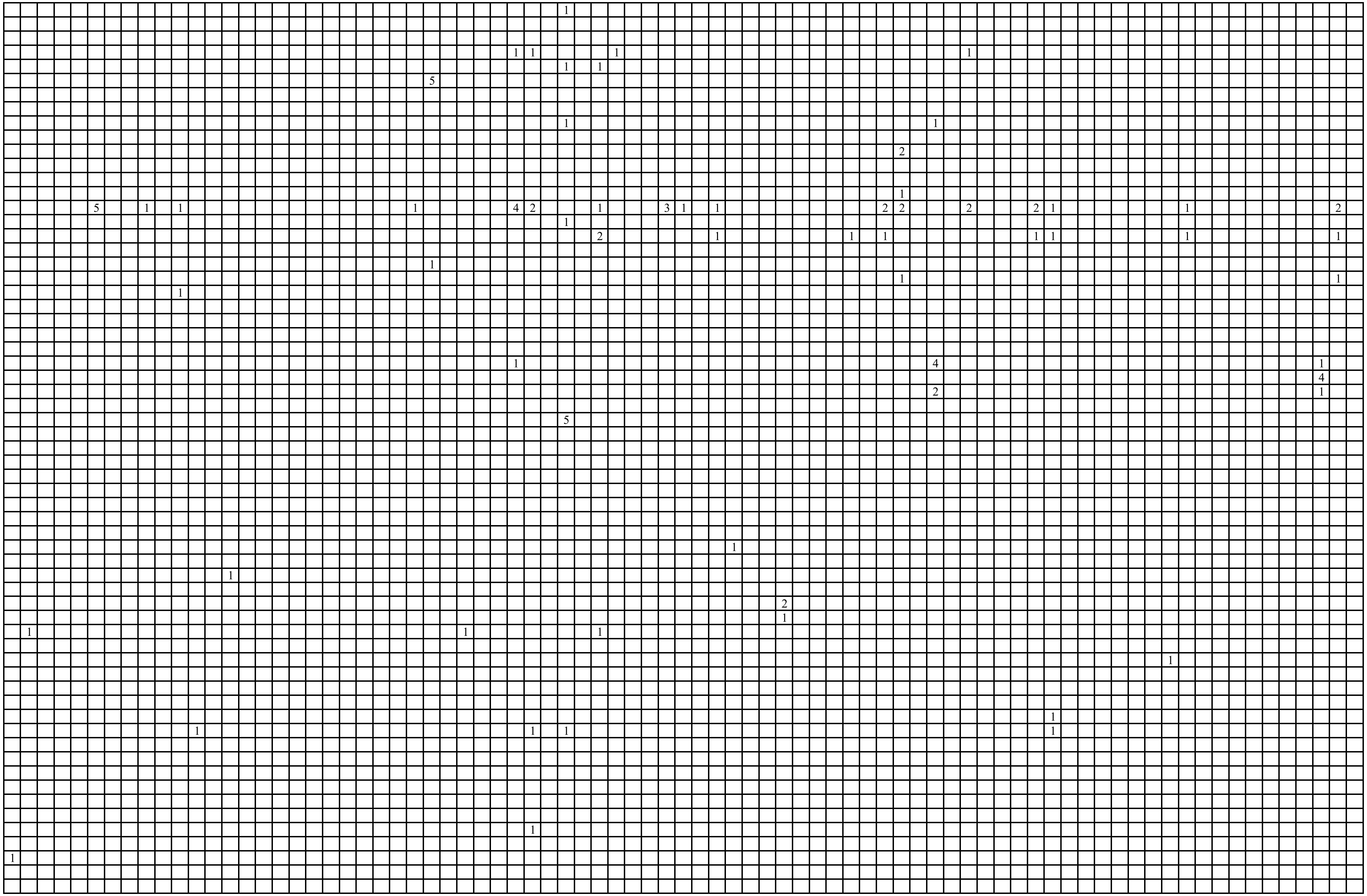
1
1 1 1 1
1 1
5
1 1
2
1
5 1 1 1 4 2 1 3 1 1 2 2 2 2 1 1 2
1
2 1 1 1 1 1 1 1
1
1 1
1
1 4 1
4
2 1
5
1
1
2
1
1 1 1
1
1
1 1 1 1
1
1

																																																									1																					
											1																																																																			
																																			1																															1													
																																											1																																1								
									1															1																																																								
				1	1	1	1																						3									1													1				1														1								1	1	
1				1						1														1							2												1									1																		2								2	1	
																																																																															1	
																																																													1																				
																																																																			1														
																																																						1																												
																																																										1																								
																																																	1																																
					1																																																																										
																																											1																																					
								1	1	1																												1			1	1											1		1																							
																																										1																																						
										1																												1																																									
								6																																																																						
								3			1																			1																												1																						
																																	1																																														
																																			1																					1																							
																																				1																																												
																														3																																																				
																														1				1																																													
																													2	2																																																				

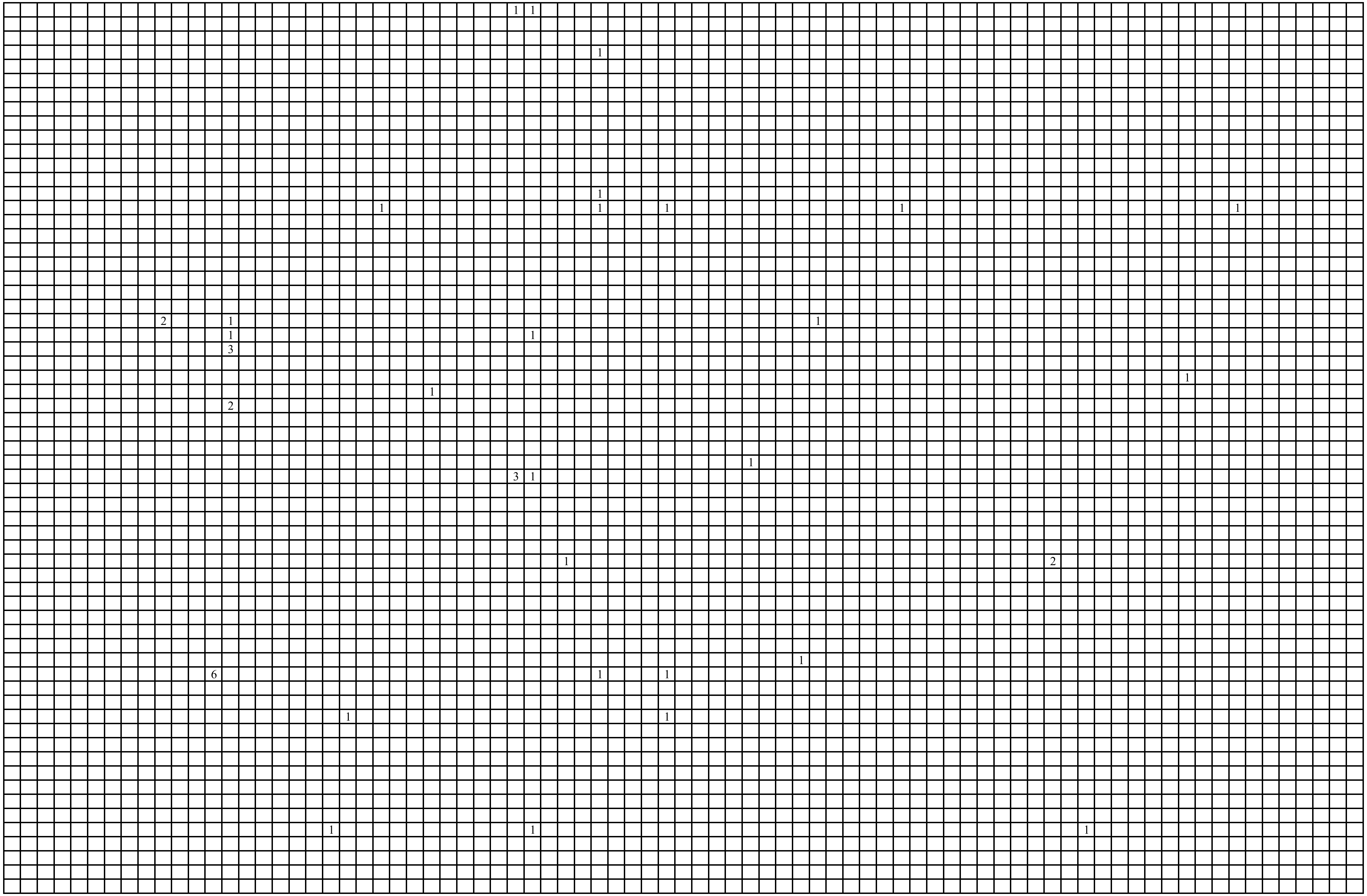
1 1
1
1
1 1 1 1 1
2 1 1
1 1
3
1
1
2
1
3 1
1 2
1
6 1 1
1 1
1 1 1

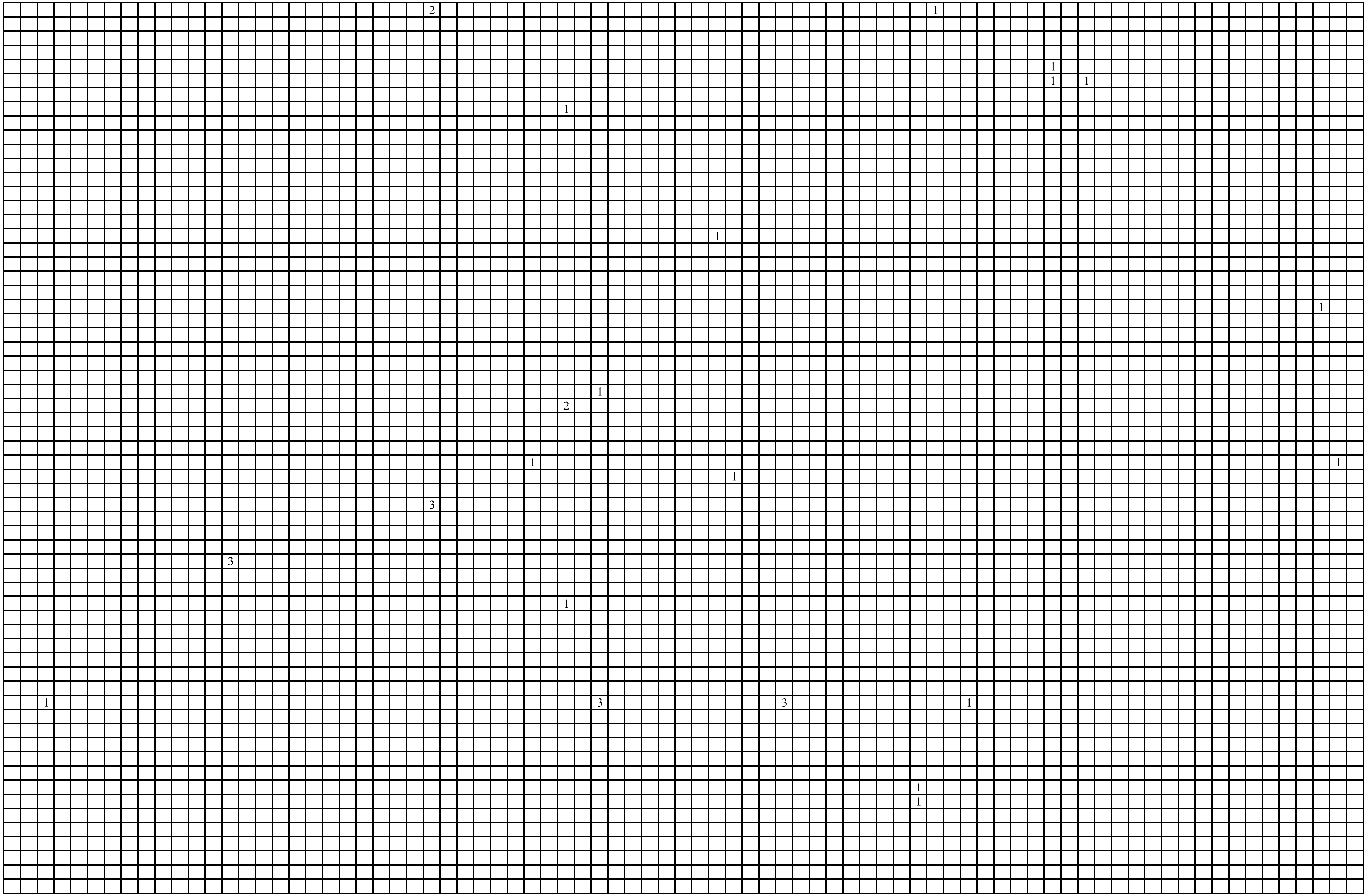
2 1
1
1 1
1
1
1
1
2
1 1
1
3
3
1
1 3 3 1
1
1

																																											1																																								
																																																						3						3																				
																																																						1																										
																																																																1																					
																								2					1								1											1																			1														
																																									1																									4	4																
																																									1																									4	6																
																																																																							1	5																
																																									1																									1	4																
																																																																					1	1	3																
																																									1																									5	4	1															
																																																																						5	5																
																																									1																									1	2																
																																																																							3																
																																																																						4	6																

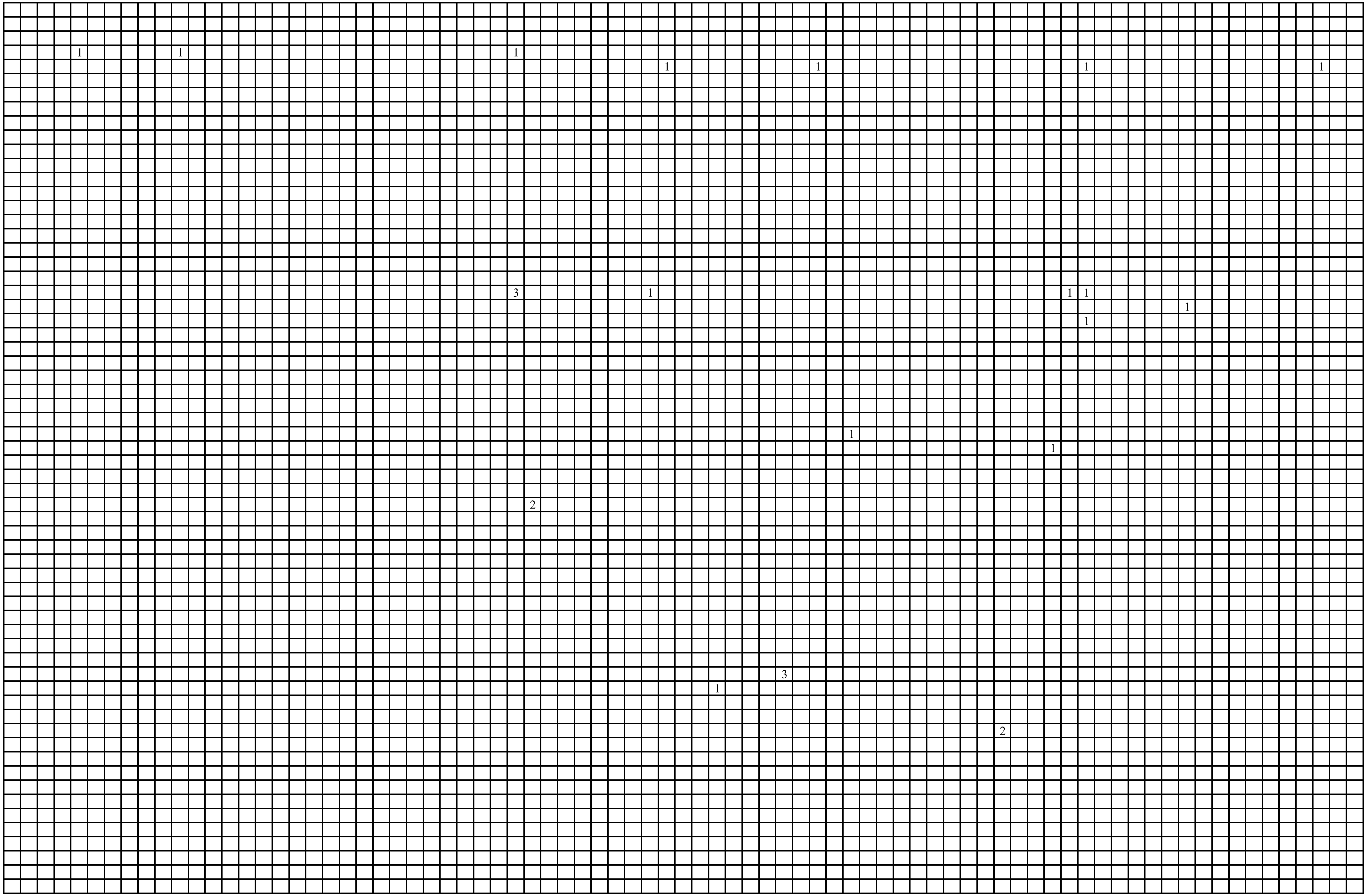
1
1
1
1
1
1
1
3
1
1
1
1
1
1
1
2
3
1
2

																																																			1																										
									1																																											1																													
																																												1				1		1																																							
1																																																																																	
																																																				1																													
																									1								1	1																																																					
																																	1																																																						
																																		1																																																					
																																1						1																																																			
																																				1																																																					
																																																				1																													
																																								1																							1																			
									1																																											1																													
																														1	1				1															1		1	1							2																						
																																																														1																	
																																												1					1		1			1											1						1										
																																											1																																																		

																																																																						1										
																																																													1																
													5																																																																		
																																																												1																		
													1																																																																		
																																1								1																																																	
																									4												1																																																					
																																								1														1									4																		
																																								1																																																
																									1															1											1											1																			
																									1															1				1																																										
																										2																																																													
																																																																								1										
																																																																											2								
																																				1																																																						
						1																																																					1																								
																																1																																																									
																																				1				1																																						2						
																																								1																																																	
																																				1																																																					
																																																																1																			
																																																																				1																
																																				1																													1																
																																		1																																																								
									1																																			2															1																							
																																								1																																																
																																								1																																																
									1														1																	1			1	1																																																		
																																																																													2							
																										1						1				1																																																												

										1																																		1																																	1	2	
								1																																1																																								
																																							1																																										
																																			1				1																																										
																															2																																																		
								1																																1																	1													10										
																																			1																																														
								1																1																																																									
										1																								1	1				1																																										
																														1			3																																																		
									4																																																																								
								1	1																																																																								
																																			1																																														
																																							1																																										
																																							1														1																											
															1																								1																																										
																																				1			6																															1											
																																							1		1																			1																					
																																											1																																						
																														1																																																		
																																							1																																										
																																							1				1																																						
																																		1																																															
																																							1																																										
																																																																														1	1	
																																							1																																										
																																							1																																										
																																																												1																					
																																				1	1																																											
							1	8		1	1		4	1							4				1					1	1				1		4		1				2							3			2			3	2			1										1						1				
																					1																																																												
																																																														1		1																

																																																																				1													
																																			1				2																																										
																																			1				1																																										
																																										1																																							
																						1																	3																																										
				1																															1										1																																		1	
				2																														1														1																																
				2																					5					1				3																				1																										
																																			1																																																	
1																														1			2																																																			
																																										1													2																								
																																																						1																									
																																																						1																									
																																										1																																					
																				1																																																														
																																																		1																														
																																																					1																										
																																																	1																															
	1																																																					13							1																		
1	1								4					1											2						1																								19															2										
									7					2											2						1				1								3						1	2					16							1								2										
													2	1											2	1									1																				3																									
																																																						5																									
									1												2				2																													4															2										
									4																																													23															3										
																																							1																																										
																																			1				1																1					4																				
					2																																						1																																					
									2																8	1													1																				7		1	1																		
																																																	1																															
																	1																																								6																							
1																																												1																																										

					1																														1																													1																
																																																															1	1																
																																							1																																									
																																																										1																						
																																																																															1	
																																																																															1	
									1																														4																																									
																																	1		1																																											1	1	
										1																																																1																						
																																																																															1	
											2																																										1																											
																1																																														1											1							
																																																																									1							
																									1																																					1										1								
																																																														1																		
																																																																									1							
																																																																									1							
																																																														1		1																
																																																																															1	
																														1																									1																									
																																																																1																
																					2																																																											
																																							1																																									
																																																							1																									
																															1																																																	
																			1																																						1																							
				1																																																																												
																								1																																																								
													5																														1														2																							
													3																																																																			
													1																																								1																											
																																			1																																						2							

																																																																									2							
					1																														1	1																1																											
																																			1																																																		
																																							1																							1																		
																																							1																							3																		
1																																																																																
1	1																																		1				1																																																
																														1										1																																															
																																			1					1																																															
																																							1																																																	
																			1																																																														
																															1																																																				
																																																					1																											
																																							1																																																	
																																															2																																	
																																			1	1			1																																																	
																																			1																																																		
						4																																																																									
1							1																																																						1	1																	
															1																							1													1																											
																																			1																																													1	
								1																																							4																									
																																																																				1										
																															1																																																				
																																																				1																	1										
								3																									3																																							2										

																																					1															1									1		1																
																																																								1																					
																																				1																																																			
																							2																																																															
																							3																																																															
																															1																										1																						
																							5																																																															
																																				1																																																			
1																							3																																																		1			1											
																					3																		1																																																
																																									1																																																	
																																												1								1																																		
																									3																																																												
		1																																																																																
																																	1																																																				
																														1	1																																																					
																																									1																																																	
																																																								2																									
																																				1																																																			
																																									1																																																	
																																												1																																																			
															1																																																																	
																																																	1																																					
																																																													2																				
																																											1																																																			
																																	1																																																				
																															1											2																																																
																									1																																																												

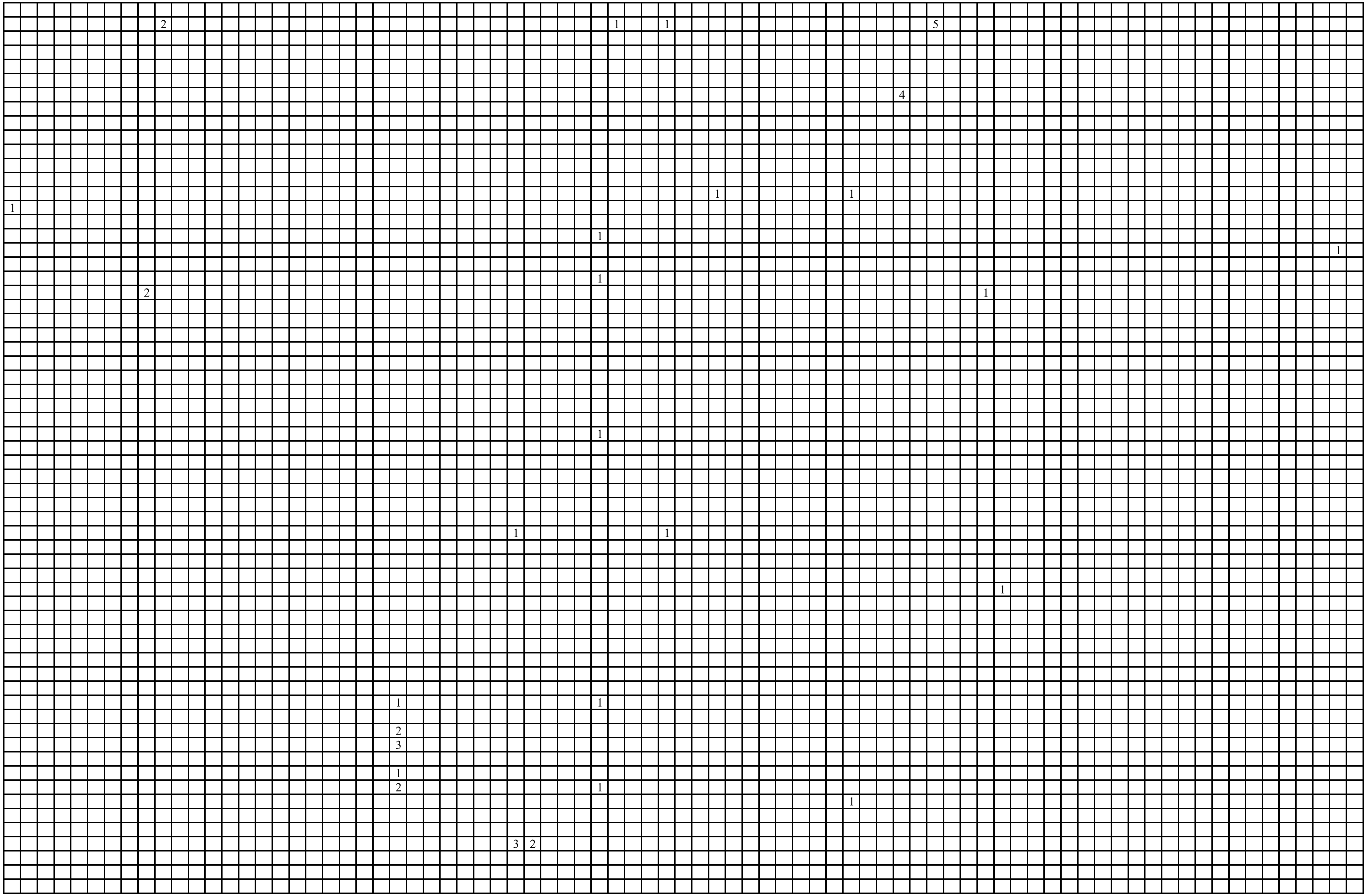
2 1 1 5
4
1 1
1
1
1
1
2 1
1
1 1
1
1 1
2
3
1
2 1
1
3 2

				1																																																	1																											
																				1																																																											
																																				1																																														
																																				1																																														
																																				1																																														
																																																												1																				
																																		1																																																
																							2																																																														
																										1																																																									
																																																																1																
																							2																																																														
																							4																													1																												

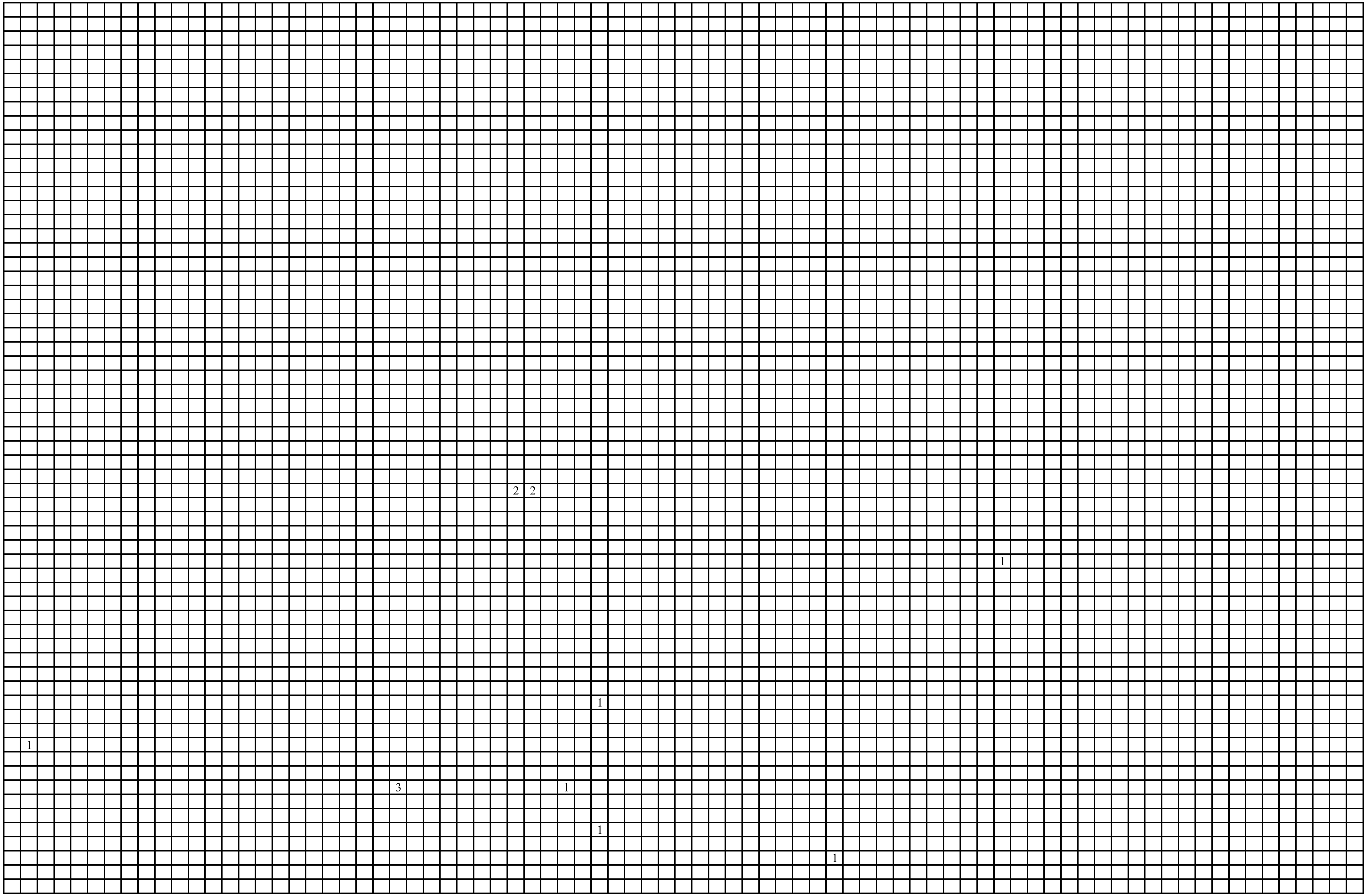

2
2
1
1
1
3
1
1
1

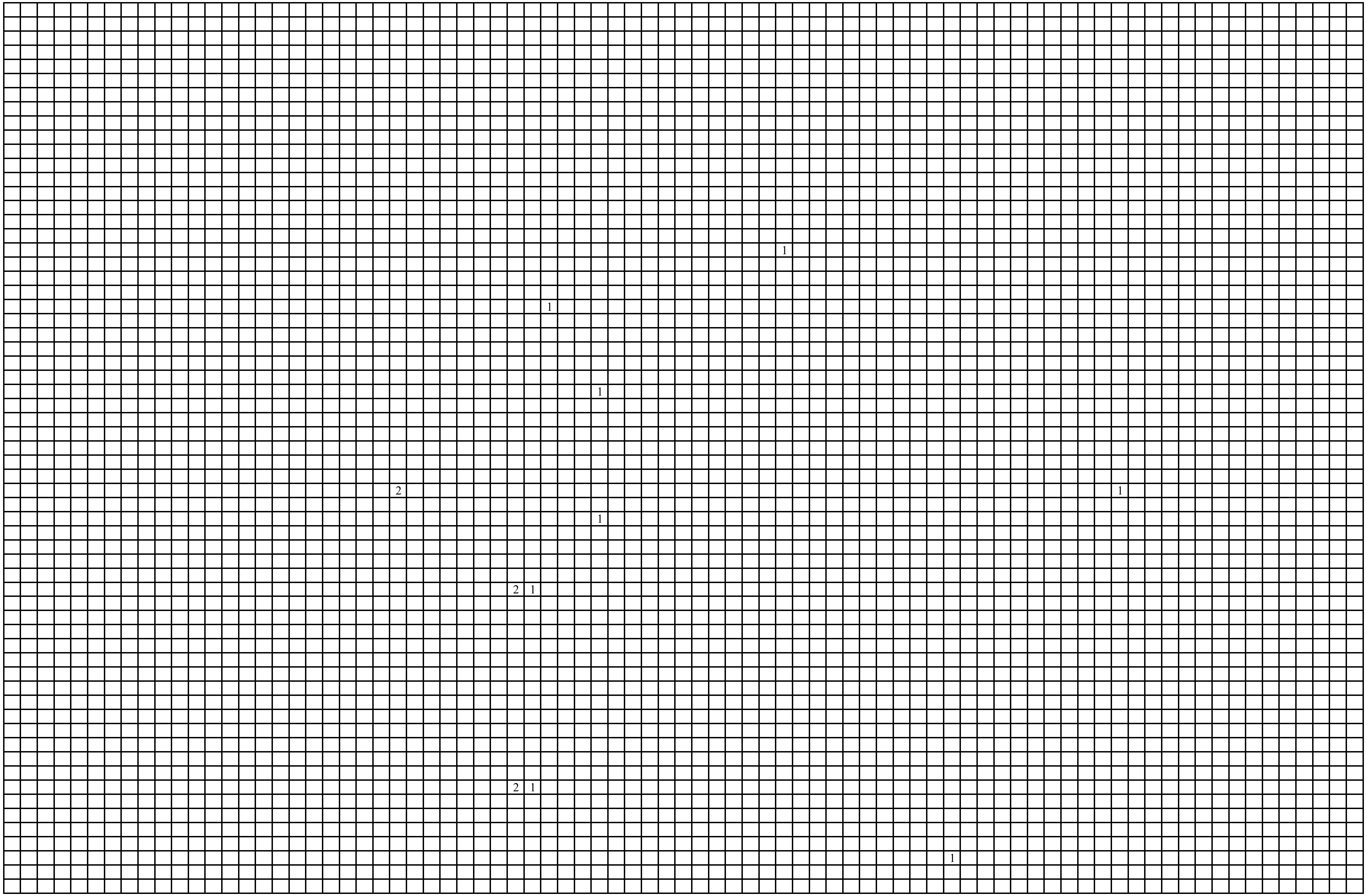
1
1
1
2
1
1
2 1
2 1
1

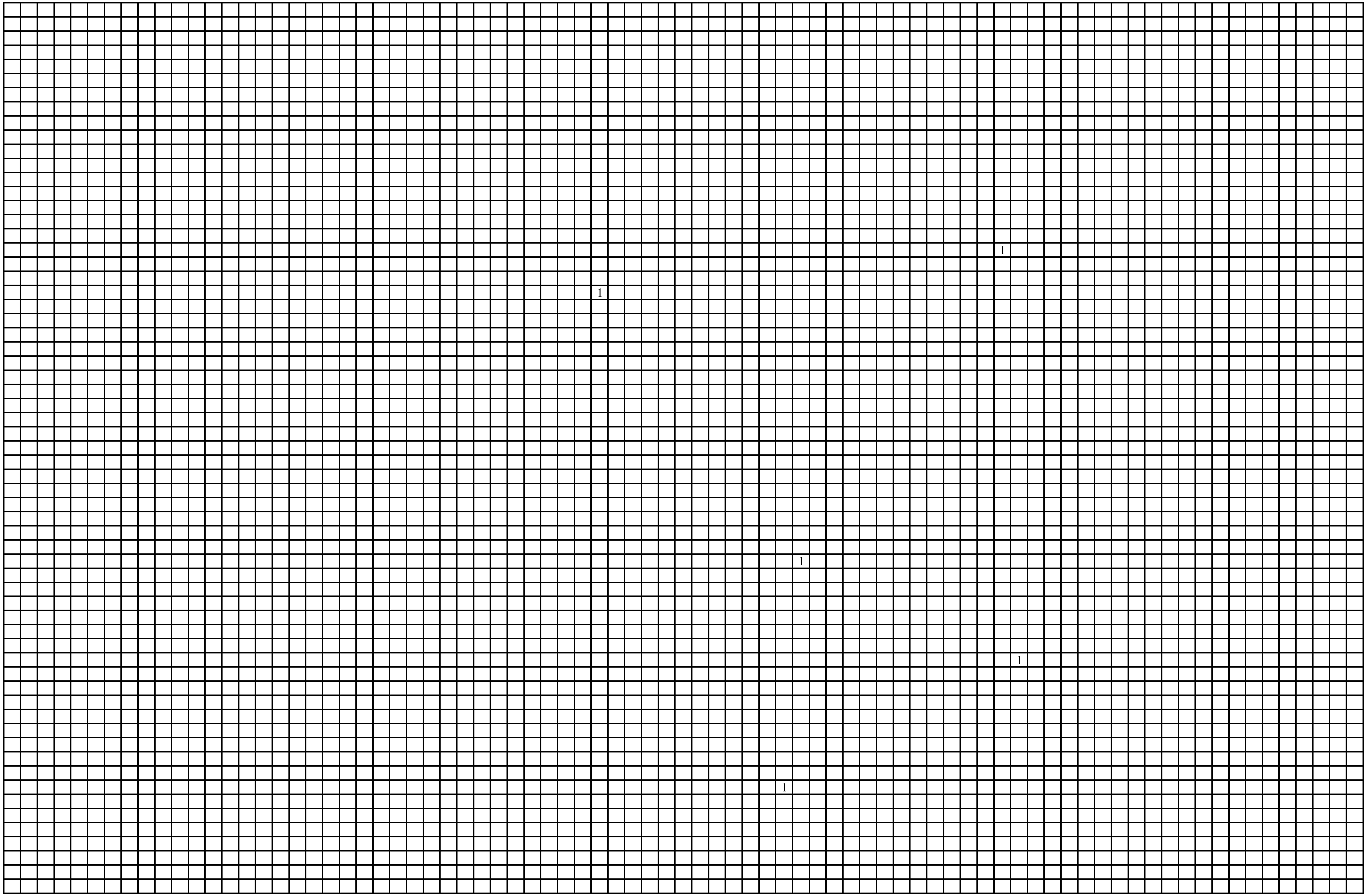
1
1
1
1
1

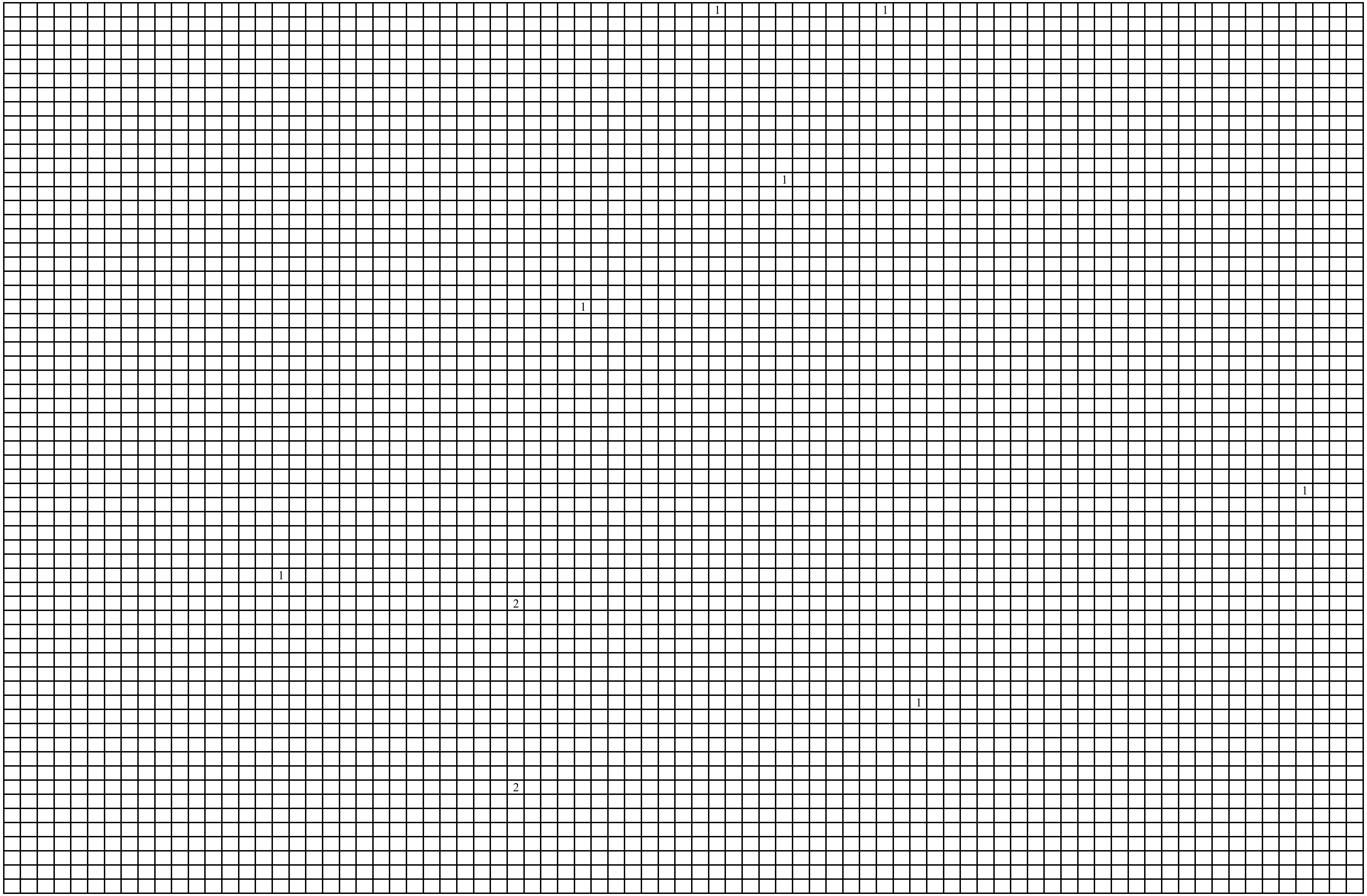
1
1
1
1
1
1
2
1
2

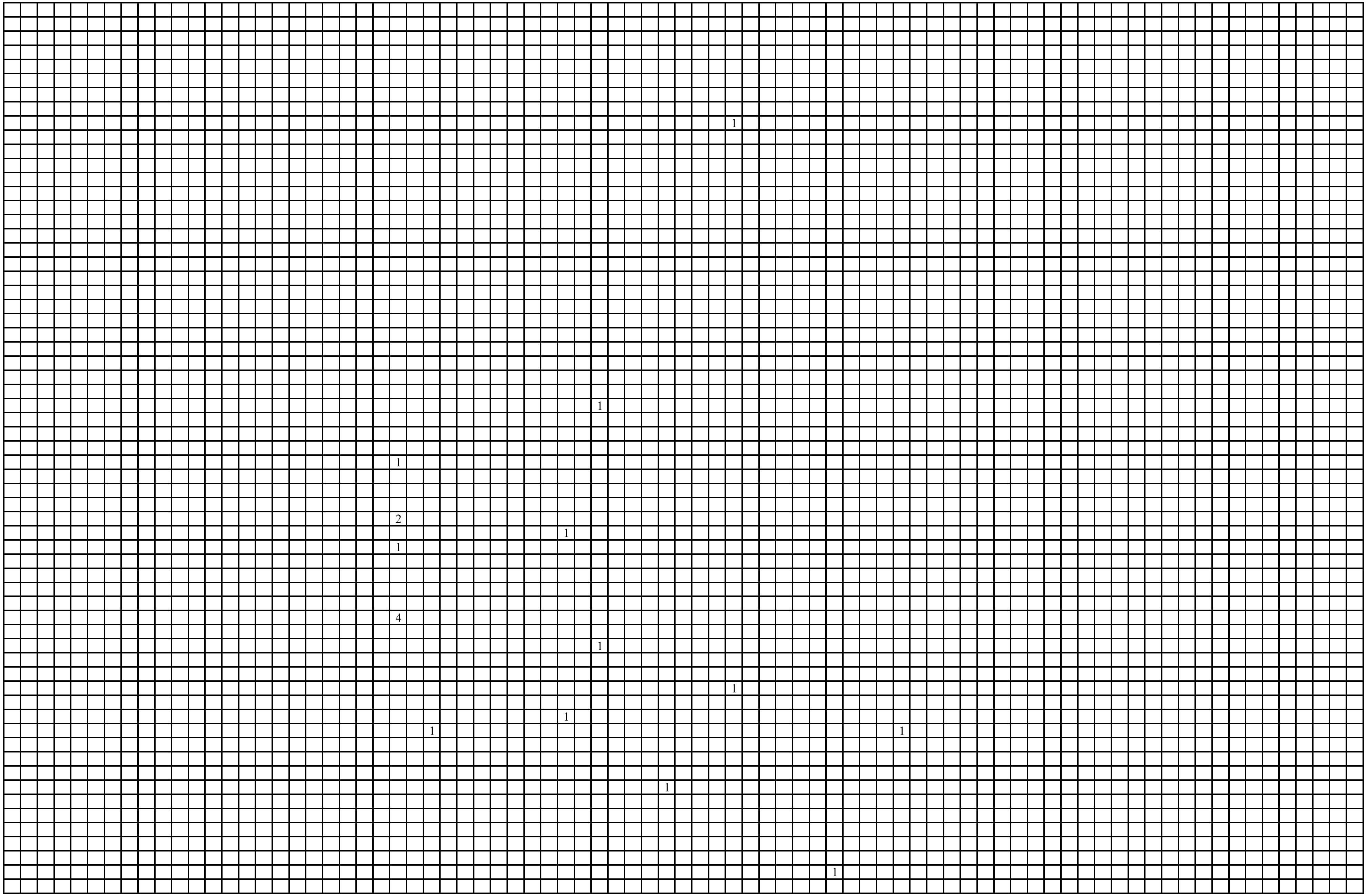
1
1
1
2
1
1
4
1
1
1
1
1
1
1
1

																																	1																				4																									1	
																																																				1																											
																									1							1																				1																									1	
																																																				1																											
																																						1																																													
																													1																																																							
																																														1											1																					
																																						1																																														
																																			1																																																		
																													1																																																								
																																																												2																				
																																																1																															
																																																					7																											
																																						1																																													
								2																																																																										
																								1																																																				1								
																																			1																																																		
																																																											1								1														
																																																							1																											
																													1																																																									
																														1																																																							
																																			1																																																		
																																			1																																																		

																																												1																											
																																		1																																													
																																						1																																										
																																		1																																														
																																						1																																										
																																		1																																														
																																		1																																														
																																														1																																	
																																		1																																														
																						3																																																												
																																																										1																						
																																		1																																														

																																						1																																															
																																										1																							1														
																																	1																																																	
																																			1				1																	1																								
																																			1																																																
																																							1																																														
																																			1																																																
																										3							1	1																							1																										
																																										1																				1	1																	
																																			1				1																																											1		
																																																						6									1																		
																																							1														1																											
																																																						1																											
																				1				1															1				1										1																											

																																							1				1										1																											
1																																											1										1																											
													2																						1																																													
					2						1						6								1		1				1																							2							5			1																	
	1		1														1		6					1		1				2	3			1									1					1		1		1	2			3	2			18	1			1						1			1					1	1	
			1		1		1												1					1	1				1		1				1							1						1		1		1	1				1			3		1											1					1		
																														1	1																																																	
																													1																																																			
		1							1																														1																					1		1										1								
																																																												2																				
					2																																																																											
																																																					1																											
																																																														1																		
																																			1																									1																				
															1																																																																	
																																	1																																															
															1																																													2																				
																													1																																																			
																													1																																																			
																													1																																																			
																													1																																																			
																														1	1																													1																				
															1																																													1																				
																																																		1				1						4																				
																													1																																	1																		
																													1																																																			
																					1									1																											1																						1	
																																									1																																							
															1																																													1																				
1																							2		2																														2																									
																																			1																																										1			
																													1																																																			
																																			1																																													
																																																									1																							
																																							1			1																																						
																																																																																1
																																			1																																													
																																							1														1																											
																																							1				1										1																											
																																			1																																													
					1				1										1																1				1			1								1		1										1	1															1	1	

																																																					1																											
																																																									1																					
																																																								1																						
																																					1																																														
																																																						2																									
																																				4																																															
																									1																																																															
																									4										1																											1																		
																																																																											2										
																																								1																		1													1										

																										TENM2	0	8
																										TIAL1	12	143
			1								2	1	1				1						1	1	1	TLE3	162	428
																										TOB1	6	78
																										TRADD	4	198
																										TRAK1	3	88
																										TRAK2	4	121
																										TRIM6	3	77
																										TRIP13	17	366
																										TRIP6	47	436
																										TXNIP	22	319
																										UBE2D1	12	858
																										UBE2D2	18	908
																										UBE2D3	8	803
																										UBE3A	97	885
																										USP34	34	149
																										VGLL2	1	2
																										VGLL3	9	70
																										VGLL4	16	47
																										VPS36	1	83
																										WASL	2	162
																										WBSCR22	8	61
																										WDR75	14	128
																										WDTC1	3	207
												1														WHSC1L1	22	156
																										WTIP	2	18
																										WWP1	56	437
														1												YWHAB	69	1815
																										ZCCHC12	7	48
																										ZMYND8	69	299
																										ZNF587B	2	8
																										AATF	29	387
																									1	ACTN4	28	394
		1	1		1						1															AES	166	573
																										AJUBA	16	117
																										AKT1	90	1137
																										ALYREF	34	460
																										ANKRD2	7	19
																										ANXA4	4	44
																										APBB1	10	284
											1															ARHGEF5	5	96
																										ARRB1	16	418
																										ARRB2	22	639
																										ATAD2	15	70
																										ATF7IP	42	112
																										ATN1	30	212
																										ATXN7L3	10	192
																										AURKB	32	1010
																										AXIN1	19	375
																										BASP1	11	283
																										BCAS3	13	105
																										BCL10	3	218
																										BCL9L	18	52
			1									1					1				1					BCOR	110	449
																										BEND3	28	123
																										BIRC2	18	600
																										BIRC5	2	232
																										BLM	54	409
																										BMI1	72	957
																										BRCA2	30	530
																					1					BRD4	178	2367
																							4	1	1	BRD7	96	706
																				5						BRMS1	36	155
																										BUD31	12	113
												1														C14orf166	20	214
												1														C1QBP	72	1022
																										CALCOCO1	10	111
																		1								CAMK2A	12	293
																										CAMK4	1	58
											2															CBFA2T2	19	62
											4	4	2													CBFA2T3	48	111
																					1		1			CBX3	185	890
															1											CBX4	29	386
																										CBX5	120	639
																										CBX6	34	365
																										CBX7	4	140
																										CBX8	32	389
																										CCAR2	53	383
																										CCDC85B	21	191
																	1			1						CCND1	54	515
											1															CCNH	14	159

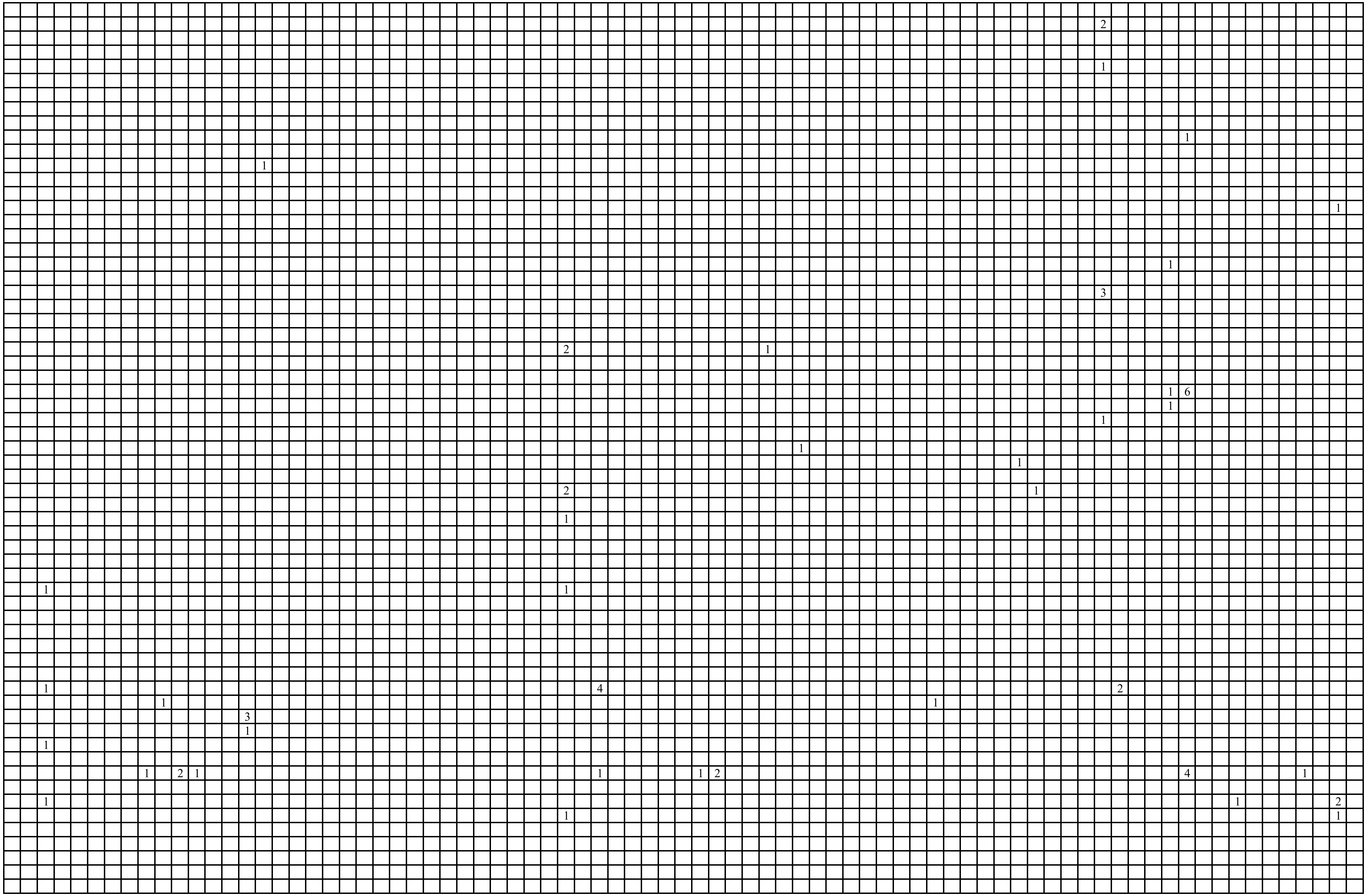
2
1
1
1
1
1
3
2 1
1 6
1
1
1
1
2 1
1
1 1
1 4 2
1 1
3
1
1
1 2 1 1 1 2 4 1
1 1 2
1 1

															1																		1							1																																								
																																																														2									4									
																													2																								1					1		1											6	1	1		1	1			1	
																											1																																																																			
																											1						2																																															
																											4																																																					
																											1																																																					
														3																																																																		
																																			6																				3							2									1									
																																																														2																		
	1	2					2	1	1					1	1						2	2		3			4		1		3	2			1	3						1	4			1	2	3	1				1				7			4	1			2	2						4		1			1	11			2
	1																										1		1		1					1											1											4			1		1	1	1		1				1	1	1			1	1			
																																									1			1																																				
																																		1								2																		2	1																			
																																										1																																						
																											3																																																					
																																																								1																								
																																																															1																	
																																																																					1											
																																																												1																				
																																																												1																				
																																	1																																		1													
		1																									1																																																					
																																																									1				1																			
																											1																						1																														1	
																																																												1																				
																																															1																								1									
																																									1																																			1				
									1																									1																																														
																																									1									1							4			1												1							1	
																																																							1					2																				
		1																																																																													1	
																										1																																																						
																																			1																							1																						
		1																																																																																
											1																						1																												1		1																	1	
																																	1																																																			
																																																												1						1														
																																																																		3														
																																																																							1									
												1																																																1						3														
																																																																		1														

																																																										1		1							1													
																																			1									1																	1							1													
	1	1											3							1								1			1				1																			1	1						1			1	1	1												1			
	2	3						1													1					2				1	2				2										1		1				2			1		1	2	1			2	2						2		1			1	1		1	
																																																		1																												1		
																																																																														1		
																																																																													1				
												2																			2																																																
																																			6		2		1		1	1					1											1			1				5										2		1				
																																																																		1															
																																										1					1											2		1	1				1									1		1				
														3																													2											7							1																		
																																												1																																				
																																												2																																				
																															1											2																	1																					
																													1																								1					2												6				1	2				
																																																																																1	
																													1																																																			
																																																																						3									
																																																																						1									
		1																			2	1					1				1					1									1																										1							
		1																																																																														
		1																																																																										1			
		2																																																			1																								1			
																																	1																																																
																																																																	2															
									1																																																																								

															1																																																																	
																																																																	1															
		1																																																																														
																																																																													1			
		1																															1																				1								1																			
		1																											1																																																			
						3																					4																															1		2		1																		
																																																												3		1												1						
		2																															1																				1																											
																																																2																																
																																	1																																															
																											4					1			6																										1																			
																											5								2						1																																							
																											1								3																																													
																											1																																																					
																																				1																					1								1															
																																	2																																															
																																																						1																							1			
		3																															1																1				1																								1			
																																																										1																						
																																										2																													1									
									1																																																																							
																																	1																																															
																											2																																																					
		1																																															1				1								1																			
																											1	2											2																					1																2				
																																																												1																				
																																																												1																				
							1			1																																																		3											1	3								
																																												2																																				
		1																																																																					1									

														2																																																										2								
																																																					1																												
																																																																							2		2							
		1												1																			1																			1	1						1																				
		1																															1																			1																												
																																																																														1	
																																																																											2				
																											1						1										1								1																		1								
		1																															1										1								1							1	1																		
																																									1	1																																					
	1							1							1																																																													1			
										1																																														1		2											10		4							
																																																																							5		2							
																											1						2				1										1																												1				
																																							1																																								
		1																																				2																																								
															1																																																	1	1						1								
																																																																								3							
		1																															1																				1																											
																																			1																																												
																																																												1																			
																																																												2										1								
									3																								1																														1																		
																																																												1																			
																																												1																																			
									4																																																																						
															1																																																																	1

																																1																																													
									1																																																																				
		1																																																																													
																								2																																																									
		1							1																							1																																														
																																	1																																									1						
											1			1															1			1																1															1																	
																																																		1																												
																																																		1																												
																																																							1				1																			
																																	1																		1	1				1				1																			
																																	2																			1								1																			
																																	1															1				2									1																			
		1																																															1			1					1				1																			
																																																	1													1																			
																																																																										1							
		3																															3																				1																											
		2																															1																																																	
		2																															2																																																	

								1																									1																1																															
	1															1																						1																																												1			
																																				1																												1																
		2																																																															1															
		1																														1												1								1								1																			
		1																														1																				1																								1			
		1																														1																			1	1								1																1			
		2						1																								2																																															
																																	1																																															
		1																														3												1							1									1																			
									1																																																								4						1									
		1																														1												1							1																												
		1																														1																1																															
									1																																																																							
																																																					1																											
																	1																1												1																																			
																	1																1												1																																			
																																	1																																															
								3																									1												2																					1													

		1																															1																1				1																											
																																																												1																				
		1																															3																																													1		
		3																															1																																															
																																	2																																															
																																																																							1									
		1																															3																																															
																																	1																											1																				
																																	1																																															
																																																												1																				
																																	1																																															
																																																														1																		
																																																														1																		
																																	1											1																2			1		1						1									
																																																												2					2						1									
																																																												2											1									
																																													1																																			
																																																													1																			
																																																											1																					
										1																																																		1																				
																																																												1											1									
														4																																														2	3																			
																																																																							1								1	
		1							1																		3		1																		1							1								1									2		1				1			
																																					1										1															1																		
																																																													1																			
		1																															1																				1																											
																																	1																				1								1																			
	1		2													3														1			1		1		2		1		1														1		2					1			1				1						1		1		1				3		1		
																																																																	1															
																								1																																																							

																								1												1																																															
																																	3											1									1														1									1	1			
		1																																																		1																								1			
		1																																																		1								1																				
																																																											1	1																			
																																				1																																												
		3																															1																																										1			1						
																													1																															2					4																
																													1																								1							1										1										
																													1																														8																				1		
																																																												2	8	2									1									
																																	1																																																
										1																																																												1								
														1							1											1																												3	1										1								1	
																																																					1																													
																													1							1																								3	2										1									
																																																				1							2	1				2		1				2		1		2					
																																																																	1						1									
									1																											1		1														1													1															
		1																																																																					1									
																																														1																																		
																		1															4																																																		
																																			1																																													
		2																															1																				1																														
		2																															1																				1							1																				
		2																					1																															1		1			1		1																				
		2																															1																				1								1																			
																																				1			1			1																		3																				
																																																												2																	1			
																																										2																																										
																																																											1											1									
																										1					1					1					1	1					1											1		2	1															1				
																																																																																	1
														1																					1																																																	

		1																																																								1		1										1										
												1											1			1	3								5																																																
														1																																																																		
																																																											1																						
																																																											1																						
																																																																														1			
																																1																														2																				
																																																																									1									
																										1																1																																											
																																1								1											1								1		3																				
																										1																																																										
		1																																																																															
																																	1																									1																						
		1																																																																					1									
																																																												1		2														1						
																																																												1															1								
		1																																																								1																						
	1							1														1					1									1											1			1					1					2				1	4															
																						2																																																											
																																													1																																						
																																				1																																																	
											3	5																																																																					
											1	3	5																																						2																					1								
																																																														1																				
		1																									1																																1		1																				
																																																														1													1							
																								1																							1								1																1					1				
		1																									1																																																									
																																																																											1						
																																																																									1										
																																																														1																				
																								1																																																						1			
		1								2		2		22	1							1				1	1				3	1			1	1	2				2			1			1	6		4			2				15	4	8	6	3											1							1	2
																										3																																																								
																										1																																																						1	
		1																						1									1																				1								1																			
														1																																																																					
		1																															2																				1																											

		1						1																									2												1							1	1																											
														1																																																																		
																																																										2			1																			
																																																										2																						
																																																											1																					
												1	1																									2									1															2																		
																																																							1																									
																																	1																																															
									1	1	1	1	1																		1	1	4																													3																	1	
																																																															1																	
																																																						1																										
																											1																											1																	1		1							
																											1																																																																																																									
																																																						1														1			1		2							
		1																																																																														
		2																								1							1																																				2	4										
																										2										1																																												
																																																												1																				
		1																																																																				1										
																																																						1						1											1		1							
																																																					1		1																6		11							
			1																							2		3																																																			1	
																										2									2																								1																					
			1																			1				6					1		2		1																								2																					
			1											1												1							1		1	1								2						1																														
			1																							2																																																						
																										3																																																						
																										4									1													1																																
			1																											1																																																		
				2																												1										1																		1							2													
																																																												2																			3	
											1																																																1														1							
																										1																																																						
		1															1																																																															
		3																																																																														
		1																															1																																															
																																																																1																

																																	1																																														
		1																															1																			1	1							1																				
		2															1	6																																			1								1																		
																	1	1						1										1																																													
																1	1	4																																																													
																	1	4															1																																													1		
											1																																																																				
																																	1																																											1				
																																										1												1	1																									
																																																													1	1																		
																																		1																												1									1									
																																												1																	1																1			
																																	1																											1																			
																																																					1								1																			
																																	1																																												1			
		1																															1											1									1							2																				
		1																																																											1																			
						1																		1																																	1																							
		1							1																																							1												1																				
		1																															1																				1	1							1																			
																																																																							1									
														1																																															1																			
														1																																															2																			
														1																					1															1																														
																																	1																				1								1																			
																																																																							1									
					1			1			1								1																												1													4	2		1																	
																																							1																																									
																																																						1			1	1																						
		1																																																																					1								2	
		1																																																			1							1									1	2	2		1							

		1																																	2																		1							2														1						
															1														3													1					1						2	1					1	2																				
																													1																																																			
																													1																																								1										
																	1							1											4												1											1		3	4																			
		1												3																					1							1																		3	6																			
																																														1						1																											
																							1					1																																																						
																																														7																																		
		1																																																																														
									1																									1																																																
																																																												1											1									
																																																																								1									
																																													1												1																							
									1																																				1												4	1			1																			
																																													1																																			
																																																																								1									
																																																						2						2												2		1							
																																														1												1																			
																																																																						1													
				1																																																																												
																																	1																																																1		
											2																																																																						
		1																							2																																																								
		1																														1												1						1	1							1																			
	1																																1																																1															
																																																																											1		2							
																																												1									1				1																							
																																																							1				1				1																1			
																					1																																1	1			1					1															1			
																										4																																																									
		1																														1											1									1																											
																																	1																																																
		2																							12							1																				1							1	1															1			
																																													1														1																			
																					1											1										1								1																							1			
																																																										1				1	1																		
																															2				1								6				1					3						1														1						1	
																																			1																																														

		1																															1												1							1	1								1																			
										2																																																						1													
										1																									1																																								2										
																																										2																																														
																																																						3																									1		
																																													1																																				
																																		1																																																		
														1																																																																			
									1																																																																						
																																																								1		1																				
																													1				2																			10																										
																																															1										1		1													1						
		2							1																							1	1																																						2							2	1		
																																				2																																																	
		1																																																																																
																																																						1																											
																															1																																																		
																																																											1																						
																								1									1		1																																																				
									1																																																																						
																																																																											1						
																																																																1															
		1																																																																																	
																																		1																																																		
		1																														1																			1																											
																																		1																																							5	2										
																													1																																																							
																																		1																																							4	1						1				
																																																																								1									
																										1																																																										
									1																								3	4																							1			2		1		1	1															

																													1												1			1														2		1	1										1	1								
																																																									1																							
																																																											1														1						
																																																																									2				
										1																							1																									8															1					1		
																																		2																																											1						
																																																1																																	
																																																									1																								
																																		1																																					3										
																													1																								4																				1							
																																																																							1		1							
																																															1																								1			1						
																																																																						1		1								
																																																										1					1																				
																																																										1																									
									1																								1																																															
																																																																						1									
																																																																							1		1						
																																																																									1						
															1																																																							1									
																																																																										1						
																																																																								1							
																																																																										1						

																																																																							1	3		1						
																																																																							1									
																										1																																																						1			
																																														1						1																		1									
																																																																											1		
																																																																								1							
																																																																							1			1						
																																1																																																	
																																																																														1		
															2																																																												2						
																																																																										1						
																																																																						1									
																																																																						1		1							
					1																																																																													
																																																																							1									
														1																																																												1								1		

																																																																							2		2							
																																																																									1							
																																																																										1							
																																																																								4		1							
																																																																								4		3							
		1																																																		1							1											1									
																																																																											1						
																																																																											1							
																																																																								1		1						1	
																																1																																																	1							
																																																																											1							
																																																																																1		
																																																																								1			1						
								2									1																1																																																						
																																																																								1									
															1																																																												1							
		1																																																																							1									
														1																																																																			
																																			1																																																							
									3																																																																										
																														2				1														1																																									
																																															1																																									

																																																																				1									
																																																																												1		
																																																1																														
																																																																									1							
															1																																																										1							
																																1																																																		
																																																																							1		1							
																																																																								1									
															1																																																																		
																																			1																																3															
															2																																																																		
																								1																																																												

																																																																				2							
																																																																									1		
																																																																		1		1							
																																																																		1									
																																																																		1		1							
																																																																				1							
																																																																		1									
									1																							1	2																																																
									1																																																																							
																																																																				1							
																																																																				1							
																																																																				1							
																																																																		1									
																																																																		1									
																																																																				1							
																																																																				3							
																																																																					1						
																																																																				1							
																																																																				1							
																																									1																							1															
								1																																																																								1
					1																																																																												
															2																																																																

																																																																							1							
																																																												1						1		2							
																																		1																																																
																																																																							1						
																																																																								1						
															3																																											1						3																
																																																																							1							
																																																																							1							
														1																																																																		
																																																														1						1		1							
															2																																																									3					1		
																																																																									1						
																																																																						1		1							
																																																																						1									
																																															1																																						
									1						2																																								2																		1							

																																																																						1		1							
																																	1																																																		
																										1																																																												
																																																																						1									
																																																																										1						
																																																																						1									
																													1																																																							
		1																										1																																															1							1
																																																										1						1															
																																																																	2															
																																																												1																				
		1																																										1																																								
		1																									2																																																										
		1																																																																																		

																										1																																																							
																							1																																															1					2			1	
																																	3																																												1								1	
																																																																											1		
																																																																											9	
																																	1																																1							1									
								1																								1																																												1						
																																																																					1									
																																									1	1																						1							1									
																																				2																														1						1									
																																																																						3		1							
																													1																																																	1									
		1																																																			1																											
		1																														1																																																		
																																																																					3	1	2							
																																																																										1			
																													1							1											1									1																	1							
																											4																																				1											1									
																								1																																																					2							1			
		1																																																																					2										
																																																																										1							
														1																																																																			
		1																																																																						1									
																													1																																																											
																																																																							2		1							
																																																																						1			1						
																																																																													2			

																																1																																													1		
														1																																																																
																																														1																																					
																																																																								1							
																																																																						4		5							
																													1			1																								1																							
																																1																																															
																																																																														1	
																																																					1				2			1											1									
																												1					1																																				1									
																												1																																1																					
																																																																									1							
																																																																					1															
																																																																															1		
																												2																																4																					
																																																																														1			
																										1																																														1			1						
																																																																											1									
																														1																																													1						
																												5																													1		2																					
																																																														1																					
																																																																															1				
																																																												1																							
																																1																																															
																																																														1																					
																																																																											1								

																																																									1																						
																																																																1																
																																					1																																																			
																																																																										1				
		1																																																									1																			
		1																																																								1																				
																															1																						1					1		1																				
																																																										1																						
																					1							1																																																										
		1																														1												1														1	1																				
		1																																									1								1																												
		1																																									1								1																												
									1																								1																																														1							
																																																																	2																		
																																																																												1							
																																																																										2		1							
																																																																															1		
																																																																										1									
		1																																																																															
																																		3																																																
		1							6																								1		1																		1	1							1																			
		1																																1		1																		1					1			1																			
		1																																1																				1	1				1																		1				
																																					1																		1				2		2																				
																															1																																					1																		
																															5																									1					2										1										
										3																				1																																			4																				
										3																				4																																			1																			1

																												3																								1	1						4																				
																												3						1																			1						2																				
		1																										1																5																								1											
		1																																																				1						1																				
		3												6	1														2			1	4		5										1		1					1	4					5		3	1	1		1									2				3		5	1
	1	2							1				10								1		2			1		2		1	2			1	4				1		3			1				4		1	3				1				1			1	1						1	1	2			1	2		6	2
	1								1	1				5								1		1		2	1		1		1					1				1			1	1				1			1				1			1	1		1	1	1						1	1				1				
																				1																																																												
		2												6								1			1						1								1		1												2										1																	
														1																																																																	
														3																																											3																							
									1																																																																							
											1																																																																					
																																																																															1	
															1																																																																	
				1																						2																																		1																			
														4										1						2		3	3																																						1							
																																																																																1
																																		1																																													
																																	1																																														
																																																																															1	
									1																																																																						2	
								1																							2																																															
																																	1										1								1			1	2			1																			
		1						1					1								1						1		1																															1	1	1						2		1				1			
																																													2																																		
																																																																									1							
																																			2																								1																				
																																																												1																				
																																		1																																													
																																			2					1																																							
														1																																																																	
																																				1																																											
		1																																																																													
																											1								1																																													
														1																2																																																	
																																															1																																
																																												6																				1							2	3							
																																																						2																1					1				
		1																																																																													
																													2																														1					1															
										2																		3																		1						1							2																				
																																																												1																				
																								1					1											1																	3			2			1		1					1	3	1	3			1				

			1						1													1													4									8																										1		1			1				
																																	1																																																			
									1																								1												1																																							
																																	1																																																			
		1																														1			1																									1											2									
																																			1																																																		
																												1																1																											1									
		1																																																																																
		1																																																																																
																																				1																						1						1																

																										CCNK	24	128
																										CCNT2	11	96
																										CDC73	27	489
																										CDK12	14	185
																										CDK13	10	206
																										CDK5	15	382
																										CDK5R1	1	116
																										CDK5RAP3	5	235
																							1			CDK7	39	734
																										CDKN1B	4	517
			3							1																CDKN1C	10	82
																										CDYL	13	139
																										CENPF	8	158
																										CEP290	4	236
																										CGGBP1	11	60
											1															CHCHD2	25	263
												1														CHCHD3	11	319
																										CHEK2	25	346
																										CHMP1A	29	148
																										CHUK	47	524
																										CIR1	4	90
																										CITED1	4	42
																										CITED2	15	36
																										COMMD1	29	272
																										COPS5	95	1586
																										CRTC1	4	46
											2															CTBP1	241	716
																										CTBP2	171	536
																										CTDSP1	9	147
											57	1														CTNNB1	283	1883
																										CTNNBIP1	1	60
																										CTR9	27	300
																										CXXC5	7	23
																										DAB2	7	188
																										DACT1	4	39
											1	1														DAXX	118	726
																									1	DCAF6	28	288
												1														DDX17	47	474
																										DDX39B	31	754
			1								1															DDX5	71	598
																										DDX54	39	424
																										DEPDC1	2	44
																										DGKQ	1	14
																										DHX33	7	78
																										DNAJA3	32	413
																										DUSP26	4	64
																										DVL3	64	338
																										EAF2	8	57
																										EAPP	3	128
																										EDF1	21	90
																										EGFR	91	3149
			1													1										EHMT2	131	411
																										EID1	5	90
																										EID2	7	28
																										ELL	17	135
																										ELL3	10	53
																										ELP3	7	135
																										ELP4	3	57
																										ENG	9	219
																										ENY2	10	225
																										EPC1	57	163
																										ERBB2	19	858
																										ERBB4	12	254
																										ERBIN	0	0
																										EZR	16	612
																										FAM83G	2	38
																										FGF1	8	96
																			1	1	1					FHL2	153	547
																										FHOD1	5	81
											1															FLNA	33	691
																										FRK	0	61
																										GMNN	22	193
																										GNL3	57	356
																										GTF2A2	2	31
																										GTF2H1	27	165
																										H2AFY2	31	294
																	2		1				1			H2AFZ	66	512
									1																	HCFC1	110	403
																										HCLS1	4	65
			4														6		6	6					4	HDAC1	861	2481
																										HDAC10	10	56

															2																																																															
																								1																																																													
										1																																																																										
										1																																																																									
		1													1																1																																																						
																																																																																	3		
																													1																																																										
																																																		1																															
1																																															1																																		1		
																																																																																		2		
															1																																																																						
								1																															1																									1					1											
																		2																																																																1		
																		1						2					3																																											1													
																													2																																	1										1								1		
																										1																																																													
																													1																																																							1		
																							4	7					9															1			2							1		1											6					1					1		7	
																								2																																																													
																			1																																																																		
																																																																			1													

																		1						1																											1									3																			1	
								1																																																																				4	
																																																																		1													
																																																		1																														
																																																																															2	
																																																																															1	
																	1																																																														1	
3		5	1						2		1	2		1		2			5							1	3		2		4				2		4	3	4	3			2	3												1			2	4	1						3	1	4		1	4		2	2	1	7		11	
1																																																												3																				
			1	1										1																					1		1	1	1	1				1															1								1		1			1			1		1		1	
																																						5																																												1	
																								1																																											2													
																										2												1													1																													2	
																																																																		2									1	2			
																																																																		1													
																																																										1																							
																																																																		2													
																						3							1				2																		1												1																		
																				1	1																																																													
																					2																																							2																		
																																																																															1	
																																																			1																													
														1														1									1																																															
																																						1																			1																					1		4	
																										1																																																								
																			1				4		2									1														2									1																				1	
																																								1																																										
																							1																																																										
																																																																													4			
																																																																															1	
																																																																													2			
																																																																		3												2	1
																	1																				1																																												
																																																																															2	
																																												1																																						
																																																																									1					1	1
																							1																																															1								
																																																																															1	
																																																	1																															

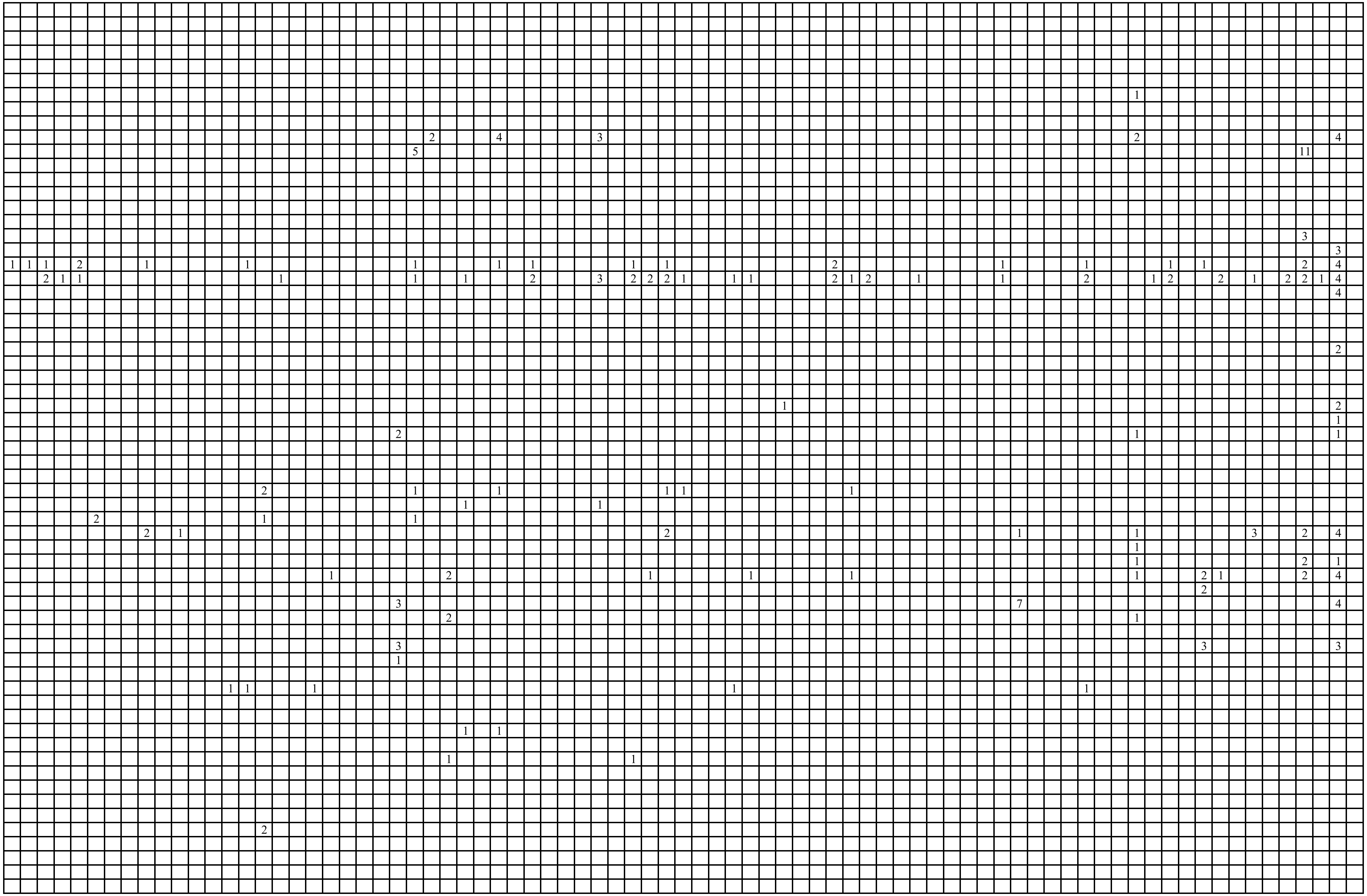

																										1																																													1					
														1																																																															
																										1																																																					
																																																																														1	
																										1																																																						
																																																																														1	
																																																										1																						
																1									1																			2																					1										1		3	
																1									1					1															2																					1										4		4	
																																1																																																
																1							1					4												1																																														
																																																																													1				
																																							1												3																3													
																2		2							2			2																						1																																
																												2																																																							
																																																															1																				
																										1	2		1								1																																				1													
																										6	3																																																											
																																																									1																						2	
																											1																																																									
																																																															4																				
																																																															3																				
																											1																																																										
																																																																								1											3	
																																																																							5											1	
		1																																																																																
1																																																																																		
																																																																																2	
		2																																		1		1																																																1	
																																																																																1	
																																				3																															1														
								1			1																1																					3																														2	

																										4			3																																																		6	
																																																																																3	
																																			1																																																																				1	3
														1																																				1																														
																													1																																																			
																													1																														1																					
																																															1																													2		2	
																										1			3														1						1					1																							
																																																													1																				
1																																																																			1														
																													3					3																																								2			2	
																																																			1																													
1																																														1																																	
																										1																																																							
																										2	2																																																				1	
																								1		13	7										1		1												2																					1							1	
																										9	1																																																				5	
																																																																																1	
																																																																																1	
																																																						1																										1	
																																																																																1	
																																																																																1	
			2																																																																													
1																				1																						1																																					
																								1																																																									
																																																																																1	
																											1																		1																																			
															1																																																																		
								1																																																																									
																																				1																																													

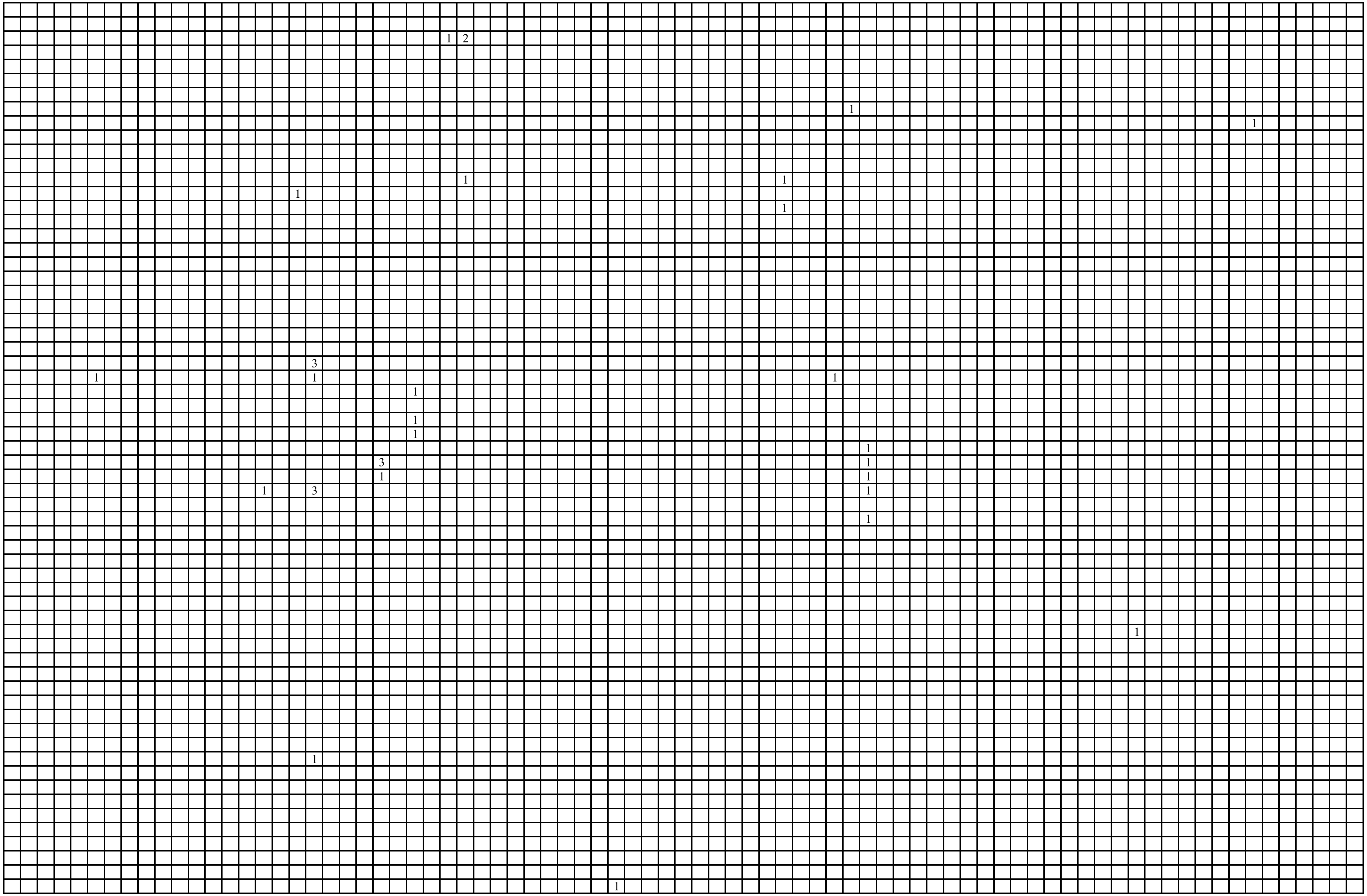
1 2
1
1
1 1
1
1
3
1 1 1
1
1
1
1
3 1
1 1
1 3 1
1
1
1
1

		1										1																																1					1							1																								
																										1																																																						1	
																																																																													2	
																																																																													2	
																																																																													2	
																																																																													3	
																																																	1																															
																																									1											1																					1								
																																																				1																															
																																																1																																				
																																																																			1													
																																																				1									1																				
																																																				1																															
																														1								1		2													1																	1											
																																																														3																				
																																																	2																																				
																														2																																																								1	
																														2																																																										
1																																																																																			

																										1																								1																													
																	3																																																														
																										2																																																										
																	2																																																														
																																																																			1				1									
																								1																																																												
																																																																															1	
																																																																															3	
																																																																			1													
																																																																			1													
														1																																																																		
																																																																			2													
																																																																			1													
																									5	4																																																											3	
																																																																																1	
																																																																																1	
																																																																							2									
		1																										1																				1																					1			1	1			2	
					1																	2					1								1											1																		2		1								
		1																																																																										1			
																																																																												2				
																																																																												2				
																																																																			1								2				2	
																	1									1											1		1					1							1																		1								1		2	
																																							1																																										2	
																													2																																																								
																													3																																																								
																											5																																																												
																																																				1																					1								
																																																			1																													
1		1	1																						1	1		4		1						1		1										2	2													1					1			2							3	
																																																																				1													

																															1																		1																															
																										1	3		3																																																					2	
																																																																															1	
																													26					1																																																			1	
																											1		2																																																									
																											1		1																																																							
		1																								2	1		22						5																																																	
																								1																1										1	1																	1												
1																																																																		3										2			8	
																																																											1									1												12	
																																																																											2			2	
																																																																					1													
	1	1																													1																																																			1	
																																			1																																																1	
		1						1															1	1					1		1				1		1	1	1											1																						1		1	3	1	1				
																															1																			1																									1	1	1			
																																																		1																															
																1													1						3		1		1									1							1													1										2	
																																			2										3																																									
1																																																																																		
																								1		11	6																																																							1	
																										7	2																																																									
																													2																																																							
																													1									1																																														
																																																																															1	
																								1																																																										
																								1																																																								1	
																																																												2							3													
																															1																			1															1													
																																																			1																											1	
																																																																				3													
								1																1		1			1								1	1												1																	1											5	
	1																																																					2	3																									1	
	4		1		1																										1				1											4							4	3																				1				
			1					2							1																							2												3																																		
																2																																																				2										1		
																											1																																																										

																													1																																																							
																							1																																																																
																													1																	1				4											1																			
																																																		1																						1			2					
								1																																									1																															
																							1																																																1																			
																																																																																1	
								1															1																																		1																						
																																																													1																
																																																																																1	
																																							1																																																
																													1						1																																																					
																							1															1																																																				
																									1																																																															
																																																									1																						
								1								1								1												1	1	1	1					2																				1					1			1							5	
																																																		1																														
																																																																					1							1						
					1			1															1																											1																						1								
																		1											1						1																																1											1	
																																																																																	1	
								1																																					1																												1						
																																																											5																3				
					3			5		1							10		2					10		2	1		1		1			1	25		1		4	1				1			1				1			6	2						1	1		1					1			5		6			2		25	
																	2		2																4																																																
																			4																6																																					3										4	
									1																										3																																																					
																			1																																																																				

																																																			1																													
																											1																	1																																				
																																																																										2					
			1	1																																			1																																										
																																																		1																													
																	2		5				1	2			1		1										1																																													5	
																			4				1				1																																																							
														2	1								1							1																																						1		1											
																			9				8				1								2									2																																							19	
																							1												2															1																													
																																																																				1													
																										1										1																																														1	
																										1																																																								1	
														1						1																																																													1	
2																										1	2		1								1		1					1																				1			1												6	
																											2		1																																						2												3	
																																																																																		1	
																																			2		1		3																																														1	
																																																																				2													
																																																																					1													
																																																								1																	1								
																																												1																													1								
															1																																																																				
								1																																																		1			1															1		1	
																			1			1		1					4				2																				2											1		2									1		5	
																			1			4							5			1	2						1														3											1		2		1										2	
							1														1			9					1						3			1															2													2												2	
																								2								1																										1																					1	
																										2	1																																																								
																								3					2			1																																																	4	
																															1	1												1																																							
																								1					1																					9											1																			1	
																																																			2																															
																								1							1				1															21											2						1					2							4	
																																																			7												3																	1	
																																																		2																															
																														2																																																				1	
																																							1					1																																		1			

																																																												1																
																																																		1																													
																																																																									1					
																											1																																																							
																										1			1																																																									
																													1																																																									
																																																																														1	
1						1																																																																											
					1																			1			1																	1																																								
																																																												1							1													
																																																			1									1																				
																																																																									1								
																																																			1									1																				
																													1																																																								1	
																										1																									1																													
																																							1																																																
																																			4																																																		
																																																			1					1																									
																																																			1																													
																																																			1																													
																											1																																																								
																																																																		1									2		1	
																																																																															3	
									1																																																																		1			
																																																																											2			
																			1					1																																																								2	
																																																																															2	
																								1																										1																													
		1																						1													1	2	1							1		2						1														1												
							1									1																								1																																												
																																																																								2								
											3																																							1	1																									3			9	
																																																																												2			4	
																																																		1																													
										2																																																											2											
																			1																																															1					1									1		2	

																			2	1						2																																									1												3	4
												1														9	6																			1																				2	1		1							1			19	
																										3	1																																																				4	
																										3																																								2	1												7	
																																																																												2				
																																																																												2			1	
																															1																																																	
																													1								1		1																												1												2	
																																																											3																					
																																																																															5	
				1								1																																																												3	1					1		
	1			1								1														1																	1																														1							
																																																		1																	1										1		1	
																										5	6																																																				1	
																																																																			1												1	
																																																			2																													
								1																1																1																		1						1																
																											1																																																		4			
																										4	1																																																		6		1	
																																																																													3			
																																																																													4			
																																																																													1			
																																														1					1																										1			
																													2							1																																												
																													1																															1																	1			
																																														1																												1					2	
																										1																																																						
																																																																															1	
																										4	1													1											3									1							2					1								
															1																																																																	
																																														1					1									1												1								
																															1																				1							1		1												1								
																										4	6																			1					3									1																				
																										3	2		2																	1																																	1	
																													2																						1									1																	1		3	
																																																			1									2																				
																										4	4																								1									1																				
																																																			1																													
																										2			5							1			4																												1					1					3		34	
																																																																			3													
																																																																			1												8	
																																																																			1													

																																																			1									1																				
																																												1						1																														
																													3																																																		1	
																																																																															1	
																																																			3																													
																																																																															1	
																										1	3																																																				1	
																																																			1																													
1																																																			1																													
																																																																										1						
																										2			1																																																		6	
																																																																															1	
																																					2		3																																	1				1				
																																																		1																														
																																																																															2	
																																																			2																												2	
		2																						1		2			3															1							4																												2	
																			1																																																												3	
																																																																															1	
																																																																															1	
																											1		3																																1						1				1									
																																					1		1																																	1								
																								1																																																								
																																																																															2	
																													1								1		1																																					1			4	
																																			2																																												2	
																																														1																																		
																																																			4																												2	
																	1																																												1																			
																																																			2																												2	
																																																																			1												1	
																								1				7									1		1					1																							1					1							12	
																										1									2																																												4	
																																																																															2	

																																														3																																		
	1																																						1												1																1										4		3	
																																			1																																												1	
																																																		1																														
																												1																																							3												14	
																										6	10																																										1											
																																																																					1											
																																														1																																		
																																			1											1																																		
																										1	3																																																					
																																														1																																		
																																								1						2																																		
																																																			1																												4	
																																																																																1
								1																												1																																												
																																																																											2					
																																																																			2												1	
																													1																																																			
															1																1									1																																								
																													1																																																			
																																																		1																														
																																																																								1								

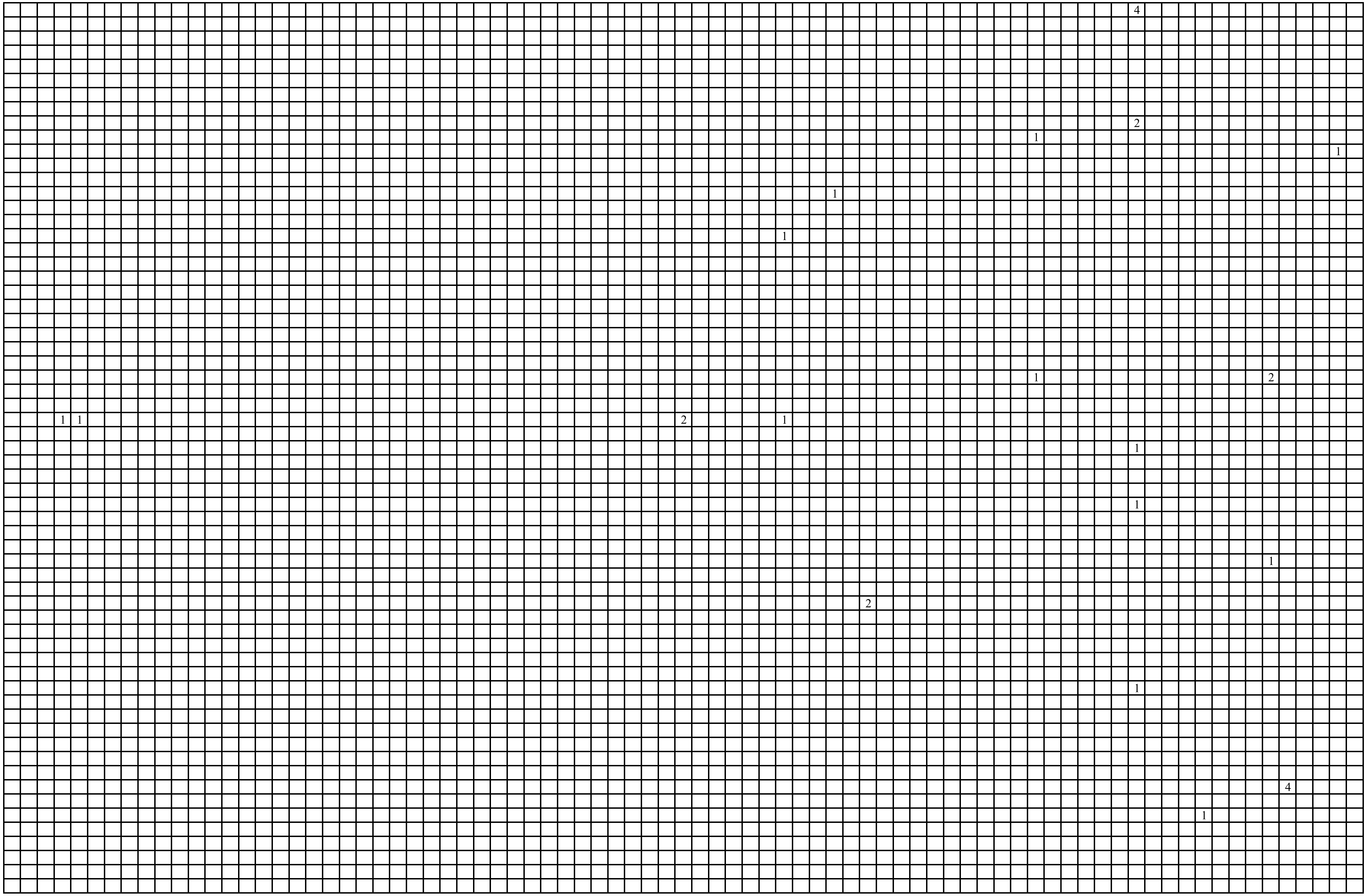
4
2
1
1
1
1
1
2
1
1
2
1
1
1
1
2
1
4
1

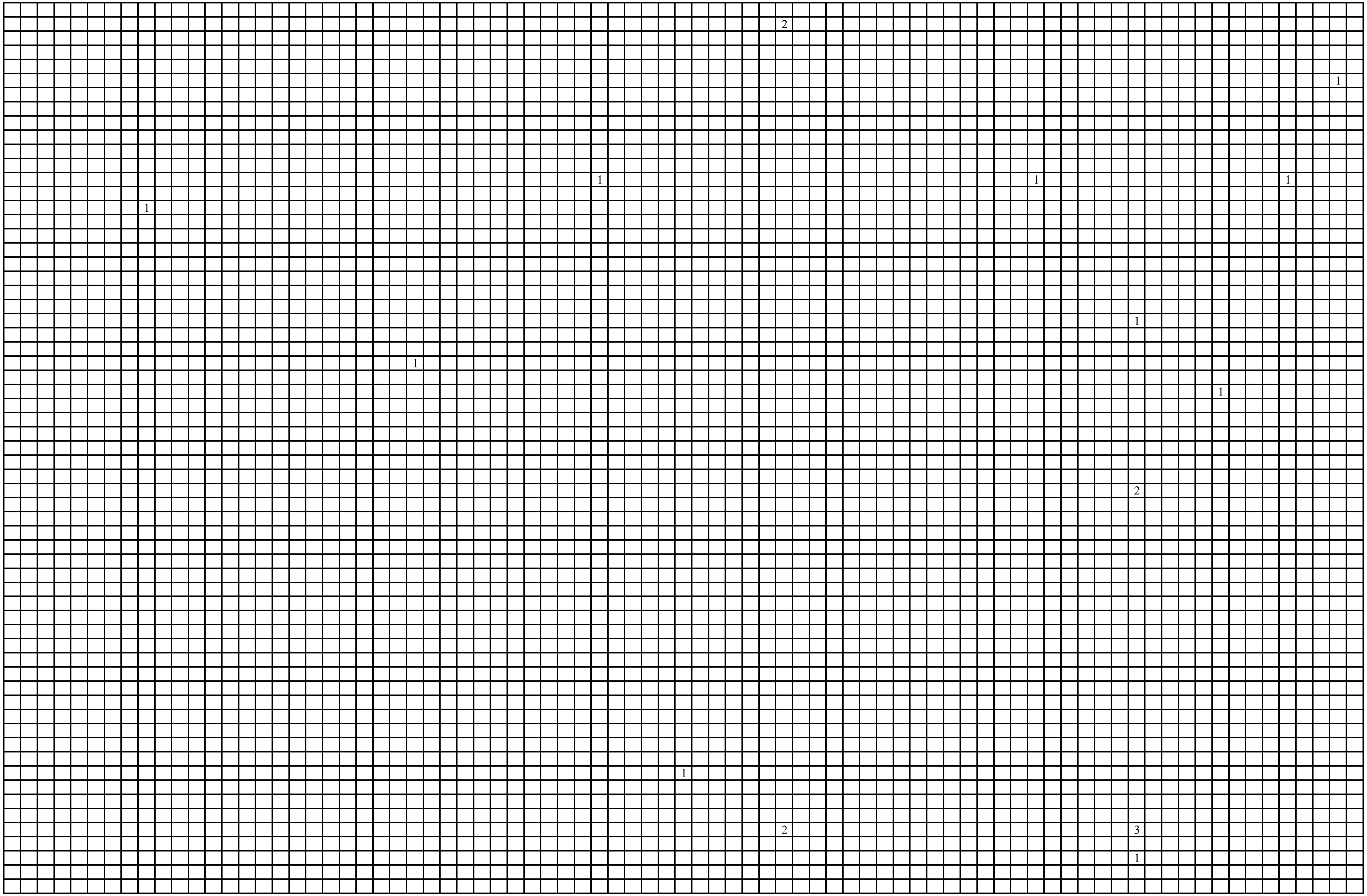
2
1
1
1
1
1
1
1
1
2
1
2
3
1

2

1

1

1

3

1

																																													1																																		
																																																													1													
																																																																					1								
																																																													1													
																																													1																																		
																																																													1													

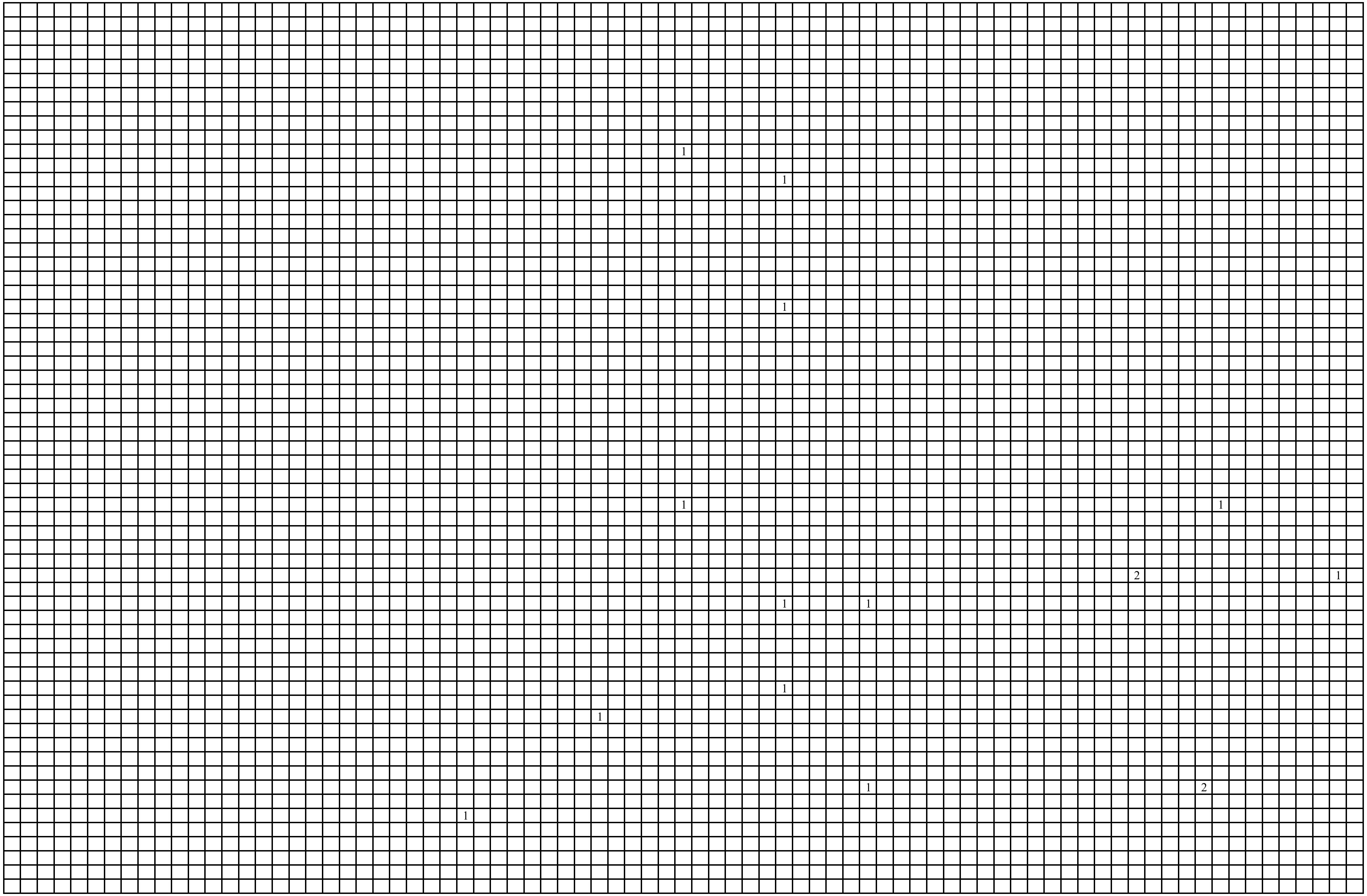
1
1
1
1
1
2
1
1
1
1
1
1
2
1

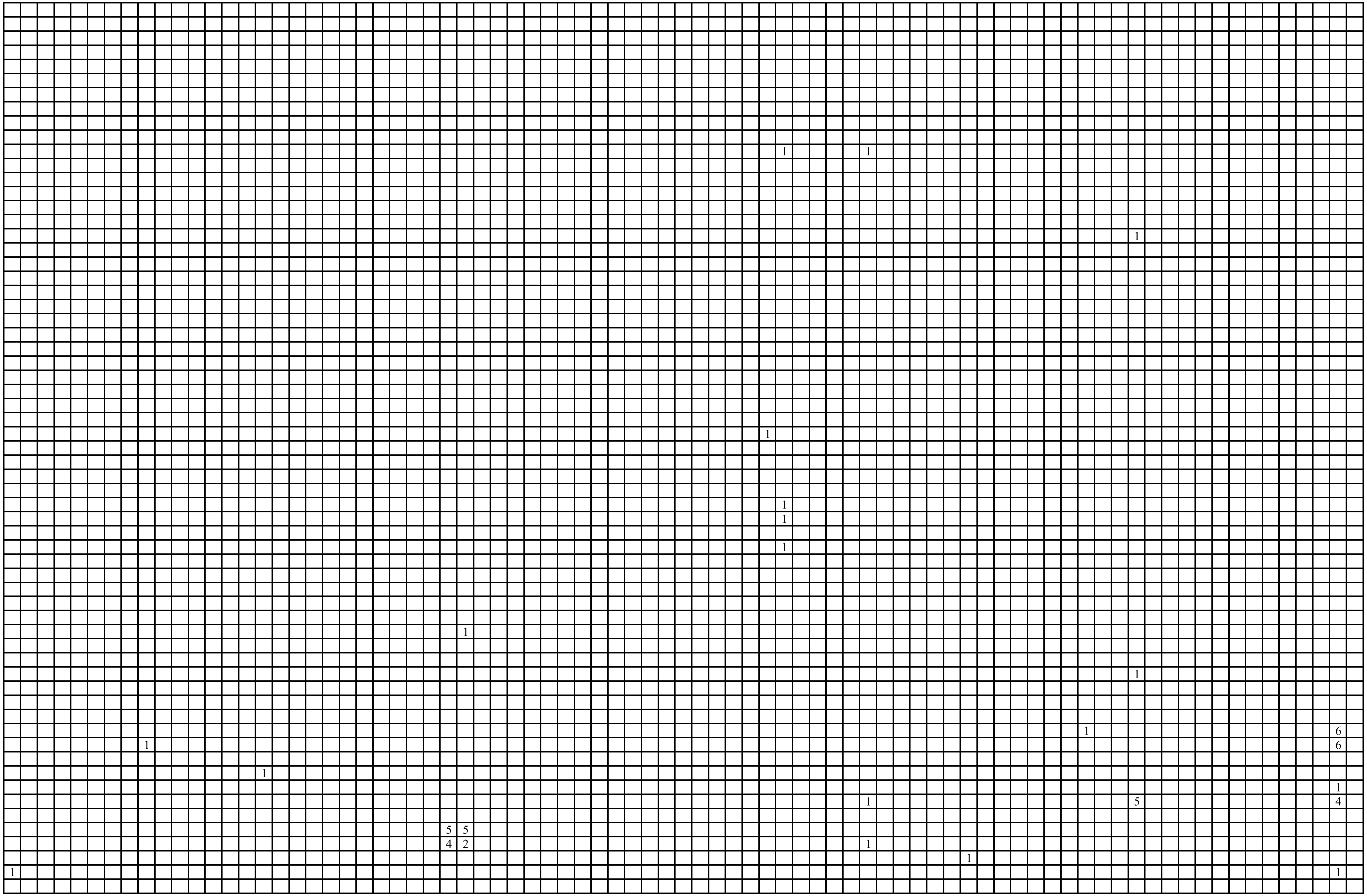
1 1
1
1
1
1
1
1
1
1 6
1 6
1
1
1 1 5 4
5 5
4 2 1
1
1 1

																										6	5									1	1		1																												1												8	
																										1	1																																																				1	
															1																						1		1																																								2	
																										3																																								2													
																																																																															1	
								1																																											1																													
															1											11	4										1							1							2																					1					1		8	
																																														1																													2					
																																			1																																													
																																																																								1								
																										3	4																	1																												1							1	
																										4																																																						
																																																																															1	
															1																																																																	
											2																	1									1			1											2																												4	
																																																																															1	
																										5																																																					6	
																																																																															3	
																																																																															1	
																															1																				1																													
											3															7	2																								9																									2			3	
																																																																					1							1			1	
																																		2																																											4			
																		1											1						1		1	1		1																																1			1				7	
																																																																															1	
																													1																																						1											1		
		1																																																	1																										1			
																																			2																																													
																																														1																																		
																											2																																																					
																																																			1																													
																										12	7												1												1																												6	
																										1	1																																																				3	
																																														1																																		
																																																																													1			

																																														1																					1																
														2																																																																			1	
																																																				4																														
																																										1																																									
																																										1																																									
																																																																																1	
																																																			1																																
																																										2																																				1				
																												1																			1						1																													3	
																											5	1																							1																													5	
																																																																																4	
																																																													1	1																			
																																																																																1	
																																																1																																		
																1																									1																																									
																																																																																3	
																																																																													1			
																														1																																																			1	
																																										1																																									
																																																																																4	
																																																1																																		
																																																																											2					
																																																1				1																														
								1																																																																									
																																																1																																		
																																																																																2	
									2																																																																								
																																																													3																1				

																																																																	3								2	
																																														1																																			
																																																																													1	
																												3										1																																																	
																																																																													2	
																																																		1																														
																																																																										1		2	
																																																																													3	
																																																										1			1																
																																																			1																													
																																														1																																			
																		1																																																														1	
																																														1																																			
																																																																															2	
																																																																														1	
																																																																														2	
																																																			1																													
																																																																														3	
		1															1									1											1										1		1																							1				1	
																												2																																																							
																																																																		1											6	
																										1	1																																																				2			11	
																											1																																												1								2			12	
								1																			1																																																				2			24	

																											1									1																																								2			32	
								1																																																																							10	
																																																			2																										1		7	
																										5	2																																																				3	
1		1						4																		2	1		18		1				6	7	1		1											2	1									1							3		1			1		1			2		8	
1			1	1			1	2			1		1	1							2			1	1	2	2		1		1	1	1		1		5		6	3			1	2					1	3	2										1	1					2		1			2		3		1			9	
1	1	1	1	1			1				1	1				1		1								1	1		1		1							1		1			1							1	1			1						1				1										1					1	
																																																		1											1																			
		1														1													1						7	4				1										3																									2				4	
																																			2																																													
																								2																1																																								
																																																																															2	
																																																	2																															
																		1																																																														
																																																			1																												3	
					5																			1					5								1							1					2	3																		1				1							3	
																																																																											1				1	
																																																																												1				
																								1																																																								
																																																																															2	
								1																																																																								
																																1																																																
																								1					3																						1									1														1						
																								1																																																								
																																																																													1		3	
											1															1	1				1						1	1	1	1				1		1																							1			1				1	2		2	
																																				4			1																																								1	
																																																																															2	
																													2																				1																															
1																																																																										2						
																	1												3										1																																									2	
																								1																																																								
																								1																																																								
																													2									1											1																															
																													2																				1																															
																																																			1		4																											6	
			1																																					1																																								
																								1											1																																													
																																														1																																		
																																																									1																			1			2	
																											3																																																							
																																																																												1			19	
																											1												1																			1																		1			17	
																																																																															5	
1	1		1											1				2								1											1	1	1	2				1					1										1					1											1				4	

											1																									3																1																												1	
																																			1																																																													
																										6	2																																																					
																			4																1		1		2																														1							4				
														1												1																																																							
																																																														1																		
																																																														1														1			1	
																													1																																																		1	
																													2																																																		1	
																																					1		2																																	1								
																																																		1																														

														1		1	2		3	1						HDAC2		405	1470
																										HDAC3		244	605
																										HDAC4		138	619
																										HDAC5		99	485
																										HDAC9		18	103
																								1		HEXIM1		58	1075
											3		5													HEXIM2		29	136
													1													HIF1AN		34	489
																										HINT1		8	133
																										HIPK2		49	220
						1																				HIST1H1B		45	527
																										HMGB1		79	512
																									1	HSPA8		160	2989
																										ICE1		2	65
																										ICE2		4	107
																										IFI27		0	11
																										IKBKAP		9	179
																										IKBKG		52	1109
																										ILK		10	446
																										ING4		27	153
																										ING5		15	176
																										IRAK1		20	524
																										IRAK3		2	35
																										IRF2BPL		16	56
																										JADE1		7	164
											2						1									JUP		31	581
			1									2														KAT2A		91	407
			6									3														KAT2B		212	622
																										KCTD1		9	54
			1								1	1					1									KDM1A		292	1189
																										KDM3A		9	63
																										KDM4A		9	73
																										KDM4C		13	78
																										KMT5A		0	0
												1														LANCL2		3	97
												2														LDB1		85	184
																										LEO1		5	166
																										LGALS9		11	729
																										LIMD1		6	160
																										LOXL2		8	447
																										LPXN		11	108
											2															MAD2L2		43	638
																										MAGEA1		28	202
																										MAGEA2		5	39
																										MAGEA2B		2	35
																										MAK		2	48
																										MALT1		6	158
																										MAML1		38	104
																										MAML2		1	13
																										MAML3		6	18
	1	1																								MAPK3		68	500
																										MAPK7		20	208
																										MDM2		891	2703
																										MDM4		104	402
																										MED1		106	508
																										MED12		38	361
																										MED13		16	244
																										MED14		36	286
																										MED16		25	196
																										MED17		28	356
																										MED21		15	348
																										MED24		32	233
																										MED27		13	224
																										MED30	TcoF.HC	4	194
																										MED4		25	714
																										MED6		22	226
												2		1									1	1	1	MEN1		135	1256
																										MKL1		10	67
																										MMS19		19	417
																										MNAT1		29	169
																										MPHOSPH8		13	102
																										MTDH		37	582
																										MUC1		12	213
																										MYCBP		12	122
																										MYO6		29	557
			1																							MYOCD		9	28
																										NAA15		12	151
																										NACC1		21	121
																										NACC2		14	58
												1														NCL		78	900
																										NDUFA13		6	207

						2																																																																										
						1						1																																																																			
																									1	2																																																										
3						1																					4		4						6																																																
										1																																																																								
2	1	1																									5																																															2								
																																																																													1		
																																																			1																															
												2																																								1																													
				1								1																																																																						
												1																																																															1				
		2	2																																																																														
							1				1	3					1								1																																																								
																																																																							1									
																											1		1																																																							
																	1																																		1																													
																											1		1																																																								
1																1	8								3				1																									1	1		1	1	1		1	1		1																
1	3	6	2		1		15					3				1	3								1		4			3		4																				12			1	1	1													1												
																											5																																																									
	1											2									1																																																										1		
																											1																																																									
												1																													3																																											

	1											1																																																																	1		
1																									1			1																																																								
																												1																																																								
								2																																																																											
																																																																							1									
2																																																																																
1																																																																																
5	3		1		2			1		2	1	3				2	1	1	1		1				3	10	2		6	1									1			2					1			1		1		2	1			1	1		1	1	1			3		2	1	1	1		5	2		1	1		5	
2			2					1		1	1	1													1		1		1																																													1		1						1	
																									1																								1		2		1	2	2	2		2	1	2				2															
	1																																																2																														
2											2																													1																									1														
			1																																																																													
			2																																																																													
																											1																																																									
						1											1																																																																
												1																	1																					1																														
																	1																																																																
																												5																																																								
																												1																																																									
																												1																																																									
					1																																																																													
						1																																																																												
		2																																																													1														
																																																																	1														
																													1																																																							
	1																																																																															
	1			1								1															1																							1		1									1			1							2	1								
																																																					1		1	1	1		1																			
																																																		1														8														
																											1																																																							
1	1																												1																						3																													
	1	1	1																																																13														1														
											1																																							1	1	1							1							1								1						
																																																																	1														
																							3																																							1								1														
		1	1																																																																													
1																																																																																
																											1																																																								
																											1																																																								
			1																																																																													
																																																			1																														
1	1																																																																															

																													1																																					1														
																										1																																																						
																										2	1																									3																												
																											1																									4																												
												1																																																																				
2														1																																																																		
	5																																																																															
																																																		1																										1				
3	1	1			1					1	1	1						1								1			1	1																	1							2	2		1	1	2	1	1	1		1	2											1	1			
2		1		1				1		2	2	2				1							1		2	4	2		1							2						1									1			1	1	1	1	1	1	1	1	1		1				1	1		1		1			1			2	
	2	1																																																																													
																																																										1																						
			1														1																																																															
																											1																																																					
																											2																																																					
																											2																																																					
					1					1																											1															2																			1								1	
					1																																																																											
					1					1																																																								1					1									
																										3	6		2																							5	2																											
	2				1																					1			1																																		1																	
2																													1																																																			
1												2																																																																				
																																			1																																										1			
																																																																													1			
1																																																																																
																																																																		1														
											1		2																	1						1																															1														
1																																																																																
												1																																																																				

																																			1																																																		
1																													1																																																							
																																																																				1												
1																																																																										1			
																																																															1																
1												1																																																						1														
3	2																																																																	1														
1	1																																																																									1						
																																																																		4														
										1		1													1																													5																												1	
									2																																									9																													
																																																				6																													
												1																																																						1			1											
																																																		1																														
1				1						3																																										3																									1	1		
																																																																				1												
1																																																																					1												
												1																																																						1														
1																																																																																	
1		1																																																																														
																																																		1																														
	4	2	2																																																																												
																																																																		1														
1												1													1												1																														1							1					1	
5	6				1																								3												6	2								2		4														1														
	2																																																																																
					1																																																																												
					1																																																																												
																										1		1																																										1														
																										1																																																											

										1																5		1																																																			
1																											1		1																														1																				
									2																										1																																																	
																																			1												1																											1						
											3																2																																																					
																																			1												1																																	
																																																						2																										
												2																																																								2											
																																																																					1											
										1	2	1																																																									1										2	
												1						1											1																																					1														
																													1																																																			
1												1															2		1																																									1									1	
4	1	1																											1																																																			
																													1																																																			
1																																																																																
1																																																																																
1																																																																	1														
										1																																																																						
						1																							1											4																																								
											1																2																																																					
1																																																																																
											1																																																										1											

											1																																																																						
																						1																																																										
																			1								5																																																							
			1																																																																														
											1																																																																						
																																									2																																													
											1																																																																						
											1																																																																						
																																																																											1					
											1																																																																						
											1																																																																						
											1																																																																						
						2																																																																												
																													1						3																																																				
																																			3																																																						
																											1								3																																																						
											1																								4																																																							
																																			4																																																						
											1															1									7																																																							
																																			7																																																						
																																			4																																																						
																																			4																																																						
																																			5																																																						
1																																																																																						

																			1																																																													
										1	1																																																																				
												1																																							2																									
									1														1						1																																																							
																												2																																																1											
																																				1												1																																								
																																				1																																																					
	1																		1																1												1																																								
										1																																																																											
																										1									1												1								1																														
1											1																																																																					1	
																																				1																																																					
																																										1																																														
																														2																																																														
1																																																																																							
																																																																							1														

												1																																																																				
												2																																																																				
	1																																																																															
																																																	1																														
																																											1																																						
	1																									2																3										2																												
																																										3																																						
																																										4																																						
																													1																																																							
1																																																																																
		1			1					1	2	2																																								1																	1										2	
		1			1						6																																									1																												
					1						4	1																																																																					
																																																														1														
												1																																																																					
																																																																	1												
																										4																																								1														
																								1																																																											
										1																																					1																			1														
																								1																																																											
1												1													1				1						1															1																														
																																			1																																																	
										1																																																																								
																																			1																																																
																																			1																																																
1	3																	1										1	2						1	1											1			3				1														1										1	

																																																				1																												
																							1																																							1																		
1	1																						1						1						1												1																														1			
																																															1																																	
																													1																																																			
			2																																																				4																									
	1																																																																															
								1																																																																								
											1														2																3																									2														
					1						1	1																																																						1														
7																													7																																																			
10																													11																																																			
2					1							1								1																																1												1		1														
					1							1																																													1									1														
																																																		1		2														1				1										
																																																																													1			
					1						1	1							1	1				1												1	1		1																											2	1		1	1	1		1							
										1	1									1							1																																																				1	
										2	1	1															3																									3																												
2												5													1				1																					1		2					1				1	1																	2	
												3																																																																				
	2	1																																	1																					1																								
			1																																																																													
												1																																																				1																
		1										1																																																																1				
	1	5	5	2																																																	2																													
		10	1	2																									1																						3																													
	3	6	1									1															1		2																						4															1														
	1	3																																																																	6								5					
																																															1																																	
																																			1												1																			1														
																																																																		2														
																										1																																																						
1			1		3																																1		1													1														1														
					4																																																																											
					3					1																																																								1														
																																																																		1														
											1	1																											1																									1		2														
		1																											1																																																			

	1																																																																															
	1																												1													2																																					
																																																																		1													
					1					1									1	1												1																																															
																					1																																																				1						
												1																																																																			
1												3																											1																																									
	1																																																																															
																													1																																																			
										1																																																																					
1																																																																															
1					1							1																									1													1																1													
																																					3																																										
1																												4																					1																													1
														3															1										1						1																																		
										1																																																																					
			1																																																															1													
					1																																																													2													
		1																														1																				3																											
																																																																		1													
			2		1							1					1								1												1																																		1								1	
											2																																																																				
																	13																													3																																	
																																																																		1													
			1																																																																												
																																																																		1													
												1																																																					1													
																													1																																																			
												1																																																																			
											1	1					1																																														2										10						
												1																																										2																2											
																											1		1																																																			
											1																																																																				
																				1					1				3																																																			
6			2	1	2		1		17		5	13							11	5					1	3	18		9		1	5				1					1	1	3				3			4	2	##	86	3				1	1	1		1				1			4			2		1					5	
1									1																		2												1													6	2																											
1									1		1																5		1																						19	6																											
																											1																																																				
																																			1												1																																	

																																			1												1																																	
										1																															2																									1					1									
					1																																				2																									1					1									
																																																																												1	
		3	2	2																																																																							1		
1	1	1	2				3				1						5																																																	3														
							3										1																																																		1					1									
							3																																		2																																										
9	5				1		1				1	2					3		9							4																								2		4														2	1				1									
																																														2																																			
2		1	2																								1																																																							
												1																	1																																																						
																									1																																																										
1																										1	2		4						1												8	1	2													1														1	1				
												1																																																																					
2	5								4			1																													1													1	1			1	1							1														
	3	4	3																								1																																																							
			1																																																	1																														
1												1																						1																																																	
									1		5										1																																																													
										1																																																																							
																																										1																																									
									3																	1																																1	1	1		1			1	2	2															
2		1							1																	1	4																															1	1		1			1	1	1								1					
		2	2						1																	1	5																										1					1			1			1	1	1	1								2					
1																													1																	1						1														1														
																										1																																			1					1														
2	2		2																								1																																		1																			
																										1	1		2																																1			1																
	1	1										3									2																																																					1						
																																																									2									1														
1	5						1														2					1	1		2																					2		2																												2
																																																				1																														
1	1	1	1									1																																						1																										1				
												1																	1																									1																														

					1																														1												1																																			
																																																				1																								
1												1																																						1																												
					1																																	1	1											3																														
																											1	1																																																		
																																																																						1						
																											1	1																									2																												
												1																														6									9																		1												
																																																			1																							1							
																																																		2																														
									1			3																							1												1																						1										2	
																											1																									2																												1	
																											4																							1																														
																														3																																																		
											1																																																																					
	2			1																																		1																																													
																																			1												1																																			
																											2																																																									
																									1																														3																										
																																																															1														
	1																																																																													
																																																			1																														
																										1	3																									7																												1	
																							4			1	5																									3																			1								2	
1	1																																																																																
																2					2														1																																																	
																											1																																																						
																					7						1																																																								
																																																																								1						
			1		1							2				1													1																																																			1	
																																																																							1									
																																																																							2								1	
																																																																							2					1			1	
4																																																																																		
3																																																																																		
									3			1														1																																							1												1	
									1																																																																							2	
1									1	1																																																																							
	26	11	5	2																									1		4																																				2														

2	26	7	6																																																																												
13	2		1										1				1				1								33																										2												1													
3																														2																																																			
4	1																			1									6													3																																						
					1																																																																											
1					1																																																													2														
																										11	5		1																									2	1	1	1	1	1			1	1	1	1															
																										4	3																														1							1															
																			1	1																																																												
																											2																																																				
					1	1																							1																																																	
					2	1					1																						1																																												1	
																								1			1																								2																											
																	1									5	2		1																				1																														
																													5																																																	
																													1																																																	
																																																																		1												1	
												1																																																																				
																				1																				1																																						
	2																																																																														
																																																				3													1						1									
		1	1																							1								1												1																																
																							1				1																																																				
																																																		1																													
																										1	1																																																				
																											5																																																				
																											2																				1																																	
																											2																																																				
																										1	1		2																																																	
5			2																																														4																													
1																									1										1												1																																
	2																									1																																																				
1											1															1	2																																																				
10	1	3						1				1															3								1														2		2																		1					1					1	
3																																																																																

	1																									1	1		1						1												1																														1			
	2						1					1																	1																																																			
																																																																														1		
	1																										1																																																					
1																																																																																
																																																																					1											
																															2																																																	
																																									1																																							
																																																												1																				
1	2	3	3	1																																																							1						1												2			
												1																																																						1					1								2	
2																													1																																																			
3																													9																																																			
1																											4																																																					
																																																							2																									
																																																				1	1																											
																																1																																																
																																																																															4	
												1																																																																			1	
2	2																																																																															
	3																																																			5																												
																											2																																																					
																																									1																																							
6																													3																																																			
						2						1																																																																				
1	1																																																																															
9	2											1																	10																							4																						1					1	
3																											1		1																							8																												
2																																																																																

																																																				1															2															
7	9	5	3		1						1															1	2																									4															1															
																																																																										2			
1	2	4	3																																																																		3									
												2																																																																				
																													1																																																					
																																																			1																												1			
																		1																																																																
																																																																											2			
1	1											1																																																																		
	1	2	2																																																																													
2																																																																																	
																													1																																																					
																																																																														1	
																																																																1															
																																																																1															
																								1																																																											
																																																																									1						
																																																			1																												
																																																		1																													
																																																					1																											
																																																					1																											
																																																																							1										

								1																							1																																																	
																		1																																		1																											
																																								1																								1														
	2																							1		1																																								2														
																																							1																																												
																																																																			1														
																																																		1																											
																		1																																																														
											1																																																																					
											1																																																																					
										1																													2																																1						
						1																																																																									

1																																																																															
																																																	1																													
																													1																																																						
																													1																																																						
																													1																																																						
																																									2	1																									1		1												
																													1																																																						
		1																																																																														
																																																												1																								
										1																																																																										
																																																			1																													

																																																				3																														
						1																																		1																																											
																														1																																																															
																																																			1																																
1																																																																																	
																														1																																																																
						1																																																																											
												1																																																																											
																																																				2																														
						1																																			1																																																										
																																																																	1										

																																																				1																												
																																										1																																						
																		1																																																															
																																						2																																										
																													1																																																							
																													1																																																							
						1																																			1			1																1																				

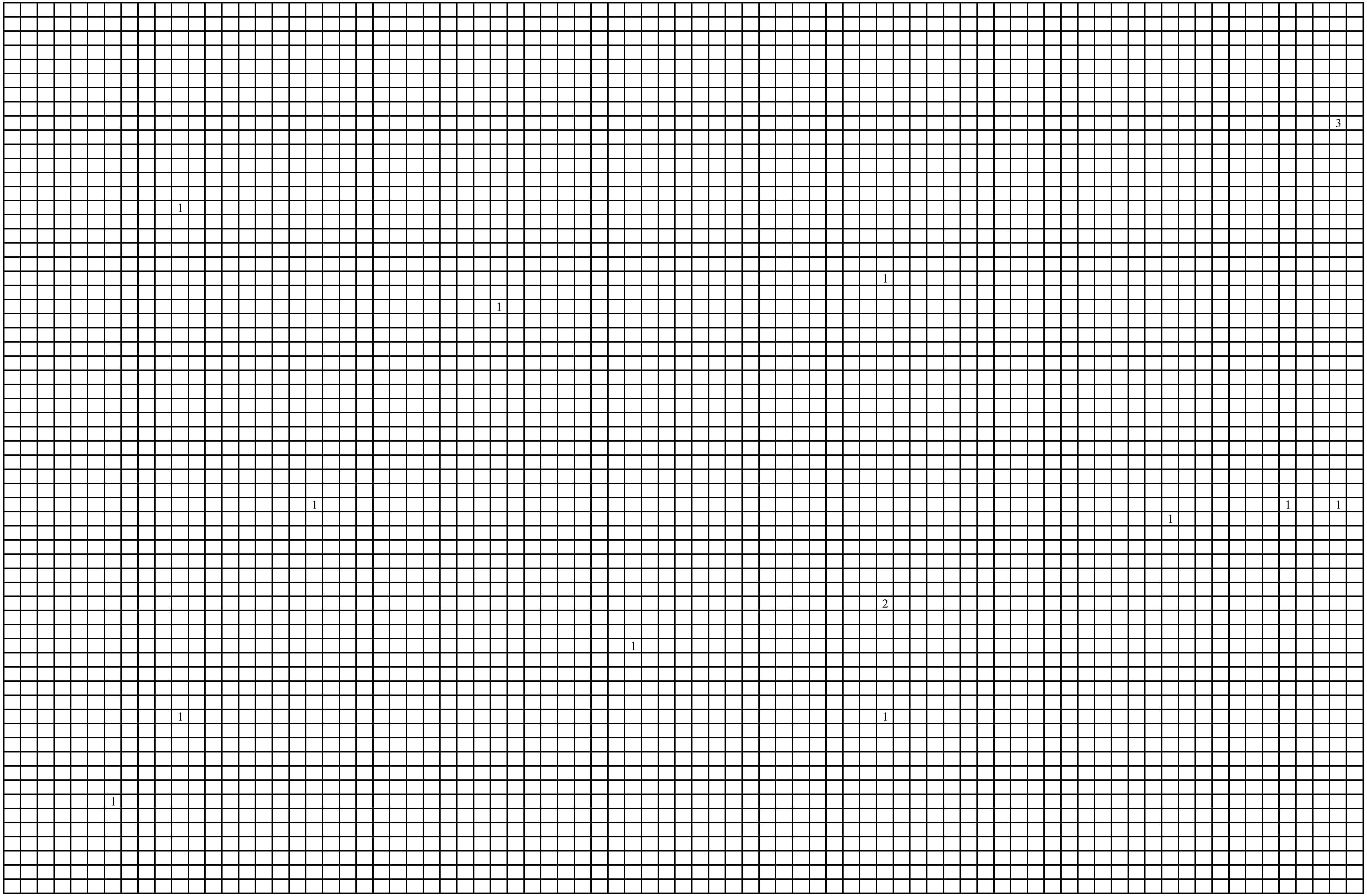

3
1
1
1
1
1
1
1
1
2
1
1
1
1

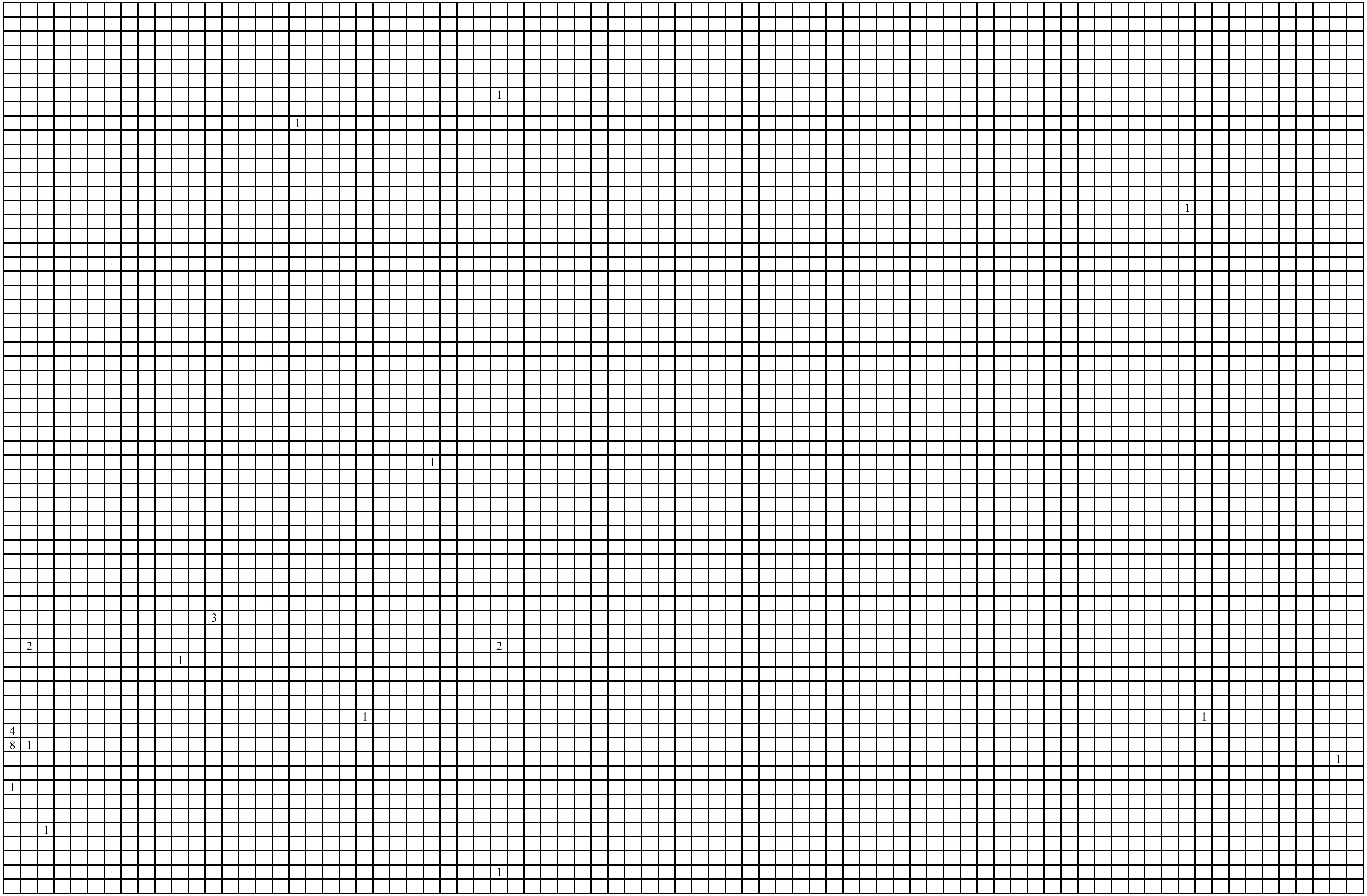
1
1
1
1
3
2
2
1
1
1
4
8
1
1
1
1
1

5	1	1	2							1	1	1																	1												1																																						1	
1		1	1																																																																												
3		1	1							1	1	1								1									1												1																																						1	
1		1	1																																																																												
																									2	2																																																								
	1																												2																																																							
5		1																								1	4		1																																													1							
										1																																																																							
3																																																																																
																													1																							1																													
1	1																																																																															
1		1	4									1																																								2																											2	
																													1																																																							
3	2																												3																																																								
1																																																																																
																													1																																																							
3																																																																		2													1	
										1		1																	1																				1																														1	
1																																																																																
		1	1				1																						1																																																							
																												1																																																								
																																																																										1				1			
																													1																																																							
									4																																																																											
5												1																	10																																																								
3																																																		1																														
										1																																																																							

1																																																																															
																																																										1														
																																																																													1	
																												1																																																						
																																																											1																		
1																																																																	1											1			
1																													3																																																						
1																																																																																
										1																																																									1												
																																																																				1														
																		1																																																																	
																	1																																																																		
																													1																																								1														
																																																																								3										
												1																																																																								
																																																																				1														
																																																				1																											1	
1																																																																		2														
																																																																									1										
																																																				1																													
										1																																																																									
																													2												1																																																	
																			1	1																																																																

1	1																																																																														
																																																																		1														
																	1																																																															
					1																																																																											
												1																																																																				
1											1																		1																						1																1												
											1																																																								1														
												2																																																																				
												1																																																					1														
1																																																																	1														
							1																																																																								
						1																																																																									
	1																																																																															
					1																																																																											
																													1																																																		
2																																																																																
							1																																																																								
		3	3	3			1																																																	2			1		1																					
		2		1																						1			1						1																		2																										
3	4	1	2																										4																						1														1														
2	1									1	1	1																	1												1																																						
4																																																																																
7					1																																																													1														
9					1																			1																																														1														
20																													4																1							2																	5									3		

18			1		1							2												1					5																																		1			1												3		
7																													3																																																	1		
2																																																																		1														
4																													1																					1																1														
2	3	6			1				1		1	1											3		1		3		5	1	1				1								1				1					25		4	3	1	1	1	1	1	2	1		1	1			1						1						
3	4	5	1	1	3							7	1												1	1			4						1												1					10		14	4	2	4	3	1	1	3	2		1	3	3		1	3	1	2	2		2			1		3	1
1	1				1							2											1			1			1									1												1		1		5	1	1	2	2	3	1	2			2	2	1		1		1		1		1					1	
			1																								2																																							1					2									
												1																																																																				
1											1	1				1						1							1											1							1		1			1		4			2		1															1		1	1		1	
																											1																																																					
											1															2																																																					
	1																																																																															
		2																											2																									1																										
		1										1															1		2																																																			
																																																			1	2																												
												1																																										3	1	3	3		3		2			3																
																																																													1																			
1	2	1																											2																							3	3																									1		
	9	3		2								1														2	1					1																				2		20			2				1											4								
2	1	1										1															3											1												1		3		4											1			1												
1	3																																																					9	2			2		3	2				2															
1	2																																																					1						1								1												
																																																		1		2		8	4	2	4	2		1	2			1	2															
	1	1	1																							1	1		1																					1		2		3			2	1	1	1	1			1																
2	12	3																											3																																																			
1	2	1								1	1	1																	3								1																																		1								1	1
1	2		3																										3																																										2									
																																																																		1														
	1	1																																																1				3							1																			
												1															1																																																					
																																																		1																														
	3																																																			3																												
							1																																																																									
																																																																															1	
																											4																																																					
	1																																																																															
																																																																		1														
10																													2																																																			
10																													3														1	1																						1		1													
																													1																																																		1	
1					1				4		2	1																																										1	1		1	1	1											1	1								1	1

1																													1													3										1																											
											2													1																	1					1					2																										1	
	1																																																																															
1																	6																																			1																											
																													1																		1																																		
											1																																					1																						1								2	

			1																							NIPBL	46	229
																										NKAP	9	193
																										NLRP12	0	12
																										NOC2L	27	296
																										NOD2	3	167
																										NOL11	9	140
																										NPAT	22	102
																										NPM1	145	1771
																										NRIP1	90	232
																										NUP62	31	387
											1															OGT	95	1055
																										PAF1	20	272
			1																							PAGR1	25	76
											1			1						1						PARK2	180	5986
																										PARK7	29	401
																										PARP10	3	58
			1																							PAXIP1	96	478
																							1			PDCD4	4	178
																										PELP1	28	256
																										PEX14	13	248
																				1						PHF12	52	161
																										PHF2	9	74
																		1								PHF21A	75	392
																										PHF8	21	212
																										PICALM	9	271
																										PID1	3	31
																										PIH1D1	15	923
						1						1		1					1							PIN1	128	518
																										PIR	2	27
																										PKN1	8	182
															1											PLK1	68	977
																										PLK3	9	54
																										PMF1	7	171
																										PPARGC1B	18	34
																										PPID	11	103
																										PPIE	16	371
																										PPM1A	4	166
																										PPP3CA	13	171
																										PRDX5	4	97
																										PRKCB	11	254
															1											PRKCI	13	414
																										PRKD2	10	281
		1																								PRKDC	98	894
																										PRMT2	14	140
																										PRMT6	41	224
																										PRPF6	33	463
																										PSEN1	3	286
																										PSIP1	32	218
																										PSMA6	17	353
																										PSMD10	16	246
															1											PTEN	37	1076
																										PTPN14	5	261
																										PYCARD	4	130
																										RAD21	33	540
																										RAI1	19	107
																										RBBP8	15	295
																										RBFOX2	24	418
															1											RBM14	46	645
																										RBM15	16	207
																										RBM15B	12	111
		1																								RBMX	40	528
																			2							RBPMS	79	489
																										RECQL5	8	173
																										RGCC	2	24
			1																							RING1	71	473
																										RNF111	22	199
											2	1														RNF14	24	77
																										RNF168	6	177
																										RNF187	23	62
																										RNF2	119	1295
																										RNF20	23	293
																										RNF25	6	90
																										RNF8	30	465
																										RPAP2	4	265
																										RPRD1A	16	177
																								1		RPRD1B	23	299
																										RPS14	46	531
																										RPS6KA1	12	260
																										RPS6KA4	6	107
																										RPS6KA5	11	128
															1											RPTOR	13	624

													1																																																																				
							1																																														2																											
																																																					1											2																
																													3																																																			
																											1																																																					
											1							1																																																														
																																																1																																
																																																					1																											
																																																		1																						3								
																																																													1																			
			1										1																																																																			
																																									1																																			1				
																																																													1																			
																																																													2																			
								3																																																																								
																						1																																																				1						
																						1																																																				1						
							1																																			1																																	1					
													8														1				3											4			1				1			1																												1
												2																																																																				
																																																																														1		
													1																																																																				
													2																																																																				

										1												1																1																										4					7					1						
1													1									1																																										3					6											
																																																																																1
																					1																																																											
																																																																1					1								1			
									1																																																																							
																																																					1																											
1	1		2		3	1	4		1	3	2	1		1	3			1		1		1	1				3			6					1							4			5		1	1		1	3		1			1	3	4	1	3		1		3					3	2		3		1	2	3				1
	1						1		1																										1							1			1		1							1		1	1	1	1	1				3					4				1	1		1				
													1																	3																																																		
						2								1																																																																		
																																										1																																						
							1																																																																									
																																												1																																				
							6																																																		1																							
																											1																																																					
																																						1																																										
													1														1																																																1					
																																																													2																			
								2	1									1				1					3										4					1																										3						1						
							1																																																																									
														1																																																																		
										2										1																																																												
																																3																												1					1															
												1				1											3																																																			1		
																											1																																																					
																																												1																		1																		
																																																1																																
																																								1																	1																							
													1																																																																			

																																																1																																
																																																																														1	
																		1																																																2													
																							1																																																								
												1																																					1												1			1												1	1	3	
										3																																																																			
												1																																																																		
												1																																																																			
								1					1																																																																			
																																										1																																						
1										1	2					1				1	1	1					1			1							1					1			1		1	1	2				1	1			1	1		1																				
	1				1		2		2	1	3			2		1		1					1	1		1				1												3			2		2						1				1	1		2		1													2	2				
												2																																										1																										
1																																																																																	
																			1																																																														
												1																																																																			
							2			1			3			1								1		1																											1							1								1			3	1				3			
							1			1			2			1										1																																										1											
								5											1																							1						2													2																
																		1								1							1									1								1			1																						
																					1																																														7				7											
										1																																																								3				3											
																																																																								1											
													1																																																																		
						1											1					1					1																		1	1		2													1	1																					
																																		1																																															
				1																																																																													
																																												1		1																1										1										
																																																																1																			

																																															1																																	
												1				1											1																																					1					1											
																			2											1																																																		
																																																																					1											
																																																		1																														
																																															1																																	
			1													1																																																1																
																																																												1																				
																														8																														1							2					2								
																											1																																																									
																																																									2																							
																														6																																																		
										1																	2																																																					
													1																																															1																				
																											2																																																									
																																																													3																			
			1															1																																														1																
																																																												1	2																			
													1																																																																			
																														1																																																		
													1																																																																			
												1				1																																																																
																																																													3																			
																																												2																																				
																																										1		3																									5							1				
																																												3																																				
																						1				1																																											1											
									1																																																					1																		

																														3																																																		
1												1				1				1		1					1																										1											1	1				1											
																1																																																				1											
																1																																																	4														
1												1				1																																																1																
						2																									1											2																																									1				
																																																													1																			
																1																										1					1		1																				2					1						
																														1																																1																			
																																	1																																								1											
																																	1																																																			
																												2																																																2								
																						1																																																												
							1																																				1																																					1				
																						1																																																											
																						1																																																												
																						1																																																												
																			1																																																																
																																																								1																											
																																			1																																																1					
										1																	1																				1																		3																			
																								1																																																													
																																													1											1																								
							1																																																																												
																																																													1																			
																																																																							1											

																																										2																																						
																																																													1																			
																																												1																															1					
																																		1																																									1					
																																																													1																			
													1														1																																																					
													1														2													1																															1									
																																										1																																						
																																																																						1										
																																																																				1												
																																																					1																											
																1					1																																1											1																
1																1																																																1																
																1				1																																																												
																1																																																				3												
							1																																																																									
													1																																																																			
1																1																																																1																

																																																								1																			
																1											1																																					1								1											
									1																																2															1			1																				
																																																								1																			
1												1				1																																																1					1											
1																1																																																															
1																1																																																1																
																																																																											1					
																																																								2																			
1												1	1			1								1	1															1														1							1																			
							2																																						1						1																												1				
																1																																																																	
																																														2																																								
																																																									3																			
																																																									5																			
																																																									1																			
																																																				2					3																			

																																																								1																			
																										1																																																												
																																																		1																									
								1																																																																							
								1																																																																							
																	2																																																																	
							1				1																																		1													1															1				
																																							1																																																		1	1		
																																																																													1					
										1		3	2			6																																																				1																
																																																																								1										
													2			1																																										6																									
																				1																																																																	
							1																																														1																						1								
																																																							1																														
							1				1					1				1		1								2												1			1								1							1				1					1							1				
																1																																																																			
									1																																																																									
																1																																																																			
																1																																																																			
2					1		1				1			1		1		1						1																							1	1					1			1	1	1											1							1				
																										1																																																											
																1																																																																			

																																																									1																
															1						1																																			1																							
																					1																																																											
															1																																																																		
																																												3																						1														
																	1																											1		2		1	1														1																			
																					5																																			1																				1	1					
																					8																																																												
									1	1			1																																											1												1											
																											1																																																								
											1										1																																							1																1						
																																																																					1											
1											1		2								1														1							1			1					1				1				1		1									1					1	1					
							2							1																												1		5			3							1		2													1											
							2																																					3			3																																					
							1																							5				1								1							2											2										2										
									3																																																																								
									1																																																																								
																																				2																									1																			
																																												1																																				
																			1																																																														
																										1																																												1											
										1			1																						1																																																
																																											2																																						
															1																																																																			
												1				1																																																																
															1																																																																				
1																1				1								1																									1											1																
										1			4	2												1	1													1		1											1				2															1								
							1																																																																									
							1																																																																									
							1																																																																									
							3			1			2	1		1											1																																												1											
																													1														1																																				
													1														1																																																				1			

																																																										1																								
																			1																																																																
									1				1			1					1																																																										
			2				2							1																									1																																		1							2				
														1																																							1						1				1									1														
																											1																																																						
														4																																																																		
																																																																		2																
																																				1										3																																			
1													2													1																																											1												
																						2																				3																																							
														1																																																									1										
																																1																													1																			
		1								1																																																											1												
							2						3																																											1																								
														2																																																																		
																																																																								1												
																																				1																																														
							2				1		3										1			1																		1							1								1					1	1			1									3			1				1				
																																												1																																					
														2																																																																		
				3										1																										5									1		2																																
																																																1																																		
														1																																																																		
							1						1																																																									1												
																												1														1						1																																			
														2																																																																		
1									4																																											2																												
														1																																																																		
																								1								5																																																	
																																											1																																						
																								1																																																		2		1						
			4				15	2		2	1		5	4		1		4									8			4	4			1			1				1		15							1	6					1		2	2						4				3	3	8		1		1		1	1			
																				2								1																																																					
																														2			3														2																															1				
																		5																																																															
1																	1																																																															

1																1																																																																
										1			1																		1																			1																														
																																							1											1						1													1											
			1																																																																									1				
																																													1																													1				1		
													1					1																															2																														1	
													1																																																																			
													2																																																																			
							4			2			2														1			1												1							5																				1		4								2	
1										3																											2																																											2
																											1			1												1																																						2
																																					3																																											
										2	1		1									2	1																								1																							1										
																1																															1										2																1							
																											3																														2																							
																											1																																																					
																																										2											2																											
																											1																																																					
																											1																																																					
							1																																																																									
1																																																																																
																																				1																																												
										1			1			2											4																																				1		4											1				
													2														7								1																										1		1		7															
																													1																																					1														
																																																																	1														1	
																																																																	5															
																		1										1																2																																				
																																							1																												2													
									1				2					3								1				1																																													4		1			
									1																																																																							
																																																												1																				
													1																																																																			
							1																																																																									
																																																												1																				
									1																					1															1												1	1											1											

																																																												3																			
																1																																		1																																
1																1																																																				1					1											
																																								1																																								
																																																																					1											
																										2	4																	3																																															
								1			1												1																														1															1							1									
																				1																																																														
																				1			1																																																												
							3													1																																																														
																1																																																																
																																						1					1																																														
																				1			1																																																												
																1																													3																																																
1							2					1				1																											4																												2	1				1											
																1				1																																1																														
																																																																											1		1	
																																																				1																											
																1																																																																		
									1				1														3																																																											
1																1				1		1																																												1					1											
									1	1			1														2																																																											
													1																																																																							
							2																																																																													
																																						1																																																	
										1			2														2																																																											
1																1																																																																		
										2																																																																										
1										1			2			1								1						1					4							2			2														2									1											
							1						1																																	1				1																									1				
																																																				1																											
1																1							1				2																											1																											
																				1																																																														
													1																													1																																														

																											3																							1																														
						1	1			1										1		5																																	1																			1						
																						1																																																										
																						1				1																																																						
													1																																																							1											
																			1																																						1				1								1											
																							1																																																										
																2						1																							1											1																							
										1																																																																					
													1																																																																		
								1		1																				2									1			2																	1					1					1											
																																																												1					1											1					
							1																																																																					2				
																1						1																																																										
													1																																																																		
							1																				2																																																										
													1																																																																			
	1		1				3			1			1			1										1																																												1											
	1								5	1															1																																																									
										4												1	1																																																							1						
										2																																																																							
										3																																																																							
										1																																																																							
													1														1																																																										
																																																			1																														
1												1				1				1		1																																					1																					
																			1																																																		1											
1																				1																																												1																
1						1														1																																	1									1																		
1																				1		1																																											2					1											
																1														3																																																									
1										1						1				1																														2																																						
																				1																																																																	
1																				1		1	1																									1					1											1																
						1														1			1																																																											
							3		4																		2																	1					1																																					
																						1																																																																
													1																																																																			

1												1				1																																																1					1											
																											1																																																					
																																																													3																			
																						1																																																										
																											2																																																					
																																																																					1											
																											3																																																					
																																																													1																			
							7						1																													1									1		2											1												2				
							1																				4													2														1																										
																																																								1					1																			
							1																																																																									
			1				2																																			1																		1																				
																									1																																																							
																											1		1																																																			
													1																																																																			
							1			1																																1			1					1										1				2					1	2						1				
																																																																								2								

										1																																																																						
							2			1										1																																	1						1					1					2											
																																																															1					1											
							1															1																																																											
										1																	1																																																							
																																																																												2				
																											1																																												2												
																																															1																																
							1																																																																										
													1																																																																			
																																											2																																					1				
																												1																																													1											
						1																																																																											
																																																																1					1											
							3																																																																										
																																		1																																													1											
																																																					2																1											
													1																																																																				
																																																																					1											
																																																															1																			
																																			1																																																		
																																																																	1															1
													1																																																																				
										1																																					1																																
										1			1																																																																			
																																																																												2				
										1																																																																					

2 1

1

1

1

1

1

1

4

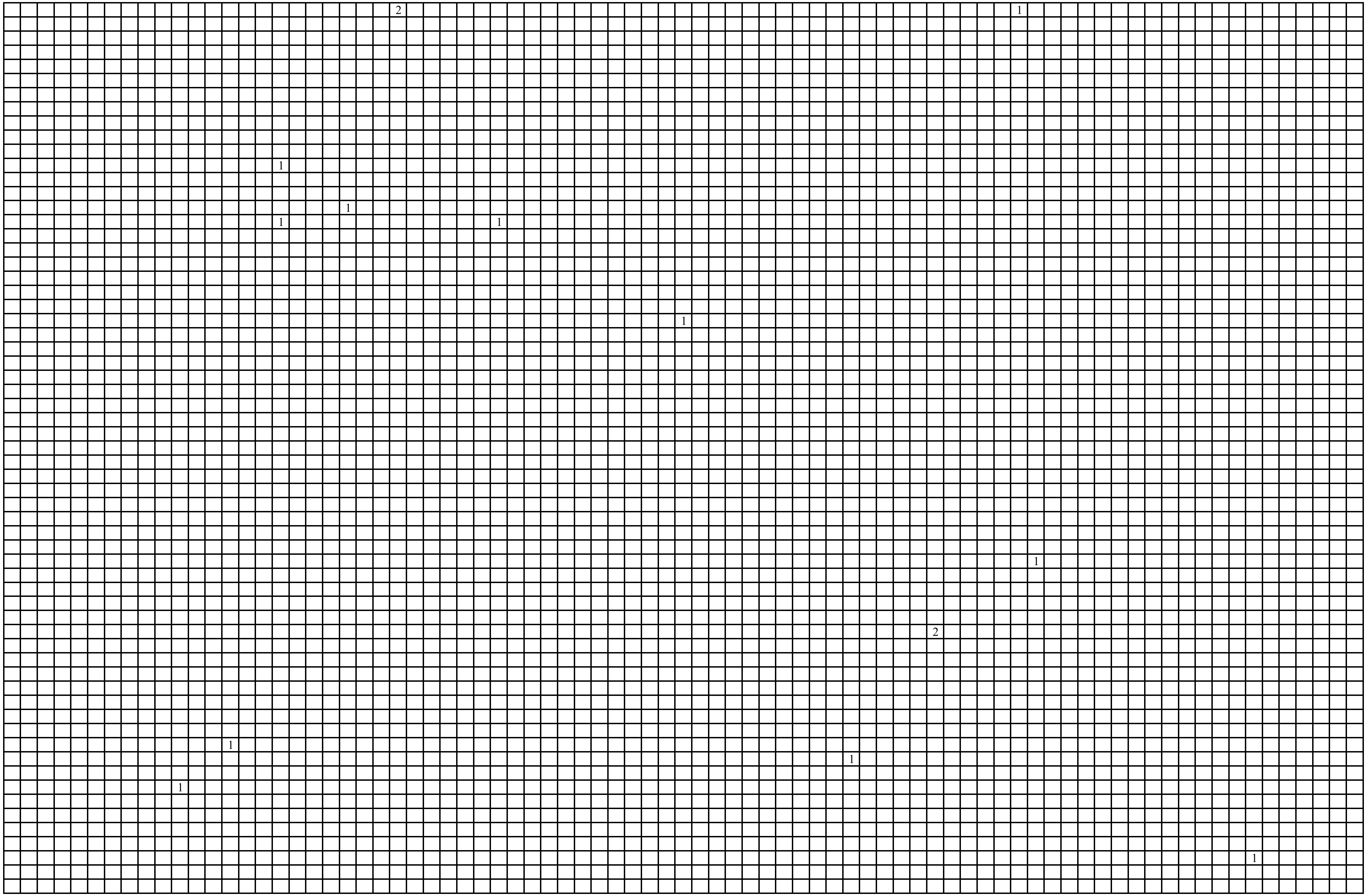
2
1
1
1
1
1
1
1
2
1
1
1
1

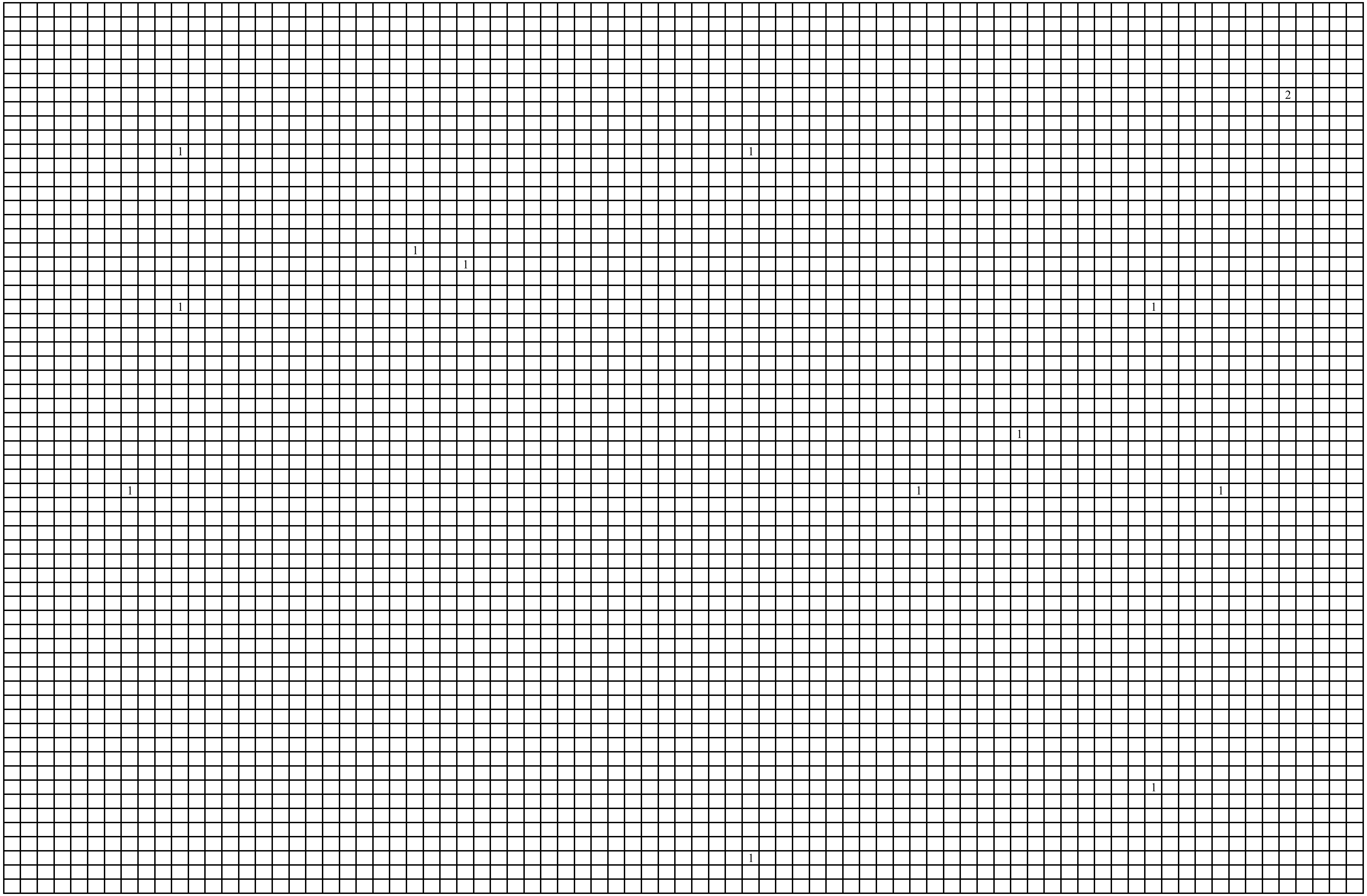
2
1
1
1
1
1
1
1
1
1
1
1
1
1

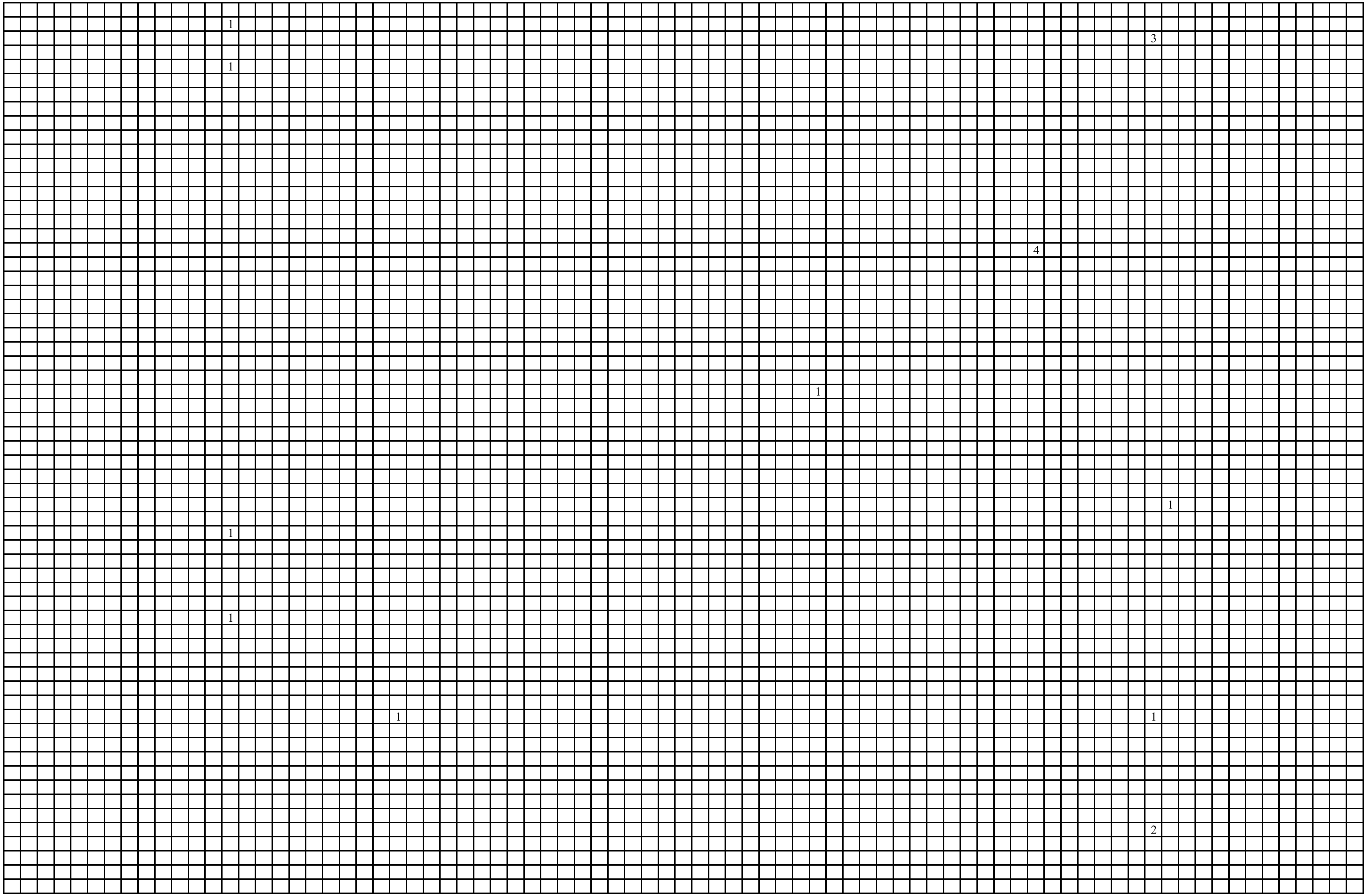
1
3
1
4
1
1
1
1
1
1
2

																										1																																																					
							1																																																																						
										1																																																																			
																														1																																																					
																																																																				1												
			1				1						4																														1			1															1																				
																																																																							1						
										1			1														1																											1																											
							1																																																																						
																																									1																																												
																																																		1																											

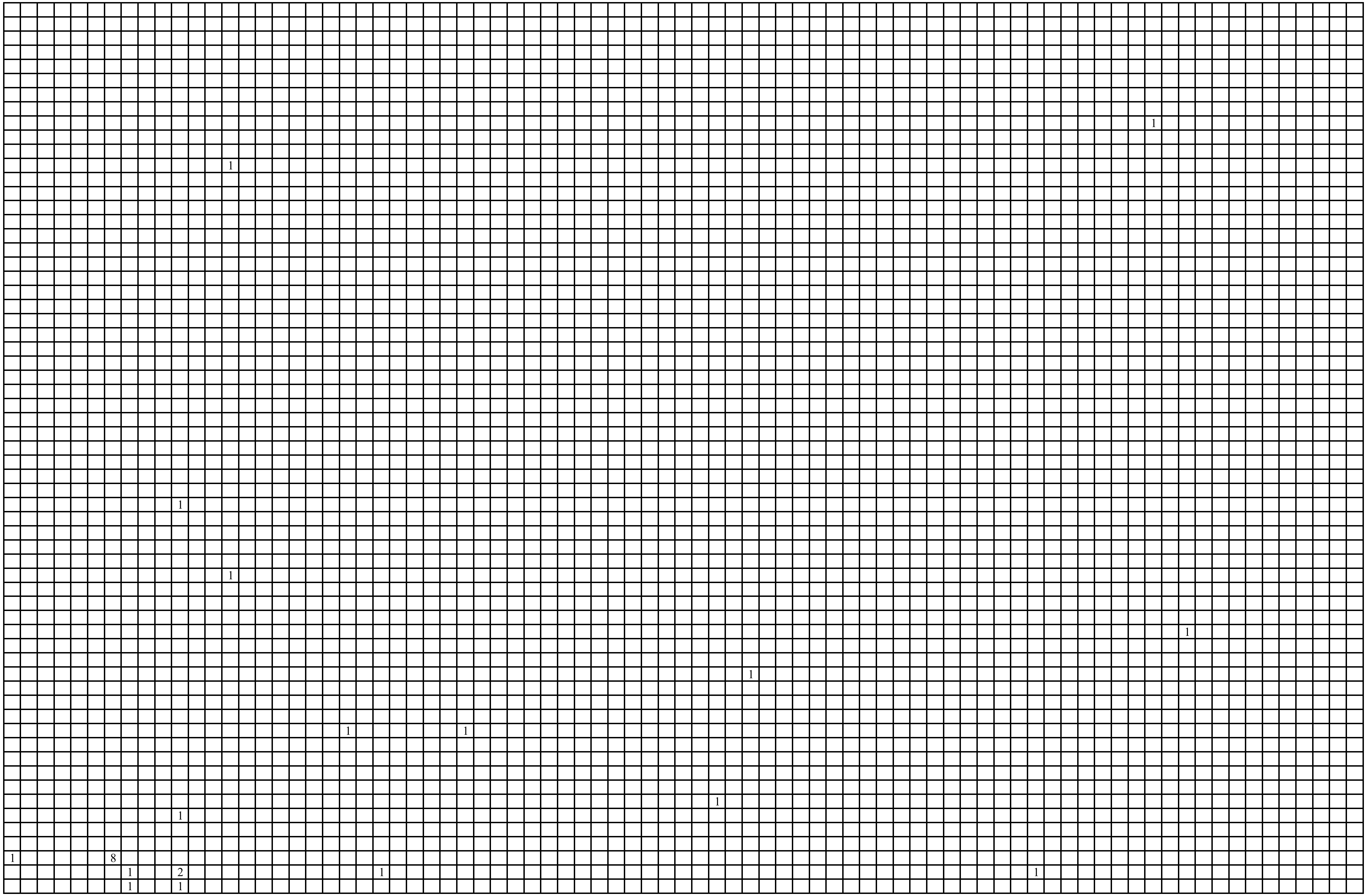
1
1
1
1
1
1
1
1
1
1
1
8
1
2
1
1
1
1

							1																				2																				1								1					1																1				
							1																				2																				1													1																1				
							1												2																																																											
										1			1																																																		1																			
											1											1																				3					1									1	1	1																						
																																																													2																			
							1																																																																									
																																															1										1																							
							2																				2			2				1													1													1									1							1				
													1									1																																																										
																						1																																																										
																						1																																																										
																						1																					2																																					
										1			1																																																		1																			
																												1					1																																									1											
																						1								1																																																			1				
													1																																																																				
														1		1		1						1			1																																																													
							1																																											1																													1
																1																																																																
			1							1																																																				1																
													1																																																																				
																						1																																			1																									
						3																																																																										
																1																																																																	
										1			2																																																																					
																				1																																																														
																																											1																																					

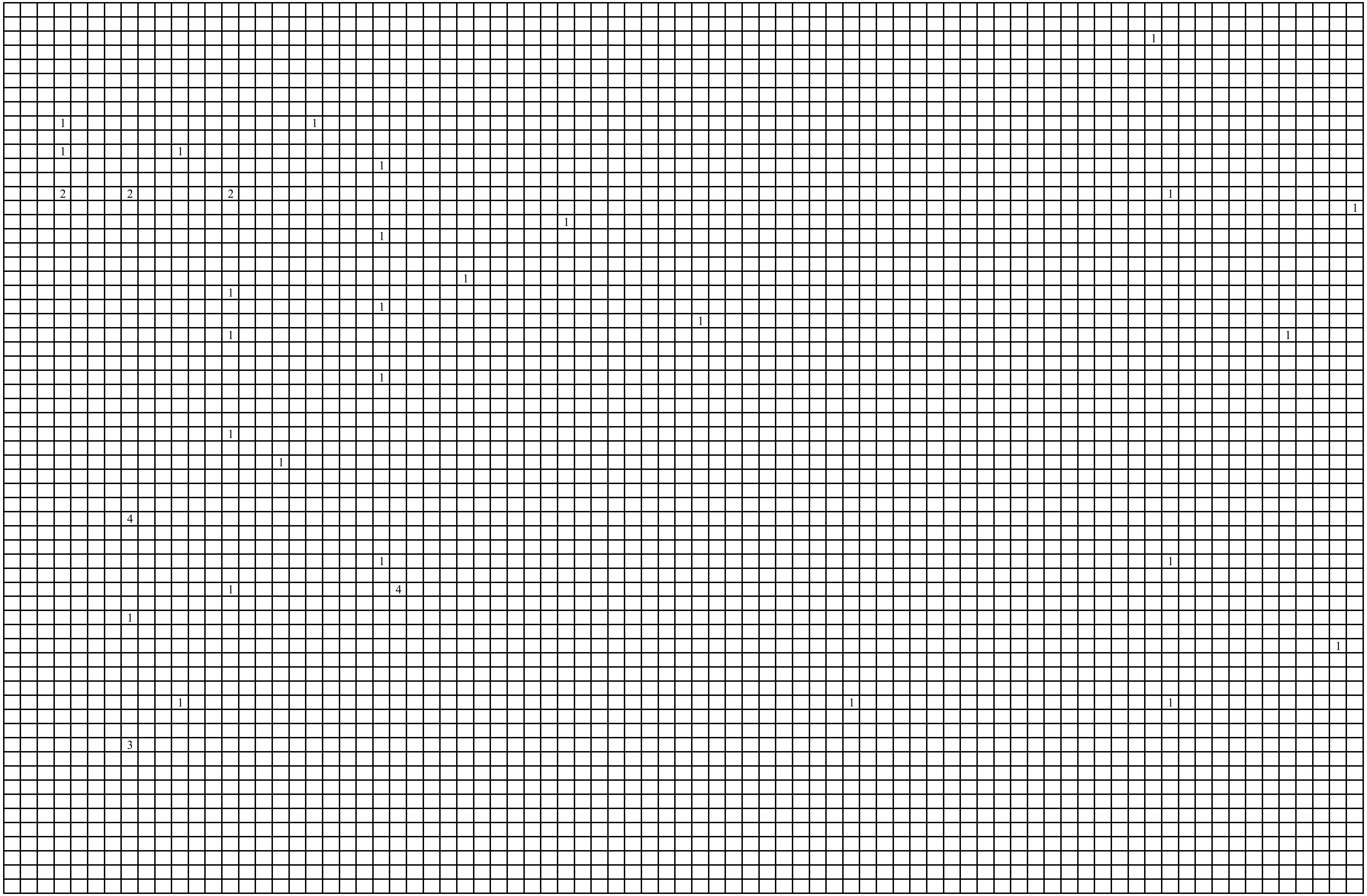
1
1 1
1 1
1
2 2 2 1
1
1
1
1
1
1
1
1 1
1
1
1
4
1 1
1 4
1
1
1 1 1
3

										1			1																																														2										1											
																																																									1															1								
																1																																																																
										1			2																																																																			
																						1																												1																														
													2																						1															1							1												1											
										1																																																																						
																																																													1																			
																																								1																																								
							2			1			3			1																																																																
																											1																																																				
																						1																																																									
								1																																																																							
																																																		1																													
																																													1																																		
																1																																					1							1			1																
1												1				1																																												1			1					1											
																1																																														1					1											
										1			1																	1												1			1		1									1	1												2											
										1																																																																						
						1														8																																				1																									
													1																																																																		
													3	1						1																																								1								1											
													1	4																																									1														1											

													3	2						1																																																	1											
													1							1																																	1																											
																																																								1																								
																						1																																																										
2										3		1		3		1		1		1		1					1							1								2			3			1		2				2			2									12			2	4		2			1		1			
1	1		1		1		5	9	1	1		3						5		1	1												2	1	1			1				2	2		1		1			1			1			1	1		1	1		1							1							1	1			2
1	1		1		1		1	3	1	1	1					1				1	1		1																	1							1	1				1	1	1		1			1		1	1													1			1		
								3					2																				1																																															
								1					1																																																															1				
								4					1																				2																																															
1								9								1		2	1	1														2				1				1												2		1	2												1						1				1	
								1																										1																																														
																																										2																																						
																																												2																	1																			
								1																									1																																															
								1																																																																								
												1																														3																																						
																																									1																																							
								2																									1																																															
								1																																																																								
								4								2											2						2																																															
							1	7															1				1																																																					
								1																																	1																																							
								2					1																																																																			
								4																																		2																																						
								1																									1									2																																						
								1					4																																																					1														
1								1				1				1				1													1																1															1																
								1																																																																								
1	1		1		1		1	1		1	1						1				1									1									1			1			1			1					1					1		1																1				
																																																								1																								
													1																																																																			
								2																						1											1																															3								
								2																																																																								
								1																																																																	1							
																		2																		1																													1															
																											1																																																					
								4																																																																								
								1																																																																								
								2																																																																								
			1																																																																									1				
																																																																					3											
																				1																																																												
														1																																																																		
																																							1	1																																								
1			2				1			1											1									1					1							1			1		1										1	1	1	1																1				

													1																																																																			
																																																													1																			
																																											1		1											4	3				3																			
								1								1																	2																								1																			1				
													1	3		1																																																																
								1																																																																							
						1																																																2																									
																						1																																																									
																						1					3																																																					
							1						1																													1						1									2			1																1				
													1																																																								1			1								

383	131	107	964	186	80	204	53	11	20	70	38	64	666	746	236	810	97	202	993	55	44	357	200	185	183	472
42	15	4	80	73	11	15	4	7	3	18	5	1	231	208	15	36	9	5	83	25	5	15	31	48	73	97
RRP8	RSF1	RTF1	RUVBL2	SAP130	SCAI	SENP1	SERTAD3	SETD6	SETSIP	SFMBT1	SFR1	SFRP4	SIN3A	SIRT1	SIRT2	SIRT7	SIVA1	SKAP1	SNW1	SRA1	STK36	STRN3	SUB1	SUDS3	SUFU	SUMO1
																1			1							
				1									5											1		
				2									3	1										2		
																						1				
									1																	
																			1							

63	482	128	352	317	141	325	219	83	128	140	103	168	120	119	171	198	182	228	104	301	15	331	162	414	147	85
5	36	18	68	14	18	68	21	11	22	20	21	33	10	5	21	79	57	28	6	4	2	10	11	36	8	1
SUPT4H1	SUPT5H	SUPT7L	SUV39H1	SYK	TADA1	TADA3	TAF10	TAF11	TAF1B	TAF2	TAF3	TAF5L	TAF7	TAF8	TAF9B	TBL1X	TBL1XR1	TCERG1	TCP10L	TDRD3	TESC	TGFB1	THOC5	THRAP3	TIMELESS	TIRAP
	1																									
	1																									
																		1								
			2								1	1				1										

291	166	1071	220	35	33	209	1228	67	234	42	490	2860	729	114	255	984	1340	565	603	955	1168	470	292	290	600	103
144	16	88	28	6	4	15	59	15	47	6	146	187	66	9	36	139	308	19	15	81	52	43	12	1	40	8
TLE1	TLE2	TNIP2	TNKS	TNNI2	TP53INP1	TPR	TRAF6	TRIB1	TRIB3	TRIM22	TRIM24	TRIM25	TRIM37	TRIM8	TRIP4	TRRAP	UBE2I	UBE2L3	UBE2N	UBE2O	UFL1	UHRF1	UIMC1	URI1	USP15	USP16
												1														
		1										1														
												1														
		1										1														
												1	1													
2																1	4									
1											1						1									
1											1					1				1						
																1										

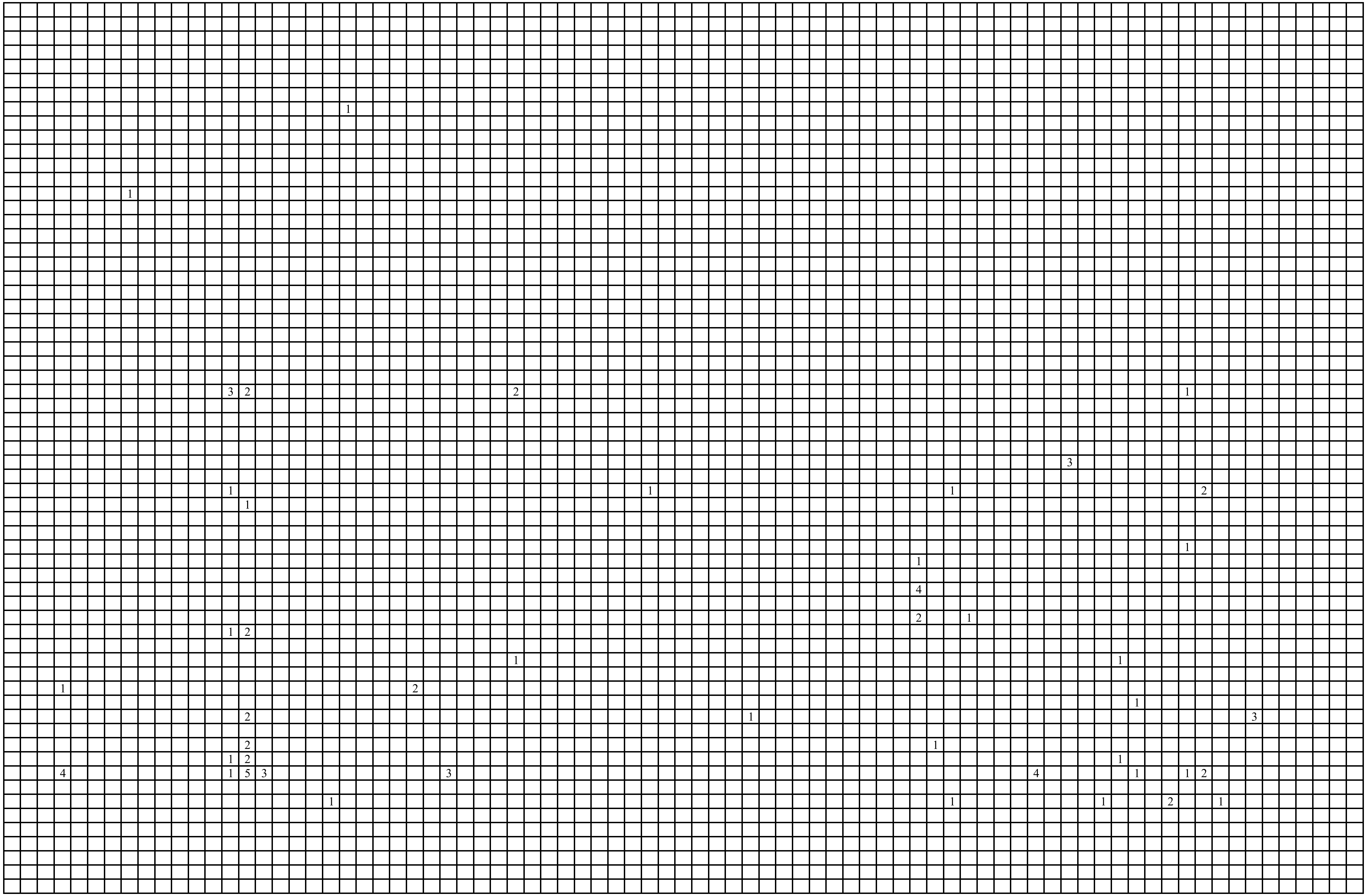
1
1
3 2 2 1
3
1 1 1 2
1
1
1
4
2 1
1 2
1 1
1 2
1
2 1 3
2 1
1 2 1
4 1 5 3 3 4 1 1 2
1 1 1 2 1

																																																																			1			1										
																																																																		1															
			2	3									4	1												1																								1																						1				7										
																														1																																				1															
																										1																																										1				3										
																																																																									1										
																																																														1																			
													3																																															1																									
				3									10												3																																																								
				2									3												2																																																								
																																														3																																			
													1																																																												3						
1	1	1	8	2									3	2					7				1			1	1	1	3	1		3	3	5		1	1		3	1		3			3						3					1				2			3		3	2				20		1				5				3
			1																											2									2																																	2										
														3					1				1											1	1									1								1	1									1										2						1			
			1									1	5																				1																															1				2	1									
																																																																								2	2									
				1										1																																																																	1	
																			1																																																				2										
																														1																														1																			1	
													1																1																									1																									
																																																								1																									
																				1																																																			1									
																																																																									1									
																																																																			1				1									
																	1																																																															
														4																																								8	2	1																										
																																																																												1					
			3																	1						2																												2																	2										
																																																																														1		
													1														1																																																					
																	1																																																															
														5											1										1																			1												3		2					3				
																												1																1										1					1						1		1						1			
														1																																																													1					
																																																																									1										
																																																																															1	
																										1																																					2	1																
																				1										1																																			1				1									
														2						1																															1							2			1						3	1									
																				1																																									1																			
																																																														1			1															
																				1																																									3																			
																																																																		1															
													1																																																									1			5								
														1											1																																												1			1								

																																																													1		3									
																				1																																												8		1																
																																																																3																		
																				1																																																													
																																																															1				1														
														1						1																																														1							1								
																																																																							2										
																																																																			1															
																																																																								1										
																																																																								1										
			1	1											2	1					1	1			1			1			1					1		1	1					1					2	2						1												2			1	1				1	1										
1	1	2	2	1											2		1				1			2			2		2						1						1				2	2						2	2					1				2		1			1	2				1	2		1			1				
																																																															1									1										
																																									1																									1									2										
																				1																																												2																			
																																																																						1										
																																			1																																														
																																			2																																														
																																			2																																														
					1																																		1																																										
														1																																									1					1					2	1		1					2	1					
																												1																																																					
																																																								1									1				1					1					
														4																		1																																				3											
																																																																	1																
																				1																																			1									1															
													1																															1		1			1															1				1			1			1				
																																																								1								1																
																																																																	1																
																			1							1																									1			1													1						1			1				
																																																						2																										
																																																						1	1																								
																																																						1																					1					
																								1																					1															1																					
																																																								1																									

																											1																														1																								
																																													1									5		1					1																			
																																												1											1											1															
																																													1																																			
																																																																		1														
				1								1											1																				1																													1						
				3								6											1																			4	2						1									1						1														
																																										1											1																					1					
														1																																			1																				3									
														4												1																																							1				1	1									
														4	5	1																																																															
														8																								1																																							1										
														1																																																																
														1			2																																																														
																																																						1																										
																																																																						4										
			1																											1		3							3																																		4										
																																																																										1					
1																																	1																																															
1																																																																																
																																																																					1													
						3																																					1											1																	2									
													1													5																																																		5									
																1																																																1				3						1				1					
								2																												1																																		1														
																																																																			1															
																																																																						1														

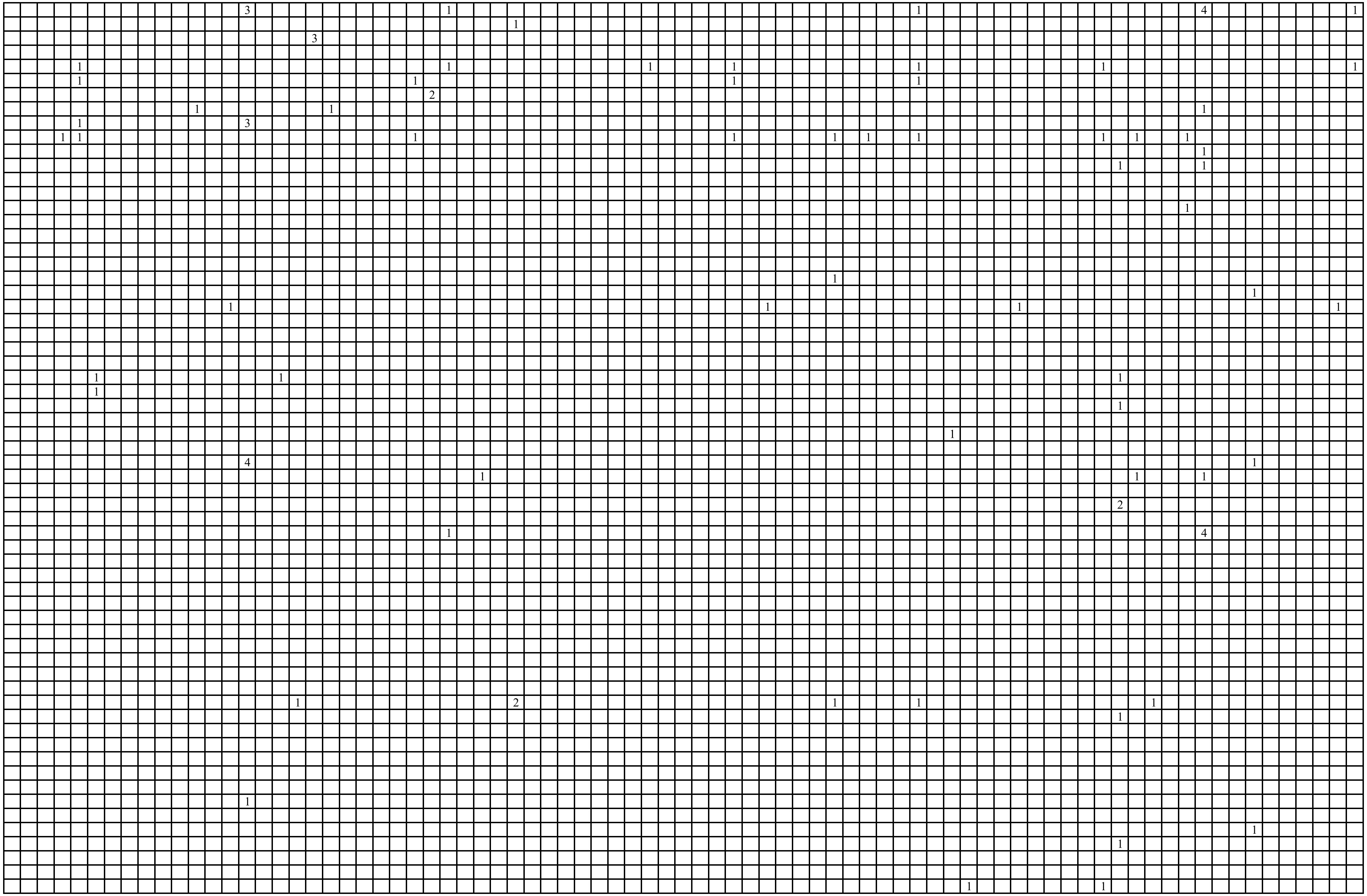
3 1 1 4 1
1
3
1 1 1 1 1 1 1
1 1 1 1
2
1 1 1
1 3
1 1 1 1 1 1 1 1 1 1
1
1 1
1
1
1
1 1 1 1
1 1 1
1
1
1
4 1
1 1 1
2
1 4
1 2 1 1 1
1
1
1
1
1 1

													3																																																1																	
										1				2																																																																
																																																																1														
																														2																																																							
																																																			1							1																								
																																																																1																	
																																																										1																								
1																									1											1													1											1																			
																																																					1									1														
																																																								1										1					1										
																																																								1										1															
																								1																														1										1															
																																										1											1										1					1										
																														1																								1																										
																																																								1	1																									
																																										1																																						
																																																									1																									
				1										1																													1											6	1											1													
																																																							1		1																								
																																																							3																										
																																																							2	2																									

																																																			1																												
	1																																																				1	1																												
				1									1																														1											1											1					2										
			1																																								1							1																				1									
													1																																																																			
																																																																		1														
																																																				1																													
													1																																																																			
				1																																							1											3																													
																																											1											2																													
																								1																			1	1										3	1										1																
																																																				3	1																1	1									
																				1																																																														
																									1																												1										1															
																																																				1																													
																																																				2																													
																																																				1																													
														2																																																																			
														1																																														1			2														
																										1																											1																1									
																										1																																															1									

							1																																																																									
																																											1											1																													
																																																																	1														
																																																				1																										
																																																				2																										
														1																																																																		
																																																				3																										
																																																						1																								
																																																															1																	
																																																																		1														
													1																															2	2																																				
													1																																											1																								
																																																																															1	
																																																															1																	
													3																																																																			
				1									1											1																																												1														
				1									1											1																																																												
	3													1					1																			1													2			1		1															2		1		1					
																																																			1					1																								
																																																																	1														
																																																																								1										
																																																					2																												
				1									1											1																																																					1									
																1															1																																																3														
																																																																					1														
			1	1									2						1					1				1																	1						1													1		1														
				1									6											1				1																																																											
										1																																																																												
																																											1											1																	3									
																																																																								1										
																																																																					1														
																																																				1																										
																								1																			1											1											1															
			1	3									1															1		2									1						1	1					1		1			2											2														1	
																																																																								1										

																																								1																																	3								1	
				1																																							1											1											1						1									
				2																																							1											1																	2									
					1																																																1											1															
																																																							1																1	2									
																																																							2	1	1																								
																																																																						2									
																																																																											1					
										2																																					2																		1														
													1																																																											1										
																			1										1										1																																																
																5										1										1																		1												1								1	1					
	1																		1				1			1	1	2															1	1	1					1	1	3													3					1	1			1	1	1				
	1		1																		1							1																							1																2				2			1		1				
			1											1					1							1																									1														2					4	1								1	
																																																		1																					2									
																																																																		1														
			1																																																																1													
																																																																				1			1					1					
																																									1																																						1									
																																																																									1									
																																																																				1													
																																																																						3		1									
																																												1											1																											
																																												1	1										1																											
				1																																							1	1										1											1	1														
				1																																							1											1											1	1				1										
													4			1			1																										1											1										1	1			1	1			1	1					
																																																																1														
																																																																		1	1														
																1																																																																
																												1																						1	2					1													1						1					
																				1																																								5																							
																																																																		1														

																																																													1														
																																																													1														
3																1															1																																																	
2																																																																		1						1						
																1			1																													1																			1									1					
													1																																												1															3									
																			1																																																														
																																																																												1					
																																																															1														
																																																										1																								
																																																														1														
																1																																										1														1	1				1					
																																																														1														
										2															1																																																														
																																																														1														
																1																																																								1										
					1																																								2																												1									
																1																																																																	
			7																									1																																											1													
																1																																										1																	1	1					
			1																																																																												1					
																																																														1														
																			1																																										1																		1					
			1										1																																												1					3					1	1								1				1	
																																																													1					2					3						9	1						1	
																																																																										2									
																1																																																																		
																																																																													1						
																							6																																			1							1			1	1							1	1					
																																																																4																			
																							1																																																														
																																																																														1					
			6																																																														1					6										
	1		1			1							6	25	1	1	3		2				3			3		3	1		1	1	6	1		1			1						1						2								4		2				5	6	3	1		3	21	1	5	1	7	4	3		2	
																										1																																																4									
														3												1																																																1									
																																																								1												1														
																																																																									1									
																																																								1																										

				1																																							1											2	1														1											
																																																															1																	
																			1												1																									1																								
																															1																																																	
1																																				1																																												
	1		1																																																					14													3											
																																																								2																								
																																																								5																								
								5						1																					1								2											1		5							2						4			1								
																																																								2																								
																																																																		1														
					2																													1																																				1		2									
					2																							1		1																																								1						1				
																									3																																																							
		1								3			1						4																																															2														
													2																	3															1									2							1					1														
																														2																								1																										
															2										1					1																								2																										
			1																																																																													
																							1																																																									
																																																																							2									
																																																																							2									
									1					2													1																									1																						2						
	1					1																				6																																													9									
																																						1																																									2	
																			3																		1		1																																									4	
				1									2						3											1							1			1									1														3		3		1			1										6	
												1							1							1																								1											1					1					7								4	
																														1																																																		
																																																																										2					3	
														3																																										1											3					1		2					3	
																																																															1			1														
																																																	1																						1									
																																																																						2										
													2		3																	3																						1								4				2		1					1									
																																																																		1														
																																																													2				1															
																																																						1												1														
																																																						3																										
						1							1																																																																			

																																											1											1																									1	
																																											1											1												1											1			
																																																																		1														
																																							1															1																										
																																																								1																								
																										1																																													2				3					
																																											3	2																																				
														5																														1																									1											
																																																																		1														
																																					1	1																											1					1										
				1																				1													1	1																												1				1										
																																																																	1															
																													1								1	1	1																										1					1										
																																																						1											1															
													1																																																										7									
																																					1	1																1																										
																																																			1															1														
																																											1	1																																				
																																																						1											1	5		1								1				
																				1																																													1						2									
																				1																																																												
																																																																							1									
																																																						1											1	1														
																																																																		1														
													1													2																																													2									
																								1													1	1			1	1												1											1															
																																																																							1									
													2																																																																			
																																																													2																			
																																																																		1														
																																																													1					1									1					
																																																						1							1																			
													2																																																	2																			
																																																													9																			
																																																						1							1																			
			1								1								7		1					1		1																																1													1		1					
																				1																																														1														
						1																																																																					1					
																																																																											1					1
													3													1																																			4										4									
				1									1											1																																																								
													3	2					3									1															16	12																						1	1				2									

													1						3							1		1															7													1										1	1			1	1									
										4													2																				1																1						1			1										
																																																																	1															
										2																	1																																																			
													3			1																	1																					1										1				1									
													1			1																																																						1								
																																		1																																1												
													1						1										1			1	11	1					1			1																												1									
														3																5			6	6	6					6			5														1														7									
																												1			1						1						1														19																							
																												1									1														1						1														1								1	
																1																																																															
			1																										1			1	6						1			1																						1						1									
2																1															1																																												1					
																																																																							1									
1													1																																																										1									
														1																																																					1													
																																																																		1													
																																				4																																												
																				1																																																											
																				1																																		1											1						2									
													5																																																													1						
				1									2											1																																																							
													4																																																																			
																																	1																																1				1									
													1																																																																			
				1																										1																																			1															
			1	1									5						2					1														1												1																1													
				1																													1					2	1			1												1										1	1					1									
																																																						1							1																			
																										1																												1										1	4					1									
																													1			1	1						1			1																								1	1													
			1	1																				1									1				1	1	1			1																						1															
				1									2											1																																																							
	1			1									1										2								1																					1														2													
																							1																																															1									

				1									1											1																			1	1	1									1											1															
																																																																	1														
																									2																																																1														
													1																																																																				
																	1								2																																																		1									
																			1																					1												1																																
													5	1																																																	2																	
													1																																																																			
													1			1															1							1																													2														
														1																																																																		
																																																						1																								
																														2																							2																										
													1								1				22																																																									
																					1				6	1																																																		1									
																					1				5																																																													
																																																																		1														
																									5																																														1																	
1																																																																																			
																																																																												1						
																									2																																																															
																							1																												1															1														
																										1																																													1					2									
						1																																						1											2	1																									
																						2																																																															
				1																																																																													
																																																																											1										
			1										4		3																																						1		1																												1	
						1							3																																																												6									
													2									2																																																															

																																																																						3									
1			12																														1				2	1				1																						1	1				1	3									
																1																																																																	
													7													2																																																	3									
																																																					1																								
																														1																																									1								1					
																										2																																																	1									
1																																				1																																																				
																										1																																																													
			1										1	1																																																									1					1									
																																																																							1														
																																																																												1										
																																																																							1								3						
																										1																																																													
2																																																																																			
																																																							1																											
																																																																															1						
																																																																								1														
																									1																																																								1									
																																																											1																								
																																																																						2															
																																																																															1						
																														2																																										1														
																																																																															1						
																																																																						2															
																									1																																																																
																																																																							1														

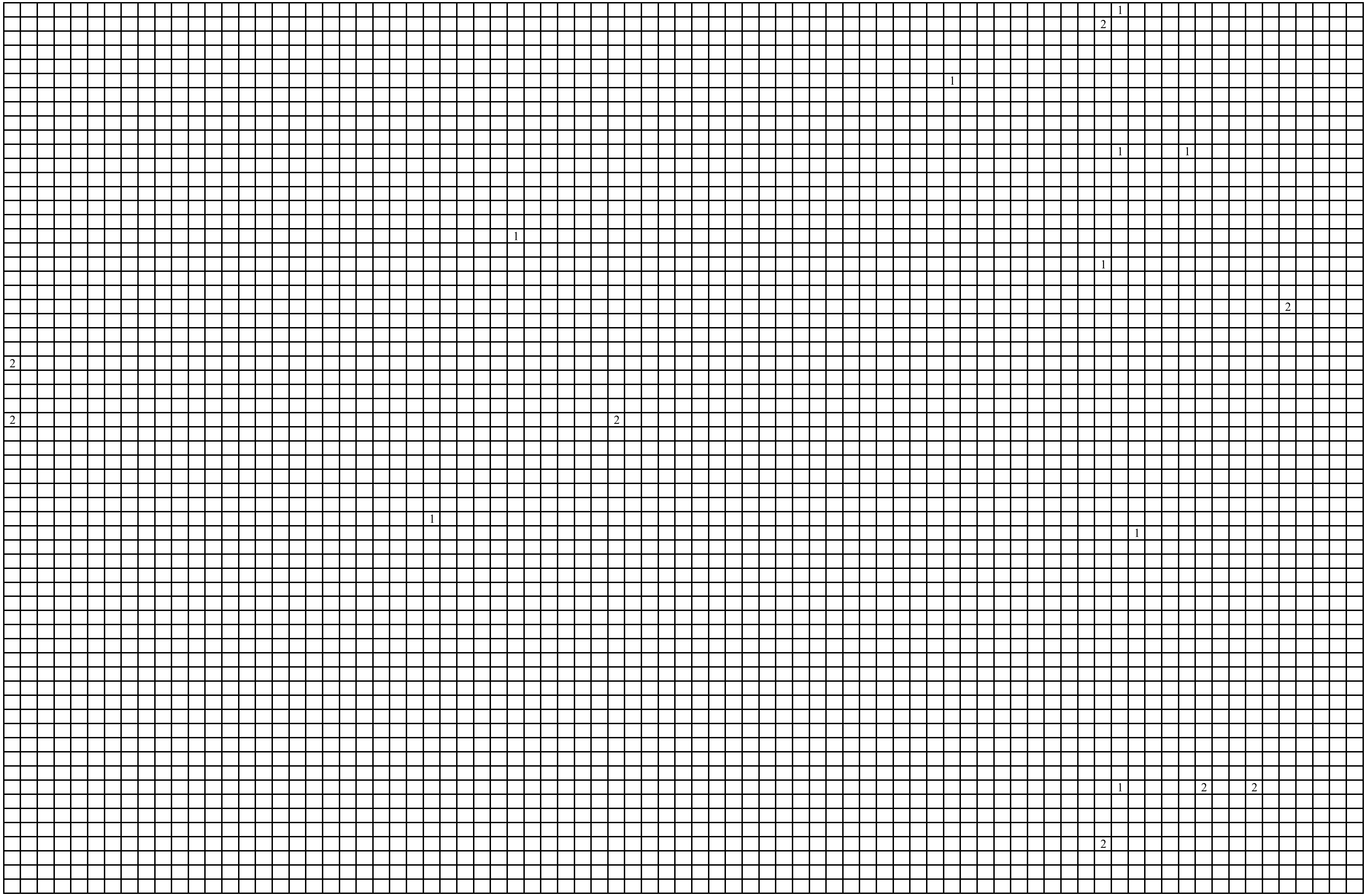

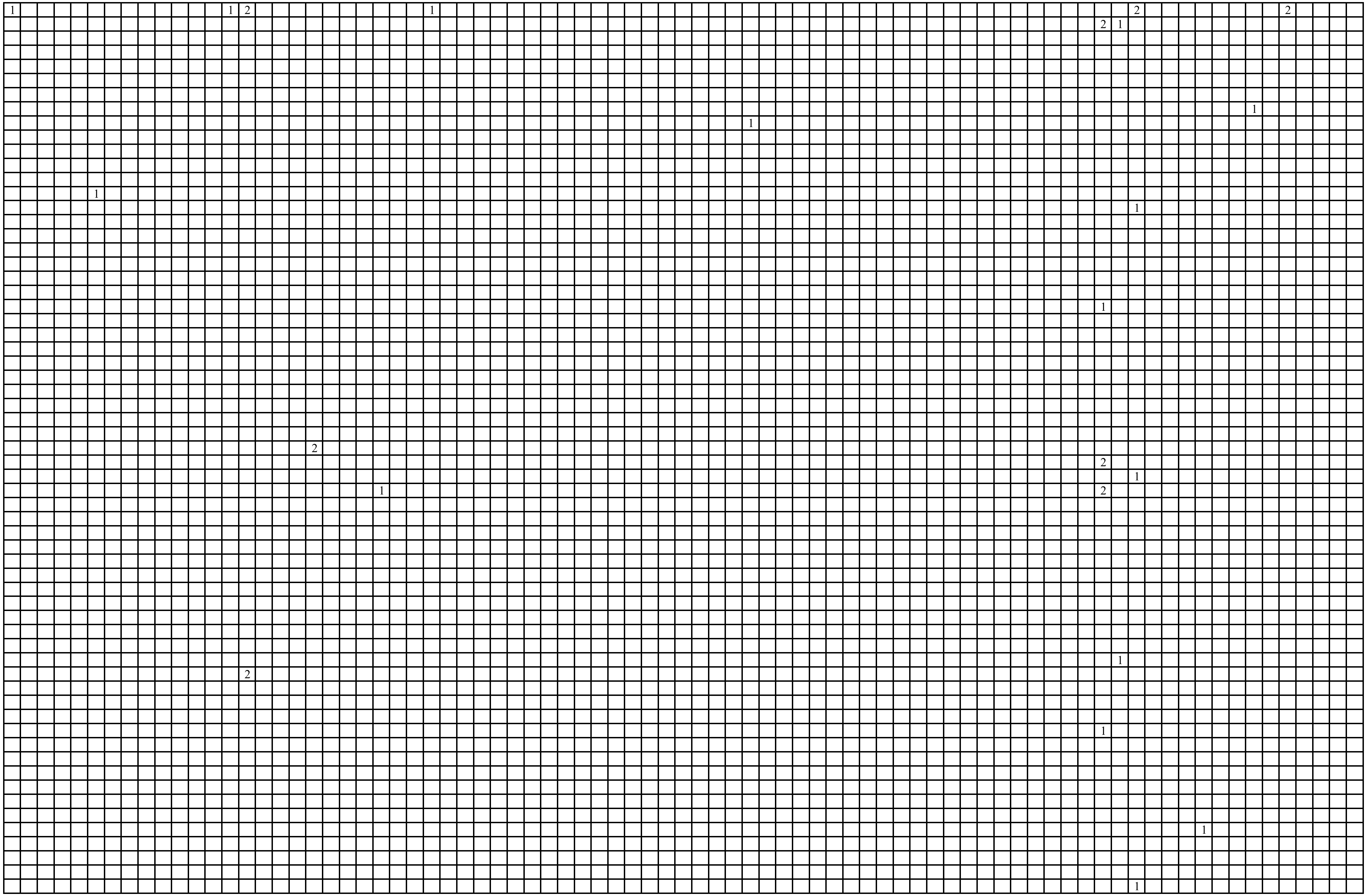

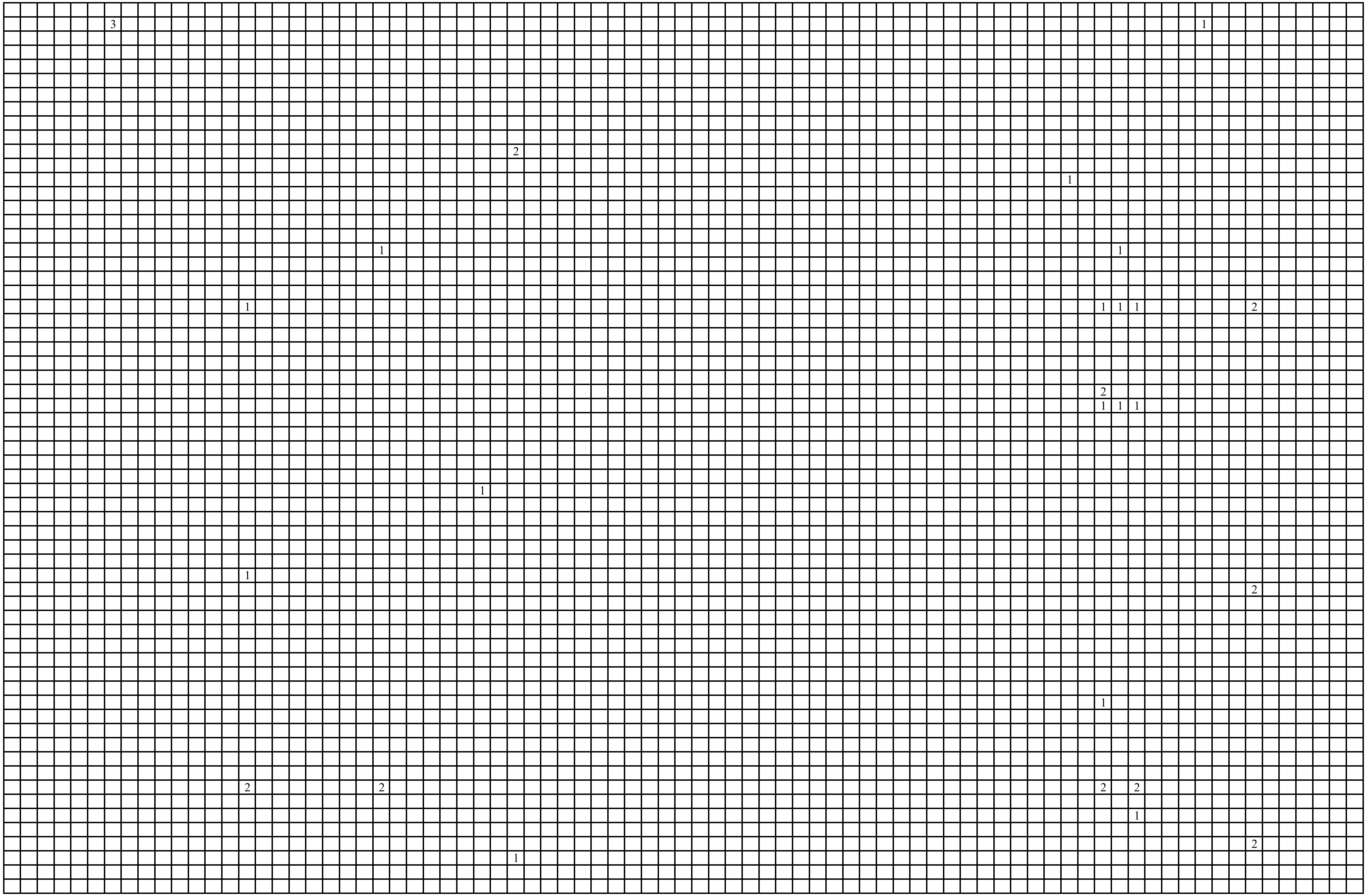
3 1
2
1
1 1
1 1 1 1 2
2
1 1 1
1
1
2
1
2 2 2 2
1
2
1

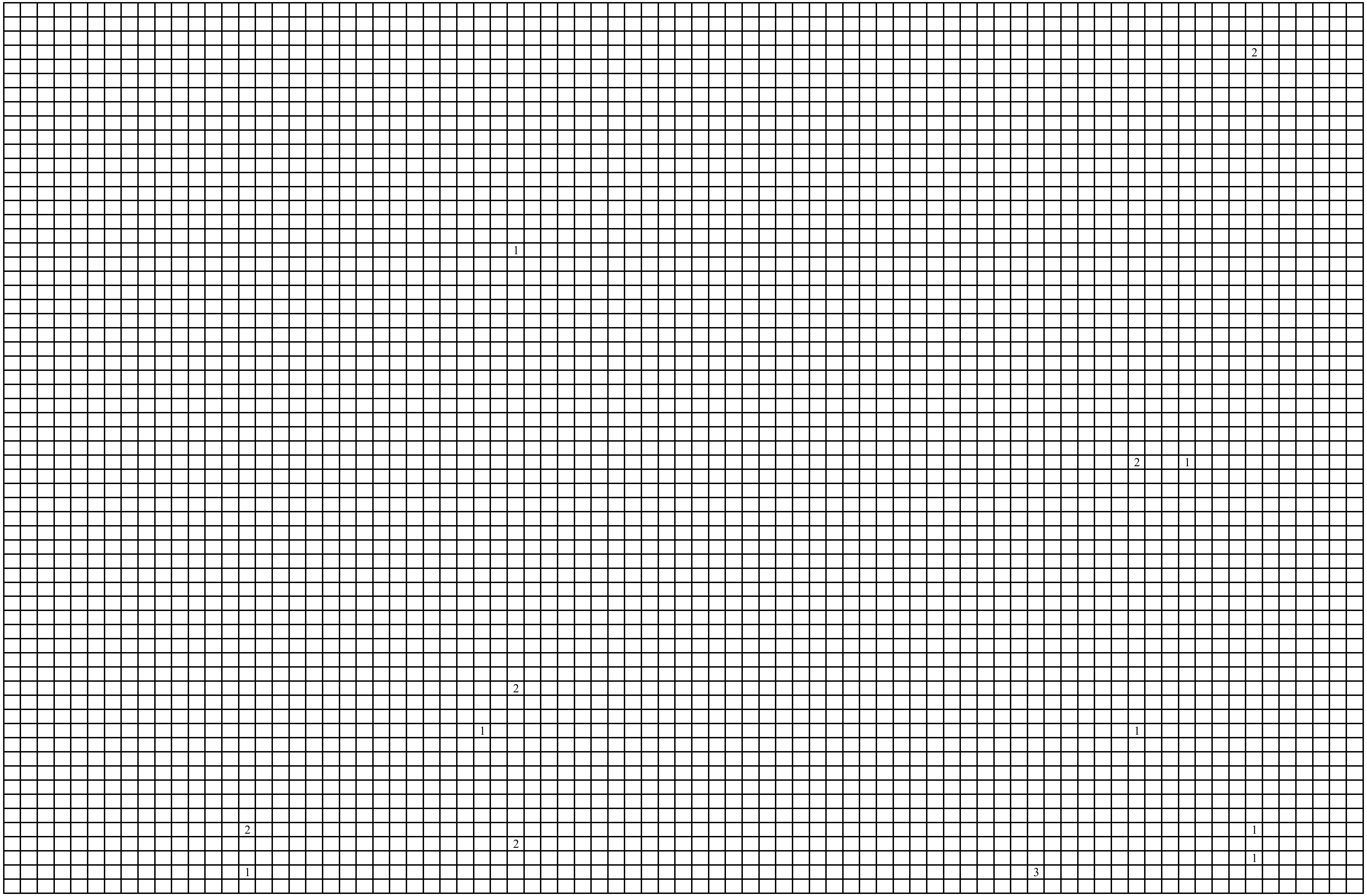
2
1
2
1
2
1
1
2
1
2
1
1
1
3
1

																																																																		1														
1																																																																									1							
																																																																													1					
																																																														2																
																																		1																																																		
														1																																																												4							
2																																																																																		
																																																																1															
																																																														1																
																																																				1				1																				
																																																														1																
																																																																	1													
																																																				1										1	1													
																									4																																																											
																									1																																																											
																									4																																												2															
																																																																										1	1					
																																																			1																										
																																																														1		1														
														1																																																																				
														2											2																																														2															

																																																																	2													
																														1																																																						
																																																																						1									
																																																																									1						
																																																																									1						
																																																																					1										
																																																																		1														
																														1																																																						
																																											1																												1										
				1									2	2										1				1																																																				1									
				1																				1						2																																																								
																																																																			1														
																																																																											2						
														5																																																			1					2									
																																																																												1					
																																																																												1					
																												1																																																									

																																																																	1														
			1										5						1																									1																											4									
													1																																																																	
			1										1													1																		1																					1					2									
													1																																																																	
																																	2																																										1			2						
																																																																												1	1					
			2											2																4														1											1		1		1							1				1									
																																																																												1				
			1										3										2																																																										
										1									1																																														1														
																										2																																												3									
																																																																									1									
																			1											1																																																											
																																																																											1									
	1																									1				1																					1																				1			1						
																																																															1									1										
			1											3																															1																																							
													1																																																																												
																																																																						1													
										1																																																									1													
				1																		1		1																																																						1					
																																																																						1														
					1																									1																																																								
																								1																																																													

																													1																																	1				2													
																																																																1															
																													1																																																										
																																																																	1														
			1																																																																														
1																																				1																																			1										1				
2																																				1																																																			
2																1																																																																		
				1									1											1				1																												1														1										
										1																																																																								
																																																																													3						
																																																																													1						
																																																															1				1							1						
																																																																				1													
																																																																										1									
																																																																										1			1						
2																																																																			1									1					
																																																										1																							
																																																																														1						
																																																																							1									1					
2																																																																																				
																									1																																																					5									
																																																						1																								
																																																						1																								
																		1																																																																	
																																						1																																									4										

																																																															1														
																												1																																																						
																																																																						1									
																																																																					1										
																																																																								1	1						
																1																																								1											1													
																			1																																																	1					1									1					
																																																															1														
																																1																																																			
																																																															1														
																																																																					1					1					
																																																																1													
				2									2											2																																																										
																																																						1																								
														1																																																																			
																									2																																																											
							1																			1																																																							
						1														1																				1			1										1											1														
				1																																							1	1									1		1								1															
																																											1	1									1																										
	1																																					1						1	1						1																								1				
				2									2											2																																																										
				1									1											1																																										1														
				1									1											1																																										1	1													
				1									2	5										1																																										3					2									

				1									3	2		1								1																																																			1					
				1									1											1																									1																															
																																	1																															1		1														
																										1																																													4									
			1	1									3	4					4					1					1		1		1							1	1	1	1	5							1			1						1			1		6	1				1	4					2				
1			1								3		2	2		1			1	1		1				1			1	1			7	1		2	2		1		1			3	2		1				2			1							1				7	4		4	2	3	2				1	2	5			1
1				1									1			1			1	1		1	1			1	1					1	1			1	1		1		1	1		1			1			1															3	2	1		2						1		1			
														3																																												2								1														
														1																																																																		
																				1																																		2				1																						
1	1																			1		1				1			1								1																												2			1			1	2	1			4				
																										1																																							3						1									
																																				1																																											1	
																																																																		1														
														1																1																																									1			1						
														1								2								2																																									1									
														2												2																																																						
																																	1												1																									1	1									
																																																																	2															
														3																																																	1								2									
																																																																		1														
			1											3		3																	1										1	1												1							2		5	1	2		2		4									
				1										2					2											1			4											1																			4		3	1	1		1											
																																																										1													1									
																																																																															1	
													1																	1					3					2			1	1																					2						1									
																																											2	1																									3		1									
														2					7																2					2											1																													
				1										2									1									1						1	1			1	1											1											1					1	2									
																																																										1																						
													1																																																																			
			1	1					1										1				1											1				1				1			1						1										1					1							1		1					1
																									3																																																							
																																																								1																								
																																																																		1			1											
													1						1														2			1																									2				3	1			2		1									
																																	1																																		1													
													1																																																																			
																																																																							1									
																																																																							2									
																																																																													1			
																			2																																																													
													4	3		1			1																																										1																			
																																																																		1														
1																																																																																
																														2																																			1															
																1																																																																
				1									3											1																																																								
				1			1						2	2										1																																														1										
													5																	1																																									1					7				
2			1										8						1				1			1		1		1						2		1	1				3	3	1					1	1																			1	1				1					

													1																	4															1																	1								1									
																																																																		1										
																			1																																																												
													1																																																																		
																										13																																																		18	1								
																										8																																																		11	1								
						1																				5																																														1			2									
						1																				1							3													1																				1							5									
														1																																																					1														
																																																																		1														
			1										3										1																																																				1	1					1	
																																																													1																				

USP22	USP7	UTP4	UXT	VHL	VPRBP	WAC	WDR61	WDR77	WWC1	WWP2	WWTR1	YAF2	YAP1	ZMIZ2
355	1592	0	240	1175	486	114	218	493	155	1190	477	376	1931	69
43	238	0	12	255	45	12	9	39	1	76	55	46	214	26
										2	1		5	
										2	1		2	
											1		2	
											1		2	
												2		
	2													
	1													
	1													
												1		
				2										

U1 snRNP			SM proteins							U2 snRNP										U2 snRNP related										A complex									LSm proteins					U5 snRNP						
SNRNP70	SNRPA	SNRPC	SNRPD1	SNRPD2	SNRPD3	SNRPE	SNRPF	SNRPG	SNRPB	SNRPA1	SNRPB2	AC008073.5	SF3A1	SF3A2	SF3A3	SF3B1	SF3B2	SF3B3	SF3B4	SMNDC1	U2AF1	U2AF2	DDX46	DHX15	CHERP	PUF60	RBM17	SR140	HTATSF1	CCAR1	PRPF40A	SF1	THRAP3	RBM10	RBM25	DDX5	RBM5	BUB3	LSM2	LSM3	LSM4	LSM6	LSM7	CD2BP2	EFTUD2	PRPF6	PRPF8	SNRNP200	SNRNP40	TXNL4A
1247	597	540	333	438	361	452	623	304	685	385	318	0	551	365	299	652	589	552	361	101	250	745	185	365	304	376	254	0	181	184	495	332	414	347	289	598	164	377	234	200	231	165	155	349	1650	463	1063	497	786	116
68	38	31	27	24	27	29	32	22	38	36	26	0	27	24	21	54	35	34	18	8	14	53	13	39	21	22	17	0	9	23	41	26	36	40	24	71	12	38	14	13	15	10	9	19	69	33	60	45	95	6
																																																	1	
																																																	1	
																																																	1	
																																																	1	
																												1																					
																																																	1	
														1																																	1			
																																				1														
																																				1														

												2		
	2													
				1										
	1													
				22										
2	7			##							1	3		
				2										

																															1				1															
																						1																												
																																						1												
			1		1				1														1													1														
																		1																																
																																																	1	
																	1																											1	1	1	1		
		1																														1																		
																																			1															

												4		
												6	1	
												1		
9	10			2	2	1		1		2		2	1	
	1						1							
	2													
	1			1										
				3										
				1										
											1			
						1								
	3					1								
													1	
								1						
													3	
	2													
					2	1								
	1													
	1													
	2													

																																																	1	
3	2	2	2	3	3	3	2	1	1	3	1		2	2	3	4	5	2	1	2	1	4		4	1	1	1			3	2	3	3	2	2	4	1	3			1	1	1	1	6	5	5	6	2	
1	1	1	1	1	1	1	1	1	1	1	1		1	1	1	1	1	1	1	1		1	1	1	1	1	1				1	1	1	1		1					1	1	1		1	1	1	1	1	
																																																		1
																										1																								
								1	1																													1	1											
										1			1			1	1								1		1		1	1		1		1			1													
	1						1				1							1																																

	1												3	
				1										
2														
	1													
1						1								1
	1						1						1	
												1	2	
	1									1				
				3										
	1													
3												6		
1														
												4		
													2	
	1												2	

																	1																																	
		1							1																																									
1	1		1	1	1	1	1	1		1	1		1	1						2				1		1	1			1		1	1	1		1	1	1	1			1	1			1		1	1	
2	1	1	2	2	2	2	1	1	1	2	2		2	2	2	2	2	2	2	1	1	2	2	2	2	1	1		2	2	2	2	2	2	2	2		2	2	1		1	1	2	2	2	2	2	2	
1		1												1								1									1					1		1		2					1		1			
																1	1								1																									
2																																													1					
																																				2														
1	1		1	1	1						1					1		1								1				1				1	1							1		1		1				
																																																1	
								1		1									1								1					1	1		1						1			1		1				
															1	1																																		
1															1									1														1									1	1	1	

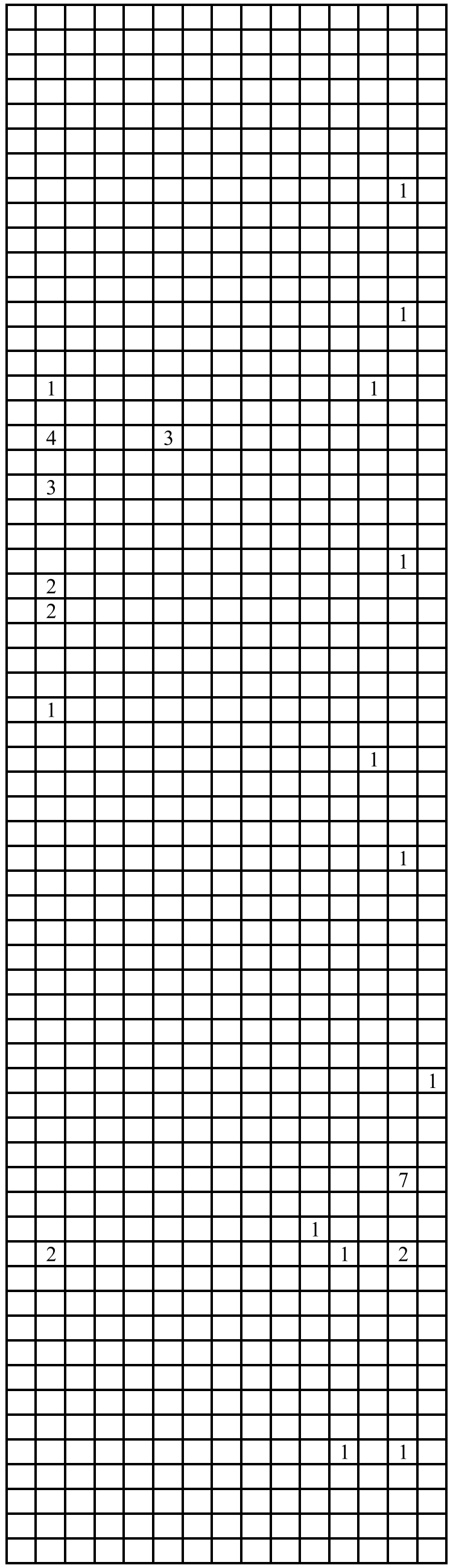

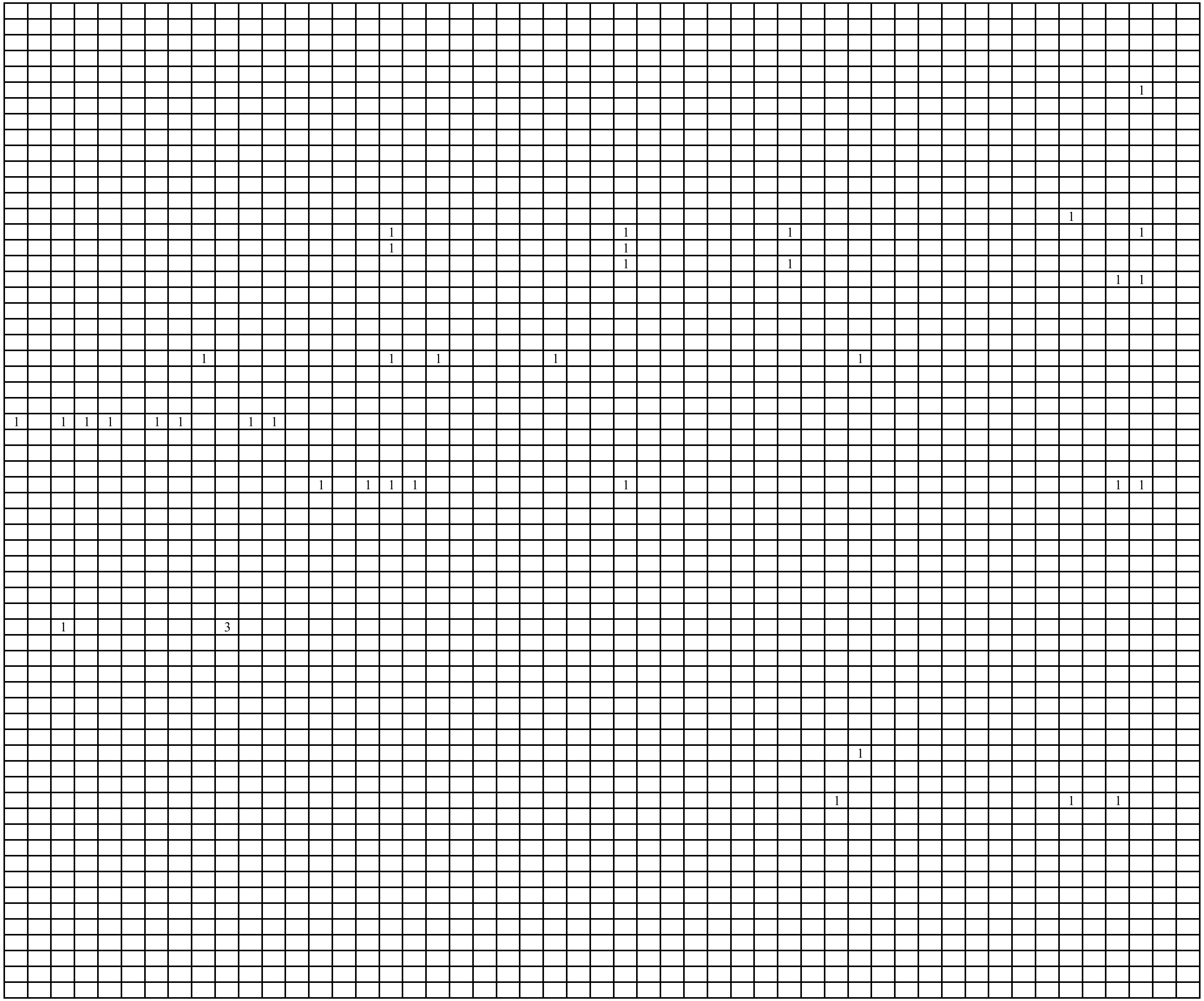

												1		
												1		
													1	
													2	
	1				1					7				
											1		1	
2	6												2	
				3										
													1	
														1

											1											1																												
										1																																								
																																			1																
										1	1																1																							
																																																	1	
											2																													1									
																																				1														
																															1																		1	
																															1																			
																																																	1	
																															1																			
		1							1																																								
																															1				1															

												1		

																																						1													
							1														1													1			1		1											
	1																																																
																																								1										
				1																																														

	1													
								1						
														1
														1

													1																						1															
									1																1		1																							1
																										1							1																	
								1								1																							2									1		1	1		
	1																						1																											
																																									1									

													1	
												1		
													1	
	1													
	1							1						
														1
	3				2					2			1	1

														1		1						1								1			2												1		2	1		
																					1	1											1																	
																																						1													
																																																1	
1																																																		
			1		1				1				1	1	1	1	1	1				1		1	1		1			1			1	1		1		1							1	1	1	1		
				1					1	1	1													1		1							1			1	1		1	1	1	1					1			

	1													1
														1
	3													
	1													
										1				
						1								
										1	1		2	1
											1		1	
	1									1				
							1			1	1		1	
	2													
	1													
	1													
	2													
											1			
	2													
										1			1	
	2													
											1		1	
	1												1	

										1								1																																
1																																																		
																																																	1	
														1																																				
																																													1					
	1	1					1		1																						1				1															
																																																	1	
																										1																								
1	1	1	1	1			1																							1	1		1	1		1			1		1	1	1	1	2	1	1	1	2	1
																																	1								1				1					
															1	1	1	1			1			1	1						1		1	1	1	1		1							2		3	2	1	
																																																1		
																						1																							1		1			
1																																																		
																																																	1	
																1																													1				1	
1	4																		1			2									1	2												2	1		1			
																																																	1	
																																												1						
3	1	2					1		1				1	1		1						2											2											1					1	

													1	
													1	
											1			
													1	
											1			
										1	1		1	
													1	
	3													
												1		
	1													
								1		1	1		3	
							1							
										1				
	1									1				
													1	
	1			1						1			2	
							1			1			1	
					2									
	1												2	
												1		
				1										
				1										
													1	
				2	5									
										1				
	1													
											1		1	
	46			5	2	4		1		1	1		1	1
													4	
										6			15	
				1										

																																													1					
																	1																												1				1	
																																																	1	
																																1													1					
																																												1						
1																																													1					
																						1																												
	1										1																																							
											1																																							
						2			1	3																																		1		3	3	3	1	
																																																	1	
							2																																									1	1	
																						1				1																			1				1	
									1																																1									
1																																																		
																																																	1	
1																								1												1													1	
1	1	1			1	2	3	2	2	1			1			1																								1				1	2		2		4	
1	1	1	1	1	1	1	1	2	1	1						1	1	1	1	1	1	2		1										1		1		1							2		2	1		
																																1																		
																																																	1	
1	1	1							1				1			1						1									1														1				1	
																																													1					
																									1																									
1																																																		
																															1																			
1					1					3			1	1		2	1	2					1	1					1	2			2	4		4		1						1	1		2			
	1																																																	

														1
				1										
	1													
	6				2		1						2	
	1													
													1	
2														
	2													
			4										4	
										1			4	
													1	
	1				1									
					1									
								2					4	
	1									4		1	1	
	5									8			2	
	1													
													2	
										1			1	
										5			6	
1										1	1	1	1	
1				1						3	1			
	2													
											1			

1																																																		
1																																																		
																									1																									
																																														1				
																																2				1														
																																							1											
1																																																		
2		1	1														1	1						1								1				1														
					1				1									1																		3									1					
																																																1		
																																				1															
1																1																																		
1																						1																1												
1																						1														1		1												
1																															1																			
1	1															1		1				1																												
1																1																																		
																						1																												
																																												1					
													1																																					
						1		1		1																				1						1								1	1				

											5		22	
											4		7	
											4		9	
											9		14	
	1							3						
	2													
										2			2	
	1													
								1						
	3													
														1
	1									1				
													1	
3														
					3									
													1	
														1
	1								1	1	1			
										1				
											1		1	

																																																1	
			1	1																													1																	
																2	2	2								2					2					1											3	2		
																		1																																
																																																1	
																																											1					
4	3	1	3	1	2	3	1	1	1	4	2		3	3	1	4	2	2	1		1	4	1		1		2							1		1	1	1	1					1	5	2	6	4	4	
																													1																1					
																																																1	
																																																1	

	1									2	1		3	
	1									2			1	
1														
4														
													2	
								1						
											1		1	
										1				
										3				1
	1													
													1	
													1	
												1		
	1			2										
								1			2		2	
1														
	2			3										
	1													
	1													

																																1																	1	
																																													1		1		1	
																																												1				1	
	1														2									1		1										1										1				
																		2																																
																																																	1	
																																		1															1	
																					1	3																												
1	1						1										1		1			1									3		1																	
																						1																												
	1																																																	
1	1																																												1					
																						2												2																
		1																																1																
																		1																																
																																								1										
																																				4														
								1																																									

												1		1
				3										
											1		1	
	1													
	1													
					1									
	2			2				4					1	
	2													
	2													
										1			1	
	1													
	1							1						
	2							1					1	

1																														1	1			1	1									1						
																1																																		
																																														1	1		
																																								1	1	2	1							
																																				3													1	
																								1														1												
																						1																												
																1	1							1										1		1								1		1	1		
																					1			1									1			1														
		1																															2																
1			1	1					1	1	1					1	1	1			1	1		1	1		1						1	1	1	1	1									1	1			

													1	
	1											1		
	2													
	1													
											1		

																						3																												
														1						1												1				1	1									1				1	
																																																1	
											2																																							
																																								1											
																																			1																
																		1																																	
																														1																					
																	1																																		
																																															1			
																																1																		
																																			1																
																																																1	
1																																																			
																						1																												

													1	
	1													
				3										
				1										
					1									

					1																																												1	
									1																																								
																							1																											
															1																																			
1																																																		

					2									
					1									
				4										
	1													

																														1																				
																																			1																
										1																																							
																																																	1	
																																																	1	
																																																	1	
																			1																															
																																			1																
																																																	1	
																																																	1	
																																			1					2											

1

2

	1													

																						1																												
													1																																					
																																																	1	

												1		
						1									
						1									
	1													

																						1																													
																		1			1			1		1																			1	1	1	1		
1																																																		

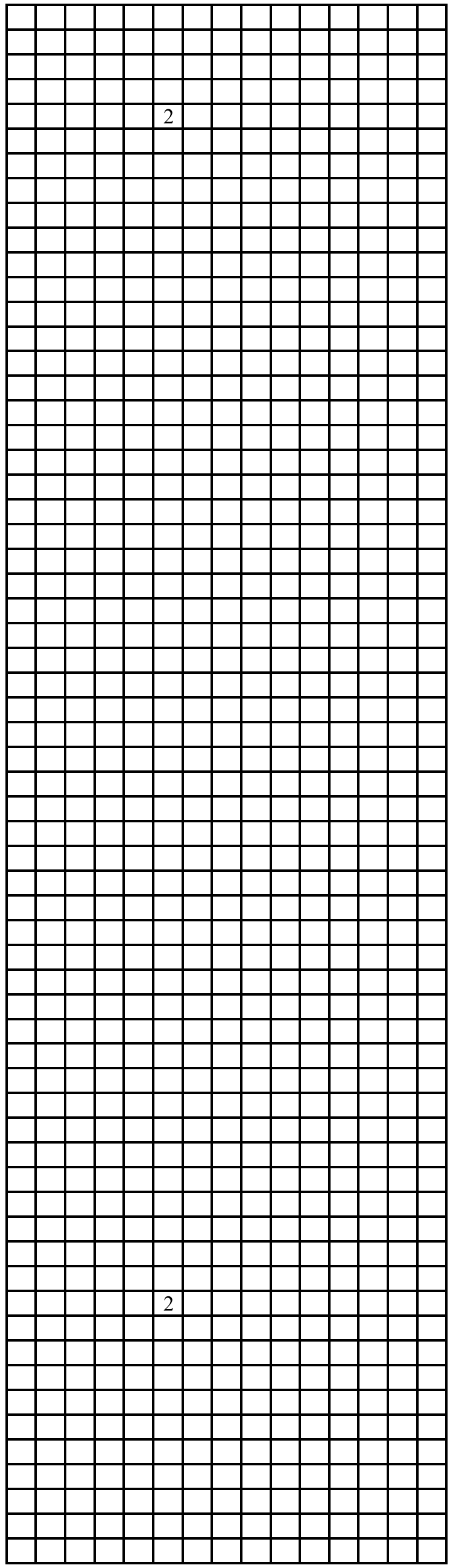
2
2

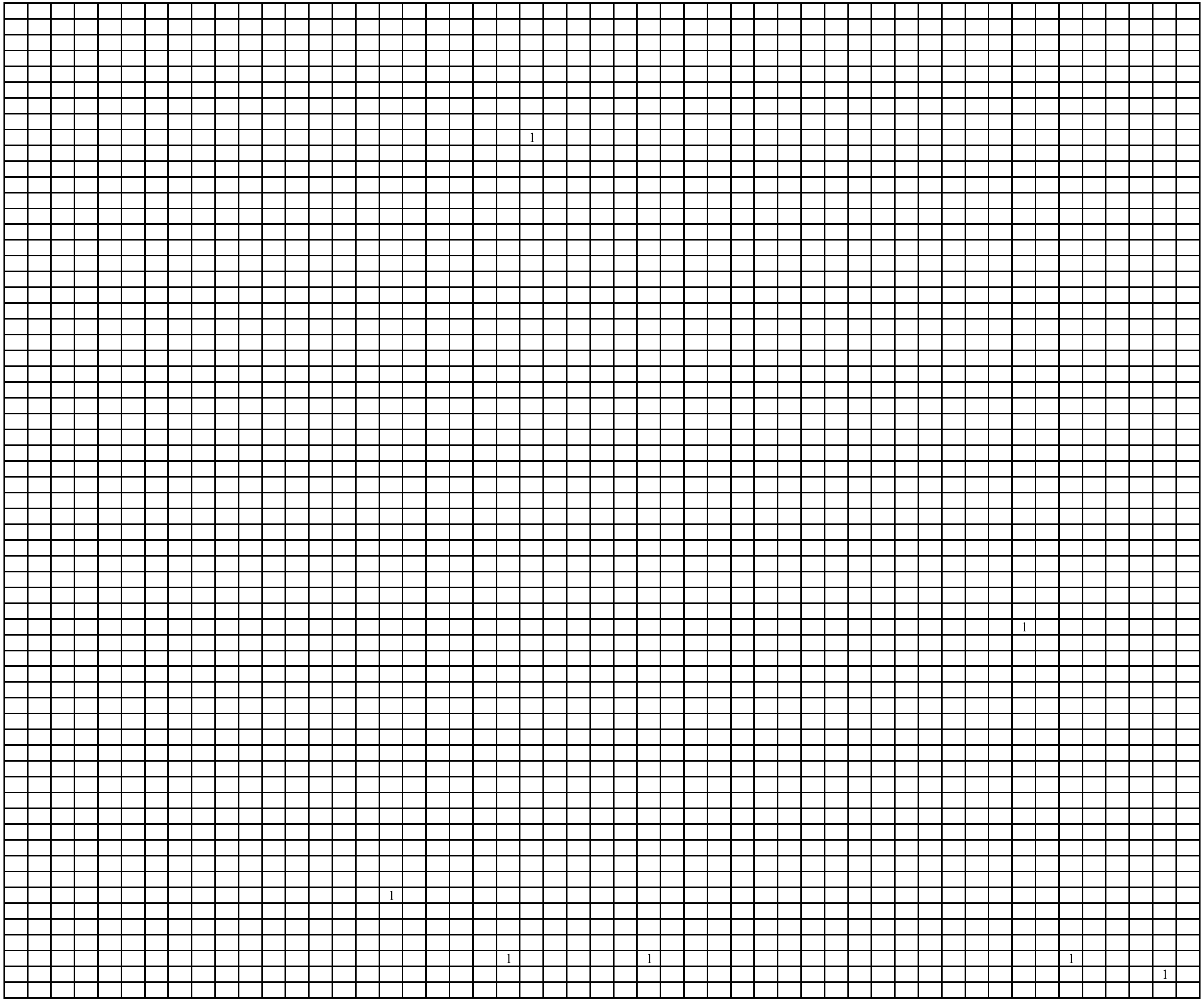
1
1
1
1
1
1
1

	4													
1														
	1													
										1				
	1													
								1						
	1													
	1													
														2
								1			1			
								1						
				1										
	1					1								
	1													

				1		1	2		1	1																									1	1		1			1				1					
				1		1			1																										1	1		1							1					
1															1																																			
			1													1								1	1																									
																																		1																
																																																	1	
			1																																															
			1		1	1				1				1		1	1								1				1																					
																															1																			
																																															1			
														1																																			1	
																																																	1	
					1					1			1																					1																
1		1		1	1		1		1				1			1	1	1													1		1												1		1	1		
																																																	1	
				1																																		1												
																																																	1	
																																				1									1				1	
																																																	1	

	1													
					1									
				1										
								1						
				1										
2														

																					1																													
																																																	1	
1																			1																															
																																																	1	
																												1																					
																						1												1																
																																																	1	
																		1																																
1																																																		
																1																																		
1																																																		
1																																																		

	1							2						
										1		1	
								3						
	1													
										1		2		
										1				
				1										
	4													
												1		
	1											1		
								1						

																																									1									
	1	1					1				1			1												1												10											1	
	1	1							1				1																																			1	
2	1	1					1		1				1			1																				1													1	
																																		1																
1																																																		
																																																1	
																																																1	
																																												1					
			1	1	1	2	1	2	2	1														8															1	2	1	1	1		1	1	2	1	1	1
																														1	1																			
					1																																													
																														1	1																			
1		1				1	1	1					1		1	1								1							1		1			1		1								1		1		
																																												1				1	
																																																1	

								1			1		1	
				1										
			2											
2	6		6	6		1		2					2	2
	4				2		1	2						
	1						1	1					1	
					1									
				1										
													2	
													2	
				1										
				1										
4	2			2										
	3													
													2	
					2									
					1		1	1						
				3										
													1	
								2						
	2													
1	1													

		1																																				1												
																2	1	1				1	1	2	1	1	1			1	1		1	2	1	1									2	3	3	2		
		1	1		1	2		1						1		2			1			1	1	2	1					1		1	2	1		6	1			1					1	1	1			
1	1		1	1	1	1	1		1	1	1		1	1	1				1								1		1		1				1		1	1	1	1	1			1						1
																																									1									
					1																																												
																		1																											1					
1																																																	1	
																																						1												
																																	1																	
																																																	1	
1	1	1	1	1	1	1	1		1	1	1		1	1	1	1	1	1	1			1	1	1	1	1				1	2	1	1		1	1		1							1	1	1	1	2	
																																				1														
																								1												1														
																																																	1	
1	1						1	1	1		1					1	2								1		1																							
1	1						1	1			1					1	1								1																									
																																																	1	
																																																	1	
																																				1									1					
1	1	1	1	1	1	1	1	1	1	1	1		1	1	1	1	1	1	1	1	1	1	1	1	1	1	1			1	4	1	1	1	2	1	1	1					1	1	1	1	1	1	1	

				2										
														1
														4
				5										1
	2													

	1	1							1										1													1													1				1
																														1					2				1										
																														1																			
																														1																			
																																																1	
																		1																																
1			1			1	1	1						1								1										1				2	1							1				1		

																						1				DDX23		SplF.core	41	551
																										NHP2L1	U4/U6 snRNP		13	253
																										PRPF3			30	414
																										PRPF4			17	331
																										RP11			0	0
																										SART1	U4/U6.U5 snRNP		27	354
																										SNRNP27			7	247
																										USP39			24	237
											1															BCAS2	PRP19 complex		44	378
																									1	CDC5L			54	1041
																										CTNNBL1			33	209
																										PLRG1			36	240
																										PQBP1			12	131
																										PRPF19			49	596
																										WBP11			12	192
																										AQR			9	249
																										BUD31			12	113
																										CRNKL1			13	230
																										ISY1			7	206
																										PPIE			16	371
																										RBM22			23	211
			1																						1	SNW1			83	993
																										YBX1			81	856
																										CWC15			9	110
																									1	HSPA8			160	2989
																1										HSPA1A			89	1931
																										XAB2			21	246
																										IK	B complex		17	220
																										MFAP1			34	325
																										PRPF4B			23	366
																										SMU1			19	243
																										WBP4			7	136
																										TFIP11			49	535
																										CCDC12	Bact complex		9	176
																										CDC40			8	165
																										CWC22			7	150
																										CWC27			3	64
																										PPIL3			8	77
																										PPIL2			14	115
																										DHX16			12	183
																										FUBP3			35	359
																										ZNF830			4	90
																										BUD13	RES complex		19	150
																										RBMX2			13	125
																								1	1	SNIP1			46	563
																										C19orf29	C complex		0	0
														1						1						C9orf78			23	644
																										CXorf56			4	89
											1															LENG1			12	150
																										SYF2			10	125
																										DGCR14			19	193
																										FAM50A			7	89
																										PPWD1			7	45
																										PPIG			15	158
																			1							DDX41			18	178
																										NOSIP			10	118
																										DHX35			3	91
																										CDK10			6	62
																										DHX38			8	166
																										FAM32A			6	33
																										FRG1			10	65
																										FAM50B			3	63
																										PRPF18			10	96
																										SLU7			10	152

																						2																																									
																																1																															
																								1	1																															2							
																													1																																			
													1																																																			
						1																1		2	1																																			1			
																								1																																						
																								1																																						
																								3	2																																						
																																1																															
1							1																																																								
																						1		3																																							
																					1																																												
																								2																																						
																								1																																						

																								1																																							
																									1																			1																			
																																3																															
2	2	4	1		4	1	1	4	5	2	3	1	4	2	1		2		1	2	7	6	1	3	1	3	4	3		2		4	1	1	1	1	1	2	1	2	1	1		4		1	1		1	3			1	3	2			2	2	2			2
1	1	1			1	1	1	1	1		1	1	1	1	1	1	1		1	1	1	3	1	1			1	1	1	1	1	1	1		1					1	1	1	1	1		1	1		1	1			1	1	1				1	1			
																					1																																											
							1									1																																															
														1										1	1																																						
																						1																																										
											1																														1																							
											1													1																																				1			
1																												1																																			
																									1																																						
	1	1	1						1																																																						

																																								1																							
																								1																																							
																																				1																												
										1																																																						
																																																								1									
1		1	1		1		1				1	2	1	1		1				2	1	1		1			1			1			1				1	2		1						1	1			1		1		1	1				1	1		1	1
2	1	2	2		2		1	2	2	1	2		2	1			1	1		1	1	2		2	1	1	2		2	1										2									1		1		1	1	1								
																																																														1	
								1	1		1		1																	2																							1										
																				1		3																																									
																						4																																									
			1		1						1		1						1				1			1			1	1			1				1																										
																								2																																							
																																																												1						
	1	1																			1									1										1			1								1				1				1				
																																								1																							
																																								1																							
1													1											2																																							
																								1																																							

																																							1																							
																																							1																							
																			1																														1													1
																							1	1																																								
																																						1	1																							
					1		3																																																							
																																						1																								
																							1																														1										
								1					1										1																																								
								1																																																						
																								1															1																							
								1																																																						
																																							1																							
																																							1																							
																							1																					1																	
																					1		1	1																												1										
																																															1																	1

																												1																																			1		
																						1							2																																				1	
																								2																				1																				
												2																																																			
		1							1													1		1																									1														
																								1																																										
																																									1									1														1	
																																2																																		
																						1		1	1																																										
																																											1																						
																1																																																
																																													1																			

3

1 1 1

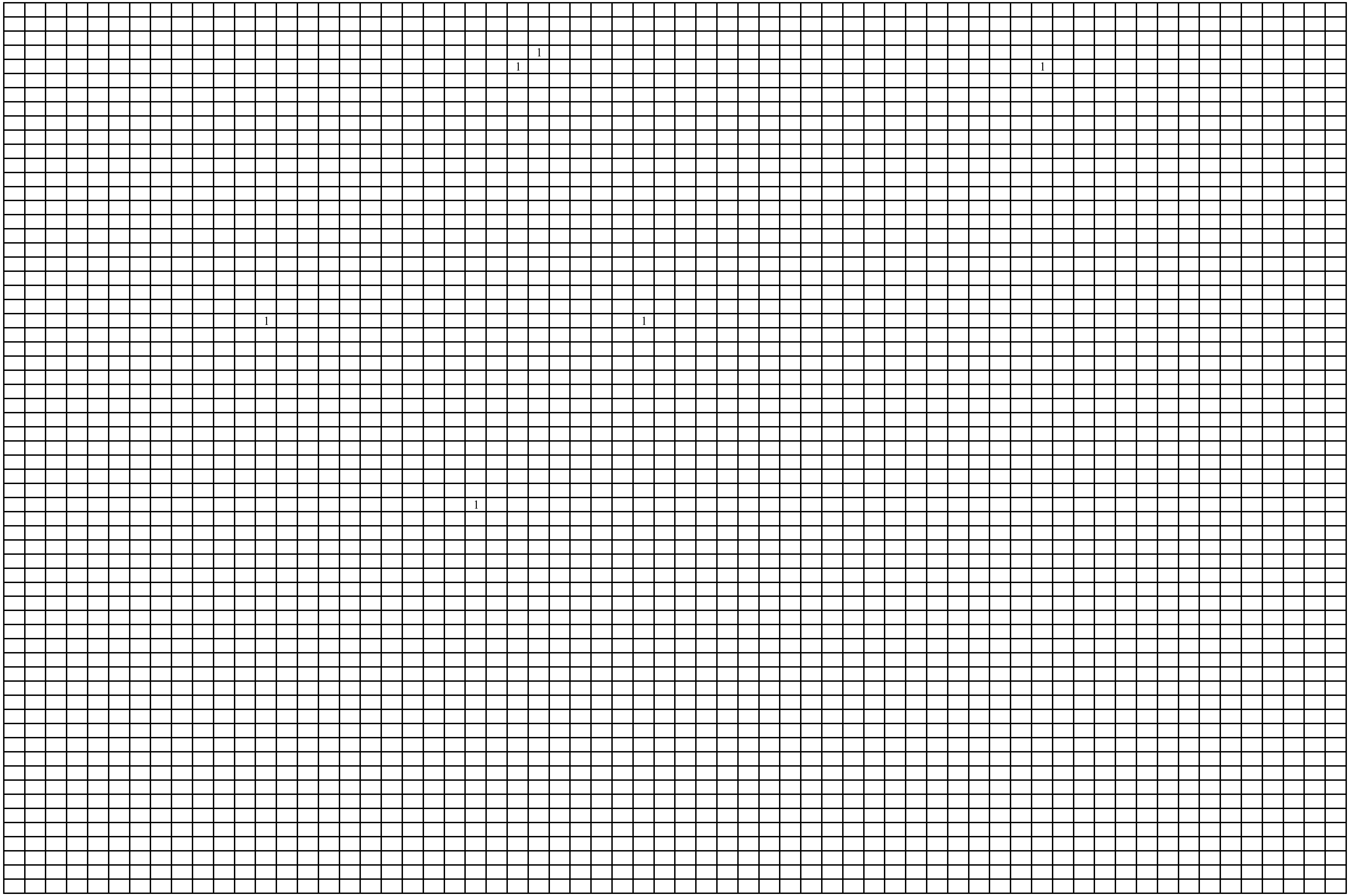
1
1
1
1
1
1

																								1																																							
																								2																																							
																																																			1												
																																																1															
																																1																															
																																3																															
																																								1																							
1								1		1						1					1			2																																							
																									1																																						
																								1																																							
																						2		1																																							
									1																																																						
																									1																																						1	
									1				1								1			1	1		1			1																																	
			1					1					1									1						1				1								1														1									

																																						1																									
																																						1																									
																									1																																						
		1																																																													
																								1																																							
																																1																															
																								1								2												1																			
					1																1			1				1	1															1																			
1									1																																																						
																																1																															
2		1	1		1		1	1			1	1	1		1		1	1	1	1	1			1					1												1			1								1											
																									1											1																											
																								1																																							
										1											1	1	1	5	7																							1															
																								3	1																																						
																								1																																							
														1					1					1																																							
																								1																																							
					1				2												1										1	1																															
																																																									1						
1																																																															
																																												1																			
1																								1					1															4																			
																																																														1	

																									1																																						
																									1																																						
																																								1																							
1																			1																																												
																						1																		1																							
																								1																																							
																					1	1		3	2						1									1				1										1									
																																																					1										
																																																		3													
																					1																																										
4								1					2					1		1													1																	2														
																																																														1	
																								1		1															2				1																			
																												1																																			
																									1																																						
1																																												1																			
																																								1																							
4												1							1		1																																										
		1			1		1		1		1											1		1	1					1																																	
																								1		1																			1																			
																								1					1																																		
																								1																																							
									1															1		1																			5																			
																																												1																			
																										2																																					
																																																3															
		1			1		1					1								1	2	7		13	7	1						3						1	1							1		1		2	1	1			1			1					
1																																																															
1																																																															
								1																																																							

																																								2																							
																				1																																										
																					1																																									
									1												1				1			1														1	1																				
																									1																																						
																																																	1															
																								2	4																			1																			
																1																																														
																								1	1																																						
																				4																																											
																								1																																							
							1																	1	1																													1									
																								1	1																																						
					1																							1	1															1																			
																																													1																			
																				3													1										1																			
																		1		3																1	1																								
																				1																							2																			
							1			1														3	1																																						
																																													1																	
																																																	1															
																								1	1																																						
									1															1	1																																						

																								1																																								
																									1																																							
																								1	1																																							
2									2															3																																							
																																																									4						
																						2			1																																							
2		2			1		1	14	8	9	13		14		2	1	3	2	4	1	7	1	4	2		3	1		3				4	1	1			1	1		1	1		1		1	1		3	1		1		1				1					
																								1																																								
																				3				1																				1																	

																					3				1																																						
																									1																					1																	
																																1																															
																									1																					1																	
									1																																			1																			
									1		1		1								1					1		1						1					1																								
																									1																																						
1																																																															
																																1																															
									1																																																							
1																																										1																					
									1																			1																									1									1
																																														1																	
										3																																																					
																						1																																									
																								1																																							
																																2																															
																																1																															
																																																	1															
													1								2							1																							1					1							1
																																								1																							
							3																	1																														1										

														1																		1					1			1										1													
																								1	1																																						
1																																																															
								1																																																							
		1																																																												
									1		1										1																																									
										1																						1																															
																						4		1																																							
											1		1																																																		
																						1																																									
																										1																																					
																						1																																									
													1											1	1																																						
								1																																																							
																								1																																							
							1		1													1		1														4																									

					1																							1	1															1																			
																													1																																				
																								2	1																																						
																						1																																										
																								1	1																																						
									1																																																						
																													1																																				
																													1																																				
																													1																																				

		1																		1																																											
																																																				1												
																						1																																										
																													2													2																					
																								1																																								
																								1																																								
																																																					2											

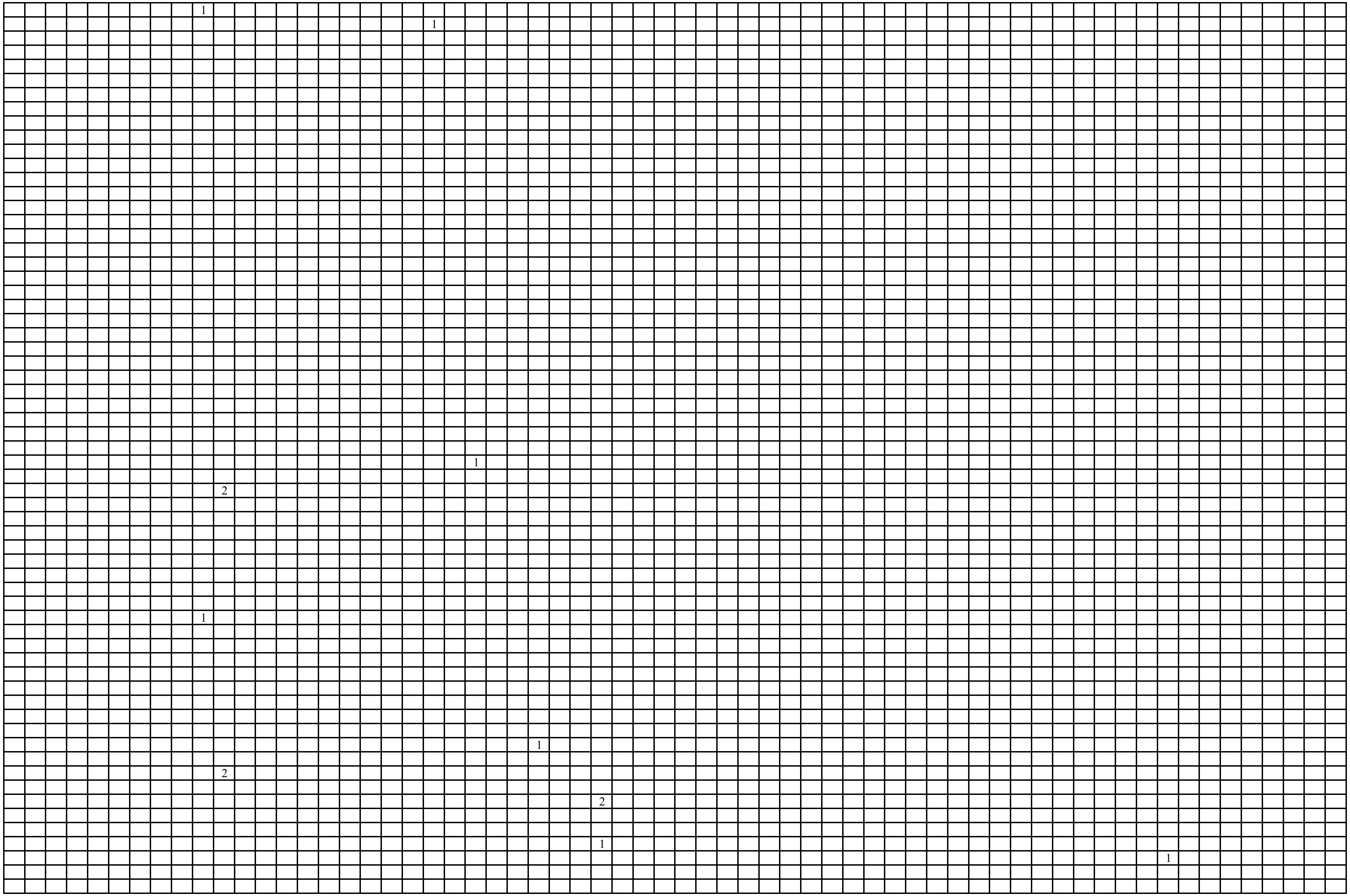
1
1
1
2
1
1
2
2
1
1

3

1

1

1

1

1

1

										1																																																			
																																																									1					
																																																		1												
																													1																																			
									1					1																																																	
																													1																																			
																						1										1																																
					1																																																										

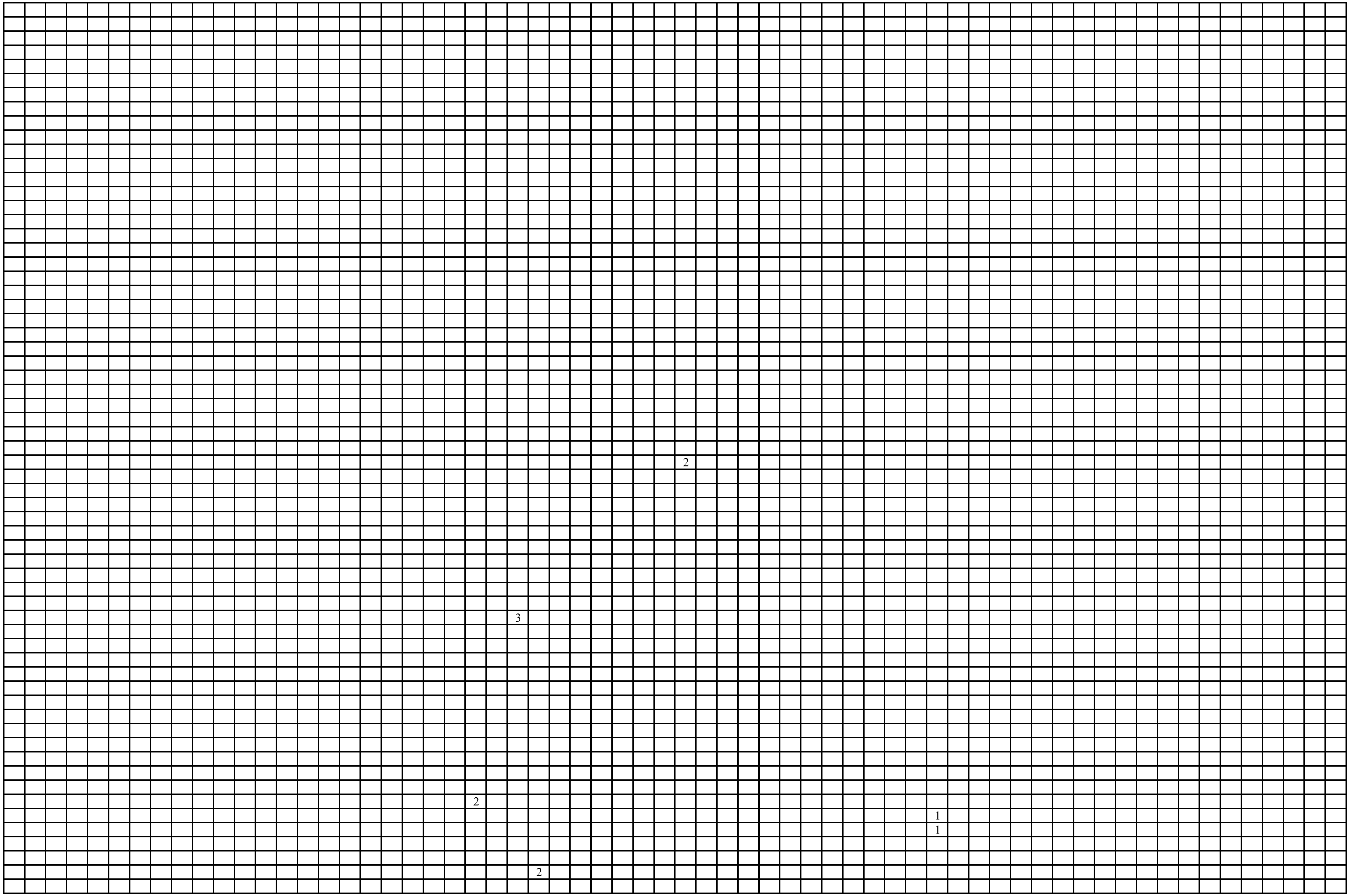
2
3
2
1
1
2

													1								1			1																														1							2	1	
								1					1											1																								1		3				1									
					1				1		1		1																																																		
																																													1																	
									1		1																																																				
										1																																																					
																						1																																									
																						1		1	1																																						
																					1																																										
																					1																																										
																								1				1																																			
	1					1			1				1																	1	1												1							1		1											
					1																																																1										
																								1																																								

																																2																															
																													1													1																					
																																										2	2			1																	
1																																																														
																																																													1	
																						1																																									
																																1																															
																								1																																							
1																																																														
																																																													1	
																						1																					2																				
																					1																																										

																																								1																							
		1																										1																																			
																								1							1																																
																									1																																						
																			1		1	1																																									
																								2																																							
																									1																																						
																								1																																							
																									1																																						
1	1	5	5		2	1	1																									4																															
																																								1																							
																													1										1																							
												3										1		1	1		1																										1										
																																													1																	
																																													1																	
																																										1				1																	

																								2	1																	1				1																	
																																										1				1																	
1		2			1				1		1		1				1				4			1	2	1	1			1							1			1												1											
		1	1					4	1	1	1	2	2		1		1		1	1	1	2		7	2	1	1		1	1		1				1	1					2	1	1														1		1			
	1		1			1	1	2			1			1	1	1				1	1			2		1	1	1	1	1				1	1		1					1	1	1									1	1				1		1			1
																								1	1																																						
																																												1																			
							1									1				1				1	2				1		1																								1								1
								1																	1																																						
																								1																																							
																								1																																							
								1																1																																							
																					2			1																																							
																									1																																						
																					7																																											
1	1		1					1	1	1	1		1		1	1	1			1	1	1	1	1	1	1		1	1			1	1	1	1				1	1			2	1						1			1	1				1		1			
																																														1																	
																					1																																											
									1																							1																															
																						1		1																1										1													
																					2																																											
																					1																																											
	1	1						1	2				1				1					2							1													1												1									
																		1				1				1								1																													
																																1																															
																																														1																	
																											1																																			
																						1																																									
1		1	1		2	1	1	1	1	1	1		1	1	1	1	1	1	1	1	1	3		1		2	1	2	1	1		1		1	1			1	1			1	1										1	1		1			1	1			

																									1																																						
																																							2																								
								1												1	1										1						1																										
	1																							2	1																			1																			
																																												1																			
																																												1																			
	1				1		1	1					1									2		1	1		1			1																																	

393	306	566	334	532	532	0	1	243	243	115	162	84	128
21	19	38	19	23	38	0	0	16	16	11	11	8	10
	EJC / mRNP							TREX					
DHX8	ACIN1	EIF4A3	MAGOH	RBM8A	RNPS1	THOC4	BAT1	THOC1	THOC2	THOC3	THOC5	THOC6	THOC7
				1									

200	91	0	474	663	249	370	680	171	603	49	78	310	680	6	256	8
22	2	0	47	71	8	37	54	23	65	6	7	41	67	1	23	1
helicase																
DDX10	DDX11	DDX12	DDX17	DDX24	DDX28	DDX31	DDX3X	DDX52	DHX30	DHX32	DHX33	DHX57	DHX9	HFM1	SKIV2L2	TDRD9
			1													

1345	378	722	990	369	777	501	178	732	568	1653	338	334	876	2029	616	489	1005	376	281	0	0	575	768	477	403	735	909
73	32	50	75	26	46	30	9	58	51	127	19	24	61	161	60	35	70	49	37	0	0	57	45	40	42	63	70
hnRNP																											
HNRNPA1	HNRNPAB	HNRNPC	FUS	HNRNPA0	HNRNPA2B1	HNRNPA3	HNRNPCL1	HNRNPD	HNRNPF	HNRNPH1	HNRNPH2	HNRNPH3	HNRNPK	HNRNPL	HNRNPM	HNRNPR	HNRNPU	HNRNPUL1	HNRNPUL2	HNRPDL	HNRPLL	ILF2	MATR3	PTBP1	RALY	SYNCRIP	ILF3
			2											1													
1			1																								
					1																						
														1													
														1		1											
		1																									

0	0	0	0
0	0	0	0
SplF.RBPs			
SFRS11	SFRS3	SFRS4	SFRS6

																									2																																				
																									2																																				
																																										1																		
																																																								1				
																																		1																												
																																								1																					
																																								1																					
																														1					1		1	1		1			1																		
																																																				1								
																													1																																
																																									1					1	1								1	2	1						
																							1																																						
																																														1																	
																																																				1								

																																		1							1							1															
																																								1																					
																																								1																					
3	3	1	2	3			1	2	1		1	1	4			4	5	1	1	2	4	3		2	1	3		4		3	4	5	4	5	3	3		7	4	3	3	3	4	3	4	3	5	3	2			4	3	4	2	3	4				
1	1	1	1	1			1	1	1	1	1	1	1			1	1	1	1	1	1	1			1	1		1		1	1	1	1	1	1	1		1	1	1	1	1	1	1	1	1	1	1	1			1	1	1	1	1	1				
																																												1																	
																																												1																	
																													1																																
																																	1																													
										1																																																			
								1																																																						
																1		1																																										
																																								1																					
																																												1																	
																																	2																													
																									2						1													1																
																													1				1	1																					1					
																																								1																					
																																							1								1															
																1																						2																							
																																								1				1																	
																																												1																	
																	1					1			1	1																							1													
																																									1																					

																																													1																	
																																									1																					
																																													1																	
																																									1																					
		1							1				1				1				1								1		1	1	1	2	1	1	1		1					1	1		1	1	2				1		1		1	1				
2	2	2	1	2	2			2	1	1	2	1	2				2	1			2	1			1		2		2		2	2	2	1	2	2	2		2	2	2	2	2	3	2	2	2	2	2	1			2	2	2	2	2	2				
																																		1																												
																																																									1					
																																									1																					
		1	1	1															1				1				2					1	1	2	1		1	1	5	1				1			2	1	2		2				2		1	2	1				
																		1	1	1			1			1															1			1						1							1					
		1	1																												1			1																												
																																													1																	
	1		1					1			1		1	1									1						1																											1						
			1															1		1		1			1				1				1									1	1		1				1	1						1						
																																																								1						
																																																		1						1						
																	1										1				1				1								1		1	1	1		1	1				1		1		1				
																																																								1						
																																												1																		
																																												1																		

																								1																																					
																																												1					1						1						
																																																	1												
																																													1																	
					2																																																								
					1																																			1				1																	
																						1																												1						1						
																						1																												1												
																						1																																								
																																														1																	
																											1				1	1		1		1	1		1	1	1		1	2																		
																																									1				1																	
																	1																																													
																																								1		1			1	1				1						2							
																															2																									1						
																																													1																	
																		1																																	1												
																																																		1						1						
																																														1																	
																	1				1						1						1					1	1					1		1	1	1					1				1					
																																									1																					
																															1														1									1								
																																							1						1												1					
																																														1																	
																																		1																													

		1																																														1														
								1																									1																													
																																													1																	
		1																																																												
																																									2			1				1														
																																																1														
																		1	1																									1									1									
																																													1																	
																	1										1													1						1	1	1					1				2	1				
																																													1																	
																																													1																	
																																													1																	
1														1									2																																							
																																									1																					

																																								1				1																	
																															1																														
																																								1				1																	
																																								1																					
																																								1																					
																																								1																					
					1																										1																														
		1																																																											
		1																															1						1													1									
																		1		1			1																																							
																		1		1		1	1																																							
			1																		1																											1													
																						1																																							

																											1				1		1											1										1							
																	1																									1								1			1									
																																		1															1				1		1							
																																																				1									
																																			1																											
																																											1																		
																						1																																							
																	1					2																													1									
																	1					1																											1													
																																												1																	
																1				1		1				1						1					1	1			1		1		1					1				1	2				
																							1																																						
																																							1																					

																																								1																					
																																													1																	
																																													1																	
																																								1				1																	
																																													1																	
																																								1																					
																																													1																	
																																								1																					
	1	1			1																												1				1							2				3														
																							1																																								
																																													1																	
																					1										1					1																					2					
																																																									1					
																																			1																												
																																																										1				
	1								1	1							1				1						1						1							1	1	1		1	1	1		1						1	1	1	1					
																							1			1																																1					
																		2					1																											1				1									
																		1																																												
		1		1													1	1	1		1	1	3			1	1		1				1	2	1	1	2			1		1		2	2	1	1	3		1			2	1	1	2		1				

																																																				1									
																							1																										1				1									
																																		1										1																	
																							1																																								
					1																																			1																					
																																													1																	
																																								1																							
																																		1																												
																																													1																	
																																		2																												
																																																1														
	1	1	1	1	1			1			1	1	1				1									2				1		1		1	1	1		1				1	1	2	1	1	1		1			1	1		1	1	1				
				1																										1	1							1						1										2		2					
																																																					1		2					
		1															1				2		1				1				1			1		2		1	2		2				1			1	1				1	1	1			1				
											1																																																		
																																									1																					
																																									1																						
																																													1																	
																																									1				1																	
																																									1																					
																															1														1																	
								1																																																						
																																									1				1																	
																																													1																	
																																													1																	
																																													1																	
																																									1				1																	
																																									1				1																	
																	1									1	1				4			4		1					2													2	3		2					
																																													1																	
																																													1																	
1																																													1																	
1								2	1	1	1	1	1																		1		1	2		2		1										1						1								
																																1																															
																																										1																					

																																														1																
																																		1																												
																																																1					1				1	2				
																																																					1							
																		2		2			1				1					1	1				1										1		1							1					
																							1																					1		1		1				1	1			1					
																					1					1					1					1									1											1					
		1			1																																																							
																																									1																					
		1						1																															1											1							1					
																																																											1				
																																							1																							
																														4										2																					
																		1		2	1		1																																						
																														1								1							2							1								
				1																			1				1				2		1							1							1									2				
1																																																														
		1		1													1	1			1		1				1				1	1	1	2	1	1	1		1	1	1	1	1	1	1	1	1	1	1	1			1	1	1		1	1				
																																																										2				
			1																																																											
																																													1																	
1		1	1		1																																																								
																											1																		1												1				
																																					1		1																							
																					1																			1																					
																																																1														
																																						1																								
																																		2																												
									1	1				1	1		1	1			2		1				2		2		2		2	1	1	2			1	1	1			7	2	3	1	3	1	1				1			2	1				
																											1					5		3			1	1	1					3	1		1										2					
																																		1																												
																																																	1				1									

																															1														1																	
																		1																1																												
1																		1		1			1			1							1																								1	1					
																																														1																
																					1																																									
		1						1																																1																						
																											3							2																												
																																													1																	
																															1														1																	
																															1																															
																		1																								1																					
																																										1																					
						1																																																									
						1																																																									
																	1				1						1							1							1					1	1	1									1					
																	1																							1	1																					
																																										1																					
								1																																																						
																																							1																							
																																														1																
1																																																														
1																					1																							1																		
		1							1		1																																																		
																																										1																					
																																										1																					
																																														1			1													
																																										1																					
																																				1								1																	
		1																									1																	1																		
																													1											1	1					1																

																																																				1						
																																								1																					
																																												1	1																
																														2					1									1																	
										1																																		1																	
																																												1																	
																																												1																	
																																																1													
																				1																												1													
																																																1													
																																								1																					
																																												1																	
																																												1				1													
																																								1																					
																				1										2	2	2			3			2	2	2		2			1		3	2	2			2	2				2				
																																								1																					
																																												1																	
													1				1		1		1																																									
																																																1													
																																																1													
																																																1													
																																		1																												
																																		3																												
																				1																				1																2				
																																																							2				
3		2	1													1											1		2		3	4		1				1	1			1		5		1		1			2		1	1		1				
																																													1											1				
																										1				1															1							1				1				
					1																																																									
		1																																																												
																																																					1								

																																													1																	
																																													1																	
																																	1	1					1																							
																																	1	2					1																							
																																						1																							
			1		1			1									1				1												1							1						1							1			1		1				
																																								1																					
									1									1																																											
																		1			1				1				1												1																					
																																													1													1				
																																													1																	
				1										1				1		2			1			1	1																					1					1									
																																																							1						
																																																1										1				
																		1																																											
																																													1																	
																		1																																											
																																								1																					
																																							1				1																		
																		2		1			2																										1													
																																								1																					
																		2					1																	1			1						1	1			1									
											1			1				2		1			1																1		1		1						1				1				1					
																							1																										1				1									
																		1																							1												1									
																		1																																											
																																									1				1																	
																		1																										1		1				1	1			1									
														1				1																							1																					
																					2																																									
																																								1																					

																		1				1																																								
																																								1																					
																																													1																
																																									1																				
															1																																														
																																									1																				
	1																2				1		1				2														1					2		3		1						1	1	2				
																																												1										1								
																																																							1			1				
																																													1																
																		1				1			1																																				
																																													1																
																	1				1		1				1				1		1			1					1					1		1						1				1				
																																									1																				
																	1														1						1						1													1						
																										1																																				
										1							1				1		1				1				1		1			1	1		1	1	1			1		1	1	1	1					1	1	1		1	1				

													1								1																																					2				
																											1																																			
																																			1																											
					1																																																				1					
																																													1																	
																																													1																	
																																																								1						
														1				1		1			1																																							
		1																2								1															1																					
		1																		1							1									1					2				1	1		1														
																																									1																					
																																													1																	
																																				1																										
					1																										3	1								3																						
																																																						1								
			1																							1																																				
																																													1													1				

																																													1																	
																							1																																									
																																									1																					
					1																																																								
																																														1										1					
																																														1																	
													2				1		1			1																																							
																																									1																					
																																									1																					
																					2																																1				1				

																				1																																1					1				
				1																																		1																		1					
																					1	1			2																																						
																																													1																
																																													1																
																																													1																
				1																																				1																					
																														1					1												1														
																																													1																
				1																																																									
																																												1												1						
				1																																																									

																																												1																	
																																												1																	
																																															1														
																																												1																	
																																												1																	
																																												1																	
					1																																																									
																				1			1			1																																				
																																												1																	
																																												1																	

																																		1																													
																																													1																		
																																		1							1																						
																																														1																	
																																														1																	
																										1																																								
					1																																																										
																																											1																					

																					1																																								
																									1														2								3														
																	2		2																					1																					
																									1																																						
																			2			1																																							
																																														1																	
																				1		1				1		1											1						1	1					1				1	1				
																																																							1						
																																								1																						
																									1																																							
																							2																																								
																																								1																					
																																														1																	

1

																																								1																					
																									1																																				
																																												1																	
				1																																																									
																									2																																				
	1																																																												
																																												1																	
																																												1																	

																																		1											1																	
	1															1										1				1					1	1							1	1	1		1					1		1		1	1				
	1															1										1				1					1	1			1				1	1	1		1					1		1		1	1				
									1							1	1			1								1			1								3	2							2		1				1			1					
																																							1	1																	1				
					1																																																								
																																													1													1				
									1																			1																																	
																																																1														
		1														1				1						1					1			1		1		1	1					1	1	1	1					1	1		1	2	1				
																																													1																	
																															1																															
1																																																														
																																					1										1															
	1			1	1								1			1	1					1				1													1			1				1								1	1	1				
																	2																																													
																																									1																					
																																													1																	
																																													1																	

																																									1																					
																																													1																	
																		1		1			1			1																																						
																		1		1			1																																		1				
																		2		2																									1																	
																					1																				1																					
																																												1																		
																																													1																	
																							1			1																																						
																										1																			1																	
		1																																																						1				
													1				1		1		1	1			1																													1				1				
																		1																																												
																																																											1				
																																												1																		
																											1																																					
																																													1																	

																																	1																							1						
																																													1																	
			1		1																																		1							2			2													
1																																																														
																											1				4		1	3		1	1				3				1					1			1	3				1				
																																													1																	
																																													1																	
					1																					1																																				
																																													1																	
			1																																						1				1																	
				1																																												1														
																																												1																		
																																													1																	
																																													1																	
																																													1																	
					1																																																									
					1																										1	1		1	1	1	1		1	1	1	1	1			1	1			1			1	1		1		1				
																																									1																					
																																																									1					

																					1		1																2																									
																																													1																					
																																													1																					
																																													1																					
	1				1												1				1						2		1			1			1				1	1	1	1	1	1		2	2	1	1	1	1			1	1	1							
1		1	1	1					1				1			5		1	1	3	1	3		1	2	3		1		2	1	1	3	1	2	1		1	1		2		1		1	1		1	1			3	1	1	1	3	2				
1	1	1	1	1	1			1		1	1	1		1				1		1		1			1	1											1								1							1						1						
																																	1								1																					
	1																																									1	1	1																				
				1																								1			1								1				1					2														
																																													1																					
																			1						1					1															1			1		1								1					1				
																																				1								1				1																
																																																							2							
																																		1										1																					
																																									1				1																			
																					3																																									
	1	1		1	1				1					1			1	1		1	1	1	1				1		1		1	1	1	1	1	1	1		1	1	1	1	1	1	1	1	1	1	1	1	1			1	1	1	1	1	1				
																																									1																					
																																		1										1									1								
																					1																				1					1	1			1	1	1		1						1		1					
		1							1																																																			
																																																											1				
																					2										1																															
				1																																																										
2																1				1	1	1			1			2						1	1				1													1						1	2						
		1													1				1		1						1								1																							1						
																																	1																													
																							1				5														1					1														
																																									1																					
																											1				1					1											1														
1	1	1	1	1				1	1	1	1	1	1				1	3		2	1		1			1	1				1	1	1		1	1	1	1	1	1	1	1	1	1	1	1	1	1	1	1			1	1	1	1	1	1				

																																																1													
															1				1						1						1											1	1		1	1						1								
																									1														1											1													
																																											1																		
																																							1				1				1													
																																						1						1									1							
																																																						1							
			2												1				2		1			1	1		1		1	1		1	1	1	1		1	1	1					2		1	1				1	1			2	1				

SFRS7	SR proteins	0	0																										
SFRS9		0	0																										
SRSF1		58	673				1																						
SRSF2		21	222														1												
SRSF5		51	457																										
TRA2B		26	398																										
SRRM2		37	509																								1		
SRRM1		27	370																										
SRRM3		1	16																										
RBM6	RNA binding proteins	16	145																										
CCDC55		0	0																										
CIRBP		18	184																										
CSDA		0	0																										
ELAVL1		215	2232	1			1					1	1			1	2												
ESRP1		3	88								1																		
ESRP2		5	72																										
EWSR1		100	941	2																									
GCFC1		0	0																										
IGF2BP3		40	581																										
KIN		3	72																										
LUC7L3		14	161																										
NONO		100	678																										
PRPF39		11	57																										
RBM15		16	207																										
RBM39		75	1342														1												
RBM9		0	0																										
SFPQ		63	605																										
TIA1		10	111																										
TIAL1		12	143																										
ZNF207		13	116																										
ADAR	RNA editing	34	339																										
ADARB1		44	543																										
C19orf43	RNA modifying	7	59																										
DBR1		2	41																										
FTO	RNA metylation	10	82																										
KIAA1429		264	3160	1	1	1				1		1	1																
METTL14		12	605																										
WTAP		25	231													1													
ALKBH5		5	51																										
METTL3		15	314									1																	
YTHDC1		58	447																										
WDR83		21	361																										
NCBP1	CBC	22	348																										
NCBP2		13	182																										
PABPN1	3´end	19	190																						1				
PABPC1		49	793																										
CPSF2		12	154																										
CPSF6		30	564																										
NUDT21		34	429																										
DIS3	RNA degradation	15	197																										
DIS3L		1	108																										
EXOSC4		10	251																										
EIF2C2	RISC	0	0																										
DICER1		13	247																										
MOV10		102	1407	1												1													
CLK1	protein kinase	9	255																										
CLK2		28	327																										
CLK3		23	367																										
CLK4		7	54																										
SRPK1		40	579																										
SRPK2		54	1013																										
DNAJC8		19	243																										
C16orf80		0	0																										

																								1																														
															1																																						
						1																																															
												1																					1																			
																				1																																		
														1																																							
												1			1					2																																		
																																		1																			
																																		1																			
												1								1																																		
																												1																									
																																						1															
												1			1																			1																			
												3																						1																			
																																		1																			
														1															1																								

																																												1										
																																			1																			
									2				1																						1																			
																1								1																														
													1																																								
		3	2	1	3	3	1		1		2		4			2		2	1	2	7	1	4	4		3	1	1	2	4		1			1		1		1	1		1	2	3	2	1	2	2	2					2
		1	1	1	1	1	1		1		1		1			1		1			3	1	1	1		1	1	1	1	1	1	1			1		1			1		1	1	1	1	1	1	1						1
																																			1																			
													1																																									
																1																																						
																																								1														
													1			1																																				1	
																																																1						
													1			2																																						
													1																																								
													1																																								
													1					1																																				
																				1			1											2									1										1
																1																																						
									1				1			2								2											1									1										
									1		1																			1		1																						
													1																																								
																1																																						
																																								1														
																																			1					1														
													1																																								
			1	1			1				1									1				1																		1												

			5				
		1		2		1	
				1	1	1	
				1			
						1	
				1			

		1																																																				
																																																						1
																																																						1
																																																						1
													1																																									
																																																						1
													1																																									
													1																						1																			1
					1	1						1		2			2		1			2	1	1	1		2		1	1	1		2							1									1		2	1						
		2	2	2	1	2	2					1		3			2		1		2	2	1	1	2		2	1	2	2	2		1						1			2	1	1		2	2	1	1	1	2		1			
													1																																									
																1																																						
																																				1																			
																																				1																			
																																			2																				1
													1																																									
													1																																									
		1		1	2	1										1		3							1											1	1											2									1
		1		1																																				1														1
		1		1			1						1											2							1				1										1									
																																																						1
													1																																									1
													1																																									
							1				1												1	1																1		1	1								1			1
																																				2																			
																1					1																																	
													1																																									
					1						1		2																	1								1					1									1			
																														1																								
																														1																								
																														1																								
		1											2			1					1			1		1																						1						

						2	
				1		2	
					1		
		1					
				1	1		
						1	
						1	
				1			

																																																					3	
															1																																							
																														1																								1
																														1																								
																														1																								
													1																																									
													1																																									
																																			1																			
																		1																	1					1														
																														1																1								
													1																																									1
																																								1														
													1																																									
													1																					1																			
					1															1														1										1									
													1																					1																			
		1											1					1																	1					1						1							
																																			1																			
															1															1																								
																																																						1
																						1								1																								
																																								1														
																1																		1																			
																1																																						1
																																			1																			
																								1																					1									
													1																																									
						1	1									1														1				2				1															
																																															1			
																																			1						3													
																																			1																			

					1		
					1		
					1		
					1		

		1																																	1					1														
													1																						1																			
																																			1																			
																1																																						
																																			1																			
																1																																						
																2																																						
					1																						1	1																										
																								1																														
													1																						1					1														1
																								1																														
																																			1																			
																1																												1									
													1																						1																			
													1																						1																			
													1																											3													
																																			1																			
																1																			1																			
		1		1			1																																2														
																																									1													
													1																																									

					1		
	2						

													1																																									
																																				1																				
																		1																																					
																																												1									
																																																	2					
						1																																																
																1																																						
																																																					1
																																				1																		

		2			1		
		2					
		1					

																																													1									
										1																1	1									1																	
					1																1					1																						1						
						1										1																																						
																		1																																				
																		1																										1										1
																																									1													
																																								1														
																																		2																				
				1									1					1			1					1																			2									
																1																																						
																1																																						
																					1					2																												
																1																																						

													1																																									
																																																		1	
													1																																									
													1																						1																			
													1																																									
													1																																									1
																																																					1
													1																																									
													1																																									
		1		1	1	1										1								1											1							1												
													1																																1			1						1
																1								1																														1
													1																						1					1					1									
													2																																2									
																					1																																	1
																																			2																			
																																1				1																			
													1																			1				1																			
																1					1			1		1									1																			
																1																																						
													1																																									
																																									1														
							1											1																																				1
						1										1							1				1	1		1													1	1										
																1																																						

					1		
				1	1		

													1																																									
																1																												1										
											1							1																										1										
																1																																						
																																			1																			
													1			1																																				1	
													1											1													1																	
													1																																							1
																1																																						1
													1																		1																1	1						
																1																			1	1																		
					1	1	1						1					1					1	1			1			1	1				1		1			1		1	1	1										
																1		1																																				
																		1																																				
		1	1		1								1			1										2																					1							1
																																										1												
																																															1						
																																			1																			
													1																																								
																																																						1
											1																																											
													1																																								
													1																										1															
													1																						1							1												
						1	1						2			3					24			1		4				1	1				2	2			1								1	1						1
																																			1																			
																																			1																			
																																			1																			
																															1				1																			1
																																			1																			
		1					1																	2											1									1			1							1
																1																																						

					1	1	
				1		2	
					1	1	
						1	
1	4	2		2	4		
					1		

													1																																									
																																		1																			
				1																										2																							
									1									1																																				
													1					2																											1								2
																																		1																			
													1																					1	1																		1
																1																									2													
																																		1																			
																																																					1
													1			1																																						
													2											1						1				2				1															
																																		1																			
													1																																								
																																			1			1															
													1																																								
																								1																														
																1																														1							
		1		1																				1						2																							1
													1																																								1
																								1																													1
		1	1	1							1		1			1		1	1	1	1			2		1	1	1		1					1	1						1	1	1	1		1	1	1					
																2																														2							
																																		1																			
																																		1																			
																																		1					1														
		1		1																				2										1											1							
													1																						1	1											1					
																					2							2						1									1									
																																		1																			
		2			1				1				1			1		2					2	2		1				1			1					1	1	1					3				2					2
																					1																									1							

				1	2		
1				1			
				1	1		
					2		
	2			2	4		
			1				
		1		4			

																1																																						
				1																				1							1									1														
													1																																									
				1			1											1																						1														1
															1																																	1					1
																									1									1																			
																																																						1
																								1										1																			
																																			1																			
																																			1																			
																																			1																			1
													1								1									1																								
													1			1					1			1		1								1					1					1		1							
		1	2			1																																						1									
																1																																						1
																																			1																			
																					1			1		1																						1						1
													1																						1			1															
																																				1			1						1									
																1						1																				1											
													1													1																											
													1																																									
													1																																									
													1																					2																			1
																																																		1				
																									3																											
													1			1					1																																
																1							1																														

					1		
					1		
	1	1		1	1		

																								1											1				1															
													1																																									1
																																			1																			
													1																																									
																																	1	1															1				1	
													1																						1																			
																																			1																			
													1																																									
													1			1																																						
					1								1							1																																		
													2								2					2				2																								
													1																																									
																1																																						
						1										1																			1																			
													1																																									1
													1																						1																			
																1																																						
																					3																			1														
																1																																						
													1																																									
		2	1			1	3		2				1								1			3		2									1						3					1	1	1						
						1																																																
																																										1								3	
													1																																									
																																			1																			
																																			1																			
																1																																						
																1																			1																			

					1		
				1	1		
				1	1		
1							

																																			2																			
																																			1					1														
													1																																							
						1							1			2					1					2					1				1																			1
							1						1			2										1					1				2																			1
																																							1															
		1		1	1	1	1													1	1			1		1																1												
																																			1																			
													1																						1																			
													1																																							
																																																				1	
						1							1																						1											1							
				1																											1																							
																							1																														
		1		1		1															1										1											1												
																								2																														
													1																																							
																																					1																	
																											1																											
													1																																							
																	1																		1																			
		1																																	1																			
																	1																																					
																	1																																					
													1																																							
																	1																											1									1
									1				1																																							
																																							1														
													1																																							
																										1																											
																										1																											
																																			1																			

		1					
		1					
		1		1	1		
						1	

																		1																							1					1								
													1																																								
																								1																														
													1																		1																							
																																			1					1														
													1																																								
													1																						1																			
																																																						1
																																																						1
																																						2				1							1							
		1		1																	1										1				1										1									
									1																																												
																																			1																			
																																			1																			
																																1																							
		1																			1														1																			
													1																																								
													1																																								
													1																						1																			
																																		1																				
		1	1										1					1			1																								1		1				1			
																																			1																			

				1	1		
				1			

																																					5																	
													1																																		1	1						
		1		1																											1																							1
													1																																									
													1																																									
													1																						1																			
		1		1																											1																							
																																			1																			
																																			1																			
									1																																													
																																																						1
																																											1					1						
																																									1													
																								1																														
																																			1																			
																																			1																			
				1																											1																							
																																								1														
		1											1																																									
																																			1																			
													1																																									
																																			1																			
																																			1																			
																																			1																			
				1																																																		

		1					
					1		
					1		

																																								1														
																																				1																			
																																									3													
												1																						1																			
		1																																																			
																2																																						
																																				1																			
																																								1														
				1												1																				1																			
												1																						1																			
																																															1					
																																				1																			
		1		1			2																								1																							
																																				1																			
																																			2																				
												1																																									
																																						1																	
																																				1																			
																																								1														

	1						
					1		
1							
					1		

																					1					1																														1	1					
													1																						1																											
																																			1																											
																																			1					1																						
																																			1																											
													1																																															
													1																																															
																																																						1						
																																			1					1																1						
																																								1																						
																																			1					1																						
																																			1																											
																																			1																											
																																																				1								
																																			1																											
																															1																															
																															1																															
													1																																															
																1																																													
																																																						1						
																																			1																					2						
																																			1																											

													1																																									
																																			1																			
																																			1																			
																																			1																			
																																			1																			1
																								1																														
																															1																							
																																			1																			
																																			1																			
					1																																																	
													1																																									
													1																																									
													1																						1																			
																																																						1
																								1																														
													1																																									
																																			1																			
													1																																									
																																																						1

	1						
	1						
				1			
	1						
				1			
					1		
	1						

													1																																									1
													1																																									
																																		1																			
																																		1																			
																																		1																			
																																				1																	
																																							1														
																																		1																			
																																		1																			
													1																					1																		1
																														1																							
																																												1						
													1																																								
																																		1																			
																																																					1
																																		1																			

					1		
				1			

																																			1																			
																																			1																			
				1																										1																							
													1																																									
																																			1																			
																																			1																			
		1											1					1			1			1		1																											1
													1																																							
													1																												1												1
													1																																							
																																			1																			
				1											1					1					1					1																							
													1																					1						1														
				1																										1																							
				1																										1				1																			
																																																						1
																																								1														
																																			1																			

	1						
	1						
		1					

																																		1																			
																																		1																			
																																		1																			
																															1				1																			
																																							1														
																																		1																			
												1																					1																			
																														1																							
			1																										1				1		1																	
																																									1												
																																																				1
																																																				1
																																		1																			
												1																																									
																																		1					1														
																																		1																			
																																		1																			
																																																				1
																																		1																			
					1	3																																														1
																																		1																			
																																		1																			

		1					
	1						
					1		
	1						
					2		
1					1		

																																			1																			
																					1					1											4											3						
																					1					1																						1						
																1																															1	1						1
		1											1										1	1											1																			
				1																															1																			
				1																																																	
																								1																													
		1		1									1			1					1					1									1										1									
																																			1																			
																																			1																			
																					1													1																			
																1																																					
													1																																									
													1																																									
																1																																					
		1														1																1																					
																																																	1					
			1	1	1								1							1																				1														1
																																			1																			
																				1				1											1																			
													1																																									
													1																		1				1																			
																																			1																			
																																								1														
																																			1																			
													1			1																			1																			
																																			1																			
																																																						1
																																			1					1														
																																			1																			
																																			1																			
				1																																																	

1							
1							
				1	1		
				1			

																																				1																			
																																				1																			
																																				1																			
																																				1																			
							1																																															
																																				1																			
		1					1																								1																							
																																				1																			
		1		1																											1																							
																																				1					1														
													1																						1																			
																																				1					1														
													1																						1																			
		1		2																															1																			1
																																									1														
																																				1					1														
																																				1					1													1
																																				1																			
				1																											1				1																			
				1																															1																			
		1		2									1																																									
																																				1																			
																																				1																			
				1									1																		1																							
																																				1																			1
																																				1																			
																																				1																			
																																																							1
																																				1					1														
				1									1																		1				1																			
																																				1																			
													1																																									
																																				1																			
																																				1																			

						1	
						1	
					1		
					1		

																					1																																	
																					1														1										1									
													1																																							
																																			1																			
																1													2																									1
																						2								1					1												1							
		1														1								2																							1							1
													1																																							
																																			1																			
													1																						1																			
																																			1																			
																																								1														
																																			1																			
																																			1																			
																					1																																	
													1																						1																			
																																			1																			
																																			1																			
																																			1																			
																																			1																			
																																			1																			
																																													1								
													1																																							
																																													1								1
			1		1	2										1					1					1																												
																1																																					
																																																					1
													1																																							
													1																		1																							

	2						
					1		
	1	1					

							1							1																													1											1									
															1																																								
															1																						1																				
																																						1																				
		1												1							1	5					4				1					1										1		1	2						
		1	2	1		1	1		1		2		1	1		2				1	3			5		1	1	1	1						1	1		1	1	1			1			1		2	1	1	1			1		1	2
				1	1	1	1			1		1			1										2			1	1		1					1		1	1		1		1	1				1	1	1			1			1
		1											1								1						1										2																				
																																						1																				
																									1																													
																																						1																				
																																						1																1			
		1																								3																													
																											1																												1
																						1			1		1																													
		1	1		1	1	1							1			1		1		1	1	1	1	1		1				1														1		1	1		1	1	1	1	1					1
															1																																								
																																																						1
																						2					1																													
																						1	1																								1										
															1												1																													
																																						1																				
															1																																								
															1																																								
		1		1			1													3						1																1		3	1		2									1	1
		1		1				1																																				1	1													1
																																																						1
																																						1																				
															1																																								
							1							1																					1																				
																						1																																	1
		1	1	1	1	1	1		1		1		2			1		1	1	1	1		1	1		1			1	1						1			1		1		1		1	1	2			1			

		1	1	3	3	1	
		1		1	1		
				1			
				1			
	1			1			
		1					
					1		
						1	
1		1					

																																																						1
			1													1					1	1				1																						1						
		3				1															1					1				1																								
													1																																									
													1																										1															
																																				1																			
																																				1																			1
		1				1					1		1					1																										1	6			1			1			3
																																				1																			

1							
				1			

0	1789	298	1142	600	0	326	230	334	59	173	56	35	50	59	199	66	13	158	41	411	622	1189	94	309	172	352	36	2743	407	702	2481	1470	605	619	1301	746	957	829	2121	178	284	491	846	515	248	2367	890	128	541	193	483	511	447	151	128	459	247	1462	167	499	228	1
0	76	46	85	73	0	28	24	0	5	8	4	5	0	2	19	4	0	2	13	131	212	292	12	37	18	68	8	985	91	150	861	405	244	138	56	208	72	90	215	10	17	173	300	72	26	178	185	19	58	20	75	38	58	11	11	61	89	125	30	33	28	0
SplF.others																																																														
Other SAPs								Minor											Lysine transferases									Histone transferases									Polycomb Group Genes					Complex Component		chromatin-related																		
DBC1	HSPA5	CARM1	PRMT1	PRMT5	CRKRS	PPM1G	ZNF326	LPAR1	PDCD7	RECQL5	RNPC3	SNRNP25	SNRNP35	SNRNP48	ZCRB1	ZMAT5	ZRSR1	ZRSR2	ASCL1	EHMT2	KAT2B	KDM1A	KDM4B	SETD1A	SETD2	SUV39H1	TET1	EP300	KAT2A	KAT5	HDAC1	HDAC2	HDAC3	HDAC4	HDAC6	SIRT1	BMI1	EED	EZH2	PHC1	PHC2	SMARCA2	SMARCA4	ASH2L	BAZ1B	BRD4	CBX3	CHD1	CTCF	DEK	HMGA1	KHDRBS1	MORF4L1	NAB2	RPS6KA5	DNMT1	MBD2	MECP2	SPEN	TAF15	TCERG1	TCERG1L
		1																													4							1	5			8	19						1									1		1		
				1																																						5	16															1				
																																										2	7				1											1				
																																																					1									
																																							1							1	1															
																															6	1																														
																															6	3				1		1	1																		1					
																						1									6	2																													1	
																				1												1						6	19																							
																																1																														
																			1									2																																		
				1															4		3	1						4	2																																	
																			3			1						1																																		
																												1																																		
																												1																																		
		1		2																1	6	1				2		8	1	2	4								1			1	4	1																		
		1		1																																							1																			

																												1																																		
																												1												1									1													
																												4															1																			
																																												1																			
																					2								1																																	
						1															4	4				2		6			3	3				2													1													
	1																																																													
																					5							5				2	1	1												1																
																				1																																												
																																1																															
																																2					1																										
																												1																										1								
	1																																																													
		2																													1					2																										
																										1		2															1																			
																																		2																												
																												2			1																															
																					1	1						1								2															1											
																					1	1						1								2																										
																						1					2	5			1	1				2						1	1																			
																					4						3	75		2	7	1	3	6	1	5															1											
	1	2	1																		5							4											2																							
																																	1																				1								
																					1							2																														1				
																																																										1				

																																	1																																																																																																																																																																																1		1						4	1	5	1	1				1			1								4						1				1	1				
			1																			1				1					4						1	1		1				1		1	6															
																													1																																																																																																																																																																					1																										4		1																										1					
																																2																																
																																1	2															1															
																																																																																																																																	4																											1	1																														
	3	4	2			1	3			1					1						2	6		3		1		14	10	11	11	5	3		1	2		2	5		1	4	9	1	2	4	4	2	1	2	2	2	3			3	1	1	2	2	3	
																														2																							2									
	1	1	3				1								1						1	1			1			1			1	2				3	4	2	7			1	1		1		1		1	1	1	1				1		1		1		
				4																								6	1		1					5						1	1																			
	1																											1					1																													
																				1											2	2														2			2								1					
						1																																																							
			2																		1			3				1																3																		
																													1																																		
																										1																																				
	2			1																		1				1																																				
																														1																																
																												3										1								1				2												
																												1											1							1																
																															1				1	4																										
																				1																						1							1													
																														1				2																	1											
																																														1																
																						1									4							1	1				1				1															
																														1			1																													
																1																																														
																					1							14			1		1			5						1				1					2											
																												5														1			2														1	
																												3								1																										
																					1																																									
																						1						1			1	1	1				1						1																	1		
																										1				4	2		1	1	3								3																			
		2																										3								2						1	1	1																		
																												1													1																					
																												2			2															1																
		2								1																		1																																		
																															1			1	3																											
																												3				1																														
																					1																																									
						1									1						1															1											1				1											
																																														1																
																															1	1	1																													

																						1																																								
																													1																																	
																					1								2																																	
																					1							8																																		
																												3																																		
																																					1									1									1							
		1								1																		11		2	4	2				1							2			1					1				3			1				
																																	5																													
																																																			1										
																															3																															
			1			1																1						2	1	4	4	3	1	1		1						4	4			1		1		1	1	1	1	1		1	1	1	1		2	
	2	3	1	1		2	1			1										2	2	1						9	4		4	2		1					1			7	5	3	1	2	2	1	1	3	4	2	1			1	1			1	2	
																												1			4		2	1																												
	2																											3			2				1							1	2																	1		
																					1							4			2																			1											
																					2							5			1																															
																					2			1							1																															
	1		1				1								2													1							1	1	1		3										4									3				
															2																																		1													
			1																																		2		1							1			1													
																					6	2						4	3	2	4					4			1			1		1																		
																															1																															
			1			1									1							1						1	1	1	4		2										1	1		1					1		1								1	
																												1																																		
																						1									4	2					2	2	3	1			3				6									1						
			1																																																										
																															3	1															3															
																																															1															
																																							1						1						1	2					1						
																																1																													
			1																			1						1														1	1							1							1				
																																1																													

																																										1								1							1				
																					1								1	1																														1	
																											1														1																				
																																																								1				
																														1																															
																															1																												1	
						1																						2			3	1					1							1		2																
						1																						1			4	3	2											1		3																
																																1	1		1																											
																																				1																										
																												1								4			2																							
	1																			3								9								4							1																	1		
																												5								8																										
																																				1																										
																														1																																
																																										1																				
							1																					3		2	2	1				1																										
							1																																					1																		
																														4					1																		3									
																										1				3																							3									
																																1					1																									
																															3															1															
																															1	1																								1					
																																1		1																												
																												1			2		4	2								1																				
			1	2																																																										
	1																														1	1														1							1									
																																														1																
																																														1							1									
			1																		3						2			1	5	6					1	1							3	1				1								1		
																																2																								1				
										1										3	1	1								2																1															
																				1																																										
																																																									1				

																					5	1						3			6					3																										
																					1	1				1					3	1																	1													
																															1																2															
																				1								4														1	3	1					1							1						
																												1														1		2																		
			1																																																											
																														2																							2									
																					2							5								3							1				1															
	1																											1														1																				
	1			1																											2																				2											
																																																			2											
																																																			1											
																												2																																		
									1											1																			1											1	1										1	
																						1									2						1	1					2			2	10											1				
																				1																											5															
																						1									1																															
	1					1														2	2	1						8		1	1	1				4							2				1															
																						1						1			5	4	1	1					1																							
																															1																															
																						1									1																															
																															1	1																														
																															1	1														1																
																															1	1														2	1															
																												3														2	1																			
																						1				2																																				
																					2																									1																
																															1	1																														
																																				1																										
				3																																																										
																																						1																								
																												1																																		

																																				3																										
																					5															2																										
				1																						2																																								
																1																																																	
																													1																																						
																													1																																						
																																																				1									
																															1																							1									
																						1																																													
																				1	1																					1							1												
																				1																						1	1	1	1				1				1					1				
												1								1																						1																				
												2								1									1													1	1																			
																				1																																													
																										1																1																				
																													1								1												1													
																																	1																														

																																										1																				
																																																				1											
									1																			1												1											1											
																						1									1																				1							1		1		
																															2																															
																															2																															
																					2										2																															
																															3																															
																			1								1															1																				
																			1																																										
																			1								1					1									1																				
																																										1																				
																													1																																
																											1																																		
																								1																																					
																			1								2	1	1													1																	1			
																			1																																										
																											3				1														1															
																																												1																	
																											1																																		
																																																			1									
																					2																																								
																																1																														
																																				2						1																				
																											1			1					1																										
																																							1																							

																				1																																										
																													2					1													1												1				
	1																																																												
																																																	1															
						1																														1																										
																																															1																
																																															1																
																															1																											1				
																													2		3		1						1				1																			
																																							1																							
																						1																																								
																															3																															
																															1																															
																															1	1																														
																																																							2								
	1						1																					2			2			1		1										2	1				1							2				
																												1						1				1	1			1				2	1				1											
																																														2	1				1							1				
																							1	1															1					12																		
																													1	4																	1																
																																						1	1							1	1									1	1				
																																						1	3			2	8			2					1											
																															2																1											3				
	1			1			1													1		1			1						2	1											1				2		1			1									1	
																												2			2														1			1														
																												1														1																				
			1																	1								1																																		
																				1																						1		1																		
																																																										1				
																				2		2			1			2			3	1	3				1				1	3	2	2	1		1		1			2					1	2	2	2		

																												1															1																			
																														1																											1					
																						1									2	1	1																			1						1					
																												1														1	1																			
																						1									1																															
																												6														1	1			1																
																												4					1																													
																												3			1																															
																												1																				1			1												
						2														1																		1							1	2												1				
						1																7									8	7							1							1					1						1	1				
																						11									12	10																														
		1		1																								1			2	2											10	23			2	1				1							2				
																															1																															
																												1		3	1								1							3	1															
							1																		1			2							1			2	1				1			3	1	3				1						1		1	1	
																					1																	1	3				1	1	1	2	2	1										2	1			
																					3																		1								1				2							1				
	1						1															1						2		1	2	2				1							3									1										
			1																									1																																		
																																											2																			
																															1		2	1	4			2	2								1			1	1					1						
																												1																																		
																																		1	1																											
																			3									4			1		1	5																												
																																			1																											
																			1			1						1						10																												
		1		1															1		1	2						2			1		3	6										2																		
																														1			1	3									3								1											
																												1														1																				
																																							1				1				1																
																				1																	1					1				1																
																				1				1					1		1											1		1														1			
			1	1																								1			5	1			1			1	1			1				3											1		1	1	
		1																													1															2																
																																																										1				
																																						1	1							1	1				1											
			2																																		1									2		1									1	1	1		
																						1								1				1																												

																																	1				1									1																
			1																			1											1																													
																																						1	1																			2				
			1																																		1																									
			1																																		1		2																							
			1																									2				1														2																
						1																									1		1														1															
																												1																																		
																						1																																								
																												1																																		
						1																																																								
																																1																														
			1																									1				1					1	1	1					1		3												1				
																						4									1	1							1		2												2			4						
																						1																																								
				1																																	1	1	1				3															1				
																																															1															
			1																									1									1									2			1									1				
																																		1	1				1																							
																																					1																									
			1																																		1		2																			1		1		
	2		2	1																								1			5				1			1	1			4	4			2						1						1		1		
																												1																																		
																												1																																		
																																												1																		
																																														1																
																												1									1	1	1							1												1				
																						1																																								
																												2											1							1												1				
																														1	1																1			1	1											
			1																																					2																				1		
																												1																														2				
																																																										1				
																					1	1									1																															
																												1																		1																
						1																3								8																							4									
	4	2	2	3		1	1											1		1	18	9		6	3			##	3	13	25	6				25	4			1			9	3		6					2					4		1	1		1	
	1	1																			2							10				1																														
																					5	1						10		1	4	1				3																										
																					1							5																																		
																												3														1																				

																				1								1														1																				
			1												2													1									2												1													
																												1									2																				1					
							1				1																																						1													
																				1														3																				1								
																												11		1	5	1	1	1																	1				1							
												2																6																									1									
	2	6	3	4																								34	4	3	19	9	5		1	1			4																2							
																				1															1				4			2	1													1						
	1																				1							5			1	2		1									2								1											
	1																					1				1		4			1												1				1			1												
																					2	4						1	1	1	1	1							1				2				1	1		1							2	2	6			
																																																									1				
	1		4	1			1																			3		2			6	2	5										3	1												3			1	1	1	
																					1					2		4			3		3	4	2																											
																										1		2			1								2							1																
	2																														1	1																														
						1																			1											2										1	1															
																																								1							6		1									1				
																												6	1		1								1					1																		
											1										4							5	1		5	2		1					2				4	1								1										
																					5				2	1		24	1		2			2					4		2		2						3													
	2																					1						8			2	1			1				1																							
																										1		2	1		1																															
																																																							1							
																					1							4			4	2	2		2	3																									
																					1	2							1										1					1																		
			2																									10			1		1	1					1			1	2			1																
																												5															3																			
																					1	2						16	1	1	4	1	5		2	3			1				3			2						2				3						
		2	2																										5			1												1																			
																												4																																		
																												2																																		
																															1	1	1	1																												
																						1								1											1		2																			

			1																																																										
																												1														1				1																	
																				1																						1							1												
																																																										1				
																																						1																							
	1																																																													
																															1	1																														
						1																						2								5																	1									
																					1			1					1													1		1							1		1									
																				1				2																				2			1						1									
																												2																																	
																				1	1			1					1	1												1		1									1									
																												4														1																			
	1																										2			1																														
																				1				1																														1									
				1																																																									
																												2														1	1			2															
																					1							3														1	1																		
																												4																																	
																					4							3																										1							
																				1																								1																	
		1																										1														1																			
																												1																																	
																														3			2		1											1															
																				1																																									
																				1				1				1																					1													
																					2																					1	2																1		
																												6			1															1															
																															3		1																								3				
																												1																																	
																					3							5	1													1								1											
																					5							3	1																																
																												10				1	1																													
		1																										1														1																			
						1														1							2			2																														
																					1							1			2																1															
																				1																																									
	1		1																			1						1										1				1	1			4						1	1					1		1		
	2					1																																																							
						1														1								1			9	4																														
																															4	3																														
																				1				1				6	1	2							1			2				1			1					1													
																												2				1						1	1								1										1					
																						1	1					1			2		26	11		2											1	2	2								1		4	1			

	1																											1			3	2	26	7												2					1								5			
																						33					1				19	13	2						1				1			2					1							1				
																						2									4	3																										1				
																						6									7	4	1						1																							
		1		2																							1								1		1	1	2			13	29			3					1							1		1		
		1																									1			1	1						1	3			12	19			3												1		1		
	1																				5	1							11		2																														
																					3								4							3																										
																											2			5																															
																																													1	1															
				1																	2																										1															
			1	1		1															1						1																															1				
		1	3	1		1																					4										1		1																			1				
																				1																																								
																				2	1		1					5		1														1																		
																					5									1																1															
																					1									1																															
																				2																											2															
																											1											1						1	1			1									2				
																																																		1									
																														1																											1				
																																			1													1													
																				1													2								1																					
																											8																											1								
																					1						1	1							1								1												2								
																											1			2												1																			
																					1																																									
																											1			1																1															
																				3	1			1				1	1																				1													
																					5							1																																		
																				1	2					1		3																					1			1										
			1							1										1								2																	1				2			1										
																					2							2																																		
																				3	1	2		1				2	1													1																				
							1																				6		4	1	5										1	1														1					
				1																1								6			3	1										1				1																
																				1	1									1			2																														
			1																	1	2			1				2	1	1		1													1				1										1			
																				1																									1				1													
																					3							26			34	10	1	3									5								4					2		2				
																											4			8	3																								2		2				

																				1	1	1						1	1				1									1	1	1								1									1	
																																							8	13																							
																						1						2		1	1		2																1							1						
																															1																					1									
																				3																																										
																					1							3		1	1		1																													
																																																					1									
																												2			1	1							2																							
																																							1																							
																				1			3																																						
																				1																																										
																														1																																
																																					1										1																
																																												1																		
				4																								2			6	1	2	3		1							1																			
																															1																															
	1						1								2									1				2									1	1					3			1			4			1										
																									1										1																										
																																				1																										
																															2																															
			1																	2		1									2	2																														
																				4		9	1			2					2	3									1		2																			
			3	1																	4										3	1			1								1																			
		1																													1				2																											
																															1				2																											
															1																																		1													
																																					1																									
				1																																																										
				1																								2																																		
																															2	2	2																													
				1																											4		3																													
																					2							3			2																															
																														2											1																					
	1			4																4		3									2	6																														
	4																																					2	3																							
																				2											2																															
																												1		1	1	1	1																													
																																															1											2				
	1			1																		10						4			12	9	2						3							2						1				4						
																					1	1									4	3																														
																															2	2																														

																																							2																	1						
			2																	1	2			1				17	1	1	3	7	9	5			2	4	3					1		1	1		1									4				
																																					1																									
															2																1															1																
			1																											1																																
																												4			14	1	2	4	2		8		4																							
	1																																																													
																						1				1															1																					
																																																					1									
																									1																																					
															1													2											1										1													
																												3		1									1																							
																				1											4	1	1			1							1									5				1						
																												3		2			1	2																												
																																2															1															
																						1																																								
																																															1															
																																						1																								
																															1																															
																																															1															
																												1																		1																
																																														1																
																																										1																				
																																										1																				
																																															1															
																										2																																				
																																																										1				
																									1										1												1															

																																															1						1									
																																															1															
																																						1																							
																												2	1		1		2						1								1															
																																						1																							
																									1																																							
																																															1															
				1																																													1													
																																				1																											
																																											1																				
						1														2																																											
				3																																																									
																																															1															
																																															1															
																												1		1																1															
																																															1															

						1																										1				2			1						1		2				1					2		1				
																															1																															
																																																					1									
																						1																																								
																						1																1									4				1											
																						1																									4															
																																							2							1	1											1				
																																															1											1				
																						1																																								
																																															1															
																												1																																		
																																		1	1												1											1				
																																			1	2																										
																																															1															
																																							1																								

																																														1															
				1																		1				2																																				
																												1																																							
																																																				1									
																																				1																										
																																1																														
																						1																																												
																																																1											2				
																																																1															
			1																																	1																										
																																							1																							
																																				2																										
																										1																																								

																																													1															
																																													1															
																																													1															
																																													1															
																					1				1																																				
																																														1															
																																														1															
																					1																																								
																									2																																				
																																			2																										
																									2																																				
																																			1																										

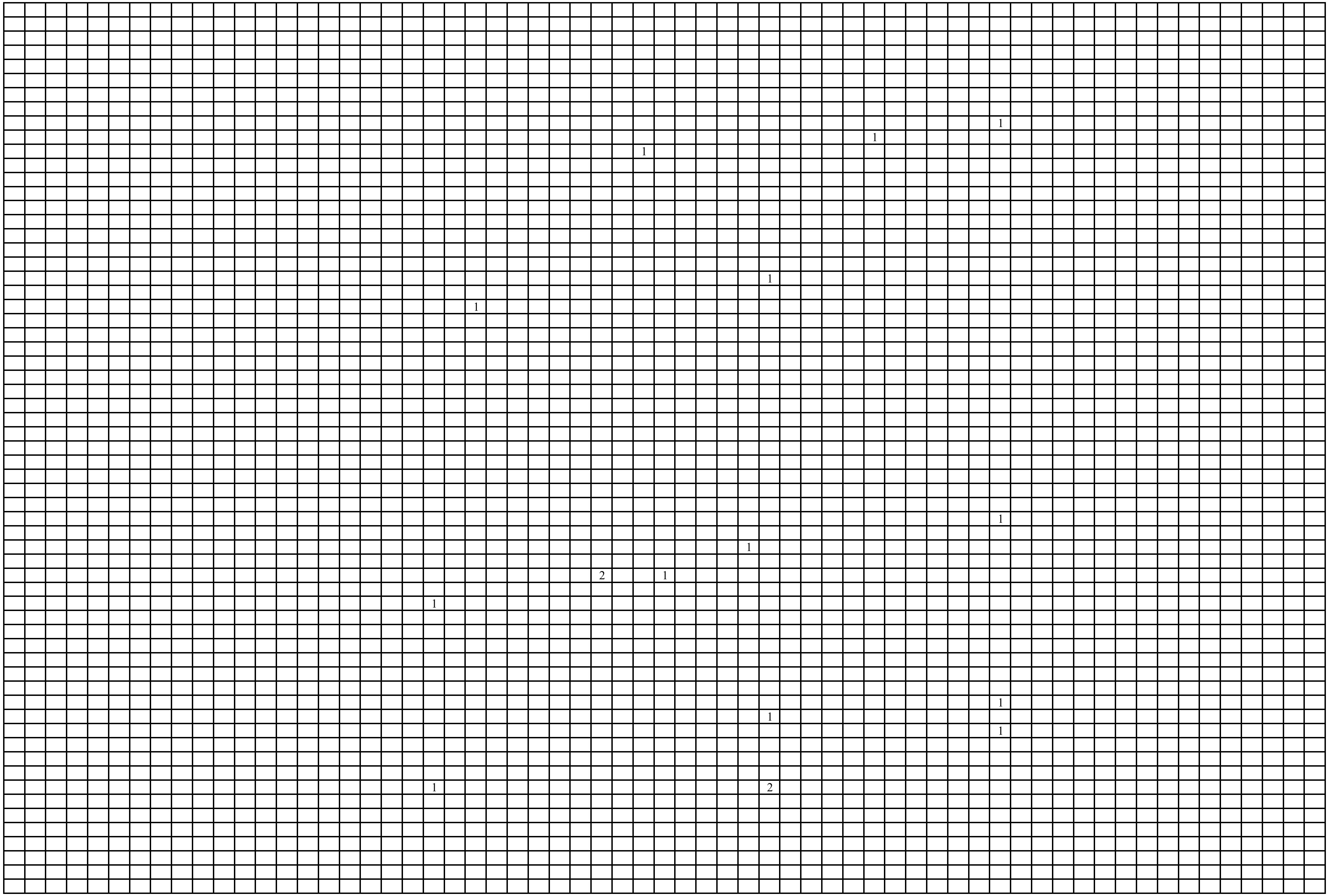
1
1
1
1
1
1
1
1
2
1
1
1
1
1
1
2

																					1				1																						1															
																			1																																												
																																														1															
																																														1															
																																																										1				
																																																										1				
																														1																																	
																					2											2																		1											
																									1																																						
																							1																																								
																															6	4				2		1	2																		1					
																									2					6	8	1		1								1													2					
																															1	1						1																									
																			1											4												2																			
																																	1		5		1	1												1					2						
																			1								1																		1											1				
																																																			1				3					
																					1									1																															

	1																					1									8	5	1	1									1				1				1	1	1				2					
																															1	1		1																												
	1																					1								2	2	3		1													1				1	1					2					
																																1		1																												
																					2								2																																	
																						2									1		1																													
																				1								1											1																			1				
			1																	2	4	1				4			1		8	5		1		2							1		1		1					1										
																																															1															
	1																														1	3													1		1				1	1										
																						1						2										1																								
																															1	1	1																													
	1																			2											4	1		1					1				3				1				1								1		
																						1									1																3															
																						3									6	3	2														1															
																															3	1													1											1					
																						1																																							
																															1								3																		1					
																				1																																					1				
			1	1																9						1					3	3						1									3												1				
																						1								1	1																															
																												1																																		
							1																			1				1	7	1						1	1						1			1	1								1	1				
																						1									1			1												1	1															
	1																																																													
																				1																3																								1			1	
																						1																																							
																																																							1						
																				1																																										
																				1		10									6	5															1															
																															3	3																									4					
																																							1																							
																										1																		1			2										1				
																																																								1					
																																															1															
																																												1																		

																										1				1											1													1								
																															1	1																												1				
																										1																											1									
																				4																			1								4											1				
																												1																																			
																												1																																			
																						1									1																																	
																																														1	1				1														
																				1											3	1						1	1								1										2	1				
																														1																																	
																						3									5	1																															
																															4	1																										1				
																														1																																	
																																													1												1				
																															1																															
															1																																																
																						1									3								1							4																
																																																								1				
																																														1											1				
																															1																															
																																														1															
																														1																			1													
																															4	1					1	1								2																
																																													1																
																				1																																			1							
																								1				1																																		
																																					1																					1				
																															2															1												2				
																						2																																										
																																																								1				
																														3																																	

																																						1																							
																															2	1	1				1								1																	
																																																				1									
																												3		2																																
																								1															1																			1				
																						1									1	1														1																
			1	2																																	1	1									1			1									1		2		
																																					1																									
																															2																															
	1																																																													
																																													1																
																								1							2																															
																															3	1																											1		
																				1																																										
																																	1																													
																						1									1					1										2											1				
																																										1																				
																															2	2																														
																																							1																							
																																																1															
		1																															1									1		1																					
																														4			2		3	3								1	1									1										
																				1		1						2	1				2				1					1	1																			
																						4						1			3	3	4	1			1					1							1													
	1																			1		1						8			1	2	1				2	1	2	1		1	1		1	2		1				1								1	1	
																												1																																		
																												1																																		
																															6	4						1	1														8				1					
																															11	7						1	2							2											10					
																															12	9						2	2							4											8					
																						4						1			24	20				5		2	2							1					1					4	3					

																						5		1				2			32	18				2		1	2				2			4					1	1				4	8	1				
																						3									10	7						1	2							2											3					
																				2				1				1			7	2												1																	
																						1									3	4														1											1					
	4		2				1												1	3	5						9		6	8	2	3	6		4	5	3	4			6	6	3		3											1			1		
	1	1	3	2			1		1	1									2		4	5		1	1		23	1	4	9	3	4	5		2			4		1	2	2	5	1		1	1	1	1		2	1				1	1	1	1	2	
			1	2			1			1	1			1					1		1	1	1	1			2	1	1	1	1	1		1			1				1	1	1	1	1	1	1	1													
																				2															3																									
	1																																		1																									
	1								1												1						5		1	4	1			3							2	4	1											1				2			
																				1							1																																	
																				2							1									3																								
																																1																													
																													2	2																															
																					2				1								2		1							1																			
																				1	2				2		1						1		1																										
		1	1																								1							1	2							1																			
			1																								1																																	1	
			1																								5								3																									
																			1		2						2			3	1	2	1																				1					1			
	1																			1							14	2	2	3		9	3		3			1			1	1	1				1			3							1				
																				3					1		2			1	2	1	1		2			1												2	1										
		1		1																							2											2																							
																											1			2																															
																									1		2				1	3																													
			1																												1	2																													
																								1			1								2			1																							
			3																1	1	1		1				1	1	1			1	1		2	2	2	2			1	1	1															1			
																					3									3	2	12	3									1													3						
	1	1	1	1			1														3		1							2	1	2	1				1				1	1		1		2	1		1						2				1	1	
																					3									1	1	2																													
																														2															1																
																											3					1	1									1			1			2													
	1						1													1							2			2				1							2	1																	1		
																																									2	2		1																		
				1																1											6		3		1	3						6																				
															1																								1										1													
															1						4																																								
																										2							1																													
																															2							2									1									1						
																																						1																								
			2	2																		2									19	10						1	1							1										1	4					
																						3					1				17	10				2		1	2			1	2			2										1	11	1				
																						1				1					5															1																
	1		1				1		1		1			1		1		1							1	1				1	4	1			1				1		1	4	1			2	3			1	2	1	1			2		2	1	1	1	

																				1		1				4					1	1															1														1	
																	1														1																															
	2																											2																		1	2				1											
																																	1							1																						
																												1																																	
																															1	1															1														1	
																						1									1					1																										
																															1											1																			
	1		1																																		1																			1						

																										CPSF6	CFIm	pAF.4C	30	564
																										CPSF7			34	387
																										NUDT1			2	87
																										CLP1	CFIIm		10	84
																										PCF11			12	97
																								1		CPSF1	CPSF		31	285
																										CPSF2			12	154
																										CPSF3			12	166
		1																								CPSF4			14	125
																										CSTF2T			17	129
																										FIP1L1			18	265
																										WDR33			11	177
																										SYMPK	CPSF-assoc		29	254
																										CSTF1	CSTF		13	114
																										CSTF2			14	184
																										CSTF3			8	133
			1																							PABPN1	pA-binding	pAF.PA	19	190
																										CPEB1			9	159
																										CPEB2			4	47
																										CPEB3			0	10
																										CPEB4			6	194
																										NGDN			18	141
																										PAPOLA	PAP, canonical		4	52
																										PAPOLB			0	2
																										PAPOLG			4	34
																										MTPAP	PAP, non-canonical		13	177
																										TENT2			0	0
																										TENT4A			0	0
																										TENT4B			0	0
																										TUT1			3	77
																										TUT4			0	0
																										TUT7			0	0
																										PARN	pA-RNAse		24	140
																										EIF1	EIFs	TlatMFs	4	37
																										EIF1AX			8	113
																										EIF1AY			3	31
																										EIF1B			0	53
																										EIF2A			11	142
																										EIF2AK1			1	39
																										EIF2AK2			33	565
																										EIF2AK3			9	206
																										EIF2AK4			4	96
																										EIF2B1			5	176
																										EIF2B2			3	154
																										EIF2B3			4	136
																										EIF2B4			5	171
																										EIF2B5			4	208
																										EIF2S1			18	290
																										EIF2S2			20	261
																										EIF2S3			15	344
																										EIF3A			27	569
																										EIF3B			25	489
																										EIF3C			15	295
																										EIF3CL			5	98
																										EIF3D			23	319
																										EIF3E			23	476
																										EIF3F			30	457
																										EIF3G			20	363
																										EIF3H			22	384
																										EIF3I			23	389
																										EIF3J			13	149
																										EIF3K			9	215
																										EIF3L			19	306
																										EIF3M			16	281
																										EIF4A1			29	429
																										EIF4A2			5	207

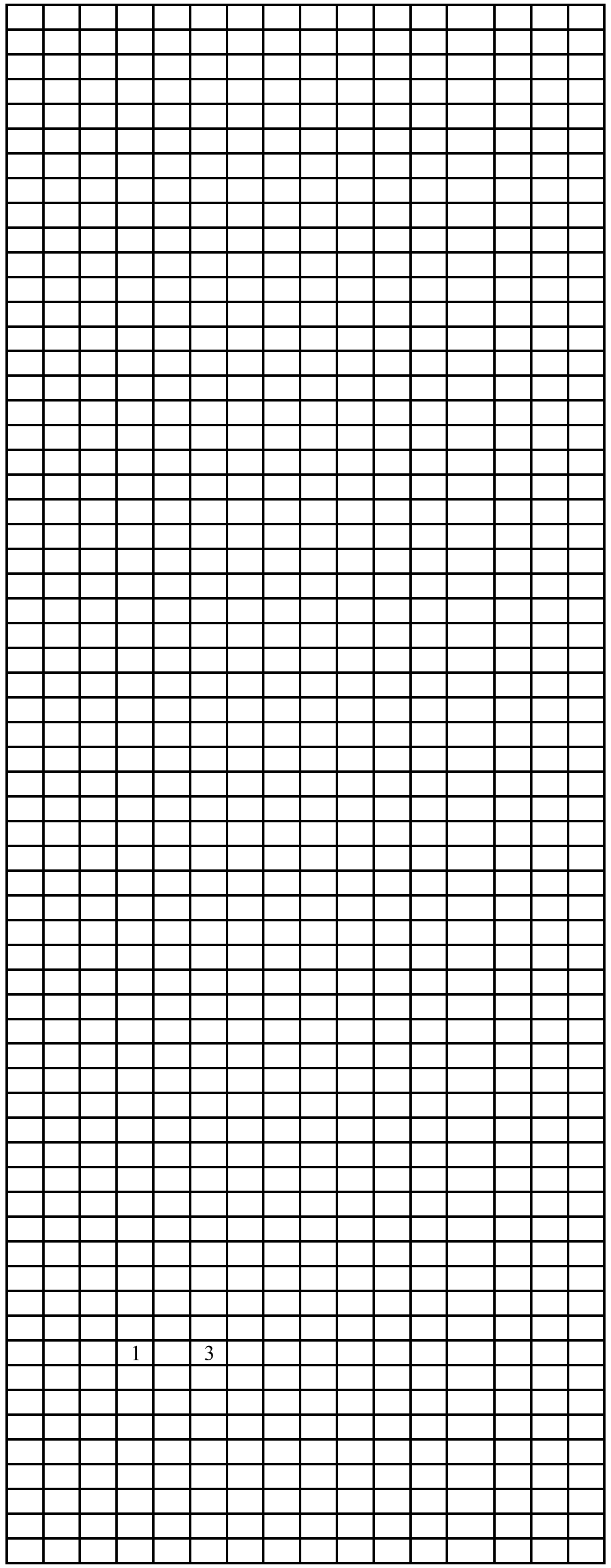
1
3

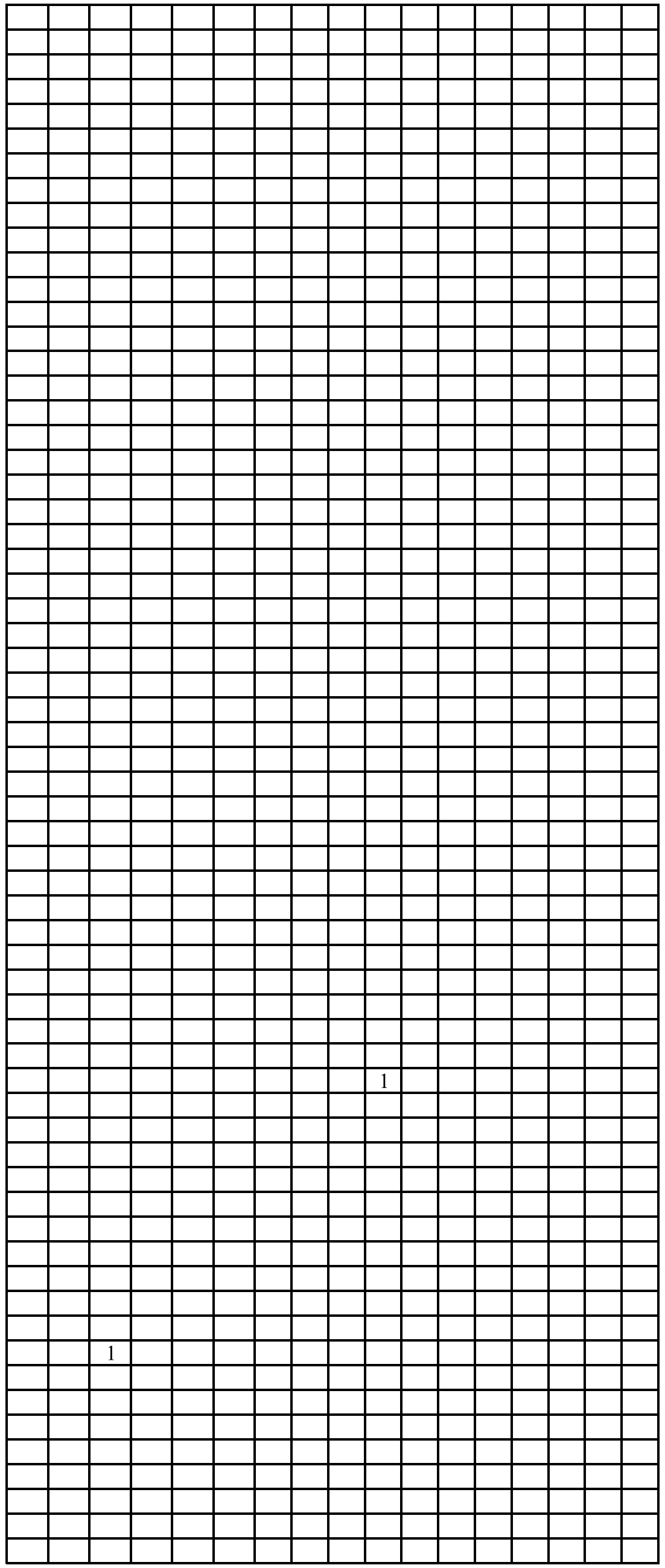
1
1

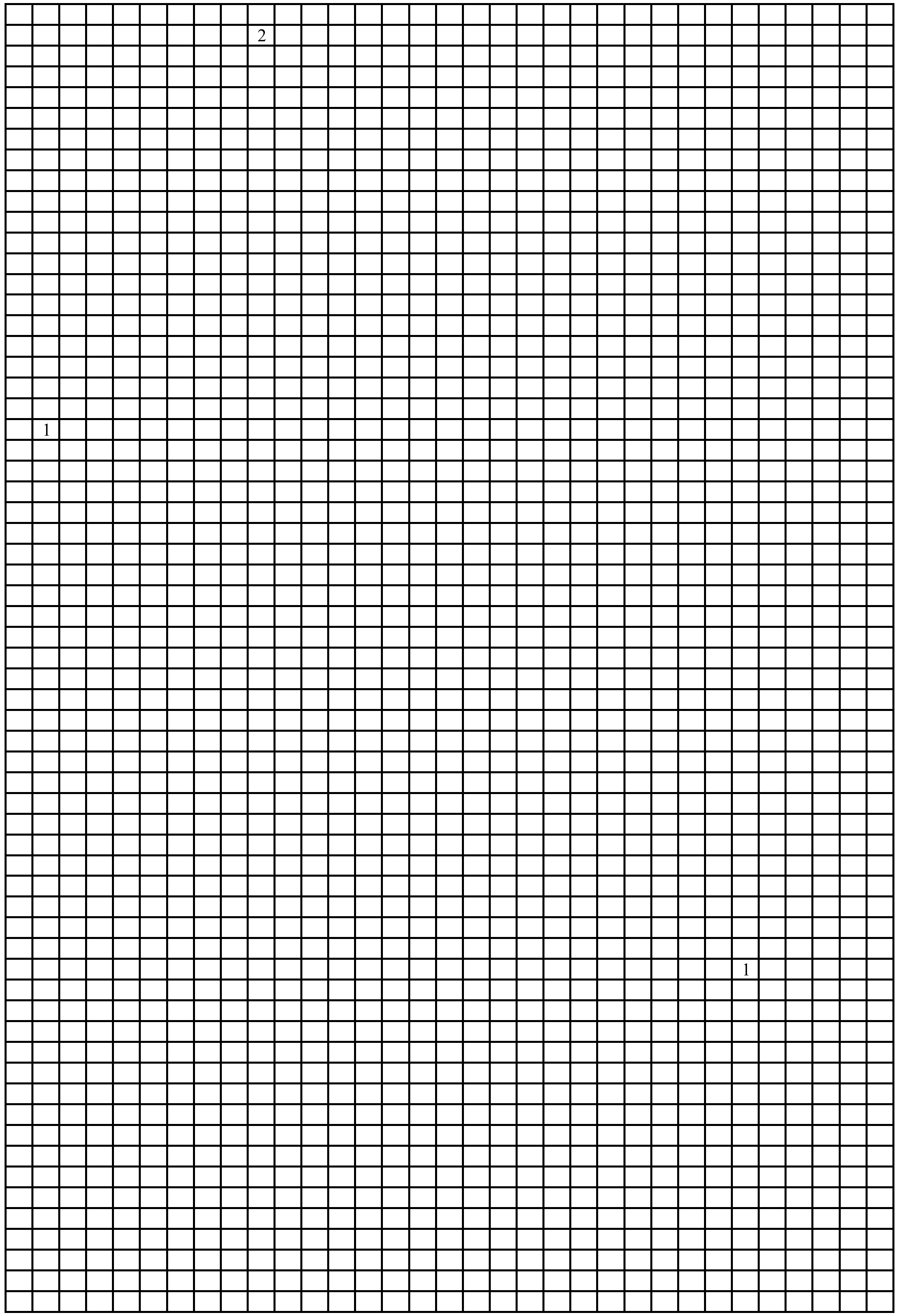
2
1
1

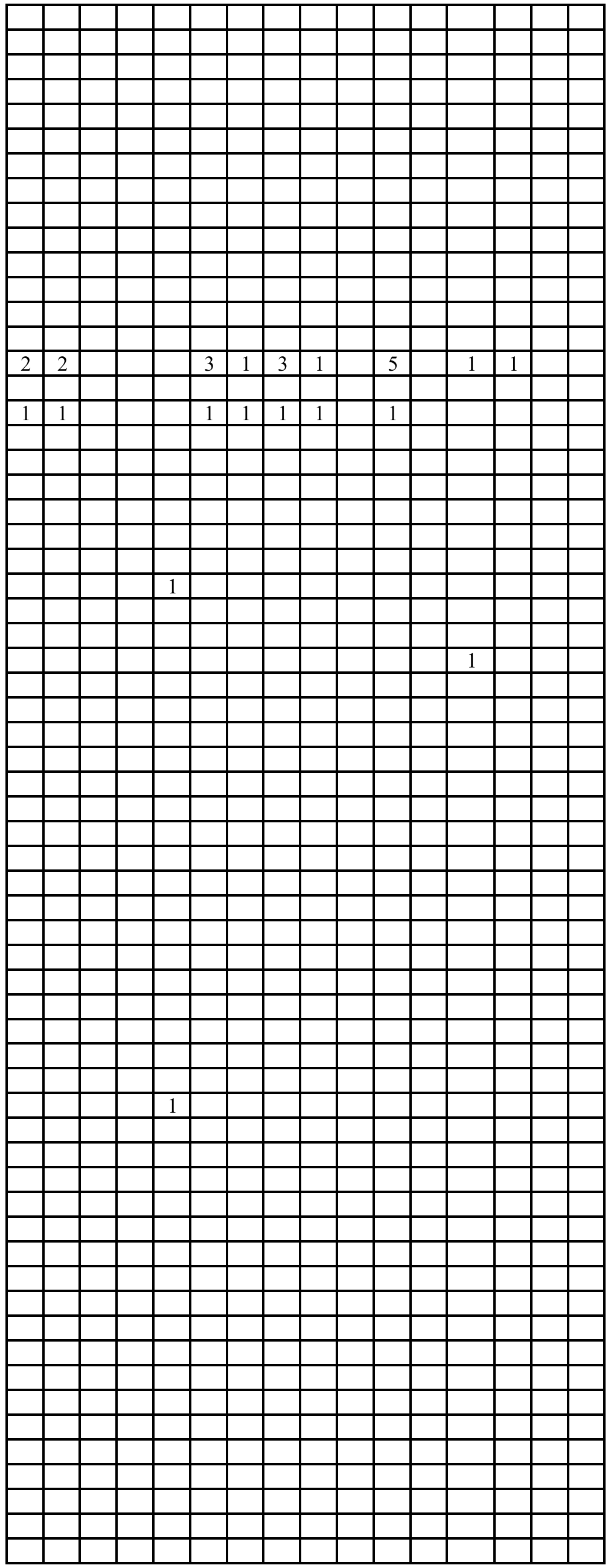
2 2 3 1 3 1 5 1 1
1 1 1 1 1 1 1
1
1
1
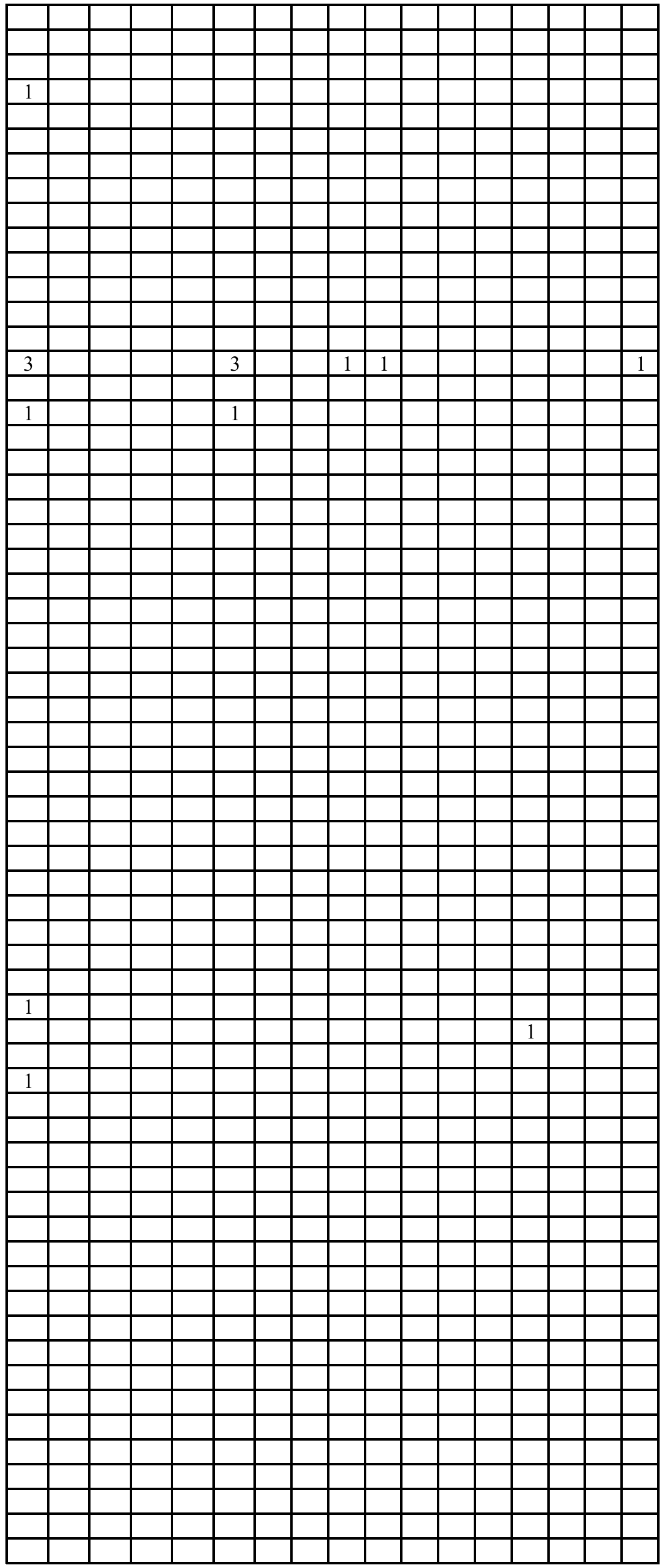
1
3 3 1 1 1
1 1
1
1
1
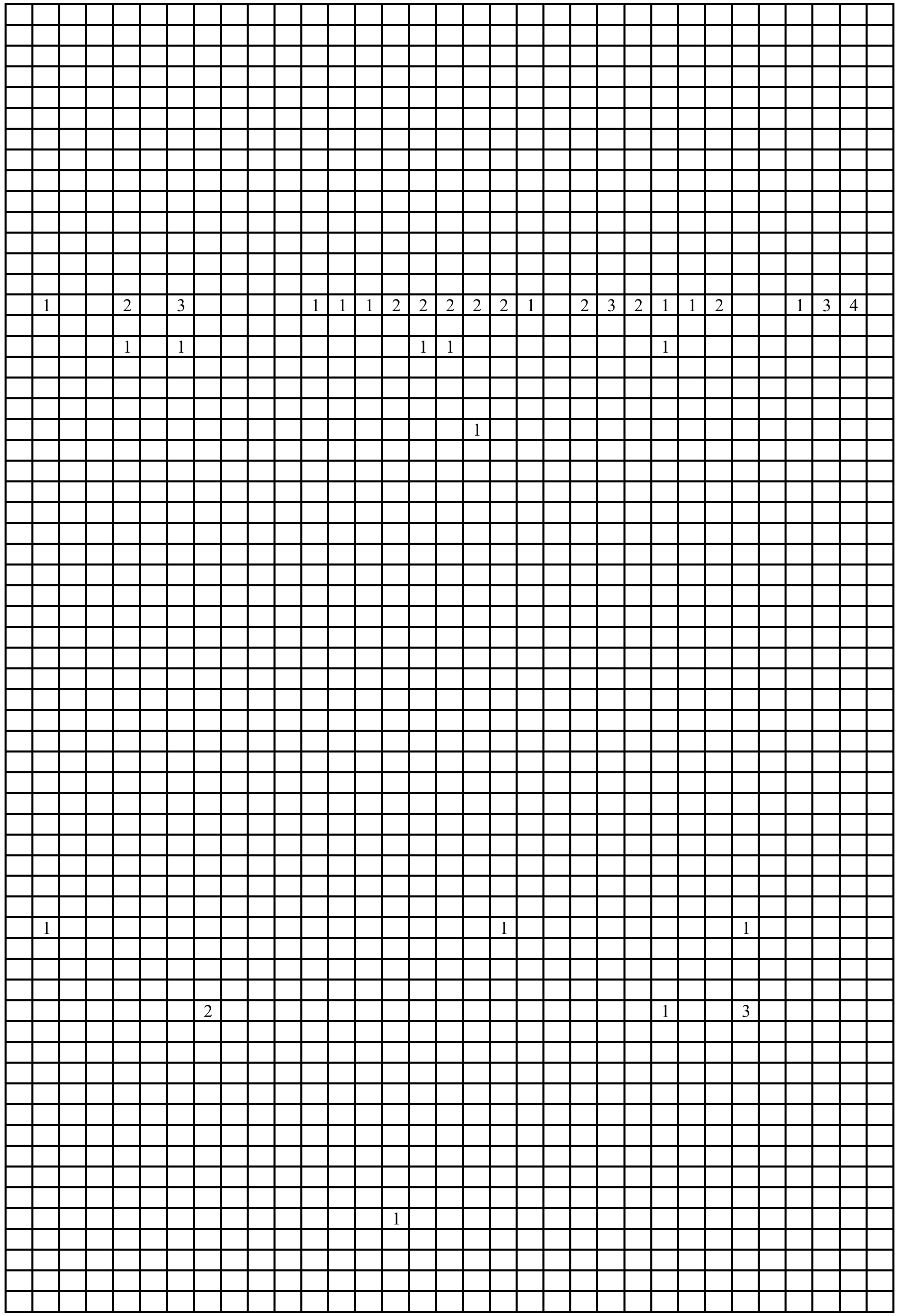
1 2 3 1 1 1 2 2 2 2 2 1 2 3 2 1 1 2 1 3 4
1 1 1 1 1
1
1 1 1
2 1 3
1

			2												
2	2			1					1	1					1
1	1			1	1	1	1	2	1	2	2	2	1	1	1
												3			
	1									1					
												1			

	1															
2					1											
								1								
	1															
	1															
						1										1

				1																												
									1																							
							1																									
1				1										1	1	1															1	
1	2			2		2		1			1			2	2	2	2	2	1	1	2	2		2	2	2	2	1		1	2	
																								1								
				1												1	1											1				
		1																														

												1			
												1			
												1			
									1						
												1			
									1						
						1						1			
										1					
1															
												1			
									2						
														1	
													1		
					1										

									1							
																1
									1							
									1							

							1																									
							1																									
														1									1								1	
									1																							
																	1														1	
																							1									

														1	
					1										
											1				
	1														

				1												

																					1											
																							1									
																					1											

1 1

1

1

1

1

1																

																										1					
																				1											
															1	1	1						1		1	1				1	

									1						
										1					
													1		
								1							

						1										
					1											
1									1				1			

																		1					1									
							2																									
1																																
1														1			1			1		1				1		1		1		
																							2								2	
																	1	1	1		1	1	1	1	1	1					1	
						1					1											1						1		2	

									1						
1	1									1					
				1	1							1		1	
1					1							2			
1	2								1						
1															

1																
1																
									1							
1																
																1
1																
		1														

																							1									
																	1								1					1		
															1																	
																		1												1	1	
					1																		1									
																							1									
																							1									

					1				2	1		4		2	
					1										
1															
1															
2															
1															
														3	
								1							
	1										2				
1															

	1			1												
	2															
																1
				1												
				1												
																1
	1															
1																
																1
						1		1								1

						1																										
																	1	1	1													
																	2		1			3			2					2		
						1																										1
		1		1		1								1	1	1															1	
																							1									
										2																						
						7									2	1		2	1			1	2	1	2	2			1	1		

												1			
					1										
													1		
1							1		1	1				1	
	1														
	1														
	2														

	1															
	1															
				1												1
								1								
									1							
						1										
						1										

						2																										
						1																										
																										1						
																	1				1											
																	1							1								1
																													1			
								2																			1					
																			1			1	1									
						3																										
						1																	3									

							1								
1	1					1	1				1	1			1
					1							1			
					1										

																3
																2
																1

											1				1																	
														1																		
						1																									1	1
							1					1																				
														1	1		1	1	1					1		1		1	1			

			1						1						
1															
	1														

																1

																		1	1						1	1			1	1		
													1																			
						2											1								1	1						
																	1	1						1	1							
																	1	1	1		1	1	1	1	1	1			1			
																	1	1	1		1	1		1	1	1			1			

	1		1	1	1	1		1			1				
					1										
														1	
1	1														

					1											

																											1					
							1																									
															1		1	1		1		1		1		1		1	1	1	1	
																										1						
																							1	1	4				1		1	

1	1				1					1					
											1				

																1
																1

															1																	
																															1	

1

1

1

1

1

1

2

1

1

1

1 1 1 1 1 3

					1										
					1										
1															
	1														
					1										
												1			
					1										
														1	

					1											
																1

																			1		1	1							1			
																			1		1	1							1			
														1																		
																												1					

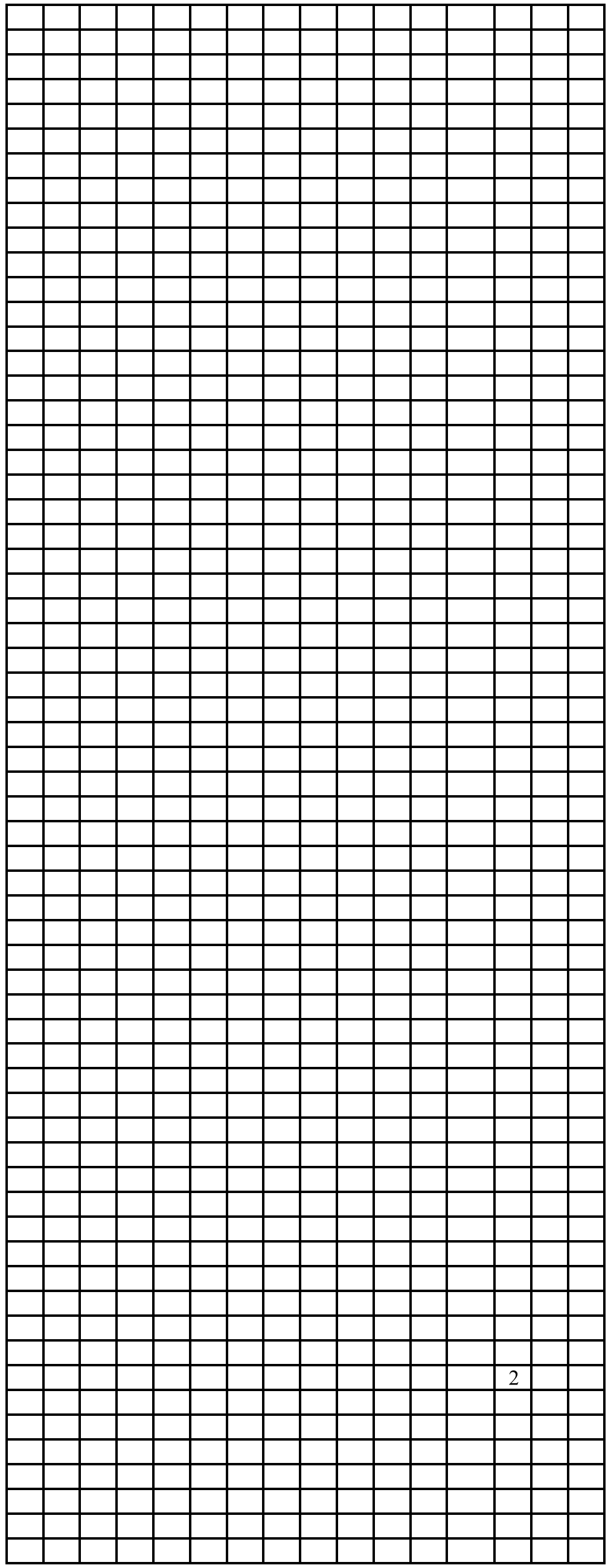
2

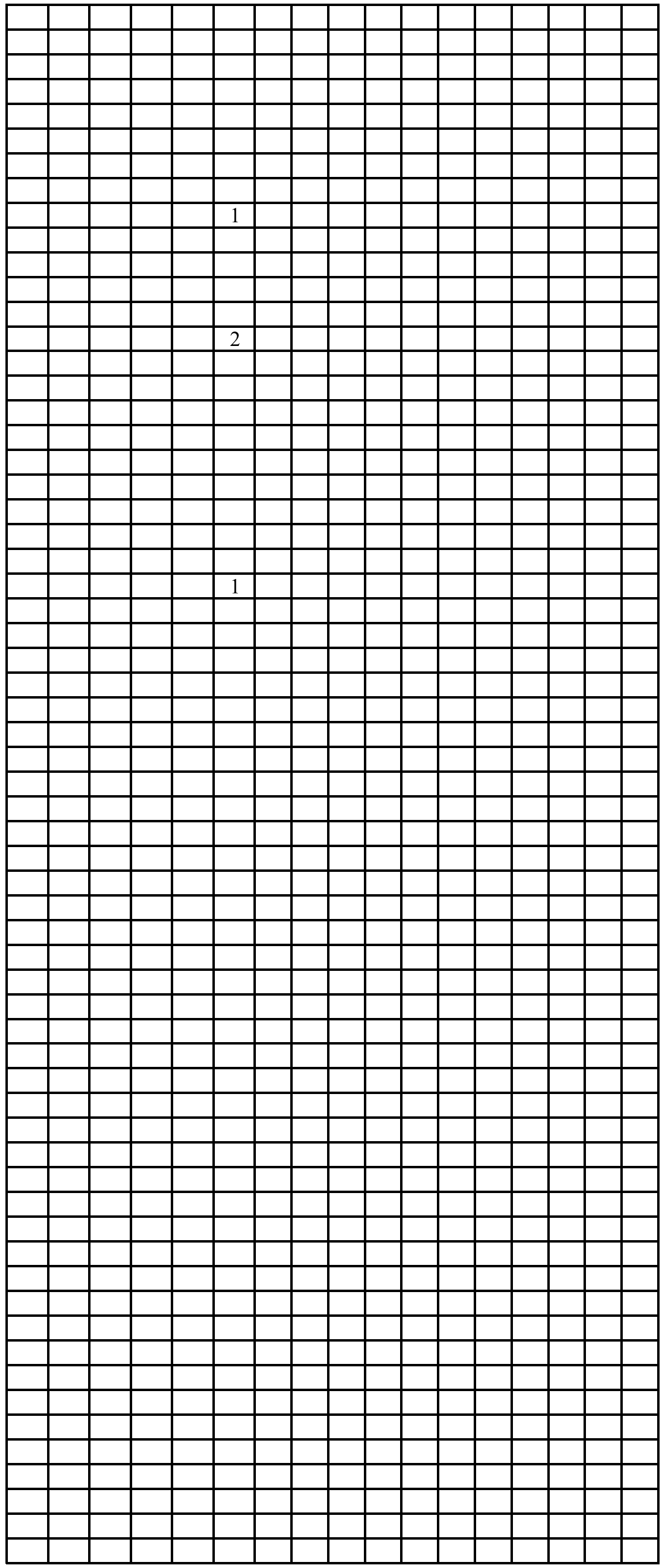
1
2
1

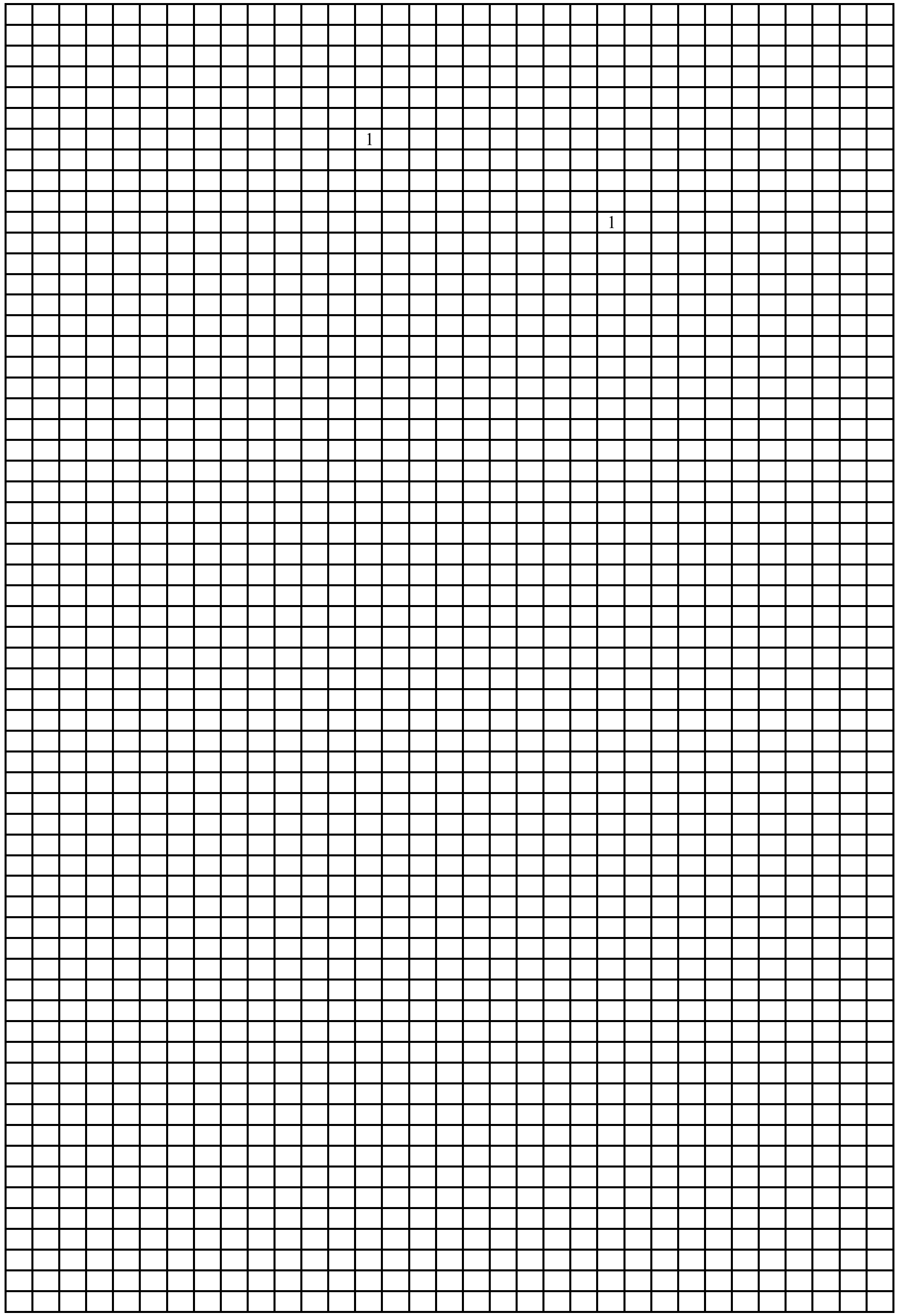
1
1

					1								1		1
1															
1															
	1			1		1		1					1		
	1			1		1									
								1							

																1

										1																						
																	1						1										
																															1		
													1																			
																1															1	
	1																															
																					1											
																					1											

1	3		1	1	1			1			1	1			1
1	1		1	1		1		1	1	1			1		1
1	1		1	1	1	1	1	1			1	1	1	1	1
												1			
1		1				1	1								
	1														
1	2		1	1	1	1	1	1	1	1	1	1	1	1	1

		1														
																1
					1				1							2
					1				1							
					1											
					1				1							
					1											
1				1												

																					1											
														1												1					1	
	1			1		1								1	2	1	1	2	1		1	1	3	1	1	1	1	1	1	1	3	
						1			1			1		1		1	1			1	1	1	1	1	1	1		1	1	1	1	
		1																														
						1					1	1		1	1	1	1	1		1	1	1	1	1		1		1	1	1	1	1
																					1											
	1					2								1	1			1	1		1		1								1	

	1														

		1							1							
1																

				1										1	1	1		2			1		1	1	1	1		1	1			

																										EIF4B			19	353
																										EIF4E			20	297
											1															EIF4EBP1			8	178
																										EIF4EBP2			2	46
																										EIF4EBP3			1	15
																						1				EIF4ENIF1			12	402
																										EIF4G1			19	457
																										EIF4G2			8	162
																										EIF4G3			5	160
																										EIF4H			5	84
																										EIF5			4	184
																										EIF5B			26	193

																										DHX29	EIcoFs	TlatMFs	0	0
																										DDX3			0	0
																										EIF6			24	320
																										PABPC1			49	793
																										PABPC2			0	0
																										PABPC3			6	74
																										PAIP1			7	197
																										PAIP2			2	86
																										PAIP2B			4	68
																										FMR1			19	433
																										CYFIP1			17	213
											1															CYFIP2			10	164
																										NUFIP			0	0

																										eEF1A1	EEFs	TlatMFs	0	0
																										eEF1A2			0	0
																										eEF1B2			15	209
																										eEF1D			40	376
																										eEF1G			27	490
																										eEF2			0	0
																										eEF2K			1	61
																										EEF2KMT			4	40
																										GFM1			8	360
																										GFM2			1	178
																										MTERF4			2	33
												1														eIF5A			24	239
																										eIF5A2			0	0
																										EEFSEC			5	39

																										RPSA			66	549
														1												RPS2			76	805
																										RPS3			84	785
																										RPS3A			65	667
																										RPS4X			38	506
																										RPS4Y			0	0
																						1				RPS5			40	387
																										RPS6			91	1178
																										RPS7			38	410
																										RPS8			70	795
																										RPS9			50	508
														1												RPS10			72	561
																										RPS11			38	519
																										RPS12			20	270
												1														RPS13			49	450
																										RPS14			46	531
																										RPS15			53	407
																										RPS15A			29	333
																										RPS16			74	869
																										RPS17			38	322
																										RPS18			34	360
																										RPS19			75	613
																										RPS20			35	459
																										RPS21			18	199
																										RPS23			27	275
																										RPS24			40	512
																										RPS25			30	353
																										RPS26			22	260
																										RPS27			23	314

					1						

			1										

		1	1													2		2	1									
	1	1				1			1									1		1								
														1														
		1				1		1			1				1		1	1	1	1	1	1				1		
						1																						

1					1	2	1		1		1
								1			1

		1	2			1	1		1	1		
			1			1	1		1		1	

			1										
		1	2	4				1			1		
				1									1
											1		
			1										
		1											
			1							1			
			1										
			2										

2	3	3	3	3		3	3	3	4	3	3	3	2	3	3	4	3	3	5	3	4	2	2	3	3	3	2	2
	1	1	1	1		1	1	1	1	1	1	1	1	1	1	1	1	1	1	1	1	1	1	1	1	1	1	1
1																							1					

1	1						1		1	1	1
2						2	1		1	2	2
			2								
							1					1
							1					
												1

			1									
		1	2									
			2			1		1				
			1									
		1							1			

		1	2										
		1	1	1									1
		1	2	2				1			2		
		1	1										
											1		
			1										

							1																					
			1																									
2	2	2	2	2		2	2	2	2	1	2	2	2	2	2	2	2	1	2	2	2	2	2	2	2	2		1
		4	2																									
2	3	1	1				1	1	1	1	2							1			1	1			1			
	3	1	1			1	1			1	1					1		1	1	1		1						
	1	1	1				1				1							1			1	1						
						1					1							1	1							1		
						1			1							1			1				1					1

											1
						1					
									1		
											1

			1									
										1		
			1		1							
									1			

			1	1									
			1										
			1										
		1	1										
		1		1									

								1					1				1							1				
		1															1	1				1						
1	1	1	1	1		1		1	1	1	1			1	1		1	1	1	1	1			1	1	1	1	

											1
		1									
	3										

									1			
			1									
									1			

																										1		
				1											1						1				1			
								1													1			1		1		
																										1		
	1	1	1	1			1		1	1				1	1		1	1		1								

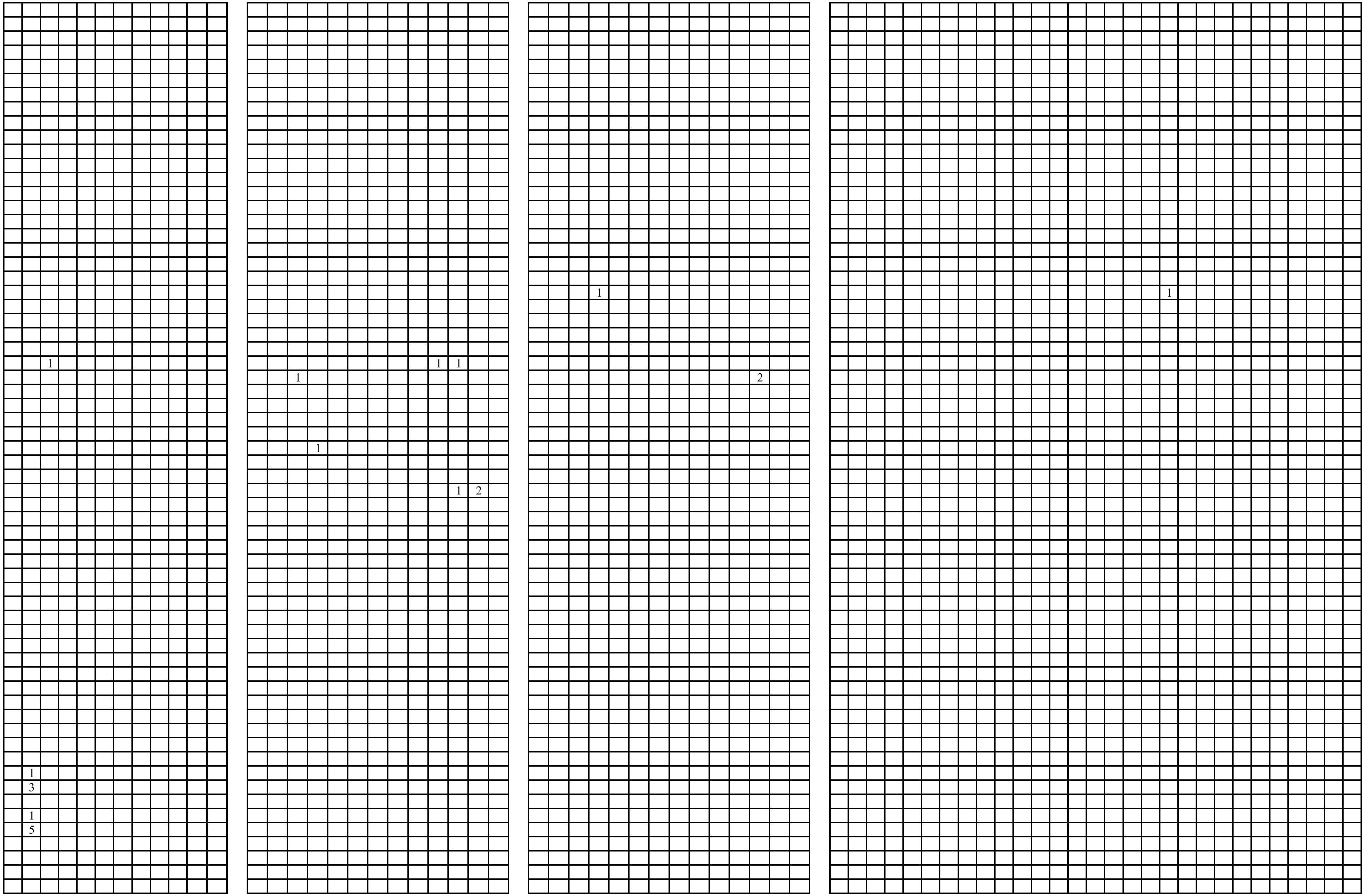
1
1
3
1
5
1 1
1
1
1 2
1
2
1

						1					
	1										
	1										

			1									
										1		
			2									

											1		
				1							1		
			1										
											1		
											1		

		1				1			1											1					1			
										1																		
1																												
1			1								1	1		1		1									1			
1	1	2	2	2		1	1	2	2	2	1			1	1	1	1	1	1	1		1			1	1		
																											1	

											1
1											
1											
								1			1

		1	1									
			1									
			2									
		1										
		2										

											1		
				1									
													1
		1		1									

1	1	1	1	1		2	2		1	1	1	1	2	1	1	1	1	1	1	1	1	2			1	1		
1	1		1	1			1	1	1		1					1	1	2		1	1	1	1	1		1	1	1
				1																								
																	1											
						1							1				1											
1	1	1	1	1		1	1	1	1	1		1	1		1	1	1	1	1	1	1	1		1	1			
							1																					
													1															
1		1	1	1			1					2	1	1	1	1	1	1	2		1	1	1					

					1	1					1
							1				

		1										
									1			
								1	2			

								2					
			4	1									
											1		

	1											1		1					1									
												1		1					1									
																					1							
							1																		1			
				1												1												
											1																	
1	1	1	1	1			1		1									1		1					1			
																		1			1							
														1														

					1						
					1				1		
						1					1
1											1

		1										
			1									
						1			2			
		1	1									
			1									
			3		1							

			1										
			2										
		1		1				2			1		
								1	1				

	1						2								2						1	1			1			
			2				2		2	1				2				1			1							
											1	1		1		1					1				1			
																						1	1					
																						1						
																						1						
							1																					
																		1										
1	1						3				1							1			1				1			
							1																					
1	1	1	1	2		1	1	1	1	1	1	1		1	1	1	1	1	1	1	1	1		1	1	1	1	1
																												1
																												1
																		1										
							1																					
1													1					1										
																				1								
									1									1										
	2	2	3	2			1	3	3	1	2			1	1	1		1	2	1	2				2	1	2	2
1																												
															1													
							1																					

		2									
				1							
2					1						
		2									

								1				
					1							
			1									
			1									
			1									
						1					1	

											2		
			1										
											1		

1		1					1		2		1									1							
3	3	1					2		1		1									1							
		1	1			1	1		1	1	1				1	1		1										
		3									1					1												
														1														
																											1	
		3																										
																				1							
															1													
										2																		
											1											1				

					1						
											1
											1

		1	1									
						1						

						1							
			1										

																	1											
																				1								
							1		1		1					1		1								1		
1	1	1	2	1		1	1	1	1	1	2	1	1	2	1	1	1		1	1	1	1	1		1		1	1

					1						
1											
						1					
						1					

										1		
										1		
		1										
			1									
		1										

			1										
													1

												1								1								
										1																		
							1																					
	1							1	1									1										
							1		1																			
1	1		1				1				1				2						1							
																1												
		1	2				3			1								1			2		1		1			
																										1		
																		1										
		1				1					1					1												
			1																									
																										1			
						1					1	1		1		1			1									
						1					1	1		1		1			1									
			1			1			1		1	1		1		1					1				1	1		
						1						1				1			1									
						1					1	1		1		1			1									
1											1					1					1							
																						1						

					1						
1											
					1						
1											

									1			
										3	3	
			1									
			1									

								1					
			1										
		1	1										

2	2	3	2	2		2	3	1	2	3	1	2	1	2	2	1	2	3	1	1	2	2	1	2	2	1	2	2
											1																	
	1										1					1		1										
2		1					1														1							
		1	1	1			1	1	1	1		1		1				1		1	1							
1	1	1	1	1		1	1	1	1	1	1	1		1	1			1		1				1	1			

							2				

		1										
									1			

											1		

1																					1							
2	1	1					1			1	1							1			1							
								1																		1		
		1					1		2	1	1				2			1			1							
									1					1		1												
		1	1							1						2												
									1						1				1		1							
										1																		
																					1							
	1	1					1		1		1										1							
							1																					
	1	1	1				2											1			2							
3											1				2													
													1															

							1						
				1									

																												1
1																												
1																					1							
		1														1												
											1										1							
							1																					
			1																									
	1		3				1				1							1			1							
																				1								

							1						

		1												1														
																					1							
							1																					
	1		1																									
																					1							
	1																											
			1													1												
											1										1							
3		1									1																	
										1	1																	

2

1

3

1

1

1

1

					1					1		

		1	1				1							1				1										
							1																					
							1				1			1							1							
1	1						1			1		1	1			1	1	2	1					1	1			
																							1					
1	1	1					1		1		2										1							
	2						1		1		1																	
2		1									1										1							
		1																										

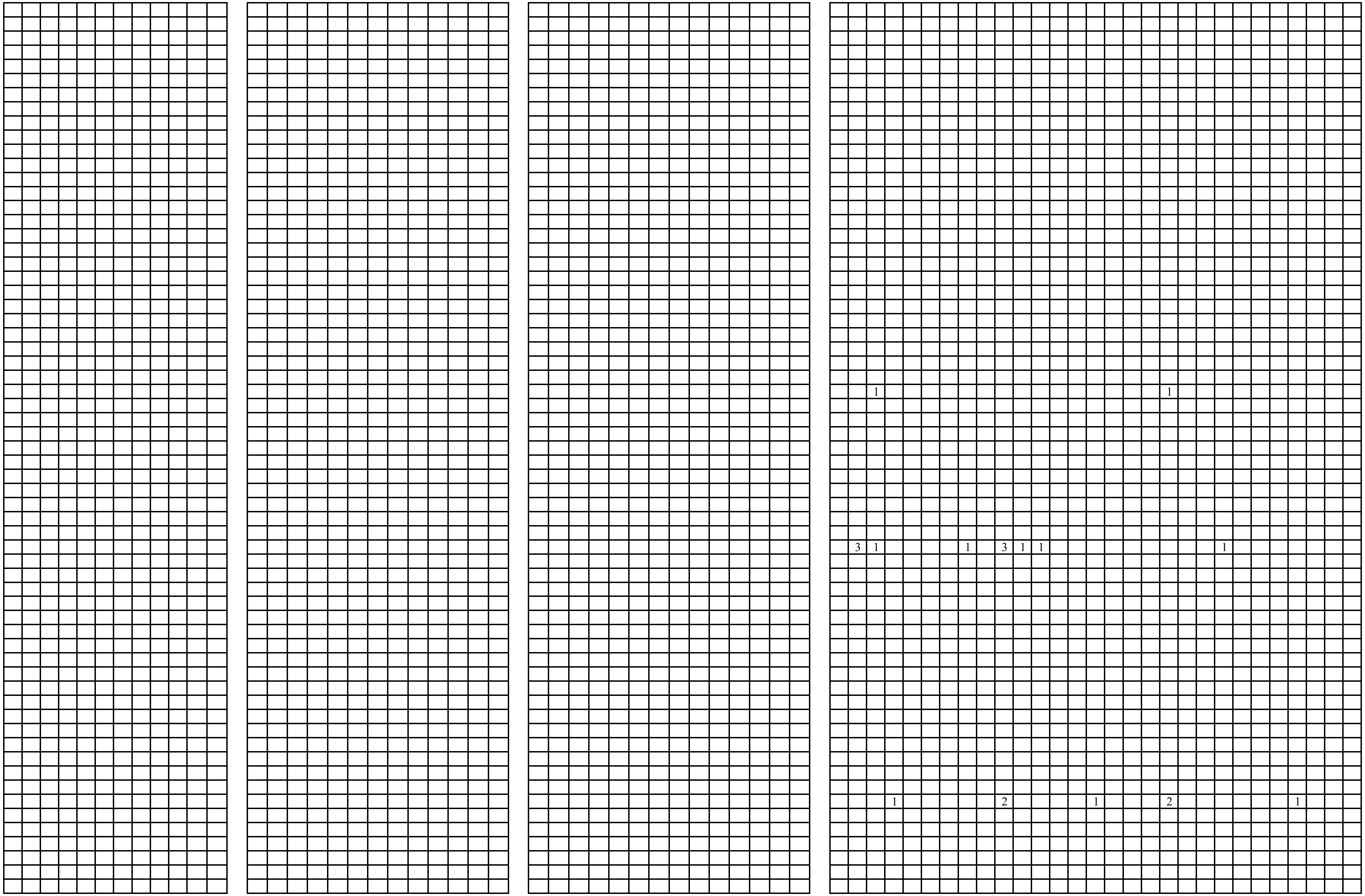
1 1
3 1 1 3 1 1 1
1 2 1 2 1

1											
	1										
1											
1											
											2

										1		
										1		
			1									
		1							1			

			1	1									
			1	1									

	1							1				1			1										1	1		
	1							1				1			1										1	1		
								1								1					2						1	
		1																1										
		1					1			1	1							1										
1		1					1			1	1							1			1							
								1																			1	
1	1	1	1	1			1	1	1	1	1	1		1	1		1	1	1	1	1	1		1	1	1	1	
							1				1																	
1				1					1	1		1		1	1		1	1						1		1		1
								1											1									1
			1			1																						1
	3																											
		1																										
3																												

											1		

		1																										
		1					1		1	1	1																	
1	1		2				2		1	1	1										1							
							1																					
4	4	1					1		1		1				1	1		2			2							
									1																			
1		1					1				1							1			1							
											1										1							
	1	2	2			1	2		2	1	1			1		1		1			2				1			
																					1							
	1		1				1			1	1																	
											1																	

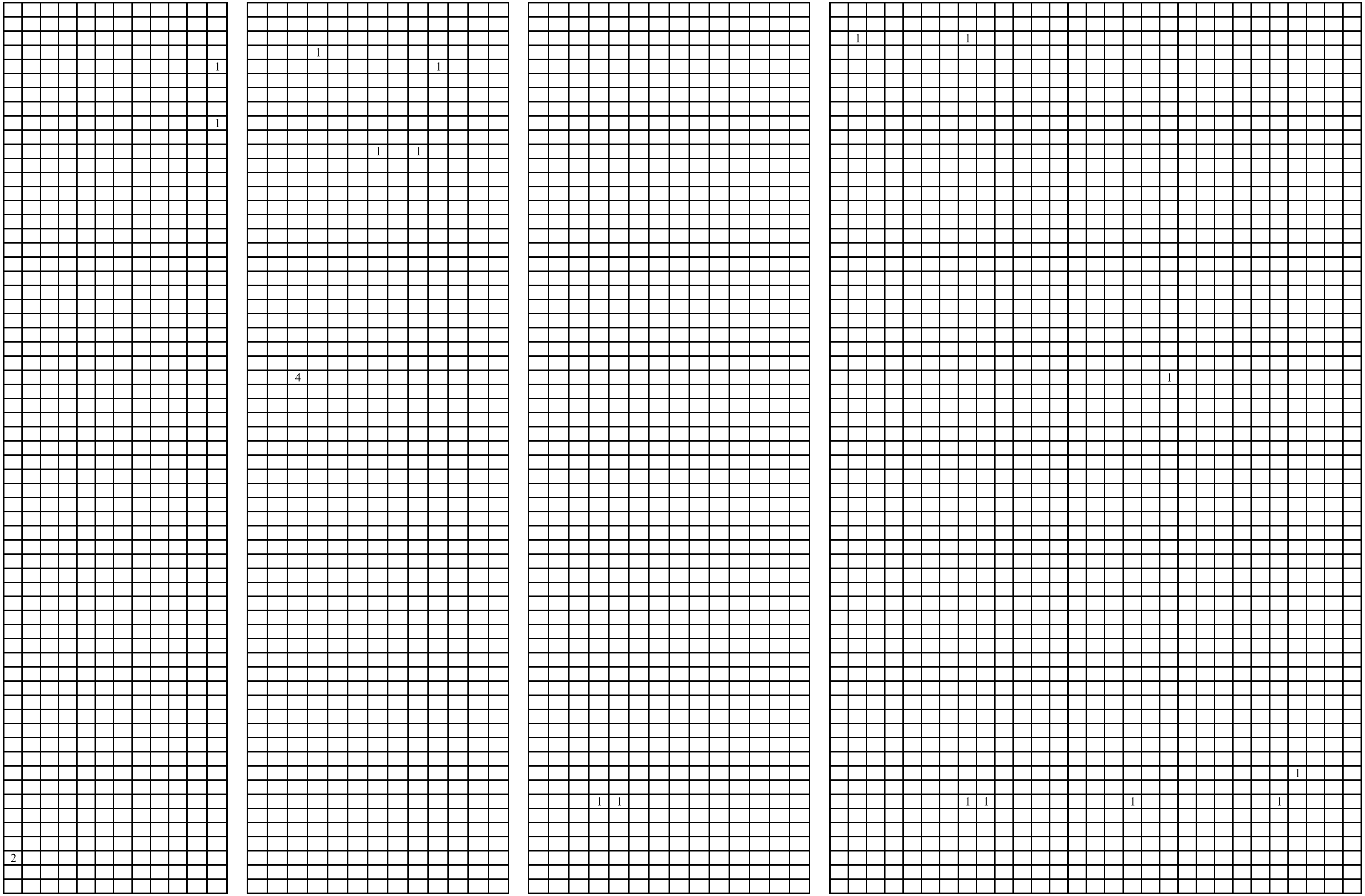
1
1
2
1
1
1
1
4
1
1
1
1
1
1
1
1
1
1

1	1				1	2	1	2		1	1
	1					1	1	1			
											1
						1					1
1											
						1					
						1					1

			1		1							
			1							1		
		2	2						2	1	1	
										1	1	
									1	1		
		1	1									
			1									
			2									

		1		2									
		1		1							2		1
		1	1	1						1	1		
				1									
		1	1	1							1		
							1						
				1									
				1									

		1		1		1	1	1	1	1		1		1				1					1	1				
2	1	1	1	2			1		2	2			1	1	1	1	2	1	1	1		1	1	1	1		2	1
1		1		1								1	1			1				1		1	1	1				
	1	1													1			1										1
	1	1						1						1								1	1	1			1	1
		1		1					1																			
1	1	1	1	1		1	1	1	1	1		1	1	1	1	1	1	1		1		1		1	1	1	1	1
1	1	1	1			1					1			1	1	1			1		1	1			1		1	
						1	2			1	1					1		1										
			1				1		1											1								
1	1	1	1	1		1	1	1	3	1	1		1		1		1	1	1	1	2	1		1		1	1	

	1					1					1

		1	6		1							

			1										

1	1	1	1	1		1		1				1			1	1		1		1		1				1		
	1	1	1	1		1	1	1	1	1	1	1	1	1	1	1	1	1	1		2	2		1	1	1		1

TlatMFs

cytoRPs

RPS27A	506	53																										
RPS28	243	20																										
RPS29	159	19																										
RPS30	0	0																										
RPL3	847	90																									1	
RPL4	927	79																									1	
RPL5	741	59																										
RPL6	643	57																										
RPL7	502	59																										
RPL7A	768	77																										
RPL8	603	63																										
RPL9	416	35																										
RPL10	797	40																										
RPL10A	513	46																										
RPL11	802	76																										
RPL12	363	33																										
RPL13	811	84													1													
RPL13A	626	68																										
RPL14	699	87													1													
RPL15	524	51																										
RPL17	596	73																										
RPL18	569	52																										
RPL18A	507	48																										
RPL19	676	60																										
RPL21	320	29																										
RPL22	395	33																										
RPL23	577	33																										
RPL23A	627	68													1													
RPL24	343	32													1													
RPL26	404	43																										
RPL27	373	41																										
RPL27A	447	43																										
RPL28	523	58																										
RPL29	258	25																										
RPL30	574	66																										
RPL31	731	69													1													
RPL32	344	32																										
RPL34	260	33																										
RPL35	791	52																										
RPL35A	358	31																										
RPL36	550	66																										
RPL36A	134	27													1													
RPL37	123	11													1													
RPL37A	436	51																										
RPL38	279	24																										
RPL39	96	24																										
RPL40	0	0																										
RPL41	11	0																										
RPLP0	677	79																										
RPLP1	262	19																										
RPLP2	347	33																										

MRPL1	233	12																										
MRPL2	262	12																										
MRPL3	204	12																										
MRPL4	483	19											1															
MRPL9	260	16																										
MRPL10	249	12														1												
MRPL11	454	44																										
MRPL12	466	26																										
MRPL13	289	11																										
MRPL14	116	8																										
MRPL15	225	12																										
MRPL16	199	11																										
MRPL17	178	11																										
MRPL18	260	11																										
MRPL19	192	10																										
MRPL20	205	12																										
MRPL21	220	10																										
MRPL22	186	14																										
MRPL23	213	12													1													
MRPL24	227	16																										
MRPL27	247	14																										
MRPL28	359	22																										

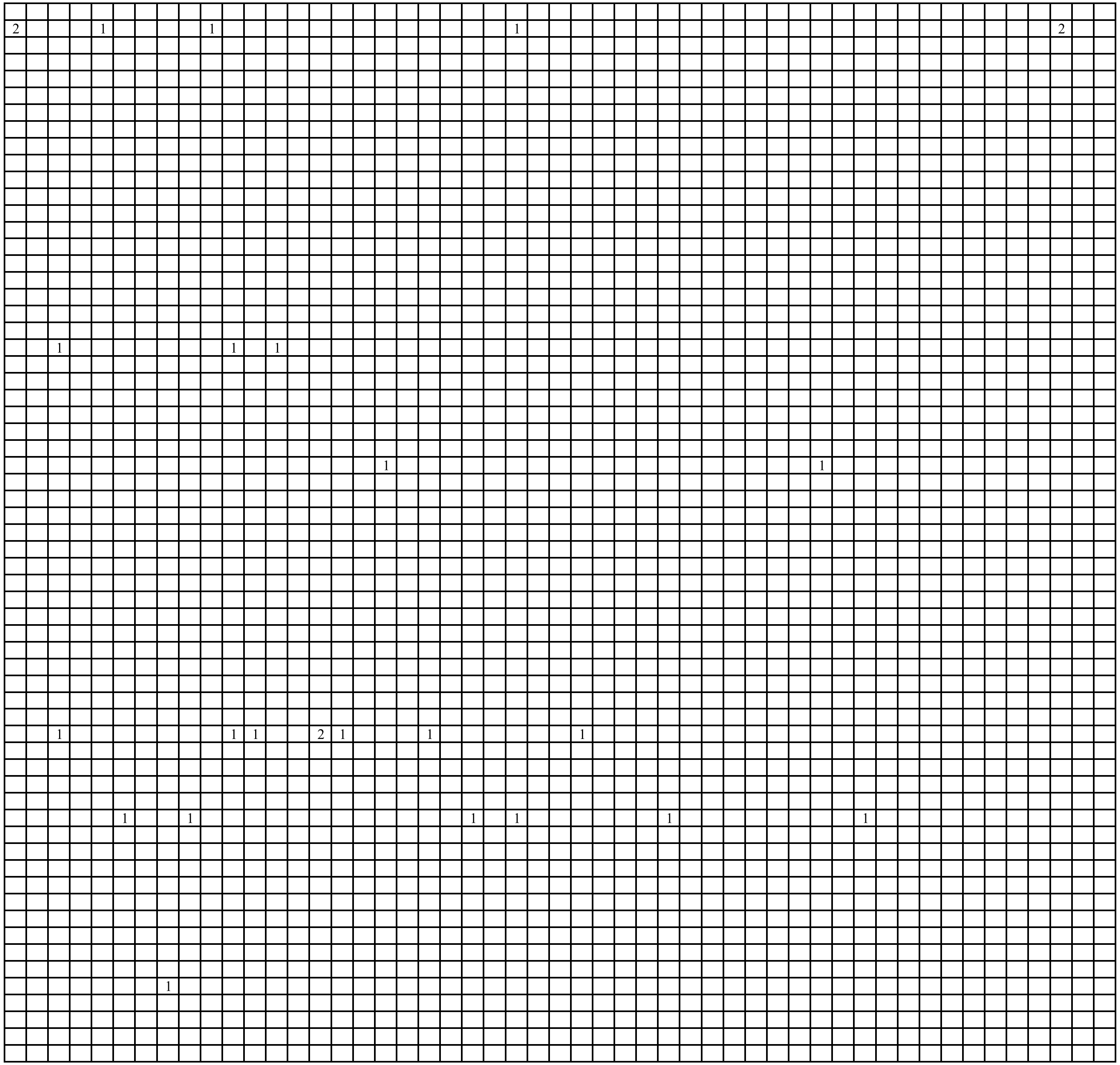
2 1 1 1 2
1 1 1
1 1
1 1 1 2 1 1 1
1 1 1 1 1 1
1
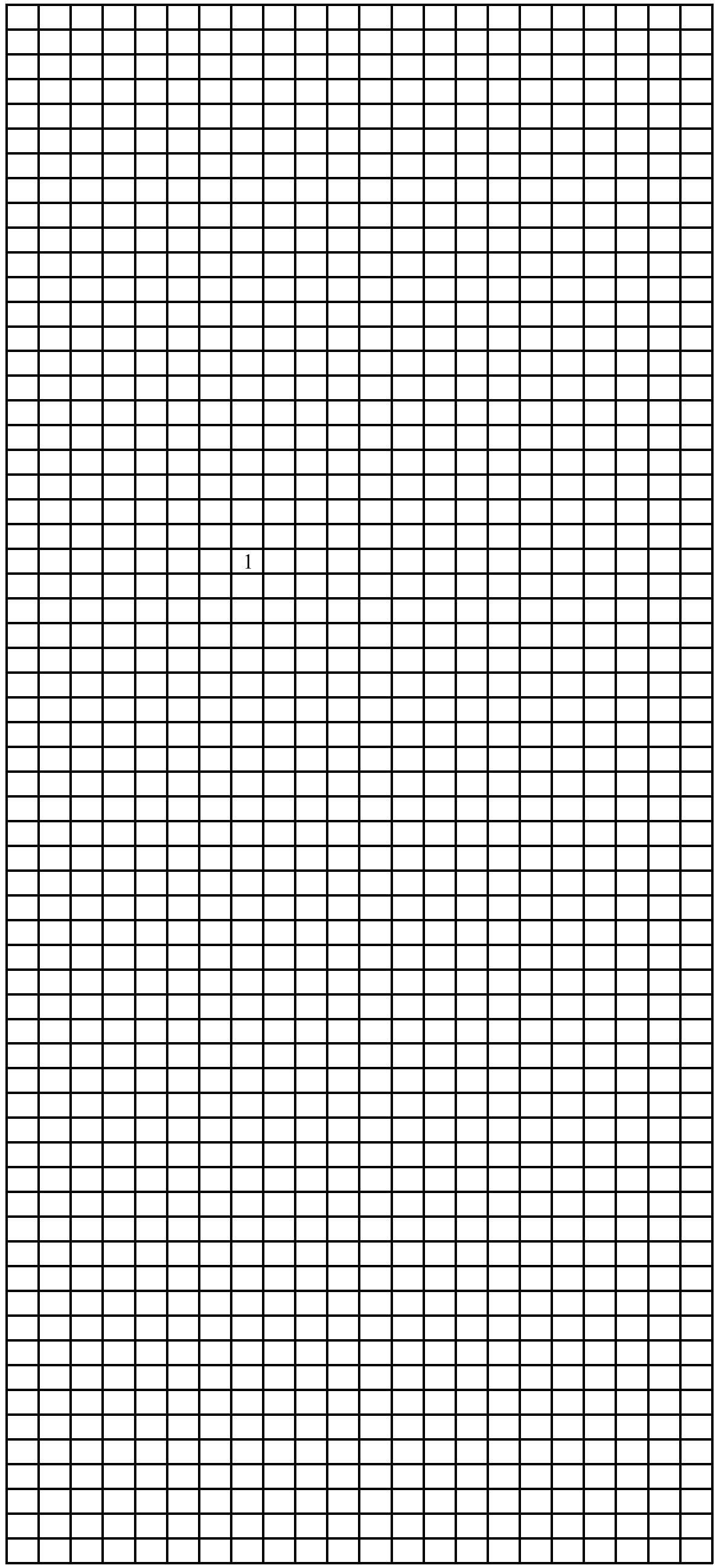
1

							1																														1	1												
																																						1												
2	2	2		3	3	3	3	3	3	3	3	2	3	5	3	4	3	3	3	3	3	3	3	3	3	3	3	3	2	3	3	3	3	3	3	2	3	3	3	3	2	1	1	3	1			2	2	3
1	1	1		1	1	1	1	1	1	1	1	1	1	1	1	1	1	1	1	1	1	1	1	1	1	1	1	1	1	1	1	1	1	1	1	1	1	1	1	1	1	1	1	1	1			1	1	1
																																												1						
																																											1							
1																																																		
											1																																							
																																																		1
																																						1												
																																							1											
																		1						1										1																
	1				1			1			1								1	1	1																		1		1									

														1						1	
1	1	1	2	1	1	1	1	1	2	1	1	1	1	1	1	1	2	1	1	1	1
1	1	1	1	1	1	1	1	1	1	1	1	1	1	1	1	1	1	1	1	1	1
		1																			
						1				1											

	1	1				1					1																																							
1	2	1		2	2	2	2	2	2	2	2	2	2	2	2	2	2	2	2	2	2	2	2	2	2	2	2	2	2	1	2	2	2	2	2	2	2	1	1	2	1	1	1	1				2	2	2
				1	1		3	1	1			1				1	1			1			1		1		1		1						1			1											1		
																				1			1													1								1									
							1		1			1				1				1			1				1		1							1			1											1		
																																										1														
																						1			1										1																			
																																										1											1							
																																												1												
				1		1		1		1		1						2	1							1							1		2		1		2	1			1	1								1		

							1															
							1															
																					1	
		1																				
2	1	2	1	1	1	1	2	1		1	2	2	3	2	2	2	1	1	2	2	1
1	1	1	1	1	1	1		1		1	1	1	1	1	1	1	1	1	1	1	1
	1						1															
						1			1										1				
						1																1	
		1												1			1					1		1		

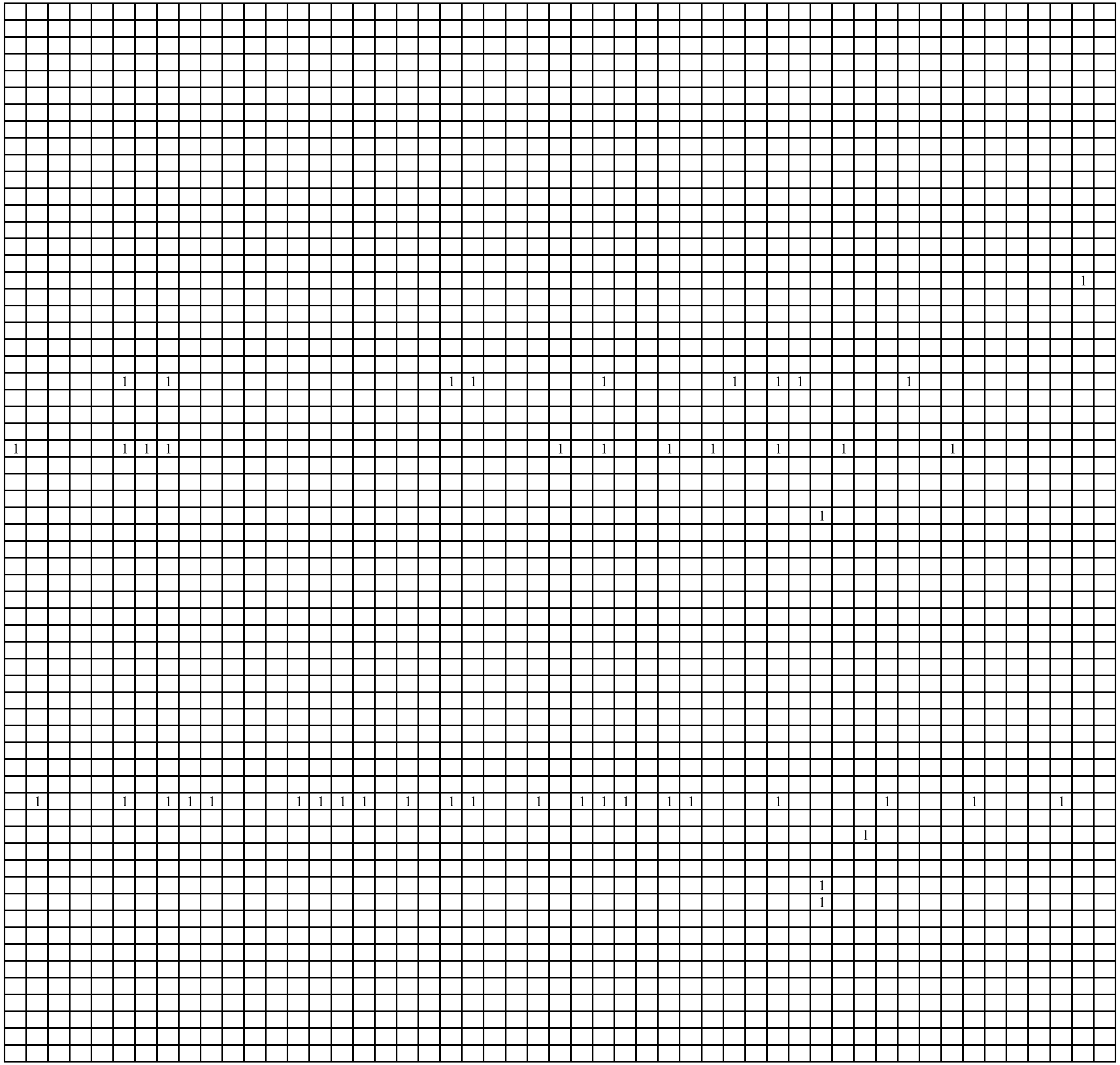
1
1 1 1 1 1 1 1 1 1
1 1 1 1 1 1 1 1 1 1 1
1
1 1 1 1 1 1 1 1 1 1 1 1 1 1 1 1 1 1 1 1 1 1
1
1
1

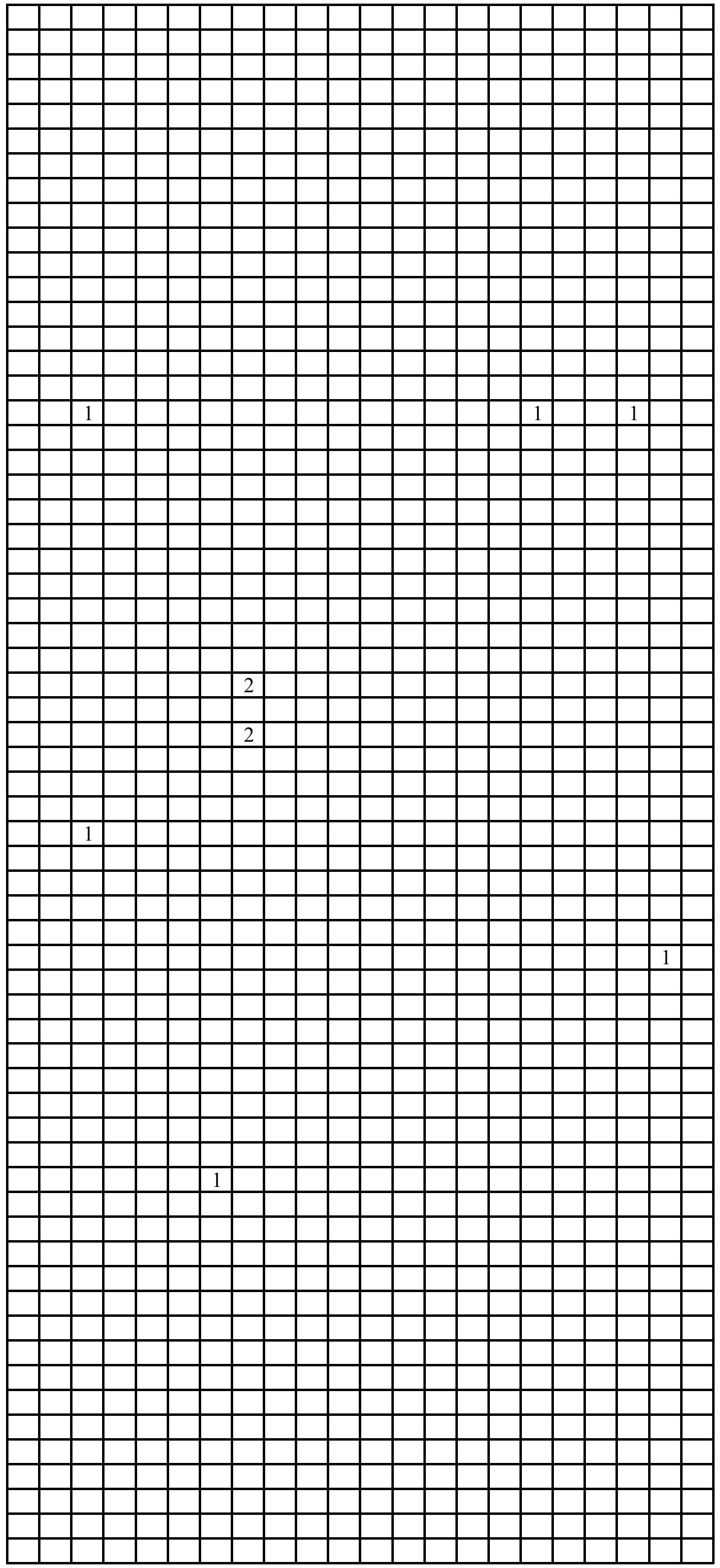
1 1 1
2
2
1
1
1

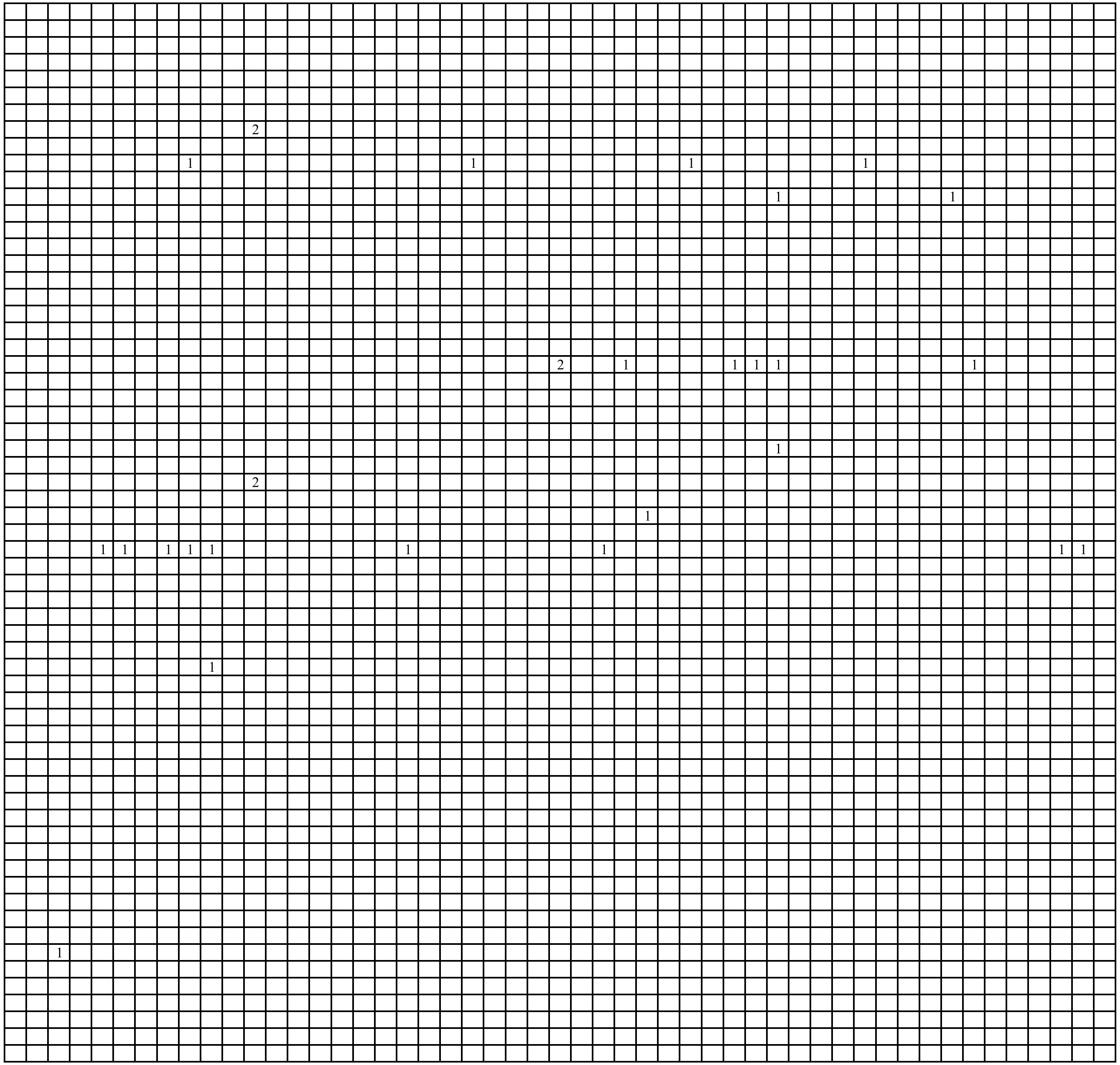

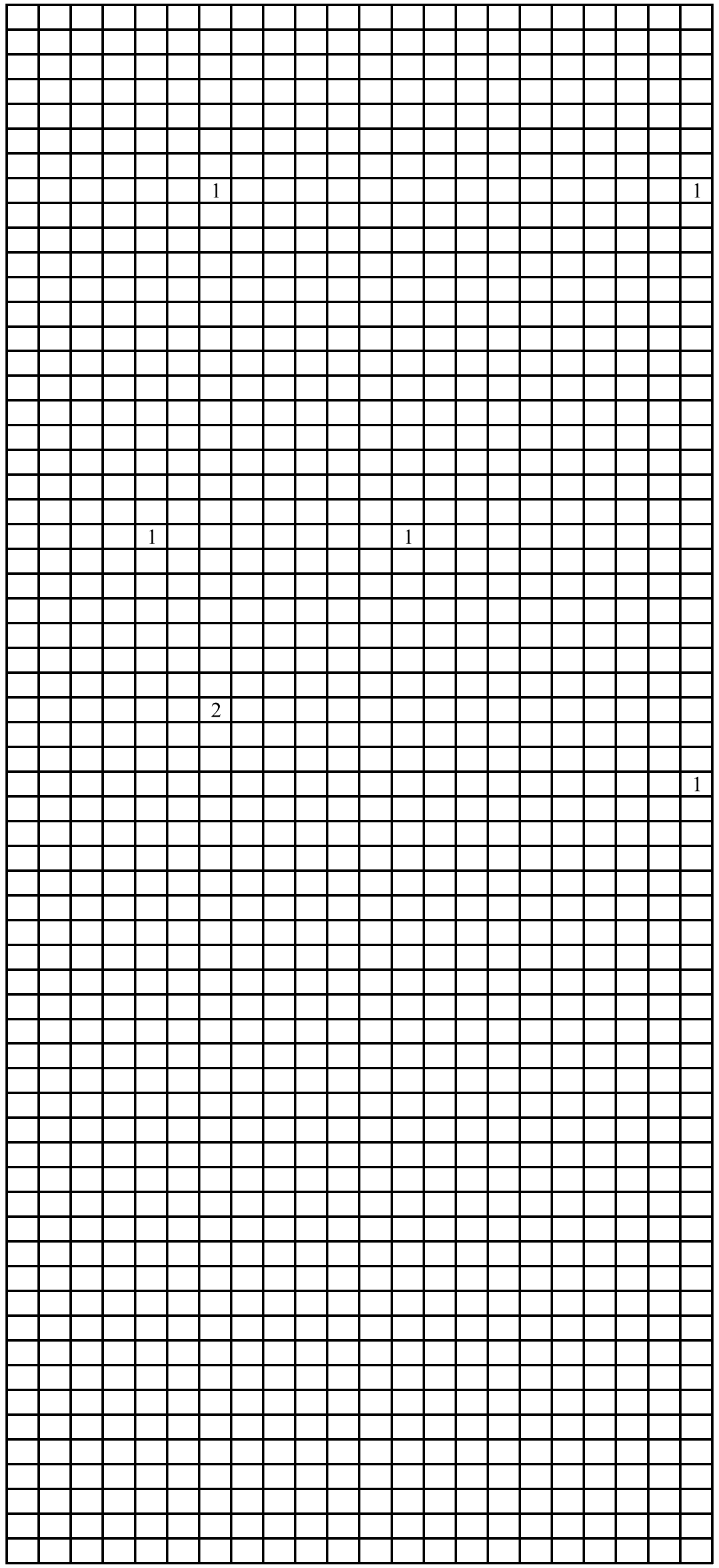

2 1

1

1

1

1

1

1

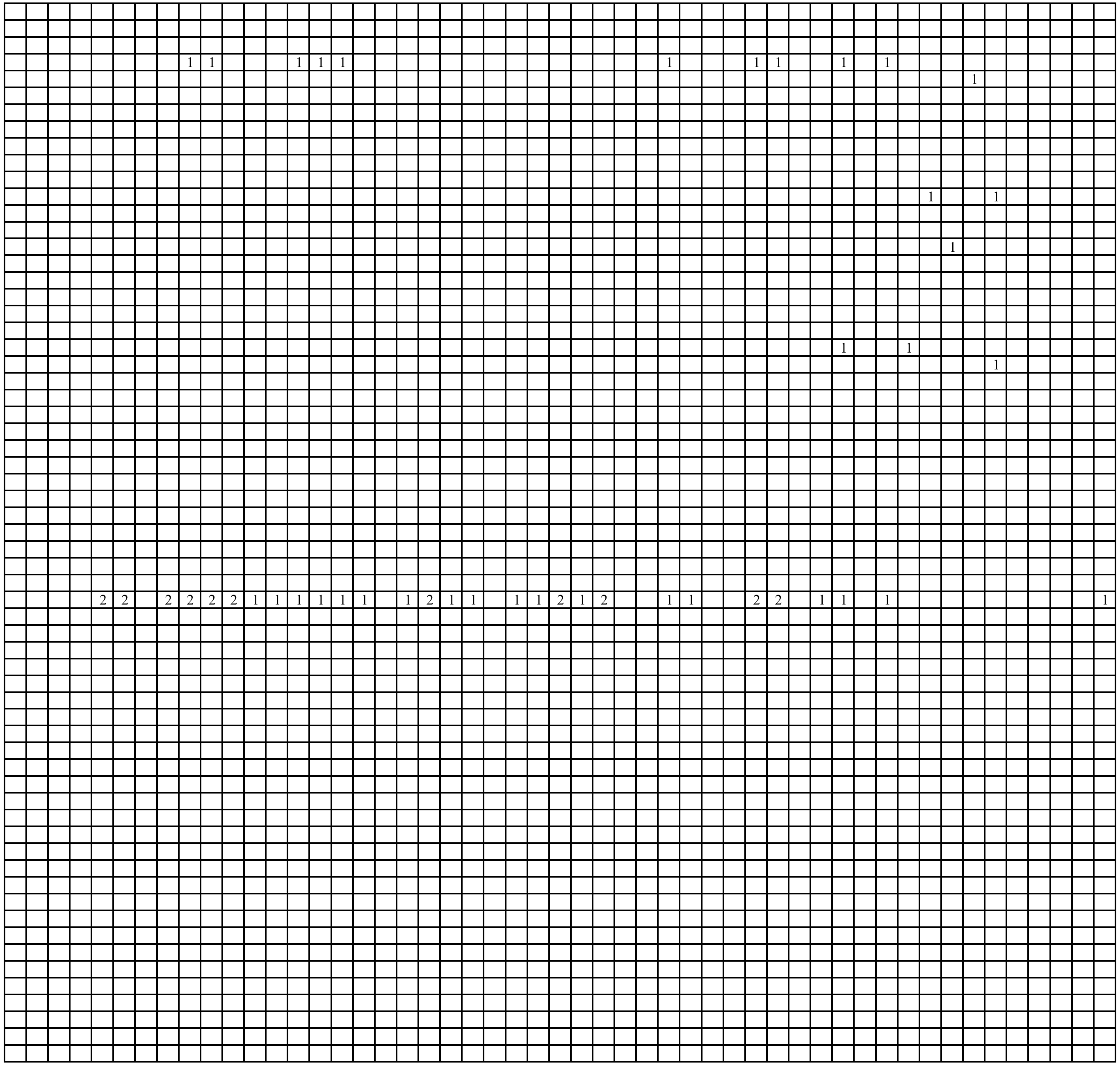
1 1 1 1 1 1 1 1 1 1
1
1 1
1
1 1
1
2 2 2 2 2 2 1 1 1 1 1 1 1 2 1 1 1 1 2 1 2 1 1 2 2 1 1 1 1
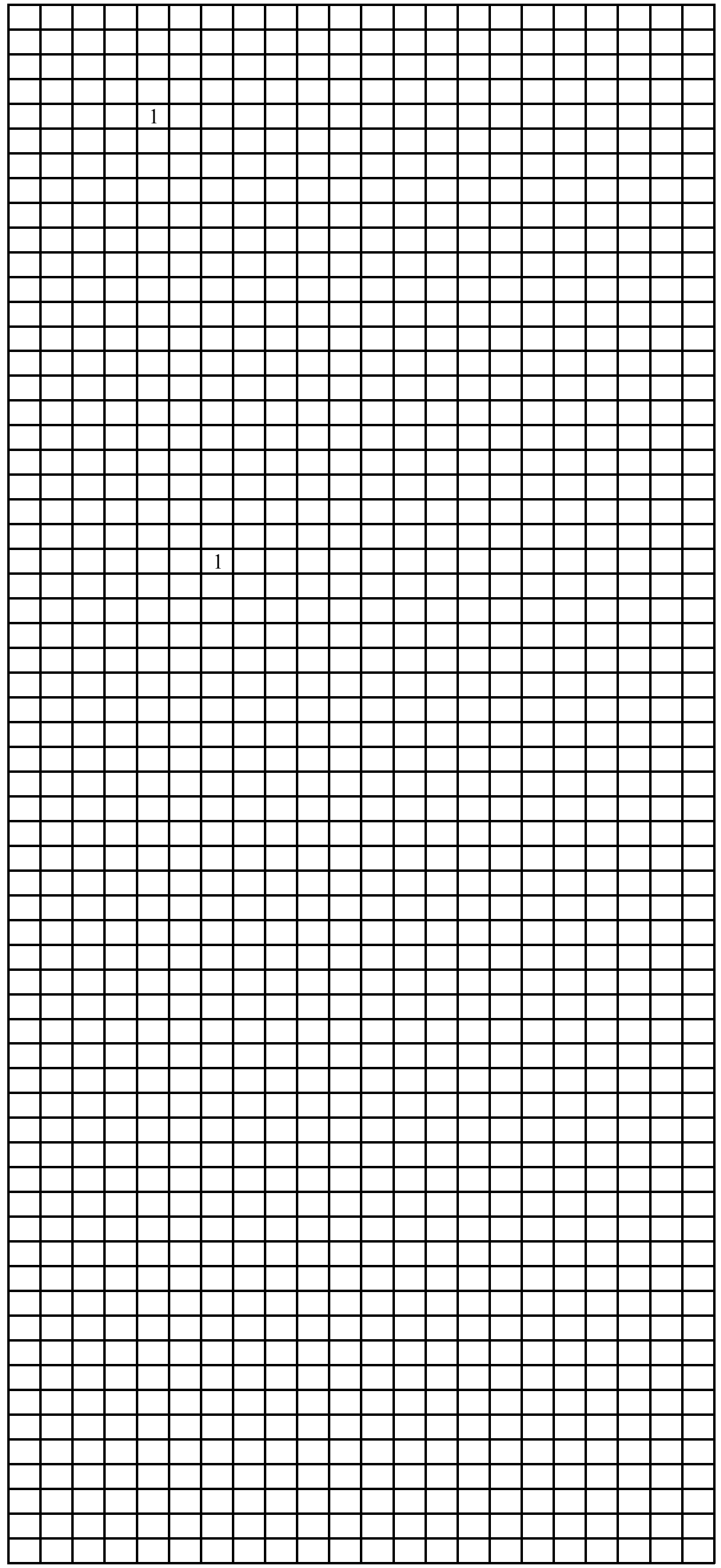
1
1

									1																																								
	1	1		2	1	1	1	1	2	1	1	1	1	1	1	1	1	3	1	1	1	1	3	1	1	1	1	1	1	1	1		2	1	2	1		2		1								1		1
	2			3	1		1			1		1	1	1		1		3		1				1	1		1	3		1	1				1	1	1	1		1				1	1			1	1	1
									1		1																																							
																																								1											
				1									1	1																												1						1		
					1												1		1			2			1						1	1			1													1		
					1																					1				1																		1		
					1	1															1											1				1			1					1						
																																												1						
1				1	1	1	1			1				1	1		2		1		1	1	1					1	1	1	1			1			2		1	1					1					

						1															
						1															
1	1		2	2		2	1			2				1				1	1		1

																																													1					
																											1																						
																																					1												
																																					1												
											1													1									1															
						1							1				1																					1												
1																												1																						
1				1	1		1	1	1	2	1		1		1	2					1																	1					1					1		
																																													1					
																										1											1												
																																		1																	

1			1		1		1							1	1	1	1	1	1		1
						1															
			1																		
							1														

				1	1				1	1				1		1	1	2	1	1	2	2	1		1							1		1	1			1		1			2					1		
				2	2	2	2	2	2	2			2	2	2	2	2	2	2	1	2	2					1		1	2				2	1	2	1		2	3			2					2	1	2
																																													1					
					1																																													
																																							1											
																			1																															
1																																																		
																			1																						1			2					1		
				1	2	1		1	1	1				1		1	1	1					2				1							1	2			2					1					1		
																																												1						
	1			1	1	1	1	2	1	1	1	1	1	1	1	1	1	1	1	1	1	1	1	1	1	1	1	1	1	1	1	1	1	1	1	1	1	1	1	1			1	1	1			1	1	1
						1										1																																		
						1								2																																				
1	1				1				1								1									1																								
				1								1															1											1		1										
																														1																				
1				2	3	6	2	1	3	2			2	10	2	2		2	3	1	2	1	2	1		3	2	1	2	2	1	1	1	1	2								1	1				3	1	1
				1											1		1											1																				1		
						3								3																																				

						2	1														
						2	1														
	1						2														
																		1			

				1	1											1	1	1	1	1			1				1								1			1										1		
				1	1				1							1											1		1						1			1										1		
1																					1																						1					1		
							1																											1																
							1																																											
																1										1																								
																										1																								
										1													1				1						1																	
2																																																		
				1	1																																											1		
1																																1																		
														1																																				
																									1																									
1																																																		

					1																
												1									
													1								

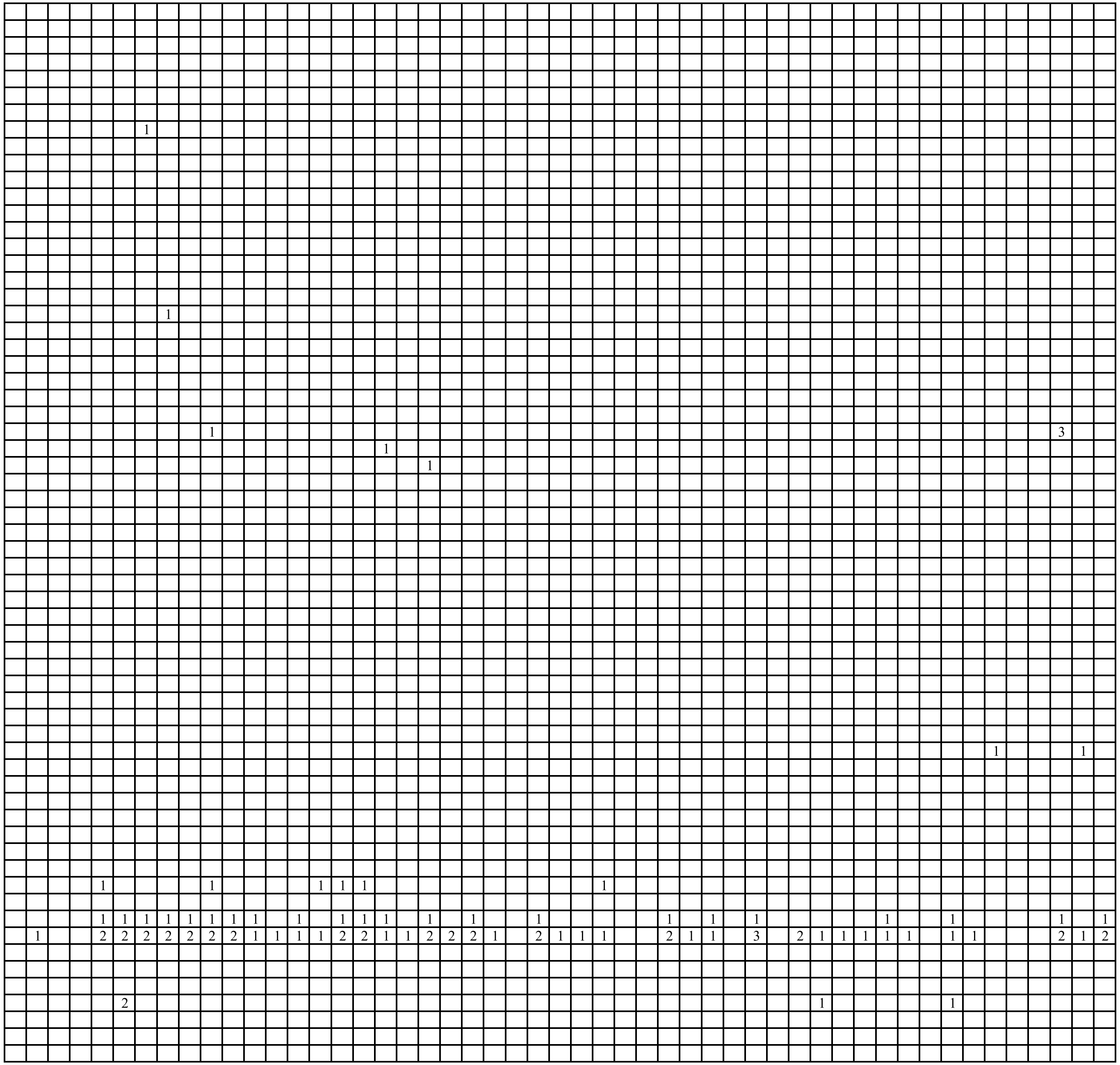

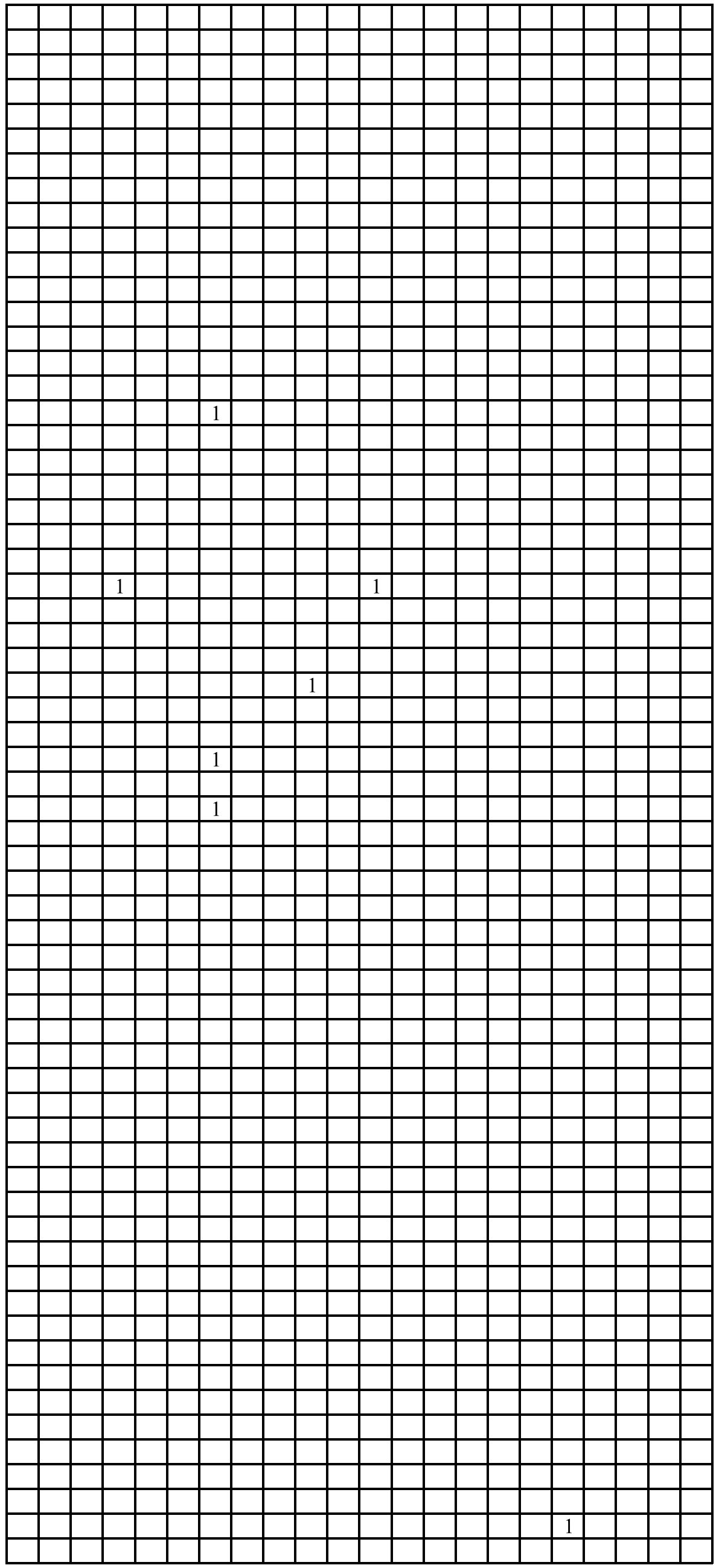

																																				1													
																																											1						
																						1																											
								1																																									
																								1																									
																1																																	
								1													1																											
															1																						1											
				1	1				1	1			1	1		1	1	3	1	1		2					1					1		2	1			1		1			1					1		
																																										1							
		1																																						1									
				2	1	1	1					1	1	2		1	1		1	1	1	2		1			2							2	2	1							2					1		1
																								1																									
1										1						1				1																													
3								1								1	1					2							1							2														
																		1																															
																	1												1			1								1						1					
																																1								1	1		1		1					
												1					1					1							1						1	1				1	2		1		1					
																																1																		
		1											1					1				1	1					1	1		1							1			1									
																																									1		1		1					
		1																																						1	1		1		1					
																																					1				1									
																																						1												
																														1																				

						1															
				1																1	1
				1																	
	1					2															
1			1		1		1	1		1	1	1			1	1	1		1		1
			1				1														
			1							1												
						1															
						1															

																																													1					
	1	1		2	2	3	1	2	2	2	1	3	2	3	1	2	4	4	1	2	1	3	3	1	1	1	2	1	2	2	1	3		2	4	2	2	2	1	1			1	1	1			2	1	1
							1																	1																										
				1															1	1																														
				1	1		1	2	1	1			1	1		1	1	3		1		1	1				1		1		1	1		2						1								1		
				1	1	1		1	1	1		1	1	1				1	1			1	1				1			1		1							1									1		
1																																																		
					1				1	1			1									1																		1										
																	1														1																			
				1	1	1	1	1	1	1	1	1		1	1	1	1	1	1	1	1	1	1	1			1	1		1			1		1													1	1	

						1															
						1															
1				1																	

1																											1												1											
				1	1		2	2		1		1		1		1	1			1			1						1					1	1			1										1		
											1																																							
								2	1	2		2				1		3		1			1		1										1					1										
									1							1					1																											1		1
				2		1														1							1									2												1		
								1																						1		1							1											
											1																											1												
								1	1							1	1	3	1	1			1									1		2						1			2					1		
																1																																		
				1					1							1		1					1				1								1							1								
																																			1															
1				1														3		1			1																	1								1		
																															1																			
					1												1										1													1										
																																1																		

						1															

2																																																		
																																			1															
				1					1							1		1					1												1					1										
				1				1						1			1			1									1			1		2	1					1										
				1	1				1											1							1		1			1		2																
				1					1											2																							2							
				1	1			2	1							1	1	3		1	2	1	1						1			1		2						1			2					1		
																																			1															
																															1																			

																					1
				1											1					1	

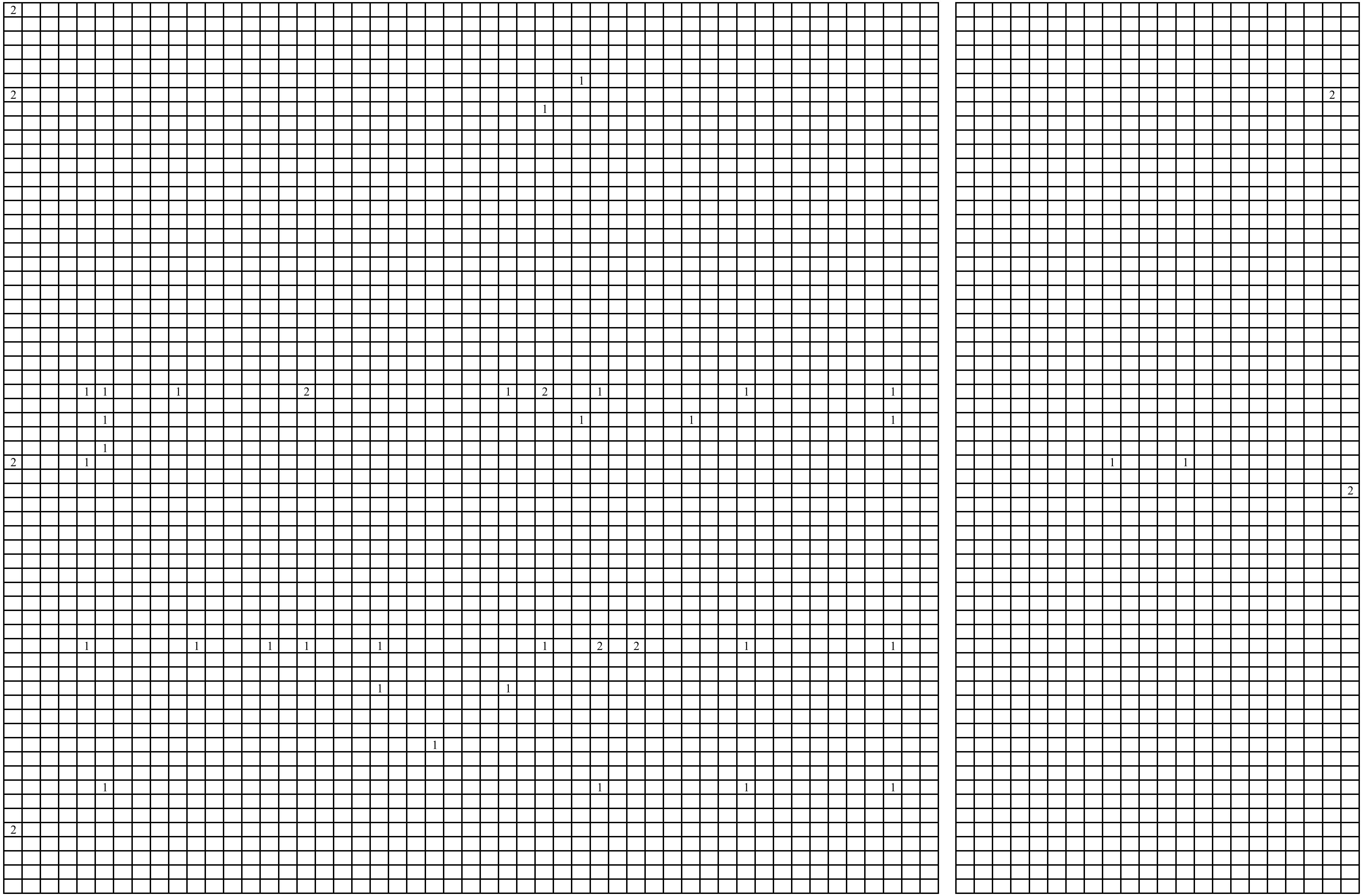

																														1																			
																	1														1																			
																															1								1										
1																1																																		
																														1																			
																														1																			
												1																		1																			
													1																																					

					1																
				1						1											

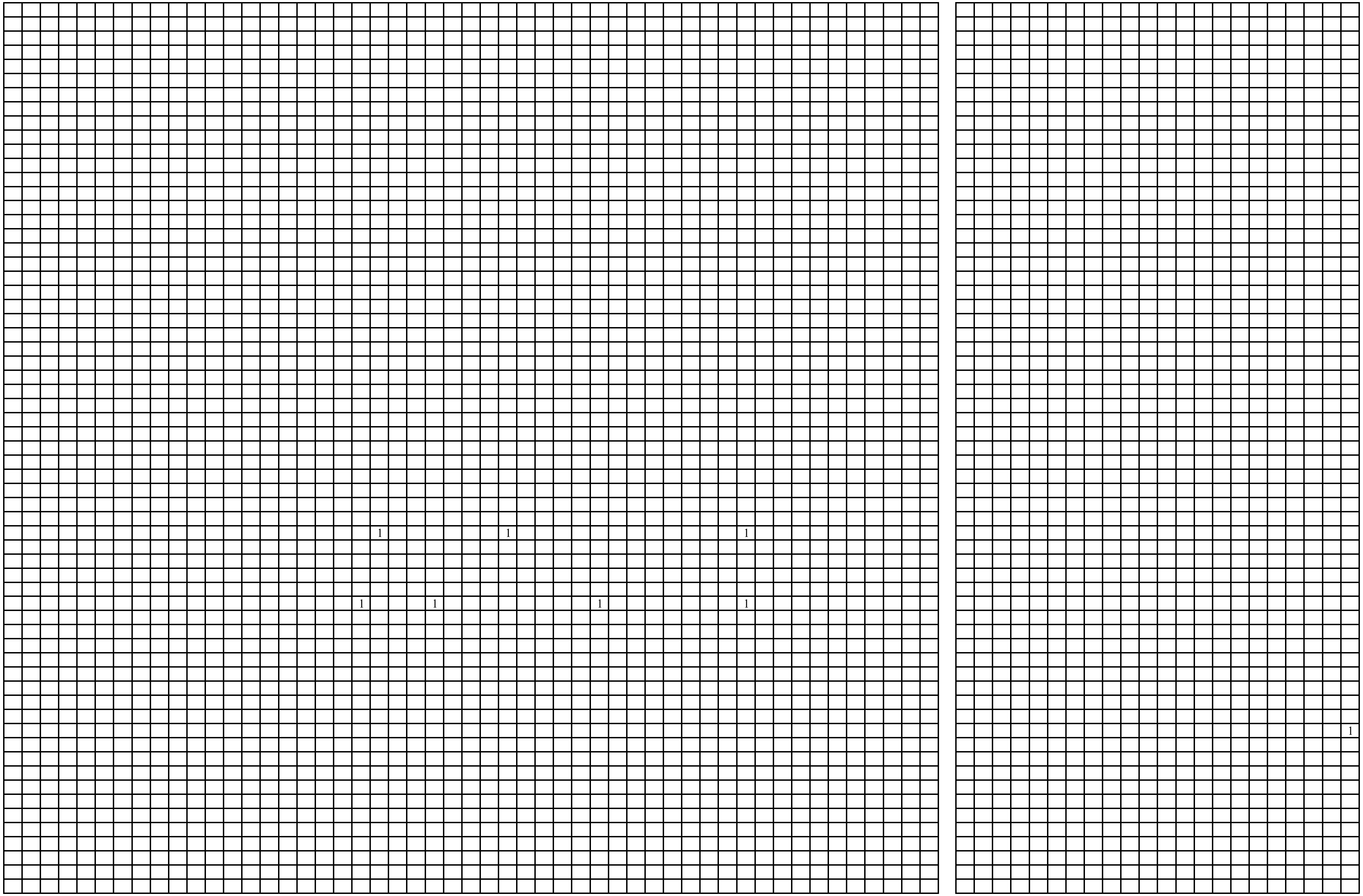
1
1
1
1
1
1
1
1

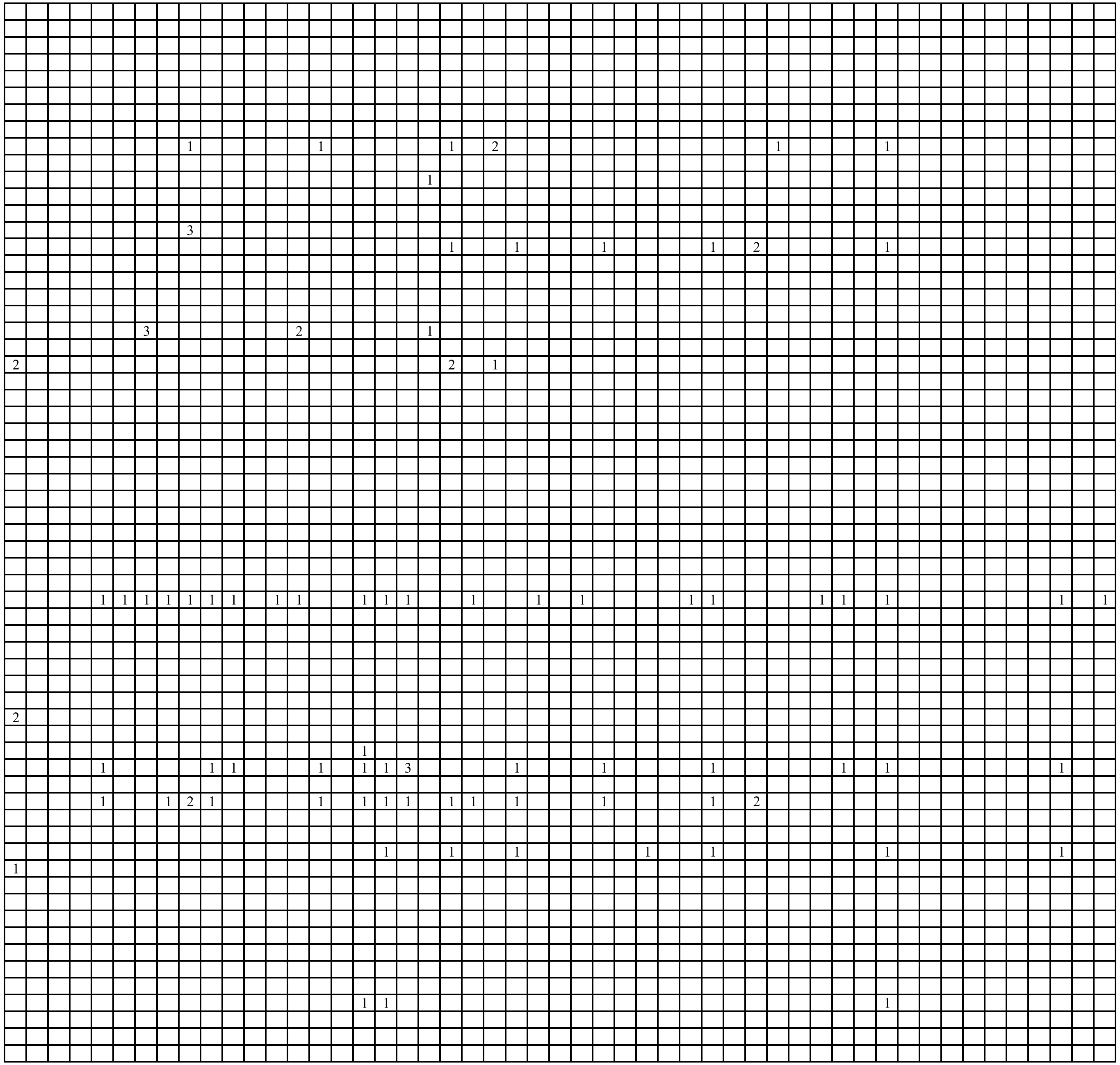
1 1 1 2 1 1
1
3
1 1 1 1 2 1
3 2 1
2 2 1
1 1 1 1 1 1 1 1 1 1 1 1 1 1 1 1 1 1 1 1 1 1
2
1
1 1 1 1 1 1 3 1 1 1 1 1 1
1 1 2 1 1 1 1 1 1 1 1 1 1 2
1 1 1 1 1 1 1
1
1 1 1
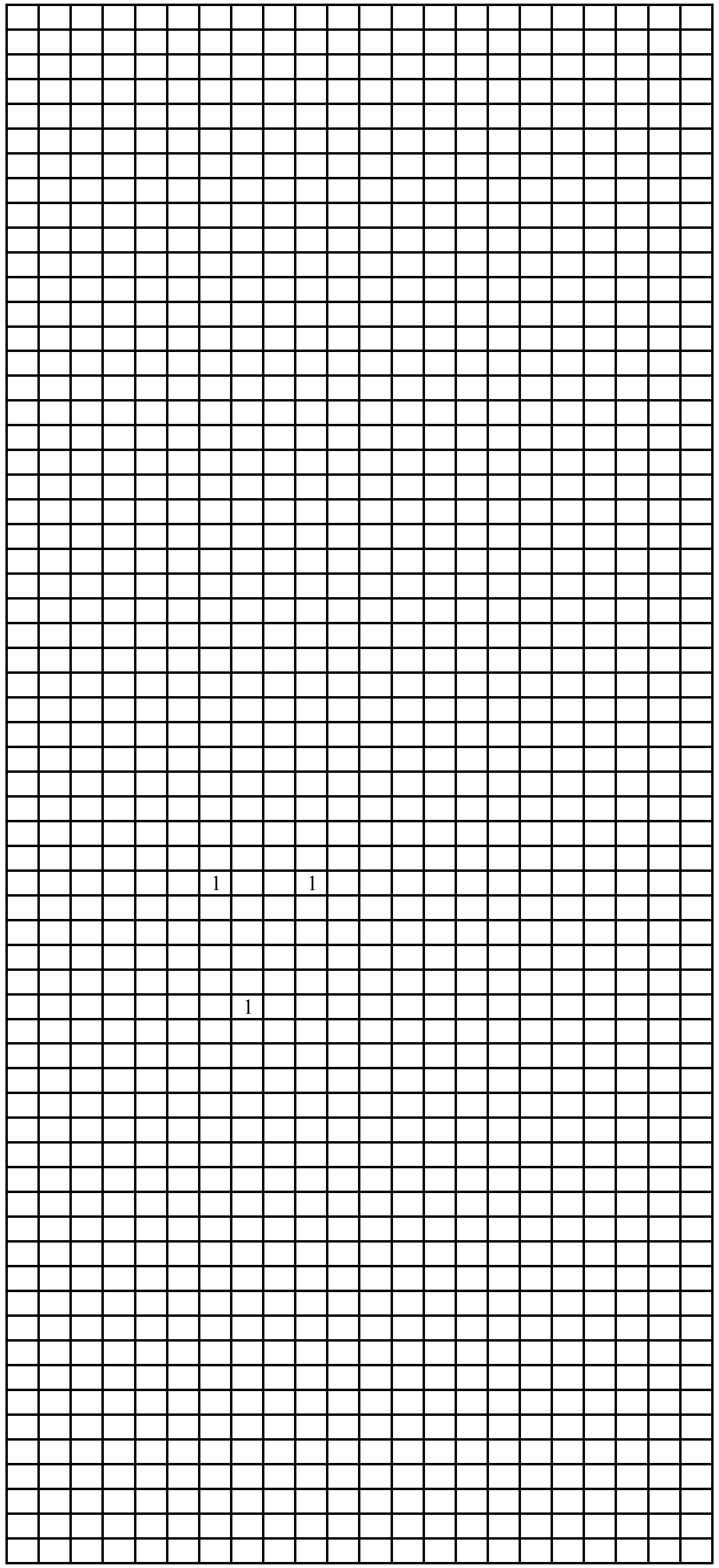
1 1
1

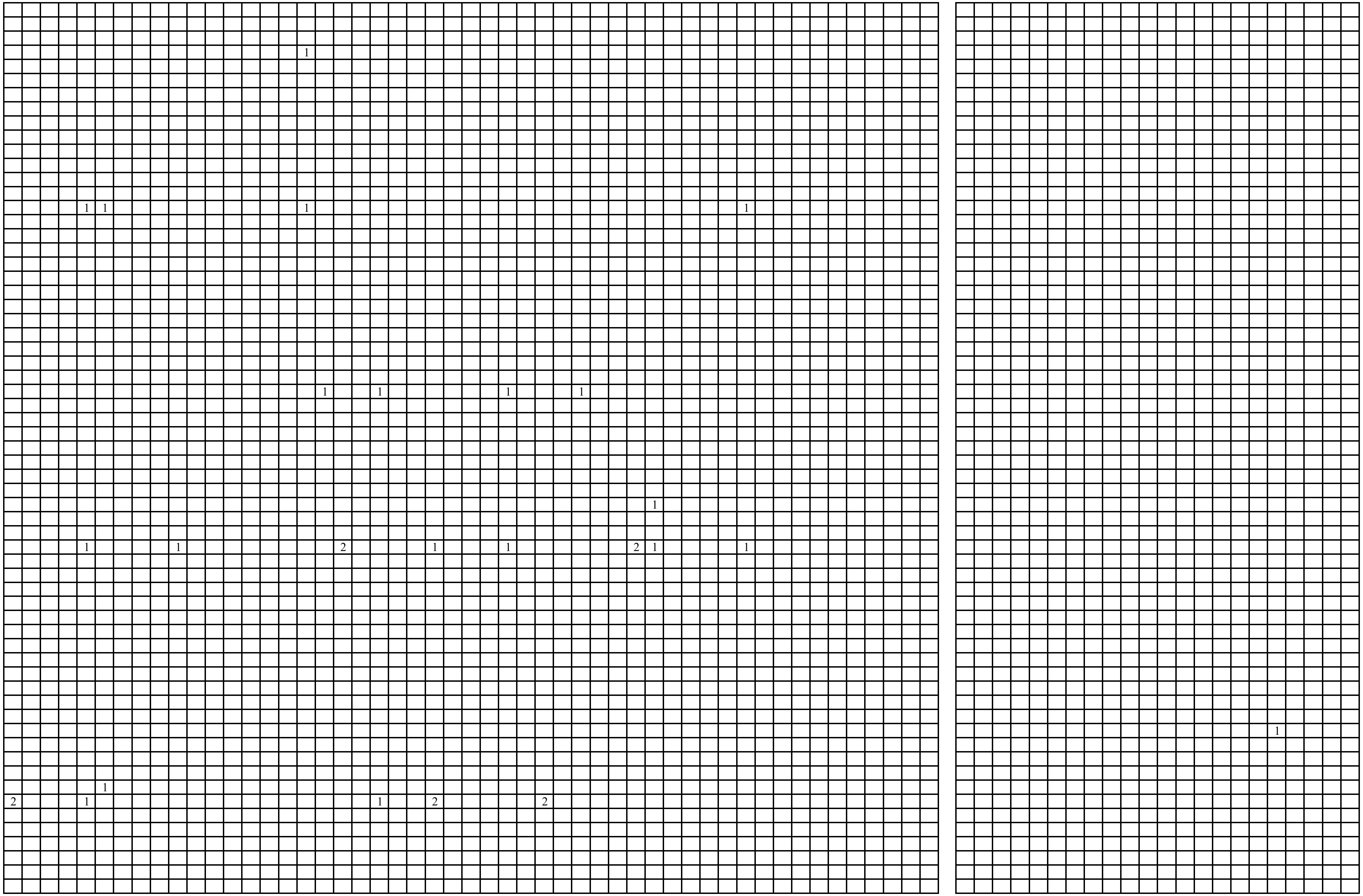
1
1 1 1 1
1 1 1 1
1
1 1 2 1 1 2 1 1
1
1
2 1 1 2 2

						1									1	1										1	1	1				1									1									1
						1									1	1										1	1	1				1									1									1
1						1																																												
					1											1	1			1	1	2																		1								1		
									1							1											1								1													1		
				1	1				1	1				1		1	1						1				1		1			1		2	1								1					1		
1						1																																												
																																							1												
		1		1	1	1	1	1	1	1	1	1	1	1	1	1	1	1	1	1	1	1	1	1	1	1	1	1	1	1	1	1	1	1	1		1	1	1		1		1	1				1	1	1
		1		1						1				1			1	1			1							1		1	1			1			1											1		
							1							1															1												1				1					
																			1														1																	
				1					1							1																								1								1		
																1																		1																
																			1																															
																																													1					
				1																																													

																					1
						1													1		
						1															

				1																1												1								1										
						1																																												
				1	1	1			1	1			1	1		1	1		1	1	1	1		1	1		1			1	1			1		1				1			1					1		1
				1	2	2			1	3			1	1		2	1	3	1	1	2	1	1						1			1		3	2	2		1		1			2					1		
1																																			1															
																		1													1																			
				2	1				1			1	1	1		1	2	1		2			2						1					1	1			1										1		
												1																														1								
														1																																				
				1	1											1	1	1		1			1									1								1								1		
																1							1																	1										
				2	1		1	2	1	2		1	1	2	1	2	1	4		2	2	1	1				2							2	1					1								2	1	1
																			1																															
				1																							1																							
				1	1			1	1	2			1	1		1	1	2		1	1	1	1				1				1	1		1	1			1		1			1					1		
													1														1																							

						1															
																			1		1
													1								
						1															
								1				1	1					1	1		1

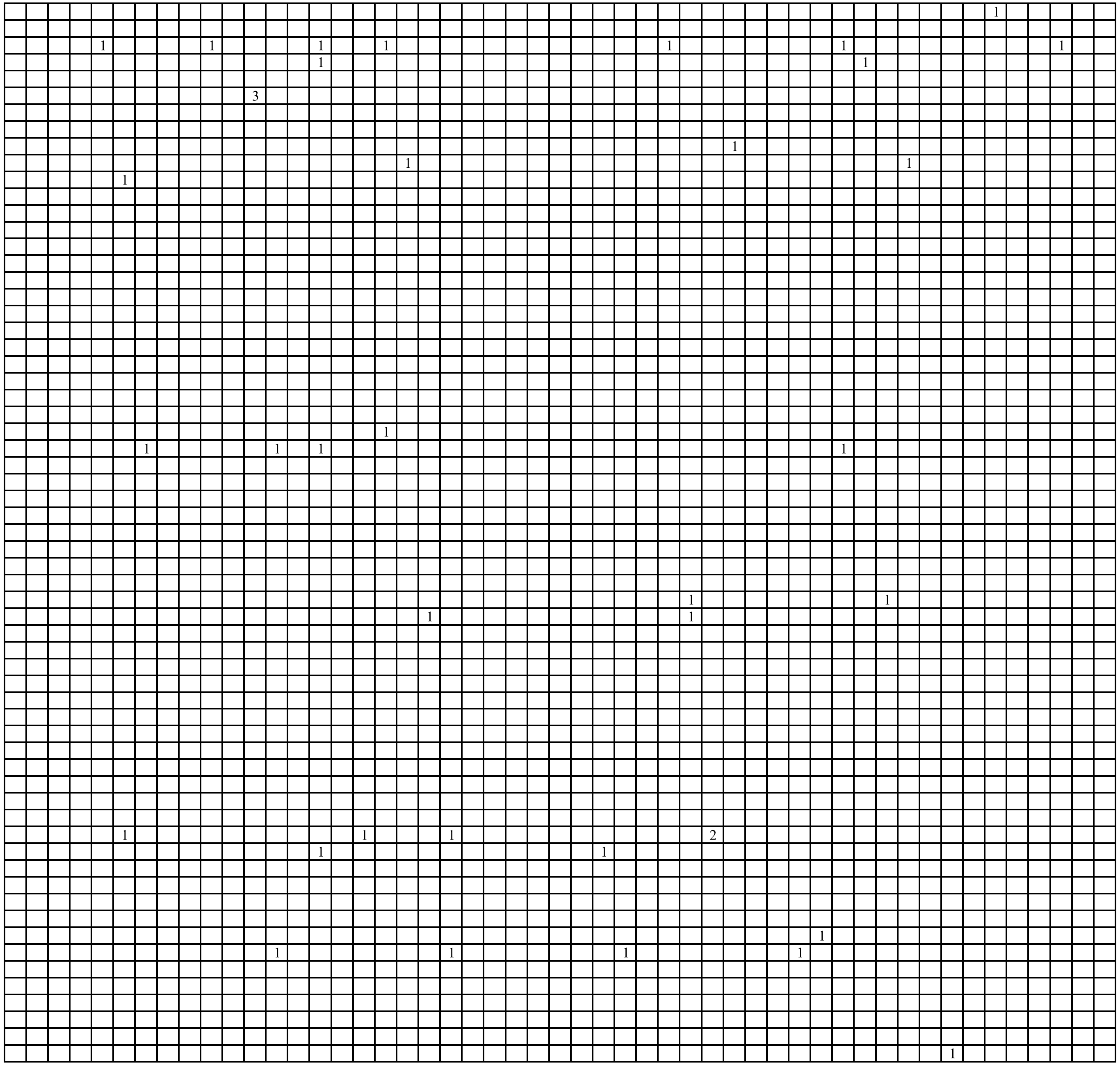
1
1 1 1 1 1 1 1
1 1
3
1
1 1
1
1
1 1 1 1
1 1
1
1 1 1 1 2
1 1
1
1 1 1 1
1

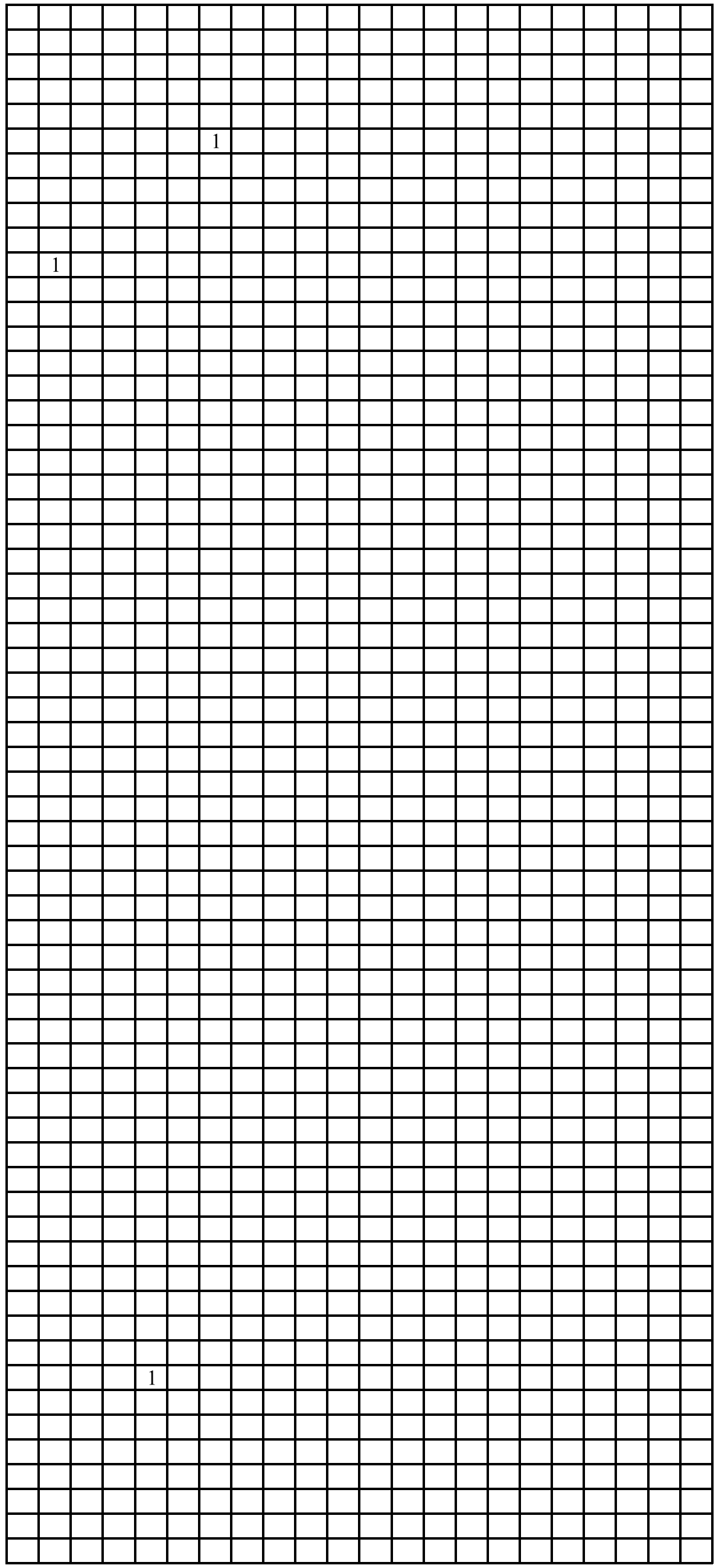
1
1
1

																																																		1
																																											1							
				1																		1				1		1			1																			
1	1	1		1	1	1	1	2	2	2	2	3	1	1	1	1	1	1		1	2	1	1	1	1		1	1			1	2	1			1	1		1	2	1		2	1				2		
	1	1								1		1														1												1		1				1	1			1	1	1
1																									1																								
		1					1			1						1		1							1				1						1			1				1			1					
									1		1		1																																					
1																																																	
							1																																											
1	1			1	1	1	1	1	1	1	1	1	1	1	1	1	1	1	1	1	1	1	1	1	1	1	1	1	1	1	1	1	1	1	1	1	1	1	1		1	1	1	1				1	1	1
								1																																										
1				1	1		1			1	1		1	1					1	1	1							1		1	1			1		2			1	1				1					1		1
				1			1			1																																							
																																				1															
							1																																											
					1														1							1			1			1		1																
1				1	1	3	2		3	3		1	2	1	1	3		1	2	3	3				1	1	1	1		3	1		1			2				2	1		1	1				1		3

																		1				
					1			1														
	1		1		1	1		1	1	1					1			1	1			
					1		1												1			1
								1														
1			1								1						1		1		
1	1	1	1	1	1	1		1		1	1	1			1	1	1		1	1	1
																					1
																	1				
			1								1			1	1						

	1																																																	
				1			1		1									1	1	1				1	1	1	1			1			1					1						1						
	1	1		2	1	1	2	1	2	1	1		1	1	1	1	2		1	2	1	1		1	2	1	1	1	1	1	1	1	1	1	1		1	1	1	1	1		1					1		

								1													
1	1	1	1			1	1	1	1		1		1		1	1		1		1	

205	125	71	88	143	9	277	305	349	168	251	307	192	264	234	217	348	179	208	365	163	186	191	49	144	0	106	339	356	183	350	345	202	407	441	240	291	251	236	205	185	263	229	117	325	347	338	302	536	410	229	412	418	184	403	362	242	348	401	345
15	10	5	3	3	2	8	13	14	11	16	10	9	14	14	8	18	6	13	12	7	8	9	5	7	0	10	29	8	11	34	48	14	58	60	28	29	16	19	22	15	23	22	10	41	45	32	26	33	39	20	68	46	17	46	51	5	11	56	36
TlatMFs																																																											
mitoRPs																																																											
MRPL30	MRPL32	MRPL33	MRPL34	MRPL35	MRPL36	MRPL37	MRPL38	MRPL39	MRPL40	MRPL41	MRPL42	MRPL43	MRPL44	MRPL45	MRPL46	MRPL47	MRPL48	MRPL49	MRPL50	MRPL51	MRPL52	MRPL53	MRPL54	MRPL55	MRPL58	MRPL57	GADD45GIP1	MRPS30	MRPS18A	MRPS2	MRPS5	MRPS6	MRPS7	MRPS9	MRPS10	MRPS11	MRPS12	MRPS14	MRPS15	MRPS16	MRPS17	MRPS18C	MRPS21	MRPS22	MRPS23	MRPS24	MRPS25	MRPS26	MRPS27	MRPS28	DAP3	MRPS31	MRPS33	MRPS34	MRPS35	CHCHD1	AURKAIP1	PTCD3	MRPS18B
																																																									1		
					1		1					1					1																																									

0	0	107	0	97	0	147	0	103	116	0	0	106
0	0	1	0	1	0	11	0	1	7	0	0	1
AARS1	RARS1	RARS2	NARS1	NARS2	DARS1	DARS2	CARS1	CARS2	QRSL1	QARS1	EPRS1	GATB

2 2 1 1

1 1

1

1	1	1	1	1	1			1	1	1	1	2	1		1	1		2	1	2	1	2	1	1		1	1		1	1	2	1	2	2	1	2	1	2	2	1	1	1	1	2	2	1	1	1	1	2	1	2	1	2	1		1	3	1
1	1	1	1	1	1		1	1	1	1	1	1	1	1	1	1	1	1	1	1	1	1	1	1		1	1		1	1	1	1	1	1	1	1	1	1	1	1	1	1	1	1	1	1	1	1	1	1	1	1	1	1	1		1	1	1
																																1																											
																																						1																					
																										2																																
								1		1					1	1								1						1	1	1	1	1	1	1			1		1		1	1	1	1	1	1	1	1	1	1		1	1			1	1

						1						
						1						

																																																											1	
																					1																																							
																										1																																		
																																1												1	2									1				1	
																																							1		1																				
2	1	1				1	1	1	1	1	2	1	1	1		2		1	1	1	1					1	2	1	1	1	1		2	1	1	1					1	1	1	3		1	1	1	1	1	1	1	1	2	1			2	2
1	1					1	1	1	1		1	1	1	1		1		1	1	1				1		1	1	1	1	1			1	1		1								1				1				1			1			1	1
1																																									1				1		1		1										
										1		1			1				1																			1																	1				
																							1							1		1			1															1				1	1			1	
							1							1					1	1							1	1																																	

						1							

							1							1													1	1																																
																											1		1																														
							1							1													1																																	
										1																																																	
									1																																																		
											1																																															

						1						
						1						
						1						
						1						

																						1																																						
																1																				1				1					1										1	1			1	
																																															1		1				1			1	
																																			2																									

1

1 1 1 1 1

1

						1												1														1																				1			1			1	
																																		1	1				1						1	1				1	1									1
																														2		1	1	1		1			1				1	2			1	1	1	2	1		2	1			1	1
																														1			1			1							1	1			1	1		1				1				1
																					1																							1														
																									1																																					

																										1																																
																																		1										1	1	1			1			1			1	1			1	1
																																																										1
											1																																															
																																1		1	1						1	1			2	2				2		2	2		2				2	2
																																					1																							
								1		2								1					1							1	1		1	3			1			1				3	2			3	2	1	3	1		2	1			1	2

																																					1																						
																																					1																		1					
																																												1														
																														1	1	1	1	1	1	1			1	1		1	1	1					1		1	1	1	1	1			1	
						1			1	1	1	1	1	1	1	1	1		1			1		1						1	1	1	2	1	2	1		1		1	1	1	1	1	1	1	1	1	1	1	1	1	1	1	1		1	1	1
								1																			1																																
																																																						1			
																																													1					1					

								1	1			1

																																																1								
								1																																																		
																																																					1	1		
										1			1					1					1										1										1					1					1		1			
																																																					1			
									1													1																																							
																																												1				1		1												
								1	1		1																					1	1			1				1							1	1		1		1	1							1	1

									2			
						1						

														1																																													
																																																										1	
								1																																																			
																																						1																						
																												1																							1							1	
																											1																							1							1	
												1							1																																								
																																																			2							
																																	1																											
																																	2																											

									1			

																														1		1	1							1				1		1					1			1				1
																										1																																	
																																												1														
																																						1																						
																																															1										
								1																																																		

					1							

							1																																																				
																																				1																									
																																																							1		
																						1																																					
																													1					1													1		1											
																			1																																										
						1										1		1	1											2	2	1	2	2	1	2		2	2	2	1	1		2	2	2	2	2	2	1	2	2	1	2	2			2	1
			1			1	1			1		1	1			1											1		1	1	2	1	1	1				1	1	1			1	1	1		1	1	1	1	1	1		1	1			1	1
																														1	2		1	1										1	1			2	1		1	1		1	1			1	1
								1																						1	1		1	1		1		1	1	1	1			1	1	1	1	1	1	1	1	1		1	1			1	1
																																						1																						1
																															1		1																						1					1

						1						

																																													1						1								
																											1			1		1	1	1				1					2					2	1	1			2	1		1	2	
														1					1		1					1		1		1	1		1	1	1	1								1		1		1		1	1	1	1	1	1				1
																																												1													
	1																																																										
																											1																																
																																														1		1	1		1				1	

		1										

																																									1																		
																																	1																										
															1				1									1	1		1											1			1			1								
																													2	2		2	2	2	2						2				2	1	1			2	2	2	2	2			2	1
																																																						2		
																																1		1	1		1			1			1				1									1				

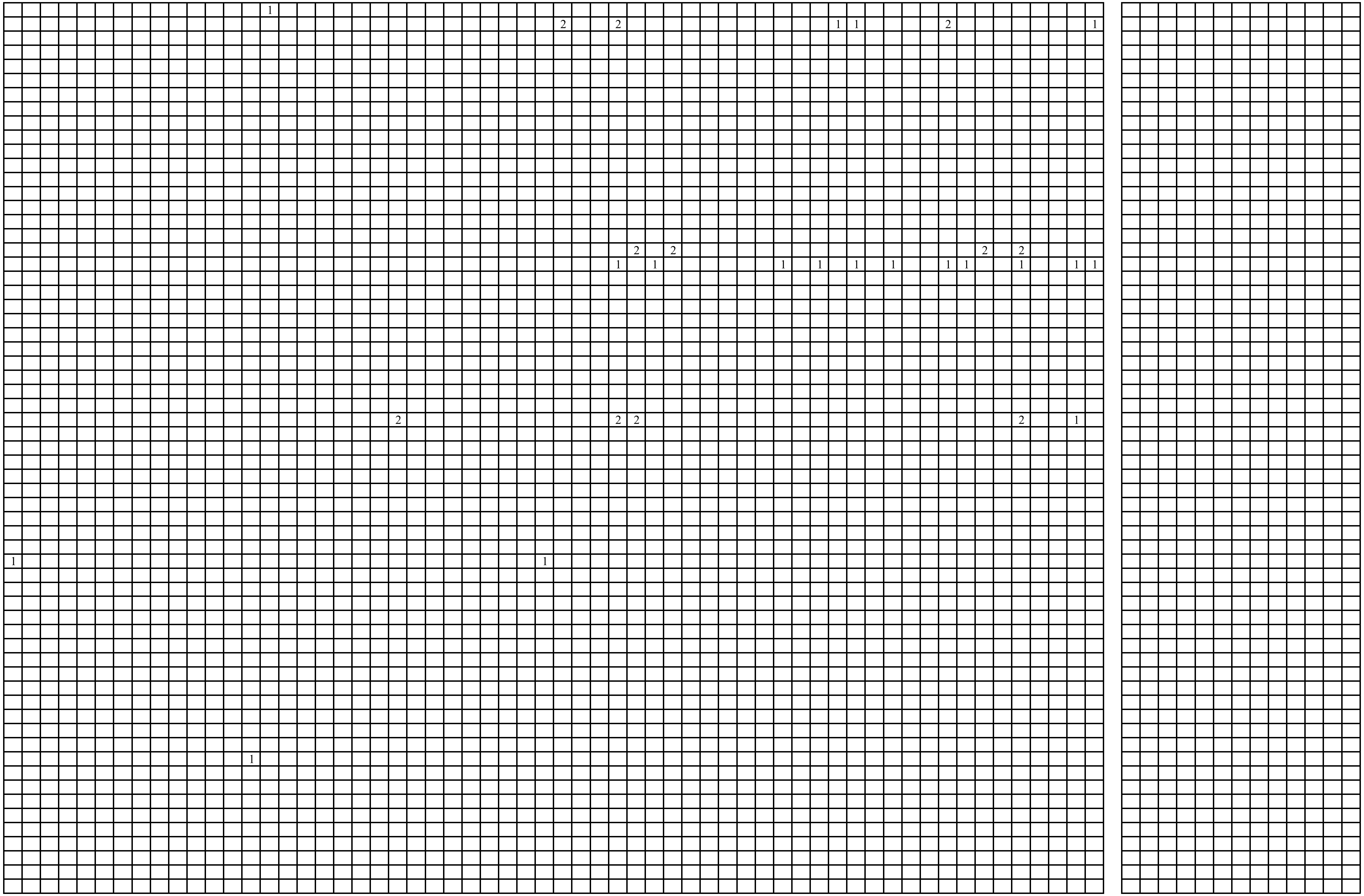

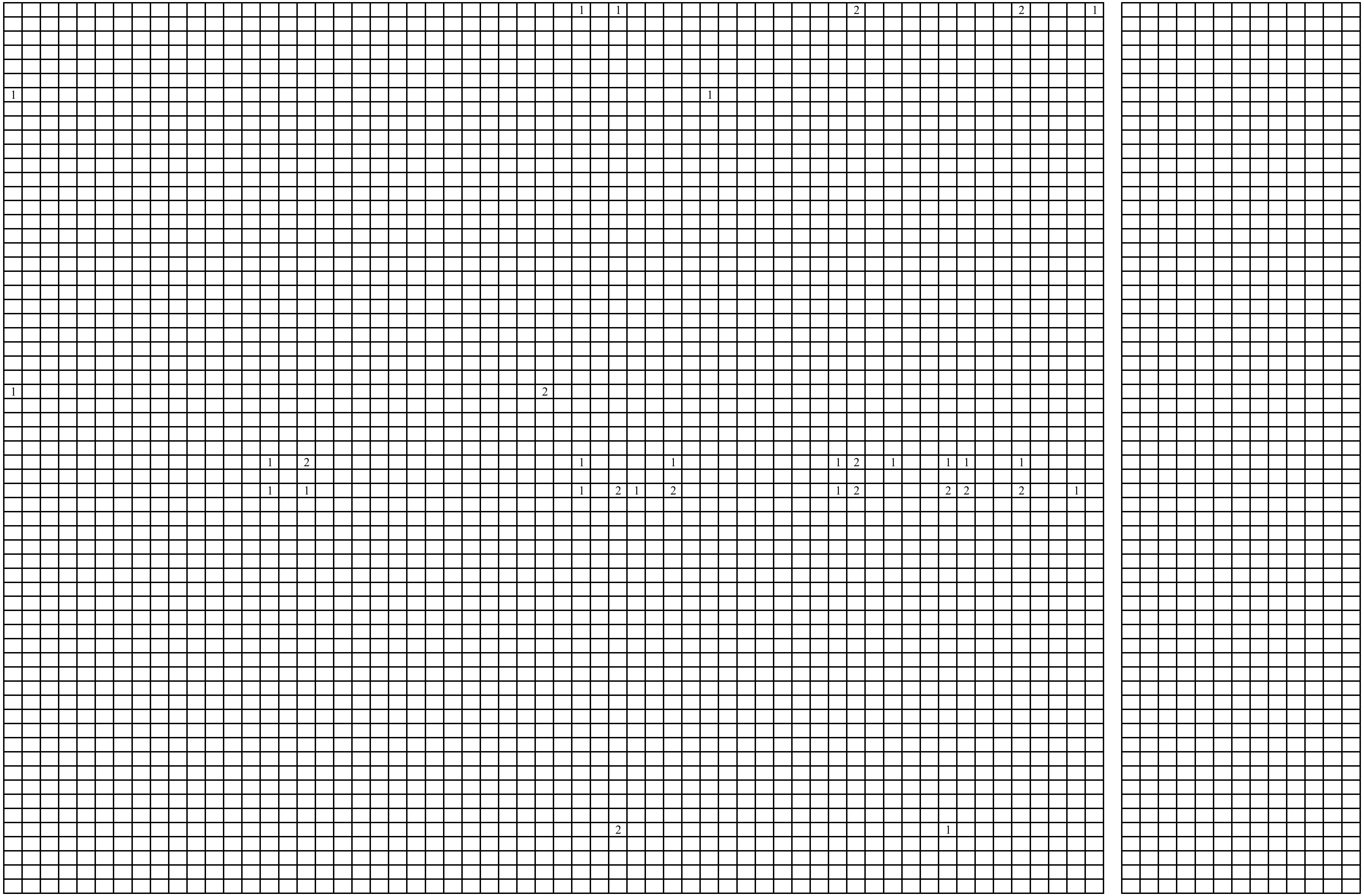

																															1	1		1	1	1	1			1			1	1			1	1				1	1	1		1			1	1
1	1												1			1											1				1		1	1																	1								
																																			2																									
																															1														2														

									1			

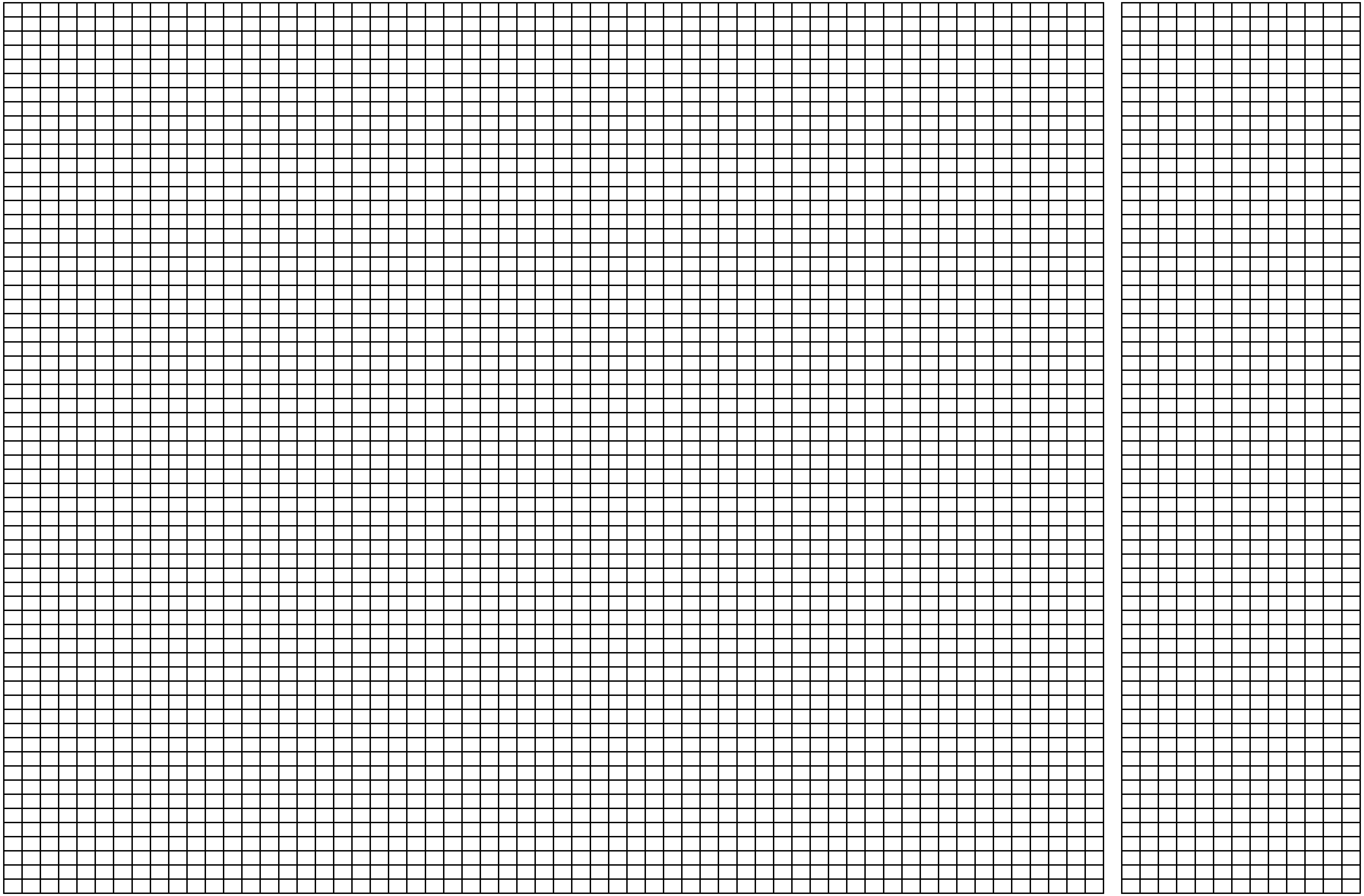

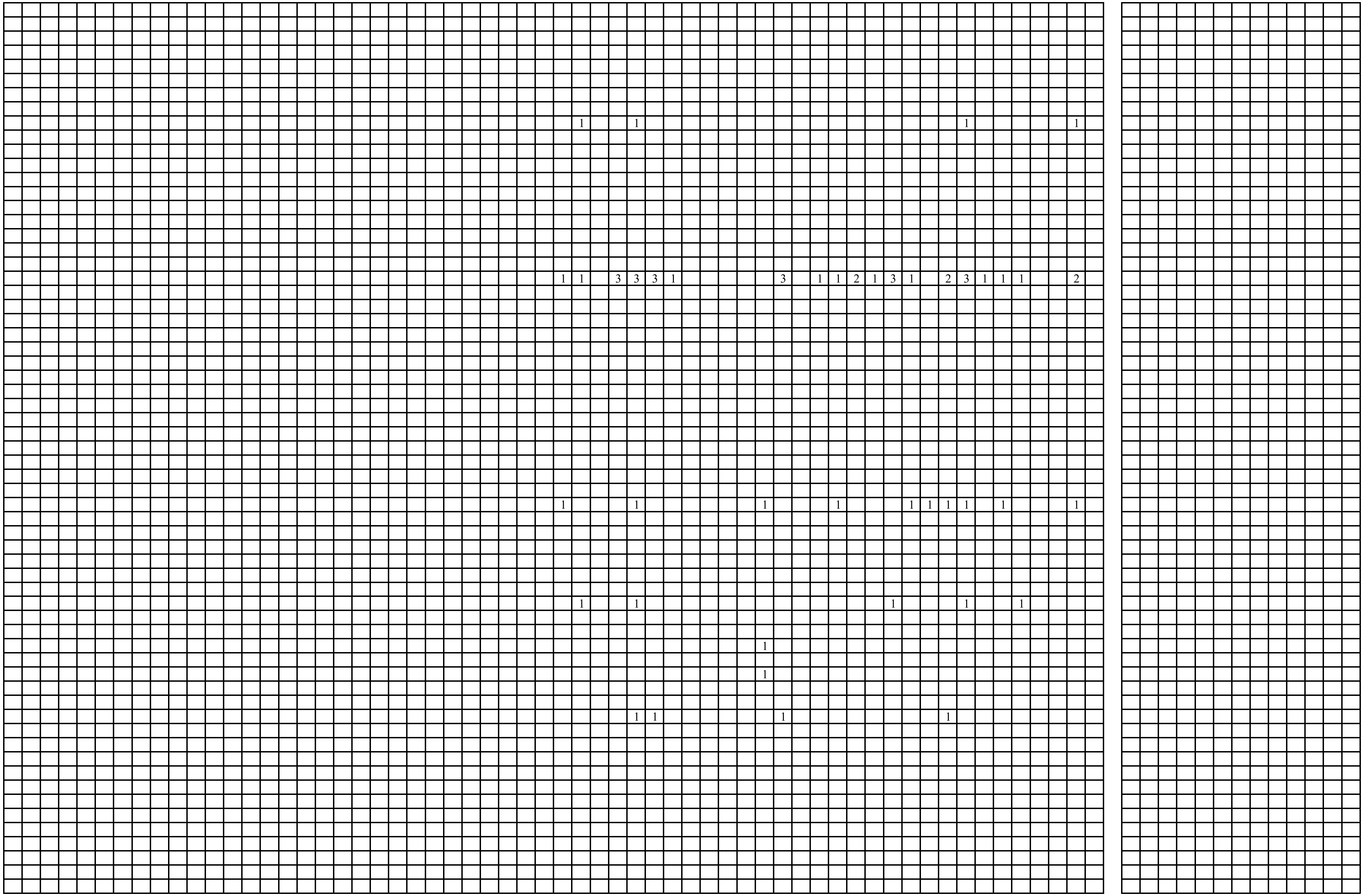

1 1 1 1
1 1 3 3 3 1 3 1 1 2 1 3 1 2 3 1 1 1 2
1 1 1 1 1 1 1 1 1 1
1 1 1 1 1
1
1
1 1 1 1

1

1

2 2 2 2 1 2 2 2 2 2 1 2 2 2 2 2 1

									1																																																															
																					1																																																								
2																																																																											
																																																1																								
							1																							1		1			1	1				1			1	1		1	1		1	1			1				1	1										1			
																														1																								2			1														

	1																														1		1	1								1	1									1			1				
																																				1																						1		
	1			1							1						1				1					1				1	1		1	2	1				1	1	1	1			1	1	1	1	1		1	1	1	2	1			1	
																																							2																				
																																													1												
																																										1						1							2					1
																																																		1								

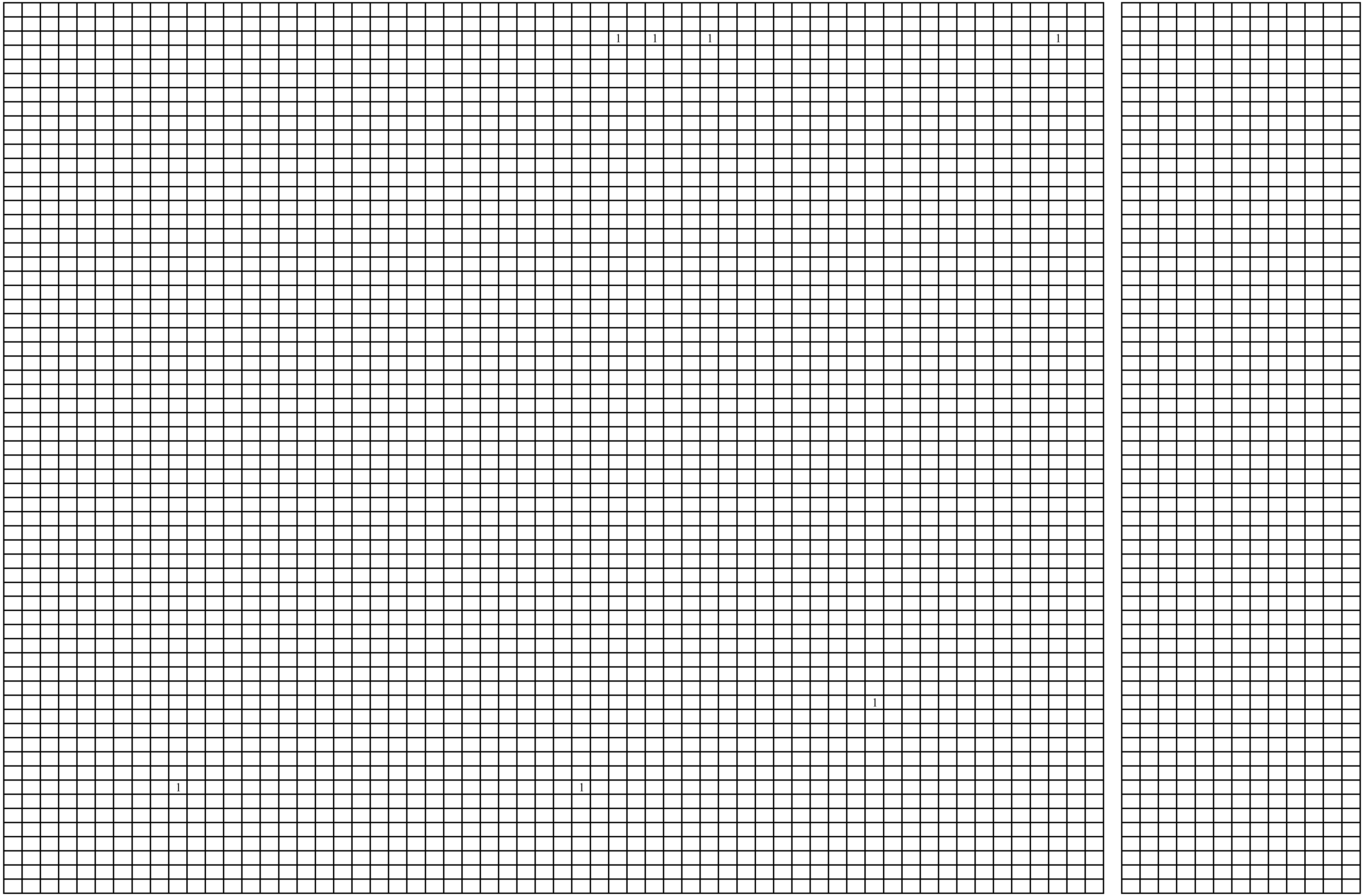
1
1
1
1
1
1
1

																																								1							1									1				1	
																												3							1																			1							1	
							1										1		1														1	1		1	1				1		1	1	1		1	1				1		1	1		1	1			1	1
							1			1																					2				1		1			1		1			2	1		1		2		2	1		1				2	
						1				1																			1				1	1		1	1				1								1	1		1	1	4				1				
																																																				1								
																																																				1								
																																																				1								
								1										1														1	1		1	1	1	1	1				1			1			1		1		1	1			1			1	
																														1																													
																																			1																									
																												3																																	
1	1						1		1		1				1	1	1		1					1		1	2	1	1				1	1	1			1	1	1		1				1	1	1	1		1	1		1	1			1	1
1	1													1												1							1	1		1						1						1							1		1				
																																																								1			
																												1																																	
		1							1	1			1	1	1		1	1						1						1						1	1	1	1	1				1	1				1	1	1			1				1	

				1								
						1						

													2																																														
													1																																1														
																																								1																		
1		1								1					1	1	1												1				1																1			1							

TlatMFs

aa-tRNA-synths

GATC	EARS2	GARS1	HARS1	HARS2	IARS1	IARS2	LARS1	LARS2	KARS1	MARS1	MARS2	FARS2	FARSA	FARSB	PARS2	SARS1	SARS2	TARS1	TARS2	TARS3	WARS2	WARS1	YARS1	YARS2	VARS1	VARS1	VARS2	Subcellular location	RBP function reported for…	pTriplo	Neuronal activity/ plasticity-responsive
69	101	0	0	207	0	283	0	158	0	0	55	156	171	144	48	0	87	0	156	0	32	0	0	266	0	0	120				
0	3	0	0	4	0	8	0	1	0	0	0	20	14	13	5	0	5	0	4	0	0	0	0	7	0	0	2				
																												Nucleoplasm	only mmu ortholog	1.000	1
																												Nucleoplasm		0.982	
																												Nucleoplasm	hsa gene	0.981	
																												Nucleoplasm, Cytosol		0.378	1
																												Nucleoplasm	hsa gene	0.557	
																												Nucleoplasm	hsa gene	0.863	
																												Nucleoplasm, Mitochondria, Cytosol	hsa gene	0.825	
																												Nucleoplasm, Nucleoli	only mmu ortholog	0.671	1
																												Nucleoplasm, Endoplasmic reticulum, Plasma membrane, Cytosol	only mmu ortholog	0.979	1
																												Nucleoplasm, Mitochondria		0.992	1
																												none reported		0.512	
																												none reported	only mmu ortholog	0.579	
																												Nucleoplasm, Nuclear speckles	hsa gene	0.772	
																												Nucleoplasm	hsa gene	0.589	
																												Nucleoplasm, Cytosol	hsa gene	0.993	1
																												Nucleoplasm, Cytosol		0.882	
																												none reported		0.320	
																												none reported		0.228	
																												none reported		0.132	
																												none reported		0.332	
																												Nucleoplasm		0.353	
																												Nucleoplasm, Mitotic spindle, Cytosol		0.247	
																												Nucleoplasm		0.692	
																												Nucleoplasm		0.622	
																									1				Nucleoplasm, Cytosol		0.279	
																												none reported		0.243	

																												Nucleoplasm		0.644	
																												Nucleoplasm, Golgi apparatus		0.981	
																												none reported		0.229	
																												Nucleoplasm, Nuclear speckles, Centrosome, Cytosol		0.266	1
																												none reported		0.553	
																												none reported		0.109	
																												none reported		0.268	
																												Nucleoplasm, Nuclear membrane, Nuclear bodies		0.820	
																												Nucleoplasm, Vesicles		0.650	
																												Nucleoplasm		0.541	
																												Nucleoplasm, Nucleoli		0.217	
																												none reported		0.342	
																												Nucleoplasm		0.715	
																												none reported		0.134	
																												none reported		0.954	1
																												none reported		0.969	1
																												none reported		0.356	
																												none reported		0.467	
																												Nucleoplasm, Nuclear bodies		0.390	
																												none reported		0.709	
																												Nucleoplasm, Nuclear speckles		0.259	
																												Nucleoplasm, Nuclear bodies		0.588	1
																												none reported		0.717	
																												Nucleoplasm, Plasma membrane		0.883	1
																												none reported		0.913	
																												Nucleoplasm		0.909	
																												none reported		NA	
																												Nucleoplasm	hsa gene	0.717	
																												Cytosol		0.209	
																												Nuclear speckles		0.469	
																												Nucleoplasm		0.257	
																												Nuclear speckles		0.246	1
																												none reported		0.986	
																												Nucleoplasm, Nucleoli		0.458	
																												Nuclear bodies	hsa gene	0.871	1
																												Nucleoplasm, Vesicles		0.523	
																												none reported		0.297	
																												none reported	hsa gene	0.538	
																												Nucleoplasm		0.794	
																												Nucleoplasm, Vesicles, Cytosol		0.245	
																												Nucleoplasm, Nuclear speckles, Cytosol		0.404	
																												Nuclear bodies, Cytosol		0.373	
																												Nucleoplasm, Nucleoli		0.764	
																												Nucleoplasm, Nuclear membrane, Cytosol		0.212	
																												Nuclear bodies, Aggresome		0.522	
																												Mitochondria, Cytosol		0.277	
																												Nucleoplasm, Cytosol		0.110	
																												Nucleoplasm, Cytosol		0.146	
																												Nucleoplasm, Nuclear bodies		0.969	1
																												Nucleoplasm		0.687	
																												gene/protein not avaialble in HPA list		0.991	
																												gene/protein not avaialble in HPA list		0.425	
																												Nucleoplasm, Vesicles		0.656	
																												Nucleoplasm, Actin filaments, Cytokinetic bridge, Cytosol		0.695	
	1																											Nucleoplasm, Nuclear bodies	only mmu ortholog	0.937	
																												Nucleoplasm, Plasma membrane, Cytosol		0.565	
																												Nucleoplasm, Plasma membrane, Cytosol		0.980	
																												Nucleoplasm		0.898	
																												Nucleoplasm		0.538	1
																												Nucleoplasm		0.940	
																												none reported		0.704	
																												Nuclear speckles		0.810	
																												Nucleoplasm, Nuclear bodies		0.498	

																												Nuclear speckles		0.150	
																												Vesicles		0.250	
																												Nucleoplasm		0.428	
																												Nucleoplasm, Golgi apparatus	hsa gene	0.912	
																												Nucleoplasm, Cytosol	only mmu ortholog	0.933	1
																												none reported		0.774	
																												Nucleoplasm, Nuclear membrane, Cytosol		0.544	1
																												none reported		0.687	
																												Nucleoplasm		0.687	
																												Nucleoplasm	hsa gene	0.502	
																												Nucleoplasm, Mitochondria, Cytosol		0.351	
																												Nucleoplasm, Nucleoli		0.307	
																												Cytosol		0.392	1
																												Nucleoplasm, Nucleoli, Nucleoli rim, Cytosol	hsa gene	0.630	1
														1										1				Nucleoplasm	hsa gene	0.299	1
																												Nucleoplasm, Mitotic chromosome		0.501	
												1																Nucleoplasm, Nucleoli	only mmu ortholog	0.766	
																												Nucleoplasm, Golgi apparatus, Cytosol		0.649	
																												Nucleoplasm, Endoplasmic reticulum, Vesicles		0.954	1
																												Nucleoplasm, Mitochondria	hsa gene	0.852	
																												Nucleoplasm, Cytosol	only mmu ortholog	NA	
																												Cytosol	mmu gene	0.441	
																												Nucleoplasm, Cytosol		0.139	
																												Nucleoplasm	hsa gene	0.938	
																												Nucleoplasm, Vesicles	only mmu ortholog	0.976	1
																												Nucleoplasm		0.062	
																												Nucleoplasm, Nuclear bodies, Centrosome		0.355	1
																												Nucleoplasm		0.480	
																												Nucleoplasm		0.260	
																												Nucleoplasm		1.000	1
																												Nucleoplasm		0.999	
																												Nucleoplasm		0.812	
																												Nucleoplasm, Golgi apparatus, Vesicles		0.866	
																												none reported		0.262	
																												Nucleoplasm		0.225	
																												Golgi apparatus		0.412	
																												none reported		0.132	
																												Nucleoplasm, Nucleoli, Centrosome		NA	
																												Nucleoplasm	only mmu ortholog	0.980	1
																												Nucleoplasm, Cytosol	hsa gene	0.891	
																												Nucleoplasm, Cytosol	mmu gene	0.776	
												1																Nucleoplasm, Cytosol		0.994	1
																												Nucleoplasm, Vesicles, Plasma membrane		0.699	
																												Nucleoplasm	hsa gene	0.327	1
																												Nucleoplasm	only mmu ortholog	0.464	1
																												Nucleoplasm	hsa gene	0.526	
																												Nucleoplasm, Actin filaments	hsa gene	0.391	
																												Nucleoplasm, Cytosol		0.873	1
																												Nucleoplasm, Golgi apparatus, Plasma membrane, Centrosome, Cytosol		0.416	
																												Nucleoplasm, Vesicles		0.275	1
																												Nucleoplasm, Nucleoli		0.585	1
																												Nucleoplasm		0.603	1
																												Nucleoplasm, Vesicles		0.933	
																												Nucleoplasm	hsa gene	0.478	
																												Nucleoplasm		0.888	1
																												Nucleoplasm, Nuclear speckles		0.307	1
																												Nucleoplasm, Nuclear bodies, Vesicles		0.467	
																												none reported		0.834	
																												Nucleoplasm, Nucleoli, Golgi apparatus, Cytosol		1.000	
																												Nucleoplasm, Mitochondria		0.585	
																												none reported		0.561	1
																												Nucleoplasm		0.318	1
																												Nucleoplasm, Cytosol		0.664	1

																												Nucleoplasm		0.319	
																												Nucleoli		0.336	
																												Nucleoplasm, Nucleoli, Cytosol		0.316	
																												none reported	hsa gene	0.268	
																												Nucleoplasm		0.234	1
																												Nucleoplasm, Cytosol		0.253	
																												Nucleoplasm	only mmu ortholog	0.853	
																												none reported		0.278	
																												Nucleoplasm, Nucleoli		0.811	
																												Nucleoplasm	only mmu ortholog	0.994	1
																												Cytosol		0.688	
																												Nucleoplasm, Cytosol		0.782	
																												Nucleoplasm, Endoplasmic reticulum		0.296	1
																												none reported		0.319	
																												Nucleoplasm, Nuclear membrane, Mitochondria		0.169	
																												Nucleoplasm, Mitochondria, Cytosol		0.836	
																												Nucleoplasm, Mitochondria		0.968	
																												Nucleoplasm, Vesicles		0.113	1
														1														Nucleoplasm, Vesicles		0.494	
													1	1														Nucleoplasm	hsa gene	0.447	
																												Nucleoplasm		0.309	
																												Nucleoplasm, Plasma membrane, Cytosol		0.363	
																												Nucleoplasm		0.742	1
																												none reported		0.637	1
																												Nucleoplasm		0.524	1
																												Nucleoplasm		0.855	1
																												Nuclear bodies		0.590	1
																												none reported		0.818	1
																												Nucleoplasm	hsa gene	0.983	1
																												Nucleoplasm		0.810	
																												Nucleoplasm	hsa gene	0.999	
																												Nucleoplasm, Vesicles		0.323	1
																												Nuclear speckles		0.994	
																												Nucleoplasm, Nucleoli rim		0.453	1
												1												1				Endoplasmic reticulum, Vesicles, Plasma membrane, Cytosol	hsa gene	0.919	
												1												1				none reported	hsa gene	0.835	
																												Cytosol	hsa gene	0.113	
																												Nucleoplasm, Centrosome		0.836	1
																												none reported		0.166	
																												Nucleoplasm, Nuclear speckles, Cytosol	hsa gene	0.853	
																		1										Nucleoplasm	mmu gene	0.900	
																												Nucleoplasm, Nucleoli, Vesicles		0.570	1
																												Nucleoplasm	hsa gene	0.936	
																												Nuclear speckles		0.553	1
																												Nucleoplasm, Nucleoli, Cytosol		0.654	
																												Nucleoplasm, Nuclear membrane, Cytosol		0.998	
				1																								Nucleoplasm, Vesicles, Mitotic spindle		0.930	
																												Nucleoplasm, Nuclear membrane, Cytosol		NA	
																		1										Nucleoplasm, Nucleoli fibrillar center		0.650	
																												Nucleoplasm, Cell Junctions		0.933	
																												Nucleoplasm		0.932	1
																												Nucleoplasm, Mitochondria		0.941	
																												none reported		0.458	
																												Nucleoplasm, Cytosol		0.615	
																												Nucleoplasm, Nuclear bodies		0.423	
																												none reported		0.891	
																												none reported		0.717	
																												none reported		0.905	
																												Nucleoplasm		0.125	
																												Nucleoplasm, Cytosol		0.270	
																												Nucleoplasm		0.235	
																												Nucleoplasm		0.488	
																												Nucleoplasm		0.227	

																												Nucleoplasm		0.220	
																												none reported		0.416	
																												none reported		0.331	
																												Nucleoplasm		0.218	
																												Nucleoplasm, Nuclear bodies, Vesicles		0.299	
																												none reported		0.968	
																												none reported		0.146	
																												Nucleoli, Vesicles		0.338	
																												none reported		0.201	
																												none reported		NA	
																												none reported		0.800	
																												Nucleoplasm, Nucleoli fibrillar center		0.669	
																												Nucleoplasm, Nuclear speckles, Vesicles		0.875	
																												Nucleoplasm, Nucleoli fibrillar center	only mmu ortholog	0.754	1
																												Nucleoplasm, Vesicles, Mitochondria	hsa gene	0.329	
																												none reported		0.458	
																												Nucleoplasm	hsa gene	0.550	
															1													Nucleoplasm, Nucleoli, Nucleoli rim, Cytosol		0.085	
																												none reported		0.657	
																												Nucleoplasm, Vesicles		0.254	
																												Nucleoplasm, Plasma membrane		0.953	
																												Nucleoplasm, Mitochondria, Cytosol		0.538	
																												Nucleoplasm, Cytosol		0.841	
																												Nucleoplasm		0.976	
																												Nuclear speckles, Cytosol		NA	
																												none reported		0.579	
																												Nucleoplasm	hsa gene	0.951	
																												Nucleoplasm		0.864	1
																												Nucleoplasm, Cytosol		NA	
																												Nucleoplasm	only mmu ortholog	0.927	
																												Nucleoplasm		0.459	
														1														Nucleoplasm, Cytosol		0.233	1
																												Nucleoplasm		NA	
						1																						Nucleoplasm, Cytosol		0.100	
																												Nucleoplasm, Vesicles	hsa gene	0.978	
																												Nucleoplasm, Golgi apparatus, Cytosol		0.749	
																												Nucleoplasm, Vesicles		0.666	1
																												Nucleoplasm		0.678	
																												Nucleoplasm, Vesicles		0.837	
																												none reported		0.481	
																												Nucleoplasm, Nuclear membrane		0.893	
																												Nucleoplasm, Nuclear bodies		0.264	
																												Vesicles		0.764	1
																												none reported		0.402	
																												Nucleoplasm, Vesicles		0.999	
																												Nucleoplasm		0.990	
																												Nucleoplasm		0.786	
												1																Nucleoplasm, Cytosol	hsa gene	0.679	
																												Nucleoli fibrillar center, Mitochondria		0.430	
																												Nucleoplasm	hsa gene	0.958	
																												Nucleoplasm	only mmu ortholog	0.966	1
																												Nucleoplasm, Cytosol	only mmu ortholog	0.936	
																												Nucleoplasm		0.415	
																												Nucleoplasm		0.576	
																												none reported		0.089	
																												none reported		NA	
																												none reported		NA	
																												Nucleoplasm, Vesicles	mmu gene	NA	
																												Nucleoplasm, Golgi apparatus	hsa gene	0.822	
																												none reported		0.991	
																												none reported		0.476	
																												Nucleoplasm, Actin filaments		0.510	
																												Nucleoplasm		0.569	

																												Nucleoplasm, Nuclear bodies		0.951	1
																												Nucleoplasm	hsa gene	0.981	
																												Nucleoplasm		0.848	1
																												none reported		0.265	
																												Nucleoplasm	hsa gene	1.000	1
																												none reported		0.995	
																												Cytosol		NA	
												1																Nucleoplasm, Nuclear bodies, Cytosol	hsa gene	0.915	
																												Nucleoplasm	only mmu ortholog	0.784	
																												Nucleoplasm, Vesicles		0.955	
																												Nucleoplasm, Cytosol	hsa gene	0.198	
																												Nucleoplasm, Endoplasmic reticulum, Vesicles	hsa gene	0.955	
																												Nucleoplasm, Vesicles		0.920	
																												Nucleoplasm, Plasma membrane, Cytosol		0.719	
																												Nucleoplasm		0.981	
																												Nucleoplasm		0.960	
																												Nucleoplasm		1.000	1
																												Nucleoplasm	hsa gene	NA	
																												none reported		0.937	
																												none reported		0.748	1
																												Nucleoplasm		0.884	
																												Nucleoplasm, Cytosol	hsa gene	0.504	
																												Nucleoplasm	mmu gene	0.121	
																												Nucleoplasm		0.135	
																												Nuclear bodies, Actin filaments		0.738	
																												none reported		0.702	
																												Nucleoplasm	only mmu ortholog	0.998	
																												Mitochondria, Cytosol	only mmu ortholog	0.384	
												1							1									Nucleoplasm, Nucleoli, Cytosol	hsa gene	0.442	
																												none reported		0.963	
																												Nucleoplasm		0.554	1
																												Nucleoplasm, Plasma membrane		0.887	1
																												Nucleoplasm, Nucleoli	hsa gene	0.780	
																												Nucleoplasm, Nucleoli, Cytosol	hsa gene	0.962	1
																												Nucleoplasm, Nucleoli fibrillar center	hsa gene	0.811	1
																												Nucleoplasm		0.905	1
																												Nucleoplasm, Nucleoli fibrillar center	hsa gene	0.871	
																												Nucleoplasm	hsa gene	0.724	
																												Nucleoplasm	only mmu ortholog	0.484	
																												Nucleoplasm		0.652	
																												none reported		0.348	
																												Nucleoplasm		0.995	
																												Nucleoplasm, Nuclear speckles, Cytosol		NA	
																												Nucleoplasm		0.273	
																												none reported		0.838	
																												none reported		0.294	
																												Nucleoplasm		1.000	
																												Nucleoplasm, Golgi apparatus, Cytosol		0.784	
																												none reported		0.580	
																												none reported		0.883	
																												Nucleoplasm, Nuclear bodies		0.658	
																												Nucleoplasm		0.687	
																												Nucleoplasm, Mitotic chromosome, Intermediate filaments		0.831	
																												Nucleoplasm, Vesicles		0.719	
																												Nucleoplasm		0.623	
																												Nuclear bodies		0.635	
																												none reported		0.824	
																												Nucleoplasm, Nuclear speckles		0.541	
																												Nucleoplasm, Nuclear membrane		0.541	1
																												Nucleoplasm		0.614	
																												Nucleoplasm		0.513	
																												Nucleoplasm		0.273	
																												Nucleoplasm		0.501	

																												Nucleoplasm		0.946	
																												Nucleoplasm, Centrosome	hsa gene	0.539	1
																												Nucleoplasm, Nucleoli fibrillar center, Cytosol		0.939	
																												Nucleoplasm, Golgi apparatus	hsa gene	0.873	
																												Nucleoplasm, Nuclear bodies, Cytosol		0.951	
																												Nucleoplasm		0.997	
																												Nucleoplasm	hsa gene	0.791	1
																												Nucleoplasm, Nuclear bodies		0.957	
																												Nucleoplasm, Cytosol		0.954	
																												none reported		0.642	
																												none reported		0.993	
																												Nucleoplasm		0.890	
																												Nucleoplasm, Cell Junctions		0.495	
																												Nucleoplasm, Cytosol		0.982	
																												Nucleoplasm, Microtubules		0.987	1
																												Nucleoplasm		0.992	
																												Nucleoplasm		0.709	
																												Nucleoplasm, Cytosol, Cytoplasmic bodies		0.624	
																												Nucleoplasm		0.201	1
																												none reported		0.263	
																												Nucleoplasm		0.403	
																												Nucleoplasm, Nuclear bodies		0.971	
																												Nucleoplasm, Cell Junctions		0.665	
																												none reported		0.554	
																												Nucleoplasm, Nucleoli		0.489	1
																												Nucleoplasm, Cytokinetic bridge, Cytosol		0.456	
																												none reported		0.255	1
																												Nucleoplasm, Nucleoli, Cytosol		0.831	
																												Nucleoplasm		0.524	
																												Nucleoplasm, Nuclear speckles, Mitochondria		0.845	
																												Rods & Rings		0.948	
																												Nucleoplasm, Nuclear membrane, Golgi apparatus		0.747	
																												Nucleoplasm		0.948	
																												none reported		0.159	
																												Nuclear speckles		0.738	
																												Nucleoplasm, Nuclear bodies, Cytosol		0.995	1
																												Nucleoli fibrillar center, Plasma membrane		0.914	
																												Nucleoplasm, Nucleoli, Cytosol		0.596	
																												Nucleoplasm		0.891	1
																												Nucleoplasm, Golgi apparatus, Vesicles		0.708	
															3													Nucleoplasm		0.971	
																												none reported		0.895	
																												none reported		0.996	1
																												Nucleoplasm, Vesicles		0.944	
																												Nucleoplasm, Golgi apparatus, Cytosol		0.255	
																												none reported		0.345	
																												none reported		0.314	
																												Nucleoplasm, Nuclear membrane, Vesicles		0.160	
																												Nucleoplasm, Vesicles, Cytosol		0.974	
																												Nuclear speckles	hsa gene	0.982	1
																												Nucleoplasm, Cytosol		0.896	1
																												Nucleoplasm		0.726	
																												Nucleoplasm		0.457	
																												Nuclear bodies		0.921	
																												Nucleoli, Nucleoli rim, Mitotic chromosome		0.794	
																												Nucleoplasm		0.656	
																												Nucleoplasm, Nucleoli rim		1.000	1
																												Nucleoplasm, Nucleoli, Nucleoli fibrillar center		1.000	
																												Nucleoplasm, Nuclear bodies		0.798	1
																												Nucleoplasm, Cytosol		0.678	
																												none reported		0.521	
																												none reported		0.687	
																												Nucleoplasm, Centriolar satellite		0.976	

																												Nucleoplasm, Nucleoli, Nuclear bodies, Cytosol		0.994	
																												Nucleoplasm, Midbody		0.961	
																												Nucleoplasm		0.119	
																												Nuclear speckles		0.854	
																												Nucleoplasm, Vesicles	mmu gene	0.337	
																												Nucleoli fibrillar center, Cytosol		1.000	
																												Nucleoli fibrillar center, Cytosol		0.336	
																												Golgi apparatus, Vesicles		0.597	1
																												none reported		0.972	
																												Nucleoli, Cytosol		0.845	
																												Nuclear bodies		0.264	
																												Nucleoplasm, Cytosol		0.973	
																												Nucleoplasm, Nuclear membrane, Cytosol		0.511	
																												Nucleoplasm, Plasma membrane		0.132	
																												Nucleoplasm, Cytosol		0.186	
																												Nucleoplasm		0.285	
																												Nucleoplasm		0.646	
																												none reported	hsa gene	0.419	
																												none reported		0.126	
																												Nucleoplasm, Cytosol		0.151	
																												none reported		0.939	
																												Nucleoplasm		1.000	
																												Nucleoplasm		0.910	
																												Nucleoplasm, Nucleoli rim		0.862	
																												Nuclear speckles		0.122	
																												Nucleoli fibrillar center		0.177	
																												Nucleoplasm, Nuclear bodies, Golgi apparatus		0.154	
																												Nucleoli fibrillar center		0.571	
																												Nucleoplasm	hsa gene	0.636	1
																												none reported		0.071	
																												none reported		NA	
																												none reported		0.375	
																												none reported		0.373	1
																												none reported		0.045	
																												Nucleoplasm, Nucleoli, Cytosol		0.465	
																												Nucleoplasm, Nucleoli, Golgi apparatus, Cytosol		NA	
																												none reported		0.015	
																												Nuclear speckles		NA	1
																												Nucleoplasm, Nuclear speckles		0.671	
																												none reported		0.076	
																												Nucleoplasm		0.204	
																												Nuclear bodies, Cytosol		0.219	
																												Nucleoplasm, Nuclear bodies		0.166	
																												Nucleoplasm, Nucleoli		NA	
																												Nucleoplasm		0.125	
																												none reported		0.299	
																												Nucleoplasm, Nucleoli fibrillar center, Nuclear bodies		0.986	
																												Nucleoplasm		0.959	1
																												Nucleoplasm, Vesicles		0.991	1
																												Nucleoplasm		0.984	
																												Nucleoplasm		1.000	
														1														Nucleoli		0.774	
																												Nucleoplasm		0.996	
																												none reported		0.948	
																												none reported		0.165	1
																												Nucleoplasm		0.791	1
																												Nucleoplasm, Nuclear bodies		0.501	
																												none reported		0.532	
																												Nucleoplasm, Nuclear speckles, Cytosol		0.880	
																												Nucleoplasm, Cytosol		NA	
																												Nucleoplasm, Plasma membrane		NA	
																												Nucleoplasm, Plasma membrane		NA	
																												none reported		NA	

																											Nucleoplasm, Vesicles		NA	
																											none reported		0.738	
																											none reported		0.235	
																											Nucleoplasm		1.000	
																											Nucleoli		0.279	
																											none reported		0.393	
																											Nucleoli, Nuclear bodies		0.245	
																											Nucleoplasm, Cytosol		0.990	1
																											none reported		0.247	
																											Nucleoplasm, Nucleoli		0.899	
																											Nucleoplasm, Nuclear membrane		0.430	1
																											Nucleoplasm		1.000	1
																											Nucleoplasm		0.816	
																											Nucleoplasm, Golgi apparatus, Vesicles, Cytosol		0.276	
																											Nucleoplasm		0.968	
																											Nucleoplasm, Golgi apparatus		0.999	
																											Nucleoplasm, Nucleoli		1.000	
																											Nucleoplasm, Cytosol		0.886	
																											Nucleoplasm, Vesicles		0.520	
																											Nuclear speckles, Microtubules, Cytosol		0.982	
																											Nucleoplasm, Mitochondria		0.802	
																											Nucleoplasm		0.973	1
																											Nucleoplasm, Cytosol	hsa gene	0.987	
																											Nucleoplasm		0.263	
																											Nucleoplasm, Nuclear speckles		0.523	1
																											Nucleoplasm, Nuclear bodies, Cytosol		0.988	1
																											Nucleoplasm	hsa gene	0.892	1
																											Nucleoplasm, Nuclear bodies, Cytosol		0.929	1
																											Vesicles		NA	
																											Nucleoplasm	hsa gene	0.757	1
																											Nucleoplasm, Cytokinetic bridge		0.964	
																											Nucleoplasm	hsa gene	0.086	
																											Nucleoplasm, Centrosome		0.624	
																											Nucleoplasm		NA	
																											none reported		NA	
																											Nucleoplasm	hsa gene	0.940	
																											Nucleoplasm, Nucleoli	hsa gene	0.292	
																											Nucleoplasm, Nucleoli	hsa gene	NA	
																											none reported		NA	
																											none reported		NA	
																											Nucleoplasm	hsa gene	0.919	
																											none reported	hsa gene	0.213	
																											Nucleoplasm		NA	
																											Nucleoplasm, Nuclear bodies	hsa gene	0.373	
																											Nucleoplasm	hsa gene	0.249	
																											Nucleoplasm	only mmu ortholog	0.650	
																											Nucleoplasm	only mmu ortholog	1.000	
																											Nucleoplasm, Cytosol		0.603	1
													1														Nucleoplasm, Vesicles	hsa gene	0.993	1
																											Nuclear speckles	hsa gene	0.787	
																											none reported		0.567	
																											Nucleoplasm		1.000	1
																											Nucleoplasm, Plasma membrane		0.784	
																											Nucleoplasm		0.245	
																											Nucleoplasm		0.603	
																											none reported		0.992	
																											Nucleoplasm, Nucleoli, Vesicles		0.256	
																											Nucleoplasm		0.320	
																											Nucleoplasm		0.272	
													2														Nucleoplasm	hsa gene	0.803	1
																											Nucleoplasm		0.947	
																											Nucleoplasm		NA	
																											Nucleoplasm, Vesicles, Cytosol		0.282	

																												Nucleoplasm, Mitochondria	hsa gene	0.389	
																												Nucleoplasm		1.000	1
																												Nucleoplasm		0.894	
																												Nuclear speckles, Golgi apparatus		0.421	
																												Nucleoplasm		0.428	1
																												Nucleoplasm		0.817	1
																												none reported		NA	
																												Nucleoplasm	hsa gene	0.583	1
																												Nucleoplasm		0.373	
																												Nucleoplasm, Cytosol		0.742	
																												Nucleoplasm, Nuclear bodies, Cytosol		0.967	1
	1			1		1																					1	Mitochondria	hsa gene	0.403	1
																												Nucleoplasm		0.771	1
																												Nucleoplasm	hsa gene	0.790	
																												Nucleoli		0.862	
																												Nucleoplasm	hsa gene	0.975	1
																												Nucleoplasm, Nucleoli fibrillar center		0.724	1
																												Nucleoplasm		0.772	
																												Nucleoplasm, Cytosol		0.733	
																												Nucleoplasm, Nucleoli, Vesicles		1.000	
																												Nucleoli fibrillar center	hsa gene	1.000	1
																												none reported		NA	
																												Nucleoplasm		NA	
																												Nucleoplasm, Cytosol	hsa gene	0.998	1
																												Nucleoplasm, Nucleoli	hsa gene	0.741	1
																												Nucleoplasm, Nucleoli, Nucleoli rim		0.481	
																												Nucleoplasm, Cytosol	hsa gene	0.822	
																												Nucleoplasm		0.796	1
																												Nuclear speckles		0.398	
																												Nucleoplasm		0.586	
						2																						Nucleoplasm, Plasma membrane, Cytosol		NA	
																												none reported		NA	
																												Nucleoplasm, Cytosol		NA	
																												Nucleoplasm	hsa gene	0.784	
																												Nucleoplasm, Nuclear membrane		0.639	
																												Cytosol		0.999	1
																												Nucleoplasm, Vesicles, Actin filaments, Mitochondria, Cytosol		0.877	1
																												Cytosol	hsa gene	0.303	1
																												Plasma membrane, Actin filaments	hsa gene	0.251	
																												Nucleoplasm, Cytosol		0.860	
																												Nucleoplasm, Cell Junctions, Cytosol		0.382	
																												Nucleoplasm, Vesicles		1.000	1
																												Nucleoplasm, Vesicles		0.964	
																												Nucleoplasm	hsa gene	0.997	
																												Nucleoplasm, Cytosol		0.822	
																												Nucleoplasm	hsa gene	0.982	1
																												Nucleoplasm, Nucleoli fibrillar center	only mmu ortholog	0.980	
																												Nucleoplasm, Nucleoli fibrillar center	hsa gene	0.857	
																												Nucleoplasm	hsa gene	0.952	1
																												Nucleoplasm, Cytosol		0.995	1
																												Nucleoplasm	hsa gene	0.583	
																												Nucleoplasm, Mitochondria	hsa gene	0.229	
																												none reported	hsa gene	NA	
																												Nucleoplasm, Cytosol	hsa gene	0.761	1
																												Nucleoplasm, Nucleoli fibrillar center	hsa gene	0.987	1
																												Nucleoplasm, Nuclear membrane, Nuclear bodies	only mmu ortholog	0.275	
																												Nucleoplasm, Nucleoli fibrillar center, Centrosome, Cytosol	hsa gene	0.151	
																												Nucleoli		0.153	
																												Nuclear speckles	hsa gene	0.169	
																												Nucleoplasm, Golgi apparatus		0.340	
																												Nucleoplasm	hsa gene	0.658	
																												Nucleoli fibrillar center, Vesicles, Cytosol		0.345	
																												none reported	hsa gene	0.053	

																												Nucleoplasm, Cytosol		0.871	
																												Nucleoli fibrillar center, Plasma membrane, Cytosol	hsa gene	0.701	1
																												none reported		0.823	1
																												Nucleoplasm, Centrosome		0.584	
																												Nucleoplasm, Vesicles	hsa gene	0.258	
																												none reported	hsa gene	NA	
																												Nucleoplasm	hsa gene	0.448	
																												Nucleoplasm, Cytosol	hsa gene	0.889	
																												Nucleoplasm, Cytosol	hsa gene	1.000	1
																												Nucleoplasm	only mmu ortholog	0.885	
																												Nucleoplasm, Cytosol	hsa gene	0.529	1
																												Golgi apparatus, Plasma membrane, Cytosol	hsa gene	0.980	
																												Nucleoplasm, Nuclear speckles		0.890	
																												Nucleoplasm		NA	
																												none reported		0.207	
																												Nucleoplasm, Cytoplasmic bodies		0.871	1
																												Nucleoplasm, Cytosol		0.339	1
																												Nucleoplasm	hsa gene	0.138	1
																												none reported		NA	1
																												Nucleoplasm, Nuclear bodies		0.919	
																												Vesicles, Plasma membrane, Cytosol	hsa gene	0.125	
																												Nucleoplasm, Plasma membrane, Focal adhesion sites, Cytosol	hsa gene	0.115	
																												Nucleoplasm	hsa gene	1.000	
																												Nuclear membrane, Intermediate filaments		0.603	
																												Nucleoplasm, Nucleoli, Nucleoli rim	hsa gene	0.674	
																												Nucleoplasm, Golgi apparatus, Vesicles		0.061	
																												none reported	hsa gene	0.303	
																												Nucleoplasm, Cytosol		0.841	1
																												Nucleoplasm, Cytosol		0.985	
																												Nucleoplasm	hsa gene	0.414	1
																												Nucleoli		0.282	
																												Nucleoplasm	only mmu ortholog	0.227	
																												Nucleoplasm, Nucleoli	hsa gene	NA	
																												Plasma membrane, Cytosol	hsa gene	0.342	
																												Nucleoplasm	hsa gene	NA	
																												Nucleoplasm, Cytosol		0.690	
												1	1	1					1								1	gene/protein not avaialble in HPA list	only mmu ortholog	0.990	
																												Nucleoli	only mmu ortholog	0.050	
																												Nucleoplasm, Golgi apparatus, Plasma membrane		0.088	
																												none reported		0.966	1
																												Endoplasmic reticulum	hsa gene	0.617	
																												Nucleoplasm		0.139	
																												none reported		0.533	
																												Nucleoplasm, Nuclear bodies, Cytosol		0.993	
																												Nuclear speckles		0.197	
																												Nucleoplasm	hsa gene	0.753	
																												Nuclear speckles	hsa gene	0.882	
																												none reported		0.113	
																												Nucleoplasm, Nucleoli	hsa gene	0.850	
																												Nucleoplasm, Cytosol	only mmu ortholog	0.770	1
																												Nucleoplasm	only mmu ortholog	0.344	
																												none reported	hsa gene	0.483	
																												Nucleoplasm, Nucleoli fibrillar center, Cytosol	hsa gene	0.080	
																												Golgi apparatus	hsa gene	0.141	
																												Nucleoplasm, Cytokinetic bridge	hsa gene	0.427	
																												Nucleoplasm, Nuclear membrane	only mmu ortholog	0.565	
						1							1	1														Nucleoplasm, Vesicles, Cytosol	hsa gene	0.771	
																												Nucleoplasm		1.000	
																												Nucleoplasm, Golgi apparatus, Vesicles, Cell Junctions		0.572	
																												none reported		0.842	1
																												Nucleoplasm		0.919	
																												Nucleoplasm		0.845	
																												Nucleoplasm		0.352	

																												Nucleoplasm, Nucleoli		0.253	
																												Nucleoplasm, Nucleoli		0.230	
																												none reported	hsa gene	NA	
																												none reported	hsa gene	0.996	
																												Nucleoplasm	hsa gene	0.673	1
																												Nucleoplasm, Nucleoli, Plasma membrane	hsa gene	0.670	
																												Nucleoplasm		0.584	
																												Nucleoplasm, Cytosol	hsa gene	0.977	
																												Nucleoplasm, Cytosol	hsa gene	0.951	
																												none reported		0.812	
																												Cytosol		0.999	
																												Nucleoplasm, Cytosol		0.901	1
																												Nucleoplasm, Cytosol		1.000	
																	1											Nucleoplasm, Nuclear bodies		0.513	
																												Nucleoplasm, Cytosol		0.998	1
																												Nucleoplasm, Cytosol		0.899	
																												Nuclear speckles, Cytosol		0.965	
																												Nucleoplasm	hsa gene	0.997	1
																												none reported		0.754	
																												Nucleoplasm, Vesicles		1.000	1
																												Nucleoplasm, Nuclear bodies		0.978	
																												Nucleoplasm, Vesicles		0.996	
																												none reported		0.233	
																												Nucleoplasm, Vesicles	hsa gene	0.896	
																												Nucleoplasm		0.931	1
																												Nucleoplasm, Vesicles, Cytosol		0.808	1
																												none reported		0.201	
																												Nucleoplasm, Nucleoli fibrillar center		0.991	
																												Nucleoplasm		0.969	
																												Nucleoplasm, Cytosol	hsa gene	0.724	
																												Nucleoplasm		0.141	1
																												Nucleoplasm, Nuclear bodies		0.459	1
																												Nucleoli fibrillar center, Mitochondria		0.103	1
																												Nucleoplasm		0.192	
																												Nucleoplasm, Cytosol	hsa gene	0.995	
																												Nucleoplasm, Nucleoli, Cytosol	hsa gene	0.998	
																												Nucleoplasm, Cytosol	hsa gene	0.992	
	1							1																				Nucleoplasm, Centrosome, Cytosol	hsa gene	1.000	
																												Nucleoplasm, Cytosol	hsa gene	0.962	
																												Nucleoplasm, Nuclear bodies, Golgi apparatus	hsa gene	0.534	
																												Nucleoplasm, Cytosol	hsa gene	0.684	1
																												Nucleoplasm, Cytosol	hsa gene	0.668	
																												Nucleoplasm, Vesicles		0.162	
																												Nucleoplasm, Cytosol	hsa gene	0.979	1
						2																						Plasma membrane, Cytosol		0.910	
																												Nucleoplasm, Cytosol	hsa gene	1.000	
																												none reported		0.636	
												1																Nucleoplasm, Cytosol		0.973	
				1																								Cytosol		0.999	
																												Nucleoplasm, Cytosol	hsa gene	0.987	
																												Nuclear speckles		0.411	
																												Nucleoplasm		NA	
																												Nuclear bodies, Cytoplasmic bodies		0.718	
																												Nucleoplasm, Cytosol		0.122	
																												Nucleoplasm, Centrosome		1.000	1
																												Nucleoplasm		0.485	1
																												Nucleoli fibrillar center		0.721	
																												none reported		0.785	
																												none reported		NA	
																												Nucleoplasm, Cytosol		0.945	
																												Nucleoplasm, Cytosol	mmu gene	0.956	1
																												Vesicles		0.498	
																												Nucleoplasm		0.966	1

																												Nucleoplasm		0.573	
																												Nucleoplasm, Golgi apparatus, Cytosol		0.560	1
																												none reported		0.994	
																												Nucleoplasm	only mmu ortholog	0.912	1
																												Nucleoplasm		0.922	
																												none reported		0.804	
																												Nucleoplasm		0.534	
																												Nucleoplasm, Nuclear bodies		0.628	1
																												Cytosol		0.704	1
																												Nuclear speckles, Golgi apparatus, Cytosol		NA	
																												Cytosol		0.918	
																												Nucleoli, Centriolar satellite		0.565	1
																												none reported		0.548	
																	1		1									Nucleoplasm, Nucleoli, Plasma membrane		0.072	
																												none reported		0.626	
																												Nucleoplasm, Golgi apparatus		0.097	1
																												Nucleoplasm	hsa gene	0.432	
																												Nucleoplasm, Nuclear bodies	hsa gene	0.458	
																												Nucleoplasm		0.280	
																												Nucleoplasm, Nuclear bodies		NA	
																												Nucleoplasm		0.185	
																												Nucleoplasm		NA	1
																												Nucleoplasm, Mitochondria		0.416	
																												Nucleoplasm		0.785	1
																												Nucleoplasm, Cytosol		1.000	1
																												Nucleoplasm, Cytosol		0.941	
																												Nucleoplasm		0.757	
																												Nucleoplasm, Plasma membrane, Cytosol		0.672	
																												Nucleoplasm		0.287	
																												none reported		0.472	
																												Nucleoplasm, Vesicles		0.943	1
																												none reported		0.243	
																												Nucleoli rim, Mitotic chromosome		0.982	
																												Nucleoplasm		0.986	1
																												Nucleoli, Cytosol		0.894	
																												Nucleoplasm		0.510	
																												Nuclear speckles		0.683	
																												Nucleoplasm, Nuclear bodies		0.745	1
																												Nucleoplasm		0.579	
																												Nucleoplasm, Nucleoli, Cytosol		0.623	
																												Nucleoplasm	hsa gene	0.633	
																												none reported		0.491	
																												none reported		0.359	
																												Nucleoplasm, Cytosol		0.612	
						1																						Nucleoplasm, Cell Junctions, Cytosol	hsa gene	0.567	
																												Cytosol	hsa gene	0.759	
																												Nucleoplasm, Cytosol	hsa gene	0.216	
																												none reported	hsa gene	0.665	
																												Nucleoplasm, Cytosol	hsa gene	0.887	
																												Nucleoplasm, Cytosol	hsa gene	0.128	
																												Nucleoplasm	hsa gene	0.259	
																												Cytosol	hsa gene	0.892	1
																												Nucleoplasm	hsa gene	0.988	
																												Nucleoplasm		0.457	
																												Endoplasmic reticulum	hsa gene	0.435	1
																												Cytosol	hsa gene	0.766	1
																												Nucleoplasm		NA	
																												Nucleoplasm		0.635	1
																												Nucleoplasm		0.705	
																												Nucleoplasm, Cytosol	hsa gene	0.825	1
																												Nucleoplasm		0.690	1
																												Nucleoplasm, Cytosol	mmu gene	0.384	
																												Nucleoplasm, Cytosol		0.984	

																												Nucleoplasm	only mmu ortholog	0.957	
																												Nucleoplasm		0.994	
																												Nucleoplasm, Vesicles, Midbody		0.682	
																												Nucleoplasm, Cytosol		0.930	
																												Nucleoplasm		0.890	
																												Nucleoplasm		1.000	
																												Nucleoplasm, Nuclear membrane	hsa gene	0.382	
											1																	Nuclear speckles		0.612	
																												Nuclear speckles	only mmu ortholog	0.941	
																												Nucleoplasm, Nucleoli fibrillar center, Cytosol		0.928	
																												Nucleoplasm, Cytosol		0.747	
																												Nucleoli, Cytosol		0.237	
																												Nucleoplasm, Nucleoli		0.409	1
																												Nucleoplasm, Cell Junctions, Cytosol		NA	
																												Nucleoli fibrillar center, Nuclear bodies		0.431	
																												Nucleoplasm, Nuclear bodies		1.000	
																												Nucleoli fibrillar center	hsa gene	0.448	1
																												Nucleoplasm, Nucleoli, Nucleoli rim	hsa gene	0.978	
																												none reported		0.447	
																												Intermediate filaments		NA	
																												Nucleoplasm, Cytosol		0.732	1
																												Vesicles		0.528	
																												none reported		0.916	1
																												Nucleoplasm, Nuclear bodies, Midbody ring		0.721	
																												Nucleoplasm, Nucleoli, Nucleoli rim	hsa gene	0.417	
																												Nucleoplasm		0.749	1
																												Nucleoplasm, Golgi apparatus, Cytosol		0.217	
																												Nucleoplasm, Cytosol		0.813	
																												Cytosol		NA	
																												Nucleoplasm, Golgi apparatus		0.051	
																												Nucleoplasm, Nucleoli, Mitochondria		0.562	1
																												Nucleoplasm, Cytosol		0.552	1
																												Nucleoplasm		0.502	
																												Nucleoplasm, Centrosome		0.298	
																												Nucleoplasm, Nuclear membrane, Vesicles		0.730	
																												Nucleoplasm		0.432	
																												Mitochondria		0.325	
																												Nucleoplasm		0.736	1
																												Nucleoplasm	hsa gene	0.977	1
																												Nucleoplasm, Vesicles		0.987	1
																												none reported		0.442	
																												Nucleoplasm		0.387	
																												none reported		0.602	
																												Nucleoplasm, Nuclear bodies, Focal adhesion sites, Cytosol		0.966	1
																												Nucleoplasm, Cytosol	hsa gene	0.711	
																												none reported	hsa gene	0.476	1
																												none reported	hsa gene	0.153	
																												Nucleoplasm, Nuclear speckles, Vesicles		0.827	
																												Nucleoplasm		0.226	
																												none reported	hsa gene	0.180	1
																												Nucleoplasm	only mmu ortholog	0.562	
																												Nucleoplasm		0.827	1
																												Nucleoplasm, Cytosol	mmu gene	0.594	1
																												Nucleoplasm, Golgi apparatus, Vesicles	only mmu ortholog	0.565	1
																												Nucleoplasm, Nucleoli, Vesicles, Cytosol		0.788	1
																												Nucleoplasm, Cytosol		0.795	
																												Nucleoplasm	only mmu ortholog	NA	
																												Nucleoplasm, Plasma membrane, Cytosol		0.726	1
																												Nucleoplasm	hsa gene	0.994	1
																												Nucleoplasm, Vesicles		0.977	
																												Nucleoplasm, Cytosol	hsa gene	0.958	
																												Nucleoplasm, Cytosol		0.836	
																												Nucleoplasm, Nucleoli		0.723	

																												Nucleoplasm, Mitotic spindle, Centrosome	hsa gene	0.807	
																												none reported		0.608	
																												Nucleoplasm		0.975	
																												Mitochondria		0.975	
																												Nucleoplasm	only mmu ortholog	0.920	1
																												none reported		0.500	
																												Nucleoplasm, Vesicles, Cytosol		0.184	
																												Nucleoplasm		0.636	1
																												none reported	hsa gene	0.270	
																												Nucleoplasm, Plasma membrane, Cytosol	hsa gene	NA	
																												Nucleoplasm	hsa gene	0.857	1
																												Nucleoplasm, Nucleoli fibrillar center, Cytosol	hsa gene	0.799	1
																												Nucleoplasm, Nucleoli		0.929	
																												Nucleoplasm		0.354	
																												Nucleoplasm, Nuclear bodies, Golgi apparatus		0.742	1
																												Nucleoplasm, Cytosol		0.465	
																												Nucleoplasm	hsa gene	0.835	
																												Nucleoli fibrillar center, Nuclear bodies		0.064	
																												none reported		0.325	
																												Nucleoplasm		0.473	
																												Nuclear speckles		0.118	
																												Nucleoplasm, Nuclear membrane, Cytosol		0.173	
																												Nucleoplasm, Nuclear bodies, Vesicles		0.270	
																												Nucleoplasm		0.791	
																												none reported		0.232	
																												Nucleoplasm	hsa gene	0.697	
																												Nucleoli	hsa gene	0.039	
													1															Nucleoplasm, Golgi apparatus	hsa gene	0.670	
																												Nucleoplasm, Vesicles, Plasma membrane	hsa gene	0.522	
																												Nucleoplasm	hsa gene	1.000	
																												Nucleoplasm, Nuclear bodies, Cytosol		0.235	1
																												Nucleoplasm, Cytosol		0.994	
																												none reported		0.966	
																												none reported	hsa gene	0.766	
																												Nucleoplasm, Plasma membrane	hsa gene	0.258	
														1														Nucleoplasm, Cytosol		0.846	
																												Nucleoplasm, Nucleoli		0.703	
																												Nucleoplasm, Nucleoli, Vesicles		0.916	
												1																Nucleoplasm, Cytokinetic bridge	hsa gene	0.215	
																												Nucleoplasm, Mitochondria, Cytosol		0.263	
																												Nucleoplasm		0.350	
																												Nucleoplasm, Golgi apparatus		0.876	1
																												Nucleoplasm	hsa gene	0.319	
																												Nucleoplasm, Nucleoli	hsa gene	0.751	
																												Nucleoplasm		0.944	1
																												Nucleoplasm	hsa gene	0.822	
																												Nucleoplasm	hsa gene	0.442	
																												Nucleoplasm, Plasma membrane	hsa gene	0.451	
																												Nucleoplasm	only mmu ortholog	0.926	
																												Nucleoplasm, Cytosol	only mmu ortholog	0.450	
																												Nucleoplasm, Nuclear speckles, Centrosome, Cytosol	hsa gene	0.453	
																												Nuclear bodies, Golgi apparatus, Vesicles	hsa gene	0.679	1
																												Nucleoplasm, Cytosol		0.994	
																												Nucleoplasm, Nucleoli		0.527	1
																												Nucleoplasm, Vesicles	hsa gene	0.237	
																												Nucleoplasm, Nucleoli, Nuclear bodies	hsa gene	0.295	1
																												Nucleoplasm		0.609	
																												Nucleoplasm		0.145	
																												Nuclear bodies		0.671	
																												Nucleoplasm		0.786	
													1															Nucleoplasm	only mmu ortholog	0.579	
																												Nucleoplasm	hsa gene	0.352	1
																												Nucleoplasm	hsa gene	0.061	

																												Nucleoplasm, Cytosol	hsa gene	0.579	
																												Nucleoplasm, Nucleoli fibrillar center, Nuclear bodies	hsa gene	1.000	1
																												Nucleoplasm, Nuclear bodies	hsa gene	NA	
																												Nucleoplasm	only mmu ortholog	0.542	
																												Nucleoplasm	hsa gene	0.897	
																												Nucleoplasm, Nucleoli, Cytosol	hsa gene	0.517	1
																												none reported	hsa gene	0.862	1
																												Nucleoplasm, Nuclear speckles	hsa gene	0.920	1
																												Nucleoplasm, Nuclear bodies	hsa gene	0.952	1
																												Nucleoplasm, Cytokinetic bridge, Centrosome	hsa gene	0.587	
																												none reported	hsa gene	0.430	
																												Nucleoplasm	hsa gene	0.689	1
																												Nucleoplasm		0.853	
																												Nucleoplasm	hsa gene	0.679	
																												Nuclear bodies, Cytosol	hsa gene	0.356	
																												Nucleoplasm	hsa gene	0.912	
																												Nucleoplasm, Cytosol	only mmu ortholog	0.501	
																												Nucleoplasm, Microtubules, Cytosol	hsa gene	0.515	1
																												Nucleoplasm, Mitochondria	hsa gene	0.583	
																												Nucleoplasm	only mmu ortholog	0.929	
																												Nucleoplasm	hsa gene	0.755	
																												none reported		0.465	
																												Nucleoplasm	hsa gene	0.126	1
																												Nuclear speckles		0.371	
																												none reported		0.016	1
																												Nucleoplasm, Cytosol		0.015	
																												Nucleoplasm, Cytosol	hsa gene	0.068	
																												Cytosol		0.081	1
																												Nucleoplasm, Vesicles		0.014	1
																												Nucleoplasm, Nucleoli, Nucleoli rim, Plasma membrane		0.252	
																												none reported	mmu gene	0.028	
																												Nucleoplasm, Actin filaments	hsa gene	0.510	
																												Nucleoplasm	hsa gene	0.401	
																												Nucleoplasm		0.172	
																												Nucleoplasm, Cytosol		0.034	
																												Nucleoplasm, Nuclear membrane, Cytosol		0.093	
																												Vesicles, Plasma membrane, Centrosome		0.064	
																												Nucleoplasm, Nucleoli	hsa gene	0.931	
																												none reported		NA	
																												Nucleoplasm, Nucleoli	mmu gene	NA	
																												Nucleoplasm, Nucleoli		NA	
																												Nucleoplasm	hsa gene	0.997	1
																												Nuclear bodies	hsa gene	0.963	
																												Nucleoplasm	hsa gene	NA	
																												Nucleoplasm, Cytosol		0.756	1
																												Nucleoplasm, Vesicles		0.735	
																												Intermediate filaments	hsa gene	0.015	
																												Nuclear speckles, Cytosol	hsa gene	0.039	
																												none reported		0.031	
																												Nucleoplasm, Nuclear bodies, Mitochondria	only mmu ortholog	0.923	1
																												Nucleoplasm, Nucleoli fibrillar center, Golgi apparatus		0.327	
																												Nucleoplasm	hsa gene	0.521	
																												Nucleoplasm	only mmu ortholog	0.264	
																												Intermediate filaments		0.747	
																												Nuclear speckles, Cytosol		0.333	
												1																Nucleoplasm, Cytosol	hsa gene	0.610	
																												Nucleoplasm, Vesicles, Plasma membrane		0.014	
																												none reported		0.123	
																												none reported		0.046	
																												Nucleoplasm, Cytosol		0.291	
																												Nucleoli fibrillar center, Cytosol		0.114	
																												Nucleoplasm, Nucleoli, Centrosome	hsa gene	0.057	
																												Nucleoplasm, Nuclear membrane		0.020	

Nucleoplasm, Intermediate filaments	hsa gene	0.949	
Nucleoplasm, Vesicles	hsa gene	0.220	
Cytosol		0.022	
Nucleoli		0.318	
none reported		0.115	
Nucleoplasm, Cytosol		0.020	
Nucleoplasm		0.040	
none reported		0.009	
Nucleoplasm, Nuclear bodies, Golgi apparatus, Vesicles	hsa gene	0.695	
Nucleoli		0.861	
Nucleoplasm, Golgi apparatus	hsa gene	0.969	
Nucleoplasm, Nucleoli fibrillar center		0.014	
Nucleoplasm, Cytosol	hsa gene	0.237	
none reported		NA	
Nucleoplasm, Nucleoli	hsa gene	0.056	
Nuclear speckles, Cytosol		0.294	
Nuclear speckles, Microtubules		0.337	
Nucleoplasm, Nuclear speckles, Vesicles, Cytosol		0.394	
none reported		0.106	
Nucleoli, Cytosol		0.902	
none reported	hsa gene	0.058	
Nucleoplasm, Golgi apparatus, Plasma membrane, Cytosol		0.418	
Nucleoplasm		0.024	
Nucleoplasm	hsa gene	0.213	
Nucleoplasm		NA	
Nucleoplasm	hsa gene	0.182	
Intermediate filaments		0.114	
Mitochondria		0.446	
Nucleoplasm		0.284	
Nucleoplasm, Plasma membrane, Cytosol	hsa gene	0.242	
Nucleoplasm, Nuclear membrane, Nuclear bodies		0.166	
Mitochondria	hsa gene	0.013	
Nucleoli rim, Nuclear bodies, Mitotic chromosome		0.564	
Nucleoplasm	hsa gene	0.418	
Golgi apparatus, Cytosol		0.033	
Nucleoplasm		0.613	
Nucleoplasm, Nuclear membrane, Vesicles, Cytosol		0.526	
Nucleoplasm, Midbody	hsa gene	0.095	
Nucleoplasm, Vesicles		0.037	
Nucleoplasm	hsa gene	0.302	
Nuclear bodies		0.060	
Nucleoplasm, Nuclear bodies		0.060	
Nucleoplasm, Mitochondria		0.087	
Nucleoplasm, Nuclear membrane	hsa gene	0.314	
Nucleoplasm	hsa gene	0.251	
Nuclear bodies	hsa gene	0.077	
Plasma membrane	hsa gene	0.210	
Vesicles, Plasma membrane		0.032	
Cytosol	hsa gene	0.221	
Mitochondria		0.052	
Nucleoplasm, Vesicles, Cytosol		0.503	
Nucleoplasm	hsa gene	0.046	
Nuclear bodies	hsa gene	0.134	
Nucleoplasm	hsa gene	0.180	
Nucleoplasm, Nuclear bodies	hsa gene	0.118	
Nucleoplasm	hsa gene	0.770	
Nucleoplasm, Golgi apparatus		0.233	
Nuclear speckles, Cytosol	hsa gene	0.414	
none reported		0.094	
none reported		0.024	
none reported	hsa gene	0.033	
Nuclear bodies, Mitochondria		0.029	
Nucleoplasm, Cytosol		0.067	

																												Nucleoplasm, Mitotic spindle, Cytosol		0.048	
																												Nucleoplasm	hsa gene	0.167	
																												Nucleoplasm, Vesicles, Rods & Rings	hsa gene	0.051	
																												Nucleoplasm	hsa gene	0.248	
																												Cytosol	hsa gene	0.019	
																												Nucleoplasm	hsa gene	0.099	
																												Nucleoplasm, Centrosome	hsa gene	0.031	
																												Nucleoli	hsa gene	NA	
																												Nucleoplasm, Nucleoli, Plasma membrane, Cytosol		0.250	
																												none reported	hsa gene	0.092	
																												Nucleoplasm	hsa gene	0.131	
																												Nucleoplasm		0.088	
																												Nucleoplasm, Nucleoli fibrillar center	hsa gene	NA	
																												Nucleoplasm, Golgi apparatus, Centrosome, Cytosol	hsa gene	0.264	
																												Nucleoplasm	hsa gene	0.745	
																												Nucleoplasm, Cytosol		0.811	
																												Nucleoplasm		0.052	
																												none reported		0.191	
																												none reported		0.088	
																												Nucleoli		0.112	
																												gene/protein not avaialble in HPA list		0.144	
																												none reported	hsa gene	0.224	
																												Nucleoplasm		0.397	
																												Nucleoplasm, Cytosol		0.161	
																												Nucleoplasm, Nucleoli, Nucleoli rim, Nuclear bodies		0.033	
																												Nucleoplasm, Cytosol		0.197	
																												none reported		0.107	
																												Nucleoplasm	hsa gene	0.216	
																												Nucleoplasm, Nuclear bodies, Cytosol	hsa gene	0.164	
																												Nucleoplasm, Cytoplasmic bodies	hsa gene	0.848	
																												Nucleoplasm, Centrosome, Cytosol	hsa gene	0.375	
																												Nucleoplasm, Nuclear membrane, Cytosol	hsa gene	0.329	
																								2				Nucleoli, Cytosol		0.129	
																												Nucleoplasm		0.028	
																												Nucleoplasm	hsa gene	0.365	
																												Nucleoplasm, Cytosol		0.153	
																												Nuclear speckles, Plasma membrane		0.230	
																												Nucleoli fibrillar center, Endoplasmic reticulum, Vesicles		0.259	
																												Nucleoli fibrillar center, Endoplasmic reticulum, Vesicles		0.182	
																												Nucleoplasm, Cytosol	hsa gene	0.931	
																												Nucleoplasm	hsa gene	0.091	
																												none reported	hsa gene	0.047	
																												Microtubules		0.020	
																												Nucleoplasm		0.486	
																												Nucleoplasm, Nuclear bodies		0.009	
																												Nucleoplasm, Nucleoli, Cytosol		0.042	
																												Nucleoplasm		0.084	
																												Nucleoplasm		0.018	
																												Nucleoplasm		0.733	
																												Nucleoplasm, Nucleoli	hsa gene	1.000	
																												Nucleoplasm		0.386	
																												Nucleoplasm, Nuclear membrane		0.101	
																												Nucleoplasm	hsa gene	0.980	
																												none reported		0.054	
																												Nucleoplasm, Cytosol		0.278	
																												Nucleoli, Plasma membrane, Microtubules, Cytosol		0.424	
																												Nucleoplasm, Nuclear membrane		0.433	
																												Nucleoli, Nucleoli rim, Intermediate filaments		0.051	
																												Centriolar satellite, Cytosol	hsa gene	0.703	
																												Nucleoplasm	hsa gene	NA	
																												Nuclear bodies		0.806	
																												Nucleoli fibrillar center, Microtubules		0.080	
																												Nucleoplasm, Nuclear speckles		0.023	

																												Nucleoplasm, Nucleoli fibrillar center		0.279	
																												Nucleoplasm		0.070	
																												Nucleoplasm	hsa gene	0.120	
																												Nucleoplasm	hsa gene	0.058	
																												Nucleoplasm		0.049	
																												none reported		0.087	
																												none reported		0.199	
																												none reported		0.244	
																												Nucleoplasm		0.016	
																												Nuclear bodies	hsa gene	0.058	
																												Nucleoplasm, Cytosol	hsa gene	0.201	
																												Nucleoplasm		0.044	
																												Nucleoplasm, Microtubules, Cytosol		0.252	
																												Nucleoplasm		0.105	
																												Nuclear speckles		0.074	
																												Nuclear membrane, Vesicles		0.074	
																												Nucleoplasm, Centriolar satellite		0.294	
																												Nucleoplasm, Nucleoli	hsa gene	0.722	
																												Nucleoplasm, Cytosol	hsa gene	NA	
																												Nucleoplasm	hsa gene	0.175	
																												none reported	hsa gene	0.026	
																												Nucleoplasm		0.214	
																												Nucleoplasm, Nucleoli, Golgi apparatus		0.063	
																												none reported	hsa gene	0.120	
																												Nucleoplasm		0.007	
																												none reported		0.048	
																												Nucleoplasm, Cytosol		0.030	
																												Nucleoli		0.327	
																												Nucleoplasm, Nuclear membrane, Cytosol		0.444	
																												none reported	hsa gene	0.289	
												1																none reported	hsa gene	0.543	
																												none reported	hsa gene	0.038	
																												none reported		0.068	
																												none reported		0.069	
																												Nucleoplasm, Cytosol	hsa gene	0.498	
																												Nucleoplasm, Cell Junctions		0.206	
												1																Nucleoplasm, Cytosol		0.494	
																												Endoplasmic reticulum		0.019	
																												none reported		0.021	
																												none reported		0.105	
																												Nucleoplasm, Nuclear bodies		0.050	
																												Nucleoplasm	hsa gene	0.945	
																												Nucleoplasm	hsa gene	0.518	
																												none reported		0.016	
																												Nuclear speckles, Cytosol	hsa gene	0.042	
																												Nucleoplasm	hsa gene	0.037	
																												Vesicles	hsa gene	0.027	
																												none reported		0.032	
																												Nucleoplasm, Vesicles, Cytosol		0.095	
																												Nucleoplasm		0.093	
																												Nucleoplasm	hsa gene	0.031	
																												Nucleoplasm, Cytosol		0.183	
																												none reported	hsa gene	0.177	
																												Nucleoplasm, Cytosol		0.261	
																												Nucleoplasm, Cytosol		0.013	
																												Vesicles, Microtubules		0.031	
																												Nucleoplasm, Nuclear speckles	hsa gene	0.015	
																												Nucleoplasm, Nucleoli	hsa gene	0.071	
																												none reported		0.060	
																												Nucleoplasm		0.039	
																												Nucleoplasm, Intermediate filaments		0.013	
																												none reported		0.127	
																												Nucleoplasm, Cytosol	hsa gene	0.008	

																												Nucleoplasm, Nuclear speckles		0.026	
																												Nucleoplasm	hsa gene	0.199	
																												Nucleoplasm		0.327	
																												Vesicles	hsa gene	0.316	
																												Golgi apparatus	hsa gene	0.118	
																												Nucleoplasm, Nuclear bodies		0.444	
																												Nucleoplasm		0.145	
																												Nucleoplasm, Cytosol		0.056	
																												Nucleoplasm		0.148	
																												Nucleoplasm, Golgi apparatus, Vesicles		0.040	
																												Nucleoli		0.234	
																												Nucleoplasm		0.076	
																												Nucleoplasm, Nucleoli, Plasma membrane	hsa gene	0.030	
																												Nuclear speckles	hsa gene	0.037	
																												Nucleoplasm, Nucleoli fibrillar center, Cytosol		0.271	
																												Nucleoplasm, Golgi apparatus	hsa gene	0.626	
																												Nucleoplasm, Nucleoli	hsa gene	0.008	
																												Nucleoplasm, Vesicles, Cytosol	hsa gene	0.700	
																												Nucleoplasm, Cytosol	hsa gene	0.054	
																												Nucleoli, Cytosol	hsa gene	0.015	
																												Nuclear speckles		0.275	
																												none reported		0.047	
																												Nucleoplasm, Cytoplasmic bodies		0.111	
																												Nucleoli fibrillar center, Vesicles		0.015	
																												Nucleoplasm		0.424	
																												Nucleoplasm	hsa gene	0.094	
																												none reported	hsa gene	0.140	
																												Nucleoli, Mitochondria		0.016	
																												Nucleoplasm, Plasma membrane, Cytosol		0.063	
																												none reported	hsa gene	0.254	
																												Nucleoplasm, Vesicles		0.049	
																												Nucleoplasm		0.224	
																												Nucleoplasm	hsa gene	0.067	
																												none reported		0.260	
																												Nucleoplasm, Vesicles		0.019	
																												Nucleoplasm, Vesicles, Cytosol		0.020	
																												Nucleoli, Mitochondria		0.008	
																												Centrosome		0.019	
																												Nucleoplasm, Intermediate filaments		0.274	
																												Centrosome	hsa gene	0.278	
																												Nuclear speckles	hsa gene	0.205	
																												Nucleoplasm		0.043	
																												Vesicles	hsa gene	0.265	
																												Nucleoplasm, Mitochondria		0.285	
																												Nucleoplasm, Nuclear membrane, Vesicles		0.021	
																												Nucleoplasm, Nucleoli		0.249	
																												Nucleoplasm, Nucleoli, Cytosol		0.521	
																												none reported		0.317	
																												none reported		0.015	
																												Nucleoplasm	hsa gene	NA	
																												Nucleoplasm		0.549	
												1																Nucleoplasm, Plasma membrane	mmu gene	0.350	
																												none reported		0.084	
																												Nucleoli fibrillar center, Cytosol		0.136	
																												Nuclear bodies, Cytosol	hsa gene	0.112	
																												Nucleoplasm		0.596	
																												Nuclear membrane		0.016	
																												Nucleoplasm	hsa gene	0.016	
																												none reported	hsa gene	0.028	
																												Nucleoplasm, Cytosol	hsa gene	0.011	
																												Nucleoplasm		0.293	
																												none reported	hsa gene	0.076	
																												none reported		0.027	

																												Nucleoplasm, Centrosome	hsa gene	0.009	
																												Nucleoplasm, Nucleoli, Cytosol		0.053	
																												none reported		0.077	
																												Nucleoplasm	hsa gene	0.163	
																												none reported		0.010	
																												Golgi apparatus		0.071	
																												none reported	hsa gene	0.244	
																												Nucleoplasm, Cytosol	hsa gene	0.273	
																												Nucleoplasm, Nucleoli fibrillar center		0.818	
																												Nucleoplasm, Nucleoli, Vesicles	hsa gene	0.829	
																												Nucleoplasm, Nucleoli	hsa gene	0.191	
																												Nuclear speckles		0.016	
																												Nucleoplasm, Nucleoli		0.535	
																												Mitochondria		0.046	
																												Cytosol		0.252	
																												Nucleoplasm, Centriolar satellite	hsa gene	0.112	
																												none reported		0.237	
																												gene/protein not avaialble in HPA list		NA	
																												none reported		0.095	
																												Nucleoplasm	hsa gene	0.402	
																												Nucleoplasm		0.123	
																												Nucleoplasm, Nucleoli	hsa gene	0.022	
																												Nucleoplasm, Mitochondria		0.451	
																												Nucleoli		0.197	
																												none reported		0.021	
																												none reported	hsa gene	NA	
																												Centriolar satellite, Cytosol		0.052	
																												none reported		0.004	
																												Nucleoplasm		0.050	
																												none reported		0.057	
																												none reported		0.028	
																												none reported		0.023	
																												Nucleoplasm		0.054	
																												none reported		0.030	
																												Nucleoplasm, Actin filaments	hsa gene	0.372	
													1															Nucleoplasm, Cytosol		0.528	
																												Centrosome, Cytosol	only mmu ortholog	0.474	
																												Nucleoplasm, Nuclear membrane		0.102	
																												Nucleoplasm, Nucleoli fibrillar center		0.008	
																												Nucleoplasm, Golgi apparatus		NA	
																												Nucleoplasm, Cytosol	hsa gene	0.638	
																												Nucleoplasm		0.070	
																												Cytosol		0.639	
																												none reported		0.105	
																												Centrosome		0.282	
																												Nucleoplasm, Vesicles	hsa gene	0.751	
																												Nucleoplasm, Nucleoli		0.250	
																												Nucleoplasm, Nucleoli	hsa gene	0.559	
																												Intermediate filaments, Cytosol	hsa gene	0.053	
																												Nucleoplasm, Cytosol		0.018	
																												Nucleoplasm	hsa gene	0.777	
																												Nucleoplasm, Centrosome		0.243	
																												Nucleoplasm, Centrosome		0.070	
																												gene/protein not avaialble in HPA list	hsa gene	0.067	
																												Nucleoplasm, Mitochondria		0.067	
																												Cytosol		0.552	
																												Nucleoplasm		0.471	
																												Nucleoplasm, Plasma membrane, Cytosol		0.446	
																												Nucleoli		0.021	
																												Nucleoplasm		0.810	
																												Nucleoplasm, Nucleoli, Cytosol		0.054	
																												Nucleoplasm, Centriolar satellite		0.261	
																												none reported		0.202	

Nucleoplasm		0.330	
none reported	hsa gene	0.255	
none reported	hsa gene	NA	
Vesicles		0.106	
Nucleoli rim	hsa gene	0.045	
none reported		0.154	
Nucleoplasm, Nuclear bodies	hsa gene	0.057	
Nucleoplasm		0.919	
Microtubules		0.042	
none reported	hsa gene	0.058	
Nucleoli		0.215	
Nucleoplasm		0.047	
none reported		0.017	
none reported		0.067	
none reported		0.055	
Actin filaments	hsa gene	0.130	
Nucleoplasm		0.106	
Cytosol		0.111	
Nucleoli		0.615	
Nucleoplasm, Nucleoli, Nucleoli rim	hsa gene	0.260	
Nucleoplasm, Mitochondria		NA	
Nucleoplasm, Nucleoli	hsa gene	0.085	
Nucleoplasm		0.019	
Nucleoplasm, Nucleoli fibrillar center, Intermediate filaments		0.029	
Nucleoplasm, Vesicles	hsa gene	0.021	
Nucleoplasm, Golgi apparatus		0.037	
none reported		0.121	
Nucleoplasm		0.194	
none reported		0.051	
none reported	hsa gene	0.050	
none reported		0.063	
Nucleoplasm, Centrosome	only mmu ortholog	0.199	1
Nucleoplasm, Nuclear bodies, Cytosol	hsa gene	0.381	
Nucleoplasm	hsa gene	0.329	
Nucleoplasm, Cytosol		0.304	
Endoplasmic reticulum	mmu gene	0.613	
Plasma membrane, Actin filaments, Cytosol	hsa gene	0.285	
none reported		0.109	
Nucleoplasm, Nucleoli fibrillar center		0.698	
Nucleoplasm, Nucleoli		NA	1
Nucleoplasm, Plasma membrane, Actin filaments		0.112	
Nucleoplasm, Cytosol		0.164	
Nucleoplasm, Cytosol		0.342	
Nucleoplasm, Cytosol	hsa gene	0.059	
none reported		0.124	
Nuclear speckles	hsa gene	0.191	
Nuclear speckles	hsa gene	0.060	
Nucleoplasm, Cytosol		0.282	
Nucleoplasm		NA	1
Nucleoplasm		NA	
Nucleoli		0.427	
Nucleoplasm, Nuclear bodies		0.953	1
Nucleoplasm, Nucleoli fibrillar center		0.932	1
Nucleoplasm	hsa gene	0.747	
Nucleoplasm	hsa gene	0.655	
Nucleoplasm, Cytosol		0.899	
Nucleoplasm	hsa gene	0.454	
Nucleoplasm	only mmu ortholog	0.798	
none reported	hsa gene	0.730	
Nucleoplasm	hsa gene	0.942	
none reported		0.768	
Nucleoplasm, Nuclear bodies, Mitochondria, Cytosol		0.735	1
Nucleoplasm	hsa gene	0.925	1

																												Nucleoplasm		0.872	1
																												Nucleoplasm, Cytosol	hsa gene	0.789	
																												Nucleoplasm, Nuclear bodies, Cytosol		0.850	1
												2																Nucleoplasm, Cytosol		0.815	
																												Nucleoplasm, Nuclear bodies	hsa gene	0.996	
																												Nucleoplasm, Plasma membrane, Cytosol		0.749	
																												none reported		0.947	
																												Nucleoplasm	hsa gene	0.644	
																												Nucleoplasm	hsa gene	0.839	
																												Nuclear speckles	only mmu ortholog	0.842	
																												Nucleoplasm	hsa gene	1.000	1
																												Nucleoplasm	hsa gene	0.132	1
																												Nucleoplasm, Nucleoli fibrillar center, Vesicles	hsa gene	0.531	
																												Nucleoplasm	hsa gene	NA	
																												Nucleoplasm, Nuclear bodies	hsa gene	0.492	
																												Nucleoplasm		0.797	
																												Nucleoplasm, Golgi apparatus	only mmu ortholog	0.803	1
																												Nucleoplasm		0.518	
																												Nucleoplasm, Nuclear bodies		0.476	1
																												none reported		0.111	
																												Nucleoplasm, Cytosol	hsa gene	0.750	1
																												Nucleoplasm, Nucleoli	hsa gene	0.949	
																												Nucleoli fibrillar center, Nuclear speckles	hsa gene	0.294	1
																												Nucleoplasm, Nucleoli, Nuclear speckles, Cytosol	hsa gene	0.877	
																												Nucleoplasm, Vesicles	hsa gene	0.608	
																												none reported	hsa gene	0.337	
																												Nucleoplasm, Vesicles	hsa gene	0.684	
																												Nucleoplasm	hsa gene	1.000	
																												Nucleoplasm	only mmu ortholog	0.964	
																												Nucleoplasm, Nuclear membrane		0.981	1
																												none reported		0.961	
																												Nucleoplasm, Cytosol	only mmu ortholog	0.964	
																												Nucleoplasm		0.646	
																												Nucleoplasm		0.520	
																												Nucleoplasm	hsa gene	0.946	
																												Vesicles		0.505	
																												Nucleoplasm, Nuclear bodies, Cytosol	hsa gene	0.941	
																												Nucleoplasm, Nuclear membrane, Cytosol	hsa gene	0.401	1
																												Nucleoplasm, Nuclear bodies		0.420	
																												none reported		0.462	
																												Nucleoplasm, Vesicles, Cytosol	hsa gene	0.240	
																												none reported		0.186	
																												Vesicles, Intermediate filaments	hsa gene	0.011	
																												Nucleoplasm		0.012	
																												Nucleoplasm, Nucleoli fibrillar center	hsa gene	0.502	
																												Nucleoplasm, Cytosol	hsa gene	0.121	
																												Mitochondria, Rods & Rings		0.026	
																												Nuclear speckles, Mitochondria	hsa gene	0.719	
																												Nucleoplasm		0.948	
																												Nucleoplasm, Cell Junctions, Cytosol	hsa gene	0.912	
																												Nucleoplasm		0.196	
																												Nucleoplasm		0.215	
																												Nucleoplasm	hsa gene	0.022	
																												Nucleoplasm, Plasma membrane	hsa gene	0.123	
																												Nucleoplasm, Nuclear membrane	mmu gene	0.939	
																												Nucleoplasm, Centrosome	mmu gene	0.687	
																												Nuclear speckles, Cytosol		0.164	
																												none reported		NA	
																												Nucleoplasm	hsa gene	0.881	
																												Nucleoplasm, Cytosol		0.062	
																												Nucleoplasm		0.745	
																												Nucleoplasm, Nuclear bodies, Vesicles	hsa gene	0.542	
																												Vesicles	hsa gene	0.049	

																												Nuclear speckles		0.122	
																												Nucleoplasm, Plasma membrane		0.546	
																												Nucleoplasm, Nucleoli		0.295	
																												Nucleoplasm	hsa gene	0.015	
																												Nucleoplasm		0.964	
												1																Nucleoplasm, Cytosol		0.213	
																												Nucleoplasm, Mitochondria, Cytosol		0.732	
																												Nucleoplasm	mmu gene	0.988	
																												Mitochondria	hsa gene	0.144	
																												Nucleoplasm, Cell Junctions		0.175	
																												Mitochondria	hsa gene	0.580	
																												Nucleoli, Cytosol		0.338	
																												Nucleoplasm, Vesicles, Plasma membrane	hsa gene	0.036	
																												Nucleoplasm		0.969	
																												Nucleoplasm		0.964	1
																												none reported	hsa gene	0.906	
																												Nucleoplasm, Cytosol		0.328	
																												Nucleoli		0.072	
																												Nucleoplasm	hsa gene	0.960	
												1																Nucleoplasm, Golgi apparatus, Vesicles	hsa gene	0.791	
																												Nucleoplasm, Golgi apparatus		0.863	
																												Nucleoplasm, Cell Junctions		0.870	
																												Nucleoplasm	hsa gene	0.259	
																												Nucleoplasm, Vesicles	hsa gene	0.574	
																												Nucleoplasm	hsa gene	0.658	
																												Nucleoplasm, Nucleoli		0.381	
																												Nucleoplasm	hsa gene	0.665	
																												Nucleoplasm, Nucleoli	hsa gene	0.094	
																												Nucleoplasm		0.019	
																												Nucleoplasm		0.960	
																												Nucleoplasm		0.609	
																												Nucleoplasm, Golgi apparatus	hsa gene	0.959	
																												Nucleoplasm		0.230	
																												Nucleoplasm, Plasma membrane, Cytosol		0.801	
																												Nucleoplasm, Microtubules, Cytokinetic bridge, Mitotic spindle	hsa gene	0.660	
																												Nucleoplasm		0.067	
																												Nucleoplasm, Nucleoli, Nucleoli rim	hsa gene	0.779	
																												Nucleoplasm		0.017	
																												Nucleoplasm		NA	
																												Nucleoplasm	hsa gene	0.992	
																												Nucleoplasm		0.988	
																												Nucleoplasm		NA	
																												Nucleoplasm, Nuclear bodies, Plasma membrane		0.067	
																												none reported	hsa gene	0.556	
																												Cytosol		0.025	
																												none reported		0.144	
																												Nucleoplasm, Nuclear bodies	hsa gene	0.093	
																												Nucleoplasm	hsa gene	0.727	
																												Nucleoplasm, Nuclear speckles, Nuclear bodies	hsa gene	0.505	
																												Nucleoplasm, Nucleoli, Mitochondria		0.453	
																												gene/protein not avaialble in HPA list		NA	
																												Nucleoplasm, Nuclear bodies		0.051	
																												Nucleoli, Nucleoli rim, Cytosol	hsa gene	0.606	
																												Nucleoplasm		0.117	
																												Nucleoplasm		0.232	
																												Nucleoplasm, Nuclear membrane, Centrosome		NA	
																												Nuclear bodies, Mitochondria, Cytosol		0.096	
																												Nucleoplasm, Plasma membrane		0.075	
																												Nucleoplasm	hsa gene	0.519	
																												Nucleoplasm, Centriolar satellite		0.798	
																												Nuclear bodies		0.418	
																												Nucleoplasm, Nucleoli fibrillar center		0.936	
																												none reported		NA	

																												Nucleoplasm, Vesicles		0.145	
																												Nucleoplasm	hsa gene	0.761	
																												Nucleoplasm	hsa gene	0.404	
																												Nucleoplasm		0.932	1
																												Mitochondria		0.777	
																												Nuclear speckles, Focal adhesion sites		0.546	
																												Nucleoplasm, Nucleoli fibrillar center	hsa gene	0.072	
																												Nucleoplasm, Centrosome		0.063	
																												Nucleoplasm	hsa gene	0.671	
																												Nucleoplasm, Cytosol	hsa gene	0.649	
																												Nucleoplasm, Golgi apparatus, Vesicles	hsa gene	0.738	
																												Nucleoplasm	hsa gene	0.933	
																												Nucleoplasm, Cytosol	hsa gene	0.435	
																												Nucleoli fibrillar center	hsa gene	0.294	
																												Nucleoplasm, Cytosol		0.524	
																												Nucleoplasm, Nuclear bodies		0.002	
																												Nucleoplasm	hsa gene	0.342	
																												none reported		0.022	
																												Nucleoplasm, Cytosol		0.446	
																												Nucleoplasm, Plasma membrane, Cytosol		0.745	
																												Nuclear speckles, Golgi apparatus		0.077	
																												Intermediate filaments, Cytosol		0.185	
																												none reported		0.018	
																												Nucleoplasm	hsa gene	0.516	
																												Nucleoplasm, Mitochondria		0.479	
																												Nucleoplasm, Cytosol	hsa gene	0.957	
																												Nucleoplasm, Nucleoli, Golgi apparatus, Cytosol	hsa gene	0.339	
																												Nucleoplasm, Cytosol		NA	
																												Nucleoplasm, Plasma membrane, Cell Junctions		0.754	
																												Nucleoplasm		0.071	
																												none reported		0.060	
																												Nucleoplasm		0.087	
																												Nucleoplasm		0.721	
																												none reported		0.162	
																												Plasma membrane	hsa gene	0.473	
																												none reported	hsa gene	0.172	
																												none reported		0.197	
																												gene/protein not avaialble in HPA list		0.152	
																												none reported		NA	
																												Nucleoli fibrillar center		0.044	
																												none reported		NA	
																												none reported		NA	
																												Nucleoplasm, Nucleoli fibrillar center		0.101	
																												Plasma membrane, Cytosol		0.566	
																												Nucleoplasm, Nuclear bodies		0.251	
																												Nucleoplasm, Nuclear speckles		0.963	
																												Nucleoplasm		0.923	
																												none reported	hsa gene	0.285	
																												Nucleoplasm	hsa gene	0.154	
																												Nucleoplasm, Nucleoli	hsa gene	0.891	
																												none reported	hsa gene	NA	
																												Nucleoplasm	hsa gene	0.071	
																												Nucleoplasm, Plasma membrane	hsa gene	0.595	
																												Nucleoplasm	hsa gene	NA	
																												Nucleoplasm	hsa gene	0.870	
														1														Nucleoplasm	hsa gene	0.876	1
																												Nuclear bodies	hsa gene	0.916	1
																												Nucleoplasm, Cytosol		0.285	
																												Nucleoplasm		0.935	
																												Nucleoplasm		0.590	1
																												Nucleoplasm	only mmu ortholog	0.925	1
																												Nucleoplasm	only mmu ortholog	0.996	
																												Nucleoplasm, Cytosol	only mmu ortholog	0.988	

																												Nucleoplasm	hsa gene	0.996	
																												Nucleoplasm, Vesicles		0.297	
																												Nucleoplasm, Nuclear bodies		0.982	
																												Nucleoplasm		0.879	1
																												Nucleoplasm, Vesicles		0.188	
													1															Cytosol		NA	
				1									2	1			1											Nucleoplasm, Vesicles	hsa gene	0.845	
														1					1									Nucleoplasm, Vesicles		0.302	
																												Nucleoplasm, Nucleoli fibrillar center, Microtubules, Cytokinetic bridge		0.985	1
																												Nucleoplasm, Cytosol		0.439	
																												Nucleoplasm		0.794	
														1														Nucleoplasm, Mitochondria, Cytosol	hsa gene	0.937	1
																												Nucleoplasm		0.723	1
																												none reported		0.278	
																												Nuclear bodies		0.962	1
																												Nucleoplasm		0.726	
																												Nucleoplasm, Vesicles		0.945	
																												Nucleoplasm, Cytosol		0.874	
																												Nucleoplasm		0.749	
																												Nucleoplasm		0.497	
																												Nucleoplasm		0.656	
																												Nucleoplasm		0.892	1
																												none reported		0.997	
																												Nucleoplasm, Vesicles		0.498	
																												Nucleoplasm, Nucleoli, Actin filaments, Cytosol		0.778	
																												Nucleoplasm		0.998	
																												Nucleoplasm	only mmu ortholog	0.978	
																												Nucleoli		0.908	
																												Nucleoplasm		0.912	1
																												Nuclear bodies		0.904	
																												Cytosol	only mmu ortholog	0.988	1
																												Nuclear bodies		0.994	
																												Nucleoplasm, Intermediate filaments, Cytosol		0.602	
																												Nucleoplasm		0.880	
																												Nucleoplasm, Cytokinetic bridge, Mitotic spindle, Cytosol		0.250	1
																												Nucleoplasm, Cell Junctions, Cytosol		0.653	
													1	1														Nucleoplasm	hsa gene	0.969	
																												Nuclear bodies		0.963	1
																												none reported		NA	
																												Nucleoplasm, Cytosol	only mmu ortholog	0.925	1
																												Nucleoplasm, Cytosol	hsa gene	0.860	
																								1				Nuclear speckles, Cytosol	hsa gene	0.336	
																												Nucleoplasm, Golgi apparatus	hsa gene	0.939	
																												Nucleoplasm, Nucleoli, Cytosol	hsa gene	0.999	
																												Nucleoplasm, Nuclear bodies		0.904	
																												Nucleoplasm, Nuclear membrane, Cytosol		0.698	1
																												Nuclear speckles		0.877	1
																												none reported		0.813	1
																												Nucleoplasm, Cytosol		0.996	
																												Nuclear speckles	mmu gene	0.859	
																												Nucleoplasm		0.935	
																												Nuclear speckles, Vesicles, Centriolar satellite	hsa gene	NA	
																												Nucleoplasm, Vesicles		0.603	
																												none reported	hsa gene	0.622	1
												1																Nucleoli, Cytosol, Rods & Rings	hsa gene	0.441	
																												Nucleoplasm, Nucleoli, Cytosol	hsa gene	0.695	
																												Nucleoplasm, Vesicles	hsa gene	0.999	
																												Nuclear speckles	hsa gene	0.999	
																												Nucleoplasm, Cytosol	hsa gene	0.791	1
																												Nucleoplasm	hsa gene	0.497	1
																												Nucleoplasm	hsa gene	0.958	
																												Nuclear speckles	hsa gene	0.634	
													1		1													Nucleoplasm	hsa gene	NA	

Nucleoplasm, Vesicles	hsa gene	1.000	1
Nucleoplasm	hsa gene	0.490	
none reported		0.131	
Nucleoplasm, Nucleoli, Vesicles, Centriolar satellite		0.079	
none reported		0.046	
Vesicles		0.100	
Nucleoplasm, Mitochondria		0.985	
none reported		0.355	
Nucleoplasm, Intermediate filaments		NA	
none reported		0.464	
Microtubules		0.998	1
Nucleoplasm		0.711	
Nucleoplasm		0.991	
Nucleoplasm, Centrosome		0.981	
Nucleoplasm, Cytosol		0.851	
Nucleoplasm, Mitochondria		0.958	1
Mitochondria	hsa gene	0.791	
Nucleoplasm, Nucleoli, Plasma membrane, Cytosol	hsa gene	0.919	
Nucleoplasm	only mmu ortholog	0.150	
