# Supplementary material for: Foxg1 and companions: Not only transcription factors
Source: Neural Regen Res. 2025 Sep 3;21(8):3426–38. doi: 10.4103/NRR.NRR-D-25-00439 (PMC13452659; doi:10.4103/NRR.NRR-D-25-00439)
Supplement: Supplementary file 3 [file NRR-21-3426_Suppl11.pdf]

Additional Table 6.2. Full TF interact grid_statistical analysis

TF-taxon	TFs	n° of interaction reports involving TF and … [real values]					n° of interaction reports involving TF and … [real values]										
		TFs	TcoFs	SplFs	pAFs	TlatMFs	SplF.core	SplF.RBP	SplF.other	pAF.4C	pAF.PA	TlatMF.EIF	TlatMF.EIcoF	TlatMF.EEF	TlatMF.cytoRP	TlatMF.mitoRP	TlatMF.aatRNAs ymth
ARID	ARID1A	127	53	54	0	0	5	8	41	0	0	0	0	0	0	0	0
	ARID1B	101	36	26	1	0	2	1	23	1	0	0	0	0	0	0	0
	ARID2	84	30	15	0	0	1	3	11	0	0	0	0	0	0	0	0
	ARID3A	67	10	4	0	2	1	2	1	0	0	1	0	0	1	0	0
	ARID3B	63	9	5	0	0	2	0	3	0	0	0	0	0	0	0	0
	ARID4A	20	37	7	0	0	0	0	7	0	0	0	0	0	0	0	0
	ARID4B	82	45	16	0	1	1	2	13	0	0	0	0	0	0	1	0
	ARID5A	33	11	1	0	0	0	1	0	0	0	0	0	0	0	0	0
	ARID5B	21	22	14	0	0	0	4	10	0	0	0	0	0	0	0	0
	JARID2	24	33	30	0	0	1	2	27	0	0	0	0	0	0	0	0
	ARID3C	4	1	0	0	0	0	0	0	0	0	0	0	0	0	0	0
bHLH	TCF25	4	22	3	0	3	2	0	1	0	0	0	0	0	2	1	0
	TCF12	110	21	8	0	0	1	4	3	0	0	0	0	0	0	0	0
	TCF3	165	43	23	0	14	2	6	15	0	0	0	0	1	8	5	0
	TCF4	143	99	10	0	3	3	2	5	0	0	1	1	0	0	1	0
	ASCL1	13	3	1	0	0	0	0	1	0	0	0	0	0	0	0	0
	ASCL2	1	2	1	0	0	0	0	1	0	0	0	0	0	0	0	0
	ASCL3	14	0	0	0	0	0	0	0	0	0	0	0	0	0	0	0
	ASCL4	11	2	0	0	0	0	0	0	0	0	0	0	0	0	0	0
	ASCL5	0	2	0	0	0	0	0	0	0	0	0	0	0	0	0	0
	MYF5	16	6	0	0	0	0	0	0	0	0	0	0	0	0	0	0
	MYF6	10	3	0	0	0	0	0	0	0	0	0	0	0	0	0	0
	MYOD1	94	58	38	1	0	2	1	35	0	1	0	0	0	0	0	0
	MYOG	25	12	3	1	0	0	0	3	1	0	0	0	0	0	0	0
	ATOH1	4	11	1	0	3	0	1	0	0	0	0	0	0	2	0	1
	ATOH7	1	0	0	0	0	0	0	0	0	0	0	0	0	0	0	0
	ATOH8	1	0	0	0	0	0	0	0	0	0	0	0	0	0	0	0
	BHLHA15	16	15	5	0	22	2	3	0	0	0	2	0	0	14	6	0
	BHLHA9	0	1	1	0	0	1	0	0	0	0	0	0	0	0	0	0
	BHLHE22	0	1	0	0	0	0	0	0	0	0	0	0	0	0	0	0
	BHLHE23	0	3	0	0	0	0	0	0	0	0	0	0	0	0	0	0
	FERD3L	11	2	0	0	0	0	0	0	0	0	0	0	0	0	0	0
	FIGLA	14	0	0	0	0	0	0	0	0	0	0	0	0	0	0	0
	HAND1	9	2	0	0	0	0	0	0	0	0	0	0	0	0	0	0
	HAND2	16	8	1	0	0	0	0	1	0	0	0	0	0	0	0	0
	LYL1	8	3	0	0	0	0	0	0	0	0	0	0	0	0	0	0
	MESP1	4	3	3	0	0	0	0	3	0	0	0	0	0	0	0	0
	MESP2	9	1	0	0	0	0	0	0	0	0	0	0	0	0	0	0
	MSC	8	4	1	0	0	0	1	0	0	0	0	0	0	0	0	0
	MSGN1	11	2	0	0	0	0	0	0	0	0	0	0	0	0	0	0
	NEUROD1	21	2	5	0	0	0	0	5	0	0	0	0	0	0	0	0
	NEUROD2	1	3	0	0	0	0	0	0	0	0	0	0	0	0	0	0
	NEUROD4	3	1	0	0	0	0	0	0	0	0	0	0	0	0	0	0
	NEUROD6	0	1	0	0	0	0	0	0	0	0	0	0	0	0	0	0
	NEUROG1	8	1	0	0	0	0	0	0	0	0	0	0	0	0	0	0
	NEUROG2	2	1	1	0	0	0	0	1	0	0	0	0	0	0	0	0
	NEUROG3	31	23	6	0	10	4	2	0	0	0	1	0	0	9	0	0
	NHLH2	13	0	0	0	0	0	0	0	0	0	0	0	0	0	0	0
	OLIG1	6	4	0	0	1	0	0	0	0	0	0	0	0	0	1	0
	OLIG2	9	1	1	0	0	0	1	0	0	0	0	0	0	0	0	0
	OLIG3	8	6	1	0	0	1	0	0	0	0	0	0	0	0	0	0
	PTF1A	4	3	3	0	0	0	0	3	0	0	0	0	0	0	0	0
	SCX	5	0	0	0	0	0	0	0	0	0	0	0	0	0	0	0
	TAL1	45	65	28	0	4	2	0	26	0	0	0	0	1	3	0	0
	TAL2	7	4	0	0	0	0	0	0	0	0	0	0	0	0	0	0
	TCF15	5	3	1	0	0	0	0	1	0	0	0	0	0	0	0	0
	TCF21	13	1	0	0	0	0	0	0	0	0	0	0	0	0	0	0
	TCF23	7	1	2	0	0	1	1	0	0	0	0	0	0	0	0	0
	TWIST1	43	31	16	0	0	0	1	15	0	0	0	0	0	0	0	0
	TWIST2	19	3	1	0	0	0	0	1	0	0	0	0	0	0	0	0
	BHLHE40	22	31	6	0	0	2	3	1	0	0	0	0	0	0	0	0
	BHLHE41	16	10	4	0	0	0	1	3	0	0	0	0	0	0	0	0
	HELT	1	1	0	0	0	0	0	0	0	0	0	0	0	0	0	0
	HES2	0	0	0	0	0	0	0	0	0	0	0	0	0	0	0	0
	HES3	0	2	0	0	0	0	0	0	0	0	0	0	0	0	0	0
	HES4	0	3	0	0	0	0	0	0	0	0	0	0	0	0	0	0
	HES5	2	1	0	0	0	0	0	0	0	0	0	0	0	0	0	0
	HES6	11	15	4	0	0	0	2	2	0	0	0	0	0	0	0	0
	HES7	0	0	1	0	0	0	1	0	0	0	0	0	0	0	0	0
	HEY1	26	23	22	1	20	11	10	1	0	1	0	0	0	20	0	0
	HEY2	15	14	6	0	0	1	0	5	0	0	0	0	0	0	0	0
	HEYL	6	6	0	0	0	0	0	0	0	0	0	0	0	0	0	0
	AHR	66	40	7	0	3	2	1	4	0	0	1	0	0	0	1	1
	AHRR	4	6	3	0	0	0	1	2	0	0	0	0	0	0	0	0
	ARNT	208	59	12	0	7	6	3	3	0	0	0	0	0	7	0	0
	ARNT2	31	12	1	0	0	1	0	0	0	0	0	0	0	0	0	0
	ARNTL	47	29	7	0	0	0	1	6	0	0	0	0	0	0	0	0
	ARNTL2	14	6	1	0	0	0	1	0	0	0	0	0	0	0	0	0
	CLOCK	42	15	6	0	0	0	1	5	0	0	0	0	0	0	0	0
	EPAS1	61	98	19	0	0	2	3	14	0	0	0	0	0	0	0	0
	HIF1A	190	465	128	5	2	9	11	108	4	1	0	0	0	0	1	1
	HIF3A	9	2	0	0	0	0	0	0	0	0	0	0	0	0	0	0
	NCOA1	271	29	19	0	0	3	1	15	0	0	0	0	0	0	0	0
	NPAS1	20	29	7	0	1	2	3	2	0	0	1	0	0	0	0	0
	NPAS2	28	5	6	0	1	1	1	4	0	0	0	0	0	1	0	0
	NPAS3	5	0	0	0	0	0	0	0	0	0	0	0	0	0	0	0
	NPAS4	5	2	0	0	0	0	0	0	0	0	0	0	0	0	0	0
	SIM1	8	3	1	0	0	0	0	1	0	0	0	0	0	0	0	0
	SIM2	25	10	2	0	0	1	1	0	0	0	0	0	0	0	0	0
	SOHLH1	3	13	2	0	0	1	0	1	0	0	0	0	0	0	0	0
	SOHLH2	0	0	0	0	0	0	0	0	0	0	0	0	0	0	0	0
	TCFL5	3	1	0	0	0	0	0	0	0	0	0	0	0	0	0	0
	MAX	232	104	29	1	6	2	4	23	0	1	0	0	1	3	2	0
	MGA	77	74	20	0	1	1	1	18	0	0	0	0	0	1	0	0
	MITF	32	25	10	0	0	0	4	6	0	0	0	0	0	0	0	0
	MLX	44	12	2	0	0	0	2	0	0	0	0	0	0	0	0	0
	MLXIP	7	8	0	0	0	0	0	0	0	0	0	0	0	0	0	0
	MLXIPL	16	12	8	0	0	0	1	7	0	0	0	0	0	0	0	0
	MNT	31	10	4	0	0	0	2	2	0	0	0	0	0	0	0	0
	MXD1	30	40	4	0	0	0	0	4	0	0	0	0	0	0	0	0
	MXD3	20	14	3	0	0	3	0	0	0	0	0	0	0	0	0	0
	MXD4	12	3	0	0	0	0	0	0	0	0	0	0	0	0	0	0
	MXI1	18	23	6	0	0	0	0	6	0	0	0	0	0	0	0	0
	MYC	456	961	585	28	375	222	219	144	19	9	46	7	9	215	95	3
	MYCL	9	33	4	0	0	0	0	4	0	0	0	0	0	0	0	0
	MYCN	120	192	210	9	169	84	82	44	7	2	7	5	2	75	78	2
	SREBF1	30	85	19	0	0	0	0	19	0	0	0	0	0	0	0	0
	SREBF2	27	21	5	0	1	0	2	3	0	0	0	0	0	0	1	0
	TFAP4	69	52	12	0	0	0	2	10	0	0	0	0	0	0	0	0
	TFE3	28	15	1	0	2	0	1	0	0	0	1	0	0	1	0	0
	TFEB	15	6	2	0	0	0	0	2	0	0	0	0	0	0	0	0
	TFEC	8	0	2	0	0	0	2	0	0	0	0	0	0	0	0	0
	USF1	56	27	14	1	0	1	3	10	1	0	0	0	0	0	0	0
	USF2	33	5	2	0	0	0	1	1	0	0	0	0	0	0	0	0
	ID1	45	18	5	0	0	3	1	1	0	0	0	0	0	0	0	0
	ID2	50	30	10	1	0	3	2	5	1	0	0	0	0	0	0	0
	ID3	40	11	2	0	0	1	0	1	0	0	0	0	0	0	0	0
	ID4	23	1	0	0	0	0	0	0	0	0	0	0	0	0	0	0
	TFAP2A	36	44	11	0	0	1	3	7	0	0	0	0	0	0	0	0
	TFAP2B	10	5	0	0	0	0	0	0	0	0	0	0	0	0	0	0
	TFAP2C	8	14	4	0	0	0	1	3	0	0	0	0	0	0	0	0
	TFAP2D	10	4	0	0	0	0	0	0	0	0	0	0	0	0	0	0
	TFAP2E	0	0	0	0	0	0	0	0	0	0	0	0	0	0	0	0

TFs	n° of interaction reports involving TF and … [expected values]										
	SplF.core	SplF.RBP	SplF.other	pAF.4C	pAF.PA	TlatMF.EIF	TlatMF.EIcoF	TlatMF.EEF	TlatMF.cytoRP	TlatMF.mitoRP	TlatMF.aatRNAs ymth
ARID1A	12.81	14.60	11.57	0.97	0.37	3.34	0.71	0.62	11.63	6.41	0.83
ARID1B	9.07	10.34	8.20	0.69	0.26	2.36	0.51	0.44	8.24	4.54	0.59
ARID2	7.65	8.72	6.91	0.58	0.22	1.99	0.43	0.37	6.94	3.83	0.50
ARID3A	4.94	5.63	4.46	0.38	0.14	1.29	0.28	0.24	4.49	2.47	0.32
ARID3B	4.76	5.42	4.30	0.36	0.14	1.24	0.26	0.23	4.32	2.38	0.31
ARID4A	3.73	4.25	3.37	0.28	0.11	0.97	0.21	0.18	3.39	1.87	0.24
ARID4B	7.83	8.92	7.08	0.60	0.23	2.04	0.44	0.38	7.11	3.92	0.51
ARID5A	5.31	6.05	4.79	0.40	0.15	1.38	0.30	0.25	4.82	2.66	0.34
ARID5B	3.00	3.42	2.71	0.23	0.09	0.78	0.17	0.14	2.72	1.50	0.19
JARID2	3.81	4.34	3.44	0.29	0.11	0.99	0.21	0.18	3.46	1.91	0.25
ARID3C	0.37	0.42	0.33	0.03	0.01	0.10	0.02	0.02	0.33	0.18	0.02
TCF25	4.28	4.88	3.87	0.33	0.12	1.12	0.24	0.21	3.89	2.14	0.28
TCF12	7.83	8.92	7.08	0.60	0.23	2.04	0.44	0.38	7.11	3.92	0.51
TCF3	11.45	13.05	10.35	0.87	0.33	2.98	0.64	0.55	10.40	5.74	0.74
TCF4	17.49	19.93	15.81	1.33	0.51	4.56	0.97	0.84	15.88	8.76	1.13
ASCL1	1.50	1.71	1.36	0.11	0.04	0.39	0.08	0.07	1.36	0.75	0.10
ASCL2	0.37	0.42	0.33	0.03	0.01	0.10	0.02	0.02	0.33	0.18	0.02
ASCL3	0.70	0.79	0.63	0.05	0.02	0.18	0.04	0.03	0.63	0.35	0.05
ASCL4	0.73	0.83	0.66	0.06	0.02	0.19	0.04	0.04	0.66	0.37	0.05
ASCL5	0.07	0.08	0.07	0.01	0.00	0.02	0.00	0.00	0.07	0.04	0.00
MYF5	1.24	1.42	1.12	0.09	0.04	0.32	0.07	0.06	1.13	0.62	0.08
MYF6	1.02	1.17	0.93	0.08	0.03	0.27	0.06	0.05	0.93	0.51	0.07
MYOD1	8.93	10.17	8.07	0.68	0.26	2.33	0.50	0.43	8.11	4.47	0.58
MYOG	3.33	3.79	3.01	0.25	0.10	0.87	0.19	0.16	3.02	1.67	0.22
ATOH1	3.00	3.42	2.71	0.23	0.09	0.78	0.17	0.14	2.72	1.50	0.19
ATOH7	0.04	0.04	0.03	0.00	0.00	0.01	0.00	0.00	0.03	0.02	0.00
ATOH8	0.07	0.08	0.07	0.01	0.00	0.02	0.00	0.00	0.07	0.04	0.00
BHLHA15	3.59	4.09	3.24	0.27	0.10	0.93	0.20	0.17	3.26	1.80	0.23
BHLHA9	0.44	0.50	0.40	0.03	0.01	0.11	0.02	0.02	0.40	0.22	0.03
BHLHE22	0.07	0.08	0.07	0.01	0.00	0.02	0.00	0.00	0.07	0.04	0.00
BHLHE23	0.73	0.83	0.66	0.06	0.02	0.19	0.04	0.04	0.66	0.37	0.05
FERD3L	0.99	1.13	0.89	0.08	0.03	0.26	0.06	0.05	0.90	0.49	0.06
FIGLA	0.70	0.79	0.63	0.05	0.02	0.18	0.04	0.03	0.63	0.35	0.05
HAND1	0.70	0.79	0.63	0.05	0.02	0.18	0.04	0.03	0.63	0.35	0.05
HAND2	1.43	1.63	1.29	0.11	0.04	0.37	0.08	0.07	1.30	0.71	0.09
LYL1	0.55	0.63	0.50	0.04	0.02	0.14	0.03	0.03	0.50	0.27	0.04
MESP1	0.40	0.46	0.36	0.03	0.01	0.10	0.02	0.02	0.37	0.20	0.03
MESP2	0.48	0.54	0.43	0.04	0.01	0.12	0.03	0.02	0.43	0.24	0.03
MSC	0.59	0.67	0.53	0.04	0.02	0.15	0.03	0.03	0.53	0.29	0.04
MSGN1	1.32	1.50	1.19	0.10	0.04	0.34	0.07	0.06	1.20	0.66	0.09
NEUROD1	1.61	1.83	1.45	0.12	0.05	0.42	0.09	0.08	1.46	0.81	0.10
NEUROD2	0.44	0.50	0.40	0.03	0.01	0.11	0.02	0.02	0.40	0.22	0.03
NEUROD4	0.37	0.42	0.33	0.03	0.01	0.10	0.02	0.02	0.33	0.18	0.02
NEUROD6	0.15	0.17	0.13	0.01	0.00	0.04	0.01	0.01	0.13	0.07	0.01
NEUROG1	0.73	0.83	0.66	0.06	0.02	0.19	0.04	0.04	0.66	0.37	0.05
NEUROG2	0.15	0.17	0.13	0.01	0.00	0.04	0.01	0.01	0.13	0.07	0.01
NEUROG3	7.13	8.13	6.45	0.54	0.21	1.86	0.40	0.34	6.48	3.57	0.46
NHLH2	1.02	1.17	0.93	0.08	0.03	0.27	0.06	0.05	0.93	0.51	0.07
OLIG1	1.24	1.42	1.12	0.09	0.04	0.32	0.07	0.06	1.13	0.62	0.08
OLIG2	0.80	0.92	0.73	0.06	0.02	0.21	0.04	0.04	0.73	0.40	0.05
OLIG3	1.32	1.50	1.19	0.10	0.04	0.34	0.07	0.06	1.20	0.66	0.09
PTF1A	0.66	0.75	0.60	0.05	0.02	0.17	0.04	0.03	0.60	0.33	0.04
SCX	0.26	0.29	0.23	0.02	0.01	0.07	0.01	0.01	0.23	0.13	0.02
TAL1	6.22	7.09	5.62	0.47	0.18	1.62	0.35	0.30	5.65	3.12	0.40
TAL2	0.48	0.54	0.43	0.04	0.01	0.12	0.03	0.02	0.43	0.24	0.03
TCF15	0.48	0.54	0.43	0.04	0.01	0.12	0.03	0.02	0.43	0.24	0.03
TCF21	0.70	0.79	0.63	0.05	0.02	0.18	0.04	0.03	0.63	0.35	0.05
TCF23	0.51	0.58	0.46	0.04	0.01	0.13	0.03	0.02	0.47	0.26	0.03
TWIST1	4.61	5.25	4.17	0.35	0.13	1.20	0.26	0.22	4.19	2.31	0.30
TWIST2	0.95	1.08	0.86	0.07	0.03	0.25	0.05	0.05	0.86	0.48	0.06
BHLHE40	5.31	6.05	4.79	0.40	0.15	1.38	0.30	0.25	4.82	2.66	0.34
BHLHE41	1.65	1.88	1.49	0.13	0.05	0.43	0.09	0.08	1.50	0.82	0.11
HELT	0.44	0.50	0.40	0.03	0.01	0.11	0.02	0.02	0.40	0.22	0.03
HES2	0.11	0.13	0.10	0.01	0.00	0.03	0.01	0.01	0.10	0.05	0.01
HES3	0.15	0.17	0.13	0.01	0.00	0.04	0.01	0.01	0.13	0.07	0.01
HES4	0.84	0.96	0.76	0.06	0.02	0.22	0.05	0.04	0.76	0.42	0.05
HES5	0.11	0.13	0.10	0.01	0.00	0.03	0.01	0.01	0.10	0.05	0.01
HES6	1.94	2.21	1.75	0.15	0.06	0.51	0.11	0.09	1.76	0.97	0.13
HES7	0.33	0.38	0.30	0.03	0.01	0.09	0.02	0.02	0.30	0.16	0.02
HEY1	5.93	6.76	5.36	0.45	0.17	1.54	0.33	0.28	5.38	2.97	0.38
HEY2	2.45	2.79	2.22	0.19	0.07	0.64	0.14	0.12	2.23	1.23	0.16
HEYL	1.32	1.50	1.19	0.10	0.04	0.34	0.07	0.06	1.20	0.66	0.09
AHR	8.27	9.42	7.47	0.63	0.24	2.15	0.46	0.40	7.51	4.14	0.54
AHRR	0.40	0.46	0.36	0.03	0.01	0.10	0.02	0.02	0.37	0.20	0.03
ARNT	15.48	17.64	13.99	1.18	0.45	4.03	0.86	0.74	14.05	7.75	1.00
ARNT2	3.77	4.30	3.41	0.29	0.11	0.98	0.21	0.18	3.42	1.89	0.24
ARNTL	5.53	6.30	4.99	0.42	0.16	1.44	0.31	0.27	5.02	2.77	0.36
ARNTL2	1.46	1.67	1.32	0.11	0.04	0.38	0.08	0.07	1.33	0.73	0.09
CLOCK	4.68	5.34	4.23	0.36	0.14	1.22	0.26	0.22	4.25	2.35	0.30
EPAS1	8.89	10.13	8.04	0.68	0.26	2.32	0.50	0.43	8.07	4.45	0.58
HIF1A	43.40	49.46	39.22	3.30	1.26	11.30	2.42	2.08	39.40	21.74	2.81
HIF3A	0.44	0.50	0.40	0.03	0.01	0.11	0.02	0.02	0.40	0.22	0.03
NCOA1	13.98	15.93	12.63	1.06	0.41	3.64	0.78	0.67	12.69	7.00	0.90
NPAS1	9.40	10.72	8.50	0.71	0.27	2.45	0.52	0.45	8.54	4.71	0.61
NPAS2	2.09	2.38	1.88	0.16	0.06	0.54	0.12	0.10	1.89	1.04	0.14
NPAS3	0.33	0.38	0.30	0.03	0.01	0.09	0.02	0.02	0.30	0.16	0.02
NPAS4	0.29	0.33	0.26	0.02	0.01	0.08	0.02	0.01	0.27	0.15	0.02
SIM1	0.44	0.50	0.40	0.03	0.01	0.11	0.02	0.02	0.40	0.22	0.03
SIM2	1.76	2.00	1.59	0.13	0.05	0.46	0.10	0.08	1.59	0.88	0.11
SOHLH1	4.17	4.75	3.77	0.32	0.12	1.09	0.23	0.20	3.79	2.09	0.27
SOHLH2	0.04	0.04	0.03	0.00	0.00	0.01	0.00	0.00	0.03	0.02	0.00
TCFL5	0.15	0.17	0.13	0.01	0.00	0.04	0.01	0.01	0.13	0.07	0.01
MAX	18.59	21.18	16.80	1.41	0.54	4.84	1.04	0.89	16.88	9.31	1.20
MGA	11.12	12.68	10.05	0.85	0.32	2.90	0.62	0.53	10.10	5.57	0.72
MITF	2.93	3.34	2.65	0.22	0.08	0.76	0.16	0.14	2.66	1.47	0.19
MLX	3.59	4.09	3.24	0.27	0.10	0.93	0.20	0.17	3.26	1.80	0.23
MLXIP	1.10	1.25	0.99	0.08	0.03	0.29	0.06	0.05	1.00	0.55	0.07
MLXIPL	2.31	2.63	2.08	0.18	0.07	0.60	0.13	0.11	2.09	1.15	0.15
MNT	1.79	2.04	1.62	0.14	0.05	0.47	0.10	0.09	1.63	0.90	0.12
MXD1	3.07	3.50	2.78	0.23	0.09	0.80	0.17	0.15	2.79	1.54	0.20
MXD3	2.63	3.00	2.38	0.20	0.08	0.69	0.15	0.13	2.39	1.32	0.17
MXD4	0.84	0.96	0.76	0.06	0.02	0.22	0.05	0.04	0.76	0.42	0.05
MXI1	3.04	3.46	2.74	0.23	0.09	0.79	0.17	0.15	2.76	1.52	0.20
MYC	203.55	231.98	183.96	15.48	5.91	53.02	11.34	9.78	184.82	101.95	13.18
MYCL	3.18	3.63	2.88	0.24	0.09	0.83	0.18	0.15	2.89	1.59	0.21
MYCN	52.32	59.63	47.29	3.98	1.52	13.63	2.91	2.51	47.51	26.21	3.39
SREBF1	6.95	7.92	6.28	0.53	0.20	1.81	0.39	0.33	6.31	3.48	0.45
SREBF2	6.22	7.09	5.62	0.47	0.18	1.62	0.35	0.30	5.65	3.12	0.40
TFAP4	7.21	8.22	6.51	0.55	0.21	1.88	0.40	0.35	6.55	3.61	0.47
TFE3	2.56	2.92	2.31	0.19	0.07	0.67	0.14	0.12	2.33	1.28	0.17
TFEB	2.34	2.67	2.12	0.18	0.07	0.61	0.13	0.11	2.13	1.17	0.15
TFEC	0.62	0.71	0.56	0.05	0.02	0.16	0.03	0.03	0.56	0.31	0.04
USF1	5.12	5.84	4.63	0.39	0.15	1.33	0.29	0.25	4.65	2.57	0.33
USF2	2.23	2.54	2.02	0.17	0.06	0.58	0.12	0.11	2.03	1.12	0.14
ID1	3.84	4.38	3.47	0.29	0.11	1.00	0.21	0.18	3.49	1.92	0.25
ID2	5.53	6.30	4.99	0.42	0.16	1.44	0.31	0.27	5.02	2.77	0.36
ID3	2.60	2.96	2.35	0.20	0.08	0.68	0.14	0.12	2.36	1.30	0.17
ID4	1.17	1.33	1.06	0.09	0.03	0.30	0.07	0.06	1.06	0.59	0.08
TFAP2A	5.85	6.67	5.29	0.45	0.17	1.52	0.33	0.28	5.32	2.93	0.38
TFAP2B	0.84	0.96	0.76	0.06	0.02	0.22	0.05	0.04	0.76	0.42	0.05
TFAP2C	1.21	1.38	1.09	0.09	0.04	0.31	0.07	0.06	1.10	0.60	0.08
TFAP2D	2.82	3.21	2.55	0.21	0.08	0.73	0.16	0.14	2.56	1.41	0.18
TFAP2E	0.04	0.04	0.03	0.00	0.00	0.01	0.00	0.00	0.03	0.02	0.00

TFs	n° of interaction reports involving TF and … [real/expected ratio]											p,chi-square test [real vs expected interactions]			
	SplF.core	SplF.RBP	SplF.other	pAF.4C	pAF.PA	TlatMF.EIF	TlatMF.EIcoF	TlatMF.EEF	TlatMF.cytoRP	TlatMF.mitoRP	TlatMF.aatRNAs ymth	all 11 groups (SplFs, pAFs, TlatMF)	only SplFs	only pAFs	only TlatMFs
ARID1A	0.4	0.5	3.5	0.0	0.0	0.0	0.0	0.0	0.0	0.0	0.0	1.75E-18	1.18E-18	2.46E-01	2.67E-04
ARID1B	0.2	0.1	2.8	1.4	0.0	0.0	0.0	0.0	0.0	0.0	0.0	9.68E-09	1.48E-09	5.26E-01	5.16E-03
ARID2	0.1	0.3	1.6	0.0	0.0	0.0	0.0	0.0	0.0	0.0	0.0	2.80E-03	2.55E-03	3.70E-01	1.53E-02
ARID3A	0.2	0.4	0.2	0.0	0.0	0.8	0.0	0.0	0.2	0.0	0.0	1.41E-01	1.68E-02	4.71E-01	2.99E-01
ARID3B	0.4	0.0	0.7	0.0	0.0	0.0	0.0	0.0	0.0	0.0	0.0	8.24E-02	2.46E-02	4.80E-01	1.20E-01
ARID4A	0.0	0.0	2.1	0.0	0.0	0.0	0.0	0.0	0.0	0.0	0.0	3.86E-02	2.62E-03	5.31E-01	2.31E-01
ARID4B	0.1	0.2	1.8	0.0	0.0	0.0	0.0	0.0	0.0	0.3	0.0	9.39E-04	2.90E-04	3.64E-01	2.69E-02
ARID5A	0.0	0.2	0.0	0.0	0.0	0.0	0.0	0.0	0.0	0.0	0.0	6.11E-03	7.80E-04	4.55E-01	8.26E-02
ARID5B	0.0	1.2	3.7	0.0	0.0	0.0	0.0	0.0	0.0	0.0	0.0	1.49E-03	1.18E-05	5.75E-01	3.56E-01
JARID2	0.3	0.5	7.9	0.0	0.0	0.0	0.0	0.0	0.0	0.0	0.0	1.00E-31	1.69E-36	5.27E-01	2.21E-01
ARID3C	0.0	0.0	0.0	0.0	0.0	0.0	0.0	0.0	0.0	0.0	0.0	9.98E-01	5.73E-01	8.45E-01	9.84E-01
TCF25	0.5	0.0	0.3	0.0	0.0	0.0	0.0	0.0	0.5	0.5	0.0	2.83E-01	1.64E-02	5.02E-01	6.44E-01
TCF12	0.1	0.4	0.4	0.0	0.0	0.0	0.0	0.0	0.0	0.0	0.0	3.44E-03	4.04E-03	3.64E-01	1.33E-02
TCF3	0.2	0.5	1.4	0.0	0.0	0.0	0.0	1.8	0.8	0.9	0.0	2.67E-02	1.06E-03	2.73E-01	3.72E-01
TCF4	0.2	0.1	0.3	0.0	0.0	0.2	1.0	0.0	0.0	0.1	0.0	4.30E-10	1.93E-08	1.75E-01	4.55E-05
ASCL1	0.0	0.0	0.7	0.0	0.0	0.0	0.0	0.0	0.0	0.0	0.0	7.97E-01	1.92E-01	6.91E-01	7.37E-01
ASCL2	0.0	0.0	3.0	0.0	0.0	0.0	0.0	0.0	0.0	0.0	0.0	9.85E-01	3.43E-01	8.45E-01	9.84E-01
ASCL3	0.0	0.0	0.0	0.0	0.0	0.0	0.0	0.0	0.0	0.0	0.0	9.68E-01	3.47E-01	7.87E-01	9.37E-01
ASCL4	0.0	0.0	0.0	0.0	0.0	0.0	0.0	0.0	0.0	0.0	0.0	9.62E-01	3.28E-01	7.82E-01	9.30E-01
ASCL5	0.0	0.0	0.0	0.0	0.0	0.0	0.0	0.0	0.0	0.0	0.0	1.00E+00	8.95E-01	9.30E-01	1.00E+00
MYF5	0.0	0.0	0.0	0.0	0.0	0.0	0.0	0.0	0.0	0.0	0.0	7.98E-01	1.51E-01	7.18E-01	8.08E-01
MYF6	0.0	0.0	0.0	0.0	0.0	0.0	0.0	0.0	0.0	0.0	0.0	8.84E-01	2.10E-01	7.43E-01	8.65E-01
MYOD1	0.2	0.1	4.3	0.0	3.9	0.0	0.0	0.0	0.0	0.0	0.0	1.40E-21	3.28E-23	9.44E-02	5.77E-03
MYOG	0.0	0.0	1.0	4.0	0.0	0.0	0.0	0.0	0.0	0.0	0.0	1.13E-01	2.84E-02	1.29E-01	2.95E-01
ATOH1	0.0	0.3	0.0	0.0	0.0	0.0	0.0	0.0	0.7	0.0	5.1	1.79E-01	2.44E-02	5.75E-01	2.94E-01
ATOH7	0.0	0.0	0.0	0.0	0.0	0.0	0.0	0.0	0.0	0.0	0.0	1.00E+00	9.46E-01	9.51E-01	1.00E+00
ATOH8	0.0	0.0	0.0	0.0	0.0	0.0	0.0	0.0	0.0	0.0	0.0	1.00E+00	8.95E-01	9.30E-01	1.00E+00
BHLHA15	0.6	0.7	0.0	0.0	0.0	2.1	0.0	0.0	4.3	3.3	0.0	1.28E-07	1.21E-01	5.39E-01	5.38E-09
BHLHA9	2.3	0.0	0.0	0.0	0.0	0.0	0.0	0.0	0.0	0.0	0.0	9.91E-01	4.46E-01	8.30E-01	9.77E-01
BHLHE22	0.0	0.0	0.0	0.0	0.0	0.0	0.0	0.0	0.0	0.0	0.0	1.00E+00	8.95E-01	9.30E-01	1.00E+00
BHLHE23	0.0	0.0	0.0	0.0	0.0	0.0	0.0	0.0	0.0	0.0	0.0	9.62E-01	3.28E-01	7.82E-01	9.30E-01
FERD3L	0.0	0.0	0.0	0.0	0.0	0.0	0.0	0.0	0.0	0.0	0.0	8.96E-01	2.22E-01	7.47E-01	8.74E-01
FIGLA	0.0	0.0	0.0	0.0	0.0	0.0	0.0	0.0	0.0	0.0	0.0	9.68E-01	3.47E-01	7.87E-01	9.37E-01
HAND1	0.0	0.0	0.0	0.0	0.0	0.0	0.0	0.0	0.0	0.0	0.0	9.68E-01	3.47E-01	7.87E-01	9.37E-01
HAND2	0.0	0.0	0.8	0.0	0.0	0.0	0.0	0.0	0.0	0.0	0.0	8.24E-01	2.10E-01	6.99E-01	7.58E-01
LYL1	0.0	0.0	0.0	0.0	0.0	0.0	0.0	0.0	0.0	0.0	0.0	9.87E-01	4.34E-01	8.10E-01	9.62E-01
MESP1	0.0	0.0	8.2	0.0	0.0	0.0	0.0	0.0	0.0	0.0	0.0	2.29E-02	4.61E-05	8.37E-01	9.81E-01
MESP2	0.0	0.0	0.0	0.0	0.0	0.0	0.0	0.0	0.0	0.0	0.0	9.93E-01	4.85E-01	8.23E-01	9.72E-01
MSC	0.0	1.5	0.0	0.0	0.0	0.0	0.0	0.0	0.0	0.0	0.0	9.92E-01	5.27E-01	8.04E-01	9.56E-01
MSGN1	0.0	0.0	0.0	0.0	0.0	0.0	0.0	0.0	0.0	0.0	0.0	7.65E-01	1.35E-01	7.10E-01	7.88E-01
NEUROD1	0.0	0.0	3.4	0.0	0.0	0.0	0.0	0.0	0.0	0.0	0.0	1.25E-01	2.38E-03	6.81E-01	7.06E-01
NEUROD2	0.0	0.0	0.0	0.0	0.0	0.0	0.0	0.0	0.0	0.0	0.0	9.95E-01	5.13E-01	8.30E-01	9.77E-01
NEUROD4	0.0	0.0	0.0	0.0	0.0	0.0	0.0	0.0	0.0	0.0	0.0	9.98E-01	5.73E-01	8.45E-01	9.84E-01
NEUROD6	0.0	0.0	0.0	0.0	0.0	0.0	0.0	0.0	0.0	0.0	0.0	1.00E+00	8.00E-01	9.01E-01	9.98E-01
NEUROG1	0.0	0.0	0.0	0.0	0.0	0.0	0.0	0.0	0.0	0.0	0.0	9.62E-01	3.28E-01	7.82E-01	9.30E-01
NEUROG2	0.0	0.0	7.6	0.0	0.0	0.0	0.0	0.0	0.0	0.0	0.0	7.90E-01	4.96E-02	9.01E-01	9.98E-01
NEUROG3	0.6	0.2	0.0	0.0	0.0	0.5	0.0	0.0	1.4	0.0	0.0	3.60E-02	1.98E-03	3.87E-01	2.92E-01
NHLH2	0.0	0.0	0.0	0.0	0.0	0.0	0.0	0.0	0.0	0.0	0.0	8.84E-01	2.10E-01	7.43E-01	8.65E-01
OLIG1	0.0	0.0	0.0	0.0	0.0	0.0	0.0	0.0	0.0	1.6	0.0	8.31E-01	1.51E-01	7.18E-01	8.64E-01
OLIG2	0.0	1.1	0.0	0.0	0.0	0.0	0.0	0.0	0.0	0.0	0.0	9.79E-01	4.63E-01	7.71E-01	9.15E-01
OLIG3	0.8	0.0	0.0	0.0	0.0	0.0	0.0	0.0	0.0	0.0	0.0	8.68E-01	2.51E-01	7.10E-01	7.88E-01
PTF1A	0.0	0.0	5.0	0.0	0.0	0.0	0.0	0.0	0.0	0.0	0.0	2.59E-01	3.84E-03	7.93E-01	9.44E-01
SCX	0.0	0.0	0.0	0.0	0.0	0.0	0.0	0.0	0.0	0.0	0.0	9.99E-01	6.77E-01	8.70E-01	9.93E-01
TAL1	0.3	0.0	4.6	0.0	0.0	0.0	0.0	3.3	0.5	0.0	0.0	1.46E-15	6.28E-19	4.19E-01	1.37E-01
TAL2	0.0	0.0	0.0	0.0	0.0	0.0	0.0	0.0	0.0	0.0	0.0	9.93E-01	4.85E-01	8.23E-01	9.72E-01
TCF15	0.0	0.0	2.3	0.0	0.0	0.0	0.0	0.0	0.0	0.0	0.0	9.88E-01	4.12E-01	8.23E-01	9.72E-01
TCF21	0.0	0.0	0.0	0.0	0.0	0.0	0.0	0.0	0.0	0.0	0.0	9.68E-01	3.47E-01	7.87E-01	9.37E-01
TCF23	2.0	1.7	0.0	0.0	0.0	0.0	0.0	0.0	0.0	0.0	0.0	9.94E-01	5.42E-01	8.17E-01	9.67E-01
TWIST1	0.0	0.2	3.6	0.0	0.0	0.0	0.0	0.0	0.0	0.0	0.0	2.02E-06	1.36E-08	4.86E-01	1.32E-01
TWIST2	0.0	0.0	1.2	0.0	0.0	0.0	0.0	0.0	0.0	0.0	0.0	9.51E-01	3.57E-01	7.52E-01	8.83E-01
BHLHE40	0.4	0.5	0.2	0.0	0.0	0.0	0.0	0.0	0.0	0.0	0.0	7.65E-02	3.69E-02	4.55E-01	8.26E-02
BHLHE41	0.0	0.5	2.0	0.0	0.0	0.0	0.0	0.0	0.0	0.0	0.0	7.45E-01	1.66E-01	6.77E-01	6.96E-01
HELT	0.0	0.0	0.0	0.0	0.0	0.0	0.0	0.0	0.0	0.0	0.0	9.95E-01	5.13E-01	8.30E-01	9.77E-01
HES2	0.0	0.0	0.0	0.0	0.0	0.0	0.0	0.0	0.0	0.0	0.0	1.00E+00	8.46E-01	9.14E-01	9.99E-01
HES3	0.0	0.0	0.0	0.0	0.0	0.0	0.0	0.0	0.0	0.0	0.0	1.00E+00	8.00E-01	9.01E-01	9.98E-01
HES4	0.0	0.0	0.0	0.0	0.0	0.0	0.0	0.0	0.0	0.0	0.0	9.38E-01	2.78E-01	7.66E-01	9.08E-01
HES5	0.0	0.0	0.0	0.0	0.0	0.0	0.0	0.0	0.0	0.0	0.0	1.00E+00	8.46E-01	9.14E-01	9.99E-01
HES6	0.0	0.9	1.1	0.0	0.0	0.0	0.0	0.0	0.0	0.0	0.0	8.35E-01	3.69E-01	6.52E-01	6.14E-01
HES7	0.0	2.7	0.0	0.0	0.0	0.0	0.0	0.0	0.0	0.0	0.0	9.93E-01	4.35E-01	8.52E-01	9.88E-01
HEY1	1.9	1.5	0.2	0.0	5.8	0.0	0.0	0.0	3.7	0.0	0.0	5.38E-09	8.90E-03	3.52E-02	1.31E-08
HEY2	0.4	0.0	2.3	0.0	0.0	0.0	0.0	0.0	0.0	0.0	0.0	2.91E-01	2.80E-02	6.12E-01	4.79E-01
HEYL	0.0	0.0	0.0	0.0	0.0	0.0	0.0	0.0	0.0	0.0	0.0	7.65E-01	1.35E-01	7.10E-01	7.88E-01
AHR	0.2	0.1	0.5	0.0	0.0	0.5	0.0	0.0	0.0	0.2	1.9	3.08E-03	9.60E-04	3.51E-01	3.81E-02
AHRR	0.0	2.2	5.5	0.0	0.0	0.0	0.0	0.0	0.0	0.0	0.0	5.15E-01	1.50E-02	8.37E-01	9.81E-01
ARNT	0.4	0.2	0.2	0.0	0.0	0.0	0.0	0.0	0.5	0.0	0.0	1.35E-06	1.69E-06	2.02E-01	3.03E-03
ARNT2	0.3	0.0	0.0	0.0	0.0	0.0	0.0	0.0	0.0	0.0	0.0	7.31E-02	7.69E-03	5.29E-01	2.26E-01
ARNTL	0.0	0.2	1.2	0.0	0.0	0.0	0.0	0.0	0.0	0.0	0.0	2.17E-02	6.15E-03	4.46E-01	7.10E-02
ARNTL2	0.0	0.6	0.0	0.0	0.0	0.0	0.0	0.0	0.0	0.0	0.0	8.24E-01	2.17E-01	6.95E-01	7.48E-01
CLOCK	0.0	0.2	1.2	0.0	0.0	0.0	0.0	0.0	0.0	0.0	0.0	6.50E-02	1.54E-02	4.83E-01	1.26E-01
EPAS1	0.2	0.3	1.7	0.0	0.0	0.0	0.0	0.0	0.0	0.0	0.0	3.91E-04	6.14E-04	3.34E-01	5.94E-03
HIF1A	0.2	0.2	2.8	1.2	0.8	0.0	0.0	0.0	0.0	0.0	0.4	7.28E-49	2.47E-39	6.53E-01	5.34E-15
HIF3A	0.0	0.0	0.0	0.0	0.0	0.0	0.0	0.0	0.0	0.0	0.0	9.95E-01	5.13E-01	8.30E-01	9.77E-01
NCOA1	0.2	0.1	1.2	0.0	0.0	0.0	0.0	0.0	0.0	0.0	0.0	2.44E-07	9.84E-06	2.26E-01	1.03E-04
NPAS1	0.2	0.3	0.2	0.0	0.0	0.4	0.0	0.0	0.0	0.0	0.0	2.69E-04	2.81E-04	3.20E-01	7.79E-03
NPAS2	0.5	0.4	2.1	0.0	0.0	0.0	0.0	0.0	0.5	0.0	0.0	7.88E-01	1.54E-01	6.40E-01	7.97E-01
NPAS3	0.0	0.0	0.0	0.0	0.0	0.0	0.0	0.0	0.0	0.0	0.0	9.98E-01	6.06E-01	8.52E-01	9.88E-01
NPAS4	0.0	0.0	0.0	0.0	0.0	0.0	0.0	0.0	0.0	0.0	0.0	9.99E-01	6.41E-01	8.61E-01	9.91E-01
SIM1	0.0	0.0	2.5	0.0	0.0	0.0	0.0	0.0	0.0	0.0	0.0	9.87E-01	3.95E-01	8.30E-01	9.77E-01
SIM2	0.6	0.5	0.0	0.0	0.0	0.0	0.0	0.0	0.0	0.0	0.0	8.30E-01	2.99E-01	6.68E-01	6.65E-01
SOHLH1	0.2	0.0	0.3	0.0	0.0	0.0	0.0	0.0	0.0	0.0	0.0	6.79E-02	1.01E-02	5.08E-01	1.76E-01
SOHLH2	0.0	0.0	0.0	0.0	0.0	0.0	0.0	0.0	0.0	0.0	0.0	1.00E+00	9.46E-01	9.51E-01	1.00E+00
TCFL5	0.0	0.0	0.0	0.0	0.0	0.0	0.0	0.0	0.0	0.0	0.0	1.00E+00	8.00E-01	9.01E-01	9.98E-01
MAX	0.1	0.2	1.4	0.0	1.9	0.0	0.0	1.1	0.2	0.2	0.0	1.29E-08	1.83E-07	1.79E-01	1.95E-04
MGA	0.1	0.1	1.8	0.0	0.0	0.0	0.0	0.0	0.1	0.0	0.0	1.46E-06	1.99E-06	2.80E-01	2.34E-03
MITF	0.0	1.2	2.3	0.0	0.0	0.0	0.0	0.0	0.0	0.0	0.0	2.24E-01	2.58E-02	5.79E-01	3.71E-01
MLX	0.0	0.5	0.0	0.0	0.0	0.0	0.0	0.0	0.0	0.0	0.0	1.37E-01	1.93E-02	5.39E-01	2.53E-01
MLXIP	0.0	0.0	0.0	0.0	0.0	0.0	0.0	0.0	0.0	0.0	0.0	8.57E-01	1.88E-01	7.34E-01	8.47E-01
MLXIPL	0.0	0.4	3.4	0.0	0.0	0.0	0.0	0.0	0.0	0.0	0.0	3.55E-02	5.77E-04	6.23E-01	5.16E-01
MNT	0.0	1.0	1.2	0.0	0.0	0.0	0.0	0.0	0.0	0.0	0.0	8.65E-01	3.90E-01	6.64E-01	6.55E-01
MXD1	0.0	0.0	1.4	0.0	0.0	0.0	0.0	0.0	0.0	0.0	0.0	2.19E-01	2.85E-02	5.70E-01	3.42E-01
MXD3	1.1	0.0	0.0	0.0	0.0	0.0	0.0	0.0	0.0	0.0	0.0	3.93E-01	6.61E-02	5.99E-01	4.36E-01
MXD4	0.0	0.0	0.0	0.0	0.0	0.0	0.0	0.0	0.0	0.0	0.0	9.38E-01	2.78E-01	7.66E-01	9.08E-01
MXI1	0.0	0.0	2.2	0.0	0.0	0.0	0.0	0.0	0.0	0.0	0.0	9.24E-02	5.63E-03	5.72E-01	3.49E-01
MYC	1.1	0.9	0.8	1.2	1.5	0.9	0.6	0.9	1.2	0.9	0.2	1.07E-03	3.93E-03	1.20E-01	7.10E-03
MYCL	0.0	0.0	1.4	0.0	0.0	0.0	0.0	0.0	0.0	0.0	0.0	2.00E-01	2.67E-02	5.63E-01	3.21E-01
MYCN	1.6	1.4	0.9	1.8	1.3	0.5	1.7	0.8	1.6	3.0	0.6	5.87E-28	9.21E-07	1.18E-01	5.28E-25
SREBF1	0.0	0.0	3.0	0.0	0.0	0.0	0.0	0.0	0.0	0.0	0.0	4.60E-08	1.52E-09	3.93E-01	2.56E-02
SREBF2	0.0	0.3	0.5	0.0	0.0	0.0	0.0	0.0	0.0	0.3	0.0	1.79E-02	3.89E-03	4.19E-01	8.26E-02
TFAP4	0.0	0.2	1.5	0.0	0.0	0.0	0.0	0.0	0.0	0.0	0.0	1.96E-03	1.02E-03	3.84E-01	2.12E-02
TFE3	0.0	0.3	0.0	0.0	0.0	1.5	0.0	0.0	0.4	0.0	0.0	5.28E-01	4.65E-02	6.04E-01	7.56E-01
TFEB	0.0	0.0	0.9	0.0	0.0	0.0	0.0	0.0	0.0	0.0	0.0	4.79E-01	8.14E-02	6.20E-01	5.07E-01
TFEC	0.0	2.8	0.0	0.0	0.0	0.0	0.0	0.0	0.0	0.0	0.0	9.08E-01	1.71E-01	7.98E-01	9.50E-01
USF1	0.2	0.5	2.2	2.6	0.0	0.0	0.0	0.0	0.0	0.0	0.0	1.82E-02	4.24E-03	2.93E-01	9.36E-02
USF2	0.0	0.4	0.5	0.0	0.0	0.0	0.0	0.0	0.0	0.0	0.0	6.27E-01	1.59E-01	6.28E-01	5.35E-01
ID1	0.8	0.2	0.3	0.0	0.0	0.0	0.0	0.0	0.0	0.0	0.0	2.84E-01	1.03E-01	5.25E-01	2.16E-01
ID2	0.5	0.3	1.0	2.4	0.0	0.0	0.0	0.0	0.0	0.0	0.0	1.25E-01	1.30E-01	3.27E-01	7.10E-02
ID3	0.4	0.0	0.4	0.0	0.0	0.0	0.0	0.0	0.0	0.0	0.0	4.61E-01	9.45E-02	6.01E-01	4.44E-01
ID4	0.0	0.0	0.0	0.0	0.0	0.0	0.0	0.0	0.0	0.0	0.0	8.29E-01	1.68E-01	7.26E-01	8.28E-01
TFAP2A	0.2	0.4	1.3	0.0	0.0	0.0	0.0	0.0	0.0	0.0	0.0	5.54E-02	3.69E-02	4.33E-01	5.64E-02
TFAP2B	0.0	0.0	0.0	0.0	0.0	0.0	0.0	0.0	0.0	0.0	0.0	9.38E-01	2.78E-01	7.66E-01	9.08E-01
TFAP2C	0.0	0.7	2.7	0.0	0.0	0.0	0.0	0.0	0.0	0.0	0.0	7.26E-01	9.78E-02	7.22E-01	8.18E-01
TFAP2D	0.0	0.0	0.0	0.0	0.0	0.0	0.0	0.0	0.0	0.0	0.0	1.71E-01	1.37E-02	5.86E-01	3.95E-01
TFAP2E	0.0	0.0	0.0	0.0	0.0	0.0	0.0	0.0	0.0	0.0	0.0	1.00E+00	9.46E-01	9.51E-01	1.00E+00

	HES1	21	23	7	0	0	0	1	6	0	0	0	0	0	0	0	0	3.55	4.04	3.21	0.27	0.10	0.92	0.20	0.17	3.22	1.78	0.23	0.0	0.2	1.9	0.0	0.0	0.0	0.0	0.0	0.0	0.0	0.0	1.26E-01	1.60E-02	5.41E-01	2.59E-01
	NHLH1	26	11	7	0	1	0	4	3	0	0	0	0	0	1	0	0	3.40	3.88	3.08	0.26	0.10	0.89	0.19	0.16	3.09	1.70	0.22	0.0	1.0	1.0	0.0	0.0	0.0	0.0	0.0	0.3	0.0	0.0	5.95E-01	1.82E-01	5.50E-01	4.70E-01
	TCF24	5	0	0	0	0	0	0	0	0	0	0	0	0	0	0	0	0.26	0.29	0.23	0.02	0.01	0.07	0.01	0.01	0.23	0.13	0.02	0.0	0.0	0.0	0.0	0.0	0.0	0.0	0.0	0.0	0.0	0.0	9.99E-01	6.77E-01	8.70E-01	9.93E-01
	USF3	0	0	0	0	0	0	0	0	0	0	0	0	0	0	0	0	0.00	0.00	0.00	0.00	0.00	0.00	0.00	0.00	0.00	0.00	0.00	na	na	na	na	na	na	na	na	na	na	na	na	na	na	na
bZIP	ATF2	93	92	5	0	1	0	1	4	0	0	0	0	1	0	0	0	14.27	16.26	12.90	1.08	0.41	3.72	0.79	0.69	12.96	7.15	0.92	0.0	0.1	0.3	0.0	0.0	0.0	0.0	1.5	0.0	0.0	0.0	1.57E-09	2.87E-08	2.21E-01	1.03E-04
	ATF7	41	8	2	0	0	0	1	1	0	0	0	0	0	0	0	0	3.07	3.50	2.78	0.23	0.09	0.80	0.17	0.15	2.79	1.54	0.20	0.0	0.3	0.4	0.0	0.0	0.0	0.0	0.0	0.0	0.0	0.0	2.87E-01	4.98E-02	5.70E-01	3.42E-01
	BACH1	92	47	17	1	2	2	6	9	0	1	0	0	0	1	1	0	10.90	12.43	9.85	0.83	0.32	2.84	0.61	0.52	9.90	5.46	0.71	0.2	0.5	0.9	0.0	3.2	0.0	0.0	0.0	0.1	0.2	0.0	1.11E-03	4.82E-03	1.29E-01	5.98E-03
	BACH2	58	10	4	1	2	0	2	2	0	1	0	0	0	1	0	1	3.95	4.50	3.57	0.30	0.11	1.03	0.22	0.19	3.59	1.98	0.26	0.0	0.4	0.6	0.0	8.7	0.0	0.0	0.0	0.3	0.0	3.9	2.39E-02	4.89E-02	7.55E-03	1.89E-01
	CREB5	58	17	2	0	0	0	1	1	0	0	0	0	0	0	0	0	8.31	9.47	7.51	0.63	0.24	2.16	0.46	0.40	7.54	4.16	0.54	0.0	0.1	0.1	0.0	0.0	0.0	0.0	0.0	0.0	0.0	0.0	4.36E-05	2.13E-05	3.50E-01	9.29E-03
	JUN	298	234	45	1	3	7	12	26	0	1	0	0	1	1	1	0	32.20	36.70	29.10	2.45	0.93	8.39	1.79	1.55	29.24	16.13	2.08	0.2	0.3	0.9	0.0	1.1	0.0	0.0	0.6	0.0	0.1	0.0	1.33E-15	1.09E-08	1.17E-01	2.18E-10
	JUNB	88	41	31	1	4	13	8	10	1	0	3	0	0	1	0	0	10.65	12.13	9.62	0.81	0.31	2.77	0.59	0.51	9.67	5.33	0.69	1.2	0.7	1.0	1.2	0.0	1.1	0.0	0.0	0.1	0.0	0.0	6.98E-02	3.78E-01	5.52E-01	1.07E-02
	JUND	47	21	5	0	0	0	1	4	0	0	0	0	0	0	0	0	3.81	4.34	3.44	0.29	0.11	0.99	0.21	0.18	3.46	1.91	0.25	0.0	0.2	1.2	0.0	0.0	0.0	0.0	0.0	0.0	0.0	0.0	1.80E-01	3.95E-02	5.27E-01	2.21E-01
	NFE2	10	15	1	0	0	0	0	1	0	0	0	0	0	0	0	0	1.17	1.33	1.06	0.09	0.03	0.30	0.07	0.06	1.06	0.59	0.08	0.0	0.0	0.9	0.0	0.0	0.0	0.0	0.0	0.0	0.0	0.0	9.05E-01	2.85E-01	7.26E-01	8.28E-01
	NFE2L1	20	28	1	0	0	0	1	0	0	0	0	0	0	0	0	0	2.45	2.79	2.22	0.19	0.07	0.64	0.14	0.12	2.23	1.23	0.16	0.0	0.4	0.0	0.0	0.0	0.0	0.0	0.0	0.0	0.0	0.0	3.91E-01	5.45E-02	6.12E-01	4.79E-01
	NFE2L2	117	195	10	0	8	0	2	8	0	0	6	0	0	0	2	0	16.50	18.81	14.91	1.25	0.48	4.30	0.92	0.79	14.98	8.27	1.07	0.0	0.1	0.5	0.0	0.0	1.4	0.0	0.0	0.0	0.2	0.0	4.23E-09	2.88E-08	1.88E-01	3.11E-04
	NFE2L3	20	6	0	0	0	0	0	0	0	0	0	0	0	0	0	0	1.13	1.29	1.03	0.09	0.03	0.30	0.06	0.05	1.03	0.57	0.07	0.0	0.0	0.0	0.0	0.0	0.0	0.0	0.0	0.0	0.0	0.0	8.43E-01	1.78E-01	7.30E-01	8.37E-01
	ATF3	107	38	16	0	1	0	1	15	0	0	0	0	1	0	0	0	6.77	7.71	6.12	0.51	0.20	1.76	0.38	0.33	6.15	3.39	0.44	0.0	0.1	2.5	0.0	0.0	0.0	0.0	3.1	0.0	0.0	0.0	1.89E-05	2.89E-06	3.99E-01	1.90E-02
	FOS	175	55	14	0	3	1	3	10	0	0	0	0	2	1	0	0	15.11	17.22	13.66	1.15	0.44	3.94	0.84	0.73	13.72	7.57	0.98	0.1	0.2	0.7	0.0	0.0	0.0	0.0	2.8	0.1	0.0	0.0	3.38E-08	2.37E-06	2.08E-01	4.86E-05
	FOSB	24	4	3	0	0	0	1	2	0	0	0	0	0	0	0	0	2.09	2.38	1.88	0.16	0.06	0.54	0.12	0.10	1.89	1.04	0.14	0.0	0.4	1.1	0.0	0.0	0.0	0.0	0.0	0.0	0.0	0.0	7.31E-01	2.36E-01	6.40E-01	5.74E-01
	FOSL1	43	11	5	0	1	0	0	5	0	0	0	0	1	0	0	0	3.55	4.04	3.21	0.27	0.10	0.92	0.20	0.17	3.22	1.78	0.23	0.0	0.0	1.6	0.0	0.0	0.0	0.0	5.9	0.0	0.0	0.0	3.60E-02	1.36E-02	5.41E-01	6.50E-02
	FOSL2	57	28	8	0	5	0	4	4	0	0	0	0	2	3	0	0	6.18	7.05	5.59	0.47	0.18	1.61	0.34	0.30	5.61	3.10	0.40	0.0	0.6	0.7	0.0	0.0	0.0	0.0	6.7	0.5	0.0	0.0	5.28E-03	1.88E-02	4.20E-01	5.71E-03
	JDP2	21	13	6	0	0	0	1	5	0	0	0	0	0	0	0	0	1.35	1.54	1.22	0.10	0.04	0.35	0.08	0.07	1.23	0.68	0.09	0.0	0.6	4.1	0.0	0.0	0.0	0.0	0.0	0.0	0.0	0.0	1.05E-01	1.36E-03	7.06E-01	7.78E-01
	MAF	25	16	5	0	0	0	1	4	0	0	0	0	0	0	0	0	2.93	3.34	2.65	0.22	0.08	0.76	0.16	0.14	2.66	1.47	0.19	0.0	0.3	1.5	0.0	0.0	0.0	0.0	0.0	0.0	0.0	0.0	3.62E-01	7.22E-02	5.79E-01	3.71E-01
	MAFA	7	4	1	0	0	0	0	1	0	0	0	0	0	0	0	0	0.70	0.79	0.63	0.05	0.02	0.18	0.04	0.03	0.63	0.35	0.05	0.0	0.0	1.6	0.0	0.0	0.0	0.0	0.0	0.0	0.0	0.0	9.80E-01	4.26E-01	7.87E-01	9.37E-01
	MAFB	39	25	27	0	42	8	12	7	0	0	1	0	0	11	30	0	9.62	10.97	8.70	0.73	0.28	2.51	0.54	0.46	8.74	4.82	0.62	0.8	1.1	0.8	0.0	0.0	0.4	0.0	0.0	1.3	6.2	0.0	2.33E-24	7.04E-01	3.15E-01	2.44E-27
	MAFF	56	9	1	0	0	0	0	1	0	0	0	0	0	0	0	0	3.62	4.13	3.27	0.28	0.11	0.94	0.20	0.17	3.29	1.81	0.23	0.0	0.0	0.3	0.0	0.0	0.0	0.0	0.0	0.0	0.0	0.0	8.96E-02	9.42E-03	5.37E-01	2.47E-01
	MAFG	51	4	0	0	0	0	0	0	0	0	0	0	0	0	0	0	2.82	3.21	2.55	0.21	0.08	0.73	0.16	0.14	2.56	1.41	0.18	0.0	0.0	0.0	0.0	0.0	0.0	0.0	0.0	0.0	0.0	0.0	1.71E-01	1.37E-02	5.86E-01	3.95E-01
	MAFK	35	5	4	0	0	0	1	3	0	0	0	0	0	0	0	0	1.87	2.13	1.69	0.14	0.05	0.49	0.10	0.09	1.69	0.93	0.12	0.0	0.5	1.8	0.0	0.0	0.0	0.0	0.0	0.0	0.0	0.0	7.15E-01	1.75E-01	6.58E-01	6.34E-01
	NRL	8	2	0	0	0	0	0	0	0	0	0	0	0	0	0	0	0.51	0.58	0.46	0.04	0.01	0.13	0.03	0.02	0.47	0.26	0.03	0.0	0.0	0.0	0.0	0.0	0.0	0.0	0.0	0.0	0.0	0.0	9.90E-01	4.59E-01	8.17E-01	9.67E-01
	BATF	39	3	1	0	0	0	0	1	0	0	0	0	0	0	0	0	1.68	1.92	1.52	0.13	0.05	0.44	0.09	0.08	1.53	0.84	0.11	0.0	0.0	0.7	0.0	0.0	0.0	0.0	0.0	0.0	0.0	0.0	7.21E-01	1.51E-01	6.74E-01	6.86E-01
	BATF2	24	2	1	0	0	0	0	1	0	0	0	0	0	0	0	0	1.39	1.58	1.26	0.11	0.04	0.36	0.08	0.07	1.26	0.70	0.09	0.0	0.0	0.8	0.0	0.0	0.0	0.0	0.0	0.0	0.0	0.0	8.38E-01	2.20E-01	7.02E-01	7.68E-01
	BATF3	66	2	1	0	3	1	0	0	0	0	0	0	3	0	0	0	4.28	4.88	3.87	0.33	0.12	1.12	0.24	0.21	3.89	2.14	0.28	0.2	0.0	0.0	0.0	0.0	0.0	0.0	14.6	0.0	0.0	0.0	1.15E-08	3.58E-03	5.02E-01	1.08E-08
	XBP1	23	20	5	0	0	1	1	3	0	0	0	0	0	0	0	0	2.41	2.75	2.18	0.18	0.07	0.63	0.13	0.12	2.19	1.21	0.16	0.4	0.4	1.4	0.0	0.0	0.0	0.0	0.0	0.0	0.0	0.0	7.31E-01	3.25E-01	6.14E-01	4.88E-01
	ATF4	51	44	10	0	3	0	1	9	0	0	1	0	0	1	1	0	6.59	7.51	5.95	0.50	0.19	1.72	0.37	0.32	5.98	3.30	0.43	0.0	0.1	1.5	0.0	0.0	0.6	0.0	0.0	0.2	0.3	0.0	1.71E-02	1.01E-03	4.06E-01	2.09E-01
	ATF5	3	5	3	0	1	0	0	3	0	0	1	0	0	0	0	0	1.06	1.21	0.96	0.08	0.03	0.28	0.06	0.05	0.96	0.53	0.07	0.0	0.0	3.1	0.0	0.0	3.6	0.0	0.0	0.0	0.0	0.0	4.15E-01	3.66E-02	7.38E-01	6.13E-01
	ATF1	18	11	4	0	0	1	0	3	0	0	0	0	0	0	0	0	2.56	2.92	2.31	0.19	0.07	0.67	0.14	0.12	2.33	1.28	0.17	0.4	0.0	1.3	0.0	0.0	0.0	0.0	0.0	0.0	0.0	0.0	5.27E-01	1.30E-01	6.04E-01	4.53E-01
	ATF6	28	8	1	0	1	0	1	0	0	0	1	0	0	0	0	0	5.85	6.67	5.29	0.45	0.17	1.52	0.33	0.28	5.32	2.93	0.38	0.0	0.1	0.0	0.0	0.0	0.7	0.0	0.0	0.0	0.0	0.0	3.74E-03	3.41E-04	4.33E-01	9.36E-02
	ATF6B	10	1	2	0	0	0	2	0	0	0	0	0	0	0	0	0	3.29	3.75	2.98	0.25	0.10	0.86	0.18	0.16	2.99	1.65	0.21	0.0	0.5	0.0	0.0	0.0	0.0	0.0	0.0	0.0	0.0	0.0	1.98E-01	2.89E-02	5.56E-01	3.01E-01
	CREB1	87	64	30	1	1	0	0	30	0	1	0	0	0	0	1	0	11.45	13.05	10.35	0.87	0.33	2.98	0.64	0.55	10.40	5.74	0.74	0.0	0.0	2.9	0.0	3.0	0.0	0.0	0.0	0.0	0.2	0.0	1.16E-13	3.79E-14	1.37E-01	1.75E-03
	CREB3	17	30	6	2	2	0	1	5	2	0	0	0	0	1	1	0	10.98	12.51	9.92	0.83	0.32	2.86	0.61	0.53	9.97	5.50	0.71	0.0	0.1	0.5	2.4	0.0	0.0	0.0	0.0	0.1	0.2	0.0	6.34E-06	6.12E-06	1.63E-01	5.66E-03
	CREB3L1	12	9	0	0	1	0	0	0	0	0	0	0	0	0	1	0	10.21	11.63	9.23	0.78	0.30	2.66	0.57	0.49	9.27	5.11	0.66	0.0	0.0	0.0	0.0	0.0	0.0	0.0	0.0	0.0	0.2	0.0	3.91E-07	1.79E-07	3.00E-01	4.58E-03
	CREB3L2	8	6	2	0	0	0	2	0	0	0	0	0	0	0	0	0	0.99	1.13	0.89	0.08	0.03	0.26	0.06	0.05	0.90	0.49	0.06	0.0	1.8	0.0	0.0	0.0	0.0	0.0	0.0	0.0	0.0	0.0	9.23E-01	2.78E-01	7.47E-01	8.74E-01
	CREB3L3	8	12	2	0	0	1	0	1	0	0	0	0	0	0	0	0	2.34	2.67	2.12	0.18	0.07	0.61	0.13	0.11	2.13	1.17	0.15	0.4	0.0	0.5	0.0	0.0	0.0	0.0	0.0	0.0	0.0	0.0	5.73E-01	1.34E-01	6.20E-01	5.07E-01
	CREB3L4	6	1	1	0	0	0	1	0	0	0	0	0	0	0	0	0	0.48	0.54	0.43	0.04	0.01	0.12	0.03	0.02	0.43	0.24	0.03	0.0	1.8	0.0	0.0	0.0	0.0	0.0	0.0	0.0	0.0	0.0	9.94E-01	5.24E-01	8.23E-01	9.72E-01
	CREBL2	6	2	2	0	0	0	2	0	0	0	0	0	0	0	0	0	0.44	0.50	0.40	0.03	0.01	0.11	0.02	0.02	0.40	0.22	0.03	0.0	4.0	0.0	0.0	0.0	0.0	0.0	0.0	0.0	0.0	0.0	8.00E-01	6.96E-02	8.30E-01	9.77E-01
	CREBZF	18	5	4	0	0	1	3	0	0	0	0	0	0	0	0	0	1.57	1.79	1.42	0.12	0.05	0.41	0.09	0.08	1.43	0.79	0.10	0.6	1.7	0.0	0.0	0.0	0.0	0.0	0.0	0.0	0.0	0.0	8.55E-01	2.95E-01	6.84E-01	7.17E-01
	CREM	18	15	6	0	0	3	0	3	0	0	0	0	0	0	0	0	3.11	3.54	2.81	0.24	0.09	0.81	0.17	0.15	2.82	1.56	0.20	1.0	0.0	1.1	0.0	0.0	0.0	0.0	0.0	0.0	0.0	0.0	4.76E-01	1.69E-01	5.68E-01	3.35E-01
	CEBPA	287	230	156	8	22	63	50	43	8	0	12	1	4	4	0	1	49.98	56.96	45.17	3.80	1.45	13.02	2.78	2.40	45.38	25.03	3.24	1.3	0.9	1.0	2.1	0.0	0.9	0.4	1.7	0.1	0.0	0.3	1.90E-12	1.14E-01	1.36E-02	5.21E-13
	CEBPB	285	389	330	21	203	124	129	77	18	3	47	3	8	136	7	2	103.29	117.72	93.35	7.85	3.00	26.90	5.75	4.96	93.79	51.74	6.69	1.2	1.1	0.8	2.3	1.0	1.7	0.5	1.6	1.5	0.1	0.3	4.62E-17	1.75E-02	2.94E-04	1.26E-15
	CEBPD	22	20	8	0	2	0	0	8	0	0	0	0	2	0	0	0	2.49	2.84	2.25	0.19	0.07	0.65	0.14	0.12	2.26	1.25	0.16	0.0	0.0	3.6	0.0	0.0	0.0	0.0	16.7	0.0	0.0	0.0	4.21E-08	4.46E-05	6.09E-01	2.34E-06
	CEBPE	39	9	0	0	0	0	0	0	0	0	0	0	0	0	0	0	2.52	2.88	2.28	0.19	0.07	0.66	0.14	0.12	2.29	1.26	0.16	0.0	0.0	0.0	0.0	0.0	0.0	0.0	0.0	0.0	0.0	0.0	2.48E-01	2.15E-02	6.07E-01	4.61E-01
	CEBPG	43	3	1	0	0	0	1	0	0	0	0	0	0	0	0	0	2.49	2.84	2.25	0.19	0.07	0.65	0.14	0.12	2.26	1.25	0.16	0.0	0.4	0.0	0.0	0.0	0.0	0.0	0.0	0.0	0.0	0.0	3.77E-01	5.17E-02	6.09E-01	4.70E-01
	DBP	15	0	0	0	0	0	0	0	0	0	0	0	0	0	0	0	0.66	0.75	0.60	0.05	0.02	0.17	0.04	0.03	0.60	0.33	0.04	0.0	0.0	0.0	0.0	0.0	0.0	0.0	0.0	0.0	0.0	0.0	9.74E-01	3.67E-01	7.93E-01	9.44E-01
	DDIT3	92	24	14	0	6	0	2	12	0	0	0	0	0	6	0	0	7.10	8.09	6.42	0.54	0.21	1.85	0.40	0.34	6.45	3.56	0.46	0.0	0.2	1.9	0.0	0.0	0.0	0.0	0.0	0.9	0.0	0.0	7.81E-03	2.55E-04	3.88E-01	2.50E-01
	HLF	6	0	0	0	0	0	0	0	0	0	0	0	0	0	0	0	0.40	0.46	0.36	0.03	0.01	0.10	0.02	0.02	0.37	0.20	0.03	0.0	0.0	0.0	0.0	0.0	0.0	0.0	0.0	0.0	0.0	0.0	9.96E-01	5.42E-01	8.37E-01	9.81E-01
	NFIL3	12	3	1	0	0	0	1	0	0	0	0	0	0	0	0	0	1.61	1.83	1.45	0.12	0.05	0.42	0.09	0.08	1.46	0.81	0.10	0.0	0.5	0.0	0.0	0.0	0.0	0.0	0.0	0.0	0.0	0.0	7.65E-01	1.79E-01	6.81E-01	7.06E-01
	TEF	4	1	1	0	1	0	1	0	0	0	0	0	0	0	1	0	0.26	0.29	0.23	0.02	0.01	0.07	0.01	0.01	0.23	0.13	0.02	0.0	3.4	0.0	0.0	0.0	0.0	0.0	0.0	0.0	7.8	0.0	5.80E-01	3.32E-01	8.70E-01	2.81E-01
CAAT-BF	NFYA	59	18	11	1	3	0	3	8	0	1	2	0	0	0	1	0	5.96	6.80	5.39	0.45	0.17	1.55	0.33	0.29	5.42	2.99	0.39	0.0	0.4	1.5	0.0	5.8	1.3	0.0	0.0	0.0	0.3	0.0	1.71E-02	9.33E-03	3.58E-02	1.64E-01
	NFYB	33	11	8	0	0	0	0	8	0	0	0	0	0	0	0	0	2.34	2.67	2.12	0.18	0.07	0.61	0.13	0.11	2.13	1.17	0.15	0.0	0.0	3.8	0.0	0.0	0.0	0.0	0.0	0.0	0.0	0.0	3.85E-03	2.29E-05	6.20E-01	5.07E-01
	NFYC	82	16	6	0	2	0	2	4	0	0	0	0	0	0	2	0	7.43	8.47	6.71	0.56	0.22	1.93	0.41	0.36	6.74	3.72	0.48	0.0	0.2	0.6	0.0	0.0	0.0	0.0	0.0	0.0	0.5	0.0	5.41E-03	1.19E-03	3.77E-01	5.71E-02
CSRNP_N	CSRNP1	3	5	2	0	0	1	1	0	0	0	0	0	0	0	0	0	1.10	1.25	0.99	0.08	0.03	0.29	0.06	0.05	1.00	0.55	0.07	0.9	0.8	0.0	0.0	0.0	0.0	0.0	0.0	0.0	0.0	0.0	9.77E-01	5.91E-01	7.34E-01	8.47E-01
	CSRNP2	0	0	1	0	0	0	1	0	0	0	0	0	0	0	0	0	0.59	0.67	0.53	0.04	0.02	0.15	0.03	0.03	0.53	0.29	0.04	0.0	1.5	0.0	0.0	0.0	0.0	0.0	0.0	0.0	0.0	0.0	9.92E-01	5.27E-01	8.04E-01	9.56E-01
	CSRNP3	0	2	0	0	0	0	0	0	0	0	0	0	0	0	0	0	0.11	0.13	0.10	0.01	0.00	0.03	0.01	0.01	0.10	0.05	0.01	0.0	0.0	0.0	0.0	0.0	0.0	0.0	0.0	0.0	0.0	0.0	1.00E+00	8.46E-01	9.14E-01	9.99E-01
DBP	YBX1	81	132	86	1	128	18	48	20	0	1	1	4	0	35	86	2	31.32	35.70	28.31	2.38	0.91	8.16	1.74	1.50	28.44	15.69	2.03	0.6	1.3	0.7	0.0	1.1	0.1	2.3	0.0	1.2	5.5	1.0	1.90E-67	2.09E-03	1.22E-01	1.31E-68
	YBX2	34	15	24	0	69	7	13	4	0	0	0	0	0	18	49	2	11.16	12.72	10.09	0.85	0.32	2.91	0.62	0.54	10.13	5.59	0.72	0.6	1.0	0.4	0.0	0.0	0.0	0.0	0.0	1.8	8.8	2.8	2.15E-70	7.32E-02	2.79E-01	2.17E-73
	YBX3	34	56	28	4	27	7	13	8	3	1	0	1	0	19	7	0	13.90	15.85	12.57	1.06	0.40	3.62	0.77	0.67	12.62	6.96	0.90	0.5	0.8	0.6	2.8	2.5	0.0	1.3	0.0	1.5	1.0	0.0	4.67E-02	6.09E-02	3.48E-02	1.32E-01
FH/WH.E2F	E2F1	100	118	31	0	1	2	1	28	0	0	0	0	0	1	0	0	16.17	18.43	14.62	1.23	0.47	4.21	0.90	0.78	14.68	8.10	1.05	0.1	0.1	1.9	0.0	0.0	0.0	0.0	0.0	0.1	0.0	0.0	3.32E-11	1.15E-09	1.92E-01	4.00E-05
	E2F2	18	6	2	0	0	0	2	0	0	0	0	0	0	0	0	0	1.98	2.25	1.79	0.15	0.06	0.51	0.11	0.09	1.79	0.99	0.13	0.0	0.9	0.0	0.0	0.0	0.0	0.0	0.0	0.0	0.0	0.0	6.65E-01	1.50E-01	6.49E-01	6.04E-01
	E2F3	28	17	3	0	0	0	2	1	0	0	0	0	0	0	0	0	3.22	3.67	2.91	0.24	0.09	0.84	0.18	0.15	2.92	1.61	0.21	0.0	0.5	0.3	0.0	0.0	0.0	0.0	0.0	0.0	0.0	0.0	3.21E-01	7.31E-02	5.61E-01	3.14E-01
	E2F4	47	91	64	0	26	26	18	20	0	0	6	2	0	8	9	1	20.49	23.35	18.52	1.56	0.59	5.34	1.14	0.98	18.61	10.26	1.33	1.3	0.8	1.1	0.0	0.0	1.1	1.8	0.0	0.4	0.9	0.8	2.25E-01	2.43E-01	1.42E-01	1.57E-01
	E2F5	11	5	1	0	0	0	0	1	0	0	0	0	0	0	0	0	1.21	1.38	1.09	0.09	0.04	0.31	0.07	0.06	1.10	0.60	0.08	0.0	0.0	0.9	0.0	0.0	0.0	0.0	0.0	0.0	0.0	0.0	8.95E-01	2.74E-01	7.22E-01	8.18E-01
	E2F6	89	93	26	0	2	0	0	26	0	0	0	0	0	2	0	0	11.75	13.39	10.61	0.89	0.34	3.06	0.65	0.56	10.66	5.88	0.76	0.0	0.0	2.4	0.0	0.0	0.0	0.0	0.0	0.2	0.0	0.0	1.98E-10	5.02E-11	2.67E-01	3.00E-03
	E2F7	12	8	3	0	0	0	2	1	0	0	0	0	0	0	0	0	1.79	2.04	1.62	0.14	0.05	0.47	0.10	0.09	1.63	0.90	0.12	0.0	1.0	0.6	0.0	0.0	0.0	0.0	0.0	0.0	0.0	0.0	8.54E-01	3.62E-01	6.64E-01	6.55E-01
	E2F8	3	2	2	0	0	0	2	0	0	0	0	0	0	0	0	0	0.95	1.08	0.86	0.07	0.03	0.25	0.05	0.05	0.86	0.48	0.06	0.0	1.8	0.0	0.0	0.0	0.0	0.0	0.0	0.0	0.0	0.0	9.26E-01	2.75E-01	7.52E-01	8.83E-01
	TFDP1	85	60	11	0	1	3	1	7	0	0	0	0	0	1	0	0	8.64	9.84	7.80	0.66	0.25	2.25	0.48	0.41	7.84	4.33	0.56	0.3	0.1	0.9	0.0	0.0	0.0	0.0	0.0	0.1	0.0	0.0	3.00E-03	2.88E-03	3.41E-01	1.56E-02
	TFDP2	37	13	2	0	1	1	0	1	0	0	0	0	0	0	0	1	3.55	4.04	3.21	0.27	0.10	0.92	0.20	0.17	3.22	1.78	0.23	0.3	0.0	0.3	0.0	0.0	0.0	0.0	0.0	0.0	0.0	4.4	8.26E-02	2.48E-02	5.41E-01	1.14E-01
	TFDP3	9	4	0	0	0	0	0	0	0	0	0	0	0	0	0	0	2.38	2.71	2.15	0.18	0.07	0.62	0.13	0.11	2.16	1.19	0.15	0.0	0.0	0.0	0.0	0.0	0.0	0.0	0.0	0.0	0.0	0.0	2.95E-01	2.68E-02	6.17E-01	4.97E-01
FH/WH.FOX	FOXA1	60	65	47	4	38	19	21	7	2	2	1	0	1	25	10	1	20.16	22.98	18.22	1.53	0.58	5.25	1.12	0.97	18.31	10.10	1.31	0.9	0.9	0.4	1.3	3.4	0.2	0.0	1.0	1.4	1.0	0.8	5.85E-02	2.81E-02	5.90E-02	2.14E-01
	FOXA2	17	5	1	0	0	0	1	0	0	0	0	0	0	0	0	0	1.10	1.25	0.99	0.08	0.03	0.29	0.06	0.05	1.00	0.55	0.07	0.0	0.8	0.0	0.0	0.0	0.0	0.0	0.0	0.0	0.0	0.0	9.34E-01	3.43E-01	7.34E-01	8.47E-01
	FOXA3	13	9	4	0	0	1	2	1	0	0	0	0	0	0	0	0	1.94	2.21	1.75	0.15	0.06	0.51	0.11	0.09	1.76	0.97	0.13	0.5	0.9	0.6	0.0	0.0	0.0	0.0	0.0	0.0	0.0	0.0	9.18E-01	6.71E-01	6.52E-01	6.14E-01
	FOXB1	17	12	6	1	14	3	3	0	1	0	1	0	1	0	11	1	4.87	5.55	4.40	0.37	0.14	1.27	0.27	0.23	4.42	2.44	0.32	0.6	0.5	0.0	2.7	0.0	0.8	0.0	4.3	0.0	4.5	3.2	1.25E-06	4.32E-02	2.71E-01	2.57E-07
	FOXB2	0	0	0	0	0	0	0	0	0	0	0	0	0	0	0	0	0.00	0.00	0.00	0.00	0.00	0.00	0.00	0.00	0.00	0.00	0.00	na	na	na	na	na	na	na	na	na	na	na	na	na	na	na
	FOXC1	88	41	39	0	0	11	21	7	0	0	0	0	0	0	0	0	9.37	10.68	8.47	0.71	0.27	2.44	0.52	0.45	8.51	4.69	0.61	1.2	2.0	0.8	0.0	0.0	0.0	0.0	0.0	0.0	0.0	0.0	1.38E-03	5.19E-03	3.21E-01	4.11E-03
	FOXC2	26	5	1	0	0	0	0	1	0	0	0	0	0	0	0	0	1.61	1.83	1.45	0.12	0.05	0.42	0.09	0.08	1.46	0.81	0.10	0.0	0.0	0.7	0.0	0.0	0.0	0.0	0.0	0.0	0.0	0.0	7.52E-01	1.66E-01	6.81E-01	7.06E-01
	FOXD1	2	4	0	0	0	0	0	0	0	0	0	0	0	0	0	0	0.22	0.25	0.20	0.02	0.01	0.06	0.01	0.01	0.20	0.11	0.01	0.0	0.0	0.0	0.0	0.0	0.0	0.0	0.0	0.0	0.0	0.0	1.00E+00	7.16E-01	8.79E-01	9.95E-01
	FOXD2	9	2	0	0	0	0	0	0	0	0	0	0	0	0	0	0	1.06	1.21	0.96	0.08	0.03	0.28	0.06	0.05	0.96	0.53	0.07	0.0	0.0	0.0	0.0	0.0	0.0	0.0	0.0	0.0	0.0	0.0	8.71E-01	1.99E-01	7.38E-01	8.56E-01
	FOXD3	9	0	0	0	0	0	0	0	0	0	0	0	0	0	0	0	1.02	1.17	0.93	0.08	0.03	0.27	0.06	0.05	0.93	0.51	0.07	0.0	0.0	0.0	0.0	0.0	0.0	0.0	0.0	0.0	0.0	0.0	8.84E-01	2.10E-01	7.43E-01	8.65E-01
	FOXD4	7	13	2	0	1	1	1	0	0	0	1	0	0	0	0	0	3.07	3.50	2.78	0.23	0.09	0.80	0.17	0.15	2.79	1.54	0.20	0.3	0.3	0.0	0.0	0.0	1.2	0.0	0.0	0.0	0.0	0.0	3.43E-01	5.07E-02	5.70E-01	4.28E-01
	FOXD4L1	7	3	1	0	0	0	1	0	0	0	0	0	0	0	0	0	1.13	1.29	1.03	0.09	0.03	0.30	0.06	0.05	1.03	0.57	0.07	0.0	0.8	0.0	0.0	0.0	0.0	0.0	0.0	0.0	0.0	0.0	9.26E-01	3.29E-01	7.30E-01	8.37E-01
	FOXD4L3	0	2	1	0	0	0	1	0	0	0	0	0	0	0	0	0	0.59	0.67	0.53	0.04	0.02	0.15	0.03	0.03	0.53	0.29	0.04	0.0	1.5	0.0	0.0	0.0	0.0	0.0	0.0	0.0	0.0	0.0	9.92E-01	5.27E-01	8.04E-01	9.56E-01
	FOXD4L4	0	0	0	0	0	0	0	0	0	0	0	0	0	0	0	0	0.04	0.04	0.03	0.00	0.00	0.01	0.00	0.00	0.03	0.02	0.00	0.0	0.0	0.0	0.0	0.0	0.0	0.0	0.0	0.0	0.0	0.0	1.00E+00	9.46E-01	9.51E-01	1.00E+00
	FOXD4L5	0	0	0	0	0	0	0	0	0	0	0	0	0	0	0	0	0.00	0.00	0.00	0.00	0.00	0.00	0.00	0.00	0.00	0.00	0.00	na	na	na	na	na	na	na	na	na	na	na	na	na	na	na
	FOXD4L6	6	8	4	0	0	0	4	0	0	0	0	0	0	0	0	0	2.78	3.17	2.51	0.21	0.08	0.72	0.15	0.13	2.52	1.39	0.18	0.0	1.3	0.0	0.0	0.0	0.0	0.0	0.0	0.0	0.0	0.0	3.64E-01	6.36E-02	5.89E-01	4.03E-01
	FOXE1	33	9	7	1	0	1	3	3	1	0	0	0	0	0	0	0	2.89	3.29	2.61	0.22	0.08	0.75	0.16	0.14	2.62	1.45	0.19	0.3	0.9	1.1	4.6	0.0	0.0	0.0	0.0	0.0	0.0	0.0	4.87E-01	5.17E-01	9.12E-02	3.79E-01
	FOXE3	0	3	0	0	0	0	0	0	0	0	0	0	0	0	0	0	0.26	0.29	0.23	0.02	0.01	0.07	0.01	0.01	0.23	0.13	0.02	0.0	0.0	0.0	0.0	0.0	0.0	0.0	0.0	0.0	0.0	0.0	9.99E-01	6.77E-01	8.70E-01	9.93E-01
	FOXF1	12	2	1	0	0	1	0	0	0	0	0	0	0	0	0	0	1.72	1.96	1.55	0.13	0.05	0.45	0.10	0.08	1.56	0.86	0.11	0.6	0.0	0.0	0.0	0.0	0.0	0.0	0.0	0.0	0.0	0.0	7.11E-01	1.48E-01	6.71E-01	6.75E-01
	FOXF2	7	6	1	0	0	0	1	0	0	0	0	0	0	0	0	0	2.45	2.79	2.22	0.19	0.07	0.64	0.14	0.12	2.23	1.23	0.16	0.0	0.4	0.0	0.0	0.0	0.0	0.0	0.0	0.0	0.0	0.0	3.91E-01	5.45E-02	6.12E-01	4.79E-01
	FOXG1	19	17	8	1	2	1	3	4	1	0	0	0	2	0	0	0	3.51	4.00	3.17	0.27	0.10	0.91	0.20	0.17	3.19	1.76	0.23	0.3	0.7	1.3	3.7	0.0	0.0	0.0	11.9	0.0	0.0	0.0	6.99E-04	3.22E-01	1.46E-01	8.31E-05
	FOXH1	45	3	1	1	1	0	1	0	1	0	0	0	0	0	0	1	4.43	5.05	4.00	0.34	0.13	1.15	0.25	0.21	4.02	2.22	0.29	0.0	0.2	0.0	3.0	0.0	0.0	0.0	0.0	0.0	0.0	3.5	1.18E-02	2.92E-03	2.31E-01	8.66E-02
	FOXI1	23	19	2	1	0	0	0	2	1	0	0	0	0	0	0	0	3.33	3.79	3.01	0.25	0.10	0.87	0.19	0.16	3.02	1.67	0.22	0.0	0.0	0.7	4.0	0.0	0.0	0.0	0.0	0.0	0.0	0.0	1.03E-01	2.40E-02	1.29E-01	2.95E-01
	FOXI2	8	5	2	2	2	0	1	1	1	1	0	0	1	0	0	1	1.90	2.17	1.72	0.14	0.06	0.50	0.11	0.09	1.73	0.95	0.12	0.0	0.5	0.6	6.9	18.1	0.0	0.0	10.9	0.0	0.0	8.1	5.82E-06	2.43E-01	4.08E-06	2.32E-03
	FOXI3	0	0	0	0	0	0	0	0	0	0	0	0	0	0	0	0	0.07	0.08	0.07	0.01	0.00	0.02	0.00	0.00	0.07	0.04	0.00	0.0	0.0	0.0	0.0	0.0	0.0	0.0	0.0	0.0	0.0	0.0	1.00E+00	8.95E-01	9.30E-01	1.00E+00
	FOXJ1	13	5	1	0	0	0	0	1	0	0	0	0	0	0	0	0	1.54	1.75	1.39	0.12	0.04	0.40	0.09	0.07	1.40	0.77	0.10	0.0	0.0	0.7	0.0	0.0	0.0	0.0	0.0	0.0	0.0	0.0	7.82E-01	1.83E-01	6.88E-01	7.27E-01
	FOXJ2	34	10	6	1	0	3	1	2	1	0	0	0	0	0	0	0	3.11	3.54	2.81	0.24	0.09	0.81	0.17	0.15	2.82	1.56	0.20	1.0	0.3	0.7	4.2	0.0	0.0	0.0	0.0	0.0	0.0	0.0	4.12E-01	3.56E-01	1.10E-01	3.35E-01
	FOXJ3	11	7	2	0	0	0	2	0	0	0	0	0	0	0	0	0	1.21	1.38	1.09	0.09	0.04	0.31	0.07	0.06	1.10	0.60	0.08	0.0	1.5	0.0	0.0	0.0	0.0	0.0	0.0	0.0	0.0	0.0	8.96E-01	2.75E-01	7.22E-01	8.18E-01
	FOXK1	68	56	17	0	1	3	3	11	0	0	1	0	0	0	0	0	10.54	12.01	9.52	0.80	0.31	2.74	0.59	0.51	9.57	5.28	0.68	0.3	0.2	1.2	0.0	0.0	0.4	0.0	0.0	0.0	0.0	0.0	5.40E-04	2.05E-03	2.93E-01	3.30E-03
	FOXK2	86	84	26	0	2	4	6	16	0	0	2	0	0	0	0	0	11.96	13.64	10.81	0.91	0.35	3.12	0.67	0.57	10.86	5.99	0.77	0.3	0.4	1.5	0.0	0.0	0.6	0.0	0.0	0.0	0.0	0.0	3.18E-04	2.40E-03	2.62E-01	1.71E-03
	FOXL1	49	32	10	2	7	2	5	3	2	0	1	0	1	0	4	1	7.43	8.47	6.71	0.56	0.22	1.93	0.41	0.36	6.74	3.72	0.48	0.3	0.6	0.4	3.5	0.0	0.5	0.0	2.8	0.0	1.1	2.1	2.37E-02	2.43E-02	4.94E-02	9.59E-02
	FOXL2	9	8	10	1	7	4	4	2	1	0	0	0	0	1	5	1	2.63	3.00	2.38	0.20	0.08	0.69	0.15	0.13	2.39	1.32	0.17	1.5	1.3	0.8	5.0	0.0	0.0	0.0	0.0	0.4	3.8	5.9	2.54E-02	5.77E-01	7.06E-02	6.65E-03
	FOXM1	19	54	15	0	1	6	2	7	0	0	0	0	0	0	0	1	7.24	8.26	6.55	0.55	0.21	1.89	0.40	0.35	6.58	3.63	0.47	0.8	0.2	1.1	0.0	0.0	0.0	0.0	0.0	0.0	0.0	2.1	3.79E-02	8.26E-02	3.83E-01	1.95E-02
	FOXN1	18	3	0	0	0	0	0	0	0	0	0	0	0	0	0	0	1.35	1.54	1.22	0.10	0.04	0.35	0.08	0.07	1.23	0.68	0.09	0.0	0.0	0.0	0.0	0.0	0.0	0.0	0.0	0.0	0.0	0.0	7.49E-01	1.27E-01	7.06E-01	7.78E-01
	FOXN2	6	16	2	0	0	1	1	0	0	0	0	0	0	0	0	0	1.06	1.21	0.96	0.08	0.03	0.28	0.06	0.05	0.96	0.53	0.07	0.9	0.8	0.0	0.0	0.0	0.0	0.0	0.0	0.0	0.0	0.0	9.80E-01	6.07E-01	7.38E-01	8.56E-01
	FOXN3	10	10	4	0	0	0	3	1	0	0	0	0	0	0	0	0	2.27	2.59	2.05	0.17	0.07	0.59	0.13	0.11	2.06	1.14	0.15	0.0	1.2	0.5	0.0	0.0	0.0	0.0	0.0	0.0	0.0	0.0	6.99E-01	2.38E-01	6.25E-01	5.25E-01
	FOXN4	1	1	0	0	0	0	0	0	0	0	0	0	0	0	0	0	0.26	0.29	0.23	0.02	0.01	0.07	0.01	0.01	0.23	0.13	0.02	0.0	0.0	0.0	0.0	0.0	0.0	0.0	0.0	0.0	0.0	0.0	9.99E-01	6.77E-01	8.70E-01	9.93E-01
	FOXO1	47	67	42	0	20	7	16	19	0	0	4	1	2	13	0	0	15.84	18.06	14.32	1.20	0.46	4.13	0.88	0.76	14.39	7.94	1.03	0.4	0.9	1.3	0.0	0.0	1.0	1.1	2.6	0.9	0.0	0.0	3.44E-02	3.51E-02	1.97E-01	4.88E-02
	FOXO3	21	62	17	0	0	0	4	13	0	0	0	0	0	0	0	0	6.18	7.05	5.59	0.47	0.18	1.61	0.34	0.30	5.61	3.10	0.40	0.0	0.6	2.3	0.0	0.0	0.0	0.0	0.0	0.0	0.0	0.0	1.10E-03	1.72E-04	4.20E-01	4.46E-02
	FOXO4	10	25	1	0	0	0	0	1	0	0	0	0	0	0	0	0	1.90	2.17	1.72	0.14	0.06	0.50	0.11	0.09	1.73	0.95	0.12	0.0	0.0	0.6	0.0	0.0	0.0	0.0	0.0	0.0	0.0	0.0	6.22E-01	1.12E-01	6.55E-01	6.24E-01
	FOXO6	6	3	1	0	0	0	0	1	0	0	0	0	0	0	0	0	0.59	0.67	0.53	0.04	0.02	0.15	0.03	0.03	0.53	0.29	0.04	0.0	0.0	1.9	0.0	0.0	0.0	0.0	0.0	0.0	0.0	0.0	9.86E-01	4.33E-01	8.04E-01	9.56E-01
	FOXP1	118	27	26	2	15	11	14	1	1	1	0	0	0	15	0	0	10.03	11.43	9.06	0.76	0.29	2.61	0.56	0.48	9.10	5.02	0.65	1.1	1.2	0.1	1.3	3.4	0.0	0.0	0.0	1.6	0.0	0.0	1.15E-02	1.98E-02	1.79E-01	2.21E-02
	FOXP2	60	20	2	0	2	1	1	0	0	0	0	0	0	0	2	0	4.24	4.84	3.84	0.32	0.12	1.11	0.24	0.20	3.85	2.13	0.27	0.2	0.2	0.0	0.0	0.0	0.0	0.0	0.0	0.0	0.9	0.0	1.15E-01	9.28E-03	5.04E-01	3.38E-01
	FOXP3	38	31	17	0	0	2	5	10	0	0	0	0	0	0	0	0	4.32	4.92	3.90	0.33	0.13	1.12	0.24	0.21	3.92	2.16	0.28	0.5	1.0	2.6	0.0	0.0	0.0	0.0	0.0	0.0	0.0	0.0	3.82E-02	4.57E-03	5.01E-01	1.60E-01
	FOXP4	90	4	2	0	2	1	1	0	0	0	0	0	0	0	2	0	4.50	5.13	4.07	0.34	0.13	1.17	0.25	0.22	4.09	2.25	0.29	0.2	0.2	0.0	0.0	0.0	0.0	0.0	0.0	0.0	0.9	0.0	8.29E-02	6.36E-03	4.92E-01	3.02E-01
	FOXQ1	25	21	14	1	1	8	3	3	1	0	0	0	0	1	0	0	5.09	5.80	4.60	0.39	0.15	1.32	0.28	0.24	4.62	2.55	0.33	1.6	0.5	0.7	2.6	0.0	0.0	0.0	0.0	0.2	0.0	0.0	2.68E-01	1.68E-01	2.90E-01	1.82E-01
	FOXR1	13	42	8	0	1	0	0	8	0	0	0	0	0	0	0	1	3.33	3.79	3.01	0.25	0.10	0.87	0.19	0.16	3.02	1.67	0.22	0.0	0.0	2.7	0.0	0.0	0.0	0.0	0.0	0.0	0.0	4.6	6.36E-03	4.52E-04	5.54E-01	1.19E-01
	FOXR2	19	40	7	0	1	0	0	7	0	0	1	0	0	0	0	0	3.55	4.04	3.21	0.27	0.10	0.92	0.20	0.17	3.22	1.78	0.23	0.0	0.0	2.2	0.0	0.0	1.1	0.0	0.0	0.0	0.0	0.0	5.40E-02	2.38E-03	5.41E-01	3.47E-01
	FOXS1	16	14	7	1	5	1	4	2	0	1	0	0	0	0	4	1	3.51	4.00	3.17	0.27	0.10	0.91	0.20	0.17	3.19	1.76	0.23	0.3	1.0	0.6	0.0	9.8	0.0	0.0	0.0	0.0	2.3	4.4	2.60E-02	3.28E-01	4.23E-03	7.67E-02
FH/WH.RFX	RFX1	31	19	6	0	0	2	0	4	0	0	0	0	0	0	0	0	3.15	3.59	2.84	0.24	0.09	0.82	0.18	0.15	2.86	1.58	0.20	0.6	0.0	1.4	0.0	0.0	0.0	0.0	0.0	0.0	0.0	0.0	3.91E-01	1.07E-01	5.65E-01	3.28E-01
	RFX2	15	3	1	0	0	0	1	0	0	0	0	0	0	0	0	0	1.13	1.29	1.03	0.09	0.03	0.30	0.06	0.05	1.03	0.57	0.07	0.0	0.8	0.0	0.0	0.0	0.0	0.0	0.0	0.0	0.0	0.0	9.26E-01	3.29E-01	7.30E-01	8.37E-01
	RFX3	19	12	5	0	0	0	2	3	0	0	0	0	0	0	0	0	2.01	2.29	1.82	0.15	0.06	0.52	0.11	0.10	1.83	1.01	0.13	0.0	0.9	1.6	0.0	0.0	0.0	0.0	0.0	0.0	0.0	0.0	7.51E-01	2.44E-01	6.46E-01	5.94E-01
	RFX4	4	0	0	0	0	0	0	0	0	0	0	0	0	0	0	0	0.15	0.17	0.13	0.01	0.00	0.04	0.01	0.01	0.13	0.07	0.01	0.0	0.0	0.0	0.0	0.0	0.0	0.0	0.0	0.0	0.0	0.0	1.00E+00	8.00E-01	9.01E-01	9.98E-01
	RFX5	16	11	4	2	2	0	2	2	2	0	0	0	0	0	2	0	2.45	2.79	2.22	0.19	0.07	0.64	0.14	0.12	2.23	1.23	0.16	0.0	0.7	0.9	10.7	0.0	0.0	0.0	0.0	0.0	1.6	0.0	7.14E-03	2.59E-01	2.56E-05	5.84E-01
	RFX6	9	7	4	1	1	4	0	0	1	0	0	1	0	0	0	0	1.87	2.13	1.69	0.14	0.05	0.49	0.10	0.09	1.69	0.93	0.12	2.1	0.0	0.0	7.0	0.0	0.0	9.6	0.0	0.0	0.0	0.0	1.25E-02	4.39E-02	2.20E-02	5.04E-02
	RFX7	6	7	1	0	0	0	1	0	0	0	0	0	0	0	0	0	0.99	1.13	0.89	0.08	0.03	0.26	0.06	0.05	0.90	0.49	0.06	0.0	0.9	0.0	0.0	0.0	0.0	0.0	0.0	0.0	0.0	0.0	9.55E-01	3.88E-01	7.47E-01	8.74E-01
	RFX8	0	1	0	0	0	0	0	0	0	0	0	0	0	0	0	0	0.04	0.04	0.03	0.00	0.00	0.01	0.00	0.00	0.03	0.02	0.00	0.0	0.0	0.0	0.0	0.0	0.0	0.0	0.0	0.0	0.0	0.0	1.00E+00	9.46E-01	9.51E-01	1.00E+00
GCM	GCM1	22	27	10	0	0	0	0	10	0	0	0	0	0	0	0	0	3.18	3.63	2.88	0.24	0.09	0.83	0.18	0.15	2.89	1.59	0.21	0.0	0.0	3.5	0.0	0.0	0.0	0.0	0.0	0.0	0.0	0.0	6.75E-04	4.91E-06	5.63E-01	3.21E-01

GCM	GCM2	18	5	0	1	0	0	0	0	1	0	0	0	0	0	0	0	1.68	1.92	1.52	0.13	0.05	0.44	0.09	0.08	1.53	0.84	0.11	0.0	0.0	0.0	7.8	0.0	0.0	0.0	0.0	0.0	0.0	0.0	1.64E-01	7.72E-02	1.44E-02	6.86E-01
GHD.CP2	GRHL1	7	0	0	0	0	0	0	0	0	0	0	0	0	0	0	0	0.33	0.38	0.30	0.03	0.01	0.09	0.02	0.02	0.30	0.16	0.02	0.0	0.0	0.0	0.0	0.0	0.0	0.0	0.0	0.0	0.0	0.0	9.98E-01	6.06E-01	8.52E-01	9.88E-01
	GRHL2	12	1	1	0	0	0	1	0	0	0	0	0	0	0	0	0	0.62	0.71	0.56	0.05	0.02	0.16	0.03	0.03	0.56	0.31	0.04	0.0	1.4	0.0	0.0	0.0	0.0	0.0	0.0	0.0	0.0	0.0	9.91E-01	5.21E-01	7.98E-01	9.50E-01
	GRHL3	8	7	3	0	0	0	0	3	0	0	0	0	0	0	0	0	0.66	0.75	0.60	0.05	0.02	0.17	0.04	0.03	0.60	0.33	0.04	0.0	0.0	5.0	0.0	0.0	0.0	0.0	0.0	0.0	0.0	0.0	2.59E-01	3.84E-03	7.93E-01	9.44E-01
	TFCP2	43	80	24	0	53	5	14	5	0	0	3	2	2	43	2	1	18.77	21.39	16.96	1.43	0.54	4.89	1.05	0.90	17.04	9.40	1.22	0.3	0.7	0.3	0.0	0.0	0.6	1.9	2.2	2.5	0.2	0.8	2.38E-11	2.63E-05	1.60E-01	3.04E-09
	TFCP2L1	5	1	1	0	0	0	0	1	0	0	0	0	0	0	0	0	0.29	0.33	0.26	0.02	0.01	0.08	0.02	0.01	0.27	0.15	0.02	0.0	0.0	3.8	0.0	0.0	0.0	0.0	0.0	0.0	0.0	0.0	9.75E-01	2.63E-01	8.61E-01	9.91E-01
	UBP1	23	12	4	0	1	0	2	2	0	0	0	0	0	1	0	0	2.78	3.17	2.51	0.21	0.08	0.72	0.15	0.13	2.52	1.39	0.18	0.0	0.6	0.8	0.0	0.0	0.0	0.0	0.0	0.4	0.0	0.0	7.14E-01	1.90E-01	5.89E-01	6.22E-01
GTF2I	GTF2I	62	99	41	0	2	5	10	26	0	0	1	0	0	0	1	0	13.39	15.26	12.10	1.02	0.39	3.49	0.75	0.64	12.16	6.71	0.87	0.4	0.7	2.1	0.0	0.0	0.3	0.0	0.0	0.0	0.1	0.0	1.78E-06	9.98E-06	2.36E-01	7.94E-04
	GTF2IRD1	17	24	7	1	0	0	4	3	0	1	0	0	0	0	0	0	4.72	5.38	4.27	0.36	0.14	1.23	0.26	0.23	4.29	2.36	0.31	0.0	0.7	0.7	0.0	7.3	0.0	0.0	0.0	0.0	0.0	0.0	3.00E-02	6.56E-02	1.60E-02	1.23E-01
	GTF2IRD2	5	1	0	0	1	0	0	0	0	0	0	0	0	1	0	0	0.48	0.54	0.43	0.04	0.01	0.12	0.03	0.02	0.43	0.24	0.03	0.0	0.0	0.0	0.0	0.0	0.0	0.0	0.0	2.3	0.0	0.0	9.88E-01	4.85E-01	8.23E-01	9.46E-01
	GTF2IRD2B	8	2	0	0	1	0	0	0	0	0	0	0	0	1	0	0	0.70	0.79	0.63	0.05	0.02	0.18	0.04	0.03	0.63	0.35	0.05	0.0	0.0	0.0	0.0	0.0	0.0	0.0	0.0	1.6	0.0	0.0	9.80E-01	3.47E-01	7.87E-01	9.73E-01
HB	ANHX	0	0	0	0	0	0	0	0	0	0	0	0	0	0	0	0	0.00	0.00	0.00	0.00	0.00	0.00	0.00	0.00	0.00	0.00	0.00	na	na	na	na	na	na	na	na	na	na	na	na	na	na	na
	CPHXL	0	0	0	0	0	0	0	0	0	0	0	0	0	0	0	0	0.00	0.00	0.00	0.00	0.00	0.00	0.00	0.00	0.00	0.00	0.00	na	na	na	na	na	na	na	na	na	na	na	na	na	na	na
	DUXB	2	3	0	0	0	0	0	0	0	0	0	0	0	0	0	0	0.62	0.71	0.56	0.05	0.02	0.16	0.03	0.03	0.56	0.31	0.04	0.0	0.0	0.0	0.0	0.0	0.0	0.0	0.0	0.0	0.0	0.0	9.79E-01	3.88E-01	7.98E-01	9.50E-01
	NANOGP8	0	0	0	0	0	0	0	0	0	0	0	0	0	0	0	0	0.00	0.00	0.00	0.00	0.00	0.00	0.00	0.00	0.00	0.00	0.00	na	na	na	na	na	na	na	na	na	na	na	na	na	na	na
HB-CUT	CUX1	59	34	14	1	1	0	5	9	1	0	0	1	0	0	0	0	8.31	9.47	7.51	0.63	0.24	2.16	0.46	0.40	7.54	4.16	0.54	0.0	0.5	1.2	1.6	0.0	0.0	2.2	0.0	0.0	0.0	0.0	3.02E-03	4.72E-03	5.00E-01	8.69E-03
	CUX2	1	0	1	0	0	0	1	0	0	0	0	0	0	0	0	0	0.33	0.38	0.30	0.03	0.01	0.09	0.02	0.02	0.30	0.16	0.02	0.0	2.7	0.0	0.0	0.0	0.0	0.0	0.0	0.0	0.0	0.0	9.93E-01	4.35E-01	8.52E-01	9.88E-01
	ONECUT1	6	1	1	0	0	0	0	1	0	0	0	0	0	0	0	0	0.48	0.54	0.43	0.04	0.01	0.12	0.03	0.02	0.43	0.24	0.03	0.0	0.0	2.3	0.0	0.0	0.0	0.0	0.0	0.0	0.0	0.0	9.88E-01	4.12E-01	8.23E-01	9.72E-01
	ONECUT2	1	0	2	0	0	0	1	1	0	0	0	0	0	0	0	0	0.22	0.25	0.20	0.02	0.01	0.06	0.01	0.01	0.20	0.11	0.01	0.0	4.0	5.0	0.0	0.0	0.0	0.0	0.0	0.0	0.0	0.0	8.04E-01	5.77E-02	8.79E-01	9.95E-01
	ONECUT3	0	0	0	0	0	0	0	0	0	0	0	0	0	0	0	0	0.15	0.17	0.13	0.01	0.00	0.04	0.01	0.01	0.13	0.07	0.01	0.0	0.0	0.0	0.0	0.0	0.0	0.0	0.0	0.0	0.0	0.0	1.00E+00	8.00E-01	9.01E-01	9.98E-01
	SATB1	80	43	21	0	0	0	3	18	0	0	0	0	0	0	0	0	6.40	7.30	5.79	0.49	0.19	1.67	0.36	0.31	5.81	3.21	0.41	0.0	0.4	3.1	0.0	0.0	0.0	0.0	0.0	0.0	0.0	0.0	8.87E-07	2.90E-08	4.12E-01	3.81E-02
	SATB2	105	19	10	0	0	0	2	8	0	0	0	0	0	0	0	0	6.55	7.46	5.92	0.50	0.19	1.71	0.36	0.31	5.95	3.28	0.42	0.0	0.3	1.4	0.0	0.0	0.0	0.0	0.0	0.0	0.0	0.0	7.58E-03	3.55E-03	4.07E-01	3.43E-02
HB-PAX	PAX3	13	13	3	0	0	0	0	3	0	0	0	0	0	0	0	0	2.49	2.84	2.25	0.19	0.07	0.65	0.14	0.12	2.26	1.25	0.16	0.0	0.0	1.3	0.0	0.0	0.0	0.0	0.0	0.0	0.0	0.0	4.05E-01	6.16E-02	6.09E-01	4.70E-01
	PAX4	1	4	2	0	0	0	2	0	0	0	0	0	0	0	0	0	0.70	0.79	0.63	0.05	0.02	0.18	0.04	0.03	0.63	0.35	0.05	0.0	2.5	0.0	0.0	0.0	0.0	0.0	0.0	0.0	0.0	0.0	9.21E-01	2.06E-01	7.87E-01	9.37E-01
	PAX6	131	51	16	0	1	2	2	12	0	0	0	0	0	0	1	0	13.98	15.93	12.63	1.06	0.41	3.64	0.78	0.67	12.69	7.00	0.90	0.1	0.1	0.9	0.0	0.0	0.0	0.0	0.0	0.0	0.1	0.0	6.82E-07	1.32E-05	2.26E-01	2.34E-04
	PAX7	51	26	4	0	0	0	0	4	0	0	0	0	0	0	0	0	4.79	5.46	4.33	0.36	0.14	1.25	0.27	0.23	4.35	2.40	0.31	0.0	0.0	0.9	0.0	0.0	0.0	0.0	0.0	0.0	0.0	0.0	3.33E-02	5.85E-03	4.78E-01	1.17E-01
HB-POU	HDX	2	7	1	0	0	0	0	1	0	0	0	0	0	0	0	0	1.06	1.21	0.96	0.08	0.03	0.28	0.06	0.05	0.96	0.53	0.07	0.0	0.0	1.0	0.0	0.0	0.0	0.0	0.0	0.0	0.0	0.0	9.31E-01	3.21E-01	7.38E-01	8.56E-01
	HMBOX1	31	20	12	0	7	6	2	4	0	0	0	1	0	3	2	1	5.63	6.42	5.09	0.43	0.16	1.47	0.31	0.27	5.12	2.82	0.36	1.1	0.3	0.8	0.0	0.0	0.0	3.2	0.0	0.6	0.7	2.7	4.99E-01	1.92E-01	4.42E-01	3.62E-01
	HNF1A	25	32	13	0	0	1	0	12	0	0	0	0	0	0	0	0	3.62	4.13	3.27	0.28	0.11	0.94	0.20	0.17	3.29	1.81	0.23	0.3	0.0	3.7	0.0	0.0	0.0	0.0	0.0	0.0	0.0	0.0	7.40E-05	4.37E-07	5.37E-01	2.47E-01
	HNF1B	84	47	6	0	4	1	2	3	0	0	0	0	0	4	0	0	7.46	8.51	6.75	0.57	0.22	1.94	0.42	0.36	6.78	3.74	0.48	0.1	0.2	0.4	0.0	0.0	0.0	0.0	0.0	0.6	0.0	0.0	1.78E-02	1.79E-03	3.76E-01	1.52E-01
	POU1F1	23	3	0	0	0	0	0	0	0	0	0	0	0	0	0	0	1.39	1.58	1.26	0.11	0.04	0.36	0.08	0.07	1.26	0.70	0.09	0.0	0.0	0.0	0.0	0.0	0.0	0.0	0.0	0.0	0.0	0.0	7.32E-01	1.21E-01	7.02E-01	7.68E-01
	POU2F1	89	26	14	0	6	3	5	6	0	0	0	0	0	6	0	0	7.98	9.09	7.21	0.61	0.23	2.08	0.44	0.38	7.24	4.00	0.52	0.4	0.6	0.8	0.0	0.0	0.0	0.0	0.0	0.8	0.0	0.0	1.91E-01	7.62E-02	3.60E-01	1.78E-01
	POU2F2	20	3	2	0	0	0	0	2	0	0	0	0	0	0	0	0	1.32	1.50	1.19	0.10	0.04	0.34	0.07	0.06	1.20	0.66	0.09	0.0	0.0	1.7	0.0	0.0	0.0	0.0	0.0	0.0	0.0	0.0	8.21E-01	1.86E-01	7.10E-01	7.88E-01
	POU2F3	2	0	0	0	0	0	0	0	0	0	0	0	0	0	0	0	0.29	0.33	0.26	0.02	0.01	0.08	0.02	0.01	0.27	0.15	0.02	0.0	0.0	0.0	0.0	0.0	0.0	0.0	0.0	0.0	0.0	0.0	9.99E-01	6.41E-01	8.61E-01	9.91E-01
	POU3F1	1	2	1	0	0	0	0	1	0	0	0	0	0	0	0	0	0.51	0.58	0.46	0.04	0.01	0.13	0.03	0.02	0.47	0.26	0.03	0.0	0.0	2.2	0.0	0.0	0.0	0.0	0.0	0.0	0.0	0.0	9.87E-01	4.23E-01	8.17E-01	9.67E-01
	POU3F2	8	3	4	0	0	2	0	2	0	0	0	0	0	0	0	0	0.77	0.88	0.69	0.06	0.02	0.20	0.04	0.04	0.70	0.38	0.05	2.6	0.0	2.9	0.0	0.0	0.0	0.0	0.0	0.0	0.0	0.0	7.44E-01	7.05E-02	7.76E-01	9.23E-01
	POU3F3	3	3	1	0	0	0	1	0	0	0	0	0	0	0	0	0	0.51	0.58	0.46	0.04	0.01	0.13	0.03	0.02	0.47	0.26	0.03	0.0	1.7	0.0	0.0	0.0	0.0	0.0	0.0	0.0	0.0	0.0	9.94E-01	5.29E-01	8.17E-01	9.67E-01
	POU3F4	0	0	1	0	0	0	1	0	0	0	0	0	0	0	0	0	0.04	0.04	0.03	0.00	0.00	0.01	0.00	0.00	0.03	0.02	0.00	0.0	24.0	0.0	0.0	0.0	0.0	0.0	0.0	0.0	0.0	0.0	1.43E-02	1.60E-05	9.51E-01	1.00E+00
	POU4F1	2	2	2	0	0	0	2	0	0	0	0	0	0	0	0	0	0.37	0.42	0.33	0.03	0.01	0.10	0.02	0.02	0.33	0.18	0.02	0.0	4.8	0.0	0.0	0.0	0.0	0.0	0.0	0.0	0.0	0.0	6.86E-01	3.50E-02	8.45E-01	9.84E-01
	POU4F2	5	2	0	0	0	0	0	0	0	0	0	0	0	0	0	0	2.85	3.25	2.58	0.22	0.08	0.74	0.16	0.14	2.59	1.43	0.18	0.0	0.0	0.0	0.0	0.0	0.0	0.0	0.0	0.0	0.0	0.0	1.63E-01	1.30E-02	5.84E-01	3.87E-01
	POU4F3	0	1	0	0	0	0	0	0	0	0	0	0	0	0	0	0	0.44	0.50	0.40	0.03	0.01	0.11	0.02	0.02	0.40	0.22	0.03	0.0	0.0	0.0	0.0	0.0	0.0	0.0	0.0	0.0	0.0	0.0	9.95E-01	5.13E-01	8.30E-01	9.77E-01
	POU5F1	70	56	21	0	22	8	7	6	0	0	2	0	0	11	9	0	13.83	15.76	12.50	1.05	0.40	3.60	0.77	0.66	12.56	6.93	0.90	0.6	0.4	0.5	0.0	0.0	0.6	0.0	0.0	0.9	1.3	0.0	9.91E-02	4.73E-03	2.28E-01	5.70E-01
	POU5F1B	0	0	0	0	0	0	0	0	0	0	0	0	0	0	0	0	0.00	0.00	0.00	0.00	0.00	0.00	0.00	0.00	0.00	0.00	0.00	na	na	na	na	na	na	na	na	na	na	na	na	na	na	na
	POU5F2	0	0	0	0	0	0	0	0	0	0	0	0	0	0	0	0	0.04	0.04	0.03	0.00	0.00	0.01	0.00	0.00	0.03	0.02	0.00	0.0	0.0	0.0	0.0	0.0	0.0	0.0	0.0	0.0	0.0	0.0	1.00E+00	9.46E-01	9.51E-01	1.00E+00
	POU6F1	1	1	0	0	0	0	0	0	0	0	0	0	0	0	0	0	0.11	0.13	0.10	0.01	0.00	0.03	0.01	0.01	0.10	0.05	0.01	0.0	0.0	0.0	0.0	0.0	0.0	0.0	0.0	0.0	0.0	0.0	1.00E+00	8.46E-01	9.14E-01	9.99E-01
	POU6F2	45	12	2	1	2	0	2	0	1	0	2	0	0	0	0	0	5.38	6.13	4.86	0.41	0.16	1.40	0.30	0.26	4.88	2.69	0.35	0.0	0.3	0.0	2.4	0.0	1.4	0.0	0.0	0.0	0.0	0.0	1.16E-02	1.49E-03	3.15E-01	1.20E-01
HB-ZF.C2H2	ADNP	50	68	21	0	2	1	0	20	0	0	1	0	0	1	0	0	8.45	9.63	7.64	0.64	0.25	2.20	0.47	0.41	7.67	4.23	0.55	0.1	0.0	2.6	0.0	0.0	0.5	0.0	0.0	0.1	0.0	0.0	3.72E-07	1.37E-08	3.46E-01	3.32E-02
	ADNP2	9	23	11	0	0	1	4	6	0	0	0	0	0	0	0	0	2.60	2.96	2.35	0.20	0.08	0.68	0.14	0.12	2.36	1.30	0.17	0.4	1.4	2.6	0.0	0.0	0.0	0.0	0.0	0.0	0.0	0.0	2.80E-01	2.98E-02	6.01E-01	4.44E-01
	HOMEZ	34	29	7	1	7	6	1	0	0	1	0	0	0	3	2	2	6.48	7.38	5.85	0.49	0.19	1.69	0.36	0.31	5.88	3.24	0.42	0.9	0.1	0.0	0.0	5.3	0.0	0.0	0.0	0.5	0.6	4.8	4.30E-03	3.34E-03	4.54E-02	6.96E-02
	NANOGNB	1	0	0	0	0	0	0	0	0	0	0	0	0	0	0	0	0.07	0.08	0.07	0.01	0.00	0.02	0.00	0.00	0.07	0.04	0.00	0.0	0.0	0.0	0.0	0.0	0.0	0.0	0.0	0.0	0.0	0.0	1.00E+00	8.95E-01	9.30E-01	1.00E+00
	TSHZ1	4	4	1	0	1	0	1	0	0	0	0	0	0	1	0	0	0.48	0.54	0.43	0.04	0.01	0.12	0.03	0.02	0.43	0.24	0.03	0.0	1.8	0.0	0.0	0.0	0.0	0.0	0.0	2.3	0.0	0.0	9.90E-01	5.24E-01	8.23E-01	9.46E-01
	TSHZ3	18	14	4	1	1	2	0	2	1	0	0	0	0	0	1	0	3.18	3.63	2.88	0.24	0.09	0.83	0.18	0.15	2.89	1.59	0.21	0.6	0.0	0.7	4.1	0.0	0.0	0.0	0.0	0.0	0.6	0.0	3.36E-01	1.14E-01	1.16E-01	4.83E-01
	ZEB1	35	68	41	0	26	4	12	25	0	0	0	1	0	21	4	0	9.40	10.72	8.50	0.71	0.27	2.45	0.52	0.45	8.54	4.71	0.61	0.4	1.1	2.9	0.0	0.0	0.0	1.9	0.0	2.5	0.8	0.0	6.89E-09	2.16E-08	3.20E-01	4.72E-04
	ZEB2	55	39	16	0	0	0	2	14	0	0	0	0	0	0	0	0	4.54	5.17	4.10	0.34	0.13	1.18	0.25	0.22	4.12	2.27	0.29	0.0	0.4	3.4	0.0	0.0	0.0	0.0	0.0	0.0	0.0	0.0	2.35E-05	2.53E-07	4.90E-01	1.39E-01
	ZFHX2	1	0	1	0	0	0	1	0	0	0	0	0	0	0	0	0	0.26	0.29	0.23	0.02	0.01	0.07	0.01	0.01	0.23	0.13	0.02	0.0	3.4	0.0	0.0	0.0	0.0	0.0	0.0	0.0	0.0	0.0	9.88E-01	3.32E-01	8.70E-01	9.93E-01
	ZFHX3	41	12	6	0	0	2	3	1	0	0	0	0	0	0	0	0	3.70	4.21	3.34	0.28	0.11	0.96	0.21	0.18	3.36	1.85	0.24	0.5	0.7	0.3	0.0	0.0	0.0	0.0	0.0	0.0	0.0	0.0	4.45E-01	2.51E-01	5.33E-01	2.37E-01
	ZFHX4	48	4	3	0	0	0	0	3	0	0	0	0	0	0	0	0	2.78	3.17	2.51	0.21	0.08	0.72	0.15	0.13	2.52	1.39	0.18	0.0	0.0	1.2	0.0	0.0	0.0	0.0	0.0	0.0	0.0	0.0	3.24E-01	4.87E-02	5.89E-01	4.03E-01
	ZHX1	51	21	7	1	0	1	4	2	1	0	0	0	0	0	0	0	4.24	4.84	3.84	0.32	0.12	1.11	0.24	0.20	3.85	2.13	0.27	0.2	0.8	0.5	3.1	0.0	0.0	0.0	0.0	0.0	0.0	0.0	2.32E-01	1.73E-01	2.14E-01	1.68E-01
	ZHX2	35	12	6	0	0	1	2	3	0	0	0	0	0	0	0	0	2.78	3.17	2.51	0.21	0.08	0.72	0.15	0.13	2.52	1.39	0.18	0.4	0.6	1.2	0.0	0.0	0.0	0.0	0.0	0.0	0.0	0.0	7.19E-01	4.35E-01	5.89E-01	4.03E-01
	ZHX3	22	13	7	0	1	0	2	5	0	0	0	0	0	1	0	0	1.98	2.25	1.79	0.15	0.06	0.51	0.11	0.09	1.79	0.99	0.13	0.0	0.9	2.8	0.0	0.0	0.0	0.0	0.0	0.6	0.0	0.0	4.24E-01	2.03E-02	6.49E-01	8.22E-01
HB.HOX	CDX1	12	7	12	0	3	2	8	2	0	0	0	1	0	0	2	0	4.39	5.00	3.97	0.33	0.13	1.14	0.24	0.21	3.99	2.20	0.28	0.5	1.6	0.5	0.0	0.0	0.0	4.1	0.0	0.0	0.9	0.0	2.52E-01	1.31E-01	4.97E-01	1.58E-01
	CDX2	10	6	7	0	0	1	0	6	0	0	0	0	0	0	0	0	1.50	1.71	1.36	0.11	0.04	0.39	0.08	0.07	1.36	0.75	0.10	0.7	0.0	4.4	0.0	0.0	0.0	0.0	0.0	0.0	0.0	0.0	2.33E-02	1.37E-04	6.91E-01	7.37E-01
	CDX4	1	5	0	0	0	0	0	0	0	0	0	0	0	0	0	0	0.37	0.42	0.33	0.03	0.01	0.10	0.02	0.02	0.33	0.18	0.02	0.0	0.0	0.0	0.0	0.0	0.0	0.0	0.0	0.0	0.0	0.0	9.98E-01	5.73E-01	8.45E-01	9.84E-01
	EVX1	0	1	0	0	0	0	0	0	0	0	0	0	0	0	0	0	0.04	0.04	0.03	0.00	0.00	0.01	0.00	0.00	0.03	0.02	0.00	0.0	0.0	0.0	0.0	0.0	0.0	0.0	0.0	0.0	0.0	0.0	1.00E+00	9.46E-01	9.51E-01	1.00E+00
	EVX2	0	0	0	0	0	0	0	0	0	0	0	0	0	0	0	0	0.33	0.38	0.30	0.03	0.01	0.09	0.02	0.02	0.30	0.16	0.02	0.0	0.0	0.0	0.0	0.0	0.0	0.0	0.0	0.0	0.0	0.0	9.98E-01	6.06E-01	8.52E-01	9.88E-01
	GBX1	2	1	0	0	0	0	0	0	0	0	0	0	0	0	0	0	0.26	0.29	0.23	0.02	0.01	0.07	0.01	0.01	0.23	0.13	0.02	0.0	0.0	0.0	0.0	0.0	0.0	0.0	0.0	0.0	0.0	0.0	9.99E-01	6.77E-01	8.70E-01	9.93E-01
	GBX2	1	0	0	0	0	0	0	0	0	0	0	0	0	0	0	0	0.15	0.17	0.13	0.01	0.00	0.04	0.01	0.01	0.13	0.07	0.01	0.0	0.0	0.0	0.0	0.0	0.0	0.0	0.0	0.0	0.0	0.0	1.00E+00	8.00E-01	9.01E-01	9.98E-01
	GSX1	0	5	0	0	0	0	0	0	0	0	0	0	0	0	0	0	1.68	1.92	1.52	0.13	0.05	0.44	0.09	0.08	1.53	0.84	0.11	0.0	0.0	0.0	0.0	0.0	0.0	0.0	0.0	0.0	0.0	0.0	5.91E-01	7.72E-02	6.74E-01	6.86E-01
	GSX2	0	0	0	0	0	0	0	0	0	0	0	0	0	0	0	0	0.11	0.13	0.10	0.01	0.00	0.03	0.01	0.01	0.10	0.05	0.01	0.0	0.0	0.0	0.0	0.0	0.0	0.0	0.0	0.0	0.0	0.0	1.00E+00	8.46E-01	9.14E-01	9.99E-01
	HOXA1	76	56	7	0	0	3	1	3	0	0	0	0	0	0	0	0	20.09	22.89	18.15	1.53	0.58	5.23	1.12	0.97	18.24	10.06	1.30	0.1	0.0	0.2	0.0	0.0	0.0	0.0	0.0	0.0	0.0	0.0	1.96E-14	3.55E-11	1.46E-01	6.22E-07
	HOXA10	14	12	4	0	0	0	1	3	0	0	0	0	0	0	0	0	1.76	2.00	1.59	0.13	0.05	0.46	0.10	0.08	1.59	0.88	0.11	0.0	0.5	1.9	0.0	0.0	0.0	0.0	0.0	0.0	0.0	0.0	7.32E-01	1.72E-01	6.68E-01	6.65E-01
	HOXA11	6	3	2	0	0	0	0	2	0	0	0	0	0	0	0	0	0.51	0.58	0.46	0.04	0.01	0.13	0.03	0.02	0.47	0.26	0.03	0.0	0.0	4.3	0.0	0.0	0.0	0.0	0.0	0.0	0.0	0.0	7.07E-01	4.51E-02	8.17E-01	9.67E-01
	HOXA13	4	2	0	0	0	0	0	0	0	0	0	0	0	0	0	0	0.51	0.58	0.46	0.04	0.01	0.13	0.03	0.02	0.47	0.26	0.03	0.0	0.0	0.0	0.0	0.0	0.0	0.0	0.0	0.0	0.0	0.0	9.90E-01	4.59E-01	8.17E-01	9.67E-01
	HOXA2	0	1	0	0	0	0	0	0	0	0	0	0	0	0	0	0	0.88	1.00	0.79	0.07	0.03	0.23	0.05	0.04	0.80	0.44	0.06	0.0	0.0	0.0	0.0	0.0	0.0	0.0	0.0	0.0	0.0	0.0	9.29E-01	2.63E-01	7.61E-01	9.00E-01
	HOXA3	2	0	0	0	0	0	0	0	0	0	0	0	0	0	0	0	0.59	0.67	0.53	0.04	0.02	0.15	0.03	0.03	0.53	0.29	0.04	0.0	0.0	0.0	0.0	0.0	0.0	0.0	0.0	0.0	0.0	0.0	9.83E-01	4.10E-01	8.04E-01	9.56E-01
	HOXA4	1	1	0	0	0	0	0	0	0	0	0	0	0	0	0	0	0.37	0.42	0.33	0.03	0.01	0.10	0.02	0.02	0.33	0.18	0.02	0.0	0.0	0.0	0.0	0.0	0.0	0.0	0.0	0.0	0.0	0.0	9.98E-01	5.73E-01	8.45E-01	9.84E-01
	HOXA5	21	10	5	1	1	3	1	1	1	0	0	0	0	1	0	0	3.33	3.79	3.01	0.25	0.10	0.87	0.19	0.16	3.02	1.67	0.22	0.9	0.3	0.3	4.0	0.0	0.0	0.0	0.0	0.3	0.0	0.0	4.25E-01	1.80E-01	1.29E-01	4.87E-01
	HOXA6	0	0	0	0	0	0	0	0	0	0	0	0	0	0	0	0	0.04	0.04	0.03	0.00	0.00	0.01	0.00	0.00	0.03	0.02	0.00	0.0	0.0	0.0	0.0	0.0	0.0	0.0	0.0	0.0	0.0	0.0	1.00E+00	9.46E-01	9.51E-01	1.00E+00
	HOXA7	2	3	0	0	0	0	0	0	0	0	0	0	0	0	0	0	0.77	0.88	0.69	0.06	0.02	0.20	0.04	0.04	0.70	0.38	0.05	0.0	0.0	0.0	0.0	0.0	0.0	0.0	0.0	0.0	0.0	0.0	9.55E-01	3.11E-01	7.76E-01	9.23E-01
	HOXA9	10	11	3	0	3	0	0	3	0	0	3	0	0	0	0	0	1.39	1.58	1.26	0.11	0.04	0.36	0.08	0.07	1.26	0.70	0.09	0.0	0.0	2.4	0.0	0.0	8.3	0.0	0.0	0.0	0.0	0.0	2.65E-03	6.74E-02	7.02E-01	6.78E-04
	HOXB1	8	6	0	0	0	0	0	0	0	0	0	0	0	0	0	0	1.24	1.42	1.12	0.09	0.04	0.32	0.07	0.06	1.13	0.62	0.08	0.0	0.0	0.0	0.0	0.0	0.0	0.0	0.0	0.0	0.0	0.0	7.98E-01	1.51E-01	7.18E-01	8.08E-01
	HOXB13	14	3	1	0	0	0	0	1	0	0	0	0	0	0	0	0	0.73	0.83	0.66	0.06	0.02	0.19	0.04	0.04	0.66	0.37	0.05	0.0	0.0	1.5	0.0	0.0	0.0	0.0	0.0	0.0	0.0	0.0	9.77E-01	4.19E-01	7.82E-01	9.30E-01
	HOXB2	12	3	1	0	0	0	0	1	0	0	0	0	0	0	0	0	1.28	1.46	1.16	0.10	0.04	0.33	0.07	0.06	1.16	0.64	0.08	0.0	0.0	0.9	0.0	0.0	0.0	0.0	0.0	0.0	0.0	0.0	8.74E-01	2.51E-01	7.14E-01	7.98E-01
	HOXB3	1	1	0	0	0	0	0	0	0	0	0	0	0	0	0	0	0.26	0.29	0.23	0.02	0.01	0.07	0.01	0.01	0.23	0.13	0.02	0.0	0.0	0.0	0.0	0.0	0.0	0.0	0.0	0.0	0.0	0.0	9.99E-01	6.77E-01	8.70E-01	9.93E-01
	HOXB4	0	1	0	0	0	0	0	0	0	0	0	0	0	0	0	0	0.22	0.25	0.20	0.02	0.01	0.06	0.01	0.01	0.20	0.11	0.01	0.0	0.0	0.0	0.0	0.0	0.0	0.0	0.0	0.0	0.0	0.0	1.00E+00	7.16E-01	8.79E-01	9.95E-01
	HOXB5	13	25	6	0	1	3	0	3	0	0	1	0	0	0	0	0	5.82	6.63	5.26	0.44	0.17	1.52	0.32	0.28	5.28	2.91	0.38	0.5	0.0	0.6	0.0	0.0	0.7	0.0	0.0	0.0	0.0	0.0	4.12E-02	1.13E-02	4.34E-01	9.58E-02
	HOXB6	3	4	5	0	0	0	2	3	0	0	0	0	0	0	0	0	2.34	2.67	2.12	0.18	0.07	0.61	0.13	0.11	2.13	1.17	0.15	0.0	0.7	1.4	0.0	0.0	0.0	0.0	0.0	0.0	0.0	0.0	6.85E-01	2.37E-01	6.20E-01	5.07E-01
	HOXB7	5	3	2	0	0	0	0	2	0	0	0	0	0	0	0	0	1.32	1.50	1.19	0.10	0.04	0.34	0.07	0.06	1.20	0.66	0.09	0.0	0.0	1.7	0.0	0.0	0.0	0.0	0.0	0.0	0.0	0.0	8.21E-01	1.86E-01	7.10E-01	7.88E-01
	HOXB8	5	0	0	0	0	0	0	0	0	0	0	0	0	0	0	0	0.59	0.67	0.53	0.04	0.02	0.15	0.03	0.03	0.53	0.29	0.04	0.0	0.0	0.0	0.0	0.0	0.0	0.0	0.0	0.0	0.0	0.0	9.83E-01	4.10E-01	8.04E-01	9.56E-01
	HOXB9	31	28	7	0	0	0	0	7	0	0	0	0	0	0	0	0	5.42	6.17	4.89	0.41	0.16	1.41	0.30	0.26	4.92	2.71	0.35	0.0	0.0	1.4	0.0	0.0	0.0	0.0	0.0	0.0	0.0	0.0	1.07E-02	1.94E-03	4.51E-01	7.66E-02
	HOXC10	1	7	0	0	0	0	0	0	0	0	0	0	0	0	0	0	0.77	0.88	0.69	0.06	0.02	0.20	0.04	0.04	0.70	0.38	0.05	0.0	0.0	0.0	0.0	0.0	0.0	0.0	0.0	0.0	0.0	0.0	9.55E-01	3.11E-01	7.76E-01	9.23E-01
	HOXC11	8	2	1	0	0	0	1	0	0	0	0	0	0	0	0	0	0.80	0.92	0.73	0.06	0.02	0.21	0.04	0.04	0.73	0.40	0.05	0.0	1.1	0.0	0.0	0.0	0.0	0.0	0.0	0.0	0.0	0.0	9.79E-01	4.63E-01	7.71E-01	9.15E-01
	HOXC12	0	1	0	0	0	0	0	0	0	0	0	0	0	0	0	0	0.18	0.21	0.17	0.01	0.01	0.05	0.01	0.01	0.17	0.09	0.01	0.0	0.0	0.0	0.0	0.0	0.0	0.0	0.0	0.0	0.0	0.0	1.00E+00	7.57E-01	8.90E-01	9.97E-01
	HOXC13	16	2	1	0	0	0	1	0	0	0	0	0	0	0	0	0	0.80	0.92	0.73	0.06	0.02	0.21	0.04	0.04	0.73	0.40	0.05	0.0	1.1	0.0	0.0	0.0	0.0	0.0	0.0	0.0	0.0	0.0	9.79E-01	4.63E-01	7.71E-01	9.15E-01
	HOXC4	7	8	6	0	0	0	2	4	0	0	0	0	0	0	0	0	2.12	2.42	1.92	0.16	0.06	0.55	0.12	0.10	1.93	1.06	0.14	0.0	0.8	2.1	0.0	0.0	0.0	0.0	0.0	0.0	0.0	0.0	5.73E-01	1.08E-01	6.37E-01	5.64E-01
	HOXC5	7	8	2	0	0	0	2	0	0	0	0	0	0	0	0	0	1.65	1.88	1.49	0.13	0.05	0.43	0.09	0.08	1.50	0.82	0.11	0.0	1.1	0.0	0.0	0.0	0.0	0.0	0.0	0.0	0.0	0.0	7.86E-01	2.08E-01	6.77E-01	6.96E-01
	HOXC6	2	5	1	0	0	0	1	0	0	0	0	0	0	0	0	0	0.59	0.67	0.53	0.04	0.02	0.15	0.03	0.03	0.53	0.29	0.04	0.0	1.5	0.0	0.0	0.0	0.0	0.0	0.0	0.0	0.0	0.0	9.92E-01	5.27E-01	8.04E-01	9.56E-01
	HOXC8	37	10	1	0	0	0	0	1	0	0	0	0	0	0	0	0	4.65	5.30	4.20	0.35	0.13	1.21	0.26	0.22	4.22	2.33	0.30	0.0	0.0	0.2	0.0	0.0	0.0	0.0	0.0	0.0	0.0	0.0	1.84E-02	2.05E-03	4.85E-01	1.29E-01
	HOXC9	13	9	1	0	0	0	1	0	0	0	0	0	0	0	0	0	2.27	2.59	2.05	0.17	0.07	0.59	0.13	0.11	2.06	1.14	0.15	0.0	0.4	0.0	0.0	0.0	0.0	0.0	0.0	0.0	0.0	0.0	4.67E-01	7.10E-02	6.25E-01	5.25E-01
	HOXD1	2	0	0	0	0	0	0	0	0	0	0	0	0	0	0	0	0.07	0.08	0.07	0.01	0.00	0.02	0.00	0.00	0.07	0.04	0.00	0.0	0.0	0.0	0.0	0.0	0.0	0.0	0.0	0.0	0.0	0.0	1.00E+00	8.95E-01	9.30E-01	1.00E+00
	HOXD10	1	1	1	0	0	0	0	1	0	0	0	0	0	0	0	0	0.48	0.54	0.43	0.04	0.01	0.12	0.03	0.02	0.43	0.24	0.03	0.0	0.0	2.3	0.0	0.0	0.0	0.0	0.0	0.0	0.0	0.0	9.88E-01	4.12E-01	8.23E-01	9.72E-01
	HOXD11	3	3	0	0	0	0	0	0	0	0	0	0	0	0	0	0	0.37	0.42	0.33	0.03	0.01	0.10	0.02	0.02	0.33	0.18	0.02	0.0	0.0	0.0	0.0	0.0	0.0	0.0	0.0	0.0	0.0	0.0	9.98E-01	5.73E-01	8.45E-01	9.84E-01
	HOXD12	7	0	0	0	0	0	0	0	0	0	0	0	0	0	0	0	0.66	0.75	0.60	0.05	0.02	0.17	0.04	0.03	0.60	0.33	0.04	0.0	0.0	0.0	0.0	0.0	0.0	0.0	0.0	0.0	0.0	0.0	9.74E-01	3.67E-01	7.93E-01	9.44E-01
	HOXD13	34	2	0	0	2	0	0	0	0	0	0	0	1	1	0	0	3.00	3.42	2.71	0.23	0.09	0.78	0.17	0.14	2.72	1.50	0.19	0.0	0.0	0.0	0.0	0.0	0.0	0.0	6.9	0.4	0.0	0.0	5.06E-02	1.04E-02	5.75E-01	1.17E-01
	HOXD3	6	2	0	0	0	0	0	0	0	0	0	0	0	0	0	0	0.51	0.58	0.46	0.04	0.01	0.13	0.03	0.02	0.47	0.26	0.03	0.0	0.0	0.0	0.0	0.0	0.0	0.0	0.0	0.0	0.0	0.0	9.90E-01	4.59E-01	8.17E-01	9.67E-01
	HOXD4	9	3	3	0	0	0	2	1	0	0	0	0	0	0	0	0	1.02	1.17	0.93	0.08	0.03	0.27	0.06	0.05	0.93	0.51	0.07	0.0	1.7	1.1	0.0	0.0	0.0	0.0	0.0	0.0	0.0	0.0	9.63E-01	4.44E-01	7.43E-01	8.65E-01
	HOXD8	1	1	0	0	0	0	0	0	0	0	0	0	0	0	0	0	0.40	0.46	0.36	0.03	0.01	0.10	0.02	0.02	0.37	0.20	0.03	0.0	0.0	0.0	0.0	0.0	0.0	0.0	0.0	0.0	0.0	0.0	9.96E-01	5.42E-01	8.37E-01	9.81E-01
	HOXD9	7	1	0	0	0	0	0	0	0	0	0	0	0	0	0	0	0.37	0.42	0.33	0.03	0.01	0.10	0.02	0.02	0.33	0.18	0.02	0.0	0.0	0.0	0.0	0.0	0.0	0.0	0.0	0.0	0.0	0.0	9.98E-01	5.73E-01	8.45E-01	9.84E-01
	MEOX1	15	17	3	0	3	1	1	1	0	0	1	2	0	0	0	0	4.32	4.92	3.90	0.33	0.13	1.12	0.24	0.21	3.92	2.16	0.28	0.2	0.2	0.3	0.0	0.0	0.9	8.3	0.0	0.0	0.0	0.0	1.99E-03	1.99E-02	5.01E-01	1.58E-03
	MEOX2	77	55	15	2	7	8	5	2	2	0	1	1	2	3	0	0	28.21	32.15	25.50	2.14	0.82	7.35	1.57	1.36	25.62	14.13	1.83	0.3	0.2	0.1	0.9	0.0	0.1	0.6	1.5	0.1	0.0	0.0	2.37E-17	1.50E-13	3.63E-01	6.11E-08
	MNX1	19	3	0	0	0	0	0	0	0	0	0	0	0	0	0	0	0.99	1.13	0.89	0.08	0.03	0.26	0.06	0.05	0.90	0.49	0.06	0.0	0.0	0.0	0.0	0.0	0.0	0.0	0.0	0.0	0.0	0.0	8.96E-01	2.22E-01	7.47E-01	8.74E-01
	PDX1	6	3	0	0	0	0	0	0	0	0	0	0	0	0	0	0	0.88	1.00	0.79	0.07	0.03	0.23	0.05	0.04	0.80	0.44	0.06	0.0	0.0	0.0	0.0	0.0	0.0	0.0	0.0	0.0	0.0	0.0	9.29E-01	2.63E-01	7.61E-01	9.00E-01
HB.LIM	ISL1	24	21	1	0	0	0	0	1	0	0	0	0	0	0	0	0	2.12	2.42	1.92	0.16	0.06	0.55	0.12	0.10	1.93	1.06	0.14	0.0	0.0	0.5	0.0	0.0	0.0	0.0	0.0	0.0	0.0	0.0	5.22E-01	8.29E-02	6.37E-01	5.64E-01
	ISL2	22	22	0	0	0	0	0	0	0	0	0	0	0	0	0	0	2.38	2.71	2.15	0.18	0.07	0.62	0.13	0.11	2.16	1.19	0.15	0.0	0.0	0.0	0.0	0.0	0.0	0.0	0.0	0.0	0.0	0.0	2.95E-01	2.68E-02	6.17E-01	4.97E-01
	LHX1	30	23	8	0	3	0	4	4	0	0	0	1	0	0	2	0	4.02	4.59	3.64	0.31	0.12	1.05	0.22	0.19	3.65	2.02	0.26	0.0	0.9	1.1	0.0	0.0	0.0	4.5	0.0	0.0	1.0	0.0	2.59E-01	1.26E-01	5.16E-01	1.65E-01
	LHX2	61	37	12	0	7	0	4	8	0	0	0	0	0	1	6	0	6.95	7.92	6.28	0.53	0.20	1.81	0.39	0.33	6.31	3.48	0.45	0.0	0.5	1.3	0.0	0.0	0.0	0.0	0.0	0.2	1.7	0.0	3.58E-02	9.26E-03	3.93E-01	9.86E-02
	LHX3	64	37	3	0	0	0	0	3	0	0	0	0	0	0	0	0	7.17	8.17	6.48	0.55	0.21	1.87	0.40	0.34	6.51	3.59	0.46	0.0	0.0	0.5	0.0	0.0	0.0	0.0	0.0	0.0	0.0	0.0	5.55E-04	1.83E-04	3.85E-01	2.17E-02
	LHX4	64	48	10	0	4	1	3	6	0	0	0	3	0	0	1	0	8.05	9.17	7.27	0.61	0.23	2.10	0.45	0.39	7.31	4.03	0.52	0.1	0.3	0.8	0.0	0.0	0.0	6.7	0.0	0.0	0.2	0.0	3.09E-05	5.11E-03	3.58E-01	5.42E-05
	LHX5	0	0	0	0	0	0	0	0	0	0	0	0	0	0	0	0	0.00	0.00	0.00	0.00	0.00	0.00	0.00	0.00	0.00	0.00	0.00	na	na	na	na	na	na	na	na	na	na	na	na	na	na	na
	LHX6	26	31	1	0	0	0	0	1	0	0	0	0	0	0	0	0	3.66	4.17	3.31	0.28	0.11	0.95	0.20	0.18	3.32	1.83	0.24	0.0	0.0	0.3	0.0	0.0	0.0	0.0	0.0	0.0	0.0	0.0	8.50E-02	8.92E-03	5.35E-01	2.42E-01
	LHX8	15	18	2	0	0	0	0	2	0	0	0	0	0	0	0	0	2.16	2.46	1.95	0.16	0.06	0.56	0.12	0.10	1.96	1.08	0.14	0.0	0.0	1.0	0.0	0.0	0.0	0.0	0.0	0.0	0.0	0.0	5.50E-01	9.92E-02	6.34E-01	5.54E-01
	LHX9	6	9	0	0	0	0	0	0	0	0	0	0	0	0	0	0	0.88	1.00	0.79	0.07	0.03	0.23	0.05	0.04	0.80	0.44	0.06	0.0	0.0	0.0	0.0	0.0	0.0	0.0	0.0	0.0	0.0	0.0	9.29E-01	2.63E-01	7.61E-01	9.00E-01
	LMX1A	9	0	0	0	0	0	0	0	0	0	0	0	0	0	0	0	0.48	0.54	0.43	0.04	0.01	0.12	0.03	0.02	0.43	0.24	0.03	0.0	0.0	0.0	0.0	0.0	0.0	0.0	0.0	0.0	0.0	0.0	9.93E-01	4.85E-01	8.23E-01	9.72E-01
	LMX1B	24	17	0	0	3	0	0	0	0	0	0	0	0	0	0	3	2.52	2.88	2.28	0.19	0.07	0.66	0.14	0.12	2.29	1.26	0.16	0.0	0.0	0.0	0.0	0.0	0.0	0.0	0.0	0.0	0.0	18.4	1.76E-09	2.15E-02	6.07E-01	2.41E-10
	BARHL1	0	3	0	0	0	0	0	0	0	0	0	0	0	0	0	0	0.11	0.13	0.10	0.01	0.00	0.03	0.01	0.01	0.10	0.05	0.01	0.0	0.0	0.0	0.0	0.0	0.0	0.0	0.0	0.0	0.0	0.0	1.00E+00	8.46E-01	9.14E-01	9.99E-01
	BARHL2	1	0	0	0	0	0	0	0	0	0	0	0	0	0	0	0	0.07	0.08	0.07	0.01	0.00	0.02	0.00	0.00	0.07	0.04	0.00	0.0	0.0	0.0	0.0	0.0	0.0	0.0	0.0	0.0	0.0	0.0	1.00E+00	8.95E-01	9.30E-01	1.00E+00
	BARX1	3	2	0	0	1	0	0	0	0	0	0	0	0	1	0	0	0.33	0.38	0.30	0.03	0.01	0.09	0.02	0.02	0.30	0.16	0.02	0.0	0.0	0.0	0.0	0.0	0.0	0.0	0.0	3.3	0.0	0.0	9.82E-01	6.06E-01	8.52E-01	8.56E-01
	BARX2	0	1	0	0	0	0	0	0	0	0	0	0	0	0	0	0	0.07	0.08	0.07	0.01	0.00	0.02	0.00	0.00	0.07	0.04	0.00	0.0	0.0	0.0	0.0	0.0	0.0	0.0	0.0	0.0	0.0	0.0	1.00E+00	8.95E-01	9.30E-01	1.00E+00
	BSX	0	1	0	0	0	0	0	0	0	0	0	0	0	0	0	0	0.04	0.04	0.03	0.00	0.00	0.01	0.00	0.00	0.03	0.02	0.00	0.0	0.0	0.0	0.0	0.0	0.0	0.0	0.0	0.0	0.0	0.0	1.00E+00	9.46E-01	9.51E-01	1.00E+00
	DBX1	0	0	0	0	0	0	0	0	0	0	0	0	0	0	0	0	0.04	0.04	0.03	0.00	0.00	0.01	0.00	0.00	0.03	0.02	0.00	0.0	0.0	0.0	0.0	0.0	0.0	0.0	0.0	0.0	0.0	0.0	1.00E+00	9.46E-01	9.51E-01	1.00E+00
	DBX2	0	0	0	0	0	0	0	0	0	0	0	0	0	0	0	0	0.26	0.29	0.23	0.02	0.01	0.07	0.01	0.01	0.23	0.13	0.02	0.0	0.0	0.0	0.0	0.0	0.0	0.0	0.0	0.0	0.0	0.0	9.99E-01	6.77E-01	8.70E-01	9.93E-01
	DLX1	2	0	0	0	0	0	0	0	0	0	0	0	0	0	0	0	0.26	0.29	0.23	0.02	0.01	0.07	0.01	0.01	0.23	0.13	0.02	0.0	0.0	0.0	0.0	0.0	0.0	0.0	0.0	0.0	0.0	0.0	9.99E-01	6.77E-01	8.70E-01	9.93E-01
	DLX2	10	2	0	0	0	0	0	0	0	0	0	0	0	0	0	0	0.77	0.88	0.69	0.06	0.02	0.20	0.04	0.04	0.70	0.38	0.05	0.0	0.0	0.0	0.0	0.0	0.0	0.0	0.0	0.0	0.0	0.0	9.55E-01	3.11E-01	7.76E-01	9.23E-01
	DLX3	1	2	0	0	0	0	0	0	0	0	0	0	0	0	0	0	0.18	0.21	0.17	0.01	0.01	0.05	0.01	0.01	0.17	0.09	0.01	0.0	0.0	0.0	0.0	0.0	0.0	0.0	0.0	0.0	0.0	0.0	1.00E+00	7.57E-01	8.90E-01	9.97E-01
	DLX4	0	0	3	0	0	1	2	0	0	0	0	0	0	0	0	0	0.22	0.25	0.20	0.02	0.01	0.06	0.01	0.01	0.20	0.11	0.01	4.6	8.0	0.0	0.0	0.0	0.0	0.0	0.0	0.0	0.0	0.0	1.11E-01	4.98E-04	8.79E-01	9.95E-01

HB.NK	DLX5	19	0	0	0	0	0	0	0	0	0	0	0	0	0	0	0	0.77	0.88	0.69	0.06	0.02	0.20	0.04	0.04	0.70	0.38	0.05	0.0	0.0	0.0	0.0	0.0	0.0	0.0	0.0	0.0	0.0	0.0	9.55E-01	3.11E-01	7.76E-01	9.23E-01
	DLX6	3	5	0	1	0	0	0	0	1	0	0	0	0	0	0	0	1.17	1.33	1.06	0.09	0.03	0.30	0.07	0.06	1.06	0.59	0.08	0.0	0.0	0.0	11.2	0.0	0.0	0.0	0.0	0.0	0.0	0.0	1.29E-01	1.68E-01	2.22E-03	8.28E-01
	EMX1	5	1	0	0	1	0	0	0	0	0	1	0	0	0	0	0	0.37	0.42	0.33	0.03	0.01	0.10	0.02	0.02	0.33	0.18	0.02	0.0	0.0	0.0	0.0	0.0	10.5	0.0	0.0	0.0	0.0	0.0	4.13E-01	5.73E-01	8.45E-01	1.03E-01
	EMX2	2	3	0	0	3	0	0	0	0	0	3	0	0	0	0	0	1.32	1.50	1.19	0.10	0.04	0.34	0.07	0.06	1.20	0.66	0.09	0.0	0.0	0.0	0.0	0.0	8.7	0.0	0.0	0.0	0.0	0.0	2.80E-03	1.35E-01	7.10E-01	3.93E-04
	EN1	32	30	6	0	3	1	2	3	0	0	1	0	0	1	1	0	4.35	4.96	3.94	0.33	0.13	1.13	0.24	0.21	3.95	2.18	0.28	0.2	0.4	0.8	0.0	0.0	0.9	0.0	0.0	0.3	0.5	0.0	5.68E-01	1.02E-01	4.99E-01	6.09E-01
	EN2	1	3	1	0	1	0	1	0	0	0	1	0	0	0	0	0	0.26	0.29	0.23	0.02	0.01	0.07	0.01	0.01	0.23	0.13	0.02	0.0	3.4	0.0	0.0	0.0	15.0	0.0	0.0	0.0	0.0	0.0	1.09E-01	3.32E-01	8.70E-01	1.94E-02
	HHEX	1	9	0	0	5	0	0	0	0	0	5	0	0	0	0	0	1.98	2.25	1.79	0.15	0.06	0.51	0.11	0.09	1.79	0.99	0.13	0.0	0.0	0.0	0.0	0.0	9.7	0.0	0.0	0.0	0.0	0.0	5.18E-07	4.95E-02	6.49E-01	5.34E-08
	HLX	2	15	2	0	0	0	1	1	0	0	0	0	0	0	0	0	1.76	2.00	1.59	0.13	0.05	0.46	0.10	0.08	1.59	0.88	0.11	0.0	0.5	0.6	0.0	0.0	0.0	0.0	0.0	0.0	0.0	0.0	8.25E-01	2.90E-01	6.68E-01	6.65E-01
	HMX1	0	0	0	0	0	0	0	0	0	0	0	0	0	0	0	0	0.00	0.00	0.00	0.00	0.00	0.00	0.00	0.00	0.00	0.00	0.00	na	na	na	na	na	na	na	na	na	na	na	na	na	na	na
	HMX2	1	2	0	0	0	0	0	0	0	0	0	0	0	0	0	0	0.22	0.25	0.20	0.02	0.01	0.06	0.01	0.01	0.20	0.11	0.01	0.0	0.0	0.0	0.0	0.0	0.0	0.0	0.0	0.0	0.0	0.0	1.00E+00	7.16E-01	8.79E-01	9.95E-01
	HMX3	2	1	1	0	0	0	1	0	0	0	0	0	0	0	0	0	0.37	0.42	0.33	0.03	0.01	0.10	0.02	0.02	0.33	0.18	0.02	0.0	2.4	0.0	0.0	0.0	0.0	0.0	0.0	0.0	0.0	0.0	9.94E-01	4.70E-01	8.45E-01	9.84E-01
	LBX1	2	1	1	0	1	0	0	1	0	0	0	0	1	0	0	0	0.99	1.13	0.89	0.08	0.03	0.26	0.06	0.05	0.90	0.49	0.06	0.0	0.0	1.1	0.0	0.0	0.0	0.0	21.1	0.0	0.0	0.0	1.03E-02	3.45E-01	7.47E-01	8.51E-04
	LBX2	0	0	0	0	0	0	0	0	0	0	0	0	0	0	0	0	0.26	0.29	0.23	0.02	0.01	0.07	0.01	0.01	0.23	0.13	0.02	0.0	0.0	0.0	0.0	0.0	0.0	0.0	0.0	0.0	0.0	0.0	9.99E-01	6.77E-01	8.70E-01	9.93E-01
	MSX1	16	4	0	0	0	0	0	0	0	0	0	0	0	0	0	0	1.06	1.21	0.96	0.08	0.03	0.28	0.06	0.05	0.96	0.53	0.07	0.0	0.0	0.0	0.0	0.0	0.0	0.0	0.0	0.0	0.0	0.0	8.71E-01	1.99E-01	7.38E-01	8.56E-01
	MSX2	36	16	10	0	20	3	6	1	0	0	2	1	2	15	0	0	7.35	8.38	6.65	0.56	0.21	1.92	0.41	0.35	6.68	3.68	0.48	0.4	0.7	0.2	0.0	0.0	1.0	2.4	5.7	2.2	0.0	0.0	4.19E-04	1.78E-02	3.79E-01	3.29E-04
	NANOG	50	54	18	0	9	6	8	4	0	0	1	0	0	1	7	0	9.99	11.38	9.03	0.76	0.29	2.60	0.56	0.48	9.07	5.00	0.65	0.6	0.7	0.4	0.0	0.0	0.4	0.0	0.0	0.1	1.4	0.0	7.23E-02	6.72E-02	3.06E-01	5.89E-02
	NKX1-1	0	0	0	0	0	0	0	0	0	0	0	0	0	0	0	0	0.04	0.04	0.03	0.00	0.00	0.01	0.00	0.00	0.03	0.02	0.00	0.0	0.0	0.0	0.0	0.0	0.0	0.0	0.0	0.0	0.0	0.0	1.00E+00	9.46E-01	9.51E-01	1.00E+00
	NKX1-2	1	0	0	0	0	0	0	0	0	0	0	0	0	0	0	0	0.07	0.08	0.07	0.01	0.00	0.02	0.00	0.00	0.07	0.04	0.00	0.0	0.0	0.0	0.0	0.0	0.0	0.0	0.0	0.0	0.0	0.0	1.00E+00	8.95E-01	9.30E-01	1.00E+00
	NKX2-1	44	34	15	0	0	2	8	5	0	0	0	0	0	0	0	0	6.77	7.71	6.12	0.51	0.20	1.76	0.38	0.33	6.15	3.39	0.44	0.3	1.0	0.8	0.0	0.0	0.0	0.0	0.0	0.0	0.0	0.0	8.06E-02	1.67E-01	3.99E-01	2.92E-02
	NKX2-2	1	3	2	0	0	0	0	2	0	0	0	0	0	0	0	0	0.18	0.21	0.17	0.01	0.01	0.05	0.01	0.01	0.17	0.09	0.01	0.0	0.0	12.1	0.0	0.0	0.0	0.0	0.0	0.0	0.0	0.0	2.04E-02	3.12E-05	8.90E-01	9.97E-01
	NKX2-3	2	1	0	0	0	0	0	0	0	0	0	0	0	0	0	0	0.15	0.17	0.13	0.01	0.00	0.04	0.01	0.01	0.13	0.07	0.01	0.0	0.0	0.0	0.0	0.0	0.0	0.0	0.0	0.0	0.0	0.0	1.00E+00	8.00E-01	9.01E-01	9.98E-01
	NKX2-4	10	1	0	0	0	0	0	0	0	0	0	0	0	0	0	0	0.44	0.50	0.40	0.03	0.01	0.11	0.02	0.02	0.40	0.22	0.03	0.0	0.0	0.0	0.0	0.0	0.0	0.0	0.0	0.0	0.0	0.0	9.95E-01	5.13E-01	8.30E-01	9.77E-01
	NKX2-5	35	10	5	0	2	0	3	2	0	0	0	0	0	2	0	0	3.33	3.79	3.01	0.25	0.10	0.87	0.19	0.16	3.02	1.67	0.22	0.0	0.8	0.7	0.0	0.0	0.0	0.0	0.0	0.7	0.0	0.0	6.65E-01	1.47E-01	5.54E-01	6.32E-01
	NKX2-6	0	0	0	0	1	0	0	0	0	0	0	0	0	1	0	0	0.04	0.04	0.03	0.00	0.00	0.01	0.00	0.00	0.03	0.02	0.00	0.0	0.0	0.0	0.0	0.0	0.0	0.0	0.0	30.1	0.0	0.0	1.63E-03	9.46E-01	9.51E-01	3.38E-05
	NKX2-8	0	0	0	0	0	0	0	0	0	0	0	0	0	0	0	0	0.00	0.00	0.00	0.00	0.00	0.00	0.00	0.00	0.00	0.00	0.00	na	na	na	na	na	na	na	na	na	na	na	na	na	na	na
	NKX3-1	18	9	5	0	2	0	1	4	0	0	0	0	0	2	0	0	2.38	2.71	2.15	0.18	0.07	0.62	0.13	0.11	2.16	1.19	0.15	0.0	0.4	1.9	0.0	0.0	0.0	0.0	0.0	0.9	0.0	0.0	6.75E-01	8.00E-02	6.17E-01	8.17E-01
	NKX3-2	6	6	3	0	0	0	0	3	0	0	0	0	0	0	0	0	0.48	0.54	0.43	0.04	0.01	0.12	0.03	0.02	0.43	0.24	0.03	0.0	0.0	7.0	0.0	0.0	0.0	0.0	0.0	0.0	0.0	0.0	6.78E-02	2.77E-04	8.23E-01	9.72E-01
	NKX6-1	2	0	0	0	0	0	0	0	0	0	0	0	0	0	0	0	0.15	0.17	0.13	0.01	0.00	0.04	0.01	0.01	0.13	0.07	0.01	0.0	0.0	0.0	0.0	0.0	0.0	0.0	0.0	0.0	0.0	0.0	1.00E+00	8.00E-01	9.01E-01	9.98E-01
	NKX6-2	0	0	0	0	0	0	0	0	0	0	0	0	0	0	0	0	0.04	0.04	0.03	0.00	0.00	0.01	0.00	0.00	0.03	0.02	0.00	0.0	0.0	0.0	0.0	0.0	0.0	0.0	0.0	0.0	0.0	0.0	1.00E+00	9.46E-01	9.51E-01	1.00E+00
	NKX6-3	0	0	0	0	0	0	0	0	0	0	0	0	0	0	0	0	0.00	0.00	0.00	0.00	0.00	0.00	0.00	0.00	0.00	0.00	0.00	na	na	na	na	na	na	na	na	na	na	na	na	na	na	na
	NOTO	7	2	1	0	0	0	1	0	0	0	0	0	0	0	0	0	1.24	1.42	1.12	0.09	0.04	0.32	0.07	0.06	1.13	0.62	0.08	0.0	0.7	0.0	0.0	0.0	0.0	0.0	0.0	0.0	0.0	0.0	8.97E-01	2.88E-01	7.18E-01	8.08E-01
	TLX1	59	37	5	0	18	0	2	3	0	0	1	0	0	9	8	0	6.59	7.51	5.95	0.50	0.19	1.72	0.37	0.32	5.98	3.30	0.43	0.0	0.3	0.5	0.0	0.0	0.6	0.0	0.0	1.5	2.4	0.0	1.31E-02	2.37E-03	4.06E-01	8.63E-02
	TLX2	34	20	8	1	22	0	7	1	0	1	0	0	0	1	21	0	5.38	6.13	4.86	0.41	0.16	1.40	0.30	0.26	4.88	2.69	0.35	0.0	1.1	0.2	0.0	6.4	0.0	0.0	0.0	0.2	7.8	0.0	8.75E-26	1.38E-02	2.57E-02	2.64E-26
	TLX3	71	35	10	0	11	2	4	4	0	0	0	0	0	0	11	0	9.70	11.05	8.76	0.74	0.28	2.53	0.54	0.47	8.80	4.86	0.63	0.2	0.4	0.5	0.0	0.0	0.0	0.0	0.0	0.0	2.3	0.0	1.27E-04	1.36E-03	3.13E-01	9.09E-04
	VAX1	3	4	0	0	0	0	0	0	0	0	0	0	0	0	0	0	0.55	0.63	0.50	0.04	0.02	0.14	0.03	0.03	0.50	0.27	0.04	0.0	0.0	0.0	0.0	0.0	0.0	0.0	0.0	0.0	0.0	0.0	9.87E-01	4.34E-01	8.10E-01	9.62E-01
	VAX2	1	1	1	0	0	0	0	1	0	0	0	0	0	0	0	0	0.40	0.46	0.36	0.03	0.01	0.10	0.02	0.02	0.37	0.20	0.03	0.0	0.0	2.7	0.0	0.0	0.0	0.0	0.0	0.0	0.0	0.0	9.87E-01	3.73E-01	8.37E-01	9.81E-01
	VENTX	19	18	1	0	0	0	1	0	0	0	0	0	0	0	0	0	4.13	4.71	3.74	0.31	0.12	1.08	0.23	0.20	3.75	2.07	0.27	0.0	0.2	0.0	0.0	0.0	0.0	0.0	0.0	0.0	0.0	0.0	4.25E-02	4.53E-03	5.10E-01	1.80E-01
HB.PD	ALX1	4	2	1	0	1	0	0	1	0	0	0	0	1	0	0	0	0.70	0.79	0.63	0.05	0.02	0.18	0.04	0.03	0.63	0.35	0.05	0.0	0.0	1.6	0.0	0.0	0.0	0.0	29.9	0.0	0.0	0.0	5.87E-04	4.26E-01	7.87E-01	2.10E-05
	ALX3	1	3	2	0	0	0	2	0	0	0	0	0	0	0	0	0	1.21	1.38	1.09	0.09	0.04	0.31	0.07	0.06	1.10	0.60	0.08	0.0	1.5	0.0	0.0	0.0	0.0	0.0	0.0	0.0	0.0	0.0	8.96E-01	2.75E-01	7.22E-01	8.18E-01
	ALX4	14	1	1	0	0	0	1	0	0	0	0	0	0	0	0	0	0.70	0.79	0.63	0.05	0.02	0.18	0.04	0.03	0.63	0.35	0.05	0.0	1.3	0.0	0.0	0.0	0.0	0.0	0.0	0.0	0.0	0.0	9.87E-01	5.02E-01	7.87E-01	9.37E-01
	ARGFX	0	1	1	0	0	0	0	1	0	0	0	0	0	0	0	0	0.29	0.33	0.26	0.02	0.01	0.08	0.02	0.01	0.27	0.15	0.02	0.0	0.0	3.8	0.0	0.0	0.0	0.0	0.0	0.0	0.0	0.0	9.75E-01	2.63E-01	8.61E-01	9.91E-01
	ARX	0	3	1	0	1	0	0	1	0	0	0	1	0	0	0	0	1.06	1.21	0.96	0.08	0.03	0.28	0.06	0.05	0.96	0.53	0.07	0.0	0.0	1.0	0.0	0.0	0.0	16.9	0.0	0.0	0.0	0.0	3.72E-02	3.21E-01	7.38E-01	4.75E-03
	CRX	97	42	7	0	1	0	0	7	0	0	0	0	1	0	0	0	10.43	11.88	9.42	0.79	0.30	2.72	0.58	0.50	9.47	5.22	0.68	0.0	0.0	0.7	0.0	0.0	0.0	0.0	2.0	0.0	0.0	0.0	4.59E-06	1.05E-05	2.95E-01	1.79E-03
	DMBX1	0	1	1	0	0	0	0	0	0	0	0	0	0	0	0	0	0.07	0.08	0.07	0.01	0.00	0.02	0.00	0.00	0.07	0.04	0.00	0.0	0.0	0.0	0.0	0.0	0.0	0.0	0.0	0.0	0.0	0.0	1.00E+00	8.95E-01	9.30E-01	1.00E+00
	DPRX	4	1	1	0	0	0	0	1	0	0	0	0	0	0	0	0	0.62	0.71	0.56	0.05	0.02	0.16	0.03	0.03	0.56	0.31	0.04	0.0	0.0	1.8	0.0	0.0	0.0	0.0	0.0	0.0	0.0	0.0	9.84E-01	4.33E-01	7.98E-01	9.50E-01
	DRGX	0	0	0	0	0	0	0	0	0	0	0	0	0	0	0	0	0.00	0.00	0.00	0.00	0.00	0.00	0.00	0.00	0.00	0.00	0.00	na	na	na	na	na	na	na	na	na	na	na	na	na	na	na
	DUX4	7	37	34	0	76	8	21	5	0	0	7	2	0	67	0	0	10.79	12.30	9.76	0.82	0.31	2.81	0.60	0.52	9.80	5.41	0.70	0.7	1.7	0.5	0.0	0.0	2.5	3.3	0.0	6.8	0.0	0.0	2.64E-71	1.01E-02	2.87E-01	1.81E-73
	DUXA	2	3	1	0	0	0	0	1	0	0	0	0	0	0	0	0	0.40	0.46	0.36	0.03	0.01	0.10	0.02	0.02	0.37	0.20	0.03	0.0	0.0	2.7	0.0	0.0	0.0	0.0	0.0	0.0	0.0	0.0	9.87E-01	3.73E-01	8.37E-01	9.81E-01
	ESX1	0	0	0	0	0	0	0	0	0	0	0	0	0	0	0	0	0.11	0.13	0.10	0.01	0.00	0.03	0.01	0.01	0.10	0.05	0.01	0.0	0.0	0.0	0.0	0.0	0.0	0.0	0.0	0.0	0.0	0.0	1.00E+00	8.46E-01	9.14E-01	9.99E-01
	GSC	41	14	1	0	0	0	0	1	0	0	0	0	0	0	0	0	2.96	3.38	2.68	0.23	0.09	0.77	0.17	0.14	2.69	1.48	0.19	0.0	0.0	0.4	0.0	0.0	0.0	0.0	0.0	0.0	0.0	0.0	2.15E-01	2.48E-02	5.77E-01	3.64E-01
	GSC2	5	7	1	0	3	0	0	1	0	0	0	0	1	0	2	0	2.20	2.50	1.98	0.17	0.06	0.57	0.12	0.11	1.99	1.10	0.14	0.0	0.0	0.5	0.0	0.0	0.0	0.0	9.5	0.0	1.8	0.0	8.44E-02	7.48E-02	6.31E-01	4.84E-02
	HESX1	4	6	2	0	0	0	0	2	0	0	0	0	0	0	0	0	0.62	0.71	0.56	0.05	0.02	0.16	0.03	0.03	0.56	0.31	0.04	0.0	0.0	3.6	0.0	0.0	0.0	0.0	0.0	0.0	0.0	0.0	7.97E-01	8.17E-02	7.98E-01	9.50E-01
	HOPX	4	4	1	0	0	0	0	1	0	0	0	0	0	0	0	0	0.66	0.75	0.60	0.05	0.02	0.17	0.04	0.03	0.60	0.33	0.04	0.0	0.0	1.7	0.0	0.0	0.0	0.0	0.0	0.0	0.0	0.0	9.82E-01	4.31E-01	7.93E-01	9.44E-01
	ISX	4	7	0	0	0	0	0	0	0	0	0	0	0	0	0	0	1.17	1.33	1.06	0.09	0.03	0.30	0.07	0.06	1.06	0.59	0.08	0.0	0.0	0.0	0.0	0.0	0.0	0.0	0.0	0.0	0.0	0.0	8.29E-01	1.68E-01	7.26E-01	8.28E-01
	LEUTX	1	0	0	0	1	0	0	0	0	0	0	0	0	0	1	0	1.68	1.92	1.52	0.13	0.05	0.44	0.09	0.08	1.53	0.84	0.11	0.0	0.0	0.0	0.0	0.0	0.0	0.0	0.0	0.0	1.2	0.0	6.70E-01	7.72E-02	6.74E-01	8.09E-01
	MIXL1	0	0	0	0	0	0	0	0	0	0	0	0	0	0	0	0	0.00	0.00	0.00	0.00	0.00	0.00	0.00	0.00	0.00	0.00	0.00	na	na	na	na	na	na	na	na	na	na	na	na	na	na	na
	NOBOX	0	1	1	0	0	0	1	0	0	0	0	0	0	0	0	0	0.18	0.21	0.17	0.01	0.01	0.05	0.01	0.01	0.17	0.09	0.01	0.0	4.8	0.0	0.0	0.0	0.0	0.0	0.0	0.0	0.0	0.0	9.60E-01	1.87E-01	8.90E-01	9.97E-01
	OTP	1	1	0	0	0	0	0	0	0	0	0	0	0	0	0	0	0.33	0.38	0.30	0.03	0.01	0.09	0.02	0.02	0.30	0.16	0.02	0.0	0.0	0.0	0.0	0.0	0.0	0.0	0.0	0.0	0.0	0.0	9.98E-01	6.06E-01	8.52E-01	9.88E-01
	OTX1	7	16	1	0	0	0	1	0	0	0	0	0	0	0	0	0	7.03	8.01	6.35	0.53	0.20	1.83	0.39	0.34	6.38	3.52	0.45	0.0	0.1	0.0	0.0	0.0	0.0	0.0	0.0	0.0	0.0	0.0	2.57E-04	5.81E-05	3.90E-01	2.42E-02
	OTX2	10	2	2	0	2	2	0	0	0	0	1	0	0	1	0	0	2.09	2.38	1.88	0.16	0.06	0.54	0.12	0.10	1.89	1.04	0.14	1.0	0.0	0.0	0.0	0.0	1.8	0.0	0.0	0.5	0.0	0.0	7.55E-01	1.19E-01	6.40E-01	8.21E-01
	PHOX2A	7	0	0	0	0	0	0	0	0	0	0	0	0	0	0	0	0.33	0.38	0.30	0.03	0.01	0.09	0.02	0.02	0.30	0.16	0.02	0.0	0.0	0.0	0.0	0.0	0.0	0.0	0.0	0.0	0.0	0.0	9.98E-01	6.06E-01	8.52E-01	9.88E-01
	PHOX2B	0	0	0	0	0	0	0	0	0	0	0	0	0	0	0	0	0.15	0.17	0.13	0.01	0.00	0.04	0.01	0.01	0.13	0.07	0.01	0.0	0.0	0.0	0.0	0.0	0.0	0.0	0.0	0.0	0.0	0.0	1.00E+00	8.00E-01	9.01E-01	9.98E-01
	PITX1	31	23	4	0	1	0	1	3	0	0	0	0	0	0	0	1	5.05	5.75	4.56	0.38	0.15	1.32	0.28	0.24	4.58	2.53	0.33	0.0	0.2	0.7	0.0	0.0	0.0	0.0	0.0	0.0	0.0	3.1	2.58E-02	8.59E-03	4.66E-01	6.62E-02
	PITX2	21	16	3	0	0	0	0	3	0	0	0	0	0	0	0	0	2.74	3.13	2.48	0.21	0.08	0.71	0.15	0.13	2.49	1.37	0.18	0.0	0.0	1.2	0.0	0.0	0.0	0.0	0.0	0.0	0.0	0.0	3.34E-01	5.03E-02	5.91E-01	4.11E-01
	PITX3	2	1	3	0	0	0	3	0	0	0	0	0	0	0	0	0	0.18	0.21	0.17	0.01	0.01	0.05	0.01	0.01	0.17	0.09	0.01	0.0	14.4	0.0	0.0	0.0	0.0	0.0	0.0	0.0	0.0	0.0	3.68E-05	6.44E-09	8.90E-01	9.97E-01
	PROP1	9	7	1	0	0	0	1	0	0	0	0	0	0	0	0	0	2.34	2.67	2.12	0.18	0.07	0.61	0.13	0.11	2.13	1.17	0.15	0.0	0.4	0.0	0.0	0.0	0.0	0.0	0.0	0.0	0.0	0.0	4.36E-01	6.39E-02	6.20E-01	5.07E-01
	PRRX1	2	0	1	0	0	0	0	1	0	0	0	0	0	0	0	0	0.33	0.38	0.30	0.03	0.01	0.09	0.02	0.02	0.30	0.16	0.02	0.0	0.0	3.4	0.0	0.0	0.0	0.0	0.0	0.0	0.0	0.0	9.81E-01	3.07E-01	8.52E-01	9.88E-01
	PRRX2	0	0	0	0	0	0	0	0	0	0	0	0	0	0	0	0	0.00	0.00	0.00	0.00	0.00	0.00	0.00	0.00	0.00	0.00	0.00	na	na	na	na	na	na	na	na	na	na	na	na	na	na	na
	RAX	2	1	0	0	0	0	0	0	0	0	0	0	0	0	0	0	0.11	0.13	0.10	0.01	0.00	0.03	0.01	0.01	0.10	0.05	0.01	0.0	0.0	0.0	0.0	0.0	0.0	0.0	0.0	0.0	0.0	0.0	1.00E+00	8.46E-01	9.14E-01	9.99E-01
	RAX2	0	0	0	0	0	0	0	0	0	0	0	0	0	0	0	0	0.00	0.00	0.00	0.00	0.00	0.00	0.00	0.00	0.00	0.00	0.00	na	na	na	na	na	na	na	na	na	na	na	na	na	na	na
	RHOXF1	1	0	0	0	0	0	0	0	0	0	0	0	0	0	0	0	0.11	0.13	0.10	0.01	0.00	0.03	0.01	0.01	0.10	0.05	0.01	0.0	0.0	0.0	0.0	0.0	0.0	0.0	0.0	0.0	0.0	0.0	1.00E+00	8.46E-01	9.14E-01	9.99E-01
	RHOXF2	21	22	2	0	0	1	1	0	0	0	0	0	0	0	0	0	5.16	5.88	4.66	0.39	0.15	1.34	0.29	0.25	4.68	2.58	0.33	0.2	0.2	0.0	0.0	0.0	0.0	0.0	0.0	0.0	0.0	0.0	1.47E-02	2.40E-03	4.62E-01	9.13E-02
	RHOXF2B	0	0	0	0	0	0	0	0	0	0	0	0	0	0	0	0	0.00	0.00	0.00	0.00	0.00	0.00	0.00	0.00	0.00	0.00	0.00	na	na	na	na	na	na	na	na	na	na	na	na	na	na	na
	SEBOX	0	0	0	0	0	0	0	0	0	0	0	0	0	0	0	0	0.00	0.00	0.00	0.00	0.00	0.00	0.00	0.00	0.00	0.00	0.00	na	na	na	na	na	na	na	na	na	na	na	na	na	na	na
	SHOX	5	0	0	0	0	0	0	0	0	0	0	0	0	0	0	0	0.33	0.38	0.30	0.03	0.01	0.09	0.02	0.02	0.30	0.16	0.02	0.0	0.0	0.0	0.0	0.0	0.0	0.0	0.0	0.0	0.0	0.0	9.98E-01	6.06E-01	8.52E-01	9.88E-01
	SHOX2	0	2	2	0	0	1	1	0	0	0	0	0	0	0	0	0	0.29	0.33	0.26	0.02	0.01	0.08	0.02	0.01	0.27	0.15	0.02	3.4	3.0	0.0	0.0	0.0	0.0	0.0	0.0	0.0	0.0	0.0	9.53E-01	1.92E-01	8.61E-01	9.91E-01
	TPRX1	1	2	0	0	0	0	0	0	0	0	0	0	0	0	0	0	0.73	0.83	0.66	0.06	0.02	0.19	0.04	0.04	0.66	0.37	0.05	0.0	0.0	0.0	0.0	0.0	0.0	0.0	0.0	0.0	0.0	0.0	9.62E-01	3.28E-01	7.82E-01	9.30E-01
	UNCX	1	1	0	0	0	0	0	0	0	0	0	0	0	0	0	0	0.11	0.13	0.10	0.01	0.00	0.03	0.01	0.01	0.10	0.05	0.01	0.0	0.0	0.0	0.0	0.0	0.0	0.0	0.0	0.0	0.0	0.0	1.00E+00	8.46E-01	9.14E-01	9.99E-01
	VSX1	27	20	3	0	0	2	0	1	0	0	0	0	0	0	0	0	2.82	3.21	2.55	0.21	0.08	0.73	0.16	0.14	2.56	1.41	0.18	0.7	0.0	0.4	0.0	0.0	0.0	0.0	0.0	0.0	0.0	0.0	4.53E-01	1.12E-01	5.86E-01	3.95E-01
	VSX2	1	8	0	0	0	0	0	0	0	0	0	0	0	0	0	0	1.17	1.33	1.06	0.09	0.03	0.30	0.07	0.06	1.06	0.59	0.08	0.0	0.0	0.0	0.0	0.0	0.0	0.0	0.0	0.0	0.0	0.0	8.29E-01	1.68E-01	7.26E-01	8.28E-01
HB.PHTF	PHTF1	1	1	0	0	0	0	0	0	0	0	0	0	0	0	0	0	0.70	0.79	0.63	0.05	0.02	0.18	0.04	0.03	0.63	0.35	0.05	0.0	0.0	0.0	0.0	0.0	0.0	0.0	0.0	0.0	0.0	0.0	9.68E-01	3.47E-01	7.87E-01	9.37E-01
HB.PROS	PROX1	14	8	5	0	0	0	0	5	0	0	0	0	0	0	0	0	1.35	1.54	1.22	0.10	0.04	0.35	0.08	0.07	1.23	0.68	0.09	0.0	0.0	4.1	0.0	0.0	0.0	0.0	0.0	0.0	0.0	0.0	7.04E-02	6.92E-04	7.06E-01	7.78E-01
	PROX2	0	1	0	0	0	0	0	0	0	0	0	0	0	0	0	0	0.07	0.08	0.07	0.01	0.00	0.02	0.00	0.00	0.07	0.04	0.00	0.0	0.0	0.0	0.0	0.0	0.0	0.0	0.0	0.0	0.0	0.0	1.00E+00	8.95E-01	9.30E-01	1.00E+00
HB.SINE	SIX1	5	22	1	0	0	0	0	1	0	0	0	0	0	0	0	0	2.34	2.67	2.12	0.18	0.07	0.61	0.13	0.11	2.13	1.17	0.15	0.0	0.0	0.5	0.0	0.0	0.0	0.0	0.0	0.0	0.0	0.0	4.28E-01	6.08E-02	6.20E-01	5.07E-01
	SIX2	0	20	0	0	0	0	0	0	0	0	0	0	0	0	0	0	1.68	1.92	1.52	0.13	0.05	0.44	0.09	0.08	1.53	0.84	0.11	0.0	0.0	0.0	0.0	0.0	0.0	0.0	0.0	0.0	0.0	0.0	5.91E-01	7.72E-02	6.74E-01	6.86E-01
	SIX3	9	5	0	0	0	0	0	0	0	0	0	0	0	0	0	0	0.84	0.96	0.76	0.06	0.02	0.22	0.05	0.04	0.76	0.42	0.05	0.0	0.0	0.0	0.0	0.0	0.0	0.0	0.0	0.0	0.0	0.0	9.38E-01	2.78E-01	7.66E-01	9.08E-01
	SIX4	22	7	1	0	1	0	0	1	0	0	0	0	0	1	0	0	1.68	1.92	1.52	0.13	0.05	0.44	0.09	0.08	1.53	0.84	0.11	0.0	0.0	0.7	0.0	0.0	0.0	0.0	0.0	0.7	0.0	0.0	8.39E-01	1.51E-01	6.74E-01	8.83E-01
	SIX5	4	7	3	0	0	0	1	2	0	0	0	0	0	0	0	0	1.02	1.17	0.93	0.08	0.03	0.27	0.06	0.05	0.93	0.51	0.07	0.0	0.9	2.2	0.0	0.0	0.0	0.0	0.0	0.0	0.0	0.0	9.34E-01	3.17E-01	7.43E-01	8.65E-01
	SIX6	1	8	0	0	0	0	0	0	0	0	0	0	0	0	0	0	0.44	0.50	0.40	0.03	0.01	0.11	0.02	0.02	0.40	0.22	0.03	0.0	0.0	0.0	0.0	0.0	0.0	0.0	0.0	0.0	0.0	0.0	9.95E-01	5.13E-01	8.30E-01	9.77E-01
HB.TALE	IRX1	0	0	1	0	0	0	1	0	0	0	0	0	0	0	0	0	0.07	0.08	0.07	0.01	0.00	0.02	0.00	0.00	0.07	0.04	0.00	0.0	12.0	0.0	0.0	0.0	0.0	0.0	0.0	0.0	0.0	0.0	4.10E-01	6.06E-03	9.30E-01	1.00E+00
	IRX2	1	5	1	0	0	0	0	1	0	0	0	0	0	0	0	0	0.22	0.25	0.20	0.02	0.01	0.06	0.01	0.01	0.20	0.11	0.01	0.0	0.0	5.0	0.0	0.0	0.0	0.0	0.0	0.0	0.0	0.0	9.41E-01	1.57E-01	8.79E-01	9.95E-01
	IRX3	0	1	0	0	0	0	0	0	0	0	0	0	0	0	0	0	0.04	0.04	0.03	0.00	0.00	0.01	0.00	0.00	0.03	0.02	0.00	0.0	0.0	0.0	0.0	0.0	0.0	0.0	0.0	0.0	0.0	0.0	1.00E+00	9.46E-01	9.51E-01	1.00E+00
	IRX4	4	1	0	0	0	0	0	0	0	0	0	0	0	0	0	0	0.26	0.29	0.23	0.02	0.01	0.07	0.01	0.01	0.23	0.13	0.02	0.0	0.0	0.0	0.0	0.0	0.0	0.0	0.0	0.0	0.0	0.0	9.99E-01	6.77E-01	8.70E-01	9.93E-01
	IRX5	0	0	0	0	0	0	0	0	0	0	0	0	0	0	0	0	0.15	0.17	0.13	0.01	0.00	0.04	0.01	0.01	0.13	0.07	0.01	0.0	0.0	0.0	0.0	0.0	0.0	0.0	0.0	0.0	0.0	0.0	1.00E+00	8.00E-01	9.01E-01	9.98E-01
	IRX6	5	3	0	0	0	0	0	0	0	0	0	0	0	0	0	0	0.40	0.46	0.36	0.03	0.01	0.10	0.02	0.02	0.37	0.20	0.03	0.0	0.0	0.0	0.0	0.0	0.0	0.0	0.0	0.0	0.0	0.0	9.96E-01	5.42E-01	8.37E-01	9.81E-01
	MEIS1	30	1	0	0	0	0	0	0	0	0	0	0	0	0	0	0	2.01	2.29	1.82	0.15	0.06	0.52	0.11	0.10	1.83	1.01	0.13	0.0	0.0	0.0	0.0	0.0	0.0	0.0	0.0	0.0	0.0	0.0	4.37E-01	4.68E-02	6.46E-01	5.94E-01
	MEIS2	35	6	3	0	0	1	1	1	0	0	0	0	0	0	0	0	4.21	4.80	3.80	0.32	0.12	1.10	0.23	0.20	3.82	2.11	0.27	0.2	0.2	0.3	0.0	0.0	0.0	0.0	0.0	0.0	0.0	0.0	1.09E-01	2.33E-02	5.06E-01	1.72E-01
	MEIS3	6	1	2	0	0	1	1	0	0	0	0	0	0	0	0	0	0.95	1.08	0.86	0.07	0.03	0.25	0.05	0.05	0.86	0.48	0.06	1.1	0.9	0.0	0.0	0.0	0.0	0.0	0.0	0.0	0.0	0.0	9.87E-01	6.48E-01	7.52E-01	8.83E-01
	MKX	1	7	4	0	0	0	2	2	0	0	0	0	0	0	0	0	0.59	0.67	0.53	0.04	0.02	0.15	0.03	0.03	0.53	0.29	0.04	0.0	3.0	3.8	0.0	0.0	0.0	0.0	0.0	0.0	0.0	0.0	5.83E-01	2.55E-02	8.04E-01	9.56E-01
	PBX1	53	17	10	0	0	0	2	8	0	0	0	0	0	0	0	0	3.84	4.38	3.47	0.29	0.11	1.00	0.21	0.18	3.49	1.92	0.25	0.0	0.5	2.3	0.0	0.0	0.0	0.0	0.0	0.0	0.0	0.0	4.71E-02	4.01E-03	5.25E-01	2.16E-01
	PBX2	41	7	5	0	0	0	4	1	0	0	0	0	0	0	0	0	3.62	4.13	3.27	0.28	0.11	0.94	0.20	0.17	3.29	1.81	0.23	0.0	1.0	0.3	0.0	0.0	0.0	0.0	0.0	0.0	0.0	0.0	2.69E-01	7.41E-02	5.37E-01	2.47E-01
	PBX3	22	10	2	0	0	1	1	0	0	0	0	0	0	0	0	0	4.21	4.80	3.80	0.32	0.12	1.10	0.23	0.20	3.82	2.11	0.27	0.2	0.2	0.0	0.0	0.0	0.0	0.0	0.0	0.0	0.0	0.0	6.54E-02	9.79E-03	5.06E-01	1.72E-01
	PBX4	25	9	5	0	3	3	1	1	0	0	2	0	0	0	1	0	3.37	3.84	3.04	0.26	0.10	0.88	0.19	0.16	3.06	1.69	0.22	0.9	0.3	0.3	0.0	0.0	2.3	0.0	0.0	0.0	0.6	0.0	5.13E-01	1.73E-01	5.52E-01	3.76E-01
	PKNOX1	33	5	1	0	0	0	1	0	0	0	0	0	0	0	0	0	3.44	3.92	3.11	0.26	0.10	0.90	0.19	0.17	3.12	1.72	0.22	0.0	0.3	0.0	0.0	0.0	0.0	0.0	0.0	0.0	0.0	0.0	1.18E-01	1.28E-02	5.48E-01	2.76E-01
	PKNOX2	10	5	1	1	0	0	1	0	1	0	0	0	0	0	0	0	1.87	2.13	1.69	0.14	0.05	0.49	0.10	0.09	1.69	0.93	0.12	0.0	0.5	0.0	7.0	0.0	0.0	0.0	0.0	0.0	0.0	0.0	2.34E-01	1.26E-01	2.20E-02	6.34E-01
	TGIF1	13	21	5	0	0	0	2	3	0	0	0	0	0	0	0	0	1.72	1.96	1.55	0.13	0.05	0.45	0.10	0.08	1.56	0.86	0.11	0.0	1.0	1.9	0.0	0.0	0.0	0.0	0.0	0.0	0.0	0.0	7.80E-01	2.16E-01	6.71E-01	6.75E-01
	TGIF2	4	8	2	1	0	0	1	1	0	1	0	0	0	0	0	0	0.77	0.88	0.69	0.06	0.02	0.20	0.04	0.04	0.70	0.38	0.05	0.0	1.1	1.4	0.0	44.9	0.0	0.0	0.0	0.0	0.0	0.0	1.94E-06	6.31E-01	5.65E-11	9.23E-01
	TGIF2LX	7	14	3	0	0	1	0	2	0	0	0	0	0	0	0	0	1.35	1.54	1.22	0.10	0.04	0.35	0.08	0.07	1.23	0.68	0.09	0.7	0.0	1.6	0.0	0.0	0.0	0.0	0.0	0.0	0.0	0.0	9.07E-01	3.45E-01	7.06E-01	7.78E-01
	TGIF2LY	3	7	2	0	2	0	0	2	0	0	2	0	0	0	0	0	1.94	2.21	1.75	0.15	0.06	0.51	0.11	0.09	1.76	0.97	0.13	0.0	0.0	1.1	0.0	0.0	4.0	0.0	0.0	0.0	0.0	0.0	2.94E-01	1.23E-01	6.52E-01	1.87E-01
HMG	HMGB1	79	83	49	2	86	17	18	14	1	1	2	0	0	74	10	0	18.73	21.35	16.93	1.42	0.54	4.88	1.04	0.90	17.01	9.38	1.21	0.9	0.8	0.8	0.7	1.8	0.4	0.0	0.0	4.4	1.1	0.0	5.28E-37	5.51E-01	4.75E-01	2.22E-40
	HMGB2	34	46	18	0	66	4	5	9	0	0	8	2	1	54	1	0	10.50	11.97	9.49	0.80	0.30	2.74	0.58	0.50	9.54	5.26	0.68	0.4	0.4	0.9	0.0	0.0	2.9	3.4	2.0	5.7	0.2	0.0	8.72E-45	1.74E-02	2.94E-01	9.73E-47
	HMGB3	18	17	9	0	3	1	3	5	0	0	0	0	0	3	0	0	2.74	3.13	2.48	0.21	0.08	0.71	0.15	0.13	2.49	1.37	0.18	0.4	1.0	2.0	0.0	0.0	0.0	0.0	0.0	1.2	0.0	0.0	7.61E-01	1.59E-01	5.91E-01	7.53E-01
	HMGB4	0	0	0	0	0	0	0	0	0	0	0	0	0	0	0	0	0.26	0.29	0.23	0.02	0.01	0.07	0.01	0.01	0.23	0.13	0.02	0.0	0.0	0.0	0.0	0.0	0.0	0.0	0.0	0.0	0.0	0.0	9.99E-01	6.77E-01	8.70E-01	9.93E-01
	HSFX4	0	0	0	0	0	0	0	0	0	0	0	0	0	0	0	0	0.00	0.00	0.00	0.00	0.00	0.00	0.00	0.00	0.00	0.00	0.00	na	na	na	na	na	na	na	na	na	na	na	na	na	na	na
	KMT2C	51	52	21	0	1	1	5	15	0	0	0	1	0	0	0	0	6.88	7.84	6.22	0.52	0.20	1.79	0.38	0.33	6.25	3.45	0.45	0.1	0.6	2.4	0.0	0.0	0.0	2.6	0.0	0.0	0.0	0.0	3.39E-04	9.80E-05	3.95E-01	2.11E-02
	MAEL	4	3	12	0	7	3	9	0	0	0	5	2	0	0	0	0	1.06	1.21	0.96	0.08	0.03	0.28	0.06	0.05	0.96	0.53	0.07	2.8	7.4	0.0	0.0	0.0	18.1	33.8	0.0	0.0	0.0	0.0	1.05E-37	1.33E-12	7.38E-01	9.10E-30
	MEIOSIN	0	0	0	0	0	0	0	0	0	0	0	0	0	0	0	0	0.00	0.00	0.00	0.00	0.00	0.00	0.00	0.00	0.00	0.00	0.00	na	na	na	na	na	na	na	na	na	na	na	na	na	na	na
	PMS1	7	18	4	0	0	2	2	0	0	0	0	0	0	0	0	0	4.46	5.09	4.03	0.34	0.13	1.16	0.25	0.21	4.05	2.24	0.29	0.4	0.4	0.0	0.0	0.0	0.0	0.0	0.0	0.0	0.0	0.0	1.01E-01	2.64E-02	4.93E-01	1.45E-01
	WDHD1	7	30	10	0	2	2	2	6	0	0	0	0	0	1	1	0	4.61	5.25	4.17	0.35	0.13	1.20	0.26	0.22	4.19	2.31	0.30	0.4	0.4	1.4	0.0	0.0	0.0	0.0	0.0	0.2	0.4	0.0	4.47E-01	1.16E-01	4.86E-01	3.98E-01
HMG.PBRM1	HMGXB4	27	30	8	0	0	0	1	7	0	0	0	0	0	0	0	0	4.65	5.30	4.20	0.35	0.13	1.21	0.26	0.22	4.22	2.33	0.30	0.0	0.2	1.7	0.0	0.0	0.0	0.0	0.0	0.0	0.0	0.0	3.99E-02	6.74E-03	4.85E-01	1.29E-01
	PBRM1	96	46	20	0	6	0	3	17	0	0	0	0	0	6	0	0	10.35	11.80	9.36	0.79	0.30	2.70	0.58	0.50	9.40	5.19	0.67	0.0	0.3	1.8	0.0	0.0	0.0	0.0	0.0	0.6	0.0	0.0	1.20E-04	9.36E-06	2.97E-01	5.42E-02
HMG.SOX	BBX	17	19	11	0	13	1	4	6	0	0	0	0	0	13	0	0	4.83	5.50	4.36	0.37	0.14	1.26	0.27	0.23	4.39	2.42	0.31	0.2	0.7	1.4	0.0	0.0	0.0	0.0	0.0	3.0	0.0	0.0	3.77E-03	1.31E-01	4.76E-01	6.77E-04
	CIC	206	109	67	1	39	29	23	15	1	0	11	0	1	26	0	1	26.23	29.90	23.71	1.99	0.76	6.83	1.46	1.26	23.82	13.14	1.70	1.1	0.8	0.6	0.5	0.0	1.6	0.0	0.8	1.1	0.0	0.6	7.54E-03	7.87E-02	2.62E-01	3.37E-03
	HBP1	14	25	10	0	8	0	4	6	0	0	0	0	0	8	0	0	5.20	5.92	4.70	0.40	0.15	1.35	0.29	0.25	4.72	2.60	0.34	0.0	0.7	1.3	0.0	0.0	0.0	0.0	0.0	1.7	0.0	0.0	1.80E-01	4.55E-02	4.60E-01	2.12E-01
	SOX1	4	3	0	0	0	0	0	0	0	0	0	0	0	0	0	0	0.33	0.38	0.30	0.03	0.01	0.09	0.02	0.02	0.30	0.16	0.02	0.0	0.0	0.0	0.0	0.0	0.0	0.0	0.0	0.0	0.0	0.0	9.98E-01	6.06E-01	8.52E-01	9.88E-01
	SOX10	31	26	2	0	0	0	0	2	0	0	0	0	0	0	0	0	2.89	3.29	2.61	0.22	0.08	0.75	0.16	0.14	2.62	1.45	0.19	0.0	0.0	0.8	0.0	0.0	0.0	0.0	0.0	0.0	0.0	0.0	2.89E-01	4.22E-02	5.82E-01	3.79E-01
	SOX11	3	1	0	0	0	0	0	0	0	0	0	0	0	0	0	0	0.22	0.25	0.20	0.02	0.01	0.06	0.01	0.01	0.20	0.11	0.01	0.0	0.0	0.0	0.0	0.0	0.0	0.0	0.0	0.0	0.0	0.0	1.00E+00	7.16E-01	8.79E-01	9.95E-01
	SOX12	1	0	1	0	0	0	1	0	0	0	0	0	0	0	0	0	0.29	0.33	0.26	0.02	0.01	0.08	0.02	0.01	0.27	0.15	0.02	0.0	3.0	0.0	0.0	0.0	0.0	0.0	0.0	0.0	0.0	0.0	9.91E-01	3.89E-01	8.61E-01	9.91E-01
	SOX13	23	10	1	0	0	0	1	0	0	0	0	0	0	0	0	0	1.72	1.96	1.55	0.13	0.05	0.45	0.10	0.08	1.56	0.86	0.11	0.0	0.5	0.0	0.0	0.0	0.0	0.0	0.0	0.0	0.0	0.0	7.17E-01	1.54E-01	6.71E-01	6.75E-01
	SOX14	7	0	0	0	0	0	0	0	0	0	0	0	0	0	0	0	0.48	0.54	0.43	0.04	0.01	0.12	0.03	0.02	0.43	0.24	0.03	0.0	0.0	0.0	0.0	0.0	0.0	0.0	0.0	0.0	0.0	0.0	9.93E-01	4.85E-01	8.23E-01	9.72E-01
	SOX15	41	17	11	0	24	0	8	3	0	0	0	1	0	1	22	0	5.42	6.17	4.89	0.41	0.16	1.41	0.30	0.26	4.92	2.71	0.35	0.0	1.3	0.6	0.0	0.0	0.0	3.3	0.0	0.2	8.1	0.0	2.14E-27	3.53E-02	4.51E-01	2.63E-29
	SOX17	30	16	4	0	4	0	1	3	0	0	0	0	1	1	2	0	2.93	3.34	2.65	0.22	0.08	0.76	0.16	0.14	2.66	1.47	0.19	0.0	0.3	1.1	0.0	0.0	0.0	0.0	7.1	0.4	1.4	0.0	2.52E-01	9.97E-02	5.79E-01	1.80E-01
	SOX18	3	0	1	0	0	0	0	1	0	0	0	0	0	0	0	0	0.22	0.25	0.20	0.02	0.01	0.06	0.01	0.01	0.20	0.11	0.01	0.0	0.0	5.0	0.0	0.0	0.0	0.0	0.0	0.0	0.0	0.0	9.41E-01	1.57E-01	8.79E-01	9.95E-01
	SOX2	247	204	106	4	105	22	47	37	1	3	8	2	2	42	49	2	60.19	68.60	54.40	4.58	1.75	15.68	3.35	2.89	54.65	30.15	3.90	0.4	0.7	0.7	0.2	1.7	0.5	0.6	0.7	0.8	1.6	0.5	2.90E-09	1.13E-08	5.46E-02	1.14E-03
	SOX21	8	3	1	0	0	0	1	0	0	0	0	0	0	0	0	0	0.51	0.58	0.46	0.04	0.01	0.13	0.03	0.02	0.47	0.26	0.03	0.0	1.7	0.0	0.0	0.0	0.0	0.0	0.0	0.0	0.0	0.0	9.94E-01	5.29E-01	8.17E-01	9.67E-01

	SOX3	10	3	0	0	0	0	0	0	0	0	0	0	0	0	0	0	0.70	0.79	0.63	0.05	0.02	0.18	0.04	0.03	0.63	0.35	0.05	0.0	0.0	0.0	0.0	0.0	0.0	0.0	0.0	0.0	0.0	0.0	9.68E-01	3.47E-01	7.87E-01	9.37E-01
	SOX30	1	2	0	0	0	0	0	0	0	0	0	0	0	0	0	0	0.73	0.83	0.66	0.06	0.02	0.19	0.04	0.04	0.66	0.37	0.05	0.0	0.0	0.0	0.0	0.0	0.0	0.0	0.0	0.0	0.0	0.0	9.62E-01	3.28E-01	7.82E-01	9.30E-01
	SOX4	14	13	3	0	1	0	1	2	0	0	0	0	0	0	1	0	3.00	3.42	2.71	0.23	0.09	0.78	0.17	0.14	2.72	1.50	0.19	0.0	0.3	0.7	0.0	0.0	0.0	0.0	0.0	0.0	0.7	0.0	4.95E-01	8.63E-02	5.75E-01	5.24E-01
	SOX5	83	24	6	1	7	1	3	2	0	1	0	0	0	5	2	0	6.84	7.80	6.18	0.52	0.20	1.78	0.38	0.33	6.21	3.43	0.44	0.1	0.4	0.3	0.0	5.0	0.0	0.0	0.0	0.8	0.6	0.0	5.02E-02	4.58E-03	5.26E-02	5.84E-01
	SOX6	55	38	14	1	4	1	6	7	0	1	0	0	0	3	1	0	6.00	6.84	5.42	0.46	0.17	1.56	0.33	0.29	5.45	3.01	0.39	0.2	0.9	1.3	0.0	5.7	0.0	0.0	0.0	0.6	0.3	0.0	1.68E-01	9.40E-02	3.65E-02	4.14E-01
	SOX7	39	16	4	0	0	0	1	3	0	0	0	0	0	0	0	0	2.93	3.34	2.65	0.22	0.08	0.76	0.16	0.14	2.66	1.47	0.19	0.0	0.3	1.1	0.0	0.0	0.0	0.0	0.0	0.0	0.0	0.0	4.15E-01	9.97E-02	5.79E-01	3.71E-01
	SOX8	3	4	2	0	0	0	0	2	0	0	0	0	0	0	0	0	0.40	0.46	0.36	0.03	0.01	0.10	0.02	0.02	0.37	0.20	0.03	0.0	0.0	5.5	0.0	0.0	0.0	0.0	0.0	0.0	0.0	0.0	5.32E-01	1.64E-02	8.37E-01	9.81E-01
	SOX9	41	33	11	0	0	1	1	9	0	0	0	0	0	0	0	0	4.72	5.38	4.27	0.36	0.14	1.23	0.26	0.23	4.29	2.36	0.31	0.2	0.2	2.1	0.0	0.0	0.0	0.0	0.0	0.0	0.0	0.0	2.16E-02	2.81E-03	4.81E-01	1.23E-01
	SRY	19	3	5	0	0	0	0	5	0	0	0	0	0	0	0	0	1.21	1.38	1.09	0.09	0.04	0.31	0.07	0.06	1.10	0.60	0.08	0.0	0.0	4.6	0.0	0.0	0.0	0.0	0.0	0.0	0.0	0.0	4.12E-02	2.50E-04	7.22E-01	8.18E-01
HMG.TCF7	LEF1	33	49	4	0	0	0	0	4	0	0	0	0	0	0	0	0	4.35	4.96	3.94	0.33	0.13	1.13	0.24	0.21	3.95	2.18	0.28	0.0	0.0	1.0	0.0	0.0	0.0	0.0	0.0	0.0	0.0	0.0	5.88E-02	9.48E-03	4.99E-01	1.56E-01
	TCF7	6	12	0	0	0	0	0	0	0	0	0	0	0	0	0	0	0.95	1.08	0.86	0.07	0.03	0.25	0.05	0.05	0.86	0.48	0.06	0.0	0.0	0.0	0.0	0.0	0.0	0.0	0.0	0.0	0.0	0.0	9.08E-01	2.35E-01	7.52E-01	8.83E-01
	TCF7L1	3	4	0	0	0	0	0	0	0	0	0	0	0	0	0	0	0.44	0.50	0.40	0.03	0.01	0.11	0.02	0.02	0.40	0.22	0.03	0.0	0.0	0.0	0.0	0.0	0.0	0.0	0.0	0.0	0.0	0.0	9.95E-01	5.13E-01	8.30E-01	9.77E-01
	TCF7L2	72	67	11	0	20	3	5	3	0	0	0	0	0	1	19	0	11.45	13.05	10.35	0.87	0.33	2.98	0.64	0.55	10.40	5.74	0.74	0.3	0.4	0.3	0.0	0.0	0.0	0.0	0.0	0.1	3.3	0.0	1.72E-09	2.71E-04	2.73E-01	2.23E-08
HMG.TFAM	TFAM	60	41	14	1	64	2	4	8	0	1	1	0	2	1	53	7	16.36	18.64	14.78	1.24	0.47	4.26	0.91	0.79	14.85	8.19	1.06	0.1	0.2	0.5	0.0	2.1	0.2	0.0	2.5	0.1	6.5	6.6	5.77E-64	1.23E-06	1.77E-01	5.33E-62
HMG.TOX	HMG20A	90	62	33	0	3	1	4	28	0	0	0	0	0	1	2	0	11.89	13.55	10.75	0.90	0.35	3.10	0.66	0.57	10.80	5.96	0.77	0.1	0.3	2.6	0.0	0.0	0.0	0.0	0.0	0.1	0.3	0.0	1.34E-09	2.28E-10	2.64E-01	5.28E-03
	HMG20B	55	56	36	0	0	2	1	33	0	0	0	0	0	0	0	0	5.60	6.38	5.06	0.43	0.16	1.46	0.31	0.27	5.08	2.80	0.36	0.4	0.2	6.5	0.0	0.0	0.0	0.0	0.0	0.0	0.0	0.0	1.05E-31	1.02E-35	4.43E-01	6.75E-02
	HMGXB3	0	0	0	0	0	0	0	0	0	0	0	0	0	0	0	0	0.33	0.38	0.30	0.03	0.01	0.09	0.02	0.02	0.30	0.16	0.02	0.0	0.0	0.0	0.0	0.0	0.0	0.0	0.0	0.0	0.0	0.0	9.98E-01	6.06E-01	8.52E-01	9.88E-01
	SMARCE1	154	81	54	0	0	5	3	46	0	0	0	0	0	0	0	0	18.37	20.93	16.60	1.40	0.53	4.78	1.02	0.88	16.68	9.20	1.19	0.3	0.1	2.8	0.0	0.0	0.0	0.0	0.0	0.0	0.0	0.0	1.42E-19	1.76E-17	1.65E-01	2.66E-06
	TOX	6	0	1	0	0	0	1	0	0	0	0	0	0	0	0	0	0.48	0.54	0.43	0.04	0.01	0.12	0.03	0.02	0.43	0.24	0.03	0.0	1.8	0.0	0.0	0.0	0.0	0.0	0.0	0.0	0.0	0.0	9.94E-01	5.24E-01	8.23E-01	9.72E-01
	TOX2	6	2	0	1	0	0	0	0	1	0	0	0	0	0	0	0	1.24	1.42	1.12	0.09	0.04	0.32	0.07	0.06	1.13	0.62	0.08	0.0	0.0	0.0	10.6	0.0	0.0	0.0	0.0	0.0	0.0	0.0	1.40E-01	1.51E-01	3.18E-03	8.08E-01
	TOX3	1	4	1	0	0	0	1	0	0	0	0	0	0	0	0	0	0.99	1.13	0.89	0.08	0.03	0.26	0.06	0.05	0.90	0.49	0.06	0.0	0.9	0.0	0.0	0.0	0.0	0.0	0.0	0.0	0.0	0.0	9.55E-01	3.88E-01	7.47E-01	8.74E-01
	TOX4	22	23	19	3	1	12	6	1	3	0	0	0	0	1	0	0	7.32	8.34	6.61	0.56	0.21	1.91	0.41	0.35	6.64	3.67	0.47	1.6	0.7	0.2	5.4	0.0	0.0	0.0	0.0	0.2	0.0	0.0	5.96E-04	1.49E-02	9.39E-04	4.07E-02
HMG.UBF	UBTF	24	61	19	0	5	3	4	12	0	0	0	0	0	5	0	0	10.14	11.55	9.16	0.77	0.29	2.64	0.56	0.49	9.20	5.08	0.66	0.3	0.3	1.3	0.0	0.0	0.0	0.0	0.0	0.5	0.0	0.0	9.87E-03	4.43E-03	3.02E-01	4.50E-02
	UBTFL1	9	2	2	0	0	2	0	0	0	0	0	0	0	0	0	0	1.10	1.25	0.99	0.08	0.03	0.29	0.06	0.05	1.00	0.55	0.07	1.8	0.0	0.0	0.0	0.0	0.0	0.0	0.0	0.0	0.0	0.0	8.83E-01	2.25E-01	7.34E-01	8.47E-01
HMG.WHSC1	NSD2	0	0	0	0	0	0	0	0	0	0	0	0	0	0	0	0	0.00	0.00	0.00	0.00	0.00	0.00	0.00	0.00	0.00	0.00	0.00	na	na	na	na	na	na	na	na	na	na	na	na	na	na	na
HMG.WHSC2	SSRP1	68	169	115	5	10	45	47	23	4	1	3	0	0	6	1	0	30.22	34.44	27.31	2.30	0.88	7.87	1.68	1.45	27.44	15.14	1.96	1.5	1.4	0.8	1.7	1.1	0.4	0.0	0.0	0.2	0.1	0.0	1.23E-07	1.95E-03	2.58E-01	3.66E-07
HMGA	HMGA1	75	107	34	0	5	5	11	18	0	0	1	0	0	3	1	0	17.67	20.14	15.97	1.34	0.51	4.60	0.98	0.85	16.05	8.85	1.14	0.3	0.5	1.1	0.0	0.0	0.2	0.0	0.0	0.2	0.1	0.0	2.84E-05	1.17E-03	1.73E-01	2.87E-04
	HMGA2	34	37	13	0	0	1	4	8	0	0	0	0	0	0	0	0	5.53	6.30	4.99	0.42	0.16	1.44	0.31	0.27	5.02	2.77	0.36	0.2	0.6	1.6	0.0	0.0	0.0	0.0	0.0	0.0	0.0	0.0	7.24E-02	4.17E-02	4.46E-01	7.10E-02
HSF	HSF1	47	127	85	4	42	38	31	16	4	0	6	1	5	27	3	0	26.23	29.90	23.71	1.99	0.76	6.83	1.46	1.26	23.82	13.14	1.70	1.4	1.0	0.7	2.0	0.0	0.9	0.7	4.0	1.1	0.2	0.0	4.17E-04	2.00E-02	9.56E-02	7.13E-04
	HSF2	16	19	8	0	0	4	2	2	0	0	0	0	0	0	0	0	4.17	4.75	3.77	0.32	0.12	1.09	0.23	0.20	3.79	2.09	0.27	1.0	0.4	0.5	0.0	0.0	0.0	0.0	0.0	0.0	0.0	0.0	3.95E-01	2.96E-01	5.08E-01	1.76E-01
	HSF4	11	8	4	0	0	0	2	2	0	0	0	0	0	0	0	0	2.49	2.84	2.25	0.19	0.07	0.65	0.14	0.12	2.26	1.25	0.16	0.0	0.7	0.9	0.0	0.0	0.0	0.0	0.0	0.0	0.0	0.0	6.68E-01	2.51E-01	6.09E-01	4.70E-01
	HSF5	0	1	0	0	0	0	0	0	0	0	0	0	0	0	0	0	0.22	0.25	0.20	0.02	0.01	0.06	0.01	0.01	0.20	0.11	0.01	0.0	0.0	0.0	0.0	0.0	0.0	0.0	0.0	0.0	0.0	0.0	1.00E+00	7.16E-01	8.79E-01	9.95E-01
	HSFX1	0	2	0	0	2	0	0	0	0	0	0	0	0	0	0	2	0.91	1.04	0.83	0.07	0.03	0.24	0.05	0.04	0.83	0.46	0.06	0.0	0.0	0.0	0.0	0.0	0.0	0.0	0.0	0.0	0.0	33.8	1.03E-10	2.49E-01	7.57E-01	1.01E-12
	HSFX3	0	0	0	0	0	0	0	0	0	0	0	0	0	0	0	0	0.00	0.00	0.00	0.00	0.00	0.00	0.00	0.00	0.00	0.00	0.00	na	na	na	na	na	na	na	na	na	na	na	na	na	na	na
	HSFY1	15	5	1	0	0	0	1	0	0	0	0	0	0	0	0	0	1.87	2.13	1.69	0.14	0.05	0.49	0.10	0.09	1.69	0.93	0.12	0.0	0.5	0.0	0.0	0.0	0.0	0.0	0.0	0.0	0.0	0.0	6.51E-01	1.26E-01	6.58E-01	6.34E-01
HTH	LCOR	27	59	19	0	4	1	2	16	0	0	3	0	0	1	0	0	9.99	11.38	9.03	0.76	0.29	2.60	0.56	0.48	9.07	5.00	0.65	0.1	0.2	1.8	0.0	0.0	1.2	0.0	0.0	0.1	0.0	0.0	7.82E-05	2.48E-05	3.06E-01	1.61E-02
	LCORL	2	10	0	0	0	0	0	0	0	0	0	0	0	0	0	0	0.88	1.00	0.79	0.07	0.03	0.23	0.05	0.04	0.80	0.44	0.06	0.0	0.0	0.0	0.0	0.0	0.0	0.0	0.0	0.0	0.0	0.0	9.29E-01	2.63E-01	7.61E-01	9.00E-01
IPT	CAMTA1	0	2	2	0	0	0	2	0	0	0	0	0	0	0	0	0	0.37	0.42	0.33	0.03	0.01	0.10	0.02	0.02	0.33	0.18	0.02	0.0	4.8	0.0	0.0	0.0	0.0	0.0	0.0	0.0	0.0	0.0	6.86E-01	3.50E-02	8.45E-01	9.84E-01
	CAMTA2	2	2	1	0	0	0	1	0	0	0	0	0	0	0	0	0	0.91	1.04	0.83	0.07	0.03	0.24	0.05	0.04	0.83	0.46	0.06	0.0	1.0	0.0	0.0	0.0	0.0	0.0	0.0	0.0	0.0	0.0	9.66E-01	4.18E-01	7.57E-01	8.91E-01
LRRFIP	LRRFIP1	5	30	5	0	0	4	0	1	0	0	0	0	0	0	0	0	4.24	4.84	3.84	0.32	0.12	1.11	0.24	0.20	3.85	2.13	0.27	0.9	0.0	0.3	0.0	0.0	0.0	0.0	0.0	0.0	0.0	0.0	1.25E-01	3.10E-02	5.04E-01	1.68E-01
	LRRFIP2	5	21	7	0	0	4	1	2	0	0	0	0	0	0	0	0	5.01	5.71	4.53	0.38	0.15	1.31	0.28	0.24	4.55	2.51	0.32	0.8	0.2	0.4	0.0	0.0	0.0	0.0	0.0	0.0	0.0	0.0	1.23E-01	6.37E-02	4.68E-01	1.01E-01
MADS	MEF2A	32	31	16	0	0	0	2	14	0	0	0	0	0	0	0	0	3.81	4.34	3.44	0.29	0.11	0.99	0.21	0.18	3.46	1.91	0.25	0.0	0.5	4.1	0.0	0.0	0.0	0.0	0.0	0.0	0.0	0.0	2.28E-06	7.21E-09	5.27E-01	2.21E-01
	MEF2B	4	1	1	0	0	0	0	1	0	0	0	0	0	0	0	0	0.33	0.38	0.30	0.03	0.01	0.09	0.02	0.02	0.30	0.16	0.02	0.0	0.0	3.4	0.0	0.0	0.0	0.0	0.0	0.0	0.0	0.0	9.81E-01	3.07E-01	8.52E-01	9.88E-01
	MEF2C	22	25	13	0	0	0	0	13	0	0	0	0	0	0	0	0	2.23	2.54	2.02	0.17	0.06	0.58	0.12	0.11	2.03	1.12	0.14	0.0	0.0	6.4	0.0	0.0	0.0	0.0	0.0	0.0	0.0	0.0	7.19E-11	9.50E-15	6.28E-01	5.35E-01
	MEF2D	34	16	23	0	0	1	2	20	0	0	0	0	0	0	0	0	4.17	4.75	3.77	0.32	0.12	1.09	0.23	0.20	3.79	2.09	0.27	0.2	0.4	5.3	0.0	0.0	0.0	0.0	0.0	0.0	0.0	0.0	2.05E-13	9.05E-17	5.08E-01	1.76E-01
	SRF	81	32	12	0	0	0	3	9	0	0	0	0	0	0	0	0	5.96	6.80	5.39	0.45	0.17	1.55	0.33	0.29	5.42	2.99	0.39	0.0	0.4	1.7	0.0	0.0	0.0	0.0	0.0	0.0	0.0	0.0	1.47E-02	5.24E-03	4.29E-01	5.22E-02
NDT80	MYRF	0	0	0	0	0	0	0	0	0	0	0	0	0	0	0	0	0.77	0.88	0.69	0.06	0.02	0.20	0.04	0.04	0.70	0.38	0.05	0.0	0.0	0.0	0.0	0.0	0.0	0.0	0.0	0.0	0.0	0.0	9.55E-01	3.11E-01	7.76E-01	9.23E-01
NF1	NFIA	124	22	5	0	0	1	2	2	0	0	0	0	0	0	0	0	6.88	7.84	6.22	0.52	0.20	1.79	0.38	0.33	6.25	3.45	0.45	0.1	0.3	0.3	0.0	0.0	0.0	0.0	0.0	0.0	0.0	0.0	4.32E-03	2.20E-03	3.95E-01	2.70E-02
	NFIB	106	24	6	0	0	0	3	3	0	0	0	0	0	0	0	0	6.44	7.34	5.82	0.49	0.19	1.68	0.36	0.31	5.85	3.23	0.42	0.0	0.4	0.5	0.0	0.0	0.0	0.0	0.0	0.0	0.0	0.0	1.12E-02	5.60E-03	4.11E-01	3.71E-02
	NFIC	128	33	11	1	0	3	4	4	0	1	0	0	0	0	0	0	8.31	9.47	7.51	0.63	0.24	2.16	0.46	0.40	7.54	4.16	0.54	0.4	0.4	0.5	0.0	4.1	0.0	0.0	0.0	0.0	0.0	0.0	3.16E-03	1.67E-02	8.21E-02	9.29E-03
	NFIX	121	33	9	0	0	0	2	7	0	0	0	0	0	0	0	0	8.12	9.26	7.34	0.62	0.24	2.12	0.45	0.39	7.38	4.07	0.53	0.0	0.2	1.0	0.0	0.0	0.0	0.0	0.0	0.0	0.0	0.0	9.92E-04	9.94E-04	3.56E-01	1.07E-02
NRF	NRF1	20	17	2	0	0	0	2	0	0	0	0	0	0	0	0	0	3.07	3.50	2.78	0.23	0.09	0.80	0.17	0.15	2.79	1.54	0.20	0.0	0.6	0.0	0.0	0.0	0.0	0.0	0.0	0.0	0.0	0.0	2.55E-01	3.89E-02	5.70E-01	3.42E-01
others	SFPQ	63	106	107	4	7	21	67	19	4	0	1	1	1	4	0	0	22.14	25.23	20.01	1.68	0.64	5.77	1.23	1.06	20.10	11.09	1.43	0.9	2.7	0.9	2.4	0.0	0.2	0.8	0.9	0.2	0.0	0.0	1.72E-17	9.09E-16	5.03E-02	1.93E-05
	AFF1	20	32	6	0	2	0	2	4	0	0	0	0	0	1	1	0	5.38	6.13	4.86	0.41	0.16	1.40	0.30	0.26	4.88	2.69	0.35	0.0	0.3	0.8	0.0	0.0	0.0	0.0	0.0	0.2	0.4	0.0	1.20E-01	1.57E-02	4.52E-01	2.64E-01
	AFF2	0	1	0	0	0	0	0	0	0	0	0	0	0	0	0	0	0.18	0.21	0.17	0.01	0.01	0.05	0.01	0.01	0.17	0.09	0.01	0.0	0.0	0.0	0.0	0.0	0.0	0.0	0.0	0.0	0.0	0.0	1.00E+00	7.57E-01	8.90E-01	9.97E-01
	AFF3	0	0	3	0	0	0	0	0	0	0	0	0	0	0	0	0	0.77	0.88	0.69	0.06	0.02	0.20	0.04	0.04	0.70	0.38	0.05	0.0	0.0	0.0	0.0	0.0	0.0	0.0	0.0	0.0	0.0	0.0	9.60E-01	3.32E-01	7.76E-01	9.23E-01
	AFF4	16	52	5	0	0	2	2	1	0	0	0	0	0	0	0	0	7.21	8.22	6.51	0.55	0.21	1.88	0.40	0.35	6.55	3.61	0.47	0.3	0.2	0.2	0.0	0.0	0.0	0.0	0.0	0.0	0.0	0.0	2.48E-03	1.41E-03	3.84E-01	2.12E-02
	AHCTF1	12	33	10	0	1	2	3	5	0	0	0	0	0	1	0	0	6.62	7.55	5.99	0.50	0.19	1.72	0.37	0.32	6.01	3.32	0.43	0.3	0.4	0.8	0.0	0.0	0.0	0.0	0.0	0.2	0.0	0.0	7.08E-02	4.67E-02	4.04E-01	6.62E-02
	AKNA	0	1	2	0	0	1	1	0	0	0	0	0	0	0	0	0	0.26	0.29	0.23	0.02	0.01	0.07	0.01	0.01	0.23	0.13	0.02	3.9	3.4	0.0	0.0	0.0	0.0	0.0	0.0	0.0	0.0	0.0	9.16E-01	1.28E-01	8.70E-01	9.93E-01
	ANKRD30A	0	2	0	0	0	0	0	0	0	0	0	0	0	0	0	0	0.29	0.33	0.26	0.02	0.01	0.08	0.02	0.01	0.27	0.15	0.02	0.0	0.0	0.0	0.0	0.0	0.0	0.0	0.0	0.0	0.0	0.0	9.99E-01	6.41E-01	8.61E-01	9.91E-01
	BCLAF1	25	94	71	2	4	25	24	22	1	1	1	3	0	0	0	0	13.28	15.14	12.00	1.01	0.39	3.46	0.74	0.64	12.06	6.65	0.86	1.9	1.6	1.8	1.0	2.6	0.3	4.1	0.0	0.0	0.0	0.0	5.53E-08	6.62E-06	3.22E-01	2.47E-05
	BLZF1	21	24	5	0	0	1	1	3	0	0	0	0	0	0	0	0	7.06	8.05	6.38	0.54	0.20	1.84	0.39	0.34	6.41	3.54	0.46	0.1	0.1	0.5	0.0	0.0	0.0	0.0	0.0	0.0	0.0	0.0	2.71E-03	1.38E-03	3.89E-01	2.36E-02
	CAPN15	2	8	0	1	0	0	0	0	0	1	0	0	0	0	0	0	1.54	1.75	1.39	0.12	0.04	0.40	0.09	0.07	1.40	0.77	0.10	0.0	0.0	0.0	0.0	22.4	0.0	0.0	0.0	0.0	0.0	0.0	1.75E-03	9.65E-02	5.69E-06	7.27E-01
	CARF	0	0	1	0	0	0	1	0	0	0	0	0	0	0	0	0	0.15	0.17	0.13	0.01	0.00	0.04	0.01	0.01	0.13	0.07	0.01	0.0	6.0	0.0	0.0	0.0	0.0	0.0	0.0	0.0	0.0	0.0	9.09E-01	1.09E-01	9.01E-01	9.98E-01
	CARHSP1	3	11	2	0	0	0	1	1	0	0	0	0	0	0	0	0	1.83	2.09	1.65	0.14	0.05	0.48	0.10	0.09	1.66	0.92	0.12	0.0	0.5	0.6	0.0	0.0	0.0	0.0	0.0	0.0	0.0	0.0	7.98E-01	2.65E-01	6.61E-01	6.44E-01
	CBFB	53	19	6	0	0	1	2	3	0	0	0	0	0	0	0	0	5.89	6.71	5.32	0.45	0.17	1.53	0.33	0.28	5.35	2.95	0.38	0.2	0.3	0.6	0.0	0.0	0.0	0.0	0.0	0.0	0.0	0.0	3.09E-02	1.51E-02	4.31E-01	5.49E-02
	CC2D1A	7	11	2	2	0	0	0	0	0	2	0	0	0	0	0	0	5.82	6.63	5.26	0.44	0.17	1.52	0.32	0.28	5.28	2.91	0.38	0.0	0.0	0.0	0.0	11.8	0.0	0.0	0.0	0.0	0.0	0.0	1.85E-06	7.49E-04	6.59E-06	5.78E-02
	CCDC88A	9	40	6	0	0	1	2	3	0	0	0	0	0	0	0	0	10.10	11.51	9.13	0.77	0.29	2.63	0.56	0.49	9.17	5.06	0.65	0.1	0.2	0.3	0.0	0.0	0.0	0.0	0.0	0.0	0.0	0.0	1.85E-05	4.18E-05	3.03E-01	2.32E-03
	CDIP1	0	6	0	0	0	0	0	0	0	0	0	0	0	0	0	0	0.66	0.75	0.60	0.05	0.02	0.17	0.04	0.03	0.60	0.33	0.04	0.0	0.0	0.0	0.0	0.0	0.0	0.0	0.0	0.0	0.0	0.0	9.74E-01	3.67E-01	7.93E-01	9.44E-01
	CEBPZ	43	43	20	0	35	5	8	7	0	0	1	1	0	33	0	0	10.61	12.09	9.59	0.81	0.31	2.76	0.59	0.51	9.63	5.31	0.69	0.5	0.7	0.7	0.0	0.0	0.4	1.7	0.0	3.4	0.0	0.0	3.18E-11	8.00E-02	2.91E-01	1.37E-12
	CENPX	0	0	0	0	0	0	0	0	0	0	0	0	0	0	0	0	0.00	0.00	0.00	0.00	0.00	0.00	0.00	0.00	0.00	0.00	0.00	na	na	na	na	na	na	na	na	na	na	na	na	na	na	na
	CIZ1	5	15	4	0	0	1	1	2	0	0	0	0	0	0	0	0	2.93	3.34	2.65	0.22	0.08	0.76	0.16	0.14	2.66	1.47	0.19	0.3	0.3	0.8	0.0	0.0	0.0	0.0	0.0	0.0	0.0	0.0	5.56E-01	2.16E-01	5.79E-01	3.71E-01
	CNBP	41	39	20	0	67	2	14	4	0	0	0	0	0	66	1	0	12.73	14.51	11.51	0.97	0.37	3.32	0.71	0.61	11.56	6.38	0.82	0.2	1.0	0.3	0.0	0.0	0.0	0.0	0.0	5.7	0.2	0.0	1.19E-54	9.29E-04	2.47E-01	1.73E-55
	CPEB1	9	20	15	12	9	2	13	0	10	2	1	1	0	7	0	0	5.82	6.63	5.26	0.44	0.17	1.52	0.32	0.28	5.28	2.91	0.38	0.3	2.0	0.0	22.6	11.8	0.7	3.1	0.0	1.3	0.0	0.0	3.81E-47	9.67E-04	3.67E-51	3.35E-01
	CREBRF	3	2	3	0	0	2	1	0	0	0	0	0	0	0	0	0	0.44	0.50	0.40	0.03	0.01	0.11	0.02	0.02	0.40	0.22	0.03	4.6	2.0	0.0	0.0	0.0	0.0	0.0	0.0	0.0	0.0	0.0	6.97E-01	3.99E-02	8.30E-01	9.77E-01
	CSDC2	1	2	0	0	0	0	0	0	0	0	0	0	0	0	0	0	0.18	0.21	0.17	0.01	0.01	0.05	0.01	0.01	0.17	0.09	0.01	0.0	0.0	0.0	0.0	0.0	0.0	0.0	0.0	0.0	0.0	0.0	1.00E+00	7.57E-01	8.90E-01	9.97E-01
	CSDE1	18	51	29	0	8	13	10	6	0	0	2	3	0	3	0	0	9.55	10.88	8.63	0.73	0.28	2.49	0.53	0.46	8.67	4.78	0.62	1.4	0.9	0.7	0.0	0.0	0.8	5.6	0.0	0.3	0.0	0.0	6.99E-03	3.46E-01	3.17E-01	7.70E-04
	DACH1	48	14	11	2	1	2	5	4	1	1	0	0	0	1	0	0	4.54	5.17	4.10	0.34	0.13	1.18	0.25	0.22	4.12	2.27	0.29	0.4	1.0	1.0	2.9	7.6	0.0	0.0	0.0	0.2	0.0	0.0	1.33E-01	4.90E-01	8.28E-03	2.54E-01
	DACH2	4	0	0	0	0	0	0	0	0	0	0	0	0	0	0	0	0.59	0.67	0.53	0.04	0.02	0.15	0.03	0.03	0.53	0.29	0.04	0.0	0.0	0.0	0.0	0.0	0.0	0.0	0.0	0.0	0.0	0.0	9.83E-01	4.10E-01	8.04E-01	9.56E-01
	FAM170A	6	4	3	0	0	3	0	0	0	0	0	0	0	0	0	0	1.57	1.79	1.42	0.12	0.05	0.41	0.09	0.08	1.43	0.79	0.10	1.9	0.0	0.0	0.0	0.0	0.0	0.0	0.0	0.0	0.0	0.0	6.71E-01	1.05E-01	6.84E-01	7.17E-01
	GABPB1	15	7	4	0	0	3	0	1	0	0	0	0	0	0	0	0	2.27	2.59	2.05	0.17	0.07	0.59	0.13	0.11	2.06	1.14	0.15	1.3	0.0	0.5	0.0	0.0	0.0	0.0	0.0	0.0	0.0	0.0	6.52E-01	1.86E-01	6.25E-01	5.25E-01
	GABPB2	4	2	3	0	0	1	1	1	0	0	0	0	0	0	0	0	0.62	0.71	0.56	0.05	0.02	0.16	0.03	0.03	0.56	0.31	0.04	1.6	1.4	1.8	0.0	0.0	0.0	0.0	0.0	0.0	0.0	0.0	9.97E-01	7.08E-01	7.98E-01	9.50E-01
	GCFC2	2	21	35	0	0	29	5	1	0	0	0	0	0	0	0	0	3.15	3.59	2.84	0.24	0.09	0.82	0.18	0.15	2.86	1.58	0.20	9.2	1.4	0.4	0.0	0.0	0.0	0.0	0.0	0.0	0.0	0.0	9.36E-42	3.13E-47	5.65E-01	3.28E-01
	GLMP	0	0	0	0	0	0	0	0	0	0	0	0	0	0	0	0	0.00	0.00	0.00	0.00	0.00	0.00	0.00	0.00	0.00	0.00	0.00	na	na	na	na	na	na	na	na	na	na	na	na	na	na	na
	GON4L	7	4	4	0	1	1	2	1	0	0	0	0	0	0	1	0	1.87	2.13	1.69	0.14	0.05	0.49	0.10	0.09	1.69	0.93	0.12	0.5	0.9	0.6	0.0	0.0	0.0	0.0	0.0	0.0	1.1	0.0	9.71E-01	7.09E-01	6.58E-01	7.77E-01
	GPBP1	2	12	3	1	4	1	2	0	0	1	3	0	0	1	0	0	3.44	3.92	3.11	0.26	0.10	0.90	0.19	0.17	3.12	1.72	0.22	0.3	0.5	0.0	0.0	10.0	3.3	0.0	0.0	0.3	0.0	0.0	1.13E-02	5.56E-02	3.79E-03	1.22E-01
	GPBP1L1	3	34	7	1	13	4	2	1	0	1	10	0	0	1	0	2	4.87	5.55	4.40	0.37	0.14	1.27	0.27	0.23	4.42	2.44	0.32	0.8	0.4	0.2	0.0	7.1	7.9	0.0	0.0	0.2	0.0	6.3	4.35E-14	8.02E-02	1.80E-02	1.04E-14
	KHSRP	30	59	33	1	1	9	13	11	0	1	0	0	1	0	0	0	10.03	11.43	9.06	0.76	0.29	2.61	0.56	0.48	9.10	5.02	0.65	0.9	1.1	1.2	0.0	3.4	0.0	0.0	2.1	0.0	0.0	0.0	1.65E-02	6.92E-01	1.14E-01	2.38E-03
	KIAA1549	0	6	1	0	1	0	1	0	0	0	0	0	0	1	0	0	3.55	4.04	3.21	0.27	0.10	0.92	0.20	0.17	3.22	1.78	0.23	0.0	0.2	0.0	0.0	0.0	0.0	0.0	0.0	0.3	0.0	0.0	1.62E-01	1.08E-02	5.41E-01	4.37E-01
	L3MBTL3	38	36	18	0	0	3	0	15	0	0	0	0	0	0	0	0	6.40	7.30	5.79	0.49	0.19	1.67	0.36	0.31	5.81	3.21	0.41	0.5	0.0	2.6	0.0	0.0	0.0	0.0	0.0	0.0	0.0	0.0	7.73E-05	6.88E-06	4.12E-01	3.81E-02
	LITAF	10	23	1	0	1	0	1	0	0	0	0	0	0	1	0	0	4.28	4.88	3.87	0.33	0.12	1.12	0.24	0.21	3.89	2.14	0.28	0.0	0.2	0.0	0.0	0.0	0.0	0.0	0.0	0.3	0.0	0.0	5.83E-02	3.64E-03	5.02E-01	2.94E-01
	LTF	12	26	10	0	0	0	7	3	0	0	0	0	0	0	0	0	6.04	6.88	5.46	0.46	0.18	1.57	0.34	0.29	5.48	3.02	0.39	0.0	1.0	0.5	0.0	0.0	0.0	0.0	0.0	0.0	0.0	0.0	4.19E-02	2.81E-02	4.26E-01	4.95E-02
	MAF1	2	12	0	0	0	0	0	0	0	0	0	0	0	0	0	0	1.94	2.21	1.75	0.15	0.06	0.51	0.11	0.09	1.76	0.97	0.13	0.0	0.0	0.0	0.0	0.0	0.0	0.0	0.0	0.0	0.0	0.0	4.70E-01	5.23E-02	6.52E-01	6.14E-01
	MLLT10	18	14	1	0	2	0	1	0	0	0	0	0	2	0	0	0	2.63	3.00	2.38	0.20	0.08	0.69	0.15	0.13	2.39	1.32	0.17	0.0	0.3	0.0	0.0	0.0	0.0	0.0	15.8	0.0	0.0	0.0	2.46E-05	4.18E-02	5.99E-01	4.84E-06
	MYEF2	17	19	15	0	6	1	6	8	0	0	0	0	0	6	0	0	5.16	5.88	4.66	0.39	0.15	1.34	0.29	0.25	4.68	2.58	0.33	0.2	1.0	1.7	0.0	0.0	0.0	0.0	0.0	1.3	0.0	0.0	3.23E-01	5.66E-02	4.62E-01	3.96E-01
	MYRFL	0	0	0	0	0	0	0	0	0	0	0	0	0	0	0	0	0.04	0.04	0.03	0.00	0.00	0.01	0.00	0.00	0.03	0.02	0.00	0.0	0.0	0.0	0.0	0.0	0.0	0.0	0.0	0.0	0.0	0.0	1.00E+00	9.46E-01	9.51E-01	1.00E+00
	NFRKB	37	67	5	1	1	2	2	1	1	0	0	0	0	1	0	0	6.88	7.84	6.22	0.52	0.20	1.79	0.38	0.33	6.25	3.45	0.45	0.3	0.3	0.2	1.9	0.0	0.0	0.0	0.0	0.2	0.0	0.0	8.66E-03	2.26E-03	4.26E-01	5.54E-02
	NKRF	40	43	31	1	34	6	16	9	0	1	2	0	0	30	2	0	10.68	12.18	9.66	0.81	0.31	2.78	0.60	0.51	9.70	5.35	0.69	0.6	1.3	0.9	0.0	3.2	0.7	0.0	0.0	3.1	0.4	0.0	1.03E-07	1.92E-01	1.25E-01	6.88E-09
	NME2	13	17	3	0	2	0	0	3	0	0	0	0	0	2	0	0	7.06	8.05	6.38	0.54	0.20	1.84	0.39	0.34	6.41	3.54	0.46	0.0	0.0	0.5	0.0	0.0	0.0	0.0	0.0	0.3	0.0	0.0	2.38E-03	2.14E-04	3.89E-01	8.73E-02
	PARP12	5	9	4	0	0	1	2	1	0	0	0	0	0	0	0	0	1.94	2.21	1.75	0.15	0.06	0.51	0.11	0.09	1.76	0.97	0.13	0.5	0.9	0.6	0.0	0.0	0.0	0.0	0.0	0.0	0.0	0.0	9.18E-01	6.71E-01	6.52E-01	6.14E-01
	PAXBP1	8	19	44	0	0	34	2	8	0	0	0	0	0	0	0	0	4.50	5.13	4.07	0.34	0.13	1.17	0.25	0.22	4.09	2.25	0.29	7.6	0.4	2.0	0.0	0.0	0.0	0.0	0.0	0.0	0.0	0.0	3.78E-39	5.92E-44	4.92E-01	1.42E-01
	PHB	55	156	122	2	106	38	57	27	1	1	9	2	5	72	12	6	45.01	51.29	40.67	3.42	1.31	11.72	2.51	2.16	40.86	22.54	2.91	0.8	1.1	0.7	0.3	0.8	0.8	0.8	2.3	1.8	0.5	2.1	2.69E-06	4.24E-02	1.81E-01	7.97E-07
	PLEK	0	2	0	0	0	0	0	0	0	0	0	0	0	0	0	0	0.40	0.46	0.36	0.03	0.01	0.10	0.02	0.02	0.37	0.20	0.03	0.0	0.0	0.0	0.0	0.0	0.0	0.0	0.0	0.0	0.0	0.0	9.96E-01	5.42E-01	8.37E-01	9.81E-01
	PLSCR1	23	13	8	2	0	1	6	1	2	0	0	0	0	0	0	0	7.43	8.47	6.71	0.56	0.22	1.93	0.41	0.36	6.74	3.72	0.48	0.1	0.7	0.1	3.5	0.0	0.0	0.0	0.0	0.0	0.0	0.0	1.42E-03	3.81E-03	4.94E-02	1.80E-02
	PRDM11	1	6	1	0	1	0	1	0	0	0	0	0	0	1	0	0	0.73	0.83	0.66	0.06	0.02	0.19	0.04	0.04	0.66	0.37	0.05	0.0	1.2	0.0	0.0	0.0	0.0	0.0	0.0	1.5	0.0	0.0	9.93E-01	4.90E-01	7.82E-01	9.74E-01
	PREB	6	7	1	0	0	0	0	1	0	0	0	0	0	0	0	0	6.99	7.96	6.32	0.53	0.20	1.82	0.39	0.34	6.35	3.50	0.45	0.0	0.0	0.2	0.0	0.0	0.0	0.0	0.0	0.0	0.0	0.0	2.72E-04	6.04E-05	3.92E-01	2.49E-02
	RAG1	11	16	0	0	0	0	0	0	0	0	0	0	0	0	0	0	2.45	2.79	2.22	0.19	0.07	0.64	0.14	0.12	2.23	1.23	0.16	0.0	0.0	0.0	0.0	0.0	0.0	0.0	0.0	0.0	0.0	0.0	2.70E-01	2.40E-02	6.12E-01	4.79E-01
	RBCK1	42	92	1	0	1	0	1	0	0	0	0	0	0	1	0	0	13.32	15.18	12.04	1.01	0.39	3.47	0.74	0.64	12.09	6.67	0.86	0.0	0.1	0.0	0.0	0.0	0.0	0.0	0.0	0.1	0.0	0.0	1.18E-09	4.15E-09	2.37E-01	4.10E-04
	SETBP1	2	7	3	0	0	0	2	1	0	0	0	0	0	0	0	0	1.35	1.54	1.22	0.10	0.04	0.35	0.08	0.07	1.23	0.68	0.09	0.0	1.3	0.8	0.0	0.0	0.0	0.0	0.0	0.0	0.0	0.0	9.40E-01	4.65E-01	7.06E-01	7.78E-01
	SLC2A4RG	2	1	0	0	0	0	0	0	0	0	0	0	0	0	0	0	0.18	0.21	0.17	0.01	0.01	0.05	0.01	0.01	0.17	0.09	0.01	0.0	0.0	0.0	0.0	0.0	0.0	0.0	0.0	0.0	0.0	0.0	1.00E+00	7.57E-01	8.90E-01	9.97E-01
	SMARCAL1	6	6	4	0	0	1	2	1	0	0	0	0	0	0	0	0	2.12	2.42	1.92	0.16	0.06	0.55	0.12	0.10	1.93	1.06	0.14	0.5	0.8	0.5	0.0	0.0	0.0	0.0	0.0	0.0	0.0	0.0	8.75E-01	5.75E-01	6.37E-01	5.64E-01
	SON	22	27	37	1	4	14	9	14	1	0	0	0	0	3	1	0	6.48	7.38	5.85	0.49	0.19	1.69	0.36	0.31	5.88	3.24	0.42	2.2	1.2	2.4	2.0	0.0	0.0	0.0	0.0	0.5	0.3	0.0	2.71E-03	3.65E-05	3.99E-01	3.32E-01
	SPZ1	1	3	1	0	0	0	0	1	0	0	0	0	0	0	0	0	1.24	1.42	1.12	0.09	0.04	0.32	0.07	0.06	1.13	0.62	0.08	0.0	0.0	0.9	0.0	0.0	0.0	0.0	0.0	0.0	0.0	0.0	8.85E-01	2.62E-01	7.18E-01	8.08E-01
	SUB1	31	43	16	3	5	3	7	6	3	0	1	0	0	4	0	0	7.32	8.34	6.61	0.56	0.21	1.91	0.41	0.35	6.64	3.67	0.47	0.4	0.8	0.9	5.4	0.0	0.5	0.0	0.0	0.6	0.0	0.0	2.79E-02	2.44E-01	9.39E-04	2.71E-01
	TAX1BP1	15	85	9	2	14	2	2	5	1	1	0	0	0	9	5	0	16.94	19.31	15.31	1.29	0.49	4.41	0.94	0.81	15.38	8.49	1.10	0.1	0.1	0.3	0.8	2.0	0.0	0.0	0.0	0.6	0.6	0.0	7.43E-07	1.83E-08	4.42E-01	4.49E-02
	TET2	38	44	20	0	4	8	8	4	0	0	2	1	0	1	0	0	6.48	7.38	5.85	0.49	0.19	1.69	0.36	0.31	5.88	3.24	0.42	1.2	1.1	0.7	0.0	0.0	1.2	2.8	0.0	0.2	0.0	0.0	3.66E-01	6.07E-01	4.09E-01	1.01E-01
	TFB1M	11	11	5	0	13	2	0	3	0	0	0	0	0	7	6	0	3.33	3.79	3.01	0.25	0.10	0.87	0.19	0.16	3.02	1.67	0.22	0.6	0.0	1.0	0.0	0.0	0.0	0.0	0.0	2.3	3.6	0.0	1.24E-02	1.15E-01	5.54E-01	3.06E-03
	THYN1	4	3	1	0	0	0	0	1	0	0	0	0	0	0	0	0	0.91	1.04	0.83	0.07	0.03	0.24	0.05	0.04	0.83	0.46	0.06	0.0	0.0	1.2	0.0	0.0	0.0	0.0	0.0	0.0	0.0	0.0	9.57E-01	3.69E-01	7.57E-01	8.91E-01
	TMF1	13	14	5	0	1	0	2	3	0	0	0	0	0	1	0	0	3.95	4.50	3.57	0.30	0.11	1.03	0.22	0.19	3.59	1.98	0.26	0.0	0.4	0.8	0.0	0.0	0.0	0.0	0.0	0.3	0.0	0.0	3.28E-01	6.60E-02	5.19E-01	3.53E-01
	XPA	9	12	5	0	0	2	1	2	0	0	0	0	0	0	0	0	5.01	5.71	4.53	0.38	0.15	1.31	0.28	0.24	4.55	2.51	0.32	0.4	0.2	0.4	0.0	0.0	0.0	0.0	0.0	0.0	0.0	0.0	7.77E-02	2.86E-02	4.68E-01	1.01E-01
	YEATS4	64	90	22	0	0	4	2	16	0	0	0	0	0	0	0	0	12.11	13.80	10.95	0.92	0.35	3.15	0.67	0.58	11.00	6.07	0.78	0.3	0.1	1.5	0.0	0.0	0.0	0.0	0.0	0.0	0.0	0.0	9.63E-06	1.32E-04	2.59E-01	4.68E-04
p53	TP53	542	1753	411	6	155	80	71	260	3	3	25	4	2	106	15	3	195.39	222.68	176.58	14.86	5.67	50.89	10.88	9.39	177.41	97.86	12.65	0.4	0.3	1.5	0.2	0.5	0.5	0.4	0.2	0.6	0.2	0.2	2.22E-69	1.62E-46	1.06E-03	2.88E-26
	TP63	35	73	38	1	6	2	21	15	1	0	0	0	0	6	0	0	9.11	10.38	8.23	0.69	0.26	2.37	0.51	0.44	8.27	4.56	0.59	0.2	2.0	1.8	1.4	0.0	0.0	0.0	0.0	0.7	0.0	0.0	4.92E-04	1.70E-05	5.27E-01	1.05E-01
	TP73	51	142	27	0	7	1	1	25	0	0	0	0	0	7	0	0	12.81	14.60	11.57	0.97	0.37	3.34	0.71	0.62	11.63	6.41	0.83	0.1	0.1	2.2	0.0	0.0	0.0	0.0	0.0	0.6	0.0	0.0	4.42E-08	3.20E-09	2.46E-01	1.73E-02
PAX	PAX1	4	0	0	0	0	0	0	0	0	0	0	0	0	0	0	0	0.18	0.21	0.17	0.01	0.01	0.05	0.01	0.01	0.17	0.09	0.01	0.0	0.0	0.0	0.0	0.0	0.0	0.0	0.0	0.0	0.0	0.0	1.00E+00	7.57E-01	8.90E-01	9.97E-01
	PAX2	28	22	0	0	0	0	0	0	0	0	0	0	0	0	0	0	3.04	3.46	2.74	0.23	0.09	0.79	0.17	0.15	2.76	1.52	0.20	0.0	0.0	0.0	0.0	0.0	0.0	0.0	0.0	0.0	0.0	0.0	1.27E-01	9.84E-03	5.72E-01	3.49E-01
	PAX5	11	16	7	0	1	1	0	6	0	0	0	0	0	1	0	0	2.67	3.04	2.41	0.20	0.08	0.70	0.15	0.13	2.43	1.34	0.17	0.4	0.0	2.5	0.0	0.0	0.0	0.0	0.0	0.4	0.0	0.0	2.23E-01	9.02E-03	5.96E-01	6.51E-01
	PAX8	38	20	6	0	0	0	2	4	0	0	0	0	0	0	0	0	4.28	4.88	3.87	0.33	0.12	1.12	0.24	0.21	3.89	2.14	0.28	0.0	0.4	1.0	0.0	0.0	0.0	0.0	0.0	0.0	0.0	0.0	1.60E-01	5.02E-02	5.02E-01	1.64E-01
	PAX9	48	32	6	0	0	2	1	3	0	0	0	0	0	0	0	0	4.87	5.55	4.40	0.37	0.14	1.27	0.27	0.23	4.42	2.44	0.32	0.4	0.2	0.7	0.0	0.0	0.0	0.0	0.0	0.0	0.0	0.0	1.21E-01	5.34E-02	4.75E-01	1.11E-01
PHDFP	TCF19	12	6	0	0	0	0	0	0	0	0	0	0	0	0	0	0	1.57	1.79	1.42	0.12	0.05	0.41	0.09	0.08	1.43	0.79	0.10	0.0	0.0	0.0	0.0	0.0	0.0	0.0	0.0	0.0	0.0	0.0	6.44E-01	9.12E-02	6.84E-01	7.17E-01
pseudogene	ZNF883	0	0	0	0	0	0	0	0	0	0	0	0	0	0	0	0	0.04	0.04	0.03	0.00	0.00	0.01	0.00	0.00	0.03	0.02	0.00	0.0	0.0	0.0	0.0	0.0	0.0	0.0	0.0	0.0	0.0	0.0	1.00E+00	9.46E-01	9.51E-01	1.00E+00
PUR	PURA	27	37	16	1	21	2	6	8	0	1	2	0	0	19	0	0	9.26	10.55	8.37	0.70	0.27	2.41	0.52	0.44	8.41	4.64	0.60	0.2	0.6	1.0	0.0	3.7	0.8	0.0	0.0	2.3	0.0	0.0	8.62E-04	2.16E-02	1.01E-01	1.47E-03
	PURB	17	24	10	1	21	2	3	5	0	1	0	0	0	21	0	0	5.53	6.30	4.99	0.42	0.16	1.44	0.31	0.27	5.02	2.77	0.36	0.4	0.5	1.0	0.0	6.2	0.0	0.0	0.0	4.2	0.0	0.0	4.32E-10	1.37E-01	2.82E-02	7.90E-11
	PURG	41	16	27	2	16	8	12	7	0	2	0	1	0	14	1	0	8.12	9.26	7.34	0.62	0.24	2.12	0.45	0.39	7.38	4.07	0.53	1.0	1.3	1.0	0.0	8.5	0.0	2.2	0.0	1.9	0.2	0.0	3.00E-03	6.60E-01	2.01E-04	3.54E-02
RFXANK	RFXANK	21	28	10	2	2	1	4	5	1	1	1	0	0	0	1	0	6.22	7.09	5.62	0.47	0.18	1.62	0.35	0.30	5.65	3.12	0.40	0.2	0.6	0.9	2.1	5.5	0.6	0.0	0.0	0.0	0.3	0.0	4.75E-02	5.51E-02	3.79E-02	1.37E-01

RHR	NFKB1	165	132	28	1	6	4	2	22	0	1	1	0	0	5	0	0	19.50	22.23	17.63	1.48	0.57	5.08	1.09	0.94	17.71	9.77	1.26	0.2	0.1	1.2	0.0	1.8	0.2	0.0	0.0	0.3	0.0	0.0	5.41E-09	1.23E-07	1.78E-01	1.14E-04
	NFKB2	56	45	2	0	3	0	2	0	0	0	0	0	0	3	0	0	6.77	7.71	6.12	0.51	0.20	1.76	0.38	0.33	6.15	3.39	0.44	0.0	0.3	0.0	0.0	0.0	0.0	0.0	0.0	0.5	0.0	0.0	4.11E-03	1.92E-04	3.99E-01	1.62E-01
	REL	72	52	16	2	9	2	5	9	1	1	4	0	2	2	1	0	18.37	20.93	16.60	1.40	0.53	4.78	1.02	0.88	16.68	9.20	1.19	0.1	0.2	0.5	0.7	1.9	0.8	0.0	2.3	0.1	0.1	0.0	3.60E-08	2.78E-07	4.70E-01	2.19E-04
	RELA	236	274	110	0	6	8	5	97	0	0	0	0	1	5	0	0	36.08	41.12	32.61	2.74	1.05	9.40	2.01	1.73	32.76	18.07	2.34	0.2	0.1	3.0	0.0	0.0	0.0	0.0	0.6	0.2	0.0	0.0	6.25E-46	5.62E-40	5.16E-02	9.62E-11
	RELB	45	28	11	0	0	1	0	10	0	0	0	0	0	0	0	0	4.79	5.46	4.33	0.36	0.14	1.25	0.27	0.23	4.35	2.40	0.31	0.2	0.0	2.3	0.0	0.0	0.0	0.0	0.0	0.0	0.0	0.0	4.99E-03	3.56E-04	4.78E-01	1.17E-01
	NFAT5	15	4	2	0	0	0	2	0	0	0	0	0	0	0	0	0	1.68	1.92	1.52	0.13	0.05	0.44	0.09	0.08	1.53	0.84	0.11	0.0	1.0	0.0	0.0	0.0	0.0	0.0	0.0	0.0	0.0	0.0	7.74E-01	2.01E-01	6.74E-01	6.86E-01
	NFATC1	61	38	16	0	1	0	2	14	0	0	0	0	0	0	0	1	7.46	8.51	6.75	0.57	0.22	1.94	0.42	0.36	6.78	3.74	0.48	0.0	0.2	2.1	0.0	0.0	0.0	0.0	0.0	0.0	0.0	2.1	1.34E-04	4.02E-05	3.76E-01	1.70E-02
	NFATC2	64	49	15	1	3	2	2	11	1	0	0	0	0	1	2	0	8.38	9.55	7.57	0.64	0.24	2.18	0.47	0.40	7.61	4.20	0.54	0.2	0.2	1.5	1.6	0.0	0.0	0.0	0.0	0.1	0.5	0.0	9.66E-03	2.05E-03	5.02E-01	6.26E-02
	NFATC3	5	9	2	0	1	0	2	0	0	0	0	1	0	0	0	0	1.61	1.83	1.45	0.12	0.05	0.42	0.09	0.08	1.46	0.81	0.10	0.0	1.1	0.0	0.0	0.0	0.0	11.2	0.0	0.0	0.0	0.0	1.20E-01	2.14E-01	6.81E-01	3.33E-02
	NFATC4	5	13	0	0	1	0	0	0	0	0	0	0	0	0	1	0	1.65	1.88	1.49	0.13	0.05	0.43	0.09	0.08	1.50	0.82	0.11	0.0	0.0	0.0	0.0	0.0	0.0	0.0	0.0	0.0	1.2	0.0	6.85E-01	8.16E-02	6.77E-01	8.15E-01
	RBPJ	97	133	36	0	0	4	5	27	0	0	0	0	0	0	0	0	14.01	15.97	12.67	1.07	0.41	3.65	0.78	0.67	12.72	7.02	0.91	0.3	0.3	2.1	0.0	0.0	0.0	0.0	0.0	0.0	0.0	0.0	8.12E-09	1.93E-07	2.25E-01	9.96E-05
	RBPJL	2	2	1	0	0	1	0	0	0	0	0	0	0	0	0	0	0.33	0.38	0.30	0.03	0.01	0.09	0.02	0.02	0.30	0.16	0.02	3.0	0.0	0.0	0.0	0.0	0.0	0.0	0.0	0.0	0.0	0.0	9.88E-01	3.61E-01	8.52E-01	9.88E-01
	EBF1	7	1	1	0	0	0	1	0	0	0	0	0	0	0	0	0	0.59	0.67	0.53	0.04	0.02	0.15	0.03	0.03	0.53	0.29	0.04	0.0	1.5	0.0	0.0	0.0	0.0	0.0	0.0	0.0	0.0	0.0	9.92E-01	5.27E-01	8.04E-01	9.56E-01
	EBF2	3	0	1	0	0	0	1	0	0	0	0	0	0	0	0	0	2.12	2.42	1.92	0.16	0.06	0.55	0.12	0.10	1.93	1.06	0.14	0.0	0.4	0.0	0.0	0.0	0.0	0.0	0.0	0.0	0.0	0.0	5.33E-01	8.75E-02	6.37E-01	5.64E-01
	EBF3	7	1	2	0	0	0	1	1	0	0	0	0	0	0	0	0	0.77	0.88	0.69	0.06	0.02	0.20	0.04	0.04	0.70	0.38	0.05	0.0	1.1	1.4	0.0	0.0	0.0	0.0	0.0	0.0	0.0	0.0	9.92E-01	6.31E-01	7.76E-01	9.23E-01
	EBF4	0	0	0	0	0	0	0	0	0	0	0	0	0	0	0	0	0.15	0.17	0.13	0.01	0.00	0.04	0.01	0.01	0.13	0.07	0.01	0.0	0.0	0.0	0.0	0.0	0.0	0.0	0.0	0.0	0.0	0.0	1.00E+00	8.00E-01	9.01E-01	9.98E-01
RUNT	RUNX1	102	99	66	5	4	13	18	35	5	0	3	1	0	0	0	0	14.75	16.81	13.33	1.12	0.43	3.84	0.82	0.71	13.39	7.39	0.95	0.9	1.1	2.6	4.5	0.0	0.8	1.2	0.0	0.0	0.0	0.0	1.78E-11	1.92E-08	1.98E-04	3.92E-04
	RUNX2	50	40	19	0	0	0	0	19	0	0	0	0	0	0	0	0	4.46	5.09	4.03	0.34	0.13	1.16	0.25	0.21	4.05	2.24	0.29	0.0	0.0	4.7	0.0	0.0	0.0	0.0	0.0	0.0	0.0	0.0	8.36E-12	7.42E-15	4.93E-01	1.45E-01
	RUNX3	38	20	7	0	0	0	0	7	0	0	0	0	0	0	0	0	3.04	3.46	2.74	0.23	0.09	0.79	0.17	0.15	2.76	1.52	0.20	0.0	0.0	2.6	0.0	0.0	0.0	0.0	0.0	0.0	0.0	0.0	4.03E-02	1.43E-03	5.72E-01	3.49E-01
SAND	AIRE	12	29	21	0	2	9	8	4	0	0	1	1	0	0	0	0	5.09	5.80	4.60	0.39	0.15	1.32	0.28	0.24	4.62	2.55	0.33	1.8	1.4	0.9	0.0	0.0	0.8	3.5	0.0	0.0	0.0	0.0	1.69E-01	1.40E-01	4.65E-01	8.64E-02
	DEAF1	14	11	0	0	0	0	0	0	0	0	0	0	0	0	0	0	1.98	2.25	1.79	0.15	0.06	0.51	0.11	0.09	1.79	0.99	0.13	0.0	0.0	0.0	0.0	0.0	0.0	0.0	0.0	0.0	0.0	0.0	4.54E-01	4.95E-02	6.49E-01	6.04E-01
	GMEB1	13	3	1	0	0	0	1	0	0	0	0	0	0	0	0	0	2.12	2.42	1.92	0.16	0.06	0.55	0.12	0.10	1.93	1.06	0.14	0.0	0.4	0.0	0.0	0.0	0.0	0.0	0.0	0.0	0.0	0.0	5.33E-01	8.75E-02	6.37E-01	5.64E-01
	GMEB2	22	10	3	0	0	1	2	0	0	0	0	0	0	0	0	0	2.31	2.63	2.08	0.18	0.07	0.60	0.13	0.11	2.09	1.15	0.15	0.4	0.8	0.0	0.0	0.0	0.0	0.0	0.0	0.0	0.0	0.0	6.82E-01	2.26E-01	6.23E-01	5.16E-01
	SP110	11	25	8	0	3	0	2	6	0	0	0	0	0	0	3	0	4.83	5.50	4.36	0.37	0.14	1.26	0.27	0.23	4.39	2.42	0.31	0.0	0.4	1.4	0.0	0.0	0.0	0.0	0.0	0.0	1.2	0.0	1.40E-01	2.16E-02	4.76E-01	2.52E-01
	SP100	61	59	20	1	9	5	6	9	1	0	0	0	1	5	3	0	9.37	10.68	8.47	0.71	0.27	2.44	0.52	0.45	8.51	4.69	0.61	0.5	0.6	1.1	1.4	0.0	0.0	0.0	2.2	0.6	0.6	0.0	3.73E-01	1.28E-01	5.33E-01	2.79E-01
	SP140	3	3	1	0	0	0	1	0	0	0	0	0	0	0	0	0	0.37	0.42	0.33	0.03	0.01	0.10	0.02	0.02	0.33	0.18	0.02	0.0	2.4	0.0	0.0	0.0	0.0	0.0	0.0	0.0	0.0	0.0	9.94E-01	4.70E-01	8.45E-01	9.84E-01
	SP140L	0	2	0	0	0	0	0	0	0	0	0	0	0	0	0	0	0.37	0.42	0.33	0.03	0.01	0.10	0.02	0.02	0.33	0.18	0.02	0.0	0.0	0.0	0.0	0.0	0.0	0.0	0.0	0.0	0.0	0.0	9.98E-01	5.73E-01	8.45E-01	9.84E-01
SMAD	SMAD1	89	102	13	0	0	3	0	10	0	0	0	0	0	0	0	0	11.16	12.72	10.09	0.85	0.32	2.91	0.62	0.54	10.13	5.59	0.72	0.3	0.0	1.0	0.0	0.0	0.0	0.0	0.0	0.0	0.0	0.0	1.46E-05	8.76E-05	2.79E-01	1.00E-03
	SMAD2	186	135	39	2	4	9	3	27	1	1	1	0	0	3	0	0	22.54	25.69	20.37	1.71	0.65	5.87	1.26	1.08	20.47	11.29	1.46	0.4	0.1	1.3	0.6	1.5	0.2	0.0	0.0	0.1	0.0	0.0	4.35E-10	2.60E-07	4.88E-01	2.35E-06
	SMAD3	306	202	64	2	7	11	5	48	2	0	3	1	0	3	0	0	33.26	37.91	30.06	2.53	0.96	8.66	1.85	1.60	30.20	16.66	2.15	0.3	0.1	1.6	0.8	0.0	0.3	0.5	0.0	0.1	0.0	0.0	7.66E-18	1.72E-12	3.00E-01	2.22E-09
	SMAD4	284	114	27	0	13	5	6	16	0	0	3	2	0	2	3	3	29.24	33.32	26.42	2.22	0.85	7.61	1.63	1.40	26.55	14.64	1.89	0.2	0.2	0.6	0.0	0.0	0.4	1.2	0.0	0.1	0.2	1.6	2.57E-14	7.60E-11	7.97E-02	6.31E-07
	SMAD5	17	35	12	0	0	5	2	5	0	0	0	0	0	0	0	0	4.06	4.63	3.67	0.31	0.12	1.06	0.23	0.20	3.69	2.03	0.26	1.2	0.4	1.4	0.0	0.0	0.0	0.0	0.0	0.0	0.0	0.0	4.33E-01	3.34E-01	5.14E-01	1.88E-01
	SMAD6	23	39	3	0	0	0	2	1	0	0	0	0	0	0	0	0	3.33	3.79	3.01	0.25	0.10	0.87	0.19	0.16	3.02	1.67	0.22	0.0	0.5	0.3	0.0	0.0	0.0	0.0	0.0	0.0	0.0	0.0	2.86E-01	6.33E-02	5.54E-01	2.95E-01
	SMAD7	46	133	18	0	0	0	0	18	0	0	0	0	0	0	0	0	9.88	11.26	8.93	0.75	0.29	2.57	0.55	0.47	8.97	4.95	0.64	0.0	0.0	2.0	0.0	0.0	0.0	0.0	0.0	0.0	0.0	0.0	3.23E-07	2.56E-07	3.08E-01	2.76E-03
	SMAD9	32	21	8	0	3	2	0	6	0	0	3	0	0	0	0	0	5.49	6.26	4.96	0.42	0.16	1.43	0.31	0.26	4.98	2.75	0.36	0.4	0.0	1.2	0.0	0.0	2.1	0.0	0.0	0.0	0.0	0.0	3.27E-02	1.30E-02	4.48E-01	6.51E-02
SNAD	SAMD11	7	3	1	0	0	0	1	0	0	0	0	0	0	0	0	0	1.83	2.09	1.65	0.14	0.05	0.48	0.10	0.09	1.66	0.92	0.12	0.0	0.5	0.0	0.0	0.0	0.0	0.0	0.0	0.0	0.0	0.0	6.68E-01	1.32E-01	6.61E-01	6.44E-01
STAT	STAT1	78	77	27	0	3	7	0	20	0	0	3	0	0	0	0	0	16.36	18.64	14.78	1.24	0.47	4.26	0.91	0.79	14.85	8.19	1.06	0.4	0.0	1.4	0.0	0.0	0.7	0.0	0.0	0.0	0.0	0.0	5.46E-08	2.46E-06	1.90E-01	8.27E-05
	STAT2	28	17	9	0	2	0	1	8	0	0	0	0	0	0	0	2	5.71	6.51	5.16	0.43	0.17	1.49	0.32	0.27	5.18	2.86	0.37	0.0	0.2	1.6	0.0	0.0	0.0	0.0	0.0	0.0	0.0	5.4	9.08E-04	2.56E-03	4.39E-01	3.94E-03
	STAT3	120	182	55	0	11	2	6	47	0	0	6	0	0	3	2	0	24.55	27.98	22.19	1.87	0.71	6.39	1.37	1.18	22.29	12.30	1.59	0.1	0.2	2.1	0.0	0.0	0.9	0.0	0.0	0.1	0.2	0.0	1.51E-16	5.34E-15	1.08E-01	1.87E-05
	STAT4	7	5	1	0	0	0	1	0	0	0	0	0	0	0	0	0	1.17	1.33	1.06	0.09	0.03	0.30	0.07	0.06	1.06	0.59	0.08	0.0	0.7	0.0	0.0	0.0	0.0	0.0	0.0	0.0	0.0	0.0	9.17E-01	3.15E-01	7.26E-01	8.28E-01
	STAT5A	25	40	13	0	2	1	1	11	0	0	0	0	0	0	1	1	5.67	6.46	5.13	0.43	0.16	1.48	0.32	0.27	5.15	2.84	0.37	0.2	0.2	2.1	0.0	0.0	0.0	0.0	0.0	0.0	0.4	2.7	4.82E-03	5.01E-04	4.40E-01	9.08E-02
	STAT5B	14	22	2	0	3	0	2	0	0	0	0	0	0	0	2	1	3.88	4.42	3.51	0.29	0.11	1.01	0.22	0.19	3.52	1.94	0.25	0.0	0.5	0.0	0.0	0.0	0.0	0.0	0.0	0.0	1.0	4.0	9.17E-02	1.28E-02	5.23E-01	2.08E-01
	STAT6	32	15	7	0	0	0	3	4	0	0	0	0	0	0	0	0	3.26	3.71	2.94	0.25	0.09	0.85	0.18	0.16	2.96	1.63	0.21	0.0	0.8	1.4	0.0	0.0	0.0	0.0	0.0	0.0	0.0	0.0	4.32E-01	1.52E-01	5.59E-01	3.08E-01
TB	TBX19	8	0	0	0	0	0	0	0	0	0	0	0	0	0	0	0	0.62	0.71	0.56	0.05	0.02	0.16	0.03	0.03	0.56	0.31	0.04	0.0	0.0	0.0	0.0	0.0	0.0	0.0	0.0	0.0	0.0	0.0	9.79E-01	3.88E-01	7.98E-01	9.50E-01
	TBXT	0	0	0	0	0	0	0	0	0	0	0	0	0	0	0	0	0.00	0.00	0.00	0.00	0.00	0.00	0.00	0.00	0.00	0.00	0.00	na	na	na	na	na	na	na	na	na	na	na	na	na	na	na
	TBX1	2	6	0	0	1	0	0	0	0	0	0	0	0	1	0	0	0.40	0.46	0.36	0.03	0.01	0.10	0.02	0.02	0.37	0.20	0.03	0.0	0.0	0.0	0.0	0.0	0.0	0.0	0.0	2.7	0.0	0.0	9.87E-01	5.42E-01	8.37E-01	9.16E-01
	TBX10	0	0	0	0	0	0	0	0	0	0	0	0	0	0	0	0	0.04	0.04	0.03	0.00	0.00	0.01	0.00	0.00	0.03	0.02	0.00	0.0	0.0	0.0	0.0	0.0	0.0	0.0	0.0	0.0	0.0	0.0	1.00E+00	9.46E-01	9.51E-01	1.00E+00
	TBX15	5	2	0	0	0	0	0	0	0	0	0	0	0	0	0	0	0.55	0.63	0.50	0.04	0.02	0.14	0.03	0.03	0.50	0.27	0.04	0.0	0.0	0.0	0.0	0.0	0.0	0.0	0.0	0.0	0.0	0.0	9.87E-01	4.34E-01	8.10E-01	9.62E-01
	TBX18	2	0	0	0	0	0	0	0	0	0	0	0	0	0	0	0	0.26	0.29	0.23	0.02	0.01	0.07	0.01	0.01	0.23	0.13	0.02	0.0	0.0	0.0	0.0	0.0	0.0	0.0	0.0	0.0	0.0	0.0	9.99E-01	6.77E-01	8.70E-01	9.93E-01
	TBX20	3	9	1	0	0	1	0	0	0	0	0	0	0	0	0	0	1.35	1.54	1.22	0.10	0.04	0.35	0.08	0.07	1.23	0.68	0.09	0.7	0.0	0.0	0.0	0.0	0.0	0.0	0.0	0.0	0.0	0.0	8.56E-01	2.39E-01	7.06E-01	7.78E-01
	TBX21	7	1	2	0	0	0	0	2	0	0	0	0	0	0	0	0	0.51	0.58	0.46	0.04	0.01	0.13	0.03	0.02	0.47	0.26	0.03	0.0	0.0	4.3	0.0	0.0	0.0	0.0	0.0	0.0	0.0	0.0	7.07E-01	4.51E-02	8.17E-01	9.67E-01
	TBX22	6	0	0	0	0	0	0	0	0	0	0	0	0	0	0	0	0.40	0.46	0.36	0.03	0.01	0.10	0.02	0.02	0.37	0.20	0.03	0.0	0.0	0.0	0.0	0.0	0.0	0.0	0.0	0.0	0.0	0.0	9.96E-01	5.42E-01	8.37E-01	9.81E-01
	TBX2	11	2	0	0	0	0	0	0	0	0	0	0	0	0	0	0	0.91	1.04	0.83	0.07	0.03	0.24	0.05	0.04	0.83	0.46	0.06	0.0	0.0	0.0	0.0	0.0	0.0	0.0	0.0	0.0	0.0	0.0	9.19E-01	2.49E-01	7.57E-01	8.91E-01
	TBX3	7	21	12	0	1	2	6	4	0	0	0	0	0	1	0	0	2.63	3.00	2.38	0.20	0.08	0.69	0.15	0.13	2.39	1.32	0.17	0.8	2.0	1.7	0.0	0.0	0.0	0.0	0.0	0.4	0.0	0.0	6.50E-01	1.20E-01	5.99E-01	6.60E-01
	TBX4	1	0	0	0	0	0	0	0	0	0	0	0	0	0	0	0	0.15	0.17	0.13	0.01	0.00	0.04	0.01	0.01	0.13	0.07	0.01	0.0	0.0	0.0	0.0	0.0	0.0	0.0	0.0	0.0	0.0	0.0	1.00E+00	8.00E-01	9.01E-01	9.98E-01
	TBX5	33	28	21	0	2	10	6	5	0	0	0	0	0	2	0	0	5.74	6.55	5.19	0.44	0.17	1.50	0.32	0.28	5.22	2.88	0.37	1.7	0.9	1.0	0.0	0.0	0.0	0.0	0.0	0.4	0.0	0.0	3.47E-01	2.01E-01	4.37E-01	1.98E-01
	TBX6	12	6	1	0	0	0	0	1	0	0	0	0	0	0	0	0	1.68	1.92	1.52	0.13	0.05	0.44	0.09	0.08	1.53	0.84	0.11	0.0	0.0	0.7	0.0	0.0	0.0	0.0	0.0	0.0	0.0	0.0	7.21E-01	1.51E-01	6.74E-01	6.86E-01
	EOMES	1	0	0	0	0	0	0	0	0	0	0	0	0	0	0	0	0.07	0.08	0.07	0.01	0.00	0.02	0.00	0.00	0.07	0.04	0.00	0.0	0.0	0.0	0.0	0.0	0.0	0.0	0.0	0.0	0.0	0.0	1.00E+00	8.95E-01	9.30E-01	1.00E+00
	TBR1	37	31	6	0	0	0	3	3	0	0	0	0	0	0	0	0	4.28	4.88	3.87	0.33	0.12	1.12	0.24	0.21	3.89	2.14	0.28	0.0	0.6	0.8	0.0	0.0	0.0	0.0	0.0	0.0	0.0	0.0	1.96E-01	7.43E-02	5.02E-01	1.64E-01
TEA	TEAD1	79	63	3	0	0	0	0	3	0	0	0	0	0	0	0	0	7.43	8.47	6.71	0.56	0.22	1.93	0.41	0.36	6.74	3.72	0.48	0.0	0.0	0.4	0.0	0.0	0.0	0.0	0.0	0.0	0.0	0.0	3.46E-04	1.27E-04	3.77E-01	1.80E-02
	TEAD2	51	31	4	0	0	0	3	1	0	0	0	0	0	0	0	0	5.34	6.09	4.83	0.41	0.15	1.39	0.30	0.26	4.85	2.68	0.35	0.0	0.5	0.2	0.0	0.0	0.0	0.0	0.0	0.0	0.0	0.0	2.63E-02	6.93E-03	4.54E-01	8.06E-02
	TEAD3	3	26	0	0	0	0	0	0	0	0	0	0	0	0	0	0	1.68	1.92	1.52	0.13	0.05	0.44	0.09	0.08	1.53	0.84	0.11	0.0	0.0	0.0	0.0	0.0	0.0	0.0	0.0	0.0	0.0	0.0	5.91E-01	7.72E-02	6.74E-01	6.86E-01
	TEAD4	17	41	1	3	0	0	1	0	0	3	0	0	0	0	0	0	5.05	5.75	4.56	0.38	0.15	1.32	0.28	0.24	4.58	2.53	0.33	0.0	0.2	0.0	0.0	20.5	0.0	0.0	0.0	0.0	0.0	0.0	8.68E-13	1.15E-03	7.38E-14	9.84E-02
TSC22	TSC22D1	21	10	3	0	1	0	2	1	0	0	0	0	0	1	0	0	4.21	4.80	3.80	0.32	0.12	1.10	0.23	0.20	3.82	2.11	0.27	0.0	0.4	0.3	0.0	0.0	0.0	0.0	0.0	0.3	0.0	0.0	1.58E-01	1.92E-02	5.06E-01	3.07E-01
	TSC22D2	9	14	2	0	0	0	1	1	0	0	0	0	0	0	0	0	2.71	3.09	2.45	0.21	0.08	0.71	0.15	0.13	2.46	1.36	0.18	0.0	0.3	0.4	0.0	0.0	0.0	0.0	0.0	0.0	0.0	0.0	4.20E-01	8.32E-02	5.94E-01	4.19E-01
	TSC22D3	23	23	4	0	0	1	1	2	0	0	0	0	0	0	0	0	3.59	4.09	3.24	0.27	0.10	0.93	0.20	0.17	3.26	1.80	0.23	0.3	0.2	0.6	0.0	0.0	0.0	0.0	0.0	0.0	0.0	0.0	3.10E-01	9.68E-02	5.39E-01	2.53E-01
	TSC22D4	19	41	4	0	0	0	4	0	0	0	0	0	0	0	0	0	7.35	8.38	6.65	0.56	0.21	1.92	0.41	0.35	6.68	3.68	0.48	0.0	0.5	0.0	0.0	0.0	0.0	0.0	0.0	0.0	0.0	0.0	6.88E-04	2.90E-04	3.79E-01	1.90E-02
TUB	TUB	4	3	0	0	0	0	0	0	0	0	0	0	0	0	0	0	0.95	1.08	0.86	0.07	0.03	0.25	0.05	0.05	0.86	0.48	0.06	0.0	0.0	0.0	0.0	0.0	0.0	0.0	0.0	0.0	0.0	0.0	9.08E-01	2.35E-01	7.52E-01	8.83E-01
	TULP1	1	1	0	0	0	0	0	0	0	0	0	0	0	0	0	0	0.11	0.13	0.10	0.01	0.00	0.03	0.01	0.01	0.10	0.05	0.01	0.0	0.0	0.0	0.0	0.0	0.0	0.0	0.0	0.0	0.0	0.0	1.00E+00	8.46E-01	9.14E-01	9.99E-01
	TULP3	36	61	15	2	14	0	6	9	0	2	2	0	1	0	9	2	15.99	18.22	14.45	1.22	0.46	4.16	0.89	0.77	14.52	8.01	1.04	0.0	0.3	0.6	0.0	4.3	0.5	0.0	1.3	0.0	1.1	1.9	2.48E-07	2.00E-06	1.21E-02	3.45E-03
	TULP4	0	7	3	0	0	0	3	0	0	0	0	0	0	0	0	0	0.80	0.92	0.73	0.06	0.02	0.21	0.04	0.04	0.73	0.40	0.05	0.0	3.3	0.0	0.0	0.0	0.0	0.0	0.0	0.0	0.0	0.0	6.46E-01	4.37E-02	7.71E-01	9.15E-01
WC.ETS	EHF	1	1	1	0	0	0	1	0	0	0	0	0	0	0	0	0	0.55	0.63	0.50	0.04	0.02	0.14	0.03	0.03	0.50	0.27	0.04	0.0	1.6	0.0	0.0	0.0	0.0	0.0	0.0	0.0	0.0	0.0	9.93E-01	5.30E-01	8.10E-01	9.62E-01
	ELF1	43	25	10	0	1	1	2	7	0	0	0	0	0	0	1	0	4.43	5.05	4.00	0.34	0.13	1.15	0.25	0.21	4.02	2.22	0.29	0.2	0.4	1.7	0.0	0.0	0.0	0.0	0.0	0.0	0.5	0.0	1.83E-01	3.44E-02	4.95E-01	2.53E-01
	ELF2	53	35	9	0	0	1	1	7	0	0	0	0	0	0	0	0	6.00	6.84	5.42	0.46	0.17	1.56	0.33	0.29	5.45	3.01	0.39	0.2	0.1	1.3	0.0	0.0	0.0	0.0	0.0	0.0	0.0	0.0	1.93E-02	8.18E-03	4.27E-01	5.08E-02
	ELF3	15	10	4	0	3	0	2	2	0	0	0	0	1	1	1	0	2.34	2.67	2.12	0.18	0.07	0.61	0.13	0.11	2.13	1.17	0.15	0.0	0.7	0.9	0.0	0.0	0.0	0.0	8.9	0.5	0.9	0.0	3.36E-01	2.84E-01	6.20E-01	1.30E-01
	ELF4	23	36	9	0	0	0	1	8	0	0	0	0	0	0	0	0	4.32	4.92	3.90	0.33	0.13	1.12	0.24	0.21	3.92	2.16	0.28	0.0	0.2	2.1	0.0	0.0	0.0	0.0	0.0	0.0	0.0	0.0	2.80E-02	2.82E-03	5.01E-01	1.60E-01
	ELF5	14	14	5	0	1	0	0	5	0	0	0	0	0	1	0	0	1.98	2.25	1.79	0.15	0.06	0.51	0.11	0.09	1.79	0.99	0.13	0.0	0.0	2.8	0.0	0.0	0.0	0.0	0.0	0.6	0.0	0.0	2.59E-01	6.69E-03	6.49E-01	8.22E-01
	ELK1	18	46	9	0	0	2	3	4	0	0	0	0	0	0	0	0	6.07	6.92	5.49	0.46	0.18	1.58	0.34	0.29	5.52	3.04	0.39	0.3	0.4	0.7	0.0	0.0	0.0	0.0	0.0	0.0	0.0	0.0	7.09E-02	6.86E-02	4.24E-01	4.82E-02
	ELK3	50	31	4	0	0	0	1	3	0	0	0	0	0	0	0	0	4.35	4.96	3.94	0.33	0.13	1.13	0.24	0.21	3.95	2.18	0.28	0.0	0.2	0.8	0.0	0.0	0.0	0.0	0.0	0.0	0.0	0.0	9.41E-02	2.09E-02	4.99E-01	1.56E-01
	ELK4	6	9	9	0	2	3	5	1	0	0	0	0	0	0	2	0	3.18	3.63	2.88	0.24	0.09	0.83	0.18	0.15	2.89	1.59	0.21	0.9	1.4	0.3	0.0	0.0	0.0	0.0	0.0	0.0	1.3	0.0	7.76E-01	4.16E-01	5.63E-01	4.99E-01
	ERF	8	17	1	0	1	0	1	0	0	0	1	0	0	0	0	0	4.72	5.38	4.27	0.36	0.14	1.23	0.26	0.23	4.29	2.36	0.31	0.0	0.2	0.0	0.0	0.0	0.8	0.0	0.0	0.0	0.0	0.0	2.46E-02	1.88E-03	4.81E-01	1.87E-01
	ERG	58	61	69	0	6	23	40	6	0	0	1	0	0	5	0	0	11.53	13.14	10.42	0.88	0.33	3.00	0.64	0.55	10.47	5.77	0.75	2.0	3.0	0.6	0.0	0.0	0.3	0.0	0.0	0.5	0.0	0.0	2.73E-13	1.52E-15	2.71E-01	3.61E-02
	ETS1	41	25	7	0	2	0	1	6	0	0	1	0	0	1	0	0	4.61	5.25	4.17	0.35	0.13	1.20	0.26	0.22	4.19	2.31	0.30	0.0	0.2	1.4	0.0	0.0	0.8	0.0	0.0	0.2	0.0	0.0	1.36E-01	1.19E-02	4.86E-01	3.53E-01
	ETS2	24	17	10	0	2	4	2	4	0	0	0	0	0	1	1	0	2.96	3.38	2.68	0.23	0.09	0.77	0.17	0.14	2.69	1.48	0.19	1.3	0.6	1.5	0.0	0.0	0.0	0.0	0.0	0.4	0.7	0.0	9.29E-01	4.55E-01	5.77E-01	7.78E-01
	ETV1	4	10	9	0	0	0	1	8	0	0	0	0	0	0	0	0	1.50	1.71	1.36	0.11	0.04	0.39	0.08	0.07	1.36	0.75	0.10	0.0	0.6	5.9	0.0	0.0	0.0	0.0	0.0	0.0	0.0	0.0	5.08E-05	3.47E-08	6.91E-01	7.37E-01
	ETV2	0	3	0	0	0	0	0	0	0	0	0	0	0	0	0	0	0.15	0.17	0.13	0.01	0.00	0.04	0.01	0.01	0.13	0.07	0.01	0.0	0.0	0.0	0.0	0.0	0.0	0.0	0.0	0.0	0.0	0.0	1.00E+00	8.00E-01	9.01E-01	9.98E-01
	ETV3	19	16	10	0	2	1	7	2	0	0	0	0	0	0	2	0	4.87	5.55	4.40	0.37	0.14	1.27	0.27	0.23	4.42	2.44	0.32	0.2	1.3	0.5	0.0	0.0	0.0	0.0	0.0	0.0	0.8	0.0	2.95E-01	9.25E-02	4.75E-01	2.53E-01
	ETV3L	1	0	0	0	0	0	0	0	0	0	0	0	0	0	0	0	0.04	0.04	0.03	0.00	0.00	0.01	0.00	0.00	0.03	0.02	0.00	0.0	0.0	0.0	0.0	0.0	0.0	0.0	0.0	0.0	0.0	0.0	1.00E+00	9.46E-01	9.51E-01	1.00E+00
	ETV4	22	12	4	0	2	0	1	3	0	0	0	0	0	0	2	0	2.93	3.34	2.65	0.22	0.08	0.76	0.16	0.14	2.66	1.47	0.19	0.0	0.3	1.1	0.0	0.0	0.0	0.0	0.0	0.0	1.4	0.0	5.30E-01	9.97E-02	5.79E-01	5.34E-01
	ETV5	3	2	3	0	0	0	2	1	0	0	0	0	0	0	0	0	1.79	2.04	1.62	0.14	0.05	0.47	0.10	0.09	1.63	0.90	0.12	0.0	1.0	0.6	0.0	0.0	0.0	0.0	0.0	0.0	0.0	0.0	8.54E-01	3.62E-01	6.64E-01	6.55E-01
	ETV6	35	29	11	0	0	1	3	7	0	0	0	0	0	0	0	0	5.12	5.84	4.63	0.39	0.15	1.33	0.29	0.25	4.65	2.57	0.33	0.2	0.5	1.5	0.0	0.0	0.0	0.0	0.0	0.0	0.0	0.0	1.04E-01	5.20E-02	4.63E-01	9.36E-02
	ETV7	18	4	2	0	0	0	1	1	0	0	0	0	0	0	0	0	1.24	1.42	1.12	0.09	0.04	0.32	0.07	0.06	1.13	0.62	0.08	0.0	0.7	0.9	0.0	0.0	0.0	0.0	0.0	0.0	0.0	0.0	9.56E-01	5.01E-01	7.18E-01	8.08E-01
	FEV	98	37	4	0	0	0	0	4	0	0	0	0	0	0	0	0	7.39	8.42	6.68	0.56	0.21	1.93	0.41	0.36	6.71	3.70	0.48	0.0	0.0	0.6	0.0	0.0	0.0	0.0	0.0	0.0	0.0	0.0	5.34E-04	2.15E-04	3.78E-01	1.85E-02
	FLI1	43	9	8	0	0	0	2	6	0	0	0	0	0	0	0	0	2.60	2.96	2.35	0.20	0.08	0.68	0.14	0.12	2.36	1.30	0.17	0.0	0.7	2.6	0.0	0.0	0.0	0.0	0.0	0.0	0.0	0.0	1.90E-01	1.36E-02	6.01E-01	4.44E-01
	GABPA	43	22	8	0	0	0	0	8	0	0	0	0	0	0	0	0	5.20	5.92	4.70	0.40	0.15	1.35	0.29	0.25	4.72	2.60	0.34	0.0	0.0	1.7	0.0	0.0	0.0	0.0	0.0	0.0	0.0	0.0	8.93E-03	1.21E-03	4.60E-01	8.91E-02
	SPDEF	11	7	1	0	0	0	0	1	0	0	0	0	0	0	0	0	2.01	2.29	1.82	0.15	0.06	0.52	0.11	0.10	1.83	1.01	0.13	0.0	0.0	0.5	0.0	0.0	0.0	0.0	0.0	0.0	0.0	0.0	5.72E-01	9.66E-02	6.46E-01	5.94E-01
	SPI1	46	16	14	0	0	0	7	7	0	0	0	0	0	0	0	0	3.44	3.92	3.11	0.26	0.10	0.90	0.19	0.17	3.12	1.72	0.22	0.0	1.8	2.3	0.0	0.0	0.0	0.0	0.0	0.0	0.0	0.0	6.57E-02	4.67E-03	5.48E-01	2.76E-01
	SPIB	9	3	1	0	0	0	0	1	0	0	0	0	0	0	0	0	0.70	0.79	0.63	0.05	0.02	0.18	0.04	0.03	0.63	0.35	0.05	0.0	0.0	1.6	0.0	0.0	0.0	0.0	0.0	0.0	0.0	0.0	9.80E-01	4.26E-01	7.87E-01	9.37E-01
	SPIC	2	1	0	0	0	0	0	0	0	0	0	0	0	0	0	0	0.18	0.21	0.17	0.01	0.01	0.05	0.01	0.01	0.17	0.09	0.01	0.0	0.0	0.0	0.0	0.0	0.0	0.0	0.0	0.0	0.0	0.0	1.00E+00	7.57E-01	8.90E-01	9.97E-01
WC.IRF	IRF1	25	35	14	0	0	3	0	11	0	0	0	0	0	0	0	0	4.61	5.25	4.17	0.35	0.13	1.20	0.26	0.22	4.19	2.31	0.30	0.7	0.0	2.6	0.0	0.0	0.0	0.0	0.0	0.0	0.0	0.0	3.77E-03	2.01E-04	4.86E-01	1.32E-01
	IRF2	18	50	9	0	3	0	0	9	0	0	0	0	0	2	0	1	5.01	5.71	4.53	0.38	0.15	1.31	0.28	0.24	4.55	2.51	0.32	0.0	0.0	2.0	0.0	0.0	0.0	0.0	0.0	0.4	0.0	3.1	1.14E-02	5.17E-04	4.68E-01	2.08E-01
	IRF3	72	37	17	0	0	0	5	12	0	0	0	0	0	0	0	0	12.62	14.39	11.41	0.96	0.37	3.29	0.70	0.61	11.46	6.32	0.82	0.0	0.3	1.1	0.0	0.0	0.0	0.0	0.0	0.0	0.0	0.0	4.39E-06	8.36E-05	2.50E-01	3.09E-04
	IRF4	37	19	3	0	0	0	0	3	0	0	0	0	0	0	0	0	3.44	3.92	3.11	0.26	0.10	0.90	0.19	0.17	3.12	1.72	0.22	0.0	0.0	1.0	0.0	0.0	0.0	0.0	0.0	0.0	0.0	0.0	1.71E-01	2.52E-02	5.48E-01	2.76E-01
	IRF5	8	14	6	0	0	0	0	6	0	0	0	0	0	0	0	0	2.56	2.92	2.31	0.19	0.07	0.67	0.14	0.12	2.33	1.28	0.17	0.0	0.0	2.6	0.0	0.0	0.0	0.0	0.0	0.0	0.0	0.0	9.07E-02	3.44E-03	6.04E-01	4.53E-01
	IRF6	9	0	0	0	0	0	0	0	0	0	0	0	0	0	0	0	0.51	0.58	0.46	0.04	0.01	0.13	0.03	0.02	0.47	0.26	0.03	0.0	0.0	0.0	0.0	0.0	0.0	0.0	0.0	0.0	0.0	0.0	9.90E-01	4.59E-01	8.17E-01	9.67E-01
	IRF7	23	29	6	1	0	1	0	5	1	0	0	0	0	0	0	0	5.53	6.30	4.99	0.42	0.16	1.44	0.31	0.27	5.02	2.77	0.36	0.2	0.0	1.0	2.4	0.0	0.0	0.0	0.0	0.0	0.0	0.0	2.03E-02	6.73E-03	3.27E-01	7.10E-02
	IRF8	29	15	1	0	0	0	0	1	0	0	0	0	0	0	0	0	2.63	3.00	2.38	0.20	0.08	0.69	0.15	0.13	2.39	1.32	0.17	0.0	0.0	0.4	0.0	0.0	0.0	0.0	0.0	0.0	0.0	0.0	3.16E-01	4.00E-02	5.99E-01	4.36E-01
	IRF9	24	2	3	0	0	0	3	0	0	0	0	0	0	0	0	0	2.05	2.34	1.85	0.16	0.06	0.53	0.11	0.10	1.86	1.03	0.13	0.0	1.3	0.0	0.0	0.0	0.0	0.0	0.0	0.0	0.0	0.0	6.22E-01	1.29E-01	6.43E-01	5.84E-01
WC.MYB	CDC5L	54	125	269	7	15	199	53	17	7	0	3	0	0	12	0	0	38.09	43.41	34.42	2.90	1.11	9.92	2.12	1.83	34.59	19.08	2.47	5.2	1.2	0.5	2.4	0.0	0.3	0.0	0.0	0.3	0.0	0.0	4.26E-153	1.04E-150	8.52E-03	1.40E-08
	DMTF1	3	3	0	0	0	0	0	0	0	0	0	0	0	0	0	0	0.29	0.33	0.26	0.02	0.01	0.08	0.02	0.01	0.27	0.15	0.02	0.0	0.0	0.0	0.0	0.0	0.0	0.0	0.0	0.0	0.0	0.0	9.99E-01	6.41E-01	8.61E-01	9.91E-01
	DNAJC1	9	24	6	0	25	0	3	3	0	0	2	0	0	22	1	0	15.29	17.43	13.82	1.16	0.44	3.98	0.85	0.73	13.89	7.66	0.99	0.0	0.2	0.2	0.0	0.0	0.5	0.0	0.0	1.6	0.1	0.0	1.46E-07	1.76E-08	2.05E-01	1.50E-02
	DNAJC2	4	20	13	0	99	2	9	2	0	0	10	2	0	86	0	1	9.26	10.55	8.37	0.70	0.27	2.41	0.52	0.44	8.41	4.64	0.60	0.2	0.9	0.2	0.0	0.0	4.1	3.9	0.0	10.2	0.0	1.7	3.80E-157	4.60E-03	3.24E-01	8.32E-160
	MIDEAS	0	0	0	0	0	0	0	0	0	0	0	0	0	0	0	0	0.00	0.00	0.00	0.00	0.00	0.00	0.00	0.00	0.00	0.00	0.00	na	na	na	na	na	na	na	na	na	na	na	na	na	na	na
	MIER1	15	24	18	0	1	0	2	16	0	0	0	0	0	0	1	0	3.29	3.75	2.98	0.25	0.10	0.86	0.18	0.16	2.99	1.65	0.21	0.0	0.5	5.4	0.0	0.0	0.0	0.0	0.0	0.0	0.6	0.0	2.48E-10	5.38E-14	5.56E-01	4.59E-01
	MIER3	1	10	8	0	0	0	1	7	0	0	0	0	0	0	0	0	1.17	1.33	1.06	0.09	0.03	0.30	0.07	0.06	1.06	0.59	0.08	0.0	0.7	6.6	0.0	0.0	0.0	0.0	0.0	0.0	0.0	0.0	5.90E-05	3.04E-08	7.26E-01	8.28E-01
	MYB	43	52	19	0	5	1	1	17	0	0	0	1	0	4	0	0	6.44	7.34	5.82	0.49	0.19	1.68	0.36	0.31	5.85	3.23	0.42	0.2	0.1	2.9	0.0	0.0	0.0	2.8	0.0	0.7	0.0	0.0	2.01E-05	1.41E-07	4.11E-01	1.95E-01
	MYBL1	0	7	0	0	0	0	0	0	0	0	0	0	0	0	0	0	0.91	1.04	0.83	0.07	0.03	0.24	0.05	0.04	0.83	0.46	0.06	0.0	0.0	0.0	0.0	0.0	0.0	0.0	0.0	0.0	0.0	0.0	9.19E-01	2.49E-01	7.57E-01	8.91E-01
	MYBL2	49	54	10	2	1	1	2	7	2	0	0	0	0	0	1	0	7.94	9.05	7.18	0.60	0.23	2.07	0.44	0.38	7.21	3.98	0.51	0.1	0.2	1.0	3.3	0.0	0.0	0.0	0.0	0.0	0.3	0.0	1.90E-03	3.09E-03	6.29E-02	2.49E-02
	NCOR1	251	159	65	2	0	6	3	56	1	1	0	0	0	0	0	0	21.19	24.14	19.15	1.61	0.61	5.52	1.18	1.02	19.24	10.61	1.37	0.3	0.1	2.9	0.6	1.6	0.0	0.0	0.0	0.0	0.0	0.0	4.75E-25	1.63E-22	4.92E-01	2.45E-07
	NCOR2	243	117	57	1	2	6	3	48	0	1	0	0	0	1	1	0	17.05	19.43	15.41	1.30	0.49	4.44	0.95	0.82	15.48	8.54	1.10	0.4	0.2	3.1	0.0	2.0	0.0	0.0	0.0	0.1	0.1	0.0	6.97E-21	2.89E-20	1.78E-01	4.52E-05
	RCOR1	151	173	78	2	4	2	2	74	2	0	1	0	0	3	0	0	22.06	25.15	19.94	1.68	0.64	5.75	1.23	1.06	20.03	11.05	1.43	0.1	0.1	3.7	1.2	0.0	0.2	0.0	0.0	0.1	0.0	0.0	1.08E-41	3.86E-41	4.02E-01	3.48E-06
	RCOR2	13	21	10	0	0	0	0	10	0	0	0	0	0	0	0	0	1.61	1.83	1.45	0.12	0.05	0.42	0.09	0.08	1.46	0.81	0.10	0.0	0.0	6.9	0.0	0.0	0.0	0.0	0.0	0.0	0.0	0.0	1.48E-08	2.26E-12	6.81E-01	7.06E-01
	RCOR3	62	46	22	0	0	1	2	19	0	0	0	0	0	0	0	0	8.60	9.80	7.77	0.65	0.25	2.24	0.48	0.41	7.81	4.31	0.56	0.1	0.2	2.4	0.0	0.0	0.0	0.0	0.0	0.0	0.0	0.0	1.52E-06	4.68E-07	3.42E-01	7.43E-03
	SMARCC1	154	74	75	0	1	5	13	57	0	0	0	0	0	1	0	0	17.82	20.31	16.10	1.35	0.52	4.64	0.99	0.86	16.18	8.93	1.15	0.3	0.6	3.5	0.0	0.0	0.0	0.0	0.0	0.1	0.0	0.0	8.00E-27	7.50E-26	1.71E-01	1.02E-05
	SMARCC2	136	82	61	0	2	4	13	44	0	0	0	0	0	2	0	0	16.87	19.22	15.24	1.28	0.49	4.39	0.94	0.81	15.32	8.45	1.09	0.2	0.7	2.9	0.0	0.0	0.0	0.0	0.0	0.1	0.0	0.0	5.17E-16	4.49E-15	1.83E-01	5.07E-05
	SNAPC4	3	19	2	0	1	0	2	0	0	0	0	0	0	1	0	0	3.00	3.42	2.71	0.23	0.09	0.78	0.17	0.14	2.72	1.50	0.19	0.0	0.6	0.0	0.0	0.0	0.0	0.0	0.0	0.4	0.0	0.0	3.98E-01	4.28E-02	5.75E-01	5.67E-01
	TADA2A	37	91	56	0	7	15	21	20	0	0	6	0	0	0	0	1	10.50	11.97	9.49	0.80	0.30	2.74	0.58	0.50	9.54	5.26	0.68	1.4	1.8	2.1	0.0	0.0	2.2	0.0	0.0	0.0	0.0	1.5	9.52E-06	3.75E-05	2.94E-01	1.29E-03
	TADA2B	18	90	13	0	0	2	1	10	0	0	0	0	0	0	0	0	6.70	7.63	6.05	0.51	0.19	1.74	0.37	0.32	6.08	3.35	0.43	0.3	0.1	1.7	0.0	0.0	0.0	0.0	0.0	0.0	0.0	0.0	6.07E-03	2.98E-03	4.02E-01	3.08E-02
	TRERF1	11	11	9	0	0	0	2	7	0	0	0	0	0	0	0	0	1.57	1.79	1.42	0.12	0.05	0.41	0.09	0.08	1.43	0.79	0.10	0.0	1.1	4.9	0.0	0.0	0.0	0.0	0.0	0.0	0.0	0.0	3.08E-03	7.97E-06	6.84E-01	7.17E-01
	ZNF541	0	2	0	0	1	0	0	0	0	0	0	0	0	1	0	0	0.11	0.13	0.10	0.01	0.00	0.03	0.01	0.01	0.10	0.05	0.01	0.0	0.0	0.0	0.0	0.0	0.0	0.0	0.0	10.0	0.0	0.0	5.72E-01	8.46E-01	9.14E-01	1.44E-01
	MIS18BP1	8	8	4	0	1	1	1	2	0	0	1	0	0	0	0	0	3.18	3.63	2.88	0.24	0.09	0.83	0.18	0.15	2.89	1.59	0.21	0.3	0.3	0.7	0.0	0.0	1.2	0.0	0.0	0.0	0.0	0.0	5.27E-01	1.60E-01	5.63E-01	4.09E-01
	MYSM1	3	17	9	0	1	3	2	4	0	0	0	0	0	1	0	0	2.27	2.59	2.05	0.17	0.07	0.59	0.13	0.11	2.06	1.14	0.15	1.3	0.8	2.0	0.0	0.0	0.0	0.0	0.0	0.5	0.0	0.0	8.83E-01	3.29E-01	6.25E-01	7.53E-01
	TERB1	0	0	0	0	0	0	0	0	0	0	0	0	0	0	0	0	0.00	0.00	0.00	0.00	0.00	0.00	0.00	0.00	0.00	0.00	0.00	na	na	na	na	na	na	na	na	na	na	na	na	na	na	na
	TERF1	35	80	14	0	10	5	2	7	0	0	1	1	1	6	1	0	17.86	20.35	16.14	1.36	0.52	4.65	0.99	0.86	16.21	8.94	1.16	0.3	0.1	0.4	0.0	0.0	0.2	1.0	1.2	0.4	0.1	0.0	2.27E-07	1.88E-07	1.71E-01	3.59E-03
	TERF2	40	85	34	1	7	11	9	14	1	0	0	1	1	4	0	1	17.82	20.31	16.10	1.35	0.52	4.64	0.99	0.86	16.18	8.93	1.15	0.6	0.4	0.9	0.7	0.0	0.0	1.0	1.2	0.2	0.0	0.9	3.21E-04	1.01E-02	4.35E-01	3.72E-04

	TTF1	16	17	5	0	32	0	4	1	0	0	0	0	0	32	0	0	4.68	5.34	4.23	0.36	0.14	1.22	0.26	0.22	4.25	2.35	0.30	0.0	0.7	0.2	0.0	0.0	0.0	0.0	0.0	7.5	0.0	0.0	3.87E-36	2.37E-02	4.83E-01	3.76E-38
	ZZZ3	35	57	12	0	1	1	0	11	0	0	0	0	0	1	0	0	7.24	8.26	6.55	0.55	0.21	1.89	0.40	0.35	6.58	3.63	0.47	0.1	0.0	1.7	0.0	0.0	0.0	0.0	0.0	0.2	0.0	0.0	1.30E-03	2.40E-04	3.83E-01	4.29E-02
ZF	ZNF750	12	24	7	0	0	0	0	7	0	0	0	0	0	0	0	0	1.76	2.00	1.59	0.13	0.05	0.46	0.10	0.08	1.59	0.88	0.11	0.0	0.0	4.4	0.0	0.0	0.0	0.0	0.0	0.0	0.0	0.0	4.27E-03	1.50E-05	6.68E-01	6.65E-01
ZF.BED	ZFTA	0	0	0	0	0	0	0	0	0	0	0	0	0	0	0	0	0.00	0.00	0.00	0.00	0.00	0.00	0.00	0.00	0.00	0.00	0.00	na	na	na	na	na	na	na	na	na	na	na	na	na	na	na
	MYT1	1	7	3	0	0	0	1	2	0	0	0	0	0	0	0	0	0.40	0.46	0.36	0.03	0.01	0.10	0.02	0.02	0.37	0.20	0.03	0.0	2.2	5.5	0.0	0.0	0.0	0.0	0.0	0.0	0.0	0.0	5.15E-01	1.50E-02	8.37E-01	9.81E-01
	MYT1L	1	0	2	0	0	2	0	0	0	0	0	0	0	0	0	0	0.26	0.29	0.23	0.02	0.01	0.07	0.01	0.01	0.23	0.13	0.02	7.8	0.0	0.0	0.0	0.0	0.0	0.0	0.0	0.0	0.0	0.0	2.30E-01	2.03E-03	8.70E-01	9.93E-01
	ST18	1	1	0	0	0	0	0	0	0	0	0	0	0	0	0	0	0.18	0.21	0.17	0.01	0.01	0.05	0.01	0.01	0.17	0.09	0.01	0.0	0.0	0.0	0.0	0.0	0.0	0.0	0.0	0.0	0.0	0.0	1.00E+00	7.57E-01	8.90E-01	9.97E-01
	L3MBTL1	30	29	5	0	3	0	1	4	0	0	0	0	0	3	0	0	5.23	5.96	4.73	0.40	0.15	1.36	0.29	0.25	4.75	2.62	0.34	0.0	0.2	0.8	0.0	0.0	0.0	0.0	0.0	0.6	0.0	0.0	1.14E-01	8.76E-03	4.58E-01	3.57E-01
	LYAR	53	49	31	1	46	5	16	10	1	0	5	0	0	40	1	1	16.25	18.52	14.68	1.24	0.47	4.23	0.90	0.78	14.75	8.14	1.05	0.3	0.9	0.7	0.8	0.0	1.2	0.0	0.0	2.7	0.1	1.0	1.22E-09	8.15E-03	4.21E-12	4.57E-10
	THAP1	20	33	12	0	4	8	3	1	0	0	0	0	0	2	2	0	6.70	7.63	6.05	0.51	0.19	1.74	0.37	0.32	6.08	3.35	0.43	1.2	0.4	0.2	0.0	0.0	0.0	0.0	0.0	0.3	0.6	0.0	1.67E-01	2.62E-02	4.02E-01	2.91E-01
	THAP10	0	1	1	0	0	0	1	0	0	0	0	0	0	0	0	0	1.10	1.25	0.99	0.08	0.03	0.29	0.06	0.05	1.00	0.55	0.07	0.0	0.8	0.0	0.0	0.0	0.0	0.0	0.0	0.0	0.0	0.0	9.34E-01	3.43E-01	7.34E-01	8.47E-01
	THAP11	21	33	6	0	1	1	3	2	0	0	0	0	0	1	0	0	6.04	6.88	5.46	0.46	0.18	1.57	0.34	0.29	5.48	3.02	0.39	0.2	0.4	0.4	0.0	0.0	0.0	0.0	0.0	0.2	0.0	0.0	4.72E-02	1.37E-02	4.26E-01	9.85E-02
ZF.C2CH	THAP12	0	0	0	0	0	0	0	0	0	0	0	0	0	0	0	0	0.00	0.00	0.00	0.00	0.00	0.00	0.00	0.00	0.00	0.00	0.00	na	na	na	na	na	na	na	na	na	na	na	na	na	na	na
	THAP2	1	9	3	0	0	3	0	0	0	0	0	0	0	0	0	0	0.80	0.92	0.73	0.06	0.02	0.21	0.04	0.04	0.73	0.40	0.05	3.7	0.0	0.0	0.0	0.0	0.0	0.0	0.0	0.0	0.0	0.0	5.14E-01	2.20E-02	7.71E-01	9.15E-01
	THAP3	8	13	4	0	15	1	1	2	0	0	0	0	0	8	7	0	2.71	3.09	2.45	0.21	0.08	0.71	0.15	0.13	2.46	1.36	0.18	0.4	0.3	0.8	0.0	0.0	0.0	0.0	0.0	3.3	5.2	0.0	1.70E-05	2.77E-01	5.94E-01	5.62E-07
	THAP4	5	4	2	0	0	1	1	0	0	0	0	0	0	0	0	0	2.89	3.29	2.61	0.22	0.08	0.75	0.16	0.14	2.62	1.45	0.19	0.3	0.3	0.0	0.0	0.0	0.0	0.0	0.0	0.0	0.0	0.0	3.53E-01	6.57E-02	5.82E-01	3.79E-01
	THAP5	0	0	0	0	0	0	0	0	0	0	0	0	0	0	0	0	0.26	0.29	0.23	0.02	0.01	0.07	0.01	0.01	0.23	0.13	0.02	0.0	0.0	0.0	0.0	0.0	0.0	0.0	0.0	0.0	0.0	0.0	9.99E-01	6.77E-01	8.70E-01	9.93E-01
	THAP6	4	5	0	0	0	0	0	0	0	0	0	0	0	0	0	0	0.77	0.88	0.69	0.06	0.02	0.20	0.04	0.04	0.70	0.38	0.05	0.0	0.0	0.0	0.0	0.0	0.0	0.0	0.0	0.0	0.0	0.0	9.55E-01	3.11E-01	7.76E-01	9.23E-01
	THAP7	12	19	7	0	12	2	1	4	0	0	0	0	0	12	0	0	5.09	5.80	4.60	0.39	0.15	1.32	0.28	0.24	4.62	2.55	0.33	0.4	0.2	0.9	0.0	0.0	0.0	0.0	0.0	2.6	0.0	0.0	1.08E-02	5.19E-02	4.65E-01	5.49E-03
	THAP8	2	7	2	0	0	1	1	0	0	0	0	0	0	0	0	0	1.21	1.38	1.09	0.09	0.04	0.31	0.07	0.06	1.10	0.60	0.08	0.8	0.7	0.0	0.0	0.0	0.0	0.0	0.0	0.0	0.0	0.0	9.64E-01	5.41E-01	7.22E-01	8.18E-01
	THAP9	0	0	0	0	2	0	0	0	0	0	0	0	0	0	2	0	0.40	0.46	0.36	0.03	0.01	0.10	0.02	0.02	0.37	0.20	0.03	0.0	0.0	0.0	0.0	0.0	0.0	0.0	0.0	0.0	9.9	0.0	5.76E-02	5.42E-01	8.37E-01	5.37E-03
	EGR1	22	22	10	0	0	0	0	10	0	0	0	0	0	0	0	0	3.00	3.42	2.71	0.23	0.09	0.78	0.17	0.14	2.72	1.50	0.19	0.0	0.0	3.7	0.0	0.0	0.0	0.0	0.0	0.0	0.0	0.0	4.26E-04	2.25E-06	5.75E-01	3.56E-01
	EGR2	58	48	7	0	0	0	0	7	0	0	0	0	0	0	0	0	6.15	7.01	5.56	0.47	0.18	1.60	0.34	0.30	5.58	3.08	0.40	0.0	0.0	1.3	0.0	0.0	0.0	0.0	0.0	0.0	0.0	0.0	4.52E-03	1.15E-03	4.22E-01	4.58E-02
	EGR3	0	0	0	0	0	0	0	0	0	0	0	0	0	0	0	0	0.04	0.04	0.03	0.00	0.00	0.01	0.00	0.00	0.03	0.02	0.00	0.0	0.0	0.0	0.0	0.0	0.0	0.0	0.0	0.0	0.0	0.0	1.00E+00	9.46E-01	9.51E-01	1.00E+00
	EGR4	0	0	0	0	0	0	0	0	0	0	0	0	0	0	0	0	0.15	0.17	0.13	0.01	0.00	0.04	0.01	0.01	0.13	0.07	0.01	0.0	0.0	0.0	0.0	0.0	0.0	0.0	0.0	0.0	0.0	0.0	1.00E+00	8.00E-01	9.01E-01	9.98E-01
	KLF1	7	17	9	0	0	2	3	4	0	0	0	0	0	0	0	0	1.57	1.79	1.42	0.12	0.05	0.41	0.09	0.08	1.43	0.79	0.10	1.3	1.7	2.8	0.0	0.0	0.0	0.0	0.0	0.0	0.0	0.0	5.65E-01	6.07E-02	6.84E-01	7.17E-01
	KLF10	8	23	3	0	1	2	0	1	0	0	0	0	0	1	0	0	1.98	2.25	1.79	0.15	0.06	0.51	0.11	0.09	1.79	0.99	0.13	1.0	0.0	0.6	0.0	0.0	0.0	0.0	0.0	0.6	0.0	0.0	8.92E-01	2.73E-01	6.49E-01	8.22E-01
	KLF11	3	11	4	0	0	0	1	3	0	0	0	0	0	0	0	0	0.95	1.08	0.86	0.07	0.03	0.25	0.05	0.05	0.86	0.48	0.06	0.0	0.9	3.5	0.0	0.0	0.0	0.0	0.0	0.0	0.0	0.0	6.16E-01	4.32E-02	7.52E-01	8.83E-01
	KLF12	54	35	16	0	58	0	8	8	0	0	0	0	0	6	52	0	8.05	9.17	7.27	0.61	0.23	2.10	0.45	0.39	7.31	4.03	0.52	0.0	0.9	1.1	0.0	0.0	0.0	0.0	0.0	0.8	12.9	0.0	6.03E-119	1.60E-02	3.58E-01	6.93E-122
	KLF13	2	9	9	0	0	0	3	6	0	0	0	0	0	0	0	0	0.95	1.08	0.86	0.07	0.03	0.25	0.05	0.05	0.86	0.48	0.06	0.0	2.8	7.0	0.0	0.0	0.0	0.0	0.0	0.0	0.0	0.0	5.85E-05	2.43E-08	7.52E-01	8.83E-01
	KLF14	0	0	0	0	0	0	0	0	0	0	0	0	0	0	0	0	0.00	0.00	0.00	0.00	0.00	0.00	0.00	0.00	0.00	0.00	0.00	na	na	na	na	na	na	na	na	na	na	na	na	na	na	na
	KLF15	63	34	18	0	59	0	9	9	0	0	4	0	0	11	44	0	11.27	12.84	10.18	0.86	0.33	2.94	0.63	0.54	10.23	5.64	0.73	0.0	0.7	0.9	0.0	0.0	1.4	0.0	0.0	1.1	7.8	0.0	1.29E-53	1.88E-03	2.77E-01	9.04E-55
	KLF16	76	62	29	0	47	7	13	9	0	0	11	1	0	17	18	0	16.50	18.81	14.91	1.25	0.48	4.30	0.92	0.79	14.98	8.27	1.07	0.4	0.7	0.6	0.0	0.0	2.6	1.1	0.0	1.1	2.2	0.0	1.07E-04	8.19E-03	1.88E-01	2.12E-04
	KLF17	0	0	0	0	0	0	0	0	0	0	0	0	0	0	0	0	0.11	0.13	0.10	0.01	0.00	0.03	0.01	0.01	0.10	0.05	0.01	0.0	0.0	0.0	0.0	0.0	0.0	0.0	0.0	0.0	0.0	0.0	1.00E+00	8.46E-01	9.14E-01	9.99E-01
	KLF2	3	12	5	0	0	0	1	4	0	0	0	0	0	0	0	0	0.80	0.92	0.73	0.06	0.02	0.21	0.04	0.04	0.73	0.40	0.05	0.0	1.1	5.5	0.0	0.0	0.0	0.0	0.0	0.0	0.0	0.0	7.22E-02	4.24E-04	7.71E-01	9.15E-01
	KLF3	107	63	12	0	1	1	0	11	0	0	0	0	0	1	0	0	10.50	11.97	9.49	0.80	0.30	2.74	0.58	0.50	9.54	5.26	0.68	0.1	0.0	1.2	0.0	0.0	0.0	0.0	0.0	0.1	0.0	0.0	2.24E-05	3.04E-05	2.94E-01	3.79E-03
	KLF4	34	44	26	0	46	0	6	20	0	0	0	1	0	20	25	0	8.09	9.22	7.31	0.61	0.23	2.11	0.45	0.39	7.34	4.05	0.52	0.0	0.7	2.7	0.0	0.0	0.0	2.2	0.0	2.7	6.2	0.0	1.89E-30	1.64E-07	3.57E-01	3.58E-27
	KLF5	79	86	20	0	9	1	5	14	0	0	0	0	0	7	2	0	9.95	11.34	8.99	0.76	0.29	2.59	0.55	0.48	9.04	4.98	0.64	0.1	0.4	1.6	0.0	0.0	0.0	0.0	0.0	0.8	0.4	0.0	1.54E-02	7.52E-04	3.07E-01	2.59E-01
	KLF6	20	25	6	0	1	0	1	5	0	0	0	0	0	0	1	0	3.62	4.13	3.27	0.28	0.11	0.94	0.20	0.17	3.29	1.81	0.23	0.0	0.2	1.5	0.0	0.0	0.0	0.0	0.0	0.0	0.6	0.0	2.53E-01	3.17E-02	5.37E-01	3.91E-01
	KLF7	0	0	3	0	0	0	3	0	0	0	0	0	0	0	0	0	0.29	0.33	0.26	0.02	0.01	0.08	0.02	0.01	0.27	0.15	0.02	0.0	9.0	0.0	0.0	0.0	0.0	0.0	0.0	0.0	0.0	0.0	1.30E-02	1.78E-05	8.61E-01	9.91E-01
	KLF8	77	68	19	0	25	0	6	13	0	0	10	0	0	11	4	0	12.44	14.18	11.24	0.95	0.36	3.24	0.69	0.60	11.30	6.23	0.81	0.0	0.4	1.2	0.0	0.0	3.1	0.0	0.0	1.0	0.6	0.0	9.31E-05	1.64E-04	2.53E-01	4.49E-03
	KLF9	29	16	7	0	4	0	4	3	0	0	0	1	0	2	1	0	3.29	3.75	2.98	0.25	0.10	0.86	0.18	0.16	2.99	1.65	0.21	0.0	1.1	1.0	0.0	0.0	0.0	5.5	0.0	0.7	0.6	0.0	5.22E-01	1.91E-01	5.56E-01	3.64E-01
	SP1	293	161	102	0	5	9	3	90	0	0	0	0	0	5	0	0	25.36	28.90	22.92	1.93	0.74	6.60	1.41	1.22	23.02	12.70	1.64	0.4	0.1	3.9	0.0	0.0	0.0	0.0	0.0	0.2	0.0	0.0	2.64E-52	1.06E-50	1.03E-01	4.36E-07
	SP2	21	4	0	0	0	0	0	0	0	0	0	0	0	0	0	0	1.61	1.83	1.45	0.12	0.05	0.42	0.09	0.08	1.46	0.81	0.10	0.0	0.0	0.0	0.0	0.0	0.0	0.0	0.0	0.0	0.0	0.0	6.26E-01	8.63E-02	6.81E-01	7.06E-01
	SP3	39	19	22	0	2	1	2	19	0	0	0	0	0	2	0	0	3.77	4.30	3.41	0.29	0.11	0.98	0.21	0.18	3.42	1.89	0.24	0.3	0.5	5.6	0.0	0.0	0.0	0.0	0.0	0.6	0.0	0.0	7.38E-13	6.15E-17	5.29E-01	5.36E-01
	SP4	18	5	1	0	0	0	1	0	0	0	0	0	0	0	0	0	1.98	2.25	1.79	0.15	0.06	0.51	0.11	0.09	1.79	0.99	0.13	0.0	0.4	0.0	0.0	0.0	0.0	0.0	0.0	0.0	0.0	0.0	6.00E-01	1.08E-01	6.49E-01	6.04E-01
	SP5	1	1	0	0	0	0	0	0	0	0	0	0	0	0	0	0	0.11	0.13	0.10	0.01	0.00	0.03	0.01	0.01	0.10	0.05	0.01	0.0	0.0	0.0	0.0	0.0	0.0	0.0	0.0	0.0	0.0	0.0	1.00E+00	8.46E-01	9.14E-01	9.99E-01
	SP6	0	0	0	0	0	0	0	0	0	0	0	0	0	0	0	0	1.21	1.38	1.09	0.09	0.04	0.31	0.07	0.06	1.10	0.60	0.08	0.0	0.0	0.0	0.0	0.0	0.0	0.0	0.0	0.0	0.0	0.0	8.14E-01	1.59E-01	7.22E-01	8.18E-01
	SP7	87	64	27	7	3	11	5	11	7	0	0	0	0	0	3	0	11.16	12.72	10.09	0.85	0.32	2.91	0.62	0.54	10.13	5.59	0.72	1.0	0.4	1.1	8.2	0.0	0.0	0.0	0.0	0.0	0.5	0.0	2.84E-10	9.21E-02	2.05E-11	6.51E-03
	SP8	3	0	0	0	0	0	0	0	0	0	0	0	0	0	0	0	0.11	0.13	0.10	0.01	0.00	0.03	0.01	0.01	0.10	0.05	0.01	0.0	0.0	0.0	0.0	0.0	0.0	0.0	0.0	0.0	0.0	0.0	1.00E+00	8.46E-01	9.14E-01	9.99E-01
	SP9	1	0	0	0	0	0	0	0	0	0	0	0	0	0	0	0	0.04	0.04	0.03	0.00	0.00	0.01	0.00	0.00	0.03	0.02	0.00	0.0	0.0	0.0	0.0	0.0	0.0	0.0	0.0	0.0	0.0	0.0	1.00E+00	9.46E-01	9.51E-01	1.00E+00
	AEBP2	13	47	22	0	1	0	1	21	0	0	0	0	0	1	0	0	3.84	4.38	3.47	0.29	0.11	1.00	0.21	0.18	3.49	1.92	0.25	0.0	0.2	6.0	0.0	0.0	0.0	0.0	0.0	0.3	0.0	0.0	3.98E-17	2.43E-21	5.25E-01	3.75E-01
	OSR1	0	5	0	0	0	0	0	0	0	0	0	0	0	0	0	0	0.22	0.25	0.20	0.02	0.01	0.06	0.01	0.01	0.20	0.11	0.01	0.0	0.0	0.0	0.0	0.0	0.0	0.0	0.0	0.0	0.0	0.0	1.00E+00	7.16E-01	8.79E-01	9.95E-01
	OVOL3	0	0	0	0	0	0	0	0	0	0	0	0	0	0	0	0	0.00	0.00	0.00	0.00	0.00	0.00	0.00	0.00	0.00	0.00	0.00	na	na	na	na	na	na	na	na	na	na	na	na	na	na	na
	ZBTB22	1	6	1	0	0	0	1	0	0	0	0	0	0	0	0	0	0.80	0.92	0.73	0.06	0.02	0.21	0.04	0.04	0.73	0.40	0.05	0.0	1.1	0.0	0.0	0.0	0.0	0.0	0.0	0.0	0.0	0.0	9.79E-01	4.63E-01	7.71E-01	9.15E-01
	ZBTB32	26	8	1	0	0	0	1	0	0	0	0	0	0	0	0	0	3.07	3.50	2.78	0.23	0.09	0.80	0.17	0.15	2.79	1.54	0.20	0.0	0.3	0.0	0.0	0.0	0.0	0.0	0.0	0.0	0.0	0.0	1.91E-01	2.19E-02	5.70E-01	3.42E-01
	ZBTB33	35	42	14	0	0	3	2	9	0	0	0	0	0	0	0	0	6.37	7.26	5.75	0.48	0.18	1.66	0.35	0.31	5.78	3.19	0.41	0.5	0.3	1.6	0.0	0.0	0.0	0.0	0.0	0.0	0.0	0.0	3.13E-02	2.45E-02	4.13E-01	3.91E-02
	ZBTB34	17	4	0	0	0	0	0	0	0	0	0	0	0	0	0	0	1.17	1.33	1.06	0.09	0.03	0.30	0.07	0.06	1.06	0.59	0.08	0.0	0.0	0.0	0.0	0.0	0.0	0.0	0.0	0.0	0.0	0.0	8.29E-01	1.68E-01	7.26E-01	8.28E-01
	ZBTB37	2	0	2	0	0	0	2	0	0	0	0	0	0	0	0	0	0.22	0.25	0.20	0.02	0.01	0.06	0.01	0.01	0.20	0.11	0.01	0.0	8.0	0.0	0.0	0.0	0.0	0.0	0.0	0.0	0.0	0.0	2.19E-01	1.79E-03	8.79E-01	9.95E-01
	ZBTB43	9	10	3	0	0	0	1	2	0	0	0	0	0	0	0	0	2.23	2.54	2.02	0.17	0.06	0.58	0.12	0.11	2.03	1.12	0.14	0.0	0.4	1.0	0.0	0.0	0.0	0.0	0.0	0.0	0.0	0.0	6.77E-01	2.05E-01	6.28E-01	5.35E-01
	ZBTB46	19	14	9	0	0	3	3	3	0	0	0	0	0	0	0	0	2.01	2.29	1.82	0.15	0.06	0.52	0.11	0.10	1.83	1.01	0.13	1.5	1.3	1.6	0.0	0.0	0.0	0.0	0.0	0.0	0.0	0.0	8.64E-01	4.80E-01	6.46E-01	5.94E-01
	ZBTB5	19	10	8	0	0	0	1	7	0	0	0	0	0	0	0	0	2.60	2.96	2.35	0.20	0.08	0.68	0.14	0.12	2.36	1.30	0.17	0.0	0.3	3.0	0.0	0.0	0.0	0.0	0.0	0.0	0.0	0.0	5.23E-02	1.42E-03	6.01E-01	4.44E-01
	ZBTB8B	0	3	2	0	0	1	0	1	0	0	0	0	0	0	0	0	0.40	0.46	0.36	0.03	0.01	0.10	0.02	0.02	0.37	0.20	0.03	2.5	0.0	2.7	0.0	0.0	0.0	0.0	0.0	0.0	0.0	0.0	9.75E-01	2.92E-01	8.37E-01	9.81E-01
	ZFPM2	19	11	7	0	1	1	0	6	0	0	0	1	0	0	0	0	1.50	1.71	1.36	0.11	0.04	0.39	0.08	0.07	1.36	0.75	0.10	0.7	0.0	4.4	0.0	0.0	0.0	12.0	0.0	0.0	0.0	0.0	6.66E-04	1.37E-04	6.91E-01	2.61E-02
	ZNF174	57	11	2	0	1	0	2	0	0	0	1	0	0	0	0	0	3.55	4.04	3.21	0.27	0.10	0.92	0.20	0.17	3.22	1.78	0.23	0.0	0.5	0.0	0.0	0.0	1.1	0.0	0.0	0.0	0.0	0.0	1.84E-01	2.03E-02	5.41E-01	3.47E-01
	ZNF396	36	7	1	0	0	0	0	1	0	0	0	0	0	0	0	0	1.87	2.13	1.69	0.14	0.05	0.49	0.10	0.09	1.69	0.93	0.12	0.0	0.0	0.6	0.0	0.0	0.0	0.0	0.0	0.0	0.0	0.0	6.39E-01	1.18E-01	6.58E-01	6.34E-01
	ZNF414	2	20	4	0	0	0	0	4	0	0	0	0	0	0	0	0	5.56	6.34	5.03	0.42	0.16	1.45	0.31	0.27	5.05	2.79	0.36	0.0	0.0	0.8	0.0	0.0	0.0	0.0	0.0	0.0	0.0	0.0	1.11E-02	2.35E-03	4.45E-01	6.92E-02
	ZNF446	85	19	1	0	0	0	0	1	0	0	0	0	0	0	0	0	5.49	6.26	4.96	0.42	0.16	1.43	0.31	0.26	4.98	2.75	0.36	0.0	0.0	0.2	0.0	0.0	0.0	0.0	0.0	0.0	0.0	0.0	4.37E-03	5.80E-04	4.48E-01	7.28E-02
	ZNF487	0	2	0	0	0	0	0	0	0	0	0	0	0	0	0	0	0.07	0.08	0.07	0.01	0.00	0.02	0.00	0.00	0.07	0.04	0.00	0.0	0.0	0.0	0.0	0.0	0.0	0.0	0.0	0.0	0.0	0.0	1.00E+00	8.95E-01	9.30E-01	1.00E+00
	ZNF511	5	2	6	0	6	5	0	1	0	0	0	6	0	0	0	0	2.16	2.46	1.95	0.16	0.06	0.56	0.12	0.10	1.96	1.08	0.14	2.3	0.0	0.5	0.0	0.0	0.0	49.9	0.0	0.0	0.0	0.0	3.67E-58	3.57E-02	6.34E-01	7.26E-61
	ZNF580	6	3	0	0	0	0	0	0	0	0	0	0	0	0	0	0	1.17	1.33	1.06	0.09	0.03	0.30	0.07	0.06	1.06	0.59	0.08	0.0	0.0	0.0	0.0	0.0	0.0	0.0	0.0	0.0	0.0	0.0	8.29E-01	1.68E-01	7.26E-01	8.28E-01
	ZNF705A	0	0	0	0	0	0	0	0	0	0	0	0	0	0	0	0	0.00	0.00	0.00	0.00	0.00	0.00	0.00	0.00	0.00	0.00	0.00	na	na	na	na	na	na	na	na	na	na	na	na	na	na	na
	ZNF740	3	9	4	0	0	0	2	2	0	0	0	0	0	0	0	0	0.88	1.00	0.79	0.07	0.03	0.23	0.05	0.04	0.80	0.44	0.06	0.0	2.0	2.5	0.0	0.0	0.0	0.0	0.0	0.0	0.0	0.0	8.62E-01	1.56E-01	7.61E-01	9.00E-01
	ZSCAN1	36	16	4	0	0	3	0	1	0	0	0	0	0	0	0	0	2.74	3.13	2.48	0.21	0.08	0.71	0.15	0.13	2.49	1.37	0.18	1.1	0.0	0.4	0.0	0.0	0.0	0.0	0.0	0.0	0.0	0.0	4.98E-01	1.33E-01	5.91E-01	4.11E-01
	BCL6	84	72	26	0	4	2	4	20	0	0	2	0	1	0	0	1	12.07	13.76	10.91	0.92	0.35	3.14	0.67	0.58	10.96	6.05	0.78	0.2	0.3	1.8	0.0	0.0	0.6	0.0	1.7	0.0	0.0	1.3	5.79E-06	1.07E-05	2.60E-01	2.42E-03
	BCL6B	6	5	1	0	0	0	0	1	0	0	0	0	0	0	0	0	1.39	1.58	1.26	0.11	0.04	0.36	0.08	0.07	1.26	0.70	0.09	0.0	0.0	0.8	0.0	0.0	0.0	0.0	0.0	0.0	0.0	0.0	8.38E-01	2.20E-01	7.02E-01	7.68E-01
	CTCF	58	67	53	0	164	9	24	20	0	0	11	1	0	133	19	0	19.80	22.56	17.89	1.51	0.57	5.16	1.10	0.95	17.97	9.91	1.28	0.5	1.1	1.1	0.0	0.0	2.1	0.9	0.0	7.4	1.9	0.0	3.66E-157	4.44E-02	1.49E-01	1.45E-160
	CTCFL	3	7	3	0	0	0	1	2	0	0	0	0	0	0	0	0	1.68	1.92	1.52	0.13	0.05	0.44	0.09	0.08	1.53	0.84	0.11	0.0	0.5	1.3	0.0	0.0	0.0	0.0	0.0	0.0	0.0	0.0	8.52E-01	3.21E-01	6.74E-01	6.86E-01
	FEZF1	0	5	1	0	0	0	0	1	0	0	0	0	0	0	0	0	1.02	1.17	0.93	0.08	0.03	0.27	0.06	0.05	0.93	0.51	0.07	0.0	0.0	1.1	0.0	0.0	0.0	0.0	0.0	0.0	0.0	0.0	9.38E-01	3.33E-01	7.43E-01	8.65E-01
	FEZF2	0	2	2	0	1	0	0	2	0	0	1	0	0	0	0	0	0.18	0.21	0.17	0.01	0.01	0.05	0.01	0.01	0.17	0.09	0.01	0.0	0.0	12.1	0.0	0.0	21.0	0.0	0.0	0.0	0.0	0.0	1.63E-05	3.12E-05	8.90E-01	1.67E-03
	GFI1	13	17	8	0	0	0	0	8	0	0	0	0	0	0	0	0	1.46	1.67	1.32	0.11	0.04	0.38	0.08	0.07	1.33	0.73	0.09	0.0	0.0	6.0	0.0	0.0	0.0	0.0	0.0	0.0	0.0	0.0	1.93E-05	1.00E-08	6.95E-01	7.48E-01
	GFI1B	11	49	26	0	0	0	2	24	0	0	0	0	0	0	0	0	4.98	5.67	4.50	0.38	0.14	1.30	0.28	0.24	4.52	2.49	0.32	0.0	0.4	5.3	0.0	0.0	0.0	0.0	0.0	0.0	0.0	0.0	2.61E-17	1.09E-20	4.70E-01	1.03E-01
	GLI1	15	86	21	0	5	4	2	15	0	0	1	0	1	2	0	1	7.03	8.01	6.35	0.53	0.20	1.83	0.39	0.34	6.38	3.52	0.45	0.6	0.2	2.4	0.0	0.0	0.5	0.0	3.0	0.3	0.0	2.2	2.11E-03	1.51E-04	3.90E-01	9.96E-02
	GLI2	9	32	6	0	0	0	2	4	0	0	0	0	0	0	0	0	3.00	3.42	2.71	0.23	0.09	0.78	0.17	0.14	2.72	1.50	0.19	0.0	0.6	1.5	0.0	0.0	0.0	0.0	0.0	0.0	0.0	0.0	4.38E-01	1.22E-01	5.75E-01	3.56E-01
	GLI3	14	26	4	0	2	0	1	3	0	0	1	0	0	1	0	0	3.55	4.04	3.21	0.27	0.10	0.92	0.20	0.17	3.22	1.78	0.23	0.0	0.2	0.9	0.0	0.0	1.1	0.0	0.0	0.3	0.0	0.0	4.28E-01	5.35E-02	5.41E-01	5.62E-01
	GLI4	29	3	6	1	31	1	3	2	0	1	0	0	0	7	23	1	4.65	5.30	4.20	0.35	0.13	1.21	0.26	0.22	4.22	2.33	0.30	0.2	0.6	0.5	0.0	7.4	0.0	0.0	0.0	1.7	9.9	3.3	1.88E-37	8.17E-02	1.51E-02	7.18E-39
	GLIS1	10	1	2	0	0	0	1	1	0	0	0	0	0	0	0	0	0.62	0.71	0.56	0.05	0.02	0.16	0.03	0.03	0.56	0.31	0.04	0.0	1.4	1.8	0.0	0.0	0.0	0.0	0.0	0.0	0.0	0.0	9.94E-01	5.82E-01	7.98E-01	9.50E-01
	GLIS2	18	24	2	1	0	2	0	0	1	0	0	0	0	0	0	0	4.06	4.63	3.67	0.31	0.12	1.06	0.23	0.20	3.69	2.03	0.26	0.5	0.0	0.0	3.2	0.0	0.0	0.0	0.0	0.0	0.0	0.0	4.75E-02	9.35E-03	1.97E-01	1.88E-01
	GLIS3	2	23	1	0	0	0	0	1	0	0	0	0	0	0	0	0	1.46	1.67	1.32	0.11	0.04	0.38	0.08	0.07	1.33	0.73	0.09	0.0	0.0	0.8	0.0	0.0	0.0	0.0	0.0	0.0	0.0	0.0	8.11E-01	2.01E-01	6.95E-01	7.48E-01
	GTF3A	0	1	0	0	0	0	0	0	0	0	0	0	0	0	0	0	0.44	0.50	0.40	0.03	0.01	0.11	0.02	0.02	0.40	0.22	0.03	0.0	0.0	0.0	0.0	0.0	0.0	0.0	0.0	0.0	0.0	0.0	9.95E-01	5.13E-01	8.30E-01	9.77E-01
	GZF1	16	8	2	0	30	1	1	0	0	0	0	0	0	30	0	0	3.15	3.59	2.84	0.24	0.09	0.82	0.18	0.15	2.86	1.58	0.20	0.3	0.3	0.0	0.0	0.0	0.0	0.0	0.0	10.5	0.0	0.0	1.25E-51	4.57E-02	5.65E-01	2.68E-54
	MTF1	3	17	29	0	30	11	15	3	0	0	0	0	0	30	0	0	4.24	4.84	3.84	0.32	0.12	1.11	0.24	0.20	3.85	2.13	0.27	2.6	3.1	0.8	0.0	0.0	0.0	0.0	0.0	7.8	0.0	0.0	1.87E-40	9.76E-08	5.04E-01	2.78E-37
	MYNN	0	4	1	0	0	0	1	0	0	0	0	0	0	0	0	0	0.48	0.54	0.43	0.04	0.01	0.12	0.03	0.02	0.43	0.24	0.03	0.0	1.8	0.0	0.0	0.0	0.0	0.0	0.0	0.0	0.0	0.0	9.94E-01	5.24E-01	8.23E-01	9.72E-01
	MZF1	10	6	1	0	1	0	1	0	0	0	0	0	0	0	1	0	1.28	1.46	1.16	0.10	0.04	0.33	0.07	0.06	1.16	0.64	0.08	0.0	0.7	0.0	0.0	0.0	0.0	0.0	0.0	0.0	1.6	0.0	9.14E-01	2.75E-01	7.14E-01	8.61E-01
	OSR2	0	1	1	0	0	0	1	0	0	0	0	0	0	0	0	0	0.29	0.33	0.26	0.02	0.01	0.08	0.02	0.01	0.27	0.15	0.02	0.0	3.0	0.0	0.0	0.0	0.0	0.0	0.0	0.0	0.0	0.0	9.91E-01	3.89E-01	8.61E-01	9.91E-01
	OVOL1	4	8	6	0	0	0	0	6	0	0	0	0	0	0	0	0	0.59	0.67	0.53	0.04	0.02	0.15	0.03	0.03	0.53	0.29	0.04	0.0	0.0	11.3	0.0	0.0	0.0	0.0	0.0	0.0	0.0	0.0	5.70E-09	2.78E-13	8.04E-01	9.56E-01
	OVOL2	20	33	17	0	1	4	5	8	0	0	0	0	0	1	0	0	5.16	5.88	4.66	0.39	0.15	1.34	0.29	0.25	4.68	2.58	0.33	0.8	0.9	1.7	0.0	0.0	0.0	0.0	0.0	0.2	0.0	0.0	3.56E-01	2.49E-01	4.62E-01	1.74E-01
	PLAG1	4	0	0	0	0	0	0	0	0	0	0	0	0	0	0	0	0.33	0.38	0.30	0.03	0.01	0.09	0.02	0.02	0.30	0.16	0.02	0.0	0.0	0.0	0.0	0.0	0.0	0.0	0.0	0.0	0.0	0.0	9.98E-01	6.06E-01	8.52E-01	9.88E-01
	PLAGL1	6	11	7	0	0	0	0	7	0	0	0	0	0	0	0	0	1.02	1.17	0.93	0.08	0.03	0.27	0.06	0.05	0.93	0.51	0.07	0.0	0.0	7.6	0.0	0.0	0.0	0.0	0.0	0.0	0.0	0.0	3.25E-06	7.44E-10	7.43E-01	8.65E-01
	PLAGL2	9	7	5	0	0	1	1	3	0	0	0	0	0	0	0	0	2.20	2.50	1.98	0.17	0.06	0.57	0.12	0.11	1.99	1.10	0.14	0.5	0.4	1.5	0.0	0.0	0.0	0.0	0.0	0.0	0.0	0.0	7.86E-01	3.55E-01	6.31E-01	5.44E-01
	PRDM1	32	33	22	0	0	0	2	20	0	0	0	0	0	0	0	0	3.88	4.42	3.51	0.29	0.11	1.01	0.22	0.19	3.52	1.94	0.25	0.0	0.5	5.7	0.0	0.0	0.0	0.0	0.0	0.0	0.0	0.0	4.54E-15	1.03E-18	5.23E-01	2.11E-01
	PRDM14	15	25	13	0	1	4	0	9	0	0	0	0	0	0	1	0	4.83	5.50	4.36	0.37	0.14	1.26	0.27	0.23	4.39	2.42	0.31	0.8	0.0	2.1	0.0	0.0	0.0	0.0	0.0	0.0	0.4	0.0	4.91E-02	5.07E-03	4.76E-01	2.00E-01
	PRDM5	17	10	4	0	6	0	0	4	0	0	0	0	0	6	0	0	2.96	3.38	2.68	0.23	0.09	0.77	0.17	0.14	2.69	1.48	0.19	0.0	0.0	1.5	0.0	0.0	0.0	0.0	0.0	2.2	0.0	0.0	1.67E-01	3.03E-02	5.77E-01	2.34E-01
	PRDM6	12	18	6	1	1	0	1	5	1	0	0	0	0	0	1	0	3.44	3.92	3.11	0.26	0.10	0.90	0.19	0.17	3.12	1.72	0.22	0.0	0.3	1.6	3.8	0.0	0.0	0.0	0.0	0.0	0.6	0.0	1.80E-01	3.40E-02	1.39E-01	4.28E-01
	RBAK	21	6	3	0	2	0	0	3	0	0	0	0	0	2	0	0	2.45	2.79	2.22	0.19	0.07	0.64	0.14	0.12	2.23	1.23	0.16	0.0	0.0	1.4	0.0	0.0	0.0	0.0	0.0	0.9	0.0	0.0	6.21E-01	6.32E-02	6.12E-01	8.06E-01
	SCRT1	1	2	0	0	0	0	0	0	0	0	0	0	0	0	0	0	0.33	0.38	0.30	0.03	0.01	0.09	0.02	0.02	0.30	0.16	0.02	0.0	0.0	0.0	0.0	0.0	0.0	0.0	0.0	0.0	0.0	0.0	9.98E-01	6.06E-01	8.52E-01	9.88E-01
	SCRT2	0	2	0	0	0	0	0	0	0	0	0	0	0	0	0	0	0.33	0.38	0.30	0.03	0.01	0.09	0.02	0.02	0.30	0.16	0.02	0.0	0.0	0.0	0.0	0.0	0.0	0.0	0.0	0.0	0.0	0.0	9.98E-01	6.06E-01	8.52E-01	9.88E-01
	SNAI1	43	156	107	2	64	29	29	49	2	0	9	1	2	45	5	2	26.64	30.36	24.07	2.03	0.77	6.94	1.48	1.28	24.19	13.34	1.72	1.1	1.0	2.0	1.0	0.0	1.3	0.7	1.6	1.9	0.4	1.2	1.60E-07	2.17E-06	3.79E-01	1.86E-04
	SNAI2	7	42	9	0	0	0	0	9	0	0	0	0	0	0	0	0	2.93	3.34	2.65	0.22	0.08	0.76	0.16	0.14	2.66	1.47	0.19	0.0	0.0	3.4	0.0	0.0	0.0	0.0	0.0	0.0	0.0	0.0	2.41E-03	2.12E-05	5.79E-01	3.71E-01
	SNAI3	0	8	5	0	0	0	1	4	0	0	0	0	0	0	0	0	0.51	0.58	0.46	0.04	0.01	0.13	0.03	0.02	0.47	0.26	0.03	0.0	1.7	8.6	0.0	0.0	0.0	0.0	0.0	0.0	0.0	0.0	1.33E-03	9.04E-07	8.17E-01	9.67E-01
	WT1	18	16	11	0	0	3	5	3	0	0	0	0	0	0	0	0	3.37	3.84	3.04	0.26	0.10	0.88	0.19	0.16	3.06	1.69	0.22	0.9	1.3	1.0	0.0	0.0	0.0	0.0	0.0	0.0	0.0	0.0	7.32E-01	8.21E-01	5.52E-01	2.88E-01
	YY1	167	210	84	4	7	11	7	66	4	0	0	0	1	5	1	0	25.36	28.90	22.92	1.93	0.74	6.60	1.41	1.22	23.02	12.70	1.64	0.4	0.2	2.9	2.1	0.0	0.0	0.0	0.8	0.2	0.1	0.0	8.99E-26	1.10E-23	8.52E-02	1.82E-06
	YY2	6	3	2	0	0	0	1	1	0	0	0	0	0	0	0	0	0.55	0.63	0.50	0.04	0.02	0.14	0.03	0.03	0.50	0.27	0.04	0.0	1.6	2.0	0.0	0.0	0.0	0.0	0.0	0.0	0.0	0.0	9.93E-01	5.26E-01	8.10E-01	9.62E-01
	ZBTB11	18	10	10	0	27	1	5	4	0	0	0	0	0	27	0	0	4.43	5.05	4.00	0.34	0.13	1.15	0.25	0.21	4.02	2.22	0.29	0.2	1.0	1.0	0.0	0.0	0.0	0.0	0.0	6.7	0.0	0.0	8.17E-25	2.65E-01	4.95E-01	1.64E-27
	ZBTB12	1	1	0	0	0	0	0	0	0	0	0	0	0	0	0	0	0.29	0.33	0.26	0.02	0.01	0.08	0.02	0.01	0.27	0.15	0.02	0.0	0.0	0.0	0.0	0.0	0.0	0.0	0.0	0.0	0.0	0.0	9.99E-01	6.41E-01	8.61E-01	9.91E-01
	ZBTB14	15	11	8	0	4	3	3	2	0	0	0	0	0	3	1	0	3.77	4.30	3.41	0.29	0.11	0.98	0.21	0.18	3.42	1.89	0.24	0.8	0.7	0.6	0.0	0.0	0.0	0.0	0.0	0.9	0.5	0.0	9.63E-01	5.69E-01	5.29E-01	8.37E-01
	ZBTB16	69	70	41	1	1	1	1	39	1	0	1	0	0	0	0	0	11.16	12.72	10.09	0.85	0.32	2.91	0.62	0.54	10.13	5.59	0.72	0.1	0.1	3.9	1.2	0.0	0.3	0.0	0.0	0.0	0.0	0.0	1.86E-21	4.44E-23	5.54E-01	2.05E-03
	ZBTB18	12	23	5	0	0	3	1	1	0	0	0	0	0	0	0	0	2.82	3.21	2.55	0.21	0.08	0.73	0.16	0.14	2.56	1.41	0.18	1.1	0.3	0.4	0.0	0.0	0.0	0.0	0.0	0.0	0.0	0.0	6.34E-01	2.90E-01	5.86E-01	3.95E-01
	ZBTB20	6	4	3	0	0	0	3	0	0	0	0	0	0	0	0	0	1.46	1.67	1.32	0.11	0.04	0.38	0.08	0.07	1.33	0.73	0.09	0.0	1.8	0.0	0.0	0.0	0.0	0.0	0.0	0.0	0.0	0.0	7.54E-01	1.46E-01	6.95E-01	7.48E-01
	ZBTB24	48	20	8	0	27	1	4	3	0	0	0	0	0	26	1	0	7.24	8.26	6.55	0.55	0.21	1.89	0.40	0.35	6.58	3.63	0.47	0.1	0.5	0.5	0.0	0.0	0.0	0.0	0.0	4.0	0.3	0.0	1.38E-11	8.65E-03	3.83E-01	3.96E-12
	ZBTB26	12	10	1	0	1	0	0	1	0	0	0	1	0	0	0	0	1.72	1.96	1.55	0.13	0.05	0.45	0.10	0.08	1.56	0.86	0.11	0.0	0.0	0.6	0.0	0.0	0.0	10.4	0.0	0.0	0.0	0.0	1.10E-01	1.44E-01	6.71E-01	4.07E-02
	ZBTB40	9	1	1	0	0	0	1	0	0	0	0	0	0	0	0	0	0.88	1.00	0.79	0.07	0.03	0.23	0.05	0.04	0.80	0.44	0.06	0.0	1.0	0.0	0.0	0.0	0.0	0.0	0.0	0.0	0.0	0.0	9.71E-01	4.33E-01	7.61E-01	9.00E-01
	ZBTB42	7	10	1	0	1	0	1	0	0	0	0	1	0	0	0	0	2.82	3.21	2.55	0.21	0.08	0.73	0.16	0.14	2.56	1.41	0.18	0.0	0.3	0.0	0.0	0.0	0.0	6.4	0.0	0.0	0.0	0.0	8.05E-02	3.20E-02	5.86E-01	8.90E-02
	ZBTB44	7	13	2	1	0	0	1	1	0	1	0	0	0	0	0	0	2.96	3.38	2.68	0.23	0.09	0.77	0.17	0.14	2.69	1.48	0.19	0.0	0.3	0.4	0.0	11.6	0.0	0.0	0.0	0.0	0.0	0.0	2.06E-02	5.82E-02	1.62E-03	3.64E-01
	ZBTB45	3	2	0	0	0	0	0	0	0	0	0	0	0	0	0	0	0.22	0.25	0.20	0.02	0.01	0.06	0.01	0.01	0.20	0.11	0.01	0.0	0.0	0.0	0.0	0.0	0.0	0.0	0.0	0.0	0.0	0.0	1.00E+00	7.16E-01	8.79E-01	9.95E-01
	ZBTB47	32	9	6	0	16	1	5	0	0	0	0	0	0	8	8	0	5.34	6.09	4.83	0.41	0.15	1.39	0.30	0.26	4.85	2.68	0.35	0.2	0.8	0.0	0.0	0.0	0.0	0.0	0.0	1.6	3.0	0.0	7.48E-03	1.39E-02	4.54E-01	1.07E-02
	ZBTB48	39	24	16	0	44	2	8	6	0	0	0	0	0	13	31	0	8.56	9.76	7.74	0.65	0.25	2.23	0.48	0.41	7.77	4.29	0.55	0.2	0.8	0.8	0.0	0.0	0.0	0.0	0.0	1.7	7.2	0.0	2.13E-33	5.68E-02	3.43E-01	1.27E-35
	ZBTB49	11	2	6	0	0	0	1	5	0	0	0	0	0	0	0	0	1.43	1.63	1.29	0.11	0.04	0.37	0.08	0.07	1.30	0.71	0.09	0.0	0.6	3.9	0.0	0.0	0.0	0.0	0.0	0.0	0.0	0.0	1.28E-01	2.09E-03	6.99E-01	7.58E-01
	ZBTB6	8	3	0	0	0	0	0	0	0	0	0	0	0	0	0	0	0.62	0.71	0.56	0.05	0.02	0.16	0.03	0.03	0.56	0.31	0.04	0.0	0.0	0.0	0.0	0.0	0.0	0.0	0.0	0.0	0.0	0.0	9.79E-01	3.88E-01	7.98E-01	9.50E-01
	ZBTB7A	64	36	28	0	11	3	10	15	0	0	3	0	0	8	0	0	7.06	8.05	6.38	0.54	0.20	1.84	0.39	0.34	6.41	3.54	0.46	0.4	1.2	2.4	0.0	0.0	1.6	0.0	0.0	1.2	0.0	0.0	2.08E-02	7.29E-04	3.89E-01	3.21E-01
	ZBTB7B	23	15	11	0	2	1	2	8	0	0	0	0	0	2	0	0	2.96	3.38	2.68	0.23	0.09	0.77	0.17	0.14	2.69	1.48	0.19	0.3	0.6	3.0	0.0	0.0	0.0	0.0	0.0	0.7	0.0	0.0	1.09E-01	1.99E-03	5.77E-01	7.10E-01
	ZBTB7C	20	0	1	0	0	1	0	0	0	0	0	0	0	0	0	0	0.84	0.96	0.76	0.06	0.02	0.22	0.05	0.04	0.76	0.42	0.05	1.2	0.0	0.0	0.0	0.0	0.0	0.0	0.0	0.0	0.0	0.0	9.71E-01	4.17E-01	7.66E-01	9.08E-01
	ZFP1	2	11	4	0	0	1	0	3	0	0	0	0	0	0	0	0	2.23	2.54	2.02	0.17	0.06	0.58	0.12	0.11	2.03	1.12	0.14	0.4	0.0	1.5	0.0	0.0	0.0	0.0	0.0	0.0	0.0	0.0	6.25E-01	1.57E-01	6.28E-01	5.35E-01
	ZFP14	0	2	0	0	0	0	0	0	0	0	0	0	0	0	0	0	0.29	0.33	0.26	0.02	0.01	0.08	0.02	0.01	0.27	0.15	0.02	0.0	0.0	0.0	0.0	0.0	0.0	0.0	0.0	0.0	0.0	0.0	9.99E-01	6.41E-01	8.61E-01	9.91E-01
	ZFP2	3	1	0	0	0	0	0	0	0	0	0	0	0	0	0	0	0.66	0.75	0.60	0.05	0.02	0.17	0.04	0.03	0.60	0.33	0.04	0.0	0.0	0.0	0.0	0.0	0.0	0.0	0.0	0.0	0.0	0.0	9.74E-01	3.67E-01	7.93E-01	9.44E-01

ZFP28	0	1	1	0	0	0	0	1	0	0	0	0	0	0	0	0	0.18	0.21	0.17	0.01	0.01	0.05	0.01	0.01	0.17	0.09	0.01	0.0	0.0	6.0	0.0	0.0	0.0	0.0	0.0	0.0	0.0	0.0	8.94E-01	1.00E-01	8.90E-01	9.97E-01
ZFP3	0	0	0	0	0	0	0	0	0	0	0	0	0	0	0	0	0.11	0.13	0.10	0.01	0.00	0.03	0.01	0.01	0.10	0.05	0.01	0.0	0.0	0.0	0.0	0.0	0.0	0.0	0.0	0.0	0.0	0.0	1.00E+00	8.46E-01	9.14E-01	9.99E-01
ZFP30	3	6	1	0	0	0	0	1	0	0	0	0	0	0	0	0	0.44	0.50	0.40	0.03	0.01	0.11	0.02	0.02	0.40	0.22	0.03	0.0	0.0	2.5	0.0	0.0	0.0	0.0	0.0	0.0	0.0	0.0	9.87E-01	3.95E-01	8.30E-01	9.77E-01
ZFP37	0	2	0	0	0	0	0	0	0	0	0	0	0	0	0	0	0.22	0.25	0.20	0.02	0.01	0.06	0.01	0.01	0.20	0.11	0.01	0.0	0.0	0.0	0.0	0.0	0.0	0.0	0.0	0.0	0.0	0.0	1.00E+00	7.16E-01	8.79E-01	9.95E-01
ZFP41	4	14	3	0	0	2	1	0	0	0	0	0	0	0	0	0	4.54	5.17	4.10	0.34	0.13	1.18	0.25	0.22	4.12	2.27	0.29	0.4	0.2	0.0	0.0	0.0	0.0	0.0	0.0	0.0	0.0	0.0	6.03E-02	1.18E-02	4.90E-01	1.39E-01
ZFP42	5	6	1	0	0	0	0	1	0	0	0	0	0	0	0	0	0.59	0.67	0.53	0.04	0.02	0.15	0.03	0.03	0.53	0.29	0.04	0.0	0.0	1.9	0.0	0.0	0.0	0.0	0.0	0.0	0.0	0.0	9.86E-01	4.33E-01	8.04E-01	9.56E-01
ZFP62	15	13	3	0	18	0	2	1	0	0	0	0	0	18	0	0	3.07	3.50	2.78	0.23	0.09	0.80	0.17	0.15	2.79	1.54	0.20	0.0	0.6	0.4	0.0	0.0	0.0	0.0	0.0	6.4	0.0	0.0	3.51E-15	8.82E-02	5.70E-01	5.25E-17
ZFP64	7	10	4	0	0	2	1	1	0	0	0	0	0	0	0	0	1.87	2.13	1.69	0.14	0.05	0.49	0.10	0.09	1.69	0.93	0.12	1.1	0.5	0.6	0.0	0.0	0.0	0.0	0.0	0.0	0.0	0.0	9.21E-01	6.42E-01	6.58E-01	6.34E-01
ZFP69	0	0	1	0	0	0	1	0	0	0	0	0	0	0	0	0	0.04	0.04	0.03	0.00	0.00	0.01	0.00	0.00	0.03	0.02	0.00	0.0	24.0	0.0	0.0	0.0	0.0	0.0	0.0	0.0	0.0	0.0	1.43E-02	1.60E-05	9.51E-01	1.00E+00
ZFP69B	1	1	1	0	0	0	1	0	0	0	0	0	0	0	0	0	0.48	0.54	0.43	0.04	0.01	0.12	0.03	0.02	0.43	0.24	0.03	0.0	1.8	0.0	0.0	0.0	0.0	0.0	0.0	0.0	0.0	0.0	9.94E-01	5.24E-01	8.23E-01	9.72E-01
ZFP82	1	5	1	0	0	0	0	1	0	0	0	0	0	0	0	0	0.44	0.50	0.40	0.03	0.01	0.11	0.02	0.02	0.40	0.22	0.03	0.0	0.0	2.5	0.0	0.0	0.0	0.0	0.0	0.0	0.0	0.0	9.87E-01	3.95E-01	8.30E-01	9.77E-01
ZFP90	1	3	0	0	2	0	0	0	0	0	0	0	0	2	0	0	1.10	1.25	0.99	0.08	0.03	0.29	0.06	0.05	1.00	0.55	0.07	0.0	0.0	0.0	0.0	0.0	0.0	0.0	0.0	2.0	0.0	0.0	8.56E-01	1.88E-01	7.34E-01	8.45E-01
ZFP91	26	24	13	0	16	0	10	3	0	0	0	0	0	14	2	0	5.56	6.34	5.03	0.42	0.16	1.45	0.31	0.27	5.05	2.79	0.36	0.0	1.6	0.6	0.0	0.0	0.0	0.0	0.0	2.8	0.7	0.0	2.13E-03	1.43E-02	4.45E-01	2.41E-03
ZFP92	0	2	0	0	0	0	0	0	0	0	0	0	0	0	0	0	0.07	0.08	0.07	0.01	0.00	0.02	0.00	0.00	0.07	0.04	0.00	0.0	0.0	0.0	0.0	0.0	0.0	0.0	0.0	0.0	0.0	0.0	1.00E+00	8.95E-01	9.30E-01	1.00E+00
ZFX	4	2	2	0	1	0	1	1	0	0	0	0	0	1	0	0	0.62	0.71	0.56	0.05	0.02	0.16	0.03	0.03	0.56	0.31	0.04	0.0	1.4	1.8	0.0	0.0	0.0	0.0	0.0	1.8	0.0	0.0	9.96E-01	5.82E-01	7.98E-01	9.69E-01
ZFY	3	5	0	0	0	0	0	0	0	0	0	0	0	0	0	0	0.51	0.58	0.46	0.04	0.01	0.13	0.03	0.02	0.47	0.26	0.03	0.0	0.0	0.0	0.0	0.0	0.0	0.0	0.0	0.0	0.0	0.0	9.90E-01	4.59E-01	8.17E-01	9.67E-01
ZIC1	17	12	0	0	0	0	0	0	0	0	0	0	0	0	0	0	5.56	6.34	5.03	0.42	0.16	1.45	0.31	0.27	5.05	2.79	0.36	0.0	0.0	0.0	0.0	0.0	0.0	0.0	0.0	0.0	0.0	0.0	1.99E-03	2.11E-04	4.45E-01	6.92E-02
ZIC2	9	5	2	0	0	0	1	1	0	0	0	0	0	0	0	0	1.57	1.79	1.42	0.12	0.05	0.41	0.09	0.08	1.43	0.79	0.10	0.0	0.6	0.7	0.0	0.0	0.0	0.0	0.0	0.0	0.0	0.0	8.84E-01	3.59E-01	6.84E-01	7.17E-01
ZIC3	8	2	0	0	0	0	0	0	0	0	0	0	0	0	0	0	0.91	1.04	0.83	0.07	0.03	0.24	0.05	0.04	0.83	0.46	0.06	0.0	0.0	0.0	0.0	0.0	0.0	0.0	0.0	0.0	0.0	0.0	9.19E-01	2.49E-01	7.57E-01	8.91E-01
ZIC4	0	0	0	0	0	0	0	0	0	0	0	0	0	0	0	0	0.07	0.08	0.07	0.01	0.00	0.02	0.00	0.00	0.07	0.04	0.00	0.0	0.0	0.0	0.0	0.0	0.0	0.0	0.0	0.0	0.0	0.0	1.00E+00	8.95E-01	9.30E-01	1.00E+00
ZIC5	2	1	0	0	0	0	0	0	0	0	0	0	0	0	0	0	0.18	0.21	0.17	0.01	0.01	0.05	0.01	0.01	0.17	0.09	0.01	0.0	0.0	0.0	0.0	0.0	0.0	0.0	0.0	0.0	0.0	0.0	1.00E+00	7.57E-01	8.90E-01	9.97E-01
ZIK1	0	0	0	0	0	0	0	0	0	0	0	0	0	0	0	0	0.11	0.13	0.10	0.01	0.00	0.03	0.01	0.01	0.10	0.05	0.01	0.0	0.0	0.0	0.0	0.0	0.0	0.0	0.0	0.0	0.0	0.0	1.00E+00	8.46E-01	9.14E-01	9.99E-01
ZIM2	5	11	0	0	0	0	0	0	0	0	0	0	0	0	0	0	2.09	2.38	1.88	0.16	0.06	0.54	0.12	0.10	1.89	1.04	0.14	0.0	0.0	0.0	0.0	0.0	0.0	0.0	0.0	0.0	0.0	0.0	4.06E-01	4.18E-02	6.40E-01	5.74E-01
ZIM3	0	2	0	0	0	0	0	0	0	0	0	0	0	0	0	0	0.11	0.13	0.10	0.01	0.00	0.03	0.01	0.01	0.10	0.05	0.01	0.0	0.0	0.0	0.0	0.0	0.0	0.0	0.0	0.0	0.0	0.0	1.00E+00	8.46E-01	9.14E-01	9.99E-01
ZKSCAN1	30	16	2	0	0	0	2	0	0	0	0	0	0	0	0	0	3.33	3.79	3.01	0.25	0.10	0.87	0.19	0.16	3.02	1.67	0.22	0.0	0.5	0.0	0.0	0.0	0.0	0.0	0.0	0.0	0.0	0.0	1.89E-01	2.75E-02	5.54E-01	2.95E-01
ZKSCAN2	2	1	2	0	0	0	1	1	0	0	0	0	0	0	0	0	0.29	0.33	0.26	0.02	0.01	0.08	0.02	0.01	0.27	0.15	0.02	0.0	3.0	3.8	0.0	0.0	0.0	0.0	0.0	0.0	0.0	0.0	9.36E-01	1.60E-01	8.61E-01	9.91E-01
ZKSCAN3	30	6	0	0	0	0	0	0	0	0	0	0	0	0	0	0	1.57	1.79	1.42	0.12	0.05	0.41	0.09	0.08	1.43	0.79	0.10	0.0	0.0	0.0	0.0	0.0	0.0	0.0	0.0	0.0	0.0	0.0	6.44E-01	9.12E-02	6.84E-01	7.17E-01
ZKSCAN4	77	18	2	0	1	1	0	1	0	0	0	0	0	1	0	0	4.79	5.46	4.33	0.36	0.14	1.25	0.27	0.23	4.35	2.40	0.31	0.2	0.0	0.2	0.0	0.0	0.0	0.0	0.0	0.2	0.0	0.0	4.61E-02	4.03E-03	4.78E-01	2.18E-01
ZKSCAN5	7	5	2	0	0	0	0	2	0	0	0	0	0	0	0	0	0.59	0.67	0.53	0.04	0.02	0.15	0.03	0.03	0.53	0.29	0.04	0.0	0.0	3.8	0.0	0.0	0.0	0.0	0.0	0.0	0.0	0.0	7.74E-01	6.92E-02	8.04E-01	9.56E-01
ZKSCAN7	13	7	0	0	0	0	0	0	0	0	0	0	0	0	0	0	0.99	1.13	0.89	0.08	0.03	0.26	0.06	0.05	0.90	0.49	0.06	0.0	0.0	0.0	0.0	0.0	0.0	0.0	0.0	0.0	0.0	0.0	8.96E-01	2.22E-01	7.47E-01	8.74E-01
ZKSCAN8	66	13	4	1	26	1	2	1	0	1	0	0	0	17	8	1	6.07	6.92	5.49	0.46	0.18	1.58	0.34	0.29	5.52	3.04	0.39	0.2	0.3	0.2	0.0	5.7	0.0	0.0	0.0	3.1	2.6	2.5	1.85E-07	3.33E-03	3.78E-02	1.41E-06
ZNF10	2	6	3	0	0	0	0	3	0	0	0	0	0	0	0	0	0.91	1.04	0.83	0.07	0.03	0.24	0.05	0.04	0.83	0.46	0.06	0.0	0.0	3.6	0.0	0.0	0.0	0.0	0.0	0.0	0.0	0.0	4.90E-01	2.16E-02	7.57E-01	8.91E-01
ZNF100	4	1	1	0	1	0	1	0	0	0	0	0	0	1	0	0	0.91	1.04	0.83	0.07	0.03	0.24	0.05	0.04	0.83	0.46	0.06	0.0	1.0	0.0	0.0	0.0	0.0	0.0	0.0	1.2	0.0	0.0	9.87E-01	4.18E-01	7.57E-01	9.71E-01
ZNF107	9	3	3	0	4	1	2	0	0	0	0	0	0	4	0	0	1.21	1.38	1.09	0.09	0.04	0.31	0.07	0.06	1.10	0.60	0.08	0.8	1.5	0.0	0.0	0.0	0.0	0.0	0.0	3.6	0.0	0.0	4.10E-01	4.94E-01	7.22E-01	1.17E-01
ZNF114	5	8	0	0	0	0	0	0	0	0	0	0	0	0	0	0	1.35	1.54	1.22	0.10	0.04	0.35	0.08	0.07	1.23	0.68	0.09	0.0	0.0	0.0	0.0	0.0	0.0	0.0	0.0	0.0	0.0	0.0	7.49E-01	1.27E-01	7.06E-01	7.78E-01
ZNF117	0	0	0	0	0	0	0	0	0	0	0	0	0	0	0	0	0.04	0.04	0.03	0.00	0.00	0.01	0.00	0.00	0.03	0.02	0.00	0.0	0.0	0.0	0.0	0.0	0.0	0.0	0.0	0.0	0.0	0.0	1.00E+00	9.46E-01	9.51E-01	1.00E+00
ZNF121	25	8	1	0	7	0	1	0	0	0	0	0	0	7	0	0	1.98	2.25	1.79	0.15	0.06	0.51	0.11	0.09	1.79	0.99	0.13	0.0	0.4	0.0	0.0	0.0	0.0	0.0	0.0	3.9	0.0	0.0	1.72E-02	1.08E-01	6.49E-01	4.61E-03
ZNF124	12	7	0	0	1	0	0	0	0	0	0	0	0	1	0	0	2.56	2.92	2.31	0.19	0.07	0.67	0.14	0.12	2.33	1.28	0.17	0.0	0.0	0.0	0.0	0.0	0.0	0.0	0.0	0.4	0.0	0.0	3.42E-01	2.03E-02	6.04E-01	6.79E-01
ZNF131	40	33	6	0	3	4	0	2	0	0	0	0	0	1	2	0	5.45	6.21	4.93	0.41	0.16	1.42	0.30	0.26	4.95	2.73	0.35	0.7	0.0	0.4	0.0	0.0	0.0	0.0	0.0	0.2	0.7	0.0	1.47E-01	1.55E-02	4.49E-01	3.38E-01
ZNF133	1	11	1	0	11	0	1	0	0	0	0	0	0	2	9	0	1.35	1.54	1.22	0.10	0.04	0.35	0.08	0.07	1.23	0.68	0.09	0.0	0.6	0.0	0.0	0.0	0.0	0.0	0.0	1.6	13.3	0.0	3.24E-18	2.51E-01	7.06E-01	1.12E-20
ZNF135	0	2	0	0	0	0	0	0	0	0	0	0	0	0	0	0	0.37	0.42	0.33	0.03	0.01	0.10	0.02	0.02	0.33	0.18	0.02	0.0	0.0	0.0	0.0	0.0	0.0	0.0	0.0	0.0	0.0	0.0	9.98E-01	5.73E-01	8.45E-01	9.84E-01
ZNF136	1	10	3	0	1	0	1	2	0	0	0	0	0	1	0	0	1.65	1.88	1.49	0.13	0.05	0.43	0.09	0.08	1.50	0.82	0.11	0.0	0.5	1.3	0.0	0.0	0.0	0.0	0.0	0.7	0.0	0.0	9.43E-01	3.28E-01	6.77E-01	8.90E-01
ZNF138	5	4	1	0	0	0	1	0	0	0	0	0	0	0	0	0	0.91	1.04	0.83	0.07	0.03	0.24	0.05	0.04	0.83	0.46	0.06	0.0	1.0	0.0	0.0	0.0	0.0	0.0	0.0	0.0	0.0	0.0	9.66E-01	4.18E-01	7.57E-01	8.91E-01
ZNF14	0	2	2	0	0	1	1	0	0	0	0	0	0	0	0	0	0.26	0.29	0.23	0.02	0.01	0.07	0.01	0.01	0.23	0.13	0.02	3.9	3.4	0.0	0.0	0.0	0.0	0.0	0.0	0.0	0.0	0.0	9.16E-01	1.28E-01	8.70E-01	9.93E-01
ZNF140	0	2	4	0	0	0	3	1	0	0	0	0	0	0	0	0	0.40	0.46	0.36	0.03	0.01	0.10	0.02	0.02	0.37	0.20	0.03	0.0	6.5	2.7	0.0	0.0	0.0	0.0	0.0	0.0	0.0	0.0	8.94E-02	4.11E-04	8.37E-01	9.81E-01
ZNF141	1	1	0	0	0	0	0	0	0	0	0	0	0	0	0	0	0.07	0.08	0.07	0.01	0.00	0.02	0.00	0.00	0.07	0.04	0.00	0.0	0.0	0.0	0.0	0.0	0.0	0.0	0.0	0.0	0.0	0.0	1.00E+00	8.95E-01	9.30E-01	1.00E+00
ZNF143	17	7	1	0	0	1	0	0	0	0	0	0	0	0	0	0	2.01	2.29	1.82	0.15	0.06	0.52	0.11	0.10	1.83	1.01	0.13	0.5	0.0	0.0	0.0	0.0	0.0	0.0	0.0	0.0	0.0	0.0	5.77E-01	9.92E-02	6.46E-01	5.94E-01
ZNF146	10	10	2	0	1	0	1	1	0	0	0	0	0	1	0	0	3.70	4.21	3.34	0.28	0.11	0.96	0.21	0.18	3.36	1.85	0.24	0.0	0.2	0.3	0.0	0.0	0.0	0.0	0.0	0.3	0.0	0.0	2.09E-01	2.04E-02	5.33E-01	4.05E-01
ZNF148	45	28	11	1	0	0	2	9	1	0	0	0	0	0	0	0	5.16	5.88	4.66	0.39	0.15	1.34	0.29	0.25	4.68	2.58	0.33	0.0	0.3	1.9	2.5	0.0	0.0	0.0	0.0	0.0	0.0	0.0	1.35E-02	2.80E-03	2.96E-01	9.13E-02
ZNF154	0	1	0	0	0	0	0	0	0	0	0	0	0	0	0	0	0.99	1.13	0.89	0.08	0.03	0.26	0.06	0.05	0.90	0.49	0.06	0.0	0.0	0.0	0.0	0.0	0.0	0.0	0.0	0.0	0.0	0.0	8.96E-01	2.22E-01	7.47E-01	8.74E-01
ZNF155	0	3	0	0	0	0	0	0	0	0	0	0	0	0	0	0	0.37	0.42	0.33	0.03	0.01	0.10	0.02	0.02	0.33	0.18	0.02	0.0	0.0	0.0	0.0	0.0	0.0	0.0	0.0	0.0	0.0	0.0	9.98E-01	5.73E-01	8.45E-01	9.84E-01
ZNF157	2	4	1	0	0	0	0	1	0	0	0	0	0	0	0	0	0.29	0.33	0.26	0.02	0.01	0.08	0.02	0.01	0.27	0.15	0.02	0.0	0.0	3.8	0.0	0.0	0.0	0.0	0.0	0.0	0.0	0.0	9.75E-01	2.63E-01	8.61E-01	9.91E-01
ZNF16	6	4	1	0	9	0	1	0	0	0	0	0	0	9	0	0	1.35	1.54	1.22	0.10	0.04	0.35	0.08	0.07	1.23	0.68	0.09	0.0	0.6	0.0	0.0	0.0	0.0	0.0	0.0	7.3	0.0	0.0	6.57E-08	2.51E-01	7.06E-01	1.16E-09
ZNF160	1	2	0	0	0	0	0	0	0	0	0	0	0	0	0	0	0.29	0.33	0.26	0.02	0.01	0.08	0.02	0.01	0.27	0.15	0.02	0.0	0.0	0.0	0.0	0.0	0.0	0.0	0.0	0.0	0.0	0.0	9.99E-01	6.41E-01	8.61E-01	9.91E-01
ZNF165	19	10	4	0	0	0	3	1	0	0	0	0	0	0	0	0	2.34	2.67	2.12	0.18	0.07	0.61	0.13	0.11	2.13	1.17	0.15	0.0	1.1	0.5	0.0	0.0	0.0	0.0	0.0	0.0	0.0	0.0	6.75E-01	2.26E-01	6.20E-01	5.07E-01
ZNF169	34	11	2	0	10	0	2	0	0	0	0	0	0	2	8	0	3.11	3.54	2.81	0.24	0.09	0.81	0.17	0.15	2.82	1.56	0.20	0.0	0.6	0.0	0.0	0.0	0.0	0.0	0.0	0.7	5.1	0.0	1.18E-04	3.70E-02	5.68E-01	3.30E-05
ZNF17	4	2	1	0	11	0	0	0	0	0	0	0	0	0	11	0	1.32	1.50	1.19	0.10	0.04	0.34	0.07	0.06	1.20	0.66	0.09	0.0	0.7	0.0	0.0	0.0	0.0	0.0	0.0	0.0	16.7	0.0	1.38E-30	2.62E-01	7.10E-01	1.52E-33
ZNF175	3	1	1	0	0	0	1	0	0	0	0	0	0	0	0	0	0.48	0.54	0.43	0.04	0.01	0.12	0.03	0.02	0.43	0.24	0.03	0.0	1.8	0.0	0.0	0.0	0.0	0.0	0.0	0.0	0.0	0.0	9.94E-01	5.24E-01	8.23E-01	9.72E-01
ZNF177	2	3	0	0	0	0	0	0	0	0	0	0	0	0	0	0	0.88	1.00	0.79	0.07	0.03	0.23	0.05	0.04	0.80	0.44	0.06	0.0	0.0	0.0	0.0	0.0	0.0	0.0	0.0	0.0	0.0	0.0	9.29E-01	2.63E-01	7.61E-01	9.00E-01
ZNF18	8	6	0	0	0	0	0	0	0	0	0	0	0	0	0	0	0.77	0.88	0.69	0.06	0.02	0.20	0.04	0.04	0.70	0.38	0.05	0.0	0.0	0.0	0.0	0.0	0.0	0.0	0.0	0.0	0.0	0.0	9.55E-01	3.11E-01	7.76E-01	9.23E-01
ZNF180	1	0	0	0	0	0	0	0	0	0	0	0	0	0	0	0	0.33	0.38	0.30	0.03	0.01	0.09	0.02	0.02	0.30	0.16	0.02	0.0	0.0	0.0	0.0	0.0	0.0	0.0	0.0	0.0	0.0	0.0	9.98E-01	6.06E-01	8.52E-01	9.88E-01
ZNF181	0	2	1	0	0	1	0	0	0	0	0	0	0	0	0	0	0.29	0.33	0.26	0.02	0.01	0.08	0.02	0.01	0.27	0.15	0.02	3.4	0.0	0.0	0.0	0.0	0.0	0.0	0.0	0.0	0.0	0.0	9.84E-01	3.16E-01	8.61E-01	9.91E-01
ZNF182	1	0	0	0	0	0	0	0	0	0	0	0	0	0	0	0	0.15	0.17	0.13	0.01	0.00	0.04	0.01	0.01	0.13	0.07	0.01	0.0	0.0	0.0	0.0	0.0	0.0	0.0	0.0	0.0	0.0	0.0	1.00E+00	8.00E-01	9.01E-01	9.98E-01
ZNF184	22	13	5	0	13	1	3	1	0	0	0	0	0	13	0	0	2.74	3.13	2.48	0.21	0.08	0.71	0.15	0.13	2.49	1.37	0.18	0.4	1.0	0.4	0.0	0.0	0.0	0.0	0.0	5.2	0.0	0.0	3.82E-07	3.68E-01	5.91E-01	6.05E-09
ZNF189	6	8	1	0	10	0	1	0	0	0	0	0	0	10	0	0	2.20	2.50	1.98	0.17	0.06	0.57	0.12	0.11	1.99	1.10	0.14	0.0	0.4	0.0	0.0	0.0	0.0	0.0	0.0	5.0	0.0	0.0	2.06E-05	7.88E-02	6.31E-01	2.17E-06
ZNF19	1	2	0	0	0	0	0	0	0	0	0	0	0	0	0	0	1.10	1.25	0.99	0.08	0.03	0.29	0.06	0.05	1.00	0.55	0.07	0.0	0.0	0.0	0.0	0.0	0.0	0.0	0.0	0.0	0.0	0.0	8.57E-01	1.88E-01	7.34E-01	8.47E-01
ZNF197	15	3	0	0	0	0	0	0	0	0	0	0	0	0	0	0	1.17	1.33	1.06	0.09	0.03	0.30	0.07	0.06	1.06	0.59	0.08	0.0	0.0	0.0	0.0	0.0	0.0	0.0	0.0	0.0	0.0	0.0	8.29E-01	1.68E-01	7.26E-01	8.28E-01
ZNF2	22	15	11	0	16	4	5	2	0	0	0	0	0	7	9	0	4.39	5.00	3.97	0.33	0.13	1.14	0.24	0.21	3.99	2.20	0.28	0.9	1.0	0.5	0.0	0.0	0.0	0.0	0.0	1.8	4.1	0.0	2.94E-03	6.03E-01	4.97E-01	1.28E-04
ZNF20	1	0	0	0	0	0	0	0	0	0	0	0	0	0	0	0	1.76	2.00	1.59	0.13	0.05	0.46	0.10	0.08	1.59	0.88	0.11	0.0	0.0	0.0	0.0	0.0	0.0	0.0	0.0	0.0	0.0	0.0	5.55E-01	6.91E-02	6.68E-01	6.65E-01
ZNF200	1	4	2	0	0	0	2	0	0	0	0	0	0	0	0	0	0.51	0.58	0.46	0.04	0.01	0.13	0.03	0.02	0.47	0.26	0.03	0.0	3.4	0.0	0.0	0.0	0.0	0.0	0.0	0.0	0.0	0.0	8.62E-01	1.10E-01	8.17E-01	9.67E-01
ZNF202	9	6	0	0	0	0	0	0	0	0	0	0	0	0	0	0	1.28	1.46	1.16	0.10	0.04	0.33	0.07	0.06	1.16	0.64	0.08	0.0	0.0	0.0	0.0	0.0	0.0	0.0	0.0	0.0	0.0	0.0	7.82E-01	1.42E-01	7.14E-01	7.98E-01
ZNF205	1	4	2	0	0	0	1	1	0	0	0	0	0	0	0	0	0.80	0.92	0.73	0.06	0.02	0.21	0.04	0.04	0.73	0.40	0.05	0.0	1.1	1.4	0.0	0.0	0.0	0.0	0.0	0.0	0.0	0.0	9.91E-01	6.33E-01	7.71E-01	9.15E-01
ZNF208	0	0	0	0	0	0	0	0	0	0	0	0	0	0	0	0	0.04	0.04	0.03	0.00	0.00	0.01	0.00	0.00	0.03	0.02	0.00	0.0	0.0	0.0	0.0	0.0	0.0	0.0	0.0	0.0	0.0	0.0	1.00E+00	9.46E-01	9.51E-01	1.00E+00
ZNF212	27	6	4	0	0	1	2	1	0	0	0	0	0	0	0	0	2.56	2.92	2.31	0.19	0.07	0.67	0.14	0.12	2.33	1.28	0.17	0.4	0.7	0.4	0.0	0.0	0.0	0.0	0.0	0.0	0.0	0.0	7.29E-01	3.70E-01	6.04E-01	4.53E-01
ZNF213	24	8	0	0	1	0	0	0	0	0	0	0	1	0	0	0	1.54	1.75	1.39	0.12	0.04	0.40	0.09	0.07	1.40	0.77	0.10	0.0	0.0	0.0	0.0	0.0	0.0	0.0	13.5	0.0	0.0	0.0	3.77E-02	9.65E-02	6.88E-01	1.34E-02
ZNF214	0	5	0	0	0	0	0	0	0	0	0	0	0	0	0	0	0.80	0.92	0.73	0.06	0.02	0.21	0.04	0.04	0.73	0.40	0.05	0.0	0.0	0.0	0.0	0.0	0.0	0.0	0.0	0.0	0.0	0.0	9.47E-01	2.94E-01	7.71E-01	9.15E-01
ZNF215	9	3	2	0	0	1	1	0	0	0	0	0	0	0	0	0	0.80	0.92	0.73	0.06	0.02	0.21	0.04	0.04	0.73	0.40	0.05	1.2	1.1	0.0	0.0	0.0	0.0	0.0	0.0	0.0	0.0	0.0	9.93E-01	6.76E-01	7.71E-01	9.15E-01
ZNF22	26	15	11	0	36	2	9	0	0	0	0	0	0	31	5	0	5.34	6.09	4.83	0.41	0.15	1.39	0.30	0.26	4.85	2.68	0.35	0.4	1.5	0.0	0.0	0.0	0.0	0.0	0.0	6.4	1.9	0.0	5.21E-28	1.57E-02	4.54E-01	1.35E-29
ZNF221	4	2	1	0	0	0	1	0	0	0	0	0	0	0	0	0	0.40	0.46	0.36	0.03	0.01	0.10	0.02	0.02	0.37	0.20	0.03	0.0	2.2	0.0	0.0	0.0	0.0	0.0	0.0	0.0	0.0	0.0	9.95E-01	4.95E-01	8.37E-01	9.81E-01
ZNF222	0	0	0	0	0	0	0	0	0	0	0	0	0	0	0	0	0.00	0.00	0.00	0.00	0.00	0.00	0.00	0.00	0.00	0.00	0.00	na	na	na	na	na	na	na	na	na	na	na	na	na	na	na
ZNF223	8	6	3	0	0	0	0	3	0	0	0	0	0	0	0	0	1.76	2.00	1.59	0.13	0.05	0.46	0.10	0.08	1.59	0.88	0.11	0.0	0.0	1.9	0.0	0.0	0.0	0.0	0.0	0.0	0.0	0.0	5.87E-01	8.15E-02	6.68E-01	6.65E-01
ZNF224	1	5	3	0	0	0	0	3	0	0	0	0	0	0	0	0	0.88	1.00	0.79	0.07	0.03	0.23	0.05	0.04	0.80	0.44	0.06	0.0	0.0	3.8	0.0	0.0	0.0	0.0	0.0	0.0	0.0	0.0	4.65E-01	1.82E-02	7.61E-01	9.00E-01
ZNF225	3	1	0	0	0	0	0	0	0	0	0	0	0	0	0	0	0.22	0.25	0.20	0.02	0.01	0.06	0.01	0.01	0.20	0.11	0.01	0.0	0.0	0.0	0.0	0.0	0.0	0.0	0.0	0.0	0.0	0.0	1.00E+00	7.16E-01	8.79E-01	9.95E-01
ZNF226	1	4	1	0	0	0	0	1	0	0	0	0	0	0	0	0	0.40	0.46	0.36	0.03	0.01	0.10	0.02	0.02	0.37	0.20	0.03	0.0	0.0	2.7	0.0	0.0	0.0	0.0	0.0	0.0	0.0	0.0	9.87E-01	3.73E-01	8.37E-01	9.81E-01
ZNF227	2	4	1	0	0	0	0	1	0	0	0	0	0	0	0	0	0.70	0.79	0.63	0.05	0.02	0.18	0.04	0.03	0.63	0.35	0.05	0.0	0.0	1.6	0.0	0.0	0.0	0.0	0.0	0.0	0.0	0.0	9.80E-01	4.26E-01	7.87E-01	9.37E-01
ZNF229	0	1	0	0	0	0	0	0	0	0	0	0	0	0	0	0	0.15	0.17	0.13	0.01	0.00	0.04	0.01	0.01	0.13	0.07	0.01	0.0	0.0	0.0	0.0	0.0	0.0	0.0	0.0	0.0	0.0	0.0	1.00E+00	8.00E-01	9.01E-01	9.98E-01
ZNF23	3	6	0	0	0	0	0	0	0	0	0	0	0	0	0	0	1.02	1.17	0.93	0.08	0.03	0.27	0.06	0.05	0.93	0.51	0.07	0.0	0.0	0.0	0.0	0.0	0.0	0.0	0.0	0.0	0.0	0.0	8.84E-01	2.10E-01	7.43E-01	8.65E-01
ZNF230	0	2	0	0	0	0	0	0	0	0	0	0	0	0	0	0	0.40	0.46	0.36	0.03	0.01	0.10	0.02	0.02	0.37	0.20	0.03	0.0	0.0	0.0	0.0	0.0	0.0	0.0	0.0	0.0	0.0	0.0	9.96E-01	5.42E-01	8.37E-01	9.81E-01
ZNF232	54	7	4	0	1	2	2	0	0	0	0	0	0	1	0	0	3.73	4.25	3.37	0.28	0.11	0.97	0.21	0.18	3.39	1.87	0.24	0.5	0.5	0.0	0.0	0.0	0.0	0.0	0.0	0.3	0.0	0.0	3.64E-01	6.82E-02	5.31E-01	3.97E-01
ZNF233	0	1	0	0	0	0	0	0	0	0	0	0	0	0	0	0	0.07	0.08	0.07	0.01	0.00	0.02	0.00	0.00	0.07	0.04	0.00	0.0	0.0	0.0	0.0	0.0	0.0	0.0	0.0	0.0	0.0	0.0	1.00E+00	8.95E-01	9.30E-01	1.00E+00
ZNF234	0	1	2	0	0	0	1	1	0	0	0	0	0	0	0	0	0.26	0.29	0.23	0.02	0.01	0.07	0.01	0.01	0.23	0.13	0.02	0.0	3.4	4.3	0.0	0.0	0.0	0.0	0.0	0.0	0.0	0.0	8.90E-01	1.04E-01	8.70E-01	9.93E-01
ZNF235	1	1	0	0	1	0	0	0	0	0	0	0	0	1	0	0	0.51	0.58	0.46	0.04	0.01	0.13	0.03	0.02	0.47	0.26	0.03	0.0	0.0	0.0	0.0	0.0	0.0	0.0	0.0	3.9	0.0	0.0	9.25E-01	4.59E-01	8.17E-01	7.25E-01
ZNF239	0	3	1	1	0	0	1	0	1	0	0	0	0	0	0	0	0.40	0.46	0.36	0.03	0.01	0.10	0.02	0.02	0.37	0.20	0.03	0.0	2.2	0.0	32.7	0.0	0.0	0.0	0.0	0.0	0.0	0.0	2.87E-04	4.95E-01	2.98E-08	9.81E-01
ZNF24	96	41	3	0	3	0	0	3	0	0	1	0	1	1	0	0	8.12	9.26	7.34	0.62	0.24	2.12	0.45	0.39	7.38	4.07	0.53	0.0	0.0	0.4	0.0	0.0	0.5	0.0	2.6	0.1	0.0	0.0	2.83E-04	4.66E-05	3.56E-01	3.35E-02
ZNF25	0	3	0	0	0	0	0	0	0	0	0	0	0	0	0	0	0.18	0.21	0.17	0.01	0.01	0.05	0.01	0.01	0.17	0.09	0.01	0.0	0.0	0.0	0.0	0.0	0.0	0.0	0.0	0.0	0.0	0.0	1.00E+00	7.57E-01	8.90E-01	9.97E-01
ZNF250	32	13	1	0	0	0	0	1	0	0	0	0	0	0	0	0	5.89	6.71	5.32	0.45	0.17	1.53	0.33	0.28	5.35	2.95	0.38	0.0	0.0	0.2	0.0	0.0	0.0	0.0	0.0	0.0	0.0	0.0	2.12E-03	3.16E-04	4.31E-01	5.49E-02
ZNF253	0	1	0	0	1	0	0	0	0	0	0	0	0	1	0	0	0.22	0.25	0.20	0.02	0.01	0.06	0.01	0.01	0.20	0.11	0.01	0.0	0.0	0.0	0.0	0.0	0.0	0.0	0.0	5.0	0.0	0.0	9.42E-01	7.16E-01	8.79E-01	6.36E-01
ZNF254	3	3	1	0	0	0	1	0	0	0	0	0	0	0	0	0	0.51	0.58	0.46	0.04	0.01	0.13	0.03	0.02	0.47	0.26	0.03	0.0	1.7	0.0	0.0	0.0	0.0	0.0	0.0	0.0	0.0	0.0	9.94E-01	5.29E-01	8.17E-01	9.67E-01
ZNF257	1	0	0	0	0	0	0	0	0	0	0	0	0	0	0	0	0.15	0.17	0.13	0.01	0.00	0.04	0.01	0.01	0.13	0.07	0.01	0.0	0.0	0.0	0.0	0.0	0.0	0.0	0.0	0.0	0.0	0.0	1.00E+00	8.00E-01	9.01E-01	9.98E-01
ZNF26	1	0	0	0	0	0	0	0	0	0	0	0	0	0	0	0	0.48	0.54	0.43	0.04	0.01	0.12	0.03	0.02	0.43	0.24	0.03	0.0	0.0	0.0	0.0	0.0	0.0	0.0	0.0	0.0	0.0	0.0	9.93E-01	4.85E-01	8.23E-01	9.72E-01
ZNF260	6	1	1	0	0	0	1	0	0	0	0	0	0	0	0	0	0.55	0.63	0.50	0.04	0.02	0.14	0.03	0.03	0.50	0.27	0.04	0.0	1.6	0.0	0.0	0.0	0.0	0.0	0.0	0.0	0.0	0.0	9.93E-01	5.30E-01	8.10E-01	9.62E-01
ZNF263	38	47	21	0	9	2	5	14	0	0	0	0	0	2	7	0	7.79	8.88	7.04	0.59	0.23	2.03	0.43	0.37	7.08	3.90	0.50	0.3	0.6	2.0	0.0	0.0	0.0	0.0	0.0	0.3	1.8	0.0	1.03E-02	1.60E-03	3.66E-01	9.27E-02
ZNF264	7	9	3	0	0	1	2	0	0	0	0	0	0	0	0	0	2.12	2.42	1.92	0.16	0.06	0.55	0.12	0.10	1.93	1.06	0.14	0.5	0.8	0.0	0.0	0.0	0.0	0.0	0.0	0.0	0.0	0.0	7.53E-01	2.75E-01	6.37E-01	5.64E-01
ZNF266	0	2	0	0	0	0	0	0	0	0	0	0	0	0	0	0	0.62	0.71	0.56	0.05	0.02	0.16	0.03	0.03	0.56	0.31	0.04	0.0	0.0	0.0	0.0	0.0	0.0	0.0	0.0	0.0	0.0	0.0	9.79E-01	3.88E-01	7.98E-01	9.50E-01
ZNF267	4	2	0	0	0	0	0	0	0	0	0	0	0	0	0	0	0.59	0.67	0.53	0.04	0.02	0.15	0.03	0.03	0.53	0.29	0.04	0.0	0.0	0.0	0.0	0.0	0.0	0.0	0.0	0.0	0.0	0.0	9.83E-01	4.10E-01	8.04E-01	9.56E-01
ZNF268	4	2	0	0	0	0	0	0	0	0	0	0	0	0	0	0	0.62	0.71	0.56	0.05	0.02	0.16	0.03	0.03	0.56	0.31	0.04	0.0	0.0	0.0	0.0	0.0	0.0	0.0	0.0	0.0	0.0	0.0	9.79E-01	3.88E-01	7.98E-01	9.50E-01
ZNF273	7	2	2	0	3	0	1	1	0	0	0	0	0	3	0	0	0.99	1.13	0.89	0.08	0.03	0.26	0.06	0.05	0.90	0.49	0.06	0.0	0.9	1.1	0.0	0.0	0.0	0.0	0.0	3.3	0.0	0.0	7.29E-01	6.02E-01	7.47E-01	3.21E-01
ZNF274	4	5	0	0	6	0	0	0	0	0	0	0	0	2	4	0	1.10	1.25	0.99	0.08	0.03	0.29	0.06	0.05	1.00	0.55	0.07	0.0	0.0	0.0	0.0	0.0	0.0	0.0	0.0	2.0	7.3	0.0	3.02E-03	1.88E-01	7.34E-01	3.18E-04
ZNF275	7	4	3	0	2	1	2	0	0	0	0	0	0	2	0	0	0.88	1.00	0.79	0.07	0.03	0.23	0.05	0.04	0.80	0.44	0.06	1.1	2.0	0.0	0.0	0.0	0.0	0.0	0.0	2.5	0.0	0.0	9.20E-01	4.05E-01	7.61E-01	7.57E-01
ZNF276	8	4	2	0	0	1	0	1	0	0	0	0	0	0	0	0	1.24	1.42	1.12	0.09	0.04	0.32	0.07	0.06	1.13	0.62	0.08	0.8	0.0	0.9	0.0	0.0	0.0	0.0	0.0	0.0	0.0	0.0	9.52E-01	4.77E-01	7.18E-01	8.08E-01
ZNF28	2	0	1	0	0	0	1	0	0	0	0	0	0	0	0	0	0.37	0.42	0.33	0.03	0.01	0.10	0.02	0.02	0.33	0.18	0.02	0.0	2.4	0.0	0.0	0.0	0.0	0.0	0.0	0.0	0.0	0.0	9.94E-01	4.70E-01	8.45E-01	9.84E-01
ZNF280A	1	1	1	0	0	0	0	1	0	0	0	0	0	0	0	0	0.59	0.67	0.53	0.04	0.02	0.15	0.03	0.03	0.53	0.29	0.04	0.0	0.0	1.9	0.0	0.0	0.0	0.0	0.0	0.0	0.0	0.0	9.86E-01	4.33E-01	8.04E-01	9.56E-01
ZNF280B	1	2	1	0	0	0	1	0	0	0	0	0	0	0	0	0	0.18	0.21	0.17	0.01	0.01	0.05	0.01	0.01	0.17	0.09	0.01	0.0	4.8	0.0	0.0	0.0	0.0	0.0	0.0	0.0	0.0	0.0	9.60E-01	1.87E-01	8.90E-01	9.97E-01
ZNF280C	5	21	8	0	0	1	0	7	0	0	0	0	0	0	0	0	2.56	2.92	2.31	0.19	0.07	0.67	0.14	0.12	2.33	1.28	0.17	0.4	0.0	3.0	0.0	0.0	0.0	0.0	0.0	0.0	0.0	0.0	4.96E-02	1.26E-03	6.04E-01	4.53E-01
ZNF280D	2	16	6	0	0	1	0	5	0	0	0	0	0	0	0	0	1.10	1.25	0.99	0.08	0.03	0.29	0.06	0.05	1.00	0.55	0.07	0.9	0.0	5.0	0.0	0.0	0.0	0.0	0.0	0.0	0.0	0.0	3.34E-02	1.62E-04	7.34E-01	8.47E-01
ZNF281	68	32	7	0	0	1	1	5	0	0	0	0	0	0	0	0	7.21	8.22	6.51	0.55	0.21	1.88	0.40	0.35	6.55	3.61	0.47	0.1	0.1	0.8	0.0	0.0	0.0	0.0	0.0	0.0	0.0	0.0	3.69E-03	2.43E-03	3.84E-01	2.12E-02
ZNF282	5	5	0	0	0	0	0	0	0	0	0	0	0	0	0	0	1.02	1.17	0.93	0.08	0.03	0.27	0.06	0.05	0.93	0.51	0.07	0.0	0.0	0.0	0.0	0.0	0.0	0.0	0.0	0.0	0.0	0.0	8.84E-01	2.10E-01	7.43E-01	8.65E-01
ZNF283	0	0	0	0	0	0	0	0	0	0	0	0	0	0	0	0	0.04	0.04	0.03	0.00	0.00	0.01	0.00	0.00	0.03	0.02	0.00	0.0	0.0	0.0	0.0	0.0	0.0	0.0	0.0	0.0	0.0	0.0	1.00E+00	9.46E-01	9.51E-01	1.00E+00
ZNF284	6	0	0	0	0	0	0	0	0	0	0	0	0	0	0	0	0.59	0.67	0.53	0.04	0.02	0.15	0.03	0.03	0.53	0.29	0.04	0.0	0.0	0.0	0.0	0.0	0.0	0.0	0.0	0.0	0.0	0.0	9.83E-01	4.10E-01	8.04E-01	9.56E-01
ZNF285	0	0	0	0	0	0	0	0	0	0	0	0	0	0	0	0	0.04	0.04	0.03	0.00	0.00	0.01	0.00	0.00	0.03	0.02	0.00	0.0	0.0	0.0	0.0	0.0	0.0	0.0	0.0	0.0	0.0	0.0	1.00E+00	9.46E-01	9.51E-01	1.00E+00
ZNF286A	8	3	3	0	1	0	3	0	0	0	0	0	0	1	0	0	1.35	1.54	1.22	0.10	0.04	0.35	0.08	0.07	1.23	0.68	0.09	0.0	1.9	0.0	0.0	0.0	0.0	0.0	0.0	0.8	0.0	0.0	8.63E-01	1.39E-01	7.06E-01	9.35E-01
ZNF286B	0	0	0	0	0	0	0	0	0	0	0	0	0	0	0	0	0.04	0.04	0.03	0.00	0.00	0.01	0.00	0.00	0.03	0.02	0.00	0.0	0.0	0.0	0.0	0.0	0.0	0.0	0.0	0.0	0.0	0.0	1.00E+00	9.46E-01	9.51E-01	1.00E+00
ZNF287	2	4	0	0	0	0	0	0	0	0	0	0	0	0	0	0	0.44	0.50	0.40	0.03	0.01	0.11	0.02	0.02	0.40	0.22	0.03	0.0	0.0	0.0	0.0	0.0	0.0	0.0	0.0	0.0	0.0	0.0	9.95E-01	5.13E-01	8.30E-01	9.77E-01
ZNF3	12	3	1	0	0	1	0	0	0	0	0	0	0	0	0	0	1.50	1.71	1.36	0.11	0.04	0.39	0.08	0.07	1.36	0.75	0.10	0.7	0.0	0.0	0.0	0.0	0.0	0.0	0.0	0.0	0.0	0.0	8.03E-01	1.99E-01	6.91E-01	7.37E-01
ZNF30	0	1	1	0	0	0	0	1	0	0	0	0	0	0	0	0	0.26	0.29	0.23	0.02	0.01	0.07	0.01	0.01	0.23	0.13	0.02	0.0	0.0	4.3	0.0	0.0	0.0	0.0	0.0	0.0	0.0	0.0	9.64E-01	2.12E-01	8.70E-01	9.93E-01
ZNF300	1	1	0	0	0	0	0	0	0	0	0	0	0	0	0	0	0.18	0.21	0.17	0.01	0.01	0.05	0.01	0.01	0.17	0.09	0.01	0.0	0.0	0.0	0.0	0.0	0.0	0.0	0.0	0.0	0.0	0.0	1.00E+00	7.57E-01	8.90E-01	9.97E-01
ZNF302	1	4	0	0	0	0	0	0	0	0	0	0	0	0	0	0	1.06	1.21	0.96	0.08	0.03	0.28	0.06	0.05	0.96	0.53	0.07	0.0	0.0	0.0	0.0	0.0	0.0	0.0	0.0	0.0	0.0	0.0	8.71E-01	1.99E-01	7.38E-01	8.56E-01
ZNF311	0	0	0	0	0	0	0	0	0	0	0	0	0	0	0	0	0.40	0.46	0.36	0.03	0.01	0.10	0.02	0.02	0.37	0.20	0.03	0.0	0.0	0.0	0.0	0.0	0.0	0.0	0.0	0.0	0.0	0.0	9.96E-01	5.42E-01	8.37E-01	9.81E-01
ZNF317	9	4	4	0	16	1	2	1	0	0	0	0	0	13	3	0	2.93	3.34	2.65	0.22	0.08	0.76	0.16	0.14	2.66	1.47	0.19	0.3	0.6	0.4	0.0	0.0	0.0	0.0	0.0	4.9	2.0	0.0	1.30E-06	2.43E-01	5.79E-01	3.52E-08
ZNF32	0	1	0	0	0	0	0	0	0	0	0	0	0	0	0	0	0.59	0.67	0.53	0.04	0.02	0.15	0.03	0.03	0.53	0.29	0.04	0.0	0.0	0.0	0.0	0.0	0.0	0.0	0.0	0.0	0.0	0.0	9.83E-01	4.10E-01	8.04E-01	9.56E-01
ZNF320	5	1	1	0	5	0	1	0	0	0	0	0	0	5	0	0	1.02	1.17	0.93	0.08	0.03	0.27	0.06	0.05	0.93	0.51	0.07	0.0	0.9	0.0	0.0	0.0	0.0	0.0	0.0	5.4	0.0	0.0	2.22E-02	3.73E-01	7.43E-01	2.13E-03
ZNF322	0	0	0	0	0	0	0	0	0	0	0	0	0	0	0	0	0.15	0.17	0.13	0.01	0.00	0.04	0.01	0.01	0.13	0.07	0.01	0.0	0.0	0.0	0.0	0.0	0.0	0.0	0.0	0.0	0.0	0.0	1.00E+00	8.00E-01	9.01E-01	9.98E-01
ZNF324	15	7	4	0	2	0	2	2	0	0	0	0	0	2	0	0	2.49	2.84	2.25	0.19	0.07	0.65	0.14	0.12	2.26	1.25	0.16	0.0	0.7	0.9	0.0	0.0	0.0	0.0	0.0	0.9	0.0	0.0	8.65E-01	2.51E-01	6.09E-01	8.00E-01
ZNF324B	39	10	6	0	22	1	5	0	0	0	0	0	0	6	14	2	5.45	6.21	4.93	0.41	0.16	1.42	0.30	0.26	4.95	2.73	0.35	0.2	0.8	0.0	0.0	0.0	0.0	0.0	0.0	1.2	5.1	5.7	2.88E-10	1.23E-02	4.49E-01	6.72E-11
ZNF329	6	2	1	0	0	0	1	0	0	0	0	0	0	0	0	0	1.21	1.38	1.09	0.09	0.04	0.31	0.07	0.06	1.10	0.60	0.08	0.0	0.7	0.0	0.0	0.0	0.0	0.0	0.0	0.0	0.0	0.0	9.07E-01	3.01E-01	7.22E-01	8.18E-01

	ZNF331	4	10	3	0	20	2	1	0	0	0	0	0	0	0	20	0	3.73	4.25	3.37	0.28	0.11	0.97	0.21	0.18	3.39	1.87	0.24	0.5	0.2	0.0	0.0	0.0	0.0	0.0	0.0	0.0	10.7	0.0	5.35E-35	3.57E-02	5.31E-01	3.54E-37
	ZNF333	1	3	1	1	0	0	0	1	0	1	0	0	0	0	0	0	0.80	0.92	0.73	0.06	0.02	0.21	0.04	0.04	0.73	0.40	0.05	0.0	0.0	1.4	0.0	42.8	0.0	0.0	0.0	0.0	0.0	0.0	3.02E-06	4.02E-01	1.60E-10	9.15E-01
	ZNF337	2	3	0	0	0	0	0	0	0	0	0	0	0	0	0	0	0.55	0.63	0.50	0.04	0.02	0.14	0.03	0.03	0.50	0.27	0.04	0.0	0.0	0.0	0.0	0.0	0.0	0.0	0.0	0.0	0.0	0.0	9.87E-01	4.34E-01	8.10E-01	9.62E-01
	ZNF33A	0	4	2	0	0	0	1	1	0	0	0	0	0	0	0	0	0.48	0.54	0.43	0.04	0.01	0.12	0.03	0.02	0.43	0.24	0.03	0.0	1.8	2.3	0.0	0.0	0.0	0.0	0.0	0.0	0.0	0.0	9.90E-01	4.45E-01	8.23E-01	9.72E-01
	ZNF33B	1	1	1	0	0	0	1	0	0	0	0	0	0	0	0	0	0.29	0.33	0.26	0.02	0.01	0.08	0.02	0.01	0.27	0.15	0.02	0.0	3.0	0.0	0.0	0.0	0.0	0.0	0.0	0.0	0.0	0.0	9.91E-01	3.89E-01	8.61E-01	9.91E-01
	ZNF34	14	7	0	0	0	0	0	0	0	0	0	0	0	0	0	0	1.94	2.21	1.75	0.15	0.06	0.51	0.11	0.09	1.76	0.97	0.13	0.0	0.0	0.0	0.0	0.0	0.0	0.0	0.0	0.0	0.0	0.0	4.70E-01	5.23E-02	6.52E-01	6.14E-01
	ZNF343	4	0	1	0	0	0	1	0	0	0	0	0	0	0	0	0	0.91	1.04	0.83	0.07	0.03	0.24	0.05	0.04	0.83	0.46	0.06	0.0	1.0	0.0	0.0	0.0	0.0	0.0	0.0	0.0	0.0	0.0	9.66E-01	4.18E-01	7.57E-01	8.91E-01
	ZNF345	1	0	2	0	0	0	2	0	0	0	0	0	0	0	0	0	0.22	0.25	0.20	0.02	0.01	0.06	0.01	0.01	0.20	0.11	0.01	0.0	8.0	0.0	0.0	0.0	0.0	0.0	0.0	0.0	0.0	0.0	2.19E-01	1.79E-03	8.79E-01	9.95E-01
	ZNF347	0	0	0	0	0	0	0	0	0	0	0	0	0	0	0	0	0.00	0.00	0.00	0.00	0.00	0.00	0.00	0.00	0.00	0.00	0.00	na	na	na	na	na	na	na	na	na	na	na	na	na	na	na
	ZNF35	6	1	5	0	1	1	4	0	0	0	0	0	1	0	0	0	1.76	2.00	1.59	0.13	0.05	0.46	0.10	0.08	1.59	0.88	0.11	0.6	2.0	0.0	0.0	0.0	0.0	0.0	11.9	0.0	0.0	0.0	7.06E-02	1.42E-01	6.68E-01	2.26E-02
	ZNF350	6	10	1	0	0	0	0	1	0	0	0	0	0	0	0	0	1.50	1.71	1.36	0.11	0.04	0.39	0.08	0.07	1.36	0.75	0.10	0.0	0.0	0.7	0.0	0.0	0.0	0.0	0.0	0.0	0.0	0.0	7.97E-01	1.92E-01	6.91E-01	7.37E-01
	ZNF354A	9	7	5	0	14	0	1	4	0	0	0	0	0	14	0	0	1.83	2.09	1.65	0.14	0.05	0.48	0.10	0.09	1.66	0.92	0.12	0.0	0.5	2.4	0.0	0.0	0.0	0.0	0.0	8.4	0.0	0.0	7.64E-17	5.71E-02	6.61E-01	1.33E-18
	ZNF354B	0	1	1	0	0	0	1	0	0	0	0	0	0	0	0	0	0.15	0.17	0.13	0.01	0.00	0.04	0.01	0.01	0.13	0.07	0.01	0.0	6.0	0.0	0.0	0.0	0.0	0.0	0.0	0.0	0.0	0.0	9.09E-01	1.09E-01	9.01E-01	9.98E-01
	ZNF354C	7	7	3	0	0	0	0	3	0	0	0	0	0	0	0	0	1.50	1.71	1.36	0.11	0.04	0.39	0.08	0.07	1.36	0.75	0.10	0.0	0.0	2.2	0.0	0.0	0.0	0.0	0.0	0.0	0.0	0.0	6.17E-01	7.41E-02	6.91E-01	7.37E-01
	ZNF358	4	1	0	0	7	0	0	0	0	0	0	0	0	7	0	0	0.80	0.92	0.73	0.06	0.02	0.21	0.04	0.04	0.73	0.40	0.05	0.0	0.0	0.0	0.0	0.0	0.0	0.0	0.0	9.6	0.0	0.0	1.30E-08	2.94E-01	7.71E-01	1.64E-10
	ZNF362	7	9	4	1	0	1	2	1	1	0	0	0	0	0	0	0	1.54	1.75	1.39	0.12	0.04	0.40	0.09	0.07	1.40	0.77	0.10	0.7	1.1	0.7	8.6	0.0	0.0	0.0	0.0	0.0	0.0	0.0	4.51E-01	8.47E-01	9.53E-03	7.27E-01
	ZNF366	0	1	0	0	0	0	0	0	0	0	0	0	0	0	0	0	0.33	0.38	0.30	0.03	0.01	0.09	0.02	0.02	0.30	0.16	0.02	0.0	0.0	0.0	0.0	0.0	0.0	0.0	0.0	0.0	0.0	0.0	9.98E-01	6.06E-01	8.52E-01	9.88E-01
	ZNF383	4	4	1	0	0	0	1	0	0	0	0	0	0	0	0	0	0.70	0.79	0.63	0.05	0.02	0.18	0.04	0.03	0.63	0.35	0.05	0.0	1.3	0.0	0.0	0.0	0.0	0.0	0.0	0.0	0.0	0.0	9.87E-01	5.02E-01	7.87E-01	9.37E-01
	ZNF384	18	14	2	0	3	2	0	0	0	0	0	0	0	3	0	0	2.67	3.04	2.41	0.20	0.08	0.70	0.15	0.13	2.43	1.34	0.17	0.7	0.0	0.0	0.0	0.0	0.0	0.0	0.0	1.2	0.0	0.0	5.78E-01	6.00E-02	5.96E-01	7.58E-01
	ZNF391	0	1	0	0	0	0	0	0	0	0	0	0	0	0	0	0	0.26	0.29	0.23	0.02	0.01	0.07	0.01	0.01	0.23	0.13	0.02	0.0	0.0	0.0	0.0	0.0	0.0	0.0	0.0	0.0	0.0	0.0	9.99E-01	6.77E-01	8.70E-01	9.93E-01
	ZNF394	10	5	4	0	0	2	1	1	0	0	0	0	0	0	0	0	0.77	0.88	0.69	0.06	0.02	0.20	0.04	0.04	0.70	0.38	0.05	2.6	1.1	1.4	0.0	0.0	0.0	0.0	0.0	0.0	0.0	0.0	9.63E-01	3.45E-01	7.76E-01	9.23E-01
	ZNF397	71	13	1	0	4	0	0	1	0	0	0	0	0	4	0	0	4.61	5.25	4.17	0.35	0.13	1.20	0.26	0.22	4.19	2.31	0.30	0.0	0.0	0.2	0.0	0.0	0.0	0.0	0.0	1.0	0.0	0.0	7.33E-02	2.16E-03	4.86E-01	5.08E-01
	ZNF398	15	4	5	0	0	2	1	2	0	0	0	0	0	0	0	0	2.20	2.50	1.98	0.17	0.06	0.57	0.12	0.11	1.99	1.10	0.14	0.9	0.4	1.0	0.0	0.0	0.0	0.0	0.0	0.0	0.0	0.0	8.79E-01	6.32E-01	6.31E-01	5.44E-01
	ZNF404	2	0	0	0	0	0	0	0	0	0	0	0	0	0	0	0	0.07	0.08	0.07	0.01	0.00	0.02	0.00	0.00	0.07	0.04	0.00	0.0	0.0	0.0	0.0	0.0	0.0	0.0	0.0	0.0	0.0	0.0	1.00E+00	8.95E-01	9.30E-01	1.00E+00
	ZNF408	34	26	5	0	5	3	2	0	0	0	0	0	0	2	3	0	8.12	9.26	7.34	0.62	0.24	2.12	0.45	0.39	7.38	4.07	0.53	0.4	0.2	0.0	0.0	0.0	0.0	0.0	0.0	0.3	0.7	0.0	5.74E-03	2.94E-04	3.56E-01	1.75E-01
	ZNF41	2	0	2	0	0	1	1	0	0	0	0	0	0	0	0	0	0.40	0.46	0.36	0.03	0.01	0.10	0.02	0.02	0.37	0.20	0.03	2.5	2.2	0.0	0.0	0.0	0.0	0.0	0.0	0.0	0.0	0.0	9.88E-01	3.89E-01	8.37E-01	9.81E-01
	ZNF410	10	7	1	0	0	1	0	0	0	0	0	0	0	0	0	0	2.27	2.59	2.05	0.17	0.07	0.59	0.13	0.11	2.06	1.14	0.15	0.4	0.0	0.0	0.0	0.0	0.0	0.0	0.0	0.0	0.0	0.0	4.62E-01	6.91E-02	6.25E-01	5.25E-01
	ZNF415	0	2	0	0	0	0	0	0	0	0	0	0	0	0	0	0	0.22	0.25	0.20	0.02	0.01	0.06	0.01	0.01	0.20	0.11	0.01	0.0	0.0	0.0	0.0	0.0	0.0	0.0	0.0	0.0	0.0	0.0	1.00E+00	7.16E-01	8.79E-01	9.95E-01
	ZNF416	2	1	1	0	0	0	1	0	0	0	0	0	0	0	0	0	0.48	0.54	0.43	0.04	0.01	0.12	0.03	0.02	0.43	0.24	0.03	0.0	1.8	0.0	0.0	0.0	0.0	0.0	0.0	0.0	0.0	0.0	9.94E-01	5.24E-01	8.23E-01	9.72E-01
	ZNF419	0	2	0	0	0	0	0	0	0	0	0	0	0	0	0	0	0.37	0.42	0.33	0.03	0.01	0.10	0.02	0.02	0.33	0.18	0.02	0.0	0.0	0.0	0.0	0.0	0.0	0.0	0.0	0.0	0.0	0.0	9.98E-01	5.73E-01	8.45E-01	9.84E-01
	ZNF420	11	13	1	0	0	0	0	1	0	0	0	0	0	0	0	0	1.68	1.92	1.52	0.13	0.05	0.44	0.09	0.08	1.53	0.84	0.11	0.0	0.0	0.7	0.0	0.0	0.0	0.0	0.0	0.0	0.0	0.0	7.21E-01	1.51E-01	6.74E-01	6.86E-01
	ZNF425	0	0	0	0	0	0	0	0	0	0	0	0	0	0	0	0	0.04	0.04	0.03	0.00	0.00	0.01	0.00	0.00	0.03	0.02	0.00	0.0	0.0	0.0	0.0	0.0	0.0	0.0	0.0	0.0	0.0	0.0	1.00E+00	9.46E-01	9.51E-01	1.00E+00
	ZNF426	2	8	1	0	0	0	1	0	0	0	0	0	0	0	0	0	2.05	2.34	1.85	0.16	0.06	0.53	0.11	0.10	1.86	1.03	0.13	0.0	0.4	0.0	0.0	0.0	0.0	0.0	0.0	0.0	0.0	0.0	5.66E-01	9.71E-02	6.43E-01	5.84E-01
	ZNF429	0	0	0	0	0	0	0	0	0	0	0	0	0	0	0	0	0.00	0.00	0.00	0.00	0.00	0.00	0.00	0.00	0.00	0.00	0.00	na	na	na	na	na	na	na	na	na	na	na	na	na	na	na
	ZNF43	0	0	0	0	0	0	0	0	0	0	0	0	0	0	0	0	0.04	0.04	0.03	0.00	0.00	0.01	0.00	0.00	0.03	0.02	0.00	0.0	0.0	0.0	0.0	0.0	0.0	0.0	0.0	0.0	0.0	0.0	1.00E+00	9.46E-01	9.51E-01	1.00E+00
	ZNF430	8	3	1	0	3	0	1	0	0	0	0	0	0	3	0	0	1.35	1.54	1.22	0.10	0.04	0.35	0.08	0.07	1.23	0.68	0.09	0.0	0.6	0.0	0.0	0.0	0.0	0.0	0.0	2.4	0.0	0.0	7.52E-01	2.51E-01	7.06E-01	5.77E-01
	ZNF431	4	1	1	0	2	0	1	0	0	0	0	0	0	2	0	0	0.99	1.13	0.89	0.08	0.03	0.26	0.06	0.05	0.90	0.49	0.06	0.0	0.9	0.0	0.0	0.0	0.0	0.0	0.0	2.2	0.0	0.0	9.34E-01	3.88E-01	7.47E-01	8.10E-01
	ZNF432	1	1	0	0	0	0	0	0	0	0	0	0	0	0	0	0	0.29	0.33	0.26	0.02	0.01	0.08	0.02	0.01	0.27	0.15	0.02	0.0	0.0	0.0	0.0	0.0	0.0	0.0	0.0	0.0	0.0	0.0	9.99E-01	6.41E-01	8.61E-01	9.91E-01
	ZNF433	0	1	0	0	0	0	0	0	0	0	0	0	0	0	0	0	0.48	0.54	0.43	0.04	0.01	0.12	0.03	0.02	0.43	0.24	0.03	0.0	0.0	0.0	0.0	0.0	0.0	0.0	0.0	0.0	0.0	0.0	9.93E-01	4.85E-01	8.23E-01	9.72E-01
	ZNF436	2	6	6	0	0	0	1	5	0	0	0	0	0	0	0	0	0.73	0.83	0.66	0.06	0.02	0.19	0.04	0.04	0.66	0.37	0.05	0.0	1.2	7.6	0.0	0.0	0.0	0.0	0.0	0.0	0.0	0.0	6.70E-04	4.50E-07	7.82E-01	9.30E-01
	ZNF439	0	7	0	0	0	0	0	0	0	0	0	0	0	0	0	0	1.32	1.50	1.19	0.10	0.04	0.34	0.07	0.06	1.20	0.66	0.09	0.0	0.0	0.0	0.0	0.0	0.0	0.0	0.0	0.0	0.0	0.0	7.65E-01	1.35E-01	7.10E-01	7.88E-01
	ZNF44	4	1	1	0	0	0	1	0	0	0	0	0	0	0	0	0	0.70	0.79	0.63	0.05	0.02	0.18	0.04	0.03	0.63	0.35	0.05	0.0	1.3	0.0	0.0	0.0	0.0	0.0	0.0	0.0	0.0	0.0	9.87E-01	5.02E-01	7.87E-01	9.37E-01
	ZNF440	2	8	2	0	0	0	1	1	0	0	0	0	0	0	0	0	1.87	2.13	1.69	0.14	0.05	0.49	0.10	0.09	1.69	0.93	0.12	0.0	0.5	0.6	0.0	0.0	0.0	0.0	0.0	0.0	0.0	0.0	7.83E-01	2.54E-01	6.58E-01	6.34E-01
	ZNF441	0	2	0	0	0	0	0	0	0	0	0	0	0	0	0	0	0.33	0.38	0.30	0.03	0.01	0.09	0.02	0.02	0.30	0.16	0.02	0.0	0.0	0.0	0.0	0.0	0.0	0.0	0.0	0.0	0.0	0.0	9.98E-01	6.06E-01	8.52E-01	9.88E-01
	ZNF442	1	0	1	0	0	0	1	0	0	0	0	0	0	0	0	0	0.07	0.08	0.07	0.01	0.00	0.02	0.00	0.00	0.07	0.04	0.00	0.0	12.0	0.0	0.0	0.0	0.0	0.0	0.0	0.0	0.0	0.0	4.10E-01	6.06E-03	9.30E-01	1.00E+00
	ZNF443	1	0	0	0	0	0	0	0	0	0	0	0	0	0	0	0	0.11	0.13	0.10	0.01	0.00	0.03	0.01	0.01	0.10	0.05	0.01	0.0	0.0	0.0	0.0	0.0	0.0	0.0	0.0	0.0	0.0	0.0	1.00E+00	8.46E-01	9.14E-01	9.99E-01
	ZNF444	39	17	6	0	1	3	1	2	0	0	0	0	0	0	0	1	5.67	6.46	5.13	0.43	0.16	1.48	0.32	0.27	5.15	2.84	0.37	0.5	0.2	0.4	0.0	0.0	0.0	0.0	0.0	0.0	0.0	2.7	3.41E-02	2.04E-02	4.40E-01	4.86E-02
	ZNF449	5	4	2	0	0	0	1	1	0	0	0	0	0	0	0	0	0.48	0.54	0.43	0.04	0.01	0.12	0.03	0.02	0.43	0.24	0.03	0.0	1.8	2.3	0.0	0.0	0.0	0.0	0.0	0.0	0.0	0.0	9.90E-01	4.45E-01	8.23E-01	9.72E-01
	ZNF45	3	2	0	0	0	0	0	0	0	0	0	0	0	0	0	0	0.33	0.38	0.30	0.03	0.01	0.09	0.02	0.02	0.30	0.16	0.02	0.0	0.0	0.0	0.0	0.0	0.0	0.0	0.0	0.0	0.0	0.0	9.98E-01	6.06E-01	8.52E-01	9.88E-01
	ZNF454	0	0	0	0	0	0	0	0	0	0	0	0	0	0	0	0	0.00	0.00	0.00	0.00	0.00	0.00	0.00	0.00	0.00	0.00	0.00	na	na	na	na	na	na	na	na	na	na	na	na	na	na	na
	ZNF460	31	17	4	0	17	1	2	1	0	0	0	0	0	0	17	0	5.71	6.51	5.16	0.43	0.17	1.49	0.32	0.27	5.18	2.86	0.37	0.2	0.3	0.2	0.0	0.0	0.0	0.0	0.0	0.0	5.9	0.0	1.05E-14	5.64E-03	4.39E-01	2.69E-15
	ZNF461	0	4	4	0	0	2	1	1	0	0	0	0	0	0	0	0	0.29	0.33	0.26	0.02	0.01	0.08	0.02	0.01	0.27	0.15	0.02	6.8	3.0	3.8	0.0	0.0	0.0	0.0	0.0	0.0	0.0	0.0	1.77E-01	1.27E-03	8.61E-01	9.91E-01
	ZNF468	0	2	1	0	0	0	1	0	0	0	0	0	0	0	0	0	0.26	0.29	0.23	0.02	0.01	0.07	0.01	0.01	0.23	0.13	0.02	0.0	3.4	0.0	0.0	0.0	0.0	0.0	0.0	0.0	0.0	0.0	9.88E-01	3.32E-01	8.70E-01	9.93E-01
	ZNF471	5	0	0	0	0	0	0	0	0	0	0	0	0	0	0	0	0.70	0.79	0.63	0.05	0.02	0.18	0.04	0.03	0.63	0.35	0.05	0.0	0.0	0.0	0.0	0.0	0.0	0.0	0.0	0.0	0.0	0.0	9.68E-01	3.47E-01	7.87E-01	9.37E-01
	ZNF479	2	1	0	0	0	0	0	0	0	0	0	0	0	0	0	0	0.29	0.33	0.26	0.02	0.01	0.08	0.02	0.01	0.27	0.15	0.02	0.0	0.0	0.0	0.0	0.0	0.0	0.0	0.0	0.0	0.0	0.0	9.99E-01	6.41E-01	8.61E-01	9.91E-01
	ZNF480	1	3	1	0	0	0	0	1	0	0	0	0	0	0	0	0	0.33	0.38	0.30	0.03	0.01	0.09	0.02	0.02	0.30	0.16	0.02	0.0	0.0	3.4	0.0	0.0	0.0	0.0	0.0	0.0	0.0	0.0	9.81E-01	3.07E-01	8.52E-01	9.88E-01
	ZNF483	41	13	4	0	3	0	1	3	0	0	0	0	0	2	1	0	2.93	3.34	2.65	0.22	0.08	0.76	0.16	0.14	2.66	1.47	0.19	0.0	0.3	1.1	0.0	0.0	0.0	0.0	0.0	0.8	0.7	0.0	7.73E-01	9.97E-02	5.79E-01	9.05E-01
	ZNF484	1	3	1	0	0	0	1	0	0	0	0	0	0	0	0	0	0.48	0.54	0.43	0.04	0.01	0.12	0.03	0.02	0.43	0.24	0.03	0.0	1.8	0.0	0.0	0.0	0.0	0.0	0.0	0.0	0.0	0.0	9.94E-01	5.24E-01	8.23E-01	9.72E-01
	ZNF485	6	2	0	0	14	0	0	0	0	0	0	0	0	3	11	0	1.13	1.29	1.03	0.09	0.03	0.30	0.06	0.05	1.03	0.57	0.07	0.0	0.0	0.0	0.0	0.0	0.0	0.0	0.0	2.9	19.4	0.0	2.18E-37	1.78E-01	7.30E-01	2.24E-40
	ZNF490	7	6	2	0	1	0	2	0	0	0	0	0	0	0	0	1	2.38	2.71	2.15	0.18	0.07	0.62	0.13	0.11	2.16	1.19	0.15	0.0	0.7	0.0	0.0	0.0	0.0	0.0	0.0	0.0	0.0	6.5	1.81E-01	9.47E-02	6.17E-01	1.15E-01
	ZNF491	0	1	1	0	0	0	0	1	0	0	0	0	0	0	0	0	1.06	1.21	0.96	0.08	0.03	0.28	0.06	0.05	0.96	0.53	0.07	0.0	0.0	1.0	0.0	0.0	0.0	0.0	0.0	0.0	0.0	0.0	9.31E-01	3.21E-01	7.38E-01	8.56E-01
	ZNF492	3	0	0	0	1	0	0	0	0	0	0	0	0	1	0	0	0.40	0.46	0.36	0.03	0.01	0.10	0.02	0.02	0.37	0.20	0.03	0.0	0.0	0.0	0.0	0.0	0.0	0.0	0.0	2.7	0.0	0.0	9.87E-01	5.42E-01	8.37E-01	9.16E-01
	ZNF493	0	0	0	0	1	0	0	0	0	0	0	0	0	1	0	0	0.15	0.17	0.13	0.01	0.00	0.04	0.01	0.01	0.13	0.07	0.01	0.0	0.0	0.0	0.0	0.0	0.0	0.0	0.0	7.5	0.0	0.0	7.93E-01	8.00E-01	9.01E-01	3.27E-01
	ZNF496	33	16	1	0	5	0	0	1	0	0	0	0	0	5	0	0	3.11	3.54	2.81	0.24	0.09	0.81	0.17	0.15	2.82	1.56	0.20	0.0	0.0	0.4	0.0	0.0	0.0	0.0	0.0	1.8	0.0	0.0	2.40E-01	2.00E-02	5.68E-01	4.71E-01
	ZNF497	12	4	0	0	0	0	0	0	0	0	0	0	0	0	0	0	1.68	1.92	1.52	0.13	0.05	0.44	0.09	0.08	1.53	0.84	0.11	0.0	0.0	0.0	0.0	0.0	0.0	0.0	0.0	0.0	0.0	0.0	5.91E-01	7.72E-02	6.74E-01	6.86E-01
	ZNF500	5	3	0	0	1	0	0	0	0	0	0	0	0	0	0	1	0.37	0.42	0.33	0.03	0.01	0.10	0.02	0.02	0.33	0.18	0.02	0.0	0.0	0.0	0.0	0.0	0.0	0.0	0.0	0.0	0.0	42.2	7.38E-06	5.73E-01	8.45E-01	9.89E-08
	ZNF501	0	0	0	0	0	0	0	0	0	0	0	0	0	0	0	0	0.51	0.58	0.46	0.04	0.01	0.13	0.03	0.02	0.47	0.26	0.03	0.0	0.0	0.0	0.0	0.0	0.0	0.0	0.0	0.0	0.0	0.0	9.90E-01	4.59E-01	8.17E-01	9.67E-01
	ZNF502	1	0	0	0	0	0	0	0	0	0	0	0	0	0	0	0	0.15	0.17	0.13	0.01	0.00	0.04	0.01	0.01	0.13	0.07	0.01	0.0	0.0	0.0	0.0	0.0	0.0	0.0	0.0	0.0	0.0	0.0	1.00E+00	8.00E-01	9.01E-01	9.98E-01
	ZNF506	0	0	0	0	0	0	0	0	0	0	0	0	0	0	0	0	0.07	0.08	0.07	0.01	0.00	0.02	0.00	0.00	0.07	0.04	0.00	0.0	0.0	0.0	0.0	0.0	0.0	0.0	0.0	0.0	0.0	0.0	1.00E+00	8.95E-01	9.30E-01	1.00E+00
	ZNF514	4	4	5	0	0	1	1	3	0	0	0	0	0	0	0	0	0.77	0.88	0.69	0.06	0.02	0.20	0.04	0.04	0.70	0.38	0.05	1.3	1.1	4.3	0.0	0.0	0.0	0.0	0.0	0.0	0.0	0.0	5.10E-01	2.08E-02	7.76E-01	9.23E-01
	ZNF517	0	5	0	0	0	0	0	0	0	0	0	0	0	0	0	0	1.65	1.88	1.49	0.13	0.05	0.43	0.09	0.08	1.50	0.82	0.11	0.0	0.0	0.0	0.0	0.0	0.0	0.0	0.0	0.0	0.0	0.0	6.08E-01	8.16E-02	6.77E-01	6.96E-01
	ZNF524	11	14	4	0	1	0	3	1	0	0	0	0	0	1	0	0	2.52	2.88	2.28	0.19	0.07	0.66	0.14	0.12	2.29	1.26	0.16	0.0	1.0	0.4	0.0	0.0	0.0	0.0	0.0	0.4	0.0	0.0	7.63E-01	1.97E-01	6.07E-01	6.88E-01
	ZNF525	0	0	0	0	0	0	0	0	0	0	0	0	0	0	0	0	0.11	0.13	0.10	0.01	0.00	0.03	0.01	0.01	0.10	0.05	0.01	0.0	0.0	0.0	0.0	0.0	0.0	0.0	0.0	0.0	0.0	0.0	1.00E+00	8.46E-01	9.14E-01	9.99E-01
	ZNF528	0	1	2	0	0	0	2	0	0	0	0	0	0	0	0	0	0.15	0.17	0.13	0.01	0.00	0.04	0.01	0.01	0.13	0.07	0.01	0.0	12.0	0.0	0.0	0.0	0.0	0.0	0.0	0.0	0.0	0.0	2.32E-02	3.67E-05	9.01E-01	9.98E-01
	ZNF529	0	1	2	0	0	0	1	1	0	0	0	0	0	0	0	0	0.22	0.25	0.20	0.02	0.01	0.06	0.01	0.01	0.20	0.11	0.01	0.0	4.0	5.0	0.0	0.0	0.0	0.0	0.0	0.0	0.0	0.0	8.04E-01	5.77E-02	8.79E-01	9.95E-01
	ZNF530	0	0	0	0	0	0	0	0	0	0	0	0	0	0	0	0	0.07	0.08	0.07	0.01	0.00	0.02	0.00	0.00	0.07	0.04	0.00	0.0	0.0	0.0	0.0	0.0	0.0	0.0	0.0	0.0	0.0	0.0	1.00E+00	8.95E-01	9.30E-01	1.00E+00
	ZNF534	0	0	0	0	0	0	0	0	0	0	0	0	0	0	0	0	0.11	0.13	0.10	0.01	0.00	0.03	0.01	0.01	0.10	0.05	0.01	0.0	0.0	0.0	0.0	0.0	0.0	0.0	0.0	0.0	0.0	0.0	1.00E+00	8.46E-01	9.14E-01	9.99E-01
	ZNF540	0	0	0	0	0	0	0	0	0	0	0	0	0	0	0	0	0.07	0.08	0.07	0.01	0.00	0.02	0.00	0.00	0.07	0.04	0.00	0.0	0.0	0.0	0.0	0.0	0.0	0.0	0.0	0.0	0.0	0.0	1.00E+00	8.95E-01	9.30E-01	1.00E+00
	ZNF543	4	1	2	0	0	0	2	0	0	0	0	0	0	0	0	0	0.77	0.88	0.69	0.06	0.02	0.20	0.04	0.04	0.70	0.38	0.05	0.0	2.3	0.0	0.0	0.0	0.0	0.0	0.0	0.0	0.0	0.0	9.28E-01	2.34E-01	7.76E-01	9.23E-01
	ZNF544	0	2	3	0	0	1	2	0	0	0	0	0	0	0	0	0	0.70	0.79	0.63	0.05	0.02	0.18	0.04	0.03	0.63	0.35	0.05	1.4	2.5	0.0	0.0	0.0	0.0	0.0	0.0	0.0	0.0	0.0	9.49E-01	2.72E-01	7.87E-01	9.37E-01
	ZNF546	2	0	0	0	0	0	0	0	0	0	0	0	0	0	0	0	0.07	0.08	0.07	0.01	0.00	0.02	0.00	0.00	0.07	0.04	0.00	0.0	0.0	0.0	0.0	0.0	0.0	0.0	0.0	0.0	0.0	0.0	1.00E+00	8.95E-01	9.30E-01	1.00E+00
	ZNF547	3	0	0	1	0	0	0	0	0	1	0	0	0	0	0	0	0.59	0.67	0.53	0.04	0.02	0.15	0.03	0.03	0.53	0.29	0.04	0.0	0.0	0.0	0.0	58.9	0.0	0.0	0.0	0.0	0.0	0.0	3.97E-09	4.10E-01	4.50E-14	9.56E-01
	ZNF548	0	1	1	0	0	0	1	0	0	0	0	0	0	0	0	0	0.15	0.17	0.13	0.01	0.00	0.04	0.01	0.01	0.13	0.07	0.01	0.0	6.0	0.0	0.0	0.0	0.0	0.0	0.0	0.0	0.0	0.0	9.09E-01	1.09E-01	9.01E-01	9.98E-01
	ZNF549	0	1	0	0	0	0	0	0	0	0	0	0	0	0	0	0	0.18	0.21	0.17	0.01	0.01	0.05	0.01	0.01	0.17	0.09	0.01	0.0	0.0	0.0	0.0	0.0	0.0	0.0	0.0	0.0	0.0	0.0	1.00E+00	7.57E-01	8.90E-01	9.97E-01
	ZNF550	9	18	2	0	2	0	0	2	0	0	0	0	0	0	2	0	3.48	3.96	3.14	0.26	0.10	0.91	0.19	0.17	3.16	1.74	0.23	0.0	0.0	0.6	0.0	0.0	0.0	0.0	0.0	0.0	1.1	0.0	2.29E-01	1.97E-02	5.46E-01	4.55E-01
	ZNF551	0	0	0	0	0	0	0	0	0	0	0	0	0	0	0	0	0.04	0.04	0.03	0.00	0.00	0.01	0.00	0.00	0.03	0.02	0.00	0.0	0.0	0.0	0.0	0.0	0.0	0.0	0.0	0.0	0.0	0.0	1.00E+00	9.46E-01	9.51E-01	1.00E+00
	ZNF554	1	4	1	0	0	0	1	0	0	0	0	0	0	0	0	0	0.48	0.54	0.43	0.04	0.01	0.12	0.03	0.02	0.43	0.24	0.03	0.0	1.8	0.0	0.0	0.0	0.0	0.0	0.0	0.0	0.0	0.0	9.94E-01	5.24E-01	8.23E-01	9.72E-01
	ZNF555	0	2	1	0	0	0	1	0	0	0	0	0	0	0	0	0	0.55	0.63	0.50	0.04	0.02	0.14	0.03	0.03	0.50	0.27	0.04	0.0	1.6	0.0	0.0	0.0	0.0	0.0	0.0	0.0	0.0	0.0	9.93E-01	5.30E-01	8.10E-01	9.62E-01
	ZNF556	2	2	1	0	3	0	1	0	0	0	0	0	0	0	3	0	0.77	0.88	0.69	0.06	0.02	0.20	0.04	0.04	0.70	0.38	0.05	0.0	1.1	0.0	0.0	0.0	0.0	0.0	0.0	0.0	7.8	0.0	2.60E-02	4.77E-01	7.76E-01	2.10E-03
	ZNF557	2	7	1	0	0	0	0	1	0	0	0	0	0	0	0	0	0.66	0.75	0.60	0.05	0.02	0.17	0.04	0.03	0.60	0.33	0.04	0.0	0.0	1.7	0.0	0.0	0.0	0.0	0.0	0.0	0.0	0.0	9.82E-01	4.31E-01	7.93E-01	9.44E-01
	ZNF558	2	4	3	0	0	1	1	1	0	0	0	0	0	0	0	0	1.21	1.38	1.09	0.09	0.04	0.31	0.07	0.06	1.10	0.60	0.08	0.8	0.7	0.9	0.0	0.0	0.0	0.0	0.0	0.0	0.0	0.0	9.91E-01	9.30E-01	7.22E-01	8.18E-01
	ZNF559	0	7	0	0	0	0	0	0	0	0	0	0	0	0	0	0	0.66	0.75	0.60	0.05	0.02	0.17	0.04	0.03	0.60	0.33	0.04	0.0	0.0	0.0	0.0	0.0	0.0	0.0	0.0	0.0	0.0	0.0	9.74E-01	3.67E-01	7.93E-01	9.44E-01
	ZNF560	0	0	0	0	0	0	0	0	0	0	0	0	0	0	0	0	0.00	0.00	0.00	0.00	0.00	0.00	0.00	0.00	0.00	0.00	0.00	na	na	na	na	na	na	na	na	na	na	na	na	na	na	na
	ZNF561	3	3	2	0	0	0	2	0	0	0	0	0	0	0	0	0	0.51	0.58	0.46	0.04	0.01	0.13	0.03	0.02	0.47	0.26	0.03	0.0	3.4	0.0	0.0	0.0	0.0	0.0	0.0	0.0	0.0	0.0	8.62E-01	1.10E-01	8.17E-01	9.67E-01
	ZNF562	4	10	1	0	0	0	1	0	0	0	0	0	0	0	0	0	1.32	1.50	1.19	0.10	0.04	0.34	0.07	0.06	1.20	0.66	0.09	0.0	0.7	0.0	0.0	0.0	0.0	0.0	0.0	0.0	0.0	0.0	8.75E-01	2.62E-01	7.10E-01	7.88E-01
	ZNF563	0	2	0	0	0	0	0	0	0	0	0	0	0	0	0	0	0.62	0.71	0.56	0.05	0.02	0.16	0.03	0.03	0.56	0.31	0.04	0.0	0.0	0.0	0.0	0.0	0.0	0.0	0.0	0.0	0.0	0.0	9.79E-01	3.88E-01	7.98E-01	9.50E-01
	ZNF564	4	8	0	0	0	0	0	0	0	0	0	0	0	0	0	0	2.01	2.29	1.82	0.15	0.06	0.52	0.11	0.10	1.83	1.01	0.13	0.0	0.0	0.0	0.0	0.0	0.0	0.0	0.0	0.0	0.0	0.0	4.37E-01	4.68E-02	6.46E-01	5.94E-01
	ZNF565	0	1	0	0	0	0	0	0	0	0	0	0	0	0	0	0	0.11	0.13	0.10	0.01	0.00	0.03	0.01	0.01	0.10	0.05	0.01	0.0	0.0	0.0	0.0	0.0	0.0	0.0	0.0	0.0	0.0	0.0	1.00E+00	8.46E-01	9.14E-01	9.99E-01
	ZNF566	0	3	1	0	0	0	0	1	0	0	0	0	0	0	0	0	0.62	0.71	0.56	0.05	0.02	0.16	0.03	0.03	0.56	0.31	0.04	0.0	0.0	1.8	0.0	0.0	0.0	0.0	0.0	0.0	0.0	0.0	9.84E-01	4.33E-01	7.98E-01	9.50E-01
	ZNF567	0	4	2	0	0	0	1	1	0	0	0	0	0	0	0	0	0.51	0.58	0.46	0.04	0.01	0.13	0.03	0.02	0.47	0.26	0.03	0.0	1.7	2.2	0.0	0.0	0.0	0.0	0.0	0.0	0.0	0.0	9.92E-01	4.89E-01	8.17E-01	9.67E-01
	ZNF568	1	3	1	0	0	0	0	1	0	0	0	0	0	0	0	0	0.26	0.29	0.23	0.02	0.01	0.07	0.01	0.01	0.23	0.13	0.02	0.0	0.0	4.3	0.0	0.0	0.0	0.0	0.0	0.0	0.0	0.0	9.64E-01	2.12E-01	8.70E-01	9.93E-01
	ZNF569	0	3	0	0	0	0	0	0	0	0	0	0	0	0	0	0	0.44	0.50	0.40	0.03	0.01	0.11	0.02	0.02	0.40	0.22	0.03	0.0	0.0	0.0	0.0	0.0	0.0	0.0	0.0	0.0	0.0	0.0	9.95E-01	5.13E-01	8.30E-01	9.77E-01
	ZNF57	2	2	3	0	0	1	2	0	0	0	0	0	0	0	0	0	0.62	0.71	0.56	0.05	0.02	0.16	0.03	0.03	0.56	0.31	0.04	1.6	2.8	0.0	0.0	0.0	0.0	0.0	0.0	0.0	0.0	0.0	9.30E-01	2.08E-01	7.98E-01	9.50E-01
	ZNF570	0	2	1	0	0	0	0	1	0	0	0	0	0	0	0	0	0.07	0.08	0.07	0.01	0.00	0.02	0.00	0.00	0.07	0.04	0.00	0.0	0.0	15.1	0.0	0.0	0.0	0.0	0.0	0.0	0.0	0.0	1.98E-01	1.27E-03	9.30E-01	1.00E+00
ZF.C2H2	ZNF571	0	0	1	2	0	0	1	0	2	0	0	0	0	0	0	0	0.26	0.29	0.23	0.02	0.01	0.07	0.01	0.01	0.23	0.13	0.02	0.0	3.4	0.0	102.7	0.0	0.0	0.0	0.0	0.0	0.0	0.0	2.25E-38	3.32E-01	1.02E-45	9.93E-01
	ZNF572	13	2	2	0	0	1	1	0	0	0	0	0	0	0	0	0	2.60	2.96	2.35	0.20	0.08	0.68	0.14	0.12	2.36	1.30	0.17	0.4	0.3	0.0	0.0	0.0	0.0	0.0	0.0	0.0	0.0	0.0	4.69E-01	9.88E-02	6.01E-01	4.44E-01
	ZNF575	2	2	1	0	0	0	1	0	0	0	0	0	0	0	0	0	0.95	1.08	0.86	0.07	0.03	0.25	0.05	0.05	0.86	0.48	0.06	0.0	0.9	0.0	0.0	0.0	0.0	0.0	0.0	0.0	0.0	0.0	9.61E-01	4.03E-01	7.52E-01	8.83E-01
	ZNF576	13	6	0	0	0	0	0	0	0	0	0	0	0	0	0	0	1.39	1.58	1.26	0.11	0.04	0.36	0.08	0.07	1.26	0.70	0.09	0.0	0.0	0.0	0.0	0.0	0.0	0.0	0.0	0.0	0.0	0.0	7.32E-01	1.21E-01	7.02E-01	7.68E-01
	ZNF577	0	0	0	0	0	0	0	0	0	0	0	0	0	0	0	0	0.18	0.21	0.17	0.01	0.01	0.05	0.01	0.01	0.17	0.09	0.01	0.0	0.0	0.0	0.0	0.0	0.0	0.0	0.0	0.0	0.0	0.0	1.00E+00	7.57E-01	8.90E-01	9.97E-01
	ZNF581	13	20	4	0	0	0	2	2	0	0	0	0	0	0	0	0	5.23	5.96	4.73	0.40	0.15	1.36	0.29	0.25	4.75	2.62	0.34	0.0	0.3	0.4	0.0	0.0	0.0	0.0	0.0	0.0	0.0	0.0	3.32E-02	8.91E-03	4.58E-01	8.69E-02
	ZNF582	0	1	0	0	0	0	0	0	0	0	0	0	0	0	0	0	0.22	0.25	0.20	0.02	0.01	0.06	0.01	0.01	0.20	0.11	0.01	0.0	0.0	0.0	0.0	0.0	0.0	0.0	0.0	0.0	0.0	0.0	1.00E+00	7.16E-01	8.79E-01	9.95E-01
	ZNF583	0	1	0	0	0	0	0	0	0	0	0	0	0	0	0	0	0.11	0.13	0.10	0.01	0.00	0.03	0.01	0.01	0.10	0.05	0.01	0.0	0.0	0.0	0.0	0.0	0.0	0.0	0.0	0.0	0.0	0.0	1.00E+00	8.46E-01	9.14E-01	9.99E-01
	ZNF584	0	2	0	0	0	0	0	0	0	0	0	0	0	0	0	0	0.40	0.46	0.36	0.03	0.01	0.10	0.02	0.02	0.37	0.20	0.03	0.0	0.0	0.0	0.0	0.0	0.0	0.0	0.0	0.0	0.0	0.0	9.96E-01	5.42E-01	8.37E-01	9.81E-01
	ZNF585A	0	3	0	0	0	0	0	0	0	0	0	0	0	0	0	0	0.15	0.17	0.13	0.01	0.00	0.04	0.01	0.01	0.13	0.07	0.01	0.0	0.0	0.0	0.0	0.0	0.0	0.0	0.0	0.0	0.0	0.0	1.00E+00	8.00E-01	9.01E-01	9.98E-01
	ZNF585B	0	1	0	0	0	0	0	0	0	0	0	0	0	0	0	0	0.22	0.25	0.20	0.02	0.01	0.06	0.01	0.01	0.20	0.11	0.01	0.0	0.0	0.0	0.0	0.0	0.0	0.0	0.0	0.0	0.0	0.0	1.00E+00	7.16E-01	8.79E-01	9.95E-01
	ZNF586	1	0	0	0	0	0	0	0	0	0	0	0	0	0	0	0	0.22	0.25	0.20	0.02	0.01	0.06	0.01	0.01	0.20	0.11	0.01	0.0	0.0	0.0	0.0	0.0	0.0	0.0	0.0	0.0	0.0	0.0	1.00E+00	7.16E-01	8.79E-01	9.95E-01
	ZNF589	3	2	2	0	0	0	2	0	0	0	0	0	0	0	0	0	0.62	0.71	0.56	0.05	0.02	0.16	0.03	0.03	0.56	0.31	0.04	0.0	2.8	0.0	0.0	0.0	0.0	0.0	0.0	0.0	0.0	0.0	9.08E-01	1.71E-01	7.98E-01	9.50E-01
	ZNF594	0	2	0	0	0	0	0	0	0	0	0	0	0	0	0	0	0.22	0.25	0.20	0.02	0.01	0.06	0.01	0.01	0.20	0.11	0.01	0.0	0.0	0.0	0.0	0.0	0.0	0.0	0.0	0.0	0.0	0.0	1.00E+00	7.16E-01	8.79E-01	9.95E-01
	ZNF595	1	1	1	0	0	0	1	0	0	0	0	0	0	0	0	0	0.26	0.29	0.23	0.02	0.01	0.07	0.01	0.01	0.23	0.13	0.02	0.0	3.4	0.0	0.0	0.0	0.0	0.0	0.0	0.0	0.0	0.0	9.88E-01	3.32E-01	8.70E-01	9.93E-01
	ZNF596	1	1	0	0	0	0	0	0	0	0	0	0	0	0	0	0	0.07	0.08	0.07	0.01	0.00	0.02	0.00	0.00	0.07	0.04	0.00	0.0	0.0	0.0	0.0	0.0	0.0	0.0	0.0	0.0	0.0	0.0	1.00E+00	8.95E-01	9.30E-01	1.00E+00
	ZNF599	0	0	0	0	0	0	0	0	0	0	0	0	0	0	0	0	0.37	0.42	0.33	0.03	0.01	0.10	0.02	0.02	0.33	0.18	0.02	0.0	0.0	0.0	0.0	0.0	0.0	0.0	0.0	0.0	0.0	0.0	9.98E-01	5.73E-01	8.45E-01	9.84E-01
	ZNF600	0	0	0	0	0	0	0	0	0	0	0	0	0	0	0	0	0.11	0.13	0.10	0.01	0.00	0.03	0.01	0.01	0.10	0.05	0.01	0.0	0.0	0.0	0.0	0.0	0.0	0.0	0.0	0.0	0.0	0.0	1.00E+00	8.46E-01	9.14E-01	9.99E-01
	ZNF605	0	1	0	0	0	0	0	0	0	0	0	0	0	0	0	0	0.07	0.08	0.07	0.01	0.00	0.02	0.00	0.00	0.07	0.04	0.00	0.0	0.0	0.0	0.0	0.0	0.0	0.0	0.0	0.0	0.0	0.0	1.00E+00	8.95E-01	9.30E-01	1.00E+00
	ZNF607	2	3	2	0	0	1	0	1	0	0	0	0	0	0	0	0	0.44	0.50	0.40	0.03	0.01	0.11	0.02	0.02	0.40	0.22	0.03	2.3	0.0	2.5	0.0	0.0	0.0	0.0	0.0	0.0	0.0	0.0	9.82E-01	3.44E-01	8.30E-01	9.77E-01
	ZNF610	0	3	0	0	0	0	0	0	0	0	0	0	0	0	0	0	0.33	0.38	0.30	0.03	0.01	0.09	0.02	0.02	0.30	0.16	0.02	0.0	0.0	0.0	0.0	0.0	0.0	0.0	0.0	0.0	0.0	0.0	9.98E-01	6.06E-01	8.52E-01	9.88E-01
	ZNF611	1	2	1	0	0	0	1	0	0	0	0	0	0	0	0	0	0.48	0.54	0.43	0.04	0.01	0.12	0.03	0.02	0.43	0.24	0.03	0.0	1.8	0.0	0.0	0.0	0.0	0.0	0.0	0.0	0.0	0.0	9.94E-01	5.24E-01	8.23E-01	9.72E-01
	ZNF613	0	3	0	0	0	0	0	0	0	0	0	0	0	0	0	0	0.22	0.25	0.20	0.02	0.01	0.06	0.01	0.01	0.20	0.11	0.01	0.0	0.0	0.0	0.0	0.0	0.0	0.0	0.0	0.0	0.0	0.0	1.00E+00	7.16E-01	8.79E-01	9.95E-01
	ZNF614	1	2	0	0	0	0	0	0	0	0	0	0	0	0	0	0	0.29	0.33	0.26	0.02	0.01	0.08	0.02	0.01	0.27	0.15	0.02	0.0	0.0	0.0	0.0	0.0	0.0	0.0	0.0	0.0	0.0	0.0	9.99E-01	6.41E-01	8.61E-01	9.91E-01
	ZNF615	0	2	2	0	0	0	1	1	0	0	0	0	0	0	0	0	0.18	0.21	0.17	0.01	0.01	0.05	0.01	0.01	0.17	0.09	0.01	0.0	4.8	6.0	0.0	0.0	0.0	0.0	0.0	0.0	0.0	0.0	6.53E-01	2.47E-02	8.90E-01	9.97E-01
	ZNF616	3	1	1	0	3	0	0	1	0	0	0	0	0	3	0	0	0.70	0.79	0.63	0.05	0.02	0.18	0.04	0.03	0.63	0.35	0.05	0.0	0.0	1.6	0.0	0.0	0.0	0.0	0.0	4.8	0.0	0.0	3.33E-01	4.26E-01	7.87E-01	8.95E-02
	ZNF619	1	1	0	0	0	0	0	0	0	0	0	0	0	0	0	0	0.07	0.08	0.07	0.01	0.00	0.02	0.00	0.00	0.07	0.04	0.00	0.0	0.0	0.0	0.0	0.0	0.0	0.0	0.0	0.0	0.0	0.0	1.00E+00	8.95E-01	9.30E-01	1.00E+00
	ZNF620	7	10	1	0	2	1	0	0	0	0	1	0	1	0	0	0	3.11	3.54	2.81	0.24	0.09	0.81	0.17	0.15	2.82	1.56	0.20	0.3	0.0	0.0	0.0	0.0	1.2	0.0	6.7	0.0	0.0	0.0	5.92E-02	2.04E-02	5.68E-01	8.60E-02
	ZNF621	0	3	0	0	0	0	0	0	0	0	0	0	0	0	0	0	0.62	0.71	0.56	0.05	0.02	0.16	0.03	0.03	0.56	0.31	0.04	0.0	0.0	0.0	0.0	0.0	0.0	0.0	0.0	0.0	0.0	0.0	9.79E-01	3.88E-01	7.98E-01	9.50E-01
	ZNF623	1	1	3	0	0	0	3	0	0	0	0	0	0	0	0	0	0.37	0.42	0.33	0.03	0.01	0.10	0.02	0.02	0.33	0.18	0.02	0.0	7.2	0.0	0.0	0.0	0.0	0.0	0.0	0.0	0.0	0.0	6.58E-02	2.37E-04	8.45E-01	9.84E-01
	ZNF624	8	4	1	0	5	0	1	0	0	0	0	0	0	5	0	0	1.83	2.09	1.65	0.14	0.05	0.48	0.10	0.09	1.66	0.92	0.12	0.0	0.5	0.0	0.0	0.0	0.0	0.0	0.0	3.0	0.0	0.0	2.44E-01	1.32E-01	6.61E-01	1.35E-01

ZNF625	0	2	0	0	0	0	0	0	0	0	0	0	0	0	0	0	0.51	0.58	0.46	0.04	0.01	0.13	0.03	0.02	0.47	0.26	0.03	0.0	0.0	0.0	0.0	0.0	0.0	0.0	0.0	0.0	0.0	0.0	9.90E-01	4.59E-01	8.17E-01	9.67E-01
ZNF626	1	1	0	0	0	0	0	0	0	0	0	0	0	0	0	0	0.11	0.13	0.10	0.01	0.00	0.03	0.01	0.01	0.10	0.05	0.01	0.0	0.0	0.0	0.0	0.0	0.0	0.0	0.0	0.0	0.0	0.0	1.00E+00	8.46E-01	9.14E-01	9.99E-01
ZNF627	0	2	0	0	0	0	0	0	0	0	0	0	0	0	0	0	1.02	1.17	0.93	0.08	0.03	0.27	0.06	0.05	0.93	0.51	0.07	0.0	0.0	0.0	0.0	0.0	0.0	0.0	0.0	0.0	0.0	0.0	8.84E-01	2.10E-01	7.43E-01	8.65E-01
ZNF641	0	4	1	0	0	0	0	1	0	0	0	0	0	0	0	0	0.77	0.88	0.69	0.06	0.02	0.20	0.04	0.04	0.70	0.38	0.05	0.0	0.0	1.4	0.0	0.0	0.0	0.0	0.0	0.0	0.0	0.0	9.74E-01	4.11E-01	7.76E-01	9.23E-01
ZNF648	17	3	0	0	0	0	0	0	0	0	0	0	0	0	0	0	2.34	2.67	2.12	0.18	0.07	0.61	0.13	0.11	2.13	1.17	0.15	0.0	0.0	0.0	0.0	0.0	0.0	0.0	0.0	0.0	0.0	0.0	3.07E-01	2.83E-02	6.20E-01	5.07E-01
ZNF649	0	7	2	0	0	0	0	2	0	0	0	0	0	0	0	0	0.48	0.54	0.43	0.04	0.01	0.12	0.03	0.02	0.43	0.24	0.03	0.0	0.0	4.7	0.0	0.0	0.0	0.0	0.0	0.0	0.0	0.0	6.60E-01	3.42E-02	8.23E-01	9.72E-01
ZNF652	3	0	0	0	0	0	0	0	0	0	0	0	0	0	0	0	0.22	0.25	0.20	0.02	0.01	0.06	0.01	0.01	0.20	0.11	0.01	0.0	0.0	0.0	0.0	0.0	0.0	0.0	0.0	0.0	0.0	0.0	1.00E+00	7.16E-01	8.79E-01	9.95E-01
ZNF653	11	6	2	0	1	0	1	1	0	0	0	0	0	1	0	0	1.94	2.21	1.75	0.15	0.06	0.51	0.11	0.09	1.76	0.97	0.13	0.0	0.5	0.6	0.0	0.0	0.0	0.0	0.0	0.6	0.0	0.0	8.73E-01	2.32E-01	6.52E-01	8.31E-01
ZNF655	13	15	1	0	3	0	1	0	0	0	1	0	0	0	1	1	6.95	7.92	6.28	0.53	0.20	1.81	0.39	0.33	6.31	3.48	0.45	0.0	0.1	0.0	0.0	0.0	0.6	0.0	0.0	0.0	0.3	2.2	9.06E-04	6.49E-05	3.93E-01	8.00E-02
ZNF66	6	0	0	0	0	0	0	0	0	0	0	0	0	0	0	0	0.29	0.33	0.26	0.02	0.01	0.08	0.02	0.01	0.27	0.15	0.02	0.0	0.0	0.0	0.0	0.0	0.0	0.0	0.0	0.0	0.0	0.0	9.99E-01	6.41E-01	8.61E-01	9.91E-01
ZNF660	2	0	1	0	0	0	1	0	0	0	0	0	0	0	0	0	0.26	0.29	0.23	0.02	0.01	0.07	0.01	0.01	0.23	0.13	0.02	0.0	3.4	0.0	0.0	0.0	0.0	0.0	0.0	0.0	0.0	0.0	9.88E-01	3.32E-01	8.70E-01	9.93E-01
ZNF662	0	4	0	0	0	0	0	0	0	0	0	0	0	0	0	0	0.37	0.42	0.33	0.03	0.01	0.10	0.02	0.02	0.33	0.18	0.02	0.0	0.0	0.0	0.0	0.0	0.0	0.0	0.0	0.0	0.0	0.0	9.98E-01	5.73E-01	8.45E-01	9.84E-01
ZNF664	2	3	2	0	0	0	2	0	0	0	0	0	0	0	0	0	1.10	1.25	0.99	0.08	0.03	0.29	0.06	0.05	1.00	0.55	0.07	0.0	1.6	0.0	0.0	0.0	0.0	0.0	0.0	0.0	0.0	0.0	9.12E-01	2.81E-01	7.34E-01	8.47E-01
ZNF665	1	0	0	0	0	0	0	0	0	0	0	0	0	0	0	0	0.07	0.08	0.07	0.01	0.00	0.02	0.00	0.00	0.07	0.04	0.00	0.0	0.0	0.0	0.0	0.0	0.0	0.0	0.0	0.0	0.0	0.0	1.00E+00	8.95E-01	9.30E-01	1.00E+00
ZNF667	0	2	1	0	0	1	0	0	0	0	0	0	0	0	0	0	0.29	0.33	0.26	0.02	0.01	0.08	0.02	0.01	0.27	0.15	0.02	3.4	0.0	0.0	0.0	0.0	0.0	0.0	0.0	0.0	0.0	0.0	9.84E-01	3.16E-01	8.61E-01	9.91E-01
ZNF669	0	8	3	0	0	0	1	2	0	0	0	0	0	0	0	0	2.82	3.21	2.55	0.21	0.08	0.73	0.16	0.14	2.56	1.41	0.18	0.0	0.3	0.8	0.0	0.0	0.0	0.0	0.0	0.0	0.0	0.0	4.47E-01	1.08E-01	5.86E-01	3.95E-01
ZNF670	0	7	2	0	0	0	0	2	0	0	0	0	0	0	0	0	1.13	1.29	1.03	0.09	0.03	0.30	0.06	0.05	1.03	0.57	0.07	0.0	0.0	2.0	0.0	0.0	0.0	0.0	0.0	0.0	0.0	0.0	8.51E-01	1.87E-01	7.30E-01	8.37E-01
ZNF672	3	2	1	0	0	0	1	0	0	0	0	0	0	0	0	0	0.40	0.46	0.36	0.03	0.01	0.10	0.02	0.02	0.37	0.20	0.03	0.0	2.2	0.0	0.0	0.0	0.0	0.0	0.0	0.0	0.0	0.0	9.95E-01	4.95E-01	8.37E-01	9.81E-01
ZNF675	1	4	2	0	0	0	1	1	0	0	0	0	0	0	0	0	0.48	0.54	0.43	0.04	0.01	0.12	0.03	0.02	0.43	0.24	0.03	0.0	1.8	2.3	0.0	0.0	0.0	0.0	0.0	0.0	0.0	0.0	9.90E-01	4.45E-01	8.23E-01	9.72E-01
ZNF676	1	0	0	0	0	0	0	0	0	0	0	0	0	0	0	0	0.04	0.04	0.03	0.00	0.00	0.01	0.00	0.00	0.03	0.02	0.00	0.0	0.0	0.0	0.0	0.0	0.0	0.0	0.0	0.0	0.0	0.0	1.00E+00	9.46E-01	9.51E-01	1.00E+00
ZNF677	3	5	2	1	0	0	2	0	0	1	0	0	0	0	0	0	0.95	1.08	0.86	0.07	0.03	0.25	0.05	0.05	0.86	0.48	0.06	0.0	1.8	0.0	0.0	36.2	0.0	0.0	0.0	0.0	0.0	0.0	2.91E-05	2.75E-01	4.65E-09	8.83E-01
ZNF678	0	1	1	0	0	0	1	0	0	0	0	0	0	0	0	0	0.11	0.13	0.10	0.01	0.00	0.03	0.01	0.01	0.10	0.05	0.01	0.0	8.0	0.0	0.0	0.0	0.0	0.0	0.0	0.0	0.0	0.0	7.68E-01	4.23E-02	9.14E-01	9.99E-01
ZNF679	1	2	0	0	0	0	0	0	0	0	0	0	0	0	0	0	0.26	0.29	0.23	0.02	0.01	0.07	0.01	0.01	0.23	0.13	0.02	0.0	0.0	0.0	0.0	0.0	0.0	0.0	0.0	0.0	0.0	0.0	9.99E-01	6.77E-01	8.70E-01	9.93E-01
ZNF680	1	0	0	0	0	0	0	0	0	0	0	0	0	0	0	0	0.15	0.17	0.13	0.01	0.00	0.04	0.01	0.01	0.13	0.07	0.01	0.0	0.0	0.0	0.0	0.0	0.0	0.0	0.0	0.0	0.0	0.0	1.00E+00	8.00E-01	9.01E-01	9.98E-01
ZNF681	0	0	0	0	0	0	0	0	0	0	0	0	0	0	0	0	0.04	0.04	0.03	0.00	0.00	0.01	0.00	0.00	0.03	0.02	0.00	0.0	0.0	0.0	0.0	0.0	0.0	0.0	0.0	0.0	0.0	0.0	1.00E+00	9.46E-01	9.51E-01	1.00E+00
ZNF682	0	1	0	0	0	0	0	0	0	0	0	0	0	0	0	0	0.11	0.13	0.10	0.01	0.00	0.03	0.01	0.01	0.10	0.05	0.01	0.0	0.0	0.0	0.0	0.0	0.0	0.0	0.0	0.0	0.0	0.0	1.00E+00	8.46E-01	9.14E-01	9.99E-01
ZNF683	1	0	0	0	0	0	0	0	0	0	0	0	0	0	0	0	0.33	0.38	0.30	0.03	0.01	0.09	0.02	0.02	0.30	0.16	0.02	0.0	0.0	0.0	0.0	0.0	0.0	0.0	0.0	0.0	0.0	0.0	9.98E-01	6.06E-01	8.52E-01	9.88E-01
ZNF684	0	1	0	0	0	0	0	0	0	0	0	0	0	0	0	0	0.73	0.83	0.66	0.06	0.02	0.19	0.04	0.04	0.66	0.37	0.05	0.0	0.0	0.0	0.0	0.0	0.0	0.0	0.0	0.0	0.0	0.0	9.62E-01	3.28E-01	7.82E-01	9.30E-01
ZNF689	20	14	10	0	16	1	8	1	0	0	0	0	0	12	4	0	3.62	4.13	3.27	0.28	0.11	0.94	0.20	0.17	3.29	1.81	0.23	0.3	1.9	0.3	0.0	0.0	0.0	0.0	0.0	3.6	2.2	0.0	1.38E-04	2.86E-02	5.37E-01	5.08E-05
ZNF691	4	4	2	0	0	1	0	1	0	0	0	0	0	0	0	0	1.32	1.50	1.19	0.10	0.04	0.34	0.07	0.06	1.20	0.66	0.09	0.8	0.0	0.8	0.0	0.0	0.0	0.0	0.0	0.0	0.0	0.0	9.39E-01	4.48E-01	7.10E-01	7.88E-01
ZNF692	6	6	6	0	1	0	5	1	0	0	0	0	0	1	0	0	2.34	2.67	2.12	0.18	0.07	0.61	0.13	0.11	2.13	1.17	0.15	0.0	1.9	0.5	0.0	0.0	0.0	0.0	0.0	0.5	0.0	0.0	6.30E-01	8.35E-02	6.20E-01	7.35E-01
ZNF695	2	6	0	0	0	0	0	0	0	0	0	0	0	0	0	0	1.21	1.38	1.09	0.09	0.04	0.31	0.07	0.06	1.10	0.60	0.08	0.0	0.0	0.0	0.0	0.0	0.0	0.0	0.0	0.0	0.0	0.0	8.14E-01	1.59E-01	7.22E-01	8.18E-01
ZNF696	3	5	2	0	0	1	1	0	0	0	0	0	0	0	0	0	2.96	3.38	2.68	0.23	0.09	0.77	0.17	0.14	2.69	1.48	0.19	0.3	0.3	0.0	0.0	0.0	0.0	0.0	0.0	0.0	0.0	0.0	3.26E-01	5.92E-02	5.77E-01	3.64E-01
ZNF7	3	5	0	0	4	0	0	0	0	0	0	0	0	4	0	0	1.06	1.21	0.96	0.08	0.03	0.28	0.06	0.05	0.96	0.53	0.07	0.0	0.0	0.0	0.0	0.0	0.0	0.0	0.0	4.2	0.0	0.0	1.78E-01	1.99E-01	7.38E-01	6.09E-02
ZNF70	9	4	1	0	11	0	1	0	0	0	0	0	0	11	0	0	1.68	1.92	1.52	0.13	0.05	0.44	0.09	0.08	1.53	0.84	0.11	0.0	0.5	0.0	0.0	0.0	0.0	0.0	0.0	7.2	0.0	0.0	6.06E-10	1.62E-01	6.74E-01	1.07E-11
ZNF701	0	0	0	0	0	0	0	0	0	0	0	0	0	0	0	0	0.51	0.58	0.46	0.04	0.01	0.13	0.03	0.02	0.47	0.26	0.03	0.0	0.0	0.0	0.0	0.0	0.0	0.0	0.0	0.0	0.0	0.0	9.90E-01	4.59E-01	8.17E-01	9.67E-01
ZNF705D	0	0	0	0	0	0	0	0	0	0	0	0	0	0	0	0	0.04	0.04	0.03	0.00	0.00	0.01	0.00	0.00	0.03	0.02	0.00	0.0	0.0	0.0	0.0	0.0	0.0	0.0	0.0	0.0	0.0	0.0	1.00E+00	9.46E-01	9.51E-01	1.00E+00
ZNF705E	0	0	0	0	0	0	0	0	0	0	0	0	0	0	0	0	0.00	0.00	0.00	0.00	0.00	0.00	0.00	0.00	0.00	0.00	0.00	na	na	na	na	na	na	na	na	na	na	na	na	na	na	na
ZNF705G	0	1	0	0	0	0	0	0	0	0	0	0	0	0	0	0	0.04	0.04	0.03	0.00	0.00	0.01	0.00	0.00	0.03	0.02	0.00	0.0	0.0	0.0	0.0	0.0	0.0	0.0	0.0	0.0	0.0	0.0	1.00E+00	9.46E-01	9.51E-01	1.00E+00
ZNF707	29	23	2	0	40	0	1	1	0	0	0	0	0	6	34	0	5.38	6.13	4.86	0.41	0.16	1.40	0.30	0.26	4.88	2.69	0.35	0.0	0.2	0.2	0.0	0.0	0.0	0.0	0.0	1.2	12.6	0.0	2.00E-75	1.71E-03	4.52E-01	5.26E-77
ZNF709	3	1	0	0	0	0	0	0	0	0	0	0	0	0	0	0	0.40	0.46	0.36	0.03	0.01	0.10	0.02	0.02	0.37	0.20	0.03	0.0	0.0	0.0	0.0	0.0	0.0	0.0	0.0	0.0	0.0	0.0	9.96E-01	5.42E-01	8.37E-01	9.81E-01
ZNF71	32	10	6	0	5	2	3	1	0	0	0	0	0	5	0	0	3.95	4.50	3.57	0.30	0.11	1.03	0.22	0.19	3.59	1.98	0.26	0.5	0.7	0.3	0.0	0.0	0.0	0.0	0.0	1.4	0.0	0.0	6.33E-01	1.90E-01	5.19E-01	5.17E-01
ZNF710	0	2	0	0	0	0	0	0	0	0	0	0	0	0	0	0	0.15	0.17	0.13	0.01	0.00	0.04	0.01	0.01	0.13	0.07	0.01	0.0	0.0	0.0	0.0	0.0	0.0	0.0	0.0	0.0	0.0	0.0	1.00E+00	8.00E-01	9.01E-01	9.98E-01
ZNF713	0	1	0	0	0	0	0	0	0	0	0	0	0	0	0	0	0.18	0.21	0.17	0.01	0.01	0.05	0.01	0.01	0.17	0.09	0.01	0.0	0.0	0.0	0.0	0.0	0.0	0.0	0.0	0.0	0.0	0.0	1.00E+00	7.57E-01	8.90E-01	9.97E-01
ZNF716	1	1	0	0	0	0	0	0	0	0	0	0	0	0	0	0	0.11	0.13	0.10	0.01	0.00	0.03	0.01	0.01	0.10	0.05	0.01	0.0	0.0	0.0	0.0	0.0	0.0	0.0	0.0	0.0	0.0	0.0	1.00E+00	8.46E-01	9.14E-01	9.99E-01
ZNF718	1	4	1	0	0	0	1	0	0	0	0	0	0	0	0	0	0.51	0.58	0.46	0.04	0.01	0.13	0.03	0.02	0.47	0.26	0.03	0.0	1.7	0.0	0.0	0.0	0.0	0.0	0.0	0.0	0.0	0.0	9.94E-01	5.29E-01	8.17E-01	9.67E-01
ZNF721	2	2	1	0	0	0	1	0	0	0	0	0	0	0	0	0	0.37	0.42	0.33	0.03	0.01	0.10	0.02	0.02	0.33	0.18	0.02	0.0	2.4	0.0	0.0	0.0	0.0	0.0	0.0	0.0	0.0	0.0	9.94E-01	4.70E-01	8.45E-01	9.84E-01
ZNF726	3	2	0	0	0	0	0	0	0	0	0	0	0	0	0	0	0.40	0.46	0.36	0.03	0.01	0.10	0.02	0.02	0.37	0.20	0.03	0.0	0.0	0.0	0.0	0.0	0.0	0.0	0.0	0.0	0.0	0.0	9.96E-01	5.42E-01	8.37E-01	9.81E-01
ZNF727	0	0	0	0	0	0	0	0	0	0	0	0	0	0	0	0	0.11	0.13	0.10	0.01	0.00	0.03	0.01	0.01	0.10	0.05	0.01	0.0	0.0	0.0	0.0	0.0	0.0	0.0	0.0	0.0	0.0	0.0	1.00E+00	8.46E-01	9.14E-01	9.99E-01
ZNF729	0	0	0	0	0	0	0	0	0	0	0	0	0	0	0	0	0.04	0.04	0.03	0.00	0.00	0.01	0.00	0.00	0.03	0.02	0.00	0.0	0.0	0.0	0.0	0.0	0.0	0.0	0.0	0.0	0.0	0.0	1.00E+00	9.46E-01	9.51E-01	1.00E+00
ZNF730	0	0	0	0	0	0	0	0	0	0	0	0	0	0	0	0	0.04	0.04	0.03	0.00	0.00	0.01	0.00	0.00	0.03	0.02	0.00	0.0	0.0	0.0	0.0	0.0	0.0	0.0	0.0	0.0	0.0	0.0	1.00E+00	9.46E-01	9.51E-01	1.00E+00
ZNF732	1	0	0	0	0	0	0	0	0	0	0	0	0	0	0	0	0.15	0.17	0.13	0.01	0.00	0.04	0.01	0.01	0.13	0.07	0.01	0.0	0.0	0.0	0.0	0.0	0.0	0.0	0.0	0.0	0.0	0.0	1.00E+00	8.00E-01	9.01E-01	9.98E-01
ZNF736	0	0	0	0	0	0	0	0	0	0	0	0	0	0	0	0	0.07	0.08	0.07	0.01	0.00	0.02	0.00	0.00	0.07	0.04	0.00	0.0	0.0	0.0	0.0	0.0	0.0	0.0	0.0	0.0	0.0	0.0	1.00E+00	8.95E-01	9.30E-01	1.00E+00
ZNF737	0	0	0	0	0	0	0	0	0	0	0	0	0	0	0	0	0.07	0.08	0.07	0.01	0.00	0.02	0.00	0.00	0.07	0.04	0.00	0.0	0.0	0.0	0.0	0.0	0.0	0.0	0.0	0.0	0.0	0.0	1.00E+00	8.95E-01	9.30E-01	1.00E+00
ZNF74	3	6	0	0	0	0	0	0	0	0	0	0	0	0	0	0	1.39	1.58	1.26	0.11	0.04	0.36	0.08	0.07	1.26	0.70	0.09	0.0	0.0	0.0	0.0	0.0	0.0	0.0	0.0	0.0	0.0	0.0	7.32E-01	1.21E-01	7.02E-01	7.68E-01
ZNF746	37	33	27	0	58	10	17	0	0	0	8	2	0	35	12	1	12.66	14.43	11.44	0.96	0.37	3.30	0.71	0.61	11.50	6.34	0.82	0.8	1.2	0.0	0.0	0.0	2.4	2.8	0.0	3.0	1.9	1.2	2.29E-12	1.97E-03	2.49E-01	3.14E-12
ZNF747	6	12	2	0	0	0	1	1	0	0	0	0	0	0	0	0	1.98	2.25	1.79	0.15	0.06	0.51	0.11	0.09	1.79	0.99	0.13	0.0	0.4	0.6	0.0	0.0	0.0	0.0	0.0	0.0	0.0	0.0	7.39E-01	2.21E-01	6.49E-01	6.04E-01
ZNF749	0	0	0	0	0	0	0	0	0	0	0	0	0	0	0	0	0.18	0.21	0.17	0.01	0.01	0.05	0.01	0.01	0.17	0.09	0.01	0.0	0.0	0.0	0.0	0.0	0.0	0.0	0.0	0.0	0.0	0.0	1.00E+00	7.57E-01	8.90E-01	9.97E-01
ZNF75A	2	1	1	0	0	0	0	1	0	0	0	0	0	0	0	0	0.33	0.38	0.30	0.03	0.01	0.09	0.02	0.02	0.30	0.16	0.02	0.0	0.0	3.4	0.0	0.0	0.0	0.0	0.0	0.0	0.0	0.0	9.81E-01	3.07E-01	8.52E-01	9.88E-01
ZNF75D	0	0	0	0	0	0	0	0	0	0	0	0	0	0	0	0	0.04	0.04	0.03	0.00	0.00	0.01	0.00	0.00	0.03	0.02	0.00	0.0	0.0	0.0	0.0	0.0	0.0	0.0	0.0	0.0	0.0	0.0	1.00E+00	9.46E-01	9.51E-01	1.00E+00
ZNF76	21	10	8	0	2	1	4	3	0	0	0	0	0	1	1	0	4.10	4.67	3.70	0.31	0.12	1.07	0.23	0.20	3.72	2.05	0.27	0.2	0.9	0.8	0.0	0.0	0.0	0.0	0.0	0.3	0.5	0.0	6.98E-01	2.76E-01	5.12E-01	5.09E-01
ZNF763	1	1	0	0	0	0	0	0	0	0	0	0	0	0	0	0	0.18	0.21	0.17	0.01	0.01	0.05	0.01	0.01	0.17	0.09	0.01	0.0	0.0	0.0	0.0	0.0	0.0	0.0	0.0	0.0	0.0	0.0	1.00E+00	7.57E-01	8.90E-01	9.97E-01
ZNF764	9	24	4	0	7	0	2	2	0	0	0	0	0	2	5	0	4.54	5.17	4.10	0.34	0.13	1.18	0.25	0.22	4.12	2.27	0.29	0.0	0.4	0.5	0.0	0.0	0.0	0.0	0.0	0.5	2.2	0.0	1.58E-01	2.29E-02	4.90E-01	2.77E-01
ZNF765	5	1	1	0	0	0	1	0	0	0	0	0	0	0	0	0	0.84	0.96	0.76	0.06	0.02	0.22	0.05	0.04	0.76	0.42	0.05	0.0	1.0	0.0	0.0	0.0	0.0	0.0	0.0	0.0	0.0	0.0	9.75E-01	4.48E-01	7.66E-01	9.08E-01
ZNF766	0	2	0	1	1	0	0	0	1	0	0	0	0	1	0	0	0.40	0.46	0.36	0.03	0.01	0.10	0.02	0.02	0.37	0.20	0.03	0.0	0.0	0.0	32.7	0.0	0.0	0.0	0.0	2.7	0.0	0.0	2.31E-04	5.42E-01	2.98E-08	9.16E-01
ZNF768	20	14	7	0	24	1	5	1	0	0	0	0	0	23	1	0	4.94	5.63	4.46	0.38	0.14	1.29	0.28	0.24	4.49	2.47	0.32	0.2	0.9	0.2	0.0	0.0	0.0	0.0	0.0	5.1	0.4	0.0	3.55E-14	5.23E-02	4.71E-01	1.11E-15
ZNF77	0	2	3	0	0	0	3	0	0	0	0	0	0	0	0	0	0.91	1.04	0.83	0.07	0.03	0.24	0.05	0.04	0.83	0.46	0.06	0.0	2.9	0.0	0.0	0.0	0.0	0.0	0.0	0.0	0.0	0.0	7.07E-01	6.66E-02	7.57E-01	8.91E-01
ZNF771	13	6	2	0	22	0	2	0	0	0	0	0	0	21	1	0	2.63	3.00	2.38	0.20	0.08	0.69	0.15	0.13	2.39	1.32	0.17	0.0	0.7	0.0	0.0	0.0	0.0	0.0	0.0	8.8	0.8	0.0	1.76E-27	6.89E-02	5.99E-01	9.68E-30
ZNF774	12	2	0	0	0	0	0	0	0	0	0	0	0	0	0	0	3.04	3.46	2.74	0.23	0.09	0.79	0.17	0.15	2.76	1.52	0.20	0.0	0.0	0.0	0.0	0.0	0.0	0.0	0.0	0.0	0.0	0.0	1.27E-01	9.84E-03	5.72E-01	3.49E-01
ZNF776	0	7	1	0	0	0	0	1	0	0	0	0	0	0	0	0	0.59	0.67	0.53	0.04	0.02	0.15	0.03	0.03	0.53	0.29	0.04	0.0	0.0	1.9	0.0	0.0	0.0	0.0	0.0	0.0	0.0	0.0	9.86E-01	4.33E-01	8.04E-01	9.56E-01
ZNF777	21	11	8	0	16	2	4	2	0	0	0	0	0	12	4	0	4.17	4.75	3.77	0.32	0.12	1.09	0.23	0.20	3.79	2.09	0.27	0.5	0.8	0.5	0.0	0.0	0.0	0.0	0.0	3.2	1.9	0.0	7.97E-03	3.53E-01	5.08E-01	6.97E-04
ZNF780A	2	5	2	0	1	0	1	1	0	0	0	0	0	1	0	0	0.95	1.08	0.86	0.07	0.03	0.25	0.05	0.05	0.86	0.48	0.06	0.0	0.9	1.2	0.0	0.0	0.0	0.0	0.0	1.2	0.0	0.0	9.96E-01	6.12E-01	7.52E-01	9.70E-01
ZNF780B	0	0	0	0	0	0	0	0	0	0	0	0	0	0	0	0	0.04	0.04	0.03	0.00	0.00	0.01	0.00	0.00	0.03	0.02	0.00	0.0	0.0	0.0	0.0	0.0	0.0	0.0	0.0	0.0	0.0	0.0	1.00E+00	9.46E-01	9.51E-01	1.00E+00
ZNF781	0	0	0	0	0	0	0	0	0	0	0	0	0	0	0	0	0.04	0.04	0.03	0.00	0.00	0.01	0.00	0.00	0.03	0.02	0.00	0.0	0.0	0.0	0.0	0.0	0.0	0.0	0.0	0.0	0.0	0.0	1.00E+00	9.46E-01	9.51E-01	1.00E+00
ZNF782	0	1	0	0	0	0	0	0	0	0	0	0	0	0	0	0	0.04	0.04	0.03	0.00	0.00	0.01	0.00	0.00	0.03	0.02	0.00	0.0	0.0	0.0	0.0	0.0	0.0	0.0	0.0	0.0	0.0	0.0	1.00E+00	9.46E-01	9.51E-01	1.00E+00
ZNF785	11	23	6	0	0	0	3	3	0	0	0	0	0	0	0	0	5.56	6.34	5.03	0.42	0.16	1.45	0.31	0.27	5.05	2.79	0.36	0.0	0.5	0.6	0.0	0.0	0.0	0.0	0.0	0.0	0.0	0.0	4.10E-02	1.71E-02	4.45E-01	6.92E-02
ZNF789	0	1	2	0	0	1	1	0	0	0	0	0	0	0	0	0	0.15	0.17	0.13	0.01	0.00	0.04	0.01	0.01	0.13	0.07	0.01	6.8	6.0	0.0	0.0	0.0	0.0	0.0	0.0	0.0	0.0	0.0	4.80E-01	9.69E-03	9.01E-01	9.98E-01
ZNF79	1	2	1	0	0	0	1	0	0	0	0	0	0	0	0	0	0.44	0.50	0.40	0.03	0.01	0.11	0.02	0.02	0.40	0.22	0.03	0.0	2.0	0.0	0.0	0.0	0.0	0.0	0.0	0.0	0.0	0.0	9.95E-01	5.13E-01	8.30E-01	9.77E-01
ZNF790	1	0	0	0	0	0	0	0	0	0	0	0	0	0	0	0	0.15	0.17	0.13	0.01	0.00	0.04	0.01	0.01	0.13	0.07	0.01	0.0	0.0	0.0	0.0	0.0	0.0	0.0	0.0	0.0	0.0	0.0	1.00E+00	8.00E-01	9.01E-01	9.98E-01
ZNF791	3	1	0	0	4	0	0	0	0	0	0	0	0	4	0	0	0.80	0.92	0.73	0.06	0.02	0.21	0.04	0.04	0.73	0.40	0.05	0.0	0.0	0.0	0.0	0.0	0.0	0.0	0.0	5.5	0.0	0.0	5.66E-02	2.94E-01	7.71E-01	8.89E-03
ZNF793	1	2	1	0	0	0	1	0	0	0	0	0	0	0	0	0	0.22	0.25	0.20	0.02	0.01	0.06	0.01	0.01	0.20	0.11	0.01	0.0	4.0	0.0	0.0	0.0	0.0	0.0	0.0	0.0	0.0	0.0	9.79E-01	2.64E-01	8.79E-01	9.95E-01
ZNF799	0	0	0	0	0	0	0	0	0	0	0	0	0	0	0	0	0.07	0.08	0.07	0.01	0.00	0.02	0.00	0.00	0.07	0.04	0.00	0.0	0.0	0.0	0.0	0.0	0.0	0.0	0.0	0.0	0.0	0.0	1.00E+00	8.95E-01	9.30E-01	1.00E+00
ZNF80	0	1	0	0	0	0	0	0	0	0	0	0	0	0	0	0	0.37	0.42	0.33	0.03	0.01	0.10	0.02	0.02	0.33	0.18	0.02	0.0	0.0	0.0	0.0	0.0	0.0	0.0	0.0	0.0	0.0	0.0	9.98E-01	5.73E-01	8.45E-01	9.84E-01
ZNF805	2	1	1	0	0	0	1	0	0	0	0	0	0	0	0	0	0.33	0.38	0.30	0.03	0.01	0.09	0.02	0.02	0.30	0.16	0.02	0.0	2.7	0.0	0.0	0.0	0.0	0.0	0.0	0.0	0.0	0.0	9.93E-01	4.35E-01	8.52E-01	9.88E-01
ZNF808	4	1	1	0	0	0	1	0	0	0	0	0	0	0	0	0	0.33	0.38	0.30	0.03	0.01	0.09	0.02	0.02	0.30	0.16	0.02	0.0	2.7	0.0	0.0	0.0	0.0	0.0	0.0	0.0	0.0	0.0	9.93E-01	4.35E-01	8.52E-01	9.88E-01
ZNF81	0	0	1	0	0	0	1	0	0	0	0	0	0	0	0	0	0.26	0.29	0.23	0.02	0.01	0.07	0.01	0.01	0.23	0.13	0.02	0.0	3.4	0.0	0.0	0.0	0.0	0.0	0.0	0.0	0.0	0.0	9.88E-01	3.32E-01	8.70E-01	9.93E-01
ZNF813	8	1	0	0	1	0	0	0	0	0	0	0	0	1	0	0	0.62	0.71	0.56	0.05	0.02	0.16	0.03	0.03	0.56	0.31	0.04	0.0	0.0	0.0	0.0	0.0	0.0	0.0	0.0	1.8	0.0	0.0	9.84E-01	3.88E-01	7.98E-01	9.69E-01
ZNF816	2	5	0	0	0	0	0	0	0	0	0	0	0	0	0	0	0.95	1.08	0.86	0.07	0.03	0.25	0.05	0.05	0.86	0.48	0.06	0.0	0.0	0.0	0.0	0.0	0.0	0.0	0.0	0.0	0.0	0.0	9.08E-01	2.35E-01	7.52E-01	8.83E-01
ZNF823	1	1	0	0	0	0	0	0	0	0	0	0	0	0	0	0	0.18	0.21	0.17	0.01	0.01	0.05	0.01	0.01	0.17	0.09	0.01	0.0	0.0	0.0	0.0	0.0	0.0	0.0	0.0	0.0	0.0	0.0	1.00E+00	7.57E-01	8.90E-01	9.97E-01
ZNF829	2	5	4	0	0	0	1	3	0	0	0	0	0	0	0	0	0.99	1.13	0.89	0.08	0.03	0.26	0.06	0.05	0.90	0.49	0.06	0.0	0.9	3.4	0.0	0.0	0.0	0.0	0.0	0.0	0.0	0.0	6.39E-01	5.04E-02	7.47E-01	8.74E-01
ZNF83	3	3	0	0	0	0	0	0	0	0	0	0	0	0	0	0	0.44	0.50	0.40	0.03	0.01	0.11	0.02	0.02	0.40	0.22	0.03	0.0	0.0	0.0	0.0	0.0	0.0	0.0	0.0	0.0	0.0	0.0	9.95E-01	5.13E-01	8.30E-01	9.77E-01
ZNF835	17	7	2	0	0	1	0	1	0	0	0	0	0	0	0	0	2.49	2.84	2.25	0.19	0.07	0.65	0.14	0.12	2.26	1.25	0.16	0.4	0.0	0.4	0.0	0.0	0.0	0.0	0.0	0.0	0.0	0.0	5.08E-01	1.10E-01	6.09E-01	4.70E-01
ZNF836	1	0	0	0	0	0	0	0	0	0	0	0	0	0	0	0	0.18	0.21	0.17	0.01	0.01	0.05	0.01	0.01	0.17	0.09	0.01	0.0	0.0	0.0	0.0	0.0	0.0	0.0	0.0	0.0	0.0	0.0	1.00E+00	7.57E-01	8.90E-01	9.97E-01
ZNF837	29	6	3	0	0	0	1	2	0	0	0	0	0	0	0	0	2.93	3.34	2.65	0.22	0.08	0.76	0.16	0.14	2.66	1.47	0.19	0.0	0.3	0.8	0.0	0.0	0.0	0.0	0.0	0.0	0.0	0.0	4.05E-01	9.44E-02	5.79E-01	3.71E-01
ZNF84	0	4	1	0	0	0	1	0	0	0	0	0	0	0	0	0	0.51	0.58	0.46	0.04	0.01	0.13	0.03	0.02	0.47	0.26	0.03	0.0	1.7	0.0	0.0	0.0	0.0	0.0	0.0	0.0	0.0	0.0	9.94E-01	5.29E-01	8.17E-01	9.67E-01
ZNF841	0	0	0	0	0	0	0	0	0	0	0	0	0	0	0	0	0.15	0.17	0.13	0.01	0.00	0.04	0.01	0.01	0.13	0.07	0.01	0.0	0.0	0.0	0.0	0.0	0.0	0.0	0.0	0.0	0.0	0.0	1.00E+00	8.00E-01	9.01E-01	9.98E-01
ZNF844	0	2	0	0	0	0	0	0	0	0	0	0	0	0	0	0	0.55	0.63	0.50	0.04	0.02	0.14	0.03	0.03	0.50	0.27	0.04	0.0	0.0	0.0	0.0	0.0	0.0	0.0	0.0	0.0	0.0	0.0	9.87E-01	4.34E-01	8.10E-01	9.62E-01
ZNF845	7	2	2	0	4	0	2	0	0	0	0	0	0	4	0	0	1.28	1.46	1.16	0.10	0.04	0.33	0.07	0.06	1.16	0.64	0.08	0.0	1.4	0.0	0.0	0.0	0.0	0.0	0.0	3.4	0.0	0.0	3.66E-01	2.67E-01	7.14E-01	1.50E-01
ZNF846	0	1	1	0	0	0	1	0	0	0	0	0	0	0	0	0	0.15	0.17	0.13	0.01	0.00	0.04	0.01	0.01	0.13	0.07	0.01	0.0	6.0	0.0	0.0	0.0	0.0	0.0	0.0	0.0	0.0	0.0	9.09E-01	1.09E-01	9.01E-01	9.98E-01
ZNF85	2	1	0	0	0	0	0	0	0	0	0	0	0	0	0	0	0.29	0.33	0.26	0.02	0.01	0.08	0.02	0.01	0.27	0.15	0.02	0.0	0.0	0.0	0.0	0.0	0.0	0.0	0.0	0.0	0.0	0.0	9.99E-01	6.41E-01	8.61E-01	9.91E-01
ZNF852	0	1	0	0	0	0	0	0	0	0	0	0	0	0	0	0	0.48	0.54	0.43	0.04	0.01	0.12	0.03	0.02	0.43	0.24	0.03	0.0	0.0	0.0	0.0	0.0	0.0	0.0	0.0	0.0	0.0	0.0	9.93E-01	4.85E-01	8.23E-01	9.72E-01
ZNF853	0	0	0	0	0	0	0	0	0	0	0	0	0	0	0	0	0.00	0.00	0.00	0.00	0.00	0.00	0.00	0.00	0.00	0.00	0.00	na	na	na	na	na	na	na	na	na	na	na	na	na	na	na
ZNF860	0	0	0	0	0	0	0	0	0	0	0	0	0	0	0	0	0.15	0.17	0.13	0.01	0.00	0.04	0.01	0.01	0.13	0.07	0.01	0.0	0.0	0.0	0.0	0.0	0.0	0.0	0.0	0.0	0.0	0.0	1.00E+00	8.00E-01	9.01E-01	9.98E-01
ZNF875	0	0	0	0	0	0	0	0	0	0	0	0	0	0	0	0	0.00	0.00	0.00	0.00	0.00	0.00	0.00	0.00	0.00	0.00	0.00	na	na	na	na	na	na	na	na	na	na	na	na	na	na	na
ZNF878	0	0	0	0	0	0	0	0	0	0	0	0	0	0	0	0	0.11	0.13	0.10	0.01	0.00	0.03	0.01	0.01	0.10	0.05	0.01	0.0	0.0	0.0	0.0	0.0	0.0	0.0	0.0	0.0	0.0	0.0	1.00E+00	8.46E-01	9.14E-01	9.99E-01
ZNF879	0	2	0	0	0	0	0	0	0	0	0	0	0	0	0	0	0.15	0.17	0.13	0.01	0.00	0.04	0.01	0.01	0.13	0.07	0.01	0.0	0.0	0.0	0.0	0.0	0.0	0.0	0.0	0.0	0.0	0.0	1.00E+00	8.00E-01	9.01E-01	9.98E-01
ZNF880	0	0	0	0	0	0	0	0	0	0	0	0	0	0	0	0	0.04	0.04	0.03	0.00	0.00	0.01	0.00	0.00	0.03	0.02	0.00	0.0	0.0	0.0	0.0	0.0	0.0	0.0	0.0	0.0	0.0	0.0	1.00E+00	9.46E-01	9.51E-01	1.00E+00
ZNF891	0	0	1	0	0	0	1	0	0	0	0	0	0	0	0	0	0.15	0.17	0.13	0.01	0.00	0.04	0.01	0.01	0.13	0.07	0.01	0.0	6.0	0.0	0.0	0.0	0.0	0.0	0.0	0.0	0.0	0.0	9.09E-01	1.09E-01	9.01E-01	9.98E-01
ZNF90	0	0	0	0	0	0	0	0	0	0	0	0	0	0	0	0	0.07	0.08	0.07	0.01	0.00	0.02	0.00	0.00	0.07	0.04	0.00	0.0	0.0	0.0	0.0	0.0	0.0	0.0	0.0	0.0	0.0	0.0	1.00E+00	8.95E-01	9.30E-01	1.00E+00
ZNF91	2	2	3	0	0	0	2	1	0	0	0	0	0	0	0	0	0.55	0.63	0.50	0.04	0.02	0.14	0.03	0.03	0.50	0.27	0.04	0.0	3.2	2.0	0.0	0.0	0.0	0.0	0.0	0.0	0.0	0.0	8.81E-01	1.30E-01	8.10E-01	9.62E-01
ZNF92	23	3	1	0	6	0	1	0	0	0	0	0	0	6	0	0	2.56	2.92	2.31	0.19	0.07	0.67	0.14	0.12	2.33	1.28	0.17	0.0	0.3	0.0	0.0	0.0	0.0	0.0	0.0	2.6	0.0	0.0	1.48E-01	4.65E-02	6.04E-01	1.46E-01
ZNF93	0	1	0	0	0	0	0	0	0	0	0	0	0	0	0	0	0.33	0.38	0.30	0.03	0.01	0.09	0.02	0.02	0.30	0.16	0.02	0.0	0.0	0.0	0.0	0.0	0.0	0.0	0.0	0.0	0.0	0.0	9.98E-01	6.06E-01	8.52E-01	9.88E-01
ZNF98	0	0	0	0	0	0	0	0	0	0	0	0	0	0	0	0	0.04	0.04	0.03	0.00	0.00	0.01	0.00	0.00	0.03	0.02	0.00	0.0	0.0	0.0	0.0	0.0	0.0	0.0	0.0	0.0	0.0	0.0	1.00E+00	9.46E-01	9.51E-01	1.00E+00
ZNF99	0	1	0	0	0	0	0	0	0	0	0	0	0	0	0	0	0.11	0.13	0.10	0.01	0.00	0.03	0.01	0.01	0.10	0.05	0.01	0.0	0.0	0.0	0.0	0.0	0.0	0.0	0.0	0.0	0.0	0.0	1.00E+00	8.46E-01	9.14E-01	9.99E-01
ZSCAN10	0	1	0	0	0	0	0	0	0	0	0	0	0	0	0	0	0.11	0.13	0.10	0.01	0.00	0.03	0.01	0.01	0.10	0.05	0.01	0.0	0.0	0.0	0.0	0.0	0.0	0.0	0.0	0.0	0.0	0.0	1.00E+00	8.46E-01	9.14E-01	9.99E-01
ZSCAN12	12	4	3	0	0	2	0	1	0	0	0	0	0	0	0	0	3.15	3.59	2.84	0.24	0.09	0.82	0.18	0.15	2.86	1.58	0.20	0.6	0.0	0.4	0.0	0.0	0.0	0.0	0.0	0.0	0.0	0.0	3.34E-01	7.43E-02	5.65E-01	3.28E-01
ZSCAN16	9	1	0	0	0	0	0	0	0	0	0	0	0	0	0	0	0.66	0.75	0.60	0.05	0.02	0.17	0.04	0.03	0.60	0.33	0.04	0.0	0.0	0.0	0.0	0.0	0.0	0.0	0.0	0.0	0.0	0.0	9.74E-01	3.67E-01	7.93E-01	9.44E-01
ZSCAN2	0	3	0	0	0	0	0	0	0	0	0	0	0	0	0	0	0.29	0.33	0.26	0.02	0.01	0.08	0.02	0.01	0.27	0.15	0.02	0.0	0.0	0.0	0.0	0.0	0.0	0.0	0.0	0.0	0.0	0.0	9.99E-01	6.41E-01	8.61E-01	9.91E-01
ZSCAN21	56	11	2	0	1	0	0	2	0	0	0	0	0	1	0	0	4.83	5.50	4.36	0.37	0.14	1.26	0.27	0.23	4.39	2.42	0.31	0.0	0.0	0.5	0.0	0.0	0.0	0.0	0.0	0.2	0.0	0.0	3.75E-02	3.00E-03	4.76E-01	2.13E-01
ZSCAN22	31	6	0	0	0	0	0	0	0	0	0	0	0	0	0	0	1.65	1.88	1.49	0.13	0.05	0.43	0.09	0.08	1.50	0.82	0.11	0.0	0.0	0.0	0.0	0.0	0.0	0.0	0.0	0.0	0.0	0.0	6.08E-01	8.16E-02	6.77E-01	6.96E-01
ZSCAN23	31	5	0	0	0	0	0	0	0	0	0	0	0	0	0	0	1.79	2.04	1.62	0.14	0.05	0.47	0.10	0.09	1.63	0.90	0.12	0.0	0.0	0.0	0.0	0.0	0.0	0.0	0.0	0.0	0.0	0.0	5.38E-01	6.53E-02	6.64E-01	6.55E-01
ZSCAN25	26	10	5	0	21	0	4	1	0	0	0	0	0	21	0	0	3.29	3.75	2.98	0.25	0.10	0.86	0.18	0.16	2.99	1.65	0.21	0.0	1.1	0.3	0.0	0.0	0.0	0.0	0.0	7.0	0.0	0.0	2.59E-20	9.92E-02	5.56E-01	1.94E-22
ZSCAN26	3	8	2	0	1	1	1	0	0	0	0	0	0	0	1	0	2.38	2.71	2.15	0.18	0.07	0.62	0.13	0.11	2.16	1.19	0.15	0.4	0.4	0.0	0.0	0.0	0.0	0.0	0.0	0.0	0.8	0.0	6.79E-01	1.33E-01	6.17E-01	6.68E-01
ZSCAN29	13	1	2	0	0	0	1	1	0	0	0	0	0	0	0	0	1.35	1.54	1.22	0.10	0.04	0.35	0.08	0.07	1.23	0.68	0.09	0.0	0.6	0.8	0.0	0.0	0.0	0.0	0.0	0.0	0.0	0.0	9.37E-01	4.53E-01	7.06E-01	7.78E-01
ZSCAN30	19	0	1	0	0	0	1	0	0	0	0	0	0	0	0	0	0.88	1.00	0.79	0.07	0.03	0.23	0.05	0.04	0.80	0.44	0.06	0.0	1.0	0.0	0.0	0.0	0.0	0.0	0.0	0.0	0.0	0.0	9.71E-01	4.33E-01	7.61E-01	9.00E-01
ZSCAN31	4	0	2	0	0	0	2	0	0	0	0	0	0	0	0	0	0.84	0.96	0.76	0.06	0.02	0.22	0.05	0.04	0.76	0.42	0.05	0.0	2.1	0.0	0.0	0.0	0.0	0.0	0.0	0.0	0.0	0.0	9.29E-01	2.55E-01	7.66E-01	9.08E-01
ZSCAN32	96	17	4	0	0	3	1	0	0	0	0	0	0	0	0	0	4.79	5.46	4.33	0.36	0.14	1.25	0.27	0.23	4.35	2.40	0.31	0.6	0.2	0.0	0.0	0.0	0.0	0.0	0.0	0.0	0.0	0.0	5.56E-02	1.32E-02	4.78E-01	1.17E-01
ZSCAN4	5	4	1	0	0	0	0	1	0	0	0	0	0	0	0	0	0.62	0.71	0.56	0.05	0.02	0.16	0.03	0.03	0.56	0.31	0.04	0.0	0.0	1.8	0.0	0.0	0.0	0.0	0.0	0.0	0.0	0.0	9.84E-01	4.33E-01	7.98E-01	9.50E-01
ZSCAN5A	12	15	5	0	1	0	0	5	0	0	0	0	0	0	1	0	2.09	2.38	1.88	0.16	0.06	0.54	0.12	0.10	1.89	1.04	0.14	0.0	0.0	2.7	0.0	0.0	0.0	0.0	0.0	0.0	1.0	0.0	2.46E-01	8.19E-03	6.40E-01	7.32E-01
ZSCAN5B	2	1	0	0	0	0	0	0	0	0	0	0	0	0	0	0	0.18	0.21	0.17	0.01	0.01	0.05	0.01	0.01	0.17	0.09	0.01	0.0	0.0	0.0	0.0	0.0	0.0	0.0	0.0	0.0	0.0	0.0	1.00E+00	7.57E-01	8.90E-01	9.97E-01
ZSCAN9	10	4	3	0	0	0	0	3	0	0	0	0	0	0	0	0	0.91	1.04	0.83	0.07	0.03	0.24	0.05	0.04	0.83	0.46	0.06	0.0	0.0	3.6	0.0	0.0	0.0	0.0	0.0	0.0	0.0	0.0	4.90E-01	2.16E-02	7.57E-01	8.91E-01
ZXDA	0	4	1	0	0	0	1	0	0	0	0	0	0	0	0	0	0.66	0.75	0.60	0.05	0.02	0.17	0.04	0.03	0.60	0.33	0.04	0.0	1.3	0.0	0.0	0.0	0.0	0.0	0.0	0.0	0.0	0.0	9.89E-01	5.13E-01	7.93E-01	9.44E-01
ZXDB	1	3	3	0	0	0	2	1	0	0	0	0	0	0	0	0	0.73	0.83	0.66	0.06	0.02	0.19	0.04	0.04	0.66	0.37	0.05	0.0	2.4	1.5	0.0	0.0	0.0	0.0	0.0	0.0	0.0	0.0	9.49E-01	2.81E-01	7.82E-01	9.30E-01
ZXDC	4	12	2	0	0	0	2	0	0	0	0	0	0	0	0	0	1.35	1.54	1.22	0.10	0.04	0.35	0.08	0.07	1.23	0.68	0.09	0.0	1.3	0.0	0.0	0.0	0.0	0.0	0.0	0.0	0.0	0.0	8.67E-01	2.58E-01	7.06E-01	7.78E-01
BCL11A	62	51	16	0	1	0	0	16	0	0	0	0	0	0	1	0	6.00	6.84	5.42	0.46	0.17	1.56	0.33	0.29	5.45	3.01	0.39	0.0	0.0	3.0	0.0	0.0	0.0	0.0	0.0	0.0	0.3	0.0	4.12E-06	5.40E-08	4.27E-01	9.55E-02

MAZ	20	19	10	1	0	2	4	4	1	0	0	0	0	0	0	0	4.10	4.67	3.70	0.31	0.12	1.07	0.23	0.20	3.72	2.05	0.27	0.5	0.9	1.1	3.2	0.0	0.0	0.0	0.0	0.0	0.0	0.0	4.09E-01	5.50E-01	2.00E-01	1.84E-01
MECOM	83	113	62	0	12	8	21	33	0	0	4	1	0	7	0	0	17.20	19.60	15.54	1.31	0.50	4.48	0.96	0.83	15.62	8.61	1.11	0.5	1.1	2.1	0.0	0.0	0.9	1.0	0.0	0.4	0.0	0.0	8.16E-06	4.49E-06	1.79E-01	8.93E-03
PATZ1	38	24	5	1	11	1	3	1	1	0	0	0	0	11	0	0	6.84	7.80	6.18	0.52	0.20	1.78	0.38	0.33	6.21	3.43	0.44	0.1	0.4	0.2	1.9	0.0	0.0	0.0	0.0	1.8	0.0	0.0	1.08E-02	2.15E-03	4.23E-01	7.38E-02
PEG3	0	4	0	0	0	0	0	0	0	0	0	0	0	0	0	0	0.55	0.63	0.50	0.04	0.02	0.14	0.03	0.03	0.50	0.27	0.04	0.0	0.0	0.0	0.0	0.0	0.0	0.0	0.0	0.0	0.0	0.0	9.87E-01	4.34E-01	8.10E-01	9.62E-01
PRDM10	10	7	6	0	10	2	3	1	0	0	0	0	0	10	0	0	2.74	3.13	2.48	0.21	0.08	0.71	0.15	0.13	2.49	1.37	0.18	0.7	1.0	0.4	0.0	0.0	0.0	0.0	0.0	4.0	0.0	0.0	3.06E-03	5.80E-01	5.91E-01	1.29E-04
PRDM13	0	1	0	0	1	0	0	0	0	0	1	0	0	0	0	0	0.37	0.42	0.33	0.03	0.01	0.10	0.02	0.02	0.33	0.18	0.02	0.0	0.0	0.0	0.0	0.0	10.5	0.0	0.0	0.0	0.0	0.0	4.13E-01	5.73E-01	8.45E-01	1.03E-01
PRDM15	14	7	3	0	23	0	3	0	0	0	0	0	0	23	0	0	3.11	3.54	2.81	0.24	0.09	0.81	0.17	0.15	2.82	1.56	0.20	0.0	0.8	0.0	0.0	0.0	0.0	0.0	0.0	8.1	0.0	0.0	7.55E-28	4.97E-02	5.68E-01	5.70E-30
PRDM16	33	36	15	0	9	3	3	9	0	0	4	1	0	4	0	0	7.50	8.55	6.78	0.57	0.22	1.95	0.42	0.36	6.81	3.76	0.49	0.4	0.4	1.3	0.0	0.0	2.0	2.4	0.0	0.6	0.0	0.0	8.53E-02	2.97E-02	3.75E-01	1.21E-01
PRDM2	14	11	5	0	1	0	1	4	0	0	0	0	0	1	0	0	1.87	2.13	1.69	0.14	0.05	0.49	0.10	0.09	1.69	0.93	0.12	0.0	0.5	2.4	0.0	0.0	0.0	0.0	0.0	0.6	0.0	0.0	6.43E-01	5.97E-02	6.58E-01	8.46E-01
PRDM4	5	4	4	0	1	1	0	3	0	0	1	0	0	0	0	0	0.88	1.00	0.79	0.07	0.03	0.23	0.05	0.04	0.80	0.44	0.06	1.1	0.0	3.8	0.0	0.0	4.4	0.0	0.0	0.0	0.0	0.0	3.40E-01	2.80E-02	7.61E-01	5.51E-01
PRDM8	0	0	0	0	0	0	0	0	0	0	0	0	0	0	0	0	0.18	0.21	0.17	0.01	0.01	0.05	0.01	0.01	0.17	0.09	0.01	0.0	0.0	0.0	0.0	0.0	0.0	0.0	0.0	0.0	0.0	0.0	1.00E+00	7.57E-01	8.90E-01	9.97E-01
PRDM9	3	2	1	0	0	1	0	0	0	0	0	0	0	0	0	0	0.29	0.33	0.26	0.02	0.01	0.08	0.02	0.01	0.27	0.15	0.02	3.4	0.0	0.0	0.0	0.0	0.0	0.0	0.0	0.0	0.0	0.0	9.84E-01	3.16E-01	8.61E-01	9.91E-01
REST	25	81	55	0	70	12	27	16	0	0	1	1	0	65	3	0	11.42	13.01	10.32	0.87	0.33	2.97	0.64	0.55	10.37	5.72	0.74	1.1	2.1	1.6	0.0	0.0	0.3	1.6	0.0	6.3	0.5	0.0	5.87E-61	1.12E-04	2.73E-01	5.11E-61
RLF	19	13	9	0	0	1	2	6	0	0	0	0	0	0	0	0	2.38	2.71	2.15	0.18	0.07	0.62	0.13	0.11	2.16	1.19	0.15	0.4	0.7	2.8	0.0	0.0	0.0	0.0	0.0	0.0	0.0	0.0	2.53E-01	1.94E-02	6.17E-01	4.97E-01
RREB1	29	31	19	0	0	2	2	15	0	0	0	0	0	0	0	0	3.62	4.13	3.27	0.28	0.11	0.94	0.20	0.17	3.29	1.81	0.23	0.6	0.5	4.6	0.0	0.0	0.0	0.0	0.0	0.0	0.0	0.0	1.85E-07	3.04E-10	5.37E-01	2.47E-01
SALL1	51	21	8	1	0	0	2	6	1	0	0	0	0	0	0	0	4.28	4.88	3.87	0.33	0.12	1.12	0.24	0.21	3.89	2.14	0.28	0.0	0.4	1.6	3.1	0.0	0.0	0.0	0.0	0.0	0.0	0.0	8.51E-02	2.80E-02	2.17E-01	1.64E-01
SALL2	21	11	3	1	0	1	1	1	1	0	0	0	0	0	0	0	4.32	4.92	3.90	0.33	0.13	1.12	0.24	0.21	3.92	2.16	0.28	0.2	0.2	0.3	3.0	0.0	0.0	0.0	0.0	0.0	0.0	0.0	6.87E-02	1.99E-02	2.21E-01	1.60E-01
SALL3	6	2	0	0	0	0	0	0	0	0	0	0	0	0	0	0	0.70	0.79	0.63	0.05	0.02	0.18	0.04	0.03	0.63	0.35	0.05	0.0	0.0	0.0	0.0	0.0	0.0	0.0	0.0	0.0	0.0	0.0	9.68E-01	3.47E-01	7.87E-01	9.37E-01
SALL4	15	4	6	0	0	0	1	5	0	0	0	0	0	0	0	0	1.46	1.67	1.32	0.11	0.04	0.38	0.08	0.07	1.33	0.73	0.09	0.0	0.6	3.8	0.0	0.0	0.0	0.0	0.0	0.0	0.0	0.0	1.40E-01	2.54E-03	6.95E-01	7.48E-01
VEZF1	23	15	5	0	0	2	1	2	0	0	0	0	0	0	0	0	3.62	4.13	3.27	0.28	0.11	0.94	0.20	0.17	3.29	1.81	0.23	0.6	0.2	0.6	0.0	0.0	0.0	0.0	0.0	0.0	0.0	0.0	3.87E-01	1.66E-01	5.37E-01	2.47E-01
WIZ	72	73	26	0	3	2	1	23	0	0	1	0	0	2	0	0	10.32	11.76	9.33	0.78	0.30	2.69	0.57	0.50	9.37	5.17	0.67	0.2	0.1	2.5	0.0	0.0	0.4	0.0	0.0	0.2	0.0	0.0	1.44E-07	1.13E-08	2.98E-01	1.72E-02
ZBTB1	22	28	14	0	26	6	5	3	0	0	1	0	0	25	0	0	5.45	6.21	4.93	0.41	0.16	1.42	0.30	0.26	4.95	2.73	0.35	1.1	0.8	0.6	0.0	0.0	0.7	0.0	0.0	5.1	0.0	0.0	2.53E-14	5.93E-01	4.49E-01	7.61E-17
ZBTB17	53	14	2	0	0	0	1	1	0	0	0	0	0	0	0	0	3.81	4.34	3.44	0.29	0.11	0.99	0.21	0.18	3.46	1.91	0.25	0.0	0.2	0.3	0.0	0.0	0.0	0.0	0.0	0.0	0.0	0.0	1.15E-01	1.74E-02	5.27E-01	2.21E-01
ZBTB2	77	68	65	1	28	24	21	20	0	1	1	2	0	8	16	1	18.29	20.85	16.53	1.39	0.53	4.77	1.02	0.88	16.61	9.16	1.18	1.3	1.0	1.2	0.0	1.9	0.2	2.0	0.0	0.5	1.7	0.8	4.42E-02	2.86E-01	1.79E-01	1.33E-02
ZBTB21	12	26	7	2	0	1	1	5	1	1	0	0	0	0	0	0	6.92	7.88	6.25	0.53	0.20	1.80	0.39	0.33	6.28	3.46	0.45	0.1	0.1	0.8	1.9	5.0	0.0	0.0	0.0	0.0	0.0	0.0	2.06E-03	3.49E-03	5.74E-02	2.63E-02
ZBTB25	11	17	1	0	0	0	0	1	0	0	0	0	0	0	0	0	2.31	2.63	2.08	0.18	0.07	0.60	0.13	0.11	2.09	1.15	0.15	0.0	0.0	0.5	0.0	0.0	0.0	0.0	0.0	0.0	0.0	0.0	4.43E-01	6.41E-02	6.23E-01	5.16E-01
ZBTB38	12	30	16	0	11	4	5	7	0	0	2	0	0	5	4	0	6.48	7.38	5.85	0.49	0.19	1.69	0.36	0.31	5.88	3.24	0.42	0.6	0.7	1.2	0.0	0.0	1.2	0.0	0.0	0.9	1.2	0.0	9.44E-01	3.79E-01	4.09E-01	9.18E-01
ZBTB39	8	5	2	0	0	0	1	1	0	0	0	0	0	0	0	0	1.10	1.25	0.99	0.08	0.03	0.29	0.06	0.05	1.00	0.55	0.07	0.0	0.8	1.0	0.0	0.0	0.0	0.0	0.0	0.0	0.0	0.0	9.74E-01	5.63E-01	7.34E-01	8.47E-01
ZBTB4	7	9	1	0	0	1	0	0	0	0	0	0	0	0	0	0	1.39	1.58	1.26	0.11	0.04	0.36	0.08	0.07	1.26	0.70	0.09	0.7	0.0	0.0	0.0	0.0	0.0	0.0	0.0	0.0	0.0	0.0	8.44E-01	2.29E-01	7.02E-01	7.68E-01
ZBTB41	0	0	3	0	5	0	3	0	0	0	0	0	0	5	0	0	0.51	0.58	0.46	0.04	0.01	0.13	0.03	0.02	0.47	0.26	0.03	0.0	5.1	0.0	0.0	0.0	0.0	0.0	0.0	10.7	0.0	0.0	2.32E-08	4.14E-03	8.17E-01	1.68E-08
ZFAT	1	4	1	0	0	0	1	0	0	0	0	0	0	0	0	0	0.40	0.46	0.36	0.03	0.01	0.10	0.02	0.02	0.37	0.20	0.03	0.0	2.2	0.0	0.0	0.0	0.0	0.0	0.0	0.0	0.0	0.0	9.95E-01	4.95E-01	8.37E-01	9.81E-01
ZFP57	0	1	2	0	0	0	1	1	0	0	0	0	0	0	0	0	0.51	0.58	0.46	0.04	0.01	0.13	0.03	0.02	0.47	0.26	0.03	0.0	1.7	2.2	0.0	0.0	0.0	0.0	0.0	0.0	0.0	0.0	9.92E-01	4.89E-01	8.17E-01	9.67E-01
ZFPM1	6	6	0	0	0	0	0	0	0	0	0	0	0	0	0	0	0.77	0.88	0.69	0.06	0.02	0.20	0.04	0.04	0.70	0.38	0.05	0.0	0.0	0.0	0.0	0.0	0.0	0.0	0.0	0.0	0.0	0.0	9.55E-01	3.11E-01	7.76E-01	9.23E-01
ZNF101	2	3	0	0	0	0	0	0	0	0	0	0	0	0	0	0	1.50	1.71	1.36	0.11	0.04	0.39	0.08	0.07	1.36	0.75	0.10	0.0	0.0	0.0	0.0	0.0	0.0	0.0	0.0	0.0	0.0	0.0	6.79E-01	1.02E-01	6.91E-01	7.37E-01
ZNF132	0	0	0	0	0	0	0	0	0	0	0	0	0	0	0	0	0.00	0.00	0.00	0.00	0.00	0.00	0.00	0.00	0.00	0.00	0.00	na	na	na	na	na	na	na	na	na	na	na	na	na	na	na
ZNF134	0	0	0	0	0	0	0	0	0	0	0	0	0	0	0	0	0.07	0.08	0.07	0.01	0.00	0.02	0.00	0.00	0.07	0.04	0.00	0.0	0.0	0.0	0.0	0.0	0.0	0.0	0.0	0.0	0.0	0.0	1.00E+00	8.95E-01	9.30E-01	1.00E+00
ZNF142	3	2	1	0	2	0	1	0	0	0	0	0	0	2	0	0	0.70	0.79	0.63	0.05	0.02	0.18	0.04	0.03	0.63	0.35	0.05	0.0	1.3	0.0	0.0	0.0	0.0	0.0	0.0	3.2	0.0	0.0	8.87E-01	5.02E-01	7.87E-01	6.06E-01
ZNF195	13	5	0	0	1	0	0	0	0	0	0	0	0	1	0	0	2.41	2.75	2.18	0.18	0.07	0.63	0.13	0.12	2.19	1.21	0.16	0.0	0.0	0.0	0.0	0.0	0.0	0.0	0.0	0.5	0.0	0.0	3.98E-01	2.54E-02	6.14E-01	7.16E-01
ZNF211	5	3	1	0	1	0	0	1	0	0	0	0	0	1	0	0	0.88	1.00	0.79	0.07	0.03	0.23	0.05	0.04	0.80	0.44	0.06	0.0	0.0	1.3	0.0	0.0	0.0	0.0	0.0	1.3	0.0	0.0	9.84E-01	3.80E-01	7.61E-01	9.72E-01
ZNF217	37	55	30	0	0	4	3	23	0	0	0	0	0	0	0	0	4.43	5.05	4.00	0.34	0.13	1.15	0.25	0.21	4.02	2.22	0.29	0.9	0.6	5.7	0.0	0.0	0.0	0.0	0.0	0.0	0.0	0.0	6.31E-17	1.67E-20	4.95E-01	1.49E-01
ZNF219	40	28	11	0	0	0	1	10	0	0	0	0	0	0	0	0	5.23	5.96	4.73	0.40	0.15	1.36	0.29	0.25	4.75	2.62	0.34	0.0	0.2	2.1	0.0	0.0	0.0	0.0	0.0	0.0	0.0	0.0	4.63E-03	4.91E-04	4.58E-01	8.69E-02
ZNF236	0	0	2	0	0	0	2	0	0	0	0	0	0	0	0	0	0.33	0.38	0.30	0.03	0.01	0.09	0.02	0.02	0.30	0.16	0.02	0.0	5.3	0.0	0.0	0.0	0.0	0.0	0.0	0.0	0.0	0.0	6.00E-01	2.17E-02	8.52E-01	9.88E-01
ZNF248	2	1	2	0	0	0	2	0	0	0	0	0	0	0	0	0	0.40	0.46	0.36	0.03	0.01	0.10	0.02	0.02	0.37	0.20	0.03	0.0	4.4	0.0	0.0	0.0	0.0	0.0	0.0	0.0	0.0	0.0	7.51E-01	5.12E-02	8.37E-01	9.81E-01
ZNF251	4	3	0	0	0	0	0	0	0	0	0	0	0	0	0	0	0.73	0.83	0.66	0.06	0.02	0.19	0.04	0.04	0.66	0.37	0.05	0.0	0.0	0.0	0.0	0.0	0.0	0.0	0.0	0.0	0.0	0.0	9.62E-01	3.28E-01	7.82E-01	9.30E-01
ZNF256	0	0	0	0	0	0	0	0	0	0	0	0	0	0	0	0	0.11	0.13	0.10	0.01	0.00	0.03	0.01	0.01	0.10	0.05	0.01	0.0	0.0	0.0	0.0	0.0	0.0	0.0	0.0	0.0	0.0	0.0	1.00E+00	8.46E-01	9.14E-01	9.99E-01
ZNF277	4	4	1	0	3	0	0	1	0	0	0	0	0	3	0	0	1.39	1.58	1.26	0.11	0.04	0.36	0.08	0.07	1.26	0.70	0.09	0.0	0.0	0.8	0.0	0.0	0.0	0.0	0.0	2.4	0.0	0.0	7.39E-01	2.20E-01	7.02E-01	5.96E-01
ZNF292	14	11	7	0	0	0	2	5	0	0	0	0	0	0	0	0	2.45	2.79	2.22	0.19	0.07	0.64	0.14	0.12	2.23	1.23	0.16	0.0	0.7	2.3	0.0	0.0	0.0	0.0	0.0	0.0	0.0	0.0	3.62E-01	4.56E-02	6.12E-01	4.79E-01
ZNF296	6	6	1	0	0	0	0	1	0	0	0	0	0	0	0	0	1.21	1.38	1.09	0.09	0.04	0.31	0.07	0.06	1.10	0.60	0.08	0.0	0.0	0.9	0.0	0.0	0.0	0.0	0.0	0.0	0.0	0.0	8.95E-01	2.74E-01	7.22E-01	8.18E-01
ZNF304	0	0	1	0	0	0	1	0	0	0	0	0	0	0	0	0	0.22	0.25	0.20	0.02	0.01	0.06	0.01	0.01	0.20	0.11	0.01	0.0	4.0	0.0	0.0	0.0	0.0	0.0	0.0	0.0	0.0	0.0	9.79E-01	2.64E-01	8.79E-01	9.95E-01
ZNF316	15	5	2	0	1	1	0	1	0	0	0	0	0	1	0	0	2.12	2.42	1.92	0.16	0.06	0.55	0.12	0.10	1.93	1.06	0.14	0.5	0.0	0.5	0.0	0.0	0.0	0.0	0.0	0.5	0.0	0.0	8.07E-01	1.78E-01	6.37E-01	7.89E-01
ZNF319	5	1	1	0	0	0	1	0	0	0	0	0	0	0	0	0	0.95	1.08	0.86	0.07	0.03	0.25	0.05	0.05	0.86	0.48	0.06	0.0	0.9	0.0	0.0	0.0	0.0	0.0	0.0	0.0	0.0	0.0	9.61E-01	4.03E-01	7.52E-01	8.83E-01
ZNF334	0	0	0	0	0	0	0	0	0	0	0	0	0	0	0	0	0.11	0.13	0.10	0.01	0.00	0.03	0.01	0.01	0.10	0.05	0.01	0.0	0.0	0.0	0.0	0.0	0.0	0.0	0.0	0.0	0.0	0.0	1.00E+00	8.46E-01	9.14E-01	9.99E-01
ZNF335	3	12	2	0	4	0	1	1	0	0	0	0	0	4	0	0	1.13	1.29	1.03	0.09	0.03	0.30	0.06	0.05	1.03	0.57	0.07	0.0	0.8	1.0	0.0	0.0	0.0	0.0	0.0	3.9	0.0	0.0	3.62E-01	5.48E-01	7.30E-01	8.68E-02
ZNF341	2	4	0	1	0	0	0	0	1	0	0	0	0	0	0	0	0.70	0.79	0.63	0.05	0.02	0.18	0.04	0.03	0.63	0.35	0.05	0.0	0.0	0.0	18.9	0.0	0.0	0.0	0.0	0.0	0.0	0.0	2.58E-02	3.47E-01	3.75E-05	9.37E-01
ZNF37A	0	2	0	0	0	0	0	0	0	0	0	0	0	0	0	0	0.11	0.13	0.10	0.01	0.00	0.03	0.01	0.01	0.10	0.05	0.01	0.0	0.0	0.0	0.0	0.0	0.0	0.0	0.0	0.0	0.0	0.0	1.00E+00	8.46E-01	9.14E-01	9.99E-01
ZNF382	1	1	0	0	0	0	0	0	0	0	0	0	0	0	0	0	0.33	0.38	0.30	0.03	0.01	0.09	0.02	0.02	0.30	0.16	0.02	0.0	0.0	0.0	0.0	0.0	0.0	0.0	0.0	0.0	0.0	0.0	9.98E-01	6.06E-01	8.52E-01	9.88E-01
ZNF407	2	2	1	0	0	0	1	0	0	0	0	0	0	0	0	0	0.40	0.46	0.36	0.03	0.01	0.10	0.02	0.02	0.37	0.20	0.03	0.0	2.2	0.0	0.0	0.0	0.0	0.0	0.0	0.0	0.0	0.0	9.95E-01	4.95E-01	8.37E-01	9.81E-01
ZNF417	39	18	8	0	0	3	1	4	0	0	0	0	0	0	0	0	7.98	9.09	7.21	0.61	0.23	2.08	0.44	0.38	7.24	4.00	0.52	0.4	0.1	0.6	0.0	0.0	0.0	0.0	0.0	0.0	0.0	0.0	2.39E-03	2.83E-03	3.60E-01	1.19E-02
ZNF418	3	2	0	0	0	0	0	0	0	0	0	0	0	0	0	0	0.80	0.92	0.73	0.06	0.02	0.21	0.04	0.04	0.73	0.40	0.05	0.0	0.0	0.0	0.0	0.0	0.0	0.0	0.0	0.0	0.0	0.0	9.47E-01	2.94E-01	7.71E-01	9.15E-01
ZNF423	16	9	3	0	0	0	0	3	0	0	0	0	0	0	0	0	1.79	2.04	1.62	0.14	0.05	0.47	0.10	0.09	1.63	0.90	0.12	0.0	0.0	1.9	0.0	0.0	0.0	0.0	0.0	0.0	0.0	0.0	5.81E-01	8.16E-02	6.64E-01	6.55E-01
ZNF438	12	7	2	0	2	0	0	2	0	0	0	0	0	0	1	1	2.63	3.00	2.38	0.20	0.08	0.69	0.15	0.13	2.39	1.32	0.17	0.0	0.0	0.8	0.0	0.0	0.0	0.0	0.0	0.0	0.8	5.9	2.00E-01	5.79E-02	5.99E-01	1.88E-01
ZNF445	32	5	2	0	6	0	2	0	0	0	1	0	0	5	0	0	2.85	3.25	2.58	0.22	0.08	0.74	0.16	0.14	2.59	1.43	0.18	0.0	0.6	0.0	0.0	0.0	1.3	0.0	0.0	1.9	0.0	0.0	4.02E-01	5.19E-02	5.84E-01	5.16E-01
ZNF462	18	27	13	0	0	1	2	10	0	0	0	0	0	0	0	0	2.93	3.34	2.65	0.22	0.08	0.76	0.16	0.14	2.66	1.47	0.19	0.3	0.6	3.8	0.0	0.0	0.0	0.0	0.0	0.0	0.0	0.0	1.85E-03	1.47E-05	5.79E-01	3.71E-01
ZNF467	50	18	7	1	11	2	5	0	0	1	0	0	0	1	10	0	6.37	7.26	5.75	0.48	0.18	1.66	0.35	0.31	5.78	3.19	0.41	0.3	0.7	0.0	0.0	5.4	0.0	0.0	0.0	0.2	3.1	0.0	1.37E-04	8.87E-03	4.33E-02	7.32E-04
ZNF469	0	1	2	0	0	0	1	1	0	0	0	0	0	0	0	0	0.26	0.29	0.23	0.02	0.01	0.07	0.01	0.01	0.23	0.13	0.02	0.0	3.4	4.3	0.0	0.0	0.0	0.0	0.0	0.0	0.0	0.0	8.90E-01	1.04E-01	8.70E-01	9.93E-01
ZNF48	25	18	10	0	30	2	7	1	0	0	1	0	0	29	0	0	5.78	6.59	5.22	0.44	0.17	1.51	0.32	0.28	5.25	2.90	0.37	0.3	1.1	0.2	0.0	0.0	0.7	0.0	0.0	5.5	0.0	0.0	1.27E-20	5.20E-02	4.36E-01	1.97E-22
ZNF507	9	7	3	0	0	0	1	2	0	0	0	0	0	0	0	0	2.38	2.71	2.15	0.18	0.07	0.62	0.13	0.11	2.16	1.19	0.15	0.0	0.4	0.9	0.0	0.0	0.0	0.0	0.0	0.0	0.0	0.0	6.20E-01	1.77E-01	6.17E-01	4.97E-01
ZNF510	3	4	0	0	0	0	0	0	0	0	0	0	0	0	0	0	0.80	0.92	0.73	0.06	0.02	0.21	0.04	0.04	0.73	0.40	0.05	0.0	0.0	0.0	0.0	0.0	0.0	0.0	0.0	0.0	0.0	0.0	9.47E-01	2.94E-01	7.71E-01	9.15E-01
ZNF512	57	34	18	2	50	6	8	4	0	2	0	0	0	47	3	0	12.26	13.97	11.08	0.93	0.36	3.19	0.68	0.59	11.13	6.14	0.79	0.5	0.6	0.4	0.0	5.6	0.0	0.0	0.0	4.2	0.5	0.0	2.31E-25	5.89E-03	3.48E-03	9.42E-25

	JAZF1	16	39	3	0	0	0	0	3	0	0	0	0	0	0	0	0	3.55	4.04	3.21	0.27	0.10	0.92	0.20	0.17	3.22	1.78	0.23	0.0	0.0	0.9	0.0	0.0	0.0	0.0	0.0	0.0	0.0	0.0	1.51E-01	2.23E-02	5.41E-01	2.59E-01
	KLF18	0	0	0	0	0	0	0	0	0	0	0	0	0	0	0	0	0.00	0.00	0.00	0.00	0.00	0.00	0.00	0.00	0.00	0.00	0.00	na	na	na	na	na	na	na	na	na	na	na	na	na	na	na
	L3MBTL4	3	1	0	0	1	0	0	0	0	0	0	0	0	1	0	0	0.84	0.96	0.76	0.06	0.02	0.22	0.05	0.04	0.76	0.42	0.05	0.0	0.0	0.0	0.0	0.0	0.0	0.0	0.0	1.3	0.0	0.0	9.67E-01	2.78E-01	7.66E-01	9.73E-01
	PRDM12	0	0	1	0	0	0	0	1	0	0	0	0	0	0	0	0	0.11	0.13	0.10	0.01	0.00	0.03	0.01	0.01	0.10	0.05	0.01	0.0	0.0	10.1	0.0	0.0	0.0	0.0	0.0	0.0	0.0	0.0	5.68E-01	1.49E-02	9.14E-01	9.99E-01
	REPIN1	15	23	11	0	13	0	3	8	0	0	0	0	0	9	4	0	4.83	5.50	4.36	0.37	0.14	1.26	0.27	0.23	4.39	2.42	0.31	0.0	0.5	1.8	0.0	0.0	0.0	0.0	0.0	2.1	1.7	0.0	6.47E-02	1.11E-02	4.76E-01	1.58E-01
	ZBTB10	37	23	6	3	3	2	3	1	3	0	0	1	0	2	0	0	6.22	7.09	5.62	0.47	0.18	1.62	0.35	0.30	5.65	3.12	0.40	0.3	0.4	0.2	6.3	0.0	0.0	2.9	0.0	0.4	0.0	0.0	4.44E-04	1.10E-02	2.16E-04	1.08E-01
	ZBTB3	20	8	1	0	2	0	1	0	0	0	1	1	0	0	0	0	2.27	2.59	2.05	0.17	0.07	0.59	0.13	0.11	2.06	1.14	0.15	0.0	0.4	0.0	0.0	0.0	1.7	7.9	0.0	0.0	0.0	0.0	1.21E-01	7.10E-02	6.25E-01	8.19E-02
	ZBTB8A	41	7	8	0	4	2	1	5	0	0	0	0	0	3	1	0	5.01	5.71	4.53	0.38	0.15	1.31	0.28	0.24	4.55	2.51	0.32	0.4	0.2	1.1	0.0	0.0	0.0	0.0	0.0	0.7	0.4	0.0	4.53E-01	5.65E-02	4.68E-01	6.10E-01
	ZNF112	2	0	0	0	0	0	0	0	0	0	0	0	0	0	0	0	0.40	0.46	0.36	0.03	0.01	0.10	0.02	0.02	0.37	0.20	0.03	0.0	0.0	0.0	0.0	0.0	0.0	0.0	0.0	0.0	0.0	0.0	9.96E-01	5.42E-01	8.37E-01	9.81E-01
	ZNF12	0	5	1	0	0	0	1	0	0	0	0	0	0	0	0	0	0.70	0.79	0.63	0.05	0.02	0.18	0.04	0.03	0.63	0.35	0.05	0.0	1.3	0.0	0.0	0.0	0.0	0.0	0.0	0.0	0.0	0.0	9.87E-01	5.02E-01	7.87E-01	9.37E-01
	ZNF207	13	26	33	0	2	19	11	3	0	0	1	0	0	1	0	0	4.24	4.84	3.84	0.32	0.12	1.11	0.24	0.20	3.85	2.13	0.27	4.5	2.3	0.8	0.0	0.0	0.9	0.0	0.0	0.3	0.0	0.0	4.54E-10	1.31E-13	5.04E-01	4.20E-01
	ZNF318	12	22	16	1	2	7	5	4	1	0	0	0	0	2	0	0	5.63	6.42	5.09	0.43	0.16	1.47	0.31	0.27	5.12	2.82	0.36	1.2	0.8	0.8	2.3	0.0	0.0	0.0	0.0	0.4	0.0	0.0	5.37E-01	6.44E-01	3.36E-01	2.11E-01
	ZNF326	24	49	51	1	5	13	27	11	1	0	1	2	0	1	1	0	8.42	9.59	7.61	0.64	0.24	2.19	0.47	0.40	7.64	4.22	0.54	1.5	2.8	1.4	1.6	0.0	0.5	4.3	0.0	0.1	0.2	0.0	1.84E-07	1.85E-08	5.04E-01	1.11E-02
	ZNF367	0	0	0	0	0	0	0	0	0	0	0	0	0	0	0	0	0.00	0.00	0.00	0.00	0.00	0.00	0.00	0.00	0.00	0.00	0.00	na	na	na	na	na	na	na	na	na	na	na	na	na	na	na
	ZNF385A	4	2	1	0	0	0	1	0	0	0	0	0	0	0	0	0	0.73	0.83	0.66	0.06	0.02	0.19	0.04	0.04	0.66	0.37	0.05	0.0	1.2	0.0	0.0	0.0	0.0	0.0	0.0	0.0	0.0	0.0	9.85E-01	4.90E-01	7.82E-01	9.30E-01
	ZNF385B	0	1	2	0	0	0	1	1	0	0	0	0	0	0	0	0	0.15	0.17	0.13	0.01	0.00	0.04	0.01	0.01	0.13	0.07	0.01	0.0	6.0	7.6	0.0	0.0	0.0	0.0	0.0	0.0	0.0	0.0	4.16E-01	6.74E-03	9.01E-01	9.98E-01
	ZNF395	0	14	3	0	0	0	1	2	0	0	0	0	0	0	0	0	1.06	1.21	0.96	0.08	0.03	0.28	0.06	0.05	0.96	0.53	0.07	0.0	0.8	2.1	0.0	0.0	0.0	0.0	0.0	0.0	0.0	0.0	9.33E-01	3.28E-01	7.38E-01	8.56E-01
	ZNF470	0	0	0	0	0	0	0	0	0	0	0	0	0	0	0	0	0.07	0.08	0.07	0.01	0.00	0.02	0.00	0.00	0.07	0.04	0.00	0.0	0.0	0.0	0.0	0.0	0.0	0.0	0.0	0.0	0.0	0.0	1.00E+00	8.95E-01	9.30E-01	1.00E+00
	ZNF473	7	0	6	0	0	1	3	2	0	0	0	0	0	0	0	0	1.10	1.25	0.99	0.08	0.03	0.29	0.06	0.05	1.00	0.55	0.07	0.9	2.4	2.0	0.0	0.0	0.0	0.0	0.0	0.0	0.0	0.0	8.47E-01	1.76E-01	7.34E-01	8.47E-01
	ZNF486	0	0	0	0	0	0	0	0	0	0	0	0	0	0	0	0	0.07	0.08	0.07	0.01	0.00	0.02	0.00	0.00	0.07	0.04	0.00	0.0	0.0	0.0	0.0	0.0	0.0	0.0	0.0	0.0	0.0	0.0	1.00E+00	8.95E-01	9.30E-01	1.00E+00
	ZNF488	0	7	3	0	0	2	0	1	0	0	0	0	0	0	0	0	0.91	1.04	0.83	0.07	0.03	0.24	0.05	0.04	0.83	0.46	0.06	2.2	0.0	1.2	0.0	0.0	0.0	0.0	0.0	0.0	0.0	0.0	9.41E-01	3.06E-01	7.57E-01	8.91E-01
	ZNF503	24	10	3	0	0	2	0	1	0	0	0	0	0	0	0	0	2.34	2.67	2.12	0.18	0.07	0.61	0.13	0.11	2.13	1.17	0.15	0.9	0.0	0.5	0.0	0.0	0.0	0.0	0.0	0.0	0.0	0.0	6.43E-01	1.91E-01	6.20E-01	5.07E-01
	ZNF527	0	1	0	0	0	0	0	0	0	0	0	0	0	0	0	0	0.07	0.08	0.07	0.01	0.00	0.02	0.00	0.00	0.07	0.04	0.00	0.0	0.0	0.0	0.0	0.0	0.0	0.0	0.0	0.0	0.0	0.0	1.00E+00	8.95E-01	9.30E-01	1.00E+00
	ZNF573	0	2	2	0	0	0	2	0	0	0	0	0	0	0	0	0	0.26	0.29	0.23	0.02	0.01	0.07	0.01	0.01	0.23	0.13	0.02	0.0	6.9	0.0	0.0	0.0	0.0	0.0	0.0	0.0	0.0	0.0	3.59E-01	5.29E-03	8.70E-01	9.93E-01
	ZNF578	1	3	0	0	0	0	0	0	0	0	0	0	0	0	0	0	0.73	0.83	0.66	0.06	0.02	0.19	0.04	0.04	0.66	0.37	0.05	0.0	0.0	0.0	0.0	0.0	0.0	0.0	0.0	0.0	0.0	0.0	9.62E-01	3.28E-01	7.82E-01	9.30E-01
	ZNF587B	2	2	0	0	0	0	0	0	0	0	0	0	0	0	0	0	0.29	0.33	0.26	0.02	0.01	0.08	0.02	0.01	0.27	0.15	0.02	0.0	0.0	0.0	0.0	0.0	0.0	0.0	0.0	0.0	0.0	0.0	9.99E-01	6.41E-01	8.61E-01	9.91E-01
	ZNF608	44	21	6	0	1	2	1	3	0	0	0	0	0	0	0	0	3.40	3.88	3.08	0.26	0.10	0.89	0.19	0.16	3.09	1.70	0.22	0.6	0.3	1.0	0.0	0.0	0.0	0.0	0.0	0.0	0.0	0.0	5.01E-01	2.57E-01	5.50E-01	2.82E-01
	ZNF609	66	26	10	0	1	1	4	5	0	0	0	0	0	1	0	0	5.78	6.59	5.22	0.44	0.17	1.51	0.32	0.28	5.25	2.90	0.37	0.2	0.6	1.0	0.0	0.0	0.0	0.0	0.0	0.2	0.0	0.0	1.55E-01	8.29E-02	4.36E-01	1.17E-01
	ZNF622	13	18	3	0	11	1	2	0	0	0	2	4	0	5	0	0	5.56	6.34	5.03	0.42	0.16	1.45	0.31	0.27	5.05	2.79	0.36	0.2	0.3	0.0	0.0	0.0	1.4	12.9	0.0	1.0	0.0	0.0	3.78E-09	2.83E-03	4.45E-01	4.33E-09
	ZNF630	0	0	0	0	0	0	0	0	0	0	0	0	0	0	0	0	0.00	0.00	0.00	0.00	0.00	0.00	0.00	0.00	0.00	0.00	0.00	na	na	na	na	na	na	na	na	na	na	na	na	na	na	na
	ZNF688	14	16	3	0	0	0	2	1	0	0	0	0	0	0	0	0	4.39	5.00	3.97	0.33	0.13	1.14	0.24	0.21	3.99	2.20	0.28	0.0	0.4	0.3	0.0	0.0	0.0	0.0	0.0	0.0	0.0	0.0	7.56E-02	1.49E-02	4.97E-01	1.52E-01
	ZNF69	5	2	0	0	0	0	0	0	0	0	0	0	0	0	0	0	0.73	0.83	0.66	0.06	0.02	0.19	0.04	0.04	0.66	0.37	0.05	0.0	0.0	0.0	0.0	0.0	0.0	0.0	0.0	0.0	0.0	0.0	9.62E-01	3.28E-01	7.82E-01	9.30E-01
	ZNF699	0	2	1	0	0	0	1	0	0	0	0	0	0	0	0	0	0.48	0.54	0.43	0.04	0.01	0.12	0.03	0.02	0.43	0.24	0.03	0.0	1.8	0.0	0.0	0.0	0.0	0.0	0.0	0.0	0.0	0.0	9.94E-01	5.24E-01	8.23E-01	9.72E-01
	ZNF700	0	0	0	0	0	0	0	0	0	0	0	0	0	0	0	0	0.00	0.00	0.00	0.00	0.00	0.00	0.00	0.00	0.00	0.00	0.00	na	na	na	na	na	na	na	na	na	na	na	na	na	na	na
	ZNF704	4	19	3	0	0	0	2	1	0	0	0	0	0	0	0	0	1.46	1.67	1.32	0.11	0.04	0.38	0.08	0.07	1.33	0.73	0.09	0.0	1.2	0.8	0.0	0.0	0.0	0.0	0.0	0.0	0.0	0.0	9.25E-01	4.47E-01	6.95E-01	7.48E-01
	ZNF705B	0	0	0	0	0	0	0	0	0	0	0	0	0	0	0	0	0.00	0.00	0.00	0.00	0.00	0.00	0.00	0.00	0.00	0.00	0.00	na	na	na	na	na	na	na	na	na	na	na	na	na	na	na
	ZNF706	1	1	1	0	0	0	1	0	0	0	0	0	0	0	0	0	0.18	0.21	0.17	0.01	0.01	0.05	0.01	0.01	0.17	0.09	0.01	0.0	4.8	0.0	0.0	0.0	0.0	0.0	0.0	0.0	0.0	0.0	9.60E-01	1.87E-01	8.90E-01	9.97E-01
	ZNF708	4	1	1	0	2	0	1	0	0	0	0	0	0	2	0	0	0.70	0.79	0.63	0.05	0.02	0.18	0.04	0.03	0.63	0.35	0.05	0.0	1.3	0.0	0.0	0.0	0.0	0.0	0.0	3.2	0.0	0.0	8.87E-01	5.02E-01	7.87E-01	6.06E-01
	ZNF714	6	2	2	0	2	0	2	0	0	0	0	0	0	2	0	0	0.62	0.71	0.56	0.05	0.02	0.16	0.03	0.03	0.56	0.31	0.04	0.0	2.8	0.0	0.0	0.0	0.0	0.0	0.0	3.5	0.0	0.0	6.46E-01	1.71E-01	7.98E-01	5.17E-01
	ZNF720	1	1	0	0	0	0	0	0	0	0	0	0	0	0	0	0	0.22	0.25	0.20	0.02	0.01	0.06	0.01	0.01	0.20	0.11	0.01	0.0	0.0	0.0	0.0	0.0	0.0	0.0	0.0	0.0	0.0	0.0	1.00E+00	7.16E-01	8.79E-01	9.95E-01
	ZNF723	0	0	0	0	0	0	0	0	0	0	0	0	0	0	0	0	0.00	0.00	0.00	0.00	0.00	0.00	0.00	0.00	0.00	0.00	0.00	na	na	na	na	na	na	na	na	na	na	na	na	na	na	na
	ZNF728	0	0	0	0	0	0	0	0	0	0	0	0	0	0	0	0	0.04	0.04	0.03	0.00	0.00	0.01	0.00	0.00	0.03	0.02	0.00	0.0	0.0	0.0	0.0	0.0	0.0	0.0	0.0	0.0	0.0	0.0	1.00E+00	9.46E-01	9.51E-01	1.00E+00
	ZNF735	0	1	0	0	0	0	0	0	0	0	0	0	0	0	0	0	0.04	0.04	0.03	0.00	0.00	0.01	0.00	0.00	0.03	0.02	0.00	0.0	0.0	0.0	0.0	0.0	0.0	0.0	0.0	0.0	0.0	0.0	1.00E+00	9.46E-01	9.51E-01	1.00E+00
	ZNF761	1	2	0	0	0	0	0	0	0	0	0	0	0	0	0	0	0.37	0.42	0.33	0.03	0.01	0.10	0.02	0.02	0.33	0.18	0.02	0.0	0.0	0.0	0.0	0.0	0.0	0.0	0.0	0.0	0.0	0.0	9.98E-01	5.73E-01	8.45E-01	9.84E-01
	ZNF773	3	2	1	0	0	0	1	0	0	0	0	0	0	0	0	0	0.48	0.54	0.43	0.04	0.01	0.12	0.03	0.02	0.43	0.24	0.03	0.0	1.8	0.0	0.0	0.0	0.0	0.0	0.0	0.0	0.0	0.0	9.94E-01	5.24E-01	8.23E-01	9.72E-01
	ZNF783	8	2	0	0	1	0	0	0	0	0	1	0	0	0	0	0	0.51	0.58	0.46	0.04	0.01	0.13	0.03	0.02	0.47	0.26	0.03	0.0	0.0	0.0	0.0	0.0	7.5	0.0	0.0	0.0	0.0	0.0	6.24E-01	4.59E-01	8.17E-01	2.66E-01
	ZNF8	3	12	7	0	0	0	1	6	0	0	0	0	0	0	0	0	1.76	2.00	1.59	0.13	0.05	0.46	0.10	0.08	1.59	0.88	0.11	0.0	0.5	3.8	0.0	0.0	0.0	0.0	0.0	0.0	0.0	0.0	5.60E-02	7.01E-04	6.68E-01	6.65E-01
	ZNF821	0	4	1	0	0	0	0	1	0	0	0	0	0	0	0	0	0.48	0.54	0.43	0.04	0.01	0.12	0.03	0.02	0.43	0.24	0.03	0.0	0.0	2.3	0.0	0.0	0.0	0.0	0.0	0.0	0.0	0.0	9.88E-01	4.12E-01	8.23E-01	9.72E-01
	ZNF827	9	11	6	0	0	0	2	4	0	0	0	0	0	0	0	0	1.10	1.25	0.99	0.08	0.03	0.29	0.06	0.05	1.00	0.55	0.07	0.0	1.6	4.0	0.0	0.0	0.0	0.0	0.0	0.0	0.0	0.0	2.35E-01	4.83E-03	7.34E-01	8.47E-01
	ZNF831	0	2	3	0	0	1	1	1	0	0	0	0	0	0	0	0	0.18	0.21	0.17	0.01	0.01	0.05	0.01	0.01	0.17	0.09	0.01	5.5	4.8	6.0	0.0	0.0	0.0	0.0	0.0	0.0	0.0	0.0	3.40E-01	4.37E-03	8.90E-01	9.97E-01
	ZNF850	2	2	1	0	0	0	0	1	0	0	0	0	0	0	0	0	0.22	0.25	0.20	0.02	0.01	0.06	0.01	0.01	0.20	0.11	0.01	0.0	0.0	5.0	0.0	0.0	0.0	0.0	0.0	0.0	0.0	0.0	9.41E-01	1.57E-01	8.79E-01	9.95E-01
	ZNF865	5	2	1	0	7	0	1	0	0	0	1	0	0	5	1	0	2.09	2.38	1.88	0.16	0.06	0.54	0.12	0.10	1.89	1.04	0.14	0.0	0.4	0.0	0.0	0.0	1.8	0.0	0.0	2.6	1.0	0.0	3.72E-01	9.22E-02	6.40E-01	3.23E-01
	ZNF888	4	1	0	0	2	0	0	0	0	0	0	0	0	2	0	0	0.37	0.42	0.33	0.03	0.01	0.10	0.02	0.02	0.33	0.18	0.02	0.0	0.0	0.0	0.0	0.0	0.0	0.0	0.0	6.0	0.0	0.0	4.52E-01	5.73E-01	8.45E-01	1.21E-01
	ZSCAN18	23	16	0	1	0	0	0	0	0	1	0	0	0	0	0	0	2.05	2.34	1.85	0.16	0.06	0.53	0.11	0.10	1.86	1.03	0.13	0.0	0.0	0.0	0.0	16.8	0.0	0.0	0.0	0.0	0.0	0.0	5.27E-03	4.42E-02	1.05E-04	5.84E-01
ZF.C3H	ZGPAT	29	22	57	0	0	52	1	4	0	0	0	0	0	0	0	0	9.00	10.26	8.13	0.68	0.26	2.34	0.50	0.43	8.17	4.51	0.58	5.8	0.1	0.5	0.0	0.0	0.0	0.0	0.0	0.0	0.0	0.0	1.70E-44	1.33E-47	3.31E-01	5.46E-03
ZF.C4_GATA	GATA1	46	57	19	5	0	3	1	15	5	0	0	0	0	0	0	0	7.35	8.38	6.65	0.56	0.21	1.92	0.41	0.35	6.68	3.68	0.48	0.4	0.1	2.3	8.9	0.0	0.0	0.0	0.0	0.0	0.0	0.0	8.35E-11	5.61E-05	2.58E-09	1.90E-02
	GATA2	75	52	12	0	1	1	1	10	0	0	0	0	0	1	0	0	8.16	9.30	7.37	0.62	0.24	2.13	0.45	0.39	7.41	4.09	0.53	0.1	0.1	1.4	0.0	0.0	0.0	0.0	0.0	0.1	0.0	0.0	1.44E-03	6.68E-04	3.55E-01	2.22E-02
	GATA3	60	49	25	3	5	4	2	19	3	0	0	0	0	1	3	1	7.54	8.59	6.81	0.57	0.22	1.96	0.42	0.36	6.84	3.78	0.49	0.5	0.2	2.8	5.2	0.0	0.0	0.0	0.0	0.1	0.8	2.0	7.82E-07	6.40E-07	1.20E-03	1.34E-01
	GATA4	105	76	79	1	12	23	26	30	1	0	2	0	2	8	0	0	16.72	19.06	15.11	1.27	0.49	4.36	0.93	0.80	15.18	8.38	1.08	1.4	1.4	2.0	0.8	0.0	0.5	0.0	2.5	0.5	0.0	0.0	5.79E-05	5.68E-05	4.61E-01	4.81E-03
	GATA5	3	3	1	0	0	0	0	1	0	0	0	0	0	0	0	0	0.40	0.46	0.36	0.03	0.01	0.10	0.02	0.02	0.37	0.20	0.03	0.0	0.0	2.7	0.0	0.0	0.0	0.0	0.0	0.0	0.0	0.0	9.87E-01	3.73E-01	8.37E-01	9.81E-01
	GATA6	11	3	2	0	0	0	1	1	0	0	0	0	0	0	0	0	0.91	1.04	0.83	0.07	0.03	0.24	0.05	0.04	0.83	0.46	0.06	0.0	1.0	1.2	0.0	0.0	0.0	0.0	0.0	0.0	0.0	0.0	9.87E-01	6.21E-01	7.57E-01	8.91E-01
	GATAD1	53	68	22	0	0	0	1	21	0	0	0	0	0	0	0	0	6.95	7.92	6.28	0.53	0.20	1.81	0.39	0.33	6.31	3.48	0.45	0.0	0.1	3.3	0.0	0.0	0.0	0.0	0.0	0.0	0.0	0.0	2.36E-09	4.91E-11	3.93E-01	2.56E-02
	GATAD2A	90	79	37	0	3	3	1	33	0	0	3	0	0	0	0	0	11.53	13.14	10.42	0.88	0.33	3.00	0.64	0.55	10.47	5.77	0.75	0.3	0.1	3.2	0.0	0.0	1.0	0.0	0.0	0.0	0.0	0.0	3.51E-14	3.66E-15	2.71E-01	2.73E-03
	GATAD2B	104	96	39	0	1	1	1	37	0	0	1	0	0	0	0	0	13.10	14.93	11.84	1.00	0.38	3.41	0.73	0.63	11.89	6.56	0.85	0.1	0.1	3.1	0.0	0.0	0.3	0.0	0.0	0.0	0.0	0.0	2.87E-17	1.38E-17	2.41E-01	4.46E-04
	MTA1	148	164	73	0	2	3	3	67	0	0	1	0	0	1	0	0	19.03	21.68	17.20	1.45	0.55	4.96	1.06	0.91	17.28	9.53	1.23	0.2	0.1	3.9	0.0	0.0	0.2	0.0	0.0	0.1	0.0	0.0	5.38E-39	1.77E-38	1.57E-01	8.45E-06
	MTA2	143	164	97	1	10	5	8	84	0	1	0	2	3	1	4	0	21.44	24.44	19.38	1.63	0.62	5.58	1.19	1.03	19.47	10.74	1.39	0.2	0.3	4.3	0.0	1.6	0.0	1.7	2.9	0.1	0.4	0.0	4.66E-53	1.16E-52	1.73E-01	3.71E-06
	MTA3	73	75	32	0	2	2	2	28	0	0	1	0	0	0	1	0	9.29	10.59	8.40	0.71	0.27	2.42	0.52	0.45	8.44	4.65	0.60	0.2	0.2	3.3	0.0	0.0	0.4	0.0	0.0	0.0	0.2	0.0	1.10E-11	2.05E-13	3.23E-01	1.76E-02
	RERE	15	43	19	0	1	2	3	14	0	0	0	0	0	1	0	0	3.88	4.42	3.51	0.29	0.11	1.01	0.22	0.19	3.52	1.94	0.25	0.5	0.7	4.0	0.0	0.0	0.0	0.0	0.0	0.3	0.0	0.0	2.97E-05	7.59E-08	5.23E-01	3.68E-01
	TRPS1	76	45	12	1	0	0	2	10	0	1	0	0	0	0	0	0	6.07	6.92	5.49	0.46	0.18	1.58	0.34	0.29	5.52	3.04	0.39	0.0	0.3	1.8	0.0	5.7	0.0	0.0	0.0	0.0	0.0	0.0	1.36E-03	1.31E-03	3.78E-02	4.82E-02
	ZGLP1	0	0	0	0	0	0	0	0	0	0	0	0	0	0	0	0	0.00	0.00	0.00	0.00	0.00	0.00	0.00	0.00	0.00	0.00	0.00	na	na	na	na	na	na	na	na	na	na	na	na	na	na	na
ZF.C4_NR	AR	339	476	178	11	29	49	43	86	11	0	3	2	0	17	6	1	61.80	70.43	55.85	4.70	1.79	16.10	3.44	2.97	56.11	30.95	4.00	0.8	0.6	1.5	2.3	0.0	0.2	0.6	0.0	0.3	0.2	0.2	9.82E-18	3.72E-07	1.37E-03	1.93E-12
	ESR1	402	651	283	14	152	74	98	111	10	4	36	8	5	76	22	5	122.76	139.91	110.94	9.33	3.56	31.97	6.84	5.90	111.46	61.49	7.95	0.6	0.7	1.0	1.1	1.1	1.1	1.2	0.8	0.7	0.4	0.6	3.40E-11	1.17E-07	7.50E-01	2.89E-07
	ESR2	195	308	130	15	82	56	42	32	13	2	21	2	5	22	30	2	87.93	100.21	79.46	6.69	2.55	22.90	4.90	4.22	79.84	44.04	5.69	0.6	0.4	0.4	1.9	0.8	0.9	0.4	1.2	0.3	0.7	0.4	3.49E-23	9.66E-17	1.36E-02	9.58E-10
	ESRRA	29	25	7	0	0	0	2	5	0	0	0	0	0	0	0	0	3.37	3.84	3.04	0.26	0.10	0.88	0.19	0.16	3.06	1.69	0.22	0.0	0.5	1.6	0.0	0.0	0.0	0.0	0.0	0.0	0.0	0.0	2.82E-01	6.38E-02	5.52E-01	2.88E-01
	ESRRB	10	17	8	0	7	2	4	2	0	0	0	0	0	7	0	0	4.43	5.05	4.00	0.34	0.13	1.15	0.25	0.21	4.02	2.22	0.29	0.5	0.8	0.5	0.0	0.0	0.0	0.0	0.0	1.7	0.0	0.0	5.00E-01	2.80E-01	4.95E-01	2.76E-01
	ESRRG	43	19	2	0	0	1	0	1	0	0	0	0	0	0	0	0	3.95	4.50	3.57	0.30	0.11	1.03	0.22	0.19	3.59	1.98	0.26	0.3	0.0	0.3	0.0	0.0	0.0	0.0	0.0	0.0	0.0	0.0	9.30E-02	1.38E-02	5.19E-01	2.02E-01
	NR3C1	187	175	51	1	46	11	13	27	0	1	0	2	1	20	21	2	64.00	72.93	57.84	4.87	1.86	16.67	3.56	3.07	58.11	32.05	4.14	0.2	0.2	0.5	0.0	0.5	0.0	0.6	0.3	0.3	0.7	0.5	6.12E-30	1.60E-24	2.18E-02	2.60E-09
	NR3C2	7	15	3	0	0	0	1	2	0	0	0	0	0	0	0	0	1.76	2.00	1.59	0.13	0.05	0.46	0.10	0.08	1.59	0.88	0.11	0.0	0.5	1.3	0.0	0.0	0.0	0.0	0.0	0.0	0.0	0.0	8.34E-01	3.07E-01	6.68E-01	6.65E-01
	PGR	42	29	9	0	1	2	1	6	0	0	0	0	0	0	1	0	4.90	5.59	4.43	0.37	0.14	1.28	0.27	0.24	4.45	2.46	0.32	0.4	0.2	1.4	0.0	0.0	0.0	0.0	0.0	0.0	0.4	0.0	1.74E-01	4.88E-02	4.73E-01	1.91E-01
	NR1D1	8	5	1	0	0	0	0	1	0	0	0	0	0	0	0	0	1.10	1.25	0.99	0.08	0.03	0.29	0.06	0.05	1.00	0.55	0.07	0.0	0.0	1.0	0.0	0.0	0.0	0.0	0.0	0.0	0.0	0.0	9.23E-01	3.09E-01	7.34E-01	8.47E-01
	NR1D2	13	11	5	0	0	0	1	4	0	0	0	0	0	0	0	0	2.63	3.00	2.38	0.20	0.08	0.69	0.15	0.13	2.39	1.32	0.17	0.0	0.3	1.7	0.0	0.0	0.0	0.0	0.0	0.0	0.0	0.0	4.24E-01	7.92E-02	5.99E-01	4.36E-01
	NR1H2	40	18	7	0	0	0	0	7	0	0	0	0	0	0	0	0	3.70	4.21	3.34	0.28	0.11	0.96	0.21	0.18	3.36	1.85	0.24	0.0	0.0	2.1	0.0	0.0	0.0	0.0	0.0	0.0	0.0	0.0	3.90E-02	2.58E-03	5.33E-01	2.37E-01
	NR1H3	41	21	9	0	0	1	0	8	0	0	0	0	0	0	0	0	3.26	3.71	2.94	0.25	0.09	0.85	0.18	0.16	2.96	1.63	0.21	0.3	0.0	2.7	0.0	0.0	0.0	0.0	0.0	0.0	0.0	0.0	2.66E-02	9.28E-04	5.59E-01	3.08E-01
	NR1H4	16	13	7	0	0	0	0	7	0	0	0	0	0	0	0	0	1.72	1.96	1.55	0.13	0.05	0.45	0.10	0.08	1.56	0.86	0.11	0.0	0.0	4.5	0.0	0.0	0.0	0.0	0.0	0.0	0.0	0.0	3.60E-03	1.14E-05	6.71E-01	6.75E-01
	NR1I2	49	14	3	0	0	0	0	3	0	0	0	0	0	0	0	0	3.62	4.13	3.27	0.28	0.11	0.94	0.20	0.17	3.29	1.81	0.23	0.0	0.0	0.9	0.0	0.0	0.0	0.0	0.0	0.0	0.0	0.0	1.39E-01	2.05E-02	5.37E-01	2.47E-01
	NR1I3	17	34	2	0	0	2	0	0	0	0	0	0	0	0	0	0	2.93	3.34	2.65	0.22	0.08	0.76	0.16	0.14	2.66	1.47	0.19	0.7	0.0	0.0	0.0	0.0	0.0	0.0	0.0	0.0	0.0	0.0	2.88E-01	4.34E-02	5.79E-01	3.71E-01
	PPARA	36	18	12	0	1	0	3	9	0	0	0	0	0	0	1	0	4.50	5.13	4.07	0.34	0.13	1.17	0.25	0.22	4.09	2.25	0.29	0.0	0.6	2.2	0.0	0.0	0.0	0.0	0.0	0.0	0.4	0.0	4.63E-02	3.40E-03	4.92E-01	2.43E-01
	PPARD	41	30	14	1	0	0	0	14	1	0	0	0	0	0	0	0	5.82	6.63	5.26	0.44	0.17	1.52	0.32	0.28	5.28	2.91	0.38	0.0	0.0	2.7	2.3	0.0	0.0	0.0	0.0	0.0	0.0	0.0	3.05E-05	1.38E-06	3.50E-01	5.78E-02
	PPARG	216	150	63	0	6	4	12	47	0	0	0	0	0	6	0	0	25.21	28.73	22.78	1.92	0.73	6.57	1.40	1.21	22.89	12.63	1.63	0.2	0.4	2.1	0.0	0.0	0.0	0.0	0.0	0.3	0.0	0.0	2.27E-15	2.63E-12	1.04E-01	9.93E-07
	RARA	137	113	25	0	1	5	3	17	0	0	0	0	0	1	0	0	13.43	15.30	12.14	1.02	0.39	3.50	0.75	0.65	12.19	6.73	0.87	0.4	0.2	1.4	0.0	0.0	0.0	0.0	0.0	0.1	0.0	0.0	9.97E-06	1.90E-04	2.35E-01	3.75E-04
	RARB	21	6	0	0	0	0	0	0	0	0	0	0	0	0	0	0	1.32	1.50	1.19	0.10	0.04	0.34	0.07	0.06	1.20	0.66	0.09	0.0	0.0	0.0	0.0	0.0	0.0	0.0	0.0	0.0	0.0	0.0	7.65E-01	1.35E-01	7.10E-01	7.88E-01
	RARG	20	10	0	0	2	0	0	0	0	0	0	0	0	1	1	0	2.16	2.46	1.95	0.16	0.06	0.56	0.12	0.10	1.96	1.08	0.14	0.0	0.0	0.0	0.0	0.0	0.0	0.0	0.0	0.5	0.9	0.0	6.09E-01	3.74E-02	6.34E-01	9.24E-01
	RORA	13	18	8	0	0	0	2	6	0	0	0	0	0	0	0	0	1.76	2.00	1.59	0.13	0.05	0.46	0.10	0.08	1.59	0.88	0.11	0.0	1.0	3.8	0.0	0.0	0.0	0.0	0.0	0.0	0.0	0.0	6.53E-02	9.01E-04	6.68E-01	6.65E-01
	RORB	13	12	0	0	0	0	0	0	0	0	0	0	0	0	0	0	1.61	1.83	1.45	0.12	0.05	0.42	0.09	0.08	1.46	0.81	0.10	0.0	0.0	0.0	0.0	0.0	0.0	0.0	0.0	0.0	0.0	0.0	6.26E-01	8.63E-02	6.81E-01	7.06E-01
	RORC	10	11	4	0	0	1	0	3	0	0	0	0	0	0	0	0	1.72	1.96	1.55	0.13	0.05	0.45	0.10	0.08	1.56	0.86	0.11	0.6	0.0	1.9	0.0	0.0	0.0	0.0	0.0	0.0	0.0	0.0	7.30E-01	1.65E-01	6.71E-01	6.75E-01
	THRA	38	65	12	0	1	1	4	7	0	0	0	0	0	1	0	0	6.04	6.88	5.46	0.46	0.18	1.57	0.34	0.29	5.48	3.02	0.39	0.2	0.6	1.3	0.0	0.0	0.0	0.0	0.0	0.3	0.0	0.0	1.02E-01	5.38E-02	4.26E-01	9.32E-02
	THRB	46	39	6	0	1	0	2	4	0	0	1	0	0	0	0	0	4.94	5.63	4.46	0.38	0.14	1.29	0.28	0.24	4.49	2.47	0.32	0.0	0.4	0.9	0.0	0.0	0.8	0.0	0.0	0.0	0.0	0.0	1.08E-01	2.56E-02	4.71E-01	1.64E-01
	VDR	98	83	14	0	0	8	0	6	0	0	0	0	0	0	0	0	9.29	10.59	8.40	0.71	0.27	2.42	0.52	0.45	8.44	4.65	0.60	0.9	0.0	0.7	0.0	0.0	0.0	0.0	0.0	0.0	0.0	0.0	1.03E-03	3.25E-03	3.23E-01	4.35E-03
	HNF4A	119	97	27	0	1	0	3	24	0	0	1	0	0	0	0	0	12.59	14.35	11.38	0.96	0.37	3.28	0.70	0.60	11.43	6.30	0.81	0.0	0.2	2.1	0.0	0.0	0.3	0.0	0.0	0.0	0.0	0.0	7.49E-09	1.89E-08	2.50E-01	6.70E-04
	HNF4G	5	7	0	0	0	0	0	0	0	0	0	0	0	0	0	0	0.62	0.71	0.56	0.05	0.02	0.16	0.03	0.03	0.56	0.31	0.04	0.0	0.0	0.0	0.0	0.0	0.0	0.0	0.0	0.0	0.0	0.0	9.79E-01	3.88E-01	7.98E-01	9.50E-01
	NR2C1	59	33	31	0	0	1	3	27	0	0	0	0	0	0	0	0	4.35	4.96	3.94	0.33	0.13	1.13	0.24	0.21	3.95	2.18	0.28	0.2	0.6	6.9	0.0	0.0	0.0	0.0	0.0	0.0	0.0	0.0	1.54E-26	8.21E-31	4.99E-01	1.56E-01
	NR2C2	105	182	163	6	121	71	63	29	4	2	21	2	4	69	22	3	52.98	60.38	47.88	4.03	1.54	13.80	2.95	2.55	48.11	26.54	3.43	1.3	1.0	0.6	1.0	1.3	1.5	0.7	1.6	1.4	0.8	0.9	1.43E-03	1.07E-03	7.09E-01	1.13E-02
	NR2E1	16	26	8	0	0	1	0	7	0	0	0	0	0	0	0	0	2.63	3.00	2.38	0.20	0.08	0.69	0.15	0.13	2.39	1.32	0.17	0.4	0.0	2.9	0.0	0.0	0.0	0.0	0.0	0.0	0.0	0.0	5.34E-02	1.52E-03	5.99E-01	4.36E-01
	NR2E3	19	2	0	0	0	0	0	0	0	0	0	0	0	0	0	0	0.95	1.08	0.86	0.07	0.03	0.25	0.05	0.05	0.86	0.48	0.06	0.0	0.0	0.0	0.0	0.0	0.0	0.0	0.0	0.0	0.0	0.0	9.08E-01	2.35E-01	7.52E-01	8.83E-01
	NR2F1	38	8	2	0	0	0	2	0	0	0	0	0	0	0	0	0	2.74	3.13	2.48	0.21	0.08	0.71	0.15	0.13	2.49	1.37	0.18	0.0	0.6	0.0	0.0	0.0	0.0	0.0	0.0	0.0	0.0	0.0	3.60E-01	5.99E-02	5.91E-01	4.11E-01
	NR2F2	37	17	4	0	0	1	0	3	0	0	0	0	0	0	0	0	6.92	7.88	6.25	0.53	0.20	1.80	0.39	0.33	6.28	3.46	0.45	0.1	0.0	0.5	0.0	0.0	0.0	0.0	0.0	0.0	0.0	0.0	1.76E-03	6.65E-04	3.94E-01	2.63E-02
	NR2F6	22	4	1	0	2	0	1	0	0	0	0	0	0	0	1	1	1.98	2.25	1.79	0.15	0.06	0.51	0.11	0.09	1.79	0.99	0.13	0.0	0.4	0.0	0.0	0.0	0.0	0.0	0.0	0.0	1.0	7.8	2.17E-01	1.08E-01	6.49E-01	1.33E-01
	RXRA	201	55	16	0	0	1	6	9	0	0	0	0	0	0	0	0	12.59	14.35	11.38	0.96	0.37	3.28	0.70	0.60	11.43	6.30	0.81	0.1	0.4	0.8	0.0	0.0	0.0	0.0	0.0	0.0	0.0	0.0	1.40E-05	3.33E-04	2.50E-01	3.18E-04
	RXRB	52	9	2	0	1	2	0	0	0	0	0	0	0	0	1	0	3.51	4.00	3.17	0.27	0.10	0.91	0.20	0.17	3.19	1.76	0.23	0.6	0.0	0.0	0.0	0.0	0.0	0.0	0.0	0.0	0.6	0.0	2.12E-01	1.99E-02	5.44E-01	4.13E-01
	RXRG	50	5	0	0	0	0	0	0	0	0	0	0	0	0	0	0	2.45	2.79	2.22	0.19	0.07	0.64	0.14	0.12	2.23	1.23	0.16	0.0	0.0	0.0	0.0	0.0	0.0	0.0	0.0	0.0	0.0	0.0	2.70E-01	2.40E-02	6.12E-01	4.79E-01
	NR4A1	61	55	31	1	5	6	13	12	1	0	0	1	0	1	3	0	10.14	11.55	9.16	0.77	0.29	2.64	0.56	0.49	9.20	5.08	0.66	0.6	1.1	1.3	1.3	0.0	0.0	1.8	0.0	0.1	0.6	0.0	1.18E-01	2.53E-01	5.47E-01	3.12E-02
	NR4A2	10	8	7	0	0	0	2	5	0	0	0	0	0	0	0	0	1.21	1.38	1.09	0.09	0.04	0.31	0.07	0.06	1.10	0.60	0.08	0.0	1.5	4.6	0.0	0.0	0.0	0.0	0.0	0.0	0.0	0.0	5.78E-02	4.33E-04	7.22E-01	8.18E-01
	NR4A3	7	3	1	0	0	0	1	0	0	0	0	0	0	0	0	0	1.87	2.13	1.69	0.14	0.05	0.49	0.10	0.09	1.69	0.93	0.12	0.0	0.5	0.0	0.0	0.0	0.0	0.0	0.0	0.0	0.0	0.0	6.51E-01	1.26E-01	6.58E-01	6.34E-01
	NR5A1	22	14	3	0	0	0	3	0	0	0	0	0	0	0	0	0	2.01	2.29	1.82	0.15	0.06	0.52	0.11	0.10	1.83	1.01	0.13	0.0	1.3	0.0	0.0	0.0	0.0	0.0	0.0	0.0	0.0	0.0	6.33E-01	1.32E-01	6.46E-01	5.94E-01
	NR5A2	19	11	1	0	0	0	1	0	0	0	0	0	0	0	0	0	1.87	2.13	1.69	0.14	0.05	0.49	0.10	0.09	1.69	0.93	0.12	0.0	0.5	0.0	0.0	0.0	0.0	0.0	0.0	0.0	0.0	0.0	6.51E-01	1.26E-01	6.58E-01	6.34E-01
	NR6A1	3	2	0	0	0	0	0	0	0	0	0	0	0	0	0	0	0.29	0.33	0.26	0.02	0.01	0.08	0.02	0.01	0.27	0.15	0.02	0.0	0.0	0.0	0.0	0.0	0.0	0.0	0.0	0.0	0.0	0.0	9.99E-01	6.41E-01	8.61E-01	9.91E-01
	NR0B1	35	7	3	0	1	3	0	0	0	0	0	0	1	0	0	0	1.72	1.96	1.55	0.13	0.05	0.45	0.10	0.08	1.56	0.86	0.11	1.7	0.0	0.0	0.0	0.0	0.0	0.0	12.1	0.0	0.0	0.0	5.65E-02	1.07E-01	6.71E-01	2.10E-02
	NR0B2	92	35	25	0	0	1	3	21	0	0	0	0	0	0	0	0	5.67	6.46	5.13	0.43	0.16	1.48	0.32	0.27	5.15	2.84	0.37	0.2	0.5	4.1	0.0	0.0	0.0	0.0	0.0	0.0	0.0	0.0	2.74E-10	1.22E-12	4.40E-01	6.41E-02
ZF.CCCH	ZFP36L1	1	10	2	0	0	0	2	0	0	0	0	0	0	0	0	0	1.21	1.38	1.09	0.09	0.04	0.31	0.07	0.06	1.10	0.60	0.08	0.0	1.5	0.0	0.0	0.0	0.0	0.0	0.0	0.0	0.0	0.0	8.96E-01	2.75E-01	7.22E-01	8.18E-01
ZF.CCHC	LIN28A	28	24	59	1	88	25	30	4	0	1	0	2	0	36	49	1	17.38	19.81	15.71	1.32	0.50	4.53	0.97	0.83	15.78	8.71	1.13	1.4	1.5	0.3	0.0	2.0	0.0	2.1	0.0	2.3	5.6	0.9	1.78E-45	1.74E-04	1.79E-01	2.56E-45
	LIN28B	19	16	31	0	22	12	13	6	0	0	0	0	0	10	12	0	9.07	10.34	8.20	0.69	0.26	2.36	0.51	0.44	8.24	4.54	0.59	1.3	1.3	0.7	0.0	0.0	0.0	0.0	0.0	1.2	2.6	0.0	3.26E-02	3.30E-01	3.29E-01	5.56E-03
ZF.CXXC	MBD1	17	30	4	0	1	0	1	3	0	0	0	0	1	0	0	0	3.40	3.88	3.08	0.26	0.10	0.89	0.19	0.16	3.09	1.70	0.22	0.0	0.3	1.0	0.0	0.0	0.0	0.0	6.1	0.0	0.0	0.0	9.22E-02	6.26E-02	5.50E-01	6.54E-02
	BAZ2A	10	20	9	0	3	1	1	7	0	0	1	0	0	1	1	0	3.22	3.67	2.91	0.24	0.09	0.84	0.18	0.15	2.92	1.61	0.21	0.3	0.3	2.4	0.0	0.0	1.2	0.0	0.0	0.3	0.6	0.0	3.10E-01	9.95E-03	5.61E-01	8.39E-01
	BAZ2B	7	8	5	0	0	2	2	1	0	0	0	0	0	0	0	0	2.38	2.71	2.15	0.18	0.07	0.62	0.13	0.11	2.16	1.19	0.15	0.8	0.7	0.5	0.0	0.0	0.0	0.0	0.0	0.0	0.0	0.0	8.57E-01	6.50E-01	6.17E-01	4.97E-01
	MBD2	89	71	52	0	2	1	8	43	0	0	0	0	0	1	1	0	9.04	10.30	8.17	0.69	0.26	2.35	0.50	0.43	8.21	4.53	0.59	0.1	0.8	5.3	0.0	0.0	0.0	0.0	0.0	0.1	0.2	0.0	2.64E-31	1.20E-34	3.30E-01	2.38E-02
	MBD3	131	98	61	0	3	3	4	54	0	0	2	0	0	0	1	0	15.22	17.35	13.76	1.16	0.44	3.96	0.85	0.73	13.82	7.62	0.99	0.2	0.2	3.9	0.0	0.0	0.5	0.0	0.0	0.0	0.1	0.0	9.75E-30	1.19E-30	2.06E-01	3.21E-04
	MBD4	5	31	15	0	10	1	6	8	0	0	0	0	0	10	0	0	4.10	4.67	3.70	0.31	0.12	1.07	0.23	0.20	3.72	2.05	0.27	0.2	1.3	2.2	0.0	0.0	0.0	0.0	0.0	2.7	0.0	0.0	1.26E-02	2.12E-02	5.12E-01	1.32E-02
	MECP2	125	204	208	18	117	91	74	43	16	2	12	0	1	76	26	2	53.49	60.97	48.35	4.07	1.55	13.93	2.98	2.57	48.57	26.79	3.46	1.7	1.2	0.9	3.9	1.3	0.9	0.0	0.4	1.6	1.0	0.6	4.88E-14	3.60E-07	3.07E-09	1.08E-03
	SETDB1	23	61	12	0	2	1	1	10	0	0	0	0	0	2	0	0	10.06	11.47	9.09	0.77	0.29	2.62	0.56	0.48	9.14	5.04	0.65	0.1	0.1	1.1	0.0	0.0	0.0	0.0	0.0	0.0	0.4	0.0	1.74E-04	1.36E-04	3.04E-01	9.21E-03
	SETDB2	3	3	2	0	0	2	0	0	0	0	0	0	0	0	0	0	1.06	1.21	0.96	0.08	0.03	0.28	0.06	0.05	0.96	0.53	0.07	1.9	0.0	0.0	0.0	0.0	0.0	0.0	0.0	0.0	0.0	0.0	8.87E-01	2.23E-01	7.38E-01	8.56E-01
ZF.DM	DMRT1	0	1	0	0	0	0	0	0	0	0	0	0	0	0	0	0	0.18	0.21	0.17	0.01	0.01	0.05	0.01	0.01	0.17	0.09	0.01	0.0	0.0	0.0	0.0	0.0	0.0	0.0	0.0	0.0	0.0	0.0	1.00E+00	7.57E-01	8.90E-01	9.97E-01
	DMRT2	0	2	0	0	0	0	0	0	0	0	0	0	0	0	0	0	0.11	0.13	0.10	0.01	0.00	0.03	0.01	0.01	0.10	0.05	0.01	0.0	0.0	0.0	0.0	0.0	0.0	0.0	0.0	0.0	0.0	0.0	1.00E+00	8.46E-01	9.14E-01	9.99E-01
	DMRT3	10	12	0	0	0	0	0	0	0	0	0	0	0	0	0	0	3.70	4.21	3.34	0.28	0.11	0.96	0.21	0.18	3.36	1.85	0.24	0.0	0.0	0.0	0.0	0.0	0.0	0.0	0.0	0.0	0.0	0.0	4.82E-02	3.61E-03	5.33E-01	2.37E-01
	DMRTA1	1	0	0	0	0	0	0	0	0	0	0	0	0	0	0	0	0.11	0.13	0.10	0.01	0.00	0.03	0.01	0.01	0.10	0.05	0.01	0.0	0.0	0.0	0.0	0.0	0.0	0.0	0.0	0.0	0.0	0.0	1.00E+00	8.46E-01	9.14E-01	9.99E-01
	DMRTA2	0	0	0	0	0	0	0	0	0	0	0	0	0	0	0	0	0.00	0.00	0.00	0.00	0.00	0.00	0.00	0.00	0.00	0.00	0.00	na	na	na	na	na	na	na	na	na	na	na	na	na	na	na

	DMRTB1	3	36	21	3	4
	DMRTC1B	0	1	0	0	0
	DMRTC2	1	0	0	0	0
ZF.MIZ	PIAS1	124	101	33	0	28
ZF.MIZ	PIAS2	81	45	6	0	0
ZF.MIZ	PIAS3	53	24	5	0	0
ZF.MIZ	PIAS4	70	50	11	0	0
ZF.MIZ	ZMIZ1	27	15	7	0	0
ZF.MIZ	ZMIZ2	26	19	3	0	0
ZF.NFX1	ZNFX1	5	7	4	0	1
ZF.NFX1	NFX1	22	67	73	1	119
ZF.NFX1	NFXL1	3	8	1	0	0

	DMRTB1	12	7	2	1	2	0	0	0	1	3	0
	DMRTC1B	0	0	0	0	0	0	0	0	0	0	0
	DMRTC2	0	0	0	0	0	0	0	0	0	0	0
ZF.MIZ	PIAS1	9	16	8	0	0	0	0	0	28	0	0
ZF.MIZ	PIAS2	1	3	2	0	0	0	0	0	0	0	0
ZF.MIZ	PIAS3	2	2	1	0	0	0	0	0	0	0	0
ZF.MIZ	PIAS4	1	5	5	0	0	0	0	0	0	0	0
ZF.MIZ	ZMIZ1	0	4	3	0	0	0	0	0	0	0	0
ZF.MIZ	ZMIZ2	1	0	2	0	0	0	0	0	0	0	0
ZF.NFX1	ZNFX1	1	3	0	0	0	0	0	0	0	1	0
ZF.NFX1	NFX1	24	44	5	0	1	16	8	1	70	24	0
ZF.NFX1	NFXL1	0	1	0	0	0	0	0	0	0	0	0

	DMRTB1	5.20	5.92	4.70	0.40	0.15	1.35	0.29	0.25	4.72	2.60	0.34
	DMRTC1B	0.04	0.04	0.03	0.00	0.00	0.01	0.00	0.00	0.03	0.02	0.00
	DMRTC2	0.07	0.08	0.07	0.01	0.00	0.02	0.00	0.00	0.07	0.04	0.00
ZF.MIZ	PIAS1	18.48	21.06	16.70	1.40	0.54	4.81	1.03	0.89	16.78	9.25	1.20
ZF.MIZ	PIAS2	9.33	10.63	8.43	0.71	0.27	2.43	0.52	0.45	8.47	4.67	0.60
ZF.MIZ	PIAS3	4.61	5.25	4.17	0.35	0.13	1.20	0.26	0.22	4.19	2.31	0.30
ZF.MIZ	PIAS4	9.22	10.51	8.33	0.70	0.27	2.40	0.51	0.44	8.37	4.62	0.60
ZF.MIZ	ZMIZ1	1.90	2.17	1.72	0.14	0.06	0.50	0.11	0.09	1.73	0.95	0.12
ZF.MIZ	ZMIZ2	2.52	2.88	2.28	0.19	0.07	0.66	0.14	0.12	2.29	1.26	0.16
ZF.NFX1	ZNFX1	2.23	2.54	2.02	0.17	0.06	0.58	0.12	0.11	2.03	1.12	0.14
ZF.NFX1	NFX1	18.22	20.77	16.47	1.39	0.53	4.75	1.01	0.88	16.55	9.13	1.18
ZF.NFX1	NFXL1	4.35	4.96	3.94	0.33	0.13	1.13	0.24	0.21	3.95	2.18	0.28

	DMRTB1	2.3	1.2	0.4	2.5	13.3	0.0	0.0	0.0	0.2	1.2	0.0	2.09E-05	4.86E-03	1.18E-06	3.90E-01
	DMRTC1B	0.0	0.0	0.0	0.0	0.0	0.0	0.0	0.0	0.0	0.0	0.0	1.00E+00	9.46E-01	9.51E-01	1.00E+00
	DMRTC2	0.0	0.0	0.0	0.0	0.0	0.0	0.0	0.0	0.0	0.0	0.0	1.00E+00	8.95E-01	9.30E-01	1.00E+00
ZF.MIZ	PIAS1	0.5	0.8	0.5	0.0	0.0	0.0	0.0	0.0	1.7	0.0	0.0	5.15E-05	4.97E-03	1.64E-01	1.60E-04
ZF.MIZ	PIAS2	0.1	0.3	0.2	0.0	0.0	0.0	0.0	0.0	0.0	0.0	0.0	8.58E-05	1.35E-04	3.22E-01	4.23E-03
ZF.MIZ	PIAS3	0.4	0.4	0.2	0.0	0.0	0.0	0.0	0.0	0.0	0.0	0.0	1.37E-01	5.23E-02	4.86E-01	1.32E-01
ZF.MIZ	PIAS4	0.1	0.5	0.6	0.0	0.0	0.0	0.0	0.0	0.0	0.0	0.0	1.05E-03	3.10E-03	3.25E-01	4.60E-03
ZF.MIZ	ZMIZ1	0.0	1.8	1.7	0.0	0.0	0.0	0.0	0.0	0.0	0.0	0.0	6.19E-01	1.11E-01	6.55E-01	6.24E-01
ZF.MIZ	ZMIZ2	0.4	0.0	0.9	0.0	0.0	0.0	0.0	0.0	0.0	0.0	0.0	5.57E-01	1.47E-01	6.07E-01	4.61E-01
ZF.NFX1	ZNFX1	0.4	1.2	0.0	0.0	0.0	0.0	0.0	0.0	0.0	0.9	0.0	8.14E-01	2.49E-01	6.28E-01	7.01E-01
ZF.NFX1	NFX1	1.3	2.1	0.3	0.0	1.9	3.4	7.9	1.1	4.2	2.6	0.0	9.30E-61	1.67E-08	1.79E-01	6.69E-57
ZF.NFX1	NFXL1	0.0	0.2	0.0	0.0	0.0	0.0	0.0	0.0	0.0	0.0	0.0	3.01E-02	3.26E-03	4.99E-01	1.56E-01
