# Supplementary material for: Foxg1 and companions: Not only transcription factors
Source: Neural Regen Res. 2025 Sep 3;21(8):3426–38. doi: 10.4103/NRR.NRR-D-25-00439 (PMC13452659; doi:10.4103/NRR.NRR-D-25-00439)
Supplement: Supplementary file 4 [file NRR-21-3426_Suppl12.pdf]

Additional Table 7.1. Filtered TF/SplF.core interaction reports & related info

TF	TF taxon	Numbers of TF/SplF.core interaction reports, real-vs-expected: Chi-square test, p evaluated over all 11 groups (3 SplFs, 2 pAFs, 6 TlatMFs)	Chi-square test, p evaluated over 3 SplF groups	Real / expected number ratio
RFX6	FH/WH.RFX	1.25E-02	4.39E-02	2.1
MAEL	HMG	1.05E-37	1.33E-12	2.8
GCFC2	others	9.36E-42	3.13E-47	9.2
PAXBP1	others	3.78E-39	5.92E-44	7.6
SON	others	2.71E-03	3.65E-05	2.2
ERG	WC.ETS	2.73E-13	1.52E-15	2.0
CDC5L	WC.MYB	4.26E-153	1.04E-150	5.2
MTF1	ZF.C2H2	1.87E-40	9.76E-08	2.6
ZNF207	ZF.C2H2	4.54E-10	1.31E-13	4.5
ZNF511	ZF.C2H2	3.67E-58	3.57E-02	2.3
ZGPAT	ZF.C3H	1.70E-44	1.33E-47	5.8
DMRTB1	ZF.DM	2.09E-05	4.86E-03	2.3

n° of filtered TF/SplF.core interaction reports

TF	U1 snRNP			SM proteins							U2 snRNP									
	SNRNP70	SNRPA	SNRPC	SNRPD1	SNRPD2	SNRPD3	SNRPE	SNRPF	SNRPG	SNRPB	SNRPA1	SNRPB2	AC004381.6	SF3A1	SF3A2	SF3A3	SF3B1	SF3B2	SF3B3	SF3B4
RFX6			1							3										
MAEL																				
GCFC2							2			1	3									
PAXBP1	1	1	1			1	2	3	2	2	1			1			1			
SON	1	1	1							1				1			1			
ERG																	2	2	2	
CDC5L	4	3	1	3	1	2	3	1	1	1	4	2		3	3	1	4	2	2	1
MTF1																	1	1		
ZNF207		1	1					1				1			1					
ZNF511																				
ZGPAT				1	1	1	2	1	2	2	1									
DMRTB1		1	1							1										1

TF	U2 snRNP related										A complex									LSm proteins				
	SMNDC1	U2AF1	U2AF2	DDX46	DHX15	CHERP	PUF60	RBM17	SR140	HTATSF1	CCAR1	PRPF40A	SF1	THRAP3	RBM10	RBM25	DDX5	RBM5	BUB3	LSM2	LSM3	LSM4	LSM6	LSM7
RFX6																								
MAEL																								
GCFC2																								
PAXBP1																					1			
SON			1									1												
ERG							2					2					1							
CDC5L		1	4	1		1		2							1		1	1	1	1				
MTF1					1										1		1							
ZNF207							1												10					
ZNF511																					1	1	2	1
ZGPAT					8															1	2	1	1	1
DMRTB1													1											

TF	U5 snRNP								U4/U6 snRNP				U4/U6.U5 snRNP		
	CD2BP2	EFTUD2	PRPF6	PRPF8	SNRNP200	SNRNP40	TXNL4A	DDX23	NHP2L1	PRPF3	PRPF4	RP11	SART1	SNRNP27	USP39
RFX6															
MAEL															
GCFC2	1		3	3	3	1		4							
PAXBP1	1	2		2		4		4							
SON		1				1									
ERG				3	2			2							
CDC5L	1	5	2	6	4	4		2		2			1		1
MTF1		1		1	1										
ZNF207						1									
ZNF511															
ZGPAT		1	1	2	1	1	1	1	1	5	5		2	1	1
DMRTB1			1				1								

TF	PRP19 complex																		
	BCAS2	CDC5L	CTNNBL1	PLRG1	PQBP1	PRPF19	WBP11	AQR	BUD31	CRNKL1	ISY1	PPIE	RBM22	SNW1	YBX1	CWC15	HSPA8	HSPA1A	XAB2
RFX6																			
MAEL															2		1		
GCFC2	1					2					1	1							
PAXBP1					1							1		1					
SON																	1	1	
ERG		2															3		
CDC5L	14	8	9	13		14		2	1	3	2	4	1	7	1	4	2		3
MTF1						1											1	1	
ZNF207																	1		
ZNF511																			
ZGPAT																			
DMRTB1	1												1	1					

TF	B complex						Bact complex									RES complex		
	IK	MFAP1	PRPF4B	SMU1	WBP4	TFIP11	CCDC12	CDC40	CWC22	CWC27	PPIL3	PPIL2	DHX16	FUBP3	ZNF830	BUD13	RBMX2	SNIP1
RFX6																		
MAEL																		
GCFC2						1												
PAXBP1																		
SON																		1
ERG																		
CDC5L	1		3				4	1	1			1	1		1	1		1
MTF1																		
ZNF207					1													
ZNF511																		
ZGPAT						4												
DMRTB1					1						1							

TF	C complex																			
	C19orf29	C9orf78	CXorf56	LENG1	SYF2	DGCR14	FAM50A	PPWD1	PPIG	DDX41	NOSIP	DHX35	CDK10	DHX38	FAM32A	FRG1	FAM50B	PRPF18	SLU7	DHX8
RFX6																				
MAEL																				
GCFC2					2															
PAXBP1																				1
SON																				1
ERG																				
CDC5L		1	1		3	1		1		1				1						3
MTF1																				
ZNF207																				
ZNF511																				
ZGPAT																				
DMRTB1																				

TF	Subcellular TF location (see Additional Table 2)	RBP function reported for… (see Additional Table 3)	pTriplo (see Additional Table 4)	Neuronal activity-plasticity responsive (see Additional Table 5)
RFX6	none reported		0.48	
MAEL	none reported	hsa gene	0.21	
GCFC2	Nucleoplasm	hsa gene	0.14	1
PAXBP1	Nucleoplasm,Cytosol		0.69	
SON	Nuclear speckles	hsa gene	0.88	
ERG	Nucleoplasm,Cytosol		0.94	
CDC5L	Nucleoplasm	hsa gene	0.99	
MTF1	Nucleoplasm		0.94	1
ZNF207	Nucleoplasm	hsa gene	0.67	
ZNF511	Nucleoplasm,Nuclear bodies,Vesicles		0.27	
ZGPAT	Nucleoplasm,Plasma membrane	hsa gene	0.59	
DMRTB1	none reported		0.35	
