# Supplementary material for: Foxg1 and companions: Not only transcription factors
Source: Neural Regen Res. 2025 Sep 3;21(8):3426–38. doi: 10.4103/NRR.NRR-D-25-00439 (PMC13452659; doi:10.4103/NRR.NRR-D-25-00439)
Supplement: Supplementary file 5 [file NRR-21-3426_Suppl13.pdf]

Additional Table 7.2. Filtered TF/SplF.RBP interaction reports & related info

TF	TF taxon	Numbers of TF/SplF.RBP interaction reports, real-vs-expected: Chi-square test, p evaluated over all 11 groups (3 SplFs, 2 pAFs, 6 TlatMFs)	Chi-square test, p evaluated over 3 SplF groups	Real / expected number ratio	Subcellular TF location (see Additioinal Table 2)	RBP function reported for… (see Additioinal Table 3)	pTriplo (see Additioinal Table 4)	Neuronal activity-plasticity responsive (see Additioinal Table 5)
FOXC1	FH/WH.FOX	1.38E-03	5.19E-03	2.0	Nucleoplasm,Cytosol		0.61	
POU3F4	HB-POU	1.43E-02	1.60E-05	24.0	Nucleoplasm	hsa gene	NA	
PITX3	HB.PD	3.68E-05	6.44E-09	14.4	none reported		0.95	
MAEL	HMG	1.05E-37	1.33E-12	7.4	none reported	hsa gene	0.21	
CPEB1	others	3.81E-47	9.67E-04	2.0	Nucleoplasm,Cytosol	hsa gene	1.00	1
SFPQ	others	1.72E-17	9.09E-16	2.7	Nucleoplasm	hsa gene	0.58	
TP63	p53	4.92E-04	1.70E-05	2.0	Nucleoplasm		1.00	
ERG	WC.ETS	2.73E-13	1.52E-15	3.0	Nucleoplasm,Cytosol		0.94	
KLF13	ZF.C2H2	5.85E-05	2.43E-08	2.8	none reported	hsa gene	0.48	1
KLF7	ZF.C2H2	1.30E-02	1.78E-05	9.0	Nucleoplasm,Cytosol		0.79	
MTF1	ZF.C2H2	1.87E-40	9.76E-08	3.1	Nucleoplasm		0.94	1
REST	ZF.C2H2	5.87E-61	1.12E-04	2.1	Nucleoplasm,Cytosol	hsa gene	0.75	1
ZBTB41	ZF.C2H2	2.32E-08	4.14E-03	5.1	Nucleoplasm,Nuclear membrane,Cytosol	hsa gene	0.40	1
ZFP69	ZF.C2H2	1.43E-02	1.60E-05	24.0	Nucleoplasm		0.17	
ZNF207	ZF.C2H2	4.54E-10	1.31E-13	2.3	Nucleoplasm	hsa gene	0.67	
ZNF326	ZF.C2H2	1.84E-07	1.85E-08	2.8	Nucleoplasm,Golgi apparatus,Vesicles	hsa gene	0.74	
ZNF528	ZF.C2H2	2.32E-02	3.67E-05	12.0	Nuclear speckles,Cytosol	hsa gene	0.04	
ZNF532	ZF.C2H2	3.40E-04	3.02E-02	3.3	Nucleoplasm,Golgi apparatus		0.86	
NFX1	ZF.NFX1	9.30E-61	1.67E-08	2.1	Nucleoplasm,Nucleoli,Plasma membrane,Cytosol	hsa gene	0.92	

n° of filtered TF/SplF.RBP interaction reports

TF	EJC / mRNP							TREX						Helicase																
	ACIN1	EIF4A3	MAGOH	RBM8A	RNPS1	THOC4	BAT1	THOC1	THOC2	THOC3	THOC5	THOC6	THOC7	DDX10	DDX11	DDX12	DDX17	DDX24	DDX28	DDX31	DDX3X	DDX52	DHX30	DHX32	DHX33	DHX57	DHX9	HFM1	SKIV2L2	TDRD9
FOXC1																	1										1			
POU3F4																														
PITX3																														
MAEL																					1									
CPEB1																							1							
SFPQ																	1									1	1			
TP63																											1			
ERG																					1									
KLF13																														
KLF7																														
MTF1																	1				1		1				1			
REST		1															1				1						1			
ZBTB41																														
ZFP69																														
ZNF207			1		1																									
ZNF326																											1			
ZNF528																														
ZNF532																														
NFX1				2													1				2		1			1	1		1	

TF	hnRNP																											
	HNRNPA1	HNRNPAB	HNRNPC	FUS	HNRNPA0	HNRNPA2B1	HNRNPA3	HNRNPCL1	HNRNPD	HNRNPF	HNRNPH1	HNRNPH2	HNRNPH3	HNRNPK	HNRNPL	HNRNPM	HNRNPR	HNRNPU	HNRNPUL1	HNRNPUL2	HNRPDL	HNRPLL	ILF2	MATR3	PTBP1	RALY	SYNCRIP	ILF3
FOXC1	1				1								1		1	1	1		1	1				1		1		1
POU3F4																		1										
PITX3																												
MAEL	1					1																					2	
CPEB1															1		1		1				1	1			1	
SFPQ	4			4		1					2													2	3		2	
TP63		5		3			1	1	1					3	1		1										2	
ERG	2	2	2			3			2	2	2		2			1		3	2	2			2	2				2
KLF13											1																	
KLF7											1				1													
MTF1	1		1			1					1					1		1						1				1
REST		1			1		1		1	1					1	1	1	1					1	1		1	2	1
ZBTB41																												
ZFP69																												
ZNF207									1							2			2									
ZNF326	4		1	3		1	1				3				1					1			1	3				1
ZNF528															1													
ZNF532															1													
NFX1	1	1		1	1	1	1		1	1	1					2		1	1				1	1			2	1

TF	SR proteins												
	SFRS11	SFRS3	SFRS4	SFRS6	SFRS7	SFRS9	SRSF1	SRSF2	SRSF5	TRA2B	SRRM2	SRRM1	SRRM3
FOXC1							1						
POU3F4													
PITX3													
MAEL													
CPEB1													
SFPQ											1	1	
TP63													
ERG													
KLF13							1						
KLF7													
MTF1							1						
REST							1		1				
ZBTB41													
ZFP69													
ZNF207													
ZNF326							1						
ZNF528													
ZNF532													
NFX1							1				1		

TF	RNA binding proteins																				
	RBM6	CCDC55	CIRBP	CSDA	ELAVL1	ESRP1	ESRP2	EWSR1	GCFC1	IGF2BP3	KIN	LUC7L3	NONO	PRPF39	RBM15	RBM39	RBM9	SFPQ	TIA1	TIAL1	ZNF207
FOXC1					2			1					1			1		1			
POU3F4																					
PITX3													1					2			
MAEL					2																
CPEB1					1					2											
SFPQ					2			3					24			1		4			
TP63													1								
ERG					2								2					2			
KLF13																					
KLF7																					
MTF1													1								
REST					1			1					1					1			
ZBTB41					1																
ZFP69																					
ZNF207								1													2
ZNF326								1								2					
ZNF528					1																
ZNF532					1																
NFX1			1		1					1											

TF	RNA editing		RNA modifying		RNA metylation								CBC		3´end					RNA degradation			RISC		
	ADAR	ADARB1	C19orf43	DBR1	FTO	KIAA1429	METTL14	WTAP	ALKBH5	METTL3	YTHDC1	WDR83	NCBP1	NCBP2	PABPN1	PABPC1	CPSF2	CPSF6	NUDT21	DIS3	DIS3L	EXOSC4	EIF2C2	DICER1	MOV10
FOXC1																			1						
POU3F4																									
PITX3																									
MAEL																2									
CPEB1																1									2
SFPQ	1	1				2	2			1								1	1						1
TP63																		1							
ERG	2																								
KLF13						1																			
KLF7											1														
MTF1						1																			
REST						1										1									
ZBTB41		1				1																			
ZFP69											1														
ZNF207																									1
ZNF326																		1							1
ZNF528																									
ZNF532						1																			
NFX1															1	6			1			1			3
