# Supplementary material for: Foxg1 and companions: Not only transcription factors
Source: Neural Regen Res. 2025 Sep 3;21(8):3426–38. doi: 10.4103/NRR.NRR-D-25-00439 (PMC13452659; doi:10.4103/NRR.NRR-D-25-00439)
Supplement: Supplementary file 6 [file NRR-21-3426_Suppl14.pdf]

Additional Table 7.3. Filtered TF/SplF.other interaction reports & related info

Numbers of TF/SplF.other interaction reports, real-vs-expected; subcellular location and related info

TF	TF taxon	Chi-square test, p evaluated over all 11 groups (3 SplFs, 2 pAFs, 6 TlatMFs)	Chi-square test, p evaluated over 3 SplF groups	Real / expected number ratio	Subcellular TF location (see Additional Table 2)	RBP function reported for… (see Additional Table 3)	pTriplo (see Additional Table 4)	Neuronal activity-plasticity responsive (see Additional Table 5)
ARID1A	ARID	1.75E-18	1.18E-18	3.5	Nucleoplasm	only mmu ortholog	1.00	1
ARID1B	ARID	9.68E-09	1.48E-09	2.8	Nucleoplasm		0.98	
ARID4A	ARID	3.86E-02	2.62E-03	2.1	Nucleoplasm	hsa gene	0.86	
ARID5B	ARID	1.49E-03	1.18E-05	3.7	Nucleoplasm,Endoplasmic reticulum,Plasma membrane,Cytosol	only mmu ortholog	0.98	1
JARID2	ARID	1.00E-31	1.69E-36	7.9	Nucleoplasm,Mitochondria		0.99	1
HIF1A	bHLH	7.28E-49	2.47E-39	2.8	Nucleoplasm,Nuclear bodies	only mmu ortholog	0.94	
MESP1	bHLH	2.29E-02	4.61E-05	8.2	Nucleoplasm,Nucleoli		0.22	
MLXIPL	bHLH	3.55E-02	5.77E-04	3.4	Nucleoplasm		0.69	
MYOD1	bHLH	1.40E-21	3.28E-23	4.3	Nucleoplasm		0.69	
SREBF1	bHLH	4.60E-08	1.52E-09	3.0	Nucleoplasm,Golgi apparatus,Cytosol		0.65	
TAL1	bHLH	1.46E-15	6.28E-19	4.6	Nucleoplasm	hsa gene	0.72	
TWIST1	bHLH	2.02E-06	1.36E-08	3.6	none reported		0.99	
USF1	bHLH	1.82E-02	4.24E-03	2.2	Nucleoplasm	hsa gene	0.94	
ATF3	bZIP	1.89E-05	2.89E-06	2.5	Nucleoplasm,Nucleoli		0.58	1
CEBPD	bZIP	4.21E-08	4.46E-05	3.6	Nucleoplasm		0.31	
CREB1	bZIP	1.16E-13	3.79E-14	2.9	Nucleoplasm	only mmu ortholog	0.99	1
NFYB	CAAT-BF	3.85E-03	2.29E-05	3.8	Nucleoplasm		0.81	
E2F6	FH/WH.E2F	1.98E-10	5.02E-11	2.4	Nucleoplasm	hsa gene	0.94	
FOXO3	FH/WH.FOX	1.10E-03	1.72E-04	2.3	Nucleoplasm		0.98	
FOXP3	FH/WH.FOX	3.82E-02	4.57E-03	2.6	Nucleoplasm,Cytosol		NA	
FOXR1	FH/WH.FOX	6.36E-03	4.52E-04	2.7	Nucleoplasm,Cytosol		0.23	1
GCM1	GCM	6.75E-04	4.91E-06	3.5	Vesicles		0.76	1
GTF2I	GTF2I	1.78E-06	9.98E-06	2.1	Nucleoplasm	only mmu ortholog	0.97	1
SATB1	HB-CUT	8.87E-07	2.90E-08	3.1	Nucleoplasm,Nuclear bodies		0.95	1
HNF1A	HB-POU	7.40E-05	4.37E-07	3.7	Nucleoplasm	only mmu ortholog	0.78	
ADNP	HB-ZF.C2H2	3.72E-07	1.37E-08	2.6	Nucleoplasm	only mmu ortholog	1.00	
ZEB1	HB-ZF.C2H2	6.89E-09	2.16E-08	2.9	Nucleoplasm,Nucleoli	hsa gene	0.78	
ZEB2	HB-ZF.C2H2	2.35E-05	2.53E-07	3.4	Nucleoplasm,Nucleoli,Cytosol	hsa gene	0.96	1
CDX2	HB.HOX	2.33E-02	1.37E-04	4.4	Nucleoplasm		1.00	
NKX2-2	HB.NK	2.04E-02	3.12E-05	12.1	none reported		0.97	
PBX1	HB.TALE	4.71E-02	4.01E-03	2.3	Nucleoplasm,Nuclear bodies,Cytosol		0.99	1
KMT2C	HMG	3.39E-04	9.80E-05	2.4	Nucleoplasm	hsa gene	0.92	
SOX9	HMG.SOX	2.16E-02	2.81E-03	2.1	Nucleoplasm		0.82	1
SRY	HMG.SOX	4.12E-02	2.50E-04	4.6	none reported		NA	
HMG20A	HMG.TOX	1.34E-09	2.28E-10	2.6	Nucleoplasm		0.77	1
HMG20B	HMG.TOX	1.05E-31	1.02E-35	6.5	Nucleoplasm	hsa gene	0.79	
SMARCE1	HMG.TOX	1.42E-19	1.76E-17	2.8	Nucleoplasm	hsa gene	0.98	1
MEF2A	MADS	2.28E-06	7.21E-09	4.1	Nucleoplasm,Cytosol		0.86	
MEF2C	MADS	7.19E-11	9.50E-15	6.4	Nucleoplasm,Vesicles		1.00	1
MEF2D	MADS	2.05E-13	9.05E-17	5.3	Nucleoplasm,Vesicles		0.96	
L3MBTL3	others	7.73E-05	6.88E-06	2.6	Nucleoplasm,Nucleoli,Nucleoli rim	hsa gene	0.67	
PAXBP1	others	3.78E-39	5.92E-44	2.0	Nucleoplasm,Cytosol		0.69	
SON	others	2.71E-03	3.65E-05	2.4	Nuclear speckles	hsa gene	0.88	
TP73	p53	4.42E-08	3.20E-09	2.2	Nucleoplasm,Golgi apparatus,Vesicles,Cell Junctions		0.57	
NFATC1	RHR	1.34E-04	4.02E-05	2.1	Nucleoplasm,Nuclear bodies		0.51	
RBPJ	RHR	8.12E-09	1.93E-07	2.1	Nucleoplasm	hsa gene	1.00	1
RELA	RHR	6.25E-46	5.62E-40	3.0	Cytosol		1.00	
RELB	RHR	4.99E-03	3.56E-04	2.3	Nucleoplasm,Cytosol		0.90	1
RUNX1	RUNT	1.78E-11	1.92E-08	2.6	Nucleoplasm,Vesicles	hsa gene	0.90	
RUNX2	RUNT	8.36E-12	7.42E-15	4.7	Nucleoplasm		0.93	1
RUNX3	RUNT	4.03E-02	1.43E-03	2.6	Nucleoplasm,Vesicles,Cytosol		0.81	1
SMAD7	SMAD	3.23E-07	2.56E-07	2.0	Nucleoplasm,Cytosol	hsa gene	0.68	1
STAT3	STAT	1.51E-16	5.34E-15	2.1	Nucleoplasm,Cytosol	hsa gene	1.00	
STAT5A	STAT	4.82E-03	5.01E-04	2.1	Nucleoplasm,Cytosol		0.97	
ELF4	WC.ETS	2.80E-02	2.82E-03	2.1	Nucleoplasm,Nuclear bodies		NA	
ETV1	WC.ETS	5.08E-05	3.47E-08	5.9	Nucleoplasm		0.29	
IRF1	WC.IRF	3.77E-03	2.01E-04	2.6	Nucleoplasm,Cytosol		0.61	
IRF2	WC.IRF	1.14E-02	5.17E-04	2.0	Nucleoplasm,Cell Junctions,Cytosol	hsa gene	0.57	
MIER1	WC.MYB	2.48E-10	5.38E-14	5.4	Nucleoplasm		0.64	1
MIER3	WC.MYB	5.90E-05	3.04E-08	6.6	Nucleoplasm		0.70	
MYB	WC.MYB	2.01E-05	1.41E-07	2.9	Nucleoplasm,Cytosol	hsa gene	0.83	1
NCOR1	WC.MYB	4.75E-25	1.63E-22	2.9	Nucleoplasm,Cytosol		0.98	
NCOR2	WC.MYB	6.97E-21	2.89E-20	3.1	Nucleoplasm	only mmu ortholog	0.96	
RCOR1	WC.MYB	1.08E-41	3.86E-41	3.7	Nucleoplasm		0.99	
RCOR2	WC.MYB	1.48E-08	2.26E-12	6.9	Nucleoplasm,Vesicles,Midbody		0.68	
RCOR3	WC.MYB	1.52E-06	4.68E-07	2.4	Nucleoplasm,Cytosol		0.93	
SMARCC1	WC.MYB	8.00E-27	7.50E-26	3.5	Nucleoplasm		0.89	
SMARCC2	WC.MYB	5.17E-16	4.49E-15	2.9	Nucleoplasm		1.00	
TADA2A	WC.MYB	9.52E-06	3.75E-05	2.1	Nuclear speckles		0.61	
TRERF1	WC.MYB	3.08E-03	7.97E-06	4.9	Nucleoplasm,Nucleoli fibrillar center,Cytosol		0.93	
ZNF750	ZF	4.27E-03	1.50E-05	4.4	none reported		0.45	
AEBP2	ZF.C2H2	3.98E-17	2.43E-21	6.0	Nucleoplasm	only mmu ortholog	0.92	1
BCL11A	ZF.C2H2	4.12E-06	5.40E-08	3.0	Nucleoplasm,Nuclear bodies		0.95	1
BCL11B	ZF.C2H2	6.52E-15	2.71E-18	5.2	Nucleoplasm,Nucleoli fibrillar center		0.93	1
EGR1	ZF.C2H2	4.26E-04	2.25E-06	3.7	Nucleoplasm		0.74	1
FEZF2	ZF.C2H2	1.63E-05	3.12E-05	12.1	none reported		0.97	
GFI1	ZF.C2H2	1.93E-05	1.00E-08	6.0	none reported	hsa gene	0.77	
GFI1B	ZF.C2H2	2.61E-17	1.09E-20	5.3	Nucleoplasm,Plasma membrane	hsa gene	0.26	
GLI1	ZF.C2H2	2.11E-03	1.51E-04	2.4	Nucleoplasm,Cytosol		0.85	
HIC1	ZF.C2H2	9.45E-06	1.21E-08	4.8	none reported	hsa gene	0.73	
INSM1	ZF.C2H2	1.43E-02	2.92E-05	6.7	none reported		0.95	
KLF13	ZF.C2H2	5.85E-05	2.43E-08	7.0	none reported	hsa gene	0.48	1
KLF4	ZF.C2H2	1.89E-30	1.64E-07	2.7	Nucleoplasm,Cytosol	mmu gene	0.59	1
MECOM	ZF.C2H2	8.16E-06	4.49E-06	2.1	Nuclear speckles	only mmu ortholog	0.84	
OVOL1	ZF.C2H2	5.70E-09	2.78E-13	11.3	Nucleoplasm	only mmu ortholog	0.93	
PLAGL1	ZF.C2H2	3.25E-06	7.44E-10	7.6	Nuclear bodies,Golgi apparatus,Vesicles	hsa gene	0.68	1
PRDM1	ZF.C2H2	4.54E-15	1.03E-18	5.7	Nucleoplasm,Nucleoli		0.53	1
PRDM14	ZF.C2H2	4.91E-02	5.07E-03	2.1	Nucleoplasm,Vesicles	hsa gene	0.24	
RREB1	ZF.C2H2	1.85E-07	3.04E-10	4.6	Nucleoli fibrillar center,Nuclear speckles	hsa gene	0.29	1
SNAI1	ZF.C2H2	1.60E-07	2.17E-06	2.0	Nucleoplasm	only mmu ortholog	0.58	
SNAI2	ZF.C2H2	2.41E-03	2.12E-05	3.4	Nucleoplasm	hsa gene	0.35	1
SNAI3	ZF.C2H2	1.33E-03	9.04E-07	8.6	Nucleoplasm	hsa gene	0.06	
SP1	ZF.C2H2	2.64E-52	1.06E-50	3.9	Nucleoplasm	hsa gene	0.99	1
SP3	ZF.C2H2	7.38E-13	6.15E-17	5.6	Nucleoplasm,Cytosol	hsa gene	0.96	
WIZ	ZF.C2H2	1.44E-07	1.13E-08	2.5	Nucleoplasm	only mmu ortholog	0.96	
YY1	ZF.C2H2	8.99E-26	1.10E-23	2.9	Nucleoplasm,Nucleoli fibrillar center,Nuclear bodies	hsa gene	1.00	1
ZBTB16	ZF.C2H2	1.86E-21	4.44E-23	3.9	none reported	hsa gene	0.86	1
ZBTB7A	ZF.C2H2	2.08E-02	7.29E-04	2.4	Nucleoplasm	only mmu ortholog	0.93	
ZFPM2	ZF.C2H2	6.66E-04	1.37E-04	4.4	Nucleoplasm	hsa gene	0.84	
ZNF217	ZF.C2H2	6.31E-17	1.67E-20	5.7	Nuclear speckles,Mitochondria	hsa gene	0.72	
ZNF219	ZF.C2H2	4.63E-03	4.91E-04	2.1	Nucleoplasm		0.95	
ZNF263	ZF.C2H2	1.03E-02	1.60E-03	2.0	Nucleoplasm,Mitotic spindle,Cytosol		0.05	
ZNF280C	ZF.C2H2	4.96E-02	1.26E-03	3.0	Nucleoplasm,Nucleoli fibrillar center	hsa gene	NA	
ZNF280D	ZF.C2H2	3.34E-02	1.62E-04	5.0	Nucleoplasm,Golgi apparatus,Centrosome,Cytosol	hsa gene	0.26	
ZNF436	ZF.C2H2	6.70E-04	4.50E-07	7.6	Nucleoplasm,Cytosol	hsa gene	0.20	
ZNF462	ZF.C2H2	1.85E-03	1.47E-05	3.8	Nucleoplasm	mmu gene	0.99	
ZNF516	ZF.C2H2	2.48E-03	1.54E-05	3.9	Nucleoplasm,Cytosol		0.33	
ZNF532	ZF.C2H2	3.40E-04	3.02E-02	2.7	Nucleoplasm,Golgi apparatus		0.86	
ZNF592	ZF.C2H2	2.71E-03	3.68E-05	3.3	Nucleoplasm	hsa gene	0.67	
GATA1	ZF.C4_GATA	8.35E-11	5.61E-05	2.3	Nucleoplasm	hsa gene	NA	
GATA3	ZF.C4_GATA	7.82E-07	6.40E-07	2.8	Nucleoplasm	hsa gene	0.88	1
GATA4	ZF.C4_GATA	5.79E-05	5.68E-05	2.0	Nuclear bodies	hsa gene	0.92	1
GATAD1	ZF.C4_GATA	2.36E-09	4.91E-11	3.3	Nucleoplasm		0.59	1

n° of filtered TF/SplF.other interaction reports (Protein kinase; Other SAPs; Minor)

TF	CLK1	CLK2	CLK3	CLK4	SRPK1	SRPK2	DNAJC8	C16orf80	DBC1	HSPA5	CARM1	PRMT1	PRMT5	CRKRS	PPM1G	ZNF326	LPAR1	PDCD7	RECQL5	RNPC3	SNRNP25	SNRNP35	SNRNP48	ZCRB1	ZMAT5	ZRSR1	ZRSR2
	Protein kinase						Other SAPs										Minor										
ARID1A											1																
ARID1B													1														
ARID4A																											
ARID5B																											
JARID2																											
HIF1A																											
MESP1																											
MLXIPL											1																
MYOD1											1		2														
SREBF1													4														
TAL1															1												
TWIST1																											
USF1												2															
ATF3																											
CEBPD																											
CREB1											1								1								
NFYB																											
E2F6							1																				
FOXO3																											
FOXP3																1											
FOXR1																											
GCM1																											
GTF2I												1															
SATB1																											
HNF1A																											
ADNP																											
ZEB1										1					1												
ZEB2																											
CDX2																											
NKX2-2																											
PBX1																											
KMT2C																											
SOX9																											
SRY																											
HMG20A															1												
HMG20B																											
SMARCE1											1		1														
MEF2A																											
MEF2C																											
MEF2D											1		1														
L3MBTL3																											
PAXBP1						2						1															
SON		2			2	4																					
TP73																											
NFATC1										1																	
RBPJ																											
RELA										2	6	3	4														
RELB																											
RUNX1										1		4	1			1											
RUNX2																											
RUNX3																											
SMAD7																											
STAT3																											
STAT5A											2	2															
ELF4																											
ETV1																											
IRF1																											
IRF2																											
MIER1															1												
MIER3																											
MYB																											
NCOR1	1																										
NCOR2										1																	
RCOR1																											
RCOR2																											
RCOR3																											
SMARCC1											1		2														
SMARCC2											1																
TADA2A										1																	
TRERF1																											
ZNF750																											
AEBP2																											
BCL11A																											
BCL11B																											
EGR1							1																				
FEZF2																											
GFI1												1															
GFI1B																											
GLI1					1							3	1														
HIC1																											
INSM1																											
KLF13																											
KLF4																1											
MECOM												1															
OVOL1																											
PLAGL1																											
PRDM1										1			4														
PRDM14										4																	
RREB1																											
SNAI1										1			1														
SNAI2																											
SNAI3																											
SP1																											
SP3																											
WIZ												1	1														
YY1												2															
ZBTB16																											
ZBTB7A																											
ZFPM2																											
ZNF217																											
ZNF219																											
ZNF263		1	1												1												
ZNF280C																											
ZNF280D																											
ZNF436		1											1														
ZNF462																											
ZNF516																											
ZNF532																											
ZNF592						1																					
GATA1																											
GATA3																											
GATA4										1																	
GATAD1																											

n° of filtered TF/SplF.other interaction reports (Lysine transferases; Histone transferases)

TF	ASCL1	EHMT2	KAT2B	KDM1A	KDM4B	SETD1A	SETD2	SUV39H1	TET1	EP300	KAT2A	KAT5	HDAC1	HDAC2	HDAC3	HDAC4	HDAC6	SIRT1
	Lysine transferases									Histone transferases								
ARID1A													4					
ARID1B																		
ARID4A													6	1				
ARID5B				1									6	2				
JARID2		1												1				
HIF1A			4						3	75		2	7	1	3	6	1	5
MESP1										1								
MLXIPL										4		1						
MYOD1		1	6	1				2		8	1	2	4					
SREBF1										6	1		1					5
TAL1			4	4				2		6			3	3				2
TWIST1			5							5				2	1	1		
USF1			1			3				1								
ATF3								1				4	2		1	1	3	
CEBPD										1			4		2	1		
CREB1										11		2	4	2				1
NFYB			2							5			1					
E2F6				1									4	2				
FOXO3										5								8
FOXP3										3		2	2	1				1
FOXR1												4					1	
GCM1										1			2		4	2		
GTF2I				3						2			1	5	6			
SATB1			5	1						3			6					3
HNF1A			2							5								3
ADNP				1									2					
ZEB1		2	2	1						8		1	1	1				4
ZEB2				1						1			5	4	1	1		
CDX2										3								
NKX2-2													2					
PBX1											2		3		1			
KMT2C					1	1												
SOX9										6								
SRY										4					1			
HMG20A				7									8	7				
HMG20B				11									12	10				
SMARCE1										1			2	2				
MEF2A	3									4			1		1	5		
MEF2C	1			1						1						10		
MEF2D	1		1	2						2			1		3	6		
L3MBTL3				4									1	1				
PAXBP1																		
SON										1								
TP73			5	1						10		1	4	1				3
NFATC1			1							5			1	2		1		
RBPJ			2	4						1	1	1	1	1				
RELA										34	4	3	19	9	5		1	1
RELB		1															1	
RUNX1								3		2			6	2	5			
RUNX2			1					2		4			3		3	4	2	
RUNX3								1		2			1					
SMAD7			1							4			4	2	2		2	3
STAT3			1	2						16	1	1	4	1	5		2	3
STAT5A										5			1					
ELF4		1	1			1					1	1						
ETV1			4							3								
IRF1			3							5	1							
IRF2			5							3	1							
MIER1		1								1			9	4				
MIER3													4	3				
MYB		1				1				6	1	2						
NCOR1				1	1					1			2		26	11		2
NCOR2										1			3	2	26	7		
RCOR1				33					1				19	13	2			
RCOR2				2									4	3				
RCOR3				6									7	4	1			
SMARCC1										1							1	
SMARCC2										1			1	1				
TADA2A			5	1							11		2					
TRERF1										2			5					
ZNF750				5									1					
AEBP2																		
BCL11A													6	4				2
BCL11B								2					6	8	1		1	
EGR1										8								
FEZF2													2					
GFI1		2		1									2	2				
GFI1B		4		9	1			2					2	3				
GLI1			4										3	1			1	
HIC1																1		5
INSM1				2									1		1			
KLF13			5							1								
KLF4										6		4	1	5				
MECOM		2	4	1				4			1		8	5		1		2
OVOL1													2	2	2			
PLAGL1			2							3			2					
PRDM1		4		3									2	6				
PRDM14																		
RREB1				3									6	3	2			
SNAI1				10						4			12	9	2			
SNAI2			1	1									4	3				
SNAI3													2	2				
SP1			3							26			34	10	1	3		
SP3										4			8	3				
WIZ		9						1					3	3				
YY1		1	2			1				17	1	1	3	7	9	5		
ZBTB16										4			14	1	2	4	2	
ZBTB7A		1											4	1	1			1
ZFPM2										2			1	1				
ZNF217		1		10									6	5				
ZNF219													3	3				
ZNF263														1				2
ZNF280C				1														
ZNF280D				1														
ZNF436				1				2										
ZNF462		4																
ZNF516				3									5	1				
ZNF532																		
ZNF592				1									3					
GATA1										4			2		3	3		
GATA3				4						1			3	3	4	1		
GATA4		1		1						8			1	2	1			
GATAD1													6	4				

n° of filtered TF/SplF.other interaction reports (Polycomb Group Genes; SWI/SNF Complex Components; Chromatin-related)

TF	BMI1	EED	EZH2	PHC1	PHC2	SMARCA2	SMARCA4	ASH2L	BAZ1B	BRD4	CBX3	CHD1	CTCF	DEK	HMGA1	KHDRBS1	MORF4L1	NAB2	RPS6KA5	DNMT1	MBD2	MECP2	SPEN	TAF15	TCERG1	TCERG1L
	Polycomb Group Genes					SWI/SNF Complex Components		Chromatin-related																		
ARID1A		1	5			8	19						1									1		1		
ARID1B						5	16															1				
ARID4A																										
ARID5B																									1	
JARID2		6	19																							
HIF1A															1											
MESP1				1									1													
MLXIPL																					1					
MYOD1			1			1	4	1																		
SREBF1						1	1																			
TAL1													1													
TWIST1										1																
USF1								3																		
ATF3							3																			
CEBPD																										
CREB1							2			1					1				3			1				
NFYB																										
E2F6	2	2	3	1			3				6									1						
FOXO3																										
FOXP3																										
FOXR1																	3									
GCM1						1																				
GTF2I		1	1							3	1				1									1		
SATB1																										
HNF1A							1				1															
ADNP	1	1					2			2	10											1				
ZEB1							2				1															
ZEB2			1																							
CDX2						2	1																			
NKX2-2																										
PBX1			1				1																			
KMT2C			1					12																		
SOX9						1	1			1																
SRY																										
HMG20A			1							1					1						1	1				
HMG20B																										
SMARCE1						10	23			2	1				1							2				
MEF2A																										
MEF2C																										
MEF2D								2																		
L3MBTL3			1		2												2			4						
PAXBP1	1		2																			1		1		
SON	1	1	1							1												1				
TP73																										
NFATC1							2								1											
RBPJ			1				2				1	1		1							2	2	6			
RELA			4																2							
RELB			4			2	1													1						
RUNX1							3	1												3			1	1	1	
RUNX2																										
RUNX3			2							1																
SMAD7																										
STAT3			1				3			2						2				3						
STAT5A							1																			
ELF4						1		1									1									
ETV1																			1							
IRF1						1									1											
IRF2																										
MIER1																										
MIER3																										
MYB	1		2			1		1					1													
NCOR1										1	2	2								1		4	1			
NCOR2										2					1								5			
RCOR1			1				1			2					1							1				
RCOR2																						1				
RCOR3			1																							
SMARCC1	1	1	2			13	29			3					1							1		1		
SMARCC2		1	3			12	19			3												1		1		
TADA2A																										
TRERF1																										
ZNF750											1															
AEBP2		8	13																							
BCL11A		1	2																		1					
BCL11B							1														2					
EGR1																		1								
FEZF2																										
GFI1																										
GFI1B					1		2																			
GLI1							1																			
HIC1		1	1												1					2						
INSM1																										
KLF13																										
KLF4						1	1														1					
MECOM							1		1		1					1										
OVOL1																										
PLAGL1																										
PRDM1																										
PRDM14		2	3																							
RREB1											1															
SNAI1			3							2						1				4						
SNAI2																										
SNAI3																										
SP1							5								4					2		2				
SP3																				2		2				
WIZ		1									3											1				
YY1	2	4	3					1		1	1		1									4				
ZBTB16	8		4																							
ZBTB7A							1									5				1						
ZFPM2			2																							
ZNF217											1															
ZNF219																					4					
ZNF263			1						1		2				1					2		1				
ZNF280C		1									4				1											
ZNF280D											4															
ZNF436																										
ZNF462			1								4											1				
ZNF516																										
ZNF532										1												1				
ZNF592			1							4																
GATA1						1	1									1										
GATA3	1					1							1													
GATA4	2	1	2	1		1	1		1	2		1				1								1	1	
GATAD1		1	1														8				1					

GATAD2A	ZF.C4_GATA	3.51E-14	3.66E-15	3.2
GATAD2B	ZF.C4_GATA	2.87E-17	1.38E-17	3.1
MTA1	ZF.C4_GATA	5.38E-39	1.77E-38	3.9
MTA2	ZF.C4_GATA	4.66E-53	1.16E-52	4.3
MTA3	ZF.C4_GATA	1.10E-11	2.05E-13	3.3
RERE	ZF.C4_GATA	2.97E-05	7.59E-08	4.0
HNF4A	ZF.C4_NR	7.49E-09	1.89E-08	2.1
NR0B2	ZF.C4_NR	2.74E-10	1.22E-12	4.1
NR1H2	ZF.C4_NR	3.90E-02	2.58E-03	2.1
NR1H3	ZF.C4_NR	2.66E-02	9.28E-04	2.7
NR1H4	ZF.C4_NR	3.60E-03	1.14E-05	4.5
NR2C1	ZF.C4_NR	1.54E-26	8.21E-31	6.9
PPARA	ZF.C4_NR	4.63E-02	3.40E-03	2.2
PPARD	ZF.C4_NR	3.05E-05	1.38E-06	2.7
PPARG	ZF.C4_NR	2.27E-15	2.63E-12	2.1
MBD2	ZF.CXXC	2.64E-31	1.20E-34	5.3
MBD3	ZF.CXXC	9.75E-30	1.19E-30	3.9
MBD4	ZF.CXXC	1.26E-02	2.12E-02	2.2

GATAD2A																																			
GATAD2B																																			
MTA1																														4					
MTA2																														5		1			
MTA3																														3					
RERE																												2				1			
HNF4A												3																1	1	1		1			
NR0B2													1															1							
NR1H2																														2				1
NR1H3																													1	2				2
NR1H4											1	1																							
NR2C1																														3					
PPARA												1																							
PPARD																												1		2					
PPARG										1																			1						
MBD2												2	2																	2					
MBD3																														3					
MBD4																														1				1

GATAD2A					11	7						1	2							2											10					
GATAD2B					12	9						2	2							4											8					
MTA1		1			24	20				5		2	2							1					1					4	3					
MTA2		2			32	18				2		1	2				2			4					1	1				4	8	1				
MTA3					10	7						1	2							2											3					
RERE		1			7	2												1																		
HNF4A		1	1	1			1	1		2	2	2	2			1	1	1															1			
NR0B2					6		3		1	3						6																				
NR1H2								2		1							1																			
NR1H3		1						1		1																										
NR1H4		1							1	2							1																			
NR2C1					3	2	12	3									1													3						
PPARA		5								3																										
PPARD		2			3	1	2	1																				1					1			
PPARG		14	2	2	3		9	3		3			1			1	1	1				1			3							1				
MBD2					19	10						1	1							1										1	4					
MBD3	1				17	10				2		1	2			1	2			2										1	11	1				
MBD4					5															1																

GATAD2A	Nucleoplasm	only mmu ortholog	0.93	1
GATAD2B	Nucleoplasm	only mmu ortholog	1.00	
MTA1	Nucleoplasm,Cytosol	only mmu ortholog	0.99	
MTA2	Nucleoplasm	hsa gene	1.00	
MTA3	Nucleoplasm,Vesicles		0.30	
RERE	Nucleoplasm,Nuclear bodies		0.98	
HNF4A	Nucleoplasm		0.88	
NR0B2	Nucleoplasm,Vesicles		0.60	
NR1H2	Nucleoplasm,Vesicles		0.94	
NR1H3	Nucleoplasm,Cytosol		0.87	
NR1H4	Nucleoplasm		0.75	
NR2C1	Nucleoplasm,Cell Junctions,Cytosol		0.65	
PPARA	Nucleoplasm		0.89	1
PPARD	none reported		1.00	
PPARG	Nucleoplasm,Vesicles		0.50	
MBD2	Nucleoplasm	hsa gene	0.50	1
MBD3	Nucleoplasm	hsa gene	0.96	
MBD4	Nuclear speckles	hsa gene	0.63	
