# Supplementary material for: Foxg1 and companions: Not only transcription factors
Source: Neural Regen Res. 2025 Sep 3;21(8):3426–38. doi: 10.4103/NRR.NRR-D-25-00439 (PMC13452659; doi:10.4103/NRR.NRR-D-25-00439)
Supplement: Supplementary file 7 [file NRR-21-3426_Suppl15.pdf]

Additional Table 7.4. Filtered TF/pAF.4C interaction reports & related info

					n° of filtered TF/pAF.4Cs interaction reports																			
		Numbers of TF/pAF.4C interaction reports, real-vs-expected			CFIm			CFIIm		CPSF							CPSF-assoc	CSTF						
TF	TF taxon	Chi-square test, p evaluated over all 11 groups (3 SplFs, 2 pAFs, 6 TlatMFs)	Chi-square test, p evaluated over 2 pAF groups	Real / expected number ratio	CPSF6	CPSF7	NUDT1	CLP1	PCF11	CPSF1	CPSF2	CPSF3	CPSF4	CSTF2T	FIP1L1	WDR33	SYMPK	CSTF1	CSTF2	CSTF3	Subcellular TF location (see Additioinal Table 2)	RBP function reported for… (see Additioinal Table 3)	pTriplo (see Additioinal Table 4)	Neuronal activity-plasticity responsive (see Additioinal Table 5)
CEBPA	bZIP	1.90E-12	1.36E-02	2.1	2	2			1					1	1					1	Nucleoplasm,Vesicles		0.49	
CEBPB	bZIP	4.62E-17	2.94E-04	2.3	1	1			1	1	1	1	2	1	2	2	2	1	1	1	Nucleoplasm	hsa gene	0.45	
YBX3	DBP	4.67E-02	3.48E-02	2.8													3				Cytosol	hsa gene	0.11	
FOXI2	FH/WH.FOX	5.82E-06	4.08E-06	6.9													1				none reported		0.20	
FOXL1	FH/WH.FOX	2.37E-02	4.94E-02	3.5							1						1				none reported		0.46	
RFX5	FH/WH.RFX	7.14E-03	2.56E-05	10.7										2							Nucleoplasm,Vesicles		0.84	
RFX6	FH/WH.RFX	1.25E-02	2.20E-02	7.0															1		none reported		0.48	
TOX4	HMG.TOX	5.96E-04	9.39E-04	5.4	1	1									1						Nucleoplasm,Nucleoli,Vesicles		1.00	
CPEB1	others	3.81E-47	3.67E-51	22.6						1				2	1		4		2		Nucleoplasm,Cytosol	hsa gene	1.00	1
PLSCR1	others	1.42E-03	4.94E-02	3.5	2																Nucleoplasm,Golgi apparatus,Plasma membrane		0.09	
SUB1	others	2.79E-02	9.39E-04	5.4															3		Nucleoplasm,Nucleoli	hsa gene	0.85	
TP53	p53	2.22E-69	1.06E-03	0.2		1										2					Nucleoplasm,Vesicles,Cytosol	hsa gene	0.77	
RFXANK	RFXANK	4.75E-02	3.79E-02	2.1													1				Nucleoplasm		0.58	
RUNX1	RUNT	1.78E-11	1.98E-04	4.5	1							1		1	1				1		Nucleoplasm,Vesicles	hsa gene	0.90	
CDC5L	WC.MYB	4.26E-153	8.52E-03	2.4	1	1					1	1				1	1			1	Nucleoplasm	hsa gene	0.99	
SP7	ZF.C2H2	2.84E-10	2.05E-11	8.2		1		1	1	1	1		1			1					none reported		0.61	
ZBED1	ZF.C2H2	1.20E-03	1.06E-02	5.0														2			Nucleoplasm,Nuclear membrane,Centrosome		NA	
ZBTB10	ZF.C2H2	4.44E-04	2.16E-04	6.3						1								1		1	Nucleoplasm		0.93	1
ZNF239	ZF.C2H2	2.87E-04	2.98E-08	32.7			1														Nucleoplasm,Nuclear bodies	hsa gene	0.12	
ZNF341	ZF.C2H2	2.58E-02	3.75E-05	18.9															1		Nucleoplasm,Nuclear bodies,Vesicles	hsa gene	0.54	
ZNF571	ZF.C2H2	2.25E-38	1.02E-45	102.7										2							Nucleoplasm,Nucleoli,Plasma membrane	hsa gene	0.03	
ZNF766	ZF.C2H2	2.31E-04	2.98E-08	32.7		1															Centrosome		0.28	
GATA1	ZF.C4_GATA	8.35E-11	2.58E-09	8.9		1			1		1		1					1			Nucleoplasm	hsa gene	NA	
GATA3	ZF.C4_GATA	7.82E-07	1.20E-03	5.2		1			1		1										Nucleoplasm	hsa gene	0.88	1
AR	ZF.C4_NR	9.82E-18	1.37E-03	2.3	1	3		1	1	1			1			1	1			1	Cytosol		NA	
ESR2	ZF.C4_NR	3.49E-23	1.36E-02	1.9	1	1		1	1	1	1	1	1			1	1	1	1	1	Nucleoplasm,Vesicles		0.30	
MECP2	ZF.CXXC	4.88E-14	3.07E-09	3.9	1	2		1	1	1	1	1	1	1	1	1	1	1	1	1	Nucleoplasm	hsa gene	NA	
DMRTB1	ZF.DM	2.09E-05	1.18E-06	2.5		1															none reported		0.35	
