# Supplementary material for: Foxg1 and companions: Not only transcription factors
Source: Neural Regen Res. 2025 Sep 3;21(8):3426–38. doi: 10.4103/NRR.NRR-D-25-00439 (PMC13452659; doi:10.4103/NRR.NRR-D-25-00439)
Supplement: Supplementary file 8 [file NRR-21-3426_Suppl16.pdf]

Additional Table 7.5. Filtered TF/pAF.PA interaction reports & related info

TF	TF taxon	Numbers of TF/pAF.PA interaction reports, real-vs-expected			n° of filtered TF/pAF.PA interaction reports																	Subcellular TF location (see Additioinal Table 2)	RBP function reported for… (see Additioinal Table 3)	pTriplo (see Additioinal Table 4)	Neuronal activity-plasticity responsive (see Additioinal Table 5)
					pA-binding						PAP, canonical			PAP, non-canonical							pA-RNAse				
		Chi-square test, p evaluated over all 11 groups (3 SplFs, 2 pAFs, 6 TlatMFs)	Chi-square test, p evaluated over 2 pAF groups	Real / expected number ratio	PABPN1	CPEB1	CPEB2	CPEB3	CPEB4	NGDN	PAPOLA	PAPOLB	PAPOLG	MTPAP	TENT2	TENT4A	TENT4B	TUT1	TUT4	TUT7	PARN				
HEY1	bHLH	5.38E-09	3.52E-02	5.8										1								Nucleoplasm,Nuclear membrane,Cytosol		0.211637839	
BACH2	bZIP	2.39E-02	7.55E-03	8.7														1				Nucleoplasm,Cytosol		0.994371195	1
NFYA	CAAT-BF	1.71E-02	3.58E-02	5.8									1									Nucleoplasm	hsa gene	0.982679253	1
YBX3	DBP	4.67E-02	3.48E-02	2.5		1																Cytosol	hsa gene	0.113400662	
FOXI2	FH/WH.FOX	5.82E-06	4.08E-06	18.1										1								none reported		0.20	
FOXS1	FH/WH.FOX	2.60E-02	4.23E-03	9.8										1								Nucleoplasm,Cytosol		0.099516344	
GTF2IRD1	GTF2I	3.00E-02	1.60E-02	7.3										1								Nucleoplasm,Cytosol	only mmu ortholog	0.936128357	
HOMEZ	HB-ZF.C2H2	4.30E-03	4.54E-02	5.3					1													Nucleoplasm,Nucleoli,Cytosol	hsa gene	0.442101619	
TLX2	HB.NK	8.75E-26	2.57E-02	6.4	1																	Nucleoplasm		0.99962619	
TGIF2	HB.TALE	1.94E-06	5.65E-11	44.9							1											Nucleoplasm,Centrosome		0.623674163	
CAPN15	others	1.75E-03	5.69E-06	22.4			1															Nucleoplasm	hsa gene	0.658028777	
CC2D1A	others	1.85E-06	6.59E-06	11.8		1			1													Nucleoli fibrillar center,Plasma membrane,Cytosol	hsa gene	0.700518567	1
CPEB1	others	3.81E-47	3.67E-51	11.8		2																Nucleoplasm,Cytosol	hsa gene	0.999805183	1
GPBP1	others	1.13E-02	3.79E-03	10.0					1													Vesicles,Plasma membrane,Cytosol	hsa gene	0.124983668	
GPBP1L1	others	4.35E-14	1.80E-02	7.1					1													Nucleoplasm,Plasma membrane,Focal adhesion sites,Cytosol	hsa gene	0.114937271	
PURB	PUR	4.32E-10	2.82E-02	6.2		1																Nucleoplasm	hsa gene	0.67	1
PURG	PUR	3.00E-03	2.01E-04	8.5					1												1	Nucleoplasm,Nucleoli,Plasma membrane	hsa gene	0.670341505	
RFXANK	RFXANK	4.75E-02	3.79E-02	5.5									1									Nucleoplasm		0.583669755	
TEAD4	TEA	8.68E-13	7.38E-14	20.5																	3	Nucleoplasm		0.534264218	
TULP3	TUB	2.48E-07	1.21E-02	4.3																	2	Nucleoplasm,Nucleoli,Plasma membrane		0.07	
GLI4	ZF.C2H2	1.88E-37	1.51E-02	7.4						1												Nucleoplasm,Cytokinetic bridge	hsa gene	0.215412751	
ZBTB44	ZF.C2H2	2.06E-02	1.62E-03	11.6																	1	Nucleoplasm	hsa gene	0.678649999	
ZKSCAN8	ZF.C2H2	1.85E-07	3.78E-02	5.7																	1	Nucleoplasm,Cytosol	hsa gene	0.609572314	
ZNF333	ZF.C2H2	3.02E-06	1.60E-10	42.8										1								Nucleoplasm,Cytosol		0.152606153	
ZNF467	ZF.C2H2	1.37E-04	4.33E-02	5.4						1												Mitochondria	hsa gene	0.143931265	
ZNF512	ZF.C2H2	2.31E-25	3.48E-03	5.6						2												Nucleoplasm		0.968704098	
ZNF547	ZF.C2H2	3.97E-09	4.50E-14	58.9														1				none reported	hsa gene	0.177170001	
ZNF677	ZF.C2H2	2.91E-05	4.65E-09	36.2																	1	Nucleoplasm,Centrosome	hsa gene	0.008842128	
ZSCAN18	ZF.C2H2	5.27E-03	1.05E-04	16.8																	1	Nucleoplasm	hsa gene	0.070590505	
TRPS1	ZF.C4_GATA	1.36E-03	3.78E-02	5.7																	1	Nucleoplasm		0.878577141	1
DMRTB1	ZF.DM	2.09E-05	1.18E-06	13.3			1							1								none reported		0.354571205	
