# Supplementary material for: Foxg1 and companions: Not only transcription factors
Source: Neural Regen Res. 2025 Sep 3;21(8):3426–38. doi: 10.4103/NRR.NRR-D-25-00439 (PMC13452659; doi:10.4103/NRR.NRR-D-25-00439)
Supplement: Supplementary file 9 [file NRR-21-3426_Suppl17.pdf]

Additional Table 7.6. Filtered TF/TlatMF.EIF interaction reports & related info

TF	TF taxon	Numbers of TlatMF/EIF interaction reports, real-vs-expected			n° of filtered TF/TllatMF.EIF interaction reports																																													Subcellular TF location (see Additioinal Table 2)	RBP function reported for… (see Additioinal Table 3)	pTriplo (see Additioinal Table 4)	Neuronal activity-plasticity responsive (see Additioinal Table 5)
		Chi-square test, p evaluated over all 11 groups (3 SplFs, 2 pAFs, 6 TlatMFs)	Chi-square test, p evaluated over 6 TlatMF groups	Real / expected number ratio	EIF1	EIF1AX	EIF1AY	EIF1B	EIF2A	EIF2AK1	EIF2AK2	EIF2AK3	EIF2AK4	EIF2B1	EIF2B2	EIF2B3	EIF2B4	EIF2B5	EIF2S1	EIF2S2	EIF2S3	EIF3A	EIF3B	EIF3C	EIF3CL	EIF3D	EIF3E	EIF3F	EIF3G	EIF3H	EIF3I	EIF3J	EIF3K	EIF3L	EIF3M	EIF4A1	EIF4A2	EIF4B	EIF4E	EIF4EBP1	EIF4EBP2	EIF4EBP3	EIF4ENIF1	EIF4G1	EIF4G2	EIF4G3	EIF4H	EIF5	EIF5B				
BHLHA15	bHLH	1.28E-07	5.38E-09	2.1										2																																				Nucleoplasm,Golgi apparatus		0.981150305	
HOXA9	HB.HOX	2.65E-03	6.78E-04	8.3																																			3											Nucleoplasm		0.613526495	
EMX2	HB.NK	2.80E-03	3.93E-04	8.7																																			3											Nucleoplasm		0.655796515	
HHEX	HB.NK	5.18E-07	5.34E-08	9.7																																			5											Nucleoplasm,Nuclear bodies		0.79772432	1
DUX4	HB.PD	2.64E-71	1.81E-73	2.5																	1	1	1						1		1	1				1														Nucleoplasm,Nucleoli,Golgi apparatus,Cytosol		NA	
HMGB2	HMG	8.72E-45	9.73E-47	2.9	1														1			1			1		1				1		1		1															Nucleoplasm,Nucleoli	hsa gene	0.29	
MAEL	HMG	1.05E-37	9.10E-30	18.1																								2								2		1												none reported	hsa gene	0.21	
GPBP1L1	others	4.35E-14	1.04E-14	7.9																		2		1			3			2					2															Nucleoplasm,Plasma membrane,Focal adhesion sites,Cytosol	hsa gene	0.114937271	
DNAJC2	WC.MYB	3.80E-157	8.32E-160	4.1															1	1		1	1	1					1		1		1	1															1	Cytosol	hsa gene	0.77	1
TADA2A	WC.MYB	9.52E-06	1.29E-03	2.2																			1	1						1	1			1	1															Nuclear speckles		0.612348106	
CTCF	ZF.C2H2	3.66E-157	1.45E-160	2.1																1		1	1		1		1		1		1		1	1	1	1														Nucleoplasm	hsa gene	0.999793048	
FEZF2	ZF.C2H2	1.63E-05	1.67E-03	21.0																																		1												none reported		0.965589063	
KLF16	ZF.C2H2	1.07E-04	2.12E-04	2.6																		1	1	1		1	1	1	1	1	1			1										1						Nucleoplasm		0.226396746	
KLF8	ZF.C2H2	9.31E-05	4.49E-03	3.1																		1	1	1		1	1		1	1	1			1										1						Nucleoplasm	only mmu ortholog	NA	
ZNF746	ZF.C2H2	2.29E-12	3.14E-12	2.4																		1	1			1	1				1			3																Nucleoplasm,Cytosol		0.53	
NFX1	ZF.NFX1	9.30E-61	6.69E-57	3.4					1										1	1	1		2			1		1	1	1	1		1	1					1					1					1	Nucleoplasm,Nucleoli,Plasma membrane,Cytosol	hsa gene	0.92	
