# Supplementary material for: Foxg1 and companions: Not only transcription factors
Source: Neural Regen Res. 2025 Sep 3;21(8):3426–38. doi: 10.4103/NRR.NRR-D-25-00439 (PMC13452659; doi:10.4103/NRR.NRR-D-25-00439)
Supplement: Supplementary file 10 [file NRR-21-3426_Suppl18.pdf]

Additioinal Table 7.7. Filtered TF/TlatMF.EIcoF interaction reports & related info

		Numbers of TlatMF/EIcoF interaction reports, real-vs-expected			n° of filtered TF/TllatMF.EIcoF interaction reports													Subcellular TF location (see Additioinal Table 2)	RBP function reported for… (see Additioinal Table 3)	pTriplo (see Additioinal Table 4)	Neuronal activity-plasticity responsive (see Additioinal Table 5)
TF	TF taxon	Chi-square test, p evaluated over all 11 groups (3 SplFs, 2 pAFs, 6 TlatMFs)	Chi-square test, p evaluated over 6 TlatMF groups	Real / expected number ratio	DHX29	DDX3	EIF6	PABPC1	PABPC2	PABPC3	PAIP1	PAIP2	PAIP2B	FMR1	CYFIP1	CYFIP2	NUFIP				
YBX1	DBP	1.90E-67	1.31E-68	2.3				2			1		1					Endoplasmic reticulum,Vesicles,Plasma membrane,Cytosol	hsa gene	0.919287932	
CUX1	HB.CUT	3.02E-03	8.69E-03	2.2										1				Nucleoplasm,Golgi apparatus	hsa gene	0.822091118	
MEOX1	HB.HOX	1.99E-03	1.58E-03	8.3										1	1			Nucleoplasm,Cytokinetic bridge,Cytosol		0.46	
LHX4	HB.LIM	3.09E-05	5.42E-05	6.7											1	2		Nuclear speckles		0.74	
MSX2	HB.NK	4.19E-04	3.29E-04	2.4				1										Nuclear speckles		0.85	
ARX	HB.PD	3.72E-02	4.75E-03	16.9											1			none reported		NA	
DUX4	HB.PD	2.64E-71	1.81E-73	3.3				2										Nucleoplasm,Nucleoli,Golgi apparatus,Cytosol		NA	
HMGB2	HMG	8.72E-45	9.73E-47	3.4			1	1										Nucleoplasm,Nucleoli	hsa gene	0.29	
KMT2C	HMG	3.39E-04	2.11E-02	2.6				1										Nucleoplasm	hsa gene	0.918768604	
MAEL	HMG	1.05E-37	9.10E-30	33.8				2										none reported	hsa gene	0.21	
SOX15	HMG.SOX	2.14E-27	2.63E-29	3.3			1											Nucleoplasm,Nucleoli,Vesicles		0.26	
BCLAF1	others	5.53E-08	2.47E-05	4.1									1	2				Nuclear speckles	hsa gene	0.17	
CSDE1	others	6.99E-03	7.70E-04	5.6							1			2				Golgi apparatus,Plasma membrane,Cytosol	hsa gene	0.98	
PURG	PUR	3.00E-03	3.54E-02	2.2									1					Nucleoplasm,Nucleoli,Plasma membrane	hsa gene	0.670341505	
DNAJC2	WC.MYB	3.80E-157	8.32E-160	3.9			1	1										Cytosol	hsa gene	0.77	1
KLF4	ZF.C2H2	1.89E-30	3.58E-27	2.2				1										Nucleoplasm,Cytosol	mmu gene	0.59	1
ZBTB2	ZF.C2H2	4.42E-02	1.33E-02	2.0			1							1				Nucleoplasm,Cytosol	only mmu ortholog	0.964055262	
ZFPM2	ZF.C2H2	6.66E-04	2.61E-02	12.0										1				Nucleoplasm	hsa gene	0.835210726	
ZNF326	ZF.C2H2	1.84E-07	1.11E-02	4.3							1		1					Nucleoplasm,Golgi apparatus,Vesicles	hsa gene	0.74	
ZNF511	ZF.C2H2	3.67E-58	7.26E-61	49.9											3	3		Nucleoplasm,Nuclear bodies,Vesicles		0.27	
ZNF622	ZF.C2H2	3.78E-09	4.33E-09	12.9			4											Nucleoplasm,Nucleoli,Golgi apparatus,Cytosol	hsa gene	0.34	
ZNF746	ZF.C2H2	2.29E-12	3.14E-12	2.8						1					1			Nucleoplasm,Cytosol		0.53	
LIN28A	ZF.CCHC	1.78E-45	2.56E-45	2.1				2										Nucleoli,Cytosol,Rods & Rings	hsa gene	0.440613494	
NFX1	ZF.NFX1	9.30E-61	6.69E-57	7.9			1	6		1								Nucleoplasm,Nucleoli,Plasma membrane,Cytosol	hsa gene	0.92	
