# Supplementary material for: Foxg1 and companions: Not only transcription factors
Source: Neural Regen Res. 2025 Sep 3;21(8):3426–38. doi: 10.4103/NRR.NRR-D-25-00439 (PMC13452659; doi:10.4103/NRR.NRR-D-25-00439)
Supplement: Supplementary file 11 [file NRR-21-3426_Suppl19.pdf]

Additioinal Table 7.8. Filtered TF/TlatMF.EEF interaction reports & related info

TF	TF taxon	Numbers of TlatMF/EEF interaction reports, real-vs-expected: Chi-square test, p evaluated over all 11 groups (3 SplFs, 2 pAFs, 6 TlatMFs)	Chi-square test, p evaluated over 6 TlatMF groups	Real / expected number ratio	n° of filtered TF/TllatMF.EEF interaction reports: eEF1A1	eEF1A2	eEF1B2	eEF1D	eEF1G	eEF2	eEF2K	EEF2KMT	GFM1	GFM2	MTERF4	eIF5A	eIF5A2	EEFSEC	Subcellular TF location (see Additioinal Table 2)	RBP function reported for… (see Additioinal Table 3)	pTriplo (see Additioinal Table 4)	Neuronal activity-plasticity responsive (see Additioinal Table 5)
ATF3	bZIP	1.89E-05	1.90E-02	3.1			1												Nucleoplasm,Nucleoli		0.58	1
BATF3	bZIP	1.15E-08	1.08E-08	14.6			1	2											Nucleoplasm,Nucleoli,Cytosol		0.32	
CEBPD	bZIP	4.21E-08	2.34E-06	16.7			1	1											Nucleoplasm		0.31	
FOS	bZIP	3.38E-08	4.86E-05	2.8				1							1				Nucleoplasm		0.60	1
FOSL2	bZIP	5.28E-03	5.71E-03	6.7				2											Nucleoplasm		0.89	1
FOXB1	FH/WH.FOX	1.25E-06	2.57E-07	4.3				1											Nucleoplasm,Mitochondria		0.94	
FOXG1	FH/WH.FOX	6.99E-04	8.31E-05	11.9				1	1										none reported		0.97	
FOXI2	FH/WH.FOX	5.82E-06	2.32E-03	10.9				1											none reported		0.20	
FOXO1	FH/WH.FOX	3.44E-02	4.88E-02	2.6			1	1											Nucleoplasm,Cytosol		0.84	
TFCP2	GHD.CP2	2.38E-11	3.04E-09	2.2			1		1										Nucleoplasm,Cytosol	hsa gene	0.68	
LBX1	HB.NK	1.03E-02	8.51E-04	21.1												1			Nucleoplasm,Nucleoli,Nuclear bodies,Cytosol		0.99	
MSX2	HB.NK	4.19E-04	3.29E-04	5.7					1							1			Nuclear speckles		0.85	
ALX1	HB.PD	5.87E-04	2.10E-05	29.9				1											Nucleoplasm,Nuclear bodies,Golgi apparatus		0.15	
CRX	HB.PD	4.59E-06	1.79E-03	2.0												1			none reported		0.37	
HMGB2	HMG	8.72E-45	9.73E-47	2.0												1			Nucleoplasm,Nucleoli	hsa gene	0.29	
TFAM	HMG.TFAM	5.77E-64	5.33E-62	2.5									2						Mitochondria	hsa gene	0.40	1
HSF1	HSF	4.17E-04	7.13E-04	4.0				4	1										Nucleoplasm,Cytosol	hsa gene	0.82	
KHSRP	others	1.65E-02	2.38E-03	2.1				1											Nucleoplasm	hsa gene	1.00	
MLLT10	others	2.46E-05	4.84E-06	15.8				2											Nucleoplasm,Cytosol		0.99	
PHB	others	2.69E-06	7.97E-07	2.3			1		1				2			1			gene/protein not avaialble in HPA list	only mmu ortholog	0.99	
REL	RHR	3.60E-08	2.19E-04	2.3												2			none reported		0.81	
IKZF1	ZF.C2H2	2.19E-02	1.08E-02	2.4				1	1										Nucleoplasm,Cytosol	hsa gene	0.79	
ZNF213	ZF.C2H2	3.77E-02	1.34E-02	13.5								1							Nucleoplasm,Nuclear membrane,Vesicles,Cytosol		0.53	
ZNF24	ZF.C2H2	2.83E-04	3.35E-02	2.6					1										Nucleoplasm	hsa gene	0.77	
ZNF532	ZF.C2H2	3.40E-04	1.20E-04	25.9												1			Nucleoplasm,Golgi apparatus		0.86	
GATA4	ZF.C4_GATA	5.79E-05	4.81E-03	2.5				1	1										Nuclear bodies	hsa gene	0.92	1
MTA2	ZF.C4_GATA	4.66E-53	3.71E-06	2.9			1		2										Nucleoplasm	hsa gene	1.00	
