# Supplementary material for: Foxg1 and companions: Not only transcription factors
Source: Neural Regen Res. 2025 Sep 3;21(8):3426–38. doi: 10.4103/NRR.NRR-D-25-00439 (PMC13452659; doi:10.4103/NRR.NRR-D-25-00439)
Supplement: Supplementary file 12 [file NRR-21-3426_Suppl2.pdf]

Additional Table 1.2. hsa-TcoF gene list (*n* = 958)

[homo sapiens Transcription coFactor genes]

NOTES

harvested from Schmeiier TcoF-DB, i.e.:
- downloaded from: https://tools.sschmeier.com/tcof/browse/?type=tcof&species=human&class=all, on 2024.11.01;
- taken into account: columns A,B,C,F

Symbol	*GeneID*	*Name*	*TcoF class*
AATF	26574	apoptosis antagonizing transcription factor	HC
ACTL6A	86	actin like 6A	2
ACTL6B	51412	actin like 6B	2
ACTN4	81	actinin alpha 4	HC
ACTR5	79913	ARP5 actin-related protein 5 homolog (yeast)	2
ACTR8	93973	ARP8 actin-related protein 8 homolog (yeast)	2
ADRB2	154	adrenoceptor beta 2	3
AES	166	amino-terminal enhancer of split	HC
AGO1	26523	argonaute 1, RISC catalytic component	1
AGO2	27161	argonaute 2, RISC catalytic component	1
AIM2	9447	absent in melanoma 2	1
AIP	9049	aryl hydrocarbon receptor interacting protein	2
AJUBA	84962	ajuba LIM protein	HC
AKAP17A	8227	A-kinase anchoring protein 17A	2
AKT1	207	v-akt murine thymoma viral oncogene homolog 1	HC
AKT2	208	v-akt murine thymoma viral oncogene homolog 2	2
ALYREF	10189	Aly/REF export factor	HC
ANKRA2	57763	ankyrin repeat family A member 2	3
ANKRD2	26287	ankyrin repeat domain 2	HC
ANP32A	8125	acidic nuclear phosphoprotein 32 family member A	2

ANXA4	307	annexin A4	HC
APBB1	322	amyloid beta precursor protein binding family B member 1	HC
ARGLU1	55082	arginine and glutamate rich 1	2
ARHGEF5	7984	Rho guanine nucleotide exchange factor 5	HC
ARRB1	408	arrestin, beta 1	HC
ARRB2	409	arrestin, beta 2	HC
ASCC1	51008	activating signal cointegrator 1 complex subunit 1	1
ASCC2	84164	activating signal cointegrator 1 complex subunit 2	3
ASCC3	10973	activating signal cointegrator 1 complex subunit 3	3
ASF1A	25842	anti-silencing function 1A histone chaperone	2
ASF1B	55723	anti-silencing function 1B histone chaperone	2
ATAD2	29028	ATPase family, AAA domain containing 2	HC
ATF7IP	55729	activating transcription factor 7 interacting protein	HC
ATF7IP2	80063	activating transcription factor 7 interacting protein 2	2
ATN1	1822	atrophin 1	HC
ATXN3	4287	ataxin 3	2
ATXN3L	92552	ataxin 3 like	3
ATXN7L3	56970	ataxin 7 like 3	HC
AURKB	9212	aurora kinase B	HC
AUTS2	26053	autism susceptibility candidate 2	1
AXIN1	8312	axin 1	HC
AXIN2	8313	axin 2	2
BAHCC1	57597	BAH domain and coiled-coil containing 1	3
BASP1	10409	brain abundant membrane attached signal protein 1	HC
BAZ1A	11177	bromodomain adjacent to zinc finger domain 1A	2
BAZ1B	9031	bromodomain adjacent to zinc finger domain 1B	2
BAZ2A	11176	bromodomain adjacent to zinc finger domain 2A	2
BBS7	55212	Bardet-Biedl syndrome 7	1
BCAS3	54828	breast carcinoma amplified sequence 3	HC
BCL10	8915	B-cell CLL/lymphoma 10	HC
BCL9	607	B-cell CLL/lymphoma 9	3
BCL9L	283149	B-cell CLL/lymphoma 9-like	HC

BCOR	54880	BCL6 corepressor	HC
BEND3	57673	BEN domain containing 3	HC
BEX1	55859	brain expressed X-linked 1	1
BIRC2	329	baculoviral IAP repeat containing 2	HC
BIRC5	332	baculoviral IAP repeat containing 5	HC
BLM	641	Bloom syndrome RecQ like helicase	HC
BMI1	648	BMI1 proto-oncogene, polycomb ring finger	HC
BRCA2	675	breast cancer 2	HC
BRD2	6046	bromodomain containing 2	1
BRD3	8019	bromodomain containing 3	1
BRD4	23476	bromodomain containing 4	HC
BRD7	29117	bromodomain containing 7	HC
BRD8	10902	bromodomain containing 8	2
BRF1	2972	BRF1, RNA polymerase III transcription initiation factor 90 kDa subunit	3
BRF2	55290	BRF2, RNA polymerase III transcription initiation factor 50 kDa subunit	3
BRMS1	25855	breast cancer metastasis suppressor 1	HC
BRMS1L	84312	breast cancer metastasis-suppressor 1-like	3
BRWD1	54014	bromodomain and WD repeat domain containing 1	2
BRWD3	254065	bromodomain and WD repeat domain containing 3	3
BTF3	689	basic transcription factor 3	3
BTG1	694	B-cell translocation gene 1, anti-proliferative	2
BTK	695	Bruton tyrosine kinase	3
BTRC	8945	beta-transducin repeat containing E3 ubiquitin protein ligase	1
BUD31	8896	BUD31 homolog	HC
C14orf166	51637	chromosome 14 open reading frame 166	HC
C14orf169	79697	chromosome 14 open reading frame 169	3
C1QBP	708	complement component 1, q subcomponent binding protein	HC
CALCOCO1	57658	calcium binding and coiled-coil domain 1	HC
CAMK1	8536	calcium/calmodulin-dependent protein kinase I	1
CAMK2A	815	calcium/calmodulin-dependent protein kinase II alpha	HC
CAMK2D	817	calcium/calmodulin-dependent protein kinase II delta	3
CAMK4	814	calcium/calmodulin-dependent protein kinase IV	HC

CAND1	55832	cullin-associated and neddylation-dissociated 1	2
CARM1	10498	coactivator associated arginine methyltransferase 1	2
CASK	8573	calcium/calmodulin-dependent serine protein kinase (MAGUK family)	2
CBFA2T2	9139	CBFA2/RUNX1 translocation partner 2	HC
CBFA2T3	863	CBFA2/RUNX1 translocation partner 3	HC
CBL	867	Cbl proto-oncogene, E3 ubiquitin protein ligase	3
CBX1	10951	chromobox 1	2
CBX3	11335	chromobox 3	HC
CBX4	8535	chromobox 4	HC
CBX5	23468	chromobox 5	HC
CBX6	23466	chromobox 6	HC
CBX7	23492	chromobox 7	HC
CBX8	57332	chromobox 8	HC
CC2D1A	54862	coiled-coil and C2 domain containing 1A	3
CCAR1	55749	cell division cycle and apoptosis regulator 1	1
CCAR2	57805	cell cycle and apoptosis regulator 2	HC
CCDC59	29080	coiled-coil domain containing 59	3
CCDC85B	11007	coiled-coil domain containing 85B	HC
CCNA1	8900	cyclin A1	3
CCNA2	890	cyclin A2	2
CCNC	892	cyclin C	3
CCND1	595	cyclin D1	HC
CCNE1	898	cyclin E1	2
CCNH	902	cyclin H	HC
CCNK	8812	cyclin K	HC
CCNT2	905	cyclin T2	HC
CDC45	8318	cell division cycle 45	2
CDC6	990	cell division cycle 6	2
CDC73	79577	cell division cycle 73	HC
CDCA7L	55536	cell division cycle associated 7 like	2
CDK1	983	cyclin-dependent kinase 1	2
CDK11A	728642	cyclin-dependent kinase 11A	2

CDK11B	984	cyclin-dependent kinase 11B	2
CDK12	51755	cyclin-dependent kinase 12	HC
CDK13	8621	cyclin-dependent kinase 13	HC
CDK2	1017	cyclin-dependent kinase 2	2
CDK5	1020	cyclin-dependent kinase 5	HC
CDK5R1	8851	cyclin-dependent kinase 5, regulatory subunit 1 (p35)	HC
CDK5RAP3	80279	CDK5 regulatory subunit associated protein 3	HC
CDK7	1022	cyclin-dependent kinase 7	HC
CDK8	1024	cyclin-dependent kinase 8	2
CDKN1B	1027	cyclin-dependent kinase inhibitor 1B	HC
CDKN1C	1028	cyclin-dependent kinase inhibitor 1C	HC
CDKN2B	1030	cyclin-dependent kinase inhibitor 2B	2
CDYL	9425	chromodomain protein, Y-like	HC
CENPF	1063	centromere protein F	HC
CENPU	79682	centromere protein U	2
CEP290	80184	centrosomal protein 290kDa	HC
CGGBP1	8545	CGG triplet repeat binding protein 1	HC
CHAF1B	8208	chromatin assembly factor 1 subunit B	2
CHCHD2	51142	coiled-coil-helix-coiled-coil-helix domain containing 2	HC
CHCHD3	54927	coiled-coil-helix-coiled-coil-helix domain containing 3	HC
CHD7	55636	chromodomain helicase DNA binding protein 7	2
CHEK1	1111	checkpoint kinase 1	2
CHEK2	11200	checkpoint kinase 2	HC
CHMP1A	5119	charged multivesicular body protein 1A	HC
CHTOP	26097	chromatin target of PRMT1	2
CHUK	1147	conserved helix-loop-helix ubiquitous kinase	HC
CIDEA	1149	cell death-inducing DFFA-like effector a	3
CIPC	85457	CLOCK-interacting pacemaker	3
CIR1	9541	corepressor interacting with RBPJ, 1	HC
CITED1	4435	/p300 interacting transactivator with Glu/Asp rich carboxy-terminal dom	HC
CITED2	10370	/p300 interacting transactivator with Glu/Asp rich carboxy-terminal dom	HC
CITED4	163732	/p300 interacting transactivator with Glu/Asp rich carboxy-terminal dom	3

CLU	1191	clusterin	1
CNBP	7555	CCHC-type zinc finger, nucleic acid binding protein	3
CNOT1	23019	CCR4-NOT transcription complex subunit 1	1
CNOT10	25904	CCR4-NOT transcription complex subunit 10	3
CNOT11	55571	CCR4-NOT transcription complex subunit 11	3
CNOT2	4848	CCR4-NOT transcription complex subunit 2	1
CNOT3	4849	CCR4-NOT transcription complex subunit 3	3
CNOT6	57472	CCR4-NOT transcription complex subunit 6	1
CNOT6L	246175	CCR4-NOT transcription complex subunit 6 like	2
CNOT7	29883	CCR4-NOT transcription complex subunit 7	2
CNOT8	9337	CCR4-NOT transcription complex subunit 8	3
COMMD1	150684	copper metabolism domain containing 1	HC
COMMD10	51397	COMM domain containing 10	3
COMMD3	23412	COMM domain containing 3	3
COMMD4	54939	COMM domain containing 4	3
COMMD5	28991	COMM domain containing 5	3
COMMD6	170622	COMM domain containing 6	1
COMMD8	54951	COMM domain containing 8	3
COMMD9	29099	COMM domain containing 9	3
COPRS	55352	coordinator of PRMT5, differentiation stimulator	2
COPS2	9318	COP9 signalosome subunit 2	2
COPS5	10987	COP9 signalosome subunit 5	HC
CRABP2	1382	cellular retinoic acid binding protein 2	2
CRK	1398	v-crk avian sarcoma virus CT10 oncogene homolog	3
CRTC1	23373	CREB regulated transcription coactivator 1	HC
CRTC2	200186	CREB regulated transcription coactivator 2	2
CRYM	1428	crystallin mu	1
CSNK2A1	1457	casein kinase 2 alpha 1	2
CSNK2A2	1459	casein kinase 2 alpha 2	2
CSRP3	8048	cysteine and glycine rich protein 3	1
CTBP1	1487	C-terminal binding protein 1	HC
CTBP2	1488	C-terminal binding protein 2	HC

CTDP1	9150	CTD phosphatase subunit 1	2
CTDSP1	58190	CTD small phosphatase 1	HC
CTDSPL2	51496	CTD small phosphatase like 2	2
CTH	1491	cystathionine gamma-lyase	2
CTNNB1	1499	catenin beta 1	HC
CTNNBIP1	56998	catenin beta interacting protein 1	HC
CTNND1	1500	catenin delta 1	2
CTNND2	1501	catenin delta 2	3
CTR9	9646	CTR9 homolog, Paf1/RNA polymerase II complex component	HC
CUL2	8453	cullin 2	2
CUL3	8452	cullin 3	3
CXXC5	51523	CXXC finger protein 5	HC
DAB2	1601	DAB2, clathrin adaptor protein	HC
DACT1	51339	dishevelled-binding antagonist of beta-catenin 1	HC
DAPK3	1613	death-associated protein kinase 3	3
DAXX	1616	death-domain associated protein	HC
DCAF6	55827	DDB1 and CUL4 associated factor 6	HC
DDX17	10521	DEAD-box helicase 17	HC
DDX39B	7919	DEAD-box helicase 39B	HC
DDX5	1655	DEAD-box helicase 5	HC
DDX54	79039	DEAD-box helicase 54	HC
DEPDC1	55635	DEP domain containing 1	HC
DGKQ	1609	diacylglycerol kinase theta	HC
DHFR	1719	dihydrofolate reductase	3
DHX33	56919	DEAH-box helicase 33	HC
DHX36	170506	DEAH-box helicase 36	2
DNAJA3	9093	DnaJ heat shock protein family (Hsp40) member A3	HC
DNAJB5	25822	DnaJ heat shock protein family (Hsp40) member B5	3
DNM2	1785	dynamin 2	3
DNTTIP2	30836	deoxynucleotidyltransferase, terminal, interacting protein 2	2
DPPA2	151871	developmental pluripotency associated 2	2
DPPA4	55211	developmental pluripotency associated 4	3

DPY30	84661	dpy-30, histone methyltransferase complex regulatory subunit	2
DTX1	1840	deltex 1	3
DUSP22	56940	dual specificity phosphatase 22	1
DUSP26	78986	dual specificity phosphatase 26 (putative)	HC
DVL2	1856	dishevelled segment polarity protein 2	1
DVL3	1857	dishevelled segment polarity protein 3	HC
DYDC1	143241	DPY30 domain containing 1	3
DYNLL1	8655	dynein light chain LC8-type 1	2
DYRK1B	9149	dual specificity tyrosine phosphorylation regulated kinase 1B	1
EAF1	85403	ELL associated factor 1	2
EAF2	55840	ELL associated factor 2	HC
EAPP	55837	E2F associated phosphoprotein	HC
ECD	11319	ecdysoneless cell cycle regulator	2
EDF1	8721	endothelial differentiation related factor 1	HC
EDRF1	26098	erythroid differentiation regulatory factor 1	3
EED	8726	embryonic ectoderm development	2
EEF1A1	1915	eukaryotic translation elongation factor 1 alpha 1	2
EFCAB6	64800	EF-hand calcium binding domain 6	3
EGFR	1956	epidermal growth factor receptor	HC
EGLN1	54583	egl-9 family hypoxia-inducible factor 1	1
EGLN2	112398	egl-9 family hypoxia-inducible factor 2	2
EGLN3	112399	egl-9 family hypoxia-inducible factor 3	2
EHMT1	79813	euchromatic histone-lysine N-methyltransferase 1	2
EHMT2	10919	euchromatic histone-lysine N-methyltransferase 2	HC
EID1	23741	EP300 interacting inhibitor of differentiation 1	HC
EID2	163126	EP300 interacting inhibitor of differentiation 2	HC
EID3	493861	EP300 interacting inhibitor of differentiation 3	3
EIF2AK2	5610	eukaryotic translation initiation factor 2 alpha kinase 2	1
ELL	8178	elongation factor for RNA polymerase II	HC
ELL2	22936	elongation factor for RNA polymerase II 2	2
ELL3	80237	elongation factor for RNA polymerase II 3	HC
ELP2	55250	elongator acetyltransferase complex subunit 2	2

ELP3	55140	elongator acetyltransferase complex subunit 3	HC
ELP4	26610	elongator acetyltransferase complex subunit 4	HC
EMSY	56946	EMSY, BRCA2 interacting transcriptional repressor	2
ENG	2022	endoglin	HC
ENY2	56943	enhancer of yellow 2 homolog (Drosophila)	HC
EPC1	80314	enhancer of polycomb homolog 1	HC
EPC2	26122	enhancer of polycomb homolog 2	3
ERBB2	2064	erb-b2 receptor tyrosine kinase 2	HC
ERBB4	2066	erb-b2 receptor tyrosine kinase 4	HC
ERBIN	55914	erbb2 interacting protein	HC
ERN1	2081	endoplasmic reticulum to nucleus signaling 1	3
ESF1	51575	ESF1 nucleolar pre-rRNA processing protein homolog	3
EWSR1	2130	EWS RNA binding protein 1	2
EYA1	2138	EYA transcriptional coactivator and phosphatase 1	2
EYA2	2139	EYA transcriptional coactivator and phosphatase 2	3
EYA3	2140	EYA transcriptional coactivator and phosphatase 3	2
EYA4	2070	EYA transcriptional coactivator and phosphatase 4	3
EZR	7430	ezrin	HC
FABP4	2167	fatty acid binding protein 4	3
FAM208A	23272	family with sequence similarity 208 member A	3
FAM58A	92002	family with sequence similarity 58 member A	3
FAM83G	644815	family with sequence similarity 83 member G	HC
FBXO5	26271	F-box protein 5	2
FBXW11	23291	F-box and WD repeat domain containing 11	1
FBXW7	55294	F-box and WD repeat domain containing 7	2
FGF1	2246	fibroblast growth factor 1	HC
FGF2	2247	fibroblast growth factor 2	1
FGFR1	2260	fibroblast growth factor receptor 1	3
FGFR2	2263	fibroblast growth factor receptor 2	2
FHIT	2272	fragile histidine triad	3
FHL2	2274	four and a half LIM domains 2	HC
FHL5	9457	four and a half LIM domains 5	3

FHOD1	29109	formin homology 2 domain containing 1	HC
FLII	2314	flightless I actin binding protein	2
FLNA	2316	filamin A	HC
FRK	2444	fyn related Src family tyrosine kinase	HC
GMCL1	64395	germ cell-less, spermatogenesis associated 1	3
GMNN	51053	geminin, DNA replication inhibitor	HC
GNL3	26354	G protein nucleolar 3	HC
GPS2	2874	G protein pathway suppressor 2	1
GSK3B	2932	glycogen synthase kinase 3 beta	2
GTF2A2	2958	general transcription factor IIA 2	HC
GTF2B	2959	general transcription factor IIB	2
GTF2E1	2960	general transcription factor IIE subunit 1	2
GTF2H1	2965	general transcription factor IIH subunit 1	HC
GTF2H2	2966	general transcription factor IIH subunit 2	2
GTF2H2C	728340	GTF2H2 family member C	3
GTF2H3	2967	general transcription factor IIH subunit 3	2
GTF2H4	2968	general transcription factor IIH subunit 4	2
GTF2H5	404672	general transcription factor IIH subunit 5	3
H2AFY2	55506	H2A histone family member Y2	HC
H2AFZ	3015	H2A histone family member Z	HC
H3F3A	3020	H3 histone, family 3A	2
H3F3B	3021	H3 histone, family 3B (H3.3B)	2
HABP4	22927	hyaluronan binding protein 4	2
HAT1	8520	histone acetyltransferase 1	2
HAX1	10456	HCLS1 associated protein X-1	1
HCFC1	3054	host cell factor C1	HC
HCFC2	29915	host cell factor C2	2
HCK	3055	HCK proto-oncogene, Src family tyrosine kinase	1
HCLS1	3059	hematopoietic cell-specific Lyn substrate 1	HC
HDAC1	3065	histone deacetylase 1	HC
HDAC10	83933	histone deacetylase 10	HC
HDAC11	79885	histone deacetylase 11	2

HDAC2	3066	histone deacetylase 2	HC
HDAC3	8841	histone deacetylase 3	HC
HDAC4	9759	histone deacetylase 4	HC
HDAC5	10014	histone deacetylase 5	HC
HDAC6	10013	histone deacetylase 6	2
HDAC7	51564	histone deacetylase 7	2
HDAC8	55869	histone deacetylase 8	2
HDAC9	9734	histone deacetylase 9	HC
HEATR1	55127	HEAT repeat containing 1	2
HELLS	3070	helicase, lymphoid-specific	3
HEXIM1	10614	hexamethylene bis-acetamide inducible 1	HC
HEXIM2	124790	hexamethylene bis-acetamide inducible 2	HC
HIF1AN	55662	hypoxia inducible factor 1 alpha subunit inhibitor	HC
HINT1	3094	histidine triad nucleotide binding protein 1	HC
HIP1	3092	huntingtin interacting protein 1	3
HIPK1	204851	homeodomain interacting protein kinase 1	2
HIPK2	28996	homeodomain interacting protein kinase 2	HC
HIPK3	10114	homeodomain interacting protein kinase 3	2
HIRA	7290	histone cell cycle regulator	2
HIST1H1B	3009	histone cluster 1, H1b	HC
HIST1H1C	3006	histone cluster 1, H1c	2
HIST1H1D	3007	histone cluster 1, H1d	2
HIST1H1E	3008	histone cluster 1, H1e	2
HMGB1	3146	high mobility group box 1	HC
HMGB3	3149	high mobility group box 3	3
HMGN3	9324	high mobility group nucleosomal binding domain 3	2
HMOX1	3162	heme oxygenase 1	3
HNRNPA2B1	3181	heterogeneous nuclear ribonucleoprotein A2/B1	2
HNRNPAB	3182	heterogeneous nuclear ribonucleoprotein A/B	2
HNRNPD	3184	heterogeneous nuclear ribonucleoprotein D	2
HNRNPUL1	11100	heterogeneous nuclear ribonucleoprotein U-like 1	2
HRAS	3265	Harvey rat sarcoma viral oncogene homolog	1

HSBP1	3281	heat shock factor binding protein 1	3
HSFX2	1E+08	heat shock transcription factor family, X-linked 2	2
HSPA1A	3303	heat shock protein family A (Hsp70) member 1A	1
HSPA1B	3304	heat shock protein family A (Hsp70) member 1B	1
HSPA5	3309	heat shock protein family A (Hsp70) member 5	2
HSPA8	3312	heat shock protein family A (Hsp70) member 8	HC
HSPH1	10808	heat shock protein family H (Hsp110) member 1	2
HTATIP2	10553	HIV-1 Tat interactive protein 2	2
HTATSF1	27336	HIV-1 Tat specific factor 1	2
ICE1	23379	interactor of little elongation complex ELL subunit 1	HC
ICE2	79664	interactor of little elongation complex ELL subunit 2	HC
IFI27	3429	interferon alpha inducible protein 27	HC
IFT74	80173	intraflagellar transport 74	3
IKBKAP	8518	kappa light polypeptide gene enhancer in B-cells, kinase complex-associ	HC
IKBKB	3551	inhibitor of kappa light polypeptide gene enhancer in B-cells, kinase beta	1
IKBKG	8517	hibitor of kappa light polypeptide gene enhancer in B-cells, kinase gamn	HC
IL16	3603	interleukin 16	2
ILK	3611	integrin linked kinase	HC
ING3	54556	inhibitor of growth family member 3	2
ING4	51147	inhibitor of growth family member 4	HC
ING5	84289	inhibitor of growth family member 5	HC
INO80B	83444	INO80 complex subunit B	2
INO80C	125476	INO80 complex subunit C	2
INO80D	54891	INO80 complex subunit D	3
INO80E	283899	INO80 complex subunit E	2
INPP5K	51763	inositol polyphosphate-5-phosphatase K	2
INSR	3643	insulin receptor	1
IRAK1	3654	interleukin 1 receptor associated kinase 1	HC
IRAK1BP1	134728	interleukin 1 receptor associated kinase 1 binding protein 1	3
IRAK2	3656	interleukin 1 receptor associated kinase 2	1
IRAK3	11213	interleukin 1 receptor associated kinase 3	HC
IRF2BP1	26145	interferon regulatory factor 2 binding protein 1	2

IRF2BP2	359948	interferon regulatory factor 2 binding protein 2	2
IRF2BPL	64207	interferon regulatory factor 2 binding protein like	HC
ITCH	83737	itchy E3 ubiquitin protein ligase	2
ITGB3BP	23421	integrin subunit beta 3 binding protein	2
JADE1	79960	jade family PHD finger 1	HC
JAK2	3717	Janus kinase 2	2
JMJD1C	221037	jumonji domain containing 1C	3
JMJD6	23210	jumonji domain containing 6	2
JMY	133746	junction mediating and regulatory protein, p53 cofactor	3
JUP	3728	junction plakoglobin	HC
KANK1	23189	KN motif and ankyrin repeat domains 1	2
KAT2A	2648	lysine acetyltransferase 2A	HC
KAT2B	8850	lysine acetyltransferase 2B	HC
KCTD1	284252	potassium channel tetramerization domain containing 1	HC
KDM1A	23028	lysine demethylase 1A	HC
KDM3A	55818	lysine demethylase 3A	HC
KDM3B	51780	lysine demethylase 3B	3
KDM4A	9682	lysine demethylase 4A	HC
KDM4B	23030	lysine demethylase 4B	2
KDM4C	23081	lysine demethylase 4C	HC
KDM4D	55693	lysine demethylase 4D	3
KDM6B	23135	lysine demethylase 6B	3
KEAP1	9817	kelch like ECH associated protein 1	2
KHDRBS2	202559	KH domain containing, RNA binding, signal transduction associated 2	3
KHDRBS3	10656	KH domain containing, RNA binding, signal transduction associated 3	2
KMT5A	387893	lysine methyltransferase 5A	HC
KMT5B	51111	lysine methyltransferase 5B	3
KMT5C	84787	lysine methyltransferase 5C	2
KPNA6	23633	karyopherin subunit alpha 6	3
LANCL2	55915	LanC like 2	HC
LBH	81606	limb bud and heart development	3
LDB1	8861	LIM domain binding 1	HC

LDB2	9079	LIM domain binding 2	2
LEO1	123169	LEO1 homolog, Paf1/RNA polymerase II complex component	HC
LGALS9	3965	lectin, galactoside-binding, soluble, 9	HC
LIMD1	8994	LIM domains containing 1	HC
LITAF	9516	lipopolysaccharide induced TNF factor	2
LMCD1	29995	LIM and cysteine rich domains 1	3
LMNA	4000	lamin A/C	2
LMO1	4004	LIM domain only 1	1
LMO2	4005	LIM domain only 2	1
LOXL2	4017	lysyl oxidase like 2	HC
LOXL3	84695	lysyl oxidase like 3	1
LPIN1	23175	lipin 1	3
LPXN	9404	leupaxin	HC
LRIF1	55791	ligand dependent nuclear receptor interacting factor 1	2
LRPPRC	10128	leucine rich pentatricopeptide repeat containing	2
MAD2L2	10459	MAD2 mitotic arrest deficient-like 2 (yeast)	HC
MAFIP	727764	MAFF interacting protein (pseudogene)	3
MAGEA1	4100	MAGE family member A1	HC
MAGEA2	4101	MAGE family member A2	HC
MAGEA2B	266740	MAGE family member A2B	HC
MAGED1	9500	MAGE family member D1	3
MAK	4117	male germ cell associated kinase	HC
MALT1	10892	MALT1 paracaspase	HC
MAML1	9794	mastermind like transcriptional coactivator 1	HC
MAML2	84441	mastermind like transcriptional coactivator 2	HC
MAML3	55534	mastermind like transcriptional coactivator 3	HC
MAP2K3	5606	mitogen-activated protein kinase kinase 3	1
MAP2K5	5607	mitogen-activated protein kinase kinase 5	3
MAP2K6	5608	mitogen-activated protein kinase kinase 6	3
MAP3K7	6885	mitogen-activated protein kinase kinase kinase 7	2
MAPK10	5602	mitogen-activated protein kinase 10	3
MAPK11	5600	mitogen-activated protein kinase 11	3

MAPK12	6300	mitogen-activated protein kinase 12	2
MAPK14	1432	mitogen-activated protein kinase 14	2
MAPK3	5595	mitogen-activated protein kinase 3	HC
MAPK7	5598	mitogen-activated protein kinase 7	HC
MAPK8	5599	mitogen-activated protein kinase 8	2
MAPK8IP1	9479	mitogen-activated protein kinase 8 interacting protein 1	3
MAPK9	5601	mitogen-activated protein kinase 9	3
MBTD1	54799	mbt domain containing 1	3
MCRS1	10445	microspherule protein 1	2
MDFI	4188	MyoD family inhibitor	3
MDM2	4193	MDM2 proto-oncogene	HC
MDM4	4194	MDM4, p53 regulator	HC
MEAF6	64769	MYST/Esa1 associated factor 6	2
MED1	5469	mediator complex subunit 1	HC
MED10	84246	mediator complex subunit 10	2
MED11	400569	mediator complex subunit 11	2
MED12	9968	mediator complex subunit 12	HC
MED12L	116931	mediator complex subunit 12 like	3
MED13	9969	mediator complex subunit 13	HC
MED13L	23389	mediator complex subunit 13 like	3
MED14	9282	mediator complex subunit 14	HC
MED15	51586	mediator complex subunit 15	2
MED16	10025	mediator complex subunit 16	HC
MED17	9440	mediator complex subunit 17	HC
MED18	54797	mediator complex subunit 18	2
MED19	219541	mediator complex subunit 19	2
MED20	9477	mediator complex subunit 20	3
MED21	9412	mediator complex subunit 21	HC
MED22	6837	mediator complex subunit 22	2
MED23	9439	mediator complex subunit 23	1
MED24	9862	mediator complex subunit 24	HC
MED25	81857	mediator complex subunit 25	1

MED27	9442	mediator complex subunit 27	HC
MED28	80306	mediator complex subunit 28	3
MED29	55588	mediator complex subunit 29	2
MED30	90390	mediator complex subunit 30	HC
MED31	51003	mediator complex subunit 31	3
MED4	29079	mediator complex subunit 4	HC
MED6	10001	mediator complex subunit 6	HC
MED7	9443	mediator complex subunit 7	1
MED8	112950	mediator complex subunit 8	2
MED9	55090	mediator complex subunit 9	3
MEGF8	1954	multiple EGF like domains 8	3
MEN1	4221	menin 1	HC
MKL1	57591	megakaryoblastic leukemia (translocation) 1	HC
MKL2	57496	MKL1/myocardin like 2	1
MMS19	64210	MMS19 homolog, cytosolic iron-sulfur assembly component	HC
MNAT1	4331	MNAT1, CDK activating kinase assembly factor	HC
MORF4L1	10933	mortality factor 4 like 1	2
MORF4L2	9643	mortality factor 4 like 2	2
MPHOSPH8	54737	M-phase phosphoprotein 8	HC
MRGBP	55257	MRG/MORF4L binding protein	2
MSL3P1	151507	male-specific lethal 3 homolog (Drosophila) pseudogene 1	3
MTDH	92140	metadherin	HC
MTOR	2475	mechanistic target of rapamycin	1
MTPN	136319	myotrophin	3
MUC1	4582	mucin 1, cell surface associated	HC
MYBBP1A	10514	MYB binding protein (P160) 1a	2
MYCBP	26292	MYC binding protein	HC
MYCBP2	23077	MYC binding protein 2, E3 ubiquitin protein ligase	3
MYO6	4646	myosin VI	HC
MYOCD	93649	myocardin	HC
N4BP2L2	10443	NEDD4 binding protein 2-like 2	2
NAA15	80155	N(alpha)-acetyltransferase 15, NatA auxiliary subunit	HC

NAA16	79612	N(alpha)-acetyltransferase 16, NatA auxiliary subunit	3
NAB1	4664	NGFI-A binding protein 1	3
NAB2	4665	NGFI-A binding protein 2	1
NACC1	112939	nucleus accumbens associated 1	HC
NACC2	138151	NACC family member 2	HC
NCK1	4690	NCK adaptor protein 1	1
NCL	4691	nucleolin	HC
NDN	4692	necdin, MAGE family member	3
NDUFA13	51079	NADH:ubiquinone oxidoreductase subunit A13	HC
NEDD4L	23327	sor cell expressed, developmentally down-regulated 4-like, E3 ubiquitin	3
NEDD8	4738	neural precursor cell expressed, developmentally down-regulated 8	3
NELFA	7469	negative elongation factor complex member A	2
NELFB	25920	negative elongation factor complex member B	2
NELFCD	51497	negative elongation factor complex member C/D	2
NELFE	7936	negative elongation factor complex member E	2
NFATC2IP	84901	nuclear factor of activated T-cells 2 interacting protein	3
NFE4	58160	nuclear factor, erythroid 4	3
NIF3L1	60491	NGG1 interacting factor 3 like 1	1
NIPBL	25836	Nipped-B homolog (Drosophila)	HC
NKAP	79576	NFKB activating protein	HC
NLK	51701	nemo-like kinase	3
NLRP12	91662	NLR family, pyrin domain containing 12	HC
NLRP3	114548	NLR family, pyrin domain containing 3	1
NMI	9111	N-myc and STAT interactor	2
NOC2L	26155	NOC2 like nucleolar associated transcriptional repressor	HC
NOD2	64127	nucleotide binding oligomerization domain containing 2	HC
NOL11	25926	nucleolar protein 11	HC
NOTCH1	4851	notch 1	1
NOTCH2	4853	notch 2	2
NOTCH3	4854	notch 3	2
NOTCH4	4855	notch 4	3
NPAT	4863	nuclear protein, co-activator of histone transcription	HC

NPM1	4869	nucleophosmin (nucleolar phosphoprotein B23, numatrin)	HC
NRBF2	29982	nuclear receptor binding factor 2	3
NRG1	3084	neuregulin 1	1
NRIP1	8204	nuclear receptor interacting protein 1	HC
NRIP2	83714	nuclear receptor interacting protein 2	3
NSD1	64324	nuclear receptor binding SET domain protein 1	1
NUP107	57122	nucleoporin 107kDa	2
NUP133	55746	nucleoporin 133kDa	2
NUP35	129401	nucleoporin 35kDa	2
NUP62	23636	nucleoporin 62kDa	HC
NUP85	79902	nucleoporin 85kDa	2
OGT	8473	O-linked N-acetylglucosamine (GlcNAc) transferase	HC
ORC2	4999	origin recognition complex subunit 2	2
PADI4	23569	peptidyl arginine deiminase, type IV	2
PAF1	54623	PAF1 homolog, Paf1/RNA polymerase II complex component	HC
PAGR1	79447	PAXIP1 associated glutamate rich protein 1	HC
PAK6	56924	p21 protein (Cdc42/Rac)-activated kinase 6	3
PARK2	5071	parkin RBR E3 ubiquitin protein ligase	HC
PARK7	11315	Parkinsonism associated deglycase	HC
PARP10	84875	poly(ADP-ribose) polymerase family member 10	HC
PARP14	54625	poly(ADP-ribose) polymerase family member 14	3
PAXIP1	22976	PAX interacting protein 1	HC
PBXIP1	57326	pre-B-cell leukemia homeobox interacting protein 1	3
PCBD1	5092	pterin-4 alpha-carbinolamine dehydratase 1	2
PCBD2	84105	pterin-4 alpha-carbinolamine dehydratase 2	1
PCGF1	84759	polycomb group ring finger 1	2
PCGF3	10336	polycomb group ring finger 3	2
PCNA	5111	proliferating cell nuclear antigen	2
PDCD4	27250	programmed cell death 4 (neoplastic transformation inhibitor)	HC
PDPK1	5170	3-phosphoinositide dependent protein kinase 1	3
PELI1	57162	pellino E3 ubiquitin protein ligase 1	3
PELP1	27043	proline, glutamate and leucine rich protein 1	HC

PER1	5187	period circadian clock 1	3
PER2	8864	period circadian clock 2	2
PEX14	5195	peroxisomal biogenesis factor 14	HC
PFDN5	5204	prefoldin subunit 5	1
PFN1	5216	profilin 1	2
PGBD1	84547	piggyBac transposable element derived 1	3
PHF1	5252	PHD finger protein 1	2
PHF10	55274	PHD finger protein 10	2
PHF12	57649	PHD finger protein 12	HC
PHF14	9678	PHD finger protein 14	3
PHF19	26147	PHD finger protein 19	2
PHF2	5253	PHD finger protein 2	HC
PHF21A	51317	PHD finger protein 21A	HC
PHF8	23133	PHD finger protein 8	HC
PHIP	55023	pleckstrin homology domain interacting protein	1
PIAS3	10401	protein inhibitor of activated STAT 3	3
PICALM	8301	phosphatidylinositol binding clathrin assembly protein	HC
PID1	55022	phosphotyrosine interaction domain containing 1	HC
PIDD1	55367	p53-induced death domain protein 1	2
PIH1D1	55011	PIH1 domain containing 1	HC
PIK3R1	5295	phosphoinositide-3-kinase regulatory subunit 1	1
PIK3R2	5296	phosphoinositide-3-kinase regulatory subunit 2	3
PIM1	5292	Pim-1 proto-oncogene, serine/threonine kinase	1
PIN1	5300	peptidylprolyl cis/trans isomerase, NIMA-interacting 1	HC
PINK1	65018	PTEN induced putative kinase 1	2
PIR	8544	pirin	HC
PKIA	5569	protein kinase (cAMP-dependent, catalytic) inhibitor alpha	3
PKIG	11142	protein kinase (cAMP-dependent, catalytic) inhibitor gamma	3
PKN1	5585	protein kinase N1	HC
PKN2	5586	protein kinase N2	2
PLCB1	23236	phospholipase C beta 1	2
PLK1	5347	polo like kinase 1	HC

PLK3	1263	polo like kinase 3	HC
PMF1	11243	polyamine-modulated factor 1	HC
PNRC1	10957	proline rich nuclear receptor coactivator 1	3
PNRC2	55629	proline rich nuclear receptor coactivator 2	2
POLR3F	10621	polymerase (RNA) III subunit F	2
POLR3G	10622	polymerase (RNA) III subunit G	2
POLR3GL	84265	polymerase (RNA) III subunit G like	2
PPARGC1B	133522	PPARG coactivator 1 beta	HC
PPHLN1	51535	periphilin 1	2
PPID	5481	peptidylprolyl isomerase D	HC
PPIE	10450	peptidylprolyl isomerase E	HC
PPM1A	5494	protein phosphatase, Mg2+/Mn2+ dependent 1A	HC
PPP1R12A	4659	protein phosphatase 1 regulatory subunit 12A	1
PPP2CA	5515	protein phosphatase 2 catalytic subunit alpha	3
PPP2R1A	5518	protein phosphatase 2 regulatory subunit A, alpha	3
PPP3CA	5530	protein phosphatase 3 catalytic subunit alpha	HC
PPP3CB	5532	protein phosphatase 3 catalytic subunit beta	1
PPP3R1	5534	protein phosphatase 3 regulatory subunit B, alpha	1
PPRC1	23082	peroxisome proliferator-activated receptor gamma, coactivator-related 1	3
PRAME	23532	preferentially expressed antigen in melanoma	1
PRDX5	25824	peroxiredoxin 5	HC
PRKAA1	5562	protein kinase AMP-activated catalytic subunit alpha 1	2
PRKAA2	5563	protein kinase AMP-activated catalytic subunit alpha 2	3
PRKCB	5579	protein kinase C beta	HC
PRKCI	5584	protein kinase C iota	HC
PRKCQ	5588	protein kinase C theta	1
PRKCZ	5590	protein kinase C zeta	3
PRKD1	5587	protein kinase D1	1
PRKD2	25865	protein kinase D2	HC
PRKDC	5591	protein kinase, DNA-activated, catalytic polypeptide	HC
PRMT1	3276	protein arginine methyltransferase 1	2
PRMT2	3275	protein arginine methyltransferase 2	HC

PRMT5	10419	protein arginine methyltransferase 5	2
PRMT6	55170	protein arginine methyltransferase 6	HC
PRMT7	54496	protein arginine methyltransferase 7	2
PRMT8	56341	protein arginine methyltransferase 8	2
PRNP	5621	prion protein	3
PRPF6	24148	pre-mRNA processing factor 6	HC
PSEN1	5663	presenilin 1	HC
PSIP1	11168	PC4 and SFRS1 interacting protein 1	HC
PSMA6	5687	proteasome subunit alpha 6	HC
PSMC1	5700	proteasome 26S subunit, ATPase 1	2
PSMC2	5701	proteasome 26S subunit, ATPase 2	2
PSMC3	5702	proteasome 26S subunit, ATPase 3	2
PSMC4	5704	proteasome 26S subunit, ATPase 4	2
PSMC5	5705	proteasome 26S subunit, ATPase 5	2
PSMC6	5706	proteasome 26S subunit, ATPase 6	2
PSMD10	5716	proteasome 26S subunit, non-ATPase 10	HC
PSMD9	5715	proteasome 26S subunit, non-ATPase 9	3
PTEN	5728	phosphatase and tensin homolog	HC
PTOV1	53635	prostate tumor overexpressed 1	2
PTPN14	5784	protein tyrosine phosphatase, non-receptor type 14	HC
PTRF	284119	polymerase I and transcript release factor	2
PTTG1	9232	pituitary tumor-transforming 1	3
PYCARD	29108	PYD and CARD domain containing	HC
PYGO1	26108	pygopus family PHD finger 1	3
RAD21	5885	RAD21 cohesin complex component	HC
RAF1	5894	Raf-1 proto-oncogene, serine/threonine kinase	3
RAI1	10743	retinoic acid induced 1	HC
RALGAPA1	253959	Ral GTPase activating protein catalytic alpha subunit 1	3
RAN	5901	RAN, member RAS oncogene family	2
RASD1	51655	RAS, dexamethasone-induced 1	3
RB1CC1	9821	RB1 inducible coiled-coil 1	2
RBBP4	5928	retinoblastoma binding protein 4	2

RBBP5	5929	retinoblastoma binding protein 5	2
RBBP7	5931	retinoblastoma binding protein 7	2
RBBP8	5932	retinoblastoma binding protein 8	HC
RBFOX2	23543	RNA binding protein, fox-1 homolog (C. elegans) 2	HC
RBL1	5933	retinoblastoma-like 1	2
RBM14	10432	RNA binding motif protein 14	HC
RBM15	64783	RNA binding motif protein 15	HC
RBM15B	29890	RNA binding motif protein 15B	HC
RBM39	9584	RNA binding motif protein 39	2
RBMX	27316	RNA binding motif protein, X-linked	HC
RBMXL1	494115	RNA binding motif protein, X-linked-like 1	3
RBPMS	11030	RNA binding protein with multiple splicing	HC
RBX1	9978	ring-box 1, E3 ubiquitin protein ligase	3
RCBTB1	55213	RCC1 and BTB domain containing protein 1	3
RECQL5	9400	RecQ like helicase 5	HC
REXO4	57109	REX4 homolog, 3'-5' exonuclease	2
RGCC	28984	regulator of cell cycle	HC
RGS14	10636	regulator of G-protein signaling 14	3
RGS20	8601	regulator of G-protein signaling 20	3
RING1	6015	ring finger protein 1	HC
RIPPLY1	92129	ripply transcriptional repressor 1	3
RITA1	84934	RBPJ interacting and tubulin associated 1	2
RLIM	51132	ring finger protein, LIM domain interacting	2
RNASEL	6041	ribonuclease L	1
RNF10	9921	ring finger protein 10	1
RNF111	54778	ring finger protein 111	HC
RNF14	9604	ring finger protein 14	HC
RNF168	165918	ring finger protein 168, E3 ubiquitin protein ligase	HC
RNF187	149603	ring finger protein 187	HC
RNF2	6045	ring finger protein 2	HC
RNF20	56254	ring finger protein 20	HC
RNF25	64320	ring finger protein 25	HC

RNF8	9025	ring finger protein 8	HC
RPAP2	79871	RNA polymerase II associated protein 2	HC
RPRD1A	55197	regulation of nuclear pre-mRNA domain containing 1A	HC
RPRD1B	58490	regulation of nuclear pre-mRNA domain containing 1B	HC
RPS14	6208	ribosomal protein S14	HC
RPS27A	6233	ribosomal protein S27a	2
RPS6KA1	6195	ribosomal protein S6 kinase A1	HC
RPS6KA3	6197	ribosomal protein S6 kinase A3	1
RPS6KA4	8986	ribosomal protein S6 kinase A4	HC
RPS6KA5	9252	ribosomal protein S6 kinase A5	HC
RPTOR	57521	regulatory associated protein of MTOR, complex 1	HC
RQCD1	9125	RCD1 required for cell differentiation1 homolog (S. pombe)	1
RRM2	6241	ribonucleotide reductase regulatory subunit M2	2
RRN3	54700	RRN3 homolog, RNA polymerase I transcription factor	2
RRP8	23378	ribosomal RNA processing 8, methyltransferase, homolog (yeast)	HC
RSF1	51773	remodeling and spacing factor 1	HC
RTF1	23168	RTF1 homolog, Paf1/RNA polymerase II complex component	HC
RUVBL1	8607	RuvB like AAA ATPase 1	2
RUVBL2	10856	RuvB like AAA ATPase 2	HC
RWDD3	25950	RWD domain containing 3	1
S100A1	6271	S100 calcium binding protein A1	2
S100A8	6279	S100 calcium binding protein A8	2
S100A9	6280	S100 calcium binding protein A9	2
SAFB	6294	scaffold attachment factor B	2
SAMD4B	55095	sterile alpha motif domain containing 4B	3
SAP130	79595	Sin3A associated protein 130kDa	HC
SAP18	10284	Sin3A associated protein 18kDa	2
SAP30BP	29115	SAP30 binding protein	2
SAV1	60485	salvador family WW domain containing protein 1	2
SCAI	286205	suppressor of cancer cell invasion	HC
SCAND1	51282	SCAN domain containing 1	2
SENP1	29843	SUMO1/sentrin specific peptidase 1	HC

SENP2	59343	SUMO1/sentrin/SMT3 specific peptidase 2	2
SERTAD1	29950	SERTA domain containing 1	3
SERTAD3	29946	SERTA domain containing 3	HC
SETD1A	9739	SET domain containing 1A	2
SETD1B	23067	SET domain containing 1B	2
SETD2	29072	SET domain containing 2	3
SETD3	84193	SET domain containing 3	3
SETD6	79918	SET domain containing 6	HC
SETD7	80854	SET domain containing lysine methyltransferase 7	2
SETSIP	646817	SET-like protein	HC
SF1	7536	splicing factor 1	2
SFMBT1	51460	Scm-like with four mbt domains 1	HC
SFR1	119392	SWI5 dependent homologous recombination repair protein 1	HC
SFRP4	6424	secreted frizzled related protein 4	HC
SFSWAP	6433	splicing factor, suppressor of white-apricot homolog	3
SGF29	112869	SAGA complex associated factor 29	2
SGK1	6446	serum/glucocorticoid regulated kinase 1	2
SIAH2	6478	siah E3 ubiquitin protein ligase 2	2
SIN3A	25942	SIN3 transcription regulator family member A	HC
SIN3B	23309	SIN3 transcription regulator family member B	3
SIRT1	23411	sirtuin 1	HC
SIRT2	22933	sirtuin 2	HC
SIRT5	23408	sirtuin 5	3
SIRT6	51548	sirtuin 6	2
SIRT7	51547	sirtuin 7	HC
SIVA1	10572	SIVA1 apoptosis inducing factor	HC
SKAP1	8631	src kinase associated phosphoprotein 1	HC
SLTM	79811	SAFB like transcription modulator	2
SMURF1	57154	SMAD specific E3 ubiquitin protein ligase 1	1
SMURF2	64750	SMAD specific E3 ubiquitin protein ligase 2	3
SMYD2	56950	SET and MYND domain containing 2	1
SMYD3	64754	SET and MYND domain containing 3	3

SNAPC2	6618	small nuclear RNA activating complex polypeptide 2	2
SNAPC5	10302	small nuclear RNA activating complex polypeptide 5	2
SNCA	6622	synuclein alpha	2
SND1	27044	staphylococcal nuclease and tudor domain containing 1	3
SNIP1	79753	Smad nuclear interacting protein 1	2
SNW1	22938	SNW domain containing 1	HC
SNX6	58533	sorting nexin 6	1
SORBS3	10174	sorbin and SH3 domain containing 3	3
SPAG8	26206	sperm associated antigen 8	3
SQSTM1	8878	sequestosome 1	2
SRA1	10011	steroid receptor RNA activator 1	HC
SRC	6714	SRC proto-oncogene, non-receptor tyrosine kinase	3
SRFBP1	153443	serum response factor binding protein 1	3
SRSF10	10772	serine/arginine-rich splicing factor 10	2
SRSF2	6427	serine/arginine-rich splicing factor 2	2
SS18	6760	synovial sarcoma translocation, chromosome 18	1
SS18L1	26039	synovial sarcoma translocation gene on chromosome 18-like 1	3
SSBP2	23635	single stranded DNA binding protein 2	3
SSBP3	23648	single stranded DNA binding protein 3	3
SSU72	29101	SSU72 homolog, RNA polymerase II CTD phosphatase	1
SSX1	6756	synovial sarcoma, X breakpoint 1	3
SSX2	6757	synovial sarcoma, X breakpoint 2	2
SSX2B	727837	synovial sarcoma, X breakpoint 2B	2
SSX3	10214	synovial sarcoma, X breakpoint 3	3
SSX4	6759	synovial sarcoma, X breakpoint 4	3
SSX4B	548313	synovial sarcoma, X breakpoint 4B	3
SSX5	6758	synovial sarcoma, X breakpoint 5	3
STAG1	10274	stromal antigen 1	2
STK3	6788	serine/threonine kinase 3	1
STK36	27148	serine/threonine kinase 36	HC
STRAP	11171	serine/threonine kinase receptor associated protein	3
STRN3	29966	striatin 3	HC

SUB1	10923	SUB1 homolog, transcriptional regulator	HC
SUDS3	64426	SDS3 homolog, SIN3A corepressor complex component	HC
SUFU	51684	SUFU negative regulator of hedgehog signaling	HC
SUMO1	7341	small ubiquitin-like modifier 1	HC
SUPT16H	11198	SPT16 homolog, facilitates chromatin remodeling subunit	2
SUPT20H	55578	SPT20 homolog, SAGA complex component	2
SUPT4H1	6827	SPT4 homolog, DSIF elongation factor subunit	HC
SUPT5H	6829	SPT5 homolog, DSIF elongation factor subunit	HC
SUPT7L	9913	SPT7-like STAGA complex gamma subunit	HC
SUV39H1	6839	suppressor of variegation 3-9 homolog 1	HC
SUV39H2	79723	suppressor of variegation 3-9 homolog 2	1
SUZ12	23512	SUZ12 polycomb repressive complex 2 subunit	2
SYK	6850	spleen tyrosine kinase	HC
TAB1	10454	TGF-beta activated kinase 1/MAP3K7 binding protein 1	2
TAB3	257397	TGF-beta activated kinase 1/MAP3K7 binding protein 3	2
TADA1	117143	transcriptional adaptor 1	HC
TADA3	10474	transcriptional adaptor 3	HC
TAF10	6881	TATA-box binding protein associated factor 10	HC
TAF11	6882	TATA-box binding protein associated factor 11	HC
TAF1B	9014	TATA-box binding protein associated factor, RNA polymerase I subunit B	HC
TAF1C	9013	TATA-box binding protein associated factor, RNA polymerase I subunit C	2
TAF2	6873	TATA-box binding protein associated factor 2	HC
TAF3	83860	TATA-box binding protein associated factor 3	HC
TAF5	6877	TATA-box binding protein associated factor 5	2
TAF5L	27097	TATA-box binding protein associated factor 5 like	HC
TAF7	6879	TATA-box binding protein associated factor 7	HC
TAF7L	54457	TATA-box binding protein associated factor 7 like	3
TAF8	129685	TATA-box binding protein associated factor 8	HC
TAF9B	51616	TATA-box binding protein associated factor 9b	HC
TARBP1	6894	TAR (HIV-1) RNA binding protein 1	3
TARDBP	23435	TAR DNA binding protein	2
TBL1X	6907	transducin (beta)-like 1X-linked	HC

TBL1XR1	79718	transducin (beta)-like 1 X-linked receptor 1	HC
TBL1Y	90665	transducin (beta)-like 1, Y-linked	3
TCEAL1	9338	transcription elongation factor A like 1	2
TCEAL2	140597	transcription elongation factor A like 2	3
TCEAL3	85012	transcription elongation factor A like 3	3
TCEAL4	79921	transcription elongation factor A like 4	3
TCEAL5	340543	transcription elongation factor A like 5	3
TCEB1	6921	transcription elongation factor B subunit 1	3
TCEB2	6923	transcription elongation factor B subunit 2	3
TCERG1	10915	transcription elongation regulator 1	HC
TCP10L	140290	t-complex 10-like	HC
TDG	6996	thymine DNA glycosylase	2
TDP2	51567	tyrosyl-DNA phosphodiesterase 2	2
TDRD3	81550	tudor domain containing 3	HC
TENM1	10178	teneurin transmembrane protein 1	3
TENM2	57451	teneurin transmembrane protein 2	3
TERF2IP	54386	TERF2 interacting protein	2
TESC	54997	tescalcin	HC
TGFB1	7040	transforming growth factor beta 1	HC
TGFB1I1	7041	transforming growth factor beta 1 induced transcript 1	1
TGS1	96764	trimethylguanosine synthase 1	2
THOC5	8563	THO complex 5	HC
THRAP3	9967	thyroid hormone receptor associated protein 3	HC
THRSP	7069	thyroid hormone responsive	2
TIAL1	7073	TIA1 cytotoxic granule-associated RNA binding protein-like 1	3
TIMELESS	8914	timeless circadian clock	HC
TIRAP	114609	toll-interleukin 1 receptor (TIR) domain containing adaptor protein	HC
TLE1	7088	transducin like enhancer of split 1	HC
TLE2	7089	transducin like enhancer of split 2	HC
TLE3	7090	transducin like enhancer of split 3	3
TLE4	7091	transducin like enhancer of split 4	2
TNIP1	10318	TNFAIP3 interacting protein 1	2

TNIP2	79155	TNFAIP3 interacting protein 2	HC
TNKS	8658	tankyrase	HC
TNNI2	7136	troponin I2, fast skeletal type	HC
TNRC18	84629	trinucleotide repeat containing 18	2
TOB1	10140	transducer of ERBB2, 1	3
TP53INP1	94241	tumor protein p53 inducible nuclear protein 1	HC
TPR	7175	translocated promoter region, nuclear basket protein	HC
TRADD	8717	TNFRSF1A-associated via death domain	3
TRAF6	7189	TNF receptor associated factor 6	HC
TRAK1	22906	trafficking protein, kinesin binding 1	3
TRAK2	66008	trafficking protein, kinesin binding 2	3
TRAPPC2	6399	trafficking protein particle complex 2	1
TRIB1	10221	tribbles pseudokinase 1	HC
TRIB3	57761	tribbles pseudokinase 3	HC
TRIM11	81559	tripartite motif containing 11	2
TRIM22	10346	tripartite motif containing 22	HC
TRIM24	8805	tripartite motif containing 24	HC
TRIM25	7706	tripartite motif containing 25	HC
TRIM29	23650	tripartite motif containing 29	2
TRIM32	22954	tripartite motif containing 32	1
TRIM37	4591	tripartite motif containing 37	HC
TRIM5	85363	tripartite motif containing 5	1
TRIM6	117854	tripartite motif containing 6	3
TRIM8	81603	tripartite motif containing 8	HC
TRIP11	9321	thyroid hormone receptor interactor 11	2
TRIP13	9319	thyroid hormone receptor interactor 13	3
TRIP4	9325	thyroid hormone receptor interactor 4	HC
TRIP6	7205	thyroid hormone receptor interactor 6	3
TRRAP	8295	transformation/transcription domain associated protein	HC
TSPYL2	64061	TSPY-like 2	2
TTC21B	79809	tetratricopeptide repeat domain 21B	1
TULP3	7289	tubby like protein 3	2

TXLNG	55787	taxilin gamma	2
TXN	7295	thioredoxin	2
TXNIP	10628	thioredoxin interacting protein	3
UBA3	9039	ubiquitin like modifier activating enzyme 3	2
UBA52	7311	ubiquitin A-52 residue ribosomal protein fusion product 1	2
UBB	7314	ubiquitin B	2
UBC	7316	ubiquitin C	2
UBE2B	7320	ubiquitin conjugating enzyme E2 B	2
UBE2D1	7321	ubiquitin conjugating enzyme E2 D1	3
UBE2D2	7322	ubiquitin conjugating enzyme E2 D2	3
UBE2D3	7323	ubiquitin conjugating enzyme E2 D3	3
UBE2I	7329	ubiquitin conjugating enzyme E2 I	HC
UBE2L3	7332	ubiquitin conjugating enzyme E2 L3	HC
UBE2N	7334	ubiquitin conjugating enzyme E2 N	HC
UBE2O	63893	ubiquitin conjugating enzyme E2 O	HC
UBE2V1	7335	ubiquitin conjugating enzyme E2 V1	1
UBE3A	7337	ubiquitin protein ligase E3A	3
UBR2	23304	ubiquitin protein ligase E3 component n-recognin 2	2
UCHL5	51377	ubiquitin C-terminal hydrolase L5	2
UFL1	23376	UFM1 specific ligase 1	HC
UHRF1	29128	ubiquitin like with PHD and ring finger domains 1	HC
UIMC1	51720	ubiquitin interaction motif containing 1	HC
URI1	8725	URI1 prefoldin-like chaperone	HC
USP15	9958	ubiquitin specific peptidase 15	HC
USP16	10600	ubiquitin specific peptidase 16	HC
USP2	9099	ubiquitin specific peptidase 2	1
USP21	27005	ubiquitin specific peptidase 21	2
USP22	23326	ubiquitin specific peptidase 22	HC
USP34	9736	ubiquitin specific peptidase 34	3
USP47	55031	ubiquitin specific peptidase 47	1
USP7	7874	ubiquitin specific peptidase 7 (herpes virus-associated)	HC
UTP4	84916	UTP4, small subunit processome component	HC

UXT	8409	ubiquitously expressed prefoldin like chaperone	HC
VGLL1	51442	vestigial like family member 1	2
VGLL2	245806	vestigial like family member 2	3
VGLL3	389136	vestigial like family member 3	3
VGLL4	9686	vestigial like family member 4	3
VHL	7428	von Hippel-Lindau tumor suppressor	HC
VPRBP	9730	Vpr (HIV-1) binding protein	HC
VPS25	84313	vacuolar protein sorting 25 homolog (S. cerevisiae)	2
VPS36	51028	vacuolar protein sorting 36 homolog (S. cerevisiae)	3
WAC	51322	WW domain containing adaptor with coiled-coil	HC
WASL	8976	Wiskott-Aldrich syndrome like	3
WBSCR22	114049	Williams-Beuren syndrome chromosome region 22	3
WDR5	11091	WD repeat domain 5	2
WDR61	80349	WD repeat domain 61	HC
WDR75	84128	WD repeat domain 75	3
WDR77	79084	WD repeat domain 77	HC
WDTC1	23038	WD and tetratricopeptide repeats 1	3
WHSC1L1	54904	Wolf-Hirschhorn syndrome candidate 1-like 1	3
WTIP	126374	Wilms tumor 1 interacting protein	3
WWC1	23286	WW and C2 domain containing 1	HC
WWOX	51741	WW domain containing oxidoreductase	2
WWP1	11059	WW domain containing E3 ubiquitin protein ligase 1	3
WWP2	11060	WW domain containing E3 ubiquitin protein ligase 2	HC
WWTR1	25937	WW domain containing transcription regulator 1	HC
XIAP	331	X-linked inhibitor of apoptosis	2
XPO1	7514	exportin 1	2
XRN2	22803	5'-3' exoribonuclease 2	2
YAF2	10138	YY1 associated factor 2	HC
YAP1	10413	Yes associated protein 1	HC
YLPM1	56252	YLP motif containing 1	2
YWHAB	7529	sine 3-monooxygenase/tryptophan 5-monooxygenase activation protein	3
YY1AP1	55249	YY1 associated protein 1	2

ZCCHC12	170261	zinc finger CCHC-type containing 12	3
ZMIZ1	57178	zinc finger MIZ-type containing 1	2
ZMIZ2	83637	zinc finger MIZ-type containing 2	HC
ZMYM2	7750	zinc finger MYM-type containing 2	2
ZMYM5	9205	zinc finger MYM-type containing 5	1
ZMYND11	10771	zinc finger MYND-type containing 11	2
ZMYND8	23613	zinc finger MYND-type containing 8	3
ZNF587B	1E+08	zinc finger protein 587B	3
ZNF638	27332	zinc finger protein 638	2
ZRANB2	9406	zinc finger RANBP2-type containing 2	2
