# Supplementary material for: Foxg1 and companions: Not only transcription factors
Source: Neural Regen Res. 2025 Sep 3;21(8):3426–38. doi: 10.4103/NRR.NRR-D-25-00439 (PMC13452659; doi:10.4103/NRR.NRR-D-25-00439)
Supplement: Supplementary file 13 [file NRR-21-3426_Suppl20.pdf]

Additioinal Table 7.9. Filtered TF/TlatMF.cytoRP interaction reports & related info

TF	TF taxon	Numbers of TlatMF/cytoRP interaction reports, real-vs-expected		
		Chi-square test, p evaluated over all 11 groups (3 SplFs, 2 pAFs, 6 TlatMFs)	Chi-square test, p evaluated over 6 TlatMF groups	Real / expected number ratio
BHLHA15	bHLH	1.28E-07	5.38E-09	4.3
HEY1	bHLH	5.38E-09	1.31E-08	3.7
TFCP2	GHD.CP2	2.38E-11	3.04E-09	2.5
ZEB1	HB-ZF.C2H2	6.89E-09	4.72E-04	2.5
MSX2	HB.NK	4.19E-04	3.29E-04	2.2
NKX2-6	HB.NK	1.63E-03	3.38E-05	30.1
DUX4	HB.PD	2.64E-71	1.81E-73	6.8
HMGB1	HMG	5.28E-37	2.22E-40	4.4
HMGB2	HMG	8.72E-45	9.73E-47	5.7
BBX	HMG.SOX	3.77E-03	6.77E-04	3.0
CEBPZ	others	3.18E-11	1.37E-12	3.4
CNBP	others	1.19E-54	1.73E-55	5.7
NKRF	others	1.03E-07	6.88E-09	3.1
TFB1M	others	1.24E-02	3.06E-03	2.3
PURA	PUR	8.62E-04	1.47E-03	2.3
PURB	PUR	4.32E-10	7.90E-11	4.2
DNAJC2	WC.MYB	3.80E-157	8.32E-160	10.2
TTF1	WC.MYB	3.87E-36	3.76E-38	7.5
LYAR	ZF.C2CH	1.22E-09	4.57E-10	2.7
THAP3	ZF.C2CH	1.70E-05	5.62E-07	3.3
THAP7	ZF.C2CH	1.08E-02	5.49E-03	2.6
CTCF	ZF.C2H2	3.66E-157	1.45E-160	7.4
E4F1	ZF.C2H2	1.74E-55	5.51E-58	2.7
GZF1	ZF.C2H2	1.25E-51	2.68E-54	10.5
KLF4	ZF.C2H2	1.89E-30	3.58E-27	2.7
MTF1	ZF.C2H2	1.87E-40	2.78E-37	7.8
PRDM10	ZF.C2H2	3.06E-03	1.29E-04	4.0
PRDM15	ZF.C2H2	7.55E-28	5.70E-30	8.1
REST	ZF.C2H2	5.87E-61	5.11E-61	6.3
ZBTB1	ZF.C2H2	2.53E-14	7.61E-17	5.1
ZBTB11	ZF.C2H2	8.17E-25	1.64E-27	6.7
ZBTB24	ZF.C2H2	1.38E-11	3.96E-12	4.0
ZBTB41	ZF.C2H2	2.32E-08	1.68E-08	10.7
ZFP62	ZF.C2H2	3.51E-15	5.25E-17	6.4
ZFP91	ZF.C2H2	2.13E-03	2.41E-03	2.8
ZKSCAN8	ZF.C2H2	1.85E-07	1.41E-06	3.1
ZNF121	ZF.C2H2	1.72E-02	4.61E-03	3.9
ZNF16	ZF.C2H2	6.57E-08	1.16E-09	7.3
ZNF184	ZF.C2H2	3.82E-07	6.05E-09	5.2
ZNF189	ZF.C2H2	2.06E-05	2.17E-06	5.0
ZNF22	ZF.C2H2	5.21E-28	1.35E-29	6.4
ZNF274	ZF.C2H2	3.02E-03	3.18E-04	2.0
ZNF317	ZF.C2H2	1.30E-06	3.52E-08	4.9
ZNF320	ZF.C2H2	2.22E-02	2.13E-03	5.4
ZNF354A	ZF.C2H2	7.64E-17	1.33E-18	8.4
ZNF358	ZF.C2H2	1.30E-08	1.64E-10	9.6
ZNF48	ZF.C2H2	1.27E-20	1.97E-22	5.5
ZNF485	ZF.C2H2	2.18E-37	2.24E-40	2.9
ZNF512	ZF.C2H2	2.31E-25	9.42E-25	4.2
ZNF574	ZF.C2H2	5.09E-62	1.66E-62	4.6
ZNF629	ZF.C2H2	2.48E-05	1.13E-06	3.7
ZNF668	ZF.C2H2	1.32E-46	1.21E-48	6.0
ZNF689	ZF.C2H2	1.38E-04	5.08E-05	3.6
ZNF70	ZF.C2H2	6.06E-10	1.07E-11	7.2
ZNF746	ZF.C2H2	2.29E-12	3.14E-12	3.0
ZNF768	ZF.C2H2	3.55E-14	1.11E-15	5.1
ZNF770	ZF.C2H2	2.58E-40	1.84E-42	8.5
ZNF771	ZF.C2H2	1.76E-27	9.68E-30	8.8
ZNF777	ZF.C2H2	7.97E-03	6.97E-04	3.2
ZSCAN25	ZF.C2H2	2.59E-20	1.94E-22	7.0
LIN28A	ZF.CCHC	1.78E-45	2.56E-45	2.3
MBD4	ZF.CXXC	1.26E-02	1.32E-02	2.7
NFX1	ZF.NFX1	9.30E-61	6.69E-57	4.2

n° of filtered TF/TlatMF.cytoRP interaction reports

TF	RPSA	RPS2	RPS3	RPS3A	RPS4X	RPS4Y	RPS5	RPS6	RPS7	RPS8	RPS9	RPS10	RPS11	RPS12	RPS13	RPS14	RPS15	RPS15A	RPS16	RPS17	RPS18	RPS19	RPS20	RPS21	RPS23	RPS24	RPS25	RPS26	RPS27	RPS27A	RPS28	RPS29	RPS30
BHLHA15			1	1													2		2	1										2			
HEY1			1				1		1			1				1		1	1	1	1	1	1				1					1	
TFCP2	1	1	1	1	1		1		1	1	1	1			1	1		1	1	1	1	1			1	1	1	1			1		
ZEB1		1	1	1	1			1		1	1				1	1		1	1		1												
MSX2			1				1			1											1					1							
NKX2-6											1																						
DUX4	1	1	2	2	2		1	1	2	2	2	1			1	1	1	1	1	1	1		1			1	1						
HMGB1	1	1	1	1	1		2	2		1	1	1	1	2	1	1	1	1	1	1	1	1	2			1	1				1	1	
HMGB2	1	1		1	1			1	1	1		1					1	1	2		1	1	1	1	1		1	1	1		2		
BBX								1								1			1														
CEBPZ		1						2								2						1	1			1							
CNBP				2				2		2	1				2				1			1											
NKRF	1	1						3				1							1			1				1							
TFB1M										1									1														
PURA	1		1					1		2		1										1											
PURB	3	3	1					2		1		1										1											
DNAJC2	1	1	1	2	1		1	1	1	1	1	2	1	1	2	1	1	1		1	1	1	1	1		1		1	1		1		
TTF1	1	1		1				1				1				2						1											
LYAR			1	2				3			1								1			2		1		1							
THAP3			1				1					1					1													1			
THAP7				1																										3			
CTCF	2	2	3	2	2		2	3	1	2	3	1	2	1	2	2	1	2	3	1	1	2	2	1	2	2	1	2	2		1	1	
E4F1				1						2					1				2							1				2			
GZF1	2		1					1														1											
KLF4				1			1			1		1	1		1		1					1				1	1					1	
MTF1			1	1	1			1	1	1	1		1		1				1		1	1											
PRDM10			1					1			1	1							1														
PRDM15	1		1					1			1	1							1			1											
REST	1	1	1	1	1			1	1	1	1	1	1		1	1		1	1	1	1	1	1		1	1	1	1				1	
ZBTB1	1				1					1	1		1		1	1		1	1						1		1		1			1	
ZBTB11	2	1	1					1			1	1							1			1											
ZBTB24			1					1		2	1	1				2			1			1											
ZBTB41																																	
ZFP62											1																						
ZFP91		1	1					1		1		1										1											
ZKSCAN8		1	1	1				2											1			2								1			
ZNF121	3											1				2																	
ZNF16	1																					1											
ZNF184												1										1											
ZNF189								1																									
ZNF22		1		3				1				1							1			1											
ZNF274																														2			
ZNF317		1		1																													
ZNF320																						1											
ZNF354A												1										1											
ZNF358	3		1									1																					
ZNF48			1					1		1	1	1																					
ZNF485																						1								1			
ZNF512	1	1		2				2		1	1	1										1											
ZNF574	4	4	1					1		1		1				1	1		2			2											
ZNF629	1		1					1				1							1			1											
ZNF668		1	2	2			1	2		2	1	1			1		1		1			2				1							
ZNF689			1	1				1							1				1														
ZNF70								1				1			1							1											
ZNF746	1	1						1			1		1	1			1	1	2	1				1		1							
ZNF768	1	1	1					1		1		2										1											
ZNF770		1		1				1			1	1																					
ZNF771		2						1		1		1																					
ZNF777	2		1									1										1											
ZSCAN25		3	1					1		3	1	1										1											
LIN28A	1	1	1	1			1					1			1	1	1			1		1	1			1		1		1			
MBD4				1				1		1											1												
NFX1		1	1	1	1		1	1	1	1	1	1	1	1	1	1	1	1	1	1		2	2		1	1	1		1		1	1	

TF	RPL3	RPL4	RPL5	RPL6	RPL7	RPL7A	RPL8	RPL9	RPL10	RPL10A	RPL11	RPL12	RPL13	RPL13A	RPL14	RPL15	RPL17	RPL18	RPL18A	RPL19	RPL21	RPL22	RPL23	RPL23A
BHLHA15	1					1														1				
HEY1							1	1			2	1				1							1	
TFCP2		1		1	1	1				1	1	1	1		1		1	1			1		1	1
ZEB1	1	1		1	1	1								1										1
MSX2					1	1				1	1	1												
NKX2-6																								
DUX4	2	2		2	2	2	2	1	1	1	1	1	1		1	2	1	1		1	1	2	1	2
HMGB1	2	1	1	1	1	2	1	1	1	1	1	1	1	1	3	1	1	1	1	3	1	1	1	1
HMGB2	3	1		1			1		1	1	1		1		3		1				1	1		1
BBX		1												1		1			2			1		
CEBPZ	1	1				1	1				1		1	1	2	1	1	2	2	1		1		
CNBP	2	2	2	2	2	2	2			2	2	2	2	2	2	2	1	2	2					1
NKRF	1	2	1		1	1	1				1		1	1	1					2				1
TFB1M	1								1															1
PURA	1	1											1	1	1	1	1			1				1
PURB	1	1				1							1											1
DNAJC2	2	2	2	2	2	2	2	1	1	1	1	2	2	1	1	2	2	2	1		2	1	1	1
TTF1	1	1				1	1			1	1		1	1	3	1	1		2					1
LYAR	2	1	1	1					1	1	2		1	1		1	1	1	2		1			2
THAP3							1						1				1							
THAP7					1								1	1					2					
CTCF	2	2	3	1	2	2	2	1	3	2	3	1	2	4	4	1	2	1	3	3	1	1	1	2
E4F1	1																1			2				
GZF1	1	1		1	2	1	1			1	1		1	1	3		1		1	1				1
KLF4										1					1				1	1				
MTF1	1	1	1		1	1	1		1	1	1				1	1			1	1				1
PRDM10						1							1											1
PRDM15	1	1				1	1					1	1	1						1				1
REST	1	1	1	1	1	1	1	1	1	1	1	1	1	1	1	1	1	1	1	1	1	1	1	1
ZBTB1	1						1				1			1	1			1						
ZBTB11	1	1		2	2		1		1		1		1	1			1			1				
ZBTB24					2	1	2		2				1		3		1			1		1		
ZBTB41	1					1							1											
ZFP62					1	1							1	1	3	1	1			1				
ZFP91	1					1							1		1					1				1
ZKSCAN8	1														3		1			1				
ZNF121																								
ZNF16	1					1							1		1					1				
ZNF184	1				1						1			1			1							
ZNF189	1	1				1											1							1
ZNF22	1	1			2	1							1	1	3		1	2	1	1				
ZNF274																								
ZNF317	1	1				1							2											1
ZNF320		1																						
ZNF354A	1						1				1		1				1							
ZNF358																	1							1
ZNF48	1	1	1			1	1			1	1		1	1		1	1	1	1		1	1		1
ZNF485													1											
ZNF512	1	2	2			1	3			1	1		2	1	3	1	1	2	1	1				
ZNF574	2	1				1			1	1	1		1	2	1		2			2				
ZNF629	1	1											1	1	1		1			1				
ZNF668	2	1		1	2	1	2		1	1	2	1	2	1	4		2	2	1	1				2
ZNF689					1						1						1		2					
ZNF70																	1			1				1
ZNF746	1	1	1	1	1	1	1		1	1			1	1	1			1			1		1	
ZNF768	1					1	1				1		1	1	3					1				1
ZNF770	1	1			1	1	2			1	1		1	1	2		1	1	1	1				1
ZNF771	1			1	2	1					1		1	1	1		1	1		1				1
ZNF777														1			1			1				
ZSCAN25	1					1									2					1				1
LIN28A	1	1		1			1	1		1	1					1	1	1						1
MBD4		1														1							1	
NFX1	2	1	1	2	1	2	1	1		1	1	1	1	2		1	2	1	1		1	2	1	1

TF	RPL24	RPL26	RPL27	RPL27A	RPL28	RPL29	RPL30	RPL31	RPL32	RPL34	RPL35	RPL35A	RPL36	RPL36A	RPL37	RPL37A	RPL38	RPL39	RPL40	RPL41	RPLP0	RPLP1	RPLP2
BHLHA15																					2		
HEY1																							
TFCP2	1		1	1				1					1				1				1		
ZEB1																					1	1	
MSX2			1				1	1			1		1										
NKX2-6																							
DUX4			1	1			2	2		1	1		1										1
HMGB1	1	1	1	1		2	1	2	1		2		1								1		1
HMGB2	3		1	1				1	1	1	1		1				1	1			1	1	1
BBX				1	1			1													1		
CEBPZ					1		1	1			1		1			2					1		
CNBP		1	2				2	1	2	1		2	3			2					2	1	2
NKRF							1	2			2					1					1		
TFB1M											1		1										
PURA								1			1										1		
PURB		1						1			1										1		
DNAJC2			2	1	1		3		2	1	1	1	1	1		1	1				2	1	2
TTF1					1		2	1			1		1			1					1		
LYAR							2	2	1							2					1		1
THAP3																							
THAP7		1							2														
CTCF	1	2	2	1	3		2	4	2	2	2	1	1			1	1	1			2	1	1
E4F1		2																					
GZF1		1		1	1		2						1								1		
KLF4	1	1		1							1			1									
MTF1			1		1							1									1		
PRDM10								1													1		
PRDM15		1			1		2	1								1					1		
REST	1	1	1	1	1	1	1	1		1	1	1		1		1	1				1	1	1
ZBTB1	1		1	1			1			1											1		
ZBTB11		1					1	1			1										1		
ZBTB24								1					1										
ZBTB41													1								1		
ZFP62					1		2						1			2					1		
ZFP91								1							1								
ZKSCAN8													1								1		
ZNF121					1																		
ZNF16								1					1										
ZNF184		1			1		2	1					1										
ZNF189		1			1		2																
ZNF22		1			1		2						1			2					1		
ZNF274																							
ZNF317		2			1								1								1		
ZNF320				1						1											1		
ZNF354A		1			2		2						1								1		
ZNF358																							
ZNF48			1	1			1		1				1			1					1		1
ZNF485																							
ZNF512		1			1		3	2	2		1		1			2					1		
ZNF574		1					1	1			1										1		
ZNF629					1								1								1		
ZNF668							2	1					1								2	1	1
ZNF689								1					1										
ZNF70					1		2						1										
ZNF746				1	1					1	1		1								1		1
ZNF768					1						1		1								1		
ZNF770				1	1		1	1			1		1			1					1		
ZNF771					1		2																
ZNF777		1			1								1								1		
ZSCAN25							2	1					1										
LIN28A		1	1			1		2			1	1				1					1		1
MBD4		1			1		1																
NFX1	1	1	1	1	1	1	1	1		1	1	1	1	1		1					1		

TF	Subcellular TF location (see Additioinal Table 2)	RBP function reported for… (see Additioinal Table 3)	pTriplo (see Additioinal Table 4)	Neuronal activity-plasticity responsive (see Additioinal Table 5)
BHLHA15	Nucleoplasm,Golgi apparatus		0.98	
HEY1	Nucleoplasm,Nuclear membrane,Cytosol		0.21	
TFCP2	Nucleoplasm,Cytosol	hsa gene	0.68	
ZEB1	Nucleoplasm,Nucleoli	hsa gene	0.78	
MSX2	Nuclear speckles		0.85	
NKX2-6	Nucleoplasm,Nuclear membrane,Cytosol		0.51	
DUX4	Nucleoplasm,Nucleoli,Golgi apparatus,Cytosol		NA	
HMGB1	Nucleoplasm	hsa gene	0.94	
HMGB2	Nucleoplasm,Nucleoli	hsa gene	0.29	
BBX	Nucleoplasm,Cytosol		0.60	1
CEBPZ	Nucleoplasm,Vesicles	hsa gene	0.26	
CNBP	Nucleoplasm,Cytosol	hsa gene	0.89	
NKRF	Nucleoplasm,Nucleoli	hsa gene	NA	
TFB1M	none reported	hsa gene	0.48	
PURA	none reported	hsa gene	1.00	
PURB	Nucleoplasm	hsa gene	0.67	1
DNAJC2	Cytosol	hsa gene	0.77	1
TTF1	Nucleoli fibrillar center	hsa gene	0.45	1
LYAR	Nucleoplasm,Nucleoli,Nucleoli rim	hsa gene	0.42	
THAP3	Nucleoplasm,Nucleoli,Mitochondria		0.56	1
THAP7	Nucleoplasm,Nuclear membrane,Vesicles		0.73	
CTCF	Nucleoplasm	hsa gene	1.00	
E4F1	Nucleoplasm	hsa gene	0.45	
GZF1	Nucleoplasm,Nucleoli	hsa gene	0.75	
KLF4	Nucleoplasm,Cytosol	mmu gene	0.59	1
MTF1	Nucleoplasm		0.94	1
PRDM10	Nucleoplasm,Nucleoli fibrillar center,Vesicles	hsa gene	0.53	
PRDM15	Nucleoplasm,Nuclear bodies	hsa gene	0.49	
REST	Nucleoplasm,Cytosol	hsa gene	0.75	1
ZBTB1	Nucleoplasm,Nuclear membrane		0.98	1
ZBTB11	Nucleoplasm	only mmu ortholog	0.54	
ZBTB24	Nucleoplasm,Cytokinetic bridge,Centrosome	hsa gene	0.59	
ZBTB41	Nucleoplasm,Nuclear membrane,Cytosol	hsa gene	0.40	1
ZFP62	Nucleoplasm,Actin filaments	hsa gene	0.51	
ZFP91	Nucleoplasm,Nucleoli	hsa gene	0.93	
ZKSCAN8	Nucleoplasm,Cytosol	hsa gene	0.61	
ZNF121	Nucleoplasm,Nucleoli,Centrosome	hsa gene	0.06	
ZNF16	Nucleoplasm,Nucleoli	hsa gene	0.06	
ZNF184	Nucleoplasm	hsa gene	0.18	
ZNF189	Intermediate filaments		0.11	
ZNF22	Nucleoplasm	hsa gene	0.30	
ZNF274	Nucleoplasm,Centrosome	hsa gene	0.03	
ZNF317	Nucleoplasm	hsa gene	0.22	
ZNF320	Nucleoplasm,Cytoplasmic bodies	hsa gene	0.85	
ZNF354A	Nucleoplasm,Nucleoli,Cytosol		0.04	
ZNF358	Nucleoplasm		0.73	
ZNF48	Mitochondria	hsa gene	0.58	
ZNF485	none reported	hsa gene	0.29	
ZNF512	Nucleoplasm		0.97	
ZNF574	Nucleoplasm,Vesicles	hsa gene	0.57	
ZNF629	Nucleoplasm,Golgi apparatus	hsa gene	0.96	
ZNF668	Nucleoplasm,Nucleoli,Nucleoli rim	hsa gene	0.78	
ZNF689	Nucleoplasm,Nucleoli fibrillar center		0.82	
ZNF70	Cytosol		0.25	
ZNF746	Nucleoplasm,Cytosol		0.53	
ZNF768	Nucleoplasm,Vesicles	hsa gene	0.75	
ZNF770	none reported	hsa gene	0.56	
ZNF771	Nucleoplasm,Nucleoli	hsa gene	0.56	
ZNF777	Nucleoplasm	hsa gene	0.78	
ZSCAN25	Nucleoplasm,Nucleoli fibrillar center		0.70	
LIN28A	Nucleoli,Cytosol,Rods & Rings	hsa gene	0.44	
MBD4	Nuclear speckles	hsa gene	0.63	
NFX1	Nucleoplasm,Nucleoli,Plasma membrane,Cytosol	hsa gene	0.92	
