# Supplementary material for: Foxg1 and companions: Not only transcription factors
Source: Neural Regen Res. 2025 Sep 3;21(8):3426–38. doi: 10.4103/NRR.NRR-D-25-00439 (PMC13452659; doi:10.4103/NRR.NRR-D-25-00439)
Supplement: Supplementary file 14 [file NRR-21-3426_Suppl21.pdf]

**Additional Table 7.10. Filtered TF/TlatMF.mitoRP interaction reports & related info**

| TF | TF taxon | Numbers of TlatMF/mitoRP interaction reports, real-vs-expected: Chi-square test, p evaluated over all 11 groups (3 SplFs, 2 pAFs, 6 TlatMFs) | Chi-square test, p evaluated over 6 TlatMF groups | Real / expected number ratio |
|---|---|---|---|---|
| BHLHA15 | bHLH | 1.28E-07 | 5.38E-09 | 3.3 |
| MYCN | bHLH | 5.87E-28 | 5.28E-25 | 3.0 |
| MAFB | bZIP | 2.33E-24 | 2.44E-27 | 6.2 |
| YBX1 | DBP | 1.90E-67 | 1.31E-68 | 5.5 |
| YBX2 | DBP | 2.15E-70 | 2.17E-73 | 8.8 |
| FOXB1 | FH/WH.FOX | 1.25E-06 | 2.57E-07 | 4.5 |
| FOXL2 | FH/WH.FOX | 2.54E-02 | 6.65E-03 | 3.8 |
| TLX2 | HB.NK | 8.75E-26 | 2.64E-26 | 7.8 |
| TLX3 | HB.NK | 1.27E-04 | 9.09E-04 | 2.3 |
| SOX15 | HMG.SOX | 2.14E-27 | 2.63E-29 | 8.1 |
| TCF7L2 | HMG.TCF7 | 1.72E-09 | 2.23E-08 | 3.3 |
| TFAM | HMG.TFAM | 5.77E-64 | 5.33E-62 | 6.5 |
| TFB1M | others | 1.24E-02 | 3.06E-03 | 3.6 |
| THAP3 | ZF.C2CH | 1.70E-05 | 5.62E-07 | 5.2 |
| E4F1 | ZF.C2H2 | 1.74E-55 | 5.51E-58 | 10.3 |
| GLI4 | ZF.C2H2 | 1.88E-37 | 7.18E-39 | 9.9 |
| KLF12 | ZF.C2H2 | 6.03E-119 | 6.93E-122 | 12.9 |
| KLF15 | ZF.C2H2 | 1.29E-53 | 9.04E-55 | 7.8 |
| KLF16 | ZF.C2H2 | 1.07E-04 | 2.12E-04 | 2.2 |
| KLF4 | ZF.C2H2 | 1.89E-30 | 3.58E-27 | 6.2 |
| ZBTB47 | ZF.C2H2 | 7.48E-03 | 1.07E-02 | 3.0 |
| ZBTB48 | ZF.C2H2 | 2.13E-33 | 1.27E-35 | 7.2 |
| ZKSCAN8 | ZF.C2H2 | 1.85E-07 | 1.41E-06 | 2.6 |
| ZNF133 | ZF.C2H2 | 3.24E-18 | 1.12E-20 | 13.3 |
| ZNF169 | ZF.C2H2 | 1.18E-04 | 3.30E-05 | 5.1 |
| ZNF17 | ZF.C2H2 | 1.38E-30 | 1.52E-33 | 16.7 |
| ZNF2 | ZF.C2H2 | 2.94E-03 | 1.28E-04 | 4.1 |
| ZNF274 | ZF.C2H2 | 3.02E-03 | 3.18E-04 | 7.3 |
| ZNF317 | ZF.C2H2 | 1.30E-06 | 3.52E-08 | 2.0 |
| ZNF324B | ZF.C2H2 | 2.88E-10 | 6.72E-11 | 5.1 |
| ZNF331 | ZF.C2H2 | 5.35E-35 | 3.54E-37 | 10.7 |
| ZNF460 | ZF.C2H2 | 1.05E-14 | 2.69E-15 | 5.9 |
| ZNF467 | ZF.C2H2 | 1.37E-04 | 7.32E-04 | 3.1 |
| ZNF485 | ZF.C2H2 | 2.18E-37 | 2.24E-40 | 19.4 |
| ZNF556 | ZF.C2H2 | 2.60E-02 | 2.10E-03 | 7.8 |
| ZNF574 | ZF.C2H2 | 5.09E-62 | 1.66E-62 | 7.4 |
| ZNF689 | ZF.C2H2 | 1.38E-04 | 5.08E-05 | 2.2 |
| ZNF707 | ZF.C2H2 | 2.00E-75 | 5.26E-77 | 12.6 |
| ZNF770 | ZF.C2H2 | 2.58E-40 | 1.84E-42 | 2.6 |
| LIN28A | ZF.CCHC | 1.78E-45 | 2.56E-45 | 5.6 |
| LIN28B | ZF.CCHC | 3.26E-02 | 5.56E-03 | 2.6 |
| NFX1 | ZF.NFX1 | 9.30E-61 | 6.69E-57 | 2.6 |

n° of filtered TF/TllatMF.mitoRP interaction reports

| TF | MRPL1 | MRPL2 | MRPL3 | MRPL4 | MRPL9 | MRPL10 | MRPL11 | MRPL12 | MRPL13 | MRPL14 | MRPL15 | MRPL16 | MRPL17 | MRPL18 | MRPL19 | MRPL20 | MRPL21 | MRPL22 | MRPL23 | MRPL24 | MRPL27 | MRPL28 | MRPL30 | MRPL32 | MRPL33 | MRPL34 | MRPL35 | MRPL36 |
|---|---|---|---|---|---|---|---|---|---|---|---|---|---|---|---|---|---|---|---|---|---|---|---|---|---|---|---|---|
| BHLHA15 | | | | | | | | | | | | | | | | | | | | | | | | | | | | |
| MYCN | 1 | 1 | 1 | 1 | 1 | 1 | 1 | 1 | 1 | 1 | 1 | 1 | 1 | 1 | 1 | 1 | 1 | 1 | 1 | 1 | 1 | 1 | 1 | 1 | 1 | 1 | 1 | 1 |
| MAFB | | | | | | | 1 | | | | 1 | | | | | | | | | | | | | | | | | |
| YBX1 | 2 | 1 | 2 | 1 | 1 | 1 | 1 | 2 | 1 | | 1 | 2 | 2 | 3 | 2 | 2 | 2 | 1 | 1 | 2 | 2 | 1 | 2 | 1 | 1 | | | |
| YBX2 | 1 | 1 | 1 | 1 | 1 | 1 | 1 | | 1 | | 1 | 1 | 1 | 1 | 1 | 1 | 1 | 1 | 1 | 1 | 1 | 1 | 1 | 1 | | | | |
| FOXB1 | | | 1 | | | | | | | | | | | 1 | | 1 | | | | 1 | 1 | | | | | | | |
| FOXL2 | | | 1 | | | | | | | | | | | | | | 1 | | | 1 | | | | | | | | |
| TLX2 | | | | | | | | | | | | | | | | | | | | | | | | | | | | |
| TLX3 | | | | | | | 1 | | | | | | | | | | | | | | | | | | | | | |
| SOX15 | | | | | | | 1 | | | | | | | | | | | | | | | | | | | | | |
| TCF7L2 | | | | | | | | | | | | | | | | | | | | | | | | | | | | |
| TFAM | 1 | | | 1 | | 1 | | 1 | | | | | | | 1 | 1 | 1 | 1 | 1 | 1 | | 1 | | | | | | |
| TFB1M | | 1 | | | | | | 2 | | | | | | | | | | | | | | | | | | | | |
| THAP3 | | | | | 1 | | | | | | | | | | | | | | | | 1 | 1 | | | | | | |
| E4F1 | | | | | | | | | | | | | | | | | | | | | | | | | | | | |
| GLI4 | 1 | | | | 1 | | | | | | | | | | | | | | | | | | | | | | | |
| KLF12 | | 1 | | | | | 2 | | | | | | | | | | | | | | | | | | | | | |
| KLF15 | 1 | | | 1 | | 1 | | 1 | 1 | | 1 | 1 | 1 | | | 1 | 1 | 1 | | 1 | | 1 | | | | 1 | | |
| KLF16 | | | | 1 | | | | 1 | | | | | | | | | | | | | | | | | | | | |
| KLF4 | | | | 1 | | | | | | | 1 | | | | | | | | | | | | | | | | | |
| ZBTB47 | | | | | | | | | | | | | | | | | | | | | | | | | | | | |
| ZBTB48 | | | | | | | | | | | | | | | | | | | | | | | | | | | | |
| ZKSCAN8 | | | | | | | | | | | | | | | | | | | | | | | | | | | | |
| ZNF133 | | | | | | | | | | | | | | | | | | | | | | | | | | | | |
| ZNF169 | | | | | | | | | | | | | | | | | | | | | | | | | | | | |
| ZNF17 | | | | | | | | | | | | | | | | | | | | | | | | | | | | |
| ZNF2 | | | | | | | | | | | | | | | | | | | | | | | | | | | | |
| ZNF274 | | | | | | | | | | | | | | | | | | | | | 2 | | 1 | | | | | |
| ZNF317 | | | | | | | | | | | | | | | | | | | | | | | 1 | | | | | |
| ZNF324B | | | | | | | | | 1 | | | | 1 | | | | | | | | | | | | | | | |
| ZNF331 | | | | | | | | | | | | | | | | | | | | | | 2 | | | | | | |
| ZNF460 | | | | | | | | | | | | | | | | | | | | | | | | | | | | |
| ZNF467 | | | | | | | | | | | | | | | | | | | | 1 | | 1 | | 1 | | | | |
| ZNF485 | | | | | 1 | | | | | | 1 | | | | | | | | | | | | 1 | 1 | | | | |
| ZNF556 | | | | | | | | | | | | | | | | | | | | | | | | | | | | |
| ZNF574 | | | | | | | | | 1 | | | | 1 | 1 | | | | | 1 | 1 | | 1 | | 1 | | | 1 | |
| ZNF689 | | | | | | | | | | | | | | | | | | | | | | | | | | | | |
| ZNF707 | | | | | | | | | | | | | | | | | | | | | | | | | | | | |
| ZNF770 | | | | | | | | | | | | | | | | | | | | | | | | | | | | |
| LIN28A | 1 | 1 | 1 | 1 | 1 | 1 | 1 | | 1 | | 1 | 1 | 1 | | | 1 | 1 | 1 | | 1 | 1 | 1 | 1 | 1 | | | | |
| LIN28B | | | | | | | | | | | | | | | | | | | | | | 1 | 1 | 1 | | | | |
| NFX1 | 1 | 1 | 1 | 1 | | | 1 | 1 | 1 | 1 | | 1 | | 1 | | 1 | 1 | | 1 | | 1 | | 1 | | 1 | | | |

| TF | MRPL37 | MRPL38 | MRPL39 | MRPL40 | MRPL41 | MRPL42 | MRPL43 | MRPL44 | MRPL45 | MRPL46 | MRPL47 | MRPL48 | MRPL49 | MRPL50 | MRPL51 | MRPL52 | MRPL53 | MRPL54 | MRPL55 | MRPL58 | MRPL57 | GADD45GIP1 | MRPS30 | MRPS18A |
|---|---|---|---|---|---|---|---|---|---|---|---|---|---|---|---|---|---|---|---|---|---|---|---|---|
| BHLHA15 | | | | | | | | | | | | | | | | | | | | | | | | |
| MYCN | | 1 | 1 | 1 | 1 | 1 | 1 | 1 | 1 | 1 | 1 | 1 | 1 | 1 | 1 | 1 | 1 | 1 | 1 | | 1 | 1 | | 1 |
| MAFB | | | 1 | | 1 | | | | | 1 | 1 | | | | | | | | 1 | | | | | |
| YBX1 | 1 | 1 | 1 | 1 | 1 | 2 | 1 | 1 | 1 | | 2 | | 1 | 1 | 1 | 1 | | | | | 1 | 2 | 1 | 1 |
| YBX2 | 1 | 1 | 1 | 1 | | 1 | 1 | 1 | 1 | | 1 | | 1 | 1 | 1 | | | | 1 | | 1 | 1 | 1 | 1 |
| FOXB1 | | 1 | | | | | | | 1 | | | | | 1 | 1 | | | | | | | 1 | 1 | |
| FOXL2 | | | | | | | | | | | | | | | | | | | | | | 1 | | 1 |
| TLX2 | | | | | | | | | | | | | | | | | | | | | | | | |
| TLX3 | | | | | | | | | | | | | | | | | | | | | | | | |
| SOX15 | | | | | | | | | | | | | | | | | | | | | | | | |
| TCF7L2 | | | | | | | | | | | | | | | | | | | | | | | | |
| TFAM | 1 | | | 1 | 1 | 1 | 1 | 1 | 1 | 1 | 1 | 1 | | 1 | | | 1 | | 1 | | | | | |
| TFB1M | | | | | | | | | | | | | | | | | | | | | | | | |
| THAP3 | | | | | | | | | | | | | | | | | | | | | | | | |
| E4F1 | | | | | | | | | | | | | | | | | | | | | | | | |
| GLI4 | | | | | | | | | 1 | | | | | 1 | | 1 | | | | | 1 | | 1 | |
| KLF12 | 1 | | | | | | | | | | 1 | | 1 | 1 | | | | | | | | | | |
| KLF15 | 1 | 1 | | | 1 | | 1 | 1 | | | 1 | | | | | | | | | | | 1 | | 1 |
| KLF16 | | | | | | | | | | | | | | | | | | | | | | | | |
| KLF4 | | | 1 | | | | | | | | | | | | | | | | | | | | | |
| ZBTB47 | | | | | | | | | | | 1 | | | | 1 | | | | | | | | | |
| ZBTB48 | | | | | | | | | | | | | | | | | | | | | | | | |
| ZKSCAN8 | | | | | | | | | | | | | | | | | | | | | | | | |
| ZNF133 | | | | | | | | | | | | | | | | | | | | | | | | |
| ZNF169 | | | | | | | | | | | | | | | | | | | | | | | | |
| ZNF17 | | | | | | | | | | | | | | | | | | | | | | | | |
| ZNF2 | | | | | | | | | | | | | | | | 2 | | | | | | | | |
| ZNF274 | | | | | | | | | | | | | | | | | | | | | | | | |
| ZNF317 | | | | | | | | | | | | | | | | | | | | | | | | 2 |
| ZNF324B | | | | | | | | | 1 | | 2 | | | | | | | | | | | | | |
| ZNF331 | | | | | | | | | 1 | | 1 | | | | | | | | | | | | | |
| ZNF460 | | | | | | | | | | | | | | | | | | | | | | | | |
| ZNF467 | | | | | | | | | | | | | | | | | | | | | | | | |
| ZNF485 | | | | | | | | 1 | | | 1 | | | | | | | | | | | 1 | | |
| ZNF556 | | | | | | | | | | | | | | | | | | | | | | | | |
| ZNF574 | | | | | | 1 | | | | | | 1 | | | | 1 | | | | | 1 | | | |
| ZNF689 | | | | | | | | | | | | | | | | | | | | | | | | |
| ZNF707 | | | | | | | | | | | | | | | | | | | | | | | | |
| ZNF770 | | | | | | | | | | | | | | | | | | | | | | | | |
| LIN28A | | 1 | | 1 | | 1 | | | | 1 | 1 | 1 | | 1 | | | | | 1 | | 1 | 2 | 1 | 1 |
| LIN28B | | | | | | | | | 1 | | | | | | | | | | | | 1 | | | |
| NFX1 | | | | | 1 | | | | | 1 | 1 | 1 | | | | | | | | | | | | 1 |

| TF | MRPS2 | MRPS5 | MRPS6 | MRPS7 | MRPS9 | MRPS10 | MRPS11 | MRPS12 | MRPS14 | MRPS15 | MRPS16 | MRPS17 | MRPS18C | MRPS21 | MRPS22 | MRPS23 | MRPS24 | MRPS25 | MRPS26 | MRPS27 | MRPS28 | DAP3 | MRPS31 | MRPS33 | MRPS34 | MRPS35 | CHCHD1 | AURKAIP1 | PTCD3 | MRPS18B |
|---|---|---|---|---|---|---|---|---|---|---|---|---|---|---|---|---|---|---|---|---|---|---|---|---|---|---|---|---|---|---|
| BHLHA15 | | | | | 2 | | | | | | | | | | | | | | | | | 2 | 1 | | | | | | | 1 |
| MYCN | 1 | 1 | 1 | 1 | 1 | 1 | 1 | 1 | 1 | 1 | 1 | 1 | 1 | 1 | 1 | 1 | 1 | 1 | 1 | 1 | 1 | 1 | 1 | 1 | 1 | 1 | | 1 | 1 | 1 |
| MAFB | 1 | 1 | 1 | 1 | 1 | 1 | 1 | | | 1 | | 1 | | 1 | 1 | 1 | 1 | 1 | 1 | 1 | 1 | 1 | 1 | | 1 | 1 | | | 1 | 1 |
| YBX1 | 1 | 1 | | 2 | 1 | 1 | 1 | | | | | 1 | 1 | 1 | 3 | | 1 | 1 | 1 | 1 | 1 | 1 | 1 | 1 | 2 | 1 | | | 2 | 2 |
| YBX2 | 1 | | | 1 | 1 | | 1 | | | | | | | | 1 | | | | 1 | | | | 1 | | | 1 | | | 1 | 1 |
| FOXB1 | | | | | | | | | | | | | | | | | | | | | | | | | | | | | | |
| FOXL2 | | | | | | | | | | | | | | | | | | | | | | | | | | | | | | |
| TLX2 | | 2 | | 1 | 1 | 1 | | 1 | | | 1 | | | | 1 | 2 | | | 1 | 1 | 1 | 2 | 1 | | 2 | 1 | | | 1 | 1 |
| TLX3 | | 1 | | | 1 | | | 1 | | | | | | | 1 | 1 | | | 1 | 1 | | 1 | | | | 1 | | | | 1 |
| SOX15 | | 1 | | 1 | 1 | | | | | | 1 | 1 | | | 2 | 2 | | | | 2 | | 2 | 2 | | 2 | | | | 2 | 2 |
| TCF7L2 | 1 | 1 | 1 | 1 | 1 | 1 | 1 | | | 1 | 1 | | 1 | 1 | 1 | | | | | 1 | | 1 | 1 | 1 | 1 | 1 | | | 1 | |
| TFAM | 1 | 1 | 1 | 2 | 1 | 2 | 1 | | 1 | | 1 | 1 | 1 | 1 | 1 | 1 | 1 | 1 | 1 | 1 | 1 | 1 | 1 | 1 | 1 | 1 | | 1 | 1 | 1 |
| TFB1M | | | | | | | | | | | | 1 | | | | 1 | | 1 | | | | | | | | | | | | |
| THAP3 | | | | 1 | | | | | | | | | | | | | 1 | | 1 | | | | | | | | | | | |
| E4F1 | 2 | 2 | | 2 | 2 | 1 | 2 | | | | | | 2 | | 2 | 2 | 2 | 1 | | | | 2 | 2 | 2 | | 2 | | | 2 | 1 |
| GLI4 | 1 | 1 | | 1 | 1 | 1 | 1 | | | | | | | | 1 | | 1 | | 1 | | 1 | 1 | 1 | 1 | 1 | 1 | | | | 1 |
| KLF12 | 2 | 2 | 1 | 2 | 2 | 1 | 2 | | 2 | 2 | 2 | 1 | 1 | | 2 | 2 | 2 | 2 | 2 | 2 | 1 | 2 | 2 | 1 | 2 | 2 | | | 2 | 1 |
| KLF15 | 1 | 2 | 1 | 1 | 1 | | | | 1 | 1 | 1 | | | 1 | 1 | 1 | | 1 | 1 | 1 | 1 | 1 | 1 | | 1 | 1 | | | 1 | 1 |
| KLF16 | 1 | 2 | | 1 | 1 | | | | | | | | | | 1 | 1 | | | 2 | 1 | | 1 | 1 | | 1 | 1 | | | 1 | 1 |
| KLF4 | 1 | 1 | | 1 | 1 | | 1 | | 1 | 1 | 1 | 1 | | | 1 | 1 | 1 | 1 | 1 | 1 | 1 | 1 | 1 | | 1 | 1 | | | 1 | 1 |
| ZBTB47 | 1 | 1 | | 1 | | | | | | | | | | | | 1 | | | 1 | | | 1 | | | | | | | | |
| ZBTB48 | 2 | 2 | | 2 | 2 | 2 | 2 | | | | | | 2 | | | | 2 | 1 | 1 | | | 2 | 2 | 2 | 2 | 2 | | | 2 | 1 |
| ZKSCAN8 | | 1 | | 1 | 1 | | 1 | | | 1 | | | 1 | | | | 1 | | | | | | | | | 1 | | | | |
| ZNF133 | 2 | | | 2 | | | | | | | | | | | | 1 | 1 | | | | | 2 | | | | | | | | 1 |
| ZNF169 | | | | | 2 | | 2 | | | | | | | | | | | | | | | | | 2 | | 2 | | | | |
| ZNF17 | | | | 1 | | 1 | | | | | | | 1 | | 1 | | 1 | | 1 | | | 1 | 1 | | | 1 | | | 1 | 1 |
| ZNF2 | | | | 2 | 2 | | | | | | | | | | | | | | | | | | | | | 2 | | | 1 | |
| ZNF274 | | | | | | | | | 1 | | | | | | | | | | | | | | | | | | | | | |
| ZNF317 | | | | | | | | | | | | | | | | | | | | | | | | | | | | | | |
| ZNF324B | | 1 | | | | | 1 | | | | | | | | | 1 | 2 | | 1 | | | 1 | 1 | | | 1 | | | | |
| ZNF331 | | 1 | | 2 | 1 | | 2 | | | | | | | | | 1 | 2 | | | | | 2 | 2 | | | 2 | | | 1 | |
| ZNF460 | 1 | 1 | | 1 | 1 | 1 | 1 | | | 1 | | | 1 | 1 | | | 1 | 1 | | | | 1 | 1 | 1 | | 1 | | | 1 | 1 |
| ZNF467 | | 1 | | 1 | 1 | | | | | | | | 1 | 1 | | | | | | | | | 1 | | | 1 | | | | |
| ZNF485 | | 1 | | 1 | 1 | | | | | | | | | | | | | | | | | 1 | | | | | | | | |
| ZNF556 | | 1 | | | | | | | | | | | | | | | 2 | | | | | | | | | | | | | |
| ZNF574 | 1 | 1 | | 1 | 2 | 1 | | | | 1 | 1 | 1 | 1 | | | 1 | 1 | 1 | 1 | 1 | | 1 | 1 | 1 | 2 | 1 | | | 1 | |
| ZNF689 | | 1 | | | 1 | | | | | | | | | | | | | | | | | | 1 | | | | | | 1 | |
| ZNF707 | 1 | 1 | | 3 | 3 | 3 | 1 | | | | | | 3 | | 1 | 1 | 2 | 1 | 3 | 1 | | 2 | 3 | 1 | 1 | 1 | | | 2 | |
| ZNF770 | | | | | | | | | | | | 1 | | | | | | 1 | | | | | | | 2 | | | | | 1 |
| LIN28A | | | | 1 | 1 | 1 | | | 1 | 1 | 1 | | 1 | | | | 1 | 1 | 1 | 1 | | 1 | 1 | | 1 | 1 | | | 1 | 1 |
| LIN28B | | | | 1 | 1 | | 1 | | | | | | 1 | | | | | | 1 | | | | | 1 | | 1 | | | | |
| NFX1 | | | | 1 | | | | | | | | | | | | | | | | 1 | | | 1 | | | | | | | |

| TF | Subcellular TF location (see Additioinal Table 2) | RBP function reported for… (see Additioinal Table 3) | pTriplo (see Additioinal Table 4) | Neuronal activity-plasticity responsive (see Additioinal Table 5) |
|---|---|---|---|---|
| BHLHA15 | Nucleoplasm,Golgi apparatus | | 0.98 | |
| MYCN | Nucleoplasm,Nucleoli | only mmu ortholog | 0.77 | |
| MAFB | Nucleoplasm,Nucleoli,Golgi apparatus,Cytosol | | 1.00 | |
| YBX1 | Endoplasmic reticulum,Vesicles,Plasma membrane,Cytosol | hsa gene | 0.92 | |
| YBX2 | none reported | hsa gene | 0.83 | |
| FOXB1 | Nucleoplasm,Mitochondria | | 0.94 | |
| FOXL2 | Nucleoplasm | hsa gene | 0.55 | |
| TLX2 | Nucleoplasm | | 1.00 | |
| TLX3 | Nucleoplasm | | 0.91 | |
| SOX15 | Nucleoplasm,Nucleoli,Vesicles | | 0.26 | |
| TCF7L2 | Nucleoplasm,Nuclear bodies,Cytosol | | 0.97 | 1 |
| TFAM | Mitochondria | hsa gene | 0.40 | 1 |
| TFB1M | none reported | hsa gene | 0.48 | |
| THAP3 | Nucleoplasm,Nucleoli,Mitochondria | | 0.56 | 1 |
| E4F1 | Nucleoplasm | hsa gene | 0.45 | |
| GLI4 | Nucleoplasm,Cytokinetic bridge | hsa gene | 0.22 | |
| KLF12 | Nucleoplasm,Cytosol | hsa gene | 0.71 | |
| KLF15 | Nucleoplasm,Nuclear speckles,Vesicles | | 0.83 | |
| KLF16 | Nucleoplasm | | 0.23 | |
| KLF4 | Nucleoplasm,Cytosol | mmu gene | 0.59 | 1 |
| ZBTB47 | Nucleoplasm | hsa gene | 0.91 | |
| ZBTB48 | Nucleoplasm,Cytosol | only mmu ortholog | 0.50 | |
| ZKSCAN8 | Nucleoplasm,Cytosol | hsa gene | 0.61 | |
| ZNF133 | Nucleoplasm,Vesicles | hsa gene | 0.22 | |
| ZNF169 | Nucleoplasm,Nuclear speckles,Vesicles,Cytosol | | 0.39 | |
| ZNF17 | none reported | | 0.11 | |
| ZNF2 | Nucleoplasm,Plasma membrane,Cytosol | hsa gene | 0.24 | |
| ZNF274 | Nucleoplasm,Centrosome | hsa gene | 0.03 | |
| ZNF317 | Nucleoplasm | hsa gene | 0.22 | |
| ZNF324B | Nucleoli,Cytosol | | 0.13 | |
| ZNF331 | Nucleoplasm | hsa gene | 0.37 | |
| ZNF460 | Nucleoplasm | | 0.21 | |
| ZNF467 | Mitochondria | hsa gene | 0.14 | |
| ZNF485 | none reported | hsa gene | 0.29 | |
| ZNF556 | Nucleoplasm | | 0.04 | |
| ZNF574 | Nucleoplasm,Vesicles | hsa gene | 0.57 | |
| ZNF689 | Nucleoplasm,Nucleoli fibrillar center | | 0.82 | |
| ZNF707 | Nucleoplasm | hsa gene | 0.40 | |
| ZNF770 | none reported | hsa gene | 0.56 | |
| LIN28A | Nucleoli,Cytosol,Rods & Rings | hsa gene | 0.44 | |
| LIN28B | Nucleoplasm,Nucleoli,Cytosol | hsa gene | 0.70 | |
| NFX1 | Nucleoplasm,Nucleoli,Plasma membrane,Cytosol | hsa gene | 0.92 | |
