# Supplementary material for: Foxg1 and companions: Not only transcription factors
Source: Neural Regen Res. 2025 Sep 3;21(8):3426–38. doi: 10.4103/NRR.NRR-D-25-00439 (PMC13452659; doi:10.4103/NRR.NRR-D-25-00439)
Supplement: Supplementary file 15 [file NRR-21-3426_Suppl22.pdf]

Additioinal Table 7.11. Filtered TF/TlatMF.aatRNAsynth interaction reports & related info

		Numbers of TlatMF/aatRNAsynth interaction reports, real-vs-expected			n° of filtered TF/TllatMF.aatRNAsynth interaction reports																																											
TF	TF taxon	Chi-square test, p evaluated over all 11 groups (3 SplFs, 2 pAFs, 6 TlatMFs)	Chi-square test, p evaluated over 6 TlatMF groups	Real / expected number ratio	AARS1	RARS1	RARS2	NARS1	NARS2	DARS1	DARS2	CARS1	CARS2	QRSL1	QARS1	EPRS1	GATB	GATC	EARS2	GARS1	HARS1	HARS2	IARS1	IARS2	LARS1	LARS2	KARS1	MARS1	MARS2	FARS2	FARSA	FARSB	PARS2	SARS1	SARS2	TARS1	TARS2	TARS3	WARS2	WARS1	YARS1	YARS2	VARS1	VARS1	VARS2	Subcellular TF location (see Additioinal Table 2)	RBP function reported for… (see Additioinal Table 3)	pTriplo (see Additioinal Table 4)	Neuronal activity-plasticity responsive (see Additioinal Table 5)
YBX2	DBP	2.15E-70	2.17E-73	2.8																										1												1				none reported	hsa gene	0.83	
FOXB1	FH/WH.FOX	1.25E-06	2.57E-07	3.2							1																																			Nucleoplasm,Mitochondria		0.94	
FOXI2	FH/WH.FOX	5.82E-06	2.32E-03	8.1							1																																			none reported		0.20	
FOXL2	FH/WH.FOX	2.54E-02	6.65E-03	5.9							1																																			Nucleoplasm	hsa gene	0.55	
FOXM1	FH/WH.FOX	3.79E-02	1.95E-02	2.1																													1													Nucleoplasm,Nucleoli,Nucleoli rim,Cytosol		0.09	
LMX1B	HB.LIM	1.76E-09	2.41E-10	18.4																													3													Nucleoplasm		0.97	
TFAM	HMG.TFAM	5.77E-64	5.33E-62	6.6									1	1			1		1			1		1																					1	Mitochondria	hsa gene	0.40	1
HSFX1	HSF	1.03E-10	1.01E-12	33.8																				2																						Nucleoplasm,Plasma membrane,Cytosol		NA	
GPBP1L1	others	4.35E-14	1.04E-14	6.3										2																																Nucleoplasm,Plasma membrane,Focal adhesion sites,Cytosol	hsa gene	0.11	
PHB	others	2.69E-06	7.97E-07	2.1							1																			1	1	1					1								1	gene/protein not avaialble in HPA list	only mmu ortholog	0.99	
NFATC1	RHR	1.34E-04	1.70E-02	2.1																															1											Nucleoplasm,Nuclear bodies		0.51	
STAT2	STAT	9.08E-04	3.94E-03	5.4																				2																						Plasma membrane,Cytosol		0.91	
GLI4	ZF.C2H2	1.88E-37	7.18E-39	3.3																										1																Nucleoplasm,Cytokinetic bridge	hsa gene	0.22	
ZKSCAN8	ZF.C2H2	1.85E-07	1.41E-06	2.5																										1																Nucleoplasm,Cytosol	hsa gene	0.61	
ZNF324B	ZF.C2H2	2.88E-10	6.72E-11	5.7																																						2				Nucleoli,Cytosol		0.13	
ZNF444	ZF.C2H2	3.41E-02	4.86E-02	2.7										1																																Nucleoplasm,Nucleoli	hsa gene	0.72	
ZNF500	ZF.C2H2	7.38E-06	9.89E-08	42.2																										1																Nucleoplasm,Cytosol		0.49	
