# Supplementary material for: Foxg1 and companions: Not only transcription factors
Source: Neural Regen Res. 2025 Sep 3;21(8):3426–38. doi: 10.4103/NRR.NRR-D-25-00439 (PMC13452659; doi:10.4103/NRR.NRR-D-25-00439)
Supplement: Supplementary file 16 [file NRR-21-3426_Suppl3.pdf]

Additional Table 1.3. hsa-SplF genes list (*n* = 304)

[homo sapiens Splicing Factor genes, i.e. genes encoding for:
- Splicing Factors, core (U1snRNP, SM proteins, U2 snRNP proteins and related ones, A complex, LSm proteins, U5 SNP, U4/U6 SNP, PPR19 complex, B complex, Bact complex, RES complex, C complex)
- Splicing Factors, RBPs (EJC/mRNP, TREX, helicases, hnRNP, SR proteins, RNA binding proteins, RNA editing, RNA modifying, RNA methylation, CBC, 3´end, RNA degradation, RISC)
- Splicing Factors, others (protein kinase, Other SAPs, Minor, Lysine transferases , chromatin-related, Histone transferases, SWI/SNF Complex Components, Polycomb Group Genes)]

NOTES

[extracted from: Rogalska et al., Science 386, 551–560 (2024). doi: 10.1126/science.adn8105. Table S1; manually adapted]

Gene.name.VT	*Gene.symbol*	*Class…family*	*Our super-grouping*	*Gene.description*
SNRNP70	SNRNP70	U1 snRNP	SplFs.core	small nuclear ribonucleoprotein U1 subunit 70 [Source:HGNC Symbol;Acc:HGNC:11150]
SNRPA	SNRPA	U1 snRNP	SplFs.core	small nuclear ribonucleoprotein polypeptide A [Source:HGNC Symbol;Acc:HGNC:11151]
SNRPC	SNRPC	U1 snRNP	SplFs.core	small nuclear ribonucleoprotein polypeptide C [Source:HGNC Symbol;Acc:HGNC:11157]
SNRPD1	SNRPD1	SM proteins	SplFs.core	small nuclear ribonucleoprotein D1 polypeptide [Source:HGNC Symbol;Acc:HGNC:11158]
SNRPD2	SNRPD2	SM proteins	SplFs.core	small nuclear ribonucleoprotein D2 polypeptide [Source:HGNC Symbol;Acc:HGNC:11159]
SNRPD3	SNRPD3	SM proteins	SplFs.core	small nuclear ribonucleoprotein D3 polypeptide [Source:HGNC Symbol;Acc:HGNC:11160]
SNRPE	SNRPE	SM proteins	SplFs.core	small nuclear ribonucleoprotein polypeptide E [Source:HGNC Symbol;Acc:HGNC:11161]
SNRPF	SNRPF	SM proteins	SplFs.core	small nuclear ribonucleoprotein polypeptide F [Source:HGNC Symbol;Acc:HGNC:11162]
SNRPG	SNRPG	SM proteins	SplFs.core	small nuclear ribonucleoprotein polypeptide G [Source:HGNC Symbol;Acc:HGNC:11163]
SNRPB	SNRPB	SM proteins	SplFs.core	small nuclear ribonucleoprotein polypeptides B and B1 [Source:HGNC Symbol;Acc:HGNC:11153]
SNRPA1	SNRPA1	U2 snRNP	SplFs.core	small nuclear ribonucleoprotein polypeptide A' [Source:HGNC Symbol;Acc:HGNC:11152]
SNRPB2	SNRPB2	U2 snRNP	SplFs.core	small nuclear ribonucleoprotein polypeptide B2 [Source:HGNC Symbol;Acc:HGNC:11155]
SF3B6	AC008073.5	U2 snRNP	SplFs.core	splicing factor 3b subunit 6 [Source:HGNC Symbol;Acc:HGNC:30096]
SF3A1	SF3A1	U2 snRNP	SplFs.core	splicing factor 3a subunit 1 [Source:HGNC Symbol;Acc:HGNC:10765]
SF3A2	SF3A2	U2 snRNP	SplFs.core	splicing factor 3a subunit 2 [Source:HGNC Symbol;Acc:HGNC:10766]
SF3A3	SF3A3	U2 snRNP	SplFs.core	splicing factor 3a subunit 3 [Source:HGNC Symbol;Acc:HGNC:10767]
SF3B1	SF3B1	U2 snRNP	SplFs.core	splicing factor 3b subunit 1 [Source:HGNC Symbol;Acc:HGNC:10768]
SF3B2	SF3B2	U2 snRNP	SplFs.core	splicing factor 3b subunit 2 [Source:HGNC Symbol;Acc:HGNC:10769]
SF3B3	SF3B3	U2 snRNP	SplFs.core	splicing factor 3b subunit 3 [Source:HGNC Symbol;Acc:HGNC:10770]
SF3B4	SF3B4	U2 snRNP	SplFs.core	splicing factor 3b subunit 4 [Source:HGNC Symbol;Acc:HGNC:10771]
SMNDC1	SMNDC1	U2 snRNP related	SplFs.core	survival motor neuron domain containing 1 [Source:HGNC Symbol;Acc:HGNC:16900]
U2AF1	U2AF1	U2 snRNP related	SplFs.core	U2 small nuclear RNA auxiliary factor 1 [Source:HGNC Symbol;Acc:HGNC:12453]
U2AF2	U2AF2	U2 snRNP related	SplFs.core	U2 small nuclear RNA auxiliary factor 2 [Source:HGNC Symbol;Acc:HGNC:23156]
DDX46	DDX46	U2 snRNP related	SplFs.core	DEAD-box helicase 46 [Source:HGNC Symbol;Acc:HGNC:18681]
DHX15	DHX15	U2 snRNP related	SplFs.core	DEAH-box helicase 15 [Source:HGNC Symbol;Acc:HGNC:2738]
CHERP	CHERP	U2 snRNP related	SplFs.core	calcium homeostasis endoplasmic reticulum protein [Source:HGNC Symbol;Acc:HGNC:16930]
PUF60	PUF60	U2 snRNP related	SplFs.core	poly(U) binding splicing factor 60 [Source:HGNC Symbol;Acc:HGNC:17042]
RBM17	RBM17	U2 snRNP related	SplFs.core	RNA binding motif protein 17 [Source:HGNC Symbol;Acc:HGNC:16944]
U2SURP	SR140	U2 snRNP related	SplFs.core	U2 snRNP associated SURP domain containing [Source:HGNC Symbol;Acc:HGNC:30855]
HTATSF1	HTATSF1	U2 snRNP related	SplFs.core	HIV-1 Tat specific factor 1 [Source:HGNC Symbol;Acc:HGNC:5276]
CCAR1	CCAR1	A complex	SplFs.core	cell division cycle and apoptosis regulator 1 [Source:HGNC Symbol;Acc:HGNC:24236]
PRPF40A	PRPF40A	A complex	SplFs.core	pre-mRNA processing factor 40 homolog A [Source:HGNC Symbol;Acc:HGNC:16463]
SF1	SF1	A complex	SplFs.core	splicing factor 1 [Source:HGNC Symbol;Acc:HGNC:12950]
THRAP3	THRAP3	A complex	SplFs.core	thyroid hormone receptor associated protein 3 [Source:HGNC Symbol;Acc:HGNC:22964]
RBM10	RBM10	A complex	SplFs.core	RNA binding motif protein 10 [Source:HGNC Symbol;Acc:HGNC:9896]
RBM25	RBM25	A complex	SplFs.core	RNA binding motif protein 25 [Source:HGNC Symbol;Acc:HGNC:23244]
DDX5	DDX5	A complex	SplFs.core	DEAD-box helicase 5 [Source:HGNC Symbol;Acc:HGNC:2746]
RBM5	RBM5	A complex	SplFs.core	RNA binding motif protein 5 [Source:HGNC Symbol;Acc:HGNC:9902]

BUB3	BUB3	A complex	SplFs.core	BUB3, mitotic checkpoint protein [Source:HGNC Symbol;Acc:HGNC:1151]
LSM2	LSM2	LSm proteins	SplFs.core	LSM2 homolog, U6 small nuclear RNA and mRNA degradation associated [Source:HGNC Symbol;Acc:HGNC:13940]
LSM3	LSM3	LSm proteins	SplFs.core	LSM3 homolog, U6 small nuclear RNA and mRNA degradation associated [Source:HGNC Symbol;Acc:HGNC:17874]
LSM4	LSM4	LSm proteins	SplFs.core	LSM4 homolog, U6 small nuclear RNA and mRNA degradation associated [Source:HGNC Symbol;Acc:HGNC:17259]
LSM6	LSM6	LSm proteins	SplFs.core	LSM6 homolog, U6 small nuclear RNA and mRNA degradation associated [Source:HGNC Symbol;Acc:HGNC:17017]
LSM7	LSM7	LSm proteins	SplFs.core	LSM7 homolog, U6 small nuclear RNA and mRNA degradation associated [Source:HGNC Symbol;Acc:HGNC:20470]
CD2BP2	CD2BP2	U5 snRNP	SplFs.core	CD2 cytoplasmic tail binding protein 2 [Source:HGNC Symbol;Acc:HGNC:1656]
EFTUD2	EFTUD2	U5 snRNP	SplFs.core	elongation factor Tu GTP binding domain containing 2 [Source:HGNC Symbol;Acc:HGNC:30858]
PRPF6	PRPF6	U5 snRNP	SplFs.core	pre-mRNA processing factor 6 [Source:HGNC Symbol;Acc:HGNC:15860]
PRPF8	PRPF8	U5 snRNP	SplFs.core	pre-mRNA processing factor 8 [Source:HGNC Symbol;Acc:HGNC:17340]
SNRNP200	SNRNP200	U5 snRNP	SplFs.core	small nuclear ribonucleoprotein U5 subunit 200 [Source:HGNC Symbol;Acc:HGNC:30859]
SNRNP40	SNRNP40	U5 snRNP	SplFs.core	small nuclear ribonucleoprotein U5 subunit 40 [Source:HGNC Symbol;Acc:HGNC:30857]
TXNL4A	TXNL4A	U5 snRNP	SplFs.core	thioredoxin like 4A [Source:HGNC Symbol;Acc:HGNC:30551]
DDX23	DDX23	U5 snRNP	SplFs.core	DEAD-box helicase 23 [Source:HGNC Symbol;Acc:HGNC:17347]
SNU13	NHP2L1	U4/U6 snRNP	SplFs.core	small nuclear ribonucleoprotein 13 [Source:HGNC Symbol;Acc:HGNC:7819]
PRPF3	PRPF3	U4/U6 snRNP	SplFs.core	pre-mRNA processing factor 3 [Source:HGNC Symbol;Acc:HGNC:17348]
PRPF4	PRPF4	U4/U6 snRNP	SplFs.core	pre-mRNA processing factor 4 [Source:HGNC Symbol;Acc:HGNC:17349]
PRPF31	RP11	U4/U6 snRNP	SplFs.core	pre-mRNA processing factor 31 [Source:HGNC Symbol;Acc:HGNC:15446]
SART1	SART1	U4/U6.U5 snRNP	SplFs.core	SART1, U4/U6.U5 tri-snRNP-associated protein 1 [Source:HGNC Symbol;Acc:HGNC:10538]
SNRNP27	SNRNP27	U4/U6.U5 snRNP	SplFs.core	small nuclear ribonucleoprotein U4/U6.U5 subunit 27 [Source:HGNC Symbol;Acc:HGNC:30240]
USP39	USP39	U4/U6.U5 snRNP	SplFs.core	ubiquitin specific peptidase 39 [Source:HGNC Symbol;Acc:HGNC:20071]
BCAS2	BCAS2	PRP19 complex	SplFs.core	BCAS2, pre-mRNA processing factor [Source:HGNC Symbol;Acc:HGNC:975]
CDC5L	CDC5L	PRP19 complex	SplFs.core	cell division cycle 5 like [Source:HGNC Symbol;Acc:HGNC:1743]
CTNNBL1	CTNNBL1	PRP19 complex	SplFs.core	catenin beta like 1 [Source:HGNC Symbol;Acc:HGNC:15879]
PLRG1	PLRG1	PRP19 complex	SplFs.core	pleiotropic regulator 1 [Source:HGNC Symbol;Acc:HGNC:9089]
PQBP1	PQBP1	PRP19 complex	SplFs.core	polyglutamine binding protein 1 [Source:HGNC Symbol;Acc:HGNC:9330]
PRPF19	PRPF19	PRP19 complex	SplFs.core	pre-mRNA processing factor 19 [Source:HGNC Symbol;Acc:HGNC:17896]
WBP11	WBP11	PRP19 complex	SplFs.core	WW domain binding protein 11 [Source:HGNC Symbol;Acc:HGNC:16461]
AQR	AQR	PRP19 complex	SplFs.core	aquarius intron-binding spliceosomal factor [Source:HGNC Symbol;Acc:HGNC:29513]
BUD31	BUD31	PRP19 complex	SplFs.core	BUD31 homolog [Source:HGNC Symbol;Acc:HGNC:29629]
CRNKL1	CRNKL1	PRP19 complex	SplFs.core	crooked neck pre-mRNA splicing factor 1 [Source:HGNC Symbol;Acc:HGNC:15762]
ISY1	ISY1	PRP19 complex	SplFs.core	ISY1 splicing factor homolog [Source:HGNC Symbol;Acc:HGNC:29201]
PPIE	PPIE	PRP19 complex	SplFs.core	peptidylprolyl isomerase E [Source:HGNC Symbol;Acc:HGNC:9258]
RBM22	RBM22	PRP19 complex	SplFs.core	RNA binding motif protein 22 [Source:HGNC Symbol;Acc:HGNC:25503]
SNW1	SNW1	PRP19 complex	SplFs.core	SNW domain containing 1 [Source:HGNC Symbol;Acc:HGNC:16696]
YBX1	YBX1	PRP19 complex	SplFs.core	Y-box binding protein 1 [Source:HGNC Symbol;Acc:HGNC:8014]
CWC15	CWC15	PRP19 complex	SplFs.core	CWC15 spliceosome associated protein homolog [Source:HGNC Symbol;Acc:HGNC:26939]
HSPA8	HSPA8	PRP19 complex	SplFs.core	heat shock protein family A (Hsp70) member 8 [Source:HGNC Symbol;Acc:HGNC:5241]
HSPA1A	HSPA1A	PRP19 complex	SplFs.core	heat shock protein family A (Hsp70) member 1A [Source:HGNC Symbol;Acc:HGNC:5232]
XAB2	XAB2	PRP19 complex	SplFs.core	XPA binding protein 2 [Source:HGNC Symbol;Acc:HGNC:14089]
IK	IK	B complex	SplFs.core	IK cytokine [Source:HGNC Symbol;Acc:HGNC:5958]
MFAP1	MFAP1	B complex	SplFs.core	microfibril associated protein 1 [Source:HGNC Symbol;Acc:HGNC:7032]
PRPF4B	PRPF4B	B complex	SplFs.core	pre-mRNA processing factor 4B [Source:HGNC Symbol;Acc:HGNC:17346]
SMU1	SMU1	B complex	SplFs.core	SMU1, DNA replication regulator and spliceosomal factor [Source:HGNC Symbol;Acc:HGNC:18247]
WBP4	WBP4	B complex	SplFs.core	WW domain binding protein 4 [Source:HGNC Symbol;Acc:HGNC:12739]
TFIP11	TFIP11	B complex	SplFs.core	tuftelin interacting protein 11 [Source:HGNC Symbol;Acc:HGNC:17165]
CCDC12	CCDC12	Bact complex	SplFs.core	coiled-coil domain containing 12 [Source:HGNC Symbol;Acc:HGNC:28332]
CDC40	CDC40	Bact complex	SplFs.core	cell division cycle 40 [Source:HGNC Symbol;Acc:HGNC:17350]
CWC22	CWC22	Bact complex	SplFs.core	CWC22 spliceosome associated protein homolog [Source:HGNC Symbol;Acc:HGNC:29322]
CWC27	CWC27	Bact complex	SplFs.core	CWC27 spliceosome associated protein homolog [Source:HGNC Symbol;Acc:HGNC:10664]
PPIL3	PPIL3	Bact complex	SplFs.core	peptidylprolyl isomerase like 3 [Source:HGNC Symbol;Acc:HGNC:9262]

PPIL2	PPIL2	Bact complex	SplFs.core	peptidylprolyl isomerase like 2 [Source:HGNC Symbol;Acc:HGNC:9261]
DHX16	DHX16	Bact complex	SplFs.core	DEAH-box helicase 16 [Source:HGNC Symbol;Acc:HGNC:2739]
FUBP3	FUBP3	Bact complex	SplFs.core	far upstream element binding protein 3 [Source:HGNC Symbol;Acc:HGNC:4005]
ZNF830	ZNF830	Bact complex	SplFs.core	zinc finger protein 830 [Source:HGNC Symbol;Acc:HGNC:28291]
BUD13	BUD13	RES complex	SplFs.core	BUD13 homolog [Source:HGNC Symbol;Acc:HGNC:28199]
RBMX2	RBMX2	RES complex	SplFs.core	RNA binding motif protein X-linked 2 [Source:HGNC Symbol;Acc:HGNC:24282]
SNIP1	SNIP1	RES complex	SplFs.core	Smad nuclear interacting protein 1 [Source:HGNC Symbol;Acc:HGNC:30587]
CACTIN	C19orf29	C complex	SplFs.core	cactin, spliceosome C complex subunit [Source:HGNC Symbol;Acc:HGNC:29938]
C9orf78	C9orf78	C complex	SplFs.core	chromosome 9 open reading frame 78 [Source:HGNC Symbol;Acc:HGNC:24932]
CXorf56	CXorf56	C complex	SplFs.core	chromosome X open reading frame 56 [Source:HGNC Symbol;Acc:HGNC:26239]
LENG1	LENG1	C complex	SplFs.core	leukocyte receptor cluster member 1 [Source:HGNC Symbol;Acc:HGNC:15502]
SYF2	SYF2	C complex	SplFs.core	SYF2 pre-mRNA splicing factor [Source:HGNC Symbol;Acc:HGNC:19824]
DGCR14	DGCR14	C complex	SplFs.core	ess-2 splicing factor homolog [Source:HGNC Symbol;Acc:HGNC:16817]
FAM50A	FAM50A	C complex	SplFs.core	family with sequence similarity 50 member A [Source:HGNC Symbol;Acc:HGNC:18786]
PPWD1	PPWD1	C complex	SplFs.core	peptidylprolyl isomerase domain and WD repeat containing 1 [Source:HGNC Symbol;Acc:HGNC:28954]
PPIG	PPIG	C complex	SplFs.core	peptidylprolyl isomerase G [Source:HGNC Symbol;Acc:HGNC:14650]
DDX41	DDX41	C complex	SplFs.core	DEAD-box helicase 41 [Source:HGNC Symbol;Acc:HGNC:18674]
NOSIP	NOSIP	C complex	SplFs.core	nitric oxide synthase interacting protein [Source:HGNC Symbol;Acc:HGNC:17946]
DHX35	DHX35	C complex	SplFs.core	DEAH-box helicase 35 [Source:HGNC Symbol;Acc:HGNC:15861]
CDK10	CDK10	C complex	SplFs.core	cyclin dependent kinase 10 [Source:HGNC Symbol;Acc:HGNC:1770]
DHX38	DHX38	C complex	SplFs.core	DEAH-box helicase 38 [Source:HGNC Symbol;Acc:HGNC:17211]
FAM32A	FAM32A	C complex	SplFs.core	family with sequence similarity 32 member A [Source:HGNC Symbol;Acc:HGNC:24563]
FRG1	FRG1	C complex	SplFs.core	FSHD region gene 1 [Source:HGNC Symbol;Acc:HGNC:3954]
FAM50B	FAM50B	C complex	SplFs.core	family with sequence similarity 50 member B [Source:HGNC Symbol;Acc:HGNC:18789]
PRPF18	PRPF18	C complex	SplFs.core	pre-mRNA processing factor 18 [Source:HGNC Symbol;Acc:HGNC:17351]
SLU7	SLU7	C complex	SplFs.core	SLU7 homolog, splicing factor [Source:HGNC Symbol;Acc:HGNC:16939]
DHX8	DHX8	C complex	SplFs.core	DEAH-box helicase 8 [Source:HGNC Symbol;Acc:HGNC:2749]
ACIN1	ACIN1	EJC / mRNP	SplF.RBPs	apoptotic chromatin condensation inducer 1 [Source:HGNC Symbol;Acc:HGNC:17066]
EIF4A3	EIF4A3	EJC / mRNP	SplF.RBPs	eukaryotic translation initiation factor 4A3 [Source:HGNC Symbol;Acc:HGNC:18683]
MAGOH	MAGOH	EJC / mRNP	SplF.RBPs	mago homolog, exon junction complex subunit [Source:HGNC Symbol;Acc:HGNC:6815]
RBM8A	RBM8A	EJC / mRNP	SplF.RBPs	RNA binding motif protein 8A [Source:HGNC Symbol;Acc:HGNC:9905]
RNPS1	RNPS1	EJC / mRNP	SplF.RBPs	RNA binding protein with serine rich domain 1 [Source:HGNC Symbol;Acc:HGNC:10080]
ALYREF	THOC4	EJC / mRNP	SplF.RBPs	Aly/REF export factor [Source:HGNC Symbol;Acc:HGNC:19071]
DDX39B	BAT1	EJC / mRNP	SplF.RBPs	DExD-box helicase 39B [Source:HGNC Symbol;Acc:HGNC:13917]
THOC1	THOC1	TREX	SplF.RBPs	THO complex 1 [Source:HGNC Symbol;Acc:HGNC:19070]
THOC2	THOC2	TREX	SplF.RBPs	THO complex 2 [Source:HGNC Symbol;Acc:HGNC:19073]
THOC3	THOC3	TREX	SplF.RBPs	THO complex 3 [Source:HGNC Symbol;Acc:HGNC:19072]
THOC5	THOC5	TREX	SplF.RBPs	THO complex 5 [Source:HGNC Symbol;Acc:HGNC:19074]
THOC6	THOC6	TREX	SplF.RBPs	THO complex 6 [Source:HGNC Symbol;Acc:HGNC:28369]
THOC7	THOC7	TREX	SplF.RBPs	THO complex 7 [Source:HGNC Symbol;Acc:HGNC:29874]
DDX10	DDX10	helicase	SplF.RBPs	DEAD-box helicase 10 [Source:HGNC Symbol;Acc:HGNC:2735]
DDX11	DDX11	helicase	SplF.RBPs	DEAD/H-box helicase 11 [Source:HGNC Symbol;Acc:HGNC:2736]
DDX12P	DDX12	helicase	SplF.RBPs	DEAD/H-box helicase 12, pseudogene [Source:HGNC Symbol;Acc:HGNC:2737]
DDX17	DDX17	helicase	SplF.RBPs	DEAD-box helicase 17 [Source:HGNC Symbol;Acc:HGNC:2740]
DDX24	DDX24	helicase	SplF.RBPs	DEAD-box helicase 24 [Source:HGNC Symbol;Acc:HGNC:13266]
DDX28	DDX28	helicase	SplF.RBPs	DEAD-box helicase 28 [Source:HGNC Symbol;Acc:HGNC:17330]
DDX31	DDX31	helicase	SplF.RBPs	DEAD-box helicase 31 [Source:HGNC Symbol;Acc:HGNC:16715]
DDX3X	DDX3X	helicase	SplF.RBPs	DEAD-box helicase 3 X-linked [Source:HGNC Symbol;Acc:HGNC:2745]
DDX52	DDX52	helicase	SplF.RBPs	DExD-box helicase 52 [Source:HGNC Symbol;Acc:HGNC:20038]
DHX30	DHX30	helicase	SplF.RBPs	DExH-box helicase 30 [Source:HGNC Symbol;Acc:HGNC:16716]
DHX32	DHX32	helicase	SplF.RBPs	DEAH-box helicase 32 (putative) [Source:HGNC Symbol;Acc:HGNC:16717]

DHX33	DHX33	helicase	SplF.RBPs	DEAH-box helicase 33 [Source:HGNC Symbol;Acc:HGNC:16718]
DHX57	DHX57	helicase	SplF.RBPs	DExH-box helicase 57 [Source:HGNC Symbol;Acc:HGNC:20086]
DHX9	DHX9	helicase	SplF.RBPs	DExH-box helicase 9 [Source:HGNC Symbol;Acc:HGNC:2750]
HFM1	HFM1	helicase	SplF.RBPs	HFM1, ATP dependent DNA helicase homolog [Source:HGNC Symbol;Acc:HGNC:20193]
SKIV2L2	SKIV2L2	helicase	SplF.RBPs	Mtr4 exosome RNA helicase [Source:HGNC Symbol;Acc:HGNC:18734]
TDRD9	TDRD9	helicase	SplF.RBPs	tudor domain containing 9 [Source:HGNC Symbol;Acc:HGNC:20122]
HNRNPA1	HNRNPA1	hnRNP	SplF.RBPs	heterogeneous nuclear ribonucleoprotein A1 [Source:HGNC Symbol;Acc:HGNC:5031]
HNRNPAB	HNRNPAB	hnRNP	SplF.RBPs	heterogeneous nuclear ribonucleoprotein A/B [Source:HGNC Symbol;Acc:HGNC:5034]
HNRNPC	HNRNPC	hnRNP	SplF.RBPs	heterogeneous nuclear ribonucleoprotein C (C1/C2) [Source:HGNC Symbol;Acc:HGNC:5035]
FUS	FUS	hnRNP	SplF.RBPs	FUS RNA binding protein [Source:HGNC Symbol;Acc:HGNC:4010]
HNRNPA0	HNRNPA0	hnRNP	SplF.RBPs	heterogeneous nuclear ribonucleoprotein A0 [Source:HGNC Symbol;Acc:HGNC:5030]
HNRNPA2B1	HNRNPA2B1	hnRNP	SplF.RBPs	heterogeneous nuclear ribonucleoprotein A2/B1 [Source:HGNC Symbol;Acc:HGNC:5033]
HNRNPA3	HNRNPA3	hnRNP	SplF.RBPs	heterogeneous nuclear ribonucleoprotein A3 [Source:HGNC Symbol;Acc:HGNC:24941]
HNRNPCL1	HNRNPCL1	hnRNP	SplF.RBPs	heterogeneous nuclear ribonucleoprotein C-like 1 [Source:HGNC Symbol;Acc:HGNC:29295]
HNRNPD	HNRNPD	hnRNP	SplF.RBPs	heterogeneous nuclear ribonucleoprotein D [Source:HGNC Symbol;Acc:HGNC:5036]
HNRNPF	HNRNPF	hnRNP	SplF.RBPs	heterogeneous nuclear ribonucleoprotein F [Source:HGNC Symbol;Acc:HGNC:5039]
HNRNPH1	HNRNPH1	hnRNP	SplF.RBPs	heterogeneous nuclear ribonucleoprotein H1 [Source:HGNC Symbol;Acc:HGNC:5041]
HNRNPH2	HNRNPH2	hnRNP	SplF.RBPs	heterogeneous nuclear ribonucleoprotein H2 [Source:HGNC Symbol;Acc:HGNC:5042]
HNRNPH3	HNRNPH3	hnRNP	SplF.RBPs	heterogeneous nuclear ribonucleoprotein H3 [Source:HGNC Symbol;Acc:HGNC:5043]
HNRNPK	HNRNPK	hnRNP	SplF.RBPs	heterogeneous nuclear ribonucleoprotein K [Source:HGNC Symbol;Acc:HGNC:5044]
HNRNPL	HNRNPL	hnRNP	SplF.RBPs	heterogeneous nuclear ribonucleoprotein L [Source:HGNC Symbol;Acc:HGNC:5045]
HNRNPM	HNRNPM	hnRNP	SplF.RBPs	heterogeneous nuclear ribonucleoprotein M [Source:HGNC Symbol;Acc:HGNC:5046]
HNRNPR	HNRNPR	hnRNP	SplF.RBPs	heterogeneous nuclear ribonucleoprotein R [Source:HGNC Symbol;Acc:HGNC:5047]
HNRNPU	HNRNPU	hnRNP	SplF.RBPs	heterogeneous nuclear ribonucleoprotein U [Source:HGNC Symbol;Acc:HGNC:5048]
HNRNPUL1	HNRNPUL1	hnRNP	SplF.RBPs	heterogeneous nuclear ribonucleoprotein U like 1 [Source:HGNC Symbol;Acc:HGNC:17011]
HNRNPUL2	HNRNPUL2	hnRNP	SplF.RBPs	heterogeneous nuclear ribonucleoprotein U like 2 [Source:HGNC Symbol;Acc:HGNC:25451]
HNRNPDL	HNRPDL	hnRNP	SplF.RBPs	heterogeneous nuclear ribonucleoprotein D like [Source:HGNC Symbol;Acc:HGNC:5037]
HNRNPLL	HNRPLL	hnRNP	SplF.RBPs	heterogeneous nuclear ribonucleoprotein L like [Source:HGNC Symbol;Acc:HGNC:25127]
ILF2	ILF2	hnRNP	SplF.RBPs	interleukin enhancer binding factor 2 [Source:HGNC Symbol;Acc:HGNC:6037]
MATR3	MATR3	hnRNP	SplF.RBPs	matrin 3 [Source:HGNC Symbol;Acc:HGNC:6912]
PTBP1	PTBP1	hnRNP	SplF.RBPs	polypyrimidine tract binding protein 1 [Source:HGNC Symbol;Acc:HGNC:9583]
RALY	RALY	hnRNP	SplF.RBPs	RALY heterogeneous nuclear ribonucleoprotein [Source:HGNC Symbol;Acc:HGNC:15921]
SYNCRIP	SYNCRIP	hnRNP	SplF.RBPs	synaptotagmin binding cytoplasmic RNA interacting protein [Source:HGNC Symbol;Acc:HGNC:16918]
ILF3	ILF3	hnRNP	SplF.RBPs	interleukin enhancer binding factor 3 [Source:HGNC Symbol;Acc:HGNC:6038]
SRSF11	SFRS11	SR proteins	SplF.RBPs	serine and arginine rich splicing factor 11 [Source:HGNC Symbol;Acc:HGNC:10782]
SRSF3	SFRS3	SR proteins	SplF.RBPs	serine and arginine rich splicing factor 3 [Source:HGNC Symbol;Acc:HGNC:10785]
SRSF4	SFRS4	SR proteins	SplF.RBPs	serine and arginine rich splicing factor 4 [Source:HGNC Symbol;Acc:HGNC:10786]
SRSF6	SFRS6	SR proteins	SplF.RBPs	serine and arginine rich splicing factor 6 [Source:HGNC Symbol;Acc:HGNC:10788]
SRSF7	SFRS7	SR proteins	SplF.RBPs	serine and arginine rich splicing factor 7 [Source:HGNC Symbol;Acc:HGNC:10789]
SRSF9	SFRS9	SR proteins	SplF.RBPs	serine and arginine rich splicing factor 9 [Source:HGNC Symbol;Acc:HGNC:10791]
SRSF1	SRSF1	SR proteins	SplF.RBPs	serine and arginine rich splicing factor 1 [Source:HGNC Symbol;Acc:HGNC:10780]
SRSF2	SRSF2	SR proteins	SplF.RBPs	serine and arginine rich splicing factor 2 [Source:HGNC Symbol;Acc:HGNC:10783]
SRSF5	SRSF5	SR proteins	SplF.RBPs	serine and arginine rich splicing factor 5 [Source:HGNC Symbol;Acc:HGNC:10787]
TRA2B	TRA2B	SR proteins	SplF.RBPs	transformer 2 beta homolog [Source:HGNC Symbol;Acc:HGNC:10781]
SRRM2	SRRM2	SR proteins	SplF.RBPs	serine/arginine repetitive matrix 2 [Source:HGNC Symbol;Acc:HGNC:16639]
SRRM1	SRRM1	SR proteins	SplF.RBPs	serine and arginine repetitive matrix 1 [Source:HGNC Symbol;Acc:HGNC:16638]
SRRM3	SRRM3	SR proteins	SplF.RBPs	serine/arginine repetitive matrix 3 [Source:HGNC Symbol;Acc:HGNC:26729]
RBM6	RBM6	RNA binding proteins	SplF.RBPs	RNA binding motif protein 6 [Source:HGNC Symbol;Acc:HGNC:9903]
NSRP1	CCDC55	RNA binding proteins	SplF.RBPs	nuclear speckle splicing regulatory protein 1 [Source:HGNC Symbol;Acc:HGNC:25305]
CIRBP	CIRBP	RNA binding proteins	SplF.RBPs	cold inducible RNA binding protein [Source:HGNC Symbol;Acc:HGNC:1982]
YBX3	CSDA	RNA binding proteins	SplF.RBPs	Y-box binding protein 3 [Source:HGNC Symbol;Acc:HGNC:2428]

ELAVL1	ELAVL1	RNA binding proteins	SplF.RBPs	ELAV like RNA binding protein 1 [Source:HGNC Symbol;Acc:HGNC:3312]
ESRP1	ESRP1	RNA binding proteins	SplF.RBPs	epithelial splicing regulatory protein 1 [Source:HGNC Symbol;Acc:HGNC:25966]
ESRP2	ESRP2	RNA binding proteins	SplF.RBPs	epithelial splicing regulatory protein 2 [Source:HGNC Symbol;Acc:HGNC:26152]
EWSR1	EWSR1	RNA binding proteins	SplF.RBPs	EWS RNA binding protein 1 [Source:HGNC Symbol;Acc:HGNC:3508]
PAXBP1	GCFC1	RNA binding proteins	SplF.RBPs	PAX3 and PAX7 binding protein 1 [Source:HGNC Symbol;Acc:HGNC:13579]
IGF2BP3	IGF2BP3	RNA binding proteins	SplF.RBPs	insulin like growth factor 2 mRNA binding protein 3 [Source:HGNC Symbol;Acc:HGNC:28868]
KIN	KIN	RNA binding proteins	SplF.RBPs	Kin17 DNA and RNA binding protein [Source:HGNC Symbol;Acc:HGNC:6327]
LUC7L3	LUC7L3	RNA binding proteins	SplF.RBPs	LUC7 like 3 pre-mRNA splicing factor [Source:HGNC Symbol;Acc:HGNC:24309]
NONO	NONO	RNA binding proteins	SplF.RBPs	non-POU domain containing octamer binding [Source:HGNC Symbol;Acc:HGNC:7871]
PRPF39	PRPF39	RNA binding proteins	SplF.RBPs	pre-mRNA processing factor 39 [Source:HGNC Symbol;Acc:HGNC:20314]
RBM15	RBM15	RNA binding proteins	SplF.RBPs	RNA binding motif protein 15 [Source:HGNC Symbol;Acc:HGNC:14959]
RBM39	RBM39	RNA binding proteins	SplF.RBPs	RNA binding motif protein 39 [Source:HGNC Symbol;Acc:HGNC:15923]
RBFOX2	RBM9	RNA binding proteins	SplF.RBPs	RNA binding fox-1 homolog 2 [Source:HGNC Symbol;Acc:HGNC:9906]
SFPQ	SFPQ	RNA binding proteins	SplF.RBPs	splicing factor proline and glutamine rich [Source:HGNC Symbol;Acc:HGNC:10774]
TIA1	TIA1	RNA binding proteins	SplF.RBPs	TIA1 cytotoxic granule associated RNA binding protein [Source:HGNC Symbol;Acc:HGNC:11802]
TIAL1	TIAL1	RNA binding proteins	SplF.RBPs	TIA1 cytotoxic granule associated RNA binding protein like 1 [Source:HGNC Symbol;Acc:HGNC:11804]
ZNF207	ZNF207	RNA binding proteins	SplF.RBPs	zinc finger protein 207 [Source:HGNC Symbol;Acc:HGNC:12998]
ADAR	ADAR	RNA editing	SplF.RBPs	adenosine deaminase, RNA specific [Source:HGNC Symbol;Acc:HGNC:225]
ADARB1	ADARB1	RNA editing	SplF.RBPs	adenosine deaminase, RNA specific B1 [Source:HGNC Symbol;Acc:HGNC:226]
C19orf43	C19orf43	RNA modifying	SplF.RBPs	telomerase RNA component interacting RNase [Source:HGNC Symbol;Acc:HGNC:28424]
DBR1	DBR1	RNA modifying	SplF.RBPs	debranching RNA lariats 1 [Source:HGNC Symbol;Acc:HGNC:15594]
FTO	FTO	RNA metylation	SplF.RBPs	FTO, alpha-ketoglutarate dependent dioxygenase [Source:HGNC Symbol;Acc:HGNC:24678]
KIAA1429	KIAA1429	RNA metylation	SplF.RBPs	vir like m6A methyltransferase associated [Source:HGNC Symbol;Acc:HGNC:24500]
METTL14	METTL14	RNA metylation	SplF.RBPs	methyltransferase like 14 [Source:HGNC Symbol;Acc:HGNC:29330]
WTAP	WTAP	RNA metylation	SplF.RBPs	WT1 associated protein [Source:HGNC Symbol;Acc:HGNC:16846]
ALKBH5	ALKBH5	RNA metylation	SplF.RBPs	alkB homolog 5, RNA demethylase [Source:HGNC Symbol;Acc:HGNC:25996]
METTL3	METTL3	RNA metylation	SplF.RBPs	methyltransferase like 3 [Source:HGNC Symbol;Acc:HGNC:17563]
YTHDC1	YTHDC1	RNA metylation	SplF.RBPs	YTH domain containing 1 [Source:HGNC Symbol;Acc:HGNC:30626]
WDR83	WDR83	RNA metylation	SplF.RBPs	WD repeat domain 83 [Source:HGNC Symbol;Acc:HGNC:32672]
NCBP1	NCBP1	CBC	SplF.RBPs	nuclear cap binding protein subunit 1 [Source:HGNC Symbol;Acc:HGNC:7658]
NCBP2	NCBP2	CBC	SplF.RBPs	nuclear cap binding protein subunit 2 [Source:HGNC Symbol;Acc:HGNC:7659]
PABPN1	PABPN1	3´end	SplF.RBPs	poly(A) binding protein nuclear 1 [Source:HGNC Symbol;Acc:HGNC:8565]
PABPC1	PABPC1	3´end	SplF.RBPs	poly(A) binding protein cytoplasmic 1 [Source:HGNC Symbol;Acc:HGNC:8554]
CPSF2	CPSF2	3´end	SplF.RBPs	cleavage and polyadenylation specific factor 2 [Source:HGNC Symbol;Acc:HGNC:2325]
CPSF6	CPSF6	3´end	SplF.RBPs	cleavage and polyadenylation specific factor 6 [Source:HGNC Symbol;Acc:HGNC:13871]
NUDT21	NUDT21	3´end	SplF.RBPs	nudix hydrolase 21 [Source:HGNC Symbol;Acc:HGNC:13870]
DIS3	DIS3	RNA degradation	SplF.RBPs	DIS3 homolog, exosome endoribonuclease and 3'-5' exoribonuclease [Source:HGNC Symbol;Acc:HGNC:20604]
DIS3L	DIS3L	RNA degradation	SplF.RBPs	DIS3 like exosome 3'-5' exoribonuclease [Source:HGNC Symbol;Acc:HGNC:28698]
EXOSC4	EXOSC4	RNA degradation	SplF.RBPs	exosome component 4 [Source:HGNC Symbol;Acc:HGNC:18189]
AGO2	EIF2C2	RISC	SplF.RBPs	argonaute 2, RISC catalytic component [Source:HGNC Symbol;Acc:HGNC:3263]
DICER1	DICER1	RISC	SplF.RBPs	dicer 1, ribonuclease III [Source:HGNC Symbol;Acc:HGNC:17098]
MOV10	MOV10	RISC	SplF.RBPs	Mov10 RISC complex RNA helicase [Source:HGNC Symbol;Acc:HGNC:7200]
CLK1	CLK1	protein kinase	SplF.others	CDC like kinase 1 [Source:HGNC Symbol;Acc:HGNC:2068]
CLK2	CLK2	protein kinase	SplF.others	CDC like kinase 2 [Source:HGNC Symbol;Acc:HGNC:2069]
CLK3	CLK3	protein kinase	SplF.others	CDC like kinase 3 [Source:HGNC Symbol;Acc:HGNC:2071]
CLK4	CLK4	protein kinase	SplF.others	CDC like kinase 4 [Source:HGNC Symbol;Acc:HGNC:13659]
SRPK1	SRPK1	protein kinase	SplF.others	SRSF protein kinase 1 [Source:HGNC Symbol;Acc:HGNC:11305]
SRPK2	SRPK2	protein kinase	SplF.others	SRSF protein kinase 2 [Source:HGNC Symbol;Acc:HGNC:11306]
DNAJC8	DNAJC8	Other SAPs	SplF.others	DnaJ heat shock protein family (Hsp40) member C8 [Source:HGNC Symbol;Acc:HGNC:15470]
CFAP20	C16orf80	Other SAPs	SplF.others	cilia and flagella associated protein 20 [Source:HGNC Symbol;Acc:HGNC:29523]
BRINP1	DBC1	Other SAPs	SplF.others	BMP/retinoic acid inducible neural specific 1 [Source:HGNC Symbol;Acc:HGNC:2687]

HSPA5	HSPA5	Other SAPs	SplF.others	heat shock protein family A (Hsp70) member 5 [Source:HGNC Symbol;Acc:HGNC:5238]
CARM1	CARM1	Other SAPs	SplF.others	coactivator associated arginine methyltransferase 1 [Source:HGNC Symbol;Acc:HGNC:23393]
PRMT1	PRMT1	Other SAPs	SplF.others	protein arginine methyltransferase 1 [Source:HGNC Symbol;Acc:HGNC:5187]
PRMT5	PRMT5	Other SAPs	SplF.others	protein arginine methyltransferase 5 [Source:HGNC Symbol;Acc:HGNC:10894]
CDK12	CRKRS	Other SAPs	SplF.others	cyclin dependent kinase 12 [Source:HGNC Symbol;Acc:HGNC:24224]
PPM1G	PPM1G	Other SAPs	SplF.others	protein phosphatase, Mg2+/Mn2+ dependent 1G [Source:HGNC Symbol;Acc:HGNC:9278]
ZNF326	ZNF326	Other SAPs	SplF.others	zinc finger protein 326 [Source:HGNC Symbol;Acc:HGNC:14104]
LPAR1	LPAR1	Minor	SplF.others	lysophosphatidic acid receptor 1 [Source:HGNC Symbol;Acc:HGNC:3166]
PDCD7	PDCD7	Minor	SplF.others	programmed cell death 7 [Source:HGNC Symbol;Acc:HGNC:8767]
RECQL5	RECQL5	Minor	SplF.others	RecQ like helicase 5 [Source:HGNC Symbol;Acc:HGNC:9950]
RNPC3	RNPC3	Minor	SplF.others	RNA binding region (RNP1, RRM) containing 3 [Source:HGNC Symbol;Acc:HGNC:18666]
SNRNP25	SNRNP25	Minor	SplF.others	small nuclear ribonucleoprotein U11/U12 subunit 25 [Source:HGNC Symbol;Acc:HGNC:14161]
SNRNP35	SNRNP35	Minor	SplF.others	small nuclear ribonucleoprotein U11/U12 subunit 35 [Source:HGNC Symbol;Acc:HGNC:30852]
SNRNP48	SNRNP48	Minor	SplF.others	small nuclear ribonucleoprotein U11/U12 subunit 48 [Source:HGNC Symbol;Acc:HGNC:21368]
ZCRB1	ZCRB1	Minor	SplF.others	zinc finger CCHC-type and RNA binding motif containing 1 [Source:HGNC Symbol;Acc:HGNC:29620]
ZMAT5	ZMAT5	Minor	SplF.others	zinc finger matrin-type 5 [Source:HGNC Symbol;Acc:HGNC:28046]
ZRSR1	ZRSR1	Minor	SplF.others	ZRSR2 pseudogene 1 [Source:HGNC Symbol;Acc:HGNC:12456]
ZRSR2	ZRSR2	Minor	SplF.others	zinc finger CCCH-type, RNA binding motif and serine/arginine rich 2 [Source:HGNC Symbol;Acc:HGNC:23019]
ASCL1	ASCL1	Lysine transferases	SplF.others	achaete-scute family bHLH transcription factor 1 [Source:HGNC Symbol;Acc:HGNC:738]
EHMT2	EHMT2	Lysine transferases	SplF.others	euchromatic histone lysine methyltransferase 2 [Source:HGNC Symbol;Acc:HGNC:14129]
KAT2B	KAT2B	Lysine transferases	SplF.others	lysine acetyltransferase 2B [Source:HGNC Symbol;Acc:HGNC:8638]
KDM1A	KDM1A	Lysine transferases	SplF.others	lysine demethylase 1A [Source:HGNC Symbol;Acc:HGNC:29079]
KDM4B	KDM4B	Lysine transferases	SplF.others	lysine demethylase 4B [Source:HGNC Symbol;Acc:HGNC:29136]
SETD1A	SETD1A	Lysine transferases	SplF.others	SET domain containing 1A [Source:HGNC Symbol;Acc:HGNC:29010]
SETD2	SETD2	Lysine transferases	SplF.others	SET domain containing 2 [Source:HGNC Symbol;Acc:HGNC:18420]
SUV39H1	SUV39H1	Lysine transferases	SplF.others	suppressor of variegation 3-9 homolog 1 [Source:HGNC Symbol;Acc:HGNC:11479]
TET1	TET1	Lysine transferases	SplF.others	tet methylcytosine dioxygenase 1 [Source:HGNC Symbol;Acc:HGNC:29484]
DNMT1	DNMT1	chromatin-related	SplF.others	DNA methyltransferase 1 [Source:HGNC Symbol;Acc:HGNC:2976]
MBD2	MBD2	chromatin-related	SplF.others	methyl-CpG binding domain protein 2 [Source:HGNC Symbol;Acc:HGNC:6917]
MECP2	MECP2	chromatin-related	SplF.others	methyl-CpG binding protein 2 [Source:HGNC Symbol;Acc:HGNC:6990]
EP300	EP300	Histone transferases	SplF.others	E1A binding protein p300 [Source:HGNC Symbol;Acc:HGNC:3373]
KAT2A	KAT2A	Histone transferases	SplF.others	lysine acetyltransferase 2A [Source:HGNC Symbol;Acc:HGNC:4201]
KAT5	KAT5	Histone transferases	SplF.others	lysine acetyltransferase 5 [Source:HGNC Symbol;Acc:HGNC:5275]
HDAC1	HDAC1	Histone transferases	SplF.others	histone deacetylase 1 [Source:HGNC Symbol;Acc:HGNC:4852]
HDAC2	HDAC2	Histone transferases	SplF.others	histone deacetylase 2 [Source:HGNC Symbol;Acc:HGNC:4853]
HDAC3	HDAC3	Histone transferases	SplF.others	histone deacetylase 3 [Source:HGNC Symbol;Acc:HGNC:4854]
HDAC4	HDAC4	Histone transferases	SplF.others	histone deacetylase 4 [Source:HGNC Symbol;Acc:HGNC:14063]
HDAC6	HDAC6	Histone transferases	SplF.others	histone deacetylase 6 [Source:HGNC Symbol;Acc:HGNC:14064]
SIRT1	SIRT1	Histone transferases	SplF.others	sirtuin 1 [Source:HGNC Symbol;Acc:HGNC:14929]
ASH2L	ASH2L	chromatin-related	SplF.others	ASH2 like, histone lysine methyltransferase complex subunit [Source:HGNC Symbol;Acc:HGNC:744]
BAZ1B	BAZ1B	chromatin-related	SplF.others	bromodomain adjacent to zinc finger domain 1B [Source:HGNC Symbol;Acc:HGNC:961]
BRD4	BRD4	chromatin-related	SplF.others	bromodomain containing 4 [Source:HGNC Symbol;Acc:HGNC:13575]
CBX3	CBX3	chromatin-related	SplF.others	chromobox 3 [Source:HGNC Symbol;Acc:HGNC:1553]
CHD1	CHD1	chromatin-related	SplF.others	chromodomain helicase DNA binding protein 1 [Source:HGNC Symbol;Acc:HGNC:1915]
CTCF	CTCF	chromatin-related	SplF.others	CCCTC-binding factor [Source:HGNC Symbol;Acc:HGNC:13723]
DEK	DEK	chromatin-related	SplF.others	DEK proto-oncogene [Source:HGNC Symbol;Acc:HGNC:2768]
HMGA1	HMGA1	chromatin-related	SplF.others	high mobility group AT-hook 1 [Source:HGNC Symbol;Acc:HGNC:5010]
KHDRBS1	KHDRBS1	chromatin-related	SplF.others	KH RNA binding domain containing, signal transduction associated 1 [Source:HGNC Symbol;Acc:HGNC:18116]
MORF4L1	MORF4L1	chromatin-related	SplF.others	mortality factor 4 like 1 [Source:HGNC Symbol;Acc:HGNC:16989]
NAB2	NAB2	chromatin-related	SplF.others	NGFI-A binding protein 2 [Source:HGNC Symbol;Acc:HGNC:7627]
RPS6KA5	RPS6KA5	chromatin-related	SplF.others	ribosomal protein S6 kinase A5 [Source:HGNC Symbol;Acc:HGNC:10434]

SMARCA2	SMARCA2	WI/SNF Complex Components	SplF.others	SWI/SNF related, matrix associated, actin dependent regulator of chromatin, subfamily a, member 2 [Source:HGNC Symbol;Acc:HGNC:11098]
SMARCA4	SMARCA4	WI/SNF Complex Components	SplF.others	SWI/SNF related, matrix associated, actin dependent regulator of chromatin, subfamily a, member 4 [Source:HGNC Symbol;Acc:HGNC:11100]
SPEN	SPEN	chromatin-related	SplF.others	spen family transcriptional repressor [Source:HGNC Symbol;Acc:HGNC:17575]
TAF15	TAF15	chromatin-related	SplF.others	TATA-box binding protein associated factor 15 [Source:HGNC Symbol;Acc:HGNC:11547]
TCERG1	TCERG1	chromatin-related	SplF.others	transcription elongation regulator 1 [Source:HGNC Symbol;Acc:HGNC:15630]
TCERG1L	TCERG1L	chromatin-related	SplF.others	transcription elongation regulator 1 like [Source:HGNC Symbol;Acc:HGNC:23533]
BMI1	BMI1	Polycomb Group Genes	SplF.others	BMI1 proto-oncogene, polycomb ring finger [Source:HGNC Symbol;Acc:HGNC:1066]
EED	EED	Polycomb Group Genes	SplF.others	embryonic ectoderm development [Source:HGNC Symbol;Acc:HGNC:3188]
EZH2	EZH2	Polycomb Group Genes	SplF.others	enhancer of zeste 2 polycomb repressive complex 2 subunit [Source:HGNC Symbol;Acc:HGNC:3527]
PHC1	PHC1	Polycomb Group Genes	SplF.others	polyhomeotic homolog 1 [Source:HGNC Symbol;Acc:HGNC:3182]
PHC2	PHC2	Polycomb Group Genes	SplF.others	polyhomeotic homolog 2 [Source:HGNC Symbol;Acc:HGNC:3183]
