# Supplementary material for: Foxg1 and companions: Not only transcription factors
Source: Neural Regen Res. 2025 Sep 3;21(8):3426–38. doi: 10.4103/NRR.NRR-D-25-00439 (PMC13452659; doi:10.4103/NRR.NRR-D-25-00439)
Supplement: Supplementary file 17 [file NRR-21-3426_Suppl4.pdf]

Additional Table 1.4. hsa-pAF genes list (n = 33)

[homo sapiens polyAdenylation Factor genes, including genes encoding for:
- polyAdenylation Factors, 4C(CFIm, CFIIm, CPSF, CSTF)-type
- polyAdenylation Factors, PolyAdenylation-type]

NOTES

manually compiled on the basis of information taken from:
- Laishram. FEBS Lett (2014). doi:10.1016/j.febslet.2014.05.029
- Tian and Manley. Nat Rev Mol Cell Biol (2016). doi:10.1038/nrm.2016.116
- www.genecards.org, accessed on 2024.10.15

pAF gene	*Category*	*Our super--grouping*
CPSF6	CFIm	pAF.4C
CPSF7	CFIm	pAF.4C
NUDT1	CFIm	pAF.4C
CLP1	CFIIm	pAF.4C
PCF11	CFIIm	pAF.4C
CPSF1	CPSF	pAF.4C
CPSF2	CPSF	pAF.4C
CPSF3	CPSF	pAF.4C
CPSF4	CPSF	pAF.4C
CSTF2T	CPSF	pAF.4C
FIP1L1	CPSF	pAF.4C
WDR33	CPSF	pAF.4C
SYMPK	CPSF-assoc	pAF.4C
CSTF1	CSTF	pAF.4C
CSTF2	CSTF	pAF.4C
CSTF3	CSTF	pAF.4C
PABPN1	pA-binding	pAF.PA
CPEB1	pA-binding, cytoplasmic	pAF.PA

CPEB2	pA-binding, cytoplasmic	pAF.PA
CPEB3	pA-binding, cytoplasmic	pAF.PA
CPEB4	pA-binding, cytoplasmic	pAF.PA
NGDN	pA-binding, cytoplasmic	pAF.PA
PAPOLA	PAP, canonical	pAF.PA
PAPOLB	PAP, canonical	pAF.PA
PAPOLG	PAP, canonical	pAF.PA
MTPAP	PAP, non-canonical	pAF.PA
TENT2	PAP, non-canonical	pAF.PA
TENT4A	PAP, non-canonical	pAF.PA
TENT4B	PAP, non-canonical	pAF.PA
TUT1	PAP, non-canonical	pAF.PA
TUT4	PAP, non-canonical	pAF.PA
TUT7	PAP, non-canonical	pAF.PA
PARN	pA-specific Ribonuclease	pAF.PA
