# Supplementary material for: Foxg1 and companions: Not only transcription factors
Source: Neural Regen Res. 2025 Sep 3;21(8):3426–38. doi: 10.4103/NRR.NRR-D-25-00439 (PMC13452659; doi:10.4103/NRR.NRR-D-25-00439)
Supplement: Supplementary file 18 [file NRR-21-3426_Suppl5.pdf]

Additional Table 1.5. hsa-TlatMF genes list (*n* = 275)

[homo sapiens Translation Machinery Factor genes list, including genes encoding for:
- Eukaryotic Initiation Factors
- Eukaryotic Initiation coFactors
- Eukaryotic Elongation Factors
- Ribosomal Proteins, cytoplasmic
- Ribosomal Proteins,, mitochondrial
- aminoacyl-tRNA-synthetases, all]

NOTE

manually compiled, on the basis of the following sources:
- Jackson et al. Nature Reviews Molecular Cell Biology (2010). doi:10.1038/nrm2838 [EIFs , EIcoFs]
- Xu et al Frontiers in Molecular Biosciences (2022) doi: 10.3389/fmolb.2021.816398 [EEFs]
- http://ribosome.med.miyazaki-u.ac.jp/rpg.cgi?mode=orglist&org=Homo%20sapiens, accessed on 2024.10.15 [RP.cyto]
- https://www.genenames.org/data/genegroup/#!/group/646, accessed on 2024.10.15[RP.mito]
- Tennakoon and Cui. Current Biology (2024). doi: 10.1016/j.cub.2024.08.029, and https://www.genecards.org, accessed on 2024.10.09 [aa.tRNA.snth]

Gene name	*TlatMF group*
EIF1	EIF
EIF1AX	EIF
EIF1AY	EIF
EIF1B	EIF
EIF2A	EIF
EIF2AK1	EIF
EIF2AK2	EIF
EIF2AK3	EIF
EIF2AK4	EIF
EIF2B1	EIF
EIF2B2	EIF
EIF2B3	EIF
EIF2B4	EIF

EIF2B5	EIF
EIF2S1	EIF
EIF2S2	EIF
EIF2S3	EIF
EIF3A	EIF
EIF3B	EIF
EIF3C	EIF
EIF3CL	EIF
EIF3D	EIF
EIF3E	EIF
EIF3F	EIF
EIF3G	EIF
EIF3H	EIF
EIF3I	EIF
EIF3J	EIF
EIF3K	EIF
EIF3L	EIF
EIF3M	EIF
EIF4A1	EIF
EIF4A2	EIF
EIF4B	EIF
EIF4E	EIF
EIF4EBP1	EIF
EIF4EBP2	EIF
EIF4EBP3	EIF
EIF4ENIF1	EIF
EIF4G1	EIF
EIF4G2	EIF
EIF4G3	EIF
EIF4H	EIF
EIF5	EIF
EIF5B	EIF

DHX29	EIcoF
DDX3	EIcoF
EIF6	EIcoF
PABPC1	EIcoF
PABPC2	EIcoF
PABPC3	EIcoF
PAIP1	EIcoF
PAIP2	EIcoF
PAIP2B	EIcoF
FMR1	EIcoF
CYFIP1	EIcoF
CYFIP2	EIcoF
NUFIP	EIcoF
EEF1A1	EEF
EEF1A2	EEF
EEF1B2	EEF
EEF1D	EEF
EEF1G	EEF
EEF2	EEF
EEF2K	EEF
EEF2KMT	EEF
GFM1	EEF
GFM2	EEF
MTERF4	EEF
EIF5A	EEF
EIF5A2	EEF
EEFSEC	EEF
RPSA	RP.cyto
RPS2	RP.cyto
RPS3	RP.cyto
RPS3A	RP.cyto
RPS4X	RP.cyto

RPS4Y	RP.cyto
RPS5	RP.cyto
RPS6	RP.cyto
RPS7	RP.cyto
RPS8	RP.cyto
RPS9	RP.cyto
RPS10	RP.cyto
RPS11	RP.cyto
RPS12	RP.cyto
RPS13	RP.cyto
RPS14	RP.cyto
RPS15	RP.cyto
RPS15A	RP.cyto
RPS16	RP.cyto
RPS17	RP.cyto
RPS18	RP.cyto
RPS19	RP.cyto
RPS20	RP.cyto
RPS21	RP.cyto
RPS23	RP.cyto
RPS24	RP.cyto
RPS25	RP.cyto
RPS26	RP.cyto
RPS27	RP.cyto
RPS27A	RP.cyto
RPS28	RP.cyto
RPS29	RP.cyto
RPS30	RP.cyto
RPL3	RP.cyto
RPL4	RP.cyto
RPL5	RP.cyto
RPL6	RP.cyto

RPL7	RP.cyto
RPL7A	RP.cyto
RPL8	RP.cyto
RPL9	RP.cyto
RPL10	RP.cyto
RPL10A	RP.cyto
RPL11	RP.cyto
RPL12	RP.cyto
RPL13	RP.cyto
RPL13A	RP.cyto
RPL14	RP.cyto
RPL15	RP.cyto
RPL17	RP.cyto
RPL18	RP.cyto
RPL18A	RP.cyto
RPL19	RP.cyto
RPL21	RP.cyto
RPL22	RP.cyto
RPL23	RP.cyto
RPL23A	RP.cyto
RPL24	RP.cyto
RPL26	RP.cyto
RPL27	RP.cyto
RPL27A	RP.cyto
RPL28	RP.cyto
RPL29	RP.cyto
RPL30	RP.cyto
RPL31	RP.cyto
RPL32	RP.cyto
RPL34	RP.cyto
RPL35	RP.cyto
RPL35A	RP.cyto

RPL36	RP.cyto
RPL36A	RP.cyto
RPL37	RP.cyto
RPL37A	RP.cyto
RPL38	RP.cyto
RPL39	RP.cyto
RPL40	RP.cyto
RPL41	RP.cyto
RPLP0	RP.cyto
RPLP1	RP.cyto
RPLP2	RP.cyto
MRPL1	RP.mito
MRPL2	RP.mito
MRPL3	RP.mito
MRPL4	RP.mito
MRPL9	RP.mito
MRPL10	RP.mito
MRPL11	RP.mito
MRPL12	RP.mito
MRPL13	RP.mito
MRPL14	RP.mito
MRPL15	RP.mito
MRPL16	RP.mito
MRPL17	RP.mito
MRPL18	RP.mito
MRPL19	RP.mito
MRPL20	RP.mito
MRPL21	RP.mito
MRPL22	RP.mito
MRPL23	RP.mito
MRPL24	RP.mito
MRPL27	RP.mito

MRPL28	RP.mito
MRPL30	RP.mito
MRPL32	RP.mito
MRPL33	RP.mito
MRPL34	RP.mito
MRPL35	RP.mito
MRPL36	RP.mito
MRPL37	RP.mito
MRPL38	RP.mito
MRPL39	RP.mito
MRPL40	RP.mito
MRPL41	RP.mito
MRPL42	RP.mito
MRPL43	RP.mito
MRPL44	RP.mito
MRPL45	RP.mito
MRPL46	RP.mito
MRPL47	RP.mito
MRPL48	RP.mito
MRPL49	RP.mito
MRPL50	RP.mito
MRPL51	RP.mito
MRPL52	RP.mito
MRPL53	RP.mito
MRPL54	RP.mito
MRPL55	RP.mito
MRPL58	RP.mito
MRPL57	RP.mito
GADD45GIP1	RP.mito
MRPS30	RP.mito
MRPS18A	RP.mito
MRPS2	RP.mito

MRPS5	RP.mito
MRPS6	RP.mito
MRPS7	RP.mito
MRPS9	RP.mito
MRPS10	RP.mito
MRPS11	RP.mito
MRPS12	RP.mito
MRPS14	RP.mito
MRPS15	RP.mito
MRPS16	RP.mito
MRPS17	RP.mito
MRPS18C	RP.mito
MRPS21	RP.mito
MRPS22	RP.mito
MRPS23	RP.mito
MRPS24	RP.mito
MRPS25	RP.mito
MRPS26	RP.mito
MRPS27	RP.mito
MRPS28	RP.mito
DAP3	RP.mito
MRPS31	RP.mito
MRPS33	RP.mito
MRPS34	RP.mito
MRPS35	RP.mito
CHCHD1	RP.mito
AURKAIP1	RP.mito
PTCD3	RP.mito
MRPS18B	RP.mito
AARS1	aa.tRNA.synth
RARS1	aa.tRNA.synth
RARS2	aa.tRNA.synth

NARS1	aa.tRNA.synth
NARS2	aa.tRNA.synth
DARS1	aa.tRNA.synth
DARS2	aa.tRNA.synth
CARS1	aa.tRNA.synth
CARS2	aa.tRNA.synth
QRSL1	aa.tRNA.synth
QARS1	aa.tRNA.synth
EPRS1	aa.tRNA.synth
GATB	aa.tRNA.synth
GATC	aa.tRNA.synth
EARS2	aa.tRNA.synth
GARS1	aa.tRNA.synth
HARS1	aa.tRNA.synth
HARS2	aa.tRNA.synth
IARS1	aa.tRNA.synth
IARS2	aa.tRNA.synth
LARS1	aa.tRNA.synth
LARS2	aa.tRNA.synth
KARS1	aa.tRNA.synth
MARS1	aa.tRNA.synth
MARS2	aa.tRNA.synth
FARS2	aa.tRNA.synth
FARSA	aa.tRNA.synth
FARSB	aa.tRNA.synth
PARS2	aa.tRNA.synth
SARS1	aa.tRNA.synth
SARS2	aa.tRNA.synth
TARS1	aa.tRNA.synth
TARS2	aa.tRNA.synth
TARS3	aa.tRNA.synth
WARS2	aa.tRNA.synth

WARS1	aa.tRNA.synth
YARS1	aa.tRNA.synth
YARS2	aa.tRNA.synth
VARS1	aa.tRNA.synth
VARS1	aa.tRNA.synth
VARS2	aa.tRNA.synth
