# Supplementary material for: Foxg1 and companions: Not only transcription factors
Source: Neural Regen Res. 2025 Sep 3;21(8):3426–38. doi: 10.4103/NRR.NRR-D-25-00439 (PMC13452659; doi:10.4103/NRR.NRR-D-25-00439)
Supplement: Supplementary file 19 [file NRR-21-3426_Suppl6.pdf]

Additional Table 2. Subcellular hsa-TF localization

[available for 1344 out of 1620 hsa-TFs]

NOTES

data harvested from https://www.proteinatlas.org/search, on 2024.10.15

hsa TFs	*Subcellular location*	*Antibody*	*Reliability, IH*	*Reliability, IF*
TCF25		HPA041699, HPA041786	Enhanced	
RFXANK	Nucleoplasm	HPA049157, HPA053330	Approved	Enhanced
PURA				
PURB	Nucleoplasm	HPA048766		Supported
PURG	Nucleoplasm,Nucleoli,Plasma membrane	HPA047746	Enhanced	Approved
NRF1	Nucleoplasm,Cytosol	HPA029329	Approved	Supported
CSRNP1	Nucleoplasm,Vesicles	HPA045207, HPA058463		Supported
CSRNP2	Nuclear speckles	HPA018503, HPA019914	Approved	Approved
CSRNP3	Nucleoplasm,Nucleoli rim	HPA017905	Approved	Approved
SFPQ	Nucleoplasm	CAB009886, HPA047513, HPA054689	Enhanced	Enhanced
LRRFIP1	Cytosol	HPA006979	Approved	Supported
LRRFIP2	Plasma membrane,Actin filaments	HPA035956, HPA042023	Uncertain	Approved
ZNFX1	Mitochondria	HPA046629	Approved	Approved
NFX1	Nucleoplasm,Nucleoli,Plasma membrane,Cytosol	HPA056310, HPA061382, HPA073519	Approved	Supported
NFXL1	Nucleoplasm	HPA059132	Approved	Approved
GTF2I	Nucleoplasm	CAB004595, HPA026638	Supported	Enhanced
GTF2IRD1	Nucleoplasm,Cytosol	HPA044254	Approved	Supported
GTF2IRD2	Nucleoplasm	HPA056679, HPA056680		Supported
GTF2IRD2B	Nucleoplasm	HPA056679, HPA056680		Supported
CAMTA1	Cytosol	HPA036342, HPA036343	Approved	Supported
CAMTA2	Nucleoplasm,Vesicles,Actin filaments,Mitochondria,Cytosol	HPA027835, HPA051147		Supported
ATF2	Nucleoplasm	CAB003769, HPA022134	Supported	Enhanced
ATF7	Nucleoplasm,Cytosol	HPA003384	Approved	Approved
BACH1	Nucleoplasm,Cytosol	HPA034949	Approved	Approved
BACH2	Nucleoplasm,Cytosol	HPA051384, HPA058384	Enhanced	Supported
CREB5	Nucleoplasm,Vesicles,Plasma membrane	HPA050020, HPA061622		Approved
JUN	Nucleoplasm	CAB003801, CAB007780, HPA059474, HPA066898, CAB080216, CAB080219, CAB080220	Enhanced	Enhanced
JUNB	Nucleoplasm	CAB004464, HPA019149	Approved	Supported
JUND	Nucleoplasm	CAB005268, HPA063029, HPA068905	Supported	Enhanced
NFE2	Nucleoplasm,Actin filaments	HPA001914	Enhanced	Approved
NFE2L1	Nucleoplasm,Cytosol	HPA063384, HPA065424	Approved	Approved
NFE2L2	cleoplasm,Golgi apparatus,Plasma membrane,Centrosome,Cyto	HPA002990, CAB020317, HPA043438	Supported	Uncertain
NFE2L3	Nucleoplasm,Vesicles	HPA055889	Enhanced	Approved
ATF3	Nucleoplasm,Nucleoli	HPA001562	Approved	Supported
FOS	Nucleoplasm	HPA018531, CAB080210	Approved	Supported
FOSB	Nucleoplasm,Vesicles	CAB010267, HPA054663	Approved	Supported

FOSL1	Nucleoplasm	CAB004396, HPA030728	Enhanced	Supported
FOSL2	Nucleoplasm	HPA004817, CAB013460, HPA061417	Supported	Enhanced
JDP2	Nucleoplasm,Nuclear speckles	HPA059511	Supported	Approved
MAF	Nucleoplasm,Nuclear bodies,Vesicles	CAB010296, HPA028289	Approved	Approved
MAFA				
MAFB	Nucleoplasm,Nucleoli,Golgi apparatus,Cytosol	HPA005653	Approved	Approved
MAFF	Nucleoplasm,Mitochondria	CAB025341, HPA055371	Approved	Approved
MAFG		CAB025573	Approved	
MAFK	Nucleoplasm	CAB026820, HPA060841	Enhanced	Enhanced
NRL	Nucleoplasm,Cytosol	CAB026066, HPA064599	Enhanced	Supported
BATF	Nucleoplasm	HPA059588, HPA064962	Approved	Enhanced
BATF2	Nucleoli	HPA062496	Approved	Approved
BATF3	Nucleoplasm,Nucleoli,Cytosol	HPA075858		Supported
XBP1				
ATF4	Nucleoplasm	CAB011596, HPA062987	Approved	Supported
ATF5	Nucleoplasm,Cytosol	HPA030187	Uncertain	Supported
ATF1	Nucleoplasm	CAB016222, HPA055069, HPA055406	Supported	Enhanced
ATF6		HPA005935	Approved	
ATF6B	Nucleoplasm,Nucleoli	HPA046871	Uncertain	Supported
CREB1	Nucleoplasm	CAB003803, HPA019150	Supported	Enhanced
CREB3	Cytosol	HPA030978		Supported
CREB3L1	Nucleoplasm,Cytosol	HPA024069, CAB026151, HPA062116	Enhanced	Approved
CREB3L2	Nucleoplasm,Endoplasmic reticulum	HPA015068, HPA015534	Enhanced	Supported
CREB3L3		HPA040671, HPA056228	Enhanced	
CREB3L4	Nucleoplasm,Nuclear membrane,Mitochondria	HPA038122	Enhanced	Approved
CREBL2	Nucleoplasm,Mitochondria,Cytosol	HPA043738	Uncertain	Approved
CREBZF	Nucleoplasm,Mitochondria	HPA021526, HPA063652	Uncertain	Approved
CREM	Nucleoplasm,Vesicles	HPA001818, CAB018352	Enhanced	Supported
CEBPA	Nucleoplasm,Vesicles	HPA052734, HPA065037, HPA067937	Approved	Approved
CEBPB	Nucleoplasm	CAB004213, HPA061355, HPA062267	Supported	Enhanced
CEBPD	Nucleoplasm	HPA067581		Approved
CEBPE	Nucleoplasm,Plasma membrane,Cytosol	HPA002928, CAB005113	Enhanced	Supported
CEBPG	Nucleoplasm	HPA012024	Enhanced	Supported
DBP				
DDIT3	Nucleoplasm	HPA058416, HPA068416	Uncertain	Supported
HLF	Nucleoplasm	HPA068156, HPA071210		Enhanced
NFIL3	Nuclear bodies	HPA003261	Approved	Approved
TEF				
TCF12	Nucleoplasm,Nuclear speckles	CAB004432, HPA065827	Approved	Supported
TCF3	Nucleoplasm	CAB018351, HPA049808, HPA062476	Approved	Enhanced
TCF4	Nucleoplasm,Cytosol	CAB020722, HPA025958	Approved	Supported
ASCL1	Nucleoplasm,Cytosol	HPA029217, HPA076307	Enhanced	Approved
ASCL2				
ASCL3		HPA027032	Uncertain	
ASCL4		HPA036116	Uncertain	

ASCL5				
MYF5	Nucleoplasm	HPA041691		Supported
MYF6	Nucleoplasm,Mitotic spindle,Cytosol	HPA065102		Supported
MYOD1	Nucleoplasm	HPA055436, HPA078647		Enhanced
MYOG	Nucleoplasm	HPA028336		Supported
ATOH1	Nucleoplasm,Cytosol	HPA065568, HPA065775		Approved
ATOH7		HPA027008	Uncertain	
ATOH8	Nucleoplasm	HPA028406		Supported
BHLHA15	Nucleoplasm,Golgi apparatus	CAB034083, HPA047834	Enhanced	Approved
BHLHA9				
BHLHE22	Nucleoplasm,Nuclear speckles,Centrosome,Cytosol	HPA064128, HPA064872, HPA067500	Enhanced	Approved
BHLHE23		HPA030575	Uncertain	
FERD3L		HPA043494	Uncertain	
FIGLA		HPA071241	Supported	
HAND1	Nucleoplasm,Nuclear membrane,Nuclear bodies	HPA040925, HPA075313	Uncertain	Approved
HAND2	Nucleoplasm,Vesicles	HPA019591, CAB025576	Approved	Approved
LYL1	Nucleoplasm	HPA072177		Approved
MESP1	Nucleoplasm,Nucleoli	HPA069981		Supported
MESP2				
MSC	Nucleoplasm	HPA062878		Supported
MSGN1				
NEUROD1		HPA003278, CAB022450	Approved	
NEUROD2				
NEUROD4		HPA015545	Enhanced	
NEUROD6		HPA074530	Enhanced	
NEUROG1	Nucleoplasm,Nuclear bodies	HPA072232		Approved
NEUROG2		CAB012342	Uncertain	
NEUROG3	Nucleoplasm,Nuclear speckles	HPA039785	Uncertain	Approved
NHLH2	Nucleoplasm,Nuclear bodies	HPA055238	Uncertain	Approved
OLIG1				
OLIG2	Nucleoplasm,Plasma membrane	HPA003254, CAB019381	Enhanced	Uncertain
OLIG3		HPA018303, CAB024865	Uncertain	
PTF1A	Nucleoplasm	CAB022612, HPA073033		Approved
SCX		HPA043183	Uncertain	
TAL1	Nucleoplasm	CAB017805, HPA047866, HPA073983	Approved	Enhanced
TAL2	Cytosol	HPA030886	Uncertain	Approved
TCF15	Nuclear speckles	HPA050998, HPA060221	Approved	Supported
TCF21	Nucleoplasm	HPA013189, HPA025769	Uncertain	Supported
TCF23	Nuclear speckles	HPA044596		Approved
TWIST1				
TWIST2	Nucleoplasm,Nucleoli	HPA062870		Supported
BHLHE40	Nuclear bodies	HPA028922	Uncertain	Supported
BHLHE41	Nucleoplasm,Vesicles	HPA056035, HPA077617		Supported
HELT		HPA036188, HPA036189	Uncertain	
HES2		HPA039462	Uncertain	

HES3	Nucleoplasm	HPA047927, HPA072697	Uncertain	Approved
HES4	Nucleoplasm,Vesicles,Cytosol	HPA062465		Approved
HES5	Nucleoplasm,Nuclear speckles,Cytosol	HPA065458, HPA076164	Enhanced	Approved
HES6	Nuclear bodies,Cytosol	HPA073997		Approved
HES7	Nucleoplasm,Nucleoli	HPA072105	Uncertain	Approved
HEY1	Nucleoplasm,Nuclear membrane,Cytosol	HPA055599, HPA063472	Enhanced	Approved
HEY2	Nuclear bodies,Aggresome	HPA030205, HPA074851	Approved	Approved
HEYL	Mitochondria,Cytosol	HPA001438, HPA076960	Approved	Approved
AHR	Nucleoplasm,Cytosol	CAB005072, HPA029722, HPA029723	Enhanced	Supported
AHRR	Nucleoplasm,Cytosol	HPA021483		Supported
ARNT	Nucleoplasm,Nuclear bodies	HPA001759, CAB004318	Supported	Supported
ARNT2	Nucleoplasm	HPA001056, CAB005081	Enhanced	Supported
ARNTL				
ARNTL2				
CLOCK	Nucleoplasm,Vesicles	HPA001867, HPA027565	Supported	Supported
EPAS1	Nucleoplasm,Actin filaments,Cytokinetic bridge,Cytosol	HPA031200, HPA069697		Supported
HIF1A	Nucleoplasm,Nuclear bodies	HPA000907, HPA001275, CAB017442	Approved	Enhanced
HIF3A	Nucleoplasm,Plasma membrane,Cytosol	HPA041141	Approved	Supported
NCOA1	Nucleoplasm,Plasma membrane,Cytosol	CAB019402, HPA070213, HPA070520	Supported	Enhanced
NPAS1	Nucleoplasm	HPA043364, HPA072259		Approved
NPAS2	Nucleoplasm	HPA019674	Uncertain	Approved
NPAS3	Nucleoplasm	HPA075337	Enhanced	Supported
NPAS4		HPA039255	Uncertain	
SIM1	Nuclear speckles	HPA024385		Approved
SIM2	Nucleoplasm,Nuclear bodies	HPA029295	Approved	Supported
SOHLH1	Nuclear speckles	HPA059439		Approved
SOHLH2	Vesicles	HPA029182		Uncertain
TCFL5	Nucleoplasm	HPA055223, HPA076419	Enhanced	Supported
MAX	Nucleoplasm,Golgi apparatus	CAB000328, HPA003474	Supported	Supported
MGA	Nucleoplasm,Cytosol	HPA042278, HPA058183	Approved	Approved
MITF		CAB002578, HPA003259	Enhanced	
MLX	Nucleoplasm,Nuclear membrane,Cytosol	CAB025329, HPA052766, HPA064438	Approved	Supported
MLXIP		HPA023084	Approved	
MLXIPL	Nucleoplasm	HPA064640		Supported
MNT	Nucleoplasm	HPA059532, HPA067578		Enhanced
MXD1	Nucleoplasm,Mitochondria,Cytosol	HPA001599	Enhanced	Uncertain
MXD3	Nucleoplasm,Nucleoli	HPA070111		Approved
MXD4	Cytosol	HPA053363		Supported
MXI1	Nucleoplasm,Nucleoli,Nucleoli rim,Cytosol	HPA035319, HPA035320, HPA056762	Approved	Supported
MYC	Nucleoplasm	CAB000084, CAB010307, HPA055893, HPA066556	Approved	Enhanced
MYCL	Nucleoplasm,Mitotic chromosome	HPA063132, HPA074515		Enhanced
MYCN	Nucleoplasm,Nucleoli	HPA057420		Approved
SREBF1	Nucleoplasm,Golgi apparatus,Cytosol	CAB005406, HPA043343, HPA043878	Uncertain	Supported
SREBF2	Nucleoplasm,Endoplasmic reticulum,Vesicles	HPA031962, HPA031963	Approved	Supported
TFAP4	Nucleoplasm,Mitochondria	HPA001912	Supported	Approved

TFE3	Nucleoplasm,Cytosol	HPA023881	Approved	Approved
TFEB	Cytosol	HPA067082		Supported
TFEC	Nucleoplasm,Cytosol	CAB032260, HPA050528, HPA063577	Approved	Supported
USF1	Nucleoplasm	CAB001480, HPA036233, HPA036535, HPA068527	Supported	Enhanced
USF2	Nucleoplasm,Vesicles	HPA029764, HPA046567		Supported
ID1	Nucleoplasm	CAB025915, HPA058518, HPA068861	Approved	Enhanced
ID2	Nucleoplasm,Nuclear bodies,Centrosome	HPA027612		Approved
ID3	Nucleoplasm	CAB021353, HPA024677	Approved	Supported
ID4	Nucleoplasm	HPA075639		Supported
TFAP2A	Nucleoplasm	CAB000326, HPA056871	Enhanced	Enhanced
TFAP2B	Nucleoplasm	HPA034683, HPA062942	Enhanced	Approved
TFAP2C	Nucleoplasm	CAB016061, CAB016079, HPA055179, HPA057076	Enhanced	Supported
TFAP2D	Nucleoplasm,Golgi apparatus,Vesicles	HPA048962		Uncertain
TFAP2E				
AR	Cytosol	CAB000001, HPA004733, CAB065764	Enhanced	Supported
ESR1	Nucleoplasm,Vesicles	CAB000037, HPA000449, HPA000450, CAB055099, CAB072858	Enhanced	Approved
ESR2	Nucleoplasm,Vesicles	HPA068406, CAB079300	Enhanced	Supported
ESRRA	cleoplasm,Nucleoli fibrillar center,Microtubules,Cytokinetic bri	HPA053785, HPA077297	Approved	Supported
ESRRB	Nucleoplasm,Cytosol	HPA074772		Supported
ESRRG	Nucleoplasm	HPA044678	Uncertain	Approved
NR3C1	Nucleoplasm,Mitochondria,Cytosol	HPA004248, CAB010435	Supported	Supported
NR3C2	Nucleoplasm	CAB009155, HPA074979	Enhanced	Supported
PGR		CAB000068, HPA004751, HPA008428, HPA017176, CAB055100	Enhanced	
NR1D1	Nuclear bodies	HPA007935	Uncertain	Supported
NR1D2	Nucleoplasm	HPA053868, HPA054798	Approved	Supported
NR1H2	Nucleoplasm,Vesicles	HPA005446, HPA056838, HPA070212	Uncertain	Enhanced
NR1H3	Nucleoplasm,Cytosol	HPA036443, CAB037109	Approved	Approved
NR1H4	Nucleoplasm	HPA047699		Approved
NR1I2	Nucleoplasm	HPA029309, HPA055121, HPA073926		Supported
NR1I3	Nucleoplasm	HPA051365		Approved
PPARA	Nucleoplasm	HPA058901, HPA067049, CAB075701	Supported	Enhanced
PPARD		CAB017635	Approved	
PPARG	Nucleoplasm,Vesicles	CAB004282, HPA051239, HPA063663	Approved	Enhanced
RARA	Nucleoplasm,Nucleoli,Actin filaments,Cytosol	HPA058282, HPA071548		Supported
RARB	Nucleoplasm	CAB002617, HPA004174	Enhanced	Enhanced
RARG	Nucleoplasm	HPA053883	Supported	Supported
RORA	Nucleoli	CAB009861, HPA074448	Approved	Supported
RORB	Nucleoplasm	HPA008393	Uncertain	Approved
RORC	Nuclear bodies	HPA065620		Supported
THRA	Cytosol	HPA009654, CAB023349	Approved	Uncertain
THRB	Nuclear bodies	CAB002008, CAB002009, HPA061035	Approved	Supported
VDR	Nucleoplasm,Intermediate filaments,Cytosol	CAB004617, HPA047740, HPA072102		Supported
HNF4A	Nucleoplasm	HPA004712, CAB019417	Enhanced	Supported
HNF4G	Nucleoplasm,Cytokinetic bridge,Mitotic spindle,Cytosol	HPA005438, HPA010610	Enhanced	Enhanced
NR2C1	Nucleoplasm,Cell Junctions,Cytosol	HPA035040, HPA035041, HPA067767	Supported	Supported

NR2C2	Nucleoplasm	HPA006313	Approved	Enhanced
NR2E1	Nuclear bodies	HPA036954, HPA055642	Uncertain	Approved
NR2E3				
NR2F1	Nucleoplasm,Cytosol	CAB019281, HPA058974		Approved
NR2F2	Nucleoplasm,Cytosol	HPA058974		Approved
NR2F6	Nuclear speckles,Cytosol	CAB079917		Supported
RXRA	Nucleoplasm,Golgi apparatus	CAB004565, CAB005352	Supported	Supported
RXRB	Nucleoplasm,Nucleoli,Cytosol	CAB002003, HPA071592, HPA075035	Supported	Supported
RXRG	Nucleoplasm,Nuclear bodies	CAB002615, HPA012933	Uncertain	Approved
NR4A1	Nucleoplasm,Nuclear membrane,Cytosol	HPA059742, HPA070142	Approved	Supported
NR4A2	Nuclear speckles	HPA000543, CAB010481	Uncertain	Supported
NR4A3		HPA043360	Uncertain	
NR5A1	Nucleoplasm,Cytosol	HPA079181		Supported
NR5A2	Nuclear speckles	HPA005455, HPA017067	Approved	Uncertain
NR6A1	Nucleoplasm	HPA031461, HPA045145		Approved
NR0B1	Nuclear speckles,Vesicles,Centriolar satellite	HPA061948, HPA067207		Supported
NR0B2	Nucleoplasm,Vesicles	HPA003983		Supported
GATA1	Nucleoplasm	HPA000232, HPA000233, CAB009195	Enhanced	Supported
GATA2	Nucleoplasm	HPA005633, CAB024847	Enhanced	Supported
GATA3	Nucleoplasm	CAB016217, HPA029730	Enhanced	Supported
GATA4	Nuclear bodies	CAB013125, HPA073899	Uncertain	Supported
GATA5	Nucleoplasm,Cytosol	HPA067583		Approved
GATA6	Nucleoplasm	CAB011581, HPA066629	Approved	Supported
GATAD1	Nucleoplasm	HPA006105, HPA072785		Enhanced
GATAD2A	Nucleoplasm	HPA006759, HPA024373	Enhanced	Enhanced
GATAD2B	Nucleoplasm	HPA017015	Approved	Enhanced
MTA1	Nucleoplasm,Cytosol	CAB004508, HPA005544	Supported	Enhanced
MTA2	Nucleoplasm	CAB005315, HPA006214, CAB017522, HPA072727	Supported	Enhanced
MTA3	Nucleoplasm,Vesicles	HPA039433	Approved	Supported
RERE	Nucleoplasm,Nuclear bodies	HPA024093	Uncertain	Supported
TRPS1	Nucleoplasm	HPA060380, HPA071767	Enhanced	Supported
ZGLP1	Nucleoplasm,Vesicles	HPA049855	Uncertain	Approved
EGR1	Nucleoplasm	CAB019427, HPA029937, HPA029938	Supported	Enhanced
EGR2	Nucleoplasm	HPA031165		Supported
EGR3	Nucleoplasm,Vesicles	HPA006206, CAB009373	Uncertain	Uncertain
EGR4		HPA028983	Enhanced	
KLF1	Nucleoplasm	HPA051850	Enhanced	Supported
KLF10				
KLF11	Nucleoplasm,Nuclear bodies,Focal adhesion sites,Cytosol	HPA058276		Supported
KLF12	Nucleoplasm,Cytosol	HPA064146, HPA075652	Approved	Approved
KLF13				
KLF14		HPA044729	Uncertain	
KLF15	Nucleoplasm,Nuclear speckles,Vesicles	HPA003286, HPA028866	Uncertain	Supported
KLF16	Nucleoplasm	HPA052481	Approved	Supported
KLF17		HPA024629	Enhanced	

KLF2	Nucleoplasm	HPA055964	Approved	Approved
KLF3	Nucleoplasm	HPA049512, HPA065054	Supported	Enhanced
KLF4	Nucleoplasm,Cytosol	HPA002926	Enhanced	Supported
KLF5	Nucleoplasm,Golgi apparatus,Vesicles	HPA040398	Approved	Supported
KLF6	Nucleoplasm,Nucleoli,Vesicles,Cytosol	CAB013529, HPA069585	Supported	Supported
KLF7	Nucleoplasm,Cytosol	HPA003423, HPA030490	Approved	Supported
KLF8	Nucleoplasm	HPA071740		Supported
KLF9	Nucleoplasm,Plasma membrane,Cytosol	HPA029308		Supported
SP1	Nucleoplasm	CAB000330, HPA001853, HPA012292	Supported	Enhanced
SP2	Nucleoplasm,Vesicles	HPA003357, CAB010245	Supported	Supported
SP3	Nucleoplasm,Cytosol	CAB004580, HPA032145, HPA032146	Supported	Supported
SP4	Nucleoplasm,Cytosol	CAB010246, HPA040395	Uncertain	Enhanced
SP5	Nucleoplasm,Nucleoli	HPA074123		Approved
SP6	Nucleoplasm,Mitotic spindle,Centrosome	HPA024516, HPA044469		Supported
SP7		HPA063202	Enhanced	
SP8	Nucleoplasm	HPA054006	Uncertain	Approved
SP9	Mitochondria	HPA062616	Uncertain	Approved
AEBP2	Nucleoplasm	HPA020893, HPA022282	Uncertain	Enhanced
OSR1		HPA015525	Uncertain	
OVOL3	Nucleoplasm,Vesicles,Cytosol	HPA047540		Approved
ZBTB22	Nucleoplasm	HPA043848		Approved
ZBTB32		HPA076730	Enhanced	
ZBTB33	Nucleoplasm,Plasma membrane,Cytosol	HPA000755, CAB001980, HPA005732	Approved	Enhanced
ZBTB34	Nucleoplasm	HPA023893	Approved	Supported
ZBTB37	Nucleoplasm,Nucleoli fibrillar center,Cytosol	HPA023438	Approved	Approved
ZBTB43	Nucleoplasm,Nucleoli	HPA016825	Enhanced	Approved
ZBTB46	Nucleoplasm	HPA013997, HPA048051	Uncertain	Approved
ZBTB5	Nucleoplasm,Nuclear bodies,Golgi apparatus	HPA021521	Approved	Approved
ZBTB8B	Nucleoplasm,Cytosol	HPA055553, HPA071427	Enhanced	Approved
ZFPM2	Nucleoplasm	HPA004094	Approved	Supported
ZNF174	Nucleoli fibrillar center,Nuclear bodies	HPA009656, HPA070692	Approved	Supported
ZNF396		HPA006686	Uncertain	
ZNF414	Nucleoplasm	HPA053769	Approved	Approved
ZNF446	Nuclear speckles	HPA052786, HPA078262	Uncertain	Supported
ZNF487	Nucleoplasm,Nuclear membrane,Cytosol	HPA037820		Approved
ZNF511	Nucleoplasm,Nuclear bodies,Vesicles	HPA035360	Uncertain	Approved
ZNF580	Nucleoplasm	HPA054058	Approved	Approved
ZNF705A				
ZNF740	Nucleoplasm	HPA035691	Approved	Approved
ZSCAN1	Nucleoli	HPA007938	Uncertain	Uncertain
BCL6	Nucleoplasm,Golgi apparatus	CAB000307, HPA004899, HPA050645	Enhanced	Supported
BCL6B	Nucleoplasm,Vesicles,Plasma membrane	HPA059147, HPA075112	Uncertain	Approved
CTCF	Nucleoplasm	HPA004122, CAB062550, CAB068181, CAB068182	Supported	Supported
CTCFL	Nucleoplasm,Nuclear bodies,Cytosol	HPA001472	Enhanced	Approved
FEZF1	Nucleoplasm,Cytosol	HPA064639, HPA073693	Approved	Supported

FEZF2				
GFI1		CAB011197	Uncertain	
GFI1B	Nucleoplasm,Plasma membrane	CAB004420, HPA006291	Enhanced	Approved
GLI1	Nucleoplasm,Cytosol	CAB009460, HPA065172, HPA068903	Uncertain	Supported
GLI2	Nucleoplasm,Nucleoli	CAB008558, HPA074275	Approved	Supported
GLI3	Nucleoplasm,Nucleoli,Vesicles	HPA005534	Approved	Supported
GLI4	Nucleoplasm,Cytokinetic bridge	HPA023131	Uncertain	Approved
GLIS1	Nucleoplasm,Mitochondria,Cytosol	HPA001409	Uncertain	Approved
GLIS2	Nucleoplasm	HPA062755, HPA065406		Enhanced
GLIS3	Nucleoplasm,Golgi apparatus	HPA056426	Approved	Approved
GTF3A	Nucleoplasm	HPA007990	Supported	Supported
GZF1	Nucleoplasm,Nucleoli	HPA024520	Uncertain	Supported
MTF1	Nucleoplasm	HPA028604, HPA028689	Uncertain	Enhanced
MYNN	Nucleoplasm	HPA045149	Approved	Supported
MZF1	Nucleoplasm	HPA001757	Approved	Supported
OSR2	Nucleoplasm,Plasma membrane	HPA052425, HPA063486	Uncertain	Approved
OVOL1	Nucleoplasm	HPA003984	Uncertain	Approved
OVOL2	Nucleoplasm,Cytosol	HPA038531	Uncertain	Approved
PLAG1	Nucleoplasm,Nuclear speckles,Centrosome,Cytosol	HPA072290		Supported
PLAGL1	Nuclear bodies,Golgi apparatus,Vesicles	HPA055706		Supported
PLAGL2	Nucleoplasm,Cytosol	HPA005900	Uncertain	Approved
PRDM1	Nucleoplasm,Nucleoli	HPA030033, HPA075427	Approved	Supported
PRDM14	Nucleoplasm,Vesicles	HPA019094	Uncertain	Supported
PRDM5	Nucleoplasm,Nucleoli,Nuclear bodies	HPA051406	Enhanced	Supported
PRDM6	Nucleoplasm	HPA030324	Uncertain	Approved
RBAK	Nucleoplasm	HPA071231		Supported
SCRT1	Nuclear bodies	HPA045265	Uncertain	Supported
SCRT2	Nucleoplasm	HPA067697		Approved
SNAI1	Nucleoplasm	CAB005883, HPA056831, HPA069985, CAB078687, CAB080398	Supported	Supported
SNAI2	Nucleoplasm	CAB011671	Supported	Supported
SNAI3	Nucleoplasm	HPA071127		Approved
WT1	Nucleoplasm,Cytosol	CAB000327, HPA035717, HPA053848	Enhanced	Enhanced
YY1	Nucleoplasm,Nucleoli fibrillar center,Nuclear bodies	HPA000597, HPA001119, CAB009392	Supported	Supported
YY2	Nucleoplasm,Nuclear bodies	HPA000597, HPA001119, HPA030335	Enhanced	Approved
ZBTB11	Nucleoplasm	HPA015328, HPA024138	Approved	Enhanced
ZBTB12	Nucleoplasm	HPA047161, HPA070639	Approved	Enhanced
ZBTB14	Nucleoplasm,Nucleoli,Cytosol	HPA050758, HPA070819	Uncertain	Supported
ZBTB16		HPA001499, CAB004540	Uncertain	
ZBTB18	Nucleoplasm,Nuclear speckles	HPA024656, HPA074019	Enhanced	Supported
ZBTB20	Nucleoplasm,Nuclear bodies	HPA015551, HPA016815	Approved	Enhanced
ZBTB24	Nucleoplasm,Cytokinetic bridge,Centrosome	HPA017872, HPA049003	Uncertain	Approved
ZBTB26		HPA021444	Uncertain	
ZBTB40	Nucleoplasm	HPA042386		Approved
ZBTB42	Nucleoplasm	HPA066961	Approved	Supported
ZBTB44	Nucleoplasm	HPA052589	Approved	Approved

ZBTB45	Nuclear bodies,Cytosol	HPA035754, HPA075485	Uncertain	Uncertain
ZBTB47	Nucleoplasm	HPA074057	Enhanced	Approved
ZBTB48	Nucleoplasm,Cytosol	HPA030417	Uncertain	Supported
ZBTB49	Nucleoplasm,Microtubules,Cytosol	HPA024450	Approved	Approved
ZBTB6	Nucleoplasm,Mitochondria	HPA054111, HPA076894	Approved	Approved
ZBTB7A	Nucleoplasm	CAB037214, CAB037285, HPA046387	Supported	Approved
ZBTB7B	Nucleoplasm	HPA006811, HPA025820	Enhanced	Enhanced
ZBTB7C				
ZFP1	Nucleoplasm	HPA044916, HPA062910	Uncertain	Approved
ZFP14	Nuclear speckles	HPA030637	Uncertain	Approved
ZFP2		HPA023364	Uncertain	
ZFP28	Nucleoplasm,Cytosol	HPA028879	Uncertain	Approved
ZFP3	Nucleoplasm,Cytosol	HPA053085, HPA057290	Approved	Approved
ZFP30	Cytosol	HPA030606	Uncertain	Approved
ZFP37	Nucleoplasm,Vesicles	HPA049112	Uncertain	Approved
ZFP41	Nucleoplasm,Nucleoli,Nucleoli rim,Plasma membrane	HPA047232, HPA056268	Uncertain	Approved
ZFP42		CAB025557	Uncertain	
ZFP62	Nucleoplasm,Actin filaments	HPA048629	Approved	Approved
ZFP64	Nucleoplasm	HPA035112, HPA058387	Uncertain	Approved
ZFP69	Nucleoplasm	HPA027808, HPA054942	Uncertain	Approved
ZFP69B	Nucleoplasm,Cytosol	HPA044289	Uncertain	Approved
ZFP82	Nucleoplasm,Nuclear membrane,Cytosol	HPA051669, HPA060470	Uncertain	Approved
ZFP90	Vesicles,Plasma membrane,Centrosome	HPA029017	Uncertain	Supported
ZFP91	Nucleoplasm,Nucleoli	HPA024037, CAB025417, HPA065325, HPA074591	Enhanced	Supported
ZFP92		HPA003030	Uncertain	
ZFX	Nucleoplasm,Nucleoli	HPA001748, HPA003877, HPA071863	Supported	Approved
ZFY	Nucleoplasm,Nucleoli	HPA001748, HPA071863	Uncertain	Approved
ZIC1	Nucleoplasm	HPA004098	Supported	Supported
ZIC2	Nuclear bodies	HPA055031		Supported
ZIC3	Nucleoplasm	HPA052936, HPA069523	Uncertain	Supported
ZIC4	Nucleoplasm,Cytosol	HPA030194	Uncertain	Approved
ZIC5	Nucleoplasm,Vesicles	HPA074045		Uncertain
ZIK1	Intermediate filaments	HPA059975	Uncertain	Approved
ZIM2	Nuclear speckles,Cytosol	HPA071856		Approved
ZIM3		HPA034575	Uncertain	
ZKSCAN1	Nucleoplasm,Nuclear bodies,Mitochondria	HPA006672	Uncertain	Approved
ZKSCAN2	Nucleoplasm,Nucleoli fibrillar center,Golgi apparatus	HPA049141	Uncertain	Approved
ZKSCAN3	Nucleoplasm	HPA009637, HPA060116	Approved	Supported
ZKSCAN4	Nucleoplasm	HPA009637	Approved	Supported
ZKSCAN5	Intermediate filaments	HPA003201	Uncertain	Approved
ZKSCAN7	Nuclear speckles,Cytosol	HPA048312, HPA049906, HPA071850	Uncertain	Supported
ZKSCAN8	Nucleoplasm,Cytosol	HPA003483	Approved	Approved
ZNF10	Nucleoplasm,Vesicles,Plasma membrane	HPA003204	Uncertain	Approved
ZNF100		HPA059135	Uncertain	
ZNF107				

ZNF114	Nucleoplasm,Cytosol	HPA025019, HPA029553	Uncertain	Approved
ZNF117	Nucleoli fibrillar center,Cytosol	HPA031148		Approved
ZNF121	Nucleoplasm,Nucleoli,Centrosome	HPA054961	Uncertain	Approved
ZNF124	Nucleoplasm,Nuclear membrane	HPA031127	Uncertain	Approved
ZNF131	Nucleoplasm,Intermediate filaments	HPA007023, HPA027748, HPA074630	Approved	Enhanced
ZNF133	Nucleoplasm,Vesicles	HPA001493	Supported	Approved
ZNF135	Cytosol	HPA006961	Approved	Approved
ZNF136	Nucleoli	HPA054120		Approved
ZNF138				
ZNF14	Nucleoplasm,Cytosol	HPA049863	Uncertain	Approved
ZNF140	Nucleoplasm	HPA003655, HPA048332	Uncertain	Approved
ZNF141				
ZNF143	Nucleoplasm,Nuclear bodies,Golgi apparatus,Vesicles	HPA003263, HPA003264, HPA074818	Supported	Supported
ZNF146	Nucleoli	HPA003358	Supported	Supported
ZNF148	Nucleoplasm,Golgi apparatus	HPA001656, CAB017486	Supported	Supported
ZNF154	Nucleoplasm,Nucleoli fibrillar center	HPA076284	Approved	Approved
ZNF155	Nucleoplasm,Cytosol	HPA003220	Uncertain	Approved
ZNF157		HPA003319	Uncertain	
ZNF16	Nucleoplasm,Nucleoli	HPA035782, HPA061835	Uncertain	Supported
ZNF160	Nuclear speckles,Cytosol	HPA023599	Uncertain	Approved
ZNF165	Nuclear speckles,Microtubules	HPA007247		Approved
ZNF169	Nucleoplasm,Nuclear speckles,Vesicles,Cytosol	HPA041769, HPA061185	Uncertain	Approved
ZNF17				
ZNF175	Nucleoli,Cytosol	HPA002717, HPA074615	Uncertain	Supported
ZNF177		HPA003141	Uncertain	
ZNF18	Nucleoplasm,Golgi apparatus,Plasma membrane,Cytosol	HPA006144	Uncertain	Approved
ZNF180	Nucleoplasm	HPA028979, HPA028980	Uncertain	Approved
ZNF181	Nucleoplasm	HPA043701, HPA062305	Uncertain	Approved
ZNF182	Nucleoplasm	HPA059574	Uncertain	Approved
ZNF184	Nucleoplasm	HPA003226, HPA064793	Uncertain	Supported
ZNF189	Intermediate filaments	HPA034814	Uncertain	Approved
ZNF19	Mitochondria	HPA063685	Approved	Approved
ZNF197	Nucleoplasm	HPA054567	Uncertain	Approved
ZNF2	Nucleoplasm,Plasma membrane,Cytosol	HPA041358	Uncertain	Approved
ZNF20	Nucleoplasm,Nuclear membrane,Nuclear bodies	HPA020887, HPA073206	Uncertain	Approved
ZNF200	Mitochondria	HPA018981, HPA059310	Uncertain	Approved
ZNF202	Nucleoli rim,Nuclear bodies,Mitotic chromosome	HPA059229		Approved
ZNF205	Nucleoplasm	HPA001791	Uncertain	Approved
ZNF208	Golgi apparatus,Cytosol	HPA077100		Uncertain
ZNF212	Nucleoplasm	HPA049807, HPA054178	Uncertain	Supported
ZNF213	Nucleoplasm,Nuclear membrane,Vesicles,Cytosol	HPA035000, HPA056750	Uncertain	Approved
ZNF214	Nucleoplasm,Midbody	HPA055391	Uncertain	Approved
ZNF215	Nucleoplasm,Vesicles	HPA047243, HPA051010	Enhanced	Approved
ZNF22	Nucleoplasm	HPA016736, HPA054893	Approved	Enhanced
ZNF221	Nuclear bodies	HPA059435	Uncertain	Approved

ZNF222	Nucleoplasm,Nuclear bodies	HPA004086	Approved	Approved
ZNF223	Nucleoplasm,Mitochondria	HPA067823, HPA079175	Approved	Approved
ZNF224	Nucleoplasm,Nuclear membrane	HPA054045	Approved	Supported
ZNF225	Nucleoplasm	HPA073778		Approved
ZNF226	Nuclear bodies	HPA006145, CAB034353, HPA069192	Uncertain	Approved
ZNF227	Plasma membrane	HPA030573	Uncertain	Approved
ZNF229	Vesicles,Plasma membrane	HPA001494	Uncertain	Uncertain
ZNF23	Cytosol	HPA023534	Uncertain	Approved
ZNF230	Mitochondria	HPA006172		Approved
ZNF232	Nucleoplasm,Vesicles,Cytosol	HPA027309, HPA028611	Uncertain	Supported
ZNF233	Nucleoplasm	HPA020975	Uncertain	Approved
ZNF234	Nuclear bodies	HPA001664	Approved	Approved
ZNF235	Nucleoplasm	HPA045582	Uncertain	Approved
ZNF239	Nucleoplasm,Nuclear bodies	HPA030843, HPA046679	Uncertain	Approved
ZNF24	Nucleoplasm	HPA024062, CAB025642	Supported	Supported
ZNF25	Nucleoplasm,Golgi apparatus	HPA030190	Uncertain	Approved
ZNF250	Nuclear speckles,Cytosol	HPA020853	Uncertain	Approved
ZNF253				
ZNF254				
ZNF257				
ZNF26	Nuclear bodies,Mitochondria	HPA018329, HPA042406	Uncertain	Approved
ZNF260	Nucleoplasm,Cytosol	HPA028806	Approved	Supported
ZNF263	Nucleoplasm,Mitotic spindle,Cytosol	HPA035085, HPA035086	Approved	Supported
ZNF264	Nucleoplasm	HPA046330		Approved
ZNF266	Nucleoplasm,Vesicles,Rods & Rings	HPA026836, HPA054110	Uncertain	Supported
ZNF267	Nucleoplasm	HPA003866	Approved	Approved
ZNF268	Cytosol	HPA001794	Approved	Approved
ZNF273	Nucleoplasm	HPA077549		Approved
ZNF274	Nucleoplasm,Centrosome	HPA028880	Uncertain	Approved
ZNF275	Nucleoli	HPA000566	Approved	Approved
ZNF276	Nucleoplasm,Nucleoli,Plasma membrane,Cytosol	HPA021309, HPA022255	Uncertain	Approved
ZNF28				
ZNF280A	Nucleoplasm	HPA044475		Approved
ZNF280B	Nucleoplasm	HPA059519	Uncertain	Approved
ZNF280C	Nucleoplasm,Nucleoli fibrillar center	HPA051494, HPA055788	Enhanced	Approved
ZNF280D	Nucleoplasm,Golgi apparatus,Centrosome,Cytosol	HPA030233	Approved	Approved
ZNF281	Nucleoplasm	HPA051228	Uncertain	Enhanced
ZNF282	Nucleoplasm,Cytosol	HPA024374	Supported	Approved
ZNF283	Nucleoplasm	HPA072977		Approved
ZNF284				
ZNF285		HPA046100, HPA049212	Uncertain	
ZNF286A	Nucleoli	HPA021594		Approved
ZNF286B				
ZNF287		HPA029290	Uncertain	
ZNF3	Nucleoplasm	HPA003719	Supported	Approved

ZNF30	Nucleoplasm,Cytosol	HPA030400	Uncertain	Approved
ZNF300	Nucleoplasm,Nucleoli,Nucleoli rim,Nuclear bodies	HPA028975, HPA051777	Uncertain	Enhanced
ZNF302	Nucleoplasm,Cytosol	HPA038377		Approved
ZNF311		HPA050541	Approved	
ZNF317	Nucleoplasm	HPA042075, HPA048625	Approved	Enhanced
ZNF32	Nucleoplasm,Nuclear bodies,Cytosol	HPA039148, HPA059640		Approved
ZNF320	Nucleoplasm,Cytoplasmic bodies	HPA043696, HPA062474	Uncertain	Approved
ZNF322	Nucleoplasm,Centrosome,Cytosol	HPA043161, HPA046692	Approved	Supported
ZNF324	Nucleoplasm,Nuclear membrane,Cytosol	HPA039774	Uncertain	Approved
ZNF324B	Nucleoli,Cytosol	HPA023400	Uncertain	Approved
ZNF329	Nucleoplasm	HPA044373, HPA077958	Uncertain	Approved
ZNF331	Nucleoplasm	HPA063157		Approved
ZNF333	Nucleoplasm,Cytosol	HPA043973, HPA054680, HPA077662	Uncertain	Approved
ZNF337	Nuclear speckles,Plasma membrane	HPA044303, HPA064219	Approved	Approved
ZNF33A	Nucleoli fibrillar center,Endoplasmic reticulum,Vesicles	HPA005538, HPA056638	Approved	Approved
ZNF33B	Nucleoli fibrillar center,Endoplasmic reticulum,Vesicles	HPA005538, HPA056638	Approved	Approved
ZNF34	Nucleoplasm,Cytosol	HPA053925, HPA060755, HPA066805	Uncertain	Enhanced
ZNF343	Nucleoplasm	HPA030587, HPA052438	Approved	Approved
ZNF345		HPA025816	Uncertain	
ZNF347	Microtubules	HPA023448	Uncertain	Approved
ZNF35	Nucleoplasm	HPA051859, HPA054738	Uncertain	Approved
ZNF350	Nucleoplasm,Nuclear bodies	HPA001521	Uncertain	Supported
ZNF354A	Nucleoplasm,Nucleoli,Cytosol	HPA052043	Approved	Supported
ZNF354B	Nucleoplasm	HPA029056	Approved	Approved
ZNF354C	Nucleoplasm	HPA030904, HPA068570	Uncertain	Supported
ZNF358	Nucleoplasm	HPA030887, HPA063624	Approved	Approved
ZNF362	Nucleoplasm,Nucleoli	HPA059239	Uncertain	Approved
ZNF366	Nucleoplasm	HPA023128, HPA023526	Uncertain	Supported
ZNF383	Nucleoplasm,Nuclear membrane	HPA063945	Approved	Supported
ZNF384	Nucleoplasm	HPA004051	Supported	Approved
ZNF391		HPA050886	Uncertain	
ZNF394	Nucleoplasm,Cytosol	HPA051421, HPA054639	Approved	Enhanced
ZNF397	Nucleoli,Plasma membrane,Microtubules,Cytosol	HPA026087	Uncertain	Approved
ZNF398	Nucleoplasm,Nuclear membrane	HPA073151		Approved
ZNF404	Nucleoli,Nucleoli rim,Intermediate filaments	HPA032014, HPA063607	Uncertain	Supported
ZNF408	Centriolar satellite,Cytosol	HPA017892	Uncertain	Approved
ZNF41	Nucleoplasm	HPA069102	Supported	Approved
ZNF410	Nuclear bodies	HPA002871		Approved
ZNF415	Nucleoli fibrillar center,Microtubules	HPA005553	Approved	Approved
ZNF416	Nucleoplasm,Nuclear speckles	HPA078491		Approved
ZNF419	Nucleoplasm,Nucleoli fibrillar center	HPA003152, HPA003274	Uncertain	Approved
ZNF420	Nucleoplasm	HPA059675	Approved	Approved
ZNF425	Nucleoplasm	HPA020087	Uncertain	Supported
ZNF426	Nucleoplasm	HPA029342	Approved	Approved
ZNF429	Nucleoplasm	HPA004139		Approved

ZNF43		HPA050271	Uncertain	
ZNF430		HPA059135	Uncertain	
ZNF431		HPA059135	Uncertain	
ZNF432	Nucleoplasm	HPA052082, HPA060274, HPA065606	Uncertain	Enhanced
ZNF433	Nuclear bodies	HPA073724	Enhanced	Approved
ZNF436	Nucleoplasm,Cytosol	HPA043817	Approved	Supported
ZNF439	Nucleoplasm	HPA047845	Approved	Uncertain
ZNF44	Nucleoplasm,Microtubules,Cytosol	HPA003146	Supported	Approved
ZNF440	Nucleoplasm	HPA047845	Approved	Approved
ZNF441	Nuclear speckles	HPA063338		Approved
ZNF442	Nuclear membrane,Vesicles	HPA045738, HPA053407	Uncertain	Approved
ZNF443	Nucleoplasm,Centriolar satellite	HPA003150, HPA018683	Approved	Approved
ZNF444	Nucleoplasm,Nucleoli	HPA056895, HPA065203	Approved	Enhanced
ZNF449	Nucleoplasm,Cytosol	HPA018079	Uncertain	Approved
ZNF45	Nucleoplasm	HPA038611, HPA075124	Uncertain	Supported
ZNF454		HPA024556	Uncertain	
ZNF460	Nucleoplasm	HPA053004, HPA066506	Uncertain	Approved
ZNF461	Nucleoplasm,Nucleoli,Golgi apparatus	HPA021139	Uncertain	Approved
ZNF468				
ZNF471	Nucleoplasm	HPA066695	Uncertain	Supported
ZNF479				
ZNF480	Nucleoplasm,Cytosol	HPA042218	Uncertain	Supported
ZNF483	Nucleoli	HPA055041		Approved
ZNF484	Nucleoplasm,Nuclear membrane,Cytosol	HPA028973	Uncertain	Approved
ZNF485		HPA059356	Uncertain	
ZNF490		HPA005576	Uncertain	
ZNF491				
ZNF492				
ZNF493				
ZNF496	Nucleoplasm,Cytosol	HPA025824		Approved
ZNF497	Nucleoplasm,Cell Junctions	HPA047872	Uncertain	Approved
ZNF500	Nucleoplasm,Cytosol	HPA035498, HPA057963	Supported	Enhanced
ZNF501	Endoplasmic reticulum	HPA044161	Uncertain	Approved
ZNF502		HPA024761, HPA029557	Uncertain	
ZNF506				
ZNF514	Nucleoplasm,Nuclear bodies	HPA028974	Approved	Approved
ZNF517	Nucleoplasm	HPA055337		Supported
ZNF524	Nucleoplasm	HPA050981	Uncertain	Approved
ZNF525				
ZNF528	Nuclear speckles,Cytosol	HPA065435		Approved
ZNF529	Nucleoplasm	HPA031311	Uncertain	Approved
ZNF530	Vesicles	HPA045868	Uncertain	Approved
ZNF534				
ZNF540	Nucleoplasm,Vesicles,Cytosol	HPA003444	Supported	Supported
ZNF543	Nucleoplasm	HPA046330		Approved

ZNF544	Nucleoplasm	HPA002732	Approved	Approved
ZNF546	Nucleoplasm,Cytosol	HPA030838		Approved
ZNF547		HPA056001	Uncertain	
ZNF548	Nucleoplasm,Cytosol	HPA066042	Uncertain	Approved
ZNF549	Nucleoplasm,Cytosol	HPA039511	Approved	Approved
ZNF550	Vesicles,Microtubules	HPA003251	Uncertain	Approved
ZNF551	Nucleoplasm,Nuclear speckles	HPA038187		Approved
ZNF554	Nucleoplasm,Nucleoli	HPA060247, HPA063358	Uncertain	Enhanced
ZNF555		HPA053956, HPA055430	Uncertain	
ZNF556	Nucleoplasm	HPA060593		Approved
ZNF557	Nucleoplasm,Intermediate filaments	HPA003151, HPA029291	Uncertain	Approved
ZNF558		HPA003151	Uncertain	
ZNF559	Nucleoplasm,Cytosol	HPA059632, HPA065485	Approved	Supported
ZNF560	Nucleoplasm,Nuclear speckles	HPA045439, HPA074438		Enhanced
ZNF561	Nucleoplasm	HPA055332, HPA062397	Uncertain	Approved
ZNF562	Nucleoplasm	HPA055332, HPA062397	Uncertain	Approved
ZNF563	Vesicles	HPA042136	Uncertain	Approved
ZNF564	Golgi apparatus	HPA043998	Uncertain	Approved
ZNF565	Nucleoplasm,Nuclear bodies	HPA044123	Uncertain	Approved
ZNF566	Nucleoplasm	HPA023456	Uncertain	Approved
ZNF567	Nucleoplasm,Cytosol	HPA023937	Uncertain	Approved
ZNF568	Nucleoplasm	HPA024541, HPA052061	Uncertain	Approved
ZNF569	Nucleoplasm,Golgi apparatus,Vesicles	HPA052994	Uncertain	Approved
ZNF57	Nucleoli	HPA061915		Approved
ZNF570	Nucleoplasm	HPA028881	Uncertain	Approved
ZNF571	Nucleoplasm,Nucleoli,Plasma membrane	HPA054762		Approved
ZNF572	Nuclear speckles	HPA066838	Approved	Approved
ZNF575	Nucleoplasm,Nucleoli fibrillar center,Cytosol	HPA051865	Approved	Approved
ZNF576	Nucleoplasm,Golgi apparatus	HPA050064	Uncertain	Approved
ZNF577	Nucleoplasm,Nucleoli	HPA041499, HPA046761	Uncertain	Supported
ZNF581	Nucleoplasm,Vesicles,Cytosol	HPA004095, HPA059657	Uncertain	Approved
ZNF582	Nucleoplasm,Cytosol	HPA028858	Uncertain	Approved
ZNF583	Nucleoli,Cytosol	HPA023805, HPA023957	Uncertain	Approved
ZNF584	Nuclear speckles	HPA043408, HPA048075	Uncertain	Approved
ZNF585A				
ZNF585B	Nucleoplasm,Cytoplasmic bodies	HPA075556		Approved
ZNF586	Nucleoli fibrillar center,Vesicles	HPA030306, HPA060782	Approved	Approved
ZNF589	Nucleoplasm	HPA003145	Uncertain	Supported
ZNF594	Nucleoplasm	HPA060283, HPA067178, HPA069360, HPA074493, HPA077596	Approved	Enhanced
ZNF595				
ZNF596	Nucleoli,Mitochondria	HPA055463	Uncertain	Approved
ZNF599	Nucleoplasm,Plasma membrane,Cytosol	HPA014701	Uncertain	Approved
ZNF600				
ZNF605	Nucleoplasm,Vesicles	HPA061970, HPA062655	Uncertain	Approved
ZNF607	Nucleoplasm	HPA049465, HPA055018	Uncertain	Approved

ZNF610	Nucleoplasm	HPA055771, HPA078134	Enhanced	Approved
ZNF611				
ZNF613	Nucleoplasm,Vesicles	HPA026833	Uncertain	Approved
ZNF614	Nucleoplasm,Vesicles,Cytosol	HPA021197	Uncertain	Approved
ZNF615	Nucleoli,Mitochondria	HPA052422	Uncertain	Approved
ZNF616	Centrosome	HPA062639, HPA071539	Approved	Approved
ZNF619	Nucleoplasm,Intermediate filaments	HPA045094	Uncertain	Approved
ZNF620	Centrosome	HPA031452, HPA059383	Uncertain	Approved
ZNF621	Nuclear speckles	HPA065518, HPA071440		Enhanced
ZNF623	Nucleoplasm	HPA044372	Uncertain	Approved
ZNF624	Vesicles	HPA023157, HPA023244	Uncertain	Approved
ZNF625	Nucleoplasm,Mitochondria	HPA074014		Approved
ZNF626	Nucleoplasm,Nuclear membrane,Vesicles	HPA045631	Uncertain	Approved
ZNF627	Nucleoplasm,Nucleoli	HPA049770, HPA073463	Uncertain	Approved
ZNF641	Nucleoplasm,Nucleoli,Cytosol	HPA035189, HPA075069	Uncertain	Supported
ZNF648		HPA053897	Uncertain	
ZNF649		HPA026541	Uncertain	
ZNF652	Nucleoplasm	HPA044408	Uncertain	Approved
ZNF653	Nucleoplasm	HPA023631, HPA023635	Uncertain	Supported
ZNF655	Nucleoplasm,Plasma membrane	HPA029534, HPA050750	Uncertain	Supported
ZNF66				
ZNF660	Nucleoli fibrillar center,Cytosol	HPA029705	Uncertain	Uncertain
ZNF662	Nuclear bodies,Cytosol	HPA039116, HPA040377	Uncertain	Approved
ZNF664	Nucleoplasm	HPA047846		Approved
ZNF665	Nuclear membrane	HPA023437	Uncertain	Approved
ZNF667	Nucleoplasm	HPA030731, HPA046673	Uncertain	Approved
ZNF669		HPA044043	Uncertain	
ZNF670	Nucleoplasm,Cytosol	HPA003142	Uncertain	Approved
ZNF672	Nucleoplasm	HPA055779, HPA062506	Approved	Enhanced
ZNF675				
ZNF676				
ZNF677	Nucleoplasm,Centrosome	HPA023106	Approved	Approved
ZNF678	Nucleoplasm,Nucleoli,Cytosol	HPA028507	Uncertain	Approved
ZNF679				
ZNF680	Nucleoplasm	HPA003173		Approved
ZNF681				
ZNF682	Golgi apparatus	HPA055998	Uncertain	Uncertain
ZNF683		HPA023865	Uncertain	
ZNF684	Nucleoplasm,Cytosol	HPA059153	Uncertain	Approved
ZNF689	Nucleoplasm,Nucleoli fibrillar center	HPA054095, HPA055385	Approved	Approved
ZNF691	Nucleoplasm,Nucleoli,Vesicles	HPA062527	Approved	Approved
ZNF692	Nucleoplasm,Nucleoli	HPA059337, HPA062792	Uncertain	Enhanced
ZNF695	Nuclear speckles	HPA024743	Uncertain	Approved
ZNF696	Nucleoplasm,Nucleoli	HPA057855, HPA072473	Uncertain	Approved
ZNF7	Mitochondria	HPA059090	Approved	Approved

ZNF70	Cytosol	HPA018821	Uncertain	Approved
ZNF701	Nucleoplasm,Centriolar satellite	HPA005532	Uncertain	Approved
ZNF705D				
ZNF705E				
ZNF705G				
ZNF707	Nucleoplasm	HPA044991	Approved	Approved
ZNF709	Nucleoplasm	HPA045573, HPA053153	Uncertain	Approved
ZNF71	Nucleoplasm,Nucleoli	HPA018124	Uncertain	Approved
ZNF710	Nucleoplasm,Mitochondria	HPA030226, HPA030227, HPA030228	Uncertain	Approved
ZNF713	Nucleoli	HPA059425, HPA062887	Uncertain	Approved
ZNF716				
ZNF718				
ZNF721	Centriolar satellite,Cytosol	HPA065313		Approved
ZNF726				
ZNF727	Nucleoplasm	HPA051027, HPA061215	Uncertain	Uncertain
ZNF729				
ZNF730				
ZNF732				
ZNF736	Nucleoplasm	HPA051027, HPA061215	Uncertain	Uncertain
ZNF737				
ZNF74	Nucleoplasm,Actin filaments	HPA003354	Approved	Supported
ZNF746	Nucleoplasm,Cytosol	HPA020272	Approved	Supported
ZNF747	Centrosome,Cytosol	HPA003203, HPA069173	Uncertain	Approved
ZNF749	Nucleoplasm,Nuclear membrane	HPA042082, HPA042091, HPA056001	Uncertain	Approved
ZNF75A	Nucleoplasm,Nucleoli fibrillar center	HPA001665	Uncertain	Approved
ZNF75D	Nucleoplasm,Golgi apparatus	HPA004705	Supported	Approved
ZNF76	Nucleoplasm,Cytosol	HPA030090, HPA030091	Approved	Approved
ZNF763	Nucleoplasm	HPA047845	Approved	Uncertain
ZNF764	Cytosol	HPA003203	Uncertain	Approved
ZNF765				
ZNF766	Centrosome	HPA044882, HPA063496	Uncertain	Supported
ZNF768	Nucleoplasm,Vesicles	HPA024630, HPA066512	Supported	Approved
ZNF77	Nucleoplasm,Nucleoli	HPA023397	Uncertain	Approved
ZNF771	Nucleoplasm,Nucleoli	HPA030730, HPA046672, HPA059398	Uncertain	Enhanced
ZNF774	Intermediate filaments,Cytosol	HPA030839, HPA030840	Uncertain	Supported
ZNF776	Nucleoplasm,Cytosol	HPA046653, HPA068553	Uncertain	Approved
ZNF777	Nucleoplasm	HPA003252	Approved	Approved
ZNF780A	Nucleoplasm,Centrosome	HPA003311	Uncertain	Approved
ZNF780B	Nucleoplasm,Centrosome	HPA003311	Uncertain	Approved
ZNF781				
ZNF782	Nucleoplasm,Mitochondria	HPA011013	Approved	Approved
ZNF785	Cytosol	HPA004010		Approved
ZNF789	Nucleoplasm	HPA029500, HPA050793	Uncertain	Enhanced
ZNF79	Nucleoplasm,Plasma membrane,Cytosol	HPA028977	Uncertain	Approved
ZNF790	Nucleoli	HPA056134, HPA060288	Uncertain	Approved

ZNF791	Nucleoplasm	HPA021537	Uncertain	Approved
ZNF793	Nucleoplasm,Nucleoli,Cytosol	HPA034781	Uncertain	Approved
ZNF799	Nucleoplasm,Centriolar satellite	HPA003150, HPA018683	Approved	Approved
ZNF80		HPA065484	Uncertain	
ZNF805	Nucleoplasm	HPA046330, HPA058895	Uncertain	Approved
ZNF808				
ZNF81		HPA001689	Approved	
ZNF813	Vesicles	HPA056406	Uncertain	Approved
ZNF816	Nucleoli rim	HPA042466	Uncertain	Approved
ZNF823				
ZNF829	Nucleoplasm,Nuclear bodies	HPA073073		Approved
ZNF83	Nucleoplasm	HPA025970	Approved	Approved
ZNF835	Microtubules	HPA051136	Uncertain	Approved
ZNF836				
ZNF837	Nucleoli	HPA042833	Uncertain	Approved
ZNF84	Nucleoplasm	HPA028860	Approved	Approved
ZNF841				
ZNF844		HPA046982	Approved	
ZNF845				
ZNF846	Actin filaments	HPA034617, HPA034618	Approved	Approved
ZNF85	Nucleoplasm	HPA044760	Approved	Supported
ZNF852	Cytosol	HPA048312, HPA071850	Uncertain	Approved
ZNF853	Nucleoli	HPA067690	Approved	Approved
ZNF860	Nucleoplasm,Nucleoli,Nucleoli rim	HPA063386	Uncertain	Approved
ZNF875	Nucleoplasm,Mitochondria	HPA036126, HPA044855	Approved	Approved
ZNF878	Nucleoplasm,Nucleoli	HPA052735	Uncertain	Approved
ZNF879	Nucleoplasm	HPA068608	Uncertain	Approved
ZNF880	Nucleoplasm,Nucleoli fibrillar center,Intermediate filaments	HPA077316		Approved
ZNF891	Nucleoplasm,Vesicles	HPA040301	Uncertain	Approved
ZNF90	Nucleoplasm,Golgi apparatus	HPA003227	Uncertain	Approved
ZNF91				
ZNF92	Nucleoplasm	HPA003173		Approved
ZNF93				
ZNF98				
ZNF99				
ZSCAN10	Nucleoplasm,Centrosome	HPA076826		Approved
ZSCAN12	Nucleoplasm,Nuclear bodies,Cytosol	HPA006637, HPA007018	Approved	Approved
ZSCAN16	Nucleoplasm	HPA007290	Supported	Approved
ZSCAN2	Nucleoplasm,Cytosol	HPA007296, HPA024331	Supported	Approved
ZSCAN21	Endoplasmic reticulum	HPA016948, HPA023591	Uncertain	Approved
ZSCAN22	Plasma membrane,Actin filaments,Cytosol	HPA006471	Uncertain	Uncertain
ZSCAN23		HPA035959, HPA035960	Uncertain	
ZSCAN25	Nucleoplasm,Nucleoli fibrillar center	HPA055127, HPA057173	Uncertain	Approved
ZSCAN26	Nucleoplasm,Nucleoli	HPA027521		Enhanced
ZSCAN29	Nucleoplasm,Plasma membrane,Actin filaments	HPA007241, HPA011109	Enhanced	Approved

ZSCAN30	Nucleoplasm,Cytosol	HPA007289	Approved	Approved
ZSCAN31	Nucleoplasm,Cytosol	HPA007124	Approved	Approved
ZSCAN32	Nucleoplasm,Cytosol	HPA005758	Uncertain	Approved
ZSCAN4		HPA006491	Uncertain	
ZSCAN5A	Nuclear speckles	HPA041263, HPA043123, HPA046836, HPA060667	Uncertain	Uncertain
ZSCAN5B	Nuclear speckles	HPA043123, HPA046836, HPA059494, HPA060667	Uncertain	Uncertain
ZSCAN9	Nucleoplasm,Cytosol	HPA054512, HPA059951	Uncertain	Approved
ZXDA	Nucleoplasm	HPA043789	Approved	Approved
ZXDB	Nucleoplasm	HPA043789	Approved	Approved
ZXDC	Nucleoli	HPA049593		Approved
BCL11A	Nucleoplasm,Nuclear bodies	CAB014891, HPA029003, HPA075941	Enhanced	Supported
BCL11B	Nucleoplasm,Nucleoli fibrillar center	HPA049117	Enhanced	Approved
BNC1	Nucleoplasm	HPA063183, HPA077428		Enhanced
BNC2	Nucleoplasm	HPA018525, HPA059419	Approved	Supported
CASZ1	Nucleoplasm,Cytosol	HPA028222, HPA029927	Approved	Supported
E4F1	Nucleoplasm	HPA069007, HPA071325, HPA072309	Enhanced	Supported
FIZ1	Nucleoplasm	HPA074026		Approved
HIC1		HPA043372	Uncertain	
HIC2	Nucleoplasm	HPA031884, HPA059399	Uncertain	Supported
HINFP		HPA061008	Uncertain	
HIVEP1	Nucleoplasm,Nuclear bodies,Mitochondria,Cytosol	HPA050724, HPA067457	Approved	Supported
HIVEP2	Nucleoplasm	HPA055954		Supported
HIVEP3	Nucleoplasm	HPA005728	Approved	Approved
IKZF1	Nucleoplasm,Cytosol	CAB009247, HPA035221, HPA035222	Enhanced	Supported
IKZF2	Nucleoplasm,Nuclear bodies,Cytosol	HPA059142	Approved	Approved
IKZF3	Nucleoplasm,Cytosol	HPA024377, HPA073266	Enhanced	Supported
IKZF4	Nucleoplasm,Nuclear bodies	HPA046270, HPA049016	Approved	Supported
IKZF5	Nucleoplasm,Plasma membrane,Cytosol	HPA015297, HPA051574		Supported
INSM1				
INSM2	Nucleoplasm	HPA044885, HPA051925	Uncertain	Approved
MAZ	Nucleoplasm	HPA048315, HPA059495, HPA065930	Approved	Enhanced
MECOM	Nuclear speckles	HPA046537	Supported	Supported
PATZ1	Nucleoplasm	HPA047893, HPA048315	Approved	Supported
PEG3	Nucleoplasm	HPA026070	Approved	Approved
PRDM10	Nucleoplasm,Nucleoli fibrillar center,Vesicles	HPA026997, HPA061749	Approved	Supported
PRDM13	Nucleoplasm	HPA062648, HPA069705, HPA070592	Uncertain	Supported
PRDM15	Nucleoplasm,Nuclear bodies	HPA024268, HPA029255, HPA029256	Uncertain	Supported
PRDM16	Nucleoplasm	HPA050343, HPA060467		Supported
PRDM2	Nucleoplasm,Golgi apparatus	HPA005809	Approved	Approved
PRDM4	Nucleoplasm	HPA024322, HPA067437	Approved	Supported
PRDM8	Nucleoplasm,Nuclear bodies	HPA057253		Supported
PRDM9		HPA059555	Uncertain	
REST	Nucleoplasm,Cytosol	HPA006079, CAB068222	Supported	Supported
RLF	Nucleoplasm,Nucleoli	HPA054653, HPA057300	Uncertain	Approved
RREB1	Nucleoli fibrillar center,Nuclear speckles	HPA001756, HPA034843	Approved	Supported

SALL1	Nucleoplasm,Nucleoli,Nuclear speckles,Cytosol	HPA049829, HPA074673	Enhanced	Approved
SALL2	Nucleoplasm,Vesicles	HPA004162	Approved	Approved
SALL3		HPA016656	Uncertain	
SALL4	Nucleoplasm,Vesicles	HPA015291, HPA015791	Enhanced	Enhanced
VEZF1	Nucleoplasm	HPA027520, HPA048315	Uncertain	Enhanced
WIZ	Nucleoplasm	HPA022923, HPA023774	Supported	Enhanced
ZBTB1	Nucleoplasm,Nuclear membrane	HPA050516, HPA054552	Approved	Supported
ZBTB17		HPA005789	Approved	
ZBTB2	Nucleoplasm,Cytosol	HPA023136, HPA023137, HPA023458, HPA023487	Approved	Approved
ZBTB21	Nucleoplasm	HPA024655, HPA031757, HPA031758	Enhanced	Enhanced
ZBTB25	Nucleoplasm	HPA054699	Uncertain	Enhanced
ZBTB38	Nucleoplasm	HPA014865, HPA059378		Enhanced
ZBTB39	Vesicles	HPA016866, HPA020306	Uncertain	Approved
ZBTB4	Nucleoplasm,Nuclear bodies,Cytosol	CAB008980, HPA064763	Supported	Supported
ZBTB41	Nucleoplasm,Nuclear membrane,Cytosol	HPA034841, HPA034842	Uncertain	Approved
ZFAT	Nucleoplasm,Nuclear bodies	HPA020989, HPA063592	Enhanced	Approved
ZFP57				
ZFPM1	Nucleoplasm,Vesicles,Cytosol	HPA046603	Enhanced	Approved
ZNF101		HPA023449, HPA055486	Uncertain	
ZNF132	Vesicles,Intermediate filaments	HPA008726	Uncertain	Approved
ZNF134	Nucleoplasm	HPA009064	Supported	Approved
ZNF142	Nucleoplasm,Nucleoli fibrillar center	HPA049594	Uncertain	Approved
ZNF195	Nucleoplasm,Cytosol	HPA030605	Uncertain	Approved
ZNF211	Mitochondria,Rods & Rings	HPA011340, HPA049967	Approved	Approved
ZNF217	Nuclear speckles,Mitochondria	HPA051857	Approved	Enhanced
ZNF219	Nucleoplasm	HPA030761, HPA056168	Approved	Supported
ZNF236	Nucleoplasm,Cell Junctions,Cytosol	HPA008600	Uncertain	Approved
ZNF248	Nucleoplasm	HPA018237, HPA018287, HPA018455	Uncertain	Approved
ZNF251	Nucleoplasm	HPA018290	Approved	Approved
ZNF256	Nucleoplasm	HPA055390	Uncertain	Supported
ZNF277	Nucleoplasm,Plasma membrane	HPA027307	Uncertain	Approved
ZNF292	Nucleoplasm,Nuclear membrane	HPA032154, HPA050618	Uncertain	Approved
ZNF296	Nucleoplasm,Centrosome	HPA046940, HPA049576	Uncertain	Approved
ZNF304	Nuclear speckles,Cytosol	HPA050531	Approved	Approved
ZNF316				
ZNF319	Nucleoplasm	HPA052584	Uncertain	Approved
ZNF334	Nucleoplasm,Cytosol	HPA050022	Uncertain	Approved
ZNF335	Nucleoplasm	HPA063999, HPA071230		Enhanced
ZNF341	Nucleoplasm,Nuclear bodies,Vesicles	HPA023240, HPA024607, HPA067108	Uncertain	Approved
ZNF37A	Vesicles	HPA003172	Approved	Approved
ZNF382	Nuclear speckles	HPA049259	Approved	Approved
ZNF407	Nucleoplasm,Plasma membrane	HPA028255, HPA041673	Uncertain	Approved
ZNF417	Nucleoplasm,Nucleoli	HPA042125, HPA043288, HPA056213	Uncertain	Approved
ZNF418	Nucleoplasm	HPA043800, HPA053762, HPA072145	Approved	Uncertain
ZNF423	Nucleoplasm	HPA015258, HPA065820		Enhanced

ZNF438	Nucleoplasm,Cytosol	HPA039843	Uncertain	Supported
ZNF445	Nucleoplasm,Mitochondria,Cytosol	HPA006678	Uncertain	Approved
ZNF462	Nucleoplasm	HPA022283	Uncertain	Supported
ZNF467	Mitochondria	HPA042907	Uncertain	Approved
ZNF469	Nucleoplasm,Cell Junctions	HPA077792		Approved
ZNF48	Mitochondria	HPA023806	Uncertain	Approved
ZNF507	Nucleoli,Cytosol	HPA030964, HPA049728	Uncertain	Supported
ZNF510	Nucleoplasm,Vesicles,Plasma membrane	HPA049139	Uncertain	Approved
ZNF512	Nucleoplasm	HPA031430	Approved	Approved
ZNF512B	Nucleoplasm	HPA006688, HPA008731	Approved	Enhanced
ZNF513		HPA051493	Uncertain	
ZNF516	Nucleoplasm,Cytosol	HPA015792	Uncertain	Approved
ZNF519	Nucleoli	HPA028982	Uncertain	Approved
ZNF521	Nucleoplasm	HPA023056, HPA023849	Enhanced	Enhanced
ZNF526	Nucleoplasm,Golgi apparatus,Vesicles	HPA023070, HPA056609	Uncertain	Approved
ZNF532	Nucleoplasm,Golgi apparatus	HPA015322	Approved	Approved
ZNF536	Nucleoplasm,Cell Junctions	HPA077341		Approved
ZNF552	Nucleoplasm	HPA043800, HPA053762, HPA072145	Uncertain	Uncertain
ZNF574	Nucleoplasm,Vesicles	HPA023196, HPA057274	Approved	Approved
ZNF579	Nucleoplasm	HPA062812	Approved	Approved
ZNF587	Nucleoplasm,Nucleoli	HPA042125, HPA043288, HPA056213	Uncertain	Approved
ZNF592	Nucleoplasm	HPA020332, HPA021600	Approved	Approved
ZNF597	Nucleoplasm,Nucleoli	HPA001556	Approved	Approved
ZNF606	Nucleoplasm	HPA041712, HPA043584	Uncertain	Supported
ZNF618	Nucleoplasm	HPA023116, HPA061732	Uncertain	Approved
ZNF628	Nucleoplasm	HPA044451	Uncertain	Approved
ZNF629	Nucleoplasm,Golgi apparatus	HPA019550, HPA056051	Uncertain	Supported
ZNF639	Nucleoplasm	HPA049023, HPA052163	Uncertain	Supported
ZNF646	Nucleoplasm,Plasma membrane,Cytosol	HPA042364, HPA066468	Uncertain	Approved
ZNF654	Nucleoplasm,Microtubules,Cytokinetic bridge,Mitotic spindle	HPA036172, HPA036173, HPA049262, HPA077939	Uncertain	Approved
ZNF658	Nucleoplasm	HPA047730, HPA048668	Uncertain	Approved
ZNF668	Nucleoplasm,Nucleoli,Nucleoli rim	HPA043048	Uncertain	Approved
ZNF671	Nucleoplasm	HPA046099, HPA056043	Uncertain	Supported
ZNF674	Nucleoplasm	HPA031026, HPA065295	Uncertain	Supported
ZNF687	Nucleoplasm	HPA021193, HPA023948	Approved	Enhanced
ZNF697	Nucleoplasm	HPA049933	Uncertain	Approved
ZNF711	Nucleoplasm	HPA030654	Enhanced	Approved
ZNF717	Nucleoplasm,Nuclear bodies,Plasma membrane	HPA044549	Uncertain	Approved
ZNF770		HPA024518	Approved	
ZNF772	Cytosol	HPA031312	Uncertain	Approved
ZNF775		HPA073171	Approved	
ZNF778	Nucleoplasm,Nuclear bodies	HPA052057, HPA057151	Uncertain	Supported
ZNF784	Nucleoplasm	HPA059676	Uncertain	Approved
ZNF786	Nucleoplasm,Nuclear speckles,Nuclear bodies	HPA021518, HPA053459	Uncertain	Approved
ZNF787	Nucleoplasm,Nucleoli,Mitochondria	HPA035140	Supported	Approved

ZNF788P				
ZNF792	Nucleoplasm,Nuclear bodies	HPA035157, HPA035158	Supported	Approved
ZNF800	Nucleoli,Nucleoli rim,Cytosol	HPA023090, HPA052194	Approved	Supported
ZNF814	Nucleoplasm	HPA043800, HPA053762, HPA072145	Approved	Uncertain
ZSCAN20	Nucleoplasm	HPA017933	Uncertain	Approved
ZBED1	Nucleoplasm,Nuclear membrane,Centrosome	HPA077591	Approved	Approved
ZBED2	Nuclear bodies,Mitochondria,Cytosol	HPA029786		Approved
ZBED3	Nucleoplasm,Plasma membrane	HPA055995	Approved	Approved
ZBED4	Nucleoplasm	HPA045341	Approved	Supported
ZBED6	Nucleoplasm,Centriolar satellite	HPA068807		Supported
DMRT1		HPA027850	Enhanced	
DMRT2	Nucleoplasm,Nucleoli,Vesicles,Centriolar satellite	HPA003498, HPA029297	Uncertain	Approved
DMRT3		HPA077685	Approved	
DMRTA1	Vesicles	HPA044764, HPA062253	Uncertain	Approved
DMRTA2	Nucleoplasm,Mitochondria	HPA043098, HPA062958	Uncertain	Approved
DMRTB1		HPA036490, HPA058553	Enhanced	
DMRTC1B	Nucleoplasm,Intermediate filaments	HPA046874, HPA046992	Uncertain	Approved
DMRTC2		HPA045016	Uncertain	
MBD1	Nucleoplasm,Vesicles	CAB009017, CAB036003, HPA068850	Approved	Approved
MYT1	Nucleoplasm,Cytosol	HPA006303	Uncertain	Supported
MYT1L	Vesicles	HPA060245		Approved
ST18		HPA044919	Uncertain	
L3MBTL1	Nucleoplasm,Nuclear bodies,Midbody ring	HPA059648		Supported
LYAR	Nucleoplasm,Nucleoli,Nucleoli rim	HPA035880, HPA035881	Enhanced	Enhanced
ZGPAT	Nucleoplasm,Plasma membrane	HPA056705		Supported
THAP1	Nucleoplasm	HPA071310		Supported
THAP10	Nucleoplasm,Golgi apparatus,Cytosol	HPA041416, HPA073220	Uncertain	Approved
THAP11	Nucleoplasm,Cytosol	HPA041434, HPA042189, HPA072537	Supported	Supported
THAP12	Cytosol	HPA008758, HPA077077	Approved	Approved
THAP2	Nucleoplasm,Golgi apparatus	HPA039790	Approved	Approved
THAP3	Nucleoplasm,Nucleoli,Mitochondria	HPA067114	Approved	Approved
THAP4	Nucleoplasm,Cytosol	HPA044982	Uncertain	Supported
THAP5	Nucleoplasm	HPA043170, HPA051488	Approved	Supported
THAP6	Nucleoplasm,Centrosome	HPA035767	Uncertain	Approved
THAP7	Nucleoplasm,Nuclear membrane,Vesicles	HPA003083	Approved	Supported
THAP8	Nucleoplasm	HPA064056, HPA070139	Approved	Enhanced
THAP9	Mitochondria	HPA037420, HPA037421	Uncertain	Approved
CDX1		HPA055196	Enhanced	
CDX2	Nucleoplasm	CAB002221, HPA045669, HPA049580	Enhanced	Enhanced
CDX4	Nucleoplasm,Nuclear speckles,Cytosol	HPA043714, HPA056528	Uncertain	Approved
EVX1	Nucleoplasm	HPA055132		Approved
EVX2		HPA041576	Uncertain	
GBX1		HPA055783	Approved	
GBX2	Nucleoplasm	HPA067809		Approved
GSX1	Nucleoplasm,Golgi apparatus,Cytosol	HPA047096		Approved

GSX2				
HOXA1				
HOXA10	Nucleoplasm,Nuclear bodies	CAB019384, HPA071918	Approved	Supported
HOXA11	Nucleoplasm	HPA006770, HPA035623	Enhanced	Supported
HOXA13	Nucleoplasm,Mitotic chromosome,Intermediate filaments	HPA046098, HPA069061		Supported
HOXA2	Nucleoplasm,Vesicles	HPA078220		Approved
HOXA3	Nucleoplasm	HPA029157	Uncertain	Supported
HOXA4	Nuclear bodies	HPA060088	Approved	Supported
HOXA5				
HOXA6	Nucleoplasm,Nuclear speckles	HPA004203, HPA077900	Approved	Supported
HOXA7	Nucleoplasm,Nuclear membrane	HPA074048		Supported
HOXA9	Nucleoplasm	HPA061982, HPA076200	Uncertain	Enhanced
HOXB1	Nucleoplasm	HPA036648, HPA044576	Uncertain	Supported
HOXB13	Nucleoplasm	CAB004438, HPA062852, HPA065019	Enhanced	Enhanced
HOXB2	Nucleoplasm	HPA047857		Supported
HOXB3	Nucleoplasm	CAB009860, HPA064990	Approved	Supported
HOXB4	Nucleoplasm,Centrosome	HPA057432	Uncertain	Supported
HOXB5	Nucleoplasm,Nucleoli fibrillar center,Cytosol	HPA043851, HPA074454		Supported
HOXB6	Nucleoplasm,Golgi apparatus	HPA042063		Supported
HOXB7	Nucleoplasm,Nuclear bodies,Cytosol	HPA049940	Uncertain	Supported
HOXB8	Nucleoplasm	HPA075905		Supported
HOXB9	Nucleoplasm	HPA004928		Supported
HOXC10	Nucleoplasm,Nuclear bodies	HPA053919	Uncertain	Supported
HOXC11	Nucleoplasm,Cytosol	HPA038291	Uncertain	Supported
HOXC12		HPA055632	Uncertain	
HOXC13		HPA051634	Enhanced	
HOXC4	Nucleoplasm	HPA053910		Supported
HOXC5	Nucleoplasm,Cell Junctions	HPA026794	Uncertain	Supported
HOXC6	Nucleoplasm,Cytosol	HPA054981		Supported
HOXC8	Nucleoplasm,Microtubules	HPA028911, CAB037020	Uncertain	Approved
HOXC9	Nucleoplasm	HPA063965, HPA072160		Enhanced
HOXD1	Nucleoplasm	HPA040062, HPA056900		Enhanced
HOXD10	Nucleoplasm,Cytosol,Cytoplasmic bodies	HPA065871		Approved
HOXD11	Nucleoplasm	HPA055155		Supported
HOXD12		HPA059444	Uncertain	
HOXD13	Nucleoplasm	HPA064064		Approved
HOXD3	Nucleoplasm,Nuclear bodies	HPA051831, HPA063671		Supported
HOXD4	Nucleoplasm,Cell Junctions	HPA054383, HPA070349	Approved	Supported
HOXD8				
HOXD9	Nucleoplasm,Nucleoli	HPA068683		Supported
MEOX1	Nucleoplasm,Cytokinetic bridge,Cytosol	HPA045214, HPA063788	Uncertain	Approved
MEOX2		HPA053793	Enhanced	
MNX1	Nucleoplasm,Nucleoli,Cytosol	HPA071717		Approved
PDX1	Nucleoplasm	CAB025873, HPA059146	Enhanced	Supported
HDX	Cytosol	HPA047189	Uncertain	Approved

HMBOX1	Nucleoplasm,Nuclear bodies,Cytosol	HPA055855, HPA058586, CAB070418	Approved	Supported
HNF1A	Nucleoplasm	CAB010430, HPA035231	Enhanced	Supported
HNF1B	Nucleoplasm,Vesicles	HPA002083, CAB068192	Enhanced	Supported
POU1F1	Nucleoplasm,Cytosol	HPA041646, HPA050624	Enhanced	Approved
POU2F1	Nucleoplasm,Endoplasmic reticulum,Vesicles	CAB002608, HPA058559, HPA064323	Supported	Enhanced
POU2F2	Nucleoplasm,Vesicles	CAB002513, HPA049056, HPA062096, HPA064404, HPA073998	Enhanced	Enhanced
POU2F3	Nucleoplasm,Plasma membrane,Cytosol	HPA019652, HPA073468	Enhanced	Supported
POU3F1	Nucleoplasm	HPA055980, HPA073824		Enhanced
POU3F2	Nucleoplasm	HPA047081, HPA056261		Supported
POU3F3	Nucleoplasm	HPA052792, HPA056039, HPA067151	Enhanced	Enhanced
POU3F4	Nucleoplasm	HPA062295		Supported
POU4F1		HPA068024	Enhanced	
POU4F2				
POU4F3	Nucleoplasm	HPA038215	Uncertain	Supported
POU5F1	Nucleoplasm,Cytosol	CAB025600, CAB026380, HPA058267, CAB079773	Approved	Supported
POU5F1B	Nucleoplasm	HPA058267		Supported
POU5F2	Nucleoplasm	HPA077615	Enhanced	Approved
POU6F1	Nuclear bodies,Actin filaments	HPA047021, HPA050411	Enhanced	Supported
POU6F2				
PHTF1	Nucleoli,Nuclear bodies	HPA059486		Approved
BARHL1		HPA004809	Enhanced	
BARHL2				
BARX1	Nucleoplasm,Vesicles	HPA055858		Approved
BARX2	Nucleoplasm,Golgi apparatus,Cytosol	CAB033311, HPA064966, HPA068414	Enhanced	Supported
BSX		HPA015654	Uncertain	
DBX1		HPA039802	Uncertain	
DBX2	Nucleoplasm,Nuclear membrane,Vesicles	HPA065045, HPA066053	Uncertain	Approved
DLX1	Nucleoplasm,Vesicles,Cytosol	HPA007175, HPA045884	Approved	Supported
DLX2	Nuclear speckles	HPA056965		Approved
DLX3	Nucleoplasm,Cytosol	HPA031164		Approved
DLX4	Nucleoplasm	HPA059933	Uncertain	Supported
DLX5	Nucleoplasm	HPA005670	Approved	Approved
DLX6	Nuclear bodies	HPA030608, HPA061460	Uncertain	Approved
EMX1	Nucleoli,Nucleoli rim,Mitotic chromosome	HPA006230, HPA006421	Approved	Supported
EMX2	Nucleoplasm	HPA003497, HPA065294	Enhanced	Approved
EN1	Nucleoplasm,Nucleoli rim	CAB014884, HPA073141	Enhanced	Approved
EN2	Nucleoplasm,Nucleoli,Nucleoli fibrillar center	HPA045646, HPA069809		Supported
HHEX	Nucleoplasm,Nuclear bodies	HPA003257, HPA051894	Approved	Approved
HLX	Nucleoplasm,Cytosol	HPA005968	Uncertain	Approved
HMX1				
HMX2		CAB013268, CAB022752	Approved	
HMX3	Nucleoplasm,Centriolar satellite	HPA069659		Approved
LBX1	Nucleoplasm,Nucleoli,Nuclear bodies,Cytosol	HPA002016, HPA002086, CAB079927	Uncertain	Approved
LBX2	Nucleoplasm,Midbody	HPA044257	Uncertain	Approved
MSX1	Nucleoplasm	CAB026198, HPA063895, HPA073604	Enhanced	Supported

MSX2	Nuclear speckles	HPA005652	Uncertain	Supported
NANOG	Nucleoplasm,Vesicles	CAB019380, HPA072117	Supported	Supported
NKX1-1	Nucleoli fibrillar center,Cytosol	HPA029367	Uncertain	Uncertain
NKX1-2	Nucleoli fibrillar center,Cytosol	HPA029367	Uncertain	Approved
NKX2-1	Golgi apparatus,Vesicles	CAB000078, CAB053633, HPA074316	Enhanced	Uncertain
NKX2-2		HPA003468	Approved	
NKX2-3	Nucleoli,Cytosol	HPA047561	Uncertain	Uncertain
NKX2-4	Nuclear bodies	HPA074996		Approved
NKX2-5	Nucleoplasm,Cytosol	HPA065034		Approved
NKX2-6	Nucleoplasm,Nuclear membrane,Cytosol	HPA025288		Approved
NKX2-8	Nucleoplasm,Plasma membrane	HPA062879	Uncertain	Approved
NKX3-1	Nucleoplasm,Cytosol	HPA025693, HPA078571	Enhanced	Supported
NKX3-2	Nucleoplasm	HPA027564	Approved	Approved
NKX6-1	Nucleoplasm	HPA036774, CAB070169	Enhanced	Approved
NKX6-2				
NKX6-3				
NOTO	Nucleoplasm,Cytosol	HPA034660, HPA063906	Uncertain	Approved
TLX1				
TLX2	Nucleoplasm	HPA062254		Approved
TLX3	Nucleoplasm	HPA030504, HPA060957, HPA069359	Enhanced	Enhanced
VAX1	Nucleoplasm,Nucleoli rim	HPA028946	Uncertain	Approved
VAX2	Nuclear speckles	HPA009708		Approved
VENTX	Nucleoli fibrillar center	HPA050955	Uncertain	Approved
ALX1	Nucleoplasm,Nuclear bodies,Golgi apparatus	HPA001598, HPA018905	Uncertain	Supported
ALX3	Nucleoli fibrillar center	HPA074290		Approved
ALX4	Nucleoplasm	HPA001903		Supported
ARGFX		HPA043049	Uncertain	
ARX				
CRX		HPA036762, HPA036763	Enhanced	
DMBX1				
DPRX		HPA043682	Uncertain	
DRGX	Nucleoplasm,Nucleoli,Cytosol	HPA043978	Uncertain	Approved
DUX4	Nucleoplasm,Nucleoli,Golgi apparatus,Cytosol	HPA058451		Approved
DUXA		HPA059358	Uncertain	
ESX1	Nuclear speckles	HPA051992, HPA073330	Enhanced	Supported
GSC	Nucleoplasm,Nuclear speckles	CAB079923, CAB079924		Supported
GSC2		HPA043011	Uncertain	
HESX1	Nucleoplasm	HPA024187	Approved	Approved
HOPX	Nuclear bodies,Cytosol	CAB018632, HPA030180	Enhanced	Approved
ISX	Nucleoplasm,Nuclear bodies	HPA060328		Supported
LEUTX	Nucleoplasm,Nucleoli	HPA041498	Uncertain	Approved
MIXL1	Nucleoplasm	HPA005662		Approved
NOBOX				
OTP	Nucleoplasm,Nucleoli fibrillar center,Nuclear bodies	HPA059342	Enhanced	Approved
OTX1	Nucleoplasm	HPA052122	Uncertain	Approved

OTX2	Nucleoplasm,Vesicles	HPA000633, HPA001123	Uncertain	Supported
PHOX2A	Nucleoplasm	HPA053810, HPA065621, HPA078004	Enhanced	Supported
PHOX2B	Nucleoplasm	HPA074325, HPA074941	Enhanced	Enhanced
PITX1	Nucleoli	HPA008743	Approved	Approved
PITX2	Nucleoplasm	HPA050074, HPA062728		Enhanced
PITX3				
PROP1		HPA049839	Enhanced	
PRRX1	Nucleoplasm	HPA051084, HPA063566	Uncertain	Supported
PRRX2	Nucleoplasm,Nuclear bodies	HPA006607, HPA026808	Approved	Approved
RAX				
RAX2	Nucleoplasm,Nuclear speckles,Cytosol	HPA052533	Uncertain	Approved
RHOXF1	Nucleoplasm,Cytosol	HPA052560, HPA056506	Approved	Enhanced
RHOXF2	Nucleoplasm,Plasma membrane	HPA003314, CAB020794	Enhanced	Approved
RHOXF2B	Nucleoplasm,Plasma membrane	HPA003314	Supported	Approved
SEBOX		HPA045689	Uncertain	
SHOX	Nucleoplasm,Vesicles	HPA035141, HPA060931		Enhanced
SHOX2				
TPRX1		HPA044922	Uncertain	
UNCX	Nucleoplasm	HPA063294		Approved
VSX1	Nucleoli	HPA079607		Approved
VSX2		HPA003436	Enhanced	
IRX1	Nucleoplasm,Golgi apparatus	HPA043160	Uncertain	Approved
IRX2	Nucleoplasm,Nucleoli	HPA050895, HPA054669	Approved	Approved
IRX3	Nucleoplasm,Cytosol	HPA066576		Approved
IRX4	Nucleoplasm,Vesicles	HPA064094		Uncertain
IRX5	Nuclear speckles,Microtubules,Cytosol	HPA047130		Approved
IRX6	Nucleoplasm,Mitochondria	HPA018332	Uncertain	Approved
MEIS1	Nucleoplasm	CAB011521, HPA056000, HPA058641	Approved	Supported
MEIS2	Nucleoplasm,Cytosol	HPA003256, CAB004999	Enhanced	Approved
MEIS3	Nucleoplasm	HPA046699	Uncertain	Approved
MKX	Nucleoplasm,Nuclear speckles	HPA006927	Uncertain	Approved
PBX1	Nucleoplasm,Nuclear bodies,Cytosol	HPA003505, HPA003881, CAB018768	Supported	Enhanced
PBX2	Nucleoplasm	HPA061478	Supported	Approved
PBX3	Nucleoplasm,Nuclear bodies,Cytosol	HPA003881	Supported	Approved
PBX4	Vesicles	HPA049859, HPA056469	Enhanced	Approved
PKNOX1	Nucleoplasm	CAB004285, HPA057215, HPA065017	Approved	Approved
PKNOX2	Nucleoplasm,Cytokinetic bridge	HPA028867, CAB034342, HPA055368, HPA066962	Supported	Enhanced
TGIF1	Nucleoplasm	HPA062160		Supported
TGIF2	Nucleoplasm,Centrosome	HPA053786, HPA064522		Enhanced
TGIF2LX	Nucleoplasm	HPA034543	Supported	Approved
TGIF2LY				
ISL1	Nucleoplasm,Nuclear speckles,Mitochondria	HPA048613, HPA057416	Approved	Approved
ISL2	Rods & Rings	HPA075192		Approved
LHX1	Nucleoplasm,Nuclear membrane,Golgi apparatus	HPA044307, HPA073521, HPA074382	Enhanced	Supported
LHX2	Nucleoplasm	HPA000838		Approved

LHX3				
LHX4	Nuclear speckles	HPA055705		Approved
LHX5	Nucleoplasm,Nuclear bodies,Cytosol	HPA053908		Approved
LHX6	Nucleoli fibrillar center,Plasma membrane	HPA047854, HPA058319	Approved	Uncertain
LHX8	Nucleoplasm,Nucleoli,Cytosol	HPA071806		Approved
LHX9	Nucleoplasm	HPA009695	Uncertain	Approved
LMX1A	Nucleoplasm,Golgi apparatus,Vesicles	HPA030088		Approved
LMX1B	Nucleoplasm	HPA073716, HPA075656		Approved
SIX1	Nucleoplasm,Nucleoli	HPA001893, CAB058690	Enhanced	Supported
SIX2	Nucleoplasm,Nuclear membrane	HPA056958		Supported
SIX3	Nucleoplasm	HPA067988		Approved
SIX4	Nucleoplasm	HPA031794	Uncertain	Approved
SIX5	Nucleoplasm,Golgi apparatus,Vesicles,Cytosol	HPA042068	Approved	Supported
SIX6	Nucleoplasm	HPA001403	Approved	Approved
PROX1	Nucleoplasm,Cytosol	HPA000842, HPA001030, HPA027298		Enhanced
PROX2		HPA045057	Uncertain	
ADNP	Nucleoplasm	HPA006371	Supported	Approved
ADNP2	Mitochondria,Cytosol	HPA007126	Uncertain	Approved
HOMEZ	Nucleoplasm,Nucleoli,Cytosol	HPA036316, HPA036317	Supported	Supported
NANOGNB		HPA060371	Uncertain	
TSHZ1	Nucleoplasm	HPA006982	Approved	Approved
TSHZ3	Nucleoplasm,Plasma membrane	HPA008834, HPA071010	Approved	Enhanced
ZEB1	Nucleoplasm,Nucleoli	HPA027524, CAB058686, CAB079943	Enhanced	Supported
ZEB2	Nucleoplasm,Nucleoli,Cytosol	HPA003456	Approved	Supported
ZFHX2	Nucleoplasm,Nucleoli fibrillar center	HPA000720, HPA005146	Uncertain	Approved
ZFHX3	Nucleoplasm	HPA059353	Uncertain	Supported
ZFHX4	Nucleoplasm,Nucleoli fibrillar center	HPA023837	Uncertain	Approved
ZHX1	Nucleoplasm	HPA055357		Supported
ZHX2	Nucleoplasm	HPA006769, HPA006996	Enhanced	Enhanced
ZHX3	Nucleoplasm	HPA046498		Supported
CUX1	Nucleoplasm,Golgi apparatus	HPA003277, HPA003317	Approved	Supported
CUX2				
ONECUT1		HPA003457	Enhanced	
ONECUT2	Nucleoplasm,Actin filaments	HPA057058	Enhanced	Supported
ONECUT3	Nucleoplasm	HPA059936		Supported
SATB1	Nucleoplasm,Nuclear bodies	HPA051769, CAB056144, HPA070534	Enhanced	Supported
SATB2	Nucleoplasm	001042, CAB023669, HPA029543, CAB062562, CAB067751, CAB068229, CAB068230, CAB06	Enhanced	Supported
PAX3	Nucleoplasm	HPA063659, HPA069000		Enhanced
PAX4				
PAX6	Nucleoplasm	HPA030775, CAB034143	Enhanced	Enhanced
PAX7				
PAX1				
PAX2	Nucleoplasm	CAB013024, HPA047704, HPA070751	Enhanced	Supported
PAX5	Nucleoplasm	CAB026269, CAB026869, HPA056394	Enhanced	Supported
PAX8	Nucleoplasm	HPA030062, CAB055097, CAB061888, HPA064554	Enhanced	Enhanced

PAX9	Nucleoplasm,Nucleoli	HPA001572, CAB016239	Enhanced	Supported
FOXA1	Nucleoplasm,Nucleoli fibrillar center	CAB011595, HPA050505	Enhanced	Supported
FOXA2	Nucleoplasm,Cell Junctions	CAB001388, HPA066846	Enhanced	Supported
FOXA3	Nucleoplasm	CAB015154, HPA054034	Uncertain	Approved
FOXB1	Nucleoplasm,Mitochondria	HPA073369		Approved
FOXB2		HPA067947	Approved	
FOXC1	Nucleoplasm,Cytosol	HPA040670		Supported
FOXC2	Nucleoplasm,Nuclear bodies	HPA056302		Supported
FOXD1				
FOXD2				
FOXD3		CAB022455	Uncertain	
FOXD4	Nucleoplasm	HPA012836	Uncertain	Approved
FOXD4L1	Nucleoplasm,Cytosol	HPA012836, HPA069839	Uncertain	Approved
FOXD4L3	Nucleoplasm	HPA012836	Uncertain	Approved
FOXD4L4	Nucleoplasm	HPA012836	Uncertain	Approved
FOXD4L5	Nucleoplasm	HPA012836	Uncertain	Approved
FOXD4L6	Nucleoplasm	HPA012836	Uncertain	Approved
FOXE1				
FOXE3				
FOXF1	Nucleoplasm	HPA028886		Approved
FOXF2	Nucleoplasm,Nuclear bodies,Vesicles	HPA004763		Supported
FOXG1				
FOXH1				
FOXI1	Nucleoli,Vesicles	HPA071469	Enhanced	Supported
FOXI2				
FOXI3				
FOXJ1		HPA005714	Enhanced	
FOXJ2	Nucleoplasm,Nucleoli fibrillar center	HPA008723	Approved	Supported
FOXJ3	Nucleoplasm,Nuclear speckles,Vesicles	HPA067284		Approved
FOXK1	Nucleoplasm,Nucleoli fibrillar center	HPA017998, HPA018864	Enhanced	Supported
FOXK2	Nucleoplasm,Vesicles,Mitochondria	HPA027523, HPA073491	Approved	Supported
FOXL1				
FOXL2	Nucleoplasm	HPA069613	Enhanced	Supported
FOXM1	Nucleoplasm,Nucleoli,Nucleoli rim,Cytosol	CAB017832	Uncertain	Supported
FOXN1				
FOXN2	Nucleoplasm,Vesicles	HPA003485	Supported	Supported
FOXN3	Nucleoplasm,Plasma membrane	HPA059209	Approved	Approved
FOXN4	Nucleoplasm,Mitochondria,Cytosol	HPA050018	Uncertain	Approved
FOXO1	Nucleoplasm,Cytosol	HPA001252, CAB022326	Approved	Supported
FOXO3	Nucleoplasm	CAB004074, HPA063104	Supported	Enhanced
FOXO4	Nuclear speckles,Cytosol	HPA039560, HPA040232	Enhanced	Supported
FOXO6				
FOXP1	Nucleoplasm	HPA003876, CAB011501	Supported	Supported
FOXP2	Nucleoplasm	HPA000382, HPA000383, HPA001679	Enhanced	Enhanced
FOXP3	Nucleoplasm,Cytosol	CAB026301, HPA045943, HPA069372	Enhanced	Supported

FOXP4	Nucleoplasm	HPA007176, HPA076256	Approved	Approved
FOXQ1	Nucleoplasm	HPA059700, CAB080326		Supported
FOXR1	Nucleoplasm,Cytosol	HPA038124		Approved
FOXR2	Nucleoplasm	HPA034487, HPA057358	Uncertain	Supported
FOXS1	Nucleoplasm,Cytosol	HPA042475	Approved	Approved
E2F1	Nucleoplasm,Centrosome	CAB000329, HPA008003, CAB019308, HPA029735	Enhanced	Supported
E2F2		CAB016313	Uncertain	
E2F3	Nucleoplasm,Nuclear speckles,Cytosol	HPA029779, HPA065012		Supported
E2F4	Nucleoplasm	HPA054128		Supported
E2F5	Nucleoplasm,Nucleoli,Vesicles	HPA055723, HPA065441		Supported
E2F6	Nucleoplasm	HPA075185		Supported
E2F7	Nuclear speckles	HPA064866		Supported
E2F8	Nucleoplasm,Nucleoli,Cytosol	HPA064882, HPA076614	Enhanced	Supported
TFDP1	Nucleoplasm,Nuclear membrane,Cytosol	CAB033605, HPA044754, HPA068909, HPA077304	Enhanced	Approved
TFDP2	Nucleoplasm,Vesicles,Mitotic spindle	CAB018396, HPA070399	Supported	Supported
TFDP3	Nucleoplasm,Nuclear membrane,Cytosol	HPA077304		Supported
RFX1	Nucleoplasm,Vesicles	HPA048722, HPA052966	Approved	Enhanced
RFX2	Nucleoplasm,Golgi apparatus,Cytosol	HPA048969	Enhanced	Approved
RFX3	Nucleoplasm,Vesicles	HPA035689	Enhanced	Approved
RFX4	Nucleoplasm	HPA050527	Enhanced	Approved
RFX5	Nucleoplasm,Vesicles	HPA018519, HPA079241	Supported	Supported
RFX6		HPA037696	Approved	
RFX7	Nucleoplasm,Nuclear membrane	HPA035462, HPA057957	Approved	Approved
RFX8	Nucleoplasm,Nuclear bodies	HPA059745		Approved
HSF1	Nucleoplasm,Cytosol	CAB004239, HPA008888	Supported	Supported
HSF2	Nucleoplasm	CAB016067, HPA031455	Approved	Supported
HSF4	Nuclear speckles	HPA048584		Supported
HSF5	Nucleoplasm	HPA016440, HPA063613	Enhanced	Approved
HSFX1	Nucleoplasm,Plasma membrane,Cytosol	HPA051700, HPA063040	Approved	Uncertain
HSFX3				
HSFY1	Nucleoplasm,Cytosol	HPA074968		Supported
CDC5L	Nucleoplasm	HPA006302, HPA011361, CAB012275	Supported	Supported
DMTF1	Nucleoplasm	HPA023846	Supported	Supported
DNAJC1	Endoplasmic reticulum	HPA013432	Approved	Approved
DNAJC2	Cytosol	HPA020454	Approved	Supported
MIDEAS	Nucleoplasm	HPA003111	Approved	Enhanced
MIER1	Nucleoplasm	HPA019589, HPA050306	Enhanced	Enhanced
MIER3	Nucleoplasm	HPA058349, HPA065946	Uncertain	Enhanced
MYB	Nucleoplasm,Cytosol	CAB017704, HPA071605	Approved	Supported
MYBL1	Nucleoplasm	HPA008791	Uncertain	Supported
MYBL2	Nucleoplasm,Cytosol	CAB017114, HPA030530, HPA055416	Uncertain	Supported
NCOR1	Nucleoplasm,Cytosol	HPA043246, HPA050288, HPA051168, CAB072830	Supported	Enhanced
NCOR2	Nucleoplasm	HPA001928	Approved	Supported
RCOR1	Nucleoplasm	CAB001956, HPA049327, HPA054241	Supported	Enhanced
RCOR2	Nucleoplasm,Vesicles,Midbody	HPA021638	Uncertain	Approved

RCOR3	Nucleoplasm,Cytosol	HPA007413, HPA007621, HPA071997	Enhanced	Enhanced
SMARCC1	Nucleoplasm	CAB011576, CAB016336, HPA024352, HPA026853	Supported	Enhanced
SMARCC2	Nucleoplasm	CAB004321, HPA021213, HPA061788	Supported	Enhanced
SNAPC4	Nucleoplasm,Nuclear membrane	HPA028939, HPA046627, HPA073243	Uncertain	Approved
TADA2A	Nuclear speckles	HPA076497		Approved
TADA2B	Nuclear speckles	HPA035770	Approved	Approved
TRERF1	Nucleoplasm,Nucleoli fibrillar center,Cytosol	HPA027771, HPA051273, HPA059943	Enhanced	Enhanced
ZNF541	Nucleoplasm,Cytosol	HPA058541, HPA076453	Enhanced	Approved
EHF	Nucleoplasm,Golgi apparatus	HPA050923, HPA060734	Enhanced	Supported
ELF1	Nucleoplasm	HPA001755, CAB005172	Enhanced	Supported
ELF2	Nucleoplasm,Nuclear bodies	HPA006057, HPA071166	Enhanced	Supported
ELF3	Nucleoplasm	HPA003316, HPA003479	Enhanced	Enhanced
ELF4	Nucleoplasm,Nuclear bodies	HPA058595, HPA060277	Enhanced	Supported
ELF5	Nucleoplasm	HPA062706		Approved
ELK1	Nucleoplasm	CAB003808, HPA036084, HPA064381	Enhanced	Enhanced
ELK3	Nucleoplasm,Mitochondria	HPA001600	Approved	Supported
ELK4	Nucleoplasm	HPA028863	Uncertain	Supported
ERF	Nucleoplasm,Cytosol	HPA058532, HPA067952	Approved	Supported
ERG	Nucleoplasm,Cytosol	CAB018377, HPA046598	Approved	Supported
ETS1	Nucleoplasm	CAB002575, HPA063230	Enhanced	Enhanced
ETS2	Nucleoplasm,Plasma membrane,Cytosol	HPA003176		Enhanced
ETV1	Nucleoplasm	HPA068389, HPA077249		Approved
ETV2				
ETV3	Nucleoplasm,Vesicles	HPA004794, HPA073211	Supported	Approved
ETV3L				
ETV4	Nucleoli rim,Mitotic chromosome	HPA005768	Approved	Supported
ETV5	Nucleoplasm	CAB009590, HPA063065, HPA073889	Approved	Supported
ETV6	Nucleoli,Cytosol	HPA000264	Enhanced	Approved
ETV7	Nucleoplasm	HPA029033	Uncertain	Supported
FEV	Nuclear speckles	HPA067679		Supported
FLI1	Nucleoplasm,Nuclear bodies	HPA065030, HPA073099	Approved	Supported
GABPA	Nucleoplasm	HPA003258, CAB004411, HPA028791	Supported	Enhanced
SPDEF	Nucleoplasm,Nucleoli,Cytosol	HPA055707		Approved
SPI1	Nucleoplasm	CAB004079, HPA044653	Enhanced	Supported
SPIB		HPA018523	Uncertain	
SPIC				
IRF1	Nucleoplasm,Cytosol	CAB011662, HPA063131, HPA070953	Supported	Approved
IRF2	Nucleoplasm,Cell Junctions,Cytosol	HPA030813, CAB032306, HPA057327	Supported	Enhanced
IRF3	Cytosol	HPA004895, CAB013018	Approved	Supported
IRF4	Nucleoplasm,Cytosol	HPA002038, HPA002698, CAB013508	Enhanced	Supported
IRF5		HPA046700, HPA076024	Enhanced	
IRF6	Nucleoplasm,Cytosol	HPA063121, HPA076162	Approved	Supported
IRF7	Nucleoplasm,Cytosol	CAB017694, HPA052757	Approved	Supported
IRF8	Nucleoplasm	HPA002267, HPA002531, CAB013480	Enhanced	Enhanced
IRF9	Cytosol	HPA001862	Approved	Supported

TEAD1	Nucleoplasm	HPA057339, HPA062471		Enhanced
TEAD2	Nucleoplasm	HPA066292, HPA069963		Supported
TEAD3		HPA028906	Uncertain	
TEAD4	Nucleoplasm	HPA056896	Uncertain	Supported
ARID1A	Nucleoplasm	HPA005456, CAB016334	Supported	Enhanced
ARID1B	Nucleoplasm	HPA016511, HPA075291	Approved	Supported
ARID2	Nucleoplasm	HPA063044, HPA078115		Supported
ARID3A	Nucleoplasm,Cytosol	HPA004793, HPA076330	Enhanced	Supported
ARID3B	Nucleoplasm	HPA050320	Enhanced	Supported
ARID4A	Nucleoplasm	HPA048899		Supported
ARID4B	Nucleoplasm,Mitochondria,Cytosol	HPA027205, HPA027333	Approved	Approved
ARID5A	Nucleoplasm,Nucleoli	HPA002860, HPA023879	Approved	Supported
ARID5B	Nucleoplasm,Endoplasmic reticulum,Plasma membrane,Cytoso	HPA015037	Uncertain	Approved
JARID2	Nucleoplasm,Mitochondria	HPA063889, CAB069890	Approved	Supported
BBX	Nucleoplasm,Cytosol	HPA006049, HPA050646	Enhanced	Supported
CIC	Nucleoplasm,Vesicles	CAB026388, HPA044341, HPA064725	Approved	Supported
HBP1	Nuclear speckles	HPA020225, CAB032824	Approved	Supported
SOX1				
SOX10	Nucleoplasm	CAB003171, HPA068898	Enhanced	Supported
SOX11	Nucleoplasm,Plasma membrane	HPA000448, HPA000536, CAB056152, CAB056153	Approved	Supported
SOX12	Nucleoplasm	HPA055052, HPA061627		Enhanced
SOX13	Nucleoplasm	HPA051790, HPA054843		Enhanced
SOX14				
SOX15	Nucleoplasm,Nucleoli,Vesicles	HPA067196, HPA074049	Enhanced	Approved
SOX17	Nucleoplasm	CAB025594, HPA068399	Enhanced	Approved
SOX18	Nucleoplasm	HPA073703		Approved
SOX2	Nucleoplasm	HPA045725, HPA062851, HPA071379, CAB079745	Enhanced	Enhanced
SOX21	Nucleoplasm	HPA048337, HPA064084	Approved	Approved
SOX3	Nucleoplasm	HPA054720, HPA075003		Enhanced
SOX30	Nucleoplasm,Vesicles,Cytosol	HPA006159, CAB009382	Enhanced	Supported
SOX4	Nucleoplasm,Mitochondria	HPA029901	Approved	Supported
SOX5	Nucleoplasm	HPA060499		Supported
SOX6	Nucleoplasm	HPA001923, HPA003908	Enhanced	Supported
SOX7	Nuclear speckles,Golgi apparatus	HPA009065	Approved	Approved
SOX8	Nucleoplasm	HPA041640, HPA058665		Enhanced
SOX9	Nucleoplasm	HPA001758, CAB022456, CAB068240, HPA070776	Enhanced	Enhanced
SRY				
HMG20A	Nucleoplasm	HPA007685, HPA008126	Supported	Approved
HMG20B	Nucleoplasm	HPA050220, HPA053157, HPA069832	Approved	Enhanced
HMGXB3	Nucleoli	HPA005967		Approved
SMARCE1	Nucleoplasm	HPA003916, CAB037318	Supported	Supported
TOX	Nucleoplasm,Nucleoli fibrillar center	HPA018322, HPA073241	Enhanced	Approved
TOX2	Nucleoplasm	HPA049900, HPA058396	Approved	Supported
TOX3	Nucleoplasm,Cytosol	HPA040240, HPA040376	Enhanced	Enhanced
TOX4	Nucleoplasm,Nucleoli,Vesicles	HPA017880, HPA027551	Approved	Approved

LEF1	Nucleoplasm	HPA002087, CAB019405	Enhanced	Approved
TCF7	Nucleoplasm	CAB019403, HPA058863, HPA070505	Enhanced	Enhanced
TCF7L1	Nucleoplasm,Cytosol	HPA071298		Supported
TCF7L2	Nucleoplasm,Nuclear bodies,Cytosol	CAB013535, HPA038800	Supported	Approved
HMGXB4	Nucleoplasm	HPA000725, HPA076681	Approved	Approved
PBRM1	Nucleoplasm	HPA015629, HPA059373	Uncertain	Enhanced
NSD2	Nucleoplasm	HPA015801, CAB068246, CAB068247	Enhanced	Supported
SSRP1	Nucleoplasm,Cytosol	HPA002697	Approved	Uncertain
UBTF	Nucleoli fibrillar center	CAB004611, HPA006385	Supported	Enhanced
UBTFL1				
TFAM	Mitochondria	HPA040648, HPA063684	Supported	Enhanced
NFYA	Nucleoplasm	CAB009250, HPA050779	Enhanced	Supported
NFYB	Nucleoplasm	CAB004211, HPA065646	Supported	Supported
NFYC	Nucleoplasm	HPA055011		Supported
MEF2A	Nucleoplasm,Cytosol	CAB004499, HPA046597, HPA056563	Approved	Supported
MEF2B	Nucleoplasm,Cell Junctions,Cytosol	HPA004734	Supported	Approved
MEF2C	Nucleoplasm,Vesicles	HPA005533, CAB068196, CAB068197	Enhanced	Supported
MEF2D	Nucleoplasm,Vesicles	HPA004807, HPA007114	Enhanced	Enhanced
SRF	Nucleoplasm	HPA001819, CAB005416	Supported	Supported
AIRE				
DEAF1	Nucleoplasm,Nucleoli fibrillar center	HPA030302	Approved	Supported
GMEB1	Nucleoplasm	HPA044811, HPA052975	Supported	Enhanced
GMEB2	Nucleoplasm,Cytosol	HPA009706, HPA067455	Approved	Enhanced
SP110	Nucleoplasm	HPA058554		Supported
SP100	Nucleoplasm,Nuclear bodies	HPA016707, HPA017384	Enhanced	Supported
SP140	Nucleoli fibrillar center,Mitochondria	HPA006162, HPA067493	Enhanced	Approved
SP140L	Nucleoplasm	HPA055864	Uncertain	Approved
NFKB1	Nucleoplasm,Cytosol	CAB004031, HPA027305	Enhanced	Supported
NFKB2	Nucleoplasm,Cytosol	HPA008422, CAB022098, HPA023900	Enhanced	Supported
REL		CAB004404	Uncertain	
RELA	Cytosol	CAB004264, CAB005030, HPA063461	Supported	Enhanced
RELB	Nucleoplasm,Cytosol	CAB007753, HPA040506	Approved	Supported
NFAT5	Nucleoplasm,Cytosol	HPA051528, HPA057304, HPA069711, HPA074040	Approved	Enhanced
NFATC1	Nucleoplasm,Nuclear bodies	CAB004513, HPA071732	Enhanced	Supported
NFATC2	Nucleoplasm,Cytosol	HPA008789, CAB018567, HPA024369	Enhanced	Supported
NFATC3	Nucleoplasm,Cytosol	HPA050665		Supported
NFATC4	Nuclear speckles,Cytosol	HPA031641, CAB032859, HPA076526	Uncertain	Supported
RBPJ	Nucleoplasm	HPA000251, HPA060647	Uncertain	Supported
RBPJL		CAB006846	Enhanced	
EBF1	Nucleoplasm,Vesicles	HPA061169		Approved
EBF2	Nucleoplasm,Nuclear bodies	HPA003954, HPA055101		Approved
EBF3	Nucleoplasm,Vesicles	HPA061169		Approved
EBF4				
STAT1	Nucleoplasm,Cytosol	HPA000931, HPA000982, CAB004049	Approved	Enhanced
STAT2	Plasma membrane,Cytosol	CAB003858, HPA018888	Approved	Supported

STAT3	Nucleoplasm,Cytosol	HPA001671, CAB003859, HPA058603, CAB068241, CAB068242	Supported	Supported
STAT4		HPA001860, CAB013108	Enhanced	
STAT5A	Nucleoplasm,Cytosol	CAB003860, HPA027873, HPA042128, HPA049883, HPA051156	Enhanced	Enhanced
STAT5B	Cytosol	CAB004298, CAB005418, HPA042128, HPA049883, HPA051156	Approved	Approved
STAT6	Nucleoplasm,Cytosol	HPA001861	Approved	Supported
TP53	Nucleoplasm,Vesicles,Cytosol	CAB002973, CAB039238, CAB039239, HPA051244, HPA063532, CAB072876	Enhanced	Enhanced
TP63	Nucleoplasm	CAB000083, HPA006288, HPA006289, HPA007009, HPA007010	Enhanced	Enhanced
TP73	Nucleoplasm,Golgi apparatus,Vesicles,Cell Junctions	CAB002514, CAB003022, HPA027314, HPA044516	Enhanced	Supported
RUNX1	Nucleoplasm,Vesicles	HPA004176, HPA037912	Enhanced	Enhanced
RUNX2	Nucleoplasm	CAB008374, HPA022040, CAB062561, CAB068226, HPA070715	Enhanced	Enhanced
RUNX3	Nucleoplasm,Vesicles,Cytosol	HPA004195, CAB025416, HPA059006	Enhanced	Supported
TBX19	Nuclear speckles	HPA005800, HPA072686	Enhanced	Approved
TBXT	Nucleoplasm	HPA003322	Uncertain	Supported
TBX1	Nuclear bodies,Cytoplasmic bodies	HPA029330		Approved
TBX10	Nucleoplasm,Cytosol	HPA047881, HPA076197	Uncertain	Approved
TBX15	Nucleoplasm,Centrosome	HPA052245, HPA053713	Uncertain	Approved
TBX18	Nucleoplasm	HPA006807		Supported
TBX20	Nucleoli fibrillar center	HPA007840		Approved
TBX21		CAB009524, CAB030043	Enhanced	
TBX22				
TBX2	Nucleoplasm,Cytosol	HPA008586, CAB013636	Approved	Approved
TBX3	Nucleoplasm,Cytosol	HPA005799, HPA075876		Supported
TBX4	Vesicles	CAB032413, HPA044457	Uncertain	Approved
TBX5	Nucleoplasm	HPA008786, HPA064683	Uncertain	Approved
TBX6	Nucleoplasm	HPA062498		Approved
MYRF	Nucleoplasm,Cytosol	HPA018310		Approved
GRHL1	Nucleoplasm,Vesicles	HPA005798, HPA006420	Enhanced	Supported
GRHL2	Nucleoplasm	HPA004820, HPA062839	Uncertain	Supported
GRHL3	Nucleoplasm	HPA059960	Enhanced	Approved
TFCP2	Nucleoplasm,Cytosol	HPA070247		Supported
TFCP2L1	Nucleoli fibrillar center,Mitochondria	HPA029708	Uncertain	Approved
UBP1	Nucleoplasm	HPA003967		Supported
SMAD1	Nucleoplasm,Cytosol	CAB005389	Approved	Supported
SMAD2	Nucleoplasm,Nucleoli,Cytosol	CAB025507, HPA067203, CAB073546	Approved	Supported
SMAD3	Nucleoplasm,Cytosol	CAB008094, HPA046386, HPA067203, CAB069409	Approved	Supported
SMAD4	Nucleoplasm,Centrosome,Cytosol	HPA019154, CAB080136	Approved	Supported
SMAD5	Nucleoplasm,Cytosol	HPA058931	Approved	Supported
SMAD6	Nucleoplasm,Nuclear bodies,Golgi apparatus	HPA061917		Supported
SMAD7	Nucleoplasm,Cytosol	CAB026212, HPA028897	Approved	Supported
SMAD9	Nucleoplasm,Cytosol	CAB009119, HPA031162	Approved	Supported
NFIA	Nucleoplasm	HPA006111, HPA008884	Enhanced	Enhanced
NFIB	Nucleoplasm,Nucleoli fibrillar center	HPA003956	Supported	Supported
NFIC	Nucleoplasm,Nucleoli fibrillar center	CAB004300, CAB004674, HPA052625, HPA063560	Supported	Enhanced
NFIX	Nucleoplasm	HPA007533	Supported	Supported
GCM1	Vesicles	HPA011343, HPA073469	Enhanced	Approved

GCM2				
HMGA1	Nucleoplasm,Nucleoli	CAB032200, HPA065612, HPA068442	Enhanced	Supported
HMGA2	Nucleoplasm,Nucleoli,Nucleoli rim	CAB017809, HPA039076	Approved	Supported
YBX1	Endoplasmic reticulum,Vesicles,Plasma membrane,Cytosol	CAB005875, HPA040304, HPA057159	Supported	Supported
YBX2		CAB012332, HPA053904, HPA056744	Enhanced	
YBX3	Cytosol	HPA034838	Approved	Supported
AFF1	Nucleoplasm,Mitochondria	HPA069947		Approved
AFF2		HPA003139	Approved	
AFF3	Nucleoplasm,Cytosol	HPA044512, HPA053379	Approved	Supported
AFF4	Nucleoplasm,Nucleoli fibrillar center	HPA023690, HPA029634	Approved	Supported
AHCTF1	Nucleoplasm,Nuclear membrane,Nuclear bodies	HPA031658	Uncertain	Supported
AKNA	Nucleoplasm,Nucleoli fibrillar center,Centrosome,Cytosol	HPA052367, HPA063993		Supported
ANHX				
ANKRD30A	Nucleoli	HPA071849		Uncertain
ARID3C				
ATMIN	Nuclear bodies	HPA054967, HPA066900	Approved	Supported
BAZ2A	Nuclear speckles	HPA005782, HPA063806		Enhanced
BAZ2B	Nucleoplasm,Cytosol	HPA019819, HPA059292	Uncertain	Approved
BCLAF1	Nuclear speckles	HPA006484, HPA006669, HPA027770	Enhanced	Enhanced
BLZF1	Nucleoplasm,Golgi apparatus	HPA025703, HPA027331, HPA067113	Supported	Supported
CAPN15	Nucleoplasm	HPA011960, HPA074218	Approved	Approved
CARF	Nucleoli fibrillar center,Vesicles,Cytosol	HPA034557, HPA043191	Approved	Approved
CARHSP1		HPA051911	Enhanced	
CBFB	Nucleoplasm,Cytosol	HPA038852	Uncertain	Approved
CC2D1A	Nucleoli fibrillar center,Plasma membrane,Cytosol	HPA005436, CAB015220	Supported	Approved
CCDC88A		HPA038101, HPA038102	Enhanced	
CDIP1	Nucleoplasm,Centrosome	HPA049352	Approved	Supported
CEBPZ	Nucleoplasm,Vesicles	HPA052065	Uncertain	Approved
CENPX				
CIZ1	Nucleoplasm	HPA020380, HPA020387	Uncertain	Enhanced
CNBP	Nucleoplasm,Cytosol	HPA063097		Approved
CPEB1	Nucleoplasm,Cytosol	HPA040396		Supported
CPHXL				
CREBRF	Nucleoplasm	HPA015329		Supported
CSDC2	Nucleoplasm,Cytosol	HPA003073, HPA076549	Uncertain	Approved
CSDE1	Golgi apparatus,Plasma membrane,Cytosol	HPA018846, HPA052221	Supported	Supported
DACH1	Nucleoplasm,Nuclear speckles	HPA012672, HPA012938, HPA073222	Approved	Supported
DACH2	Nucleoplasm	HPA000258, HPA001367	Approved	Approved
DUXB				
EOMES	Nucleoplasm,Golgi apparatus,Cytosol	HPA028896	Uncertain	Approved
FAM170A		HPA037900, HPA037902	Enhanced	
GABPB1	Nucleoplasm,Cytoplasmic bodies	HPA019653, HPA067444	Supported	Supported
GABPB2	Nucleoplasm,Cytosol	HPA028471, HPA058483	Uncertain	Supported
GCFC2	Nucleoplasm	HPA077882	Approved	Supported
GLMP		HPA029121	Uncertain	

GON4L	Nucleoplasm,Nuclear bodies	HPA006986, HPA057305, HPA074504	Uncertain	Enhanced
GPBP1	Vesicles,Plasma membrane,Cytosol	HPA037773	Approved	Approved
GPBP1L1	Nucleoplasm,Plasma membrane,Focal adhesion sites,Cytosol	HPA028593	Approved	Approved
HES1	Nucleoplasm	HPA066929		Supported
HMGB1	Nucleoplasm	HPA003506, CAB005873, HPA073663	Supported	Supported
HMGB2	Nucleoplasm,Nucleoli	HPA003506, CAB037206, HPA053314, HPA073663	Enhanced	Supported
HMGB3	Nucleoplasm,Nucleoli	HPA062583, HPA073663	Enhanced	Supported
HMGB4		HPA035699	Enhanced	
HSFX4				
JAZF1	Nucleoplasm,Nucleoli fibrillar center	CAB019271, HPA066967	Approved	Supported
KHSRP	Nucleoplasm	HPA034739, HPA056518	Supported	Enhanced
KIAA1549	Nuclear membrane,Intermediate filaments	HPA019560	Uncertain	Approved
KLF18				
KMT2C	Nucleoplasm	HPA062814, HPA074736		Supported
L3MBTL3	Nucleoplasm,Nucleoli,Nucleoli rim	HPA044382, HPA053035	Approved	Supported
L3MBTL4	Nucleoplasm,Vesicles	HPA064194, HPA069042	Approved	Approved
LCOR	Nucleoplasm	HPA031429, HPA057621, HPA077000	Approved	Enhanced
LCORL	Nucleoplasm,Nuclear membrane	HPA028794, HPA028795, HPA060033	Uncertain	Approved
LIN28A	Nucleoli,Cytosol,Rods & Rings	CAB020785, HPA063074, HPA068814	Enhanced	Supported
LIN28B	Nucleoplasm,Nucleoli,Cytosol	HPA036630, HPA061745	Enhanced	Supported
LITAF	Nucleoplasm,Golgi apparatus,Vesicles	HPA006960	Approved	Supported
LTF		CAB008646, CAB016201, HPA057177, HPA059976	Enhanced	
MAEL		HPA078602	Approved	
MAF1	Nucleoplasm,Cytosol	HPA024821, HPA058548, HPA072409	Uncertain	Supported
MBD2	Nucleoplasm	CAB037282, HPA067582, HPA067615	Approved	Supported
MBD3	Nucleoplasm	CAB013265	Supported	Supported
MBD4	Nuclear speckles	HPA002031	Supported	Supported
MECP2	Nucleoplasm	HPA000593, HPA001341, CAB037264	Enhanced	Approved
MEIOSIN	Nucleoplasm	HPA049521	Uncertain	Approved
MIS18BP1	Nucleoli,Cytosol	HPA006504	Approved	Approved
MLLT10	Nucleoplasm,Cytosol	HPA005736, HPA005747, HPA072790	Enhanced	Supported
MYEF2	Nucleoplasm	HPA004883	Supported	Supported
MYRFL	Nucleoli	HPA049340	Approved	Approved
MYSM1	Nucleoplasm,Nucleoli	HPA054291, HPA057306	Approved	Supported
NANOGP8	Nucleoplasm,Vesicles	HPA072117		Supported
NFRKB	Nucleoplasm	HPA007082, HPA007128	Approved	Enhanced
NHLH1	Golgi apparatus	HPA017943		Approved
NKRF	Nucleoplasm,Nucleoli	HPA001476		Supported
NME2	Plasma membrane,Cytosol	CAB036214, CAB036215, HPA041113, CAB080057, CAB080059	Approved	Supported
PARP12	Nucleoplasm	HPA003584, HPA063872	Approved	Approved
PAXBP1	Nucleoplasm,Cytosol	HPA003757	Approved	Approved
PHB				
PIAS1	Microtubules	HPA005743, CAB012304	Supported	Approved
PIAS2	Nucleoplasm	HPA068792, HPA077584	Enhanced	Enhanced
PIAS3	Nucleoplasm	HPA006389, HPA067383	Approved	Supported

PIAS4	Nucleoplasm,Centrosome	HPA010598, HPA076008	Enhanced	Supported
PLEK	Nucleoli	HPA031838, HPA057341	Enhanced	Approved
PLSCR1	Nucleoplasm,Golgi apparatus,Plasma membrane	HPA068923, CAB070421	Approved	Supported
PMS1	Nucleoplasm,Nuclear bodies	HPA031013		Approved
PRDM11		HPA057072	Approved	
PRDM12	Nucleoplasm	HPA043143	Uncertain	Approved
PREB	Endoplasmic reticulum	HPA013582, HPA014133	Uncertain	Approved
RAG1	Nucleoplasm	HPA043939		Supported
RBCK1				
REPIN1	Nucleoplasm	HPA036022	Approved	Supported
SAMD11	Nucleoplasm,Vesicles	HPA030110		Approved
SETBP1	Nucleoplasm,Nuclear bodies,Cytosol	HPA049022, HPA057259	Uncertain	Supported
SETDB1	Nucleoplasm,Vesicles	HPA018142, HPA058484	Uncertain	Supported
SETDB2	Nucleoplasm	CAB012190, HPA071677	Approved	Approved
SLC2A4RG	Nuclear speckles	HPA063050		Approved
SMARCAL1	Nucleoplasm	HPA020337	Uncertain	Supported
SON	Nuclear speckles	HPA023535, HPA031755, HPA031756	Supported	Enhanced
SPZ1				
SUB1	Nucleoplasm,Nucleoli	HPA001311, CAB015351	Supported	Supported
TAX1BP1	Nucleoplasm,Cytosol	HPA024432	Uncertain	Approved
TBR1		HPA051256, HPA078644, HPA078657	Enhanced	
TCF19	Nucleoplasm,Nucleoli	HPA065994		Approved
TCF24				
TERB1	Nucleoplasm,Cell Junctions,Cytosol	HPA079436		Supported
TERF1	Nucleoli fibrillar center,Nuclear bodies	HPA048379		Approved
TERF2	Nucleoplasm,Nuclear bodies	HPA001907, HPA002735, CAB010451, CAB080271	Enhanced	Enhanced
TET2	Nucleoplasm	HPA043135	Supported	Approved
TFB1M		HPA029428	Approved	
THYN1	Nucleoplasm,Nucleoli fibrillar center,Cytosol	HPA038732, HPA038733	Supported	Supported
TMF1	Golgi apparatus	HPA008729	Approved	Supported
TSC22D1	Nucleoplasm,Nuclear bodies	HPA077414		Approved
TSC22D2	Cytosol	HPA004926	Approved	Approved
TSC22D3	Nuclear speckles,Golgi apparatus,Cytosol	HPA001916, CAB034364	Approved	Approved
TSC22D4	Cytosol	HPA006757	Uncertain	Approved
TTF1	Nucleoli fibrillar center	HPA051105, HPA054837	Supported	Supported
TUB	Nucleoli,Centriolar satellite	HPA017997, HPA049019	Enhanced	Approved
TULP1				
TULP3	Nucleoplasm,Nucleoli,Plasma membrane	HPA015285, HPA018496	Uncertain	Supported
TULP4		HPA005445	Approved	
USF3	Nucleoplasm,Nucleoli,Centrosome	HPA029243, HPA029245	Uncertain	Uncertain
WDHD1	Nucleoplasm	HPA000632, HPA001122	Enhanced	Enhanced
XPA	Nucleoplasm,Cytokinetic bridge	CAB000155, HPA056856	Supported	Approved
YEATS4	Nucleoplasm,Nuclear membrane	HPA072532		Approved
ZBTB10	Nucleoplasm	HPA021554, HPA050569	Enhanced	Enhanced
ZBTB3	Mitochondria	HPA035321, HPA035322	Approved	Approved

ZBTB8A	Nuclear speckles,Focal adhesion sites	HPA031768, HPA031769, HPA031770	Approved	Approved
ZFP36L1		HPA001301	Uncertain	
ZFTA	Intermediate filaments	HPA045002		Approved
ZMIZ1	Nucleoplasm,Cytosol	HPA045144, HPA071156	Approved	Supported
ZMIZ2	Nucleoplasm,Mitochondria	HPA040716, HPA071991	Supported	Supported
ZNF112	Nucleoplasm,Nucleoli fibrillar center	HPA023856, HPA076887	Approved	Approved
ZNF12	Nucleoplasm,Centrosome	HPA063402		Supported
ZNF207	Nucleoplasm	HPA017013, HPA063908	Enhanced	Enhanced
ZNF318	Nucleoplasm,Cytosol	HPA027022, HPA027031	Approved	Supported
ZNF326	Nucleoplasm,Golgi apparatus,Vesicles	HPA028450, HPA028461, HPA028466	Supported	Approved
ZNF367	Nucleoplasm	HPA015319, HPA015785	Uncertain	Supported
ZNF385A	Nucleoplasm,Cytosol	HPA076214		Supported
ZNF385B	Nucleoli fibrillar center	HPA046086		Supported
ZNF395	Nucleoplasm,Cytosol	HPA049382, HPA076275	Uncertain	Supported
ZNF470	Nucleoplasm,Nuclear bodies	HPA029000	Uncertain	Supported
ZNF473	Nucleoplasm	HPA004191, HPA073309	Approved	Supported
ZNF486				
ZNF488	Nucleoplasm,Cytosol	HPA035957, HPA035958		Supported
ZNF503	Nucleoplasm,Plasma membrane,Cytosol	HPA026848	Approved	Approved
ZNF527	Nuclear speckles,Golgi apparatus	HPA037740, HPA037741	Uncertain	Approved
ZNF573	Intermediate filaments,Cytosol	HPA042776	Uncertain	Approved
ZNF578				
ZNF587B	Nucleoplasm	HPA043800, HPA053762, HPA072145	Uncertain	Uncertain
ZNF608	Nucleoplasm,Mitochondria	HPA005545, HPA076984	Approved	Approved
ZNF609	Nucleoplasm,Cytosol	HPA040742, HPA040832	Approved	Enhanced
ZNF622	Nucleoplasm,Nucleoli,Golgi apparatus,Cytosol	HPA036514, HPA036515	Uncertain	Supported
ZNF630	Nucleoplasm,Cytosol	HPA001053, HPA068130	Supported	Approved
ZNF688	Nucleoplasm,Plasma membrane,Cell Junctions	HPA048922	Uncertain	Approved
ZNF69	Nucleoplasm	HPA047845	Approved	Uncertain
ZNF699		HPA058287	Uncertain	
ZNF700	Nucleoplasm	HPA047845	Uncertain	Uncertain
ZNF704	Nucleoplasm	HPA024285, HPA024302	Uncertain	Approved
ZNF705B				
ZNF706	Plasma membrane	HPA042793		Approved
ZNF708				
ZNF714				
ZNF720				
ZNF723				
ZNF728	Nucleoli fibrillar center	HPA077713		Approved
ZNF735				
ZNF750		HPA021573, HPA023012	Enhanced	
ZNF761				
ZNF773	Nucleoplasm,Nucleoli fibrillar center	HPA003152	Uncertain	Approved
ZNF783	Plasma membrane,Cytosol	HPA020375	Uncertain	Approved
ZNF8	Nucleoplasm,Nuclear bodies	HPA034797, HPA034798	Uncertain	Approved

ZNF821	Nucleoplasm,Nuclear speckles	HPA036372, HPA042742	Enhanced	Supported
ZNF827	Nucleoplasm	HPA021166	Uncertain	Approved
ZNF831		HPA016839	Uncertain	
ZNF850	Nucleoplasm	HPA029299	Uncertain	Approved
ZNF865	Nucleoplasm,Nucleoli	HPA068079	Approved	Approved
ZNF883				
ZNF888				
ZSCAN18	Nucleoplasm	HPA024318	Supported	Approved
ZZZ3	Nucleoplasm,Nucleoli,Nucleoli rim	HPA053663, HPA054913, HPA066197	Uncertain	Enhanced
