# Supplementary material for: Foxg1 and companions: Not only transcription factors
Source: Neural Regen Res. 2025 Sep 3;21(8):3426–38. doi: 10.4103/NRR.NRR-D-25-00439 (PMC13452659; doi:10.4103/NRR.NRR-D-25-00439)
Supplement: Supplementary file 20 [file NRR-21-3426_Suppl7.pdf]

Additional Table 3. RBP genes list (*n* = 4775)

[RNA-Binding Protein genes list]

NOTES

manually compiled, based on sources listed below

Non-redundant RBP gene list	*RNA-binding ability experimentally verified in case of…*	*Gerstberger et al. Nature Reviews Genetics (2014). doi: 10.1038/nrg3813*	*Hentze et al. Nature Reviews Molecular Cell Biology (2018). doi: 10.1038/nrm.2017.130*	*RBPWorld. human and mouse databases accessed at: http://research.gzsys.org.cn/rbpworld/#/home, on 2024.10.18*	*Castello et al. Cell (2012). doi: 10.1016/j.cell.2012.04.031*	*Baltz et al. Molecular Cell (2012). doi: 10.1016/j.molcel.2012.05.021*	*Nabeel-Shah et al. Molecular Cell (2024). doi: 10.1016/j.molcel.2024.08.037*	*Gosztyla et al. Molecular Cell (2024). doi: 10.1016/j.molcel.2024.08.010*	*Oksuz et al. Moecular Cell (2023). doi: 10.1016/j.molcel.2023.06.012*
37500	only mmu ortholog		+						
39326	hsa gene		+						
40787	hsa gene		+						
PPIA	only mmu ortholog		+		+	+			

A1CF	hsa gene	+	+	+					
AAR2	hsa gene	+		+					
AARS	hsa gene	+	+		+				
AARS1	hsa gene			+					
AARS2	hsa gene	+		+					
AARSD1	hsa gene	+		+					
AATF	hsa gene		+	+	+	+			
ABCB7	hsa gene			+					
ABCC2	only mmu ortholog			+					
ABCD3	hsa gene			+					
ABCE1	hsa gene	+	+	+					
ABCF1	hsa gene	+	+	+	+	+			
ABCF2	only mmu ortholog		+	+	+				
ABHD12	only mmu ortholog		+						
ABT1	hsa gene	+	+	+	+	+			
AC004381.6	hsa gene	+							
AC013461.1	hsa gene		+		+				
ACAA1A	only mmu ortholog		+						
ACAA2	hsa gene		+	+	+				
ACAD9	hsa gene			+					
ACADL	only mmu ortholog		+	+					
ACADM	only mmu ortholog		+	+					
ACADS	only mmu ortholog		+	+					
ACAN	only mmu ortholog		+						
ACAT1	hsa gene		+	+					
ACD	hsa gene			+					
ACIN1	hsa gene	+	+	+	+	+			
ACLY	only mmu ortholog		+	+	+				
ACO1	hsa gene	+	+	+					
ACO2	hsa gene		+	+					

ACOT9	only mmu ortholog		+						
ACOX1	only mmu ortholog		+						
ACSL3	hsa gene			+					
ACTA2	hsa gene			+					
ACTB	only mmu ortholog			+					
ACTG1	hsa gene			+					
ACTL6A	only mmu ortholog		+						
ACTN1	hsa gene			+	+				
ACTN3	only mmu ortholog		+						
ACTN4	hsa gene		+	+	+				
ACTR2	hsa gene			+					
ACTR3	only mmu ortholog		+	+					
ACTR5	only mmu ortholog		+						
ACTR8	only mmu ortholog		+						
ADAD1	hsa gene	+		+					
ADAD2	hsa gene	+		+					
ADAM3	only mmu ortholog			+					
ADAR	hsa gene	+	+	+	+	+			
ADARB1	hsa gene	+	+	+	+	+			
ADARB2	hsa gene	+	+	+	+				
ADAT1	hsa gene	+		+					
ADAT2	hsa gene	+		+					
ADAT3	hsa gene	+		+					
ADD1	hsa gene		+	+	+				
ADD3	hsa gene		+	+					
ADGRV1	only mmu ortholog		+						
ADH5	hsa gene			+					
ADISSP	hsa gene			+					
ADK	hsa gene		+	+	+				
ADNP	only mmu ortholog		+						+

ADNP2	only mmu ortholog		+						+
AEBP2	only mmu ortholog		+						+
AEN	hsa gene	+		+	+				
AFDN	hsa gene		+	+					
AFF1	hsa gene	+		+					
AFF2	hsa gene	+		+					
AFF3	hsa gene	+		+					
AFF4	hsa gene	+	+	+					
AFG2A	only mmu ortholog			+					
AFG3L1	only mmu ortholog		+						
AFG3L2	only mmu ortholog		+						
AFP	hsa gene		+						
AGAP1	only mmu ortholog		+						
AGFG1	hsa gene	+		+	+				
AGGF1	hsa gene			+					
AGL	hsa gene			+					
AGO1	hsa gene	+	+	+					
AGO2	hsa gene	+	+	+					
AGO3	hsa gene	+	+	+					
AGO4	hsa gene	+		+					
AGPS	hsa gene			+					
AHCTF1	only mmu ortholog		+						+
AHCY	hsa gene			+					
AHCYL1	hsa gene			+					
AHCYL2	hsa gene		+						
AHDC1	only mmu ortholog		+						+
AHNAK	hsa gene		+	+	+				
AHNAK2	only mmu ortholog		+		+				
AI837181	only mmu ortholog		+	+					
AI854703	only mmu ortholog			+					

AICDA	hsa gene			+					
AIFM1	hsa gene			+					
AIM2	only mmu ortholog		+						
AIMP1	hsa gene	+	+	+					
AIMP2	hsa gene	+		+					
AK2	hsa gene			+					
AK3	hsa gene			+					
AK4	hsa gene			+					
AKAP1	hsa gene	+	+	+	+	+			
AKAP17A	hsa gene	+	+	+		+			
AKAP17B	only mmu ortholog			+					
AKAP7	hsa gene			+					
AKAP8	hsa gene	+	+	+	+	+			+
AKAP8L	hsa gene	+	+	+	+	+			+
ALB	only mmu ortholog			+					
ALDH18A1	hsa gene		+	+	+				
ALDH2	only mmu ortholog		+	+					
ALDH6A1	hsa gene		+	+	+				
ALDH7A1	hsa gene		+	+					
ALDOA	hsa gene		+	+		+			
ALDOC	hsa gene		+						
ALG13	hsa gene		+	+		+			
ALKBH1	hsa gene	+		+					
ALKBH2	hsa gene			+					
ALKBH3	hsa gene			+					
ALKBH5	hsa gene	+	+	+	+	+			
ALKBH6	hsa gene			+					
ALKBH7	hsa gene			+					
ALKBH8	hsa gene	+		+					
ALYREF	hsa gene	+	+	+					

ALYREF2	only mmu ortholog			+					
AMMECR1	only mmu ortholog		+						
AMN	only mmu ortholog		+						
AMT	only mmu ortholog		+						
ANAPC1	hsa gene			+					
ANAPC5	hsa gene			+					
ANG	hsa gene	+		+					
ANG2	only mmu ortholog			+					
ANG4	only mmu ortholog			+					
ANG5	only mmu ortholog			+					
ANG6	only mmu ortholog			+					
ANGEL1	hsa gene	+		+					
ANGEL2	hsa gene	+		+					
ANK3	hsa gene		+		+				
ANKHD1	hsa gene	+	+	+	+	+			
ANKLE2	hsa gene			+					
ANKRD17	hsa gene	+	+	+	+				
ANKRD34A	hsa gene			+					
ANKRD34B	hsa gene			+					
ANKRD34C	hsa gene			+					
ANKZF1	only mmu ortholog		+						+
ANLN	only mmu ortholog		+						
ANP32A	hsa gene		+	+		+			
ANP32B	hsa gene		+						
ANP32E	only mmu ortholog		+						
ANXA1	only mmu ortholog		+	+	+				
ANXA11	hsa gene		+	+	+	+			
ANXA2	hsa gene		+	+	+				
ANXA6	only mmu ortholog		+	+					
ANXA7	hsa gene		+	+	+	+			

AP1B1	only mmu ortholog		+						
AP2A1	hsa gene			+					
AP2A2	only mmu ortholog		+						
AP2B1	hsa gene			+					
AP2M1	only mmu ortholog		+						
AP3D1	hsa gene			+					
APEH	hsa gene		+	+	+				
APEX1	hsa gene	+	+	+	+	+			
APEX2	hsa gene			+				+	
API5	hsa gene	+	+	+	+	+			
APOB	hsa gene		+						
APOBEC1	hsa gene	+		+					
APOBEC2	hsa gene	+		+					
APOBEC3	only mmu ortholog		+	+					
APOBEC3A	hsa gene			+					
APOBEC3B	hsa gene		+	+	+	+			
APOBEC3C	hsa gene		+	+	+	+			
APOBEC3D	hsa gene			+					
APOBEC3F	hsa gene	+	+	+		+			
APOBEC3G	hsa gene	+		+					
APOBEC3H	hsa gene	+		+					
APOBEC4	hsa gene	+		+					
APOL8	only mmu ortholog		+						
APTX	hsa gene	+		+					
AQR	hsa gene	+	+	+	+	+			
ARC	hsa gene			+					
ARCN1	hsa gene		+	+	+				
ARF1	hsa gene		+	+		+			
ARF2	only mmu ortholog			+					
ARF3	hsa gene			+					

ARF4	hsa gene			+					
ARF5	hsa gene			+					
ARFGEF1	only mmu ortholog		+						
ARFIP2	hsa gene			+					
ARFRP1	hsa gene			+					
ARGLU1	only mmu ortholog		+						
ARHGAP35	hsa gene			+					
ARHGAP5	hsa gene			+	+				
ARHGDIA	only mmu ortholog		+						
ARHGDIB	only mmu ortholog		+						
ARHGEF1	hsa gene		+	+	+				
ARHGEF12	only mmu ortholog		+						
ARHGEF2	hsa gene		+						
ARHGEF28	hsa gene	+		+					
ARID1A	only mmu ortholog		+						
ARID4A	hsa gene			+					
ARID4B	hsa gene			+					
ARID5A	only mmu ortholog			+					
ARID5B	only mmu ortholog		+						+
ARL1	hsa gene			+					
ARL10	hsa gene			+					
ARL11	hsa gene			+					
ARL13A	only mmu ortholog			+					
ARL13B	only mmu ortholog			+					
ARL14	hsa gene			+					
ARL2	hsa gene			+					
ARL3	hsa gene			+					
ARL4A	hsa gene			+					
ARL4C	hsa gene			+					
ARL4D	hsa gene			+					

ARL5A	hsa gene			+					
ARL5B	hsa gene			+					
ARL5C	only mmu ortholog			+					
ARL6	hsa gene			+					
ARL6IP4	hsa gene	+	+	+	+	+			
ARL6IP5	hsa gene			+					
ARL8A	hsa gene			+					
ARL8B	hsa gene			+					
ARMC7	hsa gene			+					
ARMCX2	only mmu ortholog		+						
ARPC1B	hsa gene		+						
ARPC3	only mmu ortholog		+						
ARPC4	hsa gene			+					
ARPP19	only mmu ortholog		+						
ARPP21	hsa gene			+					
ARVCF	hsa gene			+					
AS3MT	hsa gene			+					
ASCC1	hsa gene	+		+					
ASCC3	hsa gene		+	+	+	+			
ASH1L	hsa gene	+		+					
ASH2L	hsa gene			+					
ASMT	hsa gene			+					
ASNS	hsa gene			+					
ASS1	hsa gene		+	+	+				
ASXL1	hsa gene			+					
ASXL2	hsa gene			+					
ASXL3	hsa gene			+					
ASZ1	hsa gene			+					
ATAD2B	only mmu ortholog		+						
ATF1	only mmu ortholog		+						+

ATF2	only mmu ortholog		+						+
ATG3	hsa gene			+					
ATIC	only mmu ortholog		+	+					
ATM	hsa gene			+					
ATOX1	only mmu ortholog		+						
ATP13A1	only mmu ortholog		+		+				
ATP1A1	hsa gene		+						
ATP1B1	hsa gene			+					
ATP2A2	hsa gene		+						
ATP2B1	hsa gene			+					
ATP5A1	hsa gene		+		+				
ATP5B	hsa gene		+						
ATP5C1	hsa gene		+		+				
ATP5F1	only mmu ortholog		+						
ATP5F1A	hsa gene			+					
ATP5F1B	hsa gene			+					
ATP5F1C	hsa gene			+					
ATP5J2	only mmu ortholog		+						
ATP5K	only mmu ortholog		+						
ATP5O	only mmu ortholog		+						
ATP5PD	only mmu ortholog		+						
ATP5PO	hsa gene			+					
ATP5SL	only mmu ortholog		+						
ATP6V0A1	only mmu ortholog		+						
ATP6V1A	only mmu ortholog		+						
ATR	hsa gene			+					
ATRX	only mmu ortholog		+						
ATXN1	hsa gene	+		+					
ATXN10	hsa gene			+	+				
ATXN1L	hsa gene	+		+					

ATXN2	hsa gene	+	+	+	+	+			
ATXN2L	hsa gene	+	+	+	+	+			
AUH	hsa gene	+		+					
AURKB	hsa gene			+					
AZGP1	hsa gene	+		+					
BAG3	only mmu ortholog		+		+				
BAG4	hsa gene		+	+		+			
BAG6	only mmu ortholog		+						
BARD1	hsa gene	+		+					
BASP1	hsa gene		+		+				
BAZ1B	hsa gene		+	+					
BAZ2A	hsa gene	+		+					+
BAZ2B	hsa gene	+		+					+
BCAM	hsa gene			+					
BCAP29	hsa gene			+					
BCAS2	only mmu ortholog			+					
BCCIP	hsa gene		+	+	+				
BCDIN3D	hsa gene	+		+					
BCL2L2	hsa gene			+					
BCL6	hsa gene			+					
BCL6B	hsa gene			+					
BCLAF1	hsa gene	+	+	+	+	+			
BCLAF3	hsa gene			+					
BCR	hsa gene			+					
BCS1L	hsa gene			+					
BHMT	only mmu ortholog		+						
BICC1	hsa gene	+	+	+	+				
BICD1	hsa gene			+					
BIN1	only mmu ortholog		+						
BLM	hsa gene			+					

BLVRB	only mmu ortholog		+		+				
BMP4	hsa gene			+					
BMS1	hsa gene	+	+	+	+	+			
BNC1	hsa gene			+					
BNC2	hsa gene			+					
BOLA1	only mmu ortholog		+						
BOLL	hsa gene	+		+					
BOP1	hsa gene	+	+	+	+				
BPTF	only mmu ortholog		+						+
BRCA1	hsa gene	+		+				+	
BRD1	only mmu ortholog		+						
BRD2	hsa gene		+						
BRD3	only mmu ortholog		+						
BRD4	only mmu ortholog		+						
BRD7	only mmu ortholog		+						
BRD9	only mmu ortholog		+						
BRDT	hsa gene			+					
BRF1	hsa gene			+					
BRF2	hsa gene			+					
BRIP1	hsa gene			+					
BRIX1	hsa gene	+	+	+	+	+			
BRPF3	only mmu ortholog		+						
BRSK2	hsa gene			+					
BSG	only mmu ortholog		+		+				
BSPRY	hsa gene			+	+				
BST2	hsa gene		+	+	+				
BTF3	hsa gene		+	+	+	+			
BTF3L4	only mmu ortholog		+						
BTN1A1	hsa gene			+					
BTN2A1	hsa gene			+					

BTN2A2	hsa gene			+					
BTN3A1	hsa gene			+					
BTN3A3	hsa gene			+					
BTNL1	only mmu ortholog			+					
BTNL10	only mmu ortholog			+					
BTNL3	hsa gene			+					
BTNL4	only mmu ortholog			+					
BTNL6	only mmu ortholog			+					
BTNL8	hsa gene			+					
BTNL9	hsa gene			+					
BTRC	only mmu ortholog		+						
BUB3	hsa gene			+					
BUD13	hsa gene	+	+	+	+				
BUD23	hsa gene			+					
BUD31	only mmu ortholog			+					
BYSL	hsa gene	+	+	+	+				
BZW1	hsa gene	+	+	+	+	+			
BZW2	hsa gene	+	+	+	+				
C11ORF68	hsa gene	+	+	+					
C11ORF98	hsa gene			+					
C12ORF43	hsa gene		+						
C12ORF65	hsa gene	+							
C14ORF166	hsa gene		+						
C14ORF93	hsa gene		+	+					
C15ORF52	hsa gene		+						
C17ORF85	hsa gene	+							
C19ORF47	hsa gene		+						
C19ORF66	hsa gene		+						
C1D	hsa gene	+		+					
C1ORF131	hsa gene		+	+					

C1ORF204	hsa gene		+						
C1ORF35	hsa gene		+	+					
C1ORF52	hsa gene		+	+					
C1QBP	hsa gene	+		+					
C2CD3	hsa gene			+					
C2CD5	only mmu ortholog		+						
C2ORF15	hsa gene	+	+	+					
C4B	hsa gene			+					
C4BPA	hsa gene		+	+	+				
C5ORF24	only mmu ortholog		+						
C6ORF52	hsa gene			+					
C7ORF50	hsa gene		+	+					
C9ORF114	hsa gene	+							
C9ORF129	hsa gene	+							
C9ORF40	hsa gene			+					
C9ORF78	hsa gene			+					
CAB39	hsa gene			+					
CABIN1	only mmu ortholog		+						
CACTIN	hsa gene	+	+	+					
CACYBP	hsa gene		+		+				
CAD	hsa gene			+	+				
CADM1	hsa gene		+		+				
CALD1	hsa gene		+						
CALM1	only mmu ortholog		+						
CALR	hsa gene	+	+	+	+				
CALR3	hsa gene	+		+					
CALR4	only mmu ortholog			+					
CAND1	hsa gene		+	+	+				
CANX	hsa gene	+	+	+	+	+			
CAP1	only mmu ortholog			+					

CAPG	only mmu ortholog		+	+	+				
CAPN15	hsa gene			+					
CAPN5	only mmu ortholog		+						
CAPN7	hsa gene			+					
CAPRIN1	hsa gene	+	+	+	+	+			
CAPRIN2	hsa gene	+		+					
CAPZA1	hsa gene			+					
CAPZA2	only mmu ortholog			+					
CAPZB	only mmu ortholog		+	+					
CAR2	only mmu ortholog		+						
CARHSP1	hsa gene	+	+	+					
CARLR	only mmu ortholog			+					
CARM1	hsa gene			+					
CARS	hsa gene	+							
CARS1	hsa gene			+					
CARS2	hsa gene	+		+					
CASC3	hsa gene	+	+	+	+	+			
CASP7	hsa gene			+					
CAST	hsa gene		+	+	+				
CAT	hsa gene		+						
CAVIN1	hsa gene			+					
CBX1	only mmu ortholog		+						
CBX3	only mmu ortholog		+	+					
CBX4	hsa gene			+					
CBX5	only mmu ortholog		+						
CBX6	hsa gene			+					
CBX7	only mmu ortholog			+					
CBX8	hsa gene			+	+				
CBY1	hsa gene			+					
CBY2	hsa gene			+					

CBY3	hsa gene			+					
CC2D1B	hsa gene		+						
CCAR1	hsa gene	+	+	+	+	+			
CCAR2	hsa gene	+	+	+					
CCDC115	only mmu ortholog		+						
CCDC12	hsa gene			+					
CCDC124	hsa gene		+	+	+				
CCDC137	hsa gene		+	+	+	+			
CCDC22	hsa gene			+					
CCDC25	hsa gene			+					
CCDC43	hsa gene		+		+				
CCDC47	hsa gene		+	+	+				
CCDC50	only mmu ortholog		+						
CCDC59	hsa gene	+	+	+	+				
CCDC84	only mmu ortholog		+						
CCDC86	hsa gene	+	+	+	+	+			
CCDC9	hsa gene		+	+	+	+			
CCDC9B	hsa gene			+					
CCM2	hsa gene			+					
CCNA2	only mmu ortholog		+						
CCNL1	only mmu ortholog		+						
CCNT1	hsa gene	+		+	+				
CCNT2	hsa gene	+		+					
CCRN4L	hsa gene	+							
CCT2	hsa gene		+	+					
CCT3	hsa gene		+	+	+	+			
CCT4	hsa gene		+	+	+				
CCT5	hsa gene			+	+				
CCT6A	hsa gene		+	+	+				
CCT7	only mmu ortholog			+					

CCT8	hsa gene			+	+				
CD2AP	hsa gene			+					
CD2BP2	hsa gene	+		+					
CD3EAP	hsa gene	+	+		+				
CD44	hsa gene		+	+					
CD47	hsa gene			+					
CDC27	hsa gene			+					
CDC40	hsa gene	+	+	+	+				
CDC42EP4	hsa gene		+	+	+				
CDC5L	hsa gene	+	+	+		+			+
CDC5LRT1	only mmu ortholog			+					
CDC5LRT10	only mmu ortholog			+					
CDC5LRT4	only mmu ortholog			+					
CDC5LRT5	only mmu ortholog			+					
CDC5LRT6	only mmu ortholog			+					
CDC5LRT7	only mmu ortholog			+					
CDC5LRT8	only mmu ortholog			+					
CDC5LRT9	only mmu ortholog			+					
CDC6	only mmu ortholog		+						
CDCA2	only mmu ortholog		+						
CDH2	hsa gene			+					
CDK1	hsa gene			+					
CDK11A	hsa gene			+	+				
CDK11B	hsa gene		+	+		+			
CDK12	hsa gene			+					
CDK13	hsa gene		+	+		+			
CDK5RAP1	hsa gene	+		+					
CDK9	hsa gene	+		+	+				
CDKAL1	only mmu ortholog			+					
CDKL3	only mmu ortholog			+					

CDKL5	hsa gene			+					
CDKN2A	hsa gene		+	+	+	+			
CDKN2AIP	hsa gene			+					+
CDKN2AIPNL	only mmu ortholog		+						
CDV3	hsa gene		+		+				
CEBPZ	hsa gene	+	+	+	+	+			+
CELF1	hsa gene	+	+	+	+	+			
CELF2	hsa gene	+	+	+	+	+			
CELF3	hsa gene	+		+					
CELF4	hsa gene	+		+					
CELF5	hsa gene	+		+					
CELF6	hsa gene	+		+					
CENATAC	only mmu ortholog			+					
CENPC1	only mmu ortholog		+						
CENPE	only mmu ortholog		+						
CENPF	only mmu ortholog		+						
CEP152	only mmu ortholog		+						
CEP192	only mmu ortholog		+						
CFAP20	hsa gene		+	+					
CFAP65	hsa gene		+	+					
CFDP1	only mmu ortholog		+						
CFL1	hsa gene		+	+	+				
CGGBP1	only mmu ortholog		+						+
CGN	hsa gene		+		+				
CHAF1B	only mmu ortholog			+					
CHAMP1	only mmu ortholog		+						+
CHCHD1	hsa gene		+	+		+			
CHCHD3	hsa gene		+						+
CHD1	hsa gene			+	+				
CHD1L	hsa gene			+					

CHD2	hsa gene		+	+	+	+			
CHD3	hsa gene		+	+	+				
CHD4	hsa gene			+					
CHD5	hsa gene			+					
CHD6	hsa gene			+					
CHD7	hsa gene			+					
CHD8	hsa gene			+					
CHD9	hsa gene			+					
CHERP	hsa gene	+	+	+	+	+			
CHGA	hsa gene		+						
CHMP4B	hsa gene		+						
CHORDC1	only mmu ortholog		+		+				
CHTOP	hsa gene	+	+	+		+			
CIAO1	only mmu ortholog			+					
CIR1	hsa gene			+					
CIRBP	hsa gene	+	+	+	+	+			
CIRH1A	only mmu ortholog		+		+				
CISD1	hsa gene		+					+	
CISD2	hsa gene		+	+	+			+	
CIZ1	hsa gene			+					
CKAP4	hsa gene		+	+	+	+			
CKAP5	hsa gene			+					
CKB	hsa gene			+					
CKMT1	only mmu ortholog		+						
CLASP1	only mmu ortholog		+		+				
CLASP2	hsa gene		+	+	+				
CLASRP	hsa gene	+		+					
CLGN	hsa gene			+					
CLIC1	hsa gene			+					
CLK1	hsa gene	+		+					

CLK2	hsa gene	+		+					
CLK3	hsa gene	+	+	+	+	+			
CLK4	hsa gene	+							
CLNS1A	hsa gene		+	+	+				
CLP1	hsa gene	+		+					
CLPB	hsa gene			+					
CLPX	only mmu ortholog		+						
CLTB	only mmu ortholog		+						
CLTC	hsa gene		+	+	+	+			
CLUH	hsa gene			+					
CMAS	only mmu ortholog		+						
CMIP	hsa gene			+					
CMSS1	hsa gene	+	+	+					
CMTR1	hsa gene	+		+					
CMTR2	hsa gene	+		+					
CNBP	hsa gene	+	+	+	+	+		+	
CNDP2	hsa gene		+						
CNN1	hsa gene		+						
CNN2	hsa gene		+		+				
CNN3	hsa gene		+						
CNOT1	hsa gene	+	+	+	+	+			
CNOT10	hsa gene	+		+					
CNOT11	hsa gene	+		+					
CNOT2	hsa gene	+		+					
CNOT3	hsa gene	+		+					
CNOT4	hsa gene	+	+	+	+	+		+	
CNOT6	hsa gene	+		+					
CNOT6L	hsa gene	+		+					
CNOT7	hsa gene	+		+					
CNOT8	hsa gene	+		+					

CNOT9	hsa gene		+	+					
CNP	hsa gene	+		+					
COA3	hsa gene			+					
COA6	hsa gene		+	+					
COBL	only mmu ortholog		+						
COIL	hsa gene			+					
COL14A1	hsa gene		+	+	+				
COL7A1	hsa gene			+	+				
COPA	only mmu ortholog		+	+					
COPB1	hsa gene		+						
COPB2	hsa gene			+					
COPG1	hsa gene		+	+					
COPS2	only mmu ortholog		+						
COPS5	hsa gene			+					
COQ3	hsa gene			+					
COQ5	hsa gene			+					
CORO1A	hsa gene		+	+	+				
CORO1B	only mmu ortholog		+						
COX4I1	only mmu ortholog		+		+				
COX6B1	only mmu ortholog		+						
COX7A2L	only mmu ortholog			+					
CPEB1	hsa gene	+		+					
CPEB2	hsa gene	+	+	+	+	+			
CPEB3	hsa gene	+	+	+	+	+			
CPEB4	hsa gene	+	+	+	+	+			
CPLX2	hsa gene		+						
CPNE1	hsa gene			+					
CPNE3	hsa gene		+	+	+				
CPSF1	hsa gene	+		+					
CPSF2	hsa gene	+		+					

CPSF3	hsa gene	+		+					
CPSF3L	hsa gene	+							
CPSF4	hsa gene	+	+	+	+	+		+	
CPSF4L	hsa gene	+		+				+	
CPSF6	hsa gene	+	+	+	+	+			
CPSF7	hsa gene	+	+	+	+	+			
CPT2	only mmu ortholog		+	+					
CRACR2A	hsa gene			+					
CRCP	hsa gene			+					
CREB1	only mmu ortholog		+						+
CREBBP	hsa gene			+					
CREBRF	only mmu ortholog		+						
CRIP2	only mmu ortholog		+	+					
CRIPT	hsa gene			+					
CRKL	hsa gene		+	+	+				
CRNKL1	hsa gene	+	+	+		+			
CRYAB	hsa gene		+						
CRYZ	hsa gene	+		+					
CS	hsa gene		+	+		+			
CSDC2	hsa gene	+		+					
CSDE1	hsa gene	+	+	+	+	+			
CSE1L	hsa gene		+	+					
CSKMT	hsa gene			+					
CSNK1A1	hsa gene			+					
CSNK1E	hsa gene		+	+		+			
CSNK2A1	only mmu ortholog		+						
CSNK2A2	only mmu ortholog		+						
CSNK2B	only mmu ortholog		+						
CSRP1	hsa gene		+	+	+				
CSRP2	only mmu ortholog		+						

CSTB	hsa gene		+	+	+				
CSTF1	hsa gene	+	+	+	+	+			
CSTF2	hsa gene	+	+	+	+	+			
CSTF2T	hsa gene	+	+	+	+	+			
CSTF3	hsa gene	+	+	+		+			
CT45A	only mmu ortholog			+					
CT45A1	hsa gene			+					
CT45A10	hsa gene			+					
CT45A2	hsa gene			+					
CT45A3	hsa gene			+					
CT45A5	hsa gene			+					
CT45A6	hsa gene			+					
CT45A7	hsa gene			+					
CT45A8	hsa gene			+					
CT45A9	hsa gene			+					
CT55	hsa gene			+					
CTCF	hsa gene			+			+	+	+
CTIF	hsa gene	+		+					
CTNNA1	hsa gene		+	+	+				
CTNNB1	hsa gene			+					
CTNNBL1	hsa gene			+					
CTNND1	hsa gene		+	+					
CTPS1	hsa gene			+					
CTR9	only mmu ortholog		+						
CTSD	only mmu ortholog		+						
CTTN	hsa gene		+	+					
CTU1	hsa gene	+		+					
CTU2	hsa gene	+		+					
CUL1	hsa gene			+					
CUL4B	only mmu ortholog		+						

CWC15	hsa gene	+	+	+	+				
CWC22	hsa gene	+		+					
CWC22RT1	only mmu ortholog			+					
CWC22RT2	only mmu ortholog			+					
CWC22RT3	only mmu ortholog			+					
CWC22RT4	only mmu ortholog			+					
CWC22RT5	only mmu ortholog			+					
CWC22RT6	only mmu ortholog			+					
CWC22RT7	only mmu ortholog			+					
CWC25	hsa gene	+		+					
CWC27	hsa gene	+		+					
CWF19L1	hsa gene	+		+					
CWF19L2	hsa gene	+		+					
CXADR	hsa gene			+					
CXORF23	hsa gene	+							
CXORF57	hsa gene		+						
CXXC5	hsa gene		+						+
CYB5B	only mmu ortholog		+						
CYB5R3	only mmu ortholog		+						
CYC1	only mmu ortholog		+						
CYCS	hsa gene		+		+				
CYCSP7	only mmu ortholog		+						
CYP51A1	hsa gene			+					
CYTH2	only mmu ortholog		+						
D1PAS1	only mmu ortholog			+					
DAD1	hsa gene			+					
DAG1	hsa gene			+					
DALRD3	hsa gene	+		+					
DAP3	hsa gene	+	+	+	+	+			
DAPK1	hsa gene			+					

DAPK2	hsa gene			+					
DAPK3	hsa gene			+					
DARS	hsa gene	+	+		+				
DARS1	hsa gene			+					
DARS2	hsa gene	+	+	+					
DAZ1	hsa gene	+		+					
DAZ2	hsa gene	+		+					
DAZ3	hsa gene	+		+					
DAZ4	hsa gene	+		+					
DAZAP1	hsa gene	+	+	+	+	+			
DAZL	hsa gene	+		+					
DBI	hsa gene		+	+					
DBNL	hsa gene		+						
DBR1	hsa gene	+	+	+	+				
DBT	hsa gene		+						
DCAF13	hsa gene	+	+	+	+	+			
DCAF7	only mmu ortholog		+						
DCAKD	only mmu ortholog		+						
DCD	hsa gene		+	+	+				
DCLRE1C	hsa gene		+						
DCN	hsa gene		+	+	+				
DCP1A	hsa gene	+		+					
DCP1B	hsa gene	+		+					
DCP2	hsa gene	+		+					
DCPS	hsa gene	+		+					
DCTN2	hsa gene		+						
DCTPP1	hsa gene			+					
DDB1	hsa gene			+					
DDOST	hsa gene			+					
DDR2	only mmu ortholog			+					

DDX1	hsa gene	+	+	+	+	+			
DDX10	hsa gene	+	+	+	+	+			
DDX11	hsa gene			+					
DDX17	hsa gene	+	+	+	+	+			
DDX18	hsa gene	+	+	+	+	+			
DDX19A	hsa gene	+	+	+					
DDX19B	hsa gene	+		+					
DDX20	hsa gene	+		+	+				
DDX21	hsa gene	+	+	+	+	+			
DDX23	hsa gene	+	+	+	+	+			
DDX24	hsa gene	+	+	+	+	+			
DDX25	hsa gene	+		+					
DDX26B	hsa gene	+							
DDX27	hsa gene	+	+	+	+	+			
DDX28	hsa gene	+	+	+	+	+			
DDX31	hsa gene	+	+	+	+	+			
DDX39	only mmu ortholog		+			+			
DDX39A	hsa gene	+	+	+	+				
DDX39B	hsa gene	+	+	+					
DDX3X	hsa gene	+	+	+	+	+			
DDX3Y	hsa gene	+		+					
DDX4	hsa gene	+		+					
DDX41	hsa gene	+	+	+	+	+			
DDX42	hsa gene	+	+	+	+	+			
DDX43	hsa gene	+	+	+					
DDX46	hsa gene	+	+	+		+			
DDX47	hsa gene	+	+	+	+	+			
DDX49	hsa gene	+	+	+	+				
DDX5	hsa gene	+	+	+	+	+			
DDX50	hsa gene	+	+	+	+	+			

DDX51	hsa gene	+	+	+	+	+			
DDX52	hsa gene	+	+	+	+	+			
DDX53	hsa gene	+		+					
DDX54	hsa gene	+	+	+	+	+			
DDX55	hsa gene	+	+	+	+				
DDX56	hsa gene	+	+	+	+				
DDX58	hsa gene	+							
DDX59	hsa gene	+		+					
DDX6	hsa gene	+	+	+	+	+			
DDX60	hsa gene	+		+					
DDX60L	hsa gene	+	+	+	+				
DECR1	only mmu ortholog		+		+				
DEDD2	hsa gene			+					
DEK	hsa gene	+	+	+	+	+			
DENR	hsa gene	+		+	+				
DERL2	only mmu ortholog		+						
DES	only mmu ortholog		+						
DFFB	only mmu ortholog		+						
DGCR14	hsa gene	+							
DGCR8	hsa gene	+		+	+				
DGKQ	hsa gene			+					
DHCR24	only mmu ortholog		+		+				
DHCR7	hsa gene		+	+					
DHFR	hsa gene			+					
DHFR2	hsa gene			+					
DHX15	hsa gene	+	+	+	+	+			
DHX16	hsa gene	+	+	+	+	+			
DHX29	hsa gene	+	+	+	+	+			
DHX30	hsa gene	+	+	+	+	+			
DHX32	hsa gene	+		+					

DHX33	hsa gene	+	+	+	+				
DHX34	hsa gene	+	+	+	+	+			
DHX35	hsa gene	+		+					
DHX36	hsa gene	+	+	+	+	+			
DHX37	hsa gene	+		+	+				
DHX38	hsa gene	+	+	+		+			
DHX40	hsa gene	+		+					
DHX57	hsa gene	+	+	+	+	+			
DHX58	hsa gene	+		+					
DHX8	hsa gene	+	+	+	+	+			
DHX9	hsa gene	+	+	+	+	+			
DIAPH1	hsa gene		+	+	+				
DIAPH3	hsa gene			+					
DICER1	hsa gene	+		+					
DIDO1	hsa gene		+	+	+	+			
DIEXF	hsa gene		+		+				
DIMT1	hsa gene	+	+	+					
DIS3	hsa gene	+		+	+				
DIS3L	hsa gene	+		+					
DIS3L2	hsa gene	+		+	+				
DKC1	hsa gene	+	+	+	+				
DLAT	hsa gene		+						
DLD	hsa gene		+	+					
DLG1	only mmu ortholog		+	+					
DLST	hsa gene			+					
DLX2	hsa gene			+					
DMGDH	hsa gene		+	+	+				
DNA2	hsa gene			+					
DNAAF2	hsa gene	+		+					
DNAAF6	hsa gene			+					

DNAJA1	hsa gene		+						
DNAJB1	only mmu ortholog		+						
DNAJB12	hsa gene		+						
DNAJC1	hsa gene		+						
DNAJC13	hsa gene			+					
DNAJC17	hsa gene	+		+	+				
DNAJC2	hsa gene		+	+	+				
DNAJC21	hsa gene	+	+	+	+				
DNAJC27	hsa gene			+					
DNAJC8	only mmu ortholog		+		+				
DNAJC9	only mmu ortholog		+		+				
DNASE1	hsa gene			+					
DNASE1L1	hsa gene			+					
DNASE1L2	hsa gene			+					
DNASE1L3	hsa gene			+					
DND1	hsa gene	+		+					
DNM1	hsa gene		+	+		+			
DNM1L	hsa gene			+	+				
DNM2	only mmu ortholog		+						
DNMT1	hsa gene	+		+					+
DNMT3A	only mmu ortholog		+						
DNMT3B	hsa gene	+		+					
DNMT3L	only mmu ortholog		+						
DNTTIP2	hsa gene	+	+	+	+	+			
DOCK7	hsa gene			+					
DPF2	only mmu ortholog		+						
DPPA5	hsa gene			+					
DPPA5A	only mmu ortholog			+					
DPYD	hsa gene			+					
DPYSL2	only mmu ortholog		+						

DQX1	hsa gene	+		+					
DRG1	hsa gene	+		+	+				
DRG2	hsa gene	+		+					
DROSHA	hsa gene	+	+	+	+	+			
DSG1	hsa gene			+	+				
DSP	hsa gene		+	+	+				
DST	hsa gene		+		+				
DTD1	hsa gene			+	+				
DTD2	hsa gene			+					
DTWD1	only mmu ortholog			+					
DTWD2	only mmu ortholog			+					
DUS1L	hsa gene	+		+					
DUS2	hsa gene	+		+					
DUS3L	hsa gene	+	+	+	+	+			
DUS4L	hsa gene	+		+					
DUSP11	hsa gene	+	+	+	+	+			
DUSP12	hsa gene			+					
DUSP14	hsa gene		+	+		+			
DUSP9	hsa gene		+						
DUT	hsa gene		+	+	+				
DXO	hsa gene	+		+					
DYNC1H1	hsa gene	+	+	+	+	+			
DYNC1I2	hsa gene		+		+				
DYNC1LI1	hsa gene		+	+	+				
DYNC1LI2	only mmu ortholog		+						
DYNC2H1	hsa gene		+						
DYNLL1	hsa gene	+		+					
DZIP1	hsa gene	+		+					
DZIP1L	hsa gene	+		+					
DZIP3	hsa gene	+	+	+	+				

E4F1	hsa gene			+					+
EAF1	hsa gene			+					
EAF2	hsa gene			+					
EAR1	only mmu ortholog			+					
EAR10	only mmu ortholog			+					
EAR14	only mmu ortholog			+					
EAR2	only mmu ortholog			+					
EAR6	only mmu ortholog			+					
EARS2	hsa gene	+		+					
EBNA1BP2	hsa gene	+	+	+	+	+			
EBP	hsa gene		+						
ECD	hsa gene			+					
ECHS1	only mmu ortholog		+						
ECI1	only mmu ortholog		+						
ECI2	hsa gene			+					
ECPAS	hsa gene			+					
EDC3	hsa gene	+		+					
EDC4	hsa gene	+	+	+	+				
EDDM3A	hsa gene			+					
EDDM3B	hsa gene			+					
EDF1	hsa gene		+	+	+	+			
EEA1	only mmu ortholog			+					+
EED	hsa gene	+		+	+				
EEF1A1	hsa gene	+	+	+	+	+			
EEF1A2	hsa gene	+		+					
EEF1AKMT2	hsa gene			+					
EEF1AKMT4	hsa gene			+					
EEF1B2	hsa gene	+		+					
EEF1D	hsa gene	+		+	+				
EEF1E1	hsa gene	+		+	+				

EEF1G	hsa gene	+	+	+					
EEF2	hsa gene	+	+	+	+	+			
EEF2K	hsa gene	+		+					
EEFSEC	hsa gene	+		+					
EFHD2	only mmu ortholog		+						
EFL1	hsa gene			+					
EFTUD1	hsa gene	+							
EFTUD2	hsa gene	+	+	+	+	+			
EGLN2	hsa gene			+					
EGLN3	hsa gene			+					
EHD1	hsa gene			+					
EHD2	hsa gene			+					
EHD3	hsa gene			+					
EHD4	hsa gene			+					
EHMT2	only mmu ortholog		+						
EIF1	hsa gene	+	+	+		+			
EIF1A	only mmu ortholog		+	+					
EIF1AD	hsa gene	+		+					
EIF1AD10	only mmu ortholog			+					
EIF1AD11	only mmu ortholog			+					
EIF1AD16	only mmu ortholog			+					
EIF1AD17	only mmu ortholog			+					
EIF1AD19	only mmu ortholog			+					
EIF1AD2	only mmu ortholog			+					
EIF1AD3	only mmu ortholog			+					
EIF1AD4	only mmu ortholog			+					
EIF1AD6	only mmu ortholog			+					
EIF1AD7	only mmu ortholog			+					
EIF1AD8	only mmu ortholog			+					
EIF1AX	hsa gene	+	+	+	+	+			

EIF1AY	hsa gene	+		+					
EIF1B	hsa gene	+	+	+		+			
EIF2A	hsa gene	+		+	+				
EIF2AK1	hsa gene	+		+					
EIF2AK2	hsa gene	+	+	+	+	+			
EIF2AK3	hsa gene	+							
EIF2AK4	hsa gene	+		+					
EIF2B1	hsa gene	+		+					
EIF2B2	hsa gene	+		+					
EIF2B3	hsa gene	+		+					
EIF2B4	hsa gene	+		+					
EIF2B5	hsa gene	+		+					
EIF2D	hsa gene	+	+	+	+				
EIF2S1	hsa gene	+	+	+	+	+			
EIF2S2	hsa gene	+	+	+	+	+			
EIF2S3	hsa gene	+		+					
EIF2S3B	hsa gene			+					
EIF2S3L	hsa gene	+							
EIF2S3X	only mmu ortholog		+	+					
EIF2S3Y	only mmu ortholog			+					
EIF3A	hsa gene	+	+	+	+	+			
EIF3B	hsa gene	+	+	+	+				
EIF3C	hsa gene	+	+	+	+	+			
EIF3CL	hsa gene	+		+					
EIF3D	hsa gene	+	+	+	+	+			
EIF3E	hsa gene	+	+	+	+	+			
EIF3F	hsa gene			+					
EIF3G	hsa gene	+	+	+	+	+			
EIF3H	hsa gene	+	+	+	+				
EIF3I	hsa gene	+	+	+	+				

EIF3J	hsa gene	+	+	+	+				
EIF3J1	only mmu ortholog		+	+					
EIF3J2	only mmu ortholog			+					
EIF3K	hsa gene	+		+					
EIF3L	hsa gene	+	+	+	+	+			
EIF3M	hsa gene	+		+					
EIF4A1	hsa gene	+	+	+	+	+			
EIF4A2	hsa gene	+	+	+	+	+			
EIF4A3	hsa gene	+	+	+	+	+			
EIF4A3L1	only mmu ortholog			+					
EIF4B	hsa gene	+	+	+	+	+			
EIF4E	hsa gene	+	+	+		+			
EIF4E1B	hsa gene	+		+					
EIF4E2	hsa gene	+	+	+		+			
EIF4E3	hsa gene	+		+					
EIF4EBP1	hsa gene			+					
EIF4EBP2	hsa gene			+					
EIF4EBP3	hsa gene			+					
EIF4ENIF1	hsa gene	+	+	+	+	+			
EIF4G1	hsa gene	+	+	+	+	+			
EIF4G2	hsa gene	+	+	+	+	+			
EIF4G3	hsa gene	+	+	+	+	+			
EIF4H	hsa gene	+	+	+	+	+			
EIF5	hsa gene	+	+	+	+	+			
EIF5A	hsa gene	+	+	+	+	+			
EIF5A2	hsa gene	+		+					
EIF5AL1	hsa gene	+		+					
EIF5B	hsa gene	+	+	+	+	+			
EIF6	hsa gene	+		+					
ELAC1	hsa gene	+		+					

ELAC2	hsa gene	+	+	+	+	+			
ELAPOR1	hsa gene			+					
ELAVL1	hsa gene	+	+	+	+	+			
ELAVL2	hsa gene	+	+	+	+	+			
ELAVL3	hsa gene	+	+	+					
ELAVL4	hsa gene	+		+					
ELMSAN1	only mmu ortholog		+						
ELOA	hsa gene			+					
ELOF1	hsa gene		+		+				
ELP1	hsa gene			+					
ELP2	only mmu ortholog		+	+					
ELP3	hsa gene			+					
ELP4	only mmu ortholog			+					
ELP5	hsa gene			+					
ELP6	only mmu ortholog			+					
EMC1	hsa gene			+					
EMC10	hsa gene			+					
EMD	only mmu ortholog		+						
EMG1	hsa gene	+	+	+	+	+			
EML4	hsa gene		+						
EMSY	only mmu ortholog		+						
ENDOD1	hsa gene			+					
ENDOG	hsa gene	+		+					
ENDOU	hsa gene	+		+					
ENDOV	hsa gene	+		+					
ENO1	hsa gene		+	+	+				
ENO1B	only mmu ortholog		+	+					
ENOX1	hsa gene	+		+					
ENOX2	hsa gene	+		+					
ENPP1	hsa gene			+					

ENPP2	hsa gene			+					
ENPP3	hsa gene			+					
ENSA	hsa gene		+						
ENY2	only mmu ortholog		+						
EOLA1	only mmu ortholog			+					
EP300	hsa gene			+	+				
EP400	only mmu ortholog		+						
EPB41	hsa gene		+		+				
EPB41L2	hsa gene		+						
EPHB3	only mmu ortholog		+						
EPPK1	hsa gene		+	+		+			
EPRS	hsa gene	+	+						
EPRS1	hsa gene			+					
EPS15L1	only mmu ortholog		+		+				
EPS8L3	hsa gene		+						
ERAL1	hsa gene	+	+	+	+	+			
ERCC2	hsa gene			+					
ERCC3	hsa gene			+					
ERCC6L	hsa gene			+					
ERCC6L2	hsa gene			+					
ERGIC1	only mmu ortholog		+						
ERH	hsa gene		+	+	+	+			
ERI1	hsa gene	+		+	+				
ERI2	hsa gene	+		+				+	
ERI3	hsa gene	+	+	+	+	+			
ERMAP	hsa gene			+					
ERMP1	hsa gene			+	+				
ERN1	hsa gene	+		+					
ERN2	hsa gene	+		+					
ERP29	hsa gene		+	+	+				

ERP44	hsa gene			+					
ESCO2	only mmu ortholog		+						
ESF1	hsa gene	+	+	+	+	+			
ESRP1	hsa gene	+		+					
ESRP2	hsa gene	+	+	+	+	+			
ESS2	hsa gene			+					
ESYT1	only mmu ortholog		+						
ETF1	hsa gene	+	+	+	+				
ETFA	only mmu ortholog		+	+					
ETFB	only mmu ortholog		+	+	+				
ETFBKMT	hsa gene			+					
ETHE1	hsa gene			+					
ETL4	only mmu ortholog		+						
EWSR1	hsa gene	+	+	+	+	+			
EXD1	hsa gene			+					
EXD2	hsa gene			+					
EXD3	hsa gene			+					
EXO1	hsa gene	+		+					
EXOG	hsa gene	+		+					
EXOSC1	hsa gene	+		+	+				
EXOSC10	hsa gene	+	+	+	+	+			
EXOSC2	hsa gene	+	+	+	+				
EXOSC3	hsa gene	+		+					
EXOSC4	hsa gene	+		+	+				
EXOSC5	hsa gene	+		+					
EXOSC6	hsa gene	+	+	+	+	+			
EXOSC7	hsa gene	+		+					
EXOSC8	hsa gene	+		+					
EXOSC9	hsa gene	+	+	+	+				
EYA1	hsa gene			+					

EZH2	hsa gene	+		+					
EZR	hsa gene		+	+	+	+			
F11R	hsa gene		+						
FABP5	hsa gene			+					
FADS2	hsa gene		+						
FAM103A1	hsa gene	+	+		+	+			
FAM120A	hsa gene	+	+	+	+	+			
FAM120B	hsa gene	+		+					
FAM120C	hsa gene	+	+	+	+	+			
FAM133B	hsa gene		+	+		+			
FAM168A	hsa gene			+					
FAM172A	hsa gene			+					
FAM179B	only mmu ortholog		+						
FAM193A	only mmu ortholog		+						
FAM195A	only mmu ortholog		+		+				
FAM206A	only mmu ortholog		+						
FAM207A	only mmu ortholog		+						
FAM208A	hsa gene		+						
FAM32A	hsa gene		+	+	+				
FAM46A	hsa gene	+	+		+				
FAM50A	hsa gene		+	+	+	+			
FAM60A	only mmu ortholog		+						
FAM83A	hsa gene			+					
FAM83B	hsa gene			+					
FAM83C	hsa gene			+					
FAM83D	hsa gene			+					
FAM83E	hsa gene			+					
FAM83F	hsa gene			+					
FAM83G	hsa gene			+					
FAM83H	hsa gene			+					

FAM98A	hsa gene	+	+	+	+	+			
FAM98B	hsa gene	+	+	+		+			
FAM98C	hsa gene	+		+					
FANCM	hsa gene			+					
FARS2	hsa gene	+		+					
FARSA	hsa gene	+	+	+	+	+			
FARSB	hsa gene	+	+	+	+				
FASN	hsa gene		+	+	+	+			
FASTK	hsa gene	+		+	+				
FASTKD1	hsa gene	+	+	+	+	+			
FASTKD2	hsa gene	+	+	+	+	+			
FASTKD3	hsa gene	+	+	+	+				
FASTKD5	hsa gene	+	+	+	+	+			
FAU	hsa gene	+	+	+		+			
FBH1	hsa gene			+					
FBL	hsa gene	+	+	+	+	+			
FBLL1	hsa gene	+		+		+			
FBN1	hsa gene		+						
FBRSL1	hsa gene		+	+		+			
FBXO11	only mmu ortholog		+						
FBXO17	hsa gene	+		+	+				
FBXO45	hsa gene			+					
FBXW11	only mmu ortholog		+						
FCF1	hsa gene	+	+	+	+				
FDPS	hsa gene		+	+	+				
FDXACB1	hsa gene	+		+					
FECH	only mmu ortholog		+						
FEN1	hsa gene		+	+					
FGFR1OP	hsa gene		+						
FGFR3	only mmu ortholog			+					

FHIT	hsa gene			+					
FHL1	only mmu ortholog		+						
FHOD1	hsa gene			+					
FIP1L1	hsa gene	+	+	+	+	+			
FIZ1	only mmu ortholog			+					
FKBP15	only mmu ortholog		+						
FKBP1A	hsa gene		+						
FKBP2	hsa gene		+						
FKBP3	hsa gene		+	+	+				
FKBP4	hsa gene		+	+	+				
FKBP6	hsa gene			+					
FKBP9	hsa gene			+					
FLNA	hsa gene		+	+	+	+			
FLNB	hsa gene		+	+	+	+			
FLNC	only mmu ortholog		+	+					
FLOT2	only mmu ortholog		+						
FLYWCH2	hsa gene		+	+	+				
FMR1	hsa gene	+	+	+	+	+			
FN1	hsa gene		+						
FNDC3A	hsa gene		+	+	+				
FNDC3B	hsa gene		+	+	+				
FOXK1	only mmu ortholog		+						+
FOXP4	only mmu ortholog		+						+
FRA10AC1	hsa gene			+					
FRG1	hsa gene	+	+	+	+				
FRG1B	hsa gene	+							
FSCN1	hsa gene		+	+	+				
FSD1	hsa gene			+					
FSD1L	hsa gene			+					
FSD2	hsa gene			+					

FTO	hsa gene	+		+					
FTSJ1	hsa gene	+		+					
FTSJ2	hsa gene	+			+				
FTSJ3	hsa gene	+	+	+	+	+			
FUBP1	hsa gene	+	+	+	+	+			
FUBP3	hsa gene	+	+	+	+	+			
FUS	hsa gene	+	+	+	+	+		+	
FXR1	hsa gene	+	+	+	+	+			
FXR2	hsa gene	+	+	+	+	+			
FYTTD1	hsa gene	+	+	+	+	+			
G3BP1	hsa gene	+	+	+	+	+			
G3BP2	hsa gene	+	+	+	+	+			
G6PD	hsa gene			+					
GADD45A	hsa gene			+					
GADD45B	hsa gene			+					
GADD45G	hsa gene			+					
GALNT1	only mmu ortholog		+						
GALNT2	hsa gene			+					
GANAB	hsa gene		+	+	+				
GAPDH	hsa gene	+		+	+				
GAR1	hsa gene	+	+	+	+	+			
GARS	hsa gene	+			+				
GARS1	hsa gene			+					
GART	hsa gene			+					
GAS2	hsa gene		+						
GATAD2A	only mmu ortholog		+						+
GATAD2B	only mmu ortholog		+						+
GATB	hsa gene			+					
GATC	hsa gene	+		+					
GATM	only mmu ortholog		+						

GBF1	hsa gene			+					
GCFC2	hsa gene	+		+					
GCLM	only mmu ortholog		+		+				
GCN1	hsa gene		+	+					
GCN1L1	only mmu ortholog		+			+			
GDA	hsa gene			+					
GDI2	hsa gene		+	+	+	+			
GEMIN2	hsa gene	+		+					
GEMIN4	hsa gene	+		+					
GEMIN5	hsa gene	+	+	+	+				
GEMIN6	hsa gene	+		+					
GEMIN7	hsa gene	+		+					
GEMIN8	hsa gene	+		+					
GFI1	hsa gene			+					
GFI1B	hsa gene			+					+
GFM1	hsa gene	+	+	+	+				
GFM2	hsa gene	+		+					
GGCT	hsa gene		+						
GIGYF1	only mmu ortholog		+						
GIGYF2	hsa gene		+	+	+	+			
GIMAP1	hsa gene			+					
GIMAP2	hsa gene			+					
GIMAP3	only mmu ortholog			+					
GIMAP4	hsa gene			+					
GIMAP5	hsa gene			+					
GIMAP6	hsa gene			+					
GIMAP7	hsa gene			+					
GIMAP8	hsa gene			+					
GIMAP9	only mmu ortholog			+					
GIMD1	only mmu ortholog			+					

GLA	hsa gene			+					
GLE1	hsa gene	+		+					
GLG1	only mmu ortholog		+		+				
GLMN	hsa gene			+					
GLRX3	hsa gene		+	+	+				
GLTSCR2	hsa gene	+	+		+	+			
GLUD1	only mmu ortholog		+	+					
GLYR1	only mmu ortholog		+						+
GMPR2	hsa gene		+		+				
GMPS	hsa gene			+					
GNAI2	only mmu ortholog		+						
GNAI3	hsa gene			+					
GNB2	hsa gene			+					
GNE	hsa gene		+						
GNL1	hsa gene	+		+					
GNL2	hsa gene	+	+	+	+	+			
GNL3	hsa gene	+	+	+	+	+			
GNL3L	hsa gene	+	+	+	+				
GNMT	hsa gene			+					
GOLGA4	hsa gene		+						
GOLGB1	hsa gene		+	+	+				
GOLPH3L	only mmu ortholog		+						
GOT2	hsa gene		+	+		+			
GP2	hsa gene		+						
GPANK1	hsa gene			+					
GPAT2	hsa gene			+					
GPATCH1	hsa gene	+		+					
GPATCH11	hsa gene			+					
GPATCH2	hsa gene			+					
GPATCH3	hsa gene			+					

GPATCH4	hsa gene	+	+	+	+				
GPATCH8	hsa gene	+	+	+		+			
GPBP1	hsa gene			+					
GPBP1L1	hsa gene			+					
GPC4	hsa gene			+					
GPHN	only mmu ortholog		+						
GPI1	only mmu ortholog		+						
GPKOW	hsa gene	+		+					
GPN1	hsa gene			+					
GPR161	hsa gene		+						
GPS1	only mmu ortholog		+		+				
GPX4	only mmu ortholog		+						
GRB2	hsa gene		+	+	+				
GRB7	hsa gene			+					
GRN	hsa gene		+	+	+				
GRSF1	hsa gene	+	+	+	+	+			
GRWD1	hsa gene	+	+	+	+	+			
GSPT1	hsa gene	+	+	+	+	+			
GSPT2	hsa gene	+	+	+	+	+			
GSTCD	hsa gene			+					
GSTK1	hsa gene			+					
GSTP1	hsa gene		+						
GTF2B	hsa gene			+					+
GTF2E1	hsa gene			+					
GTF2E2	hsa gene		+	+	+				
GTF2F1	hsa gene	+	+	+	+	+			
GTF2H4	only mmu ortholog		+						
GTF2I	only mmu ortholog		+		+				+
GTF2IRD1	only mmu ortholog		+						+
GTF3A	hsa gene	+		+					

GTF3C3	hsa gene			+					
GTF3C4	only mmu ortholog		+					+	
GTF3C5	hsa gene			+					
GTPBP1	hsa gene	+	+	+	+				
GTPBP10	hsa gene	+	+	+	+				
GTPBP2	hsa gene	+		+					
GTPBP3	hsa gene	+		+					
GTPBP4	hsa gene	+	+	+	+	+			
GTPBP6	hsa gene			+					
GTPBP8	hsa gene			+					
GTSF1	hsa gene			+					
GTSF1L	hsa gene			+					
GTSF2	only mmu ortholog			+					
GUF1	hsa gene	+		+					
GUSB	hsa gene			+					
GYS1	only mmu ortholog		+						
GZF1	hsa gene			+					
H1-0	hsa gene			+					
H1-10	hsa gene			+					
H1-2	hsa gene			+					
H1-3	hsa gene			+					
H1-4	hsa gene			+					
H1-5	hsa gene			+					
H1F0	hsa gene		+		+	+			
H1F2	only mmu ortholog			+					
H1F3	only mmu ortholog			+					
H1F4	only mmu ortholog			+					
H1F5	only mmu ortholog			+					
H1FX	hsa gene		+		+	+			
H2-KE6	only mmu ortholog		+						

H2AB1	hsa gene			+					
H2AB2	hsa gene			+					
H2AB3	hsa gene			+					
H2AFX	only mmu ortholog		+						
H2AFY	hsa gene		+						
H2AFY2	only mmu ortholog		+						
H2BC26	hsa gene			+					
H3C1	hsa gene			+					
H3F3B	only mmu ortholog		+						
H4C1	hsa gene			+					
H4C11	hsa gene			+					
H4C12	hsa gene			+					
H4C13	hsa gene			+					
H4C14	hsa gene			+					
H4C15	hsa gene			+					
H4C16	hsa gene			+					
H4C2	hsa gene			+					
H4C3	hsa gene			+					
H4C4	hsa gene			+					
H4C5	hsa gene			+					
H4C6	hsa gene			+					
H4C8	hsa gene			+					
H4C9	hsa gene			+					
HABP4	hsa gene	+	+	+					
HACD3	hsa gene			+					
HADH	hsa gene		+		+				
HADHA	only mmu ortholog		+	+					
HADHB	hsa gene		+	+	+				
HAGH	hsa gene			+					
HAGHL	hsa gene			+					

HAPLN1	only mmu ortholog		+						
HARS	hsa gene	+							
HARS1	hsa gene			+					
HARS2	hsa gene	+	+	+	+	+			
HAT1	hsa gene			+					
HAUS4	hsa gene			+					
HAUS6	only mmu ortholog		+						
HAUS8	hsa gene			+					
HBP1	hsa gene			+					
HBS1L	hsa gene	+		+					
HCFC1	only mmu ortholog		+	+					
HCLS1	only mmu ortholog		+						
HDAC1	only mmu ortholog		+						
HDAC2	hsa gene		+	+		+			
HDGF	hsa gene		+	+	+				
HDLBP	hsa gene	+	+	+	+	+			
HEATR1	hsa gene	+	+	+	+	+			
HEATR6	hsa gene		+	+		+			
HECA	only mmu ortholog		+						
HECTD1	hsa gene			+					
HECW1	hsa gene			+					
HELLS	hsa gene			+					
HELQ	hsa gene			+					
HELZ	hsa gene	+	+	+	+	+			
HELZ2	hsa gene	+	+	+					
HEMK1	hsa gene			+					
HENMT1	hsa gene	+		+					
HERC1	hsa gene			+					
HERC2	hsa gene			+					
HERC5	hsa gene		+	+	+				

HEXIM1	hsa gene	+		+	+				
HEXIM2	hsa gene	+		+					
HFM1	hsa gene			+					
HIBADH	hsa gene		+						
HIC1	hsa gene			+					
HIC2	hsa gene			+					+
HIF1A	only mmu ortholog		+						+
HINT1	hsa gene			+					
HINT2	hsa gene			+					
HINT3	hsa gene	+		+					
HIP1	only mmu ortholog		+		+				
HIST1H1B	hsa gene		+		+				
HIST1H1C	hsa gene		+		+	+			
HIST1H1D	hsa gene		+						
HIST1H1E	hsa gene		+						
HIST1H2BC	only mmu ortholog		+						
HIST1H2BK	only mmu ortholog		+						
HIST1H2BP	only mmu ortholog		+						
HIST1H4A	hsa gene		+						
HIST1H4B	hsa gene		+						
HIST1H4D	hsa gene		+						
HIST1H4E	hsa gene		+						
HIST1H4F	hsa gene		+						
HIST1H4H	hsa gene		+		+				
HIST1H4K	hsa gene		+						
HIST1H4L	hsa gene		+						
HIST2H2AA2	only mmu ortholog		+						
HIST2H4B	hsa gene		+			+			
HIST3H2A	only mmu ortholog		+						
HIVEP2	hsa gene			+					

HK2	only mmu ortholog		+						
HLA-A	hsa gene		+	+					
HLA-C	hsa gene			+					
HLTF	hsa gene		+	+	+				
HMCES	only mmu ortholog		+						
HMGA1	hsa gene		+	+					+
HMGA1B	only mmu ortholog			+					
HMGB1	hsa gene		+	+	+	+			
HMGB2	hsa gene		+	+	+	+			
HMGB3	hsa gene		+	+		+			
HMGN1	only mmu ortholog		+		+				
HMGN2	hsa gene		+	+		+			
HMGN5	hsa gene		+	+		+			
HMGXB4	only mmu ortholog		+						
HMMR	only mmu ortholog		+						
HN1	only mmu ortholog		+						
HN1L	only mmu ortholog		+		+				
HNF1A	only mmu ortholog			+					
HNRNPA0	hsa gene	+	+	+	+	+			
HNRNPA1	hsa gene	+	+	+	+	+			
HNRNPA1L2	hsa gene	+	+	+					
HNRNPA1L3	hsa gene			+					
HNRNPA2B1	hsa gene	+	+	+	+	+			
HNRNPA3	hsa gene	+	+	+	+	+			
HNRNPAB	hsa gene	+	+	+	+	+			
HNRNPC	hsa gene	+	+	+	+	+			
HNRNPCL1	hsa gene	+	+	+		+			
HNRNPCL2	hsa gene			+					
HNRNPCL3	hsa gene			+					
HNRNPCL4	hsa gene			+					

HNRNPD	hsa gene	+	+	+	+	+			
HNRNPDL	hsa gene	+	+	+					
HNRNPF	hsa gene	+	+	+	+	+			
HNRNPH1	hsa gene	+	+	+	+	+			
HNRNPH2	hsa gene	+	+	+	+	+			
HNRNPH3	hsa gene	+	+	+	+	+			
HNRNPK	hsa gene	+	+	+	+	+			
HNRNPL	hsa gene	+	+	+	+	+			
HNRNPLL	hsa gene	+	+	+					
HNRNPM	hsa gene	+	+	+	+	+			
HNRNPR	hsa gene	+	+	+	+	+			
HNRNPU	hsa gene	+	+	+	+	+			
HNRNPUL1	hsa gene	+	+	+	+	+			
HNRNPUL2	hsa gene	+	+	+	+	+			
HOOK1	hsa gene		+						
HOXB6	hsa gene		+	+		+			+
HP1BP3	hsa gene			+	+				
HRAS	hsa gene			+					
HRSP12	hsa gene	+			+				
HSD17B10	hsa gene		+	+		+			
HSD17B4	only mmu ortholog		+						
HSDL2	only mmu ortholog		+		+				
HSF1	hsa gene			+					
HSP90AA1	hsa gene		+	+	+	+			
HSP90AB1	hsa gene		+	+	+	+			
HSP90B1	hsa gene		+	+					
HSPA1A	hsa gene		+	+		+			
HSPA1B	hsa gene		+	+	+	+			
HSPA4	hsa gene			+	+				
HSPA5	hsa gene		+	+					

HSPA6	hsa gene			+					
HSPA8	hsa gene		+	+	+	+			
HSPA9	hsa gene		+	+	+	+			
HSPB1	hsa gene		+	+	+				
HSPBP1	only mmu ortholog		+						
HSPD1	hsa gene		+	+	+				
HSPE1	hsa gene		+	+	+				
HSPG2	only mmu ortholog		+	+					
HSPH1	only mmu ortholog		+		+				
HTATSF1	hsa gene	+	+	+	+				
HTT	hsa gene			+					
HUNK	hsa gene			+					
HUWE1	hsa gene		+	+	+				
HYOU1	hsa gene			+					
IARS	hsa gene	+							
IARS1	hsa gene			+					
IARS2	hsa gene	+		+					
IBA57	hsa gene		+	+		+			
IBTK	only mmu ortholog		+		+				
ICAM1	only mmu ortholog		+						
ICE1	only mmu ortholog		+						
ICE2	only mmu ortholog		+						
ICT1	hsa gene	+							
IDH1	hsa gene		+		+				
IDH2	hsa gene		+						
IDH3A	only mmu ortholog		+						
IDO1	only mmu ortholog		+						
IFI16	hsa gene		+	+	+				
IFI44L	hsa gene			+					
IFI47	only mmu ortholog			+					

IFIH1	hsa gene	+		+					
IFIT1	hsa gene	+		+					
IFIT1B	hsa gene	+		+					
IFIT1BL1	only mmu ortholog			+					
IFIT1BL2	only mmu ortholog			+					
IFIT2	hsa gene	+	+	+	+				
IFIT3	hsa gene	+		+					
IFIT5	hsa gene	+	+	+		+			
IFT88	hsa gene			+					
IGF2BP1	hsa gene	+	+	+	+	+			
IGF2BP2	hsa gene	+	+	+	+	+			
IGF2BP3	hsa gene	+	+	+	+	+			
IGF2R	hsa gene			+					
IGHMBP2	hsa gene	+		+				+	
IGTP	only mmu ortholog			+					
IIGP1	only mmu ortholog			+					
IIGP1C	only mmu ortholog			+					
IK	only mmu ortholog		+	+					
IKBKAP	only mmu ortholog		+						
IL6	hsa gene			+					
ILF2	hsa gene	+	+	+	+	+			
ILF3	hsa gene	+	+	+	+	+			
ILK	hsa gene			+					
IMMT	hsa gene		+	+	+				
IMP3	hsa gene	+	+	+	+	+			
IMP4	hsa gene	+		+	+				
IMPDH1	hsa gene			+					
IMPDH2	hsa gene			+					
INO80	hsa gene			+					
INO80B	only mmu ortholog		+						

INO80D	only mmu ortholog		+						
INPP5A	hsa gene			+					
INPP5B	hsa gene			+					
INPP5D	hsa gene			+					
INPP5E	hsa gene			+					
INPP5J	hsa gene			+					
INPP5K	hsa gene			+					
INPPL1	hsa gene			+					
INSM2	hsa gene			+					
INTS1	hsa gene	+		+					
INTS10	hsa gene	+		+					
INTS11	hsa gene			+					
INTS12	hsa gene	+		+					
INTS13	hsa gene			+					
INTS14	hsa gene			+					
INTS2	hsa gene	+		+					
INTS3	hsa gene	+		+					
INTS4	hsa gene	+		+					
INTS5	hsa gene	+		+					
INTS6	hsa gene	+		+					
INTS6L	hsa gene			+					
INTS7	hsa gene	+		+					
INTS8	hsa gene	+		+					
INTS9	hsa gene	+		+					
IPO11	hsa gene	+		+					
IPO13	hsa gene	+		+					
IPO4	hsa gene	+	+	+					
IPO5	hsa gene	+	+	+	+	+			
IPO7	hsa gene	+		+	+				
IPO8	hsa gene	+		+					

IPO9	hsa gene	+		+					
IQCG	hsa gene		+						
IQGAP1	only mmu ortholog		+	+	+				
IREB2	hsa gene	+	+	+	+				
IRF3	hsa gene			+					
IRF4	hsa gene			+					
IRF5	hsa gene			+					
IRF6	hsa gene			+					
IRF7	hsa gene			+					
IRF8	hsa gene			+					
IRF9	hsa gene			+					
IRGC	hsa gene			+					
IRGC1	only mmu ortholog			+					
IRGM1	only mmu ortholog			+					
IRGM2	only mmu ortholog			+					
ISG15	only mmu ortholog		+						
ISG20	hsa gene	+		+	+				
ISG20L2	hsa gene	+	+	+	+				
ISY1	hsa gene	+	+	+	+				
ITGB1	only mmu ortholog		+		+				
ITGB2	only mmu ortholog		+						
ITPR3	hsa gene			+					
IWS1	hsa gene			+					
JADE1	only mmu ortholog		+						
JAKMIP1	hsa gene	+		+					
JMJD6	hsa gene			+					
JRK	hsa gene			+					
JUN	hsa gene		+	+		+			+
JUNB	only mmu ortholog		+						+
JUP	hsa gene			+					

KANK1	hsa gene			+					
KANSL1	only mmu ortholog		+						
KANSL3	only mmu ortholog		+						
KARS	hsa gene	+			+				
KARS1	hsa gene			+					
KAT2B	hsa gene			+					
KAT5	hsa gene			+					
KAT7	only mmu ortholog		+						+
KAT8	hsa gene	+		+					
KBTBD3	hsa gene			+	+				
KCTD12	hsa gene		+	+		+			
KDM1A	hsa gene			+					
KDM2B	hsa gene			+					
KDM5A	hsa gene		+						
KEAP1	only mmu ortholog		+						
KHDC1	hsa gene	+		+					
KHDC1A	only mmu ortholog			+					
KHDC1B	only mmu ortholog			+					
KHDC1C	only mmu ortholog			+					
KHDC1L	hsa gene	+		+					
KHDC3	only mmu ortholog			+					
KHDC3L	hsa gene			+					
KHDC4	hsa gene			+					
KHDRBS1	hsa gene	+	+	+	+	+			
KHDRBS2	hsa gene	+		+					
KHDRBS3	hsa gene	+	+	+		+			
KHNYN	hsa gene	+		+					
KHSRP	hsa gene	+	+	+	+	+			
KIAA0020	hsa gene	+			+	+			
KIAA0100	hsa gene		+						

KIAA0232	only mmu ortholog		+						
KIAA0391	hsa gene	+			+				
KIAA0430	hsa gene	+	+		+				
KIAA1324	hsa gene		+		+				
KIAA1429	hsa gene		+		+	+			
KIF11	hsa gene			+	+				
KIF14	only mmu ortholog		+						
KIF1B	only mmu ortholog		+		+				
KIF1C	hsa gene		+	+	+	+			
KIF20B	only mmu ortholog		+						
KIF2A	hsa gene			+	+				
KIF5C	only mmu ortholog		+						
KIN	hsa gene	+		+					+
KLC4	hsa gene			+					
KLF2	only mmu ortholog		+						+
KLF5	only mmu ortholog		+						+
KLF8	only mmu ortholog		+				+		+
KMT2C	hsa gene		+	+					
KNOP1	hsa gene		+	+					
KNSTRN	only mmu ortholog		+						
KPNA2	hsa gene		+	+	+	+			
KPNA3	hsa gene			+	+				
KPNA4	hsa gene			+					
KPNB1	hsa gene	+	+	+		+			
KRAS	hsa gene			+					
KRI1	hsa gene		+	+	+				
KRR1	hsa gene	+	+	+	+	+			
KRT18	hsa gene		+	+	+				
KRT5	hsa gene			+					
KRT76	only mmu ortholog		+						

KRT8	only mmu ortholog		+						
KTN1	hsa gene		+	+	+				
KXD1	only mmu ortholog			+					
KYAT3	hsa gene		+	+					
L1TD1	hsa gene	+		+					
L3MBTL3	hsa gene			+					+
LACTB2	hsa gene			+					
LAGE3	hsa gene			+					
LAMA2	hsa gene		+		+				
LAMC1	only mmu ortholog		+		+				
LAMP1	only mmu ortholog		+	+					
LANCL1	hsa gene			+					
LARP1	hsa gene	+	+	+	+	+			
LARP1B	hsa gene	+	+	+	+	+			
LARP4	hsa gene	+	+	+	+	+			
LARP4B	hsa gene	+	+	+	+	+			
LARP6	hsa gene	+		+					
LARP7	hsa gene	+	+	+	+	+			
LARP7-PS	only mmu ortholog			+					
LARS	hsa gene	+							
LARS1	hsa gene			+					
LARS2	hsa gene	+		+					
LAS1L	hsa gene	+	+	+	+	+			
LASP1	hsa gene		+						
LBR	hsa gene		+	+		+			
LCMT2	hsa gene	+		+					
LCP1	only mmu ortholog		+						
LCP2	only mmu ortholog		+						
LDB3	only mmu ortholog		+						
LDHA	hsa gene		+	+					

LDHB	hsa gene		+	+					
LEMD3	hsa gene			+					
LENG8	only mmu ortholog		+						
LENG9	hsa gene	+		+	+				
LEO1	hsa gene			+					
LEPR	hsa gene		+						
LETM1	hsa gene			+					
LGALS1	hsa gene		+	+	+				
LGALS3	hsa gene		+	+	+				
LGALS9	only mmu ortholog		+						
LIG3	hsa gene			+					
LIG4	hsa gene			+					
LIMA1	hsa gene		+						
LIN28A	hsa gene	+		+					+
LIN28B	hsa gene	+	+	+		+		+	+
LIN7C	hsa gene		+						
LLPH	hsa gene		+	+	+	+			
LMAN2	only mmu ortholog		+						
LMF2	hsa gene		+						
LMNA	hsa gene		+		+				
LMNB1	only mmu ortholog		+	+					
LMNB2	only mmu ortholog		+	+					
LMO7	only mmu ortholog		+						
LONP1	hsa gene	+		+	+				
LRBA	hsa gene			+					
LRP1	hsa gene		+	+	+				
LRPPRC	hsa gene	+	+	+	+	+			
LRRC47	hsa gene	+	+	+	+	+			
LRRC59	hsa gene		+	+	+				
LRRFIP1	hsa gene	+		+					

LRRFIP2	hsa gene	+		+					
LRSAM1	hsa gene			+					
LRWD1	only mmu ortholog		+						
LSG1	hsa gene	+		+					
LSM1	hsa gene	+	+	+	+	+			
LSM10	hsa gene	+		+					
LSM11	hsa gene	+		+					
LSM12	hsa gene	+		+					
LSM14A	hsa gene	+	+	+	+	+			
LSM14B	hsa gene	+	+	+	+	+			
LSM2	hsa gene	+	+	+	+	+			
LSM3	hsa gene	+	+	+	+	+			
LSM4	hsa gene	+	+	+	+	+			
LSM5	hsa gene	+		+	+				
LSM6	hsa gene	+	+	+	+	+			
LSM7	hsa gene	+		+					
LSM8	hsa gene		+	+					
LSMD1	hsa gene	+							
LTA4H	hsa gene		+	+	+				
LTF	hsa gene			+					
LTV1	only mmu ortholog		+						
LUC7L	hsa gene	+	+	+	+				
LUC7L2	hsa gene	+	+	+	+	+			
LUC7L3	hsa gene	+	+	+	+	+			
LUZP1	only mmu ortholog		+						
LUZP4	hsa gene	+		+					
LYAR	hsa gene		+	+	+	+			
M1AP	hsa gene			+					
MACF1	hsa gene		+	+	+	+			
MACROH2A1	hsa gene			+					

MAD1L1	only mmu ortholog		+						
MAEL	hsa gene	+		+					
MAGED1	only mmu ortholog		+						
MAGED2	hsa gene			+	+				
MAGEE1	only mmu ortholog		+						
MAGOH	hsa gene	+	+	+	+	+			
MAGOHB	hsa gene	+	+	+	+				
MAK16	hsa gene	+	+	+	+				
MAMSTR	hsa gene			+					
MANF	hsa gene		+	+					
MAP1B	hsa gene		+	+					
MAP2	hsa gene		+						
MAP2K6	only mmu ortholog		+						
MAP3K20	hsa gene			+					
MAP4	hsa gene		+	+	+	+			
MAP4K4	only mmu ortholog		+						
MAP4K5	only mmu ortholog		+						
MAPK1	hsa gene			+					
MAPK14	hsa gene			+					
MAPK1IP1L	hsa gene			+					
MAPKBP1	hsa gene			+					
MAPRE1	hsa gene		+	+	+	+			
MAPT	hsa gene		+	+					
MARCKS	only mmu ortholog		+						
MARF1	hsa gene			+					
MARK2	hsa gene		+	+	+				
MARS	hsa gene	+			+				
MARS1	hsa gene			+					
MARS2	hsa gene	+		+					
MAT1A	hsa gene		+		+				

MAT2A	hsa gene		+	+					
MATN1	only mmu ortholog		+						
MATR3	hsa gene	+	+	+	+	+	+		
MAU2	only mmu ortholog		+						
MAZ	hsa gene	+	+	+	+	+	+	+	+
MBD1	hsa gene			+					+
MBD2	hsa gene			+					+
MBD3	hsa gene			+					+
MBD4	hsa gene			+					+
MBLAC1	hsa gene			+					
MBLAC2	hsa gene			+					
MBNL1	hsa gene	+	+	+	+	+		+	
MBNL2	hsa gene	+	+	+	+	+			+
MBNL3	hsa gene	+	+	+	+	+			
MCAM	only mmu ortholog		+		+				
MCAT	hsa gene		+	+	+	+			
MCCC1	hsa gene			+					
MCM3	hsa gene			+					
MCM3AP	hsa gene			+					
MCM4	hsa gene			+					
MCM5	hsa gene			+					
MCM7	only mmu ortholog		+						
MCRIP2	hsa gene		+						
MCRS1	hsa gene			+					
MCTS1	hsa gene	+		+	+				
MCTS2	hsa gene			+					
MDC1	hsa gene		+						
MDH1	hsa gene		+	+					
MDH2	hsa gene		+	+	+	+			
MDM2	hsa gene			+					

MDM4	hsa gene			+					
MDN1	only mmu ortholog		+		+				
MECOM	only mmu ortholog			+					
MECP2	hsa gene	+	+	+	+				+
MED11	hsa gene			+					
MED14	only mmu ortholog		+						
MED17	only mmu ortholog		+						
MED20	hsa gene			+					
MED31	only mmu ortholog		+						
MED6	only mmu ortholog		+						
MEFV	hsa gene			+					
MEN1	only mmu ortholog		+						
MEPCE	hsa gene	+	+	+	+	+			
METAP1	hsa gene			+	+				
METAP2	hsa gene		+	+	+				
METHIG1	only mmu ortholog			+					
METTL1	hsa gene	+		+					
METTL10	hsa gene	+							
METTL13	hsa gene			+					
METTL14	hsa gene	+		+					
METTL15	only mmu ortholog			+					
METTL16	hsa gene		+	+		+			
METTL17	hsa gene			+					
METTL18	hsa gene			+					
METTL2	only mmu ortholog			+					
METTL25B	hsa gene			+					
METTL27	only mmu ortholog			+					
METTL2A	hsa gene	+		+					
METTL2B	hsa gene	+		+					
METTL3	hsa gene	+		+					

METTL4	hsa gene			+					
METTL5	hsa gene	+		+					
METTL6	hsa gene			+					
METTL8	hsa gene		+						
MEX3A	hsa gene	+	+	+		+			
MEX3B	hsa gene	+	+	+	+	+			
MEX3C	hsa gene	+	+	+	+	+			
MEX3D	hsa gene	+	+	+	+	+			
MFAP1	hsa gene		+	+	+	+			
MFAP1A	only mmu ortholog			+					
MFAP1B	only mmu ortholog		+	+					
MFGE8	only mmu ortholog		+						
MFN1	hsa gene			+					
MFN2	hsa gene			+					
MFSD10	hsa gene			+					
MGA	only mmu ortholog		+						+
MGST1	hsa gene		+						
MICAL3	hsa gene			+					
MID1	hsa gene			+					
MID2	hsa gene			+					
MIF	only mmu ortholog		+	+	+				
MIF4GD	hsa gene	+		+					
MISP	hsa gene			+					
MKI67	hsa gene		+	+	+				
MKRN1	hsa gene	+	+	+	+				
MKRN2	hsa gene	+	+	+	+	+			
MKRN3	hsa gene	+	+	+					
MLEC	only mmu ortholog		+		+				
MLLT11	hsa gene			+					
MMAA	hsa gene		+						

MMGT1	only mmu ortholog		+						
MNDAL	only mmu ortholog		+						
MOCS3	hsa gene			+					
MOGS	hsa gene			+	+				
MORC3	hsa gene			+					
MORF4L1	hsa gene			+					
MOV10	hsa gene	+	+	+	+	+			
MOV10L1	hsa gene	+		+					
MPG	hsa gene			+	+				
MPHOSPH10	hsa gene	+	+	+	+	+			
MPHOSPH6	hsa gene	+		+	+				
MPHOSPH8	only mmu ortholog		+						
MRAS	hsa gene			+					
MRI1	hsa gene			+					
MRM1	hsa gene	+	+	+	+	+			
MRM2	hsa gene			+					
MRM3	hsa gene		+	+					
MRP63	hsa gene	+			+				
MRPL1	hsa gene	+	+	+	+	+			
MRPL10	hsa gene	+	+	+		+			
MRPL11	hsa gene	+	+	+	+	+			
MRPL12	hsa gene	+		+					
MRPL13	hsa gene	+	+	+	+	+			
MRPL14	hsa gene	+	+	+	+	+			
MRPL15	hsa gene	+	+	+	+	+			
MRPL16	hsa gene	+	+	+					
MRPL17	hsa gene	+		+	+				
MRPL18	hsa gene	+		+					
MRPL19	hsa gene	+	+	+	+				
MRPL2	hsa gene	+	+	+	+	+			

MRPL20	hsa gene	+	+	+		+			
MRPL21	hsa gene	+	+	+	+	+			
MRPL22	hsa gene	+	+	+	+	+			
MRPL23	hsa gene	+		+					
MRPL24	hsa gene	+	+	+	+				
MRPL27	hsa gene	+	+	+	+	+			
MRPL28	hsa gene	+	+	+	+				
MRPL3	hsa gene	+	+	+	+	+			
MRPL30	hsa gene	+		+					
MRPL32	hsa gene	+	+	+	+				
MRPL33	hsa gene	+		+	+				
MRPL34	hsa gene	+	+	+	+				
MRPL35	hsa gene	+		+					
MRPL36	hsa gene	+		+					
MRPL37	hsa gene	+	+	+	+				
MRPL38	hsa gene	+		+	+				
MRPL39	hsa gene	+	+	+	+				
MRPL4	hsa gene	+	+	+	+	+			
MRPL40	hsa gene	+	+	+	+	+			
MRPL41	hsa gene	+	+	+	+	+			
MRPL42	hsa gene	+	+	+	+				
MRPL43	hsa gene	+	+	+	+				
MRPL44	hsa gene	+	+	+	+	+			
MRPL45	hsa gene	+	+	+	+	+			
MRPL46	hsa gene	+		+					
MRPL47	hsa gene	+		+					
MRPL48	hsa gene	+		+	+				
MRPL49	hsa gene	+		+	+				
MRPL50	hsa gene	+		+					
MRPL51	hsa gene	+	+	+	+				

MRPL52	hsa gene	+		+					
MRPL53	hsa gene	+		+					
MRPL54	hsa gene	+	+	+		+			
MRPL55	hsa gene	+		+					
MRPL57	hsa gene		+	+					
MRPL58	hsa gene			+					
MRPL9	hsa gene	+	+	+	+	+			
MRPS10	hsa gene	+	+	+	+				
MRPS11	hsa gene	+	+	+	+	+			
MRPS12	hsa gene	+	+	+		+			
MRPS14	hsa gene	+	+	+	+	+			
MRPS15	hsa gene	+	+	+	+				
MRPS16	hsa gene	+		+					
MRPS17	hsa gene	+		+	+				
MRPS18A	hsa gene	+	+	+	+				
MRPS18B	hsa gene	+		+	+				
MRPS18C	hsa gene	+	+	+					
MRPS2	hsa gene	+		+	+				
MRPS21	hsa gene	+	+	+	+	+			
MRPS22	hsa gene	+		+					
MRPS23	hsa gene	+	+	+	+				
MRPS24	hsa gene	+	+	+	+	+			
MRPS25	hsa gene	+		+	+				
MRPS26	hsa gene	+	+	+		+			
MRPS27	hsa gene	+		+	+				
MRPS28	hsa gene	+	+	+	+	+			
MRPS30	hsa gene	+	+	+	+				
MRPS31	hsa gene	+	+	+	+	+			
MRPS33	hsa gene	+		+					
MRPS34	hsa gene	+		+					

MRPS35	hsa gene	+	+	+	+	+			
MRPS36	hsa gene	+		+					
MRPS5	hsa gene	+	+	+	+	+			
MRPS6	hsa gene	+		+					
MRPS7	hsa gene	+	+	+	+	+			
MRPS9	hsa gene	+	+	+	+	+			
MRRF	hsa gene	+		+	+				
MRTFA	hsa gene			+					
MRTFB	hsa gene			+					
MRTO4	hsa gene	+	+	+	+	+			
MRVI1	only mmu ortholog		+						
MSH2	hsa gene			+					
MSH3	hsa gene			+					
MSH4	hsa gene			+					
MSH6	hsa gene			+					
MSI1	hsa gene	+	+	+		+			
MSI2	hsa gene	+	+	+	+	+			
MSL3	hsa gene	+		+					
MSN	hsa gene		+	+					
MSR1	only mmu ortholog		+						
MST1R	hsa gene			+					
MT-CO2	only mmu ortholog		+		+				
MT1	only mmu ortholog		+						
MTA1	only mmu ortholog		+						
MTA2	hsa gene			+					
MTBP	only mmu ortholog		+						
MTCL1	hsa gene		+	+					
MTDH	hsa gene		+	+	+	+			
MTERF1	hsa gene		+	+					+
MTERF1A	only mmu ortholog			+					

MTERF1B	only mmu ortholog			+					
MTERF2	hsa gene			+					
MTERF3	hsa gene			+					+
MTERF4	hsa gene			+					+
MTERFD2	hsa gene	+							
MTFMT	hsa gene	+		+					
MTG1	hsa gene	+		+					
MTG2	hsa gene			+					
MTHFD1	hsa gene		+	+					
MTHFD1L	only mmu ortholog		+						
MTHFD2	only mmu ortholog		+						
MTHFSD	hsa gene	+	+	+	+	+			
MTIF2	hsa gene	+	+	+	+				
MTIF3	hsa gene	+		+					
MTO1	hsa gene	+	+	+		+			
MTPAP	hsa gene	+	+	+	+	+			
MTRES1	hsa gene			+					
MTREX	hsa gene			+					
MTRF1	hsa gene	+	+	+					
MTRF1L	hsa gene	+		+					
MTRFR	hsa gene			+					
MVK	only mmu ortholog			+	+				
MVP	hsa gene	+		+					
MX2	hsa gene			+					
MYADM	only mmu ortholog		+						
MYBBP1A	hsa gene		+	+	+	+			
MYBPC3	only mmu ortholog		+						
MYCBP2	hsa gene			+					
MYCN	only mmu ortholog		+						+
MYEF2	hsa gene	+	+	+		+			

MYEF2L	only mmu ortholog			+					
MYG1	hsa gene			+					
MYH10	only mmu ortholog		+	+	+				
MYH14	hsa gene		+						
MYH4	hsa gene			+					
MYH6	only mmu ortholog		+						
MYH9	hsa gene		+	+		+			
MYL12B	only mmu ortholog		+						
MYL4	only mmu ortholog		+						
MYL6	hsa gene			+					
MYLK	only mmu ortholog		+						
MYNN	hsa gene			+			+		
MYO18A	hsa gene		+	+		+			
MYO1C	hsa gene			+					
MYO5A	hsa gene		+	+		+			
MYOCD	hsa gene			+					
MYOF	only mmu ortholog		+						
MYOM1	only mmu ortholog		+						
N4BP1	hsa gene	+		+					
N4BP2	only mmu ortholog		+						
N6AMT1	hsa gene			+	+				
NA	only mmu ortholog			+	+				
NAA10	hsa gene			+					
NAA15	hsa gene		+	+	+				
NAA38	hsa gene	+		+	+	+			
NAA50	hsa gene		+	+					
NABP1	hsa gene			+					
NABP2	hsa gene			+					
NACA	only mmu ortholog		+	+					
NAE1	hsa gene			+					

NAF1	hsa gene	+	+	+	+	+			
NAGLU	hsa gene			+					
NANOS1	hsa gene	+		+					
NANOS2	hsa gene	+		+				+	
NANOS3	hsa gene	+		+					
NAP1L1	hsa gene		+	+	+	+			
NAP1L4	hsa gene		+	+	+				
NARS	hsa gene	+	+						
NARS1	hsa gene			+					
NARS2	hsa gene	+		+					
NASP	only mmu ortholog		+	+					
NAT10	hsa gene	+	+	+	+	+			
NBDY	hsa gene			+					
NBPF10	hsa gene		+	+		+			
NCAPD2	hsa gene			+					
NCAPG	hsa gene			+					
NCBP1	hsa gene	+	+	+	+	+			
NCBP2	hsa gene	+	+	+	+	+			
NCBP2L	hsa gene	+		+					
NCBP3	hsa gene		+	+					
NCEH1	only mmu ortholog		+						
NCKAP1	only mmu ortholog		+						
NCL	hsa gene	+	+	+	+	+			
NCLN	only mmu ortholog		+						
NCOA5	hsa gene		+	+	+	+			
NCOR2	only mmu ortholog		+		+				
NDC1	only mmu ortholog		+						
NDC80	only mmu ortholog		+						
NDRG1	hsa gene		+		+				
NDRG2	hsa gene		+		+				

NDRG4	hsa gene		+						
NDUFA10	only mmu ortholog		+						
NDUFA12	only mmu ortholog		+						
NDUFA2	hsa gene			+					
NDUFA4	only mmu ortholog		+						
NDUFA6	only mmu ortholog		+						
NDUFA7	only mmu ortholog		+						
NDUFA9	only mmu ortholog		+	+					
NDUFAF5	hsa gene			+					
NDUFS1	hsa gene			+					
NDUFS3	hsa gene			+					
NDUFS6	hsa gene		+						
NDUFV3	hsa gene		+	+	+	+			
NEDD4	only mmu ortholog		+	+					
NEIL3	hsa gene			+					
NEK10	hsa gene			+					
NEK9	hsa gene			+					
NELFB	hsa gene			+					
NELFCD	hsa gene			+					
NELFE	hsa gene	+	+	+					
NEMF	hsa gene			+					
NES	only mmu ortholog		+						
NFIB	only mmu ortholog		+						
NFIX	hsa gene			+					
NFRKB	only mmu ortholog		+						
NFX1	hsa gene	+	+	+	+	+			+
NFXL1	only mmu ortholog		+		+				+
NGDN	hsa gene	+	+	+	+	+			
NGRN	hsa gene		+	+	+	+			
NHERF1	hsa gene			+					

NHP2	hsa gene	+	+	+	+				
NHP2L1	hsa gene	+			+	+			
NIFK	hsa gene	+	+	+					
NIP7	hsa gene	+	+	+	+	+			
NIPBL	only mmu ortholog		+						
NKAP	hsa gene		+	+		+			
NKIRAS1	hsa gene			+					
NKIRAS2	hsa gene			+					
NKRF	hsa gene	+	+	+	+	+			+
NKTR	hsa gene		+						
NLE1	hsa gene			+	+				
NLRP1	hsa gene			+					
NLRP11	hsa gene		+	+	+				
NLRP1A	only mmu ortholog			+					
NLRP1B	only mmu ortholog			+					
NLRP2	only mmu ortholog		+						
NLRP6	hsa gene			+					
NMD3	hsa gene	+	+	+	+				
NME1	hsa gene		+	+	+				
NMT1	hsa gene		+		+				
NNT	hsa gene			+					
NOA1	hsa gene	+	+	+					
NOB1	hsa gene	+		+					
NOC2L	hsa gene	+	+	+	+	+			
NOC3L	hsa gene	+	+	+	+	+			
NOC4L	hsa gene	+	+	+	+				
NOCT	hsa gene			+					
NOL10	hsa gene	+	+	+	+	+			
NOL11	hsa gene	+	+	+	+	+			
NOL12	hsa gene	+	+	+	+	+			

NOL3	hsa gene	+		+					
NOL4	hsa gene			+					
NOL6	hsa gene	+	+	+	+				
NOL7	hsa gene	+	+	+	+				
NOL8	hsa gene	+	+	+	+	+			
NOL9	hsa gene	+		+	+				
NOLC1	hsa gene	+	+	+	+	+			
NOM1	hsa gene	+	+	+	+	+			
NOMO1	only mmu ortholog		+	+					
NONO	hsa gene	+	+	+	+	+			
NOP10	hsa gene	+		+	+				
NOP14	hsa gene	+	+	+	+	+			
NOP16	hsa gene	+	+	+	+	+			
NOP2	hsa gene	+	+	+	+	+			
NOP53	hsa gene			+					
NOP56	hsa gene	+	+	+	+	+			
NOP58	hsa gene	+	+	+	+	+			
NOP9	hsa gene	+	+	+					
NOSIP	hsa gene		+	+	+				
NOVA1	hsa gene	+	+	+		+			
NOVA2	hsa gene	+	+	+		+			
NPM1	hsa gene	+	+	+	+	+			
NPM2	hsa gene	+		+					
NPM3	hsa gene	+	+	+	+	+			
NQO1	hsa gene		+	+	+				
NR0B1	hsa gene	+		+					
NR2F1	only mmu ortholog			+					
NR3C1	hsa gene			+					+
NRAS	hsa gene			+					
NRDE2	hsa gene			+					

NSA2	hsa gene	+	+	+	+	+			
NSD1	only mmu ortholog		+						
NSF	hsa gene		+						
NSFL1C	hsa gene		+						
NSRP1	hsa gene	+	+	+					
NSUN2	hsa gene	+	+	+	+	+			
NSUN3	hsa gene	+		+					
NSUN4	hsa gene	+		+					
NSUN5	hsa gene	+	+	+	+	+			
NSUN6	hsa gene	+		+					
NSUN7	hsa gene	+		+					
NT5DC2	only mmu ortholog		+						
NTNG2	only mmu ortholog		+						
NTPCR	hsa gene		+	+	+	+			
NUAK1	hsa gene			+					
NUAK2	hsa gene			+					
NUCB1	only mmu ortholog		+		+				
NUCKS1	hsa gene		+	+		+			
NUDC	only mmu ortholog		+						
NUDT1	hsa gene			+					
NUDT16	hsa gene	+		+					
NUDT16L1	hsa gene	+	+	+	+	+			
NUDT16L2	only mmu ortholog			+					
NUDT21	hsa gene	+	+	+	+	+			
NUDT4	hsa gene			+					
NUDT4B	hsa gene			+					
NUDT5	hsa gene			+					
NUDT7	hsa gene			+					
NUF2	hsa gene			+					
NUFIP1	hsa gene	+		+					

NUFIP2	hsa gene	+	+	+	+	+			
NUGGC	hsa gene			+					
NUMA1	hsa gene			+	+				
NUP133	hsa gene			+					
NUP153	hsa gene	+		+					
NUP155	hsa gene			+	+				
NUP160	hsa gene			+					
NUP205	only mmu ortholog		+						
NUP210	only mmu ortholog		+						
NUP35	hsa gene			+					
NUP42	hsa gene			+					
NUP43	hsa gene			+					
NUP50	only mmu ortholog		+						
NUP54	only mmu ortholog		+						
NUP85	only mmu ortholog		+						
NUP88	only mmu ortholog		+	+					
NUP93	hsa gene			+					
NUP98	hsa gene			+					
NUPL2	hsa gene	+	+		+	+			
NUSAP1	hsa gene		+	+	+				
NUTF2	hsa gene	+		+					
NUTF2-PS1	only mmu ortholog			+					
NVL	hsa gene		+	+	+				
NXF1	hsa gene	+	+	+	+	+			
NXF2	hsa gene	+		+					
NXF2B	hsa gene	+		+					
NXF3	hsa gene	+		+					
NXF5	hsa gene	+		+					
NXF7	only mmu ortholog		+	+					
NXT1	hsa gene	+		+					

NXT2	hsa gene	+		+					
NYNRIN	hsa gene	+		+					
OAS1	hsa gene	+		+					
OAS1A	only mmu ortholog			+					
OAS1B	only mmu ortholog			+					
OAS1C	only mmu ortholog			+					
OAS1D	only mmu ortholog			+					
OAS1E	only mmu ortholog			+					
OAS1F	only mmu ortholog			+					
OAS1G	only mmu ortholog			+					
OAS1H	only mmu ortholog			+					
OAS2	hsa gene	+		+	+				
OAS3	hsa gene	+		+	+				
OASL	hsa gene	+	+	+	+				
OASL1	only mmu ortholog			+					
OASL2	only mmu ortholog			+					
OBFC1	hsa gene	+							
OBI1	hsa gene			+					
OBSCN	only mmu ortholog		+						
OBSL1	only mmu ortholog		+		+				
OCIAD1	only mmu ortholog		+						
OCRL	hsa gene			+					
ODF2	only mmu ortholog		+						
OGDH	only mmu ortholog		+	+					
OGN	hsa gene		+						
OGT	hsa gene			+					
OLA1	hsa gene		+	+					
OOEP	hsa gene			+					
OPA1	hsa gene			+	+				
ORC1	only mmu ortholog		+						

ORC2	only mmu ortholog		+						
ORC3	only mmu ortholog		+						
OSGEP	only mmu ortholog			+					
OSGEPL1	only mmu ortholog			+					
OTUB1	hsa gene			+					
OTUD4	hsa gene		+	+		+			
OVCA2	hsa gene			+					
OVOL1	only mmu ortholog			+					
OVOL2	only mmu ortholog			+			+		
OXCT1	hsa gene			+					
P2RX7	hsa gene		+						
P3H1	hsa gene			+					
P3H2	hsa gene			+					
P3H3	only mmu ortholog			+					
P4HA1	hsa gene			+					
P4HA2	hsa gene			+					
P4HB	hsa gene		+	+	+	+			
PA2G4	hsa gene	+	+	+	+				+
PABPC1	hsa gene	+	+	+	+	+			
PABPC1L	hsa gene	+	+	+					
PABPC1L2A	hsa gene	+		+					
PABPC1L2B	hsa gene	+		+					
PABPC2	only mmu ortholog			+					
PABPC3	hsa gene	+		+					
PABPC4	hsa gene	+	+	+	+	+			
PABPC4L	hsa gene	+	+	+		+			
PABPC5	hsa gene	+		+					
PABPC6	only mmu ortholog		+	+					
PABPN1	hsa gene	+	+	+	+	+			
PABPN1L	hsa gene	+		+					

PACSIN2	only mmu ortholog		+		+				
PAFAH1B1	only mmu ortholog		+						
PAFAH1B3	hsa gene		+						
PAICS	hsa gene		+	+					
PAIP1	hsa gene	+	+	+	+				
PAIP2	hsa gene	+		+					
PAIP2B	hsa gene	+		+					
PAK1IP1	only mmu ortholog		+		+				
PAK2	hsa gene			+					
PAN2	hsa gene	+		+					
PAN3	hsa gene	+		+					
PAPD4	hsa gene	+							
PAPD5	hsa gene	+	+		+	+			
PAPD7	hsa gene	+							
PAPOLA	hsa gene	+		+					
PAPOLB	hsa gene	+		+					
PAPOLG	hsa gene	+		+					
PARG	only mmu ortholog		+						
PARK7	hsa gene	+	+	+					
PARN	hsa gene	+	+	+	+				
PARP1	hsa gene	+	+	+	+	+			
PARP10	hsa gene			+					
PARP11	hsa gene			+					
PARP12	hsa gene	+	+	+	+				
PARP14	hsa gene			+					
PARP15	hsa gene			+					
PARP16	hsa gene			+					
PARP2	hsa gene			+					
PARP3	hsa gene			+					
PARP4	hsa gene	+		+					

PARP6	hsa gene			+					
PARP8	hsa gene			+					
PARS2	hsa gene	+		+					
PATL1	hsa gene	+	+	+	+	+			
PATL2	hsa gene	+		+					
PATZ1	hsa gene		+	+			+		+
PAWR	hsa gene		+						
PAX6	hsa gene			+					
PBRM1	only mmu ortholog		+						
PC	hsa gene		+						
PCBP1	hsa gene	+	+	+	+	+			
PCBP2	hsa gene	+	+	+	+	+			
PCBP3	hsa gene	+	+	+	+				
PCBP4	hsa gene	+	+	+	+				
PCCA	hsa gene			+					
PCDH20	hsa gene		+	+		+			
PCDHGA9	hsa gene		+			+			
PCED1B	only mmu ortholog		+						
PCF11	hsa gene	+		+	+				
PCGF1	only mmu ortholog		+						
PCGF2	only mmu ortholog		+						+
PCID2	hsa gene			+					
PCIF1	hsa gene			+					
PCMT1	hsa gene			+	+				
PCNA	hsa gene			+	+				
PCNP	hsa gene		+						
PCP4	only mmu ortholog			+					
PCSK1N	hsa gene			+					
PCSK9	hsa gene		+	+	+				
PCYT2	hsa gene			+					

PDAP1	hsa gene		+	+		+			
PDCD11	hsa gene	+	+	+	+				
PDCD4	hsa gene	+		+	+				
PDCD6	only mmu ortholog		+	+	+				
PDCD6IP	hsa gene		+	+	+				
PDCD7	hsa gene	+		+					
PDE12	hsa gene	+		+					
PDHA1	only mmu ortholog		+						
PDHB	hsa gene			+					
PDIA2	hsa gene		+						
PDIA3	hsa gene		+	+	+				
PDIA4	hsa gene		+	+	+				
PDIA5	hsa gene			+	+				
PDIA6	hsa gene			+	+				
PDK1	only mmu ortholog		+						
PDK3	only mmu ortholog		+						
PDLIM3	only mmu ortholog		+						
PDLIM5	hsa gene		+						
PDS5A	hsa gene		+	+					
PDS5B	only mmu ortholog		+						
PDXDC1	only mmu ortholog		+						
PEBP1	hsa gene		+	+	+	+			
PECAM1	only mmu ortholog		+						
PEF1	hsa gene		+	+		+			
PEG10	hsa gene	+	+	+	+	+			
PELO	hsa gene	+		+	+				
PELP1	hsa gene		+	+					+
PER2	only mmu ortholog			+					
PES1	hsa gene	+	+	+	+	+			
PET112	hsa gene	+							

PEX1	hsa gene			+					
PEX7	hsa gene			+					
PFKM	only mmu ortholog		+	+					
PFKP	only mmu ortholog		+						
PFN1	hsa gene		+	+	+	+			
PGAM1	only mmu ortholog			+	+				
PGK1	hsa gene			+	+				
PGM1	hsa gene			+					
PGRMC1	hsa gene		+	+	+				
PGRMC2	only mmu ortholog		+						
PHAX	hsa gene	+		+	+				
PHB	only mmu ortholog		+		+				
PHB1	hsa gene			+					
PHB2	only mmu ortholog		+	+					
PHF10	hsa gene		+		+				
PHF20	only mmu ortholog		+						+
PHF3	hsa gene		+		+				
PHF5A	hsa gene	+	+	+	+	+			
PHF6	hsa gene		+	+	+				
PHGDH	hsa gene		+	+					
PHIP	only mmu ortholog		+						
PHLDB1	only mmu ortholog		+						
PHRF1	hsa gene	+		+					
PI4KA	hsa gene			+					
PICALM	only mmu ortholog		+		+				
PIGS	only mmu ortholog		+						
PIH1D1	hsa gene	+		+					
PIH1D2	hsa gene	+		+					
PIH1D3	hsa gene	+							
PIN4	hsa gene	+	+	+	+	+			

PINX1	hsa gene	+		+					
PITHD1	hsa gene			+					
PITPNA	only mmu ortholog		+						
PITRM1	hsa gene			+					
PIWIL1	hsa gene	+		+					
PIWIL2	hsa gene	+		+					
PIWIL3	hsa gene	+		+					
PIWIL4	hsa gene	+		+					
PKM	hsa gene		+	+					
PKN2	hsa gene		+	+	+				
PKP1	only mmu ortholog		+						
PKP2	only mmu ortholog		+						
PKP3	hsa gene			+					
PKP4	hsa gene			+					
PLA2G1B	hsa gene		+						
PLAGL1	hsa gene			+					
PLCB1	only mmu ortholog			+					
PLCB3	only mmu ortholog		+						
PLD6	hsa gene	+		+					
PLEC	hsa gene		+	+	+				
PLEK	only mmu ortholog		+						
PLEKHA7	only mmu ortholog		+						
PLOD1	hsa gene			+					
PLOD2	hsa gene			+					
PLOD3	hsa gene			+					
PLRG1	hsa gene	+		+	+				
PMS1	hsa gene		+						
PMVK	hsa gene			+					
PNISR	hsa gene		+	+		+			
PNKD	hsa gene			+					

PNKP	only mmu ortholog		+		+				
PNLDC1	hsa gene	+		+					
PNN	hsa gene	+	+	+	+	+			
PNO1	hsa gene	+	+	+	+	+			
PNPLA6	hsa gene			+					
PNPT1	hsa gene	+	+	+	+	+			
PNRC2	hsa gene	+		+					
POC1A	hsa gene			+					
POC1B	hsa gene			+					
POGLUT1	hsa gene			+					
POGZ	only mmu ortholog		+						
POLDIP3	hsa gene	+	+	+	+	+			
POLG2	hsa gene			+					
POLL	only mmu ortholog		+						
POLQ	hsa gene			+					
POLR1A	hsa gene			+					
POLR1B	hsa gene			+					
POLR1C	hsa gene			+					
POLR1D	hsa gene			+					
POLR1E	hsa gene	+		+	+				
POLR1G	hsa gene			+					
POLR2A	hsa gene	+	+	+		+			
POLR2B	hsa gene	+	+	+	+	+			
POLR2C	hsa gene			+					
POLR2D	hsa gene	+		+					
POLR2E	hsa gene	+		+					
POLR2F	hsa gene	+		+					
POLR2G	hsa gene	+		+					
POLR2H	hsa gene	+		+					
POLR2I	hsa gene	+		+					

POLR2J	hsa gene	+		+					
POLR2J2	hsa gene	+		+					
POLR2J3	hsa gene	+		+					
POLR2K	hsa gene	+		+					
POLR2L	hsa gene	+		+					
POLR3A	hsa gene			+					
POLR3B	hsa gene			+					
POLR3C	hsa gene			+					
POLR3D	hsa gene			+					
POLR3F	hsa gene			+					
POLR3G	hsa gene			+					
POLR3GL	hsa gene			+					
POLR3H	hsa gene			+					
POLR3K	hsa gene			+					
POLRMT	hsa gene	+	+	+	+	+			
POP1	hsa gene	+	+	+	+				
POP4	hsa gene	+		+					
POP5	hsa gene	+		+					
POP7	hsa gene	+	+	+		+			
POT1A	only mmu ortholog		+						
POU1F1	hsa gene			+					
POU5F1	hsa gene		+	+	+				+
PPAN	hsa gene	+	+	+	+	+			
PPARGC1A	hsa gene	+		+					
PPARGC1B	hsa gene	+		+					
PPFIBP1	only mmu ortholog		+		+				
PPHLN1	hsa gene		+	+	+	+			
PPIA	hsa gene		+	+	+	+			
PPIB	hsa gene		+	+	+	+			
PPIC	only mmu ortholog		+						

PPID	hsa gene		+						
PPIE	hsa gene	+	+	+	+				
PPIF	hsa gene		+						
PPIG	hsa gene		+	+	+	+			
PPIH	hsa gene	+		+					
PPIL1	only mmu ortholog			+					
PPIL2	hsa gene			+					
PPIL3	hsa gene	+		+					
PPIL4	hsa gene	+	+	+	+	+			
PPM1G	hsa gene			+					
PPME1	hsa gene			+	+				
PPP1CA	hsa gene			+					
PPP1CB	hsa gene			+	+				
PPP1CC	hsa gene		+	+	+	+			
PPP1R10	hsa gene	+	+	+	+	+			
PPP1R12A	only mmu ortholog		+						
PPP1R8	hsa gene	+		+					
PPP1R9B	only mmu ortholog		+						
PPP2R1A	only mmu ortholog			+					
PPP2R1B	only mmu ortholog		+						
PPP4R1	hsa gene			+					
PPP4R2	hsa gene			+					
PPP5C	hsa gene			+					
PPRC1	hsa gene	+	+	+	+				
PPWD1	hsa gene	+		+					
PQBP1	hsa gene	+		+	+				
PRC1	hsa gene			+					
PRCC	only mmu ortholog		+		+				
PRDM10	hsa gene			+					
PRDM12	hsa gene			+			+		

PRDM14	hsa gene			+					
PRDM15	hsa gene			+			+		
PRDM2	only mmu ortholog		+		+				+
PRDM5	hsa gene			+					
PRDX1	hsa gene	+	+	+	+	+			
PRDX2	hsa gene			+	+				
PRDX3	hsa gene		+	+	+				
PRDX4	hsa gene			+					
PRDX5	hsa gene			+					
PRDX6	hsa gene			+	+				
PRIM1	hsa gene	+		+					
PRKACA	hsa gene			+					
PRKAR2A	hsa gene		+		+				
PRKCI	only mmu ortholog		+						
PRKCSH	only mmu ortholog			+					
PRKDC	hsa gene	+	+	+		+			
PRKRA	hsa gene	+	+	+		+			+
PRKRIP1	hsa gene			+					
PRMT1	hsa gene		+	+	+	+			
PRMT2	hsa gene			+					
PRMT3	hsa gene			+					+
PRMT6	hsa gene			+					
PRMT9	hsa gene			+					
PRORP	hsa gene			+					
PROSER2	hsa gene			+					
PRPF18	hsa gene	+		+					
PRPF19	hsa gene	+		+	+				
PRPF3	hsa gene	+	+	+	+	+			
PRPF31	hsa gene	+	+	+	+	+			
PRPF38A	hsa gene	+	+	+	+	+			

PRPF38B	hsa gene	+	+	+	+				
PRPF39	hsa gene	+		+					
PRPF4	hsa gene	+		+					
PRPF40A	hsa gene	+	+	+	+	+			
PRPF40B	hsa gene	+		+					
PRPF4B	hsa gene	+	+	+	+	+			
PRPF6	hsa gene	+	+	+	+				
PRPF8	hsa gene	+	+	+	+	+			
PRR12	only mmu ortholog		+						+
PRR13	hsa gene			+					
PRR3	hsa gene	+	+	+	+	+			
PRRC2A	hsa gene		+	+		+			
PRRC2B	hsa gene		+	+	+	+			
PRRC2C	hsa gene		+	+	+	+			
PRSS23	hsa gene			+					
PRSS3	only mmu ortholog		+						
PSAP	only mmu ortholog		+						
PSIP1	hsa gene	+	+	+	+	+			
PSMA1	hsa gene	+		+					
PSMA2	hsa gene			+					
PSMA3	hsa gene			+					
PSMA4	hsa gene		+						
PSMA5	only mmu ortholog		+						
PSMA6	hsa gene	+		+					
PSMA7	hsa gene			+					
PSMB1	hsa gene		+						
PSMB7	hsa gene			+					
PSMC1	hsa gene		+	+	+				
PSMC2	hsa gene		+	+					
PSMD13	hsa gene			+					

PSMD2	only mmu ortholog			+	+				
PSMD4	hsa gene		+	+	+				
PSMD6	hsa gene			+					
PSPC1	hsa gene	+	+	+	+	+			
PSTK	hsa gene	+		+					
PTBP1	hsa gene	+	+	+	+	+			
PTBP2	hsa gene	+	+	+	+	+			
PTBP3	hsa gene	+	+	+					
PTCD1	hsa gene	+	+	+	+	+			
PTCD2	hsa gene	+	+	+	+	+			
PTCD3	hsa gene	+	+	+	+	+			
PTDSS1	hsa gene		+		+				
PTGES3	hsa gene	+		+	+				
PTGES3L-AARSD	hsa gene	+							
PTMA	hsa gene		+						
PTMS	only mmu ortholog		+		+				
PTPN1	hsa gene		+	+	+				
PTPN11	hsa gene			+					
PTPN12	hsa gene		+						
PTPN6	only mmu ortholog		+						
PTPRF	hsa gene			+	+				
PTRF	hsa gene	+	+		+				
PTRH1	hsa gene	+	+	+	+				
PTRH2	hsa gene	+		+					
PTRHD1	hsa gene	+		+					
PUF60	hsa gene	+	+	+	+	+			
PUM1	hsa gene	+	+	+	+	+			
PUM2	hsa gene	+	+	+	+	+			
PUM3	hsa gene		+	+					
PURA	hsa gene	+	+	+	+	+			+

PURB	hsa gene	+	+	+	+	+			+
PURG	hsa gene	+	+	+		+			+
PUS1	hsa gene	+	+	+	+	+			
PUS10	hsa gene	+		+					
PUS3	hsa gene	+		+					
PUS7	hsa gene	+	+	+	+	+			
PUS7L	hsa gene	+		+					
PUSL1	hsa gene	+		+					
PWP1	hsa gene			+	+				
PWP2	hsa gene	+	+	+	+	+			
PYCR2	only mmu ortholog		+						
PYGB	hsa gene			+					
PYGM	only mmu ortholog		+						
PYM1	hsa gene		+	+					
PYURF	hsa gene			+					
QARS	hsa gene	+							
QARS1	hsa gene			+					
QK	only mmu ortholog		+						
QKI	hsa gene	+	+	+	+	+			
QRSL1	hsa gene	+		+					
QTRT1	hsa gene	+		+					
QTRT2	hsa gene			+					
QTRTD1	hsa gene	+							
R3HCC1	hsa gene	+		+					
R3HCC1L	hsa gene	+		+					
R3HDM1	hsa gene	+	+	+	+	+			
R3HDM2	hsa gene	+	+	+	+				
R3HDM4	hsa gene			+					
RAB10	hsa gene			+					
RAB11A	hsa gene			+					

RAB11B	hsa gene			+					
RAB12	hsa gene			+					
RAB13	hsa gene			+					
RAB14	hsa gene			+					
RAB15	hsa gene			+					
RAB18	hsa gene			+					
RAB1A	hsa gene			+					
RAB1B	hsa gene			+					
RAB21	hsa gene			+					
RAB22A	hsa gene			+					
RAB23	hsa gene			+					
RAB25	hsa gene			+					
RAB2A	hsa gene			+					
RAB2B	hsa gene			+					
RAB30	hsa gene			+					
RAB31	only mmu ortholog			+					
RAB33A	hsa gene			+					
RAB33B	hsa gene			+					
RAB34	hsa gene			+					
RAB35	hsa gene			+	+				
RAB36	hsa gene			+					
RAB37	hsa gene			+					
RAB39B	hsa gene			+					
RAB3GAP1	hsa gene			+					
RAB44	hsa gene			+					
RAB5A	hsa gene			+					
RAB5B	hsa gene			+					
RAB5C	hsa gene			+					
RAB6A	hsa gene			+					
RAB6B	hsa gene			+					

RAB7	only mmu ortholog		+	+					
RAB7A	hsa gene			+					
RAB9	only mmu ortholog			+					
RAB9A	hsa gene			+					
RAB9B	hsa gene			+					
RABL2	only mmu ortholog			+					
RABL2A	hsa gene			+					
RABL2B	hsa gene			+					
RABL3	hsa gene			+					
RACK1	hsa gene		+	+					
RAD18	hsa gene			+					
RAD21	only mmu ortholog		+	+					
RAD51AP1	hsa gene			+					
RAD54B	hsa gene			+					
RAD54L	hsa gene			+					
RAD54L2	hsa gene			+					
RADX	hsa gene			+					
RAE1	hsa gene	+		+					
RAF1	hsa gene			+					
RAI14	only mmu ortholog		+						
RAI2	hsa gene			+					
RALY	hsa gene	+	+	+	+	+			
RALYL	hsa gene	+	+	+	+				
RAMAC	hsa gene			+					
RAMACL	hsa gene			+					
RAN	hsa gene	+	+	+	+	+			
RANBP1	hsa gene			+					
RANBP10	hsa gene			+	+				
RANBP17	hsa gene	+		+					
RANBP2	hsa gene	+	+	+	+				

RANBP6	hsa gene	+		+					
RANBP9	hsa gene			+					
RANGAP1	hsa gene			+					
RAP1GAP2	only mmu ortholog		+		+				
RARG	only mmu ortholog		+						+
RARS	hsa gene	+			+				
RARS1	hsa gene			+					
RARS2	hsa gene	+	+	+		+			
RASEF	hsa gene			+					
RASL11A	hsa gene			+					
RASL11B	hsa gene			+					
RASL2-9	only mmu ortholog			+					
RAVER1	hsa gene	+	+	+	+	+			
RAVER2	hsa gene	+	+	+	+	+			
RBBP4	hsa gene			+	+				
RBBP5	hsa gene		+						
RBBP6	hsa gene	+	+	+		+			
RBBP7	hsa gene			+	+				
RBFA	hsa gene			+					
RBFOX1	hsa gene	+		+					
RBFOX2	hsa gene	+	+	+	+	+			
RBFOX3	hsa gene	+		+					
RBM10	hsa gene	+	+	+	+	+			
RBM11	hsa gene	+		+					
RBM12	hsa gene	+	+	+	+				
RBM12B	hsa gene	+	+	+	+	+			
RBM12B1	only mmu ortholog		+	+					
RBM12B2	only mmu ortholog		+	+					
RBM14	hsa gene	+	+	+	+	+			
RBM14-RBM4	hsa gene		+						

RBM15	hsa gene	+	+	+	+	+			
RBM15B	hsa gene	+	+	+	+	+			
RBM17	hsa gene	+		+	+				
RBM18	hsa gene	+		+	+				
RBM19	hsa gene	+	+	+	+	+			
RBM20	hsa gene	+	+	+					
RBM22	hsa gene	+	+	+	+	+			
RBM23	hsa gene	+	+	+					
RBM24	hsa gene	+	+	+					
RBM25	hsa gene	+	+	+	+	+			
RBM26	hsa gene	+	+	+	+	+			
RBM27	hsa gene	+	+	+	+	+			
RBM28	hsa gene	+	+	+	+	+			
RBM3	hsa gene	+	+	+	+	+			
RBM31Y	only mmu ortholog			+					
RBM33	hsa gene	+	+	+	+	+			
RBM34	hsa gene	+	+	+	+	+			
RBM38	hsa gene	+	+	+	+	+			
RBM39	hsa gene	+	+	+	+	+			
RBM4	hsa gene	+	+	+	+	+			
RBM41	hsa gene	+		+	+				
RBM42	hsa gene	+	+	+	+				
RBM43	hsa gene	+		+					
RBM44	hsa gene	+		+					
RBM45	hsa gene	+	+	+	+	+			
RBM46	hsa gene	+		+					
RBM47	hsa gene	+	+	+	+	+			
RBM48	hsa gene	+		+					
RBM4B	hsa gene	+	+	+	+	+			
RBM5	hsa gene	+	+	+	+	+		+	

RBM6	hsa gene	+	+	+	+	+			
RBM7	hsa gene	+	+	+	+	+			
RBM8A	hsa gene	+	+	+	+	+			
RBMS1	hsa gene	+	+	+	+	+			
RBMS2	hsa gene	+	+	+	+	+			
RBMS3	hsa gene	+	+	+	+				
RBMX	hsa gene	+	+	+	+	+			
RBMX2	hsa gene	+	+	+	+				
RBMXL1	hsa gene	+	+	+					
RBMXL2	hsa gene	+		+					
RBMXL3	hsa gene	+		+					
RBMY	only mmu ortholog			+					
RBMY1A1	hsa gene	+		+					
RBMY1B	hsa gene	+		+					
RBMY1C	hsa gene			+					
RBMY1D	hsa gene	+		+					
RBMY1E	hsa gene	+		+					
RBMY1F	hsa gene	+		+					
RBMY1J	hsa gene	+		+					
RBMYF1	only mmu ortholog			+					
RBMYF2	only mmu ortholog			+					
RBMYF3	only mmu ortholog			+					
RBMYF5	only mmu ortholog			+					
RBMYF6	only mmu ortholog			+					
RBMYF7	only mmu ortholog			+					
RBMYF8	only mmu ortholog			+					
RBMYF9	only mmu ortholog			+					
RBPMS	hsa gene	+	+	+	+				
RBPMS2	hsa gene	+		+	+				
RC3H1	hsa gene	+	+	+	+	+			

RC3H2	hsa gene	+	+	+	+	+			
RCAN2	hsa gene			+					
RCAN3	hsa gene			+					
RCC1	hsa gene			+	+				
RCC1L	hsa gene		+	+					
RCC2	hsa gene		+	+	+	+			
RCL1	hsa gene	+		+	+				
RDH11	hsa gene			+					
RDM1	hsa gene	+		+					
RDX	hsa gene		+	+	+				
RECQL	hsa gene			+	+				
RECQL4	hsa gene			+					
RECQL5	hsa gene			+					
REPIN1	hsa gene	+	+	+	+				+
RER1	only mmu ortholog		+						
RERG	hsa gene			+					
REXO1	hsa gene	+		+					
REXO2	hsa gene	+		+					
REXO4	hsa gene	+	+	+	+	+			+
REXO5	hsa gene			+					
RFC1	hsa gene			+					
RFC2	only mmu ortholog		+						
RFC3	hsa gene			+					
RFC4	hsa gene			+					
RFPL1	hsa gene			+					
RFPL2	hsa gene			+					
RFPL3	hsa gene			+					
RFPL4	only mmu ortholog			+					
RFPL4A	hsa gene			+					
RFPL4AL1	hsa gene			+					

RFPL4B	hsa gene			+					
RFTN1	hsa gene			+					
RFWD2	only mmu ortholog		+						
RHEB	hsa gene			+					
RHOBTB3	only mmu ortholog			+					
RIC8A	hsa gene			+					
RIDA	hsa gene		+	+					
RIF1	hsa gene		+	+					
RIGI	hsa gene			+					
RIMKLA	hsa gene			+					
RIMKLB	hsa gene			+					
RIMS1	hsa gene		+	+	+				
RIOK1	hsa gene	+		+					
RIOK2	hsa gene	+		+					
RIOK3	hsa gene	+		+	+				
RIOX1	only mmu ortholog			+					
RNASE1	hsa gene	+		+					
RNASE10	hsa gene	+		+					
RNASE11	hsa gene	+		+					
RNASE12	hsa gene	+		+					
RNASE13	hsa gene	+		+					
RNASE2	hsa gene	+		+					
RNASE2A	only mmu ortholog			+					
RNASE2B	only mmu ortholog			+					
RNASE3	hsa gene	+		+					
RNASE4	hsa gene	+		+					
RNASE6	hsa gene	+		+					
RNASE7	hsa gene	+		+					
RNASE8	hsa gene	+		+					
RNASE9	hsa gene	+		+					

RNASEH1	hsa gene	+		+					
RNASEH2A	hsa gene	+		+					
RNASEH2B	hsa gene	+		+					
RNASEH2C	hsa gene	+		+					
RNASEK	hsa gene	+		+					
RNASEL	hsa gene	+		+					
RNASET2	hsa gene	+		+					
RNASET2A	only mmu ortholog			+					
RNASET2B	only mmu ortholog			+					
RNF10	hsa gene		+		+				
RNF113A	hsa gene	+		+	+			+	
RNF113A1	only mmu ortholog			+					
RNF113A2	only mmu ortholog			+					
RNF113B	hsa gene	+		+					
RNF123	hsa gene			+					
RNF135	hsa gene			+					
RNF138	only mmu ortholog		+						
RNF17	hsa gene	+		+					
RNF213	only mmu ortholog		+						
RNF214	only mmu ortholog		+		+				
RNF31	hsa gene			+					
RNGTT	hsa gene	+		+					
RNH1	hsa gene	+	+	+	+				
RNMT	hsa gene	+		+					
RNMTL1	hsa gene	+			+	+			
RNPC3	hsa gene	+		+	+				
RNPEP	only mmu ortholog		+						
RNPS1	hsa gene	+	+	+	+	+			
RO60	hsa gene			+					
ROCK2	hsa gene		+	+		+			

ROR2	hsa gene		+						
RP1-37E16.12	hsa gene		+						
RP9	hsa gene	+	+	+	+	+			
RPA1	hsa gene			+	+				
RPF1	hsa gene	+	+	+	+	+			
RPF2	hsa gene	+	+	+	+	+			
RPGR	hsa gene		+	+	+				
RPL10	hsa gene	+	+	+	+				
RPL10-PS3	only mmu ortholog			+					
RPL10A	hsa gene	+	+	+	+	+			
RPL10L	hsa gene	+		+					
RPL11	hsa gene	+	+	+	+	+			
RPL12	hsa gene	+	+	+		+			
RPL13	hsa gene	+	+	+	+				
RPL13A	hsa gene	+	+	+	+				
RPL13A-PS1	only mmu ortholog		+						
RPL14	hsa gene	+	+	+	+	+			
RPL15	hsa gene	+	+	+	+	+			
RPL17	hsa gene	+	+	+	+	+			
RPL17-C18ORF3	hsa gene		+						
RPL18	hsa gene	+	+	+					
RPL18A	hsa gene	+	+	+	+	+			
RPL19	hsa gene	+	+	+	+				
RPL21	hsa gene	+	+	+	+	+			
RPL22	hsa gene	+	+	+	+	+			
RPL22L1	hsa gene	+		+	+				
RPL23	hsa gene	+	+	+	+				
RPL23A	hsa gene	+	+	+	+	+			
RPL24	hsa gene	+	+	+	+	+			
RPL26	hsa gene	+	+	+	+	+			

RPL26L1	hsa gene	+		+					
RPL27	hsa gene	+	+	+	+	+			
RPL27A	hsa gene	+	+	+		+			
RPL28	hsa gene	+	+	+	+				
RPL29	hsa gene	+	+	+	+	+			
RPL3	hsa gene	+	+	+	+	+			
RPL30	hsa gene	+	+	+	+	+			
RPL31	hsa gene	+	+	+	+				
RPL31-PS13	only mmu ortholog		+						
RPL32	hsa gene	+	+	+	+				
RPL34	hsa gene	+		+	+				
RPL35	hsa gene	+	+	+	+				
RPL35A	hsa gene	+	+	+	+	+			
RPL35RT	only mmu ortholog			+					
RPL36	hsa gene	+	+	+	+				
RPL36A	hsa gene	+	+	+		+			
RPL36AL	hsa gene	+		+					
RPL37	hsa gene	+	+	+					
RPL37A	hsa gene	+	+	+		+			
RPL38	hsa gene	+	+	+					
RPL39	hsa gene	+		+					
RPL39L	hsa gene	+		+					
RPL3L	hsa gene	+		+					
RPL4	hsa gene	+	+	+	+	+			
RPL41	hsa gene	+		+					
RPL5	hsa gene	+	+	+	+	+			
RPL6	hsa gene	+	+	+	+				
RPL7	hsa gene	+	+	+	+	+			
RPL7A	hsa gene	+	+	+	+	+			
RPL7L1	hsa gene	+	+	+	+	+			

RPL8	hsa gene	+	+	+	+	+			
RPL9	hsa gene	+		+	+				
RPL9-PS6	only mmu ortholog			+					
RPLP0	hsa gene	+	+	+	+	+			
RPLP1	hsa gene	+		+	+				
RPLP2	hsa gene	+	+	+					
RPN1	hsa gene		+	+	+				
RPN2	only mmu ortholog		+	+					
RPP14	hsa gene	+		+					
RPP21	hsa gene	+		+					
RPP25	hsa gene	+	+	+	+				
RPP25L	hsa gene	+	+	+					
RPP30	hsa gene	+	+	+	+				
RPP38	hsa gene	+		+					
RPP40	hsa gene	+		+					
RPRD1A	hsa gene			+					
RPRD1B	hsa gene		+	+					
RPRD2	hsa gene		+	+	+				
RPS10	hsa gene	+	+	+	+	+			
RPS11	hsa gene	+	+	+	+				
RPS12	hsa gene	+	+	+	+	+			
RPS13	hsa gene	+	+	+	+	+			
RPS13-PS1	only mmu ortholog		+						
RPS14	hsa gene	+	+	+		+			
RPS15	hsa gene	+	+	+	+	+			
RPS15A	hsa gene	+	+	+	+	+			
RPS16	hsa gene	+	+	+		+			
RPS17	hsa gene	+	+	+		+			
RPS17L	hsa gene	+							
RPS18	hsa gene	+	+	+		+			

RPS18-PS6	only mmu ortholog			+					
RPS19	hsa gene	+	+	+		+			
RPS19BP1	hsa gene	+	+	+	+	+			
RPS2	hsa gene	+	+	+	+	+			
RPS20	hsa gene	+	+	+	+	+			
RPS21	hsa gene	+	+	+	+				
RPS23	hsa gene	+	+	+	+	+			
RPS24	hsa gene	+	+	+	+	+			
RPS25	hsa gene	+	+	+		+			
RPS26	hsa gene	+	+	+	+	+			
RPS27	hsa gene	+	+	+		+			
RPS27A	hsa gene	+	+	+	+	+			
RPS27L	hsa gene	+	+	+		+			
RPS27RT	only mmu ortholog		+	+					
RPS28	hsa gene	+	+	+	+	+			
RPS29	hsa gene	+		+					
RPS3	hsa gene	+	+	+	+	+			
RPS3A	hsa gene	+	+	+	+	+			
RPS3A1	only mmu ortholog		+	+					
RPS4X	hsa gene	+	+	+	+	+			
RPS4Y1	hsa gene	+		+					
RPS4Y2	hsa gene	+		+					
RPS5	hsa gene	+	+	+	+	+			
RPS6	hsa gene	+	+	+	+				
RPS6KA3	only mmu ortholog		+						
RPS7	hsa gene	+	+	+	+	+			
RPS8	hsa gene	+	+	+	+	+			
RPS9	hsa gene	+	+	+	+				
RPSA	hsa gene	+	+	+		+			
RPSA2	hsa gene			+					

RPUSD1	hsa gene	+		+					
RPUSD2	hsa gene	+	+	+	+				
RPUSD3	hsa gene	+	+	+	+	+			
RPUSD4	hsa gene	+	+	+	+	+			
RQCD1	hsa gene	+							
RRAGA	hsa gene			+					
RRAGB	hsa gene			+					
RRBP1	hsa gene	+	+	+		+			
RREB1	hsa gene			+					+
RRNAD1	hsa gene	+							
RRP1	hsa gene	+	+	+	+	+			
RRP12	hsa gene	+	+	+	+	+			
RRP15	hsa gene	+	+	+	+				
RRP1B	hsa gene	+	+	+	+	+			
RRP36	hsa gene	+	+	+	+	+			
RRP7A	hsa gene	+	+	+	+				
RRP8	hsa gene	+	+	+	+	+			
RRP9	hsa gene	+	+	+	+	+			
RRS1	hsa gene	+	+	+	+	+			
RSF1	only mmu ortholog		+						
RSL1D1	hsa gene	+	+	+	+	+			
RSL24D1	hsa gene	+	+	+	+				
RSLCAN18	only mmu ortholog			+					
RSPRY1	hsa gene			+					
RSRC1	hsa gene	+		+					
RSRC2	hsa gene		+	+	+	+			
RTCA	hsa gene	+	+	+					
RTCB	hsa gene	+	+	+					
RTEL1	hsa gene			+					
RTF1	hsa gene	+	+	+	+				

RTF2	hsa gene			+					
RTL3	hsa gene			+					
RTN4	hsa gene		+	+	+				
RTP4	only mmu ortholog		+						
RTRAF	hsa gene			+					
RUVBL1	hsa gene	+		+	+				
RUVBL2	hsa gene	+		+					
RWDD4	hsa gene		+		+				
RYBP	hsa gene			+					
RYR1	hsa gene			+					
RYR2	hsa gene			+					
RYR3	hsa gene			+					
S100A11	only mmu ortholog		+						
S100A16	hsa gene		+	+	+				
S100A4	hsa gene		+	+	+				
S100A7	hsa gene			+	+				
S100A9	hsa gene		+	+					
SACM1L	hsa gene			+					
SAFB	hsa gene	+	+	+	+	+			+
SAFB2	hsa gene	+	+	+	+	+			+
SAGE1	hsa gene			+					
SALL1	hsa gene			+					+
SALL2	hsa gene			+					
SALL3	hsa gene			+					+
SALL4	hsa gene			+				+	
SAMD14	hsa gene			+					
SAMD4	only mmu ortholog		+	+					
SAMD4A	hsa gene	+	+	+	+				
SAMD4B	hsa gene	+	+	+	+	+			
SAMHD1	hsa gene	+		+					

SAMM50	hsa gene			+					
SAMSN1	hsa gene		+	+	+				
SAP18	hsa gene	+	+	+	+	+			
SAP18B	only mmu ortholog			+					
SAP30BP	hsa gene		+						
SAR1A	hsa gene			+					
SAR1B	hsa gene			+					
SARNP	hsa gene	+	+	+	+	+			
SARS	hsa gene	+	+						
SARS1	hsa gene			+					
SARS2	hsa gene	+	+	+		+			
SART1	hsa gene	+	+	+	+	+			
SART3	hsa gene	+	+	+	+	+			
SBDS	hsa gene	+	+	+	+				
SBNO1	hsa gene			+					
SBNO2	hsa gene			+					
SCAF1	hsa gene	+		+					
SCAF11	hsa gene	+	+	+	+	+			
SCAF4	hsa gene	+	+	+		+			
SCAF8	hsa gene	+	+	+	+				
SCAMP2	hsa gene		+						
SCAMP3	hsa gene		+		+				
SCAPER	hsa gene			+					
SCFD1	only mmu ortholog		+						
SCG3	hsa gene		+	+	+				
SCMH1	hsa gene			+					
SCML2	hsa gene			+					
SCML4	hsa gene			+					
SCNM1	hsa gene			+					
SCP2	only mmu ortholog		+						

SCYL1	only mmu ortholog		+						
SDAD1	hsa gene		+	+	+				
SDC4	only mmu ortholog		+						
SDE2	hsa gene			+					
SDHA	only mmu ortholog		+	+	+				
SDHC	only mmu ortholog		+						
SDPR	only mmu ortholog		+						
SEC22B	only mmu ortholog		+						
SEC23A	hsa gene			+					
SEC23IP	hsa gene		+	+	+				
SEC24C	only mmu ortholog		+						
SEC31A	hsa gene		+						
SEC61A1	hsa gene		+		+				
SEC61B	hsa gene		+	+	+				
SEC62	hsa gene		+						
SEC63	hsa gene		+	+	+				
SECISBP2	hsa gene	+	+	+	+				
SECISBP2L	hsa gene	+	+	+	+	+			
SELENOH	hsa gene		+	+					
SELENOO	hsa gene			+					
SENP3	hsa gene			+					
SEPSECS	hsa gene	+		+					
SEPTIN1	hsa gene			+					
SEPTIN10	only mmu ortholog			+					
SEPTIN11	hsa gene			+					
SEPTIN12	hsa gene			+					
SEPTIN14	hsa gene			+					
SEPTIN2	hsa gene			+					
SEPTIN3	hsa gene			+					
SEPTIN4	hsa gene			+					

SEPTIN5	hsa gene			+					
SEPTIN6	hsa gene			+					
SEPTIN7	hsa gene			+					
SEPTIN8	hsa gene			+					
SEPTIN9	hsa gene			+					
SERBP1	hsa gene	+	+	+	+	+			
SERF2	hsa gene			+	+				
SERPINB5	hsa gene			+					
SERPINB6	hsa gene			+					
SERPINB8	only mmu ortholog		+						
SERPINH1	hsa gene		+	+	+				
SET	only mmu ortholog		+	+					
SETD1A	hsa gene	+		+					
SETD1B	hsa gene	+		+					
SETD7	hsa gene	+		+					
SETDB1	hsa gene			+					+
SETDB2	hsa gene			+					
SETX	hsa gene	+		+					
SF1	hsa gene	+	+	+	+	+			
SF3A1	hsa gene	+	+	+	+	+			
SF3A2	hsa gene	+	+	+	+	+			
SF3A3	hsa gene	+	+	+	+	+			
SF3B1	hsa gene	+	+	+	+	+			
SF3B14	hsa gene	+				+			
SF3B2	hsa gene	+	+	+	+	+			
SF3B3	hsa gene	+		+	+				
SF3B4	hsa gene	+	+	+	+	+			
SF3B5	hsa gene	+		+					
SF3B6	hsa gene		+	+					
SFI1	only mmu ortholog		+						

SFMBT2	hsa gene			+					
SFN	hsa gene			+					
SFPQ	hsa gene	+	+	+	+	+			
SFSWAP	hsa gene	+		+					
SGF29	hsa gene			+					
SH3BGRL	only mmu ortholog		+						
SH3BP4	hsa gene			+					
SH3KBP1	only mmu ortholog		+						
SHARPIN	hsa gene			+					
SHFL	hsa gene			+					
SHMT2	hsa gene			+	+				
SHOC2	only mmu ortholog		+						
SHQ1	hsa gene	+		+					
SHROOM3	hsa gene			+					
SIDT1	hsa gene	+		+					
SIDT2	hsa gene	+		+					
SIK2	hsa gene			+					
SIN3A	hsa gene			+					
SIRT6	hsa gene			+					
SIRT7	hsa gene			+					
SKIC2	hsa gene			+					
SKIC8	hsa gene			+					
SKIV2L	hsa gene	+			+				
SKIV2L2	hsa gene	+	+		+				
SLBP	hsa gene	+	+	+	+				
SLC16A3	hsa gene		+	+	+				
SLC1A5	hsa gene			+					
SLC25A11	hsa gene		+	+		+			
SLC25A13	hsa gene			+					
SLC25A20	only mmu ortholog		+						

SLC25A3	hsa gene			+					
SLC25A4	only mmu ortholog		+	+					
SLC25A48	hsa gene		+						
SLC25A5	hsa gene		+	+	+				
SLC25A6	hsa gene			+					
SLC27A4	only mmu ortholog		+						
SLC29A1	hsa gene			+					
SLC2A3	only mmu ortholog		+						
SLC3A2	hsa gene		+	+	+				
SLC4A1AP	hsa gene	+		+					
SLC4A2	only mmu ortholog		+		+				
SLC7A2	hsa gene			+					
SLC7A5	hsa gene			+					
SLFN11	hsa gene			+					
SLFN13	hsa gene			+					
SLFN2	only mmu ortholog			+					
SLFN5	hsa gene			+					
SLFN8	only mmu ortholog			+					
SLFN9	only mmu ortholog			+					
SLIRP	hsa gene	+	+	+		+			
SLK	hsa gene			+					
SLPI	hsa gene			+					
SLTM	hsa gene	+	+	+	+	+			
SLU7	hsa gene	+		+					
SMAD1	hsa gene	+		+	+				
SMAD2	hsa gene	+		+					
SMAD3	hsa gene	+		+					
SMAD4	hsa gene	+		+					
SMAD5	hsa gene	+		+					
SMAD6	hsa gene	+		+					

SMAD7	hsa gene	+		+					
SMAD9	hsa gene	+							
SMARCA1	hsa gene			+					
SMARCA2	hsa gene			+					
SMARCA4	hsa gene			+					
SMARCA5	hsa gene			+					
SMARCAD1	hsa gene			+					
SMARCAL1	hsa gene			+					
SMARCD1	only mmu ortholog		+						
SMARCD2	only mmu ortholog		+						
SMARCE1	hsa gene			+					
SMC1A	hsa gene		+	+		+			
SMC2	only mmu ortholog		+						
SMC3	hsa gene			+					
SMC4	hsa gene			+					
SMC5	only mmu ortholog		+						
SMCHD1	only mmu ortholog		+						
SMG1	hsa gene	+	+	+	+	+			
SMG5	hsa gene	+		+					
SMG6	hsa gene	+		+					
SMG7	hsa gene	+		+					
SMG8	hsa gene	+		+					
SMG9	hsa gene	+		+					
SMN1	hsa gene	+		+					
SMN2	hsa gene	+		+					
SMNDC1	hsa gene	+	+	+	+				
SMOC1	only mmu ortholog		+		+				
SMOK2A	only mmu ortholog			+					
SMOK2B	only mmu ortholog			+					
SMOK3A	only mmu ortholog			+					

SMOK3B	only mmu ortholog			+					
SMOK3C	only mmu ortholog			+					
SMPD2	hsa gene			+					
SMPD3	hsa gene			+					
SMU1	only mmu ortholog		+	+					
SNAI1	only mmu ortholog			+					
SNAI2	hsa gene			+					
SNAI3	hsa gene			+					
SNAPC1	hsa gene			+					
SNAPC2	hsa gene			+					
SNCA	only mmu ortholog		+						
SNCG	only mmu ortholog		+						
SND1	hsa gene	+	+	+	+	+			
SNIP1	hsa gene	+	+	+	+				
SNRNP200	hsa gene	+	+	+	+	+			
SNRNP25	hsa gene	+		+					
SNRNP27	hsa gene	+		+					
SNRNP35	hsa gene	+		+					
SNRNP40	hsa gene	+	+	+	+				
SNRNP48	hsa gene	+		+					
SNRNP70	hsa gene	+	+	+	+	+			
SNRPA	hsa gene	+	+	+	+	+			
SNRPA1	hsa gene	+	+	+		+			
SNRPB	hsa gene	+	+	+		+			
SNRPB2	hsa gene	+		+	+				
SNRPC	hsa gene	+	+	+	+	+		+	
SNRPD1	hsa gene	+	+	+	+	+			
SNRPD2	hsa gene	+	+	+	+				
SNRPD3	hsa gene	+	+	+		+			
SNRPE	hsa gene	+		+	+				

SNRPF	hsa gene	+	+	+					
SNRPG	hsa gene	+	+	+	+				
SNRPN	hsa gene	+		+					
SNTB1	hsa gene		+						
SNTB2	hsa gene		+	+	+				
SNU13	hsa gene		+	+					
SNUPN	hsa gene	+		+					
SNW1	hsa gene	+	+	+	+	+			
SNX22	only mmu ortholog			+					
SOBP	hsa gene			+					
SOCS3	hsa gene			+					
SOD1	only mmu ortholog		+	+					
SON	hsa gene	+	+	+	+	+			+
SORBS1	hsa gene		+						
SORBS2	hsa gene		+	+	+				
SORL1	only mmu ortholog		+						
SOX2	hsa gene			+					+
SOX4	hsa gene			+					
SPAG7	hsa gene			+					
SPAG9	hsa gene		+		+				
SPART	hsa gene			+					
SPATA5	only mmu ortholog		+						
SPATS2	hsa gene	+	+	+	+				
SPATS2L	hsa gene	+	+	+	+	+			
SPCS2	only mmu ortholog		+		+				
SPECC1L	only mmu ortholog		+						
SPEN	hsa gene	+	+	+	+	+			+
SPI1	hsa gene			+					
SPOCD1	only mmu ortholog			+					
SPOUT1	hsa gene		+	+					

SPP1	only mmu ortholog		+						
SPRYD3	hsa gene			+					
SPRYD4	hsa gene			+					
SPRYD7	hsa gene			+					
SPSB1	hsa gene			+					
SPSB2	hsa gene			+					
SPSB3	hsa gene			+					
SPSB4	hsa gene			+					
SPTAN1	hsa gene		+	+					
SPTBN1	hsa gene		+	+		+			
SPTBN5	hsa gene		+						
SPTLC2	only mmu ortholog		+						
SQSTM1	only mmu ortholog		+		+				
SRA1	hsa gene	+		+	+				
SRBD1	hsa gene	+	+	+					
SRCAP	hsa gene			+					+
SREK1	hsa gene	+	+	+	+	+			
SREK1IP1	hsa gene			+	+				
SRF	hsa gene			+					
SRFBP1	hsa gene	+	+	+	+	+			
SRGAP1	hsa gene			+					
SRI	hsa gene			+	+				
SRL	hsa gene			+					
SRM	only mmu ortholog			+					
SRP14	hsa gene	+	+	+	+	+			
SRP19	hsa gene	+	+	+	+				
SRP54	hsa gene	+	+	+	+				
SRP54A	only mmu ortholog			+					
SRP54B	only mmu ortholog			+					
SRP54C	only mmu ortholog			+					

SRP68	hsa gene	+	+	+	+				
SRP72	hsa gene	+	+	+	+	+			
SRP9	hsa gene	+		+	+				
SRPK1	hsa gene		+	+	+	+			
SRPK2	hsa gene	+	+	+	+	+			
SRPK3	hsa gene			+					
SRPR	hsa gene	+			+				
SRPRA	hsa gene		+	+					
SRPRB	hsa gene		+	+					
SRRM1	hsa gene	+	+	+	+	+			
SRRM2	hsa gene	+	+	+	+	+			
SRRM3	hsa gene	+		+					
SRRM4	hsa gene	+		+					
SRRT	hsa gene	+	+	+	+	+			
SRSF1	hsa gene	+	+	+	+	+			
SRSF10	hsa gene	+	+	+		+			
SRSF11	hsa gene	+	+	+	+				
SRSF12	hsa gene	+	+	+		+			
SRSF2	hsa gene	+	+	+	+				
SRSF3	hsa gene	+	+	+	+	+			
SRSF4	hsa gene	+	+	+	+				
SRSF5	hsa gene	+	+	+	+				
SRSF6	hsa gene	+	+	+	+				
SRSF7	hsa gene	+	+	+	+				
SRSF8	hsa gene	+	+	+	+	+			
SRSF9	hsa gene	+	+	+	+				
SSB	hsa gene	+	+	+	+	+			
SSBP1	hsa gene		+	+	+	+			
SSR1	hsa gene		+		+				
SSRP1	hsa gene		+	+	+	+			

SSU72	hsa gene	+		+					
SSU72L1	hsa gene			+					
SSU72L2	hsa gene			+					
SSU72L3	hsa gene			+					
SSU72L4	hsa gene			+					
SSU72L5	hsa gene			+					
SSU72L6	hsa gene			+					
ST13	hsa gene		+	+					
STAP1	only mmu ortholog		+						
STAT3	hsa gene			+	+				+
STAU1	hsa gene	+	+	+	+	+			
STAU2	hsa gene	+	+	+	+	+			
STIP1	hsa gene		+	+	+				
STK10	hsa gene		+						
STK31	hsa gene			+					
STK38	hsa gene			+					
STMN1	only mmu ortholog		+						
STN1	hsa gene			+					
STOML2	only mmu ortholog		+						
STRAP	hsa gene	+	+	+	+	+			
STRBP	hsa gene	+	+	+	+	+			
STT3A	hsa gene			+					
STX18	hsa gene		+						
STX7	hsa gene		+						
STXBP1	hsa gene		+	+	+				
STXBP2	hsa gene			+					
STXBP5	only mmu ortholog		+						
SUB1	hsa gene	+	+	+	+	+			
SUCLG1	hsa gene		+	+	+				
SUCO	hsa gene		+						

SUGP1	hsa gene	+	+	+	+				
SUGP2	hsa gene	+	+	+	+	+			
SUGT1	hsa gene			+					
SUMO1	hsa gene		+	+	+				
SUMO2	hsa gene		+	+	+	+			
SUN1	hsa gene			+					
SUPT16	only mmu ortholog		+						
SUPT16H	hsa gene		+	+	+	+			
SUPT20	only mmu ortholog		+						
SUPT4A	only mmu ortholog			+					
SUPT4H1	hsa gene	+		+					
SUPT5	only mmu ortholog		+	+					
SUPT5H	hsa gene	+	+	+	+	+			
SUPT6	only mmu ortholog		+	+					
SUPT6H	hsa gene	+	+	+	+	+			
SUPV3L1	hsa gene	+	+	+	+	+			
SURF2	only mmu ortholog		+		+				
SURF6	hsa gene	+	+	+	+	+			
SUV39H1	hsa gene			+					
SUZ12	hsa gene	+		+					
SWAP70	only mmu ortholog		+						
SWT1	hsa gene	+		+					
SYBU	hsa gene			+					
SYF2	hsa gene	+	+	+	+	+			
SYMPK	hsa gene	+		+					
SYNCRIP	hsa gene	+	+	+	+	+			
SYNE1	hsa gene		+	+	+	+			
SYNE2	hsa gene		+		+				
SYNJ1	hsa gene			+					
SYNJ2	hsa gene			+					

SYNPO	only mmu ortholog		+						
SYNPO2	only mmu ortholog		+						
SYPL1	hsa gene			+					
SZRD1	hsa gene			+					
TACO1	hsa gene		+	+	+				
TADA2B	only mmu ortholog		+						
TAF15	hsa gene	+	+	+	+	+			
TAF1A	hsa gene			+					
TAF1B	hsa gene			+					
TAF4	only mmu ortholog		+						
TAF6	only mmu ortholog		+						
TAF9	hsa gene	+		+					
TAGLN	hsa gene		+						
TAGLN2	hsa gene		+	+					
TAP1	hsa gene			+					
TARBP1	hsa gene	+		+					
TARBP2	hsa gene	+		+	+				
TARDBP	hsa gene	+	+	+	+	+			
TARS	hsa gene	+	+		+				
TARS1	hsa gene			+					
TARS2	hsa gene	+		+					
TARS3	hsa gene			+					
TARSL2	hsa gene	+							
TASOR	hsa gene			+					
TATDN2	only mmu ortholog		+						
TAX1BP1	only mmu ortholog		+		+				
TBCA	hsa gene		+	+		+			
TBCE	hsa gene			+					
TBL1XR1	hsa gene			+					
TBL2	hsa gene		+	+	+				

TBL3	hsa gene	+	+	+	+				
TBRG4	hsa gene	+	+	+	+	+			
TCEA1	hsa gene		+						
TCEA3	only mmu ortholog		+						
TCEB1	only mmu ortholog		+						
TCEB3	hsa gene		+						
TCERG1	hsa gene	+	+	+	+	+			
TCERG1L	hsa gene			+					
TCF20	hsa gene		+	+	+				+
TCF25	only mmu ortholog		+	+	+				
TCOF1	hsa gene	+	+	+	+	+			
TCP1	hsa gene		+	+		+			
TDP2	hsa gene			+	+				
TDRD1	hsa gene	+		+					
TDRD10	hsa gene	+		+					
TDRD12	hsa gene	+		+					
TDRD15	hsa gene	+		+					
TDRD3	hsa gene	+	+	+	+	+			
TDRD5	hsa gene	+		+					
TDRD6	hsa gene	+		+					
TDRD7	hsa gene	+		+					
TDRD9	hsa gene	+		+					
TDRKH	hsa gene	+		+					
TEAD1	only mmu ortholog		+						+
TECR	hsa gene		+						
TEFM	hsa gene	+	+	+		+			
TELO2	only mmu ortholog		+						
TENT2	hsa gene			+					
TENT4A	hsa gene			+					
TENT4B	hsa gene			+					

TENT5A	hsa gene			+					
TENT5B	hsa gene			+					
TENT5C	hsa gene			+					
TENT5D	hsa gene			+					
TEP1	hsa gene	+		+					
TERT	hsa gene	+		+					
TES	hsa gene		+	+	+				
TET1	only mmu ortholog		+						+
TET2	only mmu ortholog		+						+
TEX10	hsa gene			+	+				
TEX13A	hsa gene	+		+				+	
TEX13B	hsa gene			+					
TEX13C	hsa gene			+					
TEX13C1	only mmu ortholog			+					
TEX13D	hsa gene			+					
TEX15	hsa gene			+					
TEX19	hsa gene			+					
TEX19.1	only mmu ortholog			+					
TEX19.2	only mmu ortholog			+					
TEX2	only mmu ortholog		+						
TF	only mmu ortholog		+						
TFAM	hsa gene	+	+	+	+	+			
TFB1M	hsa gene	+	+	+	+				
TFB2M	hsa gene	+	+	+	+	+			
TFE3	only mmu ortholog		+						+
TFIP11	hsa gene	+		+					
TFRC	hsa gene		+	+	+				
TGFB1	hsa gene			+					
TGM2	only mmu ortholog		+	+					
TGS1	hsa gene	+		+					

TGTP1	only mmu ortholog			+					
TGTP2	only mmu ortholog			+					
THG1L	hsa gene	+		+					
THOC1	hsa gene	+		+					
THOC2	hsa gene	+		+	+				
THOC2L	only mmu ortholog			+					
THOC3	hsa gene	+		+					
THOC5	hsa gene	+		+					
THOC6	hsa gene	+		+					
THOC7	hsa gene	+		+					
THOP1	only mmu ortholog		+						
THRA	only mmu ortholog			+					
THRAP3	hsa gene	+	+	+	+	+			
THTPA	hsa gene			+					
THUMPD1	hsa gene	+	+	+	+				
THUMPD2	hsa gene	+		+					
THUMPD3	hsa gene	+		+					
THYN1	hsa gene			+					
TIA1	hsa gene	+	+	+	+	+			
TIAL1	hsa gene	+	+	+	+	+			
TIMM50	hsa gene			+					
TIMM8B	only mmu ortholog		+						
TIMM9	only mmu ortholog		+						
TIPARP	hsa gene	+		+					
TJP1	only mmu ortholog		+						
TJP2	only mmu ortholog		+						
TKT	hsa gene		+	+					
TLE3	only mmu ortholog		+						
TLN1	hsa gene		+		+				
TLR13	only mmu ortholog			+					

TLR2	hsa gene			+					
TLR3	hsa gene	+		+					
TLR7	hsa gene	+		+					
TLR8	hsa gene	+		+					
TLR9	hsa gene			+					
TMA16	hsa gene		+						
TMA7	hsa gene		+						
TMCO1	only mmu ortholog		+						
TMED10	hsa gene		+						
TMED3	hsa gene			+					
TMED7	hsa gene			+					
TMED9	only mmu ortholog		+						
TMEM104	hsa gene			+					
TMEM165	hsa gene			+					
TMEM209	only mmu ortholog		+						
TMEM214	hsa gene		+	+	+				
TMEM263	hsa gene		+						
TMEM33	hsa gene			+					
TMEM57	only mmu ortholog		+						
TMEM63A	hsa gene			+					
TMEM63B	hsa gene			+					
TMEM63C	hsa gene			+					
TMF1	hsa gene			+					+
TMPO	hsa gene		+	+	+				
TMSB4X	hsa gene		+	+	+				
TMT1A	hsa gene			+					
TMT1A2	only mmu ortholog			+					
TMT1A3	only mmu ortholog			+					
TMT1B	hsa gene			+					
TMTC1	hsa gene			+					

TMX2	only mmu ortholog		+						
TNC	hsa gene			+					
TNFRSF22	only mmu ortholog			+					
TNKS	hsa gene			+					
TNKS1BP1	hsa gene		+		+				
TNKS2	hsa gene			+					
TNPO1	hsa gene	+	+	+	+	+			
TNPO2	hsa gene	+		+					
TNPO3	hsa gene	+		+	+				
TNRC6A	hsa gene	+	+	+	+	+			
TNRC6B	hsa gene	+	+	+	+				
TNRC6C	hsa gene	+		+					
TNS1	hsa gene		+	+	+				
TOB2	hsa gene			+					
TOE1	hsa gene	+		+	+				
TOP1	hsa gene	+	+	+	+	+			
TOP2A	hsa gene		+	+	+	+			
TOP3B	hsa gene	+	+	+	+	+			
TOPBP1	hsa gene			+					
TOR1AIP1	only mmu ortholog		+	+					
TOR4A	hsa gene			+					
TP53	hsa gene			+					+
TP53RK	hsa gene			+					
TPD52L2	hsa gene		+	+	+				
TPI1	hsa gene			+	+				
TPM1	hsa gene			+					
TPM2	hsa gene			+					
TPM3	hsa gene			+					
TPM3-RS7	only mmu ortholog			+					
TPM4	hsa gene			+					

TPPP	only mmu ortholog		+						
TPPP3	only mmu ortholog		+						
TPR	hsa gene	+	+	+	+				
TPRKB	only mmu ortholog			+					
TPRN	only mmu ortholog		+						
TPT1	hsa gene		+	+	+				
TPX2	hsa gene		+						
TRA2A	hsa gene	+	+	+	+	+			
TRA2B	hsa gene	+	+	+	+	+			
TRANK1	hsa gene			+					
TRAP1	hsa gene		+	+	+				
TRDMT1	hsa gene	+		+					
TRIM10	hsa gene			+					
TRIM11	hsa gene			+					
TRIM12C	only mmu ortholog			+					
TRIM14	hsa gene			+					
TRIM15	hsa gene			+					
TRIM16	hsa gene			+					
TRIM17	hsa gene			+					
TRIM2	only mmu ortholog		+						
TRIM21	hsa gene	+		+					
TRIM22	hsa gene			+					
TRIM24	only mmu ortholog		+						
TRIM25	hsa gene	+	+	+	+				
TRIM26	hsa gene			+					
TRIM27	hsa gene			+					
TRIM28	hsa gene		+	+		+	+		
TRIM30A	only mmu ortholog			+					
TRIM30C	only mmu ortholog			+					
TRIM30D	only mmu ortholog			+					

TRIM31	only mmu ortholog			+					
TRIM32	hsa gene			+					
TRIM34	hsa gene			+					
TRIM34A	only mmu ortholog			+					
TRIM34B	only mmu ortholog			+					
TRIM35	hsa gene			+					
TRIM38	hsa gene			+					
TRIM39	hsa gene			+					
TRIM4	hsa gene			+					
TRIM41	hsa gene			+					
TRIM43	hsa gene			+					
TRIM43B	hsa gene			+					
TRIM49	hsa gene			+					
TRIM49B	hsa gene			+					
TRIM49C	hsa gene			+					
TRIM49D1	hsa gene			+					
TRIM49D2	hsa gene			+					
TRIM5	hsa gene			+					
TRIM50	hsa gene			+					
TRIM51	hsa gene			+					
TRIM51G	hsa gene			+					
TRIM56	hsa gene	+	+	+	+	+			
TRIM58	hsa gene			+					
TRIM6	hsa gene			+					
TRIM60	hsa gene			+					
TRIM61	hsa gene			+	+				
TRIM62	hsa gene			+					
TRIM64	hsa gene			+					
TRIM64B	hsa gene			+					
TRIM64C	hsa gene			+					

TRIM65	hsa gene			+					
TRIM67	hsa gene			+					
TRIM68	hsa gene			+					
TRIM69	hsa gene			+					
TRIM7	hsa gene			+					
TRIM71	hsa gene	+	+	+					
TRIM72	hsa gene			+					
TRIM75	hsa gene			+					
TRIM77	hsa gene			+					
TRIM80	only mmu ortholog			+					
TRIM9	hsa gene			+					
TRIML1	hsa gene			+					
TRIML2	hsa gene			+					
TRIP12	hsa gene			+					
TRIP4	hsa gene			+					
TRIP6	hsa gene		+	+	+				
TRIR	hsa gene			+					
TRIT1	hsa gene	+		+					
TRMO	only mmu ortholog			+					
TRMT1	hsa gene	+	+	+	+				
TRMT10A	hsa gene	+	+	+					
TRMT10B	hsa gene	+		+					
TRMT10C	hsa gene	+	+	+					
TRMT11	hsa gene	+		+					
TRMT112	hsa gene	+		+					
TRMT12	hsa gene	+		+					
TRMT13	hsa gene	+		+					
TRMT1L	hsa gene	+	+	+	+				
TRMT2A	hsa gene	+	+	+	+				
TRMT2B	hsa gene	+		+					

TRMT44	hsa gene	+		+					
TRMT5	hsa gene	+		+					
TRMT6	hsa gene	+	+	+	+				
TRMT61A	hsa gene	+		+	+				
TRMT61B	hsa gene	+		+	+				
TRMT9B	hsa gene			+					
TRMU	hsa gene	+		+					
TRNAU1AP	hsa gene	+	+	+	+	+			
TRNT1	hsa gene	+		+					
TROVE2	hsa gene	+							
TRP53	only mmu ortholog			+					
TRP53RKA	only mmu ortholog			+					
TRP53RKB	only mmu ortholog			+					
TRPT1	hsa gene	+		+					
TRRAP	hsa gene			+					
TRUB1	hsa gene	+	+	+	+				
TRUB2	hsa gene	+	+	+	+	+			
TRY10	only mmu ortholog		+						
TSEN15	hsa gene	+		+					
TSEN2	hsa gene	+		+	+				
TSEN34	hsa gene	+		+					
TSEN54	hsa gene	+		+					
TSFM	hsa gene	+	+	+	+	+			
TSHZ2	hsa gene			+					
TSN	hsa gene	+		+	+				
TSNAX	hsa gene	+	+	+	+	+			
TSR1	hsa gene	+	+	+	+				
TSR2	hsa gene	+		+					
TSR3	hsa gene	+		+					
TST	hsa gene	+		+					

TTC14	hsa gene			+					
TTC21A	only mmu ortholog			+					
TTC3	hsa gene			+					
TTC39A	hsa gene			+					
TTC39B	hsa gene			+					
TTC39C	hsa gene			+					
TTC39D	only mmu ortholog			+					
TTC5	only mmu ortholog			+					
TTF1	hsa gene			+					+
TTF2	hsa gene	+		+					
TTK	hsa gene			+	+				
TTLL4	hsa gene			+					
TUBA1B	hsa gene			+					
TUBA1C	only mmu ortholog		+	+	+				
TUBA3B	only mmu ortholog		+						
TUBA4A	hsa gene			+					
TUBB	hsa gene			+	+				
TUBB4B	hsa gene			+					
TUBB5	only mmu ortholog		+	+					
TUBB6	only mmu ortholog			+					
TUBG1	only mmu ortholog		+						
TUFM	hsa gene	+	+	+	+				
TUT1	hsa gene	+		+					
TUT4	hsa gene			+					
TUT7	hsa gene			+					
TWF1	only mmu ortholog		+						
TWF2	hsa gene		+	+	+				
TWISTNB	only mmu ortholog		+		+				
TXLNA	hsa gene			+					
TXN	hsa gene		+	+	+	+			

TXN1	only mmu ortholog		+	+					
TXNDC5	hsa gene		+		+				
TXNL1	only mmu ortholog		+	+					
TXNL4A	hsa gene	+		+					
TXNL4B	hsa gene	+		+					
TYMS	hsa gene			+					
TYW1	hsa gene	+		+					
TYW1B	hsa gene			+					
TYW3	hsa gene	+		+					
TYW5	hsa gene	+		+					
U2AF1	hsa gene	+	+	+	+	+		+	
U2AF1L4	hsa gene	+		+					
U2AF2	hsa gene	+	+	+	+	+			
U2SURP	hsa gene	+	+	+	+	+			
UBA1	hsa gene	+	+	+	+	+			
UBA52	hsa gene	+		+					
UBA52RT	only mmu ortholog			+					
UBAP2	hsa gene	+	+	+	+				
UBAP2L	hsa gene	+	+	+	+	+			
UBC	hsa gene		+	+		+			
UBE2D1	only mmu ortholog		+						
UBE2I	hsa gene		+	+	+				
UBE2L3	hsa gene		+	+	+				
UBE2M	hsa gene		+						
UBE2N	hsa gene		+	+		+			
UBE2O	hsa gene		+	+	+	+			
UBE2Z	hsa gene			+					
UBE3C	hsa gene			+					
UBE4A	hsa gene			+					
UBFD1	hsa gene		+	+	+				

UBL4A	only mmu ortholog		+						
UBQLN1	hsa gene		+		+				
UBR5	hsa gene			+					
UBTF	hsa gene	+	+	+	+	+			
UBXN8	hsa gene			+					
UCHL5	hsa gene		+	+	+				
UFL1	only mmu ortholog		+	+					
UGDH	hsa gene			+					
UHMK1	hsa gene	+		+					
UHRF1	only mmu ortholog		+						
ULK1	hsa gene			+					
ULK2	hsa gene			+					
UMPS	only mmu ortholog		+						
UNC45B	only mmu ortholog		+						
UNC50	hsa gene			+					
UNK	hsa gene	+	+	+	+	+			
UNKL	hsa gene	+		+	+				
UPF1	hsa gene	+	+	+	+	+			
UPF2	hsa gene	+		+	+				
UPF3A	hsa gene	+		+					
UPF3B	hsa gene	+	+	+	+	+			
UQCRC1	only mmu ortholog		+	+					
UQCRC2	hsa gene			+					
URB1	hsa gene	+	+	+	+	+			
URB2	hsa gene	+	+	+	+				
URM1	hsa gene	+		+					
USB1	hsa gene	+		+					
USF2	only mmu ortholog		+						+
USO1	hsa gene		+	+	+				
USP10	hsa gene	+	+	+	+	+			

USP14	only mmu ortholog		+						
USP17L24	hsa gene			+					
USP24	only mmu ortholog		+						
USP3	only mmu ortholog		+						
USP36	hsa gene		+	+	+				
USP39	hsa gene	+		+					
USP42	hsa gene		+		+				
USP49	only mmu ortholog			+					
USP5	hsa gene			+					
USP54	only mmu ortholog		+						
USP9X	only mmu ortholog		+						
UTP11	hsa gene		+	+					
UTP11L	hsa gene	+			+	+			
UTP14A	hsa gene	+	+	+	+	+			
UTP14B	only mmu ortholog		+	+					
UTP14C	hsa gene	+		+					
UTP15	hsa gene	+	+	+	+	+			
UTP18	hsa gene	+	+	+	+	+			
UTP20	hsa gene	+	+	+	+	+			
UTP23	hsa gene	+	+	+	+				
UTP25	hsa gene			+					
UTP3	hsa gene	+	+	+	+	+			
UTP4	hsa gene		+	+					
UTP6	hsa gene	+		+	+				
VAPA	only mmu ortholog		+	+					
VAPB	only mmu ortholog		+						
VARS	hsa gene	+							
VARS1	hsa gene			+					
VARS2	hsa gene	+		+					
VARSL	hsa gene	+							

VASP	only mmu ortholog		+						
VBP1	hsa gene			+					
VCL	hsa gene			+					
VCP	hsa gene		+	+		+			
VDAC1	hsa gene			+					
VDAC2	only mmu ortholog		+	+					
VDAC3	only mmu ortholog		+						
VIL1	hsa gene		+						
VIM	hsa gene		+	+	+				
VIRMA	hsa gene			+					
VPS11	only mmu ortholog		+						
VPS35	only mmu ortholog		+						
VPS4B	only mmu ortholog		+						
VPS51	hsa gene			+					
VPS52	only mmu ortholog		+						
VRK2	only mmu ortholog		+						
VSIG8	hsa gene			+					
VWA5A	only mmu ortholog		+						
WAPL	only mmu ortholog		+						
WARS	hsa gene	+							
WARS1	hsa gene			+					
WARS2	hsa gene	+		+					
WASHC4	hsa gene			+					
WASHC5	only mmu ortholog		+						
WBP11	hsa gene		+	+	+	+			
WBP4	hsa gene	+		+				+	
WBSCR22	hsa gene		+			+			
WDHD1	hsa gene			+					
WDR1	only mmu ortholog		+	+					
WDR12	hsa gene	+		+					

WDR18	hsa gene			+					
WDR26	hsa gene			+					
WDR3	hsa gene	+	+	+	+				
WDR33	hsa gene		+	+	+	+			
WDR36	hsa gene	+	+	+	+				
WDR4	hsa gene	+		+					
WDR43	hsa gene	+	+	+	+				
WDR46	hsa gene	+	+	+		+			
WDR5	hsa gene	+		+					
WDR55	hsa gene			+					
WDR5B	hsa gene			+					
WDR6	hsa gene		+	+	+				
WDR61	hsa gene	+							
WDR74	hsa gene			+	+				
WDR75	hsa gene		+	+	+	+			
WDR83	hsa gene	+		+					
WIBG	hsa gene	+				+			
WIZ	only mmu ortholog		+						+
WRAP53	hsa gene	+		+					
WRN	hsa gene			+					
WT1	hsa gene			+			+		
WTAP	hsa gene			+			+		
XAB2	hsa gene	+		+					
XIRP1	hsa gene		+	+	+				
XPNPEP3	only mmu ortholog		+		+				
XPO1	hsa gene	+		+	+				
XPO4	hsa gene	+		+					
XPO5	hsa gene	+	+	+	+	+			
XPO6	hsa gene	+		+					
XPO7	hsa gene	+		+					

XPOT	hsa gene	+		+					
XRCC5	hsa gene		+	+	+	+			
XRCC6	hsa gene	+	+	+	+	+			
XRN1	hsa gene	+	+	+	+	+			
XRN2	hsa gene	+	+	+	+	+			
YAF2	hsa gene			+					
YAP1	hsa gene		+		+				
YARS	hsa gene	+	+		+				
YARS1	hsa gene			+					
YARS2	hsa gene	+	+	+	+	+			
YBEY	hsa gene			+					
YBX1	hsa gene	+	+	+	+	+			+
YBX2	hsa gene	+	+	+					+
YBX3	hsa gene	+	+	+					+
YEATS4	only mmu ortholog		+						
YES1	only mmu ortholog		+						
YJU2	only mmu ortholog			+					
YLPM1	hsa gene		+	+		+			
YME1L1	only mmu ortholog		+						
YRDC	hsa gene	+		+					
YTHDC1	hsa gene	+	+	+	+	+			
YTHDC2	hsa gene	+	+	+	+	+			
YTHDF1	hsa gene	+	+	+	+	+			
YTHDF2	hsa gene	+	+	+	+	+			
YTHDF3	hsa gene	+	+	+	+	+			
YWHAB	hsa gene		+						
YWHAE	hsa gene		+	+	+				
YWHAG	hsa gene		+	+	+				
YWHAH	only mmu ortholog		+						
YWHAQ	hsa gene			+					

YWHAZ	hsa gene		+	+	+				
YY1	hsa gene			+			+		+
ZAK	only mmu ortholog		+			+			
ZAR1	only mmu ortholog			+					
ZAR1L	only mmu ortholog			+					
ZBED4	hsa gene			+					
ZBP1	hsa gene			+					
ZBTB11	only mmu ortholog		+						
ZBTB12	hsa gene			+			+		
ZBTB14	hsa gene			+					
ZBTB16	hsa gene			+			+		
ZBTB18	hsa gene			+					
ZBTB2	only mmu ortholog		+				+		+
ZBTB20	hsa gene			+					
ZBTB24	hsa gene			+					
ZBTB32	hsa gene			+					
ZBTB34	hsa gene			+					
ZBTB37	hsa gene			+					
ZBTB40	hsa gene			+					
ZBTB41	hsa gene			+					
ZBTB44	hsa gene			+					
ZBTB45	hsa gene			+					
ZBTB47	hsa gene			+					
ZBTB48	only mmu ortholog			+			+		
ZBTB49	hsa gene			+					
ZBTB6	hsa gene			+			+		
ZBTB7A	only mmu ortholog		+						+
ZBTB7B	hsa gene			+					+
ZBTB8OS	only mmu ortholog			+					
ZC3H10	hsa gene	+	+	+	+				

ZC3H11A	hsa gene	+	+	+	+	+			
ZC3H12A	hsa gene	+		+					
ZC3H12B	hsa gene	+		+					
ZC3H12C	hsa gene	+		+					
ZC3H12D	hsa gene	+		+					
ZC3H13	hsa gene	+	+	+	+	+			
ZC3H14	hsa gene	+	+	+	+	+			
ZC3H15	hsa gene	+	+	+	+	+			
ZC3H18	hsa gene	+	+	+	+				
ZC3H3	hsa gene	+		+				+	
ZC3H4	hsa gene	+	+	+	+	+			
ZC3H6	hsa gene	+		+					
ZC3H7A	hsa gene	+	+	+	+	+			
ZC3H7B	hsa gene	+	+	+	+	+			
ZC3H8	hsa gene	+	+	+	+	+			+
ZC3HAV1	hsa gene	+	+	+	+	+		+	
ZC3HAV1L	hsa gene	+		+					
ZC3HC1	hsa gene	+		+					
ZCCHC11	hsa gene	+	+		+	+			
ZCCHC13	hsa gene	+		+					
ZCCHC14	hsa gene	+		+					
ZCCHC17	hsa gene	+	+	+	+	+			
ZCCHC2	hsa gene	+		+					
ZCCHC24	hsa gene	+	+	+	+	+			
ZCCHC3	hsa gene	+	+	+	+	+			
ZCCHC4	hsa gene			+					
ZCCHC5	hsa gene	+							
ZCCHC6	hsa gene	+	+		+	+			
ZCCHC7	hsa gene	+	+	+	+				
ZCCHC8	hsa gene	+	+	+	+				

ZCCHC9	hsa gene	+	+	+	+				
ZCRB1	hsa gene	+	+	+	+	+			
ZDBF2	only mmu ortholog		+						
ZEB1	hsa gene			+					+
ZEB2	hsa gene			+			+		+
ZFC3H1	hsa gene	+	+	+	+				
ZFHX4	hsa gene			+					
ZFP1	hsa gene			+					
ZFP106	only mmu ortholog		+	+	+				
ZFP108	only mmu ortholog			+					
ZFP111	only mmu ortholog			+					
ZFP131	only mmu ortholog			+					
ZFP141	only mmu ortholog			+					
ZFP207	only mmu ortholog		+						
ZFP212	only mmu ortholog			+					
ZFP236	only mmu ortholog			+					
ZFP251	only mmu ortholog			+					
ZFP26	only mmu ortholog			+					
ZFP275	only mmu ortholog			+					
ZFP277	only mmu ortholog		+						
ZFP296	only mmu ortholog		+						
ZFP317	only mmu ortholog			+					
ZFP319	only mmu ortholog			+					
ZFP326	only mmu ortholog		+	+					
ZFP346	only mmu ortholog			+					
ZFP354A	only mmu ortholog			+					
ZFP36	hsa gene	+	+	+	+				
ZFP367	only mmu ortholog			+					
ZFP36L1	hsa gene	+	+	+	+			+	
ZFP36L2	hsa gene	+	+	+	+				

ZFP36L3	only mmu ortholog			+					
ZFP384	only mmu ortholog		+						
ZFP385A	only mmu ortholog		+	+					
ZFP385B	only mmu ortholog		+	+					
ZFP385C	only mmu ortholog			+					
ZFP420	only mmu ortholog			+					
ZFP423	only mmu ortholog			+					
ZFP444	only mmu ortholog		+						
ZFP462	only mmu ortholog		+						
ZFP493	only mmu ortholog			+					
ZFP51	only mmu ortholog			+					
ZFP518A	only mmu ortholog		+						
ZFP560	only mmu ortholog			+					
ZFP574	only mmu ortholog			+					
ZFP579	only mmu ortholog			+					
ZFP58	only mmu ortholog			+					
ZFP59	only mmu ortholog			+					
ZFP593	only mmu ortholog		+	+					
ZFP598	only mmu ortholog		+						
ZFP60	only mmu ortholog			+					
ZFP61	only mmu ortholog			+					
ZFP618	only mmu ortholog		+						
ZFP62	hsa gene			+					
ZFP637	only mmu ortholog			+					
ZFP638	only mmu ortholog		+	+					
ZFP64	hsa gene			+			+		
ZFP646	only mmu ortholog			+					
ZFP652	only mmu ortholog			+					
ZFP655	only mmu ortholog		+						
ZFP661	only mmu ortholog			+					

ZFP672	only mmu ortholog			+					
ZFP706	only mmu ortholog		+	+					
ZFP708	only mmu ortholog			+					
ZFP709	only mmu ortholog			+					
ZFP712	only mmu ortholog			+					
ZFP768	only mmu ortholog			+					
ZFP784	only mmu ortholog			+					
ZFP800	only mmu ortholog			+					
ZFP804A	only mmu ortholog			+					
ZFP804B	only mmu ortholog			+					
ZFP810	only mmu ortholog			+					
ZFP830	only mmu ortholog		+	+					
ZFP865	only mmu ortholog			+					
ZFP867	only mmu ortholog			+					
ZFP868	only mmu ortholog			+					
ZFP869	only mmu ortholog			+					
ZFP872	only mmu ortholog			+					
ZFP93	only mmu ortholog			+					
ZFP94	only mmu ortholog			+					
ZFP974	only mmu ortholog			+					
ZFPM1	hsa gene			+					
ZFPM2	hsa gene			+	+				
ZFR	hsa gene	+	+	+	+	+			
ZFR2	hsa gene	+		+					
ZGPAT	hsa gene	+		+					+
ZGRF1	only mmu ortholog			+					
ZHX2	only mmu ortholog		+						
ZIC1	hsa gene			+					
ZIC3	hsa gene			+					
ZKSCAN1	only mmu ortholog		+						

ZKSCAN3	hsa gene			+					
ZKSCAN4	only mmu ortholog			+					
ZKSCAN8	hsa gene			+					
ZMAT1	hsa gene			+					
ZMAT2	hsa gene	+		+	+				
ZMAT3	hsa gene	+	+	+	+			+	
ZMAT4	hsa gene			+					
ZMAT5	hsa gene	+		+				+	
ZMPSTE24	hsa gene			+					
ZMYM1	hsa gene			+					
ZMYM4	only mmu ortholog		+						
ZMYND8	only mmu ortholog		+						
ZNF106	hsa gene	+	+	+					+
ZNF112	hsa gene			+					
ZNF131	hsa gene			+					+
ZNF155	hsa gene			+			+		
ZNF177	hsa gene			+					
ZNF184	hsa gene			+			+		+
ZNF2	hsa gene			+				+	
ZNF200	hsa gene			+					
ZNF205	hsa gene			+					
ZNF207	hsa gene		+	+	+	+			+
ZNF225	hsa gene			+					
ZNF226	hsa gene			+					
ZNF233	hsa gene			+					
ZNF234	hsa gene			+					
ZNF235	hsa gene			+					
ZNF236	hsa gene			+					
ZNF239	hsa gene	+		+					
ZNF266	hsa gene			+			+		

ZNF267	hsa gene			+					
ZNF275	hsa gene			+					
ZNF28	hsa gene			+					
ZNF317	hsa gene			+			+	+	
ZNF319	hsa gene			+					
ZNF320	hsa gene			+			+		
ZNF326	hsa gene	+	+	+	+	+			+
ZNF345	hsa gene			+					
ZNF346	hsa gene	+	+	+	+	+			+
ZNF367	hsa gene			+			+		
ZNF385A	hsa gene	+	+	+		+			+
ZNF385B	hsa gene			+					
ZNF385C	hsa gene			+					
ZNF385D	hsa gene			+					
ZNF408	hsa gene			+					
ZNF418	hsa gene			+					
ZNF425	hsa gene			+					
ZNF426	hsa gene			+					
ZNF468	hsa gene			+					
ZNF473	hsa gene	+		+			+	+	
ZNF491	hsa gene			+					
ZNF521	hsa gene			+					
ZNF524	hsa gene			+					
ZNF551	hsa gene			+					
ZNF552	hsa gene			+					
ZNF559	hsa gene			+					
ZNF563	hsa gene			+			+		
ZNF564	hsa gene			+					
ZNF574	hsa gene			+			+	+	
ZNF579	hsa gene	+	+	+		+			+

ZNF581	hsa gene			+					
ZNF582	hsa gene			+			+		
ZNF587B	hsa gene			+					
ZNF593	hsa gene			+	+			+	
ZNF598	hsa gene	+	+	+	+	+			+
ZNF600	hsa gene			+			+		
ZNF620	hsa gene			+					
ZNF622	hsa gene	+	+	+	+				
ZNF638	hsa gene	+	+	+	+	+			
ZNF652	hsa gene			+					
ZNF683	hsa gene			+					
ZNF701	hsa gene			+			+		
ZNF706	hsa gene			+	+			+	
ZNF74	hsa gene			+					
ZNF747	only mmu ortholog			+					
ZNF768	hsa gene	+	+	+		+	+		+
ZNF784	hsa gene			+			+		
ZNF800	hsa gene			+				+	+
ZNF804A	hsa gene			+					
ZNF804B	hsa gene			+					
ZNF808	hsa gene			+					
ZNF81	hsa gene			+					
ZNF816	hsa gene			+					
ZNF829	hsa gene			+					
ZNF830	hsa gene			+					+
ZNF836	hsa gene			+					
ZNF850	hsa gene			+					
ZNF860	hsa gene			+					
ZNF865	hsa gene			+					
ZNF878	hsa gene			+					

ZNF883	hsa gene			+					
ZNF888	hsa gene			+					
ZNF891	hsa gene			+					
ZNFX1	hsa gene	+	+	+	+	+			
ZNHIT6	hsa gene	+		+					
ZPR1	hsa gene			+					
ZRANB1	hsa gene			+					
ZRANB2	hsa gene	+	+	+	+	+			
ZRANB3	hsa gene			+					
ZRSR1	hsa gene	+							
ZRSR2	hsa gene	+		+					
ZSCAN10	only mmu ortholog			+					
ZSCAN22	hsa gene			+			+		
ZSCAN32	hsa gene			+					
ZSCAN4-PS2	only mmu ortholog			+					
ZSCAN4-PS3	only mmu ortholog			+					
ZSCAN4C	only mmu ortholog			+					
ZSCAN4D	only mmu ortholog			+					
ZSCAN4F	only mmu ortholog			+					
ZSCAN5A	hsa gene			+					
ZSCAN5B	hsa gene			+					
ZSCAN5C	hsa gene			+					
ZSWIM1	only mmu ortholog		+						
ZSWIM8	only mmu ortholog		+						
ZYX	hsa gene		+	+	+				
MLL3	hsa gene				+				
CSDA	hsa gene				+	+			
PKM2	hsa gene				+				
C17orf85	hsa gene				+	+			
C4orf14	hsa gene				+				

DIMT1L	hsa gene				+	+			
C14orf166	hsa gene				+				
AL844527.1	hsa gene				+				
C22orf28	hsa gene				+	+			
C14orf93	hsa gene				+				
C16orf88	hsa gene				+				
AC008073.5	hsa gene				+				
ROD1	hsa gene				+	+			
C14orf156	hsa gene				+				
EIF2C2	hsa gene				+	+			
EIF2C3	hsa gene				+	+			
CCDC55	hsa gene				+				
METT10D	hsa gene				+				
RP4-697K14.7	hsa gene				+				
RTCD1	hsa gene				+				
AC091153.1	hsa gene				+				
C1orf131	hsa gene				+	+			
C1orf35	hsa gene				+	+			
HNRPLL	hsa gene				+	+			
RG9MTD2	hsa gene				+				
C7orf50	hsa gene				+				
HNRPDL	hsa gene				+				
MKI67IP	hsa gene				+	+			
KIAA1967	hsa gene				+	+			
C1orf77	hsa gene				+				
C1orf52	hsa gene				+				
C3orf63	hsa gene				+				
C1orf31	hsa gene				+				
KIAA0802	hsa gene				+				
C17orf42	hsa gene				+				

RG9MTD1	hsa gene				+	+			
WBSCR16	hsa gene				+	+			
C11orf68	hsa gene				+	+			
AMAC1L3	hsa gene				+				
CCDC108	hsa gene				+				
AC010724.1	hsa gene				+				
THOC4	hsa gene				+	+			
C3orf26	hsa gene				+	+			
C15orf52	hsa gene				+				
C14orf21	hsa gene				+	+			
C9orf114	hsa gene				+	+			
RDBP	hsa gene				+				
GNB2L1	hsa gene				+	+			
SNORD84	hsa gene				+				
BX511012.1	hsa gene				+				
CR759761.2	hsa gene				+				
CR388220.8	hsa gene				+				
CR759786.10	hsa gene				+				
AC092037.3	hsa gene				+				
IFRD1	hsa gene				+				
TKTL1	hsa gene				+				
C16orf42	hsa gene				+				
ODZ1	hsa gene				+				
APBA3	hsa gene				+				
RABGAP1	hsa gene				+				
CCDC88C	hsa gene				+				
CPS1	hsa gene				+				
CNTLN	hsa gene				+				
FAM184B	hsa gene				+				
VPS13D	hsa gene				+				

ALX4	hsa gene				+				
C1orf144	hsa gene				+				
TRAF3IP2	hsa gene				+				
QSER1	hsa gene				+				
LTK	hsa gene				+				
LIMCH1	hsa gene				+				
DIP2B	hsa gene				+				
LRRC40	hsa gene				+				
MYO9A	hsa gene				+				
HES2	hsa gene				+				
CDC42	hsa gene				+				
VASH1	hsa gene				+				
ATP6AP1	hsa gene				+				
XRCC1	hsa gene				+				
GSDMB	hsa gene				+				
MOCOS	hsa gene				+				
NFKB2	hsa gene				+				+
UBE2T	hsa gene				+				
CBFA2T2	hsa gene				+				
PCDHB4	hsa gene				+				
WDR70	hsa gene				+				
AKR1B1	hsa gene				+				
ERO1LB	hsa gene				+				
TXLNG	hsa gene				+				
C11orf67	hsa gene				+				
EXOC1	hsa gene				+				
LAMB1	hsa gene				+				
CD200	hsa gene				+				
RPGRIP1	hsa gene				+				
EIF2C1	hsa gene				+	+			

PTGS1	hsa gene				+				
NUP188	hsa gene				+				
WAC	hsa gene				+				
CR925765.1	hsa gene				+				
NEFH	hsa gene				+				
PRMT5	hsa gene				+				
C20orf27	hsa gene				+				
PLP2	hsa gene				+				
PLS3	hsa gene				+				
FLT1	hsa gene				+				
FAM38A	hsa gene				+				
DNAJA3	hsa gene				+				
GSDMD	hsa gene				+				
KLHDC4	hsa gene				+				
ECH1	hsa gene				+				
C19orf53	hsa gene				+				
TIMM44	hsa gene				+				
PDCD5	hsa gene				+				
ANKRD27	hsa gene				+				
C19orf29	hsa gene				+	+			
PLIN3	hsa gene				+				
CDC37	hsa gene				+				
CCDC114	hsa gene				+				
AVL9	hsa gene				+				
RNF32	hsa gene				+				
EPHB6	hsa gene				+				
AKNA	hsa gene				+				
ENO3	hsa gene				+				
RASD1	hsa gene				+				
CPD	hsa gene				+				

SLC9A3R1	hsa gene				+				
FNBP4	hsa gene				+				
C11orf58	hsa gene				+				
PSMD9	hsa gene				+				
CCNC	hsa gene				+				
DNAH6	hsa gene				+				
ZNF142	hsa gene				+				
KYNU	hsa gene				+				
NID1	hsa gene				+				
CDC20	hsa gene				+				
VAMP4	hsa gene				+				
MESDC2	hsa gene				+				
WDR35	hsa gene				+				
RAD23B	hsa gene				+				
C9orf6	hsa gene				+				
INVS	hsa gene				+				
MXI1	hsa gene				+				
DUSP1	hsa gene				+				
KIAA0922	hsa gene				+				
KIF18A	hsa gene				+				
WIPF3	hsa gene				+				
TAF1L	hsa gene				+				
C19orf43	hsa gene				+				
ARHGAP9	hsa gene				+				
ZNF831	hsa gene				+				
IQSEC2	hsa gene				+				
TRIP10	hsa gene				+				
CTAG2	hsa gene				+				
POR	hsa gene				+				
MTERF	hsa gene				+	+			

CDHR3	hsa gene				+				
PALLD	hsa gene				+				
MAP7D3	hsa gene				+				
MAP1S	hsa gene				+				
LSP1	hsa gene				+				
HIP1R	hsa gene				+				
PPFIA1	hsa gene				+				
CC2D1A	hsa gene				+				+
KIAA0664	hsa gene				+				
KIAA0907	hsa gene				+				
INADL	hsa gene				+				
SPG20	hsa gene				+				
MYH11	hsa gene				+				
CEPT1	hsa gene				+				
TFCP2	hsa gene				+				+
ESPL1	hsa gene				+				
COX5B	hsa gene				+				
ZFHX2	hsa gene				+				
GATA4	hsa gene				+				
C9orf78	hsa gene				+				
YIPF3	hsa gene				+				
AL844220.1	hsa gene				+				
IFT172	hsa gene				+				
PREB	hsa gene				+				+
FGF2	hsa gene				+				
RAP1GDS1	hsa gene				+				
C17orf37	hsa gene				+				
FKBP10	hsa gene				+				
NLRP12	hsa gene				+				
WDTC1	hsa gene				+				

KIF2C	hsa gene				+				
MGST3	hsa gene				+				
ABL2	hsa gene				+				
S100A8	hsa gene				+				
CEP170	hsa gene				+				
C1orf55	hsa gene				+				
GMPPA	hsa gene				+				
FAM55C	hsa gene				+				
C6orf170	hsa gene				+				
CASK	hsa gene				+				
ARHGAP36	hsa gene				+				
C9orf80	hsa gene				+				
NKX6-2	hsa gene				+				
EML3	hsa gene				+				
CTAGE5	hsa gene				+				
C11orf57	hsa gene				+				
HSPB8	hsa gene				+				
SPARCL1	hsa gene				+				
FARP1	hsa gene				+				
USP16	hsa gene				+				
MTERFD1	hsa gene				+				
MYST4	hsa gene				+				
C7orf30	hsa gene				+				
WDR19	hsa gene				+				
C12orf43	hsa gene				+				
ALAS2	hsa gene				+				
DUSP23	hsa gene				+				
RAPGEF6	hsa gene				+				
ZDHHC1	hsa gene				+				
PCNT	hsa gene				+				

ABCF3	hsa gene				+				
MSLNL	hsa gene				+				
MAPKAPK2	hsa gene				+				
MEMO1	hsa gene				+				
STT3B	hsa gene				+				
WDFY3	hsa gene				+				
S100P	hsa gene				+				
C4orf29	hsa gene				+				
C9orf23	hsa gene				+	+			
GKAP1	hsa gene				+				
AP1G1	hsa gene				+				
KIAA0101	hsa gene				+				
ANPEP	hsa gene				+				
PIP4K2C	hsa gene				+				
TTC16	hsa gene				+				
DUS2L	hsa gene				+				
ZNF668	hsa gene				+				+
VKORC1	hsa gene				+				
FTH1	hsa gene				+				
IRF2BP2	hsa gene				+				
CHTF8	hsa gene				+				
ACTBL2	hsa gene				+				
DLGAP1	hsa gene				+				
ATF7	hsa gene				+				+
AKAP13	hsa gene				+				
CHCHD7	hsa gene				+				
ZNF692	hsa gene				+				
DSCAM	hsa gene				+				
MLLT3	hsa gene				+				
USP19	hsa gene				+				

NAA16	hsa gene				+				
FAM192A	hsa gene				+				
KRT2	hsa gene				+				
DMXL1	hsa gene				+				
HPSE2	hsa gene				+				
PTPRM	hsa gene				+				
C11orf80	hsa gene				+				
PPM1E	hsa gene				+				
DPP10	hsa gene				+				
C13orf26	hsa gene				+				
C8orf59	hsa gene				+				
PDE4DIP	hsa gene				+				
GADD45GIP1	hsa gene				+				
CCDC129	hsa gene				+				
PPA1	hsa gene				+				
SETD2	hsa gene				+				
C8orf33	hsa gene				+				
CCDC87	hsa gene				+				
ATL3	hsa gene				+				
FMNL1	hsa gene				+				
HPDL	hsa gene				+				
ZFP91	hsa gene				+				+
EYS	hsa gene				+				
TUBB2C	hsa gene				+				
IER5L	hsa gene				+				
KAZN	hsa gene				+				
CD55	hsa gene				+				
ZNF498	hsa gene				+				
TRIM33	hsa gene				+				
PARVA	hsa gene				+				

ZNF121	hsa gene				+		+		
ASNA1	hsa gene				+				
ASPH	hsa gene				+				
C4orf43	hsa gene				+				
ZNF277	hsa gene				+			+	+
OSTC	hsa gene				+				
MT-ATP6	hsa gene				+				
L1CAM	hsa gene				+				
ECT2L	hsa gene				+				
PFDN6	hsa gene				+				
COL5A2	hsa gene				+				
SYCE1L	hsa gene				+				
EVPLL	hsa gene				+				
ERCC6	hsa gene				+				
AKAP2	hsa gene				+				
PCDHAC1	hsa gene				+				
CCBL2	hsa gene					+			
MRPS23	hsa gene					+			
DDX39B	hsa gene					+			
MAGOH2	hsa gene					+			
HNRPDL	hsa gene					+			
SFRS7	hsa gene					+			
C2orf15	hsa gene					+			
SFRS9	hsa gene					+			
SFRS6	hsa gene					+			
SFRS2	hsa gene					+			
SFRS5	hsa gene					+			
RBM12	hsa gene					+			
SF4	hsa gene					+			
SFRS4	hsa gene					+			

SFRS11	hsa gene					+			
HIST1H1E	hsa gene					+			
ZC3H18	hsa gene					+			
C19orf47	hsa gene					+			
C16orf80	hsa gene					+			
CXorf57	hsa gene					+			
C11orf31	hsa gene					+			
PRIC285	hsa gene					+			
C1orf204	hsa gene					+			
HIST1H1D	hsa gene					+			
RPL10	hsa gene					+			
ZBTB26	hsa gene						+		
ZBTB4	hsa gene						+		
ZBTB7a	hsa gene						+		
ZNF238	hsa gene						+		
ZBTB38	hsa gene						+		
OSR2	hsa gene						+		
ZNF594	hsa gene						+		
ZNF572	hsa gene						+		
ZNF16	hsa gene						+		
ZNF654	hsa gene						+		
YY2	hsa gene						+		
ZNF76	hsa gene						+		
ZNF322	hsa gene						+		
ZNF645	hsa gene						+		
ZNF691	hsa gene						+		
ZNF32	hsa gene						+		
SP6	hsa gene						+		
HAKAI	hsa gene						+		
REST	hsa gene						+		+

GLI4	hsa gene						+		
ZNF148	hsa gene						+		+
ZNF576	hsa gene						+		
KLF17	hsa gene						+		
KLF13	hsa gene						+		
ZNF467	hsa gene						+		
Plag1	hsa gene						+		
SP1	hsa gene						+		+
Repin1	hsa gene						+		
EGR2	hsa gene						+		
GTF3a	hsa gene						+		
ZNF629	hsa gene						+		
ZNF71	hsa gene						+		
Kcmf1	hsa gene						+		
ZNF280D	hsa gene						+		+
ZIC2	hsa gene						+		
ZFP3	hsa gene						+		
KLF12	hsa gene						+		
ZNF281	hsa gene						+		+
ZNF341	hsa gene						+		
ZNF362	hsa gene						+		
ZNF384	hsa gene						+		+
ZNF513	hsa gene						+		
ZNF781	hsa gene						+		
ZNF786	hsa gene						+		
KLF14	hsa gene						+		
ZNF526	hsa gene						+		
ZNF770	hsa gene						+		
ZNF23	hsa gene						+		
ZNF41	hsa gene						+		

ZNF718	hsa gene						+		
ZNF436	hsa gene						+		
ZNF846	hsa gene						+		
ZNF37a	hsa gene						+		
ZNF554	hsa gene						+		
ZNF774	hsa gene						+		
ZNF680	hsa gene						+		
ZNF669	hsa gene						+		
ZNF610	hsa gene						+		
ZNF595	hsa gene						+		
ZNF788	hsa gene						+		
ZNF667	hsa gene						+		
ZNF45	hsa gene						+		
ZNF510	hsa gene						+		
ZNF777	hsa gene						+		
ZNF684	hsa gene						+		
ZNF331	hsa gene						+		
ZNF224	hsa gene						+		
ZNF274	hsa gene						+		
ZNF132	hsa gene						+		
ZNF133	hsa gene						+		
ZNF181	hsa gene						+		
ZNF195	hsa gene						+		
ZNF227	hsa gene						+		
ZNF250	hsa gene						+		
ZNF257	hsa gene						+		
ZNF264	hsa gene						+		
ZNF273	hsa gene						+		
ZNF454	hsa gene						+		
ZNF485	hsa gene						+		

ZNF490	hsa gene						+		
ZNF517	hsa gene						+		
ZNF528	hsa gene						+		
ZNF529	hsa gene						+		
ZNF530	hsa gene						+		
ZNF544	hsa gene						+		
ZNF547	hsa gene						+		
ZNF561	hsa gene						+		
ZNF571	hsa gene						+		
ZNF577	hsa gene						+		
ZNF583	hsa gene						+		
ZNF597	hsa gene						+		
ZNF621	hsa gene						+		
ZNF624	hsa gene						+		
ZNF677	hsa gene						+		
ZNF707	hsa gene						+		
ZNF708	hsa gene						+		
ZNF98	hsa gene						+		
ZIK1	hsa gene						+		
ZNF778	hsa gene						+		
ZNF256	hsa gene						+		
ZNF433	hsa gene						+		
ZNF214	hsa gene						+		
ZNF670	hsa gene						+		
ZNF675	hsa gene						+		
ZNF34	hsa gene						+		+
ZNF662	hsa gene						+		
ZNF268	hsa gene						+		
ZNF324	hsa gene						+		
ZNF449	hsa gene						+		

ZSCAN1	hsa gene						+		
MZF1	hsa gene						+		
PEG3	hsa gene						+		
ZSCAN1	hsa gene						+		
ZSCAN12	hsa gene						+		
ZSCAN16	hsa gene						+		
ZSCAN18	hsa gene						+		
ZCAN23	hsa gene						+		
ZNF287	hsa gene						+		
PRDM13	hsa gene						+		
ZNF978	hsa gene						+		
MYST2	hsa gene						+		
G2E3	hsa gene							+	
GIN1	hsa gene							+	
PHF7	hsa gene							+	
RNF14	hsa gene							+	
TRIM55	hsa gene							+	
ZNF22	hsa gene							+	
ARID2	hsa gene								+
ARID3B	hsa gene								+
BACH1	mmu gene								+
BHLHE40	hsa gene								+
CBX2	hsa gene								+
CEBPB	hsa gene								+
CENPB	hsa gene								+
CENPS	mmu gene								+
CENPT	hsa gene								+
CENPX	hsa gene								+
CIC	hsa gene								+
CUX1	hsa gene								+

CUX1	hsa gene								+
CXXC1	hsa gene								+
DOT1L	mmu gene								+
E2F3	hsa gene								+
E2F4	mmu gene								+
E2F6	hsa gene								+
ELF1	hsa gene								+
ELF2	hsa gene								+
ESR1	hsa gene								+
FLYWCH1	hsa gene								+
FOSL1	hsa gene								+
FOXK2	hsa gene								+
FOXL2	hsa gene								+
FOXP1	hsa gene								+
GATA1	hsa gene								+
GATA2	hsa gene								+
GATA3	hsa gene								+
GMEB2	hsa gene								+
HMBOX1	hsa gene								+
HMG20B	hsa gene								+
HMGN3	hsa gene								+
HOMEZ	hsa gene								+
HOXB4	hsa gene								+
HOXB9	hsa gene								+
IKZF1	hsa gene								+
IKZF4	hsa gene								+
IRF2	hsa gene								+
JUND	hsa gene								+
KDM2A	hsa gene								+
KDM5B	hsa gene								+

KLF4	mmu gene								+
KMT2A	hsa gene								+
KMT2B	hsa gene								+
LCOR	hsa gene								+
LEF1	hsa gene								+
LIN54	hsa gene								+
MAX	hsa gene								+
MEIS2	hsa gene								+
MNT	hsa gene								+
MYB	hsa gene								+
MYBL2	mmu gene								+
MYC	hsa gene								+
NANOG	mmu gene								+
NANOGP8	mmu gene								+
NFE2	hsa gene								+
NFIA	hsa gene								+
NFIC	hsa gene								+
NFKB1	hsa gene								+
NFYA	hsa gene								+
NFYC	hsa gene								+
NME2	hsa gene								+
NR2C2	hsa gene								+
NR2F2	hsa gene								+
NR2F6	hsa gene								+
NR5A2	mmu gene								+
OXSR1	hsa gene								+
PBX2	hsa gene								+
PCGF6	hsa gene								+
PHF1	hsa gene								+
PHF21A	hsa gene								+

PIN1	hsa gene								+
PIN1	hsa gene								+
PKNOX1	hsa gene								+
POU2F1	hsa gene								+
POU3F4	hsa gene								+
POU5F1B	mmu gene								+
RBPJ	hsa gene								+
RFX1	hsa gene								+
RLF	hsa gene								+
RUNX1	hsa gene								+
RXRA	hsa gene								+
RXRB	hsa gene								+
SATB2	hsa gene								+
SKI	hsa gene								+
SNAPC4	hsa gene								+
SP3	hsa gene								+
STAT1	hsa gene								+
STAT6	hsa gene								+
TAL1	hsa gene								+
TBP	hsa gene								+
TBX3	mmu gene								+
TCF12	hsa gene								+
TCF3	hsa gene								+
TCF4	hsa gene								+
TFAP4	hsa gene								+
TFEB	mmu gene								+
TGIF1	hsa gene								+
UBP1	hsa gene								+
USF1	hsa gene								+
VEZF1	hsa gene								+

XBP1	hsa gene								+
XPA	hsa gene								+
ZBTB33	hsa gene								+
ZFP42	mmu gene								+
ZFX	mmu gene								+
ZHX1	hsa gene								+
ZIM2	hsa gene								+
ZNF143	hsa gene								+
ZNF217	hsa gene								+
ZNF24	hsa gene								+
ZNF280A	hsa gene								+
ZNF280C	hsa gene								+
ZNF292	mmu gene								+
ZNF296	mmu gene								+
ZNF318	hsa gene								+
ZNF343	hsa gene								+
ZNF444	hsa gene								+
ZNF451	hsa gene								+
ZNF462	mmu gene								+
ZNF48	hsa gene								+
ZNF496	hsa gene								+
ZNF518B	hsa gene								+
ZNF592	hsa gene								+
ZNF609	hsa gene								+
ZNF644	hsa gene								+
ZNF655	mmu gene								+
ZNF687	hsa gene								+
ZNF740	hsa gene								+
ZNF771	hsa gene								+
ZSCAN21	mmu gene								+

ZZZ3	hsa gene								+
