# Supplementary material for: Foxg1 and companions: Not only transcription factors
Source: Neural Regen Res. 2025 Sep 3;21(8):3426–38. doi: 10.4103/NRR.NRR-D-25-00439 (PMC13452659; doi:10.4103/NRR.NRR-D-25-00439)
Supplement: Supplementary file 21 [file NRR-21-3426_Suppl8.pdf]

Additional Table 4. Distribution of triplosensitivity indices

[18641 homo sapiens genes profiled]

NOTES

data collected from: Collins et al. Cell (2022). doi: 10.1016/j.cell.2022.06.036 [Table S7]

Gene	*pTriplo*
A1BG	0.160
A1CF	0.496
A2M	0.330
A2ML1	0.350
A3GALT2	0.113
A4GALT	0.393
A4GNT	0.185
AAAS	0.629
AACS	0.414
AADAC	0.623
AADACL2	0.541
AADACL3	0.139
AADACL4	0.086
AADAT	0.314
AAED1	0.256
AAGAB	0.377
AAK1	0.735
AAMDC	0.464
AAMP	0.940
AANAT	0.270
AAR2	0.450
AARD	0.199
AARS	0.730
AARS2	0.780

AARSD1	0.400
AASDH	0.529
AASDHPPT	0.266
AASS	0.195
AATF	0.412
AATK	0.616
ABAT	0.052
ABCA1	0.646
ABCA10	0.112
ABCA12	0.575
ABCA13	0.293
ABCA2	0.605
ABCA3	0.237
ABCA4	0.444
ABCA5	0.185
ABCA6	0.213
ABCA7	0.103
ABCA8	0.379
ABCA9	0.397
ABCB1	0.744
ABCB10	0.770
ABCB11	0.648
ABCB4	0.823
ABCB5	0.122
ABCB6	0.498
ABCB8	0.611
ABCB9	0.631
ABCC1	0.778
ABCC10	0.781
ABCC11	0.293
ABCC12	0.269
ABCC2	0.176

ABCC3	0.600
ABCC4	0.282
ABCC5	0.657
ABCC6	0.547
ABCC8	0.468
ABCC9	0.995
ABCD2	0.475
ABCD3	0.556
ABCE1	0.980
ABCF1	0.961
ABCF2	0.927
ABCF3	0.623
ABCG1	0.794
ABCG2	0.217
ABCG4	0.607
ABCG5	0.168
ABCG8	0.063
ABHD1	0.188
ABHD10	0.333
ABHD11	0.356
ABHD12	0.183
ABHD12B	0.148
ABHD13	0.181
ABHD14A	0.501
ABHD14B	0.650
ABHD15	0.599
ABHD16A	0.908
ABHD16B	0.193
ABHD17A	0.615
ABHD17B	0.757
ABHD17C	0.831
ABHD2	0.454

ABHD3	0.398
ABHD4	0.254
ABHD5	0.185
ABHD6	0.549
ABHD8	0.885
ABI1	0.768
ABI2	0.749
ABI3	0.187
ABI3BP	0.389
ABL1	0.998
ABL2	0.950
ABLIM1	0.824
ABLIM2	0.280
ABLIM3	0.668
ABR	0.631
ABRA	0.243
ABRACL	0.078
ABT1	0.465
ABTB1	0.354
ABTB2	0.327
AC000003.2	0.163
AC002398.9	0.265
AC002451.1	0.194
AC002472.13	0.310
AC002985.3	0.309
AC003005.4	0.108
AC003006.7	0.021
AC003102.1	0.240
AC004017.1	0.028
AC004076.7	0.104
AC004076.9	0.027
AC004381.6	0.194

AC004466.1	0.244
AC004817.1	0.060
AC004899.1	0.149
AC005003.1	0.375
AC005008.2	0.221
AC005358.1	0.283
AC005477.1	0.132
AC005481.5	0.075
AC005544.1	0.273
AC005606.1	0.318
AC005609.1	0.071
AC005779.2	0.228
AC005841.1	0.112
AC006116.20	0.078
AC006126.3	0.267
AC006132.1	0.526
AC006372.1	0.244
AC006449.1	0.434
AC006455.1	0.269
AC006486.1	0.255
AC006547.14	0.365
AC006946.15	0.953
AC006967.1	0.134
AC007040.11	0.189
AC007375.1	0.690
AC007382.1	0.108
AC007390.5	0.141
AC007401.2	0.154
AC007405.2	0.551
AC007431.1	0.221
AC007461.1	0.159
AC007557.1	0.173

AC007773.3	0.215
AC007952.5	0.537
AC007952.6	0.441
AC007956.1	0.154
AC008060.7	0.218
AC008132.1	0.294
AC008267.1	0.158
AC008394.1	0.181
AC008443.1	0.993
AC008948.1	0.163
AC008964.1	0.191
AC009060.1	0.109
AC009403.2	0.132
AC009892.10	0.202
AC010327.2	0.292
AC010336.1	0.265
AC010441.1	0.219
AC010536.1	0.111
AC010547.9	0.136
AC010642.1	0.106
AC011239.1	0.139
AC011294.3	0.098
AC011298.1	0.243
AC011366.3	0.215
AC011484.1	0.267
AC011500.1	0.140
AC011530.4	0.195
AC011551.3	0.413
AC011755.1	0.115
AC011897.1	0.287
AC011997.1	0.241
AC012123.1	0.282

AC012215.1	0.126
AC012313.1	0.165
AC012414.1	0.231
AC013269.5	0.291
AC013449.1	0.231
AC015688.3	0.296
AC015987.2	0.148
AC016251.1	0.194
AC016586.1	0.221
AC016722.1	0.252
AC016722.2	0.253
AC016745.1	0.133
AC016757.3	0.318
AC016885.1	0.087
AC017104.2	0.097
AC018470.1	0.897
AC018755.1	0.976
AC018816.3	0.118
AC019171.1	0.354
AC019206.1	0.359
AC019294.1	0.455
AC020907.1	0.272
AC020922.1	0.277
AC021218.2	0.162
AC021860.1	0.170
AC022400.2	0.212
AC022431.2	0.159
AC022498.1	0.066
AC022532.1	0.363
AC023469.1	0.172
AC023590.1	0.909
AC023632.1	0.243

AC024257.1	0.292
AC024580.1	0.212
AC024592.12	0.308
AC024940.1	0.964
AC025263.3	0.367
AC025278.1	0.100
AC026202.1	0.218
AC026310.1	0.205
AC026703.1	0.147
AC026740.1	0.105
AC027228.1	0.190
AC027307.3	0.353
AC027763.2	0.229
AC037459.4	0.160
AC040160.1	0.357
AC040977.1	0.266
AC061992.1	0.173
AC062017.1	0.305
AC064874.1	0.115
AC068533.7	0.311
AC068620.1	0.293
AC068987.1	0.389
AC069368.3	0.308
AC069547.1	0.155
AC069547.2	0.122
AC073343.1	0.019
AC074091.13	0.290
AC074212.3	0.281
AC074389.6	0.192
AC078925.1	0.146
AC079210.1	0.197
AC079341.1	0.232

AC079354.1	0.402
AC079354.2	0.295
AC079602.1	0.232
AC079612.1	0.080
AC084121.16	0.237
AC087477.1	0.251
AC087645.1	0.094
AC090186.1	0.049
AC090616.2	0.347
AC091150.1	0.253
AC091801.1	0.056
AC092675.3	0.148
AC092687.4	0.272
AC092811.1	0.159
AC092850.1	0.307
AC092964.2	0.201
AC093157.1	0.884
AC093677.1	0.071
AC093802.1	0.064
AC096677.1	0.253
AC097381.1	0.966
AC102948.2	0.194
AC103801.2	0.082
AC104057.1	0.141
AC104472.1	0.243
AC104532.2	0.207
AC104534.3	0.628
AC104667.3	0.051
AC104809.3	0.118
AC105020.1	0.327
AC106876.2	0.172
AC107021.1	0.101

AC109829.1	0.720
AC110619.2	0.126
AC110771.1	0.058
AC110781.3	0.182
AC112693.2	0.228
AC112715.2	0.264
AC112721.1	0.064
AC114494.1	0.177
AC114546.1	0.104
AC114783.1	0.051
AC116407.2	0.314
AC117395.1	0.282
AC117834.1	0.228
AC119673.1	0.313
AC120194.1	0.157
AC124890.1	0.176
AC127496.1	0.220
AC129492.6	0.809
AC131097.4	0.200
AC131971.1	0.249
AC132186.1	0.164
AC132192.1	0.360
AC132216.1	0.465
AC132872.2	0.177
AC135048.1	0.383
AC135178.1	0.260
AC135983.2	0.427
AC136297.1	0.190
AC136604.1	0.262
AC137056.1	0.246
AC137932.1	0.175
AC138517.1	0.174

AC138647.1	0.076
AC138655.1	0.520
AC138969.4	0.185
AC139100.2	0.104
AC139426.2	0.176
AC140481.2	0.459
AC142381.1	0.198
AC145676.2	0.075
AC174470.1	0.165
AC187652.1	0.138
AC226150.4	0.067
ACAA1	0.438
ACAA2	0.973
ACACA	0.998
ACACB	0.473
ACAD10	0.153
ACAD11	0.204
ACAD8	0.095
ACAD9	0.411
ACADL	0.296
ACADM	0.702
ACADS	0.682
ACADSB	0.426
ACADVL	0.555
ACAN	0.293
ACAP1	0.319
ACAP2	0.665
ACAP3	0.334
ACAT1	0.557
ACAT2	0.348
ACBD3	0.607
ACBD4	0.527

ACBD5	0.252
ACBD6	0.412
ACBD7	0.066
ACCS	0.237
ACCSL	0.336
ACD	0.523
ACE	0.394
ACER1	0.417
ACER2	0.055
ACER3	0.566
ACHE	0.888
ACIN1	0.988
ACKR2	0.319
ACKR3	0.270
ACKR4	0.231
ACLY	0.999
ACMSD	0.212
ACN9	0.060
ACO1	0.663
ACO2	0.774
ACOT1	0.296
ACOT11	0.329
ACOT12	0.279
ACOT13	0.435
ACOT2	0.571
ACOT4	0.068
ACOT6	0.337
ACOT7	0.605
ACOT8	0.767
ACOX1	0.522
ACOX2	0.158
ACOX3	0.193

ACOXL	0.640
ACP1	0.205
ACP2	0.724
ACP5	0.292
ACP6	0.309
ACPL2	0.319
ACPP	0.230
ACPT	0.242
ACR	0.208
ACRBP	0.312
ACRV1	0.193
ACSBG1	0.780
ACSBG2	0.232
ACSF2	0.326
ACSF3	0.360
ACSL1	0.084
ACSL3	0.700
ACSL5	0.146
ACSL6	0.224
ACSM1	0.171
ACSM2A	0.331
ACSM2B	0.186
ACSM3	0.261
ACSM4	0.314
ACSM5	0.550
ACSS1	0.239
ACSS2	0.524
ACSS3	0.282
ACTA1	0.905
ACTA2	0.549
ACTB	0.988
ACTBL2	0.098

ACTC1	0.967
ACTG1	0.934
ACTG2	0.644
ACTL10	0.265
ACTL6A	0.662
ACTL6B	0.750
ACTL7A	0.118
ACTL7B	0.160
ACTL8	0.191
ACTL9	0.180
ACTN1	0.847
ACTN2	0.480
ACTN4	0.923
ACTR10	0.075
ACTR1A	0.994
ACTR1B	0.492
ACTR2	0.670
ACTR3	0.873
ACTR3B	0.195
ACTR3C	0.329
ACTR5	0.295
ACTR6	0.350
ACTR8	0.340
ACTRT2	0.136
ACTRT3	0.049
ACVR1	0.984
ACVR1B	0.966
ACVR1C	0.430
ACVR2A	0.872
ACVR2B	0.980
ACVRL1	0.854
ACY1	0.468

ACY3	0.130
ACYP1	0.413
ACYP2	0.076
AD000671.6	0.246
ADA	0.652
ADAD1	0.236
ADAD2	0.051
ADAL	0.375
ADAM10	0.969
ADAM11	0.457
ADAM12	0.476
ADAM15	0.585
ADAM17	0.904
ADAM18	0.083
ADAM19	0.391
ADAM2	0.084
ADAM20	0.117
ADAM21	0.243
ADAM22	0.671
ADAM23	0.702
ADAM28	0.327
ADAM29	0.067
ADAM30	0.972
ADAM32	0.187
ADAM33	0.970
ADAM7	0.149
ADAM8	0.322
ADAM9	0.549
ADAMDEC1	0.226
ADAMTS1	0.604
ADAMTS10	0.543
ADAMTS12	0.556

ADAMTS13	0.383
ADAMTS14	0.487
ADAMTS15	0.563
ADAMTS16	0.928
ADAMTS17	0.955
ADAMTS18	0.048
ADAMTS19	0.278
ADAMTS2	0.195
ADAMTS20	0.409
ADAMTS3	0.387
ADAMTS4	0.430
ADAMTS5	0.427
ADAMTS6	0.920
ADAMTS7	0.513
ADAMTS8	0.530
ADAMTS9	0.311
ADAMTSL1	0.198
ADAMTSL2	0.569
ADAMTSL3	0.706
ADAMTSL4	0.257
ADAMTSL4-AS1	0.313
ADAMTSL5	0.300
ADAP1	0.521
ADAP2	0.241
ADAR	0.906
ADARB1	0.904
ADARB2	0.248
ADAT1	0.877
ADAT2	0.352
ADAT3	0.354
ADC	0.427
ADCK1	0.331

ADCK2	0.498
ADCK3	0.780
ADCK4	0.610
ADCK5	0.446
ADCY1	0.974
ADCY10	0.487
ADCY2	0.301
ADCY3	0.742
ADCY5	0.974
ADCY6	0.775
ADCY7	0.626
ADCY8	0.111
ADCY9	0.703
ADCYAP1	0.377
ADCYAP1R1	0.296
ADD1	0.879
ADD2	0.540
ADD3	0.637
ADGB	0.181
ADH1A	0.073
ADH1B	0.088
ADH4	0.166
ADH5	0.356
ADH6	0.100
ADH7	0.044
ADHFE1	0.284
ADI1	0.129
ADIPOQ	0.136
ADIPOR1	0.936
ADIPOR2	0.896
ADIRF	0.248
ADK	0.719

ADM	0.224
ADM2	0.078
ADM5	0.264
ADNP	0.998
ADNP2	0.384
ADO	0.549
ADORA1	0.572
ADORA2A	0.125
ADORA2B	0.179
ADORA3	0.154
ADPGK	0.560
ADPRH	0.271
ADPRHL1	0.969
ADPRHL2	0.766
ADPRM	0.222
ADRA1A	0.270
ADRA1B	0.392
ADRA1D	0.245
ADRA2A	0.299
ADRA2B	0.379
ADRA2C	0.379
ADRB1	0.522
ADRB2	0.409
ADRB3	0.355
ADRBK1	0.962
ADRBK2	0.577
ADRM1	0.678
ADSL	0.326
ADSS	0.763
ADSSL1	0.650
ADTRP	0.228
AE000662.92	0.098

AEBP1	0.706
AEBP2	0.920
AEN	0.264
AES	0.805
AF131215.5	0.136
AF165138.7	0.198
AFAP1	0.922
AFAP1L1	0.473
AFAP1L2	0.331
AFF1	0.229
AFF3	0.761
AFF4	0.987
AFG3L2	0.616
AFM	0.232
AFMID	0.179
AFP	0.284
AFTPH	0.682
AGA	0.066
AGAP1	0.650
AGAP10	0.304
AGAP2	0.972
AGAP2-AS1	0.839
AGAP3	0.971
AGAP4	0.244
AGAP5	0.293
AGAP6	0.185
AGAP7	0.144
AGAP8	0.205
AGAP9	0.476
AGBL1	0.349
AGBL2	0.068
AGBL3	0.457

AGBL4	0.524
AGBL5	0.745
AGER	0.514
AGFG1	0.528
AGFG2	0.563
AGGF1	0.250
AGK	0.308
AGL	0.220
AGMAT	0.119
AGMO	0.058
AGO1	1.000
AGO2	1.000
AGO3	0.999
AGO4	0.984
AGPAT1	0.970
AGPAT2	0.232
AGPAT3	0.780
AGPAT4	0.361
AGPAT5	0.035
AGPAT6	0.410
AGPAT9	0.139
AGPS	0.950
AGR2	0.057
AGR3	0.035
AGRN	0.297
AGRP	0.240
AGT	0.164
AGTPBP1	0.375
AGTR1	0.124
AGTRAP	0.362
AGXT	0.128
AGXT2	0.170

AHCTF1	0.275
AHCY	0.708
AHCYL1	0.997
AHCYL2	0.951
AHDC1	0.709
AHI1	0.240
AHNAK	0.356
AHNAK2	0.067
AHR	0.110
AHRR	0.146
AHSA1	0.975
AHSA2	0.200
AHSG	0.170
AHSP	0.131
AICDA	0.155
AIDA	0.467
AIF1	0.320
AIF1L	0.202
AIFM2	0.442
AIFM3	0.497
AIG1	0.325
AIM1	0.328
AIM1L	0.323
AIM2	0.222
AIMP1	0.579
AIMP2	0.962
AIP	0.715
AIPL1	0.230
AIRE	0.201
AJAP1	0.302
AJUBA	0.649
AK1	0.312

AK2	0.460
AK3	0.179
AK4	0.628
AK5	0.720
AK7	0.404
AK8	0.320
AK9	0.763
AKAP1	0.687
AKAP10	0.527
AKAP11	0.761
AKAP12	0.503
AKAP13	0.693
AKAP2	0.344
AKAP3	0.250
AKAP5	0.239
AKAP6	0.767
AKAP7	0.103
AKAP8	0.834
AKAP8L	0.732
AKAP9	0.641
AKIP1	0.369
AKIRIN1	0.427
AKIRIN2	0.699
AKNA	0.151
AKNAD1	0.341
AKR1A1	0.309
AKR1B1	0.534
AKR1B10	0.250
AKR1B15	0.251
AKR1C1	0.161
AKR1C2	0.101
AKR1C3	0.034

AKR1C4	0.072
AKR1D1	0.355
AKR1E2	0.208
AKR7A2	0.517
AKR7A3	0.712
AKT1	0.993
AKT1S1	0.699
AKT2	0.992
AKT3	0.999
AKTIP	0.770
AL009178.1	0.149
AL020996.1	0.446
AL021546.6	0.141
AL021920.2	0.252
AL022328.1	0.086
AL031663.2	0.171
AL031666.2	0.260
AL033381.1	0.098
AL035252.1	0.128
AL049747.1	0.226
AL049840.1	0.139
AL050303.1	0.191
AL078585.1	0.104
AL079342.1	0.198
AL109927.1	0.210
AL117190.2	0.125
AL117190.3	0.203
AL118506.1	0.144
AL121761.2	0.252
AL121901.1	0.221
AL132989.1	0.418
AL133373.1	0.350

AL133481.1	0.369
AL136115.1	0.406
AL136218.1	0.122
AL136376.1	0.251
AL136531.1	0.074
AL137798.1	0.332
AL138847.1	0.099
AL139099.1	0.370
AL139147.1	0.337
AL161915.1	0.145
AL162389.1	0.129
AL162407.1	0.132
AL163636.6	0.134
AL353354.2	0.982
AL353583.1	0.189
AL353608.1	0.302
AL353791.1	0.178
AL354718.1	0.697
AL354808.2	0.059
AL354898.1	0.140
AL354993.1	0.170
AL355149.2	0.410
AL355390.1	0.151
AL356356.1	0.404
AL357673.1	0.180
AL358113.1	0.067
AL358333.1	0.194
AL358813.2	0.374
AL359091.2	0.132
AL359195.1	0.297
AL359878.1	0.132
AL360004.1	0.123

AL391421.1	0.114
AL441883.1	0.184
AL445665.1	0.375
AL445989.1	0.124
AL450307.1	0.126
AL513478.1	0.174
AL583828.1	0.203
AL589739.1	0.194
AL589765.1	0.516
AL590452.1	0.295
AL590483.1	0.173
AL590560.1	0.374
AL590708.2	0.606
AL590714.1	0.319
AL590822.1	0.191
AL590822.2	0.218
AL590867.1	0.145
AL591479.1	0.223
AL591684.1	0.569
AL592284.1	0.263
AL603965.1	0.593
AL626787.1	0.240
AL627171.2	0.255
AL627309.1	0.151
AL645728.1	0.469
AL645730.2	0.090
AL807752.1	0.134
AL953854.2	0.296
ALAD	0.802
ALAS1	0.902
ALB	0.248
ALCAM	0.548

ALDH16A1	0.276
ALDH18A1	0.771
ALDH1A1	0.457
ALDH1A2	0.376
ALDH1A3	0.318
ALDH1B1	0.184
ALDH1L1	0.156
ALDH1L2	0.206
ALDH2	0.329
ALDH3A1	0.458
ALDH3A2	0.582
ALDH3B1	0.571
ALDH3B2	0.486
ALDH4A1	0.719
ALDH5A1	0.721
ALDH6A1	0.647
ALDH7A1	0.324
ALDH8A1	0.251
ALDH9A1	0.633
ALDOA	0.312
ALDOB	0.485
ALDOC	0.650
ALG1	0.166
ALG10	0.152
ALG10B	0.132
ALG11	0.241
ALG12	0.955
ALG14	0.218
ALG1L	0.177
ALG2	0.493
ALG3	0.266
ALG5	0.300

ALG6	0.240
ALG8	0.115
ALG9	0.264
ALK	0.831
ALKBH1	0.104
ALKBH2	0.364
ALKBH3	0.346
ALKBH4	0.439
ALKBH5	0.989
ALKBH6	0.217
ALKBH7	0.548
ALKBH8	0.375
ALLC	0.103
ALMS1	0.145
ALOX12	0.319
ALOX12B	0.603
ALOX15	0.360
ALOX15B	0.169
ALOX5	0.245
ALOX5AP	0.097
ALOXE3	0.709
ALPI	0.122
ALPK1	0.215
ALPK2	0.491
ALPK3	0.609
ALPL	0.334
ALPP	0.230
ALPPL2	0.298
ALS2	0.523
ALS2CL	0.250
ALS2CR11	0.386
ALS2CR12	0.109

ALX1	0.154
ALX3	0.571
ALX4	0.636
ALYREF	0.954
AMACR	0.429
AMBN	0.206
AMBP	0.489
AMBRA1	0.961
AMD1	0.903
AMDHD1	0.249
AMDHD2	0.562
AMER2	0.623
AMER3	0.419
AMFR	0.779
AMH	0.071
AMHR2	0.540
AMICA1	0.057
AMIGO1	0.882
AMIGO2	0.414
AMIGO3	0.218
AMMECR1L	0.983
AMN	0.278
AMN1	0.248
AMOTL1	0.776
AMOTL2	0.309
AMPD1	0.398
AMPD2	0.792
AMPD3	0.537
AMPH	0.380
AMT	0.546
AMTN	0.099
AMY1A	0.257

AMY1B	0.207
AMY1C	0.284
AMY2A	0.315
AMY2B	0.189
AMZ1	0.060
AMZ2	0.459
ANAPC1	0.734
ANAPC10	0.418
ANAPC11	0.770
ANAPC13	0.296
ANAPC15	0.742
ANAPC16	0.660
ANAPC2	0.886
ANAPC4	0.410
ANAPC5	0.380
ANAPC7	0.823
ANG	0.187
ANGEL1	0.466
ANGEL2	0.371
ANGPT1	0.351
ANGPT2	0.447
ANGPT4	0.068
ANGPTL1	0.149
ANGPTL2	0.482
ANGPTL3	0.132
ANGPTL4	0.291
ANGPTL5	0.081
ANGPTL6	0.407
ANGPTL7	0.291
ANHX	0.089
ANK1	0.804
ANK2	0.977

ANK3	0.989
ANKAR	0.313
ANKDD1A	0.268
ANKDD1B	0.370
ANKEF1	0.182
ANKFN1	0.369
ANKFY1	0.407
ANKH	0.989
ANKHD1	1.000
ANKHD1-EIF4EBP	0.955
ANKIB1	0.873
ANKK1	0.558
ANKLE1	0.237
ANKLE2	0.313
ANKMY1	0.108
ANKMY2	0.078
ANKRA2	0.320
ANKRD1	0.501
ANKRD10	0.189
ANKRD11	0.234
ANKRD12	0.485
ANKRD13A	0.583
ANKRD13B	0.817
ANKRD13C	0.447
ANKRD13D	0.561
ANKRD16	0.444
ANKRD17	0.891
ANKRD18A	0.164
ANKRD18B	0.307
ANKRD2	0.340
ANKRD20A1	0.237
ANKRD20A2	0.108

ANKRD20A3	0.114
ANKRD20A4	0.139
ANKRD22	0.076
ANKRD23	0.474
ANKRD24	0.245
ANKRD26	0.394
ANKRD27	0.490
ANKRD28	0.914
ANKRD29	0.828
ANKRD30A	0.153
ANKRD30B	0.254
ANKRD31	0.304
ANKRD32	0.260
ANKRD33	0.093
ANKRD33B	0.419
ANKRD34A	0.667
ANKRD34B	0.246
ANKRD34C	0.455
ANKRD35	0.449
ANKRD36	0.335
ANKRD36C	0.397
ANKRD37	0.892
ANKRD39	0.493
ANKRD40	0.910
ANKRD42	0.193
ANKRD44	0.298
ANKRD45	0.090
ANKRD46	0.472
ANKRD49	0.400
ANKRD50	0.312
ANKRD52	0.999
ANKRD53	0.108

ANKRD54	0.596
ANKRD55	0.187
ANKRD6	0.089
ANKRD60	0.241
ANKRD61	0.962
ANKRD62	0.995
ANKRD63	0.807
ANKRD65	0.216
ANKRD66	0.164
ANKRD7	0.088
ANKRD9	0.157
ANKS1A	0.938
ANKS1B	0.857
ANKS3	0.254
ANKS4B	0.344
ANKS6	0.271
ANKUB1	0.275
ANKZF1	0.627
ANLN	0.461
ANO1	0.943
ANO10	0.282
ANO2	0.332
ANO3	0.157
ANO4	0.319
ANO5	0.057
ANO6	0.351
ANO7	0.180
ANO8	0.728
ANO9	0.141
ANP32A	0.916
ANP32B	0.863
ANP32D	0.147

ANP32E	0.937
ANPEP	0.398
ANTXR1	0.877
ANTXR2	0.387
ANTXRL	0.317
ANXA1	0.061
ANXA10	0.246
ANXA11	0.271
ANXA13	0.504
ANXA2	0.246
ANXA2R	0.154
ANXA3	0.176
ANXA4	0.426
ANXA5	0.236
ANXA6	0.446
ANXA7	0.550
ANXA8	0.580
ANXA8L1	0.425
ANXA8L2	0.349
ANXA9	0.402
AOAH	0.112
AOC1	0.548
AOC2	0.453
AOC3	0.423
AOX1	0.430
AP000275.65	0.183
AP000295.9	0.246
AP000322.53	0.136
AP000322.54	0.182
AP000349.1	0.060
AP000350.10	0.128
AP000350.4	0.111

AP000679.2	0.454
AP000695.1	0.237
AP000708.1	0.370
AP000769.1	0.241
AP000783.1	0.994
AP000867.1	0.572
AP000889.3	0.146
AP000974.1	0.145
AP001055.1	0.154
AP001468.1	0.488
AP001631.10	0.149
AP001816.1	0.381
AP001885.1	0.337
AP001888.1	0.627
AP002884.2	0.168
AP002884.3	0.267
AP003062.1	0.414
AP003068.23	0.297
AP003068.6	0.341
AP003733.1	0.350
AP003774.4	0.215
AP005482.1	0.458
AP006621.5	0.114
AP1AR	0.238
AP1B1	0.995
AP1G1	0.987
AP1G2	0.453
AP1M1	0.991
AP1M2	0.447
AP1S1	0.787
AP1S3	0.155
AP2A1	0.997

AP2A2	0.704
AP2B1	0.999
AP2M1	0.994
AP2S1	0.734
AP3B1	0.450
AP3B2	0.704
AP3D1	0.943
AP3M1	0.663
AP3M2	0.416
AP3S1	0.087
AP3S2	0.363
AP4B1	0.685
AP4E1	0.266
AP4M1	0.473
AP4S1	0.095
AP5B1	0.272
AP5M1	0.432
AP5S1	0.925
AP5Z1	0.217
APAF1	0.513
APBA1	0.704
APBA2	0.385
APBA3	0.977
APBB1	0.669
APBB1IP	0.251
APBB2	0.881
APBB3	0.714
APC	0.361
APC2	0.898
APCDD1	0.260
APCDD1L	0.261
APCS	0.085

APEH	0.803
APEX1	0.556
APH1A	0.988
APH1B	0.078
API5	0.921
APIP	0.403
APITD1	0.256
APITD1-CORT	0.102
APLF	0.383
APLNR	0.621
APLP1	0.520
APLP2	0.515
APMAP	0.137
APOA1	0.335
APOA1BP	0.667
APOA2	0.241
APOA4	0.260
APOA5	0.266
APOB	0.402
APOBEC1	0.930
APOBEC2	0.760
APOBEC3A	0.132
APOBEC3B	0.491
APOBEC3C	0.328
APOBEC3D	0.365
APOBEC3F	0.333
APOBEC3G	0.626
APOBEC3H	0.116
APOBEC4	0.295
APOBR	0.233
APOC1	0.503
APOC2	0.171

APOC3	0.217
APOC4	0.183
APOC4-APOC2	0.198
APOD	0.173
APOE	0.655
APOF	0.342
APOH	0.114
APOL1	0.099
APOL2	0.140
APOL3	0.305
APOL4	0.318
APOL5	0.205
APOL6	0.224
APOLD1	0.194
APOM	0.623
APOPT1	0.468
APP	0.669
APPBP2	0.886
APPL1	0.750
APPL2	0.534
APRT	0.100
APTX	0.288
AQP1	0.933
AQP10	0.504
AQP11	0.103
AQP12A	0.222
AQP12B	0.215
AQP2	0.919
AQP3	0.642
AQP4	0.138
AQP5	0.939
AQP6	0.203

AQP7	0.898
AQP8	0.535
AQP9	0.188
AQPEP	0.112
AQR	0.914
ARAP1	0.496
ARAP2	0.277
ARAP3	0.634
ARC	0.689
ARCN1	0.847
AREG	0.282
AREL1	0.737
ARF1	0.967
ARF3	0.985
ARF4	0.950
ARF5	0.984
ARF6	0.841
ARFGAP1	0.437
ARFGAP2	0.870
ARFGAP3	0.392
ARFGEF1	0.876
ARFGEF2	0.993
ARFIP1	0.514
ARFIP2	0.964
ARFRP1	0.245
ARG1	0.148
ARG2	0.115
ARGFX	0.071
ARGLU1	0.560
ARHGAP1	0.912
ARHGAP10	0.564
ARHGAP11A	0.284

ARHGAP12	0.603
ARHGAP15	0.141
ARHGAP17	0.690
ARHGAP18	0.206
ARHGAP19	0.278
ARHGAP19-SLIT1	0.217
ARHGAP20	0.498
ARHGAP21	0.860
ARHGAP22	0.586
ARHGAP23	0.803
ARHGAP24	0.174
ARHGAP25	0.494
ARHGAP26	0.763
ARHGAP27	0.533
ARHGAP28	0.062
ARHGAP29	0.708
ARHGAP30	0.379
ARHGAP31	0.838
ARHGAP32	0.748
ARHGAP33	0.529
ARHGAP35	0.992
ARHGAP39	0.328
ARHGAP40	0.263
ARHGAP42	0.854
ARHGAP44	0.610
ARHGAP5	0.931
ARHGAP8	0.072
ARHGAP9	0.326
ARHGDIA	0.558
ARHGDIB	0.170
ARHGDIG	0.999
ARHGEF1	0.919

ARHGEF10	0.130
ARHGEF10L	0.723
ARHGEF11	0.978
ARHGEF12	1.000
ARHGEF15	0.311
ARHGEF16	0.768
ARHGEF17	0.514
ARHGEF18	0.451
ARHGEF19	0.361
ARHGEF2	0.987
ARHGEF25	0.804
ARHGEF26	0.268
ARHGEF28	0.819
ARHGEF3	0.696
ARHGEF33	0.240
ARHGEF35	0.190
ARHGEF37	0.526
ARHGEF38	0.325
ARHGEF39	0.321
ARHGEF4	0.664
ARHGEF40	0.683
ARHGEF5	0.465
ARHGEF7	0.946
ARID1A	1.000
ARID1B	0.982
ARID2	0.981
ARID3A	0.378
ARID3B	0.557
ARID3C	0.512
ARID4A	0.863
ARID4B	0.825
ARID5A	0.671

ARID5B	0.979
ARIH1	0.996
ARIH2	0.997
ARIH2OS	0.277
ARL1	0.752
ARL10	0.470
ARL11	0.054
ARL13B	0.127
ARL14	0.232
ARL14EP	0.450
ARL14EPL	0.135
ARL15	0.338
ARL16	0.643
ARL17A	0.470
ARL17B	0.425
ARL2	0.828
ARL2BP	0.446
ARL3	0.584
ARL4A	0.509
ARL4C	0.463
ARL4D	0.144
ARL5A	0.496
ARL5B	0.266
ARL6	0.064
ARL6IP1	0.163
ARL6IP4	0.440
ARL6IP5	0.152
ARL6IP6	0.124
ARL8A	0.969
ARL8B	0.981
ARL9	0.184
ARMC1	0.722

ARMC10	0.348
ARMC12	0.188
ARMC2	0.550
ARMC3	0.183
ARMC4	0.231
ARMC5	0.805
ARMC6	0.721
ARMC7	0.105
ARMC8	0.770
ARMC9	0.306
ARMS2	0.192
ARNT	0.969
ARNT2	0.687
ARNTL	0.991
ARNTL2	0.425
ARPC1A	0.931
ARPC1B	0.493
ARPC2	0.871
ARPC3	0.185
ARPC4	0.891
ARPC4-TTLL3	0.223
ARPC5	0.293
ARPC5L	0.561
ARPP19	0.615
ARPP21	0.098
ARRB1	0.422
ARRB2	0.640
ARRDC1	0.311
ARRDC2	0.568
ARRDC3	0.841
ARRDC4	0.962
ARRDC5	0.091

ARSA	0.359
ARSB	0.495
ARSG	0.598
ARSI	0.363
ARSJ	0.229
ARSK	0.102
ART1	0.094
ART3	0.206
ART4	0.261
ART5	0.915
ARTN	0.347
ARV1	0.169
ARVCF	0.388
AS3MT	0.292
ASAH1	0.090
ASAH2	0.440
ASAH2B	0.255
ASAH2C	0.346
ASAP1	0.647
ASAP2	0.879
ASAP3	0.790
ASB1	0.480
ASB10	0.246
ASB13	0.148
ASB14	0.307
ASB15	0.924
ASB16	0.446
ASB17	0.132
ASB18	0.929
ASB2	0.439
ASB4	0.605
ASB5	0.097

ASB6	0.660
ASB7	0.596
ASB8	0.591
ASCC1	0.434
ASCC2	0.490
ASCC3	0.586
ASCL1	0.882
ASCL2	0.320
ASCL3	0.228
ASCL4	0.132
ASCL5	0.332
ASF1A	0.951
ASF1B	0.153
ASGR1	0.151
ASGR2	0.235
ASH1L	0.975
ASH2L	0.998
ASIC1	0.923
ASIC2	0.814
ASIC3	0.152
ASIC4	0.552
ASIC5	0.116
ASIP	0.412
ASL	0.244
ASNA1	0.924
ASNS	0.401
ASNSD1	0.365
ASPA	0.320
ASPDH	0.574
ASPG	0.374
ASPH	0.304
ASPHD1	0.608

ASPHD2	0.542
ASPM	0.114
ASPN	0.210
ASPRV1	0.397
ASPSCR1	0.701
ASRGL1	0.337
ASS1	0.871
ASTE1	0.545
ASTL	0.275
ASTN1	0.701
ASTN2	0.672
ASUN	0.824
ASXL1	0.687
ASXL2	0.881
ASXL3	0.541
ASZ1	0.200
ATAD1	0.387
ATAD2	0.904
ATAD2B	0.977
ATAD3A	0.759
ATAD3B	0.599
ATAD3C	0.373
ATAD5	0.694
ATAT1	0.638
ATCAY	0.316
ATE1	0.210
ATF1	0.853
ATF2	0.980
ATF3	0.585
ATF4	0.234
ATF5	0.253
ATF6	0.278

ATF6B	0.811
ATF7	0.891
ATF7IP	0.728
ATF7IP2	0.093
ATG10	0.159
ATG12	0.206
ATG13	0.897
ATG14	0.531
ATG16L1	0.667
ATG16L2	0.170
ATG2A	0.505
ATG2B	0.252
ATG3	0.287
ATG4B	0.975
ATG4C	0.121
ATG4D	0.748
ATG5	0.491
ATG7	0.624
ATG9A	0.987
ATG9B	0.398
ATHL1	0.197
ATIC	0.464
ATL1	0.905
ATL2	0.387
ATL3	0.591
ATM	0.415
ATMIN	0.418
ATN1	0.782
ATOH1	0.279
ATOH7	0.243
ATOH8	0.644
ATOX1	0.254

ATP10A	0.688
ATP10B	0.466
ATP10D	0.425
ATP11A	0.886
ATP11B	0.301
ATP12A	0.157
ATP13A1	0.994
ATP13A2	0.275
ATP13A3	0.591
ATP13A4	0.160
ATP13A5	0.183
ATP1A1	1.000
ATP1A2	0.887
ATP1A3	0.997
ATP1A4	0.378
ATP1B1	0.481
ATP1B2	0.245
ATP1B3	0.378
ATP2A1	0.368
ATP2A2	1.000
ATP2A3	0.322
ATP2B1	0.995
ATP2B2	0.995
ATP2B4	0.437
ATP2C1	0.851
ATP2C2	0.037
ATP4A	0.673
ATP4B	0.926
ATP5A1	0.976
ATP5B	0.966
ATP5C1	0.415
ATP5D	0.257

ATP5E	0.263
ATP5EP2	0.185
ATP5G1	0.776
ATP5G2	0.395
ATP5G3	0.254
ATP5H	0.195
ATP5I	0.253
ATP5J	0.296
ATP5J2	0.275
ATP5J2-PTCD1	0.348
ATP5L	0.346
ATP5L2	0.134
ATP5O	0.385
ATP5S	0.267
ATP5SL	0.539
ATP6AP1L	0.121
ATP6C	0.212
ATP6V0A1	0.979
ATP6V0A2	0.442
ATP6V0A4	0.301
ATP6V0B	0.621
ATP6V0C	0.483
ATP6V0D1	0.956
ATP6V0D2	0.177
ATP6V0E1	0.295
ATP6V0E2	0.174
ATP6V1A	0.964
ATP6V1B1	0.270
ATP6V1B2	0.669
ATP6V1C1	0.301
ATP6V1C2	0.965
ATP6V1D	0.408

ATP6V1E1	0.982
ATP6V1E2	0.075
ATP6V1F	0.227
ATP6V1G1	0.180
ATP6V1G2	0.230
ATP6V1G2-DDX39	0.300
ATP6V1G3	0.188
ATP6V1H	0.502
ATP7B	0.680
ATP8A1	0.548
ATP8A2	0.714
ATP8B1	0.991
ATP8B2	0.709
ATP8B3	0.375
ATP8B4	0.223
ATP9A	0.599
ATP9B	0.116
ATPAF1	0.687
ATPAF2	0.585
ATPIF1	0.345
ATR	0.714
ATRAID	0.367
ATRIP	0.702
ATRN	0.814
ATRNL1	0.620
ATXN1	0.935
ATXN10	0.358
ATXN1L	0.996
ATXN2	0.963
ATXN2L	0.980
ATXN3	0.476
ATXN7	0.858

ATXN7L1	0.999
ATXN7L2	0.885
ATXN7L3	0.996
ATXN7L3B	0.596
AUH	0.433
AUNIP	0.123
AUP1	1.000
AURKA	0.713
AURKAIP1	0.686
AURKB	0.270
AURKC	0.149
AUTS2	0.967
AVEN	0.115
AVIL	0.646
AVL9	0.406
AVP	0.975
AVPI1	0.173
AVPR1A	0.111
AVPR1B	0.136
AXDND1	0.122
AXIN1	0.995
AXIN2	0.472
AXL	0.910
AZGP1	0.343
AZI1	0.168
AZI2	0.351
AZIN1	0.863
AZU1	0.184
B3GALNT1	0.148
B3GALNT2	0.080
B3GALT1	0.149
B3GALT2	0.462

B3GALT4	0.440
B3GALT5	0.297
B3GALT6	0.321
B3GALTL	0.129
B3GAT1	0.124
B3GAT2	0.376
B3GAT3	0.900
B3GNT1	0.952
B3GNT2	0.311
B3GNT3	0.207
B3GNT4	0.113
B3GNT5	0.452
B3GNT6	0.256
B3GNT7	0.673
B3GNT8	0.214
B3GNT9	0.327
B3GNTL1	0.057
B4GALNT1	0.408
B4GALNT2	0.519
B4GALNT3	0.239
B4GALNT4	0.073
B4GALT1	0.122
B4GALT2	0.787
B4GALT3	0.643
B4GALT4	0.408
B4GALT5	0.489
B4GALT6	0.721
B4GALT7	0.427
B9D1	0.340
B9D2	0.104
BAALC	0.269
BAAT	0.309

BABAM1	0.716
BACE1	0.931
BACE2	0.855
BACH1	0.776
BACH2	0.994
BAD	0.674
BAG1	0.089
BAG2	0.175
BAG3	0.377
BAG4	0.389
BAG5	0.681
BAG6	0.999
BAHD1	0.906
BAI1	0.490
BAI2	0.759
BAI3	0.867
BAIAP2	0.476
BAIAP2L1	0.993
BAIAP2L2	0.252
BAIAP3	0.073
BAK1	0.865
BAMBI	0.497
BANF1	0.806
BANF2	0.130
BANK1	0.121
BANP	0.771
BAP1	0.984
BARD1	0.332
BARHL1	0.895
BARHL2	0.996
BARX1	0.944
BARX2	0.255

BASP1	0.128
BATF	0.319
BATF2	0.336
BATF3	0.316
BAX	0.749
BAZ1A	0.655
BAZ1B	0.998
BAZ2A	0.999
BAZ2B	0.791
BBC3	0.216
BBIP1	0.302
BBOX1	0.268
BBS1	0.501
BBS10	0.324
BBS12	0.255
BBS2	0.571
BBS4	0.268
BBS5	0.280
BBS7	0.226
BBS9	0.296
BBX	0.603
BCAM	0.508
BCAN	0.620
BCAP29	0.263
BCAR1	0.359
BCAR3	0.917
BCAS1	0.275
BCAS2	0.475
BCAS3	0.844
BCAS4	0.276
BCAT1	0.130
BCAT2	0.440

BCCIP	0.367
BCDIN3D	0.315
BCHE	0.039
BCKDHA	0.834
BCKDHB	0.426
BCKDK	0.676
BCL10	0.583
BCL11A	0.953
BCL11B	0.932
BCL2	0.569
BCL2A1	0.148
BCL2L1	0.459
BCL2L10	0.149
BCL2L11	0.648
BCL2L12	0.641
BCL2L13	0.950
BCL2L14	0.202
BCL2L15	0.303
BCL2L2	0.974
BCL2L2-PABPN1	0.866
BCL3	0.858
BCL6	0.670
BCL6B	0.522
BCL7A	0.561
BCL7B	0.666
BCL7C	0.636
BCL9	0.835
BCL9L	0.883
BCLAF1	0.169
BCMO1	0.043
BCO2	0.509
BCR	0.720

BCS1L	0.764
BDH1	0.198
BDH2	0.347
BDKRB1	0.081
BDKRB2	0.228
BDNF	0.915
BDP1	0.304
BEAN1	0.386
BECN1	0.998
BEGAIN	0.727
BEND3	0.915
BEND4	0.330
BEND5	0.437
BEND6	0.452
BEND7	0.300
BEST1	0.359
BEST2	0.617
BEST3	0.648
BEST4	0.593
BET1	0.135
BET1L	0.124
BFAR	0.265
BFSP1	0.176
BGLAP	0.440
BHLHA15	0.981
BHLHA9	0.229
BHLHE22	0.266
BHLHE23	0.553
BHLHE40	0.871
BHLHE41	0.523
BHMT	0.081
BHMT2	0.067

BICC1	0.834
BICD1	0.973
BICD2	0.974
BID	0.954
BIK	0.217
BIN1	0.188
BIN2	0.200
BIN3	0.378
BIRC2	0.653
BIRC3	0.466
BIRC5	0.111
BIRC6	0.186
BIRC7	0.208
BIRC8	0.033
BIVM	0.330
BIVM-ERCC5	0.164
BLACE	0.150
BLCAP	0.575
BLID	0.098
BLK	0.117
BLM	0.635
BLMH	0.764
BLNK	0.290
BLOC1S1	0.195
BLOC1S2	0.568
BLOC1S3	0.275
BLOC1S4	0.186
BLOC1S5	0.205
BLOC1S5-TXNDC	0.357
BLOC1S6	0.329
BLVRA	0.567
BLVRB	0.216

BLZF1	0.340
BMF	0.137
BMI1	0.981
BMP1	0.743
BMP10	0.634
BMP2	0.846
BMP2K	0.655
BMP3	0.311
BMP4	0.950
BMP5	0.366
BMP6	0.630
BMP7	0.839
BMP8A	0.488
BMP8B	0.437
BMPER	0.212
BMPR1A	0.970
BMPR1B	0.447
BMPR2	0.971
BMS1	0.591
BNC1	0.747
BNC2	0.655
BNIP1	0.248
BNIP2	0.270
BNIP3	0.431
BNIP3L	0.302
BNIPL	0.334
BOC	0.382
BOD1	0.941
BOD1L1	0.240
BOD1L2	0.124
BOK	0.245
BOLA1	0.279

BOLA2	0.411
BOLA2B	0.292
BOLA3	0.420
BOLL	0.268
BOP1	0.376
BORA	0.259
BPGM	0.721
BPHL	0.461
BPI	0.150
BPIFA1	0.394
BPIFA2	0.294
BPIFA3	0.176
BPIFB1	0.247
BPIFB2	0.363
BPIFB3	0.284
BPIFB4	0.212
BPIFB6	0.293
BPIFC	0.504
BPNT1	0.222
BPTF	0.998
BRAF	1.000
BRAP	0.672
BRAT1	0.313
BRCA1	0.620
BRCA2	0.416
BRD1	0.974
BRD2	0.711
BRD3	0.971
BRD4	0.996
BRD7	0.663
BRD8	0.886
BRD9	0.396

BRDT	0.073
BRE	0.509
BRF1	0.507
BRF2	0.318
BRI3	0.158
BRI3BP	0.215
BRICD5	0.080
BRINP1	0.271
BRINP2	0.673
BRINP3	0.080
BRIP1	0.372
BRIX1	0.133
BRK1	0.660
BRMS1	0.720
BRMS1L	0.595
BROX	0.381
BRPF1	0.998
BRPF3	0.999
BRSK1	0.954
BRSK2	0.749
BRWD1	0.888
BSCL2	0.223
BSDC1	0.690
BSG	0.139
BSN	0.916
BSND	0.163
BSPH1	0.248
BSPRY	0.374
BST1	0.063
BST2	0.196
BSX	0.345
BTAF1	0.698

BTBD1	0.611
BTBD10	0.515
BTBD11	0.883
BTBD16	0.352
BTBD17	0.542
BTBD18	0.655
BTBD19	0.226
BTBD2	0.881
BTBD3	0.248
BTBD6	0.707
BTBD7	0.448
BTBD8	0.020
BTBD9	0.727
BTC	0.146
BTD	0.449
BTF3	0.608
BTF3L4	0.649
BTG1	0.361
BTG2	0.548
BTG3	0.478
BTG4	0.271
BTLA	0.068
BTN1A1	0.189
BTN2A1	0.293
BTN2A2	0.334
BTN3A1	0.424
BTN3A2	0.267
BTN3A3	0.128
BTNL2	0.405
BTNL3	0.234
BTNL8	0.221
BTNL9	0.158

BTRC	0.363
BUB1	0.402
BUB1B	0.339
BUB3	0.607
BUD13	0.255
BUD31	0.757
BVES	0.056
BX088651.1	0.171
BX255923.1	0.144
BX649567.1	0.323
BX842679.1	0.431
BYSL	0.596
BZRAP1	0.438
BZW1	0.980
BZW2	0.119
C10orf10	0.222
C10orf105	0.067
C10orf107	0.079
C10orf11	0.183
C10orf111	0.086
C10orf112	0.157
C10orf113	0.173
C10orf115	0.230
C10orf118	0.360
C10orf12	0.564
C10orf120	0.157
C10orf126	0.295
C10orf128	0.079
C10orf129	0.291
C10orf131	0.276
C10orf137	0.283
C10orf2	0.797

C10orf25	0.224
C10orf32	0.302
C10orf32-ASMT	0.261
C10orf35	0.154
C10orf40	0.213
C10orf53	0.162
C10orf54	0.317
C10orf55	0.252
C10orf62	0.224
C10orf67	0.212
C10orf68	0.204
C10orf71	0.452
C10orf76	0.621
C10orf82	0.335
C10orf85	0.151
C10orf88	0.317
C10orf90	0.154
C10orf91	0.157
C10orf95	0.193
C10orf99	0.078
C11orf1	0.180
C11orf16	0.120
C11orf21	0.111
C11orf24	0.400
C11orf30	0.867
C11orf31	0.211
C11orf34	0.130
C11orf35	0.436
C11orf42	0.279
C11orf44	0.111
C11orf45	0.947
C11orf48	0.250

C11orf49	0.481
C11orf52	0.258
C11orf53	0.300
C11orf54	0.181
C11orf57	0.383
C11orf58	0.679
C11orf63	0.112
C11orf65	0.120
C11orf68	0.793
C11orf70	0.054
C11orf71	0.194
C11orf72	0.100
C11orf73	0.616
C11orf74	0.168
C11orf80	0.336
C11orf82	0.093
C11orf83	0.277
C11orf84	0.712
C11orf85	0.249
C11orf86	0.258
C11orf87	0.272
C11orf88	0.127
C11orf89	0.248
C11orf91	0.259
C11orf94	0.090
C11orf96	0.312
C12orf10	0.660
C12orf23	0.228
C12orf29	0.228
C12orf36	0.136
C12orf39	0.183
C12orf4	0.343

C12orf40	0.946
C12orf42	0.186
C12orf43	0.197
C12orf44	0.788
C12orf45	0.158
C12orf49	0.217
C12orf5	0.518
C12orf50	0.155
C12orf52	0.479
C12orf54	0.139
C12orf55	0.282
C12orf56	0.138
C12orf57	0.295
C12orf60	0.107
C12orf61	0.180
C12orf65	0.195
C12orf66	0.165
C12orf68	0.402
C12orf71	0.266
C12orf73	0.218
C12orf74	0.133
C12orf75	0.168
C12orf76	0.317
C12orf77	0.077
C12orf79	0.199
C13orf35	0.081
C13orf45	0.083
C14orf1	0.685
C14orf105	0.350
C14orf119	0.616
C14orf132	0.256
C14orf142	0.433

C14orf144	0.070
C14orf159	0.400
C14orf164	0.384
C14orf166	0.403
C14orf166B	0.204
C14orf177	0.137
C14orf178	0.131
C14orf180	0.433
C14orf183	0.059
C14orf2	0.229
C14orf23	0.270
C14orf28	0.137
C14orf37	0.165
C14orf39	0.169
C14orf64	0.223
C14orf79	0.146
C14orf80	0.273
C14orf93	0.554
C15orf26	0.127
C15orf27	0.331
C15orf32	0.164
C15orf37	0.332
C15ORF37	0.211
C15orf38	0.385
C15orf38-AP3S2	0.251
C15orf39	0.236
C15orf40	0.297
C15orf41	0.099
C15orf43	0.232
C15orf48	0.077
C15orf52	0.092
C15orf53	0.039

C15orf54	0.113
C15orf56	0.310
C15orf57	0.545
C15orf59	0.660
C15orf60	0.238
C15orf61	0.212
C15orf62	0.497
C15orf65	0.179
C16orf11	0.262
C16orf13	0.207
C16orf3	0.074
C16orf45	0.267
C16orf46	0.069
C16orf47	0.242
C16orf52	0.264
C16orf54	0.252
C16orf58	0.412
C16orf59	0.077
C16orf62	0.427
C16orf70	0.894
C16orf71	0.149
C16orf72	0.149
C16orf74	0.160
C16orf78	0.170
C16orf80	0.368
C16orf86	0.399
C16orf87	0.203
C16orf89	0.035
C16orf90	0.930
C16orf91	0.111
C16orf92	0.201
C16orf93	0.593

C16orf95	0.189
C16orf96	0.369
C16orf97	0.157
C16orf98	0.499
C17orf100	0.486
C17orf102	0.311
C17orf103	0.296
C17orf104	0.480
C17orf105	0.221
C17orf107	0.794
C17orf112	0.176
C17orf47	0.247
C17orf49	0.581
C17orf50	0.170
C17orf51	0.283
C17orf53	0.376
C17orf58	0.212
C17orf59	0.452
C17orf61-PLSCR3	0.313
C17orf62	0.090
C17orf64	0.095
C17orf66	0.085
C17orf67	0.162
C17orf70	0.449
C17orf72	0.401
C17orf74	0.119
C17orf75	0.620
C17orf77	0.079
C17orf78	0.133
C17orf80	0.277
C17orf82	0.181
C17orf85	0.738

C17orf89	0.425
C17orf96	0.671
C17orf97	0.248
C17orf98	0.213
C17orf99	0.420
C18orf21	0.312
C18orf25	0.762
C18orf32	0.328
C18orf42	0.133
C18orf54	0.044
C18orf56	0.047
C18orf63	0.270
C18orf8	0.589
C19orf10	0.658
C19orf12	0.902
C19orf18	0.019
C19orf24	0.248
C19orf25	0.378
C19orf26	0.337
C19orf33	0.312
C19orf35	0.116
C19orf38	0.073
C19orf40	0.153
C19orf43	0.821
C19orf44	0.418
C19orf45	0.401
C19orf47	0.801
C19orf48	0.248
C19orf52	0.679
C19orf53	0.297
C19orf54	0.329
C19orf55	0.376

C19orf57	0.403
C19orf59	0.159
C19orf60	0.292
C19orf66	0.694
C19orf67	0.352
C19orf68	0.923
C19orf69	0.261
C19orf70	0.480
C19orf71	0.211
C19orf73	0.350
C19orf77	0.217
C19orf80	0.375
C19orf81	0.377
C19orf82	0.127
C1D	0.186
C1GALT1	0.225
C1orf100	0.135
C1orf101	0.219
C1orf105	0.184
C1orf106	0.140
C1orf109	0.415
C1orf110	0.217
C1orf111	0.094
C1orf112	0.207
C1orf115	0.168
C1orf116	0.178
C1orf122	0.430
C1orf123	0.471
C1orf127	0.160
C1orf131	0.168
C1orf134	0.337
C1orf141	0.290

C1orf143	0.283
C1orf145	0.175
C1orf146	0.844
C1orf147	0.151
C1orf158	0.084
C1orf159	0.315
C1orf162	0.101
C1orf167	0.112
C1orf168	0.390
C1orf170	0.492
C1orf172	0.539
C1orf173	0.049
C1orf174	0.500
C1orf177	0.092
C1orf180	0.118
C1orf185	0.174
C1orf186	0.323
C1orf189	0.126
C1orf192	0.071
C1orf194	0.134
C1orf195	0.063
C1orf198	0.199
C1orf200	0.200
C1orf204	0.526
C1orf21	0.412
C1orf210	0.218
C1orf213	0.247
C1orf216	0.203
C1orf222	0.129
C1orf226	0.376
C1orf227	0.214
C1orf228	0.440

C1orf229	0.168
C1orf233	0.713
C1orf234	0.153
C1orf27	0.156
C1orf35	0.550
C1orf43	0.560
C1orf50	0.262
C1orf51	0.690
C1orf52	0.219
C1orf53	0.094
C1orf54	0.210
C1orf56	0.498
C1orf61	0.348
C1orf63	0.057
C1orf64	0.207
C1orf65	0.147
C1orf68	0.789
C1orf74	0.221
C1orf85	0.569
C1orf86	0.288
C1orf87	0.144
C1orf94	0.340
C1orf95	0.240
C1QA	0.295
C1QB	0.272
C1QBP	0.731
C1QC	0.306
C1QL1	0.286
C1QL2	0.432
C1QL3	0.486
C1QL4	0.520
C1QTNF1	0.989

C1QTNF1-AS1	0.992
C1QTNF2	0.191
C1QTNF3	0.150
C1QTNF4	0.324
C1QTNF5	0.747
C1QTNF6	0.170
C1QTNF7	0.156
C1QTNF8	0.098
C1QTNF9	0.382
C1QTNF9B	0.242
C1R	0.702
C1RL	0.493
C1S	0.521
C2	0.689
C20orf112	0.724
C20orf141	0.090
C20orf144	0.191
C20orf166	0.132
C20orf173	0.235
C20orf187	0.103
C20orf194	0.268
C20orf195	0.195
C20orf196	0.227
C20orf197	0.130
C20orf201	0.326
C20orf202	0.063
C20orf24	0.225
C20orf26	0.419
C20orf27	0.927
C20orf62	0.261
C20orf78	0.306
C20orf85	0.107

C20orf96	0.071
C21orf119	0.579
C21orf128	0.069
C21orf2	0.204
C21orf33	0.293
C21orf37	0.173
C21orf49	0.154
C21orf54	0.151
C21orf58	0.163
C21orf59	0.192
C21orf62	0.325
C21orf67	0.168
C21orf88	0.163
C21orf90	0.061
C21orf91	0.202
C22orf15	0.092
C22orf23	0.339
C22orf24	0.106
C22orf26	0.178
C22orf29	0.334
C22orf31	0.360
C22orf34	0.132
C22orf39	0.313
C22orf42	0.151
C22orf43	0.199
C22orf46	0.366
C2CD2	0.689
C2CD2L	0.965
C2CD3	0.448
C2CD4A	0.958
C2CD4B	0.265
C2CD4C	0.329

C2CD4D	0.564
C2CD5	0.463
C2orf15	0.161
C2ORF15	0.108
C2orf16	0.117
C2orf27A	0.230
C2orf27B	0.190
C2orf40	0.119
C2orf42	0.318
C2orf43	0.306
C2orf44	0.162
C2orf47	0.311
C2orf48	0.242
C2orf49	0.247
C2orf50	0.108
C2orf53	0.218
C2orf54	0.132
C2orf57	0.113
C2orf61	0.133
C2orf62	0.186
C2orf66	0.293
C2orf68	0.390
C2orf69	0.401
C2orf70	0.179
C2orf71	0.051
C2orf73	0.059
C2orf74	0.149
C2orf76	0.106
C2orf78	0.550
C2orf80	0.067
C2orf81	0.785
C2orf82	0.496

C2orf83	0.126
C2orf88	0.117
C2orf91	0.115
C3	0.730
C3AR1	0.055
C3orf14	0.090
C3orf17	0.330
C3orf18	0.150
C3orf20	0.402
C3orf22	0.142
C3orf27	0.192
C3orf30	0.175
C3orf33	0.102
C3orf35	0.155
C3orf36	0.083
C3orf38	0.211
C3orf49	0.211
C3orf52	0.501
C3orf55	0.132
C3orf56	0.180
C3orf58	0.350
C3orf62	0.211
C3orf65	0.080
C3orf67	0.357
C3orf70	0.222
C3orf72	0.117
C3orf80	0.510
C3orf83	0.109
C3orf84	0.239
C4A	0.496
C4B	0.849
C4BPA	0.381

C4BPB	0.160
C4orf17	0.225
C4orf19	0.159
C4orf21	0.109
C4orf22	0.110
C4orf26	0.164
C4orf27	0.195
C4orf29	0.299
C4orf3	0.059
C4orf32	0.167
C4orf33	0.099
C4orf36	0.234
C4orf40	0.041
C4orf45	0.167
C4orf46	0.131
C4orf47	0.936
C4orf48	0.319
C4orf50	0.163
C4orf51	0.158
C4orf6	0.033
C5	0.331
C5AR1	0.339
C5AR2	0.159
C5orf15	0.424
C5orf20	0.138
C5orf22	0.275
C5orf24	0.528
C5orf27	0.086
C5orf28	0.107
C5orf30	0.311
C5orf34	0.183
C5orf38	0.998

C5orf42	0.073
C5orf45	0.965
C5orf46	0.201
C5orf47	0.177
C5orf48	0.182
C5orf49	0.039
C5orf50	0.121
C5orf51	0.243
C5orf52	0.139
C5orf54	0.245
C5orf55	0.187
C5orf56	0.092
C5orf58	0.155
C5orf63	0.123
C5orf64	0.203
C6	0.178
C6orf1	0.421
C6orf10	0.215
C6orf100	0.112
C6orf106	0.877
C6orf118	0.018
C6orf120	0.959
C6orf123	0.098
C6orf132	0.416
C6orf136	0.889
C6orf141	0.601
C6orf15	0.297
C6orf163	0.158
C6orf164	0.150
C6orf165	0.410
C6ORF165	0.209
C6orf195	0.062

C6orf201	0.308
C6orf203	0.249
C6orf211	0.141
C6orf222	0.301
C6orf223	0.256
C6orf226	0.179
C6orf25	0.228
C6orf47	0.867
C6orf48	0.111
C6ORF50	0.142
C6orf52	0.222
C6orf57	0.152
C6orf58	0.177
C6orf62	0.590
C6orf89	0.247
C6orf99	0.193
C7	0.487
C7orf10	0.571
C7orf13	0.142
C7orf25	0.289
C7orf26	0.470
C7orf31	0.131
C7orf33	0.312
C7orf34	0.189
C7orf41	0.206
C7orf43	0.648
C7orf49	0.283
C7orf50	0.349
C7orf55	0.488
C7orf55-LUC7L2	0.736
C7orf57	0.096
C7orf60	0.475

C7orf61	0.284
C7orf62	0.016
C7orf63	0.109
C7orf65	0.043
C7orf66	0.079
C7orf69	0.136
C7orf71	0.354
C7orf72	0.315
C7orf73	0.138
C7orf76	0.106
C8A	0.232
C8B	0.131
C8G	0.095
C8orf12	0.106
C8orf17	0.073
C8orf22	0.083
C8orf31	0.248
C8orf33	0.935
C8orf34	0.141
C8orf37	0.079
C8orf4	0.091
C8orf44	0.267
C8orf46	0.209
C8orf47	0.194
C8orf48	0.068
C8orf49	0.279
C8orf56	0.140
C8orf58	0.246
C8orf59	0.224
C8orf74	0.149
C8orf76	0.362
C8orf82	0.281

C8orf86	0.156
C8orf87	0.195
C9	0.164
C9orf114	0.787
C9orf116	0.194
C9orf117	0.305
C9orf129	0.334
C9orf131	0.358
C9orf135	0.221
C9orf139	0.112
C9orf141	0.074
C9orf142	0.580
C9orf147	0.294
C9orf152	0.116
C9orf153	0.166
C9orf156	0.353
C9orf16	0.559
C9orf163	0.967
C9orf169	0.990
C9orf170	0.138
C9orf171	0.235
C9orf172	0.787
C9orf173	0.962
C9orf24	0.169
C9orf3	0.509
C9orf37	0.255
C9orf38	0.064
C9orf40	0.165
C9orf41	0.345
C9orf43	0.236
C9orf47	0.211
C9orf50	0.364

C9orf53	0.144
C9orf57	0.162
C9orf62	0.076
C9orf64	0.151
C9orf66	0.034
C9orf69	0.387
C9orf72	0.150
C9orf78	0.436
C9orf84	0.289
C9orf85	0.181
C9orf89	0.401
C9orf9	0.390
C9orf91	0.121
C9orf92	0.175
C9orf96	0.319
CA1	0.168
CA10	0.557
CA11	0.479
CA12	0.103
CA13	0.538
CA14	0.509
CA2	0.939
CA3	0.142
CA4	0.139
CA5A	0.237
CA6	0.329
CA7	0.695
CA8	0.369
CA9	0.853
CAAP1	0.290
CAB39	0.956
CAB39L	0.332

CABIN1	0.993
CABLES1	0.678
CABLES2	0.172
CABP1	0.972
CABP4	0.284
CABP5	0.174
CABP7	0.387
CABS1	0.128
CABYR	0.101
CACFD1	0.336
CACHD1	0.507
CACNA1A	1.000
CACNA1B	1.000
CACNA1C	1.000
CACNA1D	0.978
CACNA1E	1.000
CACNA1G	0.990
CACNA1H	0.263
CACNA1I	0.895
CACNA1S	0.703
CACNA2D1	0.385
CACNA2D2	0.954
CACNA2D3	0.644
CACNB1	0.722
CACNB2	0.439
CACNB3	0.653
CACNB4	0.858
CACNG1	0.180
CACNG2	0.876
CACNG3	0.865
CACNG4	0.363
CACNG5	0.220

CACNG6	0.225
CACNG7	0.458
CACNG8	0.626
CACTIN	0.977
CACUL1	0.462
CACYBP	0.730
CAD	0.999
CADM1	0.919
CADM2	0.355
CADM3	0.882
CADM4	0.969
CADPS	0.944
CADPS2	0.704
CAGE1	0.134
CALB1	0.190
CALB2	0.999
CALCA	0.885
CALCB	0.153
CALCOCO1	0.584
CALCOCO2	0.588
CALCR	0.665
CALCRL	0.446
CALD1	0.890
CALHM1	0.098
CALHM2	0.225
CALHM3	0.191
CALM1	0.901
CALM2	0.674
CALM3	0.978
CALML3	0.188
CALML4	0.189
CALML5	0.223

CALML6	0.322
CALN1	0.043
CALR	0.405
CALR3	0.132
CALU	0.558
CALY	0.532
CAMK1	0.625
CAMK1D	0.981
CAMK1G	0.422
CAMK2A	0.998
CAMK2B	0.992
CAMK2D	0.888
CAMK2G	0.997
CAMK2N1	0.383
CAMK2N2	0.675
CAMK4	0.626
CAMKK1	0.532
CAMKK2	0.308
CAMKMT	0.900
CAMKV	0.888
CAMLG	0.259
CAMP	0.136
CAMSAP1	0.871
CAMSAP2	0.430
CAMSAP3	0.882
CAMTA1	0.999
CAMTA2	0.877
CAND1	0.925
CAND2	0.481
CANT1	0.511
CANX	0.999
CAP1	0.641

CAP2	0.262
CAPG	0.284
CAPN1	0.942
CAPN10	0.283
CAPN11	0.288
CAPN12	0.373
CAPN13	0.418
CAPN14	0.444
CAPN15	0.658
CAPN2	0.611
CAPN3	0.836
CAPN5	0.529
CAPN7	0.399
CAPN8	0.392
CAPN9	0.246
CAPNS1	0.396
CAPNS2	0.350
CAPRIN1	0.829
CAPRIN2	0.385
CAPS	0.209
CAPS2	0.078
CAPSL	0.093
CAPZA1	0.800
CAPZA2	0.462
CAPZA3	0.088
CAPZB	0.577
CARD10	0.585
CARD11	0.156
CARD14	0.957
CARD16	0.040
CARD17	0.103
CARD18	0.070

CARD6	0.116
CARD8	0.377
CARD9	0.335
CARF	0.345
CARHSP1	0.053
CARKD	0.952
CARM1	0.999
CARNS1	0.664
CARS	0.634
CARS2	0.251
CARTPT	0.520
CASC1	0.147
CASC10	0.234
CASC3	0.919
CASC4	0.328
CASC5	0.295
CASD1	0.431
CASKIN1	0.636
CASKIN2	0.586
CASP1	0.106
CASP10	0.441
CASP12	0.062
CASP14	0.746
CASP16	0.141
CASP2	0.705
CASP3	0.650
CASP4	0.146
CASP5	0.062
CASP6	0.688
CASP7	0.716
CASP8	0.629
CASP9	0.557

CASQ1	0.369
CASQ2	0.407
CASR	0.871
CASS4	0.448
CAST	0.127
CASZ1	0.899
CAT	0.471
CATSPER1	0.452
CATSPER2	0.478
CATSPER3	0.216
CATSPER4	0.191
CATSPERB	0.376
CATSPERD	0.110
CATSPERG	0.274
CAV1	0.607
CAV2	0.561
CAV3	0.978
CBFA2T2	0.842
CBFA2T3	0.122
CBFB	0.871
CBL	0.918
CBLB	0.718
CBLC	0.366
CBLL1	0.896
CBLN1	0.861
CBLN2	0.584
CBLN3	0.256
CBLN4	0.341
CBR1	0.234
CBR3	0.122
CBR4	0.294
CBS	0.406

CBWD1	0.208
CBWD2	0.358
CBWD3	0.293
CBWD5	0.155
CBWD6	0.196
CBX1	1.000
CBX2	0.725
CBX3	0.832
CBX4	0.990
CBX5	0.999
CBX6	0.843
CBX7	0.656
CBX8	0.918
CBY1	0.400
CBY3	0.989
CC2D1A	0.701
CC2D1B	0.342
CC2D2A	0.286
CC2D2B	0.275
CCAR1	0.992
CCAR2	0.934
CCBE1	0.209
CCBL1	0.560
CCBL2	0.296
CCDC101	0.598
CCDC102A	0.294
CCDC102B	0.040
CCDC103	0.236
CCDC104	0.214
CCDC105	0.292
CCDC106	0.388
CCDC107	0.463

CCDC108	0.210
CCDC109B	0.171
CCDC11	0.129
CCDC110	0.079
CCDC112	0.356
CCDC113	0.214
CCDC114	0.239
CCDC115	0.277
CCDC116	0.257
CCDC117	0.464
CCDC12	0.261
CCDC121	0.566
CCDC122	0.224
CCDC124	0.367
CCDC125	0.207
CCDC126	0.116
CCDC127	0.383
CCDC129	0.229
CCDC13	0.207
CCDC130	0.328
CCDC132	0.405
CCDC134	0.401
CCDC135	0.359
CCDC136	0.337
CCDC137	0.337
CCDC138	0.173
CCDC14	0.376
CCDC140	0.373
CCDC141	0.344
CCDC142	0.457
CCDC144A	0.393
CCDC144NL	0.118

CCDC146	0.524
CCDC147	0.299
CCDC148	0.211
CCDC149	0.445
CCDC15	0.179
CCDC150	0.043
CCDC151	0.598
CCDC152	0.098
CCDC153	0.505
CCDC154	0.100
CCDC155	0.420
CCDC157	0.329
CCDC158	0.328
CCDC159	0.337
CCDC166	0.396
CCDC167	0.137
CCDC168	0.250
CCDC169	0.134
CCDC169-SOHLH2	0.407
CCDC170	0.109
CCDC171	0.073
CCDC172	0.243
CCDC173	0.220
CCDC174	0.321
CCDC175	0.228
CCDC176	0.469
CCDC177	0.498
CCDC178	0.114
CCDC179	0.064
CCDC18	0.263
CCDC180	0.452
CCDC181	0.093

CCDC19	0.307
CCDC23	0.194
CCDC24	0.326
CCDC25	0.234
CCDC27	0.191
CCDC28A	0.185
CCDC28B	0.361
CCDC3	0.279
CCDC30	0.235
CCDC33	0.274
CCDC34	0.168
CCDC36	0.280
CCDC37	0.170
CCDC38	0.229
CCDC39	0.127
CCDC40	0.197
CCDC41	0.227
CCDC42	0.256
CCDC42B	0.284
CCDC43	0.439
CCDC47	0.681
CCDC50	0.195
CCDC51	0.223
CCDC53	0.165
CCDC54	0.044
CCDC57	0.301
CCDC58	0.225
CCDC59	0.222
CCDC6	0.944
CCDC60	0.214
CCDC61	0.418
CCDC62	0.305

CCDC63	0.198
CCDC64	0.581
CCDC64B	0.132
CCDC65	0.138
CCDC66	0.326
CCDC67	0.328
CCDC68	0.137
CCDC69	0.207
CCDC7	0.133
CCDC70	0.084
CCDC71	0.610
CCDC71L	0.261
CCDC73	0.297
CCDC74A	0.190
CCDC74B	0.135
CCDC77	0.032
CCDC78	0.081
CCDC79	0.518
CCDC8	0.292
CCDC80	0.175
CCDC81	0.170
CCDC82	0.174
CCDC83	0.090
CCDC84	0.381
CCDC85A	0.150
CCDC85B	0.453
CCDC85C	0.457
CCDC86	0.653
CCDC87	0.238
CCDC88A	0.823
CCDC88B	0.171
CCDC88C	0.266

CCDC89	0.924
CCDC9	0.595
CCDC90B	0.141
CCDC91	0.153
CCDC92	0.624
CCDC93	0.276
CCDC94	0.604
CCDC96	0.617
CCDC97	0.380
CCER1	0.251
CCHCR1	0.291
CCIN	0.216
CCK	0.229
CCKAR	0.236
CCKBR	0.459
CCL1	0.070
CCL11	0.036
CCL13	0.032
CCL14	0.132
CCL15	0.131
CCL15-CCL14	0.171
CCL16	0.124
CCL17	0.320
CCL18	0.069
CCL19	0.223
CCL2	0.129
CCL20	0.043
CCL21	0.269
CCL22	0.150
CCL23	0.128
CCL24	0.271
CCL25	0.347

CCL26	0.160
CCL27	0.511
CCL28	0.290
CCL3	0.284
CCL3L1	0.462
CCL3L3	0.351
CCL4	0.163
CCL4L1	0.511
CCL4L2	0.469
CCL5	0.034
CCL7	0.043
CCL8	0.045
CCM2	0.767
CCM2L	0.143
CCNA1	0.301
CCNA2	0.199
CCNB1	0.215
CCNB1IP1	0.088
CCNB2	0.044
CCNC	0.786
CCND1	0.974
CCND2	1.000
CCND3	0.881
CCNDBP1	0.709
CCNE1	0.781
CCNE2	0.342
CCNF	0.361
CCNG1	0.304
CCNG2	0.178
CCNH	0.428
CCNI	0.464
CCNI2	0.350

CCNJ	0.919
CCNJL	0.096
CCNK	0.997
CCNL1	0.555
CCNL2	0.374
CCNO	0.729
CCNT1	0.775
CCNT2	0.936
CCNY	0.554
CCNYL1	0.683
CCP110	0.311
CCPG1	0.150
CCR1	0.228
CCR10	0.558
CCR2	0.168
CCR3	0.138
CCR4	0.127
CCR5	0.092
CCR6	0.978
CCR7	0.279
CCR8	0.156
CCR9	0.337
CCRL2	0.187
CCRN4L	0.195
CCS	0.625
CCSAP	0.543
CCSER1	0.196
CCSER2	0.537
CCT2	0.793
CCT3	0.960
CCT4	0.426
CCT5	0.536

CCT6A	0.760
CCT6B	0.193
CCT7	0.971
CCT8	0.705
CCT8L2	0.222
CCZ1	0.191
CCZ1B	0.193
CD101	0.329
CD109	0.081
CD14	0.460
CD151	0.167
CD160	0.279
CD163	0.335
CD163L1	0.353
CD164	0.257
CD164L2	0.238
CD180	0.130
CD19	0.338
CD1A	0.250
CD1B	0.224
CD1C	0.131
CD1D	0.212
CD1E	0.299
CD2	0.299
CD200	0.206
CD200R1	0.085
CD200R1L	0.122
CD207	0.149
CD209	0.601
CD22	0.474
CD226	0.216
CD244	0.152

CD247	0.144
CD248	0.352
CD27	0.143
CD274	0.134
CD276	0.364
CD28	0.177
CD2AP	0.439
CD2BP2	0.470
CD300A	0.080
CD300C	0.054
CD300E	0.027
CD300LB	0.065
CD300LD	0.055
CD300LF	0.041
CD300LG	0.332
CD302	0.108
CD320	0.514
CD33	0.368
CD34	0.258
CD36	0.114
CD37	0.248
CD38	0.157
CD3D	0.145
CD3E	0.116
CD3EAP	0.532
CD3G	0.206
CD4	0.623
CD40	0.659
CD44	0.142
CD46	0.374
CD47	0.574
CD48	0.129

CD5	0.298
CD52	0.152
CD53	0.138
CD55	0.186
CD58	0.600
CD59	0.350
CD5L	0.185
CD6	0.205
CD63	0.442
CD68	0.232
CD69	0.139
CD7	0.075
CD70	0.286
CD72	0.490
CD74	0.294
CD79A	0.193
CD79B	0.267
CD80	0.253
CD81	0.328
CD82	0.225
CD83	0.184
CD84	0.255
CD86	0.273
CD8A	0.045
CD8B	0.193
CD9	0.181
CD93	0.191
CD96	0.178
CD97	0.334
CDA	0.119
CDADC1	0.273
CDAN1	0.340

CDC123	0.202
CDC14A	0.489
CDC14B	0.890
CDC16	0.198
CDC20	0.267
CDC20B	0.363
CDC23	0.978
CDC25A	0.753
CDC25B	0.996
CDC25C	0.176
CDC26	0.625
CDC27	0.984
CDC34	0.976
CDC37	0.542
CDC37L1	0.337
CDC40	0.937
CDC42	1.000
CDC42BPA	0.999
CDC42BPB	0.994
CDC42BPG	0.318
CDC42EP1	0.268
CDC42EP2	0.332
CDC42EP3	0.195
CDC42EP4	0.292
CDC42EP5	0.193
CDC42SE1	0.239
CDC42SE2	0.275
CDC45	0.248
CDC5L	0.988
CDC6	0.298
CDC7	0.163
CDC73	0.949

CDCA2	0.085
CDCA3	0.755
CDCA4	0.140
CDCA5	0.310
CDCA7	0.199
CDCA7L	0.310
CDCA8	0.219
CDCP1	0.348
CDCP2	0.192
CDH1	0.276
CDH10	0.154
CDH11	0.388
CDH12	0.195
CDH13	0.245
CDH15	0.083
CDH16	0.205
CDH17	0.390
CDH18	0.062
CDH19	0.023
CDH2	0.751
CDH20	0.440
CDH22	0.805
CDH23	0.364
CDH24	0.420
CDH26	0.204
CDH3	0.056
CDH4	0.374
CDH5	0.697
CDH6	0.685
CDH7	0.419
CDH8	0.320
CDH9	0.146

CDHR1	0.319
CDHR2	0.302
CDHR3	0.385
CDHR4	0.243
CDHR5	0.371
CDIP1	0.584
CDIPT	0.499
CDK1	0.609
CDK10	0.329
CDK11A	0.597
CDK11B	0.800
CDK12	0.992
CDK13	0.550
CDK14	0.386
CDK15	0.402
CDK17	0.936
CDK18	0.530
CDK19	0.925
CDK2	0.934
CDK20	0.177
CDK2AP1	0.186
CDK2AP2	0.824
CDK3	0.542
CDK4	0.896
CDK5	0.911
CDK5R1	0.359
CDK5R2	0.890
CDK5RAP1	0.282
CDK5RAP2	0.232
CDK5RAP3	0.537
CDK6	0.993
CDK7	0.509

CDK8	0.905
CDK9	0.914
CDKAL1	0.548
CDKL1	0.239
CDKL2	0.180
CDKL3	0.427
CDKL4	0.055
CDKN1A	0.626
CDKN1B	0.355
CDKN1C	0.671
CDKN2A	0.136
CDKN2AIP	0.200
CDKN2AIPNL	0.342
CDKN2B	0.140
CDKN2C	0.569
CDKN2D	0.434
CDKN3	0.206
CDNF	0.292
CDO1	0.286
CDON	0.337
CDPF1	0.230
CDR2	0.285
CDR2L	0.251
CDRT1	0.371
CDRT15	0.225
CDRT15L2	0.189
CDRT4	0.095
CDS1	0.201
CDS2	0.278
CDSN	0.849
CDT1	0.098
CDV3	0.572

CDX1	0.348
CDX2	0.995
CDYL	0.844
CDYL2	0.624
CEACAM1	0.224
CEACAM16	0.235
CEACAM18	0.960
CEACAM19	0.908
CEACAM21	0.050
CEACAM3	0.169
CEACAM4	0.055
CEACAM5	0.232
CEACAM6	0.132
CEACAM7	0.136
CEACAM8	0.167
CEBPA	0.494
CEBPB	0.447
CEBPD	0.309
CEBPE	0.363
CEBPG	0.742
CEBPZ	0.258
CECR1	0.977
CECR2	0.984
CECR5	0.947
CECR6	0.996
CEL	0.735
CELA1	0.332
CELA2A	0.817
CELA2B	0.749
CELA3A	0.497
CELA3B	0.524
CELF1	0.991

CELF2	0.910
CELF3	0.966
CELF4	0.939
CELF5	0.989
CELF6	0.500
CELSR1	0.705
CELSR2	0.353
CELSR3	0.930
CEMP1	0.095
CEND1	0.123
CENPA	0.438
CENPB	1.000
CENPBD1	0.260
CENPC	0.251
CENPE	0.513
CENPF	0.101
CENPH	0.102
CENPJ	0.234
CENPK	0.038
CENPL	0.208
CENPM	0.191
CENPN	0.089
CENPO	0.197
CENPP	0.197
CENPQ	0.082
CENPT	0.431
CENPV	0.551
CENPW	0.099
CEP104	0.636
CEP112	0.331
CEP120	0.350
CEP128	0.285

CEP135	0.385
CEP152	0.258
CEP164	0.500
CEP170	0.458
CEP170B	0.998
CEP19	0.145
CEP192	0.409
CEP250	0.793
CEP290	0.125
CEP350	0.927
CEP41	0.306
CEP44	0.354
CEP55	0.118
CEP57	0.456
CEP57L1	0.456
CEP63	0.308
CEP68	0.258
CEP70	0.276
CEP72	0.210
CEP76	0.192
CEP78	0.123
CEP85	0.811
CEP85L	0.147
CEP89	0.233
CEP95	0.399
CEP97	0.262
CEPT1	0.683
CER1	0.066
CERCAM	0.221
CERK	0.542
CERKL	0.077
CERS1	0.428

CERS2	0.645
CERS3	0.092
CERS4	0.286
CERS5	0.240
CERS6	0.418
CES1	0.313
CES2	0.500
CES3	0.379
CES5A	0.628
CETN1	0.043
CETN3	0.130
CETP	0.452
CFB	0.855
CFC1	0.306
CFC1B	0.397
CFD	0.135
CFDP1	0.211
CFH	0.374
CFHR1	0.096
CFHR2	0.118
CFHR3	0.156
CFHR4	0.072
CFHR5	0.102
CFI	0.445
CFL1	0.902
CFL2	0.192
CFLAR	0.631
CFTR	0.337
CGA	0.182
CGB	0.219
CGB1	0.230
CGB2	0.185

CGB5	0.212
CGB7	0.389
CGGBP1	0.523
CGN	0.617
CGNL1	0.161
CGREF1	0.146
CGRRF1	0.267
CH17-132F21.1	0.204
CH25H	0.413
CHAC1	0.457
CHAC2	0.117
CHAD	0.267
CHADL	0.825
CHAF1A	0.685
CHAF1B	0.422
CHAMP1	0.360
CHAT	0.488
CHCHD1	0.556
CHCHD10	0.192
CHCHD2	0.256
CHCHD3	0.204
CHCHD4	0.682
CHCHD5	0.283
CHCHD6	0.234
CHCHD7	0.420
CHD1	0.937
CHD1L	0.243
CHD2	0.986
CHD3	0.999
CHD4	1.000
CHD5	0.986
CHD6	0.992

CHD7	0.995
CHD8	1.000
CHD9	0.989
CHDH	0.298
CHEK1	0.533
CHEK2	0.715
CHERP	0.998
CHFR	0.323
CHGA	0.816
CHGB	0.651
CHI3L1	0.161
CHI3L2	0.221
CHIA	0.222
CHIC2	0.305
CHID1	0.292
CHIT1	0.191
CHKA	0.759
CHKB	0.132
CHKB-CPT1B	0.119
CHL1	0.090
CHML	0.060
CHMP1A	0.186
CHMP1B	0.261
CHMP2A	0.278
CHMP2B	0.220
CHMP3	0.987
CHMP4A	0.604
CHMP4B	0.842
CHMP4C	0.964
CHMP5	0.572
CHMP6	0.475
CHMP7	0.784

CHN1	0.718
CHN2	0.193
CHODL	0.139
CHORDC1	0.101
CHP1	0.902
CHP2	0.688
CHPF	0.671
CHPF2	0.373
CHPT1	0.113
CHRAC1	0.120
CHRD	0.619
CHRDL2	0.247
CHRFAM7A	0.282
CHRM1	0.644
CHRM2	0.417
CHRM3	0.330
CHRM4	0.791
CHRM5	0.179
CHRNA1	0.228
CHRNA10	0.171
CHRNA2	0.113
CHRNA3	0.410
CHRNA4	0.246
CHRNA5	0.113
CHRNA6	0.223
CHRNA7	0.580
CHRNA9	0.320
CHRNB1	0.514
CHRNB2	0.574
CHRNB3	0.262
CHRNB4	0.336
CHRNE	0.767

CHRNG	0.218
CHST1	0.900
CHST10	0.107
CHST11	0.627
CHST12	0.097
CHST13	0.225
CHST14	0.641
CHST15	0.365
CHST2	0.463
CHST3	0.477
CHST4	0.226
CHST5	0.284
CHST6	0.233
CHST8	0.727
CHST9	0.090
CHSY1	0.208
CHSY3	0.422
CHTF18	0.068
CHTF8	0.563
CHTOP	0.690
CHUK	0.981
CHURC1	0.474
CHURC1-FNTB	0.363
CIAO1	0.916
CIAPIN1	0.670
CIB1	0.196
CIB2	0.194
CIB3	0.246
CIB4	0.199
CIC	0.993
CIDEA	0.150
CIDEB	0.658

CIDEC	0.289
CIITA	0.434
CILP	0.436
CILP2	0.265
CINP	0.383
CIR1	0.478
CIRBP	0.496
CIRH1A	0.501
CISD1	0.149
CISD2	0.554
CISD3	0.677
CISH	0.598
CIT	0.831
CITED2	0.323
CITED4	0.345
CIZ1	0.448
CKAP2	0.257
CKAP2L	0.119
CKAP4	0.439
CKAP5	0.999
CKB	0.706
CKLF	0.127
CKLF-CMTM1	0.108
CKM	0.427
CKMT1A	0.449
CKMT1B	0.301
CKMT2	0.864
CKS1B	0.591
CKS2	0.188
CLASP1	0.961
CLASP2	0.790
CLASRP	0.935

CLC	0.174
CLCA1	0.265
CLCA2	0.348
CLCA4	0.181
CLCC1	0.131
CLCF1	0.866
CLCN1	0.863
CLCN2	0.276
CLCN3	0.865
CLCN6	0.587
CLCN7	0.864
CLCNKA	0.289
CLCNKB	0.395
CLDN1	0.093
CLDN10	0.210
CLDN11	0.841
CLDN12	0.053
CLDN14	0.262
CLDN15	0.122
CLDN16	0.054
CLDN17	0.079
CLDN18	0.127
CLDN19	0.270
CLDN20	0.116
CLDN22	0.074
CLDN23	0.252
CLDN25	0.079
CLDN3	0.483
CLDN4	0.244
CLDN5	0.596
CLDN6	0.188
CLDN7	0.338

CLDN8	0.103
CLDN9	0.114
CLDND1	0.249
CLDND2	0.989
CLEC10A	0.213
CLEC11A	0.365
CLEC12A	0.063
CLEC12B	0.142
CLEC14A	0.345
CLEC16A	0.660
CLEC17A	0.149
CLEC18A	0.506
CLEC18B	0.371
CLEC18C	0.354
CLEC1A	0.145
CLEC1B	0.058
CLEC2A	0.341
CLEC2B	0.128
CLEC2D	0.184
CLEC2L	0.130
CLEC3A	0.026
CLEC3B	0.570
CLEC4A	0.125
CLEC4C	0.958
CLEC4D	0.123
CLEC4E	0.027
CLEC4F	0.066
CLEC4G	0.509
CLEC4M	0.274
CLEC5A	0.140
CLEC6A	0.140
CLEC7A	0.052

CLEC9A	0.331
CLECL1	0.101
CLGN	0.174
CLHC1	0.076
CLIC1	0.399
CLIC3	0.213
CLIC4	0.559
CLIC5	0.654
CLIC6	0.553
CLINT1	0.863
CLIP1	0.913
CLIP2	0.732
CLIP3	0.578
CLIP4	0.342
CLK1	0.506
CLK2	1.000
CLK3	0.979
CLK4	0.303
CLLU1OS	0.102
CLMN	0.389
CLMP	0.404
CLN3	0.315
CLN5	0.252
CLN6	0.343
CLN8	0.067
CLNK	0.068
CLNS1A	0.239
CLOCK	0.656
CLP1	0.671
CLPB	0.599
CLPP	0.980
CLPS	0.309

CLPSL1	0.265
CLPSL2	0.186
CLPTM1	0.999
CLPTM1L	0.306
CLPX	0.974
CLRN1	0.350
CLRN2	0.029
CLRN3	0.153
CLSPN	0.295
CLSTN1	0.586
CLSTN2	0.266
CLSTN3	0.632
CLTA	0.626
CLTB	0.438
CLTC	1.000
CLTCL1	0.345
CLU	0.301
CLUAP1	0.908
CLUH	0.856
CLUL1	0.065
CLVS1	0.343
CLVS2	0.210
CLYBL	0.471
CMA1	0.047
CMAS	0.708
CMBL	0.945
CMC1	0.218
CMC2	0.081
CMIP	0.796
CMKLR1	0.345
CMPK1	0.842
CMPK2	0.231

CMSS1	0.272
CMTM1	0.074
CMTM2	0.179
CMTM3	0.344
CMTM4	0.562
CMTM5	0.363
CMTM6	0.269
CMTM7	0.174
CMTM8	0.182
CMTR1	0.992
CMTR2	0.923
CMYA5	0.783
CNBD1	0.224
CNBD2	0.535
CNBP	0.889
CNDP1	0.375
CNDP2	0.228
CNEP1R1	0.344
CNFN	0.418
CNGA1	0.953
CNGA3	0.432
CNGA4	0.299
CNGB1	0.479
CNGB3	0.268
CNIH1	0.466
CNIH2	0.744
CNIH3	0.349
CNIH4	0.276
CNKSR1	0.517
CNKSR3	0.523
CNN1	0.517
CNN2	0.090

CNN3	0.711
CNNM1	0.526
CNNM2	0.999
CNNM3	0.621
CNNM4	0.722
CNOT1	0.999
CNOT10	0.601
CNOT11	0.573
CNOT2	0.984
CNOT3	0.847
CNOT4	0.950
CNOT6	0.524
CNOT6L	0.998
CNOT7	0.856
CNOT8	0.734
CNP	0.978
CNPPD1	0.493
CNPY1	0.250
CNPY2	0.472
CNPY3	0.528
CNPY4	0.506
CNR1	0.231
CNR2	0.310
CNRIP1	0.146
CNST	0.265
CNTD1	0.309
CNTD2	0.342
CNTF	0.595
CNTFR	0.899
CNTLN	0.077
CNTN1	0.393
CNTN2	0.851

CNTN3	0.128
CNTN4	0.178
CNTN5	0.923
CNTN6	0.054
CNTNAP1	0.661
CNTNAP2	0.382
CNTNAP3	0.186
CNTNAP3B	0.095
CNTNAP5	0.198
CNTRL	0.164
CNTROB	0.457
COA1	0.176
COA3	0.752
COA4	0.187
COA5	0.287
COA6	0.495
COASY	0.585
COBL	0.518
COBLL1	0.308
COCH	0.381
COG1	0.421
COG2	0.442
COG3	0.807
COG4	0.559
COG5	0.273
COG6	0.223
COG7	0.374
COG8	0.718
COIL	0.392
COL10A1	0.191
COL11A1	0.425
COL11A2	0.320

COL12A1	0.729
COL13A1	0.349
COL14A1	0.218
COL15A1	0.768
COL16A1	0.403
COL17A1	0.342
COL18A1	0.540
COL19A1	0.294
COL1A1	0.999
COL1A2	0.403
COL20A1	0.123
COL21A1	0.167
COL22A1	0.343
COL23A1	0.226
COL24A1	0.105
COL25A1	0.357
COL27A1	0.426
COL28A1	0.180
COL2A1	1.000
COL3A1	0.512
COL4A1	0.976
COL4A2	0.996
COL4A3	0.639
COL4A3BP	0.616
COL4A4	0.667
COL5A1	0.645
COL5A2	0.376
COL5A3	0.186
COL6A1	0.475
COL6A2	0.232
COL6A3	0.366
COL6A5	0.427

COL6A6	0.374
COL7A1	0.763
COL8A1	0.665
COL8A2	0.648
COL9A1	0.349
COL9A2	0.208
COL9A3	0.499
COLCA1	0.270
COLCA2	0.501
COLEC10	0.210
COLEC11	0.339
COLEC12	0.871
COLGALT1	0.536
COLGALT2	0.357
COLQ	0.264
COMMD1	0.335
COMMD10	0.174
COMMD2	0.189
COMMD3	0.322
COMMD3-BMI1	0.610
COMMD4	0.453
COMMD5	0.211
COMMD6	0.237
COMMD7	0.229
COMMD8	0.098
COMMD9	0.253
COMP	0.358
COMT	0.478
COMTD1	0.259
COPA	0.997
COPB1	0.712
COPB2	0.700

COPE	0.816
COPG1	0.944
COPG2	0.285
COPRS	0.264
COPS2	0.996
COPS3	0.950
COPS4	0.963
COPS5	0.998
COPS6	0.996
COPS7A	0.975
COPS7B	0.696
COPS8	0.189
COPZ1	0.449
COPZ2	0.286
COQ10A	0.603
COQ10B	0.317
COQ2	0.195
COQ3	0.135
COQ4	0.606
COQ5	0.235
COQ6	0.540
COQ7	0.220
COQ9	0.477
CORIN	0.216
CORO1A	0.735
CORO1B	0.648
CORO1C	0.557
CORO2A	0.281
CORO2B	0.528
CORO6	0.457
CORO7	0.105
CORO7-PAM16	0.071

CORT	0.202
COTL1	0.094
COX10	0.287
COX11	0.277
COX14	0.438
COX15	0.335
COX16	0.118
COX17	0.473
COX18	0.258
COX19	0.078
COX20	0.381
COX4I1	0.404
COX4I2	0.072
COX5A	0.574
COX5B	0.405
COX6A1	0.275
COX6A1P2	0.130
COX6A2	0.642
COX6B1	0.337
COX6B2	0.262
COX6C	0.261
COX7A1	0.249
COX7A2	0.129
COX7A2L	0.076
COX7B2	0.081
COX7C	0.160
COX8A	0.231
COX8C	0.075
CP	0.549
CPA1	0.602
CPA2	0.895
CPA3	0.164

CPA4	0.739
CPA5	0.336
CPA6	0.199
CPAMD8	0.242
CPB1	0.811
CPB2	0.250
CPD	0.608
CPE	0.661
CPEB1	1.000
CPEB2	0.238
CPEB3	0.906
CPEB4	0.981
CPED1	0.183
CPLX1	0.104
CPLX2	0.461
CPLX3	0.387
CPLX4	0.179
CPM	0.392
CPN1	0.418
CPN2	0.348
CPNE1	0.508
CPNE2	0.387
CPNE3	0.104
CPNE4	0.491
CPNE5	0.298
CPNE6	0.600
CPNE7	0.034
CPNE8	0.932
CPNE9	0.452
CPO	0.209
CPOX	0.658
CPPED1	0.065

CPQ	0.323
CPS1	0.342
CPSF1	0.503
CPSF2	0.659
CPSF3	0.794
CPSF3L	0.711
CPSF4	0.987
CPSF4L	0.174
CPSF6	0.974
CPSF7	0.993
CPT1A	0.661
CPT1B	0.197
CPT1C	0.394
CPT2	0.737
CPVL	0.212
CPXM1	0.113
CPXM2	0.640
CPZ	0.035
CR1	0.097
CR2	0.194
CR769776.1	0.208
CRABP1	0.277
CRABP2	0.654
CRADD	0.619
CRAMP1L	0.492
CRAT	0.746
CRB1	0.292
CRB2	0.184
CRB3	0.380
CRBN	0.194
CRCP	0.495
CRCT1	0.629

CREB1	0.994
CREB3	0.688
CREB3L1	0.782
CREB3L2	0.296
CREB3L3	0.319
CREB3L4	0.169
CREB5	0.699
CREBBP	1.000
CREBL2	0.836
CREBRF	0.885
CREBZF	0.968
CREG1	0.294
CREG2	0.113
CRELD1	0.788
CRELD2	0.617
CREM	0.113
CRH	0.861
CRHBP	0.166
CRHR1	0.238
CRHR2	0.334
CRIM1	0.583
CRIP1	0.227
CRIP2	0.304
CRIP3	0.160
CRIPAK	0.942
CRIPT	0.171
CRISP1	0.073
CRISP2	0.028
CRISP3	0.052
CRISPLD1	0.179
CRISPLD2	0.088
CRK	0.997

CRKL	0.944
CRLF1	0.798
CRLF3	0.286
CRLS1	0.359
CRMP1	0.749
CRNKL1	0.480
CRNN	0.150
CROCC	0.327
CROT	0.283
CRP	0.244
CRTAC1	0.447
CRTAM	0.103
CRTAP	0.264
CRTC1	0.625
CRTC2	0.924
CRTC3	0.433
CRX	0.375
CRY1	0.616
CRY2	0.716
CRYAA	0.296
CRYAB	0.586
CRYBA2	0.387
CRYBA4	0.227
CRYBB1	0.319
CRYBB2	0.556
CRYBB3	0.061
CRYBG3	0.149
CRYGA	0.104
CRYGB	0.198
CRYGC	0.216
CRYGD	0.190
CRYGN	0.194

CRYGS	0.221
CRYL1	0.306
CRYM	0.343
CRYZ	0.131
CRYZL1	0.336
CS	1.000
CSAD	0.782
CSDC2	0.529
CSDE1	0.980
CSE1L	0.932
CSF1	0.868
CSF1R	0.828
CSF2	0.731
CSF2RB	0.307
CSF3	0.434
CSF3R	0.105
CSGALNACT1	0.055
CSGALNACT2	0.611
CSH1	0.205
CSH2	0.337
CSHL1	0.311
CSK	0.931
CSMD1	0.052
CSMD2	0.210
CSMD3	0.174
CSN1S1	0.029
CSN2	0.043
CSN3	0.049
CSNK1A1	1.000
CSNK1A1L	0.163
CSNK1D	0.858
CSNK1E	0.998

CSNK1G1	0.851
CSNK1G2	0.997
CSNK1G3	0.876
CSNK2A1	0.935
CSNK2A2	0.998
CSNK2A3	0.158
CSNK2B	0.996
CSNK2B-LY6G5B-11	0.641
CSPG4	0.671
CSPG5	0.400
CSPP1	0.068
CSRNP1	0.323
CSRNP2	0.994
CSRNP3	0.453
CSRP1	0.130
CSRP2	0.523
CSRP2BP	0.404
CSRP3	0.699
CST1	0.071
CST11	0.049
CST2	0.099
CST3	0.315
CST4	0.141
CST5	0.055
CST6	0.849
CST7	0.063
CST8	0.040
CST9	0.066
CST9L	0.030
CSTA	0.106
CSTB	0.246
CSTF1	0.976

CSTF2T	0.578
CSTF3	0.428
CT62	0.069
CTA-299D3.8	0.113
CTAGE1	0.110
CTAGE15	0.194
CTAGE4	0.251
CTAGE5	0.189
CTAGE6	0.179
CTAGE8	0.240
CTAGE9	0.216
CTB-102L5.4	0.247
CTB-129P6.11	0.154
CTB-133G6.1	0.636
CTB-134H23.2	0.232
CTB-167G5.5	0.072
CTB-186H2.3	0.134
CTB-50L17.14	0.171
CTB-54O9.9	0.075
CTB-58E17.5	0.403
CTB-60B18.6	0.219
CTB-78H18.1	0.290
CTBP1	1.000
CTBP2	0.741
CTC-236F12.4	0.130
CTC-241N9.1	0.953
CTC-260F20.3	0.242
CTC-326K19.6	0.135
CTC-349C3.1	0.340
CTC-360G5.1	0.336
CTC-398G3.6	0.322
CTC-479C5.12	0.266

CTC-487M23.8	0.431
CTC-490E21.12	0.434
CTC-534A2.2	0.313
CTC-554D6.1	0.183
CTC-786C10.1	0.182
CTC1	0.509
CTCF	1.000
CTCFL	0.235
CTD-2006C1.10	0.112
CTD-2014B16.3	0.079
CTD-2021H9.3	0.193
CTD-2049J23.3	0.306
CTD-2054N24.2	0.099
CTD-2105E13.6	0.400
CTD-2116N17.1	0.592
CTD-2140B24.4	0.011
CTD-2144E22.5	0.047
CTD-2162K18.4	0.123
CTD-2192J16.20	0.248
CTD-2192J16.22	0.199
CTD-2207O23.10	0.174
CTD-2207O23.12	0.262
CTD-2207O23.3	0.360
CTD-2210P24.4	0.229
CTD-2215E18.1	0.064
CTD-2228K2.5	0.178
CTD-2267D19.3	0.281
CTD-2278I10.6	0.189
CTD-2287O16.3	0.060
CTD-2330K9.3	0.456
CTD-2349B8.1	0.106
CTD-2368P22.1	0.021

CTD-2369P2.10	0.185
CTD-2369P2.12	0.304
CTD-2370N5.3	0.195
CTD-2410N18.5	0.478
CTD-2510F5.6	0.103
CTD-2528L19.4	0.121
CTD-2535L24.2	0.214
CTD-2545G14.7	0.343
CTD-2545M3.6	0.557
CTD-2547L24.3	0.185
CTD-2561J22.3	0.125
CTD-2583A14.10	0.277
CTD-2583A14.9	0.111
CTD-2587H24.4	0.557
CTD-2600O9.1	0.151
CTD-2616J11.11	0.901
CTD-2616J11.4	0.908
CTD-3105H18.14	0.101
CTD-3105H18.18	0.084
CTD-3138B18.4	0.033
CTD-3148I10.1	0.628
CTD-3148I10.9	0.409
CTD-3193O13.9	0.404
CTD-3214H19.16	0.395
CTD-3214H19.4	0.231
CTD-3222D19.2	0.291
CTDNEP1	0.922
CTDP1	0.324
CTDSP1	0.780
CTDSP2	0.865
CTDSPL	0.742
CTDSPL2	0.805

CTF1	0.216
CTGF	0.300
CTH	0.126
CTHRC1	0.166
CTIF	0.778
CTLA4	0.664
CTNNA1	0.998
CTNNA2	0.715
CTNNA3	0.173
CTNNAL1	0.527
CTNNB1	1.000
CTNNBIP1	0.563
CTNNBL1	0.718
CTNND1	0.993
CTNND2	0.797
CTNS	0.323
CTPS1	0.914
CTR9	0.942
CTRB1	0.739
CTRB2	0.402
CTRC	0.535
CTRL	0.286
CTSA	0.891
CTSB	0.221
CTSC	0.222
CTSD	0.671
CTSE	0.289
CTSF	0.456
CTSG	0.246
CTSK	0.366
CTSL	0.529
CTSO	0.091

CTSS	0.379
CTSV	0.489
CTSW	0.224
CTSZ	0.567
CTTN	0.601
CTTNBP2	0.290
CTTNBP2NL	0.488
CTU1	0.539
CTU2	0.072
CTXN1	0.323
CTXN2	0.172
CTXN3	0.183
CUBN	0.393
CUEDC1	0.268
CUEDC2	0.519
CUL1	1.000
CUL2	0.213
CUL3	0.997
CUL4A	1.000
CUL5	0.891
CUL7	0.817
CUTA	0.584
CUTC	0.521
CUX1	0.822
CUX2	0.991
CUZD1	0.622
CWC22	0.330
CWC25	0.615
CWC27	0.345
CWF19L1	0.372
CWF19L2	0.335
CWH43	0.217

CX3CL1	0.441
CX3CR1	0.156
CXADR	0.533
CXCL1	0.026
CXCL10	0.146
CXCL11	0.300
CXCL12	0.369
CXCL13	0.277
CXCL14	0.346
CXCL16	0.343
CXCL17	0.217
CXCL2	0.172
CXCL3	0.032
CXCL5	0.041
CXCL6	0.027
CXCL9	0.062
CXCR1	0.076
CXCR2	0.155
CXCR4	0.192
CXCR5	0.422
CXCR6	0.270
CXXC1	0.948
CXXC11	0.199
CXXC4	0.821
CXXC5	0.554
CYB561	0.513
CYB561A3	0.760
CYB561D1	0.404
CYB561D2	0.538
CYB5A	0.121
CYB5B	0.489
CYB5D1	0.431

CYB5D2	0.196
CYB5R1	0.458
CYB5R2	0.990
CYB5R3	0.820
CYB5R4	0.169
CYB5RL	0.359
CYBA	0.094
CYBRD1	0.488
CYC1	0.611
CYCS	0.741
CYFIP1	0.876
CYFIP2	0.824
CYGB	0.419
CYHR1	0.715
CYLC2	0.014
CYLD	0.984
CYP11A1	0.803
CYP11B1	0.807
CYP11B2	0.612
CYP17A1	0.766
CYP19A1	0.229
CYP1A1	0.251
CYP1A2	0.132
CYP1B1	0.345
CYP20A1	0.176
CYP21A2	0.844
CYP24A1	0.084
CYP26A1	0.455
CYP26B1	0.968
CYP26C1	0.266
CYP27A1	0.381
CYP27B1	0.391

CYP27C1	0.078
CYP2A13	0.336
CYP2A6	0.346
CYP2A7	0.135
CYP2B6	0.426
CYP2C18	0.104
CYP2C19	0.076
CYP2C8	0.111
CYP2C9	0.053
CYP2D6	0.345
CYP2E1	0.241
CYP2F1	0.392
CYP2J2	0.204
CYP2R1	0.180
CYP2S1	0.349
CYP2U1	0.197
CYP2W1	0.431
CYP39A1	0.227
CYP3A4	0.457
CYP3A43	0.645
CYP3A5	0.350
CYP3A7	0.254
CYP46A1	0.689
CYP4A11	0.318
CYP4A22	0.204
CYP4B1	0.347
CYP4F11	0.361
CYP4F12	0.254
CYP4F2	0.481
CYP4F22	0.406
CYP4F3	0.205
CYP4F31P	0.268

CYP4V2	0.068
CYP4X1	0.099
CYP4Z1	0.271
CYP51A1	0.216
CYP7A1	0.201
CYP7B1	0.106
CYP8B1	0.230
CYR61	0.364
CYS1	0.979
CYSLTR2	0.160
CYSTM1	0.218
CYTH1	0.753
CYTH2	0.898
CYTH3	1.000
CYTH4	0.351
CYTIP	0.143
CYTL1	0.085
CYYR1	0.032
D2HGDH	0.959
DAAM1	0.972
DAAM2	0.729
DAB1	0.936
DAB2	0.636
DAB2IP	0.910
DACH1	0.890
DACT1	0.536
DACT2	0.213
DACT3	0.817
DAD1	0.128
DAG1	0.973
DAGLA	0.855
DAGLB	0.250

DAK	0.786
DALRD3	0.611
DAND5	0.216
DAO	0.329
DAP	0.211
DAP3	0.641
DAPK1	0.906
DAPK2	0.684
DAPK3	0.673
DAPL1	0.319
DAPP1	0.261
DARC	0.212
DARS	0.716
DARS2	0.468
DAW1	0.107
DAXX	0.678
DAZAP1	0.993
DAZAP2	0.485
DAZL	0.289
DBF4B	0.418
DBH	0.237
DBI	0.232
DBN1	0.772
DBNDD1	0.061
DBNDD2	0.678
DBNL	0.823
DBP	0.637
DBR1	0.311
DBT	0.733
DBX1	0.314
DBX2	0.160
DCAF10	0.785

DCAF11	0.899
DCAF12	0.152
DCAF13	0.090
DCAF15	0.994
DCAF16	0.087
DCAF17	0.288
DCAF4	0.137
DCAF4L1	0.466
DCAF4L2	0.108
DCAF5	0.954
DCAF6	0.489
DCAF7	0.956
DCAF8	1.000
DCAKD	0.712
DCBLD1	0.435
DCBLD2	0.599
DCC	0.677
DCD	0.648
DCDC1	0.213
DCDC2	0.201
DCDC2B	0.278
DCDC2C	0.182
DCHS1	0.594
DCHS2	0.122
DCK	0.485
DCLK1	0.990
DCLK2	0.871
DCLK3	0.578
DCLRE1A	0.278
DCLRE1B	0.765
DCLRE1C	0.342
DCN	0.204

DCP1B	0.187
DCP2	0.532
DCPS	0.630
DCST1	0.286
DCST2	0.266
DCSTAMP	0.333
DCT	0.098
DCTD	0.283
DCTN1	0.930
DCTN2	1.000
DCTN3	0.261
DCTN4	0.868
DCTN5	0.544
DCTN6	0.448
DCTPP1	0.591
DCUN1D1	0.844
DCUN1D2	0.900
DCUN1D3	0.981
DCUN1D4	0.503
DCUN1D5	0.641
DCXR	0.686
DDA1	0.929
DDAH1	0.696
DDAH2	0.572
DDB1	1.000
DDB2	0.763
DDC	0.515
DDC8	0.250
DDHD1	0.593
DDHD2	0.319
DDI1	0.062
DDI2	0.957

DDIT3	0.524
DDIT4	0.373
DDIT4L	0.125
DDN	0.863
DDO	0.220
DDOST	0.453
DDR1	0.582
DDR2	0.958
DDRGK1	0.285
DDT	0.414
DDTL	0.376
DDX1	0.998
DDX10	0.619
DDX11	0.353
DDX17	0.996
DDX18	0.614
DDX19A	0.957
DDX19B	0.784
DDX20	0.496
DDX21	0.978
DDX23	1.000
DDX24	0.787
DDX25	0.487
DDX27	0.507
DDX28	0.536
DDX31	0.548
DDX39A	0.845
DDX39B	0.999
DDX4	0.691
DDX41	0.759
DDX42	1.000
DDX43	0.276

DDX46	0.998
DDX47	0.283
DDX49	0.551
DDX5	0.628
DDX50	0.885
DDX51	0.156
DDX52	0.360
DDX54	0.642
DDX55	0.447
DDX56	0.354
DDX58	0.590
DDX59	0.521
DDX6	0.999
DDX60	0.275
DDX60L	0.164
DEAF1	0.991
DEC1	0.067
DECR1	0.517
DECR2	0.942
DEDD	0.989
DEDD2	0.509
DEF6	0.669
DEF8	0.377
DEFA1	0.081
DEFA1B	0.242
DEFA3	0.078
DEFA4	0.016
DEFA5	0.027
DEFA6	0.054
DEFB1	0.065
DEFB103A	0.358
DEFB103B	0.242

DEFB118	0.012
DEFB119	0.031
DEFB123	0.091
DEFB124	0.048
DEFB125	0.077
DEFB126	0.048
DEFB131	0.097
DEFB132	0.067
DEFB134	0.040
DEFB4A	0.068
DEGS1	0.395
DEGS2	0.314
DEK	0.374
DENND1A	0.820
DENND1B	0.174
DENND1C	0.199
DENND2A	0.126
DENND2C	0.414
DENND2D	0.356
DENND3	0.147
DENND4A	0.979
DENND4B	0.922
DENND4C	0.069
DENND5A	0.409
DENND5B	0.956
DENND6A	0.493
DENND6B	0.080
DENR	0.831
DEPDC1	0.106
DEPDC1B	0.234
DEPDC4	0.090
DEPDC5	0.767

DEPDC7	0.074
DEPTOR	0.311
DERA	0.308
DERL1	0.339
DERL2	0.847
DERL3	0.088
DES	0.861
DESI1	0.504
DESI2	0.536
DET1	0.584
DEXI	0.101
DFFA	0.520
DFFB	0.476
DFNA5	0.181
DFNB31	0.561
DFNB59	0.125
DGAT1	0.310
DGAT2	0.226
DGCR14	0.512
DGCR2	0.584
DGCR6	0.515
DGCR6L	0.344
DGCR8	0.996
DGKA	0.856
DGKB	0.254
DGKD	0.745
DGKE	0.290
DGKG	0.460
DGKH	0.801
DGKI	0.834
DGKQ	0.202
DGKZ	0.764

DGUOK	0.538
DHCR24	0.496
DHCR7	0.515
DHDDS	0.703
DHDH	0.099
DHFR	0.614
DHFRL1	0.220
DHH	0.902
DHODH	0.981
DHPS	0.764
DHRS1	0.837
DHRS11	0.633
DHRS12	0.271
DHRS13	0.414
DHRS2	0.382
DHRS3	0.282
DHRS4	0.458
DHRS4-AS1	0.126
DHRS4L2	0.480
DHRS7	0.249
DHRS7B	0.361
DHRS7C	0.493
DHRS9	0.113
DHTKD1	0.109
DHX15	0.994
DHX16	0.965
DHX29	0.740
DHX30	1.000
DHX32	0.634
DHX33	0.582
DHX34	0.621
DHX35	0.502

DHX36	0.696
DHX37	0.683
DHX38	0.987
DHX40	0.853
DHX57	0.577
DHX58	0.640
DHX8	1.000
DHX9	1.000
DIABLO	0.271
DIAPH1	0.866
DIAPH3	0.146
DICER1	0.990
DIDO1	0.745
DIEXF	0.409
DIMT1	0.168
DIO1	0.312
DIO2	0.267
DIO3	0.439
DIP2A	0.256
DIP2B	0.959
DIP2C	0.816
DIRAS1	0.522
DIRAS2	0.306
DIRAS3	0.602
DIRC1	0.149
DIRC2	0.173
DIS3	0.176
DIS3L	0.289
DIS3L2	0.715
DISC1	0.432
DISP1	0.543
DISP2	0.307

DIXDC1	0.696
DKFZP434E1119	0.447
DKFZP434H0512	0.193
DKFZP434O1614	0.067
DKFZP667F0711	0.138
DKFZP761J1410	0.773
DKFZP779J2370	0.080
DKFZP779L1853	0.523
DKK1	0.264
DKK2	0.226
DKK3	0.399
DKK4	0.111
DKKL1	0.292
DLAT	0.810
DLC1	0.967
DLD	0.737
DLEC1	0.307
DLEU1	0.189
DLEU2L	0.198
DLEU7	0.293
DLG1	0.827
DLG2	0.997
DLG4	0.991
DLG5	0.598
DLGAP1	0.406
DLGAP2	0.143
DLGAP3	0.952
DLGAP4	0.900
DLGAP5	0.063
DLK1	0.971
DLK2	0.519
DLL1	0.516

DLL3	0.447
DLL4	0.974
DLST	0.940
DLX1	0.974
DLX2	0.982
DLX3	0.896
DLX4	0.726
DLX5	0.457
DLX6	0.921
DMAP1	0.811
DMBT1	0.288
DMBX1	0.373
DMC1	0.586
DMGDH	0.958
DMKN	0.603
DMP1	0.317
DMPK	0.615
DMRT1	0.131
DMRT2	0.079
DMRT3	0.046
DMRTA1	0.100
DMRTA2	0.985
DMRTB1	0.355
DMRTC2	0.464
DMTF1	0.457
DMTN	0.604
DMWD	0.564
DMXL1	0.331
DMXL2	0.764
DNA2	0.076
DNAAF1	0.069
DNAAF2	0.408

DNAAF3	0.556
DNAH1	0.335
DNAH10	0.342
DNAH10OS	0.242
DNAH11	0.192
DNAH12	0.218
DNAH14	0.103
DNAH17-AS1	0.139
DNAH2	0.703
DNAH3	0.392
DNAH5	0.193
DNAH6	0.781
DNAH7	0.277
DNAH8	0.668
DNAH9	0.305
DNAI1	0.346
DNAI2	0.121
DNAJA1	0.924
DNAJA2	0.999
DNAJA3	0.473
DNAJA4	0.472
DNAJB1	0.538
DNAJB11	0.679
DNAJB12	0.631
DNAJB13	0.149
DNAJB14	0.437
DNAJB2	0.600
DNAJB4	0.417
DNAJB5	0.519
DNAJB6	0.652
DNAJB7	0.096
DNAJB8	0.218

DNAJB9	0.273
DNAJC1	0.435
DNAJC11	0.806
DNAJC12	0.152
DNAJC13	0.459
DNAJC14	0.931
DNAJC15	0.037
DNAJC16	0.206
DNAJC17	0.639
DNAJC18	0.188
DNAJC19	0.234
DNAJC2	0.766
DNAJC21	0.139
DNAJC22	0.609
DNAJC24	0.314
DNAJC25	0.342
DNAJC25-GNG10	0.281
DNAJC27	0.369
DNAJC28	0.147
DNAJC3	0.334
DNAJC30	0.668
DNAJC4	0.314
DNAJC5	0.628
DNAJC5B	0.204
DNAJC5G	0.107
DNAJC6	0.545
DNAJC7	0.998
DNAJC8	0.963
DNAJC9	0.426
DNAL1	0.140
DNAL4	0.328
DNALI1	0.386

DNASE1	0.216
DNASE1L2	0.167
DNASE1L3	0.470
DNASE2	0.236
DNASE2B	0.190
DND1	0.837
DNER	0.739
DNHD1	0.389
DNLZ	0.146
DNM1	0.966
DNM1L	0.802
DNM2	0.995
DNM3	0.938
DNMBP	0.863
DNMT1	0.993
DNMT3A	0.988
DNMT3B	0.732
DNMT3L	0.352
DNPEP	0.366
DNPH1	0.420
DNTT	0.234
DNTTIP1	0.772
DNTTIP2	0.544
DOC2A	0.304
DOC2B	0.224
DOCK1	0.467
DOCK10	0.728
DOCK2	0.878
DOCK3	0.997
DOCK4	0.571
DOCK5	0.578
DOCK6	0.599

DOCK7	0.865
DOCK8	0.066
DOCK9	0.966
DOHH	0.529
DOK1	0.923
DOK2	0.340
DOK3	0.128
DOK4	0.412
DOK5	0.325
DOK6	0.323
DOK7	0.182
DOLK	0.742
DOLPP1	0.693
DONSON	0.260
DOPEY1	0.717
DOPEY2	0.137
DOT1L	0.912
DPAGT1	0.672
DPCD	0.232
DPCR1	0.204
DPEP1	0.215
DPEP2	0.255
DPEP3	0.222
DPF1	0.958
DPF2	1.000
DPF3	0.395
DPH1	0.459
DPH2	0.634
DPH3	0.239
DPH5	0.348
DPH6	0.363
DPH7	0.654

DPM1	0.578
DPM2	0.601
DPM3	0.242
DPP10	0.337
DPP3	0.797
DPP4	0.257
DPP6	0.119
DPP7	0.327
DPP8	0.927
DPP9	0.977
DPPA2	0.900
DPPA3	0.832
DPPA4	0.914
DPPA5	0.101
DPRX	0.045
DPT	0.076
DPY19L1	0.277
DPY19L2	0.171
DPY19L3	0.264
DPY19L4	0.220
DPY30	0.438
DPYD	0.233
DPYS	0.230
DPYSL2	0.994
DPYSL3	0.838
DPYSL4	0.656
DPYSL5	0.994
DQX1	0.997
DR1	0.854
DRAM1	0.265
DRAP1	0.806
DRAXIN	0.505

DRC1	0.310
DRD1	0.787
DRD2	0.800
DRD3	0.309
DRD4	0.091
DRD5	0.185
DRG1	0.964
DRG2	0.725
DRGX	0.465
DROSHA	0.824
DSC1	0.790
DSC2	0.183
DSC3	0.357
DSCAM	0.697
DSCAML1	0.539
DSCC1	0.086
DSCR3	0.384
DSCR4	0.153
DSCR8	0.120
DSE	0.679
DSEL	0.094
DSG1	0.488
DSG2	0.982
DSG3	0.970
DSG4	0.891
DSN1	0.273
DSP	0.913
DSPP	0.627
DST	0.945
DSTN	0.343
DSTYK	0.861
DTD1	0.492

DTD2	0.261
DTHD1	0.239
DTL	0.306
DTNA	0.535
DTNB	0.571
DTNBP1	0.272
DTWD1	0.215
DTWD2	0.101
DTX1	0.769
DTX2	0.379
DTX3	0.897
DTX3L	0.506
DTX4	0.275
DTYMK	0.371
DUOX1	0.216
DUOX2	0.102
DUOXA1	0.213
DUOXA2	0.045
DUPD1	0.186
DUS1L	0.701
DUS2	0.895
DUS3L	0.444
DUS4L	0.298
DUSP1	0.613
DUSP10	0.649
DUSP11	0.314
DUSP12	0.514
DUSP13	0.167
DUSP14	0.537
DUSP15	0.180
DUSP16	0.885
DUSP18	0.630

DUSP19	0.070
DUSP2	0.732
DUSP22	0.934
DUSP23	0.313
DUSP26	0.501
DUSP27	0.599
DUSP28	0.967
DUSP3	0.855
DUSP4	0.838
DUSP5	0.318
DUSP6	0.496
DUSP7	0.959
DUSP8	0.703
DUT	0.103
DUXA	0.015
DVL1	0.521
DVL2	0.976
DVL3	0.855
DXO	0.613
DYDC1	0.061
DYDC2	0.065
DYM	0.264
DYNAP	0.034
DYNC1H1	1.000
DYNC1I1	0.494
DYNC1I2	0.560
DYNC1LI1	0.461
DYNC1LI2	0.983
DYNC2H1	0.135
DYNC2LI1	0.056
DYNLL1	0.758
DYNLL2	0.915

DYNLRB1	0.399
DYNLRB2	0.032
DYNLT1	0.382
DYRK1A	0.985
DYRK1B	0.996
DYRK2	0.996
DYRK3	0.327
DYRK4	0.152
DYSF	0.083
DYTN	0.270
DYX1C1	0.134
DZANK1	0.172
DZIP1	0.193
DZIP1L	0.296
DZIP3	0.284
E2F1	0.836
E2F2	0.166
E2F3	0.853
E2F4	0.900
E2F5	0.570
E2F6	0.936
E2F7	0.553
E2F8	0.654
E4F1	0.454
EAF1	0.556
EAF2	0.135
EAPP	0.115
EARS2	0.835
EBAG9	0.138
EBF1	1.000
EBF2	0.978
EBF3	0.996

EBF4	0.233
EBI3	0.099
EBLN1	0.413
EBLN2	0.238
EBNA1BP2	0.386
EBPL	0.220
ECD	0.662
ECE1	0.848
ECE2	0.396
ECEL1	0.154
ECH1	0.630
ECHDC1	0.176
ECHDC2	0.438
ECHDC3	0.403
ECHS1	0.389
ECI1	0.437
ECI2	0.620
ECM1	0.244
ECM2	0.249
ECSCR	0.211
ECSIT	0.603
ECT2	0.158
ECT2L	0.257
EDAR	0.197
EDARADD	0.033
EDC3	0.947
EDC4	0.980
EDDM3A	0.176
EDEM1	0.232
EDEM2	0.423
EDEM3	0.462
EDF1	0.942

EDIL3	0.240
EDN1	0.378
EDN2	0.293
EDN3	0.309
EDNRA	0.995
EDNRB	0.336
EEA1	0.715
EED	0.731
EEF1A1	0.711
EEF1A2	0.971
EEF1B2	0.251
EEF1D	0.484
EEF1E1	0.402
EEF1E1-BLOC1S5	0.203
EEF1G	0.911
EEF2	0.972
EEF2K	0.793
EEFSEC	0.707
EEPD1	0.414
EFCAB1	0.061
EFCAB10	0.227
EFCAB11	0.150
EFCAB12	0.289
EFCAB13	0.318
EFCAB14	0.476
EFCAB2	0.068
EFCAB3	0.511
EFCAB4A	0.199
EFCAB4B	0.561
EFCAB5	0.462
EFCAB6	0.290
EFCAB7	0.145

EFCAB8	0.304
EFCAB9	0.170
EFCC1	0.276
EFEMP1	0.553
EFEMP2	0.764
EFHB	0.098
EFHC1	0.388
EFHD1	0.141
EFHD2	0.304
EFNA1	0.619
EFNA2	0.589
EFNA3	0.839
EFNA4	0.316
EFNA5	0.871
EFNB2	0.859
EFNB3	0.758
EFR3A	0.095
EFR3B	0.627
EFS	0.892
EFTUD1	0.490
EFTUD2	1.000
EGF	0.562
EGFL7	0.227
EGFL8	0.800
EGFLAM	0.365
EGFR	0.880
EGLN1	0.903
EGLN2	0.844
EGLN3	0.999
EGR1	0.736
EGR2	0.977
EGR3	0.987

EGR4	0.442
EHBP1	0.752
EHBP1L1	0.417
EHD1	0.282
EHD2	0.743
EHD3	0.341
EHD4	0.641
EHF	0.097
EHHADH	0.228
EHMT1	0.997
EHMT2	0.992
EI24	0.835
EID1	0.237
EID2	0.574
EID2B	0.456
EID3	0.246
EIF1	0.606
EIF1AD	0.831
EIF1B	0.401
EIF2A	0.598
EIF2AK1	0.967
EIF2AK2	0.796
EIF2AK3	0.667
EIF2AK4	0.898
EIF2B1	0.200
EIF2B2	0.668
EIF2B3	0.797
EIF2B4	0.977
EIF2B5	0.532
EIF2D	0.772
EIF2S1	0.861
EIF2S2	0.966

EIF2S3L	0.191
EIF3A	0.965
EIF3B	0.513
EIF3C	0.285
EIF3CL	0.365
EIF3D	0.879
EIF3E	0.523
EIF3F	0.692
EIF3G	0.938
EIF3H	0.338
EIF3I	0.976
EIF3J	0.980
EIF3K	0.660
EIF3M	0.380
EIF4A1	0.892
EIF4A2	0.280
EIF4A3	1.000
EIF4B	0.967
EIF4E	0.956
EIF4E1B	0.402
EIF4E2	0.979
EIF4E3	0.213
EIF4EBP1	0.388
EIF4EBP2	0.681
EIF4EBP3	0.379
EIF4ENIF1	0.957
EIF4G1	0.984
EIF4G2	0.824
EIF4G3	0.992
EIF4H	0.950
EIF5	0.845
EIF5A	0.911

EIF5A2	0.187
EIF5AL1	0.338
EIF5B	0.987
EIF6	0.588
ELAC1	0.855
ELAC2	0.171
ELANE	0.207
ELAVL1	0.999
ELAVL2	0.758
ELAVL3	0.999
ELAVL4	0.991
ELF1	0.432
ELF2	0.458
ELF3	0.280
ELF5	0.185
ELFN1	0.967
ELFN2	0.410
ELK3	0.416
ELK4	0.785
ELL	0.513
ELL2	0.642
ELL3	0.463
ELMO1	0.720
ELMO2	0.937
ELMO3	0.368
ELMOD1	0.562
ELMOD2	0.314
ELMOD3	0.451
ELMSAN1	0.756
ELN	0.629
ELOF1	0.469
ELOVL1	0.810

ELOVL2	0.356
ELOVL3	0.783
ELOVL4	0.505
ELOVL5	0.387
ELOVL6	0.686
ELOVL7	0.406
ELP2	0.465
ELP3	0.411
ELP4	0.261
ELP5	0.579
ELP6	0.308
ELSPBP1	0.097
ELTD1	0.038
EMB	0.217
EMC1	0.366
EMC10	0.623
EMC2	0.348
EMC3	0.771
EMC4	0.820
EMC6	0.413
EMC7	0.510
EMC8	0.799
EMC9	0.531
EMCN	0.128
EME1	0.250
EMG1	0.574
EMID1	0.273
EMILIN1	0.606
EMILIN2	0.869
EMILIN3	0.295
EML1	0.668
EML2	0.533

EML3	0.817
EML4	0.312
EML5	0.328
EML6	0.599
EMP1	0.234
EMP2	0.106
EMP3	0.269
EMR1	0.040
EMR2	0.181
EMR3	0.247
EMX1	0.794
EMX2	0.656
EN1	1.000
EN2	1.000
ENAH	0.957
ENAM	0.240
ENC1	0.639
ENDOD1	0.307
ENDOG	0.501
ENDOU	0.431
ENDOV	0.323
ENG	0.832
ENGASE	0.950
ENHO	0.566
ENKD1	0.483
ENKUR	0.230
ENO1	0.099
ENO2	0.700
ENO3	0.459
ENO4	0.415
ENOPH1	0.707
ENOSF1	0.336

ENOX1	0.162
ENPEP	0.190
ENPP1	0.456
ENPP2	0.593
ENPP3	0.366
ENPP4	0.408
ENPP5	0.108
ENPP6	0.970
ENPP7	0.220
ENSA	0.563
ENTHD1	0.285
ENTHD2	0.818
ENTPD1	0.412
ENTPD2	0.309
ENTPD3	0.326
ENTPD4	0.270
ENTPD5	0.238
ENTPD6	0.223
ENTPD7	0.321
ENTPD8	0.347
ENY2	0.734
EOGT	0.357
EOMES	0.560
EP300	0.999
EP400	1.000
EP400NL	0.224
EPAS1	0.695
EPB41	0.696
EPB41L1	0.838
EPB41L2	0.522
EPB41L3	0.689
EPB41L4A	0.262

EPB41L4A-AS2	0.090
EPB41L4B	0.970
EPB41L5	0.428
EPB42	0.521
EPC1	0.932
EPC2	0.984
EPCAM	0.132
EPDR1	0.162
EPG5	0.394
EPGN	0.148
EPHA1	0.514
EPHA10	0.469
EPHA2	0.779
EPHA3	0.516
EPHA4	0.936
EPHA5	0.454
EPHA6	0.673
EPHA7	0.547
EPHA8	0.416
EPHB1	0.959
EPHB2	0.871
EPHB3	0.811
EPHB4	0.786
EPHB6	0.604
EPHX1	0.553
EPHX2	0.650
EPHX3	0.428
EPHX4	0.058
EPM2A	0.129
EPM2AIP1	0.782
EPN1	0.862
EPN2	0.795

EPN3	0.402
EPO	0.791
EPOR	0.638
EPPIN	0.251
EPPK1	0.215
EPRS	0.700
EPS15	0.546
EPS15L1	0.862
EPS8	0.732
EPS8L1	0.490
EPS8L2	0.402
EPS8L3	0.533
EPSTI1	0.026
EPT1	0.574
EPX	0.333
EPYC	0.126
EQTN	0.076
ERAL1	0.617
ERAP1	0.330
ERAP2	0.097
ERBB2	0.939
ERBB2IP	0.688
ERBB3	0.824
ERBB4	0.792
ERC1	0.247
ERC2	0.934
ERCC1	0.927
ERCC2	0.404
ERCC3	0.989
ERCC4	0.342
ERCC5	0.388
ERCC6	0.512

ERCC6-PGBD3	0.328
ERCC6L2	0.398
ERCC8	0.225
EREG	0.158
ERF	1.000
ERG	0.941
ERGIC1	0.295
ERGIC2	0.104
ERGIC3	0.514
ERH	0.936
ERI1	0.204
ERI2	0.358
ERI3	0.964
ERICH1	0.059
ERICH2	0.133
ERLEC1	0.220
ERLIN1	0.387
ERLIN2	0.573
ERMAP	0.155
ERMARD	0.118
ERMN	0.163
ERMP1	0.129
ERN1	0.956
ERN2	0.389
ERO1L	0.356
ERO1LB	0.101
ERP27	0.381
ERP29	0.637
ERP44	0.668
ERRFI1	0.490
ERV3-1	0.168
ERVFRD-1	0.207

ERVMER34-1	0.221
ERVV-1	0.053
ERVV-2	0.072
ERVW-1	0.107
ESAM	0.282
ESCO1	0.778
ESCO2	0.091
ESD	0.254
ESF1	0.252
ESM1	0.342
ESPL1	0.778
ESPN	0.433
ESPNL	0.269
ESR1	0.845
ESR2	0.302
ESRP1	0.945
ESRP2	0.551
ESRRA	0.985
ESRRB	0.439
ESRRG	0.794
ESYT1	0.631
ESYT2	0.163
ESYT3	0.173
ETAA1	0.132
ETF1	1.000
ETFA	0.801
ETFB	0.855
ETFDH	0.238
ETHE1	0.425
ETNK1	0.551
ETNK2	0.397
ETNPPL	0.306

ETS1	0.757
ETS2	0.672
ETV1	0.287
ETV2	0.472
ETV3	0.943
ETV3L	0.243
ETV4	0.982
ETV5	0.986
ETV6	0.894
ETV7	0.510
EVA1A	0.305
EVA1B	0.248
EVA1C	0.350
EVC	0.177
EVC2	0.117
EVI2A	0.116
EVI2B	0.145
EVI5	0.592
EVI5L	0.941
EVL	0.558
EVPL	0.484
EVPLL	0.497
EVX1	0.273
EVX2	0.838
EWSR1	0.995
EXD1	0.406
EXD2	0.332
EXD3	0.542
EXO1	0.029
EXO5	0.125
EXOC1	0.123
EXOC2	0.174

EXOC3	0.295
EXOC3L1	0.405
EXOC3L2	0.453
EXOC3L4	0.959
EXOC4	0.289
EXOC5	0.795
EXOC6	0.602
EXOC6B	0.689
EXOC7	0.552
EXOC8	0.851
EXOG	0.410
EXOSC1	0.380
EXOSC10	0.532
EXOSC2	0.566
EXOSC3	0.313
EXOSC4	0.617
EXOSC5	0.616
EXOSC6	0.577
EXOSC7	0.733
EXOSC8	0.163
EXOSC9	0.060
EXPH5	0.334
EXT1	0.760
EXT2	0.535
EXTL1	0.223
EXTL2	0.951
EXTL3	0.403
EYA1	0.610
EYA2	0.828
EYA3	0.846
EYA4	0.530
EYS	0.443

EZH1	0.984
EZH2	1.000
EZR	0.711
F10	0.413
F11	0.149
F11R	0.355
F12	0.362
F13A1	0.486
F13B	0.162
F2	0.398
F2R	0.625
F2RL1	0.217
F2RL2	0.214
F2RL3	0.316
F3	0.210
F5	0.506
F7	0.196
FA2H	0.676
FAAH	0.676
FABP1	0.068
FABP12	0.087
FABP2	0.066
FABP3	0.477
FABP4	0.148
FABP5	0.161
FABP6	0.209
FABP7	0.276
FABP9	0.172
FADD	0.523
FADS1	0.737
FADS2	0.644
FADS3	0.706

FADS6	0.302
FAF1	0.839
FAF2	0.939
FAH	0.282
FAHD1	0.137
FAHD2A	0.317
FAHD2B	0.214
FAIM	0.101
FAIM2	0.332
FAIM3	0.219
FAM101A	0.230
FAM101B	0.178
FAM102A	0.282
FAM102B	0.395
FAM103A1	0.220
FAM104A	0.295
FAM105A	0.269
FAM105B	0.609
FAM106A	0.408
FAM107A	0.169
FAM107B	0.080
FAM109A	0.300
FAM109B	0.321
FAM110A	0.384
FAM110B	0.386
FAM110C	0.949
FAM110D	0.467
FAM111A	0.146
FAM111B	0.190
FAM114A1	0.478
FAM114A2	0.382
FAM115A	0.682

FAM115C	0.166
FAM117A	0.517
FAM117B	0.669
FAM118A	0.110
FAM118B	0.451
FAM120A	0.952
FAM120AOS	0.345
FAM120B	0.151
FAM122A	0.561
FAM124A	0.356
FAM124B	0.159
FAM126A	0.251
FAM126B	0.236
FAM129A	0.256
FAM129B	0.528
FAM129C	0.180
FAM131A	0.570
FAM131B	0.789
FAM131C	0.346
FAM132A	0.298
FAM132B	0.167
FAM133B	0.234
FAM134A	0.372
FAM134B	0.374
FAM134C	0.722
FAM135A	0.289
FAM135B	0.200
FAM136A	0.371
FAM13A	0.279
FAM13B	0.516
FAM13C	0.216
FAM149A	0.210

FAM149B1	0.473
FAM150A	0.075
FAM150B	0.073
FAM151A	0.080
FAM151B	0.273
FAM153B	0.195
FAM153C	0.172
FAM154A	0.061
FAM154B	0.259
FAM155A	0.467
FAM159A	0.153
FAM159B	0.050
FAM160A1	0.687
FAM160A2	0.851
FAM160B1	0.593
FAM160B2	0.459
FAM161A	0.115
FAM161B	0.318
FAM162A	0.302
FAM162B	0.070
FAM163A	0.366
FAM163B	0.119
FAM166A	0.874
FAM166B	0.285
FAM167A	0.288
FAM167B	0.168
FAM168A	0.592
FAM168B	0.614
FAM169A	0.650
FAM169B	0.893
FAM170A	0.207
FAM170B	0.131

FAM171A1	0.636
FAM171A2	0.573
FAM171B	0.399
FAM172A	0.289
FAM173A	0.206
FAM173B	0.128
FAM174A	0.068
FAM174B	0.273
FAM175A	0.111
FAM175B	0.304
FAM177A1	0.100
FAM177B	0.194
FAM178A	0.488
FAM178B	0.241
FAM179A	0.243
FAM179B	0.221
FAM180A	0.129
FAM180B	0.462
FAM181A	0.277
FAM181B	0.221
FAM182B	0.116
FAM183A	0.151
FAM183B	0.125
FAM184A	0.452
FAM184B	0.240
FAM185A	0.527
FAM186A	0.704
FAM186B	0.358
FAM187A	0.362
FAM187B	0.099
FAM188A	0.272
FAM188B	0.822

FAM188B2	0.325
FAM189A1	0.346
FAM189A2	0.337
FAM189B	0.652
FAM192A	0.737
FAM193A	0.704
FAM193B	0.700
FAM194A	0.173
FAM194B	0.409
FAM195A	0.227
FAM195B	0.170
FAM196A	0.391
FAM196B	0.465
FAM198A	0.489
FAM198B	0.111
FAM19A1	0.256
FAM19A2	0.117
FAM19A3	0.046
FAM19A4	0.118
FAM19A5	0.098
FAM200A	0.343
FAM200B	0.148
FAM203A	0.168
FAM203B	0.388
FAM204A	0.219
FAM205A	0.405
FAM206A	0.276
FAM207A	0.409
FAM208A	0.907
FAM208B	0.468
FAM209A	0.109
FAM209B	0.121

FAM20A	0.385
FAM20B	0.808
FAM20C	0.836
FAM210A	0.127
FAM210B	0.238
FAM211A	0.552
FAM211B	0.958
FAM212A	0.541
FAM212B	0.169
FAM213A	0.490
FAM213B	0.297
FAM214A	0.411
FAM214B	0.868
FAM215A	0.342
FAM216A	0.206
FAM216B	0.053
FAM217A	0.111
FAM217B	0.304
FAM218A	0.079
FAM219A	0.364
FAM219B	0.444
FAM21A	0.341
FAM21B	0.257
FAM21C	0.312
FAM21D	0.522
FAM220A	0.848
FAM221A	0.126
FAM221B	0.608
FAM222A	0.919
FAM222B	0.288
FAM227A	0.315
FAM227B	0.238

FAM228A	0.051
FAM228B	0.144
FAM229A	0.509
FAM229B	0.163
FAM230A	0.113
FAM24A	0.093
FAM24B	0.159
FAM25A	0.191
FAM25B	0.350
FAM25C	0.392
FAM25E	0.345
FAM25G	0.358
FAM26D	0.147
FAM26E	0.126
FAM26F	0.126
FAM27A	0.544
FAM27B	0.113
FAM27D1	0.289
FAM27E1	0.131
FAM27E2	0.268
FAM27E3	0.181
FAM32A	0.668
FAM35A	0.677
FAM3B	0.591
FAM3C	0.517
FAM3D	0.180
FAM43A	0.157
FAM43B	0.657
FAM45A	0.205
FAM46A	0.713
FAM46B	0.155
FAM46C	0.985

FAM47E	0.350
FAM47E-STBD1	0.478
FAM49A	0.347
FAM49B	0.267
FAM50B	0.527
FAM53A	0.202
FAM53B	0.452
FAM53C	0.657
FAM57A	0.137
FAM57B	0.612
FAM60A	0.975
FAM63A	0.645
FAM63B	0.214
FAM64A	0.134
FAM65A	0.677
FAM65B	0.342
FAM65C	0.360
FAM69A	0.691
FAM69B	0.145
FAM69C	0.110
FAM71A	0.169
FAM71B	0.128
FAM71C	0.170
FAM71E1	0.285
FAM71E2	0.281
FAM71F1	0.141
FAM71F2	0.196
FAM72A	0.390
FAM72B	0.227
FAM72C	0.340
FAM72D	0.321
FAM73A	0.235

FAM73B	0.511
FAM76A	0.625
FAM76B	0.294
FAM78A	0.154
FAM78B	0.394
FAM81A	0.479
FAM81B	0.153
FAM83A	0.225
FAM83B	0.342
FAM83C	0.225
FAM83D	0.176
FAM83E	0.079
FAM83F	0.587
FAM83G	0.674
FAM83H	0.664
FAM84A	0.352
FAM84B	0.171
FAM86A	0.461
FAM86B1	0.114
FAM86B2	0.127
FAM86C1	0.329
FAM89A	0.201
FAM89B	0.329
FAM8A1	0.152
FAM90A1	0.173
FAM91A1	0.432
FAM92A1	0.278
FAM92B	0.090
FAM96A	0.394
FAM96B	0.373
FAM98A	0.467
FAM98B	0.318

FAM98C	0.320
FAN1	0.392
FANCA	0.018
FANCC	0.415
FANCD2	0.274
FANCD2OS	0.137
FANCE	0.357
FANCF	0.101
FANCG	0.493
FANCI	0.120
FANCL	0.168
FANCM	0.172
FANK1	0.230
FAP	0.225
FAR1	0.810
FAR2	0.299
FARP1	0.678
FARP2	0.250
FARS2	0.444
FARSA	0.590
FARSB	0.751
FAS	0.334
FASLG	0.419
FASN	0.896
FASTK	0.716
FASTKD1	0.196
FASTKD2	0.488
FASTKD3	0.237
FASTKD5	0.447
FAT1	0.126
FAT2	0.302
FAT3	0.490

FAT4	0.318
FAU	0.433
FAXC	0.286
FAXDC2	0.278
FBF1	0.392
FBL	0.939
FBLIM1	0.360
FBLL1	0.497
FBLN1	0.310
FBLN2	0.362
FBLN5	0.649
FBLN7	0.370
FBN1	0.985
FBN2	0.580
FBN3	0.172
FBP1	0.201
FBP2	0.258
FBRS	0.901
FBRSL1	0.372
FBXL12	0.454
FBXL13	0.304
FBXL14	0.463
FBXL15	0.631
FBXL16	0.471
FBXL17	0.784
FBXL18	0.419
FBXL19	1.000
FBXL2	0.829
FBXL20	0.864
FBXL22	0.233
FBXL3	0.887
FBXL4	0.314

FBXL5	0.309
FBXL6	0.285
FBXL7	0.579
FBXL8	0.568
FBXO10	0.149
FBXO11	0.998
FBXO15	0.166
FBXO16	0.120
FBXO17	0.396
FBXO18	0.878
FBXO2	0.610
FBXO21	0.906
FBXO22	0.693
FBXO24	0.389
FBXO25	0.089
FBXO27	0.391
FBXO28	0.749
FBXO3	0.828
FBXO30	0.387
FBXO31	0.746
FBXO32	0.530
FBXO33	0.574
FBXO34	0.460
FBXO36	0.114
FBXO38	0.821
FBXO39	0.185
FBXO4	0.342
FBXO40	0.547
FBXO41	0.695
FBXO42	0.704
FBXO43	0.148
FBXO44	0.482

FBXO45	0.787
FBXO46	0.425
FBXO47	0.262
FBXO48	0.160
FBXO5	0.432
FBXO6	0.326
FBXO7	0.226
FBXO8	0.218
FBXO9	0.343
FBXW10	0.329
FBXW11	1.000
FBXW12	0.293
FBXW2	0.816
FBXW4	0.466
FBXW5	0.193
FBXW7	0.910
FBXW8	0.331
FBXW9	0.330
FCAMR	0.205
FCAR	0.030
FCER1A	0.100
FCER1G	0.170
FCER2	0.079
FCF1	0.261
FCGBP	0.537
FCGR1A	0.163
FCGR1B	0.092
FCGR2A	0.340
FCGR2B	0.245
FCGR3A	0.159
FCGR3B	0.101
FCGRT	0.424

FCHO1	0.564
FCHO2	0.678
FCHSD1	0.431
FCHSD2	0.795
FCN1	0.057
FCN2	0.311
FCN3	0.247
FCRL1	0.099
FCRL2	0.139
FCRL3	0.159
FCRL4	0.417
FCRL5	0.415
FCRL6	0.112
FCRLA	0.132
FCRLB	0.489
FDCSP	0.036
FDFT1	0.146
FDPS	0.753
FDX1	0.471
FDX1L	0.291
FDXACB1	0.174
FDXR	0.369
FECH	0.486
FEM1A	0.560
FEM1B	0.914
FEM1C	0.819
FEN1	0.749
FER	0.632
FER1L6	0.479
FERD3L	0.109
FERMT1	0.277
FERMT2	0.932

FERMT3	0.571
FES	0.579
FETUB	0.201
FEV	0.683
FEZ1	0.655
FEZ2	0.187
FEZF1	0.994
FEZF2	0.966
FFAR1	0.312
FFAR2	0.227
FFAR3	0.416
FFAR4	0.482
FGA	0.138
FGB	0.242
FGD2	0.138
FGD3	0.237
FGD4	0.260
FGD5	0.762
FGD6	0.360
FGF1	0.419
FGF10	0.668
FGF11	0.839
FGF12	1.000
FGF14	0.923
FGF17	0.859
FGF18	0.992
FGF19	0.567
FGF2	0.729
FGF20	0.170
FGF21	0.252
FGF22	0.179
FGF23	0.525

FGF3	0.639
FGF4	0.906
FGF5	0.173
FGF6	0.268
FGF7	0.467
FGF9	0.889
FGFBP1	0.096
FGFBP2	0.199
FGFBP3	0.140
FGFR1	1.000
FGFR1OP	0.941
FGFR1OP2	0.394
FGFR2	0.946
FGFR3	0.701
FGFR4	0.323
FGFRL1	0.194
FGG	0.205
FGGY	0.310
FGL1	0.066
FGL2	0.110
FGR	0.478
FH	0.200
FHAD1	0.170
FHDC1	0.387
FHIT	0.336
FHL2	0.553
FHL3	0.230
FHL5	0.076
FHOD1	0.516
FHOD3	0.502
FIBCD1	0.303
FIBIN	0.061

FIBP	0.548
FICD	0.611
FIG4	0.321
FIGLA	0.268
FIGN	0.964
FIGNL1	0.283
FILIP1	0.618
FILIP1L	0.599
FIP1L1	0.827
FIS1	0.438
FITM1	0.499
FITM2	0.302
FIZ1	0.798
FJX1	0.212
FKBP10	0.459
FKBP11	0.393
FKBP14	0.149
FKBP15	0.979
FKBP1A	0.348
FKBP1B	0.295
FKBP1C	0.072
FKBP2	0.794
FKBP3	0.254
FKBP4	0.357
FKBP5	0.308
FKBP6	0.310
FKBP7	0.073
FKBP8	0.888
FKBP9	0.560
FKBPL	0.922
FKRP	0.574
FKSG48	0.310

FKSG52	0.154
FKSG61	0.229
FKSG62	0.128
FKSG63	0.399
FKSG68	0.230
FKTN	0.115
FLAD1	0.771
FLCN	0.704
FLG	0.112
FLG2	0.290
FLI1	0.745
FLII	0.628
FLJ00104	0.228
FLJ00273	0.403
FLJ00388	0.116
FLJ00418	0.948
FLJ14816	0.410
FLJ20306	0.102
FLJ20373	0.383
FLJ27365	0.318
FLJ30594	0.104
FLJ45079	0.165
FLNB	0.908
FLNC	0.813
FLOT1	0.725
FLOT2	0.758
FLRT1	0.339
FLRT2	0.957
FLRT3	0.397
FLT1	0.938
FLT3	0.580
FLT3LG	0.874

FLT4	0.342
FLVCR1	0.311
FLVCR2	0.559
FLYWCH1	0.258
FLYWCH2	0.320
FMN1	0.399
FMN2	0.667
FMNL1	0.651
FMNL2	0.807
FMNL3	0.655
FMO1	0.448
FMO2	0.143
FMO3	0.237
FMO4	0.225
FMO5	0.229
FMO6P	0.245
FMOD	0.354
FN1	0.550
FN3K	0.748
FN3KRP	0.490
FNBP1	0.267
FNBP1L	0.871
FNBP4	0.888
FNDC1	0.426
FNDC3A	0.777
FNDC3B	0.906
FNDC4	0.851
FNDC5	0.618
FNDC7	0.336
FNDC8	0.210
FNDC9	0.233
FNIP1	0.911

FNIP2	0.932
FNTA	0.767
FNTB	0.865
FOCAD	0.197
FOLH1	0.292
FOLR1	0.330
FOLR2	0.273
FOLR3	0.090
FOLR4	0.157
FOPNL	0.177
FOS	0.603
FOSB	0.933
FOSL1	0.478
FOSL2	0.888
FOXA1	0.650
FOXA2	0.933
FOXA3	0.932
FOXB1	0.941
FOXB2	0.458
FOXC1	0.615
FOXC2	0.423
FOXD1	0.891
FOXD2	0.717
FOXD3	0.905
FOXD4	0.125
FOXD4L1	0.270
FOXD4L2	0.457
FOXD4L3	0.235
FOXD4L4	0.488
FOXD4L5	0.227
FOXD4L6	0.220
FOXE1	0.416

FOXE3	0.331
FOXF1	0.218
FOXF2	0.299
FOXG1	0.968
FOXH1	0.146
FOXI1	0.338
FOXI2	0.201
FOXJ1	0.800
FOXJ2	0.669
FOXJ3	0.875
FOXK1	0.754
FOXK2	0.329
FOXL1	0.458
FOXL2	0.550
FOXM1	0.085
FOXN1	0.657
FOXN2	0.254
FOXN3	0.953
FOXN4	0.538
FOXO1	0.841
FOXO3	0.976
FOXO6	0.579
FOXP1	0.951
FOXP2	0.864
FOXP4	0.927
FOXQ1	0.459
FOXR1	0.233
FOXRED1	0.428
FOXRED2	0.166
FOXS1	0.100
FP15737	0.156
FPGS	0.791

FPGT	0.066
FPGT-TNNI3K	0.076
FPR1	0.024
FPR2	0.039
FPR3	0.051
FRA10AC1	0.210
FRAS1	0.166
FRAT1	0.597
FRAT2	0.162
FREM1	0.057
FREM2	0.286
FREM3	0.347
FRG1	0.126
FRG1B	0.138
FRG2	0.342
FRG2B	0.310
FRG2C	0.357
FRK	0.515
FRMD1	0.908
FRMD3	0.540
FRMD4A	0.769
FRMD4B	0.249
FRMD5	0.979
FRMD6	0.372
FRMD8	0.519
FRMPD1	0.610
FRMPD2	0.266
FRRS1	0.157
FRRS1L	0.171
FRS2	0.867
FRS3	0.946
FRY	0.513

FRYL	0.781
FRZB	0.091
FSBP	0.196
FSCB	0.115
FSCN1	0.589
FSCN2	0.351
FSCN3	0.144
FSD1	0.215
FSD1L	0.374
FSD2	0.357
FSHB	0.300
FSHR	0.061
FSIP1	0.175
FSIP2	0.149
FST	0.976
FSTL1	0.478
FSTL3	0.993
FSTL4	0.359
FSTL5	0.047
FTCD	0.158
FTCDNL1	0.296
FTH1	0.361
FTL	0.216
FTMT	0.127
FTO	0.762
FTSJ2	0.369
FTSJ3	0.507
FUBP1	0.878
FUBP3	0.824
FUCA1	0.458
FUCA2	0.205
FUK	0.585

FUOM	0.183
FURIN	0.861
FUS	0.941
FUT1	0.345
FUT10	0.099
FUT11	0.574
FUT2	0.287
FUT3	0.106
FUT4	0.299
FUT5	0.127
FUT6	0.165
FUT7	0.068
FUT8	0.206
FUT9	0.335
FUZ	0.302
FXN	0.186
FXR1	0.897
FXR2	0.999
FXYD1	0.428
FXYD2	0.077
FXYD3	0.257
FXYD4	0.099
FXYD5	0.186
FXYD6	0.128
FXYD6-FXYD2	0.131
FXYD7	0.226
FYB	0.281
FYCO1	0.782
FYN	0.984
FYTTD1	0.115
FZD1	0.342
FZD10	0.182

FZD2	0.987
FZD3	0.446
FZD4	0.496
FZD5	0.856
FZD6	0.942
FZD7	0.502
FZD8	0.800
FZD9	0.373
FZR1	0.982
G0S2	0.301
G2E3	0.665
G3BP1	0.946
G3BP2	0.940
G6PC	0.499
G6PC2	0.101
G6PC3	0.593
GAA	0.976
GAB1	0.688
GAB2	0.475
GAB4	0.066
GABARAP	0.635
GABARAPL1	0.640
GABARAPL2	0.985
GABARAPL3	0.433
GABBR1	0.886
GABBR2	0.991
GABPA	0.579
GABPB1	0.871
GABPB2	0.339
GABRA1	0.536
GABRA2	0.434
GABRA4	0.454

GABRA5	0.327
GABRA6	0.144
GABRB1	0.644
GABRB2	0.647
GABRB3	0.993
GABRD	0.532
GABRG1	0.089
GABRG2	0.593
GABRG3	0.396
GABRP	0.158
GABRR1	0.235
GAD1	0.863
GAD2	0.812
GADD45A	0.462
GADD45B	0.308
GADD45G	0.731
GADD45GIP1	0.632
GADL1	0.115
GAK	0.442
GAL	0.792
GAL3ST1	0.552
GAL3ST2	0.144
GAL3ST3	0.278
GAL3ST4	0.215
GALC	0.110
GALE	0.454
GALK1	0.666
GALK2	0.720
GALM	0.620
GALNS	0.261
GALNT1	0.287
GALNT10	0.708

GALNT11	0.109
GALNT12	0.199
GALNT13	0.251
GALNT14	0.255
GALNT15	0.331
GALNT16	0.585
GALNT18	0.617
GALNT2	0.610
GALNT3	0.213
GALNT4	0.424
GALNT5	0.183
GALNT6	0.279
GALNT7	0.886
GALNT8	0.352
GALNT9	0.046
GALNTL5	0.034
GALNTL6	0.389
GALP	0.063
GALR1	0.143
GALR2	0.787
GALR3	0.290
GALT	0.720
GAMT	0.490
GAN	0.308
GANAB	0.999
GANC	0.450
GAP43	0.355
GAPDH	0.727
GAPDHS	0.341
GAPT	0.079
GAPVD1	0.885
GAR1	0.228

GAREM	0.663
GAREML	0.584
GARNL3	0.727
GARS	0.659
GART	0.890
GAS1	0.425
GAS2	0.221
GAS2L1	0.741
GAS2L2	0.416
GAS2L3	0.115
GAS6	0.257
GAS7	0.862
GAS8	0.871
GAST	0.159
GATA2	0.870
GATA3	0.876
GATA4	0.916
GATA5	0.285
GATA6	0.935
GATAD1	0.590
GATAD2A	0.925
GATAD2B	0.996
GATC	0.169
GATM	0.294
GATS	0.341
GATSL1	0.534
GATSL2	0.410
GATSL3	0.489
GBA	0.686
GBA2	0.736
GBAS	0.507
GBE1	0.303

GBF1	0.938
GBGT1	0.349
GBP1	0.176
GBP2	0.127
GBP3	0.168
GBP4	0.080
GBP5	0.137
GBP6	0.407
GBP7	0.245
GBX1	0.294
GBX2	1.000
GC	0.385
GCA	0.080
GCAT	0.474
GCC1	0.566
GCC2	0.189
GCDH	0.458
GCFC2	0.138
GCG	0.534
GCGR	0.378
GCH1	0.989
GCHFR	0.251
GCK	0.985
GCKR	0.406
GCLC	0.604
GCLM	0.405
GCM1	0.764
GCM2	0.402
GCN1L1	0.895
GCNT1	0.114
GCNT2	0.311
GCNT3	0.063

GCNT4	0.081
GCNT6	0.297
GCNT7	0.248
GCOM1	0.167
GCSAM	0.163
GCSAML	0.057
GCSH	0.045
GDA	0.665
GDAP1	0.074
GDAP1L1	0.341
GDAP2	0.377
GDE1	0.251
GDF1	0.352
GDF10	0.568
GDF11	0.975
GDF15	0.236
GDF2	0.227
GDF3	0.865
GDF5	0.740
GDF5OS	0.170
GDF6	0.875
GDF7	0.499
GDF9	0.191
GDI2	0.873
GDNF	0.335
GDPD1	0.069
GDPD3	0.346
GDPD4	0.367
GDPD5	0.363
GDPGP1	0.278
GEM	0.692
GEMIN2	0.138

GEMIN4	0.594
GEMIN5	0.536
GEMIN6	0.117
GEMIN7	0.460
GEN1	0.305
GET4	0.972
GFAP	0.875
GFER	0.628
GFI1	0.766
GFI1B	0.258
GFM1	0.211
GFM2	0.545
GFOD1	0.574
GFOD2	0.330
GFPT1	0.916
GFPT2	0.304
GFRA1	0.361
GFRA2	0.424
GFRA3	0.735
GFRA4	0.969
GFRAL	0.182
GGA1	0.851
GGA2	0.255
GGA3	0.498
GGACT	0.279
GGCT	0.288
GGCX	0.587
GGH	0.183
GGN	0.417
GGNBP1	0.208
GGNBP2	0.964
GGPS1	0.246

GGT1	0.303
GGT2	0.394
GGT5	0.349
GGT6	0.294
GGT7	0.786
GGTLC1	0.370
GGTLC3	0.431
GH1	0.343
GH2	0.320
GHDC	0.393
GHITM	0.160
GHR	0.511
GHRH	0.314
GHRHR	0.306
GHRL	0.135
GHSR	0.637
GID4	0.875
GID8	0.889
GIF	0.302
GIGYF1	0.798
GIGYF2	0.895
GIMAP1	0.181
GIMAP2	0.285
GIMAP4	0.119
GIMAP5	0.585
GIMAP6	0.247
GIMAP7	0.071
GIMAP8	0.166
GIN1	0.368
GINM1	0.130
GINS1	0.042
GINS2	0.084

GINS3	0.117
GINS4	0.155
GIP	0.263
GIPC1	0.678
GIPC2	0.452
GIPC3	0.100
GIPR	0.352
GIT1	0.926
GIT2	0.925
GJA1	0.575
GJA10	0.251
GJA3	0.560
GJA4	0.318
GJA5	0.206
GJA8	0.445
GJA9	0.262
GJB2	0.094
GJB3	0.356
GJB4	0.581
GJB5	0.331
GJB6	0.067
GJB7	0.105
GJC1	0.821
GJC2	0.953
GJC3	0.281
GJD2	0.797
GJD3	0.673
GJD4	0.093
GK2	0.036
GK5	0.076
GKAP1	0.150
GKN1	0.358

GKN2	0.118
GLB1	0.779
GLB1L	0.381
GLB1L2	0.167
GLB1L3	0.105
GLCCI1	0.216
GLCE	0.752
GLDC	0.068
GLDN	0.324
GLE1	0.858
GLG1	0.512
GLI1	0.846
GLI2	0.703
GLI3	0.916
GLI4	0.215
GLIPR1	0.063
GLIPR1L1	0.042
GLIPR1L2	0.077
GLIPR2	0.314
GLIS1	0.263
GLIS2	0.350
GLIS3	0.876
GLMN	0.959
GLO1	0.480
GLOD4	0.163
GLP1R	0.573
GLP2R	0.271
GLRA1	0.687
GLRA3	0.282
GLRB	0.071
GLRX	0.062
GLRX2	0.168

GLRX3	0.514
GLRX5	0.217
GLS	0.854
GLS2	0.761
GLT1D1	0.034
GLT6D1	0.174
GLT8D1	0.430
GLT8D2	0.328
GLTP	0.462
GLTPD1	0.506
GLTPD2	0.553
GLTSCR1	0.748
GLTSCR1L	0.902
GLTSCR2	0.515
GLUD1	0.987
GLUL	0.734
GLYAT	0.251
GLYATL1	0.065
GLYATL2	0.094
GLYCTK	0.364
GLYR1	0.996
GM2A	0.324
GMCL1	0.428
GMDS	0.585
GMEB1	0.969
GMEB2	0.724
GMFB	0.343
GMFG	0.242
GMIP	0.505
GML	0.349
GMNC	0.295
GMNN	0.413

GMPPA	0.466
GMPPB	0.699
GMPR	0.336
GMPR2	0.729
GMPS	0.905
GNA11	0.812
GNA12	0.998
GNA13	0.829
GNA14	0.153
GNA15	0.557
GNAI1	0.962
GNAI2	0.660
GNAI3	0.785
GNAL	0.952
GNAO1	0.995
GNAQ	0.993
GNAS	0.973
GNAT1	0.316
GNAT2	0.296
GNAT3	0.209
GNAZ	0.896
GNB1	1.000
GNB1L	0.254
GNB2	0.861
GNB2L1	0.982
GNB3	0.379
GNB4	0.261
GNB5	0.248
GNE	0.795
GNG10	0.173
GNG11	0.186
GNG12	0.472

GNG13	0.044
GNG2	0.264
GNG3	0.495
GNG4	0.091
GNG5	0.139
GNG7	0.282
GNG8	0.277
GNGT1	0.245
GNGT2	0.146
GNL1	0.985
GNL2	0.615
GNL3	0.716
GNLY	0.075
GNMT	0.652
GNPAT	0.446
GNPDA1	0.463
GNPDA2	0.149
GNPNAT1	0.294
GNPTAB	0.421
GNPTG	0.161
GNRH1	0.111
GNRH2	0.045
GNRHR	0.916
GNS	0.312
GOLGA1	0.729
GOLGA2	0.849
GOLGA3	0.434
GOLGA4	0.551
GOLGA5	0.296
GOLGA6A	0.329
GOLGA6B	0.200
GOLGA6C	0.282

GOLGA6D	0.230
GOLGA6L1	0.164
GOLGA6L10	0.117
GOLGA6L2	0.425
GOLGA6L4	0.206
GOLGA6L6	0.120
GOLGA7	0.359
GOLGA7B	0.770
GOLGA8A	0.260
GOLGA8B	0.224
GOLGA8F	0.275
GOLGA8G	0.233
GOLGA8H	0.077
GOLGA8I	0.186
GOLGA8J	0.121
GOLGA8K	0.169
GOLGA8M	0.155
GOLGA8N	0.258
GOLGA8O	0.127
GOLGA8Q	0.188
GOLGA8R	0.120
GOLGA8T	0.120
GOLGB1	0.595
GOLIM4	0.243
GOLM1	0.555
GOLPH3	0.998
GOLPH3L	0.342
GOLT1A	0.263
GOLT1B	0.391
GON4L	0.919
GOPC	0.589
GORAB	0.273

GORASP1	0.480
GORASP2	0.825
GOSR1	0.277
GOSR2	0.521
GOT1	0.518
GOT1L1	0.169
GOT2	0.548
GP1BA	0.326
GP1BB	0.532
GP2	0.798
GP5	0.124
GP6	0.033
GP9	0.147
GPA33	0.280
GPAA1	0.573
GPALPP1	0.295
GPAM	0.091
GPANK1	0.537
GPAT2	0.409
GPATCH1	0.559
GPATCH11	0.300
GPATCH2	0.233
GPATCH2L	0.370
GPATCH3	0.610
GPATCH4	0.593
GPATCH8	0.741
GPBAR1	0.166
GPBP1	0.125
GPBP1L1	0.115
GPC1	0.605
GPC2	0.571
GPC5	0.064

GPC6	0.219
GPCPD1	0.101
GPD1	0.654
GPD1L	0.579
GPD2	0.528
GPER1	0.118
GPHA2	0.309
GPHN	0.852
GPI	0.885
GPIHBP1	0.168
GPLD1	0.229
GPM6A	0.434
GPN1	0.498
GPN2	0.805
GPN3	0.698
GPNMB	0.221
GPR1	0.125
GPR107	0.333
GPR108	0.409
GPR110	0.177
GPR111	0.150
GPR113	0.134
GPR114	0.279
GPR115	0.354
GPR116	0.397
GPR12	0.668
GPR123	0.246
GPR124	0.338
GPR125	0.203
GPR126	0.431
GPR128	0.395
GPR132	0.151

GPR133	0.162
GPR135	0.108
GPR137	0.742
GPR137B	0.150
GPR137C	0.422
GPR139	0.399
GPR141	0.098
GPR142	0.038
GPR144	0.313
GPR146	0.081
GPR148	0.085
GPR149	0.146
GPR15	0.073
GPR150	0.268
GPR151	0.170
GPR152	0.207
GPR153	0.295
GPR155	0.257
GPR156	0.381
GPR157	0.301
GPR158	0.310
GPR160	0.208
GPR161	0.318
GPR162	0.442
GPR17	0.168
GPR171	0.069
GPR176	0.271
GPR179	0.235
GPR18	0.141
GPR180	0.148
GPR182	0.327
GPR183	0.176

GPR19	0.296
GPR20	0.073
GPR21	0.381
GPR22	0.211
GPR25	0.372
GPR26	0.718
GPR27	0.183
GPR3	0.547
GPR31	0.067
GPR32	0.605
GPR35	0.108
GPR37	0.290
GPR37L1	0.361
GPR39	0.079
GPR4	0.678
GPR42	0.567
GPR45	0.108
GPR52	0.357
GPR55	0.220
GPR56	0.278
GPR6	0.419
GPR61	0.824
GPR62	0.630
GPR63	0.103
GPR65	0.049
GPR68	0.245
GPR75	0.171
GPR75-ASB3	0.219
GPR83	0.115
GPR84	0.117
GPR85	0.694
GPR87	0.089

GPR88	0.861
GPR89A	0.141
GPR89B	0.375
GPR89C	0.476
GPR97	0.123
GPR98	0.498
GPRC5A	0.155
GPRC5B	0.281
GPRC5C	0.349
GPRC5D	0.223
GPRC6A	0.300
GPRIN1	0.508
GPRIN2	0.289
GPRIN3	0.124
GPS1	0.949
GPS2	0.579
GPSM1	0.146
GPSM2	0.195
GPSM3	0.336
GPT	0.324
GPT2	0.494
GPX1	0.358
GPX2	0.274
GPX3	0.516
GPX4	0.968
GPX5	0.195
GPX6	0.265
GPX7	0.509
GPX8	0.272
GRAMD1A	0.704
GRAMD1B	0.858
GRAMD1C	0.325

GRAMD2	0.409
GRAMD3	0.162
GRAMD4	0.724
GRAP	0.434
GRAP2	0.317
GRAPL	0.515
GRASP	0.890
GRB10	0.432
GRB14	0.337
GRB2	0.689
GRB7	0.492
GREB1	0.125
GREB1L	0.956
GREM1	0.378
GREM2	0.171
GRHL1	0.999
GRHL2	0.990
GRHL3	0.786
GRHPR	0.573
GRIA1	0.983
GRIA2	0.994
GRIA4	0.679
GRID1	0.783
GRID2	0.909
GRID2IP	0.206
GRIK1	0.510
GRIK1-AS2	0.166
GRIK2	0.502
GRIK3	0.983
GRIK4	0.612
GRIK5	0.883
GRIN1	1.000

GRIN2A	1.000
GRIN2B	1.000
GRIN2C	0.369
GRIN2D	0.997
GRIN3A	0.382
GRIN3B	0.068
GRINA	0.397
GRIP1	0.847
GRK1	0.354
GRK4	0.936
GRK5	0.896
GRK6	0.774
GRK7	0.418
GRM1	0.667
GRM2	0.798
GRM3	0.836
GRM4	0.740
GRM5	0.774
GRM6	0.031
GRM7	0.576
GRM8	0.442
GRN	0.341
GRP	0.351
GRPEL1	0.384
GRPEL2	0.237
GRSF1	0.483
GRTP1	0.875
GRWD1	0.377
GRXCR1	0.113
GRXCR2	0.188
GS1-259H13.10	0.332
GSAP	0.308

GSC	0.671
GSC2	0.076
GSDMA	0.464
GSDMB	0.435
GSDMC	0.238
GSDMD	0.504
GSE1	0.621
GSG1	0.176
GSG1L	0.581
GSG2	0.261
GSK3A	1.000
GSK3B	0.997
GSKIP	0.331
GSN	0.627
GSPT1	0.995
GSR	0.410
GSS	0.739
GSTA1	0.223
GSTA2	0.370
GSTA3	0.410
GSTA4	0.546
GSTCD	0.263
GSTK1	0.305
GSTM1	0.342
GSTM2	0.370
GSTM3	0.206
GSTM4	0.354
GSTM5	0.154
GSTO1	0.466
GSTO2	0.532
GSTP1	0.076
GSTT1	0.301

GSTT2	0.426
GSTT2B	0.543
GSTZ1	0.336
GSX1	0.784
GSX2	0.580
GTDC1	0.122
GTF2A1	0.609
GTF2A1L	0.076
GTF2A2	0.251
GTF2B	0.694
GTF2E1	0.516
GTF2E2	0.147
GTF2F1	0.599
GTF2F2	0.831
GTF2H1	0.971
GTF2H2	0.330
GTF2H2C	0.251
GTF2H3	0.116
GTF2H4	0.511
GTF2H5	0.228
GTF2I	0.966
GTF2IRD1	0.936
GTF2IRD2	0.415
GTF2IRD2B	0.576
GTF3A	0.319
GTF3C1	0.983
GTF3C2	0.973
GTF3C3	0.561
GTF3C4	0.605
GTF3C5	0.587
GTF3C6	0.146
GTPBP1	0.973

GTPBP10	0.157
GTPBP2	0.999
GTPBP3	0.634
GTPBP4	0.330
GTPBP8	0.186
GTSCR1	0.173
GTSE1	0.148
GTSF1	0.343
GTSF1L	0.177
GUCA1A	0.274
GUCA1B	0.130
GUCA1C	0.137
GUCA2A	0.242
GUCA2B	0.368
GUCD1	0.960
GUCY1A2	0.387
GUCY1A3	0.470
GUCY1B3	0.575
GUCY2C	0.338
GUCY2D	0.309
GUF1	0.165
GUK1	0.524
GULP1	0.656
GUSB	0.473
GVQW1	0.364
GXYLT1	0.350
GXYLT2	0.207
GYG1	0.254
GYLTL1B	0.353
GYPA	0.184
GYPB	0.097
GYPC	0.137

GYPE	0.158
GYS1	0.847
GYS2	0.157
GZF1	0.751
GZMA	0.164
GZMB	0.093
GZMH	0.177
GZMK	0.198
GZMM	0.907
H1F0	0.566
H1FNT	0.179
H1FOO	0.149
H1FX	0.246
H2AFJ	0.376
H2AFV	0.902
H2AFX	0.338
H2AFY	0.979
H2AFY2	0.918
H2AFZ	0.820
H3F3A	0.661
H3F3B	0.731
H3F3C	0.358
H6PD	0.954
HAAO	0.462
HABP2	0.483
HABP4	0.393
HACE1	0.392
HACL1	0.333
HADH	0.591
HADHA	0.555
HADHB	0.853
HAGH	0.457

HAGHL	0.175
HAL	0.133
HAMP	0.447
HAND1	0.820
HAND2	0.650
HAO1	0.120
HAO2	0.961
HAP1	0.146
HAPLN1	0.339
HAPLN2	0.634
HAPLN3	0.197
HAPLN4	0.450
HARBI1	0.216
HARS	0.586
HARS2	0.785
HAS1	0.928
HAS2	0.330
HAS3	0.939
HAT1	0.873
HAUS1	0.217
HAUS2	0.361
HAUS3	0.144
HAUS4	0.297
HAUS5	0.443
HAUS6	0.111
HAUS8	0.671
HAVCR1	0.159
HAVCR2	0.133
HAX1	0.781
HBA1	0.237
HBA2	0.259
HBB	0.018

HBCBP	0.308
HBD	0.016
HBE1	0.139
HBEGF	0.479
HBG1	0.257
HBG2	0.125
HBM	0.055
HBP1	0.787
HBQ1	0.107
HBS1L	0.527
HBZ	0.419
HCAR1	0.121
HCAR2	0.162
HCAR3	0.096
HCFC1R1	0.056
HCFC2	0.862
HCG27	0.245
HCK	0.347
HCLS1	0.088
HCN1	1.000
HCN2	0.751
HCN3	0.535
HCN4	0.989
HCRT	0.531
HCRTR1	0.314
HCRTR2	0.327
HCST	0.346
HDAC1	0.999
HDAC10	0.199
HDAC11	0.617
HDAC2	0.961
HDAC3	1.000

HDAC4	0.874
HDAC5	0.981
HDAC7	0.964
HDAC9	0.988
HDC	0.359
HDDC2	0.154
HDDC3	0.618
HDGF	0.534
HDGFL1	0.113
HDGFRP2	0.818
HDGFRP3	0.795
HDHD2	0.294
HDHD3	0.365
HDLBP	0.783
HEATR1	0.058
HEATR2	0.515
HEATR3	0.897
HEATR4	0.253
HEATR5A	0.157
HEATR5B	0.367
HEATR6	0.243
HEBP1	0.347
HEBP2	0.437
HECA	0.603
HECTD1	0.994
HECTD2	0.913
HECTD3	0.788
HECTD4	0.991
HECW1	0.958
HECW2	1.000
HEG1	0.731
HELB	0.171

HELLS	0.560
HELQ	0.945
HELT	0.297
HELZ	0.956
HELZ2	0.183
HEMGN	0.159
HEMK1	0.283
HENMT1	0.364
HEPACAM	0.356
HEPACAM2	0.370
HEPHL1	0.386
HEPN1	0.146
HERC1	0.986
HERC2	0.764
HERC3	0.776
HERC4	0.852
HERC5	0.184
HERC6	0.408
HERPUD1	0.547
HERPUD2	0.646
HES1	0.225
HES2	0.538
HES3	0.794
HES4	0.245
HES5	0.404
HES6	0.373
HES7	0.764
HESX1	0.204
HEXA	0.500
HEXB	0.549
HEXDC	0.352
HEXIM1	0.725

HEXIM2	0.504
HEY1	0.212
HEY2	0.522
HEYL	0.277
HFE	0.165
HFE2	0.825
HFM1	0.110
HGD	0.456
HGF	0.834
HGFAC	0.970
HGS	0.916
HGSNAT	0.135
HHAT	0.557
HHATL	0.656
HHEX	0.798
HHIP	0.760
HHIPL1	0.264
HHIPL2	0.362
HHLA1	0.155
HHLA2	0.289
HHLA3	0.186
HIAT1	0.769
HIATL1	0.424
HIATL2	0.156
HIBADH	0.571
HIBCH	0.366
HIC1	0.730
HIC2	0.942
HID1	0.322
HIF1A	0.937
HIF1AN	0.951
HIF3A	0.565

HIGD1A	0.357
HIGD1B	0.203
HIGD1C	0.106
HIGD2A	0.226
HIGD2B	0.247
HILPDA	0.161
HINFP	0.768
HINT1	0.241
HINT2	0.560
HINT3	0.279
HIP1	0.591
HIP1R	0.560
HIPK1	0.999
HIPK2	0.997
HIPK3	0.342
HIPK4	0.427
HIRA	0.990
HIRIP3	0.657
HIST1H1A	0.046
HIST1H1B	0.159
HIST1H1C	0.027
HIST1H1D	0.062
HIST1H1E	0.050
HIST1H1T	0.026
HIST1H2AA	0.270
HIST1H2AB	0.056
HIST1H2AC	0.280
HIST1H2AD	0.062
HIST1H2AE	0.098
HIST1H2AG	0.160
HIST1H2AH	0.174
HIST1H2AI	0.356

HIST1H2AJ	0.555
HIST1H2AK	0.128
HIST1H2AL	0.126
HIST1H2AM	0.134
HIST1H2BA	0.212
HIST1H2BB	0.120
HIST1H2BC	0.083
HIST1H2BD	0.077
HIST1H2BE	0.035
HIST1H2BF	0.073
HIST1H2BG	0.066
HIST1H2BH	0.085
HIST1H2BI	0.118
HIST1H2BJ	0.109
HIST1H2BK	0.195
HIST1H2BL	0.193
HIST1H2BM	0.158
HIST1H2BN	0.154
HIST1H2BO	0.191
HIST1H3A	0.048
HIST1H3B	0.145
HIST1H3C	0.126
HIST1H3D	0.063
HIST1H3E	0.106
HIST1H3F	0.125
HIST1H3G	0.080
HIST1H3H	0.118
HIST1H3I	0.358
HIST1H3J	0.268
HIST1H4A	0.112
HIST1H4B	0.043
HIST1H4C	0.071

HIST1H4D	0.030
HIST1H4E	0.062
HIST1H4F	0.064
HIST1H4H	0.032
HIST1H4I	0.088
HIST1H4J	0.258
HIST1H4K	0.140
HIST2H2AA3	0.455
HIST2H2AA4	0.415
HIST2H2AB	0.277
HIST2H2AC	0.370
HIST2H2BE	0.604
HIST2H2BF	0.109
HIST2H3A	0.413
HIST2H3C	0.442
HIST2H3D	0.435
HIST2H3PS2	0.436
HIST2H4A	0.369
HIST2H4B	0.363
HIST3H2A	0.262
HIST3H2BB	0.119
HIST3H3	0.096
HIST4H4	0.180
HIVEP1	0.735
HIVEP2	0.925
HIVEP3	0.872
HJURP	0.140
HK1	0.977
HK2	0.240
HK3	0.188
HKDC1	0.313
HKR1	0.242

HLA-A	0.592
HLA-B	0.582
HLA-C	0.504
HLA-DMA	0.515
HLA-DMB	0.329
HLA-DOA	0.155
HLA-DOB	0.219
HLA-DPA1	0.246
HLA-DPB1	0.499
HLA-DQA1	0.216
HLA-DQA2	0.159
HLA-DQB1	0.256
HLA-DQB2	0.222
HLA-DRA	0.281
HLA-DRB1	0.289
HLA-DRB5	0.199
HLA-E	0.629
HLA-F	0.567
HLA-G	0.335
HLCS	0.602
HLF	0.855
HLTF	0.329
HLX	0.678
HM13	0.446
HMBOX1	0.915
HMBS	0.661
HMCES	0.707
HMCN1	0.816
HMCN2	0.253
HMG20A	0.771
HMG20B	0.790
HMGA1	0.741

HMGA2	0.481
HMGB1	0.940
HMGB2	0.292
HMGCL	0.567
HMGCLL1	0.134
HMGCR	0.993
HMGCS1	0.962
HMGCS2	0.996
HMGN1	0.110
HMGN2	0.400
HMGN3	0.198
HMGN4	0.206
HMGXB3	0.862
HMGXB4	0.650
HMHA1	0.263
HMHB1	0.075
HMMR	0.097
HMOX1	0.372
HMOX2	0.359
HMSD	0.072
HMX1	0.521
HMX2	0.687
HMX3	0.976
HN1	0.205
HN1L	0.532
HNF1A	0.784
HNF1B	0.955
HNF4A	0.880
HNF4G	0.250
HNMT	0.072
HNRNPA0	0.641
HNRNPA1	0.875

HNRNPA1L2	0.242
HNRNPA2B1	0.500
HNRNPA3	0.865
HNRNPAB	0.432
HNRNPC	0.687
HNRNPCL1	0.388
HNRNPD	0.884
HNRNPDL	0.307
HNRNPF	0.948
HNRNPH1	0.522
HNRNPH3	0.916
HNRNPK	0.986
HNRNPL	0.988
HNRNPLL	0.467
HNRNPM	0.966
HNRNPR	0.997
HNRNPU	0.800
HNRNPUL1	0.998
HNRNPUL2	0.880
HNRNPUL2-BSCL2	0.286
HOGA1	0.507
HOMER1	0.998
HOMER2	0.714
HOMER3	0.815
HOMEZ	0.442
HOOK1	0.303
HOOK2	0.507
HOOK3	0.865
HOPX	0.219
HORMAD1	0.505
HORMAD2	0.267
HOXA1	0.883

HOXA10	0.658
HOXA11	0.687
HOXA13	0.831
HOXA2	0.719
HOXA3	0.623
HOXA4	0.635
HOXA5	0.824
HOXA6	0.541
HOXA7	0.541
HOXA9	0.614
HOXB1	0.513
HOXB13	0.273
HOXB2	0.501
HOXB3	0.946
HOXB4	0.539
HOXB5	0.939
HOXB6	0.873
HOXB7	0.951
HOXB8	0.997
HOXB9	0.791
HOXC10	0.957
HOXC11	0.954
HOXC12	0.642
HOXC13	0.993
HOXC4	0.890
HOXC5	0.495
HOXC6	0.982
HOXC8	0.987
HOXC9	0.992
HOXD1	0.709
HOXD10	0.624
HOXD11	0.201

HOXD12	0.263
HOXD13	0.403
HOXD3	0.971
HOXD4	0.665
HOXD8	0.554
HOXD9	0.489
HP	0.397
HP1BP3	0.969
HPCA	0.886
HPCAL1	0.406
HPCAL4	0.702
HPD	0.184
HPDL	0.986
HPGD	0.118
HPGDS	0.065
HPN	0.903
HPR	0.250
HPS1	0.581
HPS3	0.457
HPS4	0.321
HPS5	0.413
HPS6	0.369
HPSE	0.141
HPSE2	0.210
HPX	0.186
HR	0.828
HRAS	1.000
HRASLS	0.036
HRASLS2	0.109
HRASLS5	0.334
HRC	0.327
HRCT1	0.239

HRG	0.309
HRH1	0.183
HRH2	0.336
HRH3	0.463
HRH4	0.209
HRK	0.400
HRNR	0.057
HRSP12	0.102
HS1BP3	0.245
HS2ST1	0.635
HS3ST1	0.158
HS3ST2	0.602
HS3ST3A1	0.199
HS3ST3B1	0.310
HS3ST4	0.436
HS3ST5	0.490
HS3ST6	0.571
HS6ST1	0.469
HS6ST3	0.717
hsa-mir-1199	0.427
hsa-mir-150	0.134
HSBP1	0.094
HSBP1L1	0.136
HSCB	0.453
HSD11B1	0.626
HSD11B1L	0.523
HSD11B2	0.426
HSD17B1	0.895
HSD17B11	0.456
HSD17B12	0.368
HSD17B13	0.945
HSD17B14	0.452

HSD17B2	0.976
HSD17B3	0.318
HSD17B4	0.469
HSD17B6	0.378
HSD17B7	0.521
HSD17B8	0.823
HSD3B1	0.962
HSD3B2	0.335
HSD3B7	0.666
HSDL1	0.957
HSDL2	0.537
HSF1	0.822
HSF2	0.796
HSF2BP	0.332
HSF4	0.398
HSF5	0.586
HSH2D	0.190
HSP90AA1	0.559
HSP90AB1	0.771
HSP90B1	0.890
HSPA12A	0.820
HSPA12B	0.940
HSPA13	0.341
HSPA14	0.655
HSPA1A	0.416
HSPA1B	0.395
HSPA1L	0.361
HSPA2	0.916
HSPA4	0.915
HSPA4L	0.263
HSPA5	0.669
HSPA6	0.377

HSPA8	0.915
HSPA9	0.883
HSPB1	0.304
HSPB11	0.671
HSPB2	0.649
HSPB2-C11orf52	0.241
HSPB3	0.128
HSPB6	0.356
HSPB7	0.326
HSPB8	0.181
HSPB9	0.348
HSPBAP1	0.646
HSPBP1	0.722
HSPD1	0.987
HSPE1	0.728
HSPE1-MOB4	0.807
HSPG2	0.575
HSPH1	0.297
HTATIP2	0.410
HTN1	0.113
HTN3	0.105
HTR1A	0.315
HTR1B	0.854
HTR1D	0.153
HTR1E	0.144
HTR1F	0.235
HTR2A	0.455
HTR2B	0.080
HTR3A	0.213
HTR3B	0.270
HTR3C	0.158
HTR3E	0.188

HTR4	0.651
HTR5A	0.082
HTR5A-AS1	0.074
HTR6	0.857
HTR7	0.488
HTRA1	0.600
HTRA2	1.000
HTRA3	0.319
HTRA4	0.198
HTT	0.997
HUG1	0.819
HUNK	0.777
HUS1	0.186
HUS1B	0.043
HVCN1	0.469
HYAL1	0.358
HYAL2	0.702
HYAL3	0.407
HYAL4	0.947
HYDIN	0.592
HYI	0.377
HYKK	0.367
HYLS1	0.206
HYOU1	0.797
HYPK	0.519
IAH1	0.486
IAPP	0.126
IARS	0.305
IARS2	0.454
IBA57	0.616
IBA57-AS1	0.188
IBSP	0.493

IBTK	0.579
ICA1	0.122
ICA1L	0.224
ICAM1	0.338
ICAM2	0.459
ICAM3	0.074
ICAM4	0.151
ICAM5	0.460
ICK	0.721
ICMT	0.910
ICOS	0.256
ICOSLG	0.382
ICT1	0.388
ID1	0.062
ID2	0.355
ID3	0.480
ID4	0.260
IDE	0.826
IDH1	0.184
IDH2	0.432
IDH3A	0.917
IDH3B	0.259
IDI1	0.295
IDI2	0.065
IDNK	0.249
IDO1	0.114
IDUA	0.237
IER2	0.138
IER3	0.201
IER3IP1	0.083
IER5	0.205
IER5L	0.237

IFFO1	0.275
IFFO2	0.654
IFI16	0.304
IFI27	0.146
IFI27L1	0.190
IFI27L2	0.363
IFI30	0.241
IFI35	0.542
IFI44	0.082
IFI44L	0.041
IFI6	0.254
IFIH1	0.115
IFIT1	0.410
IFIT1B	0.226
IFIT2	0.387
IFIT3	0.291
IFIT5	0.564
IFITM1	0.043
IFITM10	0.218
IFITM2	0.261
IFITM3	0.244
IFITM5	0.046
IFLTD1	0.898
IFNA1	0.239
IFNA13	0.273
IFNA2	0.141
IFNA21	0.081
IFNA5	0.190
IFNA6	0.182
IFNA8	0.171
IFNAR1	0.424
IFNAR2	0.606

IFNB1	0.158
IFNE	0.143
IFNG	0.468
IFNGR1	0.293
IFNGR2	0.512
IFNK	0.065
IFNL1	0.122
IFNL2	0.318
IFNL3	0.144
IFNLR1	0.681
IFNW1	0.136
IFRD1	0.195
IFRD2	0.666
IFRG15	0.716
IFT122	0.556
IFT140	0.463
IFT172	0.567
IFT20	0.667
IFT27	0.317
IFT43	0.258
IFT46	0.141
IFT52	0.313
IFT57	0.220
IFT74	0.154
IFT80	0.285
IFT81	0.315
IFT88	0.379
IGDCC3	0.342
IGDCC4	0.528
IGF1	0.732
IGF1R	0.818
IGF2	0.551

IGF2BP1	0.978
IGF2BP2	0.811
IGF2BP3	0.960
IGF2R	0.636
IGFALS	0.414
IGFBP1	0.218
IGFBP2	0.906
IGFBP3	0.566
IGFBP4	0.547
IGFBP5	0.739
IGFBP6	0.420
IGFBP7	0.495
IGFBPL1	0.183
IGFL1	0.131
IGFL2	0.325
IGFL3	0.377
IGFL4	0.278
IGFLR1	0.318
IGFN1	0.614
IGHMBP2	0.586
IGIP	0.349
IGJ	0.069
IGLL1	0.285
IGLL5	0.052
IGLON5	0.838
IGSF10	0.306
IGSF11	0.147
IGSF21	0.349
IGSF22	0.240
IGSF23	0.263
IGSF3	0.635
IGSF5	0.274

IGSF6	0.095
IGSF8	0.357
IGSF9	0.561
IGSF9B	0.995
IHH	0.876
IK	0.989
IKBIP	0.078
IKBKAP	0.537
IKBKB	0.939
IKBKE	0.791
IKZF1	0.789
IKZF2	0.850
IKZF3	0.815
IKZF4	0.996
IKZF5	0.749
IL10	0.322
IL10RA	0.406
IL10RB	0.554
IL11	0.370
IL11RA	0.455
IL12A	0.277
IL12B	0.497
IL12RB1	0.452
IL12RB2	0.295
IL13	0.180
IL15	0.206
IL15RA	0.262
IL16	0.352
IL17B	0.147
IL17C	0.058
IL17D	0.185
IL17F	0.184

IL17RA	0.938
IL17RB	0.099
IL17RC	0.502
IL17RD	0.296
IL17RE	0.637
IL17REL	0.145
IL18	0.313
IL18BP	0.193
IL18R1	0.190
IL18RAP	0.138
IL19	0.431
IL1A	0.182
IL1B	0.166
IL1F10	0.199
IL1R1	0.702
IL1R2	0.102
IL1RAP	0.967
IL1RL1	0.280
IL1RL2	0.363
IL1RN	0.204
IL2	0.371
IL20	0.222
IL20RA	0.522
IL20RB	0.273
IL21	0.371
IL21R	0.333
IL22	0.151
IL22RA1	0.727
IL22RA2	0.236
IL23A	0.365
IL23R	0.588
IL24	0.138

IL25	0.091
IL26	0.129
IL27	0.339
IL27RA	0.132
IL2RA	0.190
IL2RB	0.328
IL3	0.109
IL31	0.171
IL31RA	0.288
IL32	0.965
IL33	0.048
IL34	0.824
IL36A	0.093
IL36B	0.333
IL36G	0.576
IL36RN	0.382
IL37	0.451
IL4	0.368
IL4I1	0.261
IL4R	0.210
IL5	0.128
IL5RA	0.185
IL6	0.360
IL6R	0.404
IL6ST	0.862
IL7	0.371
IL7R	0.182
IL8	0.035
IL9	0.241
ILDR1	0.368
ILDR2	0.654
ILF2	0.989

ILF3	0.998
ILK	0.267
ILKAP	0.922
ILVBL	0.681
IMMP1L	0.151
IMMP2L	0.198
IMMT	0.269
IMP3	0.307
IMP4	0.243
IMPA1	0.404
IMPA2	0.316
IMPACT	0.096
IMPAD1	0.444
IMPDH1	0.580
IMPDH2	0.987
IMPG1	0.059
IMPG2	0.264
INA	0.693
INADL	0.686
INCA1	0.397
INCENP	0.892
INF2	0.672
ING1	0.993
ING2	0.551
ING3	0.900
ING4	0.730
ING5	0.533
INHA	0.464
INHBA	0.924
INHBB	0.849
INHBC	0.422
INHBE	0.477

INIP	0.102
INMT	0.438
INMT-FAM188B	0.375
INO80	0.999
INO80B	0.928
INO80C	0.223
INO80D	0.814
INO80E	0.510
INPP1	0.306
INPP4A	0.880
INPP4B	0.967
INPP5A	0.687
INPP5B	0.589
INPP5D	0.390
INPP5E	0.960
INPP5F	0.425
INPP5J	0.459
INPP5K	0.396
INPPL1	0.808
INS	0.732
INS-IGF2	0.310
INSC	0.437
INSIG1	0.155
INSIG2	0.526
INSL3	0.106
INSL4	0.017
INSL5	0.131
INSL6	0.006
INSM1	0.947
INSM2	0.644
INSR	0.879
INSRR	0.668

INTS1	0.264
INTS10	0.359
INTS12	0.400
INTS2	0.818
INTS3	0.999
INTS4	0.359
INTS5	0.920
INTS6	0.989
INTS7	0.647
INTS8	0.656
INTS9	0.515
INTU	0.261
INVS	0.405
IP6K1	0.983
IP6K2	0.982
IP6K3	0.619
IPCEF1	0.439
IPMK	0.606
IPO11	0.184
IPO13	0.999
IPO4	0.979
IPO5	0.901
IPO7	0.933
IPO8	0.959
IPO9	0.995
IPP	0.064
IPPK	0.852
IQCA1	0.108
IQCB1	0.202
IQCC	0.312
IQCD	0.301
IQCE	0.349

IQCF1	0.183
IQCF3	0.154
IQCF5	0.361
IQCF6	0.381
IQCG	0.041
IQCH	0.545
IQCJ	0.233
IQCJ-SCHIP1	0.468
IQCK	0.095
IQGAP1	0.482
IQGAP2	0.497
IQGAP3	0.225
IQSEC1	0.807
IQSEC3	0.169
IQUB	0.355
IRAK1BP1	0.067
IRAK2	0.437
IRAK3	0.441
IRAK4	0.258
IREB2	0.979
IRF1	0.612
IRF2	0.567
IRF2BP1	0.986
IRF2BP2	0.442
IRF2BPL	0.533
IRF3	0.759
IRF4	0.216
IRF5	0.665
IRF6	0.887
IRF7	0.128
IRF8	0.259
IRF9	0.892

IRGC	0.282
IRGM	0.221
IRGQ	0.563
IRS1	0.256
IRS2	0.870
IRX1	0.999
IRX2	1.000
IRX3	0.886
IRX4	0.520
IRX5	0.982
IRX6	0.802
ISCA1	0.491
ISCA2	0.530
ISCU	0.357
ISG15	0.252
ISG20	0.195
ISG20L2	0.862
ISL1	0.845
ISL2	0.948
ISLR	0.283
ISLR2	0.548
ISM1	0.507
ISM2	0.208
ISOC1	0.219
ISOC2	0.409
ISPD	0.367
IST1	0.975
ISX	0.166
ISY1	0.885
ISY1-RAB43	0.787
ISYNA1	0.469
ITCH	0.973

ITFG1	0.330
ITFG2	0.207
ITFG3	0.955
ITGA1	0.714
ITGA10	0.279
ITGA11	0.271
ITGA2	0.768
ITGA2B	0.251
ITGA3	0.776
ITGA4	0.207
ITGA5	0.631
ITGA6	0.353
ITGA7	0.380
ITGA8	0.172
ITGA9	0.465
ITGAD	0.261
ITGAL	0.239
ITGAM	0.207
ITGAV	0.280
ITGAX	0.122
ITGB1	0.947
ITGB1BP1	0.458
ITGB2	0.510
ITGB3	0.698
ITGB3BP	0.260
ITGB4	0.630
ITGB5	0.677
ITGB6	0.173
ITGB7	0.532
ITGB8	0.283
ITGBL1	0.234
ITIH1	0.283

ITIH2	0.223
ITIH3	0.426
ITIH4	0.342
ITIH5	0.260
ITK	0.326
ITLN1	0.198
ITLN2	0.269
ITM2B	0.205
ITM2C	0.135
ITPA	0.365
ITPK1	0.770
ITPKA	0.848
ITPKB	0.308
ITPKC	0.814
ITPR1	0.945
ITPR2	0.725
ITPR3	0.719
ITPRIP	0.153
ITPRIPL1	0.348
ITPRIPL2	0.328
ITSN1	0.997
ITSN2	0.358
IVD	0.482
IVL	0.307
IVNS1ABP	0.759
IWS1	0.823
IYD	0.187
IZUMO1	0.326
IZUMO2	0.113
IZUMO3	0.023
IZUMO4	0.095
JAG1	0.952

JAG2	0.998
JAGN1	0.477
JAK1	0.990
JAK2	0.461
JAK3	0.573
JAKMIP1	0.843
JAKMIP2	0.983
JAKMIP3	0.454
JAM2	0.154
JAM3	0.099
JARID2	0.992
JAZF1	0.936
JDP2	0.307
JHDM1D	0.587
JKAMP	0.445
JMJD1C	0.933
JMJD4	0.397
JMJD6	0.923
JMJD7	0.529
JMJD8	0.093
JMY	0.789
JOSD1	0.780
JOSD2	0.749
JPH1	0.417
JPH2	0.308
JPH3	0.403
JPH4	0.693
JRKL	0.381
JSRP1	0.260
JTB	0.431
JUN	0.327
JUNB	0.464

JUND	0.526
JUP	0.901
KAAG1	0.406
KALRN	1.000
KANK1	0.096
KANK2	0.762
KANK3	0.237
KANK4	0.492
KANSL1	0.956
KANSL1L	0.312
KANSL2	0.778
KANSL3	0.967
KARS	0.465
KAT2A	0.997
KAT2B	0.807
KAT5	1.000
KAT6A	0.980
KAT6B	0.991
KAT7	1.000
KAT8	0.989
KATNA1	0.373
KATNAL1	0.716
KATNAL2	0.293
KATNB1	0.673
KATNBL1	0.204
KAZALD1	0.453
KAZN	0.850
KB-1507C5.2	0.203
KB-1980E6.3	0.296
KBTBD11	0.126
KBTBD12	0.308
KBTBD13	0.210

KBTBD2	0.899
KBTBD3	0.141
KBTBD4	0.848
KBTBD6	0.527
KBTBD7	0.341
KBTBD8	0.309
KCMF1	0.975
KCNA1	0.932
KCNA10	0.183
KCNA2	1.000
KCNA3	0.901
KCNA4	0.934
KCNA5	0.577
KCNA6	0.842
KCNA7	0.719
KCNAB1	0.832
KCNAB2	0.457
KCNAB3	0.265
KCNB1	1.000
KCNB2	0.957
KCNC1	0.999
KCNC2	0.222
KCNC3	0.347
KCNC4	0.652
KCND2	0.858
KCND3	1.000
KCNE1	0.038
KCNE2	0.076
KCNE3	0.110
KCNE4	0.257
KCNF1	0.916
KCNG1	0.557

KCNG2	0.219
KCNG3	0.465
KCNG4	0.114
KCNH1	0.997
KCNH2	0.878
KCNH3	0.968
KCNH4	0.977
KCNH5	0.963
KCNH6	0.453
KCNH7	0.994
KCNH8	0.527
KCNIP1	0.685
KCNIP2	0.503
KCNIP3	0.465
KCNIP4	0.176
KCNJ1	0.989
KCNJ10	0.595
KCNJ11	0.785
KCNJ12	0.765
KCNJ13	0.692
KCNJ14	0.230
KCNJ15	0.650
KCNJ16	0.302
KCNJ2	0.948
KCNJ3	0.983
KCNJ4	0.956
KCNJ5	1.000
KCNJ6	0.964
KCNJ8	0.981
KCNJ9	0.945
KCNK1	0.440
KCNK10	0.895

KCNK12	0.987
KCNK13	0.230
KCNK15	0.451
KCNK16	0.599
KCNK17	0.495
KCNK18	0.524
KCNK2	0.566
KCNK3	0.946
KCNK4	0.882
KCNK5	0.624
KCNK6	0.540
KCNK7	0.781
KCNK9	0.937
KCNMA1	1.000
KCNMB1	0.197
KCNMB2	0.434
KCNMB3	0.064
KCNMB4	0.641
KCNN1	0.645
KCNN2	0.978
KCNN3	0.962
KCNN4	0.772
KCNQ1	0.928
KCNQ2	0.834
KCNQ3	0.531
KCNQ4	0.943
KCNQ5	0.778
KCNRG	0.187
KCNS1	0.603
KCNS2	0.643
KCNS3	0.478
KCNT1	0.490

KCNT2	0.737
KCNU1	0.740
KCNV1	0.685
KCNV2	0.064
KCTD1	0.998
KCTD10	0.702
KCTD11	0.449
KCTD12	0.386
KCTD14	0.243
KCTD15	0.660
KCTD16	0.841
KCTD17	0.747
KCTD18	0.183
KCTD19	0.501
KCTD20	0.343
KCTD21	0.289
KCTD3	0.391
KCTD4	0.311
KCTD5	0.955
KCTD6	0.385
KCTD7	0.529
KCTD8	0.109
KCTD9	0.610
KDELC1	0.161
KDELC2	0.279
KDELR1	0.814
KDELR2	0.227
KDELR3	0.258
KDM1A	1.000
KDM1B	0.569
KDM2A	1.000
KDM2B	0.987

KDM3A	0.960
KDM3B	0.999
KDM4A	1.000
KDM4B	0.957
KDM4C	0.204
KDM4D	0.258
KDM4E	0.437
KDM5A	0.140
KDM5B	0.489
KDM6B	0.930
KDM8	0.520
KDR	0.994
KDSR	0.519
KEAP1	0.959
KEL	0.254
KERA	0.189
KHDC1	0.089
KHDC1L	0.154
KHDC3L	0.085
KHDRBS1	0.991
KHDRBS2	0.100
KHDRBS3	0.533
KHK	0.376
KHNYN	0.874
KHSRP	1.000
KIAA0020	0.050
KIAA0040	0.207
KIAA0087	0.215
KIAA0100	0.798
KIAA0101	0.125
KIAA0125	0.228
KIAA0141	0.222

KIAA0195	0.683
KIAA0196	0.331
KIAA0226	0.980
KIAA0226L	0.199
KIAA0232	0.882
KIAA0247	0.320
KIAA0319	0.221
KIAA0319L	0.876
KIAA0355	0.695
KIAA0368	0.844
KIAA0391	0.167
KIAA0408	0.235
KIAA0430	0.842
KIAA0513	0.076
KIAA0556	0.486
KIAA0586	0.250
KIAA0753	0.469
KIAA0754	0.562
KIAA0825	0.142
KIAA0895	0.399
KIAA0895L	0.406
KIAA0907	0.935
KIAA0922	0.442
KIAA0930	0.905
KIAA0947	0.635
KIAA1009	0.172
KIAA1024	0.281
KIAA1024L	0.150
KIAA1033	0.161
KIAA1045	0.415
KIAA1107	0.185
KIAA1109	0.619

KIAA1143	0.255
KIAA1147	0.478
KIAA1161	0.238
KIAA1191	0.456
KIAA1199	0.235
KIAA1211	0.323
KIAA1211L	0.815
KIAA1217	0.751
KIAA1239	0.719
KIAA1244	0.788
KIAA1257	0.090
KIAA1279	0.626
KIAA1324	0.480
KIAA1324L	0.268
KIAA1328	0.415
KIAA1377	0.105
KIAA1407	0.233
KIAA1429	0.723
KIAA1430	0.893
KIAA1432	0.152
KIAA1456	0.019
KIAA1462	0.974
KIAA1467	0.280
KIAA1468	0.627
KIAA1522	0.341
KIAA1524	0.179
KIAA1549	0.603
KIAA1549L	0.559
KIAA1551	0.091
KIAA1586	0.139
KIAA1598	0.361
KIAA1614	0.381

KIAA1644	0.222
KIAA1658	0.226
KIAA1671	0.996
KIAA1683	0.335
KIAA1715	0.149
KIAA1731	0.375
KIAA1737	0.364
KIAA1755	0.147
KIAA1919	0.198
KIAA1958	0.203
KIAA1984	0.230
KIAA2013	0.386
KIAA2018	0.531
KIAA2026	0.106
KIDINS220	0.437
KIF11	0.800
KIF12	0.185
KIF13A	0.604
KIF13B	0.855
KIF14	0.136
KIF15	0.485
KIF16B	0.710
KIF17	0.378
KIF18A	0.314
KIF18B	0.481
KIF19	0.214
KIF1A	0.803
KIF1B	0.998
KIF1C	0.970
KIF20A	0.378
KIF20B	0.223
KIF21A	0.997

KIF21B	0.765
KIF22	0.645
KIF23	0.803
KIF24	0.203
KIF25	0.136
KIF26A	0.308
KIF26B	0.506
KIF27	0.153
KIF2A	0.575
KIF2B	0.038
KIF2C	0.391
KIF3A	0.708
KIF3B	0.965
KIF3C	0.627
KIF4B	0.178
KIF5A	0.921
KIF5B	0.976
KIF5C	0.705
KIF6	0.424
KIF7	0.248
KIF9	0.653
KIFAP3	0.544
KIFC1	0.398
KIFC2	0.177
KIFC3	0.915
KIN	0.364
KIR2DL1	0.159
KIR2DL3	0.289
KIR2DL4	0.365
KIR3DL1	0.221
KIR3DL2	0.160
KIR3DL3	0.131

KIR3DX1	0.083
KIRREL	0.644
KIRREL2	0.388
KIRREL3	0.750
KISS1	0.221
KISS1R	0.508
KIT	0.698
KITLG	0.281
KL	0.415
KLB	0.387
KLC1	0.971
KLC2	0.938
KLC3	0.395
KLC4	0.576
KLF1	0.387
KLF10	0.602
KLF11	0.966
KLF12	0.711
KLF13	0.476
KLF14	0.153
KLF15	0.827
KLF16	0.226
KLF17	0.180
KLF2	0.562
KLF3	0.827
KLF4	0.594
KLF5	0.565
KLF6	0.788
KLF7	0.795
KLF9	0.726
KLHDC1	0.126
KLHDC10	0.931

KLHDC2	0.427
KLHDC3	0.999
KLHDC4	0.202
KLHDC7A	0.265
KLHDC7B	0.124
KLHDC8A	0.549
KLHDC8B	0.430
KLHDC9	0.251
KLHL1	0.097
KLHL10	0.530
KLHL11	0.816
KLHL12	0.889
KLHL14	0.550
KLHL17	0.321
KLHL18	0.808
KLHL2	0.638
KLHL20	0.729
KLHL21	0.437
KLHL22	0.789
KLHL23	0.195
KLHL24	0.649
KLHL25	0.163
KLHL26	0.583
KLHL28	0.399
KLHL29	0.870
KLHL3	0.666
KLHL30	0.241
KLHL31	0.570
KLHL32	0.394
KLHL33	0.238
KLHL35	0.381
KLHL36	0.556

KLHL38	0.299
KLHL40	0.368
KLHL41	0.430
KLHL42	0.501
KLHL5	0.236
KLHL6	0.293
KLHL7	0.564
KLHL8	0.180
KLHL9	0.589
KLK1	0.946
KLK10	0.388
KLK11	0.701
KLK12	0.196
KLK13	0.401
KLK14	0.378
KLK15	0.353
KLK2	0.201
KLK3	0.581
KLK4	0.341
KLK5	0.458
KLK6	0.543
KLK7	0.358
KLK8	0.601
KLK9	0.602
KLKB1	0.131
KLLN	0.285
KLRB1	0.033
KLRC1	0.156
KLRC2	0.120
KLRC3	0.108
KLRC4	0.158
KLRC4-KLRK1	0.182

KLRD1	0.094
KLRF1	0.102
KLRF2	0.208
KLRG1	0.222
KLRG2	0.128
KLRK1	0.085
KM-PA-2	0.386
KMO	0.236
KMT2A	0.985
KMT2B	0.993
KMT2C	0.919
KMT2D	0.999
KMT2E	0.994
KNCN	0.045
KNDC1	0.377
KNG1	0.156
KNOP1	0.283
KNSTRN	0.113
KNTC1	0.097
KPNA1	0.997
KPNA2	0.420
KPNA3	0.864
KPNA4	0.995
KPNA5	0.540
KPNA6	1.000
KPNA7	0.326
KPNB1	1.000
KPRP	0.998
KPTN	0.181
KRAS	0.987
KRBA1	0.410
KRBA2	0.572

KRBOX1	0.094
KRCC1	0.235
KREMEN1	0.323
KREMEN2	0.482
KRI1	0.340
KRIT1	0.541
KRR1	0.096
KRT1	0.883
KRT10	0.953
KRT12	0.303
KRT13	0.257
KRT14	0.779
KRT15	0.323
KRT16	0.469
KRT17	0.467
KRT18	0.459
KRT19	0.107
KRT2	0.815
KRT20	0.396
KRT222	0.214
KRT23	0.488
KRT24	0.290
KRT25	0.527
KRT26	0.281
KRT27	0.438
KRT28	0.523
KRT3	0.436
KRT31	0.569
KRT32	0.410
KRT33A	0.197
KRT33B	0.459
KRT34	0.349

KRT35	0.332
KRT36	0.244
KRT37	0.580
KRT38	0.393
KRT39	0.348
KRT4	0.363
KRT40	0.334
KRT5	0.402
KRT6A	0.333
KRT6B	0.261
KRT6C	0.395
KRT7	0.736
KRT71	0.277
KRT72	0.418
KRT73	0.341
KRT74	0.237
KRT75	0.348
KRT76	0.407
KRT77	0.816
KRT78	0.463
KRT79	0.570
KRT8	0.492
KRT80	0.435
KRT81	0.569
KRT82	0.141
KRT83	0.209
KRT84	0.052
KRT85	0.153
KRT86	0.298
KRT9	0.518
KRTAP1-1	0.419
KRTAP1-3	0.498

KRTAP1-5	0.475
KRTAP10-1	0.186
KRTAP10-12	0.193
KRTAP10-2	0.317
KRTAP10-3	0.182
KRTAP10-4	0.156
KRTAP10-6	0.314
KRTAP10-7	0.379
KRTAP10-9	0.226
KRTAP11-1	0.246
KRTAP12-1	0.339
KRTAP13-1	0.093
KRTAP13-2	0.093
KRTAP13-4	0.096
KRTAP16-1	0.380
KRTAP17-1	0.560
KRTAP19-3	0.065
KRTAP19-8	0.118
KRTAP2-1	0.501
KRTAP2-2	0.526
KRTAP2-3	0.647
KRTAP2-4	0.449
KRTAP20-2	0.039
KRTAP20-4	0.077
KRTAP21-2	0.118
KRTAP24-1	0.087
KRTAP26-1	0.115
KRTAP29-1	0.438
KRTAP3-1	0.297
KRTAP3-2	0.677
KRTAP3-3	0.407
KRTAP4-1	0.143

KRTAP4-12	0.294
KRTAP4-6	0.121
KRTAP4-7	0.366
KRTAP4-9	0.271
KRTAP5-1	0.070
KRTAP5-10	0.314
KRTAP5-11	0.288
KRTAP5-2	0.189
KRTAP5-3	0.589
KRTAP5-4	0.246
KRTAP5-5	0.139
KRTAP5-6	0.071
KRTAP5-7	0.182
KRTAP5-8	0.369
KRTAP5-9	0.166
KRTAP8-1	0.266
KRTAP9-2	0.244
KRTAP9-3	0.349
KRTAP9-4	0.328
KRTAP9-8	0.209
KRTCAP2	0.380
KRTCAP3	0.358
KRTDAP	0.391
KSR1	0.893
KSR2	0.996
KTI12	0.405
KTN1	0.389
KXD1	0.682
KY	0.701
KYNU	0.062
L1TD1	0.255
L2HGDH	0.114

L34079.2	0.249
L3HYPDH	0.469
L3MBTL1	0.721
L3MBTL2	0.768
L3MBTL3	0.674
L3MBTL4	0.145
LA16c-431H6.6	0.253
LACC1	0.326
LACE1	0.553
LACRT	0.155
LACTB	0.272
LACTB2	0.981
LACTBL1	0.243
LAD1	0.447
LAG3	0.211
LAIR1	0.247
LAIR2	0.191
LALBA	0.445
LAMA1	0.122
LAMA2	0.472
LAMA3	0.409
LAMA4	0.310
LAMA5	0.226
LAMB1	0.598
LAMB2	0.356
LAMB3	0.351
LAMB4	0.368
LAMC1	0.809
LAMC2	0.352
LAMC3	0.332
LAMP1	0.580
LAMP3	0.357

LAMP5	0.101
LAMTOR1	0.898
LAMTOR2	0.846
LAMTOR3	0.244
LAMTOR4	0.592
LAMTOR5	0.315
LANCL1	0.324
LANCL2	0.344
LAP3	0.397
LAPTM4A	0.194
LAPTM4B	0.263
LAPTM5	0.069
LARGE	0.850
LARP1	1.000
LARP1B	0.359
LARP4	0.850
LARP4B	0.990
LARP6	0.617
LARP7	0.194
LARS	0.664
LARS2	0.482
LASP1	0.695
LAT	0.430
LAT2	0.149
LATS1	0.986
LATS2	0.940
LAX1	0.254
LAYN	0.398
LBH	0.344
LBP	0.397
LBR	0.117
LBX1	0.994

LBX2	0.961
LCA5	0.090
LCA5L	0.246
LCAT	0.775
LCE1A	0.641
LCE1B	0.308
LCE1C	0.307
LCE1D	0.751
LCE1E	0.661
LCE1F	0.970
LCE2A	0.966
LCE2B	0.951
LCE2C	0.914
LCE2D	0.957
LCE3A	0.346
LCE3D	0.581
LCE3E	0.524
LCE4A	0.908
LCE5A	0.826
LCE6A	0.338
LCK	0.870
LCLAT1	0.484
LCMT1	0.337
LCMT2	0.449
LCN1	0.187
LCN10	0.134
LCN15	0.323
LCN2	0.151
LCN6	0.154
LCN8	0.084
LCOR	0.784
LCORL	0.639

LCP1	0.355
LCP2	0.397
LCT	0.386
LCTL	0.248
LDB1	0.901
LDB2	0.856
LDB3	0.612
LDHA	0.339
LDHAL6A	0.236
LDHAL6B	0.206
LDHB	0.596
LDHC	0.138
LDHD	0.395
LDLR	0.352
LDLRAD1	0.266
LDLRAD2	0.240
LDLRAD3	0.291
LDLRAD4	0.680
LDLRAP1	0.210
LDOC1L	0.402
LEAP2	0.190
LECT1	0.210
LECT2	0.262
LEF1	0.583
LEFTY1	0.530
LEFTY2	0.232
LEKR1	0.262
LELP1	0.878
LEMD1	0.137
LEMD2	0.717
LEMD3	0.621
LENEP	0.195

LENG1	0.388
LENG8	0.490
LENG9	0.155
LEO1	0.897
LEP	0.564
LEPR	0.837
LEPRE1	0.565
LEPREL1	0.466
LEPREL4	0.546
LEPROT	0.082
LEPROTL1	0.317
LETM1	0.730
LETM2	0.153
LETMD1	0.297
LFNG	0.314
LGALS1	0.323
LGALS12	0.204
LGALS14	0.179
LGALS16	0.228
LGALS2	0.285
LGALS3	0.488
LGALS3BP	0.318
LGALS4	0.611
LGALS7	0.497
LGALS7B	0.444
LGALS8	0.089
LGALS9B	0.381
LGALS9C	0.229
LGALSL	0.544
LGI1	0.975
LGI2	0.282
LGI3	0.397

LGI4	0.271
LGMN	0.829
LGR4	0.692
LGR5	0.410
LGR6	0.349
LGSN	0.144
LHB	0.549
LHCGR	0.108
LHFP	0.312
LHFPL2	0.492
LHFPL3	0.137
LHFPL4	0.636
LHFPL5	0.328
LHPP	0.450
LHX1	0.747
LHX2	0.948
LHX3	0.159
LHX4	0.738
LHX5	0.995
LHX6	0.914
LHX8	0.596
LHX9	0.891
LIAS	0.159
LIF	0.836
LIFR	0.509
LIG1	0.637
LIG3	0.882
LIG4	0.153
LILRA1	0.086
LILRA2	0.029
LILRA3	0.141
LILRA4	0.073

LILRA5	0.049
LILRA6	0.262
LILRB1	0.234
LILRB2	0.235
LILRB3	0.147
LILRB4	0.894
LILRB5	0.118
LIM2	0.911
LIMA1	0.602
LIMCH1	0.877
LIMD1	0.650
LIMD2	0.424
LIME1	0.273
LIMK1	0.951
LIMK2	0.858
LIMS1	0.744
LIMS2	0.232
LIMS3	0.341
LIMS3L	0.186
LIN28A	0.441
LIN28B	0.695
LIN37	0.413
LIN52	0.614
LIN54	0.887
LIN7A	0.169
LIN7B	0.801
LIN7C	0.514
LIN9	0.949
LINC00346	0.161
LINC00692	0.157
LINC00696	0.149
LINC00908	0.091

LINC00923	0.903
LINC00935	0.351
LINC00955	0.257
LINC00998	0.213
LINC00999	0.213
LINGO1	0.917
LINGO2	0.143
LINGO3	0.301
LINGO4	0.239
LINS	0.158
LIPA	0.401
LIPC	0.476
LIPE	0.836
LIPF	0.145
LIPG	0.460
LIPH	0.280
LIPI	0.149
LIPJ	0.273
LIPK	0.437
LIPM	0.632
LIPN	0.153
LIPT1	0.309
LIPT2	0.220
LITAF	0.061
LIX1	0.104
LIX1L	0.368
LL22NC03-63E9.3	0.207
LL22NC03-75H12.2	0.032
LLGL1	0.511
LLGL2	0.328
LLPH	0.111
LMAN1	0.225

LMAN1L	0.324
LMAN2	0.278
LMAN2L	0.422
LMBR1	0.279
LMBR1L	0.492
LMBRD1	0.247
LMBRD2	0.350
LMCD1	0.332
LMF1	0.258
LMF2	0.087
LMLN	0.205
LMNA	0.841
LMNB1	0.285
LMNB2	0.827
LMO1	0.735
LMO2	0.526
LMO3	0.481
LMO4	0.837
LMO7	0.723
LMOD1	0.663
LMOD2	0.994
LMOD3	0.219
LMTK2	0.985
LMTK3	0.901
LMX1A	0.708
LMX1B	0.971
LNP1	0.285
LNPEP	0.788
LNX1	0.718
LNX2	0.690
LOH12CR1	0.463
LOH12CR2	0.216

LONP1	0.920
LONP2	0.695
LONRF1	0.261
LONRF2	0.229
LOR	1.000
LOX	0.802
LOXHD1	0.360
LOXL1	0.702
LOXL2	0.709
LOXL3	0.992
LOXL4	0.661
LPA	0.120
LPAR1	0.738
LPAR2	0.386
LPAR3	0.334
LPAR5	0.253
LPAR6	0.170
LPCAT1	0.934
LPCAT2	0.281
LPCAT3	0.921
LPCAT4	0.460
LPGAT1	0.488
LPHN1	0.843
LPHN2	0.683
LPHN3	0.635
LPIN1	0.586
LPIN2	0.185
LPIN3	0.488
LPL	0.157
LPO	0.382
LPP	0.379
LPPR1	0.370

LPPR3	0.059
LPPR4	0.483
LPPR5	0.272
LPXN	0.622
LRAT	0.175
LRBA	0.305
LRCH1	0.519
LRCH3	0.096
LRCH4	0.506
LRFN1	0.603
LRFN2	0.363
LRFN3	0.516
LRFN4	0.668
LRFN5	0.226
LRG1	0.199
LRGUK	0.185
LRIF1	0.192
LRIG1	0.498
LRIG2	0.138
LRIG3	0.438
LRIT1	0.078
LRIT2	0.095
LRIT3	0.230
LRMP	0.028
LRP1	1.000
LRP10	0.643
LRP11	0.489
LRP12	0.667
LRP1B	0.885
LRP2	0.628
LRP2BP	0.912
LRP3	0.508

LRP4	0.736
LRP5	0.817
LRP5L	0.197
LRP6	0.974
LRP8	0.921
LRPPRC	0.127
LRR1	0.146
LRRC1	0.682
LRRC10	0.290
LRRC10B	0.639
LRRC14	0.258
LRRC14B	0.080
LRRC15	0.102
LRRC16A	0.781
LRRC16B	0.517
LRRC17	0.239
LRRC18	0.100
LRRC19	0.079
LRRC2	0.189
LRRC20	0.182
LRRC23	0.295
LRRC24	0.192
LRRC25	0.201
LRRC26	0.321
LRRC27	0.082
LRRC28	0.066
LRRC29	0.222
LRRC3	0.156
LRRC30	0.064
LRRC31	0.229
LRRC32	0.628
LRRC34	0.078

LRRC36	0.280
LRRC37A	0.305
LRRC37A3	0.309
LRRC37B	0.179
LRRC38	0.149
LRRC39	0.273
LRRC3B	0.196
LRRC3C	0.247
LRRC3DN	0.326
LRRC4	0.739
LRRC40	0.114
LRRC41	0.949
LRRC42	0.367
LRRC43	0.254
LRRC45	0.277
LRRC46	0.383
LRRC47	0.778
LRRC48	0.345
LRRC49	0.396
LRRC4B	0.746
LRRC4C	0.319
LRRC52	0.133
LRRC53	0.116
LRRC55	0.236
LRRC56	0.293
LRRC57	0.650
LRRC58	0.427
LRRC59	0.630
LRRC6	0.122
LRRC61	0.164
LRRC63	0.254
LRRC66	0.196

LRRC69	0.108
LRRC7	0.841
LRRC70	0.082
LRRC71	0.310
LRRC72	0.138
LRRC73	0.518
LRRC8A	0.806
LRRC8B	0.919
LRRC8C	0.407
LRRC8D	0.997
LRRC8E	0.386
LRRC9	0.226
LRRCC1	0.080
LRRD1	0.390
LRRFIP1	0.303
LRRFIP2	0.251
LRRIQ1	0.043
LRRIQ3	0.031
LRRIQ4	0.206
LRRK1	0.453
LRRK2	0.296
LRRN1	0.275
LRRN2	0.344
LRRN3	0.190
LRRN4	0.274
LRRN4CL	0.208
LRRTM1	0.726
LRRTM2	0.833
LRRTM3	0.574
LRRTM4	0.134
LRSAM1	0.665
LRTM1	0.128

LRTM2	0.333
LRTOMT	0.173
LRWD1	0.367
LSAMP	0.869
LSG1	0.218
LSM1	0.617
LSM10	0.310
LSM11	0.379
LSM12	0.952
LSM14A	0.721
LSM14B	0.824
LSM2	0.618
LSM3	0.385
LSM4	0.531
LSM5	0.182
LSM6	0.295
LSM7	0.687
LSMD1	0.169
LSMEM1	0.042
LSMEM2	0.237
LSP1	0.702
LSR	0.270
LSS	0.500
LST1	0.254
LST3	0.127
LTA	0.505
LTA4H	0.182
LTB	0.312
LTB4R	0.372
LTB4R2	0.432
LTBP1	0.207
LTBP2	0.635

LTBP3	0.828
LTBP4	0.479
LTBR	0.625
LTC4S	0.987
LTF	0.303
LTK	0.380
LTN1	0.311
LTV1	0.356
LUC7L	0.398
LUC7L2	0.593
LUC7L3	0.945
LUM	0.093
LURAP1	0.213
LURAP1L	0.081
LUZP1	0.596
LUZP2	0.070
LUZP6	0.172
LUZPP1	0.232
LXN	0.117
LY6D	0.999
LY6E	0.447
LY6G5B	0.440
LY6G5C	0.297
LY6G6C	0.601
LY6G6D	0.269
LY6G6E	0.157
LY6G6F	0.164
LY6H	0.132
LY6K	0.118
LY75	0.180
LY75-CD302	0.145
LY86	0.105

LY9	0.159
LY96	0.143
LYAR	0.417
LYG1	0.106
LYG2	0.324
LYL1	0.541
LYN	0.408
LYNX1	0.991
LYPD1	0.227
LYPD2	0.078
LYPD3	0.325
LYPD4	0.260
LYPD5	0.601
LYPD6	0.195
LYPD6B	0.493
LYPD8	0.398
LYPLA1	0.306
LYPLA2	0.891
LYPLAL1	0.176
LYRM1	0.244
LYRM2	0.095
LYRM4	0.157
LYRM5	0.139
LYRM7	0.037
LYRM9	0.125
LYSMD1	0.833
LYSMD2	0.170
LYSMD3	0.272
LYSMD4	0.189
LYST	0.466
LYVE1	0.232
LYZ	0.074

LYZL1	0.180
LYZL2	0.179
LYZL4	0.364
LYZL6	0.126
LZIC	0.251
LZTFL1	0.212
LZTR1	0.400
LZTS1	0.368
LZTS2	0.375
LZTS3	0.451
M1AP	0.988
M6PR	0.417
MAATS1	0.163
MAB21L1	0.775
MAB21L2	0.999
MAB21L3	0.407
MACC1	0.190
MACF1	0.978
MACROD1	0.809
MACROD2	0.848
MAD1L1	0.986
MAD2L1	0.134
MAD2L1BP	0.679
MAD2L2	0.705
MADCAM1	0.143
MADD	0.815
MAEA	0.992
MAEL	0.213
MAF	0.467
MAF1	0.841
MAFA	0.834
MAFB	1.000

MAFF	0.585
MAFG	0.561
MAFK	0.318
MAG	0.653
MAGEF1	0.575
MAGEL2	0.354
MAGI1	0.752
MAGI2	0.961
MAGI3	0.984
MAGOH	0.937
MAGOHB	0.400
MAK	0.155
MAK16	0.295
MAL	0.283
MAL2	0.373
MALL	0.373
MALT1	0.696
MAMDC2	0.265
MAMDC4	0.164
MAML1	0.995
MAML2	0.665
MAML3	0.790
MAMSTR	0.350
MAN1A1	0.232
MAN1A2	0.995
MAN1B1	0.430
MAN1C1	0.632
MAN2A1	0.411
MAN2A2	0.435
MAN2B1	0.457
MAN2B2	0.405
MAN2C1	0.323

MANBA	0.277
MANBAL	0.305
MANEA	0.203
MANEAL	0.324
MANF	0.501
MANSC1	0.190
MANSC4	0.096
MAP10	0.093
MAP1A	0.840
MAP1B	0.991
MAP1LC3A	0.539
MAP1LC3B	0.438
MAP1LC3B2	0.120
MAP1LC3C	0.042
MAP1S	0.352
MAP2	0.563
MAP2K1	1.000
MAP2K2	1.000
MAP2K3	0.469
MAP2K4	0.955
MAP2K5	0.915
MAP2K6	0.828
MAP2K7	0.989
MAP3K1	0.922
MAP3K10	0.959
MAP3K11	0.906
MAP3K12	0.999
MAP3K13	0.769
MAP3K19	0.393
MAP3K2	0.896
MAP3K3	0.996
MAP3K4	0.375

MAP3K5	0.984
MAP3K6	0.538
MAP3K7	0.819
MAP3K7CL	0.265
MAP3K8	0.725
MAP3K9	0.472
MAP4	0.721
MAP4K1	0.897
MAP4K2	0.408
MAP4K3	0.485
MAP4K4	0.993
MAP4K5	0.332
MAP6	0.522
MAP6D1	0.291
MAP7	0.535
MAP7D1	0.478
MAP9	0.053
MAPK1	0.999
MAPK10	0.596
MAPK11	0.545
MAPK12	0.472
MAPK13	0.699
MAPK14	0.963
MAPK15	0.342
MAPK1IP1L	0.326
MAPK3	0.947
MAPK4	0.853
MAPK6	0.857
MAPK7	0.942
MAPK8	0.966
MAPK8IP1	0.697
MAPK8IP2	1.000

MAPK8IP3	0.966
MAPK9	0.222
MAPKAP1	0.980
MAPKAPK2	0.882
MAPKAPK3	0.785
MAPKAPK5	0.687
MAPKBP1	0.607
MAPRE1	0.952
MAPRE2	0.981
MAPRE3	0.975
MAPT	0.178
MARC1	0.291
MARC2	0.635
MARCH1	0.333
MARCH10	0.462
MARCH11	0.420
MARCH2	0.338
MARCH3	0.527
MARCH4	0.482
MARCH5	0.950
MARCH6	0.956
MARCH7	0.557
MARCH8	0.451
MARCH9	0.875
MARCKS	0.229
MARCKSL1	0.345
MARCO	0.118
MARK1	0.977
MARK2	1.000
MARK3	0.899
MARK4	0.982
MARS	0.709

MARS2	0.618
MARVELD1	0.242
MARVELD2	0.392
MARVELD3	0.487
MAS1	0.088
MAS1L	0.505
MASP1	0.076
MASP2	0.486
MAST1	0.991
MAST2	0.319
MAST3	0.991
MAST4	0.857
MASTL	0.249
MAT1A	0.617
MAT2A	0.998
MAT2B	0.613
MATK	0.980
MATN1	0.122
MATN2	0.362
MATN3	0.190
MATN4	0.495
MATR3	0.993
MAU2	0.992
MAVS	0.981
MAX	0.912
MAZ	0.839
MB	0.592
MB21D1	0.100
MB21D2	0.321
MBD1	0.999
MBD2	0.497
MBD3	0.958

MBD3L1	0.224
MBD3L2	0.220
MBD3L3	0.082
MBD4	0.634
MBD5	0.259
MBD6	0.878
MBIP	0.169
MBL2	0.201
MBLAC1	0.341
MBLAC2	0.158
MBNL1	0.914
MBNL2	0.636
MBOAT1	0.230
MBOAT2	0.377
MBOAT4	0.321
MBOAT7	0.175
MBP	0.123
MBTD1	1.000
MBTPS1	0.878
MC1R	0.078
MC2R	0.181
MC3R	0.162
MC4R	0.079
MC5R	0.055
MCAM	0.502
MCAT	0.629
MCC	0.263
MCCC1	0.688
MCCC2	0.361
MCCD1	0.243
MCEE	0.509
MCF2L	0.218

MCF2L2	0.598
MCFD2	0.225
MCHR1	0.175
MCHR2	0.054
MCIDAS	0.632
MCL1	0.353
MCM10	0.148
MCM2	0.587
MCM3	0.519
MCM3AP	0.138
MCM4	0.256
MCM5	0.559
MCM6	0.713
MCM7	0.698
MCM8	0.291
MCM9	0.575
MCMBP	0.913
MCMDC2	0.364
MCOLN1	0.857
MCOLN2	0.395
MCOLN3	0.412
MCPH1	0.066
MCRS1	0.989
MCTP1	0.492
MCTP2	0.017
MCU	0.725
MCUR1	0.197
MDC1	0.847
MDFI	0.712
MDFIC	0.166
MDGA1	0.685
MDGA2	0.852

MDH1	0.783
MDH1B	0.188
MDH2	0.824
MDK	0.453
MDM1	0.221
MDM2	0.935
MDM4	0.937
MDN1	0.590
MDP1	0.474
MDS2	0.273
ME1	0.679
ME2	0.549
ME3	0.282
MEA1	0.656
MEAF6	0.809
MECOM	0.842
MECR	0.819
MED1	0.951
MED10	0.198
MED11	0.481
MED12L	0.972
MED13	0.945
MED13L	1.000
MED15	0.974
MED16	0.330
MED17	0.137
MED18	0.124
MED19	0.672
MED20	0.698
MED21	0.399
MED22	0.451
MED23	0.429

MED24	0.820
MED25	0.839
MED26	0.561
MED27	0.381
MED28	0.164
MED29	0.500
MED30	0.200
MED31	0.555
MED4	0.395
MED6	0.282
MED7	0.351
MED8	0.639
MED9	0.592
MEDAG	0.441
MEF2A	0.860
MEF2B	0.382
MEF2BNB	0.392
MEF2BNB-MEF2B	0.384
MEF2C	1.000
MEF2D	0.964
MEFV	0.041
MEGF10	0.185
MEGF11	0.221
MEGF6	0.257
MEGF8	0.900
MEGF9	0.488
MEGT1	0.236
MEI1	0.265
MEI4	0.172
MEIG1	0.065
MEIOB	0.221
MEIS1	0.973

MEIS2	0.987
MEIS3	0.263
MELK	0.144
MEMO1	0.937
MEN1	0.998
MEOX1	0.456
MEOX2	0.255
MEP1A	0.503
MEP1B	0.506
MEPCE	0.982
MEPE	0.242
MERTK	0.539
MESDC1	0.742
MESDC2	0.362
MESP1	0.217
MESP2	0.342
MEST	0.349
MET	0.992
METAP1	0.798
METAP1D	0.269
METAP2	0.904
METRN	0.264
METRNL	0.153
METTL1	0.593
METTL10	0.266
METTL11B	0.462
METTL13	0.695
METTL14	0.522
METTL15	0.186
METTL16	0.895
METTL17	0.669
METTL18	0.367

METTL20	0.119
METTL21A	0.478
METTL21B	0.538
METTL21C	0.129
METTL22	0.047
METTL23	0.247
METTL24	0.293
METTL25	0.103
METTL2A	0.151
METTL2B	0.095
METTL3	0.969
METTL4	0.032
METTL5	0.570
METTL6	0.318
METTL7A	0.179
METTL7B	0.394
METTL8	0.140
METTL9	0.556
MEX3A	0.688
MEX3B	0.505
MEX3C	0.933
MEX3D	0.823
MFAP1	0.938
MFAP2	0.373
MFAP3	0.210
MFAP3L	0.396
MFAP4	0.473
MFAP5	0.095
MFF	0.229
MFGE8	0.115
MFHAS1	0.383
MFI2	0.402

MFN1	0.228
MFN2	0.826
MFNG	0.376
MFSD1	0.045
MFSD10	0.952
MFSD11	0.430
MFSD12	0.303
MFSD2A	0.389
MFSD2B	0.134
MFSD3	0.411
MFSD4	0.262
MFSD5	0.814
MFSD6	0.627
MFSD6L	0.213
MFSD7	0.216
MFSD8	0.220
MFSD9	0.216
MGA	0.933
MGAM	0.283
MGARP	0.124
MGAT1	0.286
MGAT2	0.371
MGAT3	0.332
MGAT4A	0.304
MGAT4B	0.967
MGAT4C	0.149
MGAT5	0.836
MGAT5B	0.226
MGC10955	0.171
MGC4294	0.126
MGEA5	0.977
MGLL	0.686

MGME1	0.302
MGMT	0.290
MGP	0.223
MGRN1	0.703
MGST1	0.114
MGST2	0.339
MGST3	0.209
MIA	0.397
MIA-RAB4B	0.428
MIA2	0.297
MIA3	0.481
MIB1	0.470
MIB2	0.374
MICA	0.509
MICAL1	0.510
MICAL2	0.353
MICAL3	1.000
MICALCL	0.155
MICALL1	0.498
MICALL2	0.356
MICB	0.326
MICU1	0.620
MICU2	0.384
MICU3	0.090
MIDN	0.199
MIEF1	0.464
MIEF2	0.707
MIEN1	0.677
MIER1	0.635
MIER2	0.446
MIER3	0.705
MIF	0.077

MIF4GD	0.340
MIIP	0.360
MILR1	0.161
MINA	0.547
MINK1	0.995
MINOS1-NBL1	0.956
MINPP1	0.646
MIOS	0.109
MIOX	0.047
MIP	0.751
MIPEP	0.590
MIPOL1	0.206
MIR3654	0.346
MIS12	0.557
MIS18A	0.162
MIS18BP1	0.237
MISP	0.243
MITD1	0.223
MITF	0.774
MIXL1	0.125
MKI67	0.211
MKI67IP	0.280
MKKS	0.285
MKL1	0.528
MKL2	0.642
MKLN1	0.805
MKNK1	0.764
MKNK2	0.653
MKRN1	0.716
MKRN2	0.649
MKRN3	0.320
MKS1	0.471

MKX	0.523
MLANA	0.054
MLC1	0.068
MLEC	0.920
MLF1	0.091
MLF1IP	0.079
MLF2	0.887
MLH1	0.839
MLH3	0.693
MLIP	0.305
MLK4	0.735
MLKL	0.214
MLLT1	0.967
MLLT10	0.985
MLLT11	0.192
MLLT3	0.942
MLLT4	0.999
MLLT6	0.992
MLNR	0.182
MLPH	0.294
MLST8	0.559
MLTK	0.742
MLX	0.544
MLXIP	0.687
MLXIPL	0.687
MLYCD	0.318
MMAA	0.334
MMAB	0.800
MMACHC	0.163
MMADHC	0.306
MMD	0.174
MMD2	0.109

MME	0.090
MMEL1	0.281
MMP1	0.058
MMP10	0.310
MMP11	0.140
MMP13	0.284
MMP14	0.970
MMP15	0.604
MMP16	0.892
MMP17	0.097
MMP19	0.205
MMP2	0.747
MMP20	0.263
MMP21	0.286
MMP23B	0.207
MMP24	0.522
MMP25	0.130
MMP26	0.111
MMP27	0.180
MMP28	0.464
MMP3	0.287
MMP7	0.168
MMP8	0.093
MMP9	0.144
MMRN1	0.102
MMRN2	0.421
MMS19	0.836
MMS22L	0.266
MN1	0.921
MNAT1	0.494
MND1	0.075
MNDA	0.025

MNS1	0.076
MNT	0.502
MNX1	0.831
MOAP1	0.582
MOB1A	0.707
MOB1B	0.521
MOB2	0.368
MOB3A	0.226
MOB3B	0.288
MOB3C	0.162
MOB4	0.953
MOBP	0.290
MOCOS	0.365
MOCS1	0.363
MOCS2	0.160
MOCS3	0.673
MOG	0.413
MOGAT1	0.251
MOGAT2	0.297
MOGAT3	0.220
MOGS	0.671
MOK	0.260
MON1A	0.380
MON1B	0.175
MON2	0.744
MORC1	0.244
MORC2	0.999
MORC3	0.992
MORF4L1	0.847
MORN1	0.433
MORN2	0.095
MORN3	0.175

MORN4	0.371
MORN5	0.340
MOS	0.763
MOSPD3	0.410
MOV10	0.551
MOV10L1	0.230
MOXD1	0.166
MPC1	0.068
MPC2	0.323
MPDU1	0.363
MPDZ	0.091
MPEG1	0.079
MPG	0.104
MPHOSPH10	0.707
MPHOSPH6	0.090
MPHOSPH8	0.095
MPHOSPH9	0.209
MPI	0.448
MPL	0.271
MPLKIP	0.119
MPND	0.438
MPO	0.323
MPP2	0.625
MPP3	0.629
MPP4	0.164
MPP5	0.580
MPP6	0.715
MPP7	0.445
MPPE1	0.372
MPPED1	0.427
MPPED2	0.803
MPRIP	0.925

MPST	0.676
MPV17	0.240
MPV17L	0.298
MPV17L2	0.611
MPZ	0.677
MPZL1	0.509
MPZL2	0.167
MPZL3	0.132
MR1	0.421
MRAP	0.146
MRAP2	0.088
MRAS	0.778
MRC1	0.570
MRC1L1	0.374
MRC2	0.922
MRE11A	0.435
MREG	0.303
MRFAP1	0.212
MRFAP1L1	0.115
MRGBP	0.726
MRGPRD	0.207
MRGPRE	0.076
MRGPRF	0.317
MRGPRG	0.103
MRGPRX2	0.194
MRGPRX3	0.412
MRGPRX4	0.300
MRI1	0.770
MRM1	0.230
MRO	0.095
MROH1	0.441
MROH2A	0.284

MROH2B	0.234
MROH6	0.185
MROH7	0.070
MROH7-TTC4	0.101
MROH8	0.306
MROH9	0.248
MRP63	0.398
MRPL1	0.330
MRPL10	0.754
MRPL11	0.335
MRPL12	0.676
MRPL13	0.420
MRPL14	0.457
MRPL15	0.315
MRPL16	0.470
MRPL17	0.405
MRPL18	0.288
MRPL19	0.217
MRPL2	0.690
MRPL20	0.324
MRPL21	0.496
MRPL22	0.347
MRPL23	0.318
MRPL24	0.633
MRPL27	0.621
MRPL28	0.990
MRPL3	0.106
MRPL30	0.184
MRPL32	0.210
MRPL33	0.191
MRPL34	0.376
MRPL35	0.134

MRPL36	0.147
MRPL37	0.550
MRPL38	0.566
MRPL39	0.123
MRPL4	0.331
MRPL40	0.476
MRPL41	0.153
MRPL42	0.160
MRPL43	0.806
MRPL44	0.240
MRPL45	0.704
MRPL46	0.302
MRPL47	0.158
MRPL48	0.190
MRPL49	0.813
MRPL50	0.285
MRPL51	0.261
MRPL52	0.251
MRPL53	0.233
MRPL54	0.965
MRPL55	0.550
MRPL9	0.668
MRPS10	0.455
MRPS11	0.232
MRPS12	0.606
MRPS14	0.220
MRPS15	0.615
MRPS16	0.158
MRPS17	0.272
MRPS18A	0.563
MRPS18B	0.557
MRPS18C	0.929

MRPS2	0.375
MRPS21	0.192
MRPS22	0.304
MRPS23	0.408
MRPS24	0.469
MRPS25	0.298
MRPS26	0.263
MRPS27	0.332
MRPS28	0.293
MRPS30	0.156
MRPS31	0.521
MRPS33	0.352
MRPS34	0.165
MRPS35	0.170
MRPS36	0.372
MRPS5	0.161
MRPS6	0.313
MRPS7	0.418
MRPS9	0.426
MRRF	0.426
MRS2	0.193
MRTO4	0.594
MRVI1	0.345
MS4A1	0.085
MS4A10	0.489
MS4A12	0.139
MS4A13	0.274
MS4A14	0.103
MS4A15	0.282
MS4A18	0.351
MS4A2	0.115
MS4A3	0.240

MS4A4A	0.153
MS4A4E	0.119
MS4A5	0.140
MS4A6A	0.095
MS4A6E	0.195
MS4A7	0.065
MS4A8	0.328
MSANTD1	0.256
MSANTD2	0.683
MSANTD3	0.545
MSANTD3-TMEFF	0.439
MSANTD4	0.288
MSC	0.715
MSGN1	0.134
MSH2	0.199
MSH3	0.488
MSH4	0.404
MSH5	0.581
MSH5-SAPCD1	0.285
MSH6	0.264
MSI1	0.938
MSI2	0.979
MSL1	0.987
MSL2	0.985
MSLN	0.069
MSLNL	0.042
MSMB	0.188
MSMO1	0.162
MSMP	0.468
MSR1	0.017
MSRA	0.116
MSRB1	0.286

MSRB2	0.440
MSRB3	0.428
MSS51	0.637
MST1	0.654
MST1R	0.663
MSTN	0.255
MSTO1	0.234
MSX1	0.119
MSX2	0.854
MT1A	0.134
MT1B	0.115
MT1E	0.197
MT1F	0.229
MT1G	0.164
MT1H	0.203
MT1HL1	0.083
MT1M	0.160
MT1X	0.437
MT2A	0.296
MT3	0.399
MT4	0.186
MTA1	0.988
MTA2	0.996
MTA3	0.297
MTAP	0.358
MTBP	0.110
MTCH1	0.485
MTCH2	0.392
MTDH	0.230
MTERF	0.079
MTERFD1	0.402
MTERFD2	0.591

MTERFD3	0.233
MTF1	0.944
MTF2	0.970
MTFMT	0.160
MTFP1	0.145
MTFR1	0.235
MTFR1L	0.788
MTFR2	0.111
MTG1	0.592
MTG2	0.715
MTHFD1	0.849
MTHFD1L	0.175
MTHFD2	0.604
MTHFD2L	0.578
MTHFR	0.477
MTHFS	0.351
MTHFSD	0.135
MTIF2	0.147
MTIF3	0.278
MTL5	0.301
MTMR10	0.517
MTMR11	0.445
MTMR12	0.994
MTMR14	0.541
MTMR2	0.533
MTMR3	0.920
MTMR4	0.998
MTMR6	0.601
MTMR7	0.208
MTMR9	0.175
MTNR1A	0.051
MTNR1B	0.127

MTO1	0.244
MTOR	1.000
MTPAP	0.761
MTPN	0.405
MTR	0.417
MTRF1	0.198
MTRF1L	0.419
MTRNR2L1	0.120
MTRNR2L12	0.156
MTRNR2L2	0.224
MTRNR2L3	0.306
MTRNR2L4	0.031
MTRNR2L5	0.120
MTRNR2L8	0.255
MTRNR2L9	0.028
MTRR	0.355
MTSS1	0.923
MTSS1L	0.999
MTTP	0.181
MTUS1	0.176
MTUS2	0.569
MTX1	0.517
MTX2	0.100
MTX3	0.168
MUC1	0.467
MUC13	0.180
MUC15	0.079
MUC16	0.121
MUC17	0.189
MUC2	0.473
MUC20	0.399
MUC21	0.213

MUC22	0.554
MUC3A	0.194
MUC4	0.024
MUC5B	0.285
MUC6	0.324
MUC7	0.096
MUC8	0.095
MUCL1	0.234
MUL1	0.381
MUM1	0.447
MURC	0.354
MUS81	0.711
MUSK	0.491
MUSTN1	0.265
MUT	0.088
MUTYH	0.952
MVB12A	0.239
MVB12B	0.624
MVD	0.358
MVK	0.824
MVP	0.670
MX1	0.481
MX2	0.438
MXD1	0.351
MXD3	0.307
MXD4	0.392
MXI1	0.630
MXRA7	0.191
MXRA8	0.548
MYADM	0.159
MYADML2	0.224
MYB	0.825

MYBBP1A	0.176
MYBL1	0.690
MYBL2	0.384
MYBPC1	0.346
MYBPC2	0.505
MYBPC3	0.868
MYBPH	0.508
MYBPHL	0.952
MYC	0.299
MYCBP	0.777
MYCBP2	0.957
MYCBPAP	0.117
MYCL	0.501
MYCN	0.766
MYCT1	0.118
MYD88	0.430
MYEF2	0.414
MYEOV	0.215
MYEOV2	0.116
MYF5	0.353
MYF6	0.247
MYH1	0.446
MYH10	1.000
MYH11	0.647
MYH13	0.304
MYH14	0.893
MYH15	0.481
MYH2	0.425
MYH3	0.267
MYH4	0.226
MYH6	0.949
MYH7	0.971

MYH7B	0.540
MYH8	0.295
MYH9	0.996
MYL1	0.164
MYL10	0.171
MYL12A	0.060
MYL12B	0.200
MYL2	0.373
MYL3	0.294
MYL4	0.487
MYL5	0.221
MYL6	0.711
MYL6B	0.387
MYL7	0.738
MYL9	0.183
MYLIP	0.632
MYLK	0.913
MYLK2	0.417
MYLK3	0.990
MYLK4	0.507
MYLPF	0.270
MYNN	0.822
MYO10	0.852
MYO15A	0.500
MYO16	0.887
MYO18A	0.983
MYO18B	0.596
MYO19	0.601
MYO1A	0.459
MYO1B	0.814
MYO1C	0.707
MYO1D	0.739

MYO1E	0.495
MYO1F	0.227
MYO1G	0.240
MYO1H	0.516
MYO3A	0.430
MYO3B	0.413
MYO5A	0.988
MYO5B	0.824
MYO5C	0.567
MYO6	0.442
MYO7A	0.864
MYO7B	0.298
MYO9A	0.746
MYO9B	0.988
MYOC	0.274
MYOCD	0.354
MYOD1	0.692
MYOF	0.625
MYOG	0.622
MYOM1	0.349
MYOM2	0.047
MYOM3	0.550
MYOT	0.253
MYOZ1	0.234
MYOZ2	0.132
MYOZ3	0.224
MYPN	0.479
MYPOP	0.664
MYRF	0.822
MYRFL	0.282
MYRIP	0.606
MYSM1	0.409

MYT1	0.732
MYT1L	0.528
MYZAP	0.088
MZB1	0.134
MZF1	0.442
MZT1	0.110
MZT2A	0.279
MZT2B	0.422
N4BP1	0.879
N4BP2	0.226
N4BP2L1	0.315
N4BP2L2	0.271
N4BP3	0.086
N6AMT1	0.174
N6AMT2	0.094
NAA11	0.080
NAA15	0.844
NAA16	0.319
NAA20	0.574
NAA25	0.976
NAA30	0.729
NAA35	0.895
NAA38	0.135
NAA40	0.724
NAA50	0.828
NAA60	0.999
NAAA	0.349
NAALAD2	0.164
NAALADL1	0.326
NAALADL2	0.105
NAB1	0.821
NAB2	0.876

NABP1	0.201
NABP2	0.939
NACA	0.473
NACA2	0.215
NACAD	0.563
NACC1	1.000
NACC2	0.808
NADK	0.757
NADK2	0.414
NADSYN1	0.698
NAE1	0.572
NAF1	0.466
NAGA	0.467
NAGK	0.470
NAGLU	0.735
NAGPA	0.047
NAGS	0.675
NAIF1	0.660
NAIP	0.352
NALCN	0.998
NAMPT	0.510
NAMPTL	0.095
NANOG	0.337
NANOGNB	0.963
NANOS1	0.496
NANOS2	0.192
NANOS3	0.322
NANP	0.290
NANS	0.454
NAP1L1	0.721
NAP1L4	0.471
NAP1L5	0.278

NAPA	0.657
NAPB	0.305
NAPEPLD	0.446
NAPG	0.153
NAPRT1	0.191
NAPSA	0.368
NARF	0.074
NARFL	0.295
NARG2	0.314
NARS	0.495
NARS2	0.140
NASP	0.950
NAT1	0.041
NAT10	0.615
NAT14	0.353
NAT16	0.631
NAT2	0.050
NAT6	0.486
NAT8	0.264
NAT8L	0.671
NAT9	0.340
NAV1	0.926
NAV2	0.997
NAV3	0.827
NBAS	0.132
NBEA	0.996
NBEAL1	0.262
NBEAL2	0.512
NBL1	0.982
NBN	0.275
NBPF1	0.281
NBPF10	0.079

NBPF11	0.118
NBPF12	0.123
NBPF14	0.071
NBPF15	0.084
NBPF16	0.100
NBPF20	0.067
NBPF24	0.176
NBPF3	0.185
NBPF4	0.260
NBPF6	0.225
NBPF8	0.078
NBPF9	0.119
NBR1	0.989
NCALD	0.569
NCAM1	0.904
NCAM2	0.170
NCAN	0.473
NCAPD2	0.448
NCAPD3	0.082
NCAPG	0.088
NCAPG2	0.110
NCAPH	0.333
NCAPH2	0.123
NCBP1	0.980
NCBP2	0.862
NCCRP1	0.772
NCDN	0.993
NCEH1	0.393
NCF1	0.180
NCF2	0.126
NCF4	0.208
NCK1	0.917

NCK2	0.446
NCKAP1	0.559
NCKAP1L	0.414
NCKAP5	0.120
NCKAP5L	0.810
NCKIPSD	0.780
NCL	0.613
NCLN	0.606
NCMAP	0.148
NCOA1	0.980
NCOA2	0.995
NCOA3	0.829
NCOA4	0.580
NCOA5	0.979
NCOA6	0.818
NCOA7	0.469
NCOR1	0.984
NCOR2	0.957
NCR1	0.098
NCR2	0.240
NCR3	0.158
NCR3LG1	0.265
NCS1	0.696
NCSTN	0.968
NDC1	0.403
NDC80	0.092
NDE1	0.219
NDEL1	0.963
NDFIP1	0.426
NDFIP2	0.461
NDN	0.390
NDNF	0.281

NDNL2	0.376
NDOR1	0.965
NDRG1	0.497
NDRG2	0.448
NDRG3	0.314
NDRG4	0.873
NDST1	0.905
NDST2	0.910
NDST3	0.391
NDST4	0.278
NDUFA10	0.926
NDUFA11	0.265
NDUFA12	0.585
NDUFA13	0.321
NDUFA2	0.695
NDUFA3	0.148
NDUFA4	0.069
NDUFA4L2	0.490
NDUFA5	0.064
NDUFA6	0.519
NDUFA7	0.454
NDUFA8	0.647
NDUFA9	0.489
NDUFAB1	0.698
NDUFAF1	0.171
NDUFAF2	0.089
NDUFAF3	0.242
NDUFAF4	0.865
NDUFAF5	0.210
NDUFAF6	0.255
NDUFAF7	0.194
NDUFB1	0.086

NDUFB10	0.311
NDUFB2	0.318
NDUFB3	0.147
NDUFB4	0.402
NDUFB5	0.156
NDUFB6	0.141
NDUFB7	0.699
NDUFB8	0.328
NDUFB9	0.464
NDUFC1	0.162
NDUFC2	0.285
NDUFC2-KCTD14	0.180
NDUFS1	0.430
NDUFS2	0.884
NDUFS3	0.820
NDUFS4	0.265
NDUFS5	0.505
NDUFS6	0.155
NDUFS7	0.835
NDUFS8	0.580
NDUFV1	0.510
NDUFV2	0.316
NDUFV3	0.295
NEB	0.149
NEBL	0.172
NECAB1	0.262
NECAB2	0.045
NECAB3	0.446
NECAP1	0.747
NECAP2	0.327
NEDD1	0.339
NEDD4	0.555

NEDD4L	0.671
NEDD8	0.942
NEDD9	0.423
NEFH	0.514
NEFM	0.370
NEGR1	0.536
NEIL1	0.354
NEIL2	0.410
NEIL3	0.059
NEK1	0.480
NEK10	0.329
NEK11	0.408
NEK2	0.150
NEK3	0.361
NEK4	0.548
NEK5	0.231
NEK6	0.539
NEK7	0.907
NEK8	0.437
NEK9	0.809
NELFA	0.790
NELFB	0.998
NELFCD	0.957
NELFE	0.932
NELL1	0.221
NELL2	0.292
NEMF	0.510
NENF	0.195
NEO1	0.679
NES	0.261
NET1	0.366
NETO1	0.267

NETO2	0.162
NEU1	0.725
NEU2	0.234
NEU3	0.525
NEU4	0.970
NEURL	0.300
NEURL1B	0.815
NEURL2	0.537
NEURL4	0.996
NEUROD1	0.954
NEUROD2	0.969
NEUROD4	0.356
NEUROD6	0.467
NEUROG1	0.390
NEUROG2	0.709
NEUROG3	0.259
NEXN	0.130
NF1	0.999
NF2	0.939
NFAM1	0.044
NFASC	0.736
NFAT5	1.000
NFATC1	0.513
NFATC2	0.998
NFATC2IP	0.658
NFATC3	0.899
NFATC4	0.965
NFE2	0.391
NFE2L1	0.873
NFE2L2	0.416
NFE2L3	0.275
NFIA	0.982

NFIB	0.980
NFIC	0.857
NFIL3	0.590
NFIX	0.952
NFKB1	0.977
NFKB2	0.951
NFKBIA	0.919
NFKBIB	0.893
NFKBID	0.355
NFKBIE	0.734
NFKBIL1	0.715
NFKBIZ	0.546
NFRKB	0.227
NFS1	0.836
NFU1	0.380
NFX1	0.919
NFXL1	0.150
NFYA	0.983
NFYB	0.810
NFYC	0.999
NGB	0.377
NGDN	0.393
NGEF	0.661
NGF	0.416
NGFR	0.932
NGLY1	0.488
NGRN	0.172
NHEJ1	0.526
NHLH1	0.412
NHLH2	0.588
NHLRC1	0.496
NHLRC2	0.463

NHLRC3	0.201
NHLRC4	0.109
NHP2	0.363
NHP2L1	0.819
NHSL1	0.598
NICN1	0.347
NID1	0.240
NID2	0.476
NIF3L1	0.307
NIM1	0.754
NIN	0.539
NINJ1	0.212
NINJ2	0.055
NINL	0.126
NIP7	0.575
NIPA1	0.525
NIPA2	0.400
NIPAL1	0.280
NIPAL2	0.241
NIPAL3	0.390
NIPAL4	0.777
NIPBL	0.918
NIPSNAP1	0.524
NIPSNAP3A	0.156
NIPSNAP3B	0.113
NISCH	0.948
NIT1	0.632
NIT2	0.240
NKAIN1	0.503
NKAIN2	0.385
NKAIN3	0.207
NKAIN4	0.080

NKAPL	0.278
NKD1	0.728
NKD2	0.280
NKG2-E	0.137
NKG7	0.983
NKIRAS1	0.260
NKIRAS2	0.595
NKPD1	0.575
NKTR	0.834
NKX1-1	1.000
NKX1-2	0.336
NKX2-1	0.597
NKX2-2	0.972
NKX2-3	0.845
NKX2-4	0.264
NKX2-5	0.973
NKX2-6	0.511
NKX2-8	0.132
NKX3-1	0.186
NKX3-2	0.285
NKX6-1	0.646
NKX6-2	0.419
NKX6-3	0.126
NLE1	0.576
NLGN1	0.526
NLGN2	0.993
NLK	0.998
NLN	0.795
NLRC4	0.191
NLRC5	0.569
NLRP1	0.669
NLRP10	0.333

NLRP11	0.122
NLRP12	0.033
NLRP13	0.015
NLRP14	0.106
NLRP2	0.052
NLRP3	0.252
NLRP4	0.036
NLRP5	0.029
NLRP6	0.130
NLRP7	0.139
NLRP8	0.024
NLRP9	0.106
NLRX1	0.694
NMB	0.262
NMBR	0.038
NMD3	0.163
NME1	0.443
NME1-NME2	0.422
NME2	0.342
NME3	0.501
NME4	0.952
NME5	0.119
NME6	0.444
NME7	0.521
NME8	0.091
NME9	0.268
NMI	0.087
NMNAT1	0.495
NMNAT2	0.971
NMNAT3	0.275
NMRAL1	0.603
NMRK1	0.360

NMRK2	0.604
NMT1	0.995
NMT2	0.552
NMU	0.259
NMUR1	0.203
NMUR2	0.168
NNAT	0.486
NNMT	0.252
NNT	0.898
NOA1	0.331
NOB1	0.979
NOBOX	0.299
NOC2L	0.315
NOC3L	0.176
NOC4L	0.095
NOD1	0.466
NOD2	0.277
NODAL	0.691
NOG	0.829
NOL10	0.304
NOL11	0.250
NOL12	0.381
NOL3	0.557
NOL4	0.828
NOL6	0.773
NOL7	0.303
NOL8	0.245
NOL9	0.735
NOLC1	0.562
NOM1	0.292
NOMO1	0.224
NOMO2	0.206

NOMO3	0.294
NOP10	0.387
NOP14	0.953
NOP16	0.458
NOP2	0.760
NOP56	0.190
NOP58	0.181
NOP9	0.627
NOS1	0.933
NOS1AP	0.734
NOS2	0.550
NOS3	0.730
NOSIP	0.504
NOSTRIN	0.302
NOTCH1	0.569
NOTCH2	0.906
NOTCH2NL	0.856
NOTCH3	0.863
NOTCH4	0.705
NOTO	0.151
NOTUM	0.662
NOV	0.448
NOVA1	0.903
NOVA2	0.988
NOX3	0.191
NOX4	0.092
NOX5	0.260
NOXA1	0.300
NOXO1	0.212
NOXRED1	0.446
NPAP1	0.104
NPAS1	0.898

NPAS2	0.538
NPAS3	0.940
NPAS4	0.704
NPAT	0.515
NPB	0.337
NPBWR1	0.066
NPBWR2	0.204
NPC1	0.974
NPC1L1	0.360
NPC2	0.579
NPDC1	0.280
NPEPL1	0.418
NPEPPS	0.846
NPFF	0.406
NPFFR1	0.295
NPFFR2	0.185
NPHP1	0.332
NPHP3	0.249
NPHP4	0.272
NPHS1	0.287
NPHS2	0.365
NPIPA1	0.261
NPIPA2	0.229
NPIPA3	0.326
NPIPA5	0.096
NPIPA7	0.131
NPIPA8	0.229
NPIPB11	0.258
NPIPB15	0.173
NPIPB3	0.162
NPIPB4	0.229
NPIPB5	0.308

NPIPB6	0.231
NPIPB7	0.228
NPIPB8	0.168
NPIPB9	0.202
NPL	0.240
NPLOC4	0.926
NPM1	0.797
NPM2	0.345
NPM3	0.511
NPNT	0.226
NPPA	0.490
NPPB	0.410
NPPC	0.240
NPR1	0.958
NPR2	0.674
NPR3	0.424
NPRL2	0.764
NPSR1	0.222
NPTN	0.933
NPTX1	0.881
NPTX2	0.994
NPTXR	0.641
NPVF	0.242
NPW	0.398
NPY	0.260
NPY1R	0.292
NPY2R	0.257
NPY4R	0.250
NPY5R	0.217
NQO1	0.148
NQO2	0.955
NR0B2	0.603

NR1D1	0.962
NR1D2	0.726
NR1H2	0.945
NR1H3	0.874
NR1H4	0.749
NR1I2	0.497
NR1I3	0.656
NR2C1	0.653
NR2C2	0.969
NR2C2AP	0.717
NR2E1	0.963
NR2F1	0.925
NR2F2	0.860
NR2F6	0.336
NR3C1	0.937
NR3C2	0.723
NR4A1	0.698
NR4A2	0.877
NR4A3	0.813
NR5A1	0.996
NR5A2	0.859
NR6A1	0.935
NRAP	0.453
NRARP	0.782
NRAS	1.000
NRBF2	0.375
NRBP1	0.983
NRBP2	0.490
NRCAM	0.826
NRD1	0.575
NRDE2	0.571
NREP	0.063

NRF1	0.995
NRG1	0.799
NRG2	0.988
NRG3	0.168
NRGN	0.380
NRIP1	0.637
NRIP2	0.062
NRIP3	0.291
NRL	0.664
NRM	0.353
NRN1	0.808
NRN1L	0.306
NRP1	0.815
NRP2	0.714
NRROS	0.385
NRSN1	0.088
NRSN2	0.122
NRTN	0.361
NRXN1	0.537
NRXN2	0.957
NRXN3	0.873
NSA2	0.289
NSD1	1.000
NSF	0.844
NSFL1C	0.354
NSG1	0.479
NSG2	0.332
NSL1	0.414
NSMAF	0.479
NSMCE1	0.264
NSMCE2	0.992
NSMCE4A	0.464

NSMF	0.538
NSRP1	0.430
NSUN2	0.986
NSUN3	0.263
NSUN4	0.620
NSUN5	0.703
NSUN6	0.493
NSUN7	0.260
NT5C	0.210
NT5C1A	0.367
NT5C1B	0.179
NT5C1B-RDH14	0.112
NT5C2	0.941
NT5C3A	0.499
NT5C3B	0.300
NT5DC1	0.511
NT5DC2	0.484
NT5DC3	0.444
NT5DC4	0.244
NT5E	0.331
NT5M	0.265
NTAN1	0.313
NTF3	0.315
NTF4	0.547
NTHL1	0.108
NTM	0.992
NTMT1	0.710
NTN1	0.992
NTN3	0.374
NTN4	0.942
NTN5	0.457
NTNG1	0.715

NTNG2	0.859
NTPCR	0.522
NTRK1	0.658
NTRK2	0.981
NTRK3	0.934
NTS	0.115
NTSR1	0.134
NTSR2	0.088
NUAK1	0.521
NUAK2	0.238
NUB1	0.654
NUBP1	0.236
NUBP2	0.357
NUBPL	0.341
NUCB1	0.478
NUCB2	0.310
NUCKS1	0.945
NUDC	0.944
NUDCD1	0.326
NUDCD2	0.212
NUDT1	0.097
NUDT12	0.059
NUDT13	0.528
NUDT14	0.975
NUDT15	0.473
NUDT16	0.305
NUDT16L1	0.840
NUDT17	0.283
NUDT18	0.810
NUDT19	0.114
NUDT2	0.405
NUDT21	0.997

NUDT22	0.732
NUDT3	0.812
NUDT4	0.918
NUDT5	0.310
NUDT6	0.408
NUDT7	0.059
NUDT8	0.110
NUDT9	0.257
NUF2	0.109
NUFIP1	0.497
NUFIP2	0.964
NUGGC	0.365
NUMA1	0.852
NUMB	0.616
NUMBL	0.996
NUP107	0.569
NUP133	0.464
NUP153	0.549
NUP155	0.095
NUP160	0.495
NUP188	0.999
NUP205	0.557
NUP210	0.372
NUP210L	0.488
NUP214	0.733
NUP35	0.105
NUP37	0.220
NUP43	0.436
NUP50	0.987
NUP54	0.523
NUP62	0.436
NUP85	0.783

NUP88	0.383
NUP93	0.635
NUP98	0.793
NUPL1	0.519
NUPL2	0.371
NUPR1	0.415
NUPR1L	0.239
NUS1	0.736
NUSAP1	0.135
NUTF2	0.991
NUTM1	0.295
NUTM2A	0.338
NUTM2B	0.462
NUTM2D	0.418
NUTM2E	0.230
NUTM2F	0.308
NUTM2G	0.217
NVL	0.666
NWD1	0.514
NXF1	0.998
NXN	0.671
NXNL1	0.241
NXNL2	0.234
NXPE1	0.217
NXPE2	0.254
NXPE3	0.515
NXPE4	0.118
NXPH1	0.149
NXPH2	0.568
NXPH3	0.648
NXPH4	0.927
NXT1	0.573

NYAP1	0.652
NYAP2	0.391
NYNRIN	0.502
OAF	0.337
OARD1	0.415
OAS1	0.120
OAS2	0.179
OAS3	0.100
OASL	0.191
OAT	0.310
OAZ1	0.209
OAZ2	0.356
OAZ3	0.126
OBFC1	0.329
OBP2A	0.236
OBP2B	0.129
OBSCN	0.306
OBSL1	0.409
OC90	0.062
OCA2	0.089
OCEL1	0.199
OCIAD1	0.228
OCIAD2	0.114
OCLM	0.051
OCLN	0.383
OCM	0.141
OCSTAMP	0.129
ODAM	0.044
ODC1	0.322
ODF1	0.336
ODF2	0.705
ODF2L	0.172

ODF3	0.072
ODF3B	0.125
ODF3L1	0.253
ODF3L2	0.082
ODF4	0.211
OGDH	0.994
OGDHL	0.189
OGFOD1	0.491
OGFOD2	0.360
OGFOD3	0.109
OGFR	0.295
OGFRL1	0.251
OGG1	0.554
OGN	0.126
OIP5	0.109
OIT3	0.455
OLA1	0.774
OLAH	0.039
OLFM1	0.783
OLFM2	0.736
OLFM3	0.236
OLFM4	0.125
OLFML1	0.131
OLFML2A	0.180
OLFML2B	0.300
OLFML3	0.294
OLIG1	0.717
OLIG2	0.883
OLIG3	0.913
OLR1	0.214
OMA1	0.341
OMD	0.102

OMG	0.388
OMP	0.111
ONECUT1	0.476
ONECUT2	0.510
ONECUT3	0.569
OOEP	0.199
OPA1	0.999
OPA3	0.447
OPALIN	0.173
OPCML	1.000
OPLAH	0.333
OPN1SW	0.342
OPN3	0.052
OPN4	0.134
OPN5	0.311
OPRD1	0.460
OPRK1	0.109
OPRL1	0.439
OPRM1	0.143
OPTC	0.167
OPTN	0.602
OR10A2	0.132
OR10A3	0.097
OR10A4	0.033
OR10A6	0.048
OR10A7	0.178
OR10AD1	0.097
OR10C1	0.323
OR10D3	0.382
OR10G2	0.214
OR10G3	0.298
OR10G4	0.064

OR10G6	0.092
OR10H1	0.191
OR10H2	0.408
OR10H4	0.077
OR10H5	0.413
OR10J1	0.059
OR10J3	0.092
OR10J5	0.069
OR10P1	0.120
OR10Q1	0.054
OR10V1	0.189
OR11A1	0.340
OR11G2	0.063
OR11H12	0.067
OR11H4	0.008
OR11H6	0.025
OR11L1	0.051
OR12D2	0.291
OR12D3	0.213
OR13A1	0.081
OR13C2	0.116
OR13C3	0.041
OR13C5	0.128
OR13C9	0.214
OR13D1	0.053
OR13G1	0.034
OR13J1	0.152
OR14A16	0.019
OR14A2	0.081
OR14I1	0.067
OR14J1	0.043
OR14K1	0.037

OR1B1	0.069
OR1C1	0.021
OR1D2	0.092
OR1D5	0.216
OR1E1	0.216
OR1E2	0.090
OR1F1	0.018
OR1G1	0.170
OR1J1	0.895
OR1J2	0.910
OR1J4	0.949
OR1K1	0.095
OR1L1	0.133
OR1L3	0.033
OR1L4	0.022
OR1L8	0.934
OR1N1	0.930
OR1Q1	0.194
OR1S2	0.199
OR2A1	0.237
OR2A12	0.043
OR2A14	0.047
OR2A25	0.018
OR2A4	0.248
OR2A42	0.297
OR2A5	0.054
OR2A7	0.235
OR2AE1	0.087
OR2AG2	0.077
OR2AJ1	0.045
OR2AK2	0.038
OR2AT4	0.333

OR2B11	0.040
OR2B2	0.100
OR2B3	0.200
OR2B6	0.234
OR2C1	0.061
OR2C3	0.036
OR2D2	0.028
OR2D3	0.049
OR2F1	0.062
OR2F2	0.188
OR2G2	0.041
OR2G3	0.083
OR2G6	0.024
OR2H1	0.368
OR2H2	0.315
OR2J2	0.232
OR2J3	0.201
OR2K2	0.098
OR2L13	0.035
OR2L2	0.019
OR2L3	0.037
OR2L5	0.029
OR2M3	0.023
OR2M4	0.020
OR2M5	0.020
OR2S2	0.124
OR2T10	0.078
OR2T11	0.073
OR2T2	0.103
OR2T27	0.127
OR2T29	0.256
OR2T3	0.102

OR2T33	0.049
OR2T34	0.155
OR2T4	0.025
OR2T5	0.217
OR2T7	0.054
OR2T8	0.022
OR2V1	0.080
OR2V2	0.054
OR2W1	0.320
OR2W3	0.026
OR3A1	0.132
OR3A2	0.065
OR3A3	0.092
OR4A16	0.002
OR4C3	0.004
OR4D1	0.304
OR4D5	0.072
OR4D6	0.130
OR4E2	0.077
OR4F16	0.297
OR4F17	0.138
OR4F21	0.128
OR4F29	0.206
OR4F3	0.260
OR4K1	0.031
OR4K15	0.183
OR4M1	0.137
OR4M2	0.081
OR4N2	0.033
OR4N4	0.115
OR4Q3	0.027
OR51A7	0.025

OR51B4	0.072
OR51B5	0.068
OR51E1	0.243
OR51E2	0.037
OR51G1	0.047
OR51G2	0.052
OR51H1P	0.119
OR51I1	0.077
OR51I2	0.150
OR51J1	0.118
OR51Q1	0.150
OR51S1	0.039
OR52B6	0.102
OR52D1	0.031
OR52E2	0.025
OR52E4	0.018
OR52H1	0.041
OR52I1	0.032
OR52I2	0.081
OR52K2	0.036
OR52L1	0.074
OR52N1	0.114
OR52N2	0.046
OR52N4	0.023
OR52N5	0.032
OR52W1	0.103
OR56A1	0.129
OR56A3	0.044
OR56A4	0.159
OR56B1	0.067
OR56B4	0.030
OR5A1	0.021

OR5AC2	0.027
OR5AK2	0.017
OR5AS1	0.020
OR5AU1	0.065
OR5B12	0.042
OR5B2	0.050
OR5B21	0.157
OR5C1	0.101
OR5D16	0.003
OR5D18	0.010
OR5H14	0.046
OR5H2	0.016
OR5H6	0.042
OR5J2	0.003
OR5K1	0.013
OR5K2	0.031
OR5K3	0.034
OR5K4	0.026
OR5M11	0.023
OR5M3	0.025
OR5M8	0.053
OR5P2	0.067
OR5P3	0.110
OR5T2	0.000
OR5V1	0.174
OR6A2	0.106
OR6B1	0.064
OR6B2	0.221
OR6B3	0.282
OR6C2	0.103
OR6C3	0.038
OR6C74	0.163

OR6F1	0.012
OR6K3	0.206
OR6M1	0.129
OR6S1	0.107
OR6T1	0.167
OR6V1	0.183
OR6X1	0.237
OR7A5	0.346
OR7C1	0.445
OR7D2	0.051
OR7D4	0.103
OR7E24	0.050
OR8A1	0.123
OR8B12	0.130
OR8B2	0.160
OR8B3	0.128
OR8D1	0.160
OR8D2	0.133
OR8D4	0.133
OR8G5	0.063
OR8H1	0.025
OR8S1	0.142
OR9A2	0.151
OR9A4	0.070
OR9G4	0.023
OR9Q1	0.160
OR9Q2	0.046
ORAI1	0.281
ORAI2	0.323
ORAI3	0.485
ORAOV1	0.325
ORC1	0.126

ORC2	0.756
ORC3	0.313
ORC4	0.517
ORC5	0.573
ORC6	0.192
ORM1	0.101
ORM2	0.304
ORMDL1	0.320
ORMDL2	0.607
ORMDL3	0.877
OS9	0.673
OSBP	0.994
OSBP2	0.707
OSBPL10	0.653
OSBPL11	0.627
OSBPL1A	0.384
OSBPL2	0.746
OSBPL3	0.977
OSBPL5	0.106
OSBPL6	0.834
OSBPL7	0.390
OSBPL8	0.714
OSBPL9	0.853
OSCAR	0.201
OSCP1	0.213
OSER1	0.271
OSGEP	0.694
OSGEPL1	0.426
OSGIN1	0.190
OSGIN2	0.348
OSM	0.422
OSMR	0.569

OSR1	0.500
OSR2	0.451
OST4	0.366
OSTC	0.141
OSTF1	0.172
OSTM1	0.333
OSTN	0.147
OTOA	0.560
OTOF	0.187
OTOG	0.226
OTOGL	0.383
OTOL1	0.147
OTOP1	0.191
OTOP2	0.360
OTOP3	0.205
OTOS	0.051
OTP	0.986
OTUB1	0.951
OTUB2	0.541
OTUD1	0.428
OTUD3	0.353
OTUD4	0.916
OTUD6B	0.162
OTUD7A	0.808
OTUD7B	0.872
OTX1	0.959
OTX2	0.991
OVCA2	0.261
OVCH1	0.164
OVCH1-AS1	0.321
OVGP1	0.205
OVOL1	0.926

OVOL2	0.450
OVOL3	0.184
OXA1L	0.096
OXCT1	0.558
OXCT2	0.211
OXER1	0.073
OXGR1	0.064
OXLD1	0.805
OXNAD1	0.367
OXR1	0.471
OXSM	0.375
OXSR1	0.935
OXT	0.999
OXTR	0.308
P2RX1	0.225
P2RX2	0.103
P2RX3	0.403
P2RX4	0.129
P2RX5	0.078
P2RX5-TAX1BP3	0.212
P2RX6	0.362
P2RX7	0.111
P2RY1	0.338
P2RY11	0.324
P2RY12	0.130
P2RY13	0.033
P2RY14	0.075
P2RY2	0.137
P2RY6	0.161
P4HA1	0.682
P4HA2	0.655
P4HA3	0.274

P4HB	0.944
P4HTM	0.452
PA2G4	1.000
PAAF1	0.127
PABPC1	0.828
PABPC1L	0.148
PABPC3	0.191
PABPC4	0.988
PABPC4L	0.147
PABPN1	0.888
PABPN1L	0.067
PACRG	0.074
PACRGL	0.111
PACS1	0.995
PACS2	0.616
PACSIN1	0.720
PACSIN2	0.547
PACSIN3	0.584
PADI1	0.393
PADI2	0.222
PADI3	0.190
PADI4	0.087
PAEP	0.317
PAF1	0.970
PAFAH1B1	0.998
PAFAH1B2	0.979
PAFAH1B3	0.417
PAFAH2	0.514
PAG1	0.283
PAGR1	0.586
PAH	0.168
PAICS	0.472

PAIP1	0.790
PAIP2	0.996
PAIP2B	0.275
PAK1	0.738
PAK1IP1	0.366
PAK2	0.993
PAK4	0.815
PAK6	0.718
PAK7	0.748
PALB2	0.312
PALD1	0.358
PALLD	0.445
PALM	0.221
PALM2	0.681
PALM2-AKAP2	0.151
PALM3	0.429
PALMD	0.335
PAM	0.207
PAM16	0.273
PAMR1	0.102
PAN3	0.913
PANK1	0.785
PANK2	0.988
PANK3	0.397
PANK4	0.869
PANX1	0.601
PANX2	0.200
PANX3	0.157
PAOX	0.155
PAPD4	0.367
PAPD5	0.984
PAPD7	1.000

PAPL	0.303
PAPLN	0.150
PAPOLA	0.976
PAPOLB	0.081
PAPOLG	0.293
PAPPA	0.516
PAPPA-AS1	0.166
PAPPA2	0.552
PAPSS1	0.386
PAPSS2	0.635
PAQR3	0.143
PAQR4	0.179
PAQR5	0.297
PAQR6	0.599
PAQR7	0.213
PAQR8	0.318
PAQR9	0.740
PARD3	0.446
PARD3B	0.309
PARD6A	0.906
PARD6B	0.431
PARD6G	0.449
PARG	0.835
PARK2	0.593
PARK7	0.433
PARL	0.293
PARM1	0.544
PARN	0.762
PARP1	0.869
PARP10	0.661
PARP11	0.325
PARP14	0.747

PARP15	0.193
PARP16	0.352
PARP2	0.236
PARP3	0.432
PARP4	0.333
PARP6	0.775
PARP8	0.308
PARP9	0.523
PARPBP	0.108
PARS2	0.040
PARVA	0.574
PARVB	0.429
PARVG	0.240
PASK	0.529
PATE1	0.141
PATE2	0.127
PATE3	0.064
PATE4	0.110
PATL1	0.985
PATL2	0.295
PATZ1	1.000
PAWR	0.668
PAX1	0.842
PAX2	0.919
PAX3	0.848
PAX4	0.265
PAX5	0.845
PAX6	1.000
PAX7	0.995
PAX8	0.352
PAX9	0.253
PAXBP1	0.690

PAXIP1	0.852
PAXIP1-AS2	0.138
PBK	0.312
PBLD	0.484
PBOV1	0.147
PBRM1	1.000
PBX1	0.988
PBX2	0.892
PBX3	0.929
PBXIP1	0.385
PC	0.952
PCBD1	0.409
PCBD2	0.370
PCBP1	0.711
PCBP2	0.992
PCBP3	0.656
PCBP4	0.521
PCCA	0.694
PCCB	0.391
PCDH1	0.900
PCDH10	0.495
PCDH12	0.357
PCDH15	0.225
PCDH17	0.392
PCDH18	0.161
PCDH20	0.128
PCDH7	0.660
PCDH8	0.720
PCDH9	0.713
PCDHA1	0.153
PCDHA10	0.172
PCDHA11	0.148

PCDHA12	0.173
PCDHA13	0.202
PCDHA2	0.102
PCDHA3	0.101
PCDHA4	0.121
PCDHA5	0.179
PCDHA6	0.134
PCDHA7	0.126
PCDHA8	0.127
PCDHA9	0.109
PCDHAC1	0.227
PCDHAC2	0.794
PCDHB1	0.147
PCDHB10	0.105
PCDHB11	0.062
PCDHB12	0.100
PCDHB13	0.095
PCDHB14	0.104
PCDHB15	0.062
PCDHB16	0.057
PCDHB17	0.220
PCDHB2	0.116
PCDHB3	0.086
PCDHB4	0.218
PCDHB5	0.075
PCDHB6	0.100
PCDHB7	0.098
PCDHB8	0.119
PCDHGA1	0.149
PCDHGA10	0.147
PCDHGA11	0.131
PCDHGA12	0.188

PCDHGA2	0.123
PCDHGA3	0.167
PCDHGA4	0.270
PCDHGA5	0.176
PCDHGA6	0.261
PCDHGA7	0.254
PCDHGA8	0.173
PCDHGA9	0.249
PCDHGB1	0.255
PCDHGB2	0.249
PCDHGB3	0.253
PCDHGB4	0.217
PCDHGB6	0.324
PCDHGB7	0.141
PCDHGC3	0.648
PCDHGC4	0.792
PCDHGC5	0.455
PCDP1	0.215
PCED1A	0.238
PCED1B	0.444
PCF11	0.938
PCGF1	0.998
PCGF2	0.663
PCGF3	0.668
PCGF5	0.728
PCGF6	0.420
PCID2	0.728
PCIF1	0.965
PCK1	0.299
PCK2	0.461
PCLO	0.121
PCM1	0.036

PCMT1	0.931
PCMTD1	0.596
PCMTD2	0.473
PCNA	0.537
PCNP	0.596
PCNT	0.240
PCNX	0.391
PCNXL2	0.363
PCNXL3	0.973
PCNXL4	0.225
PCOLCE	0.271
PCOLCE2	0.313
PCP2	0.178
PCP4	0.252
PCP4L1	0.175
PCSK1	0.376
PCSK2	0.951
PCSK4	0.457
PCSK5	0.701
PCSK6	0.347
PCSK7	0.865
PCSK9	0.598
PCTP	0.197
PCYOX1	0.452
PCYOX1L	0.198
PCYT1A	0.638
PCYT2	0.649
PDAP1	0.814
PDC	0.028
PDCD1	0.884
PDCD10	0.919
PDCD11	0.340

PDCD1LG2	0.213
PDCD2	0.254
PDCD2L	0.438
PDCD4	0.431
PDCD5	0.304
PDCD6	0.503
PDCD6IP	0.721
PDCD7	0.400
PDCL	0.445
PDCL2	0.232
PDCL3	0.069
PDDC1	0.364
PDE10A	0.797
PDE11A	0.167
PDE12	0.524
PDE1A	0.279
PDE1B	0.793
PDE1C	0.265
PDE2A	0.830
PDE3A	0.582
PDE3B	0.415
PDE4A	0.831
PDE4B	0.973
PDE4C	0.669
PDE4D	1.000
PDE4DIP	0.490
PDE5A	0.676
PDE6A	0.207
PDE6B	0.346
PDE6C	0.646
PDE6D	0.686
PDE6G	0.139

PDE6H	0.267
PDE7A	0.784
PDE7B	0.518
PDE8A	0.823
PDE8B	0.619
PDE9A	0.828
PDF	0.445
PDGFA	0.822
PDGFB	0.724
PDGFC	0.703
PDGFD	0.323
PDGFRA	0.664
PDGFRB	0.815
PDGFRL	0.101
PDHA2	0.040
PDHB	0.835
PDHX	0.331
PDIA2	0.996
PDIA3	0.889
PDIA4	0.413
PDIA5	0.644
PDIA6	0.565
PDIK1L	0.910
PDILT	0.337
PDK1	0.525
PDK2	0.677
PDK4	0.194
PDLIM1	0.363
PDLIM2	0.590
PDLIM3	0.163
PDLIM4	0.669
PDLIM5	0.397

PDLIM7	0.261
PDP1	0.659
PDP2	0.388
PDPK1	0.993
PDPN	0.140
PDPR	0.378
PDRG1	0.155
PDS5A	0.996
PDS5B	0.940
PDSS1	0.485
PDSS2	0.304
PDX1	0.524
PDXDC1	0.675
PDXK	0.852
PDXP	0.396
PDYN	0.099
PDZD2	0.837
PDZD3	0.553
PDZD7	0.459
PDZD8	0.533
PDZD9	0.183
PDZK1	0.578
PDZK1IP1	0.143
PDZRN3	0.721
PDZRN4	0.088
PEA15	0.840
PEAK1	0.594
PEAR1	0.546
PEBP1	0.528
PEBP4	0.469
PECR	0.314
PEF1	0.491

PEG10	0.847
PEG3	0.132
PELI1	0.361
PELI2	0.921
PELI3	0.686
PELO	0.328
PELP1	0.889
PEMT	0.620
PENK	0.114
PEPD	0.605
PER1	0.770
PER2	0.807
PER3	0.543
PERP	0.476
PES1	0.956
PET100	0.289
PET112	0.452
PET117	0.394
PEX1	0.501
PEX10	0.507
PEX11A	0.098
PEX11B	0.264
PEX11G	0.105
PEX12	0.458
PEX13	0.334
PEX14	0.603
PEX16	0.535
PEX19	0.529
PEX2	0.213
PEX26	0.938
PEX3	0.391
PEX5	0.804

PEX5L	0.740
PEX6	0.817
PEX7	0.223
PF4	0.032
PF4V1	0.047
PFAS	0.673
PFDN1	0.363
PFDN2	0.582
PFDN4	0.428
PFDN5	0.450
PFDN6	0.984
PFKFB2	0.581
PFKFB3	0.347
PFKFB4	0.352
PFKL	0.445
PFKM	0.763
PFKP	0.278
PFN1	0.701
PFN2	0.422
PFN3	0.293
PFN4	0.046
PGA3	0.392
PGA4	0.301
PGA5	0.333
PGAM1	0.472
PGAM2	0.283
PGAM5	0.391
PGAP1	0.319
PGAP2	0.311
PGAP3	0.250
PGBD1	0.159
PGBD2	0.143

PGBD3	0.575
PGBD4	0.219
PGBD5	0.283
PGC	0.436
PGD	0.671
PGF	0.512
PGGT1B	0.357
PGK2	0.045
PGLS	0.463
PGLYRP1	0.174
PGLYRP2	0.326
PGLYRP3	0.940
PGLYRP4	0.325
PGM1	0.968
PGM2	0.325
PGM2L1	0.331
PGM3	0.549
PGM5	0.738
PGP	0.452
PGPEP1	0.426
PGPEP1L	0.114
PGR	0.278
PGRMC2	0.507
PGS1	0.373
PHACTR1	0.629
PHACTR2	0.452
PHACTR3	0.561
PHACTR4	0.217
PHAX	0.300
PHB	0.990
PHB2	0.741
PHC1	0.930

PHC2	0.442
PHC3	0.889
PHF1	0.928
PHF10	0.205
PHF11	0.461
PHF12	0.997
PHF13	0.680
PHF14	0.186
PHF15	0.858
PHF17	0.485
PHF19	0.968
PHF2	0.995
PHF20	0.894
PHF20L1	0.791
PHF21A	0.991
PHF21B	0.360
PHF23	0.719
PHF3	0.819
PHF5A	0.973
PHF7	0.239
PHGDH	0.985
PHGR1	0.054
PHIP	0.987
PHKB	0.427
PHKG1	0.571
PHKG2	0.895
PHLDA1	0.359
PHLDA2	0.379
PHLDA3	0.305
PHLDB1	0.798
PHLDB2	0.589
PHLDB3	0.223

PHLPP1	0.854
PHLPP2	0.364
PHOSPHO1	0.456
PHOSPHO2	0.077
PHOX2A	0.984
PHOX2B	1.000
PHPT1	0.507
PHRF1	0.268
PHTF1	0.245
PHTF2	0.279
PHYH	0.259
PHYHD1	0.219
PHYHIP	0.848
PHYHIPL	0.603
PHYKPL	0.199
PI15	0.258
PI16	0.120
PI3	0.169
PI4K2A	0.991
PI4K2B	0.473
PI4KA	0.841
PI4KB	0.999
PIANP	0.504
PIAS1	0.998
PIAS2	0.711
PIAS3	0.991
PIAS4	0.981
PIBF1	0.250
PICALM	0.429
PICK1	0.654
PID1	0.205
PIDD	0.432

PIEZO1	0.076
PIEZO2	0.202
PIF1	0.192
PIFO	0.192
PIGB	0.069
PIGC	0.715
PIGF	0.100
PIGG	0.258
PIGH	0.129
PIGK	0.249
PIGL	0.277
PIGM	0.292
PIGN	0.302
PIGO	0.672
PIGP	0.164
PIGQ	0.207
PIGR	0.278
PIGS	0.595
PIGT	0.491
PIGU	0.425
PIGV	0.552
PIGW	0.260
PIGX	0.256
PIGY	0.353
PIGZ	0.248
PIH1D1	0.843
PIH1D2	0.192
PIK3AP1	0.817
PIK3C2A	0.523
PIK3C2B	0.686
PIK3C2G	0.196
PIK3C3	0.172

PIK3CA	0.985
PIK3CB	0.641
PIK3CD	0.782
PIK3CG	0.634
PIK3IP1	0.612
PIK3R1	1.000
PIK3R2	0.999
PIK3R3	0.097
PIK3R4	0.609
PIK3R5	0.354
PIK3R6	0.271
PIKFYVE	0.473
PILRA	0.374
PILRB	0.825
PIM1	0.843
PIM3	0.756
PIN1	0.963
PINK1	0.398
PINLYP	0.337
PINX1	0.127
PIP	0.106
PIP4K2A	0.361
PIP4K2B	1.000
PIP4K2C	0.649
PIP5K1A	0.611
PIP5K1B	0.194
PIP5K1C	0.903
PIP5KL1	0.309
PIPOX	0.564
PIRT	0.366
PISD	0.517
PITHD1	0.568

PITPNA	0.721
PITPNB	0.539
PITPNC1	0.946
PITPNM1	0.509
PITPNM2	0.658
PITPNM3	0.786
PITRM1	0.182
PITX1	0.774
PITX2	0.996
PITX3	0.948
PIWIL1	0.337
PIWIL2	0.205
PIWIL3	0.917
PIWIL4	0.369
PJA2	0.367
PKD1	0.515
PKD1L1	0.284
PKD2	0.741
PKD2L1	0.619
PKD2L2	0.261
PKDCC	0.984
PKDREJ	0.219
PKHD1	0.446
PKHD1L1	0.200
PKIA	0.263
PKIB	0.122
PKIG	0.394
PKLR	0.598
PKM	0.979
PKMYT1	0.218
PKN1	0.884
PKN2	0.563

PKN3	0.406
PKNOX1	0.757
PKNOX2	0.964
PKP1	0.801
PKP2	0.727
PKP3	0.341
PKP4	0.680
PLA1A	0.274
PLA2G10	0.366
PLA2G12A	0.255
PLA2G12B	0.373
PLA2G15	0.651
PLA2G16	0.486
PLA2G1B	0.854
PLA2G2A	0.282
PLA2G2C	0.099
PLA2G2D	0.163
PLA2G2E	0.158
PLA2G2F	0.204
PLA2G3	0.206
PLA2G4A	0.542
PLA2G4B	0.324
PLA2G4C	0.535
PLA2G4D	0.245
PLA2G4E	0.590
PLA2G4F	0.356
PLA2G5	0.329
PLA2G6	0.396
PLA2G7	0.138
PLA2R1	0.196
PLAA	0.459
PLAC1L	0.172

PLAC8	0.075
PLAC8L1	0.207
PLAC9	0.447
PLAG1	0.453
PLAGL1	0.679
PLAGL2	0.994
PLAT	0.683
PLAU	0.538
PLAUR	0.501
PLB1	0.171
PLBD1	0.111
PLBD2	0.686
PLCB1	0.992
PLCB2	0.302
PLCB3	0.978
PLCB4	0.388
PLCD1	0.743
PLCD3	0.601
PLCD4	0.655
PLCE1	0.748
PLCG1	0.713
PLCG2	0.419
PLCH1	0.237
PLCH2	0.459
PLCL1	0.635
PLCL2	0.972
PLCXD2	0.548
PLCXD3	0.402
PLCZ1	0.416
PLD1	0.831
PLD2	0.529
PLD3	0.721

PLD4	0.046
PLD5	0.235
PLD6	0.406
PLEC	0.407
PLEK	0.050
PLEK2	0.249
PLEKHA1	0.531
PLEKHA3	0.411
PLEKHA4	0.593
PLEKHA5	0.562
PLEKHA6	0.652
PLEKHA7	0.758
PLEKHA8	0.277
PLEKHB1	0.117
PLEKHB2	0.311
PLEKHD1	0.434
PLEKHF1	0.229
PLEKHF2	0.782
PLEKHG1	0.874
PLEKHG2	0.397
PLEKHG3	0.265
PLEKHG4	0.673
PLEKHG4B	0.127
PLEKHG5	0.631
PLEKHG6	0.318
PLEKHG7	0.074
PLEKHH1	0.235
PLEKHH2	0.848
PLEKHH3	0.634
PLEKHJ1	0.183
PLEKHM1	0.564
PLEKHM2	0.596

PLEKHM3	0.296
PLEKHN1	0.759
PLEKHO1	0.441
PLEKHO2	0.252
PLEKHS1	0.460
PLG	0.375
PLGLB1	0.384
PLGLB2	0.292
PLGRKT	0.064
PLIN1	0.327
PLIN2	0.098
PLIN3	0.376
PLIN4	0.296
PLIN5	0.193
PLK1	0.803
PLK2	0.429
PLK3	0.666
PLK4	0.224
PLK5	0.185
PLLP	0.248
PLN	0.066
PLOD1	0.337
PLOD2	0.536
PLOD3	0.402
PLRG1	0.776
PLS1	0.334
PLSCR1	0.088
PLSCR2	0.254
PLSCR4	0.165
PLSCR5	0.789
PLTP	0.404
PLVAP	0.358

PLXDC1	0.170
PLXDC2	0.377
PLXNA1	0.650
PLXNA2	0.742
PLXNA4	0.811
PLXNB1	0.811
PLXNB2	0.415
PLXNC1	0.968
PLXND1	0.747
PM20D1	0.669
PM20D2	0.256
PMAIP1	0.162
PMCH	0.170
PMEL	0.410
PMEPA1	0.732
PMF1	0.533
PMF1-BGLAP	0.293
PMFBP1	0.155
PML	0.714
PMM1	0.511
PMM2	0.855
PMP2	0.098
PMP22	0.676
PMPCA	0.712
PMPCB	0.259
PMS1	0.373
PMS2	0.484
PMVK	0.745
PNISR	0.745
PNKD	0.354
PNKP	0.589
PNLDC1	0.186

PNLIP	0.489
PNLIPRP1	0.958
PNLIPRP3	0.192
PNMA1	0.471
PNMA2	0.341
PNMAL1	0.327
PNMAL2	0.481
PNMT	0.565
PNN	0.573
PNO1	0.409
PNOC	0.243
PNP	0.459
PNPLA1	0.685
PNPLA2	0.365
PNPLA3	0.268
PNPLA5	0.311
PNPLA6	0.842
PNPLA7	0.416
PNPLA8	0.320
PNPO	0.590
PNPT1	0.049
PNRC1	0.418
PNRC2	0.425
POC1A	0.467
POC1B	0.185
POC1B-GALNT4	0.430
POC5	0.337
PODN	0.509
PODNL1	0.419
PODXL	0.201
PODXL2	0.751
POFUT1	0.926

POFUT2	0.348
POGK	0.969
POGLUT1	0.549
POGZ	0.975
POLA2	0.463
POLB	0.455
POLD1	0.721
POLD2	0.863
POLD3	0.446
POLD4	0.476
POLDIP2	0.953
POLDIP3	0.424
POLE	0.387
POLE2	0.167
POLE3	0.566
POLE4	0.026
POLG	0.489
POLG2	0.423
POLH	0.798
POLI	0.517
POLK	0.732
POLL	0.590
POLM	0.422
POLN	0.281
POLQ	0.089
POLR1A	0.325
POLR1B	0.903
POLR1C	0.560
POLR1D	0.329
POLR1E	0.182
POLR2A	1.000
POLR2B	1.000

POLR2C	0.978
POLR2D	0.746
POLR2E	0.994
POLR2F	0.619
POLR2G	0.735
POLR2H	0.740
POLR2I	0.613
POLR2J	0.379
POLR2J2	0.121
POLR2K	0.179
POLR2L	0.260
POLR2M	0.148
POLR3A	0.287
POLR3B	0.834
POLR3C	0.617
POLR3D	0.508
POLR3E	0.834
POLR3F	0.374
POLR3G	0.274
POLR3GL	0.221
POLR3H	0.612
POLR3K	0.257
POLRMT	0.322
POM121	0.582
POM121C	0.327
POM121L12	0.027
POM121L2	0.333
POM121L7	0.414
POMC	0.895
POMGNT1	0.398
POMGNT2	0.235
POMK	0.137

POMP	0.359
POMT1	0.347
POMT2	0.348
POMZP3	0.230
PON1	0.313
PON2	0.330
PON3	0.191
POP1	0.252
POP4	0.265
POP5	0.416
POP7	0.768
POPDC2	0.293
POPDC3	0.251
POR	0.739
POSTN	0.155
POT1	0.496
POTEB	0.216
POTEB2	0.341
POTEC	0.244
POTED	0.296
POTEE	0.241
POTEF	0.237
POTEG	0.273
POTEH	0.224
POTEI	0.146
POTEJ	0.214
POTEM	0.405
POU1F1	0.198
POU2AF1	0.392
POU2F1	0.955
POU2F2	0.920
POU2F3	0.719

POU3F1	0.981
POU3F2	0.960
POU3F3	1.000
POU4F1	0.937
POU4F2	0.748
POU4F3	0.884
POU5F1	0.504
POU5F1B	0.121
POU5F2	0.135
POU6F1	0.738
POU6F2	0.702
PP13004	0.094
PP13439	0.167
PP2D1	0.269
PPA1	0.500
PPA2	0.257
PPAN	0.312
PPAN-P2RY11	0.246
PPAP2A	0.571
PPAP2B	0.424
PPAP2C	0.540
PPAPDC1A	0.306
PPAPDC1B	0.433
PPAPDC2	0.214
PPAPDC3	0.113
PPARA	0.892
PPARD	0.997
PPARG	0.498
PPARGC1A	0.799
PPARGC1B	0.624
PPAT	0.852
PPBP	0.032

PPCDC	0.495
PPCS	0.743
PPDPF	0.118
PPEF2	0.568
PPFIA1	0.854
PPFIA2	0.900
PPFIA3	0.825
PPFIA4	0.205
PPFIBP1	0.661
PPFIBP2	0.406
PPHLN1	0.297
PPIA	0.886
PPIAL4A	0.295
PPIAL4B	0.437
PPIAL4D	0.266
PPIAL4G	0.311
PPIB	0.502
PPIC	0.248
PPID	0.326
PPIE	0.571
PPIF	0.692
PPIG	0.955
PPIH	0.557
PPIL1	0.232
PPIL2	0.868
PPIL3	0.540
PPIL4	0.299
PPIL6	0.223
PPIP5K1	0.818
PPIP5K2	0.451
PPL	0.589
PPM1A	0.983

PPM1B	0.998
PPM1D	0.389
PPM1E	0.960
PPM1F	0.346
PPM1G	0.992
PPM1H	0.605
PPM1J	0.113
PPM1K	0.472
PPM1L	0.822
PPM1M	0.297
PPM1N	0.335
PPME1	0.666
PPOX	0.912
PPP1CA	0.998
PPP1CB	1.000
PPP1CC	0.994
PPP1R10	0.995
PPP1R11	0.780
PPP1R12A	0.775
PPP1R12B	0.738
PPP1R12C	0.766
PPP1R13B	0.858
PPP1R13L	0.910
PPP1R14A	0.475
PPP1R14B	0.504
PPP1R14C	0.532
PPP1R14D	0.252
PPP1R15A	0.140
PPP1R15B	0.211
PPP1R16A	0.329
PPP1R16B	0.566
PPP1R17	0.137

PPP1R18	0.409
PPP1R1A	0.819
PPP1R1B	0.581
PPP1R1C	0.266
PPP1R2	0.147
PPP1R21	0.152
PPP1R26	0.449
PPP1R27	0.203
PPP1R32	0.451
PPP1R35	0.289
PPP1R36	0.266
PPP1R37	0.744
PPP1R3A	0.241
PPP1R3B	0.177
PPP1R3C	0.318
PPP1R3D	0.590
PPP1R3E	0.398
PPP1R3G	0.278
PPP1R42	0.206
PPP1R7	0.863
PPP1R8	1.000
PPP1R9A	0.559
PPP1R9B	0.993
PPP2CA	1.000
PPP2CB	0.968
PPP2R1A	0.995
PPP2R1B	0.628
PPP2R2A	0.973
PPP2R2B	0.761
PPP2R2C	0.699
PPP2R3A	0.780
PPP2R3C	0.125

PPP2R4	0.891
PPP2R5A	0.920
PPP2R5B	0.864
PPP2R5C	0.986
PPP2R5D	0.989
PPP2R5E	0.638
PPP3CA	0.998
PPP3CB	0.999
PPP3CC	0.511
PPP3R1	0.694
PPP3R2	0.120
PPP4C	0.995
PPP4R1	0.724
PPP4R1L	0.457
PPP4R2	0.402
PPP4R4	0.242
PPP5C	1.000
PPP5D1	0.333
PPP6C	0.732
PPP6R1	0.699
PPP6R2	0.633
PPP6R3	0.952
PPRC1	0.876
PPT1	0.466
PPT2	0.701
PPT2-EGFL8	0.360
PPTC7	0.933
PPWD1	0.488
PPY	0.363
PQLC1	0.139
PQLC2	0.561
PQLC3	0.146

PRAC1	0.254
PRADC1	0.251
PRAM1	0.124
PRAME	0.202
PRAMEF1	0.090
PRAMEF10	0.121
PRAMEF11	0.103
PRAMEF12	0.061
PRAMEF13	0.141
PRAMEF15	0.206
PRAMEF17	0.176
PRAMEF19	0.111
PRAMEF2	0.082
PRAMEF21	0.315
PRAMEF22	0.194
PRAMEF26	0.091
PRAMEF3	0.188
PRAMEF7	0.189
PRAMEF9	0.319
PRAP1	0.235
PRB2	0.111
PRB3	0.097
PRB4	0.063
PRC1	0.228
PRCC	0.873
PRCP	0.403
PRDM1	0.527
PRDM10	0.531
PRDM11	0.966
PRDM12	0.761
PRDM14	0.237
PRDM15	0.492

PRDM16	0.797
PRDM2	0.803
PRDM4	0.518
PRDM5	0.295
PRDM6	0.609
PRDM7	0.284
PRDM8	0.476
PRDM9	0.111
PRDX1	0.426
PRDX2	0.869
PRDX3	0.547
PRDX5	0.650
PRDX6	0.615
PREB	0.617
PRED58	0.143
PRELID1	0.745
PRELID2	0.095
PRELP	0.409
PREP	0.645
PREPL	0.905
PREX1	0.762
PREX2	0.460
PRF1	0.139
PRG2	0.263
PRG3	0.086
PRG4	0.280
PRH2	0.220
PRICKLE1	0.581
PRICKLE2	0.493
PRIM1	0.808
PRIM2	0.159
PRIMA1	0.602

PRIMPOL	0.169
PRKAA1	0.924
PRKAA2	0.775
PRKAB1	0.419
PRKAB2	0.684
PRKACA	1.000
PRKACB	0.688
PRKACG	0.197
PRKAG1	1.000
PRKAG2	0.943
PRKAG3	0.330
PRKAR1A	1.000
PRKAR1B	0.350
PRKAR2A	0.688
PRKAR2B	0.963
PRKCA	0.974
PRKCB	0.844
PRKCD	0.960
PRKCDBP	0.207
PRKCE	0.950
PRKCG	0.787
PRKCH	0.862
PRKCI	0.907
PRKCQ	0.797
PRKCSH	0.546
PRKCZ	0.941
PRKD1	0.533
PRKD2	0.970
PRKD3	0.643
PRKDC	0.733
PRKG1	0.913
PRKG2	0.759

PRKRA	0.678
PRKRIP1	0.524
PRKRIR	0.953
PRL	0.622
PRLHR	0.275
PRLR	0.688
PRM1	0.041
PRM2	0.031
PRM3	0.121
PRMT1	1.000
PRMT10	0.352
PRMT2	0.511
PRMT3	0.228
PRMT5	0.992
PRMT6	0.207
PRMT7	0.617
PRMT8	0.948
PRND	0.114
PRNP	0.202
PRNT	0.067
PROB1	0.901
PROC	0.791
PROCA1	0.295
PROCR	0.318
PRODH	0.528
PRODH2	0.390
PROK1	0.152
PROK2	0.112
PROKR1	0.154
PROKR2	0.073
PROL1	0.168
PROM1	0.332

PROM2	0.236
PROP1	0.165
PROS1	0.463
PROSC	0.368
PROSER1	0.289
PROSER2	0.255
PROX1	0.990
PROX2	0.247
PROZ	0.298
PRPF18	0.320
PRPF19	1.000
PRPF3	1.000
PRPF31	0.958
PRPF38A	0.547
PRPF38B	0.745
PRPF39	0.782
PRPF4	0.965
PRPF40A	0.861
PRPF40B	0.874
PRPF4B	0.997
PRPF6	0.872
PRPF8	0.989
PRPH	0.062
PRPH2	0.246
PRPS1L1	0.102
PRPSAP1	0.351
PRPSAP2	0.402
PRR11	0.215
PRR12	0.999
PRR13	0.197
PRR14	0.733
PRR14L	0.831

PRR15	0.646
PRR15L	0.248
PRR16	0.031
PRR18	0.296
PRR19	0.319
PRR20A	0.302
PRR20B	0.322
PRR20C	0.201
PRR20D	0.335
PRR20E	0.319
PRR21	0.975
PRR22	0.092
PRR23A	0.201
PRR23B	0.331
PRR23C	0.250
PRR24	0.217
PRR25	0.093
PRR26	0.080
PRR3	0.589
PRR4	0.148
PRR5	0.223
PRR5-ARHGAP8	0.045
PRR5L	0.217
PRR7	0.460
PRR9	0.961
PRRC1	0.183
PRRC2A	0.885
PRRC2B	0.810
PRRC2C	0.854
PRRG2	0.344
PRRG4	0.349
PRRT1	0.728

PRRT2	0.394
PRRT3	0.270
PRRT4	0.415
PRRX1	0.791
PRRX2	0.501
PRSS1	0.215
PRSS12	0.100
PRSS16	0.547
PRSS21	0.454
PRSS22	0.562
PRSS23	0.537
PRSS27	0.284
PRSS3	0.894
PRSS33	0.237
PRSS35	0.139
PRSS36	0.677
PRSS37	0.214
PRSS38	0.316
PRSS42	0.314
PRSS45	0.261
PRSS46	0.354
PRSS48	0.126
PRSS50	0.435
PRSS53	0.663
PRSS54	0.389
PRSS55	0.207
PRSS56	0.404
PRSS57	0.279
PRSS58	0.040
PRSS8	0.455
PRTFDC1	0.520
PRTG	0.150

PRTN3	0.085
PRUNE	0.607
PRUNE2	0.227
PRX	0.643
PSAP	0.578
PSAPL1	0.189
PSAT1	0.527
PSCA	0.150
PSD	0.601
PSD2	0.250
PSD3	0.340
PSD4	0.571
PSEN1	0.912
PSEN2	0.770
PSENEN	0.988
PSG1	0.206
PSG2	0.215
PSG3	0.183
PSG4	0.231
PSG5	0.228
PSG8	0.210
PSIP1	0.539
PSKH1	0.948
PSKH2	0.108
PSMA1	0.988
PSMA2	0.899
PSMA3	0.810
PSMA4	0.973
PSMA5	0.995
PSMA6	0.998
PSMA7	0.956
PSMA8	0.322

PSMB1	0.914
PSMB10	0.467
PSMB11	0.237
PSMB2	0.913
PSMB3	0.956
PSMB4	0.375
PSMB5	0.987
PSMB6	0.931
PSMB7	0.940
PSMB8	0.865
PSMB9	0.684
PSMC1	0.970
PSMC2	1.000
PSMC3	0.998
PSMC3IP	0.372
PSMC4	0.998
PSMC5	1.000
PSMC6	0.989
PSMD1	0.967
PSMD11	0.991
PSMD12	0.861
PSMD13	0.266
PSMD14	0.858
PSMD2	0.953
PSMD3	0.990
PSMD4	0.990
PSMD5	0.485
PSMD6	0.753
PSMD7	0.527
PSMD8	0.677
PSMD9	0.301
PSME1	0.452

PSME2	0.686
PSME3	0.996
PSME4	0.855
PSMF1	0.388
PSMG1	0.200
PSMG2	0.141
PSMG3	0.945
PSMG4	0.406
PSORS1C1	0.366
PSORS1C2	0.765
PSPC1	0.762
PSPH	0.429
PSPN	0.218
PSRC1	0.965
PSTK	0.433
PSTPIP1	0.194
PSTPIP2	0.140
PTAFR	0.811
PTAR1	0.464
PTBP1	0.665
PTBP2	0.812
PTBP3	0.595
PTCD1	0.530
PTCD2	0.162
PTCD3	0.080
PTCH1	0.962
PTCH2	0.384
PTCHD2	0.168
PTCHD3	0.069
PTCHD4	0.323
PTCRA	0.152
PTDSS2	0.291

PTEN	0.985
PTER	0.128
PTF1A	0.909
PTGDR	0.077
PTGDR2	0.292
PTGDS	0.191
PTGER1	0.277
PTGER2	0.217
PTGER3	0.356
PTGER4	0.831
PTGES	0.171
PTGES2	0.599
PTGES3	0.892
PTGES3L	0.347
PTGES3L-AARSD1	0.193
PTGFR	0.114
PTGFRN	0.560
PTGIR	0.216
PTGIS	0.295
PTGR1	0.092
PTGR2	0.281
PTGS1	0.502
PTGS2	0.553
PTH1R	0.811
PTH2	0.259
PTH2R	0.087
PTHLH	0.663
PTK2	1.000
PTK2B	0.902
PTK6	0.494
PTK7	0.761
PTMA	0.290

PTMS	0.304
PTN	0.189
PTOV1	0.422
PTP4A1	0.569
PTP4A2	0.808
PTP4A3	0.501
PTPDC1	0.343
PTPLA	0.098
PTPLAD1	0.453
PTPLAD2	0.068
PTPLB	0.338
PTPMT1	0.463
PTPN1	0.965
PTPN11	1.000
PTPN12	0.605
PTPN13	0.605
PTPN14	0.742
PTPN18	0.286
PTPN2	0.838
PTPN20A	0.313
PTPN20B	0.391
PTPN21	0.246
PTPN22	0.335
PTPN23	0.732
PTPN3	0.823
PTPN4	0.839
PTPN5	0.574
PTPN6	0.784
PTPN7	0.474
PTPN9	0.967
PTPRA	1.000
PTPRB	0.821

PTPRC	0.275
PTPRCAP	0.442
PTPRD	0.818
PTPRE	0.567
PTPRF	0.912
PTPRG	0.812
PTPRH	0.809
PTPRJ	0.215
PTPRK	0.970
PTPRM	0.646
PTPRN	0.807
PTPRN2	0.357
PTPRO	0.540
PTPRQ	0.477
PTPRR	0.603
PTPRS	0.940
PTPRT	0.917
PTPRU	0.799
PTPRZ1	0.844
PTRF	0.423
PTRH1	0.390
PTRH2	0.360
PTRHD1	0.291
PTS	0.339
PTTG1	0.087
PTTG1IP	0.213
PTTG2	0.158
PTX3	0.482
PTX4	0.893
PUF60	0.997
PUM1	0.997
PUM2	0.719

PURA	0.996
PURB	0.673
PURG	0.670
PUS1	0.923
PUS10	0.495
PUS3	0.420
PUS7	0.135
PUS7L	0.308
PUSL1	0.260
PVALB	0.157
PVR	0.380
PVRIG	0.489
PVRL1	0.671
PVRL2	0.876
PVRL3	0.643
PVRL4	0.606
PWP1	0.295
PWP2	0.621
PWWP2A	0.663
PWWP2B	0.348
PXDC1	0.301
PXDN	0.145
PXDNL	0.320
PXK	0.273
PXMP2	0.220
PXMP4	0.209
PXN	0.314
PXT1	0.265
PYCARD	0.366
PYCR1	0.483
PYCR2	0.711
PYCRL	0.227

PYDC1	0.432
PYGB	0.323
PYGL	0.375
PYGM	0.476
PYGO1	0.218
PYGO2	0.924
PYHIN1	0.112
PYROXD1	0.208
PYROXD2	0.475
PYURF	0.276
PYY	0.259
PZP	0.249
QARS	0.804
QDPR	0.177
QKI	0.839
QPCT	0.498
QPCTL	0.460
QPRT	0.158
QRFP	0.262
QRFPR	0.185
QRICH1	0.999
QRICH2	0.430
QRSL1	0.289
QSER1	0.437
QSOX1	0.450
QSOX2	0.299
QTRT1	0.488
QTRTD1	0.579
R3HCC1	0.457
R3HCC1L	0.454
R3HDM1	0.663
R3HDM2	0.998

R3HDM4	0.126
R3HDML	0.291
RAB10	0.980
RAB11A	1.000
RAB11B	0.650
RAB11FIP1	0.467
RAB11FIP2	0.773
RAB11FIP3	0.990
RAB11FIP4	0.737
RAB11FIP5	0.494
RAB12	0.408
RAB13	0.348
RAB14	0.947
RAB15	0.373
RAB17	0.166
RAB18	0.567
RAB19	0.094
RAB1A	0.933
RAB1B	0.994
RAB20	0.866
RAB21	0.445
RAB22A	0.974
RAB23	0.183
RAB24	0.675
RAB25	0.286
RAB26	0.207
RAB27A	0.090
RAB27B	0.259
RAB28	0.310
RAB2A	0.952
RAB2B	0.819
RAB30	0.592

RAB31	0.672
RAB32	0.199
RAB33B	0.452
RAB34	0.568
RAB35	0.981
RAB36	0.327
RAB37	0.039
RAB38	0.289
RAB39A	0.080
RAB3A	0.642
RAB3B	0.710
RAB3C	0.576
RAB3D	0.559
RAB3GAP1	0.537
RAB3GAP2	0.438
RAB3IL1	0.281
RAB3IP	0.411
RAB40B	0.470
RAB40C	0.430
RAB42	0.412
RAB43	0.840
RAB44	0.288
RAB4A	0.650
RAB4B	0.924
RAB4B-EGLN2	0.313
RAB5A	0.454
RAB5B	0.961
RAB5C	0.921
RAB6A	0.636
RAB6B	0.986
RAB6C	0.330
RAB7A	0.945

RAB7L1	0.529
RAB8A	0.986
RAB8B	0.449
RABAC1	0.570
RABEP1	0.903
RABEP2	0.532
RABEPK	0.122
RABGAP1	0.980
RABGAP1L	0.452
RABGEF1	0.865
RABGGTA	0.541
RABGGTB	0.485
RABIF	0.235
RABL2A	0.171
RABL2B	0.358
RABL3	0.294
RABL5	0.217
RABL6	0.774
RAC1	0.754
RAC2	0.821
RAC3	0.742
RACGAP1	0.617
RAD1	0.367
RAD17	0.269
RAD18	0.444
RAD21	0.801
RAD21L1	0.096
RAD23A	0.949
RAD23B	0.950
RAD50	0.593
RAD51	0.454
RAD51AP1	0.079

RAD51AP2	0.102
RAD51B	0.449
RAD51C	0.188
RAD51D	0.435
RAD51L3-RFFL	0.360
RAD52	0.113
RAD54B	0.448
RAD54L	0.138
RAD54L2	0.998
RAD9A	0.661
RAD9B	0.296
RADIL	0.982
RAE1	0.970
RAET1E	0.207
RAET1G	0.251
RAET1L	0.151
RAF1	0.579
RAG1	0.139
RAG2	0.117
RAI1	0.382
RAI14	0.395
RALA	0.851
RALB	0.563
RALBP1	0.713
RALGAPA1	0.762
RALGAPA2	0.579
RALGAPB	0.884
RALGDS	0.918
RALGPS1	0.990
RALGPS2	0.212
RALY	0.555
RALYL	0.431

RAMP1	0.422
RAMP2	0.400
RAMP3	0.239
RAN	1.000
RANBP1	0.932
RANBP10	0.871
RANBP17	0.163
RANBP2	0.836
RANBP3	0.933
RANBP3L	0.187
RANBP6	0.409
RANBP9	0.955
RANGAP1	0.738
RANGRF	0.515
RAP1A	0.932
RAP1B	0.947
RAP1GAP	0.611
RAP1GAP2	0.644
RAP1GDS1	0.486
RAP2A	0.871
RAP2B	0.312
RAPGEF1	0.838
RAPGEF2	0.994
RAPGEF3	0.481
RAPGEF4	0.931
RAPGEF5	0.744
RAPGEF6	0.749
RAPGEFL1	0.358
RAPH1	0.693
RAPSN	0.273
RARA	0.778
RARB	0.998

RARG	0.978
RARRES1	0.105
RARRES2	0.185
RARRES3	0.249
RARS	0.384
RARS2	0.270
RASA1	0.939
RASA2	0.518
RASA3	0.119
RASA4	0.387
RASA4B	0.350
RASAL1	0.469
RASAL2	0.933
RASAL3	0.422
RASD1	0.606
RASD2	0.854
RASEF	0.478
RASGEF1A	0.586
RASGEF1B	0.684
RASGEF1C	0.149
RASGRF1	0.940
RASGRF2	0.628
RASGRP1	0.656
RASGRP2	0.602
RASGRP3	0.315
RASGRP4	0.408
RASIP1	0.882
RASL10A	0.774
RASL10B	0.723
RASL11A	0.434
RASL11B	0.205
RASL12	0.593

RASSF1	0.429
RASSF2	0.101
RASSF3	0.126
RASSF4	0.105
RASSF5	0.666
RASSF6	0.091
RASSF7	0.353
RASSF8	0.840
RASSF9	0.095
RAVER1	0.730
RAVER2	0.557
RAX	0.532
RAX2	0.880
RB1	0.945
RB1CC1	0.269
RBAK	0.145
RBAK-RBAKDN	0.134
RBBP4	1.000
RBBP5	0.996
RBBP6	0.986
RBBP8	0.131
RBBP8NL	0.227
RBBP9	0.181
RBCK1	0.533
RBFA	0.110
RBFOX1	0.343
RBFOX2	0.922
RBFOX3	0.985
RBKS	0.703
RBL1	0.697
RBL2	0.802
RBM11	0.087

RBM12	0.749
RBM12B	0.415
RBM12B-AS1	0.149
RBM14	0.985
RBM14-RBM4	0.742
RBM15	0.857
RBM15B	0.807
RBM17	0.653
RBM18	0.330
RBM19	0.189
RBM20	0.894
RBM22	0.978
RBM23	0.632
RBM24	0.539
RBM25	0.863
RBM26	0.779
RBM27	0.857
RBM28	0.315
RBM33	0.757
RBM34	0.270
RBM38	0.382
RBM39	0.997
RBM4	0.713
RBM42	0.940
RBM43	0.184
RBM44	0.143
RBM45	0.323
RBM46	0.506
RBM47	0.763
RBM48	0.576
RBM4B	0.685
RBM5	0.983

RBM6	0.805
RBM7	0.685
RBM8A	0.789
RBMS1	0.629
RBMS2	0.607
RBMS3	0.450
RBMXL1	0.323
RBMXL2	0.316
RBP1	0.336
RBP2	0.183
RBP3	0.218
RBP4	0.887
RBP5	0.139
RBP7	0.195
RBPJ	0.997
RBPJL	0.754
RBPMS	0.610
RBPMS2	0.886
RBX1	0.987
RC3H1	0.949
RC3H2	0.952
RCAN1	0.188
RCAN2	0.646
RCAN3	0.282
RCBTB1	0.221
RCBTB2	0.361
RCC1	0.468
RCC2	0.965
RCCD1	0.159
RCE1	0.729
RCHY1	0.626
RCL1	0.218

RCN1	0.161
RCN2	0.341
RCN3	0.202
RCOR1	0.994
RCOR2	0.682
RCOR3	0.930
RCSD1	0.302
RCVRN	0.257
RD3	0.191
RD3L	0.950
RDH10	0.948
RDH11	0.381
RDH12	0.612
RDH13	0.154
RDH14	0.163
RDH16	0.426
RDH5	0.339
RDH8	0.206
RDM1	0.106
RDX	0.630
REC8	0.273
RECK	0.424
RECQL	0.474
RECQL4	0.081
RECQL5	0.631
REEP1	0.141
REEP2	0.778
REEP3	0.299
REEP4	0.353
REEP5	0.266
REEP6	0.328
REG1A	0.060

REG1B	0.046
REG3A	0.126
REG3G	0.087
REG4	0.950
REL	0.812
RELA	0.999
RELB	0.901
RELL1	0.149
RELL2	0.408
RELN	0.979
RELT	0.088
REM1	0.090
REM2	0.763
REN	0.273
REP15	0.147
REPIN1	0.404
REPS1	0.703
RER1	0.809
RERE	0.982
RERG	0.305
RERGL	0.071
RESP18	0.291
REST	0.750
RET	0.925
RETN	0.070
RETNLB	0.122
RETSAT	0.509
REV1	0.964
REV3L	0.809
REXO1	0.724
REXO1L1	0.177
REXO1L10P	0.554

REXO1L11P	0.488
REXO2	0.422
REXO4	0.552
RFC1	0.942
RFC2	0.828
RFC3	0.822
RFC4	0.105
RFC5	0.333
RFESD	0.089
RFFL	0.525
RFK	0.331
RFNG	0.376
RFPL1	0.394
RFPL2	0.137
RFPL3	0.273
RFPL3S	0.175
RFPL4A	0.400
RFPL4AL1	0.548
RFPL4B	0.061
RFT1	0.412
RFTN1	0.630
RFTN2	0.186
RFWD2	0.810
RFWD3	0.296
RFX1	0.978
RFX2	0.749
RFX3	0.666
RFX4	0.678
RFX5	0.837
RFX6	0.481
RFX7	0.893
RFX8	0.264

RFXANK	0.584
RFXAP	0.236
RGCC	0.270
RGL1	0.907
RGL2	0.721
RGL3	0.592
RGL4	0.223
RGMA	0.436
RGMB	0.490
RGP1	0.671
RGPD1	0.520
RGPD2	0.212
RGPD3	0.139
RGPD4	0.142
RGPD5	0.549
RGPD6	0.367
RGPD8	0.316
RGR	0.071
RGS1	0.166
RGS10	0.401
RGS11	0.959
RGS12	0.995
RGS13	0.080
RGS14	0.412
RGS16	0.410
RGS17	0.447
RGS18	0.059
RGS19	0.638
RGS2	0.103
RGS20	0.183
RGS22	0.181
RGS3	0.688

RGS4	0.199
RGS5	0.162
RGS6	0.806
RGS7	0.752
RGS7BP	0.422
RGS8	0.611
RGS9	0.248
RGS9BP	0.346
RHAG	0.196
RHBDD1	0.164
RHBDD2	0.428
RHBDD3	0.332
RHBDF1	0.323
RHBDF2	0.321
RHBDL1	0.266
RHBDL2	0.203
RHBDL3	0.306
RHBG	0.394
RHCE	0.263
RHCG	0.664
RHD	0.392
RHEB	0.972
RHEBL1	0.331
RHNO1	0.112
RHO	0.216
RHOA	0.997
RHOB	0.420
RHOBTB1	0.791
RHOBTB2	0.819
RHOBTB3	0.271
RHOC	0.202
RHOD	0.515

RHOF	0.198
RHOG	0.373
RHOH	0.297
RHOJ	0.211
RHOQ	0.832
RHOT1	0.943
RHOT2	0.328
RHOU	0.319
RHOV	0.913
RHPN1	0.128
RHPN2	0.519
RIBC2	0.090
RIC3	0.198
RIC8A	0.241
RIC8B	0.882
RICTOR	0.944
RIF1	0.543
RIIAD1	0.208
RILP	0.303
RILPL1	0.848
RILPL2	0.311
RIMBP2	0.171
RIMBP3	0.337
RIMBP3B	0.269
RIMBP3C	0.582
RIMKLA	0.485
RIMKLB	0.501
RIMS1	0.955
RIMS2	0.911
RIMS3	0.511
RIMS4	0.852
RIN1	0.528

RIN2	0.323
RIN3	0.403
RING1	0.955
RINL	0.372
RINT1	0.148
RIOK1	0.170
RIOK2	0.280
RIOK3	0.384
RIPK1	0.926
RIPK2	0.605
RIPK3	0.361
RIPK4	0.477
RIPPLY2	0.117
RIT1	0.995
RIT2	0.206
RLBP1	0.169
RLF	0.949
RLN1	0.116
RLN2	0.079
RLN3	0.094
RLTPR	0.332
RMDN1	0.275
RMDN2	0.285
RMDN3	0.583
RMI1	0.495
RMI2	0.062
RMND1	0.410
RMND5A	0.833
RMND5B	0.304
RNASE1	0.433
RNASE10	0.196
RNASE11	0.224

RNASE12	0.130
RNASE13	0.119
RNASE2	0.153
RNASE3	0.292
RNASE4	0.154
RNASE6	0.485
RNASE7	0.291
RNASE8	0.296
RNASE9	0.237
RNASEH1	0.196
RNASEH2A	0.222
RNASEH2B	0.285
RNASEH2C	0.487
RNASEK	0.252
RNASEK-C17orf49	0.333
RNASEL	0.360
RNASET2	0.975
RND1	0.741
RND2	0.651
RND3	0.776
RNF10	0.751
RNF103	0.983
RNF103-CHMP3	0.977
RNF11	0.557
RNF111	0.587
RNF112	0.421
RNF113B	0.343
RNF114	0.336
RNF115	0.689
RNF121	0.396
RNF122	0.082
RNF123	0.771

RNF125	0.160
RNF126	0.537
RNF13	0.478
RNF130	0.140
RNF133	0.086
RNF135	0.111
RNF138	0.166
RNF139	0.774
RNF14	0.688
RNF141	0.251
RNF144A	0.986
RNF144B	0.241
RNF145	0.456
RNF146	0.430
RNF148	0.093
RNF149	0.048
RNF150	0.401
RNF151	0.172
RNF152	0.275
RNF157	0.334
RNF165	0.989
RNF166	0.246
RNF167	0.313
RNF168	0.291
RNF169	0.778
RNF17	0.796
RNF170	0.474
RNF175	0.144
RNF180	0.276
RNF181	0.483
RNF182	0.149
RNF183	0.170

RNF185	0.575
RNF186	0.119
RNF19A	0.338
RNF19B	0.757
RNF2	0.898
RNF20	0.854
RNF207	0.395
RNF208	0.999
RNF212	0.100
RNF213	0.711
RNF214	0.770
RNF215	0.371
RNF216	0.416
RNF217	0.167
RNF219	0.300
RNF220	0.791
RNF222	0.421
RNF223	0.295
RNF224	0.975
RNF24	0.869
RNF25	0.848
RNF26	0.702
RNF31	1.000
RNF32	0.358
RNF34	0.864
RNF38	0.974
RNF39	0.601
RNF4	0.633
RNF40	0.994
RNF41	1.000
RNF43	0.615
RNF44	0.414

RNF5	0.677
RNF6	0.342
RNF7	0.647
RNF8	0.865
RNFT1	0.206
RNFT2	0.954
RNGTT	0.763
RNH1	0.720
RNLS	0.512
RNMT	0.373
RNMTL1	0.484
RNPC3	0.214
RNPEP	0.403
RNPEPL1	0.179
RNPS1	0.903
ROBO1	0.602
ROBO2	0.282
ROBO4	0.326
ROCK1	0.729
ROCK2	0.993
ROGDI	0.428
ROM1	0.420
ROMO1	0.422
ROPN1	0.291
ROPN1B	0.155
ROPN1L	0.154
ROR1	0.984
ROR2	0.538
RORA	0.908
RORB	0.912
RORC	0.904
ROS1	0.304

RP1	0.450
RP1-102H19.8	0.265
RP1-139D8.6	0.145
RP1-228P16.5	0.265
RP1-27O5.3	0.417
RP1-309K20.6	0.406
RP1-317E23.6	0.396
RP1-32I10.10	0.810
RP1-4G17.5	0.384
RP1-66C13.4	0.312
RP11-1026M7.2	0.195
RP11-1035H13.3	0.405
RP11-1055B8.6	0.084
RP11-1055B8.7	0.396
RP11-105C20.2	0.196
RP11-1070N10.3	0.219
RP11-1084J3.4	0.370
RP11-108K14.8	0.252
RP11-1099M24.7	0.197
RP11-10A14.4	0.189
RP11-10J21.3	0.078
RP11-1102P16.1	0.184
RP11-1105G2.3	0.247
RP11-1118M6.1	0.244
RP11-111M22.2	0.810
RP11-113D6.10	0.571
RP11-113D6.6	0.509
RP11-1167A19.2	0.358
RP11-116D17.1	0.116
RP11-1212A22.4	0.452
RP11-1220K2.2	0.195
RP11-122A3.2	0.136

RP11-123K3.4	0.571
RP11-125O5.2	0.305
RP11-126K1.2	0.244
RP11-127H5.1	0.305
RP11-12J10.3	0.248
RP11-131H24.4	0.202
RP11-133K1.2	0.209
RP11-139J15.7	0.038
RP11-1407O15.2	0.562
RP11-144F15.1	0.289
RP11-145E5.5	0.246
RP11-146D12.2	0.372
RP11-147C23.1	0.157
RP11-155D18.14	0.277
RP11-156E8.1	0.148
RP11-156P1.2	0.463
RP11-159D12.5	0.279
RP11-159G9.5	0.799
RP11-15E18.4	0.163
RP11-160N1.10	0.053
RP11-162A12.2	0.153
RP11-162P23.2	0.072
RP11-166B2.1	0.218
RP11-166N6.3	0.288
RP11-169F17.1	0.085
RP11-171N4.2	0.064
RP11-178C3.1	0.345
RP11-178L8.4	0.227
RP11-17M16.1	0.159
RP11-180C1.1	0.129
RP11-181C3.1	0.130
RP11-182J1.16	0.186

RP11-187E13.1	0.063
RP11-190A12.7	0.253
RP11-195B21.3	0.308
RP11-195F19.5	0.386
RP11-196G11.1	0.289
RP11-1C1.5	0.252
RP11-204N11.1	0.109
RP11-20I23.1	0.079
RP11-210M15.2	0.148
RP11-211G3.3	0.229
RP11-212D19.4	0.256
RP11-215A19.2	0.086
RP11-216L13.17	0.171
RP11-219A15.1	0.577
RP11-219B4.5	0.116
RP11-219B4.6	0.132
RP11-231C14.4	0.355
RP11-234B24.6	0.179
RP11-247C2.2	0.283
RP11-248J23.6	0.503
RP11-248J23.7	0.153
RP11-257K9.8	0.415
RP11-268J15.5	0.524
RP11-272B17.2	0.023
RP11-276H1.3	0.071
RP11-279O9.4	0.107
RP11-287D1.3	0.356
RP11-293I14.2	0.302
RP11-293M10.1	0.165
RP11-295D22.1	0.254
RP11-295K3.1	0.223
RP11-296A16.1	0.279

RP11-297M9.1	0.161
RP11-297N6.4	0.162
RP11-298I3.5	0.455
RP11-302B13.5	0.586
RP11-302M6.4	0.942
RP11-307N16.6	0.470
RP11-315D16.2	0.119
RP11-321F6.1	0.154
RP11-321M21.3	0.077
RP11-321N4.5	0.355
RP11-322E11.6	0.118
RP11-322L20.1	0.146
RP11-324D17.1	0.164
RP11-332O19.5	0.272
RP11-342M21.2	0.177
RP11-343C2.11	0.781
RP11-343C2.12	0.243
RP11-343C2.7	0.218
RP11-343C2.9	0.222
RP11-345J4.3	0.353
RP11-345J4.5	0.566
RP11-347C12.1	0.230
RP11-347C12.3	0.562
RP11-351M8.1	0.142
RP11-362K2.2	0.110
RP11-366L20.2	0.245
RP11-368I7.4	0.073
RP11-382J12.1	0.339
RP11-385D13.1	0.308
RP11-386G21.1	0.100
RP11-386G21.2	0.143
RP11-389E17.1	0.080

RP11-399J13.3	0.374
RP11-3B7.1	0.169
RP11-403P17.5	0.327
RP11-404L6.2	0.132
RP11-404P21.8	0.158
RP11-407N17.3	0.167
RP11-408E5.4	0.044
RP11-410N8.4	0.236
RP11-41O4.1	0.086
RP11-422N16.3	0.387
RP11-426L16.10	0.125
RP11-429E11.3	0.902
RP11-432B6.3	0.351
RP11-433C9.2	0.225
RP11-434D12.1	0.378
RP11-438J1.1	0.475
RP11-444E17.6	0.642
RP11-446E24.4	0.266
RP11-447L10.1	0.229
RP11-451M19.3	0.168
RP11-455G16.1	0.199
RP11-458D21.5	0.724
RP11-45M22.4	0.296
RP11-463C8.4	0.312
RP11-463D19.2	0.302
RP11-463J10.2	0.133
RP11-467N20.5	0.149
RP11-468E2.2	0.532
RP11-468E2.4	0.537
RP11-468E2.6	0.361
RP11-474G23.1	0.515
RP11-477N12.3	0.406

RP11-47I22.3	0.163
RP11-47I22.4	0.186
RP11-480I12.4	0.308
RP11-481A20.11	0.149
RP11-497E19.2	0.172
RP11-500M8.7	0.154
RP11-503N18.3	0.119
RP11-505K9.4	0.901
RP11-507M3.1	0.180
RP11-508N12.4	0.299
RP11-512M8.5	0.240
RP11-514O12.4	0.110
RP11-514P8.6	0.610
RP11-514P8.7	0.141
RP11-51F16.8	0.347
RP11-51L5.7	0.345
RP11-520P18.5	0.499
RP11-527L4.2	0.219
RP11-529K1.3	0.276
RP11-536G4.1	0.303
RP11-542C16.2	0.428
RP11-542P2.1	0.194
RP11-552F3.12	0.203
RP11-552I14.1	0.129
RP11-553A10.1	0.138
RP11-565P22.6	0.249
RP11-574K11.31	0.637
RP11-57H12.6	0.518
RP11-595B24.2	0.341
RP11-597K23.2	0.169
RP11-598P20.5	0.557
RP11-599B13.6	0.554

RP11-5A19.5	0.660
RP11-613M10.8	0.181
RP11-613M10.9	0.414
RP11-618P17.4	0.306
RP11-625H11.1	0.072
RP11-637O19.3	0.133
RP11-644F5.10	0.205
RP11-650K20.3	0.178
RP11-65D24.2	0.088
RP11-661C8.3	0.071
RP11-664D7.4	0.097
RP11-664I21.6	0.318
RP11-676J12.7	0.235
RP11-680G10.1	0.079
RP11-683L23.1	0.094
RP11-685N3.1	0.242
RP11-690P14.4	0.397
RP11-691N7.6	0.347
RP11-697E2.6	0.181
RP11-6L6.2	0.049
RP11-701P16.2	0.065
RP11-712L6.5	0.150
RP11-723G8.2	0.038
RP11-723O4.6	0.423
RP11-724O16.1	0.254
RP11-729L2.2	0.279
RP11-73M18.2	0.432
RP11-758M4.1	0.363
RP11-761B3.1	0.087
RP11-762I7.5	0.878
RP11-763F8.1	0.039
RP11-766F14.2	0.233

RP11-770J1.4	0.686
RP11-77K12.1	0.110
RP11-77K12.7	0.171
RP11-795F19.5	0.043
RP11-796G6.2	0.293
RP11-80A15.1	0.158
RP11-812E19.9	0.100
RP11-817J15.3	0.059
RP11-81K2.1	0.353
RP11-826N14.2	0.156
RP11-830F9.6	0.229
RP11-834C11.12	0.561
RP11-849F2.7	0.264
RP11-849H4.2	0.507
RP11-863K10.7	0.179
RP11-865B13.1	0.149
RP11-867G23.8	0.383
RP11-872D17.8	0.236
RP11-87C12.2	0.254
RP11-886H22.1	0.889
RP11-894J14.5	0.261
RP11-89K11.1	0.173
RP11-89N17.1	0.219
RP11-934B9.3	0.366
RP11-93B14.6	0.088
RP11-944C7.1	0.126
RP11-94B19.4	0.159
RP11-96O20.4	0.907
RP11-977G19.10	0.454
RP11-986E7.7	0.044
RP11-998D10.1	0.196
RP11-9B6.1	0.262

RP13-279N23.2	0.266
RP13-512J5.1	0.055
RP13-672B3.2	0.138
RP13-996F3.4	0.288
RP13-996F3.5	0.289
RP1L1	0.088
RP3-369A17.5	0.112
RP3-422G23.4	0.156
RP3-468K18.5	0.106
RP4-559A3.7	0.333
RP4-576H24.4	0.065
RP4-583P15.15	0.208
RP4-734P14.4	0.110
RP4-758J18.2	0.259
RP5-1021I20.4	0.182
RP5-1052I5.2	0.453
RP5-966M1.6	0.226
RP9	0.096
RPA1	0.797
RPA2	0.484
RPA3	0.133
RPA3-AS1	0.151
RPAIN	0.451
RPAP1	0.562
RPAP2	0.976
RPAP3	0.424
RPE	0.588
RPE65	0.217
RPF1	0.238
RPF2	0.102
RPGRIP1	0.217
RPGRIP1L	0.437

RPH3A	0.275
RPH3AL	0.317
RPIA	0.220
RPL10A	0.606
RPL10L	0.893
RPL11	0.378
RPL12	0.290
RPL13	0.095
RPL13A	0.335
RPL14	0.368
RPL15	0.420
RPL17	0.204
RPL17-C18orf32	0.317
RPL18	0.499
RPL18A	0.435
RPL19	0.642
RPL21	0.365
RPL22	0.638
RPL22L1	0.097
RPL23	0.570
RPL23A	0.189
RPL24	0.389
RPL26	0.297
RPL26L1	0.259
RPL27	0.403
RPL27A	0.289
RPL28	0.173
RPL29	0.247
RPL3	0.516
RPL30	0.731
RPL31	0.176
RPL32	0.277

RPL34	0.260
RPL35	0.305
RPL35A	0.066
RPL36	0.308
RPL36AL	0.537
RPL37	0.114
RPL37A	0.130
RPL38	0.158
RPL39L	0.059
RPL3L	0.319
RPL4	0.643
RPL41	0.351
RPL5	0.529
RPL6	0.440
RPL7	0.491
RPL7A	0.421
RPL7L1	0.437
RPL8	0.985
RPL9	0.147
RPLP0	0.354
RPLP1	0.198
RPLP2	0.078
RPN1	0.471
RPN2	0.667
RPP14	0.420
RPP21	0.443
RPP25	0.448
RPP25L	0.676
RPP30	0.163
RPP38	0.201
RPP40	0.136
RPRD1A	0.752

RPRD1B	0.544
RPRD2	0.974
RPRM	0.558
RPRML	0.769
RPS10	0.442
RPS10-NUDT3	0.848
RPS11	0.604
RPS12	0.326
RPS13	0.441
RPS14	0.816
RPS15	0.825
RPS15A	0.618
RPS16	0.405
RPS17	0.202
RPS17L	0.316
RPS18	0.572
RPS19	0.518
RPS19BP1	0.340
RPS2	0.513
RPS20	0.282
RPS21	0.426
RPS23	0.653
RPS24	0.227
RPS25	0.731
RPS26	0.655
RPS27	0.303
RPS27A	0.123
RPS27L	0.095
RPS28	0.606
RPS29	0.350
RPS3	0.601
RPS3A	0.206

RPS5	0.529
RPS6	0.075
RPS6KA1	0.702
RPS6KA2	0.876
RPS6KA4	0.984
RPS6KA5	0.888
RPS6KB1	1.000
RPS6KB2	0.770
RPS6KC1	0.531
RPS6KL1	0.342
RPS7	0.152
RPS8	0.659
RPS9	0.196
RPSA	0.603
RPSAP58	0.103
RPTN	0.143
RPTOR	0.970
RPUSD1	0.134
RPUSD2	0.452
RPUSD3	0.313
RPUSD4	0.271
RQCD1	0.994
RRAD	0.701
RRAGA	0.231
RRAGC	0.989
RRAGD	0.781
RRAS	0.609
RRAS2	0.999
RRBP1	0.464
RREB1	0.294
RRH	0.091
RRM1	0.955

RRM2	0.315
RRM2B	0.407
RRN3	0.189
RRNAD1	0.530
RRP1	0.462
RRP12	0.244
RRP15	0.150
RRP1B	0.664
RRP36	0.540
RRP7A	0.545
RRP8	0.465
RRP9	0.805
RRS1	0.245
RSAD1	0.261
RSAD2	0.108
RSBN1	0.847
RSBN1L	0.739
RSC1A1	0.150
RSF1	0.649
RSG1	0.422
RSL1D1	0.254
RSL24D1	0.101
RSPH1	0.234
RSPH10B	0.326
RSPH10B2	0.602
RSPH3	0.250
RSPH4A	0.336
RSPH6A	0.258
RSPH9	0.326
RSPO1	0.482
RSPO2	0.111
RSPO3	0.213

RSPO4	0.155
RSPRY1	0.988
RSRC1	0.322
RSRC2	0.424
RSU1	0.372
RTBDN	0.409
RTCA	0.646
RTCB	0.548
RTDR1	0.189
RTEL1	0.371
RTEL1-TNFRSF6B	0.130
RTF1	0.999
RTFDC1	0.458
RTKN	0.503
RTKN2	0.122
RTL1	0.330
RTN1	0.443
RTN2	0.278
RTN3	0.391
RTN4	0.470
RTN4IP1	0.262
RTN4R	0.591
RTN4RL1	0.957
RTN4RL2	0.845
RTP1	0.053
RTP2	0.212
RTP3	0.168
RTP4	0.209
RTTN	0.335
RUFY1	0.483
RUFY2	0.552
RUFY3	0.572

RUFY4	0.337
RUNDC1	0.829
RUNDC3A	0.953
RUNDC3B	0.220
RUNX1	0.896
RUNX1T1	0.978
RUNX2	0.931
RUNX3	0.808
RUSC1	0.890
RUSC2	0.912
RUVBL1	0.952
RUVBL2	0.998
RWDD1	0.495
RWDD2A	0.218
RWDD3	0.536
RWDD4	0.171
RXFP1	0.267
RXFP2	0.304
RXFP3	0.260
RXFP4	0.134
RXRA	0.939
RXRB	0.999
RXRG	0.904
RYBP	0.506
RYK	0.853
RYR1	0.836
RYR2	0.999
RYR3	0.863
S100A1	0.264
S100A10	0.169
S100A11	0.044
S100A12	0.113

S100A13	0.401
S100A14	0.233
S100A16	0.261
S100A2	0.170
S100A3	0.132
S100A4	0.112
S100A5	0.053
S100A6	0.242
S100A7	0.182
S100A7A	0.220
S100A8	0.153
S100A9	0.137
S100B	0.338
S100P	0.040
S100PBP	0.561
S100Z	0.134
S1PR1	0.998
S1PR2	0.358
S1PR3	0.296
S1PR4	0.359
S1PR5	0.238
SAA1	0.279
SAA2	0.221
SAA4	0.079
SAAL1	0.261
SAC3D1	0.530
SACM1L	0.701
SACS	0.351
SAE1	0.584
SAFB	0.731
SAFB2	0.449
SAG	0.161

SALL1	0.877
SALL2	0.608
SALL3	0.337
SALL4	0.684
SAMD1	0.849
SAMD10	0.305
SAMD11	0.162
SAMD12	0.257
SAMD13	0.102
SAMD14	0.367
SAMD15	0.163
SAMD3	0.239
SAMD4A	0.989
SAMD4B	0.995
SAMD5	0.173
SAMD7	0.304
SAMD8	0.616
SAMD9	0.105
SAMD9L	0.153
SAMHD1	0.361
SAMM50	0.504
SAMSN1	0.123
SAP130	0.939
SAP18	0.470
SAP25	0.312
SAP30	0.289
SAP30BP	0.507
SAP30L	0.353
SAPCD1	0.255
SAPCD2	0.382
SAR1A	0.984
SAR1B	0.468

SARDH	0.191
SARM1	0.806
SARNP	0.822
SARS	0.760
SARS2	0.699
SART1	0.993
SART3	0.807
SASH1	0.543
SASS6	0.226
SAT2	0.785
SATB1	0.951
SATB2	0.981
SAV1	0.296
SAYSD1	0.219
SBDS	0.546
SBF1	0.227
SBF2	0.408
SBK1	0.656
SBK2	0.526
SBK3	0.503
SBNO1	0.724
SBNO2	0.963
SBSN	0.543
SBSPON	0.159
SC5D	0.085
SCAF1	0.959
SCAF11	0.717
SCAF4	0.871
SCAF8	0.957
SCAI	0.942
SCAMP1	0.590
SCAMP2	0.514

SCAMP3	0.610
SCAMP4	0.442
SCAMP5	0.734
SCAND1	0.269
SCAND3	0.232
SCAP	0.579
SCAPER	0.829
SCARA3	0.623
SCARA5	0.512
SCARB1	0.359
SCARB2	0.540
SCARF1	0.243
SCARF2	0.807
SCCPDH	0.163
SCD	0.768
SCD5	0.329
SCEL	0.620
SCFD1	0.451
SCFD2	0.295
SCG2	0.367
SCG3	0.388
SCG5	0.549
SCGB1A1	0.113
SCGB1C1	0.183
SCGB1D2	0.184
SCGB1D4	0.171
SCGB2A1	0.122
SCGB2A2	0.238
SCGB2B2	0.186
SCGB3A1	0.076
SCGB3A2	0.030
SCGN	0.371

SCHIP1	0.565
SCIMP	0.178
SCIN	0.061
SCLT1	0.068
SCLY	0.456
SCMH1	0.892
SCML4	0.210
SCN10A	0.513
SCN11A	0.445
SCN1A	0.949
SCN1B	0.644
SCN2A	1.000
SCN2B	0.368
SCN3A	0.956
SCN3B	0.367
SCN4A	0.786
SCN4B	0.204
SCN5A	0.995
SCN7A	0.315
SCN8A	1.000
SCN9A	0.516
SCNM1	0.459
SCNN1A	0.297
SCNN1B	0.391
SCNN1D	0.196
SCNN1G	0.438
SCO1	0.127
SCO2	0.123
SCOC	0.118
SCP2	0.571
SCP2D1	0.149
SCPEP1	0.274

SCRG1	0.115
SCRIB	0.967
SCRN1	0.348
SCRN2	0.432
SCRN3	0.271
SCRT1	0.671
SCRT2	0.786
SCT	0.077
SCTR	0.337
SCUBE1	0.520
SCUBE2	0.200
SCUBE3	0.970
SCXA	0.404
SCYL1	0.716
SCYL2	0.441
SCYL3	0.586
SDAD1	0.322
SDC1	0.369
SDC2	0.555
SDC3	0.655
SDC4	0.202
SDCBP	0.285
SDCBP2	0.118
SDCCAG3	0.599
SDCCAG8	0.175
SDE2	0.481
SDF2	0.346
SDF2L1	0.521
SDF4	0.413
SDHA	0.727
SDHAF1	0.679
SDHAF2	0.523

SDHB	0.231
SDHC	0.167
SDHD	0.322
SDIM1	0.091
SDK1	0.166
SDK2	0.557
SDPR	0.201
SDR16C5	0.488
SDR39U1	0.698
SDR42E1	0.057
SDR42E2	0.236
SDR9C7	0.826
SDS	0.351
SDSL	0.389
SEC11A	0.363
SEC11C	0.409
SEC13	0.642
SEC14L1	0.750
SEC14L2	0.514
SEC14L3	0.598
SEC14L4	0.329
SEC14L5	0.152
SEC14L6	0.227
SEC16A	0.946
SEC16B	0.297
SEC22A	0.254
SEC22C	0.378
SEC23A	0.337
SEC23B	0.273
SEC23IP	0.484
SEC24A	0.593
SEC24B	0.857

SEC24C	0.995
SEC24D	0.666
SEC31A	0.566
SEC31B	0.235
SEC61A1	0.977
SEC61A2	0.333
SEC61B	0.362
SEC61G	0.085
SEC62	0.756
SEC63	0.483
SECISBP2	0.092
SECISBP2L	0.539
SECTM1	0.076
SEH1L	0.389
SEL1L	0.726
SEL1L2	0.164
SEL1L3	0.111
SELE	0.188
SELENBP1	0.240
SELK	0.318
SELL	0.176
SELM	0.321
SELO	0.182
SELP	0.270
SELPLG	0.199
SELRC1	0.187
SELT	0.649
SELV	0.377
SEMA3A	0.716
SEMA3C	0.110
SEMA3D	0.203
SEMA3E	0.115

SEMA3F	0.898
SEMA3G	0.279
SEMA4A	0.280
SEMA4B	0.249
SEMA4C	0.957
SEMA4D	0.309
SEMA4F	0.085
SEMA4G	0.501
SEMA5A	0.088
SEMA5B	0.216
SEMA6A	0.938
SEMA6B	0.410
SEMA6C	0.418
SEMA6D	0.589
SEMA7A	0.452
SEMG1	0.225
SEMG2	0.191
SENP1	0.892
SENP2	0.698
SENP3	0.999
SENP5	0.489
SENP6	0.599
SENP7	0.734
SENP8	0.186
SEP15	0.234
SEPHS1	1.000
SEPHS2	0.765
SEPN1	0.440
SEPP1	0.194
SEPSECS	0.245
SEPT1	0.390
SEPT10	0.983

SEPT11	0.889
SEPT12	0.122
SEPT14	0.272
SEPT2	0.804
SEPT3	0.847
SEPT4	0.592
SEPT5	0.777
SEPT7	0.970
SEPT8	0.805
SEPT9	0.545
SEPW1	0.247
SERAC1	0.130
SERBP1	0.844
SERF1A	0.280
SERF1B	0.401
SERF2	0.314
SERGEF	0.287
SERHL2	0.245
SERINC1	0.293
SERINC2	0.310
SERINC3	0.266
SERINC4	0.174
SERINC5	0.212
SERP1	0.331
SERP2	0.245
SERPINA1	0.167
SERPINA10	0.165
SERPINA11	0.134
SERPINA12	0.462
SERPINA3	0.056
SERPINA4	0.137
SERPINA5	0.181

SERPINA6	0.373
SERPINA9	0.060
SERPINB1	0.204
SERPINB10	0.124
SERPINB12	0.080
SERPINB13	0.332
SERPINB2	0.047
SERPINB3	0.142
SERPINB4	0.080
SERPINB5	0.121
SERPINB6	0.878
SERPINB7	0.370
SERPINB8	0.153
SERPINB9	0.969
SERPINC1	0.681
SERPIND1	0.410
SERPINE1	0.303
SERPINE2	0.351
SERPINE3	0.351
SERPINF1	0.260
SERPINF2	0.319
SERPING1	0.715
SERPINH1	0.332
SERPINI1	0.410
SERPINI2	0.338
SERTAD1	0.525
SERTAD2	0.561
SERTAD3	0.343
SERTAD4	0.353
SERTM1	0.203
SESN1	0.544
SESN2	0.677

SESN3	0.637
SESTD1	0.968
SET	0.936
SETBP1	0.993
SETD1A	0.997
SETD1B	0.997
SETD2	0.986
SETD3	0.867
SETD4	0.151
SETD5	0.965
SETD6	0.641
SETD7	0.557
SETD8	0.907
SETD9	0.068
SETDB1	1.000
SETDB2	0.490
SETMAR	0.459
SETX	0.883
SEZ6	0.620
SEZ6L	0.729
SEZ6L2	0.686
SF1	0.977
SF3A1	0.999
SF3A2	0.596
SF3A3	0.945
SF3B1	1.000
SF3B14	0.528
SF3B2	0.999
SF3B3	1.000
SF3B4	0.999
SF3B5	0.288
SFI1	0.312

SFMBT1	0.934
SFMBT2	0.630
SFN	0.717
SFPQ	0.583
SFR1	0.273
SFRP1	0.433
SFRP2	0.394
SFRP4	0.144
SFRP5	0.811
SFSWAP	0.700
SFT2D1	0.124
SFT2D2	0.178
SFT2D3	0.055
SFTA2	0.131
SFTA3	0.129
SFTPA1	0.060
SFTPA2	0.091
SFTPB	0.348
SFTPC	0.457
SFTPD	0.215
SFXN1	0.230
SFXN2	0.297
SFXN3	0.260
SFXN4	0.236
SFXN5	0.158
SGCA	0.353
SGCB	0.106
SGCD	0.217
SGCE	0.679
SGCG	0.176
SGCZ	0.022
SGIP1	0.932

SGK1	0.363
SGK2	0.834
SGK223	0.283
SGK3	0.477
SGMS1	0.677
SGMS2	0.596
SGOL1	0.060
SGOL2	0.146
SGPL1	0.863
SGPP1	0.480
SGPP2	0.463
SGSH	0.982
SGSM1	0.224
SGSM2	0.478
SGSM3	0.327
SGTA	0.778
SGTB	0.502
SH2B1	0.957
SH2B2	0.421
SH2B3	0.280
SH2D1B	0.195
SH2D2A	0.285
SH2D3A	0.383
SH2D3C	0.641
SH2D4A	0.111
SH2D4B	0.210
SH2D5	0.254
SH2D6	0.375
SH2D7	0.261
SH3BGR	0.113
SH3BGRL2	0.092
SH3BGRL3	0.402

SH3BP1	0.469
SH3BP2	0.769
SH3BP4	0.214
SH3BP5	0.280
SH3BP5L	0.145
SH3D19	0.135
SH3D21	0.251
SH3GL1	0.278
SH3GL2	0.164
SH3GL3	0.317
SH3GLB1	0.680
SH3GLB2	0.746
SH3PXD2A	0.882
SH3PXD2B	0.524
SH3RF1	0.634
SH3RF2	0.462
SH3RF3	0.595
SH3TC1	0.126
SH3TC2	0.194
SH3YL1	0.143
SHANK1	0.985
SHANK2	0.563
SHANK3	0.455
SHARPIN	0.617
SHB	0.602
SHBG	0.179
SHC1	0.799
SHC2	0.268
SHC3	0.397
SHC4	0.605
SHCBP1	0.071
SHCBP1L	0.208

SHD	0.109
SHE	0.555
SHF	0.130
SHFM1	0.163
SHH	1.000
SHISA2	0.277
SHISA3	0.303
SHISA4	0.536
SHISA5	0.294
SHISA6	0.454
SHISA7	0.636
SHISA8	0.615
SHISA9	0.481
SHKBP1	0.723
SHMT1	0.667
SHMT2	0.628
SHOC2	0.955
SHOX2	0.738
SHPK	0.298
SHPRH	0.736
SHQ1	0.471
SHROOM1	0.303
SHROOM3	0.498
SI	0.026
SIAE	0.348
SIAH1	0.963
SIAH2	0.816
SIAH3	0.266
SIDT1	0.112
SIDT2	0.503
SIGIRR	0.130
SIGLEC1	0.879

SIGLEC10	0.410
SIGLEC11	0.313
SIGLEC12	0.931
SIGLEC14	0.932
SIGLEC15	0.134
SIGLEC5	0.153
SIGLEC6	0.959
SIGLEC7	0.297
SIGLEC8	0.963
SIGLEC9	0.263
SIGLECL1	0.334
SIGMAR1	0.622
SIK1	0.892
SIK2	0.983
SIK3	0.963
SIKE1	0.158
SIL1	0.505
SIM1	0.810
SIM2	0.498
SIMC1	0.447
SIN3A	0.996
SIN3B	0.961
SIPA1	0.616
SIPA1L1	0.859
SIPA1L2	0.555
SIPA1L3	0.892
SIRPA	0.361
SIRPB1	0.056
SIRPB2	0.110
SIRPD	0.042
SIRPG	0.064
SIRT1	0.914

SIRT2	0.676
SIRT3	0.281
SIRT4	0.235
SIRT5	0.548
SIRT6	0.465
SIRT7	0.607
SIT1	0.181
SIVA1	0.610
SIX1	0.899
SIX2	0.430
SIX3	1.000
SIX4	0.816
SIX5	0.276
SIX6	0.968
SKA1	0.089
SKA2	0.257
SKA3	0.127
SKAP1	0.425
SKAP2	0.149
SKI	0.407
SKIDA1	0.472
SKIL	0.830
SKIV2L	0.908
SKIV2L2	0.863
SKOR1	0.504
SKOR2	0.531
SKP1	0.989
SKP2	0.858
SLA	0.543
SLA2	0.223
SLAIN1	0.451
SLAIN2	0.665

SLAMF1	0.170
SLAMF6	0.189
SLAMF7	0.196
SLAMF8	0.300
SLAMF9	0.101
SLBP	0.156
SLC10A1	0.190
SLC10A2	0.014
SLC10A4	0.399
SLC10A5	0.108
SLC10A6	0.130
SLC10A7	0.296
SLC11A1	0.126
SLC11A2	0.791
SLC12A1	0.305
SLC12A2	0.919
SLC12A3	0.152
SLC12A4	0.759
SLC12A5	0.986
SLC12A6	0.774
SLC12A7	0.411
SLC12A8	0.459
SLC12A9	0.344
SLC13A1	0.026
SLC13A2	0.383
SLC13A3	0.512
SLC13A4	0.542
SLC13A5	0.427
SLC14A1	0.365
SLC14A2	0.222
SLC15A1	0.245
SLC15A2	0.193

SLC15A3	0.335
SLC15A4	0.063
SLC15A5	0.434
SLC16A1	0.074
SLC16A10	0.199
SLC16A11	0.473
SLC16A12	0.762
SLC16A13	0.205
SLC16A14	0.055
SLC16A3	0.039
SLC16A4	0.377
SLC16A5	0.063
SLC16A6	0.480
SLC16A7	0.166
SLC16A8	0.363
SLC16A9	0.350
SLC17A1	0.251
SLC17A2	0.267
SLC17A3	0.312
SLC17A4	0.330
SLC17A5	0.043
SLC17A6	0.099
SLC17A7	0.969
SLC17A8	0.312
SLC17A9	0.283
SLC18A1	0.137
SLC18A2	0.405
SLC18A3	0.840
SLC18B1	0.074
SLC19A1	0.246
SLC19A2	0.278
SLC19A3	0.122

SLC1A1	0.092
SLC1A2	0.503
SLC1A3	0.337
SLC1A4	0.246
SLC1A5	0.583
SLC1A6	0.585
SLC1A7	0.225
SLC20A1	0.617
SLC20A2	0.835
SLC22A1	0.133
SLC22A10	0.125
SLC22A11	0.132
SLC22A12	0.210
SLC22A13	0.247
SLC22A14	0.187
SLC22A15	0.181
SLC22A16	0.115
SLC22A17	0.500
SLC22A18	0.301
SLC22A18AS	0.258
SLC22A2	0.086
SLC22A23	0.768
SLC22A24	0.256
SLC22A25	0.178
SLC22A3	0.295
SLC22A31	0.157
SLC22A4	0.329
SLC22A5	0.271
SLC22A6	0.344
SLC22A7	0.364
SLC22A8	0.332
SLC22A9	0.195

SLC23A1	0.403
SLC23A2	0.592
SLC23A3	0.197
SLC24A1	0.431
SLC24A2	0.083
SLC24A3	0.673
SLC24A4	0.291
SLC24A5	0.142
SLC25A1	0.382
SLC25A10	0.137
SLC25A11	0.910
SLC25A12	0.864
SLC25A13	0.513
SLC25A15	0.269
SLC25A16	0.126
SLC25A17	0.069
SLC25A18	0.921
SLC25A19	0.221
SLC25A2	0.050
SLC25A20	0.190
SLC25A21	0.097
SLC25A21-AS1	0.105
SLC25A22	0.198
SLC25A23	0.588
SLC25A24	0.095
SLC25A25	0.243
SLC25A26	0.117
SLC25A27	0.117
SLC25A28	0.913
SLC25A29	0.096
SLC25A3	0.535
SLC25A30	0.314

SLC25A31	0.149
SLC25A32	0.211
SLC25A33	0.106
SLC25A34	0.188
SLC25A35	0.518
SLC25A36	0.299
SLC25A37	0.218
SLC25A38	0.344
SLC25A39	0.212
SLC25A4	0.488
SLC25A40	0.137
SLC25A41	0.208
SLC25A42	0.590
SLC25A44	0.591
SLC25A45	0.226
SLC25A46	0.210
SLC25A47	0.174
SLC25A48	0.150
SLC25A51	0.304
SLC25A52	0.116
SLC26A1	0.240
SLC26A11	0.940
SLC26A2	0.225
SLC26A3	0.313
SLC26A4	0.117
SLC26A5	0.421
SLC26A6	0.498
SLC26A7	0.194
SLC26A8	0.088
SLC26A9	0.954
SLC27A1	0.417
SLC27A2	0.491

SLC27A3	0.376
SLC27A4	0.556
SLC27A5	0.222
SLC27A6	0.237
SLC28A1	0.221
SLC28A2	0.050
SLC28A3	0.236
SLC29A1	0.628
SLC29A2	0.373
SLC29A3	0.375
SLC29A4	0.235
SLC2A1	0.558
SLC2A10	0.414
SLC2A11	0.124
SLC2A12	0.466
SLC2A13	0.546
SLC2A14	0.243
SLC2A2	0.254
SLC2A3	0.145
SLC2A4	0.546
SLC2A4RG	0.197
SLC2A5	0.854
SLC2A6	0.200
SLC2A7	0.043
SLC2A8	0.291
SLC2A9	0.110
SLC30A1	0.465
SLC30A10	0.294
SLC30A2	0.699
SLC30A3	0.788
SLC30A4	0.064
SLC30A5	0.389

SLC30A6	0.143
SLC30A7	0.934
SLC30A8	0.221
SLC30A9	0.338
SLC31A1	0.579
SLC31A2	0.146
SLC32A1	0.951
SLC33A1	0.416
SLC34A1	0.272
SLC34A2	0.107
SLC34A3	0.863
SLC35A1	0.403
SLC35A3	0.245
SLC35A4	0.353
SLC35A5	0.231
SLC35B1	0.653
SLC35B2	0.526
SLC35B3	0.140
SLC35B4	0.133
SLC35C1	0.218
SLC35C2	0.484
SLC35D1	0.211
SLC35D2	0.196
SLC35D3	0.170
SLC35E1	0.813
SLC35E2	0.419
SLC35E2B	0.759
SLC35E3	0.193
SLC35E4	0.313
SLC35F1	0.581
SLC35F2	0.039
SLC35F3	0.396

SLC35F4	0.115
SLC35F5	0.125
SLC35F6	0.585
SLC35G1	0.229
SLC35G2	0.414
SLC35G3	0.300
SLC35G5	0.073
SLC35G6	0.325
SLC36A1	0.158
SLC36A2	0.263
SLC36A3	0.238
SLC36A4	0.134
SLC37A1	0.438
SLC37A2	0.814
SLC37A3	0.038
SLC37A4	0.198
SLC38A1	0.625
SLC38A10	0.589
SLC38A11	0.088
SLC38A2	0.341
SLC38A4	0.457
SLC38A6	0.133
SLC38A7	0.263
SLC38A8	0.007
SLC38A9	0.193
SLC39A1	0.360
SLC39A10	0.451
SLC39A11	0.151
SLC39A12	0.172
SLC39A13	0.654
SLC39A14	0.356
SLC39A2	0.223

SLC39A3	0.439
SLC39A4	0.086
SLC39A5	0.445
SLC39A6	0.436
SLC39A7	0.666
SLC39A8	0.295
SLC39A9	0.344
SLC3A1	0.902
SLC3A2	0.325
SLC40A1	0.600
SLC41A1	0.897
SLC41A2	0.532
SLC41A3	0.164
SLC43A1	0.554
SLC43A2	0.256
SLC43A3	0.287
SLC44A1	0.456
SLC44A2	0.314
SLC44A3	0.450
SLC44A4	0.258
SLC44A5	0.278
SLC45A1	0.258
SLC45A2	0.216
SLC45A3	0.355
SLC45A4	0.075
SLC46A1	0.473
SLC46A2	0.186
SLC46A3	0.319
SLC47A1	0.441
SLC47A2	0.160
SLC48A1	0.278
SLC4A1	0.806

SLC4A10	0.763
SLC4A11	0.125
SLC4A1AP	0.407
SLC4A2	0.738
SLC4A3	0.306
SLC4A4	0.949
SLC4A5	0.281
SLC4A7	0.223
SLC4A8	0.964
SLC4A9	0.131
SLC50A1	0.436
SLC51A	0.173
SLC51B	0.145
SLC52A1	0.254
SLC52A2	0.325
SLC52A3	0.101
SLC5A1	0.732
SLC5A10	0.182
SLC5A11	0.498
SLC5A12	0.205
SLC5A2	0.384
SLC5A3	0.855
SLC5A4	0.974
SLC5A5	0.198
SLC5A6	0.569
SLC5A7	0.186
SLC5A8	0.128
SLC5A9	0.060
SLC6A1	0.853
SLC6A11	0.440
SLC6A12	0.074
SLC6A13	0.052

SLC6A15	0.095
SLC6A16	0.239
SLC6A17	0.797
SLC6A18	0.040
SLC6A19	0.106
SLC6A2	0.563
SLC6A20	0.285
SLC6A3	0.335
SLC6A4	0.552
SLC6A5	0.201
SLC6A6	0.537
SLC6A7	0.389
SLC6A9	0.586
SLC7A1	0.803
SLC7A10	0.316
SLC7A11	0.098
SLC7A13	0.018
SLC7A14	0.430
SLC7A2	0.075
SLC7A4	0.342
SLC7A5	0.326
SLC7A6	0.583
SLC7A6OS	0.445
SLC7A7	0.142
SLC7A8	0.314
SLC7A9	0.260
SLC8A1	0.350
SLC8A2	0.930
SLC8A3	0.618
SLC8B1	0.403
SLC9A1	0.932
SLC9A2	0.922

SLC9A3	0.168
SLC9A3R1	0.130
SLC9A3R2	0.371
SLC9A4	0.470
SLC9A5	0.517
SLC9A8	0.325
SLC9A9	0.232
SLC9B1	0.237
SLC9B2	0.126
SLC9C1	0.338
SLC9C2	0.543
SLCO1A2	0.209
SLCO1B1	0.062
SLCO1B3	0.038
SLCO1B7	0.100
SLCO1C1	0.037
SLCO2A1	0.491
SLCO2B1	0.204
SLCO3A1	0.735
SLCO4A1	0.265
SLCO4C1	0.039
SLCO5A1	0.392
SLCO6A1	0.019
SLFN11	0.232
SLFN12	0.137
SLFN12L	0.253
SLFN13	0.122
SLFN14	0.539
SLFN5	0.570
SLFNL1	0.362
SLIRP	0.151
SLIT1	0.875

SLIT2	0.817
SLIT3	0.525
SLITRK1	0.518
SLITRK3	0.342
SLITRK5	0.440
SLITRK6	0.081
SLK	0.559
SLMAP	0.917
SLMO1	0.148
SLMO2	0.503
SLN	0.089
SLPI	0.371
SLTM	0.734
SLU7	0.588
SLURP1	0.334
SLX1A	0.424
SLX1B	0.810
SLX4	0.928
SLX4IP	0.252
SMAD1	0.995
SMAD2	0.998
SMAD3	0.992
SMAD4	1.000
SMAD5	0.962
SMAD6	0.534
SMAD7	0.684
SMAD9	0.668
SMAGP	0.159
SMAP1	0.618
SMAP2	0.348
SMARCA2	0.831
SMARCA4	1.000

SMARCA5	0.995
SMARCAD1	0.902
SMARCAL1	0.753
SMARCB1	0.733
SMARCC1	0.890
SMARCC2	1.000
SMARCD1	1.000
SMARCD2	0.582
SMARCD3	0.804
SMARCE1	0.975
SMC1B	0.452
SMC2	0.486
SMC3	1.000
SMC4	0.417
SMC5	0.552
SMC6	0.455
SMCHD1	0.971
SMCO1	0.490
SMCO2	0.220
SMCO3	0.085
SMCO4	0.080
SMCP	0.185
SMCR8	0.581
SMDT1	0.362
SMEK1	0.987
SMEK2	0.676
SMG1	0.839
SMG5	0.956
SMG6	0.893
SMG7	0.897
SMG8	0.391
SMG9	0.793

SMIM1	0.239
SMIM11	0.257
SMIM12	0.436
SMIM13	0.237
SMIM14	0.288
SMIM15	0.223
SMIM17	0.031
SMIM18	0.068
SMIM19	0.220
SMIM2	0.119
SMIM20	0.182
SMIM21	0.119
SMIM22	0.232
SMIM3	0.076
SMIM4	0.515
SMIM5	0.102
SMIM6	0.061
SMIM7	0.132
SMIM8	0.136
SMKR1	0.189
SMLR1	0.110
SMN1	0.227
SMN2	0.246
SMNDC1	0.753
SMO	0.694
SMOC1	0.500
SMOC2	0.287
SMOX	0.993
SMPD1	0.687
SMPD2	0.655
SMPD3	0.911
SMPD4	0.408

SMPDL3A	0.104
SMPDL3B	0.330
SMR3A	0.172
SMR3B	0.100
SMTN	0.604
SMTNL1	0.255
SMTNL2	0.358
SMU1	1.000
SMUG1	0.229
SMURF1	0.931
SMURF2	0.974
SMYD1	0.389
SMYD2	0.672
SMYD3	0.185
SMYD4	0.217
SMYD5	0.715
SNAI1	0.579
SNAI2	0.352
SNAI3	0.061
SNAP23	0.555
SNAP25	0.761
SNAP29	0.295
SNAP47	0.568
SNAP91	0.498
SNAPC1	0.097
SNAPC2	0.378
SNAPC3	0.211
SNAPC4	0.382
SNAPC5	0.303
SNAPIN	0.562
SNCA	0.312
SNCAIP	0.446

SNCB	0.589
SNCG	0.458
SND1	0.994
SNED1	0.692
SNF8	0.754
SNIP1	0.649
SNN	0.104
SNPH	0.447
SNRK	0.883
SNRNP200	1.000
SNRNP25	0.246
SNRNP27	0.727
SNRNP35	0.338
SNRNP40	0.795
SNRNP48	0.333
SNRNP70	0.974
SNRPA	0.858
SNRPA1	0.333
SNRPB	0.256
SNRPB2	0.898
SNRPC	0.365
SNRPD1	0.885
SNRPD2	0.959
SNRPD3	1.000
SNRPE	0.696
SNRPF	0.605
SNRPG	0.677
SNRPN	0.221
SNTA1	0.252
SNTB1	0.250
SNTB2	0.944
SNTG1	0.085

SNTG2	0.159
SNTN	0.141
SNUPN	0.613
SNURF	0.047
SNW1	0.995
SNX1	0.807
SNX10	0.179
SNX11	0.341
SNX13	0.419
SNX14	0.282
SNX15	0.912
SNX16	0.396
SNX17	0.808
SNX18	0.312
SNX19	0.152
SNX2	0.394
SNX20	0.110
SNX21	0.448
SNX22	0.240
SNX24	0.318
SNX25	0.969
SNX27	0.943
SNX29	0.208
SNX3	0.609
SNX30	0.094
SNX31	0.216
SNX32	0.180
SNX33	0.548
SNX4	0.327
SNX5	0.146
SNX6	0.322
SNX7	0.387

SNX8	0.088
SNX9	0.732
SOAT1	0.470
SOAT2	0.476
SOBP	0.823
SOCS1	0.279
SOCS2	0.570
SOCS3	0.754
SOCS4	0.260
SOCS5	0.497
SOCS6	0.825
SOCS7	0.911
SOD1	0.678
SOD2	0.333
SOD3	0.555
SOGA1	0.642
SOGA2	0.273
SOGA3	0.779
SOHLH1	0.150
SOHLH2	0.250
SON	0.882
SORBS1	0.841
SORBS2	0.537
SORBS3	0.391
SORCS1	0.223
SORCS2	0.196
SORCS3	0.499
SORD	0.645
SORL1	0.177
SORT1	0.724
SOS1	0.999
SOS2	0.792

SOST	0.691
SOSTDC1	0.129
SOWAHA	0.520
SOWAHB	0.571
SOWAHC	0.343
SOX1	0.567
SOX10	1.000
SOX11	0.784
SOX12	0.245
SOX13	0.603
SOX14	0.992
SOX15	0.256
SOX17	0.320
SOX18	0.272
SOX2	0.803
SOX21	0.947
SOX30	0.282
SOX4	0.389
SOX5	1.000
SOX6	0.894
SOX7	0.421
SOX8	0.428
SOX9	0.817
SP1	0.994
SP100	0.459
SP110	0.141
SP140	0.103
SP140L	0.192
SP2	0.977
SP3	0.958
SP4	0.836
SP5	0.723

SP6	0.807
SP7	0.608
SP8	0.975
SP9	0.975
SPA17	0.134
SPACA1	0.279
SPACA3	0.121
SPACA4	0.087
SPACA7	0.062
SPAG1	0.598
SPAG16	0.141
SPAG17	0.965
SPAG4	0.296
SPAG5	0.248
SPAG6	0.381
SPAG7	0.586
SPAG8	0.495
SPAG9	0.967
SPAM1	0.950
SPARC	0.358
SPARCL1	0.259
SPAST	0.813
SPATA12	0.231
SPATA13	0.535
SPATA16	0.155
SPATA17	0.074
SPATA18	0.249
SPATA19	0.038
SPATA2	0.917
SPATA20	0.262
SPATA21	0.123
SPATA22	0.185

SPATA24	0.440
SPATA25	0.299
SPATA2L	0.169
SPATA3	0.107
SPATA31A1	0.238
SPATA31A2	0.229
SPATA31A3	0.164
SPATA31A5	0.300
SPATA31A6	0.073
SPATA31A7	0.132
SPATA31D1	0.095
SPATA31E1	0.085
SPATA32	0.220
SPATA33	0.148
SPATA4	0.056
SPATA5	0.481
SPATA5L1	0.140
SPATA6	0.084
SPATA6L	0.886
SPATA7	0.146
SPATA8	0.074
SPATA9	0.126
SPATC1	0.242
SPATC1L	0.192
SPATS1	0.504
SPATS2	0.662
SPATS2L	0.502
SPC24	0.195
SPC25	0.111
SPCS1	0.463
SPCS2	0.779
SPCS3	0.116

SPDEF	0.623
SPDL1	0.093
SPDYA	0.299
SPDYC	0.367
SPDYE1	0.118
SPDYE2	0.388
SPDYE2B	0.427
SPDYE3	0.323
SPDYE4	0.257
SPECC1	0.598
SPECC1L	0.960
SPECC1L-ADORA2	0.276
SPEF1	0.982
SPEF2	0.201
SPEG	0.781
SPEM1	0.181
SPEN	0.997
SPERT	0.300
SPESP1	0.156
SPG11	0.317
SPG20	0.419
SPG20OS	0.080
SPG21	0.463
SPG7	0.177
SPHK1	0.570
SPHK2	0.490
SPHKAP	0.175
SPI1	0.633
SPIB	0.491
SPIC	0.359
SPICE1	0.964
SPIDR	0.376

SPIN1	0.809
SPINK1	0.155
SPINK13	0.129
SPINK2	0.927
SPINK4	0.065
SPINK5	0.605
SPINK6	0.087
SPINK7	0.111
SPINK8	0.090
SPINK9	0.102
SPINT1	0.283
SPINT2	0.248
SPINT3	0.132
SPIRE1	0.639
SPIRE2	0.138
SPN	0.158
SPNS1	0.735
SPNS2	0.160
SPNS3	0.129
SPO11	0.251
SPOCD1	0.143
SPOCK1	0.467
SPOCK2	0.336
SPOCK3	0.059
SPON2	0.046
SPOP	1.000
SPOPL	0.339
SPP1	0.231
SPP2	0.080
SPPL2A	0.084
SPPL2C	0.112
SPPL3	0.718

SPR	0.440
SPRED1	0.825
SPRED2	0.924
SPRED3	0.733
SPRN	0.111
SPRR1A	0.094
SPRR1B	0.880
SPRR2A	0.071
SPRR2B	0.087
SPRR2D	0.083
SPRR2E	0.104
SPRR2F	0.059
SPRR2G	0.225
SPRR3	0.931
SPRR4	0.094
SPRTN	0.910
SPRY1	0.236
SPRY2	0.584
SPRY4	0.726
SPRYD3	0.820
SPRYD4	0.824
SPRYD7	0.359
SPSB1	1.000
SPSB2	0.375
SPSB3	0.491
SPSB4	0.700
SPTA1	0.170
SPTAN1	1.000
SPTB	0.624
SPTBN1	0.999
SPTBN2	0.744
SPTBN4	0.879

SPTBN5	0.301
SPTLC1	0.110
SPTLC2	0.632
SPTLC3	0.224
SPTSSA	0.305
SPTSSB	0.068
SPTY2D1	0.686
SPZ1	0.113
SQLE	0.430
SQRDL	0.186
SQSTM1	0.968
SRA1	0.600
SRBD1	0.068
SRC	0.999
SRCAP	1.000
SRCIN1	0.991
SRCRB4D	0.443
SRD5A1	0.861
SRD5A3	0.236
SREBF1	0.649
SREBF2	0.954
SREK1	0.671
SREK1IP1	0.138
SRF	0.997
SRFBP1	0.160
SRGAP1	0.764
SRGAP2	0.985
SRGAP3	0.857
SRGN	0.283
SRI	0.191
SRL	0.769
SRM	0.936

SRMS	0.179
SRP14	0.744
SRP19	0.327
SRP54	0.511
SRP68	0.867
SRP72	0.356
SRP9	0.575
SRPK1	0.637
SRPK2	0.967
SRPR	0.714
SRPRB	0.362
SRR	0.491
SRRD	0.354
SRRM1	0.931
SRRM2	0.382
SRRM3	0.400
SRRM4	0.647
SRRM5	0.365
SRRT	0.996
SRSF1	0.762
SRSF10	0.370
SRSF11	0.865
SRSF12	0.175
SRSF2	0.567
SRSF3	0.657
SRSF4	0.887
SRSF5	0.624
SRSF6	0.599
SRSF7	0.599
SRSF9	0.903
SRXN1	0.234
SS18	0.621

SS18L1	0.905
SS18L2	0.146
SSB	0.703
SSBP1	0.391
SSBP2	0.531
SSBP3	0.846
SSBP3-AS1	0.293
SSBP4	0.191
SSC5D	0.490
SSFA2	0.163
SSH1	0.456
SSH2	0.855
SSH3	0.566
SSMEM1	0.223
SSNA1	0.984
SSPN	0.186
SSR1	0.138
SSR2	0.276
SSR3	0.342
SSRP1	0.998
SSSCA1	0.970
SST	0.435
SSTR1	0.452
SSTR2	0.292
SSTR3	0.261
SSTR4	0.115
SSTR5	0.239
SSU72	0.873
SSUH2	0.227
SSX2IP	0.220
ST13	0.474
ST14	0.560

ST18	0.916
ST20	0.098
ST20-MTHFS	0.186
ST3GAL1	0.415
ST3GAL2	0.318
ST3GAL3	0.462
ST3GAL4	0.090
ST3GAL5	0.377
ST3GAL6	0.573
ST5	0.848
ST6GAL1	0.408
ST6GAL2	0.303
ST6GALNAC1	0.292
ST6GALNAC2	0.351
ST6GALNAC3	0.229
ST6GALNAC4	0.397
ST6GALNAC5	0.270
ST6GALNAC6	0.625
ST7	0.593
ST7-OT4	0.301
ST7L	0.359
ST8SIA1	0.466
ST8SIA2	0.380
ST8SIA3	0.550
ST8SIA4	0.155
ST8SIA5	0.167
ST8SIA6	0.241
STAB1	0.397
STAB2	0.427
STAC	0.238
STAC2	0.694
STAC3	0.382

STAG1	0.998
STAG3	0.544
STAM	0.687
STAM2	0.951
STAMBP	0.789
STAMBPL1	0.262
STAP1	0.122
STAP2	0.427
STAR	0.704
STARD10	0.552
STARD13	0.753
STARD3	0.664
STARD3NL	0.197
STARD4	0.135
STARD5	0.296
STARD6	0.157
STARD7	0.600
STARD9	0.581
STAT1	0.979
STAT2	0.910
STAT3	1.000
STAT4	0.636
STAT5A	0.973
STAT5B	0.999
STAT6	0.987
STATH	0.027
STAU1	0.910
STAU2	0.753
STC1	0.773
STC2	0.597
STEAP1	0.154
STEAP1B	0.174

STEAP2	0.036
STEAP3	0.788
STEAP4	0.227
STH	0.082
STIL	0.184
STIM1	0.960
STIM2	0.847
STIP1	0.994
STK10	0.700
STK11	0.526
STK11IP	0.322
STK16	0.647
STK17A	0.455
STK17B	0.375
STK24	0.652
STK25	0.826
STK3	0.771
STK31	0.339
STK32A	0.499
STK32B	0.301
STK32C	0.688
STK33	0.332
STK35	0.261
STK36	0.856
STK38	0.849
STK38L	0.937
STK39	0.474
STK4	0.782
STK40	0.911
STMN1	0.310
STMN2	0.914
STMN3	0.326

STMN4	0.513
STMND1	0.306
STOM	0.521
STOML1	0.316
STOML2	0.929
STOML3	0.200
STON1	0.047
STON1-GTF2A1L	0.033
STON2	0.420
STOX1	0.247
STOX2	0.962
STPG1	0.321
STPG2	0.142
STRA13	0.664
STRA6	0.464
STRA8	0.397
STRADA	0.747
STRADB	0.458
STRAP	0.799
STRBP	0.993
STRC	0.554
STRIP1	0.919
STRIP2	0.399
STRN	0.441
STRN3	0.871
STRN4	0.972
STT3A	0.931
STT3B	0.997
STUB1	0.346
STX10	0.407
STX11	0.171
STX12	0.848

STX16	0.500
STX16-NPEPL1	0.433
STX17	0.421
STX18	0.279
STX19	0.033
STX1A	0.562
STX1B	0.914
STX2	0.151
STX3	0.232
STX4	0.343
STX5	0.688
STX6	0.236
STX7	0.186
STX8	0.254
STXBP1	0.961
STXBP2	0.970
STXBP3	0.339
STXBP4	0.378
STXBP5	0.675
STXBP5L	0.245
STXBP6	0.260
STYK1	0.367
STYX	0.696
STYXL1	0.318
SUB1	0.850
SUCLA2	0.640
SUCLG1	0.423
SUCLG2	0.293
SUCNR1	0.197
SUCO	0.630
SUDS3	0.557
SUFU	0.991

SUGP1	0.906
SUGP2	0.894
SUGT1	0.438
SULF1	0.538
SULF2	0.449
SULT1A1	0.414
SULT1A2	0.315
SULT1A3	0.529
SULT1A4	0.585
SULT1B1	0.081
SULT1C2	0.251
SULT1C3	0.299
SULT1C4	0.175
SULT1E1	0.248
SULT2A1	0.515
SULT2B1	0.179
SULT4A1	0.823
SULT6B1	0.123
SUMF1	0.308
SUMF2	0.564
SUMO1	0.760
SUMO2	0.963
SUMO3	0.818
SUMO4	0.174
SUN1	0.993
SUN2	0.646
SUN3	0.127
SUN5	0.307
SUOX	0.708
SUPT16H	1.000
SUPT20H	0.263
SUPT3H	0.229

SUPT4H1	0.547
SUPT5H	1.000
SUPT6H	1.000
SUPT7L	0.489
SUPV3L1	0.898
SURF1	0.616
SURF2	0.344
SURF4	0.784
SURF6	0.498
SUSD1	0.367
SUSD2	0.421
SUSD3	0.115
SUSD4	0.545
SUSD5	0.131
SUV39H2	0.896
SUV420H1	0.991
SUV420H2	0.756
SUZ12	0.986
SV2A	0.257
SV2B	0.532
SV2C	0.387
SVEP1	0.589
SVIL	0.522
SVIP	0.050
SVOP	0.394
SVOPL	0.113
SWAP70	0.317
SWI5	0.382
SWSAP1	0.402
SWT1	0.200
SYBU	0.696
SYCE1	0.151

SYCE1L	0.054
SYCE2	0.301
SYCE3	0.128
SYCN	0.874
SYCP1	0.492
SYCP2	0.222
SYCP2L	0.195
SYCP3	0.133
SYDE1	0.627
SYDE2	0.338
SYF2	0.206
SYK	0.701
SYMPK	0.981
SYN3	0.758
SYNC	0.335
SYNCRIP	0.996
SYNDIG1	0.330
SYNDIG1L	0.181
SYNE1	0.999
SYNE2	0.238
SYNE3	0.267
SYNE4	0.222
SYNGAP1	0.984
SYNGR1	0.343
SYNGR2	0.281
SYNGR3	0.696
SYNGR4	0.169
SYNJ1	0.708
SYNJ2	0.496
SYNJ2BP	0.305
SYNM	0.135
SYNPO	0.751

SYNPO2	0.222
SYNPO2L	0.446
SYNPR	0.090
SYNRG	0.907
SYPL1	0.202
SYPL2	0.328
SYS1	0.419
SYS1-DBNDD2	0.237
SYT1	0.681
SYT10	0.031
SYT11	0.616
SYT12	0.320
SYT13	0.429
SYT14	0.656
SYT15	0.298
SYT16	0.576
SYT17	0.793
SYT2	0.834
SYT3	0.690
SYT4	0.247
SYT5	0.668
SYT6	0.441
SYT7	0.969
SYT8	0.178
SYT9	0.562
SYTL1	0.160
SYTL2	0.921
SYTL3	0.144
SYVN1	0.995
SZRD1	0.467
SZT2	0.857
T	0.341

TAAR6	0.208
TAB1	0.861
TAB2	0.887
TAC1	0.107
TAC3	0.277
TAC4	0.184
TACC1	0.526
TACC2	0.677
TACC3	0.907
TACO1	0.590
TACR1	0.244
TACR2	0.207
TACR3	0.172
TACSTD2	0.460
TADA1	0.448
TADA2A	0.612
TADA2B	0.941
TADA3	0.715
TAF10	0.396
TAF11	0.106
TAF12	0.820
TAF13	0.199
TAF15	0.688
TAF1A	0.165
TAF1B	0.355
TAF1C	0.160
TAF1L	0.437
TAF2	0.304
TAF3	0.811
TAF4	0.983
TAF4B	0.346
TAF5	0.985

TAF5L	0.857
TAF6	0.963
TAF6L	0.658
TAF7	0.123
TAF8	0.413
TAF9	0.769
TAGAP	0.547
TAGLN	0.218
TAGLN2	0.371
TAGLN3	0.224
TAL1	0.717
TAL2	0.209
TALDO1	0.271
TAMM41	0.193
TANC1	0.567
TANC2	0.672
TANGO2	0.443
TANGO6	0.529
TANK	0.713
TAOK1	0.993
TAOK2	0.995
TAOK3	0.960
TAP1	0.649
TAP2	0.409
TAPBP	0.433
TAPBPL	0.494
TAPT1	0.214
TARBP1	0.267
TARBP2	0.840
TARDBP	0.910
TARM1	0.255
TARS	0.432

TARS2	0.696
TARSL2	0.109
TAS1R1	0.174
TAS1R2	0.063
TAS1R3	0.169
TAS2R1	0.044
TAS2R10	0.105
TAS2R13	0.084
TAS2R14	0.107
TAS2R19	0.126
TAS2R20	0.166
TAS2R3	0.123
TAS2R30	0.212
TAS2R31	0.149
TAS2R38	0.165
TAS2R39	0.179
TAS2R4	0.066
TAS2R40	0.149
TAS2R42	0.125
TAS2R43	0.094
TAS2R46	0.148
TAS2R5	0.217
TAS2R50	0.117
TAS2R60	0.180
TAS2R8	0.100
TAS2R9	0.105
TASP1	0.487
TAT	0.252
TATDN1	0.370
TATDN2	0.467
TATDN3	0.461
TAX1BP1	0.770

TAX1BP3	0.354
TBATA	0.130
TBC1D1	0.711
TBC1D10A	0.424
TBC1D10B	0.982
TBC1D10C	0.279
TBC1D12	0.528
TBC1D13	0.684
TBC1D14	0.671
TBC1D15	0.291
TBC1D16	0.331
TBC1D17	0.586
TBC1D19	0.281
TBC1D2	0.430
TBC1D20	0.552
TBC1D21	0.249
TBC1D22A	0.983
TBC1D22B	0.953
TBC1D23	0.513
TBC1D24	0.234
TBC1D26	0.316
TBC1D28	0.314
TBC1D29	0.180
TBC1D2B	0.238
TBC1D3	0.547
TBC1D30	0.573
TBC1D31	0.119
TBC1D32	0.123
TBC1D3B	0.405
TBC1D3C	0.087
TBC1D3F	0.279
TBC1D3G	0.361

TBC1D3H	0.497
TBC1D4	0.770
TBC1D5	0.477
TBC1D7	0.163
TBC1D8	0.255
TBC1D9	0.721
TBC1D9B	0.982
TBCA	0.273
TBCB	0.592
TBCC	0.344
TBCCD1	0.592
TBCD	0.459
TBCE	0.105
TBCEL	0.653
TBCK	0.936
TBK1	0.805
TBKBP1	0.818
TBL1XR1	0.702
TBL2	0.339
TBL3	0.382
TBP	0.263
TBPL1	0.630
TBPL2	0.152
TBR1	0.994
TBRG1	0.492
TBRG4	0.700
TBX1	0.718
TBX10	0.122
TBX15	1.000
TBX18	0.485
TBX19	0.411
TBX2	0.945

TBX20	0.721
TBX21	0.785
TBX3	0.956
TBX4	0.498
TBX5	0.966
TBX6	0.573
TBXA2R	0.257
TBXAS1	0.069
TC2N	0.464
TCAIM	0.526
TCAP	0.717
TCEA1	0.676
TCEA2	0.480
TCEA3	0.529
TCEANC2	0.161
TCEB1	0.895
TCEB2	0.531
TCEB3	0.876
TCEB3B	0.113
TCEB3C	0.350
TCEB3CL	0.406
TCEB3CL2	0.569
TCERG1	0.985
TCERG1L	0.168
TCF12	0.772
TCF15	0.469
TCF19	0.230
TCF20	0.483
TCF21	0.257
TCF23	0.246
TCF24	0.132
TCF25	0.579

TCF3	0.589
TCF4	0.993
TCF7	0.373
TCF7L1	0.742
TCF7L2	0.967
TCFL5	0.428
TCHH	0.255
TCHHL1	0.343
TCHP	0.220
TCIRG1	0.252
TCL1A	0.133
TCL1B	0.215
TCN1	0.225
TCN2	0.254
TCOF1	0.674
TCP1	0.662
TCP10	0.323
TCP10L	0.059
TCP11	0.196
TCP11L1	0.166
TCP11L2	0.380
TCTA	0.459
TCTE1	0.269
TCTE3	0.052
TCTEX1D1	0.076
TCTEX1D2	0.180
TCTEX1D4	0.399
TCTN1	0.450
TCTN2	0.087
TCTN3	0.372
TDG	0.754
TDGF1	0.131

TDO2	0.103
TDP1	0.339
TDP2	0.419
TDRD1	0.855
TDRD10	0.426
TDRD12	0.392
TDRD15	0.251
TDRD3	0.449
TDRD5	0.717
TDRD6	0.244
TDRD7	0.511
TDRD9	0.227
TDRKH	0.320
TDRP	0.031
TEAD1	0.912
TEAD2	0.922
TEAD3	0.804
TEAD4	0.534
TEC	0.368
TECPR1	0.964
TECPR2	0.644
TECR	0.548
TECRL	0.102
TECTA	0.600
TECTB	0.244
TEDDM1	0.146
TEF	0.818
TEFM	0.333
TEK	0.317
TEKT1	0.208
TEKT2	0.367
TEKT3	0.183

TEKT4	0.088
TEKT5	0.066
TELO2	0.962
TEN1	0.634
TENC1	0.741
TENM2	0.973
TENM3	0.396
TENM4	0.846
TEP1	0.217
TEPP	0.309
TERF1	0.431
TERF2	1.000
TERF2IP	0.583
TERT	0.723
TES	0.281
TESC	0.341
TESK1	0.943
TESK2	0.469
TESPA1	0.230
TET1	0.935
TET2	0.344
TET3	0.934
TEX10	0.623
TEX101	0.215
TEX12	0.234
TEX14	0.542
TEX15	0.175
TEX19	0.098
TEX2	0.440
TEX22	0.178
TEX26	0.097
TEX261	0.326

TEX264	0.625
TEX29	0.125
TEX30	0.196
TEX33	0.207
TEX35	0.189
TEX36	0.242
TEX37	0.025
TEX38	0.216
TEX40	0.514
TEX9	0.146
TF	0.538
TFAM	0.403
TFAP2A	1.000
TFAP2B	0.999
TFAP2C	0.812
TFAP2D	0.866
TFAP2E	0.262
TFAP4	0.852
TFB1M	0.483
TFB2M	0.070
TFCP2	0.679
TFCP2L1	0.430
TFDP1	0.998
TFDP2	0.930
TFEB	0.441
TFEC	0.139
TFF1	0.284
TFF2	0.223
TFF3	0.134
TFG	0.453
TFIP11	0.599
TFPI	0.335

TFPI2	0.338
TFPT	0.507
TFR2	0.386
TFRC	0.194
TG	0.176
TGDS	0.328
TGFA	0.413
TGFB1	1.000
TGFB1I1	0.502
TGFB2	0.991
TGFB3	0.979
TGFBI	0.741
TGFBR1	1.000
TGFBR2	0.481
TGFBR3	0.191
TGFBR3L	0.854
TGFBRAP1	0.546
TGIF1	0.086
TGIF2	0.624
TGIF2-C20orf24	0.610
TGM1	0.463
TGM2	0.144
TGM3	0.238
TGM4	0.511
TGM5	0.760
TGM7	0.987
TGOLN2	0.369
TGS1	0.083
TH	0.706
THADA	0.365
THAP1	0.749
THAP10	0.217

THAP11	0.813
THAP2	0.051
THAP3	0.562
THAP4	0.552
THAP5	0.502
THAP6	0.298
THAP7	0.730
THAP8	0.432
THAP9	0.325
THBD	0.594
THBS1	0.562
THBS2	0.262
THBS3	0.495
THBS4	0.566
THEG	0.052
THEGL	0.271
THEM4	0.285
THEM5	0.650
THEM6	0.477
THEMIS	0.339
THEMIS2	0.369
THG1L	0.413
THNSL1	0.111
THNSL2	0.137
THOC1	0.529
THOC3	0.234
THOC5	0.541
THOC6	0.212
THOC7	0.283
THOP1	0.720
THPO	0.420
THRA	0.988

THRAP3	0.996
THRB	0.994
THRSP	0.107
THSD1	0.301
THSD4	0.507
THSD7A	0.265
THSD7B	0.151
THTPA	0.487
THUMPD1	0.552
THUMPD2	0.263
THUMPD3	0.199
THY1	0.208
THYN1	0.080
TIA1	0.851
TIAF1	0.647
TIAL1	0.993
TIAM1	0.962
TIAM2	0.470
TICAM1	0.483
TICAM2	0.241
TICRR	0.054
TIE1	0.453
TIFA	0.236
TIFAB	0.215
TIGD1	0.348
TIGD2	0.198
TIGD3	0.541
TIGD4	0.098
TIGD5	0.342
TIGD6	0.359
TIGD7	0.032
TIGIT	0.173

TIMD4	0.123
TIMELESS	0.485
TIMM10	0.749
TIMM10B	0.498
TIMM13	0.688
TIMM17A	0.352
TIMM21	0.194
TIMM22	0.155
TIMM23	0.366
TIMM23B	0.253
TIMM44	0.855
TIMM50	0.727
TIMM8B	0.275
TIMM9	0.367
TIMMDC1	0.410
TIMP2	0.537
TIMP3	0.842
TIMP4	0.371
TINAG	0.098
TINAGL1	0.667
TINF2	0.613
TIPARP	0.732
TIPIN	0.299
TIPRL	0.540
TIRAP	0.401
TJAP1	0.741
TJP1	0.916
TJP2	0.271
TJP3	0.977
TK1	0.170
TK2	0.527
TKT	0.582

TKTL2	0.079
TLCD1	0.333
TLCD2	0.134
TLDC1	0.071
TLDC2	0.167
TLE1	0.876
TLE2	0.237
TLE3	0.895
TLE4	0.840
TLE6	0.308
TLK1	0.986
TLK2	1.000
TLL1	0.640
TLL2	0.473
TLN1	0.999
TLN2	0.844
TLR1	0.146
TLR10	0.146
TLR2	0.207
TLR3	0.031
TLR4	0.034
TLR5	0.099
TLR6	0.317
TLR9	0.256
TLX1	0.939
TLX1NB	0.403
TLX2	1.000
TLX3	0.910
TM2D1	0.181
TM2D2	0.304
TM2D3	0.058
TM4SF1	0.169

TM4SF18	0.107
TM4SF19	0.257
TM4SF20	0.084
TM4SF4	0.357
TM4SF5	0.371
TM6SF1	0.232
TM6SF2	0.176
TM7SF2	0.783
TM7SF3	0.398
TM9SF1	0.840
TM9SF2	0.813
TM9SF3	0.882
TM9SF4	0.674
TMA16	0.197
TMA7	0.387
TMBIM1	0.339
TMBIM4	0.298
TMBIM6	0.655
TMC1	0.397
TMC2	0.833
TMC3	0.219
TMC4	0.465
TMC5	0.471
TMC6	0.403
TMC7	0.177
TMC8	0.177
TMCC1	0.797
TMCC2	0.439
TMCC3	0.171
TMCO1	0.313
TMCO2	0.176
TMCO3	0.909

TMCO4	0.370
TMCO5A	0.109
TMCO6	0.493
TMED1	0.358
TMED10	0.895
TMED2	0.670
TMED3	0.327
TMED4	0.647
TMED5	0.248
TMED6	0.487
TMED7	0.451
TMED7-TICAM2	0.247
TMED8	0.535
TMED9	0.328
TMEFF1	0.235
TMEFF2	0.293
TMEM100	0.258
TMEM101	0.492
TMEM102	0.535
TMEM104	0.280
TMEM105	0.158
TMEM106A	0.454
TMEM106B	0.185
TMEM106C	0.523
TMEM107	0.701
TMEM108	0.573
TMEM109	0.305
TMEM110	0.451
TMEM110-MUSTN	0.266
TMEM114	0.037
TMEM115	0.889
TMEM116	0.306

TMEM117	0.446
TMEM119	0.245
TMEM120B	0.384
TMEM121	0.782
TMEM123	0.135
TMEM125	0.316
TMEM126A	0.890
TMEM126B	0.885
TMEM127	0.359
TMEM128	0.202
TMEM129	0.936
TMEM130	0.951
TMEM131	0.859
TMEM132A	0.393
TMEM132B	0.502
TMEM132C	0.130
TMEM132D	0.360
TMEM132E	0.560
TMEM133	0.172
TMEM134	0.495
TMEM135	0.366
TMEM136	0.425
TMEM138	0.395
TMEM139	0.298
TMEM140	0.115
TMEM141	0.288
TMEM143	0.487
TMEM144	0.086
TMEM145	0.484
TMEM147	0.805
TMEM14A	0.148
TMEM14B	0.249

TMEM14C	0.169
TMEM14E	0.284
TMEM150A	0.580
TMEM150B	0.235
TMEM150C	0.391
TMEM151A	0.850
TMEM151B	0.826
TMEM154	0.051
TMEM155	0.027
TMEM156	0.106
TMEM158	0.287
TMEM159	0.187
TMEM160	0.357
TMEM161A	0.241
TMEM161B	0.282
TMEM163	0.578
TMEM165	0.419
TMEM167A	0.156
TMEM167B	0.241
TMEM168	0.169
TMEM169	0.114
TMEM17	0.122
TMEM170A	0.107
TMEM170B	0.370
TMEM171	0.246
TMEM173	0.617
TMEM174	0.556
TMEM175	0.169
TMEM176A	0.370
TMEM176B	0.233
TMEM177	0.279
TMEM178A	0.444

TMEM178B	0.845
TMEM179	0.681
TMEM179B	0.477
TMEM18	0.120
TMEM180	0.239
TMEM181	0.328
TMEM182	0.386
TMEM183A	0.588
TMEM184A	0.948
TMEM184B	0.409
TMEM184C	0.358
TMEM185B	0.846
TMEM186	0.951
TMEM189	0.550
TMEM189-UBE2V	0.621
TMEM19	0.243
TMEM190	0.326
TMEM192	0.056
TMEM194A	0.793
TMEM194B	0.249
TMEM196	0.078
TMEM198	0.440
TMEM199	0.468
TMEM2	0.043
TMEM200A	0.164
TMEM200B	0.293
TMEM200C	0.189
TMEM201	0.595
TMEM202	0.116
TMEM203	1.000
TMEM204	0.968
TMEM205	0.541

TMEM206	0.171
TMEM207	0.060
TMEM208	0.703
TMEM209	0.218
TMEM210	0.260
TMEM211	0.128
TMEM212	0.125
TMEM213	0.106
TMEM214	0.526
TMEM215	0.467
TMEM216	0.264
TMEM217	0.426
TMEM218	0.268
TMEM219	0.458
TMEM220	0.137
TMEM221	0.124
TMEM222	0.635
TMEM223	0.342
TMEM225	0.111
TMEM229A	0.999
TMEM229B	0.280
TMEM230	0.128
TMEM231	0.257
TMEM232	0.141
TMEM233	0.151
TMEM234	0.377
TMEM235	0.165
TMEM236	0.201
TMEM237	0.202
TMEM238	0.355
TMEM239	0.127
TMEM240	0.468

TMEM241	0.202
TMEM242	0.132
TMEM243	0.153
TMEM244	0.145
TMEM245	0.591
TMEM246	0.418
TMEM247	0.082
TMEM248	0.408
TMEM249	0.133
TMEM25	0.244
TMEM251	0.428
TMEM252	0.072
TMEM253	0.357
TMEM254	0.140
TMEM255B	0.091
TMEM256	0.444
TMEM256-PLSCR3	0.470
TMEM258	0.426
TMEM259	0.465
TMEM26	0.088
TMEM260	0.128
TMEM261	0.248
TMEM30A	0.467
TMEM30B	0.396
TMEM30C	0.186
TMEM33	0.183
TMEM37	0.272
TMEM38A	0.249
TMEM38B	0.129
TMEM39A	0.557
TMEM39B	0.668
TMEM40	0.115

TMEM41A	0.288
TMEM41B	0.157
TMEM42	0.412
TMEM43	0.340
TMEM44	0.155
TMEM45A	0.742
TMEM45B	0.365
TMEM5	0.207
TMEM50A	0.238
TMEM50B	0.186
TMEM51	0.148
TMEM52	0.349
TMEM52B	0.222
TMEM53	0.304
TMEM54	0.511
TMEM55A	0.275
TMEM55B	0.870
TMEM56	0.392
TMEM57	0.976
TMEM59	0.529
TMEM59L	0.494
TMEM60	0.265
TMEM61	0.125
TMEM62	0.607
TMEM63A	0.314
TMEM63B	0.886
TMEM63C	0.797
TMEM64	0.281
TMEM65	0.190
TMEM66	0.321
TMEM67	0.135
TMEM68	0.147

TMEM69	0.102
TMEM70	0.345
TMEM71	0.083
TMEM72	0.173
TMEM74	0.166
TMEM74B	0.137
TMEM75	0.048
TMEM78	0.135
TMEM79	0.402
TMEM80	0.413
TMEM81	0.198
TMEM82	0.038
TMEM86A	0.504
TMEM86B	0.241
TMEM87A	0.428
TMEM87B	0.232
TMEM88	0.534
TMEM88B	0.365
TMEM89	0.238
TMEM8A	0.894
TMEM8B	0.705
TMEM8C	0.156
TMEM9	0.432
TMEM91	0.191
TMEM92	0.186
TMEM95	0.137
TMEM97	0.419
TMEM98	0.456
TMEM99	0.313
TMEM9B	0.356
TMF1	0.141
TMIE	0.224

TMIGD1	0.165
TMIGD2	0.112
TMOD1	0.739
TMOD2	0.216
TMOD3	0.252
TMOD4	0.566
TMPO	0.267
TMPPE	0.395
TMPRSS11A	0.142
TMPRSS11B	0.196
TMPRSS11BNL	0.181
TMPRSS11D	0.930
TMPRSS11E	0.386
TMPRSS11F	0.166
TMPRSS12	0.215
TMPRSS13	0.366
TMPRSS15	0.235
TMPRSS2	0.642
TMPRSS3	0.349
TMPRSS4	0.346
TMPRSS5	0.393
TMPRSS6	0.406
TMPRSS7	0.353
TMPRSS9	0.203
TMSB10	0.290
TMTC1	0.525
TMTC2	0.316
TMTC3	0.283
TMTC4	0.281
TMUB1	0.532
TMUB2	0.563
TMX1	0.274

TMX2	0.679
TMX2-CTNND1	0.332
TMX3	0.041
TMX4	0.103
TNC	0.696
TNF	0.940
TNFAIP1	0.923
TNFAIP2	0.981
TNFAIP3	0.815
TNFAIP6	0.061
TNFAIP8	0.237
TNFAIP8L1	0.284
TNFAIP8L2	0.263
TNFAIP8L3	0.128
TNFRSF10A	0.265
TNFRSF10B	0.356
TNFRSF10C	0.147
TNFRSF10D	0.189
TNFRSF11A	0.981
TNFRSF11B	0.314
TNFRSF12A	0.074
TNFRSF13B	0.133
TNFRSF13C	0.554
TNFRSF14	0.428
TNFRSF17	0.018
TNFRSF18	0.128
TNFRSF19	0.464
TNFRSF1A	0.598
TNFRSF1B	0.307
TNFRSF21	0.356
TNFRSF25	0.430
TNFRSF4	0.312

TNFRSF6B	0.112
TNFRSF8	0.355
TNFRSF9	0.141
TNFSF10	0.260
TNFSF11	0.327
TNFSF12	0.324
TNFSF13	0.414
TNFSF13B	0.163
TNFSF14	0.409
TNFSF15	0.335
TNFSF18	0.162
TNFSF4	0.224
TNFSF8	0.440
TNFSF9	0.309
TNIK	0.999
TNIP1	0.482
TNIP2	0.545
TNIP3	0.177
TNK1	0.461
TNK2	0.340
TNKS	0.703
TNKS1BP1	0.566
TNKS2	0.842
TNN	0.485
TNNC1	0.555
TNNC2	0.342
TNNI1	0.607
TNNI2	0.198
TNNI3	0.947
TNNI3K	0.176
TNNT1	0.404
TNNT2	0.852

TNNT3	0.968
TNP1	0.172
TNP2	0.106
TNPO1	0.987
TNPO2	0.999
TNPO3	0.927
TNR	0.455
TNRC18	0.831
TNRC6A	0.910
TNRC6B	0.985
TNRC6C	0.975
TNS1	0.405
TNS3	0.770
TNS4	0.291
TNXB	0.470
TOB1	0.823
TOB2	0.756
TOE1	0.981
TOLLIP	0.715
TOM1	0.882
TOM1L1	0.423
TOM1L2	0.636
TOMM20	0.476
TOMM20L	0.111
TOMM22	0.841
TOMM34	0.355
TOMM40	0.980
TOMM40L	0.728
TOMM5	0.441
TOMM6	0.291
TOMM7	0.085
TOMM70A	0.722

TONSL	0.145
TOP1	0.954
TOP1MT	0.316
TOP2A	0.564
TOP2B	0.981
TOP3A	0.952
TOP3B	0.872
TOPAZ1	0.707
TOPBP1	0.755
TOPORS	0.735
TOR1A	0.722
TOR1AIP1	0.190
TOR1AIP2	0.236
TOR1B	0.244
TOR2A	0.496
TOR3A	0.266
TOR4A	0.469
TOX	0.724
TOX2	0.772
TOX3	0.733
TOX4	1.000
TP53	0.771
TP53AIP1	0.870
TP53BP1	0.985
TP53BP2	0.937
TP53I11	0.415
TP53I13	0.397
TP53I3	0.218
TP53INP1	0.498
TP53INP2	0.664
TP53RK	0.435
TP53TG3B	0.294

TP53TG3C	0.309
TP53TG3D	0.188
TP53TG5	0.294
TP63	1.000
TP73	0.572
TPBG	0.257
TPBGL	0.078
TPCN1	0.956
TPCN2	0.456
TPD52	0.169
TPD52L1	0.181
TPD52L2	0.177
TPD52L3	0.042
TPGS1	0.972
TPGS2	0.185
TPH1	0.526
TPH2	0.741
TPI1	0.515
TPK1	0.357
TPM1	0.754
TPM2	0.569
TPM3	0.645
TPM4	0.430
TPMT	0.401
TPO	0.074
TPP1	0.415
TPP2	0.908
TPPP	0.765
TPPP2	0.113
TPPP3	0.452
TPR	0.489
TPRA1	0.334

TPRG1	0.230
TPRG1L	0.196
TPRKB	0.215
TPRN	0.943
TPRX1	0.235
TPRX2P	0.453
TPRXL	0.292
TPSAB1	0.254
TPSD1	0.195
TPSG1	0.932
TPST1	0.440
TPST2	0.604
TPT1	0.333
TPTE	0.076
TPTE2	0.174
TPX2	0.277
TRA2A	0.383
TRA2B	0.888
TRABD	0.103
TRABD2A	0.203
TRABD2B	0.926
TRADD	0.722
TRAF1	0.607
TRAF2	0.940
TRAF3	0.956
TRAF3IP1	0.629
TRAF3IP2	0.338
TRAF3IP3	0.154
TRAF4	0.740
TRAF5	0.644
TRAF6	0.941
TRAF7	0.647

TRAFD1	0.622
TRAIP	0.433
TRAK1	0.376
TRAK2	0.713
TRAM1	0.567
TRAM1L1	0.103
TRAM2	0.416
TRANK1	0.332
TRAP1	0.359
TRAPPC1	0.519
TRAPPC10	0.829
TRAPPC11	0.170
TRAPPC12	0.243
TRAPPC13	0.298
TRAPPC2L	0.216
TRAPPC2P1	0.331
TRAPPC3	0.713
TRAPPC3L	0.277
TRAPPC4	0.431
TRAPPC5	0.463
TRAPPC6A	0.467
TRAPPC6B	0.236
TRAPPC8	0.417
TRAPPC9	0.404
TRAT1	0.054
TRDMT1	0.199
TRDN	0.068
TREH	0.536
TREM1	0.075
TREM2	0.254
TREML1	0.067
TREML2	0.144

TRERF1	0.928
TREX1	0.429
TRH	0.270
TRHDE	0.433
TRHR	0.435
TRIAP1	0.251
TRIB1	0.611
TRIB2	0.663
TRIB3	0.113
TRIM10	0.414
TRIM11	0.569
TRIM13	0.695
TRIM14	0.428
TRIM15	0.423
TRIM16	0.419
TRIM16L	0.405
TRIM17	0.088
TRIM2	0.982
TRIM21	0.120
TRIM22	0.054
TRIM23	0.687
TRIM24	0.996
TRIM25	0.492
TRIM26	0.916
TRIM27	0.983
TRIM28	0.945
TRIM29	0.643
TRIM3	0.983
TRIM31	0.393
TRIM32	0.273
TRIM33	0.999
TRIM34	0.128

TRIM35	0.624
TRIM36	0.245
TRIM37	0.482
TRIM38	0.214
TRIM39	0.999
TRIM39-RPP21	0.821
TRIM4	0.450
TRIM40	0.439
TRIM41	0.998
TRIM42	0.242
TRIM43	0.364
TRIM44	0.313
TRIM45	0.211
TRIM46	0.933
TRIM47	0.318
TRIM49	0.226
TRIM49B	0.113
TRIM49C	0.244
TRIM49D1	0.410
TRIM5	0.407
TRIM50	0.626
TRIM51	0.091
TRIM52	0.991
TRIM54	0.529
TRIM55	0.533
TRIM56	0.663
TRIM58	0.022
TRIM59	0.291
TRIM6	0.141
TRIM6-TRIM34	0.078
TRIM60	0.097
TRIM61	0.128

TRIM62	0.603
TRIM63	0.638
TRIM64B	0.379
TRIM65	0.612
TRIM66	0.720
TRIM67	0.521
TRIM68	0.145
TRIM69	0.396
TRIM7	0.946
TRIM71	0.621
TRIM72	0.654
TRIM73	0.334
TRIM74	0.544
TRIM8	0.946
TRIM9	0.982
TRIML1	0.020
TRIML2	0.026
TRIO	0.936
TRIOBP	0.313
TRIP10	0.649
TRIP11	0.422
TRIP12	0.575
TRIP13	0.639
TRIP4	0.632
TRIP6	0.645
TRIQK	0.054
TRIT1	0.499
TRMT1	0.605
TRMT10A	0.156
TRMT10B	0.398
TRMT10C	0.535
TRMT11	0.139

TRMT112	0.315
TRMT12	0.650
TRMT13	0.447
TRMT1L	0.195
TRMT2A	0.672
TRMT44	0.078
TRMT5	0.218
TRMT6	0.577
TRMT61A	0.556
TRMT61B	0.346
TRMU	0.300
TRNAU1AP	0.267
TRNP1	0.273
TRNT1	0.315
TROAP	0.222
TROVE2	0.711
TRPA1	0.130
TRPC1	0.620
TRPC3	0.778
TRPC4	0.586
TRPC4AP	0.612
TRPC6	0.149
TRPC7	0.638
TRPM1	0.495
TRPM2	0.222
TRPM3	0.846
TRPM4	0.506
TRPM5	0.220
TRPM6	0.603
TRPM7	0.243
TRPM8	0.278
TRPS1	0.879

TRPT1	0.699
TRPV1	0.244
TRPV2	0.399
TRPV4	0.529
TRPV5	0.637
TRPV6	0.777
TRRAP	0.731
TRUB1	0.240
TRUB2	0.528
TSACC	0.130
TSC1	0.930
TSC2	0.432
TSC22D1	0.628
TSC22D2	0.704
TSC22D4	0.918
TSEN15	0.364
TSEN2	0.212
TSEN34	0.318
TSEN54	0.247
TSFM	0.411
TSG101	0.961
TSGA10	0.328
TSGA13	0.097
TSHB	0.319
TSHR	0.147
TSHZ1	0.554
TSHZ2	0.593
TSHZ3	0.887
TSKS	0.415
TSKU	0.384
TSLP	0.127
TSN	0.732

TSNARE1	0.171
TSNAX	0.754
TSNAX-DISC1	0.313
TSNAXIP1	0.299
TSPAN1	0.134
TSPAN11	0.895
TSPAN12	0.531
TSPAN13	0.094
TSPAN14	0.193
TSPAN15	0.399
TSPAN16	0.066
TSPAN17	0.392
TSPAN18	0.369
TSPAN19	0.040
TSPAN2	0.237
TSPAN3	0.230
TSPAN31	0.488
TSPAN32	0.070
TSPAN33	0.277
TSPAN4	0.097
TSPAN5	0.396
TSPAN8	0.200
TSPAN9	0.125
TSPEAR	0.214
TSPO	0.272
TSPO2	0.228
TSPYL1	0.314
TSPYL4	0.813
TSPYL5	0.515
TSPYL6	0.038
TSR1	0.364
TSR3	0.320

TSSC1	0.214
TSSC4	0.421
TSSK1B	0.240
TSSK2	0.360
TSSK3	0.508
TSSK4	0.516
TSSK6	0.629
TST	0.322
TSTA3	0.554
TSTD1	0.865
TSTD2	0.629
TTBK1	0.993
TTBK2	0.839
TTC1	0.379
TTC12	0.245
TTC13	0.129
TTC14	0.151
TTC16	0.281
TTC17	0.471
TTC18	0.267
TTC19	0.222
TTC21B	0.109
TTC22	0.070
TTC23	0.062
TTC23L	0.238
TTC24	0.178
TTC26	0.153
TTC27	0.055
TTC28	0.898
TTC29	0.084
TTC3	0.491
TTC30A	0.232

TTC30B	0.340
TTC31	0.981
TTC32	0.378
TTC33	0.376
TTC36	0.273
TTC37	0.284
TTC38	0.389
TTC39A	0.679
TTC39B	0.183
TTC39C	0.383
TTC4	0.141
TTC40	0.338
TTC5	0.150
TTC6	0.146
TTC7A	0.107
TTC7B	0.481
TTC8	0.126
TTC9	0.161
TTC9B	0.613
TTC9C	0.289
TTF1	0.448
TTF2	0.491
TTI1	0.726
TTI2	0.150
TTK	0.396
TTLL1	0.276
TTLL10	0.329
TTLL11	0.692
TTLL12	0.682
TTLL13	0.181
TTLL2	0.175
TTLL3	0.140

TTLL4	0.634
TTLL5	0.336
TTLL6	0.446
TTLL7	0.823
TTLL8	0.068
TTLL9	0.312
TTN	0.797
TTPA	0.226
TTPAL	0.376
TTR	0.254
TTYH1	0.409
TTYH2	0.096
TTYH3	0.132
TUB	0.565
TUBA1A	1.000
TUBA1B	1.000
TUBA1C	0.703
TUBA3C	0.100
TUBA3D	0.280
TUBA3E	0.256
TUBA4A	0.863
TUBA8	0.951
TUBAL3	0.116
TUBB	0.970
TUBB1	0.236
TUBB2A	0.996
TUBB2B	0.998
TUBB3	1.000
TUBB4A	1.000
TUBB4B	1.000
TUBB6	0.637
TUBB8	0.342

TUBD1	0.666
TUBE1	0.298
TUBG1	0.999
TUBG2	0.779
TUBGCP2	0.465
TUBGCP3	0.248
TUBGCP4	0.676
TUBGCP5	0.385
TUBGCP6	0.309
TUFM	0.886
TUFT1	0.545
TULP1	0.548
TULP2	0.146
TULP3	0.072
TULP4	0.626
TUSC1	0.050
TUSC2	0.841
TUSC3	0.094
TUSC5	0.246
TUT1	0.846
TVP23A	0.156
TVP23B	0.315
TVP23C	0.363
TVP23C-CDRT4	0.195
TWF1	0.239
TWF2	0.875
TWIST1	0.986
TWIST2	0.458
TWISTNB	0.233
TWSG1	0.209
TXK	0.506
TXLNA	0.975

TXLNB	0.306
TXN	0.376
TXN2	0.459
TXNDC11	0.357
TXNDC12	0.353
TXNDC15	0.286
TXNDC16	0.295
TXNDC17	0.357
TXNDC2	0.218
TXNDC5	0.223
TXNDC8	0.115
TXNDC9	0.432
TXNIP	0.220
TXNL1	0.386
TXNL4A	0.433
TXNL4B	0.913
TXNRD1	0.638
TXNRD2	0.432
TXNRD3	0.286
TXNRD3NB	0.117
TYK2	0.372
TYMP	0.074
TYMS	0.232
TYR	0.155
TYRO3	0.805
TYROBP	0.097
TYRP1	0.065
TYSND1	0.557
TYW1	0.590
TYW3	0.234
TYW5	0.359
U2AF1	0.974

U2AF1L4	0.558
U2AF2	1.000
U2SURP	0.896
UACA	0.677
UAP1	0.298
UAP1L1	0.315
UBA2	0.997
UBA3	0.344
UBA5	0.237
UBA52	0.859
UBA6	0.250
UBA7	0.241
UBAC1	0.646
UBAC2	0.271
UBALD1	0.364
UBALD2	0.486
UBAP1	0.804
UBAP1L	0.319
UBAP2	0.122
UBAP2L	0.997
UBASH3A	0.393
UBASH3B	0.319
UBB	0.754
UBBP4	0.071
UBC	0.278
UBD	0.223
UBE2B	0.877
UBE2C	0.228
UBE2D1	0.746
UBE2D2	0.997
UBE2D3	0.977
UBE2D4	0.443

UBE2E1	0.902
UBE2E2	0.829
UBE2E3	0.468
UBE2F	0.774
UBE2F-SCLY	0.173
UBE2G1	0.864
UBE2G2	0.687
UBE2H	0.982
UBE2I	0.991
UBE2J1	0.227
UBE2J2	0.811
UBE2K	0.801
UBE2L3	1.000
UBE2L6	0.470
UBE2M	0.809
UBE2N	0.942
UBE2O	0.997
UBE2Q1	1.000
UBE2Q2	0.424
UBE2QL1	0.999
UBE2R2	0.967
UBE2S	0.647
UBE2T	0.187
UBE2U	0.241
UBE2V1	0.654
UBE2V2	0.240
UBE2W	0.824
UBE2Z	0.995
UBE3A	0.937
UBE3B	0.701
UBE3C	0.859
UBE3D	0.308

UBE4A	0.808
UBE4B	0.998
UBFD1	0.820
UBIAD1	0.247
UBL3	0.612
UBL4B	0.173
UBL5	0.478
UBL7	0.632
UBLCP1	0.370
UBN1	0.477
UBN2	0.759
UBOX5	0.570
UBP1	0.958
UBQLN1	0.980
UBQLN3	0.106
UBQLN4	0.998
UBQLNL	0.067
UBR1	0.597
UBR2	0.994
UBR3	0.915
UBR4	0.985
UBR5	0.978
UBR7	0.779
UBTD1	0.805
UBTD2	0.671
UBTF	1.000
UBXN1	0.718
UBXN10	0.215
UBXN11	0.229
UBXN2A	0.207
UBXN2B	0.155
UBXN4	0.582

UBXN6	0.390
UBXN7	0.955
UCHL1	0.648
UCHL3	0.533
UCHL5	0.460
UCK1	0.560
UCK2	0.903
UCKL1	0.784
UCMA	0.259
UCN	0.430
UCN2	0.195
UCN3	0.148
UCP1	0.176
UCP2	0.107
UCP3	0.073
UEVLD	0.542
UFC1	0.786
UFD1L	0.938
UFL1	0.305
UFM1	0.093
UFSP1	0.418
UFSP2	0.252
UGCG	0.553
UGDH	0.585
UGGT1	0.261
UGGT2	0.154
UGP2	0.761
UGT1A1	0.643
UGT1A10	0.407
UGT1A3	0.210
UGT1A4	0.280
UGT1A5	0.295

UGT1A6	0.590
UGT1A7	0.489
UGT1A8	0.164
UGT1A9	0.300
UGT2A1	0.071
UGT2A2	0.052
UGT2A3	0.218
UGT2B10	0.083
UGT2B11	0.065
UGT2B15	0.351
UGT2B17	0.219
UGT2B28	0.086
UGT2B4	0.026
UGT2B7	0.277
UGT3A1	0.102
UGT3A2	0.274
UGT8	0.333
UHMK1	0.630
UHRF1BP1	0.243
UHRF1BP1L	0.095
UHRF2	0.861
UIMC1	0.802
ULBP1	0.196
ULBP2	0.157
ULBP3	0.069
ULK1	0.990
ULK2	0.648
ULK3	0.610
ULK4	0.227
UMOD	0.678
UMODL1	0.149
UMPS	0.359

UNC119	0.511
UNC119B	0.723
UNC13A	0.952
UNC13B	0.802
UNC13C	0.114
UNC13D	0.189
UNC45A	0.314
UNC45B	0.386
UNC50	0.559
UNC5A	0.979
UNC5B	0.163
UNC5C	0.386
UNC5CL	0.679
UNC5D	0.488
UNC79	1.000
UNC80	0.699
UNC93A	0.154
UNCX	1.000
UNG	0.530
UNK	0.989
UNKL	0.485
UPB1	0.253
UPF1	1.000
UPF2	0.904
UPF3A	0.102
UPK1A	0.150
UPK1B	0.189
UPK2	0.133
UPK3A	0.081
UPK3B	0.168
UPK3BL	0.188
UPP1	0.217

UPP2	0.539
UQCC1	0.504
UQCC2	0.465
UQCR10	0.152
UQCR11	0.252
UQCRB	0.224
UQCRC1	0.608
UQCRC2	0.482
UQCRFS1	0.552
UQCRH	0.447
UQCRQ	0.311
URAD	0.685
URB1	0.416
URB2	0.422
URGCP	0.539
URGCP-MRPS24	0.280
URI1	0.947
URM1	0.297
UROC1	0.386
UROD	0.759
UROS	0.383
USB1	0.508
USE1	0.274
USF1	0.938
USF2	0.976
USH1C	0.252
USH1G	0.458
USH2A	0.163
USHBP1	0.338
USMG5	0.295
USO1	0.758
USP1	0.813

USP10	0.451
USP12	0.957
USP13	0.772
USP14	0.529
USP15	0.975
USP16	0.567
USP17L15	0.161
USP17L2	0.141
USP18	0.404
USP19	0.999
USP2	0.537
USP20	0.693
USP21	0.983
USP22	0.941
USP24	0.998
USP25	0.703
USP28	0.360
USP29	0.088
USP3	0.692
USP30	0.730
USP31	0.527
USP32	0.931
USP33	0.932
USP34	0.928
USP35	0.744
USP36	0.603
USP37	0.910
USP38	0.262
USP39	0.779
USP4	0.595
USP40	0.427
USP42	0.984

USP43	0.693
USP44	0.399
USP45	0.289
USP46	0.811
USP47	0.818
USP48	0.937
USP49	0.880
USP5	1.000
USP53	0.980
USP54	0.312
USP6	0.345
USP6NL	0.194
USP7	0.976
USP8	0.313
USPL1	0.556
UST	0.657
UTF1	0.546
UTP11L	0.485
UTP14C	0.485
UTP15	0.762
UTP18	0.578
UTP20	0.413
UTP23	0.241
UTP3	0.728
UTP6	0.138
UTRN	0.363
UTS2	0.140
UTS2B	0.087
UTS2R	0.239
UVRAG	0.752
UVSSA	0.949
UXS1	0.577

VAC14	0.989
VAMP1	0.362
VAMP2	0.597
VAMP3	0.131
VAMP4	0.229
VAMP5	0.159
VAMP8	0.199
VANGL1	0.272
VANGL2	0.725
VAPA	0.464
VAPB	0.792
VARS	0.943
VARS2	0.596
VASH1	0.750
VASH2	0.572
VASN	0.164
VASP	0.842
VAT1	0.795
VAT1L	0.177
VAV1	0.259
VAV2	0.449
VAV3	0.151
VAX1	0.862
VAX2	0.122
VCAM1	0.470
VCAN	0.554
VCL	0.958
VCP	1.000
VCPIP1	0.917
VCPKMT	0.204
VDAC1	0.930
VDAC2	0.790

VDAC3	0.867
VDR	0.602
VEGFA	0.446
VEGFB	0.474
VEGFC	0.527
VENTX	0.177
VEPH1	0.530
VEZF1	1.000
VEZT	0.595
VGF	0.953
VGLL2	0.879
VGLL3	0.361
VGLL4	0.328
VHL	0.325
VHLL	0.169
VIL1	0.579
VILL	0.407
VIM	0.507
VIMP	0.096
VIP	0.109
VIPAS39	0.565
VIPR1	0.305
VIPR2	0.113
VIT	0.143
VKORC1	0.736
VKORC1L1	0.607
VLDLR	0.094
VMAC	0.485
VMO1	0.297
VMP1	0.304
VN1R1	0.108
VN1R2	0.035

VN1R4	0.024
VNN1	0.154
VNN2	0.026
VNN3	0.045
VOPP1	0.142
VPRBP	0.998
VPREB3	0.049
VPS11	0.743
VPS13A	0.411
VPS13B	0.645
VPS13C	0.067
VPS13D	0.647
VPS16	0.277
VPS18	0.822
VPS25	0.859
VPS26A	0.676
VPS26B	0.243
VPS28	0.521
VPS29	0.942
VPS33A	0.588
VPS33B	0.202
VPS35	0.931
VPS36	0.530
VPS37A	0.157
VPS37B	0.201
VPS37C	0.441
VPS37D	0.540
VPS39	0.999
VPS41	0.373
VPS45	0.584
VPS4A	0.999
VPS4B	0.636

VPS51	0.926
VPS52	0.924
VPS53	0.277
VPS54	0.495
VPS72	0.730
VPS8	0.420
VPS9D1	0.266
VRK1	0.559
VRK2	0.189
VRK3	0.391
VRTN	0.457
VSIG10	0.227
VSIG10L	0.528
VSIG2	0.283
VSIG8	0.677
VSNL1	0.922
VSTM1	0.176
VSTM2A	0.156
VSTM2B	0.159
VSTM2L	0.682
VSTM4	0.480
VSTM5	0.212
VSX1	0.279
VSX2	0.393
VTA1	0.275
VTCN1	0.307
VTI1A	0.213
VTI1B	0.288
VTN	0.599
VWA1	0.409
VWA2	0.262
VWA3A	0.247

VWA3B	0.339
VWA5A	0.225
VWA5B1	0.220
VWA5B2	0.690
VWA7	0.320
VWA8	0.249
VWA9	0.563
VWC2	0.351
VWC2L	0.546
VWCE	0.343
VWDE	0.087
VWF	0.394
WAC	0.871
WAPAL	0.951
WARS	0.536
WARS2	0.984
WASF1	0.924
WASF2	0.932
WASF3	0.615
WASL	0.998
WBP1	0.650
WBP11	0.955
WBP1L	0.489
WBP2	0.463
WBP2NL	0.249
WBP4	0.560
WBSCR16	0.727
WBSCR17	0.643
WBSCR22	0.338
WBSCR27	0.190
WBSCR28	0.045
WDFY1	0.202

WDFY2	0.705
WDFY3	0.815
WDFY4	0.263
WDHD1	0.249
WDPCP	0.265
WDR1	0.192
WDR11	0.211
WDR12	0.358
WDR16	0.100
WDR17	0.052
WDR18	0.407
WDR19	0.482
WDR20	0.896
WDR24	0.484
WDR25	0.458
WDR26	0.997
WDR27	0.085
WDR3	0.239
WDR31	0.161
WDR33	0.987
WDR34	0.254
WDR35	0.204
WDR36	0.056
WDR37	0.258
WDR38	0.187
WDR4	0.337
WDR41	0.132
WDR43	0.406
WDR45B	0.294
WDR46	0.499
WDR47	0.281
WDR48	0.978

WDR49	0.120
WDR5	0.989
WDR52	0.299
WDR53	0.305
WDR54	0.195
WDR55	0.717
WDR59	0.534
WDR5B	0.566
WDR6	0.799
WDR60	0.123
WDR61	0.998
WDR62	0.278
WDR63	0.332
WDR64	0.077
WDR65	0.392
WDR66	0.584
WDR7	0.592
WDR70	0.909
WDR72	0.102
WDR73	0.212
WDR74	0.213
WDR75	0.151
WDR76	0.260
WDR77	0.586
WDR78	0.211
WDR81	0.548
WDR82	0.973
WDR83	0.741
WDR83OS	0.762
WDR86	0.180
WDR87	0.399
WDR88	0.419

WDR89	0.327
WDR90	0.133
WDR91	0.640
WDR92	0.300
WDR93	0.236
WDR96	0.221
WDSUB1	0.199
WDTC1	0.984
WDYHV1	0.420
WEE1	0.956
WEE2	0.259
WFDC1	0.045
WFDC10A	0.114
WFDC10B	0.295
WFDC11	0.182
WFDC12	0.389
WFDC13	0.279
WFDC2	0.220
WFDC3	0.518
WFDC5	0.685
WFDC6	0.280
WFDC8	0.548
WFIKKN1	0.197
WFIKKN2	0.251
WFS1	0.131
WHAMM	0.465
WHSC1	0.986
WHSC1L1	0.991
WIBG	0.502
WIF1	0.279
WIPF1	0.533
WIPF2	0.529

WIPF3	0.274
WIPI1	0.445
WIPI2	0.492
WISP1	0.231
WISP2	0.233
WISP3	0.202
WIZ	0.964
WLS	0.404
WNK1	0.659
WNK2	0.981
WNK4	0.647
WNT1	0.868
WNT10A	0.281
WNT10B	0.590
WNT11	0.424
WNT16	0.252
WNT2	0.232
WNT2B	0.459
WNT3	0.999
WNT3A	0.637
WNT4	0.765
WNT5A	0.736
WNT5B	0.266
WNT6	0.482
WNT7A	0.544
WNT7B	0.681
WNT8A	0.214
WNT8B	0.423
WNT9A	0.375
WNT9B	0.351
WRAP53	0.375
WRAP73	0.230

WRB	0.222
WRN	0.466
WRNIP1	0.473
WSB1	0.389
WSB2	0.598
WSCD1	0.305
WSCD2	0.280
WT1	0.579
WTAP	0.905
WTIP	0.489
WWC1	0.713
WWC2	0.990
WWOX	0.109
WWP1	0.990
WWP2	0.999
WWTR1	0.841
XAB2	0.975
XAF1	0.201
XBP1	0.268
XCL1	0.159
XCL2	0.152
XCR1	0.275
XDH	0.324
XIRP1	0.428
XIRP2	0.210
XKR3	0.498
XKR4	0.822
XKR6	0.815
XKR7	0.279
XKR8	0.509
XKR9	0.059
XPA	0.427

XPC	0.667
XPNPEP1	0.817
XPNPEP3	0.314
XPO1	1.000
XPO4	0.881
XPO5	0.995
XPO6	0.711
XPO7	0.996
XPOT	0.894
XPR1	0.918
XRCC1	0.717
XRCC2	0.024
XRCC3	0.339
XRCC4	0.476
XRCC5	0.893
XRCC6	0.986
XRCC6BP1	0.244
XRN1	0.218
XRN2	0.673
XRRA1	0.355
XXbac-BPG181M17	0.300
XXbac-BPG32J3.2(	0.221
XXcos-LUCA11.5	0.315
XXYLT1	0.149
XYLB	0.351
XYLT1	0.415
XYLT2	0.836
YAE1D1	0.116
YAF2	0.691
YAP1	0.919
YARS	0.872
YARS2	0.254

YBEY	0.140
YBX1	0.919
YBX2	0.835
YBX3	0.113
YDJC	0.480
YEATS2	0.681
YEATS4	0.565
YES1	0.295
YIF1A	0.754
YIF1B	0.489
YIPF1	0.210
YIPF2	0.732
YIPF3	0.800
YIPF4	0.243
YIPF5	0.214
YIPF7	0.045
YJEFN3	0.236
YKT6	0.722
YLPM1	0.841
YME1L1	0.365
YOD1	0.616
YPEL1	0.497
YPEL2	0.210
YPEL3	0.565
YPEL4	0.336
YPEL5	0.492
YRDC	0.787
YTHDC1	0.829
YTHDC2	0.306
YTHDF1	0.466
YTHDF2	0.883
YTHDF3	0.605

YWHAB	0.858
YWHAE	0.639
YWHAG	0.996
YWHAH	0.936
YWHAQ	0.975
YWHAZ	0.992
YY1	1.000
YY1AP1	0.269
Z98049.1	0.143
ZADH2	0.555
ZAP70	0.863
ZAR1	0.270
ZAR1L	0.345
ZBBX	0.125
ZBED2	0.096
ZBED3	0.075
ZBED4	0.519
ZBED5	0.514
ZBED6	0.798
ZBED6CL	0.155
ZBP1	0.200
ZBTB1	0.981
ZBTB10	0.932
ZBTB11	0.542
ZBTB12	0.897
ZBTB14	0.517
ZBTB16	0.862
ZBTB17	0.961
ZBTB18	0.920
ZBTB2	0.964
ZBTB20	0.952
ZBTB21	0.646

ZBTB22	0.636
ZBTB24	0.587
ZBTB25	0.520
ZBTB26	0.430
ZBTB3	0.777
ZBTB32	0.270
ZBTB34	0.857
ZBTB37	0.799
ZBTB38	0.946
ZBTB39	0.505
ZBTB4	0.941
ZBTB40	0.689
ZBTB41	0.401
ZBTB42	0.853
ZBTB43	0.929
ZBTB44	0.679
ZBTB45	0.356
ZBTB46	0.354
ZBTB47	0.912
ZBTB48	0.501
ZBTB49	0.515
ZBTB5	0.742
ZBTB6	0.583
ZBTB7A	0.929
ZBTB7B	0.755
ZBTB7C	0.465
ZBTB8A	0.546
ZBTB8B	0.465
ZBTB8OS	0.655
ZBTB9	0.779
ZC2HC1A	0.156
ZC2HC1B	0.157

ZC2HC1C	0.306
ZC3H10	0.993
ZC3H11A	0.993
ZC3H12A	0.506
ZC3H12C	0.883
ZC3H12D	0.332
ZC3H13	0.653
ZC3H14	0.654
ZC3H15	0.734
ZC3H18	0.482
ZC3H3	0.522
ZC3H4	0.801
ZC3H6	0.688
ZC3H7A	0.986
ZC3H7B	0.991
ZC3H8	0.351
ZC3HAV1	0.524
ZC3HAV1L	0.156
ZC3HC1	0.494
ZCCHC10	0.270
ZCCHC11	0.979
ZCCHC14	0.972
ZCCHC17	0.577
ZCCHC2	0.635
ZCCHC24	0.535
ZCCHC3	0.240
ZCCHC4	0.312
ZCCHC6	0.970
ZCCHC7	0.564
ZCCHC8	0.488
ZCCHC9	0.403
ZCRB1	0.242

ZCWPW1	0.405
ZCWPW2	0.276
ZDBF2	0.285
ZDHHC1	0.579
ZDHHC11	0.370
ZDHHC12	0.344
ZDHHC13	0.203
ZDHHC14	0.844
ZDHHC16	0.466
ZDHHC17	0.762
ZDHHC18	0.366
ZDHHC19	0.137
ZDHHC2	0.067
ZDHHC20	0.569
ZDHHC21	0.178
ZDHHC22	0.672
ZDHHC23	0.315
ZDHHC24	0.595
ZDHHC3	0.481
ZDHHC4	0.256
ZDHHC5	0.982
ZDHHC6	0.237
ZDHHC7	0.137
ZDHHC8	0.678
ZEB1	0.780
ZEB2	0.962
ZER1	0.988
ZFAND1	0.918
ZFAND2A	0.165
ZFAND2B	0.653
ZFAND3	0.686
ZFAND4	0.247

ZFAND5	0.451
ZFAND6	0.304
ZFAT	0.420
ZFC3H1	0.791
ZFHX2	0.811
ZFHX3	0.905
ZFHX4	0.871
ZFP1	0.126
ZFP14	0.371
ZFP2	0.016
ZFP28	0.015
ZFP3	0.068
ZFP30	0.081
ZFP36	0.349
ZFP36L1	0.622
ZFP36L2	0.346
ZFP37	0.014
ZFP41	0.252
ZFP42	0.028
ZFP57	0.462
ZFP62	0.510
ZFP64	0.401
ZFP69	0.172
ZFP69B	0.034
ZFP82	0.093
ZFP90	0.064
ZFP91	0.931
ZFP91-CNTF	0.513
ZFPL1	0.793
ZFPM1	0.240
ZFPM2	0.835
ZFR	0.961

ZFR2	0.240
ZFYVE1	0.296
ZFYVE16	0.469
ZFYVE19	0.456
ZFYVE20	0.492
ZFYVE21	0.653
ZFYVE26	0.256
ZFYVE27	0.499
ZFYVE28	0.239
ZFYVE9	0.967
ZG16	0.595
ZG16B	0.277
ZGLP1	0.188
ZGPAT	0.595
ZHX1	0.724
ZHX1-C8ORF76	0.242
ZHX2	0.484
ZHX3	0.652
ZIC1	0.997
ZIC2	0.963
ZIC4	0.756
ZIC5	0.735
ZIK1	0.015
ZIM2	0.039
ZIM3	0.031
ZKSCAN1	0.923
ZKSCAN2	0.327
ZKSCAN3	0.521
ZKSCAN4	0.264
ZKSCAN5	0.747
ZKSCAN7	0.333
ZKSCAN8	0.610

ZMAT2	0.992
ZMAT3	0.230
ZMAT4	0.590
ZMAT5	0.236
ZMIZ1	0.851
ZMIZ2	0.958
ZMPSTE24	0.290
ZMYM1	0.236
ZMYM2	0.520
ZMYM4	0.997
ZMYM5	0.100
ZMYM6	0.514
ZMYM6NB	0.224
ZMYND10	0.477
ZMYND11	0.330
ZMYND12	0.419
ZMYND15	0.210
ZMYND19	0.913
ZMYND8	0.999
ZNF10	0.014
ZNF100	0.123
ZNF101	0.186
ZNF106	0.675
ZNF107	0.046
ZNF112	0.072
ZNF114	0.291
ZNF117	0.114
ZNF12	0.063
ZNF121	0.057
ZNF124	0.020
ZNF131	0.949
ZNF132	0.011

ZNF133	0.220
ZNF134	0.012
ZNF135	0.022
ZNF136	0.318
ZNF138	0.115
ZNF14	0.020
ZNF140	0.040
ZNF141	0.009
ZNF142	0.502
ZNF143	0.695
ZNF146	0.861
ZNF148	0.969
ZNF154	0.014
ZNF155	0.237
ZNF16	0.056
ZNF160	0.294
ZNF165	0.337
ZNF169	0.394
ZNF17	0.106
ZNF174	0.064
ZNF175	0.902
ZNF177	0.058
ZNF18	0.418
ZNF180	0.024
ZNF181	0.213
ZNF184	0.182
ZNF189	0.114
ZNF19	0.446
ZNF195	0.121
ZNF197	0.284
ZNF2	0.242
ZNF20	0.166

ZNF200	0.013
ZNF202	0.564
ZNF205	0.418
ZNF207	0.671
ZNF208	0.033
ZNF211	0.026
ZNF212	0.613
ZNF213	0.526
ZNF214	0.095
ZNF215	0.037
ZNF217	0.719
ZNF219	0.948
ZNF22	0.302
ZNF221	0.060
ZNF222	0.060
ZNF223	0.087
ZNF224	0.314
ZNF225	0.251
ZNF226	0.077
ZNF227	0.210
ZNF229	0.032
ZNF23	0.221
ZNF230	0.052
ZNF232	0.503
ZNF233	0.046
ZNF234	0.134
ZNF235	0.180
ZNF236	0.912
ZNF239	0.118
ZNF24	0.770
ZNF248	0.196
ZNF25	0.233

ZNF250	0.414
ZNF251	0.215
ZNF253	0.094
ZNF254	0.024
ZNF256	0.022
ZNF257	0.033
ZNF259	0.289
ZNF26	0.029
ZNF260	0.067
ZNF263	0.048
ZNF264	0.167
ZNF266	0.051
ZNF267	0.248
ZNF268	0.019
ZNF273	0.099
ZNF274	0.031
ZNF276	0.250
ZNF277	0.123
ZNF28	0.092
ZNF280A	0.131
ZNF280B	0.088
ZNF280D	0.264
ZNF281	0.745
ZNF282	0.811
ZNF283	0.052
ZNF284	0.191
ZNF285	0.088
ZNF286A	0.112
ZNF286B	0.144
ZNF287	0.224
ZNF292	0.939
ZNF296	0.687

ZNF3	0.397
ZNF30	0.161
ZNF300	0.033
ZNF302	0.197
ZNF304	0.164
ZNF311	0.107
ZNF317	0.216
ZNF318	0.649
ZNF319	0.881
ZNF32	0.164
ZNF320	0.848
ZNF321P	0.144
ZNF322	0.375
ZNF324	0.329
ZNF324B	0.129
ZNF326	0.738
ZNF329	0.028
ZNF330	0.386
ZNF331	0.365
ZNF333	0.153
ZNF334	0.062
ZNF335	0.745
ZNF337	0.230
ZNF33A	0.259
ZNF33B	0.182
ZNF34	0.931
ZNF341	0.542
ZNF343	0.091
ZNF345	0.047
ZNF346	0.678
ZNF347	0.020
ZNF35	0.486

ZNF350	0.009
ZNF354A	0.042
ZNF354B	0.084
ZNF354C	0.018
ZNF358	0.733
ZNF362	1.000
ZNF365	0.362
ZNF366	0.386
ZNF367	0.933
ZNF37A	0.049
ZNF382	0.122
ZNF383	0.101
ZNF384	0.980
ZNF385A	0.435
ZNF385B	0.294
ZNF385C	0.393
ZNF385D	0.155
ZNF391	0.054
ZNF394	0.278
ZNF395	0.524
ZNF396	0.325
ZNF397	0.424
ZNF398	0.433
ZNF404	0.051
ZNF407	0.546
ZNF408	0.703
ZNF410	0.806
ZNF414	0.473
ZNF415	0.080
ZNF416	0.023
ZNF417	0.295
ZNF418	0.015

ZNF419	0.279
ZNF420	0.070
ZNF423	0.964
ZNF425	0.120
ZNF426	0.058
ZNF428	0.584
ZNF429	0.049
ZNF43	0.087
ZNF430	0.199
ZNF431	0.244
ZNF432	0.016
ZNF433	0.058
ZNF436	0.201
ZNF438	0.213
ZNF439	0.044
ZNF44	0.252
ZNF440	0.105
ZNF441	0.074
ZNF442	0.074
ZNF443	0.294
ZNF444	0.722
ZNF445	0.732
ZNF446	0.118
ZNF45	0.175
ZNF451	0.396
ZNF454	0.026
ZNF460	0.214
ZNF461	0.063
ZNF462	0.988
ZNF467	0.144
ZNF468	0.120
ZNF469	0.175

ZNF470	0.002
ZNF471	0.007
ZNF473	0.342
ZNF474	0.115
ZNF479	0.048
ZNF48	0.580
ZNF480	0.030
ZNF483	0.327
ZNF484	0.444
ZNF485	0.289
ZNF486	0.022
ZNF487	0.173
ZNF488	0.446
ZNF490	0.543
ZNF491	0.038
ZNF492	0.068
ZNF493	0.069
ZNF496	0.498
ZNF497	0.206
ZNF500	0.494
ZNF501	0.019
ZNF502	0.021
ZNF503	0.745
ZNF506	0.105
ZNF507	0.338
ZNF510	0.036
ZNF511	0.270
ZNF512	0.969
ZNF512B	0.964
ZNF513	0.906
ZNF514	0.050
ZNF516	0.328

ZNF517	0.945
ZNF518B	0.025
ZNF519	0.072
ZNF521	0.960
ZNF524	0.518
ZNF525	0.016
ZNF526	0.791
ZNF527	0.077
ZNF528	0.042
ZNF529	0.037
ZNF530	0.027
ZNF532	0.863
ZNF534	0.032
ZNF536	0.870
ZNF540	0.095
ZNF541	0.747
ZNF543	0.093
ZNF544	0.031
ZNF546	0.183
ZNF547	0.177
ZNF548	0.261
ZNF549	0.013
ZNF550	0.031
ZNF551	0.015
ZNF552	0.259
ZNF554	0.071
ZNF555	0.060
ZNF556	0.039
ZNF557	0.013
ZNF558	0.127
ZNF559	0.008
ZNF560	0.026

ZNF561	0.199
ZNF562	0.327
ZNF563	0.316
ZNF564	0.118
ZNF565	0.444
ZNF566	0.145
ZNF567	0.056
ZNF568	0.148
ZNF569	0.040
ZNF57	0.234
ZNF570	0.076
ZNF571	0.030
ZNF572	0.037
ZNF573	0.185
ZNF574	0.574
ZNF575	0.271
ZNF576	0.626
ZNF577	0.008
ZNF578	0.018
ZNF579	0.658
ZNF580	0.791
ZNF581	0.700
ZNF582	0.054
ZNF583	0.015
ZNF584	0.275
ZNF585A	0.047
ZNF585B	0.111
ZNF586	0.015
ZNF587	0.381
ZNF587B	0.516
ZNF589	0.424
ZNF592	0.665

ZNF593	0.728
ZNF594	0.094
ZNF595	0.140
ZNF596	0.016
ZNF597	0.094
ZNF598	0.617
ZNF599	0.063
ZNF600	0.254
ZNF605	0.049
ZNF606	0.019
ZNF607	0.224
ZNF608	0.479
ZNF609	0.957
ZNF610	0.067
ZNF611	0.260
ZNF613	0.019
ZNF614	0.020
ZNF615	0.008
ZNF616	0.019
ZNF618	0.960
ZNF619	0.274
ZNF620	0.278
ZNF621	0.205
ZNF622	0.339
ZNF623	0.043
ZNF624	0.265
ZNF625	0.285
ZNF625-ZNF20	0.170
ZNF626	0.021
ZNF627	0.249
ZNF628	0.609
ZNF629	0.959

ZNF639	0.230
ZNF641	0.521
ZNF644	0.872
ZNF646	0.801
ZNF648	0.317
ZNF649	0.015
ZNF653	0.549
ZNF654	0.660
ZNF655	0.350
ZNF658	0.067
ZNF66	0.084
ZNF660	0.136
ZNF662	0.112
ZNF664	0.596
ZNF665	0.016
ZNF667	0.016
ZNF668	0.779
ZNF669	0.028
ZNF670	0.011
ZNF671	0.017
ZNF672	0.293
ZNF675	0.076
ZNF676	0.027
ZNF677	0.009
ZNF678	0.053
ZNF679	0.077
ZNF680	0.163
ZNF681	0.010
ZNF682	0.071
ZNF683	0.244
ZNF684	0.273
ZNF687	0.992

ZNF688	0.754
ZNF689	0.818
ZNF69	0.071
ZNF691	0.829
ZNF692	0.191
ZNF695	0.016
ZNF696	0.535
ZNF697	0.988
ZNF699	0.060
ZNF7	0.046
ZNF70	0.252
ZNF700	0.087
ZNF701	0.112
ZNF703	0.425
ZNF704	0.721
ZNF705A	0.232
ZNF705B	0.162
ZNF705D	0.237
ZNF705G	0.095
ZNF706	0.473
ZNF707	0.402
ZNF708	0.172
ZNF709	0.123
ZNF71	0.022
ZNF710	0.451
ZNF713	0.197
ZNF714	0.197
ZNF716	0.021
ZNF717	0.067
ZNF720	0.152
ZNF721	0.052
ZNF724P	0.023

ZNF726	0.004
ZNF727	0.050
ZNF728	0.044
ZNF729	0.057
ZNF730	0.028
ZNF732	0.023
ZNF736	0.054
ZNF737	0.030
ZNF738	0.103
ZNF74	0.372
ZNF740	0.697
ZNF746	0.528
ZNF747	0.474
ZNF749	0.102
ZNF750	0.447
ZNF75A	0.008
ZNF76	0.638
ZNF763	0.070
ZNF764	0.639
ZNF765	0.105
ZNF766	0.282
ZNF768	0.751
ZNF77	0.250
ZNF770	0.556
ZNF771	0.559
ZNF772	0.025
ZNF773	0.101
ZNF774	0.053
ZNF775	0.144
ZNF776	0.018
ZNF777	0.777
ZNF778	0.093

ZNF780A	0.243
ZNF780B	0.070
ZNF781	0.067
ZNF782	0.067
ZNF783	0.566
ZNF784	0.727
ZNF785	0.552
ZNF786	0.505
ZNF787	0.453
ZNF788	0.139
ZNF789	0.471
ZNF79	0.446
ZNF790	0.021
ZNF791	0.810
ZNF792	0.051
ZNF793	0.054
ZNF799	0.261
ZNF8	0.251
ZNF80	0.202
ZNF800	0.606
ZNF804A	0.039
ZNF804B	0.055
ZNF805	0.330
ZNF808	0.255
ZNF812	0.070
ZNF813	0.106
ZNF814	0.117
ZNF816	0.045
ZNF821	0.963
ZNF823	0.154
ZNF827	0.923
ZNF829	0.057

ZNF83	0.919
ZNF830	0.624
ZNF831	0.285
ZNF835	0.042
ZNF836	0.058
ZNF837	0.215
ZNF839	0.356
ZNF84	0.047
ZNF841	0.017
ZNF843	0.280
ZNF844	0.067
ZNF845	0.055
ZNF846	0.130
ZNF85	0.106
ZNF850	0.154
ZNF852	0.111
ZNF853	0.615
ZNF860	0.260
ZNF862	0.172
ZNF865	0.891
ZNF878	0.085
ZNF879	0.019
ZNF880	0.029
ZNF891	0.021
ZNF90	0.037
ZNF91	0.121
ZNF92	0.194
ZNF93	0.051
ZNF98	0.050
ZNF99	0.063
ZNFX1	0.791
ZNHIT1	0.624

ZNHIT2	0.726
ZNHIT3	0.477
ZNHIT6	0.245
ZNRD1	0.201
ZNRF1	0.622
ZNRF2	0.313
ZNRF3	0.897
ZNRF4	0.114
ZP1	0.254
ZP2	0.182
ZP3	0.351
ZPBP	0.122
ZPBP2	0.166
ZPLD1	0.189
ZRANB1	0.980
ZRANB2	0.607
ZRANB3	0.477
ZRSR1	0.281
ZSCAN1	0.039
ZSCAN10	0.199
ZSCAN12	0.381
ZSCAN16	0.329
ZSCAN18	0.071
ZSCAN2	0.304
ZSCAN20	0.232
ZSCAN21	0.613
ZSCAN22	0.285
ZSCAN23	0.109
ZSCAN25	0.698
ZSCAN29	0.112
ZSCAN30	0.164
ZSCAN31	0.342

ZSCAN32	0.059
ZSCAN4	0.124
ZSCAN5A	0.191
ZSCAN5B	0.060
ZSCAN5C	0.025
ZSCAN5D	0.074
ZSCAN9	0.282
ZSWIM1	0.490
ZSWIM2	0.110
ZSWIM3	0.369
ZSWIM4	0.796
ZSWIM5	0.848
ZSWIM6	0.955
ZSWIM7	0.233
ZSWIM8	0.998
ZUFSP	0.633
ZW10	0.265
ZWILCH	0.226
ZWINT	0.144
ZXDC	0.427
ZYG11A	0.301
ZYG11B	0.979
ZYX	0.203
ZZEF1	0.739
ZZZ3	0.978
