# Supplementary material for: Foxg1 and companions: Not only transcription factors
Source: Neural Regen Res. 2025 Sep 3;21(8):3426–38. doi: 10.4103/NRR.NRR-D-25-00439 (PMC13452659; doi:10.4103/NRR.NRR-D-25-00439)
Supplement: Supplementary file 22 [file NRR-21-3426_Suppl9.pdf]

## Additional Table 5. Gene relatedness to neuronal activity/plasticity

[information referring to 22,711 human Biogrid interactor genes]

*NOTES*

data collected from: from: Joy & Carmichel. Communications Biology (2024). doi: 10.1038/s42003-024-06723-3 [Supplementary Data S2]

| *category acronym* | *definition* |
|---|---|
| IEGs.up | Immediate early genes upregulated |
| IEGs.down | Immediate early genes downregulated |
| LRGs.up | Late response (secondary) genes upregulated |
| LRGs.down | Late response (secondary) genes downregulated |
| Engr.up | Genes upregulated in Fos/Arc enriched neurons during engram formation |
| Engr.down | Genes downregulated in Fos/Arc enriched neurons during engram formation |
| RemEngr.up | Genes upregulated in Fos/Arc enriched neurons during engram consolidation or long-term storage |
| RemEngr.down | Genes downregulated in Fos/Arc enriched neurons during engram consolidation or long-term storage |
| neuronal activity/plasticity related | fulfilling at least one of the previous conditions |

| *Non-redundant human Biogrid A/B interactor gene* | *IEGs.up* | *IEGs.down* | *LRGs.up* | *LRGs.down* | *Engr.up* | *Engr.down* | *RemEngr.up* | *RemEngr.down* | *Neuronal activity / plasticity-related* |
|---|---|---|---|---|---|---|---|---|---|
| "BRCA2(IV)" | | | | | | | | | |
| "E(z)" | | | | | | | | | |
| "His2A:CG31618" | | | | | | | | | |
| "His2B:CG17949" | | | | | | | | | |
| "His3:CG31613" | | | | | | | | | |
| "His4:CG31611" | | | | | | | | | |
| "l(1)G0148" | | | | | | | | | |
| "Su(fu)" | | | | | | | | | |
| "Su(H)" | | | | | | | | | |
| "Su(var)205" | | | | | | | | | |
| "tI(GAU)Q" | | | | | | | | | |
| "tV(UAC)Q" | | | | | | | | | |
| 1200014J11Rik | + | | | + | + | | + | | + |
| 1700006A11Rik | | | | | | | | | |
| 1700009N14Rik | | | | | | | | | |
| 1810009A15Rik | | | | | | | | | |
| 1810026J23Rik | | | | | | | | | |
| 1810055G02Rik | | | | | | | | | |
| 2010300C02Rik | | | | | | | | | |
| 2310003H01Rik | | | | | | | | | |
| 2310033P09Rik | | | | | | | | | |
| 2310035C23Rik | | | | | | | | | |
| 2510003E04Rik | | | | | | | | | |
| 2700060E02Rik | | | | | | | | | |
| 2810004N23Rik | | | | | | | | | |
| 4932438A13Rik | | | + | | + | | | | + |
| 5830415F09Rik | | | | | | | | | |
| A1BG | | | | | | | | | |
| A1CF | | | | | | | | | |
| A1i3 | | | | | | | | | |
| A2M | | | | | | | | | |

| | | | | | | | | | |
|---|---|---|---|---|---|---|---|---|---|
| A2ML1 | | | | | | | | | |
| A3GALT2 | | | | | | | | | |
| A49R | | | | | | | | | |
| A4GALT | | | | | | | | | |
| A4GNT | | | | | | | | | |
| A55R | | | | | | | | | |
| AAAS | | | | | | | | | |
| AAC3 | | | | | | | | | |
| AACS | | | | | | | | | |
| AADAC | | | | | | | | | |
| AADACL2 | | | | | | | | | |
| AADACL3 | | | | | | | | | |
| AADAT | | | | | | | | | |
| AAED1 | | | | | | | | | |
| AAGAB | | | | | | | | | |
| AAK1 | | | | | | | | | |
| AAMDC | | | | | | + | | | + |
| AAMP | | | | | | | | | |
| AANAT | | | | | | | | | |
| AAR2 | | | | | | | | | |
| AARD | | | | | | | | | |
| AARS | | | | | + | | | | + |
| AARS2 | | | | | | | | | |
| AARSD1 | | | | + | | | | | + |
| AASDH | | | | | | | | | |
| AASDHPPT | | | | | | | | | |
| AASS | | | | | | | | | |
| AATF | | | | | | + | | | + |
| AATK | | | | | | + | | | + |
| ABAT | | | | | | | | | |
| ABCA1 | | | | + | | | | | + |
| ABCA10 | | | | | | | | | |
| ABCA12 | | | | | | | | | |
| ABCA13 | | | | | | | | | |
| ABCA17P | | | | | | | | | |
| ABCA2 | | | | | | | | | |
| ABCA3 | | | | | | | | | |
| ABCA4 | | | | | | | | | |
| ABCA5 | + | | | | + | | | | + |
| ABCA6 | | | | | | | | | |
| ABCA7 | | | | | | | | | |
| ABCA8 | | | | | | | | | |
| ABCA9 | | | | | + | | | | + |
| ABCB1 | | | | | | | | | |
| ABCB10 | | | | | | | | | |
| ABCB11 | | | | | | | | | |
| ABCB4 | | | | | | | | | |
| ABCB5 | | | | | | | | | |
| ABCB6 | | | | | | | | | |
| ABCB7 | | | | | | | | | |
| ABCB8 | | | | | | | | | |
| ABCB9 | | | | | | | | | |
| ABCC1 | | | | | | | | | |

| | | | | | | | | | |
|---|---|---|---|---|---|---|---|---|---|
| ABCC10 | | | | | | | | | |
| ABCC11 | | | | | | | | | |
| ABCC12 | | | | | | | | | |
| ABCC13 | | | | | | | | | |
| ABCC2 | | | | | | | | | |
| ABCC3 | | | | | | | | | |
| ABCC4 | | | | | | | | | |
| ABCC5 | | | | | | | | | |
| ABCC6 | | | | | | | | | |
| ABCC8 | | | | | | | | | |
| ABCC9 | | | | | | | | | |
| ABCD1 | | | | | | | | | |
| ABCD2 | | | | | | | | | |
| ABCD3 | | | | | | | | | |
| ABCD4 | | | | | | | | | |
| ABCE1 | | | | | | | | | |
| ABCF1 | | | | | | | | | |
| ABCF2 | | | | | | | | | |
| ABCF3 | | | | | | | + | | + |
| ABCG1 | | + | | | | + | | | + |
| ABCG2 | | | | | | + | | | + |
| ABCG4 | | | | | + | + | | | + |
| ABCG5 | | | | | | | | | |
| ABCG8 | | | | | | | | | |
| ABHD1 | | | | | | | | | |
| ABHD10 | | | | | | | | | |
| ABHD11 | | | | | | | | | |
| ABHD12 | | | | | | + | | | + |
| ABHD12B | | | | | | | | | |
| ABHD13 | | | | | | | | | |
| ABHD14A | | | | | | | | | |
| ABHD14A-ACY1 | | | | | | | | | |
| ABHD14B | | | | | | | | | |
| ABHD15 | | | | | | | | | |
| ABHD15-AS1 | | | | | | | | | |
| ABHD16A | | | | | | | | | |
| ABHD16B | | | | | | | | | |
| ABHD17A | | | | | | | | | |
| ABHD17B | | | | | | | | | |
| ABHD17C | | | | | | | | | |
| ABHD2 | + | | | | + | | | | + |
| ABHD3 | | | | | | | | | |
| ABHD4 | | | | | | + | | | + |
| ABHD5 | | | | | | | | | |
| ABHD6 | | | | | | | | | |
| ABHD8 | | | | | | + | | | + |
| ABI1 | | | | | | | | | |
| ABI2 | | | | | | | | | |
| ABI3 | | | | | | | | | |
| ABI3BP | | | | | | | | | |
| ABL1 | | | | | | | | | |
| ABL2 | | | | | | | | | |
| ABLIM1 | | + | | | | + | | | + |

| | | | | | | | | | |
|---|---|---|---|---|---|---|---|---|---|
| ABLIM2 | | | | | | | | | |
| ABLIM3 | + | | + | | | + | | | + |
| ABO | | | | | | | | | |
| ABR | + | | | | + | | | | + |
| ABRA | | | | | | | | | |
| ABRACL | | | | | | | | | |
| Abt1 | | | | | | | | | |
| ABTB1 | | | | | | | | | |
| ABTB2 | | | | | | | | | |
| ABZ2 | | | | | | | | | |
| ACAA1 | | | | | | | | | |
| ACAA2 | | | | | | | | | |
| ACACA | | | | | | | | | |
| ACACB | | | | | | | | | |
| ACAD10 | | | | | + | | | | + |
| ACAD11 | | | | | | | | | |
| ACAD8 | | | | | | + | | | + |
| ACAD9 | | + | | | | | | | + |
| ACADL | | | | | | | | | |
| ACADM | | | | | | | | | |
| ACADS | | | | | | | | | |
| ACADSB | | | | | | | | | |
| ACADVL | | | | | + | | | | + |
| ACAN | | | | | + | | | | + |
| ACAP1 | | | | | | | | | |
| ACAP2 | | | | + | + | | | | + |
| ACAP3 | | | | | | | | | |
| ACAT1 | | | | | | | | | |
| ACAT2 | | | | | | | | | |
| ACBD3 | | | | | | | | | |
| ACBD4 | | | | | | | | | |
| ACBD5 | | | | | | | | | |
| ACBD6 | | | | | | + | | | + |
| ACBD7 | | | | | | | | | |
| ACC1 | | | | | | | | | |
| ACCS | | | | | | | | | |
| ACCSL | | | | | | | | | |
| ACD | | | | | | | | | |
| ACE | | | | | | | | | |
| ACE2 | | | | | | | | | |
| ACER1 | | | | | | | | | |
| ACER2 | | | | | | | | | |
| ACER3 | | | | | | | | | |
| ACF | | | | | | | | | |
| ACHE | | | | | + | | | | + |
| ACIN1 | | | | | | | | | |
| ACKR1 | | | | | | | | | |
| ACKR2 | | | | | | | | | |
| ACKR3 | + | | | + | + | | | | + |
| ACKR4 | | | | | | | | | |
| ACLY | | | | | | | | | |
| ACM1 | | | | | | | | | |
| ACMSD | | + | + | | | + | | | + |

| | | | | | | | | | |
|---|---|---|---|---|---|---|---|---|---|
| ACN9 | | | | | | | | | |
| Acnat1 | | | | | | | | | |
| ACO1 | | | | | | | | | |
| ACO2 | | | | | | | | | |
| ACOT1 | | | | | | | | | |
| ACOT11 | | | | | | | | | |
| ACOT12 | | | | | | | | | |
| ACOT13 | | + | | | | + | | + | + |
| ACOT2 | + | | + | + | + | | | | + |
| ACOT4 | | | | | | | | | |
| ACOT6 | | | | | | | | | |
| ACOT7 | | | | | | | | | |
| ACOT8 | | | | | | | | | |
| ACOT9 | | | | | | | | | |
| ACOX1 | | | | | | | | | |
| ACOX2 | | | | | | | | | |
| ACOX3 | | | | | | | | | |
| ACOXL | | | | | | | | | |
| ACP1 | | | | | | | | | |
| ACP2 | | | | | | | | | |
| ACP5 | | | | | | | | | |
| ACP6 | | | | | | | | | |
| ACPP | | | | | | | | | |
| ACPT | | | | | | | | | |
| ACR | | | | | | | | | |
| ACRBP | | | | | | | | | |
| ACRC | | | | | | | | | |
| ACRV1 | | | | + | | | | | + |
| ACS1 | | | | | | | | | |
| ACS2 | | | | | | | | | |
| ACSBG1 | | | | | | | | + | + |
| ACSBG2 | | | | | | | | | |
| ACSF2 | | | | | | | | | |
| ACSF3 | | | | | | | | | |
| ACSL1 | | | | | | | | | |
| ACSL3 | | | | | | | | | |
| ACSL4 | + | | + | | | | | | + |
| ACSL5 | | | | | | | | | |
| ACSL6 | | | | | | | | | |
| ACSM1 | | | | | | | | | |
| Acsm2 | + | | | + | + | | + | | + |
| ACSM2A | | | | | | | | | |
| ACSM2B | | | | | | | | | |
| ACSM3 | | | | | | | | | |
| ACSM4 | | | | | | | | | |
| ACSM5 | | | | | | | | | |
| ACSS1 | | | | | | | | | |
| ACSS2 | | | | | | | | | |
| ACSS3 | | | | | | | | | |
| ACT | | | | | | | | | |
| ACT1 | | | | | | | | | |
| Act5C | | | | | | | | | |
| ACTA1 | | | | | | | | | |

| | | | | | | | | | |
|---|---|---|---|---|---|---|---|---|---|
| ACTA2 | | | | | | | | | |
| ACTA2-AS1 | | | | | | | | | |
| ACTB | | | | | | | | | |
| ACTBL2 | | | | | | | | | |
| ACTC1 | | | | | | | | | |
| ACTG1 | | | | | | + | | | + |
| ACTG1P14 | | | | | | | | | |
| ACTG2 | | | | | | | | | |
| ACTL6A | | | | + | | | | | + |
| ACTL6B | | | | | | + | | | + |
| ACTL7A | | | | | | | | | |
| ACTL7B | | | | | | | | | |
| ACTL8 | | | | | | | | | |
| ACTL9 | | | | | | | | | |
| ACTN1 | | | | | + | | | | + |
| ACTN2 | + | | | | + | | | | + |
| ACTN3 | | | | | | | | | |
| ACTN4 | | | | | + | | | | + |
| ACTR10 | | | | | | | | | |
| ACTR1A | | | | | | | | | |
| ACTR1B | | | | | | | | | |
| ACTR2 | | | | | | | | | |
| ACTR3 | | | | | | | | | |
| ACTR3B | | | | | | | | | |
| ACTR3C | | | | | | | | | |
| ACTR5 | | | | | | | | | |
| ACTR6 | | | | | | | | | |
| ACTR8 | | | | | | | | | |
| ACTRT1 | | | | | | | | | |
| ACTRT2 | | | | | | | | | |
| ACTRT3 | | | | | | | | | |
| ACVR1 | | | | | | | | | |
| ACVR1B | | | | | + | + | | | + |
| ACVR1C | | | | | + | | | | + |
| ACVR2A | | | | | | | | | |
| ACVR2B | | | | | | | | | |
| ACVRL1 | | | | | | | | | |
| ACY1 | | | | | | | | | |
| ACY3 | | | | | | | | | |
| ACYP1 | | | | | | + | | | + |
| ACYP2 | | | | | | | | | |
| ADA | | | | | | | | | |
| ADA2 | | | | | | | | | |
| ADAD2 | | | | | | | | | |
| ADAL | | + | | | | + | | | + |
| ADAM10 | | | | | | | | | |
| ADAM11 | | | | | | + | | | + |
| ADAM12 | | | | | | | | | |
| ADAM15 | | | | | | | | | |
| ADAM17 | | | | | | | | | |
| ADAM18 | | | | | | | | | |
| ADAM19 | | | | | | | | | |
| ADAM1A | | | | | | | | | |

| | | | | | | | | | |
|---|---|---|---|---|---|---|---|---|---|
| ADAM2 | | | | | | | | | |
| ADAM20 | | | | | | | | | |
| ADAM20P1 | | | | | | | | | |
| ADAM21 | | + | + | | | + | | | + |
| ADAM22 | | | | | | | | | |
| ADAM23 | | | | | | | | | |
| ADAM28 | | | | | | | | | |
| ADAM29 | | | | | | | | | |
| ADAM30 | | | | | | | | | |
| ADAM32 | | | | | | | | | |
| ADAM33 | | | | | | | | | |
| ADAM5 | | | | | | | | | |
| ADAM7 | | | | | | | | | |
| ADAM8 | | | | | | + | | | + |
| ADAM9 | | | | | | | | | |
| ADAMTS1 | | | | | | + | | | + |
| ADAMTS10 | | | | | | | | | |
| ADAMTS12 | | | | | | | | | |
| ADAMTS13 | | | | | | | | | |
| ADAMTS14 | | | | | | | | | |
| ADAMTS15 | | | + | | | | | | + |
| ADAMTS16 | | | | | | | | | |
| ADAMTS17 | | | | | | + | | | + |
| ADAMTS18 | | | | | | | | | |
| ADAMTS19 | | | | | | | | | |
| ADAMTS2 | | | | | | | | | |
| ADAMTS20 | | | | | | | | | |
| ADAMTS3 | | | | | | | | | |
| ADAMTS4 | | | | | | | | | |
| ADAMTS5 | + | | | + | + | | | | + |
| ADAMTS6 | | | | | | | | | |
| ADAMTS7 | | | | | | | | + | + |
| ADAMTS8 | | | | | | | | | |
| ADAMTS9 | | | | | | | | | |
| ADAMTS9-AS2 | | | | | | | | | |
| ADAMTSL1 | | | | | | | | | |
| ADAMTSL2 | + | | | | + | | | | + |
| ADAMTSL3 | | | | | | | | | |
| ADAMTSL4 | | | | | | | | | |
| ADAMTSL5 | | | | | | | | | |
| ADAP1 | | | + | | | | | | + |
| ADAP2 | | | | | | | | | |
| ADAR | | | + | | | | | | + |
| ADARB1 | | | | | | | | | |
| ADARB2 | | | | | | | | | |
| ADARB2-AS1 | | | | | | | | | |
| ADAT1 | + | | | | | + | | | + |
| ADAT2 | | | | | | | | | |
| ADAT3 | | | | | | | | | |
| ADCK1 | | | | | | + | | | + |
| ADCK2 | | | | | | + | | | + |
| ADCK3 | | | | | | | | | |
| ADCK4 | | | | | | | | | |

| | | | | | | | | | |
|---|---|---|---|---|---|---|---|---|---|
| ADCK5 | | | | | | | | | |
| ADCY1 | | | | | | + | | | + |
| ADCY10 | | | | | | | | | |
| ADCY2 | | | | | | | | | |
| ADCY3 | | | | | | | | | |
| ADCY4 | | | | | | | | | |
| ADCY5 | + | | | + | + | | | | + |
| ADCY6 | | | | | | | | | |
| ADCY7 | | | | | | | | | |
| Adcy8 | | | | | | | | | |
| ADCY9 | | | | | | + | | | + |
| ADCYAP1 | | | | | | | | | |
| ADCYAP1R1 | | + | | | | + | | | + |
| ADD1 | | + | | | | + | | | + |
| ADD2 | | | | | | + | | | + |
| ADD3 | | | | | | | | | |
| ADD37 | | | | | | | | | |
| ade1 | | | | | | | | | |
| ade10 | | | | | | | | | |
| ADE13 | | | | | | | | | |
| ADE16 | | | | | | | | | |
| ADE17 | | | | | | | | | |
| ADE2 | | | | | | | | | |
| ADE3 | | | | | | | | | |
| ADE4 | | | | | | | | | |
| ADE5,7 | | | | | | | | | |
| ade6 | | | | | | | | | |
| ADGB | | | | | | | | | |
| ADH1 | | | | | | | | | |
| ADH1A | | | | | | | | | |
| ADH1B | | | | | | | | | |
| ADH1C | | | | | | | | | |
| ADH4 | | | | | | | | | |
| ADH5 | | | | | | | | | |
| ADH6 | | | | | | | | | |
| ADH7 | | | | | | | | | |
| ADHFE1 | | | | | | | | | |
| ADI1 | | | | | | | | | |
| ADIPOQ | | | | | | | | | |
| ADIPOR1 | | | | | | | | | |
| ADIPOR2 | | | | | + | | | | + |
| ADIRF | | | | | | | | | |
| ADK | | | | | | | | | |
| adk1 | | | | | | | | | |
| ADM | | | | | | | | | |
| ADM2 | | | | | | | | | |
| ADM5 | | | | | | | | | |
| ADNP | | | | | | | | | |
| ADNP2 | | | | | | | | | |
| ADO | + | | | + | + | | + | | + |
| ADORA1 | + | | | | + | + | | | + |
| ADORA2A | | | | | | | | | |
| ADORA2B | | | | | | | | | |

| | | | | | | | | | |
|---|---|---|---|---|---|---|---|---|---|
| ADORA3 | | | | | | | | | |
| ADPGK | | | | | + | | | | + |
| ADPGK-AS1 | | | | | | | | | |
| ADPRH | | | + | | | + | | | + |
| ADPRHL1 | | | | | | | | | |
| ADPRHL2 | | | | | | | | | |
| ADPRM | | | | | | | | | |
| ADRA1A | | | | + | | | | | + |
| ADRA1B | | | | | | | | | |
| ADRA1D | | | | | | | | | |
| ADRA2A | | | | | | | | | |
| ADRA2B | | | | | | | | | |
| ADRA2C | | | | | | | | | |
| ADRB1 | + | | | | | | | | + |
| ADRB2 | | | | | | | | | |
| ADRB3 | | | | | | | | | |
| ADRBK1 | | | | | | | | | |
| ADRBK2 | | | | | | | | | |
| ADRM1 | | | | | + | | | | + |
| ADSL | | | | | | | | | |
| ADSS | | | | | | | | | |
| ADSSL1 | | | | | | | | | |
| ADTRP | | + | | + | | | | | + |
| ADY3 | | | | | | | | | |
| ADY4 | | | | | | | | | |
| AEBP1 | + | | | | + | | | | + |
| AEBP2 | + | | | + | + | | | | + |
| AEN | | | | | | | | | |
| AES | | | | | | + | | | + |
| AFAP1 | | | | | | | | | |
| AFAP1-AS1 | | | | | | | | | |
| AFAP1L1 | | | | | | | | | |
| AFAP1L2 | | | | | | | | | |
| AFD1 | | | | | | | | | |
| AFF1 | | | | | | | | | |
| AFF2 | | | | | | | | | |
| AFF3 | | | | | + | | | | + |
| AFF4 | | | | | + | | | | + |
| AFG3L1P | | | | | | | | | |
| AFG3L2 | | | | | + | | | | + |
| AFM | | | | | | | | | |
| AFMID | | | | | | | | | |
| AFP | | | | | | | | | |
| Afti | | | | | | | | | |
| AFTPH | | | | | | | | | |
| AGA | | | | | | | | | |
| AGAP1 | | | | | | | | | |
| AGAP11 | | | | | | | | | |
| AGAP2 | | | | | | + | | | + |
| AGAP3 | | | + | | | | | | + |
| AGAP4 | | | | | | | | | |
| AGAP5 | | | | | | | | | |
| AGAP6 | | | | | | | | | |

| | | | | | | | | | |
|---|---|---|---|---|---|---|---|---|---|
| AGAP7P | | | | | | | | | |
| AGAP9 | | | | | | | | | |
| AGBL1 | | | | | | | | | |
| AGBL2 | | | | | | | | | |
| AGBL3 | | | | | | + | | | + |
| AGBL4 | | | | | | | | | |
| AGBL5 | | | | | | | | | |
| AGER | | | | | | | | | |
| AGFG1 | | | + | | | | | | + |
| AGFG2 | | | | | | | | | |
| AGGF1 | | | | | | | | | |
| AGK | | | | | | | | | |
| AGL | + | | | + | + | | + | | + |
| AGMAT | | | | | | | | | |
| AGMO | | | | | | | | | |
| AGO1 | | | | | | + | | | + |
| AGO2 | + | | | | + | | | | + |
| AGO3 | + | | | | + | | | | + |
| AGO4 | | | | | | | | | |
| AGPAT1 | | | | | | | | | |
| AGPAT2 | | | | | | | | | |
| AGPAT3 | | | | | | | | | |
| AGPAT4 | | | | | | | | | |
| AGPAT4-IT1 | | | | | | | | | |
| AGPAT5 | | | | | | | | | |
| AGPAT6 | | | | | | | | | |
| AGPAT9 | | | | | | | | | |
| AGPS | | | | | | | | | |
| AGR2 | | | | | | | | | |
| AGR3 | | | | | | | | | |
| Agrn | | | | | | + | | | + |
| AGRP | | | | | | | | | |
| ags1 | | | | | | | | | |
| AGT | | | | | | | | | |
| AGTPBP1 | | | | | | | | | |
| AGTR1 | | | | | | | | | |
| Agtr1a | | | | | | | | | |
| AGTR2 | | | | | | | | | |
| AGTRAP | | | | | | | | | |
| AGXT | | | | | | | | | |
| AGXT2 | | | | | | | | | |
| AHA1 | | | | | | | | | |
| AHC1 | | | | | | | | | |
| AHCTF1 | | | | | | | | | |
| AHCTF1P1 | | | | | | | | | |
| AHCY | | | | | | | | | |
| AHCYL1 | + | | | | + | | | | + |
| AHCYL2 | | | | | | + | | | + |
| AHDC1 | | | | | | | | | |
| AHI1 | | | | | | | | | |
| AHNAK | + | | | | + | | | | + |
| AHNAK2 | | | | | | | | | |
| AHR | | | | | | | | | |

| | | | | | | | | | |
|---|---|---|---|---|---|---|---|---|---|
| AHRR | | | | | | | | | |
| AHSA1 | | | + | | | | | | + |
| AHSA2 | | | | | | | | | |
| AHSG | | | | | | | | | |
| AHSP | | | | | | | | | |
| AI314180 | | | | | + | | | | + |
| AI837181 | | | | | | | | | |
| AICDA | | | | | | | | | |
| AIDA | | | | | + | | | | + |
| AIF1 | | | | | + | | | | + |
| Aif1l | | | | | | | | | |
| AIFM1 | | | | | | | | | |
| AIFM2 | | | | | | | | | |
| AIFM3 | | | | | | | | | |
| AIG1 | | | | | | | | | |
| AIM1 | | | | | | | | | |
| AIM17 | | | | | | | | | |
| AIM1L | | | | | + | | | | + |
| AIM2 | | | | | | | | | |
| AIM46 | | | | | | | | | |
| AIM7 | | | | | | | | | |
| AIM9 | | | | | | | | | |
| AIMP1 | | | | | | | | | |
| AIMP2 | | | | | | | | | |
| AIP | | | | | | | | | |
| AIP1 | | | | | | | | | |
| AIPL1 | | | | | | | | | |
| AIR1 | | | | | | | | | |
| AIR2 | | | | | | | | | |
| AIRE | | | | | | | | | |
| AIRN | | | | | | | | | |
| AJAP1 | | | | | | + | | | + |
| AJUBA | | | | | | | | | |
| AK1 | | | | | | | | | |
| AK2 | | | | | | | | | |
| AK3 | | | | | | | | | |
| AK4 | | | | | | | | | |
| AK4P1 | | | | | | | | | |
| AK5 | | + | | | | + | | | + |
| AK6 | | | | | | | | | |
| AK7 | | | | | | | | | |
| AK8 | | | | | | | | | |
| AK9 | | | | | | | | | |
| AKAP1 | | | | | | + | | | + |
| AKAP10 | | | | | | | | | |
| AKAP11 | | + | | | | + | | | + |
| AKAP12 | | | | | | | | | |
| AKAP13 | | + | | | + | + | | | + |
| AKAP14 | | | | | | | | | |
| AKAP17A | | | | | | | | | |
| AKAP2 | | | | | | | | | |
| AKAP3 | | | | | | | | | |
| AKAP4 | | | | | | | | | |

| | | | | | | | | | |
|---|---|---|---|---|---|---|---|---|---|
| AKAP5 | | | | | | | | | |
| AKAP6 | | | | | | | | | |
| Akap7 | | | | | | | | | |
| AKAP8 | | | | | | | | | |
| AKAP8L | | | | | | | | | |
| AKAP9 | | + | | | | + | | | + |
| AKIP1 | | | | | | | | | |
| AKIRIN1 | | | | | | | | | |
| AKIRIN2 | | | | | | | | | |
| AKNA | | | | | | | | | |
| AKNAD1 | | | | | | | | | |
| AKR1A1 | | + | | | | + | | | + |
| AKR1B1 | | | | | | | | | |
| AKR1B10 | | | | | | | | | |
| AKR1B15 | | | | | | | | | |
| Akr1b3 | | | | | | | | | |
| AKR1C1 | | | | | | | | | |
| AKR1C2 | | | | | | | | | |
| AKR1C3 | | | | | | | | | |
| AKR1C4 | | | | | | | | | |
| AKR1C8P | | | | | | | | | |
| AKR1D1 | | | | | | | | | |
| AKR1E2 | | | | | | | | | |
| AKR7A2 | | | | | | | | | |
| AKR7A3 | | | | | | | | | |
| Akr7a5 | | | | | | | | | |
| AKR7L | | | | | | | | | |
| AKT1 | | | | | + | + | | | + |
| AKT1S1 | | | | | | | | | |
| AKT2 | | | | | | | | | |
| AKT3 | | | | | | + | | | + |
| AKTIP | | | | | | | | | |
| ALA1 | | | | | | | | | |
| ALAD | | | | | | + | | | + |
| ALAS1 | | | | | | | | | |
| ALAS2 | | | | | | | | | |
| ALB | | | | | | | | | |
| ALB1 | | | | | | | | | |
| ALCAM | + | | + | | | | | | + |
| ALD4 | | | | | | | | | |
| ALD6 | | | | | | | | | |
| ALDH16A1 | | | | | | | | | |
| ALDH18A1 | | | + | | | | | | + |
| ALDH1A1 | | | | | | | | | |
| ALDH1A2 | | | | | | | | | |
| ALDH1A3 | | | | | | | | | |
| Aldh1a7 | | | | | | | | | |
| ALDH1B1 | | | | | | | | | |
| ALDH1L1 | | | | | | | | | |
| ALDH1L2 | | | | | | | | | |
| ALDH2 | | | | | | + | | | + |
| ALDH2C4 | | | | | | | | | |
| ALDH3A1 | | | | | | | | | |

| | | | | | | | | | |
|---|---|---|---|---|---|---|---|---|---|
| ALDH3A2 | | | | | | | | | |
| ALDH3B1 | | | | | | | | | |
| ALDH3B2 | | | | | | | | | |
| ALDH4A1 | | | | | | | | | |
| ALDH5A1 | | | | | | | | | |
| ALDH6A1 | | | | | | + | | | + |
| ALDH7A1 | | | | | | | | | |
| ALDH8A1 | | | | | | | | | |
| ALDH9A1 | | | | | | + | | | + |
| ALDOA | | | | | | | | | |
| ALDOB | | | | | | | | | |
| ALDOC | | | | | | | | | |
| ALG1 | | | | | | | | | |
| ALG10 | | | | | | | | | |
| ALG10B | | | | | | | | | |
| ALG11 | | | | | | | | | |
| ALG12 | | | | | | | | | |
| ALG13 | | | | | + | | | | + |
| ALG14 | | | | | | | | | |
| ALG1L | | | | | | | | | |
| ALG1L2 | | | | | | | | | |
| ALG2 | | | | | | | + | | + |
| ALG3 | | | | | | | | | |
| ALG5 | | | | | | | | | |
| ALG6 | | | | | | + | | | + |
| ALG8 | | | | | | | | | |
| ALG9 | | | | | | | | | |
| ALG9-IT1 | | | | | | | | | |
| alien | | | | | | | | | |
| ALK | | | | | | | | | |
| ALKBH1 | | | | | | | | | |
| ALKBH2 | | | | | | | | | |
| ALKBH3 | | | | | | + | | | + |
| ALKBH4 | | | | | | | | | |
| ALKBH5 | | | + | | + | | | | + |
| ALKBH6 | | | | | | | | | |
| ALKBH7 | | | | | | | | | |
| ALKBH8 | | | | | | | | | |
| ALL1 | | | | | | | | | |
| ALLC | | | | | | | | | |
| ALMS1 | | | | | | | | | |
| ALOX12 | | | | + | | | | | + |
| ALOX12B | | + | | | | + | | | + |
| ALOX12P2 | | | | | | | | | |
| ALOX15 | | | | | | | | | |
| ALOX15B | | | | | | | | | |
| ALOX5 | | | | | | | | | |
| ALOX5AP | | | | | | | | | |
| ALOXE3 | | | + | | | | | | + |
| alp14 | | | | | | | | | |
| alp4 | | | | | | | | | |
| alp6 | | | | | | | | | |
| ALPI | | | | | | | | | |

| | | | | | | | | | |
|---|---|---|---|---|---|---|---|---|---|
| ALPK1 | | | | | + | | | | + |
| ALPK2 | | | | | | | | | |
| ALPK3 | | | | | | | | | |
| ALPL | | | | | | | | | |
| ALPP | | | | | | | | | |
| ALPPL2 | | | | | | | | | |
| ALR1 | | | | | | | | | |
| ALS2 | | | | | | | | | |
| ALS2CL | | | | | | | | | |
| ALS2CR11 | | | | | | | | | |
| ALS2CR12 | | | | | | | | | |
| ALX1 | | | | | | | | | |
| ALX3 | | | | | | | | | |
| ALX4 | + | | | + | + | | + | | + |
| ALY1 | | | | | | | | | |
| ALYREF | | | | | | | | | |
| Alyref2 | | | | | | | | | |
| AMACR | | | | | | | | | |
| AMBN | | | | | | | | | |
| AMBP | | | | | | | | | |
| AMBRA1 | | | | | | | | | |
| AMD1 | | | + | | | | | | + |
| AMDHD1 | | | | | | | | | |
| AMDHD2 | | | | | | + | | | + |
| AMELY | | | | | | | | | |
| AMER1 | | | | | | | | | |
| AMER2 | | | | | | | | | |
| AMER3 | | + | | | | + | | | + |
| AMFR | | | | | | | | | |
| AMH | | | | | | | | | |
| AMHR2 | | | | | | | | | |
| AMICA1 | | | | | | | | | |
| AMIGO1 | | | | | | | | | |
| AMIGO2 | | | | | | | | | |
| AMIGO3 | | | | | | | | | |
| AMMECR1 | + | | | + | + | | + | | + |
| AMMECR1L | | | | | | | | | |
| AMN | | | | | | | | | |
| AMN1 | | | | | | | | | |
| AMOT | | | | | | | | | |
| AMOTL1 | + | | | + | + | | | | + |
| AMOTL2 | | | | | | | | | |
| AMPD1 | | | | | | | | | |
| AMPD2 | | | | | | | | | |
| AMPD3 | | | | | | | | | |
| AMPH | | | | | | | | | |
| AMS1 | | | | | | | | | |
| AMT | | | | | | | | | |
| AMTN | | | | | | | | | |
| AMY1A | | | | | | | | | |
| AMY1B | | | | | | | | | |
| AMY1C | | | | | | | | | |
| AMY2A | | | | | | | | | |

| | | | | | | | | | |
|---|---|---|---|---|---|---|---|---|---|
| AMY2B | | | | | | | | | |
| AMZ1 | | | | | | | | | |
| Amz2 | | | | | | | | | |
| AMZ2P1 | | | | | | | | | |
| ANAPC1 | | | | | | | | | |
| ANAPC10 | | | | | | | | | |
| ANAPC11 | | | | | | | | | |
| Anapc13 | | | | | | | | | |
| ANAPC15 | | | | | | | | | |
| Anapc16 | | | | | | | | | |
| ANAPC2 | | | | | | | | | |
| ANAPC4 | | | | | | | | | |
| ANAPC5 | | | | | | | | | |
| ANAPC7 | | | | + | | | | | + |
| anc1 | | | | | | | | | |
| Ance | | | | | | | | | |
| ANG | | | | | | | | | |
| ANGEL1 | | | | | | | | | |
| ANGEL2 | | | | | | | | | |
| ANGPT1 | | | | | | | | | |
| ANGPT2 | + | | | + | + | | | | + |
| ANGPT4 | | | | | | | | | |
| ANGPTL1 | | + | + | | | + | | | + |
| ANGPTL2 | | | | | | | | | |
| ANGPTL3 | | + | + | | | + | | | + |
| ANGPTL4 | | | | | | | | | |
| ANGPTL5 | | | | | | | | | |
| ANGPTL6 | | | | | | | | | |
| ANGPTL7 | | | | | | | | | |
| ANIB1 | | | | | | | | | |
| ANK1 | | | | | | | | | |
| ANK2 | | | | | | | | | |
| ANK3 | | | | | | | | | |
| ANKAR | | | | | | | | | |
| ANKDD1A | | | | | | | | | |
| ANKDD1B | | | | | | | | | |
| ANKEF1 | | | | | | | | | |
| ANKFY1 | | | | + | | | | | + |
| ANKH | | | | | | | | | |
| ANKHD1 | | | | | | | | | |
| ANKHD1-EIF4EBP3 | | | | | | | | | |
| ANKIB1 | | | | + | | | | | + |
| ANKK1 | | | | | | | | | |
| ANKLE1 | | | | | | | | | |
| ANKLE2 | | | | | | | | | |
| ANKMY1 | | | | | | | | | |
| Ankmy2 | | | | | | | | | |
| ANKRA2 | | | | | | | | | |
| ANKRD1 | | | | | | | | | |
| ANKRD10 | | | | | | | | | |
| ANKRD11 | | | | | | | | | |
| ANKRD12 | | | | | | | | | |
| ANKRD13A | | | | | + | | | | + |

| | | | | | | | | | |
|---|---|---|---|---|---|---|---|---|---|
| ANKRD13B | | | | | + | | | | + |
| ANKRD13C | | | | | | | | | |
| ANKRD13D | | | | | | | | | |
| ANKRD16 | | | | | | | | | |
| ANKRD17 | | | | | | | | | |
| ANKRD18A | | | | | | | | | |
| ANKRD18B | | | | | | | | | |
| ANKRD18CP | | | | | | | | | |
| ANKRD19P | | | | | | | | | |
| ANKRD2 | | | | | | | | | |
| ANKRD20A1 | | | | | | | | | |
| ANKRD20A12P | | | | | | | | | |
| ANKRD20A2 | | | | | | | | | |
| ANKRD20A3 | | | | | | | | | |
| ANKRD20A4 | | | | | | | | | |
| ANKRD20A5P | | | | | | | | | |
| ANKRD20A8P | | | | | | | | | |
| ANKRD22 | | | | | | | | | |
| ANKRD23 | | | | | | | | | |
| ANKRD24 | | | | | | | | | |
| Ankrd26 | | | | | | + | | | + |
| ANKRD26P1 | | | | | | | | | |
| ANKRD27 | | | | | | + | | | + |
| ANKRD28 | | | | | | | | | |
| ANKRD29 | | | | | | | | | |
| ANKRD30A | | | | | | | | | |
| ANKRD30B | | | | | | | | | |
| ANKRD30BL | | | | | | | | | |
| ANKRD31 | | | | | | | | | |
| ANKRD32 | | | | | | + | | | + |
| ANKRD33 | | | | | | | | | |
| ANKRD33B | + | | | | + | | | | + |
| ANKRD34A | | | | | | | | | |
| ANKRD34B | | | | | | | | | |
| ANKRD34C | | | | | | | | | |
| ANKRD35 | | | | | | | | | |
| ANKRD36 | | | | | | | | | |
| ANKRD36B | | | | | | | | | |
| ANKRD36BP1 | | | | | | | | | |
| ANKRD36C | | | | | | | | | |
| ANKRD37 | | | | | | | | | |
| ANKRD39 | | | | | | | | | |
| ANKRD40 | | | | | | | | | |
| ANKRD42 | | | | | | | | | |
| ANKRD44 | + | | | + | + | | + | | + |
| ANKRD45 | | | | | | + | + | | + |
| ANKRD46 | | | | + | | + | | | + |
| ANKRD49 | | | | | | | | | |
| ANKRD50 | | | + | | | | | | + |
| ANKRD52 | | | | | | | | | |
| ANKRD53 | | | | | | | | | |
| ANKRD54 | | | | | | + | | | + |
| ANKRD55 | | | | | + | | | | + |

| | | | | | | | | | |
|---|---|---|---|---|---|---|---|---|---|
| ANKRD6 | | | | | | + | | | + |
| ANKRD62 | | | | | | | | | |
| ANKRD65 | | | | | | | | | |
| ANKRD7 | | | | | | | | | |
| ANKRD9 | | | | | | | | | |
| ANKS1A | | | | | | | | | |
| ANKS1B | | | | | | | | | |
| ANKS3 | | | | | | | | | |
| ANKS4B | | | | | | | | | |
| ANKS6 | | | | | | + | | | + |
| ANKUB1 | | | | | | | | | |
| ANKZF1 | | | | | | | | | |
| Anln | | | | | | | | | |
| ANO1 | | | | | | | | | |
| ANO10 | | | | | | | | | |
| ANO2 | | | | | | | | | |
| ANO3 | | + | | | | + | | | + |
| ANO4 | | | | | | | | | |
| ANO5 | | | | | | | | | |
| ANO6 | | | | | | | | | |
| ANO7 | | | | | | | | | |
| ANO8 | + | | | + | + | | | | + |
| ANO9 | | | | | | | | | |
| ANP32A | | | | | | | | | |
| ANP32A-IT1 | | | | | | | | | |
| ANP32B | + | | | + | + | | | | + |
| ANP32C | | | | | | | | | |
| ANP32E | | | + | | | | | | + |
| ANPEP | | | | | | | | | |
| ANTXR1 | | | | | | | | | |
| ANTXR2 | | | | | | | | | |
| ANXA1 | | | | | | | | | |
| ANXA10 | | | | | | | | | |
| ANXA11 | | | | | | | | | |
| ANXA13 | | | | | | | | | |
| ANXA2 | | | | | | | | | |
| ANXA2P2 | | | | | | | | | |
| ANXA2R | | | | | | | | | |
| ANXA3 | | | | | | | | | |
| ANXA4 | | | | | | | | | |
| ANXA5 | | | | | | | | | |
| ANXA6 | | | | | | + | | | + |
| ANXA7 | | | | | | | | | |
| ANXA8 | | | | | | | | | |
| ANXA8L1 | | | | | | | | | |
| ANXA9 | | | | | | | | | |
| AOAH | | | | | | | | | |
| AOC1 | | | | | | | | | |
| AOC2 | | | | | | | | | |
| AOC3 | | + | + | | | + | | | + |
| AOC4P | | | | | | | | | |
| AOX1 | | | | | | | | | |
| AP1AR | | | | | | | | | |

| | | | | | | | | | |
|---|---|---|---|---|---|---|---|---|---|
| AP1B1 | | | | | + | + | | | + |
| AP1G1 | | | + | | | | | | + |
| AP1G2 | | | | | | | | | |
| AP1M1 | | | | | | | | | |
| AP1M2 | | | | | | | | | |
| AP1S1 | | | | | | | | | |
| AP1S2 | | | | | | | | | |
| AP1S3 | | | | | | | | | |
| AP2A1 | | | | | | | | | |
| AP2A2 | | | | | + | | | | + |
| AP2B1 | + | | | | + | | | | + |
| AP2M1 | | | | | | | | | |
| AP2S1 | | | | | | | | | |
| AP3B1 | | | | | | | | | |
| AP3B2 | | | | | + | | | | + |
| AP3D1 | | | | | | | | | |
| AP3M1 | | | | | | | | | |
| AP3M2 | | | | | | | | | |
| AP3S1 | | | | | | | | | |
| AP3S2 | | | | | | + | | | + |
| AP4B1 | | | | | | | | | |
| AP4E1 | | | | | | | | | |
| AP4M1 | | | | | | + | | | + |
| AP4S1 | | | | | | | | | |
| AP5B1 | | | | | | | | | |
| AP5M1 | | | | | | | | | |
| AP5S1 | | | | | | | | | |
| AP5Z1 | | | | | | | | | |
| APAF1 | | | | | | + | | | + |
| APBA1 | | | | | | | | | |
| APBA2 | | | | | | | | | |
| APBA3 | | | | | | | | | |
| APBB1 | | | | | | | | | |
| APBB1IP | | + | | + | | | | | + |
| APBB2 | | | | | | | | | |
| APBB3 | | | | | | | | | |
| APC | | | | | | | | | |
| APC11 | | | | | | | | | |
| APC2 | | | | | | | | | |
| APCDD1 | | | | | | | | | |
| APCDD1L | | | | | | | | | |
| APCS | | | | | | | | | |
| APE1 | | | | | | | | | |
| APE2 | | | | | | | | | |
| APE3 | | | | | | | | | |
| APEH | | | | | | | | | |
| APEX1 | | | | | | | | | |
| APEX2 | | | | | | | | | |
| APH1A | | | | | | | | | |
| APH1B | | | | | | | | | |
| API5 | | | | | | | | | |
| API5P1 | | | | | | | | | |
| APIP | | | | | | | | | |

| | | | | | | | | | |
|---|---|---|---|---|---|---|---|---|---|
| APITD1 | | | | | | | | | |
| APITD1-CORT | | | | | | | | | |
| APJ1 | | | | | | | | | |
| apl3 | | | | | | | | | |
| APL4 | | | | | | | | | |
| APL5 | | | | | | | | | |
| APL6 | | | | | | | | | |
| APLF | | | | | | | | | |
| APLN | | | | | | | | | |
| APLNR | | | | | | | | | |
| APLP1 | | | | | | | + | | + |
| APLP2 | | | | | | | | | |
| APM2 | | | | | | | | | |
| APM4 | | | | | | | | | |
| APMAP | | | | | | | | | |
| APOA1 | | | | | | | | | |
| APOA1BP | | | | | | | | | |
| APOA2 | | | | | | | | | |
| APOA4 | | | | | | | | | |
| APOA5 | | | | | | | | | |
| APOB | | | | | | | | | |
| APOBEC1 | | | | | | | | | |
| APOBEC2 | | | | | | | | | |
| APOBEC3A | | | | | | | | | |
| APOBEC3B | | | | | | | | | |
| APOBEC3C | | | | | | | | | |
| APOBEC3D | | | | | | | | | |
| APOBEC3F | | | | | | | | | |
| APOBEC3G | | | | | | | | | |
| APOBEC3H | | | | | | | | | |
| APOBEC4 | | + | + | | | + | | | + |
| APOBR | | | | | | | | | |
| APOC1 | | | | | | | | | |
| APOC2 | | | | | | | | | |
| APOC3 | | | | | | | | | |
| APOC4 | | | | | | | | | |
| APOD | | | | | + | | | | + |
| APOE | | | | | | | | | |
| APOF | | | | | | | | | |
| APOH | | | | | | | | | |
| APOL1 | | | | | | | | | |
| APOL2 | | | | | | | | | |
| APOL3 | | | | | | | | | |
| APOL4 | | | | | | | | | |
| APOL5 | | | | | | | | | |
| APOL6 | | | | | | | | | |
| APOLD1 | | | | | | | + | | + |
| APOM | | | | | | | | | |
| APOO | | | | | | | | | |
| APOOL | | | | | | | | | |
| APOPT1 | | | | | | | | | |
| APP | | | | | | | + | | + |
| APP1 | | | | | | | | | |

| | | | | | | | | | |
|---|---|---|---|---|---|---|---|---|---|
| APPBP2 | | | | | | | | | |
| APPL1 | | | + | | | | | | + |
| APPL2 | | | | | | + | | | + |
| APRT | | | | | | | | | |
| APTX | | | | | | | | | |
| AQN-1 | | | | | | | | | |
| AQP1 | | | | | | | | | |
| AQP10 | | | | | | | | | |
| AQP11 | | | | | | | | | |
| AQP12B | | | | | | | | | |
| AQP2 | | | | | | | | | |
| AQP3 | + | | | + | + | | | | + |
| AQP4 | | | | | | | | | |
| AQP4-AS1 | | | | | | | | | |
| AQP5 | | | | | | | | | |
| AQP6 | | | | | | | | | |
| AQP7 | + | | | + | + | | | | + |
| AQP7P3 | | | | | | | | | |
| AQP8 | | | | | | | | | |
| AQP9 | | | | | | | | | |
| AQPEP | | | | | | | | | |
| AQR | | | | | | | | | |
| AQY1 | | | | | | | | | |
| AR | | | | | | | | | |
| ARAF | | + | | | | + | | | + |
| ARAP1 | | | | | | | | | |
| ARAP1-AS2 | | | | | | | | | |
| ARAP2 | | | | | | | | | |
| ARAP3 | | | | | | | | | |
| ARC | + | | | | + | | | | + |
| ARC35 | | | | | | | | | |
| arc4 | | | | | | | | | |
| ARC40 | | | | | | | | | |
| ARCN1 | | | | | | | | | |
| ARD1 | | | | | | | | | |
| ARE2 | | | | | | | | | |
| AREG | | | | | | | | | |
| AREL1 | | | | | | | | | |
| ARF1 | | | | | | | | | |
| ARF3 | | | | | | | | | |
| ARF4 | | | | | | | | | |
| ARF5 | + | | | + | + | | | | + |
| ARF6 | | | + | | | | | | + |
| ARFGAP1 | | | | | | | | | |
| ARFGAP2 | | | | | | | | | |
| ARFGAP3 | | | | | | | | | |
| ARFGEF1 | | | | | | | | | |
| ARFGEF2 | | | | | | | | | |
| ARFIP1 | | | | | | | | | |
| ARFIP2 | | | | | | | | | |
| ARFRP1 | | | | | | | | | |
| ARG1 | | | | | | | | | |
| arg11 | | | | | | | | | |

| | | | | | | | | | |
|---|---|---|---|---|---|---|---|---|---|
| arg12 | | | | | | | | | |
| ARG2 | | | | | | | | | |
| ARG3 | | | | | | | | | |
| ARG4 | | | | | | | | | |
| arg5 | | | | | | | | | |
| ARG5,6 | | | | | | | | | |
| ARGFX | | | | | | | | | |
| ARGLU1 | + | | + | + | + | | | | + |
| ARHGAP1 | | | | | | | | | |
| ARHGAP10 | | | | | | | | | |
| ARHGAP11A | | | | | | | | | |
| ARHGAP11B | | | | | | | | | |
| ARHGAP12 | | | | | | + | | | + |
| ARHGAP15 | | | | | | | | | |
| ARHGAP17 | | | | | | | | | |
| Arhgap18 | | | + | | | | | | + |
| ARHGAP19 | | | | | | | | | |
| ARHGAP20 | | + | + | | | + | | | + |
| ARHGAP21 | | + | | | | + | | | + |
| ARHGAP22 | | | | | | | | | |
| ARHGAP23 | | | | | | | | | |
| ARHGAP24 | | | | | | | | | |
| ARHGAP25 | | | | | | | | | |
| ARHGAP26 | | | | | | | | | |
| ARHGAP27 | | | | | | | | | |
| ARHGAP28 | | | | | | | | | |
| ARHGAP29 | | | | | | | | | |
| ARHGAP30 | | | | | | | | | |
| ARHGAP31 | | | | | | | | | |
| ARHGAP32 | | | + | | + | | | | + |
| ARHGAP33 | | | | | | | | | |
| ARHGAP35 | | | | | | | | | |
| ARHGAP36 | | | | | | | | | |
| ARHGAP39 | | | | | + | + | | | + |
| ARHGAP4 | | | | | | | | | |
| ARHGAP40 | | | | | | | | | |
| ARHGAP42 | | | | | | | | | |
| ARHGAP44 | | | | | | | | | |
| ARHGAP5 | | | | | | | | | |
| Arhgap6 | | | | | | | | | |
| ARHGAP8 | | | | | | | | | |
| ARHGAP9 | | | | | | | | | |
| ARHGDIA | | | | | | | | | |
| ARHGDIB | | | | | | | | | |
| ARHGDIG | | | | | + | | | | + |
| ARHGEF1 | | | | | | | | | |
| ARHGEF10 | | | | | | | | | |
| ARHGEF10L | | | | | | | | | |
| ARHGEF11 | | | | | | | | | |
| ARHGEF12 | | | | | | | | | |
| ARHGEF15 | | | | | | | | | |
| ARHGEF16 | | | | | | | | | |
| Arhgef17 | | | | | | | | | |

| | | | | | | | | | |
|---|---|---|---|---|---|---|---|---|---|
| ARHGEF18 | | | | | | | | | |
| ARHGEF19 | | | | | | | | | |
| ARHGEF2 | | | | | | | | | |
| ARHGEF25 | | | | | | | | | |
| ARHGEF26 | | | | | | | | | |
| ARHGEF28 | | | | | | | | | |
| ARHGEF3 | | | | | | | | | |
| ARHGEF33 | | | | | | | | | |
| ARHGEF34P | | | | | | | | | |
| ARHGEF35 | | | | | | | | | |
| ARHGEF37 | | | | | | | | | |
| ARHGEF38 | | | | | | | | | |
| ARHGEF39 | | | | | | | | | |
| ARHGEF4 | | | | | | | | | |
| ARHGEF40 | | | | | | | | | |
| ARHGEF5 | | | | | | | | | |
| ARHGEF6 | | | | | | | | | |
| ARHGEF7 | + | | | | + | | | | + |
| ARHGEF9 | | | | | | | | | |
| ARID1A | | | | | + | | | | + |
| ARID1B | | | | | | | | | |
| ARID2 | | | | | | | | | |
| ARID3A | + | | | + | + | | | | + |
| ARID3B | | | | | | | | | |
| ARID3C | | | | | | | | | |
| ARID4A | | | | | | | | | |
| ARID4B | | | | | | | | | |
| ARID5A | | | | | + | | | | + |
| ARID5B | + | | + | | + | | | | + |
| ARIH1 | | | + | | | | | | + |
| ARIH2 | | | | | + | | | | + |
| ARIH2OS | | | | | | | | | |
| ark1 | | | | | | | | | |
| arl-13 | | | | | | | | | |
| ARL1 | | + | | | | + | | | + |
| ARL10 | | | | | | | | | |
| ARL11 | | | | | | | | | |
| ARL13A | | | | | | | | | |
| ARL13B | | | | | | | | | |
| ARL14 | | | | | | | | | |
| ARL14EP | | | | | | | | | |
| ARL14EPL | + | | | + | + | | | | + |
| ARL15 | | | | | | | | | |
| ARL16 | | | | | | + | | | + |
| ARL17A | | | | | | | | | |
| ARL2 | | | | | | | | | |
| ARL2-SNX15 | | | | | | | | | |
| ARL2BP | | | + | | | | | | + |
| ARL3 | | | | | | | | | |
| ARL4A | | | + | | | | | | + |
| ARL4C | | | | | | | | | |
| ARL4D | | | | | | | | | |
| ARL5A | | | | | | | | | |

| | | | | | | | | | |
|---|---|---|---|---|---|---|---|---|---|
| ARL5B | + | | + | | + | | | | + |
| ARL6 | | | | | | | | | |
| ARL6IP1 | | + | | | | + | | | + |
| ARL6IP4 | | | | | | | | | |
| ARL6IP5 | | | | | | | | | |
| ARL6IP6 | | | | | | | | | |
| ARL8A | | | | | | | | | |
| ARL8B | | + | | | | + | | | + |
| ARL9 | | | | | | | | | |
| ARMC1 | | | | | | | | | |
| ARMC10 | | | | | | | | | |
| ARMC12 | | | | | | | | | |
| ARMC2 | | | | | | + | | | + |
| ARMC3 | | | | | | | | | |
| ARMC4 | | | | | | | | | |
| ARMC5 | | | | | | | | | |
| ARMC6 | | | | | | | | | |
| ARMC7 | | | | | | | | | |
| ARMC8 | | | | | | | | | |
| ARMC9 | | | | | | + | | | + |
| ARMCX1 | | | | | | + | | | + |
| ARMCX2 | | | | | | | | | |
| ARMCX3 | | | | | | | | | |
| ARMCX4 | | | | | | | | | |
| ARMCX5 | | | + | | | | | | + |
| ARMCX5-GPRASP2 | | | | | | | | | |
| ARMCX6 | | | | | | | | | |
| ARMS2 | | | | | | | | | |
| ARNT | | + | | | | + | | | + |
| ARNT2 | | | | | | | | | |
| ARNTL | | | | | | | | | |
| ARNTL2 | | | | | | | | | |
| ARO1 | | | | | | | | | |
| ARO10 | | | | | | | | | |
| ARO2 | | | | | | | | | |
| ARO4 | | | | | | | | | |
| ARO8 | | | | | | | | | |
| ARP2 | | | | | | | | | |
| ARP3 | | | | | | | | | |
| ARP9 | | | | | | | | | |
| ARPC1A | | | | | | | | | |
| ARPC1B | + | | | + | + | | + | | + |
| ARPC2 | + | | | | + | | | | + |
| ARPC3 | | | | | | | | | |
| ARPC3P1 | | | | | | | | | |
| ARPC4 | | | | | | | | | |
| ARPC4-TTLL3 | | | | | | | | | |
| ARPC5 | | | | | | | | | |
| ARPC5L | | | | | + | | | | + |
| ARPIN | | | | | | | | | |
| ARPP19 | + | | | | + | | | | + |
| ARPP21 | | | + | | + | | | | + |
| ARR3 | | | | | | | | | |

| | | | | | | | | | |
|---|---|---|---|---|---|---|---|---|---|
| ARRB1 | | | | | | | | | |
| ARRB2 | | | | | | | | | |
| ARRDC1 | | | | | + | | | | + |
| ARRDC1-AS1 | | | | | | | | | |
| ARRDC2 | | | | | | | | | |
| ARRDC3 | | | | | | | | | |
| ARRDC4 | | | | | | | | | |
| ARRDC5 | | | | | | | | | |
| ARSA | | | | | | + | | | + |
| ARSB | | | | | | + | | | + |
| ARSD | | | | | | | | | |
| ARSE | | | | | | | | | |
| ARSF | | | | | | | | | |
| ARSG | | | | | | | | | |
| ARSH | | | | | | | | | |
| ARSI | + | | | + | + | | | | + |
| ARSJ | | | | | | | | | |
| ARSK | | | | | | | | | |
| ART1 | | | | | | | | | |
| ART3 | | | | | | | | | |
| ART4 | | | | | | | | | |
| ART5 | | | | | | | | | |
| ARTN | | | | | | | | | |
| ARV1 | | | | | | | | | |
| ARVCF | | | | | | | | | |
| ARVD4 | | | | | | | | | |
| ARX | | | | | | | | | |
| Arxes1 | | | | | | | | | |
| AS3MT | | | | | | | | | |
| ASAH1 | | | | | | | | | |
| ASAH2 | | | | | | | | | |
| ASAH2B | | | | | | | | | |
| ASAP1 | | | | | | | | | |
| ASAP2 | | | | | + | | | | + |
| ASAP3 | | | | | | | | | |
| ASB1 | | | | | | | | | |
| ASB10 | | | | | | | | | |
| ASB11 | | | | | + | | | | + |
| ASB12 | | | | | | | | | |
| ASB13 | | | | | | | | | |
| ASB14 | | | | | | | | | |
| ASB15 | | | | | | | | | |
| ASB16 | | | | | | | | | |
| ASB16-AS1 | | | | | | | | | |
| ASB17 | | | | | | | | | |
| ASB18 | | | | | | | | | |
| ASB2 | | | | | | | | | |
| ASB3 | | | | | | | | | |
| ASB4 | | | | | | | | | |
| ASB5 | | | | | | | | | |
| ASB6 | | | | | + | | | | + |
| ASB7 | | | | | | | | | |
| ASB8 | | | | | | + | | | + |

| | | | | | | | | | |
|---|---|---|---|---|---|---|---|---|---|
| ASB9 | | | | | | | | | |
| ASC1 | | | | | | | | | |
| ASCC1 | | | | | | + | | | + |
| ASCC2 | | | | | | | | | |
| ASCC3 | | | + | | | | | | + |
| ASCL1 | | | | | | | | | |
| ASCL2 | | | | | | | | | |
| ASCL3 | | | | | | | | | |
| ASCL4 | | | | | | | | | |
| ASCL5 | | | | | | | | | |
| ASF1 | | | | | | | | | |
| ASF1A | | | + | | | | | | + |
| ASF1B | | | | | | | | | |
| ASG1 | | | | | | | | | |
| ASGR1 | | | | | | | | | |
| ASGR2 | | | | | | | | | |
| ASH1L | | | | + | | | | | + |
| ASH1L-AS1 | | | | | | | | | |
| ASH2L | | | | + | | | | | + |
| ASI1 | | | | | | | | | |
| ASIC1 | | | | | | | | | |
| ASIC2 | | | + | | | | | | + |
| ASIC3 | | | | | | | | | |
| ASIC4 | + | | + | | + | | | | + |
| ASIC5 | | | | | | | | | |
| ASIP | | | | | | | | | |
| ASL | | | | | | | | | |
| asl1 | | | | | | | | | |
| ASM4 | | | | | | | | | |
| ASMT | | | | | | | | | |
| ASMTL | | | | | | | | | |
| ASMTL-AS1 | | | | | | | | | |
| ASN1 | | | | | | | | | |
| ASN2 | | | | | | | | | |
| ASNA1 | | | + | | | | | | + |
| ASNS | | | | | | | | | |
| ASNSD1 | | | | | | + | | | + |
| asp | | | | | | | | | |
| ASP1 | | | | | | | | | |
| ASPA | | | | | | | | + | + |
| ASPDH | | | | | | | | | |
| ASPG | | | | | | | | | |
| ASPH | | | | | | | | | |
| ASPHD1 | | | | | | | | | |
| ASPHD2 | | | | | | | | | |
| ASPM | | | | | | | | | |
| ASPN | | | | | | | | | |
| ASPRV1 | | | | | | | | | |
| ASPSCR1 | | | | | | | | | |
| ASR1 | | | | | | | | | |
| ASRGL1 | | | | + | | | | | + |
| ASS1 | | | | | | | | | |
| ASTE1 | | | | | | | | | |

| | | | | | | | | | |
|---|---|---|---|---|---|---|---|---|---|
| ASTL | | | | | | | | | |
| ASTN1 | | | | | | + | | | + |
| ASTN2 | | | | | | | | | |
| ASUN | | | | | | | | | |
| ASXL1 | | | + | | | | | | + |
| ASXL2 | | | | | | | | | |
| ASXL3 | | | | | | | | | |
| ASZ1 | | | | | | | | | |
| AT1G04360 | | | | | | | | | |
| AT1G04410 | | | | | | | | | |
| AT1G07920 | | | | | | | | | |
| AT1G07930 | | | | | | | | | |
| AT1G07940 | | | | | | | | | |
| AT1G09310 | | | | | | | | | |
| AT1G18030 | | | | | | | | | |
| AT1G34750 | | | | | | | | | |
| AT1G43900 | | | | | | | | | |
| AT1G59660 | | | | | | | | | |
| AT1G79630 | | | | | | | | | |
| AT1G80930 | | | | | | | | | |
| AT2G01720 | | | | | | | | | |
| AT2G04520 | | | | | | | | | |
| AT2G25070 | | | | | | | | | |
| AT2G30170 | | | | | | | | | |
| AT2G30620 | | | | | | | | | |
| AT2G31610 | | | | | | | | | |
| AT2G36950 | | | | | | | | | |
| AT2G40650 | | | | | | | | | |
| AT2G47610 | | | | | | | | | |
| AT3G02750 | | | | | | | | | |
| AT3G03773 | | | | | | | | | |
| AT3G05640 | | | | | | | | | |
| AT3G05900 | | | | | | | | | |
| AT3G06270 | | | | | | | | | |
| AT3G08530 | | | | | | | | | |
| AT3G09440 | | | | | | | | | |
| AT3G11130 | | | | | | | | | |
| AT3G12630 | | | | | | | | | |
| AT3G15260 | | | | | | | | | |
| AT3G17250 | | | | | | | | | |
| AT3G49660 | | | | | | | | | |
| AT3G50520 | | | | | | | | | |
| AT3G53230 | | | | | | | | | |
| AT3G53870 | | | | | | | | | |
| AT3G62870 | | | | | | | | | |
| AT4G24110 | | | | | | | | | |
| AT4G24690 | | | | | | | | | |
| AT4G28400 | | | | | | | | | |
| AT4G31860 | | | | | | | | | |
| AT4G36130 | | | | | | | | | |
| AT4G36820 | | | | | | | | | |
| AT4G38520 | | | | | | | | | |
| AT5G02490 | | | | | | | | | |

| | | | | | | | | | |
|---|---|---|---|---|---|---|---|---|---|
| AT5G03340 | | | | | | | | | |
| AT5G04120 | | | | | | | | | |
| AT5G06750 | | | | | | | | | |
| AT5G10740 | | | | | | | | | |
| AT5G24940 | | | | | | | | | |
| AT5G35210 | | | | | | | | | |
| AT5G35530 | | | | | | | | | |
| AT5G35680 | | | | | | | | | |
| AT5G46630 | | | | | | | | | |
| AT5G53140 | | | | | | | | | |
| AT5G60390 | | | | | | | | | |
| AT5G66080 | | | | | | | | | |
| ATAD1 | | | | | | | | | |
| ATAD2 | | | | | | | | | |
| ATAD2B | | | | | | | | | |
| ATAD3A | | | | | | | | | |
| ATAD3B | | | | | | | | | |
| ATAD3C | | | | | | | | | |
| ATAD5 | | | | | | | | | |
| ATAT1 | | | | | | | | | |
| ATCAY | | | + | | | | | | + |
| atd1 | | | | | | | | | |
| ATE1 | | + | | | | + | | | + |
| ATF1 | | | | | | | | | |
| ATF2 | | | + | | | | | | + |
| ATF3 | + | | | | + | | | | + |
| ATF4 | | | + | | + | | | | + |
| ATF5 | | | | | | | | | |
| ATF6 | | | | | | | | | |
| ATF6B | | | | | | | | | |
| ATF7 | | | | | | | | | |
| ATF7IP | | | | | | | | | |
| ATF7IP2 | | | | | | | | | |
| ATG1 | | | | | | | | | |
| ATG10 | | | | | | | | | |
| ATG101 | | | | | | | | | |
| ATG11 | | | | | | | | | |
| ATG12 | | | + | | | | | | + |
| ATG13 | | | | | | | | | |
| ATG14 | | | | | | | | | |
| ATG15 | | | | | | | | | |
| ATG16L1 | | | | | | | | | |
| ATG16L2 | | | + | | | | | | + |
| ATG18 | | | | | | | | | |
| ATG19 | | | | | | | | | |
| ATG21 | | | | | | | | | |
| ATG23 | | | | | | | | | |
| ATG2A | | | | | | | | | |
| ATG2B | | | | | | | | | |
| ATG3 | | | | | | | | | |
| ATG32 | | | | | | | | | |
| ATG4 | | | | | | | | | |
| ATG4A | | | | | | | | | |

| | | | | | | | | | |
|---|---|---|---|---|---|---|---|---|---|
| ATG4B | | | | | | + | | | + |
| ATG4C | | | | | | + | | | + |
| ATG4D | | | | | | | | | |
| ATG5 | | | | | | | | | |
| ATG7 | | | | | | | | | |
| ATG8 | | | | | | | | | |
| ATG9A | | | | | | | | | |
| ATG9B | | | | | | | | | |
| ATHL1 | | | | | | | | | |
| ATIC | | | | | | | | | |
| ATL1 | | + | | | + | + | | | + |
| ATL2 | | | | | | | | | |
| ATL3 | | | | | | | | | |
| ATM | | | | | | | | | |
| ATMIN | | | | | | | | | |
| ATMS1 | | | | | | | | | |
| ATN1 | | | | | | | | | |
| ATOH1 | | | | | | | | | |
| ATOH7 | | | | | | | | | |
| ATOH8 | | | | | | | | | |
| ATOX1 | | | | | | | | | |
| ATP1 | | | | | | | | | |
| ATP10 | | | | | | | | | |
| ATP10A | | | | | | | | | |
| ATP10B | | | | | | | | | |
| ATP10D | | | + | | | | | | + |
| ATP11 | | | | | | | | | |
| ATP11A | | | | | | | | | |
| ATP11B | | | | | | | | | |
| ATP11C | | | | | | | | | |
| ATP12A | | | | | | | | | |
| ATP13A1 | | | | | | | | | |
| ATP13A2 | | | | | | | | | |
| ATP13A3 | | | + | | | | | | + |
| ATP13A4 | | | | | | | | | |
| ATP13A5 | | | | | | | | | |
| ATP1A1 | | | + | | | | | | + |
| ATP1A2 | | | | + | | | | | + |
| ATP1A3 | | | | | + | | | | + |
| ATP1A4 | | + | + | | | + | | | + |
| ATP1B1 | | | + | | + | | | | + |
| ATP1B2 | | | | | | + | | | + |
| ATP1B3 | | | | | | | | | |
| ATP1B4 | | | | | | | | | |
| ATP2 | | | | | | | | | |
| ATP2A1 | | | | | | | | | |
| ATP2A2 | | | + | | | | | | + |
| ATP2A3 | | | | | | | | | |
| ATP2B1 | | | | | | | | | |
| ATP2B2 | | + | | | | + | | | + |
| ATP2B3 | | | | | | | | | |
| ATP2B4 | | | | | | + | | | + |
| ATP2C1 | | | + | | | | | | + |

| | | | | | | | | | |
|---|---|---|---|---|---|---|---|---|---|
| ATP2C2 | | | | | | | | | |
| ATP3 | | | | | | | | | |
| ATP4 | | | | | | | | | |
| ATP4A | | | | | | | | | |
| ATP4B | | | | | | | | | |
| ATP5 | | | | | | | | | |
| ATP5A1 | | | | | | | | | |
| ATP5B | | | | | | | | | |
| ATP5C1 | | + | | | | + | | | + |
| ATP5D | | | | | + | | | | + |
| ATP5E | | | | | | | | + | + |
| ATP5EP2 | | | | | | | | | |
| ATP5F1 | | | | | | | | | |
| ATP5G1 | | | | | | | | | |
| ATP5G2 | | | | | | + | | | + |
| ATP5G3 | | | | | | + | | | + |
| ATP5H | | | | | | | | | |
| ATP5I | | | | | | | | | |
| ATP5J | | | | | | | | | |
| ATP5J2 | | + | | | | + | | | + |
| ATP5J2-PTCD1 | | | | | | | | | |
| ATP5L | | | | | | | | | |
| ATP5L2 | | | | | | | | | |
| ATP5O | | | | | | | | | |
| ATP5S | | | | | | | | | |
| ATP5SL | | | | | | | | | |
| ATP6 | | | | | | | | | |
| ATP6AP1 | | | | | | | | | |
| ATP6AP2 | | | | | | | | | |
| ATP6V0A1 | | | | | | | | | |
| ATP6V0A2 | | | | | | | | | |
| ATP6V0A4 | | | | | | | | | |
| ATP6V0B | | | | | | + | | + | + |
| ATP6V0C | | + | + | | | + | | + | + |
| ATP6V0D1 | | | | | | | | | |
| ATP6V0D2 | + | | | + | + | | + | | + |
| Atp6v0e | | | | | | | | | |
| ATP6V0E1 | | | | | | | | | |
| ATP6V0E2 | | | | | | + | | | + |
| ATP6V1A | | | | | | + | | | + |
| ATP6V1B1 | | | | | | | | | |
| ATP6V1B2 | | | | | + | | | | + |
| ATP6V1C1 | | | | | | | | | |
| ATP6V1C2 | | | | | | | | | |
| ATP6V1D | | + | | | | + | | | + |
| ATP6V1E1 | | | | | | | | | |
| ATP6V1E2 | | | | | | | | | |
| ATP6V1F | | | | | | | | | |
| ATP6V1G1 | + | | + | | | | | | + |
| ATP6V1G2 | | + | + | | | + | | | + |
| ATP6V1G2-DDX39B | | | | | | | | | |
| ATP6V1G3 | | + | + | | | + | | | + |
| ATP6V1H | | | | | | | | | |

| | | | | | | | | | |
|---|---|---|---|---|---|---|---|---|---|
| ATP7 | | | | | | | | | |
| ATP7A | | | + | | + | | | | + |
| ATP7B | | | | | | | | | |
| ATP8 | | | | | | | | | |
| ATP8A1 | | + | + | | | + | | | + |
| ATP8A2 | | | | | | | | | |
| ATP8B1 | | | | | | | | | |
| Atp8b2 | | | | | | | | | |
| ATP8B3 | | | | | | | | | |
| ATP8B4 | + | | | + | + | | + | | + |
| ATP9A | | | | | | + | | | + |
| ATP9B | | | | | | | | | |
| ATPAF1 | | | | | | | | | |
| ATPAF2 | | | | | | | | | |
| ATPIF1 | | + | | | | + | | | + |
| ATR | | | | | | | | | |
| ATRAID | | | | | | | | | |
| ATRIP | | | | | | | | | |
| ATRN | | | | | | | | | |
| ATRNL1 | | | | | | | | | |
| ATRX | | | | | | | | | |
| ATXN1 | | | + | | | | | | + |
| ATXN10 | | | | | | | | | |
| ATXN1L | | | | | | | | | |
| ATXN2 | | | | | + | | | | + |
| ATXN2L | | | | | | | | | |
| ATXN3 | | | | | | | | | |
| ATXN3L | | | | | | | | | |
| ATXN7 | | | + | | | | | | + |
| ATXN7L1 | | | | | | | | | |
| ATXN7L2 | | | | | | | | | |
| ATXN7L3 | | | | | | | | | |
| ATXN7L3B | | | | | | | | | |
| AUH | | | + | | | | | | + |
| AUNIP | | | | | | | | | |
| AUP1 | | | | | | | | | |
| AURKA | | | | | | | | | |
| AURKAIP1 | | + | | | | + | | | + |
| AURKB | | | | | | | | | |
| AURKC | | | | | | | | | |
| aut12 | | | | | | | | | |
| AUTS2 | | | | | | | | | |
| AVEN | | | | | | | | | |
| AVIL | | | | | | | | | |
| AVL9 | | | | | + | + | | | + |
| AVP | | | | | | | | | |
| AVPI1 | | | | | | | | | |
| AVPR1A | | | | | | | | | |
| AVPR1B | | | | | | | | | |
| AVPR2 | | | | | | | | | |
| AVT6 | | | | | | | | | |
| AVT7 | | | | | | | | | |
| AWAT2 | | | | | | | | | |

| | | | | | | | | | |
|---|---|---|---|---|---|---|---|---|---|
| AXDND1 | | | | | | | | | |
| AXIN1 | | | | | | | | | |
| AXIN2 | | | | | | | | | |
| AXL | | | | | | | | | |
| axl-1 | | | | | | | | | |
| AYR1 | | | | | | | | | |
| AZGP1 | | | | | | | | | |
| AZI2 | | | | | | | | | |
| AZIN1 | | | | | | | | | |
| AZIN2 | | | | | | | | | |
| AZU1 | | | | | | | | | |
| B1R | | | | | | | | | |
| B2M | | + | + | | | + | | + | + |
| B3GALNT1 | | | | | | + | | | + |
| B3GALNT2 | | | | | | | | | |
| B3GALT1 | | | | | | + | | | + |
| B3GALT2 | | | | + | | + | | | + |
| B3GALT4 | | | | | | | | | |
| B3GALT5 | | + | | | | + | | | + |
| B3GALT6 | | | | | | | | | |
| B3GALTL | | | | | | | | | |
| B3GAT1 | | | | | | + | | | + |
| B3GAT2 | | | | | | | | | |
| B3GAT3 | | | | | | | | | |
| B3GNT2 | | | | | | | | | |
| B3GNT3 | | | | | | | | | |
| B3GNT4 | | | | | | | | | |
| B3GNT5 | | | | | | | | | |
| B3GNT7 | | | | | | | | | |
| B3GNT8 | | | | | | | | | |
| B3GNT9 | | | | | | | | | |
| B3GNTL1 | | | | | | | | | |
| B4GALNT1 | | | | | | | | | |
| B4GALNT2 | | | | | | | | | |
| B4GALNT3 | | | | | | | | | |
| B4GALNT4 | | | | | | | | | |
| B4GALT1 | | | | | + | | | | + |
| B4GALT1-AS1 | | | | | | | | | |
| B4GALT2 | | | | | + | | | | + |
| B4GALT3 | | | | | | + | | | + |
| B4GALT4 | | | | | | | | | |
| B4GALT5 | | | | | | | | | |
| B4GALT6 | | | + | | | | | | + |
| B4GALT7 | | | | | | | | | |
| B4GAT1 | | | | | | | | | |
| B9D1 | | | | | | | | | |
| B9D2 | | | | | | | | | |
| BAALC | | | | | | | | | |
| BAALC-AS2 | | | | | | | | | |
| BAAT | | | | | | | | | |
| BABAM1 | | | | | | | | | |
| BACE1 | | | | | | | | | |
| BACE2 | | | | | | | | | |

| | | | | | | | | | |
|---|---|---|---|---|---|---|---|---|---|
| BACH1 | | | | | | | | | |
| BACH2 | | | + | | | | | | + |
| BAD | | | | | | | | | |
| BAG1 | | | + | | | | | | + |
| bag101 | | | | | | | | | |
| bag102 | | | | | | | | | |
| BAG2 | | | | | | | | | |
| BAG3 | + | | | | + | | | | + |
| BAG4 | | | | | | | | | |
| BAG5 | | | | | | | | | |
| BAG6 | | | | | | | | | |
| BAG7 | | | | | | | | | |
| BAGE | | | | | | | | | |
| BAGE2 | | | | | | | | | |
| BAHCC1 | | | | | | | | | |
| BAHD1 | | | | | | | | | |
| BAI1 | | | | | + | | | | + |
| BAI2 | | | | | | | | | |
| BAI3 | | | | | | | | | |
| BAIAP2 | + | | + | | + | | | | + |
| BAIAP2L1 | | | | | | | | | |
| BAIAP2L2 | | | | | | | | | |
| BAIAP3 | + | | | + | + | | | | + |
| BAK1 | | | | | | | | | |
| bam | | | | | | | | | |
| BAMBI | | | | | | | | | |
| BANF1 | | | | | | | | | |
| BANF2 | | | | | | | | | |
| BANK1 | | | | | | | | | |
| Banp | | + | | | | + | | | + |
| BAP1 | | | + | | | | | | + |
| BAP2 | | | | | | | | | |
| BARD1 | | | | | | | | | |
| BARHL1 | | | | | | | | | |
| BARHL2 | + | | | + | + | | | | + |
| BARX1 | | | | | | | | | |
| BARX2 | | | | | | | | | |
| BASP1 | | | | | | | | | |
| BAT1 | | | | | | | | | |
| BATF | | | | | | | | | |
| BATF2 | | | | | | | | | |
| BATF3 | | | | | | | | | |
| BAX | | | | | | | | | |
| BAZ1A | + | | + | | + | | | | + |
| BAZ1B | | | | | | | | | |
| BAZ2A | | | | | | | | | |
| BAZ2B | | | + | | | | | | + |
| BBC1 | | | | | | | | | |
| BBC3 | | | | | | | | | |
| BBIP1 | | | | | | | | | |
| BBLF2-BBLF3 | | | | | | | | | |
| BBOX1 | | | | | | | | | |
| BBS1 | | | | | | | | | |

| | | | | | | | | | |
|---|---|---|---|---|---|---|---|---|---|
| BBS10 | | | | | | | | | |
| BBS12 | | | | | | | | | |
| BBS2 | | | | | | + | | | + |
| BBS4 | | | | | | | | | |
| BBS5 | | | | | | | | | |
| BBS7 | | | | | | | | | |
| BBS9 | | | | | | | | | |
| BBX | | | + | | | | | | + |
| BC048507 | | | | | | | | | |
| BC055324 | | | | | | | | | |
| BCAM | | | | | | | | | |
| BCAN | | | | | | + | | | + |
| BCAP29 | | | | | | | | | |
| BCAP31 | | | | | | | | | |
| BCAR1 | | | | | | | | | |
| BCAR3 | | | | | | | | | |
| BCAS1 | | | | | | | | | |
| BCAS2 | | | | | + | | | | + |
| BCAS3 | | | | | | | | | |
| BCAS4 | | | | | | | | | |
| BCAT1 | | | | | | | | | |
| BCAT2 | | | | | | | | | |
| BCAT4 | | | | | | | | | |
| BCCIP | | | | | | | | | |
| bcd | | | | | | | | | |
| BCDIN3D | | | | | | | | | |
| BCH1 | | | | | | | | | |
| BCHE | | | | | | | | | |
| BCKDHA | | | | | | | | | |
| BCKDHB | | | | | | | | | |
| BCKDK | | | | | | | | | |
| BCL10 | | | | | | | | | |
| BCL11A | | + | | | | + | | | + |
| BCL11B | + | + | | + | + | + | | | + |
| BCL2 | | | | | | | | | |
| BCL2A1 | | | | | | | | | |
| BCL2L1 | | | | | | | | | |
| BCL2L10 | | | | | | | | | |
| Bcl2l11 | | | | | | | | | |
| BCL2L12 | | | | | | | | | |
| BCL2L13 | | | | | | | | | |
| BCL2L14 | | | | | | | | | |
| BCL2L15 | | | | | | | + | | + |
| BCL2L2 | | | | | | | | | |
| BCL2L2-PABPN1 | | | | | | | | | |
| BCL3 | | | | | | | | | |
| BCL6 | | | | | | | | | |
| BCL6B | | | | | | | | | |
| BCL7A | | | | | | + | | | + |
| Bcl7b | | | | | | | | | |
| BCL7C | | | | | | | | | |
| BCL9 | | | | | | + | | | + |
| BCL9L | | | | | | + | | | + |

| | | | | | | | | | |
|---|---|---|---|---|---|---|---|---|---|
| BCLAF1 | | | | | | | | | |
| BCO1 | | | | | | | | | |
| BCOR | | | + | | | | | | + |
| BCORL1 | | | | | + | | | | + |
| BCR | | | | | | | | | |
| BcRF1 | | | | | | | | | |
| BCS1L | | | | | | + | | | + |
| BCY1 | | | | | | | | | |
| BCYRN1 | | | | | | | | | |
| BDH1 | | | | | | | | | |
| BDH2 | | | | | | | | | |
| BDKRB1 | | | | | | | | | |
| BDKRB2 | | | | | | | | | |
| BDNF | + | | + | | + | | | | + |
| BDNF-AS | | | | | | | | | |
| BDP1 | | | | | + | | | | + |
| BEAN1 | | | | | | | | | |
| BECN1 | | | | | | | | | |
| BECN1P1 | | | | | | | | | |
| Becn2 | | | | | | | | | |
| BEGAIN | | | | | | | | | |
| BEM4 | | | | | | | | | |
| BEND2 | | | | | | | | | |
| BEND3 | | | | | | | | | |
| BEND3P3 | | | | | | | | | |
| BEND4 | | | | | + | | | | + |
| BEND5 | | | | | | | | | |
| BEND6 | | | | | | + | | | + |
| BEND7 | | | | | | | | | |
| BEST1 | | | | | | | | | |
| BEST2 | | | | | | | | | |
| BEST3 | | | | | | | | | |
| BEST4 | | | | | | | | | |
| BET1 | | | | | | | | | |
| BET1L | | | | | | | | | |
| BET2 | | | | | | | | | |
| BET3 | | | | | | | | | |
| BET4 | | | | | | | | | |
| betaTub56D | | | | | | | | | |
| BEX1 | | | + | | | | | | + |
| BEX2 | | | | | | | | | |
| BEX4 | | | | | | | | | |
| BEX5 | | | | | | | | | |
| BFAR | | | | | | | | | |
| BFR1 | | | | | | | | | |
| BFRF1 | | | | | | | | | |
| BFSP1 | | | | | | | | | |
| BFSP2 | | | | | | | | | |
| BGL2 | | | | | | | | | |
| BGLAP | | | | | | | | | |
| BGLF1 | | | | | | | | | |
| BGLF2 | | | | | | | | | |
| BGLF4 | | | | | | | | | |

| | | | | | | | | | |
|---|---|---|---|---|---|---|---|---|---|
| BGLF5 | | | | | | | | | |
| BGLT3 | | | | | | | | | |
| BGN | | | | | | | | | |
| bgs4 | | | | | | | | | |
| BHLHA15 | | | | | | | | | |
| BHLHA9 | | | | | | | | | |
| BHLHB9 | | | | | | | | | |
| BHLHE22 | | | | | | + | | | + |
| BHLHE23 | | | | | | | | | |
| BHLHE40 | + | | | | | + | | | + |
| BHLHE41 | | | | | | | | | |
| BHMT | | | | | | | | | |
| BHMT2 | | | | | | | | | |
| BICC1 | | | | | | | | | |
| BICD1 | | + | + | | + | + | | | + |
| Bicd2 | | | | | | + | | | + |
| BID | | | | | | | | | |
| BIK | | | | | | | | | |
| BIL1 | | | | | | | | | |
| BIN1 | | | | | | | | | |
| BIN2 | + | | | + | + | | | | + |
| BIN3 | | | | | | | | | |
| BIN3-IT1 | | | | | | | | | |
| BIO2 | | | | | | | | | |
| BIP1 | | | | | | | | | |
| BIP2 | | | | | | | | | |
| BIRC2 | | | | | | + | | | + |
| BIRC3 | | | | | | | | | |
| BIRC5 | | | | | | | | | |
| BIRC6 | | | | | | | | | |
| BIRC7 | | | | | | | | | |
| BIRC8 | | | | | | | | | |
| BIT61 | | | | | | | | | |
| BIVM | | | | | | | | | |
| BIVM-ERCC5 | | | | | | | | | |
| BKRF1 | | | | | | | | | |
| BKRF4 | | | | | | | | | |
| BLCAP | | | | | | | | | |
| BLID | | | | | | | | | |
| BLK | | | | | | | | | |
| BLM | | | | | | | | | |
| BLM10 | | | | | | | | | |
| BLMH | + | | | | + | | | | + |
| BLNK | | | | | + | | | | + |
| BLOC1S1 | | | | | | | | | |
| BLOC1S2 | | | | | | | | | |
| BLOC1S3 | | | | | | | | | |
| BLOC1S4 | | | | | | | | | |
| BLOC1S5 | | | | | | | | | |
| BLOC1S5-TXNDC5 | | | | | | | | | |
| BLOC1S6 | | | | | | | | | |
| BLRF1 | | | | | | | | | |
| BLVRA | | | | | | | | | |

| | | | | | | | | | |
|---|---|---|---|---|---|---|---|---|---|
| BLVRB | | | | | + | | | | + |
| BLZF1 | | | | | | | | | |
| BMF | | | | | | | | | |
| BMH1 | | | | | | | | | |
| BMH2 | | | | | | | | | |
| Bmi1 | | | | | | | | | |
| BMP1 | | | | | | | | | |
| BMP10 | | | | | | | | | |
| BMP15 | | | | | | | | | |
| BMP2 | | | | | | | | | |
| BMP2K | | | | | | | | | |
| BMP3 | | | | | | | | | |
| BMP4 | + | | | + | + | | + | | + |
| BMP5 | | | | | | | | | |
| BMP6 | | | | | | | | | |
| BMP7 | | | | | | | | | |
| BMP8A | | | | | | | | | |
| BMP8B | | | | | | | | | |
| BMPER | | | | | | | | | |
| BMPR1A | | | | | | | | | |
| BMPR1B | | | | | | | | | |
| BMPR2 | | | | | | | | | |
| BMS1 | | | + | | | | | | + |
| BMS1P5 | | | | | | | | | |
| BMX | | | | | | | | | |
| BNA1 | | | | | | | | | |
| BNC1 | | | | | | | | | |
| BNC2 | | | | | | | | | |
| BNIP1 | | | | | | | | | |
| BNIP2 | | | | | | | | | |
| BNIP3 | | | | | | | | | |
| BNIP3L | | + | | | | + | | | + |
| BNIP3P6 | | | | | | | | | |
| BNIPL | | | | | | | | | |
| BOC | | | | | | | | | |
| BOD1 | | | + | | | | | | + |
| BOD1L1 | | | | | | | | | |
| BOD1L2 | | | | | | | | | |
| BOI1 | | | | | | | | | |
| BOK | | | | | | | | | |
| BOLA1 | | | | | | | | | |
| BOLA2 | | | | | | | | | |
| BOLA2-SMG1P6 | | | | | | | | | |
| BOLA2B | | | | | | | | | |
| BOLA3 | | | | | | | | | |
| BOLL | | | | | | | | | |
| BOP1 | | | | | | | | | |
| BORA | | | | | | | | | |
| BOS1 | | | | | | | | | |
| BPGM | | | | | | | | | |
| BPHL | | + | | | | + | | | + |
| BPI | | | | | | | | | |
| BPIFA1 | | | | | | | | | |

| | | | | | | | | | |
|---|---|---|---|---|---|---|---|---|---|
| BPIFA2 | | | | | | | | | |
| BPIFA3 | | | | | | | | | |
| BPIFB1 | | | | | | | | | |
| BPIFB2 | | | | | | | | | |
| BPIFB3 | | | | | | | | | |
| BPIFB4 | | | | | | | | | |
| BPIFB6 | | | | | | | | | |
| BPIFC | + | | | + | + | | | | + |
| BPLF1 | | | | | | | | | |
| BPNT1 | | | | | | | | | |
| BPTF | | | | | | | | | |
| BPY2 | | | | | | | | | |
| BRAF | | | + | | | | | | + |
| BRAP | | | | | | | | | |
| BRAT1 | | | | | | | | | |
| BRCA1 | | | | | | | | | |
| BRCA2 | | | | | | | | | |
| BRCA3 | | | | | | | | | |
| BRCC3 | | | | | | | | | |
| BRD1 | | | | | | | | | |
| BRD2 | + | | | | + | | | | + |
| BRD3 | | | | | | | | | |
| BRD4 | | | | | | | | | |
| BRD7 | | | | | | | | | |
| BRD8 | | | | | | | | | |
| BRD9 | | | | | | + | | | + |
| BRDT | | | | | | + | | | + |
| BRE | | | | | | | | | |
| BRE-AS1 | | | | | | | | | |
| BRE1 | | | | | | | | | |
| BRE5 | | | | | | | | | |
| BRF1 | | | | | | | | | |
| BRF2 | | | | | | | | | |
| BRI3 | | | + | | | + | | | + |
| BRI3BP | | | + | | | | | | + |
| BRICD5 | | | | | | | | | |
| BRINP1 | + | | + | | + | | | | + |
| BRINP2 | | | | | | | | | |
| BRINP3 | | | | | | | | | |
| BRIP1 | | | | | | | | | |
| BRIX1 | | | | | + | | | | + |
| BRK1 | | | | | | | | | |
| BRL1 | | | | | | | | | |
| BRLF1 | | | | | | | | | |
| BRMS1 | | | | | | | | | |
| BRMS1L | | | | | | | | | |
| BROX | | | | | | | | | |
| BRPF1 | | | | | | | | | |
| BRPF3 | | | | | | | | | |
| BRRF1 | | | | | | | | | |
| BRSK1 | | | | | | | | | |
| BRSK2 | | | | | | | | | |
| BRWD1 | | | | | | | | | |

| | | | | | | | | | |
|---|---|---|---|---|---|---|---|---|---|
| Brwd3 | | | | | | | | | |
| BSC1 | | | | | | | | | |
| BSCL2 | | | | | | | | | |
| Bsdc1 | | | | | | | | | |
| BSG | | | | | | | | | |
| BSN | | | + | | + | | | | + |
| BSND | | | | | | | | | |
| BSPRY | | | | | | | | | |
| BST1 | | | | | | | | | |
| BST2 | | | | | | | | | |
| BSX | | | | | | | | | |
| BTAF1 | + | | + | | | | | | + |
| BTBD1 | | | | | | | | | |
| BTBD10 | | | + | | | | | | + |
| BTBD11 | | | | | | | | | |
| BTBD16 | | | | | | | | | |
| BTBD17 | | | | | | | | | |
| BTBD18 | | | | | | | | | |
| BTBD2 | | | | | | + | | | + |
| BTBD3 | | | | | | | | | |
| BTBD6 | | | | | | + | | | + |
| btbd6a | | | | | | | | | |
| BTBD7 | | | | | | | | | |
| BTBD8 | | | | | | | | | |
| BTBD9 | + | | + | + | + | + | + | | + |
| BTC | | | | | | | | | |
| BTD | | | | | | | | | |
| BTF3 | | | | | | | | | |
| BTF3L4 | | | | | | | | | |
| BTG1 | + | | | | | + | | | + |
| BTG2 | + | | | | + | | | | + |
| BTG3 | | | + | | | | | | + |
| BTG4 | | | | | | | | | |
| BTK | | | | | | | | | |
| BTLA | | | | | | | | | |
| BTN1A1 | | | | | | | | | |
| BTN2 | | | | | | | | | |
| BTN2A1 | | | | | | | | | |
| BTN2A2 | | | | | | | | | |
| BTN2A3P | | | | | | | | | |
| BTN3A1 | | | | | | | | | |
| BTN3A2 | | | | | | | | | |
| BTN3A3 | | | | | | | | | |
| BTNL2 | | | | | | | | | |
| BTNL3 | | | | | | | | | |
| BTNL8 | | | | | | | | | |
| BTNL9 | | | | | | | | | |
| BTRC | | | | | | | | | |
| BUB1 | | | | | | | | | |
| BUB1B | | | | | | | | | |
| BUB2 | | | | | | | | | |
| BUB3 | | | | | | | | | |
| Bud13 | | | | | | | | | |

| | | | | | | | | | |
|---|---|---|---|---|---|---|---|---|---|
| BUD2 | | | | | | | | | |
| BUD3 | | | | | | | | | |
| BUD31 | | | | | | | | | |
| BUR2 | | | | | | | | | |
| but2 | | | | | | | | | |
| BVES | | | | | | | | | |
| BYRF1 | | | | | | | | | |
| BYSL | | | | | | | | | |
| BZLF1 | | | | | | | | | |
| BZLF2 | | | | | | | | | |
| BZRAP1 | | | | | | | | | |
| BZW1 | | | | | + | | | | + |
| BZW2 | | | | | | | | | |
| BZZ1 | | | | | | | | | |
| c-raf | | | | | | | | | |
| C10orf10 | | | | | | | | | |
| C10orf105 | | | | | | | | | |
| C10orf107 | | | | | | | | | |
| C10orf11 | | | | | | | | | |
| C10orf111 | | | | | | | | | |
| C10orf12 | | | | | | | | | |
| C10orf120 | | | | | | | | | |
| C10orf128 | | | | | | | | | |
| C10orf131 | | | | | | | | | |
| C10orf2 | | | | | | | | | |
| C10orf25 | | | | | | | | | |
| C10orf32 | | | | | | | | | |
| C10orf35 | | | | | | | | | |
| C10orf53 | | | | | | | | | |
| C10orf54 | | | | | | | | | |
| C10orf55 | | | | | | | | | |
| C10orf62 | | | | | | | | | |
| C10orf67 | | | | | | | | | |
| C10orf71 | | | | | | | | | |
| C10orf76 | | | | | | | | | |
| C10orf82 | | | | | | | | | |
| C10orf88 | | | | | | | | | |
| C10orf90 | | | | | | | | | |
| C10orf91 | | | | | | | | | |
| C10orf95 | | | | | | | | | |
| C11orf1 | | | | | | | | | |
| C11orf16 | | | | | | | | | |
| C11orf21 | | | | | | | | | |
| C11orf24 | | | | | | | | | |
| C11orf30 | | | | | | | | | |
| C11orf31 | | | | | | | | | |
| C11orf42 | | | | | | | | | |
| C11orf45 | | | | | | | | | |
| C11orf49 | | | | | | | | | |
| C11orf52 | | | | | | | | | |
| C11orf53 | | | | | | | | | |
| C11orf54 | | | | | | | | | |
| C11orf57 | | | | | | | | | |

| | | | | | | | | | |
|---|---|---|---|---|---|---|---|---|---|
| C11orf58 | | | | | | | | | |
| C11orf63 | | | | | | | | | |
| C11orf65 | | | | | | | | | |
| C11orf68 | | | | | | | | | |
| C11orf70 | | | | | | | | | |
| C11orf71 | | | | | | | | | |
| C11orf72 | | | | | | | | | |
| C11orf73 | | | | | | | | | |
| C11orf74 | | | | | | | | | |
| C11orf80 | | | | | | | | | |
| C11orf84 | | | | | | | | | |
| C11orf85 | | | | | | | | | |
| C11orf87 | | | | | | | | | |
| C11orf91 | | | | | | | | | |
| C11orf96 | | | | | | | | | |
| C11orf98 | | | | | | | | | |
| C12orf10 | | | | | | | | | |
| C12orf29 | | | | | | | | | |
| C12orf4 | | | | | | | | | |
| C12orf40 | | | | | | | | | |
| C12orf43 | | | | | | | | | |
| C12orf45 | | | | | | | | | |
| C12orf49 | | | | | | | | | |
| C12orf5 | | | | | | | | | |
| C12orf50 | | | | | | | | | |
| C12orf56 | | | | | | | | | |
| C12orf57 | | | | | | | | | |
| C12orf60 | | | | | | | | | |
| C12orf65 | | | | | | | | | |
| C12orf66 | | | | | | | | | |
| C12orf73 | | | | | | | | | |
| C12orf74 | | | | | | | | | |
| C12orf75 | | | | | | | | | |
| C12orf76 | | | | | | | | | |
| C14orf1 | | | | | | | | | |
| C14orf105 | | | | | | | | | |
| C14orf119 | | | | | | | | | |
| C14orf142 | | | | | | | | | |
| C14orf159 | | | | | | | | | |
| C14orf166 | | | | | | | | | |
| C14orf169 | | | | | | | | | |
| C14orf178 | | | | | | | | | |
| C14orf180 | | | | | | | | | |
| C14orf183 | | | | | | | | | |
| C14orf2 | | | | | | | | | |
| C14orf37 | | | | | | | | | |
| C14orf39 | | | | | | | | | |
| C14orf79 | | | | | | | | | |
| C14orf80 | | | | | | | | | |
| C14orf93 | | | | | | | | | |
| C15orf26 | | | | | | | | | |
| C15orf27 | | | | | | | | | |
| C15orf32 | | | | | | | | | |

| | | | | | | | | | |
|---|---|---|---|---|---|---|---|---|---|
| C15orf38-AP3S2 | | | | | | | | | |
| C15orf39 | | | | | | | | | |
| C15orf40 | | | | | | | | | |
| C15orf41 | | | | | | | | | |
| C15orf43 | | | | | | | | | |
| C15orf48 | | | | | | | | | |
| C15orf52 | | | | | | | | | |
| C15orf53 | | | | | | | | | |
| C15orf54 | | | | | | | | | |
| C15orf57 | | | | | | | | | |
| C15orf59 | | | | | | | | | |
| C15orf61 | | | | | | | | | |
| C15orf62 | | | | | | | | | |
| C15orf65 | | | | | | | | | |
| C16orf13 | | | | | | | | | |
| C16orf45 | | | | | | | | | |
| C16orf46 | | | | | | | | | |
| C16orf47 | | | | | | | | | |
| C16orf54 | | | | | | | | | |
| C16orf58 | | | | | | | | | |
| C16orf59 | | | | | | | | | |
| C16orf62 | | | | | | | | | |
| C16orf70 | | | | | | | | | |
| C16orf71 | | | | | | | | | |
| C16orf72 | | | | | | | | | |
| C16orf74 | | | | | | | | | |
| C16orf78 | | | | | | | | | |
| C16orf87 | | | | | | | | | |
| C16orf89 | | | | | | | | | |
| C16orf90 | | | | | | | | | |
| C16orf91 | | | | | | | | | |
| C16orf92 | | | | | | | | | |
| C16orf93 | | | | | | | | | |
| C16orf95 | | | | | | | | | |
| C16orf96 | | | | | | | | | |
| C17orf102 | | | | | | | | | |
| C17orf104 | | | | | | | | | |
| C17orf105 | | | | | | | | | |
| C17orf47 | | | | | | | | | |
| C17orf49 | | | | | | | | | |
| C17orf50 | | | | | | | | | |
| C17orf51 | | | | | | | | | |
| C17orf53 | | | | | | | | | |
| C17orf59 | | | | | | | | | |
| C17orf62 | | | | | | | | | |
| C17orf64 | | | | | | | | | |
| C17orf67 | | | | | | | | | |
| C17orf70 | | | | | | | | | |
| C17orf75 | | | | | | | | | |
| C17orf78 | | | | | | | | | |
| C17orf80 | | | | | | | | | |
| C17orf82 | | | | | | | | | |
| C17orf85 | | | | | | | | | |

| | | | | | | | | | |
|---|---|---|---|---|---|---|---|---|---|
| C17orf89 | | | | | | | | | |
| C17orf96 | | | | | | | | | |
| C17orf97 | | | | | | | | | |
| C17orf98 | | | | | | | | | |
| C17orf99 | | | | | | | | | |
| C18orf21 | | | | | | | | | |
| C18orf25 | | | | | | | | | |
| C18orf32 | | | | | | | | | |
| C18orf42 | | | | | | | | | |
| C18orf54 | | | | | | | | | |
| C18orf63 | | | | | | | | | |
| C18orf8 | | | | | | | | | |
| C19orf10 | | | | | | | | | |
| C19orf12 | | | | | | | | | |
| C19orf18 | | | | | | | | | |
| C19orf24 | | | | | | | | | |
| C19orf25 | | | | | | | | | |
| C19orf26 | | | | | | | | | |
| C19orf33 | | | | | | | | | |
| C19orf35 | | | | | | | | | |
| C19orf38 | | | | | | | | | |
| C19orf40 | | | | | | | | | |
| C19orf43 | | | | | | | | | |
| C19orf44 | | | | | | | | | |
| C19orf45 | | | | | | | | | |
| C19orf47 | | | | | | | | | |
| C19orf48 | | | | | | | | | |
| C19orf52 | | | | | | | | | |
| C19orf53 | | | | | | | | | |
| C19orf54 | | | | | | | | | |
| C19orf57 | | | | | | | | | |
| C19orf60 | | | | | | | | | |
| C19orf66 | | | | | | | | | |
| C19orf68 | | | | | | | | | |
| C19orf70 | | | | | | | | | |
| C19orf71 | | | | | | | | | |
| C19orf73 | | | | | | | | | |
| C19orf80 | | | | | | | | | |
| C1D | | | | | | | | | |
| C1GALT1 | | | | | | | | | |
| C1GALT1C1 | | | | | | + | | | + |
| C1orf101 | | | | | | | | | |
| C1orf105 | | | | | | | | | |
| C1orf106 | | | | | | | | | |
| C1orf109 | | | | | | | | | |
| C1orf110 | | | | | | | | | |
| C1orf111 | | | | | | | | | |
| C1orf112 | | | | | | | | | |
| C1orf115 | | | | | | | | | |
| C1orf116 | | | | | | | | | |
| C1orf122 | | | | | | | | | |
| C1orf123 | | | | | | | | | |
| C1orf127 | | | | | | | | | |

| | | | | | | | | | |
|---|---|---|---|---|---|---|---|---|---|
| C1orf131 | | | | | | | | | |
| C1orf141 | | | | | | | | | |
| C1orf146 | | | | | | | | | |
| C1orf158 | | | | | | | | | |
| C1orf159 | | | | | | | | | |
| C1orf162 | | | | | | | | | |
| C1orf167 | | | | | | | | | |
| C1orf168 | | | | | | | | | |
| C1orf174 | | | | | | | | | |
| C1orf177 | | | | | | | | | |
| C1orf185 | | | | | | | | | |
| C1orf186 | | | | | | | | | |
| C1orf189 | | | | | | | | | |
| C1orf194 | | | | | | | | | |
| C1orf198 | | | | | | | | | |
| C1orf21 | | | | | | | | | |
| C1orf210 | | | | | | | | | |
| C1orf216 | | | | | | | | | |
| C1orf220 | | | | | | | | | |
| C1orf226 | | | | | | | | | |
| C1orf228 | | | | | | | | | |
| C1orf233 | | | | | | | | | |
| C1orf27 | | | | | | | | | |
| C1orf35 | | | | | | | | | |
| C1orf43 | | | | | | | | | |
| C1orf50 | | | | | | | | | |
| C1orf52 | | | | | | | | | |
| C1orf53 | | | | | | | | | |
| C1orf54 | | | | | | | | | |
| C1orf56 | | | | | | | | | |
| C1orf61 | | | | | | | | | |
| C1orf64 | | | | | | | | | |
| C1orf68 | | | | | | | | | |
| C1orf74 | | | | | | | | | |
| C1orf85 | | | | | | | | | |
| C1orf86 | | | | | | | | | |
| C1orf87 | | | | | | | | | |
| C1orf94 | | | | | | | | | |
| C1orf95 | | | | | | | | | |
| C1QA | | | | | | | | | |
| C1QB | | + | | | | | | | + |
| C1QBP | | | | | | + | | | + |
| C1QC | | | | | + | | | | + |
| C1QL1 | | | | | | | | | |
| C1QL2 | | | | | | | | | |
| C1QL3 | | | + | | | | | | + |
| C1QL4 | | | | | | | | | |
| C1QTNF1 | | | | | | | | | |
| C1QTNF2 | | | | | | | | | |
| C1QTNF3 | | | | | | | | | |
| C1QTNF4 | | | | | | + | | | + |
| C1QTNF5 | | | | | | | | | |
| C1QTNF6 | | | | | | | | | |

| | | | | | | | | | |
|---|---|---|---|---|---|---|---|---|---|
| C1QTNF7 | | | | | | | | | |
| C1QTNF8 | | | | | | | | | |
| C1QTNF9 | | | | | | | | | |
| C1QTNF9B | | | | | | | | | |
| C1QTNF9B-AS1 | | | | | | | | | |
| C1R | | | | | | | | | |
| C1RL | | | | | | | | | |
| C1RL-AS1 | | | | | | | | | |
| C1S | | | | | | | | | |
| C2 | | | | | | | | | |
| C20orf141 | | | | | | | | | |
| C20orf166-AS1 | | | | | | | | | |
| C20orf173 | | | | | | | | | |
| C20orf194 | | | | | | | | | |
| C20orf195 | | | | | | | | | |
| C20orf196 | | | | | | | | | |
| C20orf197 | | | | | | | | | |
| C20orf202 | | | | | | | | | |
| C20orf203 | | | | | | | | | |
| C20orf24 | | | | | | | | | |
| C20orf27 | | | | | | | | | |
| C20orf85 | | | | | | | | | |
| C20orf96 | | | | | | | | | |
| C21orf2 | | | | | | | | | |
| C21orf33 | | | | | | | | | |
| C21orf58 | | | | | | | | | |
| C21orf59 | | | | | | | | | |
| C21orf62-AS1 | | | | | | | | | |
| C21orf91 | | | | | | | | | |
| C22orf15 | | | | | | | | | |
| C22orf23 | | | | | | | | | |
| C22orf29 | | | | | | | | | |
| C22orf31 | | | | | | | | | |
| C22orf34 | | | | | | | | | |
| C22orf39 | | | | | | | | | |
| C22orf42 | | | | | | | | | |
| C22orf46 | | | | | | | | | |
| C2CD2 | | | | | | | | | |
| C2CD2L | | | | | | | | | |
| C2CD3 | | | | + | | | | | + |
| C2CD4A | | | | | | | | | |
| C2CD4B | | | | | | | | | |
| C2CD4C | + | | | + | + | + | | | + |
| C2CD4D | | | | | | | | | |
| C2CD5 | | + | | | | + | | | + |
| C2orf15 | | | | | | | | | |
| C2orf16 | | | | | | | | | |
| C2orf27A | | | | | | | | | |
| C2orf27B | | | | | | | | | |
| C2orf40 | | | | | | | | | |
| C2orf42 | | | | | | | | | |
| C2orf43 | | | | | | | | | |
| C2orf44 | | | | | | | | | |

| | | | | | | | | | |
|---|---|---|---|---|---|---|---|---|---|
| C2orf47 | | | | | | | | | |
| C2orf48 | | | | | | | | | |
| C2orf49 | | | | | | | | | |
| C2orf50 | | | | | | | | | |
| C2orf54 | | | | | | | | | |
| C2orf57 | | | | | | | | | |
| C2orf61 | | | | | | | | | |
| C2orf66 | | | | | | | | | |
| C2orf68 | | | | | | | | | |
| C2orf69 | | | | | | | | | |
| C2orf71 | | | | | | | | | |
| C2orf72 | | | | | | | | | |
| C2orf73 | | | | | | | | | |
| C2orf74 | | | | | | | | | |
| C2orf76 | | | | | | | | | |
| C2orf78 | | | | | | | | | |
| C2orf80 | | | | | | | | | |
| C2orf82 | | | | | | | | | |
| C2orf83 | | | | | | | | | |
| C2orf88 | | | | | | | | | |
| C3 | | | | | | | | | |
| C3AR1 | + | | | + | + | | + | | + |
| C3orf14 | | | | | | | | | |
| C3orf17 | | | | | | | | | |
| C3orf18 | | | | | | | | | |
| C3orf20 | | | | | | | | | |
| C3orf33 | | | | | | | | | |
| C3orf35 | | | | | | | | | |
| C3orf36 | | | | | | | | | |
| C3orf38 | | | | | | | | | |
| C3orf49 | | | | | | | | | |
| C3orf52 | | | | | | | | | |
| C3orf56 | | | | | | | | | |
| C3orf58 | | | | | | | | | |
| C3orf62 | | | | | | | | | |
| C3orf67 | | | | | | | | | |
| C3orf70 | | | | | | | | | |
| C3P1 | | | | | | | | | |
| C4A | | | | | | | | | |
| C4B | | | | | | | | | |
| C4B_2 | | | | | | | | | |
| C4BPA | | | | | | | | | |
| C4BPB | | | | | | | | | |
| C4orf17 | | | | | | | | | |
| C4orf19 | | | | | | | | | |
| C4orf22 | | | | | | | | | |
| C4orf26 | | | | | | | | | |
| C4orf27 | | | | | | | | | |
| C4orf29 | | | | | | | | | |
| C4orf3 | | | | | | | | | |
| C4orf32 | | | | | | | | | |
| C4orf33 | | | | | | | | | |
| C4orf36 | | | | | | | | | |

| | | | | | | | | | |
|---|---|---|---|---|---|---|---|---|---|
| C4orf45 | | | | | | | | | |
| C4orf46 | | | | | | | | | |
| C4orf47 | | | | | | | | | |
| C4orf48 | | | | | | | | | |
| C4orf50 | | | | | | | | | |
| C4orf51 | | | | | | | | | |
| C5 | | | | | | | | | |
| C5AR1 | | | | | | | | | |
| C5AR2 | + | | | + | + | | | | + |
| C5orf15 | | | | | | | | | |
| C5orf17 | | | | | | | | | |
| C5orf22 | | | | | | | | | |
| C5orf24 | | | | | | | | | |
| C5orf28 | | | | | | | | | |
| C5orf30 | | | | | | | | | |
| C5orf34 | | | | | | | | | |
| C5orf38 | | | | | | | | | |
| C5orf42 | | | | | | | | | |
| C5orf45 | | | | | | | | | |
| C5orf46 | | | | | | | | | |
| C5orf49 | | | | | | | | | |
| C5orf51 | | | | | | | | | |
| C5orf56 | | | | | | | | | |
| C5orf58 | | | | | | | | | |
| C5orf60 | | | | | | | | | |
| C5orf63 | | | | | | | | | |
| C5orf64 | | | | | | | | | |
| C6 | | | | | | | | | |
| C6orf1 | | | | | | | | | |
| C6orf10 | | | | | | | | | |
| C6orf106 | | | | | | | | | |
| C6orf118 | | | | | | | | | |
| C6orf120 | | | | | | | | | |
| C6orf132 | | | | | | | | | |
| C6orf136 | | | | | | | | | |
| C6orf141 | | | | | | | | | |
| C6orf15 | | | | | | | | | |
| C6orf163 | | | | | | | | | |
| C6orf165 | | | | | | | | | |
| C6orf195 | | | | | | | | | |
| C6orf201 | | | | | | | | | |
| C6orf203 | | | | | | | | | |
| C6orf211 | | | | | | | | | |
| C6orf222 | | | | | | | | | |
| C6orf223 | | | | | | | | | |
| C6orf226 | | | | | | | | | |
| C6orf25 | | | | | | | | | |
| C6orf47 | | | | | | | | | |
| C6orf48 | | | | | | | | | |
| C6orf57 | | | | | | | | | |
| C6orf58 | | | | | | | | | |
| C6orf62 | | | | | | | | | |
| C6orf89 | | | | | | | | | |

| | | | | | | | | | |
|---|---|---|---|---|---|---|---|---|---|
| C6orf99 | | | | | | | | | |
| C7 | | | | | | | | | |
| C7orf25 | | | | | | | | | |
| C7orf26 | | | | | | | | | |
| C7orf31 | | | | | | | | | |
| C7orf33 | | | | | | | | | |
| C7orf34 | | | | | | | | | |
| C7orf43 | | | | | | | | | |
| C7orf49 | | | | | | | | | |
| C7orf50 | | | | | | | | | |
| C7orf55 | | | | | | | | | |
| C7orf55-LUC7L2 | | | | | | | | | |
| C7orf57 | | | | | | | | | |
| C7orf60 | | | | | | | | | |
| C7orf61 | | | | | | | | | |
| C7orf62 | | | | | | | | | |
| C7orf69 | | | | | | | | | |
| C7orf72 | | | | | | | | | |
| C7orf73 | | | | | | | | | |
| C7orf76 | | | | | | | | | |
| C8A | | | | | | | + | | + |
| C8B | | | | | | | | | |
| C8G | | | | | | | | | |
| C8orf22 | | | | | | | | | |
| C8orf31 | | | | | | | | | |
| C8orf33 | | | | | | | | | |
| C8orf34 | | | | | | | | | |
| C8orf37 | | | | | | | | | |
| C8orf4 | | | | | | | | | |
| C8orf44 | | | | | | | | | |
| C8orf46 | | | | | | | | | |
| C8orf48 | | | | | | | | | |
| C8orf58 | | | | | | | | | |
| C8orf59 | | | | | | | | | |
| C8orf74 | | | | | | | | | |
| C8orf76 | | | | | | | | | |
| C8orf82 | | | | | | | | | |
| C8orf86 | | | | | | | | | |
| C8orf88 | | | | | | | | | |
| C9 | | | | | | | | | |
| C9orf106 | | | | | | | | | |
| C9orf114 | | | | | | | | | |
| C9orf116 | | | | | | | | | |
| C9orf117 | | | | | | | | | |
| C9orf131 | | | | | | | | | |
| C9orf139 | | | | | | | | | |
| C9orf142 | | | | | | | | | |
| C9orf152 | | | | | | | | | |
| C9orf153 | | | | | | | | | |
| C9orf156 | | | | | | | | | |
| C9orf16 | | | | | | | | | |
| C9orf163 | | | | | | | | | |
| C9orf170 | | | | | | | | | |

| | | | | | | | | | |
|---|---|---|---|---|---|---|---|---|---|
| C9orf171 | | | | | | | | | |
| C9orf172 | | | | | | | | | |
| C9orf173 | | | | | | | | | |
| C9orf24 | | | | | | | | | |
| C9orf3 | | | | | | | | | |
| C9orf40 | | | | | | | | | |
| C9orf41 | | | | | | | | | |
| C9orf43 | | | | | | | | | |
| C9orf50 | | | | | | | | | |
| C9orf57 | | | | | | | | | |
| C9orf62 | | | | | | | | | |
| C9orf64 | | | | | | | | | |
| C9orf69 | | | | | | | | | |
| C9orf72 | | | | | | | | | |
| C9orf78 | | | | | | | | | |
| C9orf84 | | | | | | | | | |
| C9orf85 | | | | | | | | | |
| C9orf89 | | | | | | | | | |
| C9orf9 | | | | | | | | | |
| C9orf91 | | | | | | | | | |
| CA1 | | | | | | | | | |
| CA10 | | | | | | | | | |
| CA11 | | | | | | | | | |
| CA12 | | | | | | | | | |
| CA13 | | | | | | | | | |
| CA14 | | | | | | | | | |
| CA2 | | | | | | | | | |
| CA3 | | | | | | | | | |
| CA4 | | | | | | | | | |
| CA5A | | | | | | | | | |
| CA5B | | | | | | | | | |
| CA6 | | | | | | | | | |
| CA8 | | | | | | | | | |
| CA9 | | | | | | | | | |
| CAAP1 | | | | | | | | | |
| CAB39 | | | | | | | | | |
| CAB39L | | | | | | | | | |
| CABIN1 | | | | | | | | | |
| CABLES1 | | | | | | | | | |
| CABLES2 | | | | | + | | | | + |
| CABP1 | | | | | + | | | | + |
| CABP2 | | | | | | | | | |
| CABP4 | | | | | | | | | |
| CABP5 | | | | | | | | | |
| CABP7 | | | | | | | | | |
| CABS1 | | | | | | | | | |
| CABYR | | | | | | + | | | + |
| CAC2 | | | | | | | | | |
| CACFD1 | | | | | | | | | |
| CACHD1 | | | | | | | | | |
| CACNA1A | | | | | | | | | |
| CACNA1B | | | | | | | | | |
| CACNA1C | | | + | | | | | | + |

| | | | | | | | | | |
|---|---|---|---|---|---|---|---|---|---|
| CACNA1D | | | | | | | | | |
| CACNA1E | | | | | | | | | |
| CACNA1F | + | | | + | + | | | | + |
| CACNA1G | | | | | | | | | |
| CACNA1H | | | | | | + | | | + |
| CACNA1I | | | | | | | | | |
| CACNA1S | | | | | | | | | |
| CACNA2D1 | | | | | | | | | |
| CACNA2D2 | | | | | | | | | |
| CACNA2D3 | | | | | | | | | |
| Cacnb1 | | | | | | | | | |
| CACNB2 | | | | + | | | | | + |
| CACNB3 | | | | | | | | | |
| CACNB4 | | | | | | | | | |
| CACNG1 | | | | | | | | | |
| CACNG2 | | | | | | + | | | + |
| CACNG3 | | | | | | | | | |
| CACNG4 | | | | | | | | | |
| CACNG5 | | | + | | | | | | + |
| CACNG6 | | | | | | | | | |
| CACNG7 | | | | | | | | | |
| CACNG8 | | | | | | | | | |
| CACTIN | | | | | | | | | |
| CACTIN-AS1 | | | | | | | | | |
| CACUL1 | | | | | | | | | |
| CACYBP | | | | | + | | | | + |
| CAD | | | | | | | | | |
| CAD1 | | | | | | | | | |
| CADM1 | | | | + | | | | | + |
| CADM2 | | | | | | | | | |
| CADM3 | | | | | | | | | |
| CADM4 | | | | | + | | | | + |
| CADPS | | | | | | | | | |
| CADPS2 | | | | | | + | | | + |
| CAF16 | | | | | | | | | |
| CAF40 | | | | | | | | | |
| CAGE1 | | | | | | | | | |
| CAK1 | | | | | | | | | |
| CALB1 | | + | | | | + | | | + |
| CALB2 | | | | | | | | | |
| CALCA | | | | | | | | | |
| CALCB | | | | | | | | | |
| CALCOCO1 | | | | | | + | | | + |
| CALCOCO2 | | | | | | | | | |
| CALCR | | | | | | | | | |
| CALCRL | + | | | + | + | | + | | + |
| CALD1 | + | | | | + | | | | + |
| CALHM1 | | | | | | | | | |
| CALHM2 | | | | | | | | | |
| CALHM3 | | | | | | | | | |
| CALM | | | | | | | | | |
| CALM1 | | | | | | | | | |
| CALM1P1 | | | | | | | | | |

| | | | | | | | | | |
|---|---|---|---|---|---|---|---|---|---|
| CALM2 | | | | | | + | | | + |
| CALM3 | | | | | | + | | | + |
| CALML3 | | | | | | | | | |
| CALML4 | | | | | | | | | |
| CALML5 | | | | | | | | | |
| CALML6 | | | | | | | | | |
| Calmodulin | | | | | | | | | |
| CALN1 | + | | | + | + | | | | + |
| CALR | | | | | | | | | |
| CALR3 | | | | | | | | | |
| CALU | | | | | | | | | |
| CALY | | | | | | | | | |
| Cam | | | | | | | | | |
| CAM1 | | | | | | | | | |
| cam2 | | | | | | | | | |
| CAMK1 | | | | | | | | | |
| CAMK1D | | | | | | | | | |
| CAMK1G | + | | + | | | | | | + |
| CAMK2A | | | | | | + | | | + |
| CAMK2B | | | | | | + | | | + |
| CAMK2D | | | + | | + | | | | + |
| CAMK2G | | | | | | | | | |
| Camk2n1 | | | | | | + | | | + |
| CAMK2N2 | | | | | | | | | |
| CAMK4 | | | | | + | | | | + |
| CAMKK1 | + | | | | + | | | | + |
| CAMKK2 | | | | | | + | | | + |
| CAMKMT | | | | | | | | | |
| CAMKV | | | | | | + | | | + |
| CAMLG | | | | | | | | | |
| CAMP | | | | | | | | | |
| CAMSAP1 | | | | | | | | | |
| CAMSAP2 | | | | | | | | | |
| CAMSAP3 | | | | | + | | | | + |
| CAMTA1 | | + | | | | + | | | + |
| CAMTA2 | | | | | | + | | | + |
| CAND1 | | | | | | | | | |
| CAND2 | | | | | | | | | |
| CANT1 | | | | | | | | | |
| CANX | | | | | | | | | |
| CAP1 | | | | | + | | | | + |
| CAP2 | | | + | | | | | | + |
| CAPG | | | | | | | | | |
| CAPN1 | | | | | | | | | |
| CAPN10 | | | | | | | | | |
| CAPN11 | | | | | | | | | |
| CAPN12 | | | | | | | | | |
| CAPN13 | | | | | | | | | |
| CAPN14 | | | | | | | | | |
| CAPN15 | | | | | | | | | |
| CAPN2 | | | | | | | | | |
| CAPN3 | | | | | | + | | | + |
| CAPN5 | | | | | | | | | |

| | | | | | | | | | |
|---|---|---|---|---|---|---|---|---|---|
| CAPN6 | | | | | | | | | |
| CAPN7 | | | | | | | | | |
| CAPN8 | | | | | | | | | |
| CAPN9 | | | | | | | | | |
| CAPNS1 | | | + | | | | | | + |
| CAPNS2 | | | | | | | | | |
| CAPRIN1 | | | | | | | | | |
| CAPRIN2 | | | | | | | | | |
| CAPS | | | | | | | | | |
| CAPSL | | | | | | | | | |
| Capza1 | | | | | | | | | |
| CAPZA2 | | | | | | | | | |
| CAPZA3 | | | | | | | | | |
| CAPZB | | | | | | | | | |
| Car1 | | | | | | | | | |
| Car2 | | | | | | | | | |
| Car3 | | | | | | | | | |
| CARD10 | | | | | | | | | |
| CARD11 | | | | | | | | | |
| CARD14 | + | | | | + | | | | + |
| CARD16 | | | | | | | | | |
| CARD17 | | | | | | | | | |
| CARD18 | | | | | | | | | |
| CARD6 | | | | | | | | | |
| CARD8 | | | | | | | | | |
| CARD9 | | | | | | | | | |
| CARF | | | | | | | | | |
| CARHSP1 | | | | | | | | | |
| CARKD | | | | | | | | | |
| CARM1 | | | | | | | | | |
| CARNS1 | | | | | | | | | |
| CARS | | | | | + | | | | + |
| CARS-AS1 | | | | | | | | | |
| CARS2 | | | | | | | | | |
| CARTPT | | | | | | | | | |
| CASC1 | | | | | | | | | |
| CASC10 | | | | | | | | | |
| CASC18 | | | | | | | | | |
| CASC2 | | | | | | | | | |
| CASC3 | | | | | | | | | |
| CASC4 | + | | | | + | | | | + |
| Casc5 | | | | | | | | | |
| CASC9 | | | | | | | | | |
| CASD1 | | | | | | | | | |
| CASK | | | | | | | | | |
| Caskin1 | | | | | | | | | |
| CASKIN2 | | | | | | | | | |
| CASP1 | | | | | | | | | |
| CASP10 | | | | | | | | | |
| CASP12 | | | | | | | | | |
| CASP14 | | | | | | | | | |
| CASP16 | | | | | | | | | |
| CASP2 | | | | | | | | | |

| | | | | | | | | | |
|---|---|---|---|---|---|---|---|---|---|
| CASP3 | | | | | | | | | |
| CASP4 | | | | | | | | | |
| CASP5 | | | | | | | | | |
| CASP6 | | | | | | | | | |
| CASP7 | | | | | | | | | |
| CASP8 | | | | | | | | | |
| CASP8AP2 | | | | | | | | | |
| CASP9 | | | | | | | | | |
| CASQ1 | | | | | | | | | |
| CASQ2 | | | | | | | | | |
| CASR | | | | | | | | | |
| CASS4 | | | | | | | | | |
| CAST | | | | | | | | | |
| CASZ1 | | | | | | | | | |
| CAT | | | | | | + | | | + |
| CAT2 | | | | | | | | | |
| CAT5 | | | | | | | | | |
| CATIP | | | | | | | | | |
| CATSPER1 | | | | | | | | | |
| CATSPER2 | | | | | | | | | |
| CATSPER4 | | | | | | | | | |
| CATSPERB | | | | | | | | | |
| CATSPERD | | | | | | | | | |
| CATSPERG | | | | | | | | | |
| CAV1 | | | | | | | | | |
| CAV2 | | | | | | | | | |
| CAV3 | | | | | | | | | |
| CB5-B | | | | | | | | | |
| CB5-E | | | | | | | | | |
| cbc1 | | | | | | | | | |
| CBF2 | | | | | | | | | |
| CBF5 | | | | | | | | | |
| CBFA2T2 | | | | | | | | | |
| CBFA2T3 | | | | | | | | | |
| CBFB | | | | | | | | | |
| CBK1 | | | | | | | | | |
| CBL | | | | | | | | | |
| CBLB | | | | | | | | | |
| CBLC | | | | | | | | | |
| CBLL1 | | + | + | | | + | | | + |
| CBLN1 | | | | | | | | | |
| CBLN4 | | | + | | | | | | + |
| CBP | | | | | | | | | |
| cbp-1 | | | | | | | | | |
| CBP3 | | | | | | | | | |
| CBR1 | | | | | | | | | |
| CBR3 | | | | | | | | | |
| CBR4 | | | | | | | | | |
| CBS | | | | | | | | | |
| CBS2 | | | | | | | | | |
| CBWD1 | | | | | | | | | |
| CBWD2 | | | | | | | | | |
| CBWD3 | | | | | | | | | |

| | | | | | | | | | |
|---|---|---|---|---|---|---|---|---|---|
| CBWD5 | | | | | | | | | |
| CBWD6 | | | | | | | | | |
| CBWD7 | | | | | | | | | |
| CBX1 | | | | | | | | | |
| Cbx2 | | | | | | | | | |
| CBX3 | | | | | | | | | |
| CBX4 | | | | | | + | | | + |
| CBX5 | | | | | | | | | |
| CBX6 | | | | | | | | | |
| CBX7 | | | | | | | | | |
| CBX8 | | | | | | | | | |
| CBY1 | | | | | | + | | | + |
| CC2D1A | | | | | + | + | | | + |
| CC2D1B | | | | | | | | | |
| CC2D2A | | | | | | | | | |
| CC2D2B | | | | | | | | | |
| CCAR1 | | | | | | | | | |
| CCAR2 | | | | | | | | | |
| CCAT1 | | | | | | | | | |
| CCBE1 | | | | | | | | | |
| CCBL1 | | | | | | | | | |
| CCBL2 | | | | | | | | | |
| CCC2 | | | | | | | | | |
| CCDC101 | | | | | | | | | |
| CCDC102A | | | | | | | | | |
| CCDC102B | | | | | | | | | |
| CCDC103 | | | | | | | | | |
| CCDC105 | | | | | | | | | |
| CCDC106 | | | | | | | | | |
| CCDC107 | | | | | | | | | |
| CCDC108 | | | | | | | | | |
| CCDC109B | | | | | | | | | |
| CCDC110 | | | | | | | | | |
| CCDC112 | | | | | | | | | |
| CCDC113 | | | | | | | | | |
| CCDC114 | | | | | | | | | |
| CCDC115 | | | | | | | | | |
| CCDC116 | | | | | | | | | |
| CCDC117 | | | | | | | | | |
| CCDC12 | | | | | | | | | |
| CCDC120 | | | | | | | | | |
| CCDC121 | | | | | | | | | |
| CCDC122 | | | | | | | | | |
| CCDC124 | | | | | | | | | |
| CCDC125 | | | | | | | | | |
| CCDC126 | | | | | | | | | |
| CCDC127 | | | + | | | | | | + |
| CCDC129 | | | | | | | | | |
| CCDC13 | | | | | | + | | | + |
| CCDC130 | | | | | | + | | | + |
| CCDC132 | | + | | | | + | | | + |
| CCDC134 | | | | | | | | | |
| CCDC136 | | | | | | | | | |

| | | | | | | | | | |
|---|---|---|---|---|---|---|---|---|---|
| CCDC137 | | | | | | | | | |
| CCDC138 | | | | | | | | | |
| CCDC14 | | | | | | | | | |
| CCDC140 | | | | | | | | | |
| CCDC141 | | | + | | | | | + | + |
| CCDC142 | | | | | | | | | |
| CCDC144A | | | | | | | | | |
| CCDC144B | | | | | | | | | |
| CCDC144CP | | | | | | | | | |
| CCDC144NL | | | | | | | | | |
| CCDC144NL-AS1 | | | | | | | | | |
| CCDC146 | | | | | | | | | |
| CCDC148 | | | | | | | | | |
| CCDC149 | | | | | | | | | |
| Ccdc15 | | | | | | | | | |
| CCDC150 | | + | + | | | + | | | + |
| CCDC151 | | | | | | | | | |
| CCDC152 | | | | | | | | | |
| CCDC153 | | | | | | | | | |
| CCDC154 | | | | | | | | | |
| CCDC155 | | | | | | | | | |
| CCDC157 | | | | | | | | | |
| CCDC158 | | | | | | | | | |
| CCDC159 | | | | | | | | | |
| CCDC160 | | | | | | | | | |
| CCDC167 | | | | | | | | | |
| CCDC168 | | | | | | | | | |
| CCDC169 | | | | | | | | | |
| CCDC17 | | | | | | | | | |
| CCDC170 | | | | | | | | | |
| CCDC171 | | | | | | | | | |
| CCDC172 | | | | | | | | | |
| CCDC173 | | | | | | | | | |
| CCDC174 | | | | | | | | | |
| CCDC175 | | | | | | | | | |
| CCDC176 | | | | | | | | | |
| CCDC177 | | | | | | + | | | + |
| CCDC178 | | | | | | | | | |
| CCDC179 | | | | | | | | | |
| CCDC18 | | | | | | | | | |
| CCDC180 | | | | | | | | | |
| CCDC181 | | | | | | | | | |
| CCDC182 | | | | | | | | | |
| CCDC183 | | | | | | | | | |
| CCDC184 | + | | | | | | | | + |
| CCDC185 | | | | | | | | | |
| CCDC186 | | | | | | | | | |
| Ccdc22 | | | | | | | | | |
| CCDC23 | | | | | | | | | |
| CCDC24 | | | | | | | | | |
| CCDC25 | | | | | | | | | |
| CCDC26 | | | | | | | | | |
| CCDC27 | | | | | | | | | |

| | | | | | | | | | |
|---|---|---|---|---|---|---|---|---|---|
| CCDC28A | | | + | | | | | | + |
| CCDC28B | | | | | | | | | |
| CCDC3 | | | | | | | | | |
| CCDC30 | | | | | | | | | |
| CCDC33 | + | | | | + | | | | + |
| CCDC34 | | | | | | | | | |
| CCDC36 | | | | | | | | | |
| CCDC37 | | | | | | | | | |
| CCDC38 | | + | + | | | + | | + | + |
| CCDC39 | | | | | | | | | |
| CCDC40 | | | | | | | | | |
| CCDC42 | | | | | | | | | |
| CCDC43 | | | | | | | | | |
| CCDC47 | | | | | | + | | | + |
| CCDC50 | + | | | + | + | + | + | | + |
| CCDC51 | | | | | | | | | |
| CCDC53 | | | | | | | | | |
| CCDC54 | | | | | | | | | |
| CCDC57 | | | | | | | | | |
| CCDC58 | | | | | | | | | |
| CCDC59 | | | | | + | | | | + |
| CCDC6 | | | | + | | | | | + |
| CCDC60 | | | | | | | | | |
| CCDC61 | | | | | | | | | |
| CCDC62 | | | | + | | | | | + |
| CCDC63 | | | | + | | | | | + |
| Ccdc64 | | | | | | | | | |
| Ccdc64b | | | | | | | | | |
| CCDC65 | | | | | | | | | |
| CCDC66 | | | | | | | | | |
| CCDC67 | | | | | | | | | |
| CCDC68 | | | | | | | | | |
| CCDC69 | | | | | | | | | |
| CCDC7 | | | | | | | | | |
| CCDC70 | | | | | | | | | |
| CCDC71 | | | | | | | | | |
| CCDC71L | | | | | | | | | |
| CCDC73 | | | | | | | | | |
| CCDC74A | | | | | | | | | |
| CCDC74B | | | | | | | | | |
| Ccdc77 | | | | | | | | | |
| CCDC78 | | | | | | | | | |
| CCDC79 | | | | | | | | | |
| CCDC8 | | | | | | | | | |
| CCDC80 | | | | | | | | | |
| CCDC82 | | | | | | | | | |
| CCDC83 | | | | | | | | | |
| CCDC84 | | | | | | | | | |
| CCDC85A | | | | + | | + | | | + |
| CCDC85B | | | | | | + | | | + |
| CCDC85C | + | | | + | + | | | | + |
| CCDC86 | | | | + | | | | | + |
| CCDC87 | | | | | | | | | |

| | | | | | | | | | |
|---|---|---|---|---|---|---|---|---|---|
| CCDC88A | | + | | | | + | | | + |
| CCDC88B | | | | | | | | | |
| CCDC88C | | | | | + | | | | + |
| CCDC89 | | | | | | | | | |
| Ccdc9 | + | | | + | + | | | | + |
| CCDC90B | | | | | | | | | |
| CCDC91 | | | | | | | | | |
| CCDC92 | | | | | | | | | |
| CCDC93 | | | | | | | | | |
| CCDC94 | | | | | | | | | |
| CCDC96 | | | | | | | | | |
| CCDC97 | | | | | | + | | | + |
| CCE1 | | | | | | | | | |
| CCER1 | | | | | | | | | |
| CCHCR1 | | | | | | | | | |
| CCIN | | | | | | | | | |
| CCK | | | + | | + | + | | | + |
| CCKAR | | | | | | | | | |
| CCKBR | | | | | | | | | |
| CCL1 | | | | | | | | | |
| CCL11 | | | | | | | | | |
| CCL13 | | | | | | | | | |
| CCL14 | | | | | | | | | |
| CCL15 | | | | | | | | | |
| CCL16 | | | | | | | | | |
| CCL17 | | | | | | | | | |
| CCL18 | | | | | | | | | |
| CCL19 | | | | | | | | | |
| CCL2 | | | | | | | | | |
| CCL20 | | | | | | | | | |
| CCL21 | | | | | | | | | |
| CCL22 | | | | | | | | | |
| CCL23 | | | | | | | | | |
| CCL24 | | | | | | | | | |
| CCL25 | | | | | | + | | | + |
| CCL26 | | | | | | | | | |
| CCL27 | | | | | | | | | |
| CCL28 | | | | | | | | | |
| CCL3 | | | | | | | | | |
| CCL3L1 | | | | | | | | | |
| CCL3L3 | | | | | | | | | |
| CCL4 | | | | | | | | | |
| CCL4L1 | | | | | | | | | |
| CCL4L2 | | | | | | | | | |
| CCL5 | | | | | | | | | |
| CCL7 | | | | | | | | | |
| CCL8 | | | | | | | | | |
| CCM2 | | | | | | | | | |
| CCM2L | | | | | | | | | |
| CCNA1 | | | | | | | | | |
| CCNA2 | | | | | | | | | |
| CCNB1 | | | | | | | | | |
| CCNB1IP1 | | | | | | | | | |

| | | | | | | | | | |
|---|---|---|---|---|---|---|---|---|---|
| CCNB2 | | | | | | | | | |
| CCNB3 | | | | | | | | | |
| CCNC | | | | + | | | | | + |
| CCND1 | | | | | | | | | |
| CCND2 | | | | | | + | | | + |
| CCND3 | | | + | | | + | | | + |
| CCNDBP1 | | | | | | | | | |
| CCNE1 | | | | | | | | | |
| CCNE2 | | | | | | | | | |
| CCNF | | | | | + | | | | + |
| CCNG1 | | | | | | | | | |
| CCNG2 | | | | | | + | | | + |
| CCNH | | + | | | | + | | | + |
| CCNI | | | | | | | | | |
| CCNI2 | | | | | | | | | |
| CCNJ | | | | | | | | | |
| CCNJL | | | | | | | | | |
| CCNK | | | | | | | | | |
| CCNL1 | + | | | | | | | | + |
| CCNL2 | | | + | | | | | | + |
| CCNO | | | | | | | | | |
| CCNT1 | + | | | | | | | | + |
| CCNT2 | + | | | | + | | | | + |
| CCNY | | | | | | | | | |
| CCNYL1 | | | | | | | | | |
| CCNYL2 | | | | | | | | | |
| CCP1 | | | | | | | | | |
| CCP110 | | | | | | + | | | + |
| CCPG1 | | | | | | | | | |
| CCR1 | | | | | | | | | |
| CCR10 | | | | | | | | | |
| CCR2 | | | | | | | | | |
| CCR3 | | | | | | | | | |
| CCR4 | + | | | + | + | | + | | + |
| CCR5 | | | | + | | | | | + |
| CCR6 | | | | | | | | | |
| CCR7 | | | | | | | | | |
| CCR8 | | | | | | | | | |
| CCR9 | | | | | | | + | | + |
| CCRL2 | | | | | | | | | |
| CCRN4L | | | | | + | | | | + |
| CCS | | | | | | | | | |
| CCS1 | | | | | | | | | |
| CCSAP | | | | | | | | | |
| CCSER1 | | | | | | | | | |
| CCSER2 | | | | | | | | | |
| CCT2 | | | | | + | | | | + |
| CCT3 | | | | | | | | | |
| CCT4 | | | | | | | | | |
| CCT5 | | | | | | | | | |
| CCT6 | | | | | | | | | |
| CCT6A | | | | | | | | | |
| CCT6B | | | | | | | | | |

| | | | | | | | | | |
|---|---|---|---|---|---|---|---|---|---|
| CCT6P1 | | | | | | | | | |
| CCT7 | | | | | | | | | |
| CCT8 | | | | | + | | | | + |
| CCT8L1P | | | | | | | | | |
| CCT8L2 | | | | | | | | | |
| Cctgamma | | | | | | | | | |
| CCZ1 | | | | | | | | | |
| CCZ1B | | | | | | | | | |
| CD101 | | | | | | | | | |
| CD109 | | + | | | | + | | | + |
| CD14 | | | | | | | | | |
| CD151 | | | | | | | | | |
| CD160 | | | | | | | | | |
| CD163 | | | | | | | | | |
| CD163L1 | | | | | | | | | |
| CD164 | | | | | | | | | |
| CD164L2 | | | | | | | | | |
| CD177 | | | | | | | | | |
| CD180 | | | | | | | | | |
| CD19 | | | | | | | | | |
| CD1A | | | | | | | | | |
| CD1B | | | | | | | | | |
| CD1D | | | | | | | | | |
| CD1E | | | | | | | | | |
| CD2 | | | | | | | | | |
| CD200 | | + | | | | + | | | + |
| CD200R1 | | | | | | | | | |
| CD200R1L | | | | | | | | | |
| CD207 | | | | | | | | | |
| CD209 | | | | | | | | | |
| CD22 | | | | | | | | | |
| CD226 | | | | | | | | | |
| CD24 | | | | | | | | | |
| Cd244 | | | | | | | | | |
| CD247 | | | | | | | | | |
| CD248 | | | | | | | | | |
| CD27 | | | | | | | | | |
| CD274 | + | | | + | + | | + | | + |
| CD276 | | | | | | | | | |
| CD28 | | | | | | | | | |
| CD2AP | | | | | | | | | |
| CD2BP2 | | | | | | | | | |
| CD300A | | | | | | | | | |
| CD300C | | | | | | | | | |
| CD300E | | | | | | | | | |
| CD300LB | | | | | | | | | |
| CD300LD | | | | | | | | | |
| CD300LF | + | | | + | + | | | | + |
| CD300LG | + | | | + | + | | + | | + |
| CD302 | | + | + | | | + | | | + |
| CD320 | | | | | | | | | |
| CD33 | | | | | | | | | |
| CD34 | | | | | | | | | |

| | | | | | | | | | |
|---|---|---|---|---|---|---|---|---|---|
| CD36 | | | | | | | | | |
| CD37 | | | | | | | | | |
| CD38 | + | | | + | + | | + | | + |
| CD3D | + | | | + | + | | + | | + |
| Cd3e | | | | | | | | | |
| CD3EAP | | | | | | | | | |
| CD3G | | | | | | | | | |
| CD4 | | | | | | | | | |
| CD40 | | | | | | | | | |
| CD40LG | | | | | | | | | |
| CD44 | | | | | | | | | |
| CD46 | | | | | | | | | |
| CD47 | | + | | | | + | | | + |
| CD48 | | | | | | | | | |
| CD5 | | | | | | | | | |
| CD52 | | | | | | | | | |
| CD53 | | + | + | | | + | | | + |
| CD55 | | | | | | | | | |
| CD58 | | | | | | | | | |
| CD59 | | | | | | | | | |
| CD6 | | | | | | | | | |
| CD63 | | | | | + | | | | + |
| CD68 | | | + | | | | | | + |
| CD69 | | | | | | | | | |
| CD7 | | | | | | | | | |
| CD70 | | | | | | | | | |
| CD72 | | | | | | | | | |
| CD74 | + | | | + | + | | | | + |
| CD79A | | | | | | | | | |
| CD79B | | | | | | | | | |
| CD80 | | | | | | | | | |
| CD81 | | | | | | | | | |
| CD82 | | | | | + | | | | + |
| CD83 | | | | | | | | | |
| CD84 | | + | + | | | + | | | + |
| CD86 | | | | | | | | | |
| CD8A | | + | + | | | + | | | + |
| CD8B | | | | | | | | | |
| CD9 | | | | | | | | | |
| CD93 | | | | | | | | | |
| CD96 | | | | | | | | | |
| CD97 | | | | | | | | | |
| CD99 | | | | | | | | | |
| CD99L2 | | | | | | | | | |
| CD99P1 | | | | | | | | | |
| CDA | | | | | | | | | |
| CDADC1 | | | | | | | | | |
| CDAN1 | | | | | | + | | | + |
| CDC10 | | | | | | | | | |
| CDC11 | | | | | | | | | |
| CDC12 | | | | | | | | | |
| CDC123 | | | | | | + | | | + |
| CDC14A | | | | | | | | | |

| | | | | | | | | | |
|---|---|---|---|---|---|---|---|---|---|
| CDC14B | | | + | + | | | | | + |
| CDC14C | | | | | | | | | |
| CDC16 | | | | | + | | | | + |
| CDC19 | | | | | | | | | |
| cdc2 | | | | | | | | | |
| CDC20 | | | | | | | | | |
| CDC20B | | | | | | | | | |
| CDC21 | | | | | | | | | |
| CDC23 | | | | | | | | | |
| CDC25A | | | | | | | | | |
| CDC25B | | | | | | | | | |
| CDC25C | | | | | | | | | |
| CDC26 | | | | | | | | | |
| CDC27 | | | | | | | | | |
| CDC28 | | | | | | | | | |
| CDC3 | | | | | | | | | |
| cdc31 | | | | | | | | | |
| CDC33 | | | | | | | | | |
| CDC34 | | | | | | | | | |
| CDC37 | | | | + | | | | | + |
| CDC37L1 | | | | | | | | | |
| CDC39 | | | | | | | | | |
| CDC4 | | | | | | | | | |
| CDC40 | | + | | | | + | | | + |
| CDC42 | | | | | | | | | |
| CDC42BPA | | | + | | | | | | + |
| CDC42BPB | | | | | | | | | |
| CDC42BPG | | | | | | | | | |
| CDC42EP1 | | | | | | | | + | + |
| CDC42EP2 | | | | | | | | | |
| CDC42EP3 | + | | | | | | | | + |
| CDC42EP4 | | | | | | | | | |
| CDC42EP5 | | | | | | | | | |
| CDC42SE1 | | | | | | | | | |
| CDC42SE2 | | | | | | | + | | + |
| CDC45 | | | | | | | | | |
| CDC48 | | | | | | | | | |
| CDC5 | | | | | | | | | |
| CDC50 | | | | | | | | | |
| CDC53 | | | | | | | | | |
| CDC5L | | | | | | | | | |
| CDC6 | | | | | | | | | |
| CDC60 | | | | | | | | | |
| CDC7 | | | | | | | | | |
| CDC73 | | | | | | | | | |
| CDC9 | | | | | | | | | |
| CDCA2 | | | | | | | | | |
| CDCA3 | | | | | | | | | |
| CDCA4 | | | | | | | | | |
| CDCA5 | | | | | | | | | |
| CDCA7 | | | | | | | | | |
| CDCA7L | | | | | | | | | |
| CDCA8 | + | | | + | + | | | | + |

| | | | | | | | | | |
|---|---|---|---|---|---|---|---|---|---|
| CDCP1 | | | | | | | | | |
| CDCP2 | | | | | | | | | |
| CDH1 | | | | | | | | | |
| CDH10 | | | | | + | | | | + |
| CDH11 | | | + | | | | | | + |
| CDH12 | | | | + | | | | | + |
| CDH13 | | + | | | | + | | | + |
| CDH15 | | | | | | | | | |
| CDH16 | | | | | | | | | |
| CDH17 | | | | | | | | | |
| CDH18 | | | | | | | | | |
| CDH19 | | | | | | | | | |
| CDH2 | | | | | | | | | |
| CDH20 | | | | | | | | | |
| CDH22 | | | | | | | | | |
| CDH23 | | | | | | | | | |
| CDH24 | | + | | | | + | | | + |
| CDH26 | | | | | | | | | |
| CDH3 | | | | | | | | | |
| cdh3-b | | | | | | | | | |
| CDH4 | | | | | | | | | |
| CDH5 | | | | | | | | | |
| CDH6 | | | | | | | | | |
| CDH7 | | | | | | | | | |
| CDH8 | | | | | | + | | | + |
| CDH9 | | | | | | | | | |
| CDHR1 | | | | | | | | | |
| CDHR2 | | | | | | | | | |
| CDHR3 | | | | | | | | | |
| CDHR4 | | | | | | | | | |
| CDHR5 | | | | | | | | | |
| CDIP1 | | | | | | | | | |
| CDIPT | | | | | | | | | |
| CDK1 | | | | | | | | | |
| cdk1-a | | | | | | | | | |
| cdk1-b | | | | | | | | | |
| CDK10 | | | | | | | | | |
| CDK11A | | | | | | | | | |
| CDK11B | + | | | + | + | | | | + |
| CDK12 | | | + | | | | | | + |
| CDK13 | | | | | | | | | |
| CDK14 | | | | | | + | | | + |
| CDK15 | | | | | | | | | |
| CDK16 | | | + | | | | | | + |
| CDK17 | | | | | | | | | |
| CDK18 | | | | | + | | | | + |
| CDK19 | | | + | | | | | | + |
| CDK2 | | | | | | | | | |
| CDK20 | | | | | | | | | |
| CDK2AP1 | | | + | | + | | | | + |
| CDK2AP2 | | | | | | | | | |
| CDK3 | | | | | | | | | |
| CDK4 | | | | | | | | | |

| | | | | | | | | | |
|---|---|---|---|---|---|---|---|---|---|
| CDK5 | | | | | | | | | |
| CDK5R1 | + | | + | + | | + | | | + |
| CDK5R2 | | | + | | | | | | + |
| CDK5RAP1 | | | | | | | | | |
| CDK5RAP2 | | | | | | | | | |
| CDK5RAP3 | | | | | | | | | |
| CDK6 | + | | | | + | | | | + |
| CDK7 | | | | | | | | | |
| CDK8 | | | | | | | | | |
| CDK9 | | | | | | | | | |
| CDKAL1 | | | | | | | | | |
| CDKL1 | | | | | | + | | | + |
| CDKL2 | | | | | | | | | |
| CDKL3 | | | | | | | | | |
| CDKL4 | | | | | | | | | |
| CDKL5 | | | | | | | | | |
| CDKN1A | + | | + | | + | | | | + |
| CDKN1B | | | + | | | | | | + |
| CDKN1C | | | | | | | | + | + |
| CDKN2A | | | | | | | | | |
| CDKN2AIP | | | | | | | | | |
| CDKN2AIPNL | | | | | | | | | |
| CDKN2B | | | | | | | | | |
| CDKN2B-AS1 | | | | | | | | | |
| CDKN2C | | | | | | | | | |
| CDKN2D | | | | | | | | | |
| CDKN3 | | | | | | | | | |
| cdknx | | | | | | | | | |
| CDNF | | | | | | | | | |
| CDO1 | | | | | | | | | |
| CDON | | | | | | | | | |
| CDPF1 | | | | | | | | | |
| CDR1 | | + | | | | + | | | + |
| CDR2 | | | | | | | | | |
| CDR2L | | | | | | | | | |
| CDRT1 | | | | | | | | | |
| CDRT15 | | | | | | | | | |
| CDRT15L2 | | | | | | | | | |
| CDRT4 | | | | | | | | | |
| CDS1 | | | | | | | | | |
| CDS2 | | | | | | + | | | + |
| CDSN | | | | | | | | | |
| CDT1 | | | | | | | | | |
| CDV3 | | | | | | | + | | + |
| CDX1 | | | | | | | | | |
| CDX2 | | | | | | | | | |
| CDX4 | | | | | | | | | |
| CDY1 | | | | | | | | | |
| CDY1B | | | | | | | | | |
| CDY2A | | | | | | | | | |
| CDY2B | | | | | | | | | |
| CDYL | | | | | | | | | |
| CDYL2 | | | | | | | | | |

| | | | | | | | | | |
|---|---|---|---|---|---|---|---|---|---|
| CEACAM1 | | | | | | | | | |
| CEACAM16 | | | | | | | | | |
| CEACAM19 | | | | | | | | | |
| CEACAM21 | | | | | | | | | |
| CEACAM3 | | | | | | | | | |
| CEACAM5 | | | | | | | | | |
| CEACAM6 | | | | | | | | | |
| CEACAM7 | | | | | | | | | |
| CEACAM8 | | | | | | | | | |
| CEBPA | | | | | | | | | |
| CEBPB | | | | | | | | | |
| CEBPD | | | | | | | | | |
| CEBPE | | | | | | | | | |
| CEBPG | | | + | | | | | | + |
| CEBPZ | | | | | | | | | |
| CEBPZOS | | | | | | | | | |
| CECR1 | | | | | | | | | |
| CECR2 | | | | | | | | | |
| CECR5 | | | | | | | | | |
| CECR5-AS1 | | | | | | | | | |
| CECR6 | | | | | | | | | |
| CECR7 | | | | | | | | | |
| ced-12 | | | | | | | | | |
| ced-3 | | | | | | | | | |
| ced-4 | | | | | | | | | |
| ced-5 | | | | | | | | | |
| ced-9 | | | | | | | | | |
| CEF1 | | | | | | | | | |
| CEL | | | | | | | | | |
| CELA1 | | | | | | | | | |
| CELA2A | | | | | | | | | |
| CELA2B | | | | | | | | | |
| CELA3A | | | | | | | | | |
| CELA3B | | | | | | | | | |
| CELF1 | | | | | | | | | |
| CELF2 | | | | | | | | | |
| CELF3 | | | | | | | | | |
| CELF4 | | + | | | | + | | | + |
| CELF5 | | | | | | + | | | + |
| CELF6 | | | | | | | | | |
| CELSR1 | | | | | | | | | |
| CELSR2 | | | | | + | + | | | + |
| CELSR3 | | | + | | | | | | + |
| CEMIP | | | | | | | | | |
| CEMP1 | | | | | | | | | |
| CEND1 | | | | | | | | | |
| CENPA | + | | + | | + | | | | + |
| CENPB | | + | | | | | | | + |
| CENPBD1 | | | | | | | | | |
| CENPBD1P1 | | | | | | | | | |
| CENPC | | | | | | | | | |
| CENPE | | | | | | | | | |
| CENPF | | | | | | | | | |

| | | | | | | | | | |
|---|---|---|---|---|---|---|---|---|---|
| CENPH | | | | | | | | | |
| CENPI | | | | | | | | | |
| CENPJ | | | | | | | | | |
| CENPK | | | | | | | | | |
| CENPL | | | | | | | | | |
| CENPM | | | | | | | | | |
| CENPN | | | | | | | | | |
| CENPO | | | | | | | | | |
| CENPP | | | | | | | | | |
| CENPQ | | | + | | | | | | + |
| CENPT | | | | | | | | | |
| CENPU | | | | | | | | | |
| CENPV | | | | | | + | | | + |
| CENPVP1 | | | | | | | | | |
| CENPW | | | | | | | | | |
| CEP104 | | | | | | | | | |
| CEP112 | | | | | | | | | |
| Cep120 | | | | | | | | | |
| CEP128 | | | | | | | | | |
| Cep131 | | | | | | | | | |
| Cep135 | | | | | | | | | |
| Cep152 | | | | | | | | | |
| Cep162 | | | | | | + | | | + |
| CEP164 | | | | | | | | | |
| Cep170 | | | | | | | | | |
| CEP170B | | | | | | | | | |
| CEP170P1 | | | | | | | | | |
| CEP19 | | | | | | | | | |
| CEP192 | | | | | | | | | |
| CEP250 | | | | | | | | | |
| CEP290 | | | | | | | | | |
| CEP295 | | | | | | + | | | + |
| CEP3 | | | | | | | | | |
| Cep350 | | | | | | | | | |
| CEP41 | | | | | | | | | |
| CEP44 | | | | | | + | | | + |
| Cep55 | | | | | | | | | |
| CEP57 | | | | | | | | | |
| CEP57L1 | | | | | | | | | |
| CEP63 | | | | | | | | | |
| CEP68 | | | | | | | | | |
| CEP70 | | + | | | | + | | | + |
| CEP72 | | | | | | | | | |
| Cep76 | | | | | | | | | |
| Cep78 | | | | | | | | | |
| CEP83 | | | | | | | | | |
| CEP85 | | | | | | | | | |
| Cep85l | | | | | | | | | |
| CEP89 | | | | | | | | | |
| CEP95 | | | | | | | | | |
| CEP97 | | | | | | | | | |
| CEPT1 | | + | | | | + | | | + |
| CER1 | | | | | | | | | |

| | | | | | | | | | |
|---|---|---|---|---|---|---|---|---|---|
| CERCAM | | | | | | | | | |
| CERK | | | + | | | | | | + |
| CERKL | | | | | | | | | |
| CERS1 | | | | | | + | | | + |
| CERS2 | | | | | | | | | |
| CERS3 | | | | | | | | | |
| CERS4 | | | | | | | | | |
| CERS5 | | | | | | | | | |
| CERS6 | | | | | | | | | |
| CERS6-AS1 | | | | | | | | | |
| CES1 | | | | | | | | | |
| CES1P1 | | | | | | | | | |
| CES2 | | | | | | | | | |
| CES3 | | | | | | | | | |
| Ces3a | | | | | | | | | |
| CES4A | | | | | | | | | |
| CETN1 | | | | | | | | | |
| CETN2 | | | | | | | | | |
| CETN3 | | | | | | | | | |
| CETP | | | | | | | | | |
| CFAP126 | | | | | | | | | |
| CFAP20 | | | | | | | | | |
| CFAP221 | | | | | | | | | |
| CFAP36 | | + | | | | + | | | + |
| CFAP43 | | | | | | | | | |
| CFAP44 | | | | | | | | | |
| CFAP45 | | | | | | | | | |
| CFAP46 | | | | | | | | | |
| CFAP52 | | | | | | | | | |
| CFAP53 | | | | | | | | | |
| CFAP54 | | | | | | | | | |
| CFAP57 | | | | | | | | | |
| CFAP58 | | | | | | | | | |
| CFAP61 | | | | | | | | | |
| CFAP69 | | | | | | | | | |
| CFAP70 | | | | | | | | | |
| CFAP74 | | | | | | | | | |
| CFAP97 | | | | | | | | | |
| CFAP99 | | | | | | | | | |
| CFB | | | | | | | | | |
| CFC1 | | | | | | | | | |
| CFD | | | | | | | | | |
| CFDP1 | | | | | | | | | |
| CFH | | | | | | | | | |
| CFHR1 | | | | | | | | | |
| CFHR2 | | | | | | | | | |
| CFHR3 | | | | | | | | | |
| CFHR4 | | | | | | | | | |
| CFHR5 | | | | | | | | | |
| CFI | | | | | | | | | |
| CFL1 | | | | | | | | | |
| CFL2 | | | | | | | | | |
| CFLAR | | | | + | | | | | + |

| | | | | | | | | | |
|---|---|---|---|---|---|---|---|---|---|
| CFP | | | | | | | | | |
| cfr1 | | | | | | | | | |
| CFTR | | | | | | | | | |
| CG1218 | | | | | | | | | |
| CG18769 | | | | | | | | | |
| CG5525 | | | | | | | | | |
| CG7033 | | | | | | | | | |
| CG8258 | | | | | | | | | |
| CGA | | | | | | | | | |
| CGB | | | | | | | | | |
| CGB1 | | | | | | | | | |
| CGB2 | | | | | | | | | |
| CGB5 | | | | | | | | | |
| CGB7 | | | | | | | | | |
| CGB8 | | | | | | | | | |
| CGGBP1 | | | + | | | | | | + |
| cgn | | | | | | | | | |
| CGNL1 | | | | | | | | + | + |
| cgr1 | | | | | | | | | |
| CGREF1 | | | | | + | | | | + |
| CGRRF1 | | | | | | | | | |
| CH25H | | | | | | | | | |
| CHAC1 | | | | | | | | | |
| CHAC2 | | | | | | | | | |
| CHAD | | | | | | | | | |
| CHADL | | | | | | | | | |
| CHAF1A | | | | | | | | | |
| CHAF1B | | | | | | | | | |
| CHAMP1 | | | | | | | | | |
| CHAT | | | | | | | | | |
| CHC1 | | | | | | | | | |
| CHCHD1 | | | | | | | | | |
| CHCHD10 | | | | | | | | | |
| CHCHD2 | | | | | | | | + | + |
| CHCHD2P9 | | | | | | | | | |
| CHCHD3 | | | | | | | | | |
| CHCHD4 | | | | | | | | | |
| CHCHD5 | | | | | | | | | |
| CHCHD6 | | | | | | | | | |
| CHCHD7 | | | | | | | | | |
| CHD1 | | | | | | | | | |
| CHD1L | | | | | | | | | |
| CHD2 | | | | | | | | | |
| CHD3 | | | | | | + | | | + |
| CHD4 | | | | | | | | | |
| CHD5 | | | | | | | | | |
| CHD6 | | + | | | | + | | | + |
| CHD7 | | | | | | | | | |
| CHD8 | | | | | | | | | |
| CHD9 | | | | | | | | | |
| CHDC2 | | | | | | | | | |
| CHDH | | | | | | | | | |
| CHEK1 | | | | | + | | + | | + |

| | | | | | | | | | |
|---|---|---|---|---|---|---|---|---|---|
| CHEK2 | | | | | | | | | |
| cher | | | | | | | | | |
| CHERP | | | | | | | | | |
| CHFR | | | | | | | | | |
| CHGA | | | | | + | + | | | + |
| CHGB | | | | | + | | | | + |
| CHI3L1 | | | | | | | | | |
| CHI3L2 | | | | | | | | | |
| CHIA | | | | | | | | | |
| CHIC1 | + | | | + | + | | + | | + |
| CHIC2 | | | | | | | | | |
| CHID1 | | | | | | | | | |
| CHIT1 | | | | | | | | | |
| chk1 | | | | | | | | | |
| CHKA | | | | | | | | | |
| CHKB | | + | + | | | + | | | + |
| CHL1 | | | | | | + | | | + |
| CHM | | | | | | | | | |
| CHML | | | + | | + | | | | + |
| CHMP1A | | | | | | | | | |
| CHMP1B | | | | | | | | | |
| CHMP2A | | | | | | | | | |
| CHMP2B | | | | | | | | | |
| CHMP3 | | | | | | | | | |
| CHMP4A | | | | | | | | | |
| CHMP4B | | | | | | | | | |
| Chmp4bl1 | | | | | | | | | |
| CHMP4BP1 | | | | | | | | | |
| CHMP4C | | | | | | | | | |
| CHMP5 | | | | | | | | | |
| CHMP6 | | | | | | | | | |
| CHMP7 | | | | | | | | | |
| chn-1 | | | | | | | | | |
| CHN1 | | | | | | + | | | + |
| CHN2 | | | | | | | | | |
| CHO1 | | | | | | | | | |
| CHODL | | | | | | | | | |
| CHORDC1 | | | | | | | | | |
| CHP1 | | | + | | | | | | + |
| CHP2 | | | | | | | | | |
| CHPF | | | | | | | | | |
| CHPF2 | | | | | | | | | |
| CHPT1 | | | | | | | | | |
| CHRAC1 | | + | | | | | | | + |
| CHRD | | | | | | + | | | + |
| CHRDL1 | | | | | | | | | |
| CHRDL2 | | | | | | | | | |
| CHRFAM7A | | | | | | | | | |
| CHRM1 | | | | | | + | | | + |
| CHRM2 | | | | | | | | | |
| CHRM3 | | | | | | | | | |
| CHRM4 | | | | | | | | | |
| CHRM5 | | | | | | | | | |

| | | | | | | | | | |
|---|---|---|---|---|---|---|---|---|---|
| CHRNA1 | | | | | | | | | |
| CHRNA10 | | | | | | | | | |
| CHRNA3 | | | | | | | | | |
| CHRNA4 | | | | | | | | | |
| CHRNA5 | | | | | | | | | |
| CHRNA6 | | | | | | | | | |
| CHRNA7 | | | + | | | | | | + |
| CHRNA9 | | | | | | | | | |
| CHRNB1 | | | | | | | | | |
| CHRNB2 | | | | | | | | | |
| CHRNB3 | | | | | | | | | |
| CHRNB4 | | | | | | | | | |
| CHRND | | | | | | | | | |
| CHRNE | | | | | | | | | |
| CHRNG | | | | | | | | | |
| CHST1 | | + | | | | + | | | + |
| CHST10 | | | | | | + | | | + |
| CHST11 | | | | | | | | | |
| CHST12 | | | | | | + | | | + |
| CHST13 | | | | | | | | | |
| CHST14 | | | | | | | | | |
| CHST15 | | | + | | | + | | | + |
| CHST2 | | | + | | | + | | | + |
| CHST3 | + | | | + | + | | + | | + |
| CHST4 | | | | | | | | | |
| CHST5 | | | | | | | | | |
| CHST6 | | | | | | | | | |
| CHST7 | | | | | | | | | |
| CHST8 | | | | | | | | | |
| CHST9 | | | | | | + | | | + |
| CHSY1 | | | | | | | | | |
| CHSY3 | | | | | | | | | |
| CHTF18 | | | | | | | | | |
| CHTF8 | | | | | | | | | |
| CHTOP | | | | | | | | | |
| CHUK | + | | + | | | | | | + |
| CHURC1 | | | | | + | | | | + |
| CHURC1-FNTB | | | | | | | | | |
| ci | | | | | | | | | |
| CIAO1 | | | | | | | | | |
| CIAPIN1 | + | | | | + | | | | + |
| CIAPIN1P | | | | | | | | | |
| CIART | | | | | | | | | |
| cib | | | | | | | | | |
| CIB1 | | | | | | | | | |
| CIB2 | | | | | | | | | |
| CIB3 | | | | | | | | | |
| CIB4 | | | | | | | | | |
| CIC | | | | | + | | | | + |
| CIDEA | | | | | | | | | |
| CIDEB | | | | | | | | | |
| CIDEC | | | | | | | | | |
| CIDECP | | | | | | | | | |

| | | | | | | | | | |
|---|---|---|---|---|---|---|---|---|---|
| CIITA | | | | | | | | | |
| CIK1 | | | | | | | | | |
| CILP | | | | | | | | | |
| CILP2 | | | | | | | | | |
| CINP | | | | | + | | | | + |
| CIPC | | | | | | | | | |
| CIR1 | | | | | | + | | | + |
| CIRBP | | | | | | | | | |
| CIRH1A | | | | | | | | | |
| CISD1 | | + | | | | + | | | + |
| CISD2 | | | | | | | | | |
| CISD3 | | + | | | | + | | | + |
| CISH | | | | | | | | | |
| CIT | | | | | | | | | |
| CIT1 | | | | | | | | | |
| CIT2 | | | | | | | | | |
| CITED1 | | | | | | | | | |
| CITED2 | + | | + | | | | | | + |
| CITED4 | | | | | | | | | |
| CIZ1 | | | | | | | | | |
| cka1 | | | | | | | | | |
| CKAP2 | | | | + | | | | | + |
| CKAP2L | | | | | | | | | |
| CKAP4 | | | + | | | | | | + |
| CKAP5 | | + | | | | + | | | + |
| CKB | | | | | + | | | | + |
| ckb1 | | | | | | | | | |
| CKI1 | | | | | | | | | |
| CKLF | | | | | | | | | |
| CKM | | | | | | | | | |
| CKMT1A | | | | | | | | | |
| CKMT1B | | | | | | | | | |
| CKMT2 | | | | | | | | | |
| CKMT2-AS1 | | | | | | | | | |
| CKS1 | | | | | | | | | |
| CKS1B | | | | | | | | | |
| CKS1BP3 | | | | | | | | | |
| CKS1BP7 | | | | | | | | | |
| CKS2 | | | | | | | | | |
| CLAM | | | | | | | | | |
| CLASP1 | | | | | | + | | | + |
| CLASP2 | | + | | | | + | | | + |
| CLASRP | | | | | | | | | |
| CLB1 | | | | | | | | | |
| CLC | | | | | | | | | |
| CLC1 | | | | | | | | | |
| CLCA1 | | | | | | | | | |
| CLCA2 | | | | | | | | | |
| CLCA3P | | | | | | | | | |
| CLCA4 | | | | | | | | | |
| CLCC1 | | | | | | | | | |
| CLCF1 | | | | | | | | | |
| CLCN1 | | | | | | | | | |

| | | | | | | | | | |
|---|---|---|---|---|---|---|---|---|---|
| CLCN2 | | | | | | + | | | + |
| CLCN3 | | | | | | | | | |
| CLCN4 | | | | | | | | | |
| CLCN5 | | | + | | | | | | + |
| CLCN6 | | | | | | | | | |
| CLCN7 | | | | | | | | | |
| CLCNKA | | | | | | | | | |
| CLCNKB | | | | | | | | | |
| CLDN1 | | | | | | | | | |
| CLDN10 | | | | | | | | | |
| CLDN11 | | | | | | | | | |
| CLDN12 | | | | | | + | | | + |
| CLDN14 | | | | | | | | | |
| CLDN15 | | | | | | | | | |
| CLDN16 | | | | | | | | | |
| CLDN17 | | | | | | | | | |
| CLDN18 | | | | | | | | | |
| CLDN19 | | | | | | | | | |
| CLDN2 | | | | | | | | | |
| CLDN20 | | | | | | | | | |
| CLDN22 | | | | | | | | | |
| CLDN23 | | | | | | | | | |
| CLDN3 | | | | | | | | | |
| CLDN4 | | | | | | | | | |
| CLDN5 | | | | | | | | | |
| CLDN6 | | | | | | | | | |
| CLDN7 | | | | | | | | | |
| CLDN8 | | | | | | | | | |
| CLDN9 | | | | | | | | | |
| CLDND1 | | | | | | | | | |
| CLDND2 | | | | | | | | | |
| CLEC10A | | | | | + | | | | + |
| CLEC11A | | | | | | | | | |
| CLEC12A | | | | | | | | | |
| CLEC12B | | | | | | | | | |
| CLEC14A | | | | | | | | | |
| CLEC16A | | | | | | | | | |
| CLEC17A | | | | | | | | | |
| CLEC18A | | | | | | | | | |
| CLEC18C | | | | | | | | | |
| CLEC19A | | | | | | | | | |
| CLEC1A | | | | | | | | | |
| CLEC1B | | | | | | | | | |
| CLEC2A | | | | | | | | | |
| CLEC2B | | | | | | | | | |
| CLEC2D | | | | | | | | | |
| CLEC2L | | | | | | | | | |
| CLEC3A | | | | | | | | | |
| CLEC3B | | | | | | | | | |
| CLEC4A | | | | | | | | | |
| CLEC4C | | | | | | | | | |
| CLEC4D | | | | | | | | | |
| CLEC4E | | | | | | | | | |

| | | | | | | | | | |
|---|---|---|---|---|---|---|---|---|---|
| CLEC4F | | | | | | | | | |
| CLEC4G | | | | | | | | | |
| CLEC4M | | | | | | | | | |
| CLEC5A | | | | | | | | | |
| CLEC7A | | | | | | | | | |
| Clec9a | | | | | | | | | |
| CLECL1 | | | | | | | | | |
| CLGN | | | | | | | | | |
| CLHC1 | | | | | | | | | |
| CLIC1 | | | | | | | | | |
| CLIC2 | | | | | | | | | |
| CLIC3 | | | | | | | | | |
| Clic4 | | | | | | | | | |
| CLIC5 | | | | | | | | | |
| CLIC6 | | | | | | | | | |
| CLINT1 | | | | | | | | | |
| clint1-a | | | | | | | | | |
| Clip1 | | | + | | | | | | + |
| CLIP2 | | | | | | | | | |
| CLIP3 | | | | | | | | | |
| CLIP4 | | | | | | | | | |
| CLK1 | | | | | | | | | |
| CLK2 | | | | | | | | | |
| CLK3 | | | | | | | | | |
| CLK4 | | | | | | | | | |
| CLLU1OS | | | | | | | | | |
| CLMN | | | | | | | | | |
| CLMP | | | | | | | | | |
| CLN3 | | | | | | | | | |
| CLN5 | | | | | | | | | |
| CLN6 | | | | | | + | | | + |
| CLN8 | | | | | + | | | | + |
| CLNK | | | | | | | | | |
| CLNS1A | | | | | | + | | | + |
| CLOCK | | | | | | | | | |
| CLP1 | | | | | | | | | |
| CLPB | | | | | | | | | |
| CLPP | | + | | | | + | | | + |
| CLPS | | | | | | | | | |
| CLPSL1 | | | | | | | | | |
| CLPSL2 | | | | | | | | | |
| CLPTM1 | | | | | | | | | |
| CLPTM1L | | | | | | | | | |
| CLPX | | | | | + | | | | + |
| clr5 | | | | | | | | | |
| CLRN1 | + | | | + | + | | + | | + |
| CLRN2 | | | | | | | | | |
| CLRN3 | | | | | | | | | |
| CLSPN | | | | | | | | | |
| CLSTN1 | | + | | | + | + | | | + |
| CLSTN2 | | | | | | | | | |
| CLSTN3 | | | + | | + | | | | + |
| CLTA | | | | | | | | | |

| | | | | | | | | | |
|---|---|---|---|---|---|---|---|---|---|
| CLTB | + | | | | + | | | | + |
| CLTC | + | | | | + | | | | + |
| CLTCL1 | | | | | | | | | |
| CLU | | | | | | | | | |
| CLUAP1 | | | | | | | | | |
| CLUH | | | | | | + | | | + |
| CLUHP3 | | | | | | | | | |
| CLUL1 | | | | | | | | | |
| CLVS1 | | | | | | | | | |
| CLVS2 | | | | | | | | | |
| CLYBL | | | | | | | | | |
| CMA1 | | | | | | | | | |
| CMAHP | | | | | | | | | |
| CMAS | | | | | | | | | |
| cmb1 | | | | | | | | | |
| CMBL | | | | | | | | | |
| CMC1 | | | | | | | | | |
| CMC2 | | | | | | | | | |
| CMC4 | | | | | | | | | |
| CMD1 | | | | | | | | | |
| CMIP | | | | + | | | | | + |
| CMK1 | | | | | | | | | |
| CMKLR1 | | | | | | | | | |
| CMPK1 | | | | | | | | | |
| CMSS1 | | | | | | | | | |
| CMTM1 | | | | | | | | | |
| CMTM2 | | | | | | | | | |
| CMTM3 | | | | | | | | | |
| CMTM4 | | | + | | | | | | + |
| CMTM5 | | | | | | | | | |
| CMTM6 | | | | | | | | | |
| CMTM7 | | | | | | | | | |
| CMTM8 | + | | | + | + | | + | | + |
| CMTR1 | | | | | | | | | |
| CMTR2 | | | | | | | | | |
| CMYA5 | | | | | | | | | |
| CNBD1 | | | | | | | | | |
| CNBP | | | | | | | | | |
| CNDP1 | | | | | | | | | |
| CNDP2 | | | | | | | | | |
| CNEP1R1 | | | | | | | | | |
| CNFN | | | | | | | | | |
| CNGA1 | | | | | | | | | |
| CNGA3 | | | | | | | | | |
| CNGA4 | | | | | | | | | |
| CNGB1 | | | | | | | | | |
| CNGB3 | | | | | | | | | |
| CNIH1 | | | | | | | | | |
| CNIH2 | | | + | | | | | | + |
| CNIH3 | | | | | | | | | |
| CNIH4 | | | | | | + | | | + |
| CNKSR1 | | | | | | | | | |
| CNKSR2 | | | | | | | | | |

| | | | | | | | | | |
|---|---|---|---|---|---|---|---|---|---|
| CNKSR3 | | | | | | | | | |
| CNN1 | | | | | | | | | |
| CNN2 | | | | | | | + | | + |
| CNN3 | | | | | | | | | |
| CNNM1 | + | | | | + | | | | + |
| CNNM2 | | | + | | + | | | | + |
| CNNM3 | | | | | | | | | |
| CNNM4 | | | | | | | | | |
| cno | | | | | | | | | |
| CNOT1 | | | | | | | | | |
| CNOT10 | | | | | | | | | |
| CNOT11 | | | | | + | | | | + |
| CNOT2 | | | | | | | | | |
| CNOT3 | | | | | | | | | |
| CNOT4 | | | + | | | | | | + |
| CNOT6 | | | | | | | | | |
| CNOT6L | | | + | | | | | | + |
| CNOT7 | | + | + | | | + | | | + |
| CNOT8 | | | | | | | | | |
| CNP | | | | | + | | | | + |
| CNPPD1 | | | | | | | | | |
| CNPY2 | | + | | | | + | | | + |
| CNPY3 | | | | | | + | | | + |
| CNPY4 | | | | | | | | | |
| CNR1 | | | | | | | | | |
| CNR2 | | | | | | | | | |
| CNRIP1 | | | | | | | | | |
| CNST | | | | | + | | | | + |
| CNTD1 | | | | | | | + | | + |
| CNTF | | | | | | | | | |
| CNTFR | | | | | | | | | |
| CNTLN | | | | | | | | | |
| CNTN1 | | | | | | | | | |
| CNTN2 | | | | | | + | | | + |
| CNTN4 | | | | | | | | | |
| CNTN5 | | | | | | | | | |
| CNTN6 | | | | | | | | | |
| CNTNAP1 | | | | | | + | | | + |
| CNTNAP2 | | | | | | | | | |
| CNTNAP3 | | | | | | | | | |
| CNTNAP3B | | | | | | | | | |
| CNTNAP4 | | | | | | | | | |
| CNTNAP5 | | | | | | | | | |
| CNTRL | | | | | | | | | |
| CNTROB | | | | | | | | | |
| cnx1 | | | | | | | | | |
| COA1 | | | | | | | | | |
| COA3 | | + | | | | + | | | + |
| COA4 | | | | | | | | | |
| COA5 | + | | | + | + | | | | + |
| COA6 | | | | | | | | | |
| COA7 | | | | | | | | | |
| COASY | | | | | | | | | |

| | | | | | | | | | |
|---|---|---|---|---|---|---|---|---|---|
| COBL | | | | | + | | | | + |
| Cobll1 | | | | | | | | | |
| COCH | + | | + | | | | | | + |
| COG1 | | | | | | | | | |
| COG2 | | | | | | | | | |
| COG3 | | | | | | | | | |
| COG4 | | | | | | | | | |
| COG5 | | | | | | | | | |
| COG6 | | | | | | | | | |
| COG7 | | | | | | | | | |
| COG8 | | | | | | | | | |
| COIL | | | | | | | | | |
| COL10A1 | | | | | | | | | |
| COL11A1 | | | | | | | | | |
| COL11A2 | | | | | | | | | |
| COL12A1 | | | | | | | | | |
| COL13A1 | | | | | | | | | |
| COL14A1 | | | | + | | | | | + |
| COL15A1 | | | | | | | | | |
| COL16A1 | | | | | | | | | |
| COL17A1 | | | | | | | | | |
| COL18A1 | | | | | | | | | |
| COL19A1 | | + | | + | | | | | + |
| COL1A1 | | | | | | | | | |
| COL1A2 | | | | | | | | | |
| COL20A1 | | | | | | | | | |
| COL21A1 | | | | | | | | | |
| COL22A1 | | | | | | | | | |
| COL23A1 | + | | | + | + | | | | + |
| COL24A1 | | | | | | | | | |
| COL25A1 | | | | + | | + | | | + |
| COL26A1 | | | + | | | | | | + |
| COL27A1 | + | | | + | + | | | | + |
| COL28A1 | | | | | | | | | |
| COL2A1 | | | | | | | | | |
| COL3A1 | | | | | | | | | |
| COL4A1 | | | | | | | | | |
| COL4A2 | | | | | | | | | |
| COL4A3 | | | | | | | | + | + |
| COL4A3BP | | | | | | | | | |
| COL4A4 | | | | | | | | | |
| COL4A5 | | | | | | | | + | + |
| COL4A6 | | | | | | | | | |
| COL5A1 | + | | | + | + | | | | + |
| COL5A2 | | | | | | | | | |
| COL5A3 | | | | | | | | | |
| COL6A1 | + | | | + | + | | | | + |
| COL6A2 | + | | | + | + | | | | + |
| COL6A3 | | | | | | | | | |
| COL6A4P1 | | | | | | | | | |
| COL6A5 | | | | | | | | | |
| COL6A6 | | | | | | | | | |
| COL7A1 | + | | | + | + | | | | + |

| | | | | | | | | | |
|---|---|---|---|---|---|---|---|---|---|
| COL8A1 | | | | | | | | | |
| COL8A2 | | | | | | | | | |
| COL9A1 | + | | | + | + | | | | + |
| COL9A2 | | | | | | | | | |
| COL9A3 | | | | | + | | | | + |
| COLCA2 | | | | | | | | | |
| COLEC10 | | | | | | | | | |
| COLEC11 | | | | | | | | | |
| COLEC12 | | | | + | | | | | + |
| COLGALT1 | | | | | | | | | |
| COLGALT2 | | | | | | | | | |
| COLQ | + | | | + | + | | | | + |
| COMMD1 | | | | | | | | | |
| COMMD10 | | | | | | | | | |
| COMMD2 | | | | | | | | | |
| COMMD3 | | + | | | | + | | | + |
| COMMD3-BMI1 | | | | | | | | | |
| COMMD4 | | | | | | | | | |
| COMMD5 | | | | | | | | | |
| COMMD6 | | | | | | | | | |
| COMMD7 | | | | | | | | | |
| COMMD8 | | | | | | | | | |
| COMMD9 | | | | | | | | | |
| COMP | | | | | | | | | |
| COMT | | | | | | + | | | + |
| COMTD1 | | | | | | | | | |
| COP1 | | | | | | | | | |
| COPA | | | | | | | | | |
| COPB1 | | | | | | | | | |
| COPB2 | | + | | + | | + | | | + |
| COPD | | | | | | | | | |
| COPE | | | | | | | | | |
| COPG1 | | | | | | | | | |
| COPG2 | | | | | | | | | |
| COPRS | | | | | | | | | |
| COPS2 | | | | | | | | | |
| COPS3 | | | | | | | | | |
| COPS4 | | | | | | | | | |
| COPS5 | | | | | + | | | | + |
| COPS6 | | + | | | | + | | | + |
| COPS7A | | | | | | | | | |
| COPS7B | | | | | | | | | |
| COPS8 | | | | | | | | | |
| COPZ1 | | | | | | | | | |
| COPZ2 | | | | | | | | | |
| COQ10A | | | | | | | | | |
| COQ10B | | + | + | | + | + | | | + |
| COQ2 | | | | | | | | | |
| COQ3 | | | | | | | | | |
| COQ4 | | | | | | | | | |
| COQ5 | | | | | | | | | |
| COQ6 | | | | | | | | | |
| COQ7 | | | | | | | | | |

| | | | | | | | | | | |
|---|---|---|---|---|---|---|---|---|---|---|
| COQ8 | | | | | | | | | | |
| COQ9 | | | | | | | | | | |
| COR1 | | | | | | | | | | |
| CORIN | | | | | | | | | | |
| CORO1A | | | | | | | | | | |
| CORO1B | | | | | | | | | | |
| CORO1C | + | | + | | | | | | + |
| CORO2A | | | | | | | | | | |
| CORO2B | | | + | | | | | | + |
| CORO6 | | | | | | | | | | |
| CORO7 | | | | | | | | | | |
| CORO7-PAM16 | | | | | | | | | | |
| CORT | | | | | | | | | | |
| COS111 | | | | | | | | | | |
| COS12 | | | | | | | | | | |
| COTL1 | | | | | | | | | | |
| COX1 | | | | | | | | | | |
| COX10 | | | | | | | | | | |
| COX11 | | | | | | | | | | |
| COX14 | | | | | | | | | | |
| COX15 | | | | | | | | | | |
| COX16 | | | | | | + | | | + |
| COX17 | | | | | | | | | | |
| COX18 | | | | | | | | | | |
| COX19 | | | | | | | | | | |
| COX2 | | | | | | | | | | |
| COX20 | | | | | | | | | | |
| COX3 | | | | | | | | | | |
| cox4 | | | | | | | | | | |
| COX4I1 | | | | | | | | | | |
| COX4I1P1 | | | | | | | | | | |
| COX4I2 | | | | | | | | | | |
| cox5 | | | | | | | | | | |
| COX5A | | | | | | | | | | |
| COX5B | | | | | | | | | | |
| COX6A1 | | | | | | | | | | |
| COX6A2 | | | | | | | | | | |
| COX6B1 | | | | | | | | | | |
| COX6B2 | | | | | | | | | | |
| COX6C | | | | | | | | | | |
| COX7A1 | | | | | | | | | | |
| COX7A2 | | | | | | | | | | |
| COX7A2L | | + | + | | | + | | | + |
| COX7A2P2 | | | | | | | | | | |
| COX7B | | | | | | | | | | |
| COX7B2 | | | | | | | | | | |
| COX7C | | + | + | | | + | | + | + |
| COX8A | | | | | | | | | | |
| CP | | | | | | | | | | |
| Cp190 | | | | | | | | | | |
| CPA1 | | | | | | | | | | |
| CPA2 | | | | | | | | | | |
| CPA3 | | | | | | | | | | |

| | | | | | | | | | |
|---|---|---|---|---|---|---|---|---|---|
| CPA4 | | | | | | | + | | + |
| CPA5 | | | | | | | | | |
| CPA6 | | | | | | | | | |
| CPAMD8 | | | | | | | | | |
| CPB2 | | | | | | | | | |
| cpc2 | | | | | | | | | |
| CPD | | | | | | | | | |
| CPE | | + | | | + | + | | | + |
| CPEB1 | + | | | | | | | | + |
| CPEB2 | | | | | | | | | |
| CPEB3 | | | | | | | | | |
| CPEB4 | | | | | | | | | |
| CPED1 | | | | | | | | | |
| CPLX1 | | | + | | | | | | + |
| CPLX2 | + | | + | + | + | + | | | + |
| CPLX3 | | | | | | | | | |
| CPLX4 | | | | | | | | | |
| CPM | | | | | | | | | |
| CPN1 | | | | | | | | | |
| CPN2 | | | | | | | | | |
| CPNE1 | | | | | | | | | |
| CPNE2 | | | | | | | | | |
| CPNE3 | | | + | | | | | | + |
| CPNE4 | | + | | + | | + | | | + |
| CPNE5 | | | | | | | | | |
| CPNE6 | | | | | | + | | | + |
| CPNE7 | | | + | | | | | | + |
| CPNE8 | + | | | | | | | | + |
| CPNE9 | | | | | | | | | |
| CPOX | | | | | | | | | |
| CPPED1 | | | | | | | | | |
| CPQ | | | | | | | | | |
| CPR1 | | | | | | | | | |
| CPR2 | | | | | | | | | |
| CPR5 | | | | | | | | | |
| CPR6 | | | | | | | | | |
| CPS1 | | | | | | | | | |
| CPSF1 | | | | | | | | | |
| CPSF2 | | | | | + | + | | | + |
| CPSF3 | | | | | | | | | |
| CPSF3L | | | | | | | | | |
| CPSF4 | | | | | | + | | | + |
| CPSF4L | | | | | | | | | |
| CPSF6 | | | + | | | | | | + |
| CPSF7 | | | | | | | | | |
| CPT1A | | | | | | | | | |
| CPT1B | | | | | | | | | |
| CPT1C | | | | | | | | | |
| CPT2 | | | | | | | | | |
| CPTP | | | | | | | | | |
| CPVL | | | | | | | | | |
| CPXCR1 | | | | | | | | | |
| CPXM1 | | | | | | | | | |

| | | | | | | | | | |
|---|---|---|---|---|---|---|---|---|---|
| CPXM2 | | | | | | | | | |
| cpy1 | | | | | | | | | |
| CPZ | | | | | | | | | |
| CR1 | | | | | | | | | |
| CR1L | | | | | | | | | |
| CR2 | | | | | | | | | |
| CRA1 | | | | | | | | | |
| CRABP1 | | | | | | | | | |
| CRABP2 | | | + | | | | | | + |
| CRACR2A | | | | | | | | | |
| CRACR2B | | | | | | | | | |
| CRADD | | | | | | | | | |
| CRAMP1L | | | | | | | | | |
| CRAT | | | | | | | | | |
| CRB1 | | | | | | | | | |
| CRB2 | | | | | | | | | |
| CRB3 | | | | | | | | | |
| CRBN | | | | | | | | | |
| CRCP | | | | | | | | | |
| CRCT1 | | | | | | | | | |
| CREB1 | | | + | | | | | | + |
| CREB3 | | | | | | | | | |
| CREB3L1 | | | | | | | | | |
| CREB3L2 | + | | | + | + | | | | + |
| CREB3L3 | | | | | | | | | |
| CREB3L4 | | | | | | | | | |
| CREB5 | | | | | | | | | |
| CREBBP | | | | | | | | | |
| CREBL2 | | | | | | | | | |
| CREBRF | | | | | | | | | |
| CREBZF | | | | | | | | | |
| CREG1 | | + | + | | | + | | | + |
| CREG2 | | | | | | + | | | + |
| CRELD1 | | | | | | | | | |
| CRELD2 | | | | | | | | | |
| CREM | + | | + | | | | | | + |
| CRH | + | | + | | + | | | | + |
| CRH1 | | | | | | | | | |
| CRHBP | | | + | | | | | | + |
| CRHR1 | | | | | | | | | |
| Crhr2 | | | | | | | | | |
| CRIM1 | | | | | | | | | |
| CRIP1 | | | | | | | | | |
| CRIP2 | | | | | | + | | | + |
| CRIP3 | | | | | | | | | |
| CRIPAK | | | | | | | | | |
| CRIPT | | | | | | | | | |
| CRISP1 | | | | | | | | | |
| CRISP2 | | | | | | | | | |
| CRISP3 | | | | | | | | | |
| CRISPLD1 | | | | | | | | | |
| CRISPLD2 | | | | | | | | | |
| CRK | | | | | | | | | |

| | | | | | | | | | |
|---|---|---|---|---|---|---|---|---|---|
| CRKL | | | | | | | | | |
| CRLF1 | | | | | | + | | | + |
| CRLF2 | | | | | | | | | |
| CRLF3 | | | | | | | | | |
| CRLS1 | | | | | | | | | |
| CRM1 | | | | | | | | | |
| CRMP1 | | | | | | + | | | + |
| CRNDE | | | | | | | | | |
| Crnkl1 | | | | | | | | | |
| CRNN | | | | | | | | | |
| CROCC | | | | | | | | | |
| CROCCP2 | | | | | | | | | |
| CROCCP3 | | | | | | | | | |
| CROCCP4 | | | | | | | | | |
| CROT | | | | | | | | | |
| CRP | | | | | | | | | |
| CRP1 | | | | | | | | | |
| CRS5 | | | | | | | | | |
| CRSP5 | | | | | | | | | |
| CRTAC1 | | | | | | | | | |
| CRTAP | | | | | | | | | |
| CRTC1 | | | | | | | | | |
| CRTC2 | | | | | | | | | |
| CRTC3 | | | | + | | | | | + |
| CRTC3-AS1 | | | | | | | | | |
| CRU3 | | | | | | | | | |
| CRX | | | | | | | | | |
| CRY1 | | | | | + | | | | + |
| CRY2 | | | + | | | | | | + |
| CRYAA | | | | | | | | | |
| CRYAB | | | | | | | | | |
| CRYBA1 | | | | | | | | | |
| CRYBA2 | | | | | | | | | |
| CRYBA4 | | | | | | | | | |
| CRYBB1 | | | | | | | | | |
| CRYBB2 | | | | | | | | | |
| CRYBB2P1 | | | | | | | | | |
| CRYBB3 | | | | | | | | | |
| CRYBG3 | | | | | + | | | | + |
| CRYGA | | | | | | | | | |
| CRYGB | | | | | | | | | |
| CRYGC | | | | | | | | | |
| CRYGD | | | | | | | | | |
| CRYGN | | | | | | | | | |
| CRYGS | | | | | | | | | |
| CRYL1 | | | | | | | | | |
| CRYM | | | | | | | | | |
| CRYZ | | | | | | + | | | + |
| CRYZL1 | | + | | | | + | | | + |
| CS | | | + | | | | | | + |
| CSAD | | | | | | | | | |
| CSAG1 | | | | | | | | | |
| CSAG3 | | | | | | | | | |

| | | | | | | | | | |
|---|---|---|---|---|---|---|---|---|---|
| CSDC2 | | | + | | | | | | + |
| CSDE1 | | | | | | | | | |
| CSE1 | | | | | | | | | |
| CSE1L | | | | | | | | | |
| CSE4 | | | | | | | | | |
| CSF1 | | | | | | + | | | + |
| CSF1R | | + | | | + | | | | + |
| CSF2 | | | | | | | | | |
| CSF2RA | | | | | | | | | |
| CSF2RB | | | | | | | | | |
| CSF2RBP1 | | | | | | | | | |
| CSF3 | | | | | | | | | |
| CSF3R | | | | + | | | | | + |
| CSGALNACT1 | | | | | | + | | | + |
| CSGALNACT2 | | | | | | | | | |
| CSH1 | | | | | | | | | |
| CSH2 | | | | | | | | | |
| CSHL1 | | | | | | | | | |
| CSK | | | | | | | | | |
| csk1 | | | | | | | | | |
| CSM1 | | | | | | | | | |
| CSMD1 | | | | | | | | | |
| CSMD2 | | | | | | | | | |
| CSMD3 | | | | + | | + | | | + |
| CSN1S1 | | | | | | | | | |
| CSN2 | | | | | | | | | |
| CSN3 | | | | | | | | | |
| CSN9 | | | | | | | | | |
| CSNK1A1 | | | | | | | | | |
| CSNK1A1L | | | | | | | | | |
| CSNK1D | | | + | | | | | | + |
| CSNK1E | | | | | | + | | | + |
| CSNK1G1 | | | | | | | | | |
| CSNK1G2 | | | | | | | | | |
| CSNK1G2-AS1 | | | | | | | | | |
| Csnk1g3 | | | | | | | | | |
| CSNK2A1 | | | + | | | | | | + |
| CSNK2A2 | | | | | | | | | |
| CSNK2A3 | | | | | | | | | |
| CSNK2B | | | | | | | | | |
| CSPG4 | | | | | | | | | |
| CSPG4P10 | | | | | | | | | |
| CSPG5 | | | | | | | | | |
| CSPP1 | | | | | | | | | |
| CSR2 | | | | | | | | | |
| CSRNP1 | | | + | | + | | | | + |
| CSRNP2 | | | | | | | | | |
| CSRNP3 | | | + | | | + | | | + |
| CSRP1 | | | | | + | | | | + |
| CSRP2 | | | | | | | | | |
| CSRP2BP | | | | | | | | | |
| CSRP3 | | | | | | | | | |
| CST1 | | | | | | | | | |

| | | | | | | | | | |
|---|---|---|---|---|---|---|---|---|---|
| CST11 | | | | | | | | | |
| CST2 | | | | | | | | | |
| CST3 | | | | | + | | | | + |
| CST4 | | | | | | | | | |
| CST5 | | | | | | | | | |
| CST6 | | | | | | | | | |
| CST7 | | | | | | | | | |
| CST8 | | | | | | | | | |
| CST9 | | | | | | | | | |
| CST9L | | | | | | | | | |
| CSTA | | | | | | | | | |
| CSTB | | | | | | | | | |
| CSTF1 | | | | | | | | | |
| CSTF2 | | | | | + | | | | + |
| CSTF2T | | | | | | | | | |
| CSTF3 | | | | | | | | | |
| CSTL1 | | | | | | | | | |
| csul | | | | | | | | | |
| CT45A1 | | | | | | | | | |
| CT45A10 | | | | | | | | | |
| CT45A2 | | | | | | | | | |
| CT45A3 | | | | | | | | | |
| CT45A5 | | | | | | | | | |
| CT45A6 | | | | | | | | | |
| CT45A8 | | | | | | | | | |
| CT45A9 | | | | | | | | | |
| CT47A1 | | | | | | | | | |
| CT47A10 | | | | | | | | | |
| CT47A11 | | | | | | | | | |
| CT47A12 | | | | | | | | | |
| CT47A2 | | | | | | | | | |
| CT47A3 | | | | | | | | | |
| CT47A4 | | | | | | | | | |
| CT47A5 | | | | | | | | | |
| CT47A6 | | | | | | | | | |
| CT47A7 | | | | | | | | | |
| CT47A8 | | | | | | | | | |
| CT47A9 | | | | | | | | | |
| CT55 | | | | | | | | | |
| CT62 | | | | | | | | | |
| CTA1 | | | | | | | | | |
| CTAG1A | | | | | | | | | |
| CTAG1B | | | | | | | | | |
| CTAG2 | | | | | | | | | |
| CTAGE1 | | | | | | | | | |
| CTAGE15 | | | | | | | | | |
| CTAGE4 | | | | | | | | | |
| CTAGE5 | | | | | + | | | | + |
| CTAGE6 | | | | | | | | | |
| CTAGE7P | | | | | | | | | |
| CTAGE8 | | | | | | | | | |
| CTBP1 | | | | | | | + | | + |
| CTBP1-AS | | | | | | | | | |

| | | | | | | | | | |
|---|---|---|---|---|---|---|---|---|---|
| CTBP1-AS2 | | | | | | | | | |
| CTBP2 | | | | | | | | | |
| CTBS | | | | | | | | | |
| CTC1 | | | | | | + | | | + |
| CTCF | | | | | | | | | |
| CTCFL | + | | | + | + | | + | | + |
| CTDNEP1 | | | + | | | | | | + |
| CTDP1 | | | | | | + | | | + |
| CTDSP1 | | | | | | | | | |
| CTDSP2 | | | | | | | | | |
| CTDSPL | | | | | | | | | |
| CTDSPL2 | | | | | | | | | |
| CTF1 | | | | | | | | | |
| CTGF | | | | | | | | | |
| CTGLF10P | | | | | | | | | |
| CTGLF11P | | | | | | | | | |
| CTH | | | | | | | | | |
| CTHRC1 | | | | | | | | | |
| CTIF | | | | | | | | | |
| CTK1 | | | | | | | | | |
| CTLA4 | | | | | | | | | |
| CTNNA1 | | | | | | | | | |
| CTNNA2 | | | | | | + | | | + |
| CTNNA3 | | | | | | | | | |
| CTNNAL1 | | | + | | | | | | + |
| CTNNB1 | | | | | | | | | |
| ctnnb1-a | | | | | | | | | |
| CTNNBIP1 | | | | | | | | | |
| CTNNBL1 | | | | | | | | | |
| CTNND1 | | | | | | | | | |
| CTNND2 | | | | | | | | | |
| CTNS | | | | | | | | | |
| Ctps | | | | | | | | | |
| CTPS1 | | | | | | | | | |
| CTPS2 | | | | | | | | | |
| CTR1 | | | | | | | | | |
| CTR9 | | | | | | | | | |
| CTRB1 | | | | | | | | | |
| CTRB2 | | | | | | | | | |
| CTRC | | | | | | | | | |
| CTRL | | | | | | | | | |
| CTSA | | | | | | | | | |
| CTSB | + | | | | | | | | + |
| CTSC | | | | | | | | | |
| CTSD | | + | | | + | + | | | + |
| CTSE | | | | | | | | | |
| CTSF | | | | | | + | | | + |
| CTSG | | | | | | | | | |
| CTSH | | | | | | | | | |
| CTSK | | | | | | | | | |
| CTSL | | | | + | | | | | + |
| CTSL3P | | | | | | | | | |
| CTSO | | | | | | + | | | + |

| | | | | | | | | | |
|---|---|---|---|---|---|---|---|---|---|
| CTSS | | | | | | | | | |
| CTSV | | | | | | | | | |
| CTSW | | | | | | | | | |
| CTSZ | | | | | + | | | | + |
| CTT1 | | | | | | | | | |
| CTTN | | | | | | | | | |
| CTTNBP2 | | | | + | | | | | + |
| CTTNBP2NL | | | + | | | | | | + |
| CTU1 | | | | | | | | | |
| CTU2 | + | | | | + | | | | + |
| CTXN1 | | | | | | + | | | + |
| CTXN3 | | | | | | | | | |
| CUBN | | | | | | | | | |
| CUE1 | | | | | | | | | |
| CUE4 | | | | | | | | | |
| CUE5 | | | | | | | | | |
| CUEDC1 | | | | | | | | | |
| CUEDC2 | | | | | | | | | |
| CUL1 | | | | | | | | | |
| CUL2 | | | | + | | | | | + |
| CUL3 | | | + | | + | | | | + |
| CUL4A | | | | + | | | | | + |
| CUL4B | | | + | | | | | | + |
| CUL5 | | | | | | | | | |
| CUL7 | | | | | | | | | |
| CUL9 | | | | | | | | | |
| CUP9 | | | | | | | | | |
| cut6 | | | | | | | | | |
| cut7 | | | | | | | | | |
| CUTA | | | | | | | | | |
| CUTC | | | | | | | | | |
| CUX1 | | | | | | | | | |
| CUX2 | | | | | | | | | |
| CUZD1 | | | | | | | | | |
| CWC15 | | | | | | | | | |
| CWC21 | | | | | | | | | |
| CWC22 | | | | | | + | | | + |
| CWC25 | | | | | | | | | |
| CWC27 | | | | | + | | | | + |
| CWF19L1 | | | | | | | | | |
| CWF19L2 | | | | | | | | | |
| CWH43 | | | | | | | | | |
| CWP1 | | | | | | | | | |
| CWP2 | | | | | | | | | |
| CX3CL1 | | | + | | | + | | | + |
| CX3CR1 | + | | | + | + | | + | | + |
| CXADR | | | | | | | | | |
| CXCL1 | | | | | | | | | |
| CXCL10 | | | | | | | | | |
| CXCL11 | | | | | | | | | |
| CXCL12 | + | + | | | + | + | | | + |
| CXCL13 | | | | | | | | | |
| CXCL14 | | | + | | | | | | + |

| | | | | | | | | | |
|---|---|---|---|---|---|---|---|---|---|
| CXCL16 | | | | | | + | | | + |
| CXCL17 | | | | | | | | | |
| CXCL2 | | | | | | | | | |
| CXCL3 | | | | | | | | | |
| CXCL5 | | + | + | | | + | | | + |
| CXCL6 | | | | | | | | | |
| CXCL8 | | | | | | | | | |
| CXCL9 | + | | | + | + | | | | + |
| CXCR1 | | | | | | | | | |
| CXCR2 | | | | | | | | | |
| CXCR3 | | | | | | | | | |
| CXCR4 | + | | | + | + | | | | + |
| CXCR5 | | | | | | | | + | + |
| CXorf21 | | | | | | | | | |
| CXorf22 | | | | | | | | | |
| CXorf23 | | | | | | | | | |
| CXorf36 | | | | | | | | | |
| CXorf38 | | | | | | | | | |
| CXorf40A | | | | | | | | | |
| CXorf51A | | | | | | | | | |
| CXorf51B | | | | | | | | | |
| CXorf56 | | | | | | | | | |
| CXorf57 | | | | | | | | | |
| CXorf58 | | | | | | | | | |
| CXorf65 | | | | | | | | | |
| CXorf66 | | | | | | | | | |
| CXorf67 | | | | | | | | | |
| Cxx1a | | | | | | | | + | + |
| CXXC1 | | | | | | | | | |
| CXXC4 | | | + | | | | | | + |
| CXXC5 | | | | | | | | | |
| CYB5 | | | | | | | | | |
| CYB561 | | | | | | | | | |
| CYB561A3 | | | | | | | | | |
| CYB561D1 | | | | | | | | | |
| CYB561D2 | | | | | | | | | |
| CYB5A | | | | | | | | | |
| CYB5B | | | | | | | | | |
| CYB5D1 | | | | | | | | | |
| CYB5D2 | | | | | | | | | |
| CYB5R1 | | | | | + | | | | + |
| CYB5R2 | | | | | | | | | |
| CYB5R3 | + | | | + | + | | | | + |
| CYB5R4 | | | | | | | | | |
| CYBA | | | | | | | | | |
| CYBB | | | | | | | | | |
| CYBRD1 | | | | | | | | | |
| CYC1 | | | | | | | | | |
| CYC2 | | | | | | | | | |
| CYCS | | | | | | | | + | + |
| CYFIP1 | | | | | | | | | |
| CYFIP2 | | | | | | | | | |
| CYGB | | | | | | + | | | + |

| | | | | | | | | | |
|---|---|---|---|---|---|---|---|---|---|
| CYHR1 | | | | | | | | | |
| CYLC1 | | | | | | | | | |
| CYLC2 | | | | | | | | | |
| CYLD | | | | | | | | | |
| CYP11A1 | + | | | | + | | | | + |
| CYP11B1 | | | | | | | | | |
| CYP11B2 | | | | | | | | | |
| CYP17A1 | | | | | | | | | |
| CYP19A1 | | | | | | | | | |
| CYP1A1 | | | | | | | | | |
| CYP1A2 | | | | | | | | | |
| CYP1B1 | | | | | | | | | |
| CYP20A1 | | | | | | | | | |
| CYP21A2 | | | | | | | | | |
| CYP24A1 | | | | | | | | | |
| CYP26A1 | | | | | | | | | |
| CYP26B1 | | | | | | | | | |
| CYP27A1 | | | | | | | | | |
| CYP27B1 | | | | | | | | | |
| CYP27C1 | | | | | | | | | |
| CYP2A13 | | | | | | | | | |
| CYP2A6 | | | | | | | | | |
| CYP2A7 | | | | | | | | | |
| CYP2B4 | | | | | | | | | |
| CYP2B6 | | | | | | | | | |
| CYP2C18 | | | | | | | | | |
| CYP2C19 | | | | | | | | | |
| CYP2C2 | | | | | | | | | |
| CYP2C8 | | | | | | | | | |
| Cyp2c80 | | | | | | | | | |
| CYP2C9 | | | | | | | | | |
| CYP2D6 | | | | | | | | | |
| CYP2E1 | | | | | | | | | |
| CYP2J2 | | | | | | | | | |
| CYP2S1 | | | | | | | | + | + |
| CYP2T1P | | | | | | | | | |
| CYP2U1 | | | | | | | | | |
| CYP2W1 | | | | | | | | | |
| CYP39A1 | | | | | | | | | |
| CYP3A4 | | | | | | | | | |
| CYP3A43 | | | | | | | | | |
| CYP3A5 | | | | | | | | | |
| CYP3A7 | | | | | | | | | |
| CYP46A1 | | | | | | | | | |
| CYP4A11 | | | | | | | | | |
| CYP4A22 | | | | | | | | | |
| CYP4B1 | | | | | | | | | |
| CYP4F11 | | | | | | | | | |
| CYP4F12 | | | | | | | | | |
| CYP4F2 | | | | | | | | | |
| CYP4F22 | | | | | | | | | |
| CYP4F3 | | | | | | | | | |
| CYP4F8 | | | | | | | | | |

| | | | | | | | | | |
|---|---|---|---|---|---|---|---|---|---|
| CYP4V2 | | | | | | | | | |
| CYP4X1 | | | | | | | | | |
| CYP51A1 | | | | | | | | | |
| Cyp6a2 | | | | | | | | | |
| CYP71 | | | | | | | | | |
| CYP71B7 | | | | | | | | | |
| CYP7B1 | | | | | | | | | |
| CYP81F4 | | | | | | | | | |
| CYP8B1 | | | | | | | | | |
| Cyr61 | + | | | | + | | | | + |
| CYS1 | | | | | | + | | | + |
| cys12 | | | | | | | | | |
| CYS3 | | | | | | | | | |
| CYS4 | | | | | | | | | |
| CYSLTR1 | | | | | | | | | |
| CYSLTR2 | | | | | | | | | |
| CYSRT1 | | | | | | | | | |
| CYSTM1 | | | | | | | | | |
| CYT1 | | | | | | | | | |
| CYTB | | | | | | | | | |
| CYTH1 | | | | | | | | | |
| CYTH2 | | | | | | | | | |
| CYTH3 | | | | | | | | | |
| CYTH4 | | | | | | | | | |
| CYTIP | + | | | + | + | | | | + |
| CYTL1 | | | | | | | | | |
| CYYR1 | + | | | + | + | | | | + |
| D10Bwg1379e | | | | | | | | | |
| D15Ertd621e | | | | | | | | | |
| D16Ertd472e | | | + | | | | | | + |
| D2HGDH | | | | | | | | | |
| D430042O09Rik | | | | | | | | | |
| D8Ertd738e | | | | | | | | | |
| D8Ertd82e | | | | | | | | | |
| DAAM1 | | | | | | | | | |
| DAAM2 | | | | | | | | | |
| DAB1 | | | + | | | | | | + |
| DAB2 | | | | | | | | | |
| DAB2IP | | | | | | | | | |
| DACH1 | | | | | | | | | |
| DACH2 | | | | | | | | | |
| DACT1 | | | | | | | | | |
| Dact2 | | | | | | | | | |
| Dact3 | | | | | | | | | |
| DAD1 | | | | | | | | | |
| DAD4 | | | | | | | | | |
| daf-12 | | | | | | | | | |
| daf-4 | | | | | | | | | |
| DAG1 | | | | | | | | | |
| DAGLA | | | | | | | | | |
| DAGLB | | | | | + | | | | + |
| DAK | | | | | | | | | |
| DAK1 | | | | | | | | | |

| | | | | | | | | | |
|---|---|---|---|---|---|---|---|---|---|
| DAL2 | | | | | | | | | |
| DAL5 | | | | | | | | | |
| DAL80 | | | | | | | | | |
| DALRD3 | | | | | | | | | |
| DAN1 | | | | | | | | | |
| DANCR | | | | | | | | | |
| DAO | | | | | | | | | |
| DAO1 | | | | | | | | | |
| DAOA | | | | | | | | | |
| DAP | | | | | | | | | |
| DAP2 | | | | | | | | | |
| DAP3 | | | | | | | | | |
| DAPK1 | | | | | | + | | | + |
| DAPK2 | | | | | | | | | |
| DAPK3 | | | | | | | | | |
| DAPL1 | | | | | | | | | |
| DAPP1 | | | | | | | | | |
| DARS | | | | | | | | | |
| DARS-AS1 | | | | | | | | | |
| DARS2 | | | | | | | | | |
| DAW1 | | | | | | | | | |
| DAXX | | | | | | | | | |
| DAZ1 | | | | | | | | | |
| DAZ2 | | | | | | | | | |
| DAZ3 | | | | | | | | | |
| DAZ4 | | | | | | | | | |
| DAZAP1 | | | | | | | | | |
| DAZAP2 | | | | | | | | | |
| DAZL | | | | | | | | | |
| DBET | | | | | | | | | |
| DBF4 | | | + | + | | | | | + |
| DBF4B | | | | | | | | | |
| DBH | | | | | | | | | |
| DBI | | | | | | | | | |
| DBN1 | | | + | | | | | | + |
| DBNDD1 | | | | | | | | | |
| DBNDD2 | | | | | | | | | |
| DBNL | | | | | | | | | |
| DBP | | | | | | + | | | + |
| DBP10 | | | | | | | | | |
| dbp2 | | | | | | | | | |
| dbp3 | | | | | | | | | |
| DBR1 | | | | | | | | | |
| DBT | + | | | + | + | | + | | + |
| DBX1 | | | | | | | | | |
| DBX2 | | | | | | | | | |
| DCAF10 | | | | | | | | | |
| DCAF11 | | | | | | | | | |
| DCAF12 | | | | | | | | | |
| DCAF12L1 | | | | | | + | | | + |
| DCAF12L2 | + | | | + | + | | | | + |
| DCAF13 | | | | | | | | | |
| DCAF15 | | | | | | | | | |

| | | | | | | | | | |
|---|---|---|---|---|---|---|---|---|---|
| DCAF16 | | | | | | | | | |
| DCAF17 | | + | | + | | + | | | + |
| DCAF4 | | | | | | | | | |
| DCAF4L1 | | | | | | | | | |
| DCAF4L2 | | | | | | | | | |
| DCAF5 | | | | | | | | | |
| DCAF6 | | | | | | | | | |
| DCAF7 | | | | | | | | | |
| DCAF8 | | | | | | + | | | + |
| DCAF8L1 | | | | | | | | | |
| DCAF8L2 | | | | | | | | | |
| DCAKD | | | | | | + | | | + |
| DCANP1 | | | | | | | | | |
| DCBLD1 | | | | | | | | | |
| DCBLD2 | | | | | | | | | |
| DCC | | | | | | + | | | + |
| DCD | | | | | | | | | |
| DCDC1 | | | | | | | | | |
| DCDC2 | | | | | | | | | |
| DCDC2B | | | | | | | | | |
| DCDC2C | | | | | | | | | |
| DCDC5 | | | | | | | | | |
| DCG1 | | | | | | | | | |
| DCHS1 | | | | | | | | | |
| DCHS2 | | | | | | | | | |
| DCK | | | | | | | | | |
| DCLK1 | + | | + | | + | | | | + |
| DCLK2 | | | + | | | | | | + |
| DCLK3 | | | | | | + | | | + |
| DCLRE1A | | | | | | + | | | + |
| DCLRE1B | | | | | | | | | |
| DCLRE1C | | | | | | | | | |
| DCN | | | | | | | | | |
| DCN1 | | | | | | | | | |
| dco | | | | | | | | | |
| DCP1 | | | | | | | | | |
| DCP1A | | | | | | | | | |
| DCP1B | | | | | | | | | |
| DCP2 | | | | | | | | | |
| DCPS | | | | | | | | | |
| DCS1 | | | | | | | | | |
| DCST1 | | | | | | | | | |
| DCST2 | | | | | | | | | |
| DCSTAMP | | | | | | | | | |
| DCT | | | | | | | | | |
| DCTD | | | | | | | | | |
| DCTN1 | | | | | | | | | |
| DCTN2 | | + | | | | + | | | + |
| Dctn3 | | | | | | | | | |
| DCTN4 | | | | | | | | | |
| DCTN5 | + | | + | | | | | | + |
| DCTN6 | | + | | | | + | | | + |
| DCTPP1 | | | | | | | | | |

| | | | | | | | | | |
|---|---|---|---|---|---|---|---|---|---|
| DCUN1D1 | | | | | | | | | |
| DCUN1D2 | | | | | | | | | |
| DCUN1D3 | | | | | | | | | |
| DCUN1D4 | + | | | | + | | | | + |
| DCUN1D5 | | | | | | | | | |
| DCX | | | | | | | | | |
| DCXR | | | | | | | | | |
| DDA1 | | | | | | | | | |
| DDAH1 | | | | | | | | | |
| DDAH2 | | | | | | | | | |
| DDB_G0286429 | | | | | | | | | |
| DDB1 | | | | | | | | | |
| DDB2 | | | | | | | | | |
| DDC | | | | + | | | | | + |
| DDHD1 | + | | + | | + | | | | + |
| DDHD2 | | + | | | | + | | | + |
| DDI1 | | | | | | | | | |
| DDI2 | | | | | | | | | |
| DDIAS | | | | | | | | | |
| DDIT3 | | | | | + | | | | + |
| DDIT4 | | | | | | | | | |
| DDIT4L | + | | | + | + | | + | | + |
| DDN | | | | | | + | | | + |
| DDO | | | | | | + | | | + |
| DDOST | | | | | | | | | |
| DDR1 | + | | + | | + | | | | + |
| DDR1-AS1 | | | | | | | | | |
| DDR2 | | | | | | | | | |
| DDRGK1 | | | | | | | | | |
| DDT | | | | | | | | | |
| DDTL | | | | | | | | | |
| DDX1 | | | | | | | | | |
| DDX10 | | | | | | | | | |
| DDX11 | | | | | | | | | |
| DDX11L8 | | | | | | | | | |
| DDX12P | | | | | | | | | |
| DDX17 | | | | | | | | | |
| DDX18 | | | | | | | | | |
| DDX19A | | | | | | | | | |
| DDX19B | | | | | | | | | |
| DDX20 | | | | | | | | | |
| Ddx21 | | | | | | | | | |
| DDX23 | | | | | | | | | |
| DDX24 | | | | | | | | | |
| DDX25 | | | | | | | | | |
| DDX26B | | | | | | | | | |
| DDX27 | | | | | | | | | |
| DDX28 | | | | | | | | | |
| DDX31 | | | | | | | | | |
| Ddx39 | | | | + | | | | | + |
| DDX39A | | | | | | | | | |
| DDX39B | | | | | | | | | |
| DDX3P3 | | | | | | | | | |

| | | | | | | | | | |
|---|---|---|---|---|---|---|---|---|---|
| DDX3X | | | | | | | | | |
| DDX3Y | | | + | | | | | | + |
| DDX4 | | | | | | | | | |
| Ddx41 | | | | | | | | | |
| DDX42 | | | | | | | | | |
| DDX43 | | | | | | | | | |
| DDX46 | | | | | | | | | |
| DDX47 | | | | | + | | | | + |
| DDX49 | | | | | | | | | |
| DDX5 | | | | | | | | | |
| DDX50 | | | | | | | | | |
| DDX51 | | | | | + | | | | + |
| DDX52 | | | | | | | | | |
| DDX53 | | | | | | | | | |
| DDX54 | | | | | | | | | |
| DDX55 | | | | | | | | | |
| DDX56 | | | | | | | | | |
| DDX58 | | | | | | | | | |
| DDX59 | | | | | | | | | |
| DDX6 | | | | | | | | | |
| DDX60 | | | | | | | | | |
| DDX60L | | | | | | | | | |
| DEAF1 | | | | | | | | | |
| DEC1 | | | | | | | | | |
| DECR1 | | | | | | | | | |
| DECR2 | | | | | | | | | |
| DED81 | | | | | | | | | |
| DEDD | | | | | | | | | |
| DEDD2 | | | | | | | | | |
| DEF1 | | | | | | | | | |
| DEF6 | | | | | | | | | |
| DEF8 | | | | | | | | | |
| DEFA1 | | | | | | | | | |
| DEFA1B | | | | | | | | | |
| DEFA3 | | | | | | | | | |
| DEFA5 | | | | | | | | | |
| DEFA6 | | | | | | | | | |
| DEFB1 | | | | | | | | | |
| DEFB103A | | | | | | | | | |
| DEFB103B | | | | | | | | | |
| DEFB104A | | | | | | | | | |
| DEFB104B | | | | | | | | | |
| DEFB105A | | | | | | | | | |
| DEFB105B | | | | | | | | | |
| DEFB106A | | | | | | | | | |
| DEFB107A | | | | | | | | | |
| DEFB108B | | | | | | | | | |
| DEFB109P1B | | | | | | | | | |
| DEFB110 | | | | | | | | | |
| DEFB112 | | | | | | | | | |
| DEFB114 | | | | | | | | | |
| DEFB115 | | | | | | | | | |
| DEFB116 | | | | | | | | | |

| | | | | | | | | | |
|---|---|---|---|---|---|---|---|---|---|
| DEFB118 | | | | | | | | | |
| DEFB119 | | | | | | | | | |
| DEFB121 | | | | | | | | | |
| DEFB123 | | | | | | | | | |
| DEFB124 | | | | | | | | | |
| DEFB125 | | | | | | | | | |
| DEFB126 | | | | | | | | | |
| DEFB127 | | | | | | | | | |
| DEFB128 | | | | | | | | | |
| DEFB129 | | | | | | | | | |
| DEFB130 | | | | | | | | | |
| DEFB131 | | | | | | | | | |
| DEFB132 | | | | | | | | | |
| DEFB135 | | | | | | | | | |
| DEFB136 | | | | | | | | | |
| DEFB4A | | | | | | | | | |
| DEGS1 | | | | | | | | | |
| DEGS2 | | | | | | | | | |
| DEK | | | | | | | | | |
| DENND1A | | | | | | | | | |
| DENND1B | | | | | | | | | |
| DENND1C | | | | | | | | | |
| DENND2A | | | | | | | | | |
| DENND2C | | | | + | | | | | + |
| DENND2D | | | | | | | | | |
| DENND3 | | | | | | | | | |
| DENND4A | | | | | | | | | |
| DENND4B | | | | | | | | | |
| DENND4C | | | | | | | | | |
| DENND5A | | | | | | | | | |
| DENND5B | | | | | | | | | |
| Dennd6a | | | | | | | | | |
| DENND6B | | | | | | | | | |
| DENR | | | | | | | | | |
| DEPDC1 | | | | | | | | | |
| DEPDC1B | | | | | | | | | |
| DEPDC5 | | | | | | | | | |
| DEPDC7 | | | | | | | | | |
| DEPTOR | + | | | + | + | | | | + |
| DERA | | | | | | | | | |
| DERL1 | | | + | | | | | | + |
| DERL2 | | | | | | + | | | + |
| DERL3 | | | | | | | | | |
| DES | | | | | | | | | |
| DESI1 | | | | | | | | | |
| DESI2 | | | | | | | | | |
| DET1 | | | | | | | | | |
| DEXI | | | | | | | | | |
| DFFA | | | | | | | | | |
| DFFB | | | | | | | | | |
| DFG10 | | | | | | | | | |
| DFNA5 | | | | | | | | | |
| DFNB26 | | | | | | | | | |

| | | | | | | | | | |
|---|---|---|---|---|---|---|---|---|---|
| DFNB31 | | | | | | | | | |
| DFNB59 | | | | | | | | | |
| dfr1 | | | | | | | | | |
| DGAT1 | | | | | | | | | |
| DGAT2 | | | | | | | | | |
| DGAT2L6 | | | | | + | | | | + |
| DGCR14 | | | | | | | | | |
| DGCR2 | | + | | | | + | | | + |
| DGCR6 | | | | | | | | | |
| DGCR6L | | | | | | | | | |
| DGCR8 | | | | | | | | | |
| DGCR9 | | | | | | | | | |
| DGK1 | | | | | | | | | |
| DGKA | | | | | | | | | |
| DGKB | | | | | | | | | |
| DGKD | | | | | | | | | |
| DGKE | | | | + | | | | | + |
| DGKG | | | | | | | | | |
| DGKH | | | | | | | | | |
| DGKI | | | | | | | | | |
| DGKK | | | | | | | | | |
| DGKQ | | | | | | | | | |
| DGKZ | | | | | | + | | | + |
| DGKZP1 | | | | | | | | | |
| DGUOK | | | | | | | | | |
| DHCR24 | | | | | | | | | |
| DHCR7 | | | | | | | | | |
| DHDDS | | | | | | | | | |
| DHDH | | | | | | | | | |
| DHFR | | | | | | | | | |
| DHFRL1 | | | | | | | | | |
| DHFRP1 | | | | | | | | | |
| DHH | | | | | | | | | |
| DHH1 | | | | | | | | | |
| DHODH | | | | | | | | | |
| DHPS | | | | | | | | | |
| DHR2 | | | | | | | | | |
| DHRS1 | | | | | | | | | |
| DHRS11 | | | | | | | | | |
| DHRS12 | | | | | | | | | |
| DHRS13 | | | | | | | | | |
| DHRS2 | | | | | | | | | |
| DHRS3 | | | | | | | | | |
| DHRS4 | | | | | | | | | |
| DHRS4L1 | | | | | | | | | |
| DHRS4L2 | | | | | | | | | |
| DHRS7 | | | | | | | | | |
| DHRS7B | | | | | | + | | | + |
| DHRS7C | | | | | | | | | |
| DHRS9 | | | | | | | | | |
| DHRSX | | | | | | | | | |
| DHTKD1 | | | | | | | | | |
| DHX15 | | | | | | | | | |

| | | | | | | | | | |
|---|---|---|---|---|---|---|---|---|---|
| DHX16 | | | | | | | | | |
| DHX29 | | | | | | | | | |
| DHX30 | | | | | | | | | |
| DHX32 | | | | | | | | | |
| DHX33 | | | | | | | | | |
| DHX34 | | | | | | | | | |
| DHX35 | | | | | | | | | |
| DHX36 | | | | | | | | | |
| DHX37 | | | | | | + | | | + |
| DHX38 | | | | | | | | | |
| DHX40 | + | | | + | + | | + | | + |
| DHX57 | | | | | | | | | |
| DHX58 | | | | | | | | | |
| DHX8 | | | | | + | | | | + |
| DHX9 | | | | + | | | | | + |
| DIABLO | | | | | | | | | |
| Diap1 | | | | | | | | | |
| Diap3 | | | | | | | | | |
| DIAPH1 | | | | | | | | | |
| DIAPH2 | | | | | | | | | |
| DIAPH3 | | | | | | | | | |
| DIB1 | | | | | | | | | |
| DICER1 | | | | | | | | | |
| DICER1-AS1 | | | | | | | | | |
| DID2 | | | | | | | | | |
| DID4 | | | | | | | | | |
| DIDO1 | | | + | + | | | | | + |
| DIE2 | | | | | | | | | |
| DIEXF | | | | | | + | | | + |
| DIG1 | | | | | | | | | |
| DIG2 | | | | | | | | | |
| DIMT1 | | | | | | | | | |
| DIO1 | | | | | | | | | |
| DIO2 | | | + | | | | | | + |
| DIO3 | | | | | | | | | |
| dip2 | | | | | | | | | |
| DIP2A | | | | | | | | | |
| DIP2B | | | | | | | | | |
| DIP2C | | | + | | | | | | + |
| DIRAS1 | | | | | | + | | | + |
| DIRAS2 | | | + | | | | | | + |
| DIRAS3 | | | | | | | | | |
| DIRC1 | | | | | | | | | |
| DIRC2 | | | | | + | | | | + |
| DIRC3 | | | | | | | | | |
| DIS3 | | | | | | | | | |
| DIS3L | | | | | | | | | |
| DIS3L2 | | | | | | | | | |
| DISC1 | | | | | | | | | |
| DISC1FP1 | | | | | | | | | |
| DISP1 | | | | | | | | | |
| DISP2 | | | | | | | | | |
| DIXDC1 | | | | | | + | | | + |

| | | | | | | | | | |
|---|---|---|---|---|---|---|---|---|---|
| DKC1 | | | | | | | | | |
| DKFZP434K028 | | | | | | | | | |
| DKK1 | + | | | + | + | | + | | + |
| DKK2 | + | | | + | + | | + | | + |
| DKK3 | | | | | + | + | | | + |
| DKK4 | | | | | | | | | |
| DKKL1 | | | | | | | | | |
| Dl | | | | | | | | | |
| DLAT | | | | | | | | | |
| DLC1 | + | | + | | | | | | + |
| dld | | | | | | | | | |
| dld1 | | | | | | | | | |
| DLD3 | | | | | | | | | |
| DLEC1 | | | | | | | | | |
| DLEU1 | | | | | | | | | |
| DLEU2 | | | | | | | | | |
| DLEU7 | | | | | | | | | |
| DLG1 | | | | | | | | | |
| DLG2 | | | | | | | | | |
| DLG3 | + | | + | | | + | | | + |
| DLG4 | | | | | | | | | |
| DLG5 | | | | | | | | | |
| DLGAP1 | | | | | | | | | |
| DLGAP1-AS2 | | | | | | | | | |
| DLGAP2 | | | | | | | | | |
| DLGAP3 | | | | | | | | | |
| DLGAP4 | | | | | + | | | | + |
| DLGAP4-AS1 | | | | | | | | | |
| DLGAP5 | | | | + | | | + | | + |
| DLK1 | + | | | + | + | | + | | + |
| DLK2 | + | | + | | | | | | + |
| DLL1 | | | | | | | | | |
| DLL3 | | + | + | | | + | | | + |
| Dll4 | | | | | | | | | |
| DLST | | | | | | | | | |
| DLSTP1 | | | | | | | | | |
| DLX1 | | | | | | | | | |
| DLX2 | | + | | | | | | | + |
| DLX3 | + | | | + | + | | + | | + |
| DLX4 | | | | | | | | | |
| DLX5 | | | | | | | | | |
| DLX6 | | | | | | | | | |
| DLX6-AS1 | | | | | | | | | |
| DMA2 | | | | | | | | | |
| DMAP1 | | | | | | | | | |
| DMBT1 | | | | | | | | | |
| DMBX1 | + | | | + | + | | + | | + |
| DMC1 | | | | | | | | | |
| Dmd | | + | | + | | + | | | + |
| DMGDH | | | | | | | | | |
| DMKN | | | | | | | | | |
| DMP1 | + | | | + | + | | + | | + |
| DMPK | | | | | | | | | |

| | | | | | | | | | |
|---|---|---|---|---|---|---|---|---|---|
| DMRT1 | | | | | | | | | |
| DMRT2 | | | | | | | | | |
| DMRT3 | | | | | | | | | |
| DMRTA1 | | | | | | | | | |
| DMRTB1 | | | | | | | | | |
| DMRTC1 | | | | | | | | | |
| DMRTC1B | | | | | | | | | |
| DMRTC2 | | | | | | | | | |
| DMT2 | | | | | | | | | |
| DMTF1 | | | | | | | | | |
| DMTN | | | | | | | + | | + |
| DMWD | | | | | | | | | |
| DMXL1 | | | | | + | | | | + |
| DMXL2 | | | | | | | | | |
| DNA2 | | | | | | | | | |
| DNAAF1 | | | | | | | | | |
| DNAAF2 | | | | | | | | | |
| Dnaaf3 | | | | | | | | | |
| DNAAF5 | | | | | | | | | |
| dnaB | | | | | | | | | |
| DNAH1 | | | | | | | | | |
| DNAH10 | | | | | | | | | |
| DNAH11 | | | | | | | | + | + |
| DNAH12 | | | | | | | | | |
| DNAH14 | | | | | | | | | |
| DNAH17 | | | | | | | | | |
| DNAH2 | | | | | | | | | |
| DNAH3 | | | | | | | | | |
| DNAH5 | | | | | | | | | |
| DNAH6 | | | | | | | | + | + |
| DNAH7 | | | | | | | | | |
| DNAH8 | | | | | | | | | |
| DNAH9 | | | | | | | | | |
| DNAI1 | | | | | | | | | |
| DNAI2 | | | | | | | | | |
| DNAJA1 | | | | | | | | | |
| DNAJA2 | | | | | | | | | |
| DNAJA3 | | | | | | | | | |
| DNAJA4 | + | | + | | | | | | + |
| DNAJB1 | + | | | | + | | | | + |
| DNAJB11 | | | | | | | | | |
| DNAJB12 | | | | | | | | | |
| DNAJB13 | | | | | | | | | |
| DNAJB14 | | | | + | | | | | + |
| DNAJB2 | | | | | | | | | |
| DNAJB3 | | | | | | | | | |
| DNAJB4 | | | | | | | | | |
| DNAJB5 | + | | + | | + | | | | + |
| DNAJB6 | | | + | | | | | | + |
| DNAJB7 | | | | | | | | | |
| DNAJB8 | | | | | | | | | |
| DNAJB9 | | | | | | | | | |
| DNAJC1 | + | | + | | + | | | | + |

| | | | | | | | | | |
|---|---|---|---|---|---|---|---|---|---|
| DNAJC10 | | | | | | | | | |
| DNAJC11 | | | | | | | | | |
| DNAJC12 | | | | + | | | | | + |
| DNAJC13 | | | | | | | | | |
| DNAJC14 | | | | | | | | | |
| DNAJC15 | | + | + | | | + | | | + |
| DNAJC16 | | | | | | | | | |
| DNAJC17 | | | | | | | | | |
| DNAJC18 | | | | | | | | | |
| DNAJC19 | | | | | | | | | |
| DNAJC2 | | | | | + | | | | + |
| DNAJC21 | | | | | | | | | |
| DNAJC22 | | | | | | | | | |
| DNAJC24 | | + | | | | + | | | + |
| DNAJC25 | | | | | | | | | |
| DNAJC25-GNG10 | | | | | | | | | |
| DNAJC27 | | | | | | | | | |
| DNAJC28 | | | | | | | | | |
| DNAJC3 | | | | | | | | | |
| DNAJC3-AS1 | | | | | | | | | |
| DNAJC30 | | | | | | | | | |
| DNAJC4 | | | | | | | | | |
| DNAJC5 | | | + | | | | | | + |
| DNAJC5B | | | | | | | | | |
| DNAJC5G | | | | | | | | | |
| DNAJC6 | | | | | | | | | |
| DNAJC7 | | | | | | | | | |
| DNAJC8 | | | + | | | | | | + |
| DNAJC9 | | | | | | | | | |
| DNAL1 | | | | | | | | | |
| DNAL4 | | | | | | | | | |
| DNALI1 | | | | | + | | | | + |
| DNASE1 | | | | | | | | | |
| DNASE1L1 | | | | | | | | | |
| DNASE1L2 | | | | | | | | | |
| DNASE1L3 | | | | | | | | | |
| DNASE2 | | | | | | | | | |
| DNASE2B | | | | | | | | | |
| DND1 | | | | | | | | | |
| DNER | | | | | + | | + | | + |
| DNHD1 | | | | | | | | | |
| DNLZ | | | | | | | | | |
| DNM1 | | + | | | + | + | | | + |
| DNM1L | | | | | | | | | |
| DNM1P34 | | | | | | | | | |
| DNM2 | | | | | | | | | |
| DNM3 | | | | + | | | | | + |
| DNMBP | | | | | | | | | |
| DNMT1 | | | | | | | | | |
| DNMT3A | | | | | | | | | |
| DNMT3B | | | | | | | | | |
| DNMT3L | | | | | | | | | |
| DNPEP | | | | | | | | | |

| | | | | | | | | | |
|---|---|---|---|---|---|---|---|---|---|
| DNPH1 | | | | | | | | | |
| DNTT | | | | | | | | | |
| DNTTIP1 | | | | | | | | | |
| DNTTIP2 | | | | | | | | | |
| DOA4 | | | | | | | | | |
| DOC2A | | | | | | | | | |
| DOC2B | | | | | | + | | | + |
| DOCK1 | | | | | | | | + | + |
| DOCK10 | + | | | | + | + | | | + |
| Dock11 | | | | | | | | | |
| DOCK2 | | | | | | | | | |
| DOCK3 | | | | | | | | | |
| DOCK4 | | | + | | | | | | + |
| DOCK5 | | | | | | | | | |
| DOCK6 | | | | | | | | | |
| DOCK7 | | | | | | | | | |
| DOCK8 | | | | | | | | | |
| DOCK9 | | | | | + | + | | | + |
| DOHH | | | | | | | | | |
| DOK1 | | | | | | | | | |
| DOK2 | | | | | | | | | |
| DOK3 | | | | | | | | | |
| DOK4 | | | | | | | | | |
| DOK5 | + | | | + | + | | | | + |
| DOK6 | | | | | | | | | |
| DOK7 | | | | | | | | | |
| DOLK | | | | | | | | | |
| DOLPP1 | | | | | | | | | |
| DOM34 | | | | | | | | | |
| DONSON | | | | | | | | | |
| DOPEY1 | | | | | | | | | |
| DOPEY2 | | | | | | | | | |
| DOT1L | + | | + | | + | | | | + |
| DPAGT1 | | | | | | | | | |
| DPB11 | | | | | | | | | |
| DPB3 | | | | | | | | | |
| DPB4 | | | | | | | | | |
| DPCD | | | | | | | | | |
| DPCR1 | + | | | + | + | | | | + |
| DPEP1 | | | | | | | | | |
| DPEP2 | | | | | | | | | |
| DPEP3 | | | | | | | | | |
| DPF1 | | | | | | + | | | + |
| DPF2 | | | | | | | | | |
| DPF3 | | | | | | | | | |
| DPH1 | | | | | | | | | |
| DPH2 | | | | | | | | | |
| DPH3 | | | | | + | | | | + |
| DPH5 | | | | | | | | | |
| DPH6 | | | | + | | | | | + |
| DPH7 | | | | | | + | | | + |
| DPM1 | | | | | | | | | |
| DPM2 | | | | | | | | | |

| | | | | | | | | | |
|---|---|---|---|---|---|---|---|---|---|
| DPM3 | | | | | | | | | |
| DPP1 | | | | | | | | | |
| DPP10 | | | | | | | | | |
| DPP3 | | | | | | | | | |
| DPP4 | | | | | | | | | |
| DPP6 | | | | | | | | | |
| DPP7 | | | | | | | | | |
| DPP8 | | + | | | | | | | + |
| DPP9 | | | | | | | | | |
| DPPA2 | | | | | | | | | |
| DPPA2P2 | | | | | | | | | |
| Dppa3 | | | | | | | | | |
| DPPA4 | | | | | | | | | |
| DPPA5 | | | | | | | | | |
| DPRX | | | | | | | | | |
| DPS1 | | | | | | | | | |
| DPT | | | | | | | | | |
| DPY19L1 | | | | | | + | | | + |
| DPY19L2 | | | | | | | | | |
| DPY19L2P1 | | | | | | | | | |
| DPY19L2P3 | | | | | | | | | |
| DPY19L2P4 | | | | | | | | | |
| DPY19L3 | | | | | + | | | | + |
| DPY19L4 | | | | | | + | | | + |
| DPY30 | | | | | | | | | |
| DPYD | | | | | | | | | |
| DPYS | | | | | | | | | |
| DPYSL2 | | | | | | + | | | + |
| DPYSL3 | | | | | | | | | |
| DPYSL4 | | | | | | | + | | + |
| DPYSL5 | + | | | | + | + | | | + |
| DQX1 | | | | | | | | | |
| DR1 | | | | | | | | | |
| DRAM1 | | | | | | | | | |
| DRAM2 | | | | | | | | | |
| DRAP1 | + | | | + | + | | | | + |
| DRC1 | | | | | | + | | | + |
| DRC7 | | | | | | | | | |
| DRD1 | | | | | | | | | |
| DRD2 | | | | | | | | | |
| DRD3 | | | | | | | | | |
| DRD4 | | | | | | | | | |
| DRD5 | | | | | | | | | |
| DRD5P2 | | | | | | | | | |
| DREB2A | | | | | | | | | |
| DRG1 | | + | | | | + | | | + |
| DRG2 | | | | | | | | | |
| DRICH1 | | | | | | | | | |
| DRN1 | | | | | | | | | |
| DROSHA | | + | | | | + | | | + |
| DRP2 | | | | | | | | | |
| drs1 | | | | | | | | | |
| DSC1 | | | | | | | | | |

| | | | | | | | | | |
|---|---|---|---|---|---|---|---|---|---|
| DSC2 | | | | | | | | | |
| DSC3 | | | | | | | | | |
| DSCAM | | | | | | + | | | + |
| DSCAML1 | | | | | | + | | | + |
| DSCC1 | | | | | | | | | |
| DSCR3 | | | | | | | | | |
| DSCR4 | | | | | | | | | |
| DSCR8 | | | | | | | | | |
| DSCR9 | | | | | | | | | |
| DSE | | | | | | | | | |
| DSEL | | | | | | | | | |
| DSG1 | | | | | | | | | |
| Dsg1a | | | | | | | | | |
| DSG2 | | | | | | | | | |
| DSG3 | | | | | | | | | |
| DSG4 | | | | | | | | | |
| DSK2 | | | | | | | | | |
| DSN1 | | | | | | | | | |
| DSP | + | + | | | + | + | | | + |
| DSPP | | | | | | | | | |
| DSS4 | | | | | | | | | |
| DST | | | | | | | | | |
| DSTN | | | | | | | | | |
| DSTYK | | | | | | | | | |
| DTD1 | | | + | | | + | | | + |
| DTD2 | | | | | | | | | |
| DTHD1 | | | | | | | | | |
| DTL | | | | | | | | | |
| DTNA | + | | | | + | | | | + |
| DTNB | | + | | + | | | | | + |
| DTNBP1 | | | | | | | | | |
| DTWD1 | | | | | | | | | |
| DTWD2 | | | | | | | | | |
| DTX1 | | | | | | | | | |
| DTX2 | | | | | | | | | |
| DTX2P1-UPK3BP1-PMS2P | | | | | | | | | |
| DTX3 | | | + | | | | | | + |
| DTX3L | | | | | | | | | |
| DTX4 | | | | | | | | | |
| DTYMK | | | | | | + | | | + |
| DUG1 | | | | | | | | | |
| DUN1 | | | | | | | | | |
| DUOX1 | | | | | | | | | |
| DUOX2 | | | | | | | | | |
| DUOXA1 | | | | | | | | | |
| DUOXA2 | | | | | | | | | |
| DUPD1 | | | | | | | | | |
| DUS1L | | | | | | | | | |
| DUS2 | | | | | | + | | | + |
| DUS3L | | | | | | | | | |
| DUS4L | | | | | | | | | |
| DUSP1 | + | | | | + | | | | + |
| DUSP10 | + | | + | + | + | | + | | + |

| | | | | | | | | | |
|---|---|---|---|---|---|---|---|---|---|
| DUSP11 | | | | | | | | | |
| DUSP12 | | | | | | | | | |
| DUSP13 | | | | | | | | | |
| DUSP14 | + | | + | | | + | | | + |
| DUSP15 | | | | | | | | | |
| DUSP16 | | | + | | | | | | + |
| DUSP18 | | | | | | | | | |
| DUSP19 | | | | | | | | | |
| DUSP2 | | | | | | | | | |
| DUSP21 | | | | | | | | | |
| Dusp22 | | | | | | + | | | + |
| DUSP23 | | | | | | | | | |
| DUSP26 | | | | | | + | | | + |
| DUSP27 | | | | | | | | | |
| DUSP28 | | | | | | | | | |
| DUSP3 | | | | | | | | | |
| DUSP4 | | | | | | | | | |
| DUSP5 | + | | + | | + | | | | + |
| DUSP5P1 | | | | | | | | | |
| DUSP6 | + | | + | | + | | | | + |
| DUSP7 | + | | | + | + | | | | + |
| DUSP8 | | | | | + | | | | + |
| Dusp9 | | | | | | | | | |
| DUT | | | | | | + | | | + |
| DUX1 | | | | | | | | | |
| DUX3 | | | | | | | | | |
| DUX4 | | | | | | | | | |
| DUX4L8 | | | | | | | | | |
| DUX4L9 | | | | | | | | | |
| DUXA | | | | | | | | | |
| DUXAP10 | | | | | | | | | |
| DUXAP9 | | | | | | | | | |
| DUXB | | | | | | | | | |
| dvc-1 | | | | | | | | | |
| DVL1 | | | | | | | | | |
| DVL1P1 | | | | | | | | | |
| DVL2 | | | | | | + | | | + |
| DVL3 | | | | | | | | | |
| DWF1 | | | | | | | | | |
| DXO | | | | | | | | | |
| DYDC1 | | | | | | | | | |
| DYDC2 | | | | | | | | | |
| DYM | | | | | | | | | |
| DYN2 | | | | | | | | | |
| DYNC1H1 | | | | | + | | | | + |
| DYNC1I1 | | | | | | | | | |
| DYNC1I2 | | | | | | | | | |
| DYNC1LI1 | | | | | | | | | |
| DYNC1LI2 | | | | | | | | | |
| DYNC2H1 | | + | | | | + | | | + |
| DYNC2LI1 | | | | | | + | | | + |
| DYNLL1 | | | | | | | | | |
| DYNLL2 | | | | | | | | | |

| | | | | | | | | | |
|---|---|---|---|---|---|---|---|---|---|
| DYNLRB1 | | | | | | | | | |
| DYNLRB2 | | | | | | | | | |
| DYNLT1 | | | | | | | | | |
| Dynlt1b | | | | | | | | | |
| DYNLT3 | | + | | | | + | | | + |
| DYRK1A | | | + | | | | | | + |
| DYRK1B | | | | | | | | | |
| DYRK2 | | | | | | | | | |
| DYRK3 | | | | | + | | | | + |
| DYRK4 | | | | | | | | | |
| DYSF | | | | | | | | | |
| DYT10 | | | | | | | | | |
| DYTN | | | | | | | | | |
| DYX1C1 | | | | | | | | | |
| DZANK1 | | | | | | | | | |
| DZIP1 | | | | | | | | | |
| DZIP1L | | | | | | | | | |
| DZIP3 | | + | | | + | + | | | + |
| E | | | | | | | | | |
| E1 | | | | | | | | | |
| E2 | | | | | | | | | |
| E2F1 | + | | | + | + | | | | + |
| E2F2 | | | | | | | | | |
| E2F3 | | | | | | | | | |
| E2F4 | | | | | | | | | |
| E2F5 | | | + | | | | | | + |
| E2F6 | | | | | | | | | |
| E2F7 | + | | | + | + | | | | + |
| E2F8 | | | | | | | | | |
| E3L | | | | | | | | | |
| E4F1 | | | | | | | | | |
| E5 | | | | | | | | | |
| E5a | | | | | | | | | |
| E5b | | | | | | | | | |
| E6 | | | | | | | | | |
| E7 | | | | | | | | | |
| EAF1 | | | | | + | | | | + |
| EAF2 | | | | | | | | | |
| eag | | | | | | | | | |
| EAPP | | | | | | | | | |
| ear | | | | | | | | | |
| EARS2 | | | | | | | | | |
| EB1A | | | | | | | | | |
| EBAG9 | | | | | | | | | |
| EBF1 | + | | | + | + | | + | | + |
| EBF2 | | | | | | | | | |
| EBF3 | | | | | | | | | |
| EBF4 | | | | | | | | | |
| ebi | | | | | | | | | |
| EBI3 | | | | | | | | | |
| EBLN1 | | | | | | | | | |
| EBLN2 | | | | | | | | | |
| EBNA-LP | | | | | | | | | |

| | | | | | | | | | |
|---|---|---|---|---|---|---|---|---|---|
| EBNA1BP2 | | | | | | | | | |
| EBNA3A | | | | | | | | | |
| EBNA3B | | | | | | | | | |
| EBNA3C | | | | | | | | | |
| EBP | | | | | | | | | |
| EBPL | | | | | | + | | | + |
| ECD | | | | | | | | | |
| ECE1 | | | | | | | | | |
| ECE2 | | | | | | | | | |
| ECEL1 | | | | | | | | | |
| ECH1 | | | | | | | | | |
| echA1 | | | | | | | | | |
| ECHDC1 | | | | | | + | | | + |
| ECHDC2 | | | | + | | | | | + |
| ECHDC3 | | | | | | | | | |
| ECHS1 | | | | | | | | | |
| ECI1 | | | | + | | | | | + |
| ECI2 | | | | | | | | | |
| ECM1 | | | | | | | | | |
| ECM10 | | | | | | | | | |
| ECM11 | | | | | | | | | |
| ECM19 | | | | | | | | | |
| ECM2 | | | | | | | | | |
| ECM32 | | | | | | | | | |
| ecm33 | | | | | | | | | |
| ECO1 | | | | | | | | | |
| EcR | | | | | | | | | |
| ECSCR | | | | | | | | | |
| ECSIT | | | | | | | | | |
| ECT2 | | | | | | | | | |
| ECT2L | | | | | | | | | |
| EDA | | | | | | | | | |
| EDA2R | | | | | | | | | |
| EDAR | | | | | | | | | |
| EDARADD | | | | | | | | | |
| EDC3 | | | | | | | | | |
| Edc4 | | | | | + | | | | + |
| EDDM3A | | | | | | | | | |
| EDDM3B | | | | | | | | | |
| EDE1 | | | | | | | | | |
| EDEM1 | | | | | + | | | | + |
| EDEM2 | | | | | | | | | |
| EDEM3 | | | + | | | | | | + |
| EDF1 | | | | | | | | | |
| EDIL3 | | | | | | | | | |
| EDN1 | | | | | | | | | |
| EDN3 | | | | | | | | | |
| EDNRA | | | | | + | | | | + |
| EDNRB | + | | | + | + | | + | | + |
| EDRF1 | | | | | | | | | |
| EEA1 | | | | | | | | | |
| EED | | | | | | | | | |
| EEF1A1 | | | | | | | | | |

| | | | | | | | | | |
|---|---|---|---|---|---|---|---|---|---|
| EEF1A1P3 | | | | | | | | | |
| EEF1A1P4 | | | | | | | | | |
| EEF1A1P5 | | | | | | | | | |
| EEF1A1P6 | | | | | | | | | |
| EEF1A2 | | | | | | | | | |
| EEF1B2 | | | | | | | | | |
| EEF1B2P5 | | | | | | | | | |
| EEF1B2P6 | | | | | | | | | |
| EEF1D | | | | | | | | | |
| EEF1DP3 | | | | | | | | | |
| EEF1E1 | | | + | | | | | | + |
| EEF1E1-BLOC1S5 | | | | | | | | | |
| EEF1G | | | | | | | | | |
| EEF2 | | | | | | | | | |
| EEF2K | | | | | | + | | | + |
| EEF2KMT | | | | | | | | | |
| EEFSEC | | | | | | | | | |
| EEPD1 | | | | | | + | | | + |
| Ef1alpha100E | | | | | | | | | |
| EFB1 | | | | | | | | | |
| EFCAB1 | | | | | | | | | |
| EFCAB11 | | | | | | | | | |
| EFCAB12 | | | | | | | | | |
| EFCAB13 | | | | | | | | | |
| EFCAB14 | | | | | | | | | |
| EFCAB2 | | | | | | | | | |
| EFCAB3 | | | | | | | | | |
| EFCAB5 | | | | | | | | | |
| EFCAB6 | | | | | | | | | |
| EFCAB7 | | | | | | | | | |
| EFCC1 | | | | | | | | | |
| EFEMP1 | | | | | | | | | |
| EFEMP2 | | | | | | | | | |
| efg1 | | | | | | | | | |
| EFHB | | | | | | | | | |
| EFHC1 | | | | | | | | | |
| EFHC2 | | | | | | | | | |
| EFHD1 | | | | | | | | | |
| EFHD2 | + | | + | | + | | | | + |
| EFM1 | | | | | | | | | |
| EFM3 | | | | | | | | | |
| EFNA1 | | | | | | | | | |
| EFNA2 | | | | | | | | | |
| EFNA3 | | | | | | | | | |
| EFNA4 | | | | | | | | | |
| EFNA5 | + | | | + | + | | | | + |
| EFNB1 | | | | | | | | | |
| EFNB2 | | | | | | | | | |
| EFNB3 | | | | | | + | | | + |
| efr3 | | | | | | | | | |
| EFR3A | | | | | + | | | | + |
| EFR3B | | | | | | | | | |
| EFS | | | | | | | | | |

| | | | | | | | | | |
|---|---|---|---|---|---|---|---|---|---|
| EFT1 | | | | | | | | | |
| EFT2 | | | | | | | | | |
| eft201 | | | | | | | | | |
| EFTUD1 | | | | | | | | | |
| EFTUD2 | | | | | | | | | |
| EGD1 | | | | | | | | | |
| EGD2 | | | | | | | | | |
| EGF | | | | | | | | | |
| EGFL6 | | | | | | | | | |
| EGFL7 | | | | | | | | | |
| EGFL8 | | | | | | | | | |
| EGFLAM | + | | | + | + | | + | | + |
| EGFR | | | | + | | | | | + |
| EGLN1 | | | + | | | | | | + |
| EGLN2 | | | | | | | | | |
| EGLN3 | | | | | | | | | |
| EGR1 | + | | | | + | | | | + |
| EGR2 | + | | | | + | | | | + |
| EGR3 | + | | + | | + | | | | + |
| EGR4 | | | | | | | | | |
| EHBP1 | | | | | | | | | |
| EHBP1L1 | | | | | | + | | | + |
| EHD1 | | + | | | | + | | | + |
| EHD2 | | | | | | | | | |
| EHD3 | | | + | | | | | | + |
| EHD4 | | | + | | | | | | + |
| EHF | + | | | + | + | | + | | + |
| EHHADH | | | | | | | | | |
| EHMT1 | | | | | | | | | |
| EHMT2 | | | | | | | | | |
| EHT1 | | | | | | | | | |
| EI24 | | | | | | | | | |
| EID1 | | | | | | | | | |
| EID2 | | | | | | | | | |
| EID2B | | | | | | | | | |
| EID3 | | | | | | | | | |
| EIF1 | | | | | | | | | |
| Eif1a | | | | | | | | | |
| EIF1AD | | | | | | | | | |
| EIF1AX | | | | | | | | | |
| EIF1AY | | | | | | | | | |
| EIF1B | | | | | | | | | |
| EIF2A | | | | | | | | | |
| EIF2AK1 | | | | | | | + | | + |
| EIF2AK2 | | | | + | | | | | + |
| EIF2AK3 | | | | | | | | | |
| EIF2AK4 | | | | | | | | | |
| EIF2B1 | | | | | | | | | |
| EIF2B2 | | | | | | | | | |
| EIF2B3 | | | | | | | | | |
| EIF2B4 | | | | | | | | | |
| EIF2B5 | | | | | | | | | |
| EIF2D | | | | | | | | | |

| | | | | | | | | | |
|---|---|---|---|---|---|---|---|---|---|
| EIF2S1 | | | | | | | | | |
| EIF2S2 | | | | | + | | | | + |
| EIF2S3 | | | | | | | | | |
| Eif2s3x | | | | | | | | | |
| Eif2s3y | | | | | | | | | |
| EIF3A | | | | | | | | | |
| EIF3B | | | | | + | | | | + |
| EIF3C | | | | | + | | | | + |
| EIF3CL | | | | | | | | | |
| EIF3D | | | | | | | | | |
| EIF3E | | | | | | | | | |
| EIF3F | | | | | | | | | |
| EIF3FP1 | | | | | | | | | |
| EIF3FP3 | | | | | | | | | |
| EIF3G | | | | | | | | | |
| EIF3H | | | | | | | | | |
| EIF3I | | | | | | | | | |
| EIF3J | | | | | | | | | |
| EIF3K | | | | | | | | | |
| EIF3L | | | | | | | | | |
| EIF3M | | | | | | | | | |
| EIF4A1 | | | | | | | | | |
| EIF4A2 | | | | | | | | | |
| EIF4A3 | | | | | | | | | |
| EIF4B | | | | | | | | | |
| EIF4E | | | | | | | | | |
| EIF4E1B | | | | | | | | | |
| EIF4E2 | + | | | + | + | + | | | + |
| EIF4E3 | + | | | + | + | | + | | + |
| EIF4EBP1 | | | + | | | | | | + |
| EIF4EBP2 | | | + | | | | | | + |
| EIF4EBP3 | | | | | | | | | |
| EIF4ENIF1 | | | | | | | | | |
| EIF4G1 | | | | | | | | | |
| EIF4G2 | | | + | | + | | | | + |
| EIF4G3 | | + | | | | + | | | + |
| EIF4H | | | + | | | | | | + |
| EIF5 | | | | | + | | | | + |
| EIF5A | | | | | | | | | |
| EIF5A2 | | | | | | | | | |
| EIF5AL1 | | | | | | | | | |
| EIF5B | | | | | + | | | | + |
| EIF6 | | | | | | | | | |
| EIN3 | | | | | | | | | |
| eis1 | | | | | | | | | |
| ELA1 | | | | | | | | | |
| ELAC1 | | | | | | + | | | + |
| ELAC2 | | | | | + | | | | + |
| ELANE | | | | | | | | | |
| ELAVL1 | | | | | | + | | | + |
| ELAVL2 | | | | | | | | | |
| ELAVL3 | | | | | | | | | |
| ELAVL4 | + | | | | + | | | | + |

| | | | | | | | | | |
|---|---|---|---|---|---|---|---|---|---|
| elc-1 | | | | | | | | | |
| ELC1 | | | | | | | | | |
| ELF1 | | | | | | | | | |
| ELF2 | | | | | | | | | |
| ELF3 | | | | | | | | | |
| ELF4 | | | | | | | | | |
| ELF5 | | | | | | | | | |
| ELFN1 | | | | | | | | | |
| ELFN2 | | | | | | + | | | + |
| ELK1 | | | | | | + | | | + |
| ELK3 | | | | | | | | | |
| ELK4 | | | + | | | | | | + |
| ELL | | | | | | | | | |
| ELL2 | | | + | | + | | | | + |
| ELL3 | | | | | | | | | |
| ELMO1 | | | | | + | | | | + |
| ELMO2 | | | + | | | | | | + |
| ELMO3 | | | | | | | | | |
| ELMOD1 | | + | | | | + | | + | + |
| ELMOD2 | | | | | | | | | |
| ELMOD3 | | | | | | | | | |
| ELMSAN1 | | | | | | | | | |
| ELN | | | | | | | | | |
| ELOF1 | | | | | | | | | |
| ELOVL1 | | | | | | | | | |
| ELOVL2 | | | | | + | | | | + |
| ELOVL3 | | | | | | | | | |
| ELOVL4 | | | + | | | | | | + |
| ELOVL5 | | | + | | | + | | | + |
| ELOVL6 | | | | | | | | | |
| ELOVL7 | | | | | | | | | |
| ELP2 | | + | + | | | + | | | + |
| ELP3 | | | | | | + | | | + |
| ELP4 | | | | | | | | | |
| ELP5 | | | | | | | | | |
| ELP6 | | | | | | | | | |
| ELSPBP1 | | | | | | | | | |
| ELTD1 | | | | | | | | | |
| EMB | | | | | | | | | |
| EMB2296 | | | | | | | | | |
| EMBP1 | | | | | | | | | |
| EMC1 | | | | | + | | + | | + |
| EMC10 | | | + | | | + | | | + |
| EMC2 | | | | | | | | | |
| EMC3 | | | | | | | | | |
| EMC4 | | | | | | + | | | + |
| Emc6 | | | | | | | | | |
| EMC7 | | | | | | | | | |
| EMC8 | | | | | | + | | | + |
| EMC9 | | | | | | | | | |
| EMCN | | | | | | | | | |
| EMD | | | + | | | | | | + |
| EME1 | | | | | | | | | |

| | | | | | | | | | |
|---|---|---|---|---|---|---|---|---|---|
| EME2 | | | | | | | | | |
| EMG1 | | | | | | | | | |
| EMI2 | | | | | | | | | |
| EMID1 | | | | | | | | | |
| EMILIN1 | | | | | | | | | |
| EMILIN2 | | | | | | | | | |
| EMILIN3 | | | | | | | | | |
| EML1 | | | + | | | + | | | + |
| EML2 | | | | | | | | | |
| EML3 | | | | | | | | | |
| EML4 | | | | | | | | | |
| EML5 | | | | | | | | | |
| EML6 | | | | | | | | | |
| EMP1 | | | + | | | | | | + |
| EMP2 | + | | | + | + | | | | + |
| EMP3 | | | | | | | | | |
| EMP47 | | | | | | | | | |
| EMR2 | | | | | | | | | |
| EMR4P | | | | | | | | | |
| EMX1 | | | | | | | | | |
| EMX2 | | | | | | | | | |
| EN1 | + | | | + | + | | + | | + |
| EN2 | | | | | | | | | |
| ENAH | | | + | | | | | | + |
| ENAM | | | | | | | | | |
| ENC1 | | | + | | | + | | | + |
| end4 | | | | | | | | | |
| EndoA | | | | | | | | | |
| ENDOD1 | | | | | | + | | | + |
| ENDOG | | | | | | | | | |
| ENDOU | | | + | | | | | | + |
| ENDOV | | | | | | | | | |
| ENG | | | | | | | | | |
| eng1 | | | | | | | | | |
| ENGASE | | | | | | | | | |
| ENHO | | | | | | | | | |
| ENKD1 | | | | | | | | | |
| ENKUR | | | | | | | | | |
| ENO1 | | + | + | | | + | | + | + |
| eno101 | | | | | | | | | |
| ENO2 | | | + | | | | | | + |
| ENO3 | | | | | | | | | |
| ENO4 | | | | | | | | | |
| ENOPH1 | | | | | | | | | |
| ENOSF1 | | | | | | | | | |
| ENOX1 | | | | | | | | | |
| ENOX2 | | | | | | | | | |
| ENP1 | | | | | | | | | |
| ENPEP | | | | | | | | | |
| ENPP1 | | | | | | | | | |
| ENPP2 | | + | + | | | + | | + | + |
| ENPP3 | | | | | | | | | |
| ENPP4 | | | | | | | + | | + |

| | | | | | | | | | |
|---|---|---|---|---|---|---|---|---|---|
| ENPP5 | | + | | | | + | | | + |
| ENPP6 | + | | | + | + | | + | | + |
| ENPP7 | | | | | | | | | |
| ENPP7P4 | | | | | | | | | |
| ENSA | | | | | | | | | |
| ent1 | | | | | | | | | |
| ENTHD1 | | | | | | | | | |
| ENTHD2 | | | | | | | | | |
| ENTPD1 | | | | | | | | | |
| ENTPD2 | | | | | | | | | |
| ENTPD3 | | | | | | | | | |
| ENTPD4 | | | | | | | | | |
| ENTPD5 | | | | | | | | | |
| ENTPD6 | | | | | | | | | |
| ENTPD7 | | | | | | | | | |
| ENTPD8 | | | | | | | | | |
| env | | | | | | | | | |
| ENY2 | | | | | | | | | |
| EOGT | | | | | | | | | |
| EOMES | + | | | + | + | | + | | + |
| EOS | | | | | | | | | |
| EP300 | | | | | | | | | |
| EP400 | | | | | | | | | |
| EP400NL | | | | | | | | | |
| EPAS1 | | | | | | | | | |
| Epb4.1 | | | | | | | | | |
| Epb4.1l2 | | | | | | | | | |
| Epb4.1l3 | | | | | | | | | |
| EPB41 | | | | | | | | | |
| EPB41L1 | | | | | | | | | |
| EPB41L2 | | | | | | | | | |
| EPB41L3 | | | | | | | | | |
| EPB41L4A | | | | | | | | | |
| EPB41L4A-AS1 | | | | | | | | | |
| EPB41L4A-AS2 | | | | | | | | | |
| EPB41L4B | | | | | | | | | |
| EPB41L5 | | | | | | | | | |
| EPB42 | | | | | | | | | |
| EPC1 | | | | | + | | | | + |
| epc2 | | | | | | | | | |
| EPCAM | | | | | | | | | |
| EPDR1 | | + | | | | + | | | + |
| EPG5 | | | | | | | | | |
| EPGN | | | | | | | | | |
| EPHA1 | | | | | | | | | |
| EPHA10 | + | | | | + | | | | + |
| EPHA2 | | | | | | | | | |
| EPHA3 | | | + | | | | | | + |
| EPHA4 | | | | | | | | | |
| EPHA5 | | | | | + | | | | + |
| EPHA6 | | | | | | | | | |
| EPHA7 | | | | | + | | | | + |
| EPHA8 | | | | | | | | | |

| | | | | | | | | | |
|---|---|---|---|---|---|---|---|---|---|
| EPHB1 | | | | | | | | | |
| EPHB2 | + | | | + | + | | + | | + |
| EPHB3 | | | | | | | | | |
| EPHB4 | | | | | | | | | |
| EPHB6 | | | | | | | | | |
| EPHX1 | | | | | | | | | |
| EPHX2 | | | | | | | | | |
| EPHX3 | | | | | | | | | |
| EPHX4 | | | | | | | | | |
| EPM2A | | | | | | | | | |
| EPM2AIP1 | | | + | | | | | | + |
| EPN1 | | | | | | | | | |
| EPN2 | | | | | | + | | | + |
| EPN3 | | | | | | | | | |
| EPO | | | | | | | | | |
| EPOR | | | | | | + | | | + |
| EPPIN | | | | | | | | | |
| EPPK1 | | | | | | | | | |
| EPRS | + | | + | | + | | | | + |
| EPS1 | | | | | | | | | |
| EPS15 | | | | | | | | | |
| EPS15L1 | | | | | | | | | |
| EPS15P1 | | | | | | | | | |
| EPS8 | | | + | | | | | | + |
| EPS8L1 | | | | | | | | | |
| EPS8L2 | | | | | | | | | |
| EPS8L3 | | | | | | | | | |
| EPSTI1 | | | | | | | | | |
| EPT1 | | | + | | | | | | + |
| EPX | | | | | | | | | |
| EPYC | | | | | | | | | |
| ERAL1 | | | | | | | | | |
| ERAP1 | | | | | | | | | |
| ERAP2 | | | | | | | | | |
| ERAS | | | | | | | | | |
| ERBB2 | | | | | | | | | |
| ERBB2IP | | | | | | | | | |
| ERBB3 | | | | | | | | | |
| ERBB4 | | | | | | | | | |
| ERC1 | + | | + | | + | | | | + |
| ERC2 | | | | | | | | | |
| ERCC1 | | | | | | | | | |
| ERCC2 | | | | | | | | | |
| ERCC3 | | | | | | | | | |
| ERCC4 | | | | | | + | | | + |
| ERCC5 | | | | | | | | | |
| ERCC6 | | | | | | | | | |
| ERCC6L | | | | | | | | | |
| ERCC6L2 | | | | | | | | | |
| ERCC8 | | | | | | | | | |
| ERD10 | | | | | | | | | |
| ERD2 | | | | | | | | | |
| EREG | | | | | | | | | |

| | | | | | | | | | |
|---|---|---|---|---|---|---|---|---|---|
| ERF | | | + | | | | | | + |
| ERG | | | | | | | | | |
| ERG10 | | | | | | | | | |
| ERG13 | | | | | | | | | |
| ERG20 | | | | | | | | | |
| ERG26 | | | | | | | | | |
| ERG28 | | | | | | | | | |
| ERG6 | | | | | | | | | |
| ERG9 | | | | | | | | | |
| ERGIC1 | | | + | | | | | | + |
| ERGIC2 | | | | | | | | | |
| ERGIC3 | | | | | | | | | |
| ERH | | | | | | | | | |
| erh1 | | | | | | | | | |
| ERI1 | | | | | | | | | |
| ERI2 | | | | | | + | | | + |
| ERI3 | | | | | | | | | |
| ERICH1 | | | | | | | | | |
| ERICH2 | | | | | | | | | |
| ERICH3 | | | | | | | | | |
| ERICH4 | | | | | | | | | |
| ERICH5 | | | | + | | | | | + |
| ERICH6 | | | | | | | | | |
| ERICH6B | | | | | | | | | |
| ERLEC1 | | | | | | | | | |
| ERLIN1 | | | | | | | | | |
| ERLIN2 | | | | | | | | | |
| ERMAP | | | | | + | | | | + |
| ERMARD | | | | | | | | | |
| ERMN | | | | | | | | | |
| ERMP1 | + | | | + | + | | + | | + |
| ERN1 | | | | | | | | | |
| Ern2 | | | | | | | | | |
| ERO1L | | | + | | | | | | + |
| ERO1LB | | | | | | | | | |
| ERP1 | | | | | | | | | |
| ERP27 | | | | | | | | | |
| ERP29 | | | | | | | | + | + |
| ERP44 | | | | | | | | | |
| Errfi1 | + | | + | | + | | | | + |
| ERV1 | | | | | | | | | |
| ERV14 | | | | | | | | | |
| ERV29 | | | | | | | | | |
| ERV3-1 | | | | | | | | | |
| ERVFC1-1 | | | | | | | | | |
| ERVFRD-1 | | | | | | | | | |
| ERVK-10 | | | | | | | | | |
| ERVK-19 | | | | | | | | | |
| ERVK-21 | | | | | | | | | |
| ERVK-6 | | | | | | | | | |
| ERVK13-1 | | | | | | | | | |
| ERVK3-1 | | | | | | | | | |
| ERVMER34-1 | | | | | | | | | |

| | | | | | | | | | |
|---|---|---|---|---|---|---|---|---|---|
| ERVV-1 | | | | | | | | | |
| ESAM | | | | | | | | | |
| esc | | | | | | | | | |
| ESC2 | | | | | | | | | |
| ESCO1 | | | | | | | | | |
| ESCO2 | | | | | | | | | |
| ESD | | | | | + | | | | + |
| ESF1 | | | | + | + | | | | + |
| ESM1 | | | | | | | | | |
| Espl1 | | | | | | | | | |
| Espn | | | | | | | | | |
| ESPNL | | | | | | | | | |
| ESPNP | | | | | | | | | |
| ESR1 | | | | | | | | | |
| ESR2 | | | | | | | | | |
| ESRP1 | | | | | | | | | |
| ESRP2 | | | | | | | | | |
| ESRRA | | | | | | + | | | + |
| Esrrb | | | | | | | | | |
| ESRRG | | | | | | | | | |
| EST1 | | | | | | | | | |
| ESX1 | + | | | + | + | | + | | + |
| ESYT1 | | | | | | | | | |
| ESYT2 | | | | | | | | | |
| ESYT3 | | | | | | + | | | + |
| ETAA1 | | | | | | | | | |
| ETF1 | | | + | | + | | | | + |
| ETFA | | | | | | | | | |
| ETFB | | | | | | | | | |
| ETFDH | | | | | | | | | |
| ETHE1 | | | | | | | | | |
| Etl4 | | | | | | | | | |
| ETNK1 | | | | | | | | | |
| ETNK2 | | | | | | | | | |
| ETNPPL | | | | | | | | | |
| ETR1 | | | | | | | | | |
| ETS1 | | | | | | | | | |
| ETS2 | | | | | | | | | |
| ETV1 | | | | | | | | | |
| ETV2 | | | | | | | | | |
| ETV3 | + | | + | | + | | | | + |
| ETV3L | | | | | | | | | |
| ETV4 | | | | | | | | | |
| ETV5 | + | | + | | | | | | + |
| ETV6 | | | | | | | | | |
| ETV7 | | | | | | | | | |
| EUG1 | | | | | | | | | |
| EVA1A | | | | | | | | | |
| EVA1B | | | | | | | | | |
| EVA1C | | | | | | | | | |
| EVC | | | | | + | | | | + |
| EVC2 | | | | | | + | | | + |
| EVI2A | | | | | | | | | |

| | | | | | | | | | |
|---|---|---|---|---|---|---|---|---|---|
| EVI2B | | | | | | | | | |
| EVI5 | | | | | | | | | |
| EVI5L | | | | | | | | | |
| EVL | | | | | | | | | |
| EVPL | | | | | | | | | |
| EVPLL | | | | | | | | | |
| EVX1 | | | | | | | | | |
| EVX2 | | | | | | | | | |
| EWSR1 | | | | | | | | | |
| EXD1 | | | | | | | | | |
| EXD2 | | | | | | | | | |
| EXD3 | | | | | | | | | |
| EXO1 | | | | | | | | | |
| exo2 | | | | | | | | | |
| EXO5 | | | | | | | | | |
| EXO84 | | | | | | | | | |
| EXOC1 | | | | | | + | | | + |
| EXOC2 | | | | | | | | | |
| EXOC3 | | | | | | | | | |
| EXOC3-AS1 | | | | | | | | | |
| EXOC3L1 | | | | | | | | | |
| EXOC3L2 | | | | | | | | | |
| EXOC3L4 | | | | | | | | | |
| EXOC4 | | | | | | + | | | + |
| EXOC5 | | | | | | | | | |
| EXOC6 | | | + | | | | | | + |
| EXOC6B | | | | | | | | | |
| EXOC7 | | | | | | | | | |
| EXOC8 | | | | | | + | | | + |
| EXOG | | | | | | | | | |
| EXOSC1 | | | | | | | | | |
| EXOSC10 | | | | | | | | | |
| EXOSC2 | | | | + | | | | | + |
| EXOSC3 | | | | | | | | | |
| EXOSC4 | | | | | | | | | |
| EXOSC5 | | | | | | | | | |
| EXOSC6 | | | | | | | | | |
| EXOSC7 | | | | | | | | | |
| EXOSC8 | | | | | | | | | |
| EXOSC9 | | | | | | | | | |
| EXPH5 | | | | | | | | | |
| EXT1 | | | + | | + | | | | + |
| EXT2 | | | | | | + | | | + |
| EXTL1 | | | | | | | | | |
| EXTL2 | + | | + | | | + | | | + |
| EXTL3 | | | | | | | | | |
| EYA1 | | | | | | | | | |
| EYA2 | | | | | | | | | |
| EYA3 | | | | | | | | | |
| EYA4 | | | | | | | | | |
| EYS | | | | | | | | | |
| EZH1 | | | | | | | | | |
| EZH2 | | | | | | + | | | + |

| | | | | | | | | | |
|---|---|---|---|---|---|---|---|---|---|
| ezh2-a | | | | | | | | | |
| EZR | | | + | | | | | | + |
| F10 | | | | | | | | | |
| F11 | | | | | | | | | |
| F11R | | | | | | | | | |
| F12 | | | | | | | | | |
| F13A1 | | | | | | | | | |
| F13B | | | | | | | | | |
| F1L | | | | | | | | | |
| F2 | | | | | | | | | |
| F2R | | | | | | | | | |
| F2RL1 | | | | | | | + | | + |
| F2RL3 | | | | | | | | | |
| F3 | | | | | | | | | |
| F5 | | + | + | | | + | | + | + |
| F7 | | | | | | | | | |
| F8 | | | | | | + | | | + |
| F8a | | | + | | | | | | + |
| F8A1 | | | | | | | | | |
| F8A2 | | | | | | | | | |
| F9 | + | | | + | + | | + | | + |
| FA2H | | | | | | | | | |
| FAA1 | | | | | | | | | |
| FAA2 | | | | | | | | | |
| FAAH | | | | | | + | | | + |
| FAAH2 | | | | | | | | | |
| FABP1 | | | | | | | | | |
| FABP12 | | + | + | | | + | | | + |
| FABP2 | | | | | | | | | |
| FABP3 | | | | | | | | | |
| FABP4 | | | | | | | | | |
| FABP5 | | | | | | | | | |
| FABP6 | | | | | | | | | |
| FABP7 | | | | | | | | | |
| FAD1 | | | | | | | | | |
| FADD | | | | | | | | | |
| FADS1 | | | | | | | | | |
| FADS2 | | | | | | | | | |
| FADS3 | | | | | | | | | |
| FADS6 | | | | | | | | | |
| FAF1 | | | | | | | | | |
| FAF2 | | | | | | | | | |
| FAH | | | | | | | | | |
| FAHD1 | | | | | | | | | |
| FAHD2A | | | | | | + | | | + |
| FAHD2B | | | | | | | | | |
| FAIM | | | | | | | | | |
| FAIM2 | | | | | | + | | | + |
| FAIM3 | | | | | | | | | |
| Fam101a | | | | | | | | | |
| FAM101B | | | | | | + | | | + |
| FAM102A | | | | | | | | | |
| FAM102B | | | | | | | | | |

| | | | | | | | | | |
|---|---|---|---|---|---|---|---|---|---|
| FAM103A1 | | | | | | | | | |
| FAM103A2P | | | | | | | | | |
| FAM104A | | | | | | | | | |
| FAM104B | | | | | | | | | |
| FAM105A | | | | | | | | | |
| FAM106A | | | | | | | | | |
| FAM107A | | | | | | | | | |
| Fam107b | | | + | | | | | | + |
| FAM109A | | | | | | | | | |
| FAM109B | | | | | | | | | |
| FAM110A | | | | | | | | | |
| FAM110B | | | | | | | | | |
| FAM110C | | | | | | | | | |
| FAM110D | | | | | | | | | |
| FAM111A | | | | | | | | | |
| FAM111B | | | | | | | | | |
| FAM114A1 | | | | | | | | | |
| FAM114A2 | | | | | | | | | |
| FAM115A | | | | | | | | | |
| FAM115C | | | | | | | | | |
| FAM117A | | | | | | | | | |
| FAM117B | | | | | | | | | |
| FAM118A | | | | | | | | | |
| FAM118B | | | | | | | | | |
| FAM120A | | | | | | | | | |
| FAM120AOS | | | | | | | | | |
| FAM120B | | | | | + | | | | + |
| FAM120C | | | | | | | | | |
| FAM122A | | | | | | | | | |
| FAM122B | | | | | | | | | |
| FAM122C | | | | | | | | | |
| FAM124A | + | | | + | + | | | | + |
| FAM124B | | | | | | | | | |
| FAM126A | | | | | | + | | | + |
| FAM126B | | | | | + | | | | + |
| FAM127A | | | | | | | | | |
| FAM127B | | | | | | | | | |
| FAM127C | | | | | | | | | |
| FAM129A | | | | | | | | | |
| Fam129b | | | | | + | | | | + |
| FAM129C | | | | | | | | | |
| FAM131A | | | + | | | | + | | + |
| FAM131B | | | | | | | | | |
| FAM131C | | | | | | | | | |
| FAM132B | | | | | | | | | |
| FAM133A | | | | | | | | | |
| FAM133B | | | | | | | | | |
| FAM134A | | + | | | | + | + | | + |
| FAM134B | | | | + | | | | | + |
| FAM134C | | | | | | | | | |
| FAM135A | | | | | | | | | |
| FAM135B | | | | | | | | | |
| FAM136A | | | | | | | | | |

| | | | | | | | | | |
|---|---|---|---|---|---|---|---|---|---|
| FAM13A | | + | | | | + | | | + |
| FAM13A-AS1 | | | | | | | | | |
| FAM13B | | | | | | | | | |
| FAM13C | + | | | | | | | | + |
| FAM149A | | | | | | | | | |
| FAM149B1 | | | | | | | | | |
| FAM150A | | | | | | | | | |
| FAM150B | | | | | | | | | |
| FAM151A | | | | | | | | | |
| FAM151B | | | | | | | | | |
| FAM153A | | | | | | | | | |
| FAM153B | | | | | | | | | |
| FAM153C | | | | | | | | | |
| FAM154A | | | | | | | | | |
| FAM154B | | | | | | | | | |
| FAM155A | | | | | | | | | |
| FAM156A | | | | | | | | | |
| FAM156B | | | | | | | | | |
| FAM157A | | | | | | | | | |
| FAM157B | | | | | | | | | |
| FAM159A | | | | | | | | | |
| FAM160A1 | | | | | | | | | |
| FAM160A2 | | | | | | | | | |
| FAM160B1 | | | | | | | | | |
| FAM160B2 | | | | | | + | | | + |
| FAM161A | | | | | | | | | |
| FAM161B | | | | | | | | | |
| FAM162A | | | | | | | | | |
| FAM163A | | | | | | | | | |
| FAM163B | | + | | | | + | | | + |
| FAM166A | | | | | | | | | |
| FAM166B | | | | | | | | | |
| FAM167A | | | | | | | | | |
| FAM168A | | | | | + | | | | + |
| FAM168B | | | | | | | | | |
| FAM169A | | | | | | | | | |
| FAM169B | | | | | | | | | |
| FAM170A | | | | | | | | | |
| FAM171A1 | | | | | | | | | |
| FAM171A2 | | | | | | | | | |
| FAM171B | | + | | | | + | | | + |
| FAM172A | | | | | | | | | |
| FAM173A | | | | | | | | | |
| FAM173B | | | | | | | | | |
| FAM174A | | | | | | | | | |
| FAM175A | | | | | | | | | |
| FAM175B | | | | | | | | | |
| FAM177A1 | | | | | | | | | |
| FAM177B | | | | | | | | | |
| FAM178A | | | | | | | | | |
| FAM178B | | | | | | | | | |
| FAM179A | | | | | | | | | |
| FAM179B | | | | | | | | | |

| | | | | | | | | | |
|---|---|---|---|---|---|---|---|---|---|
| FAM180B | | | | | | | | | |
| FAM181A | | | | | | | | | |
| FAM181B | | | | | | | | | |
| FAM182A | | | | | | | | | |
| FAM184A | | | | | | | | | |
| FAM184B | | | | | | + | | | + |
| FAM185A | | | | | | | | | |
| FAM186A | | | | | | | | | |
| FAM186B | | | | | | | | | |
| FAM187B | | | | | | | | | |
| FAM188A | | | | | | | | | |
| FAM188B | | | | | | | | | |
| FAM189A1 | | | | | | | | | |
| FAM189A2 | | | | | | | | | |
| FAM189B | | | | | | | | | |
| FAM192A | | | | | | | | | |
| FAM193A | | | | | | | | | |
| FAM193B | | | + | | | + | | | + |
| FAM195A | | | | | | | | | |
| FAM195B | | | | | | | | | |
| FAM196A | | | | | | | | | |
| FAM196B | | | | | | | | | |
| FAM198A | | | | | | | | | |
| FAM198B | | | + | | | | | | + |
| FAM199X | | | | | | | | | |
| FAM19A1 | | | + | | | | | | + |
| FAM19A2 | | | | | | | | | |
| FAM19A3 | | | | | | | | | |
| FAM19A4 | | | | | | | | | |
| FAM19A5 | | | + | | | | | | + |
| FAM200A | | | | | | | | | |
| FAM200B | | | | | | | | | |
| FAM204A | | | | | | | | | |
| FAM205A | | | | | | | | | |
| FAM205CP | | | | | | | | | |
| FAM206A | | | | + | | | | | + |
| FAM207A | | | | | + | | | | + |
| FAM208A | | | | | | | | | |
| FAM208B | | | | | | | | | |
| FAM209A | | | | | | | | | |
| FAM20A | | | | | | | | | |
| FAM20B | | | | | | | | | |
| FAM20C | | | + | | | | | | + |
| FAM210A | | | | | | | | | |
| FAM210B | | | | | | | | | |
| FAM212A | | | | | | | | | |
| FAM212B | | | | | | | | | |
| FAM213A | | + | | | | + | | | + |
| FAM213B | | | | | | | | | |
| FAM214A | | + | | + | | | | | + |
| FAM214B | | | | | + | | | | + |
| FAM216A | | | | | | | | | |
| FAM216B | | | | | | | | | |

| | | | | | | | | | |
|---|---|---|---|---|---|---|---|---|---|
| FAM217A | + | | | + | + | | + | | + |
| FAM217B | | | | | | | | | |
| FAM218A | | | | | | | | | |
| FAM219A | | | | | + | | | | + |
| FAM219B | | | | | | | | | |
| FAM21A | | | | | | | | | |
| FAM21C | | | | | | | | | |
| FAM220A | | | | | | + | | | + |
| FAM221A | | | | | | | | | |
| FAM221B | | | | | | | | | |
| FAM222A | | | | | | | | | |
| FAM222B | | | | | | | | | |
| FAM227A | | | | | | | | | |
| FAM227B | | | | | | | | | |
| FAM228A | | | | | | | | | |
| FAM228B | | | | | | | | | |
| FAM229A | | | | | | | | | |
| FAM229B | | + | + | + | | + | | | + |
| FAM24A | | | | | | | | | |
| FAM24B | | | | | | | | | |
| FAM25A | | | | | | | | | |
| FAM25C | | | | | | | | | |
| FAM25G | | | | | | | | | |
| FAM26D | | + | + | | | + | | | + |
| FAM26E | | | | | | | | | |
| FAM26F | | | | | | | | | |
| FAM27E3 | | | | | | | | | |
| FAM32A | | | | | | | | | |
| FAM35A | | | | | | + | | | + |
| FAM3A | | | | | | | | | |
| FAM3B | | | | | | | | | |
| FAM3C | | + | + | | | + | | | + |
| FAM3D | | | | | | | | | |
| FAM43A | + | | | + | + | | | | + |
| FAM43B | | | | | | + | | | + |
| FAM45A | | | | | | | | | |
| FAM45B | | | | | | | | | |
| FAM46A | + | | + | + | + | | + | | + |
| FAM46B | | | + | | | | | | + |
| FAM46C | | | | | | | | | |
| FAM46D | | | | | | | | | |
| FAM47A | | | | | | | | | |
| FAM47B | | | | | | | | | |
| FAM47C | | | | | | | | | |
| FAM47E | | | | | | | | | |
| FAM47E-STBD1 | | | | | | | | | |
| FAM49A | | | | | | | | | |
| FAM49B | | | | | | | | | |
| FAM50A | + | | | + | + | | | | + |
| FAM50B | | | | | | | | | |
| FAM53A | | | | | | + | | | + |
| FAM53B | + | | | | + | | | | + |
| FAM53C | | | | | | | | | |

| | | | | | | | | | |
|---|---|---|---|---|---|---|---|---|---|
| FAM57A | | | | | | + | | | + |
| FAM57B | | | | | | + | | | + |
| FAM58A | | | | | | | | | |
| FAM60A | | | | | | | | | |
| FAM63A | | | | | | + | | | + |
| FAM63B | | | | | | | | | |
| FAM64A | | | | | | | | | |
| FAM65A | | | | | | | | | |
| FAM65B | | | | | | | | | |
| FAM65C | | | | | | | | | |
| FAM66E | | | | | | | | | |
| FAM69A | | | | | | | | | |
| FAM69B | | | | | | | | | |
| FAM69C | | | | | | | | | |
| FAM71A | | | | | | | | | |
| FAM71B | | | | | | | | | |
| FAM71C | | | | | | | | | |
| FAM71D | | | | | | | | | |
| FAM71E1 | | | | | | | | | |
| FAM71E2 | | | | | | | | | |
| FAM71F1 | | | | | | | | | |
| FAM71F2 | | | | | | | | | |
| FAM72A | | | | | | | | | |
| FAM72B | | | | | | | | | |
| FAM72C | | | | | | | | | |
| FAM72D | | | | | | | | | |
| FAM73A | | | | | | | | | |
| FAM73B | | | | | | | | | |
| FAM74A1 | | | | | | | | | |
| FAM74A4 | | | | | | | | | |
| FAM76A | | | | + | | | | | + |
| FAM76B | | | | | | + | | | + |
| FAM78A | | | | | | | | | |
| FAM78B | | | | | | | | | |
| FAM81A | | | | | | | | | |
| FAM81B | | | | | | | | | |
| FAM83A | | | | | | | | | |
| FAM83B | | | | | | | | | |
| FAM83C | | | | | | | | | |
| FAM83D | | | | | + | | | | + |
| FAM83F | | | | | | | | | |
| FAM83G | | | | | | | | | |
| FAM83H | | | | | | | | | |
| FAM83H-AS1 | | | | | | | | | |
| FAM84A | | | | | | | | | |
| FAM84B | | | | | | | | | |
| FAM86B1 | | | | | | | | | |
| FAM86B2 | | | | | | | | | |
| FAM86B3P | | | | | | | | | |
| FAM86C1 | | | | | | | | | |
| FAM86JP | | | | | | | | | |
| FAM89A | | | | | | | | | |
| FAM89B | | | | | | | | | |

| | | | | | | | | | |
|---|---|---|---|---|---|---|---|---|---|
| FAM8A1 | | | | | | | | | |
| FAM90A1 | | | | | | | | | |
| FAM90A26 | | | | | | | | | |
| FAM90A27P | | | | | | | | | |
| FAM90A5P | | | | | | | | | |
| FAM91A1 | | | | | | | | | |
| FAM92A1 | | | | | | | | | |
| FAM92B | | | | | | | | | |
| FAM96A | | | | | | | | | |
| FAM96B | | | | | | | | | |
| FAM98A | | | | | + | | | | + |
| FAM98B | | | | | | | | | |
| FAM98C | | | | | | | | | |
| FAM9A | | | | | | | | | |
| FAM9B | | | | | | | | | |
| FAM9C | | | | | | | | | |
| FAN1 | | | | | | | | | |
| FANCA | | | | | | | | | |
| FANCB | | | | | | | | | |
| FANCC | | | | | | | | | |
| FANCD2 | | | | | | | | | |
| FANCD2OS | | | | | | | | | |
| FANCE | | | | | | | | | |
| FANCF | | | | | | | | | |
| FANCG | | | | | | | | | |
| FANCI | | | | | | | | | |
| FANCL | | | | | | | | | |
| FANCM | | | | | + | | | | + |
| FANK1 | | | | | | | | | |
| FAP | | | | | | | | | |
| FAP7 | | | | | | | | | |
| FAR1 | | | + | | | | | | + |
| FAR10 | | | | | | | | | |
| FAR11 | | | | | | | | | |
| FAR2 | | | | | | | | | |
| FAR3 | | | | | | | | | |
| FAR7 | | | | | | | | | |
| FAR8 | | | | | | | | | |
| FARP1 | | | | | | | | | |
| FARP2 | | | | | | | | | |
| FARS2 | | | | | | | | | |
| FARSA | | | | | | + | | | + |
| FARSB | | | | | | | | | |
| FAS | | | | | | | | | |
| FAS1 | | | | | | | | | |
| FAS2 | | | | | | | | | |
| FASLG | | | | | | | | | |
| FASN | | | | | | | | | |
| FASTK | | | | | | | | | |
| FASTKD1 | | | | | | + | | | + |
| FASTKD2 | | | | | | | | | |
| FASTKD3 | | | | | | | | | |
| FASTKD5 | | | | | | | | | |

| | | | | | | | | | |
|---|---|---|---|---|---|---|---|---|---|
| FAT1 | | | | | | | | | |
| Fat2 | | | | | | | | | |
| FAT3 | | | | | | | | | |
| FAT4 | | | | | | + | | | + |
| FATE1 | | | | | | | | | |
| FAU | | | | | | | | | |
| FAU1 | | | | | | | | | |
| FAUP1 | | | | | | | | | |
| FAXC | | | | | | | | | |
| FAXDC2 | | | | | | | | | |
| FBA1 | | | | | | | | | |
| FBF1 | | | | | | | | | |
| FBL | | | | | | | | | |
| FBLIM1 | | | | | | | | | |
| FBLL1 | | | | | | | | | |
| FBLN1 | | | | | | + | | | + |
| FBLN2 | | | | | | | | | |
| FBLN5 | | | | | | | | | |
| FBLN7 | + | | | + | + | | + | | + |
| FBN1 | | | | | | | | | |
| FBN2 | | | | | | | | | |
| fbn2b | | | | | | | | | |
| FBN3 | | | | | | | | | |
| FBP1 | | | | | | | | | |
| FBP2 | | | | | | | | | |
| FBRS | | | | | | | | | |
| FBRSL1 | | | | | | | | | |
| FBXL12 | | | | | | | | | |
| FBXL13 | | | | | | | | | |
| FBXL14 | | | | | | | | | |
| FBXL15 | | | | | | | | | |
| FBXL16 | | | | | | | | | |
| FBXL17 | | | | | | | | | |
| FBXL18 | | | | | | | | | |
| FBXL19 | | | | | | | | | |
| FBXL19-AS1 | | | | | | | | | |
| FBXL2 | | | | | | | | | |
| Fbxl20 | | | | | | | | | |
| FBXL21 | | | | | | | | | |
| FBXL22 | | | | | + | | | | + |
| FBXL3 | | | | | | | | | |
| FBXL4 | | | | | | | | | |
| FBXL5 | | | | | | | | | |
| FBXL6 | | | | | | | | | |
| FBXL7 | | | | | | | | | |
| FBXL8 | | | | | | | | | |
| FBXO10 | | | + | | | | | | + |
| FBXO11 | | | | | | | | | |
| FBXO15 | | | | | | | | | |
| FBXO16 | | | | | | | | | |
| FBXO17 | | | | | | | | | |
| FBXO18 | | | | | | | | | |
| FBXO2 | + | | | + | + | | | | + |

| | | | | | | | | | |
|---|---|---|---|---|---|---|---|---|---|
| FBXO21 | | | | | | | | | |
| FBXO22 | | | | | | | | | |
| FBXO22-AS1 | | | | | | | | | |
| FBXO24 | | | | | | | | | |
| FBXO25 | | + | | | | + | | | + |
| FBXO27 | | | | | | | | | |
| FBXO28 | | | + | | | | | | + |
| FBXO3 | | | | | | + | | | + |
| FBXO30 | | | | | | | | | |
| FBXO31 | | | | | | + | | | + |
| FBXO32 | | | | | | | | | |
| FBXO33 | + | | | | + | | | | + |
| FBXO34 | | | | | | | | | |
| FBXO36 | | | | | | | | | |
| FBXO38 | | | | | | | | | |
| FBXO39 | | | | | | | | | |
| FBXO4 | | | | | | | | | |
| FBXO40 | | | | | | | | | |
| FBXO41 | | | | | | + | | | + |
| FBXO42 | | | + | | | | | | + |
| FBXO43 | | | | | | | | | |
| FBXO44 | | | | | + | | | | + |
| FBXO45 | | | | | | | | | |
| FBXO46 | | | | | | | | | |
| FBXO47 | | | | | | | | | |
| FBXO48 | | | | | | | | | |
| FBXO5 | | | | | | | | | |
| FBXO6 | | | | | | | | | |
| FBXO7 | | | | | | | | | |
| FBXO8 | | + | | | | + | | | + |
| FBXO9 | | | | | | | | | |
| FBXW10 | | | | | | | | | |
| FBXW11 | | | | | | | | | |
| FBXW12 | | | | | | | | | |
| Fbxw14 | | | | | | | | | |
| Fbxw15 | | | | | | | | | |
| FBXW2 | | | + | | | + | | | + |
| FBXW4 | | | | | | | | | |
| FBXW4P1 | | | | | | | | | |
| FBXW5 | | | | | | | | | |
| FBXW7 | | | | | + | | | | + |
| FBXW8 | | | | | | | | | |
| FBXW9 | | | | | | | | | |
| FCAMR | | | | | | | | | |
| FCAR | | | | | | | | | |
| FCER1A | | | | | | | | | |
| FCER1G | | | | | | | | | |
| FCER2 | | | | | | | | | |
| FCF1 | | | | | | | | | |
| FCGBP | | | | + | | | | | + |
| Fcgr1 | | | | | | | | | |
| FCGR1A | | | | | | | | | |
| FCGR1B | | | | | | | | | |

| | | | | | | | | | |
|---|---|---|---|---|---|---|---|---|---|
| FCGR1C | | | | | | | | | |
| FCGR2A | | | | | | | | | |
| FCGR2B | | | | | | | | | |
| FCGR2C | | | | | | | | | |
| FCGR3A | | | | | | | | | |
| FCGR3B | | | | | | | | | |
| FCGRT | | | | | | | | | |
| FCHO1 | | | | | | | | | |
| FCHO2 | | | | | | | | | |
| FCHSD1 | | | | | | | | | |
| FCHSD2 | | | | | | | | | |
| FCN1 | | | | | | | | | |
| FCN2 | | | | | | | | | |
| FCN3 | | | | | | | | | |
| Fcna | | | | | | | | | |
| FCP1 | | | | | | | | | |
| FCRL1 | | | | | | | | | |
| FCRL2 | | | | | | | | | |
| FCRL3 | | | | | | | | | |
| FCRL4 | | | | | | | | | |
| FCRL5 | | + | + | | | + | | | + |
| FCRL6 | | + | + | | | + | | | + |
| FCRLA | | | | | | | | | |
| FCRLB | | | | | | | | | |
| FDCSP | | | | | | | | | |
| FDFT1 | | | | | | | | | |
| FDPS | | | | | | | | | |
| FDPSP2 | | | | | | | | | |
| FDPSP4 | | | | | | | | | |
| FDX1 | | | | | | | | | |
| FDX1L | | | | | | | | | |
| FDXACB1 | | | | | | | | | |
| FDXR | | | | | + | | | | + |
| FECD3 | | | | | | | | | |
| FECH | | | | | | | | | |
| fem-1 | | | | | | | | | |
| fem-2 | | | | | | | | | |
| fem-3 | | | | | | | | | |
| FEM1A | | | | | | + | | | + |
| FEM1B | | | + | | | | | | + |
| FEM1C | | | | | | | | | |
| FEN1 | | | | | | | | | |
| fen1-a | | | | | | | | | |
| FER | | | | | | + | | | + |
| FER1L4 | | | | | | | | | |
| FER1L5 | | | | | | | | | |
| FER1L6 | | | | | | | | | |
| FERD3L | | | | | | | | | |
| FERMT1 | | | | | | | | | |
| FERMT2 | | | | | | | | | |
| FERMT3 | | | | | | | | | |
| FES | | | | | + | | | | + |
| FES1 | | | | | | | | | |

| | | | | | | | | | |
|---|---|---|---|---|---|---|---|---|---|
| FETUB | | | | | | | | | |
| FEV | | | | | | | | | |
| FEZ1 | | + | | | | + | | | + |
| FEZ2 | | | | | | | | | |
| FEZF1 | | | | | | | | | |
| FEZF2 | | | | | | | | | |
| FFAR1 | | | | | | | | | |
| FFAR2 | | | | | | | | | |
| FFAR3 | | | | | | | | | |
| fft3 | | | | | | | | | |
| FGA | | | | | | | | | |
| FGB | | | | | | | | | |
| FGD1 | | | | | | | | | |
| FGD2 | | | | | | | | | |
| FGD3 | | | | | | | | | |
| FGD4 | | | | + | | | | | + |
| FGD5 | | | | | | | | | |
| FGD6 | | | | | | | | | |
| FGF1 | | | | | | | | | |
| FGF10 | + | | | + | + | | | | + |
| FGF11 | + | | | + | + | | | | + |
| FGF12 | | | | | | | | | |
| FGF13 | + | | | + | + | | | | + |
| FGF14 | | | | | | | | | |
| FGF16 | | | | | | | | | |
| FGF17 | | | | | | | | | |
| FGF18 | | | | | | | | | |
| FGF19 | | | | | | | | | |
| FGF2 | | | | | | | | | |
| FGF20 | | | | | | | | | |
| FGF21 | | | | | | | | | |
| FGF22 | | | | | | | | | |
| FGF23 | | | | | | | | | |
| FGF3 | | | | | | | | | |
| FGF4 | | | | | | | | | |
| FGF5 | | | | | | | | | |
| FGF6 | | | | | | | | | |
| FGF7 | | | | | | | | | |
| FGF8 | | | | | | | | | |
| FGF9 | + | | | + | + | | | | + |
| FGFBP1 | | | | | | | | | |
| FGFBP2 | | | | | | | | | |
| FGFBP3 | | | | | | | | | |
| FGFR1 | + | | + | | + | | | | + |
| FGFR1OP | | | | | | | | | |
| FGFR1OP2 | | | | | | | | | |
| FGFR2 | | | + | | + | | | | + |
| FGFR3 | | | | | | | | | |
| FGFR4 | | | | | | | | | |
| FGFRL1 | | | | | | | | | |
| FGG | | | | | | | | | |
| FGGY | | | | | | | | | |
| FGL1 | | | | | | | | | |

| | | | | | | | | | |
|---|---|---|---|---|---|---|---|---|---|
| FGL2 | | | | | | | | | |
| FGR | | | | | | | | | |
| FH | | | | | | | | | |
| Fh1 | | | | | | | | | |
| FHAD1 | + | | | | + | + | | | + |
| FHDC1 | | | | | | | | | |
| FHIT | | | | | | | | | |
| FHL1 | | | | | | | | | |
| FHL2 | | | | | | | | | |
| FHL3 | | | | | | | | | |
| Fhl4 | | | | | | | | | |
| FHL5 | | | | | | | | | |
| fhn1 | | | | | | | | | |
| FHOD1 | | | | | | | | | |
| FHOD3 | | | | | | | | | |
| fib1 | | | | | | | | | |
| FIBCD1 | | | | | | + | | | + |
| FIBIN | | | | | | | | | |
| FIBP | | | | | | | | | |
| FICD | | | | | | | | | |
| FIE | | | | | | | | | |
| FIG4 | | | | | | | | | |
| FIGF | | | | | | | | | |
| FIGLA | | | | | | | | | |
| Fign | + | | | + | + | | | | + |
| FIGNL1 | | | | | | | | | |
| FIGNL2 | | | | | | | | | |
| FILIP1 | | | | | | | | | |
| FILIP1L | | | | | | | | | |
| FIP1L1 | | | | | + | | | | + |
| FIRRE | | | | | | | | | |
| FIS1 | | | | | | | | | |
| FITM1 | | | | | | | | | |
| FITM2 | | | | | | | | | |
| FIZ1 | | | | | | | | | |
| FJX1 | + | | + | | | + | | | + |
| FKBP10 | | | | | | | | | |
| FKBP11 | | | | | | | | | |
| FKBP14 | | | | | | | | | |
| FKBP15 | | | | | | | | | |
| FKBP1A | | | | | | | | | |
| FKBP1B | | | | | + | + | | | + |
| FKBP2 | | | | | | | | | |
| FKBP3 | | | | | | | | | |
| fkbp39 | | | | | | | | | |
| FKBP4 | | | | | | | | | |
| FKBP5 | | | | | | | | | |
| FKBP6 | | | | | | | | | |
| FKBP7 | | | | | | | | | |
| FKBP8 | | | | | | | | | |
| FKBP9 | | | | | | | | | |
| FKBP9P1 | | | | | | | | | |
| FKBPL | | | | | | | | | |

| | | | | | | | | | |
|---|---|---|---|---|---|---|---|---|---|
| FKRP | | | | | | | | | |
| FKS1 | | | | | | | | | |
| FKSG29 | | | | | | | | | |
| FKTN | | | | | | | | | |
| FLAD1 | | | | | | | | | |
| FLC2 | | | | | | | | | |
| FLCN | | | + | | | | | | + |
| FLD1 | | | | | | | | | |
| FLG | | | | | | | | | |
| FLG2 | + | | | + | + | | | | + |
| FLI1 | + | | | + | + | | | | + |
| FLII | | | | | | | | | |
| FLJ20712 | | | | | | | | | |
| FLJ22447 | | | | | | | | | |
| FLJ25613 | | | | | | | | | |
| FLJ25758 | | | | | | | | | |
| FLJ38576 | | | | | | | | | |
| FLJ38668 | | | | | | | | | |
| FLJ42627 | | | | | | | | | |
| FLJ44635 | | | | | | | | | |
| FLJ45950 | | | | | | | | | |
| FLJ90680 | | | | | | | | | |
| FLNA | + | | | | + | | | | + |
| FLNB | | | + | | | | | | + |
| FLNB-AS1 | | | | | | | | | |
| FLNC | | | | | | | | | |
| FLO5 | | | | | | | | | |
| FLOT1 | | | | | | + | | | + |
| FLOT2 | | | | | | | | | |
| FLRT1 | | | | | | | | | |
| FLRT2 | | | | | | | | | |
| FLRT3 | | | + | | | | | | + |
| FLT1 | | | | | | | | + | + |
| FLT3 | | | | + | | | | | + |
| FLT3LG | | | | | | | | | |
| FLT4 | + | | | + | + | | | | + |
| FLVCR1 | | | | | | | | | |
| FLVCR2 | | | | | | | | | |
| FLYWCH1 | | | | | | | | | |
| FLYWCH2 | | | | | | | | | |
| fma2 | | | | | | | | | |
| FMC1 | | | | | | | | | |
| FMN1 | | | | | | | | | |
| FMN2 | | | | | | | | | |
| FMNL1 | + | | + | | + | | | | + |
| FMNL2 | | | | | | | | | |
| FMNL3 | | | | | | | | | |
| FMO1 | | | | | | | | | |
| FMO2 | | | | | | | | | |
| FMO3 | | | | | | | | | |
| FMO4 | | | | | | | | | |
| FMO5 | + | | | + | + | | + | | + |
| FMOD | | | | | | | | | |

| | | | | | | | | | |
|---|---|---|---|---|---|---|---|---|---|
| FMR1 | | | | | | | | | |
| FMR1-AS1 | | | | | | | | | |
| FMR1NB | | | | | | | | | |
| FN1 | | + | | + | | + | | | + |
| fn1a | | | | | | | | | |
| FN3K | | | | | | | | | |
| FN3KRP | | | | | | | | | |
| FNBP1 | | | | | + | | | | + |
| FNBP1L | | | | | | | | | |
| FNBP4 | | | | | | | | | |
| FNDC1 | | | | | | | | | |
| FNDC3A | | | | | | + | | | + |
| FNDC3B | | | + | | | | | | + |
| FNDC4 | | | | | | | | | |
| FNDC5 | | | | | | | | | |
| FNDC7 | | | | | | | | | |
| FNDC8 | | | | | | | | | |
| FNDC9 | | | | | | | | | |
| FNIP1 | | | + | | | | | | + |
| FNIP2 | | | | | | | | | |
| FNTA | | | | | | | | | |
| FNTB | | | | | | | | | |
| FOCAD | | | | | | | | | |
| FOLH1 | | | | | | | | | |
| FOLH1B | | | | | | | | | |
| FOLR1 | | | | | | | | | |
| FOLR2 | | | | | | | | | |
| FOLR3 | | | | | | | | | |
| FOPNL | | | | | | | | | |
| FOS | + | | | | + | | | | + |
| FOSB | | | | | | | | | |
| FOSL1 | | | | | | | | | |
| FOSL2 | + | | + | | + | | | | + |
| FOXA1 | | | | | | | | | |
| FOXA2 | | | | | | | | | |
| FOXA3 | + | | | + | + | | + | | + |
| FOXB1 | | | | | | | | | |
| FOXC1 | | | | | | | | | |
| FOXC2 | | | | | | | | | |
| FOXD1 | | | | | | | | | |
| FOXD2 | | | | | | | | | |
| FOXD3 | | | | | | | | | |
| FOXD4 | | | | | | | | | |
| FOXD4L1 | | | | | | | | | |
| FOXD4L3 | | | | | | | | | |
| FOXD4L4 | | | | | | | | | |
| FOXD4L6 | | | | | | | | | |
| FOXE1 | | | | | | | | | |
| FOXE3 | | | | | | | | | |
| FOXF1 | | | | | | | | | |
| FOXF2 | | | | | | | | | |
| FOXG1 | | | | | | | | | |
| FOXH1 | | | | | | | | | |

| | | | | | | | | | |
|---|---|---|---|---|---|---|---|---|---|
| FOXI1 | | | | | | | | | |
| FOXI2 | | | | | | | | | |
| FOXI3 | | | | | | | | | |
| FOXJ1 | | | | | | | | | |
| FOXJ2 | | | | | | | | | |
| FOXJ3 | | | | | | | | | |
| FOXK1 | | | | | | + | | | + |
| FOXK2 | | | | | | | | | |
| FOXL1 | | | | | | | | | |
| FOXL2 | | | | | | | | | |
| FOXL2NB | | | | | | | | | |
| FOXM1 | | | | | | | | | |
| FOXN1 | | | | | | | | | |
| FOXN2 | | | | | | | | | |
| FOXN3 | | | | | | | | | |
| FOXN4 | | | | | | | | | |
| FOXO1 | | | | | | | | | |
| FOXO3 | | | | | | | | | |
| FOXO3B | | | | | | | | | |
| FOXO4 | | | | | | | | | |
| FOXO6 | | | | | | | | | |
| FOXP1 | | | | | | | | | |
| FOXP2 | + | | | + | + | | | | + |
| FOXP3 | | | | | | | | | |
| FOXP4 | | | | | | | | | |
| FOXP4-AS1 | | | | | | | | | |
| FOXQ1 | | | | | | | | | |
| FOXR1 | | | | + | | | | | + |
| FOXR2 | | | | | | | | | |
| FOXRED1 | | | | | | | | | |
| FOXRED2 | | | | | | + | | | + |
| FOXS1 | | | | | | | | | |
| FPGS | | | | | | | | | |
| FPGT | | | | | | | | | |
| FPGT-TNNI3K | | | | | | | | | |
| FPR1 | | | | | | | | | |
| FPR2 | | | | | | | | | |
| FPR4 | | | | | | | | | |
| FRA10AC1 | | | | | | | | | |
| FRA10F | | | | | | | | | |
| FRAS1 | | | | | | | | | |
| FRAT1 | | | | | | | | | |
| FRAT2 | | | | | | | | | |
| FRD1 | | | | | | | | | |
| FRE1 | | | | | | | | | |
| FREM1 | | | | | | | | | |
| FREM2 | | | | | | | | | |
| FREM3 | | | | | | | | | |
| FRG1 | | | | | | | | | |
| FRG1B | | | | | | | | | |
| FRG2 | | | | | | | | | |
| FRK | | | | | | | + | | + |
| FRMD1 | | | | | | | | | |

| | | | | | | | | | |
|---|---|---|---|---|---|---|---|---|---|
| FRMD3 | | | | | | | | | |
| FRMD4A | | | | | | | | | |
| FRMD4B | | | | | | + | | | + |
| FRMD5 | | | | | | + | | | + |
| FRMD6 | + | | + | | + | | | | + |
| FRMD7 | | | | | | | | | |
| FRMD8 | | | | | | | | | |
| FRMD8P1 | | | | | | | | | |
| FRMPD1 | | | | | | | | | |
| FRMPD2 | | | | | | | | | |
| FRMPD3 | | | + | + | | | | | + |
| FRMPD4 | | | | | | | | | |
| FRQ1 | | | | | | | | | |
| FRRS1 | | | | | | | | | |
| FRRS1L | | | | | | | | + | + |
| FRS1 | | | | | | | | | |
| FRS2 | | | | | | | | | |
| FRS3 | | | | | | | | | |
| FRY | | | | | | | | | |
| FRYL | | | | | | | | | |
| FRZB | | | | | | + | | | + |
| FSBP | | | | | | | | | |
| FSCB | | | | | | | | | |
| FSCN1 | | | + | | | | | | + |
| FSCN2 | | | | | | | | | |
| FSCN3 | | | | | | | | | |
| FSD1 | | | | | | | | | |
| FSD1L | | | | | | | | | |
| FSD2 | | | | | | | | | |
| FSHB | | | | | | | | | |
| FSHR | | | | | | | | | |
| FSIP1 | | | | | | | | | |
| FSIP2 | | | | | | | | | |
| FST | | | | | | | | | |
| FSTL1 | | | + | | | | | | + |
| FSTL3 | | | | | | | | | |
| FSTL4 | | | | + | | | | | + |
| FSTL5 | | | | | | | | | |
| FTCD | | | | | | | | | |
| FTH1 | | | | | | | | | |
| FTH1P23 | | | | | | | | | |
| FTH1P3 | | | | | | | | | |
| FTHL17 | | | | | | | | | |
| FTL | | | | | | | | | |
| Ftl1 | | | | | | | | | |
| FTMT | | | | | | | | | |
| FTO | | | | | | | | | |
| FTSJ1 | | | | | | | | | |
| FTSJ2 | | | | | | | | | |
| FTSJ3 | | | | | | | | | |
| FTX | | | | | | | | | |
| FUBP1 | | | + | | | | | | + |
| FUBP3 | | | | | | | | | |

| | | | | | | | | | |
|---|---|---|---|---|---|---|---|---|---|
| FUCA1 | | | | | | | | | |
| FUCA2 | | | | | | | | | |
| FUK | | | | | | + | | | + |
| FUM1 | | | | | | | | | |
| FUN12 | | | | | | | | | |
| FUN26 | | | | | | | | | |
| FUNDC1 | | | | | | | | | |
| FUNDC2 | | | | | | + | | | + |
| FUNDC2P2 | | | | | | | | | |
| FUOM | | | | | | | | | |
| FUR4 | | | | | | | | | |
| FURIN | | | + | | | | | | + |
| FUS | | | | | | + | | | + |
| FUS3 | | | | | | | | | |
| FUT1 | | | | | | | | | |
| FUT10 | | | | | | | | | |
| FUT11 | | | | | | | | | |
| FUT2 | | | | | | | | | |
| FUT3 | | | | | | | | | |
| FUT4 | | | | | | | | | |
| FUT7 | | | | | | | | | |
| FUT8 | | + | + | | | + | | + | + |
| FUT9 | | | | | | | | | |
| futsch | | | | | | | | | |
| FUZ | | | | | | | | | |
| FXN | | | | + | | | | | + |
| FXR1 | | | | | | | | | |
| FXR2 | | | | | | | | | |
| FXYD1 | | | | | | | | | |
| FXYD2 | | | | | | | | | |
| FXYD3 | | | | | | | | | |
| FXYD5 | | | | | | | | | |
| FXYD6 | | | + | | | + | | | + |
| FXYD6-FXYD2 | | | | | | | | | |
| FXYD7 | | | | | | | | | |
| FYB | | | | | | | | | |
| FYCO1 | | | | | | | | | |
| FYN | | | | | | | | | |
| FYTTD1 | | | + | | + | | | | + |
| fyu1 | | | | | | | | | |
| FZD1 | | | | | | | | | |
| FZD10 | | | | | | | | | |
| FZD10-AS1 | | | | | | | | | |
| FZD2 | | | | | | | | | |
| FZD3 | | | + | | + | | | | + |
| FZD4 | | | | | | | | | |
| FZD5 | | | | | | | | | |
| FZD6 | | | | | | | | | |
| FZD7 | | | | | | | | | |
| FZD8 | | | | | | | | | |
| FZD9 | | | | | | | | | |
| FZO1 | | | | | | | | | |
| FZR1 | | | + | | | + | | | + |

| | | | | | | | | | |
|---|---|---|---|---|---|---|---|---|---|
| G0S2 | | | | | | | | | |
| G2E3 | | | | | | | | | |
| G3BP1 | | | | | | | | | |
| G3BP2 | | | | | | | | | |
| G6PC | | | | | | | | | |
| G6pc2 | | | | | | | | | |
| G6PC3 | | | | | | | | | |
| G6PD | | | | | | | | | |
| GAA | | | | | | | | | |
| GAB1 | | | | | | | | | |
| GAB2 | | | | | | | | | |
| GAB3 | + | | | + | + | | | | + |
| GAB4 | | | | | | | | | |
| GABARAP | | | | | | | | | |
| GABARAPL1 | | | + | | | | | | + |
| GABARAPL2 | | | | | | | | | |
| GABARAPL3 | | | | | | | | | |
| GABBR1 | | | | | | | | | |
| GABBR2 | | | | | | | | | |
| GABPA | | | | | | | | | |
| GABPB1 | | | + | | | | | | + |
| GABPB1-AS1 | | | | | | | | | |
| GABPB2 | | | | | | + | | | + |
| GABRA1 | | + | | | | + | | | + |
| GABRA2 | | | + | | | | | | + |
| GABRA3 | | | | | | | | | |
| GABRA4 | | | + | | | | | | + |
| GABRA5 | | | | | | | | | |
| GABRA6 | | | | | | | | | |
| GABRB1 | | | + | | | | | | + |
| GABRB2 | | | | | | | | | |
| GABRB3 | | | | | | + | | | + |
| GABRD | | | | | | | | | |
| GABRE | | | | | | | | | |
| GABRG1 | | | | | | | | | |
| GABRG2 | | | | | | | | | |
| GABRG3 | | | | | | | | | |
| GABRP | | | | | | | | | |
| GABRQ | | | | | | | | | |
| GABRR1 | | | | | | | | | |
| GACAT2 | | | | | | | | | |
| GAD1 | + | | + | | + | + | | | + |
| GAD2 | | | | | + | | | | + |
| GADD45A | | | | | | | | | |
| Gadd45b | + | | | | + | | | | + |
| GADD45G | + | | | | + | | | | + |
| GADD45GIP1 | | | | | | | | | |
| GADL1 | + | | | + | + | | | | + |
| GAFA3 | | | | | | | | | |
| gag | | | | | | | | | |
| gag-pol | | | | | | | | | |
| GAGE1 | | | | | | | | | |
| GAGE10 | | | | | | | | | |

| | | | | | | | | | |
|---|---|---|---|---|---|---|---|---|---|
| GAGE12B | | | | | | | | | |
| GAGE12C | | | | | | | | | |
| GAGE12D | | | | | | | | | |
| GAGE12E | | | | | | | | | |
| GAGE12F | | | | | | | | | |
| GAGE12G | | | | | | | | | |
| GAGE12I | | | | | | | | | |
| GAGE12J | | | | | | | | | |
| GAGE13 | | | | | | | | | |
| GAGE2A | | | | | | | | | |
| GAGE2B | | | | | | | | | |
| GAGE2C | | | | | | | | | |
| GAGE2D | | | | | | | | | |
| GAGE2E | | | | | | | | | |
| GAGE5 | | | | | | | | | |
| GAGE6 | | | | | | | | | |
| GAGE7 | | | | | | | | | |
| GAGE8 | | | | | | | | | |
| GAK | | | | | | | | | |
| GAL | | | | | | | | | |
| GAL11 | | | | | | | | | |
| GAL3 | | | | | | | | | |
| GAL3ST1 | | | | | | | | | |
| GAL3ST2 | | | | | | | | | |
| GAL3ST3 | | | | | | + | | | + |
| GAL3ST4 | | + | + | | | + | | | + |
| GAL4 | | | | | | | | | |
| GALC | | | | | | | | | |
| GALE | | | | | | | | | |
| GALK1 | | | | | | | | | |
| GALK2 | | | | | | | | | |
| GALM | | | | | | | | | |
| GALNS | | | | | | | | | |
| GALNT1 | | | | | | | | | |
| GALNT10 | | | | | | | | | |
| GALNT11 | | | | | | | | | |
| GALNT12 | | | | | | | | | |
| GALNT13 | | | | | | | | | |
| GALNT14 | | | + | | | | | | + |
| GALNT15 | | | | | | | | | |
| GALNT16 | | | | | | + | | | + |
| GALNT18 | | | | | | | | | |
| GALNT2 | | | | | + | | | | + |
| GALNT3 | | | | | | | | | |
| GALNT4 | | | | | | | | | |
| GALNT5 | | | | | | | | | |
| GALNT6 | | | | | | | | | |
| GALNT7 | | | | | | | | | |
| GALNT8 | | | | | | | | | |
| GALNT9 | | | | | + | | | | + |
| GALNTL5 | | | | | | | | | |
| GALNTL6 | | | | | | | | | |
| GALP | | | | | | | | | |

| | | | | | | | | | |
|---|---|---|---|---|---|---|---|---|---|
| GALR1 | | | | | | | | | |
| GALR2 | | | | | | | | | |
| GALR3 | | | | | | | | | |
| GALT | | | | | | | | | |
| GAMT | | | | | | | | | |
| GAMTP1 | | | | | | | | | |
| GAN | | | | | | | | | |
| GANAB | | | | | | | | | |
| GANC | | | | | | | | | |
| GAP43 | | | | | | | | | |
| GAPC1 | | | | | | | | | |
| GAPC2 | | | | | | | | | |
| GAPDH | | | | | | | | | |
| GAPDHP1 | | | | | | | | | |
| GAPDHP65 | | | | | | | | | |
| GAPDHS | | | | | | | | | |
| GAPVD1 | | | | | | | | | |
| GAR1 | | | | | | | | | |
| gar2 | | | | | | | | | |
| GAREM | | | | | | | | | |
| GAREML | | | | | | | | | |
| GARNL3 | | | | + | | | + | | + |
| GARS | | | + | | | | | | + |
| GART | | | | | | | | | |
| GAS1 | | | | | | | | | |
| GAS2 | | | | | | | | | |
| GAS2L1 | | | | | | | | | |
| GAS2L2 | | | | | | | | | |
| GAS2L3 | | | | | | | | | |
| GAS5 | | | | | | | | | |
| GAS6 | | | | | | | | | |
| GAS7 | | | | | | | | | |
| GAS8 | | | + | | | | | | + |
| GAS8-AS1 | | | | | | | | | |
| GAST | | | | | | | | | |
| GATA1 | | | | | | | | | |
| GATA2 | | | | | | | | | |
| GATA3 | + | | | + | + | | + | | + |
| GATA4 | | | | | | | + | | + |
| GATA5 | | | | | | | | | |
| GATA6 | | | | | | | | | |
| GATAD1 | + | | | + | + | | + | | + |
| GATAD2A | | | | | + | | | | + |
| GATAD2B | | | | | | | | | |
| GATB | | | | | + | | | | + |
| GATC | | | | | | | | | |
| Gatm | | | | | | | | | |
| GATS | | | | | | | | | |
| GATSL2 | | | | | | | | | |
| GATSL3 | | | | | | | | | |
| GBA | | | | | | | | | |
| GBA2 | | | | | | | | | |
| GBA3 | | | | | | | | | |

| | | | | | | | | | |
|---|---|---|---|---|---|---|---|---|---|
| GBAP1 | | | | | | | | | |
| GBAS | | | | | | | | | |
| GBE1 | | | | + | | | | | + |
| GBF1 | | | | | | | | | |
| GBGT1 | | | | | | | | | |
| GBP1 | | | | | | | | | |
| GBP2 | | | | | | | | | |
| GBP3 | | | | | | | | | |
| Gbp4 | | | | | | | | | |
| GBP5 | + | | | + | + | | + | | + |
| GBP6 | | | | | | | | | |
| GBP7 | | | | | | | | | |
| GBX1 | | | | | | | | | |
| GBX2 | | | | | | | | | |
| GC | | | | | | | | | |
| GCA | | | | | | | | | |
| GCAT | | | | | | | | | |
| GCC1 | | | | | | | | | |
| GCC2 | | + | | + | | + | | | + |
| GCD11 | | | | | | | | | |
| GCD2 | | | | | | | | | |
| GCD6 | | | | | | | | | |
| GCD7 | | | | | | | | | |
| GCDH | | | | | | | | | |
| GCFC2 | | | | | | + | | | + |
| GCG | | | | | | | | | |
| gcg1 | | | | | | | | | |
| GCGR | | | | | | | | | |
| GCH1 | | | | | | | | | |
| GCHFR | | | | | | | | | |
| GCK | | | | | | | | | |
| GCKR | | | | | | | | | |
| GCLC | | | | | | | | | |
| GCLM | | | | | | | | | |
| GCM1 | + | | | + | + | | | | + |
| GCM2 | | | | | | | | | |
| GCN1 | | | | | | | | | |
| GCN1L1 | | | | + | + | | | | + |
| GCN20 | | | | | | | | | |
| GCN4 | | | | | | | | | |
| GCN5 | | | | | | | | | |
| GCNT1 | | | | | | | | | |
| GCNT2 | | | | | + | | | | + |
| GCNT3 | | | | | | | | | |
| Gcnt4 | | | | | | | + | | + |
| GCNT6 | | | | | | | | | |
| GCNT7 | | | | | | | | | |
| GCOM1 | | | | | | | | | |
| GCOM2 | | | | | | | | | |
| GCS1 | | | | | | | | | |
| GCSAM | + | | | + | + | | + | | + |
| GCSAML | | | | | | | | | |
| GCSH | | | | | | | | | |

| | | | | | | | | | |
|---|---|---|---|---|---|---|---|---|---|
| GDA | | | | | | | | | |
| GDA1 | | | | | | | | | |
| GDAP1 | | | | | | | | | |
| GDAP1L1 | | | | | | | | | |
| GDAP2 | | | | | | | | | |
| GDE1 | | | | | + | | | | + |
| GDF1 | | | | | | | | | |
| GDF10 | | | + | | | | | | + |
| GDF11 | | | | | | | | | |
| GDF15 | | | | | | | | | |
| GDF2 | | | | | | | | | |
| GDF3 | | | | | | | | | |
| GDF5 | | | | | | | | | |
| GDF5OS | | | | | | | | | |
| GDF6 | | | | | | | | | |
| GDF9 | | | | | | | | | |
| GDH1 | | | | | | | | | |
| GDH2 | | | | | | | | | |
| GDI1 | | | | | | | | | |
| GDI2 | | | | | | | + | | + |
| GDI2P1 | | | | | | | | | |
| GDNF | | | | | | | | | |
| GDPD1 | | | | | | | | | |
| GDPD2 | | | | | | | | | |
| GDPD3 | | | | | | | | | |
| GDPD4 | | | | | | | | | |
| GDPD5 | | | | | | | | | |
| GDPGP1 | | | | | | | | | |
| GDU1 | | | | | | | | | |
| GDU2 | | | | | | | | | |
| GDU3 | | | | | | | | | |
| GDU5 | | | | | | | | | |
| GDU7 | | | | | | | | | |
| GEA1 | | | | | | | | | |
| GEM | | | | | + | | | | + |
| GEMIN2 | | | | | | | | | |
| GEMIN4 | | | | | | + | | | + |
| GEMIN5 | | | | | + | | | | + |
| GEMIN6 | | | | | | | | | |
| GEMIN7 | | | | | | | | | |
| GEMIN8 | | | | | + | | | | + |
| GEMIN8P4 | | | | | | | | | |
| GEN1 | | | | | | | | | |
| GET3 | | | | | | | | | |
| GET4 | | | | | | | | | |
| GFA1 | | | | | | | | | |
| GFAP | | | | | + | | | | + |
| GFER | | | | | | | | | |
| GFI1 | | | | | | | | | |
| GFI1B | | | | | | | | | |
| GFM1 | | | | | | | | | |
| GFM2 | | + | | | | + | | | + |
| GFOD1 | + | | + | | | | | | + |

| | | | | | | | | | |
|---|---|---|---|---|---|---|---|---|---|
| GFOD2 | | | | | + | | | | + |
| GFPT1 | | | | | | | | | |
| GFPT2 | | | | | | | | | |
| GFRA1 | | | | | | | | | |
| GFRA2 | | | | | | + | + | | + |
| GFRA3 | | | | | | | | | |
| GFRA4 | | | | | | | | | |
| GFRAL | | | | | | | | | |
| GGA1 | | | | | | | | | |
| GGA2 | | | | | | | | | |
| GGA3 | | | + | | | | | | + |
| GGACT | | | | | | + | | | + |
| GGCT | | | | | | | | | |
| GGCX | | | | | | | | | |
| GGH | | | | | | + | | | + |
| GGN | | | | | | | | | |
| GGNBP1 | | | | | | | | | |
| GGNBP2 | | + | | | | + | | | + |
| GGPS1 | | | | | | | | | |
| GGT1 | | | | | | | | | |
| GGT2 | | | | | | | | | |
| GGT5 | + | | | + | + | | + | | + |
| GGT6 | | | | | | | | | |
| GGT7 | | | | | | | | | |
| GGTA1P | | | | | | | | | |
| GGTLC1 | | | | | | | | | |
| GGTLC2 | | | | | | | | | |
| GGTLC3 | | | | | | | | | |
| GH1 | | | | | | | | | |
| GH2 | | | | | | | | | |
| GHDC | | | | | | | | | |
| GHET1 | | | | | | | | | |
| GHITM | | | | | | | + | | + |
| GHR | | | + | | | | | | + |
| GHRH | | | | | | | | | |
| GHRHR | | | | | | | | | |
| GHRL | | | | | | | | | |
| GHSR | | | | | | | | | |
| ght5 | | | | | | | | | |
| GID4 | + | | | + | + | | | | + |
| GID8 | | | | | | | | | |
| GIF | | | | | | | | | |
| GIGYF1 | | | | | | | | | |
| GIGYF2 | | | | | | | | | |
| GIM3 | | | | | | | | | |
| GIMAP1 | | | | | | | | | |
| GIMAP2 | | | | | | | | | |
| GIMAP4 | | | | | | | | | |
| GIMAP5 | | | | | | | | | |
| GIMAP6 | | + | + | | | + | | | + |
| GIMAP7 | | | | | | | | | |
| GIMAP8 | | + | + | | | + | | | + |
| GIN1 | | | | | | | | | |

| | | | | | | | | | |
|---|---|---|---|---|---|---|---|---|---|
| GIN4 | | | | | | | | | |
| GINM1 | | | | | | | | | |
| GINS1 | | | | | | | | | |
| GINS2 | | | | | | | | | |
| GINS3 | | | | | | | | | |
| GINS4 | | | | | | | | | |
| GIP | | | | | | | | | |
| GIP2 | | | | | | | | | |
| GIPC1 | | | | | | | | | |
| GIPC2 | | | | | | | | | |
| GIPC3 | | | | | | | | | |
| GIPR | | | | | | | | | |
| GIR2 | | | | | | | | | |
| GIS1 | | | | | | | | | |
| gish | | | | | | | | | |
| GIT1 | | | | | | | | | |
| git11 | | | | | | | | | |
| GIT2 | | | | | | | | | |
| git5 | | | | | | | | | |
| GJA1 | | | | | + | | | | + |
| GJA10 | | | | | | | | | |
| GJA3 | | | | | | | | | |
| GJA4 | | | | | | | | | |
| GJA5 | | | | | | | | | |
| GJA8 | | | | | | | + | | + |
| GJA9 | | | | | | | | | |
| GJB1 | | | | | | | | | |
| GJB2 | | | | | | | | | |
| GJB3 | | | | | | | | | |
| GJB4 | | | | | | | | | |
| GJB5 | | | | | | | | | |
| GJB6 | | | | | | | | | |
| GJB7 | | | | | | | | | |
| GJC1 | | | | | | | | | |
| GJC2 | | | | | | | | | |
| GJC3 | | | | | | | | | |
| GJD2 | | | | | | | | | |
| GJD3 | | | | | | | | | |
| GJD4 | | + | + | | | + | | | + |
| GK | | | | | | | | | |
| GK2 | | | | | | | | | |
| GK3P | | | | | | | | | |
| GK5 | | | | | | | | | |
| GKAP1 | | | | | | | | | |
| GKN1 | | | | | | | | | |
| GKN2 | | | | | | | | | |
| GLA | | | | | | | | | |
| GLB1 | | | | | | | | | |
| GLB1L | | | | | | | | | |
| GLB1L2 | | | | | | | | | |
| GLB1L3 | | | | | | | | | |
| GLC3 | | | | | | | | | |
| GLC7 | | | | | | | | | |

| | | | | | | | | | |
|---|---|---|---|---|---|---|---|---|---|
| GLCCI1 | | | + | | + | | | | + |
| GLCE | + | | | | + | | | | + |
| gld1 | | | | | | | | | |
| GLDC | | | | | + | | | | + |
| GLE1 | | | | | | | | | |
| GLG1 | | | | | | | | | |
| GLI1 | | | | | | | | | |
| GLI2 | | | | | | | | | |
| GLI3 | | | | | | | | | |
| GLI4 | | | | | | | | | |
| GLIDR | | | | | | | | | |
| GLIPR1 | | | | | | | | | |
| GLIPR1L2 | + | | | + | + | | | | + |
| GLIPR2 | | | | | | | | | |
| GLIS1 | | | | | | | | | |
| GLIS2 | | | | | | | | | |
| Glis3 | + | | | | + | | | | + |
| GLK1 | | | | | | | | | |
| GLMN | | | | | | | | | |
| GLN1 | | | | | | | | | |
| GLN3 | | | | | | | | | |
| GLO1 | | + | | | | + | | | + |
| GLO2 | | | | | | | | | |
| GLO3 | | | | | | | | | |
| GLO4 | | | | | | | | | |
| GLOD4 | | | | | | + | | | + |
| GLOD5 | | | | | | | | | |
| GLP1R | | | | | | | | | |
| GLP2R | | | | | | + | | | + |
| GLRA1 | | | | | | | | | |
| GLRA2 | | | | | | | | | |
| GLRA3 | | | | | | | | | |
| GLRA4 | | | | | | | | | |
| GLRB | | | | | | + | | | + |
| GLRX | | | | | | | | | |
| GLRX2 | | | | | | | | | |
| GLRX3 | | + | + | | | + | | | + |
| GLRX5 | | | + | | | | | | + |
| GLS | | | | | | | | | |
| GLS2 | | | | | + | | | | + |
| GLT1 | | | | | | | | | |
| GLT1D1 | | | | | | | | | |
| GLT6D1 | | | | | | | | | |
| GLT8D1 | | | | | | | | | |
| GLT8D2 | | | | | | + | | | + |
| GLTP | + | | | + | + | | | | + |
| GLTPD2 | | | | | | | | | |
| GLTSCR1 | | | | | | | | | |
| GLTSCR1L | | | | | | | | | |
| GLTSCR2 | + | | + | | + | | | | + |
| GLUD1 | | | | | | | | | |
| GLUD1P3 | | | | | | | | | |
| GLUD2 | | | | | | | | | |

| | | | | | | | | | |
|---|---|---|---|---|---|---|---|---|---|
| GLUL | + | | | | | | | | + |
| GLY1 | | | | | | | | | |
| GLYAT | | | | | | | | | |
| GLYATL1 | | | | | | | | | |
| GLYATL3 | | | | | | | | | |
| GLYCTK | | | | | | | | | |
| GLYR1 | | | | | | | | | |
| Gm10068 | | | | | | | | | |
| Gm16470 | | | | | | | | | |
| Gm2890 | | | | | | | | | |
| GM2A | | | | | | | | | |
| Gm4779 | | | | | | | | | |
| Gm5745 | | | | | | | | | |
| Gm8129 | | | | | | | | | |
| Gm8991 | | | | | | | | | |
| Gm9174 | | | | | | | | | |
| GMC2 | | | | | | | | | |
| Gmcl1 | | | | | | | | | |
| GMCL1P1 | | | | | | | | | |
| GMDS | | | | | | | | | |
| GMDS-AS1 | | | | | | | | | |
| GMEB1 | | | | | | | | | |
| GMEB2 | | | | | | | | | |
| GMFB | | | | | | | | | |
| GMFG | | | | | | | | | |
| GMH1 | | | | | | | | | |
| gmh4 | | | | | | | | | |
| GMIP | | | | | | | | | |
| GML | | | | | | | | | |
| GMNC | | | | | | | | | |
| GMNN | | | | | | | | | |
| GMPPA | | | | | | | | | |
| GMPPB | | | | | | | | | |
| GMPR | | | | | | + | | | + |
| GMPR2 | | | | | | | | | |
| GMPS | | | | | + | | | | + |
| GNA11 | | | | | | | | | |
| GNA12 | | | | | | | | | |
| GNA13 | | | | | | + | | | + |
| GNA14 | | | | | | | | | |
| GNA15 | | | | | | | | | |
| GNAI1 | | | | | | | | | |
| GNAI2 | | | | | | | | | |
| GNAI3 | | | | | | | | | |
| GNAL | | + | | | | + | | | + |
| GNAO1 | | | | | + | | | | + |
| GNAQ | | | | | | | | | |
| GNAS | | | | | | | | | |
| GNAS-AS1 | | | | | | | | | |
| GNAT1 | | | | | | | | | |
| GNAT2 | | | | | | | | | |
| GNAT3 | | | | | | | | | |
| GNAZ | | | | | | | | | |

| | | | | | | | | | |
|---|---|---|---|---|---|---|---|---|---|
| GNB1 | | | + | | | | | | + |
| GNB1L | | | | | | | | | |
| GNB2 | | | | | | + | | | + |
| GNB2L1 | | | | | | | | | |
| GNB3 | | | | | | | | | |
| GNB4 | | | | | | | | | |
| GNB5 | | | | | | | | | |
| GND1 | | | | | | | | | |
| Gne | | | | | | | | | |
| GNG10 | | | | | | | | | |
| GNG11 | | | | | | | | | |
| GNG12 | | | | | | | | | |
| GNG12-AS1 | | | | | | | | | |
| GNG13 | | | | | | | | | |
| GNG2 | | + | | | | + | | | + |
| GNG3 | | | | | + | | | | + |
| GNG4 | | | | | | | | | |
| GNG5 | | | | | | | | | |
| GNG7 | | | | | | | | | |
| GNG8 | | | | | | | | | |
| GNGT1 | | | | | | | | | |
| GNGT2 | | | | | | | | | |
| GNL1 | | | | | | | | | |
| GNL2 | | | | | | | | | |
| GNL3 | | | | | | | | | |
| GNL3L | | | | | | | | | |
| GNL3LP1 | | | | | | | | | |
| GNLY | | | | | | | | | |
| GNMT | | | | | | | | | |
| GNN | | | | | | | | | |
| GNPAT | | | | | | | | | |
| GNPDA1 | | | | | | | | | |
| GNPDA2 | | | | | | + | | | + |
| GNPNAT1 | | | | | | | | | |
| GNPTAB | | | | | | | | | |
| GNPTG | | | + | | | | | | + |
| GNRH1 | | | | | | | | | |
| GNRH2 | | | | | | | | | |
| GNRHR | | | | | | | | | |
| GNS | | | + | | | | | | + |
| GOLGA1 | | | | | | | | | |
| GOLGA2 | | | | | | | | | |
| GOLGA2P10 | | | | | | | | | |
| GOLGA2P11 | | | | | | | | | |
| GOLGA2P5 | | | | | | | | | |
| GOLGA2P7 | | | | | | | | | |
| GOLGA3 | | | | + | | | | | + |
| GOLGA4 | | | | | | | | | |
| GOLGA5 | | | | | | + | | | + |
| GOLGA6A | | | | | | | | | |
| GOLGA6B | | | | | | | | | |
| GOLGA6C | | | | | | | | | |
| GOLGA6D | | | | | | | | | |

| | | | | | | | | | |
|---|---|---|---|---|---|---|---|---|---|
| GOLGA6L1 | | | | | | | | | |
| GOLGA6L10 | | | | | | | | | |
| GOLGA6L2 | | | | | | | | | |
| GOLGA6L5P | | | | | | | | | |
| GOLGA6L6 | | | | | | | | | |
| GOLGA6L7P | | | | | | | | | |
| GOLGA6L9 | | | | | | | | | |
| GOLGA7 | | | | | | | | | |
| GOLGA7B | | | | | | + | | | + |
| GOLGA8A | | | | | | | | | |
| GOLGA8B | | | | | | | | | |
| GOLGA8CP | | | | | | | | | |
| GOLGA8DP | | | | | | | | | |
| GOLGA8EP | | | | | | | | | |
| GOLGA8F | | | | | | | | | |
| GOLGA8G | | | | | | | | | |
| GOLGA8IP | | | | | | | | | |
| GOLGA8J | | | | | | | | | |
| GOLGA8K | | | | | | | | | |
| GOLGA8M | | | | | | | | | |
| GOLGA8N | | | | | | | | | |
| GOLGA8O | | | | | | | | | |
| GOLGA8Q | | | | | | | | | |
| GOLGA8R | | | | | | | | | |
| GOLGB1 | | + | | | | + | | | + |
| GOLIM4 | | | | | | | | | |
| GOLM1 | | | | | | + | | | + |
| GOLPH3 | | | | | | | | | |
| GOLPH3L | | | | | | | | | |
| GOLT1A | | | | | | | | | |
| GOLT1B | | | | | | | | | |
| GON4L | | | | | | | | | |
| GOPC | | | | | + | | | | + |
| GORAB | | | | | | | | | |
| GORASP1 | | | | | | + | | | + |
| GORASP2 | | | | | | | | | |
| GOS1 | | | | | | | | | |
| GOSR1 | | | | | | | | | |
| GOSR2 | | | + | | | | | | + |
| GOT1 | | | | | | | | | |
| GOT1L1 | | | | | | | | | |
| GOT2 | | | | | | | | | |
| GP1BA | | | | | | | | | |
| GP1BB | | | | | | | | | |
| GP2 | | | | | | | | | |
| GP5 | | | | | | | | | |
| GP6 | | | | | | | | | |
| GP9 | | | | | | | | | |
| GPA1 | | | | | | | | | |
| GPA2 | | | | | | | | | |
| GPA33 | | | | | | | | | |
| GPAA1 | | | | | | | | | |
| GPALPP1 | | | | | | | | | |

| | | | | | | | | | |
|---|---|---|---|---|---|---|---|---|---|
| GPAM | | | | | | | | | |
| GPANK1 | | | | | | | | | |
| GPAT2 | | | | | | | | | |
| GPATCH1 | | | | | | | | | |
| GPATCH11 | | | | | | | | | |
| GPATCH2 | | | | | | + | | | + |
| GPATCH2L | | | | | | | | | |
| GPATCH3 | | | | | | | | | |
| GPATCH4 | | | | | | | | | |
| GPATCH8 | | + | | | | + | | | + |
| GPB1 | | | | | | | | | |
| GPBAR1 | | | | | | | | | |
| GPBP1 | | | | | | | | | |
| GPBP1L1 | | | | | | | | | |
| GPC1 | | | | | + | | | | + |
| GPC2 | | | | | | | | | |
| GPC3 | + | | | + | + | | + | | + |
| GPC4 | | | | | | | | | |
| GPC5 | | | | | | | | | |
| GPC6 | + | | | + | + | | | | + |
| GPCPD1 | | | | | | | | | |
| GPD1 | + | | | | | | | | + |
| GPD1L | | | | | | + | | | + |
| GPD2 | | | | | | | | | |
| gpd3 | | | | | | | | | |
| GPER1 | | | | | | | | | |
| GPH1 | | | | | | | | | |
| GPHA2 | | | | | | | | | |
| GPHB5 | | | | | | | | | |
| GPHN | | | + | | | | | | + |
| GPI | | | | | | | | | |
| Gpi1 | | | | | | | | | |
| GPI14 | | | | | | | | | |
| GPIHBP1 | | | | | | | | | |
| GPKOW | | | | | | | | | |
| GPLD1 | | | | | | | | | |
| GPM1 | | | | | | | | | |
| GPM6A | | | | | | | + | | + |
| GPM6B | | + | + | | | + | | | + |
| GPN1 | | | | | | | | | |
| GPN2 | | | | | | | | | |
| GPN3 | | | | | | | | | |
| GPNMB | | | | | + | | | | + |
| GPP1 | | | | | | | | | |
| GPR101 | | | | | | | | | |
| GPR107 | | | | | | | | | |
| GPR108 | | | | | | | | | |
| GPR110 | | | | | | | | | |
| GPR111 | | | | | | | | | |
| GPR112 | | | | | | | | | |
| GPR113 | | | | | | | | | |
| GPR114 | | | | | | | | | |
| GPR115 | | | | | | | | | |

| | | | | | | | | | |
|---|---|---|---|---|---|---|---|---|---|
| GPR116 | | | | | | | | | |
| GPR119 | | | | | | | | | |
| GPR12 | | | | | | | | | |
| GPR123 | | | | | | | | | |
| GPR124 | | | | | | | | | |
| GPR125 | | | | | | | | | |
| GPR126 | | | | | | | | | |
| GPR128 | | | | | | | | | |
| GPR133 | | | | | | | | | |
| GPR135 | | | | | | | | | |
| GPR137 | | | | | | | | | |
| GPR137B | | | + | | | | | | + |
| GPR137C | | | | | | | | | |
| GPR141 | | | | | | | | | |
| GPR142 | | | | | | | | | |
| GPR143 | | | | | | | | | |
| GPR146 | | | | | | | | | |
| GPR148 | | | | | | | | | |
| GPR149 | + | | | + | + | | + | | + |
| GPR15 | | | | | | | | | |
| GPR150 | | | | | | | | | |
| GPR151 | | | | | | | | | |
| GPR152 | | | | | | | | | |
| GPR153 | + | | + | | | | | | + |
| GPR155 | | + | | | | + | | | + |
| GPR156 | | | | | | | | | |
| GPR157 | | | | | | | | | |
| GPR158 | | | | | | | | | |
| GPR160 | | | | | | | | | |
| GPR161 | | + | | | | + | | | + |
| GPR162 | | | | | | | | | |
| GPR17 | | | | | | | | + | + |
| GPR171 | | | | | | | | | |
| GPR173 | | | | | | | | | |
| GPR176 | | | | | + | | | | + |
| GPR179 | | | | | | | | | |
| GPR18 | | | | | | | | | |
| GPR180 | | | | | | | | | |
| GPR182 | | | | | | | | | |
| GPR183 | | | | | | | | | |
| GPR19 | | | | | | | | | |
| GPR20 | | | | | | | | | |
| GPR21 | | | | | | | | | |
| GPR22 | + | | | | + | | | | + |
| GPR25 | | | | | | | | | |
| GPR26 | | | | | | | | | |
| GPR27 | | | | | | | | | |
| GPR3 | | | | | | | | | |
| GPR34 | | + | + | | | + | | | + |
| GPR35 | | + | + | | | + | | | + |
| GPR37 | | | | | | + | | | + |
| GPR37L1 | | | | | | | | | |
| GPR39 | | | | | | | | | |

| | | | | | | | | | |
|---|---|---|---|---|---|---|---|---|---|
| GPR42 | | | | | | | | | |
| GPR45 | | | | | | | | | |
| GPR50 | | | | | | | | | |
| GPR50-AS1 | | | | | | | | | |
| GPR52 | | | | | | | | | |
| GPR55 | | | | | | | | | |
| GPR56 | | | | | | | | | |
| GPR6 | | | | | | | | | |
| GPR61 | | | | | | + | | | + |
| GPR63 | | | | | | | | | |
| GPR64 | + | | | + | + | | + | | + |
| GPR65 | | | | | | | | | |
| GPR75-ASB3 | | | | | | | | | |
| GPR78 | | | | | | | | | |
| GPR83 | + | | | + | + | | + | | + |
| GPR84 | | | | | | | | | |
| GPR85 | | + | + | | | + | | | + |
| GPR87 | | | | | | | | | |
| GPR88 | + | | + | | | | | | + |
| GPR89A | | | | | | | | | |
| GPR89B | | | | | | | | | |
| GPR97 | | | | | | | | | |
| GPR98 | | | | | | | | + | + |
| GPRASP1 | | | | | | | | | |
| GPRASP2 | | + | | | | + | | | + |
| GPRC5A | | | | | | | | | |
| GPRC5B | | | | | | | | | |
| GPRC5C | | | | | | + | | | + |
| GPRC5D | | | | | | | | | |
| GPRIN1 | | | | | | + | | | + |
| GPRIN2 | | | | | | | | | |
| GPRIN3 | | | | | | | | | |
| GPS1 | | | | | | | | | |
| GPS2 | | | | | | | | | |
| GPSM1 | | | | | | | | | |
| GPSM2 | | | | | | | | | |
| GPSM3 | | | | | | | | | |
| GPT | | | | | | | | | |
| GPT2 | + | | + | | | | | | + |
| GPX1 | | | | | | | | | |
| GPX2 | | | | | | | | | |
| GPX3 | | | | | | | | | |
| GPX4 | | | | | | | | | |
| GPX7 | | | | | | | | | |
| GPX8 | | | | | | | | | |
| GRAMD1A | | | | | | | | | |
| GRAMD1B | + | | + | | + | | | | + |
| GRAMD1C | | | | | | | | | |
| GRAMD2 | | | | | | | | | |
| GRAMD3 | | | | | | | | | |
| GRAMD4 | | | | | | | | | |
| GRAP | + | | | + | + | | + | | + |
| GRAP2 | | | | | | | | | |

| | | | | | | | | | |
|---|---|---|---|---|---|---|---|---|---|
| GRAPL | | | | | | | | | |
| Grasp | + | | + | | + | | | | + |
| GRB10 | | | | | | | | | |
| GRB14 | | + | | | | + | | | + |
| GRB2 | | | | | | | | | |
| GRB7 | | | | | | | | | |
| GRE2 | | | | | | | | | |
| GRE3 | | | | | | | | | |
| GREB1 | | | | | | | | | |
| GREB1L | | | | | | | | | |
| GREM1 | | | | | | | | | |
| GREM2 | | | | | + | | | | + |
| grf1 | | | | | | | | | |
| GRHL1 | | | | | | | | | |
| GRHL2 | | | | | | | | | |
| GRHL3 | | | | | | | | | |
| GRHPR | | | | | | + | | | + |
| GRIA1 | | | | | | | | | |
| GRIA2 | | | | + | | | | | + |
| GRIA3 | | | | | | | | | |
| GRIA4 | | | | | + | | | | + |
| GRID1 | | | | | | | | | |
| GRID2 | | | | | | | | | |
| GRID2IP | | | | | | | | | |
| GRIK1 | | | | | | | | | |
| GRIK2 | | | + | | | | | | + |
| GRIK3 | | | | | | + | | | + |
| GRIK4 | | | | | | | | | |
| GRIK5 | | | | | | | | | |
| GRIN1 | | | | | | | | | |
| GRIN2A | + | | | | + | | | | + |
| GRIN2B | | | | + | | | | | + |
| GRIN2C | | | | | | | | + | + |
| GRIN2D | | | | | | | | | |
| GRIN3A | | | | | | | | | |
| GRIN3B | | | | | | | | | |
| GRINA | | | | | | | | | |
| GRIP1 | | | | | | | | | |
| GRIP2 | | | | | | | | | |
| GRIPAP1 | | | | | | | | | |
| GRK1 | | | | | | | | | |
| GRK4 | | | | | | | | | |
| GRK5 | | | + | | | | | | + |
| GRK6 | | | | | | | | | |
| GRK7 | | | | | | | | | |
| GRL | | | | | | | | | |
| GRM1 | | | | + | | | | | + |
| Grm2 | | | | | | | | | |
| GRM3 | | | | | | | | | |
| Grm4 | | | | | | | | | |
| Grm5 | | | | | | + | | | + |
| Grm6 | | | | | | | | | |
| GRM7 | | | | | | | | | |

| | | | | | | | | | |
|---|---|---|---|---|---|---|---|---|---|
| Grm8 | | | | | | | | | |
| GRN | | | | | | | | | |
| GRP | | | | | | | | | |
| GRPEL1 | | | | | | | | | |
| GRPEL2 | | | | | | | | | |
| GRPR | | | | | | | | | |
| GRR1 | | | | | | | | | |
| GRS1 | | | | | | | | | |
| GRSF1 | | | | | | | | | |
| GRTP1 | | | | | | | | | |
| GRWD1 | | | | | | | | | |
| GRX2 | | | | | | | | | |
| GRX3 | | | | | | | | | |
| GRX4 | | | | | | | | | |
| GRX6 | | | | | | | | | |
| GRX7 | | | | | | | | | |
| GRXCR1 | | | | | | | | | |
| GRXCR2 | | | | | | | | | |
| GS1-259H13.2 | | | | | | | | | |
| GSAP | | | | | | | | | |
| GSC | | | | | | | | | |
| GSC2 | | | | | | | | | |
| GSDMA | | | | | | | | | |
| GSDMB | | | | | | | | | |
| GSDMC | | | | | | | | | |
| GSDMD | | | | | | | | | |
| GSE1 | | | | | | + | | | + |
| GSF2 | | | | | | | | | |
| GSG1 | | | | | | | | | |
| GSG1L | | | | | + | | | | + |
| GSG2 | | | | | | | | | |
| GSH1 | | | | | | | | | |
| GSH2 | | | | | | | | | |
| gsk-3 | | | | | | | | | |
| GSK3A | | | | | | | | | |
| GSK3B | + | | | + | + | | + | | + |
| GSKIP | | | | | | | | | |
| GSN | | | | | | | | | |
| GSP1 | | | | | | | | | |
| GSPT1 | | | | | | | | | |
| GSPT2 | | | | | | | | | |
| GSR | | | | | | | | | |
| GSS | | | | | | + | | | + |
| gst2 | | | | | | | | | |
| GSTA1 | | | | | | | | | |
| GSTA2 | | | | | | | | | |
| GSTA3 | | + | + | | | + | | + | + |
| GSTA4 | | | | | | | | | |
| GSTA5 | | | | | | | | | |
| GSTCD | | | | | | | | | |
| GSTK1 | | | | | | | | | |
| GSTM1 | | | | | | | | | |
| GSTM2 | | | | | | | | | |

| | | | | | | | | | |
|---|---|---|---|---|---|---|---|---|---|
| GSTM3 | | | | | | | | | |
| GSTM4 | | | | | | | | | |
| GSTM5 | | + | | | | + | | | + |
| Gstm7 | | | | | | + | | | + |
| GSTO1 | | | | | | | | | |
| GSTO2 | | | | | | | | | |
| GSTP1 | | | | | | | | | |
| GSTT1 | | | | | | | | | |
| GSTT2 | | | | | | | | | |
| GSTT2B | | | | | | | | | |
| GSTZ1 | | | | | | | | | |
| GSX1 | | | | | | | | | |
| GSX2 | | | | | | | | | |
| GSY1 | | | | | | | | | |
| GSY2 | | | | | | | | | |
| GTDC1 | | | | | | | | | |
| GTF2A1 | | | | | | | | | |
| GTF2A1L | | | | | | | | | |
| GTF2A2 | | | | | | | | | |
| GTF2B | | | | | | | | | |
| GTF2E1 | | | | | | + | | | + |
| GTF2E2 | | | | | | | | | |
| GTF2F1 | | | | | | | | | |
| GTF2F2 | | | | | | | | | |
| GTF2H1 | | | | | | | | | |
| GTF2H2 | | | | | | + | | | + |
| GTF2H2B | | | | | | | | | |
| GTF2H2C | | | | | | | | | |
| GTF2H2C_2 | | | | | | | | | |
| GTF2H3 | | | | | | | | | |
| GTF2H4 | | | | | | | | | |
| GTF2H5 | | | | | | | | | |
| GTF2I | | + | | | | + | | | + |
| GTF2IP1 | | | | | | | | | |
| GTF2IRD1 | | | | | | | | | |
| GTF2IRD1P1 | | | | | | | | | |
| GTF2IRD2 | | | | | | | | | |
| GTF2IRD2B | | | | | | | | | |
| GTF3A | | | | | | | | | |
| GTF3C1 | | | | | | | | | |
| GTF3C2 | | | | | | | | | |
| GTF3C2-AS1 | | | | | | | | | |
| GTF3C3 | | | | | | | | | |
| GTF3C4 | | | | | | | | | |
| GTF3C5 | | | | | | + | | | + |
| GTF3C6 | | | | | | | | | |
| GTO1 | | | | | | | | | |
| GTPBP1 | | | | | | | | | |
| GTPBP10 | | | | | | | | | |
| GTPBP2 | + | | + | | + | | | | + |
| GTPBP3 | | | | | | | | | |
| GTPBP4 | | | | | | + | | | + |
| GTPBP6 | | | | | | | | | |

| | | | | | | | | | |
|---|---|---|---|---|---|---|---|---|---|
| GTPBP8 | | | | | | | | | |
| GTS1 | | | | | | | | | |
| GTSE1 | | | | | | | | | |
| GTSF1 | | | | | | | | | |
| GTSF1L | | | | | | | | | |
| GTT1 | | | | | | | | | |
| GTT3 | | | | | | | | | |
| GUA1 | | | | | | | | | |
| gua2 | | | | | | | | | |
| GUCA1A | | | | | | | | | |
| GUCA1B | | | | | | | | | |
| GUCA1C | | | | | | | | | |
| GUCA2A | | | | | | | | | |
| GUCA2B | | | | | | | | | |
| GUCD1 | | | | | | | | | |
| GUCY1A2 | | | | | | | | | |
| GUCY1A3 | | | | | | + | | | + |
| GUCY1B2 | | | | | | | | | |
| GUCY1B3 | | + | | | | + | | | + |
| GUCY2C | | | | | | | | | |
| GUCY2D | | | | | | | | | |
| GUCY2EP | | | | | | | | | |
| GUCY2F | | | | | | | | | |
| GUF1 | | | | | | + | | | + |
| Gug | | | | | | | | | |
| GUK1 | | | | | | | | | |
| GULP1 | | | | | | | | | |
| GUS1 | | | | | | | | | |
| GUSB | | | | | | | | | |
| GUSBP1 | | | | | | | | | |
| GUSBP3 | | | | | | | | | |
| GUSBP4 | | | | | | | | | |
| GUSBP5 | | | | | | | | | |
| GUT2 | | | | | | | | | |
| GVINP1 | | | | | | | | | |
| GVP36 | | | | | | | | | |
| gw | | | | | | | | | |
| GXYLT1 | | | + | | | | | | + |
| GXYLT2 | | | | | | | | | |
| GYG1 | | | | | | | | | |
| GYG2 | | | | | | | | | |
| Gyk | | | | | | | | | |
| GYLTL1B | | | | | | | | | |
| GYP5 | | | | | | | | | |
| GYPA | | | | | | | | | |
| GYPB | | | | | | | | | |
| GYPC | | | | | | | | | |
| GYS1 | | | | | | | | | |
| GYS2 | + | | | + | + | | | | + |
| GZF1 | | | | | | | | | |
| GZF3 | | | | | | | | | |
| GZMA | | | | | | | | | |
| GZMB | | | | | | | | | |

| | | | | | | | | | |
|---|---|---|---|---|---|---|---|---|---|
| GZMH | | | | | | | | | |
| GZMK | | | | | | | | | |
| GZMM | | | | | | | | | |
| H | | | | | | | | | |
| H13 | | | | | | | | | |
| H19 | | | | | | | | | |
| H1F0 | | | | | | | | | |
| H1FNT | | | | | | | | | |
| H1FOO | | | | | | | | | |
| H1FX | | | | | | | | | |
| H1FX-AS1 | | | | | | | | | |
| H2-Aa | | | | | | | | | |
| H2-K1 | | | + | | | | | | + |
| H2AFB1 | | | | | | | | | |
| H2AFB2 | | | | | | | | | |
| H2AFB3 | | | | | | | | | |
| H2AFJ | | | | | | | | | |
| H2AFV | | + | | | | + | | | + |
| H2AFX | | | | | | | | | |
| H2AFY | | | + | | | | | | + |
| H2AFY2 | | | | | | | | | |
| H2AFZ | | | | | + | | | | + |
| H2B | | | | | | | | | |
| H2BFM | | | | | | | | | |
| H2BFS | | | | | | | | | |
| H2BFWT | | | | | | | | | |
| H3.1 | | | | | | | | | |
| H3F3A | | | | | | | | | |
| H3F3AP6 | | | | | | | | | |
| H3F3B | + | | | | | | | | + |
| H3F3C | | | | | | | | | |
| H4 | | | | | | | | | |
| h4-A | | | | | | | | | |
| H6PD | | | | | | | | | |
| HAAO | | | | | | | | | |
| HAB1 | | | | | | | | | |
| HABP2 | | | | | | | | | |
| HABP4 | | | + | | | | | | + |
| HACE1 | | | | | | | | | |
| HACL1 | | | | | | | | | |
| HADH | | | | | + | | | | + |
| HADHA | | | | | | | | | |
| HADHB | | | | | | | | | |
| HAGH | | | | | | + | | | + |
| HAGHL | | + | | | | + | | | + |
| HAL | | | | | | | | | |
| HAMP | | | | | | | | | |
| HAND1 | | | | | | | | | |
| HAND2 | | | | | | | | | |
| HAND2-AS1 | | | | | | | | | |
| HAO1 | | | | | | | | | |
| HAO2 | | | | | | | | | |
| HAP1 | | | | | | + | | | + |

| | | | | | | | | | |
|---|---|---|---|---|---|---|---|---|---|
| HAPLN1 | | | | | | | | | |
| HAPLN2 | | | | | | | | | |
| HAPLN3 | | | | | | | | | |
| HAPLN4 | | | | | + | | | | + |
| HARBI1 | | | | | | | | | |
| HARS | | | | | + | | | | + |
| HARS2 | | | | | | | | | |
| HAS1 | | | | | | | | | |
| HAS2 | | | | | | | | | |
| HAS3 | | | | | | | | | |
| HAT1 | | | | | | | | | |
| HAT2 | | | | | | | | | |
| Haus1 | | | | | | | | | |
| Haus2 | | | | | | | | | |
| Haus3 | | | | | | | | | |
| Haus4 | | | | | | | | | |
| HAUS5 | | | | | | | | | |
| Haus6 | | | | | | + | | | + |
| Haus7 | | | | | | | | | |
| Haus8 | | | | | | | | | |
| HAVCR1 | | | | | | | | | |
| HAVCR2 | | | | | | | | | |
| HAX1 | | | | | | | | | |
| HB-1 | | | | | | | | | |
| Hba-a1 | | | | | | | | | |
| HBA1 | | | | | | | | | |
| HBA2 | | | | | | | | | |
| HBB | | | | | | | | | |
| Hbb-bh1 | | | | | | | | | |
| HBD | | | | | | | | | |
| HBE1 | | | | | | | | | |
| HBEGF | | | | | | | | | |
| HBG1 | | | | | | | | | |
| HBG2 | | | | | | | | | |
| HBM | | | | | | | | | |
| HBP1 | | | | | | | | | |
| HBQ1 | | | | | | | | | |
| HBS1 | | | | | | | | | |
| HBS1L | | | | | | | | | |
| HBZ | | | | | | | | | |
| HCAR1 | | | | | | | | | |
| HCCS | | | | | | | | | |
| HCFC1 | | | | | | | | | |
| HCFC1R1 | | | | | | | | | |
| HCFC2 | | | + | | | | | | + |
| HCG11 | | | | | | | | | |
| HCG18 | | | | | | | | | |
| HCG27 | | | | | | | | | |
| HCK | | | | | | | | | |
| HCLS1 | | | | | | | | | |
| HCN1 | | | + | | + | | | | + |
| HCN2 | | | | | | | | | |
| HCN3 | | | | | | | | | |

| | | | | | | | | | | |
|---|---|---|---|---|---|---|---|---|---|---|
| HCN4 | | | | | | | | | | |
| HCP5 | | | | | | | | | | |
| HCR1 | | | | | | | | | | |
| HCRT | | | | | | | | | | |
| HCRTR1 | | | | | | | | | | |
| HCRTR2 | | | | | | | | | | |
| HCST | | | | | | | | | | |
| HCT | | | | | | | | | | |
| HCVgp1 | | | | | | | | | | |
| HCVgp2 | | | | | | | | | | |
| hda14 | | | | | | | | | | |
| HDA3 | | | | | | | | | | |
| HDAC1 | | | | | | | | | | |
| HDAC10 | | | | | | | | | | |
| HDAC11 | | | | | | | | | | |
| HDAC2 | | | | | | | | | | |
| HDAC3 | | | | | | | | | | |
| HDAC4 | | | | | | | | | | |
| HDAC5 | | | | | | | | | | |
| HDAC6 | | | | | | | | | | |
| HDAC7 | | | | | | | | | | |
| HDAC8 | | | | | | | | | | |
| HDAC9 | | | | | | | | | | |
| HDC | | | | | | | | | | |
| HDDC2 | | | | | | | | | | |
| HDDC3 | | | | | | | | | | |
| HDGF | | | | | | | | | | |
| HDGFL1 | | | | | | | | | | |
| HDGFRP2 | | | | | | | | | | |
| Hdgfrp3 | | | | | | | | | | |
| HDHD1 | | | | | | | | | | |
| HDHD2 | | | | | | | | | | |
| HDHD3 | | | | | | | | | | |
| HDL3 | | | | | | | | | | |
| HDLBP | | | | | | | | | | |
| HDX | | | | | | | | | | |
| HEATR1 | | | | | | | | | | |
| Heatr2 | | | | | | | | | | |
| HEATR3 | | | | | | | | | | |
| HEATR4 | | | | | | | | | | |
| HEATR5A | | | | | | | | | | |
| HEATR5B | | | | | | | | | | |
| HEATR6 | | + | | | | + | | | | + |
| HEATR9 | | | | | | | | | | |
| HEBP1 | | | | | | + | | | | + |
| HEBP2 | | | | | | | | | | |
| HECA | | | | | | | | | | |
| HECTD1 | | | | | | | | | | |
| HECTD2 | + | | | + | + | | | | | + |
| HECTD3 | | | | | | + | | | | + |
| HECTD4 | | | | | | | | | | |
| HECW1 | | | | | | | | | | |
| HECW2 | | | | | | | | | | |

| | | | | | | | | | |
|---|---|---|---|---|---|---|---|---|---|
| HEG1 | | | | | | | | | |
| HEL1 | | | | | | | | | |
| HELB | | | + | | | | | | + |
| HELLS | | | | | | | | | |
| HELQ | | | | | | | | | |
| HELT | | | | | | | | | |
| HELZ | | | | | | | | | |
| HELZ2 | | | | | | | | | |
| HEM1 | | | | | | | | | |
| HEM12 | | | | | | | | | |
| HEM15 | | | | | | | | | |
| HEMGN | | | | | | | | | |
| HEMK1 | | | | | | + | | | + |
| HENMT1 | | | | | | | | | |
| HEPACAM | | | | | | | | | |
| HEPACAM2 | | | | | | | | | |
| HEPH | | | | | | | | | |
| HEPHL1 | | | | | | | | | |
| HEPN1 | | | | | | | | | |
| HERC1 | | | | + | | | | | + |
| HERC2 | + | | | | + | | | | + |
| HERC2P2 | | | | | | | | | |
| HERC2P3 | | | | | | | | | |
| HERC2P4 | | | | | | | | | |
| HERC2P9 | | | | | | | | | |
| HERC3 | | | | | | | | | |
| HERC4 | | | | | | | | | |
| HERC5 | | | | | | | | | |
| HERC6 | | | | | | | | | |
| HERPUD1 | | | | | | | | | |
| HERPUD2 | | | | | | | | | |
| HES1 | | | | | | | | | |
| HES2 | | | | | | | | | |
| HES3 | | | | | | | | | |
| HES4 | | | | | | | | | |
| HES5 | | | | | | | | | |
| HES6 | | | | | | | | | |
| HES7 | | | | | | | | | |
| HESX1 | | | | | | | | | |
| HEXA | | | | | | | | | |
| HEXB | | | | | | | | | |
| HEXDC | | | | | | | | | |
| HEXIM1 | | | + | | | | | | + |
| HEXIM2 | | | | | | | | | |
| HEY | | | | | | | | | |
| HEY1 | | | | | | | | | |
| HEY2 | | | | | | | | | |
| HEYL | | | | | | | | | |
| HFE | | | | | | | | | |
| HFE2 | | | | | | | | | |
| HFI1 | | | | | | | | | |
| HFM1 | | | | | | | | | |
| HGD | | | | | | | | | |

| | | | | | | | | | |
|---|---|---|---|---|---|---|---|---|---|
| HGF | | | | | | | | | |
| HGFAC | | | | | | | | | |
| HGH1 | | | | | | | | | |
| HGS | | | | | | | | | |
| HGSNAT | | | | | | + | | | + |
| HHAT | | | | | | | | | |
| HHATL | | | | | | | | | |
| HHEX | + | | | + | + | | + | | + |
| HHF1 | | | | | | | | | |
| HHF2 | | | | | | | | | |
| HHIP | | | | | + | | | | + |
| HHIPL1 | | | | | | | | | |
| HHIPL2 | | | | | | | | | |
| HHLA1 | | | | | | | | | |
| HHLA2 | | | | | | | | | |
| HHLA3 | | | | | | | | | |
| HHO1 | | | | | | | | | |
| HHT1 | | | | | | | | | |
| HHT2 | | | | | | | | | |
| HHV8_gs01 | | | | | | | | | |
| HHV8GK18_gp78 | | | | | | | | | |
| HHV8GK18_gp79 | | | | | | | | | |
| HHV8GK18_gp80 | | | | | | | | | |
| HHV8GK18_gp81 | | | | | | | | | |
| HHV8GK18_gp82 | | | | | | | | | |
| HHV8GK18_gp83 | | | | | | | | | |
| HIAT1 | | | + | | | | | | + |
| HIATL1 | | | | | | | | | |
| HIATL2 | | | | | | | | | |
| HIBADH | | | | | | + | | | + |
| HIBCH | | | | | | + | | | + |
| HIC1 | | | | | | | | | |
| HIC2 | | | | | | | | | |
| HID1 | | | | | | | + | | + |
| HIF1A | | | | | | | | | |
| HIF1A-AS2 | | | | | | | | | |
| HIF1AN | | | | | | | | | |
| HIF3A | | | | | | | | | |
| HIGD1A | | | | | | | | | |
| HIGD1B | | | | | | | | | |
| HIGD1C | | | | | | | | | |
| HIGD2A | | + | | | | + | | | + |
| HIGD2B | | | | | | | | | |
| HILPDA | | | | | | | | | |
| HILS1 | | | | | | | | | |
| HINFP | | | | | | | | | |
| HINT1 | | | | | + | + | | + | + |
| HINT2 | | | | | | | | | |
| HINT3 | | | | + | | | | | + |
| HIP1 | | | | | | | | | |
| HIP1R | | | | | | | | | |
| HIPK1 | | | | | | | | | |
| HIPK2 | | | | | + | | | | + |

| | | | | | | | | | |
|---|---|---|---|---|---|---|---|---|---|
| HIPK3 | | | | | | | | | |
| HIPK4 | | | | | | | | | |
| HIRA | | | + | | | | | | + |
| HIRIP3 | | | | | | | | | |
| HIS2 | | | | | | | | | |
| HIS4 | | | | | | | | | |
| HIST1H1A | | | | | | | | | |
| HIST1H1B | | | | | | | | | |
| HIST1H1C | | | | | | | | | |
| HIST1H1D | | | | | | | | | |
| HIST1H1E | | | | | | | | | |
| HIST1H1T | | | | | | | | | |
| HIST1H2A4 | | | | | | | | | |
| HIST1H2A9 | | | | | | | | | |
| HIST1H2AA | | | | | | | | | |
| HIST1H2AB | | | | | | | | | |
| HIST1H2AC | | | | | | | | | |
| HIST1H2AD | | | | | | | | | |
| HIST1H2AE | | | | | | | | | |
| Hist1h2af | | | | | | | | | |
| HIST1H2AG | | | | | | | | | |
| HIST1H2AH | | | | | | | | | |
| HIST1H2AI | | | | | | | | | |
| HIST1H2AJ | | | | | | | | | |
| HIST1H2AK | | | | | | | | | |
| HIST1H2AL | | | | | | | | | |
| HIST1H2AM | | | | | | | | | |
| HIST1H2B7 | | | | | | | | | |
| HIST1H2BA | | | | | | | | | |
| HIST1H2BB | | | | | | | | | |
| HIST1H2BC | | + | + | | | + | | + | + |
| HIST1H2BD | | | | | | | | | |
| HIST1H2BE | | | | | | | | | |
| HIST1H2BF | | | | | | | | | |
| HIST1H2BG | | | | | | | | | |
| HIST1H2BH | | | | | | | | | |
| HIST1H2BI | | | | | | | | | |
| HIST1H2BJ | | | | | | | | | |
| HIST1H2BK | | | | | | | | | |
| HIST1H2BL | | | | | | | | | |
| HIST1H2BM | | | | | | | | | |
| HIST1H2BN | | | | | | | | | |
| HIST1H2BO | | | | | | | | | |
| Hist1h2bp | | | | | | | | | |
| HIST1H3A | | | | | | | | | |
| HIST1H3B | | | | | | | | | |
| HIST1H3C | | | | | | | | | |
| HIST1H3D | | | | | | | | | |
| HIST1H3E | | | | | | | | | |
| HIST1H3F | | | | | | | | | |
| hist1h3g | | | | | | | | | |
| HIST1H3H | | | | | | | | | |
| HIST1H3I | | | | | | | | | |

| | | | | | | | | | |
|---|---|---|---|---|---|---|---|---|---|
| HIST1H3J | | | | | | | | | |
| HIST1H47 | | | | | | | | | |
| HIST1H4A | | | | | | | | | |
| HIST1H4B | | | | | | | | | |
| HIST1H4C | | | | | | | | | |
| HIST1H4D | | | | | | | | | |
| HIST1H4E | | | | | | | | | |
| HIST1H4F | | | | | | | | | |
| HIST1H4G | | | | | | | | | |
| HIST1H4H | | | + | | | | | + | + |
| HIST1H4I | | | | | | | | | |
| HIST1H4J | | | | | | | | | |
| HIST1H4K | | | | | | | | | |
| HIST1H4L | | | | | | | | | |
| Hist2h2aa1 | | | | | | | | | |
| HIST2H2AA3 | | | | | | | | | |
| HIST2H2AA4 | | | | | | | | | |
| HIST2H2AB | | | | | | | | | |
| HIST2H2AC | | | | | | | | | |
| HIST2H2BA | | | | | | | | | |
| Hist2h2bb | | | | | + | | | | + |
| HIST2H2BC | | | | | | | | | |
| HIST2H2BD | | | | | | | | | |
| HIST2H2BE | | | | | | | | | |
| HIST2H2BF | | | | | | | | | |
| HIST2H3A | | | | | | | | | |
| HIST2H3C | | | | | | | | | |
| HIST2H3D | | | | | | | | | |
| HIST2H3PS2 | | | | | | | | | |
| HIST2H4A | | | | | | | | | |
| HIST2H4B | | | | | | | | | |
| HIST3H2A | | + | | | | + | | | + |
| Hist3h2ba | | | | | | | | | |
| HIST3H2BB | | | | | | | | | |
| Hist3h2bb-ps | | | | | | | | | |
| HIST3H3 | | | | | | | | | |
| HIST4H4 | | | | | | | | | |
| HIV2gp2 | | | | | | | | | |
| HIV2gp3 | | | | | | | | | |
| HIV2gp4 | | | | | | | | | |
| HIV2gp5 | | | | | | | | | |
| HIV2gp7 | | | | | | | | | |
| HIVEP1 | | | + | | | | | | + |
| HIVEP2 | | | + | | + | | | | + |
| HIVEP3 | + | | | | + | | | | + |
| HJURP | | | | | | | | | |
| HK1 | | | + | | | | | | + |
| HK2 | + | | | + | + | | | | + |
| HK3 | | | | | | | | | |
| HKDC1 | | | | | | | | | |
| HKR1 | | | | | | | | | |
| HLA-A | | | | | | | | | |
| HLA-B | | | | | | | | | |

| | | | | | | | | | |
|---|---|---|---|---|---|---|---|---|---|
| HLA-C | | | | | | | | | |
| HLA-DMA | | | | | | | | | |
| HLA-DMB | | | | | | | | | |
| HLA-DOA | | | | | | | | | |
| HLA-DOB | | | | | | | | | |
| HLA-DPA1 | | | | | | | | | |
| HLA-DPB1 | | | | | | | | | |
| HLA-DQA1 | | | | | | | | | |
| HLA-DQA2 | | | | | | | | | |
| HLA-DQB1 | | | | | | | | | |
| HLA-DQB2 | | | | | | | | | |
| HLA-DRA | | | | | | | | | |
| HLA-DRB1 | | | | | | | | | |
| HLA-DRB3 | | | | | | | | | |
| HLA-DRB4 | | | | | | | | | |
| HLA-DRB5 | | | | | | | | | |
| HLA-E | | | | | | | | | |
| HLA-F | | | | | | | | | |
| HLA-G | | | | | | | | | |
| HLA-H | | | | | | | | | |
| HLA-L | | | | | | | | | |
| HLCS | | | | | | | | | |
| HLF | | | + | | | + | | | + |
| HLJ1 | | | | | | | | | |
| HLR1 | | | | | | | | | |
| HLTF | | | | | | + | | | + |
| HLX | | | | | | | | | |
| HM13 | | | | | | | | | |
| HM13-AS1 | | | | | | | | | |
| HMBOX1 | | | | | | | | | |
| HMBS | | | | | | | | | |
| HMCES | | + | | | | | | | + |
| HMCN1 | | | | | | | | | |
| HMCN2 | | | | | | | | | |
| HMG20A | | | | | | | + | | + |
| HMG20B | | | | | | | | | |
| HMGA1 | | | | | + | | | | + |
| HMGA1P3 | | | | | | | | | |
| HMGA2 | | | | | | | | | |
| HMGB1 | | | | | | | | | |
| HMGB1P1 | | | | | | | | | |
| HMGB1P10 | | | | | | | | | |
| HMGB2 | | | | | | | | | |
| HMGB3 | | | | | | | | | |
| HMGB3P1 | | | | | | | | | |
| HMGB4 | | | | | | | | | |
| HMGCL | | | | | | | | | |
| HMGCLL1 | | | | | | | | | |
| HMGCR | | | | | | | | | |
| HMGCS1 | | | | | | | | | |
| HMGCS2 | | | | | | | | | |
| HMGN1 | | | + | | | | | | + |
| HMGN1P30 | | | | | | | | | |

| | | | | | | | | | |
|---|---|---|---|---|---|---|---|---|---|
| HMGN2 | | | | | | | | | |
| HMGN2P46 | | | | | | | | | |
| HMGN3 | | + | | | | + | | | + |
| HMGN4 | | | | | | | | | |
| HMGN5 | | | + | | | | | | + |
| Hmgn5b | | | | | | | | | |
| HMGXB3 | | | | | | | | | |
| HMGXB4 | | | | | | | | | |
| HMHA1 | | | | | | | | | |
| HMMR | | | | | | | | | |
| HMO1 | | | | | | | | | |
| HMOX1 | | | | | + | | | | + |
| HMOX2 | | | | | | | | | |
| HMP19 | | | | | | | | | |
| HMS1 | | | | | | | | | |
| HMS2 | | | | | | | | | |
| HMT1 | | | | | | | | | |
| hmt2 | | | | | | | | | |
| HMX1 | | | | | | | | | |
| HMX2 | | | | | | | | | |
| HMX3 | | | | | | | | | |
| HN1 | | | | | | | | | |
| HN1L | | | | | | | | | |
| HNF1A | | | | | | | | | |
| HNF1A-AS1 | | | | | | | | | |
| HNF1B | | | | | | | | | |
| HNF4A | | | | | | | | | |
| HNF4G | + | | | + | + | | + | | + |
| HNMT | | | | | | | | | |
| HNP1 | | | | | | | | | |
| HNRNPA0 | | | + | | | | | | + |
| HNRNPA1 | | | | | | | | | |
| HNRNPA1L2 | | | | | | | | | |
| HNRNPA1P10 | | | | | | | | | |
| HNRNPA1P33 | | | | | | | | | |
| HNRNPA1P70 | | | | | | | | | |
| HNRNPA2B1 | | | | | + | | | | + |
| HNRNPA3 | | | | | | | | | |
| HNRNPA3P1 | | | | | | | | | |
| HNRNPAB | | | | | | | | | |
| HNRNPC | | | | | | | | | |
| HNRNPCL1 | | | | | | | | | |
| HNRNPCL2 | | | | | | | | | |
| HNRNPCL3 | | | | | | | | | |
| HNRNPCL4 | | | | | | | | | |
| HNRNPCP2 | | | | | | | | | |
| HNRNPD | | | | | | | | | |
| HNRNPDL | | | | | | | | | |
| HNRNPF | | | | | | | | | |
| HNRNPH1 | | | | | | | | | |
| HNRNPH1P1 | | | | | | | | | |
| HNRNPH2 | | | | | | | + | | + |
| HNRNPH3 | | | | | | | | | |

| | | | | | | | | | |
|---|---|---|---|---|---|---|---|---|---|
| HNRNPK | | | | | | + | + | | + |
| HNRNPL | | | | | | | | | |
| HNRNPLL | | | | | + | | | | + |
| HNRNPM | | | | | | | | | |
| HNRNPR | | | | | | | | | |
| HNRNPU | | | | | | | | | |
| HNRNPU-AS1 | | | | | | | | | |
| HNRNPUL1 | | | | | | | | | |
| HNRNPUL2 | | | | | | | | | |
| HNRNPUL2-BSCL2 | | | | | | | | | |
| Hnrpd | | | | | | | | | |
| hob1 | | | | | | | | | |
| hob3 | | | | | | | | | |
| HOC1 | | | | | | | | | |
| HOG1 | | | | | | | | | |
| HOGA1 | | | | | | | | | |
| HOM2 | | | | | | | | | |
| HOM6 | | | | | | | | | |
| HOMER1 | + | | + | | + | | | | + |
| HOMER2 | | | | | | | | | |
| HOMER3 | | | | | | | | | |
| HOMEZ | | | | | | | | | |
| HOOK1 | | + | | | | + | | | + |
| HOOK2 | | | | | | | | | |
| HOOK3 | | | | | | | | | |
| HOPX | | | | | | | | | |
| HORMAD1 | | | | | | | | | |
| HORMAD2 | | | | | | | | | |
| HORMAD2-AS1 | | | | | | | | | |
| HOTAIR | | | | | | | | | |
| HOTAIRM1 | | | | | | | | | |
| HOTTIP | | | | | | | | | |
| HOXA-AS2 | | | | | | | | | |
| HOXA-AS3 | | | | | | | | | |
| HOXA1 | | | | | | | | | |
| HOXA10 | | | | | | | | | |
| HOXA10-AS | | | | | | | | | |
| HOXA10-HOXA9 | | | | | | | | | |
| HOXA11 | | | | | | | | | |
| HOXA11-AS | | | | | | | | | |
| HOXA13 | | | | | | | | | |
| HOXA2 | | | | | | | | | |
| HOXA3 | | | | | | | | | |
| HOXA4 | | | | | | | | | |
| HOXA5 | | | | | | | | | |
| HOXA6 | | | | | | | | | |
| HOXA7 | + | | | + | + | | | | + |
| HOXA9 | | | | | | | | | |
| HOXB-AS3 | | | | | | | | | |
| HOXB1 | | | | | | | | | |
| HOXB13 | | | | | | | | | |
| HOXB2 | | | | | | | | | |
| HOXB3 | | | | | | | | | |

| | | | | | | | | | |
|---|---|---|---|---|---|---|---|---|---|
| HOXB4 | + | | | + | + | | | | + |
| HOXB5 | | | | | | | | | |
| HOXB6 | | | | | | | | | |
| HOXB7 | | | | | | | | | |
| HOXB8 | | | | | | | | | |
| HOXB9 | + | | | + | + | | + | | + |
| HOXC-AS3 | | | | | | | | | |
| HOXC10 | | | | | | | | | |
| HOXC11 | | | | | | | | | |
| HOXC12 | | | | | | | | | |
| HOXC13 | | | | | | | | | |
| HOXC4 | | | | | | | | | |
| HOXC5 | | | | | | | | | |
| HOXC6 | | | | | | | | | |
| HOXC8 | + | | | + | + | | + | | + |
| HOXC9 | | | | | | | | | |
| HOXD1 | | | | | | | | | |
| HOXD10 | | | | | | | | | |
| HOXD11 | + | | | + | + | | + | | + |
| HOXD12 | | | | | | | | | |
| HOXD13 | | | | | | | | | |
| HOXD3 | | | | | | | | | |
| HOXD4 | | | | | | | | | |
| HOXD8 | | | | | | | | | |
| HOXD9 | + | | | + | + | | + | | + |
| HP | | | | | | | | | |
| HP1BP3 | | | | | | | | | |
| HPCA | | | | | | + | | | + |
| HPCAL1 | | | | | | | | | |
| HPCAL4 | + | | | | + | | | | + |
| HPD | | | | | | | | | |
| HPDL | | | | | | | | | |
| HPF1 | | | | | | | | | |
| HPGD | | | + | | | | | | + |
| HPGDS | | | | + | | | | | + |
| HPM1 | | | | | | | | | |
| HPN | | | | | | | | | |
| HPR | | | | | | | | | |
| HPRT1 | | | | | | | | | |
| HPS1 | | | | | | | | | |
| HPS3 | | | | | | | | | |
| HPS4 | | + | | | | + | | | + |
| HPS5 | | | | | | | | | |
| HPS6 | | | | | | | | | |
| HPSE | | | | | | | | | |
| HPSE2 | | | | | | | | | |
| HPX | | | | | | | | | |
| HR | | | | | | | | | |
| Hras | | | + | | | | | | + |
| HRASLS | | | | | | | | | |
| HRASLS2 | | | | | | | | | |
| HRASLS5 | | | | | | | | | |
| HRC | + | | | + | + | | | | + |

| | | | | | | | | | |
|---|---|---|---|---|---|---|---|---|---|
| HRCT1 | | | | | | | | | |
| HRD1 | | | | | | | | | |
| HRES1 | | | | | | | | | |
| HRG | | | | | | | | | |
| HRH1 | | | | | | | | | |
| HRH2 | | | | | | | | | |
| HRH3 | | | | + | | | | | + |
| HRK | | | | | | + | | | + |
| HRNR | | | | | | | | | |
| HRP1 | | | | | | | | | |
| HRR25 | | | | | | | | | |
| HRSP12 | | | | | | + | | | + |
| HRT1 | | | | | | | | | |
| HS1BP3 | | | | | | | | | |
| HS2ST1 | | | | | | | | | |
| HS3ST1 | | | | | | | | | |
| HS3ST2 | | | | | | | | | |
| HS3ST3A1 | | | | | | | | | |
| HS3ST3B1 | | | | | | | | | |
| HS3ST4 | | | | | + | | | | + |
| HS3ST5 | | | | | | | | | |
| HS3ST6 | | | | | | | | | |
| HS6ST1 | | | | | + | | | | + |
| HS6ST2 | | | + | | | | | | + |
| HS6ST3 | | | | | | | | | |
| HSBP1 | | | | | | | | | |
| HSBP1L1 | | | | | | | | | |
| HSC70-1 | | | | | | | | | |
| Hsc70-ps1 | | | | | | | | | |
| HSC82 | | | | | | | | | |
| HSCB | | | | | | | | | |
| HSD11B1 | | | | | | | | | |
| HSD11B1L | | | | | | | | | |
| HSD11B2 | | | | | | | | | |
| HSD17B1 | | | | | | | | | |
| HSD17B10 | | | | | | | | | |
| HSD17B11 | | | | | | | | | |
| HSD17B12 | + | | + | | + | | | | + |
| HSD17B13 | | | | | | | | | |
| HSD17B14 | | | | | | | | | |
| HSD17B1P1 | | | | | | | | | |
| HSD17B2 | | | | | | | | | |
| HSD17B3 | | | | | | | | | |
| HSD17B4 | | | | | | | | | |
| HSD17B6 | | | | | | | | | |
| Hsd17b7 | | | | | | | | | |
| HSD17B7P2 | | | | | | | | | |
| HSD17B8 | | | | | | | | | |
| HSD3B1 | | | | | | | | | |
| HSD3B2 | | + | | + | | | | | + |
| HSD3B7 | | | | | | | | | |
| HSDL1 | | | | | | | | | |
| HSDL2 | | | | | | | | | |

| | | | | | | | | | |
|---|---|---|---|---|---|---|---|---|---|
| HSF1 | | | | | | | | | |
| HSF2 | + | | | | + | | | | + |
| HSF2BP | | | | | | | | | |
| HSF4 | | | | | | | | | |
| HSF5 | | | | | | | | | |
| HSFX1 | | | | | | | | | |
| HSFX2 | | | | | | | | | |
| HSFY1 | | | | | | | | | |
| HSFY2 | | | | | | | | | |
| HSH155 | | | | | | | | | |
| HSH2D | | | | | | | | | |
| HSL1 | | | | | | | | | |
| hsp10 | | | | | | | | | |
| HSP104 | | | | | | | | | |
| HSP12 | | | | | | | | | |
| HSP150 | | | | | | | | | |
| hsp16 | | | | | | | | | |
| Hsp22 | | | | | | | | | |
| HSP26 | | | | | | | | | |
| HSP30 | | | | | | | | | |
| HSP32 | | | | | | | | | |
| HSP42 | | | | | | | | | |
| Hsp60 | | | | | | | | | |
| HSP70 | | | | | | | | | |
| Hsp70b | | | | | | | | | |
| HSP81-2 | | | | | | | | | |
| HSP81-3 | | | | | | | | | |
| Hsp81.4 | | | | | | | | | |
| HSP82 | | | | | | | | | |
| HSP83_ANOGA | | | | | | | | | |
| hsp90 | | | | | | | | | |
| HSP90.1 | | | | | | | | | |
| HSP90AA1 | + | | | | + | | | | + |
| HSP90AA2P | | | | | | | | | |
| HSP90AA4P | | | | | | | | | |
| HSP90AA5P | | | | | | | | | |
| HSP90AB1 | | | | | | | | | |
| HSP90AB2P | | | | | | | | | |
| HSP90AB3P | | | | | | | | | |
| HSP90AB4P | | | | | | | | | |
| HSP90AB6P | | | | | | | | | |
| HSP90B1 | + | | | | + | | | | + |
| HSP90B2P | | | | | | | | | |
| HSP90B3P | | | | | | | | | |
| HSPA12A | | | | | | | | | |
| HSPA12B | | | | + | | | | | + |
| HSPA13 | | | | | | | | | |
| HSPA14 | | | | | | | | | |
| HSPA1A | | | | | | | | | |
| HSPA1B | | | + | | | | | + | + |
| HSPA1L | | | | | | | | | |
| HSPA2 | | | + | | | | | | + |
| HSPA4 | | | + | | + | | | | + |

| | | | | | | | | | |
|---|---|---|---|---|---|---|---|---|---|
| HSPA4L | | | + | | + | | | | + |
| HSPA5 | + | | + | | + | | | | + |
| HSPA6 | | | | | | | | | |
| HSPA7 | | | | | | | | | |
| HSPA8 | | | + | | + | | | | + |
| HSPA9 | | | | | | | | | |
| HSPB1 | | | | | | | | | |
| HSPB11 | | | | | | | | | |
| HSPB2 | | | | | | | | | |
| HSPB2-C11orf52 | | | | | | | | | |
| HSPB3 | | | | | | | | | |
| HSPB6 | | | | | + | | | | + |
| HSPB7 | + | | | + | + | | | | + |
| HSPB8 | | | + | | + | | | | + |
| HSPB9 | | | | | | | | | |
| HSPBAP1 | | | | | + | | | | + |
| HSPBP1 | | | | | | | | | |
| HSPD1 | | | | | + | | | | + |
| HSPD1P11 | | | | | | | | | |
| HSPE1 | | | | | + | | | | + |
| HSPE1-MOB4 | | | | | | | | | |
| HSPG2 | | | | | | | | | |
| HSPH1 | + | | + | | + | | | | + |
| HTA1 | | | | | | | | | |
| HTA2 | | | | | | | | | |
| HTATIP2 | | | | | | | | | |
| HTATSF1 | + | | | | | + | | | + |
| HTB1 | | | | | | | | | |
| HTB2 | | | | | | | | | |
| HTL1 | | | | | | | | | |
| HTN1 | | | | | | | | | |
| HTN3 | | | | | | | | | |
| HTR1A | | | | | | + | | | + |
| HTR1B | | | | | | | | | |
| HTR1D | + | | | + | + | | + | | + |
| HTR1E | | | | | | | | | |
| HTR2A | | | | | | | | | |
| HTR2B | | | | | | | | | |
| HTR2C | | | | | | | | | |
| HTR3A | | | | | | | | | |
| HTR3B | | | | | | | | | |
| HTR3C | | | | | | | | | |
| HTR3D | | | | | | | | | |
| HTR3E | | | | | | | | | |
| HTR4 | | | | | | | | | |
| HTR5A | | | | | | | | | |
| HTR5BP | | | | | | | | | |
| HTR6 | | | | | | | | | |
| HTR7 | | | | | | | | | |
| HTRA1 | | | | | | | | | |
| HTRA2 | | | | | | | | | |
| HTRA3 | | | | | | | | | |
| HTRA4 | | | | | | | | | |

| | | | | | | | | | |
|---|---|---|---|---|---|---|---|---|---|
| HTT | | | | | | | | | |
| HTZ1 | | | | | | | | | |
| HUA1 | | | | | | | | | |
| HUA2 | | | | | | | | | |
| HUL4 | | | | | | | | | |
| HULC | | | | | | | | | |
| HUNK | | | + | | | | | | + |
| HUS1 | | | | + | | | | | + |
| HUS1B | | | | | | | | | |
| hus5 | | | | | | | | | |
| HUWE1 | | | | | | | | | |
| HVCN1 | | | | | | | | | |
| HXK1 | | | | | | | | | |
| HXK2 | | | | | | | | | |
| HXT1 | | | | | | | | | |
| HXT3 | | | | | | | | | |
| HXT5 | | | | | | | | | |
| HXT6 | | | | | | | | | |
| HYAL1 | | | | | | | | | |
| HYAL2 | | | | | | | | | |
| HYAL3 | | | | | | | | | |
| HYAL4 | | | | | | | | | |
| HYDIN | | | | | | | | | |
| HYDIN2 | | | | | | | | | |
| HYI | | | | | | | | | |
| HYKK | | | | | | | | | |
| HYLS1 | | | | | | | | | |
| HYM1 | | | | | | | | | |
| HYOU1 | | | | | | | | | |
| HYP2 | | | | | | | | | |
| HYPK | | | | | | | | | |
| HYPM | | | | | | | | | |
| HYR1 | | | | | | | | | |
| IAH1 | | | | | | | | | |
| IAPP | | | | | | | | | |
| IAR1 | | | | | | | | | |
| IARS | | | | | | | | | |
| IARS2 | | | | | | | | | |
| IBA57 | | | | | | | | | |
| IBD8 | | | | | | | | | |
| IBSP | + | | | + | + | | + | | + |
| IBTK | | | | | | | | | |
| ICA1 | | | | | | | | | |
| ICA1L | | | | | | | | | |
| ICAM1 | | | | | | | | | |
| ICAM2 | | | | | | | | | |
| ICAM3 | | | | | | | | | |
| ICAM4 | | | | | | | | | |
| ICAM5 | | | | | | + | | | + |
| ICE1 | | | | | | | | | |
| ICE2 | | | | | | + | | | + |
| ICK | | | | | | + | | | + |
| ICL1 | | | | | | | | | |

| | | | | | | | | | |
|---|---|---|---|---|---|---|---|---|---|
| ICMT | | | | | | | | | |
| ICOS | | | | | | | | | |
| ICOSLG | | | | | | | | | |
| ICT1 | | | | | | + | | | + |
| ID1 | | | | | | | | | |
| ID2 | | | | | + | | | | + |
| ID3 | | | | | | | | | |
| ID4 | | | | | | | | | |
| IDE | | | | | | + | | | + |
| IDH1 | | | | | | | | | |
| IDH2 | | | + | | | | | | + |
| IDH3A | | | | | | | | | |
| IDH3B | | | | | | | | | |
| IDH3G | | | | | | | | | |
| IDI1 | | | | | + | | | | + |
| IDI2 | | | | | | | | | |
| IDI2-AS1 | | | | | | | | | |
| IDO1 | | | | | | | | | |
| IDO2 | | | | | | | | | |
| IDP1 | | | | | | | | | |
| IDP2 | | | | | | | | | |
| IDS | | | | | | | | | |
| IDUA | | | | | | | | | |
| iec1 | | | | | | | | | |
| IER2 | + | | | | | | | | + |
| IER3 | | | | | | | | | |
| IER3IP1 | | + | | | + | + | | | + |
| IER5 | + | | + | | + | | | | + |
| IER5L | | | | | | | | | |
| IFFO1 | | | | | | | | | |
| IFFO2 | | | | | | + | | | + |
| IFI16 | | | | | | | | | |
| Ifi202b | | | | | | | | | |
| Ifi204 | | | | + | | | | | + |
| IFI27 | | | | | + | | | | + |
| IFI27L1 | | | | | | | | | |
| IFI30 | | | | | | | | | |
| IFI35 | | | | | | | | | |
| IFI44 | + | | | + | + | | + | | + |
| IFI44L | | | | | | | | | |
| IFI6 | | | | | | | | | |
| IFIH1 | | | | | | | | | |
| IFIT1 | + | | | + | + | | + | | + |
| IFIT1B | | | | | | | | | |
| IFIT2 | | | | | | | | | |
| IFIT3 | | | | | + | | | | + |
| IFIT5 | | | | | | | | | |
| IFITM1 | | | | | | | | | |
| IFITM10 | | | | | | + | | | + |
| IFITM2 | | | | | | | | | |
| IFITM3 | | | | | | | | + | + |
| IFITM5 | | | | | | | | | |
| IFN1@ | | | | | | | | | |

| | | | | | | | | | |
|---|---|---|---|---|---|---|---|---|---|
| IFNA1 | | | | | | | | | |
| IFNA10 | | | | | | | | | |
| IFNA13 | | | | | | | | | |
| IFNA14 | | | | | | | | | |
| IFNA16 | | | | | | | | | |
| IFNA17 | | | | | | | | | |
| IFNA2 | | | | | | | | | |
| IFNA21 | | | | | | | | | |
| IFNA4 | | | | | | | | | |
| IFNA5 | | | | | | | | | |
| IFNA6 | | | | | | | | | |
| IFNA7 | | | | | | | | | |
| IFNA8 | | | | | | | | | |
| IFNAR1 | | | | | | + | | | + |
| IFNAR2 | | | | | | | | | |
| IFNB1 | | | | | | | | | |
| IFNE | | | | | | | | | |
| IFNG | | | | | | | | | |
| IFNGR1 | | | | | | | | | |
| IFNGR2 | | | + | | | + | | | + |
| IFNK | | | | | | | | | |
| IFNL1 | | | | | | | | | |
| IFNL2 | | | | | | | | | |
| IFNL3 | | | | | | | | | |
| IFNLR1 | | | | | | | | | |
| IFNW1 | | | | | | | | | |
| IFRD1 | + | | + | | + | | | | + |
| IFRD2 | | | | | | | | | |
| IFT122 | | | | | | | | | |
| IFT140 | | | | | | + | | | + |
| IFT172 | | | | | | | | | |
| IFT20 | | | + | | | + | | | + |
| IFT22 | | | | | | | | | |
| IFT27 | | | | | | | | | |
| IFT43 | | | | | | | | | |
| IFT46 | | | | | | | | | |
| IFT52 | | | | | | | | | |
| IFT57 | | | | | | | | | |
| IFT74 | | | | | | + | | | + |
| IFT80 | | | | | | | | | |
| IFT81 | | | | | | + | | | + |
| IFT88 | | | | | | | | | |
| IGBP1 | | | | | | | | | |
| IGBP1P1 | | | | | | | | | |
| IGDCC3 | | | | | | | | | |
| IGDCC4 | | | | | | | | | |
| IGF1 | + | | + | + | + | | + | | + |
| IGF1R | | | | + | | | | | + |
| IGF2 | | | | | | | | | |
| IGF2-AS | | | | | | | | | |
| IGF2BP1 | + | | | + | + | | | | + |
| IGF2BP2 | | | + | | + | | | | + |
| IGF2BP3 | | | | | | | | | |

| | | | | | | | | | |
|---|---|---|---|---|---|---|---|---|---|
| IGF2R | | | | | | | | | |
| IGFALS | | | | | | | | | |
| IGFBP1 | | | | | | | | | |
| IGFBP2 | | | | | | | | + | + |
| IGFBP3 | | | | | | | | | |
| IGFBP4 | | | | | + | | | | + |
| IGFBP5 | | | + | | | + | | | + |
| IGFBP6 | | | | | | | | | |
| Igfbp7 | | | | | | | | | |
| IGFBPL1 | | | | | | | | | |
| IGFL1 | | | | | | | | | |
| IGFL2 | | | | | | | | | |
| IGFL3 | | | | | | | | | |
| IGFL4 | | | | | | | | | |
| IGFN1 | | | | | | | | | |
| IgG-2a | | | | | | | | | |
| IGH | | | | | | | | | |
| Igh-6 | | | | | | | | | |
| Igh-Np | | | | | | | | | |
| IGHA1 | | | | | | | | | |
| IGHA2 | | | | | | | | | |
| IGHD | | | | | | | | | |
| IGHD6-19 | | | | | | | | | |
| IGHE | | | | | | | | | |
| IGHG1 | | | | | | | | | |
| IGHG2 | | | | | | | | | |
| IGHG3 | | | | | | | | | |
| IGHG4 | | | | | | | | | |
| IGHM | | | | | | | | | |
| IGHMBP2 | | | | | | | | | |
| IGHV1-18 | | | | | | | | | |
| IGHV1-2 | | | | | | | | | |
| IGHV1-24 | | | | | | | | | |
| IGHV1-3 | | | | | | | | | |
| IGHV1-45 | | | | | | | | | |
| IGHV1-46 | | | | | | | | | |
| IGHV1-69 | | | | | | | | | |
| IGHV1OR15-1 | | | | | | | | | |
| IGHV1OR15-9 | | | | | | | | | |
| IGHV2-5 | | | | | | | | | |
| IGHV3-13 | | | | | | | | | |
| IGHV3-15 | | | | | | | | | |
| IGHV3-23 | | | | | | | | | |
| IGHV3-30 | | | | | | | | | |
| IGHV3-33 | | | | | | | | | |
| IGHV3-48 | | | | | | | | | |
| IGHV3-49 | | | | | | | | | |
| IGHV3-53 | | | | | | | | | |
| IGHV3-64D | | | | | | | | | |
| IGHV3-66 | | | | | | | | | |
| IGHV3-7 | | | | | | | | | |
| IGHV3-72 | | | | | | | | | |
| IGHV3-73 | | | | | | | | | |

| | | | | | | | | | |
|---|---|---|---|---|---|---|---|---|---|
| IGHV3OR15-7 | | | | | | | | | |
| IGHV3OR16-8 | | | | | | | | | |
| IGHV4-31 | | | | | | | | | |
| IGHV4-39 | | | | | | | | | |
| IGHV4-4 | | | | | | | | | |
| IGHV4OR15-8 | | | | | | | | | |
| IGHV5-10-1 | | | | | | | | | |
| IGHV5-51 | | | | | | | | | |
| IGIP | | | | | | | | | |
| IGJ | | | | | | | | | |
| IGKC | | | | | | | | | |
| IGKV@ | | | | | | | | | |
| IGKV1-12 | | | | | | | | | |
| IGKV1-16 | | | | | | | | | |
| IGKV1-17 | | | | | | | | | |
| IGKV1-27 | | | | | | | | | |
| IGKV1-33 | | | | | | | | | |
| IGKV1-39 | | | | | | | | | |
| IGKV1-5 | | | | | | | | | |
| IGKV1-8 | | | | | | | | | |
| IGKV1D-12 | | | | | | | | | |
| IGKV1D-13 | | | | | | | | | |
| IGKV1D-16 | | | | | | | | | |
| IGKV1D-33 | | | | | | | | | |
| IGKV1D-39 | | | | | | | | | |
| IGKV2-24 | | | | | | | | | |
| IGKV2-28 | | | | | | | | | |
| IGKV2-29 | | | | | | | | | |
| IGKV2-30 | | | | | | | | | |
| IGKV2-40 | | | | | | | | | |
| IGKV2D-24 | | | | | | | | | |
| IGKV2D-26 | | | | | | | | | |
| IGKV2D-28 | | | | | | | | | |
| IGKV2D-29 | | | | | | | | | |
| IGKV2D-30 | | | | | | | | | |
| IGKV2D-40 | | | | | | | | | |
| IGKV3-11 | | | | | | | | | |
| IGKV3-15 | | | | | | | | | |
| IGKV3-20 | | | | | | | | | |
| IGKV3D-20 | | | | | | | | | |
| IGKV3D-7 | | | | | | | | | |
| IGKV3OR2-268 | | | | | | | | | |
| IGKV4-1 | | | | | | | | | |
| IGL | | | | | | | | | |
| IGLC1 | | | | | | | | | |
| IGLC2 | | | | | | | | | |
| IGLC3 | | | | | | | | | |
| IGLC6 | | | | | | | | | |
| IGLC7 | | | | | | | | | |
| IGLJ3 | | | | | | | | | |
| IGLL1 | | | | | | | | | |
| IGLL5 | | | | | | | | | |
| IGLV1-44 | | | | | | | | | |

| | | | | | | | | | |
|---|---|---|---|---|---|---|---|---|---|
| IGLV1-47 | | | | | | | | | |
| IGLV1-51 | | | | | | | | | |
| IGLV2-11 | | | | | | | | | |
| IGLV2-14 | | | | | | | | | |
| IGLV2-23 | | | | | | | | | |
| IGLV3-10 | | | | | | | | | |
| IGLV3-19 | | | | | | | | | |
| IGLV3-21 | | | | | | | | | |
| IGLV3-25 | | | | | | | | | |
| IGLV3-9 | | | | | | | | | |
| IGLV4-3 | | | | | | | | | |
| IGLV4-60 | | | | | | | | | |
| IGLV6-57 | | | | | | | | | |
| IGO1 | | | | | | | | | |
| IGSF1 | | | | | | | | | |
| IGSF10 | | | | | + | | | | + |
| IGSF11 | | | | | | | | | |
| IGSF21 | | | | | | | | | |
| IGSF3 | | | | | | | | | |
| IGSF5 | | | | | | | | | |
| IGSF6 | | | | | | | | | |
| IGSF8 | | | | | | + | | | + |
| IGSF9 | | | | | | | | | |
| IGSF9B | | | | | + | | | | + |
| IHH | | | | | | | | | |
| IK | | | | | | | | | |
| Ikbip | | | | + | | | | | + |
| IKBKAP | | | | | | | | | |
| IKBKB | | | | | | | | | |
| IKBKE | | | | | + | | | | + |
| IKBKG | | | | | | | | | |
| IKZF1 | | | | | | | | | |
| IKZF2 | | | | + | | | | | + |
| IKZF3 | | | | | | | | | |
| IKZF4 | | | | | | | | | |
| IKZF5 | | | | | | | | | |
| IL10 | | | | | | | | | |
| IL10RA | | | | | | | | | |
| IL10RB | | | | | | | | | |
| IL11 | | | | | | | | | |
| IL11RA | | | | | | | | | |
| IL12A | | | | | | | | | |
| IL12B | | | | | | | | | |
| IL12RB1 | | | | | | | | | |
| IL12RB2 | | | | | | | | | |
| IL13 | | | | | | | | | |
| IL13RA1 | | | | | | | | | |
| IL13RA2 | | | | | | | | | |
| IL15 | | | | | | | | | |
| IL15RA | | | | | | | | | |
| IL16 | | | | | | + | | | + |
| IL17A | | | | | | | | | |
| IL17B | | | | | | | | | |

| | | | | | | | | | |
|---|---|---|---|---|---|---|---|---|---|
| IL17D | | | | | | | | | |
| IL17F | | | | | | | | | |
| IL17RA | + | | | + | + | | | | + |
| IL17RB | | | | | | | | | |
| IL17RC | | | | | | | | | |
| IL17RD | | | | | | | | | |
| IL17RE | | | | | | | | | |
| IL17REL | | | | | | | | | |
| IL18 | | | | | | | | | |
| IL18BP | | | | | | | | | |
| IL18R1 | | | | | | | | | |
| IL18RAP | | | | | | | | | |
| IL19 | | | | | | | | | |
| IL1A | | | | | + | | | | + |
| IL1B | | | | | | | | | |
| IL1F10 | | | | | | | | | |
| IL1R1 | | | | | | | | | |
| Il1r2 | | | | | | | | | |
| IL1RAP | | + | | + | | + | | | + |
| IL1RAPL1 | | | | | | | | | |
| IL1RAPL2 | | | | | | | | | |
| IL1RL1 | | | | | | | | | |
| IL1RL2 | | | | | | | | | |
| IL1RN | | | | | | | | | |
| IL2 | | | | | | | | | |
| IL20 | | | | | | | | | |
| IL20RA | | | | | | | | | |
| IL20RB | + | | | | + | + | | | + |
| IL20RB-AS1 | | | | | | | | | |
| IL21 | | | | | | | | | |
| IL21R | | | | | | | + | | + |
| IL21R-AS1 | | | | | | | | | |
| IL22 | | | | | | | | | |
| IL22RA1 | | | | | | | | | |
| IL22RA2 | | | | | | | | | |
| IL23A | | | | | | | | | |
| IL23R | | | | | | | | | |
| IL24 | | | | | | | | | |
| IL25 | | | | | | | | | |
| IL26 | | | | | | | | | |
| IL27 | | | | | | | | | |
| IL27RA | | | | | | | | | |
| IL2RA | | | | | | | | | |
| IL2RB | | | | | | | | | |
| IL2RG | | | | | | | | | |
| IL3 | | | | | | | | | |
| IL31 | | | | | | | | | |
| IL31RA | | | | | | | | | |
| IL32 | | | | | | | | | |
| IL33 | | | | | | + | | | + |
| IL34 | | | | | | | | | |
| IL36A | | | | | | | | | |
| IL36B | | | | | | | | | |

| | | | | | | | | | |
|---|---|---|---|---|---|---|---|---|---|
| IL36G | | | | | | | | | |
| IL36RN | | | | | | | | | |
| IL37 | | | | | | | | | |
| IL3RA | | | | | | | | | |
| IL4 | | | | | | | | | |
| IL4I1 | | | | | | | | | |
| IL4R | | | | | | | | | |
| IL5 | | | | | | | | | |
| IL5RA | | | | | | | | | |
| IL6 | | | | | | | | | |
| IL6R | | | | | | | | | |
| IL6ST | | | | | | + | | | + |
| IL7 | + | | | + | + | | + | | + |
| IL7R | | | | | | | | | |
| IL9 | | | | | | | | | |
| IL9R | | | | | | | | | |
| ILDR1 | | | | | | | | | |
| ILDR2 | | | | | | | | | |
| ILF2 | | | | | | | | | |
| ILF3 | | | | | | | | | |
| ILK | | | | | | | | | |
| ILKAP | | | | | | | | | |
| ILM1 | | | | | | | | | |
| ILS1 | | | | | | | | | |
| ilv1 | | | | | | | | | |
| ILV2 | | | | | | | | | |
| ILV5 | | | | | | | | | |
| ILV6 | | | | | | | | | |
| ILVBL | | | | | | | | | |
| IMA1 | | | | | | | | | |
| IMD2 | | | | | | | | | |
| IMD3 | | | | | | | | | |
| IME2 | | | | | | | | | |
| IML3 | | | | | | | | | |
| IMMP1L | | | | | | | | | |
| IMMP2L | | | | | | | | | |
| IMMT | | | | | | | | | |
| imp1 | | | | | | | | | |
| imp2 | | | | | | | | | |
| IMP3 | | | | | | | | | |
| IMP4 | | | | | | | | | |
| IMPA1 | | | | | | | | | |
| IMPA2 | | | | | | | | | |
| IMPACT | | | | | + | | | | + |
| IMPAD1 | | | | | | | | | |
| IMPDH1 | | | | | | | | | |
| IMPDH2 | | | | | | | | | |
| IMPG1 | | | | | | | | | |
| INA | | | + | | + | | | | + |
| INADL | | | | | + | | | | + |
| INCA1 | | | | | | | | | |
| INCENP | | | | | | | | | |
| INE1 | | | | | | | | | |

| | | | | | | | | | |
|---|---|---|---|---|---|---|---|---|---|
| INF2 | | | | | | + | | | + |
| ING1 | | | | | | | | | |
| ING2 | | | + | | | | | | + |
| ING3 | | | + | | | + | | | + |
| ING4 | | | | | | | | | |
| ING5 | | | | | | + | | | + |
| INH1 | | | | | | | | | |
| INHA | | | | | | | | | |
| INHBA | + | | + | | + | | | | + |
| INHBB | + | | | + | + | | | | + |
| INHBE | | | | | | | | | |
| INIP | | | | | | | | | |
| INMT | | | | | | | | | |
| INO1 | | | | | | | | | |
| INO4 | | | | | | | | | |
| INO80 | | | | | | | | | |
| INO80B | | | | | | | | | |
| INO80B-WBP1 | | | | | | | | | |
| INO80C | | | | | | | | | |
| INO80D | | | | | | | | | |
| INO80E | | | | | | | | | |
| INPP1 | | | | | | + | | | + |
| INPP4A | | | | | | | | | |
| INPP4B | | | | | | | | | |
| INPP5A | | | + | | | | | | + |
| INPP5B | | | | | | | | | |
| INPP5D | | | | | | | | | |
| INPP5E | | | | | | | | | |
| INPP5F | | | | | + | | + | | + |
| INPP5J | | | | | | | | | |
| INPP5K | | | | | | + | | | + |
| INPPL1 | | | | | | | | | |
| INS | | | | | | | | | |
| INS-IGF2 | | | | | | | | | |
| Ins2 | | | | | | | | | |
| INSC | | | | | | | | | |
| INSIG1 | | | | | | | | | |
| INSIG2 | | | | | | | | | |
| INSL3 | | | | | | | | | |
| INSL4 | | | | | | | | | |
| INSL5 | | | | | | | | | |
| INSL6 | | | | | | | | | |
| INSM1 | | | | | | | | | |
| INSM2 | | | | | | | | | |
| INSR | | | | | | | | | |
| INSRR | | | | | | | | | |
| INTS1 | | | | | | | | | |
| INTS10 | | | | | | | | | |
| INTS12 | | | + | | | | | | + |
| INTS2 | | | | | | | | | |
| INTS3 | | | | | | | | | |
| INTS4 | | | | | | + | | | + |
| INTS4P2 | | | | | | | | | |

| | | | | | | | | | |
|---|---|---|---|---|---|---|---|---|---|
| INTS5 | | | | | | | | | |
| INTS6 | | | | | | | | | |
| INTS6-AS1 | | | | | | | | | |
| INTS7 | | | | | | | | | |
| INTS8 | | | | | | + | | | + |
| INTS9 | | | | | | | | | |
| INTU | | | | | | | | | |
| INVS | | | | | | | | | |
| IP6K1 | | | | | | | | | |
| IP6K2 | | | | | | | | | |
| IP6K3 | | | | | | | | | |
| IPCEF1 | | | | | | + | | | + |
| IPK1 | | | | | | | | | |
| IPL1 | | | | | | | | | |
| IPMK | | | | | | | | | |
| IPO11 | | | | | | | | | |
| IPO13 | | | | | | | | | |
| IPO4 | | | | | | | | | |
| IPO5 | + | | | | + | | | | + |
| IPO7 | | | | | | | | | |
| IPO8 | | + | | | | + | | | + |
| IPO9 | | | | + | | | | | + |
| IPP | | | | | | | | | |
| IPP1 | | | | | | | | | |
| IPPK | | | | | | | | | |
| IPW | | | | | | | | | |
| IQCA1 | | | | | | | | | |
| IQCA1P1 | | | | | | | | | |
| IQCB1 | | | | | + | | | | + |
| IQCC | | | | | | | | | |
| IQCD | | | | | | | | | |
| IQCE | | | | | | + | | | + |
| IQCF1 | | | | | | | | | |
| IQCF2 | | | | | | | | | |
| IQCF3 | | | | | | | | | |
| IQCF5 | | | | | | | | | |
| IQCG | | | | | | | | | |
| IQCH | | | | | | | | | |
| IQCJ | | | | | | | | | |
| IQCJ-SCHIP1 | | | | | | | | | |
| IQCK | | | | | | | | | |
| IQGAP1 | | | | | | | | | |
| IQGAP2 | | | + | | | | | | + |
| IQGAP3 | | | | | | | | | |
| IQSEC1 | | | | | | | | | |
| IQSEC2 | | + | | | | + | | | + |
| IQSEC3 | + | | | | + | | | | + |
| IQUB | | | | | | | | | |
| IRAK1 | | | | | | | | | |
| IRAK1BP1 | | | | | | | | | |
| IRAK2 | | | | | | | | | |
| IRAK3 | | | | | | | | | |
| IRAK4 | | | | | | | | | |

| | | | | | | | | | |
|---|---|---|---|---|---|---|---|---|---|
| IRC25 | | | | | | | | | |
| IRE1 | | | | | | | | | |
| IREB2 | | | | | | | | | |
| IRF1 | | | | | | | | | |
| IRF2 | | | | | | | | | |
| IRF2BP1 | | | | | | + | | | + |
| IRF2BP2 | | | + | | | | | | + |
| IRF2BPL | | | | | | | | | |
| IRF3 | | | | | | | | | |
| IRF4 | | | | | | | | | |
| IRF5 | | | | | | | | | |
| IRF6 | | | | | | | | | |
| IRF7 | | | | | | | | | |
| IRF8 | | | | | | | | | |
| IRF9 | | | | + | | | | | + |
| IRG1 | | | | | | | | | |
| IRGC | | | | | | | | | |
| IRGM | | | | | | | | | |
| IRGQ | | | | | | | | | |
| IRS1 | | | | | | | | | |
| IRS2 | + | | | | | | | | + |
| IRS4 | | | | | | | | | |
| IRX1 | | | | | | | | | |
| IRX2 | | | | | | | | | |
| IRX3 | | | | | | | | | |
| IRX4 | | | | | | | | | |
| IRX5 | | | | | | | | | |
| IRX6 | | | | | | | | | |
| ISA1 | | | | | | | | | |
| ISC10 | | | | | | | | | |
| ISCA1 | | | | | | | | | |
| ISCA2 | | | | | | | | | |
| ISCU | | | | | | | | | |
| ISG15 | | | | | | | | | |
| ISG20 | | | | | | | | | |
| ISG20L2 | | | | | | | | | |
| ish1 | | | | | | | | | |
| ISL1 | | | | | | | | | |
| ISL2 | | | | | | | | | |
| ISLR | | | | | | | | | |
| ISLR2 | | | | | | + | | | + |
| ISM1 | | | | | | | | | |
| ISM2 | | | | | | | | | |
| ISOC1 | | | + | | | + | | | + |
| ISOC2 | | | | | | | | | |
| ISPD | | | | | | | | | |
| IST1 | | | | | | | | | |
| IST2 | | | | | | | | | |
| ISU1 | | | | | | | | | |
| ISU2 | | | | | | | | | |
| ISW1 | | | | | | | | | |
| ISX | | | | | | | | | |
| ISY1 | | | + | | | | | | + |

| | | | | | | | | | |
|---|---|---|---|---|---|---|---|---|---|
| ISY1-RAB43 | | | | | | | | | |
| ISYNA1 | | | | | | | | | |
| ITCH | | | | + | | | | | + |
| ITFG1 | | | | | | | + | | + |
| ITFG2 | | | | | | | | | |
| ITFG3 | | | | | | | | | |
| ITGA1 | | | | | | | | | |
| ITGA10 | | | | | | + | | | + |
| ITGA11 | | | | | | | | | |
| ITGA2 | | | | | | | | | |
| ITGA2B | | | | | | | | | |
| ITGA3 | | | | | | | | | |
| ITGA4 | | | | | | | | | |
| ITGA5 | | | | | | | | | |
| ITGA6 | | | | | | | | | |
| itga6b | | | | | | | | | |
| ITGA7 | | | | | | | | | |
| ITGA8 | | | | | | + | | | + |
| ITGA9 | | | | | | | | | |
| ITGAD | | | | | | | | | |
| ITGAE | | | | | | | | | |
| ITGAL | | | | | | | | | |
| ITGAM | + | | | + | + | | + | | + |
| ITGAV | | | + | | + | | | | + |
| ITGAX | | | | | | | | | |
| ITGB1 | | | | | | | | | |
| ITGB1BP1 | | | | | | | | | |
| Itgb1bp2 | | | | | | | | | |
| ITGB2 | | | | | | | | | |
| ITGB3 | + | | | + | + | | + | | + |
| ITGB3BP | | + | + | + | | + | | | + |
| ITGB4 | | | | | | | | | |
| ITGB5 | | | | | | | | | |
| ITGB6 | + | | | + | + | | + | | + |
| ITGB7 | | | | | | | | | |
| ITGB8 | | | + | | | | | | + |
| ITGBL1 | | | | | | | | | |
| ITIH1 | | | | | | | | | |
| ITIH2 | | | | | | | | | |
| ITIH3 | | | | | | | | + | + |
| ITIH4 | | | | | | | | | |
| ITIH5 | + | | | + | + | | | | + |
| ITIH6 | | | | | | | | | |
| ITK | | | | | | | | | |
| ITLN1 | | | | | | | | | |
| ITLN2 | | | | | | | | | |
| ITM2A | | | | | | | | | |
| ITM2B | | | | | | | | | |
| ITM2C | | | | | | + | | | + |
| ITPA | | | | | | | | | |
| ITPK1 | | | | | | | | | |
| ITPKA | | | | | | + | | | + |
| ITPKB | | | | | | + | | | + |

| | | | | | | | | | |
|---|---|---|---|---|---|---|---|---|---|
| ITPKC | | | | | + | | | | + |
| ITPR1 | | | | | | | | | |
| Itpr2 | | | | | | | | | |
| ITPR3 | | | | | | | | | |
| ITPRIP | | | | | | | | | |
| ITPRIPL1 | + | | | + | + | | + | | + |
| ITPRIPL2 | + | | | + | + | | | | + |
| ITR1 | | | | | | | | | |
| ITSN1 | | | | | | | | | |
| ITSN2 | | | | | | | | | |
| ITT1 | | | | | | | | | |
| IVD | | | | | | + | | | + |
| IVL | | | | | | | | | |
| IVNS1ABP | + | | + | | + | | | | + |
| IWS1 | | | + | + | | | | | + |
| IXR1 | | | | | | | | | |
| IYD | | | | | | | | | |
| IZUMO1 | | | | | | | | | |
| IZUMO2 | | | | | | | | | |
| IZUMO3 | | | | | | | | | |
| IZUMO4 | | | | | | | | | |
| JADE1 | | | | | | | | | |
| JADE2 | | | | | | | | | |
| JADE3 | | | | | | | | | |
| JAG1 | | | | | | | | | |
| JAG2 | | | | | | | | | |
| JAGN1 | | | | | | | | | |
| JAK1 | | | | | | | | | |
| JAK2 | | | | | | | | | |
| JAK3 | | | | | | | | | |
| JAKMIP1 | | | | | | | | | |
| JAKMIP2 | | | | | | | | | |
| JAKMIP3 | | | | | | + | | | + |
| JAM2 | | | | | | | | | |
| JAM3 | | | | | | | | | |
| JARID2 | | | + | + | | | | | + |
| JAZ6 | | | | | | | | | |
| JAZF1 | | | | | | | | | |
| JDP2 | | | | | + | | | | + |
| JKAMP | | + | | | | + | | | + |
| JMJD1C | + | | + | | + | | | | + |
| JMJD4 | | | | | | | | | |
| JMJD6 | | | | | | | | | |
| JMJD7 | | | | | | | | | |
| JMJD7-PLA2G4B | | | | | | | | | |
| JMJD8 | | | | | | | | | |
| JMY | | | | | | | | | |
| JOSD1 | | | | | | + | | | + |
| JOSD2 | | | | | | | | | |
| JPH1 | | | | | | | | | |
| JPH2 | | | | | | | | | |
| JPH3 | | | | | | | | | |
| JPH4 | | | | | | + | | | + |

| | | | | | | | | | |
|---|---|---|---|---|---|---|---|---|---|
| JPX | | | | | | | | | |
| JRK | | | | | | | | | |
| JRKL | | | | | | | | | |
| JSRP1 | | | | | | | | | |
| JTB | | | | | | | | | |
| JUN | + | | + | | + | | | | + |
| JUNB | + | | | | + | | | | + |
| JUND | | | | | | | | | |
| JUP | | | | | + | | | | + |
| K07C5.3 | | | | | | | | | |
| K1 | | | | | | | | | |
| K2 | | | | | | | | | |
| K3 | | | | | | | | | |
| K3L | | | | | | | | | |
| K4 | | | | | | | | | |
| K4.1 | | | | | | | | | |
| K4.2 | | | | | | | | | |
| K5 | | | | | | | | | |
| K6 | | | | | | | | | |
| K8 | | | | | | | | | |
| K8.1 | | | | | | | | | |
| KAAG1 | | | | | | | | | |
| KAL1 | | | | | | | | | |
| KALRN | | | | | | | | | |
| KANK1 | | | | | | | | | |
| KANK2 | + | | | + | + | | | | + |
| KANK3 | | | | | | | | | |
| KANK4 | | | | | | | | | |
| KANSL1 | | | | | | | | | |
| KANSL1L | | | | | | | | | |
| KANSL2 | | | | | | | | | |
| KANSL3 | | | | | | | | | |
| KANTR | | | | | | | | | |
| KAP104 | | | | | | | | | |
| KAP123 | | | | | | | | | |
| KAP95 | | | | | | | | | |
| KAR2 | | | | | | | | | |
| KARS | | | | | | | | | |
| KAT2A | | | | | | | | | |
| KAT2B | | | | | | | | | |
| KAT5 | | | | | | | | | |
| KAT6A | | | | | | | | | |
| KAT6B | | | | | | | | | |
| KAT7 | | | | | | | | | |
| KAT8 | | | | | + | | | | + |
| KATNA1 | | | | | | | | | |
| KATNAL1 | | | | | | | | | |
| KATNAL2 | | | | | | | | | |
| KATNB1 | | | | | | | | | |
| KATNBL1 | | | | | | | | | |
| KAZALD1 | | | | | | | | | |
| KAZN | | | | | | + | | | + |
| Kb15 | | | | | | | | | |

| | | | | | | | | | |
|---|---|---|---|---|---|---|---|---|---|
| Kb23 | | | | | | | | | |
| KBTBD11 | | | | | | + | | | + |
| KBTBD11-OT1 | | | | | | | | | |
| KBTBD12 | | | | | | | | | |
| KBTBD13 | | | | | | | | | |
| KBTBD2 | | | | | | | | | |
| KBTBD3 | | | + | | | | | | + |
| KBTBD4 | | | | | | | | | |
| KBTBD6 | | | | | | | | | |
| KBTBD7 | | | | | | | | | |
| KBTBD8 | | | | | | | | | |
| KCMF1 | | | | | | | | | |
| KCNA1 | | | | + | + | | | | + |
| KCNA10 | | | | | | | | | |
| KCNA2 | | | | | | | | | |
| KCNA3 | | | | | | | | | |
| KCNA4 | + | | + | | + | | | | + |
| KCNA5 | | | | | | | | | |
| KCNA6 | | | + | | + | | | | + |
| KCNAB1 | | + | | | | + | | | + |
| KCNAB2 | | | | | | + | | | + |
| KCNAB3 | | | + | | | | | | + |
| KCNB1 | | | | | | | | | |
| KCNB2 | | | | | | + | | | + |
| KCNC1 | | | | | + | | | | + |
| KCNC2 | | | | | | | | | |
| KCNC3 | | | | | + | | | | + |
| KCNC4 | | | + | | | | | | + |
| KCND1 | | | | | | | | | |
| KCND2 | | | | | | | | | |
| KCND3 | | | | | | | | | |
| KCNE1 | | | | | | | | | |
| KCNE1L | | | | | | | | | |
| KCNE2 | | | | | | | | | |
| KCNE3 | | | | | | | | | |
| KCNE4 | | | | | | | | | |
| KCNF1 | + | | + | | + | | | | + |
| KCNG1 | | | | | | | | | |
| KCNG2 | | | | | | + | | | + |
| KCNG3 | | | | | | | | | |
| KCNG4 | | | | | | | | | |
| KCNH1 | | | | | | | | | |
| KCNH2 | | | | | | | | | |
| KCNH3 | | | | | | + | | | + |
| KCNH4 | | | | | | | | | |
| KCNH5 | | | | | | | | | |
| KCNH6 | | | | | | | | | |
| KCNH7 | | | | | | | | | |
| KCNH8 | | | | | | | | | |
| KCNIP1 | | | | | | | | | |
| KCNIP2 | | | | | | + | | | + |
| KCNIP3 | | | + | | | | | | + |
| KCNIP4 | | | | | | | | | |

| | | | | | | | | | |
|---|---|---|---|---|---|---|---|---|---|
| KCNJ1 | | | | | | | | | |
| KCNJ10 | | | | | | | | | |
| KCNJ11 | | | | | | | | | |
| KCNJ12 | + | | | + | + | | | | + |
| KCNJ15 | + | | | + | + | | + | | + |
| KCNJ16 | + | | | + | + | | | | + |
| KCNJ18 | | | | | | | | | |
| KCNJ2 | + | | | + | + | | + | | + |
| KCNJ3 | | | + | | | | | | + |
| KCNJ4 | | | + | | + | | | | + |
| KCNJ5 | | | | | | | | | |
| KCNJ6 | | | | | | | | | |
| KCNJ8 | | | | | | | | | |
| KCNJ9 | | | | | | + | | | + |
| KCNK1 | | | + | | + | | | | + |
| KCNK10 | | | + | | | | | | + |
| KCNK12 | | | | | | | | | |
| KCNK13 | | | | | | | | | |
| KCNK16 | | | | | | | | | |
| KCNK17 | | | | | | | | | |
| KCNK18 | | | | | | | | | |
| KCNK2 | + | | | + | + | | | | + |
| KCNK3 | | | | | | | | | |
| KCNK4 | | | | | | | | | |
| KCNK5 | | | | | | | | | |
| KCNK7 | | | | | | | | | |
| KCNK9 | | | | | | | | | |
| KCNMA1 | | | | | | | | | |
| KCNMB1 | + | | | + | + | | | | + |
| KCNMB2 | | | | | | | | | |
| KCNMB3 | | | | | | | | | |
| KCNMB4 | | | | | | | | | |
| KCNN1 | | | | | | | | | |
| KCNN2 | | | + | | | | | | + |
| KCNN3 | | | | | | + | | | + |
| KCNN4 | | | | | | | | | |
| KCNQ1 | | | | | | | | | |
| KCNQ1OT1 | | | | | | | | | |
| KCNQ2 | | | | | | + | | | + |
| KCNQ3 | | | | | | + | | | + |
| KCNQ4 | | | | | | | | | |
| KCNQ5 | | | | | | | | | |
| KCNRG | | | | | | | | | |
| KCNS1 | | | | | | | | | |
| KCNS2 | | | + | | | | | | + |
| KCNS3 | | | | | | | | | |
| KCNT2 | | | | | | + | | | + |
| KCNV1 | | | | | | | | | |
| KCNV2 | | | | | | | | | |
| KCS5 | | | | | | | | | |
| KCS6 | | | | | | | | | |
| KCTD1 | | | | | | | | | |
| KCTD10 | | | | | | | + | | + |

| | | | | | | | | | |
|---|---|---|---|---|---|---|---|---|---|
| KCTD11 | | | | | | | | | |
| KCTD12 | + | | | | + | | | | + |
| KCTD13 | | | | | | + | | | + |
| KCTD14 | + | | | + | + | | + | | + |
| KCTD15 | | | | | | | | | |
| KCTD16 | | | | | | | | | |
| KCTD17 | | | | | | | | | |
| KCTD18 | | | | | | | | | |
| KCTD19 | | | | | | | | | |
| KCTD2 | | | | | | | | | |
| KCTD20 | | | | | | | | | |
| KCTD21 | | | | | | | | | |
| KCTD3 | | | | | | | | | |
| KCTD4 | | | | | | | | | |
| KCTD5 | | | | | | | | | |
| KCTD6 | | | | | | | | | |
| KCTD7 | | | | | | | | | |
| KCTD8 | | | | | | | | | |
| KCTD9 | | | | | | | | | |
| KCTD9P2 | | | | | | | | | |
| KCTD9P3 | | | | | | | | | |
| KDELC1 | | | | | | | | | |
| KDELC2 | | | | | | | | | |
| KDELR1 | | | | | | | | | |
| KDELR2 | + | | | + | + | | + | | + |
| KDELR3 | | | | | | | | | |
| KDF1 | | | | | | | | | |
| KDM1A | | | | | | | | | |
| KDM1B | | | | | | | | | |
| KDM2A | | | | | | | | | |
| KDM2B | | | | | | | | | |
| KDM3A | | | | | | | | | |
| KDM3B | | | | | | | | | |
| KDM4A | | | | | | | | | |
| KDM4A-AS1 | | | | | | | | | |
| KDM4B | | | | | | | | | |
| KDM4C | | + | | | | + | | | + |
| KDM4D | | | | | | | | | |
| KDM5A | | | | | + | | | | + |
| KDM5B | | | | | | | | | |
| KDM5C | | | | | | | | | |
| KDM5D | | | | | | | | | |
| KDM6A | | | | | | | | | |
| KDM6B | + | | | | + | + | | | + |
| KDM7A | + | | | | | | | | + |
| KDM8 | | | | | | | | | |
| KDR | | | | | | | | | |
| KDSR | | + | | + | | + | | | + |
| KEAP1 | | | + | | | | | | + |
| KEL | | | | | | | | | |
| KERA | | | | | | | | | |
| KGD1 | | | | | | | | | |
| KGD2 | | | | | | | | | |

| | | | | | | | | | |
|---|---|---|---|---|---|---|---|---|---|
| KHDC1 | | | | | | | | | |
| KHDC3L | | | | | | | | | |
| KHDRBS1 | | | | | | | | | |
| KHDRBS2 | | | | | | | | | |
| KHDRBS3 | | | | | | | | | |
| KHK | | | | | | | | | |
| KHNYN | | | | | | | | | |
| KHSRP | | | | | | | | | |
| KIAA0020 | | | | | | | | | |
| KIAA0040 | | | | | | | | | |
| KIAA0087 | | | | | | | | | |
| KIAA0100 | | | | | | | | | |
| KIAA0101 | | | | | | | | | |
| KIAA0141 | | | | | | | | | |
| KIAA0195 | | | | | | | | | |
| KIAA0196 | | | | | | | | | |
| KIAA0226 | | | | | | | | | |
| KIAA0226L | | | | | | | | | |
| KIAA0232 | | | | | | | | | |
| KIAA0319 | | | | | | | | | |
| KIAA0319L | | | | | | | | | |
| KIAA0355 | | | | | | | | | |
| KIAA0368 | | | | | | | | | |
| KIAA0391 | | | | | | | | | |
| KIAA0408 | | | | | | | | | |
| KIAA0430 | | | | | | | | | |
| KIAA0509 | | | | | | | | | |
| KIAA0513 | | | | | | | | | |
| KIAA0556 | | | | | | | | | |
| KIAA0586 | | | | | | | | | |
| KIAA0753 | | | | | | | | | |
| KIAA0754 | | | | | | | | | |
| KIAA0825 | | | | | | | | | |
| KIAA0895 | | | | | | | | | |
| KIAA0895L | | | | | | | | | |
| KIAA0907 | | | | | | | | | |
| KIAA0922 | | | | | | | | | |
| KIAA0930 | | | | | | | | | |
| KIAA1024 | | | | | | | | | |
| KIAA1033 | | | | | | | | | |
| KIAA1045 | | | | | | | | | |
| KIAA1107 | | | | | | | | | |
| KIAA1109 | | | | | | | | | |
| KIAA1143 | | | | | | | | | |
| KIAA1147 | | | | | | | | | |
| KIAA1161 | | | | | | | | | |
| KIAA1191 | | | | | | | | | |
| KIAA1210 | | | | | | | | | |
| KIAA1211 | | | | | | | | | |
| KIAA1211L | | | | | | | | | |
| KIAA1217 | | | | | | | | | |
| KIAA1244 | | | | | | | | | |
| KIAA1257 | | | | | | | | | |

| | | | | | | | | | |
|---|---|---|---|---|---|---|---|---|---|
| KIAA1279 | | | | | | | | | |
| KIAA1324 | | | | | | | | | |
| KIAA1324L | | | | | | | | | |
| KIAA1328 | | | | | | | | | |
| KIAA1377 | | | | | | | | | |
| KIAA1407 | | | | | | | | | |
| KIAA1429 | | | | | | | | | |
| KIAA1456 | | | | | | | | | |
| KIAA1462 | | | | | | | | | |
| KIAA1467 | | | | | | | | | |
| KIAA1468 | | | | | | | | | |
| KIAA1522 | | | | | | | | | |
| KIAA1524 | | | | | | | | | |
| KIAA1549 | | | | | | | | | |
| KIAA1549L | | | | | | | | | |
| KIAA1551 | | | | | | | | | |
| KIAA1586 | | | | | | | | | |
| KIAA1598 | | | | | | | | | |
| KIAA1614 | | | | | | | | | |
| KIAA1644 | | | | | | | | | |
| KIAA1671 | | | | | | | | | |
| KIAA1683 | | | | | | | | | |
| KIAA1715 | | | | | | | | | |
| KIAA1731NL | | | | | | | | | |
| KIAA1804 | | | | | | | | | |
| KIAA1841 | | | | | | | | | |
| KIAA1875 | | | | | | | | | |
| KIAA1919 | | | | | | | | | |
| KIAA1958 | | | | | | | | | |
| KIAA2012 | | | | | | | | | |
| KIAA2013 | | | | | | | | | |
| KIAA2018 | | | | | | | | | |
| KIAA2022 | | | | | | | | | |
| KIAA2026 | | | | | | | | | |
| KIDINS220 | | | | | | | | | |
| KIF11 | | | | | | | | | |
| KIF12 | | | | | | | | | |
| KIF13A | | | | | | | | | |
| KIF13B | | | | | | + | | | + |
| KIF14 | | | | | | | | | |
| KIF15 | | | | | | | | | |
| KIF16B | | | | | | | | | |
| KIF17 | | | | | | | | | |
| KIF18A | | | + | + | | | | | + |
| Kif18b | | | | | | | | | |
| KIF19 | | | | | | | | | |
| Kif19a | | | | | | | | | |
| KIF1A | + | | | | + | | | | + |
| KIF1B | | | + | | | | | | + |
| KIF1C | | | | | | | | | |
| Kif20a | | | | | | | | | |
| KIF20B | | | | | | | | | |
| KIF21A | | | | | | | | | |

| | | | | | | | | | |
|---|---|---|---|---|---|---|---|---|---|
| KIF21B | | | | | | | | | |
| KIF22 | | | | | | | | | |
| KIF23 | | | | | | | | | |
| KIF24 | | | | | | | | | |
| KIF25 | | | | | | | | | |
| KIF25-AS1 | | | | | | | | | |
| KIF26A | | | | | | | | | |
| KIF26B | | | | | | + | | | + |
| KIF27 | | | | | | | | | |
| KIF2A | | | | | + | | | | + |
| KIF2B | | | | | | | | | |
| KIF2C | | | | | | | | | |
| KIF3A | | | | | | | | | |
| KIF3B | | | | | | | | | |
| KIF3C | | | | | | + | | | + |
| Kif4 | | | | | | | | | |
| KIF4A | | | | | | | | | |
| KIF4B | | | | | | | | | |
| KIF5A | | | | | + | | | | + |
| KIF5B | | | | | | | | | |
| KIF5C | | | | | | | | | |
| KIF6 | | | | | | | | | |
| KIF7 | | | | | | | | | |
| KIF9 | | | | | | | | | |
| KIFAP3 | | + | | | | + | | | + |
| KIFC1 | | | | | | | | | |
| KIFC2 | | | | | | | | | |
| KIFC3 | | | | | | | | | |
| Kifc5b | | | | | | | | | |
| KIN | | | | | | | | | |
| KIN2 | | | | | | | | | |
| KIN3 | | | | | | | | | |
| KIR2DL1 | | | | | | | | | |
| KIR2DL2 | | | | | | | | | |
| KIR2DL3 | | | | | | | | | |
| KIR2DL4 | | | | | | | | | |
| KIR2DL5A | | | | | | | | | |
| KIR2DS1 | | | | | | | | | |
| KIR2DS2 | | | | | | | | | |
| KIR2DS3 | | | | | | | | | |
| KIR2DS5 | | | | | | | | | |
| KIR3DL1 | | | | | | | | | |
| KIR3DL2 | | | | | | | | | |
| KIR3DL3 | | | | | | | | | |
| KIR3DP1 | | | | | | | | | |
| KIR3DS1 | | | | | | | | | |
| KIR3DX1 | | | | | | | | | |
| KIRREL | | | | | | | | | |
| KIRREL2 | | | | | | | | | |
| KIRREL3 | | | | | | | | | |
| KIRREL3-AS3 | | | | | | | | | |
| KISS1 | | | | | | | | | |
| KISS1R | | | | | | | | | |

| | | | | | | | | | |
|---|---|---|---|---|---|---|---|---|---|
| KIT | | | | | | | | | |
| KITLG | | | | | | | | | |
| KIZ | | | | | | | | | |
| KL | | | | + | | | | | + |
| KLB | | | | | | | | | |
| KLC1 | | | | | | | | | |
| KLC2 | | | | | | | | | |
| KLC3 | | | | | | | | | |
| KLC4 | | | | | | | | | |
| KLF1 | | | | | | | | | |
| KLF10 | | | | | | | | | |
| KLF11 | + | | | + | + | | | | + |
| KLF12 | | | | | | | | | |
| KLF13 | | + | | | | + | | + | + |
| KLF15 | | | | | | | | | |
| KLF16 | | | | | | | | | |
| KLF17 | | | | | + | | | | + |
| KLF2 | | | | | | | | | |
| KLF3 | | | | | | + | | | + |
| KLF4 | + | | + | | + | | | | + |
| KLF5 | | | + | | | | | | + |
| KLF6 | | | | | + | | | | + |
| KLF7 | | | | | | | | | |
| KLF8 | | | | | | | | | |
| KLF9 | | | + | | | | | | + |
| KLHDC1 | | | | | | | | | |
| KLHDC10 | | | | | | | | | |
| KLHDC2 | | | | | | + | | | + |
| KLHDC3 | | | | | | | | | |
| KLHDC4 | | | | | | | | | |
| KLHDC7A | | | | | | | | + | + |
| KLHDC7B | | | | | | | | | |
| KLHDC8A | | | | | | | | | |
| KLHDC8B | | | | | | | | | |
| KLHDC9 | | | | | | | | | |
| KLHL1 | | | | | | | | | |
| KLHL10 | | | | | | | | | |
| KLHL11 | | | | | | | | | |
| KLHL12 | | | | | | | | | |
| KLHL13 | | | | | | | | | |
| KLHL14 | | | | | | | | | |
| KLHL15 | | | | | | | | | |
| KLHL17 | | | | | | | | | |
| KLHL18 | | | | | | | | | |
| KLHL2 | | | | | | | | | |
| KLHL20 | | | | | | | | | |
| KLHL21 | | | | | | | | | |
| KLHL22 | | | | | | | | | |
| KLHL23 | | | | | | | | | |
| KLHL24 | | | | | | | | | |
| KLHL25 | | | | | | | | | |
| KLHL26 | | | | | | | | | |
| KLHL28 | | | | | | | | | |

| | | | | | | | | | |
|---|---|---|---|---|---|---|---|---|---|
| KLHL29 | | | | | | | | | |
| KLHL3 | | | | | | + | | | + |
| KLHL30 | | | | | | | | | |
| KLHL30-AS1 | | | | | | | | | |
| KLHL32 | | | | | | | | | |
| KLHL33 | | | | | | | | | |
| KLHL34 | | | | | | | | | |
| KLHL35 | | | | | | | | | |
| KLHL36 | | | | | | | | | |
| KLHL38 | | | | | | | | | |
| KLHL4 | | | | | | | | | |
| KLHL40 | | | | | | | | | |
| KLHL41 | | | | | | | | | |
| KLHL42 | | | | | | | | | |
| KLHL5 | | | | | | | | | |
| KLHL6 | | | | | | | | | |
| KLHL7 | | | | | | | | | |
| KLHL8 | | | | | | | | | |
| KLHL9 | | | | | | | | | |
| KLK1 | | | | | | | | | |
| KLK10 | | + | | | | + | | | + |
| KLK11 | | + | | | | + | | | + |
| KLK12 | | + | | | | + | | | + |
| KLK13 | | | | | | | | | |
| KLK14 | | | | | | | | | |
| KLK15 | | | | | | | | | |
| KLK2 | | | | | | | | | |
| KLK3 | | | | | | | | | |
| KLK4 | | | | | | | | | |
| KLK5 | | | | | | | | | |
| KLK6 | | | | | | | | | |
| KLK7 | | | | | | | | | |
| KLK8 | | | | | | | | | |
| KLK9 | | | | | | | | | |
| KLKB1 | | | | | | | | | |
| KLKP1 | | | | | | | | | |
| KLLN | | | | | | | | | |
| KLRAP1 | | | | | | | | | |
| KLRB1 | | | | | | | | | |
| KLRC1 | | | | | | | | | |
| KLRC2 | | | | | | | | | |
| KLRC3 | | | | | | | | | |
| KLRC4 | | | | | | | | | |
| KLRD1 | | | | | | | | | |
| KLRF1 | | | | | | | | | |
| KLRG1 | | | | | | | | | |
| KLRG2 | | | | | | | | | |
| KLRK1 | | | | | | | | | |
| KMO | | | | | | | | | |
| KMT2A | | | | | | | | | |
| KMT2B | | | | | | | | | |
| KMT2C | | | | | | | | | |
| KMT2D | | | | | | | | | |

| | | | | | | | | | |
|---|---|---|---|---|---|---|---|---|---|
| KMT2E | | | | | | | | | |
| KNDC1 | | | | | | + | | | + |
| KNG1 | | | | | | | | | |
| KNOP1 | | | | | | | | | |
| Knstrn | | | | | | | | | |
| KNTC1 | | | | | | | | | |
| Kpna1 | + | | | | + | | | | + |
| KPNA2 | | | | | | | | | |
| KPNA3 | | | | | | | | | |
| KPNA4 | | | | | | | | | |
| KPNA5 | | | | | | | | | |
| KPNA6 | | | + | | | | | | + |
| KPNA7 | | | | | | | | | |
| KPNB1 | | | | | | | | | |
| kpnb2 | | | | | | | | | |
| KPRP | | | | | | | | | |
| KPTN | | | | | | | | | |
| KRAS | + | | | | | | | | + |
| KRBA1 | | | | | | + | | | + |
| KRBA2 | | | | | | | | | |
| KRBOX4 | | | | | | | | | |
| KRCC1 | | | | | | | | | |
| KRE2 | | | | | | | | | |
| KRE6 | | | | | | | | | |
| KREMEN1 | | | | | | | | | |
| KREMEN2 | | | | | | | | | |
| KRI1 | | | | | | | | | |
| KRIT1 | | | | | | | | | |
| KRR1 | | | | | | | | | |
| KRS1 | | | | | | | | | |
| KRT1 | | | | | | | | | |
| KRT10 | | | | | | | | | |
| KRT12 | | | | | | + | | | + |
| KRT13 | | | | | | | | | |
| KRT14 | | | | | | | | | |
| KRT15 | | | | | | | | | |
| KRT16 | | | | | | | | | |
| KRT17 | | | | | | | | | |
| KRT18 | | | | | | | | | |
| KRT18P11 | | | | | | | | | |
| KRT18P15 | | | | | | | | | |
| KRT18P31 | | | | | | | | | |
| KRT19 | | | | | | | | | |
| KRT19P3 | | | | | | | | | |
| KRT2 | | | | | | | | | |
| KRT20 | | + | + | | | + | | | + |
| KRT222 | | | | | | | | | |
| KRT23 | | | | | | | | | |
| KRT24 | | | | | | | | | |
| KRT25 | | | | | | | | | |
| KRT26 | | | | | | | | | |
| KRT27 | | | | | | | | | |
| KRT28 | | | | | | | | | |

| | | | | | | | | | |
|---|---|---|---|---|---|---|---|---|---|
| KRT3 | | | | | | | | | |
| KRT31 | | | | | | | | | |
| KRT32 | | | | | | | | | |
| KRT33A | | | | | | | | | |
| KRT33B | | | | | | | | | |
| KRT34 | | | | | | | | | |
| KRT35 | | | | | | | | | |
| KRT36 | | | | | | | | | |
| KRT37 | | | | | | | | | |
| KRT38 | | | | | | | | | |
| KRT39 | | | | | | | | | |
| KRT4 | | | | | | | | | |
| KRT40 | | | | | | | | | |
| KRT43P | | | | | | | | | |
| KRT5 | + | | | + | + | | | | + |
| KRT6A | | | | | | | | | |
| KRT6B | | | | | | | | | |
| KRT6C | | | | | | | | | |
| KRT7 | | | | | | | | | |
| KRT71 | | | | | | | | | |
| KRT72 | | | | | | | | | |
| KRT73 | | + | | | | + | | | + |
| KRT74 | | | | | | | | | |
| KRT75 | | | | | | | | | |
| KRT76 | | | | | | | | | |
| KRT77 | | | | | | | | | |
| KRT78 | | | | | | | | | |
| KRT79 | | | | | | | | | |
| KRT8 | | | | | | | | | |
| KRT80 | | | | | | | | | |
| KRT81 | | | | | | | | | |
| KRT82 | | | | | | | | | |
| KRT83 | | | | | | | | | |
| KRT84 | | | | | | | | | |
| KRT85 | | | | | | | | | |
| KRT86 | | | | | | | | | |
| KRT87P | | | | | | | | | |
| KRT8P12 | | | | | | | | | |
| KRT8P3 | | | | | | | | | |
| KRT8P34 | | | | | | | | | |
| KRT8P39 | | | | | | | | | |
| KRT8P48 | | | | | | | | | |
| KRT9 | | | | | | | | | |
| KRTAP1-1 | | | | | | | | | |
| KRTAP1-3 | | | | | | | | | |
| KRTAP1-5 | | | | | | | | | |
| KRTAP10-1 | | | | | | | | | |
| KRTAP10-10 | | | | | | | | | |
| KRTAP10-11 | | | | | | | | | |
| KRTAP10-2 | | | | | | | | | |
| KRTAP10-3 | | | | | | | | | |
| KRTAP10-4 | | | | | | | | | |
| KRTAP10-5 | | | | | | | | | |

| | | | | | | | | | |
|---|---|---|---|---|---|---|---|---|---|
| KRTAP10-6 | | | | | | | | | |
| KRTAP10-7 | | | | | | | | | |
| KRTAP10-8 | | | | | | | | | |
| KRTAP10-9 | | | | | | | | | |
| KRTAP11-1 | | | | | | | | | |
| KRTAP12-1 | | | | | | | | | |
| KRTAP12-2 | | | | | | | | | |
| KRTAP12-3 | | | | | | | | | |
| KRTAP12-4 | | | | | | | | | |
| KRTAP13-1 | | | | | | | | | |
| KRTAP13-2 | | | | | | | | | |
| KRTAP13-3 | | | | | | | | | |
| KRTAP13-4 | | | | | | | | | |
| KRTAP15-1 | | | | | | | | | |
| KRTAP16-1 | | | | | | | | | |
| KRTAP17-1 | | | | | | | | | |
| KRTAP19-1 | | | | | | | | | |
| KRTAP19-2 | | | | | | | | | |
| KRTAP19-3 | | | | | | | | | |
| KRTAP19-4 | | | | | | | | | |
| KRTAP19-5 | | | | | | | | | |
| KRTAP19-6 | | | | | | | | | |
| KRTAP19-7 | | | | | | | | | |
| KRTAP2-2 | | | | | | | | | |
| KRTAP2-3 | | | | | | | | | |
| KRTAP2-4 | | | | | | | | | |
| KRTAP21-2 | | | | | | | | | |
| KRTAP22-1 | | | | | | | | | |
| KRTAP23-1 | | | | | | | | | |
| KRTAP26-1 | | | | | | | | | |
| KRTAP3-1 | | | | | | | | | |
| KRTAP3-2 | | | | | | | | | |
| KRTAP3-3 | | | | | | | | | |
| KRTAP4-1 | | | | | | | | | |
| KRTAP4-11 | | | | | | | | | |
| KRTAP4-12 | | | | | | | | | |
| KRTAP4-2 | | | | | | | | | |
| KRTAP4-4 | | | | | | | | | |
| KRTAP4-5 | | | | | | | | | |
| KRTAP4-7 | | | | | | | | | |
| KRTAP5-1 | | | | | | | | | |
| KRTAP5-10 | | | | | | | | | |
| KRTAP5-11 | | | | | | | | | |
| KRTAP5-2 | | | | | | | | | |
| KRTAP5-3 | | | | | | | | | |
| KRTAP5-4 | | | | | | | | | |
| KRTAP5-6 | | | | | | | | | |
| KRTAP5-7 | | | | | | | | | |
| KRTAP5-8 | | | | | | | | | |
| KRTAP5-9 | | | | | | | | | |
| KRTAP6-1 | | | | | | | | | |
| KRTAP6-2 | | | | | | | | | |
| KRTAP6-3 | | | | | | | | | |

| | | | | | | | | | |
|---|---|---|---|---|---|---|---|---|---|
| KRTAP7-1 | | | | | | | | | |
| KRTAP8-1 | | | | | | | | | |
| KRTAP9-1 | | | | | | | | | |
| KRTAP9-2 | | | | | | | | | |
| KRTAP9-3 | | | | | | | | | |
| KRTAP9-4 | | | | | | | | | |
| KRTAP9-7 | | | | | | | | | |
| KRTAP9-8 | | | | | | | | | |
| KRTAP9-9 | | | | | | | | | |
| KRTCAP2 | | + | | | | + | | | + |
| KRTCAP3 | | | | | | | | | |
| Ksr1 | | | | | | | | | |
| KSR2 | | | | | | + | | | + |
| KSS1 | | | | | | | | | |
| KTI12 | | | | | | | | | |
| KTN1 | | | | | | | | | |
| KTR1 | | | | | | | | | |
| KTR3 | | | | | | | | | |
| KTR4 | | | | | | | | | |
| KXD1 | | | | | | | | | |
| KY | | | | | | | | | |
| KYNU | + | | | + | + | | + | | + |
| L1CAM | | | | | | + | | | + |
| L1RE1 | | | | | | | | | |
| L1TD1 | | | | | | | | | |
| L2 | | | | | | | | | |
| L2HGDH | | | | | | | | | |
| L3HYPDH | | | | | | | | | |
| L3MBTL1 | | | | | | | | | |
| L3MBTL2 | | | | | | | | | |
| L3MBTL3 | | | | | | | | | |
| L3MBTL4 | | | | | | | | | |
| LAC1 | | | | | | | | | |
| LACC1 | | | | | | | | | |
| LACE1 | | | | | | | | | |
| LACRT | | | | | | | | | |
| LACTB | | | | | | | | | |
| LACTB2 | | | | | | | | | |
| LACTBL1 | | | | | | | | | |
| LAD1 | | | | | | | | | |
| LAG1 | | | | | | | | | |
| LAG2 | | | | | | | | | |
| LAG3 | + | | | + | + | | + | | + |
| LAGE3 | | | | | | | | | |
| LAIR1 | | | | | | | | | |
| LAIR2 | | | | | | | | | |
| LALBA | | | | | | | | | |
| LAMA1 | | | | | | | | | |
| LAMA2 | | | | | | | | | |
| LAMA3 | | | | | | | | | |
| LAMA4 | | | | | | | | | |
| LAMA5 | | | | | | | | | |
| LAMB1 | | | | | | | | | |

| | | | | | | | | | |
|---|---|---|---|---|---|---|---|---|---|
| LAMB2 | | | | | | | | | |
| LAMB3 | | | | | | | | | |
| LAMB4 | | | | | | | | | |
| LAMC1 | | | | | | | | | |
| LAMC2 | | | | | | | | | |
| LAMC3 | | | | | | | | | |
| LAMP1 | | | | | | | | | |
| LAMP2 | | | + | | | | | | + |
| LAMP3 | | | | | | | | | |
| LAMP5 | | | | | | + | | | + |
| LAMTOR1 | | | | | | | | | |
| LAMTOR2 | + | | | | | | | | + |
| LAMTOR3 | | | + | | | | | | + |
| LAMTOR4 | | | | | | | | | |
| LAMTOR5 | | | | | | | | | |
| LANCL1 | | | | | | | | | |
| LANCL1-AS1 | | | | | | | | | |
| LANCL2 | | | | | | | | | |
| LANCL3 | | | | | | | | | |
| lap | | | | | | | | | |
| LAP2 | | | | | | | | | |
| LAP3 | | | | | | | | | |
| LAPTM4A | | | | | | | | | |
| LAPTM4B | | | | | | | | | |
| LAPTM5 | | | | | | | | | |
| LARGE | | | | | | | | | |
| LARP1 | | | | | | | | | |
| LARP1B | | | | | | | | | |
| LARP4 | | | + | | + | | | | + |
| LARP4B | | | | | | | | | |
| LARP4P | | | | | | | | | |
| LARP6 | | | | | + | | | | + |
| LARP7 | | | | | | | | | |
| LARS | | | | | | | | | |
| LARS2 | | | | | | | | | |
| LAS17 | | | | | | | | | |
| LAS1L | | | | | | | | | |
| LASP1 | | | + | | | + | | | + |
| LAT | | | | | | | | | |
| lat1 | | | | | | | | | |
| LAT2 | | | | | | | | | |
| LATS1 | | | | | | | | | |
| LATS2 | | | | | | | | | |
| LAX1 | | | | | | | | | |
| LAYN | | | | | | | | | |
| LBH | + | | + | + | + | | + | | + |
| LBHD1 | | | | | | | | | |
| LBP | | | | | | | | | |
| LBR | | | | | | | | | |
| LBX1 | | | | | | | | | |
| LBX1-AS1 | | | | | | | | | |
| LBX2 | | | | | | | | | |
| LCA10 | | | | | | | | | |

| | | | | | | | | | |
|---|---|---|---|---|---|---|---|---|---|
| LCA5 | | | | | | | | | |
| LCA5L | | | | | | | | | |
| LCAT | | | | | | | | | |
| LCB1 | | | | | | | | | |
| LCB3 | | | | | | | | | |
| LCE1A | | | | | | | | | |
| LCE1B | | | | | | | | | |
| LCE1C | | | | | | | | | |
| LCE1D | | | | | | | | | |
| LCE1E | | | | | | | | | |
| LCE1F | | | | | | | | | |
| LCE2A | | | | | | | | | |
| LCE2B | | | | | | | | | |
| LCE2C | | | | | | | | | |
| LCE2D | | | | | | | | | |
| LCE3A | | | | | | | | | |
| LCE3B | | | | | | | | | |
| LCE3C | | | | | | | | | |
| LCE3D | | | | | | | | | |
| LCE3E | | | | | | | | | |
| LCE4A | | | | | | | | | |
| LCE5A | | | | | | | | | |
| lcf1 | | | | | | | | | |
| lcf2 | | | | | | | | | |
| LCK | | | | | | | | | |
| LCLAT1 | | | | | | | | | |
| LCMT1 | | | | | | | | | |
| LCMT2 | | | | | + | | | | + |
| LCN1 | | | | | | | | | |
| LCN10 | | | | | | | | | |
| LCN12 | | | | | | | | | |
| LCN15 | | | | | | | | | |
| LCN1P1 | | | | | | | | | |
| LCN2 | | | | | | | | | |
| LCN6 | | | | | | | | | |
| LCN8 | | | | | | | | | |
| LCN9 | | | | | | | | | |
| LCNL1 | | | | | | | | | |
| LCOR | | | | | | | | | |
| LCORL | | | | | | | | | |
| LCP1 | | | | + | | | | | + |
| LCP2 | | | | | | | | | |
| LCT | + | | | | + | | | | + |
| LCTL | | | | | | | | | |
| LDB1 | | | | | | | | | |
| LDB17 | | | | | | | | | |
| LDB18 | | | | | | | | | |
| LDB19 | | | | | | | | | |
| LDB2 | | | | | | | | | |
| LDB3 | | | | | | | | | |
| LDHA | | | | | | + | | | + |
| LDHAL6A | | | | | | | | | |
| LDHAL6B | | | | | | | | | |

| | | | | | | | | | |
|---|---|---|---|---|---|---|---|---|---|
| LDHB | | | | | | + | | | + |
| LDHC | | | | | | | | | |
| LDHD | | | | | | | | | |
| LDLR | | | | | | | | | |
| LDLRAD1 | | | | | | | | | |
| LDLRAD2 | | | | | | | | | |
| LDLRAD3 | | | | | | | | | |
| LDLRAD4 | | | | | | | | | |
| LDLRAP1 | | | | | | | | | |
| LDOC1 | | | | | | | | | |
| LDOC1L | | | | | | | | | |
| LEAP2 | | | | | | | | | |
| LECT1 | | | | | | | | | |
| LECT2 | | | | | | | | | |
| LEF1 | + | | | + | + | | | | + |
| LEFTY1 | | | | | | | | | |
| LEFTY2 | | | | | | | | | |
| LEKR1 | | | | | | | | | |
| LELP1 | | | | | | | | | |
| LEMD1 | | | | | | | | | |
| LEMD2 | | | | | | | | | |
| LEMD3 | | | | | | | | | |
| LENEP | | | | | | | | | |
| LENG1 | | | + | | | | | | + |
| LENG8 | | | | | | | | | |
| LENG9 | | | | | | | | | |
| LEO1 | | | | | | | | | |
| LEP | | | | | | | | | |
| LEPR | | | | | | | | | |
| LEPREL2 | | | | | | | | | |
| LEPROT | | + | | | | + | | | + |
| LEPROTL1 | | | | | | | | | |
| LETM1 | | | | | | | | | |
| LETM2 | | | | | | | | | |
| LETMD1 | | | | | | | | | |
| LEU1 | | | | | | | | | |
| leu2 | | | | | | | | | |
| leu3 | | | | | | | | | |
| LEU4 | | | | | | | | | |
| LEUTX | | | | | | | | | |
| LF1 | | | | | | | | | |
| LFNG | | | | | | | | + | + |
| LGALS1 | | | | | | | | | |
| LGALS12 | | | | | | | | | |
| LGALS13 | | | | | | | | | |
| LGALS14 | | | | | | | | | |
| LGALS2 | | | | | | | | | |
| LGALS3 | | | | | | | | | |
| LGALS3BP | | | | | | | | | |
| LGALS4 | | | | | | | | | |
| LGALS7 | | | | | | | | | |
| LGALS7B | | | | | | | | | |
| LGALS8 | | | | | | | | | |

| | | | | | | | | | |
|---|---|---|---|---|---|---|---|---|---|
| LGALS9 | | | | | | | | | |
| LGALS9B | | | | | | | | | |
| LGALS9C | | | | | | | | | |
| LGALSL | | | | | | | | | |
| LGI1 | | | + | | | + | | | + |
| LGI2 | | | | | | | | | |
| LGI3 | | | | | | | | | |
| LGI4 | | | | | | | | | |
| LGMN | | | | | | | | | |
| LGR4 | | | | | | | | | |
| LGR5 | + | | | + | + | | + | | + |
| LGR6 | | | | | | | | | |
| LGSN | | | | | | | | | |
| LGTN | | | | | | | | | |
| LHB | | | | | | | | | |
| LHCGR | | | | | | | | | |
| LHFP | | | | | | | | | |
| LHFPL1 | | | | | | | | | |
| LHFPL2 | | | | | | | | | |
| LHFPL3 | | | | | | | | | |
| LHFPL4 | | | + | | | | | | + |
| LHFPL5 | | | | | | | | | |
| LHPP | | | | | | | | | |
| LHS1 | | | | | | | | | |
| LHX1 | | | | | | | | | |
| LHX2 | | | | | | | | | |
| LHX3 | | | | | | | | | |
| LHX4 | | | | | | | | | |
| LHX6 | | | | | | | | | |
| LHX8 | | | | | | | | | |
| LHX9 | + | | | + | + | | + | | + |
| LIAS | | | | | | | | | |
| lid | | | | | | | | | |
| LIF | + | | | + | + | | + | | + |
| LIF1 | | | | | | | | | |
| LIFR | | | | | | | | | |
| LIFR-AS1 | | | | | | | | | |
| LIG1 | | | | | | | | | |
| LIG3 | | + | | | | | | | + |
| LIG4 | | | | | | | | | |
| LILRA1 | | | | | | | | | |
| LILRA3 | | | | | | | | | |
| LILRA5 | | | | | | | | | |
| LILRA6 | | | | | | | | | |
| LILRB1 | | | | | | | | | |
| LILRB2 | | | | | | | | | |
| LILRB3 | | | | | | | | | |
| LILRB4 | | | | | | | | | |
| LILRB5 | | | | | | | | | |
| LIM2 | | | | | | | | | |
| LIMA1 | | | | | | | | | |
| LIMCH1 | | | | | | | | | |
| LIMD1 | | | | | | | | | |

| | | | | | | | | | |
|---|---|---|---|---|---|---|---|---|---|
| LIMD2 | | | | | | + | | | + |
| LIME1 | | | | | | | | | |
| LIMK1 | | | | | | | | | |
| LIMK2 | | | | | | | | | |
| LIMS1 | | | | | | | | | |
| LIMS2 | | | | | | | | | |
| LIMS3 | | | | | | | | | |
| LIMS3L | | | | | | | | | |
| LIN28A | | | | | | | | | |
| LIN28B | | | | | | | | | |
| LIN37 | | | | | | | | | |
| LIN52 | | | | | | | | | |
| LIN54 | | | + | | + | | | | + |
| LIN7A | | | + | | | | | | + |
| LIN7B | | | | | | | | | |
| LIN7C | | | | | | | | | |
| LIN9 | | | | + | | | | | + |
| LINC00083 | | | | | | | | | |
| LINC00086 | | | | | | | | | |
| LINC00087 | | | | | | | | | |
| LINC00092 | | | | | | | | | |
| LINC00094 | | | | | | | | | |
| LINC00113 | | | | | | | | | |
| LINC00152 | | | | | | | | | |
| LINC00173 | | | | | | | | | |
| LINC00189 | | | | | | | | | |
| LINC00238 | | | | | | | | | |
| LINC00239 | | | | | | | | | |
| LINC00240 | | | | | | | | | |
| LINC00242 | | | | | | | | | |
| LINC00260 | | | | | | | | | |
| LINC00263 | | | | | | | | | |
| LINC00265 | | | | | | | | | |
| LINC00266-1 | | | | | | | | | |
| LINC00269 | | | | | | | | | |
| LINC00272 | | | | | | | | | |
| LINC00273 | | | | | | | | | |
| LINC00294 | | | | | | | | | |
| LINC00299 | | | | | | | | | |
| LINC00305 | | | | | | | | | |
| LINC00308 | | | | | | | | | |
| LINC00311 | | | | | | | | | |
| LINC00312 | | | | | | | | | |
| LINC00322 | | | | | | | | | |
| LINC00324 | | | | | | | | | |
| LINC00336 | | | | | | | | | |
| LINC00339 | | | | | | | | | |
| LINC00341 | | | | | | | | | |
| LINC00342 | | | | | | | | | |
| LINC00346 | | | | | | | | | |
| LINC00452 | | | | | | | | | |
| LINC00460 | | | | | | | | | |
| LINC00461 | | | | | | | | | |

| | | | | | | | | | |
|---|---|---|---|---|---|---|---|---|---|
| LINC00467 | | | | | | | | | |
| LINC00470 | | | | | | | | | |
| LINC00471 | | | | | | | | | |
| LINC00472 | | | | | | | | | |
| LINC00473 | | | | | | | | | |
| LINC00482 | | | | | | | | | |
| LINC00491 | | | | | | | | | |
| LINC00493 | | | | | | | | | |
| LINC00511 | | | | | | | | | |
| LINC00518 | | | | | | | | | |
| LINC00521 | | | | | | | | | |
| LINC00523 | | | | | | | | | |
| LINC00526 | | | | | | | | | |
| LINC00528 | | | | | | | | | |
| LINC00537 | | | | | | | | | |
| LINC00588 | | | | | | | | | |
| LINC00598 | | | | | | | | | |
| LINC00602 | | | | | | | | | |
| LINC00624 | | | | | | | | | |
| LINC00629 | | | | | | | | | |
| LINC00630 | | | | | | | | | |
| LINC00632 | | | | | | | | | |
| LINC00636 | | | | | | | | | |
| LINC00638 | | | | | | | | | |
| LINC00649 | | | | | | | | | |
| LINC00656 | | | | | | | | | |
| LINC00657 | | | | | | | | | |
| LINC00659 | | | | | | | | | |
| LINC00663 | | | | | | | | | |
| LINC00668 | | | | | | | | | |
| LINC00669 | | | | | | | | | |
| LINC00671 | | | | | | | | | |
| LINC00673 | | | | | | | | | |
| LINC00696 | | | | | | | | | |
| LINC00839 | | | | | | | | | |
| LINC00842 | | | | | | | | | |
| LINC00847 | | | | | | | | | |
| LINC00852 | | | | | | | | | |
| LINC00854 | | | | | | | | | |
| LINC00857 | | | | | | | | | |
| LINC00887 | | | | | | | | | |
| LINC00894 | | | | | | | | | |
| LINC00898 | | | | | | | | | |
| LINC00905 | | | | | | | | | |
| LINC00908 | | | | | | | | | |
| LINC00909 | | | | | | | | | |
| LINC00910 | | | | | | | | | |
| LINC00938 | | | | | | | | | |
| LINC00941 | | | | | | | | | |
| LINC00963 | | | | | | | | | |
| LINC00964 | | | | | | | | | |
| LINC00969 | | | | | | | | | |
| LINC00982 | | | | | | | | | |

| | | | | | | | | | |
|---|---|---|---|---|---|---|---|---|---|
| LINC00998 | | | | | | | | | |
| LINC01000 | | | | | | | | | |
| LINC01018 | | | | | | | | | |
| LINC01024 | | | | | | | | | |
| LINC01108 | | | | | | | | | |
| LINC01128 | | | | | | | | | |
| LINC01137 | | | | | | | | | |
| LINC01139 | | | | | | | | | |
| LINC01158 | | | | | | | | | |
| LINC01194 | | | | | | | | | |
| LINC01198 | | | | | | | | | |
| LINC01232 | | | | | | | | | |
| LINC01234 | | | | | | | | | |
| LINC01257 | | | | | | | | | |
| LINC01268 | | | | | | | | | |
| LINC01270 | | | | | | | | | |
| LINC01296 | | | | | | | | | |
| LINC01347 | | | | | | | | | |
| LINC01420 | | | | | | | | | |
| LINC01451 | | | | | | | | | |
| LINC01465 | | | | | | | | | |
| LINC01468 | | | | | | | | | |
| LINC01483 | | | | | | | | | |
| LINC01485 | | | | | | | | | |
| LINC01537 | | | | | | | | | |
| LINC01541 | | | | | | | | | |
| LINC01547 | | | | | | | | | |
| LINC01549 | | | | | | | | | |
| LINC01553 | | | | | | | | | |
| LINC01554 | | | | | | | | | |
| LINC01556 | | | | | | | | | |
| LINC01558 | | | | | | | | | |
| LINC01561 | | | | | | | | | |
| LINC01565 | | | | | | | | | |
| LINC01572 | | | | | | | | | |
| LINC01587 | | | | | | | | | |
| LINC01588 | | | | | | | | | |
| LINGO1 | + | | + | | + | | | | + |
| LINGO2 | | | | | | | | | |
| LINGO4 | + | | | + | + | | + | | + |
| LINS | | | + | | + | | | | + |
| LIP1 | | | | | | | | | |
| LIP5 | | | | | | | | | |
| LIPA | | | | | | | | | |
| LIPC | | | | | | | | | |
| LIPE | | | | | | + | | | + |
| LIPF | | | | | | | | | |
| LIPG | | | | | | | | | |
| LIPH | | | | | | | | | |
| LIPI | | | | | | | | | |
| LIPK | | | | | | | | | |
| LIPM | | | | | | | | | |
| LIPT1 | | | | | | | | | |

| | | | | | | | | | |
|---|---|---|---|---|---|---|---|---|---|
| LIPT2 | | | | | | | | | |
| LITAF | | | | | | | | | |
| LIX1 | | | | | | | | | |
| LIX1L | | | | | | | | | |
| LKAAEAR1 | | | | | | | | | |
| LLGL1 | | | | | | | | | |
| LLGL2 | | | | | | | | | |
| LLPH | | | + | | + | | | | + |
| LMAN1 | | | | | | | | | |
| LMAN1L | | | | | | | | | |
| LMAN2 | | | | | | + | | | + |
| LMAN2L | | | | + | | | | | + |
| LMBR1 | | | | | | | | | |
| LMBR1L | | | | | | | | | |
| LMBRD1 | | | | | | | + | | + |
| LMBRD2 | | | | | | | | | |
| LMCD1 | | | | | | | | | |
| LMF1 | | | | | | | | | |
| LMF2 | | | | | | | | | |
| LMLN | | | | | | | | | |
| LMNA | | | + | | | | | | + |
| LMNB1 | | | | | | | | | |
| LMNB2 | | | | | | + | | | + |
| LMNTD1 | | | | | | | | | |
| LMNTD2 | | | | | | | | | |
| LMO1 | | | | | | | | | |
| LMO2 | | | + | | | | | | + |
| LMO3 | | + | | | | + | | | + |
| LMO4 | | | | | | | | | |
| LMO7 | | | | | + | | | | + |
| LMOD1 | | | | | | | | | |
| LMOD2 | | | | | | | | | |
| LMOD3 | | | | | | | | | |
| LMP1 | | | | | | | | | |
| LMP2 | | | | | | | | | |
| LMP2A | | | | | | | | | |
| LMTK2 | | | | | | | | | |
| LMTK3 | | | | | | | | | |
| LMX1A | | | | | | | | | |
| LMX1B | | | | | | | | | |
| LNP1 | | | | | | | | | |
| LNPEP | | | | | | | | | |
| LNX1 | | | | | | | | | |
| LNX2 | | | | | | | | | |
| lnx2b | | | | | | | | | |
| LOC100128274 | | | | | | | | | |
| LOC100128400 | | | | | | | | | |
| LOC100128429 | | | | | | | | | |
| LOC100128554 | | | | | | | | | |
| LOC100128588 | | | | | | | | | |
| LOC100129118 | | | | | | | | | |
| LOC100129492 | | | | | | | | | |
| LOC100129917 | | | | | | | | | |

| | | | | | | | | | |
|---|---|---|---|---|---|---|---|---|---|
| LOC100129924 | | | | | | | | | |
| LOC100130075 | | | | | | | | | |
| LOC100130238 | | | | | | | | | |
| LOC100130417 | | | | | | | | | |
| LOC100130691 | | | | | | | | | |
| LOC100131294 | | | | | | | | | |
| LOC100131303 | | | | | | | | | |
| LOC100131689 | | | | | | | | | |
| LOC100132057 | | | | | | | | | |
| LOC100132735 | | | | | | | | | |
| LOC100133091 | | | | | | | | | |
| LOC100133315 | | | | | | | | | |
| LOC100133669 | | | | | | | | | |
| LOC100144595 | | | | | | | | | |
| LOC100158003 | | | | | | | | | |
| LOC100190986 | | | | | | | | | |
| LOC100272216 | | | | | | | | | |
| LOC100287015 | | | | | | | | | |
| LOC100287042 | | | | | | | | | |
| LOC100288069 | | | | | | | | | |
| LOC100288637 | | | | | | | | | |
| LOC100288778 | | | | | | | | | |
| LOC100289097 | | | | | | | | | |
| LOC100289290 | | | | | | | | | |
| LOC100289511 | | | | | | | | | |
| LOC100294362 | | | | | | | | | |
| LOC100342572 | | | | | | | | | |
| LOC100348511 | | | | | | | | | |
| LOC100355886 | | | | | | | | | |
| LOC100359517 | | | | | | | | | |
| LOC100359583 | | | | | | | | | |
| LOC100359600 | | | | | | | | | |
| LOC100360057 | | | | | | | | | |
| LOC100360154 | | | | | | | | | |
| LOC100360508 | | | | | | | | | |
| LOC100360522 | | | | | | | | | |
| LOC100360573 | | | | | | | | | |
| LOC100361008 | | | | | | | | | |
| LOC100361067 | | | | | | | | | |
| LOC100361427 | | | | | | | | | |
| LOC100361858 | | | | | | | | | |
| LOC100362640 | | | | | | | | | |
| LOC100363176 | | | | | | | | | |
| LOC100363620 | | | | | | | | | |
| LOC100363737 | | | | | | | | | |
| LOC100363860 | | | | | | | | | |
| LOC100363878 | | | | | | | | | |
| LOC100363904 | | | | | | | | | |
| LOC100364368 | | | | | | | | | |
| LOC100364825 | | | | | | | | | |
| LOC100365196 | | | | | | | | | |
| LOC100365839 | | | | | | | | | |
| LOC100365943 | | | | | | | | | |

| | | | | | | | | | |
|---|---|---|---|---|---|---|---|---|---|
| LOC100366016 | | | | | | | | | |
| LOC100366048 | | | | | | | | | |
| LOC100419906 | | | | | | | | | |
| LOC100499484-C9ORF17 | | | | | | | | | |
| LOC100505478 | | | | | | | | | |
| LOC100505498 | | | | | | | | | |
| LOC100505666 | | | | | | | | | |
| LOC100505817 | | | | | | | | | |
| LOC100506055 | | | | | | | | | |
| LOC100506127 | | | | | | | | | |
| LOC100506422 | | | | | | | | | |
| LOC100506548 | | | | | | | | | |
| LOC100506562 | | | | | | | | | |
| LOC100506688 | | | | | | | | | |
| LOC100506753 | | | | | | | | | |
| LOC100506797 | | | | | | | | | |
| LOC100506990 | | | | | | | | | |
| LOC100507373 | | | | | | | | | |
| LOC100507462 | | | | | | | | | |
| LOC100507537 | | | | | | | | | |
| LOC100507577 | | | | | | | | | |
| LOC100507703 | | | | | | | | | |
| LOC100507855 | | | | | | | | | |
| LOC100508781 | | | | | | | | | |
| LOC100509457 | | | | | | | | | |
| LOC100630923 | | | | | | | | | |
| LOC100653049 | | | | | | | | | |
| LOC100760824 | | | | | | | | | |
| LOC100911597 | | | | | | | | | |
| LOC100912032 | | | | | | | | | |
| LOC100996437 | | | | | | | | | |
| LOC100996747 | | | | | | | | | |
| LOC101060022 | | | | | | | | | |
| LOC101060321 | | | | | | | | | |
| LOC101060389 | | | | | | | | | |
| LOC101060521 | | | | | | | | | |
| LOC101060621 | | | | | | | | | |
| LOC101927359 | | | | | | | | | |
| LOC101928279 | | | | | | | | | |
| LOC101928405 | | | | | | | | | |
| LOC101928464 | | | | | | | | | |
| LOC101928728 | | | | | | | | | |
| LOC101928841 | | | | | | | | | |
| LOC101929876 | | | | | | | | | |
| LOC101929977 | | | | | | | | | |
| LOC101930047 | | | | | | | | | |
| LOC101930123 | | | | | | | | | |
| LOC101930400 | | | | | | | | | |
| LOC102723553 | | | | | | | | | |
| LOC102723555 | | | | | | | | | |
| LOC102723729 | | | | | | | | | |
| LOC102724023 | | | | | | | | | |
| LOC102724159 | | | | | | | | | |

| | | | | | | | | | |
|---|---|---|---|---|---|---|---|---|---|
| LOC102724334 | | | | | | | | | |
| LOC102724428 | | | | | | | | | |
| LOC102724488 | | | | | | | | | |
| LOC102724539 | | | | | | | | | |
| LOC102724560 | | | | | | | | | |
| LOC102724594 | | | | | | | | | |
| LOC102724652 | | | | | | | | | |
| LOC107983993 | | | | | | | | | |
| LOC113230 | | | | | | | | | |
| LOC142937 | | | | | | | | | |
| LOC145837 | | | | | | | | | |
| LOC146880 | | | | | | | | | |
| LOC147791 | | | | | | | | | |
| LOC148413 | | | | | | | | | |
| LOC148696 | | | | | | | | | |
| LOC149950 | | | | | | | | | |
| LOC153684 | | | | | | | | | |
| LOC154449 | | | | | | | | | |
| LOC155060 | | | | | | | | | |
| LOC202181 | | | | | | | | | |
| LOC254896 | | | | | | | | | |
| LOC255308 | | | | | | | | | |
| LOC255654 | | | | | | | | | |
| LOC260334 | | | | | | | | | |
| LOC283335 | | | | | | | | | |
| LOC283575 | | | | | | | | | |
| LOC283788 | | | | | | | | | |
| LOC283922 | | | | | | | | | |
| LOC284014 | | | | | | | | | |
| LOC284023 | | | | | | | | | |
| LOC284379 | | | | | | | | | |
| LOC284412 | | | | | | | | | |
| LOC284454 | | | | | | | | | |
| LOC284685 | | | | | | | | | |
| LOC284930 | | | | | | | | | |
| LOC285423 | | | | | | | | | |
| LOC285556 | | | | | | | | | |
| LOC285696 | | | | | | | | | |
| LOC286987 | | | | | | | | | |
| LOC290704 | | | | | | | | | |
| LOC297756 | | | | | | | | | |
| LOC300303 | | | | | | | | | |
| LOC313641 | | | | | | | | | |
| LOC338667 | | | | | | | | | |
| LOC338797 | | | | | | | | | |
| LOC344382 | | | | | | | | | |
| LOC366310 | | | | | | | | | |
| LOC388882 | | | | | | | | | |
| LOC389842 | | | | | | | | | |
| LOC389895 | | | | | | | | | |
| LOC389906 | | | | | | | | | |
| LOC390760 | | | | | | | | | |
| LOC390937 | | | | | | | | | |

| | | | | | | | | | |
|---|---|---|---|---|---|---|---|---|---|
| LOC392364 | | | | | | | | | |
| LOC396905 | | | | | | | | | |
| LOC399491 | | | | | | | | | |
| LOC400043 | | | | | | | | | |
| LOC400499 | | | | | | | | | |
| LOC400682 | | | | | | | | | |
| LOC400927 | | | | | | | | | |
| LOC400927-CSNK1E | | | | | | | | | |
| LOC401010 | | | | | | | | | |
| LOC401052 | | | | | | | | | |
| LOC401068 | | | | | | | | | |
| LOC401296 | | | | | | | | | |
| LOC401442 | | | | | | | | | |
| LOC403312 | | | | | | | | | |
| LOC407835 | | | | | | | | | |
| LOC407840 | | | | | | | | | |
| LOC440300 | | | | | | | | | |
| LOC440434 | | | | | | | | | |
| LOC440570 | | | | | | | | | |
| LOC440786 | | | | | | | | | |
| LOC440910 | | | | | | | | | |
| LOC441155 | | | | | | | | | |
| LOC441178 | | | | | | | | | |
| LOC442497 | | | | | | | | | |
| LOC494141 | | | | | | | | | |
| LOC498555 | | | | | | | | | |
| LOC501222 | | | | | | | | | |
| LOC541472 | | | | | | | | | |
| LOC541473 | | | | | | | | | |
| LOC554206 | | | | | | | | | |
| LOC554207 | | | | | | | | | |
| LOC554223 | | | | | | | | | |
| LOC576779 | | | | | | | | | |
| LOC613037 | | | | | | | | | |
| LOC613266 | | | | | | | | | |
| LOC641367 | | | | | | | | | |
| LOC642852 | | | | | | | | | |
| LOC642943 | | | | | | | | | |
| LOC643201 | | | | | | | | | |
| LOC644422 | | | | | | | | | |
| LOC644669 | | | | | | | | | |
| LOC644961 | | | | | | | | | |
| LOC645086 | | | | | | | | | |
| LOC645513 | | | | | | | | | |
| LOC646377 | | | | | | | | | |
| LOC646644 | | | | | | | | | |
| LOC646762 | | | | | | | | | |
| LOC646938 | | | | | | | | | |
| LOC649305 | | | | | | | | | |
| LOC652276 | | | | | | | | | |
| LOC653602 | | | | | | | | | |
| LOC679835 | | | | | | | | | |
| LOC680039 | | | | | | | | | |

| | | | | | | | | | |
|---|---|---|---|---|---|---|---|---|---|
| LOC680161 | | | | | | | | | |
| LOC681754 | | | | | | | | | |
| LOC686499 | | | | | | | | | |
| LOC688869 | | | | | | | | | |
| LOC689805 | | | | | | | | | |
| LOC727751 | | | | | | | | | |
| LOC727803 | | | | | | | | | |
| LOC727896 | | | | | | | | | |
| LOC728024 | | | | | | | | | |
| LOC728026 | | | | | | | | | |
| LOC728175 | | | | | | | | | |
| LOC728323 | | | | | | | | | |
| LOC728637 | | | | | | | | | |
| LOC728743 | | | | | | | | | |
| LOC728763 | | | | | | | | | |
| LOC728819 | | | | | | | | | |
| LOC729324 | | | | | | | | | |
| LOC729603 | | | | | | | | | |
| LOC729732 | | | | | | | | | |
| LOC729774 | | | | | | | | | |
| LOC729867 | | | | | | | | | |
| LOC730668 | | | | | | | | | |
| LOC81691 | | | | | | | | | |
| LOC90768 | | | | | | | | | |
| LOC90784 | | | | | | | | | |
| LOC93432 | | | | | | | | | |
| LOC93622 | | | | | | | | | |
| LOH12CR1 | | | | | | | | | |
| LONP1 | | | | | + | | | | + |
| LONP2 | | | | | + | | | | + |
| LONRF1 | | | | | + | | | | + |
| LONRF2 | | | | | | | | | |
| LONRF3 | | | | | | | | | |
| LOR | | | | | | | | | |
| LOS1 | | | | | | | | | |
| LOX | | | | | | | | | |
| LOXHD1 | | | | | | | | | |
| LOXL1 | | | | | | | | | |
| LOXL2 | + | | | + | + | | + | | + |
| LOXL3 | | | | | | | | | |
| LOXL4 | | | | | | | | | |
| LPA | | | | | | | | | |
| LPAL2 | | | | | | | | | |
| LPAR1 | | | + | | | | | | + |
| LPAR2 | | | | | | | | | |
| LPAR3 | + | | | + | + | | | | + |
| LPAR4 | | | | | | | | | |
| LPAR5 | | | | | | | | | |
| LPAR6 | | + | + | | | + | | | + |
| LPCAT1 | | | + | | | | | | + |
| LPCAT2 | | | | | | | | | |
| LPCAT3 | | | | | | | | | |
| LPCAT4 | | | | | | | | | |

| | | | | | | | | | |
|---|---|---|---|---|---|---|---|---|---|
| LPD1 | | | | | | | | | |
| LPGAT1 | | + | | | | + | | | + |
| Lphn1 | | | | | | | | | |
| LPHN2 | | | | | | | | | |
| LPHN3 | | | | | + | | | | + |
| LPIN1 | | | | | | + | | | + |
| LPIN2 | | | | | + | | | | + |
| LPIN3 | | | | | | | | | |
| LPL | | | | | | | | | |
| LPO | | | | | | | | | |
| LPP | | | | | | | | | |
| LPPR1 | | | | | | | | | |
| LPPR2 | | | | | | | | | |
| LPPR3 | | | | | | | | | |
| LPPR4 | | | | | | + | | | + |
| LPXN | | | | | | | | | |
| lqf | | | | | | | | | |
| LRAT | | | | | | | | | |
| LRBA | | | | | | + | | | + |
| LRCH1 | | | | | | | | | |
| LRCH2 | | | | | | | | | |
| LRCH3 | | | | | | | | | |
| LRCH4 | | | | | | | | | |
| LRFN1 | | | | | | | | | |
| LRFN2 | | | | | | | | | |
| LRFN3 | | | | | | | | | |
| LRFN4 | | | | | | | | | |
| LRFN5 | | | | | | | | | |
| LRG1 | | | | | | | | | |
| LRGUK | | | | | | | | | |
| LRIF1 | | + | + | | | + | | | + |
| LRIG1 | | | | | | | | | |
| LRIG2 | | | + | | + | | | | + |
| LRIG3 | | | | | | | | | |
| LRIT1 | | | | | | | | | |
| LRIT3 | | | | | | | | | |
| LRMP | | | | | | | | | |
| LRP1 | | + | + | | + | + | | | + |
| LRP10 | | | | | | | | | |
| LRP11 | | | | | | | | | |
| LRP12 | | | | | | | | | |
| LRP1B | | | | | | | | | |
| LRP2 | | + | | | | + | | | + |
| LRP2BP | | | | | | | | | |
| LRP3 | | | | | | | | | |
| LRP4 | | | | | | | | | |
| LRP5 | | | | | | | | | |
| LRP5L | | | | | | | | | |
| LRP6 | | | | | | | | | |
| LRP8 | | | | | | | | | |
| LRPAP1 | | | | | | | | | |
| LRPPRC | | | | | | | | | |
| LRR1 | | | | | | | | | |

| | | | | | | | | | |
|---|---|---|---|---|---|---|---|---|---|
| LRRC1 | | | | | | | | | |
| LRRC10 | | | | | | | | | |
| LRRC10B | | | | | | | | | |
| LRRC14 | | | | | | | | | |
| LRRC14B | | | | | | | | | |
| LRRC15 | | | | | | | | | |
| LRRC16A | | | | | | + | | | + |
| LRRC16B | | | | | | | | | |
| LRRC17 | | + | + | | + | + | | + | + |
| LRRC18 | | | | | | | | | |
| LRRC19 | + | | | + | + | | + | | + |
| LRRC2 | | | | | | | | | |
| LRRC20 | | | | | | | | | |
| LRRC23 | | | | | | | | + | + |
| LRRC24 | | | | | | + | | | + |
| LRRC25 | | | | | | | | | |
| LRRC27 | | | | | | | | | |
| LRRC28 | | | | | | | | | |
| LRRC29 | | | | | | | | | |
| LRRC3 | | | | | | | | | |
| LRRC31 | | | | | | | | | |
| LRRC32 | | | | | | | | | |
| LRRC34 | | | | | | | | | |
| LRRC36 | | | | | | | | | |
| LRRC37A | | | | | | | | | |
| LRRC37A11P | | | | | | | | | |
| LRRC37A2 | | | | | | | | | |
| LRRC37A3 | | | | | | | | | |
| LRRC37A4P | | | | | | | | | |
| LRRC37B | | | | | | | | | |
| LRRC37BP1 | | | | | | | | | |
| LRRC39 | | | | | | + | | | + |
| LRRC3B | | | | | | | | | |
| LRRC3C | | | | | | | | | |
| LRRC3DN | | | | | | | | | |
| LRRC4 | | | | | + | | | | + |
| LRRC40 | | | | | | | | | |
| LRRC41 | | | | | | | | | |
| LRRC42 | | | | | | + | | | + |
| LRRC43 | | | | | | | | | |
| LRRC45 | | | | | | | | | |
| LRRC46 | | | | | | | | | |
| LRRC47 | | | | | | | | | |
| LRRC48 | | | | | | | | | |
| LRRC49 | | | | | | | | | |
| LRRC4B | | | | | | + | | | + |
| LRRC4C | | | | + | + | | | | + |
| LRRC52 | | | | | | | | | |
| LRRC53 | | | | | | | | | |
| LRRC55 | | | | | | | | | |
| LRRC56 | | | | | | | | | |
| LRRC57 | | + | | | | + | | | + |
| LRRC58 | | | | | + | | | | + |

| | | | | | | | | | |
|---|---|---|---|---|---|---|---|---|---|
| LRRC59 | | | | | | | | | |
| LRRC6 | | | | | | | | | |
| LRRC61 | | | | | | | | | |
| LRRC63 | + | | | + | + | | + | | + |
| LRRC66 | | | | | | | | | |
| LRRC69 | | | | | | | | | |
| LRRC7 | | | | | | | | | |
| LRRC70 | | | | | | | | | |
| LRRC71 | | | | | | | | | |
| LRRC73 | | | | | | | | | |
| LRRC74A | | | | | | | | | |
| LRRC75A-AS1 | | | | | | | | | |
| LRRC75B | | | | | | | | | |
| LRRC8A | | | | | | | | | |
| LRRC8B | | | | | | | | | |
| LRRC8C | | | | | | | | | |
| LRRC8D | | | | | + | | | | + |
| LRRC8E | | | | + | | | | | + |
| LRRC9 | | | | | | | | | |
| Lrrcc1 | | | | | | + | | | + |
| LRRD1 | | | | | | | | | |
| LRRFIP1 | | | | | | + | | | + |
| LRRFIP2 | | | | | | | | | |
| LRRIQ1 | | | | | | | | | |
| LRRIQ3 | | | | | | | | | |
| LRRIQ4 | | | | | | | | | |
| LRRK1 | | | | | | | | | |
| LRRK2 | + | | + | | | | | | + |
| LRRN1 | | | | + | | + | | | + |
| LRRN2 | | + | | | | + | | | + |
| LRRN3 | | | + | | | + | | | + |
| LRRN4 | | | | | | | | | |
| LRRN4CL | | | | | | | | | |
| LRRTM1 | | | | | | | | | |
| LRRTM2 | | | | + | | + | | | + |
| LRRTM3 | | | | | | | | | |
| LRRTM4 | | + | | | | + | | | + |
| lrs1 | | | | | | | | | |
| LRS4 | | | | | | | | | |
| LRSAM1 | | | | | | | | | |
| LRTM1 | | | | | | | | | |
| LRTM2 | | | + | | | | | | + |
| LRTOMT | | | | | | | | | |
| LRWD1 | | | | | | | | | |
| LSAMP | | | | | | | | | |
| LSB3 | | | | | | | | | |
| LSB5 | | | | | | | | | |
| lsd90 | | | | | | | | | |
| LSG1 | | | | | | | | | |
| LSM1 | | | | | | | | | |
| LSM10 | | | | | | | | | |
| LSM11 | | | | | + | | | | + |
| LSM12 | | | + | | | + | | | + |

| | | | | | | | | | |
|---|---|---|---|---|---|---|---|---|---|
| Lsm14a | | | | | | | | | |
| LSM14B | | | | | | | | | |
| LSM2 | | | | | | | | | |
| LSM3 | | | | | | | | | |
| LSM4 | | | | | | | | | |
| LSM5 | | | | | | | | | |
| LSM6 | | | | | | | | | |
| LSM7 | | | | | | | | | |
| LSM8 | | + | | | | + | | | + |
| LSMEM1 | | | | | | | | | |
| LSMEM2 | | | | | | | | | |
| LSP1 | | | | | | | | | |
| LSP1P3 | | | | | | | | | |
| LSR | | | | | | | | | |
| LSS | | | | | | | | | |
| LST1 | | | | | | | | | |
| LTA | | | | | | | | | |
| LTA4H | | | | | | | | | |
| LTB | | | | | | | | | |
| LTB4R | | | | | | | | | |
| LTB4R2 | | | | | | | | | |
| LTBP1 | + | + | | + | + | | + | | + |
| LTBP2 | | | | | | | | | |
| LTBP3 | | | | | | | | | |
| LTBP4 | | | | | | | | | |
| LTBR | | | | | | | | | |
| LTC4S | | | | | | | | | |
| LTF | | | | | | | | | |
| LTK | | | | | | + | | | + |
| LTN1 | | | | | | | | | |
| LTV1 | | | | | | | | | |
| LUC7L | | | | | + | | | | + |
| LUC7L2 | | | + | | | | | | + |
| LUC7L3 | | + | | | | + | | | + |
| LUCAT1 | | | | | | | | | |
| LUM | | | | | | | | | |
| LURAP1 | | | | | | | | | |
| LURAP1L | | | | | | | | | |
| LUZP1 | | | + | | | | | | + |
| LUZP2 | | | | | | | | | |
| LUZP4 | | | | | | | | | |
| LUZP6 | | | | | | | | | |
| lwr | | | | | | | | | |
| LXN | | | | | | + | | | + |
| Ly6a | | | | | | | | | |
| LY6D | | | | | | | | | |
| LY6E | | | | | | | | | |
| LY6G5B | | | | | | | | | |
| LY6G5C | | | | | | | | | |
| LY6G6C | | | | | | | | | |
| LY6G6D | | | | | | | | | |
| LY6G6F | | | | | | | | | |
| LY6H | | | | | | | | | |

| | | | | | | | | | |
|---|---|---|---|---|---|---|---|---|---|
| LY6K | | | | | | | | | |
| LY86 | | | | | | | | | |
| LY9 | | | | | | | | | |
| LY96 | | | | | | | + | | + |
| LYAR | | | | | | | | | |
| LYG1 | | | | | | | | | |
| LYG2 | | | | | | | | | |
| LYL1 | | | | | | | | | |
| LYN | | | | | | | | | |
| LYNX1 | | | | | | | | | |
| LYPD1 | | | | | | + | | | + |
| LYPD2 | | | | | | | | | |
| LYPD3 | | | | | | | | | |
| LYPD4 | | | | | | | | | |
| LYPD5 | | | | | | | | | |
| LYPD6 | | | | | | | | | |
| LYPD6B | | | | | | | | | |
| LYPLA1 | | | | | | | | | |
| LYPLA2 | | | + | | | | | | + |
| LYPLA2P1 | | | | | | | | | |
| Lyplal1 | | | | | | | | | |
| LYRM1 | | | | | | | | | |
| LYRM2 | | | | | | | | | |
| LYRM4 | | | | | | + | | | + |
| LYRM5 | | + | + | | | + | | | + |
| LYRM7 | | | | | | | | | |
| lys1 | | | | | | | | | |
| LYS12 | | | | | | | | | |
| LYS20 | | | | | | | | | |
| LYS21 | | | | | | | | | |
| lys3 | | | | | | | | | |
| lys4 | | | | | | | | | |
| LYS9 | | | | | | | | | |
| LYSMD1 | | | | | | | | | |
| LYSMD2 | | | | | | | | | |
| LYSMD3 | | | | | | | | | |
| LYSMD4 | | | | | | | | | |
| LYST | | | | | | | | | |
| LYVE1 | | | | | | | | | |
| LYZ | | | | | | | | | |
| LYZL1 | | | | | | | | | |
| LYZL2 | | | | | | | | | |
| LYZL4 | | | | | | | | | |
| LYZL6 | | | | | | | | | |
| LZIC | | | | | | | | | |
| LZTFL1 | | | | | | | | | |
| LZTR1 | | | | | | | | | |
| LZTS1 | | | | | + | | | | + |
| LZTS2 | | | | | | | | | |
| LZTS3 | | | | | | + | | | + |
| M | | | | | | | | | |
| M1AP | | | | | | | | | |
| M6PR | | | + | | | | | | + |

| | | | | | | | | | |
|---|---|---|---|---|---|---|---|---|---|
| MAATS1 | | | | | | | | | |
| MAB21L1 | | | | | | | | | |
| MAB21L2 | | | | | | | | | |
| MAB21L3 | | | | | | | | | |
| MAC1 | | | | | | | | | |
| MACC1 | | | | | | | | | |
| MACF1 | | | | | | | | | |
| MACROD1 | | | | | | + | | | + |
| MACROD2 | | | + | | | | | | + |
| MAD1 | | | | | | | | | |
| MAD1L1 | | | | | | | | | |
| mad2 | | | | | | | | | |
| MAD2L1 | | | | | | | | | |
| MAD2L1BP | | | | | | | | | |
| MAD2L2 | | | | | | | | | |
| MADCAM1 | | | | | | | | | |
| MADD | | | | | | | | | |
| mae2 | | | | | | | | | |
| MAEA | | | | | | | | | |
| MAEL | | | | | | | | | |
| MAF | | | | | | | | | |
| MAF1 | | + | | | | + | | | + |
| MAFA | | | | | | | | | |
| MAFB | | | | | | | | | |
| MAFF | | | | | | | | | |
| MAFG | | | | | + | | | | + |
| MAFIP | | | | | | | | | |
| MAFK | | | + | | | | | | + |
| MAG | | | | | | | | + | + |
| MAGEA1 | | | | | | | | | |
| MAGEA10 | | | | | | | | | |
| MAGEA11 | | | | | | | | | |
| MAGEA12 | | | | | | | | | |
| MAGEA2 | | | | | | | | | |
| MAGEA2B | | | | | | | | | |
| MAGEA3 | | | | | | | | | |
| MAGEA4 | | | | | | | | | |
| MAGEA6 | | | | | | | | | |
| MAGEA8 | | | | | | | | | |
| MAGEA9 | | | | | | | | | |
| MAGEA9B | | | | | | | | | |
| MAGEB1 | | | | | | | | | |
| MAGEB10 | | | | | | | | | |
| MAGEB16 | | | | | | | | | |
| MAGEB17 | | | | | | | | | |
| MAGEB18 | | | | | | | | | |
| MAGEB2 | | | | | | | | | |
| MAGEB3 | | | | | | | | | |
| MAGEB4 | | | | | | | | | |
| MAGEB5 | | | | | | | | | |
| MAGEB6 | | | | | | | | | |
| MAGEC1 | | | | | | | | | |
| MAGEC2 | | | | | | | | | |

| | | | | | | | | | |
|---|---|---|---|---|---|---|---|---|---|
| MAGEC3 | | | | | | | | | |
| MAGED1 | | | | | | | | | |
| MAGED2 | | | | | | | | | |
| MAGED4 | | | | | | | | | |
| MAGED4B | | | | | | | | | |
| MAGEE1 | | | | | | | | | |
| MAGEE2 | | | | | | + | | | + |
| MAGEF1 | | | | | | | | | |
| MAGEH1 | | | | | | | | | |
| MAGEL2 | | | + | | | | | | + |
| MAGI1 | | | + | + | | + | | | + |
| MAGI2 | | | | | | | | | |
| MAGI3 | | | | + | | | | | + |
| MAGIX | | | | | | | | | |
| MAGOH | | | | | | | | | |
| MAGOH2P | | | | | | | | | |
| MAGOHB | | | | | | | | | |
| MAGT1 | | | | | | + | | | + |
| MAK | | | | | | | | | |
| MAK16 | | | | | | | | | |
| MAK31 | | | | | | | | | |
| MAL | | | | + | | | | + | + |
| MAL2 | | + | | | | + | | | + |
| mal3 | | | | | | | | | |
| MAL31 | | | | | | | | | |
| MALAT1 | | | | | | | | | |
| MALL | | | | | | | | | |
| MALSU1 | | | | | | | | | |
| MALT1 | | | | | | | | | |
| MAMDC2 | | | | | | | | | |
| MAMDC4 | | | | | | | | | |
| MAML1 | | | + | | | | | | + |
| MAML2 | + | + | | + | + | + | + | | + |
| MAML3 | | | | | | | | | |
| MAMLD1 | | | | | + | | | | + |
| MAN1A1 | | | | | | | | | |
| MAN1A2 | | | | | | | | | |
| MAN1B1 | | | + | | | | | | + |
| MAN1C1 | | | | | | | | | |
| MAN2A1 | | | + | | | + | | | + |
| MAN2A2 | | | | | | | | | |
| MAN2B1 | | | | | | | | | |
| MAN2B2 | | | | | | | | | |
| MAN2C1 | | | | | | | | | |
| MANBA | | | | | | | | | |
| MANBAL | | | | | | | | | |
| MANEA | | | | | | + | | | + |
| MANEAL | | | | | | | | | |
| MANF | | | | | | | | | |
| MANSC1 | | | | | | | | | |
| MANSC4 | | | | | | | | | |
| MAOA | | | | + | | | | | + |
| MAOB | | | | | | | | | |

| | | | | | | | | | |
|---|---|---|---|---|---|---|---|---|---|
| MAP10 | | | | | | | | | |
| MAP1A | | | | | | | | | |
| MAP1B | | | | | | | | | |
| MAP1LC3A | | | | | | | | | |
| MAP1LC3B | | | | | | | | | |
| MAP1LC3B2 | | | | | | | | | |
| MAP1LC3C | | | | | | | | | |
| MAP1S | | | | | | | | | |
| MAP2 | | | | | | | | | |
| MAP2K1 | | | | | | | | | |
| MAP2K2 | | | | | | | | | |
| MAP2K3 | | | | | | | | | |
| MAP2K4 | | | + | | | | | | + |
| MAP2K5 | | | | | | | | | |
| MAP2K6 | | | | | | | | | |
| MAP2K7 | | | | | | + | | | + |
| MAP3K1 | + | | | + | + | | + | | + |
| MAP3K10 | | | | | | | | | |
| MAP3K11 | | | | | | | | | |
| MAP3K12 | | | | | | | | | |
| MAP3K13 | | | | | | | | | |
| MAP3K14 | | | | | | | | | |
| MAP3K15 | | | | | | | | | |
| MAP3K19 | | | | | | | | | |
| MAP3K2 | | | + | | | | | | + |
| MAP3K3 | | | | | | | | | |
| MAP3K4 | | | | | | + | | | + |
| MAP3K5 | | | | | + | + | | | + |
| MAP3K6 | | | | | | | | | |
| MAP3K7 | | | | | | | | | |
| MAP3K7CL | | | | | | | | | |
| MAP3K8 | | | | | | | | | |
| MAP3K9 | | | | | | | | | |
| MAP4 | | | + | | | | | | + |
| MAP4K1 | | | | | | | | | |
| MAP4K2 | | | | | | | | | |
| Map4k3 | | | | | | + | | | + |
| MAP4K4 | | | | | | + | | | + |
| MAP4K5 | | | | | | | | | |
| Map6 | | | | | | + | | | + |
| MAP6D1 | | | | | | | | | |
| MAP7 | | | | | | + | | | + |
| MAP7D1 | | | | | | | | | |
| MAP7D2 | | | | | | | | | |
| MAP7D3 | | | | | | | | | |
| MAP9 | | | | | | | | | |
| MAPK1 | | + | | | | + | | | + |
| MAPK10 | + | | | | + | | | | + |
| MAPK11 | | | | | | | | | |
| MAPK12 | | | | | | | | | |
| MAPK13 | | | | | | | | | |
| MAPK14 | | | | | | | | | |
| MAPK15 | | | | | | | | | |

| | | | | | | | | | |
|---|---|---|---|---|---|---|---|---|---|
| MAPK1IP1L | | | | | | | | | |
| MAPK3 | | | | | | | | | |
| MAPK4 | + | | + | | + | | | | + |
| MAPK6 | | | + | | + | | | | + |
| MAPK7 | | | | | | | | | |
| MAPK8 | | | + | | | | | | + |
| MAPK8IP1 | | | | | | | | | |
| MAPK8IP2 | | | | | + | | | | + |
| MAPK8IP3 | | | | | | | | | |
| MAPK9 | | | + | | | | | | + |
| MAPKAP1 | | | | | | | | | |
| MAPKAPK2 | | | | | + | | | | + |
| MAPKAPK3 | | | | | | | | | |
| MAPKAPK5 | | | | | | | | | |
| MAPKBP1 | | | | | | | | | |
| MAPRE1 | | | | | | | | | |
| MAPRE2 | | | | | | | | | |
| MAPRE3 | | | | | | | | | |
| MAPT | | | | | | + | | | + |
| MARC1 | | | | | | | | | |
| MARC2 | | | | | | | | | |
| MARCH1 | | | | | | | | | |
| MARCH10 | | | | | | | | | |
| MARCH2 | | | | | | | | | |
| MARCH3 | | | | | | | | | |
| MARCH4 | | | | | | | | | |
| MARCH5 | | | | | | | | | |
| MARCH6 | | | | | | | | | |
| MARCH7 | | | | | | | | | |
| MARCH8 | | | | | | | | | |
| MARCH9 | | | | | | | | | |
| MARCKS | | | + | | | | | | + |
| MARCKSL1 | | | | | | | | | |
| MARCO | | | | | | | | | |
| MARK1 | | | | | | | | | |
| MARK2 | | | | | | | | | |
| MARK3 | | | | | | | | | |
| MARK4 | | | | | | | | | |
| MARS | | | | | | | | | |
| MARS2 | | | | | | | | | |
| MARVELD1 | | | | | | | | | |
| MARVELD2 | | | | | | | | | |
| MARVELD3 | | | | | | | | | |
| MAS1 | | | | | | | | | |
| MAS1L | | | | | | | | | |
| mas5 | | | | | | | | | |
| MASP1 | | | | | | | | | |
| MASP2 | | | | | | | | | |
| MAST1 | | | | | | | | | |
| MAST2 | | | | | | | | | |
| MAST3 | | | | | | + | | | + |
| MAST4 | + | | | | + | | | | + |
| MASTL | | | | + | | | | | + |

| | | | | | | | | | |
|---|---|---|---|---|---|---|---|---|---|
| MAT1A | | | | | | | | | |
| MAT2A | | | | | | | | | |
| MAT2B | | + | | | | + | | | + |
| MAT3 | | | | | | | | | |
| MATK | | | | | + | + | | | + |
| MATN1 | | | | | | | | | |
| MATN1-AS1 | | | | | | | | | |
| MATN2 | | | | | | | | | |
| MATN3 | | | | | | | | | |
| MATN4 | | | | | | | | | |
| MATR3 | | + | + | | | + | | | + |
| Mau2 | | | | | | | | | |
| MAVS | | | | | | | | | |
| MAX | | | | | | | | | |
| MAZ | | | | | | | | | |
| MB | | | | | | | | | |
| MB21D1 | | | | + | | | | | + |
| MB21D2 | | | + | | | | | | + |
| MBD1 | | | | | | | | | |
| MBD2 | + | | | | + | | | | + |
| MBD3 | | | | | | | | | |
| MBD3L1 | | | | | | | | | |
| MBD3L2 | | | | | | | | | |
| MBD3L3 | | | | | | | | | |
| MBD3L5 | | | | | | | | | |
| MBD4 | | | | | | | | | |
| MBD5 | | | | + | | + | | | + |
| MBD6 | | | | | | | | | |
| MBF1 | | | | | | | | | |
| MBIP | | | | | | | | | |
| MBL2 | | | | | | | | | |
| MBLAC1 | | | | | | | | | |
| MBLAC2 | | + | | | | + | | | + |
| MBNL1 | | | | | | | | | |
| MBNL2 | + | | + | | + | | | | + |
| MBNL3 | | | | | | | | | |
| MBOAT1 | | | | | | | | | |
| MBOAT2 | | | | | | | | | |
| MBOAT7 | | | | | | | | | |
| MBP | | | + | | | | | | + |
| MBR1 | | | | | | | | | |
| MBTD1 | | | | | | | | | |
| MBTPS1 | | | | | | | | | |
| MBTPS2 | | | | | | | | | |
| MC1R | + | | | + | + | | + | | + |
| MC2R | | | | | | | | | |
| MC3R | | | | | | | | | |
| MC4R | | | | | | | | | |
| MC5R | | | | | | | | | |
| MCA1 | | | | | | | | | |
| MCAM | | | | | | | | | |
| MCAT | | | | | | | | | |
| MCC | | | | | | | | | |

| | | | | | | | | | |
|---|---|---|---|---|---|---|---|---|---|
| MCCC1 | | | | | | | | | |
| MCCC2 | | | | | | | | | |
| MCCD1 | | | | | | | | | |
| MCD1 | | | | | | | | | |
| MCEE | | | | | | | | | |
| MCEMP1 | | | | | | | | | |
| MCF2 | | | | | | | | | |
| MCF2L | | | | | | + | | | + |
| MCF2L2 | | | | | | | | | |
| MCFD2 | | | | | | | | | |
| MCHR1 | | | | | | | | | |
| MCHR2 | | | | | | | | | |
| MCIDAS | + | | | + | + | | | | + |
| MCL1 | | | | | + | | | | + |
| MCM10 | | | | | | | | | |
| MCM2 | | | | | | | | | |
| MCM22 | | | | | | | | | |
| MCM3 | | | | | | | | | |
| MCM3AP | | | | | | | | | |
| MCM3AP-AS1 | | | | | | | | | |
| MCM4 | | | | | | | | | |
| MCM5 | | | | | | | | | |
| MCM6 | + | | | | + | | | | + |
| MCM7 | | | | | | | | | |
| MCM8 | | | | | | | | | |
| MCM9 | | | | | | | | | |
| MCMBP | | | | | | | | | |
| MCMDC2 | | | | + | | | | | + |
| MCOLN1 | | | | | | | | | |
| MCOLN2 | | | | | | | | | |
| MCOLN3 | | | | | | | | | |
| mcp2 | | | | | | | | | |
| mcp60 | | | | | | | | | |
| MCPH1 | | | | | | | | | |
| MCRS1 | | | | | | | | | |
| MCTP1 | | + | | | | + | | | + |
| MCTP2 | | | | | | | | | |
| MCTS1 | | + | + | | | + | | | + |
| MCTS2P | | | | | | | | | |
| MCU | | | | | | | | | |
| mcu-1 | | | | | | | | | |
| MCUR1 | | | | | | | | | |
| MDC1 | | | | | | | | | |
| MDFI | | | | | | | | | |
| MDFIC | | | | | | | | | |
| MDG1 | | | | | | | | | |
| MDGA1 | | | | | | | | | |
| MDGA2 | | | | | | | | | |
| MDH1 | | + | | | | + | | | + |
| MDH1B | | | | | | | | | |
| MDH2 | | | | | | | | | |
| MDJ1 | | | | | | | | | |
| MDK | | | | | | | | | |

| | | | | | | | | | |
|---|---|---|---|---|---|---|---|---|---|
| MDM1 | | | | | | | | | |
| mdm10 | | | | | | | | | |
| MDM2 | | | + | | | + | | | + |
| MDM30 | | | | | | | | | |
| MDM38 | | | | | | | | | |
| MDM4 | | + | | | | + | | | + |
| MDN1 | + | | | | + | | | | + |
| MDP1 | | | + | | | | | | + |
| MDY2 | | | | | | | | | |
| ME1 | | | | | + | | | | + |
| ME2 | | | | | | | | | |
| ME3 | | | | | | | | | |
| MEA1 | | | | | | | | | |
| MEAF6 | | | | | | | | | |
| MEC1 | | | | | | | | | |
| MECOM | | | | | | | | | |
| MECP2 | | | | | | | | | |
| MECR | | | | | | | | | |
| MED1 | | | | | | | | | |
| MED10 | | | | | | | | | |
| MED11 | | | | | | | | | |
| MED12 | | | | | | + | | | + |
| MED12L | | | | | | | | | |
| MED13 | | | + | | | | | | + |
| MED13L | | | | | | | | | |
| MED14 | + | | | | + | | | | + |
| MED15 | | | | | | | | | |
| MED16 | | | | | | | | | |
| MED17 | | | | | | | | | |
| MED18 | | | | | | | + | | + |
| MED19 | | | | | | | | | |
| MED20 | | | | | | | | | |
| MED21 | | | | + | | | | | + |
| MED22 | | | | | | | | | |
| MED23 | | | | | | | | | |
| MED24 | | | | | | | | | |
| MED25 | | | | | | | | | |
| MED26 | | | | | | | | | |
| MED27 | | | | | | | | | |
| MED28 | | | | | | | | | |
| MED29 | | | | | | | | | |
| MED30 | | | | | | | | | |
| MED31 | | | | | | | | | |
| MED4 | | | | | | | | | |
| MED6 | | | | | | | | | |
| MED7 | | | | | | | | | |
| MED8 | | | | | + | | | | + |
| MED9 | | | | | | | | | |
| MEDAG | | | | | | | | | |
| MEF2A | | | | | | | | | |
| MEF2B | | | | | | | | | |
| MEF2BNB | | | | | | | | | |
| MEF2BNB-MEF2B | | | | | | | | | |

| | | | | | | | | | |
|---|---|---|---|---|---|---|---|---|---|
| MEF2C | | | + | + | | | | | + |
| MEF2D | | | | | | | | | |
| MEFV | | | | | | | | | |
| MEG3 | | | | | | | | | |
| MEG8 | | | | | | | | | |
| MEGF10 | | | | | | | | | |
| MEGF11 | | | | | + | | | | + |
| MEGF6 | | | | | + | | | | + |
| MEGF8 | | | | | | | | | |
| MEGF9 | | | | | | | | | |
| MEI1 | | | | | | | | | |
| MEI4 | | | | + | | | | | + |
| MEIG1 | | | | | | | | | |
| MEIOB | | | | | | | | | |
| MEIS1 | + | | | + | + | | + | | + |
| MEIS2 | | | | | | | | | |
| MEIS3 | | | | | | | | | |
| MELK | | | | | | | | | |
| MEMO1 | | | | | | | | | |
| MEMO1P1 | | | | | | | | | |
| MEN1 | | | | | | | | | |
| MEOX1 | | | | | | | | | |
| MEOX2 | + | | | + | + | | + | | + |
| MEP1 | | | | | | | | | |
| MEP1A | | | | | | | | | |
| MEP1B | | | | | | | | | |
| MEPCE | | | | | | | | | |
| MEPE | | | | | | | | | |
| MER1 | | | | | | | | | |
| MERTK | | | | | + | | | | + |
| MES1 | | | | | | | | | |
| MESDC1 | | | | | | | | | |
| MESDC2 | | | | | | | | | |
| MESP1 | | | | | | | | | |
| MESP2 | | | | | | | | | |
| MEST | + | | + | | + | | | | + |
| MET | | | | | | | | | |
| met11 | | | | | | | | | |
| MET17 | | | | | | | | | |
| MET18 | | | | | | | | | |
| met26 | | | | | | | | | |
| MET30 | | | | | | | | | |
| MET32 | | | | | | | | | |
| MET6 | | | | | | | | | |
| MET8 | | | | | | | | | |
| METAP1 | | | | | | | | | |
| METAP1D | | | | | | | | | |
| METAP2 | | | | | | | | | |
| METRN | | | | | | | | | |
| METRNL | | | | | | | | | |
| METTL1 | | | | | | | | | |
| METTL10 | | | | | | | | | |
| METTL12 | | | | | | | | | |

| | | | | | | | | | |
|---|---|---|---|---|---|---|---|---|---|
| METTL13 | | | | | | | | | |
| METTL14 | | | | | | | | | |
| METTL15 | | | | | | + | | | + |
| METTL16 | | | | | | | | | |
| METTL17 | | | | | | | | | |
| METTL18 | | | | | | | | | |
| METTL20 | | | | | | | | | |
| METTL21A | | | | | | | | | |
| METTL21B | | | | | | | | | |
| METTL21C | | | | | | | | | |
| METTL21EP | | | | | | | | | |
| METTL22 | | | | | | + | | | + |
| METTL23 | | | | | | | | | |
| METTL24 | | | | | | | | | |
| METTL25 | | | | | | | | | |
| METTL2A | | | | | | | | | |
| METTL2B | | | | | | | | | |
| METTL3 | | | | | | | | | |
| METTL5 | | + | + | | | + | | | + |
| METTL6 | | | | | | | | | |
| METTL7A | | | | | | | | | |
| METTL7B | | | | | | | | | |
| METTL8 | | | | | | + | | | + |
| METTL9 | | | | | | | | | |
| MEX3A | | | | | | | | | |
| MEX3B | | | | | | | | | |
| MEX3C | | | | | | | | | |
| MEX3D | | | | | | | | | |
| mex67 | | | | | | | | | |
| MFAP1 | | | | | | | | | |
| Mfap1a | | | | | | | | | |
| MFAP2 | | | | | | | | | |
| MFAP3 | | | | | | | | | |
| MFAP3L | | | + | | | | | | + |
| MFAP4 | | | | | | | | | |
| MFAP5 | | | | | | | | | |
| MFF | | | | | | | | | |
| MFGE8 | | | | | | + | | | + |
| MFHAS1 | | | | | | | | | |
| MFI2 | | | | | | | | | |
| MFN1 | | | | | | | | | |
| MFN2 | | + | | | | + | | | + |
| MFNG | | | | | | | | | |
| MFRP | | | | | | | | | |
| MFSD1 | | | | | | | | | |
| MFSD10 | | | | | | | | | |
| MFSD11 | | + | | | | + | | | + |
| MFSD12 | | | | | | | | | |
| MFSD2A | + | | | | + | | | | + |
| MFSD2B | | | | | | | | | |
| MFSD3 | | | | | | | | | |
| MFSD4 | | | | | | | | | |
| MFSD5 | | | | | | | | | |

| | | | | | | | | | |
|---|---|---|---|---|---|---|---|---|---|
| MFSD6 | | | | + | | | | | + |
| MFSD7 | | | | | | | | | |
| MFSD8 | | | | | | | | | |
| MFSD9 | | | | | | | | | |
| MFT1 | | | | | | | | | |
| MGA | | + | | | | + | | | + |
| MGAM | + | | | + | + | | | | + |
| MGARP | | | | | | | | | |
| MGAT1 | | | | | | | | | |
| MGAT2 | | | | | | | | | |
| MGAT3 | | | | | | | | | |
| MGAT4A | + | | | | + | | | | + |
| MGAT4B | | | | | | | | | |
| MGAT4C | + | | | + | + | | + | | + |
| MGAT4D | | | | | | | | | |
| MGAT5 | | | | | | | | | |
| MGAT5B | | | | | | + | | | + |
| MGC15885 | | | | | | | | | |
| MGC16025 | | | | | | | | | |
| MGC27345 | | | | | | | | | |
| MGC34796 | | | | | | | | | |
| MGC39584 | | | | | | | | | |
| MGC45922 | | | | | | | | | |
| MGC50722 | | | | | | | | | |
| MGC53867 | | | | | | | | | |
| MGEA5 | | | | | | | | | |
| MGLL | | | | | | + | | | + |
| MGME1 | | | | | | | | | |
| MGMT | | | | | | | | | |
| Mgp | | | | | | | | | |
| MGRN1 | | | | | | + | | | + |
| MGS | | | | | | | | | |
| MGS1 | | | | | | | | | |
| MGST1 | | | | | | | | | |
| MGST2 | | | | | | | | | |
| MGST3 | | | | | | + | | | + |
| MHR1 | | | | | | | | | |
| MIA | | | | | | | | | |
| MIA2 | | | | | | | | | |
| MIA3 | | | + | | + | | | | + |
| MIA40 | | | | | | | | | |
| MIAT | | | | | | | | | |
| MIATNB | | | | | | | | | |
| MIB1 | | | | | | | | | |
| MIB2 | | | | | | | | | |
| MIC19 | | | | | | | | | |
| MICA | | | | | | | | | |
| MICAL1 | | | | | | | | | |
| MICAL2 | | | | | | | | | |
| Mical3 | | | | | | | | | |
| MICALCL | | | | | | | | | |
| MICALL1 | | | | | | | | | |
| MICALL2 | | | | | | | | | |

| | | | | | | | | | |
|---|---|---|---|---|---|---|---|---|---|
| MICB | | | | | | | | | |
| MICU1 | + | | | | + | | | | + |
| MICU2 | | | | | | | | | |
| MICU3 | | | | | | | | | |
| MID1 | | | | | | | | | |
| MID1IP1 | | | | | | | | | |
| MID2 | | | | | | | | | |
| Midn | + | | | | + | | | | + |
| MIEF1 | | | | | | | | | |
| MIEF2 | | | | | | + | | | + |
| MIEN1 | | | | | | | | | |
| MIER1 | | | | | | + | | | + |
| MIER2 | | | | | | | | | |
| MIER3 | | | | | | | | | |
| MIF | | | | | | | | | |
| MIF-AS1 | | | | | | | | | |
| MIF4GD | | | | | | | | | |
| MIG1 | | | | | | | | | |
| MIIP | | | | | | | | | |
| MILR1 | | | | | | | | | |
| MIM1 | | | | | | | | | |
| MINA | | | | | | | | | |
| MINK1 | | | | | | + | | | + |
| MINOS1 | | | | | | | | | |
| MINOS1-NBL1 | | | | | | | | | |
| MINPP1 | | | | | | | | | |
| MIOS | | | | | | | | | |
| MIOX | | | | | | | | | |
| MIP | | | | | | | | | |
| MIPEP | | | | | | | | | |
| MIPOL1 | | | | | | | | | |
| MIR1 | | | | | | | | | |
| MIR1-1 | | | | | | | | | |
| MIR1-2 | | | | | | | | | |
| MIR100 | | | | | | | | | |
| MIR100HG | | | | | | | | | |
| MIR101-2 | | | | | | | | | |
| MIR103A1 | | | | | | | | | |
| MIR103A2 | | | | | | | | | |
| MIR106A | | | | | | | | | |
| MIR106B | | | | | | | | | |
| MIR107 | | | | | | | | | |
| MIR10B | | | | | | | | | |
| MIR1180 | | | | | | | | | |
| MIR1181 | | | | | | | | | |
| MIR1185-1 | | | | | | | | | |
| MIR1185-2 | | | | | | | | | |
| MIR1193 | | | | | | | | | |
| MIR122 | | | | | | | | | |
| MIR1236 | | | | | | | | | |
| MIR1248 | | | | | | | | | |
| MIR1257 | | | | | | | | | |
| MIR125B1 | | | | | | | | | |

| | | | | | | | | | |
|---|---|---|---|---|---|---|---|---|---|
| MIR125B2 | | | | | | | | | |
| MIR127 | | | | | | | | | |
| MIR1275 | | | | | | | | | |
| MIR128-1 | | | | | | | | | |
| MIR128-2 | | | | | | | | | |
| MIR1292 | | | | | | | | | |
| MIR1301 | | | | | | | | | |
| MIR130A | | | | | | | | | |
| MIR132 | | | | | | | | | |
| MIR138-1 | | | | | | | | | |
| MIR138-2 | | | | | | | | | |
| MIR139 | | | | | | | | | |
| MIR140 | | | | | | | | | |
| MIR141 | | | | | | | | | |
| MIR142 | | | | | | | | | |
| MIR143 | | | | | | | | | |
| MIR144 | | | | | | | | | |
| MIR145 | | | | | | | | | |
| MIR1469 | | | | | | | | | |
| MIR152 | | | | | | | | | |
| MIR155 | | | | | | | | | |
| MIR15A | | | | | | | | | |
| MIR15B | | | | | | | | | |
| MIR16-1 | | | | | | | | | |
| MIR16-2 | | | | | | | | | |
| MIR17 | | | | | | | | | |
| MIR17HG | | | | | | | | | |
| MIR181A1HG | | | | | | | | | |
| MIR182 | | | | | | | | | |
| MIR183 | | | | | | | | | |
| MIR188 | | | | | | | | | |
| MIR18A | | | | | | | | | |
| MIR18B | | | | | | | | | |
| MIR190B | | | | | | | | | |
| MIR196B | | | | | | | | | |
| MIR199A1 | | | | | | | | | |
| MIR199A2 | | | | | | | | | |
| MIR19A | | | | | | | | | |
| MIR19B1 | | | | | | | | | |
| MIR19B2 | | | | | | | | | |
| MIR200A | | | | | | | | | |
| MIR200B | | | | | | | | | |
| MIR200C | | | | | | | | | |
| MIR203A | | | | | | | | | |
| MIR204 | | | | | | | | | |
| MIR205 | | | | | | | | | |
| MIR205HG | | | | | | | | | |
| MIR206 | | | | | | | | | |
| MIR20A | | | | | | | | | |
| MIR20B | | | | | | | | | |
| MIR21 | | | | | | | | | |
| MIR214 | | | | | | | | | |
| MIR216A | | | | | | | | | |

| | | | | | | | | | |
|---|---|---|---|---|---|---|---|---|---|
| MIR217 | | | | | | | | | |
| MIR22 | | | | | | | | | |
| MIR221 | | | | | | | | | |
| MIR222 | | | | | | | | | |
| MIR223 | | | | | | | | | |
| MIR22HG | | | | | | | | | |
| MIR23B | | | | | | | | | |
| MIR24-1 | | | | | | | | | |
| MIR25 | | | | | | | | | |
| MIR26A2 | | | | | | | | | |
| MIR26B | | | | | | | | | |
| MIR27A | | | | | | | | | |
| MIR27B | | | | | | | | | |
| MIR299 | | | | | | | | | |
| MIR29A | | | | | | | | | |
| MIR29B1 | | | | | | | | | |
| MIR29B2 | | | | | | | | | |
| MIR29C | | | | | | | | | |
| MIR302A | | | | | | | | | |
| MIR30A | | | | | | | | | |
| MIR30B | | | | | | | | | |
| MIR30C1 | | | | | | | | | |
| MIR31 | | | | | | | | | |
| MIR3118-1 | | | | | | | | | |
| MIR3187 | | | | | | | | | |
| MIR3194 | | | | | | | | | |
| MIR323A | | | | | | | | | |
| MIR324 | | | | | | | | | |
| MIR326 | | | | | | | | | |
| MIR331 | | | | | | | | | |
| MIR340 | | | | | | | | | |
| MIR34A | | | | | | | | | |
| MIR34B | | | | | | | | | |
| MIR34C | | | | | | | | | |
| MIR363 | | | | | | | | | |
| MIR3648-1 | | | | | | | | | |
| MIR3648-2 | | | | | | | | | |
| MIR3652 | | | | | | | | | |
| MIR3654 | | | | | | | | | |
| MIR3677 | | | | | | | | | |
| MIR3687-1 | | | | | | | | | |
| MIR3687-2 | | | | | | | | | |
| MIR3713 | | | | | | | | | |
| MIR375 | | | | | | | | | |
| MIR376C | | | | | | | | | |
| MIR378A | | | | | | | | | |
| MIR3910-1 | | | | | | | | | |
| MIR3910-2 | | | | | | | | | |
| MIR3927 | | | | | | | | | |
| MIR429 | | | | | | | | | |
| MIR4435-1HG | | | | | | | | | |
| MIR4449 | | | | | | | | | |
| MIR4487 | | | | | | | | | |

| | | | | | | | | | |
|---|---|---|---|---|---|---|---|---|---|
| MIR4497 | | | | | | | | | |
| MIR4500HG | | | | | | | | | |
| MIR451A | | | | | | | | | |
| MIR4534 | | | | | | | | | |
| MIR454 | | | | | | | | | |
| MIR4634 | | | | | | | | | |
| MIR4658 | | | | | | | | | |
| MIR4665 | | | | | | | | | |
| MIR4668 | | | | | | | | | |
| MIR4688 | | | | | | | | | |
| MIR4732 | | | | | | | | | |
| MIR4744 | | | | | | | | | |
| MIR484 | | | | | | | | | |
| MIR5010 | | | | | | | | | |
| MIR5193 | | | | | | | | | |
| MIR520D | | | | | | | | | |
| MIR548AJ1 | | | | | | | | | |
| MIR548E | | | | | | | | | |
| MIR570 | | | | | | | | | |
| MIR579 | | | | | | | | | |
| MIR589 | | | | | | | | | |
| MIR600HG | | | | | | | | | |
| MIR6087 | | | | | | | | | |
| MIR612 | | | | | | | | | |
| MIR614 | | | | | | | | | |
| MIR6505 | | | | | | | | | |
| MIR652 | | | | | | | | | |
| MIR657 | | | | | | | | | |
| MIR663A | | | | | | | | | |
| MIR663AHG | | | | | | | | | |
| MIR663B | | | | | | | | | |
| MIR671 | | | | | | | | | |
| MIR6775 | | | | | | | | | |
| MIR6791 | | | | | | | | | |
| MIR6800 | | | | | | | | | |
| MIR6816 | | | | | | | | | |
| MIR6871 | | | | | | | | | |
| MIR6891 | | | | | | | | | |
| MIR7-1 | | | | | | | | | |
| MIR7-2 | | | | | | | | | |
| MIR7-3 | | | | | | | | | |
| MIR769 | | | | | | | | | |
| MIR7705 | | | | | | | | | |
| MIR7843 | | | | | | | | | |
| MIR877 | | | | | | | | | |
| MIR9-1 | | | | | | | | | |
| MIR9-2 | | | | | | | | | |
| MIR9-3 | | | | | | | | | |
| MIR92A1 | | | | | | | | | |
| MIR92A2 | | | | | | | | | |
| MIR93 | | | | | | | | | |
| MIR98 | | | | | | | | | |
| MIR99AHG | | | | | | | | | |

| | | | | | | | | | | |
|---|---|---|---|---|---|---|---|---|---|---|
| MIRLET7A1 | | | | | | | | | | |
| MIRLET7A2 | | | | | | | | | | |
| MIRLET7A3 | | | | | | | | | | |
| MIRLET7B | | | | | | | | | | |
| MIRLET7BHG | | | | | | | | | | |
| MIRLET7C | | | | | | | | | | |
| MIRLET7D | | | | | | | | | | |
| MIRLET7E | | | | | | | | | | |
| MIRLET7F1 | | | | | | | | | | |
| MIRLET7F2 | | | | | | | | | | |
| MIRLET7G | | | | | | | | | | |
| MIRLET7I | | | | | | | | | | |
| MIS1 | | | | | | | | | | |
| MIS12 | | | | | | | | | | |
| MIS18A | | | | | | | | | | |
| MIS18BP1 | | | | | | | | | | |
| MISP | | | | | | | | | | |
| MITD1 | | | | | | | | | | |
| MITF | | | | | | | | | | |
| MKI67 | | | | | | | | | | |
| MKKS | | | | | | | | | | |
| MKL1 | + | | | | + | | | | | + |
| MKL2 | | | | | | | | | | |
| MKLN1 | | | | | | | | | | |
| MKNK1 | | | | + | | | | | | + |
| MKNK1-AS1 | | | | | | | | | | |
| MKNK2 | | | + | | | | | | | + |
| MKRN1 | | | | | | | | | | |
| MKRN2 | | | | | | | | | | |
| MKRN2OS | | | | | | | | | | |
| MKRN3 | | | | | | | | | | |
| MKRN4P | | | | | | | | | | |
| MKS1 | | | | | | | | | | |
| MKX | | | | | | + | | | | + |
| MLANA | | | | | | | | | | |
| MLC1 | | | | | | | | | | |
| MLEC | | | | | | | | | | |
| MLF1 | | | | | | | | | | |
| MLF2 | | | | | | | | | | |
| MLH1 | | | | | | | | | | |
| MLH3 | | | | | | | | | | |
| MLIP | | | | | | | | | | |
| MLKL | | | | | | | | | | |
| MLLT1 | | | | | | | | | | |
| MLLT10 | | | | | | | | | | |
| MLLT11 | | | | | | | | | | |
| MLLT3 | | | + | | | | | | | + |
| MLLT4 | | | | | | | | | | |
| MLLT4-AS1 | | | | | | | | | | |
| MLLT6 | | | | | | | | | | |
| MLN | | | | | | | | | | |
| MLNR | | | | | | | | | | |
| mlo3 | | | | | | | | | | |

| | | | | | | | | | |
|---|---|---|---|---|---|---|---|---|---|
| MLP1 | | | | | | | | | |
| MLP2 | | | | | | | | | |
| MLPH | + | | | + | + | | + | | + |
| MLST8 | | | | | | | | | |
| MLX | | | + | | | | | | + |
| MLXIP | | | | | | | | | |
| Mlxipl | | | | | | | | | |
| MLYCD | | | | | | | | | |
| MMAA | | | | | | | | | |
| MMAB | + | | | + | + | | | | + |
| MMACHC | | | | | | | | | |
| MMADHC | | | | | | | | | |
| MMD | | + | | | | + | + | | + |
| MMD2 | | + | | | | + | | | + |
| MME | | | | + | | | | | + |
| MMEL1 | | | | | | | | | |
| MMF1 | | | | | | | | | |
| MMGT1 | | | | | | | | | |
| MMP1 | | | | | | | | | |
| MMP10 | | | | | | | | | |
| MMP11 | | | | | | | | | |
| MMP12 | | | | | | | | | |
| MMP13 | | | | | | | | | |
| MMP14 | | | | | | | | | |
| MMP15 | | | | | | | | | |
| MMP16 | | + | | | | + | | | + |
| MMP17 | | | | | | + | | | + |
| MMP19 | | | | | | | | | |
| MMP2 | | | | | | | | | |
| MMP20 | | | | | | | | | |
| MMP21 | | | | | | | | | |
| MMP23B | | | | | | | | | |
| MMP24 | | | | | | | | | |
| MMP25 | | | | | | | | | |
| MMP26 | | | | | | | | | |
| MMP27 | | | | | | | | | |
| MMP28 | | | | | | | | | |
| MMP3 | | | | | | | | | |
| MMP7 | | | | | | | | | |
| MMP8 | | | | | | | | | |
| MMP9 | | | | | | | | | |
| MMRN1 | | | | | | | | | |
| MMRN2 | + | | | + | + | | + | | + |
| MMS19 | | | | | | | | | |
| MMS2 | | | | | | | | | |
| MMS21 | | | | | | | | | |
| MMS22L | | | | | | | | | |
| MMS4 | | | | | | | | | |
| MMZ1 | | | | | | | | | |
| MN1 | | | | | + | | | | + |
| MNAT1 | | | | | | | | | |
| MND1 | | | | | | | | | |
| MNDA | | | | | | | | | |

| | | | | | | | | | |
|---|---|---|---|---|---|---|---|---|---|
| MNN11 | | | | | | | | | |
| MNN2 | | | | | | | | | |
| mnn9 | | | | | | | | | |
| MNS1 | | | | + | | | | | + |
| MNT | | | | | | | | | |
| MNX1 | | | | | | | | | |
| MOAP1 | | | | | | | | | |
| MOB1A | | | | | | | | | |
| MOB1B | | | | | | | | | |
| MOB2 | | | | | | | | | |
| MOB3A | | | | | | | | | |
| MOB3B | | | | | | | | | |
| MOB3C | | | | | | | | | |
| MOB4 | + | | | + | + | | + | | + |
| MOBP | | | | | + | | | | + |
| MOCOS | | | | | | + | | | + |
| MOCS1 | | | | | | | | | |
| MOCS2 | | | | | | | | | |
| MOCS3 | | | | | | | | | |
| moe1 | | | | | | | | | |
| MOG | | | | | | | | + | + |
| MOGAT1 | | | | | | | | | |
| MOGAT2 | | | | | | | | | |
| MOGAT3 | | | | | | | | | |
| MOGS | | | | | | | | | |
| MOK | | | | | | | | | |
| MON1 | | | | | | | | | |
| MON1A | | | | | | | | | |
| MON1B | + | | | + | + | | + | | + |
| MON2 | | | | | | | | | |
| MORC1 | | | | | | | | | |
| MORC2 | | | | | | | | | |
| MORC3 | | | | | | | | | |
| MORC4 | | | | | | | | | |
| MORF4 | | | | | | | | | |
| MORF4L1 | | | | | | | | | |
| MORF4L1P2 | | | | | | | | | |
| MORF4L2 | | | | | | | | | |
| MORN1 | | | | | | | | | |
| MORN2 | | | | | | | | | |
| MORN3 | | | | | | | | | |
| MORN4 | | | | | | | | | |
| MORN5 | | | | | | | | | |
| MOS | | | | | | | | | |
| MOSPD1 | | | | | | | | | |
| MOSPD2 | | | | + | | | | | + |
| MOSPD3 | | | | | | | | | |
| MOT2 | | | | | | | | | |
| MOV10 | | | | | | | | | |
| MOV10L1 | | | | | | | | | |
| MOXD1 | | | | | | | | | |
| MPA43 | | | | | | | | | |
| MPC1 | | | | | | + | | | + |

| | | | | | | | | | |
|---|---|---|---|---|---|---|---|---|---|
| MPC2 | | | | | | | | | |
| MPD1 | | | | | | | | | |
| MPDU1 | | + | | | | + | | | + |
| MPDZ | | | | | | | | | |
| MPEG1 | | | | | | | | | |
| MPG | | | | | | | | | |
| mpg1 | | | | | | | | | |
| MPH1 | | | | | | | | | |
| MPHOSPH10 | | | | | | | | | |
| MPHOSPH6 | | | | | | | | | |
| MPHOSPH8 | | | | | | | | | |
| Mphosph9 | | + | | | | + | | | + |
| MPI | | | | | | | | | |
| MPL | | | | | | | | | |
| MPLKIP | | | | | | | | | |
| MPND | | | | | | | | | |
| MPO | | | | | | | | | |
| MPP1 | | | | | | | | | |
| MPP2 | | | | | | + | | | + |
| MPP3 | | | | | | | | | |
| MPP4 | | | | | | | | | |
| MPP5 | | | | | | | | | |
| MPP6 | | | + | | | | | | + |
| MPP7 | | | | | | | | | |
| MPPE1 | | | | | | | | | |
| MPPED1 | | | | | | | | | |
| MPPED2 | | | | | | | | | |
| MPRIP | | | | | | | | | |
| MPS1 | | | | | | | | | |
| MPS3 | | | | | | | | | |
| MPST | | | | | | | | | |
| MPTX1 | | | | | | | | | |
| MPV17 | | | | | | | | | |
| MPV17L | | | | | | + | | | + |
| MPV17L2 | | | | | | | | | |
| MPZ | | | | | | | | | |
| MPZL1 | | | | | | | | | |
| MPZL2 | | + | + | | | + | | | + |
| MPZL3 | | | + | | | | | | + |
| MR1 | | | | | | | | | |
| mra1 | | | | | | | | | |
| MRAP | | | | | | | | | |
| MRAP2 | | | | | | | | | |
| MRAS | | | | | | | | | |
| MRC1 | | | | | | | | | |
| MRC2 | + | | | + | + | | | | + |
| MRE11 | | | | | | | | | |
| MRE11A | | | | | | + | | | + |
| MREG | | | + | | | | | | + |
| MRFAP1 | | | | | | | | | |
| MRFAP1L1 | | | | | | | | | |
| MRGBP | | | | | | | | | |
| MRGPRE | | | | | | | | | |

| | | | | | | | | | |
|---|---|---|---|---|---|---|---|---|---|
| MRGPRF | | | | | | | | | |
| MRGPRG | | | | | | | | | |
| MRGPRG-AS1 | | | | | | | | | |
| MRGPRX1 | | | | | | | | | |
| MRGPRX3 | | | | | | | | | |
| MRGPRX4 | | | | | | | | | |
| MRI1 | | | | | | | | | |
| MRM1 | | | | | | | | | |
| MRO | + | | | + | + | | | | + |
| MROH1 | | | | | | | | | |
| MROH2A | | | | | | | | | |
| MROH2B | | | | | | | | | |
| MROH5 | + | | | + | + | | | | + |
| Mroh6 | | | | | | | | | |
| MROH7 | | | | | | | | | |
| MROH7-TTC4 | | | | | | | | | |
| MROH8 | | | | | | | | | |
| MROH9 | | | | | | | | | |
| MRP4 | | | | | | | | | |
| MRP8 | | | | | | | | | |
| MRPL1 | | | | | | | | | |
| MRPL10 | | | | | | | | | |
| MRPL11 | | | | | | | | | |
| MRPL12 | | | | | | | | | |
| MRPL13 | | | | | | | | | |
| MRPL14 | | | | | | | | | |
| MRPL15 | | | | | | | | | |
| MRPL16 | | | | | | | | | |
| MRPL17 | | | | | | | | | |
| MRPL18 | | | | | | | | | |
| MRPL19 | | | | | | | | | |
| MRPL2 | | | | | | | | | |
| MRPL20 | | | | | | | | | |
| MRPL21 | | | | | | | | | |
| MRPL22 | | | | | | | | | |
| MRPL23 | | | | | | | | | |
| MRPL24 | | | | | | + | | | + |
| MRPL27 | | | | | | | | | |
| MRPL28 | | | | | | | | | |
| MRPL3 | | | | | | | | | |
| MRPL30 | | | | | | + | | | + |
| MRPL32 | | | | | | | | | |
| MRPL33 | | | | | | | | | |
| MRPL34 | | | | | | | | | |
| MRPL35 | | | | | | | | | |
| MRPL36 | | | | | | | | | |
| MRPL37 | | | | | | | | | |
| MRPL38 | | | | | | | | | |
| MRPL39 | | | | | | | | | |
| MRPL4 | | | | | | | | | |
| MRPL40 | | | | | | | | | |
| MRPL41 | | | | | | | | | |
| MRPL42 | | | | | | | | | |

| | | | | | | | | | |
|---|---|---|---|---|---|---|---|---|---|
| MRPL43 | | | | | | | | | |
| MRPL44 | | | | | | | | | |
| MRPL45 | | | | | | | | | |
| MRPL46 | | | | | | | | | |
| MRPL47 | | | | | | | | | |
| MRPL48 | | | | | | | | | |
| MRPL49 | | | | | | | | | |
| MRPL50 | | | | | | | | | |
| MRPL51 | | | | | | | | | |
| MRPL52 | | | | | | | | | |
| MRPL53 | | | | | | | | | |
| MRPL54 | | | | | | | | | |
| MRPL55 | | + | | | | + | | | + |
| MRPL57 | | | | | | | | | |
| MRPL9 | | | | | | | | | |
| MRPS10 | | | | | | | | | |
| MRPS11 | | | | | | | | | |
| MRPS12 | | | | | | | | | |
| MRPS14 | | | | | | | | | |
| MRPS15 | | | | | | | | | |
| MRPS16 | | | | | | + | | | + |
| MRPS17 | | | | | | | | | |
| MRPS18A | | | | | | | | | |
| MRPS18B | | | | | | | | | |
| MRPS18C | | | | | | | | | |
| MRPS2 | | | | | | | | | |
| MRPS21 | | | | | | | | | |
| MRPS22 | | | | | | + | | | + |
| MRPS23 | | | | | | | | | |
| MRPS24 | | | | | | | | | |
| MRPS25 | | | | | | | | | |
| MRPS26 | | | | | | | | | |
| MRPS27 | | | | | | + | | | + |
| MRPS28 | | | | | | | | | |
| MRPS30 | | | | | | | | | |
| MRPS31 | | | | | | | | | |
| MRPS33 | | | | | | + | | | + |
| MRPS34 | | | | | | | | | |
| MRPS35 | | | | | | + | | | + |
| MRPS36 | | | | | | | | | |
| MRPS36P4 | | | | | | | | | |
| MRPS5 | | + | | | | + | | | + |
| MRPS6 | | | | | + | | | | + |
| MRPS7 | | | | | | | | | |
| MRPS9 | | + | | | | + | | | + |
| MRRF | | | | | | | | | |
| MRRFP1 | | | | | | | | | |
| MRS2 | | | | | | | | | |
| MRT4 | | | | | | | | | |
| MRTO4 | | | | | | | | | |
| MRVI1 | | | | | | | | | |
| MS2 | | | | | | | | | |
| MS4A1 | | | | | | | | | |

| | | | | | | | | | |
|---|---|---|---|---|---|---|---|---|---|
| MS4A10 | | | | | | | | | |
| MS4A12 | | | | | | | | | |
| MS4A13 | | | | | | | | | |
| MS4A14 | | | | | | | | | |
| MS4A15 | | | | | | | | | |
| MS4A2 | | | | | | | | | |
| MS4A3 | | | | | | | | | |
| MS4A4A | | | | | | | | | |
| MS4A5 | | | | | | | | | |
| MS4A6A | | | | | | | | | |
| Ms4a6d | | | | | | | | | |
| MS4A6E | | | | | | | | | |
| MS4A7 | | | | | | | | | |
| MSANTD1 | | | | | | | | | |
| MSANTD2 | | | | | | | | | |
| MSANTD3 | | | | | | | | | |
| MSANTD4 | | | | | | | | | |
| MSB1 | | | | | | | | | |
| MSB3 | | | | | | | | | |
| MSB4 | | | | | | | | | |
| MSC | | | | | | | | | |
| MSGN1 | | | | | | | | | |
| MSH2 | | | | | | | | | |
| MSH3 | | | | | | | | | |
| MSH4 | | | | | | | | | |
| MSH5 | | | | | | | | | |
| MSH6 | | | | | | | | | |
| MSI1 | | | | | | | | | |
| MSI2 | | | | | | | | | |
| MSK1 | | | | | | | | | |
| msl-1 | | | | | | | | | |
| msl-2 | | | | | | | | | |
| msl-3 | | | | | | | | | |
| MSL1 | | | | | | | | | |
| MSL2 | | | | | | | | | |
| MSL3 | | | | | | | | | |
| MSL3P1 | | | | | | | | | |
| MSL5 | | | | | | | | | |
| MSLN | | | | | | | | | |
| MSLNL | | | | | | | | | |
| MSMB | | | | | | | | | |
| MSMO1 | | | | | | | | | |
| MSMP | | | | | | | | | |
| MSN | | | | | + | | | + | + |
| MSN2 | | | | | | | | | |
| MSR1 | | | | | | | | | |
| MSRA | | | | | | | | | |
| MSRB1 | | | | | | | | + | + |
| MSRB2 | | | | | | | | | |
| MSRB3 | + | | | + | + | | | | + |
| MSS4 | | | | | | | | | |
| MSS51 | | | | | | | | | |
| MST1 | | | | | | | | | |

| | | | | | | | | | |
|---|---|---|---|---|---|---|---|---|---|
| MST1L | | | | | | | | | |
| MST1R | | | | | | | | | |
| MST27 | | | | | | | | | |
| MSTN | | | | | | | | | |
| MSTO1 | | | | | | | | | |
| MSTO2P | | | | | | | | | |
| MSX1 | | | | | | | | | |
| MSX2 | | | | | | | | | |
| MT1A | | | | | | | | | |
| MT1B | | | | | | | | | |
| MT1DP | | | | | | | | | |
| MT1E | | | | | | | | | |
| MT1F | | | | | | | | | |
| MT1G | | | | | | | | | |
| MT1H | | | | | | | | | |
| MT1HL1 | | | | | | | | | |
| MT1IP | | | | | | | | | |
| MT1L | | | | | | | | | |
| MT1M | | | | | | | | | |
| MT1P3 | | | | | | | | | |
| MT1X | | | | | | | | | |
| MT2A | | | | | | | | | |
| MT2P1 | | | | | | | | | |
| MT3 | | | | | | | | | |
| MTA1 | | | | | | | | | |
| MTA2 | | | | | | | | | |
| MTA3 | | | | | | | | | |
| MTAP | | | | | | | | | |
| MTBP | | | | | | | | | |
| MTC1 | | | | | | | | | |
| MTC2 | | | | | | | | | |
| MTC3 | | | | | | | | | |
| MTC4 | | | | | | | | | |
| MTCH1 | | | | | | | | | |
| MTCH2 | | + | | | | + | | | + |
| MTCL1 | | | | | | | | | |
| MTCO2P1 | | | | | | | | | |
| MTCP1 | | | | | | | | | |
| MTDH | | | + | | + | | | | + |
| MTERF1 | | | | | | | | | |
| MTERF2 | | | | | | | | | |
| MTERF3 | | | | | | | | | |
| MTERF4 | | | | | | | | | |
| MTF1 | | | + | | | | | | + |
| MTF2 | | | | | | | | | |
| MTFMT | | | | | | + | | | + |
| MTFP1 | | + | | | | + | | | + |
| MTFR1 | | | | | | | | | |
| MTFR1L | | | | | | | | | |
| MTFR2 | | | | | + | | | | + |
| MTG1 | | | | | | | | | |
| MTG2 | | | | | | | | | |
| MTHFD1 | | | | | | | | | |

| | | | | | | | | | |
|---|---|---|---|---|---|---|---|---|---|
| MTHFD1L | | | + | | | | | | + |
| MTHFD2 | | | | | + | | | | + |
| MTHFD2L | | | | | | | | | |
| MTHFR | | | | | | + | | | + |
| MTHFS | | | | | | | | | |
| MTHFSD | | | | | | | | | |
| MTIF2 | | + | | | | + | | | + |
| MTIF3 | | | | | | | | | |
| MTL5 | | | | | | | | | |
| MTM1 | | | | + | | + | | | + |
| MTMR1 | | | | | | | | | |
| MTMR10 | | | | | | | | | |
| MTMR11 | | | + | | | | | | + |
| MTMR12 | | | | | | | | | |
| MTMR14 | | | | | | | | | |
| MTMR2 | | | | | | | | | |
| MTMR3 | | | | | | | | | |
| MTMR4 | | | | | | | | | |
| MTMR6 | | | | | | | | | |
| MTMR7 | | | | | | | | | |
| MTMR8 | | | | | | | | | |
| MTMR9 | | | | | | | | | |
| MTNR1A | | | | | | | | | |
| MTNR1B | | | | | | | | | |
| MTO1 | | | | | | | | | |
| MTO3 | | | | | | | | | |
| MTOR | | | | | | | | | |
| MTPAP | | | | | | | | | |
| MTPN | | | | | | | | | |
| MTQ2 | | | | | | | | | |
| MTR | | | | | | | | | |
| MTR10 | | | | | | | | | |
| MTRF1 | | | | | | | | | |
| MTRF1L | | | | | | | | | |
| MTRNR2L1 | | | | | | | | | |
| MTRNR2L8 | | | | | | | | | |
| MTRR | | | | | | + | | | + |
| mts4 | | | | | | | | | |
| MTSS1 | | | | | | | | | |
| MTSS1L | | | + | | + | | | | + |
| MTTP | | | | | | | | | |
| MTURN | | | | | | | | | |
| MTUS1 | | | | | | | | | |
| MTUS2 | | | | + | | + | | | + |
| Mtx1 | | | | | | | | | |
| MTX2 | | | | | | | | | |
| MTX3 | | | | | | + | | | + |
| MUC1 | | | | | | | | | |
| MUC12 | | | | | | | | | |
| MUC13 | | | | | | | + | | + |
| MUC15 | | | | | | | | | |
| MUC16 | | | | | | | | | |
| MUC17 | | | | | | | | | |

| | | | | | | | | | |
|---|---|---|---|---|---|---|---|---|---|
| MUC19 | | | | | | | | | |
| MUC2 | | | | | | | | | |
| MUC20 | | | | | | | | | |
| MUC3A | | | | | | | | | |
| MUC4 | | | | | | | | | |
| MUC5AC | | | | | | | | | |
| MUC5B | | | | | | | | | |
| MUC6 | | | | | | | | | |
| MUC7 | | | | | | | | | |
| MUCL1 | | | | | | | | | |
| MUD2 | | | | | | | | | |
| Mug1 | | | | | | | | | |
| mug161 | | | | | | | | | |
| Mug2 | | | | | | | | | |
| MUL1 | | | | | + | | | | + |
| MUM1 | | | | | | | | | |
| MUM1L1 | | | | | | | | | |
| MUM4 | | | | | | | | | |
| MUP1 | | | | | | | | | |
| MURC | | | | | | | | | |
| MUS81 | | | | | | | | | |
| MUSK | | | | | | | | | |
| MUSTN1 | | | | | | | | | |
| MUT | | | | | | + | | | + |
| MUTYH | | | | | | | | | |
| MVB12A | | | | | | | | | |
| MVB12B | | | | | | | | | |
| MVD | | | | | | | | | |
| MVK | | | | | | | | | |
| MVP | | | | + | | | | | + |
| MVP1 | | | | | | | | | |
| MX1 | | | | | | | | | |
| MX2 | | | | | + | | | | + |
| MXD1 | | | | | | | | | |
| MXD3 | | | | | | | | | |
| MXD4 | | | | | | + | | | + |
| MXI1 | | | + | | | + | | | + |
| MXR2 | | | | | | | | | |
| MXRA5 | | | | | | | | | |
| MXRA7 | | | | | | | | | |
| MXRA8 | | | | | | | | | |
| MYADM | | | | | + | | | | + |
| MYADML2 | | | | | | | | | |
| MYB | + | | | + | + | | | | + |
| MYBBP1A | | | | | | | | | |
| MYBL1 | + | | | + | + | | + | | + |
| MYBL2 | | | | | | | | | |
| MYBPC1 | | | | | | | | | |
| MYBPC2 | | | | | | | | | |
| MYBPC3 | | | | | | | | | |
| MYBPH | | | | | | | | | |
| MYBPHL | | | | | | | | | |
| MYC | | | | | + | | | | + |

| | | | | | | | | | |
|---|---|---|---|---|---|---|---|---|---|
| MYCBP | | | | | | | | | |
| Mycbp2 | | | | | | | | | |
| MYCBPAP | | | | | | | | | |
| MYCL | | | | | | | | | |
| MYCN | | | | | | | | | |
| MYCT1 | | | | | | | | | |
| MYD88 | | | | | | | | | |
| MYEF2 | | + | | | | + | | | + |
| MYEOV | | | | | | | | | |
| MYEOV2 | | | | | | | | | |
| MYF5 | | | | | | | | | |
| MYF6 | | | | | | | | | |
| MYH1 | | | | | | | | | |
| MYH10 | | | | | + | | | | + |
| MYH11 | | | | | | | | | |
| MYH13 | | | | | | | | | |
| MYH14 | | | | | | | | | |
| MYH15 | | | | | | | | | |
| MYH16 | | | | | | | | | |
| MYH2 | | | | | | | | | |
| MYH3 | | | | | | | | | |
| MYH4 | | | | | | | | | |
| MYH6 | | | | | | | | | |
| MYH7 | | | | | | | | | |
| MYH7B | | | | | | | | | |
| MYH8 | + | | | + | + | | | | + |
| MYH9 | + | | | | + | | | | + |
| MYL1 | | | | | | | | | |
| MYL10 | | | | | | | | | |
| MYL12A | | | | | + | | | | + |
| MYL12B | | + | | | | + | | | + |
| MYL2 | | | | | | | | | |
| MYL3 | | | | | | | | | |
| MYL4 | | | | | | | | | |
| MYL5 | | | | | | | | | |
| MYL6 | | | | | | | | | |
| MYL6B | | | | | | | | | |
| Myl6l | | | | | | | | | |
| MYL7 | | | | | | | | | |
| MYL9 | | | | | | | | | |
| MYLIP | | | | | | | | | |
| MYLK | | | | | | | | | |
| MYLK2 | | | | | | | | | |
| MYLK3 | + | | | | + | | | | + |
| MYLK4 | + | | | + | + | | | | + |
| MYLPF | | | | | | | | | |
| MYNN | | | | | | | | | |
| myo1 | | | | | | | | | |
| MYO10 | | | + | | | | | | + |
| MYO15A | | | | | | | | | |
| MYO15B | | | | | | | | | |
| MYO16 | | | | | | | | | |
| MYO18A | | | | | | | | | |

| | | | | | | | | | |
|---|---|---|---|---|---|---|---|---|---|
| MYO18B | | | | | | | | | |
| MYO19 | | | | | | | | | |
| MYO1A | | | | | | | | | |
| MYO1B | | | + | | | | | | + |
| MYO1C | | | | | | | | | |
| MYO1D | | | | | | | | | |
| MYO1E | | | | | + | | | | + |
| MYO1F | | | | | | | | | |
| MYO1G | | | | | | | | | |
| MYO1H | | | | | | | | | |
| MYO2 | | | | | | | | | |
| Myo3a | | | | | | | | | |
| MYO3B | | | | | | | | | |
| MYO5 | | | | | | | | | |
| MYO5A | | | | | | | | | |
| MYO5B | | | | | | + | | | + |
| MYO5C | | | | | | | | | |
| MYO6 | | | | | | | | | |
| MYO7A | | | | | | | | | |
| MYO7B | | | | | | | | | |
| MYO9A | | | | | | | | | |
| MYO9B | | | | | | | | | |
| MYOC | | | | | | | | | |
| MYOCD | + | | | + | + | | + | | + |
| MYOD1 | | | | | | | | | |
| MYOF | | | | | | | | | |
| MYOG | | | | | | | | | |
| MYOM1 | | | | | | | | | |
| MYOM2 | | | | | | | | | |
| MYOM3 | | | | | | | | | |
| MYOT | | | | | | | | | |
| MYOZ1 | | | | | | | | | |
| MYOZ2 | | | | | | | | | |
| MYOZ3 | + | | | + | + | | + | | + |
| MYPN | | | | | | | | | |
| MYPOP | | | | | | + | | | + |
| MYRF | | | | | | | | | |
| MYRFL | | | | | | | | | |
| MYRIP | | | | | | | | | |
| MYSM1 | | + | | | | + | | | + |
| MYT1 | | | | | | + | | | + |
| MYT1L | | | | | | | | | |
| MYZAP | | | | | | | | | |
| MZB1 | | | | | | | | | |
| MZF1 | | | | | | | | | |
| MZM1 | | | | | | | | | |
| Mzt1 | | | | | | | | | |
| Mzt2 | | | | | | + | | | + |
| MZT2A | | | | | | | | | |
| MZT2B | | | | | | | | | |
| N | | | | | | | | | |
| N4BP1 | | | | | | | | | |
| N4BP2 | | | | | | | | | |

| | | | | | | | | | |
|---|---|---|---|---|---|---|---|---|---|
| N4BP2L1 | | | | | | | | | |
| N4BP2L2 | | | | + | | | | | + |
| N4BP3 | | | | | | | | | |
| N6AMT1 | | | | | | | | | |
| N6AMT2 | | | | | | | | | |
| NAA10 | | | | | | | | | |
| Naa11 | | | | | | | | | |
| Naa15 | | | | | + | | | | + |
| NAA16 | | | | | + | + | | | + |
| NAA20 | | | | | | | | | |
| NAA25 | | | + | | | | | | + |
| NAA30 | | | | | | | | | |
| NAA35 | | + | | | | + | | | + |
| NAA38 | | + | | | | + | | | + |
| NAA40 | | | | | | | | | |
| Naa50 | | | | | | | | | |
| NAA60 | | | | | | | | | |
| NAAA | | | | | + | | | | + |
| NAALAD2 | | | | | | | | | |
| NAALADL1 | | | | | | | | | |
| NAALADL2 | | | | | | | | | |
| NAB1 | | | + | | | | | | + |
| NAB2 | + | | | | | | | | + |
| NAB3 | | | | | | | | | |
| NABP1 | | | | | | | | | |
| NABP2 | | | | | | + | | | + |
| NACA | | | | | | | | | |
| NACA2 | | | | | | | | | |
| NACAD | | | | | | | | | |
| NACAP1 | | | | | | | | | |
| NACC1 | | | | | | | | | |
| NACC2 | | | | | | | | | |
| NADK | | | | | | + | | | + |
| NADK2 | | | | | | | | | |
| NADSYN1 | | | | | | | | | |
| NAE1 | | | | | | | | | |
| NAF1 | | | | | | | | | |
| NAGA | | | | | | | | | |
| NAGK | | | | | | | | | |
| NAGLU | | | | | | | | | |
| NAGPA | | | | | + | | | | + |
| NAGS | | | | | | | | | |
| NAIF1 | | | | | | | | | |
| NAIP | | | | | | | | | |
| NALCN | | | | | | | | | |
| NAM7 | | | | | | | | | |
| NAM8 | | | | | | | | | |
| NAMPT | | | | | | | | | |
| NAMPTP1 | | | | | | | | | |
| nan1 | | | | | | | | | |
| NANOG | | | | | | | | | |
| NANOGNB | | | | | | | | | |
| NANOS1 | | | | | | | | | |

| | | | | | | | | | |
|---|---|---|---|---|---|---|---|---|---|
| NANOS2 | | | | | | | | | |
| NANOS3 | | | | | | | | | |
| NANP | | | | | | | | | |
| NANS | | | | | | | | | |
| NAP1 | | | | | | | | | |
| NAP1L1 | | | | | | | | | |
| NAP1L2 | | | | | | | | | |
| NAP1L3 | | | | | | | | | |
| NAP1L4 | | | | | | | | | |
| NAP1L5 | | | + | | | | | | + |
| NAP1L6 | | | | | | | | | |
| nap2 | | | | | | | | | |
| NAPA | | | | | | | | | |
| NAPB | | | | | | | | | |
| NAPEPLD | | | | | | | | | |
| NAPG | | + | | | | + | | | + |
| NAPRT | | | | | | | | | |
| NAPSA | | | | | | | | | |
| NAPSB | | | | | | | | | |
| NARF | | | | | | | | | |
| NARFL | | | | | | | | | |
| NARR | | | | | | | | | |
| NARS | | | | | | | | | |
| NARS2 | | | | | | | | | |
| NAS2 | | | | | | | | | |
| NASP | | | | | | | | | |
| NAT1 | | | | | | | | | |
| NAT10 | | | | | | | | | |
| NAT14 | | | | | | | | | |
| NAT16 | | | | | | | | | |
| NAT2 | | | | | | | | | |
| NAT3 | | | | | | | | | |
| NAT6 | | | | | | | | | |
| NAT8 | | | | | | | | | |
| NAT8B | | | | | | | | | |
| NAT8L | | | + | | | + | | | + |
| NAT9 | | | | | | | | | |
| NATD1 | | | | | | | | | |
| NAV1 | | + | | | | + | | | + |
| NAV2 | | | | | | | | | |
| NAV3 | | | | | | | | | |
| NBAS | | | | | | | | | |
| NBEA | | | | | | + | | | + |
| NBEAL1 | | | | | | | | | |
| NBEAL2 | | | | | + | | | | + |
| NBEAP1 | | | | | | | | | |
| NBL1 | | | | | | | | | |
| NBN | | | | | | | | | |
| NBP2 | | | | | | | | | |
| NBPF1 | | | | | | | | | |
| NBPF10 | | | | | | | | | |
| NBPF11 | | | | | | | | | |
| NBPF12 | | | | | | | | | |

| | | | | | | | | | |
|---|---|---|---|---|---|---|---|---|---|
| NBPF14 | | | | | | | | | |
| NBPF15 | | | | | | | | | |
| NBPF19 | | | | | | | | | |
| NBPF20 | | | | | | | | | |
| NBPF22P | | | | | | | | | |
| NBPF25P | | | | | | | | | |
| NBPF26 | | | | | | | | | |
| NBPF3 | | | | | | | | | |
| NBPF4 | | | | | | | | | |
| NBPF6 | | | | | | | | | |
| NBPF8 | | | | | | | | | |
| NBPF9 | | | | | | | | | |
| NBR1 | | + | | | | + | | | + |
| NBR2 | | | | | | | | | |
| NCALD | | | | | | + | | | + |
| NCAM1 | | | | | | | | | |
| NCAM2 | | | + | + | | + | | | + |
| NCAN | + | | + | | + | | | | + |
| NCAPD2 | | | | | | | | | |
| NCAPD3 | | | | | | | | | |
| NCAPG | | | | | | | | | |
| NCAPG2 | | | | | | | | | |
| NCAPH | | | | | | | | | |
| NCAPH2 | | | | | | | | | |
| NCB2 | | | | | | | | | |
| NCBP1 | | | | | + | | | | + |
| NCBP2 | | | | | | | | | |
| NCBP2-AS2 | | | | | | | | | |
| NCCRP1 | | | | | | | | | |
| NCDN | | | | | | + | + | | + |
| NCE102 | | | | | | | | | |
| nce103 | | | | | | | | | |
| NCEH1 | | | | | | | | | |
| NCF1 | | | | | | | | | |
| NCF1C | | | | | | | | | |
| NCF2 | | | | | | | | | |
| NCF4 | | | | | | | | | |
| NCK1 | | | | | | | | | |
| NCK2 | | | | | | | + | | + |
| NCKAP1 | | | | | | | | | |
| NCKAP1L | | | | | | | | | |
| NCKAP5 | | | | | | + | | | + |
| NCKAP5L | | | | | | | | | |
| NCKIPSD | | | | | | | | | |
| NCL | | | | | | | | | |
| NCL1 | | | | | | | | | |
| NCLN | | | | | | | | | |
| NCOA1 | | | | | | | | | |
| NCOA2 | | | | | | | | | |
| NCOA3 | | | | | | | | | |
| NCOA4 | | | | | | | | | |
| NCOA5 | | | | | | | | | |
| NCOA6 | | | | | | | | | |

| | | | | | | | | | |
|---|---|---|---|---|---|---|---|---|---|
| NCOA7 | | | | | | | | | |
| NCOR1 | | | | | | | | | |
| NCOR2 | | | | | | | | | |
| NCP1 | | | | | | | | | |
| NCR1 | | | | | | | | | |
| NCR2 | | | | | | | | | |
| NCR3 | | | | | | | | | |
| NCR3LG1 | | | | | | | | | |
| NCS1 | | | + | | | | | | + |
| NCS2 | | | | | | | | | |
| NCSTN | | | | | | | | | |
| ND1 | | | | | | | | | |
| ND2 | | | | | | | | | |
| ND3 | | | | | | | | | |
| ND4 | | | | | | | | | |
| ND4L | | | | | | | | | |
| ND5 | | | | | | | | | |
| ND6 | | | | | | | | | |
| nda2 | | | | | | | | | |
| nda3 | | | | | | | | | |
| Ndc1 | | | | | | | | | |
| NDC80 | | | | | | | | | |
| Nde1 | | | | | | | | | |
| nde2 | | | | | | | | | |
| NDEL1 | + | | | | + | | | | + |
| Ndfip1 | | | | | | | | | |
| NDFIP2 | | | | | | | | | |
| NDN | | + | | | | + | | | + |
| NDNF | | + | + | | | + | | | + |
| NDNL2 | | | | | | | | | |
| NDOR1 | | | | | | | | | |
| NDP | + | | | + | + | | | | + |
| NDRG1 | | | | | | | | | |
| NDRG2 | | | + | | | | | | + |
| NDRG3 | | | | | | + | | | + |
| NDRG4 | | | | | | | | | |
| NDST1 | | | | | | | | | |
| NDST2 | | | | | | | | | |
| NDST3 | | | | | | | | | |
| NDST4 | | | | | | | | | |
| NDT80 | | | | | | | | | |
| NDUFA1 | | + | | | | + | | | + |
| NDUFA10 | | | | | | | | | |
| NDUFA11 | | | | | | | | | |
| NDUFA12 | | | | | | | | | |
| NDUFA13 | | | | | | | | | |
| NDUFA2 | | | | | | | | | |
| NDUFA3 | | + | | | | + | | | + |
| NDUFA4 | | | | | | | | | |
| NDUFA4L2 | | | | | | | | | |
| NDUFA4P1 | | | | | | | | | |
| NDUFA5 | | | | | | + | | | + |
| NDUFA6 | | | | | | | | | |

| | | | | | | | | | |
|---|---|---|---|---|---|---|---|---|---|
| NDUFA7 | | | | | | | | | |
| NDUFA8 | | | | | | | | | |
| NDUFA9 | | | | | | | | | |
| NDUFAB1 | | | | | | | | | |
| NDUFAF1 | | | | | | | | | |
| NDUFAF2 | | | | | | | | | |
| NDUFAF3 | | | | | | + | | | + |
| NDUFAF4 | | | | | | | | | |
| NDUFAF5 | | | | | | | | | |
| NDUFAF6 | | | | | | | | | |
| NDUFAF7 | | | | | | + | | | + |
| NDUFB1 | | | | | | | | | |
| NDUFB10 | | | | | | | | | |
| NDUFB11 | | + | | | | + | | | + |
| NDUFB2 | | | | | | | | | |
| NDUFB3 | | + | | | | + | | | + |
| NDUFB4 | | | | | | | | | |
| NDUFB5 | | | | | | | | | |
| NDUFB6 | | + | | | | + | | | + |
| NDUFB7 | | | | | | | | | |
| NDUFB8 | | | | | | | | | |
| NDUFB9 | | | | | | | | | |
| NDUFC1 | | | | | | | | | |
| NDUFC2 | | + | + | | | + | | | + |
| NDUFC2-KCTD14 | | | | | | | | | |
| NDUFS1 | | | | | | | | | |
| NDUFS2 | | | | | | | | | |
| NDUFS3 | | + | | | | + | | | + |
| NDUFS4 | | | | | | | | | |
| NDUFS5 | | + | | | | + | | | + |
| NDUFS6 | | | | | | | | | |
| NDUFS7 | | | | | | | | | |
| NDUFS8 | | | | | | | | | |
| NDUFV1 | | | | | | | | | |
| NDUFV2 | | + | | | | + | | | + |
| NDUFV3 | | + | | | | + | | | + |
| Ndufv3-ps1 | | | | | | | | | |
| NEAT1 | | | | | | | | | |
| NEB | | | | | | | | | |
| NEBL | | | | | | | | | |
| NECAB1 | | | | | | | | | |
| NECAB2 | | | | | | | | | |
| NECAB3 | | | | | | | | | |
| NECAP1 | | | | | | | | | |
| NECAP2 | | | | | | | | | |
| Nedd1 | | | | | | | | | |
| NEDD4 | | | | | | + | | | + |
| NEDD4L | | | | | | + | | | + |
| NEDD8 | | | | | | | | | |
| NEDD8-MDP1 | | | | | | | | | |
| NEDD9 | | | + | | + | | | | + |
| nef | | | | | | | | | |
| NEFH | | | + | | | | | | + |

| | | | | | | | | | |
|---|---|---|---|---|---|---|---|---|---|
| NEFL | | | + | | + | | | | + |
| NEFM | + | | + | + | + | | | | + |
| NEGR1 | | | | | | + | | | + |
| NEIL1 | | | | | | | | | |
| NEIL2 | | | | | | | | | |
| NEIL3 | | | | | | | | | |
| NEK1 | | | | | | | | | |
| NEK10 | | | | | | | | | |
| NEK11 | | | | | + | | | | + |
| NEK2 | | | | | | | | | |
| NEK3 | | | | | | | | | |
| NEK4 | | | | | | | | | |
| NEK5 | | | | | | | | | |
| NEK6 | | | | | | + | | | + |
| NEK7 | | | | | | | | | |
| NEK8 | | | | | | | | | |
| NEK9 | | | | | | | | | |
| NEL1 | | | | | | | | | |
| NELFA | | | | | | | | | |
| NELFB | | | | | | | | | |
| NELFCD | | | | | | + | | | + |
| NELFE | | | | | | | | | |
| NELL1 | | | | | | | | | |
| NELL2 | | | | | | + | + | | + |
| NEMF | | | | | | | | | |
| NENF | | | | | | | | | |
| NEO1 | | | | | | | | | |
| NEPNP | | | | | | | | | |
| NES | | | | | | | | | |
| Net1 | | | | | | | | | |
| NETO1 | | | | | | | | | |
| NETO2 | | | | | | | | | |
| NEU1 | | | | | | + | | | + |
| NEU2 | | | | | | | | | |
| NEU3 | | | | | | | | | |
| NEU4 | | + | + | | | + | | + | + |
| neur | | | | | | | | | |
| NEURL1 | | | | | | | | | |
| NEURL1B | + | | | + | + | | | | + |
| NEURL2 | | | | | | | | | |
| NEURL3 | | | | | | | | | |
| NEURL4 | | | | | | | | | |
| NEUROD1 | | | | | | + | | | + |
| NEUROD2 | | | | | | + | | | + |
| NEUROD4 | | | | | | | | | |
| NEUROD6 | | | | | | | | | |
| NEUROG1 | | | | | | | | | |
| Neurog2 | | | | | | | | | |
| NEUROG3 | | | | | | | | | |
| NEXN | | | | | | | | | |
| NEXN-AS1 | | | | | | | | | |
| NF1 | | | | | | | | | |
| NF2 | | | | | | | | | |

| | | | | | | | | | |
|---|---|---|---|---|---|---|---|---|---|
| NFAM1 | | | | | | | | | |
| NFASC | | | | | | | | | |
| NFAT5 | | | | | | | | | |
| NFATC1 | | | | | | | | | |
| NFATC2 | | | | | | + | | | + |
| Nfatc2ip | | | | | | | | | |
| NFATC3 | | | | | | | | | |
| NFATC4 | | | | | | | | | |
| NFE2 | | | | | | | | | |
| NFE2L1 | | | + | | | | | | + |
| NFE2L2 | | | | | | | | | |
| NFE2L3 | | | | | + | | | | + |
| NFE4 | | | | | | | | | |
| NFI1 | | | | | | | | | |
| NFIA | | | | | | + | | | + |
| NFIB | | | | | | | | | |
| NFIC | | | | | | | | | |
| NFIL3 | | | + | | | | | | + |
| NFIX | | + | | | | + | | | + |
| NFKB1 | | | | | | | | | |
| NFKB2 | | | | | | | | | |
| NFKBIA | + | | | + | + | | | | + |
| NFKBIB | | | | | | | | | |
| NFKBID | | | + | | | | | | + |
| NFKBIE | | | | | | | | | |
| NFKBIL1 | | | | | | | | | |
| NFKBIZ | | | + | | | | | | + |
| NFRKB | | | | | | | | | |
| NFS1 | | | | | | | | | |
| NFU1 | | | | | | | | | |
| NFX1 | | | | | | | | | |
| NFXL1 | | | | | | | | | |
| NFYA | | | + | | | | | | + |
| NFYB | | | | | | | | | |
| NFYC | | | | | | | | | |
| NFYC-AS1 | | | | | | | | | |
| NGB | | | | | | | | | |
| NGDN | | | | | | | | | |
| NGEF | | | + | | | | | | + |
| NGF | | | | | | | | | |
| NGFR | | | | | | | | | |
| Ngfrap1 | | | | | | | | | |
| NGG1 | | | | | | | | | |
| NGLY1 | | | | | | | | | |
| NGR1 | | | | | | | | | |
| NGRN | | + | | | | + | | | + |
| NHEJ1 | | | | | | | | | |
| NHLH1 | | | | | | | | | |
| NHLH2 | + | | | + | + | | | | + |
| NHLRC1 | | | | | | | | | |
| NHLRC2 | | | | | | | | | |
| NHLRC3 | | | | | | | | | |
| NHLRC4 | | | | | | | | | |

| | | | | | | | | | |
|---|---|---|---|---|---|---|---|---|---|
| NHP2 | | | | | | + | | | + |
| NHP2L1 | | + | + | | | + | | | + |
| nhp6 | | | | | | | | | |
| NHS | | | | | | | | | |
| Nhsl1 | | | | | | | | | |
| NHSL2 | | | | | | + | | | + |
| NHX1 | | | | | | | | | |
| NICN1 | | | + | | | + | | | + |
| NID1 | | | | | | | | | |
| NID2 | | | | | | | | | |
| NIF3L1 | | | | | | | | | |
| NIFK | | | | | | | | | |
| NIM1K | | + | | + | | | | | + |
| NIN | | | | | | | | | |
| ninaE | | | | | | | | | |
| NINJ1 | | | | | | | | | |
| NINJ2 | | | | | | | | | |
| NINL | | | | | | | | | |
| NIP1 | | | | | | | | | |
| NIP7 | | | | | | | | | |
| NIPA1 | | | | | | | | | |
| NIPA2 | | | | | | | | | |
| NIPAL1 | | | | | | | | | |
| NIPAL2 | | | | | | | | | |
| NIPAL3 | | | | | | + | | | + |
| NIPAL4 | | | | | | | | | |
| NIPBL | | | | | | | | | |
| NIPSNAP1 | | | | | | | | | |
| NIPSNAP3A | | | | | | | | | |
| NIPSNAP3B | | | | | | | | | |
| NISCH | | | | | | | | | |
| NIT1 | | | | | | + | | | + |
| NIT2 | | | | | | | | | |
| NKAIN1 | | | | | | | | | |
| NKAIN3 | | | | | | | | | |
| NKAIN4 | | | | | | + | | | + |
| NKAP | | | | | | | | | |
| NKAPL | | | | | | | | | |
| NKAPP1 | | | | | | | | | |
| NKD1 | | | | | | | | | |
| NKD2 | | | | | | | | | |
| NKG7 | | | | | | | | | |
| NKIRAS1 | | | | | | | | | |
| NKIRAS2 | | | | | | | | | |
| NKP1 | | | | | | | | | |
| NKP2 | | | | | | | | | |
| NKRF | | | | | | | | | |
| NKTR | | | | | | | | | |
| NKX1-1 | | | | | | | | | |
| NKX1-2 | | | | | | | | | |
| NKX2-1 | + | | | + | + | | + | | + |
| NKX2-2 | | | | | | | | | |
| NKX2-3 | | | | | | | | | |

| | | | | | | | | | |
|---|---|---|---|---|---|---|---|---|---|
| NKX2-4 | | | | | | | | | |
| NKX2-5 | | | | | | | | | |
| NKX2-6 | | | | | | | | | |
| NKX3-1 | | | | | | | | | |
| NKX3-2 | | | | | | | | | |
| NKX6-1 | | | | | | | | | |
| NKX6-2 | | | | | | | | | |
| NKX6-3 | | | | | | | | | |
| NLE1 | | | | | | | | | |
| NLGN1 | | | | | | | | | |
| NLGN2 | | | | | | | | | |
| NLGN3 | | | | | | + | | | + |
| NLGN4X | | | | | | | | | |
| NLGN4Y | | | | | | | | | |
| NLK | | | | + | + | | | | + |
| NLN | | | | | | | | | |
| NLRC3 | | | | + | | | | | + |
| NLRC4 | | | | | | | | | |
| NLRC5 | | | | | | | | | |
| NLRP1 | | | | | | | | | |
| NLRP10 | | | | | | | | | |
| NLRP11 | | | | | | | | | |
| NLRP12 | | | | | | | | | |
| NLRP13 | | | | | | | | | |
| NLRP14 | | | | | | | | | |
| NLRP2 | | | | | | | | | |
| NLRP3 | | | | | | | | | |
| NLRP4 | | | | | | | | | |
| Nlrp4c | | | | | | | | | |
| NLRP5 | | | | | | | | | |
| NLRP6 | | | | | | | | | |
| NLRP7 | | | | | | | | | |
| NLRP8 | | | | | | | | | |
| NLRP9 | | | | | | | | | |
| NLRX1 | | + | | | | | | | + |
| NMA1 | | | | | | | | | |
| NMB | | | | | | | | | |
| NMBR | | | | | | | | | |
| NMD3 | | | | | | | | | |
| NME1 | | | | | | | | | |
| NME1-NME2 | | | | | | | | | |
| NME2 | | | | | | | | | |
| NME2P1 | | | | | | | | | |
| NME3 | | | | | | | | | |
| NME4 | | | | | | | | | |
| NME5 | | | | | | | | | |
| NME6 | | | | | | | | | |
| NME7 | | | | | | | | | |
| NME8 | | | | | | | | | |
| NMI | | | | | | | | | |
| NMNAT1 | | | | | | | | | |
| NMNAT2 | | | | | | + | | | + |
| NMNAT3 | | | | | | | | | |

| | | | | | | | | | |
|---|---|---|---|---|---|---|---|---|---|
| NMRAL1 | | | | | | | | | |
| NMRK1 | | | | | | | | | |
| NMRK2 | | | | | | | | | |
| NMS | | | | | | | | | |
| NMT1 | | | + | | | | | | + |
| NMT2 | | | + | | | | | | + |
| NMU | | | | | | | | | |
| NMUR1 | | | | | | | | | |
| NMUR2 | | | | | | | | | |
| NNAT | | | | | | | | | |
| NNMT | | | + | | | | | | + |
| NNT | | | | | | | | | |
| NNT-AS1 | | | | | | | | | |
| NOA1 | | | | | | | | | |
| NOB1 | | | | | | | | | |
| NOBOX | | | | | | | | | |
| NOC2L | | | | | | | | | |
| NOC3L | | | | | | | | | |
| NOC4L | | | | | | | | | |
| NOD1 | | | | | | | | | |
| NOD2 | | | | | | | | | |
| NODAL | | | | | | | | | |
| NOG | | | | | | | | | |
| NOL10 | | | | | | | | | |
| NOL11 | | | | | | | | | |
| NOL12 | | | | + | | + | | | + |
| NOL3 | | | | | + | | | | + |
| NOL4 | | | | | | | | | |
| NOL4L | | | | | | | | | |
| NOL6 | | | | | | | | | |
| NOL7 | | | | | | | | | |
| NOL8 | | | | | | | | | |
| NOL9 | | | | | | | | | |
| NOLC1 | | | | | | | | | |
| NOM1 | | | | | | | | | |
| NOMO1 | | | | | | | | | |
| NOMO2 | | | | | | | | | |
| NOMO3 | | | | | | | | | |
| NONO | | | | | | | | | |
| NOP1 | | | | | | | | | |
| NOP10 | | + | | | | + | | | + |
| NOP14 | | | | | | | | | |
| NOP14-AS1 | | | | | | | | | |
| NOP15 | | | | | | | | | |
| NOP16 | | | | | + | | | | + |
| NOP2 | | | | | | | | | |
| NOP56 | | | | | | | | | |
| NOP58 | | | | | | | | | |
| NOP9 | | | | | | | | | |
| NOS1 | | | | | | + | | | + |
| NOS1AP | | | | | | | | | |
| NOS2 | | | | | | | | | |
| NOS3 | | | | | | | | | |

| | | | | | | | | | |
|---|---|---|---|---|---|---|---|---|---|
| NOSIP | | | | | | | | | |
| NOSTRIN | | | | | | | | | |
| NOTCH1 | + | | | | + | + | | | + |
| NOTCH2 | | | | | | | | | |
| NOTCH2NL | | | | | | | | | |
| NOTCH3 | | | | | | | | | |
| Notch4 | | | | | | | | | |
| NOTO | | | | | | | | | |
| NOTUM | | | | | | | | | |
| NOV | | | | | | | | | |
| NOVA1 | | | | | | | | | |
| NOVA2 | | | | | | | | | |
| NOX1 | | | | | | | | | |
| NOX3 | | | | | | | | | |
| NOX4 | | | | | | | | | |
| NOX5 | | | | | | | | | |
| NOXA1 | | | | | | | | | |
| Noxo1 | | | | | | | | | |
| NOXRED1 | | | | | | | | | |
| NPAP1 | | | | | | | | | |
| NPAS1 | | | | | | | | | |
| NPAS2 | | | | | + | | | | + |
| NPAS3 | | | | | | | | | |
| NPAS4 | | | | | | | | | |
| NPAT | | | | | | | | | |
| NPB | | | | | | | | | |
| NPBWR1 | | | | | | | | | |
| NPBWR2 | | | | | | | | | |
| NPC1 | | | | | | | | | |
| NPC1L1 | | | | | | | | | |
| NPC2 | | | | | | | | | |
| NPDC1 | | | | | | | | | |
| NPEPL1 | | | | | | | | | |
| NPEPPS | | | | | | | | | |
| NPFF | | | | | | | | | |
| NPFFR1 | | | | | | | | | |
| NPFFR2 | | | | | | | | | |
| NPHP1 | | | | | | + | | | + |
| NPHP3 | | | | | + | | | | + |
| NPHP4 | | | | | + | | | | + |
| NPHS1 | | | | | | | | | |
| NPHS2 | | | | | | | | | |
| NPIPA1 | | | | | | | | | |
| NPIPA2 | | | | | | | | | |
| NPIPA3 | | | | | | | | | |
| NPIPB11 | | | | | | | | | |
| NPIPB15 | | | | | | | | | |
| NPIPB3 | | | | | | | | | |
| NPIPB5 | | | | | | | | | |
| NPIPB6 | | | | | | | | | |
| NPIPB9 | | | | | | | | | |
| NPL | | | | | | | | | |
| NPL3 | | | | | | | | | |

| | | | | | | | | | |
|---|---|---|---|---|---|---|---|---|---|
| NPLOC4 | | | | | | | | | |
| NPM1 | | | | | | | | | |
| NPM1P13 | | | | | | | | | |
| NPM2 | | | | | | | | | |
| NPM3 | | | | | | | | | |
| NPNT | | | | | | | | | |
| npp-10 | | | | | | | | | |
| NPP1 | | | | | | | | | |
| NPPA | | | | | | | | | |
| NPPB | | | | | | | | | |
| NPPC | | | + | | | | | | + |
| NPR1 | | | | | | | | | |
| NPR2 | | | | | | | | | |
| NPR3 | | | + | | | | | | + |
| NPRL2 | | | | | | | | | |
| NPRL3 | | | | | | | | | |
| NPSR1 | | | | | | | | | |
| NPTN | | | | | | | | | |
| NPTX1 | | | | | | + | | | + |
| NPTX2 | + | | + | | + | | | | + |
| Nptxr | | | | | | | | + | + |
| NPVF | | | | | | | | | |
| NPW | | | | | | | | | |
| NPY | + | | + | | + | | | | + |
| NPY1R | | | | | | | | | |
| NPY2R | | + | | | | + | | | + |
| NPY4R | | | | | | | | | |
| NPY5R | | | | | | | | | |
| NQO1 | | | | | | | | | |
| NQO2 | | | | | | | | | |
| NR0B1 | | | | | | | | | |
| NR0B2 | | | | | | | | | |
| NR1D1 | | | | | | + | | | + |
| NR1D2 | | | | | | | | | |
| NR1H2 | | | | | | | | | |
| NR1H3 | | | | | | | | | |
| NR1H4 | | | | | | | | | |
| NR1I2 | | | | | | | | | |
| NR1I3 | | | | | | | | | |
| NR2C1 | | | | | | | | | |
| NR2C2 | | | | | | | | | |
| NR2C2AP | | | | | | | | | |
| NR2E1 | + | | | + | + | | | | + |
| NR2E3 | | | | | | | | | |
| NR2F1 | | | | | | + | | | + |
| NR2F1-AS1 | | | | | | | | | |
| NR2F2 | | | | | | | | | |
| NR2F2-AS1 | | | | | | | | | |
| NR2F6 | | | | | | | | | |
| NR3C1 | | | | + | | | | | + |
| NR3C2 | | | | | | + | | | + |
| NR4A1 | + | | + | | + | | | | + |
| NR4A2 | + | | | | + | | | | + |

| | | | | | | | | | |
|---|---|---|---|---|---|---|---|---|---|
| NR4A3 | + | | | | + | | | | + |
| NR5A1 | | | | | | | | | |
| NR5A2 | | | | | | | | | |
| NR6A1 | | | | | | | | | |
| NRAP | | | | | | | | | |
| nrarp | | | | | | | | | |
| NRAS | | | | + | | | | | + |
| NRBF2 | | | | | | | | | |
| NRBP1 | | | | | | | | | |
| NRBP2 | | | | | | | | | |
| NRCAM | | | | | | | | | |
| NRD1 | + | | + | | | | | | + |
| NRDE2 | | | | | | | | | |
| NREP | | | | | | | | | |
| NRF1 | | | + | | | | | | + |
| NRG1 | | | | | | | | | |
| NRG2 | | | | | | | | | |
| NRG3 | | | | | | | | | |
| NRG4 | | | | | | | | | |
| NRGN | | | | | | | | | |
| NRIP1 | | | | | | | | | |
| Nrip2 | | | | | | | | | |
| Nrip3 | | | + | | + | | | | + |
| Nrk | | | | | | | | | |
| NRL | + | | | + | + | | | | + |
| NRM | | | | | | | | | |
| NRN1 | + | | + | | + | | | | + |
| NRN1L | | | | | | | | | |
| NRON | | | | | | | | | |
| NRP1 | + | | + | | + | | | | + |
| NRP2 | + | | | | + | | | | + |
| NRPD1B | | | | | | | | | |
| NRROS | | | | | | | | | |
| nrs1 | | | | | | | | | |
| NRSN1 | | | | | | | | | |
| NRSN2 | | | | | + | + | | | + |
| NRTN | | | | | | | | | |
| NRXN1 | | | | | | + | | | + |
| NRXN2 | | | | | + | | | | + |
| NRXN3 | | | | | + | | | | + |
| NSA2 | | | | | | | | | |
| NSD1 | | | | | | | | | |
| NSDHL | | | | | | + | | | + |
| NSE5 | | | | | | | | | |
| NSF | | | | | | + | + | | + |
| Nsf2 | | | | | | | | | |
| NSFL1C | | | | | | | | | |
| NSG1 | | | | | | | | | |
| NSL1 | | | | | | | | | |
| NSMAF | | | + | | | | | | + |
| NSMCE1 | | | | | | | | | |
| NSMCE2 | | | | | | | | | |
| NSMCE4A | | | | | | | | | |

| | | | | | | | | | |
|---|---|---|---|---|---|---|---|---|---|
| NSMF | | | | | | | | | |
| NSP1 | | | | | | | | | |
| nsp10 | | | | | | | | | |
| nsp10ab | | | | | | | | | |
| nsp11 | | | | | | | | | |
| nsp11a | | | | | | | | | |
| nsp12 | | | | | | | | | |
| nsp12ab | | | | | | | | | |
| nsp13 | | | | | | | | | |
| nsp13ab | | | | | | | | | |
| nsp14 | | | | | | | | | |
| nsp14ab | | | | | | | | | |
| nsp15 | | | | | | | | | |
| nsp15ab | | | | | | | | | |
| nsp16 | | | | | | | | | |
| nsp16ab | | | | | | | | | |
| nsp1ab | | | | | | | | | |
| nsp2 | | | | | | | | | |
| nsp2ab | | | | | | | | | |
| nsp3 | | | | | | | | | |
| nsp3ab | | | | | | | | | |
| nsp4 | | | | | | | | | |
| nsp4ab | | | | | | | | | |
| nsp5 | | | | | | | | | |
| nsp5ab | | | | | | | | | |
| nsp6 | | | | | | | | | |
| nsp6ab | | | | | | | | | |
| nsp7 | | | | | | | | | |
| nsp7ab | | | | | | | | | |
| nsp8 | | | | | | | | | |
| nsp8ab | | | | | | | | | |
| nsp9 | | | | | | | | | |
| nsp9ab | | | | | | | | | |
| NSR1 | | | | | | | | | |
| NSRP1 | | | | | | | | | |
| NSUN2 | | | | | | | | | |
| NSUN3 | | | | | | | | | |
| NSUN4 | | | | | | | | | |
| NSUN5 | | | | | | | | | |
| NSUN5P1 | | | | | | | | | |
| NSUN5P2 | | | | | | | | | |
| NSUN6 | | | | | | | | | |
| NSUN7 | | | | | | | | | |
| NT5C | | | | | | | | | |
| NT5C1A | | | | | | | | | |
| NT5C1B | | | | | | | | | |
| NT5C2 | | | | | | | | | |
| NT5C3A | | | | | | | | | |
| NT5C3B | | | | | | | | | |
| NT5DC1 | | | | | | | | | |
| NT5DC2 | | | | | | | | | |
| NT5DC3 | + | | | | | | | | + |
| NT5DC4 | | | | | | | | | |

| | | | | | | | | | |
|---|---|---|---|---|---|---|---|---|---|
| NT5E | | | | | | | | | |
| NT5M | | | | | | | | | |
| NTA1 | | | | | | | | | |
| NTAN1 | | | | | | | | | |
| NTC20 | | | | | | | | | |
| NTF2A | | | | | | | | | |
| NTF2B | | | | | | | | | |
| NTF3 | | | | | | | | | |
| NTF4 | | | | | | | | | |
| NTG2 | | | | | | | | | |
| NTH1 | | | | | | | | | |
| NTHL1 | | | | | | | | | |
| NTM | | | | | | | | + | + |
| NTMT1 | | | | | | | | | |
| NTN1 | | | | | | | | | |
| Ntn3 | | | | | | | | | |
| NTN4 | | | | | | | | | |
| NTN5 | | | | | | | | | |
| NTNG1 | | | | | | | | | |
| NTNG2 | | | | | | | | | |
| ntp1 | | | | | | | | | |
| NTPCR | | | | | | | | | |
| NTR2 | | | | | | | | | |
| NTRK1 | | | | | | | | | |
| NTRK2 | + | | + | | + | | | | + |
| NTRK3 | + | | | | | | | | + |
| NTS | | | | | | | | | |
| NTSR1 | | | | | | | | | |
| NTSR2 | | | | | | | | | |
| NUAK1 | | | | | | | | | |
| NUAK2 | + | | | + | + | | + | | + |
| NUB1 | | | | | | + | | | + |
| NUBP1 | | | | | | | | | |
| NUBP2 | | | | | | | | | |
| NUBPL | | | | | | | | | |
| NUCB1 | | | | | | | | | |
| NUCB2 | | | | | | | | | |
| NUCKS1 | | | | | | | | | |
| NUDC | | | | | | | | | |
| NUDCD1 | | | | | | | | | |
| NUDCD2 | | | | | | | | | |
| NUDCD3 | | | | | | + | | | + |
| NUDT1 | | | | | | | | | |
| NUDT10 | | | | | | + | | | + |
| NUDT11 | | | | | | | | | |
| NUDT12 | | | | | | | | | |
| NUDT13 | | | | | | | | | |
| NUDT14 | | | | | | | | | |
| NUDT15 | | | | | | | | | |
| NUDT16 | | | | | | | | | |
| NUDT16L1 | | | | | | | | | |
| NUDT16P1 | | | | | | | | | |
| NUDT18 | | | | | | | | | |

| | | | | | | | | | |
|---|---|---|---|---|---|---|---|---|---|
| NUDT19 | | | | | | | | | |
| NUDT2 | | | | | | | | | |
| NUDT21 | | | | | | | | | |
| NUDT22 | | | | | | | | | |
| NUDT3 | | | | | | | | | |
| NUDT4 | | | + | | | | | | + |
| NUDT4P1 | | | | | | | | | |
| NUDT5 | | | | | | | | | |
| NUDT6 | | | | | | | | | |
| NUDT7 | + | | | + | + | | | | + |
| NUDT8 | | | | | | | | | |
| NUDT9 | | | | | | | | | |
| NUDT9P1 | | | | | | | | | |
| NUF2 | | + | + | | | + | | | + |
| NUFIP1 | | | | | | | | | |
| NUFIP2 | | | | | | | | | |
| NUGGC | | | | | | | | | |
| NUMA1 | | | | | | | | | |
| NUMB | | | | | | | | | |
| NUMBL | | | | | + | | | | + |
| NUP1 | | | | | | | | | |
| NUP100 | | | | | | | | | |
| NUP107 | | | | | | | | | |
| NUP116 | | | | | | | | | |
| NUP120 | | | | | | | | | |
| NUP133 | | | | | | | | | |
| NUP145 | | | | | | | | | |
| NUP153 | | | | | | | | | |
| NUP155 | | | | | | | | | |
| NUP159 | | | | | | | | | |
| NUP160 | | | | | | | | | |
| NUP170 | | | | | | | | | |
| Nup188 | | | | | | | | | |
| NUP2 | | | | | | | | | |
| NUP205 | | | | | | | | | |
| NUP210 | | | | | | | | | |
| NUP210L | | | | | | | | | |
| NUP210P1 | | | | | | | | | |
| NUP214 | | | | | | | | | |
| NUP35 | | | | | | | | | |
| NUP37 | | | | | | | | | |
| NUP43 | | | | | | | | | |
| NUP50 | | | + | | | | | | + |
| NUP53 | | | | | | | | | |
| NUP54 | | | + | | | | | | + |
| NUP57 | | | | | | | | | |
| NUP62 | | | | | | | | | |
| NUP62CL | | | | | | | | | |
| NUP82 | | | | | | | | | |
| NUP85 | | | | | | | | | |
| Nup88 | | | | | | | | | |
| NUP93 | | | | | | | | | |
| NUP98 | | | | | | | | | |

| | | | | | | | | | |
|---|---|---|---|---|---|---|---|---|---|
| Nup98-96 | | | | | | | | | |
| NUPL1 | | | | | | | | | |
| NUPL2 | | | | | | + | | | + |
| NUPR1 | | | | | | | | | |
| NUS1 | | | | | | | | | |
| NUSAP1 | | + | + | | | + | | | + |
| NUTF2 | | | | | | | | | |
| NUTM1 | | | | | | | | | |
| NUTM2A | | | | | | | | | |
| NUTM2A-AS1 | | | | | | | | | |
| NUTM2B | | | | | | | | | |
| NUTM2D | | | | | | | | | |
| NUTM2F | | | | | | | | | |
| NUTM2G | | | | | | | | | |
| NVL | | | | | | | | | |
| NWD1 | | | | | | | | | |
| NWD2 | | | | | | | | | |
| nxf-1 | | | | | | | | | |
| NXF1 | | | | | | | | | |
| NXF2 | | | | | | | | | |
| NXF2B | | | | | | | | | |
| NXF3 | | | | | | | | | |
| NXF4 | | | | | | | | | |
| NXF5 | | | | | | | | | |
| NXN | | | | | | | | | |
| NXNL1 | + | | | + | + | | + | | + |
| NXNL2 | | | | | | | | | |
| NXPE2 | | | | | | | | | |
| NXPE3 | | | | | | | | | |
| NXPE4 | | | | | | | | | |
| NXPH1 | | | | | | | | | |
| NXPH2 | | | | | | | | | |
| NXPH3 | + | | | + | + | | | | + |
| NXPH4 | | | | | | | | | |
| NXT1 | | | | | | | | | |
| NXT2 | | | | | | | | | |
| NYAP1 | | | | | | | | | |
| NYAP2 | | | | | | | | | |
| NYNRIN | | | | | | | | | |
| NYX | | | | | | | | | |
| OAF | | | | | | | | | |
| OAF3 | | | | | | | | | |
| OARD1 | | | | | | | | | |
| OAS1 | | | | | | | | | |
| OAS2 | | | | | | | | | |
| OAS3 | | | | | | | | | |
| OASL | | | | | | | | | |
| OAT | | | | | | | | | |
| Oaz1 | | + | + | | | + | | + | + |
| OAZ2 | | | | | | | | | |
| OAZ3 | | | | | | | | | |
| OBFC1 | | | | | | | | | |
| OBP2A | | | | | | | | | |

| | | | | | | | | | |
|---|---|---|---|---|---|---|---|---|---|
| OBP2B | | | | | | | | | |
| obr1 | | | | | | | | | |
| OBSCN | | | | | | | | | |
| OBSL1 | | | | | | | | | |
| OC90 | | | | | | | | | |
| OCA2 | | | | | | | | | |
| OCA6 | | | | | | | | | |
| oca8 | | | | | | | | | |
| OCEL1 | | | | | | | | | |
| OCIAD1 | | | | | | | | | |
| OCIAD2 | | | | | | | | | |
| OCLM | | | | | | | | | |
| OCLN | | | | | | + | | | + |
| OCM | | | | | | | | | |
| OCM2 | | | | | | | | | |
| OCRL | | | | | | | | | |
| ODAM | | | | | | | | | |
| ODC1 | | | | | | | | | |
| ODF1 | | | | | | | | | |
| ODF2 | | + | | | | + | | | + |
| ODF2L | | | | | | | | | |
| ODF3 | | | | | | | | | |
| ODF3B | | | | | | | | | |
| ODF3L1 | | | | | | | | | |
| ODF3L2 | | | | | | | | | |
| ODF4 | | | | | | | | | |
| OFCC1 | | | | | | | | | |
| OFD1 | | | | | | | | | |
| oga1 | | | | | | | | | |
| OGDH | | | | | | | | | |
| OGDHL | | | | | | | | | |
| OGFOD1 | | | | | | | | | |
| OGFOD2 | | | | | | | | | |
| OGFOD3 | | | | | | | | | |
| OGFR | | | | | | | | | |
| OGFRL1 | | | | | | | | | |
| OGG1 | | | | | | | | | |
| ogm1 | | | | | | | | | |
| OGN | | | | | | + | | | + |
| OGT | | | | | | | | | |
| OIP5 | | | | | | | | | |
| OIP5-AS1 | | | | | | | | | |
| OIT3 | | | | | | | | | |
| OLA1 | | | | | | | | | |
| OLAH | | | | | | | | | |
| OLEO1 | | | | | | | | | |
| OLEO2 | | | | | | | | | |
| OLFM1 | | | | | | + | | | + |
| OLFM2 | | | | | + | + | | | + |
| OLFM3 | | | | | | | | | |
| OLFM4 | | | | | | | | | |
| OLFML2A | | | | | | | | | |
| OLFML2B | | | | | | + | | | + |

| | | | | | | | | | |
|---|---|---|---|---|---|---|---|---|---|
| OLFML3 | | | | | + | | | | + |
| OLI1 | | | | | | | | | |
| OLIG1 | | | | | | | | | |
| OLIG2 | | | | | | | | + | + |
| OLIG3 | | | | | | | | | |
| OLR1 | | | | | | | | | |
| OM45 | | | | | | | | | |
| OMA1 | | | | | | | | | |
| OMD | | | | | | | | | |
| OMG | | + | + | | | + | | | + |
| OMP | | | | | | | | | |
| ompA | | | | | | | | | |
| ONECUT1 | | | | | | | | | |
| ONECUT2 | | | | | | | | | |
| ONECUT3 | | | | | | | | | |
| OOEP | | | | | | | | | |
| OOSP2 | | | | | | | | | |
| OPA1 | | | | | | | | | |
| OPA3 | | | | | | | | | |
| OPA6 | | | | | | | | | |
| OPALIN | | | | | | | | | |
| OPCML | | | | | | + | | | + |
| OPHN1 | | | | | | | | | |
| OPI3 | | | | | | | | | |
| OPLAH | | | | | | | | | |
| OPN1LW | | | | | | | | | |
| OPN1SW | | | | | | | | | |
| OPN3 | + | | + | | | | | | + |
| OPN4 | | | | | | | | | |
| OPN5 | | | | | | | | | |
| OPRD1 | | | | | | | | | |
| OPRK1 | | | | | | | | | |
| OPRL1 | | | | | | + | | | + |
| OPRM1 | + | | | + | + | | | | + |
| OPTC | + | | | + | + | | + | | + |
| OPTN | | | | | | | | | |
| OPY1 | | | | | | | | | |
| OPY2 | | | | | | | | | |
| OR10A4 | | | | | | | | | |
| OR10A5 | | | | | | | | | |
| OR10AD1 | | | | | | | | | |
| OR10AG1 | | | | | | | | | |
| OR10G2 | | | | | | | | | |
| OR10G3 | | | | | | | | | |
| OR10G4 | | | | | | | | | |
| OR10G7 | | | | | | | | | |
| OR10G8 | | | | | | | | | |
| OR10G9 | | | | | | | | | |
| OR10H1 | | | | | | | | | |
| OR10H2 | | | | | | | | | |
| OR10H3 | | | | | | | | | |
| OR10J5 | | | | | | | | | |
| OR10K1 | | | | | | | | | |

| | | | | | | | | | |
|---|---|---|---|---|---|---|---|---|---|
| OR10S1 | | | | | | | | | |
| OR10V1 | | | | | | | | | |
| OR11G2 | | | | | | | | | |
| OR12D3 | | | | | | | | | |
| OR13A1 | | | | | | | | | |
| OR13C3 | | | | | | | | | |
| OR13C4 | | | | | | | | | |
| OR13C5 | | | | | | | | | |
| OR13C8 | | | | | | | | | |
| OR13D1 | | | | | | | | | |
| OR13G1 | | | | | | | | | |
| OR13J1 | | | | | | | | | |
| OR14C36 | | | | | | | | | |
| OR14I1 | | | | | | | | | |
| OR14J1 | | | | | | | | | |
| OR14K1 | | | | | | | | | |
| OR1A1 | | | | | | | | | |
| OR1A2 | | | | | | | | | |
| OR1C1 | | | | | | | | | |
| OR1D2 | | | | | | | | | |
| OR1D4 | | | | | | | | | |
| OR1D5 | | | | | | | | | |
| OR1E1 | | | | | | | | | |
| OR1E2 | | | | | | | | | |
| OR1F12 | | | | | | | | | |
| OR1G1 | | | | | | | | | |
| OR1I1 | | | | | | | | | |
| OR1L3 | | | | | | | | | |
| OR1M1 | | | | | | | | | |
| OR1N1 | | | | | | | | | |
| OR2A1-AS1 | | | | | | | | | |
| OR2A2 | | | | | | | | | |
| OR2A25 | | | | | | | | | |
| OR2A4 | | | | | | | | | |
| OR2AE1 | | | | | | | | | |
| OR2AJ1 | | | | | | | | | |
| OR2C1 | | | | | | | | | |
| OR2F2 | | | | | | | | | |
| OR2G6 | | | | | | | | | |
| OR2H1 | | | | | | | | | |
| OR2H2 | | | | | | | | | |
| OR2K2 | | | | | | | | | |
| OR2L13 | | | | | | | | | |
| OR2L5 | | | | | | | | | |
| OR2M7 | | | | | | | | | |
| OR2S2 | | | | | | | | | |
| OR2T1 | | | | | | | | | |
| OR2T10 | | | | | | | | | |
| OR2T11 | | | | | | | | | |
| OR2T12 | | | | | | | | | |
| OR2T2 | | | | | | | | | |
| OR2T29 | | | | | | | | | |
| OR2T33 | | | | | | | | | |

| | | | | | | | | | |
|---|---|---|---|---|---|---|---|---|---|
| OR2T35 | | | | | | | | | |
| OR2T5 | | | | | | | | | |
| OR2T6 | | | | | | | | | |
| OR2W1 | | | | | | | | | |
| OR2W3 | | | | | | | | | |
| OR3A1 | | | | | | | | | |
| OR3A2 | | | | | | | | | |
| OR4A15 | | | | | | | | | |
| OR4A16 | | | | | | | | | |
| OR4B1 | | | | | | | | | |
| OR4C15 | | | | | | | | | |
| OR4D1 | | | | | | | | | |
| OR4D2 | | | | | | | | | |
| OR4D6 | | | | | | | | | |
| OR4E2 | | | | | | | | | |
| OR4F16 | | | | | | | | | |
| OR4F17 | | | | | | | | | |
| OR4F21 | | | | | | | | | |
| OR4F29 | | | | | | | | | |
| OR4F3 | | | | | | | | | |
| OR4K1 | | | | | | | | | |
| OR4K14 | | | | | | | | | |
| OR4K17 | | | | | | | | | |
| OR4K3 | | | | | | | | | |
| OR4L1 | | | | | | | | | |
| OR4N2 | | | | | | | | | |
| OR4N4 | | | | | | | | | |
| OR4N5 | | | | | | | | | |
| OR4Q3 | | | | | | | | | |
| OR4S2 | | | | | | | | | |
| OR4X1 | | | | | | | | | |
| OR4X2 | | | | | | | | | |
| OR51A4 | | | | | | | | | |
| OR51B2 | | | | | | | | | |
| OR51B4 | | | | | | | | | |
| OR51B5 | | | | | | | | | |
| OR51E1 | | | | | | | | | |
| OR51E2 | | | | | | | | | |
| OR51F2 | | | | | | | | | |
| OR51L1 | | | | | | | | | |
| OR51M1 | | | | | | | | | |
| OR51V1 | | | | | | | | | |
| OR52B2 | | | | | | | | | |
| OR52E6 | | | | | | | | | |
| OR52I1 | | | | | | | | | |
| OR52K2 | | | | | | | | | |
| OR52L1 | | | | | | | | | |
| OR52N1 | | | | | | | | | |
| OR52N4 | | | | | | | | | |
| OR52N5 | | | | | | | | | |
| OR52P1P | | | | | | | | | |
| OR52W1 | | | | | | | | | |
| OR56A3 | | | | | | | | | |

| | | | | | | | | | |
|---|---|---|---|---|---|---|---|---|---|
| OR56A4 | | | | | | | | | |
| OR56B4 | | | | | | | | | |
| OR5A2 | | | | | | | | | |
| OR5AC2 | | | | | | | | | |
| OR5AR1 | | | | | | | | | |
| OR5AS1 | | | | | | | | | |
| OR5B2 | | | | | | | | | |
| OR5B3 | | | | | | | | | |
| OR5C1 | | | | | | | | | |
| OR5F1 | | | | | | | | | |
| OR5H1 | | | | | | | | | |
| OR5H8 | | | | | | | | | |
| OR5K2 | | | | | | | | | |
| OR5L1 | | | | | | | | | |
| OR5M10 | | | | | | | | | |
| OR5M11 | | | | | | | | | |
| OR5M3 | | | | | | | | | |
| OR5M8 | | | | | | | | | |
| OR5T1 | | | | | | | | | |
| OR6A2 | | | | | | | | | |
| OR6B1 | | | | | | | | | |
| OR6B3 | | | | | | | | | |
| OR6C1 | | | | | | | | | |
| OR6C4 | | | | | | | | | |
| OR6C70 | | | | | | | | | |
| OR6C75 | | | | | | | | | |
| OR6J1 | | | | | | | | | |
| OR6K3 | | | | | | | | | |
| OR6M1 | | | | | | | | | |
| OR6N1 | | | | | | | | | |
| OR6S1 | | | | | | | | | |
| OR6T1 | | | | | | | | | |
| OR6X1 | | | | | | | | | |
| OR7A10 | | | | | | | | | |
| OR7A17 | | | | | | | | | |
| OR7A5 | | | | | | | | | |
| OR7C1 | | | | | | | | | |
| OR7C2 | | | | | | | | | |
| OR7D2 | | | | | | | | | |
| OR7D4 | | | | | | | | | |
| OR7E24 | | | | | | | | | |
| OR7E91P | | | | | | | | | |
| OR7G1 | | | | | | | | | |
| OR7G3 | | | | | | | | | |
| OR8B2 | | | | | | | | | |
| OR8B8 | | | | | | | | | |
| OR8D2 | | | | | | | | | |
| OR8D4 | | | | | | | | | |
| OR8G1 | | | | | | | | | |
| OR8G2 | | | | | | | | | |
| OR8G5 | | | | | | | | | |
| OR8H2 | | | | | | | | | |
| OR8U1 | | | | | | | | | |

| | | | | | | | | | |
|---|---|---|---|---|---|---|---|---|---|
| OR8U8 | | | | | | | | | |
| OR9A4 | | | | | | | | | |
| OR9G1 | | | | | | | | | |
| OR9I1 | | | | | | | | | |
| OR9Q2 | | | | | | | | | |
| ORAI1 | + | | | + | + | | | | + |
| ORAI2 | | + | | | | + | | | + |
| ORAI3 | | | | | | | | | |
| ORAOV1 | | | | | | | | | |
| ORC1 | | + | + | | | + | | | + |
| ORC2 | | | | | | | | | |
| ORC3 | | | | | | | | | |
| ORC4 | | | | | | | | | |
| ORC5 | | | | | | + | | | + |
| ORC6 | | | | | | | | | |
| ORF0 | | | | | | | | | |
| ORF1 | | | | | | | | | |
| ORF10 | | | | | | | | | |
| ORF11 | | | | | | | | | |
| ORF14 | | | | | | | | | |
| ORF16 | | | | | | | | | |
| ORF17 | | | | | | | | | |
| ORF17.5 | | | | | | | | | |
| ORF1a | | | | | | | | | |
| ORF1ab | | | | | | | | | |
| ORF2 | | | | | | | | | |
| ORF20 | | | | | | | | | |
| ORF21 | | | | | | | | | |
| ORF23 | | | | | | | | | |
| ORF24 | | | | | | | | | |
| ORF25 | | | | | | | | | |
| ORF26 | | | | | | | | | |
| ORF27 | | | | | | | | | |
| ORF28 | | | | | | | | | |
| ORF29 | | | | | | | | | |
| ORF3 | | | | | | | | | |
| ORF30 | | | | | | | | | |
| ORF31 | | | | | | | | | |
| ORF34 | | | | | | | | | |
| ORF35 | | | | | | | | | |
| ORF36 | | | | | | | | | |
| ORF38 | | | | | | | | | |
| ORF39 | | | | | | | | | |
| ORF3a | | | | | | | | | |
| ORF3b | | | | | | | | | |
| ORF4 | | | | | | | | | |
| ORF42 | | | | | | | | | |
| ORF43 | | | | | | | | | |
| ORF45 | | | | | | | | | |
| ORF46 | | | | | | | | | |
| ORF47 | | | | | | | | | |
| ORF48 | | | | | | | | | |
| ORF49 | | | | | | | | | |

| | | | | | | | | | |
|---|---|---|---|---|---|---|---|---|---|
| ORF4a | | | | | | | | | |
| ORF4b | | | | | | | | | |
| ORF5 | | | | | | | | | |
| ORF50 | | | | | | | | | |
| ORF52 | | | | | | | | | |
| ORF55 | | | | | | | | | |
| ORF56 | | | | | | | | | |
| ORF57 | | | | | | | | | |
| ORF58 | | | | | | | | | |
| ORF59 | | | | | | | | | |
| ORF6 | | | | | | | | | |
| ORF60 | | | | | | | | | |
| ORF62 | | | | | | | | | |
| ORF63 | | | | | | | | | |
| ORF64 | | | | | | | | | |
| ORF67 | | | | | | | | | |
| ORF70 | | | | | | | | | |
| ORF7a | | | | | | | | | |
| ORF7b | | | | | | | | | |
| ORF8 | | | | | | | | | |
| ORF8a | | | | | | | | | |
| ORF8b | | | | | | | | | |
| ORF9b | | | | | | | | | |
| ORF9c | | | | | | | | | |
| ORM1 | | | | | | | | | |
| ORM2 | | | | | | | | | |
| ORMDL1 | | | | | | | | | |
| ORMDL2 | | | | | | | | | |
| ORMDL3 | | | + | | | | | | + |
| ORT1 | | | | | | | | | |
| Os03g0232600 | | | | | | | | | |
| Os05g0125000 | | | | | | | | | |
| OS9 | | | | | | | | | |
| OSBP | | | | | | | | | |
| OSBP2 | | + | | | | + | | | + |
| OSBPL10 | | | | | | | | | |
| OSBPL11 | | | | | | | | | |
| OSBPL1A | | | | | | | | | |
| OSBPL2 | | | | | | | | | |
| OSBPL3 | | | + | | | + | | | + |
| OSBPL5 | | | + | | | | | | + |
| OSBPL6 | + | | + | | + | | | | + |
| OSBPL7 | | | | | | | | | |
| OSBPL8 | | | | | | | | | |
| OSBPL9 | | | | | | | | | |
| OSCAR | | | | | | | | | |
| OSCP1 | | | | | | + | | | + |
| OSER1 | | | | | | | | | |
| OSGEP | | | | | | | | | |
| OSGEPL1 | | + | + | | | + | | | + |
| OSGIN1 | | | | | | | | | |
| OSGIN2 | + | | + | | + | | | | + |
| OSH2 | | | | | | | | | |

| | | | | | | | | | |
|---|---|---|---|---|---|---|---|---|---|
| OSH3 | | | | | | | | | |
| OSM | | | | | | | | | |
| OSMR | | | | | | | | | |
| OSR1 | | | | | | | | | |
| OSR2 | | | | | | | | | |
| OST1 | | | | | | | | | |
| OST2 | | | | | | | | | |
| OST4 | | | | | | | | | |
| OSTC | | | | | | | | | |
| OSTCP1 | | | | | | | | | |
| OSTF1 | | | | | + | | | | + |
| OSTM1 | | | | | | | | | |
| OSTN | | | | | | | | | |
| OTC | | | | | | | | | |
| OTOA | | | | | | | | | |
| OTOF | + | | | + | + | + | | | + |
| OTOG | | | | | | | | | |
| OTOGL | | | | | | | | | |
| OTOL1 | | | | | | | | | |
| OTOP1 | | | | | | | | | |
| OTOP2 | | | | | | | | | |
| OTOP3 | | | | | | | | | |
| OTOR | | | | | | | | | |
| OTOS | | | | | | | | | |
| OTP | | | | | | | | | |
| OTU1 | | | | | | | | | |
| otub-1 | | | | | | | | | |
| OTUB1 | | | | | | | | | |
| OTUB2 | | | | | | | | | |
| OTUD1 | | | + | | | | | | + |
| OTUD3 | | | | | + | | | | + |
| OTUD4 | | | | | | | | | |
| OTUD4P1 | | | | | | | | | |
| OTUD5 | | | | | | | | | |
| OTUD6A | | | | | | | | | |
| OTUD6B | | | | | | | | | |
| OTUD7A | + | | | | + | | | | + |
| OTUD7B | | | | | | + | | | + |
| OTULIN | | | | | | | | | |
| OTX1 | + | | | + | + | | | | + |
| OTX2 | | | | | | | | + | + |
| OVAL | | | | | | | | | |
| OVCA2 | + | | | | + | | | | + |
| OVCH1 | | | | | | | | | |
| OVCH2 | | | | | | | | | |
| OVGP1 | | | | | | | | | |
| OVOL1 | | | | | | | | | |
| OVOL2 | | | | | | | | | |
| OVOS | | | | | | | | | |
| OVOS2 | | | | | | | | | |
| OXA1L | | | | | | + | | | + |
| OXCT1 | | | | | | | | | |
| OXCT2 | | | | | | | | | |

| | | | | | | | | | |
|---|---|---|---|---|---|---|---|---|---|
| OXER1 | | | | | | | | | |
| OXGR1 | | | | | | | | | |
| OXLD1 | | | | | | | | | |
| OXNAD1 | | | | | | | | | |
| OXR1 | | | | | | | | | |
| OXSM | | | | + | | | + | | + |
| OXSR1 | | | | | | | | | |
| OXT | + | | | + | + | | + | | + |
| OXTR | | | | | | | | | |
| OYE2 | | | | | | | | | |
| P2RX1 | | | | | | | | | |
| P2RX2 | | | | | | | | | |
| P2RX3 | + | | | + | + | | | | + |
| P2RX4 | | | | | | + | | | + |
| P2RX5 | | | | | | | | | |
| P2RX5-TAX1BP3 | | | | | | | | | |
| P2RX6 | | | | | | | | | |
| P2rx7 | | | | | | | | | |
| P2RY1 | | | | | | | | | |
| P2RY10 | | | | | | | | | |
| P2RY11 | | | | | | | | | |
| P2RY12 | | | | | | | | | |
| P2RY13 | | | | | | | | | |
| P2RY14 | | | | | | | | | |
| P2RY2 | | | | | | | | | |
| P2RY6 | | | | | | | | | |
| P2RY8 | | | | | | | | | |
| P3H1 | | | | | | | | | |
| P3H2 | | | | | | | | | |
| P3H4 | | | | | | | | | |
| P4HA1 | + | | + | | + | | | | + |
| P4ha2 | | | | | + | | | | + |
| P4HA3 | | | | | | | | | |
| P4HB | | | | | | | | | |
| P4HTM | | | | | | + | | | + |
| PA2G4 | | | | | | | | | |
| PAAF1 | | | | | | | | | |
| PAB1 | | | | | | | | | |
| pab2 | | | | | | | | | |
| pabp | | | | | | | | | |
| PABPC1 | | | | | | | | | |
| PABPC1L | | | | | | | | | |
| PABPC1L2A | | | | | | | | | |
| PABPC1P1 | | | | | | | | | |
| PABPC3 | | | | | | | | | |
| PABPC4 | | | | | | | | | |
| PABPC4L | | | | | | | | | |
| PABPC5 | | | | | | | | | |
| PABPN1 | | | + | | | | | | + |
| PABPN1L | | | | | | | | | |
| PAC1 | | | | | | | | | |
| PACRG | | | | | | | | | |
| PACRGL | | | | | | | | | |

| | | | | | | | | | |
|---|---|---|---|---|---|---|---|---|---|
| PACS1 | | | | | | + | | | + |
| PACS2 | | | | | | | | | |
| PACSIN1 | | + | | | | + | | | + |
| PACSIN2 | | | | | | | | | |
| PACSIN3 | | | | | | | | | |
| PADI1 | | | | | | | | | |
| PADI2 | | | | | | | | | |
| PADI3 | | | | | | | | | |
| PADI4 | | | | | | | | | |
| PADI6 | | | | | | | | | |
| PAEP | | | | | | | | | |
| PAF1 | | | | | | | | | |
| PAFAH1B1 | | | + | | + | | | | + |
| PAFAH1B2 | | | | | | | | | |
| PAFAH1B3 | | | | | | | | | |
| PAFAH2 | | | | | | | | | |
| PAG1 | | | | | | | | | |
| PAGE1 | | | | | | | | | |
| PAGE2 | | | | | | | | | |
| PAGE2B | | | | | | | | | |
| PAGE3 | | | | | | | | | |
| PAGE4 | | | | | | | | | |
| PAGE5 | | | | | | | | | |
| PAGR1 | | | | | | | | | |
| PAH | | | | | | | | | |
| PAH1 | | | | | | | | | |
| PAICS | | | + | | | | | | + |
| PAIP1 | | | | | | | | | |
| PAIP2 | | | | | | + | | | + |
| PAIP2B | | | | | | + | | | + |
| PAK1 | | | + | | | | + | | + |
| PAK1IP1 | | | | | | | | | |
| PAK2 | | | | | | | | | |
| Pak3 | | | | | | | | | |
| PAK4 | + | | | + | + | | | | + |
| PAK6 | + | | + | | + | | | | + |
| PAK7 | | | + | | | | | | + |
| PAL1 | | | | | | | | | |
| PAL2 | | | | | | | | | |
| PAL4 | | | | | | | | | |
| PALB2 | + | | | + | + | | + | | + |
| PALD1 | | | | | | | | | |
| PALLD | | | | | | | | | |
| PALM | | | | | | | | | |
| PALM2 | | | | | | | | | |
| PALM2-AKAP2 | | | | | | | | | |
| PALM3 | | | | | | | | | |
| PALMD | | | | | | | | | |
| PAM | | | + | | + | | | | + |
| pam1 | | | | | | | | | |
| PAM16 | | | | | | | | | |
| PAM18 | | | | | | | | | |
| PAMR1 | | | | | | | | | |

| | | | | | | | | | |
|---|---|---|---|---|---|---|---|---|---|
| pan1 | | | | | | | | | |
| PAN2 | | | | | | | | | |
| PAN3 | | | | | | | | | |
| PANK1 | | | | | | | | | |
| PANK2 | | | | | | | | | |
| PANK3 | | | + | | | | | | + |
| PANK4 | | | + | | | | | | + |
| PANX1 | | | | | | | | | |
| PANX2 | | | | | | + | | | + |
| PANX3 | | | | | | | | | |
| PAOX | | | | | | | | | |
| PAP1 | | | | | | | | | |
| PAPD4 | | | | | | | | | |
| PAPD5 | | | | | | | | | |
| PAPD7 | | | + | | | | | | + |
| PAPL | | | | | | | | | |
| PAPLN | | | | | | | | | |
| PAPOLA | | | + | | | | | | + |
| PAPOLB | | | | | | | | | |
| PAPOLG | | | | | | | | | |
| PAPPA | | | | | + | | | | + |
| PAPPA2 | | | | | | | | | |
| PAPSS1 | | | | | | | | | |
| PAPSS2 | | | | | | | | | |
| PAQR3 | | | | + | | | | | + |
| PAQR4 | | | + | | | | | | + |
| PAQR5 | | | | | | | | | |
| PAQR6 | | | | | | | | | |
| PAQR7 | | | + | | | + | | | + |
| PAQR8 | + | | | | | | | | + |
| PAQR9 | | | | | | | | | |
| PAR32 | | | | | | | | | |
| PARD3 | | | | | | | | | |
| PARD3B | | | | | | | | | |
| PARD6A | | | | | | | | | |
| PARD6B | + | | | + | + | | | | + |
| PARD6G | | | | | | | | | |
| Parg | | | | | | | | | |
| park | | | | | | | | | |
| PARK2 | | | | | | + | | | + |
| PARK7 | | | | | | + | | | + |
| PARL | | | + | | | | | | + |
| PARM1 | | | | | | | | | |
| PARN | | | | | | | | | |
| PARP1 | | | | | | | | | |
| PARP10 | | | | | | | | | |
| PARP11 | | | | | | | | | |
| PARP12 | | | | | | | | | |
| PARP14 | | | | | | | | | |
| PARP15 | | | | | | | | | |
| PARP16 | | | | | | | | | |
| Parp2 | | | | | | | | | |
| PARP3 | | | | | | | | | |

| | | | | | | | | | |
|---|---|---|---|---|---|---|---|---|---|
| PARP4 | | | | | | | | | |
| PARP6 | | | | | | | | | |
| PARP8 | | | | | | | | | |
| PARP9 | | | | | | | | | |
| PARPBP | | | | | | | | | |
| PARS2 | | | | | | | | | |
| PARVA | | | | | | | | | |
| PARVB | | | | | | | | | |
| PARVG | | | | | | | | | |
| PASD1 | | | | | | | | | |
| PASK | | | | | | | | | |
| PAT1 | | | | | | | | | |
| PATE1 | | | | | | | | | |
| PATE2 | | | | | | | | | |
| PATL1 | | | | | | | | | |
| PATL2 | | | | | | | | | |
| PATZ1 | | | | | | + | | | + |
| PAWR | | | + | | | | | | + |
| PAX1 | + | | | + | + | | | | + |
| PAX2 | | | | | | | | | |
| PAX3 | + | | | + | + | | | | + |
| PAX4 | | | | | | | | | |
| PAX5 | | | | | | | | | |
| PAX6 | | | | | | + | | | + |
| PAX7 | | | | | | | | | |
| PAX8 | | | | | | | | | |
| PAX9 | | | | | | | | | |
| PAXBP1 | | | | | | | | | |
| PAXIP1 | | | | | | | | | |
| PBDC1 | | | | | | | | | |
| PBK | | | | | | | | | |
| PBLD | | | | | | | | | |
| PBP1 | | | | | | | | | |
| PBP2 | | | | | | | | | |
| PBRM1 | | | | | | | | | |
| Pbsn | | | | | | | | | |
| PBX1 | | | | | | + | | | + |
| PBX2 | | | | | | + | | | + |
| PBX3 | | | + | | | | | | + |
| PBX4 | | | | | | | | | |
| PBXIP1 | | | | | | | | | |
| PC | | | | | | | | | |
| PCA1 | | | | | | | | | |
| PCAP | | | | | | | | | |
| PCAT1 | | | | | | | | | |
| PCAT6 | | | | | | | | | |
| PCBD1 | | | | | | | | | |
| PCBD2 | | | | | | | | | |
| PCBP1 | | | | | | | | | |
| PCBP2 | | | | | | | | | |
| PCBP2-OT1 | | | | | | | | | |
| PCBP3 | | | | | | | | | |
| PCBP4 | + | | + | | | + | | | + |

| | | | | | | | | | |
|---|---|---|---|---|---|---|---|---|---|
| PCCA | | | | | | | | | |
| PCCB | | | | | | + | | | + |
| PCDH1 | | | | | + | | | | + |
| PCDH10 | | | | | + | | | | + |
| PCDH11X | | | | | + | | | | + |
| PCDH11Y | | | | | | | | | |
| PCDH12 | | | | | | | | | |
| Pcdh15 | | | | | | | | | |
| PCDH17 | | | + | | + | | | | + |
| PCDH18 | | | + | | | | | | + |
| PCDH19 | | | | | | + | | | + |
| PCDH20 | | | | | | | | | |
| PCDH7 | | | + | | + | | | | + |
| PCDH8 | + | | + | | + | | | | + |
| PCDH9 | | | + | | | | | | + |
| PCDHA1 | | | | | | | | | |
| PCDHA10 | | | | | | | | | |
| PCDHA11 | | | | | | | | | |
| PCDHA12 | | | | | | | | | |
| PCDHA13 | | | | | | | | | |
| PCDHA2 | | | | | | | | | |
| PCDHA3 | | | | | | | | | |
| PCDHA4 | | | | | | | | | |
| PCDHA5 | | | | | | | | | |
| PCDHA6 | | | | | | | | | |
| PCDHA7 | | | | | | | | | |
| PCDHA8 | | | | | | | | | |
| PCDHA9 | | | | | | | | | |
| PCDHAC1 | | | | | | | | | |
| PCDHAC2 | | | | | | | | | |
| PCDHB1 | | | | | | | | | |
| PCDHB10 | | | | | | | | | |
| PCDHB11 | | | | | | | | | |
| PCDHB12 | | | | | | | | | |
| PCDHB13 | | | | | | | | | |
| PCDHB14 | | | | | | | | | |
| PCDHB15 | | | | | | | | | |
| PCDHB16 | | | | | | | | | |
| PCDHB18 | | | | | | | | | |
| PCDHB2 | | | | | | | | | |
| PCDHB3 | | | | | | | | | |
| PCDHB4 | | | | | | | | | |
| PCDHB5 | | | | | | | | | |
| PCDHB6 | | | | | | | | | |
| PCDHB7 | | | | | | | | | |
| PCDHB8 | | | | | | | | | |
| PCDHB9 | | | | | | + | | | + |
| PCDHGA1 | | | | | | | | | |
| PCDHGA10 | | | | | | | | | |
| PCDHGA11 | | | | | | | | | |
| PCDHGA12 | | | | | | | | | |
| PCDHGA2 | | | | | | | | | |
| PCDHGA3 | | | | | | | | | |

| | | | | | | | | | |
|---|---|---|---|---|---|---|---|---|---|
| PCDHGA4 | | | | | | | | | |
| PCDHGA5 | | | | | | | | | |
| PCDHGA6 | | | | | | | | | |
| PCDHGA7 | | | | | | | | | |
| PCDHGA8 | | | | | | | | | |
| PCDHGA9 | | | | | | | | | |
| PCDHGB1 | | | | | | | | | |
| PCDHGB2 | | | | | | | | | |
| PCDHGB3 | | | | | | | | | |
| PCDHGB4 | | | | | | | | | |
| PCDHGB5 | | | | | | | | | |
| PCDHGB6 | | | | | | | | | |
| PCDHGB7 | | | | | | | | | |
| PCDHGC3 | | | | | | | | | |
| PCDHGC4 | | | | | | | | | |
| PCDHGC5 | | | | | | | | | |
| PCED1A | | | | | | | | | |
| PCED1B | | | | | | | | | |
| PCED1B-AS1 | | | | | | | | | |
| PCF11 | | | | | | | | | |
| PCGF1 | | | | | | | | | |
| PCGF2 | | | | + | | | | | + |
| PCGF3 | | | | | | | | | |
| PCGF5 | | | | | | | | | |
| PCGF6 | | | | | | | | | |
| PCID2 | | | | | | | | | |
| PCIF1 | | | | | | | | | |
| PCK1 | | | | | | | | | |
| PCK2 | | | | | | | | | |
| PCL6 | | | | | | | | | |
| PCLO | | + | | | + | + | | | + |
| PCM1 | | | | | | | | | |
| PCMT1 | | | | | | | | | |
| PCMTD1 | | | | | | | | | |
| PCMTD2 | | + | | | | + | | | + |
| pcn1 | | | | | | | | | |
| PCNA | | | | | | | | | |
| PCNA1 | | | | | | | | | |
| PCNA2 | | | | | | | | | |
| PCNP | + | | | | | | | | + |
| PCNT | | | | | | + | | | + |
| PCNX | | | + | | + | | | | + |
| PCNXL2 | | | | | | + | | | + |
| PCNXL3 | | | | | | | | | |
| PCNXL4 | | | | | | | | | |
| PCOLCE | | | | | | | | | |
| PCOLCE-AS1 | | | | | | | | | |
| PCOLCE2 | | | | | + | | | | + |
| PCP2 | | + | + | | | + | | | + |
| PCP4 | | + | | | | + | | | + |
| PCP4L1 | | | | | | | | | |
| PCSK1 | + | | | | + | | | | + |
| PCSK1N | | | | | | | | | |

| | | | | | | | | | |
|---|---|---|---|---|---|---|---|---|---|
| Pcsk2 | | | | | | | + | | + |
| PCSK4 | | | | | | | | | |
| PCSK5 | | | | | | | | | |
| PCSK6 | | | | | | | | | |
| PCSK7 | | | | | | | | | |
| PCSK9 | | | | | | | | | |
| PCTP | | | | | | | | | |
| Pcx | | | | | | | | | |
| PCYOX1 | | | | | | | | | |
| PCYOX1L | | | | | | | | | |
| Pcyt1a | | | | | | | | | |
| PCYT1B | + | | | + | + | | + | | + |
| PCYT2 | | | | | | | | | |
| pda1 | | | | | | | | | |
| PDAP1 | | | | | | | | | |
| pdb1 | | | | | | | | | |
| PDC | | | | | | | | | |
| PDC1 | | | | | | | | | |
| PDCD1 | | | | | | | | | |
| PDCD10 | | | | | | | | | |
| PDCD11 | | | | | | | | | |
| PDCD1LG2 | | | | | | | | | |
| PDCD2 | | | | | | | | | |
| PDCD2L | | | | | | | | | |
| PDCD4 | | + | | | | + | | | + |
| PDCD5 | | | | | | | | | |
| PDCD6 | | | | | + | | | | + |
| PDCD6IP | | | | | | | | | |
| PDCD7 | | | | | | | | | |
| PDCL | | | | | | | | | |
| PDCL2 | | | | | | | | | |
| PDCL3 | | | | | | | | | |
| PDDC1 | | | | | | | | | |
| PDE10A | | | + | | | + | | | + |
| PDE11A | | | | | | | | | |
| PDE12 | | | | | | | | | |
| PDE1A | | | | | | + | | | + |
| PDE1B | | | | | | + | | | + |
| PDE1C | | | | | | | | | |
| PDE2 | | | | | | | | | |
| PDE2A | | | | | | | | | |
| PDE3A | + | | | + | + | | + | | + |
| PDE3B | | | | | | | | | |
| PDE4A | + | | | | + | + | | | + |
| PDE4B | | | | | | | | | |
| PDE4C | | | | | | | | | |
| PDE4D | | | | | | | | | |
| PDE4DIP | | | | | | | | | |
| PDE5A | | | | | | | | | |
| PDE6A | | | | | | | | | |
| PDE6B | | | | | | | | | |
| PDE6C | | | | | | | | | |
| PDE6D | | | | | | | | | |

| | | | | | | | | | |
|---|---|---|---|---|---|---|---|---|---|
| PDE6G | | | | | | | | | |
| PDE6H | | | | | | | | | |
| PDE7A | | | | | | | | | |
| PDE7B | | | | | | | | | |
| Pde8a | | | | | | | | | |
| PDE8B | | | | | | | | | |
| PDE9A | | | | | | | | | |
| PDF | | | | | | + | | | + |
| PDGFA | + | | | | + | | | | + |
| pdgfab | | | | | | | | | |
| PDGFB | | | | | | | | | |
| PDGFC | | | | | | | | | |
| PDGFD | | | | | | | | | |
| PDGFRA | | | | | | | | + | + |
| PDGFRB | | | | | | | | | |
| PDGFRL | | | | | | | | | |
| PDHA1 | | | | | | | + | | + |
| PDHA2 | | | | | | | | | |
| PDHB | | | | | | | | | |
| PDHX | | | | | | | | | |
| PDI1 | | | | | | | | | |
| PDIA2 | | | | | | | | | |
| PDIA3 | | | | | | | | | |
| PDIA4 | | | | | | | | | |
| PDIA5 | | | | | | | | | |
| PDIA6 | | | | | | | | | |
| PDIK1L | | | | | | | | | |
| PDILT | | | | | | | | | |
| PDK1 | | | | | | + | | | + |
| PDK2 | | | | | | + | | | + |
| PDK3 | | | | | | | | | |
| PDK4 | + | | | + | + | | + | | + |
| PDLIM1 | | | + | | | | | | + |
| Pdlim2 | | | | | | | | | |
| PDLIM3 | | | | | | | | | |
| PDLIM4 | | | | | | | | | |
| PDLIM5 | | | + | | | | | | + |
| PDLIM7 | | | + | | | | | | + |
| PDP1 | | | | | | | | | |
| PDP2 | | | | | | | | | |
| PDPK1 | | | | | | | | | |
| PDPK2P | | | | | | | | | |
| PDPN | | | | | | | | | |
| PDPR | | | | | | | | | |
| PDR | | | | | | | | | |
| PDR1 | | | | | | | | | |
| PDR15 | | | | | | | | | |
| PDR5 | | | | | | | | | |
| PDRG1 | | | | | + | | | | + |
| PDS1 | | | | | | | | | |
| PDS5 | | | | | | | | | |
| PDS5A | | | | | | | | | |
| PDS5B | | | | | | | | | |

| | | | | | | | | | |
|---|---|---|---|---|---|---|---|---|---|
| PDSS1 | | | | | | | | | |
| PDSS2 | | | | | | | | | |
| PDX1 | | | | | | | | | |
| PDX1.1 | | | | | | | | | |
| PDX3 | | | | | | | | | |
| PDXDC1 | | | | | | | | | |
| PDXDC2P | | | | | | | | | |
| PDXK | | | + | | | | | | + |
| PDXP | | | | | | | | | |
| PDYN | | | | | | | | | |
| PDZD11 | | | | | | | | | |
| PDZD2 | | | | | + | | | | + |
| PDZD3 | | | | | | | | | |
| PDZD4 | | | | | | | | | |
| PDZD7 | | | | | | | | | |
| PDZD8 | | | + | | + | | | | + |
| PDZD9 | | | | | | | | | |
| PDZK1 | | | | | | | | | |
| PDZK1IP1 | | | | | | | | | |
| PDZK1P2 | | | | | | | | | |
| PDZRN3 | | | + | | | | | | + |
| PDZRN4 | | | | | | | | | |
| PE_PGRS29 | | | | | | | | | |
| PEA15 | | | | | | | | | |
| PEAK1 | | | | | | | | | |
| PEAR1 | | | | | | | | | |
| PEBP1 | | | + | | | | | | + |
| PEBP4 | | | | | | | | | |
| PECAM1 | | | | | | | | | |
| PECR | | | | | | | | | |
| PEF1 | | | | | | | | | |
| PEG10 | | | | | | | | | |
| PEG3 | | | + | | + | | | | + |
| PELI1 | | | + | | | | | | + |
| PELI2 | | | | | | | | | |
| PELI3 | | | | | | | | | |
| PELO | | | | | | | | | |
| PELP1 | | | | | | | | | |
| PEMT | | | | | | | | | |
| PENK | | | + | | + | | | | + |
| PEP3 | | | | | | | | | |
| PEP8 | | | | | | | | | |
| PEPD | | | | | | | | | |
| per | | | | | | | | | |
| PER1 | + | | | | + | | | | + |
| PER2 | + | | + | | + | | | | + |
| PER3 | | + | | + | | + | | | + |
| PERM1 | | | | | | | | | |
| PERP | | | | | | | | | |
| PES1 | | | | | | | | | |
| PET10 | | | | | | | | | |
| PET100 | | | | | | | | | |
| PET117 | | | | | | | | | |

| | | | | | | | | | |
|---|---|---|---|---|---|---|---|---|---|
| PET9 | | | | | | | | | |
| PEX1 | | | | | | | | | |
| PEX10 | | | | | | | | | |
| PEX11A | | | | | | | | | |
| PEX11B | | | | | | | | | |
| PEX11E | | | | | | | | | |
| PEX11G | | | | | | | | | |
| PEX12 | | | | | | | | | |
| PEX13 | | | + | | | | | | + |
| PEX14 | | | | | | | | | |
| PEX16 | | | | | | | | | |
| PEX19 | | | | | | | | | |
| PEX2 | | | | | | | | | |
| PEX25 | | | | | | | | | |
| PEX26 | | | | | | | | | |
| PEX3 | | | | | | | | | |
| PEX5 | | | + | | | | | | + |
| PEX5L | | | | | | | | | |
| PEX6 | | | | | | | | | |
| PEX7 | | | | | | | | | |
| PEX8 | | | | | | | | | |
| PF11_0336 | | | | | | | | | |
| PF4 | | | | | | | | | |
| PF4V1 | | | | | | | | | |
| PFA3 | | | | | | | | | |
| PFA4 | | | | | | | | | |
| PFAS | | | | | | | | | |
| PFDN1 | | + | | | | + | | | + |
| PFDN2 | | | | | | | | | |
| PFDN4 | | | | | | | | | |
| PFDN5 | | + | | | | + | | | + |
| PFDN6 | | | | | | | | | |
| PFI0930c | | | | | | | | | |
| pfk1 | | | | | | | | | |
| PFKFB1 | | | | | | | | | |
| PFKFB2 | | | | | | | | | |
| PFKFB3 | | | | | | | | | |
| PFKFB4 | | | | | | | | | |
| PFKL | | | | | | | | | |
| PFKM | | | | | | + | + | | + |
| PFKP | | | | | | | | | |
| PFN1 | | | | | | | | | |
| PFN2 | | | | | | | | | |
| PFN3 | | | | | | | | | |
| PfPK6 | | | | | | | | | |
| PfRh5 | | | | | | | | | |
| PGA3 | | | | | | | | | |
| PGA4 | | | | | | | | | |
| PGA5 | | | | | | | | | |
| PGAM1 | + | | + | | + | | | | + |
| PGAM1P5 | | | | | | | | | |
| PGAM2 | | | | | | + | | | + |
| PGAM4 | | | | | | | | | |

| | | | | | | | | | |
|---|---|---|---|---|---|---|---|---|---|
| PGAM5 | | | | | | | | | |
| PGAP1 | | | | | + | | | | + |
| PGAP2 | | | | | | | | | |
| PGAP3 | | | | | | | | | |
| PGBD1 | | | | | | | | | |
| PGBD2 | | | | | | | | | |
| PGBD3 | | | | | | | | | |
| PGBD4 | | | | | | | | | |
| PGBD5 | | | + | | | | | | + |
| PGC | | | | | | | | | |
| PGD | | | | | | | | | |
| PGF | | | | | | | | | |
| PGGT1B | | | | | | | | | |
| PGI1 | | | | | | | | | |
| PGK1 | | | | | | | | | |
| Pgk1-rs7 | | | | | | | | | |
| PGK2 | | | | | | | | | |
| PGLS | | | | | | | | | |
| PGLYRP1 | | | | | | | | | |
| PGLYRP2 | | | | | | | | | |
| PGLYRP3 | | | | | | | | | |
| PGLYRP4 | | | | | | | | | |
| PGM1 | | | | | | | | | |
| PGM2 | | | | | | | | | |
| PGM2L1 | | | | | | + | | | + |
| PGM3 | | | | | | | | | |
| PGM5 | | | | | | | | | |
| PGP | | | | | | | | | |
| PGPEP1 | | | | | | | | | |
| PGR | | | | | | | | | |
| PGRMC1 | | | | | | | | | |
| PGRMC2 | | | | | | | | | |
| PGS1 | | | | | | | | | |
| PHACTR1 | | | | | | + | | | + |
| PHACTR2 | | | | | | | | | |
| PHACTR3 | | | + | | | | | | + |
| PHACTR4 | | | | | | | | | |
| PHAX | | | | | | | | | |
| PHB | | | | | | | | | |
| PHB1 | | | | | | | | | |
| PHB2 | | | | | | | | | |
| PHC1 | | | | | | | | | |
| PHC1P1 | | | | | | | | | |
| PHC2 | | | | | | | | | |
| PHC3 | | | | | | | | | |
| PHEX | + | | | + | + | | + | | + |
| PHF1 | | | | | | | | | |
| PHF10 | | | + | | + | | | | + |
| PHF11 | | | | | | | | | |
| PHF12 | | | | | | | | | |
| PHF13 | | | | | + | | | | + |
| PHF14 | | | | + | | | | | + |
| PHF19 | | | | | | | | | |

| | | | | | | | | | |
|---|---|---|---|---|---|---|---|---|---|
| PHF2 | | | | | | | | | |
| PHF20 | | | | | | | | | |
| PHF20L1 | | | | | | | | | |
| PHF21A | | | | | | + | | | + |
| PHF21B | + | | + | | + | | | | + |
| PHF23 | | | | | + | | | | + |
| PHF3 | | | | | | | | | |
| PHF5A | | | | | | | | | |
| PHF6 | | | | | | | | | |
| PHF7 | | | | | | | | | |
| PHF8 | | | | | | | | | |
| PHGDH | | | | | | | | | |
| PHGK | | | | | | | | | |
| PHIP | | | | | | | | | |
| PHKA1 | | | | | | | | | |
| PHKA1P1 | | | | | | | | | |
| PHKA2 | | | | | | | | | |
| PHKA2-AS1 | | | | | | | | | |
| PHKB | | | | | | | | | |
| PHKBP2 | | | | | | | | | |
| PHKG1 | | | | | | | | + | + |
| PHKG2 | | | | | | + | | | + |
| PHLDA1 | + | | + | | | | | | + |
| PHLDA2 | | | | | | | | | |
| PHLDA3 | | | | | | | | | |
| PHLDB1 | | | | | | + | | | + |
| PHLDB2 | | | | | | | | | |
| PHLDB3 | | | | | | | | | |
| PHLPP1 | | | | | | | | | |
| PHLPP2 | | | | | | | | | |
| PHM6 | | | | | | | | | |
| PHM8 | | | | | | | | | |
| pho | | | | | | | | | |
| PHO13 | | | | | | | | | |
| PHO8 | | | | | | | | | |
| PHO85 | | | | | | | | | |
| PHO86 | | | | | | | | | |
| PHO88 | | | | | | | | | |
| PHOSPHO1 | | | | | | | | | |
| PHOSPHO2 | | | | | | | | | |
| PHOSPHO2-KLHL23 | | | | | | | | | |
| PHOX2A | | | | | | | | | |
| PHOX2B | | | | | | | | | |
| PHPT1 | | | | | | | | | |
| PHRF1 | | | | | | | | | |
| PHS1 | | | | | | | | | |
| PHTF1 | | | | | | | | | |
| PHTF2 | | | | | | | | | |
| Phum_PHUM577390 | | | | | | | | | |
| PHYA | | | | | | | | | |
| PHYH | | | | | | | | | |
| PHYHD1 | | | | | | | | | |
| PHYHIP | | | | | | + | | | + |

| | | | | | | | | | |
|---|---|---|---|---|---|---|---|---|---|
| PHYHIPL | | | | | | | | | |
| PHYKPL | | | | | | | | | |
| PI15 | | | | | | | | | |
| PI16 | | | | | | | | | |
| PI3 | | | | | | | | | |
| PI4K2A | | | | | | | | | |
| PI4K2B | | | | | | | | | |
| PI4KA | | | | | + | | | | + |
| PI4KAP1 | | | | | | | | | |
| PI4KAP2 | | | | | | | | | |
| PI4KB | | | | | | | | | |
| PIANP | | | + | | | + | | | + |
| PIAS1 | | | + | | | | | | + |
| PIAS2 | | | | | | | | | |
| PIAS3 | | | | | | | | | |
| PIAS4 | | | | | | | | | |
| PIB1 | | | | | | | | | |
| PIBF1 | | | | | | | | | |
| PICALM | | | | | | | | | |
| PICK1 | | | | | | | | | |
| PID1 | | | | | | | | | |
| PIDD1 | | | | | | | | | |
| PIEZO1 | | | | | | | | | |
| PIEZO2 | | | | | | | | | |
| PIF1 | | | | | | | | | |
| PIFO | | | | | | | | | |
| PIGA | + | | | + | + | | + | | + |
| PIGB | | | | | | + | | | + |
| PIGC | | | | | | | | | |
| Pigf | | | | | | | | | |
| PIGG | | | | | | | | | |
| PIGH | | | | | | | | | |
| PIGK | | + | | | | + | | | + |
| PIGL | | | | | | | | | |
| PIGM | | | | | | | | | |
| PIGN | | | | | | | | | |
| PIGO | | | | | | | | | |
| PIGP | | | | | | | | | |
| PIGQ | | | | | | | + | | + |
| PIGR | + | | | + | + | | + | | + |
| PIGS | | | | | | | | | |
| PIGT | | | | | | | | | |
| PIGU | | | | | | | | | |
| PIGV | | | | | | | | | |
| PIGW | | | | | | | | | |
| PIGX | | | | | | | | | |
| PIGY | | | | | | | | | |
| PIGZ | | | | | | | | | |
| PIH1D1 | | | | | | | | | |
| PIH1D2 | | | | | | | | | |
| PIH1D3 | | | | | | | | | |
| PIK3AP1 | | | | | | | | | |
| PIK3C2A | | | | | | | | | |

| | | | | | | | | | |
|---|---|---|---|---|---|---|---|---|---|
| PIK3C2B | | | | | | | | | |
| PIK3C2G | | | | | | | | | |
| PIK3C3 | | | | | | | | | |
| PIK3CA | | | | | | | | | |
| PIK3CB | | | | | | | | | |
| PIK3CD | | | | | | | | | |
| PIK3CG | | | | | | | | | |
| PIK3IP1 | | | | + | | + | | | + |
| PIK3R1 | | | | | | | | | |
| PIK3R2 | | | | | | | | | |
| PIK3R3 | | | | | | | | | |
| PIK3R4 | | | | | | | | | |
| PIK3R5 | + | | | + | + | | | | + |
| PIK3R6 | | | | | | | | | |
| PIKFYVE | | | | | | | | | |
| PIL1 | | | | | | | | | |
| PILRA | | | | | | | | | |
| PILRB | | | | | | | | | |
| PIM1 | | | | | | | | | |
| PIM2 | | | | | | + | | | + |
| PIM3 | + | | | | | | | | + |
| PIN1 | | | | | | | | | |
| pin1-a | | | | | | | | | |
| pin1-b | | | | | | | | | |
| PIN4 | | | | | | | | | |
| PIN4P1 | | | | | | | | | |
| PINK1 | | | | | | | | | |
| PINX1 | | | | | | | | | |
| PIP | | | | | | | | | |
| PIP4K2A | | | | | | | | | |
| PIP4K2B | | | | | | | | | |
| PIP4K2C | | | | | + | | + | | + |
| PIP5K1A | | | | | | | | | |
| PIP5K1B | | | | | | + | | | + |
| PIP5K1C | | | + | | + | | | | + |
| PIP5K1P1 | | | | | | | | | |
| PIP5KL1 | | | | | | | | | |
| PIPOX | | | | | | | | | |
| PIPSL | | | | | | | | | |
| PIR | | | | | | | | | |
| PIR121 | | | | | | | | | |
| PIRT | + | | | + | + | | + | | + |
| PIS1 | | | | | | | | | |
| PISD | | | | | | | | | |
| PITHD1 | | | | | | | | | |
| PITPNA | | | + | | + | | | | + |
| PITPNB | | | | | | | | | |
| PITPNC1 | | | | | | + | | | + |
| PITPNM1 | | | | | | | | | |
| Pitpnm2 | | | | | | | | | |
| PITPNM3 | | | | | | | | | |
| PITRM1 | | | | | | + | | | + |
| PITX1 | | | | | | | | | |

| | | | | | | | | | |
|---|---|---|---|---|---|---|---|---|---|
| PITX2 | | | | | | | | | |
| PITX3 | | | | | | | | | |
| PIWIL1 | + | | | + | + | | + | | + |
| PIWIL2 | | | | | + | | | | + |
| PIWIL3 | | | | | | | | | |
| PIWIL4 | | | | | | | | | |
| PJA1 | | | | | | | | | |
| PJA2 | | | | | | | + | | + |
| PKD1 | | | | | | | | | |
| PKD1L1 | | | | | | | | | |
| PKD1L2 | | | | | | | | | |
| PKD1L3 | | | | | | | | | |
| PKD1P1 | | | | | | | | | |
| PKD1P6 | | | | | | | | | |
| PKD2 | | | | | | | | | |
| PKD2L1 | | | | | | | | | |
| PKD2L2 | | | | | | | | | |
| PKDCC | | | | | | | | | |
| PKDREJ | | | | | | | | | |
| PKHD1 | | | | | | | | | |
| PKHD1L1 | | | | | | | | | |
| PKIA | | | | | | + | | | + |
| PKIB | | | | | | | | | |
| PKIG | | | | | | | | | |
| PKLR | | | | | | | | | |
| PKM | | | | | | | | | |
| PKMYT1 | | | | | | | | | |
| pkn-1 | | | | | | | | | |
| PKN1 | | | | | | + | | | + |
| PKN2 | | | + | | | | | | + |
| PKN3 | | | | | | | | | |
| PKNOX1 | | | + | | | | | | + |
| PKNOX2 | | | | | | | | | |
| PKP1 | | | | | | | | | |
| PKP2 | | | | | | | | | |
| PKP3 | | | | | | | | | |
| PKP4 | | | | | | | | | |
| PLA1A | | | | | | | | | |
| PLA2G10 | | | | | | | | | |
| PLA2G12A | | | | | | + | | | + |
| PLA2G12B | | | | | | | | | |
| PLA2G15 | | | + | | | | | | + |
| PLA2G16 | | | | | | | | | |
| PLA2G1B | | | | | | | | | |
| PLA2G2A | | | | | | | | | |
| PLA2G2D | | | | | | | | | |
| PLA2G2E | | | | | | | | | |
| PLA2G2F | | | | | | | | | |
| PLA2G3 | | | | | | | | | |
| PLA2G4A | | | | | | | | | |
| PLA2G4B | | | | | | | | | |
| PLA2G4C | | | | | | | | | |
| PLA2G4D | | | | | | | | | |

| | | | | | | | | | |
|---|---|---|---|---|---|---|---|---|---|
| PLA2G4E | | | | | | + | | | + |
| PLA2G4F | | | | | | | | | |
| PLA2G5 | | | | | | | | | |
| PLA2G6 | | | | | | | | | |
| PLA2G7 | | | | | | | | | |
| PLA2R1 | | | | | | | | | |
| PLAA | | | | | | | | | |
| PLAC1 | | | | | | | | | |
| PLAC4 | | | | | | | | | |
| PLAC8 | | | | | | | | | |
| PLAC8L1 | | | | | | | | | |
| PLAC9 | | | | | | | | | |
| PLAG1 | | | | | | | | | |
| PLAGL1 | + | | + | | + | | | | + |
| PLAGL2 | | | | | | | | | |
| PLAT | + | | + | | | | | | + |
| PLAU | | | | | | | | | |
| PLAUR | | | | | | | | | |
| PLB1 | | | | | | | | | |
| PLBD1 | | | | | | | | | |
| PLBD2 | | | | | | | | | |
| PLCB1 | | | + | | | + | | | + |
| PLCB2 | | | | | | | | | |
| PLCB3 | | | | | | | | | |
| PLCB4 | | | | | | | | | |
| PLCD1 | | | | | | | | | |
| PLCD3 | | | | | | | | | |
| PLCD4 | | | | | | | | | |
| PLCE1 | | | | | + | | | | + |
| PLCG1 | | | | | | + | | | + |
| PLCG2 | | | | | | + | | | + |
| PLCH1 | | | | | | | | | |
| PLCH2 | | | | | | | | | |
| PLCL1 | | | | | | | | | |
| PLCL2 | | | | | | | | | |
| PLCXD1 | | | | | + | | | | + |
| PLCXD2 | | | | | | | | | |
| PLCXD3 | | | | | | | | | |
| PLCZ1 | | | | | | | | | |
| PLD1 | | | | | | | | | |
| PLD2 | | | | | | | | | |
| PLD3 | | | | | | | | | |
| PLD4 | | | | | + | | | | + |
| PLD5 | | | | | | | | | |
| PLD6 | | | | | | | | | |
| PLEC | | | | | | + | | | + |
| PLEK | | | | | | | | | |
| PLEK2 | | | | | | | | | |
| PLEKHA1 | | | | | | | | | |
| PLEKHA2 | | | | | + | | | | + |
| PLEKHA3 | | | | | | | | | |
| PLEKHA4 | | | | | | | | | |
| PLEKHA5 | | | | | | | | | |

| | | | | | | | | | |
|---|---|---|---|---|---|---|---|---|---|
| PLEKHA6 | | | | | | + | | | + |
| PLEKHA7 | | | | | | | | | |
| PLEKHA8 | | | | | | | | | |
| PLEKHA8P1 | | | | | | | | | |
| PLEKHB1 | | | | | | + | | | + |
| PLEKHB2 | | | | | | | + | | + |
| PLEKHD1 | | | | | | | | | |
| PLEKHF1 | | | | | | | | | |
| PLEKHF2 | | | | | | | | | |
| PLEKHG1 | | | | | | | | | |
| PLEKHG2 | | | | | | | | | |
| PLEKHG3 | | | | | | | | | |
| PLEKHG4 | | | | | | | | | |
| PLEKHG4B | | | | | | | | | |
| Plekhg5 | | | | | + | | | | + |
| PLEKHG6 | | | | | | | | | |
| PLEKHG7 | | | | | | | | | |
| PLEKHH1 | | | | | | | | | |
| PLEKHH2 | | | | | + | | | | + |
| PLEKHH3 | | | | | | | | | |
| PLEKHJ1 | | | | | | | | | |
| PLEKHM1 | | | | | | | | | |
| PLEKHM1P | | | | | | | | | |
| PLEKHM2 | | | + | | | | | | + |
| PLEKHM3 | | | | | | | | | |
| PLEKHN1 | | | | | | | | | |
| PLEKHO1 | | | | | | | | | |
| PLEKHO2 | | | | | | | | | |
| PLEKHS1 | | | | | | | | | |
| PLG | | | | | | | | | |
| PLGRKT | | | | | | + | | | + |
| PLIN1 | | | | | | | | | |
| PLIN2 | | | | | | | | | |
| PLIN3 | | | | | | | | | |
| PLIN4 | | | | | | | | | |
| PLIN5 | | | | | | | | | |
| PLK1 | | | | | | | | | |
| PLK2 | + | | | | + | | | | + |
| PLK3 | | | | | + | | | | + |
| PLK4 | | | | | | | | | |
| PLK5 | + | | | | + | + | | | + |
| PLLP | | | | | | | | | |
| PLN | | | | | | | | | |
| PLOD1 | | | | | | | | | |
| PLOD2 | | | | | | | | | |
| PLOD3 | | | | | | | | | |
| PLP1 | + | | + | + | + | | | | + |
| PLP2 | | | | | | | | | |
| plr1 | | | | | | | | | |
| PLRG1 | | | | | | | | | |
| PLS1 | | | + | | | | | | + |
| PLS3 | | | | | | | + | | + |
| PLSCR1 | | | | | | | | | |

| | | | | | | | | | |
|---|---|---|---|---|---|---|---|---|---|
| PLSCR2 | | | | | | | | | |
| PLSCR3 | | | | | | | | | |
| PLSCR4 | | | | | | | | | |
| PLSCR5 | | | | | | | | | |
| PLTP | | | | | | | | | |
| PLVAP | | | | | | | | | |
| PLXDC1 | | | | | | | | | |
| PLXDC2 | | | + | | | | | | + |
| PLXNA1 | | | | | | | | | |
| PLXNA2 | | | | | | | | | |
| PLXNA3 | | | | | | | | | |
| PLXNA4 | | | + | | | | | | + |
| PLXNB1 | | | | | | | | | |
| PLXNB2 | | | | | | | | | |
| PLXNB3 | | | | | | | | + | + |
| PLXNC1 | | | | | | | | | |
| PLXND1 | | | | | | | | | |
| PM20D1 | | | | | | | | | |
| PM20D2 | | | | | | | | | |
| PMA1 | | | | | | | | | |
| PMAIP1 | | | | | | | | | |
| PMCH | | | | | | | | | |
| PMEL | | | | | | | | | |
| PMEPA1 | | | + | | + | | | | + |
| PMF1 | | | | | | | | | |
| PMF1-BGLAP | | | | | | | | | |
| PMFBP1 | | | | | | | | | |
| PML | | | | | | | | | |
| PMM1 | | + | | | | + | | | + |
| PMM2 | | | | | | | | | |
| PMP2 | | | | | | | | | |
| PMP22 | | | | | | | | | |
| PMPCA | | | | | | | | | |
| PMPCB | | | | | | | | | |
| PMS1 | | | | | | | | | |
| PMS2 | | | | | | + | | | + |
| PMS2CL | | | | | | | | | |
| PMS2L2 | | | | | | | | | |
| PMS2P1 | | | | | | | | | |
| PMS2P3 | | | | | | | | | |
| PMS2P5 | | | | | | | | | |
| PMS2P9 | | | | | | | | | |
| PMT1 | | | | | | | | | |
| PMT2 | | | | | | | | | |
| PMT3 | | | | | | | | | |
| PMT4 | | | | | | | | | |
| PMT5 | | | | | | | | | |
| PMVK | | | + | | | | | | + |
| PNCK | | | | | | + | | | + |
| PNISR | | | | | | | | | |
| PNKD | | | + | | | | | | + |
| PNKP | | | | | | | | | |
| PNLDC1 | | | | | | | | | |

| | | | | | | | | | |
|---|---|---|---|---|---|---|---|---|---|
| PNLIP | | | | | | | | | |
| PNLIPRP1 | | | | | | | | | |
| PNLIPRP2 | | | | | | | | | |
| PNMA1 | | | | | | | | | |
| PNMA2 | | | | | | + | | | + |
| PNMA3 | | | | | | | | | |
| PNMA5 | | | | | | | | | |
| PNMA6A | | | | | | | | | |
| PNMAL1 | | + | | | | + | | | + |
| PNMAL2 | | | | | | + | | | + |
| PNMT | | | | | | | | | |
| PNN | | | + | | | | | | + |
| PNO1 | | | | | | | | | |
| PNOC | | | | | | | | | |
| PNP | | | | | | | | | |
| PNPLA1 | | + | + | | | + | | | + |
| PNPLA2 | | | | | | | | | |
| PNPLA3 | | | | | | | | | |
| PNPLA4 | | | | | | | | | |
| PNPLA5 | | | | | | | | | |
| PNPLA6 | | | | | | | | | |
| PNPLA7 | | | | | | | | | |
| PNPLA8 | | | | | | | | | |
| PNPO | | | | | | | | | |
| PNPT1 | | | | | | | | | |
| PNRC1 | | | | | | | | | |
| PNRC2 | | | | | | | | | |
| pnut | | | | | | | | | |
| POB3 | | | | | | | | | |
| POC1A | | | | | + | | | | + |
| Poc1b | | | | | | | | | |
| POC1B-GALNT4 | | | | | | | | | |
| POC4 | | | | | | | | | |
| POC5 | | | | | | | | | |
| PODN | | | | | | | | | |
| PODNL1 | | | | | | | | | |
| PODXL | | | | | | | | | |
| PODXL2 | | | | | | | | | |
| POF1B | | | | | | | | | |
| POFUT1 | | + | | + | | + | | | + |
| POFUT2 | | | | | | | | | |
| POG1 | | | | | | | | | |
| POGK | | | | | | | | | |
| POGLUT1 | | + | | | | + | | | + |
| POGZ | | | | | | | | | |
| POL1 | | | | | | | | | |
| POL30 | | | | | | | | | |
| pol5 | | | | | | | | | |
| POLA1 | | | | + | + | | | | + |
| POLA2 | | | | | + | | | | + |
| POLB | | | | | | | | | |
| POLD1 | | | | | | | | | |
| POLD2 | | | | | | | | | |

| | | | | | | | | | |
|---|---|---|---|---|---|---|---|---|---|
| POLD3 | | | | | | | | | |
| POLD4 | | | | | | | | | |
| POLDIP2 | | | | | | | | | |
| POLDIP3 | | | | | | | | | |
| POLE | | | | | | | | | |
| POLE2 | | | | | | | | | |
| POLE3 | | | | | | | | | |
| POLE4 | | | | | | | | | |
| POLG | | | | | | | | | |
| POLG2 | | | | | + | | | | + |
| POLH | | | | | | | | | |
| Poli | | | | | | | | | |
| POLK | | | | | | | | | |
| POLL | | | | | | | | | |
| POLM | | | | | | | | | |
| POLN | | | | | | | | | |
| POLQ | | | | | | | | | |
| POLR1A | | | | | | | | | |
| POLR1B | | | | + | | | | | + |
| POLR1C | | | | | | | | | |
| POLR1D | | | | | | | | | |
| POLR1E | | | | | | + | | | + |
| POLR2A | | | | | | | | | |
| POLR2B | | | | | | | | | |
| POLR2C | | | | | | | | | |
| POLR2D | | | | | | | | | |
| POLR2E | | | | | | + | | | + |
| POLR2F | | | | | | | | | |
| POLR2G | | | | | | | | | |
| POLR2H | | | | | | | | | |
| POLR2I | | + | | | | + | | | + |
| POLR2J | | + | | | | + | | | + |
| POLR2J2 | | | | | | | | | |
| POLR2J3 | | | | | | | | | |
| POLR2J4 | | | | | | | | | |
| POLR2K | | | | | | | | | |
| POLR2L | + | | | | + | | | | + |
| POLR2M | | | | | | | | | |
| POLR3A | | | | | | | | | |
| POLR3B | | | | | | | | | |
| POLR3C | | | | | | | | | |
| POLR3D | | | | | | | | | |
| POLR3E | | | | | | | | | |
| POLR3F | | | | | | | | | |
| POLR3G | | | | | | | | | |
| POLR3GL | | | | | | | | | |
| POLR3H | | | | | | | | | |
| POLR3K | | | | | | | | | |
| POLRMT | | | | | | | | | |
| POM121 | | | | | | | | | |
| POM121B | | | | | | | | | |
| POM121C | | | | | | | | | |
| POM121L1P | | | | | | | | | |

| | | | | | | | | | |
|---|---|---|---|---|---|---|---|---|---|
| POM121L4P | | | | | | | | | |
| POM121L8P | | | | | | | | | |
| POM152 | | | | | | | | | |
| POM33 | | | | | | | | | |
| POM34 | | | | | | | | | |
| POMC | | | | | | | | | |
| POMGNT1 | | | | | | | | | |
| POMGNT2 | | | | | | | | | |
| POMK | | | | | | | | | |
| POMP | | | | | | | | | |
| POMT1 | | | | | | | | | |
| POMT2 | | | | | | | | | |
| POMZP3 | | | | | | | | | |
| PON1 | | | | | | | | | |
| PON2 | | | | | | + | | | + |
| PON3 | | | | | | | | | |
| pop-1 | | | | | | | | | |
| POP1 | | | | | | | | | |
| POP2 | | | | | | | | | |
| POP3 | | | | | | | | | |
| POP4 | | | | | | | | | |
| POP5 | | | + | | | | | | + |
| POP6 | | | | | | | | | |
| POP7 | | | | | | | | | |
| POPDC2 | | | | | | | | | |
| POPDC3 | | | + | | + | | | | + |
| POR | | | | | | | | | |
| POR1 | | | | | | | | | |
| PORCN | | | | | | | | | |
| POS5 | | | | | | | | | |
| POSTN | + | | | | + | | | | + |
| POT1 | | | | | | | | | |
| POTEA | | | | | | | | | |
| POTEB | | | | | | | | | |
| POTEB2 | | | | | | | | | |
| POTEB3 | | | | | | | | | |
| POTEC | | | | | | | | | |
| POTED | | | | | | | | | |
| POTEE | | | | | | | | | |
| POTEF | | | | | | | | | |
| POTEG | | | | | | | | | |
| POTEH | | | | | | | | | |
| POTEI | | | | | | | | | |
| POTEJ | | | | | | | | | |
| POTEKP | | | | | | | | | |
| POTEM | | | | | | | | | |
| POU1F1 | | | | | | | | | |
| POU2AF1 | | | | | | | | | |
| POU2F1 | | | | | | | | | |
| POU2F2 | | | | | | | | | |
| POU2F3 | | | | | | | | | |
| POU3F1 | | | | | | | | | |
| POU3F2 | | | | | | | | | |

| | | | | | | | | | |
|---|---|---|---|---|---|---|---|---|---|
| Pou3f3 | | | + | | | | | | + |
| Pou3f4 | | | | | | | | | |
| POU4F1 | | | | | | | | | |
| POU4F2 | + | | | + | + | | + | | + |
| POU4F3 | | | | | | | | | |
| POU5F1 | | | | | | | | | |
| POU5F2 | | | | | | | | | |
| POU6F1 | | | | | | | | | |
| POU6F2 | | | | | | | | | |
| POX1 | | | | | | | | | |
| PP14571 | | | | | | | | | |
| PP2D1 | | | | | | | | | |
| PP7080 | | | | | | | | | |
| PPA1 | | | | | | | | | |
| PPA2 | | | | | | | | | |
| PPAN | + | | | | + | | | | + |
| PPAN-P2RY11 | | | | | | | | | |
| PPAP2A | | | | | + | | | | + |
| PPAP2B | | | | | | + | | | + |
| PPAP2C | | | | | | | | | |
| PPAPDC1A | | | | | | | | | |
| PPAPDC1B | | | | | | | | | |
| PPAPDC2 | | | | | | | | | |
| PPAPDC3 | | | | | | | | | |
| PPARA | + | | | + | + | | + | | + |
| PPARD | | | | | | | | | |
| PPARG | | | | | | | | | |
| PPARGC1A | | | | | | | | | |
| PPARGC1B | | | | | | | | | |
| PPAT | | | + | | | | | | + |
| PPBP | | | | | | | | | |
| PPC1 | | | | | | | | | |
| PPC2 | | | | | | | | | |
| PPCDC | | | | | | | | | |
| PPCS | | | | | | | | | |
| PPDPF | | | | | | | | | |
| PPE34 | | | | | | | | | |
| PPEF1 | | | | | | | | | |
| PPEF2 | | | | | | | | | |
| PPFIA1 | | | | | | | | | |
| PPFIA2 | | | | | | | | | |
| PPFIA3 | | | | | | | | | |
| PPFIA4 | | | | | | | | | |
| PPFIBP1 | | | | | | + | | | + |
| PPFIBP2 | | | | | | | | | |
| PPH21 | | | | | | | | | |
| PPH22 | | | | | | | | | |
| PPH3 | | | | | | | | | |
| PPHLN1 | | | | | | | | | |
| PPI1 | | | | | | | | | |
| PPIA | | | | | | | | | |
| PPIAL4B | | | | | | | | | |
| PPIAL4C | | | | | | | | | |

| | | | | | | | | | |
|---|---|---|---|---|---|---|---|---|---|
| PPIAL4D | | | | | | | | | |
| PPIAL4E | | | | | | | | | |
| PPIAL4F | | | | | | | | | |
| PPIAL4G | | | | | | | | | |
| PPIAP11 | | | | | | | | | |
| PPIAP22 | | | | | | | | | |
| PPIB | | | | | | | | | |
| PPIC | | | | | | | | | |
| PPID | | | | | | | | | |
| PPIE | | | | | | | | | |
| PPIF | | | | | | | | | |
| PPIG | | | | | | | | | |
| PPIH | | | | | | | | | |
| PPIL1 | | | | | | | | | |
| PPIL2 | | | | | | | | | |
| PPIL3 | | | | | | | | | |
| PPIL4 | | | | | + | | | | + |
| PPIL6 | | | | | | | | | |
| PPIP5K1 | | | | | | | | | |
| PPIP5K2 | | | | | | + | | | + |
| PPL | | | | | | | | | |
| PPM1A | | | | | | | | | |
| PPM1B | | | + | | | | | | + |
| PPM1D | | | | | | | | | |
| PPM1E | | | | | | | | | |
| PPM1F | | | | | | + | | | + |
| PPM1G | | | | | | | | | |
| PPM1H | | | + | | + | | | + | + |
| PPM1J | | | | | | | | | |
| PPM1K | | | | | | + | | | + |
| PPM1L | | | | | | | | | |
| PPM1M | | | | | | | | | |
| PPM1N | | | | | | | | | |
| PPME1 | | | + | | | | | | + |
| PPOX | | | | | | | | | |
| PPP1CA | | | | | | | | | |
| PPP1CB | | | | | | | | | |
| PPP1CC | | | | | | | | | |
| PPP1R10 | | | | | | | | | |
| PPP1R11 | | | | | | | | | |
| PPP1R12A | | | + | | | | | | + |
| PPP1R12B | | | | | + | | | | + |
| PPP1R12C | | | | | | | | | |
| PPP1R13B | | | | | | | | | |
| PPP1R13L | | | | | | | | | |
| PPP1R14A | | | | | | | | | |
| PPP1R14B | | | | | | | | | |
| PPP1R14BP3 | | | | | | | | | |
| PPP1R14C | | | | | | + | | | + |
| PPP1R14D | | | | | | | | | |
| PPP1R15A | + | | | | | | | | + |
| PPP1R15B | | | | | + | | | | + |
| PPP1R16A | | | | | | | | | |

| | | | | | | | | | |
|---|---|---|---|---|---|---|---|---|---|
| PPP1R16B | + | | | | + | | | | + |
| PPP1R17 | | | | | | | | | |
| PPP1R18 | | | | | | | | | |
| PPP1R1A | | + | | | + | + | | | + |
| PPP1R1B | | | | | | | | | |
| PPP1R1C | | | | | | | | | |
| PPP1R2 | | | + | | | | | | + |
| PPP1R21 | | | | | | | | | |
| PPP1R26 | | | | | | | | | |
| PPP1R27 | | | | | | | | | |
| PPP1R2P3 | | | | | | | | | |
| PPP1R2P4 | | | | | | | | | |
| PPP1R2P9 | | | | | | | | | |
| PPP1R32 | | | | | | | | | |
| PPP1R35 | | | | | | | | | |
| PPP1R36 | | | | | | | | | |
| PPP1R37 | | | | | | | | | |
| PPP1R3A | | | | | | | | | |
| PPP1R3B | | | | | | | | | |
| PPP1R3C | | | | | | | | | |
| PPP1R3D | | | | | | | | | |
| PPP1R3E | | | | | | | | | |
| PPP1R3F | | | | | | | | | |
| PPP1R3G | | | | | | | | | |
| PPP1R42 | | | | | | | | | |
| PPP1R7 | | | | | | | | | |
| PPP1R8 | | | | | | | | | |
| PPP1R9A | | + | | | | + | | | + |
| PPP1R9B | | | | | | + | | | + |
| PPP2CA | | | | | | | | | |
| PPP2CB | | | | | | | | | |
| PPP2R1A | | | | | | | | | |
| PPP2R1B | | | | | | | | | |
| PPP2R2A | | | | | | | | | |
| PPP2R2B | | | | | + | | | | + |
| PPP2R2C | | | + | | | | | | + |
| PPP2R2D | | | | | + | | | | + |
| PPP2R3A | | | | | | | | | |
| PPP2R3B | | | | | | | | | |
| PPP2R3C | | | | | | | | | |
| PPP2R4 | | + | | | | + | | | + |
| PPP2R5A | | | + | | | | | | + |
| PPP2R5B | | | | | | | | | |
| PPP2R5C | | | | | | | | | |
| PPP2R5D | | | | | | | | | |
| PPP2R5E | | | | | | | | | |
| PPP3CA | | | | | | | | | |
| PPP3CB | | + | | | | + | | | + |
| PPP3CB-AS1 | | | | | | | | | |
| PPP3CC | | | | | | | | | |
| PPP3R1 | | | + | | | | | | + |
| PPP3R2 | | | | | | | | | |
| PPP4C | | | | | | | | | |

| | | | | | | | | | |
|---|---|---|---|---|---|---|---|---|---|
| PPP4R1 | | | | | | | | | |
| PPP4R1L | | | | | | | | | |
| PPP4R2 | | | | | | | | | |
| PPP4R4 | | | | | + | | | | + |
| Ppp5c | | | + | | | | | | + |
| PPP5D1 | | | | | | | | | |
| PPP6C | | | | | | | | | |
| PPP6R1 | | | | | | | | | |
| PPP6R2 | | | | | | | | | |
| PPP6R3 | | | | | | | | | |
| PPRC1 | | | | | | | | | |
| PPS1 | | | | | | | | | |
| PPT1 | | | | | | | | | |
| PPT2 | | | | | | | | | |
| PPT2-EGFL8 | | | | | | | | | |
| PPTC7 | | | + | | | | | | + |
| PPWD1 | | | | | | | | | |
| PPY | | | | | | | | | |
| PPZ1 | | | | | | | | | |
| PQBP1 | | | | | | | | | |
| PQLC1 | | | | | | | | | |
| PQLC2 | | | | | + | | | | + |
| PQLC2L | | | | | | | | | |
| PQLC3 | | | | | | | | | |
| pr-set7 | | | | | | | | | |
| PR39 | | | | | | | | | |
| PRA1 | | | | | | | | | |
| PRADC1 | | | | | | | | | |
| PRAF2 | | + | | | | + | | | + |
| PRAM1 | | | | | | | | | |
| PRAME | + | | | + | + | | + | | + |
| PRAMEF10 | | | | | | | | | |
| PRAMEF12 | | | | | | | | | |
| PRAMEF15 | | | | | | | | | |
| PRAMEF17 | | | | | | | | | |
| PRAMEF2 | | | | | | | | | |
| PRAMEF20 | | | | | | | | | |
| PRAMEF22 | | | | | | | | | |
| PRAMEF25 | | | | | | | | | |
| PRAMEF4 | | | | | | | | | |
| PRAMEF5 | | | | | | | | | |
| PRAMEF8 | | | | | | | | | |
| PRAMEF9 | | | | | | | | | |
| PRAP1 | | | | | | | | | |
| PRB1 | | | | | | | | | |
| PRB2 | | | | | | | | | |
| PRB3 | | | | | | | | | |
| PRB4 | | | | | | | | | |
| PRC1 | | | | | | | | | |
| PRC1-AS1 | | | | | | | | | |
| PRCC | | | | | | | | | |
| PRCD | | | | | | | | | |
| PRCP | | | | | | | | | |

| | | | | | | | | | |
|---|---|---|---|---|---|---|---|---|---|
| PRD | | | | | | | | | |
| PRD1 | | | | | | | | | |
| PRDM1 | + | | | + | + | | | | + |
| PRDM10 | | | | | | | | | |
| PRDM11 | | + | | + | | | | | + |
| PRDM12 | | | | | | | | | |
| PRDM13 | | | | | | | | | |
| PRDM14 | | | | | | | | | |
| PRDM15 | | | | | | | | | |
| PRDM16 | | | | | | | | | |
| PRDM2 | | | + | | | | | | + |
| PRDM4 | | | | | | | | | |
| PRDM5 | + | + | | | + | + | | | + |
| PRDM6 | | | | | | | | | |
| PRDM7 | | | | | | | | | |
| PRDM8 | | | | | | + | | | + |
| PRDM9 | | | | | | | | | |
| PRDX1 | | | | | | | | | |
| PRDX2 | | | | | | | | | |
| PRDX3 | | | | | | | | | |
| PRDX4 | | | | | | | | | |
| PRDX5 | | | | | | | | | |
| PRDX6 | | | | | | | | | |
| PRE1 | | | | | | | | | |
| PRE10 | | | | | | | | | |
| PRE2 | | | | | | | | | |
| PRE3 | | | | | | | | | |
| PRE4 | | | | | | | | | |
| PRE5 | | | | | | | | | |
| PRE6 | | | | | | | | | |
| PRE7 | | | | | | | | | |
| PRE8 | | | | | | | | | |
| PRE9 | | | | | | | | | |
| Preb | | | | | | | | | |
| PRELID1 | | | | | | | | | |
| PRELID2 | | | | | | | | | |
| PRELP | | | | | | | | | |
| PREP | | | | | | | | | |
| PREPL | | | | | | | | | |
| PREX1 | | | | | + | | | | + |
| PREX2 | | | | + | | + | | | + |
| PRF1 | | | | | | | | | |
| PRG2 | | | | | | | | | |
| PRG3 | | | | | | | | | |
| PRG4 | | | | | | | | | |
| PRH1 | | | | | | | | | |
| PRH2 | | | | | | | | | |
| PRICKLE1 | | | | | | | | | |
| PRICKLE2 | + | | | | | | | | + |
| PRICKLE3 | | | | | | | | | |
| PRICKLE4 | | | | | | | | | |
| PRIM1 | | | | | | | | | |
| PRIM2 | | | | + | + | | | | + |

| | | | | | | | | | |
|---|---|---|---|---|---|---|---|---|---|
| PRIMA1 | | | | | | | | | |
| PRIMPOL | | | | | | | | | |
| PRKAA1 | | | | | | | | | |
| PRKAA2 | | | | | | | | | |
| PRKAB1 | | | | | | | | | |
| PRKAB2 | | | | | + | | | | + |
| PRKACA | | | | | | | | | |
| PRKACB | | | | | | | | | |
| PRKACG | | | | | | | | | |
| PRKAG1 | | | | | | | | | |
| PRKAG2 | | | + | | | + | | | + |
| PRKAG3 | + | | | + | + | | + | | + |
| PRKAR1A | | | + | | | | | | + |
| PRKAR1B | | | + | | + | | + | | + |
| PRKAR2A | | | | | | | | | |
| PRKAR2A-AS1 | | | | | | | | | |
| PRKAR2B | | | | | | | | | |
| PRKCA | | | + | | | | | | + |
| PRKCB | | | | | | | | | |
| PRKCD | | | | | | | | | |
| PRKCDBP | | | | | | | | | |
| PRKCE | | | | | | | | | |
| PRKCG | | | | | | | | | |
| PRKCH | | | | | | | | | |
| PRKCI | | | | | | | | | |
| PRKCQ | | | | | | | | | |
| PRKCQ-AS1 | | | | | | | | | |
| PRKCSH | | | | | | | | | |
| PRKCZ | | | | | | | | | |
| PRKD1 | + | | | | + | | | | + |
| PRKD2 | | | | | | | | | |
| PRKD3 | | | | | | | | | |
| PRKDC | | | | | | + | | | + |
| PRKG1 | | | | | | | | | |
| PRKG2 | | | + | | | | | | + |
| PRKRA | | | | | | | | | |
| PRKRIP1 | | | | | | | | | |
| PRKRIR | | | | | | | | | |
| PRKX | | | | | | | | | |
| PRKY | | | | | | | | | |
| PRL | | | | | | | | | |
| PRL1 | | | | | | | | | |
| PRLHR | | | | | | | | | |
| PRLR | | | | | + | | | | + |
| PRM1 | | | | | | | | | |
| PRM15 | | | | | | | | | |
| PRM2 | | | | | | | | | |
| PRM3 | | | | | | | | | |
| PRM5 | | | | | | | | | |
| PRM8 | | | | | | | | | |
| PRMT1 | | | | | | | | | |
| PRMT2 | | | | | | | | | |
| PRMT3 | | | | | | | | | |

| | | | | | | | | | |
|---|---|---|---|---|---|---|---|---|---|
| PRMT5 | | | | | | | | | |
| PRMT6 | | | | | | | | | |
| PRMT7 | | | | | | | | | |
| PRMT8 | + | | + | | + | | | | + |
| PRMT9 | | | | | | | | | |
| PRND | | | | | | | | | |
| PRNP | | | | | | | | | |
| PRO1596 | | | | | | | | | |
| PRO3 | | | | | | | | | |
| PROC | | | | | | | | | |
| PROCA1 | | | | | | | | | |
| PROCR | | | | | | | | | |
| PRODH | | | | | | | | | |
| PROK1 | | | | | | | | | |
| PROK2 | | | | | | | | | |
| PROKR1 | | | | | | | | | |
| PROKR2 | | | | | | | | | |
| PROL1 | | | | | | | | | |
| prom | | | | | | | | | |
| PROM1 | | | | | | | | | |
| PROM2 | | | | | | | | | |
| PROP1 | + | | | + | + | | + | | + |
| PROS1 | | | | | | | | | |
| PROSC | + | | | | + | | | | + |
| Proser1 | | | | | | | | | |
| PROSER2 | | | | | | | | | |
| PROSER3 | | | | | | | | | |
| PROX1 | + | | + | | + | | | | + |
| Prox2 | | | | | | | | | |
| PROZ | | | | | | | | | |
| PRP11 | | | | | | | | | |
| PRP2 | | | | | | | | | |
| PRP3 | | | | | | | | | |
| PRP38 | | | | | | | | | |
| PRP39 | | | | | | | | | |
| PRP4 | | | | | | | | | |
| PRP43 | | | | | | | | | |
| PRP45 | | | | | | | | | |
| PRP46 | | | | | | | | | |
| PRP8 | | | | | | | | | |
| PRP9 | | | | | | | | | |
| PRPF18 | | | | | | | | | |
| PRPF19 | | | | | | | | | |
| PRPF3 | | | | | | | | | |
| PRPF31 | | | | | | | | | |
| PRPF38A | | | | | | | | | |
| PRPF38B | + | | + | | + | | | | + |
| PRPF39 | | | | | | | | | |
| PRPF4 | | | | | | | | | |
| PRPF40A | | | | | | | | | |
| PRPF40B | | | | | | | | | |
| PRPF4B | | | | | | | | | |
| PRPF6 | | | | | | | | | |

| | | | | | | | | | |
|---|---|---|---|---|---|---|---|---|---|
| PRPF8 | | | | | | | | | |
| PRPH | | | | | | | | | |
| PRPH2 | | | | | | | | | |
| PRPS1 | | | | | | | | | |
| PRPS1L1 | | | | | | | | | |
| PRPS2 | | | | | | + | | | + |
| PRPSAP1 | | | | | | | | | |
| PRPSAP2 | | + | | | | | | | + |
| PRR11 | | | | | | | | | |
| PRR12 | | | | | | | | | |
| PRR13 | | | | | | | | | |
| PRR14 | | | | | | | | | |
| PRR14L | | | | | | | | | |
| PRR15 | | | | | | | | | |
| PRR15L | | | | | | | | | |
| PRR16 | | | | | | | | | |
| PRR19 | | | | | | | | | |
| PRR20A | | | | | | | | | |
| PRR20B | | | | | | | | | |
| PRR20C | | | | | | | | | |
| PRR20D | | | | | | | | | |
| PRR20E | | | | | | | | | |
| PRR22 | | | | | | | | | |
| PRR23B | | | | | | | | | |
| PRR23D1 | | | | | | | | | |
| PRR23D2 | | | | | | | | | |
| PRR25 | | | | | | | | | |
| PRR27 | | | | | | | | | |
| PRR3 | | | | | | | | | |
| PRR30 | | | | | | | | | |
| PRR32 | | | | | | | | | |
| PRR34 | | | | | | | | | |
| PRR35 | | | | | | | | | |
| PRR36 | | | | | | | | | |
| PRR4 | | | | | | | | | |
| PRR5 | | | | | | | | | |
| PRR5-ARHGAP8 | | | | | | | | | |
| PRR5L | | | | | | | | | |
| PRR7 | | | | | | | | | |
| PRRC1 | | | | | | | | | |
| PRRC2A | + | | | | | | | | + |
| PRRC2B | | | | | | | | | |
| PRRC2C | | | | | | | | | |
| PRRG1 | | | | | | | | | |
| PRRG2 | | | | | | | | | |
| PRRG3 | + | | | + | + | + | | | + |
| PRRG4 | | | | | | | | | |
| PRRT1 | | + | | | | + | | | + |
| PRRT2 | | | | | | + | | | + |
| PRRT3 | | | | | | + | | | + |
| PRRT4 | | | | | | | | | |
| PRRX1 | + | | | + | + | | | | + |
| PRRX2 | | | | | | | | | |

| | | | | | | | | | |
|---|---|---|---|---|---|---|---|---|---|
| PRS1 | | | | | | | | | |
| PRSS1 | | | | | | | | | |
| PRSS12 | | | | | | | | | |
| PRSS16 | | | | | | | | | |
| PRSS2 | | | | | | | | | |
| PRSS21 | | | | | | | | | |
| PRSS22 | | | | | | | | | |
| PRSS23 | | | | | | | | | |
| PRSS27 | | | | | | | | | |
| PRSS3 | | | | | | | | | |
| PRSS33 | | | | | | | | | |
| PRSS35 | | | | | | | | | |
| PRSS37 | | | | | | | | | |
| PRSS38 | | | | | | | | | |
| PRSS3P2 | | | | | | | | | |
| PRSS41 | | | | | | | | | |
| PRSS42 | | | | | | | | | |
| PRSS45 | | | | | | | | | |
| PRSS46 | | | | | | | | | |
| PRSS48 | | | | | | | | | |
| PRSS50 | | | | | | | | | |
| PRSS53 | | | | | | | | | |
| PRSS56 | | | | | | | | | |
| PRSS58 | | | | | | | | | |
| PRSS8 | | | | | | | | | |
| PRT1 | | | | | | | | | |
| PRTFDC1 | | | | | | | | | |
| PRTG | | | | | | + | | | + |
| PRTN3 | | | | | | | | | |
| PRUNE | | | | | | | | | |
| PRUNE2 | | | | | | | | | |
| prw1 | | | | | | | | | |
| PRX | | | | | | | | | |
| PRX1 | | | | | | | | | |
| PRY | | | | | | | | | |
| pry-1 | | | | | | | | | |
| PRY1 | | | | | | | | | |
| PRY2 | | | | | | | | | |
| PSA1 | | | | | | | | | |
| PSAP | | | | | | | | | |
| PSAPL1 | | | | | | | | | |
| PSAT1 | | | | | + | | | | + |
| PSAT1P1 | | | | | | | | | |
| PSCA | | | | | | | | | |
| PSD | | | | | | | | | |
| PSD1 | | | | | | | | | |
| PSD2 | | | | | | | | | |
| PSD3 | | | | | + | | | | + |
| PSD4 | | | | | | | | | |
| PSEN1 | | | | | | | | | |
| PSEN2 | | | | | | | | | |
| PSENEN | | + | | | | + | | | + |
| PSG1 | | | | | | | | | |

| | | | | | | | | | |
|---|---|---|---|---|---|---|---|---|---|
| PSG11 | | | | | | | | | |
| PSG2 | | | | | | | | | |
| PSG3 | | | | | | | | | |
| PSG4 | | | | | | | | | |
| PSG5 | | | | | | | | | |
| PSG6 | | | | | | | | | |
| PSG7 | | | | | | | | | |
| PSG8 | | | | | | | | | |
| PSG9 | | | | | | | | | |
| PSH1 | | | | | | | | | |
| psi1 | | | | | | | | | |
| PSIP1 | | + | | | | + | | | + |
| PSKH1 | | | | | | | | | |
| PSKH2 | | | | | | | | | |
| PSMA1 | | | | | | | | | |
| PSMA2 | | + | | | + | + | | | + |
| PSMA3 | | | | | + | | | | + |
| PSMA3-AS1 | | | | | | | | | |
| Psma3l | | | | | | | | | |
| PSMA4 | | | | | | | | | |
| PSMA5 | | | | | | | | | |
| PSMA6 | | | | | | | | | |
| PSMA7 | | | | | | | | | |
| PSMA8 | | | | | | | | | |
| PSMB1 | | | | | | | | | |
| PSMB10 | | | | | | | | | |
| PSMB11 | | | | | | | | | |
| PSMB2 | | | | | | | | | |
| PSMB3 | | | | | + | | | | + |
| PSMB4 | | | | | + | | | | + |
| PSMB5 | | | | | | | | | |
| PSMB6 | | | | | + | + | | + | + |
| PSMB7 | | | | | | | | | |
| PSMB8 | | | | | | | | | |
| PSMB9 | | | | | | | | | |
| PSMC1 | | | + | | | | | | + |
| PSMC2 | | | | | | | | | |
| PSMC3 | | | | | | | | | |
| Psmc3ip | | | | | | | | | |
| PSMC4 | | | | | + | | | | + |
| PSMC5 | | | | | + | | | | + |
| PSMC6 | | | | | + | | | | + |
| PSMD1 | | | | | + | | | | + |
| PSMD10 | | | | | | | | | |
| PSMD11 | | | | | + | | | | + |
| PSMD12 | | | | | + | | | | + |
| PSMD13 | | | | | + | | | | + |
| psmd14 | | + | | | + | + | | | + |
| PSMD2 | | | | | + | | | | + |
| PSMD3 | | | | | + | | | | + |
| PSMD4 | | + | | | + | + | | | + |
| PSMD5 | | | | | | | | | |
| PSMD6 | | | | | + | | | | + |

| | | | | | | | | | |
|---|---|---|---|---|---|---|---|---|---|
| PSMD6-AS2 | | | | | | | | | |
| PSMD7 | | | | | + | | | | + |
| PSMD8 | | | | | | | | | |
| PSMD9 | | | | | | | | | |
| PSME1 | | | | | | | | | |
| PSME2 | | | | | | | | | |
| PSME3 | | | + | | | | | | + |
| PSME4 | | | | | + | | | | + |
| PSMF1 | | | | | | | | | |
| PSMG1 | | | | | | | | | |
| PSMG2 | | | | | | | | | |
| PSMG3 | | | | | | | | | |
| PSMG3-AS1 | | | | | | | | | |
| PSMG4 | | | | | | | | | |
| PSORS1C1 | | | | | | | | | |
| PSORS1C2 | | | | | | | | | |
| PSORS1C3 | | | | | | | | | |
| PSP1 | | | | | | | | | |
| PSPC1 | | | | | | | | | |
| PSPH | | | | | | | | | |
| PSPN | | | | | | | | | |
| PSRC1 | | | | | | + | | | + |
| pss1 | | | | | | | | | |
| PSTK | | + | | | | + | | | + |
| PSTPIP1 | | | | | | | | | |
| PSTPIP2 | | | | | | + | | | + |
| psu1 | | | | | | | | | |
| PSY3 | | | | | | | | | |
| PTAFR | | | | | | | | | |
| PTAR1 | | | | | | | | | |
| PTBP1 | | | | | | | | | |
| PTBP2 | | | | | | | | | |
| PTBP3 | | | | | | | | | |
| PTC1 | | | | | | | | | |
| PTC2 | | | | | | | | | |
| PTC3 | | | | | | | | | |
| PTC5 | | | | | | | | | |
| PTCD1 | | | | | | | | | |
| PTCD2 | | | | | | | | | |
| PTCD3 | | | | | | | | | |
| PTCH1 | | | | | | | | | |
| PTCH2 | | | | | | | | | |
| PTCHD1 | + | | | + | + | | | | + |
| PTCHD2 | | | | | | + | | | + |
| PTCHD3 | | | | | | | | | |
| PTCRA | | | | + | | | | | + |
| PTDSS1 | | | | | | | | | |
| PTDSS2 | | | + | | | | | | + |
| PTEN | | | + | | | | | | + |
| PTER | | + | + | | | + | | | + |
| PTF1A | | | | | | | | | |
| PTGDR | + | | | + | + | | + | | + |
| PTGDR2 | | | | | | | | | |

| | | | | | | | | | |
|---|---|---|---|---|---|---|---|---|---|
| PTGDS | | | | | | | | + | + |
| PTGER1 | | | | | | | | | |
| PTGER2 | | | | | | | | | |
| PTGER3 | | | + | | | | | | + |
| PTGER4 | + | | | | | | | | + |
| PTGES | | | | | | | | | |
| PTGES2 | | | | | | | | | |
| PTGES3 | | | | | + | | | | + |
| PTGES3L | | | | | | | | | |
| PTGES3L-AARSD1 | | | | | | | | | |
| PTGFR | + | | | + | + | | + | | + |
| PTGFRN | | | | | | | | | |
| PTGIR | | | | | | | | | |
| PTGIS | | | | | | | | | |
| PTGR1 | | | | | | | | | |
| PTGR2 | | | | | | | | | |
| PTGS1 | | | | | | | | | |
| PTGS2 | + | | + | | + | | | | + |
| PTH | | | | | | | | | |
| PTH1R | | | | | | | | | |
| PTH2 | | | | | | | | | |
| PTH2R | | | | | | | | | |
| pth4 | | | | | | | | | |
| PTHLH | | | | | | | | | |
| PTK2 | | | | | | | | | |
| PTK2B | + | | + | | | + | | | + |
| PTK6 | | | | | | | | | |
| PTK7 | | | | | | | | | |
| PTMA | | | | | | | | | |
| PTMS | + | | + | + | + | | | | + |
| PTN | | | | | | | | | |
| PTOV1 | | | | | | | | | |
| PTP1 | | | | | | | | | |
| PTP2 | | | | | | | | | |
| PTP4A1 | | | | | | | + | | + |
| PTP4A2 | | | | | + | | | | + |
| PTP4A3 | | | | | | | | | |
| ptpA | | | | | | | | | |
| PTPDC1 | | | | | | | | | |
| PTPLA | | | | | | | | | |
| PTPLAD1 | | | | | | | | | |
| PTPLAD2 | | | | | | | | | |
| PTPLB | | | + | | | | | | + |
| PTPMT1 | | | | | | + | | | + |
| PTPN1 | | | | | | | | | |
| PTPN11 | | | | | | | | | |
| PTPN12 | | | | | | | | | |
| PTPN13 | | | | | + | | | | + |
| PTPN14 | | | | | | | | | |
| PTPN18 | | | | | | | | | |
| PTPN2 | | | | | | | | | |
| PTPN20B | | | | | | | | | |
| PTPN21 | | | | | | | | | |

| | | | | | | | | | |
|---|---|---|---|---|---|---|---|---|---|
| PTPN22 | | | | | | | | | |
| PTPN23 | | | | | | | | | |
| PTPN3 | | + | | | | + | | | + |
| PTPN4 | | | | | | | | | |
| PTPN5 | | | + | | | | | | + |
| PTPN6 | | | | | | | | | |
| PTPN7 | + | | | + | + | | | | + |
| PTPN9 | | | | | | | | | |
| PTPRA | | | | | | | | | |
| PTPRB | | | | | | | | | |
| PTPRC | | | | | | | | | |
| PTPRCAP | | | | | | | | | |
| PTPRD | | | | | | | | | |
| PTPRE | | | | | | | | | |
| PTPRF | | | | | | | | | |
| PTPRG | | | | | | + | | | + |
| PTPRH | | | | | | | | | |
| PTPRJ | | | | | | | | | |
| PTPRK | | | + | | | | | | + |
| PTPRM | | | | | | | | | |
| PTPRN | + | | + | | + | | | | + |
| PTPRN2 | | | | | + | | | | + |
| PTPRO | | | | | + | | | | + |
| PTPRQ | | | | | | | | | |
| PTPRR | | | | | | | | | |
| PTPRS | | | | | | + | | | + |
| PTPRT | | | | | + | | | | + |
| PTPRU | | | | | | | | | |
| PTPRZ1 | | | | | + | | | | + |
| ptr3 | | | | | | | | | |
| PTRF | | | | | | | | | |
| PTRH1 | | | | | | | | | |
| PTRH2 | | | | | | | | | |
| PTRHD1 | | | | | | | | | |
| PTS | | | | | | + | | | + |
| PTTG1 | + | | | + | + | | | | + |
| PTTG1IP | + | | + | | + | | | | + |
| PTTG2 | | | | | | | | | |
| PTX3 | | | | | | | | | |
| PTX4 | | | | | | | | | |
| pub1 | | | | | | | | | |
| PUB13 | | | | | | | | | |
| puc | | | | | | | | | |
| PUF60 | | | + | | | | | | + |
| PUM1 | | | | | | | | | |
| PUM2 | | | | | | | | | |
| PUP1 | | | | | | | | | |
| PUP2 | | | | | | | | | |
| PUP3 | | | | | | | | | |
| PURA | | | | | | | | | |
| PURB | | | + | | | | | | + |
| PURG | | | | | | | | | |
| PUS1 | | | | | | + | | | + |

| | | | | | | | | | |
|---|---|---|---|---|---|---|---|---|---|
| PUS10 | | | | | | + | | | + |
| PUS3 | | | | | | | | | |
| PUS7 | | | | | | | | | |
| PUS7L | | | | | | | | | |
| PUSL1 | | | | | | | | | |
| PVALB | | | | | | | | + | + |
| PVR | + | | + | | + | | | | + |
| PVRIG | | | | | | | | | |
| PVRL1 | | | | | | | | | |
| PVRL2 | | | | | | | | | |
| PVRL3 | | + | + | | + | + | | | + |
| PVRL4 | | | | | | | | | |
| PVT1 | | | | | | | | | |
| PWAR5 | | | | | | | | | |
| PWARSN | | | | | | | | | |
| pwi1 | | | | | | | | | |
| PWP1 | | | | + | | | | | + |
| PWP2 | | | | | | | | | |
| PWWP2A | | | + | | | | | | + |
| PWWP2B | | | | | | | | | |
| PXA1 | | | | | | | | | |
| PXA2 | | | | | | | | | |
| Pxdc1 | | | | | | | | | |
| PXDN | | | | | + | | | | + |
| PXDNL | | | | | | | | | |
| PXK | | | | | | | | | |
| PXMP2 | | | | | | | | | |
| PXMP4 | | | | | | | | | |
| PXN | | | | | | | | | |
| PXN-AS1 | | | | | | | | | |
| PXR1 | | | | | | | | | |
| PXT1 | | | | | | | | | |
| PXYLP1 | | | | | | | | | |
| PYC1 | | | | | | | | | |
| PYCARD | | | | | | | | | |
| PYCR1 | | | | | | | | | |
| PYCR2 | | | | + | | | | | + |
| PYCRL | | | | | | | | | |
| PYDC1 | | | | | | | | | |
| PYGB | | | | | | | | | |
| PYGL | | | | | | | | | |
| PYGM | | | | | | | | | |
| PYGO1 | | | + | | | + | | | + |
| PYGO2 | | | | | | | | | |
| PYHIN1 | | | | | | | | | |
| pyk1 | | | | | | | | | |
| pyr1 | | | | | | | | | |
| PYROXD1 | | | | | | | | | |
| PYROXD2 | | | | | | | | | |
| PYURF | | | | | | | | | |
| PYY | | | | | | | | | |
| PZP | | | | | | | | | |
| QARS | | | | | | | | | |

| | | | | | | | | | |
|---|---|---|---|---|---|---|---|---|---|
| qcr1 | | | | | | | | | |
| QCR2 | | | | | | | | | |
| qcr7 | | | | | | | | | |
| qcr8 | | | | | | | | | |
| qcr9 | | | | | | | | | |
| QDPR | | | | | | | | | |
| Qk | + | | + | | + | | | | + |
| qkgA-1 | | | | | | | | | |
| QKI | | | | | | | | | |
| QPCT | + | | | + | + | | + | | + |
| QPCTL | | | | | | | | | |
| QPRT | | | | | | | | | |
| QRFP | | | | | | | + | | + |
| QRFPR | | | | | | | | | |
| QRICH1 | | | | | | | | | |
| QRICH2 | | | | | | | | | |
| QRSL1 | | + | | | | + | | | + |
| QSER1 | | | | | | | | | |
| QSOX1 | | | | | | | | | |
| QSOX2 | | | | | | | | | |
| QTRT1 | | | | | | | | | |
| QTRTD1 | | | | | | | | | |
| R3HCC1 | + | | | + | + | | | | + |
| R3HCC1L | | | | | | | | | |
| R3HDM1 | | | + | | + | | | | + |
| R3HDM2 | | | + | | | | | | + |
| R3HDM4 | | + | | | | + | | | + |
| R3HDML | | | | | | | | | |
| Rab1 | | | | | | | | | |
| RAB10 | | | | | | | | | |
| RAB11A | + | | | | + | | | | + |
| Rab11b | | | + | | | | | | + |
| RAB11FIP1 | | | | | | | | | |
| RAB11FIP2 | | | | | | | | | |
| RAB11FIP3 | + | | | | + | | | | + |
| RAB11FIP4 | | | | | | | | | |
| RAB11FIP5 | | | | | | | | | |
| RAB12 | | | | | | | | | |
| RAB13 | | | | | | | | | |
| RAB14 | | | | | + | | | | + |
| RAB15 | | | | | | | + | | + |
| RAB17 | | | | | | | | | |
| RAB18 | | | | | | | | | |
| RAB19 | | | | | | | | | |
| RAB1A | | | | | | | | | |
| RAB1B | | | | | | | | | |
| RAB1C | | | | | | | | | |
| RAB20 | | | | | | | | | |
| RAB21 | + | | | + | + | | + | | + |
| RAB22A | | | | | | | | | |
| RAB23 | | | | | | + | | | + |
| RAB24 | | | | | | | | | |
| RAB25 | | | | | | | | | |

| | | | | | | | | | |
|---|---|---|---|---|---|---|---|---|---|
| RAB26 | | | | | | + | | | + |
| RAB27A | | | | | | + | | | + |
| RAB27B | | | | | | + | | | + |
| RAB28 | | | | | | + | | | + |
| RAB29 | | | | | | | | | |
| RAB2A | | + | | | | + | | | + |
| RAB2B | | | | | | | | | |
| Rab3 | | | | | | | | | |
| RAB30 | | | | | | | | | |
| RAB31 | | | | | | | | | |
| RAB32 | | | | | | | | | |
| RAB33A | | | | | + | | | | + |
| RAB33B | | | | + | | | | | + |
| RAB34 | | | | | | | | | |
| RAB35 | | | | | | + | | | + |
| RAB36 | | | | | | | | | |
| RAB37 | | | | | | | | | |
| RAB38 | | | | | | | | | |
| RAB39A | | | | | | | | | |
| RAB39B | | | | | | | | | |
| RAB3A | | | | | | + | | | + |
| RAB3B | | | | | | + | | | + |
| RAB3C | | | + | | | | | | + |
| RAB3D | | | | | | + | | | + |
| RAB3GAP1 | | | | | + | | | | + |
| RAB3GAP2 | | | | | | | | | |
| RAB3IL1 | | | | | | | | | |
| RAB3IP | | | + | | | | | | + |
| RAB40A | | | | | | | | | |
| RAB40AL | | | | | | | | | |
| RAB40B | | | | | | | | | |
| RAB40C | | | | | | | | | |
| RAB41 | | | | | | | | | |
| RAB42 | | | | | | | | | |
| RAB43 | | | | | | | | | |
| RAB44 | | | | | | | | | |
| RAB4A | | | | | | | | | |
| RAB4B | | | | | | | | | |
| RAB4B-EGLN2 | | | | | | | | | |
| Rab5 | | | | | | | | | |
| RAB5A | | | | | | | + | | + |
| RAB5B | | | | | | | | | |
| RAB5C | | | | | | | | | |
| RAB6A | | | | | | | | | |
| RAB6B | | | + | | | | | | + |
| RAB6C | | | | | | | | | |
| Rab7 | | | | | | | | | |
| RAB7A | | | | | | | | | |
| RAB7B | | | | | | | | | |
| RAB8A | | | | | | | | | |
| RAB8B | | | | | | | | | |
| RAB9A | | | | | | | | | |
| RAB9B | | | | | | | | | |

| | | | | | | | | | |
|---|---|---|---|---|---|---|---|---|---|
| RABAC1 | | | | | | | | | |
| RABEP1 | | | | | | | | | |
| RABEP2 | | | | | | | | | |
| RABEPK | | | | | | + | | | + |
| RABGAP1 | | + | | | | + | | | + |
| RABGAP1L | | | | | | | | | |
| RABGEF1 | | | + | | | | | | + |
| RABGGTA | | | | | | | | | |
| RABGGTB | | | | | | | | | |
| RABIF | | | | | | | | | |
| Rabl2 | | | | | | + | | | + |
| RABL2A | | | | | | | | | |
| RABL2B | | | | | | | | | |
| RABL3 | + | | | + | + | | + | | + |
| RABL6 | | | | | | | | | |
| RAC1 | | + | | | | + | | | + |
| RAC2 | | | | | | | | | |
| RAC3 | | | | | | | | | |
| RACGAP1 | | | | | + | | | | + |
| RAD1 | | | | | | | | | |
| rad13 | | | | | | | | | |
| RAD16 | | | | | | | | | |
| RAD17 | | | | | + | | | | + |
| RAD18 | | | | | | | | | |
| RAD21 | | | | | | | | | |
| RAD21L1 | | | | | | | | | |
| RAD23 | | | | | | | | | |
| RAD23A | | | | | | | | | |
| RAD23B | | | | | + | | | | + |
| rad24 | | | | | | | | | |
| rad25 | | | | | | | | | |
| RAD27 | | | | | | | | | |
| rad3 | | | | | | | | | |
| RAD50 | | | | | | | | | |
| RAD51 | | | | | | | | | |
| rad51-b | | | | | | | | | |
| RAD51AP1 | | | | | | | | | |
| RAD51AP2 | | | | | | | | | |
| RAD51B | | | | | | | | | |
| RAD51C | | | | | | | | | |
| RAD51D | | | | | | | | | |
| RAD52 | | | | | | | | | |
| RAD53 | | | | | | | | | |
| RAD54 | | | | | | | | | |
| RAD54B | | | | | | | | | |
| RAD54L | | | | | | | | | |
| RAD54L2 | | | | | | | | | |
| RAD55 | | | | | | | | | |
| RAD57 | | | | | | | | | |
| RAD6 | | | | | | | | | |
| rad9 | | | | | | | | | |
| RAD9A | | | | | | | | | |
| RAD9B | | | | | | | | | |

| | | | | | | | | | |
|---|---|---|---|---|---|---|---|---|---|
| RADIL | | | | | | | | | |
| RAE1 | | | | | | | | | |
| RAET1E | | | | | | | | | |
| RAET1G | | | | | | | | | |
| RAET1L | | | | | | | | | |
| RAF1 | | | | | | | | | |
| RAG1 | | | | | | | | | |
| RAG2 | | | | | | | | | |
| RAI1 | | | | | | | | | |
| RAI14 | | | | | | | | | |
| RAI2 | | | | | + | | | | + |
| RALA | | | | | + | | | | + |
| RALB | | | | | | | | | |
| RALBP1 | | | | | | + | | | + |
| RALGAPA1 | | | | | | | | | |
| RALGAPA2 | | | | | | | | | |
| RALGAPB | | | | | | | | | |
| RALGDS | | | | | | | | | |
| RALGPS1 | | | | | | | | | |
| RALGPS2 | | | | | | + | | | + |
| RALY | | | | | | | | | |
| RALY-AS1 | | | | | | | | | |
| RALYL | | | | | | | | | |
| RAM2 | | | | | | | | | |
| RAMP1 | | + | | | | + | | | + |
| RAMP2 | | | | | | | | | |
| RAMP3 | | | | | | | | | |
| RAN | | + | | | | + | | | + |
| RANBP1 | | | | | | | | | |
| RANBP10 | | | | | | | | | |
| RANBP17 | | | | | | | | | |
| RANBP2 | | | | | + | | | | + |
| RANBP3 | | | | | | | | | |
| RANBP3L | | | | | | | | | |
| RANBP6 | | | | | | | | | |
| RANBP9 | | | | | | | | | |
| RANGAP1 | | | | | | | | | |
| RANGRF | | | | | | | | | |
| RANP1 | | | | | | | | | |
| RAP1A | | | | + | | | | | + |
| RAP1B | + | | | | + | | | | + |
| RAP1BL | | | | | | | | | |
| RAP1GAP | | | | | | | | | |
| RAP1GAP2 | | | + | | | | | | + |
| RAP1GDS1 | | | | | + | | | | + |
| RAP2A | | | | | | | | | |
| RAP2B | | | | | | | | | |
| RAP2C | | | | | | | | | |
| RAPGEF1 | | | | | + | | | | + |
| RAPGEF2 | | | | | | + | | | + |
| RAPGEF3 | | | | | | | | | |
| RAPGEF4 | | | | | | | | | |
| RAPGEF5 | | | | | | | | | |

| | | | | | | | | | |
|---|---|---|---|---|---|---|---|---|---|
| RAPGEF6 | + | | | | + | | | | + |
| RAPGEFL1 | | | | | | + | | | + |
| RAPH1 | | | | | | | | | |
| RAPSN | | | | | | | | | |
| RAPTOR1 | | | | | | | | | |
| rar1 | | | | | | | | | |
| RARA | | | | | | | | | |
| RARB | | | | | | | | | |
| RARG | | | | | | | | | |
| RARRES1 | | | | | | | | | |
| RARRES2 | | | | | | | | | |
| RARRES3 | | | | | | | | | |
| RARS | | | | | | | | | |
| RARS2 | | | | | | | | | |
| RAS1 | | | | | | | | | |
| RAS2 | | | | | | | | | |
| RASA1 | | + | | | | + | | | + |
| RASA2 | | | | | | | | | |
| RASA3 | | | + | | | | | | + |
| RASA4 | | | | | + | | | | + |
| RASA4B | | | | | | | | | |
| RASAL1 | | | | | | + | | | + |
| RASAL2 | | | | | | | | | |
| RASAL3 | | | | | + | | | | + |
| RASD1 | | | | | | | | | |
| RASD2 | | | | | | | | | |
| RASEF | | | | | | | | | |
| RASGEF1A | | | | | | | | | |
| RASGEF1B | | | + | | + | | | | + |
| RASGEF1C | | | | | | | | | |
| RASGRF1 | | | | | | | | | |
| RASGRF2 | | | | | | + | | | + |
| RASGRP1 | | | + | | | | | | + |
| RASGRP2 | | | | | | | | | |
| RASGRP3 | | | | | | | | | |
| RASGRP4 | | | | | | | | | |
| RASIP1 | | | | | | | | | |
| RASL10A | | | | | | | | | |
| RASL10B | | | | | | | | | |
| RASL11A | | | | | | | | | |
| RASL11B | | | | | | | | | |
| RASL12 | | | | + | | | | | + |
| RASSF1 | + | | | | | | | | + |
| RASSF10 | | | | | | | | | |
| RASSF2 | | | | | | | | | |
| RASSF3 | | | + | | | | | | + |
| RASSF4 | | | | | | | | | |
| RASSF5 | | | | | | | | | |
| RASSF6 | | | | | | | | | |
| RASSF7 | | | | | | | | | |
| RASSF8 | | | | | | | | | |
| RASSF9 | | | | | | | | | |
| RAVER1 | | + | + | | | + | | | + |

| | | | | | | | | | |
|---|---|---|---|---|---|---|---|---|---|
| RAVER2 | | | | | | | | | |
| RAX | | | | | | | | | |
| RB1 | | | | | | | | | |
| RB1CC1 | | | | + | | | | | + |
| RBAK | | | | | | | | | |
| RBAK-RBAKDN | | | | | | | | | |
| RBBP4 | | | | | | | | | |
| RBBP4P1 | | | | | | | | | |
| RBBP5 | | | + | | | | | | + |
| RBBP6 | | | + | | | | | | + |
| RBBP7 | + | | | | + | | | | + |
| RBBP8 | | | | | | | | | |
| RBBP8NL | | | | | | | | | |
| RBBP9 | | | | | | | | | |
| RBCK1 | | | | | | | | | |
| RBFA | | | | | | | | | |
| RBFOX1 | | | | | | + | | | + |
| RBFOX2 | | | + | + | | | | | + |
| RBFOX3 | | | | | | + | | | + |
| RBG1 | | | | | | | | | |
| RBKS | | | | | | | | | |
| RBL1 | | | | | | | | | |
| RBL2 | | | | | | | | | |
| RBM10 | | | | | | | | | |
| RBM11 | | | | | | | | | |
| RBM12 | | | + | | | | | | + |
| RBM12B | | | | | | | | | |
| RBM14 | | | | | | | | | |
| RBM14-RBM4 | | | | | | | | | |
| RBM15 | + | | + | | + | | | | + |
| RBM15B | | | | | + | | | | + |
| RBM17 | | | | | | | | | |
| RBM18 | | | | | | | | | |
| RBM19 | | | | | | | | | |
| RBM20 | | | | | | | | | |
| RBM22 | | | | | | | | | |
| RBM23 | | | | | | | | | |
| RBM24 | | | | | | | | | |
| RBM25 | + | | | + | + | | | | + |
| RBM26 | | | | | | | | | |
| RBM27 | | | | | | | | | |
| RBM28 | | | | | | | | | |
| RBM3 | | | + | | | | | | + |
| RBM33 | | | + | | | | | | + |
| RBM34 | | | | | + | | | | + |
| RBM38 | | | | | | | | | |
| Rbm39 | | | | | | | | | |
| RBM4 | | | | | | | | | |
| RBM41 | | | | | | | | | |
| RBM42 | | | | | | | | | |
| RBM43 | | | | | | | | | |
| RBM44 | | | | | | | | | |
| RBM45 | | | | | + | | | | + |

| | | | | | | | | | |
|---|---|---|---|---|---|---|---|---|---|
| RBM46 | + | | | + | + | | + | | + |
| RBM47 | | | | | | | | | |
| RBM48 | | | | | | | | | |
| RBM4B | + | | | | + | | | | + |
| RBM5 | | + | | | | + | | | + |
| RBM6 | | | + | | + | | | | + |
| RBM7 | | | | | | | | | |
| RBM8A | | | | + | | | | | + |
| RBMS1 | | | + | | | | | | + |
| RBMS2 | | | | | | | | | |
| RBMS3 | + | | | + | + | | + | | + |
| RBMX | | + | | | | + | | | + |
| RBMX2 | | | | | | | | | |
| RBMXL1 | | | | | | | | | |
| RBMXL2 | | | | | | | | | |
| RBMXL3 | | | | | | | | | |
| RBMXP1 | | | | | | | | | |
| Rbmxrtl | | | | | | | | | |
| RBMY1A1 | | | | | | | | | |
| RBMY1B | | | | | | | | | |
| RBMY1C | | | | | | | | | |
| RBMY1D | | | | | | | | | |
| RBMY1E | | | | | | | | | |
| RBMY1F | | | | | | | | | |
| RBMY1J | | | | | | | | | |
| RBMY2BP | | | | | | | | | |
| RBMY2FP | | | | | | | | | |
| RBP1 | | | | | | | | | |
| RBP2 | | | | | | | | | |
| RBP3 | | | | | | | | | |
| RBP4 | | | | | | | | | |
| RBP5 | | | | | | | | | |
| RBP7 | | | | | | | | | |
| RBPJ | | | + | | | | | | + |
| RBPJL | | | | | | | | | |
| RBPJP3 | | | | | | | | | |
| RBPMS | | + | + | | | + | | | + |
| RBPMS2 | | | | | | | | | |
| RBSN | | | | | | | | | |
| RBX1 | | | | | | | | | |
| Rc3h1 | | | | | | | | | |
| RC3H2 | | | | | | | | | |
| RCAN1 | | | | | + | | | | + |
| RCAN2 | | | + | | | | | | + |
| RCAN3 | | | | | | + | | | + |
| RCBTB1 | | | | | | | | | |
| RCBTB2 | | | | | | | | | |
| RCC1 | | | | | | | | | |
| RCC2 | | | + | | | | | | + |
| RCCD1 | | | | | | | | | |
| RCD1 | | | | | | | | | |
| RCE1 | | | | | | | | | |
| RCHY1 | | | | | | | | | |

| | | | | | | | | | |
|---|---|---|---|---|---|---|---|---|---|
| RCL1 | | | | | | | | | |
| RCN1 | | | | | | | | | |
| RCN2 | | + | | | | + | | | + |
| RCN3 | | | | | | | | | |
| RCOM_2159910 | | | | | | | | | |
| RCOR1 | | | | | | | | | |
| RCOR2 | | | | | | | | | |
| RCOR3 | | | | | | | | | |
| RCSD1 | | | | + | | | | | + |
| rct1 | | | | | | | | | |
| RCVRN | | | | | | | | | |
| RD3 | | | | | | | | | |
| RD3L | | | | | | | | | |
| rdgC | | | | | | | | | |
| RDH10 | | | | | | | | | |
| RDH11 | | | | | | | | | |
| RDH12 | | + | | + | | | | | + |
| RDH13 | | | | | | | | | |
| RDH14 | | | | | | | | | |
| RDH16 | | | | | | | | | |
| RDH5 | | | | | | | | | |
| RDH8 | | | | | | | | | |
| RDM1 | | | | | | | | | |
| RDS1 | | | | | | | | | |
| Rdx | | | | | | | | | |
| REC107 | | | | | | | | | |
| REC114 | | | | | | | | | |
| REC8 | | | | | | | | | |
| RECK | | | | | | | | | |
| RECQL | | | | | | | | | |
| Recql4 | | | | | | | | | |
| RECQL5 | | | | | | | | | |
| REEP1 | | | | | | | | | |
| REEP2 | | | | | + | | | | + |
| REEP3 | | | | | | | | | |
| REEP4 | | | | | | | | | |
| REEP5 | | | | | | | | | |
| REEP6 | | | | | | | | | |
| REG1A | | | | | | | | | |
| REG1B | | | | | | | | | |
| REG3A | | | | | | | | | |
| REG3G | | | | | | | | | |
| REG4 | | | | | | | | | |
| REL | | | | | | | | | |
| RELA | | | | | | | | | |
| RELB | | | | | + | | | | + |
| RELL1 | | | | | | | | | |
| RELL2 | | | + | | + | | | | + |
| RELN | | | | | + | | | | + |
| RELT | | | + | | | | | | + |
| REM1 | | | | | | | | | |
| REM2 | | | | | | | | | |
| REN | | | | | | | | | |

| | | | | | | | | | |
|---|---|---|---|---|---|---|---|---|---|
| RENBP | | | | | | | | | |
| REP15 | | | | | | | | | |
| REPIN1 | | | | | | | | | |
| REPS1 | | | | | | | | | |
| REPS2 | | | | | | | | | |
| RER1 | | | | | | | | | |
| RERE | | | | | | | | | |
| RERG | | | + | | | | | | + |
| REST | + | | | + | + | | + | | + |
| RET | | | | | | | | | |
| RET2 | | | | | | | | | |
| RETN | | | | | | | | | |
| RETNLB | | | | | | | | | |
| RETSAT | | | | | | | | | |
| rev | | | | | | | | | |
| REV1 | | | | | | | | | |
| REV3L | | | | | | | | | |
| REXO1 | | | | | | | | | |
| REXO1L1P | | | | | | | | | |
| REXO1L6P | | | | | | | | | |
| REXO2 | | | | | | | | | |
| REXO4 | | | | | | | | | |
| RFA1 | | | | | | | | | |
| RFC1 | | | | | | | | | |
| RFC2 | | | | | | | | | |
| RFC3 | | | | | | | | | |
| RFC4 | | | | | | | | | |
| RFC5 | | | | | | | | | |
| RFESD | | | | | | | | | |
| RFFL | | | | | | | | | |
| RFK | | | | | | | | | |
| RFNG | | | | | | | | | |
| RFPL1 | | | | | | | | | |
| RFPL2 | | | | | | | | | |
| RFPL3 | | | | | | | | | |
| RFPL4A | | | | | | | | | |
| RFPL4B | | | | | | | | | |
| RFS1 | | | | | | | | | |
| RFT1 | | | | | | | | | |
| RFTN1 | | | | | | + | | | + |
| RFTN2 | | | | | | | | | |
| RFWD2 | + | | | | + | | | | + |
| RFWD3 | | | | | | | | | |
| RFX1 | | | | | | | | | |
| RFX2 | | | | | | | | | |
| RFX3 | | | | | | + | | | + |
| RFX4 | | | | | | | | | |
| RFX5 | | | | | | | | | |
| RFX6 | | | | | | | | | |
| RFX7 | | | | | | | | | |
| RFX8 | | | | | | | | | |
| RFXANK | | | | | | | | | |
| RFXAP | | | + | | | | | | + |

| | | | | | | | | | |
|---|---|---|---|---|---|---|---|---|---|
| rga3 | | | | | | | | | |
| rga7 | | | | | | | | | |
| RGAG1 | | | | | | | | | |
| RGAG4 | | | | | | + | | | + |
| RGCC | | | | | | | | | |
| RGD1 | | | | | | | | | |
| RGD1304704 | | | | | | | | | |
| RGD1308106 | | | | | | | | | |
| RGD1562284 | | | | | | | | | |
| RGD1563551 | | | | | | | | | |
| RGD1566197 | | | | | | | | | |
| RGD2 | | | | | | | | | |
| RGL1 | | | + | | | + | | | + |
| RGL2 | | | | | | | | | |
| RGL3 | | | | | | | | | |
| RGL4 | | | | | | | | | |
| RGMA | | | | | | | | | |
| RGMB | | | | | | | | | |
| RGN | | | | | | | | | |
| RGP1 | | | | | | | | | |
| RGPD1 | | | | | | | | | |
| RGPD2 | | | | | | | | | |
| RGPD3 | | | | | | | | | |
| RGPD4 | | | | | | | | | |
| RGPD5 | | | | | | | | | |
| RGPD6 | | | | | | | | | |
| RGPD8 | | | | | | | | | |
| RGR | | | | | | | | | |
| RGS1 | | | | | | | | | |
| RGS10 | | | + | | | | | | + |
| RGS11 | | | | | | | | | |
| Rgs12 | | | | | + | | | | + |
| Rgs13 | | | | | | | | | |
| RGS14 | | | | | | + | | | + |
| RGS16 | | | | | | | | | |
| RGS17 | + | | | + | + | | | | + |
| RGS18 | | | | | | | | | |
| RGS19 | | | | | | | | | |
| RGS2 | | | + | | + | | | | + |
| RGS20 | + | | | + | + | | | | + |
| RGS22 | | | | | | | | | |
| RGS3 | | | | | | | | | |
| RGS4 | | | + | | | | | | + |
| RGS5 | | | | | | | | | |
| RGS6 | | | | | | | | | |
| RGS7 | | | + | | + | | | | + |
| RGS7BP | + | | + | | + | | | | + |
| RGS8 | | | | | | | | | |
| RGS9 | | | | | | | | | |
| RGS9BP | + | | | + | + | | + | | + |
| RGSL1 | | | | | | | | | |
| RHAG | | | | | | | | | |
| RHBDD1 | | | | | | | | | |

| | | | | | | | | | |
|---|---|---|---|---|---|---|---|---|---|
| RHBDD2 | | | | | | | | | |
| RHBDD3 | | | | | | | | | |
| RHBDF1 | | | | | | | | | |
| RHBDF2 | | | | | | | | | |
| RHBDL1 | | | | | | + | | | + |
| RHBDL2 | | | | | | | | | |
| RHBDL3 | | | | | | | | | |
| RHBG | | | | | | | | | |
| RHCG | | | | | | | | | |
| RHD | | | | | | | | | |
| RHEB | + | | + | | + | | | | + |
| RHEBL1 | | | | | | | | | |
| RHM1 | | | | | | | | | |
| RHM3 | | | | | | | | | |
| RHNO1 | | | | | | | | | |
| RHO | | | | | | | | | |
| RHO1 | | | | | | | | | |
| RHO2 | | | | | | | | | |
| RHO5 | | | | | | | | | |
| RHOA | + | | | + | + | | | | + |
| RHOB | | | | | | | | | |
| RHOBTB1 | | | | | | | | | |
| RHOBTB2 | | | | | | + | | | + |
| rhobtb2a | | | | | | | | | |
| RHOBTB3 | | | | | | + | | | + |
| RHOC | | | | | | | | | |
| RHOD | | | | | | | | | |
| RHOF | | | | | | | | | |
| RHOG | | | | | + | | | | + |
| RHOH | | | | | | | | | |
| RHOJ | | | | | | | | | |
| RHOQ | | | + | | + | | | | + |
| RHOT1 | | | | | | | | | |
| RHOT2 | | | + | | | + | | | + |
| RHOU | | | | | | | | | |
| RHOV | | | | | | | | | |
| Rhox5 | | | | | | | | | |
| RHOXF1 | | | | | | | | | |
| RHOXF2 | | | | | | | | | |
| RHPN1 | | | | | | | | | |
| RHPN1-AS1 | | | | | | | | | |
| RHPN2 | | | | | | | | | |
| RHPN2P1 | | | | | | | | | |
| RIBC1 | | | | | | | | | |
| RIBC2 | | | | | | | | | |
| RIC1 | | | | | | | | | |
| RIC3 | | | | | | | | | |
| RIC8A | | | | | | | | | |
| Ric8b | | | | | | | | | |
| RICTOR | | | | | | | | | |
| RIF1 | | | | + | | | | | + |
| RIIAD1 | | | | | | | | | |
| RILP | | | | | | | | | |

| | | | | | | | | | |
|---|---|---|---|---|---|---|---|---|---|
| RILPL1 | | | | | | | | | |
| RILPL2 | | | | | | | | | |
| RIM11 | | | | | | | | | |
| RIM15 | | | | | | | | | |
| RIM4 | | | | | | | | | |
| RIMBP2 | | | | | | | | | |
| RIMBP3 | | | | | | | | | |
| RIMBP3C | | | | | | | | | |
| RIMKLA | | | | | | | | | |
| RIMKLB | | | | | | | | | |
| RIMS1 | | | | | | | | | |
| RIMS2 | | | + | | | | | | + |
| RIMS3 | | | | | | | | | |
| RIMS4 | | | | | | | | | |
| RIN1 | | | | | | | | | |
| RIN2 | | | | | | | | | |
| RIN3 | | | | | | | | | |
| RING1 | | | | | | | | | |
| RINL | | | | | | | | | |
| RINT1 | | | | + | | | | | + |
| RIO1 | | | | | | | | | |
| RIOK1 | | | | | | | | | |
| RIOK2 | | | | | | | | | |
| RIOK3 | | | | | + | | | | + |
| rip1 | | | | | | | | | |
| RIPK1 | | | | | | | | | |
| RIPK2 | | | + | | | | | | + |
| RIPK3 | | | | | | | | | |
| RIPK4 | | | | | | | | | |
| RIPPLY1 | + | | | + | + | | | | + |
| RIPPLY2 | | | | | | | | | |
| RIPPLY3 | | | | + | | | | | + |
| RIT1 | | | | | | | | | |
| RIT2 | | + | | | | + | | | + |
| RITA1 | | | | | | | | | |
| RKR1 | | | | | | | | | |
| RL1 | | | | | | | | | |
| RL2 | | | | | | | | | |
| RLBP1 | | | | | | | | | |
| RLF | | | | | | | | | |
| RLIM | | | + | | | | | | + |
| rlim-a | | | | | | | | | |
| RLN1 | | | | | | | | | |
| RLN2 | | | | | | | | | |
| RLN3 | | | | | | | | | |
| rlp7 | | | | | | | | | |
| RLTPR | | | | | | | | | |
| RMA1 | | | | | | | | | |
| RMD9 | | | | | | | | | |
| RMDN1 | | | | | | | | | |
| RMDN2 | | | | | | | | | |
| Rmdn3 | | | | | | | | | |
| RMI1 | | | | | | | | | |

| | | | | | | | | | |
|---|---|---|---|---|---|---|---|---|---|
| RMI2 | | | | | | | | | |
| RMND1 | | | | | | | | | |
| RMND5A | | | | | | + | | | + |
| RMND5B | | | | | | + | | | + |
| RMRP | | | | | | | | | |
| RN5S1@ | | | | | | | | | |
| RN7SK | | | | | | | | | |
| RN7SL1 | | | | | | | | | |
| RN7SL2 | | | | | | | | | |
| RN7SL3 | | | | | | | | | |
| RN7SL4P | | | | | | | | | |
| RN7SL5P | | | | | | | | | |
| rna1 | | | | | | | | | |
| RNA15 | | | | | | | | | |
| RNA18S5 | | | | | | | | | |
| RNA28S5 | | | | | | | | | |
| RNA5-8SP3 | | | | | | | | | |
| RNA5S1 | | | | | | | | | |
| RNA5S10 | | | | | | | | | |
| RNA5S11 | | | | | | | | | |
| RNA5S12 | | | | | | | | | |
| RNA5S13 | | | | | | | | | |
| RNA5S15 | | | | | | | | | |
| RNA5S16 | | | | | | | | | |
| RNA5S17 | | | | | | | | | |
| RNA5S2 | | | | | | | | | |
| RNA5S3 | | | | | | | | | |
| RNA5S4 | | | | | | | | | |
| RNA5S5 | | | | | | | | | |
| RNA5S6 | | | | | | | | | |
| RNA5S7 | | | | | | | | | |
| RNA5S8 | | | | | | | | | |
| RNA5S9 | | | | | | | | | |
| RNA5SP103 | | | | | | | | | |
| RNA5SP115 | | | | | | | | | |
| RNA5SP117 | | | | | | | | | |
| RNA5SP145 | | | | | | | | | |
| RNA5SP162 | | | | | | | | | |
| RNA5SP191 | | | | | | | | | |
| RNA5SP202 | | | | | | | | | |
| RNA5SP259 | | | | | | | | | |
| RNA5SP352 | | | | | | | | | |
| RNA5SP359 | | | | | | | | | |
| RNA5SP452 | | | | | | | | | |
| RNA5SP506 | | | | | | | | | |
| RNA5SP53 | | | | | | | | | |
| RNA5SP74 | | | | | | | | | |
| RNASE1 | | | | | | | | | |
| RNASE10 | | | | | | | | | |
| RNASE11 | | | | | | | | | |
| RNASE12 | | | | | | | | | |
| RNASE13 | | | | | | | | | |
| RNASE2 | | | | | | | | | |

| | | | | | | | | | |
|---|---|---|---|---|---|---|---|---|---|
| RNASE3 | | | | | | | | | |
| RNASE4 | | | | | | | | | |
| RNASE6 | | | | | | | | | |
| RNASE7 | | | | | | | | | |
| RNASE9 | | | | | | | | | |
| RNASEH1 | | | | | | | | | |
| RNASEH1P1 | | | | | | | | | |
| RNASEH2A | | | | | | | | | |
| RNASEH2B | | | | | | | | | |
| RNASEH2C | | | | | | | | | |
| RNASEK | | | | | | | | + | + |
| RNASEK-C17orf49 | | | | | | | | | |
| RNASEL | | | | + | | | | | + |
| RNASET2 | | | | | | | | | |
| RND1 | | | | + | | | | | + |
| RND2 | | | | | | | | | |
| RND3 | | | + | | + | | | | + |
| rnf-113 | | | | | | | | | |
| rnf-5 | | | | | | | | | |
| RNF10 | | | | | | | | | |
| RNF103 | | | | | | | | | |
| RNF103-CHMP3 | | | | | | | | | |
| RNF11 | | | | | | | | | |
| RNF111 | | | | | | | | | |
| Rnf112 | | | | | | | | | |
| RNF113A | | | | | | | | | |
| RNF113B | | | | | | | | | |
| RNF114 | | | | | | | | | |
| RNF115 | | | | | | | | | |
| RNF121 | | | | | | | | | |
| RNF122 | | | | | | | | | |
| RNF123 | | | | | + | + | | | + |
| RNF125 | | | | | | | | | |
| RNF126 | | | | | | | | | |
| RNF128 | + | | | | + | | | | + |
| RNF13 | | | | | | | | | |
| RNF130 | | | | + | | | | | + |
| RNF133 | | | | | | | | | |
| RNF135 | | | | | | | | | |
| RNF138 | | | | | | | | | |
| RNF139 | | | | | | | | | |
| RNF14 | | | | | | | | | |
| RNF141 | | | | | | | | | |
| RNF144A | | | | | | | | | |
| RNF144B | | | | | | | | | |
| RNF145 | | | | | | | | | |
| RNF146 | | | | | | | | | |
| RNF148 | | | | | | | | | |
| RNF149 | | | | | | | | | |
| RNF150 | | | | | | | | | |
| RNF151 | | | | | | | | | |
| RNF152 | + | | | + | + | | | | + |
| Rnf157 | | | | | | + | | | + |

| | | | | | | | | | |
|---|---|---|---|---|---|---|---|---|---|
| RNF165 | + | + | | + | + | | | | + |
| RNF166 | | | | | | | | | |
| RNF167 | | + | | | | + | | | + |
| RNF168 | | | | | | | | | |
| RNF169 | | | | | | | | | |
| RNF17 | | | | | | | | | |
| RNF170 | | + | | | | + | | | + |
| RNF175 | | | | | | | | | |
| RNF180 | | | | | | | | | |
| RNF181 | | | | | | | | | |
| RNF182 | | | | | | + | | | + |
| RNF183 | | | | | | | | | |
| RNF185 | | | | | | | | | |
| RNF186 | | | | | | | | | |
| RNF187 | | | | | | | | | |
| RNF19A | + | | | | + | | | | + |
| RNF19B | | | | | | | | | |
| RNF2 | | | | | | | | | |
| RNF20 | | | | | | | | | |
| RNF207 | | | | | | | | | |
| RNF208 | | | | | | | | | |
| RNF212 | | | | | | | | | |
| RNF213 | | | | | | | | | |
| RNF214 | | | | | | | | | |
| RNF215 | | | | | | | | | |
| RNF216 | | | | | | | | | |
| RNF216P1 | | | | | | | | | |
| RNF217 | | | + | | + | | | | + |
| RNF219 | | | | | | | | | |
| RNF220 | | | | | | | | | |
| RNF222 | | | | | | | | | |
| RNF223 | | | | | | | | | |
| RNF224 | | | | | | | | | |
| RNF24 | | | | | | | | | |
| RNF25 | | | | | | | | | |
| RNF26 | | | | | | | | | |
| RNF31 | | | | | | | | | |
| RNF32 | | | | | | | | | |
| RNF34 | | | | | | | | | |
| RNF38 | | | + | | | | | | + |
| RNF39 | | | | | | | | | |
| RNF4 | | | | | + | | | | + |
| RNF40 | | | | | | | | | |
| RNF41 | | | | | | | | | |
| RNF43 | | | | | | | | | |
| RNF44 | | | | | | | | | |
| RNF5 | | | | | | | | | |
| RNF6 | | | | | | | | | |
| RNF7 | | | | | | | | | |
| RNF8 | | | | | | | | | |
| RNFT1 | + | | | | + | | | | + |
| RNFT2 | | | | | | | | | |
| RNGTT | | | | | | | | | |

| | | | | | | | | | |
|---|---|---|---|---|---|---|---|---|---|
| RNH1 | | | | | + | | | | + |
| RNH70 | | | | | | | | | |
| RNLS | | | | | | | | | |
| RNMT | | | | | | | | | |
| RNMTL1 | | | | | | | | | |
| RNPC3 | | | + | + | | | | | + |
| RNPEP | | | | | | + | | | + |
| RNPEPL1 | | | | | | | | | |
| RNPS1 | | | | | | | | | |
| RNQ1 | | | | | | | | | |
| RNR1 | | | | | | | | | |
| RNR2 | | | | | | | | | |
| RNR4 | | | | | | | | | |
| RNU1-1 | | | | | | | | | |
| RNU1-11P | | | | | | | | | |
| RNU1-18P | | | | | | | | | |
| RNU1-19P | | | | | | | | | |
| RNU1-2 | | | | | | | | | |
| RNU1-27P | | | | | | | | | |
| RNU1-28P | | | | | | | | | |
| RNU1-3 | | | | | | | | | |
| RNU1-4 | | | | | | | | | |
| RNU11 | | | | | | | | | |
| RNU12 | | | | | | | | | |
| RNU2-1 | | | | | | | | | |
| RNU2-2P | | | | | | | | | |
| RNU2-4P | | | | | | | | | |
| RNU3P1 | | | | | | | | | |
| RNU4-1 | | | | | | | | | |
| RNU4-2 | | | | | | | | | |
| RNU4ATAC | | | | | | | | | |
| RNU5A-1 | | | | | | | | | |
| RNU5B-1 | | | | | | | | | |
| RNU5E-6P | | | | | | | | | |
| RNU5F-1 | | | | | | | | | |
| RNU6-1 | | | | | | | | | |
| RNU6-50P | | | | | | | | | |
| RNU6-6P | | | | | | | | | |
| RNU6-8 | | | | | | | | | |
| RNU6ATAC | | | | | | | | | |
| RNU7-1 | | | | | | | | | |
| RNVU1-10 | | | | | | | | | |
| RNVU1-14 | | | | | | | | | |
| RNVU1-17 | | | | | | | | | |
| RNVU1-18 | | | | | | | | | |
| RNVU1-19 | | | | | | | | | |
| RNVU1-6 | | | | | | | | | |
| RNVU1-7 | | | | | | | | | |
| RNY1 | | | | | | | | | |
| RNY3 | | | | | | | | | |
| RNY4 | | | | | | | | | |
| RNY5P1 | | | | | | | | | |
| ROBO1 | | | | | | | | | |

| | | | | | | | | | |
|---|---|---|---|---|---|---|---|---|---|
| ROBO2 | | + | | | | + | | | + |
| ROBO3 | | | | | | | | | |
| ROBO4 | | | | | | | | | |
| ROCK1 | | | | | | | | | |
| ROCK1P1 | | | | | | | | | |
| ROCK2 | | | | | + | | | | + |
| ROGDI | | | | | | | | | |
| ROM1 | | | | | | | | | |
| ROM2 | | | | | | | | | |
| ROMO1 | | | | | | | | | |
| ROP2 | | | | | | | | | |
| ROPN1 | | | | | | | | | |
| ROPN1B | | | | | | | | | |
| ROPN1L | | | | | | | | | |
| roq | | | | | | | | | |
| ROR1 | | | | | | | | | |
| ROR2 | | | | | | | | | |
| RORA | | | | | | | | | |
| RORB | + | | + | + | + | | | | + |
| RORC | | | | | | | | | |
| ROS1 | | | | | | | | | |
| RP1 | | | | | | | | | |
| RP1L1 | | | | | | | | | |
| RP2 | | | | | | | | | |
| RP9 | | | | | | | | | |
| RP9P | | | | | | | | | |
| RPA1 | | | | | + | | | | + |
| RPA12 | | | | | | | | | |
| RPA2 | | | | | | | | | |
| RPA3 | | | | | | | | | |
| RPA4 | | | | | | | | | |
| RPA43 | | | | | | | | | |
| RPA49 | | | | | | | | | |
| RPAIN | | | | | | | | | |
| RPAP1 | | | | | | | | | |
| RPAP2 | | | | | | | | | |
| RPAP3 | | | | | | | | | |
| RPB2 | | | | | | | | | |
| RPB3 | | | | | | | | | |
| RPB4 | | | | | | | | | |
| rpb5 | | | | | | | | | |
| rpb8 | | | | | | | | | |
| RPC10 | | | | | | | | | |
| RPC11 | | | | | | | | | |
| RPC37 | | | | | | | | | |
| RPC40 | | | | | | | | | |
| RPD3 | | | | | | | | | |
| RPE | | | | | | | | | |
| RPE65 | | | | | | | | | |
| RPEL1 | | | | | | | | | |
| RPF1 | | | | | | | | | |
| RPF2 | | | | | | | | | |
| RPG1 | | | | | | | | | |

| | | | | | | | | | |
|---|---|---|---|---|---|---|---|---|---|
| RPGR | | | | | | | | | |
| RPGRIP1 | + | | | + | + | | | | + |
| RPGRIP1L | | | | | | | | | |
| RPH3A | | | | | | + | | | + |
| RPH3AL | | | | | | | | | |
| RPIA | | | | | | | | | |
| RpII215 | | | | | | | | | |
| RPL10 | | | | | | | | | |
| rpl1001 | | | | | | | | | |
| rpl101 | | | | | | | | | |
| RPL10A | | | | | | | | | |
| RPL10AP3 | | | | | | | | | |
| RPL10L | | | | | | | | | |
| RPL10P16 | | | | | | | | | |
| RPL11 | | | | | | | | | |
| rpl1101 | | | | | | | | | |
| RPL11A | | | | | | | | | |
| RPL12 | | + | | | | + | | | + |
| rpl1201 | | | | | | | | | |
| RPL12A | | | | | | | | | |
| RPL12B | | | | | | | | | |
| RPL12P1 | | | | | | | | | |
| RPL12P14 | | | | | | | | | |
| RPL12P19 | | | | | | | | | |
| RPL12P2 | | | | | | | | | |
| RPL12P32 | | | | | | | | | |
| RPL12P35 | | | | | | | | | |
| RPL12P6 | | | | | | | | | |
| RPL13 | | | | | | | | | |
| RPL13A | | | | | | | | | |
| RPL13AP17 | | | | | | | | | |
| RPL13AP2 | | | | | | | | | |
| RPL13AP3 | | | | | | | | | |
| RPL13P12 | | | | | | | | | |
| RPL14 | | | | | | | | | |
| RPL15 | | | | | | | | | |
| rpl1502 | | | | | | | | | |
| rpl1602 | | | | | | | | | |
| RPL16A | | | | | | | | | |
| RPL16B | | | | | | | | | |
| RPL17 | | | | | | | | | |
| RPL17-C18orf32 | | | | | | | | | |
| Rpl17-ps1 | | | | | | | | | |
| rpl1701 | | | | | | | | | |
| rpl1702 | | | | | | | | | |
| RPL17A | | | | | | | | | |
| RPL17B | | | | | | | | | |
| RPL18 | | | | | | | | | |
| rpl1802 | | | | | | | | | |
| RPL18A | | | | | | | | | |
| RPL18AP3 | | | | | | | | | |
| RPL18B | | | | | | | | | |
| RPL18P11 | | | | | | | | | |

| | | | | | | | | | |
|---|---|---|---|---|---|---|---|---|---|
| RPL18P4 | | | | | | | | | |
| RPL19 | | | | | | | | | |
| rpl1901 | | | | | | | | | |
| rpl1902 | | | | | | | | | |
| RPL1A | | | | | | | | | |
| RPL1B | | | | | | | | | |
| rpl2002 | | | | | | | | | |
| RPL20A | | | | | | | | | |
| RPL20B | | | | | | | | | |
| RPL21 | | | | | | | | | |
| rpl2101 | | | | | | | | | |
| rpl2102 | | | | | | | | | |
| RPL21B | | | | | | | | | |
| RPL21P16 | | | | | | | | | |
| RPL21P19 | | | | | | | | | |
| RPL21P97 | | | | | | | | | |
| RPL22 | | | | | | | | | |
| RPL22L1 | | | | | + | | | | + |
| RPL23 | | | | | | | | | |
| rpl2301 | | | | | | | | | |
| RPL23A | | + | | | | + | | | + |
| RPL23AP32 | | | | | | | | | |
| RPL23AP42 | | | | | | | | | |
| RPL23AP5 | | | | | | | | | |
| RPL23AP53 | | | | | | | | | |
| RPL23AP64 | | | | | | | | | |
| RPL24 | | | | | | | | | |
| rpl2401 | | | | | | | | | |
| RPL24P4 | | | | | | | | | |
| rpl2501 | | | | | | | | | |
| RPL26 | | | | | | | | | |
| RPL26L1 | | | | | | | | | |
| RPL26P30 | | | | | | | | | |
| RPL26P32 | | | | | | | | | |
| RPL27 | | | | | | | | | |
| rpl2701 | | | | | | | | | |
| rpl2702 | | | | | | | | | |
| RPL27A | | + | | | | + | | | + |
| RPL27AP6 | | | | | | | | | |
| RPL28 | | | | | | | | | |
| rpl2801 | | | | | | | | | |
| rpl2802 | | | | | | | | | |
| RPL29 | | | | | | | | | |
| RPL29P11 | | | | | | | | | |
| RPL29P30 | | | | | | | | | |
| RPL3 | | | | | | | | | |
| RPL30 | | | | | | | | | |
| rpl3001 | | | | | | | | | |
| rpl3002 | | | | | | | | | |
| rpl301 | | | | | | | | | |
| RPL30P8 | | | | | | | | | |
| RPL31 | | | | | | | | | |
| Rpl31-ps9 | | | | | | | | | |

| | | | | | | | | | |
|---|---|---|---|---|---|---|---|---|---|
| RPL32 | | | | | | | | | |
| rpl3201 | | | | | | | | | |
| rpl3202 | | | | | | | | | |
| RPL32P18 | | | | | | | | | |
| RPL32P3 | | | | | | | | | |
| RPL34 | | | | | | | | | |
| Rpl35 | | | | | | | | | |
| RPL35A | | | | | | | | | |
| RPL35B | | | | | | | | | |
| RPL35P5 | | | | | | | | | |
| RPL36 | | | | | | | | | |
| rpl3602 | | | | | | | | | |
| RPL36A | | | | | | | | | |
| RPL36A-HNRNPH2 | | | | | | | | | |
| RPL36AL | | | | | | | | | |
| RPL36AP37 | | | | | | | | | |
| RPL36P14 | | | | | | | | | |
| RPL37 | | | | | | | | | |
| rpl3702 | | | | | | | | | |
| RPL37A | | | | | | | | | |
| RPL37AP8 | | | | | | | | | |
| RPL38 | | | | | | | | | |
| rpl3801 | | | | | | | | | |
| rpl3802 | | | | | | | | | |
| RPL39 | | + | | | | + | | | + |
| RPL39L | | | | | | | | | |
| RPL39P5 | | | | | | | | | |
| RPL3L | | | | | | | | | |
| RPL4 | | | | | | | | | |
| rpl401 | | | | | | | | | |
| rpl402 | | | | | | | | | |
| RPL40A | | | | | | | | | |
| RPL40B | | | | | | | | | |
| RPL41 | + | | | | | | | | + |
| RPL41A | | | | | | | | | |
| rpl4301 | | | | | | | | | |
| rpl44 | | | | | | | | | |
| RPL4A | | | | | | | | | |
| RPL4P5 | | | | | | | | | |
| RPL5 | | | | | | | | | |
| rpl501 | | | | | | | | | |
| RPL6 | | | | | | | | | |
| RPL6A | | | | | | | | | |
| RPL6B | | | | | | | | | |
| RPL6P1 | | | | | | | | | |
| RPL6P27 | | | | | | | | | |
| RPL7 | | | | | | | | | |
| rpl701 | | | | | | | | | |
| rpl702 | | | | | | | | | |
| RPL7A | | + | | | | + | | | + |
| RPL7AP27 | | | | | | | | | |
| RPL7AP66 | | | | | | | | | |
| RPL7AP9 | | | | | | | | | |

| | | | | | | | | | |
|---|---|---|---|---|---|---|---|---|---|
| RPL7B | | | | | | | | | |
| RPL7L1 | | | | + | | | | | + |
| RPL7P23 | | | | | | | | | |
| RPL7P32 | | | | | | | | | |
| RPL8 | | + | | | | + | | | + |
| rpl801 | | | | | | | | | |
| RPL8A | | | | | | | | | |
| RPL8B | | | | | | | | | |
| RPL9 | | | | | | | | | |
| rpl901 | | | | | | | | | |
| rpl902 | | | | | | | | | |
| RPL9A | | | | | | | | | |
| RPLP0 | | | | | | | | | |
| RPLP0P2 | | | | | | | | | |
| RPLP0P3 | | | | | | | | | |
| RPLP0P6 | | | | | | | | | |
| RPLP1 | | | | | | | | | |
| RPLP2 | | | | | | | | | |
| RPLP2P3 | | | | | | | | | |
| RPN1 | | | + | | + | | | | + |
| rpn10 | | | | | | | | | |
| RPN11 | | | | | | | | | |
| RPN13 | | | | | | | | | |
| RPN14 | | | | | | | | | |
| RPN1A | | | | | | | | | |
| RPN2 | | | | | | | | | |
| rpn3 | | | | | | | | | |
| RPN5 | | | | | | | | | |
| RPN6 | | | | | | | | | |
| RPN7 | | | | | | | | | |
| RPN8 | | | | | | | | | |
| RPN9 | | | | | | | | | |
| RPO21 | | | | | | | | | |
| RPP0 | | | | | | | | | |
| RPP14 | | | | | | | | | |
| rpp201 | | | | | | | | | |
| rpp202 | | | | | | | | | |
| rpp203 | | | | | | | | | |
| RPP21 | | | | | | | | | |
| RPP25 | | | | | | | | | |
| RPP25L | | | | | | | | | |
| RPP30 | | | | | | | | | |
| RPP38 | | | | | | | | | |
| RPP40 | + | | | + | + | | | | + |
| RPRD1A | | | | | | | | | |
| RPRD1B | | | | | | | | | |
| RPRD2 | | | | | | | | | |
| RPRM | | | | + | | | | | + |
| RPRML | + | | | | + | | | | + |
| rps001 | | | | | | | | | |
| rps002 | | | | | | | | | |
| RPS10 | | | | | | | | | |
| RPS10-NUDT3 | | | | | | | | | |

| | | | | | | | | | |
|---|---|---|---|---|---|---|---|---|---|
| rps1001 | | | | | | | | | |
| rps1002 | | | | | | | | | |
| rps101 | | | | | | | | | |
| rps102 | | | | | | | | | |
| RPS10P11 | | | | | | | | | |
| RPS10P13 | | | | | | | | | |
| RPS10P22 | | | | | | | | | |
| RPS10P4 | | | | | | | | | |
| RPS10P5 | | | | | | | | | |
| RPS10P7 | | | | | | | | | |
| RPS11 | | | | | | | | | |
| rps1102 | | | | | | | | | |
| RPS12 | | | | | + | | | | + |
| Rps12l1 | | | | | | | | | |
| RPS13 | | | | | | | | | |
| RPS14 | | | | | | | | | |
| rps1401 | | | | | | | | | |
| RPS14A | | | | | | | | | |
| RPS14P3 | | | | | | | | | |
| RPS15 | | | | | | | | | |
| rps1501 | | | | | | | | | |
| rps1502 | | | | | | | | | |
| RPS15A | | | | | | | | | |
| RPS15AP21 | | | | | | | | | |
| RPS15AP25 | | | | | | | | | |
| RPS15P2 | | | | | | | | | |
| RPS15P5 | | | | | | | | | |
| RPS16 | | | | | | | | | |
| rps1602 | | | | | | | | | |
| RPS17 | | | | | | | | | |
| rps1701 | | | | | | | | | |
| rps1702 | | | | | | | | | |
| RPS17P16 | | | | | | | | | |
| RPS18 | | | | | | | | | |
| rps1801 | | | | | | | | | |
| RPS18P12 | | | | | | | | | |
| RPS18P5 | | | | | | | | | |
| RPS19 | | + | | | | + | | | + |
| rps1901 | | | | | | | | | |
| RPS19BP1 | | | | | | | | | |
| RPS19P3 | | | | | | | | | |
| RPS2 | | | | | | | | | |
| RPS20 | | | | | | | | | |
| RPS20P14 | | | | | | | | | |
| RPS21 | | | | | | | | | |
| rps2201 | | | | | | | | | |
| RPS22A | | | | | | | | | |
| RPS22B | | | | | | | | | |
| RPS23 | | + | | | | + | | | + |
| RPS24 | | | | | | | | | |
| rps2401 | | | | | | | | | |
| rps2402 | | | | | | | | | |
| RPS24A | | | | | | | | | |

| | | | | | | | | | |
|---|---|---|---|---|---|---|---|---|---|
| RPS24B | | | | | | | | | |
| RPS25 | | | | | | | | | |
| rps2502 | | | | | | | | | |
| RPS25P8 | | | | | | | | | |
| RPS26 | | | | | | | | | |
| rps2601 | | | | | | | | | |
| rps2602 | | | | | | | | | |
| RPS26A | | | | | | | | | |
| RPS26P11 | | | | | | | | | |
| RPS26P50 | | | | | | | | | |
| RPS26P54 | | | | | | | | | |
| RPS27 | | | | | | | | | |
| RPS27A | | | | | | | | | |
| RPS27L | | | | | | | | | |
| RPS27P17 | | | | | | | | | |
| RPS28 | | + | | | | + | | | + |
| RPS28A | | | | | | | | | |
| RPS29 | | + | | | | | | | + |
| RPS2P32 | | | | | | | | | |
| RPS2P40 | | | | | | | | | |
| RPS2P46 | | | | | | | | | |
| RPS2P5 | | | | | | | | | |
| RPS3 | | | | | | | | | |
| RPS31 | | | | | | | | | |
| RPS3A | | | | | | | | | |
| Rps3a1 | | | | | | | | | |
| Rps3a2 | | | | | | | | | |
| RPS3P6 | | | | | | | | | |
| RPS3P7 | | | | | | | | | |
| rps401 | | | | | | | | | |
| rps402 | | | | | | | | | |
| rps403 | | | | | | | | | |
| RPS4A | | | | | | | | | |
| RPS4B | | | | | | | | | |
| RPS4X | | | | | | | | | |
| RPS4XP17 | | | | | | | | | |
| RPS4XP21 | | | | | | | | | |
| RPS4Y1 | | | | | | | | | |
| RPS4Y2 | | | | | | | | | |
| RPS5 | | | | | | | | | |
| rps502 | | | | | | | | | |
| RPS6 | | + | | | | + | | | + |
| rps601 | | | | | | | | | |
| RPS6KA1 | | | | | | | | | |
| RPS6KA2 | | | | | | | | | |
| Rps6ka3 | | | + | | | | | | + |
| RPS6KA4 | | | | | | | | | |
| RPS6KA5 | | | | | | | | | |
| RPS6KA6 | | | | | | | | | |
| RPS6KB1 | | | | | | | | | |
| RPS6KB2 | | | | | + | | | | + |
| RPS6KC1 | | | | | | | | | |
| RPS6KL1 | | | | | | | | | |

| | | | | | | | | | |
|---|---|---|---|---|---|---|---|---|---|
| RPS7 | | | | | | | | | |
| RPS7A | | | | | | | | | |
| RPS7B | | | | | | | | | |
| RPS7P4 | | | | | | | | | |
| RPS8 | | + | | | | + | | | + |
| rps801 | | | | | | | | | |
| rps802 | | | | | | | | | |
| RPS8A | | | | | | | | | |
| RPS8B | | | | | | | | | |
| RPS9 | | | | | | | | | |
| rps901 | | | | | | | | | |
| rps902 | | | | | | | | | |
| RPS9A | | | | | | | | | |
| RPSA | | | | | | | | | |
| RPSAP11 | | | | | | | | | |
| RPSAP12 | | | | | | | | | |
| RPSAP14 | | | | | | | | | |
| RPSAP15 | | | | | | | | | |
| RPSAP18 | | | | | | | | | |
| RPSAP19 | | | | | | | | | |
| RPSAP26 | | | | | | | | | |
| RPSAP29 | | | | | | | | | |
| RPSAP52 | | | | | | | | | |
| RPSAP55 | | | | | | | | | |
| RPSAP58 | | | | | | | | | |
| RPSAP61 | | | | | | | | | |
| RPSAP8 | | | | | | | | | |
| RPSAP9 | | | | | | | | | |
| RPT1 | | | | | | | | | |
| RPT2 | | | | | | | | | |
| RPT3 | | | | | | | | | |
| RPT4 | | | | | | | | | |
| RPT5 | | | | | | | | | |
| RPT6 | | | | | | | | | |
| RPTN | | | | | | | | | |
| RPTOR | | | | | | | | | |
| RPUSD1 | | | | | | | | | |
| RPUSD2 | | | | | | | | | |
| RPUSD3 | | | | | | | | | |
| RPUSD4 | | | | | | | | | |
| RQCD1 | | | | | | | | | |
| RRAD | | | | | | | | | |
| RRAGA | | | | | | | | | |
| RRAGB | | | | | | + | | | + |
| RRAGC | | | + | | | | | | + |
| RRAGD | | | | | | | | | |
| RRAS | | | | | | | | | |
| RRAS2 | | | | | | | | | |
| RRBP1 | | | | | | | | | |
| RRD1 | | | | | | | | | |
| RRD2 | | | | | | | | | |
| RREB1 | + | | | | + | | | | + |
| RRF1 | | | | | | | | | |

| | | | | | | | | | |
|---|---|---|---|---|---|---|---|---|---|
| Rrh | | | | | | | | | |
| RRI1 | | | | | | | | | |
| RRM1 | | | + | | | | | | + |
| RRM2 | | | | | | | | | |
| RRM2B | + | | | + | + | | | | + |
| RRM3 | | | | | | | | | |
| RRN11 | | | | | | | | | |
| RRN3 | | | | | | | | | |
| RRN3P1 | | | | | | | | | |
| RRN3P2 | | | | | | | | | |
| RRNAD1 | | | | | | | | | |
| RRP1 | | | | | | | | | |
| RRP12 | | | | | + | | | | + |
| RRP15 | | | | | | | | | |
| RRP1B | + | | | | + | | | | + |
| RRP36 | | | | | | | | | |
| RRP4 | | | | | | | | | |
| RRP7A | | | | | | | | | |
| RRP7B | | | | | | | | | |
| RRP8 | | | | | | | | | |
| RRP9 | | | | | | | | | |
| RRS1 | | | | | | | | | |
| RS1 | | | | | | | | | |
| RSA1 | | | | | | | | | |
| RSAD1 | | | | | | | | | |
| Rsad2 | + | | | + | + | | | | + |
| RSBN1 | | | | | | | | | |
| RSBN1L | | | | + | | + | | | + |
| RSC1A1 | | | | | | | | | |
| RSC30 | | | | | | | | | |
| RSC8 | | | | | | | | | |
| rsd1 | | | | | | | | | |
| RSF1 | | | | | | | | | |
| RSG1 | | | | | | | | | |
| RSL1D1 | | | | | | | | | |
| RSL24D1 | | | | | | | | | |
| RSM26 | | | | | | | | | |
| RSM27 | | | | | | | | | |
| RSP5 | | | | | | | | | |
| RSPH1 | | | | | | | | | |
| RSPH10B | | | | | | | | | |
| RSPH10B2 | | | | | | | | | |
| RSPH14 | | | | | | | | | |
| RSPH3 | | | | | | | | | |
| RSPH4A | | | | | | | | | |
| RSPH6A | | | | | | | | | |
| RSPH9 | | | | | | + | | | + |
| RSPO1 | | | | | | | | | |
| RSPO2 | | | | | | | | | |
| RSPO3 | | | + | | | | | | + |
| RSPO4 | | | | | | | | | |
| RSPRY1 | | | | | | | | | |
| RSR1 | | | | | | | | | |

| | | | | | | | | | | |
|---|---|---|---|---|---|---|---|---|---|---|
| RSR4 | | | | | | | | | | |
| RSRC1 | | | | | | + | | | + |
| RSRC2 | | | | | | | | | | |
| RSRP1 | | | | | | | | | | |
| RSU1 | | | | | | | | | | |
| RTBDN | | | | | | | | | | |
| RTCA | | | | | | | | | | |
| RTCB | | | | | | | | | | |
| RTEL1 | | | | | | | | | | |
| RTEL1-TNFRSF6B | | | | | | | | | | |
| RTF1 | | | | | + | | | | + |
| RTFDC1 | | | | | | | | | | |
| RTK1 | | | | | | | | | | |
| RTKN | | | | | | | | | | |
| RTKN2 | | | | | | | | | | |
| RTL1 | | | | | + | | | | + |
| RTN1 | | | | | | + | | + | + |
| RTN2 | | | | | | | | | | |
| RTN3 | | | | | | | + | | + |
| RTN4 | | + | | | | + | | | + |
| RTN4IP1 | | | | | | | | | | |
| RTN4R | | | | | | | | | | |
| RTN4RL1 | + | | | | + | | | | + |
| RTN4RL2 | + | | | | + | | | | + |
| RTP1 | | | | | | | | | | |
| RTP2 | | | | | | | | | | |
| RTP3 | | | | | | | | | | |
| RTP4 | | | | | | | | | | |
| RTP5 | | | | | | | | | | |
| RTT109 | | | | | | | | | | |
| RTTN | | | | | | | | | | |
| RUB1 | | | | | | | | | | |
| RUFY1 | | | | | | + | | | + |
| RUFY2 | | | | | | | | | | |
| RUFY3 | | + | | | | + | | | + |
| RUFY4 | | | | | | | | | | |
| RUNDC1 | | | | | | | | | | |
| RUNDC3A | | | | | | | | | | |
| RUNDC3B | | | | | | | | | | |
| RUNX1 | | | | | | | | | | |
| RUNX1T1 | | | | + | | | | | + |
| RUNX2 | + | | | + | + | | | | + |
| runx2a | | | | | | | | | | |
| runx2b | | | | | | | | | | |
| RUNX3 | + | | | + | + | | | | + |
| RUP1 | | | | | | | | | | |
| RUSC1 | | | + | | + | | | | + |
| RUSC1-AS1 | | | | | | | | | | |
| RUSC2 | | | | | + | | | | + |
| RUVBL1 | | | | | | | | | | |
| RUVBL2 | | | | | | | | | | |
| RVB1 | | | | | | | | | | |
| RVB2 | | | | | | | | | | |

| | | | | | | | | | |
|---|---|---|---|---|---|---|---|---|---|
| RVS161 | | | | | | | | | |
| RVS167 | | | | | | | | | |
| RWDD1 | | + | | | | + | | | + |
| RWDD2A | | | | | | | | | |
| RWDD2B | | | | | | | | | |
| RWDD3 | | | | | | | | | |
| RWDD4 | | | | | | | | | |
| RXFP1 | | | | | | | | | |
| RXFP2 | | | | | | | | | |
| RXFP3 | | | | | | | | | |
| RXFP4 | | | | | | | | | |
| RXRA | | | | | | | | | |
| RXRB | | | | | | | | | |
| RXRG | | | | | | | | | |
| RYBP | | | | | | | | | |
| ryh1 | | | | | | | | | |
| RYK | | | | | | | | | |
| RYR1 | + | | | | + | + | | | + |
| RYR2 | | | | | | | | | |
| RYR3 | | | | | | | | | |
| S | | | | | | | | | |
| S100A1 | | | | | | | | | |
| S100A10 | | | | | + | | | + | + |
| S100A11 | | | | | | | | | |
| S100A12 | | | | | | | | | |
| S100A13 | | | | | | | | | |
| S100A14 | | | | | | | | | |
| S100A16 | | | | | | | | | |
| S100A2 | | | | | | | | | |
| S100A3 | | | | | | | | | |
| S100A4 | | | | | | | | | |
| S100A5 | | | | | | | | | |
| S100A6 | | | | | | | | | |
| S100A7 | | | | | | | | | |
| S100A7A | | | | | | | | | |
| S100A7L2 | | | | | | | | | |
| S100A8 | | | | | | | | | |
| S100A9 | | | | | | | | | |
| S100B | | | | | | | | | |
| S100G | | | | | | | | | |
| S100P | | | | | | | | | |
| S100PBP | | | | | | | | | |
| S100Z | | | | | | | | | |
| S1PR1 | | | | | | | | | |
| S1PR2 | | | | | | | | | |
| S1PR3 | | | | | | | | | |
| S1PR4 | | | | | | | | | |
| S1PR5 | | | | | | | | | |
| SAA1 | | | | | | | | | |
| SAA2 | | | | | | | | | |
| SAA4 | + | | | + | + | | | | + |
| SAAL1 | | | | | | + | | | + |
| SAC1 | | | | | | | | | |

| | | | | | | | | | |
|---|---|---|---|---|---|---|---|---|---|
| SAC3D1 | | | | | | + | | | + |
| SAC6 | | | | | | | | | |
| SAC7 | | | | | | | | | |
| SACM1L | | | | | | | | | |
| SACS | | | | | | | | | |
| SAE1 | | | | | | | | | |
| SAE2 | | | | | | | | | |
| SAFB | | | | | | | | | |
| SAFB2 | | | | | | | | | |
| SAG | | | | + | | | | | + |
| SAGE1 | | | | | | | | | |
| SAH1 | | | | | | | | | |
| SALL1 | | | | | | | | | |
| SALL2 | | | | | | | | | |
| SALL3 | | | | | | | | | |
| SALL4 | | | | | | | | | |
| SAM-2 | | | | | | | | | |
| SAM1 | | | | | | | | | |
| SAM2 | | | | | | | | | |
| SAMD1 | | | | | | | | | |
| SAMD10 | | | | | | + | | | + |
| SAMD11 | | | | | | | | | |
| SAMD12 | | | | | | | | | |
| SAMD13 | | | | | | | | | |
| SAMD15 | | | | | | | | | |
| SAMD3 | | | | | | | | | |
| Samd4 | + | | + | | + | | | | + |
| SAMD4A | | | | | | | | | |
| SAMD4B | | | | | | | | | |
| SAMD5 | | | | | | | | | |
| SAMD7 | | | | | | | | | |
| Samd8 | | | | | | | | | |
| SAMD9 | | | | | | | | | |
| SAMD9L | | | | | | | | | |
| SAMHD1 | | | | + | | | | | + |
| SAMM50 | | + | | | | + | | | + |
| SAMSN1 | | | | | | | | | |
| sap1 | | | | | | | | | |
| SAP130 | | | | | | | | | |
| SAP18 | | | | | | | | | |
| SAP25 | | | | | | | | | |
| SAP30 | | | | | | | | | |
| SAP30BP | | | | | | | | | |
| SAP30L | | | | | | | | | |
| SAP30L-AS1 | | | | | | | | | |
| SAP4 | | | | | | | | | |
| SAPCD1 | | | | | | | | | |
| SAPCD2 | | | | | | | | | |
| SAR1 | | | | | | | | | |
| SAR1A | | | + | | | | + | | + |
| SAR1B | | | | | | | | | |
| SARAF | | | | | | | | | |
| SARDH | | | | | | | | | |

| | | | | | | | | | |
|---|---|---|---|---|---|---|---|---|---|
| SARM1 | | | | | | | | | |
| SARNP | | | | | | | | + | + |
| SARS | | + | | | | + | | | + |
| SARS2 | | | | | | | | | |
| SART1 | | | | | | | | | |
| SART3 | | | | | | | | | |
| SAS3 | | | | | | | | | |
| SASH1 | | | | | | | | | |
| SASH3 | | | | | | | | | |
| SASS6 | | | | | | | | | |
| SAT1 | + | | + | | | | | | + |
| SAT2 | | | | | | | | | |
| SATB1 | | | | | | + | | | + |
| SATB1-AS1 | | | | | | | | | |
| SATB2 | | | | | | | | | |
| SATL1 | + | | | + | + | | | | + |
| SAV1 | | | | | | | | | |
| sax-3 | | | | | | | | | |
| SAY1 | | | | | | | | | |
| SAYSD1 | | | | | | | | | |
| SBA1 | | | | | | | | | |
| SBDS | | | + | | | | | | + |
| SBDSP1 | | | | | | | | | |
| SBF1 | | + | | | | + | | | + |
| SBF2 | | | | | | + | | | + |
| SBH1 | | | | | | | | | |
| SBK1 | | | | | | | | | |
| SBK2 | | | | | | | | | |
| SBK3 | | | | | | | | | |
| SBNO1 | | | | | | | | | |
| SBNO2 | | | | | | | | | |
| sbp-1 | | | | | | | | | |
| SBP1 | | | | | | | | | |
| sbr | | | | | | | | | |
| SBSN | | | | | | | | | |
| SBSPON | | + | + | | | + | | | + |
| SC5D | | | | | | | | | |
| SCAF1 | | | | | | | | | |
| SCAF11 | | | | | | | | | |
| SCAF4 | | | | | | | | | |
| SCAF8 | | | | | | | | | |
| Scai | | | | | | | | | |
| SCAMP1 | | | + | | | | | | + |
| SCAMP2 | | | | | | | | | |
| SCAMP3 | | | | | | | | | |
| SCAMP4 | | | | | | | | | |
| SCAMP5 | | | | | | | | | |
| SCAND1 | | | | | | | | | |
| SCAND2P | | | | | | | | | |
| Scap | | | | | | | | | |
| SCAPER | | | | | | | | | |
| SCARA3 | | | | | | | | | |
| SCARA5 | | | | | | | | | |

| | | | | | | | | | |
|---|---|---|---|---|---|---|---|---|---|
| SCARB1 | | | | | | | | | |
| SCARB2 | | | + | | | | | | + |
| SCARF1 | | | | | | | | | |
| SCARF2 | | | | | | | | | |
| SCARNA1 | | | | | | | | | |
| SCARNA10 | | | | | | | | | |
| SCARNA11 | | | | | | | | | |
| SCARNA12 | | | | | | | | | |
| SCARNA13 | | | | | | | | | |
| SCARNA16 | | | | | | | | | |
| SCARNA17 | | | | | | | | | |
| SCARNA2 | | | | | | | | | |
| SCARNA20 | | | | | | | | | |
| SCARNA22 | | | | | | | | | |
| SCARNA23 | | | | | | | | | |
| SCARNA27 | | | | | | | | | |
| SCARNA3 | | | | | | | | | |
| SCARNA4 | | | | | | | | | |
| SCARNA5 | | | | | | | | | |
| SCARNA6 | | | | | | | | | |
| SCARNA7 | | | | | | | | | |
| SCARNA8 | | | | | | | | | |
| SCARNA9 | | | | | | | | | |
| SCCPDH | | | + | | | | | | + |
| SCD | | | | | | | | | |
| SCD5 | | | | | | | | | |
| SCD6 | | | | | | | | | |
| SCDP1 | | | | | | | | | |
| SCE1 | | | | | | | | | |
| SCEL | | | | | | | | | |
| SCFD1 | | | | | | | | | |
| SCFD2 | + | | | + | + | | | | + |
| SCG2 | + | | + | | + | | | | + |
| SCG3 | | | | | | + | | | + |
| SCG5 | | | | | | | | | |
| SCGB1A1 | | | | | | | | | |
| SCGB1B2P | | | | | | | | | |
| SCGB1C1 | | | | | | | | | |
| SCGB1C2 | | | | | | | | | |
| SCGB1D1 | | | | | | | | | |
| SCGB1D2 | | | | | | | | | |
| SCGB1D4 | | | | | | | | | |
| SCGB2A1 | | | | | | | | | |
| SCGB2A2 | | | | | | | | | |
| SCGB2B2 | | | | | | | | | |
| SCGB3A1 | | | | | | | | | |
| SCGB3A2 | | | | | | | | | |
| SCGN | | | | | | | | | |
| SCHIP1 | + | | | | + | | | | + |
| SCIMP | | | | | | | | | |
| SCIN | | | | | | | | | |
| SCJ1 | | | | | | | | | |
| SCL1 | | | | | | | | | |

| | | | | | | | | | |
|---|---|---|---|---|---|---|---|---|---|
| SCLT1 | | | + | + | | | | | + |
| SCLY | | | | | | | | | |
| SCMH1 | | | | | | | | | |
| SCML1 | | | | | | | | | |
| SCML2 | | | | | | | | | |
| SCML4 | | | | | | | | | |
| Scn10a | | | | | | | | | |
| SCN11A | | | | | | | | | |
| SCN1A | | + | | + | | | | | + |
| SCN1B | | | | | | | | | |
| Scn2a | | | | | | | | | |
| SCN2B | | | | | | + | | | + |
| SCN3A | | + | | | | + | | | + |
| SCN3B | | + | | | | + | | | + |
| SCN4A | | | | | | | | | |
| SCN4B | | + | | | | + | | | + |
| SCN5A | | | | | | | | | |
| SCN7A | | | | | | | | | |
| SCN8A | | | | | | | | | |
| SCN9A | | | | | | | | | |
| SCNM1 | | | | | | | | | |
| SCNN1A | | | | | | + | | | + |
| SCNN1B | | | | | | | | | |
| SCNN1D | | | | | | | | | |
| SCNN1G | | | | | | | | | |
| SCO1 | | | | | | | | | |
| SCO2 | | | | | | | | | |
| SCOC | | | | | | | | | |
| SCOC-AS1 | | | | | | | | | |
| SCP160 | | | | | | | | | |
| SCP2 | | | | | | | | | |
| SCP2D1 | | | | | | | | | |
| SCPEP1 | | | + | | + | | | | + |
| SCRG1 | | | | | | | | | |
| SCRIB | | | | | | | | | |
| SCRN1 | | | | | | | | | |
| SCRN2 | | | | | | | | | |
| SCRN3 | | | | | | + | | | + |
| SCRT1 | | | | | | | | | |
| SCRT2 | | | | | | | | | |
| SCS2 | | | | | | | | | |
| SCS22 | | | | | | | | | |
| SCS3 | | | | | | | | | |
| SCT | | | | | | | | | |
| SCTR | | | | | | | | | |
| Scube1 | | | | | | | | | |
| SCUBE2 | | + | | + | | + | | | + |
| SCUBE3 | | | | | | + | | | + |
| SCX | | | | | | | | | |
| SCYL1 | | | | | | | | | |
| SCYL2 | | | | | | + | | | + |
| SCYL3 | | | | | | | | | |
| SDAD1 | | | | | | | | | |

| | | | | | | | | | |
|---|---|---|---|---|---|---|---|---|---|
| SDC1 | | | | | | | | | |
| SDC2 | | | + | | | | | | + |
| SDC3 | | | | | + | + | | | + |
| SDC4 | | | | | + | | | | + |
| SDCBP | | | | | + | + | | | + |
| SDCBP2 | | | | | | | | | |
| SDCBP2-AS1 | | | | | | | | | |
| SDCCAG3 | | | + | | | | | | + |
| Sdccag8 | | | | | | | | | |
| SDE2 | | | | | + | | | | + |
| SDF2 | | | | | | | | | |
| SDF2L1 | | | | | | | | | |
| SDF4 | | | | | | | | | |
| SDG26 | | | | | | | | | |
| sdh1 | | | | | | | | | |
| SDHA | | | | | | | + | | + |
| SDHAF1 | | | | | | | | | |
| SDHAF2 | | | | | | | | | |
| SDHAP1 | | | | | | | | | |
| SDHAP2 | | | | | | | | | |
| SDHB | | | | | | | | | |
| SDHC | | | | | | | | | |
| SDHD | | | | | | | | | |
| SDIM1 | | | | | | | | | |
| SDK1 | | | | | | | | | |
| SDK2 | + | | | | + | | | | + |
| sdo1 | | | | | | | | | |
| SDPR | | | | | | | | | |
| SDR16C5 | | | | | | | | | |
| SDR39U1 | | | | | | | | | |
| SDR42E1 | | | | | | | | | |
| SDR42E2 | | | | | | | | | |
| SDR9C7 | | | | | | | | | |
| SDS | | | | | | | | | |
| SDS22 | | | | | | | | | |
| sds23 | | | | | | | | | |
| SDSL | | | | | | | | | |
| SDT1 | | | | | | | | | |
| SEC11A | | | | | | | | | |
| SEC11B | | | | | | | | | |
| SEC11C | | | | | | | | | |
| SEC13 | | | | | | | | | |
| SEC14 | | | | | | | | | |
| SEC14L1 | | | | | | + | | | + |
| SEC14L2 | | | | | | + | | | + |
| SEC14L3 | | | | | | | | | |
| SEC14L4 | | | | | | | | | |
| SEC14L5 | | | | | | | | | |
| SEC16A | | | | | | | | | |
| SEC16B | | | | | | | | | |
| SEC18 | | | | | | | | | |
| SEC21 | | | | | | | | | |
| SEC22A | | + | | | | + | | | + |

| | | | | | | | | | |
|---|---|---|---|---|---|---|---|---|---|
| SEC22B | | | | | | | | | |
| SEC22C | + | | + | + | + | | | | + |
| SEC23 | | | | | | | | | |
| SEC23A | | + | | | | + | | | + |
| SEC23B | | | | | | | | | |
| SEC23IP | | | | | | | | | |
| SEC24 | | | | | | | | | |
| SEC24A | | | | | | | | | |
| SEC24B | | | | | | | | | |
| SEC24C | | | | | | | | | |
| SEC24D | | | | | | | | | |
| SEC26 | | | | | | | | | |
| SEC27 | | | | | | | | | |
| SEC28 | | | | | | | | | |
| SEC31 | | | | | | | | | |
| SEC31A | | | | | | | | | |
| SEC31B | | | | | | | | | |
| SEC4 | | | | | | | | | |
| SEC53 | | | | | | | | | |
| SEC6 | | | | | | | | | |
| SEC61 | | | | | | | | | |
| SEC61A1 | | | | | + | | | | + |
| SEC61A2 | + | | | | + | | | | + |
| SEC61B | | | | | | | | | |
| Sec61beta | | | | | | | | | |
| SEC61G | | | | + | | + | | | + |
| SEC62 | | | | | | | | | |
| SEC63 | | | + | | | | | | + |
| SEC9 | | | | | | | | | |
| SECISBP2 | | | | | | | | | |
| SECISBP2L | | | | | | | | | |
| SECTM1 | | | | | | | | | |
| SED1 | | | | | | | | | |
| SED5 | | | | | | | | | |
| SEH1L | | | | | | | | | |
| SEL1L | | | | | | | | | |
| Sel1l2 | | | | | | | | | |
| SEL1L3 | | | | | | | | | |
| SELE | | | | | | | | | |
| SELENBP1 | | | | | | | | | |
| SELK | | | | | | | | | |
| SELL | | | | | | | | | |
| SELM | | | | | | + | | | + |
| SELO | | | | | | + | | | + |
| SELP | | | | | | | | | |
| SELPLG | | | | | + | | | | + |
| SELT | | | | | | | | | |
| SELV | | | | | | | | | |
| SEMA3A | + | | | + | + | | + | | + |
| SEMA3B | | | | | | | | | |
| SEMA3C | | | | | | | | | |
| SEMA3D | | | | | | | | | |
| SEMA3E | | | | | + | | | | + |

| | | | | | | | | | |
|---|---|---|---|---|---|---|---|---|---|
| SEMA3F | | | | | | | | | |
| sema3fa | | | | | | | | | |
| SEMA3G | | | | | | | | | |
| SEMA4A | | | | | + | | | | + |
| SEMA4B | | | | | | | | | |
| SEMA4C | | | | | | | | | |
| SEMA4D | | | | | | | | | |
| SEMA4F | | | | | | + | | | + |
| SEMA4G | | | | | | | | | |
| SEMA5B | | | | | | + | | | + |
| SEMA6A | | | | | | | | | |
| SEMA6B | | | | | | + | | | + |
| SEMA6C | | | | | | | | | |
| SEMA6D | | + | | | | + | | | + |
| SEMA7A | | | | | | | | | |
| SEMG1 | | | | | | | | | |
| SEMG2 | | | | | | | | | |
| SEN15 | | | | | | | | | |
| SENP1 | | | | | | | | | |
| SENP2 | | | | | | | | | |
| SENP3 | | | | | | | | | |
| SENP3-EIF4A1 | | | | | | | | | |
| SENP5 | | | | | | | | | |
| SENP6 | | + | | | | + | | | + |
| SENP7 | | | | | | | | | |
| SENP8 | | | | | | | | | |
| Sep1 | | | | | | | | | |
| SEP15 | | | | | | | | | |
| SEPHS1 | | | | | | | | | |
| SEPHS2 | | | | | | | | | |
| SEPN1 | | | | | | | | | |
| SEPP1 | | | | | | | | | |
| SEPSECS | | | | | | | | | |
| SEPT1 | | | | | | | | | |
| SEPT10 | | | | | | | | | |
| SEPT11 | | | | | | | | | |
| SEPT12 | | | | | | | | | |
| SEPT14 | | | | | | | | | |
| SEPT2 | | | | | | | | | |
| SEPT3 | | | | | | | | | |
| SEPT4 | | | | | | | | | |
| SEPT5 | | | | | | | | | |
| SEPT6 | | | | | | | | | |
| SEPT7 | | | | | | | | | |
| SEPT7P2 | | | | | | | | | |
| SEPT8 | | | | | | | | | |
| SEPT9 | | | | | | | | | |
| SEPW1 | | | | | | | | | |
| ser3 | | | | | | | | | |
| SER33 | | | | | | | | | |
| SERAC1 | | | | | | | | | |
| SERBP1 | | | | | | | | | |
| SERBP1P5 | | | | | | | | | |

| | | | | | | | | | |
|---|---|---|---|---|---|---|---|---|---|
| SERF1A | | | | | | | | | |
| SERF1B | | | | | | | | | |
| SERF2 | | | | | | | | | |
| SERF2-C15ORF63 | | | | | | | | | |
| SERGEF | | | | | | | | | |
| SERHL | | | | | | | | | |
| SERHL2 | | | | | | | | | |
| SERINC1 | | | | | | | + | | + |
| SERINC2 | | | | | + | | | | + |
| SERINC3 | | | + | | | | + | | + |
| SERINC5 | | | | | | | | | |
| SERP1 | | | + | | | | | | + |
| SERP2 | | | | | | | | | |
| SERPINA1 | | | | | | | | | |
| SERPINA10 | | | | | + | | | | + |
| SERPINA11 | | | | | | | | | |
| SERPINA12 | | | | | | | | | |
| SERPINA2 | | | | | | | | | |
| SERPINA3 | | | | | | | | | |
| Serpina3c | | | | | | | | | |
| Serpina3j | | | | | | | | | |
| Serpina3k | | | | | | | | | |
| SERPINA4 | | | | | | | | | |
| SERPINA5 | | | | | | | | | |
| SERPINA6 | | | | | | | | | |
| SERPINA7 | | | | | | | | | |
| SERPINA9 | | | | | | | | | |
| SERPINB1 | | | | | | | | | |
| SERPINB10 | | | | | | | | | |
| SERPINB11 | | | | | | | | | |
| SERPINB12 | | | | | | | | | |
| SERPINB13 | | | | | | | + | | + |
| Serpinb1a | | | | | | | | | |
| SERPINB2 | | | + | | | | | | + |
| SERPINB3 | | | | | | | | | |
| SERPINB4 | | | | | | | | | |
| SERPINB5 | | | | | | | | | |
| SERPINB6 | | | | | | | | | |
| SERPINB7 | | | | | | | | | |
| SERPINB8 | + | | | + | + | | | | + |
| SERPINB9 | | | | | | | | | |
| SERPINB9P1 | | | | | | | | | |
| SERPINC1 | | | | | | | | | |
| SERPIND1 | | | | | | | | | |
| SERPINE1 | | | | | | | | | |
| SERPINE2 | | | + | | | + | | | + |
| SERPINF1 | | | | | | | | | |
| SERPINF2 | | | | | | | | | |
| SERPING1 | | | | | | | | | |
| SERPINH1 | | | | | | | | | |
| SERPINI1 | | + | | | | | | | + |
| SERPINI2 | | | | | | | | | |
| SERTAD1 | | | | | | | | | |

| | | | | | | | | | |
|---|---|---|---|---|---|---|---|---|---|
| SERTAD2 | | | | | | | | | |
| SERTAD3 | | | | | | | | | |
| SERTAD4 | | | | | | | | | |
| SERTAD4-AS1 | | | | | | | | | |
| SERTM1 | | | | | | | | | |
| SES1 | | | | | | | | | |
| SESN1 | | + | | | | + | | | + |
| SESN2 | | | | | | | | | |
| SESN3 | | | | | | | | | |
| Sestd1 | | | | | | | | | |
| SET | + | | | + | + | | | | + |
| SET1 | | | | | | | | | |
| SET3 | | | | | | | | | |
| SETBP1 | | | | | | | | | |
| SETD1A | | | | | | | | | |
| SETD1B | | | | | | | | | |
| SETD2 | | | | | | | | | |
| SETD3 | | | | | | | | | |
| SETD4 | | | | | | | | | |
| SETD5 | | + | | | | + | | | + |
| SETD6 | | | | | | + | | | + |
| SETD7 | | | | | | | | | |
| SETD8 | | | | | | | | | |
| SETD9 | | | | | | | | | |
| SETDB1 | | | | | | + | | | + |
| SETDB2 | | | | | | | | | |
| SETMAR | | | | | | | | | |
| SETSIP | | | | | | | | | |
| SETX | | | | | | | | | |
| SEY1 | | | | | | | | | |
| SEZ6 | | | | | | + | | | + |
| SEZ6L | | | | | | | | | |
| SEZ6L2 | | | | | | | | | |
| SF1 | | | + | | | | | | + |
| SF3A1 | | | | | | | | | |
| SF3A2 | | | | | | | | | |
| SF3A3 | | | | | | | | | |
| SF3B1 | | | | | | | | | |
| SF3B2 | | + | | | | + | | | + |
| SF3B3 | | | | | | + | | | + |
| SF3B4 | | | | | | | | | |
| SF3B5 | | | | | | | | | |
| SF3B6 | | | | | | | | | |
| SFB2 | | | | | | | | | |
| SFI1 | | | | | | | | | |
| SFMBT1 | | | | | | | | | |
| SFMBT2 | | | | | + | | | | + |
| SFN | | | | | | | | | |
| SFPQ | | | | | | | | | |
| SFR1 | | | | | | | | | |
| SFRP1 | | | | | | | | | |
| SFRP2 | | | + | | | | | | + |
| SFRP4 | | | | | | | | | |

| | | | | | | | | | |
|---|---|---|---|---|---|---|---|---|---|
| SFRP5 | | | | | | | | | |
| SFSWAP | | | | | | | | | |
| SFT2 | | | | | | | | | |
| SFT2D1 | | | | | | | | | |
| SFT2D2 | | | | | | | | | |
| SFT2D3 | | | | | | | | | |
| SFTA2 | | | | | | | | | |
| SFTPA1 | | | | | | | | | |
| SFTPA2 | | | | | | | | | |
| SFTPB | + | | | + | + | | + | | + |
| SFTPC | | | | | | | | | |
| SFTPD | | | | | | | | | |
| SFXN1 | | | | | | + | | | + |
| SFXN2 | | | | | | | | | |
| SFXN3 | + | | | + | + | | | | + |
| SFXN4 | | | | | | | | | |
| SFXN5 | | | | | | | | | |
| SGA1 | | | | | | | | | |
| SGCA | | | | | | | | | |
| SGCB | | | | | | | | | |
| SGCD | | | | | | | | | |
| SGCE | | | | | | | | | |
| SGCG | | | | | | | | | |
| SGCZ | | | | | | | | | |
| SGD1 | | | | | | | | | |
| SGIP1 | | | + | | + | | | | + |
| SGK1 | + | | | | + | | | | + |
| SGK2 | | + | + | | | + | | | + |
| SGK223 | | | | | | | | | |
| SGK3 | | | | | + | | | | + |
| SGK494 | | | | | | | | | |
| SGMS1 | | | | | | | | | |
| SGMS1-AS1 | | | | | | | | | |
| SGMS2 | | | | | | | | | |
| SGOL1 | | | | | | | | | |
| SGOL2 | | | | | | | | | |
| SGPL1 | | | | | | | | | |
| SGPP1 | | | | | | | | | |
| SGPP2 | | | | | | | | | |
| SGS1 | | | | | | | | | |
| SGSH | | | | | | | | | |
| SGSM1 | | | + | | | | | | + |
| SGSM2 | | | | | | | | | |
| SGSM3 | | | | | | | | | |
| SGT1 | | | | | | | | | |
| SGT2 | | | | | | | | | |
| SGTA | | | | | | | | | |
| SGTB | | | + | | | | | | + |
| SGV1 | | | | | | | | | |
| SH2B1 | | | | | | | | | |
| SH2B2 | | | | | | | | | |
| SH2B3 | | | | | | | | | |
| SH2D1A | | | | | | | | | |

| | | | | | | | | | |
|---|---|---|---|---|---|---|---|---|---|
| SH2D1B | | | | | | | | | |
| Sh2d1b1 | | | | | | | | | |
| SH2D2A | + | | | + | + | | | | + |
| SH2D3A | | | | | | | | | |
| SH2D3C | | | | + | | | | | + |
| SH2D4A | | | | | | | | | |
| SH2D5 | | | | | | | | | |
| SH3BGR | | | | | | | | | |
| SH3BGRL | | | | | | | | | |
| SH3BGRL2 | | | | | | | | | |
| SH3BGRL3 | | | | | | | | | |
| SH3BP1 | | | | | | | | | |
| SH3BP2 | | | | | | | | | |
| SH3BP4 | | | | | | | | | |
| SH3BP5 | | | | | | + | | | + |
| SH3BP5-AS1 | | | | | | | | | |
| SH3BP5L | | | | | | + | | | + |
| SH3D19 | | | | | | | | | |
| Sh3d21 | | | | | | | | | |
| SH3GL1 | | | | | | | | | |
| SH3GL1P1 | | | | | | | | | |
| SH3GL1P2 | | | | | | | | | |
| SH3GL2 | | | + | | | | | | + |
| SH3GL3 | | | | | | + | | | + |
| SH3GLB1 | | | | | | | | | |
| SH3GLB2 | | | | | | + | | | + |
| SH3KBP1 | | | | | | | | | |
| SH3PXD2A | | | | | | | | | |
| SH3PXD2B | | | | | | | | | |
| SH3RF1 | | | | | | | | | |
| SH3RF2 | | | | | | | | | |
| SH3RF3 | | | + | | | + | | | + |
| SH3TC1 | | | | | | | | | |
| SH3YL1 | | | | | | | | | |
| SHANK1 | | | | | + | | | | + |
| SHANK2 | | | | | | | | | |
| SHANK3 | | | | | | | | | |
| SHARPIN | | | | | | | | | |
| SHB | | | | | | | | | |
| SHBG | | | | | | | | | |
| SHC1 | | | | | | | | | |
| SHC2 | | | | | | | | | |
| SHC3 | | | | | | | | | |
| SHC4 | + | | | + | + | | + | | + |
| SHCBP1 | | | | | | | | | |
| SHCBP1L | | | | | | | | | |
| SHD | | | | | | | | | |
| shd1 | | | | | | | | | |
| SHE | | | | | | | | | |
| SHF | | | | | | | | | |
| SHFM1 | | | | | | | | | |
| SHH | | | | | | | | | |
| shi | | | | | | | | | |

| | | | | | | | | | |
|---|---|---|---|---|---|---|---|---|---|
| SHISA2 | | | | | | | | | |
| SHISA3 | + | | | + | + | | | | + |
| SHISA4 | | + | | | + | + | | | + |
| SHISA5 | | | | | | | | | |
| SHISA6 | | | | | | + | | | + |
| SHISA7 | | | | | | + | | | + |
| SHISA8 | | | | | | | | | |
| SHISA9 | | | + | | | | | | + |
| SHKBP1 | | | | | | | | | |
| SHM1 | | | | | | | | | |
| SHM2 | | | | | | | | | |
| SHMT1 | | + | | + | | | | | + |
| SHMT2 | | | | | | | | | |
| SHOC2 | | | | | | | | | |
| SHOX | | | | | | | | | |
| SHOX2 | | | | | | | | | |
| SHP1 | | | | | | | | | |
| SHPK | | | | | | | | | |
| SHPRH | | | | | | + | | | + |
| SHQ1 | | | | | | | | | |
| SHR3 | | | | | | | | | |
| SHROOM1 | | | | | | | | | |
| SHROOM2 | | | | | | + | | | + |
| SHROOM3 | | | | | + | | | | + |
| SHROOM4 | | | | | | | | | |
| SHS1 | | | | | | | | | |
| SI | | | | | | | | | |
| SIAE | | | | | | | | | |
| SIAH1 | | | | | | | | | |
| Siah1a | | | | | | | | | |
| SIAH1P1 | | | | | | | | | |
| SIAH2 | | | + | | + | | | | + |
| SIAH3 | | | | | | | | | |
| SIC1 | | | | | | | | | |
| SIDT1 | | + | | | | + | | | + |
| Sidt2 | | | | | | | | | |
| SIGIRR | | | | | | | | | |
| SIGLEC1 | | | | | + | | | | + |
| SIGLEC10 | | | | | | | | | |
| SIGLEC11 | | | | | | | | | |
| SIGLEC12 | | | | | | | | | |
| SIGLEC15 | | | | | | | | | |
| SIGLEC16 | | | | | | | | | |
| SIGLEC5 | | | | | | | | | |
| SIGLEC6 | | | | | | | | | |
| SIGLEC7 | | | | | | | | | |
| SIGLEC8 | | | | | | | | | |
| SIGLEC9 | | | | | | | | | |
| Siglece | | | | | | | | | |
| SIGLECL1 | | | | | | | | | |
| SIGMAR1 | | | | | | | | | |
| SIK1 | + | | + | | + | | | | + |
| SIK2 | | | + | | + | | | | + |

| | | | | | | | | | |
|---|---|---|---|---|---|---|---|---|---|
| SIK3 | + | | | | + | | | | + |
| SIK3-IT1 | | | | | | | | | |
| SIKE1 | | | | | | | | | |
| SIL1 | | | | | | | | | |
| SIM1 | | | | | | | | | |
| SIM2 | | | | | | | | | |
| SIMC1 | | | | | | | | | |
| SIN3A | | | | | | | | | |
| SIN3B | | | | | | | | | |
| sina | | | | | | | | | |
| SIP1 | | | | | | | | | |
| SIP18 | | | | | | | | | |
| SIP2 | | | | | | | | | |
| SIPA1 | | | | | | | | | |
| SIPA1L1 | | | + | | | | | | + |
| SIPA1L2 | + | | + | | | + | | | + |
| SIPA1L3 | | | | | | | | | |
| sir1 | | | | | | | | | |
| SIRPA | | + | + | | | + | | | + |
| SIRPB1 | | | | | | | | | |
| SIRPD | | | | | | | | | |
| SIRPG | | | | | | | | | |
| SIRT1 | | | | | | | | | |
| SIRT2 | | | | | | | | | |
| SIRT3 | | | | | | | | | |
| SIRT4 | | | + | | | | | | + |
| SIRT5 | | | | | | | | | |
| SIRT6 | | | | | | | | | |
| SIRT7 | | | | | | | | | |
| SIS1 | | | | | | | | | |
| SIT1 | | | | | | | | | |
| SIT4 | | | | | | | | | |
| SIVA1 | | | | | | | | | |
| SIW14 | | | | | | | | | |
| SIX1 | | | | | | | | | |
| SIX2 | + | | | + | + | | + | | + |
| SIX3 | + | | | + | + | | | | + |
| SIX4 | | | | | | | | | |
| SIX5 | | | | | | | | | |
| SIX6 | | | | | | | | | |
| SIZ1 | | | | | | | | | |
| SKA1 | | | | | | | | | |
| SKA2 | | | | | | | | | |
| SKA3 | | | | | | | | | |
| SKAP1 | | | | | | | | | |
| SKAP2 | | + | | | | + | | | + |
| SKG3 | | | | | | | | | |
| SKI | + | | | | + | | | | + |
| SKI6 | | | | | | | | | |
| SKI8 | | | | | | | | | |
| SKIDA1 | | | | | | + | | | + |
| Skil | | | | | + | + | | | + |
| SKIV2L | | | | | | | | | |

| | | | | | | | | | |
|---|---|---|---|---|---|---|---|---|---|
| SKIV2L2 | | | | | | | + | | + |
| SKN7 | | | | | | | | | |
| SKO1 | | | | | | | | | |
| SKOR1 | | | | | | | | | |
| SKOR2 | | | | | | | | | |
| SKP1 | | | | | | | | | |
| Skp1a | | | | | | | | | |
| SKP1P2 | | | | | | | | | |
| SKP2 | | | | | | | | | |
| sks2 | | | | | | | | | |
| SKY1 | | | | | | | | | |
| SLA | | | | | | | | | |
| SLA1 | | | | | | | | | |
| SLA2 | | | | | | | | | |
| Slain1 | | | | | | + | | | + |
| SLAIN2 | | | | | | | | | |
| SLAMF1 | | | | + | | | | | + |
| SLAMF6 | | | | | | | | | |
| SLAMF7 | | | | | + | | | | + |
| SLAMF8 | | | | | | | | | |
| SLAMF9 | | | | | | | | | |
| SLBP | | | | | | | | | |
| SLC10A1 | | | | | | | | | |
| SLC10A2 | | | | | | | | | |
| SLC10A3 | | | | | | | | | |
| SLC10A4 | | | | | | | | | |
| SLC10A6 | | | | | | | | | |
| SLC10A7 | | | | | | | | | |
| SLC11A2 | | | | | | | | | |
| Slc12a1 | | | | | | | | | |
| SLC12A2 | | | | | | | | | |
| SLC12A3 | | | | | | | | | |
| SLC12A4 | | | | | | | | + | + |
| SLC12A5 | | | | | | + | | | + |
| SLC12A6 | | | | | | | | | |
| SLC12A7 | | | | | | | | | |
| SLC12A8 | | | | | | | | | |
| SLC12A9 | | | | | | | | | |
| SLC13A1 | | | | | | | | | |
| SLC13A2 | | | | | | | | | |
| SLC13A3 | | | | | | | | | |
| SLC13A4 | | | | | | | | | |
| SLC13A5 | | | | | | | | | |
| SLC14A1 | + | | | + | + | | + | | + |
| SLC14A2 | | | | | | | | | |
| SLC15A1 | | | | | | | | | |
| SLC15A2 | | + | + | | | + | | | + |
| SLC15A3 | | | | | | | | | |
| SLC15A4 | | | | | | | | | |
| SLC16A1 | + | | | | + | | | | + |
| SLC16A10 | | | | | | | | | |
| SLC16A11 | | | | | | + | | | + |
| SLC16A12 | | | | | | | | | |

| | | | | | | | | | |
|---|---|---|---|---|---|---|---|---|---|
| SLC16A13 | | | | | | | | | |
| SLC16A14 | | | | | | | | | |
| SLC16A2 | | | | | | + | | | + |
| SLC16A3 | | | | | | | | | |
| SLC16A4 | | | | | | | | | |
| SLC16A5 | | | | | | | | | |
| SLC16A6 | | | + | | | | | | + |
| SLC16A7 | | | | | | | | | |
| SLC16A8 | | | | | | | | | |
| SLC16A9 | | | | | | | | | |
| SLC17A1 | | | | | | | | | |
| SLC17A2 | | | | | | | | | |
| SLC17A4 | | | | | | | | | |
| SLC17A5 | | | | | | + | | | + |
| SLC17A6 | | | | | | | | | |
| SLC17A7 | | | | | | | | | |
| SLC17A8 | | | | + | | | | | + |
| SLC17A9 | | | | | | | | | |
| SLC18A1 | | | | | | | | | |
| SLC18A2 | | | | | | | | | |
| SLC18A3 | | | | | | | | | |
| SLC18B1 | | | | | | | | | |
| SLC19A1 | | | | | | | | | |
| SLC19A2 | | | | | | | | | |
| SLC19A3 | | | | | | | | | |
| SLC1A1 | | | + | | | | | | + |
| SLC1A2 | | + | | | | + | | | + |
| SLC1A3 | | | | | | + | | | + |
| SLC1A4 | | | + | | | | | | + |
| SLC1A5 | | | | | | | | | |
| SLC1A6 | | | | | | | | | |
| SLC1A7 | | | | | | | | | |
| SLC20A1 | | | + | | | | | | + |
| SLC20A2 | | | | | | | | | |
| SLC22A1 | | | | | | | | | |
| SLC22A10 | | | | | | | | | |
| SLC22A11 | | | | | | | | | |
| SLC22A12 | + | | | + | + | | | | + |
| SLC22A13 | | | | | | | | | |
| SLC22A14 | | | | | | | | | |
| SLC22A15 | | | | + | | | | | + |
| SLC22A16 | | | | | | | | | |
| SLC22A17 | | | | | | | | | |
| SLC22A18 | | | | | | | | | |
| SLC22A18AS | | | | | | | | | |
| SLC22A2 | | | | | | | | | |
| SLC22A20 | | | | | | | | | |
| SLC22A23 | | | | | | | | | |
| SLC22A24 | | | | | | | | | |
| SLC22A25 | | | | | | | | | |
| SLC22A3 | | | | | | | | | |
| SLC22A4 | | | | | | | | | |
| SLC22A5 | | | | | | | | | |

| | | | | | | | | | |
|---|---|---|---|---|---|---|---|---|---|
| SLC22A6 | | | | | | | | | |
| SLC22A8 | | | | | | | | | |
| SLC22A9 | | | | | | | | | |
| SLC23A1 | | | | + | | | | | + |
| SLC23A2 | | | | | | | | | |
| SLC23A3 | | | | | | | | | |
| SLC24A1 | | | | | | | | | |
| SLC24A2 | | + | | | | + | | | + |
| SLC24A3 | | | | | | + | | | + |
| SLC24A4 | | | | | | | | | |
| SLC24A5 | | | | | | | | | |
| SLC25A1 | | | | | | | | | |
| SLC25A10 | | | | | | | | | |
| SLC25A11 | | | | | | | | | |
| SLC25A12 | | + | | | | + | | | + |
| SLC25A13 | | | | | | | | | |
| SLC25A14 | | + | | | | + | | | + |
| SLC25A15 | | | | | | | | | |
| SLC25A16 | | | | | | | | | |
| SLC25A17 | | | | | | | | | |
| SLC25A18 | | | | | | | | | |
| SLC25A19 | | | | | | | | | |
| SLC25A2 | | | | | | | | | |
| SLC25A20 | | | | | | | | | |
| SLC25A21 | | | | | | | | | |
| SLC25A21-AS1 | | | | | | | | | |
| SLC25A22 | | | + | | | | | | + |
| SLC25A23 | | | | | | + | | | + |
| SLC25A24 | | | | | | | | | |
| SLC25A25 | | | | | | | | | |
| SLC25A26 | | | | | | | | | |
| SLC25A27 | | | | | | | | | |
| SLC25A28 | | | | | | | | | |
| SLC25A29 | | | | | | | | | |
| SLC25A3 | + | | | | + | | | | + |
| SLC25A30 | | | | | | | | | |
| SLC25A31 | | | | | | | | | |
| SLC25A32 | | | | | | | | | |
| SLC25A33 | | | | | | | | | |
| SLC25A34 | + | | | + | + | | + | | + |
| SLC25A35 | | | | | | | | | |
| SLC25A36 | | | | | | | | | |
| SLC25A37 | | | | | | | | | |
| SLC25A38 | | | | | | | | | |
| SLC25A39 | | | | | | | | | |
| SLC25A4 | | | | | | | | | |
| SLC25A40 | | | | | | | | | |
| SLC25A41 | | | | | | | | | |
| SLC25A42 | + | | | + | + | | + | | + |
| SLC25A43 | | | | | | | | | |
| SLC25A44 | | | | | | | | | |
| SLC25A45 | | | | | | | | | |
| SLC25A46 | | | | | | | + | | + |

| | | | | | | | | | |
|---|---|---|---|---|---|---|---|---|---|
| SLC25A47 | | | | | | | | | |
| SLC25A48 | | | | | | | | | |
| SLC25A5 | | | | | | | | | |
| SLC25A5-AS1 | | | | | | | | | |
| SLC25A51 | | | | | | | | | |
| SLC25A52 | | | | | | | | | |
| SLC25A53 | | | | | | | | | |
| SLC25A6 | | | | | | | | | |
| SLC26A1 | | | | | | | | | |
| SLC26A10 | + | | | | + | + | | | + |
| SLC26A11 | | | | | | | | | |
| SLC26A2 | | | | | + | + | | | + |
| SLC26A3 | | | | | | | | | |
| SLC26A4 | | | | | | | | | |
| SLC26A4-AS1 | | | | | | | | | |
| SLC26A5 | | | | | | | | | |
| SLC26A6 | | | | | | | | | |
| SLC26A7 | | | | | | | | | |
| SLC26A8 | | | | | | | | | |
| SLC26A9 | | | | | | | | | |
| SLC27A1 | | | | | | | | | |
| SLC27A2 | | | | | | | | | |
| SLC27A3 | | | | | + | + | | | + |
| SLC27A4 | | | + | | | | | | + |
| SLC27A5 | | | | | | | | | |
| SLC27A6 | | | | | | | | | |
| SLC28A1 | | | | | | | | | |
| SLC28A2 | | | | | | | | | |
| SLC28A3 | | | | | | | | | |
| SLC29A1 | | | | | | | | | |
| SLC29A2 | | | | | | | | | |
| SLC29A3 | | | | | | | | | |
| SLC29A4 | | | | | | + | | | + |
| SLC2A1 | + | | + | | + | | | | + |
| SLC2A10 | | | | | | | | | |
| SLC2A11 | | | | | | | | | |
| SLC2A12 | | | | | | | | | |
| SLC2A13 | | | + | | | | | | + |
| SLC2A14 | | | | | | | | | |
| SLC2A2 | | | | | | | | | |
| SLC2A3 | + | | + | | + | | | | + |
| SLC2A4 | | | | | | | | | |
| SLC2A4RG | | | | | | | | | |
| SLC2A5 | | | | | | | | | |
| SLC2A6 | | | | | | | | | |
| SLC2A7 | | | | | | | | | |
| SLC2A8 | | | | | | | | | |
| SLC2A9 | | | | | | | | | |
| SLC30A1 | | | + | | | | | | + |
| SLC30A10 | | | | | | | | | |
| SLC30A2 | | | | | | | | | |
| SLC30A3 | | | | | | | | | |
| SLC30A4 | | | | | | | | | |

| | | | | | | | | | |
|---|---|---|---|---|---|---|---|---|---|
| SLC30A5 | | | | | | | | | |
| SLC30A6 | | | | | | | | | |
| SLC30A7 | | | | | | | | | |
| SLC30A8 | | | | | | | | | |
| SLC30A9 | | | | | | | + | | + |
| SLC31A1 | | | | | | | | | |
| SLC31A2 | | | | | | | | | |
| SLC32A1 | | | | | | | | | |
| SLC33A1 | | | + | | | | | | + |
| SLC34A1 | | | | | | | | | |
| SLC34A2 | | | | | | | | | |
| SLC34A3 | | | | | | | | | |
| SLC35A1 | | + | + | | | + | | | + |
| SLC35A2 | | | | | | | | | |
| SLC35A3 | | | | | | + | | | + |
| SLC35A4 | | | | | | | | | |
| SLC35A5 | | | | | | | | | |
| SLC35B1 | | | | | | | | | |
| SLC35B2 | | | | | | | | | |
| SLC35B3 | | | | | | | | | |
| SLC35B4 | | | | | | | | | |
| SLC35C1 | | | | | | | | | |
| SLC35C2 | | | | | | | | | |
| SLC35D1 | + | | | + | + | | + | | + |
| SLC35D2 | | | | | | | | | |
| SLC35D3 | | | | | | | | | |
| SLC35E1 | | | | | | | | | |
| SLC35E2 | | | | | | | | | |
| SLC35E2B | | | | | | | | | |
| SLC35E3 | | | | | | | | | |
| SLC35E4 | | | | | | | | | |
| SLC35F1 | | | | | | | | | |
| SLC35F2 | | | | | | | | | |
| SLC35F3 | | | | | | | | | |
| SLC35F5 | | | | | | | | | |
| SLC35F6 | | | | | | | | | |
| SLC35G1 | | | | | | | | | |
| SLC35G2 | | | | | | | | | |
| SLC35G5 | | | | | | | | | |
| SLC36A1 | | | | | | | | | |
| SLC36A3 | | | | | | | | | |
| SLC37A1 | | | | | | | | | |
| SLC37A3 | | | | | | | | | |
| SLC37A4 | | | | | | | | | |
| SLC38A1 | | | + | | | | | | + |
| SLC38A10 | | | + | | | | | | + |
| SLC38A11 | | | | | | | | | |
| SLC38A2 | | | + | | + | | | | + |
| SLC38A3 | | | | | | | | | |
| SLC38A4 | | | | | | | | | |
| SLC38A5 | + | | | | + | | | | + |
| SLC38A6 | | | | | | | | | |
| SLC38A7 | | | | | | + | | | + |

| | | | | | | | | | |
|---|---|---|---|---|---|---|---|---|---|
| SLC38A9 | | | | | + | | | | + |
| SLC39A1 | | | | | | | | | |
| SLC39A10 | | | + | | | + | | | + |
| SLC39A11 | | | | | | | | | |
| SLC39A12 | | | | | | | | | |
| SLC39A13 | | | | | | + | | | + |
| SLC39A14 | | | | + | | | | | + |
| SLC39A2 | | | | | | | | | |
| SLC39A3 | | | + | | | + | | | + |
| SLC39A4 | | | | | | | | | |
| SLC39A5 | | | | | | | | | |
| SLC39A6 | | | | | | | | | |
| SLC39A7 | | | | | | | | | |
| SLC39A8 | + | | | + | + | | + | | + |
| SLC39A9 | | | | | | | | | |
| SLC3A1 | | | | | | | | | |
| SLC3A2 | | | | | + | | | | + |
| SLC40A1 | | | | | | | | | |
| SLC41A1 | | | | | | | | | |
| SLC41A2 | | | | | | | | | |
| SLC41A3 | | | | | | | | | |
| SLC43A1 | | | | | | | | | |
| SLC43A2 | | | | | | | | | |
| SLC43A3 | + | | | + | + | | | | + |
| SLC44A1 | | | | | | | | | |
| SLC44A2 | | | | | | | | | |
| SLC44A3 | | | | | | | | | |
| SLC44A4 | | | | | | | | | |
| SLC44A5 | | | | | | + | | | + |
| SLC45A1 | | | | | | | | | |
| SLC45A2 | | | | | | | | | |
| SLC45A3 | | | | | | | | | |
| SLC45A4 | | | | | | | | | |
| SLC46A1 | | | | | | | | | |
| SLC46A3 | | | | | | | | | |
| SLC47A1 | + | | | + | + | | + | | + |
| SLC47A2 | | | | | | | | | |
| SLC48A1 | | + | | | | + | | | + |
| SLC4A1 | | | | | | | | | |
| SLC4A10 | | | | | | | | | |
| SLC4A11 | | | | | | | | | |
| SLC4A1AP | | | | | | | | | |
| SLC4A2 | | | | | | | | | |
| SLC4A3 | | | | | | | | | |
| SLC4A4 | + | | | | + | | | | + |
| SLC4A5 | | + | + | | | + | | + | + |
| SLC4A7 | | | | | | | | | |
| SLC4A8 | | | | | | | | | |
| SLC50A1 | | | | | | + | | | + |
| SLC51A | | | | | | | | | |
| SLC51B | | | | | | | | | |
| SLC52A1 | | | | | | | | | |
| SLC52A2 | | | | | | | | | |

| | | | | | | | | | |
|---|---|---|---|---|---|---|---|---|---|
| SLC52A3 | | | | | | | | | |
| SLC5A1 | | | | | | | | | |
| SLC5A10 | | | | | | | | | |
| SLC5A11 | | | | | | | | | |
| SLC5A12 | | | | | | | | | |
| SLC5A2 | | | | | | | | | |
| SLC5A3 | | | | | | | | | |
| SLC5A4 | | | | | | | | | |
| SLC5A5 | | | | | | | | | |
| SLC5A6 | | | | | | | | | |
| SLC5A7 | | | | | | | | | |
| SLC5A8 | | | | | | | | | |
| SLC5A9 | | | | | | | | | |
| SLC6A1 | | | | | | + | | | + |
| SLC6A12 | | | | | | | | | |
| SLC6A13 | | | | | | | | | |
| SLC6A14 | | | | | | | | | |
| SLC6A15 | | | | | | + | | | + |
| SLC6A17 | + | | + | | + | | | | + |
| SLC6A19 | | | | | | | | | |
| SLC6A2 | | | | | | | | | |
| SLC6A20 | | | | | | | | | |
| SLC6A3 | | | | | | | | | |
| SLC6A4 | | | | | | | | | |
| SLC6A5 | | | | | | | | | |
| SLC6A6 | + | | + | | + | | | | + |
| Slc6a8 | + | | | | + | | | | + |
| SLC6A9 | | | | | | | | | |
| SLC7A1 | | | + | | | | | | + |
| SLC7A10 | | | | | | | | + | + |
| SLC7A11 | | | | | + | | | | + |
| SLC7A11-AS1 | | | | | | | | | |
| SLC7A13 | + | | | + | + | | + | | + |
| SLC7A14 | | | | | | | | | |
| SLC7A2 | | | | | | | | | |
| SLC7A3 | | | | | + | | | | + |
| SLC7A4 | | | | | | | | | |
| SLC7A5 | | | | | + | | | | + |
| SLC7A5P2 | | | | | | | | | |
| SLC7A6 | | | | | | | | | |
| SLC7A6OS | | | + | | | | | | + |
| SLC7A7 | | | | | | | | | |
| SLC7A8 | | | + | | | | | | + |
| SLC7A9 | | | | | | | | | |
| SLC8A1 | | | | | | | | | |
| SLC8A2 | | | | | | | | | |
| SLC8A3 | | | | | | | | | |
| SLC8B1 | | | | | | + | | | + |
| SLC9A1 | | | | | | | | | |
| SLC9A2 | | + | | | | + | | | + |
| SLC9A3 | | | | | | | | | |
| SLC9A3R1 | | | | | | | | | |
| SLC9A3R2 | | | | | | | | | |

| | | | | | | | | | |
|---|---|---|---|---|---|---|---|---|---|
| SLC9A4 | | + | | | | + | | | + |
| SLC9A5 | | | + | | | | | | + |
| SLC9A6 | | | + | | | | | | + |
| SLC9A7 | | | | | | | | | |
| SLC9A8 | | | | | | + | | | + |
| SLC9A9 | | | | | | | | | |
| SLC9B1 | | | | | | | | | |
| SLC9B2 | | | | | | | | | |
| SLC9C1 | | | | | | | | | |
| SLCO1B1 | | | | | | | | | |
| SLCO1B3 | | | | | | | | | |
| SLCO1C1 | | | | | | | | | |
| SLCO2B1 | | | | | | | | | |
| SLCO3A1 | | | | | | | | | |
| SLCO4A1 | | | | | | | | | |
| SLCO4C1 | | | | | | | | | |
| SLCO5A1 | | | | | | | | | |
| SLCO6A1 | | | | | | | | | |
| sld5 | | | | | | | | | |
| SLF1 | | | | | | | | | |
| SLFN11 | | | | | | | | | |
| SLFN12 | | | | | | | | | |
| SLFN12L | | | | | | | | | |
| SLFN13 | | | | | | | | | |
| SLFN14 | | | | | | | | | |
| SLFN5 | | | | | | | | | |
| SLFNL1 | | | | | | | | | |
| SLFNL1-AS1 | | | | | | | | | |
| SLG1 | | | | | | | | | |
| SLI15 | | | | | | | | | |
| SLIRP | | | | | | | | | |
| SLIT1 | | | | | | | | | |
| SLIT1-AS1 | | | | | | | | | |
| SLIT2 | | | + | | | + | | | + |
| SLIT3 | | | | | | + | | | + |
| SLITRK1 | | | | | | | | | |
| SLITRK2 | | | | | | | | | |
| SLITRK3 | | | | | | | | | |
| Slitrk4 | | | | | | | | | |
| SLITRK5 | | | | | | | | | |
| SLITRK6 | | | | | | | | | |
| SLK | | | + | | | | | | + |
| SLK19 | | | | | | | | | |
| SLM1 | | | | | | | | | |
| SLM2 | | | | | | | | | |
| SLM4 | | | | | | | | | |
| SLMAP | | | | | | | | | |
| SLMO1 | | | | | | | | | |
| SLMO2 | | | | | | | | | |
| SLMO2-ATP5E | | | | | | | | | |
| SLN | | | | | | | | | |
| slp1 | | | | | | | | | |
| SLPI | | | | | | | | | |

| | | | | | | | | | |
|---|---|---|---|---|---|---|---|---|---|
| SLT2 | | | | | | | | | |
| SLTM | | + | | | | + | | | + |
| SLU7 | | | | | | | | | |
| SLURP1 | | | | | | | | | |
| SLX1A | | | | | | | | | |
| SLX1B | | | | | | | | | |
| SLX4 | | | | | | | | | |
| SLX4IP | | | | | | | | | |
| SLX5 | | | | | | | | | |
| slx8 | | | | | | | | | |
| SLY41 | | | | | | | | | |
| SMA4 | | | | | | | | | |
| SMA5 | | | | | | | | | |
| SMAD1 | | | | | | | | | |
| SMAD1-AS1 | | | | | | | | | |
| smad1-b | | | | | | | | | |
| SMAD2 | | | | | | | | | |
| SMAD3 | | | | | | | | | |
| SMAD4 | | | | | | | | | |
| SMAD5 | | | | | | | | | |
| SMAD6 | | | | | | | | | |
| SMAD7 | + | | + | | + | | | | + |
| SMAD9 | | | | | | | | | |
| SMAGP | | | | | | | | | |
| SMAP1 | | | + | | | | | | + |
| SMAP2 | | | | | + | | | | + |
| SMARCA1 | | | | | | | | | |
| SMARCA2 | | | | | | | | | |
| SMARCA4 | | | | | + | | | | + |
| SMARCA5 | + | | + | | | | | | + |
| SMARCAD1 | | | | | | | | | |
| SMARCAL1 | | | | | | | | | |
| SMARCB1 | | | | | | + | | | + |
| SMARCC1 | | | | | | | | | |
| SMARCC2 | | | | | | | | | |
| SMARCD1 | | | | | | | | | |
| SMARCD2 | | | | | | | | | |
| SMARCD3 | | | | | | | | | |
| Smarce1 | | | + | | | | | | + |
| SMC1A | | | | | | | | | |
| SMC1B | | | | | | | | | |
| SMC2 | | | | | | + | | | + |
| SMC3 | | | | | | | | | |
| SMC4 | | | | | | | | | |
| SMC5 | | | | | | | | | |
| SMC6 | | | | | | | | | |
| SMCHD1 | | | | | | | | | |
| SMCO1 | | | | | | | | | |
| SMCO2 | | | | | | | | | |
| SMCO3 | | | | | | | | | |
| SMCO4 | | | | | | | | | |
| SMCP | | | | | | | | | |
| SMCR8 | | | | | | | | | |

| | | | | | | | | | |
|---|---|---|---|---|---|---|---|---|---|
| smd1 | | | | | | | | | |
| smd2 | | | | | | | | | |
| SMDT1 | | | | | | | | | |
| SMEK1 | | | + | | | | | | + |
| SMEK2 | | | | | | | | | |
| SMF3 | | | | | | | | | |
| SMG1 | | | | | | | | | |
| SMG1P1 | | | | | | | | | |
| SMG1P2 | | | | | | | | | |
| SMG1P3 | | | | | | | | | |
| SMG1P5 | | | | | | | | | |
| SMG1P6 | | | | | | | | | |
| SMG5 | | | | | | | | | |
| SMG6 | | | | | | | | | |
| SMG7 | | | | | | | | | |
| SMG8 | | | | | | | | | |
| SMG9 | | | | | | | | | |
| smid | | | | | | | | | |
| SMIM1 | | | | | | | | | |
| SMIM11 | | | | | | | | | |
| SMIM12 | | | | | | | | | |
| SMIM13 | | | | | | | | | |
| SMIM14 | + | | | | + | | | | + |
| SMIM15 | | | | | | | | | |
| SMIM17 | | | | | | | | | |
| SMIM19 | | | | | | | | | |
| SMIM2 | | | | | | | | | |
| SMIM20 | | | | | | + | | | + |
| SMIM21 | | | | | | | | | |
| SMIM24 | | | | | | | | | |
| SMIM3 | | | | | | | | | |
| SMIM4 | | | | | | + | | | + |
| SMIM5 | | | | | | | | | |
| SMIM7 | | | | | | + | | | + |
| SMIM8 | | | | | | | | | |
| SMKR1 | | | | | | | | | |
| SMLR1 | | | | | | | | | |
| SMN1 | | + | + | | | + | | + | + |
| SMN2 | | | | | | | | | |
| SMNDC1 | | | | | | | | | |
| SMO | | | | | | | | | |
| SMOC1 | | | | | | | | | |
| SMOC2 | | | | | | | | | |
| SMOX | | | | | + | | | | + |
| SMP1 | | | | | | | | | |
| SMPD1 | | | | | | | | | |
| SMPD2 | | | | | | | | | |
| SMPD3 | | | | | | | | | |
| SMPD4 | | | | | | | | | |
| SMPDL3A | | | | | | | | | |
| SMPDL3B | | | | | | | | | |
| SMPX | | | | | | | | | |
| Smr | | | | | | | | | |

| | | | | | | | | | |
|---|---|---|---|---|---|---|---|---|---|
| SMR3A | | | | | | | | | |
| SMR3B | | | | | | | | | |
| SMS | | | | | | + | | | + |
| smt3 | | | | | | | | | |
| SMTN | | | | + | + | | | | + |
| SMTNL1 | | | | | | | | | |
| SMTNL2 | | | | | | | | | |
| SMU1 | | | | | | | | | |
| SMUG1 | | | | | | | | | |
| SMURF1 | | | | | | | | | |
| SMURF2 | | | | | | | | | |
| SMYD1 | | | | | | | | | |
| SMYD2 | | + | | | | + | | | + |
| SMYD3 | | | | | | | | | |
| SMYD4 | | | | | | | | | |
| SMYD5 | | | | | | + | | | + |
| SNA3 | | | | | | | | | |
| SNAI1 | | | | | | | | | |
| SNAI2 | + | | | + | + | | | | + |
| SNAI3 | | | | | | | | | |
| SNAI3-AS1 | | | | | | | | | |
| SNAP23 | | | | | | | | | |
| SNAP23P | | | | | | | | | |
| SNAP25 | | | | | | | | | |
| SNAP29 | | | | | | | | | |
| SNAP47 | | | | | | | | | |
| SNAP91 | | | | | + | | | | + |
| SNAPC1 | | | | | | | | | |
| SNAPC2 | | | | | | | | | |
| SNAPC3 | | + | | | | + | | | + |
| SNAPC4 | | | | | | | | | |
| SNAPC5 | | | | | | + | | | + |
| SNAPIN | | | | | | + | | | + |
| SNAR-B2 | | | | | | | | | |
| SNC1 | | | | | | | | | |
| SNC2 | | | | | | | | | |
| SNCA | | | | | | | | | |
| SNCAIP | | | | | | | | | |
| SNCB | | | | | | + | | | + |
| SNCG | | | | | | | | | |
| SND1 | | + | | | | | | | + |
| SNED1 | | | | | | | | | |
| SNF1 | | | | | | | | | |
| snf22 | | | | | | | | | |
| SNF4 | | | | | | | | | |
| SNF8 | + | | | | + | | | | + |
| SNG1 | | | | | | | | | |
| SNHG1 | | | | | | | | | |
| SNHG11 | | | | | + | | | | + |
| SNHG12 | | | | | | | | | |
| SNHG19 | | | | | | | | | |
| SNHG21 | | | | | | | | | |
| SNHG3 | | | | | | | | | |

| | | | | | | | | | |
|---|---|---|---|---|---|---|---|---|---|
| SNHG4 | | | | | | | | | |
| SNHG5 | | | | | | | | | |
| SNHG6 | | | | | | | | | |
| SNHG7 | | | | | | | | | |
| SNHG8 | | | | | | | | | |
| SNIP1 | | | | | | | | | |
| SNN | | | | | | | | | |
| SNN1 | | | | | | | | | |
| SNORA10 | | | | | | | | | |
| SNORA11D | | | | | | | | | |
| SNORA12 | | | | | | | | | |
| SNORA13 | | | | | | | | | |
| SNORA14A | | | | | | | | | |
| SNORA16A | | | | | | | | | |
| SNORA17 | | | | | | | | | |
| SNORA18 | | | | | | | | | |
| SNORA21 | | | | | | | | | |
| SNORA23 | | | | | | | | | |
| SNORA24 | | | | | | | | | |
| SNORA26 | | | | | | | | | |
| SNORA30 | | | | | | | | | |
| SNORA32 | | | | | | | | | |
| SNORA33 | | | | | | | | | |
| SNORA34 | | | | | | | | | |
| SNORA4 | | | | | | | | | |
| SNORA40 | | | | | | | | | |
| SNORA41 | | | | | | | | | |
| SNORA43 | | | | | | | | | |
| SNORA45B | | | | | | | | | |
| SNORA52 | | | | | | | | | |
| SNORA53 | | | | | | | | | |
| SNORA54 | | | | | | | | | |
| SNORA57 | | | | | | | | | |
| SNORA5A | | | | | | | | | |
| SNORA5C | | | | | | | | | |
| SNORA6 | | | | | | | | | |
| SNORA61 | | | | | | | | | |
| SNORA63 | | | | | | | | | |
| SNORA64 | | | | | | | | | |
| SNORA66 | | | | | | | | | |
| SNORA67 | | | | | | | | | |
| SNORA71D | | | | | | | | | |
| SNORA73A | | | | | | | | | |
| SNORA73B | | | | | | | | | |
| SNORA74A | | | | | | | | | |
| SNORA74B | | | | | | | | | |
| SNORA75 | | | | | | | | | |
| SNORA76C | | | | | | | | | |
| SNORA77 | | | | | | | | | |
| SNORA78 | | | | | | | | | |
| SNORA79 | | | | | | | | | |
| SNORA7A | | | | | | | | | |
| SNORA7B | | | | | | | | | |

| | | | | | | | | | |
|---|---|---|---|---|---|---|---|---|---|
| SNORA8 | | | | | | | | | |
| SNORA80A | | | | | | | | | |
| SNORA80E | | | | | | | | | |
| SNORA81 | | | | | | | | | |
| SNORD10 | | | | | | | | | |
| SNORD101 | | | | | | | | | |
| SNORD104 | | | | | | | | | |
| SNORD109B | | | | | | | | | |
| SNORD116-1 | | | | | | | | | |
| SNORD116-18 | | | | | | | | | |
| SNORD116-21 | | | | | | | | | |
| SNORD116-22 | | | | | | | | | |
| SNORD116-24 | | | | | | | | | |
| SNORD116-29 | | | | | | | | | |
| SNORD116-4 | | | | | | | | | |
| SNORD116-5 | | | | | | | | | |
| SNORD116-6 | | | | | | | | | |
| SNORD116-8 | | | | | | | | | |
| SNORD118 | | | | | | | | | |
| SNORD12B | | | | | | | | | |
| SNORD12C | | | | | | | | | |
| SNORD13 | | | | | | | | | |
| SNORD13P1 | | | | | | | | | |
| SNORD14C | | | | | | | | | |
| SNORD14D | | | | | | | | | |
| SNORD14E | | | | | | | | | |
| SNORD15A | | | | | | | | | |
| SNORD15B | | | | | | | | | |
| SNORD16 | | | | | | | | | |
| SNORD17 | | | | | | | | | |
| SNORD18B | | | | | | | | | |
| SNORD18C | | | | | | | | | |
| SNORD19 | | | | | | | | | |
| SNORD19B | | | | | | | | | |
| SNORD1C | | | | | | | | | |
| SNORD2 | | | | | | | | | |
| SNORD20 | | | | | | | | | |
| SNORD21 | | | | | | | | | |
| SNORD22 | | | | | | | | | |
| SNORD23 | | | | | | | | | |
| SNORD24 | | | | | | | | | |
| SNORD25 | | | | | | | | | |
| SNORD26 | | | | | | | | | |
| SNORD27 | | | | | | | | | |
| SNORD28 | | | | | | | | | |
| SNORD29 | | | | | | | | | |
| SNORD30 | | | | | | | | | |
| SNORD31 | | | | | | | | | |
| SNORD33 | | | | | | | | | |
| SNORD34 | | | | | | | | | |
| SNORD35A | | | | | | | | | |
| SNORD35B | | | | | | | | | |
| SNORD36A | | | | | | | | | |

| | | | | | | | | | |
|---|---|---|---|---|---|---|---|---|---|
| SNORD36B | | | | | | | | | |
| SNORD36C | | | | | | | | | |
| SNORD38A | | | | | | | | | |
| SNORD3A | | | | | | | | | |
| SNORD3B-1 | | | | | | | | | |
| SNORD3B-2 | | | | | | | | | |
| SNORD3C | | | | | | | | | |
| SNORD41 | | | | | | | | | |
| SNORD42A | | | | | | | | | |
| SNORD43 | | | | | | | | | |
| SNORD44 | | | | | | | | | |
| SNORD49A | | | | | | | | | |
| SNORD5 | | | | | | | | | |
| SNORD50A | | | | | | | | | |
| SNORD50B | | | | | | | | | |
| SNORD54 | | | | | | | | | |
| SNORD55 | | | | | | | | | |
| SNORD58A | | | | | | | | | |
| SNORD58B | | | | | | | | | |
| SNORD58C | | | | | | | | | |
| SNORD59A | | | | | | | | | |
| SNORD6 | | | | | | | | | |
| SNORD60 | | | | | | | | | |
| SNORD61 | | | | | | | | | |
| SNORD62A | | | | | | | | | |
| SNORD62B | | | | | | | | | |
| SNORD63 | | | | | | | | | |
| SNORD69 | | | | | | | | | |
| SNORD7 | | | | | | | | | |
| SNORD73A | | | | | | | | | |
| SNORD74 | | | | | | | | | |
| SNORD75 | | | | | | | | | |
| SNORD76 | | | | | | | | | |
| SNORD8 | | | | | | | | | |
| SNORD80 | | | | | | | | | |
| SNORD83A | | | | | | | | | |
| SNORD83B | | | | | | | | | |
| SNORD87 | | | | | | | | | |
| SNORD88B | | | | | | | | | |
| SNORD88C | | | | | | | | | |
| SNORD9 | | | | | | | | | |
| SNORD95 | | | | | | | | | |
| SNPH | | | | | | + | | | + |
| SNRK | | | | | | | | | |
| SNRNP200 | | | | | | | | | |
| SNRNP25 | | | | | | | | | |
| SNRNP27 | | | | | | | | | |
| SNRNP35 | | | | | | | | | |
| SNRNP40 | | | | | | | | | |
| SNRNP48 | | | | | + | | | | + |
| SNRNP70 | | | | | | | | | |
| SNRPA | | | | | + | | | | + |
| SNRPA1 | | | | | + | | | | + |

| | | | | | | | | | |
|---|---|---|---|---|---|---|---|---|---|
| SNRPB | | | | | | + | | | + |
| SNRPB2 | | | | | | | | | |
| SNRPC | | | | | | | | | |
| SNRPD1 | | | | | | | | | |
| SNRPD2 | | | | | | | | | |
| SNRPD2P1 | | | | | | | | | |
| SNRPD3 | | | | | | | | | |
| SNRPE | | | | | | | | | |
| SNRPEP2 | | | | | | | | | |
| SNRPF | | | | | | | | | |
| SNRPG | | | | | | | | | |
| SNRPGP15 | | | | | | | | | |
| SNRPN | | | + | | | | | | + |
| SNT1 | | | | | | | | | |
| SNTA1 | | | | | | | | | |
| SNTB1 | | | | | | | | | |
| SNTB2 | | | | | | | | | |
| SNTG1 | | | | | | | | | |
| SNTG2 | | | | | | | | | |
| SNU114 | | | | | | | | | |
| SNU13 | | | | | | | | | |
| SNU56 | | | | | | | | | |
| SNUPN | | | | | | | | | |
| SNURF | | | | | | | | | |
| SNW1 | | | | | | | | | |
| SNX1 | | | | | | | | | |
| SNX10 | | | | | | + | | | + |
| SNX11 | | | | | | | | | |
| SNX12 | | | + | | | | | | + |
| SNX13 | | | | | | | | | |
| SNX14 | | | | | | | | | |
| SNX15 | | | | | | | | | |
| SNX16 | | | | | | | | | |
| SNX17 | | | | | | | | | |
| SNX18 | | | + | | | | | | + |
| SNX19 | + | | | | | | | | + |
| SNX2 | | | | | | | | | |
| SNX20 | | | | | | | | | |
| SNX21 | | | | | | | | | |
| SNX22 | | | | | | | | | |
| SNX24 | | | | | | | | | |
| SNX25 | | | | | | | | | |
| SNX27 | | + | | | | + | | | + |
| SNX29 | | | | | | | | | |
| SNX3 | | | + | | | | | | + |
| SNX30 | | | | | | | | | |
| SNX31 | | | | | | | | | |
| SNX32 | | | | | | | | | |
| SNX33 | | | | | | | | | |
| SNX4 | | | | | | | | | |
| SNX5 | | | + | | | | | | + |
| SNX6 | | | + | | | | | | + |
| SNX7 | | | | | | | | | |

| | | | | | | | | | |
|---|---|---|---|---|---|---|---|---|---|
| SNX8 | | | | | | | | | |
| SNX9 | | | | | | | | | |
| snz1 | | | | | | | | | |
| SOAT1 | | | + | | | | | | + |
| SOAT2 | | | | | | | | | |
| SOBP | | | | | | + | | | + |
| SOCS1 | | | | | | | | | |
| SOCS2 | + | | | | | | | | + |
| SOCS3 | | | | | | | | | |
| Socs4 | | | | | | | | | |
| Socs5 | | | | | | | | | |
| Socs6 | | | + | | | | | | + |
| SOCS7 | | | | | | + | | | + |
| SOD1 | | | | | + | | | | + |
| SOD2 | | | | | | | | | |
| SOD3 | | | | | | | | | |
| SOF1 | | | | | | | | | |
| SOG1 | | | | | | | | | |
| Soga1 | | | | | + | | | | + |
| SOGA3 | | + | | | | + | | | + |
| SOHLH1 | | | | | | | | | |
| SOHLH2 | | | | | | | | | |
| SON | | | | | | | | | |
| SORBS1 | | | | | | | | | |
| SORBS2 | | | | | | | | | |
| SORBS3 | | | | | | | | | |
| SORCS1 | | | | | + | | | | + |
| SORCS2 | | | | | | + | | | + |
| SORCS3 | | | | | + | | | | + |
| SORD | | | | | | | | | |
| SORL1 | | | | | | + | | | + |
| SORT1 | | | | | + | + | | | + |
| SOS1 | | | | | | | | | |
| SOS2 | | | | | | | | | |
| SOST | | | | | | | | | |
| SOSTDC1 | | | | | | | | + | + |
| SOWAHA | | | | | | + | | | + |
| SOWAHB | | | | | + | | | | + |
| SOWAHC | | | | | | | | | |
| SOX1 | | | | | | | | | |
| SOX10 | | | | | | | | + | + |
| SOX11 | | | | | | | | | |
| SOX12 | | | | | | | | | |
| SOX13 | | | | | | | | | |
| SOX14 | | | | | | | | | |
| SOX15 | | | | | | | | | |
| SOX17 | | | | | | | | | |
| SOX18 | | | | | | | | | |
| SOX2 | | | + | | | | | | + |
| SOX2-OT | | | | | | | | | |
| SOX21 | | | | | | | | | |
| SOX21-AS1 | | | | | | | | | |
| SOX3 | | | | | | | | | |

| | | | | | | | | | |
|---|---|---|---|---|---|---|---|---|---|
| SOX30 | | | | | | | | | |
| SOX4 | | | | | | | | | |
| SOX5 | | | | | | + | | | + |
| SOX6 | | | | | | | | | |
| SOX7 | | | | | | | | | |
| SOX8 | + | | | + | + | + | + | | + |
| SOX9 | + | | + | + | + | | + | | + |
| SP1 | | | | | + | | | | + |
| SP100 | | | | + | | | | | + |
| SP110 | + | | | + | + | | + | | + |
| SP140 | | + | + | | | + | | + | + |
| SP140L | | | | | | | | | |
| SP2 | | | | | | | | | |
| SP3 | | | | | | | | | |
| SP4 | | | | | | | | | |
| SP5 | | | | | | | | | |
| SP6 | | | | | | | | | |
| SP7 | | | | | | | | | |
| SP8 | | | | | | | | | |
| SP9 | | | | | | | | | |
| SPA17 | | | | | | | | | |
| SPAC1006.07 | | | | | | | | | |
| SPAC14C4.11 | | | | | | | | | |
| SPAC1527.03 | | | | | | | | | |
| SPAC1635.01 | | | | | | | | | |
| SPAC167.05 | | | | | | | | | |
| SPAC1687.21 | | | | | | | | | |
| SPAC1751.03 | | | | | | | | | |
| SPAC17H9.12c | | | | | | | | | |
| SPAC17H9.13c | | | | | | | | | |
| SPAC18G6.06 | | | | | | | | | |
| SPAC1952.08c | | | | | | | | | |
| SPAC19A8.07c | | | | | | | | | |
| SPAC19G12.05 | | | | | | | | | |
| SPAC19G12.09 | | | | | | | | | |
| SPAC1B3.01c | | | | | | | | | |
| SPAC1F12.04c | | | | | | | | | |
| SPAC1F12.07 | | | | | | | | | |
| SPAC1F12.10c | | | | | | | | | |
| SPAC1F8.07c | | | | | | | | | |
| SPAC22A12.16 | | | | | | | | | |
| SPAC23A1.17 | | | | | | | | | |
| SPAC23D3.12 | | | | | | | | | |
| SPAC23D3.13c | | | | | | | | | |
| SPAC23G3.06 | | | | | | | | | |
| SPAC23G3.12c | | | | | | | | | |
| SPAC24C9.06c | | | | | | | | | |
| SPAC24C9.12c | | | | | | | | | |
| SPAC24H6.01c | | | | | | | | | |
| SPAC24H6.13 | | | | | | | | | |
| SPAC25B8.12c | | | | | | | | | |
| SPAC25G10.01 | | | | | | | | | |
| SPAC25G10.08 | | | | | | | | | |

| | | | | | | | | | |
|---|---|---|---|---|---|---|---|---|---|
| SPAC27D7.11c | | | | | | | | | |
| SPAC27F1.06c | | | | | | | | | |
| SPAC29A4.02c | | | | | | | | | |
| SPAC29E6.06c | | | | | | | | | |
| SPAC30C2.04 | | | | | | | | | |
| SPAC323.04 | | | | | | | | | |
| SPAC3A11.10c | | | | | | | | | |
| SPAC3C7.13c | | | | | | | | | |
| SPAC458.02c | | | | | | | | | |
| SPAC4G9.12 | | | | | | | | | |
| SPAC56F8.03 | | | | | | | | | |
| SPAC57A7.12 | | | | | | | | | |
| SPAC589.10c | | | | | | | | | |
| SPAC5D6.13 | | | | | | | | | |
| SPAC5H10.10 | | | | | | | | | |
| SPAC694.02 | | | | | | | | | |
| SPAC821.05 | | | | | | | | | |
| SPAC823.04 | | | | | | | | | |
| SPAC959.03c | | | | | | | | | |
| SPAC977.14c | | | | | | | | | |
| SPAC977.15 | | | | | | | | | |
| SPACA1 | | | | | | | | | |
| SPACA3 | | | | | | | | | |
| SPACA4 | | | | | | | | | |
| SPACA6P | | | | | | | | | |
| SPACA7 | | | | | | | | | |
| SPACUNK4.10 | | | | | | | | | |
| SPACUNK4.16c | | | | | | | | | |
| SPACUNK4.17 | | | | | | | | | |
| SPAG1 | | | | | | | | | |
| SPAG11A | | | | | | | | | |
| SPAG11B | | | | | | | | | |
| SPAG16 | | | | | | | | | |
| SPAG17 | | | | | | | | | |
| SPAG4 | | | | | + | | | | + |
| Spag5 | | | | | | + | | | + |
| SPAG5-AS1 | | | | | | | | | |
| SPAG6 | | | | | | | | | |
| SPAG7 | | | | | | | | | |
| SPAG8 | | | | | | | | | |
| SPAG9 | | | | | | | | | |
| SPAM1 | | | | | | | | | |
| SPANXA1 | | | | | | | | | |
| SPANXA2 | | | | | | | | | |
| SPANXB1 | | | | | | | | | |
| SPANXC | | | | | | | | | |
| SPANXD | | | | | | | | | |
| SPANXN2 | | | | | | | | | |
| SPANXN3 | | | | | | | | | |
| SPANXN4 | | | | | | | | | |
| SPANXN5 | | | | | | | | | |
| SPARC | | | | | | | | | |
| SPARCL1 | | | | | | | | | |

| | | | | | | | | | |
|---|---|---|---|---|---|---|---|---|---|
| SPAST | | | | | | | | | |
| SPATA1 | | | | + | | | | | + |
| SPATA12 | | | | | | | | | |
| SPATA13 | + | | | | + | | | | + |
| SPATA16 | | | | | | | | | |
| SPATA17 | | | | | | | | | |
| SPATA18 | | | | | | | | | |
| SPATA19 | | | | | | | | | |
| SPATA2 | | | | | | | | | |
| SPATA20 | | | | | | | | | |
| SPATA21 | | | | | | | | | |
| SPATA22 | | | | | | | | | |
| SPATA24 | | | | | | | | | |
| SPATA25 | | | | | | | | | |
| SPATA2L | | | | | | | | | |
| SPATA3 | | | | | | | | | |
| SPATA31A1 | | | | | | | | | |
| SPATA31A3 | | | | | | | | | |
| SPATA31A6 | | | | | | | | | |
| SPATA31A7 | | | | | | | | | |
| SPATA31D1 | | | | | | | | | |
| SPATA31D3 | | | | | | | | | |
| SPATA31D4 | | | | | | | | | |
| SPATA31E1 | | | | | | | | | |
| SPATA32 | | | | | | | | | |
| SPATA33 | | | | | | | | | |
| SPATA4 | | | | | | | | | |
| SPATA45 | | | | | | | | | |
| SPATA5 | | | | | | | | | |
| SPATA5L1 | | | | | | | | | |
| SPATA6 | | | | | | | | | |
| SPATA6L | | | | | | | | | |
| SPATA7 | | | | | | | | | |
| SPATA8 | | | | | | | | | |
| SPATA9 | | | | | | | | | |
| SPATC1 | | | | | | | | | |
| SPATC1L | | | | | | | | | |
| SPATS1 | | | | | | | | | |
| SPATS2 | | | | | | | | | |
| SPATS2L | | | | | + | | | | + |
| spb1 | | | | | | | | | |
| SPBC106.03 | | | | | | | | | |
| SPBC11C11.06c | | | | | | | | | |
| SPBC13E7.07 | | | | | | | | | |
| SPBC15D4.09c | | | | | | | | | |
| SPBC16A3.02c | | | | | | | | | |
| SPBC16H5.08c | | | | | | | | | |
| SPBC16H5.12c | | | | | | | | | |
| SPBC1703.07 | | | | | | | | | |
| SPBC1703.13c | | | | | | | | | |
| SPBC1711.05 | | | | | | | | | |
| SPBC19F5.04 | | | | | | | | | |
| SPBC19G7.10c | | | | | | | | | |

| | | | | | | | | | |
|---|---|---|---|---|---|---|---|---|---|
| SPBC216.03 | | | | | | | | | |
| SPBC24C6.04 | | | | | | | | | |
| SPBC25B2.10 | | | | | | | | | |
| SPBC26H8.11c | | | | | | | | | |
| SPBC29A10.16c | | | | | | | | | |
| SPBC2F12.05c | | | | | | | | | |
| SPBC2G5.01 | | | | | | | | | |
| SPBC2G5.05 | | | | | | | | | |
| SPBC30D10.02 | | | | | | | | | |
| SPBC30D10.03c | | | | | | | | | |
| SPBC32F12.10 | | | | | | | | | |
| SPBC36B7.05c | | | | | | | | | |
| SPBC405.06 | | | | | | | | | |
| SPBC4C3.03 | | | | | | | | | |
| SPBC4C3.07 | | | | | | | | | |
| SPBC530.07c | | | | | | | | | |
| SPBC646.07c | | | | | | | | | |
| SPBC646.10c | | | | | | | | | |
| SPBC660.16 | | | | | | | | | |
| SPBC8D2.18c | | | | | | | | | |
| SPBP23A10.11c | | | | | | | | | |
| SPBP4H10.15 | | | | | | | | | |
| SPBP8B7.18c | | | | | | | | | |
| SPC1 | | | | | | | | | |
| SPC2 | | | | | | | | | |
| SPC24 | + | | | + | + | | + | | + |
| SPC25 | | | | | | | | | |
| SPCC1183.07 | | | | | | | | | |
| SPCC11E10.07c | | | | | | | | | |
| SPCC1281.06c | | | | | | | | | |
| SPCC1450.13c | | | | | | | | | |
| SPCC1620.06c | | | | | | | | | |
| SPCC1672.07 | | | | | | | | | |
| SPCC1827.03c | | | | | | | | | |
| SPCC1827.05c | | | | | | | | | |
| SPCC1827.06c | | | | | | | | | |
| SPCC306.08c | | | | | | | | | |
| SPCC330.03c | | | | | | | | | |
| SPCC338.06c | | | | | | | | | |
| SPCC584.01c | | | | | | | | | |
| SPCC622.14 | | | | | | | | | |
| SPCC70.03c | | | | | | | | | |
| SPCC825.01 | | | | | | | | | |
| SPCC970.03 | | | | | | | | | |
| SPCP1E11.08 | | | | | | | | | |
| SPCPB16A4.02c | | | | | | | | | |
| SPCS1 | | | | | | | | | |
| SPCS2 | | | | | | | | | |
| SPCS2P4 | | | | | | | | | |
| SPCS3 | | | | | | | | | |
| SPDEF | | | | | | | | | |
| SPDL1 | | | | | | | | | |
| SPDYA | | | | | | | | | |

| | | | | | | | | | |
|---|---|---|---|---|---|---|---|---|---|
| Spdyb | | | | | | | | | |
| SPDYC | | | | | | | | | |
| spdyc-b | | | | | | | | | |
| SPDYE1 | | | | | | | | | |
| SPDYE2 | | | | | | | | | |
| SPDYE2B | | | | | | | | | |
| SPDYE3 | | | | | | | | | |
| SPDYE4 | | | | | | | | | |
| SPDYE6 | | | | | | | | | |
| SPDYE7P | | | | | | | | | |
| SPECC1 | | | | | | | | | |
| SPECC1L | | | | | | | | | |
| SPECC1L-ADORA2A | | | | | | | | | |
| SPEF1 | | | | | | | | | |
| SPEF2 | | | | | | | | + | + |
| SPEG | | | | | | | | | |
| SPEM1 | | | | | | | | | |
| SPEN | | | | | | | | | |
| SPERT | | | | | | | | | |
| SPESP1 | | | | | | | | | |
| SPF1 | | | | | | | | | |
| spf30 | | | | | | | | | |
| SPG11 | | | | | | + | | | + |
| SPG20 | | | | | | | | | |
| SPG21 | | | | | | | | | |
| SPG7 | | | | | | | | | |
| SPHK1 | | | | | | | | | |
| SPHK2 | | | | | | + | | | + |
| SPHKAP | | + | + | | | + | | | + |
| SPI | | | | | | | | | |
| SPI1 | | | | | | | | | |
| SPIB | | | | | | | | | |
| SPIC | | | | | | | | | |
| SPICE1 | | | | | | | | | |
| SPIDR | | | | | | | | | |
| SPIN1 | | | + | | | | | | + |
| SPIN2A | | | | | | | | | |
| SPIN2B | | | | | | | | | |
| SPIN3 | | | | | | | | | |
| SPIN4 | | | | | | | | | |
| SPINK1 | | | | | | | | | |
| SPINK14 | | | | | | | | | |
| SPINK2 | | | | | | | | | |
| Spink3 | | | | | | | | | |
| SPINK4 | | | | | | | | | |
| SPINK5 | | | | | | | | | |
| SPINK6 | | | | | | | | | |
| SPINK7 | | | | | | | | | |
| SPINK9 | | | | | | | | | |
| SPINT1 | | | | | | | | | |
| SPINT2 | | | | | | | | | |
| SPINT3 | | | | | | | | | |
| SPIRE1 | | | | | | | | | |

| | | | | | | | | | |
|---|---|---|---|---|---|---|---|---|---|
| Spire2 | | | | | | | | | |
| SPN | | | | | | | | | |
| SPNS1 | | | | | | | | | |
| SPNS2 | | | | | | | | | |
| SPNS3 | | | | | | | | | |
| SPO11 | | | | | | | | | |
| SPOCD1 | | | | | | | | | |
| SPOCK1 | | | | | + | | | | + |
| SPOCK2 | | | | | | + | | | + |
| SPOCK3 | | | | + | | | | | + |
| SPON1 | | | | | | + | | | + |
| SPON2 | | | | | | | | | |
| SPOP | + | | | + | + | | | | + |
| SPOPL | | | | | | | | | |
| SPP1 | | | | | | | | | |
| SPP2 | | | | | | | | | |
| spp27 | | | | | | | | | |
| SPP381 | | | | | | | | | |
| SPP382 | | | | | | | | | |
| SPPL2A | | | | | | | | | |
| SPPL2B | | | | | | | | | |
| SPPL2C | | | | | | | | | |
| SPPL3 | | | | | + | | | | + |
| SPR | | | | | | | | | |
| SPRED1 | | | + | | | | | | + |
| SPRED2 | | | + | | | | | | + |
| SPRED3 | | | | | | | | | |
| SPRN | | | | | | | | | |
| SPRR1A | | | | | | | | | |
| SPRR1B | | | | | | | | | |
| SPRR2A | | | | | | | | | |
| SPRR2B | | | | | | | | | |
| SPRR2C | | | | | | | | | |
| SPRR2D | | | | | | | | | |
| SPRR2E | | | | | | | | | |
| SPRR2F | | | | | | | | | |
| SPRR2G | | | | | | | | | |
| SPRR3 | | | | | | | | | |
| SPRR4 | | | | | | | | | |
| SPRTN | | | | | | | | | |
| SPRY1 | | | | | | | | | |
| SPRY2 | + | | | + | + | | | | + |
| SPRY3 | | | | | | | | | |
| SPRY4 | | | + | | | | | | + |
| SPRYD3 | | | | | | | | | |
| SPRYD4 | | | | | | | | | |
| SPRYD7 | | | | | | | | | |
| SPS1 | | | | | | | | | |
| SPS100 | | | | | | | | | |
| SPSB1 | + | | + | | + | | | | + |
| SPSB2 | | | | | | | | | |
| SPSB3 | | | | | | | | | |
| SPSB4 | | | | | | | | | |

| | | | | | | | | | |
|---|---|---|---|---|---|---|---|---|---|
| SPT15 | | | | | | | | | |
| SPT16 | | | | | | | | | |
| SPT2 | | | | | | | | | |
| SPT23 | | | | | | | | | |
| SPT4 | | | | | | | | | |
| spt5 | | | | | | | | | |
| SPT7 | | | | | | | | | |
| SPT8 | | | | | | | | | |
| SPTA1 | | | | | | | | | |
| SPTAN1 | | | | | | | | | |
| SPTB | | | | | | | | | |
| SPTBN1 | | + | | | | + | | | + |
| SPTBN2 | + | | | | + | | | | + |
| SPTBN4 | | | | | | | | | |
| SPTBN5 | | | | | | | | | |
| SPTLC1 | | | + | | | | | | + |
| Sptlc2 | | | | | | | | | |
| SPTLC3 | | | | | | | | | |
| SPTSSA | | | | | | | | | |
| SPTY2D1 | | | + | | | | | | + |
| SPX | | | | | | | | | |
| SPZ1 | | | | | | | | | |
| SQLE | | | | | | | | | |
| SQRDL | | | | | | | | | |
| SQSTM1 | | | | | + | | | | + |
| SQT1 | | | | | | | | | |
| SRA1 | | | | | | | | | |
| SRBD1 | | | | | | | | | |
| SRC | | | | | | + | | | + |
| SRCAP | | | | | | | | | |
| SRCIN1 | | | | | | | | | |
| SRD5A1 | | | | | | | | | |
| SRD5A2 | | | | | | | | | |
| SRD5A3 | + | | | + | + | + | + | | + |
| SREBF1 | | | | | | | | | |
| SREBF2 | | | + | | | | | | + |
| SREK1 | | | | | | | | | |
| SREK1IP1 | | | | | | | | | |
| SRF | | | | | | | | | |
| SRFBP1 | | | | | | | | | |
| SRGAP1 | | | | | | | | | |
| SRGAP2 | | | + | | | | | | + |
| SRGAP2B | | | | | | | | | |
| SRGAP2C | | | | | | | | | |
| SRGAP3 | | + | + | | | + | | | + |
| SRGN | | | | | | | | | |
| srgp-1 | | | | | | | | | |
| SRI | | | | | | | | | |
| SRL | | | | | | | | | |
| SRL2 | | | | | | | | | |
| SRM | | | | | | | | | |
| SRMS | | | | | | | | | |
| SRP1 | | | | | | | | | |

| | | | | | | | | | |
|---|---|---|---|---|---|---|---|---|---|
| SRP102 | | | | | | | | | |
| SRP14 | | | | | | | | | |
| SRP19 | | | | | | | | | |
| srp2 | | | | | | | | | |
| SRP21 | | | | | | | | | |
| SRP54 | | | | | | | | | |
| Srp54a | | + | + | | | + | | | + |
| SRP68 | | | | | | | | | |
| SRP72 | | | | | | | | | |
| SRP9 | | | | | | | | | |
| SRP9P1 | | | | | | | | | |
| SRPK1 | | | | | | | | | |
| SRPK2 | | | | | | | | | |
| SRPK3 | | | | | | | | | |
| SRPR | | + | | | | + | | | + |
| SRPRB | | | | | | | | | |
| SRPX | | | | | | | | | |
| SRR | | | + | | | | | | + |
| SRRD | | | | | | | | | |
| SRRM1 | | | | | | | | | |
| SRRM2 | | | | | | | | | |
| SRRM3 | | | | + | | | | | + |
| SRRM4 | | | | | | | | | |
| SRRM5 | | | | | | | | | |
| SRRT | | | | | | | | | |
| SRSF1 | | + | | | | + | | | + |
| SRSF10 | | | | | | | | | |
| SRSF11 | | | | | | | | | |
| SRSF12 | | | + | | | | | | + |
| SRSF2 | | | | | | | | | |
| SRSF3 | | | | | | | | | |
| SRSF4 | | | | | | | | | |
| SRSF5 | | | | | | | | | |
| SRSF6 | | | | | | | | | |
| SRSF7 | | | | | | | | | |
| SRSF8 | | | | | | | | | |
| SRSF9 | | | | | | + | | | + |
| SRV2 | | | | | | | | | |
| SRXN1 | + | | + | | + | | | | + |
| SRY | | | | | | | | | |
| SS18 | | | | | | | | | |
| SS18L1 | | | | | | | | | |
| SS18L2 | | | | | | | | | |
| SSA1 | | | | | | | | | |
| SSA2 | | | | | | | | | |
| SSA3 | | | | | | | | | |
| SSB | | | | | + | | | | + |
| SSB1 | | | | | | | | | |
| SSB2 | | | | | | | | | |
| SSBP1 | | | | | | | | | |
| SSBP2 | | | | | | + | | | + |
| SSBP3 | + | | | + | + | | | | + |
| SSBP4 | | | | | | | | | |

| | | | | | | | | | |
|---|---|---|---|---|---|---|---|---|---|
| SSC1 | | | | | | | | | |
| SSC4D | | | | | | | | | |
| SSC5D | | | | | | | | | |
| SSE1 | | | | | | | | | |
| SSE2 | | | | | | | | | |
| SSFA2 | | | | | | | | | |
| SSH1 | | + | | | | | | | + |
| SSH2 | | | | | + | | | | + |
| SSH3 | | | | | | | | | |
| SSM4 | | | | | | | | | |
| SSMEM1 | | | | | | | | | |
| SSN2 | | | | | | | | | |
| SSN3 | | | | | | | | | |
| SSNA1 | | | | | | | | | |
| SSO1 | | | | | | | | | |
| SSO2 | | | | | | | | | |
| SSP1 | | | | | | | | | |
| SSP120 | | | | | | | | | |
| ssp2 | | | | | | | | | |
| SSPN | | | | | + | | | | + |
| SSPO | | | | | | | | | |
| SSR1 | | | + | | | | | | + |
| SSR2 | | | | | | | | | |
| SSR3 | | | | | | | | | |
| SSR4 | | | | | | | | | |
| SSR4P1 | | | | | | | | | |
| SSRP1 | | + | | | | + | | | + |
| SSS1 | | | | | | | | | |
| SSSCA1 | | | | | | | | | |
| SST | | | | | + | | | | + |
| sst2 | | | | | | | | | |
| Sstr1 | | | | | | | | | |
| SSTR2 | | | | | | | | | |
| SSTR3 | | | | | + | | | | + |
| SSTR4 | | | | | | | | | |
| SSTR5 | | | | | | | | | |
| SSU72 | | | | | | | | | |
| SSUH2 | | | | | | | | | |
| SSX1 | | | | | | | | | |
| SSX2 | | | | | | | | | |
| SSX2B | | | | | | | | | |
| SSX2IP | | | | | | | | | |
| SSX3 | | | | | | | | | |
| SSX4 | | | | | | | | | |
| SSX4B | | | | | | | | | |
| SSX5 | | | | | | | | | |
| SSX6 | | | | | | | | | |
| SSX7 | | | | | | | | | |
| SSX9 | | | | | | | | | |
| SSZ1 | | | | | | | | | |
| ST13 | | | + | | | | | | + |
| ST13P4 | | | | | | | | | |
| ST13P5 | | | | | | | | | |

| | | | | | | | | | |
|---|---|---|---|---|---|---|---|---|---|
| ST14 | | | | | | | | | |
| ST18 | | | | | | + | | | + |
| ST20 | | | | | | | | | |
| ST20-AS1 | | | | | | | | | |
| ST3 | | | | | | | | | |
| ST3GAL1 | | | | | | + | | | + |
| ST3GAL2 | | | | | | | | | |
| ST3GAL3 | | | | | | | | | |
| ST3GAL4 | | | | | | | | | |
| ST3GAL5 | | | | | | | | | |
| ST3GAL6 | | | | | | | | | |
| ST5 | | | | | | + | | | + |
| ST6GAL1 | | | | | | | | | |
| ST6GAL2 | | | | | | | | | |
| ST6GALNAC1 | | | | | | | | | |
| ST6GALNAC2 | | | | | | | | | |
| ST6GALNAC3 | | | | | | | | | |
| ST6GALNAC4 | | | | | | | | | |
| ST6GALNAC5 | | + | | | | + | | | + |
| ST6GALNAC6 | | | | | | + | | | + |
| ST7 | | | | | | | | | |
| ST7-OT3 | | | | | | | | | |
| ST7-OT4 | | | | | | | | | |
| ST7L | | + | | | | + | | | + |
| ST8SIA1 | | | | | | | | | |
| ST8SIA2 | | | | | | | | | |
| ST8SIA3 | | | | | | | | | |
| ST8SIA4 | | | | | | | | | |
| ST8SIA5 | | | | | | | | | |
| ST8SIA6 | | | | | | | | | |
| STAB1 | | | | | | | | | |
| STAB2 | | | | | + | | | | + |
| STAC | | | + | | | | | | + |
| STAC2 | | | | | | | | | |
| STAC3 | | | | | | | | | |
| STAG1 | | | | | | | | | |
| STAG2 | | | | | | | | | |
| STAG3 | | | | | | | | | |
| STAG3L1 | | | | | | | | | |
| STAG3L3 | | | | | | | | | |
| STAG3L4 | | | | | | | | | |
| STAG3L5P | | | | | | | | | |
| STAM | | | | | | | | | |
| STAM2 | | | | | | + | | | + |
| STAMBP | | | | | | | | | |
| STAMBPL1 | | | | | | | | | |
| STAP1 | | | | | | | | | |
| STAP2 | | | | | | | | | |
| STAR | | | | | | | | | |
| STARD10 | | | | | | | | | |
| STARD13 | | | | | | + | | | + |
| STARD3 | | | | | | | | | |
| STARD3NL | | | | | | | | | |

| | | | | | | | | | |
|---|---|---|---|---|---|---|---|---|---|
| STARD4 | | | + | | | | | | + |
| STARD5 | | | | | | + | | | + |
| STARD6 | | | | | | | | | |
| STARD7 | | | + | | | | | | + |
| STARD7-AS1 | | | | | | | | | |
| STARD8 | | | | | | | | | |
| STARD9 | | | | | | | | | |
| STAT1 | | | | | | + | | | + |
| STAT2 | | | | | | | | | |
| STAT3 | | | | | | | | | |
| STAT4 | | | | | | | | | |
| STAT5A | | | | | | | | | |
| STAT5B | | | | | | | | | |
| STAT6 | | | | | | | | | |
| Stat92E | | | | | | | | | |
| STATH | | | | | | | | | |
| STAU1 | | | | | | | | | |
| STAU2 | | | | | | | | | |
| STBD1 | | | | | | | | | |
| STC1 | + | | | + | + | | + | | + |
| STC2 | | | + | | | | | | + |
| STE11 | | | | | | | | | |
| ste13 | | | | | | | | | |
| STE20 | | | | | | | | | |
| STE23 | | | | | | | | | |
| STE4 | | | | | | | | | |
| STE7 | | | | | | | | | |
| STEAP1 | | | | | | | | | |
| STEAP1B | | | | | | | | | |
| STEAP2 | | | | | | | | | |
| STEAP3 | | | | | | | | | |
| STEAP4 | | | | | | | | | |
| STF1 | | | | | | | | | |
| STH | | | | | | | | | |
| STH1 | | | | | | | | | |
| STI1 | | | | | | | | | |
| STIL | | | | | | | | | |
| STIM1 | | | | | | | | | |
| STIM2 | | | | | | | | | |
| STIP1 | | | + | | | | | | + |
| STK10 | | | | | | | | | |
| STK11 | | | | | | | | | |
| STK11IP | | | | | | | | | |
| STK16 | | | | | | | | | |
| STK17A | | | | | | | | | |
| STK17B | | | | | | | | | |
| STK19 | | | | | | | | | |
| STK24 | | | | | | | | | |
| STK25 | | | | | | | | | |
| STK26 | | | | | | | | | |
| STK3 | | | | | | | | | |
| STK31 | | | | | | | | | |
| STK32A | | | | | | | | | |

| | | | | | | | | | |
|---|---|---|---|---|---|---|---|---|---|
| STK32B | | | | | | | | | |
| STK32C | | | + | | | + | | | + |
| STK33 | | | | | | | | | |
| STK35 | | | | | | | | | |
| STK36 | | | | | | | | | |
| STK38 | | | | | | | | | |
| STK38L | | | | | | | | | |
| STK39 | | | | | | | | | |
| STK4 | | | | | | | | | |
| STK40 | + | | | | + | | | | + |
| STKLD1 | | | | | | | | | |
| STM1 | | | | | | | | | |
| STMN1 | | | | | | | | | |
| STMN2 | | | | | + | | | | + |
| STMN3 | | + | | | | + | | | + |
| STMN4 | + | | | | + | | | | + |
| STOM | | | | | | | | | |
| STOML1 | | | | | | | | | |
| STOML2 | | | | | | | | | |
| STOML3 | | | | | | | | | |
| STON1 | | | | | | | | | |
| STON1-GTF2A1L | | | | | | | | | |
| STON2 | | | | | | | | | |
| STOX1 | | | | | | | | | |
| STOX2 | | | | | | | | | |
| STP2 | | | | | | | | | |
| STPG1 | | | | | | | | | |
| STPG2 | | | | | | | | | |
| STRA13 | | | | | | + | | | + |
| STRA6 | | | | | | | | | |
| STRA8 | + | | | + | + | | | | + |
| STRADA | | | | | | | | | |
| STRADB | | | | | | | | | |
| STRAP | | | | | | | | | |
| STRBP | | | | | | | | | |
| STRC | | | | | | | | | |
| STRIP1 | | | | | | | + | | + |
| STRIP2 | | | | | | | | | |
| STRN | | | | | | | | | |
| STRN3 | | | | | | | | | |
| STRN4 | | | | + | | | | | + |
| STS | | | | | | | | | |
| STT3A | | | | | + | | | | + |
| STT3B | | | | | | | | | |
| stt4 | | | | | | | | | |
| STUB1 | | | | | | | | | |
| STX10 | | | | | | | | | |
| STX11 | | | | | | | | | |
| STX12 | | | | | | | | | |
| STX16 | | | | | | | | | |
| STX16-NPEPL1 | | | | | | | | | |
| STX17 | | | | | | | | | |
| STX18 | | | | | | | | | |

| | | | | | | | | | |
|---|---|---|---|---|---|---|---|---|---|
| STX19 | | | | | | | | | |
| STX1A | | | | | | | | | |
| STX1B | | | | | | | + | | + |
| STX2 | | | | | | | | | |
| STX3 | | | | | | | | | |
| STX4 | | | | | | | | | |
| STX5 | | | | | | | | | |
| STX6 | | | | | | | | | |
| STX7 | | | | | | | | | |
| STX8 | | | | | | | | | |
| STXBP1 | | + | | | | + | | | + |
| STXBP2 | | | | | | | | | |
| STXBP3 | | | | | | | | | |
| STXBP4 | | | | + | | + | | | + |
| STXBP5 | | | + | | | | | | + |
| STXBP5L | | | | | + | | | | + |
| STXBP6 | | | | | | + | | | + |
| STYK1 | | + | + | | | + | | + | + |
| STYX | | | | | | | | | |
| STYXL1 | | | | | | | | | |
| sua1 | | | | | | | | | |
| SUB1 | | | | | | | | | |
| suc1 | | | | | | | | | |
| SUC2 | | | | | | | | | |
| SUCLA2 | | | | | | | | | |
| SUCLG1 | | | | | | | | | |
| SUCLG2 | + | | | + | + | | + | | + |
| SUCNR1 | | | | | | | | | |
| SUCO | + | | | | + | | | | + |
| SUDS3 | | | | | | | | | |
| SUFU | | | | | | | | | |
| SUGCT | | | | | | | | | |
| SUGP1 | | + | | | | | | | + |
| SUGP2 | | | | | + | | | | + |
| SUGT1 | | | | | | | | | |
| SUI2 | | | | | | | | | |
| SUI3 | | | | | | | | | |
| SULF1 | | | | | | | | | |
| SULF2 | | | | | | | | | |
| SULT1A1 | | | | | | | | | |
| SULT1A2 | | | | | | | | | |
| SULT1A3 | | | | | | | | | |
| SULT1A4 | | | | | | | | | |
| SULT1B1 | | | | | | | | | |
| SULT1C2 | | | | | | | | | |
| SULT1C3 | | | | | | | | | |
| SULT1C4 | | | | | | | | | |
| SULT1E1 | | | | | | | | | |
| SULT2A1 | | | | | | | | | |
| SULT2B1 | + | | | | + | | | | + |
| SULT4A1 | | | + | | | | + | | + |
| SULT6B1 | | | | | | | | | |
| sum1 | | | | | | | | | |

| | | | | | | | | | |
|---|---|---|---|---|---|---|---|---|---|
| sum3 | | | | | | | | | |
| SUMF1 | | | | | | + | | | + |
| SUMF2 | | | | | | | | | |
| SUMO1 | | | | | | | | | |
| SUMO1P1 | | | | | | | | | |
| SUMO1P3 | | | | | | | | | |
| SUMO2 | | + | | | | + | | | + |
| SUMO2P1 | | | | | | | | | |
| SUMO3 | | + | | | | + | | | + |
| SUMO4 | | | | | | | | | |
| SUN1 | | | | | | | | | |
| SUN2 | | | | | | | | | |
| SUN3 | | | | | | | | | |
| SUN5 | | | | | | | | | |
| SUOX | | | | | | | | | |
| SUP35 | | | | | | | | | |
| SUP45 | | | | | | | | | |
| SUPT16H | | | | | | | | | |
| SUPT16HP1 | | | | | | | | | |
| SUPT20H | | | | | | | | | |
| SUPT20HL1 | | | | | | | | | |
| SUPT3H | | | | | | | | | |
| SUPT4H1 | | | | | | | | | |
| Supt5 | | | | | + | | | | + |
| SUPT5H | | | | | | | | | |
| Supt6 | | | | | | | | | |
| SUPT6H | | | | | | | | | |
| SUPT7L | | | | | | | | | |
| SUPV3L1 | | | | | | | | | |
| SURF1 | | | | | | | | | |
| SURF2 | | | | | | | | | |
| SURF4 | | | | | | | | | |
| SURF6 | | | | | | | | | |
| SUSD1 | | | | | | + | | | + |
| SUSD2 | | | | | | | | | |
| SUSD3 | | | | | | | | | |
| SUSD4 | | | | | | | | | |
| SUSD5 | | | | | | | | | |
| SUSD6 | | | | | | | | | |
| SUV3 | | | | | | | | | |
| SUV39H1 | | | | | | | | | |
| SUV39H2 | | | + | | | | | | + |
| SUV420H1 | | | + | | | | | | + |
| SUV420H2 | | | | | | + | | | + |
| SUZ12 | | | | | | | | | |
| SUZ12P1 | | | | | | | | | |
| SV2A | | | | | | | | | |
| SV2B | | + | | | | + | | | + |
| SV2C | + | | | + | + | | | | + |
| SV40gp6 | | | | | | | | | |
| SVEP1 | | | | | | | | | |
| SVF1 | | | | | | | | | |
| SVIL | + | | + | | + | | | | + |

| | | | | | | | | | |
|---|---|---|---|---|---|---|---|---|---|
| SVIP | | | | | | + | | | + |
| SVOP | | | + | | | | | | + |
| SVOPL | | | | | | | | | |
| svp | | | | | | | | | |
| SWAP70 | | | | | | | | | |
| SWC5 | | | | | | | | | |
| SWC7 | | | | | | | | | |
| SWE1 | | | | | | | | | |
| SWI5 | | + | | | | + | | | + |
| SWR1 | | | | | | | | | |
| SWSAP1 | | | | | | | | | |
| SWT1 | | | | + | | + | | | + |
| SXM1 | | | | | | | | | |
| SYAP1 | | | | | | | | | |
| SYBU | | | | | | | | | |
| SYCE1 | | | | | | | | | |
| SYCE1L | | | | | | | | | |
| Syce2 | | | | | | | | | |
| SYCE3 | | | | | | | | | |
| SYCN | | | | | | | | | |
| SYCP1 | | | | | | | | | |
| SYCP2 | | + | + | | | + | | | + |
| SYCP2L | | | | | | | | | |
| SYCP3 | | | | | | | | | |
| syd-2 | | | | | | | | | |
| SYDE1 | | | | | | | | | |
| SYDE2 | | | | | | | | | |
| SYF2 | | | | | | | | | |
| SYH1 | | | | | | | | | |
| SYK | | | | | | | | | |
| SYMPK | | | | | | | | | |
| Syn | | | | | | | | | |
| SYN1 | | | + | | | | | | + |
| SYN2 | | | + | | | | | | + |
| SYN3 | | | | | + | | | | + |
| SYNC | | | | | | | | | |
| SYNCRIP | | | | | | | | | |
| SYNDIG1 | | | | | | | | | |
| SYNE1 | | | | | + | | | | + |
| SYNE2 | | | | | | + | | | + |
| SYNE3 | | | | | | | | | |
| SYNE4 | | | | | | | | | |
| SYNGAP1 | | | | | + | | | | + |
| SYNGR1 | | | + | | | | | | + |
| SYNGR2 | | | | | | | | | |
| SYNGR3 | + | | | | + | | | | + |
| SYNGR4 | | | | | | | | | |
| Synj | | | | | | | | | |
| SYNJ1 | | | | | + | | | | + |
| SYNJ2 | | | | | | | | | |
| SYNJ2BP | | | | | | | | | |
| SYNJ2BP-COX16 | | | | | | | | | |
| SYNM | | | | | | | | | |

| | | | | | | | | | |
|---|---|---|---|---|---|---|---|---|---|
| SYNPO | + | | | | + | | | | + |
| SYNPO2 | | | | | | | | | |
| SYNPO2L | + | | | + | + | | | | + |
| SYNPR | | | | | | | | | |
| SYNRG | | | | | | | | | |
| SYP | | | | | | + | | | + |
| SYP1 | | | | | | | | | |
| SYPL1 | | | | | | | | | |
| SYPL2 | | | | | | | | | |
| SYS1 | | | | | | | | | |
| SYT1 | | | | | | | | | |
| SYT10 | | | | | | + | | | + |
| SYT11 | | | | | + | | | | + |
| SYT12 | | | | | | | | | |
| SYT13 | | | + | | | + | + | | + |
| SYT14 | | | | | | | | | |
| SYT15 | | | | | | | | | |
| SYT16 | | | | | | | | | |
| SYT17 | | | | | | | | | |
| SYT2 | | | | | | | | | |
| SYT3 | | | | | | | | | |
| SYT4 | + | | + | | | | | | + |
| SYT5 | | | | | | | | | |
| SYT6 | | | | | | | | | |
| SYT7 | | | | | | | | | |
| SYT8 | | | | | | | | | |
| SYT9 | | | | | | | | | |
| SYTL1 | | | | | | | | | |
| SYTL2 | | | | | | | | | |
| SYTL3 | | | | | | | | | |
| SYTL4 | | | | | | | | | |
| SYTL5 | | | | | | | | | |
| SYVN1 | | | | | | | | | |
| SZRD1 | | | | | | | | | |
| SZT2 | | | | | | | | | |
| T | | | | | | | | | |
| T-cp1 | | | | | | | | | |
| TAAR1 | | | | | | | | | |
| TAAR2 | | | | | | | | | |
| TAAR3 | | | | | | | | | |
| TAAR9 | | | | | | | | | |
| TAB1 | | | | | | | | | |
| TAB2 | | | | | | | | | |
| TAB3 | | | | | + | | | | + |
| TAC1 | | | + | | | | | | + |
| TAC3 | | | | | | | | | |
| TAC4 | | | | | | | | | |
| TACC1 | + | | | | + | | | | + |
| TACC2 | | | | | | | | | |
| TACC3 | | | | | | | | | |
| TACO1 | | | | | | | | | |
| TACR1 | | | | | | | | | |
| TACR2 | | | | | | | | | |

| | | | | | | | | | |
|---|---|---|---|---|---|---|---|---|---|
| TACR3 | | | | | | | | | |
| TACSTD2 | | | | | | | | | |
| TADA1 | | | | | | | | | |
| TADA2A | | | | | | | | | |
| TADA2B | | | | | | | | | |
| TADA3 | | | | | | + | | | + |
| TAE1 | | | | | | | | | |
| TAF1 | | | | | | | | | |
| TAF10 | | | | | | | | | |
| TAF11 | | | | | | | | | |
| TAF12 | | | | | | | | | |
| TAF13 | | | + | | | | | | + |
| TAF14 | | | | | | | | | |
| TAF15 | | | | + | | | | | + |
| TAF1A | | | | | | | | | |
| TAF1B | | | | | | | | | |
| TAF1C | | | | | | | | | |
| TAF1D | | | | | | | | | |
| TAF1L | | | | | | | | | |
| TAF2 | | | | | | | | | |
| TAF3 | | | | | | | | | |
| TAF4 | | | | | | | | | |
| TAF4B | | | | | | | | | |
| TAF5 | | | | | | | | | |
| TAF5L | | | | | | | | | |
| TAF6 | | | | | | | | | |
| TAF6L | | | | | | | | | |
| TAF7 | + | | | | | | | | + |
| TAF7L | | | | | | | | | |
| TAF8 | | | | | | | | | |
| TAF9 | | | | | | | | | |
| TAF9B | | | | | | | | | |
| Tagap | | | | | | | | | |
| TAGLN | | | | | | | | | |
| TAGLN2 | + | | | + | + | | + | | + |
| TAGLN3 | | | + | | | | | | + |
| TAH1 | | | | | | | | | |
| TAH11 | | | | | | | | | |
| TAH18 | | | | | | | | | |
| Tak1 | | | | | | | | | |
| TAL1 | | | | | | | | | |
| TAL2 | | | | | | | | | |
| TALDO1 | | | | | | | | | |
| tam10 | | | | | | | | | |
| TAMM41 | | | | | | | | | |
| TANC1 | + | | + | | + | | | | + |
| TANC2 | | | | | | | | | |
| TANGO2 | | + | | | | + | | | + |
| TANGO6 | | | | | | | | | |
| TANK | | | + | | | | | | + |
| TAOK1 | | | | | | | | | |
| TAOK2 | | | | | | + | | | + |
| TAOK3 | | | | | | | | | |

| | | | | | | | | | |
|---|---|---|---|---|---|---|---|---|---|
| TAP1 | | | | | | | | | |
| TAP2 | | | | | | | | | |
| TAPBP | | | | | | | | | |
| TAPBPL | | | | | + | | | | + |
| Tapt1 | | | | | | | | | |
| TARBP1 | | | | | | | | | |
| TARBP2 | | | | | | + | | | + |
| TARDBP | | | + | | | | | | + |
| TARP | | | | | | | | | |
| TARS | | | | | + | | | | + |
| TARS2 | | | | | | | | | |
| TARSL2 | | | | | | | | | |
| TAS1R1 | | | | | | | | | |
| TAS1R2 | | | | | | | | | |
| TAS1R3 | | | | | + | | | | + |
| TAS2R10 | | | | | | | | | |
| TAS2R13 | | | | | | | | | |
| TAS2R14 | | | | | | | | | |
| TAS2R19 | | | | | | | | | |
| TAS2R20 | | | | | | | | | |
| TAS2R3 | | | | | | | | | |
| TAS2R31 | | | | | | | | | |
| TAS2R38 | | | | | | | | | |
| TAS2R39 | | | | | | | | | |
| TAS2R41 | | | | | | | | | |
| TAS2R42 | | | | | | | | | |
| TAS2R5 | | | | | | | | | |
| TAS2R50 | | | | | | | | | |
| TAS2R60 | | | | | | | | | |
| TAS2R62P | | | | | | | | | |
| TAS2R7 | | | | | | | | | |
| TAS2R8 | | | | | | | | | |
| TASP1 | | | | | | | | | |
| TAT | | | | | | | | | |
| TATDN1 | | | | | | | | | |
| TATDN2 | | | | | | | | | |
| TATDN3 | | | | | | | | | |
| TAX1BP1 | | | | | + | | | | + |
| TAX1BP3 | | | | | | | | | |
| TAZ | | | | | | + | | | + |
| TBATA | + | | | + | + | | | | + |
| tbc-8 | | | | | | | | | |
| TBC1D1 | | | + | | | | | | + |
| TBC1D10A | | | | | | | | | |
| TBC1D10B | | | | + | | | | | + |
| TBC1D10C | | | | | | | | | |
| TBC1D12 | | | | | | | | | |
| TBC1D13 | | | | | | | | | |
| TBC1D14 | | | | | | | | | |
| TBC1D15 | | | | | | | | | |
| TBC1D16 | | | | | | | | | |
| TBC1D17 | | | | | | | | | |
| TBC1D19 | | | | | | | | | |

| | | | | | | | | | |
|---|---|---|---|---|---|---|---|---|---|
| TBC1D2 | | | | | | | | | |
| TBC1D20 | | | | | | | | | |
| TBC1D21 | | | | | | | | | |
| TBC1D22A | | | | | | | | | |
| TBC1D22B | | | | | | | | | |
| TBC1D23 | | | | | | | | | |
| TBC1D24 | | | | | | | | | |
| TBC1D25 | | | | | | | | | |
| TBC1D26 | | | | | | | | | |
| TBC1D27 | | | | | | | | | |
| TBC1D28 | | | | | | | | | |
| TBC1D2B | | | | | | | | | |
| TBC1D3 | | | | | | | | | |
| TBC1D30 | | | | | | | | | |
| TBC1D31 | | | | | | | | | |
| TBC1D32 | | | | | | | | | |
| TBC1D3B | | | | | | | | | |
| TBC1D3C | | | | | | | | | |
| TBC1D3F | | | | | | | | | |
| TBC1D3H | | | | | | | | | |
| TBC1D3K | | | | | | | | | |
| TBC1D3L | | | | | | | | | |
| TBC1D3P2 | | | | | | | | | |
| TBC1D4 | | | | | | | | | |
| TBC1D5 | | | | | | | | | |
| TBC1D7 | | | | | | | | | |
| TBC1D8 | | | | | | | | | |
| TBC1D8B | | | | | | + | | | + |
| TBC1D9 | | | + | | + | | | | + |
| TBC1D9B | | | | | | | | | |
| TBCA | | | | | | | | | |
| TBCB | | | | | | | | | |
| TBCC | | | | | | | | | |
| TBCCD1 | | | | | | | | | |
| TBCD | | | | | | | | | |
| TBCE | | | | | | | | | |
| TBCEL | | | | | | | | | |
| TBCK | | | | | | | | | |
| TBK1 | | | | | | | | | |
| TBKBP1 | | | | | | | | | |
| TBL1X | | | | | | | | | |
| TBL1XR1 | | | | | | | | | |
| TBL1Y | | | | | | | | | |
| TBL2 | | | | | | | | | |
| TBL3 | | | | | | | | | |
| TBP | | | | | | | | | |
| TBPL1 | | | + | | | | | | + |
| TBPL2 | | | | | | | | | |
| TBR1 | | | | | | | | | |
| TBRG1 | | | | | | | | | |
| TBRG4 | | | | | | | | | |
| TBX1 | | | | | | | | | |
| TBX10 | | | | | | | | | |

| | | | | | | | | | |
|---|---|---|---|---|---|---|---|---|---|
| TBX15 | + | | | + | + | | + | | + |
| TBX18 | + | | | + | + | | + | | + |
| TBX19 | | | | | | | | | |
| TBX2 | | | | | | | | | |
| TBX20 | | | | | | | | | |
| TBX21 | | | | | | | | | |
| TBX22 | | | | | | | | | |
| TBX3 | | | | | | | + | | + |
| TBX4 | | | | | | | | | |
| TBX5 | + | | | + | + | | | | + |
| TBX6 | | | | | | | | | |
| TBXA2R | | | | | | | | | |
| TBXAS1 | | | | | | | | | |
| TC2N | | | | | | | | | |
| TCAIM | | | | | | | | | |
| TCAP | | | | | | | | | |
| TCB1 | | | | | | | | | |
| TCB3 | | | | | | | | | |
| TCD1 | | | | | | | | | |
| TCEA1 | | | | | | | | | |
| TCEA2 | | | | | | | | | |
| TCEA3 | | | | | | | | | |
| TCEAL1 | | | | | | | | | |
| TCEAL2 | | | | | | | | | |
| Tceal3 | | | | | | + | | | + |
| TCEAL4 | | | | | | | | | |
| TCEAL5 | | | + | | | | | | + |
| TCEAL6 | | | | | | | | | |
| TCEAL7 | | | | | | | | | |
| TCEAL8 | | | | | | | | | |
| TCEANC | | | | | | | | | |
| TCEANC2 | | | | | | + | | | + |
| TCEB1 | | | | | | | | | |
| TCEB1P3 | | | | | | | | | |
| TCEB2 | | | | | | | | | |
| TCEB3 | | | | | | | | | |
| TCEB3B | | | | | | | | | |
| TCEB3C | | | | | | | | | |
| TCEB3CL | | | | | | | | | |
| TCEB3CL2 | | | | | | | | | |
| tcer-1 | | | | | | | | | |
| TCERG1 | | | | | | | | | |
| TCERG1L | | | + | | | | | | + |
| TCF12 | | | | | | | | | |
| TCF15 | | | | | | | | | |
| TCF19 | | | | | | | | | |
| TCF20 | | | | | | | | | |
| TCF21 | | | | | | | | | |
| TCF23 | | | | | + | | | | + |
| TCF24 | | | | | | | | | |
| TCF25 | | | | | | | | | |
| TCF3 | | | | | | | | | |
| TCF4 | | + | | | | + | | | + |

| | | | | | | | | | |
|---|---|---|---|---|---|---|---|---|---|
| TCF7 | | | | | | | | | |
| TCF7L1 | | | | | | | | | |
| tcf7l1a | | | | | | | | | |
| TCF7L2 | + | | | + | + | | | | + |
| TCFL5 | | | | | | | | | |
| TCHH | | | | | | | | | |
| TCHHL1 | | | | | | | | | |
| TCHP | | | | | | | | | |
| TCIRG1 | | | | + | | | | | + |
| TCL1A | | | | | | | | | |
| TCL1B | | | | | | | | | |
| TCL6 | | | | | | | | | |
| TCN1 | | | | | | | | | |
| TCN2 | | | | | | | | | |
| TCOF1 | + | | | | | | | | + |
| Tcp-1eta | | | | | | | | | |
| Tcp-1zeta | | | | | | | | | |
| TCP1 | | | | | | | | | |
| TCP10 | | | | | | | | | |
| TCP10L | | | | | | | | | |
| TCP10L2 | | | | | | | | | |
| TCP11 | | | | | | | | | |
| TCP11L1 | | | | | | | | | |
| TCP11L2 | + | | | + | + | | + | | + |
| TCP11X2 | | | | | | | | | |
| TCPT | | | | | | | | | |
| TCTA | | | | | + | + | | | + |
| TCTE3 | | | | | | | | | |
| TCTEX1D1 | | | | | | | | | |
| TCTEX1D2 | | | | | | + | | | + |
| TCTEX1D4 | | | | | | | | | |
| TCTN1 | | | | | | | | | |
| TCTN2 | | | | | | | | | |
| TCTN3 | | | | | | | | | |
| TDA2 | | | | | | | | | |
| TDG | | | | | | | + | | + |
| TDGF1 | | | | | | | | | |
| TDH | | | | | | | | | |
| TDH1 | | | | | | | | | |
| TDH2 | | | | | | | | | |
| TDH3 | | | | | | | | | |
| TDO2 | | | | | | | | | |
| TDP1 | | | | | | + | | | + |
| TDP2 | | | | | | | | | |
| TDRD1 | + | | | + | + | | | | + |
| TDRD10 | | | | | | | | | |
| TDRD12 | | | | | | | | | |
| TDRD15 | | | | | | | | | |
| TDRD3 | | | | | | | | | |
| TDRD5 | | | | | | | | | |
| TDRD6 | | | | | | | | | |
| TDRD7 | | | | | | | | | |
| TDRD9 | | | | | | | | | |

| | | | | | | | | | |
|---|---|---|---|---|---|---|---|---|---|
| TDRKH | | | | | | | | | |
| Tdrp | | | | | | | | | |
| TEA1 | | | | | | | | | |
| TEAD1 | | | + | | | | | | + |
| TEAD2 | | | | | | | | | |
| TEAD3 | | | | | | | | | |
| TEAD4 | | | | | | | | | |
| TEC | | | | | | | | | |
| TECPR1 | | | | | | + | | | + |
| Tecpr2 | | | | | | + | | | + |
| TECR | | | | | | | | | |
| TECRL | | | | | | | | | |
| TECTA | + | | | | + | | | | + |
| TECTB | | | | | | | | | |
| TED1 | | | | | | | | | |
| TEDDM1 | | | | | | | | | |
| TEF | | + | | | | + | | | + |
| TEF1 | | | | | | | | | |
| tef102 | | | | | | | | | |
| TEF2 | | | | | | | | | |
| tef3 | | | | | | | | | |
| TEF4 | | | | | | | | | |
| TEFM | | | | | | | | | |
| TEK | | | | | | | | | |
| TEKT1 | | | | | | | | | |
| TEKT2 | | | | | | | | | |
| TEKT3 | | | | | | | | | |
| TEKT4 | | | | | | | | | |
| TEKT4P2 | | | | | | | | | |
| TEKT5 | | | | | | | | | |
| TEL2 | | | | | | | | | |
| TELO2 | | | | | | + | | | + |
| Ten1 | | | | | | | | | |
| TENC1 | | | | | | | | | |
| TENM1 | | | | | | + | | | + |
| TENM2 | | | | | | | | | |
| TENM3 | | | | | | | | | |
| TENM4 | | | | | | | | | |
| TEP1 | | | | | | | | | |
| TEPP | | | | | | | | | |
| TER94 | | | | | | | | | |
| TERC | | | | | | | | | |
| TERF1 | | | | | | | | | |
| TERF2 | | | | | | | | | |
| TERF2IP | | | | | | | | | |
| TERT | | | | | | | | | |
| TES | | | + | | | | | | + |
| TESC | | | | | | | | | |
| TESK1 | | | | | | | | | |
| TESK2 | | | | | | | | | |
| TESPA1 | | | | | | | | | |
| TET1 | | | | | | | | | |
| TET2 | | | | | | | | | |

| | | | | | | | | | |
|---|---|---|---|---|---|---|---|---|---|
| TET3 | + | | | | + | | | | + |
| TEX1 | | | | | | | | | |
| TEX10 | | | | | | | | | |
| TEX101 | | | | | | | | | |
| TEX11 | | | | | | | | | |
| TEX12 | | | | | | | | | |
| TEX13A | | | | | | | | | |
| TEX13B | | | | | | | | | |
| TEX14 | | | | | | | | | |
| TEX15 | | | | | | | | | |
| Tex16 | | | | | | | | | |
| TEX19 | | | | | | | | | |
| TEX2 | | | | | | | | | |
| TEX22 | | | | | | | | | |
| TEX261 | | | | | | + | | | + |
| TEX264 | | | | | | | | | |
| TEX28 | | | | | | | | | |
| TEX29 | | | | | | | | | |
| TEX30 | | | | | + | | | | + |
| TEX33 | | | | | | | | | |
| TEX35 | | | | | | | | | |
| TEX36 | | | | | | | | | |
| TEX37 | | | | | | | | | |
| TEX40 | | | | | | | | | |
| TEX43 | | | | | | | | | |
| TEX9 | | | | | | | | | |
| TF | | | | | | | | | |
| TFAM | | | | | | + | | | + |
| TFAP2A | + | | | + | + | | | | + |
| TFAP2B | | | | | | | | | |
| TFAP2C | | | | | | | | | |
| TFAP2D | | | | | | | | | |
| TFAP2E | | | | | | | | | |
| TFAP4 | | | | | | | | | |
| TFB1 | | | | | | | | | |
| TFB1M | | | | | | | | | |
| TFB2 | | | | | | | | | |
| TFB2M | | | | | | | | | |
| TFCP2 | | | | | | | | | |
| TFCP2L1 | | | | | | | | | |
| TFDP1 | | | | | | | | | |
| TFDP2 | | | | | | | | | |
| TFDP3 | | | | | | | | | |
| TFE3 | | | | | | | | | |
| TFEB | | | | | | | | | |
| TFEC | | | | | | | | | |
| TFF1 | | | | | | | | | |
| TFF2 | | | | | | | | | |
| TFF3 | | | | | | | | | |
| TFG | | | | | | | | | |
| tfiis | | | | | | | | | |
| TFIP11 | | | | | + | | | | + |
| TFP1 | | | | | | | | | |

| | | | | | | | | | |
|---|---|---|---|---|---|---|---|---|---|
| TFPI | | | | | | | | | |
| TFPI2 | | | | | | | | | |
| TFPT | | | | | | | | | |
| TFR2 | | | | | | | | | |
| TFRC | + | | | | | | | | + |
| TG | | | | | | | | | |
| TGDS | | | | | | | | | |
| TGFA | | | | | | + | | | + |
| TGFB1 | | | | | | | | | |
| TGFB1I1 | | | | | | | | | |
| TGFB2 | | | + | | | | | | + |
| TGFB3 | | | | | | | | | |
| TGFBI | | | | | | | | | |
| TGFBR1 | | | | | | | | | |
| TGFBR2 | | | | | | | | | |
| TGFBR3 | | | | | | | | | |
| TGFBRAP1 | | | | | | + | | | + |
| TGIF1 | | | | | | | | | |
| TGIF2 | | | | | | | | | |
| TGIF2-C20orf24 | | | | | | | | | |
| TGIF2LX | | | | | | | | | |
| TGIF2LY | | | | | | | | | |
| TGM1 | | | | | | | | | |
| TGM2 | | | | | | | | | |
| TGM3 | | | | | | | | | |
| TGM4 | | | | | | | | | |
| TGM5 | | | | | | | | | |
| TGM6 | | | | | | | | | |
| TGM7 | | | | | | | | | |
| TGOLN2 | | | | | | | | | |
| TGS1 | | | | | | | | | |
| th | | | | | | | | | |
| THADA | | | | | | | | | |
| THAP1 | | | + | | | | | | + |
| THAP10 | | | | | | | | | |
| THAP11 | | | | | | | | | |
| THAP2 | | | | | | | | | |
| THAP3 | | + | | | | + | | | + |
| THAP4 | | | | | | + | | | + |
| THAP5 | | | | | | | | | |
| THAP6 | | | | | | | | | |
| THAP7 | | | | | | | | | |
| THAP8 | | | | | | | | | |
| THAP9 | | | | | | | | | |
| THBD | | | | | | | | | |
| THBS1 | + | | | + | + | | | | + |
| THBS2 | | | | | | | | | |
| THBS3 | | | | | | | | | |
| THBS4 | | | | | | | | | |
| THEG | | | | | | | | | |
| THEGL | | | | | | | | | |
| THEM4 | | | | | | + | | | + |
| THEM5 | | | | | | | | | |

| | | | | | | | | | |
|---|---|---|---|---|---|---|---|---|---|
| Them6 | | | | | | | | | |
| THEMIS | | | | | | | | | |
| THEMIS2 | | | | | | | | | |
| thf1 | | | | | | | | | |
| THG1L | | | | | | | | | |
| thi2 | | | | | | | | | |
| THI20 | | | | | | | | | |
| THI7 | | | | | | | | | |
| THNSL1 | | | | | | | | | |
| THNSL2 | | | | | | | | | |
| THO1 | | | | | | | | | |
| THO2 | | | | | | | | | |
| THOC1 | | | | | | | | | |
| THOC2 | | | | | | | | | |
| THOC3 | | | | | | | | | |
| THOC5 | | | | | | | | | |
| THOC6 | | | | | | | | | |
| THOC7 | | | | | | | | | |
| THOP1 | | | | | | | | | |
| THP2 | | | | | | | | | |
| THPO | | | | | | | | | |
| THR1 | | | | | | | | | |
| THRA | | + | | | | + | | | + |
| THRAP3 | | | | | | | | | |
| THRB | | | | | | | | | |
| THRIL | | | | | | | | | |
| THRSP | | | | | | | | | |
| THS1 | | | | | | | | | |
| THSD1 | | | | | | | | | |
| THSD4 | | | | | | | | | |
| THSD7A | + | | | + | + | | | | + |
| THSD7B | | | | | | | | | |
| THTPA | | | | | | | | | |
| THUMPD1 | | | | | | | | | |
| THUMPD2 | | | | | | + | | | + |
| THUMPD3 | | | | + | | | | | + |
| THUMPD3-AS1 | | | | | | | | | |
| THY1 | | | | | | | | | |
| THYN1 | | | | | | | | | |
| TIA1 | | + | | | | + | | | + |
| TIAF1 | | | | | | | | | |
| TIAL1 | | | | | | | | | |
| TIAM1 | | | | | + | | | | + |
| Tiam2 | | | | | | | | | |
| TICAM1 | | | | | | | | | |
| TICAM2 | | | | | | | | | |
| TICRR | | | | | | | | | |
| TIE1 | | | | | | | | | |
| TIF1 | | | | | | | | | |
| TIF11 | | | | | | | | | |
| tif211 | | | | | | | | | |
| tif212 | | | | | | | | | |
| tif213 | | | | | | | | | |

| | | | | | | | | | |
|---|---|---|---|---|---|---|---|---|---|
| tif224 | | | | | | | | | |
| tif225 | | | | | | | | | |
| tif32 | | | | | | | | | |
| tif33 | | | | | | | | | |
| TIF34 | | | | | | | | | |
| tif35 | | | | | | | | | |
| tif45 | | | | | | | | | |
| TIF4631 | | | | | | | | | |
| tif471 | | | | | | | | | |
| TIF5 | | | | | | | | | |
| tif51 | | | | | | | | | |
| tif512 | | | | | | | | | |
| TIFA | | | | | | | + | | + |
| TIGD1 | | | | | | | | | |
| TIGD2 | | | | | | | | | |
| TIGD3 | | + | + | | | + | | | + |
| TIGD4 | | | | | | | | | |
| TIGD5 | | | | | | | | | |
| TIGD6 | | | | | | | | | |
| TIGD7 | | | | | | | | | |
| TIGIT | + | | | + | + | | + | | + |
| TIM10 | | | | | | | | | |
| tim11 | | | | | | | | | |
| tim16 | | | | | | | | | |
| TIM23 | | | | | | | | | |
| TIMD4 | | | | | | | | | |
| TIMELESS | | | | | | | | | |
| TIMM10 | | | | | | | | | |
| TIMM10B | | + | | | | + | | | + |
| TIMM13 | | | | | | | | | |
| TIMM17A | | | | | | | | | |
| TIMM17B | | | | | | | | | |
| TIMM21 | | | | | | + | | | + |
| TIMM22 | | | | | | | | | |
| TIMM23 | | | | | | | | | |
| TIMM23B | | | | | | | | | |
| TIMM44 | | | | | | | | | |
| TIMM50 | | | | | | | | | |
| TIMM8A | | | | | | | | | |
| TIMM8B | | | | | | | | | |
| TIMM9 | | | | | | | | | |
| TIMMDC1 | | | | | | | | | |
| TIMP1 | | | | | | | | | |
| TIMP2 | + | | | | | + | | | + |
| TIMP3 | | | | | | | | | |
| TIMP4 | | | | | | | | | |
| TINAG | | | | | | | | | |
| TINAGL1 | | | | | | | | | |
| TINCR | | | | | | | | | |
| TINF2 | | | | | | | | | |
| TIP1 | | | | | | | | | |
| TIP41 | | | | | | | | | |
| TIPARP | + | + | + | | + | + | | | + |

| | | | | | | | | | |
|---|---|---|---|---|---|---|---|---|---|
| TIPIN | | | | | | | | | |
| TIPRL | | | | | | | | | |
| TIRAP | | | | + | | | | | + |
| TJAP1 | | | | | | | | | |
| TJP1 | | | | | | | | | |
| TJP2 | | | + | | + | | | | + |
| TJP3 | | | | | | | | | |
| TK1 | | | | | | | | | |
| TK2 | | | | | | | | | |
| TKL1 | | | | | | | | | |
| TKT | | | | | | | | | |
| TKTL1 | | | | | | | | | |
| TKTL2 | | | | | | | | | |
| TLCD1 | | | | | | | | | |
| TLCD2 | | | | | | | | | |
| TLDC1 | | | | | | | | | |
| TLDC2 | | | | | | | | | |
| TLE1 | | | | | | | | | |
| TLE2 | | | | | | | | | |
| TLE3 | | | | | | | | | |
| TLE4 | | | | | | + | | | + |
| TLE6 | | | | | + | | | | + |
| TLG2 | | | | | | | | | |
| TLK1 | | | | | | | | | |
| TLK2 | | | | | | | | | |
| TLL1 | + | | | + | + | | | | + |
| TLL2 | | | | | | | | | |
| TLN1 | | | | | | | | | |
| TLN2 | | | | | | + | | | + |
| TLR1 | | | | | | | | | |
| TLR10 | | | | | | | | | |
| TLR2 | | | | | | | | | |
| TLR3 | | | | | | | | | |
| TLR4 | | | | | | | | | |
| TLR5 | | | | | | | | | |
| TLR6 | | | | | | | | | |
| TLR7 | | | | | | | | | |
| TLR8 | | | | | | | | | |
| TLR9 | | | | | | | | | |
| TLX1 | | | | | | | | | |
| TLX2 | | | | | | | | | |
| TLX3 | | | | | | | | | |
| TM2D1 | | | | | | | | | |
| TM2D2 | | | | | | | | | |
| TM2D3 | | | | | | | | | |
| TM4SF1 | | | | | | | | | |
| TM4SF18 | | | | | | | | | |
| TM4SF19 | | | | | | | | | |
| TM4SF20 | | | | | | | | | |
| TM4SF4 | | | | | | | | | |
| TM4SF5 | | | | | | | | | |
| TM6SF1 | | | | + | | | | | + |
| TM6SF2 | | | | | | | | | |

| | | | | | | | | | |
|---|---|---|---|---|---|---|---|---|---|
| TM7SF2 | | | | | | | | | |
| TM7SF3 | | | | | | + | | | + |
| TM9SF1 | | | | | | | | | |
| TM9SF2 | | | | | | | | | |
| TM9SF3 | | | | | | | | | |
| TM9SF4 | | | | | | | | | |
| TMA16 | | | | + | + | | | | + |
| TMA20 | | | | | | | | | |
| TMA22 | | | | | | | | | |
| TMA7 | | | | | | | | | |
| TMBIM1 | | | | | | + | | | + |
| TMBIM4 | | | | | + | | | | + |
| TMBIM6 | | + | | | | + | | | + |
| TMC1 | | | | | | | | | |
| TMC2 | | | | | | | | | |
| TMC3 | | | | | | | | | |
| TMC4 | | | | | | | | | |
| TMC5 | | | | | | | | | |
| TMC6 | | | | | | | | | |
| TMC7 | + | | | + | + | + | + | | + |
| TMC8 | | | | | | | | | |
| TMCC1 | | | | | | | | | |
| TMCC1-AS1 | | | | | | | | | |
| TMCC2 | | | | | | | | | |
| TMCC3 | | | | | | | | | |
| TMCO1 | | | | | | + | | | + |
| TMCO2 | | | | | | | | | |
| TMCO3 | | | | | | | | | |
| TMCO4 | | | | | | | | | |
| TMCO5A | | | | | | | | | |
| TMCO6 | | | | | | | | | |
| TMED1 | | | | | | | | | |
| TMED10 | | | | | | | | | |
| TMED10P1 | | | | | | | | | |
| TMED2 | | | | | | | | | |
| TMED3 | | | | | | + | | | + |
| TMED4 | | + | | | | + | | | + |
| TMED5 | | | | | | | | | |
| TMED6 | | | | | | | | | |
| TMED7 | | | | | | | | | |
| TMED7-TICAM2 | | | | | | | | | |
| TMED8 | | | | | | | | | |
| TMED9 | | | | | | | | | |
| TMEFF1 | | | | | | | | | |
| TMEFF2 | | | | | | | | | |
| TMEM100 | | | | | | | | | |
| TMEM101 | | | | | | + | | | + |
| TMEM102 | | | | | | | | | |
| TMEM104 | | | | | | | | | |
| TMEM106A | | | | | | | | | |
| TMEM106B | | | | | | | | | |
| TMEM106C | | | | | | | | | |
| TMEM107 | | | | | | | | | |

| | | | | | | | | | |
|---|---|---|---|---|---|---|---|---|---|
| TMEM108 | | | | | | | | | |
| TMEM109 | | | | | | + | | | + |
| TMEM11 | | | | | | | | | |
| TMEM110 | | | | | | | | | |
| TMEM114 | | | | | | | | | |
| TMEM115 | | | | | | | | | |
| TMEM116 | | | | | | | | | |
| TMEM117 | | | | | | | | | |
| TMEM119 | | | | | | | | | |
| TMEM120A | | | | | | | | | |
| TMEM120B | | | | | | | | | |
| TMEM121 | | | | | | | | | |
| TMEM123 | | | | | | | | | |
| Tmem125 | | | | | | | | | |
| TMEM126A | | | | | | | | | |
| TMEM126B | | | | | | | | | |
| TMEM127 | | | | | | | | | |
| TMEM128 | | | | | | | | | |
| TMEM129 | | | | | | | | | |
| TMEM130 | | | | | | | | | |
| TMEM131 | | | | | | | | | |
| TMEM132A | | | | | | + | | | + |
| TMEM132B | | | | | + | | | | + |
| TMEM132C | + | | | + | + | | + | | + |
| TMEM132D | | | | | | | | | |
| TMEM132E | | | | | | | | | |
| TMEM134 | | | | | | + | | | + |
| TMEM135 | | | | | | | | | |
| TMEM136 | | | | | | | | | |
| TMEM138 | | | | | | | | | |
| TMEM139 | | | | | | | | | |
| TMEM140 | | | | | | | | | |
| TMEM141 | | | | | | | | | |
| TMEM143 | | | | | | | | | |
| TMEM144 | | | | | | | | | |
| TMEM145 | | | | | | | | | |
| TMEM147 | | | | | | | | | |
| TMEM14A | | | | | | + | | | + |
| TMEM14B | | | | | | | | | |
| TMEM14C | | | | | | | | | |
| TMEM14D | | | | | | | | | |
| TMEM150A | | | | | | | | | |
| TMEM150B | | | | | | | | | |
| TMEM150C | | | | | | + | | | + |
| TMEM151A | | | | | | | + | | + |
| TMEM151B | | | | | | | | | |
| TMEM154 | | | | | | | | | |
| TMEM155 | | | | | | | | | |
| TMEM156 | | | | | | | | | |
| TMEM158 | | | | | | | | | |
| TMEM159 | | | | | | | | | |
| TMEM160 | | | | | | | | | |
| TMEM161A | | | | | | | | | |

| | | | | | | | | | |
|---|---|---|---|---|---|---|---|---|---|
| TMEM161B | | + | | | | + | | | + |
| TMEM163 | | | | | | | | | |
| TMEM164 | | | | | | | | | |
| TMEM165 | | | | | | | | | |
| TMEM167A | | | | | | | | | |
| TMEM167B | | | | | | | | | |
| TMEM168 | | | | | | | | | |
| TMEM169 | | | | | | | | | |
| TMEM17 | | | | | | | | | |
| TMEM170A | | | | | | | | | |
| TMEM171 | | | | | | | | | |
| TMEM173 | | | | | | | | | |
| TMEM174 | | | | | | | | | |
| TMEM175 | | | | | | | | | |
| TMEM176A | | | | | | | | | |
| TMEM176B | | | | | | | | | |
| TMEM177 | | | | | | | | | |
| TMEM178A | | | | | | | | | |
| TMEM178B | | | | | | | | | |
| TMEM179B | | | | | | | | | |
| TMEM18 | | | | | | | | | |
| TMEM180 | | | | | | | | | |
| TMEM181 | | | | | | | | | |
| TMEM182 | | | | | | | | | |
| TMEM183A | | | | | | | | | |
| TMEM183B | | | | | | | | | |
| TMEM184A | | | | | | | | | |
| TMEM184B | | | + | | + | | | | + |
| TMEM184C | | + | + | | | + | | | + |
| TMEM185A | | | | | | | | | |
| TMEM185B | | | | | | | | | |
| TMEM186 | | | | | | | | | |
| TMEM187 | | | | | | | | | |
| TMEM189 | | | | | | | | | |
| TMEM189-UBE2V1 | | | | | | | | | |
| TMEM19 | | | | | | | | | |
| TMEM190 | | | | | | | | | |
| TMEM192 | | | | | | | | | |
| TMEM194A | | | | | | | | | |
| TMEM194B | | | | | | | | | |
| TMEM196 | | | | | | | | | |
| TMEM199 | | | | | | | | | |
| TMEM2 | + | | | | + | | | | + |
| TMEM200A | | | | | | | | | |
| TMEM200B | | | | | | | | | |
| TMEM200C | | | | | | | | | |
| TMEM201 | | | | | | | | | |
| TMEM202 | | | | | | | | | |
| TMEM203 | | | | | | | | | |
| TMEM204 | | | | | | | | | |
| TMEM205 | | | | | | | | | |
| TMEM206 | | | + | | | | | | + |
| TMEM207 | | | | | | | | | |

| | | | | | | | | | |
|---|---|---|---|---|---|---|---|---|---|
| TMEM208 | | | | | | | | | |
| TMEM209 | | | | | | | | | |
| TMEM212 | | | | | | | | | |
| TMEM213 | | | | | | | | | |
| TMEM214 | | | | | | | | | |
| TMEM216 | | | | | | | | | |
| TMEM218 | | | | | | | | | |
| TMEM219 | | | | | | | | | |
| TMEM220 | | | | | | | | | |
| TMEM221 | | | | | | | | | |
| TMEM222 | | | | | | | | | |
| TMEM223 | | | | | | | | | |
| TMEM225 | | | | | | | | | |
| TMEM229B | | | + | | | | | | + |
| TMEM230 | | | | | | + | | | + |
| TMEM231 | | | | | | | | | |
| TMEM232 | | | | | | | | | |
| TMEM233 | | | | | | | | | |
| TMEM234 | | | | | | | | | |
| TMEM236 | | | | | | | + | | + |
| TMEM237 | | | | | | | | | |
| TMEM238 | | | | | | | | | |
| TMEM239 | | | | | | | | | |
| TMEM241 | | | | | | | | | |
| TMEM242 | | | | | | | | | |
| TMEM243 | | | | | | | | | |
| TMEM245 | | | | | | | | | |
| TMEM246 | | | | | | | | | |
| TMEM248 | | | | | | | | | |
| TMEM25 | | | | | + | | | | + |
| TMEM251 | | | | | + | | | | + |
| TMEM252 | | | | | | | | | |
| TMEM253 | | | | | | | | | |
| TMEM254 | | | | | | | | | |
| Tmem254b | | | | | | | | | |
| TMEM255A | | | | | | | | | |
| TMEM255B | | | | | | | | | |
| TMEM256 | | | | | | | | | |
| TMEM257 | | | | | | | | | |
| TMEM258 | | + | | | | + | | | + |
| TMEM259 | | | | | | | | | |
| TMEM260 | | | | | | | | | |
| TMEM261 | | | | | | | | | |
| TMEM262 | | | | | | | | | |
| TMEM263 | | | | | | | | | |
| TMEM27 | | | | | | | | | |
| TMEM30A | | + | + | | | + | | | + |
| TMEM30B | | | | | | | | | |
| TMEM31 | | | | | | | | | |
| TMEM33 | | + | | | | + | | | + |
| TMEM35 | | | | | | | | | |
| TMEM37 | | | | | | | | | |
| TMEM38A | | | | | | | | | |

| | | | | | | | | | |
|---|---|---|---|---|---|---|---|---|---|
| TMEM38B | + | | + | + | + | | | | + |
| TMEM39A | | | | | | | | | |
| TMEM39B | | | | | | | | | |
| TMEM40 | | | | | | | | | |
| TMEM41A | | | | | | | | | |
| TMEM41B | | | | | | | | | |
| TMEM42 | | | | | | | | | |
| TMEM43 | | | | | | | | | |
| TMEM44 | | | | | | | | | |
| TMEM45A | | | | | | | | | |
| TMEM45B | | + | + | | | + | | | + |
| TMEM47 | + | | + | + | + | | | | + |
| TMEM5 | | | | | | | | | |
| TMEM50A | | + | | | | + | | | + |
| TMEM50B | | | | | | | | | |
| TMEM51 | | | | | | | | | |
| TMEM52 | | | | | | | | | |
| TMEM52B | | | | | | | | | |
| TMEM53 | | | | | | | | | |
| TMEM54 | | | | | | | | | |
| Tmem55a | | | | | | | | | |
| TMEM55B | | | | | | | | | |
| TMEM56 | | | + | | | | | | + |
| TMEM57 | | | | | | | | | |
| TMEM59 | | | | | | | | | |
| TMEM59L | | | | | | + | | | + |
| TMEM60 | | | | | | | | | |
| TMEM61 | | | | | | | | | |
| TMEM62 | | | | | | + | | | + |
| TMEM63A | | | | | | | | + | + |
| Tmem63b | | | | | | | | | |
| TMEM63C | | | | | | | | | |
| TMEM64 | | | + | | | | | | + |
| TMEM65 | | | | | | | | | |
| Tmem66 | | | | | | | | | |
| TMEM67 | | | | | | | | | |
| TMEM68 | | | | | | | | | |
| TMEM69 | | | | | | | | | |
| TMEM70 | | | | | | | | | |
| TMEM71 | | | | | | | | | |
| TMEM72 | | | | | | | | | |
| TMEM74 | | | + | | | | | | + |
| TMEM74B | | | | | | | | | |
| TMEM75 | | | | | | | | | |
| TMEM78 | | | | | | | | | |
| TMEM79 | | | | | | | | | |
| TMEM80 | | | | | | | | | |
| TMEM81 | | | | | | | | | |
| TMEM82 | | | | | | | | | |
| TMEM86A | | | | | | | | | |
| TMEM86B | | | | | | | | | |
| TMEM87A | | | | | | | | | |
| TMEM87B | | | | | | | | | |

| | | | | | | | | | |
|---|---|---|---|---|---|---|---|---|---|
| TMEM88 | | | | | | | | | |
| TMEM88B | | | | | | | | | |
| TMEM89 | | | | | | | | | |
| TMEM8A | | | | | | | | | |
| TMEM8B | | | | | | | | | |
| TMEM9 | | + | | | | + | | | + |
| TMEM91 | | | | | | | | | |
| TMEM92 | | | | | | | | | |
| TMEM95 | | | | | | | | | |
| TMEM97 | | | | | | | | | |
| TMEM98 | | | | | | | | | |
| TMEM9B | | + | + | | | + | | | + |
| TMF1 | | | | | | | | | |
| TMIE | + | | | + | + | | | | + |
| TMIGD1 | | | | | | | | | |
| TMIGD2 | | | | | | | | | |
| TMLHE | | | | | | | | | |
| TMOD1 | | | | | | | | | |
| TMOD2 | | | | | | + | | | + |
| TMOD3 | | | | | | | | | |
| TMOD4 | | | | | | | | | |
| TMPO | | | | | | | | | |
| TMPPE | | | | | + | + | | | + |
| TMPRSS11A | | | | | | | | | |
| TMPRSS11B | | | | | | | | | |
| TMPRSS11D | | | | | | | | | |
| TMPRSS11E | | | | | | | | | |
| TMPRSS11F | | | | | | | | | |
| TMPRSS12 | | | | | | | | | |
| TMPRSS13 | | | | | | | | | |
| TMPRSS15 | | | | | | | | | |
| TMPRSS2 | | | | | | | | | |
| TMPRSS3 | | | | | | | | | |
| TMPRSS4 | | | | | | | | | |
| TMPRSS5 | | | | | | | | | |
| TMPRSS6 | | | | | | | | | |
| TMPRSS7 | | | | | | | | | |
| TMPRSS9 | | | | | | | | | |
| TMSB10 | | | | | | | | | |
| TMSB15A | | | | | | | | | |
| TMSB15B | | | | | | | | | |
| Tmsb4x | | | | | | + | | | + |
| TMSB4XP1 | | | | | | | | | |
| TMSB4XP2 | | | | | | | | | |
| TMSB4XP4 | | | | | | | | | |
| TMSB4XP6 | | | | | | | | | |
| TMSB4XP8 | | | | | | | | | |
| TMSB4Y | | | | | | | | | |
| TMTC1 | | | | | + | | | | + |
| TMTC2 | | | + | | | | | | + |
| TMTC3 | | | | | | | | | |
| TMTC4 | | | | + | | | | | + |
| TMUB1 | | | | | | | | | |

| | | | | | | | | | |
|---|---|---|---|---|---|---|---|---|---|
| TMUB2 | | | | | | | | | |
| TMX1 | | | | | | | + | | + |
| TMX2 | | + | | | | + | | | + |
| TMX2-CTNND1 | | | | | | | | | |
| TMX3 | | | | | | | | | |
| TMX4 | | | | | | | | | |
| TNA1 | | | | | | | | | |
| TNAP | | | | | | | | | |
| TNC | | | | | | | | | |
| TNF | | | | | | | | | |
| TNFAIP1 | | | | | | | | | |
| TNFAIP2 | | | | | | | | | |
| TNFAIP3 | | | | | | | | | |
| TNFAIP6 | + | | | | | | + | | + |
| TNFAIP8 | | | | | | | | | |
| TNFAIP8L1 | | | | | | | | | |
| TNFAIP8L2 | | | | | | | | | |
| TNFAIP8L2-SCNM1 | | | | | | | | | |
| TNFAIP8L3 | | | | | | | | | |
| TNFRSF10A | | | | | | | | | |
| TNFRSF10B | | | | | | | | | |
| TNFRSF10C | | | | | | | | | |
| TNFRSF10D | | | | | | | | | |
| TNFRSF11A | + | | | + | + | | | | + |
| TNFRSF11B | | | | | | | | | |
| TNFRSF12A | | | | | | | | | |
| TNFRSF13B | | | | | | | | | |
| TNFRSF13C | + | | | + | + | | | | + |
| TNFRSF14 | | | | | | | | | |
| TNFRSF17 | | | | | | | | | |
| TNFRSF18 | | | | | | | | | |
| TNFRSF19 | | | | | | | | | |
| TNFRSF1A | | | | | | | | | |
| TNFRSF1B | | | | | | | | | |
| TNFRSF21 | | | | | | | | | |
| TNFRSF25 | | | | | | | | | |
| TNFRSF4 | | | | | | | | | |
| TNFRSF6B | | | | | | | | | |
| TNFRSF8 | | | | | | | | | |
| TNFRSF9 | | | | | | | | | |
| TNFSF10 | | | | | | | | | |
| TNFSF11 | | | | | | | | | |
| TNFSF12 | | | | | | | | | |
| TNFSF12-TNFSF13 | | | | | | | | | |
| TNFSF13 | | | | | | | | | |
| TNFSF13B | | | | | | | | | |
| TNFSF14 | | | | | | | | | |
| TNFSF15 | + | | | + | + | | + | | + |
| TNFSF18 | | | | | | | | | |
| TNFSF8 | | | | | | | | | |
| TNFSF9 | | | | | | | | | |
| TNIK | | | | | | + | | | + |
| TNIP1 | | | | | | | | | |

| | | | | | | | | | |
|---|---|---|---|---|---|---|---|---|---|
| TNIP2 | | | | | | | | | |
| TNIP3 | | | | | | | | | |
| TNK1 | | | | | | | | | |
| TNK2 | | | | | | | | | |
| TNKS | | | | | | | | | |
| TNKS1BP1 | | | | | | | | | |
| TNKS2 | | | | | | | | | |
| TNMD | | | | | | | | | |
| TNN | | | | | + | | | | + |
| TNNC1 | | | | | | | | | |
| TNNC2 | | | | | | | | | |
| TNNI1 | | | | | | | | | |
| TNNI2 | | | | | | | | | |
| TNNI3 | | | | | | | | | |
| TNNI3K | | | | | | | | | |
| TNNT1 | + | | | + | + | | | | + |
| TNNT2 | + | | | + | + | | | | + |
| TNNT3 | | | | | | | | | |
| TNP1 | | | | | | | | | |
| TNP2 | | | | | | | | | |
| TNPO1 | | | | | | | | | |
| TNPO2 | | | | | | | | | |
| TNPO3 | | | | | | | | | |
| TNR | | | | | + | | | | + |
| TNRC17 | | | | | | | | | |
| TNRC18 | | | | | | | | | |
| TNRC18P1 | | | | | | | | | |
| TNRC6A | | | | | | + | | | + |
| TNRC6B | | | | | | | | | |
| TNRC6C | | + | | | | + | | | + |
| TNS1 | | | | | + | | | | + |
| TNS3 | | | | | | | | | |
| TNS4 | | | | | | | | | |
| TNXB | | | | | | | | | |
| toa2 | | | | | | | | | |
| TOB1 | | | | | | | | | |
| TOB2 | | | | | | | | | |
| TOE1 | | | | | | | | | |
| TOG1 | | | | | | | | | |
| TOLLIP | | | | | | | | | |
| TOM1 | | | | | | | | | |
| TOM1L1 | | | | | | | | | |
| TOM1L2 | | | | | | | | | |
| TOM20 | | | | | | | | | |
| TOM22 | | | | | | | | | |
| TOM40 | | | | | | | | | |
| TOM70 | | | | | | | | | |
| TOM71 | | | | | | | | | |
| TOMM20 | | | | | | | | | |
| TOMM20L | | | | | | | | | |
| TOMM22 | | | | | | | | | |
| TOMM34 | | | | | | | | | |
| TOMM40 | | | | | + | | | | + |

| | | | | | | | | | |
|---|---|---|---|---|---|---|---|---|---|
| TOMM40L | | | | | | | | | |
| TOMM5 | | | | | | | | | |
| TOMM6 | | | | | | | | | |
| TOMM7 | | | | | | | | | |
| TOMM70A | | | | | | | | | |
| TON1A | | | | | | | | | |
| TONSL | | + | | | | + | | | + |
| TOP1 | + | | | | + | | | | + |
| TOP1MT | | | | | | | | | |
| TOP1P1 | | | | | | | | | |
| Top2 | | | | | | | | | |
| TOP2A | | | | + | | | | | + |
| TOP2B | | | | | | | | | |
| TOP3A | | | | | | | | | |
| TOP3B | | | | | | | | | |
| TOPAZ1 | | | | | | | | | |
| TOPBP1 | | | | | + | + | | | + |
| TOPORS | | | | | | | | | |
| TOPP8 | | | | | | | | | |
| tor1 | | | | | | | | | |
| TOR1A | | | | | | + | | | + |
| Tor1aip1 | | | + | | | | | | + |
| TOR1AIP2 | | | + | | | | | | + |
| TOR1B | | | | | | | | | |
| tor2 | | | | | | | | | |
| TOR2A | | + | | | | + | | | + |
| TOR3A | | | | | + | | | | + |
| TOR4A | | | | | | | | | |
| TOX | | | | | | + | | | + |
| TOX2 | | | | | | | | | |
| TOX3 | | | | | | | | | |
| TOX4 | | | | | | | | | |
| TP53 | | | | | | | | | |
| TP53AIP1 | | | | | | | | | |
| TP53BP1 | | | | | | | | | |
| TP53BP2 | | | | | | | | | |
| TP53I11 | | | | | | | | | |
| TP53I13 | | | | | | | | | |
| TP53I3 | | | | | | | | | |
| TP53INP1 | | | | | | | | | |
| TP53INP2 | | | | | | | | | |
| TP53RK | | | | | | | | | |
| TP53TG1 | | | | | | | | | |
| TP53TG3 | | | | | | | | | |
| TP53TG3B | | | | | | | | | |
| TP53TG3C | | | | | | | | | |
| TP53TG5 | | | | | | | | | |
| TP63 | | | | | | | | | |
| TP73 | | | | | | | | | |
| TP73-AS1 | | | | | | | | | |
| TPBG | + | | + | + | + | | | | + |
| TPCN1 | | | | | | | | | |
| TPCN2 | | | | | | | | | |

| | | | | | | | | | |
|---|---|---|---|---|---|---|---|---|---|
| TPD52 | | | | | | | | | |
| TPD52L1 | | | | | | | | | |
| TPD52L2 | | | | | | | | | |
| TPD52L3 | | | | | | | | | |
| TPGS1 | | | | | | | | | |
| TPGS2 | | | | | | | | | |
| TPH1 | | | | | | | | | |
| TPH2 | | | | | | | | | |
| TPI1 | | | | | | + | | | + |
| TPI1P1 | | | | | | | | | |
| TPI1P2 | | | | | | | | | |
| TPK1 | | | | | | | | | |
| TPK2 | | | | | | | | | |
| TPK3 | | | | | | | | | |
| TPM1 | | | | | | | | | |
| TPM2 | | | | | | | | | |
| TPM3 | | | + | | | | | | + |
| TPM3P4 | | | | | | | | | |
| TPM3P9 | | | | | | | | | |
| TPM4 | | | | | | + | | | + |
| TPMT | | | | | | | | | |
| TPP1 | | | | | | + | | | + |
| TPP2 | | | | | | | | | |
| TPPP | | | | | | + | | | + |
| TPPP2 | | | | | | | | | |
| TPPP3 | | | | | | + | | | + |
| TPR | | | | | | | | | |
| tpr1 | | | | | | | | | |
| TPRA1 | | | | | + | | | | + |
| TPRG1 | | | | | | | | | |
| TPRG1L | | | | | | | | | |
| TPRKB | | | | | | | | | |
| TPRN | | | | | | | | | |
| TPRX1 | | | | | | | | | |
| TPRX2P | | | | | | | | | |
| TPRXL | | | | | | | | | |
| TPS1 | | | | | | | | | |
| TPS2 | | | | | | | | | |
| TPS3 | | | | | | | | | |
| TPSAB1 | | | | | | | | | |
| TPSB2 | | | | | | | | | |
| TPSG1 | | | | | | | | | |
| TPST1 | | | | | | | | | |
| TPST2 | | | | | | | | | |
| TPT1 | + | | + | | + | | | | + |
| TPT1P8 | | | | | | | | | |
| TPTE | | | | | | | | | |
| TPTE2 | | | | | | | | | |
| tpx1 | | | | | | | | | |
| TPX2 | | | | | | | | | |
| tpx2-a | | | | | | | | | |
| TRA | | | | | | | | | |
| tra-1 | | | | | | | | | |

| | | | | | | | | | |
|---|---|---|---|---|---|---|---|---|---|
| TRA2A | | | | | | | | | |
| TRA2B | | | | | | | | | |
| TRABD | | | | | | | | | |
| TRABD2A | | | | | | | | | |
| TRAC | | | | | | | | | |
| TRADD | | | | | | | | | |
| TRAF1 | | | | | | | | | |
| TRAF2 | | | | | | | | | |
| TRAF3 | | | | | | | | | |
| TRAF3IP1 | + | | | + | + | | | | + |
| TRAF3IP2 | | | | | | | | | |
| TRAF3IP3 | | | | | | | | | |
| TRAF4 | | | | | | | | | |
| TRAF5 | | | | | | | | | |
| TRAF6 | | | | | | | | | |
| TRAF7 | | | | | | | | | |
| TRAFD1 | | | | | + | | | | + |
| TRAIP | | | | | | + | | | + |
| TRAJ56 | | | | | | | | | |
| TRAK1 | | | + | | | | | | + |
| TRAK2 | | | | | | | | | |
| TRAM1 | | | | | | | | | |
| TRAM1L1 | | | | | | | | | |
| TRAM2 | | | | | | | | | |
| TRAM2-AS1 | | | | | | | | | |
| TRANK1 | | | | | | | | | |
| TRAP | | | | | | | | | |
| TRAP1 | | | | | | | | | |
| TRAPPC1 | | + | | | | + | | | + |
| TRAPPC10 | | | | | | | | | |
| TRAPPC11 | | + | | | | + | | | + |
| TRAPPC12 | | | | | | + | | | + |
| TRAPPC13 | | | | | | | | | |
| TRAPPC2 | | | | | | | | | |
| TRAPPC2B | | | | | | | | | |
| TRAPPC2L | | + | | | | + | | | + |
| TRAPPC3 | | | | | | | | | |
| TRAPPC3L | | | | | | | | | |
| TRAPPC4 | | | | | | | | | |
| TRAPPC5 | | | | | | | | | |
| TRAPPC6A | | | | | | | | | |
| TRAPPC6B | | | | | | | | | |
| TRAPPC8 | | | | | | | | | |
| TRAPPC9 | | | | | | | | | |
| TRAT1 | | | | | | | | | |
| TRAV20 | | | | | | | | | |
| TRAV38-2DV8 | | | | | | | | | |
| TRB | | | | | | | | | |
| TRBC1 | | | | | | | | | |
| TRBC2 | | | | | | | | | |
| TRBV12-3 | | | | | | | | | |
| TRBV13 | | | | | | | | | |
| TRBV2 | | | | | | | | | |

| | | | | | | | | | |
|---|---|---|---|---|---|---|---|---|---|
| TRDMT1 | | | | | | | | | |
| TRDN | | | | | | | | | |
| TRE17 | | | | | | | | | |
| TREH | | | | | | | | | |
| TREM1 | | | | | | | + | | + |
| TREM2 | | | | | | | | | |
| TREML1 | | | | | | | | | |
| TREML2 | + | | | + | + | | + | | + |
| TRERF1 | | | | | | | | | |
| TREX1 | | | | | | | | | |
| TREX2 | | | | | | | | | |
| TRGV3 | | | | | | | | | |
| TRGV7 | | | | | | | | | |
| TRH | | | | | | | | | |
| TRHDE | | | | | | | | | |
| TRHR | | | | | | | | | |
| TRIAP1 | | | | | | | | | |
| TRIB1 | + | | + | | + | | | | + |
| TRIB2 | | | | | | | | | |
| TRIB3 | | | + | | | | | | + |
| TRIL | | | | | | | | | |
| TRIM10 | | | | | | | | | |
| TRIM11 | | | | | | | | | |
| TRIM13 | | | | | | | | | |
| TRIM14 | | | | | | | | | |
| TRIM15 | | | | | | | | | |
| TRIM16 | | | | | | | | | |
| TRIM16L | | | | | | | | | |
| TRIM17 | | | | | | | | | |
| TRIM2 | | | | | | | | | |
| TRIM21 | | | | | | | | | |
| TRIM22 | | | | | | | | | |
| TRIM23 | | | | | | | | | |
| TRIM24 | | | | | | | | | |
| TRIM25 | + | | | + | + | | | | + |
| TRIM26 | + | | | + | + | | | | + |
| TRIM27 | | | | | | + | | | + |
| TRIM28 | + | | | | + | | | | + |
| TRIM29 | | | | | | | | | |
| TRIM3 | | | | | | | | | |
| Trim30a | | + | + | | | + | | | + |
| TRIM31 | | | | | | | | | |
| TRIM32 | | | | | | | + | | + |
| TRIM33 | | | | | | | | | |
| TRIM34 | | | | | | | | | |
| TRIM35 | | | | | | | + | | + |
| TRIM36 | | | | | | | | | |
| TRIM37 | | | | | | | | | |
| TRIM38 | | | | | | | | | |
| TRIM39 | | | | | + | | | | + |
| TRIM39-RPP21 | | | | | | | | | |
| TRIM4 | | | | | | | | | |
| TRIM40 | | | | | | | | | |

| | | | | | | | | | |
|---|---|---|---|---|---|---|---|---|---|
| TRIM41 | | | | | | | | | |
| TRIM42 | | | | | | | | | |
| TRIM43 | | | | | | | | | |
| TRIM43B | | | | + | | | | | + |
| TRIM44 | | | | | | | | | |
| TRIM45 | | | | | | | | | |
| TRIM46 | | | | | | + | | | + |
| TRIM47 | | | | | | | | | |
| TRIM48 | | | | | | | | | |
| TRIM49 | | | | | | | | | |
| TRIM49C | | | | | | | | | |
| TRIM49D2P | | | | | | | | | |
| TRIM5 | | | | | | | | | |
| TRIM50 | | | | + | | | | | + |
| TRIM51 | | | | | | | | | |
| TRIM52 | | | | | | | | | |
| TRIM52-AS1 | | | | | | | | | |
| TRIM54 | | | | | | | | | |
| TRIM55 | | | | | | | | | |
| TRIM56 | | | | | | | | | |
| TRIM58 | + | | | + | + | | | | + |
| TRIM59 | | | | | | | | | |
| TRIM6 | | | | | | | | | |
| TRIM6-TRIM34 | | | | | | | | | |
| TRIM60 | | | | | | | | | |
| TRIM61 | | | | | | | | | |
| TRIM62 | | | | | | | | | |
| TRIM63 | | | | | | | | | |
| TRIM64 | | | | | | | | | |
| TRIM64C | | | | | | | | | |
| TRIM65 | | | | | | | | | |
| TRIM66 | | | + | | | | | | + |
| TRIM67 | + | | | + | + | | | | + |
| TRIM68 | | | | | | | | | |
| TRIM69 | | | | | | | | | |
| TRIM7 | | | | | + | | | | + |
| TRIM71 | | | | | | | | | |
| TRIM72 | | | | | | | | | |
| TRIM73 | | | | | | | | | |
| TRIM74 | | | | | | | | | |
| TRIM75P | | | | | | | | | |
| TRIM77 | | | | | | | | | |
| TRIM8 | | | | | | | | | |
| TRIM9 | + | | + | | + | | | | + |
| TRIML1 | | | | | | | | | |
| TRIML2 | | | | | | | | | |
| TRIO | | | | | + | | | | + |
| TRIOBP | | | | | | | | | |
| TRIP10 | | | | | | | | | |
| TRIP11 | | | | | + | | | | + |
| TRIP12 | | | | | + | | | | + |
| TRIP13 | | | | | | | | | |
| TRIP4 | | | | | | | | | |

| | | | | | | | | | |
|---|---|---|---|---|---|---|---|---|---|
| TRIP6 | | | | | | | | | |
| TRIQK | | | | | | | | | |
| TRIT1 | | | | | | | | | |
| TRM8 | | | | | | | | | |
| TRMT1 | | | | | | | | | |
| TRMT10A | + | | | + | + | | + | | + |
| TRMT10B | | | | | | | | | |
| TRMT10C | | | | | | | | | |
| TRMT11 | | | | | | | | | |
| TRMT112 | | | | | | | | | |
| TRMT12 | | | | | | | | | |
| TRMT13 | | | | | | + | | | + |
| TRMT1L | | | | | | | | | |
| TRMT2A | | | | | | + | | | + |
| TRMT2B | | | | | | | | | |
| TRMT44 | | | | | | | | | |
| TRMT5 | | | | | | | | | |
| TRMT6 | | | | | + | | | | + |
| TRMT61A | | | | | | | | | |
| TRMT61B | | | | | | | | | |
| TRMU | | | | | | + | | | + |
| TRN-GTT2-1 | | | | | | | | | |
| TRNAU1AP | | | | | | + | | | + |
| TRNE | | | | | | | | | |
| TRNI | | | | | | | | | |
| TRNP1 | | | | | + | | | | + |
| TRNT1 | | | | | | | | | |
| TRNV | | | | | | | | | |
| TRO | | + | | | | + | | | + |
| TROAP | | | | | | | | | |
| TROVE2 | | | | | | + | | | + |
| TRP2 | | | | | | | | | |
| TRP5 | | | | | | | | | |
| Trp53 | | | | | | | | | |
| Trp53bp1 | | | | | | | | | |
| Trp53bp2 | | | | | | | | | |
| Trp53inp2 | | | | | | | | | |
| TRPA1 | | | | | | | | | |
| TRPC1 | | | | | | | | | |
| TRPC2 | | | | | | | | | |
| TRPC3 | | | | | | | | | |
| TRPC4 | | | | | | | | | |
| TRPC4AP | | | | | | | | | |
| TRPC5 | | | | | | | | | |
| TRPC5OS | | | | | | | | | |
| TRPC6 | + | | | | + | | | | + |
| TRPC7 | | | | | | | | | |
| TRPM1 | | | | | | | | | |
| TRPM2 | | | | | | + | | | + |
| TRPM3 | | | | | + | + | | | + |
| TRPM4 | | | | | | | | | |
| TRPM5 | | | | | | | | | |
| Trpm6 | | | | | | | | | |

| | | | | | | | | | |
|---|---|---|---|---|---|---|---|---|---|
| TRPM7 | | | | | | | | | |
| TRPM8 | | | | | | | | | |
| TRPS1 | | + | + | + | | + | | | + |
| TRPT1 | | | | | | | | | |
| TRPV1 | | | | | | | | | |
| TRPV2 | | | | + | | | | | + |
| TRPV3 | | | | | | | | | |
| TRPV4 | | + | + | | | + | | + | + |
| TRPV5 | | | | | | | | | |
| TRPV6 | | | | | | | | | |
| TRR | | | | | | | | | |
| TRRAP | | | | | | | | | |
| TRS-AGA2-4 | | | | | | | | | |
| trs1 | | | | | | | | | |
| TRS20 | | | | | | | | | |
| TRUB1 | | | | | | | | | |
| TRUB2 | | | | | | + | | | + |
| TRX1 | | | | | | | | | |
| TRX2 | | | | | | | | | |
| TRX3 | | | | | | | | | |
| Try10 | | | | | | | | | |
| TRY2P | | | | | | | | | |
| Try5 | | | | | | | | | |
| TSA1 | | | | | | | | | |
| TSACC | | | | | | | | | |
| TSC1 | | | | | | | | | |
| TSC10 | | | | | | | | | |
| Tsc2 | | | | | | | | | |
| TSC22D1 | | | + | | | | | | + |
| TSC22D2 | + | | + | | + | | | | + |
| TSC22D3 | | | | | | | | | |
| TSC22D4 | | | | | | | | | |
| TSEN15 | | | | | | + | | | + |
| TSEN2 | | | | | | + | | | + |
| TSEN34 | | | | | | + | | | + |
| TSEN54 | | | | | | | | | |
| TSFM | | | | | | | | | |
| TSG101 | | | | | | | | | |
| TSGA10 | | | | | | | | | |
| TSGA10IP | | | | | | | | | |
| TSGA13 | | | | | | | | | |
| TSHB | | | | | | | | | |
| TSHR | | | | | | | | | |
| TSHZ1 | | | + | | | | | | + |
| TSHZ2 | | | | | | | | | |
| TSHZ3 | + | | | + | + | | + | | + |
| TSKS | | | | | | | | | |
| TSKU | | | | | | | | | |
| TSL1 | | | | | | | | | |
| TSLP | | | | | | | | | |
| TSN | | | | | | | | | |
| TSNARE1 | | | | | | | | | |
| TSNAX | | | + | | | | | | + |

| | | | | | | | | | |
|---|---|---|---|---|---|---|---|---|---|
| TSNAX-DISC1 | | | | | | | | | |
| TSNAXIP1 | | | | | | | | | |
| TSPAN1 | | | | | | | | | |
| TSPAN10 | | | | | | | | | |
| TSPAN11 | | | | | | | | | |
| TSPAN12 | | | + | | | | | | + |
| TSPAN13 | | | + | | | | | | + |
| TSPAN14 | | | + | | | | | | + |
| TSPAN15 | | | | | | | | | |
| TSPAN16 | | | | | | | | | |
| TSPAN17 | | | + | | + | | | | + |
| TSPAN18 | | | | | | + | | | + |
| TSPAN2 | | | | | | | | | |
| TSPAN3 | | | | | | | | | |
| TSPAN31 | + | | | | + | | | | + |
| TSPAN32 | | | | | | | | | |
| TSPAN33 | | | | | | + | | | + |
| TSPAN4 | | | | | | | | | |
| TSPAN5 | + | | | | + | | | | + |
| TSPAN6 | | + | + | | | + | | | + |
| TSPAN7 | | | | | | + | | | + |
| TSPAN8 | | | | | | | | | |
| TSPAN9 | | | + | | | | | | + |
| TSPEAR | | | | | | | | | |
| TSPEAR-AS2 | | | | | | | | | |
| TSPO | | | | | | | | | |
| TSPO2 | | | | | | | | | |
| TSPY1 | | | | | | | | | |
| TSPY10 | | | | | | | | | |
| TSPY2 | | | | | | | | | |
| TSPY3 | | | | | | | | | |
| TSPY8 | | | | | | | | | |
| TSPYL1 | | | + | | | | | | + |
| TSPYL2 | | | | | | | | | |
| TSPYL4 | | | | | | | | | |
| TSPYL5 | | | | | + | | | | + |
| TSPYL6 | | | | | | | | | |
| TSR1 | | | | | | | | | |
| TSR2 | | | | | | | | | |
| TSR3 | | | | | | | | | |
| TSSC1 | | | | | | | | | |
| TSSC4 | | + | | | | + | | | + |
| TSSK1B | | | | | | | | | |
| TSSK2 | | | | | | | | | |
| TSSK3 | | | | | | | | | |
| TSSK4 | | | | | | | | | |
| Tssk5 | | | | | | | | | |
| TSSK6 | | | | | | | | | |
| TST | | | | | | | | | |
| TSTA3 | | | | | | | | | |
| TSTD2 | | | | | | | | | |
| TTBK1 | | | | | | | | | |
| TTBK2 | | | | | | | | | |

| | | | | | | | | | |
|---|---|---|---|---|---|---|---|---|---|
| TTC1 | | | | | | | | | |
| TTC12 | | | | | | | | | |
| TTC13 | | | | | | | | | |
| TTC14 | | | | | | | | | |
| TTC16 | | | | | | | | | |
| TTC17 | | | | | | | | | |
| TTC19 | | | + | | | | | | + |
| TTC21A | | | | | | | | | |
| TTC21B | | | | | | + | | | + |
| Ttc22 | | | | | | | | | |
| TTC23 | | | | | | | | | |
| TTC23L | | | | | | | | | |
| TTC24 | | | | | | | | | |
| TTC25 | | | | | | | | | |
| TTC26 | | | | | | + | | | + |
| TTC27 | | | | | | | | | |
| TTC28 | | | | | | | | | |
| TTC29 | | | | | | | | | |
| TTC3 | | | | | | | | | |
| TTC30A | | | | | | | | | |
| TTC30B | | | | | | | | | |
| TTC31 | | | | | | | | | |
| TTC32 | | | | | | | | | |
| TTC33 | | | | | | | | | |
| TTC34 | | | | | | | | | |
| TTC36 | | | | | | | | | |
| TTC37 | | | | | | | | | |
| TTC38 | | | | | | | | | |
| TTC39A | | | | | | | | | |
| TTC39B | | | | | | | | | |
| TTC39C | | | | | | | | | |
| TTC4 | | | | | | | | | |
| TTC5 | | | | | | + | | | + |
| TTC6 | | | | | | | | | |
| TTC7A | | | | | | | | | |
| TTC7B | | | | | | | | | |
| TTC8 | | | | + | | | | | + |
| TTC9 | | | | | | | | | |
| TTC9B | | | | | | | | | |
| TTC9C | | | | | | | | | |
| TTF1 | | | | + | | | | | + |
| TTF2 | | | | | | | | | |
| TTI1 | | | | | | | | | |
| TTI2 | | | | | | | | | |
| TTK | | | | | | | | | |
| TTL | | | | | | | | | |
| TTLL1 | | | | | | | | | |
| TTLL10 | | | | | | | | | |
| TTLL11 | | | | | + | | | | + |
| Ttll12 | | | | | | | | | |
| TTLL13 | | | | | | | | | |
| TTLL2 | | | | | | | | | |
| TTLL3 | | | | | | | | | |

| | | | | | | | | | |
|---|---|---|---|---|---|---|---|---|---|
| TTLL4 | | | | | | | | | |
| TTLL5 | | | | | | | | | |
| TTLL6 | | | | | | | | | |
| TTLL7 | | | | | + | | | | + |
| TTLL9 | | | | | | | | | |
| TTN | | | | | + | | | | + |
| TTN-AS1 | | | | | | | | | |
| TTPA | | | | | | | | | |
| TTPAL | | | | | | | | | |
| TTR | | + | + | | | + | | + | + |
| TTTY14 | | | | | | | | | |
| TTTY15 | | | | | | | | | |
| TTYH1 | | | | | + | | | | + |
| TTYH2 | | | | | | | | | |
| TTYH3 | | | + | | | | | | + |
| TUB | | | | | | + | | | + |
| TUB1 | | | | | | | | | |
| TUB2 | | | | | | | | | |
| TUB3 | | | | | | | | | |
| TUBA1A | | + | | | | + | | | + |
| TUBA1B | | + | + | | | + | | + | + |
| TUBA1C | | | | | | | | | |
| Tuba3a | | | | | | | | | |
| TUBA3C | | | | | | | | | |
| TUBA3D | | | | | | | | | |
| TUBA3E | | | | | | | | | |
| TUBA4A | | | + | | | | | | + |
| TUBA4B | | | | | | | | | |
| TUBA8 | | | | | | | | | |
| TUBAL3 | + | | | + | + | | + | | + |
| TUBAP2 | | | | | | | | | |
| TUBB | | | | | | | | | |
| TUBB1 | | | | | | | | | |
| TUBB2A | | | | | | | | | |
| Tubb2b | | | | | | | | | |
| TUBB3 | | | | | + | | | | + |
| TUBB4A | | | | | | | | | |
| TUBB4B | | | | | + | | | | + |
| Tubb4b-ps1 | | | | | | | | | |
| Tubb5 | | + | + | | + | | | | + |
| TUBB6 | | | | | | | | | |
| TUBB7P | | | | | | | | | |
| TUBB8 | | | | | | | | | |
| TUBBP2 | | | | | | | | | |
| TUBBP5 | | | | | | | | | |
| TUBD1 | | | | | | | | | |
| Tube1 | | | | | | | | | |
| TUBG1 | | | | | | | | | |
| TUBG2 | | | | | | + | | | + |
| Tubgcp2 | | | | | | | | | |
| tubgcp3 | | | | | | | | | |
| TUBGCP4 | | | | | + | | | | + |
| TUBGCP5 | | | | | | | | | |

| | | | | | | | | | |
|---|---|---|---|---|---|---|---|---|---|
| TUBGCP6 | | | | | | + | | | + |
| tuf1 | | | | | | | | | |
| TUFM | | | | | | | | | |
| TUFT1 | | | | | | | | | |
| TUG1 | | | | | | | | | |
| TUL1 | | | | | | | | | |
| TULP1 | | | | | | | | | |
| TULP2 | | | | | | | | | |
| TULP3 | | | | | | | | | |
| TULP4 | | | | | | | | | |
| TUM1 | | | | | | | | | |
| TUP1 | | | | | | | | | |
| tus | | | | | | | | | |
| TUS1 | | | | | | | | | |
| TUSC1 | | | | | | | | | |
| TUSC2 | | | | | | + | | | + |
| TUSC3 | | | | | | | | | |
| TUSC5 | | | | | | | | | |
| TUT1 | | | | | | | | | |
| TVP15 | | | | | | | | | |
| TVP23A | | | | | | | | | |
| TVP23B | | | | | | | | | |
| TVP23C | | | | | | | | | |
| TVP23C-CDRT4 | | | | | | | | | |
| TWF1 | | | | | | + | | | + |
| TWF2 | | | | | | | | | |
| TWIST1 | | | | | | | | | |
| twist1-a | | | | | | | | | |
| TWIST2 | | | | | | | | | |
| TWISTNB | | | | | | | | | |
| TWSG1 | | | | | | | | | |
| TXK | | | | | | | | | |
| TXLNA | | | | | | | | | |
| TXLNB | | | | | | | | | |
| TXLNG | | | | | | | | | |
| TXLNGY | | | | | | | | | |
| TXN | | | | | | | | | |
| Txn1 | | | | | | | | | |
| TXN2 | | | | | | | | | |
| TXNDC11 | | | | | | | | | |
| TXNDC12 | | | | | | + | | | + |
| TXNDC15 | | + | | | + | + | | | + |
| TXNDC16 | | | | | | | | | |
| TXNDC17 | | | | | | | | | |
| TXNDC2 | | | | | | | | | |
| TXNDC5 | | | | | | + | | | + |
| TXNDC8 | | | | | | | | | |
| TXNDC9 | | | | | | | | | |
| TXNIP | | | | | | | | | |
| TXNL1 | | | | | | | | | |
| TXNL4A | | | | | | | | | |
| TXNL4B | | | | | + | | | | + |
| TXNRD1 | | | | | | | | | |

| | | | | | | | | | |
|---|---|---|---|---|---|---|---|---|---|
| TXNRD2 | | | | | | | | | |
| TXNRD3 | | | | | | | | | |
| TXNRD3NB | | | | | | | | | |
| TYK2 | | | | | | | | | |
| TYMP | | | | | | | | | |
| TYMS | | | | | | | | | |
| TYMSOS | | | | | | | | | |
| TYR | | | | | | | | | |
| TYR1 | | | | | | | | | |
| TYRL | | | | | | | | | |
| TYRO3 | | | | | + | | | | + |
| TYROBP | | | | | + | | | | + |
| TYRP1 | | | | | | | | | |
| TYS1 | | | | | | | | | |
| TYSND1 | | | | | | | | | |
| TYW1 | | | | | | | | | |
| TYW1B | | | | | | | | | |
| Tyw3 | | | | | | | | | |
| TYW5 | | | | | | | | | |
| U14 | | | | | | | | | |
| U19 | | | | | | | | | |
| U24 | | | | | | | | | |
| U2AF1 | | | | | | | | | |
| U2AF1L4 | | | | | | | | | |
| U2AF2 | | | + | | | | | | + |
| U2SURP | | | | | | | | | |
| U90 | | | | | | | | | |
| U95 | | | | | | | | | |
| UACA | | | | | | | | | |
| UAP1 | | | + | | | | | | + |
| UAP1L1 | | | | | | | | | |
| Uba1 | | | | | | | | | |
| UBA2 | | | | | | | | | |
| UBA3 | | | | | | | | | |
| UBA4 | | | | | | | | | |
| UBA5 | | | | | | | | | |
| UBA52 | | | | | | | | | |
| UBA6 | | | | | | | | | |
| UBA7 | | | | | + | | | | + |
| UBAC1 | | | | | | | | | |
| UBAC2 | | | | | | | | | |
| UBALD1 | | | | | | + | | | + |
| UBALD2 | | | | | | | | | |
| UBAP1 | | | | | + | | | | + |
| UBAP2 | | | | | | | | | |
| UBAP2L | | | | | | | | | |
| UBASH3A | | + | | | | + | | | + |
| UBASH3B | | | + | | | + | | | + |
| UBB | | | | | | | | | |
| UBBP4 | | | | | | | | | |
| UBC | + | | | | + | | | | + |
| UBC1 | | | | | | | | | |
| UBC10 | | | | | | | | | |

| | | | | | | | | | |
|---|---|---|---|---|---|---|---|---|---|
| UBC12 | | | | | | | | | |
| UBC13 | | | | | | | | | |
| UBC35 | | | | | | | | | |
| UBC36 | | | | | | | | | |
| UBC4 | | | | | | | | | |
| UBC5 | | | | | | | | | |
| UBC8 | | | | | | | | | |
| UBC9 | | | | | | | | | |
| UBD | | | | | | | | | |
| UBE2A | | | | | | | | | |
| UBE2B | | | | | | | | | |
| UBE2C | | | | | | | | | |
| UBE2D1 | | | | | | + | | | + |
| UBE2D2 | | | | | | | | | |
| Ube2d2a | | | | | | | | | |
| UBE2D3 | | | + | | | | | | + |
| UBE2D4 | | | | | | | | | |
| UBE2DNL | | | | | | | | | |
| UBE2E1 | | | | | | | | | |
| UBE2E2 | | | | | | | | | |
| UBE2E3 | | | | | | | | | |
| UBE2F | | | | | | | | | |
| UBE2G1 | + | | | | + | | | | + |
| UBE2G2 | | | | | | | | | |
| UBE2H | | | + | | | | | | + |
| UBE2I | | | | | | | | | |
| UBE2J1 | | | + | | | | | | + |
| UBE2J2 | | | | | | | | | |
| UBE2K | | | | | + | | | | + |
| UBE2L1 | | | | | | | | | |
| UBE2L3 | | | | | | | | | |
| UBE2L6 | | | | | | + | | | + |
| UBE2M | | | | | | | | | |
| UBE2MP1 | | | | | | | | | |
| UBE2N | | | | | | | | | |
| ube2nb | | | | | | | | | |
| UBE2NL | | | | | | | | | |
| Ube2o | | | | | | | | | |
| UBE2Q1 | | | | | | | | | |
| UBE2Q2 | | | | | | | | | |
| UBE2Q2P2 | | | | | | | | | |
| UBE2QL1 | | | | | | | | | |
| UBE2R2 | | | | | | | | | |
| UBE2S | | | | | | | | | |
| UBE2T | | | | | | | | | |
| UBE2U | + | | | + | + | | + | | + |
| UBE2V1 | | | | | | | | | |
| UBE2V2 | | | | | | | | | |
| UBE2W | | | | | | | | | |
| UBE2Z | | | + | | | + | | | + |
| UBE3A | | | + | | + | | | | + |
| UBE3B | | | | | | | | | |
| UBE3C | | | | | | | | | |

| | | | | | | | | | |
|---|---|---|---|---|---|---|---|---|---|
| UBE3D | | | | | | | | | |
| UBE4A | | | | | | | | | |
| UBE4B | | | | | | | | | |
| UBFD1 | + | | + | | + | + | | | + |
| UBI4 | | | | | | | | | |
| UBIAD1 | | | | | | | | | |
| UBL3 | | | | | | | | | |
| UBL4A | | | | | | | | | |
| UBL4B | | | | | | | | | |
| UBL5 | | | | | | | | | |
| UBL7 | | | | | | | | | |
| UBLCP1 | | | | | | | | | |
| UBN1 | | | + | | | | | | + |
| UBN2 | | | | | | | | | |
| UBOX5 | | | | | | | | | |
| UBP1 | | | | | | | | | |
| UBP10 | | | | | | | | | |
| UBP11 | | | | | | | | | |
| UBP12 | | | | | | | | | |
| UBP13 | | | | | | | | | |
| UBP15 | | | | | | | | | |
| UBP2 | | | | | | | | | |
| UBP3 | | | | | | | | | |
| UBP6 | | | | | | | | | |
| UBQLN1 | | | + | | | | | | + |
| UBQLN2 | | | | | | | | | |
| UBQLN3 | | | | | | | | | |
| UBQLN4 | | | | | | | | | |
| UBQLNL | | | | | | | | | |
| UBR1 | | | | | | + | | | + |
| UBR2 | | | | | | | | | |
| UBR3 | | | | | + | | | | + |
| UBR4 | | | | | + | | | | + |
| UBR5 | | | | | | | | | |
| UBR7 | | | | | | | | | |
| UBTD1 | | | | | | | | | |
| UBTD2 | | | | | | | | | |
| UBTF | | | | | | + | | | + |
| UBTFL1 | | | | | | | | | |
| UBTFL6 | | | | | | | | | |
| UBX3 | | | | | | | | | |
| UBXN1 | | | | | + | | | | + |
| UBXN10 | | | | | | | | | |
| UBXN11 | | | | | | | | | |
| UBXN2A | | | | | | | | | |
| UBXN2B | | | | + | | | | | + |
| UBXN4 | | | | | | | | | |
| UBXN6 | | | | | | | | | |
| UBXN7 | | | | | | | | | |
| UBXN8 | | | | | + | | | | + |
| UCA1 | | | | | | | | | |
| Uch | | | | | | | | | |
| UCHL1 | | | + | | + | | | | + |

| | | | | | | | | | |
|---|---|---|---|---|---|---|---|---|---|
| UCHL3 | | | | | | | | | |
| UCHL5 | | | | | + | | | | + |
| UCK1 | | | | | | + | | | + |
| UCK2 | | | | | | | | | |
| UCKL1 | | | | + | | | | | + |
| UCMA | | | | | | | | | |
| Ucn | | | | | | | | | |
| UCN2 | | | | | | | + | | + |
| UCN3 | | | | | | | | | |
| UCP1 | | | | | | | | | |
| UCP2 | | | | | | | | | |
| UCP3 | | | | | | | | | |
| UEV1D-4 | | | | | | | | | |
| UEVLD | | | | | | | | | |
| UFC1 | | | | | | | | | |
| UFD1L | | | | | + | | | | + |
| UFD2 | | | | | | | | | |
| Ufl1 | | | | + | | | | | + |
| UFM1 | | | | | | | | | |
| UFSP1 | | | | | | | | | |
| UFSP2 | | + | | + | | + | | | + |
| UGCG | | | | | | | | | |
| UGDH | | | | | | | | | |
| UGGT1 | | | | | | | | | |
| UGGT2 | | | | | | | | | |
| UGP1 | | | | | | | | | |
| UGP2 | | | | | | | | | |
| UGT1A1 | | | | | | | | | |
| UGT1A10 | | | | | | | | | |
| UGT1A3 | | | | | | | | | |
| UGT1A4 | | | | | | | | | |
| UGT1A5 | | | | | | | | | |
| UGT1A6 | | | | | | | | | |
| UGT1A7 | | | | | | | | | |
| UGT1A8 | | | | | | | | | |
| UGT1A9 | | | | | | | | | |
| UGT2A1 | | | | | | | | | |
| UGT2A2 | | | | | | | | | |
| UGT2A3 | | | | | | | | | |
| UGT2B10 | | | | | | | | | |
| UGT2B11 | | | | | | | | | |
| UGT2B15 | | | | | | | | | |
| UGT2B17 | | | | | | | | | |
| UGT2B28 | | | | | | | | | |
| UGT2B4 | | | | | | | | | |
| UGT2B7 | | | | | | | | | |
| UGT3A1 | | | | | | | | | |
| UGT3A2 | | | | | | | | | |
| UGT8 | | | | | | | | | |
| UHMK1 | | | | | | | | | |
| UHRF1 | | | | | | | | | |
| UHRF1BP1 | | | | | | | | | |
| UHRF1BP1L | | | | | + | | | | + |

| | | | | | | | | | |
|---|---|---|---|---|---|---|---|---|---|
| UHRF2 | | | | | | | | | |
| UIMC1 | | + | | | | + | | | + |
| UIP3 | | | | | | | | | |
| UL1 | | | | | | | | | |
| UL10 | | | | | | | | | |
| UL114 | | | | | | | | | |
| UL12 | | | | | | | | | |
| UL122 | | | | | | | | | |
| UL123 | | | | | | | | | |
| UL13 | | | | | | | | | |
| UL135 | | | | | | | | | |
| UL138 | | | | | | | | | |
| UL14 | | | | | | | | | |
| UL144 | | | | | | | | | |
| UL145 | | | | | | | | | |
| UL17 | | | | | | | | | |
| UL18 | | | | | | | | | |
| UL19 | | | | | | | | | |
| UL21 | | | | | | | | | |
| UL21A | | | | | | | | | |
| UL22 | | | | | | | | | |
| UL24 | | | | | | | | | |
| UL25 | | | | | | | | | |
| UL26 | | | | | | | | | |
| UL27 | | | | | | | | | |
| UL28 | | | | | | | | | |
| UL29 | | | | | | | | | |
| UL30 | | | | | | | | | |
| UL31 | | | | | | | | | |
| UL35 | | | | | | | | | |
| UL36 | | | | | | | | | |
| UL37 | | | | | | | | | |
| UL38 | | | | | | | | | |
| UL39 | | | | | | | | | |
| UL40 | | | | | | | | | |
| UL41 | | | | | | | | | |
| UL42 | | | | | | | | | |
| UL44 | | | | | | | | | |
| UL45 | | | | | | | | | |
| UL46 | | | | | | | | | |
| UL47 | | | | | | | | | |
| UL48 | | | | | | | | | |
| UL49 | | | | | | | | | |
| UL5 | | | | | | | | | |
| UL50 | | | | | | | | | |
| UL51 | | | | | | | | | |
| UL52 | | | | | | | | | |
| UL54 | | | | | | | | | |
| UL55 | | | | | | | | | |
| UL56 | | | | | | | | | |
| UL6 | | | | | | | | | |
| UL7 | | | | | | | | | |
| UL76 | | | | | | | | | |

| | | | | | | | | | |
|---|---|---|---|---|---|---|---|---|---|
| UL82 | | | | | | | | | |
| UL84 | | | | | | | | | |
| UL87 | | | | | | | | | |
| UL9 | | | | | | | | | |
| UL97 | | | | | | | | | |
| ULBP1 | | | | | | | | | |
| ULBP2 | | | | | | | | | |
| ULBP3 | | | | | | | | | |
| ULK1 | | | | | | | | | |
| ULK2 | | | | | | | | | |
| ULK3 | + | | | + | + | | | | + |
| ULK4 | | | | | | | | | |
| ULP1 | | | | | | | | | |
| UM03200.1 | | | | | | | | | |
| UMAD1 | | | | | | | | | |
| UMOD | | | | | | | | | |
| UMODL1 | | | | | | | | | |
| UMPS | | | | | | | | | |
| UNC119 | | | | | | | | | |
| UNC119B | | | | | | | | | |
| UNC13A | | | | | | | | | |
| UNC13B | | | + | | + | | | | + |
| UNC13C | | | | | | | | | |
| UNC13D | | | | | | | | | |
| UNC45A | | | | | | | | | |
| Unc45b | | | | | | | | | |
| UNC50 | | | | | | | | | |
| UNC5A | | | + | | | | | | + |
| UNC5B | | | | | | | | | |
| UNC5C | | | | | | | | | |
| UNC5CL | | | | | | | | | |
| UNC5D | | | | | | | | | |
| UNC79 | | | | | | | | | |
| UNC80 | | + | | | | + | | | + |
| UNC93A | | | | | | | | | |
| UNC93B1 | | | | | | | | | |
| UNC93B3 | | | | | | | | | |
| UNCX | | | | | | | | | |
| UNG | | | | | | | | | |
| UNG1 | | | | | | | | | |
| UNK | | | | | | | | | |
| UNKL | | | | | | | | | |
| UPB1 | | | | | | | | | |
| UPF1 | | | | | | | | | |
| UPF2 | | | | | | | | | |
| UPF3A | | | | | | | | | |
| UPF3B | | + | | | | + | | | + |
| UPK1A | | | | | | | | | |
| UPK1B | | | | | | | | | |
| UPK2 | | | | | | | | | |
| UPK3A | | | | | | | | | |
| UPK3B | | | | | | | | | |
| UPK3BL | | | | | | | | | |

| | | | | | | | | | |
|---|---|---|---|---|---|---|---|---|---|
| UPL2 | | | | | | | | | |
| UPP1 | | | | | | | | | |
| UPP2 | | | | | | | | | |
| UPRT | | | | | | | | | |
| UQCC1 | | | | | | | | | |
| UQCC2 | | | | | | | | | |
| UQCC3 | | + | | | | + | | | + |
| UQCR10 | | + | | | | + | | | + |
| UQCR11 | | + | | | | + | | | + |
| UQCRB | | + | + | | | + | | | + |
| UQCRC1 | | | | | | | | | |
| UQCRC2 | | | | | | | | | |
| UQCRFS1 | | | | | | | | | |
| UQCRFS1P1 | | | | | | | | | |
| UQCRH | | | | | | | | | |
| UQCRHL | | | | | | | | | |
| UQCRQ | | + | | | | + | | | + |
| URA1 | | | | | | | | | |
| URA2 | | | | | | | | | |
| ura4 | | | | | | | | | |
| URAD | | | | | | | | | |
| URB1 | | | | | | | | | |
| URB1-AS1 | | | | | | | | | |
| URB2 | | | | | | | | | |
| URGCP | | | | | | | | | |
| URH1 | | | | | | | | | |
| URI1 | | | | | | | | | |
| URK1 | | | | | | | | | |
| URM1 | | | | | | | | | |
| UROC1 | | | | | | | | | |
| UROD | | | | | | + | | | + |
| UROS | | + | | | | + | | | + |
| US1 | | | | | | | | | |
| US10 | | | | | | | | | |
| US11 | | | | | | | | | |
| US2 | | | | | | | | | |
| US3 | | | | | | | | | |
| US34A | | | | | | | | | |
| US6 | | | | | | | | | |
| USA1 | | | | | | | | | |
| USB1 | | | | | | | | | |
| USE1 | | | | | | | | | |
| USF1 | | | | | | | | | |
| USF2 | | | + | | | | | | + |
| USH1C | | | | | | | | | |
| USH1G | + | | | + | + | | + | | + |
| USH2A | | | | | | | | | |
| USHBP1 | | | | | | | | | |
| USMG5 | | | | | | + | | + | + |
| USO1 | | | | | | | | | |
| USP1 | + | | | | | + | | | + |
| USP10 | | | | | | | | | |
| USP11 | | | + | | | | | | + |

| | | | | | | | | | |
|---|---|---|---|---|---|---|---|---|---|
| USP12 | | | | | | | | | |
| USP12P1 | | | | | | | | | |
| USP13 | | | | | | | | | |
| USP14 | | | | | + | | | | + |
| USP15 | | | | | | | | | |
| USP16 | | | | | + | | | | + |
| USP17L | | | | | | | | | |
| USP17L1 | | | | | | | | | |
| USP17L10 | | | | | | | | | |
| USP17L11 | | | | | | | | | |
| USP17L12 | | | | | | | | | |
| USP17L17 | | | | | | | | | |
| USP17L18 | | | | | | | | | |
| USP17L19 | | | | | | | | | |
| USP17L2 | | | | | | | | | |
| USP17L20 | | | | | | | | | |
| USP17L21 | | | | | | | | | |
| USP17L22 | | | | | | | | | |
| USP17L23 | | | | | | | | | |
| USP17L24 | | | | | | | | | |
| USP17L27 | | | | | | | | | |
| USP17L28 | | | | | | | | | |
| USP17L29 | | | | | | | | | |
| USP17L3 | | | | | | | | | |
| USP17L5 | | | | | | | | | |
| USP17L7 | | | | | | | | | |
| USP17L9P | | | | | | | | | |
| USP18 | | | | | | | | | |
| USP19 | | | | | | | | | |
| USP2 | | | + | | | | | | + |
| USP2-AS1 | | | | | | | | | |
| USP20 | | | | | | | | | |
| USP21 | | | | | | | | | |
| USP22 | | | | | | | | | |
| USP24 | | | | | | | | | |
| USP25 | | | | | | | | | |
| USP26 | | | | | | | | | |
| USP27X | | | | | | | | | |
| USP28 | | | | | | | | | |
| USP29 | | | | | | | | | |
| USP3 | | | + | | | | | | + |
| USP30 | | | | | | + | | | + |
| USP31 | | | | | | | | | |
| USP32 | | | | + | | | | | + |
| USP32P2 | | | | | | | | | |
| USP33 | | | | | | | | | |
| USP34 | | | | | | | | | |
| USP35 | + | | | + | + | | | | + |
| USP36 | + | | + | | + | | | | + |
| USP37 | | | | | | | | | |
| USP38 | | | | | | | | | |
| USP39 | | | | | | | | | |
| USP4 | | | | | | | | | |

| | | | | | | | | | |
|---|---|---|---|---|---|---|---|---|---|
| USP40 | + | | | | + | | | | + |
| USP41 | | | | | | | | | |
| USP42 | | | | | | | | | |
| USP43 | | | | | | | | | |
| USP44 | | | | | | | | | |
| USP45 | | | | | + | + | | | + |
| USP46 | | | | | | | | | |
| USP46-AS1 | | | | | | | | | |
| USP47 | | | | | | | | | |
| USP48 | | + | + | | | + | | | + |
| USP49 | | | | | | | | | |
| USP5 | | | | | | | + | | + |
| USP50 | | | | | | | | | |
| USP51 | | | | | | | | | |
| USP53 | | | | | | | | | |
| USP54 | | | | | | + | | | + |
| USP6 | | | | | | | | | |
| USP6NL | | | | | | | | | |
| USP7 | | | | | | | | | |
| USP8 | | | + | | | | | | + |
| USP9X | | | + | | | | | | + |
| USP9Y | | | | | | | | | |
| USPL1 | | | | | | | | | |
| UST | | | | | | | | | |
| UTF1 | | | | | | | | | |
| UTP11L | | | | | | | | | |
| UTP14A | | | | | | | | | |
| UTP14C | | | | | | | | | |
| UTP15 | | | | | | | | | |
| UTP18 | | | | | | | | | |
| UTP20 | | | | | | | | | |
| utp22 | | | | | | | | | |
| UTP23 | | | | | | | | | |
| UTP25 | | | | | | | | | |
| UTP3 | | | | | | | | | |
| UTP6 | | | | | | + | | | + |
| UTRN | | | | | | | | | |
| UTS2 | | | | | | | | | |
| UTS2B | | | | | | | | | |
| Uts2r | | | | | | | | | |
| UTY | | | | | | | | | |
| UVRAG | | | | | | | | | |
| UVSSA | | | | | | | | | |
| UXS1 | | | | | | | | | |
| UXT | | | | | | | | | |
| VAC14 | | | | | | | | | |
| VAMP1 | | | | | | | | | |
| VAMP2 | | | + | | | | + | | + |
| VAMP3 | | | | | | | | | |
| VAMP4 | | | + | | | | | | + |
| VAMP5 | | | | | | | | | |
| VAMP7 | | | | | | | | | |
| VAMP721 | | | | | | | | | |

| | | | | | | | | | |
|---|---|---|---|---|---|---|---|---|---|
| VAMP8 | | | | | | | | | |
| VAN1 | | | | | | | | | |
| Vangl1 | | | | | | | | | |
| VANGL2 | | | | | | | | | |
| VAPA | | | | | | | | | |
| VAPB | | | | | | | | | |
| VARS | | | | | | | | | |
| VARS2 | | | | | | + | | | + |
| VAS1 | | | | | | | | | |
| VASH1 | | | | | | | | | |
| VASH2 | | | | | | | | | |
| VASN | | | | | | | | | |
| VASP | | | | | | | | | |
| VAT1 | | | | | | | | | |
| VAT1L | | | | | | | | | |
| VAV1 | | | | | | | | | |
| VAV2 | | | | | | | | | |
| VAV3 | | | | | | | | | |
| VAX1 | | | | | | | | | |
| VAX2 | | | | | | | | | |
| VBP1 | | + | | | | + | | | + |
| VCAM1 | | + | + | | | + | | | + |
| VCAN | | | | | | | | | |
| VCL | + | | | | + | | | | + |
| VCP | | | | | + | | | | + |
| VCPIP1 | | | | | | | | | |
| VCPKMT | | | | | | | | | |
| VCX | | | | | | | | | |
| VCX2 | | | | | | | | | |
| VCY | | | | | | | | | |
| VCY1B | | | | | | | | | |
| VDAC1 | | | | | | | | | |
| VDAC1P1 | | | | | | | | | |
| VDAC2 | | + | | | | + | | | + |
| VDAC3 | | + | | | | + | | | + |
| VDR | | | | | | | | | |
| VEGFA | | | + | | + | | | | + |
| VEGFB | | | | | | | | | |
| VEGFC | | | | | | | | | |
| VENTX | | | | | | | | | |
| ventx2.1-b | | | | | | | | | |
| VEPH1 | | | | | | | | | |
| VEZF1 | | | | | | | | | |
| VEZT | | | | | | | | | |
| VGF | + | | + | | + | | | | + |
| vgl1 | | | | | | | | | |
| VGLL1 | | | | | | | | | |
| VGLL2 | | | | | | | | | |
| VGLL3 | | | | | | | | | |
| VGLL4 | | | + | | | | | | + |
| VHL | | | | | | | | | |
| VHLL | | | | | | | | | |
| VHR1 | | | | | | | | | |

| | | | | | | | | | |
|---|---|---|---|---|---|---|---|---|---|
| VHS3 | | | | | | | | | |
| VID24 | | | | | | | | | |
| vif | | | | | | | | | |
| VIL1 | | | | | | | | | |
| VILL | | | | | | | | | |
| VIM | | | | | | | | | |
| VIMP | | | | | | | | | |
| VIP | + | | | | + | | | | + |
| VIPAS39 | | | | | | | | | |
| VIPR1 | | | | | | | | | |
| VIPR2 | | | | | | | | | |
| vIRF-1 | | | | | | | | | |
| vIRF-2 | | | | | | | | | |
| vIRF-3 | | | | | | | | | |
| vIRF-4 | | | | | | | | | |
| VIT | | | | | | | | | |
| VKORC1 | | | | | | | | | |
| VKORC1L1 | | | | | | | | | |
| VLDLR | | | + | | | | | | + |
| VMA1 | | | | | | | | | |
| VMA13 | | | | | | | | | |
| VMA16 | | | | | | | | | |
| VMA2 | | | | | | | | | |
| VMA21 | | | | | | | | | |
| VMA3 | | | | | | | | | |
| VMA4 | | | | | | | | | |
| VMA5 | | | | | | | | | |
| VMA6 | | | | | | | | | |
| VMA8 | | | | | | | | | |
| VMAC | | | | | | | | | |
| Vmn1r41 | | | | | | | | | |
| Vmp1 | | | | | | | | | |
| VN1R1 | | | | | | | | | |
| VN1R10P | | | | | | | | | |
| VN1R2 | | | | | | | | | |
| VN1R5 | | | | | | | | | |
| VNN1 | | | | | | | | | |
| VNN2 | | | | | | | | | |
| VNN3 | | | | | | | | | |
| VOA1 | | | | | | | | | |
| VOPP1 | | | | | | | + | | + |
| VPH1 | | | | | | | | | |
| vpr | | | | | | | | | |
| VPRBP | | | | | | | | | |
| VPREB1 | | | | | | | | | |
| VPS1 | | | | | | | | | |
| VPS11 | | | | | | | | | |
| VPS13A | | | | | | | | | |
| VPS13B | | | | | | | | | |
| VPS13C | | | | | | | | | |
| VPS13D | | | | | | | | | |
| VPS15 | | | | | | | | | |
| VPS16 | | | | | | | | | |

| | | | | | | | | | |
|---|---|---|---|---|---|---|---|---|---|
| VPS18 | | | | | | | | | |
| VPS20 | | | | | | | | | |
| VPS21 | | | | | | | | | |
| VPS24 | | | | | | | | | |
| VPS25 | | | | | | | | | |
| VPS26A | | | | | | | | | |
| VPS26B | | | | | | | | | |
| VPS28 | | | | | | | | | |
| VPS29 | | | | | | | | | |
| VPS30 | | | | | | | | | |
| VPS33A | | | | | | | | | |
| VPS33B | | | | | | | | | |
| VPS34 | | | | | | | | | |
| VPS35 | | | | | | | | | |
| VPS36 | | | | | | + | | | + |
| VPS37A | | | | | + | | | | + |
| VPS37B | | | | | | | | | |
| VPS37C | | | | | | | | | |
| VPS37D | | | | | | | | | |
| VPS38 | | | | | | | | | |
| VPS39 | | | | | | | | | |
| VPS4 | | | | | | | | | |
| VPS41 | | + | | | | + | | | + |
| VPS45 | | | | | | | | | |
| VPS4A | | | | | | | | | |
| VPS4B | | | | | | | | | |
| VPS51 | | | | | | | | | |
| VPS52 | | | | | | | | | |
| VPS53 | | | | | | | | | |
| VPS54 | | | | | | | | | |
| VPS55 | | | | | | | | | |
| VPS60 | | | | | | | | | |
| VPS64 | | | | | | | | | |
| VPS70 | | | | | | | | | |
| VPS72 | | | | | | | | | |
| VPS73 | | | | | | | | | |
| VPS74 | | | | | | | | | |
| VPS8 | | | | | | | | | |
| VPS9 | | | | | | | | | |
| VPS9D1 | | | | | | | | | |
| VPS9D1-AS1 | | | | | | | | | |
| vpu | | | | | | | | | |
| vpx | | | | | | | | | |
| VRG4 | | | | | | | | | |
| VRK1 | | | | | | | | | |
| VRK2 | | | | | | | | | |
| VRK3 | | | | | | | | | |
| vrs1 | | | | | | | | | |
| VRTN | | | | | | | | | |
| VSIG1 | | | | | | | | | |
| VSIG10 | | | | | | | | | |
| VSIG10L | | | | | | | | | |
| VSIG2 | | | | | | | | | |

| | | | | | | | | | |
|---|---|---|---|---|---|---|---|---|---|
| VSIG4 | | | | | | | | | |
| VSIG8 | | | | | | | | | |
| VSNL1 | | | | | | | | | |
| VSTM1 | | | | | | | | | |
| VSTM2A | | + | | | | + | | | + |
| VSTM2B | | | | | | | | | |
| VSTM2L | | | | | | | | | |
| VSTM4 | | | | | | | | | |
| VSX1 | | | | | | | | | |
| VSX2 | | | | | | | | | |
| VTA1 | | + | | | | | | | + |
| vtc4 | | | | | | | | | |
| VTCN1 | | | | | | | | | |
| VTI1 | | | | | | | | | |
| VTI1A | | | | | | | | | |
| VTI1B | | + | | | | + | | | + |
| VTN | | | | | | | | | |
| VTRNA1-1 | | | | | | | | | |
| VTRNA1-2 | | | | | | | | | |
| VTRNA2-1 | | | | | | | | | |
| VTRNA3-1P | | | | | | | | | |
| VWA1 | | | | | | | | | |
| VWA2 | | | | | | | | | |
| VWA3A | + | | | | + | + | | | + |
| VWA3B | | | | | | | | | |
| VWA5A | | | | | | | | | |
| VWA5B1 | + | | | | + | | | | + |
| VWA5B2 | | | | | | | | | |
| VWA7 | + | | | + | + | | | | + |
| VWA8 | | | | | | | | | |
| VWA9 | | | | | | | | | |
| VWC2 | | | | | | | | | |
| VWC2L | | | | | | | | | |
| VWCE | | | | | | | | | |
| VWDE | | | | | | | | | |
| VWF | | | | | | | | + | + |
| VWFP1 | | | | | | | | | |
| WAC | | | | | | | | | |
| WAPAL | | | + | | | | | | + |
| WAR1 | | | | | | | | | |
| WARS | | | | | | | | | |
| WARS2 | | | | | | | | | |
| WAS | | | | + | | | | | + |
| WASF1 | | + | | | | + | | | + |
| WASF2 | | | | | | | | | |
| WASF3 | | | | | | | | | |
| WASF4P | | | | | | | | | |
| Wash | | | | | | | | | |
| WASH1 | | | | | | | | | |
| WASH2P | | | | | | | | | |
| WASH3P | | | | | | | | | |
| WASH4P | | | | | | | | | |
| WASH6P | | | | | | | | | |

| | | | | | | | | | |
|---|---|---|---|---|---|---|---|---|---|
| WASL | | | | | | | | | |
| WBP1 | | | | | | | | | |
| WBP11 | | | | | | | | | |
| WBP1L | | | | | | | | | |
| WBP2 | | | | | | | | | |
| WBP2NL | | | | | | | | | |
| WBP4 | | | | | | | | | |
| WBP5 | + | | | | | | | | + |
| WBSCR16 | | | | | | | | | |
| WBSCR17 | | | | | | + | | | + |
| WBSCR22 | | | | | | | | | |
| WBSCR27 | | | | | | | | | |
| WBSCR28 | | | | | | | | | |
| WDFY1 | | | + | | | | | | + |
| WDFY2 | | | | | | | | | |
| WDFY3 | | | | | | + | | | + |
| WDFY4 | | | | | | | | | |
| WDHD1 | | | | | | | | | |
| WDPCP | | | | | | | | | |
| WDR1 | | | | | | | | | |
| WDR11 | | | | | | | | | |
| WDR12 | | | | | | | | | |
| Wdr13 | | | | | | | | | |
| WDR17 | | | | | | | | | |
| WDR18 | | | | + | | | | | + |
| WDR19 | | | | | | | | | |
| WDR20 | | | | | | | | | |
| WDR24 | | | | | | | | | |
| WDR25 | | | | | | | | | |
| WDR26 | | | | | | | | | |
| WDR27 | | | | | | | | | |
| WDR3 | | | | | | | | | |
| WDR31 | | | | | | | | | |
| WDR33 | | + | | | | + | | | + |
| WDR34 | | + | | + | | | | | + |
| WDR35 | | | | | | | | | |
| WDR36 | | | | | | | | | |
| WDR37 | + | | | + | + | | + | | + |
| WDR4 | | | | | | | | | |
| WDR41 | | | | | | + | | | + |
| WDR43 | | | | | | | | | |
| WDR44 | | | | | | | | | |
| WDR45 | | | | | | | | | |
| WDR45B | | | | | | + | | | + |
| WDR46 | | | | | | | | | |
| WDR47 | | | | | | | | | |
| WDR48 | | | | | | | | | |
| WDR49 | | | | | | | | | |
| WDR5 | | | | | | | | | |
| WDR53 | | | | | | | | | |
| WDR54 | | | | | | | | | |
| WDR55 | | | | | | | | | |
| WDR59 | | | | | | | | | |

| | | | | | | | | | |
|---|---|---|---|---|---|---|---|---|---|
| WDR5B | | | | | | | | | |
| WDR6 | | | | | | + | | | + |
| WDR60 | | | | | | | | | |
| WDR61 | | | | | | | | | |
| WDR62 | | | | | | | | | |
| WDR63 | | | | | | | | | |
| WDR64 | | | | | | | | | |
| WDR66 | | | | | | + | | | + |
| WDR7 | | | | | | | | | |
| WDR70 | | | | | | | | | |
| WDR72 | | | | | | | | | |
| WDR73 | | | | | | | | | |
| WDR74 | | | | | | | | | |
| WDR75 | | | | | | | | | |
| WDR76 | | | | | | | | | |
| WDR77 | | | | | | | | | |
| WDR78 | | | | | | | | | |
| WDR81 | | | | | | | | | |
| WDR82 | | | | | | | | | |
| WDR82P1 | | | | | | | | | |
| WDR83 | | | | | | | | | |
| WDR83OS | | | | | | | | | |
| WDR86 | | | | | | | | + | + |
| WDR87 | | | | | | | | | |
| WDR88 | | | | | | | | | |
| WDR89 | | | | | | | | | |
| WDR90 | | | | | + | | | | + |
| WDR91 | | | | | | | | | |
| WDR92 | | | | | | | | | |
| WDSUB1 | | | | | | | | | |
| WDTC1 | | | | | | | | | |
| WDYHV1 | | | | | | | | | |
| WEE1 | | | | | | | | | |
| wee1B | | | | | | | | | |
| WEE2 | | | | | | | | | |
| WEE2-AS1 | | | | | | | | | |
| WFDC1 | | | | | | | | | |
| WFDC10A | | | | | | | | | |
| WFDC10B | | | | | | | | | |
| WFDC11 | | | | | | | | | |
| WFDC12 | | | | | | | | | |
| WFDC13 | + | | | + | + | | + | | + |
| WFDC2 | | | | | | | | | |
| WFDC3 | | | | | | | | | |
| WFDC5 | | | | | | | | | |
| WFDC6 | | | | | | | | | |
| WFDC8 | | | | | | | | | |
| WFDC9 | | | | | | | | | |
| WFIKKN1 | | | | | | | | | |
| WFS1 | | | | | | | | | |
| wg | | | | | | | | | |
| WHAMM | | | | | | | | | |
| WHAMMP1 | | | | | | | | | |

| | | | | | | | | | |
|---|---|---|---|---|---|---|---|---|---|
| WHAMMP2 | | | | | | | | | |
| WHAMMP3 | | | | | | | | | |
| WHI3 | | | | | | | | | |
| WHSC1 | | | + | | | | | | + |
| WHSC1L1 | | | | | | | | | |
| WIBG | | | | | | | | | |
| WIF1 | | | | | | | | | |
| WIPF1 | | | | | | | | | |
| WIPF2 | | | | | | | | | |
| WIPF3 | | | | | | | | | |
| WIPI1 | | | | | | | | | |
| WIPI2 | | | | | | | | | |
| WISP1 | + | | | + | + | | + | | + |
| WISP2 | | | | | | | | | |
| WISP3 | | | | | | | | | |
| WIZ | | | | | | | | | |
| WLS | | | + | | | | | | + |
| WNK1 | | | | | | | | | |
| WNK2 | | | | | | + | | | + |
| WNK3 | | | | | | | | | |
| WNK4 | | | | | | | | | |
| WNT1 | | | | | | | | | |
| WNT10A | | | | | | | | | |
| WNT10B | | | | | | | | | |
| WNT11 | | | | | | | | | |
| WNT16 | | | | | | | | | |
| WNT2 | | | | | | | | | |
| WNT2B | | | | | | | | | |
| WNT3 | | | | | | | | | |
| WNT3A | | | | | | | | | |
| WNT4 | + | | | + | + | | | | + |
| WNT5A | | | | | | | | | |
| WNT5B | | | | | | | | | |
| WNT6 | | | | | | | | | |
| WNT7A | | | | | | | | | |
| WNT7B | | | | | | | | | |
| WNT8A | | | | | | | | | |
| WNT8B | | | | | | | | | |
| WNT9A | | | | | | | | | |
| WNT9B | | | | | | | | | |
| WRAP53 | | | | | | | | | |
| WRAP73 | | | | | | | | | |
| WRB | | | | | | | | | |
| WRN | | | | | | | | | |
| WRNIP1 | | | | | | | | | |
| WSB1 | | | | | + | | | | + |
| WSB2 | | | | | + | | | | + |
| WSS1 | | | | | | | | | |
| WT1 | | | | | | | | | |
| WT1-AS | | | | | | | | | |
| WTAP | | | | | | | | | |
| wtf14 | | | | | | | | | |
| WTH3DI | | | | | | | | | |

| | | | | | | | | | |
|---|---|---|---|---|---|---|---|---|---|
| WTIP | | | | | | | | | |
| WTM2 | | | | | | | | | |
| WWC1 | | | | | | + | | | + |
| WWC2 | | | | | | | | | |
| WWC3 | | | | | | | | | |
| WWOX | | + | | | | | | | + |
| WWP1 | | | | | | | | | |
| WWP2 | | | | | | | | | |
| WWTR1 | | | | | | | | | |
| WWTR1-AS1 | | | | | | | | | |
| XAB2 | | | | | | | | | |
| XAF1 | | | | | | | | | |
| XAGE1B | | | | | | | | | |
| XAGE1E | | | | | | | | | |
| XAGE2 | | | | | | | | | |
| XAGE3 | | | | | | | | | |
| XBP1 | | | | | | | | | |
| XBP1P1 | | | | | | | | | |
| XCL1 | | | | | | | | | |
| XCL2 | | | | | | | | | |
| XCR1 | | + | | + | | | | | + |
| XDH | | | | | | | | | |
| XIAP | | | | | | | | | |
| XIRP1 | | | | | | | | | |
| XIRP2 | | | | | | | | | |
| XIST | | | | | | | | | |
| XK | | | | | | | | | |
| XKR3 | | | | | | | | | |
| XKR4 | + | | | + | + | + | + | | + |
| XKR5 | | | | | | | | | |
| XKR6 | | | | | | | | | |
| XKR7 | | | | | | | | | |
| XKR8 | | | | | | | | | |
| XKRX | | | | | | | | | |
| XKRY | | | | | | | | | |
| XKRY2 | | | | | | | | | |
| xlf1 | | | | | | | | | |
| xlmo1 | | | | | | | | | |
| XOG1 | | | | | | | | | |
| XPA | | | | | | | | | |
| XPC | | | | | | | | | |
| XPNPEP1 | | | | | | | | | |
| XPNPEP2 | | | | | | | | | |
| XPNPEP3 | | | | | | | | | |
| XPO1 | | | | | | | | | |
| XPO4 | | | | | | | | | |
| XPO5 | | | | | | | | | |
| XPO6 | | | | | | | | | |
| XPO7 | | | | | | | | | |
| XPOT | | | + | | | | | | + |
| XPR1 | | | | | | | | | |
| XRCC1 | | | | | | | | | |
| XRCC2 | | | + | | | | | | + |

| | | | | | | | | | |
|---|---|---|---|---|---|---|---|---|---|
| XRCC3 | | | | | | | | | |
| XRCC4 | | | | | | | | | |
| XRCC5 | | | | | | | | | |
| XRCC6 | | | | | | | | | |
| XRCC6BP1 | | | | | | | | | |
| XRN1 | | | | | | | | | |
| XRN2 | | | | | | | | | |
| XRRA1 | | | | | | | | | |
| XRS2 | | | | | | | | | |
| XXYLT1 | | | | | | | | | |
| XYLB | | | | | | | | | |
| XYLT1 | | | | | | | | | |
| XYLT2 | + | | + | | | | | | + |
| YAE1D1 | | | | | | | | | |
| YAF2 | | | | | | | | | |
| YAF9 | | | | | | | | | |
| YAL044W-A | | | | | | | | | |
| YAP1 | | | | | | | | | |
| YAP3 | | | | | | | | | |
| YARS | | | | | | | | | |
| YARS2 | | | | | | | | | |
| YAT1 | | | | | | | | | |
| YBEY | | | | | | | | | |
| YBL086C | | | | | | | | | |
| YBP2 | | | | | | | | | |
| YBR013C | | | | | | | | | |
| YBR056W | | | | | | | | | |
| YBR139W | | | | | | | | | |
| YBR197C | | | | | | | | | |
| YBR242W | | | | | | | | | |
| YBR284W | | | | | | | | | |
| YBX1 | | | | | | | | | |
| YBX2 | | | | | | | | | |
| YBX3 | | | | | | | | | |
| YCH1 | | | | | | | | | |
| YCK1 | | | | | | | | | |
| YCK2 | | | | | | | | | |
| YCK3 | | | | | | | | | |
| YCL042W | | | | | | | | | |
| YCP4 | | | | | | | | | |
| YCR007C | | | | | | | | | |
| YCR050C | | | | | | | | | |
| YCR051W | | | | | | | | | |
| YCR087C-A | | | | | | | | | |
| YDJ1 | | | | | | | | | |
| YDJC | | | | | | | | | |
| YDL057W | | | | | | | | | |
| YDL124W | | | | | | | | | |
| YDR134C | | | | | | | | | |
| YDR341C | | | | | | | | | |
| YDR476C | | | | | | | | | |
| YEATS2 | | | + | | | | | | + |
| YEATS4 | | | | | | | | | |

| | | | | | | | | | |
|---|---|---|---|---|---|---|---|---|---|
| YEF1 | | | | | | | | | |
| YEF3 | | | | | | | | | |
| YES1 | | | | | | | | | |
| YES1P1 | | | | | | | | | |
| YET1 | | | | | | | | | |
| YFH1 | | | | | | | | | |
| YFL051C | | | | | | | | | |
| YFR035C | | | | | | | | | |
| YFT2 | | | | | | | | | |
| YGL082W | | | | | | | | | |
| YGL176C | | | | | | | | | |
| YGL262W | | | | | | | | | |
| YGR015C | | | | | | | | | |
| YGR021W | | | | | | | | | |
| YGR053C | | | | | | | | | |
| YGR054W | | | | | | | | | |
| YGR067C | | | | | | | | | |
| YGR130C | | | | | | | | | |
| YGR201C | | | | | | | | | |
| YHB1 | | | | | | | | | |
| YHL018W | | | | | | | | | |
| YHR020W | | | | | | | | | |
| YHR022C | | | | | | | | | |
| YHR045W | | | | | | | | | |
| YHR127W | | | | | | | | | |
| YHR140W | | | | | | | | | |
| YIF1 | | | | | | | | | |
| YIF1A | | | | | | | | | |
| YIF1B | | | | | | | | | |
| YIH1 | | | | | | | | | |
| YIL001W | | | | | | | | | |
| YIL108W | | | | | | | | | |
| YIL152W | | | | | | | | | |
| YIL165C | | | | | | | | | |
| YIPF1 | | | | | | | | | |
| YIPF2 | | | | | | | | | |
| YIPF3 | | + | | | | + | | | + |
| Yipf4 | | | | | | | | | |
| YIPF5 | | | | | + | | | | + |
| YIPF6 | | | | | | | | | |
| YJEFN3 | | | | | | | | | |
| YJL107C | | | | | | | | | |
| YJL118W | | | | | | | | | |
| YJL144W | | | | | | | | | |
| YJR003C | | | | | | | | | |
| YJR011C | | | | | | | | | |
| YJR039W | | | | | | | | | |
| YJR084W | | | | | | | | | |
| YJR107W | | | | | | | | | |
| YJR154W | | | | | | | | | |
| YKL063C | | | | | | | | | |
| YKL071W | | | | | | | | | |
| YKR011C | | | | | | | | | |

| | | | | | | | | | |
|---|---|---|---|---|---|---|---|---|---|
| YKR023W | | | | | | | | | |
| YKT6 | | | | | | | | | |
| YLPM1 | | + | | | | + | | | + |
| YLR031W | | | | | | | | | |
| YLR036C | | | | | | | | | |
| YLR108C | | | | | | | | | |
| YLR118C | | | | | | | | | |
| YLR177W | | | | | | | | | |
| YLR224W | | | | | | | | | |
| YLR297W | | | | | | | | | |
| YMD8 | | | | | | | | | |
| YME1L1 | | | | | | | | | |
| YML053C | | | | | | | | | |
| YML108W | | | | | | | | | |
| YMR111C | | | | | | | | | |
| YMR122W-A | | | | | | | | | |
| YMR160W | | | | | | | | | |
| YMR226C | | | | | | | | | |
| YMR31 | | | | | | | | | |
| YMR315W | | | | | | | | | |
| YNL146W | | | | | | | | | |
| YNL217W | | | | | | | | | |
| YNL247W | | | | | | | | | |
| YOD1 | | | | | | | | | |
| YOP1 | | | | | | | | | |
| YOR192C-A | | | | | | | | | |
| YOR292C | | | | | | | | | |
| YOR385W | | | | | | | | | |
| YPEL1 | | | | | | | | | |
| YPEL2 | | | | | | | | | |
| YPEL3 | | | | | | | | | |
| Ypel5 | | | | | + | + | | | + |
| YPF1 | | | | | | | | | |
| YPK1 | | | | | | | | | |
| YPK2 | | | | | | | | | |
| YPK9 | | | | | | | | | |
| YPL067C | | | | | | | | | |
| YPL077C | | | | | | | | | |
| YPL108W | | | | | | | | | |
| YPL199C | | | | | | | | | |
| YPR148C | | | | | | | | | |
| YPR153W | | | | | | | | | |
| YPR159C-A | | | | | | | | | |
| YPR174C | | | | | | | | | |
| YPT1 | | | | | | | | | |
| YPT10 | | | | | | | | | |
| YPT35 | | | | | | | | | |
| YPT52 | | | | | | | | | |
| YPT53 | | | | | | | | | |
| YPT6 | | | | | | | | | |
| YRA1 | | | | | | | | | |
| YRB1 | | | | | | | | | |
| YRB30 | | | | | | | | | |

| | | | | | | | | | |
|---|---|---|---|---|---|---|---|---|---|
| YRDC | | | | | | | | | |
| yrs1 | | | | | | | | | |
| YSA1 | | | | | | | | | |
| YSY6 | | | | | | | | | |
| YTA12 | | | | | | | | | |
| YTH1 | | | | | | | | | |
| YTHDC1 | | | | | | | | | |
| YTHDC2 | | | | | | | | | |
| YTHDF1 | | | | | | | | | |
| YTHDF2 | | | | | | | | | |
| YTHDF3 | | | + | | | | | | + |
| YUH1 | | | | | | | | | |
| YVH1 | | | | | | | | | |
| YWHAB | | | | | | | | | |
| YWHAE | | | | | | | | | |
| YWHAEP7 | | | | | | | | | |
| YWHAG | + | | + | | + | | | | + |
| YWHAH | | + | | | | + | | | + |
| YWHAQ | | | | | | | | | |
| YWHAZ | | | | | + | | | | + |
| YY1 | + | | | + | + | | | | + |
| YY1AP1 | | | | | | | | | |
| YY2 | | | | | | | | | |
| ZACN | | | | | | | | | |
| ZADH2 | | | | | | + | | | + |
| ZAK | + | | | + | + | | | | + |
| ZAN | | | | | | | | | |
| ZAP70 | | + | | | | + | | | + |
| ZAR1 | | | | | | | | | |
| ZAR1L | | | | | | | | | |
| ZBBX | | | | | | | | | |
| ZBED1 | | | | | | | | | |
| ZBED2 | | | | | | | | | |
| ZBED3 | | | | | | | | | |
| ZBED4 | | | | | | | | | |
| ZBED5 | | | | | | | | | |
| ZBED6 | | | | | | | | | |
| ZBED6CL | | | | | | | | | |
| ZBED8 | | | | | | | | | |
| ZBED9 | | | | | | | | | |
| ZBP1 | | | | | | | | | |
| ZBTB1 | | | + | | | | | | + |
| ZBTB10 | | | + | | | | | | + |
| ZBTB11 | | | | | | | | | |
| ZBTB12 | | | | | | | | | |
| ZBTB14 | | | | | | + | | | + |
| ZBTB16 | + | | | | + | | | | + |
| ZBTB17 | | | | | | | | | |
| ZBTB18 | | + | | | | + | | | + |
| ZBTB2 | | | | | | | | | |
| ZBTB20 | | | | | | + | | | + |
| ZBTB21 | | | | | | | | | |
| ZBTB22 | | | | | + | | | | + |

| | | | | | | | | | |
|---|---|---|---|---|---|---|---|---|---|
| ZBTB24 | | | | | | | | | |
| ZBTB25 | | | | | | | | | |
| ZBTB26 | | | | | | | | | |
| ZBTB3 | | | | | | | | | |
| ZBTB32 | | | | | | | | | |
| ZBTB33 | | | | | | | | | |
| ZBTB34 | + | | | + | + | | | | + |
| ZBTB37 | | | | | | + | | | + |
| ZBTB38 | | | | | | | | | |
| ZBTB39 | | | | | | | | | |
| ZBTB4 | | | | | | | | | |
| ZBTB40 | | | | | | + | | | + |
| ZBTB41 | | | + | | | | | | + |
| ZBTB42 | | | | | | | | | |
| ZBTB43 | | | | | | | | | |
| ZBTB44 | | | | | | | | | |
| ZBTB45 | | | | | | | | | |
| ZBTB46 | | | | | | | | | |
| ZBTB47 | | | | | | | | | |
| ZBTB48 | | | | | | | | | |
| ZBTB49 | | | | | | + | | | + |
| ZBTB5 | | | | | | + | | | + |
| ZBTB6 | | | | | | | | | |
| ZBTB7A | | | | | | | | | |
| ZBTB7B | | | | | | | | | |
| ZBTB7C | | | | | | | | | |
| ZBTB8A | | | | | | | | | |
| Zbtb8b | | | | | | | | | |
| ZBTB8OS | | | | | | | | | |
| ZBTB9 | | | | | | | | | |
| ZC2HC1A | | | | | | | | | |
| ZC2HC1B | | | | | | | | | |
| ZC2HC1C | | | | | | | | | |
| ZC3H10 | | | | | | | | | |
| ZC3H11A | | | + | | | | | | + |
| ZC3H11B | | | | | | | | | |
| ZC3H12A | | | | | | | | | |
| ZC3H12B | | | | | | | | | |
| ZC3H12C | | | + | | | | | | + |
| ZC3H12D | | | | | | | | | |
| ZC3H13 | + | | | + | + | | | | + |
| ZC3H14 | | | | | | | | | |
| ZC3H15 | | | | | | | | | |
| ZC3H18 | | | | | | | | | |
| ZC3H3 | | | | | | | | | |
| ZC3H4 | | | | | | | | | |
| ZC3H6 | | | | | | + | | | + |
| ZC3H7A | | | | | | | | | |
| ZC3H7B | | | | | | | | | |
| ZC3H8 | | | | | | | | | |
| ZC3HAV1 | | + | + | | | + | | | + |
| ZC3HAV1L | + | | | + | + | | | | + |
| ZC3HC1 | | | | + | | + | | | + |

| | | | | | | | | | |
|---|---|---|---|---|---|---|---|---|---|
| ZC4H2 | | | | | | | | | |
| ZCCHC10 | | | | | | | | | |
| ZCCHC11 | | | | + | | | | | + |
| ZCCHC12 | | | + | | | | | | + |
| ZCCHC13 | | | | | | | | | |
| ZCCHC14 | | | | | | | | | |
| ZCCHC16 | | | | | | | | | |
| ZCCHC17 | | | | | | | | | |
| ZCCHC18 | | | | | | + | | | + |
| ZCCHC2 | | | | | | + | | | + |
| ZCCHC24 | | | | | | | | | |
| ZCCHC3 | | | | | | | | | |
| ZCCHC4 | | | | | | | | | |
| ZCCHC6 | | | | | | | | | |
| ZCCHC7 | | + | | | | + | | | + |
| ZCCHC8 | | | | | | | | | |
| ZCCHC9 | | | + | | | | | | + |
| ZCRB1 | | | | | | | | | |
| ZCWPW1 | | | | | | | | | |
| ZCWPW2 | | | | | | | | | |
| ZDBF2 | + | | + | | + | | | | + |
| ZDHHC1 | | | | | | | | | |
| ZDHHC11 | | | | | | | | | |
| ZDHHC11B | | | | | | | | | |
| ZDHHC12 | | | | | | | | | |
| ZDHHC13 | | | | | | | | | |
| ZDHHC14 | | | | | | | | | |
| ZDHHC15 | | | | | | | | | |
| ZDHHC16 | | | | | | | | | |
| ZDHHC17 | | | | | | | | | |
| ZDHHC18 | | | | | | | | | |
| ZDHHC19 | | | | | | | | | |
| ZDHHC2 | | | | | | | | | |
| ZDHHC20 | | | | | | | | | |
| ZDHHC21 | | | | | | | | | |
| ZDHHC22 | | | | | | | | | |
| ZDHHC23 | | | | | | | | | |
| ZDHHC24 | | | | | | | | | |
| ZDHHC3 | | | | | | + | | | + |
| ZDHHC4 | | | | | | | | | |
| ZDHHC5 | + | | | | + | | | | + |
| ZDHHC6 | | | | | | | | | |
| ZDHHC7 | | | | | | | | | |
| ZDHHC8 | | | | | | | | | |
| ZDHHC9 | + | | | + | + | | | | + |
| ZEB1 | | | | | | | | | |
| ZEB2 | | + | | | | + | | | + |
| ZEO1 | | | | | | | | | |
| ZER1 | | | | | | + | | | + |
| ZFAND1 | | | | | | + | | | + |
| ZFAND2A | | | | | + | | | | + |
| ZFAND2B | | | | | | | | | |
| ZFAND3 | | | | | | | | | |

| | | | | | | | | | |
|---|---|---|---|---|---|---|---|---|---|
| ZFAND4 | | | | | | | | | |
| ZFAND5 | | | + | | + | | | | + |
| ZFAND6 | | + | | | | + | | | + |
| ZFAS1 | | | | | | | | | |
| ZFAT | | | | | | | | | |
| ZFC3H1 | | | | | + | | | | + |
| ZFHX2 | + | | | | + | | | | + |
| ZFHX3 | + | | | + | + | | | | + |
| ZFHX4 | | | | | | | | | |
| Zfml | | | | | | | | | |
| ZFP1 | | | | | | + | | | + |
| Zfp128 | | | | | | | | | |
| ZFP14 | | | | | | | | | |
| ZFP2 | | | | | | + | | | + |
| Zfp275 | | | | | + | | | | + |
| ZFP28 | | | | | | | | | |
| ZFP3 | | | | | | | | | |
| ZFP30 | | | | | | + | | | + |
| Zfp326 | | | | | | | | | |
| ZFP36 | | | | | | | | | |
| ZFP36L1 | | | | | | | | + | + |
| ZFP36L2 | | | + | | | | | | + |
| ZFP37 | + | | | + | + | | | | + |
| ZFP41 | | | | | | | | | |
| ZFP42 | | | | | | | | | |
| Zfp512 | | | | | | | | | |
| Zfp518a | | | | | | | | | |
| Zfp521 | | | | | | | | | |
| ZFP57 | | | | | | | | | |
| Zfp574 | | | | | | | | | |
| Zfp598 | | | | | | | | | |
| ZFP62 | | | | | | | | | |
| Zfp622 | + | | | + | + | | | | + |
| ZFP64 | | | | | | | | | |
| ZFP69 | | | | | | | | | |
| ZFP69B | | | | | | | | | |
| Zfp748 | | | | | | | | | |
| Zfp780b | + | | | + | + | | + | | + |
| Zfp788 | | | | | | | | | |
| ZFP82 | | | | | | | | | |
| ZFP90 | | | | | | | | | |
| ZFP91 | | | | | | | | | |
| ZFP91-CNTF | | | | | | | | | |
| ZFP92 | | | | | | | | | |
| ZFPL1 | | | | | | | | | |
| ZFPM1 | | | | | | | | | |
| ZFPM2 | | | | | | | | | |
| ZFR | | | | | | | | | |
| ZFR2 | | | | | | + | | | + |
| ZFX | | | | | | | | | |
| ZFY | | | | | | | | | |
| ZFYVE1 | | | | | | | | | |
| ZFYVE16 | | | | | | | | | |

| | | | | | | | | | |
|---|---|---|---|---|---|---|---|---|---|
| ZFYVE19 | | | | | | | | | |
| ZFYVE20 | | | | | | | | | |
| ZFYVE21 | | | | | | | | | |
| ZFYVE26 | | | | | | + | | | + |
| ZFYVE27 | | | | | | | | | |
| ZFYVE28 | | | | | | | | | |
| ZFYVE9 | | | | | | | | | |
| ZG16 | | | | | | | | | |
| ZG16B | | | | | | | | | |
| ZGPAT | | | | | | | | | |
| ZGRF1 | | | | | | | | | |
| ZHX1 | | | | | | | | | |
| ZHX1-C8orf76 | | | | | | | | | |
| ZHX2 | | | | | | | | | |
| ZHX3 | | | | | | | | | |
| ZIC1 | + | | | + | + | | | | + |
| ZIC2 | | | | | | | | | |
| ZIC3 | | | | | | | | | |
| ZIC4 | + | | | + | + | | + | | + |
| ZIC5 | | | | | | | | | |
| ZIK1 | | | | | | | | | |
| ZIM2 | | | | | | | | | |
| ZIM3 | | | | | | | | | |
| ZKSCAN1 | | + | | | | | | | + |
| ZKSCAN2 | | | | | | | | | |
| ZKSCAN3 | | | | | | | | | |
| ZKSCAN4 | | | | | | | | | |
| ZKSCAN5 | | | | | | | | | |
| ZKSCAN7 | | | | | | | | | |
| ZKSCAN8 | | | | | | | | | |
| ZMAT1 | | | | | | + | | | + |
| ZMAT2 | | | | | | | | | |
| ZMAT3 | | | | | | | | | |
| ZMAT4 | | | | | | | | | |
| ZMAT5 | | | | | | | | | |
| ZMIZ1 | | | | | | | | | |
| ZMIZ1-AS1 | | | | | | | | | |
| ZMIZ2 | | + | | | | | | | + |
| ZMPSTE24 | | | | | | | | | |
| ZMYM1 | | | | | | | | | |
| ZMYM2 | | | | | | | | | |
| ZMYM3 | | | | | | | | | |
| ZMYM4 | | | | | | | | | |
| ZMYM5 | | | | | | | | | |
| ZMYM6 | | | | | | | | | |
| ZMYM6NB | | | | | | | | | |
| ZMYND10 | | | | | | | | | |
| ZMYND11 | | | | | | | | | |
| ZMYND12 | | | | | | | | | |
| ZMYND15 | | | | | | | | | |
| ZMYND19 | | | | | | | | | |
| ZMYND8 | | | | | | | | | |
| ZNF10 | | | | | | | | | |

| | | | | | | | | | | |
|---|---|---|---|---|---|---|---|---|---|---|
| ZNF100 | | | | | | | | | | |
| ZNF101 | | | | | | | | | | |
| ZNF106 | | | | | | | | | | |
| ZNF107 | | | | | | | | | | |
| ZNF112 | | | | | | | | | | |
| ZNF114 | | | | | | | | | | |
| ZNF117 | | | | | | | | | | |
| ZNF12 | | | | | | | | | | |
| ZNF121 | | | | | | | | | | |
| ZNF124 | | | | | | | | | | |
| ZNF131 | | | | | | | | | | |
| ZNF133 | | | | | | | | | | |
| ZNF134 | | | | | | | | | | |
| ZNF135 | | | | | | | | | | |
| ZNF136 | | | | | | | | | | |
| ZNF137P | | | | | | | | | | |
| ZNF138 | | | | | | | | | | |
| ZNF14 | | | | | | | | | | |
| ZNF140 | | | | | | | | | | |
| ZNF141 | | | | | | | | | | |
| ZNF142 | | | | | | | | | | |
| ZNF143 | | | | | | | | | | |
| ZNF146 | | | | | | | | | | |
| ZNF148 | | | | | | | | | | |
| ZNF154 | | | | | | | | | | |
| ZNF155 | | | | | | | | | | |
| ZNF157 | | | | | | | | | | |
| ZNF16 | | | | | | | | | | |
| ZNF160 | | | | | | | | | | |
| ZNF165 | | | | | | | | | | |
| ZNF169 | | | | | | | | | | |
| ZNF17 | | | | | | | | | | |
| ZNF174 | | | | | | | | | | |
| ZNF175 | | | | | | | | | | |
| ZNF177 | | | | | | | | | | |
| ZNF18 | | | | | | | | | | |
| ZNF180 | | | | | | | | | | |
| ZNF181 | | | | | | | | | | |
| ZNF182 | | | | | | | | | | |
| ZNF184 | | | | | | | | | | |
| ZNF185 | | | | | | | | | | |
| ZNF189 | | | | | | | | | | |
| ZNF19 | | | | | | | | | | |
| ZNF195 | | | | | | | | | | |
| ZNF197 | | | | | | | | | | |
| ZNF2 | | | | | | | | | | |
| ZNF20 | | | | | | | | | | |
| ZNF200 | | | | | | | | | | |
| ZNF202 | | | | | | | | | | |
| ZNF205 | | | | | | | | | | |
| ZNF205-AS1 | | | | | | | | | | |
| ZNF207 | | | | | | | | | | |
| ZNF208 | | | | | | | | | | |

| | | | | | | | | | | |
|---|---|---|---|---|---|---|---|---|---|---|
| ZNF211 | | | | | | | | | | |
| ZNF212 | | | | | | | | | | |
| ZNF213 | | | | | | | | | | |
| ZNF214 | | | | | | | | | | |
| ZNF215 | | | | | | | | | | |
| ZNF217 | | | | | | | | | | |
| ZNF219 | | | | | | | | | | |
| ZNF22 | | | | | | | | | | |
| ZNF221 | | | | | | | | | | |
| ZNF223 | | | | | | | | | | |
| ZNF224 | | | | | | | | | | |
| ZNF225 | | | | | | | | | | |
| ZNF226 | | | | | | | | | | |
| ZNF227 | | | | | | | | | | |
| ZNF229 | | | | | | | | | | |
| ZNF23 | | | | | | | | | | |
| ZNF230 | | | | | | | | | | |
| ZNF232 | | | | | | | | | | |
| ZNF233 | | | | | | | | | | |
| ZNF234 | | | | | | | | | | |
| ZNF235 | | | | | | | | | | |
| ZNF236 | | | | | | | | | | |
| ZNF239 | | | | | | | | | | |
| ZNF24 | | | | | | | | | | |
| ZNF248 | | | | | | | | | | |
| ZNF25 | | | | | | | | | | |
| ZNF250 | | | | | | | | | | |
| ZNF251 | | | | | | | | | | |
| ZNF252P-AS1 | | | | | | | | | | |
| ZNF253 | | | | | | | | | | |
| ZNF254 | | | | | | | | | | |
| ZNF256 | | | | | | | | | | |
| ZNF257 | | | | | | | | | | |
| ZNF26 | | | | | | | | | | |
| ZNF260 | | | | | | | | | | |
| ZNF263 | | | | | | | | | | |
| ZNF264 | | | | | | | | | | |
| ZNF266 | | | | | | | | | | |
| ZNF267 | | | | | | | | | | |
| ZNF268 | | | | | | | | | | |
| ZNF273 | | | | | | | | | | |
| ZNF274 | | | | | | | | | | |
| ZNF275 | | | | | | | | | | |
| ZNF276 | | | | | | | | | | |
| ZNF277 | | | | | | | | | | |
| ZNF28 | | | | | | | | | | |
| ZNF280A | | | | | | | | | | |
| ZNF280B | | | | | | | | | | |
| ZNF280C | | | | | | | | | | |
| ZNF280D | | | | | | | | | | |
| ZNF281 | | | | | | | | | | |
| ZNF282 | | | | | | | | | | |
| ZNF283 | | | | | | | | | | |

| | | | | | | | | | |
|---|---|---|---|---|---|---|---|---|---|
| ZNF284 | | | | | | | | | |
| ZNF285 | | | | | | | | | |
| ZNF286A | | | | | | | | | |
| ZNF286B | | | | | | | | | |
| ZNF287 | | | | | | | | | |
| ZNF292 | | | | | | | | | |
| ZNF296 | | | | | | | | | |
| ZNF3 | | | | | | | | | |
| ZNF30 | | | | | | | | | |
| ZNF300 | | | | | | | | | |
| ZNF302 | | | | | | | | | |
| ZNF304 | | | | | | | | | |
| ZNF311 | | | | | | | | | |
| ZNF316 | | | | | | | | | |
| ZNF317 | | | | | | | | | |
| ZNF318 | | | | | | | | | |
| ZNF319 | | | | | | | | | |
| ZNF32 | | | | | | | | | |
| ZNF320 | | | | | | | | | |
| ZNF321P | | | | | | | | | |
| ZNF322 | | | | | | | | | |
| ZNF324 | | | | | | | | | |
| ZNF324B | | | | | | | | | |
| ZNF326 | | | | | | | | | |
| ZNF329 | | | | | | | | | |
| ZNF330 | | | | | | | | | |
| ZNF331 | | | | | | | | | |
| ZNF333 | | | | | | | | | |
| ZNF334 | | | | | | | | | |
| ZNF335 | | | | | | | | | |
| ZNF337 | | | | | | | | | |
| ZNF33A | | | | | | | | | |
| ZNF33B | | | | | | | | | |
| ZNF34 | | | | | | | | | |
| ZNF341 | | | | | | | | | |
| ZNF343 | | | | | | | | | |
| ZNF345 | | | | | | | | | |
| ZNF346 | | | | | | | | | |
| ZNF35 | | | | | | | | | |
| ZNF350 | | | | | | | | | |
| ZNF354A | | | | | | | | | |
| ZNF354B | | | | | | | | | |
| ZNF354C | | | | | | | | | |
| ZNF355P | | | | | | | | | |
| ZNF358 | | | | | | | | | |
| ZNF362 | | | | | | | | | |
| ZNF365 | | | | | | | | | |
| ZNF366 | | | | | | | | | |
| ZNF37A | | | | | | | | | |
| ZNF37BP | | | | | | | | | |
| ZNF382 | | | | | | | | | |
| ZNF383 | | | | | | | | | |
| ZNF384 | | | | | | | | | |

| | | | | | | | | | |
|---|---|---|---|---|---|---|---|---|---|
| ZNF385A | | | | | | | | | |
| ZNF385B | | | | | | | | | |
| ZNF385C | | | | | | | | | |
| ZNF385D | | | | | | | | | |
| ZNF391 | | | | | | | | | |
| ZNF394 | | | | | | | | | |
| ZNF395 | | | | | | | | | |
| ZNF396 | | | | | | | | | |
| ZNF397 | | | | | | | | | |
| ZNF398 | | | | | | | | | |
| ZNF404 | | | | | | | | | |
| ZNF407 | | | | | | | | | |
| ZNF408 | | | | | | | | | |
| ZNF41 | | | | | | | | | |
| ZNF410 | | | | | | | | | |
| ZNF414 | | | | | | | | | |
| ZNF415 | | | | | | | | | |
| ZNF416 | | | | | | | | | |
| ZNF417 | | | | | | | | | |
| ZNF418 | | | | | | | | | |
| ZNF419 | | | | | | | | | |
| ZNF420 | | | | | | | | | |
| ZNF423 | | | | | | | | | |
| ZNF425 | | | | | | | | | |
| ZNF426 | | | | | | | | | |
| ZNF428 | | | | | | | | | |
| ZNF43 | | | | | | | | | |
| ZNF430 | | | | | | | | | |
| ZNF431 | | | | | | | | | |
| ZNF432 | | | | | | | | | |
| ZNF433 | | | | | | | | | |
| ZNF436 | | | | | | | | | |
| ZNF438 | | | | | | | | | |
| ZNF439 | | | | | | | | | |
| ZNF44 | | | | | | | | | |
| ZNF440 | | | | | | | | | |
| ZNF441 | | | | | | | | | |
| ZNF442 | | | | | | | | | |
| ZNF443 | | | | | | | | | |
| ZNF444 | | | | | | | | | |
| ZNF445 | | | | | | | | | |
| ZNF446 | | | | | | | | | |
| ZNF449 | | | | | | | | | |
| ZNF45 | | | | | | | | | |
| ZNF451 | | | | | | | | | |
| ZNF454 | | | | | | | | | |
| ZNF460 | | | | | | | | | |
| ZNF461 | | | | | | | | | |
| ZNF462 | | | | | | | | | |
| ZNF467 | | | | | | | | | |
| ZNF468 | | | | | | | | | |
| ZNF469 | | | | | | | | | |
| ZNF470 | | | | | | | | | |

| | | | | | | | | | |
|---|---|---|---|---|---|---|---|---|---|
| ZNF471 | | | | | | | | | |
| ZNF473 | | | | | | | | | |
| ZNF474 | | | | | | | | | |
| ZNF479 | | | | | | | | | |
| ZNF48 | | | | | | | | | |
| ZNF480 | | | | | | | | | |
| ZNF483 | | | | | | | | | |
| ZNF484 | | | | | | | | | |
| ZNF485 | | | | | | | | | |
| ZNF486 | | | | | | | | | |
| ZNF487 | | | | | | | | | |
| ZNF488 | | | | | | | | | |
| ZNF490 | | | | | | | | | |
| ZNF491 | | | | | | | | | |
| ZNF492 | | | | | | | | | |
| ZNF493 | | | | | | | | | |
| ZNF496 | | | | | | | | | |
| ZNF497 | | | | | | | | | |
| ZNF500 | | | | | | | | | |
| ZNF501 | | | | | | | | | |
| ZNF502 | | | | | | | | | |
| ZNF503 | | | | | | | | | |
| ZNF506 | | | | | | | | | |
| ZNF507 | | | | | | | | | |
| ZNF510 | | | | | | | | | |
| ZNF511 | | | | | | | | | |
| ZNF512 | | | | | | | | | |
| ZNF512B | | + | | | | + | | | + |
| ZNF513 | | | | | | | | | |
| ZNF514 | | | | | | | | | |
| ZNF516 | | | | | | | | | |
| ZNF517 | | | | | | | | | |
| ZNF518A | | | | | | | | | |
| ZNF518B | | | | | | | | | |
| ZNF519 | | | | | | | | | |
| ZNF521 | | | | | | | | | |
| ZNF524 | | | | | | | | | |
| ZNF525 | | | | | | | | | |
| ZNF526 | | | | | | | | | |
| ZNF527 | | | | | | | | | |
| ZNF528 | | | | | | | | | |
| ZNF529 | | | | | | | | | |
| ZNF530 | | | | | | | | | |
| ZNF532 | | | | | | | | | |
| ZNF534 | | | | | | | | | |
| ZNF536 | | | | | | | | | |
| ZNF540 | | | | | | | | | |
| ZNF541 | | | | | | | | | |
| ZNF543 | | | | | | | | | |
| ZNF544 | | | | | | | | | |
| ZNF546 | | | | | | | | | |
| ZNF547 | | | | | | | | | |
| ZNF548 | | | | | | | | | |

| | | | | | | | | | |
|---|---|---|---|---|---|---|---|---|---|
| ZNF549 | | | | | | | | | |
| ZNF550 | | | | | | | | | |
| ZNF551 | | | | | | | | | |
| ZNF552 | | | | | | | | | |
| ZNF554 | | | | | | | | | |
| ZNF555 | | | | | | | | | |
| ZNF556 | | | | | | | | | |
| ZNF557 | | | | | | | | | |
| ZNF558 | | | | | | | | | |
| ZNF559 | | | | | | | | | |
| ZNF559-ZNF177 | | | | | | | | | |
| ZNF561 | | | | | | | | | |
| ZNF562 | | | | | | | | | |
| ZNF563 | | | | | | | | | |
| ZNF564 | | | | | | | | | |
| ZNF565 | | | | | | | | | |
| ZNF566 | | | | | | | | | |
| ZNF567 | | | | | | | | | |
| ZNF568 | | | | | | | | | |
| ZNF569 | | | | | | | | | |
| ZNF57 | | | | | | | | | |
| ZNF570 | | | | | | | | | |
| ZNF571 | | | | | | | | | |
| ZNF572 | | | | | | | | | |
| ZNF573 | | | | | | | | | |
| ZNF574 | | | | | | | | | |
| ZNF575 | | | | | | | | | |
| ZNF576 | | | | | | | | | |
| ZNF577 | | | | | | | | | |
| ZNF578 | | | | | | | | | |
| ZNF579 | | | | | | | | | |
| ZNF580 | | | | | | | | | |
| ZNF581 | | | | | | | | | |
| ZNF582 | | | | | | | | | |
| ZNF583 | | | | | | | | | |
| ZNF584 | | | | | | | | | |
| ZNF585A | | | | | | | | | |
| ZNF585B | | | | | | | | | |
| ZNF586 | | | | | | | | | |
| ZNF587 | | | | | | | | | |
| ZNF587B | | | | | | | | | |
| ZNF589 | | | | | | | | | |
| ZNF592 | | | | | | | | | |
| ZNF593 | | | | | | | | | |
| ZNF594 | | | | | | | | | |
| ZNF595 | | | | | | | | | |
| ZNF596 | | | | | | | | | |
| ZNF597 | | | | | | | | | |
| ZNF598 | | | | | | | | | |
| ZNF599 | | | | | | | | | |
| ZNF600 | | | | | | | | | |
| ZNF605 | | | | | | | | | |
| ZNF606 | | | | | | | | | |

| | | | | | | | | | |
|---|---|---|---|---|---|---|---|---|---|
| ZNF607 | | | | | | | | | |
| ZNF608 | | | | | | | | | |
| ZNF609 | | | | | | | | | |
| ZNF610 | | | | | | | | | |
| ZNF611 | | | | | | | | | |
| ZNF613 | | | | | | | | | |
| ZNF614 | | | | | | | | | |
| ZNF615 | | | | | | | | | |
| ZNF616 | | | | | | | | | |
| ZNF618 | | | | | | | | | |
| ZNF619 | | | | | | | | | |
| ZNF620 | | | | | | | | | |
| ZNF621 | | | | | | | | | |
| ZNF622 | | | | | | | | | |
| ZNF623 | | | | | | | | | |
| ZNF624 | | | | | | | | | |
| ZNF625 | | | | | | | | | |
| ZNF626 | | | | | | | | | |
| ZNF627 | | | | | | | | | |
| ZNF628 | | | | | | | | | |
| ZNF629 | | | | | | | | | |
| ZNF638 | | | | | | | | | |
| ZNF639 | | | | | | | | | |
| ZNF641 | | | | | | | | | |
| ZNF644 | | | | | | | | | |
| ZNF645 | | | | | | | | | |
| ZNF646 | | | | | | | | | |
| ZNF648 | | | | | | | | | |
| ZNF649 | | | | | | | | | |
| ZNF652 | | | | | | | | | |
| ZNF653 | | | | | | | | | |
| ZNF654 | | | | | | | | | |
| ZNF655 | | | | | | | | | |
| ZNF658 | | | | | | | | | |
| ZNF658B | | | | | | | | | |
| ZNF66 | | | | | | | | | |
| ZNF660 | | | | | | | | | |
| ZNF662 | | | | | | | | | |
| ZNF664 | | | | | | | | | |
| ZNF664-FAM101A | | | | | | | | | |
| ZNF665 | | | | | | | | | |
| ZNF667 | | | | | | | | | |
| ZNF667-AS1 | | | | | | | | | |
| ZNF668 | | | | | | | | | |
| ZNF669 | | | | | | | | | |
| ZNF670 | | | | | | | | | |
| ZNF671 | | | | | | | | | |
| ZNF672 | | | | | | | | | |
| ZNF674 | | | | | | | | | |
| ZNF675 | | | | | | | | | |
| ZNF676 | | | | | | | | | |
| ZNF677 | | | | | | | | | |
| ZNF678 | | | | | | | | | |

| | | | | | | | | | |
|---|---|---|---|---|---|---|---|---|---|
| ZNF679 | | | | | | | | | |
| ZNF680 | | | | | | | | | |
| ZNF681 | | | | | | | | | |
| ZNF682 | | | | | | | | | |
| ZNF683 | | | | | | | | | |
| ZNF684 | | | | | | | | | |
| ZNF687 | | | | | | | | | |
| ZNF688 | | | | | | | | | |
| ZNF689 | | | | | | | | | |
| ZNF69 | | | | | | | | | |
| ZNF691 | | | | | | | | | |
| ZNF692 | | | | | | | | | |
| ZNF695 | | | | | | | | | |
| ZNF696 | | | | | | | | | |
| ZNF697 | | | | | | | | | |
| ZNF699 | | | | | | | | | |
| ZNF7 | | | | | | | | | |
| ZNF70 | | | | | | | | | |
| ZNF701 | | | | | | | | | |
| ZNF702P | | | | | | | | | |
| ZNF703 | | | | | | | | | |
| ZNF704 | | | | | | | | | |
| ZNF705D | | | | | | | | | |
| ZNF705G | | | | | | | | | |
| ZNF706 | | | | | | | | | |
| ZNF707 | | | | | | | | | |
| ZNF708 | | | | | | | | | |
| ZNF709 | | | | | | | | | |
| ZNF71 | | | | | | | | | |
| ZNF710 | | | | | | | | | |
| ZNF711 | | | | | | | | | |
| ZNF713 | | | | | | | | | |
| ZNF714 | | | | | | | | | |
| ZNF716 | | | | | | | | | |
| ZNF717 | | | | | | | | | |
| ZNF718 | | | | | | | | | |
| ZNF720 | | | | | | | | | |
| ZNF721 | | | | | | | | | |
| ZNF724P | | | | | | | | | |
| ZNF726 | | | | | | | | | |
| ZNF727 | | | | | | | | | |
| ZNF728 | | | | | | | | | |
| ZNF729 | | | | | | | | | |
| ZNF730 | | | | | | | | | |
| ZNF732 | | | | | | | | | |
| ZNF735 | | | | | | | | | |
| ZNF736 | | | | | | | | | |
| ZNF737 | | | | | | | | | |
| ZNF738 | | | | | | | | | |
| ZNF74 | | | | | | | | | |
| ZNF740 | | | | | | | | | |
| ZNF746 | | | | | | | | | |
| ZNF747 | | | | | | | | | |

| | | | | | | | | | |
|---|---|---|---|---|---|---|---|---|---|
| ZNF749 | | | | | | | | | |
| ZNF750 | | | | | | | | | |
| ZNF75A | | | | | | | | | |
| ZNF75D | | | | | | | | | |
| ZNF76 | | | | | | | | | |
| ZNF761 | | | | | | | | | |
| ZNF763 | | | | | | | | | |
| ZNF764 | | | | | | | | | |
| ZNF765 | | | | | | | | | |
| ZNF766 | | | | | | | | | |
| ZNF767P | | | | | | | | | |
| ZNF768 | | | | | | | | | |
| ZNF77 | | | | | | | | | |
| ZNF770 | | | | | | | | | |
| ZNF771 | | | | | | | | | |
| ZNF772 | | | | | | | | | |
| ZNF773 | | | | | | | | | |
| ZNF774 | | | | | | | | | |
| ZNF775 | | | | | | | | | |
| ZNF776 | | | | | | | | | |
| ZNF777 | | | | | | | | | |
| ZNF778 | | | | | | | | | |
| ZNF780A | | | | | | | | | |
| ZNF780B | | | | | | | | | |
| ZNF781 | | | | | | | | | |
| ZNF782 | | | | | | | | | |
| ZNF783 | | | | | | | | | |
| ZNF784 | | | | | | | | | |
| ZNF785 | | | | | | | | | |
| ZNF786 | | | | | | | | | |
| ZNF787 | | | | | | | | | |
| ZNF788 | | | | | | | | | |
| ZNF789 | | | | | | | | | |
| ZNF79 | | | | | | | | | |
| ZNF790 | | | | | | | | | |
| ZNF791 | | | | | | | | | |
| ZNF792 | | | | | | | | | |
| ZNF793 | | | | | | | | | |
| ZNF799 | | | | | | | | | |
| ZNF8 | | | | | | | | | |
| ZNF80 | | | | | | | | | |
| ZNF800 | | | | | | | | | |
| ZNF804A | | | | | | | | | |
| ZNF804B | | | | | | | | | |
| ZNF805 | | | | | | | | | |
| ZNF808 | | | | | | | | | |
| ZNF81 | | | | | | | | | |
| ZNF813 | | | | | | | | | |
| ZNF814 | | | | | | | | | |
| ZNF816 | | | | | | | | | |
| ZNF821 | | | | | | | | | |
| ZNF823 | | | | | | | | | |
| ZNF827 | | | | | | | | | |

| | | | | | | | | | |
|---|---|---|---|---|---|---|---|---|---|
| ZNF829 | | | | | | | | | |
| ZNF83 | | | | | | | | | |
| ZNF830 | | | | | | | | | |
| ZNF831 | | | | | | | | | |
| ZNF835 | | | | | | | | | |
| ZNF836 | | | | | | | | | |
| ZNF837 | | | | | | | | | |
| ZNF839 | | | | | | | | | |
| ZNF84 | | | | | | | | | |
| ZNF840P | | | | | | | | | |
| ZNF841 | | | | | | | | | |
| ZNF843 | | | | | | | | | |
| ZNF844 | | | | | | | | | |
| ZNF845 | | | | | | | | | |
| ZNF846 | | | | | | | | | |
| ZNF85 | | | | | | | | | |
| ZNF850 | | | | | | | | | |
| ZNF852 | | | | | | | | | |
| ZNF860 | | | | | | | | | |
| ZNF862 | | | | | | | | | |
| ZNF865 | | | | | | | | | |
| ZNF878 | | | | | | | | | |
| ZNF879 | | | | | | | | | |
| ZNF880 | | | | | | | | | |
| ZNF883 | | | | | | | | | |
| ZNF888 | | | | | | | | | |
| ZNF891 | | | | | | | | | |
| ZNF90 | | | | | | | | | |
| ZNF91 | | | | | | | | | |
| ZNF92 | | | | | | | | | |
| ZNF93 | | | | | | | | | |
| ZNF98 | | | | | | | | | |
| ZNF99 | | | | | | | | | |
| ZNFX1 | | | | | | | | | |
| ZNHIT1 | | | | | | | | | |
| ZNHIT2 | | | | | | | | | |
| ZNHIT3 | | | | | | | | | |
| ZNHIT6 | | | | | | | | | |
| ZNRD1 | | | | | | | | | |
| ZNRD1-AS1 | | | | | | | | | |
| ZNRF1 | | | | | | | | | |
| ZNRF2 | | | | | | | | | |
| ZNRF2P1 | | | | | | | | | |
| ZNRF3 | | | | | | | | | |
| ZNRF4 | | | | | | | | | |
| ZP1 | | | | | | | | | |
| ZP2 | | | | | | | | | |
| ZP3 | | | | | | | | | |
| ZPBP | | | | | | | | | |
| ZPBP2 | | | | | | | | | |
| ZPLD1 | | | | | | | | | |
| Zpr1 | | | | | | | | | |
| ZRANB1 | | | | | | | | | |

| | | | | | | | | | |
|---|---|---|---|---|---|---|---|---|---|
| ZRANB2 | | | | | | | | | |
| ZRANB3 | | | | | | | | | |
| ZRC1 | | | | | | | | | |
| ZRSR1 | | | | | | | | | |
| ZRSR2 | | | | | | | | | |
| ZRT1 | | | | | | | | | |
| ZSCAN1 | | | | | | | | | |
| ZSCAN10 | | | | | | | | + | + |
| ZSCAN12 | | | | | | | | | |
| ZSCAN16 | | | | | | | | | |
| ZSCAN16-AS1 | | | | | | | | | |
| ZSCAN18 | | | | | | | | | |
| ZSCAN2 | | | | | | | | | |
| ZSCAN20 | | | | | | | | | |
| ZSCAN21 | | | | | | | | | |
| ZSCAN22 | | | | | | | | | |
| ZSCAN23 | | | | | | | | | |
| ZSCAN25 | | | | | | | | | |
| ZSCAN26 | | + | | | | + | | | + |
| ZSCAN29 | | | | | | | | | |
| ZSCAN30 | | | | | | | | | |
| ZSCAN31 | | | | | | | | | |
| ZSCAN32 | | | | | | | | | |
| ZSCAN4 | | | | | | | | | |
| ZSCAN5A | | | | | | | | | |
| ZSCAN5B | | | | | | | | | |
| ZSCAN5D | | | | | | | | | |
| ZSCAN9 | | | | | | | | | |
| ZSWIM1 | | | | | | | | | |
| Zswim2 | | | | | | | | | |
| ZSWIM3 | | | | | | | | | |
| ZSWIM4 | | | | | | | | | |
| ZSWIM5 | | | | | | + | | | + |
| ZSWIM6 | | | | | | | | | |
| ZSWIM7 | | | | | | | | | |
| Zswim8 | | | | | | | | | |
| ZUFSP | | | | | | | | | |
| ZUO1 | | | | | | | | | |
| ZW10 | | | | | | | | | |
| ZWILCH | | | | | | + | | | + |
| ZWINT | | | | | | | | | |
| ZXDA | + | | | + | + | | + | | + |
| ZXDB | | | | | | | | | |
| ZXDC | | | | | | | | | |
| ZYG11A | | | | | | | | | |
| ZYG11B | | | | | | | | | |
| ZYX | | | | | | | | | |
| ZZEF1 | | | | | | | | | |
| ZZZ3 | | | | | | | | | |
